# Genome-wide comparison of microRNAs and their targeted transcripts among leaf, flower and fruit of sweet orange

**DOI:** 10.1186/1471-2164-15-695

**Published:** 2014-08-20

**Authors:** Yuanlong Liu, Lun Wang, Dijun Chen, Xiaomeng Wu, Ding Huang, Lingling Chen, Li Li, Xiuxin Deng, Qiang Xu

**Affiliations:** Key Laboratory of Horticultural Plant Biology of Ministry of Education, Huazhong Agricultural University, Wuhan, 430070 China; Center for Bioinformatics, College of Life Science and Technology, Huazhong Agricultural University, Wuhan, 430070 China; Robert W. Holley Center for Agriculture and Health, Cornell University, Ithaca, NY 14853 USA

**Keywords:** *Citrus sinensis*, miRNA, Degraded transcript, Genome-wide comparison, Fruit ripening

## Abstract

**Background:**

In plants, microRNAs (miRNAs) regulate gene expression mainly at the post-transcriptional level. Previous studies have demonstrated that miRNA-mediated gene silencing pathways play vital roles in plant development. Here, we used a high-throughput sequencing approach to characterize the miRNAs and their targeted transcripts in the leaf, flower and fruit of sweet orange.

**Results:**

A total of 183 known miRNAs and 38 novel miRNAs were identified. An in-house script was used to identify all potential secondary siRNAs derived from miRNA-targeted transcripts using sRNA and degradome sequencing data. Genome mapping revealed that these miRNAs were evenly distributed across the genome with several small clusters, and 69 pre-miRNAs were co-localized with simple sequence repeats (SSRs). Noticeably, the loop size of pre-miR396c was influenced by the repeat number of CUU unit. The expression pattern of miRNAs among different tissues and developmental stages were further investigated by both qRT-PCR and RNA gel blotting. Interestingly, Csi-miR164 was highly expressed in fruit ripening stage, and was validated to target a NAC transcription factor. This study depicts a global picture of miRNAs and their target genes in the genome of sweet orange, and focused on the comparison among leaf, flower and fruit tissues.

**Conclusions:**

This study provides a global view of miRNAs and their target genes in different tissue of sweet orange, and focused on the identification of miRNA involved in the regulation of fruit ripening. The results of this study lay a foundation for unraveling key regulators of orange fruit development and ripening on post-transcriptional level.

**Electronic supplementary material:**

The online version of this article (doi:10.1186/1471-2164-15-695) contains supplementary material, which is available to authorized users.

## Background

Micro RNAs (miRNAs), which are typically 20–24 nucleotides (nt) in length, are derived from primary miRNA transcripts (pri-miRNAs) that contain a stem-loop secondary structure. The pri-miRNA is processed in the nucleus by DCL1, a Dicer-like protein, to create a miRNA-miRNA* duplex, where miRNA* is a passenger strand complementary to the miRNA. The duplex is then separated by helicase and the mature miRNA is incorporated into an ARGONAUTE 1 (AGO1) protein to form an RNA-induced silencing complex (RISC) [1, 2]. Target genes that contain a sequence with almost complete complementary to the miRNA are cleaved by the RISC at a specific site opposite to the 10th or 11th nucleotide in the miRNA. The miRNA* species is thought to be degraded [1, 3]. However, recent research demonstrated that AGO-associated miRNA393* guides the silencing of a golgi-localized SNARE gene, indicating that miRNA* species may be capable of functioning as miRNAs [4].

Typically, miRNA-guided cleavage represses target gene expression; however, a group of 22 nt miRNAs also trigger secondary siRNA biogenesis in plants [5]. Some 22 nt miRNAs are generated from bulged duplexes comprising a 22 nt miRNA and a 21 nt miRNA*; these duplexes are processed by DCL1. By contrast, DCL2 generates 22 nt miRNAs by processing perfect duplexes comprising a 22 nt miRNA and a 22 nt miRNA*. The latter type of 22 nt miRNA may trigger secondary siRNA biogenesis. During this process, the miRNA is incorporated into AGO1 which guides 5’ cleavage of the target, and then RNA-dependent RNA polymerase 6 (RDR6) synthesizes a double-stranded RNA fragment that is subsequently cut into secondary siRNAs. With the exception of the 24 nt secondary siRNAs (triggered by miR2775), most secondary siRNAs are 21 nt in length [5, 6]. The mechanism of biogenesis of trans-acting siRNAs (tasiRNAs) generated from *TAS3* differs from that described above. A complex comprising miR390 and AGO7 binds to *TAS3* at two sites and guides the cleavage of the tasiRNA precursor. The cleavage product is then converted to double-stranded RNA by RDR6 and DCL4, and tasiRNAs are generated one by one from the cleavage site to the 5’ side. All of the secondary siRNAs described above may incorporate into AGOs and function similarly as miRNAs [6, 7].

Traditional cloning and sequencing methods have been used to identify miRNAs in model plants, including *Arabidopsis*, rice and poplar. Comparison of the sequences of miRNAs from these species has revealed that most are highly conserved [8]. Nevertheless, a number of non-conserved miRNAs may be family-specific or species-specific. Generally, non-conserved miRNAs are expressed at a lower level than conserved miRNAs. Recently, high-throughput sequencing has been used for the identification and expression profiling of miRNAs in many horticultural plants, including tomato, grape, papaya, radish and trifoliate orange [9–13]. Accurate identification of miRNAs from large amounts of sequencing data is challenging; therefore, minimal criteria for annotation of miRNAs in plants were consolidated [14]. A miRNA-mediated degraded fragments sequencing (degradome sequencing) approach was also reported to be capable of efficient characterization of miRNA target genes [15]. This method involves ligation of the 3’ fragment generated by RISC, which contains a 5’-monophosphate, to a 5’ RACE adaptor. Reverse transcription is then performed using oligo(dT) as a 3’ adaptor and the cDNAs formed by second strand synthesis are digested with *MmeI* to generate 20 bp signatures that are sequenced and used to identify miRNA target pairs [15–17].

As described above, miRNAs act as regulators of plant development [18, 19]; miR156, miR164 and miR166, in particular, play important roles in regulating leaf development [20–24]. Additionally, miR156, miR159, miR319 and miR172 are involved in flowering regulation and phase changing from vegetative growth to reproductive growth [21, 25–29]. Overexpression of osmiR397 leads to increasing rice size and promoting panicle branching [30]. The understanding of the roles of miRNA in the regulation of reproductive growth is further improved by recent research on non-conserved (novel) miRNAs; for example, miR4376 in the Solanaceae regulates a Ca^2+^-ATPase involved in tomato reproductive growth [31].

The first report of miRNAs in the Rutaceae family was in *Poncirus trifoliata* [32] and high-throughput sequencing was recently used to identify miRNAs and their targets in this species [33, 34]. Our group previously identified miRNAs in sweet orange (*Citrus sinensis*) by using EST assembly as reference to dig out miRNA seqeunces [35]. Here, based on our recently published sweet orange genome [36], we performed a genome-wide characterization of sRNAs (the sRNAome) and their targets (the degradome) in the leaves, flowers and fruits of sweet orange (*C. sinensis* [L.] Osbeck). A total of 183 known and 38 novel miRNAs were identified; the tissue specifically expressed miRNAs were validated with the aim of identifying miRNAs involved in the regulation of fruit development and maturation.

## Results and discussion

### Sequencing of sRNAs and the degradome

**Figure 1:**
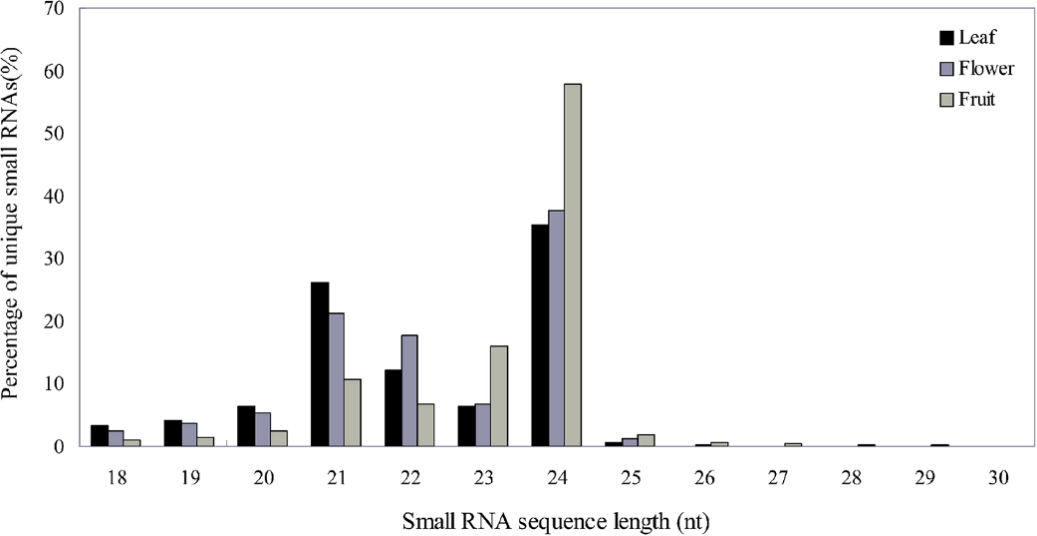
**Lengths of the sweet orange sRNAs identified in leaf, flower and fruit tissues.** The sRNAome sequencing data are expressed as a percentage of the total number of the unique sRNAs identified in each sample type.

The Illumina sequencing data of sRNAs from leaf, flower and fruit showed that 24 nt sRNAs are the most abundant and very few longer sRNAs (Figure 1). The greater abundance of 24 nt sRNAs rather than 21 nt sRNAs in all three tissues agrees with the results observed for other dicots [37, 38]. After discarding low quality reads and sequence counts lower than 3 in each library, clean reads were produced. The clean reads were then compared with the sequence of the *C. sinensis* genome [36]. Overall, perfect matches with the *C. sinensis* genome were obtained for 61.21%, 58.56% and 76.30% of unique sRNAs from leaf, flower and fruit, respectively (Table 1). The sRNA tags were annotated with sequences from the Rfam database to eliminate non-coding RNAs, including rRNA, tRNA, snRNA and snoRNA. A search of the miRBase database (v16) identified 2699, 1985 and 771 unique matches for the sRNAs identified in leaf, flower and fruit, respectively (Table 1). A BLAST search of miRBase identified 183 known miRNAs based on sequence similarity and the presence of a stem-loop structure in the precursor. The sRNA clean reads that were not known miRNAs were mapped to the genome and were used to predict novel miRNAs according to the structure and expression criteria [14]. Finally, 38 candidate novel miRNAs with a clear precursor containing a stem-loop secondary structure were identified (Additional files 1 and 2).

**Table 1:** **Characteristics of the sRNAs identified in orange leaf, flower and fruit**

		Total sRNAs	Unique sRNAs
Leaf	Raw reads	18961890	
	Clean reads^a^	10174438 (100%)	586073 (100%)
	Match genome	6234563 (61.28%)	358731 (61.21%)
	Match miRbase^b^	1068582 (10.50%)	2699 (0.46%)
Flower	Raw reads	17841995	
	Clean reads^a^	9252302 (100%)	612511 (100%)
	Match genome	5172633 (55.91%)	358709 (58.56%)
	Match miRbase^b^	559470 (6.05%)	1985 (0.32%)
Fruit	Raw reads^c^	4598696	
	Clean reads^a^	1785395 (100%)	202965 (100%)
	Match genome	1415319 (79.27%)	154864 (76.30%)
	Match miRbase^b^	55653 (3.12%)	771 (0.38%)

^a^ Low quality sequences containing ambiguous nucleotides, sequences shorter than 18 nt, and sequence counts lower than three were removed.

^b^ Small RNAs matching miRbase (v16) with a maximum of two mismatches (if gap exist, number of gaps plus mismatches ≤ 5).

^c^ The raw reads of fruit sRNA library were from our previous research (Xu et al. [35]).

To identify the target genes of miRNAs, a high-throughput experimental approach was used to sequence the degradome libraries of the leaf, flower and fruit tissues. After discarding low quality sequences, more than 17 million clean reads were obtained for each tissue. The cDNA sequences annotated from orange genome platform [36] was used as the mRNA reference dataset and the combination of 183 known miRNAs and 38 novel miRNAs was used as the miRNA reference dataset. Overall, perfect matches with the mRNA reference dataset were obtained for 72.86%, 67.12% and 56.42% of unique tags from the leaf, flower and fruit samples, respectively (Table 2). In addition, 55257, 62365 and 19393 degraded mRNA fragments of miRNA targets were identified in leaf, flower and fruit, respectively. The miRNA reference dataset and the degraded fragments were then used to generate miRNA-mRNA pairs. A total of 405 targets were identified for 107 miRNAs which expressed in leaf. In addition, 265 targets were identified for 166 miRNAs in flower and 322 targets were identified for 118 miRNAs in fruit (Additional file 3). Confidence evaluation of degradome data was performed as reported previously [16, 39]. The target transcripts were divided into three classes (category I, category II and category III; Additional file 4). In category I, the miRNA-guided cleavage fragment was the most abundant tag matching the transcript; therefore, category I is most reliable for the detection of miRNA-targeted genes. In category II, the miRNA-guided cleavage fragment was not the most abundant fragment; however, it still formed a clear peak in the T-plot. The remaining target transcripts were classified into category III (Additional file 4).

**Table 2:** **Characteristics of the degradome sequences from orange leaf, flower and fruit**

		Total RNAs	Unique RNAs
Leaf	Raw reads	22826037	
	Clean reads^a^	20566571 (100%)	1448803 (100%)
	Match mRNA reference dataset	17683257 (85.98%)	1055651 (72.86%)
Flower	Raw reads	19030542	
	Clean reads^a^	17629275 (100%)	1093131 (100%)
	Match mRNA reference dataset	14577696 (82.69%)	733751 (67.12%)
Fruit	Raw reads	21213198	
	Clean reads^a^	18284467 (100%)	331940 (100%)
	Match mRNA reference dataset	16047135 (87.76%)	187295 (56.42%)

^a^Low quality sequences, sequences shorter than 18 nt, and adaptor contaminants were removed.

### Known miRNAs and their targets in the leaf, flower and fruit tissues

A total of 183 known miRNAs, including 53 isoforms with high expression level, belonging to 58 families, were identified. The sweet orange genome sequence was used to predict RNA secondary structures; all of the precursors of the 183 known miRNAs had regular stem-loop secondary structures (Additional file 1).

**Figure 2:**
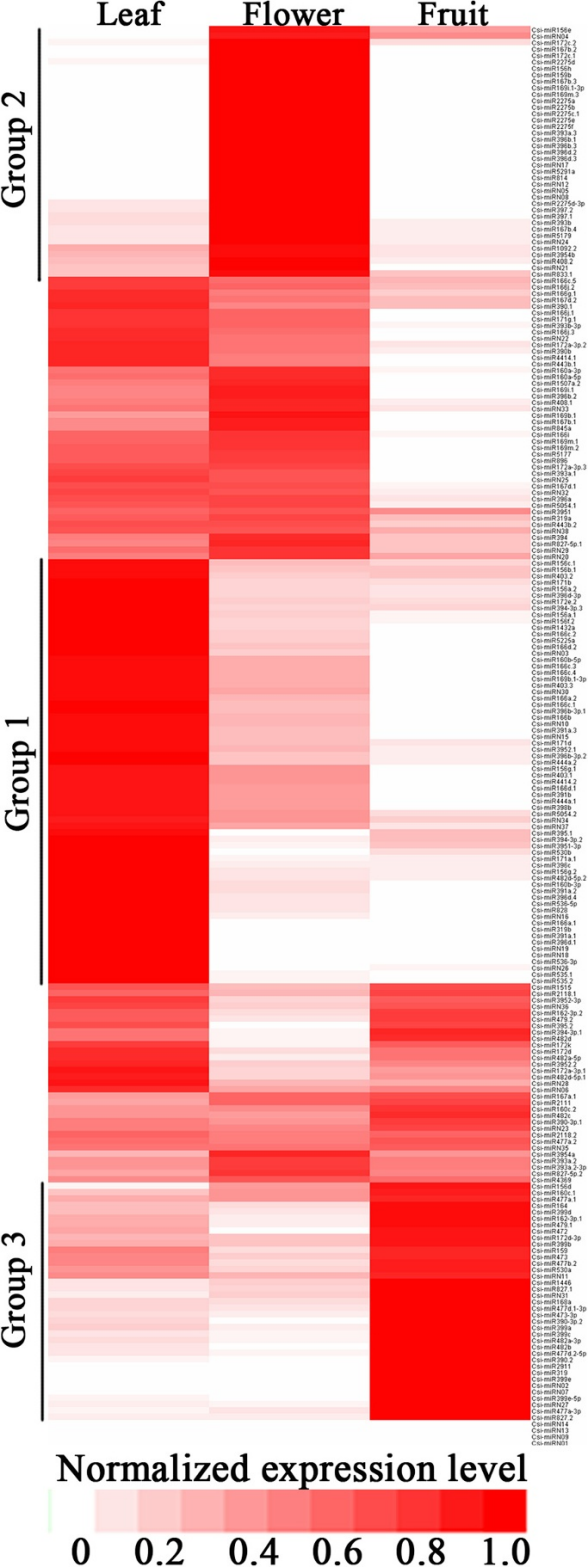
**Heat map of the normalized expression level (TPM) of all miRNAs.** miRNAs belong to group 1 are highly expressed in leaf; miRNAs belong to group 2 are highly expressed in flower; miRNAs belong to group 3 are highly expressed in fruit. The colorbar is shown at the bottom of the figure.

Comparison of the normalized expression levels of miRNAs in the leaf, flower and fruit tissues revealed that 60 known miRNAs displayed higher expression (>2 fold change) in leaf than those in flower or fruit; 33 known miRNAs showed higher expression in flower; and 32 known miRNAs displayed higher expression in fruit (Figure 2 and Additional file 2). For example, csi-miR477a-3p, csi-miR827.1 and csi-miR168a showed much higher expression levels (>4 fold change) in fruit. In addition, some miRNAs, including csi-miR166j.1, csi-miR166j.3, csi-miR4414.1, csi-miR391b, csi-miR1432a, the csi-miR169 family and the csi-miR171 family, were expressed in leaf and flower but were not detected in fruit.

The targets of known and conserved miRNA families were identified from the degradome data. The target genes were divided into three categories as described above. A total of 395 targets of 100 known miRNAs were identified in leaf degradome data; of these targets, 259 were classified into category I, 38 into category II, and 98, into category III. In flower, 241 targets of 142 known miRNAs were identified; 105, 26 and 110 were classified into categories I, II and III, respectively. In fruit, 301 targets of 101 known miRNAs were identified; of these targets, 107, 66 and 128 were grouped into categories I, II and III, respectively (Additional files 3 and 4;).Most of the conserved miRNAs have similar targets to *Arabidopsis thaliana* [28]. Multiple targets were also observed for these miRNAs. For example, the miR168 family controls feedback regulation of AGO 1 as previously reported [40, 41].

The miRNAs and their targeted transcripts from leaf, flower and fruit tissues indicated that a single miRNA may target genes in a tissue-specific manner. In most circumstance, conserved known miRNAs had the same or homologous targets as other plant species, and most of them were grouped into category I. However, a minority of the conserved miRNAs had different targets in specific tissues. An interesting example was csi-miR168a; the finding that this miRNA targeted AGO1 as a feedback regulator in all three tissues is consistent with previous reports [40, 41]. Additionally, csi-miR168a was found to specifically target a CUP-SHAPED COTYLEDON 2 (*CUC2)* gene in leaves and a pantothenate kinase gene in fruit, as highly supported by the degradome data from different tissues (Additional file 5a). Therefore, csi-miR168a is not only involved in stabilizing the accumulation of AGO1, but also has additional roles in the leaf and fruit biology. Another example is that csi-miR159 family was detected to target the transcription factor GAMYB in leaf and flower in consistency with previous reports [42, 43]. However, in fruit, degraded fragments of *GAMYB* generated by csi-miR159 were absent and this miRNA was found to targete *Cs8g05120* which was annotated as the LRR-containing protein DRT100 with potential roles in DNA damage repair (Additional file 5b). A previous study in rice also demonstrated that members from the same miRNA family can have different expression patterns, and this expression difference affects the selectiveness of target gene [44]. These observations indicated that conserved miRNAs may fulfill additional regulatory roles in specific tissue in addition to the conserved and general functions across plant species.

### Novel miRNAs and their targets in the leaf, flower and fruit tissues

A total of 38 candidate novel miRNAs with a clear precursor containing a stem-loop secondary structure were identified (Additional file 1). The complementary miRNA* sequences for each candidate novel miRNAs were also detected, although most were present at a lower level than their corresponding miRNAs. Unfortunately, we were unable to determine if biogenesis of these candidate novel miRNAs is dependent on DCL1 because of the lack of availability of a *C. sinensis* DCL1 mutant strain.

More than half of the novel miRNAs identified was expressed at high levels (greater than 10 TPM). 11 novel miRNAs showed higher expression in leaf than flower or fruit; 7 novel miRNAs showed higher expression in flower than leaf or fruit; and 4 novel miRNAs displayed higher expression in fruit than leaf or flower. For example, csi-miRN27 and csi-miRN31 showed higher expression in fruit than leaf or flower (Figure 2 and Additional file 2).

From the degradome data, 10 targets of seven novel miRNAs were identified in leaf; of these targets, 5, 2 and 3 were classified into categories I, II and III, respectively. A total of 24 targets of 24 novel miRNAs in flower were identified; three of these targets were classified into category I, two were grouped into category II, and the remaining 19 were classified into category III. In fruit, 21 targets of 17 miRNAs were identified, of which four were classified into category I, three were grouped into category II, and 14 were classified into category III (Additional file 3). In summary, nine target genes of novel miRNAs were grouped into category I, some of which had multiple predicted functions. *Cs8g09620*, which is annotated as an flowering-related (SRF-type transcription factor family) protein, was identified as a target of csi-miRN7; and *Cs1g12230*, which is annotated as a light control (FAR1-RELATED SEQUENCE 6 ) protein, was identified as a target of csi-miRN20. Some of the identified targets were not sufficiently annotated; for example, *Cs4g15010* (category II), targeted by csi-miRN28, was annotated as a putative uncharacterized protein. (Additional files 3, 4 and 6 and Table 3).

**Table 3:** **Target genes grouped into category I of novel miRNAs in orange**

		Category			
Novel miRNA	Target gene	in leaves	in flowers	in fruit	Annotation
Csi-miRN02	Cs1g08400	I	N/A	N/A	Chaperonin CPN60-1
Csi-miRN02	Cs3g18790	I	II	I	Alkylated DNA repair protein alkB homolog 8
Csi-miRN02	Cs8g12570	I	N/A	N/A	LRR receptor-like serine/threonine-protein kinase EFR
Csi-miRN07	Cs8g09620	III	I	N/A	SRF-type transcription factor family protein
Csi-miRN11	Cs8g13560	I	III	I	No annotation
Csi-miRN20	Cs1g12230	N/A	N/A	I	Protein FAR1-RELATED SEQUENCE 6
Csi-miRN20	Cs3g05320	I	III	I	No annotation
Csi-miRN37	orange1.1 t02400	II	I	N/A	Indole-3-acetic acid-induced protein ARG7

N/A means not available in the degradome data. Target transcripts were divided into three classes. In category I, the miRNA-guided cleavage fragment was the most abundant tag matching the transcript; In category II, the miRNA-guided cleavage fragment was not the most abundant fragment; however, it still formed a clear peak in the T-plot. The remaining target transcripts were classified into category III.

Of the novel miRNAs that were considered to be younger (recently evolved) than the conserved miRNAs, only three (csi-miRN2, csi-miRN11 and csi-miRN20) had common targets or homologous genes in all tissues. Most of the novel miRNAs appeared to target different transcripts in different tissues; for example, csi-miRN10 targeted *Cs9g04330* in flower and *Cs4g05310* in fruit (Additional file 5c). These results suggested that the functions of novel miRNAs may differ in leaf, flower and fruit tissues and might fulfill a more specific role when compared with known and conserved miRNAs.

### Identification of secondary siRNAs in *C. sinensis*

An in-house script was developed to predict secondary siRNAs that might be triggered by 22 nt miRNAs in citrus. After the prediction, we examined the abundances of the secondary siRNAs using sRNA sequencing data. Seven miRNAs were predicted to trigger the secondary siRNAs generation (see in Additional file 7), four of them were identified in at least two tissues. Csi-miRN20 could be identified in all three tissues; csi-miR482a-3p in both leaves and fruits, csi-miR3954a and csi-miR482c in both leaves and flowers. Secondary siRNAs were named using the convention of “miRNA-target-serial number”; for example, csi-miR3954a-Cs1g09635.1-9 refers to the ninth phased siRNA generated from *Cs1g09635.1* and triggered by csi-miR3954a. Moreover, we identified these secondary siRNAs’ target genes using degradome sequencing data (Additional files 7 and 8). Most of the secondary siRNA and target pairs had a high penalty score and only a few targets were classified into categories I and II. The analysis indicated that Csi-miR3954a-Cs1g09635.1-9 targeted triose-phosphate transporter gene (category I), csi-miR482a-3p-Cs5g18480.1-18 targeted the cleavage and polyadenylation specificity factor (CPSF) gene (category I), and csi-miRN20-Cs3g05320.1-24 targeted a signal peptidase gene (category I). These findings suggest that these phased siRNAs may play regulatory roles in plant development.

Here, multiple lines of evidence suggest the existence of both conserved and orange-specific *TAS* gene families. The homologous *TAS3* gene (*Cs9g01780*) was identified as a target of csi-miR390 in orange. This is consistent with previous studies from TAS1-4 genes in *Arabidopsis* [6, 37, 45], MdTAS3 and MdTAS4 in apple [46]and TAS3 gene containing two miR390 binding sites in tobacco [47]. Noticeably, a potential orange-specific TAS gene targeted by cis-miR3954a was observed with multiple supports. Firstly, csi-miR3954a was a citrus-specific miRNA which showed abundant expression in all three tissues; Secondly, this miRNA was found to be 22 nt in length and was also found to contain a 5’U; this characteristic structure was believed to trigger phased siRNA biogenesis[5]; Thirdly, the target gene (*Cs1g09635*) of csi-miR3954a could not be functionally annotated and was predicted to be a non-coding transcript; Fourthly, the secondary siRNAs, derived from csi-miR3954 target genes, were detected in the small RNA libraries. One of these secondary siRNAs, named csi-miR3954a-Cs1g09635.1-7, was abundant in leaf and flower and targeted *Cs4g11930*. Therefore, we hypothesized that targets of csi-miR3954a are potential novel *TAS* target genes. A further evidence to determine if this is dependent on RNA-dependent RNA polymerase (RDR) is difficult in sweet orange due to the lack of RDR mutants. A recent example in tobacco observed that the 22 nt nta-miR6019 targets the *N* gene and triggers the generation of nta-siRNAI and nta-siRNAII which was dependent on RDR6 as well as nta-siR2142 (generated from tobacco *TAS3*), and concluded that the target of nta-miR6019 might be a novel *TAS* gene [48].

### The distruibution of miRNAs in the *C. sinensis*genome

**Figure 3:**
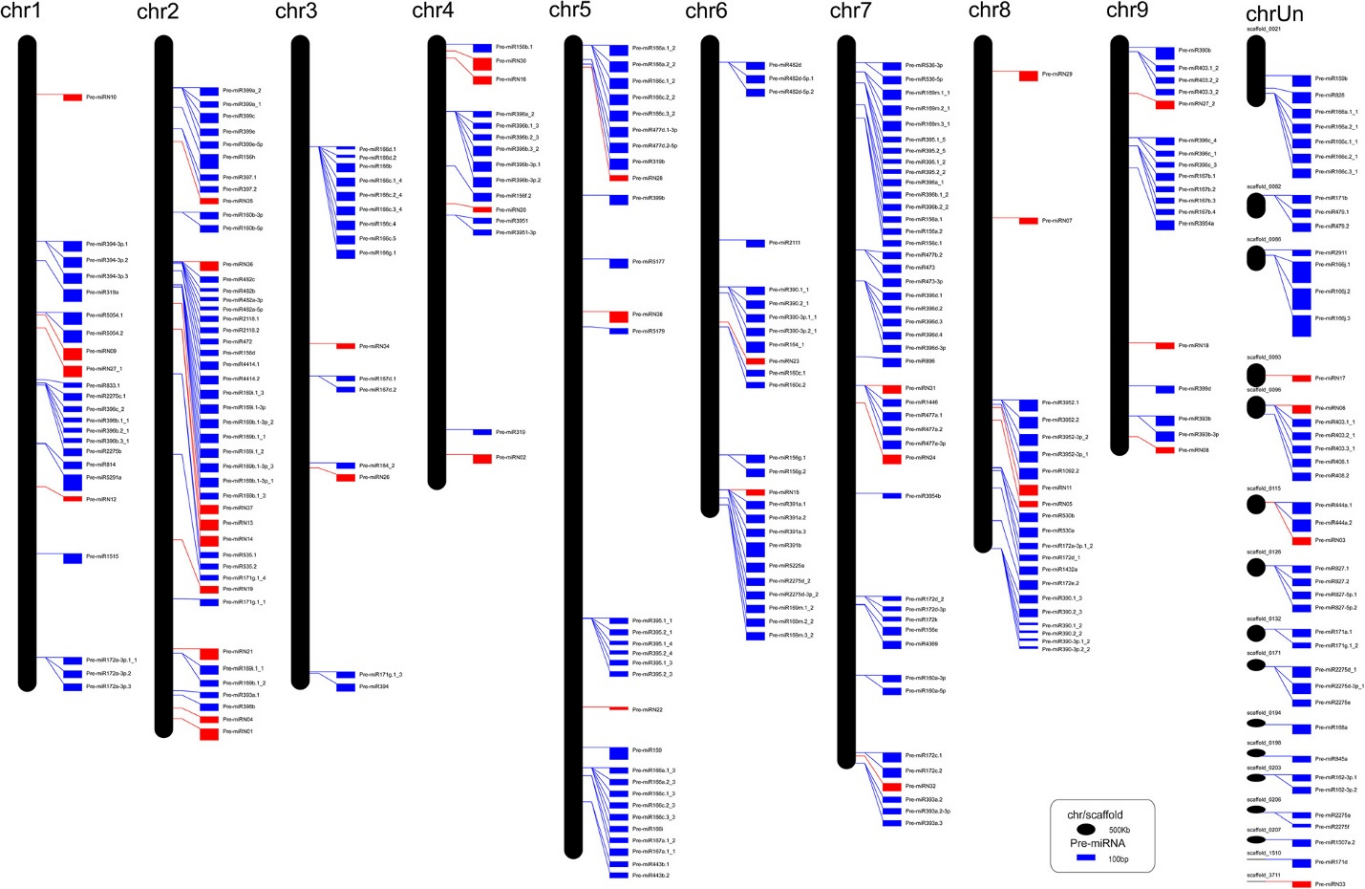
**The distribution of pre-miRNAs in the** ***C. sinensis*** **genome.** Precursors of known miRNAs are shown as blue rectangle and precursors of novel miRNAs are shown as red rectangle. The width of rectangle indicated the length of the precursors while the height of oblong indicated the length of the chromosome/scaffold. The chromosome/scaffold and precursors scale are showed at the bottom of the figure. The chromosomal locations of the pre-miRNAs are indicated.

Both novel and known miRNAs were equally distributed in the citrus genome (Figure 3); however, some members of conserved miRNA families were located in the same region of a single chromosome. For example, csi-miR169c and csi-miR169i were located in the same region of chromosome 2.

**Figure 4:**
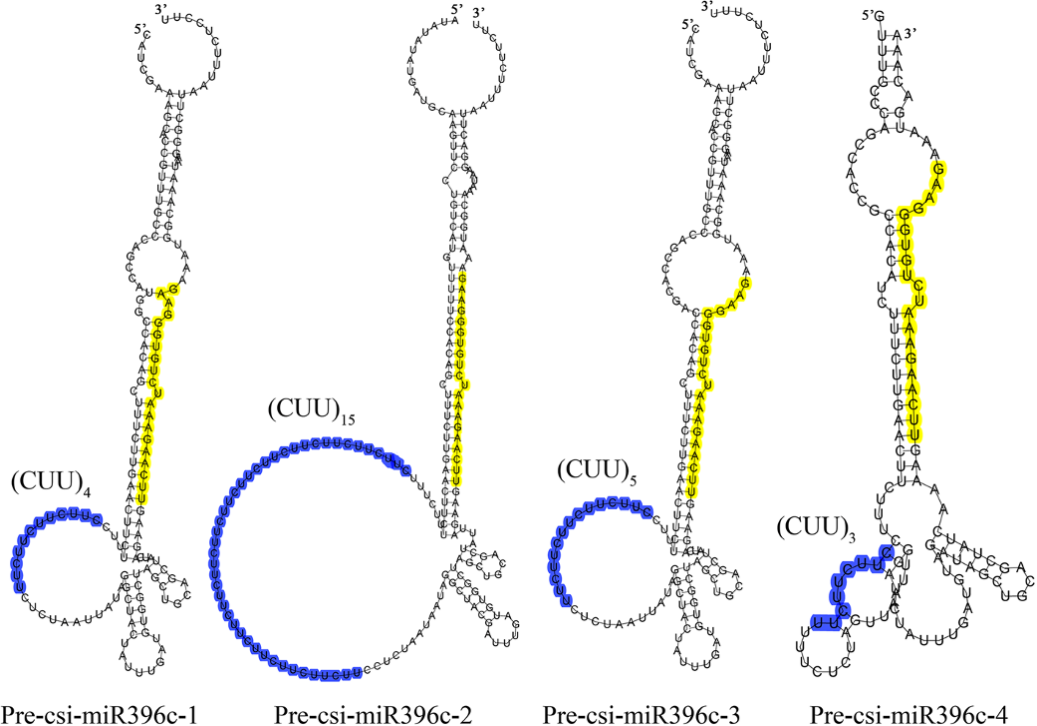
**Co-localization of pre-Csi-miR396c and simple sequence repeat (SSR).** The mature miR396c sequence is highlighted in yellow and the SSRs are highlighted in blue. The different secondary structures of pre-Csi-miR396c caused by the different number of SSR units are shown.

We analyzed all simple sequence repeat (SSR) loci in the orange genome and examined their co-localization with pre-miRNAs. Overall, 69 csi-pre-miRNA were identified to co-localize with SSR region. 77.6% of the co-localized SSRs were binucleotide or trinucleotide sequences and the most frequent repeat unit was UA, consistent with the notion that most trinucleotide SSRs contain the base U in plants [49]. The longest SSR, consisting of (CUU)_15_ (45 nt), was located within the pre-miR396c sequence (Table 4). Notably, csi-miR396c had four precursors containing different sized trinucleotide SSRs. (CUU)_4_, (CUU)_5_ and (CUU)_3_ were located within the pre-csi-miR396c-1, pre-csi-miR396c-3 and pre-csi-miR396c-4 sequences, respectively. To date, little is known about the functions of SSRs co-localized with pre-miRNAs. Here, the length of the (CUU)_n_ repeat influenced the shape of the loop in the secondary structures of the csi-miR396c precursors (Figure 4). A previous report demonstrated that SSRs located in UTRs and introns can affect gene expression and expression level [50]. Interestingly, analysis of the maize zma-miR396 family showed that zma-miR396e and zma-miR396f bearing SSRs (CU)_n_ and (CUU)_n_ had similar expression pattern among different tissues which was distinctly different from the expression pattern of the other miR396 members without SSRs (expression data is form the publication of Zhang et al. [51]. Additional file 9). Changes in the organization of the loop may directly influence the expression of mature miRNA and may cause unexpected changes in their evolution.

**Table 4:** **Numbers of Simple Sequence Repeats (SSRs) and their co-localization with pre-miRNAs in sweet orange**

Repeat unit	Repeat type	Frequency of SSR region co-localize with pre-miRNAs
A	Mononucleotide	1
G	Mononucleotide	1
T	Mononucleotide	5
AC	Dinucleotide	2
AG	Dinucleotide	5
AT	Dinucleotide	2
CT	Dinucleotide	3
GA	Dinucleotide	7
GT	Dinucleotide	1
TA	Dinucleotide	12
TC	Dinucleotide	3
AAG	Trinucleotide	1
ACA	Trinucleotide	1
ATA	Trinucleotide	1
ATT	Trinucleotide	3
CAG	Trinucleotide	1
CTT	Trinucleotide	7
GAT	Trinucleotide	2
GTG	Trinucleotide	1
TAA	Trinucleotide	5
TCA	Trinucleotide	1
TGC	Trinucleotide	1
TGG	Trinucleotide	2
TTC	Trinucleotide	5
CATG	Tetranucleotide	1
TCAT	Tetranucleotide	1
TGCA	Tetranucleotide	1
TTAA	Tetranucleotide	2
TTCT	Tetranucleotide	4
TTTG	Tetranucleotide	1
TATAAT	Hexanucleotide	2

Isoforms of miRNAs that originated from a long pre-miRNA (greater than 350 nt in length) were also identified. Csi-miR166j.1 and csi-miR166j.3 were derived from the same precursor and were positioned 195 nt apart. Similarly, csi-miR477d.1 andcsi-miR477d.2 were positioned 126 nt apart in the same precursor. However, the expression modes of the mature miRNAs originating from these two precursors were distinctly different. Csi-miR166j.1 and csi-miR166j.3 had extremely similar patterns of expression in different tissues and both the mature miRNAs were derived from 3’ strand of the miRNA-miRNA* duplexes (Additional file 10a). In contrast, Csi-miR477d.1 and csi-miR477d.2 were produced from different strands of the duplexes, i.e., csi-miR477d.1-3p was produced from 3’ strand while csi-miR477d.2-5p was produced from 5’ strand of the duplexes (Additional file 10b). A study in grapevine showed that miRC13 and miRC15 are located 224 nt apart on chromosome 17, but these two miRNAs exhibit different expression patterns [52].

### Characterization of highly expressed miRNAs in fruit

**Figure 5:**
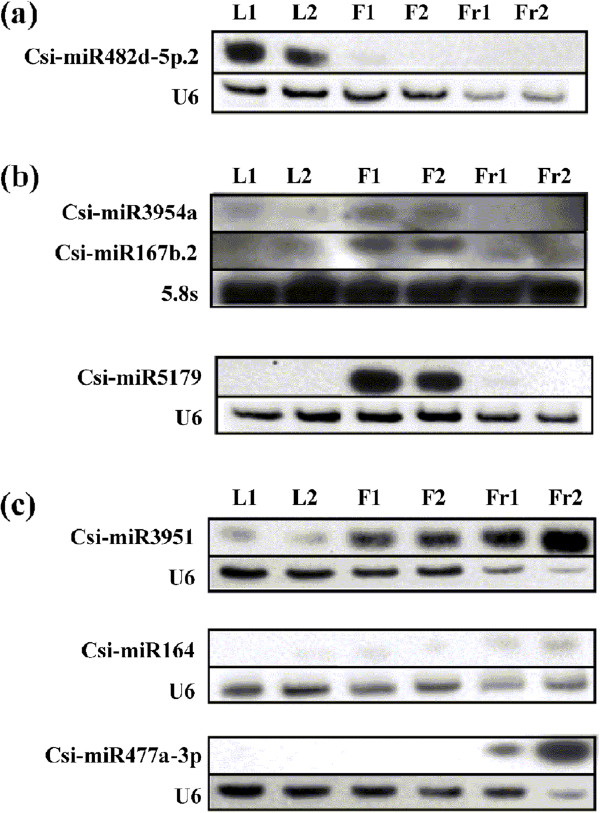
**Expression levels of selected known miRNAs in leaf, flower and fruit.** Total RNAs from mixed leaf, mixed flower and fruit in 170DAF were extracted for RNA gel blot analyses; reverse complement probes were used to detect the indicated miRNAs. U6 RNA or 5.8 s rRNA were used as loading controls. Two different genotypes of orange were used. Orange genotype 1 is a common sweet orange while genotype 2 is a red flesh orange. **(a)** miR482d-5p.2 was specifically expressed in leaf. **(b)** miR3954a and miR167b.2 were expressed at higher levels in flower than the other tissues; miR5179 was specifically expressed in flower **(c)** miR3951 and miR164 were expressed at higher levels in fruit than the other tissues; miR477a-3p was specifically expressed in fruit. L1, leaf of orange genotype 1; L2, leaf of orange genotype 2; F1, flower of orange genotype 1; F2, flower of orange genotype 2; Fr1, fruit of orange genotype 1; Fr2, fruit of orange genotype 2.

**Figure 6:**
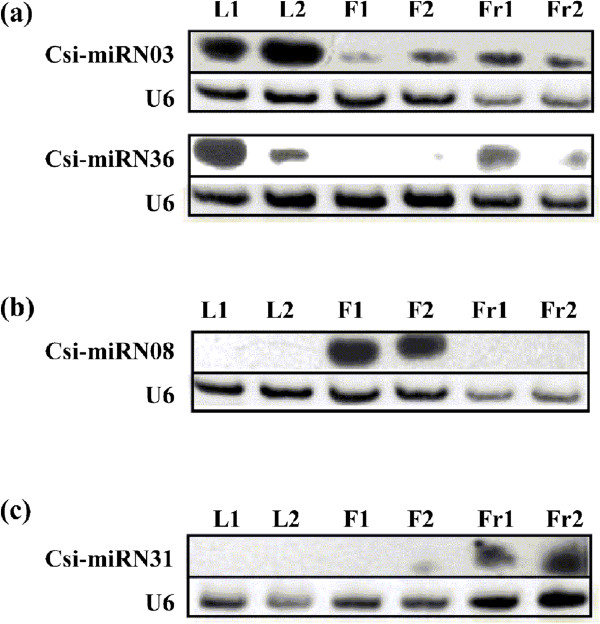
**Expression levels of selected novel miRNAs in leaf, flower and fruit.** Total RNAs from mixed leaf, mixed flower and fruit in 170DAF were extracted for RNA gel blot analysis. Reverse complement probes were used to detect specific miRNAs. U6 RNA was used as a loading control for each membrane. Two different genotypes of orange were used. Orange genotype 1 is a common sweet orange while genotype 2 is a red flesh orange. **(a)** miRN03 and miRN36 were expressed at higher levels in leaf than flower or fruit. **(b)** miRN08 was specifically expressed in flower. **(c)** miRN31 was specifically expressed in fruit. L1, leaf of orange genotype 1; L2, leaf of orange genotype 2; F1, flower of orange genotype 1; F2, flower of orange genotype 2; Fr1, fruit of orange genotype 1; Fr2, fruit of orange genotype 2.

**Figure 7:**
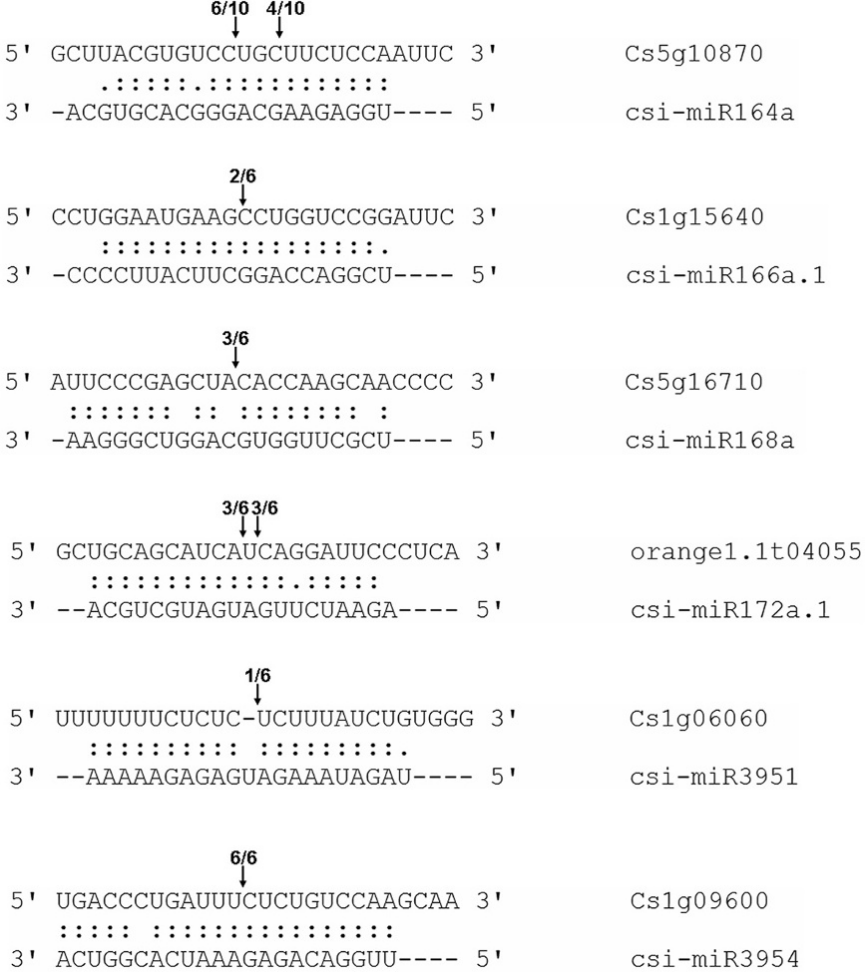
**Target validation of selected sweet orange miRNAs.** 5’ RACE analysis was carried out for each selected target gene which identified by degradome sequencing. Arrows indicated the cleavage sites of targets and the numbers showed the frequency of the clones sequenced. The Cleavage sites outside of the displayed sequence are not shown.

Candidate miRNAs that showed remarkably differential expression levels in fruit as revealed by the sequencing data were further confirmed by Quantitative RT-PCR (qRT-PCR) and RNA gel blotting analyses. Totally, 65 known- and 15 novel- miRNAs were chose for qRT-PCR confirmation; 55 of them (about two thirds) are consistent with the sequencing data (Additional files 2 and 11). The results showed that csi-miR164, csi-miR3951, csi-miR477a-3p and csi-miRN31 have remarkably higher expression in fruits compared with other tissues. These results were verified by both qRT-PCR and RNA gel blotting (Figure 5, Figure 6 and Additional file 11). Two targets of csi-miR164 were identified in fruit; the first was annotated as a NAC transcription factor gene which was confirmed by 5’ RACE (Figure 7) and the second was annotated as a thymidine diphospho-glucose 4-6-dehydratase gene. Csi-miR3951 also had two targets in fruit; the first was *orange1.1 t05622* and the second was *Cs1g06060* (verified by 5’ RACE, see in Figure 7), which were annotated as polyubiquitin 12 and polyubiquitin 3, respectively. These targets may be involved in protein degradation. *Cs6g19680*, which was annotated as the developmental protein SEPALLATA 2 (SEP2), was identified as a target of csi-miR477a-3p (degradome data). Targets of csi-miRN31 were not detected by the degradome analysis. The target prediction revealed that csi-miRN34 targeted transmembrane emp24 domain-containing gene.

**Figure 8:**
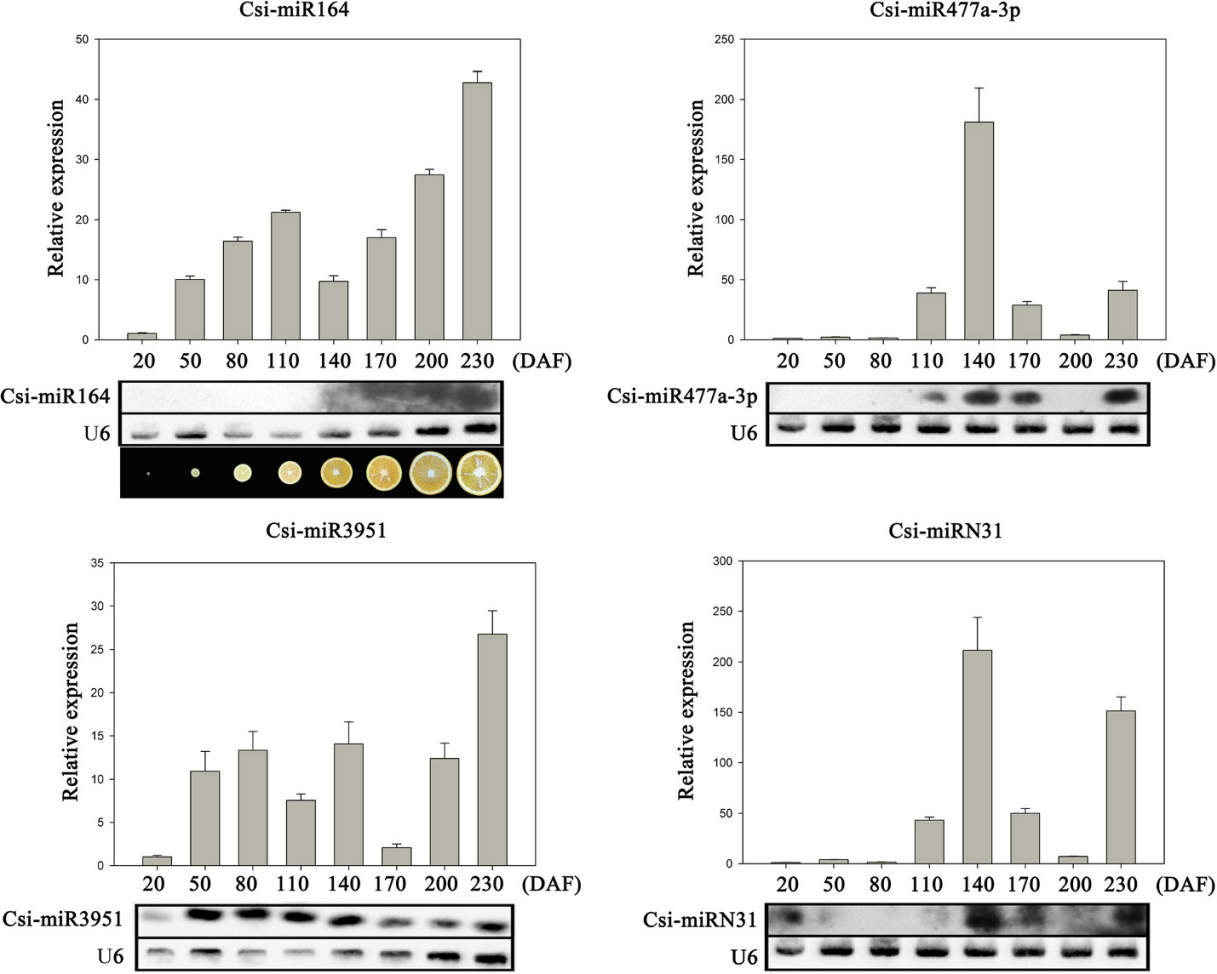
**Expression pattern of four candidate miRNAs during fruit development and ripening.** Total RNAs were isolated from fruit samples at different developmental stages, ranging from 20–230 days after flowering (DAF). Reverse complement probes were used to detect specific miRNAs by RNA gel blotting. U4 and U6 were used as loading controls in qRT-PCR and RNA gel blot analyses, respectively. Data from the qRT-PCR experiments are represented as the mean plus SD of n = 3 biological replicates. Internal appearance of orange fruits during 8 developmental stages was shown at the bottom.

Expression pattern of the four potential miRNAs were then further investigated on eight stages of fruit development and ripening (Figure 8). The expression pattern suggested three of them are probably involved in the regulation of fruit development and ripening. Both csi-miRN31 and csi-miR477a-3p may have important role in the color break stage (140 DAF). Csi-miR164 showed significantly higher expression level at the final stage (fruit ripening stage), with a steady increase in expression level during fruit ripening (170–230 DAF). In depth function of the former two miRNAs are now under progress in other research. This study focused on csi-miR164a which may have particular function at the fruit ripening stages. At this stage, sweet orange is undergoing maturation with extensive molecular repertoire on both transcriptional and posttranscriptional levels. Therefore, csi-miR164, as a known miRNA, may have new function in orange fruit ripening on posttranscriptional level.

### Csi-mi164 functions via target a NAC transcription factor

**Figure 9:**
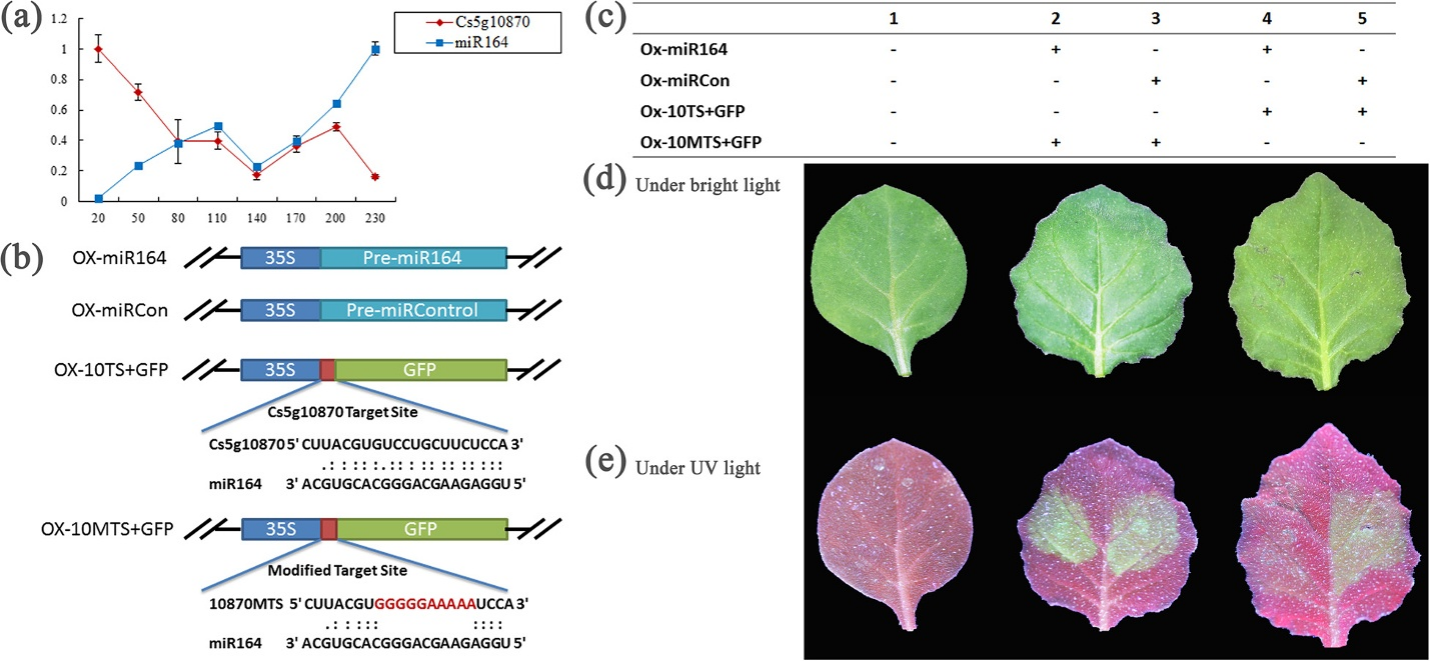
**Csi-miR164 targeted** ***Cs5g10870*** **was verified** ***in vivo.*** **(a)** Expression levels of mi164 and *Cs5g10870* were detected during the fruit development by qRT-PCR. U4 and *ACTIN* were used as loading controls in qRT-PCR Data from the qRT-PCR experiments are represented as the mean plus SD of n = 3 biological replicates. **(b)** Four overexpression vectors were constructed for transient expression system in tobacco. Vector ox-miR164 overexpress csi-miR164, ox-miRCon overexpress a control miRNA, ox-10TS + GFP overexpress a *GFP* gene carrying target site of miR164 in *Cs5g10870*, ox-10MTS + GFP overexpress a *GFP* gene carrying a modified target site. **(c)** Vectors used in tobacco co-expression assays in each lane were shown in the table. **(d)** Co-infiltrated leaves and control leaves were photographed at the 3rd day after infiltration under bright light. **(e)** Same leaves were photographed at the 3rd day after infiltration under UV light (wavelength = 365 nm).

As mentioned above, csi-miR164 was highly active during fruit ripening and 5’RACE analysis showed csi-miR164 targeted *Cs5g10870*, a NAC transcription factor. To confirm the interaction between csi-miR164 and *Cs5g10870* in orange fruit, we detected the expression level of *Cs5g10870* by qRT-PCR at fruit developmental stages. As showed in Figure 9a, the expression level of csi-miR164 and *Cs5g10870* displayed complementary expression pattern. Furthermore, a transient expression system was used to confirm that csi-miR164 degraded *Cs5g10870 in vivo*. Overexpression vectors of csi-miR164 and a control miRNA were constructed respectively. Target site of miR164 in *Cs5g10870* and a modified target site (inactivated target site) were inserted into a green fluorescent protein (*GFP*) gene overexpression vector respectively as shown in Figure 9b. In this assays, we used *Agrobacterium tumefaciens* infiltration to co-express csi-miR164, control miRNA, *GFP* gene carrying target site and *GFP* gene carrying modified target site pair by pair (Figure 9c). As a result, csi-miR164 targeted the target region of *Cs5g10870* and repressed the expression of GFP obviously (Figure 9e).

*Cs5g10870* was categorized as a NAC domain-containing transcription factor. Plant NAC transcription factor was one of the largest families involved in diverse biological processes [53–56]. More, recent publications reveal that NAC transcript factors function as regulators in fruit ripening. NACs in banana(*MaNACs*) were supposed to be involved in banana fruit ripening by interaction with ethylene signaling components [57]. Interestingly, the transcripts of *MaNAC4* decreased during banana ripening as well as *Cs5g10870* decreased during orange ripening. So far in *C. sinensis*, two NACs were reported and suggested to be related to fruit development, fruit senescence and fruit response to postharvest stresses [58, 59]. As csi-miR164 was higher expressed in fruit and was more active at the late stages during the orange fruit development, we suggested that csi-miR164 might be involved as a regulator in orange ripening by interacting with a NAC transcription factor. Further functional investigations of csi-miR164 and *Cs5g10870* are required to confirm this hypothesis.

## Conclusions

Our study provides a genome-wide comparison of miRNAs and their target genes among leaf, flower and fruit of sweet orange with the aim of identification of regulators involved in fruit development and ripening. Comprehensive genome analysis uncovered 183 known- and 38 novel- miRNAs, and revealed their genomic characteristics such as isoform miRNAs, co-localization with SSR locus and secondary siRNAs. Comparative analysis showed that some known miRNAs, such as csi-miR168a and csi-miR159 in this study, may fulfill specific function in specific tissues via target different genes. This kind of tissue specific regulation was even obvious for novel miRNAs. Three miRNAs, csi-miRN31, csi-miR477a-3p and csi-164a, were identified to be highly expressed in fruit and probably important regulators of fruit ripening. Csi-miR164 was further validated to functions by target NAC transcription factor. The results of this study provide three promising miRNAs for understanding the posttranscriptional regulation of orange fruit development and ripening.

## Methods

### Plant materials

The ‘Anliu’ sweet orange (*C. sinensis* [L.] Osbeck) was planted at the Institute of Citrus Research located in Guilin, Guangxi Province, China. Leaves (young and mature stages) and flowers (early flower bud, flower bud and mature flower) used in sRNAome and degradome sequencing were mixed as a pool from three trees respectively. Fruit samples used in degradome sequencing were collected at 170 days after flowering (DAF) as previously published [35]. To detect the expression pattern of key miRNAs in the fruit development, we also collected fruit samples (from three trees as replicates) at developmental stages, including 20 DAF, 50 DAF, 80 DAF, 110 DAF, 140 DAF, 170 DAF, 200 DAF and 230 DAF. Fruit samples were separated into peel and pulp after collection. Pulp without seeds was used in all analysis in this research. All samples were frozen in liquid nitrogen immediately after collection and kept at -80°C until use. The *Nicotiana benthamiana* was grown in a growth chamber controlled at 14 h light, 10 h dark, 25°C cycles.

### SRNAome sequencing and degradome sequencing

Total RNAs were extracted from different samples as Xu et al. described [35] and used in sRNAome sequencing and degradome sequencing. The sRNA libraries were constructed and sequenced for flower and leaf in this study, fruit sRNA data were derived from our published data with the same strategy [35]. Degradome sequencing strategy was applied to all the three tissues as described previously [16, 39]. All these high-throughput sequencing were performed by Beijing Genomics Institute (BGI) (Shenzhen, China). The Illumina 1 G Genome Analyzer was used for the sRNA sequencing while the Illumina HiSeq 2000 was used for the degordome sequencing.

### Bioinformatic analysis

All sRNAome raw data were processed by removing adaptors, low quality tags as well as contaminants at pre-analysis. Then, the clean sRNA sequences were compared to the Rfam database (http://rfam.sanger.ac.uk/) to exclude rRNA, tRNA, snRNA and snoRNA. The rest sRNA sequences were used to search known miRNAs by BLASTN against miRBase 16.0 (http://www.mirbase.org/). SRNAs with two or less mismatches compared to known miRNAs annotated in miRBase 16.0 were considered as known miRNAs in *C. sinensis*. To identify novel miRNA, we performed a bioinformatics approach according to criteria described previously [14]. To predict the secondary structure of miRNAs, we used RNAfold in the Vienna RNA Package downloaded from vienna RNA web servers (http://rna.tbi.univie.ac.at/). Genome sequence of *C. sinensis* annotated by our group (http://citrus.hzau.edu.cn/orange/index.php) [36] was used as reference.

Degradome raw data was trimmed by pre-analysis as similar as sRNAome raw data. Then, clean degradome data were processed using the CleaveLand pipeline [16] (http://axtell-lab-psu.weebly.com/cleaveland.html). Subsequently, the transcripts targeted by miRNAs were grouped into three categories as previously reported [39].

To predict all potential secondary siRNA in orange, we performed a bioinformatics approach as follows. First of all, we obtained all miRNAs with length of 22 nt from sRNAome data and their target transcripts from degradome data. Then, 21 nt standard secondary siRNAs were generated from the cleavage site of target transcripts to 3’ end of targets one by one. We named secondary siRNA as miRNA-target-serial number. Such as csi-miR3954a-Cs1g09635.1-9, which meant the ninth phased siRNA generated form Cs1g09635.1 and triggered by csi-miR3954a. Finally, we could obtain the digital expressions (TPM) of these secondary siRNAs from the sRNAome data and target transcripts of these secondary siRNAs from degradome data through CleaveLand pipeline (Flow chart see in Additional file 12).

### qRT-PCR and RNA gel blotting analyses

To confirm miRNAs’ expression, stem-loop qRT-PCR and RNA gel blot analysis were both used in our research. RNAs used in both experiments were the same as RNAs used in high-throughput sequencing.

Stem-loop qRT-PCR was described to be a highly sensitive method for detection of miRNAs [60, 61]. We performed stem-loop qRT-PCR mainly according to previous publication in three biological replicates. All primers used in qRT-PCR analysis were provided in Additional file 13. Additionally, we improved a much more efficient loading control gene U4 instead of traditional U6 as Kou et al. described [62]. U4 showed more stably expressed at leaves, flowers and fruit samples at developmental stages of ‘Anliu’ sweet orange than U6 after experiment.

For RNA gel blot analysis, 10 μg total RNA from different samples were loaded to a denaturing 15% polyacrylamide gel and transferred to Hybond-N + membranes (Amersham, GE Healthcare). The membranes were cross linked by ultraviolet cross linker and then used for bloting. Reverse complement DNA oligonucletides were used as probes to detect specific miRNAs. Probes were labeled the by Biotin 3’ End DNA Labeling Kit (Thermo) and then used for bloting. Chemiluminescent Hybridization and Detection Kit (Thermo) were used for hybridization and signal detection. At the beginning, 5.8 s rRNA was used as a loading control. Instead, U6 was used as a loading control later.

### 5’ RACE

RNA Ligase-Mediated 5’ RACE (RLM-RACE) was performed with the GeneRacer kit (Invitrogen) as described in the product manual. Briefly, 10 μg total RNA was ligated to the 5’ adaptor. The ligated mRNA was reverse transcribed by oligo (dT) primer. To obtain the 5’ end products, PCR was proformed using 5’ adaptor primers and 3’ gene-specific primers. Finally, the products were cloned, sequenced and analysed.

### Transient expression system in tobacco

To verify the interaction between miRNAs and their targets *in vivo*, we used *Agrobacterium tumefaciens* infiltration to co-express miRNAs and their targets in tobacco. Transient expression in tobacco was applied as described previously [63].

## Availability of supporting data

The sRNA sequence data and the degradome data supporting the results of this article have been submitted to Gene Expression Omnibus (GEO) under accession NO. GSE46765 and GSE18207 at website: http://www.ncbi.nlm.nih.gov/geo/query/acc.cgi?token=bvcnfememqckehi&acc=GSE46765 and http://www.ncbi.nlm.nih.gov/geo/query/acc.cgi?token=bvcnfememqckehi&acc=GSE18207.

Accession NO. GSE46765 will be released on Dec 30, 2014.

## Electronic supplementary material

Additional file 1: **Predicted secondary structures of known and novel miRNAs.** The mature miRNA sequences are highlighted in yellow. For novel miRNAs, the miRNA* sequences are highlighted in gray. (PDF 4 MB)

Additional file 2: **Normalized count of all miRNAs in orange leaf, flower and fruit.** (PDF 19 KB)

Additional file 3: **Targets of miRNAs identified using degradome sequencing.** (PDF 28 KB)

Additional file 4: **T-plots of the miRNA targets in different tissues.** Densities of the 5’ positions of degradome tags matching each target gene are shown as T-plots. The miRNA-mediated degradome tag is highlighted in red. (PDF 14 MB)

Additional file 5: **T-plots of diverse targets of miRNAs in different tissues.** Densities of the 5’ positions of degradome tags matching each target gene are shown as T-plots. The miRNA-mediated degradome tag is highlighted in red. (a) miR168a targets *AGO1* in all three tissues; additionally, miR168a targets CUC2 in leaf and a pantothenate kinase gene in fruit; (b) miR159 targets GAMYB in leaf and flower; but it targets DRT100 in fruit; (c) miRN10 targets different transcripts in flower and fruit. (PDF 552 KB)

Additional file 6: **Annotations of all target genes.** (PDF 153 KB)

Additional file 7: **Normalized count of secondary siRNAs and their targets identified using degradome sequencing.** (PDF 36 KB)

Additional file 8: **T-plots of the targets of secondary siRNAs in different tissues.** Densities of the 5’ positions of degradome tags matching each target gene are shown as T-plots. The miRNA mediated degradome tag is highlighted in red. (PDF 4 MB)

Additional file 9: **Comparative analysis of the presence of SSRs in zma-miR396 and the resulting expression pattern among different tissues in maize.** Sequences data were collected from miRBase, the expression data were come from the publication of Zhang et al. (PDF 488 KB)

Additional file 10: **Clusters of miRNAs.** Two miRNAs located within a single precursor are shown. The mature miRNA sequences are highlighted in yellow and the miRNA* sequences are highlighted in gray. The mismatched nucleotide is highlighted in red. Data in the tables show the digital expression levels (TPM) of miRNAs in different tissues. (a) Csi-miR166j.1 and Csi-miR166j.3. (b) Csi-miR477d.1-3p and Csi-miR477d.2-5p. (PDF 615 KB)

Additional file 11: **Confirmation of the expression levels of 80 selected miRNAs in different tissues by qRT-PCR.** A total of 65 known miRNAs and 15 novel miRNAs were selected according to their differential expression in leaf (L), flower (F) and fruit (Fr), which was derived from high-throughput sequencing. The expression levels of these miRNAs were confirmed using stem-loop qRT-PCR. U4 was used as a loading control gene in qRT-PCR. The data are represented as the mean plus SD of n = 3 biological replicates. (PDF 2 MB)

Additional file 12: **Flow chart to identify the phased siRNAs and their target transcripts.** (PDF 227 KB)

Additional file 13: **Primers used in miRNA stem-loop RT-PCR.** (PDF 38 KB)

## Abbreviations

AGO: Argonaute protein
miRNA: Micro RNA
nt: Nucleotide
pri-miRNA: Primary miRNA
per-miRNA: Precursor of miRNA
RISC: RNA-induced silencing complex
RDR6: RNA-dependent RNA polymerase
sRNA: Small RNA
SSR: Simple sequence repeat
tasiRNA: Trans-acting siRNA.
