# Supplementary material for: Genome-wide comparison of microRNAs and their targeted transcripts among leaf, flower and fruit of sweet orange
Source: BMC Genomics. 2014 Aug 20;15(1):695. doi: 10.1186/1471-2164-15-695 (PMC4158063; doi:10.1186/1471-2164-15-695)

Additional file 1: Predicted secondary structures of known and novel miRNAs. The mature miRNA sequences are highlighted in yellow. For novel miRNAs, the miRNA* sequences are highlighted in gray.

Secondary structure for miR1092.2

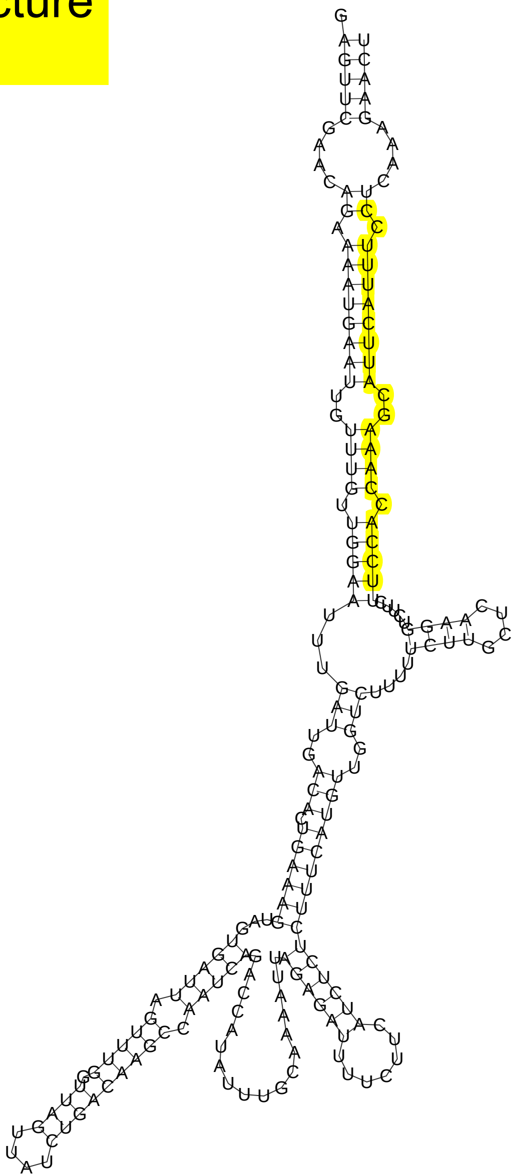

Secondary structure for miR1432a

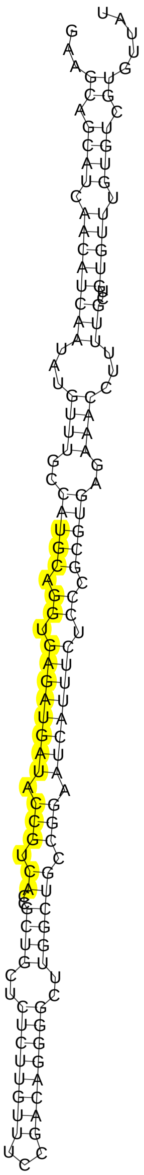

Secondary structure for miR1446

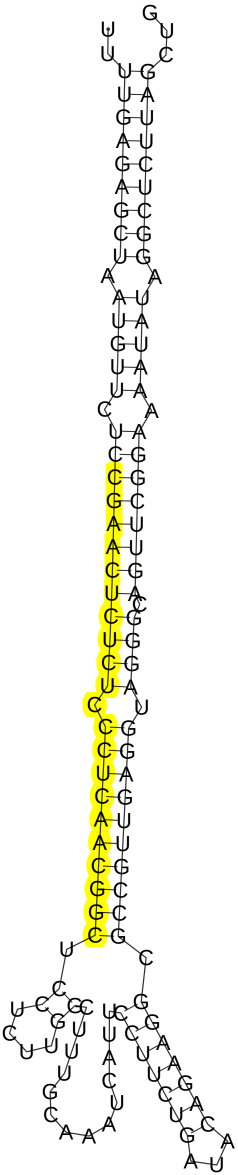

Secondary structure for miR1507a.2

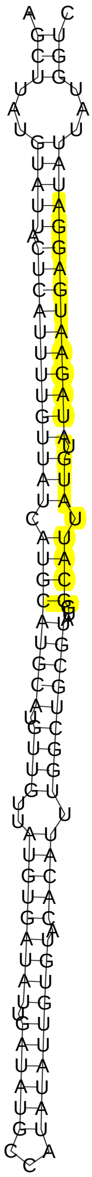

Secondary structure for miR1515

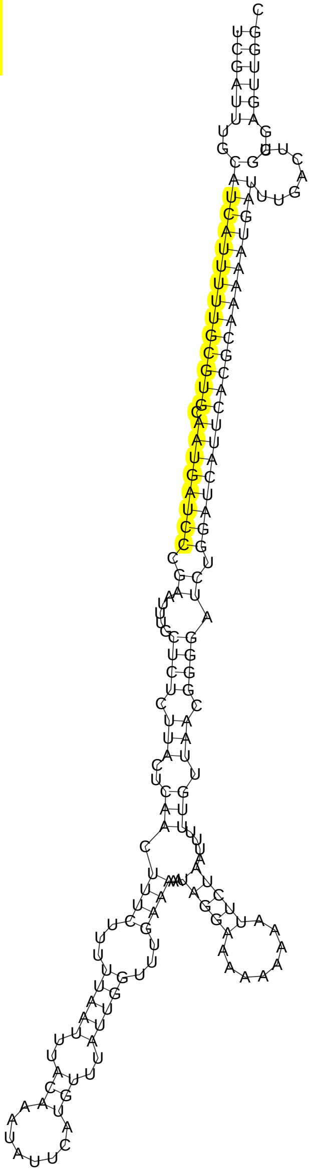

Secondary structure
for miR156a.1

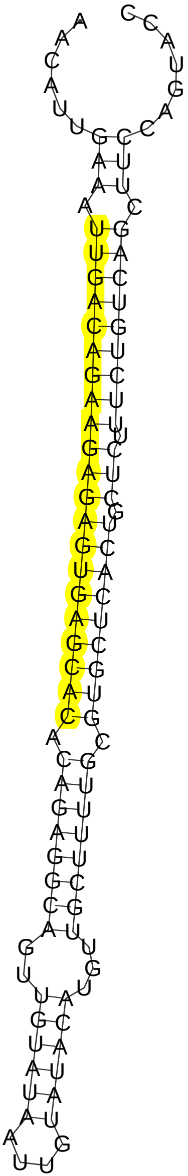

Secondary structure
for miR156a.2

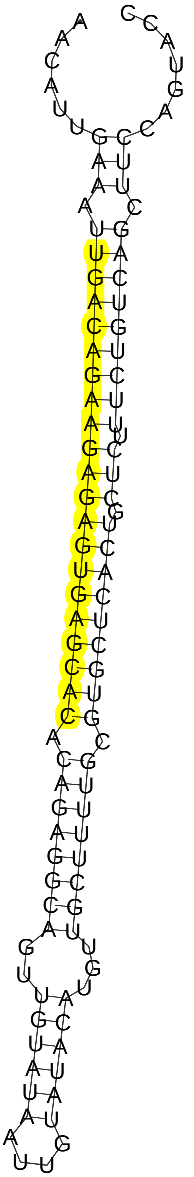

Secondary structure
for miR156b.1

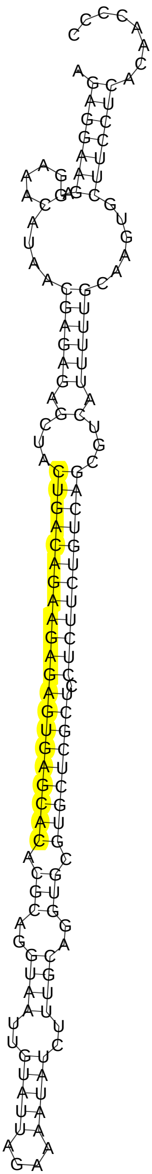

Secondary structure for miR156c.1

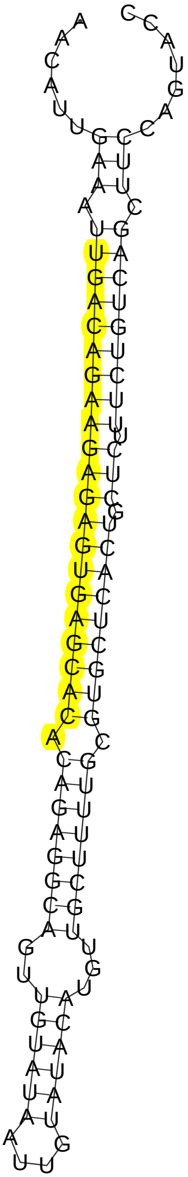

Secondary structure for miR156d

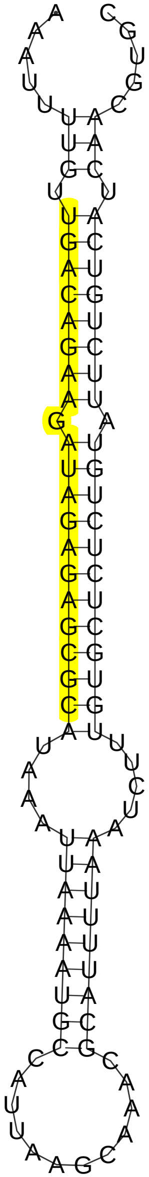

Secondary structure for miR156e

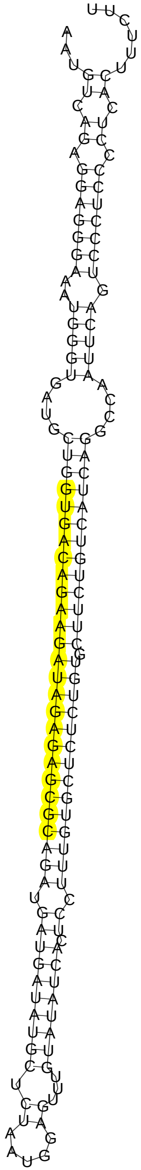

Secondary structure for miR156f.2

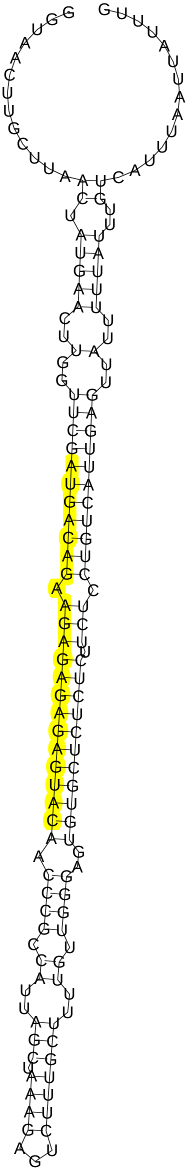

Secondary structure for miR156g.1

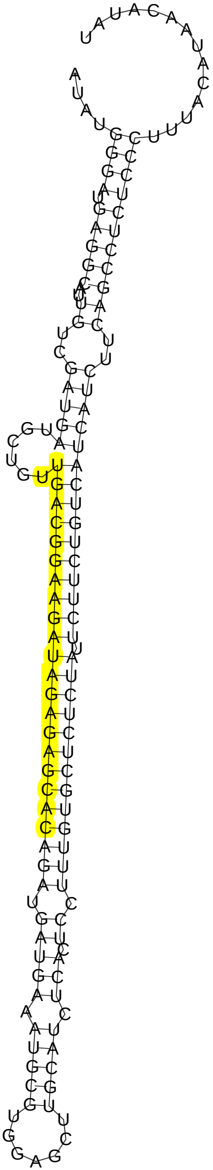

Secondary structure for miR156g.2

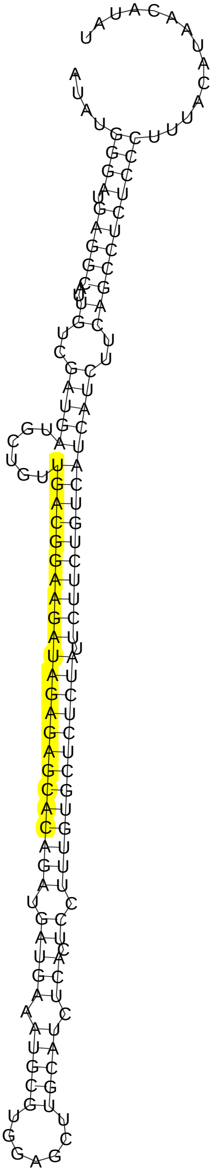

Secondary structure for miR156h

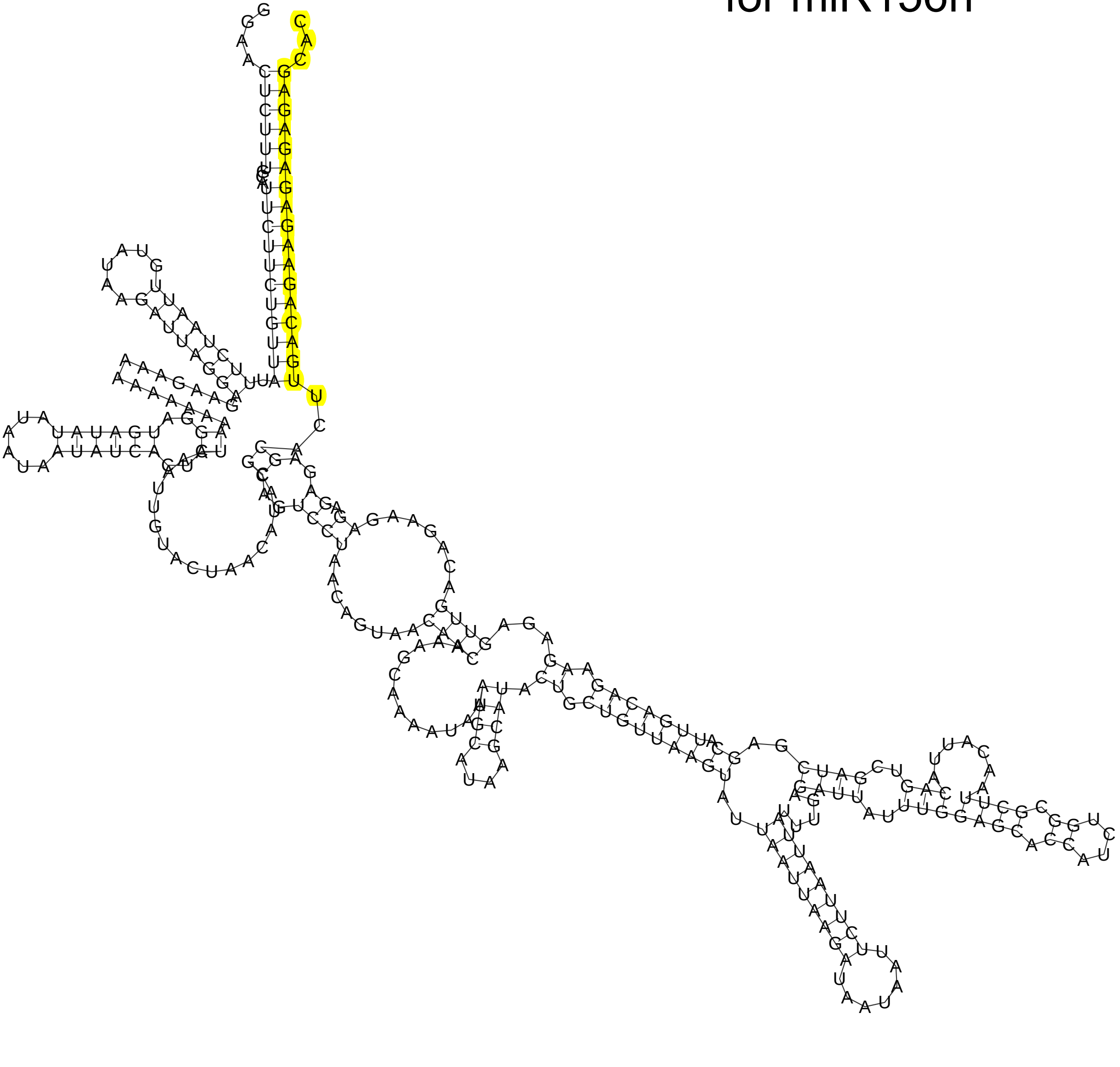

Secondary structure for miR159

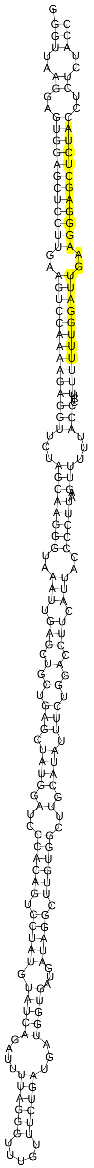

Secondary structure for miR159b

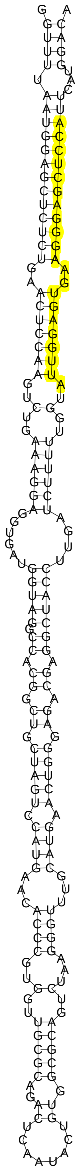

Secondary structure for miR160a-3p

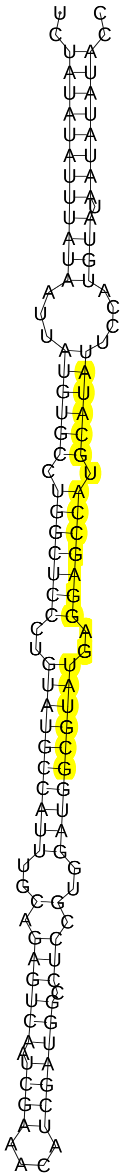

Secondary structure for miR160a-5p

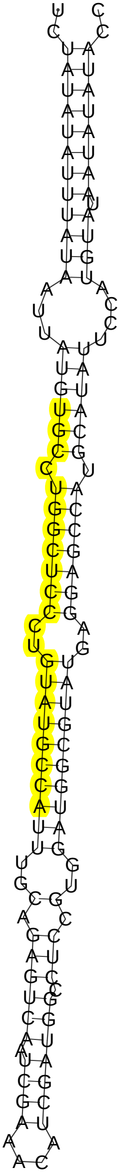

Secondary structure for miR160b-3p

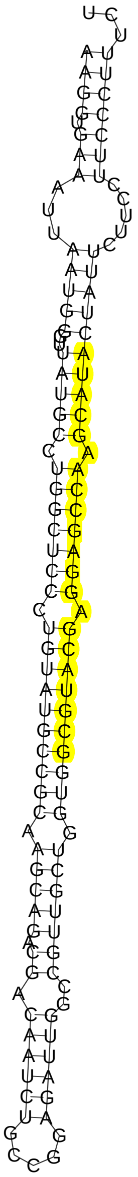

Secondary structure for miR160b-5p

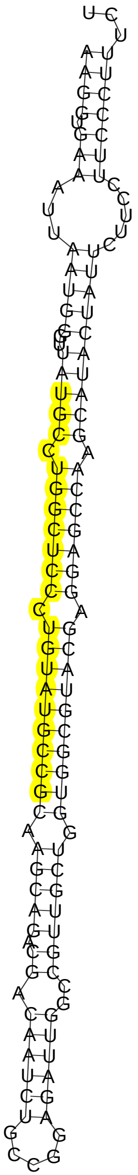

Secondary structure
for miR160c.1

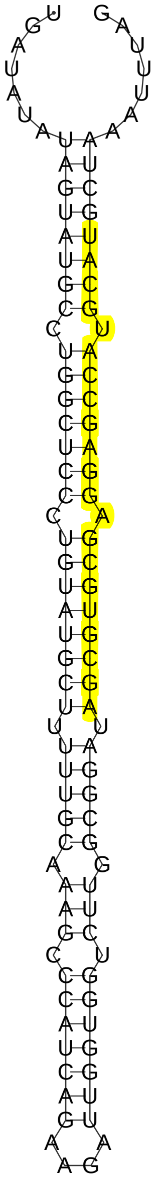

Secondary structure for miR160c.2

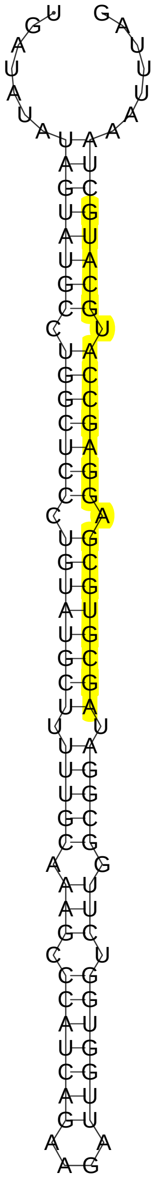

Secondary structure for miR162-3p.1

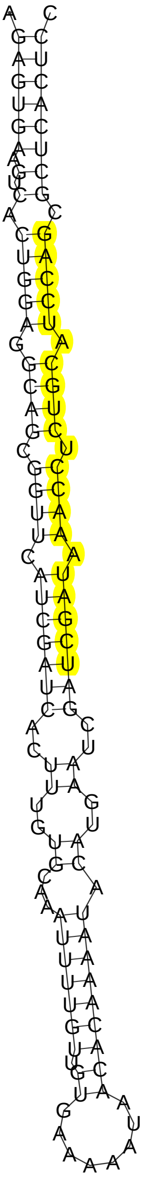

Secondary structure for miR162-3p.2

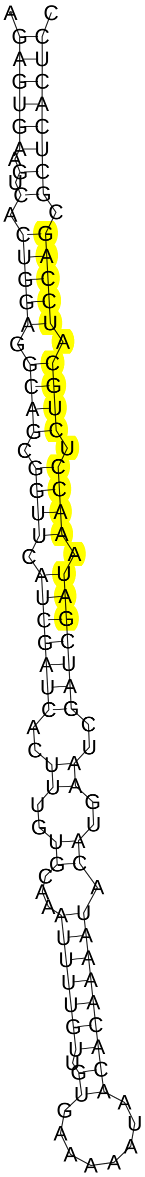

Secondary structure I
for miR164

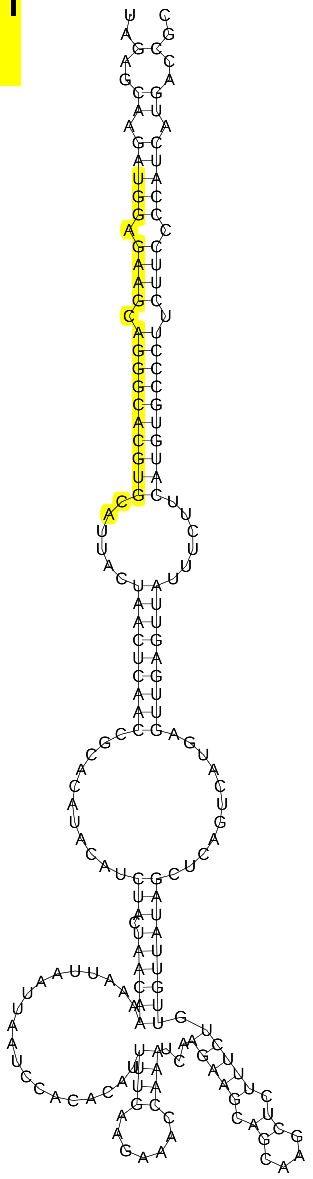

Secondary structure II for miR164

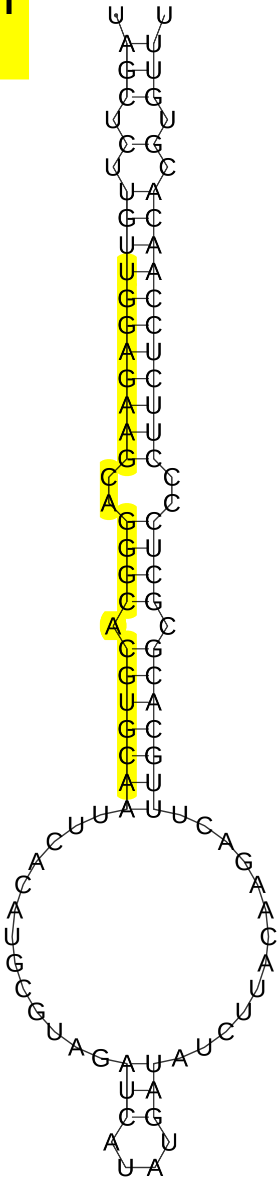

Secondary structure I for miR166a.1

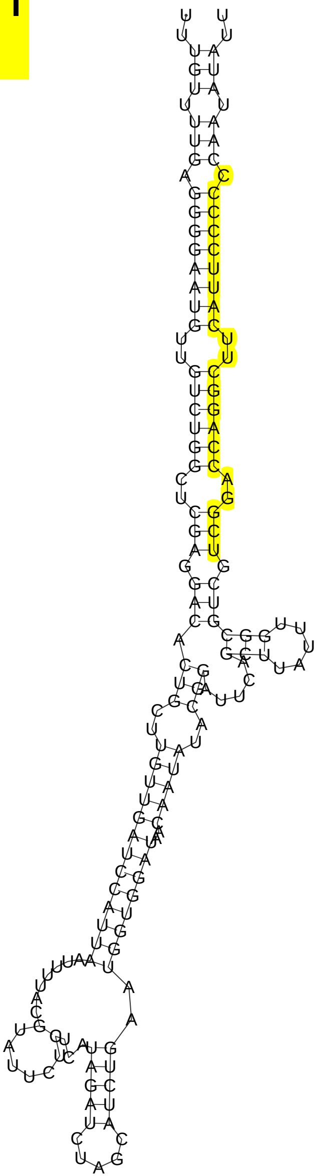

Secondary structure II for miR166a.1

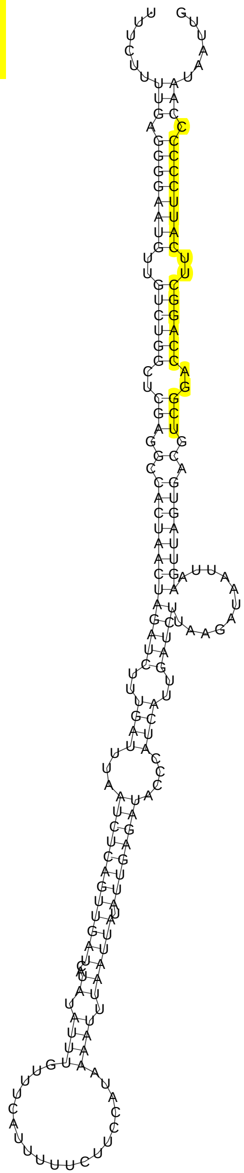

Secondary structure III for miR166a.1

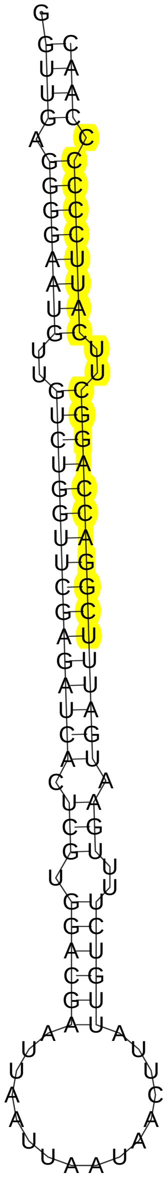

Secondary structure I
for miR166a.2

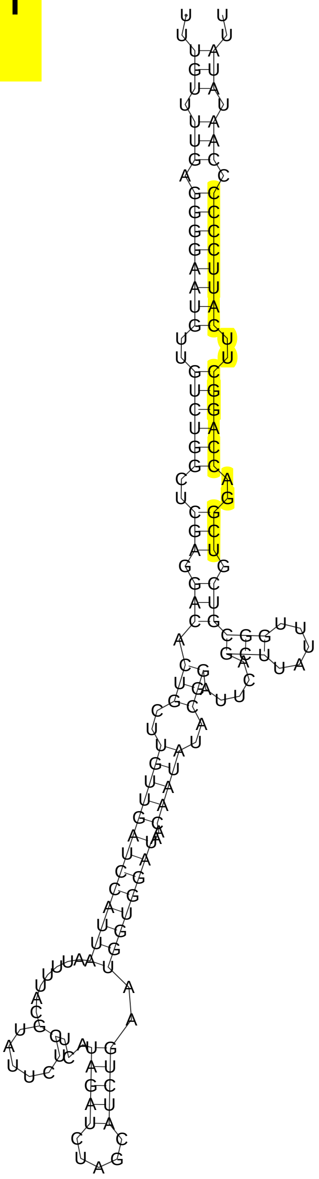

Secondary structure II for miR166a.2

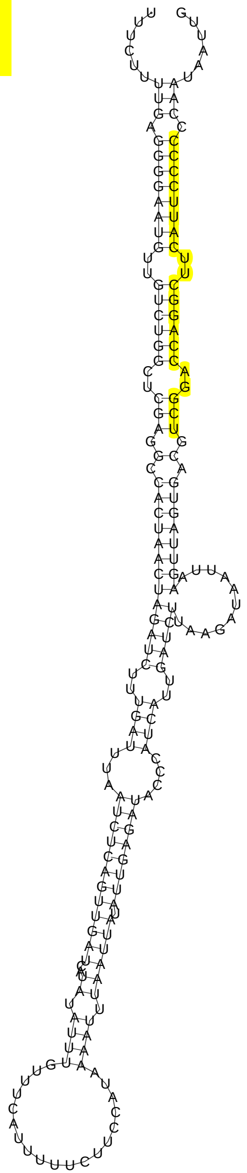

Secondary structure III for miR166a.2

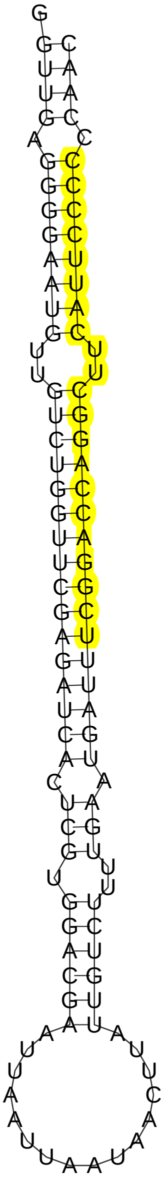

Secondary structure for miR166b

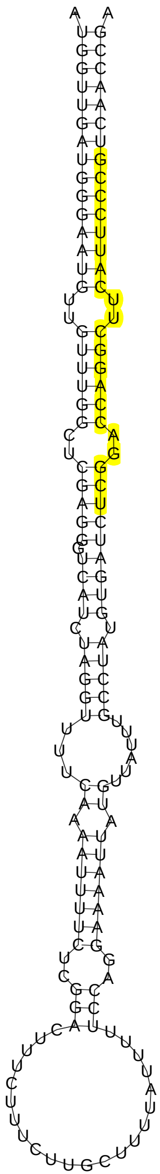

Secondary structure I for miR166c.1

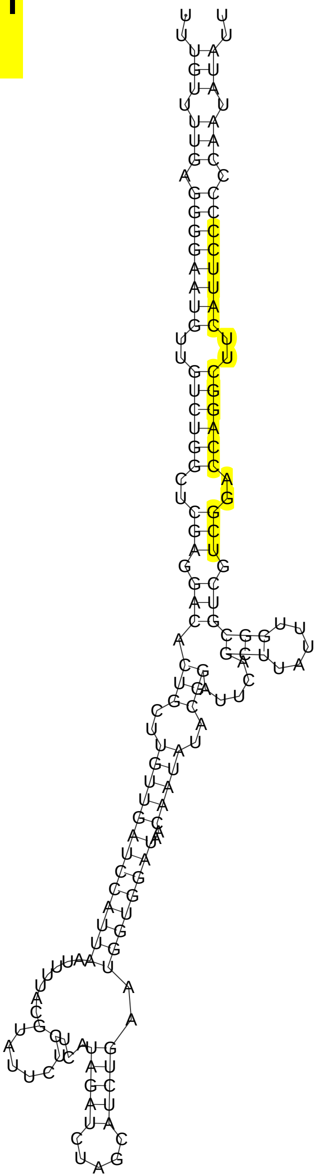

Secondary structure II for miR166c.1

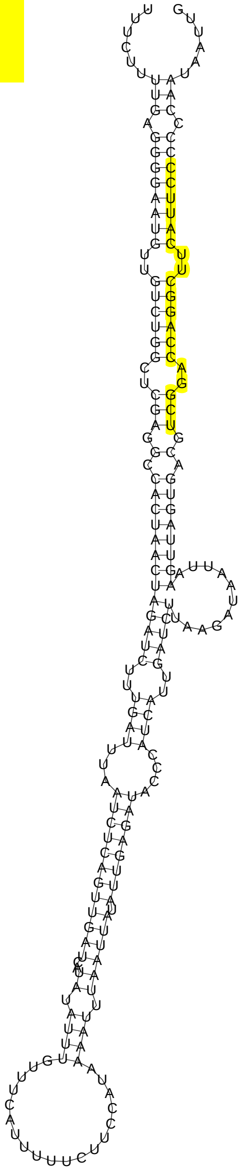

Secondary structure III for miR166c.1

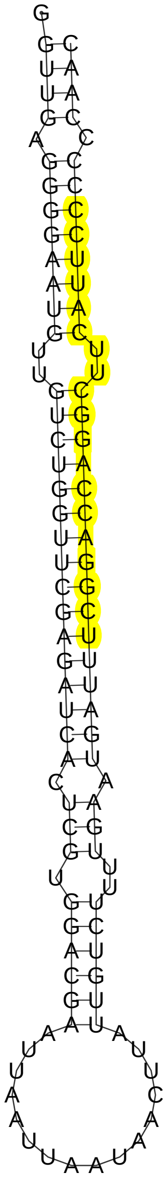

Secondary structure IV for miR166c.1

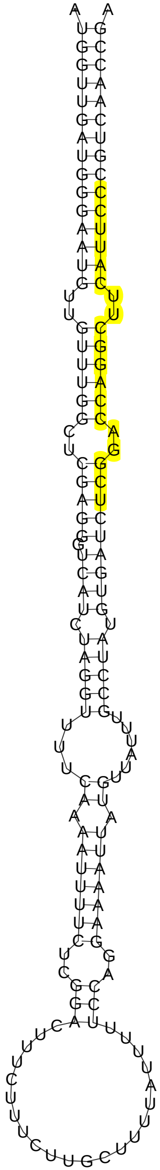

Secondary structure I for miR166c.2

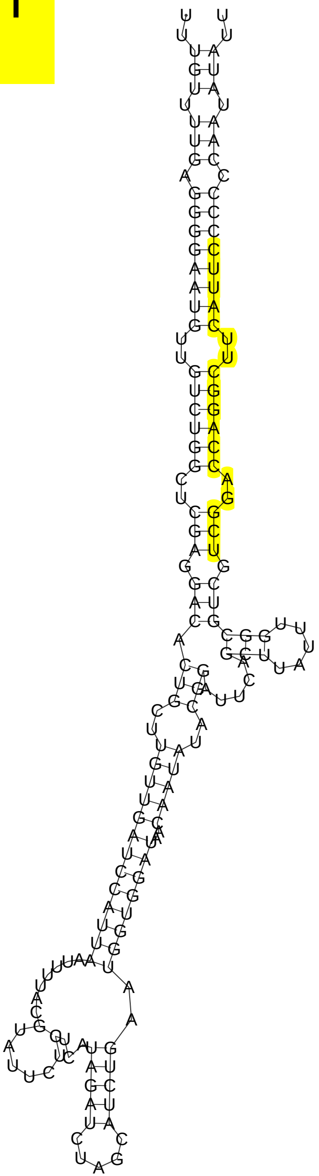

Secondary structure II for miR166c.2

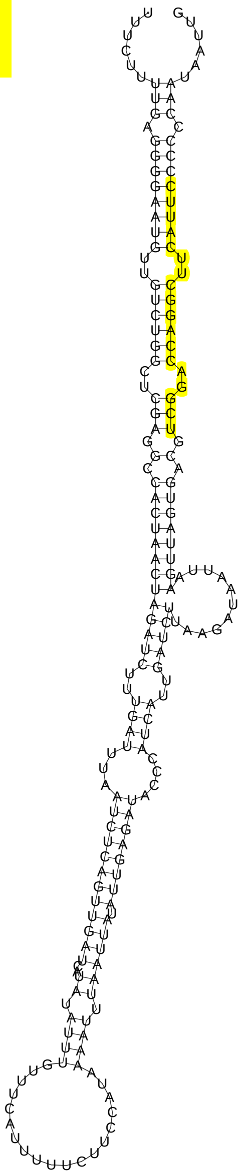

Secondary structure III
for miR166c.2

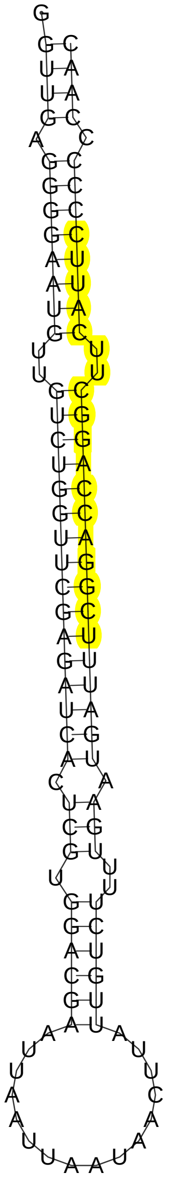

Secondary structure IV for miR166c.2

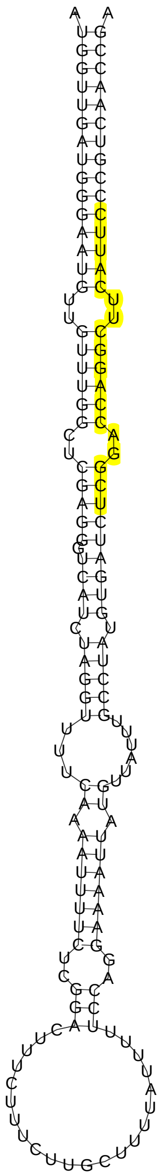

Secondary structure I for miR166c.3

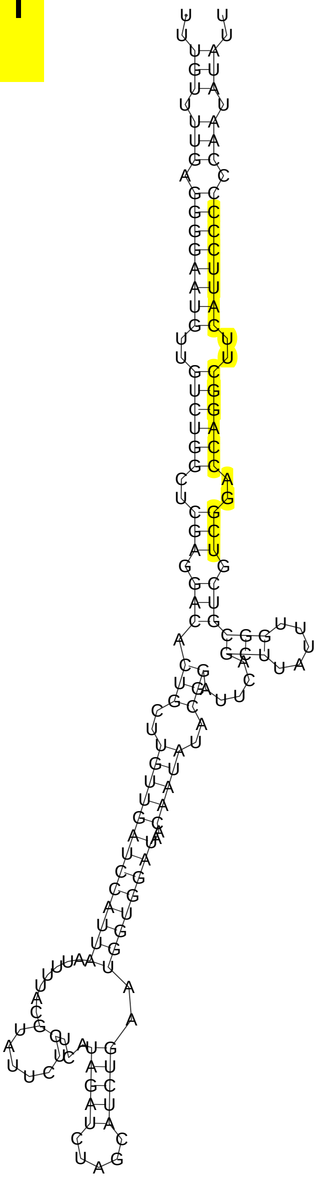

Secondary structure II for miR166c.3

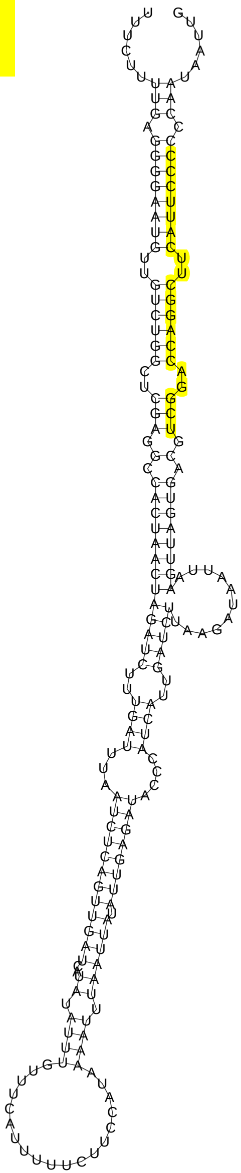

Secondary structure III for miR166c.3

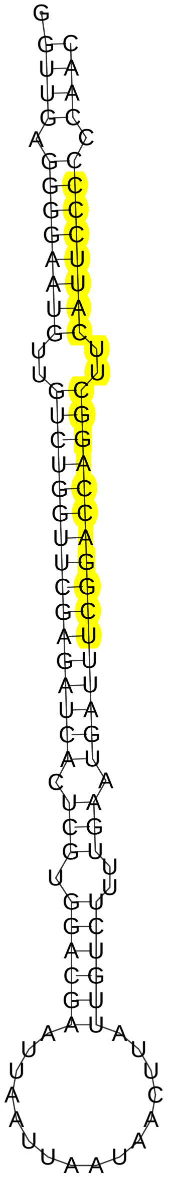

Secondary structure IV for miR166c.3

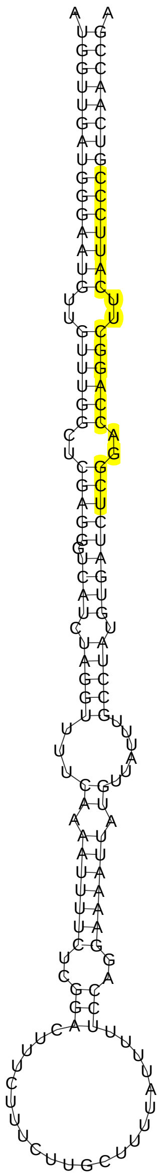

Secondary structure for miR166c.4

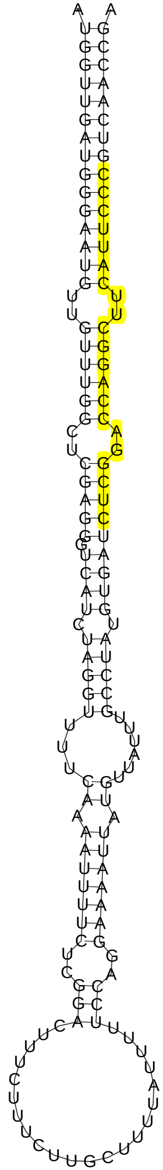

Secondary structure
for miR166c.5

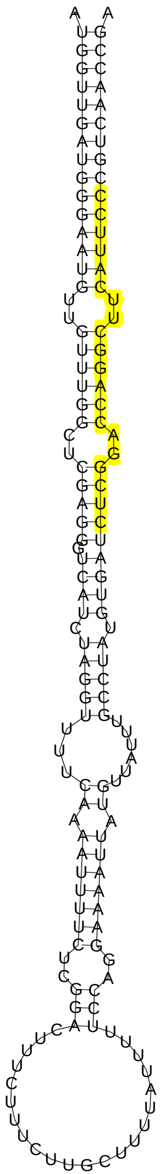

Secondary structure for miR166d.1

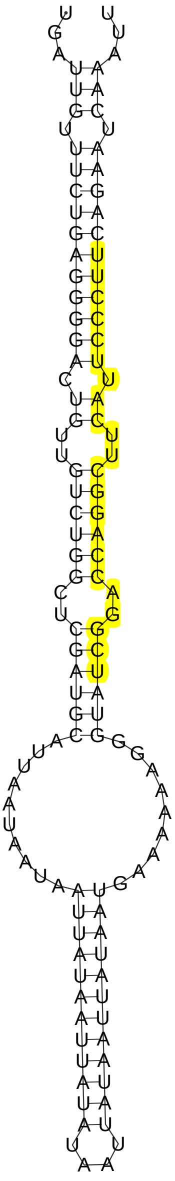

Secondary structure for miR166d.2

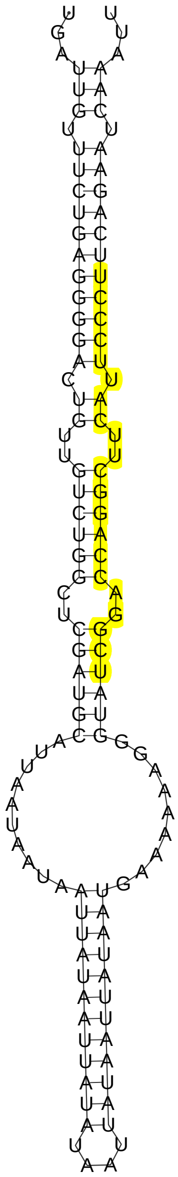

Secondary structure for miR166g.1

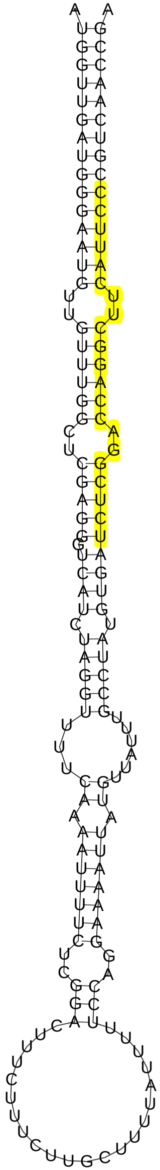

Secondary structure for miR166i

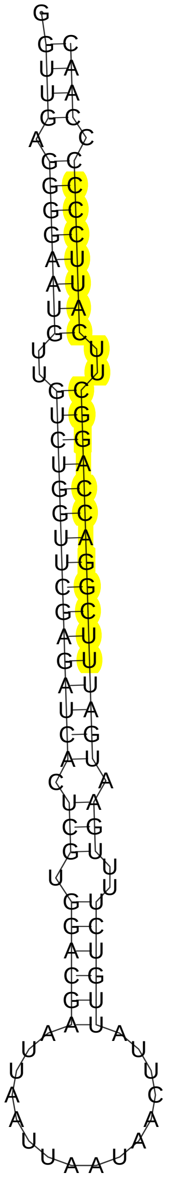

Secondary structure for miR166j.1

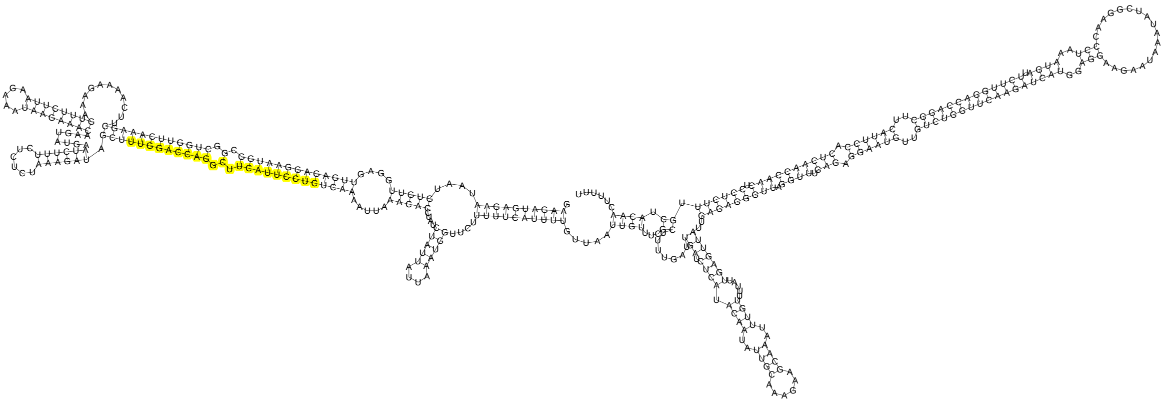

Secondary structure for miR166j.2

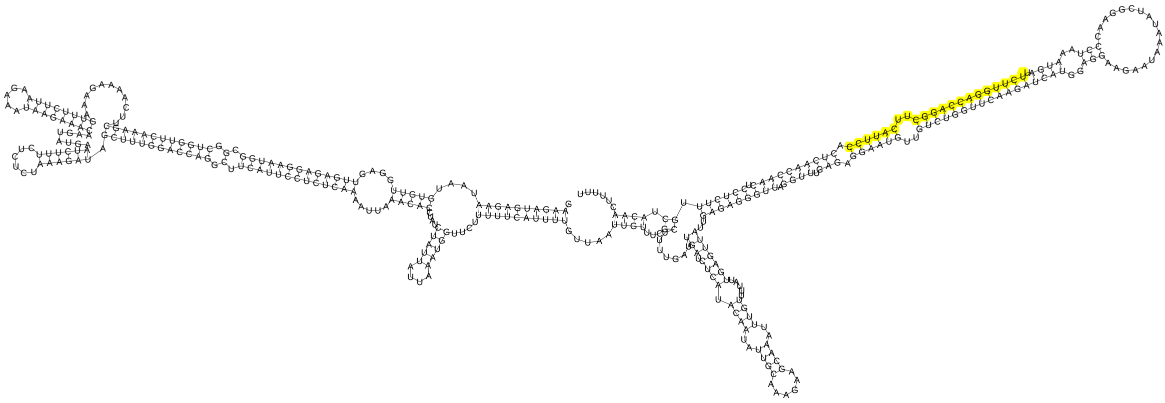

Secondary structure for miR166j.3

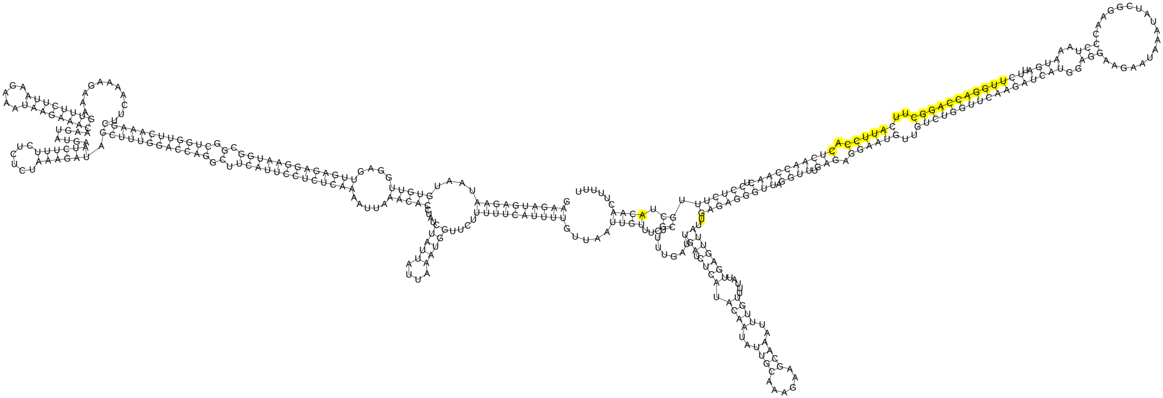

Secondary structure I for miR167a.1

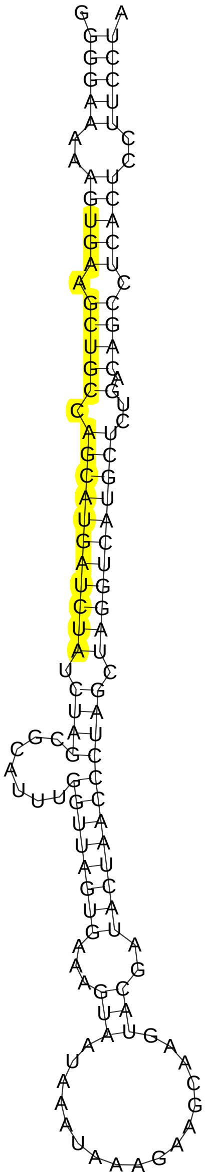

Secondary structure II for miR167a.1

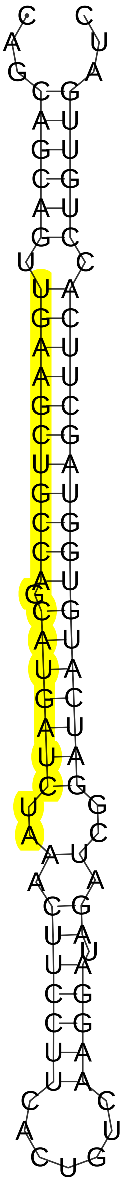

Secondary structure for miR167b.1

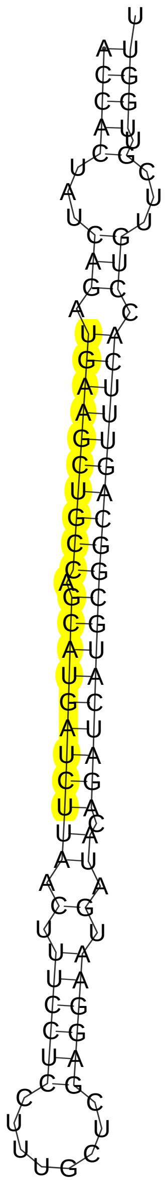

Secondary structure for miR167b.2

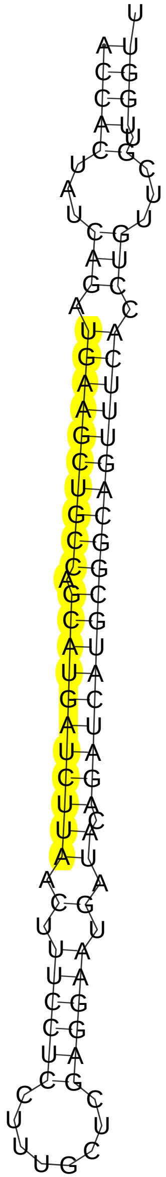

Secondary structure for miR167b.3

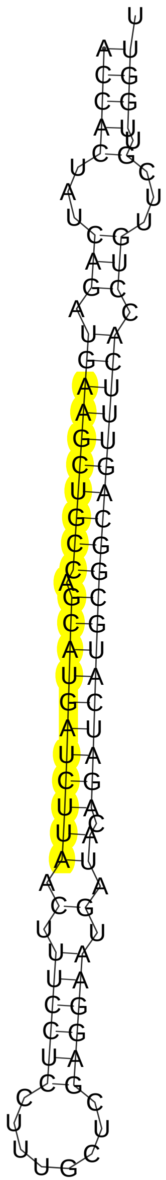

Secondary structure for miR167b.4

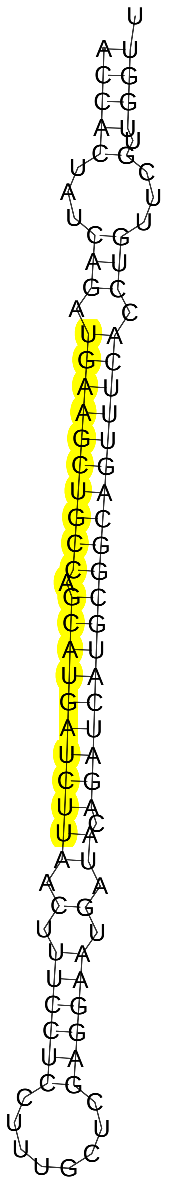

Secondary structure for miR167d.1

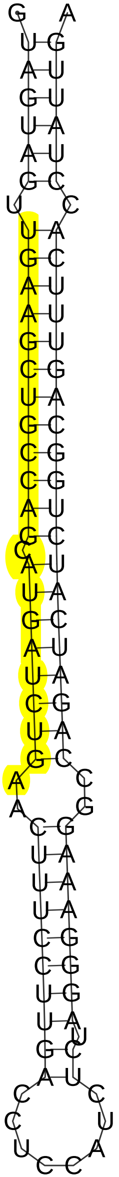

Secondary structure for miR167d.2

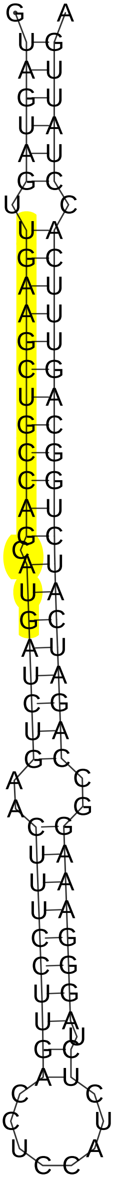

Secondary structure for miR168a

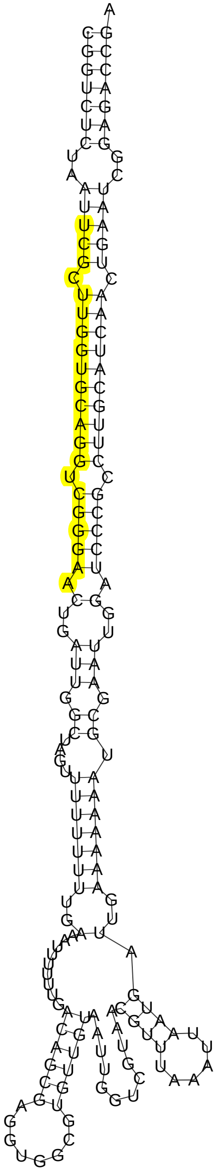

Secondary structure I
for miR169b.1

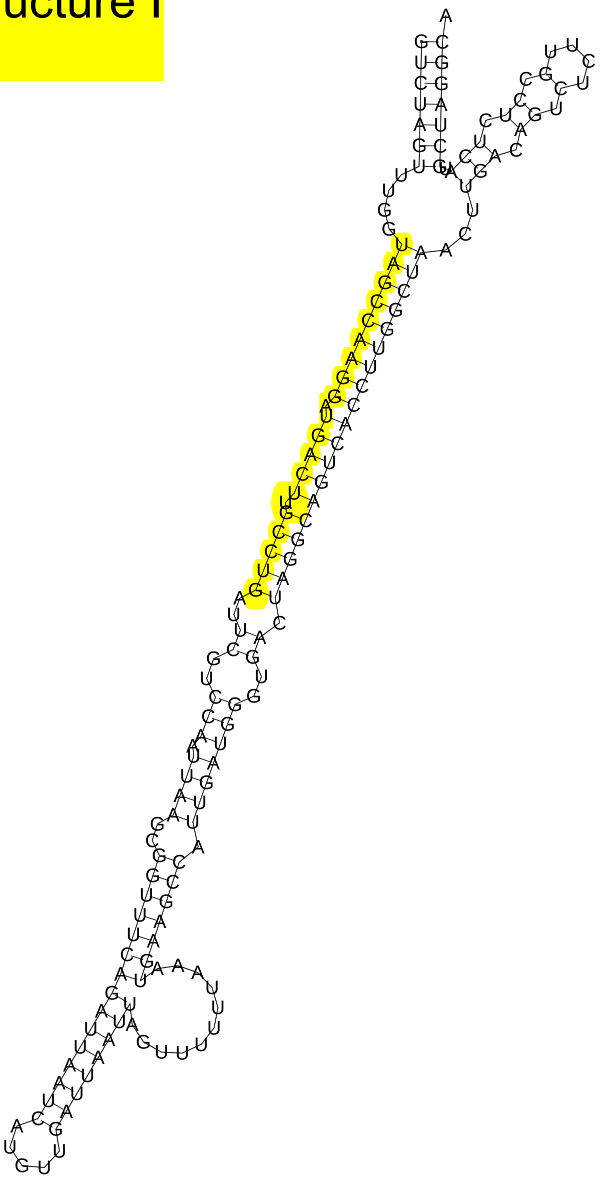

Secondary structure II for miR169b.1

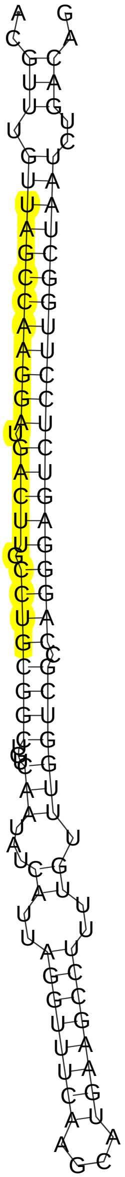

Secondary structure III for miR169b.1

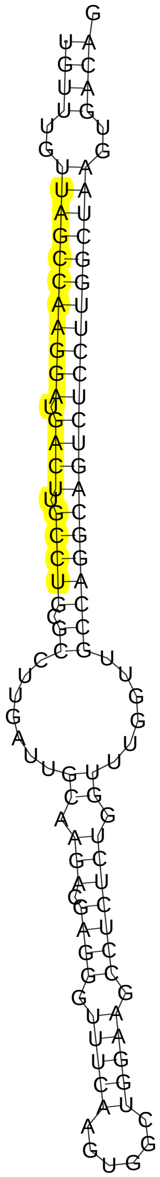

Secondary structure I
for miR169b.1-3p

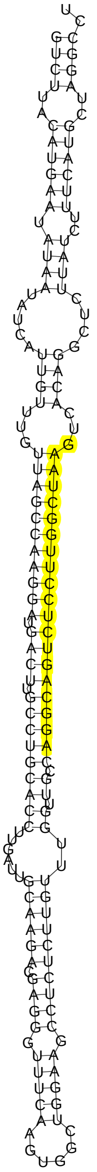

Secondary structure II
for miR169b.1-3p

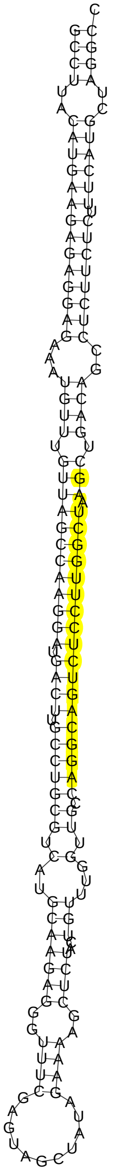

Secondary structure III for miR169b.1-3p

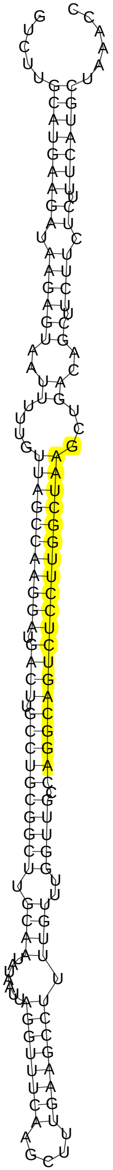

Secondary structure I for miR169i.1

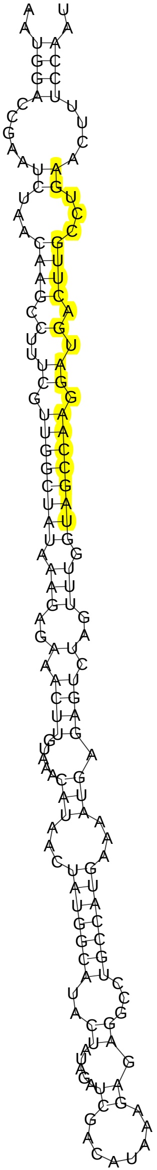

Secondary structure II
for miR169i.1

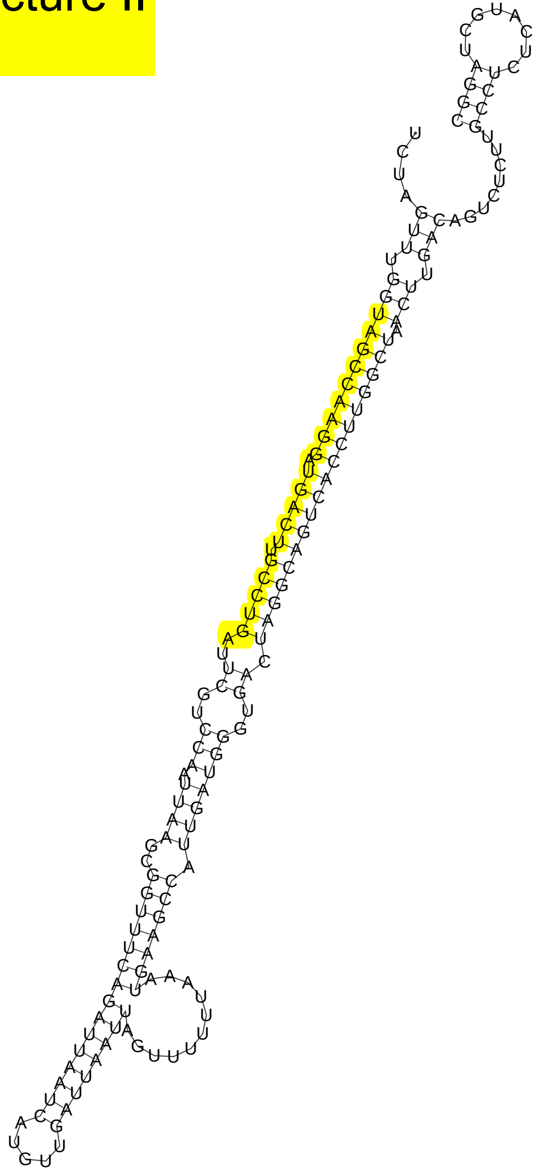

Secondary structure III
for miR169i.1

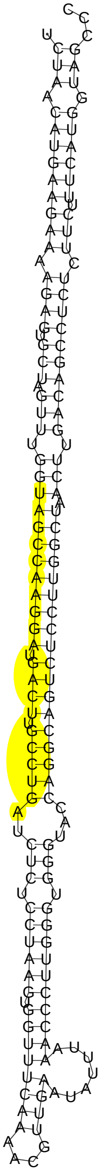

Secondary structure for miR169i.1-3p

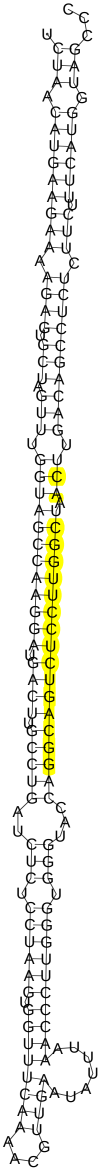

Secondary structure I for miR169m.1

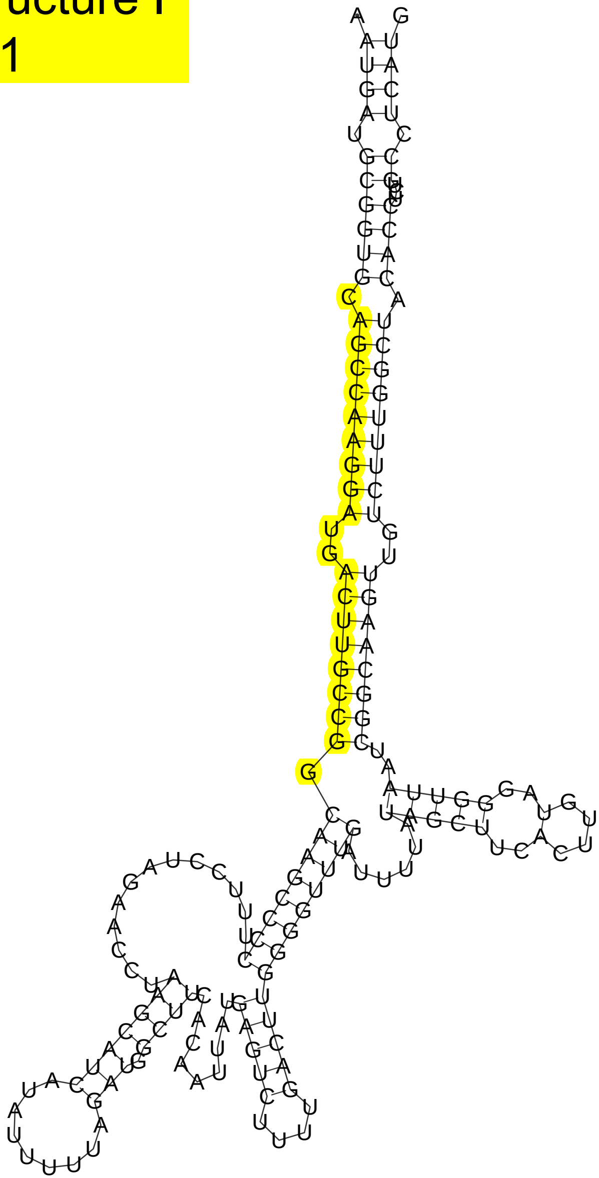

Secondary structure II for miR169m.1

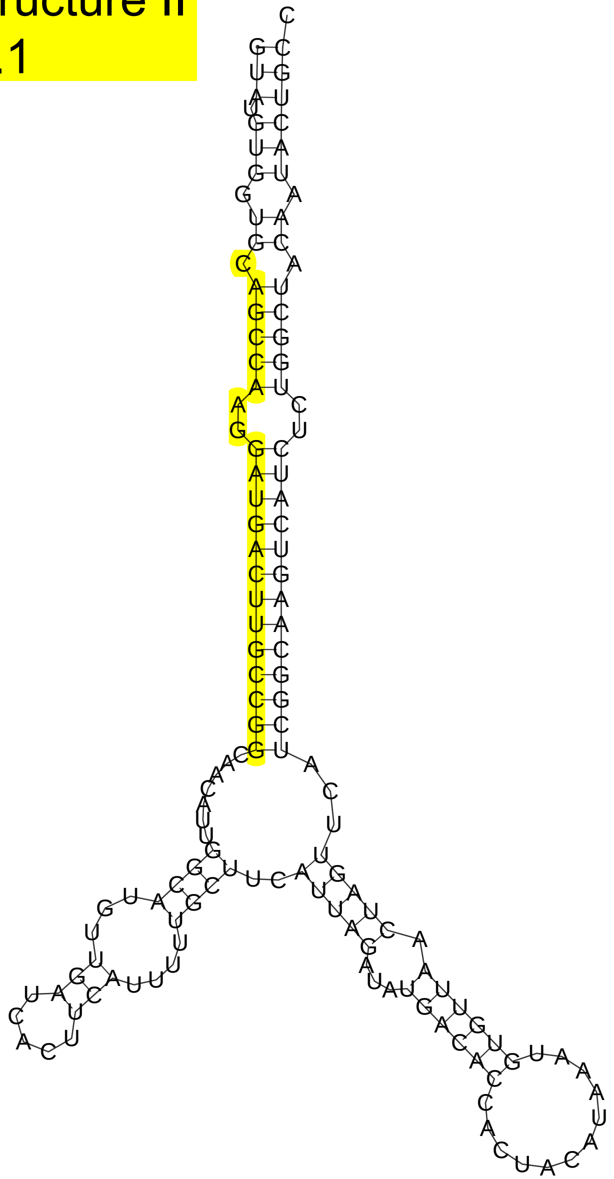

Secondary structure I
for miR169m.2

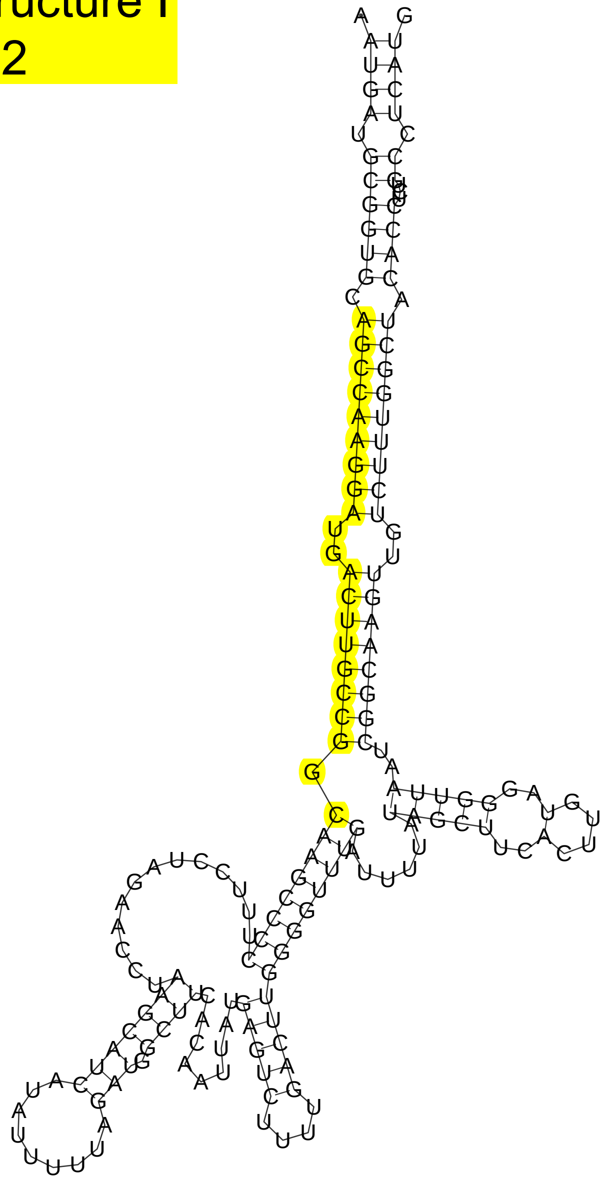

Secondary structure II for miR169m.2

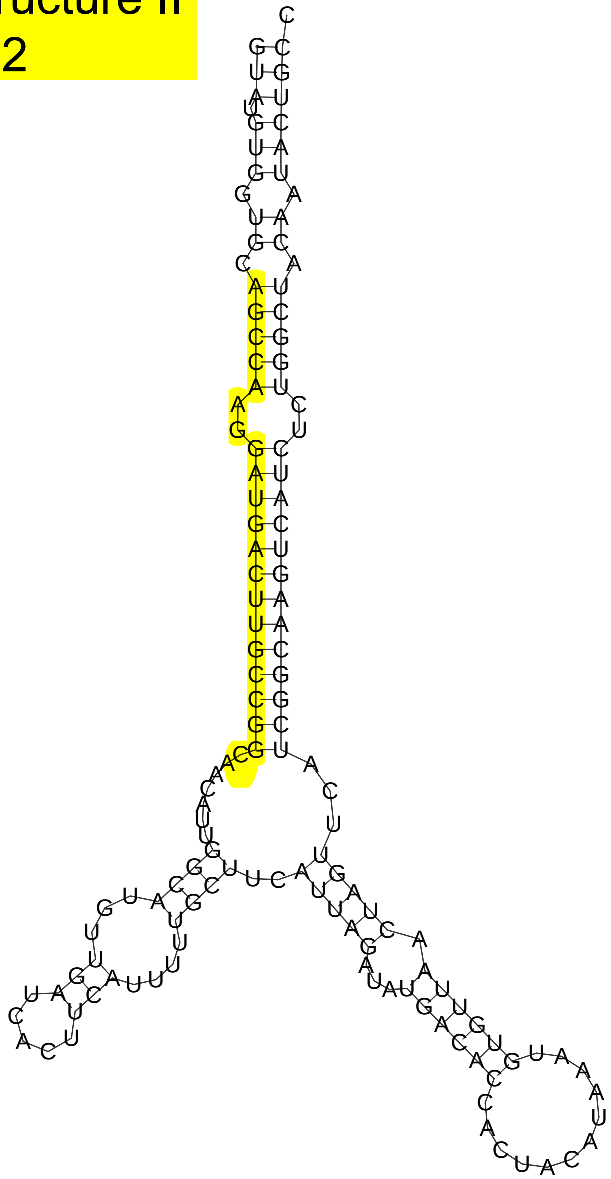

Secondary structure I for miR169m.3

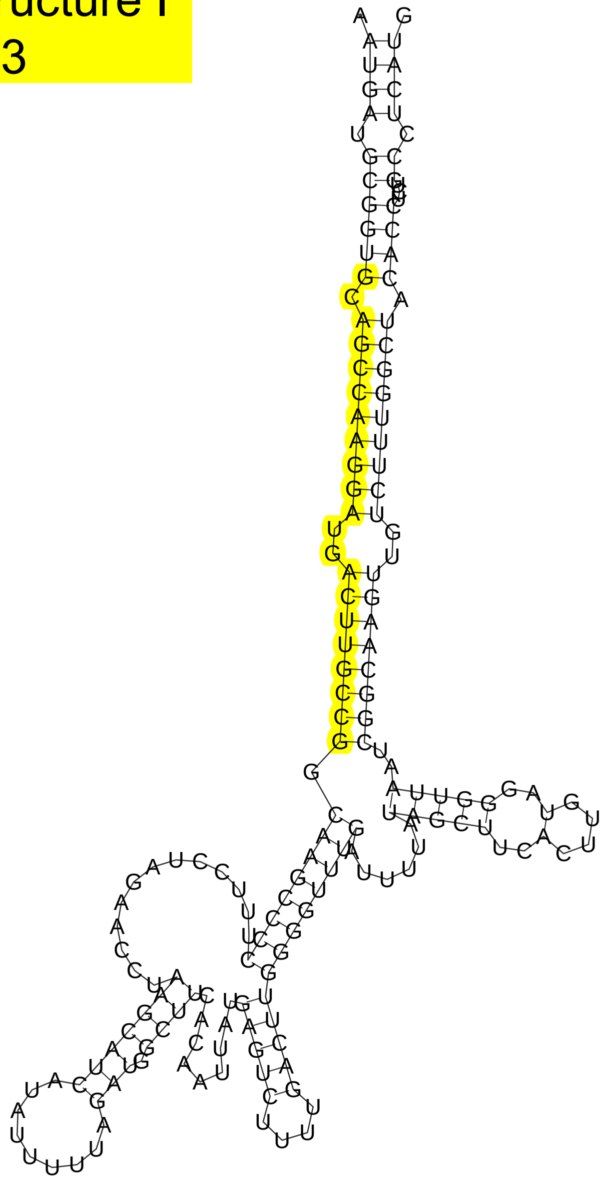

Secondary structure II
for miR169m.3

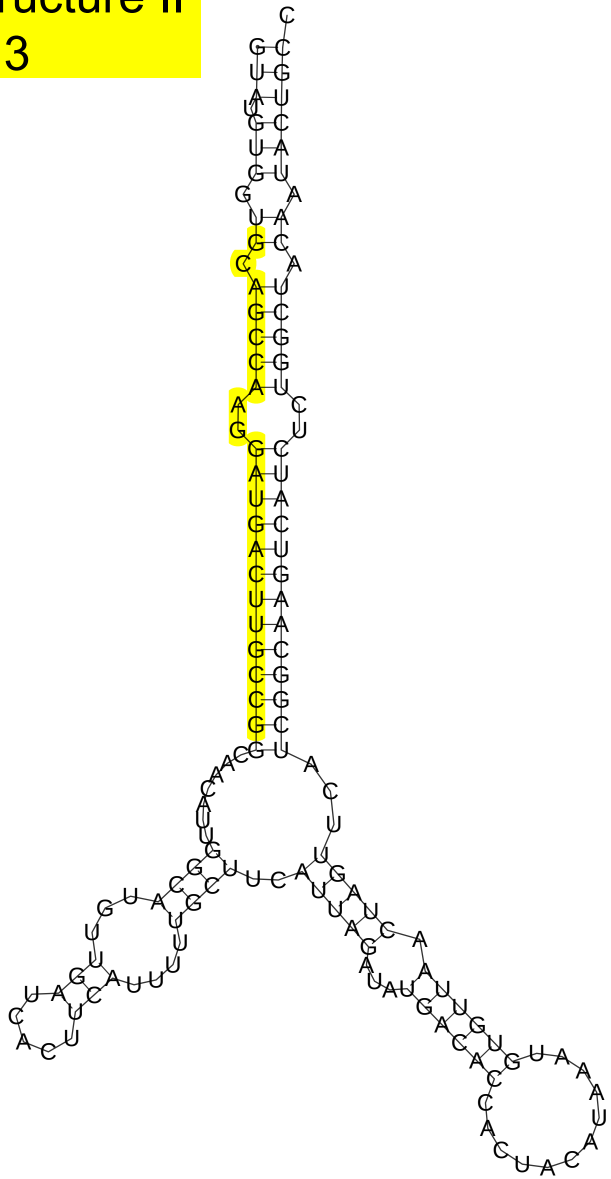

Secondary structure for miR171a.1

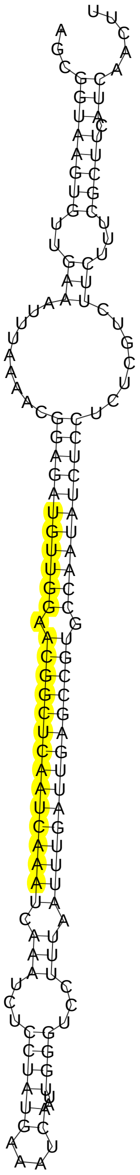

Secondary structure
for miR171b

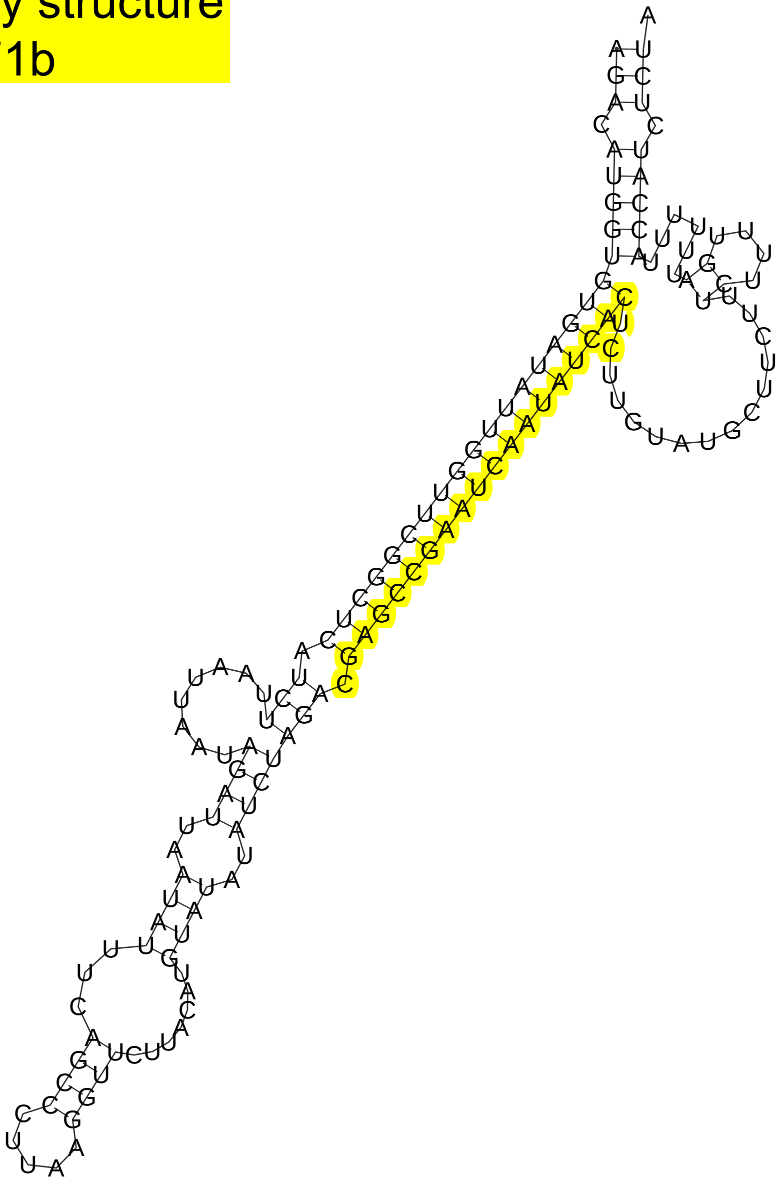

Secondary structure for miR171d

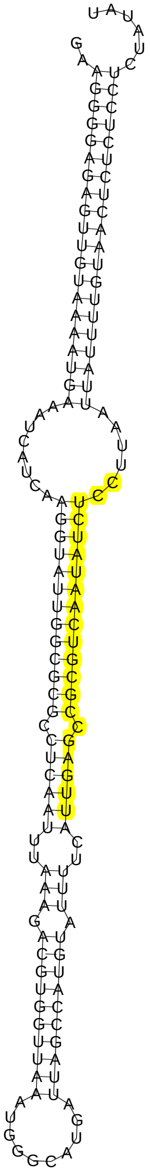

Secondary structure I for miR171g.1

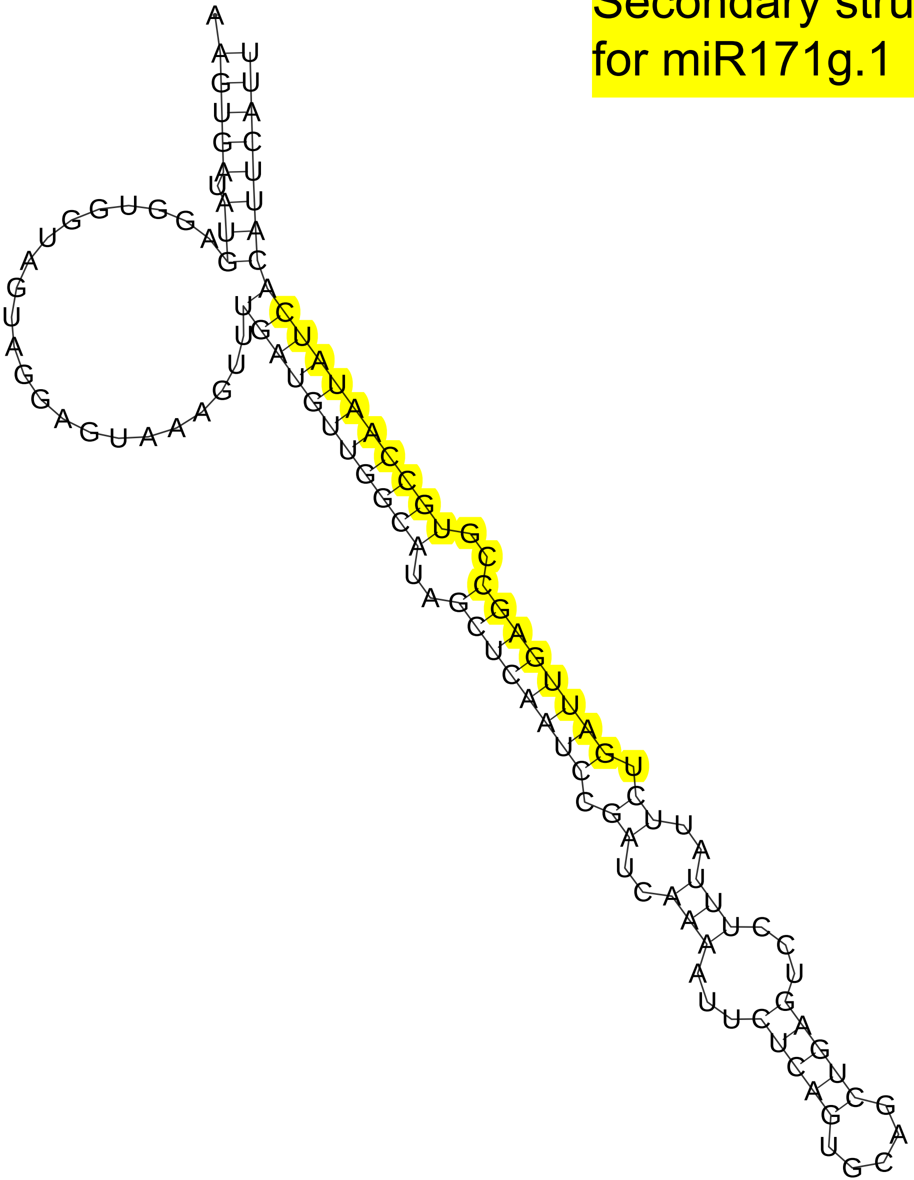

Secondary structure II for miR171g.1

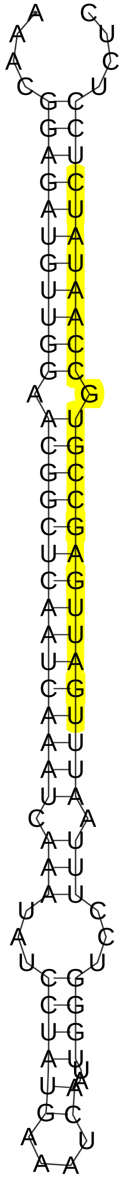

Secondary structure III for miR171g.1

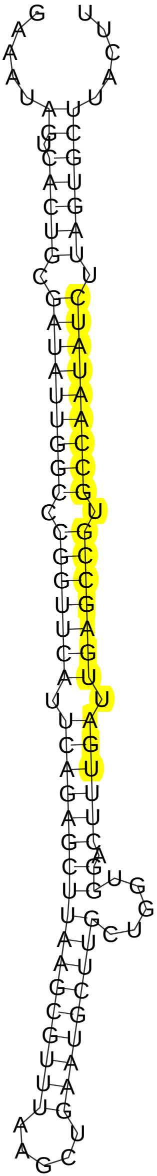

Secondary structure IV for miR171g.1

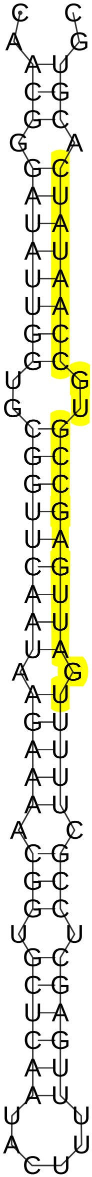

Secondary structure I
for miR172a-3p.1

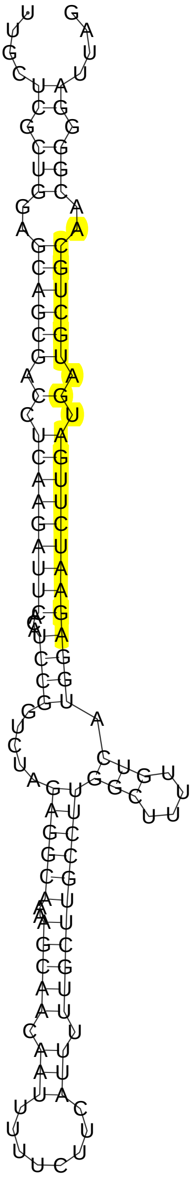

Secondary structure II
for miR172a-3p.1

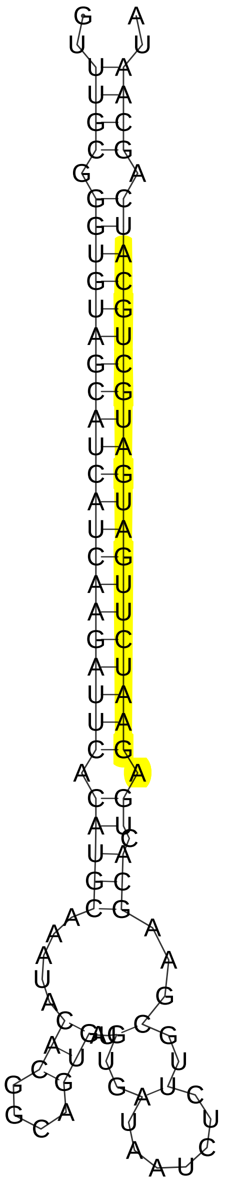

Secondary structure for miR172a-3p.2

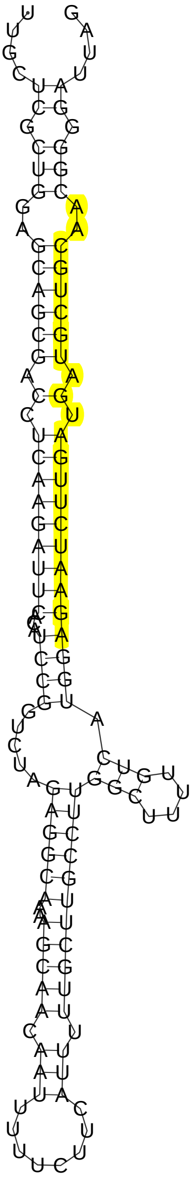

Secondary structure for miR172a-3p.3

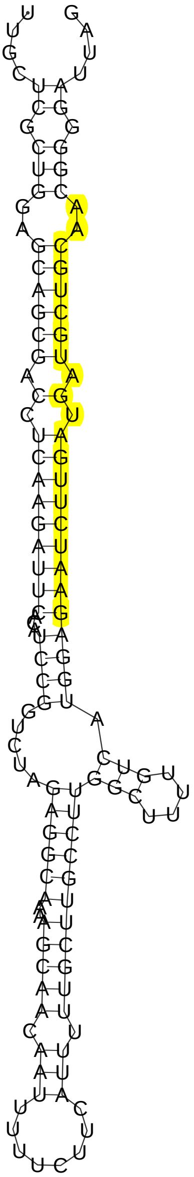

Secondary structure for miR172c.1

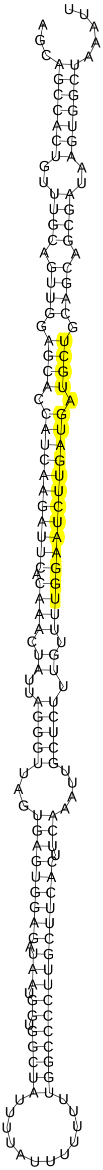

Secondary structure for miR172c.2

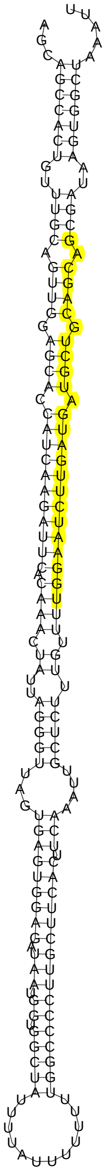

Secondary structure I for miR172d

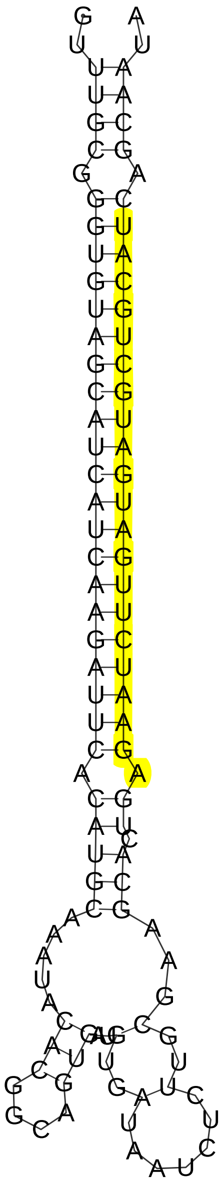

Secondary structure II for miR172d

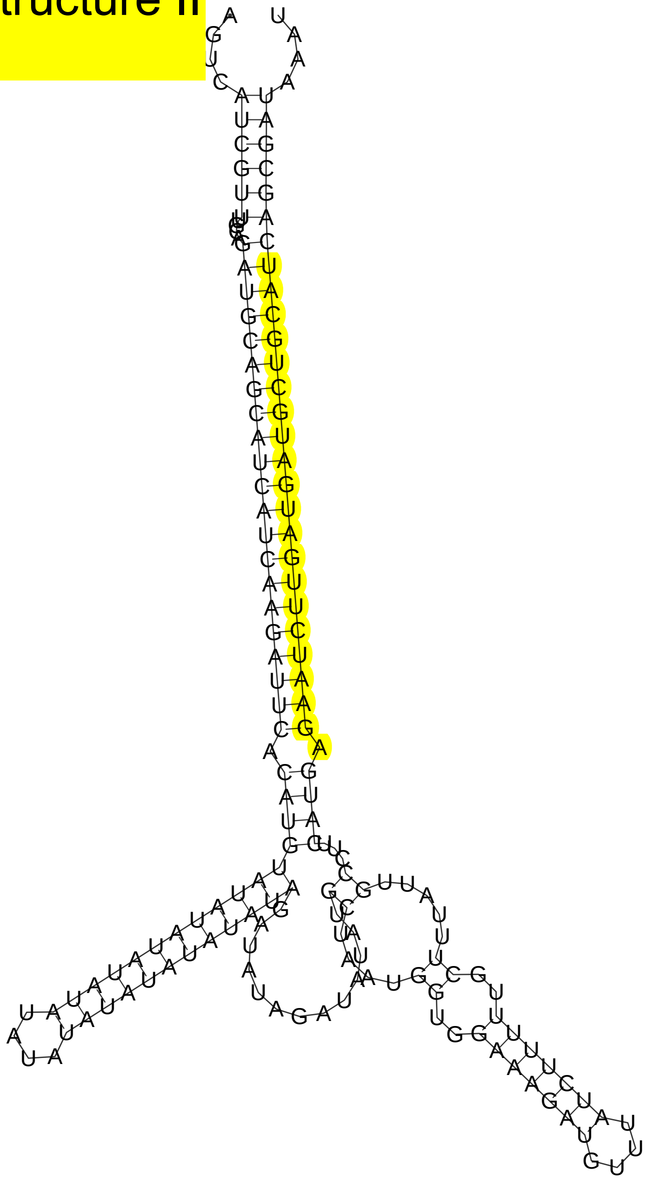

Secondary structure for miR172d-3p

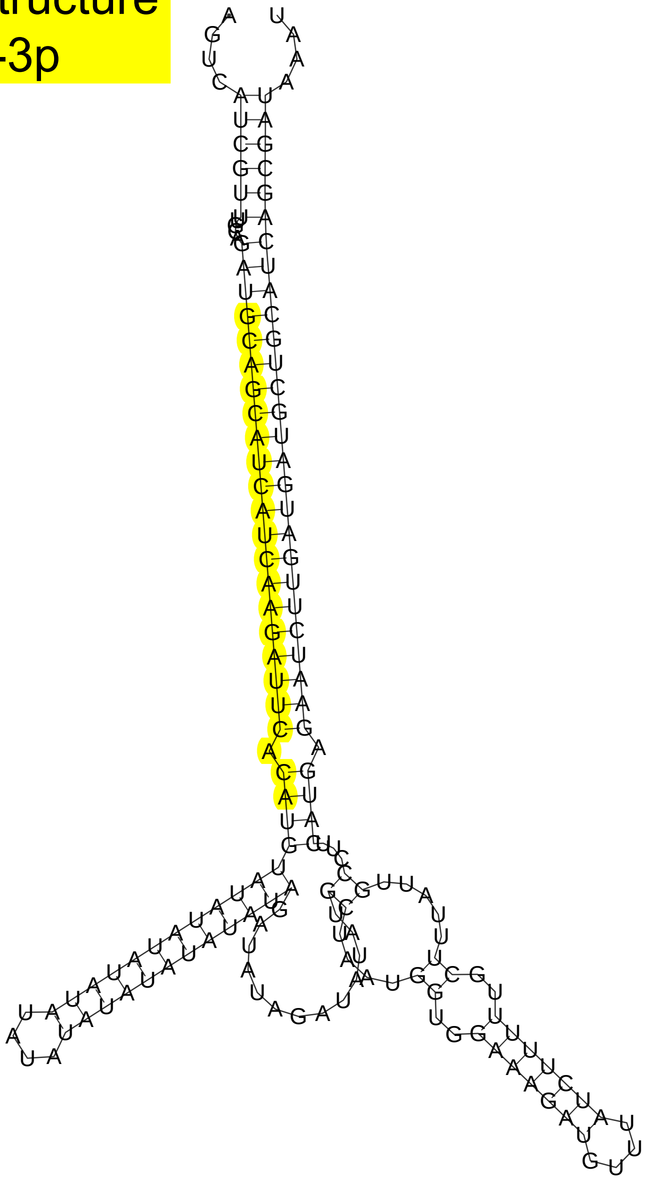

Secondary structure for miR172e.2

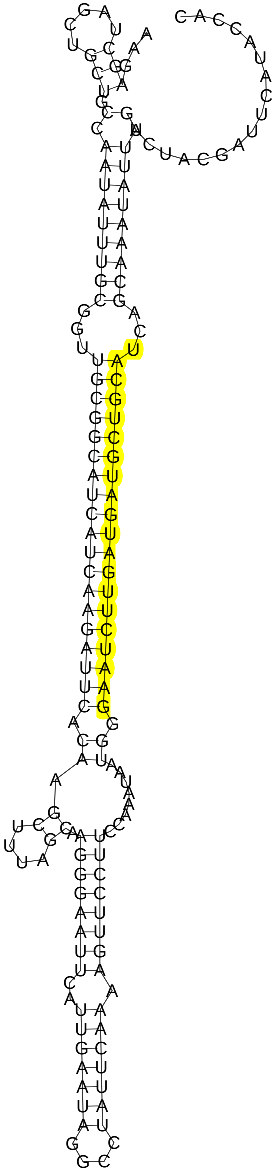

Secondary structure for miR172k

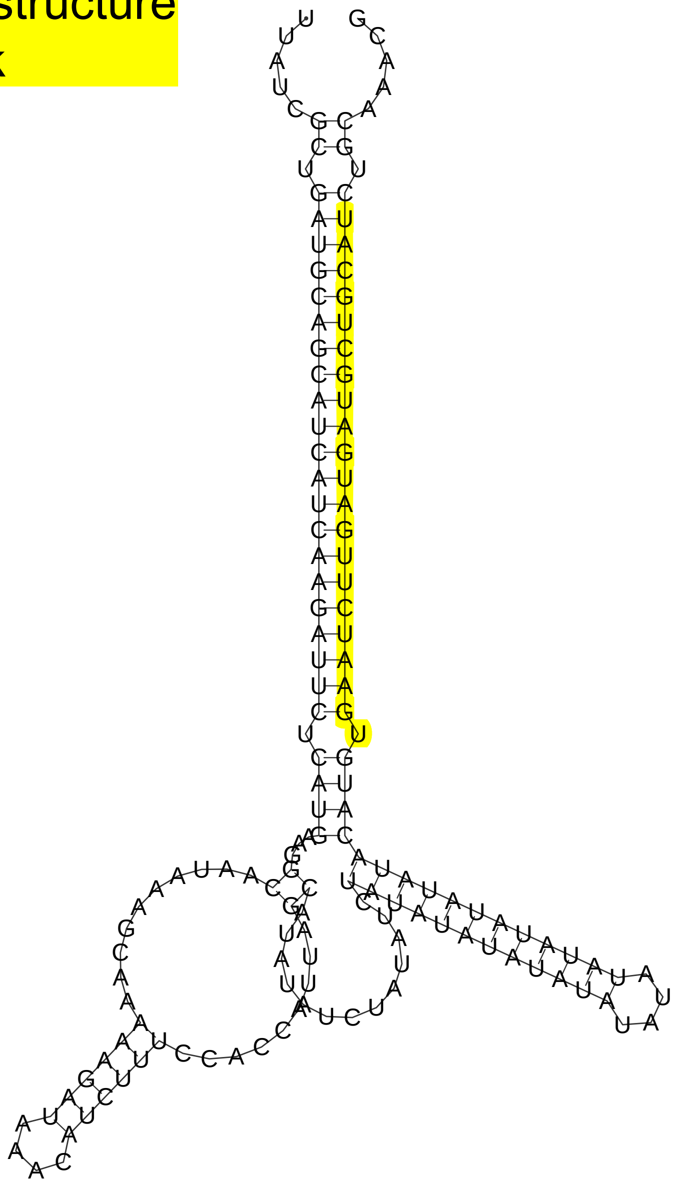

Secondary structure for miR2111

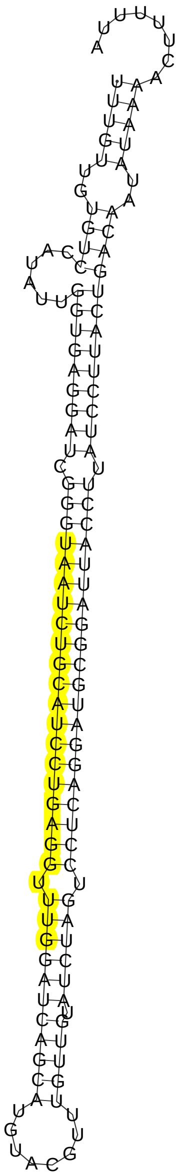

Secondary structure for miR2118.1

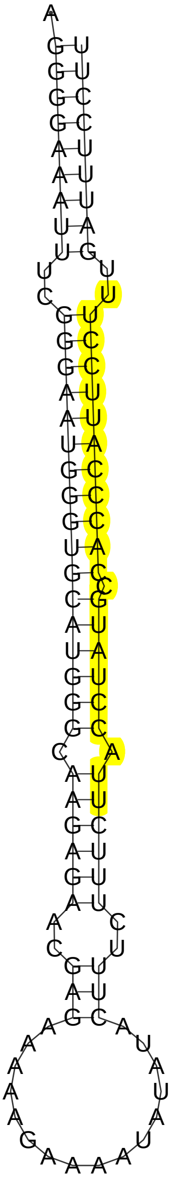

Secondary structure for miR2118.2

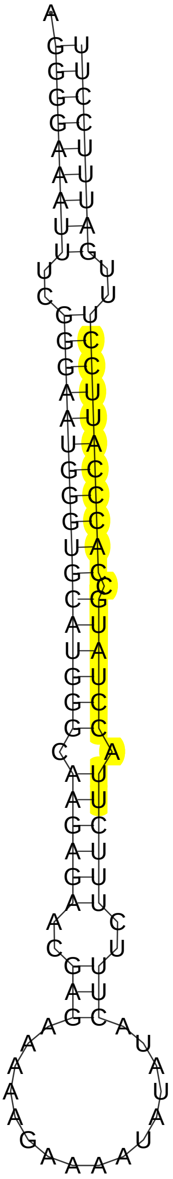

Secondary structure for miR2275a

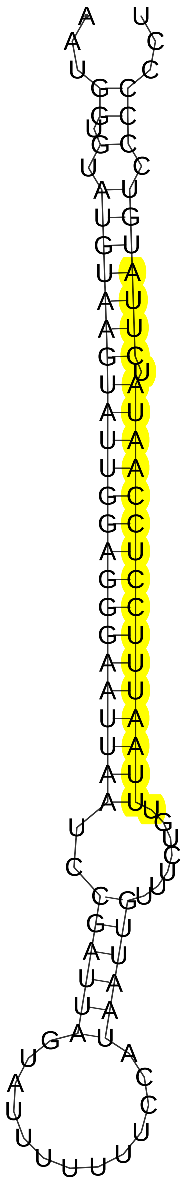

Secondary structure for miR2275b

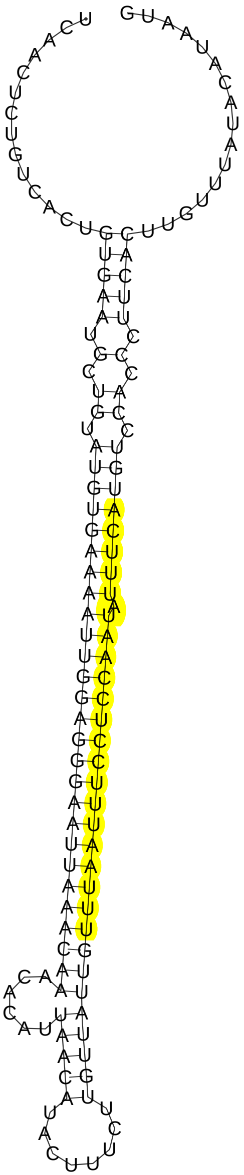

Secondary structure for miR2275c.1

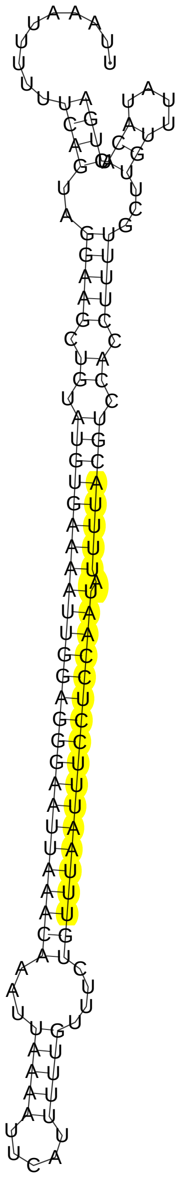

Secondary structure I for miR2275d

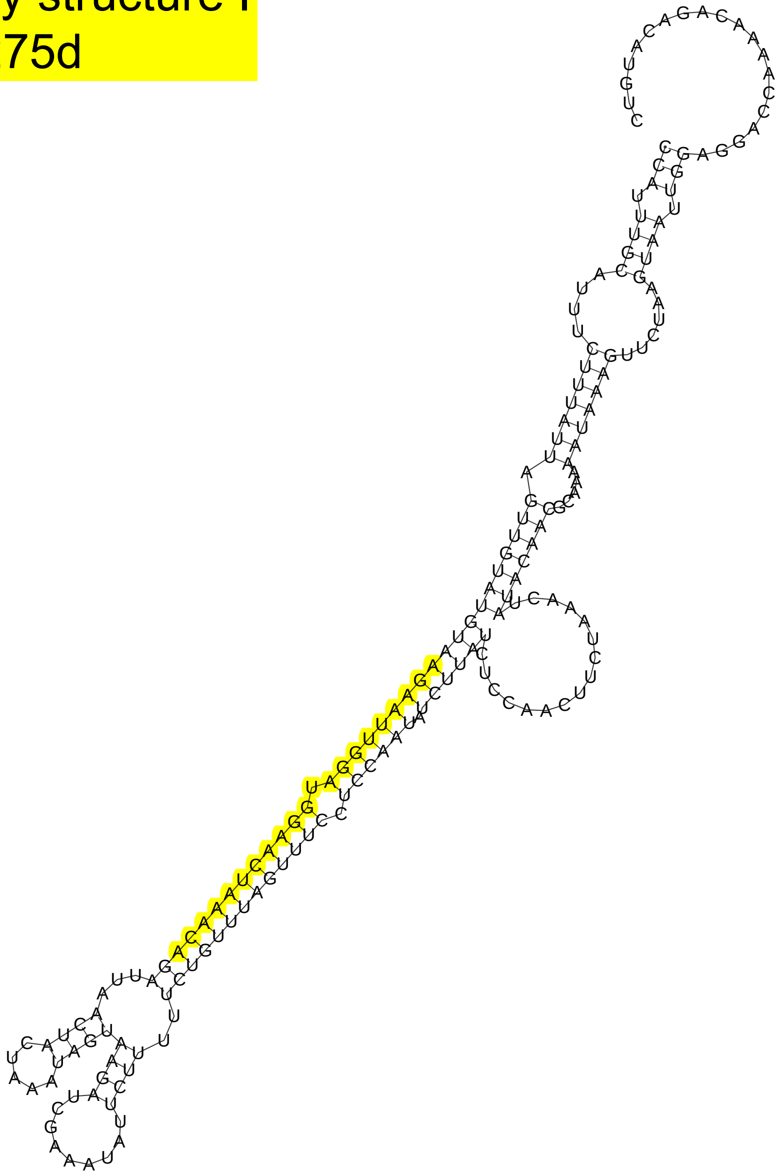

Secondary structure II for miR2275d

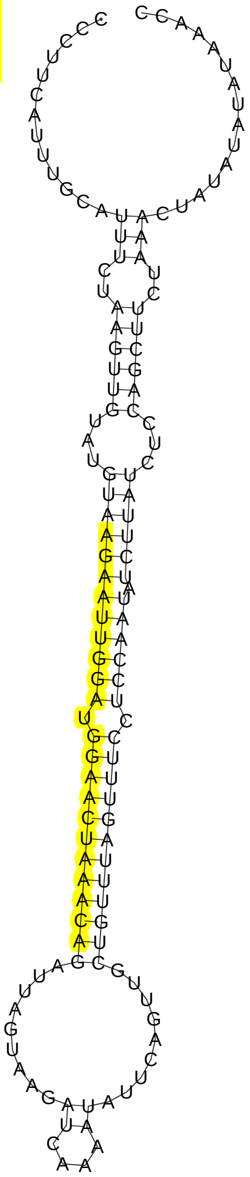

Secondary structure I for miR2275d-3p

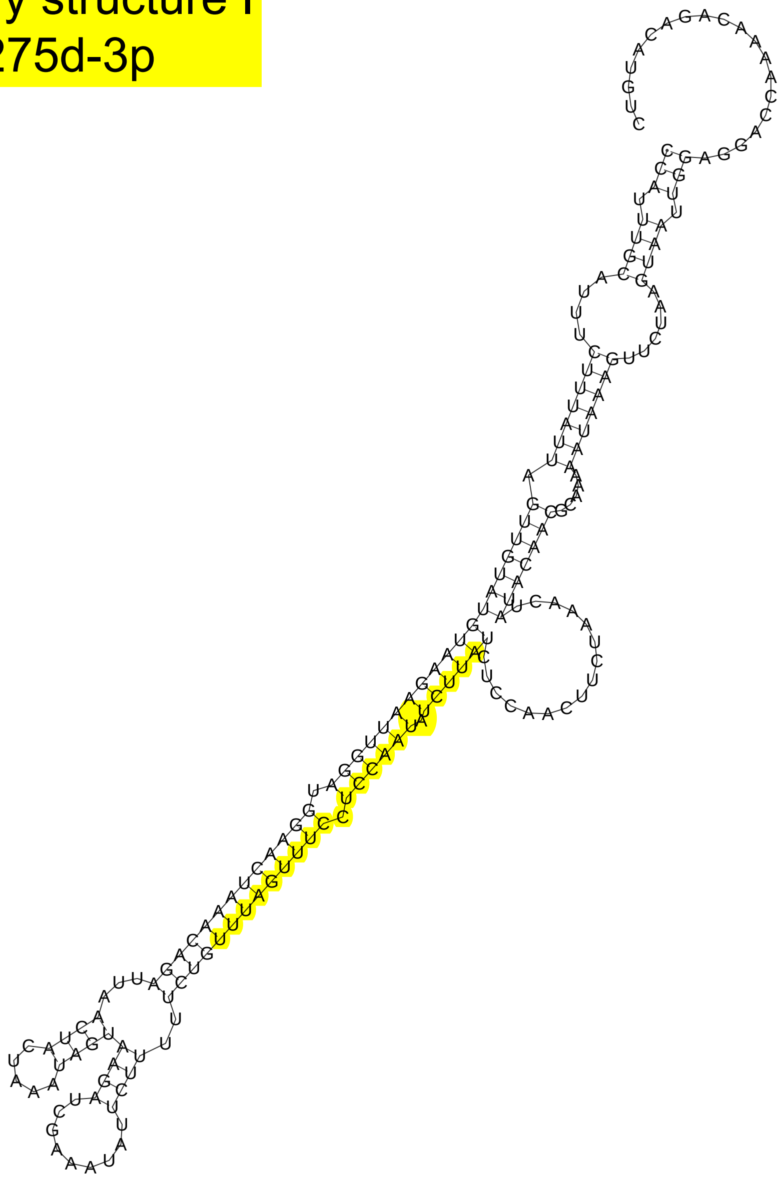

Secondary structure I for miR2275d-3p

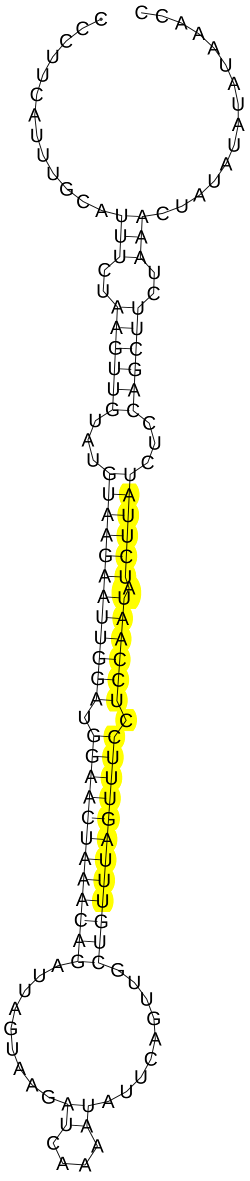

Secondary structure for miR2275e

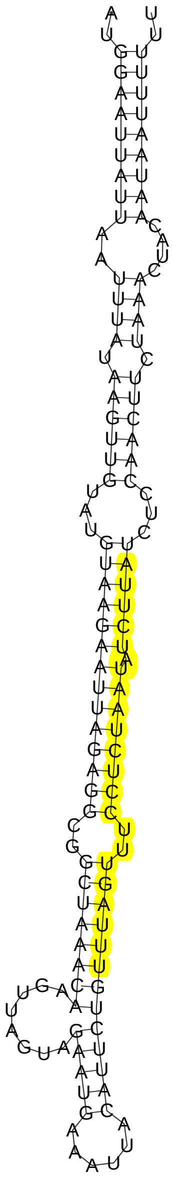

Secondary structure for miR2275f

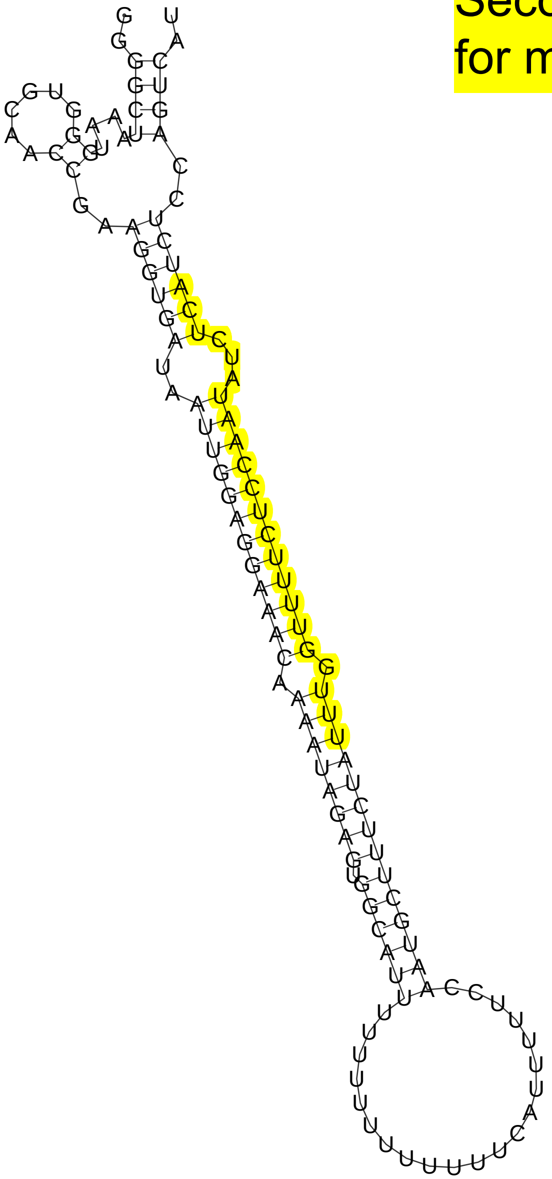

Secondary structure for miR2911

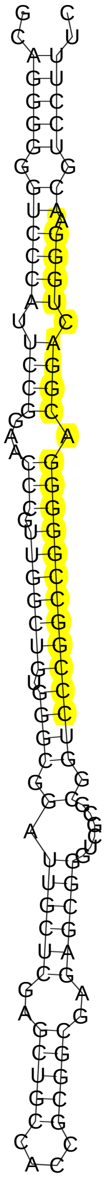

Secondary structure for miR319

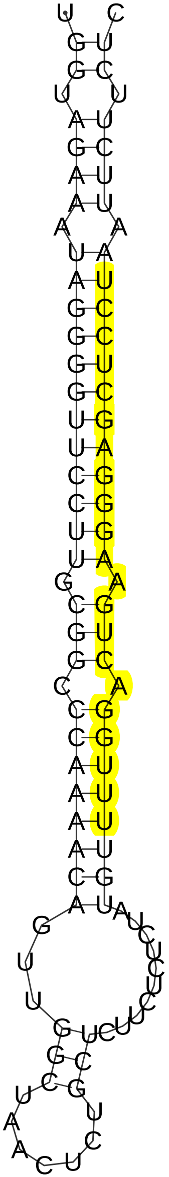

Secondary structure for miR319a

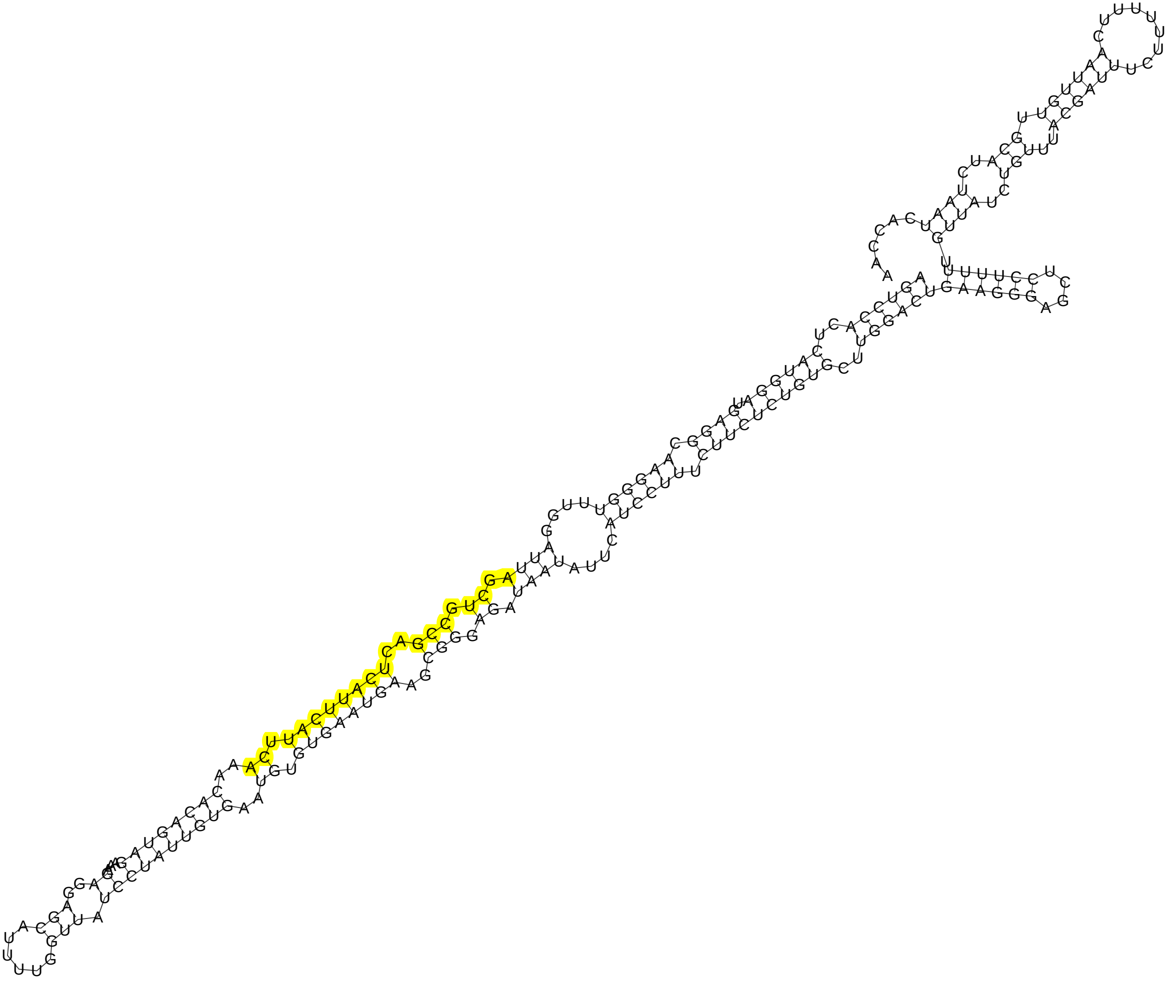

Secondary structure for miR319b

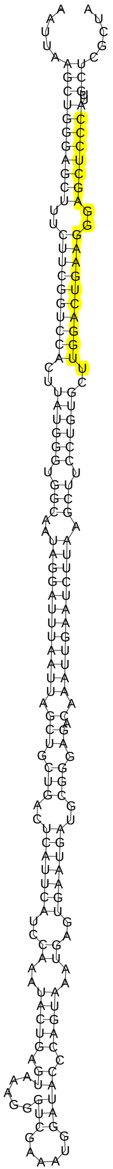

Secondary structure I for miR390.1

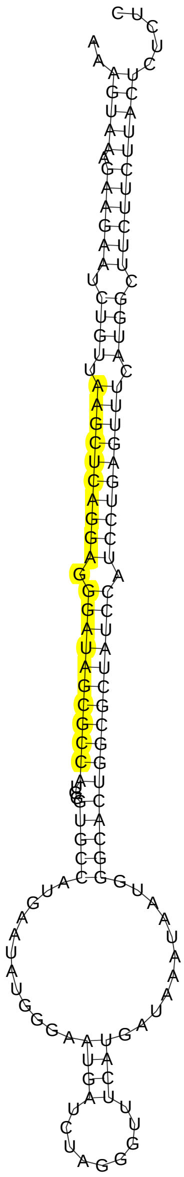

Secondary structure II for miR390.1

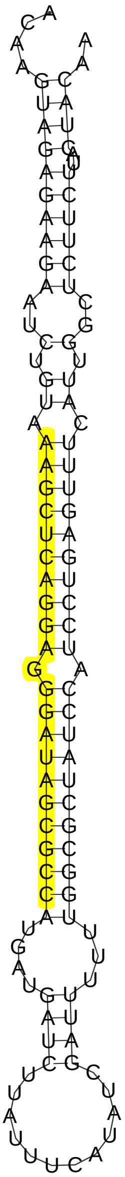

Secondary structure III for miR390.1

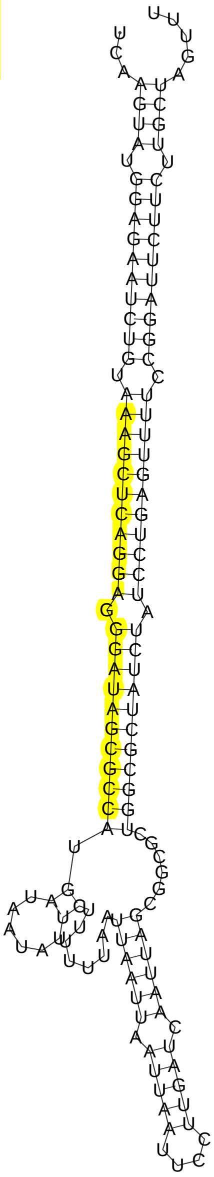

Secondary structure I for miR390.2

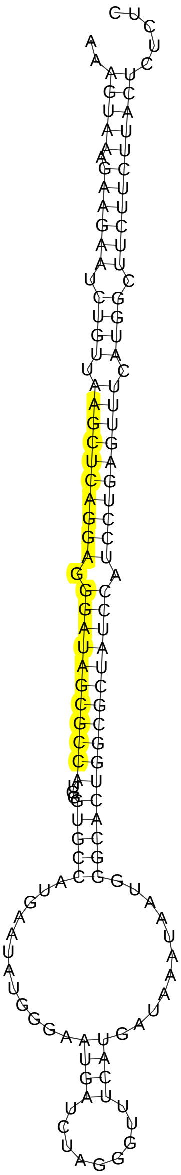

Secondary structure II for miR390.2

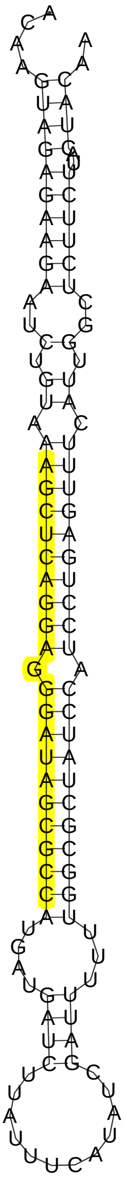

Secondary structure III
for miR390.2

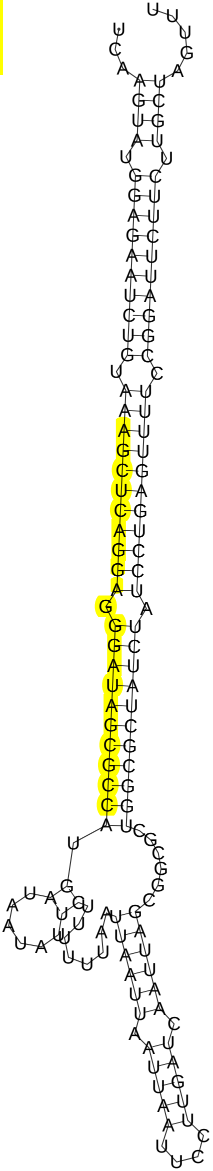

Secondary structure I for miR390-3p.1

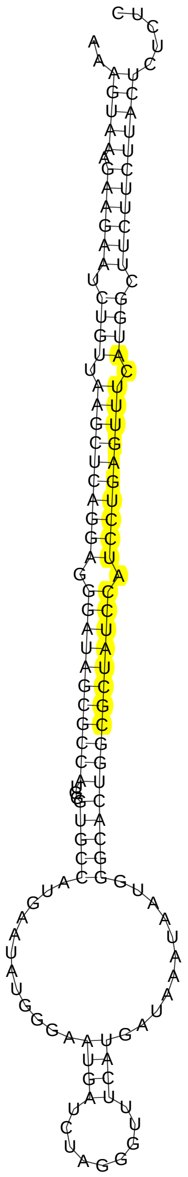

Secondary structure II
for miR390-3p.1

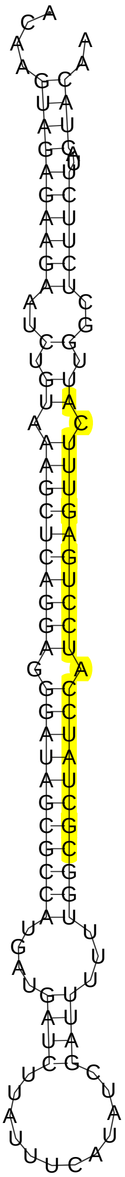

Secondary structure I for miR390-3p.2

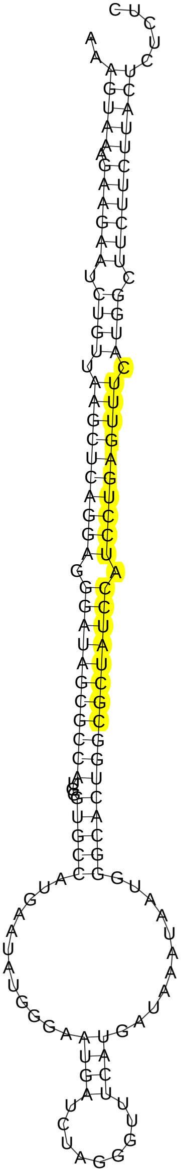

Secondary structure II
for miR390-3p.2

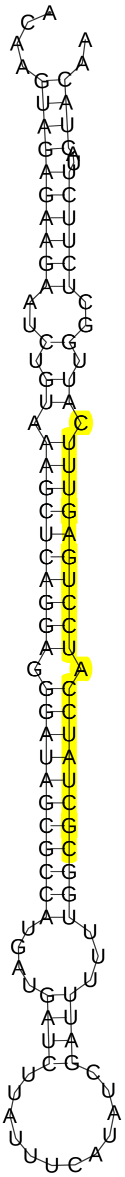

Secondary structure for miR390b

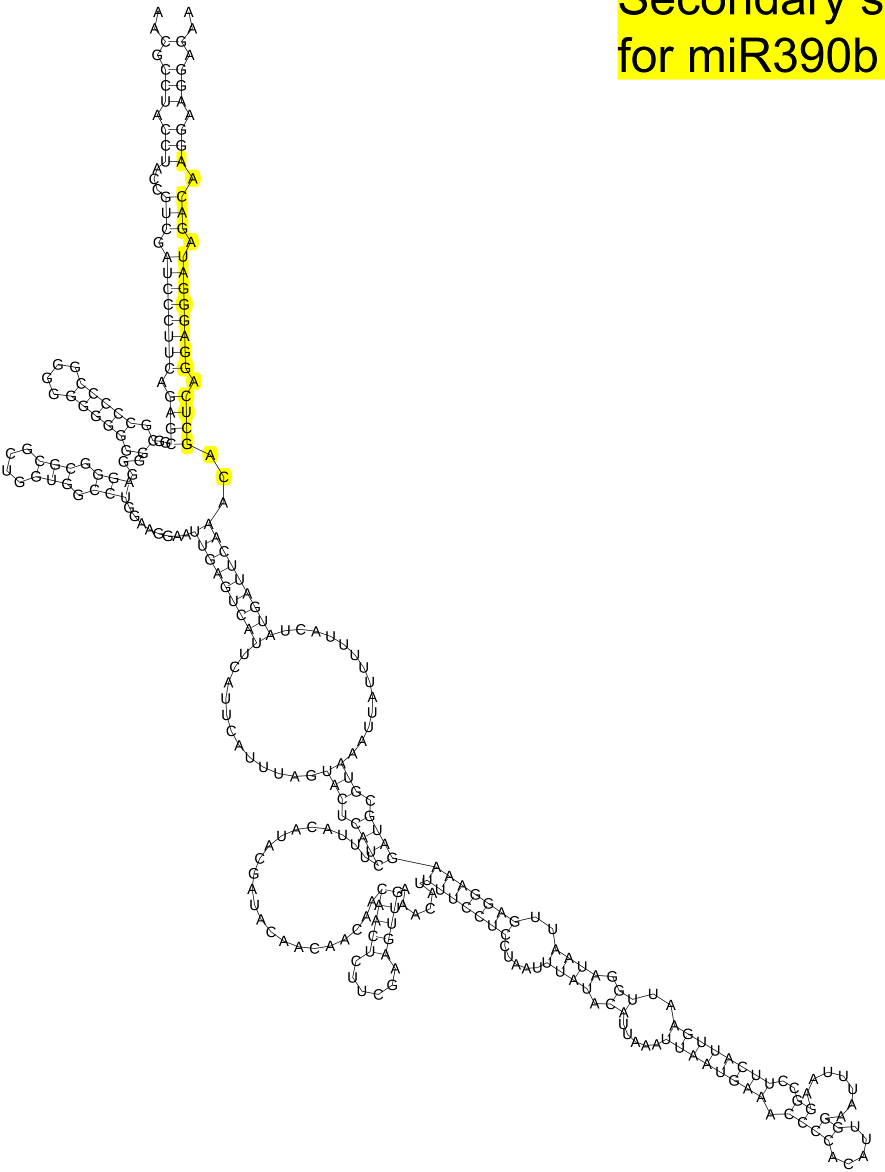

Secondary structure for miR391a.1

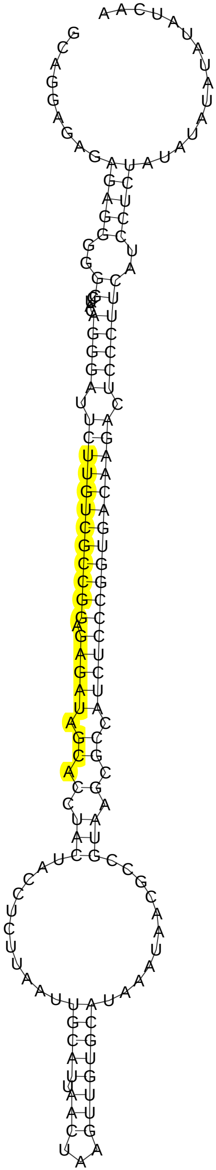

Secondary structure for miR391a.2

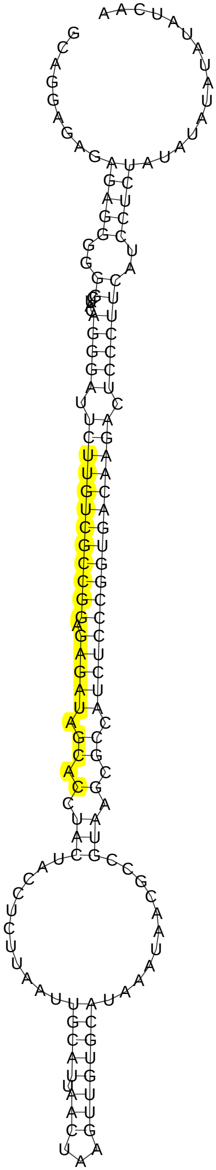

Secondary structure for miR391a.3

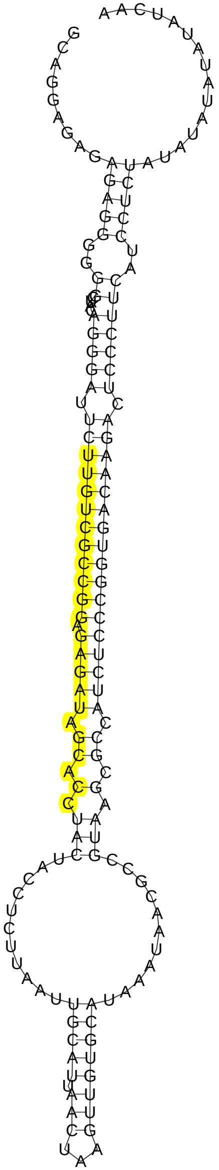

Secondary structure for miR391b

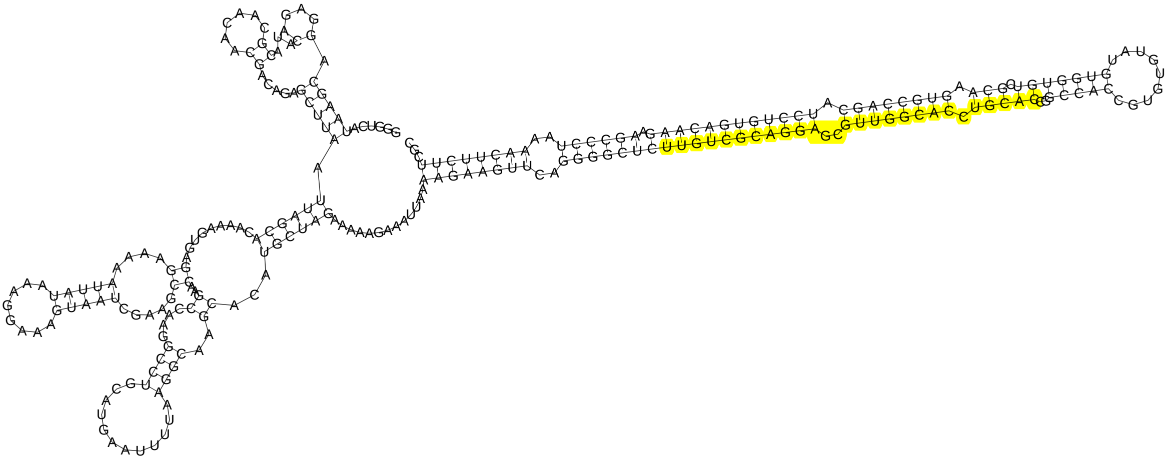

Secondary structure for miR393a.1

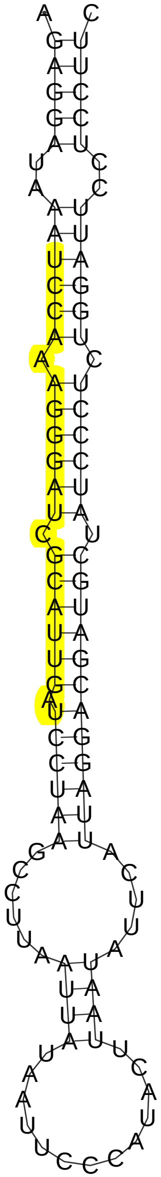

Secondary structure for miR393a.2

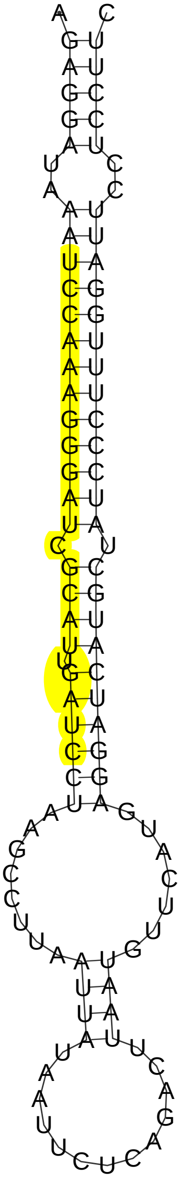

Secondary structure for miR393a.2-3p

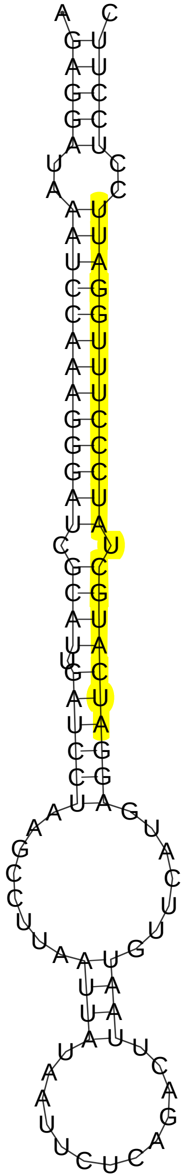

Secondary structure for miR393a.3

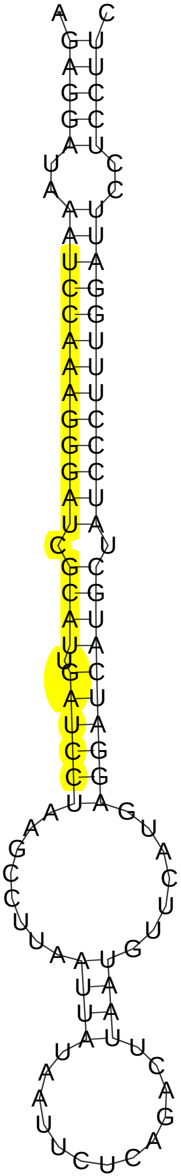

Secondary structure for miR393b

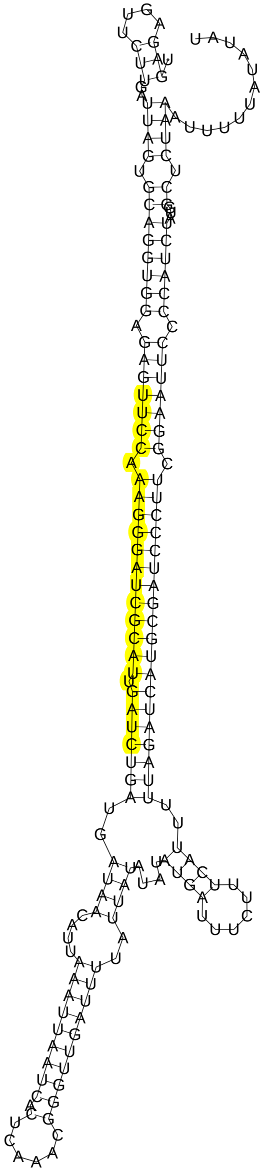

Secondary structure
for miR393b-3p

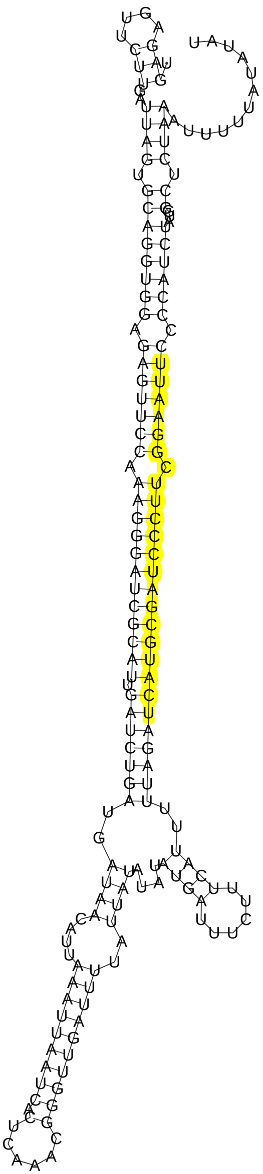

Secondary structure for miR394

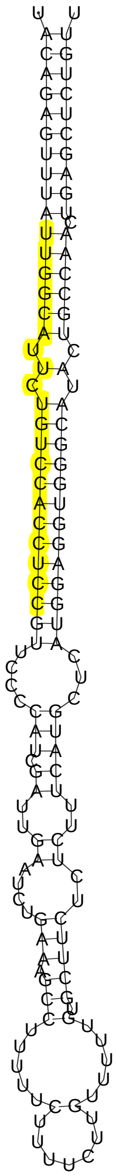

Secondary structure
for miR394-3p.1

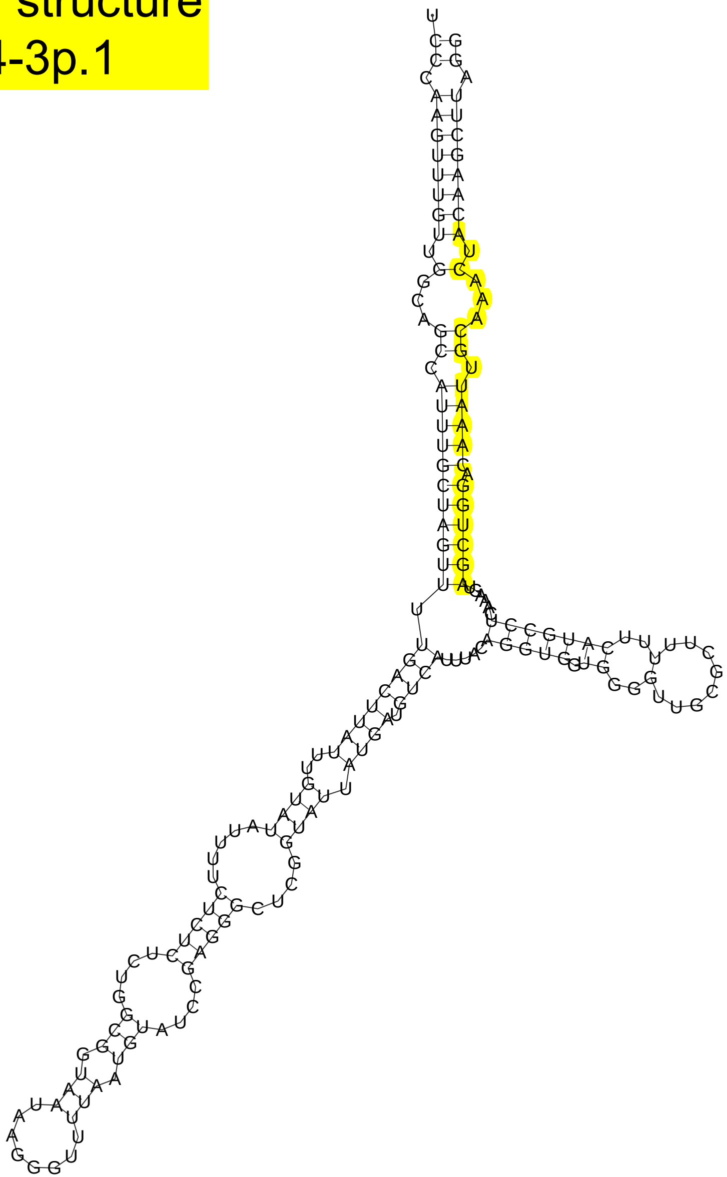

Secondary structure for miR394-3p.2

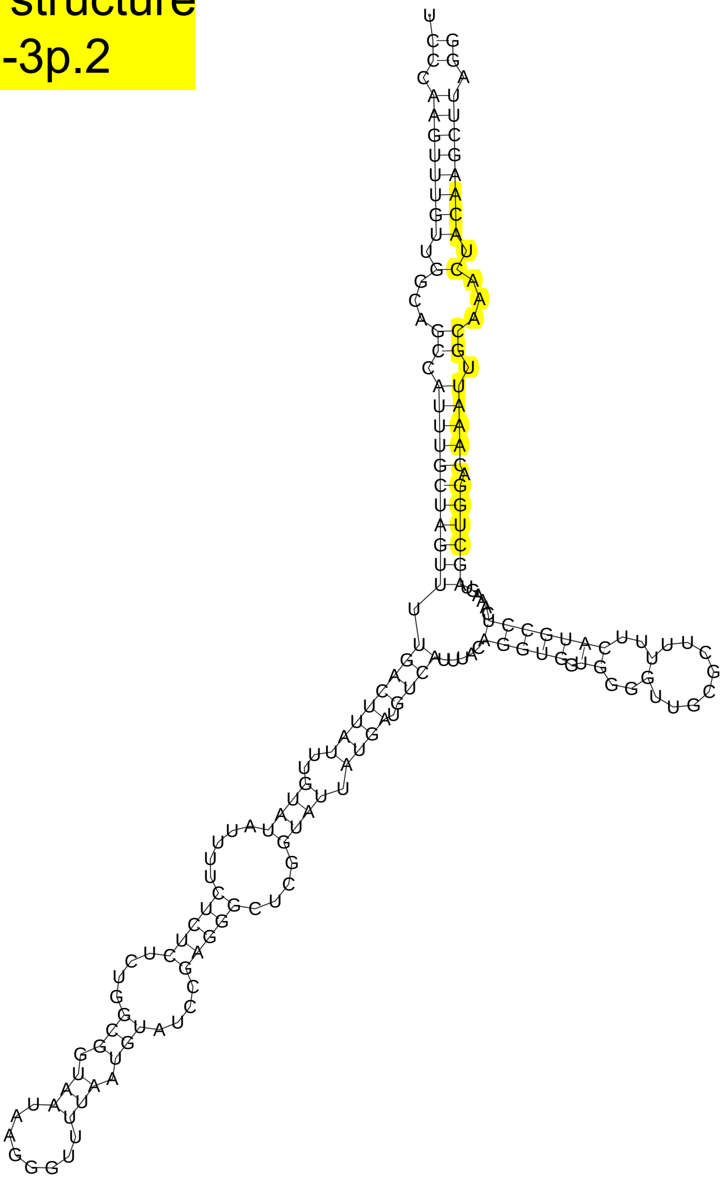

Secondary structure for miR394-3p.3

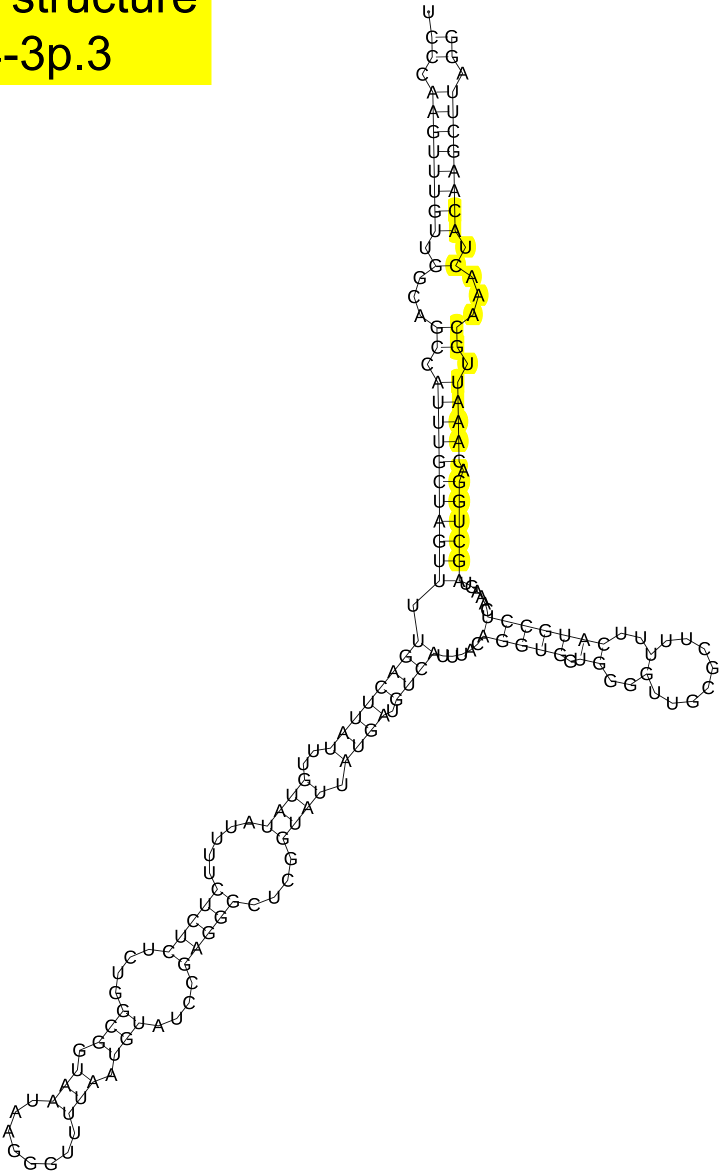

Secondary structure I for miR395.1

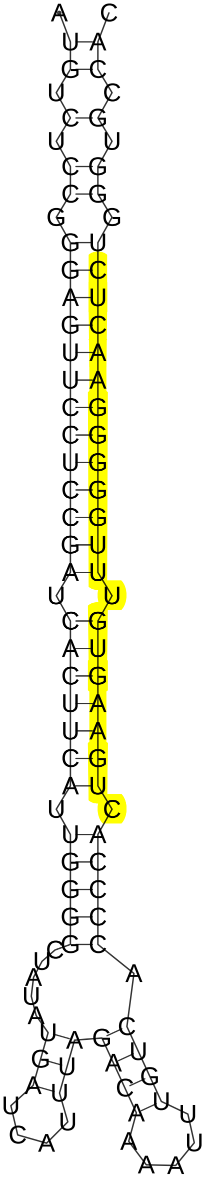

Secondary structure II
for miR395.1

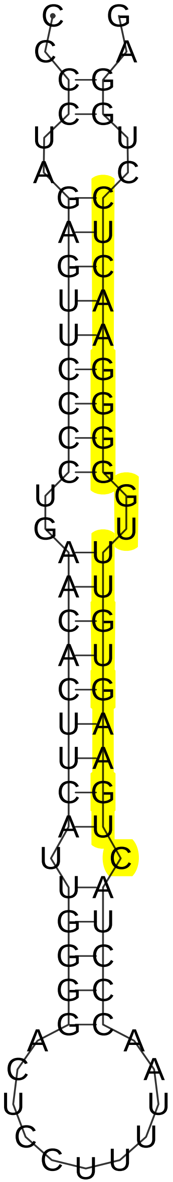

Secondary structure III for miR395.1

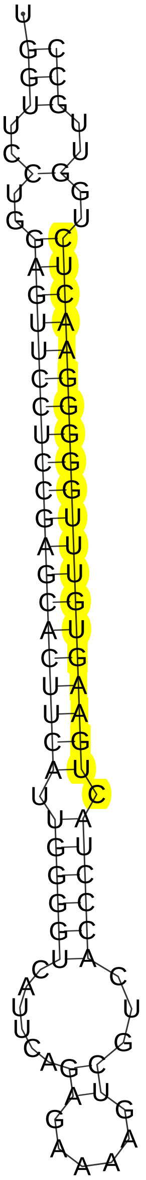

Secondary structure IV for miR395.1

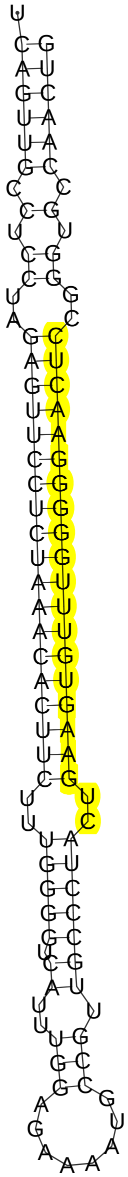

Secondary structure V for miR395.1

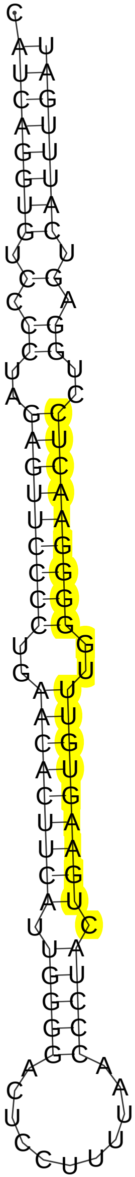

Secondary structure I for miR395.2

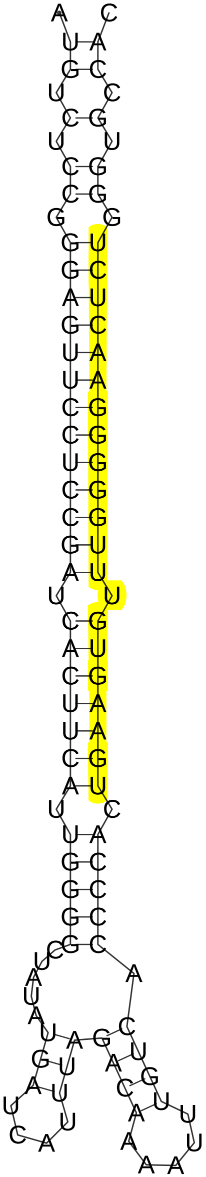

Secondary structure II for miR395.2

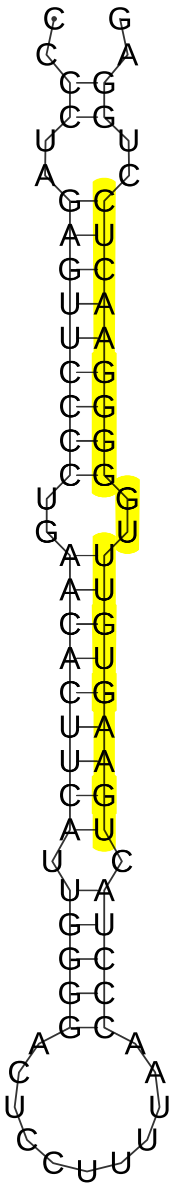

Secondary structure III for miR395.2

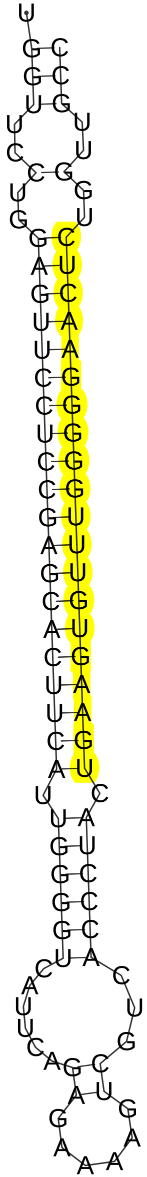

Secondary structure IV for miR395.2

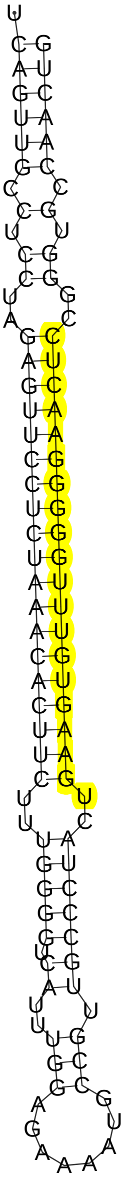

Secondary structure V for miR395.2

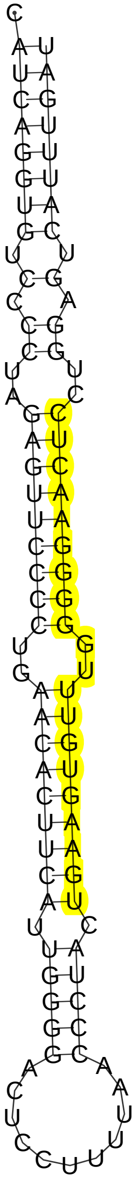

Secondary structure for miR3951

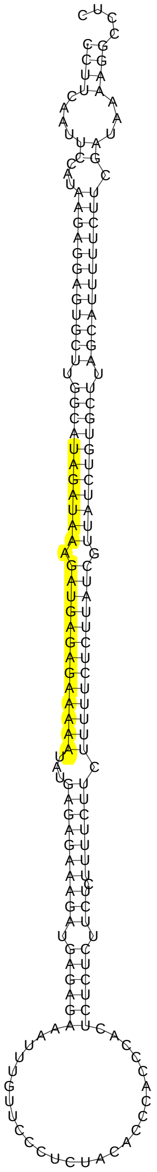

Secondary structure for miR3951-3p

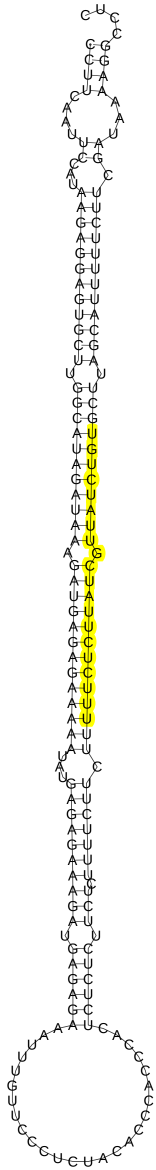

Secondary structure for miR3952.1

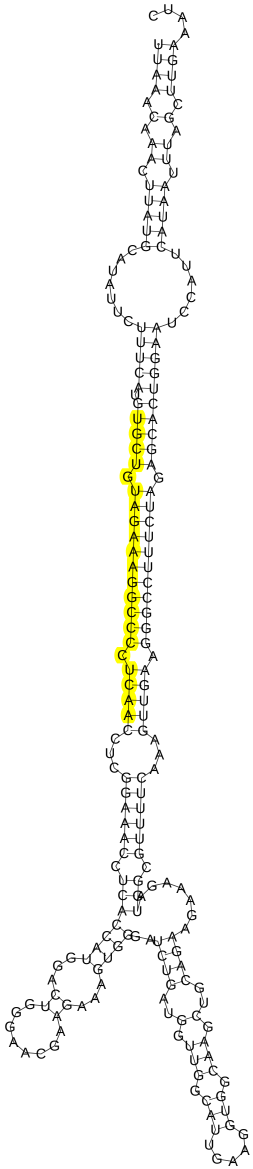

Secondary structure for miR3952.2

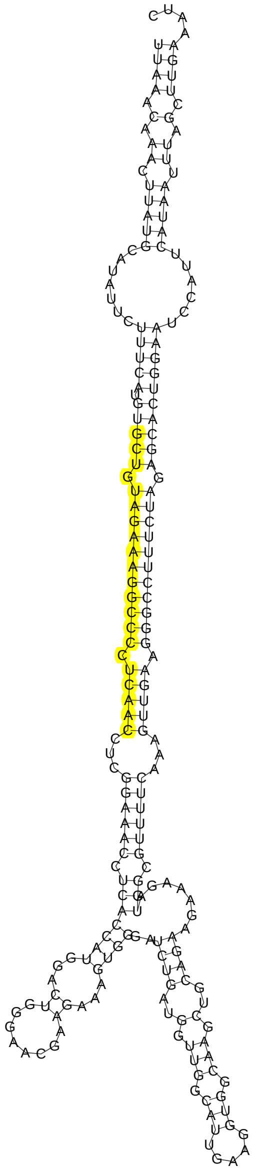

Secondary structure II
for miR3952-3p

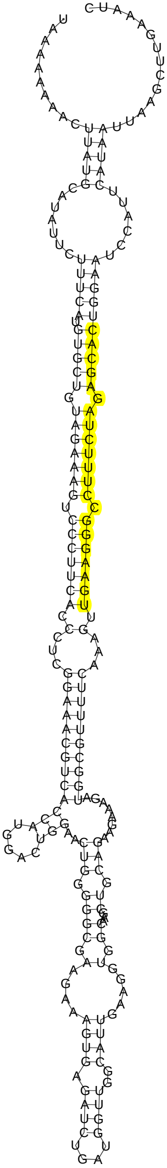

Secondary structure I for miR3952-3p

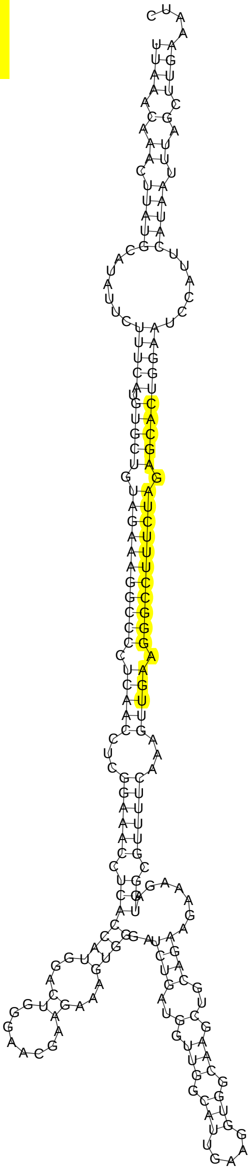

Secondary structure for miR3954a

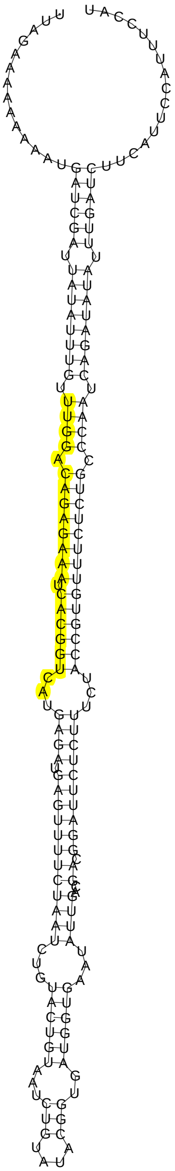

Secondary structure for miR3954b

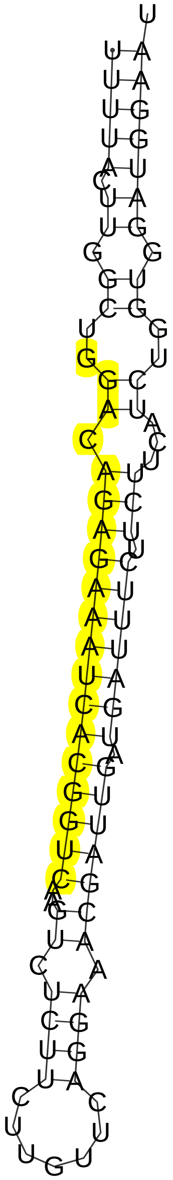

Secondary structure I for miR396a

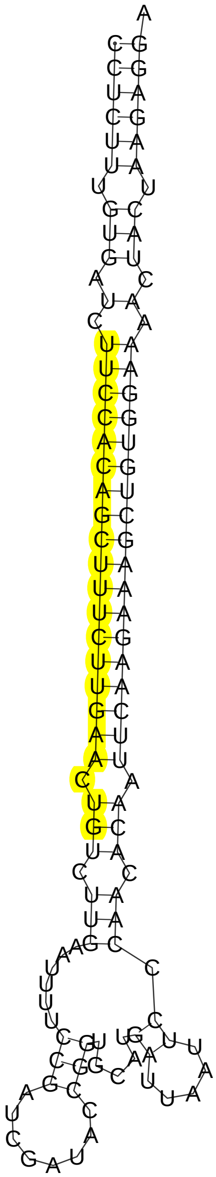

Secondary structure II for miR396a

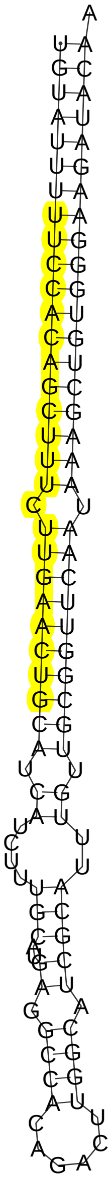

Secondary structure I for miR396b.1

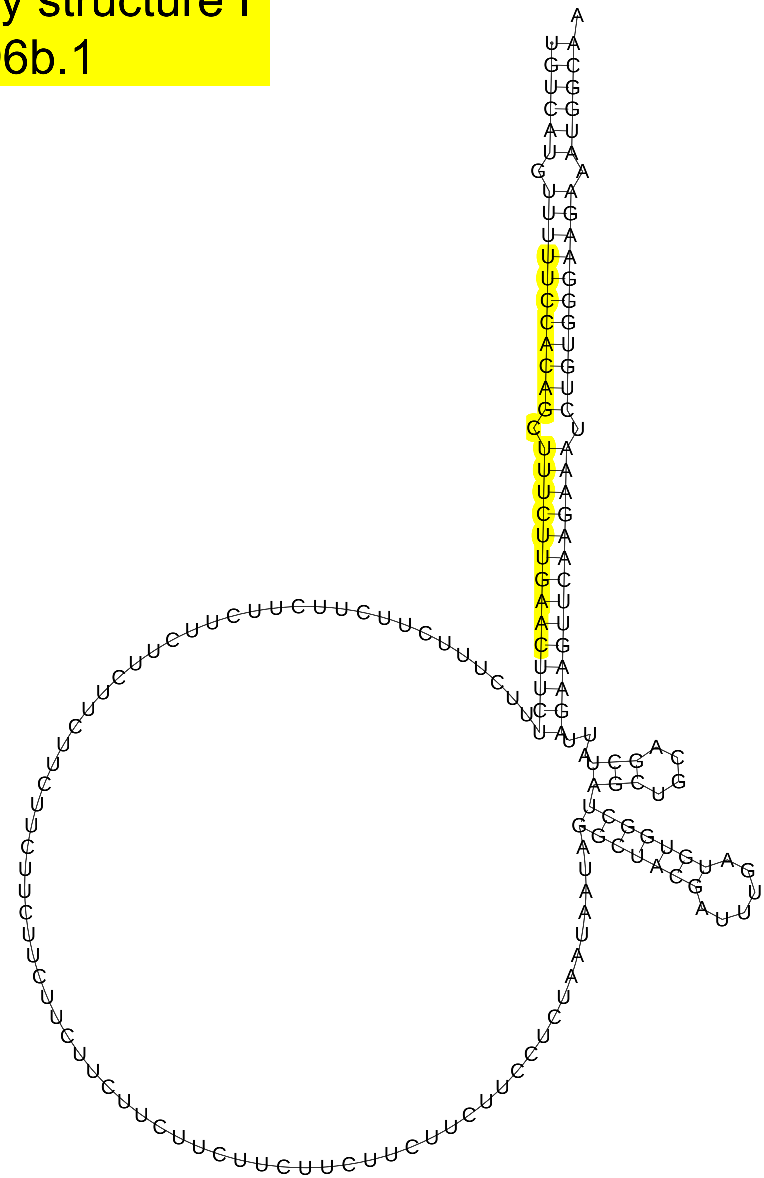

Secondary structure II for miR396b.1

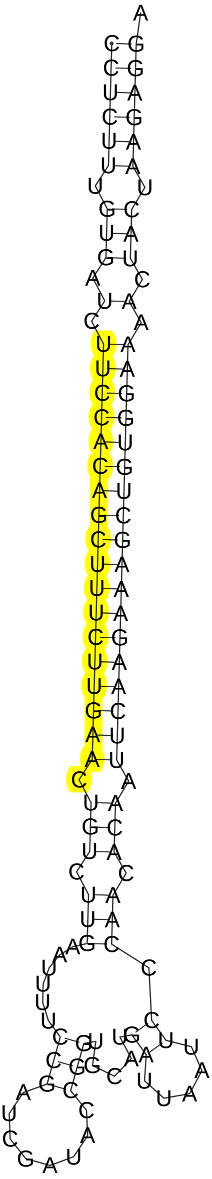

Secondary structure III for miR396b.1

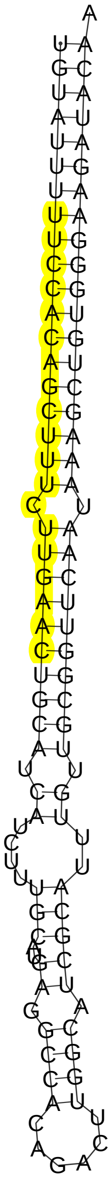

Secondary structure I
for miR396b.2

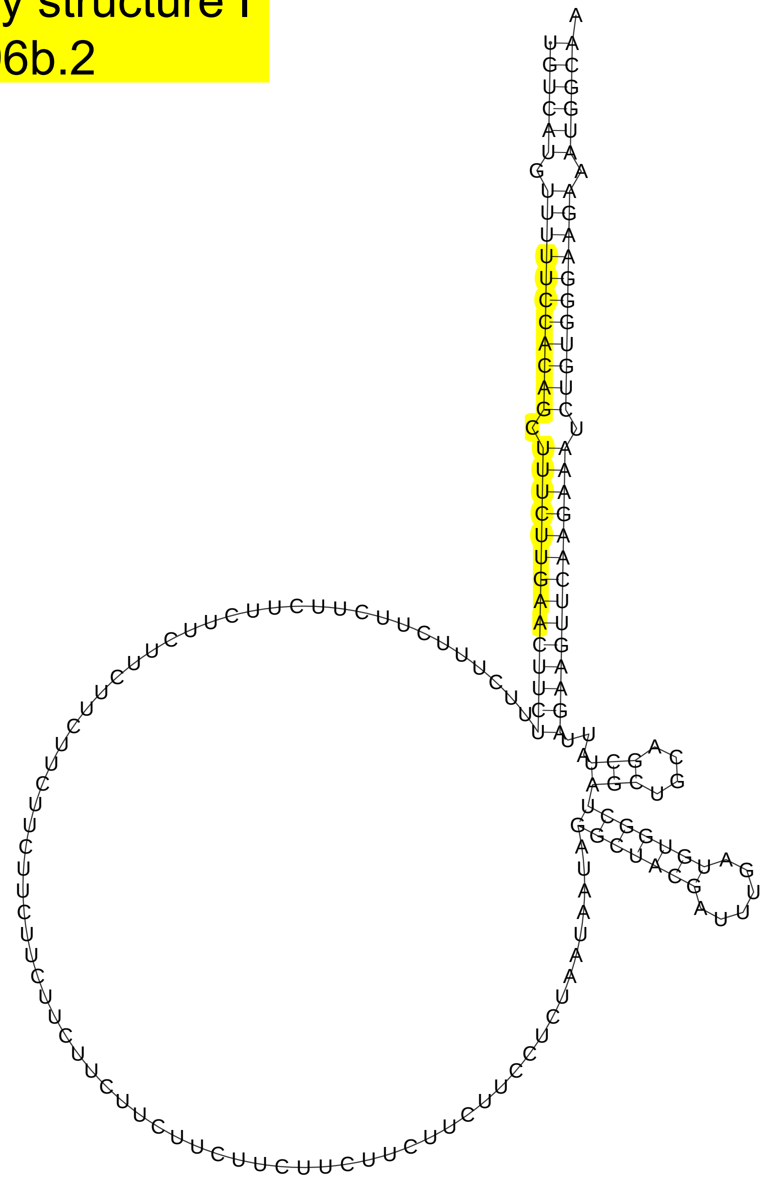

Secondary structure II for miR396b.2

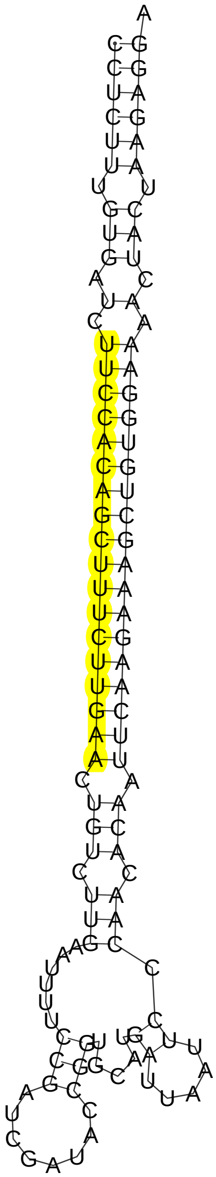

Secondary structure III for miR396b.2

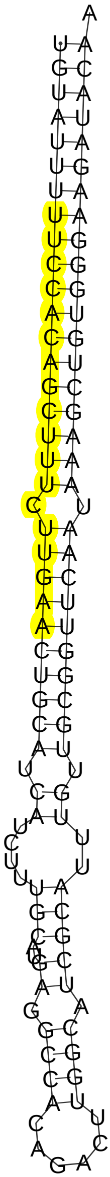

Secondary structure I
for miR396b.3

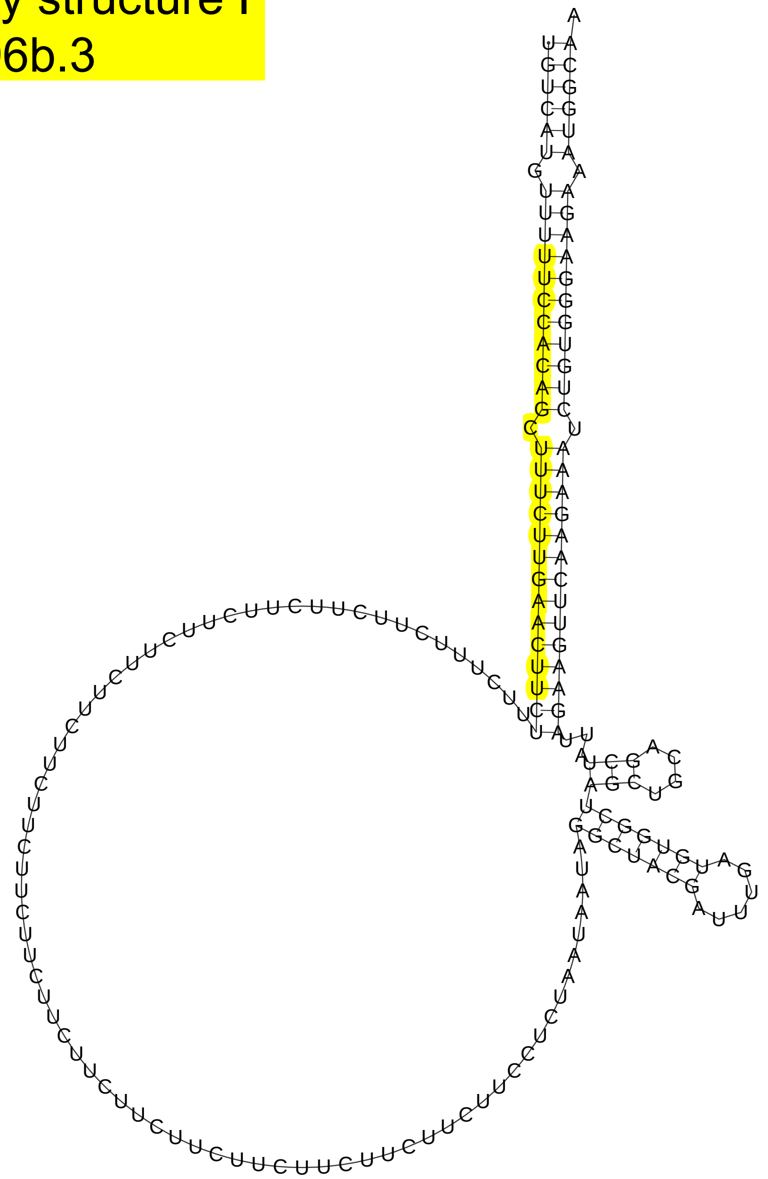

Secondary structure II for miR396b.3

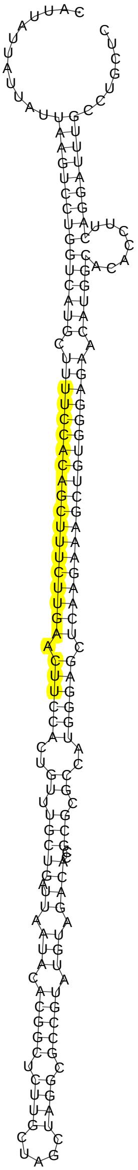

Secondary structure for miR396b-3p.1

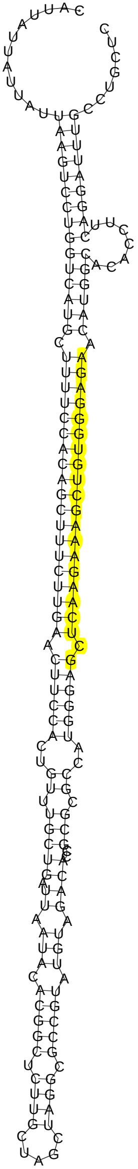

Secondary structure for miR396b-3p.2

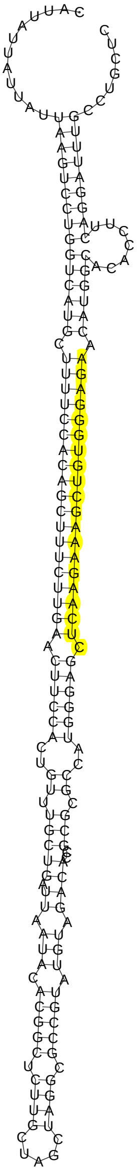

Secondary structure I
for miR396c

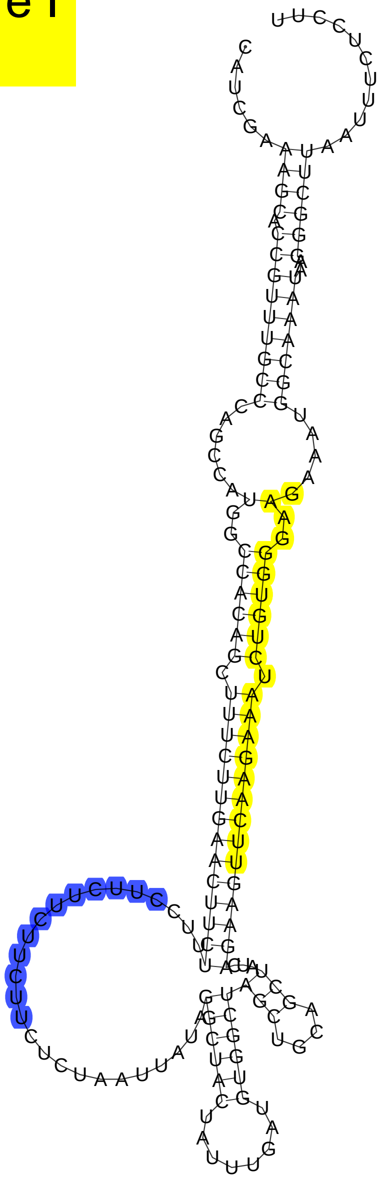

Secondary structure II for miR396c

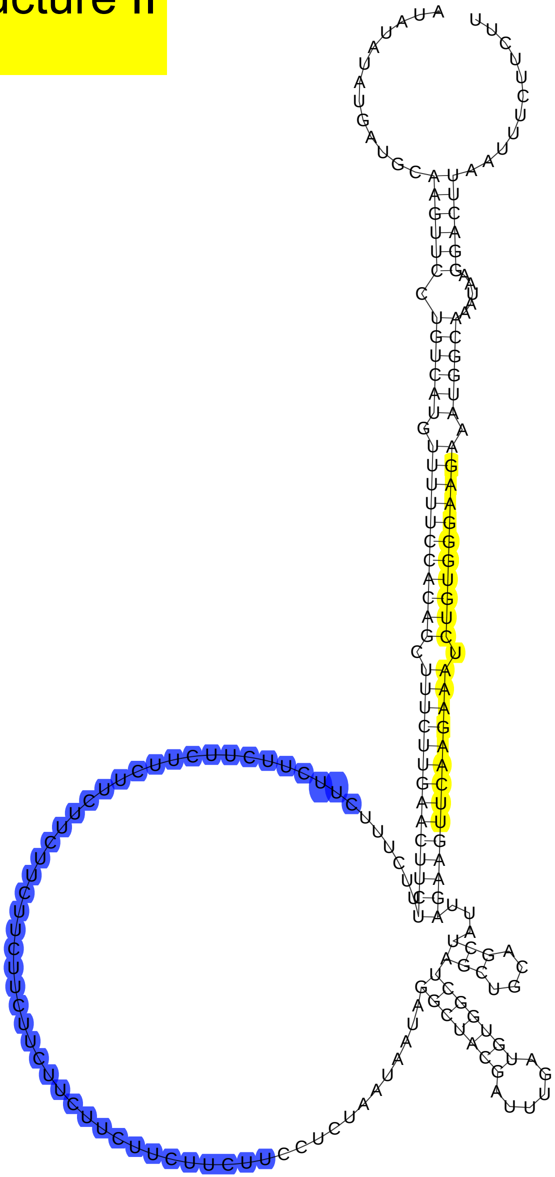

Secondary structure III for miR396c

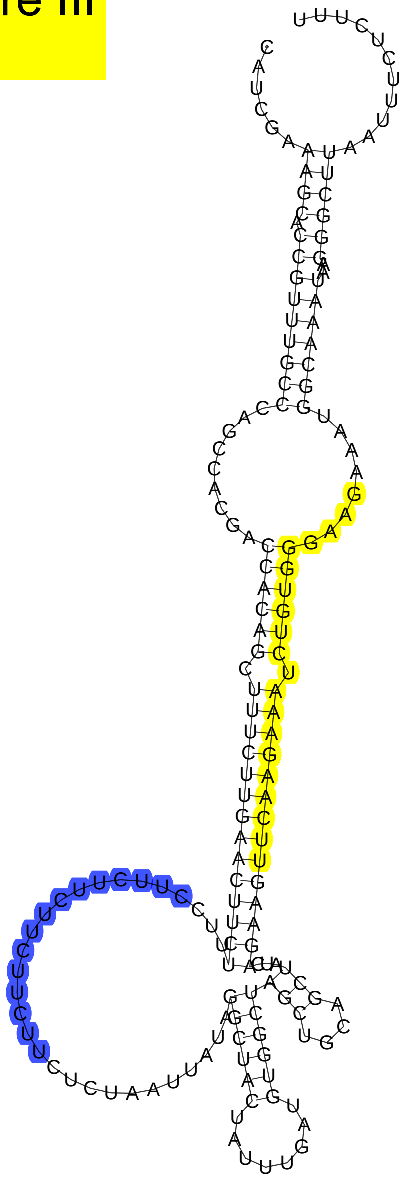

Secondary structure IV for miR396c

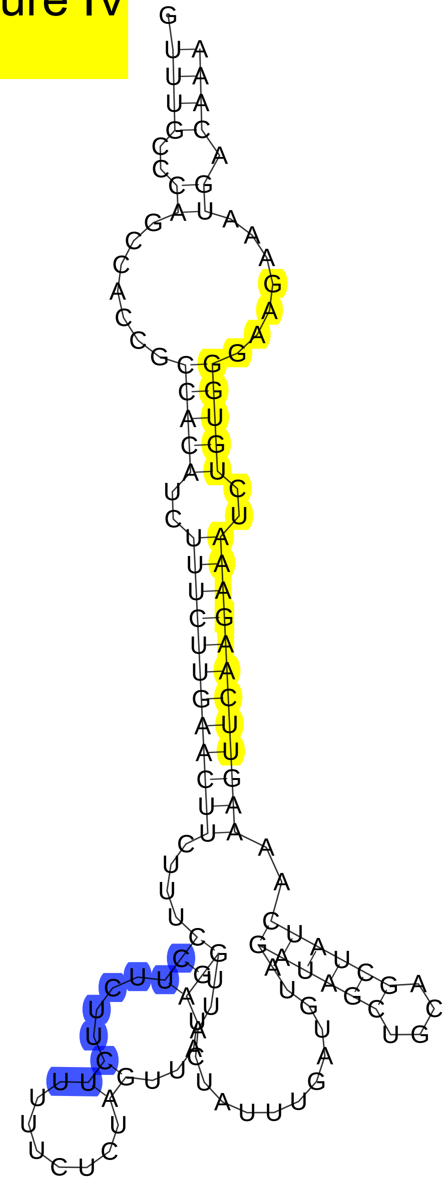

Secondary structure for miR396d.1

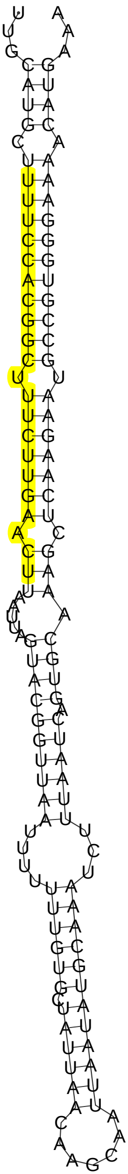

Secondary structure for miR396d.2

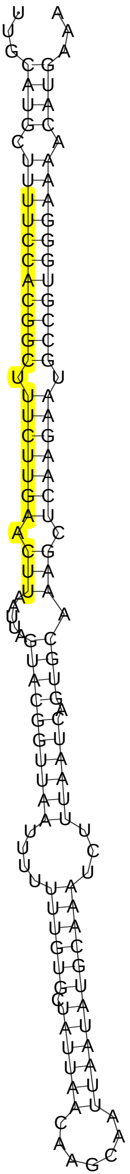

Secondary structure for miR396d.3

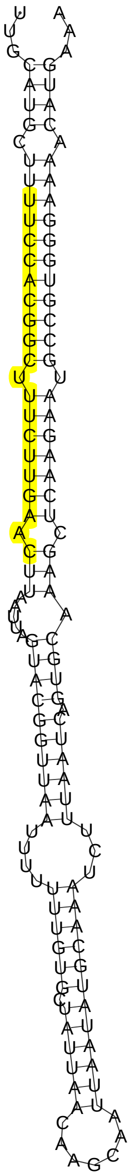

Secondary structure for miR396d.4

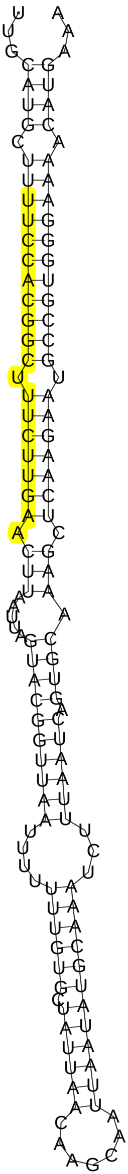

Secondary structure for miR396d-3p

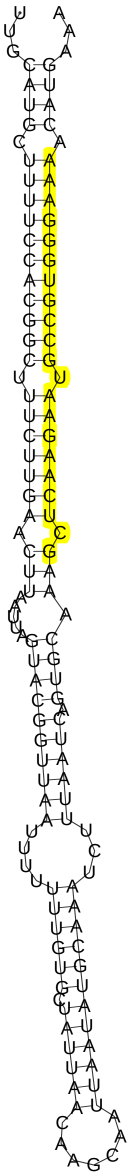

Secondary structure for miR397.1

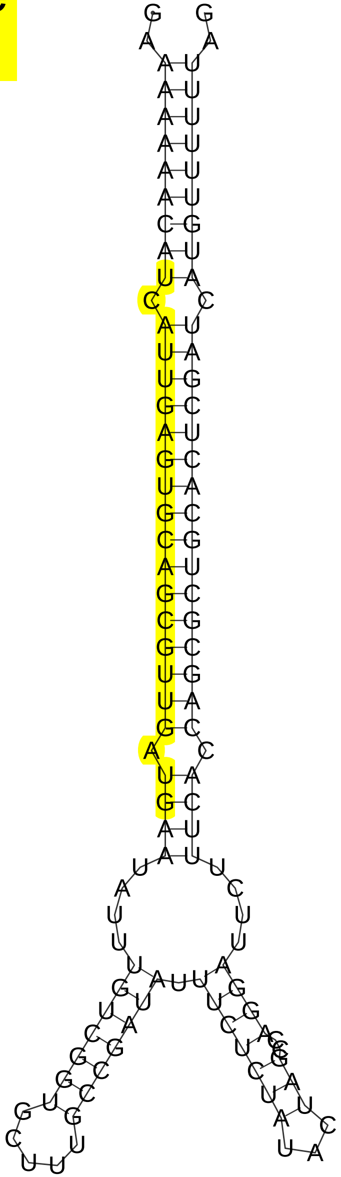

Secondary structure for miR397.2

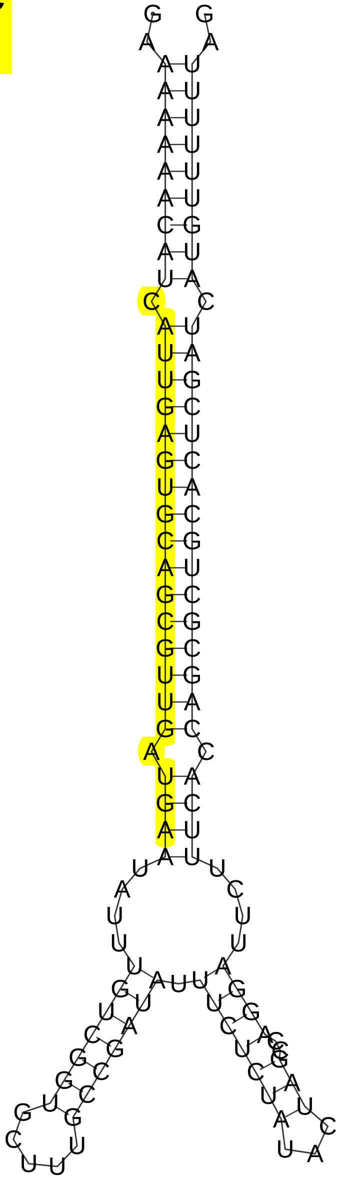

Secondary structure for miR398b

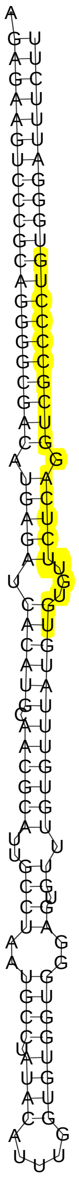

Secondary structure I for miR399a

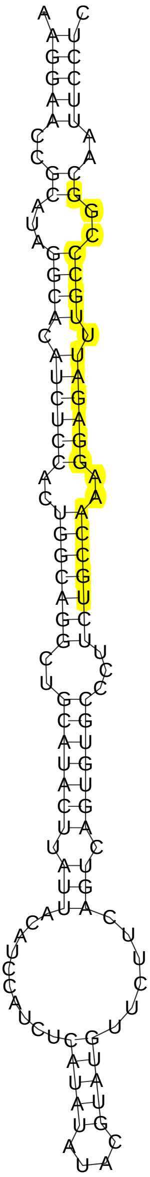

Secondary structure II for miR399a

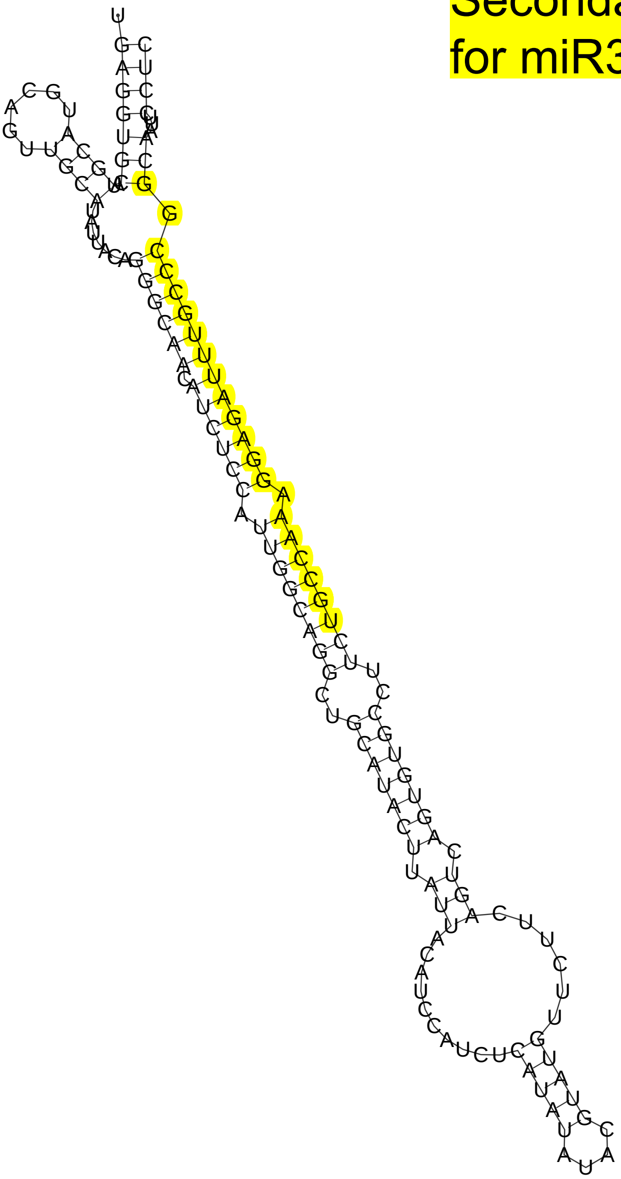

Secondary structure for miR399b

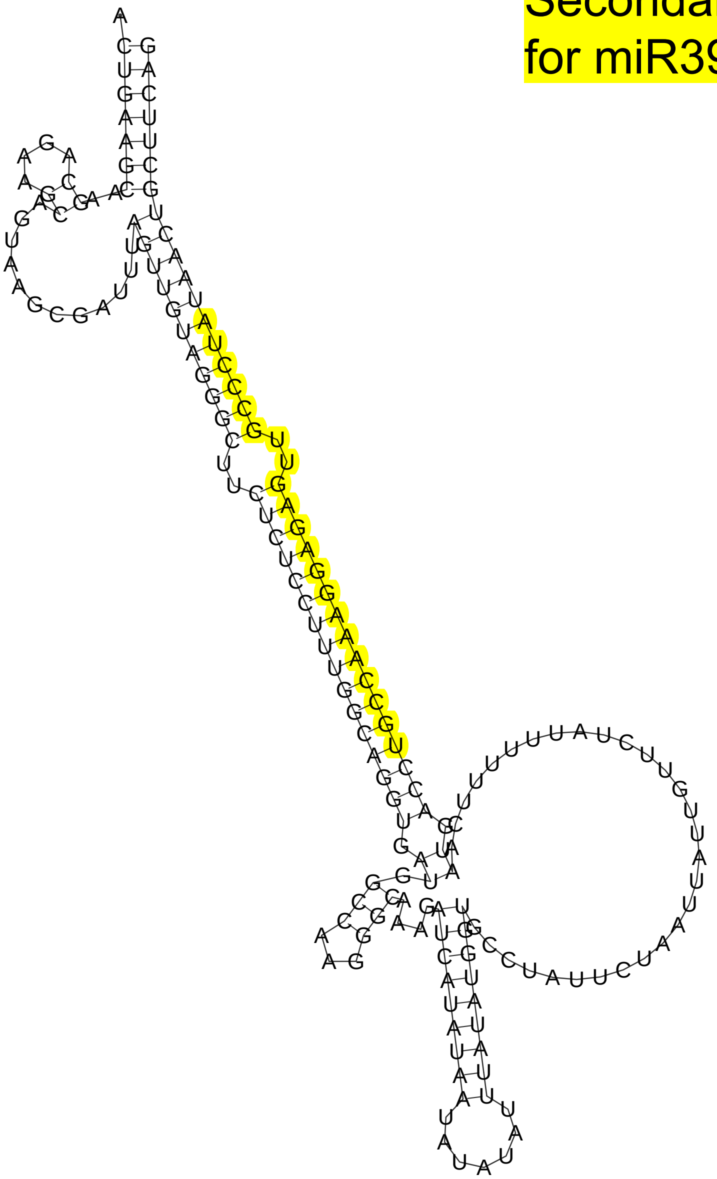

Secondary structure for miR399c

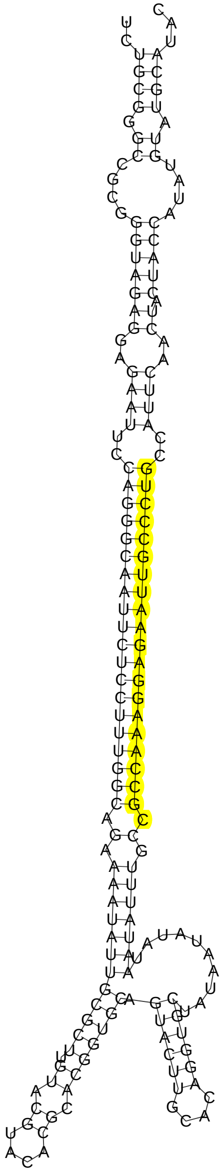

Secondary structure for miR399d

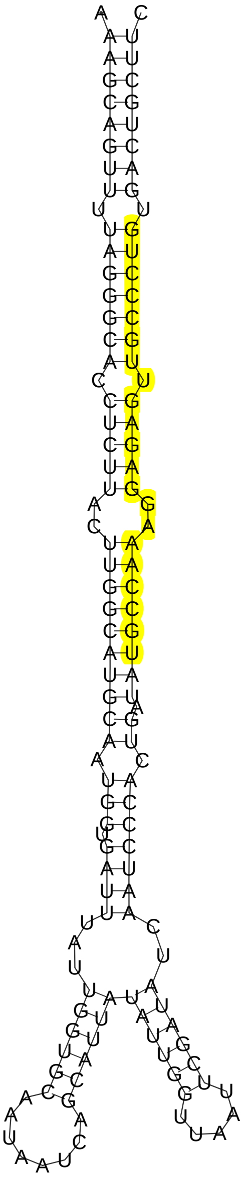

Secondary structure for miR399e

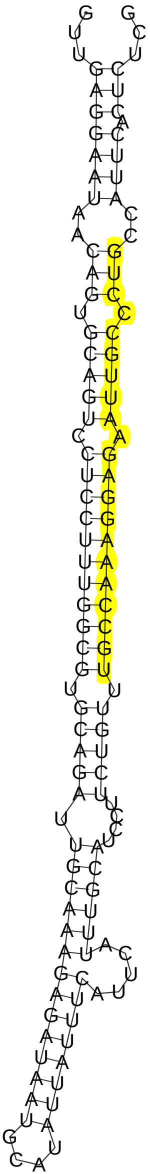

Secondary structure for miR399e-5p

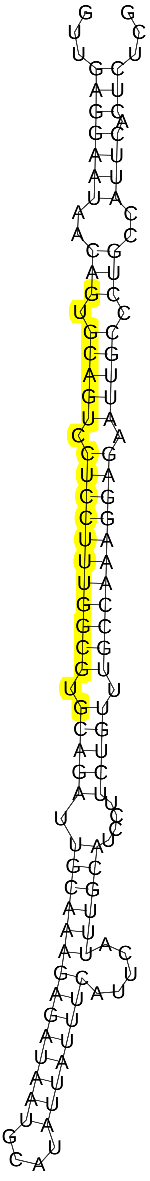

Secondary structure I for miR403.1

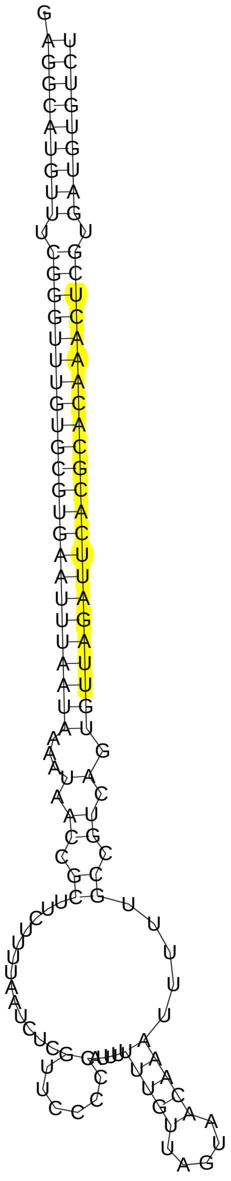

Secondary structure II for miR403.1

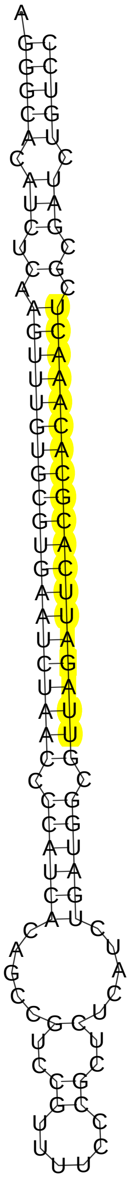

Secondary structure I for miR403.2

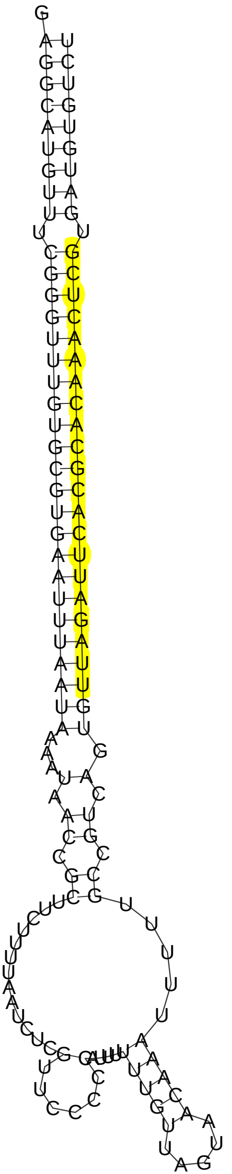

Secondary structure II for miR403.2

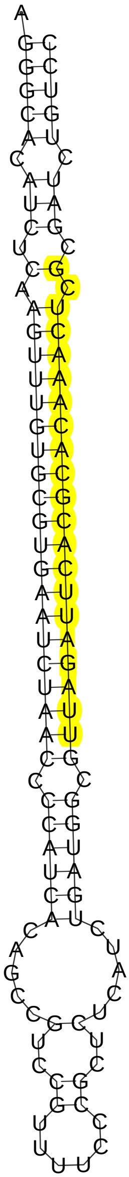

Secondary structure I
for miR403.3

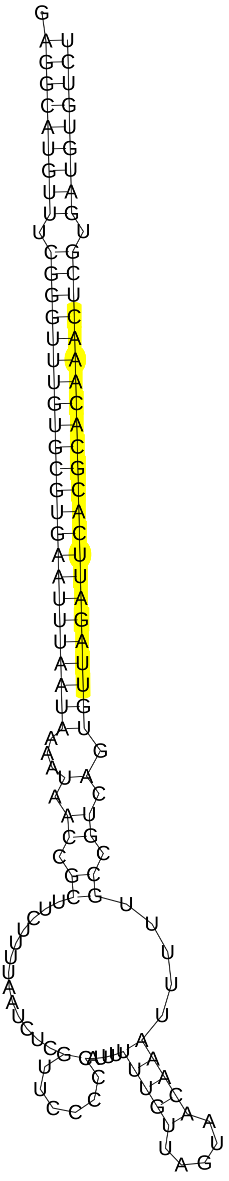

Secondary structure II for miR403.3

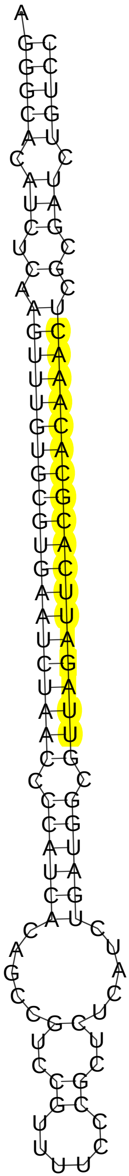

Secondary structure for miR408.1

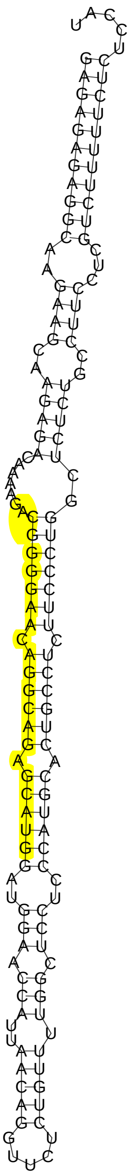

Secondary structure for miR408.2

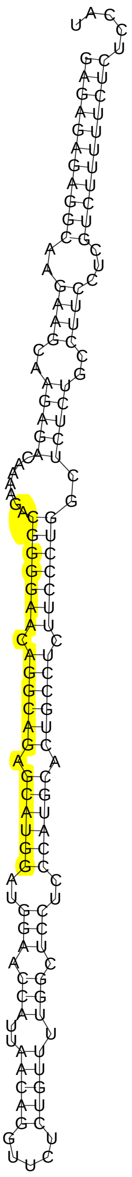

Secondary structure for miR4369

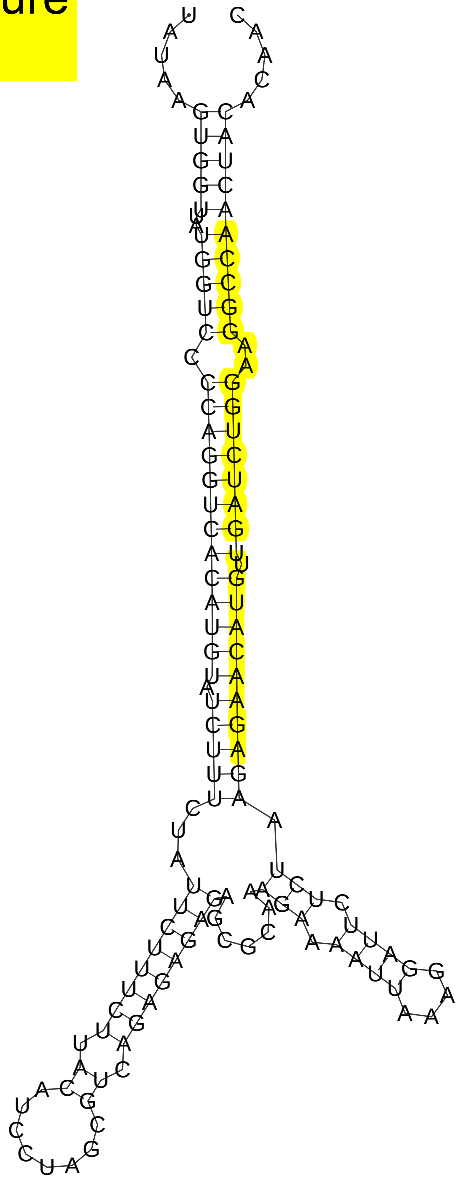

Secondary structure for miR4414.1

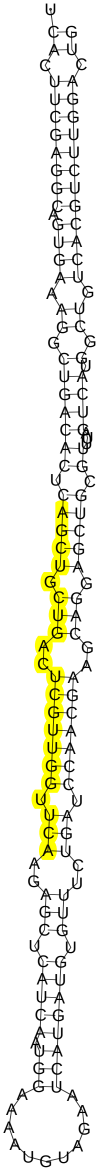

Secondary structure for miR4414.2

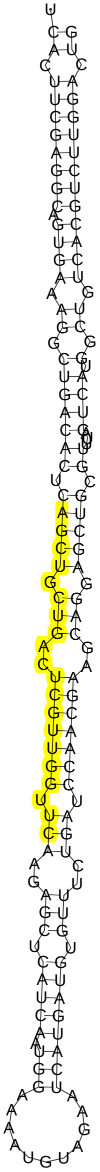

Secondary structure for miR443b.1

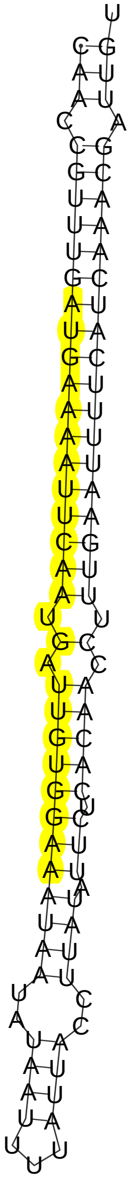

Secondary structure for miR443b.2

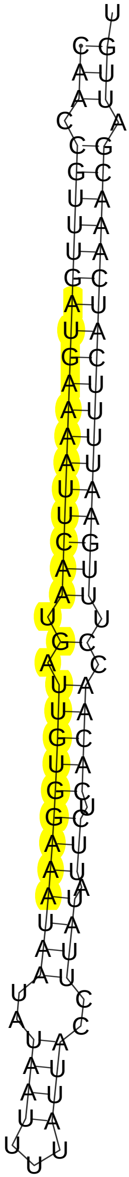

Secondary structure for miR444a.1

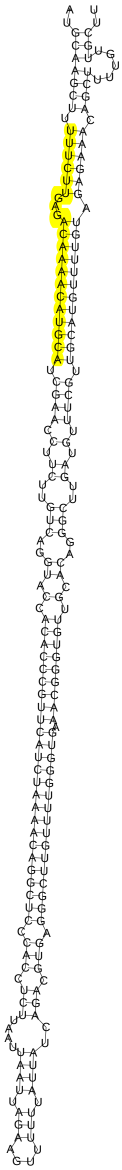

Secondary structure for miR444a.2

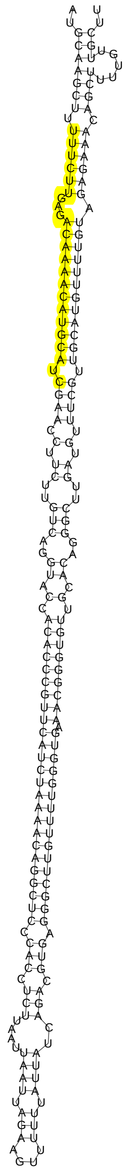

Secondary structure for miR472

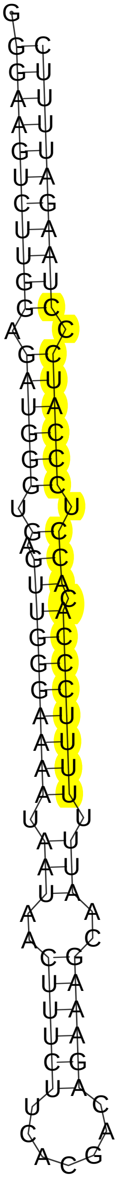

Secondary structure for miR473

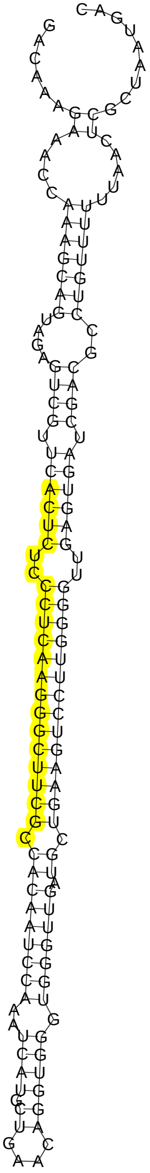

Secondary structure for miR473-3p

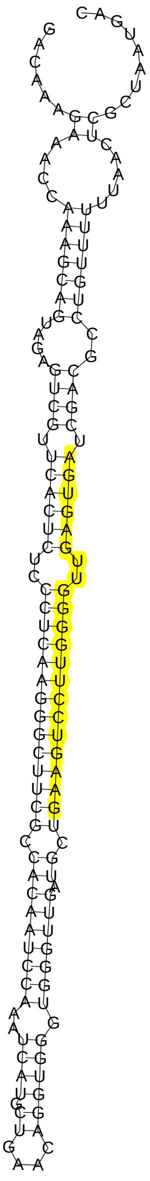

Secondary structure for miR477a.1

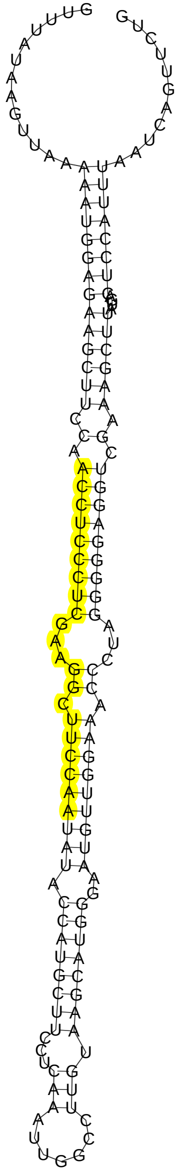

Secondary structure for miR477a.2

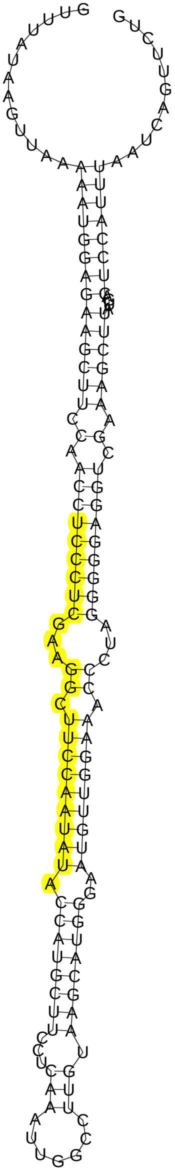

Secondary structure for miR477a-3p

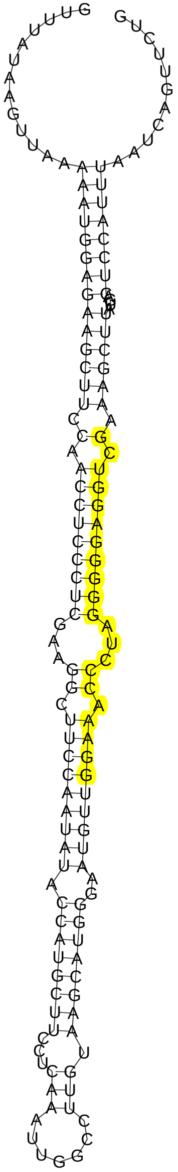

Secondary structure for miR477b.2

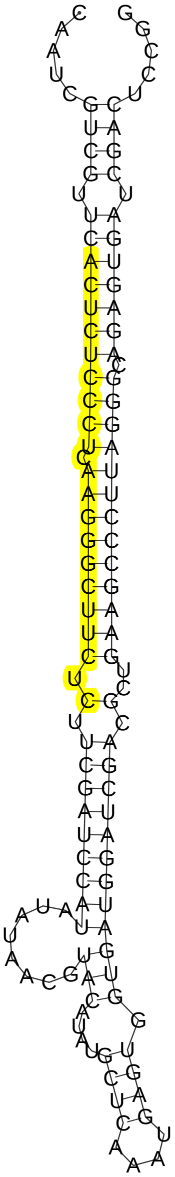

Secondary structure for miR477d.1-3p

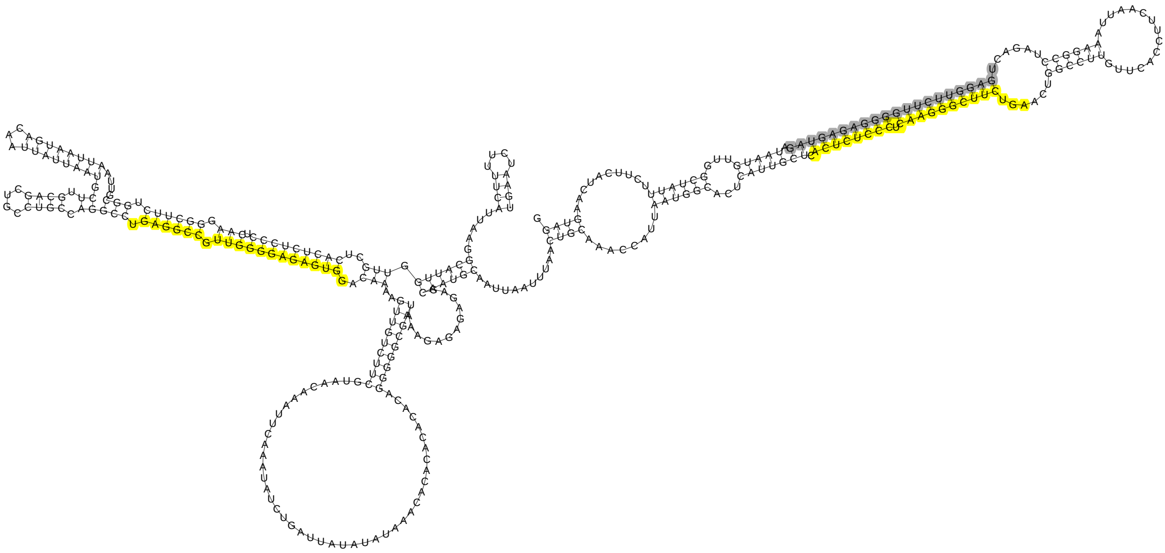

Secondary structure for miR477d.2-5p

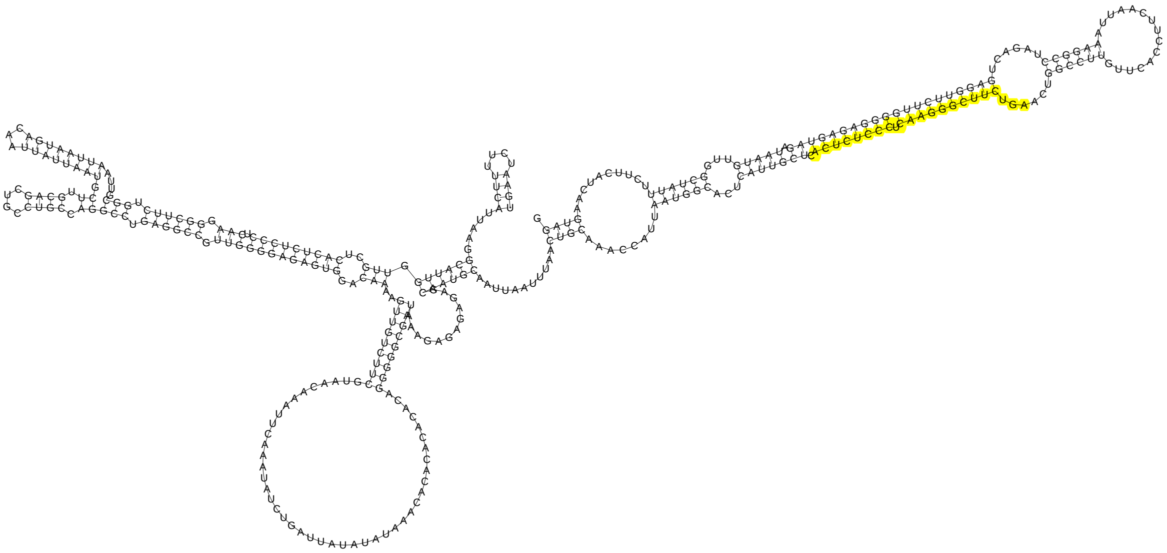

Secondary structure for miR479.1

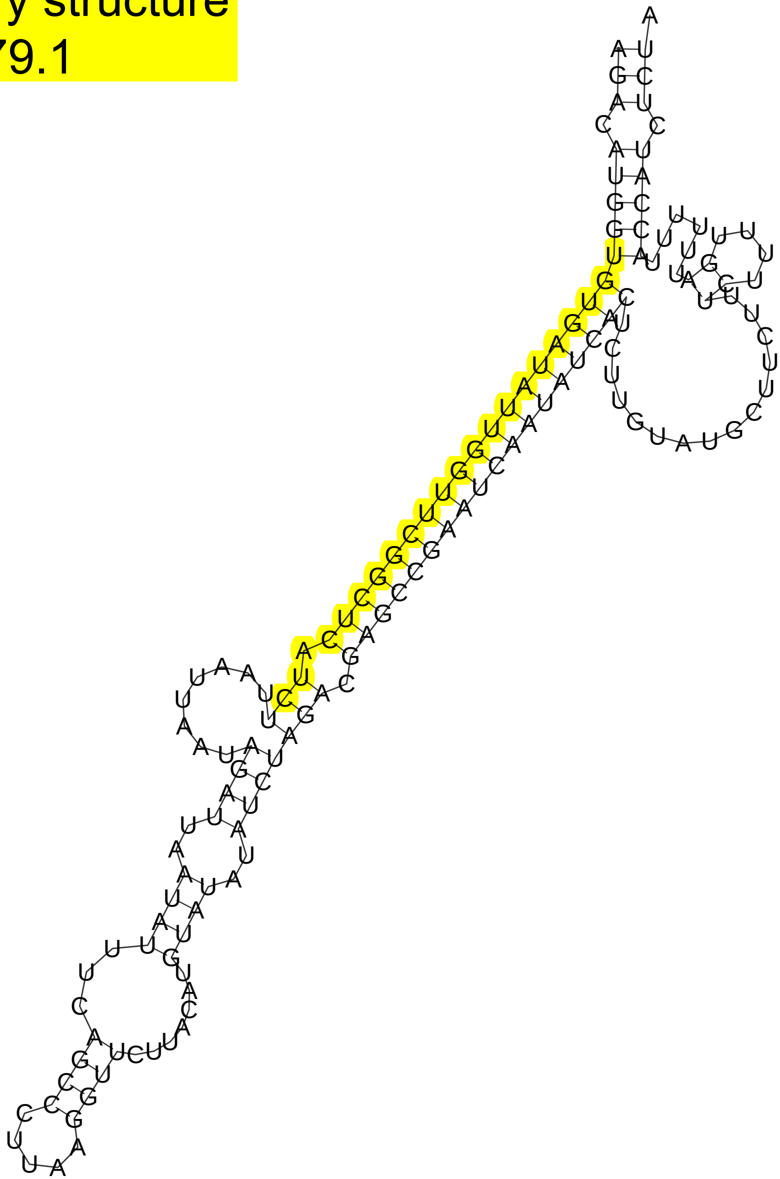

Secondary structure for miR479.2

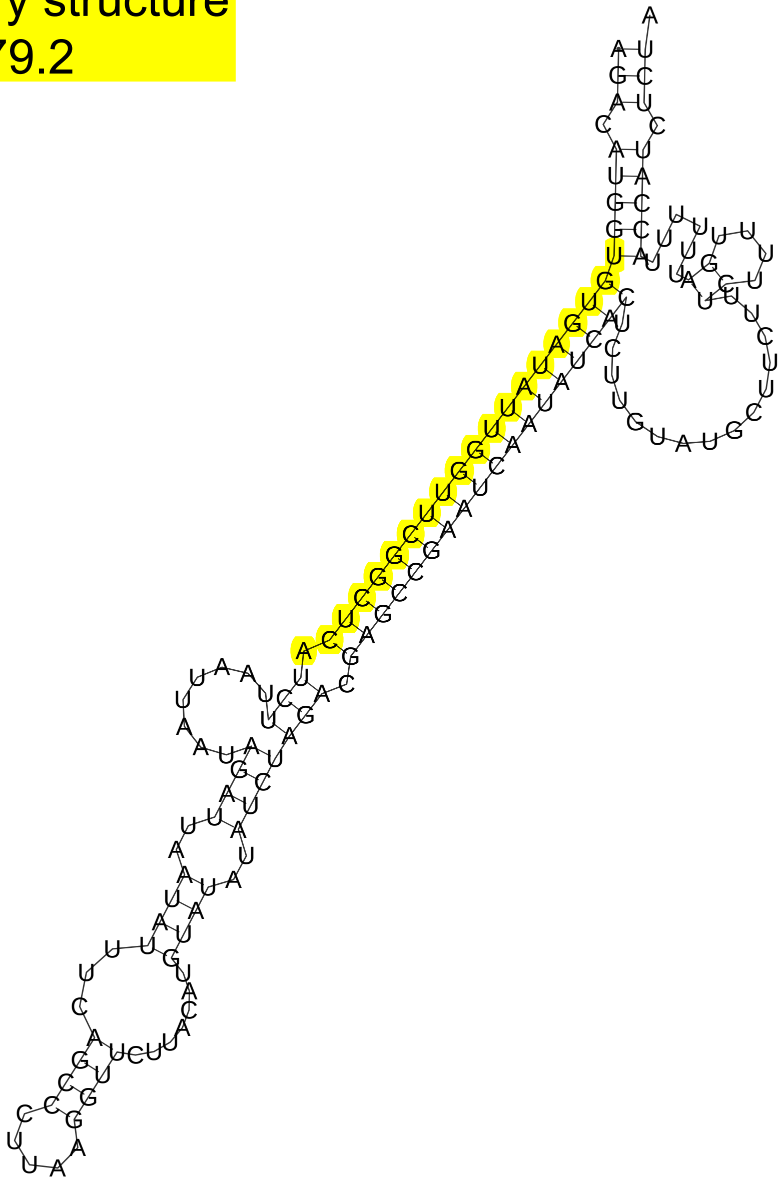

Secondary structure for miR482a-3p

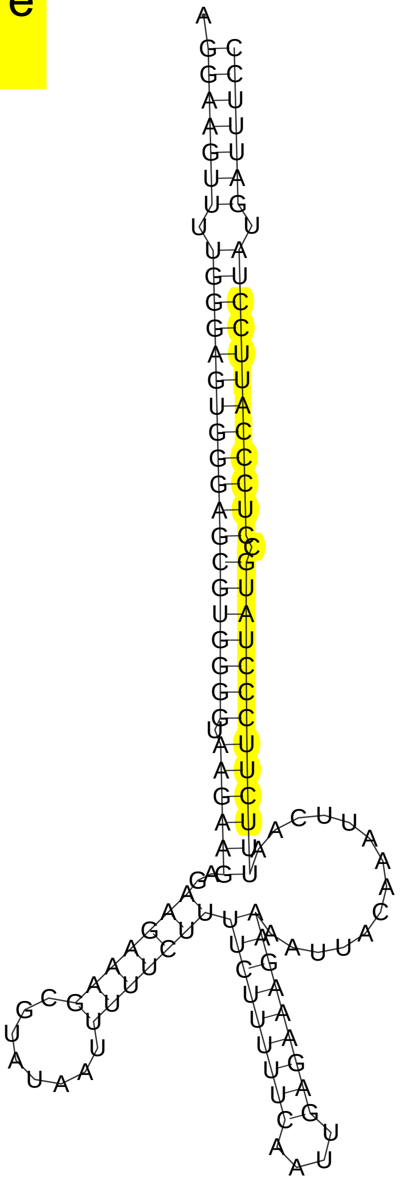

Secondary structure for miR482a-5p

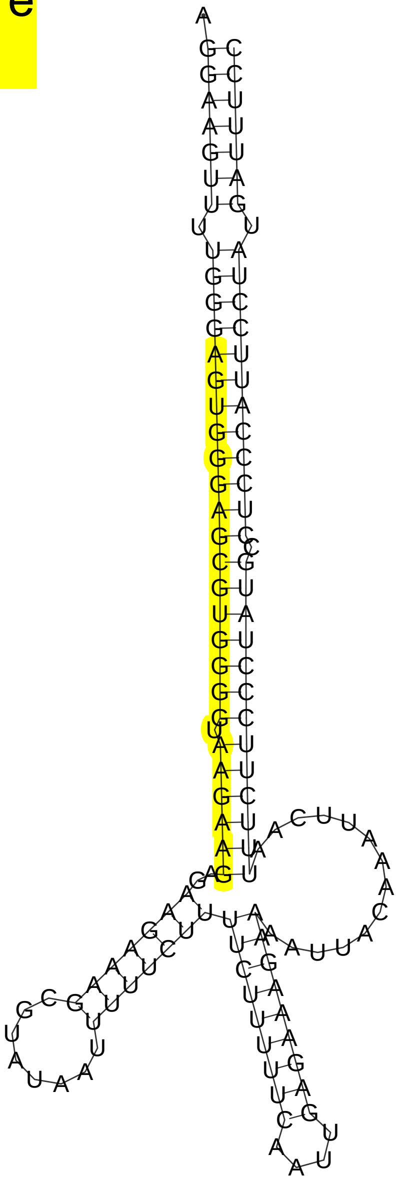

Secondary structure for miR482b

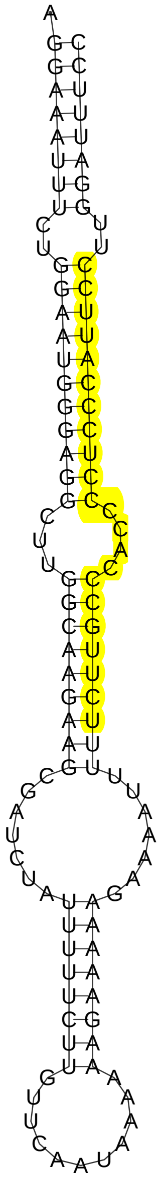

Secondary structure for miR482c

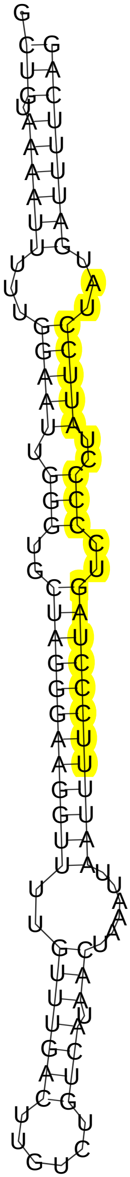

Secondary structure for miR482d

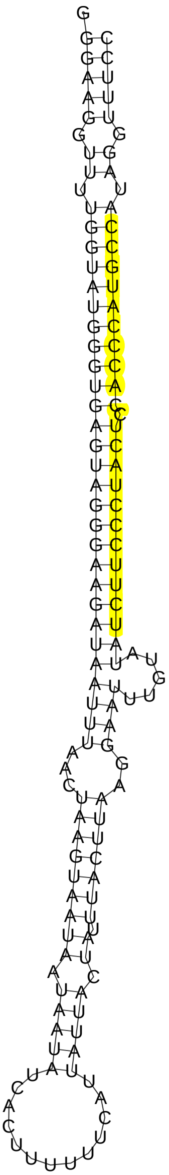

Secondary structure for miR482d-5p.1

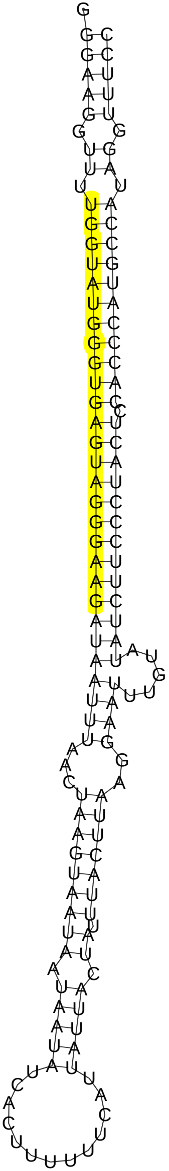

Secondary structure for miR482d-5p.2

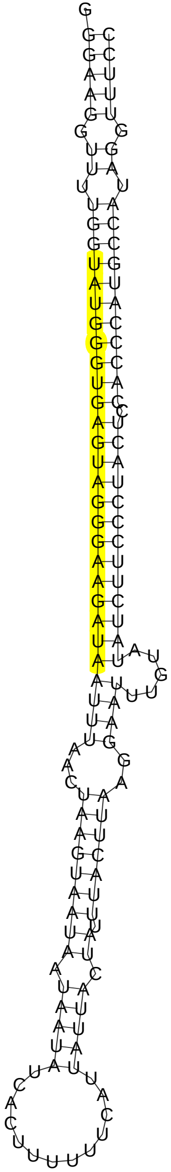

Secondary structure for miR5054.1

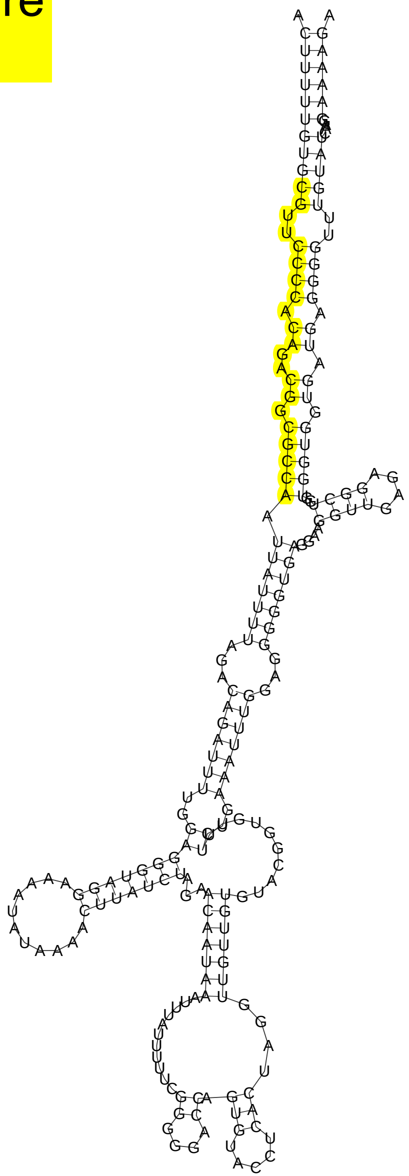

Secondary structure for miR5054.2

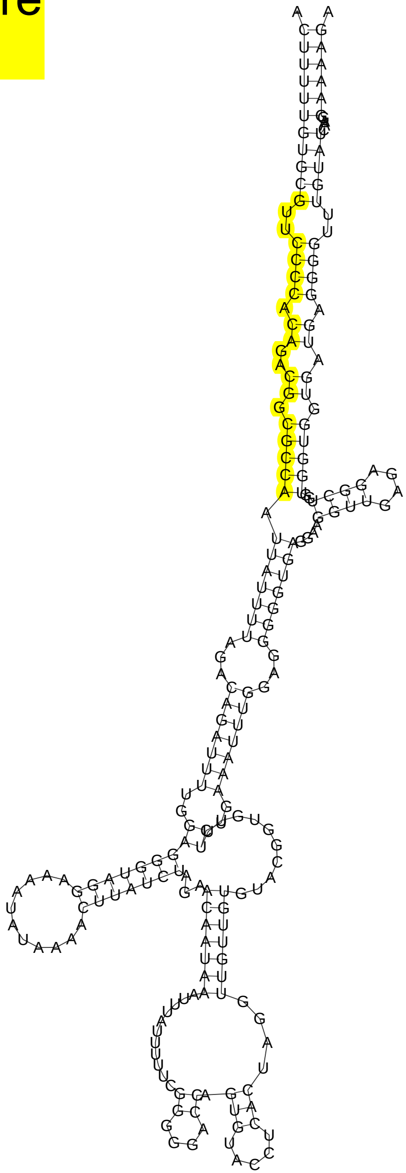

Secondary structure for miR5177

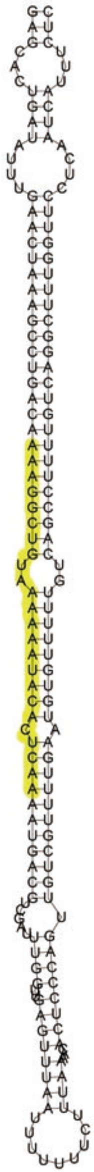

Secondary structure for miR5179

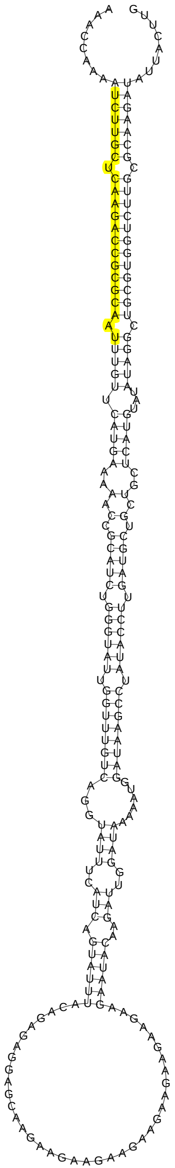

Secondary structure for miR5225a

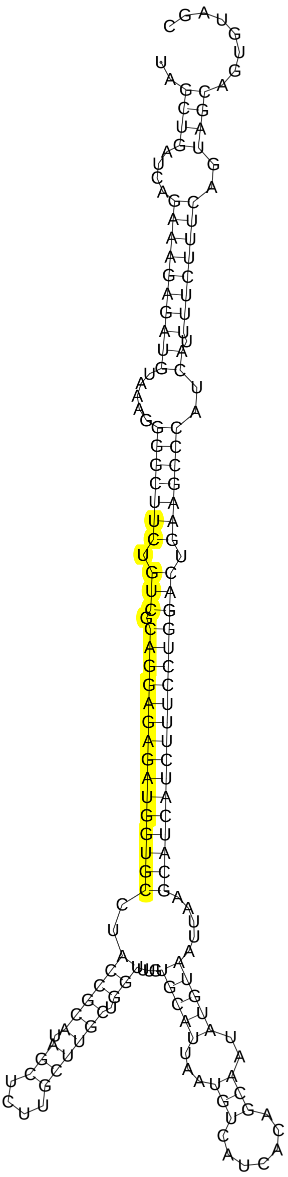

Secondary structure for miR5291a

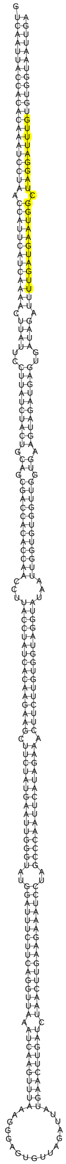

Secondary structure for miR530a

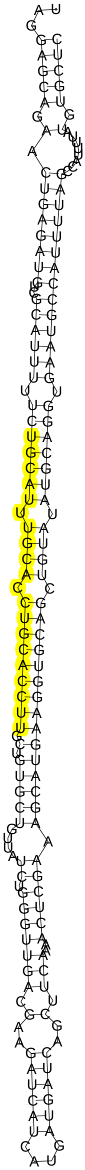

Secondary structure for miR530b

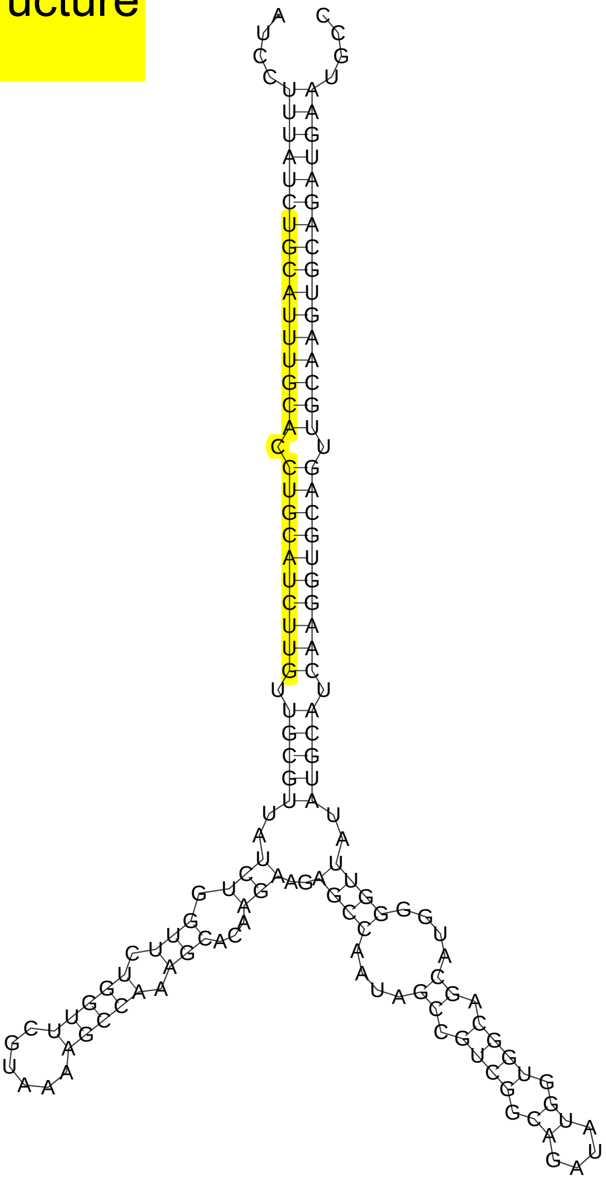

Secondary structure for miR535.1

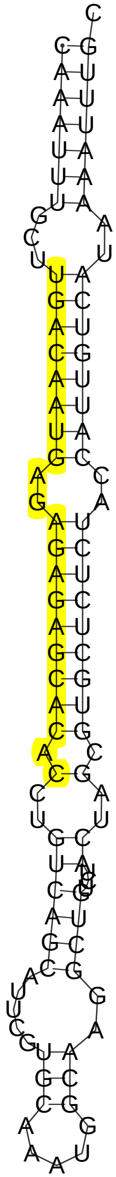

Secondary structure for miR535.2

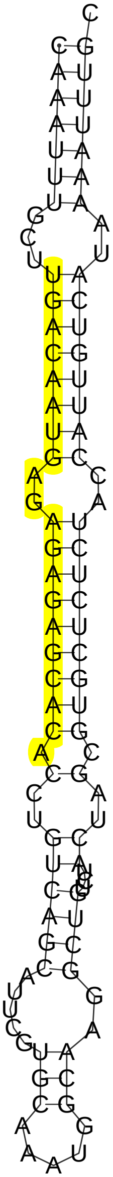

Secondary structure for miR536-3p

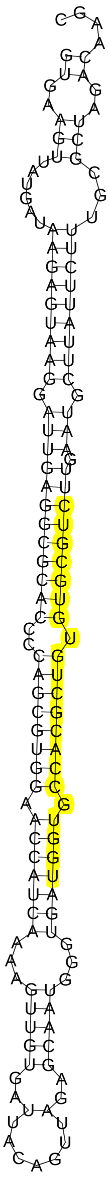

Secondary structure for miR536-5p

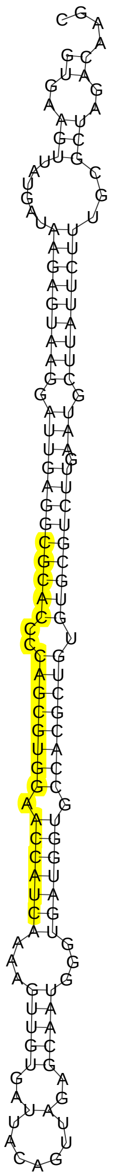

Secondary structure for miR814

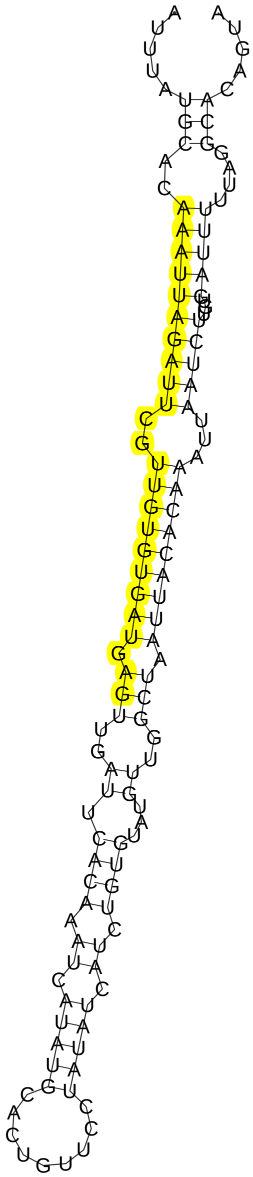

Secondary structure for miR827.1

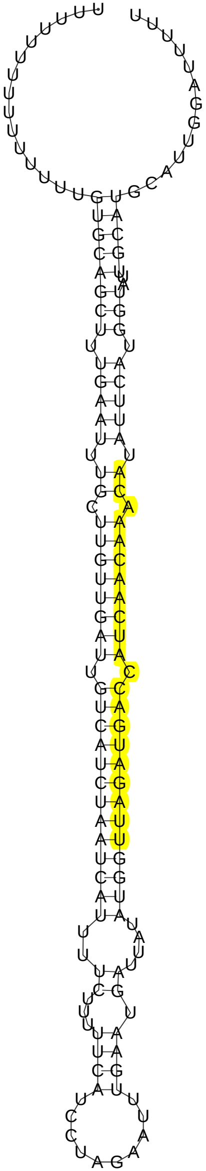

Secondary structure for miR827.2

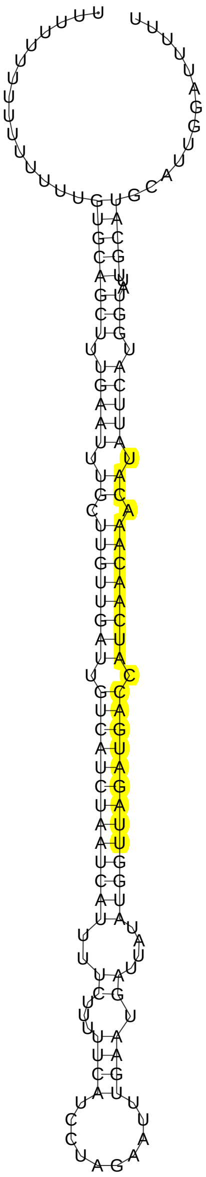

Secondary structure for miR827-5p.1

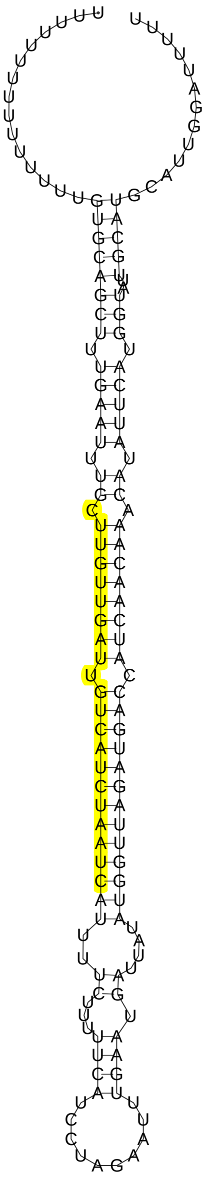

Secondary structure for miR827-5p.2

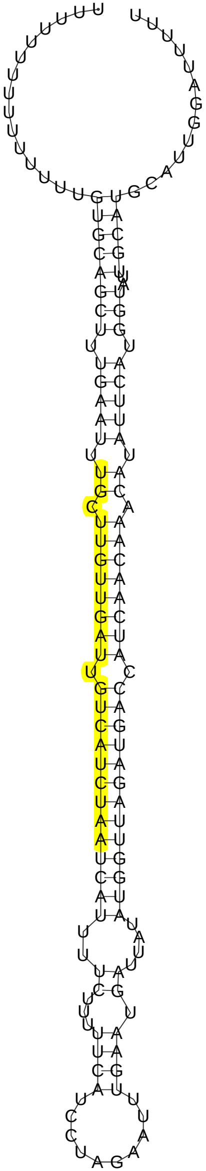

Secondary structure for miR828

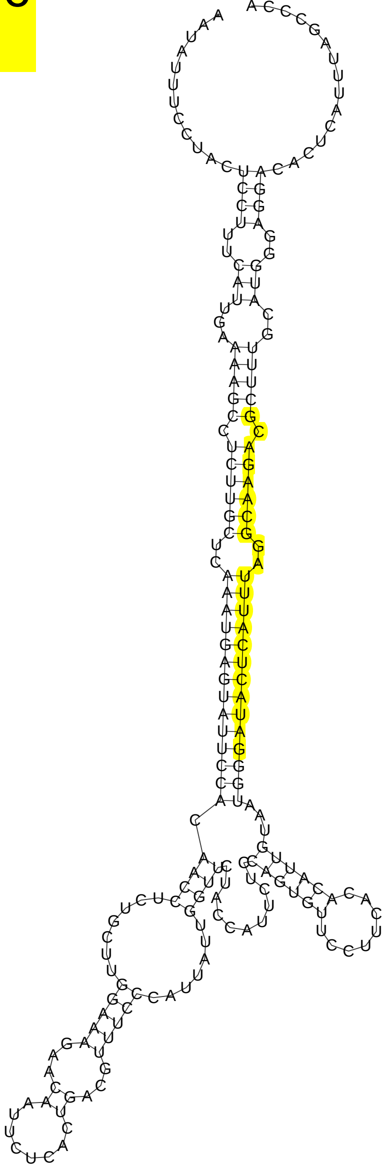

Secondary structure for miR833.1

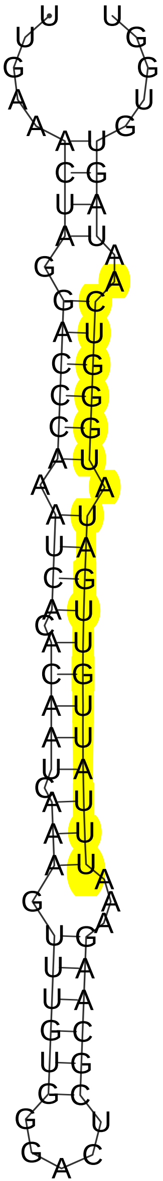

Secondary structure for miR845a

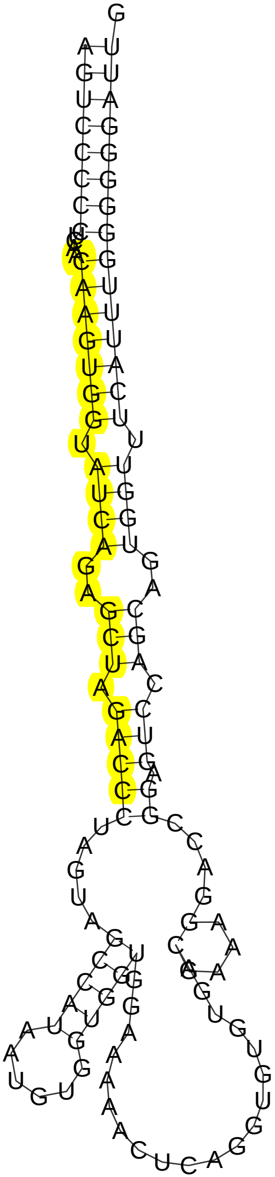

Secondary structure for miR896

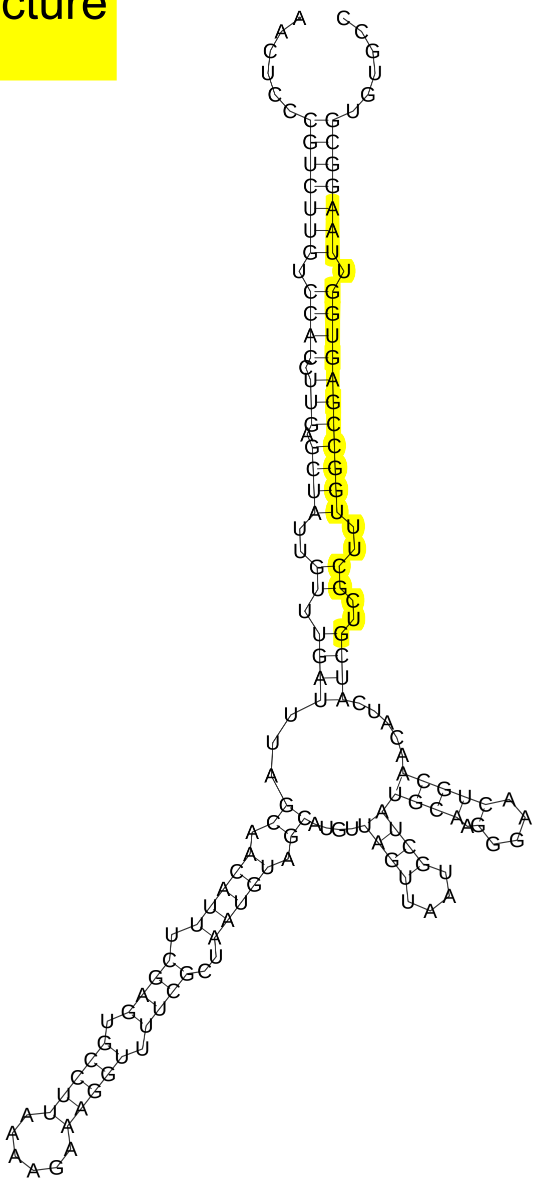

Secondary structure for miRN01

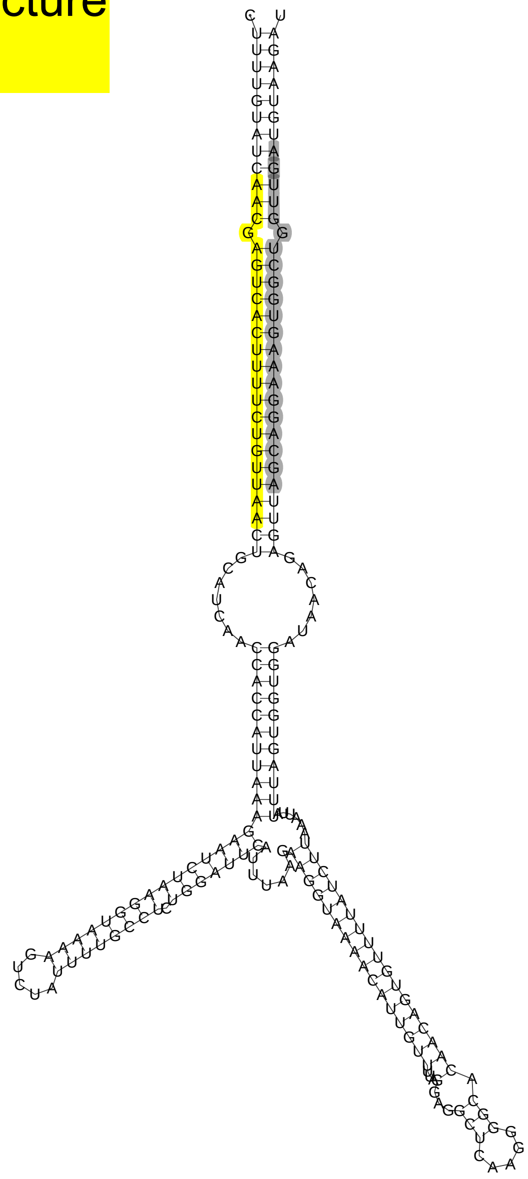

Secondary structure for miRN02

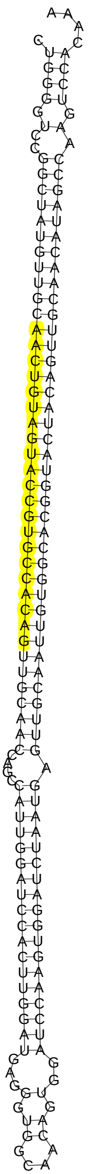

Secondary structure for miRN03

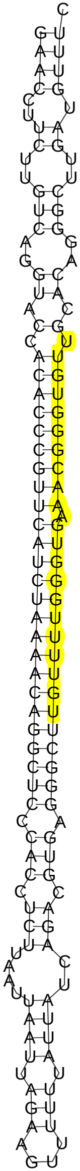

Secondary structure for miRN04

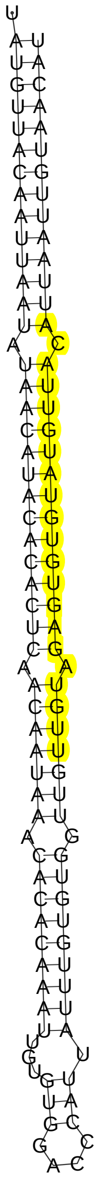

Secondary structure for miRN05

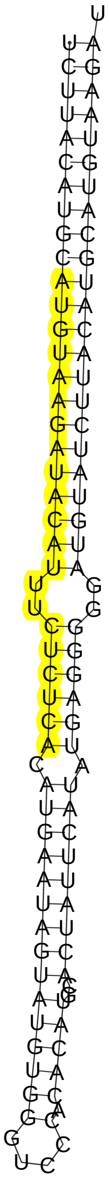

Secondary structure for miRN06

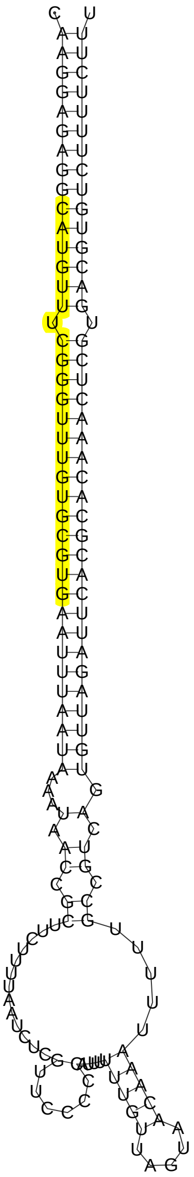

Secondary structure for miRN07

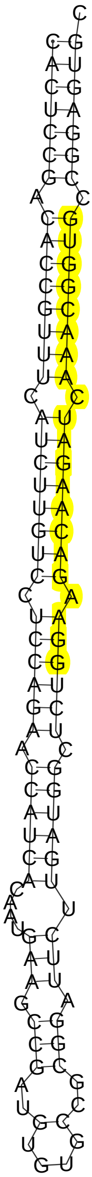

Secondary structure for miRN08

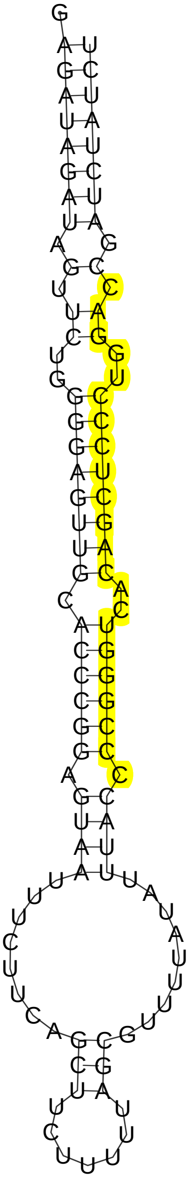

Secondary structure for miRN09

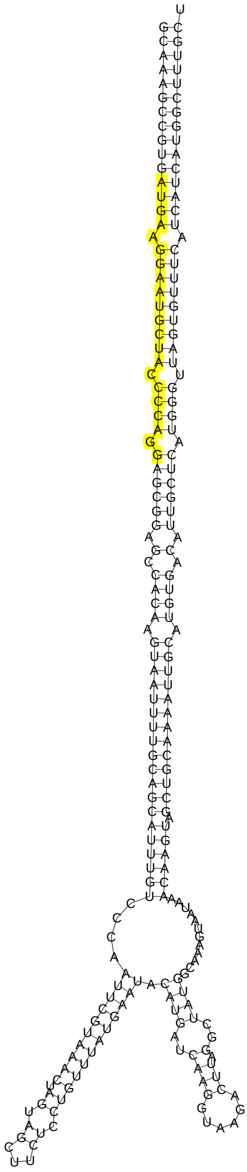

Secondary structure for miRN10

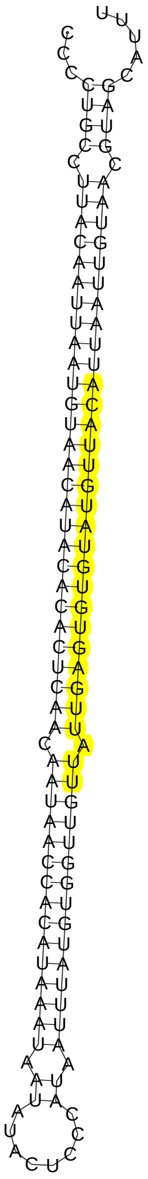

Secondary structure for miRN11

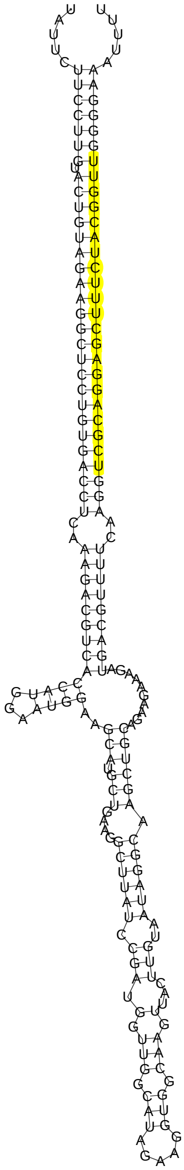

Secondary structure for miRN12

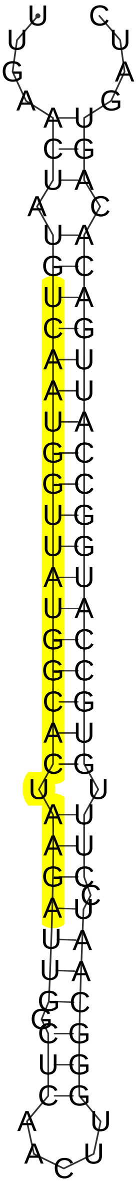

Secondary structure for miRN13

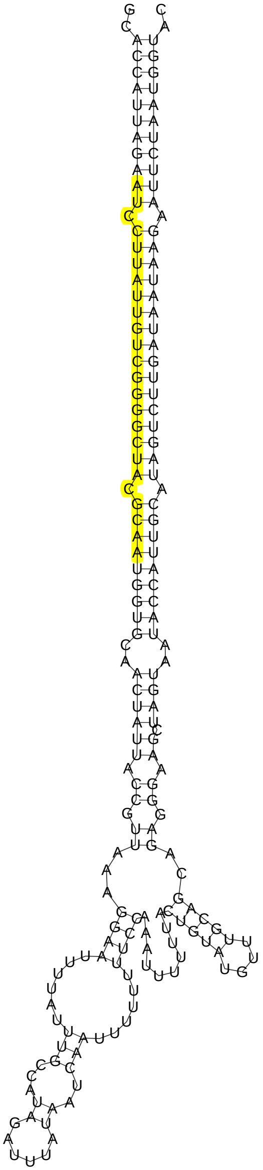

Secondary structure for miRN14

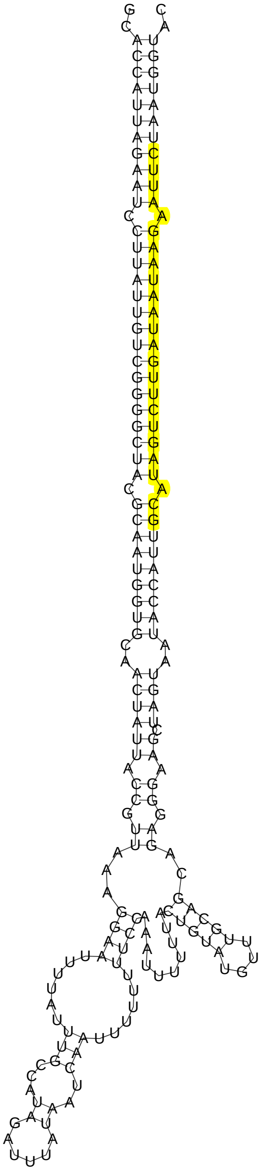

Secondary structure for miRN15

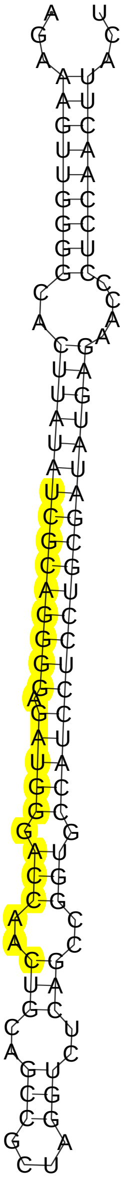

Secondary structure for miRN16

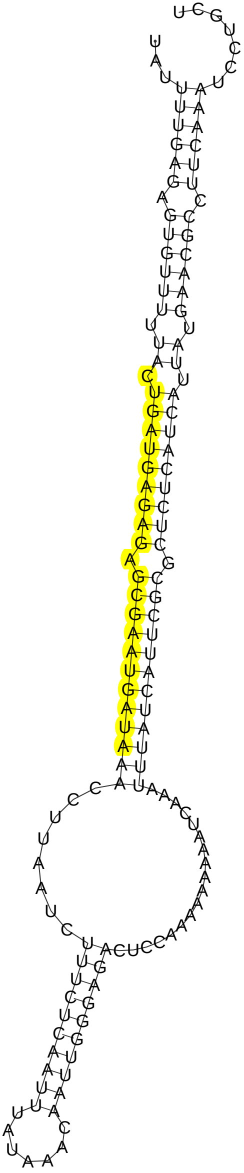

Secondary structure for miRN17

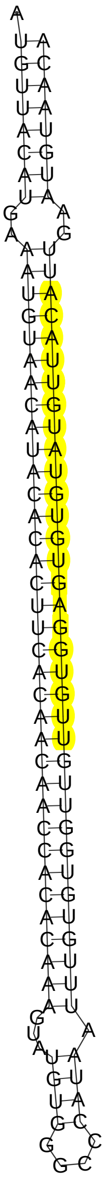

Secondary structure for miRN18

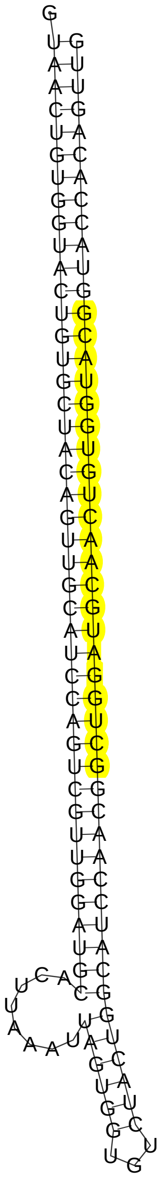

Secondary structure for miRN19

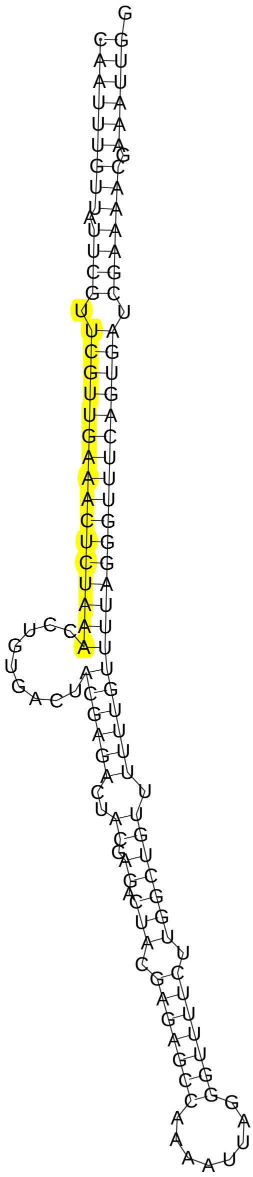

Secondary structure for miRN20

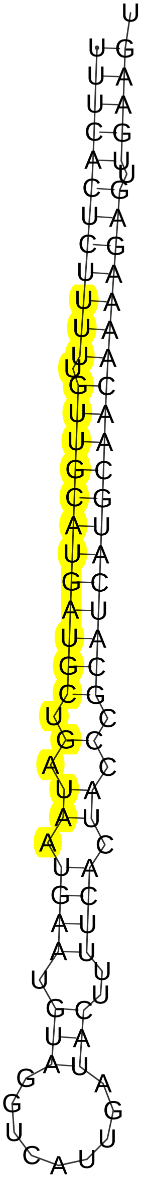

Secondary structure for miRN21

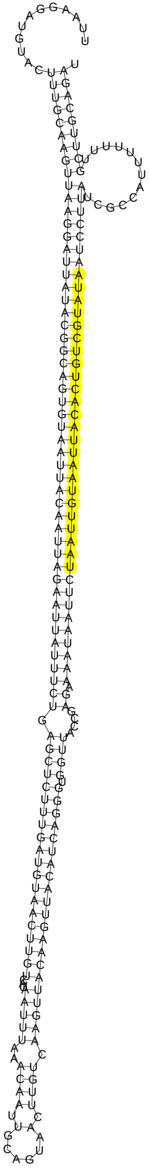

Secondary structure for miRN22

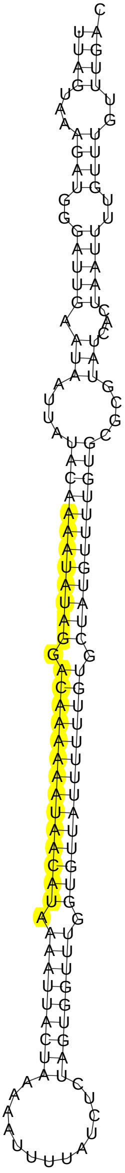

Secondary structure for miRN23

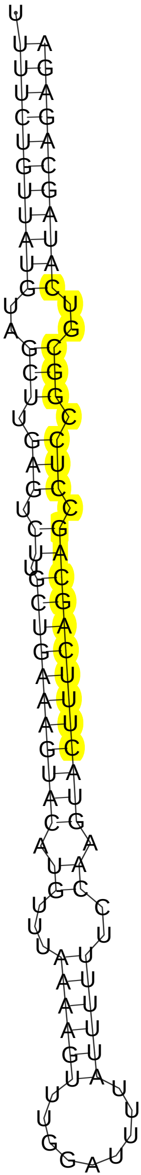

Secondary structure for miRN24

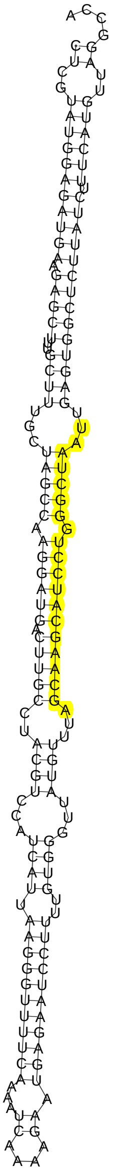

Secondary structure for miRN25

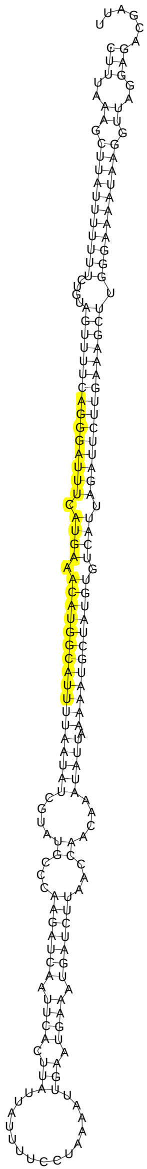

Secondary structure for miRN26

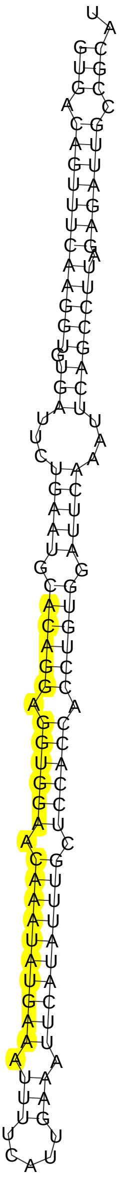

Secondary structure I for miRN27

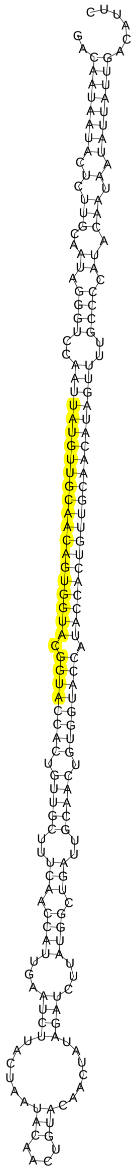

Secondary structure II for miRN27

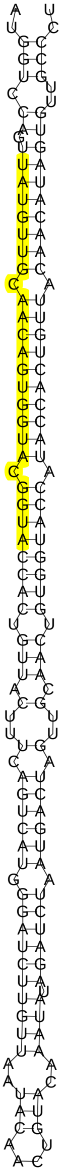

Secondary structure for miRN28

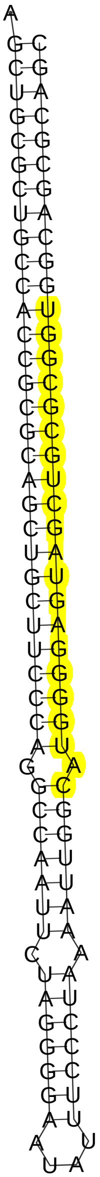

Secondary structure for miRN29

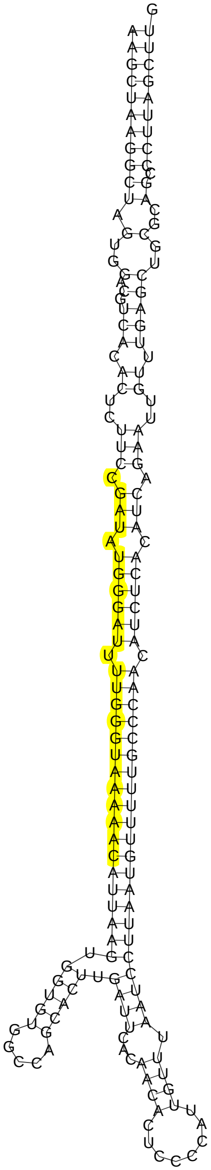

Secondary structure for miRN30

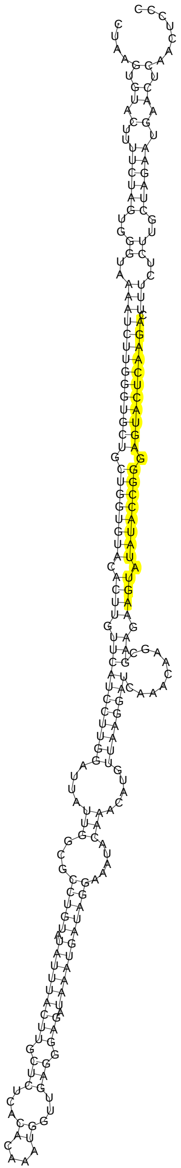

Secondary structure for miRN31

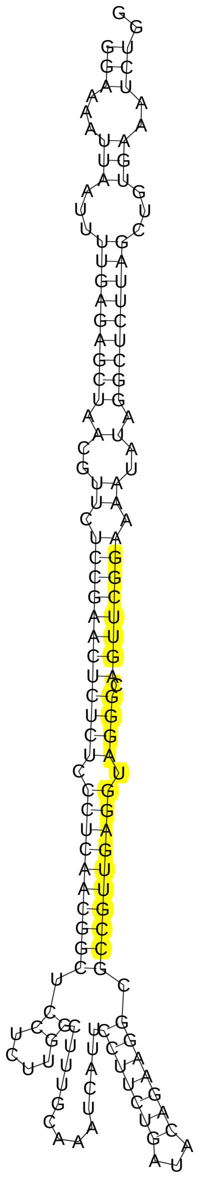

Secondary structure for miRN32

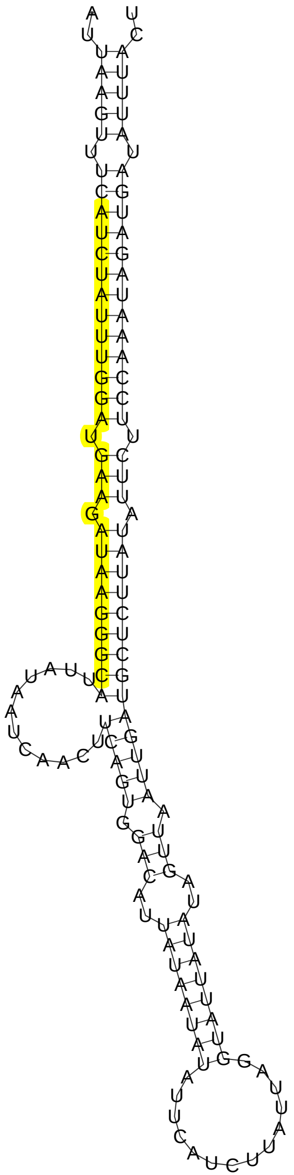

Secondary structure for miRN33

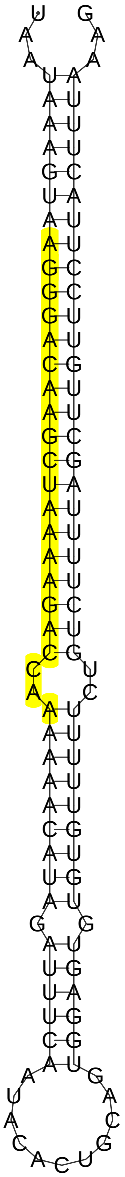

Secondary structure for miRN34

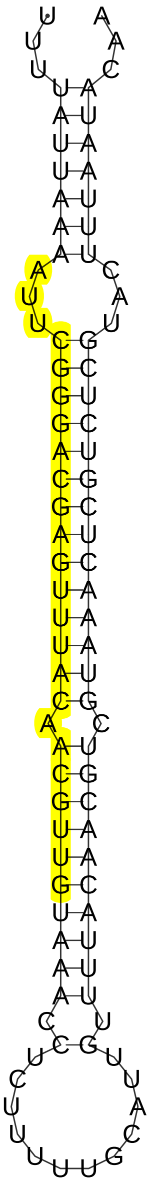

Secondary structure for miRN35

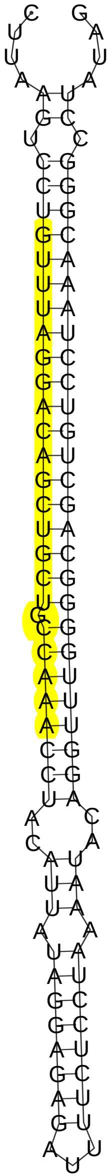

Secondary structure for miRN36

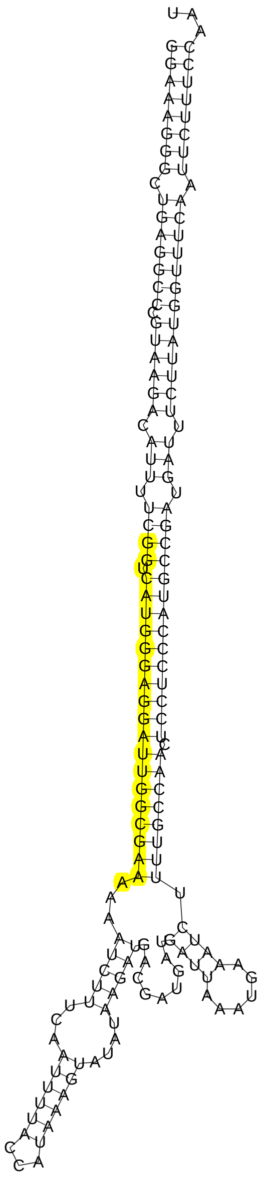

Secondary structure for miRN37

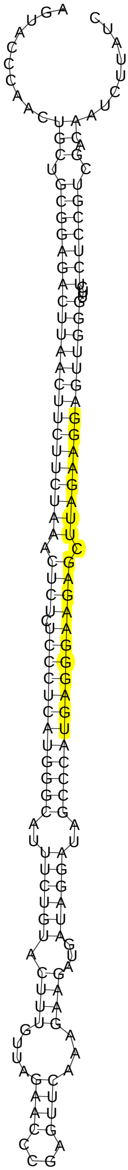

Secondary structure for miRN38

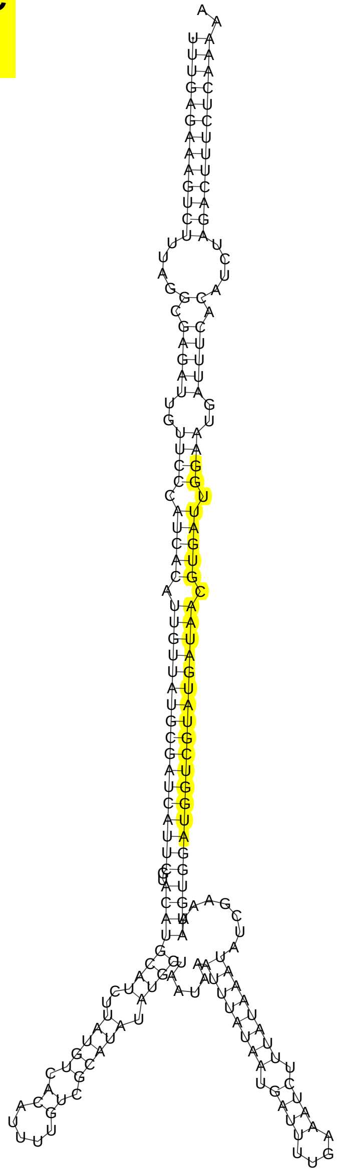

Supplement: Supplementary file 1 — Additional file 1: Predicted secondary structures of known and novel miRNAs. The mature miRNA sequences are highlighted in yellow. For novel miRNAs, the miRNA* sequences are highlighted in gray. (PDF 4 MB) [file 12864_2014_6413_MOESM1_ESM.pdf]
