# Supplementary material for: Genome-wide comparison of microRNAs and their targeted transcripts among leaf, flower and fruit of sweet orange
Source: BMC Genomics. 2014 Aug 20;15(1):695. doi: 10.1186/1471-2164-15-695 (PMC4158063; doi:10.1186/1471-2164-15-695)
Supplement: Supplementary file 2 — Additional file 2: Normalized count of all miRNAs in orange leaf, flower and fruit. (PDF 19 KB) [file 12864_2014_6413_MOESM2_ESM.pdf]

Additional file 2: Normailzed count[a] of all miRNAs in leaf, flower and fruit of sweet orange

Name	Normailzed count[a] in leaf	Normailzed count[a] in flower	Normailzed count[a] in fruit	sRNA sequence	Family	Higher expressed in
Csi-miR1092.2[b]	32.73	91.87	8.96	TTCCACCAAAGCATTCATTTCC	miR1092	Flower
Csi-miR1432a	50.22	8.86	0	TGCAGGTGAGATGATACCGTCA	miR1432	Leaf
Csi-miR1446	59.36	120.83	592.03	CGAACTCTCTCCCTCAACGGC	miR1446	Fruit
Csi-miR1507a.2	1.28	2.16	0	ATGGCATTATGTATAGAATGAGGA	miR1507	
Csi-miR1515	19.56	7.67	19.6	TCATTTTTGCGTGCAATGATCC	miR1515	
Csi-miR156a.1	1514.38	279.61	62.17	TTGACAGAAGAGAGTGAGCAC	miR156	Leaf
**Csi-miR156a.2**	**2718.97**	**487.01**	**283.97**	**TGACAGAAGAGAGTGAGCAC**	**miR156**	**Leaf**
Csi-miR156b.1	1628.79	378.5	407.19	CTGACAGAAGAGAGTGAGCAC	miR156	Leaf
Csi-miR156c.1	329.16	93.71	54.89	TGACAGAAGAGAGTGAGCACA	miR156	Leaf
Csi-miR156d	2.46	37.29	91.3	TGACAGAAGATAGAGAGCGC	miR156	Flower
Csi-miR156e	54.65	2967.91	1224.38	GTGACAGAAGATAGAGAGCGC	miR156	Flower
Csi-miR156f.2	22.41	3.57	1.12	ATGACAGAAGAGAGAGAGTAC	miR156	Leaf
Csi-miR156g.1	27031.96	12066.94	497.37	TTGACGGAAGATAGAGAGCAC	miR156	Leaf
**Csi-miR156g.2**	**8893.56**	**846.28**	**586.98**	**TGACGGAAGATAGAGAGCAC**	**miR156**	**Leaf**
Csi-miR156h	0	1.19	0	TTGACAGAAGAGAGAGAGCAC	miR156	Flower
Csi-miR159	165.61	40.31	272.77	TTTGGATTGAAGGGAGCTCTA	miR159	
Csi-miR159b	0	9.51	0	ATTGGAGTGAAGGGAGCTCCA	miR159	Flower
Csi-miR160a-3p	87.87	135.43	5.6	GCGTATGAGGAGCCATGCATA	miR160	
**Csi-miR160a-5p**	**11.11**	**16.64**	**0**	**TGCCTGGCTCCCTGTATGCCA**	**miR160**	
Csi-miR160b-3p	647.21	87.98	0	GCGTACGAGGAGCCAAGCATA	miR160	Leaf
**Csi-miR160b-5p**	**4.52**	**1.51**	**0**	**TGCCTGGCTCCCTGTATGCCG**	**miR160**	**Leaf**
Csi-miR160c.1	0.88	1.51	3.36	AGCGTGCGAGGAGCCATGCAT	miR160	Fruit
**Csi-miR160c.2**	**0.59**	**0.65**	**1.12**	**AGCGTGCGAGGAGCCATGCATG**	**miR160**	
Csi-miR162-3p.1	115.58	23.13	341.1	TCGATAAACCTCTGCATCCAG	miR162	Fruit
**Csi-miR162-3p.2**	**5.01**	**1.08**	**6.16**	**GATAAACCTCTGCATCCAG**	**miR162**	

Csi-miR164	423.51	246.32	1651.18	TGGAGAAGCAGGGCACGTGCA	miR164	Fruit
**Csi-miR166a.1**	**0.79**	**0**	**0**	**TCGGACCAGGCTTCATTCCCCC**	**miR166**	**Leaf**
Csi-miR166a.2	6211.15	1848.08	101.38	TCGGACCAGGCTTCATTCCCC	miR166	Leaf
Csi-miR166b	1250.09	355.05	1.68	TCGGACCAGGCTTCATTCCCG	miR166	Leaf
Csi-miR166c.1	1468.68	376.01	2.24	TCGGACCAGGCTTCATTCC	miR166	Leaf
**Csi-miR166c.2**	**1542.89**	**289.87**	**0**	**TCGGACCAGGCTTCATTC**	**miR166**	**Leaf**
**Csi-miR166c.3**	**589.52**	**191.74**	**0**	**TCGGACCAGGCTTCATTCCC**	**miR166**	**Leaf**
**Csi-miR166c.4**	**20.05**	**6.92**	**0**	**CTCGGACCAGGCTTCATTCCC**	**miR166**	**Leaf**
**Csi-miR166c.5**	**24.37**	**17.83**	**9.52**	**CTCGGACCAGGCTTCATTCC**	**miR166**	
**Csi-miR166d.1**	**17.49**	**7.24**	**0**	**TCGGACCAGGCTTCATTCCCTT**	**miR166**	**Leaf**
Csi-miR166d.2	5560.5	1229.96	2.24	TCGGACCAGGCTTCATTCCCT	miR166	Leaf
Csi-miR166g.1	5454.16	3336.79	1349.28	TCTCGGACCAGGCTTCATTCC	miR166	
Csi-miR166i	67.23	89.28	2.8	TTCGGACCAGGCTTCATTCCC	miR166	
Csi-miR166j.1	581.56	431.35	0	TTGGACCAGGCTTCATTCCTC	miR166	
**Csi-miR166j.2**	**3.44**	**2.81**	**1.12**	**TCTTGGACCAGGCTTCATTCC**	**miR166**	
**Csi-miR166j.3**	**863.44**	**600.5**	**0**	**TTGGACCAGGCTTCATTCCAC**	**miR166**	
Csi-miR167a.1	279.23	453.62	520.33	TGAAGCTGCCAGCATGATCTA	miR167	
**Csi-miR167b.1**	**119.91**	**249.45**	**5.04**	**TGAAGCTGCCAGCATGATCT**	**miR167**	**Flower**
Csi-miR167b.2	132.88	8029.68	55.45	TGAAGCTGCCAGCATGATCTTA	miR167	Fruit
**Csi-miR167b.3**	**0**	**14.27**	**0**	**AAGCTGCCAGCATGATCTTA**	**miR167**	**Fruit**
**Csi-miR167b.4**	**9.14**	**84.52**	**6.72**	**TGAAGCTGCCAGCATGATCTT**	**miR167**	**Fruit**
Csi-miR167d.1	14326.29	14164.04	938.17	TGAAGCTGCCAGCATGATCTGA	miR167	
**Csi-miR167d.2**	**111.36**	**73.5**	**34.73**	**TGAAGCTGCCAGCATGATCTG**	**miR167**	
Csi-miR168a	1151.61	962.36	7139.6	TCGCTTGGTGCAGGTCGGGAA	miR168	Fruit
Csi-miR169b.1	2.06	4.76	0	TAGCCAAGGATGACTTGCCTG	miR169	Flower
**Csi-miR169b.1-3p**	**6.98**	**2.38**	**0**	**AGGCAGTCTCCTTGGCTAAG**	**miR169**	**Leaf**
Csi-miR169i.1	3.54	6.38	0	TAGCCAAGGATGACTTGCCTGA	miR169	
**Csi-miR169i.1-3p**	**0**	**1.62**	**0**	**GGCAGTCTCCTTGGCTAAC**	**miR169**	**Flower**
Csi-miR169m.1	45.8	62.58	0	CAGCCAAGGATGACTTGCCGG	miR169	

**Csi-miR169m.2**	**0.69**	**0.86**	**0**	**AGCCAAGGATGACTTGCCGGC**	**miR169**	
**Csi-miR169m.3**	**0**	**0.43**	**0**	**GCAGCCAAGGATGACTTGCCG**	**miR169**	**Flower**
Csi-miR171a.1	113.03	4.86	7.84	TGTTGGAACGGCTCAATCAAA	miR171	Leaf
Csi-miR171b	175.44	27.45	20.72	CGAGCCGAATCAATATCACTC	miR171	Leaf
Csi-miR171d	16.02	4.32	1.68	TTGAGCCGCGTCAATATCTCC	miR171	Leaf
Csi-miR171g.1	71.65	55.55	0	TGATTGAGCCGTGCCAATATC	miR171	
**Csi-miR172a-3p.1**	**61.82**	**11.02**	**25.76**	**AGAATCTTGATGATGCTGCA**	**miR172**	**Leaf**
Csi-miR172a-3p.2	1237.32	778.73	151.79	AGAATCTTGATGATGCTGCAA	miR172	
**Csi-miR172a-3p.3**	**28.21**	**29.07**	**0**	**GAATCTTGATGATGCTGCAA**	**miR172**	
Csi-miR172c.1	0.49	27.45	0	TGGAATCTTGATGATGCT	miR172	Flower
**Csi-miR172c.2**	**1.57**	**60.74**	**7.84**	**TGGAATCTTGATGATGCTGCAG**	**miR172**	**Flower**
Csi-miR172d	6368.41	879.13	4072.49	AGAATCTTGATGATGCTGCAT	miR172	
**Csi-miR172d-3p**	**0.69**	**0.54**	**2.24**	**GCAGCATCATCAAGATTCACA**	**miR172**	**Fruit**
Csi-miR172e.2	247.29	45.72	30.25	GAATCTTGATGATGCTGCAT	miR172	Leaf
Csi-miR172k	13.56	0.76	11.2	TGAATCTTGATGATGCTGCAT	miR172	Leaf
Csi-miR2111	0.79	1.41	1.68	TAATCTGCATCCTGAGGTTTG	miR2111	
Csi-miR2118.1	15.53	7.24	19.04	TTACCTATGCCACCCATTCCTT	miR2118	
**Csi-miR2118.2**	**2.26**	**1.84**	**2.24**	**TTACCTATGCCACCCATTCC**	**miR2118**	
Csi-miR2275a	0	2.27	0	TTTAATTTCCTCCAATATCTTA	miR2275	Flower
Csi-miR2275b	0	5.62	0	TTTAATTTCCTCCAATATTTCA	miR2275	Flower
Csi-miR2275c.1	0	11.56	0	TTTAATTTCCTCCAATATTTTA	miR2275	Flower
Csi-miR2275d	0.2	5.94	0	AGAATTGGATGGAACTAAACA	miR2275	Flower
**Csi-miR2275d-3p**	**0.88**	**10.48**	**0**	**TTTAGTTTCCTCCAATATCTTA**	**miR2275**	**Flower**
Csi-miR2275e	0	2.16	0	TTTAGTTTCCTCTAATATCTTA	miR2275	Flower
Csi-miR2275f	0	1.3	0	TTTGGTTTTCTCCAATATCTCA	miR2275	Flower
Csi-miR2911	0	0	34.17	CCCGGCCGGGGGACGGACTGGGA	miR2911	Fruit
Csi-miR319	0	0	3.36	TTTGGACTGAAGGGAGCTCCT	miR319	Fruit
Csi-miR319a	4.32	4.97	1.68	AGCTGCCGACTCATTCATTCA	miR319	
Csi-miR319b	0.79	0	0	TTGGACTGAAGGGAGCTCCC	miR319	Leaf

Csi-miR390.1	200.5	109.38	58.81	AAGCTCAGGAGGGATAGCGCC	miR390	
Csi-miR390.2	14.05	6.81	660.36	AGCTCAGGAGGGATAGCGCC	miR390	Fruit
Csi-miR390-3p.1	5.31	4.11	7.84	CGCTATCCATCCTGAGTTTCA	miR390	
**Csi-miR390-3p.2**	**1.47**	**0**	**8.96**	**CGCTATCCATCCTGAGTTTC**	**miR390**	**Fruit**
Csi-miR390b	80	49.5	5.6	CAGCTCAGGAGGGATAGACAA	miR390	
Csi-miR391a.1	2.06	0	0	TTGTCGCCGGAGAGATAGCA	miR391	Leaf
**Csi-miR391a.2**	**2.95**	**0.43**	**0**	**TTGTCGCCGGAGAGATAGCAC**	**miR391**	**Leaf**
**Csi-miR391a.3**	**2.36**	**0.65**	**0**	**TTGTCGCCGGAGAGATAGCACC**	**miR391**	**Leaf**
Csi-miR391b	15.73	6.48	0	TTGTCGCAGGAGCGTTGGCACC	miR391	Leaf
Csi-miR393a.1	1.67	1.51	0	TCCAAAGGGATCGCATTGAT	miR393	
**Csi-miR393a.2**	**1.38**	**2.59**	**1.68**	**TCCAAAGGGATCGCATTGATC**	**miR393**	
**Csi-miR393a.2-3p**	**1.38**	**2.59**	**1.68**	**ATCATGCTATCCCTTTGGATT**	**miR393**	
**Csi-miR393a.3**	**0**	**0.97**	**0**	**TCCAAAGGGATCGCATTGATCC**	**miR393**	**Flower**
Csi-miR393b	10.22	73.93	3.92	TTCCAAAGGGATCGCATTGATC	miR393	Flower
**Csi-miR393b-3p**	**27.62**	**20.97**	**1.68**	**TCATGCGATCCCTTCGGAATT**	**miR393**	
Csi-miR394	23.49	37.94	10.64	TTGGCATTCTGTCCACCTCC	miR394	
**Csi-miR394-3p.1**	**27.72**	**2.49**	**43.13**	**AGCTGGACAAATTGCAAACTA**	**miR394**	
**Csi-miR394-3p.2**	**27.91**	**2.27**	**7.28**	**CTGGACAAATTGCAAACTACA**	**miR394**	**Leaf**
**Csi-miR394-3p.3**	**16.71**	**2.59**	**2.8**	**GCTGGACAAATTGCAAACTAC**	**miR394**	**Leaf**
Csi-miR395.1	30.17	0	8.4	CTGAAGTGTTTGGGGGAACTC	miR395	Leaf
**Csi-miR395.2**	**5.31**	**0**	**5.6**	**TGAAGTGTTTGGGGGAACTC**	**miR395**	
Csi-miR3951	291.22	312.25	210.6	TAGATAAAGATGAGAGAAAAA	miR3951	
**Csi-miR3951-3p**	**1445.39**	**46.58**	**309.18**	**TTTCTCTTATCGTTATCTGT**	**miR3951**	**Leaf**
Csi-miR3952.1	245.91	71.55	20.72	TGCTGTAGAAAGGCCCCTCAA	miR3952	Leaf
**Csi-miR3952.2**	**176.13**	**37.94**	**94.1**	**GCTGTAGAAAGGCCCCTCAAC**	**miR3952**	**Leaf**
**Csi-miR3952-3p**	**379.87**	**86.46**	**325.42**	**TGAAGGGCCTTTCTAGAGCAC**	**miR3952**	
Csi-miR3954a	13410.18	41460.6	19926.12	TTGGACAGAGAAATCACGGTCA	miR3954	Flower
Csi-miR3954b	150.08	478.15	57.13	GGACAGAGAAATCACGGTCA	miR3954	Flower
Csi-miR396a	161.88	169.9	24.08	TTCCACAGCTTTCTTGAACTG	miR396	

Csi-miR396b.1	0	44.64	0	TTCCACAGCTTTCTTGAAC	miR396	Flower
**Csi-miR396b.2**	**170.23**	**310.84**	**3.36**	**TTCCACAGCTTTCTTGAA**	**miR396**	
**Csi-miR396b.3**	**0**	**39.88**	**0**	**TTCCACAGCTTTCTTGAACTT**	**miR396**	**Flower**
**Csi-miR396b-3p.1**	**142.32**	**36.96**	**0**	**GCTCAAGAAAGCTGTGGGAGA**	**miR396**	**Leaf**
**Csi-miR396b-3p.2**	**59.17**	**14.59**	**3.36**	**CTCAAGAAAGCTGTGGGAGA**	**miR396**	**Leaf**
Csi-miR396c	123.64	7.13	7.84	TTCAAGAAATCTGTGGGAAG	miR396	Leaf
Csi-miR396d.1	0.88	0	0	TTTCCACGGCTTTCTTGAACT	miR396	Leaf
**Csi-miR396d.2**	**0**	**0.97**	**0**	**TTCCACGGCTTTCTTGAACTT**	**miR396**	**Flower**
**Csi-miR396d.3**	**0**	**1.73**	**0**	**TTCCACGGCTTTCTTGAAC**	**miR396**	**Flower**
**Csi-miR396d.4**	**318.05**	**35.67**	**1.12**	**TTCCACGGCTTTCTTGAA**	**miR396**	**Leaf**
**Csi-miR396d-3p**	**11.7**	**2.05**	**1.12**	**GCTCAAGAATGCCGTGGGAAA**	**miR396**	**Leaf**
Csi-miR397.1	22.7	169.58	0	TCATTGAGTGCAGCGTTGATG	miR397	Flower
**Csi-miR397.2**	**2.26**	**23.13**	**0**	**CATTGAGTGCAGCGTTGATGA**	**miR397**	**Flower**
Csi-miR398b	27.81	11.89	0	TGTGTTCTCAGGTCGCCCCTG	miR398	Leaf
Csi-miR399a	12.48	3.57	64.97	TGCCAAAGGAGATTTGCCCGG	miR399	Fruit
Csi-miR399b	15.53	11.24	43.13	TGCCAAAGGAGAGTTGCCCTA	miR399	Fruit
Csi-miR399c	3.15	0.65	29.69	CGCCAAAGGAGAATTGCCCTG	miR399	Fruit
Csi-miR399d	1.57	0.65	5.6	TGCCAAAGGAGAGTTGCCCTG	miR399	Fruit
Csi-miR399e	0	0	1.68	TGCCAAAGGAGAATTGCCCTG	miR399	Fruit
Csi-miR399e-5p	1.08	0.22	25.76	GTGCAGTCCTCCTTTGGCGTG	miR399	Fruit
Csi-miR403.1	819.41	373.42	5.04	TTAGATTCACGCACAAACT	miR403	Leaf
**Csi-miR403.2**	**125.31**	**27.02**	**27.44**	**TTAGATTCACGCACAAACTCG**	**miR403**	**Leaf**
**Csi-miR403.3**	**189.3**	**60.96**	**2.24**	**TTAGATTCACGCACAAAC**	**miR403**	**Leaf**
Csi-miR408.1	125.31	220.59	16.8	ACGGGGAACAGGCAGAGCATG	miR408	
**Csi-miR408.2**	**62.41**	**249.99**	**14.56**	**CGGGGAACAGGCAGAGCATGG**	**miR408**	**Flower**
Csi-miR4369	1.77	2.59	2.24	AGAACATGTTGATCTGGAAGGCCA	miR4369	
Csi-miR4414.1	257.31	153.58	0	AGCTGCTGACTCGTTGGTTCA	miR4414	
**Csi-miR4414.2**	**101.43**	**45.29**	**0**	**AGCTGCTGACTCGTTGGTTC**	**miR4414**	**Leaf**
Csi-miR443b.1	2.06	1.19	0	ATGAAAATTCAATGATTGTGGAA	miR443	

**Csi-miR443b.2**	**2.06**	**2.05**	**0.56**	**ATGAAAATTCAATGATTGTGGAAA**	**miR443**	
Csi-miR444a.1	102.71	41.5	0.56	TTTCTTGAGACAAAACATGCA	miR444	Leaf
**Csi-miR444a.2**	**262.23**	**61.39**	**15.68**	**TTTCTTGAGACAAAACATGCATC**	**miR444**	**Leaf**
Csi-miR472	1.08	0	2.8	TTTTCCCACACCTCCCATCCC	miR472	Fruit
Csi-miR473	39.81	15.56	69.45	ACTCTCCCTCAAGGGCTTCGC	miR473	Fruit
**Csi-miR473-3p**	**5.31**	**2.92**	**40.89**	**GAAGTCCTTGGGGTTGAGTGA**	**miR473**	**Fruit**
Csi-miR477a.1	77.15	97.06	208.36	ACCTCCCTCGAAGGCTTCCAA	miR477	Fruit
**Csi-miR477a.2**	**1.47**	**1.3**	**1.68**	**TCCCTCGAAGGCTTCCAATATA**	**miR477**	
**Csi-miR477a-3p**	**5.41**	**10.38**	**114.26**	**GGAAACCCTAGGGGGAGGTCG**	**miR477**	**Fruit**
Csi-miR477b.2	5.31	1.62	10.08	ACTCTCCCTCAAGGGCTTCTC	miR477	
Csi-miR477d.1-3p	2.85	1.95	19.04	TGAGGCCGTTGGGGAGAGTGG	miR477	Fruit
Csi-miR477d.2-5p	1.28	0.65	13.44	ACTCTCCCTCAAGGGCTTCTGA	miR477	Fruit
Csi-miR479.1	378.89	65.06	1141.48	TGTGATATTGGTTCGGCTCATC	miR479	Fruit
**Csi-miR479.2**	**23.2**	**3.57**	**27.44**	**TGTGATATTGGTTCGGCTCA**	**miR479**	
**Csi-miR482a-3p**	**9.63**	**1.73**	**82.33**	**TCTTCCCTATGCCTCCCATTCC**	**miR482**	**Fruit**
Csi-miR482a-5p	361.99	29.18	236.36	AGTGGGAGCGTGGGGTAAGAAG	miR482	
Csi-miR482b	0.98	0	10.64	TCTTGCCCACCCCTCCCATTCC	miR482	Fruit
Csi-miR482c	5.41	4.97	10.64	TTCCCTAGTCCCCCTATTCCTA	miR482	
Csi-miR482d	4.03	0.32	6.16	TCTTCCCTACTCCACCCATGCC	miR482	
Csi-miR482d-5p.1	915.53	157.26	438.56	TGGTATGGGTGAGTAGGGAAG	miR482	Leaf
**Csi-miR482d-5p.2**	**1025.81**	**105.16**	**54.89**	**TATGGGTGAGTAGGGAAGATA**	**miR482**	**Leaf**
Csi-miR5054.1	5.5	6.05	0.56	CGTTCCCCACAGACGGCGCCA	miR5054	
**Csi-miR5054.2**	**3.64**	**1.62**	**0.56**	**GTTCCCCACAGACGGCGCCA**	**miR5054**	**Leaf**
Csi-miR5177	5.41	6.48	0	AAAGGCTGTAAAAAATACACTCAA	miR5177	
Csi-miR5179	43.93	447.35	23.52	TCTTGCTCAAGACCGCGCAAT	miR5179	Flower
Csi-miR5225a	2.26	0.43	0	TCTGTCGCAGGAGAGATGGTGC	miR5225	Leaf
Csi-miR5291a	0	0.86	0	TTGATGAATGGCTAGGATTTG	miR5291	Flower
Csi-miR530a	1.87	0.76	3.92	TGCATTTGCACCTGCACCTTG	miR530	Fruit
Csi-miR530b	17.1	0.32	2.24	TGCATTTGCACCTGCATCTTG	miR530	Leaf

Csi-miR535.1	674.53	31.99	0	TGACAATGAGAGAGAGCACAC	miR535	Leaf
**Csi-miR535.2**	**18.28**	**0.65**	**0**	**TGACAATGAGAGAGAGCACA**	**miR535**	**Leaf**
**Csi-miR536-3p**	**6.49**	**0**	**0**	**TGGTGCCACGCTGTGTGCGTC**	**miR536**	**Leaf**
Csi-miR536-5p	3.73	0.32	0	CGCACCCCAGCGTGGAACCATC	miR536	Leaf
Csi-miR814	0	10.16	0	AAATTAGATTCGTTGTGTGATGAG	miR814	Flower
Csi-miR827.1	7.96	14.16	81.21	TTAGATGACCATCAACAAACA	miR827	Fruit
**Csi-miR827.2**	**1.28**	**1.95**	**24.08**	**TTAGATGACCATCAACAAACAT**	**miR827**	**Fruit**
Csi-miR827-5p.1	93.08	169.26	44.81	CTTGTTGATTGTCATCTAATC	miR827	
**Csi-miR827-5p.2**	**103.69**	**229.67**	**142.27**	**TGCTTGTTGATTGTCATCTAA**	**miR827**	
Csi-miR828	3.24	0.32	0	GATACTCATTTAGGCAAGACG	miR828	Leaf
Csi-miR833.1	2.26	9.73	2.24	ATTTATTGTTGATATGGGTCA	miR833	Flower
Csi-miR845a	1.87	3.67	0	AACAAGTGGTATCAGAGCTAGACC	miR845	
Csi-miR896	1.38	1.62	0	GTCGCTTTGGCCGAGTGGTTAA	miR896	
Csi-miRN01[c]	0	0	0	AACGAGTCACTTTTCTGTTAA		
Csi-miRN02	0	0	17.36	AACTGTAGTACCGTGCCACAG		Fruit
Csi-miRN03	85.02	17.4	0	TGTTTTGGGTGAAACGGGTGTT		Leaf
Csi-miRN04	0	22.91	11.2	TTGTAGAGTGTGTATGTTACA		Flower
Csi-miRN05	0	0.54	0	ATGTAAGATACATTTCTCTCA		Flower
Csi-miRN06	43.93	19.13	25.76	CATGTTTCGGGTTTGTGCGTG		
Csi-miRN07	0	0	64.41	GGAAGACAAGATCAAACGGTG		Fruit
Csi-miRN08	0	19.45	0	CCCGGGTCACAGCTCCCTGGAC		Flower
Csi-miRN09[c]	0	0	0	ATGAAGGAATGCTACCCCAGG		
Csi-miRN10	1.87	0.54	0	TTATTGAGTGTGTATGTTACA		Leaf
Csi-miRN11	2121.89	1274.17	3892.14	TCGCAGGAGCTTTCTACGGTT		
Csi-miRN12	0	43.88	0	TCAATGGTTATGGCACTAAGA		Flower
Csi-miRN13[c]	0	0	0	ATCCTTATTGTCGGGGCTACGCAA		
Csi-miRN14[c]	0	0	0	GCATAGTCTTGATAATAAGAATTC		
Csi-miRN15	55.04	15.13	0	TCGCAGGGGAGATGGACCAAC		Leaf
Csi-miRN16	229.01	17.51	3.36	CTGATGAGAGAGCGAATGATA		Leaf

Csi-miRN17	0	1.08	0	TTGTGGAGTGTGTATGTTACA	Flower
Csi-miRN18	1.08	0	0	GCTGGATGCAACTGTGGTACG	Leaf
Csi-miRN19	0.88	0	0	TTCGTTCGTTGAAACTCTAAA	Leaf
Csi-miRN20	79.71	121.7	54.89	TTTTGTTGCATGATGCTGATAA	
Csi-miRN21	0.49	2.27	0	TAATTGTAATTACACTGTCGTATA	Flower
Csi-miRN22	7.27	4.76	0	AAATATAGGACAAAAAATAACATA	
Csi-miRN23	14.45	13.51	20.72	CTTTCAGCAGCCTCCGGCGTC	
Csi-miRN24	2.95	33.4	2.24	AGCAAGCATCCTGGGCTAAT	Flower
Csi-miRN25	15.53	13.94	0	AGGGATTTCATGAAACATGGCATT	
Csi-miRN26	848.2	8.32	23.52	ACAGGAGGTGGAACAAATATGAAA	Leaf
Csi-miRN27	0.79	0.32	15.12	TATGTTGCAACAGTGGTACGGTA	Fruit
Csi-miRN28	7.86	2.49	2.8	CATGGGGAGTAGCTGCGCGGT	Leaf
Csi-miRN29	2.75	3.89	1.12	CGATATGGGATTTTGGGTAAAAAC	
Csi-miRN30	1.18	0.43	0	AGTATATACCGGGAGTACTCAAGA	Leaf
Csi-miRN31	24.96	85.6	435.76	CCGTTGAGGTAGGGCAGTTCGG	Fruit
Csi-miRN32	139.86	129.59	15.12	ATCTATTTGGATGAAGATAAGGGC	
Csi-miRN33	37.25	59.01	6.72	AGGGACAAGCTAAAAGACCAA	
Csi-miRN34	98.58	44.64	21.84	ATTCGGGACGAGTTTACAACGTTG	Leaf
Csi-miRN35	2.95	2.92	3.36	GTTTAGGACAGCTGCTGCCAAA	
Csi-miRN36	4757.41	1220.13	4312.77	GGTCATGGGAGGATTGGCGAAA	
Csi-miRN37	101.04	37.4	11.76	TGAGGGAAGAGCTTAGAAGG	Leaf
Csi-miRN38	2.26	2.27	1.12	ATGGTCGTATGATAACGTGATTGG	

miRNA isoforms and miRNA* species with high expression levels are indicated using bold text.

[a] The miRNA counts were normalized to the number of transcripts per million.

[b] The csi-miR1092.1 isoform was removed because its abundance was very low.

[c] These four novel miRNAs were not identified in our sequencing data; however, they were identified in other *C. sinensis* sequencing data (data not shown).
