# Supplementary material for: Genome-wide comparison of microRNAs and their targeted transcripts among leaf, flower and fruit of sweet orange
Source: BMC Genomics. 2014 Aug 20;15(1):695. doi: 10.1186/1471-2164-15-695 (PMC4158063; doi:10.1186/1471-2164-15-695)
Supplement: Supplementary file 3 — Additional file 3: Targets of miRNAs identified using degradome sequencing. (PDF 28 KB) [file 12864_2014_6413_MOESM3_ESM.pdf]

Additional file 3: Targets of miRNAs identified using degradome sequencing in sweet orange.

a. Targets of miRNAs identified in leaf using degradome sequencing

miRNA	Target	Category
Csi-miR1092.2	——	——
Csi-miR1432a	——	——
Csi-miR1446	——	——
Csi-miR1507a.2	——	——
Csi-miR1515	——	——
Csi-miR156a.1	Cs2g05730	1
Csi-miR156a.1	Cs2g23550	1
Csi-miR156a.1	Cs7g10830	1
Csi-miR156a.1	orange1.1t02265	1
Csi-miR156a.1	Cs5g11880	3
Csi-miR156a.1	Cs8g17370	3
Csi-miR156a.2	Cs2g05730	1
Csi-miR156a.2	Cs2g23550	1
Csi-miR156a.2	Cs7g10830	1
Csi-miR156a.2	orange1.1t02265	1
Csi-miR156a.2	Cs8g17370	3
Csi-miR156b.1	Cs2g05730	1
Csi-miR156b.1	Cs2g23550	1
Csi-miR156b.1	Cs7g10830	1
Csi-miR156b.1	orange1.1t02265	1
Csi-miR156c.1	Cs2g05730	1
Csi-miR156c.1	Cs2g23550	1
Csi-miR156c.1	Cs7g10830	1
Csi-miR156c.1	orange1.1t02265	1
Csi-miR156d	Cs2g05730	1
Csi-miR156d	Cs2g23550	1
Csi-miR156d	Cs7g10830	1
Csi-miR156d	orange1.1t02265	1
Csi-miR156e	Cs2g05730	1
Csi-miR156e	Cs2g23550	1
Csi-miR156e	Cs7g10830	1
Csi-miR156e	orange1.1t02265	1
Csi-miR156f.2	Cs2g05730	1
Csi-miR156f.2	Cs2g23550	1
Csi-miR156f.2	Cs7g10830	1
Csi-miR156f.2	orange1.1t02265	1
Csi-miR156f.2	Cs8g19900	2
Csi-miR156g.1	Cs2g05730	1
Csi-miR156g.1	Cs2g23550	1
Csi-miR156g.1	Cs7g10830	1
Csi-miR156g.1	orange1.1t02265	1
Csi-miR156g.2	Cs2g05730	1

Csi-miR156g.2	Cs2g23550	1
Csi-miR156g.2	Cs7g10830	1
Csi-miR156g.2	orange1.1t02265	1
Csi-miR156h	Cs2g05730	1
Csi-miR156h	Cs2g23550	1
Csi-miR156h	Cs7g10830	1
Csi-miR156h	orange1.1t02265	1
Csi-miR159	Cs6g10950	1
Csi-miR159	Cs3g06390	2
Csi-miR159b	Cs3g06390	2
Csi-miR160a-3p	——	——
Csi-miR160a-5p	Cs3g18940	1
Csi-miR160a-5p	Cs6g11800	1
Csi-miR160a-5p	Cs7g25670	1
Csi-miR160a-5p	Cs8g16440	1
Csi-miR160b-3p	——	——
Csi-miR160b-5p	Cs3g18940	1
Csi-miR160b-5p	Cs6g11800	1
Csi-miR160b-5p	Cs7g25670	1
Csi-miR160b-5p	Cs8g16440	1
Csi-miR160c.1	——	——
Csi-miR160c.2	——	——
Csi-miR162-3p.1	——	——
Csi-miR162-3p.2	——	——
Csi-miR164	Cs5g10870	1
Csi-miR164	Cs7g01530	3
Csi-miR166a.1	Cs2g09770	1
Csi-miR166a.1	Cs4g19310	1
Csi-miR166a.1	Cs8g16510	1
Csi-miR166a.1	Cs1g15640	3
Csi-miR166a.2	Cs2g09770	1
Csi-miR166a.2	Cs4g19310	1
Csi-miR166a.2	Cs8g16510	1
Csi-miR166a.2	Cs1g15640	3
Csi-miR166b	Cs2g09770	1
Csi-miR166b	Cs4g19310	1
Csi-miR166b	Cs8g16510	1
Csi-miR166b	Cs1g15640	3
Csi-miR166c.1	Cs2g09770	1
Csi-miR166c.1	Cs4g19310	1
Csi-miR166c.1	Cs8g16510	1
Csi-miR166c.1	Cs1g15640	3
Csi-miR166c.2	Cs2g09770	1
Csi-miR166c.2	Cs4g19310	1
Csi-miR166c.2	Cs8g16510	1

Csi-miR166c.2	Cs1g15640	3
Csi-miR166c.2	Cs4g06030	3
Csi-miR166c.3	Cs2g09770	1
Csi-miR166c.3	Cs4g19310	1
Csi-miR166c.3	Cs8g16510	1
Csi-miR166c.3	Cs1g15640	3
Csi-miR166c.4	Cs2g09770	1
Csi-miR166c.4	Cs4g19310	1
Csi-miR166c.4	Cs8g16510	1
Csi-miR166c.4	Cs1g15640	3
Csi-miR166c.5	Cs2g09770	1
Csi-miR166c.5	Cs4g19310	1
Csi-miR166c.5	Cs8g16510	1
Csi-miR166c.5	Cs1g15640	3
Csi-miR166d.1	Cs2g09770	1
Csi-miR166d.1	Cs4g19310	1
Csi-miR166d.1	Cs8g16510	1
Csi-miR166d.1	Cs1g15640	3
Csi-miR166d.2	Cs2g09770	1
Csi-miR166d.2	Cs4g19310	1
Csi-miR166d.2	Cs8g16510	1
Csi-miR166d.2	Cs1g15640	3
Csi-miR166g.1	Cs2g09770	1
Csi-miR166g.1	Cs4g19310	1
Csi-miR166g.1	Cs8g16510	1
Csi-miR166g.1	Cs1g15640	3
Csi-miR166i	Cs2g09770	1
Csi-miR166i	Cs4g19310	1
Csi-miR166i	Cs8g16510	1
Csi-miR166i	Cs1g15640	3
Csi-miR166j.1	Cs2g09770	1
Csi-miR166j.1	Cs4g19310	1
Csi-miR166j.1	Cs8g16510	1
Csi-miR166j.1	Cs1g15640	3
Csi-miR166j.2	Cs1g15640	3
Csi-miR166j.3	Cs2g09770	1
Csi-miR166j.3	Cs4g19310	1
Csi-miR166j.3	Cs8g16510	1
Csi-miR166j.3	Cs1g15640	3
Csi-miR167a.1	Cs2g15130	1
Csi-miR167a.1	Cs6g16030	2
Csi-miR167a.1	Cs2g09440	3
Csi-miR167b.1	Cs2g15130	1
Csi-miR167b.1	Cs1g09030	2
Csi-miR167b.1	Cs2g09440	2

Csi-miR167b.1	Cs6g16030	2
Csi-miR167b.2	Cs1g09030	2
Csi-miR167b.2	Cs6g16030	2
Csi-miR167b.2	Cs2g09440	3
Csi-miR167b.3	Cs2g15130	1
Csi-miR167b.3	Cs6g16030	2
Csi-miR167b.3	Cs2g09440	3
Csi-miR167b.4	Cs2g15130	1
Csi-miR167b.4	Cs1g09030	2
Csi-miR167b.4	Cs6g16030	2
Csi-miR167b.4	Cs2g09440	3
Csi-miR167d.1	Cs6g16030	2
Csi-miR167d.1	Cs2g09440	3
Csi-miR167d.2	Cs2g15130	1
Csi-miR167d.2	Cs6g16030	2
Csi-miR167d.2	Cs2g09440	3
Csi-miR168a	orange1.1t00591	1
Csi-miR168a	Cs5g16710	3
Csi-miR169b.1	Cs1g17780	1
Csi-miR169b.1	Cs2g30350	1
Csi-miR169b.1	Cs6g13560	1
Csi-miR169b.1	Cs7g01720	1
Csi-miR169b.1-3p	——	——
Csi-miR169i.1	Cs1g17780	1
Csi-miR169i.1	Cs2g30350	1
Csi-miR169i.1	Cs6g13560	1
Csi-miR169i.1	Cs7g01720	1
Csi-miR169i.1-3p	——	——
Csi-miR169m.1	Cs1g17780	1
Csi-miR169m.1	Cs2g30350	1
Csi-miR169m.1	Cs6g13560	1
Csi-miR169m.1	Cs7g01720	1
Csi-miR169m.2	Cs1g17780	1
Csi-miR169m.2	Cs2g30350	1
Csi-miR169m.2	Cs7g01720	1
Csi-miR169m.3	Cs1g17780	1
Csi-miR169m.3	Cs2g30350	1
Csi-miR171a.1	——	——
Csi-miR171b	——	——
Csi-miR171d	Cs5g08980	1
Csi-miR171d	orange1.1t00199	1
Csi-miR171d	orange1.1t00200	1
Csi-miR171g.1	Cs5g08980	1
Csi-miR171g.1	orange1.1t00199	1
Csi-miR171g.1	orange1.1t00200	1

Csi-miR172a-3p.1	Cs7g27790	1
Csi-miR172a-3p.1	Cs8g17390	1
Csi-miR172a-3p.1	orange1.1t04055	1
Csi-miR172a-3p.1	Cs6g11940	3
Csi-miR172a-3p.2	Cs7g27790	1
Csi-miR172a-3p.2	Cs8g17390	1
Csi-miR172a-3p.2	orange1.1t04055	1
Csi-miR172a-3p.3	Cs7g27790	1
Csi-miR172a-3p.3	Cs8g17390	1
Csi-miR172a-3p.3	orange1.1t04055	1
Csi-miR172a-3p.3	Cs2g21180	3
Csi-miR172c.1	Cs3g06140	1
Csi-miR172c.1	Cs7g27790	1
Csi-miR172c.1	Cs8g17390	1
Csi-miR172c.1	orange1.1t04055	1
Csi-miR172c.1	Cs7g23640	2
Csi-miR172c.1	Cs2g21180	3
Csi-miR172c.1	Cs6g11940	3
Csi-miR172c.1	Cs7g31800	3
Csi-miR172c.2	Cs7g27790	1
Csi-miR172c.2	Cs8g17390	1
Csi-miR172c.2	orange1.1t04055	1
Csi-miR172c.2	Cs7g31800	3
Csi-miR172d	Cs7g27790	1
Csi-miR172d	Cs8g17390	1
Csi-miR172d	orange1.1t04055	1
Csi-miR172d-3p	Cs5g21000	3
Csi-miR172d-3p	Cs9g03090	3
Csi-miR172e.2	Cs7g27790	1
Csi-miR172e.2	Cs8g17390	1
Csi-miR172e.2	orange1.1t04055	1
Csi-miR172e.2	Cs9g07740	3
Csi-miR172k	Cs7g27790	1
Csi-miR172k	Cs8g17390	1
Csi-miR172k	orange1.1t04055	1
Csi-miR2111	——	——
Csi-miR2118.1	orange1.1t00557	1
Csi-miR2118.1	orange1.1t00149	2
Csi-miR2118.1	orange1.1t02171	3
Csi-miR2118.1	orange1.1t02175	3
Csi-miR2118.2	Cs2g09590	1
Csi-miR2118.2	Cs2g30960	1
Csi-miR2118.2	Cs2g30970	1
Csi-miR2118.2	Cs3g12720	1
Csi-miR2118.2	Cs3g12760	1

Csi-miR2118.2	Cs3g12850	1
Csi-miR2118.2	Cs5g18480	1
Csi-miR2118.2	Cs5g19310	1
Csi-miR2118.2	Cs5g19850	1
Csi-miR2118.2	Cs5g19920	1
Csi-miR2118.2	orange1.1t00557	1
Csi-miR2118.2	Cs8g10560	2
Csi-miR2118.2	orange1.1t00149	2
Csi-miR2118.2	orange1.1t02171	3
Csi-miR2118.2	orange1.1t02175	3
Csi-miR2275a	——	——
Csi-miR2275b	——	——
Csi-miR2275c.1	——	——
Csi-miR2275d	Cs5g35220	1
Csi-miR2275d	Cs8g03105	1
Csi-miR2275d-3p	——	——
Csi-miR2275e	——	——
Csi-miR2275f	——	——
Csi-miR2911	——	——
Csi-miR319	Cs3g06390	2
Csi-miR319a	——	——
Csi-miR319b	Cs3g06390	2
Csi-miR390.1	Cs9g01780	2
Csi-miR390.2	Cs9g01780	2
Csi-miR390-3p.1	——	——
Csi-miR390-3p.2	——	——
Csi-miR390b	Cs9g01780	2
Csi-miR391a.1	——	——
Csi-miR391a.2	——	——
Csi-miR391a.3	——	——
Csi-miR391b	——	——
Csi-miR393a.1	Cs2g14270	1
Csi-miR393a.1	Cs7g31800	2
Csi-miR393a.2	Cs2g14270	1
Csi-miR393a.2-3p	——	——
Csi-miR393a.3	Cs2g14270	1
Csi-miR393b	Cs2g14270	1
Csi-miR393b-3p	——	——
Csi-miR394	Cs7g10850	2
Csi-miR394	Cs2g02980	3
Csi-miR394-3p.1	——	——
Csi-miR394-3p.2	——	——
Csi-miR394-3p.3	——	——
Csi-miR395.1	——	——
Csi-miR395.2	Cs5g34330	1

Csi-miR3951	Cs1g06060	1
Csi-miR3951	orange1.1t05622	1
Csi-miR3951-3p	——	——
Csi-miR3952.1	——	——
Csi-miR3952.2	——	——
Csi-miR3952-3p	orange1.1t02252	2
Csi-miR3954a	Cs1g09600	2
Csi-miR3954a	Cs7g22460	2
Csi-miR3954a	Cs1g09635	3
Csi-miR3954a	Cs5g04670	3
Csi-miR3954b	Cs2g29120	1
Csi-miR3954b	Cs1g09600	2
Csi-miR3954b	Cs1g09635	2
Csi-miR3954b	Cs7g22460	2
Csi-miR3954b	Cs1g09665	3
Csi-miR3954b	Cs5g04670	3
Csi-miR396a	Cs1g21350	1
Csi-miR396a	Cs1g22520	1
Csi-miR396a	Cs3g23180	1
Csi-miR396a	Cs5g01380	1
Csi-miR396a	Cs7g15220	1
Csi-miR396a	Cs7g27670	1
Csi-miR396a	Cs7g27680	1
Csi-miR396a	orange1.1t00172	1
Csi-miR396a	orange1.1t03122	1
Csi-miR396a	Cs4g05000	3
Csi-miR396a	Cs5g09850	3
Csi-miR396b.1	Cs1g21350	1
Csi-miR396b.1	Cs1g22520	1
Csi-miR396b.1	Cs3g23180	1
Csi-miR396b.1	Cs5g01380	1
Csi-miR396b.1	Cs7g15220	1
Csi-miR396b.1	Cs7g27670	1
Csi-miR396b.1	Cs7g27680	1
Csi-miR396b.1	orange1.1t00172	1
Csi-miR396b.1	orange1.1t02555	1
Csi-miR396b.1	orange1.1t03122	1
Csi-miR396b.1	Cs6g19380	2
Csi-miR396b.1	Cs4g05000	3
Csi-miR396b.1	Cs5g06510	3
Csi-miR396b.1	Cs5g09850	3
Csi-miR396b.1	Cs5g24150	3
Csi-miR396b.2	Cs1g21350	1
Csi-miR396b.2	Cs3g23180	1
Csi-miR396b.2	Cs5g01380	1

Csi-miR396b.2	Cs7g15220	1
Csi-miR396b.2	Cs7g27670	1
Csi-miR396b.2	Cs7g27680	1
Csi-miR396b.2	orange1.1t00172	1
Csi-miR396b.2	orange1.1t02555	1
Csi-miR396b.2	orange1.1t03122	1
Csi-miR396b.2	Cs6g19380	2
Csi-miR396b.2	Cs4g05000	3
Csi-miR396b.2	Cs4g06230	3
Csi-miR396b.2	Cs5g05620	3
Csi-miR396b.2	Cs5g06510	3
Csi-miR396b.2	Cs5g09850	3
Csi-miR396b.2	Cs5g24150	3
Csi-miR396b.2	Cs6g19000	3
Csi-miR396b.2	Cs8g03020	3
Csi-miR396b.2	Cs8g17370	3
Csi-miR396b.3	Cs1g21350	1
Csi-miR396b.3	Cs1g22520	1
Csi-miR396b.3	Cs3g23180	1
Csi-miR396b.3	Cs5g01380	1
Csi-miR396b.3	Cs7g15220	1
Csi-miR396b.3	orange1.1t00172	1
Csi-miR396b.3	orange1.1t02555	1
Csi-miR396b.3	orange1.1t03122	1
Csi-miR396b.3	Cs5g09850	3
Csi-miR396b-3p.1	——	——
Csi-miR396b-3p.2	Cs2g01990	3
Csi-miR396c	——	——
Csi-miR396d.1	Cs1g21350	1
Csi-miR396d.1	Cs1g22520	1
Csi-miR396d.1	Cs3g23180	1
Csi-miR396d.1	Cs5g01380	1
Csi-miR396d.1	Cs7g15220	1
Csi-miR396d.1	orange1.1t00172	1
Csi-miR396d.1	orange1.1t02555	1
Csi-miR396d.1	orange1.1t03122	1
Csi-miR396d.1	Cs4g12200	3
Csi-miR396d.1	Cs5g09850	3
Csi-miR396d.1	Cs8g17370	3
Csi-miR396d.2	Cs1g21350	1
Csi-miR396d.2	Cs1g22520	1
Csi-miR396d.2	Cs3g23180	1
Csi-miR396d.2	Cs5g01380	1
Csi-miR396d.2	Cs7g15220	1
Csi-miR396d.2	orange1.1t00172	1

Csi-miR396d.2	orange1.1t02555	1
Csi-miR396d.2	orange1.1t03122	1
Csi-miR396d.2	Cs2g09620	3
Csi-miR396d.2	Cs5g09850	3
Csi-miR396d.2	Cs8g17370	3
Csi-miR396d.3	Cs1g21350	1
Csi-miR396d.3	Cs1g22520	1
Csi-miR396d.3	Cs3g23180	1
Csi-miR396d.3	Cs5g01380	1
Csi-miR396d.3	Cs7g15220	1
Csi-miR396d.3	orange1.1t00172	1
Csi-miR396d.3	orange1.1t02555	1
Csi-miR396d.3	orange1.1t03122	1
Csi-miR396d.3	Cs1g15720	3
Csi-miR396d.3	Cs2g21180	3
Csi-miR396d.3	Cs4g05000	3
Csi-miR396d.3	Cs4g12200	3
Csi-miR396d.3	Cs5g09850	3
Csi-miR396d.3	Cs8g17370	3
Csi-miR396d.4	Cs1g21350	1
Csi-miR396d.4	Cs1g22520	1
Csi-miR396d.4	Cs3g23180	1
Csi-miR396d.4	Cs5g01380	1
Csi-miR396d.4	Cs7g15220	1
Csi-miR396d.4	Cs7g27670	1
Csi-miR396d.4	Cs7g27680	1
Csi-miR396d.4	orange1.1t00172	1
Csi-miR396d.4	orange1.1t02555	1
Csi-miR396d.4	orange1.1t03122	1
Csi-miR396d.4	Cs6g19380	2
Csi-miR396d.4	Cs1g15720	3
Csi-miR396d.4	Cs2g21180	3
Csi-miR396d.4	Cs4g05000	3
Csi-miR396d.4	Cs4g10880	3
Csi-miR396d.4	Cs4g12200	3
Csi-miR396d.4	Cs5g01840	3
Csi-miR396d.4	Cs5g06510	3
Csi-miR396d.4	Cs5g09850	3
Csi-miR396d.4	Cs6g17090	3
Csi-miR396d.4	Cs8g03230	3
Csi-miR396d.4	Cs8g17370	3
Csi-miR396d-3p	——	——
Csi-miR397.1	orange1.1t03059	3
Csi-miR397.2	orange1.1t03059	3
Csi-miR398b	——	——

Csi-miR399a	——	——
Csi-miR399b	——	——
Csi-miR399c	——	——
Csi-miR399d	——	——
Csi-miR399e	——	——
Csi-miR399e-5p	——	——
Csi-miR403.1	Cs2g10760	1
Csi-miR403.1	Cs4g01460	1
Csi-miR403.1	Cs4g19605	1
Csi-miR403.1	Cs3g01650	2
Csi-miR403.1	Cs2g10770	3
Csi-miR403.2	Cs2g10760	1
Csi-miR403.2	Cs2g10770	3
Csi-miR403.3	Cs2g10760	1
Csi-miR403.3	Cs4g01460	1
Csi-miR403.3	Cs4g19605	1
Csi-miR403.3	Cs3g01650	2
Csi-miR403.3	Cs2g10770	3
Csi-miR408.1	——	——
Csi-miR408.2	——	——
Csi-miR4369	——	——
Csi-miR4414.1	——	——
Csi-miR4414.2	——	——
Csi-miR443b.1	——	——
Csi-miR443b.2	——	——
Csi-miR444a.1	Cs9g07680	3
Csi-miR444a.1	orange1.1t02003	3
Csi-miR444a.2	——	——
Csi-miR472	Cs2g30590	1
Csi-miR472	Cs7g26730	1
Csi-miR473	——	——
Csi-miR473-3p	Cs1g08770	3
Csi-miR477a.1	——	——
Csi-miR477a.2	——	——
Csi-miR477a-3p	Cs7g09520	2
Csi-miR477b.2	——	——
Csi-miR477d.1-3p	——	——
Csi-miR477d.2-5p	——	——
Csi-miR479.1	——	——
Csi-miR479.2	——	——
Csi-miR482a-3p	Cs5g18480	1
Csi-miR482a-3p	Cs5g19920	1
Csi-miR482a-5p	——	——
Csi-miR482b	Cs1g11740	1
Csi-miR482c	Cs9g06846	1

Csi-miR482d	Cs1g11740	1
Csi-miR482d-5p.1	——	——
Csi-miR482d-5p.2	——	——
Csi-miR5054.1	——	——
Csi-miR5054.2	——	——
Csi-miR5177	——	——
Csi-miR5179	——	——
Csi-miR5225a	——	——
Csi-miR5291a	——	——
Csi-miR530a	——	——
Csi-miR530b	Cs6g11650	3
Csi-miR535.1	orange1.1t02265	1
Csi-miR535.1	Cs2g05730	2
Csi-miR535.1	Cs4g05470	3
Csi-miR535.2	Cs2g05730	1
Csi-miR535.2	Cs7g10830	1
Csi-miR535.2	orange1.1t02265	1
Csi-miR535.2	Cs4g05470	3
Csi-miR536-3p	orange1.1t01470	1
Csi-miR536-3p	orange1.1t04955	1
Csi-miR536-3p	orange1.1t05081	1
Csi-miR536-5p	——	——
Csi-miR814	——	——
Csi-miR827.1	——	——
Csi-miR827.2	——	——
Csi-miR827-5p.1	——	——
Csi-miR827-5p.2	Cs4g14890	3
Csi-miR828	——	——
Csi-miR833.1	Cs8g03110	3
Csi-miR845a	——	——
Csi-miR896	——	——
Csi-miRN01	——	——
Csi-miRN02	Cs1g08400	1
Csi-miRN02	Cs3g18790	1
Csi-miRN02	Cs8g12570	1
Csi-miRN02	orange1.1t01714	3
Csi-miRN03	——	——
Csi-miRN04	——	——
Csi-miRN05	Cs4g19940	2
Csi-miRN06	——	——
Csi-miRN07	Cs8g09620	3
Csi-miRN08	——	——
Csi-miRN09	——	——
Csi-miRN10	——	——
Csi-miRN11	Cs8g13560	1

Csi-miRN12	Cs4g06030	3
Csi-miRN13	——	——
Csi-miRN14	——	——
Csi-miRN15	——	——
Csi-miRN16	——	——
Csi-miRN17	——	——
Csi-miRN18	——	——
Csi-miRN19	——	——
Csi-miRN20	Cs3g05320	1
Csi-miRN21	——	——
Csi-miRN22	——	——
Csi-miRN23	——	——
Csi-miRN24	——	——
Csi-miRN25	——	——
Csi-miRN26	——	——
Csi-miRN27	——	——
Csi-miRN28	——	——
Csi-miRN29	——	——
Csi-miRN30	——	——
Csi-miRN31	——	——
Csi-miRN32	——	——
Csi-miRN33	——	——
Csi-miRN34	——	——
Csi-miRN35	——	——
Csi-miRN36	——	——
Csi-miRN37	orange1.1t02400	2
Csi-miRN38	——	——

b. Targets of miRNAs identified in flower using degradome sequencing

miRNA	Target	Category
Csi-miR1092.2	Cs8g11330	3
Csi-miR1432a	Cs1g21570	3
Csi-miR1446	Cs7g23010	1
Csi-miR1507a.2	——	——
Csi-miR1515	——	——
Csi-miR156a.1	Cs2g23550	1
Csi-miR156a.1	Cs2g05730	1
Csi-miR156a.2	Cs2g23550	1
Csi-miR156a.2	Cs2g05730	1
Csi-miR156b.1	Cs2g23550	1
Csi-miR156b.1	Cs2g05730	1
Csi-miR156c.1	Cs2g23550	1
Csi-miR156c.1	Cs2g05730	1
Csi-miR156d	Cs2g23550	1

Csi-miR156d	Cs2g05730	1
Csi-miR156e	Cs2g23550	1
Csi-miR156e	Cs2g05730	1
Csi-miR156f.2	Cs2g23550	1
Csi-miR156f.2	Cs2g05730	1
Csi-miR156f.2	Cs8g19900	2
Csi-miR156g.1	Cs2g23550	1
Csi-miR156g.1	Cs2g05730	1
Csi-miR156g.2	Cs2g23550	1
Csi-miR156g.2	Cs2g05730	1
Csi-miR156h	Cs2g23550	1
Csi-miR156h	Cs2g05730	1
Csi-miR159	Cs3g06390	1
Csi-miR159b	Cs3g06390	1
Csi-miR160a-3p	Cs9g06430	3
Csi-miR160a-5p	——	——
Csi-miR160b-3p	——	——
Csi-miR160b-5p	——	——
Csi-miR160c.1	——	——
Csi-miR160c.2	——	——
Csi-miR162-3p.1	orange1.1t00584	2
Csi-miR162-3p.2	orange1.1t00584	2
Csi-miR164	Cs5g10870	1
Csi-miR166a.1	Cs8g16510	1
Csi-miR166a.1	Cs2g09770	3
Csi-miR166a.2	Cs8g16510	1
Csi-miR166a.2	Cs2g09770	3
Csi-miR166b	Cs8g16510	1
Csi-miR166b	Cs2g09770	3
Csi-miR166c.1	Cs8g16510	1
Csi-miR166c.1	Cs2g09770	2
Csi-miR166c.2	Cs8g16510	1
Csi-miR166c.2	Cs2g09770	2
Csi-miR166c.3	Cs8g16510	1
Csi-miR166c.3	Cs2g09770	2
Csi-miR166c.4	Cs8g16510	1
Csi-miR166c.4	Cs2g09770	2
Csi-miR166c.5	Cs8g16510	1
Csi-miR166c.5	Cs2g09770	2
Csi-miR166d.1	Cs8g16510	1
Csi-miR166d.1	Cs2g09770	3
Csi-miR166d.2	Cs8g16510	1
Csi-miR166d.2	Cs2g09770	3
Csi-miR166g.1	Cs8g16510	1
Csi-miR166g.1	Cs2g09770	2

Csi-miR166i	Cs8g16510	1
Csi-miR166i	Cs2g09770	2
Csi-miR166j.1	Cs8g16510	1
Csi-miR166j.1	Cs2g09770	3
Csi-miR166j.2	Cs4g14480	3
Csi-miR166j.3	Cs8g16510	1
Csi-miR166j.3	Cs2g09770	3
Csi-miR167a.1	Cs2g15130	1
Csi-miR167a.1	Cs8g08320	3
Csi-miR167b.1	Cs2g15130	1
Csi-miR167b.1	Cs8g08320	3
Csi-miR167b.2	Cs1g09030	3
Csi-miR167b.3	Cs2g15130	1
Csi-miR167b.4	Cs2g15130	1
Csi-miR167b.4	Cs8g08320	3
Csi-miR167d.1	Cs8g08320	3
Csi-miR167d.2	Cs2g15130	1
Csi-miR167d.2	Cs8g08320	3
Csi-miR168a	Cs5g16710	3
Csi-miR169b.1	Cs6g13560	1
Csi-miR169b.1-3p	Cs9g16380	3
Csi-miR169b.1-3p	Cs2g28180	3
Csi-miR169i.1	Cs6g13560	1
Csi-miR169i.1-3p	orange1.1t00423	3
Csi-miR169m.1	Cs6g13560	1
Csi-miR169m.1	Cs3g24090	1
Csi-miR169m.2	Cs3g24090	1
Csi-miR169m.3	Cs7g06000	3
Csi-miR171a.1	Cs2g24890	3
Csi-miR171b	Cs4g09320	3
Csi-miR171d	Cs9g19410	3
Csi-miR171g.1	orange1.1t00199	1
Csi-miR171g.1	orange1.1t00200	1
Csi-miR172a-3p.1	orange1.1t04055	3
Csi-miR172a-3p.1	Cs8g17390	3
Csi-miR172a-3p.2	orange1.1t04055	3
Csi-miR172a-3p.2	Cs8g17390	3
Csi-miR172a-3p.3	orange1.1t04055	3
Csi-miR172a-3p.3	Cs8g17390	3
Csi-miR172c.1	Cs3g06140	1
Csi-miR172c.1	orange1.1t04055	3
Csi-miR172c.1	Cs8g17390	3
Csi-miR172c.1	Cs1g21180	3
Csi-miR172c.2	orange1.1t04055	3
Csi-miR172c.2	Cs8g17390	3

Csi-miR172d	orange1.1t04055	3
Csi-miR172d	Cs8g17390	3
Csi-miR172d-3p	Cs9g03090	1
Csi-miR172e.2	orange1.1t04055	2
Csi-miR172e.2	Cs8g17390	3
Csi-miR172k	orange1.1t04055	3
Csi-miR172k	Cs8g17390	3
Csi-miR2111	Cs5g29630	3
Csi-miR2118.1	Cs6g12050	3
Csi-miR2118.2	Cs3g12850	1
Csi-miR2118.2	Cs3g12760	1
Csi-miR2118.2	Cs3g12720	1
Csi-miR2118.2	orange1.1t00149	2
Csi-miR2275a	orange1.1t02313	3
Csi-miR2275b	Cs2g10790	3
Csi-miR2275c.1	Cs2g10790	3
Csi-miR2275d	Cs8g02270.1	3
Csi-miR2275d	Cs2g06980	3
Csi-miR2275d-3p	——	——
Csi-miR2275e	——	——
Csi-miR2275f	Cs4g12820	3
Csi-miR2911	——	——
Csi-miR319	Cs3g06390	1
Csi-miR319a	Cs1g06290	3
Csi-miR319b	Cs3g06390	1
Csi-miR390.1	Cs9g01780	1
Csi-miR390.2	Cs9g01780	1
Csi-miR390-3p.1	——	——
Csi-miR390-3p.2	Cs8g04530	3
Csi-miR390b	Cs9g01780	1
Csi-miR391a.1	Cs5g17560	3
Csi-miR391a.2	——	——
Csi-miR391a.3	——	——
Csi-miR391b	——	——
Csi-miR393a.1	Cs7g31800	3
Csi-miR393a.2	orange1.1t02367	3
Csi-miR393a.2-3p	Cs9g05070	3
Csi-miR393a.3	orange1.1t02367	3
Csi-miR393b	orange1.1t02367	3
Csi-miR393b-3p	Cs5g31220	3
Csi-miR394	Cs5g08980	3
Csi-miR394-3p.1	Cs3g09210	3
Csi-miR394-3p.2	Cs8g14600	3
Csi-miR394-3p.3	——	——
Csi-miR395.1	orange1.1t01708	3

Csi-miR395.2	Cs5g34330	3
Csi-miR3951	orange1.1t05622	1
Csi-miR3951-3p	Cs2g03130	3
Csi-miR3952.1	Cs8g13286	2
Csi-miR3952.2	Cs8g13286	2
Csi-miR3952-3p	Cs8g13286	3
Csi-miR3954a	Cs1g09600	1
Csi-miR3954a	Cs5g04670	3
Csi-miR3954a	Cs1g09635	3
Csi-miR3954b	Cs1g09600	1
Csi-miR3954b	Cs2g29120	1
Csi-miR3954b	Cs1g09635	3
Csi-miR3954b	Cs5g04670	3
Csi-miR396a	Cs6g15330	1
Csi-miR396a	Cs7g15220	1
Csi-miR396a	orange1.1t03122	1
Csi-miR396a	Cs1g22520	1
Csi-miR396a	Cs5g09850	2
Csi-miR396a	Cs3g23180	3
Csi-miR396b.1	Cs6g15330	1
Csi-miR396b.1	Cs7g15220	1
Csi-miR396b.1	orange1.1t03122	1
Csi-miR396b.1	Cs1g22520	1
Csi-miR396b.1	Cs5g09850	2
Csi-miR396b.1	Cs3g23180	3
Csi-miR396b.2	Cs6g15330	1
Csi-miR396b.2	Cs7g15220	1
Csi-miR396b.2	orange1.1t03122	1
Csi-miR396b.2	Cs1g22520	1
Csi-miR396b.2	orange1.1t02555	1
Csi-miR396b.2	Cs5g09850	2
Csi-miR396b.2	Cs3g23180	3
Csi-miR396b.2	Cs8g17370	3
Csi-miR396b.3	Cs6g15330	1
Csi-miR396b.3	Cs7g15220	1
Csi-miR396b.3	orange1.1t03122	1
Csi-miR396b.3	Cs1g22520	1
Csi-miR396b.3	Cs5g09850	2
Csi-miR396b.3	Cs3g23180	3
Csi-miR396b-3p.1	Cs9g04080	3
Csi-miR396b-3p.2	Cs9g04080	3
Csi-miR396c	Cs2g09460	3
Csi-miR396d.1	Cs6g15330	1
Csi-miR396d.1	Cs7g15220	1
Csi-miR396d.1	orange1.1t03122	1

Csi-miR396d.1	Cs1g22520	1
Csi-miR396d.1	Cs5g09850	2
Csi-miR396d.1	Cs3g23180	3
Csi-miR396d.2	Cs6g15330	1
Csi-miR396d.2	Cs7g15220	1
Csi-miR396d.2	orange1.1t03122	1
Csi-miR396d.2	Cs1g22520	1
Csi-miR396d.2	Cs5g09850	2
Csi-miR396d.2	Cs3g23180	3
Csi-miR396d.3	Cs6g15330	1
Csi-miR396d.3	Cs7g15220	1
Csi-miR396d.3	orange1.1t03122	1
Csi-miR396d.3	Cs1g22520	1
Csi-miR396d.3	Cs5g09850	2
Csi-miR396d.3	Cs3g23180	3
Csi-miR396d.4	orange1.1t03122	1
Csi-miR396d.4	Cs7g15220	1
Csi-miR396d.4	Cs6g15330	1
Csi-miR396d.4	Cs1g22520	1
Csi-miR396d.4	orange1.1t02555	1
Csi-miR396d.4	Cs5g09850	2
Csi-miR396d.4	Cs3g23180	3
Csi-miR396d.4	Cs8g17370	3
Csi-miR396d-3p	Cs8g05290	3
Csi-miR397.1	Cs6g07450	2
Csi-miR397.2	Cs6g07410	1
Csi-miR398b	Cs1g13240	1
Csi-miR399a	Cs3g22510	3
Csi-miR399b	orange1.1t01536	3
Csi-miR399c	——	——
Csi-miR399d	Cs2g30260	3
Csi-miR399e	——	——
Csi-miR399e-5p	Cs1g02320	3
Csi-miR403.1	Cs2g10760	1
Csi-miR403.2	Cs2g10760	1
Csi-miR403.3	Cs2g10760	1
Csi-miR403.3	Cs8g07240	3
Csi-miR408.1	Cs2g13550	3
Csi-miR408.2	Cs6g02740	3
Csi-miR4369	——	——
Csi-miR4414.1	——	——
Csi-miR4414.2	Cs3g26540	1
Csi-miR4414.2	orange1.1t02428	1
Csi-miR443b.1	——	——
Csi-miR443b.2	——	——

Csi-miR444a.1	orange1.1t02003	2
Csi-miR444a.2	——	——
Csi-miR472	Cs7g26730	3
Csi-miR473	——	——
Csi-miR473-3p	orange1.1t02213	3
Csi-miR477a.1	——	——
Csi-miR477a.2	——	——
Csi-miR477a-3p	Cs7g09520	2
Csi-miR477b.2	Cs8g17030	3
Csi-miR477d.1-3p	Cs2g02350	3
Csi-miR477d.2-5p	Cs3g10900	3
Csi-miR479.1	Cs5g24810	3
Csi-miR479.2	Cs6g21570	3
Csi-miR482a-3p	——	——
Csi-miR482a-5p	——	——
Csi-miR482b	Cs1g13430	3
Csi-miR482c	Cs9g06846	3
Csi-miR482d	Cs6g10170	3
Csi-miR482d-5p.1	——	——
Csi-miR482d-5p.2	——	——
Csi-miR5054.1	——	——
Csi-miR5054.2	Cs7g07710	3
Csi-miR5177	——	——
Csi-miR5179	Cs1g24860	3
Csi-miR5225a	——	——
Csi-miR5291a	Cs1g02730	3
Csi-miR530a	Cs3g18550	3
Csi-miR530b	Cs4g05330	3
Csi-miR530b	Cs6g11650	3
Csi-miR535.1	Cs4g05470	3
Csi-miR535.2	Cs2g05730	1
Csi-miR535.2	Cs4g05470	3
Csi-miR536-3p	——	——
Csi-miR536-5p	Cs1g15460	3
Csi-miR814	——	——
Csi-miR827.1	——	——
Csi-miR827.2	——	——
Csi-miR827-5p.1	Cs1g24800	3
Csi-miR827-5p.2	Cs9g10630	3
Csi-miR828	orange1.1t00180	3
Csi-miR833.1	Cs9g02420	2
Csi-miR845a	——	——
Csi-miR896	——	——
Csi-miRN01	Cs5g31745	3
Csi-miRN02	Cs3g18790	2

Csi-miRN03	Cs5g26760	3
Csi-miRN04	orange1.1t00471	3
Csi-miRN05	Cs4g19940	3
Csi-miRN06	——	——
Csi-miRN07	Cs8g09620	1
Csi-miRN08	——	——
Csi-miRN09	——	——
Csi-miRN10	Cs9g04330	3
Csi-miRN11	Cs8g13560	3
Csi-miRN12	Cs4g06030	2
Csi-miRN13	——	——
Csi-miRN14	Cs7g14990	3
Csi-miRN15	——	——
Csi-miRN16	Cs7g06555	3
Csi-miRN17	orange1.1t00471	3
Csi-miRN18	Cs7g24200	3
Csi-miRN19	Cs5g32800	3
Csi-miRN20	Cs3g05320	3
Csi-miRN21	——	——
Csi-miRN22	——	——
Csi-miRN23	Cs1g07330	3
Csi-miRN24	Cs4g01970	3
Csi-miRN25	——	——
Csi-miRN26	Cs9g18000	3
Csi-miRN27	——	——
Csi-miRN28	Cs3g17940	3
Csi-miRN29	——	——
Csi-miRN30	——	——
Csi-miRN31	——	——
Csi-miRN32	——	——
Csi-miRN33	Cs4g07210	3
Csi-miRN34	——	——
Csi-miRN35	——	——
Csi-miRN36	——	——
Csi-miRN37	orange1.1t02400	1
Csi-miRN38	——	——

c. Targets of miRNAs identified in fruit using degradome sequencing

miRNA	Target	Category
Csi-miR1092.2	Cs8g11330	3
Csi-miR1432a	——	——
Csi-miR1446	Cs2g01340	3
Csi-miR1507a.2	——	——
Csi-miR1515	——	——

Csi-miR156a.1	Cs2g23550	1
Csi-miR156a.2	Cs2g23550	1
Csi-miR156a.2	Cs7g11770	1
Csi-miR156a.2	Cs5g12260	1
Csi-miR156a.2	Cs3g10870	1
Csi-miR156a.2	Cs7g11770	1
Csi-miR156a.2	Cs5g12260	1
Csi-miR156a.2	Cs3g10870	1
Csi-miR156a.2	Cs2g17270	3
Csi-miR156a.2	orange1.1t01983	3
Csi-miR156a.2	orange1.1t01983	3
Csi-miR156b.1	Cs2g23550	1
Csi-miR156b.1	Cs7g11770	1
Csi-miR156b.1	Cs5g12260	1
Csi-miR156b.1	Cs3g10870	1
Csi-miR156b.1	orange1.1t01983	3
Csi-miR156c.1	Cs2g23550	1
Csi-miR156c.1	Cs7g11770	1
Csi-miR156c.1	Cs5g12260	1
Csi-miR156c.1	Cs3g10870	1
Csi-miR156c.1	Cs2g17270	3
Csi-miR156d	Cs2g23550	1
Csi-miR156d	Cs7g11770	1
Csi-miR156e	Cs2g23550	1
Csi-miR156e	Cs7g11770	1
Csi-miR156e	Cs4g07790	3
Csi-miR156f.2	Cs2g23550	1
Csi-miR156f.2	Cs7g11770	1
Csi-miR156f.2	Cs3g10870	1
Csi-miR156f.2	Cs5g12260	1
Csi-miR156f.2	Cs8g19900	2
Csi-miR156f.2	orange1.1t01983	3
Csi-miR156g.1	Cs2g23550	1
Csi-miR156g.1	Cs7g11770	1
Csi-miR156g.2	Cs2g23550	1
Csi-miR156g.2	Cs7g11770	1
Csi-miR156h	Cs2g23550	1
Csi-miR156h	Cs1g26030	1
Csi-miR156h	Cs7g11770	1
Csi-miR156h	Cs5g12260	1
Csi-miR156h	Cs3g10870	1
Csi-miR156h	orange1.1t01983	3
Csi-miR159	Cs8g05120	1
Csi-miR159	Cs3g10900	3
Csi-miR159	Cs6g10950	3

Csi-miR159b	——	——
Csi-miR160a-3p	——	——
Csi-miR160a-5p	——	——
Csi-miR160b-3p	——	——
Csi-miR160b-5p	——	——
Csi-miR160c.1	——	——
Csi-miR160c.2	——	——
Csi-miR162-3p.1	——	——
Csi-miR162-3p.2	Cs7g27400	3
Csi-miR164	Cs5g10870	1
Csi-miR164	Cs8g18140	3
Csi-miR166a.1	Cs8g16510	1
Csi-miR166a.2	Cs8g16510	1
Csi-miR166b	Cs8g16510	1
Csi-miR166c.1	Cs8g16510	1
Csi-miR166c.2	Cs8g16510	1
Csi-miR166c.2	Cs4g06030	2
Csi-miR166c.3	Cs8g16510	1
Csi-miR166c.4	Cs8g16510	1
Csi-miR166c.5	Cs8g16510	1
Csi-miR166d.1	Cs8g16510	1
Csi-miR166d.2	Cs8g16510	1
Csi-miR166g.1	Cs8g16510	1
Csi-miR166i	Cs8g16510	1
Csi-miR166j.1	Cs8g16510	1
Csi-miR166j.2	——	——
Csi-miR166j.3	Cs8g16510	1
Csi-miR166j.3	Cs4g04520	3
Csi-miR167a.1	Cs2g15130	2
Csi-miR167a.1	Cs8g08320	3
Csi-miR167b.1	Cs2g15130	2
Csi-miR167b.1	Cs8g08320	3
Csi-miR167b.1	Cs2g09440	3
Csi-miR167b.2	——	——
Csi-miR167b.3	Cs2g15130	2
Csi-miR167b.3	Cs8g07440	3
Csi-miR167b.4	Cs2g15130	2
Csi-miR167b.4	Cs8g08320	3
Csi-miR167d.1	Cs8g08320	3
Csi-miR167d.2	Cs2g15130	2
Csi-miR167d.2	Cs8g08320	3
Csi-miR168a	Cs5g05510	1
Csi-miR168a	Cs5g16710	2
Csi-miR169b.1	Cs7g27875	1
Csi-miR169b.1	Cs6g13560	2

Csi-miR169b.1-3p	Cs2g28180	2
Csi-miR169b.1-3p	Cs9g16380	3
Csi-miR169i.1	Cs6g13560	2
Csi-miR169i.1-3p	Cs4g06620	3
Csi-miR169i.1-3p	Cs7g12040	3
Csi-miR169m.1	Cs6g13560	2
Csi-miR169m.1	Cs8g17280	3
Csi-miR169m.2	——	——
Csi-miR169m.3	——	——
Csi-miR171a.1	——	——
Csi-miR171b	——	——
Csi-miR171d	——	——
Csi-miR171g.1	orange1.1t00199	1
Csi-miR171g.1	orange1.1t00200	1
Csi-miR172a-3p.1	Cs2g04660	2
Csi-miR172a-3p.1	Cs7g02590	3
Csi-miR172a-3p.1	orange1.1t04055	3
Csi-miR172a-3p.2	Cs2g04660	2
Csi-miR172a-3p.2	orange1.1t04055	3
Csi-miR172a-3p.3	Cs2g04660	2
Csi-miR172a-3p.3	orange1.1t04055	3
Csi-miR172a-3p.3	Cs2g21180	3
Csi-miR172c.1	Cs2g21180	3
Csi-miR172c.1	Cs3g06140	1
Csi-miR172c.1	Cs2g04660	2
Csi-miR172c.1	Cs7g19400	3
Csi-miR172c.1	orange1.1t04055	3
Csi-miR172c.1	Cs1g21180	3
Csi-miR172c.1	Cs7g32470	3
Csi-miR172c.1	orange1.1t00903	3
Csi-miR172c.2	orange1.1t04055	3
Csi-miR172d	orange1.1t04055	3
Csi-miR172d-3p	Cs9g03090	2
Csi-miR172d-3p	Cs5g08980	3
Csi-miR172d-3p	Cs7g07030	3
Csi-miR172d-3p	Cs5g21000	3
Csi-miR172e.2	Cs6g08190	2
Csi-miR172e.2	orange1.1t04055	3
Csi-miR172e.2	Cs1g23980	3
Csi-miR172k	Cs4g17900	1
Csi-miR172k	orange1.1t04055	3
Csi-miR172k	orange1.1t00603	3
Csi-miR2111	——	——
Csi-miR2118.1	orange1.1t00557	2
Csi-miR2118.2	orange1.1t01829	1

Csi-miR2118.2	Cs5g19440	1
Csi-miR2118.2	Cs3g12720	1
Csi-miR2118.2	Cs3g12760	1
Csi-miR2118.2	Cs3g12850	1
Csi-miR2118.2	Cs3g13390	1
Csi-miR2118.2	orange1.1t02518	1
Csi-miR2118.2	Cs3g13320	1
Csi-miR2118.2	orange1.1t00557	2
Csi-miR2118.2	Cs3g13340	2
Csi-miR2118.2	Cs3g13740	2
Csi-miR2275a	——	——
Csi-miR2275b	——	——
Csi-miR2275c.1	——	——
Csi-miR2275d	——	——
Csi-miR2275d-3p	——	——
Csi-miR2275e	——	——
Csi-miR2275f	——	——
Csi-miR2911	——	——
Csi-miR319	——	——
Csi-miR319a	——	——
Csi-miR319b	——	——
Csi-miR390.1	Cs9g01780	2
Csi-miR390.2	Cs9g01780	2
Csi-miR390-3p.1	——	——
Csi-miR390-3p.2	——	——
Csi-miR390b	Cs9g01780	2
Csi-miR391a.1	Cs5g17560	2
Csi-miR391a.2	——	——
Csi-miR391a.3	——	——
Csi-miR391b	——	——
Csi-miR393a.1	——	——
Csi-miR393a.2	——	——
Csi-miR393a.2-3p	orange1.1t01772	3
Csi-miR393a.3	——	——
Csi-miR393b	——	——
Csi-miR393b-3p	——	——
Csi-miR394	orange1.1t03542	3
Csi-miR394-3p.1	Cs3g09210	3
Csi-miR394-3p.2	Cs8g14600	3
Csi-miR394-3p.3	——	——
Csi-miR395.1	——	——
Csi-miR395.2	——	——
Csi-miR3951	orange1.1t05622	2
Csi-miR3951	Cs1g06060	2
Csi-miR3951-3p	Cs6g09300	3

Csi-miR3952.1	Cs8g13286	3
Csi-miR3952.2	Cs8g13286	3
Csi-miR3952-3p	Cs8g13286	3
Csi-miR3954a	Cs5g04670	2
Csi-miR3954a	Cs1g09600	3
Csi-miR3954b	Cs5g04670	2
Csi-miR3954b	Cs2g28990	3
Csi-miR3954b	Cs1g09600	3
Csi-miR396a	Cs5g01380	1
Csi-miR396a	Cs7g15220	1
Csi-miR396a	Cs7g12930	1
Csi-miR396a	orange1.1t03122	2
Csi-miR396a	Cs5g19200	2
Csi-miR396a	Cs3g23180	3
Csi-miR396a	orange1.1t02254	3
Csi-miR396a	Cs3g07260	3
Csi-miR396b.1	Cs5g01380	1
Csi-miR396b.1	Cs7g15220	1
Csi-miR396b.1	Cs7g12930	1
Csi-miR396b.1	orange1.1t02555	1
Csi-miR396b.1	orange1.1t03122	2
Csi-miR396b.1	Cs5g19200	2
Csi-miR396b.1	Cs3g23180	3
Csi-miR396b.1	orange1.1t02254	3
Csi-miR396b.1	Cs3g07260	3
Csi-miR396b.2	Cs5g01380	1
Csi-miR396b.2	orange1.1t02555	1
Csi-miR396b.2	Cs7g15220	1
Csi-miR396b.2	Cs7g12930	1
Csi-miR396b.2	Cs4g07730	1
Csi-miR396b.2	orange1.1t03122	2
Csi-miR396b.2	Cs5g19200	2
Csi-miR396b.2	Cs7g23200	2
Csi-miR396b.2	Cs9g05650	2
Csi-miR396b.2	Cs3g23180	3
Csi-miR396b.2	orange1.1t02254	3
Csi-miR396b.2	Cs3g07260	3
Csi-miR396b.2	Cs9g19220	3
Csi-miR396b.2	Cs6g06540	3
Csi-miR396b.2	Cs6g04030	3
Csi-miR396b.2	Cs8g04510	3
Csi-miR396b.2	Cs8g17370	3
Csi-miR396b.2	Cs1g21890	3
Csi-miR396b.2	Cs5g23130	3
Csi-miR396b.2	Cs7g11670	3

Csi-miR396b.2	Cs5g05620	3
Csi-miR396b.2	orange1.1t01932	3
Csi-miR396b.2	Cs6g20570	3
Csi-miR396b.2	orange1.1t02489	3
Csi-miR396b.2	orange1.1t02492	3
Csi-miR396b.2	Cs2g08090	3
Csi-miR396b.2	Cs5g24150	3
Csi-miR396b.2	Cs9g17470	3
Csi-miR396b.2	Cs7g18780	3
Csi-miR396b.3	Cs5g01380	1
Csi-miR396b.3	orange1.1t02555	1
Csi-miR396b.3	Cs7g15220	1
Csi-miR396b.3	Cs7g12930	1
Csi-miR396b.3	Cs5g19200	2
Csi-miR396b.3	orange1.1t03122	2
Csi-miR396b.3	Cs3g23180	3
Csi-miR396b.3	Cs9g19220	3
Csi-miR396b.3	orange1.1t02254	3
Csi-miR396b-3p.1	——	——
Csi-miR396b-3p.2	Cs8g05290	2
Csi-miR396c	Cs5g07360	2
Csi-miR396c	Cs2g27200	3
Csi-miR396d.1	Cs5g01380	1
Csi-miR396d.1	orange1.1t02555	1
Csi-miR396d.1	Cs7g15220	1
Csi-miR396d.1	Cs7g12930	1
Csi-miR396d.1	Cs5g19200	2
Csi-miR396d.1	orange1.1t03122	2
Csi-miR396d.1	Cs4g12200	2
Csi-miR396d.1	Cs3g23180	3
Csi-miR396d.1	Cs9g19220	3
Csi-miR396d.2	Cs5g01380	1
Csi-miR396d.2	orange1.1t02555	1
Csi-miR396d.2	Cs7g15220	1
Csi-miR396d.2	Cs5g19200	2
Csi-miR396d.2	orange1.1t03122	2
Csi-miR396d.2	Cs3g23180	3
Csi-miR396d.2	Cs8g17370	3
Csi-miR396d.2	Cs9g19220	3
Csi-miR396d.2	Cs2g09620	3
Csi-miR396d.3	Cs5g01380	1
Csi-miR396d.3	orange1.1t02555	1
Csi-miR396d.3	Cs7g15220	1
Csi-miR396d.3	Cs7g12930	1
Csi-miR396d.3	orange1.1t03122	2

Csi-miR396d.3	Cs4g12200	2
Csi-miR396d.3	Cs9g19220	2
Csi-miR396d.3	Cs5g07120	2
Csi-miR396d.3	Cs1g15720	2
Csi-miR396d.3	Cs3g23180	3
Csi-miR396d.3	Cs5g19200	3
Csi-miR396d.3	Cs8g17370	3
Csi-miR396d.3	orange1.1t02254	3
Csi-miR396d.3	Cs8g15450	3
Csi-miR396d.3	Cs9g14020	3
Csi-miR396d.4	Cs5g01380	1
Csi-miR396d.4	orange1.1t02555	1
Csi-miR396d.4	Cs7g15220	1
Csi-miR396d.4	Cs7g12930	1
Csi-miR396d.4	orange1.1t03122	2
Csi-miR396d.4	Cs5g19200	2
Csi-miR396d.4	Cs1g15720	2
Csi-miR396d.4	Cs4g12200	2
Csi-miR396d.4	orange1.1t02739	2
Csi-miR396d.4	Cs5g07120	2
Csi-miR396d.4	Cs3g23180	3
Csi-miR396d.4	Cs8g17370	3
Csi-miR396d.4	Cs6g06560	3
Csi-miR396d.4	orange1.1t02254	3
Csi-miR396d.4	Cs9g14020	3
Csi-miR396d.4	Cs3g07260	3
Csi-miR396d.4	Cs9g19220	3
Csi-miR396d.4	Cs8g15450	3
Csi-miR396d.4	Cs6g06540	3
Csi-miR396d.4	Cs4g18150	3
Csi-miR396d.4	Cs6g04030	3
Csi-miR396d.4	Cs1g16550	3
Csi-miR396d-3p	Cs8g05290	2
Csi-miR397.1	——	——
Csi-miR397.2	——	——
Csi-miR398b	Cs1g13240	1
Csi-miR399a	——	——
Csi-miR399b	——	——
Csi-miR399c	——	——
Csi-miR399d	Cs4g08850	3
Csi-miR399e	——	——
Csi-miR399e-5p	orange1.1t03762	3
Csi-miR403.1	——	——
Csi-miR403.2	——	——
Csi-miR403.3	Cs9g07320	3

Csi-miR403.3	Cs8g07240	3
Csi-miR403.3	Cs3g01650	3
Csi-miR408.1	——	——
Csi-miR408.2	——	——
Csi-miR4369	——	——
Csi-miR4414.1	——	——
Csi-miR4414.2	——	——
Csi-miR443b.1	——	——
Csi-miR443b.2	——	——
Csi-miR444a.1	orange1.1t01329	2
Csi-miR444a.2	Cs8g15380	3
Csi-miR472	Cs2g30590	1
Csi-miR472	Cs1g13430	1
Csi-miR472	orange1.1t01918	1
Csi-miR473	——	——
Csi-miR473-3p	——	——
Csi-miR477a.1	——	——
Csi-miR477a.2	——	——
Csi-miR477a-3p	Cs6g19680	3
Csi-miR477b.2	Cs3g10900	3
Csi-miR477d.1-3p	——	——
Csi-miR477d.2-5p	Cs3g10900	3
Csi-miR479.1	——	——
Csi-miR479.2	——	——
Csi-miR482a-3p	orange1.1t01829	1
Csi-miR482a-3p	Cs5g19440	1
Csi-miR482a-3p	Cs1g15550	1
Csi-miR482a-3p	orange1.1t01918	1
Csi-miR482a-3p	Cs3g13390	1
Csi-miR482a-3p	orange1.1t02518	1
Csi-miR482a-3p	Cs3g13320	1
Csi-miR482a-3p	Cs3g13340	2
Csi-miR482a-3p	Cs3g13740	2
Csi-miR482a-5p	——	——
Csi-miR482b	Cs1g13430	1
Csi-miR482c	——	——
Csi-miR482d	Cs5g21975	3
Csi-miR482d-5p.1	Cs1g02710	3
Csi-miR482d-5p.2	——	——
Csi-miR5054.1	——	——
Csi-miR5054.2	Cs2g09030	2
Csi-miR5177	——	——
Csi-miR5179	——	——
Csi-miR5225a	——	——
Csi-miR5291a	——	——

Csi-miR530a	——	——
Csi-miR530b	Cs4g14880	3
Csi-miR530b	Cs6g11650	3
Csi-miR535.1	——	——
Csi-miR535.2	Cs7g11770	1
Csi-miR535.2	orange1.1t04075	3
Csi-miR536-3p	——	——
Csi-miR536-5p	——	——
Csi-miR814	——	——
Csi-miR827.1	Cs5g10180	3
Csi-miR827.2	Cs5g10180	3
Csi-miR827-5p.1	Cs1g24800	3
Csi-miR827-5p.2	Cs8g15030	2
Csi-miR828	orange1.1t00180	3
Csi-miR833.1	Cs9g02420	2
Csi-miR833.1	Cs5g27500	2
Csi-miR845a	——	——
Csi-miR896	——	——
Csi-miRN01	Cs8g10430	2
Csi-miRN02	Cs3g18790	1
Csi-miRN02	Cs8g09160	3
Csi-miRN03	orange1.1t02280	3
Csi-miRN04	Cs2g04030	3
Csi-miRN05	——	——
Csi-miRN06	——	——
Csi-miRN07	Cs6g11600	3
Csi-miRN07	Cs1g18270	3
Csi-miRN08	——	——
Csi-miRN09	——	——
Csi-miRN10	Cs4g05310	3
Csi-miRN11	Cs8g13560	1
Csi-miRN12	——	——
Csi-miRN13	——	——
Csi-miRN14	Cs7g14990	3
Csi-miRN15	——	——
Csi-miRN16	——	——
Csi-miRN17	Cs5g04990	3
Csi-miRN18	——	——
Csi-miRN19	Cs5g32800	3
Csi-miRN20	Cs3g05320	1
Csi-miRN20	Cs1g12230	1
Csi-miRN21	——	——
Csi-miRN22	——	——
Csi-miRN23	Cs1g07330	3
Csi-miRN24	Cs1g01190	3

Csi-miRN25	——	——
Csi-miRN26	——	——
Csi-miRN27	Cs4g18880	3
Csi-miRN28	Cs4g15010	2
Csi-miRN29	——	——
Csi-miRN30	——	——
Csi-miRN31	——	——
Csi-miRN32	——	——
Csi-miRN33	——	——
Csi-miRN34	——	——
Csi-miRN35	——	——
Csi-miRN36	——	——
Csi-miRN37	Cs3g18880	2
Csi-miRN37	Cs7g08960	3
Csi-miRN38	——	——
