# Supplementary material for: Genome-wide comparison of microRNAs and their targeted transcripts among leaf, flower and fruit of sweet orange
Source: BMC Genomics. 2014 Aug 20;15(1):695. doi: 10.1186/1471-2164-15-695 (PMC4158063; doi:10.1186/1471-2164-15-695)
Supplement: Supplementary file 5 — Additional file 5: T-plots of diverse targets of miRNAs in different tissues. Densities of the 5’ positions of degradome tags matching each target gene are shown as T-plots. The miRNA-mediated degradome tag is highlighted in red. (a) miR168a targets AGO1 in all three tissues; additionally, miR168a targets CUC2 in leaf and a pantothenate kinase gene in fruit; (b) miR159 targets GAMYB in leaf and flower; but it targets DRT100 in fruit; (c) miRN10 targets different transcripts in flower and fruit. (PDF 552 KB) [file 12864_2014_6413_MOESM5_ESM.pdf]

Additional file 5. T-plots of diverse targets of miRNAs in different tissues. Densities of the 5' positions of degradome tags matching each target gene are shown as T-plots. The miRNA-mediated degradome tag is highlighted in red. (a) miR168a targets AGO1 in all three tissues; additionally, miR168a targets CUC2 in leaf and a pantothenate kinase gene in fruit; (b) miR159 targets GAMYB in leaf and flower; but it targets DRT100 in fruit; (c) miRN10 targets different transcripts in flower and fruit.

# (a)miR168a

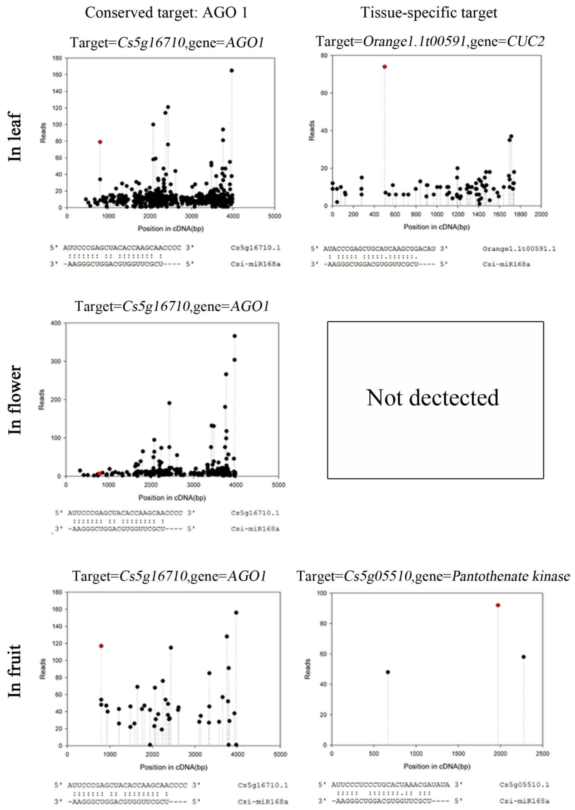

Conserved target: AGO 1
Tissue-specific target
In leaf
In flower
In fruit
Target=Cs5g16710,gene=AGO1
Target=Orange1.1t00591,gene=CUC2
Target=Cs5g16710,gene=AGO1
Not dectected
Target=Cs5g16710,gene=AGO1
Target=Cs5g05510,gene=Pantothenate kinase
Reads
Position in cDNA(bp)
5' AUUCCCGAGCUACACCAAGCAACCCC 3' Cs5g16710.1
3' -AAGGGCUGGACGUGGUUCGCU---- 5' Csi-miR168a
5' AUACCCGAGCUGCAUCAAGCGGACAU 3' Orange1.1t00591.1
3' -AAGGGCUGGACGUGGUUCGCU---- 5' Csi-miR168a
5' AUUCCCUCCCUGCACUAAACGAUAUA 3' Cs5g05510.1
3' -AAGGGCUGGACGUGGUUCGCU---- 5' Csi-miR168a

## (b)miR159

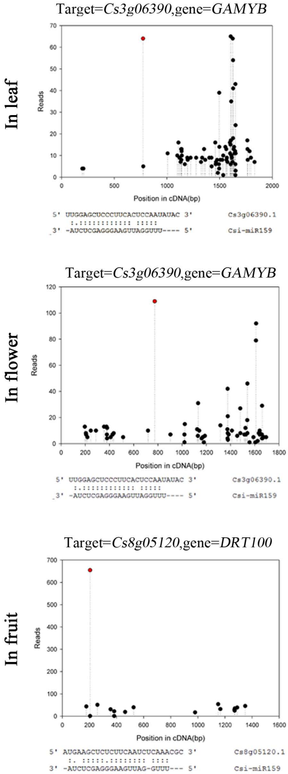
Target=*Cs3g06390*,gene=*GAMYB*
In leaf
Reads
Position in cDNA(bp)
5' UUGGAGCUCCCUUCACUCCAAUAUAC 3'
:.::::::::::::: :::::
3' -AUCUCGAGGGAAGUUAGGUUU---- 5'
Cs3g06390.1
Csi-miR159
Target=*Cs3g06390*,gene=*GAMYB*
In flower
Reads
Position in cDNA(bp)
5' UUGGAGCUCCCUUCACUCCAAUAUAC 3'
:.::::::::::::: :::::
3' -AUCUCGAGGGAAGUUAGGUUU---- 5'
Cs3g06390.1
Csi-miR159
Target=*Cs8g05120*,gene=*DRT100*
In fruit
Reads
Position in cDNA(bp)
5' AUGAAGCUCUCUUCAAUCUCAAACGC 3'
:. :::::.::::::::: ::::
3' -AUCUCGAGGGAAGUUAG-GUUU--- 5'
Cs8g05120.1
Csi-miR159

(c)miRN10

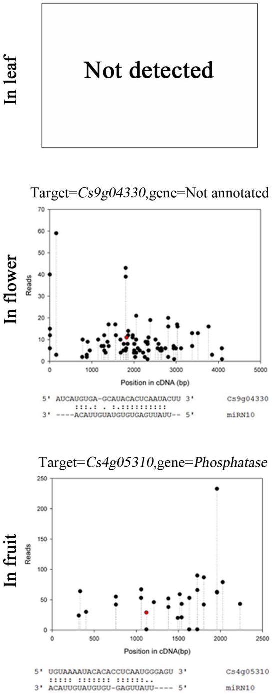
In leaf
Not detected
In flower
Target=Cs9g04330,gene=Not annotated
Reads
0
10
20
30
40
50
60
70
0
1000
2000
3000
4000
5000
Position in cDNA (bp)
5' AUCAUGUGA-GCAUACACUCAAUACUU 3'
::::.: . :.:::::::::::
3' ----ACAUUGUAUGUGUGAGUUAUU-- 5'
Cs9g04330
miRN10
In fruit
Target=Cs4g05310,gene=Phosphatase
Reads
0
50
100
150
200
250
0
500
1000
1500
2000
2500
Position in cDNA(bp)
5' UGUAAAAUACACACCUCAAUGGGAGU 3'
::::: ::::::: :::::::..
3' ACAUUGUAUGUGU-GAGUUAUU---- 5'
Cs4g05310
miRN10
