# Supplementary material for: Genome-wide comparison of microRNAs and their targeted transcripts among leaf, flower and fruit of sweet orange
Source: BMC Genomics. 2014 Aug 20;15(1):695. doi: 10.1186/1471-2164-15-695 (PMC4158063; doi:10.1186/1471-2164-15-695)
Supplement: Supplementary file 6 — Additional file 6: Annotations of all target genes. (PDF 153 KB) [file 12864_2014_6413_MOESM6_ESM.pdf]

Additional file 6: Annotations of all miRNA target genes in sweet orange

Target gene	Annotation
orange1.1t05622	Polyubiquitin 3; Polyubiquitin-B; Ubiquitin-60S ribosomal protein L40; Polyubiquitin-C; Polyubiquitin (Fragment); Polyubiquitin-A; Polyubiquitin-G; Polyubiquitin-D; Polyubiquitin-F; Polyubiquitin-H;Polyubiquitin containing 7 ubiquitin monomers
orange1.1t04055	Floral homeotic protein APETALA2, putative (Fragment);Floral homeotic protein APETALA 2; AP2-like ethylene-responsive transcription factor TOE3; Ethylene-responsive transcription factor RAP2-7; AP2-like ethylene-responsive transcription factor ANT; AP2-like ethylene-responsive transcription factor BBM; AP2-like ethylene-responsive transcription factor SMZ; AP2-like ethylene-responsive transcription factor SNZ; AP2/ERF and B3 domain-containing protein Os05g0549800; Ethylene-responsive transcription factor SHINE 3; AP2/ERF and B3 domain-containing transcription factor ARF14; Pathogenesis-related genes transcriptional activator PTI6; AP2/ERF and B3 domain-containing transcription repressor RAV2
orange1.1t03122	Putative uncharacterized protein At3g13960 (Fragment)
orange1.1t02555	Putative uncharacterized protein Sb01g009330
orange1.1t02428	Transcription factor TCP11;Proliferating cell nuclear antigen gene-controlling element binding factor (Fragment)
orange1.1t02400	Indole-3-acetic acid-induced protein ARG7, putative;Auxin-induced protein 6B
orange1.1t02367	Transcription factor bHLH62; Transcription factor BPE; Transcription factor BEE 2; Transcription factor ALC; Transcription factor SPATULA;Putative uncharacterized protein Sb02g027430
orange1.1t02313	Pentatricopeptide repeat-containing protein, putative;Pentatricopeptide repeat-containing protein At2g41720; Putative pentatricopeptide repeat-containing protein At1g13800
orange1.1t02213	Putative uncharacterized protein At2g41950/T6D20.6
orange1.1t02003	Putative phi-1-like phosphate-induced protein
orange1.1t01708	Putative uncharacterized protein Sb03g000730;Probable ubiquitin-conjugating enzyme E2 23; Baculoviral IAP repeat-containing protein 6; Probable ubiquitin-conjugating enzyme protein 17; Ubiquitin-conjugating enzyme E2 O; Ubiquitin-conjugating enzyme E2 4; Ubiquitin-conjugating enzyme E2-17 kDa; SUMO-conjugating enzyme UBC9; Ubiquitin-conjugating enzyme E2 T; Ubiquitin-conjugating enzyme E2 Z
orange1.1t01536	Beta-fructofuranosidase, putative, expressed
orange1.1t00584	Endoribonuclease Dicer homolog 1; Dicer-like protein 4; Endoribonuclease Dicer; Endoribonuclease Dcr-1; Ribonuclease 3-like protein 3; Protein Dicer;Helicase, C-terminal; Argonaute and Dicer protein, PAZ; Ribonuclease III,
orange1.1t00471	Hydroquinone glucosyltransferase; UDP-glycosyltransferase 72B1; Anthocyanidin 3-O-glucosyltransferase 5; Baicalein 7-O-glucuronosyltransferase; Anthocyanidin 3-O-glucosyltransferase 2 (Fragment); Zeatin O-glucosyltransferase; Zeatin O-xylosyltransferase;UDP-glucoronosyl/UDP-glucosyl transferase family protein
orange1.1t00423	Proline iminopeptidase;Prolyl aminopeptidase 2. Serine peptidase. MEROPS family S33
orange1.1t00200	Scarecrow-like protein 6; DELLA protein RGA1; DELLA protein RGA;Scarecrow transcription factor family protein
orange1.1t00199	Scarecrow-like protein 6;GRAS family transcription factor containing protein, expressed

orange1.1t00180	T-complex protein 1 subunit alpha (Fragment);T-complex protein 1 subunit alpha; Thermosome subunit beta; Thermosome subunit alpha; Thermosome subunit; Thermosome subunit beta (Fragment); T-complex protein 1 subunit
orange1.1t00149	Chromosome undetermined scaffold_137, whole genome shotgun sequence;Protein bem46; Abhydrolase domain-containing protein 13; Uncharacterized membrane protein YNL320W; Uncharacterized protein Rv2307c/MT2364; Uncharacterized protein yfhR
Cs9g19410	Scarecrow-like protein 8; Scarecrow-like transcription factor PAT1; Chitin-inducible gibberellin-responsive protein 2; DELLA protein RGL2; DELLA protein GAI; DELLA protein GAIP-B; Protein SCARECROW 1;Chitin-inducible gibberellin-responsive protein, putative
Cs9g18000	Putative uncharacterized protein Sb10g020830
Cs9g16380	Protein kinase domain containing protein, expressed;Receptor-like serine/threonine-protein kinase At4g25390; Putative receptor-like protein kinase At1g80870; Proline-rich receptor-like protein kinase PERK7; Serine/threonine-protein kinase At3g07070; Nodulation receptor kinase; Probable LRR receptor-like serine/threonine-protein kinase At1g06840; Putative leucine-rich repeat receptor-like protein kinase At2g19210; Putative leucine-rich repeat receptor-like serine/threonine-protein kinase At2g14440; Leucine-rich repeat receptor-like serine/threonine-protein kinase At2g14510; G-type lectin S-receptor-like serine/threonine-protein kinase At1g34300; Senescence-induced receptor-like serine/threonine-protein kinase; Probable leucine-rich repeat receptor-like serine/threonine-protein kinase At5g15730; Probable LRR receptor-like protein kinase At1g51890; Probable receptor-like protein kinase At1g49730; Receptor-like protein kinase At5g59670; Putative proline-rich receptor-like protein kinase PERK11; U-box domain-contalike 2; Leucine-rich repeat receptor protein kinase EXS; Somatic embryogenesis receptor kinase 4;ERL1a AtERECTA-like receptor [illegible]
Cs9g06430	SNF2 family ATP-dependent chromatin-remodeling factor snf21;Probable global transcription activator SNF2L2; Transcription activator BRG1; ATP-dependent helicase brm; Transcription regulatory protein SNF2; Chromatin structure-remodeling complex subunit snf21; Nuclear protein STH1/NPS1; SWI/SNF chromatin-remodeling complex subunit snf22; ATP-dependent helicase BRM; Chromatin-remodeling complex ATPase chain Iswi; Putative chromatin-remodeling complex ATPase chain; Probable chromatin-remodeling complex ATPase chain; ISWI chromatin-remodeling complex ATPase ISW2; SWI/SNF-related matrix-associated actin-dependent regulator of chromatin subfamily A member 5; Chromatin-remodeling complex ATPase chain isw-1; Chromo domain-containing protein 1; Chromodomain-helicase-DNA-binding protein 2; Chromodomain helicase hrp1; ATP-dependent DNA helicase DDM1; Lymphoid-specific helicase; Chromodomain-helicase-DNA-binding protein 3 [illegible]
Cs9g05070	3-dehydroquinate dehydratase/shikimate 5-dehydrogenase (Fragment);Bifunctional 3-dehydroquinate dehydratase/shikimate dehydrogenase, chloroplastic; Pentafunctional AROM polypeptide; Shikimate dehydrogenase
Cs9g04330	Putative uncharacterized protein Sb03g013910;Kinesin-like protein KIF2C; Kinesin-related protein 6; Diatom spindle kinesin 1; Chromosome-associated kinesin KIF4; Osmotic avoidance abnormal protein 3; Kinesin-II 95 kDa subunit
Cs9g04080	Putative uncharacterized protein (Fragment);Epoxide hydrolase 2; Soluble epoxide hydrolase; Fluoroacetate dehalogenase; Haloalkane dehalogenase; Probable oxidoreductase ephD; Abhydrolase domain-containing protein 8; Non-haem bromoperoxidase BPO-A2; Arylesterase; Non-heme chloroperoxidase
Cs9g03090	Armadillo/beta-catenin-like repeat-containing protein;Importin-5; Ran-binding protein 6; Importin subunit beta-3

Cs9g02420	DNA-directed RNA polymerase subunit (Fragment);DNA-directed RNA polymerase II subunit RPB9; DNA-directed RNA polymerase subunit M; DNA-directed RNA polymerase III subunit RPC10
Cs8g19900	ATP-dependent zinc metalloprotease FTSH 7, chloroplastic; ATP-dependent zinc metalloprotease FTSH, chloroplastic (Fragment); ATP-dependent zinc metalloprotease FtsH;Putative cell division protein FtsH3 [Oryza sativa (ISS)
Cs8g17390	Floral homeotic protein APETALA2, putative, expressed;Ethylene-responsive transcription factor RAP2-7; Floral homeotic protein APETALA 2; AP2-like ethylene-responsive transcription factor TOE3; AP2-like ethylene-responsive transcription factor ANT; AP2-like ethylene-responsive transcription factor BBM; AP2-like ethylene-responsive transcription factor SMZ; AP2-like ethylene-responsive transcription factor SNZ; AP2/ERF and B3 domain-containing transcription factor ARF14; AP2/ERF and B3 domain-containing protein Os05g0549800; Ethylene-responsive transcription factor SHINE 3; AP2/ERF and B3 domain-containing transcription repressor RAV2; Pathogenesis-related genes transcriptional activator PTI6
Cs8g17370	Putative uncharacterized protein Sb02g044060;L-ascorbate peroxidase 1, cytosolic; L-ascorbate peroxidase, cytosolic; L-ascorbate peroxidase 3, peroxisomal; Probable L-ascorbate peroxidase 4; Cytochrome c peroxidase, mitochondrial; Putative heme-binding peroxidase; Putative cytochrome c peroxidase, mitochondrial; L-ascorbate peroxidase T, chloroplastic; Probable L-ascorbate peroxidase 7, chloroplastic; L-ascorbate peroxidase S, chloroplastic/mitochondrial; Putative L-ascorbate peroxidase 6; Catalase-peroxidase
Cs8g17030	Legumin B; Legumin B (Fragment); Legumin A; Legumin K (Fragment);Putative vicilin storage protein (Globulin-like)
Cs8g16510	Homeobox-leucine zipper protein REVOLUTA; Homeobox-leucine zipper protein HOX9; Homeobox-leucine zipper protein PROTODERMAL FACTOR 2; Homeobox-leucine zipper protein MERISTEM L1; Homeobox-leucine zipper protein ANTHOCYANINLESS 2;Class III homeodomain-leucine zipper protein C3HDZ1
Cs8g14600	Putative uncharacterized protein Sb0067s002250
Cs8g11330	Putative uncharacterized protein;Probable pectate lyase 1; Putative pectate lyase 14; Pectate lyase; Major pollen allergen Jun a 1; Sugi basic protein; Major pollen allergen Cha o 1; Major pollen allergen Jun v 1; Pollen allergen Amb a 1.1; Major pollen allergen Cup a 1; Pectate trisaccharide-lyase; Pectate lyase B
Cs8g09620	SRF-type transcription factor family protein, expressed;Agamous-like MADS-box protein AGL61; MADS-box transcription factor 57; MADS-box protein AGL24; MADS-box protein JOINTLESS; Floral homeotic protein AGAMOUS
Cs8g08320	Cysteine proteinase RD19a; Probable cysteine proteinase A494; Cathepsin L; Putative cysteine proteinase CG12163; Cruzipain; Viral cathepsin; Thiol protease aleurain; Thiol protease aleurain-like; Cathepsin F; Germination-specific cysteine protease 1; Cysteine proteinase B; Xylem cysteine proteinase 2; Cathepsin L1; Oryzain alpha chain; Cysteine proteinase A; Cysteine proteinase COT44 (Fragment); Pro-cathepsin H; Cathepsin W; Digestive cysteine proteinase 1; Crustapain;Papain-like cysteine proteinase-like protein 1
Cs8g07240	Putative uncharacterized protein Sb03g036900;40S ribosomal protein S25-2
Cs8g05290	UDP-glucose: chalcononaringenin 2'-O-glucosyltransferase;UDP-glycosyltransferase 73B3; UDP-glucosyl transferase 73B2; Anthocyanin 3'-O-beta-glucosyltransferase; Abscisate beta-glucosyltransferase; Anthocyanidin 3-O-glucosyltransferase 4 (Fragment); Hydroquinone glucosyltransferase
Cs8g04530	Putative uncharacterized protein Sb01g014910

Cs8g02270.2	Putative uncharacterized protein Sb01g030180;Probable protein phosphatase 2C 60; Protein phosphatase 2C 35
Cs7g31800	Ribulose bisphosphate carboxylase/oxygenase activase 1, chloroplastic; Ribulose bisphosphate carboxylase/oxygenase activase, chloroplastic; Ribulose bisphosphate carboxylase/oxygenase activase A, chloroplastic; Ribulose bisphosphate carboxylase/oxygenase activase B, chloroplastic; Ribulose bisphosphate carboxylase/oxygenase activase; Ribulose bisphosphate carboxylase/oxygenase activase, chloroplastic (Fragments);Chloroplast ribulose-1,5-bisphosphate carboxylase/oxygenase activase large protein isoform
Cs7g26730	TMV resistance protein N; Putative disease resistance protein At4g11170; Probable WRKY transcription factor 19; Protein SUPPRESSOR OF npr1-1, CONSTITUTIVE 1; Protein DA1-related 4; Protein PHLOEM PROTEIN 2-LIKE A5; Vesicle-associated protein 1-4; Protein popC; Leucine-rich repeat-containing protein 7; Leucine-rich repeat and IQ domain-containing protein 4; Leucine-rich repeat protein soc-2 homolog; Malignant fibrous histiocytoma-amplified sequence 1; Leucine-rich repeat and death domain-containing protein 1;Leucine-rich repeat containing protein, putative
Cs7g24200	Syntaxin-22; Syntaxin-23; Syntaxin-21; Putative syntaxin-24; Syntaxin-7;Putative uncharacterized protein Sb03g009820
Cs7g15220	Putative uncharacterized protein At3g13960 (Fragment)
Cs7g14990	Armadillo/beta-catenin-like repeat-containing protein
Cs7g09520	cDNA, clone: J100060F14, full insert sequence
Cs7g07710	DNA topoisomerase 1; DNA topoisomerase 1 type prokaryotic;DNA topoisomerase, type IA, core
Cs7g06555	Whole genome shotgun assembly, reference scaffold set, scaffold scaffold_18;Enhancer of rudimentary homolog; Protein enhancer of rudimentary
Cs7g06000	Aspartate aminotransferase (Precursor);Aspartate aminotransferase, chloroplastic; Aspartate aminotransferase P2, mitochondrial (Fragment); Aspartate aminotransferase, cytoplasmic; Aspartate aminotransferase 1; Aspartate aminotransferase, cytoplasmic isozyme 1; Aspartate aminotransferase, mitochondrial; Probable aspartate aminotransferase, cytoplasmic; Aromatic-amino-acid aminotransferase; Tyrosine aminotransferase; Homogentisate phytyltransferase 1, chloroplastic; Probable homogentisate phytyltransferase 1, chloroplastic; Putative aspartate aminotransferase, cytoplasmic 2
Cs6g21570	Transmembrane emp24 domain-containing protein 10, putative
Cs6g15330	Putative uncharacterized protein Sb01g009330
Cs6g13560	Nuclear transcription factor Y subunit A-10; Transcriptional activator HAP2; Nuclear transcription factor Y subunit alpha;CCAAT-binding transcription factor subunit B family protein, expressed
Cs6g12050	Protein kinase domain containing protein, expressed;Probable receptor-like protein kinase At5g47070; Receptor-like serine/threonine-protein kinase At3g01300; Protein kinase 2A, chloroplastic; Serine/threonine-protein kinase At5g01020; Probable serine/threonine-protein kinase NAK; Probable serine/threonine-protein kinase RLCKVII; Probable serine/threonine-protein kinase Cx32, chloroplastic; Putative receptor-like protein kinase At1g72540; Proline-rich receptor-like protein kinase PERK13; Putative proline-rich receptor-like protein kinase PERK11; Probable receptor-like serine/threonine-protein kinase At4g34500; Probable serine/threonine-protein kinase At1g01540; Receptor-like protein kinase FERONIA; Probable LRR receptor-like serine/threonine-protein kinase At1g51810; Nodulation receptor kinase; Somatic embryogenesis receptor kinase 2; Receptor-like protein kinase At3g21340; BRASSINOSTEROID INSENSITIVE 1-associated receptor kinase 1

Cs6g11650	V-type proton ATPase subunit G; V-type proton ATPase subunit G1;Vacuolar (H+)-ATPase G subunit; KH, prokaryotic type
Cs6g10170	Similar to Nuclear protein SkiP (Ski-interacting protein) (Predicted);SNW domain-containing protein 1; Puff-specific protein Bx42; Pre-mRNA-processing protein 45; Uncharacterized protein T27F2.1; Protein snwA
Cs6g07450	Laccase-7; Laccase-9; Laccase-8; Laccase-12; Laccase-24; Laccase-3; Laccase-14; Laccase-25; Laccase-5; Laccase-6; Putative laccase-17; Laccase-23; Laccase-13; Laccase-12/13; Laccase-4; Laccase-2; Laccase-10; Laccase-17; Laccase-16; Laccase-11; Laccase-1; Laccase-19; Laccase-15; Laccase-22; Laccase-20; Laccase-18; Laccase-21; L-ascorbate oxidase;Putative uncharacterized protein Sb03g038550
Cs6g07410	Laccase-7; Laccase-9; Laccase-8; Laccase-24; Laccase-12; Laccase-14; Laccase-3; Laccase-25; Laccase-5; Laccase-6; Laccase-23; Putative laccase-17; Laccase-12/13; Laccase-13; Laccase-4; Laccase-2; Laccase-11; Laccase-10; Laccase-17; Laccase-16; Laccase-1; Laccase-19; Laccase-15; Laccase-22; Laccase-20; Laccase-18; Laccase-21; L-ascorbate oxidase;Putative uncharacterized protein Sb03g038550
Cs6g02740	Catalytic protein serine threonine phosphatase;Probable protein phosphatase 2C 33; Protein phosphatase 2C 3; Protein phosphatase 1K, mitochondrial
Cs5g34330	Putative uncharacterized protein (Fragment)
Cs5g32800	Glutathione S-transferase; Glutathione S-transferase PARB; Glutathione S-transferase APIC; Glutathione S-transferase F8, chloroplastic; Probable glutathione S-transferase GSTF1; Probable elongation factor 1-gamma 1; Protein gstA; Glutathione S-transferase 2 (Fragment); Maleylacetoacetate isomerase; Glutathione S-transferase zeta class;Putative glutathione-s-transferase theta (Fragment)
Cs5g31745	LSM2 homolog, U6 small nuclear RNA associated (S. cerevisiae), isoform CRA_b;U6 snRNA-associated Sm-like protein LSm2; Probable U6 snRNA-associated Sm-like protein LSm2; U7 snRNA-associated Sm-like protein LSm10
Cs5g31220	Putative uncharacterized protein At2g26310/T1D16.5
Cs5g29630	Dolichyl-diphosphooligosaccharide--protein glycosyltransferase, putative, expressed
Cs5g26760	Tubulin alpha-5 chain-like protein;Tubulin alpha-3 chain; Tubulin alpha chain
Cs5g24810	Chromosome region maintenance protein 1/exportin, putative;Exportin-1; Exportin-5
Cs5g17560	Regulator of nonsense transcripts 3A;Chromosome 2 SCAF14990, whole genome shotgun sequence. (Fragment)
Cs5g16710	Protein argonaute 1; Protein argonaute-3; Protein argonaute-2 (Fragment); Putative protein tag-76;Eukaryotic translation initiation factor 2c, putative
Cs5g10870	NAC domain-containing protein 100; Protein CUP-SHAPED COTYLEDON 2; Protein BEARSKIN1; Protein SOMBRERO; Putative NAC domain-containing protein 94; Protein FEZ;NAC domain-containing protein 21/22, putative
Cs5g09850	Growth-regulating factor, putative,expressed (Fragment)
Cs5g08980	Scarecrow-like protein 6;GRAS family transcription factor containing protein, expressed
Cs4g19940	Beta-adaptin-like protein A; AP-4 complex subunit beta; AP-4 complex subunit beta-1; AP-2 complex subunit beta; AP-1 complex subunit beta-1; Beta-adaptin-like protein C; AP-1 complex subunit beta; Beta-adaptin-like protein B;Singapore isolate B (sub-type 7) whole genome shotgun sequence assembly,

Cs4g14480	Chloroplastic group IIA intron splicing facilitator CRS1, chloroplastic; Uncharacterized CRM domain-containing protein At3g25440, chloroplastic; CRS2-associated factor 1, chloroplastic; CRS2-associated factor 1, mitochondrial;Putative uncharacterized protein OSJNBa0079H23.10
Cs4g12820	DEAD (Asp-Glu-Ala-Asp) box polypeptide 47 isoform 1 variant (Fragment);DEAD-box ATP-dependent RNA helicase 10; Probable ATP-dependent RNA helicase DDX47; Putative ATP-dependent RNA helicase T26G10.1; ATP-dependent rRNA helicase rrp3; ATP-dependent RNA helicase DBP8
Cs4g09320	Putative uncharacterized protein Sb03g002730
Cs4g07210	MAP kinase-activating protein C22orf5, putative, expressed;Transmembrane protein 184C; Transmembrane protein 184 homolog DDB_G0279555
Cs4g06030	Putative uncharacterized protein Sb02g025980;Thermospermine synthase ACAULIS5; Probable spermidine synthase; Spermidine synthase
Cs4g05470	Bifunctional inhibitor/lipid-transfer protein/seed storage 2S albumin-like protein
Cs4g05330	F-box/LRR-repeat protein At4g14103;Putative uncharacterized protein
Cs4g01970	Eukaryotic initiation factor iso-4F subunit p82-34; Zinc finger CCCH domain-containing protein 43; Eukaryotic translation initiation factor 4G; Eukaryotic translation initiation factor 4 gamma 1; von Willebrand factor A domain-containing protein DDB_G0286969;Putative eukaryotic translation initiation factor 4 gamma (Fragment)
Cs3g26540	Basic region/leucine zipper motif 27-containing protein;Protein FD; ABSCISIC ACID-INSENSITIVE 5-like protein 2; bZIP transcription factor TRAB1; Protein ABSCISIC ACID-INSENSITIVE 5
Cs3g24090	Chloroplast thylakoidal processing peptidase-like protein;Probable thylakoidal processing peptidase 2, chloroplastic; Thylakoidal processing peptidase 1, chloroplastic; Chloroplast processing peptidase; Probable signal peptidase I-1; Signal peptidase I; Probable signal peptidase I; Signal peptidase I T; Signal peptidase I P; Signal peptidase I V; Mitochondrial inner membrane protease subunit 1
Cs3g23180	Cysteine proteinase RD21a; Oryzain alpha chain; Oryzain beta chain; Low-temperature-induced cysteine proteinase (Fragment); Cysteine protease 1; Germination-specific cysteine protease 1; Actinidain; Cysteine proteinase COT44 (Fragment); Probable cysteine proteinase At3g19400; Xylem cysteine proteinase 1; KDEL-tailed cysteine endopeptidase CEP2; Thiol protease SEN102; Vignain; Cysteine proteinase EP-B 2; Chymopapain; Zingipain-2; Caricain; Zingipain-1; Ervatamin-B; Fruit bromelain; Papain; Ananain; Cathepsin L; Cathepsin S; Papaya proteinase 4; Ervatamin-C; Oryzain gamma chain; Thiol protease aleurain; Cathepsin L1; Digestive cysteine proteinase 1; Crustapain; P34 probable thiol protease;Cysteine protease component of [illegible]
Cs3g22510	Protein SENSITIVE TO PROTON RHIZOTOXICITY 1; Zinc finger protein STOP1 homolog; Zinc finger protein STAR3; Zinc finger protein NUTCRACKER; Protein TRANSPARENT TESTA 1; Zinc finger protein MAGPIE; Zinc finger protein JACKDAW; ATM interactor; PR domain zinc finger protein 10;Putative uncharacterized protein Sb01g001950
Cs3g18790	Alkylated DNA repair protein alkB homolog 8; tRNA (uracil-5-)-methyltransferase TRM9; Putative methyltransferase KIAA1456; Putative methyltransferase KIAA1456 homolog; Uncharacterized protein C13D6.03c;S-adenosylmethionine-dependent methyltransferase, putative

Cs3g18550	Pollen-specific protein C13; Olee1-like protein; Anther-specific protein LAT52; Pollen allergen Phl p 11; Major pollen allergen Lol p 11; Pollen allergen Che a 1; Major pollen allergen; Major pollen allergen Lig v 1; Major pollen allergen Pla l 1;Putative pollen Ole e 1 allergen and extensin family protein
Cs3g17940	Alcohol dehydrogenase-like 2; Alcohol dehydrogenase 1; Alcohol dehydrogenase class-P; Alcohol dehydrogenase class-3 chain L; S-(hydroxymethyl)glutathione dehydrogenase; Alcohol dehydrogenase class-3 chain H;Alcohol dehydrogenase 2, putative, expressed
Cs3g12850	Probable disease resistance protein At1g52660; Putative disease resistance protein At3g15700;Disease resistance protein RFL1, putative
Cs3g12760	Probable disease resistance protein At1g52660; Putative disease resistance protein At3g15700;Disease resistance protein RFL1, putative
Cs3g12720	Probable disease resistance protein At1g52660; Putative disease resistance protein At3g15700;Disease resistance protein RFL1, putative
Cs3g10900	Putative uncharacterized protein At4g30980 (Fragment);Transcription factor UNE12; Transcription factor BEE 2; Transcription factor BPE; Transcription factor SPATULA; Transcription factor ALC; Putative transcription factor bHLH086; Transcription factor LAX PANICLE; Transcription factor IND
Cs3g09210	Nicastrin;Putative uncharacterized protein (Fragment)
Cs3g06390	Putative transcription factor GAMYB (Fragment);Transcription factor GAMYB
Cs3g06140	Zinc finger protein ZPR1; Zinc finger protein ZPR1 homolog; Uncharacterized ZPR1-like protein PH1223;Similar to uniprot|P53303 Saccharomyces cerevisiae YGR211w Zinc-finger protein
Cs2g30260	2-oxoglutarate/malate translocator, chloroplastic; Putative malate transporter yflS; Inner membrane protein ybhI; Uncharacterized transporter HI_0020; L-tartrate/succinate antiporter; Citrate carrier;2-oxoglutarate/malate translocator, chloroplast, putative, expressed
Cs2g29120	Late embryogenesis abundant (LEA) hydroxyproline-rich glycoprotein
Cs2g28180	Alcohol dehydrogenase zinc-binding domain protein;Quinone oxidoreductase-like protein At1g23740, chloroplastic; Reticulon-4-interacting protein 1, mitochondrial; Reticulon-4-interacting protein 1 homolog, mitochondrial; Quinone-oxidoreductase homolog, chloroplastic; Zinc-type alcohol dehydrogenase-like protein C16A3.02c; Putative quinone-oxidoreductase homolog, chloroplastic; Protein TOXD; Quinone oxidoreductase; Probable quinone oxidoreductase; Synaptic vesicle membrane protein VAT-1 homolog
Cs2g24890	Ribosome-recycling factor;Ribosome-recycling factor RRF; Ribosome-releasing factor
Cs2g23550	Squamosa promoter-binding-like protein 4; Squamosa promoter-binding protein 1; Protein LIGULELESS 1; Putative squamosa promoter-binding-like protein 19;Putative squamosa promoter-binding protein (Fragment)
Cs2g15130	Auxin response factor 6; Putative auxin response factor 21;Putative uncharacterized protein Sb04g004430
Cs2g13550	ATP synthase subunit beta, mitochondrial; ATP synthase subunit beta-3, mitochondrial; ATP synthase subunit beta;ATP synthase, H+ transporting, mitochondrial F1 complex, beta polypeptide, isoform CRA_a
Cs2g10790	Ankyrin repeat BTB/POZ domain-containing protein;Regulatory protein NPR3
Cs2g10760	Protein argonaute 2; Protein argonaute-2; Protein argonaute-2 (Fragment); Putative protein tag-76;Eukaryotic translation initiation factor 2c, putative
Cs2g09770	Homeobox-leucine zipper protein ATHB-14; Homeobox-leucine zipper protein REVOLUTA;Class III homeodomain-leucine zipper protein C3HDZ1
Cs2g09460	Metallo-beta-lactamase domain-containing protein;Putative hydrolase C777.06c

Cs2g06980	Zinc finger, RING-type; Transcription factor jumonji, jmjC; Zinc finger, C2H2-type;Lysine-specific demethylase 3B; Probable JmjC domain-containing histone demethylation protein 2C; Protein hairless
Cs2g05730	Putative uncharacterized protein At3g60030 (Fragment);Squamosa promoter-binding protein 1; Squamosa promoter-binding-like protein 3; Protein LIGULELESS 1; Putative squamosa promoter-binding-like protein 19
Cs2g03130	Calcium-dependent calmodulin-independent protein kinase isoform 1;Calcium-dependent protein kinase 21; Calcium-dependent protein kinase isoform 2; Calcium/calmodulin-dependent serine/threonine-protein kinase 1; CDPK-related protein kinase
Cs2g02350	Putative uncharacterized protein OSJNBa0072H09.10
Cs1g24860	Floral homeotic protein DEFICIENS; Floral homeotic protein PMADS 1; Floral homeotic protein APETALA 3; MADS-box transcription factor 16; MADS-box protein CMB2; Floral homeotic protein GLOBOSA; Floral homeotic protein FBP1; Floral homeotic protein PISTILLATA; Developmental protein SEPALLATA 3; Agamous-like MADS-box protein AGL9 homolog; Protein TRANSPARENT TESTA 16; Agamous-like MADS-box protein AGL6; Floral homeotic protein AGAMOUS; Transcription factor CAULIFLOWER;MADS box transcription factor TM6 (Fragment)
Cs1g24800	Putative uncharacterized protein F24M12.90
Cs1g21570	NADH dehydrogenase [ubiquinone] iron-sulfur protein 4, mitochondrial; NADH-ubiquinone oxidoreductase 21 kDa subunit, mitochondrial; NADH-ubiquinone oxidoreductase 18 kDa subunit (Fragment);NADH dehydrogenase (Ubiquinone) Fe-S protein 4, (NADH-coenzyme Q reductase)
Cs1g21180	Putative nuclear matrix constituent protein 1-like protein;DNA double-strand break repair rad50 ATPase, putative
Cs1g15460	VHS domain-containing protein At3g16270; Uncharacterized protein C17orf56 homolog;Singapore isolate B (sub-type 7) whole genome shotgun sequence assembly, scaffold_1
Cs1g13430	LRR and NB-ARC domain-containing disease resistance protein;Probable disease resistance protein At5g63020; Disease resistance protein RPS5; Putative disease resistance protein At4g10780
Cs1g13240	Putative uncharacterized protein Sb10g002915 (Fragment);Cytochrome c oxidase subunit 5b-1, mitochondrial; Putative cytochrome c oxidase subunit 5b-like; Cytochrome c oxidase subunit 5
Cs1g09030	Mitogen-activated protein kinase kinase kinase ANP1; Mitogen-activated protein kinase kinase kinase A; MAP kinase kinase kinase mkh1; Serine/threonine-protein kinase BCK1/SLK1/SSP31; Protein kinase byr2; SPS1/STE20-related protein kinase YSK4; Probable WRKY transcription factor 19; Cytokinesis protein sepH; Cell division control protein 7; Serine/threonine-protein kinase sepA; Serine/threonine-protein kinase svkA; Probable serine/threonine-protein kinase DDB_G0284251; Mitogen-activated protein kinase kinase kinase kinase 5;Mitogen activated protein kinase kinase kinase 3, mapkkk3, mekk3, putative
Cs1g07330	Metallothionein class I type 3 (Fragment);Metallothionein-like protein type 3; Metallothionein-like protein 1
Cs1g06290	Vesicle-associated protein 4-1;Putative uncharacterized protein Sb02g036280
Cs1g02730	DNA replication ATP-dependent helicase dna2-like protein;DNA2-like helicase; DNA replication ATP-dependent helicase dna2; DNA-binding protein SMUBP-2; Regulator of nonsense transcripts 1; Regulator of nonsense transcripts 1 homolog; Uncharacterized protein FLJ44066; Putative regulator of nonsense transcripts 1; Uncharacterized ATP-dependent helicase MJ0104; Putative helicase mov-10-B.1; Helicase SEN1; Probable helicase senataxin

Cs1g02320	Alanine--glyoxylate aminotransferase 2 homolog 1, mitochondrial; Alanine--glyoxylate aminotransferase 2, mitochondrial; Alanine--glyoxylate aminotransferase 2-like 1; Uncharacterized aminotransferase YhxA; Acetylornithine aminotransferase; 2,2-dialkylglycine decarboxylase; Uncharacterized aminotransferase BpOF4_10225; Acetylornithine/acetyl-lysine aminotransferase; 4-aminobutyrate aminotransferase GabT; Acetylornithine aminotransferase, chloroplastic/mitochondrial; Diaminobutyrate--2-oxoglutarate aminotransferase;Alanine-glyoxylate aminotransferase 2, mitochondrial, putative, expressed
orange1.1t04075	Putative uncharacterized protein Sb02g013790;F-box protein SKIP2; F-box/LRR-repeat protein 16; Putative F-box/LRR-repeat protein 8
orange1.1t03762	Protein Dr1 homolog; Negative cofactor 2 complex subunit beta; Protein Dr1; Nuclear transcription factor Y subunit B-8; Nuclear transcription factor Y subunit B; Nuclear transcription factor Y subunit beta; Transcriptional activator hap3; Nuclear transcription factor Y subunit beta (Fragment);Chromosome undetermined SCAF14488, whole genome shotgun sequence. (Fragment)
orange1.1t03542	Protein IQ-DOMAIN 14;Putative uncharacterized protein Sb03g042570
orange1.1t02739	Putative uncharacterized protein
orange1.1t02518	Leucine-rich repeat containing protein, putative (Fragment);Putative disease resistance protein RGA3; Disease resistance protein RGA2; Probable disease resistance protein RXW24L; Polygalacturonase
orange1.1t02492	Putative uncharacterized protein At4g28590 (Fragment)
orange1.1t02489	Dihydrodipicolinate reductase 2, chloroplastic; Probable dihydrodipicolinate reductase 1, chloroplastic;Dihydrodipicolinate reductase family protein, putative, expressed
orange1.1t02280	Putative Werner helicase-interacting protein (Fragment);ATPase WRNIP1; ATPase WRNIP1 homolog C26H5.02c; Replication-associated recombination protein A; DNA-dependent ATPase MGS1; Uncharacterized AAA domain-containing protein Rv2559c/MT2636; Uncharacterized protein TM_0508; Uncharacterized AAA domain-containing protein YrvN; Holliday junction ATP-dependent DNA helicase RuvB
orange1.1t02254	Putative uncharacterized protein Sb02g028470
orange1.1t01983	UDP-glycosyltransferase 73C3; UDP-glucosyl transferase 73B2; Anthocyanin 3'-O-beta-glucosyltransferase; Abscisate beta-glucosyltransferase; Anthocyanidin 3-O-glucosyltransferase 4 (Fragment); Zeatin O-xylosyltransferase; Zeatin O-glucosyltransferase; Hydroquinone glucosyltransferase;UDP-glucoronosyl/UDP-glucosyl transferase family protein
orange1.1t01932	Putative uncharacterized protein Sb08g009580;Stromal 70 kDa heat shock-related protein, chloroplastic; Stromal 70 kDa heat shock-related protein, chloroplastic (Fragment); Chaperone protein dnaK2; Chaperone protein dnaK; Chaperone protein DnaK 2
orange1.1t01918	Leucine-rich repeat containing protein, putative;Putative disease resistance protein RGA1; Disease resistance protein RGA2
orange1.1t01829	TIR-NBS-LRR-TIR type disease resistance protein (Fragment);TMV resistance protein N; Putative disease resistance protein At4g11170; Protein SUPPRESSOR OF npr1-1, CONSTITUTIVE 1; Probable WRKY transcription factor 19; Protein PHLOEM PROTEIN 2-LIKE A8; Vesicle-associated protein 1-4; Probable disease resistance protein At5g66900
orange1.1t01772	DNA-damage-repair/toleration protein DRT111, chloroplastic; Cysteine desulfurase;Putative isopenicillin N epimerase, identical
orange1.1t01329	H+-or Na+-translocating f-type, v-type and A-type ATPase superfamily;ATP synthase subunit epsilon, mitochondrial

orange1.1t00903	Isoamylase N-terminal domain containing protein, expressed
orange1.1t00603	Similarity to hypothetical protein SPAC922.05c - Schizosaccharomyces pombe;UNC93-like protein C922.05c; Uncharacterized membrane protein
Cs9g19220	Putative uncharacterized protein Sb06g000660;Heat shock protein 83; Heat shock cognate protein 80; Heat shock protein HSP 90-alpha; Heat shock cognate 90 kDa protein; Heat shock-like 85 kDa protein; Heat shock protein 82 (Fragment); Endoplasmin homolog; Endoplasmin
Cs9g17470	cDNA FLJ61714, highly similar to Tripeptidyl-peptidase 2 (EC 3.4.14.10) (Fragment);Tripeptidyl-peptidase 2; Putative subtilase-type proteinase F21H12.6; Tripeptidyl-peptidase 2 homolog; Subtilisin DY; Subtilisin; Subtilisin BPN'; Thermophilic serine proteinase; Subtilisin E; Subtilisin NAT; Subtilisin Carlsberg; Major intracellular serine protease; Serine protease AprX; Minor extracellular protease vpr; Subtilisin J; Subtilisin amylosacchariticus
Cs9g14020	Ndufb11, NADH dehydrogenase 1 beta subcomplex subunit
Cs9g07320	Proteasome subunit beta type-2-A; Probable proteasome subunit beta type-2; Proteasome component C11;Putative beta4 proteasome subunit (Fragment)
Cs9g05650	Galactokinase; N-acetylgalactosamine kinase; Protein GAL3; Galactokinase (Fragment);Putative uncharacterized protein Sb01g002480
Cs8g18140	Thymidine diphospho-glucose 4-6-dehydratase homolog (Fragment);UDP-glucuronic acid decarboxylase 1; Putative UDP-glucose 4-epimerase; dTDP-glucose 4,6-dehydratase; Uncharacterized UDP-glucose epimerase ytcB
Cs8g17280	Arogenate dehydratase/prephenate dehydratase 2, chloroplastic; Arogenate dehydratase 3, chloroplastic; P-protein; Prephenate dehydratase; Putative prephenate dehydratase;Putative uncharacterized protein Sb01g038740
Cs8g15450	Cyclin-dependent protein kinase CDC28 regulatory subunit CKS1;Cyclin-dependent kinases regulatory subunit 1; Probable cyclin-dependent kinases regulatory subunit
Cs8g15380	Putative uncharacterized protein OSJNBa0001O14.7
Cs8g15030	TGACG-sequence-specific DNA-binding protein TGA-2.1; Transcription factor HBP-1b(c1) (Fragment); Transcription factor HBP-1b(c38); Transcription factor PERIANTHIA;CAMP response element binding (CREB) protein
Cs8g10430	Putative uncharacterized protein Sb03g010370
Cs8g09160	Putative uncharacterized protein
Cs8g07440	Sec14 cytosolic factor; CRAL-TRIO domain-containing protein YKL091C; SEC14-like protein 1; Protein real-time; Retinal-binding protein;Putative phosphatidylinositol/ phosphatidylcholine transfer protein (Fragment)
Cs8g05120	DNA-damage-repair/toleration protein DRT100; Leucine-rich repeat receptor-like serine/threonine-protein kinase At1g17230; LRR receptor-like serine/threonine-protein kinase FLS2; Polygalacturonase inhibitor 1; Leucine-rich repeat receptor-like tyrosine-protein kinase At2g41820; Putative leucine-rich repeat receptor-like serine/threonine-protein kinase At2g24130; Leucine-rich repeat receptor-like protein kinase PEPR2; Probable LRR receptor-like serine/threonine-protein kinase At4g08850; Receptor-like protein kinase 2; Probable leucine-rich repeat receptor-like protein kinase IMK3; Probably inactive leucine-rich repeat receptor-like protein kinase At2g25790; Leucine-rich repeat receptor protein kinase EXS; Phytosulfokine receptor 1; Protein BRASSINOSTEROID INSENSITIVE 1; Brassinosteroid LRR receptor kinase; Putative receptor-like protein kinase At3g47110; Receptor protein kinase CLAVATA1; LRR receptor-like serine/threonine-protein kinase EFR; Systemin receptor SR160; LRR receptor-like serine/threonine-protein kilike 2; Leucine-rich repeat receptor protein kinase EXS; Somatic em

Cs8g04510	Pentatricopeptide repeat-containing protein At5g02860; Pentatricopeptide repeat-containing protein At2g31400, chloroplastic; Pentatricopeptide repeat-containing protein At1g63080, mitochondrial;Pentatricopeptide repeat-containing protein, putative
Cs7g32470	Serine/arginine-rich splicing factor 10; Polyadenylate-binding protein, cytoplasmic and nuclear; Probable splicing factor, arginine/serine-rich 4; Multiple RNA-binding domain-containing protein 1; Cold-inducible RNA-binding protein B; RNA-binding motif protein, X-linked-like-2; Heterogeneous nuclear ribonucleoprotein G; Eukaryotic translation initiation factor 3 subunit G; Cold-inducible RNA-binding protein; Heterogeneous nuclear ribonucleoprotein G-like 1; Heterogeneous nuclear ribonucleoprotein G retrogene-like; Probable RNA-binding protein 19;Putative uncharacterized protein Sb0514s002010
Cs7g27875	Haloacid dehalogenase-like hydrolase-like protein
Cs7g27400	Putative uncharacterized protein At5g45410 (Fragment)
Cs7g23200	GTP-binding protein hflx, putative;GTP-binding protein At3g49725,
Cs7g19400	Calnexin homolog; Calnexin; Calmegin; Calreticulin-2; Calreticulin; Calreticulin-1; Calreticulin-3;Putative papillar cell-specific calnexin
Cs7g18780	Oxygen-evolving enhancer protein 2, chloroplastic; Oxygen-evolving enhancer protein 2, chloroplastic (Fragment); Putative oxygen-evolving enhancer protein 2-2;Chloroplast photosystem II oxygen-evolving complex 23 kDa polypeptide (Fragment)
Cs7g12930	Endosomal targeting BRO1-like domain-containing protein
Cs7g12040	Phosphatidylinositol kinase family-like protein (Fragment);Probable phosphatidylinositol 4-kinase type 2-beta At1g26270; Uncharacterized PI3/PI4-kinase family protein C343.19
Cs7g11770	Squamosa promoter-binding-like protein 6; Putative squamosa promoter-binding-like protein 19; Protein LIGULELESS 1; Squamosa promoter-binding protein 1;Transcription factor squamosa promoter binding protein-like
Cs7g11670	Probable 6-phosphogluconolactonase 1; Probable 6-phosphogluconolactonase 3, chloroplastic; 6-phosphogluconolactonase; Putative 6-phosphogluconolactonase; GDH/6PGL endoplasmic bifunctional protein; 6-phosphogluconolactonase-like protein 1;Glucosamine/galactosamine-6-phosphate isomerase family protein
Cs7g08960	F-box/kelch-repeat protein At1g23390;F-box/Kelch repeat-containing F-box family protein
Cs7g07030	Ubiquitin carboxyl-terminal hydrolase 19; Ubiquitin carboxyl-terminal hydrolase 17-like protein 5; Probable ubiquitin carboxyl-terminal hydrolase 16; Inactive ubiquitin carboxyl-terminal hydrolase 17-like protein 8; Putative ubiquitin carboxyl-terminal hydrolase 17-like protein 1;Ubiquitin carboxyl-terminal hydrolase family protein
Cs7g02590	Intron-binding protein aquarius; NFX1-type zinc finger-containing protein 1; Regulator of nonsense transcripts 1; Helicase required for RNAi-mediated heterochromatin assembly 1; DNA polymerase alpha-associated DNA helicase A;cDNA FLJ75449, highly similar to Homo sapiens aquarius homolog (mouse) (AQR), mRNA
Cs6g20570	Plasma membrane ATPase 1; ATPase 4, plasma membrane-type; Plasma membrane ATPase 2 (Fragment); Probable plasma membrane ATPase; Putative ATPase, plasma membrane-like; Putative cation-transporting ATPase MJ1226; Probable proton ATPase 1A; Putative Pol polyprotein from transposon element Bs1; Calcium-transporting ATPase 1;N.plumbaginifolia H+-translocating ATPase mRNA

Cs6g19680	Developmental protein SEPALLATA 2; MADS-box protein CMB1; Agamous-like MADS-box protein AGL9 homolog; MADS-box transcription factor 1; Agamous-like MADS-box protein AGL3; Floral homeotic protein APETALA 1; Transcription factor CAULIFLOWER; Floral homeotic protein AGAMOUS;MADS-box transcription factor SEP-like 3 (Fragment)
Cs6g11600	Arabidopsis thaliana genomic DNA, chromosome 5, P1 clone:MOK16
Cs6g10950	RNA-directed DNA polymerase (Reverse transcriptase); Chromo; Zinc finger, CCHC-type; Peptidase aspartic, active site; Polynucleotidyl transferase, Ribonuclease H fold;Transposon Ty3-G Gag-Pol polyprotein; Retrotransposable element Tf2 155 kDa protein type 1; Retrovirus-related Pol polyprotein from transposon 297; Retrovirus-related Pol polyprotein from transposon opus; Retrovirus-related Pol polyprotein from transposon gypsy; Enzymatic polyprotein; Uncharacterized mitochondrial protein AtMg00860; Retrotransposon-like protein 1; RNA-directed DNA polymerase homolog
Cs6g09300	PRA1 family protein B4; PRA1 family protein E; PRA1 family protein D; Prenylated Rab acceptor protein 1;Putative uncharacterized protein Sb01g001896 (Fragment)
Cs6g08190	Glucoamylase;Chromosome undetermined scaffold_48, whole genome shotgun sequence
Cs6g06560	Putative oxysterol-binding protein (Fragment);Oxysterol-binding protein-related protein 3A; Oxysterol-binding protein 8; Oxysterol-binding protein homolog C2F12.05c
Cs6g06540	Putative uncharacterized protein Sb03g039500;Uncharacterized ribonuclease sll1290
Cs6g04030	Singapore isolate B (sub-type 7) whole genome shotgun sequence assembly, scaffold_24;Zinc finger CCCH domain-containing protein 11; Zinc finger CCCH domain-containing protein 15 homolog; Translation machinery-associated protein 46; mRNA export protein 33
Cs5g27500	Putative reverse transcriptase/RNA-dependent DNA polymerase
Cs5g24150	Putative uncharacterized protein Sb02g010890
Cs5g23130	Leucine-rich repeat receptor-like protein kinase (Fragment);LRR receptor-like serine/threonine-protein kinase FLS2; Probable leucine-rich repeat receptor-like protein kinase At1g35710; Leucine-rich repeat receptor protein kinase EXS; Leucine-rich repeat receptor-like serine/threonine-protein kinase At1g17230; Receptor-like protein kinase 2; Probable LRR receptor-like serine/threonine-protein kinase At4g36180; Leucine-rich repeat receptor-like protein kinase PEPR1; Brassinosteroid LRR receptor kinase; Systemin receptor SR160; Leucine-rich repeat receptor-like protein kinase TDR; Putative leucine-rich repeat receptor-like serine/threonine-protein kinase At2g24130; Receptor protein kinase CLAVATA1; LRR receptor-like serine/threonine-protein kinase EFR; Serine/threonine-protein kinase BRI1-like 2; LRR receptor-like serine/threonine-protein kinase ERECTA; Putative receptor-like protein kinase At3g47110; Tyrosine-sulfated glycopeptide receptor 1; Phytosulfokine receptor 2; Leucine-rich repeat receptor-like tyrosinelike 2; Leucine-rich repeat receptor protein kinase EXS; Somatic embryogenesis receptor kinase 4;ERL1a
Cs5g21975	Leucine-rich repeat containing protein, putative (Fragment);Putative disease resistance protein RGA4; Disease resistance protein RGA2; Probable disease resistance protein At1g61180

Cs5g21000	Allyl alcohol dehydrogenase, putative;NADP-dependent alkenal double bond reductase P2; (+)-pulegone reductase; Zinc-type alcohol dehydrogenase-like protein PB24D3.08c; Putative NADP-dependent oxidoreductase yfmJ; Putative NADP-dependent oxidoreductase yncB; Prostaglandin reductase 1; Zinc-binding alcohol dehydrogenase domain-containing protein 2; Probable quinone oxidoreductase; Quinone oxidoreductase
Cs5g19440	TIR-NBS-LRR-TIR type disease resistance protein (Fragment);TMV resistance protein N; Putative disease resistance protein At4g11170; Protein SUPPRESSOR OF npr1-1, CONSTITUTIVE 1; Probable WRKY transcription factor 52; Protein DA1-related 4
Cs5g19200	Novel protein similar to H.sapiens LGTN, ligatin (LGTN, zgc:63669);Eukaryotic translation initiation factor 2D; Translation machinery-
Cs5g12260	Squamosa promoter-binding-like protein 6; Putative squamosa promoter-binding-like protein 19; Protein LIGULELESS 1; Squamosa promoter-binding protein 1;Transcription factor squamosa promoter binding protein-like
Cs5g10180	Major Facilitator Superfamily with SPX domain-containing protein;SPX domain-containing membrane protein At4g22990; SPX domain-containing protein 5; Major facilitator superfamily domain-containing protein 8
Cs5g07360	Putative uncharacterized protein At4g32920 (Fragment)
Cs5g07120	Argininosuccinate synthase, chloroplast, putative, expressed;Argininosuccinate synthase, chloroplastic; Argininosuccinate synthase; Argininosuccinate synthase (Fragment)
Cs5g05620	U3 small nucleolar ribonucleoprotein protein MPP10; U3 small nucleolar RNA-associated protein MPP10;cDNA FLJ32300 fis, clone PROST2002227, highly similar to U3 small nucleolar ribonucleoprotein protein MPP10 (Fragment)
Cs5g05510	Pantothenate kinase 2; Uncharacterized protein At2g17340; Pantothenate kinase 2, mitochondrial; Type II pantothenate kinase;cDNA FLJ53892, highly similar to Pantothenate kinase 4 (EC 2.7.1.33)
Cs5g04990	Calmodulin binding protein-like protein (Fragment)
Cs5g01380	Putative uncharacterized protein Sb01g009330
Cs4g18880	Protein EARLY FLOWERING 3;Putative uncharacterized protein Sb03g025560
Cs4g18150	Putative uncharacterized protein At3g24150
Cs4g17900	Putative uncharacterized protein At1g52565
Cs4g15010	Putative uncharacterized protein (Fragment);Probable cytokinin riboside 5'-monophosphate phosphoribohydrolase LOGL6; Cytokinin riboside 5'-monophosphate phosphoribohydrolase LOG7; Cytokinin riboside 5'-monophosphate phosphoribohydrolase LOG; Retrovirus-related Pol polyprotein from transposon opus; LOG family protein PA4923; Retrovirus-related Pol polyprotein from transposon 17.6; Retrovirus-related Pol polyprotein from transposon gypsy; Retrotransposable element Tf2 155 kDa protein type 1; Pol polyprotein (Fragment); Pol polyprotein; Uncharacterized mitochondrial protein AtMg00860; Gag-Pol polyprotein; Transposon Ty3-I Gag-Pol polyprotein; Pro-Pol polyprotein; Putative cytokinin riboside 5'-monophosphate phosphoribohydrolase LOG9
Cs4g14880	Lactoylglutathione lyase family protein / glyoxalase I family protein
Cs4g12200	Histone H2A (Fragment);Probable histone H2A.1; Histone H2A.6; Protein H2A.7; Histone H2A, gonadal; Late histone H2A.3, gonadal; Histone H2A type
Cs4g08850	Putative uncharacterized protein AT4g16400
Cs4g07790	Probable amino acid permease 7; Amino acid permease 2; Lysine histidine transporter 2;Putative uncharacterized protein Sb04g029540
Cs4g07730	Putative uncharacterized protein (Fragment);Putative disease resistance RPP13-like protein 1; Disease resistance protein RGA2

Cs4g06620	Alpha-aminoadipic semialdehyde synthase; Alpha-aminoadipic semialdehyde synthase, mitochondrial; Saccharopine dehydrogenase [NADP+, L-glutamate-forming]; Probable saccharopine dehydrogenase [NADP+, L-glutamate-forming]; Saccharopine dehydrogenase [NAD+, L-lysine-forming];Lysine-ketoglutarate reductase/saccharopine dehydrogenase bifunctional enzyme
Cs4g05310	Bifunctional purple acid phosphatase 26; Fe(3+)-Zn(2+) purple acid phosphatase 12; Purple acid phosphatase 2; Probable purple acid phosphatase 20; Iron/zinc purple acid phosphatase-like protein;Iron(III)-zinc(II) purple acid phosphatase, putative
Cs4g04520	Auxin response factor 5; Putative auxin response factor 21;Putative uncharacterized protein Sb04g003240
Cs3g18880.2	Chaperone protein DnaJ; DnaJ homolog subfamily A member 1 homolog;Co-chaperone that stimulates the ATPase activity of the HSP70 protein Ssc1p
Cs3g13740	Putative disease resistance protein RGA3;Leucine-rich repeat containing protein, putative
Cs3g13390	Leucine-rich repeat containing protein, putative;Putative disease resistance protein RGA3; Disease resistance protein RGA2; Probable disease resistance protein At5g43730
Cs3g13340	Leucine-rich repeat containing protein, putative;Putative disease resistance protein RGA4; Disease resistance protein RGA2
Cs3g13320	Leucine-rich repeat containing protein, putative;Putative disease resistance protein RGA4; Disease resistance protein RGA2; Probable disease resistance RPP8-like protein 4
Cs3g10870	Sec-independent protein translocase protein TatA;Twin arginine-targeting protein translocase, TatA/E family, putative
Cs3g07260	Cyclin-dependent kinase F-4; Serine/threonine-protein kinase MAK; Serine/threonine-protein kinase ICK; Serine/threonine-protein kinase MHK; Probable serine/threonine-protein kinase DDB_G0268078; MAPK/MAK/MRK overlapping kinase; Sporulation protein kinase pit1; Cell division control protein 2 homolog A; Cell division control protein 2 homolog 2; Cyclin-dependent kinase 5 homolog; Serine/threonine-protein kinase ppk23; Cyclin-dependent kinase 7 (Fragment);Serine/threonine-protein kinase Mak (Male germ cell-associated kinase)-like protein
Cs3g01650	cDNA FLJ56105, highly similar to Nuclear valosin-containing protein-like;Cell division control protein 48 homolog C; Uncharacterized AAA domain-containing protein C16E9.10c; Putative ribosome biogenesis ATPase nvl; Nuclear valosin-containing protein-like; Ribosome biogenesis ATPase RIX7; Cell division cycle protein 48 homolog; Cell division control protein 48 homolog E; Cell division control protein 48 homolog D; VCP-like ATPase; Cell division control protein 48 homolog A; Transitional endoplasmic reticulum ATPase; Cell division control protein 48; Protein SAV; Transitional endoplasmic reticulum ATPase homolog 2; Cell division cycle protein 48; Protein CdcH; Spermatogenesis-associated protein 5; ATPase family gene 2 protein; Cell division control protein 48 homolog B; Spermatogenesis-associated protein 5-like protein 1; Peroxisomal biogenesis factor 6; Peroxisome assembly factor 2; Putative cell division cycle ATPase; Peroxisome biogenesis protein 6; Peroxisomal ATPase PEX6; Peroxisome biogenesis factor 1
Cs2g30590	Disease resistance protein RPP13 variant;Disease resistance protein RPP13; Putative disease resistance RPP13-like protein 3; Probable disease resistance RPP8-like protein 4; Disease susceptibility protein LOV1; Inactive disease susceptibility protein LOV1; Putative inactive disease susceptibility protein LOV1; Putative late blight resistance protein homolog R1B-17

Cs2g28990	CBL-interacting serine/threonine-protein kinase 3; CBL-interacting protein kinase 32; Pentatricopeptide repeat-containing protein At5g21222; Putative CBL-interacting protein kinase 27;CBL-interacting serine/threonine-protein kinase, putative
Cs2g27200	Acetylglucosaminyltransferase/ transferase, transferring glycosyl groups
Cs2g21180	B-cell receptor-associated protein 31-like containing protein, expressed
Cs2g17270	cDNA, clone: J100068E06, full insert sequence
Cs2g09620	Putative uncharacterized protein (Putative leucine-rich repeat protein)
Cs2g09440	Auxin response factor 6; Putative auxin response factor 21;Putative uncharacterized protein Sb04g004430
Cs2g09030	F-box/LRR-repeat protein 3; Uncharacterized F-box/LRR-repeat protein C02F5.7; EIN3-binding F-box protein 1; F-box protein SKP2A;Leucine Rich Repeat family protein, expressed
Cs2g08090	50S ribosomal protein L14;Putative uncharacterized protein Sb08g021680
Cs2g04660	Putative uncharacterized protein Sb03g002380;Shaggy-related protein kinase eta; Shaggy-related protein kinase zeta; Shaggy-related protein kinase iota; Shaggy-related protein kinase epsilon; Shaggy-related protein kinase gamma; Shaggy-related protein kinase alpha; Shaggy-related protein kinase NtK-1; Glycogen synthase kinase-3 homolog MsK-1; Shaggy-related protein kinase theta; Shaggy-related protein kinase kappa; Shaggy-related protein kinase delta; Shaggy-related protein kinase beta; Glycogen synthase kinase-3 beta; Glycogen synthase kinase-3 alpha; Protein kinase shaggy; Glycogen synthase kinase-3; Putative glycogen synthase kinase-3 homolog; Protein kinase gsk3; Serine/threonine-protein kinase RIM11/MSD1; Glycogen synthase kinase 3; Probable serine/threonine-protein kinase glkA; Mitogen-activated protein kinase 10; Mitogen-activated protein kinase homolog NTF6; Cyclin-dependent kinase 5 homolog; Cyclin-dependent kinase A-1
Cs2g04030.2	Putative uncharacterized protein F15D2.11
Cs2g01340	Putative uncharacterized protein Sb02g027720;Uncharacterized protein At4g26485; Ferredoxin-fold anticodon-binding domain-containing protein 1 homolog; UPF0617 protein C1919.13c; Ferredoxin-fold anticodon-binding domain-containing protein 1
Cs1g26030	Putative uncharacterized protein Sb01g030190;NEP1-interacting protein 1; NEP1-interacting protein-like 1; RING-H2 finger protein ATL51; E3 ubiquitin-protein ligase ATL76; Putative RING-H2 finger protein ATL12; RING finger
Cs1g23980	Putative uncharacterized protein Sb01g026920;U1 small nuclear ribonucleoprotein 70 kDa; U1 small nuclear ribonucleoprotein 70 kDa homolog; U11/U12 small nuclear ribonucleoprotein 35 kDa protein; RNA-binding motif protein, X-linked-like-3; Cleavage stimulation factor subunit 2; Nucleolin; Heterogeneous nuclear ribonucleoprotein G; Transformer-2 protein homolog beta; Cold-inducible RNA-binding protein; Transformer-2 protein homolog alpha; Putative RNA-binding protein 3; Cleavage stimulation factor subunit 2 tau variant; Cold-inducible RNA-binding protein A; Transformer-2 sex-determining
Cs1g21890	Putative uncharacterized protein Sb03g037610
Cs1g18270	cDNA, clone: J100074O19, full insert sequence;Transmembrane 9 superfamily member 4; Putative phagocytic receptor 1a; Transmembrane 9 superfamily protein C1105.08
Cs1g16550	Pectinesterase (Precursor);Pectinesterase 3; Pectinesterase/pectinesterase inhibitor U1; Probable pectinesterase/pectinesterase inhibitor 34; Putative pectinesterase/pectinesterase inhibitor 22

Cs1g15720	NADP-dependent malic enzyme; NADP-dependent malic enzyme, chloroplastic; NADP-dependent malic enzyme, chloroplastic (Fragment); NADP-dependent malic enzyme (Fragment); NADP-dependent malic enzyme, mitochondrial; NAD-dependent malic enzyme, mitochondrial; NAD-dependent malic enzyme, mitochondrial (Fragment); NAD-dependent malic enzyme;NADP-dependent malic enzyme
Cs1g15550	Leucine-rich repeat containing protein, putative;Putative disease resistance protein RGA3; Disease resistance protein RGA2; Probable disease resistance protein At4g27220
Cs1g12230	Protein FAR1-RELATED SEQUENCE 6; Protein FAR-RED IMPAIRED RESPONSE 1; Protein FAR-RED ELONGATED HYPOCOTYL 3;Putative uncharacterized protein Sb01g001615 (Fragment)
Cs1g06060	Polyubiquitin 12; Ubiquitin-60S ribosomal protein L40; Polyubiquitin (Fragment); Polyubiquitin-A; Polyubiquitin-H; Polyubiquitin-F; Polyubiquitin-J; Polyubiquitin-I; Polyubiquitin-G; Polyubiquitin-D; Polyubiquitin-C; Polyubiquitin-B;Ubiquitin-like protein (Fragment)
Cs1g02710	Multidrug resistance-associated protein 2, 6 (Mrp2, 6), abc-transoprter, putative (Fragment);ABC transporter C family member 9; Putative ABC transporter C family member 15; Multidrug resistance-associated protein 1; Uncharacterized ABC transporter ATP-binding protein/permease C359.05; Metal resistance protein YCF1; Canalicular multispecific organic anion transporter 1; ATP-binding cassette transporter abc2; ATP-dependent bile acid permease; ATP-binding cassette sub-family C member 11; Bile pigment transporter 1; ABC transporter ATP-binding protein/permease VMR1
Cs1g01190	Putative uncharacterized protein P0441A12.55
orange1.1t05081	Putative uncharacterized protein Sb01g020050
orange1.1t04955	Putative uncharacterized protein
orange1.1t03059	Delta-1-pyrroline-5-carboxylate synthase; Delta-1-pyrroline-5-carboxylate synthase A; Delta-1-pyrroline-5-carboxylate synthase B; Probable delta-1-pyrroline-5-carboxylate synthase; Probable gamma-glutamyl phosphate reductase; Gamma-glutamyl phosphate reductase;Putative delta-1-pyrroline 5-carboxylase synthetase P5C1
orange1.1t02265	Squamosa promoter-binding-like protein 17; Protein LIGULELESS 1; Squamosa promoter-binding protein 2; Putative squamosa promoter-binding-like protein 19;Squamosa promoter binding protein-homologue 4 (Fragment)
orange1.1t02252	SWR1 complex protein, SNF2 family DNA-dependent ATPase (Fragment);Helicase SWR1; Helicase SRCAP; Helicase domino; Probable ATP-dependent helicase PF08_0048; E1A-binding protein p400; Putative DNA helicase INO80; DNA helicase INO80
orange1.1t02175	Putative TIR-NBS-LRR class disease resistance protein;Probable WRKY transcription factor 19; TMV resistance protein N; Putative disease resistance protein At4g11170; E3 ubiquitin-protein ligase ipaH3; Leucine-rich repeat-containing protein 40; Outer membrane protein yopM; Protein flightless-1 homolog; Probable E3 ubiquitin-protein ligase ipaH4.5
orange1.1t02171	TIR-NBS-LRR-TIR type disease resistance protein (Fragment);Protein SUPPRESSOR OF npr1-1, CONSTITUTIVE 1; TMV resistance protein N; Putative disease resistance protein At4g11170; Probable WRKY transcription factor 19; Protein PHLOEM PROTEIN 2-LIKE A8; Vesicle-associated protein 1-4; Leucine-rich repeat-containing protein 40; Disease resistance protein RPP13
orange1.1t01714	Flowering time control protein FCA; CUGBP Elav-like family member 1-A; ELAV-like protein 3; Sex-lethal homolog; Polyadenylate-binding protein 2;Flowering time control protein FCA, putative

orange1.1t01470	Putative uncharacterized protein Sb01g020050
orange1.1t00591	Protein CUP-SHAPED COTYLEDON 2; Protein FEZ; Putative NAC domain-containing protein 94; NAC domain-containing protein 78; Protein BEARSKIN1; Protein SOMBRERO;Putative uncharacterized protein
orange1.1t00172	Putative uncharacterized protein At3g13960 (Fragment)
Cs9g07740	Eukaryotic translation initiation factor 2c, putative;Protein argonaute 10; Protein argonaute-3; Protein argonaute-2 (Fragment); Putative protein tag-76
Cs9g07680	Putative low molecular mass early light-induced protein,chloroplast (ELIP);Early light-induced protein, chloroplastic; Desiccation stress protein DSP-22, chloroplastic; High molecular mass early light-inducible protein HV58, chloroplastic; Low molecular mass early light-inducible protein HV90, chloroplastic; Carotene biosynthesis-related protein CBR, chloroplastic
Cs8g16440	Auxin response factor 18; Putative auxin response factor 14;Putative uncharacterized protein At2g28350 (Fragment)
Cs8g12570	LRR receptor-like serine/threonine-protein kinase EFR; Probable LRR receptor-like serine/threonine-protein kinase At3g47570; Putative receptor-like protein kinase At3g47110; LRR receptor-like serine/threonine-protein kinase FLS2; Probable leucine-rich repeat receptor-like protein kinase At5g63930; Leucine-rich repeat receptor-like serine/threonine-protein kinase BAM2; Putative leucine-rich repeat receptor-like serine/threonine-protein kinase At2g24130; Receptor-like protein kinase 2; Leucine-rich repeat receptor-like protein kinase PEPR2; Leucine-rich repeat receptor protein kinase EXS; LRR receptor-like serine/threonine-protein kinase ERECTA; Phytosulfokine receptor 1; Serine/threonine-protein kinase BRI1-like 2; Brassinosteroid LRR receptor kinase; Systemin receptor SR160; Receptor protein kinase CLAVATA1; Protein BRASSINOSTEROID INSENSITIVE 1; Leucine-rich repeat receptor-like tyrosine-protein kinase At2g41820; LRR receptor-like serine/threonine-protein kinase FEI 1;Serine-threonine protein kinase, plant-tlike 2; Leuci
Cs8g10560	Leucine-rich repeat containing protein, putative;Putative disease resistance protein At4g11170; TMV resistance protein N; Protein SUPPRESSOR OF npr1-1, CONSTITUTIVE 1; Protein PHLOEM PROTEIN 2-LIKE A5; Vesicle-associated protein 1-4; Probable WRKY transcription factor 52; Protein DA1-
Cs8g03230	Malate dehydrogenase, glyoxysomal; Probable malate dehydrogenase, glyoxysomal; Malate dehydrogenase 1, glyoxysomal; Malate dehydrogenase, mitochondrial; Malate dehydrogenase 1, mitochondrial; Malate dehydrogenase, chloroplastic; Probable malate dehydrogenase, mitochondrial; Malate dehydrogenase;Glyoxisomal malate dehydrogenase
Cs8g03110	K(+) efflux antiporter 3, chloroplastic; Glutathione-regulated potassium-efflux system protein kefB; Glutathione-regulated potassium-efflux system protein kefC;Glutathione-regulated potassium-efflux system protein KefB-like protein (Fragment)
Cs8g03105	Chloroplast methionine sulfoxide reductase B2 (Precursor);Peptide methionine sulfoxide reductase B3, chloroplastic; Peptide methionine sulfoxide reductase B5; Uncharacterized protein C216.04c; Peptide methionine sulfoxide reductase MsrB; Methionine-R-sulfoxide reductase B1; Methionine-R-sulfoxide reductase B3, mitochondrial
Cs8g03020	Putative uncharacterized protein Sb04g002885 (Fragment)

Cs7g27790	Ethylene-responsive transcription factor RAP2-7; Floral homeotic protein APETALA 2; AP2-like ethylene-responsive transcription factor TOE3; AP2-like ethylene-responsive transcription factor SMZ; AP2-like ethylene-responsive transcription factor SNZ; AP2-like ethylene-responsive transcription factor BBM; AP2-like ethylene-responsive transcription factor ANT;Ethylene-responsive transcription factor RAP2-7
Cs7g27680	Serine/threonine-protein kinase/endoribonuclease IRE2; Probable serine/threonine-protein kinase ireA; Serine/threonine-protein kinase 17B; Probable serine/threonine-protein kinase irlA;Endoplasmic reticulum (ER) to nucleus signalling 2
Cs7g27670	Putative uncharacterized protein (Fragment)
Cs7g25670	Putative uncharacterized protein At2g28350 (Fragment);Auxin response factor 18; Putative auxin response factor 15
Cs7g23640	Putative uncharacterized protein Sb01g005790
Cs7g10850	Putative uncharacterized protein Sb03g044270;F-box only protein 6; F-box/kelch-repeat protein At3g61590
Cs7g10830	Squamosa promoter-binding-like protein 2; Protein LIGULELESS 1; Putative squamosa promoter-binding-like protein 19; Squamosa promoter-binding protein 1;Transcription factor squamosa promoter binding protein-like
Cs7g01720	Nuclear transcription factor Y subunit A-1; Transcriptional activator HAP2; Nuclear transcription factor Y subunit alpha;CCAAT-box transcription factor complex WHAP5, putative, expressed
Cs7g01530	Putative uncharacterized protein T8G24.2 (Fragment)
Cs6g19380	Cytochrome P450 monooxygenase CYP707A17 (Fragment);Abscisic acid 8'-hydroxylase 1; Cytochrome P450 85A1; Ent-kaurenoic acid oxidase 2; Taxane 13-alpha-hydroxylase; 3-epi-6-deoxocathasterone 23-monooxygenase; Taxadiene 5-alpha hydroxylase; Putative cytochrome P450 120; Abietadienol/abietadienal oxidase
Cs6g19000	Putative nuclear matrix constituent protein 1-like protein;DNA double-strand break repair rad50 ATPase, putative
Cs6g17090	Eukaryotic aspartyl protease family protein, expressed;Aspartic proteinase nepenthesin-1
Cs6g16030	Auxin response factor 8; Putative auxin response factor 21;Putative uncharacterized protein Sb04g004430
Cs6g11940	Phenylalanine ammonia-lyase (Fragment);Phenylalanine ammonia-lyase; Phenylalanine ammonia-lyase class 2; Phenylalanine ammonia-lyase G4 (Fragment); Phenylalanine ammonia-lyase class 1 (Fragment)
Cs6g11800	Putative uncharacterized protein At2g28350 (Fragment);Auxin response factor 18; Putative auxin response factor 15
Cs5g35220	Chloroplast methionine sulfoxide reductase B2 (Precursor);Peptide methionine sulfoxide reductase B3, chloroplastic; Peptide methionine sulfoxide reductase B5; Uncharacterized protein C216.04c; Peptide methionine sulfoxide reductase MsrB; Methionine-R-sulfoxide reductase B3, mitochondrial; Methionine-R-sulfoxide reductase B3
Cs5g19920	Leucine-rich repeat containing protein, putative;TMV resistance protein N; Putative disease resistance protein At4g11170; Probable WRKY transcription factor 19; Protein SUPPRESSOR OF npr1-1, CONSTITUTIVE 1; Protein PHLOEM PROTEIN 2-LIKE A5; Vesicle-associated protein 1-4

Cs5g19850	Leucine-rich repeat containing protein, putative;Putative disease resistance protein At4g11170; TMV resistance protein N; Protein SUPPRESSOR OF npr1-1, CONSTITUTIVE 1; Probable WRKY transcription factor 16; Protein DA1-related 4; Protein PHLOEM PROTEIN 2-LIKE A8; Vesicle-associated protein 1-4; Probable disease resistance protein At1g15890
Cs5g19310	Leucine-rich repeat containing protein, putative;TMV resistance protein N; Putative disease resistance protein At4g11170; Protein SUPPRESSOR OF npr1-1, CONSTITUTIVE 1; Probable WRKY transcription factor 19; Protein DA1-related 4; Protein PHLOEM PROTEIN 2-LIKE A5; Vesicle-associated protein 1-4; Protein popC; Probable disease resistance protein At1g15890
Cs5g18480	TMV resistance protein N; Putative disease resistance protein At4g11170; Probable WRKY transcription factor 19; Protein SUPPRESSOR OF npr1-1, CONSTITUTIVE 1; Protein DA1-related 4; Protein PHLOEM PROTEIN 2-LIKE A8; Vesicle-associated protein 1-4;Putative TIR-NBS-LRR class disease resistance protein
Cs5g11880	ATP synthase subunit gamma, mitochondrial; ATP synthase gamma chain;H+-transporting ATP synthase (Fragment)
Cs5g06510	Leucine-rich repeat receptor-like serine/threonine/tyrosine-protein kinase SOBIR1; Leucine-rich repeat receptor-like protein kinase PXL2; Leucine-rich repeat receptor protein kinase EXS; Probable leucine-rich repeat receptor-like protein kinase At5g63930; LRR receptor-like serine/threonine-protein kinase FEI 1; Leucine-rich repeat receptor-like serine/threonine-protein kinase BAM2; Probable LRR receptor-like serine/threonine-protein kinase At5g45780; Receptor protein kinase CLAVATA1; Systemin receptor SR160; Brassinosteroid LRR receptor kinase; Serine/threonine-protein kinase BRI1-like 1; Receptor-like protein kinase BRI1-like 3; LRR receptor-like serine/threonine-protein kinase HSL2; Protein BRASSINOSTEROID INSENSITIVE 1; Probably inactive leucine-rich repeat receptor-like protein kinase At5g48380; Leucine-rich repeat receptor-like protein kinase TDR; Receptor-like serine/threonine-protein kinase At1g78530; Inactive leucine-rich repeat receptor-like protein kinase CORYNE; Tyrosine-sulfated glycopeptide recep
Cs5g01840	Luminal-binding protein 5; Heat shock 70 kDa protein cognate 3; 78 kDa glucose-regulated protein; Heat shock 70 kDa protein C; 78 kDa glucose-regulated protein homolog; Heat shock 70 kDa protein D; Probable heat shock protein ssa2; Heat shock cognate 70 kDa protein 1; Heat shock 70 kDa protein; Heat shock-related 70 kDa protein 2; Heat shock 70 kDa protein A;Endoplasmic reticulum HSC70-cognate binding protein (Precursor)
Cs4g19310	Homeobox-leucine zipper protein ATHB-8; Homeobox-leucine zipper protein REVOLUTA; Homeobox-leucine zipper protein PROTODERMAL FACTOR 2; Homeobox-leucine zipper protein MERISTEM L1; Homeobox-leucine zipper protein GLABRA 2; Homeobox-leucine zipper protein ANTHOCYANINLESS 2;Class III homeodomain-leucine zipper protein C3HDZ1
Cs4g14890	Putative nuclease HARBI1;Putative uncharacterized protein P0512G09.2-1

Cs4g10880	RNA recognition motif family protein, expressed;tRNA selenocysteine 1-associated protein 1; RNA-binding post-transcriptional regulator csx1; Uncharacterized RNA-binding protein C23E6.01c; Protein NAM8; Polyadenylate-binding protein, cytoplasmic and nuclear; Nucleolysin TIAR; Polyadenylate-binding protein 1; Negative growth regulatory protein NGR1; Embryonic polyadenylate-binding protein; Nuclear and cytoplasmic polyadenylated RNA-binding protein PUB1; Embryonic polyadenylate-binding protein B; 28 kDa ribonucleoprotein, chloroplastic; Embryonic polyadenylate-
Cs4g06230	LRR receptor-like serine/threonine-protein kinase ERECTA; LRR receptor-like serine/threonine-protein kinase ERL1; Leucine-rich repeat receptor-like serine/threonine-protein kinase BAM1; Probable leucine-rich repeat receptor-like protein kinase At2g33170; Receptor-like protein kinase 2; Leucine-rich repeat receptor-like protein kinase TDR; Leucine-rich repeat receptor-like protein kinase PXL2; Probable LRR receptor-like serine/threonine-protein kinase At1g34110; Protein BRASSINOSTEROID INSENSITIVE 1; Systemin receptor SR160; Brassinosteroid LRR receptor kinase; Phytosulfokine receptor 1; Putative leucine-rich repeat receptor-like serine/threonine-protein kinase At2g24130; Tyrosine-sulfated glycopeptide receptor 1; Leucine-rich repeat receptor-like tyrosine-protein kinase At2g41820; LRR receptor-like serine/threonine-protein kinase FEI 1; Probably inactive leucine-rich repeat receptor-like protein kinase At2g25790; BRASSINOSTEROID INSENSITIVE 1-associated receptor kinase 1; Serine/threonine-protein kinase BRI1-like 2; Leucine-rich repeat receptor protein kinase EXS; Somatic embryogenesis receptor kinase 4;ERL1a AtERECTA-like receptor S/T protein kinase protein (Fragment)
Cs4g05000	Ubiquitin-associated /TS-N domain-containing protein
Cs4g01460	Transcription factor bHLH66; Transcription factor BEE 2; Transcription factor BPE; Transcription factor ALC; Transcription factor SPATULA; Putative transcription factor bHLH086;Helix-loop-helix DNA-binding domain containing protein
Cs3g18940	Auxin response factor 17; Putative auxin response factor 15;Putative uncharacterized protein At2g28350 (Fragment)
Cs2g30970	TMV resistance protein N; Putative disease resistance protein At4g11170; Protein SUPPRESSOR OF npr1-1, CONSTITUTIVE 1; Probable WRKY transcription factor 19; Protein DA1-related 4; Protein PHLOEM PROTEIN 2-LIKE A8; Vesicle-associated protein 1-4; Leucine-rich repeat and death domain-containing protein 1; Leucine-rich repeat protein SHOC-2; Leucine-rich repeat protein soc-2 homolog; Leucine-rich repeat-containing protein 58; Malignant fibrous histiocytoma-amplified sequence 1; Leucine-rich repeat receptor-like protein kinase TDR;Leucine-rich repeat containing protein, putative
Cs2g30960	TMV resistance protein N; Putative disease resistance protein At4g11170; Protein SUPPRESSOR OF npr1-1, CONSTITUTIVE 1; Probable WRKY transcription factor 19; Protein PHLOEM PROTEIN 2-LIKE A8; Vesicle-associated protein 1-4;Leucine-rich repeat containing protein, putative
Cs2g30350	Nuclear transcription factor Y subunit A-1; Nuclear transcription factor Y subunit alpha; Transcriptional activator HAP2;CCAAT-box transcription factor complex WHAP5, putative, expressed
Cs2g14270	TIR1/AFB auxin receptor protein PintaAFB4A (Fragment);Protein AUXIN SIGNALING F-BOX 2; Transport inhibitor response 1-like protein Os04g0395600; Protein TRANSPORT INHIBITOR RESPONSE 1; GRR1-like protein 1; F-box protein FBX14; Coronatine-insensitive protein 1
Cs2g10770	Protein argonaute 2; Protein argonaute-2; Protein argonaute-2 (Fragment); Putative protein tag-76;Eukaryotic translation initiation factor 2c, putative

Cs2g09590	Cation-chloride cotransporter 1; Solute carrier family 12 member 4; Sodium/chloride cotransporter 3;Putative uncharacterized protein Sb05g003370
Cs2g02980	APRL1-Zea mays adenosine 5'-phosphosulfate reductase-like;5'-adenylylsulfate reductase 1, chloroplastic; Probable 5'-adenylylsulfate reductase 1, chloroplastic; Phosphoadenosine phosphosulfate reductase; Phosphoadenosine phosphosulfate reductase (Fragment); Probable phosphoadenosine phosphosulfate reductase
Cs2g01990	Scarecrow-like protein 13; Scarecrow-like transcription factor PAT1; Chitin-inducible gibberellin-responsive protein 2; DELLA protein GAI1; DELLA protein GAI; DELLA protein GAIP; Protein SCARECROW; DELLA protein RGA; DELLA protein GAIP-B;Chitin-inducible gibberellin-responsive protein 2, putative, expressed
Cs1g21350	Putative uncharacterized protein Sb01g009330
Cs1g17780	Nuclear transcription factor Y subunit A-3; Nuclear transcription factor Y subunit alpha; Transcriptional activator HAP2;CCAAT-binding transcription factor subunit B family protein, expressed
Cs1g15640	Homeobox-leucine zipper protein ATHB-15; Homeobox-leucine zipper protein REVOLUTA; Homeobox-leucine zipper protein PROTODERMAL FACTOR 2; Homeobox-leucine zipper protein GLABRA 2;Class III homeodomain-leucine zipper protein C3HDZ1
Cs1g11740	Putative uncharacterized protein (Fragment)
Cs1g08770	26S protease regulatory subunit 6B homolog; 26S protease regulatory subunit 6B; Probable 26S protease regulatory subunit 6B; 26S proteasome regulatory subunit 4 homolog A; 26S proteasome regulatory subunit 4 homolog B; Proteasome-activating nucleotidase;Putative uncharacterized protein GLEAN_07999
Cs1g08400	Chaperonin CPN60-1, mitochondrial, putative, expressed;Chaperonin CPN60-like 1, mitochondrial
