# Supplementary material for: Genome-wide comparison of microRNAs and their targeted transcripts among leaf, flower and fruit of sweet orange
Source: BMC Genomics. 2014 Aug 20;15(1):695. doi: 10.1186/1471-2164-15-695 (PMC4158063; doi:10.1186/1471-2164-15-695)
Supplement: Supplementary file 7 — Additional file 7: Normalized count of secondary siRNAs and their targets identified using degradome sequencing. (PDF 36 KB) [file 12864_2014_6413_MOESM7_ESM.pdf]

Additional file 7: Normalized count of secondary siRNAs and their targets identified using degradome sequencing in sweet orange.

a. Normailzed count[a] of secondary siRNAs in leaf and their targets identified using degradome sequencing

Secondary siRNA	Normalized count[a] in leaf	Target	Category
Csi-miR167b.2-Cs2g09440.1-8	0.39	Cs4g19850.1	3
Csi-miR167b.2-Cs2g09440.1-9	0.39	Cs2g12460.1	3
Csi-miR167b.2-Cs2g09440.1-13	0.49	Cs3g15060.1	3
Csi-miR482a-3p-Cs5g18480.1-15	0.29	Cs7g01530.1	3
Csi-miR482a-3p-Cs5g18480.1-18	4.42	Cs3g05730.1	1
Csi-miR482a-3p-Cs5g18480.1-18	4.42	Cs3g06120.1	1
Csi-miR482a-3p-Cs5g18480.1-20	0.29	Cs9g03090.1	3
Csi-miR482a-3p-Cs5g18480.1-41	4.03	Cs3g22270.2	2
Csi-miR482a-3p-Cs5g18480.1-77	1.18	Cs7g19320.2	3
Csi-miR482a-3p-Cs5g19920.1-36	0.59	Cs4g03050.1	3
Csi-miR482a-3p-Cs5g19920.1-36	0.59	Cs4g03140.1	3
Csi-miR482c-Cs9g06846.1-5	1.67	Cs9g08050.1	3
Csi-miR482c-Cs9g06846.1-10	0.88	Cs2g30790.2	2
Csi-miR482c-Cs9g06846.1-15	2.36	Cs1g16450.1	2
Csi-miR482c-Cs9g06846.1-22	31.84	Cs5g19660.1	3
Csi-miR2118.1-orange1.1t02171.1-12	0.29	Cs7g21420.1	2
Csi-miR2118.1-orange1.1t02171.1-21	0.39	Cs9g08950.1	3
Csi-miR2118.1-orange1.1t02171.1-30	0.39	Cs1g22190.1	2
Csi-miR2118.1-orange1.1t02175.1-85	0.39	Cs6g16200.1	3
Csi-miR2118.1-orange1.1t02175.1-85	0.39	Cs3g06440.1	3
Csi-miR3954a-Cs1g09600.1-7	3.15	Cs2g11250.1	3
Csi-miR3954a-Cs1g09600.1-9	0.39	Cs2g04220.1	3
Csi-miR3954a-Cs1g09600.1-9	0.39	Cs3g07100.1	3
Csi-miR3954a-Cs1g09635.1-7	14.35	Cs4g11930.1	3
Csi-miR3954a-Cs1g09635.1-9	0.39	Cs2g11990.1	1
Csi-miR3954a-Cs1g09635.1-9	0.39	Cs5g11880.1	3
Csi-miR3954a-Cs5g04670.1-10	0.39	Cs4g17040.1	2
Csi-miR3954a-Cs5g04670.1-13	0.39	Cs7g13090.1	3
Csi-miR3954a-Cs5g04670.1-16	0.39	Cs9g03100.1	3
Csi-miR3954a-Cs5g04670.1-21	1.18	Cs3g25700.1	3
Csi-miR3954a-Cs7g22460.1-2	1.18	Cs3g07280.1	2
Csi-miR3954a-Cs7g22460.1-2	1.18	Cs6g18000.1	3
Csi-miR3954a-Cs7g22460.1-8	0.69	Cs9g06660.1	3
Csi-miR3954a-Cs7g22460.1-15	0.49	Cs7g06320.1	3
Csi-miRN20-Cs3g05320.1-4	1.67	Cs5g18400.1	2
Csi-miRN20-Cs3g05320.1-23	87.57	Cs7g19800.1	3
Csi-miRN20-Cs3g05320.1-24	2.95	Cs1g12760.1	1
Csi-miRN20-Cs3g05320.1-26	20.35	Cs1g15370.1	3
Csi-miRN20-Cs3g05320.1-26	20.35	Cs3g15060.1	3
Csi-miRN20-Cs3g05320.1-26	20.35	Cs6g18830.1	3
Csi-miRN20-Cs3g05320.1-36	0.59	orange1.1t00530.2	3

Csi-miRN20-Cs3g05320.1-65	10.42	Cs3g01450.1	3
Csi-miRN20-Cs3g05320.1-65	10.42	orange1.1t01632.1	3

b. Normailzed count[a] of secondary siRNAs in flower and their targets identified using degradome sequencing

Secondary siRNA	Normalized count[a] in flowe	Target	Category
Csi-miR482c-Cs9g06846.1-5	0.76	Cs9g08050.1	3
Csi-miR482c-Cs9g06846.1-6	7.35	Cs7g31270.1	3
Csi-miR482c-Cs9g06846.1-15	1.73	Cs1g16450.1	3
Csi-miR3954a-Cs1g09635.1-5	0.32	Cs1g01010.1	3
Csi-miR3954a-Cs1g09635.1-5	0.32	orange1.1t03220.1	3
Csi-miR3954a-Cs1g09635.1-5	0.32	Cs5g24790.1	3
Csi-miR3954a-Cs1g09635.1-7	33.61	Cs4g11930.1	3
Csi-miR3954a-Cs1g09635.1-7	33.61	Cs9g18330.1	2
Csi-miR3954a-Cs1g09635.1-9	0.43	Cs5g11880.1	2
Csi-miR3954a-Cs1g09635.1-9	0.43	Cs2g17600.1	2
Csi-miR3954a-Cs1g09635.1-9	0.43	Cs2g17570.1	3
Csi-miR3954a-Cs5g04670.1-21	0.32	Cs5g09880.1	3
Csi-miRN20-Cs3g05320.1-14	0.54	Cs8g19960.1	2
Csi-miRN20-Cs3g05320.1-16	2.81	Cs9g03400.1	3
Csi-miRN20-Cs3g05320.1-19	0.43	Cs7g05730.1	1
Csi-miRN20-Cs3g05320.1-4	1.08	Cs5g05630.1	2
Csi-miRN20-Cs3g05320.1-4	1.08	Cs5g18400.1	2
Csi-miRN20-Cs3g05320.1-9	0.32	Cs3g26640.1	3
Csi-miRN20-Cs3g05320.1-24	2.81	orange1.1t00622.1	2
Csi-miRN20-Cs3g05320.1-24	2.81	orange1.1t01948.1	3
Csi-miRN20-Cs3g05320.1-24	2.81	Cs1g12760.1	2
Csi-miRN20-Cs3g05320.1-26	26.48	Cs3g11480.1	3
Csi-miRN20-Cs3g05320.1-26	26.48	Cs6g18830.1	3

c. Normailzed count[a] of secondary siRNAs in fruit and their targets identified using degradome sequencing

Secondary siRNA	Normalized count[a] in flowe	Target	Category
csi-miR482a-3p-Cs1g15550.1-4	12.88	Cs8g11330.1	3
csi-miR482a-3p-Cs3g13340.1-20	1.68	Cs2g11470.1	3
csi-miR482a-3p-Cs3g13340.1-20	1.68	Cs1g07080.1	3
csi-miR482a-3p-Cs3g13340.1-35	3.92	orange1.1t02629.1	3
csi-miR482a-3p-orange1.1t01918.1-6	3.92	Cs6g02560.1	3
csi-miR482a-3p-orange1.1t01918.1-29	2.8	Cs2g21880.1	3
csi-miR482a-3p-orange1.1t01918.1-29	2.8	orange1.1t03281.1	2
csi-miR482a-3p-orange1.1t01918.1-29	2.8	Cs8g02370.1	1
csi-miR827.2-Cs5g10180.1-14	8.4	Cs7g18380.1	3
csi-miRN20-Cs3g05320.1-12	1.68	Cs1g17610.1	3
csi-miRN20-Cs3g05320.1-12	1.68	Cs2g28240.1	3

csi-miRN20-Cs3g05320.1-23	10.64	Cs7g19680.1	3
csi-miRN20-Cs3g05320.1-26	6.16	Cs6g18830.1	3
csi-miRN20-Cs3g05320.1-26	6.16	Cs2g16940.1	3

Normailzed count[a]: The counts of secondary siRNAs were normalized to transcripts per million (TPM)
