# Supplementary material for: Genome-wide comparison of microRNAs and their targeted transcripts among leaf, flower and fruit of sweet orange
Source: BMC Genomics. 2014 Aug 20;15(1):695. doi: 10.1186/1471-2164-15-695 (PMC4158063; doi:10.1186/1471-2164-15-695)
Supplement: Supplementary file 8 — Additional file 8: T-plots of the targets of secondary siRNAs in different tissues. Densities of the 5’ positions of degradome tags matching each target gene are shown as T-plots. The miRNA mediated degradome tag is highlighted in red. (PDF 4 MB) [file 12864_2014_6413_MOESM8_ESM.pdf]

Additional file 8: T-plots of the targets of secondary siRNAs in different tissues. Densities of the 5' positions of degradome tags matching each target gene are shown as T-plots. The miRNA mediated degradome tag is highlighted in red.

T-plots for targets of secondary siRNAs in leaf

Csi-miR167b.2-Cs2g09440.1-13, target=Cs3g15060.1 gene=Cs3g15060
Category:3
Score:4.5
Cleavage site:423

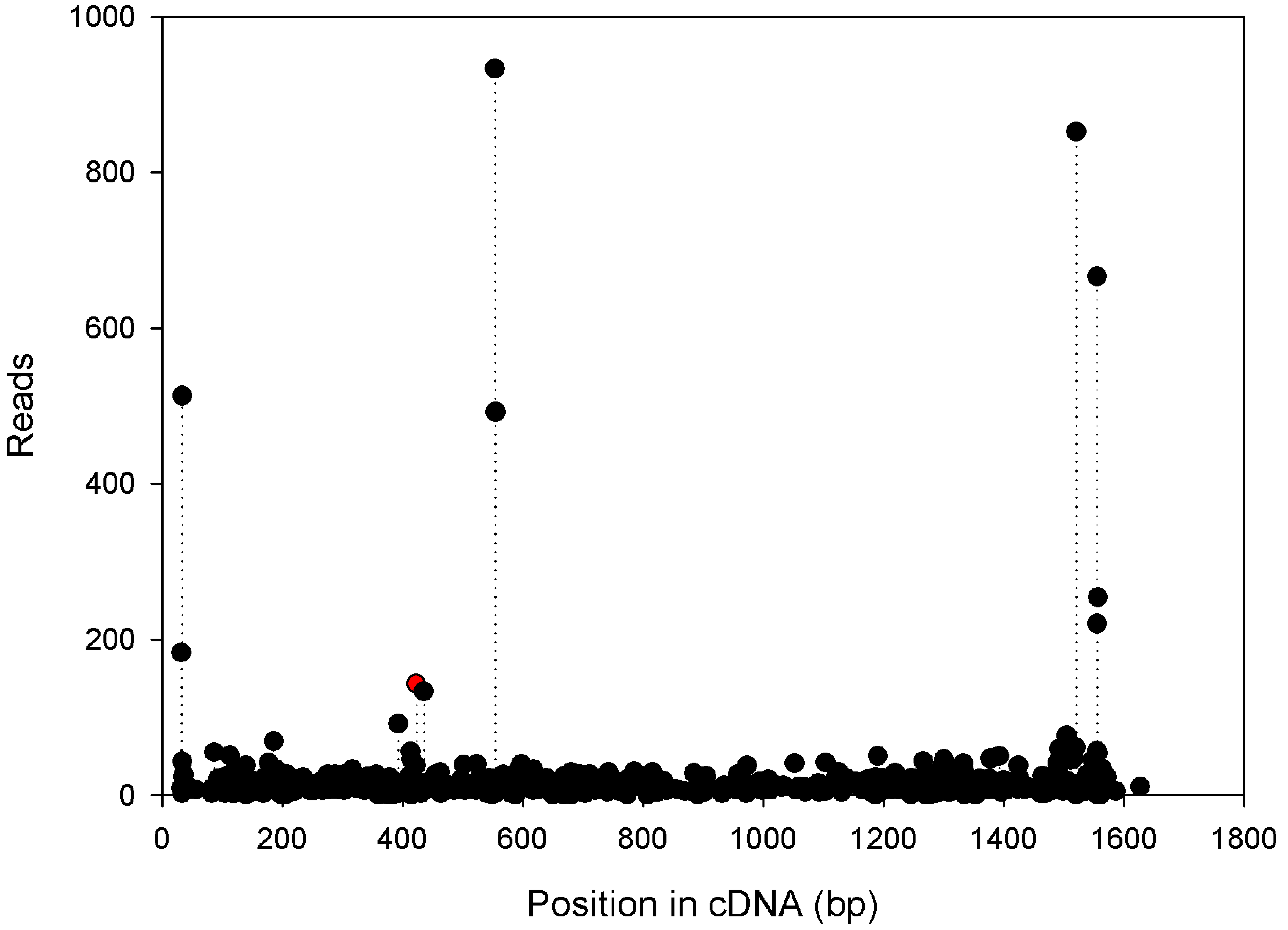


```
5' UUAGUGGCGAAGUUGAUCAGAAGAAA 3'        Cs3g15060.1
     : :::  ::.:::::::::::.
3' --UGACCAAUUUAACUAGUCUUU--- 5'        Csi-miR167b.2-Cs2g09440.1-13
```

Csi-miR167b.2-Cs2g09440.1-8, target=Cs4g19850.1 gene=Cs4g19850
Category:3
Score:5
Cleavage site:1244

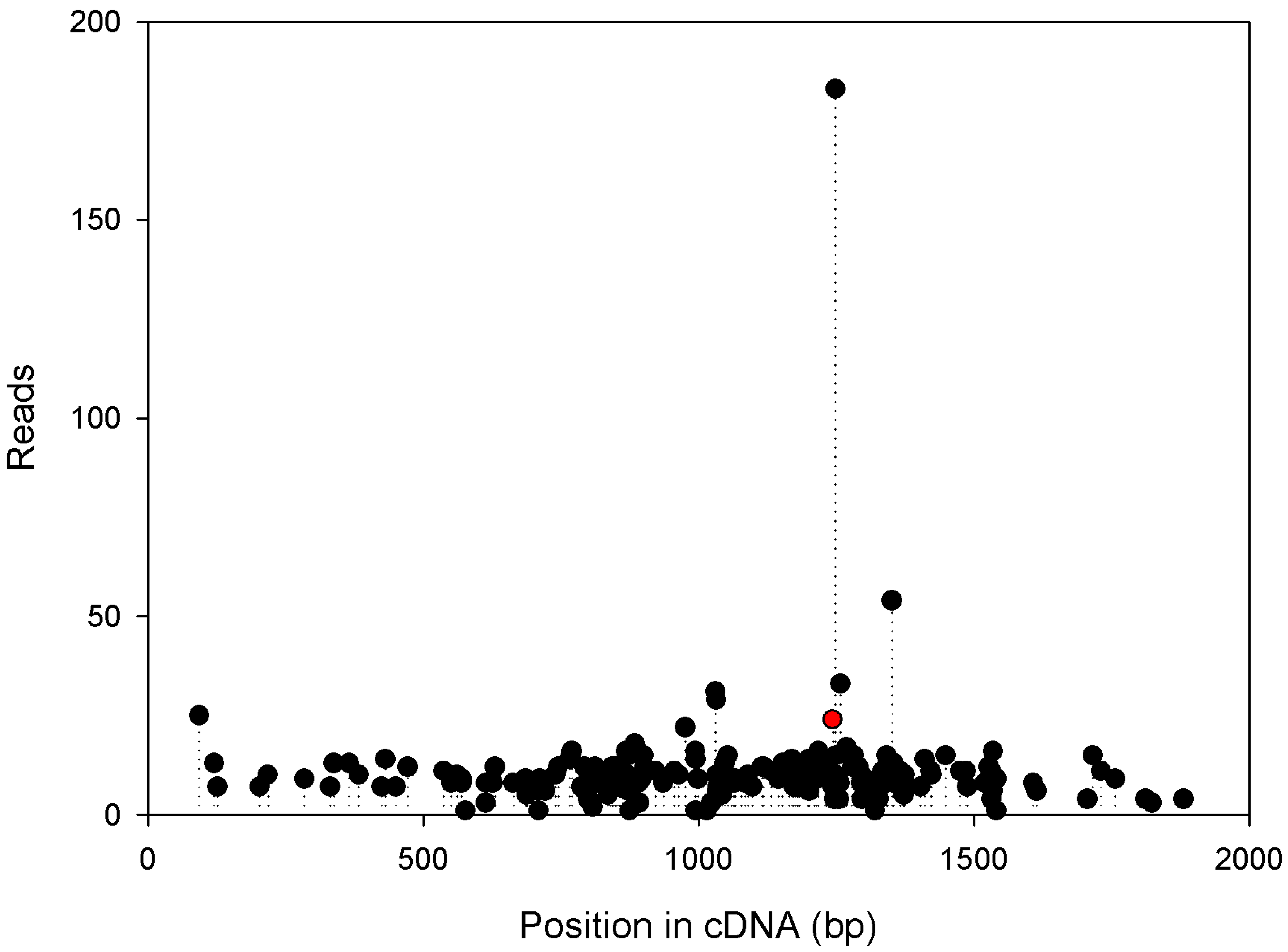


```
5' CAUCGACUUCAAAAUUGGAGCUGAGU 3'        Cs4g19850.1
       . :.::  :::::::.:::::
3' ----UCGGAGACUUAACCUUGACUC- 5'        Csi-miR167b.2-Cs2g09440.1-8
```

Csi-miR167b.2-Cs2g09440.1-9, target=Cs2g12460.1 gene=Cs2g12460
Category:3
Score:5
Cleavage site:1047

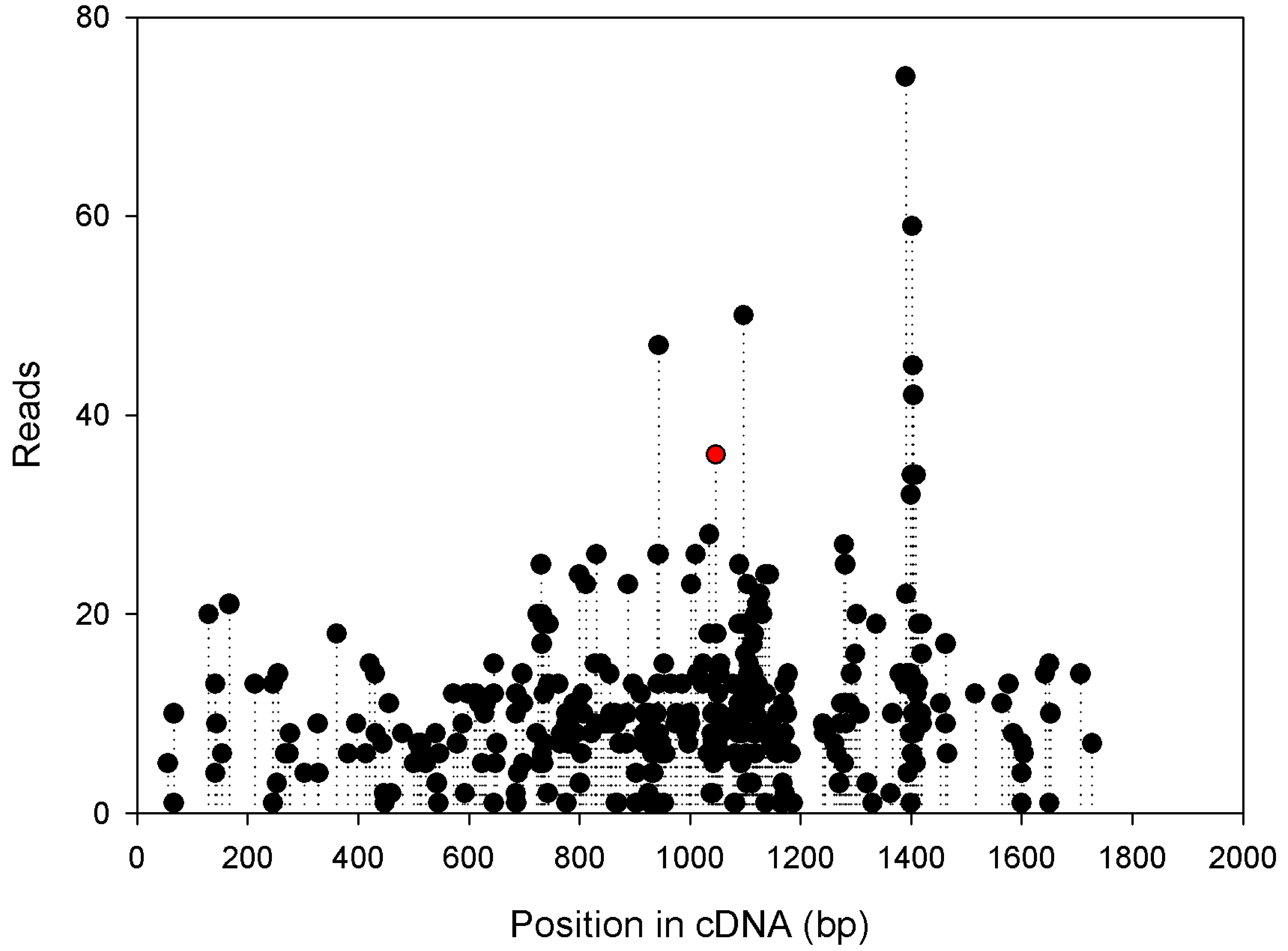


```
5' AAACAUCAGCCGCUGCUGUUGGUGUU 3'        Cs2g12460.1
     ::::: : ::::::::::: :
3' --AGUAGU-GUCGACGACAACCUC-- 5'        Csi-miR167b.2-Cs2g09440.1-9
```

Csi-miR2118.1-Orange1.1t02171.1-12, target=Cs7g21420.1 gene=Cs7g21420
Category:2
Score:4.5
Cleavage site:2399

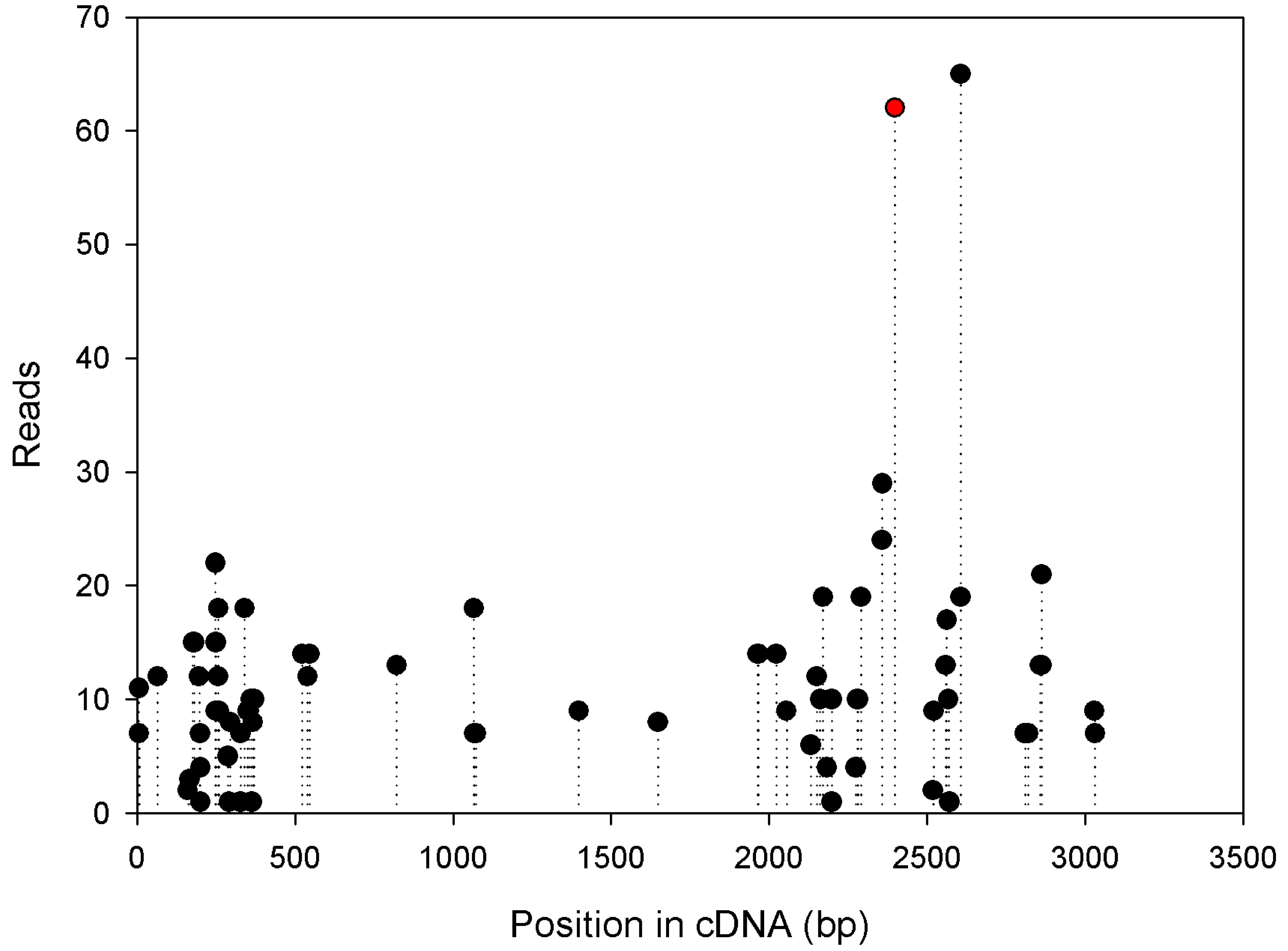


```
5' ACGCUUUGUC-AUACUUACAUCAUCAC 3'       Cs7g21420.1
         ....::  ::::.:::::::::
3' -----GGUAGAAAUGAGUGUAGUAGU- 5'       Csi-miR2118.1-Orange1.1t02171.1-12
```

Csi-miR2118.1-Orange1.1t02171.1-21, target=Cs9g08950.1 gene=Cs9g08950
Category:3
Score:4.5
Cleavage site:624

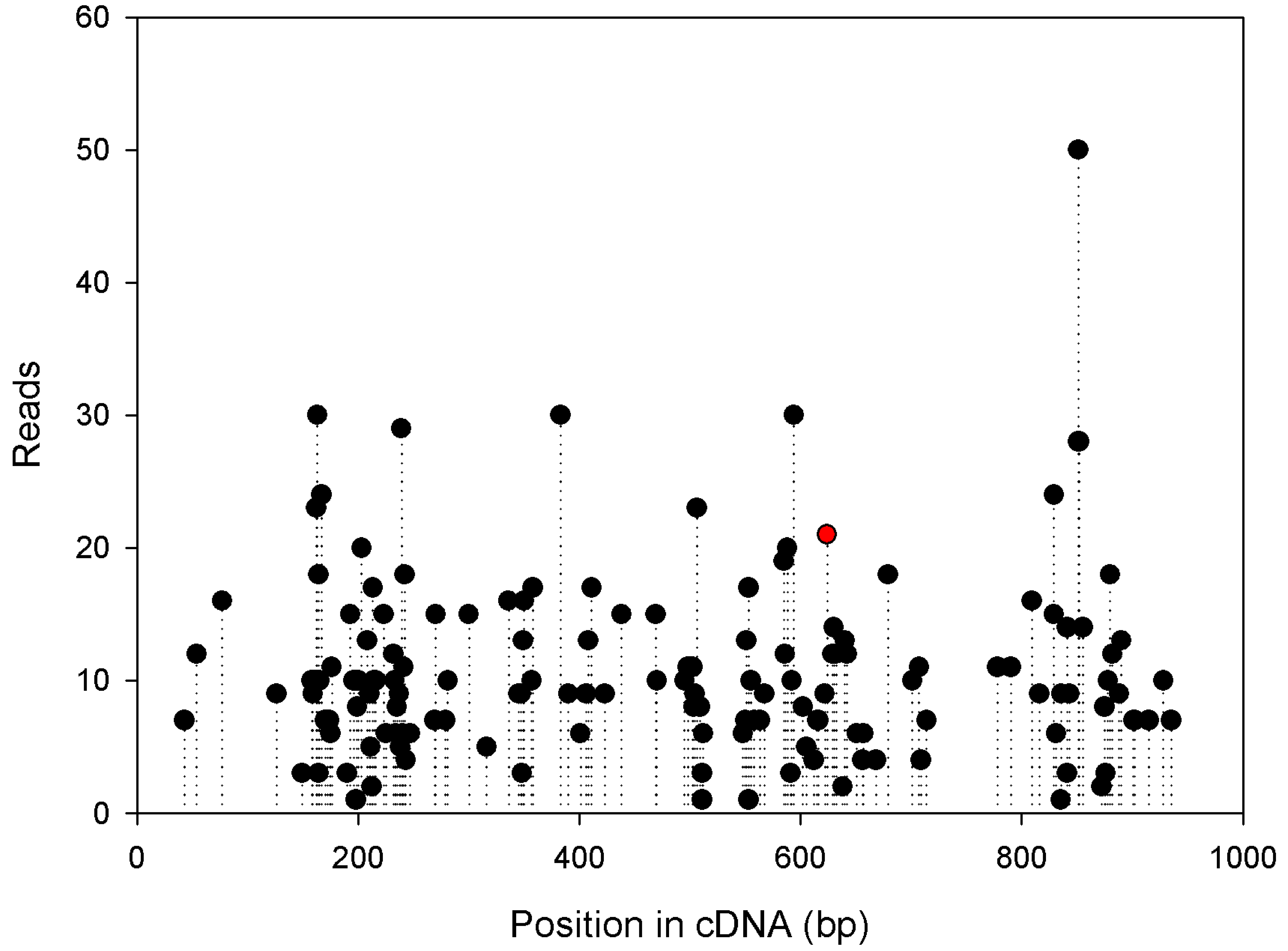


```
5' UUGGUGCAUUGCUUUUCUUUAUUGGG 3'        Cs9g08950.1
   :..: .:: ::::::::::::
3' AGUCCUGUUACGAAAAGAAAU----- 5'        Csi-miR2118.1-Orange1.1t02171.1-21
```

Csi-miR2118.1-Orange1.1t02171.1-30, target=Cs1g22190.1 gene=Cs1g22190
Category:2
Score:5
Cleavage site:1501

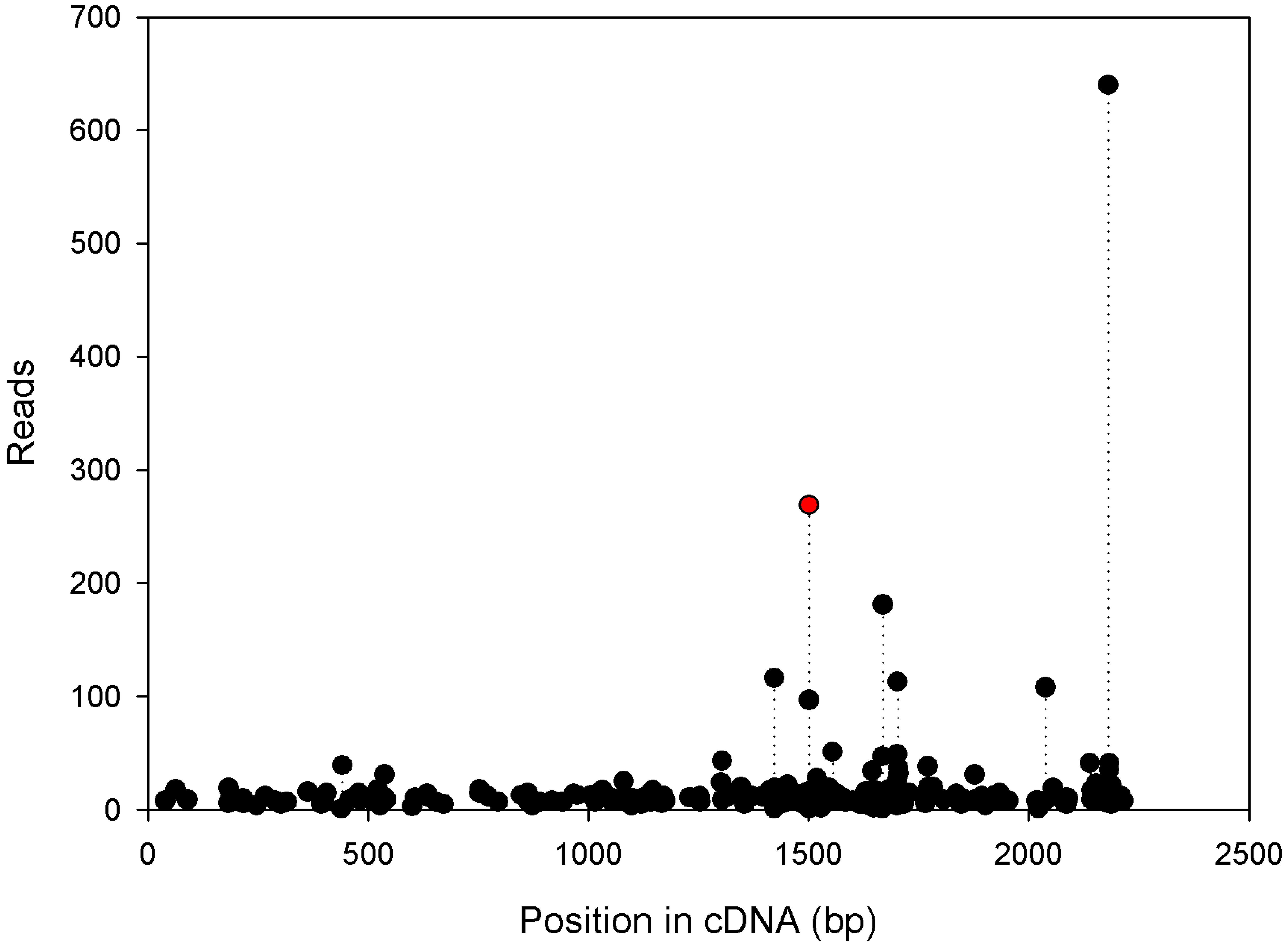


```
5' UUCGUUGUUGGCCUCUUGCUCAGAUG 3'        Cs1g22190.1
      ::::.. : ::::: :::::::
3' ----AACGGACCGAGAAAGAGUCUA- 5'        Csi-miR2118.1-Orange1.1t02171.1-30
```

Csi-miR2118.1-Orange1.1t02175.1-85, target=Cs3g06440.1 gene=Cs3g06440
Category:3
Score:5
Cleavage site:1273

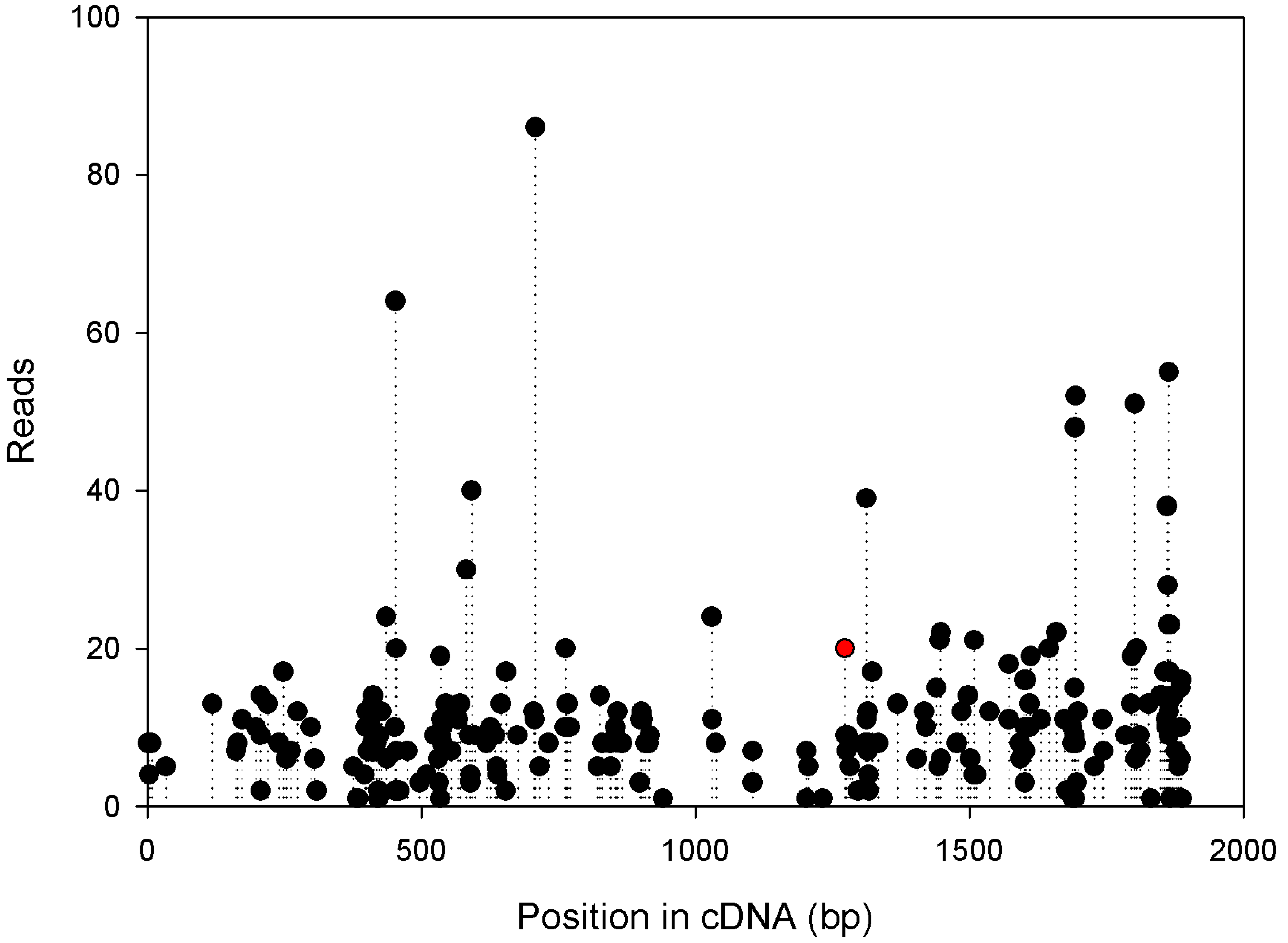


```
5' ACCCUUAUAUAG-CAAUGUUAUCGGGG 3'       Cs3g06440.1
        :: :: : :::::::::: ::
3' -----AUUUAACGGUUACAAUAGGCC- 5'       Csi-miR2118.1-Orange1.1t02175.1-85
```

Csi-miR2118.1-Orange1.1t02175.1-85, target=Cs6g16200.1 gene=Cs6g16200
Category:3
Score:5
Cleavage site:811

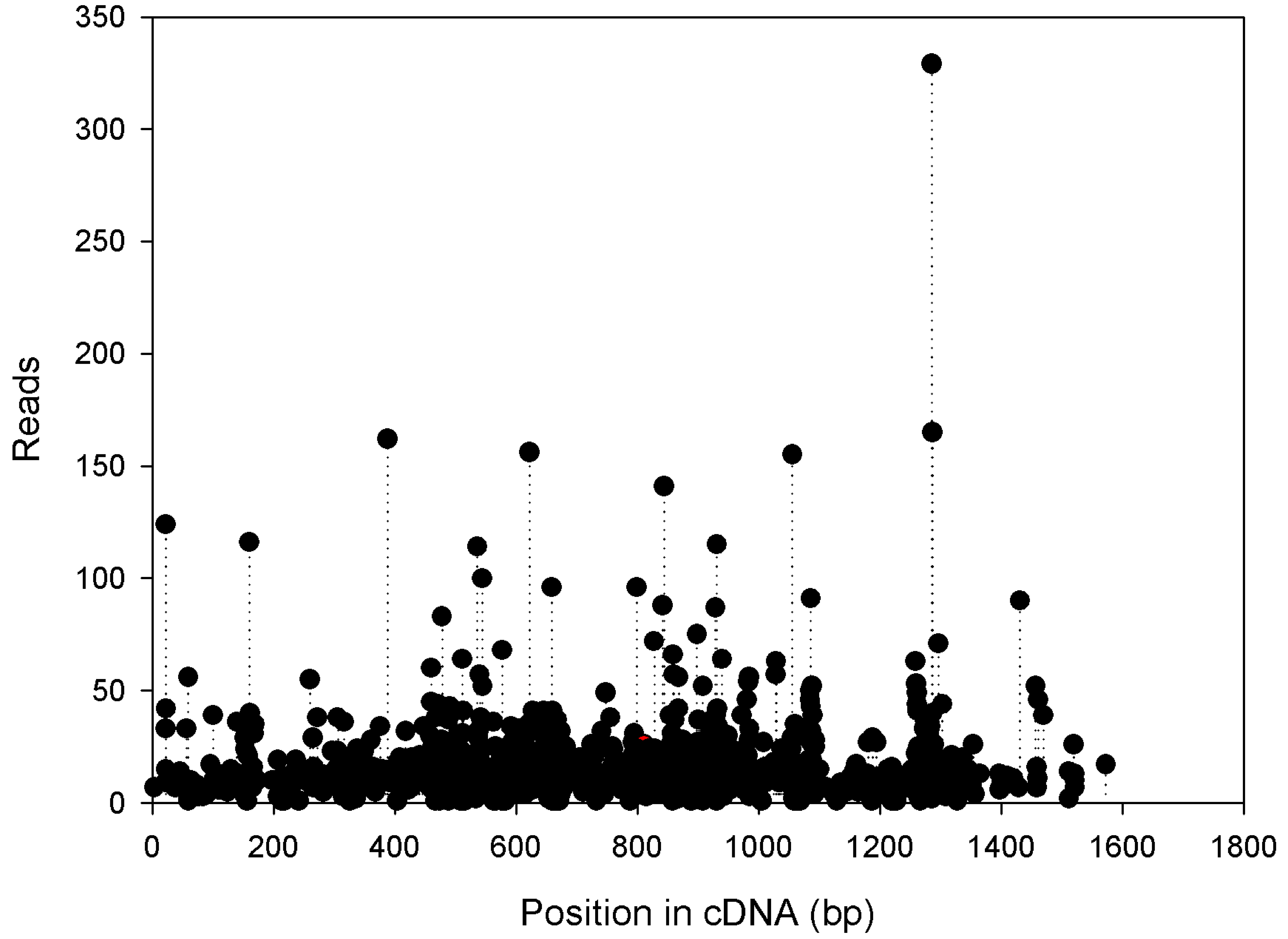


```
5' CAACCCUGAGUU-CCAAUGUUAUCCCA 3'       Cs6g16200.1
         :.:.:: ::::::::::::
3' ------AUUUAACGGUUACAAUAGGCC 5'       Csi-miR2118.1-Orange1.1t02175.1-85
```

Csi-miR3954a-Cs1g09600.1-7, target=Cs2g11250.1 gene=Cs2g11250
Category:3
Score:4.5
Cleavage site:2603

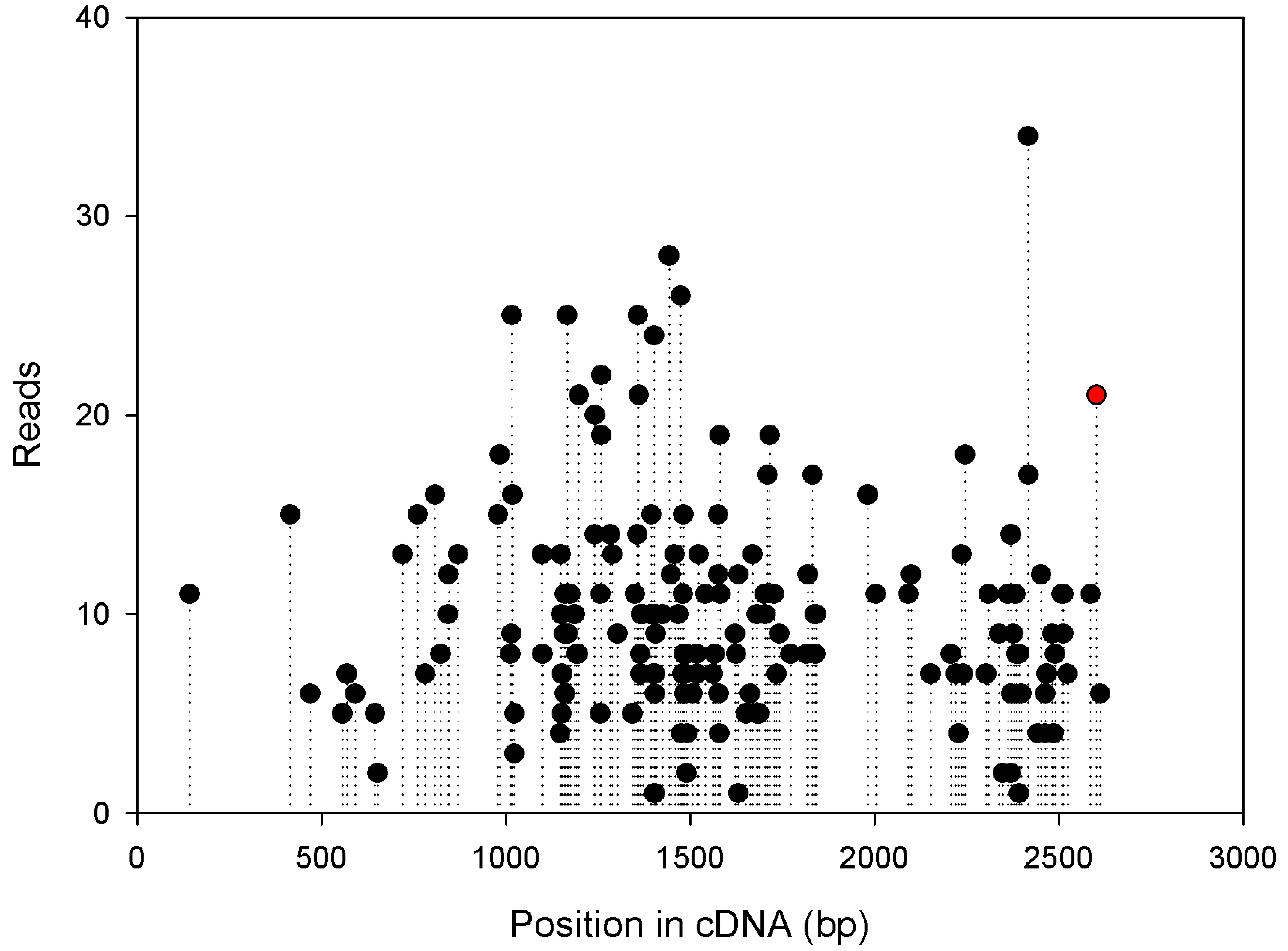


```
5' GUAAUUA-GUUCUGCUCUUCAGAAUAA 3'       Cs2g11250.1
       .:: :::::.:.::::::::
3' ---CGAUCCAAGAUGGGAAGUCUU--- 5'       Csi-miR3954a-Cs1g09600.1-7
```

Csi-miR3954a-Cs1g09600.1-9, target=Cs2g04220.1 gene=Cs2g04220
Category:3
Score:4.5
Cleavage site:1133

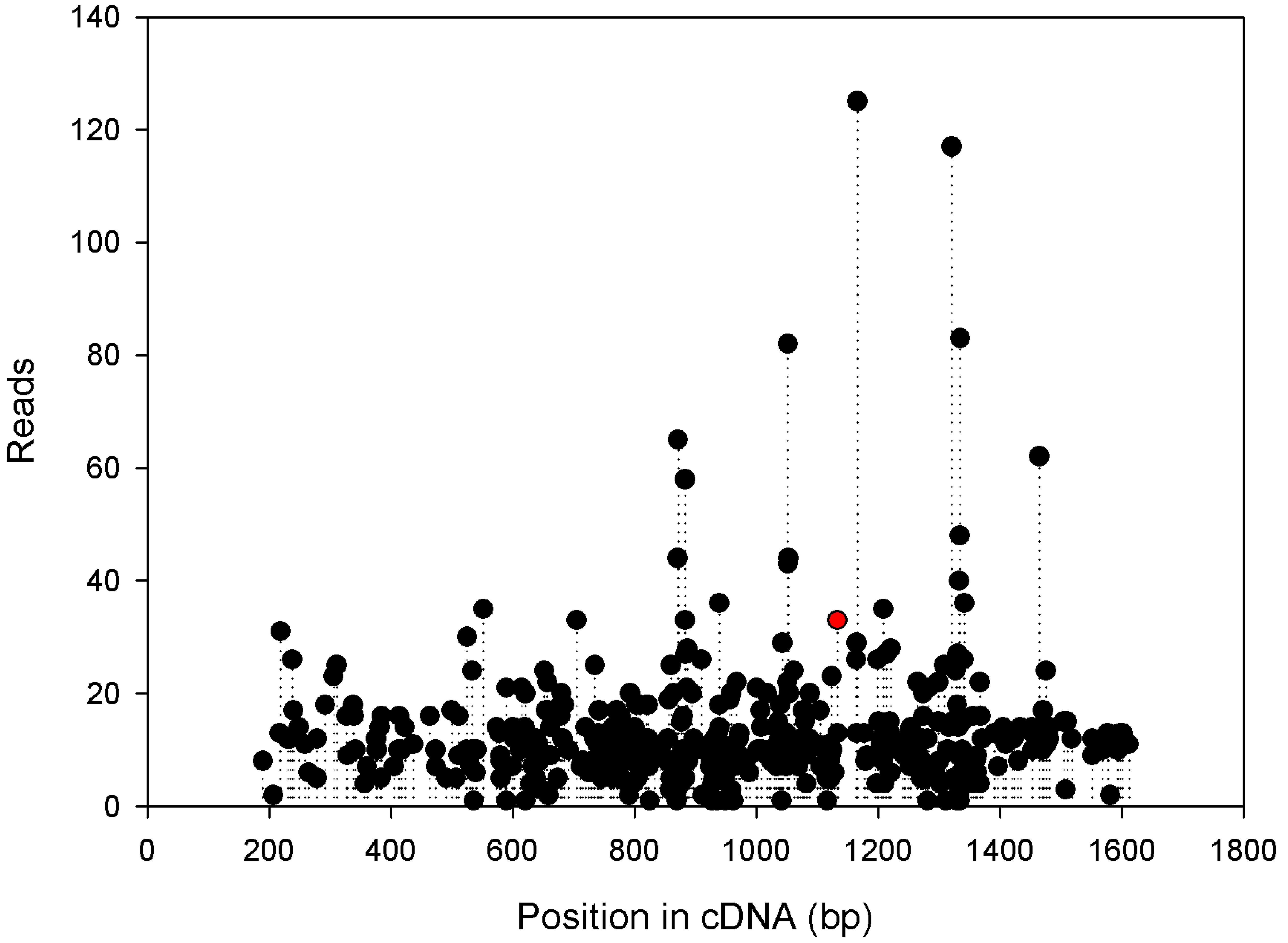


```
5' CACGCUAUGGUUU-UAGAGUGACAAAU 3'       Cs2g04220.1
         :::.::: ::::.: ::::::
3' ------UACUAAAGAUCUUAGUGUUUA 5'       Csi-miR3954a-Cs1g09600.1-9
```

Csi-miR3954a-Cs1g09600.1-9, target=Cs3g07100.1 gene=Cs3g07100
Category:3
Score:4.5
Cleavage site:963

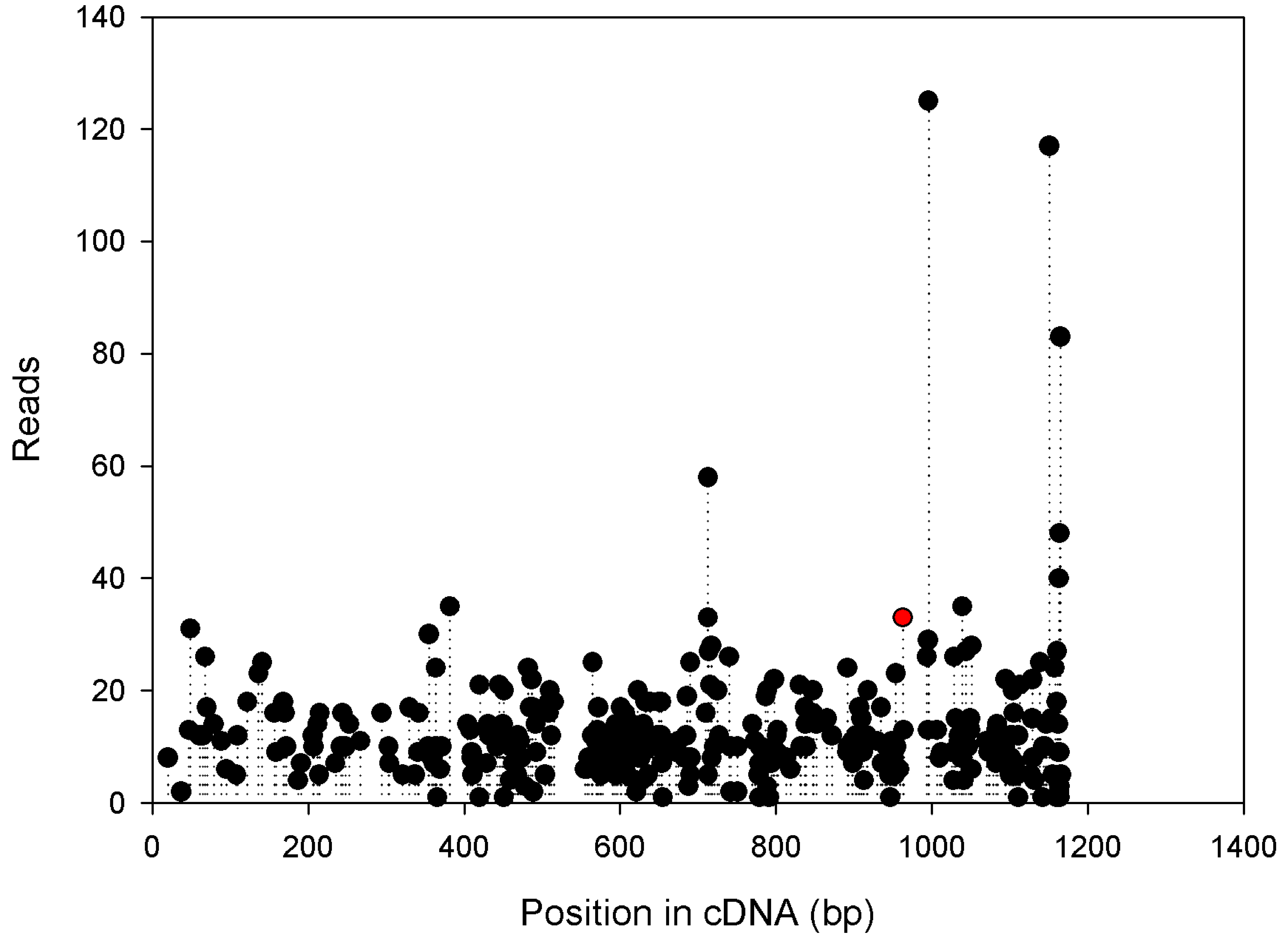


```
5' CACGCUAUGGUUU-UAGAGUGACAAAU 3'       Cs3g07100.1
         :::.::: ::::.: ::::::
3' ------UACUAAAGAUCUUAGUGUUUA 5'       Csi-miR3954a-Cs1g09600.1-9
```

Csi-miR3954a-Cs1g09635.1-7, target=Cs4g11930.1 gene=Cs4g11930
Category:3
Score:3.5
Cleavage site:504

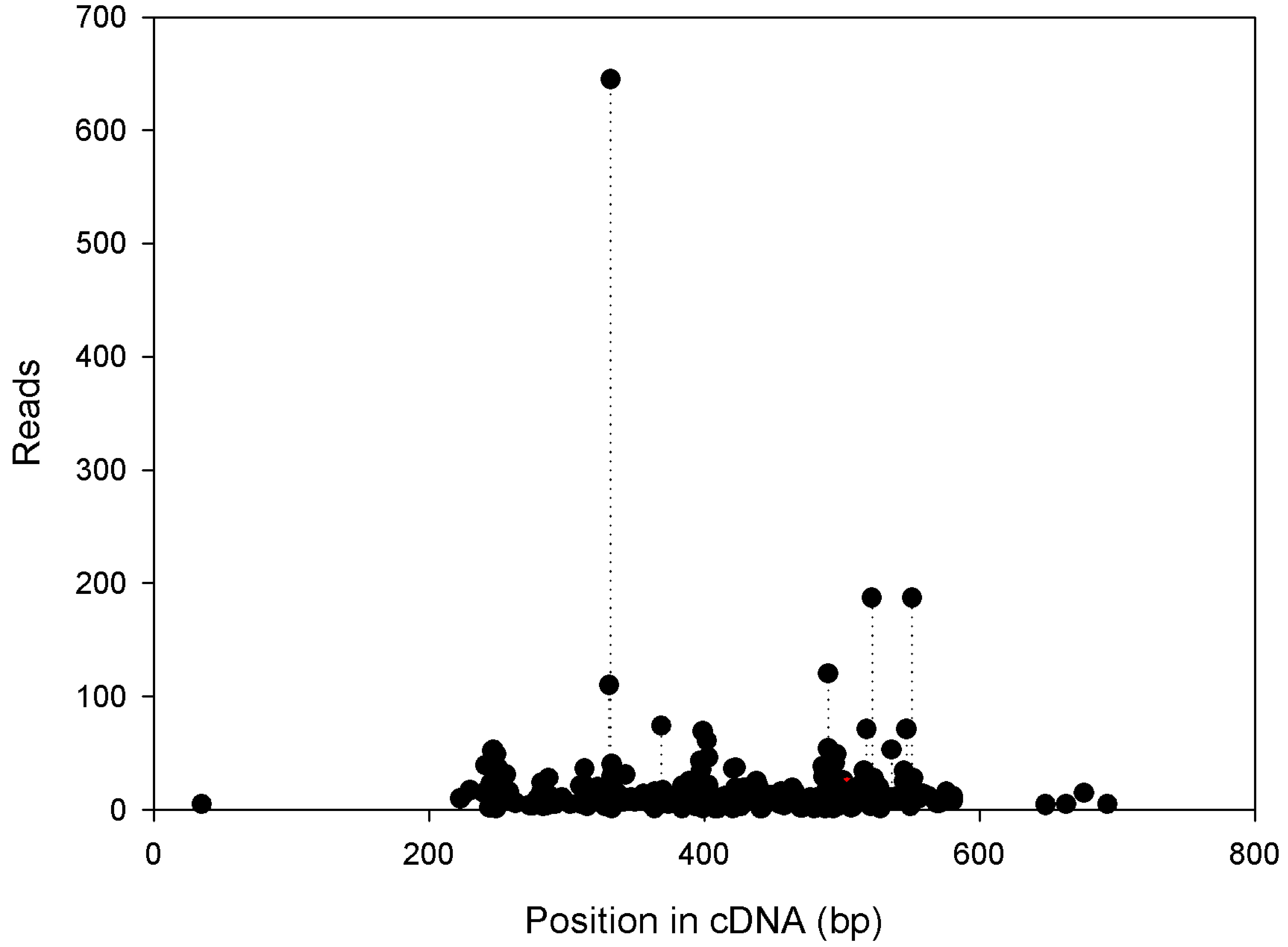


```
5' UCUCCUGAGGUUUGACCUUUGAGAAU 3'        Cs4g11930.1
       ::::::::..:::::::: ::::
3' ----GACUCCAGGCUGGAAAGUCUU- 5'        Csi-miR3954a-Cs1g09635.1-7
```

Csi-miR3954a-Cs1g09635.1-9, target=Cs2g11990.1 gene=Cs2g11990
Category:1
Score:4.5
Cleavage site:478

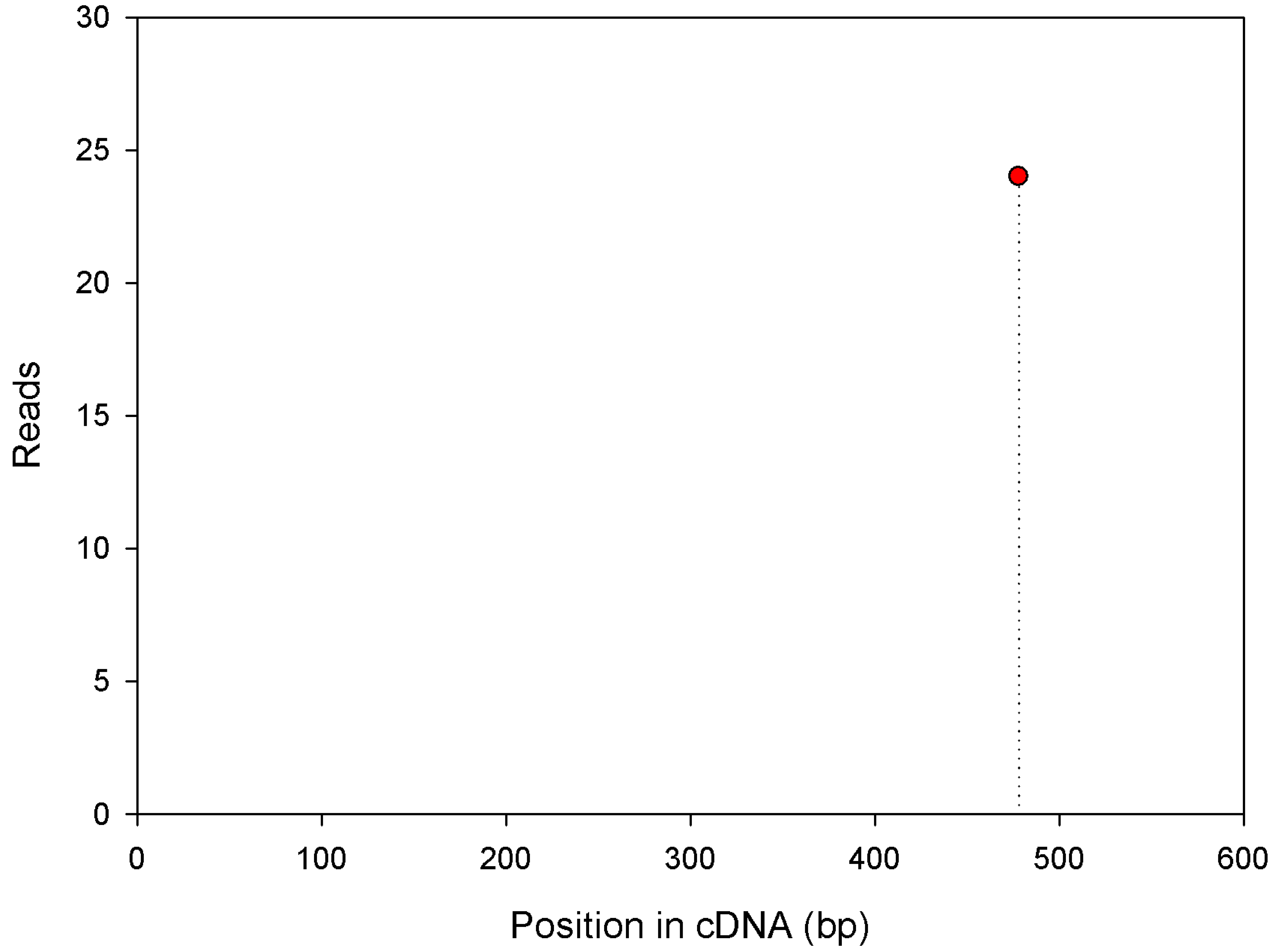


```
5' AGUACUACUUCAAUCACAAAGGCUCA 3'        Cs2g11990.1
     :  :::  :.  ::::::::::::
3' --AAGAU-AGUUUAGUGUUUCCGU-- 5'        Csi-miR3954a-Cs1g09635.1-9
```

Csi-miR3954a-Cs1g09635.1-9, target=Cs5g11880.1 gene=Cs5g11880
Category:3
Score:5
Cleavage site:407

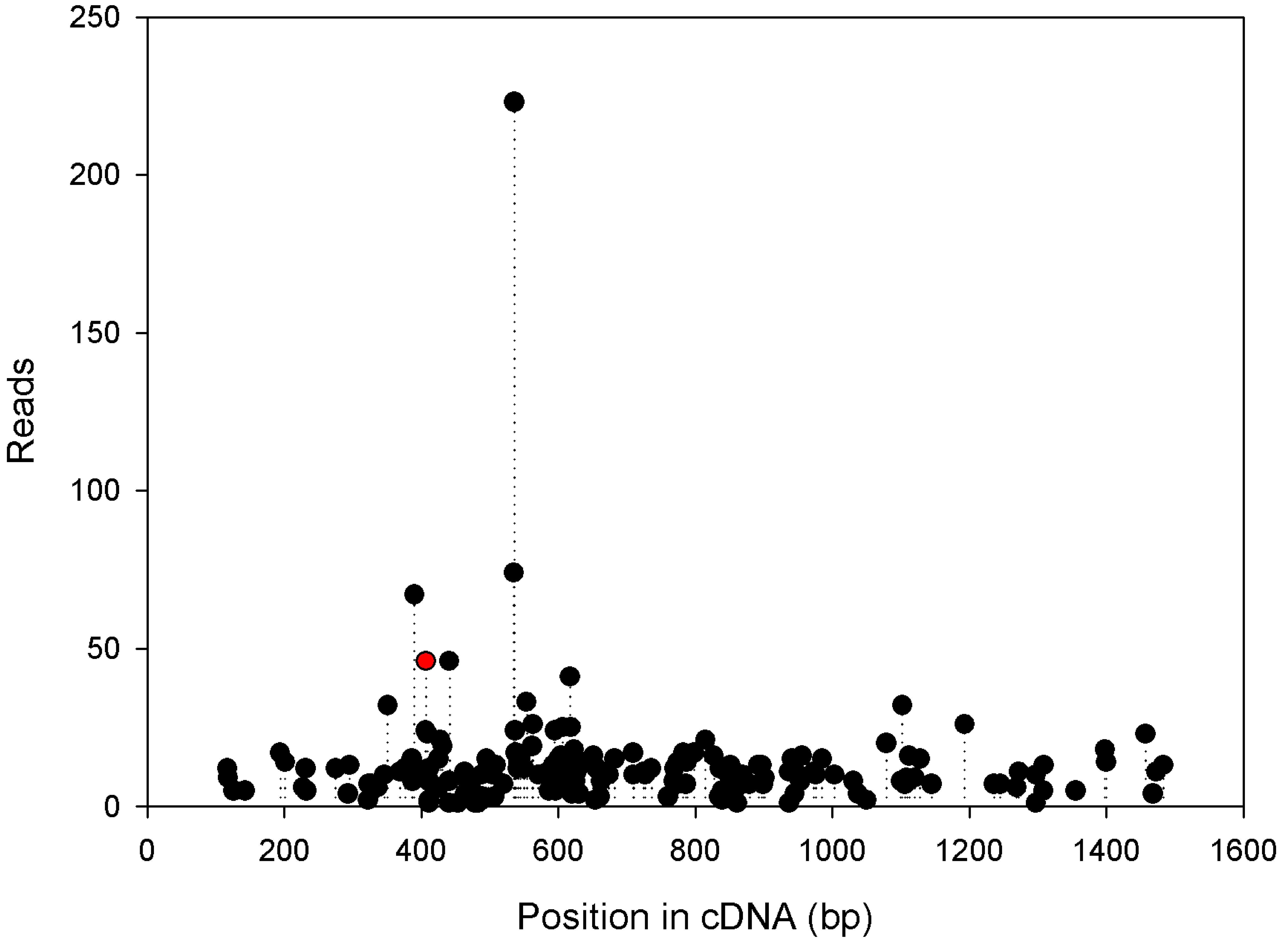


```
5' AAUAUUC-AGAAAAUUACAAAGGCUAU 3'       Cs5g11880.1
        ::: :  ::::.::::::::
3' ----AAGAUAGUUUAGUGUUUCCGU-- 5'       Csi-miR3954a-Cs1g09635.1-9
```

Csi-miR3954a-Cs5g04670.1-10, target=Cs4g17040.1 gene=Cs4g17040
Category:2
Score:5
Cleavage site:1227

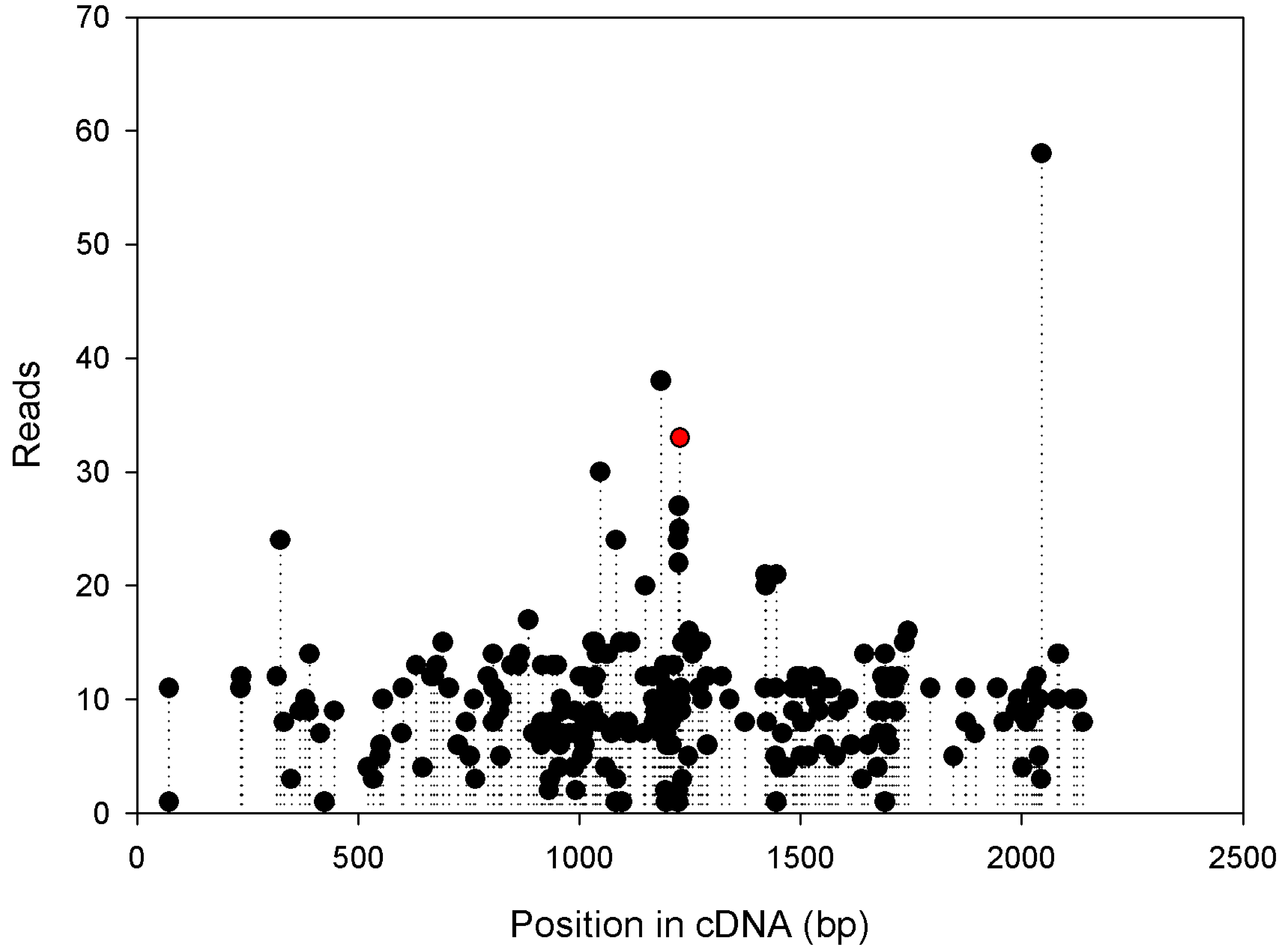


```
5' AAAUUGAACCGUAUUUUCUG-AAUUCU 3'       Cs4g17040.1
       ::::: :::::::::: ::.
3' ----ACUUGUCAUAAAAGACGUUGU-- 5'       Csi-miR3954a-Cs5g04670.1-10
```

Csi-miR3954a-Cs5g04670.1-13, target=Cs7g13090.1 gene=Cs7g13090
Category:3
Score:5
Cleavage site:1413

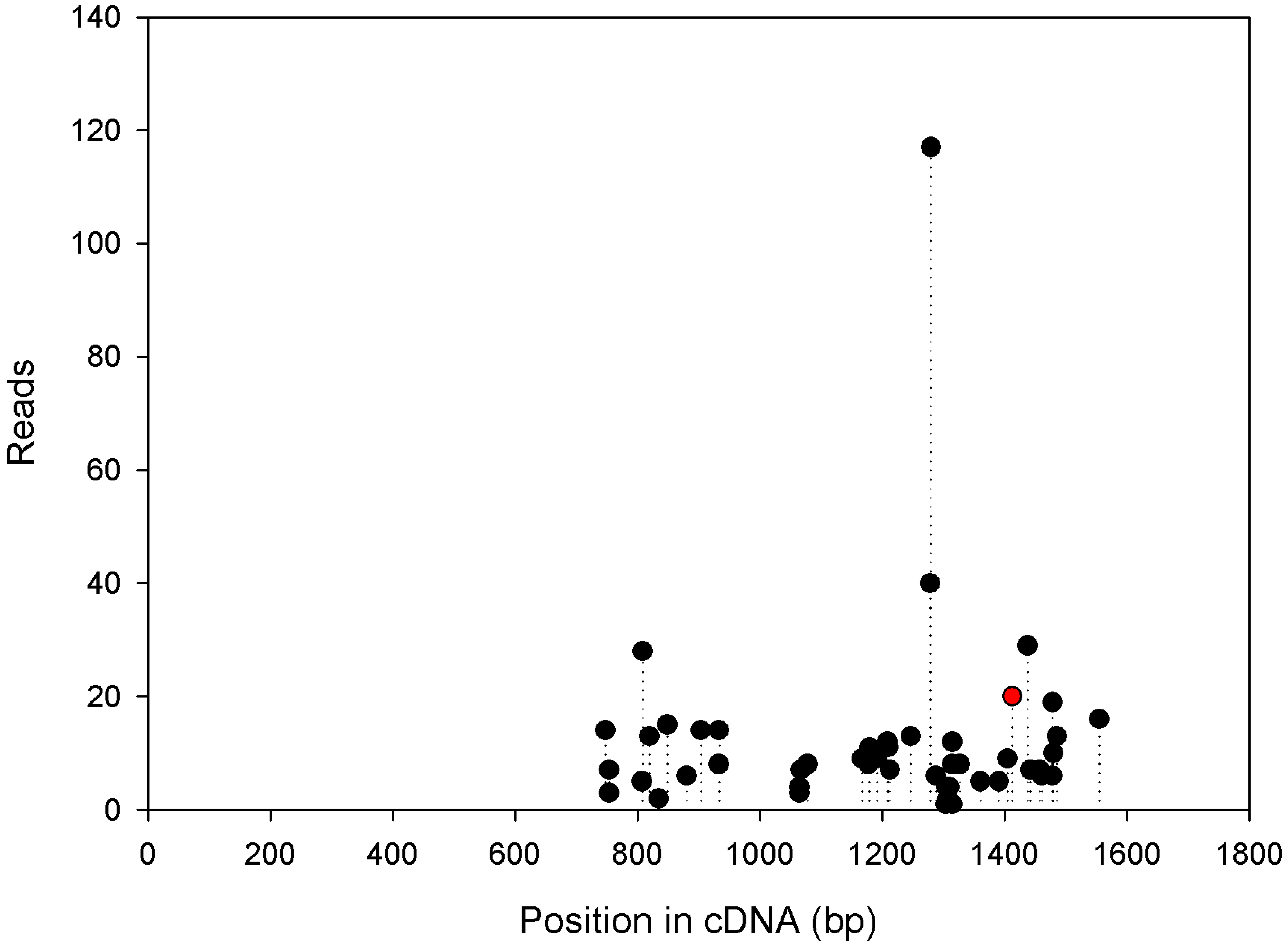


```
5' UUAUUGGAUU-UGAUUUGUAUAAUUGA 3'       Cs7g13090.1
          . ::. :::::: ::::::::
3' -----UGUAGUACUAAAGAUAUUAAC- 5'       Csi-miR3954a-Cs5g04670.1-13
```

Csi-miR3954a-Cs5g04670.1-16, target=Cs9g03100.1 gene=Cs9g03100
Category:3
Score:5
Cleavage site:2657

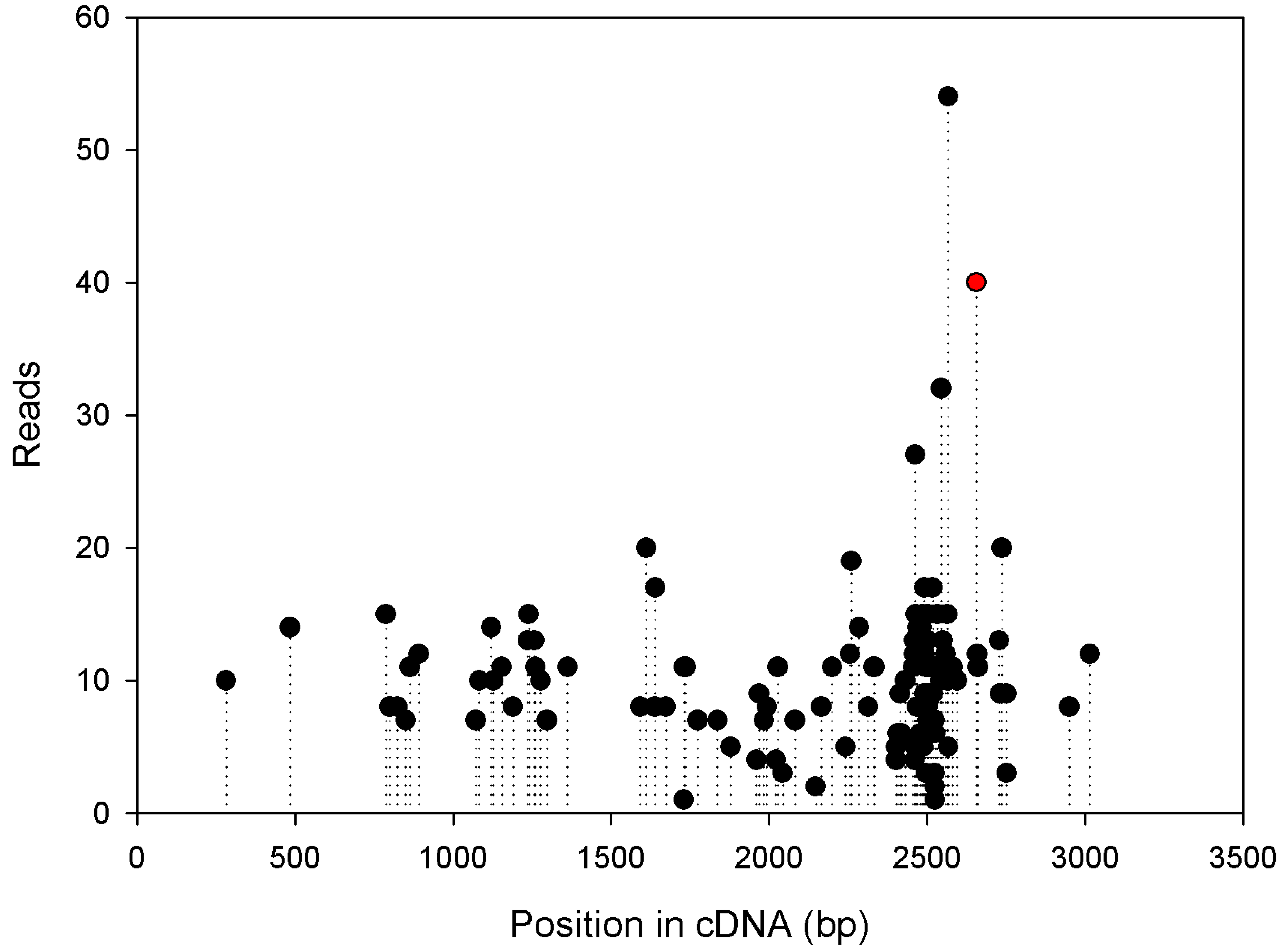


```
5' GAAAUUGA-GUGGAAAAGCUGGUUUUC 3'       Cs9g03100.1
     :: :::: ::..::::::::
3' -UUCAACUACAUUUUUUCGACA----- 5'       Csi-miR3954a-Cs5g04670.1-16
```

Csi-miR3954a-Cs5g04670.1-21, target=Cs3g25700.1 gene=Cs3g25700
Category:3
Score:5
Cleavage site:989

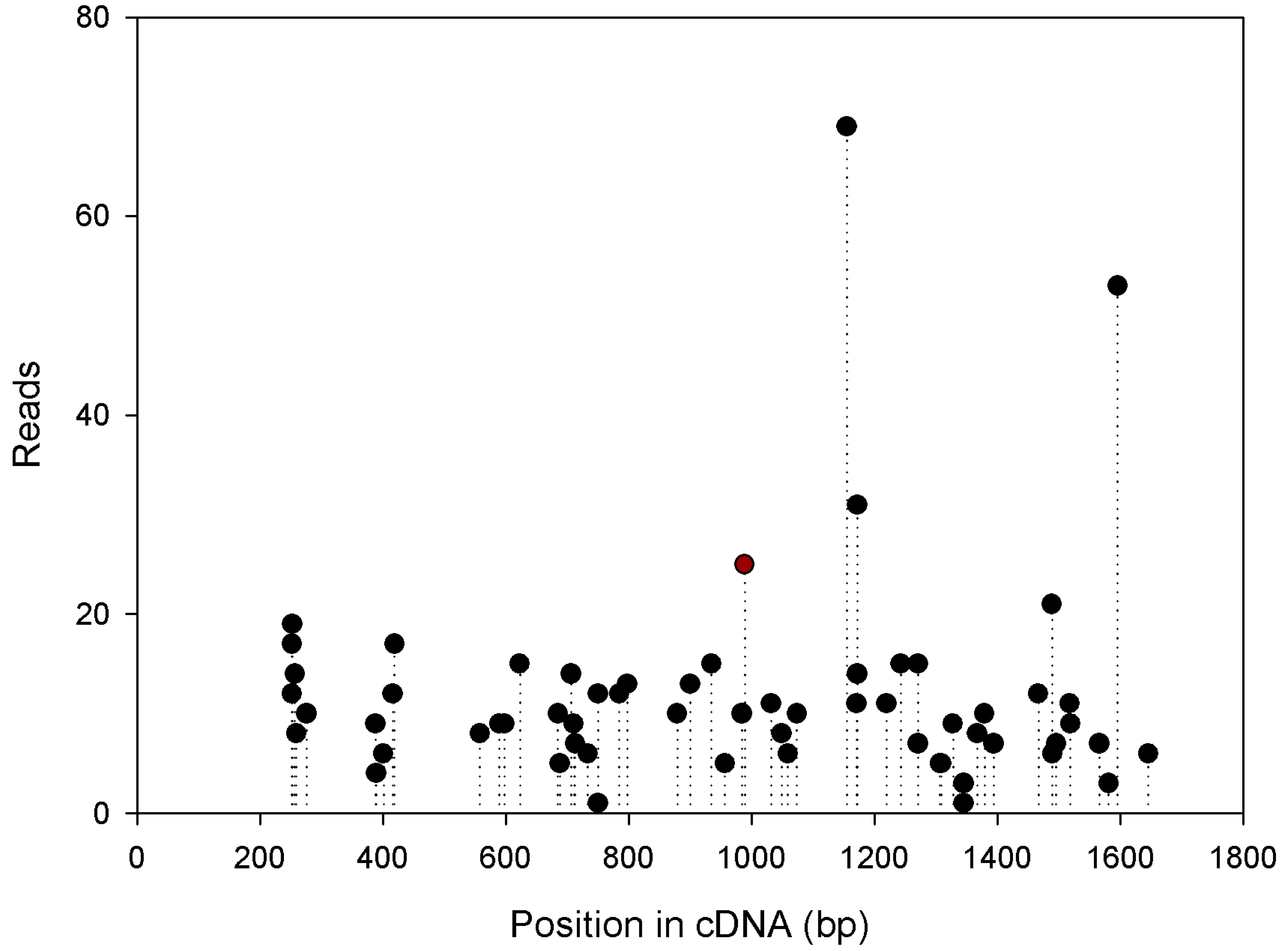


```
5' CAGCUCAAUCAGAUGCCAAAU-CUGAG 3'       Cs3g25700.1
        :::::::::::::::  :::
3' -----CUUAGUCUACGGUUUACCACU- 5'       Csi-miR3954a-Cs5g04670.1-21
```

Csi-miR3954a-Cs7g22460.1-15, target=Cs7g06320.1 gene=Cs7g06320
Category:3
Score:5
Cleavage site:109

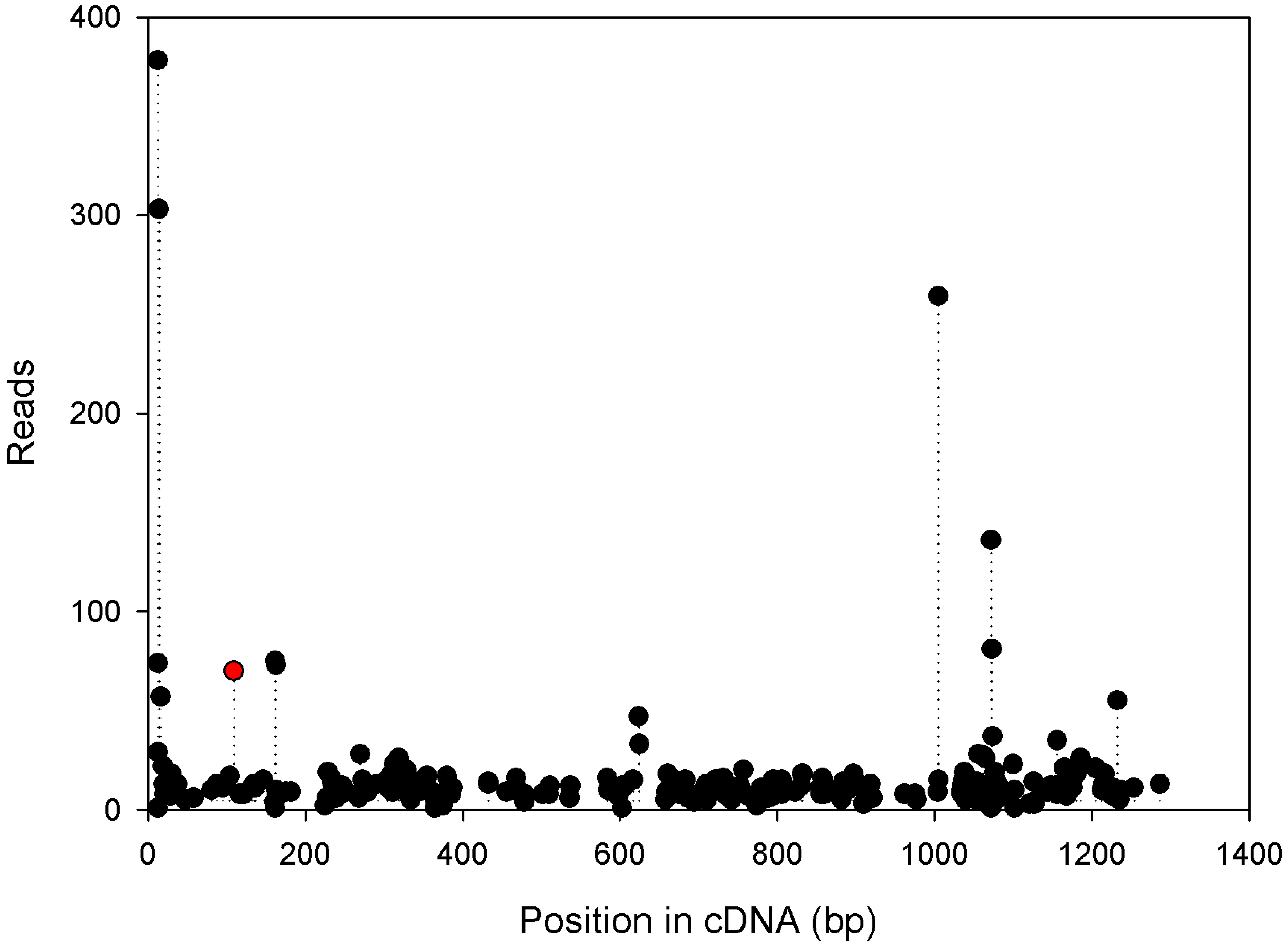


```
5' CGCAUAUAUUUUCUUUUUCAAGUUCU 3'        Cs7g06320.1
    : : ::::::: ::::::::
3' ACCUUUAUAAAAAAAAAAAGUU----- 5'        Csi-miR3954a-Cs7g22460.1-15
```

Csi-miR3954a-Cs7g22460.1-2, target=Cs3g07280.1 gene=Cs3g07280
Category:2
Score:4
Cleavage site:2828

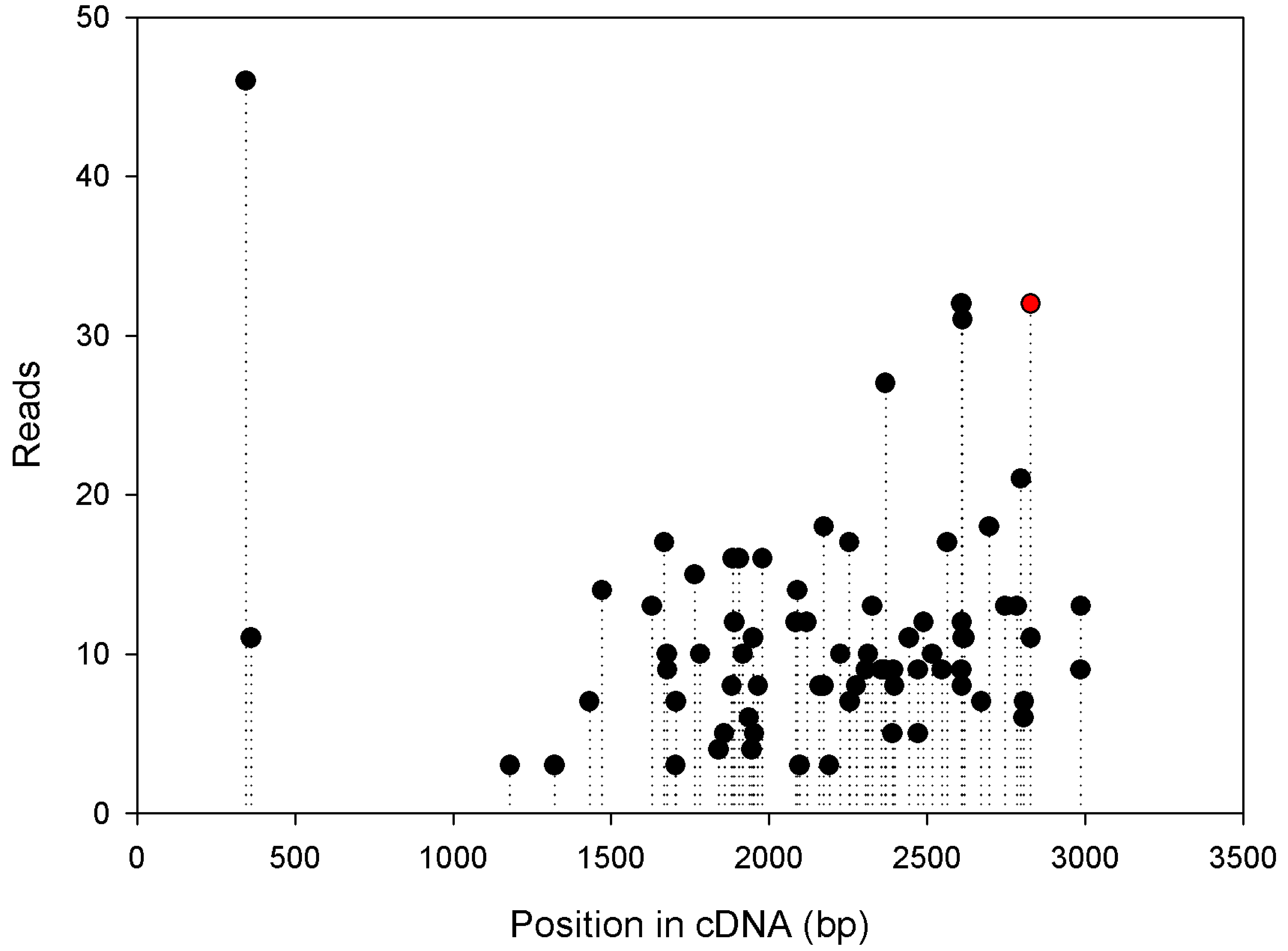


```
5' UACUG-AAAUUUAGCUGUAUGATGCCA 3'        Cs3g07280.1
   .:: . : :::::::::::::
3' GUGCUGUGUAAAUCGACAUAC------ 5'        Csi-miR3954a-Cs7g22460.1-2
```

Csi-miR3954a-Cs7g22460.1-2, target=Cs6g18000.1 gene=Cs6g18000
Category:3
Score:5
Cleavage site:371

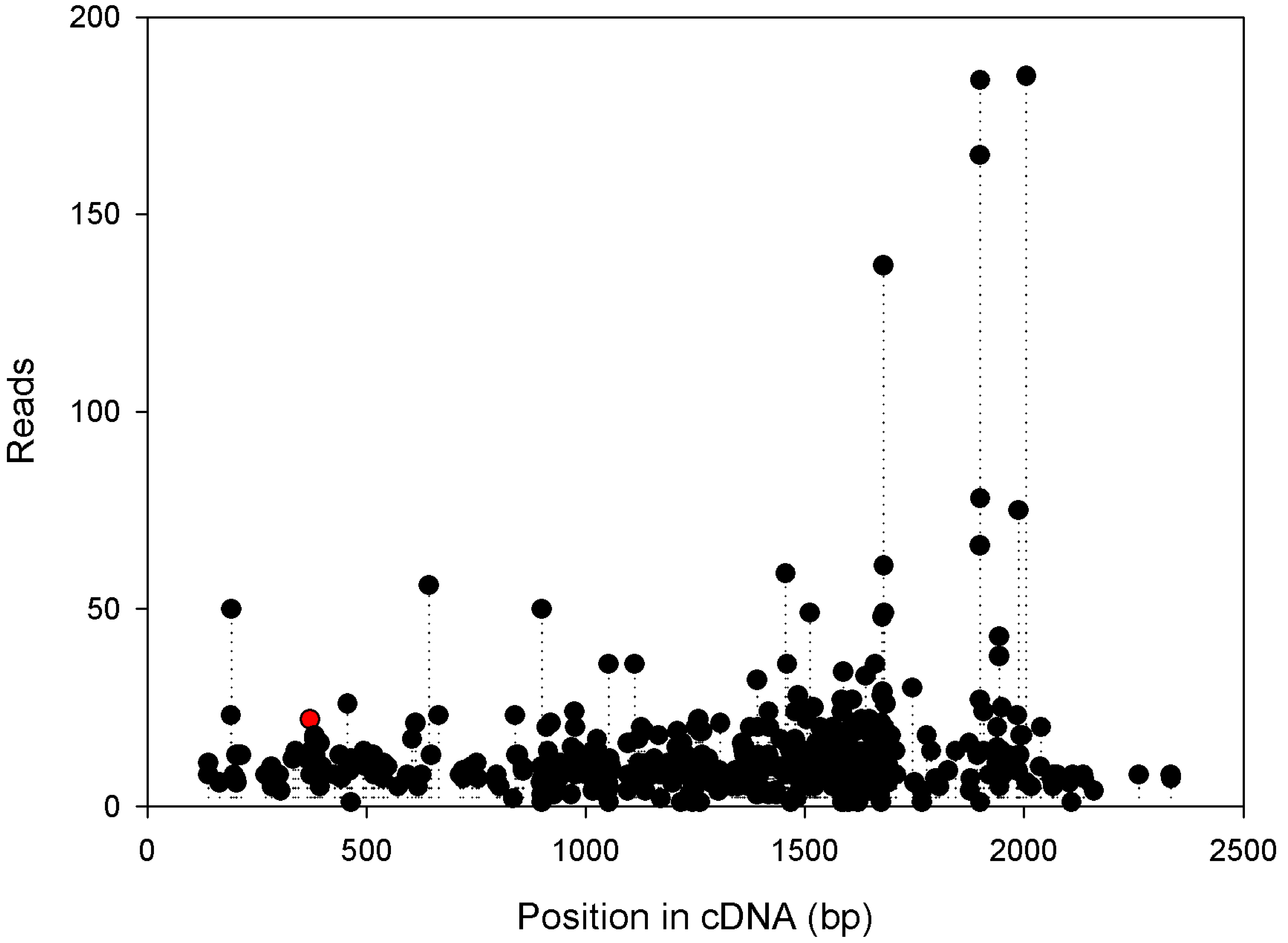


```
5' UGUCUGAAAGAUUUGGCUGUAUGCAU 3'        Cs6g18000.1
      .  .:: :  ::::.:::::::::
3' --GUGCUGUGUAAAUCGACAUAC--- 5'        Csi-miR3954a-Cs7g22460.1-2
```

Csi-miR3954a-Cs7g22460.1-8, target=Cs9g06660.1 gene=Cs9g06660
Category:3
Score:4
Cleavage site:798

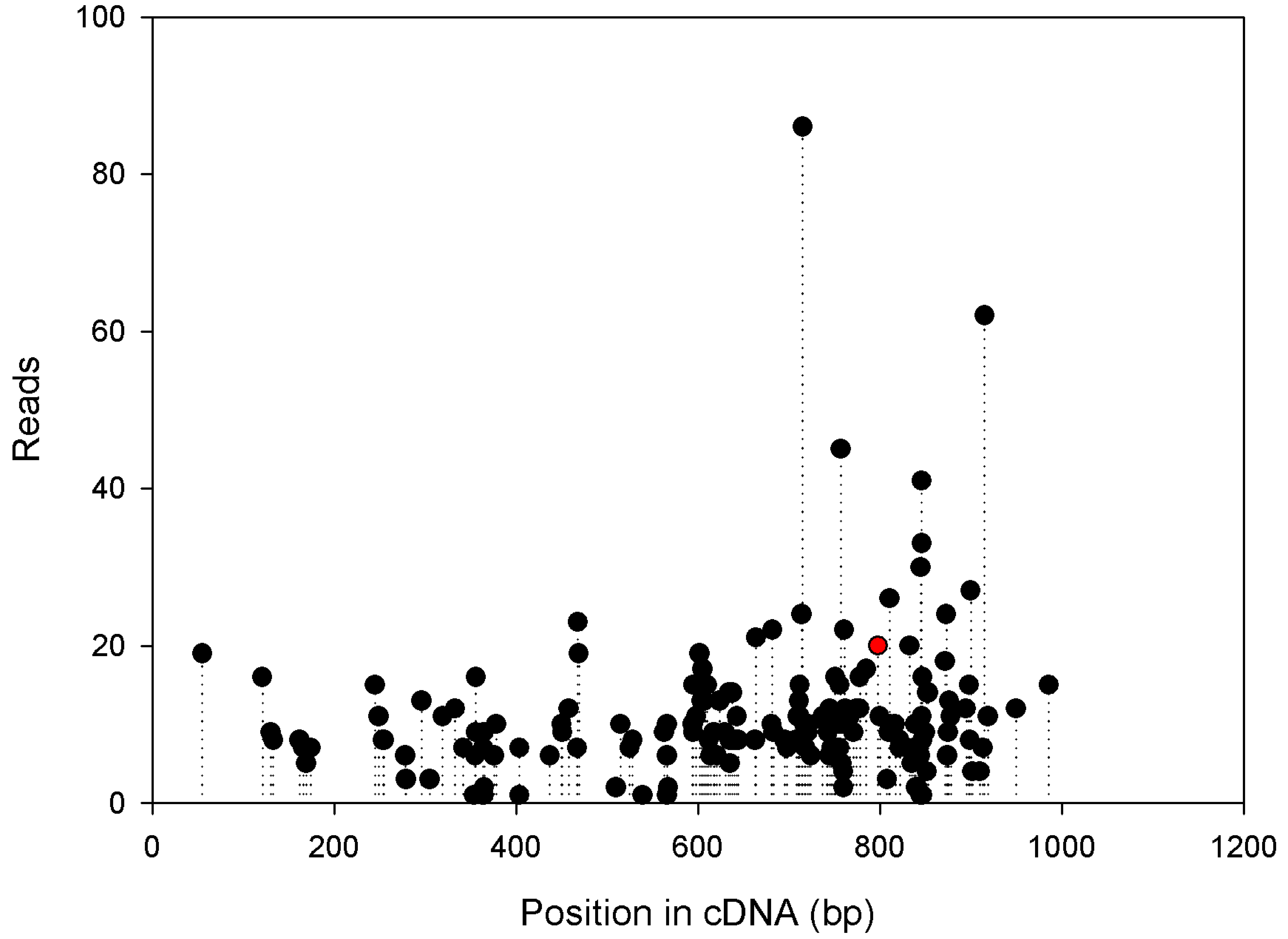


```
5' UUGUGAUCAUUUACUUGUUUGUAUUC 3'        Cs9g06660.1
          :::  :::::::::..:::::
3' -----UAGACAAUGAACAAGUAUAAG 5'        Csi-miR3954a-Cs7g22460.1-8
```

Csi-miR482a-3p-Cs5g18480.1-15, target=Cs7g01530.1 gene=Cs7g01530
Category:3
Score:5
Cleavage site:1936

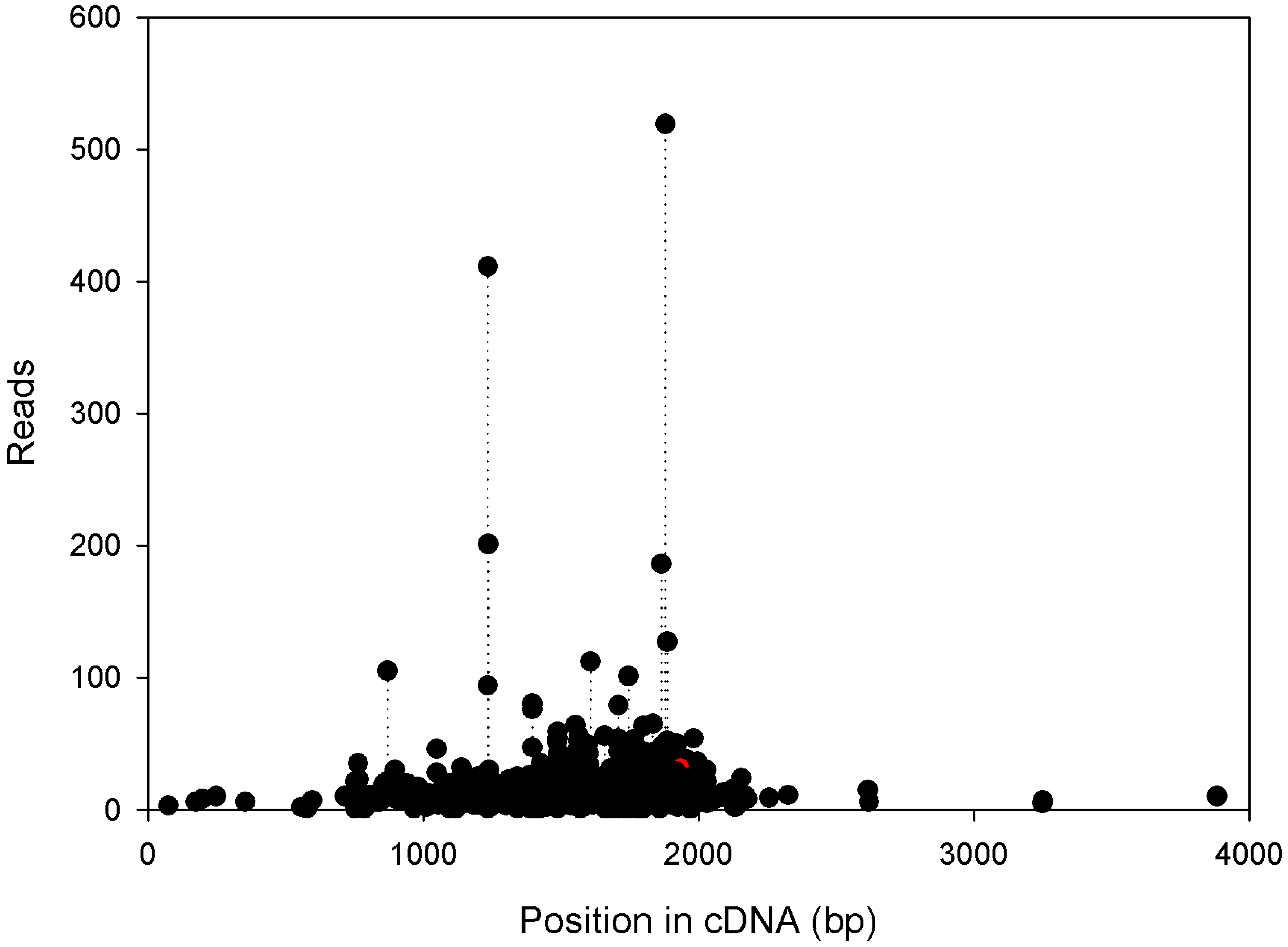


```
5' UUGU-AUGAUCCCACUUCCACUUUUGU 3'       Cs7g01530.1
   ::.: ::.:::: :::::::
3' AAUACUAUUAGGAUGAAGGUU------ 5'       Csi-miR482a-3p-Cs5g18480.1-15
```

Csi-miR482a-3p-Cs5g18480.1-18, target=Cs3g05730.1 gene=Cs3g05730
Category:1
Score:5
Cleavage site:732

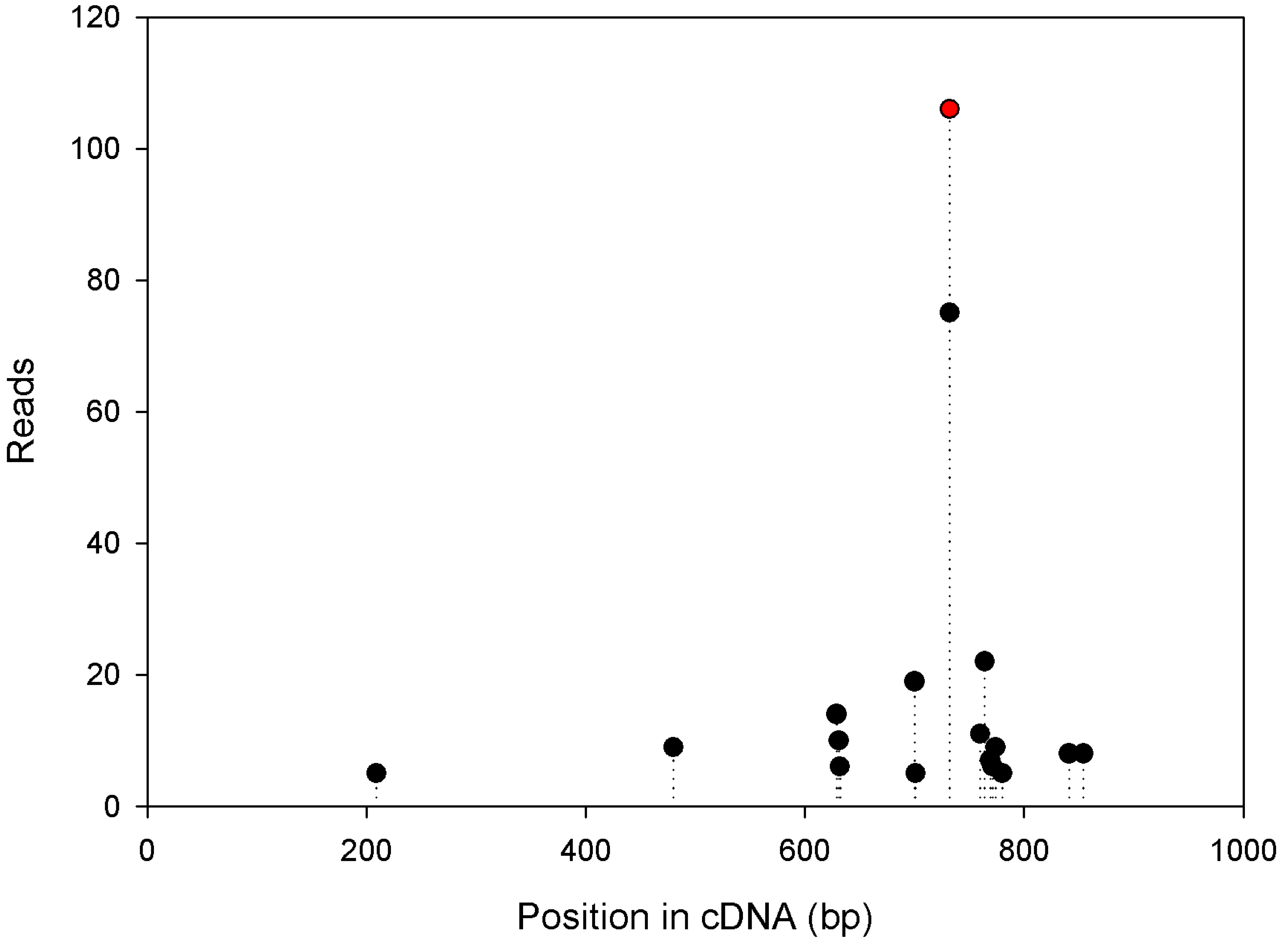


```
5' CUUGAAUCUUCAGCCU-GUACAGCUUA 3'       Cs3g05730.1
    ::.: ::.:::.::: :::::
3' -AAUUAAGGAGUUGGAGCAUGU----- 5'       Csi-miR482a-3p-Cs5g18480.1-18
```

Csi-miR482a-3p-Cs5g18480.1-18, target=Cs3g06120.1 gene=Cs3g06120
Category:1
Score:5
Cleavage site:2364

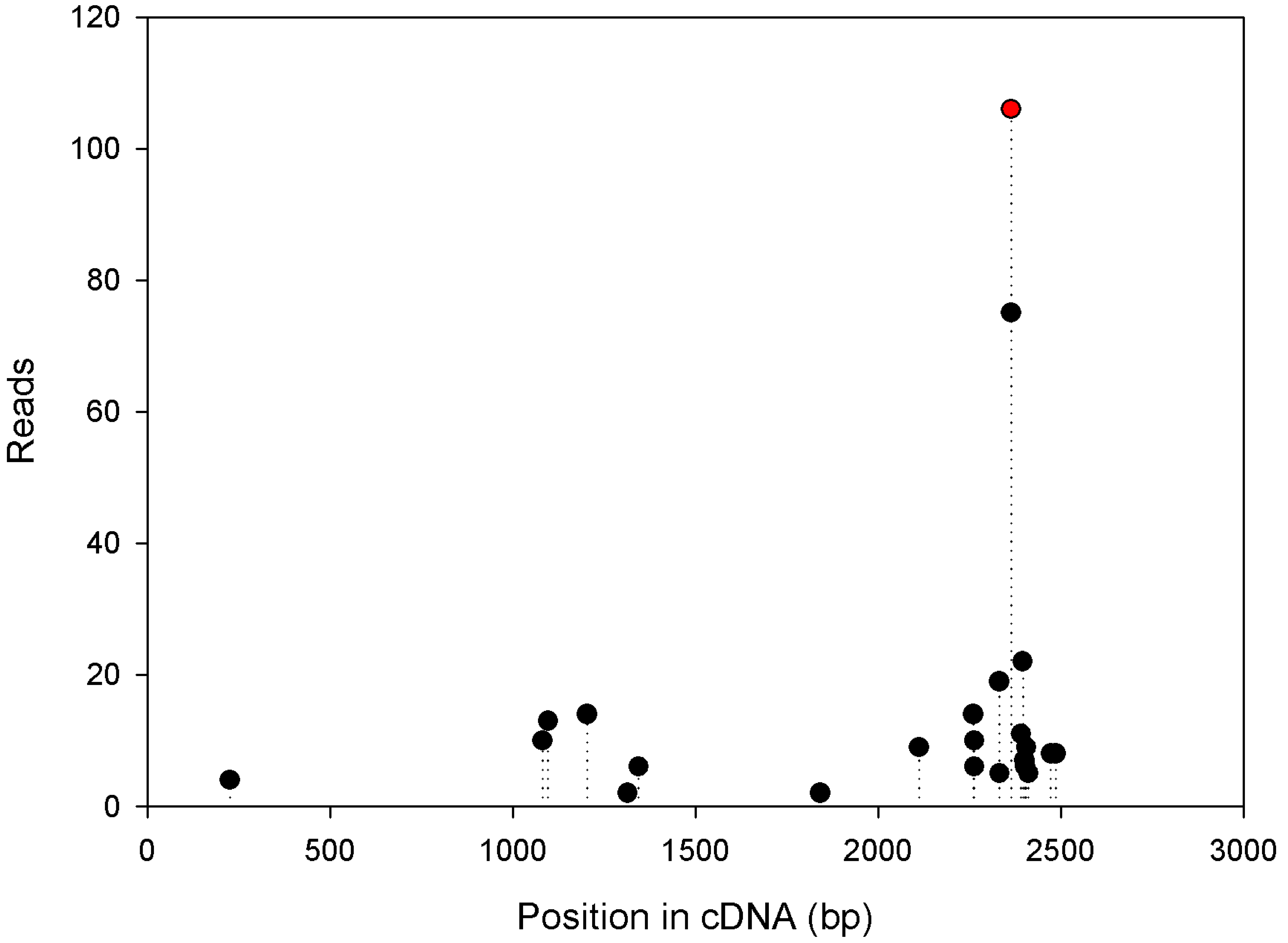


```
5' CUUGAAUCUUCAGCCU-GUACAGCUUA 3'       Cs3g06120.1
    ::.: ::.:::.::: :::::
3' -AAUUAAGGAGUUGGAGCAUGU----- 5'       Csi-miR482a-3p-Cs5g18480.1-18
```

Csi-miR482a-3p-Cs5g18480.1-20, target=Cs9g03090.1 gene=Cs9g03090
Category:3
Score:3.5
Cleavage site:3813

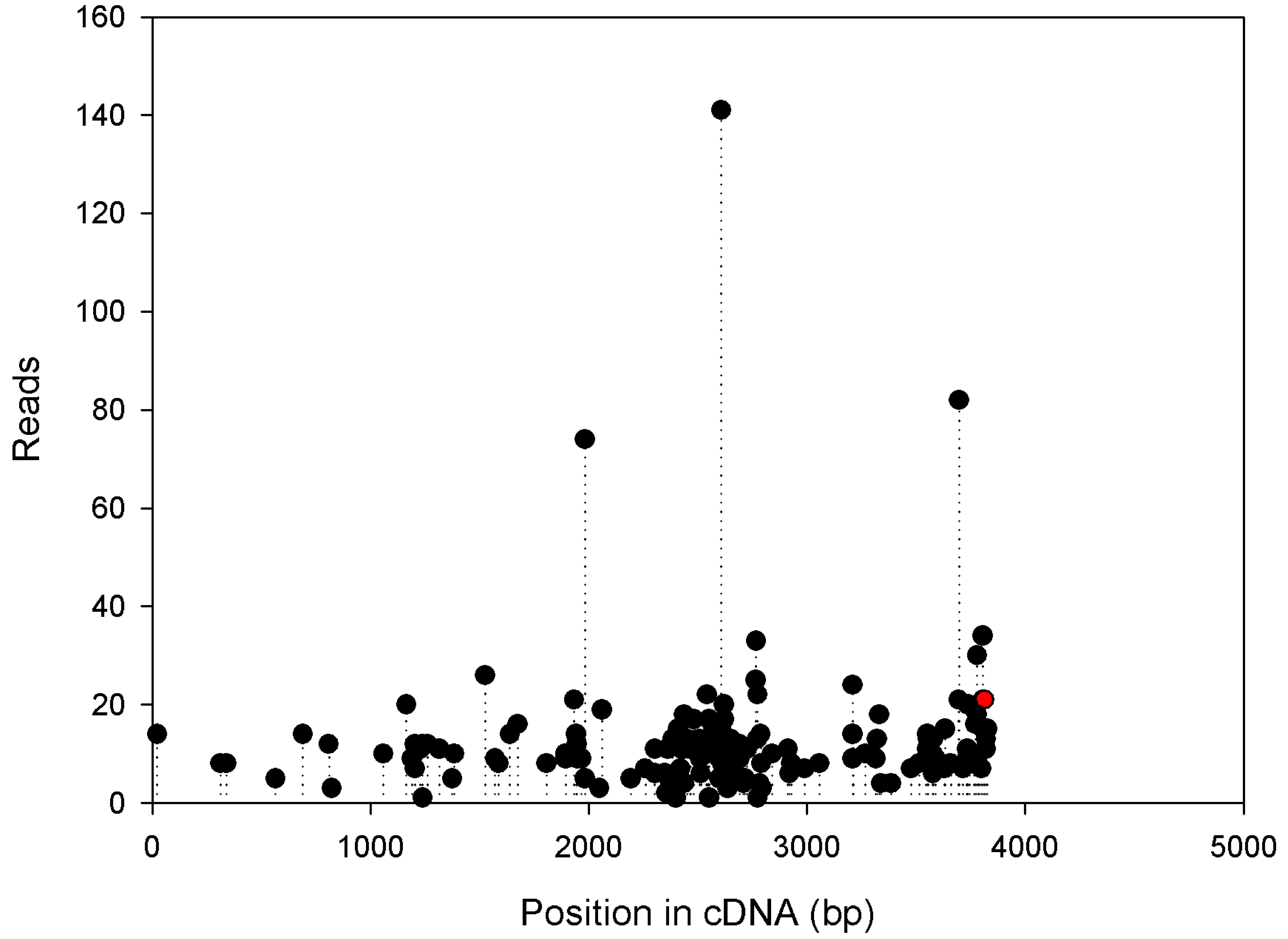


```
5' UUGUGAUUGUAUUUAAACUGAAAUUU 3'        Cs9g03090.1
       :::   :.:::::::::::::
3' ---ACUUCCGUAAAUUUGACUUUU-- 5'        Csi-miR482a-3p-Cs5g18480.1-20
```

Csi-miR482a-3p-Cs5g18480.1-41, target=Cs3g22270.2 gene=Cs3g22270
Category:2
Score:5
Cleavage site:2399

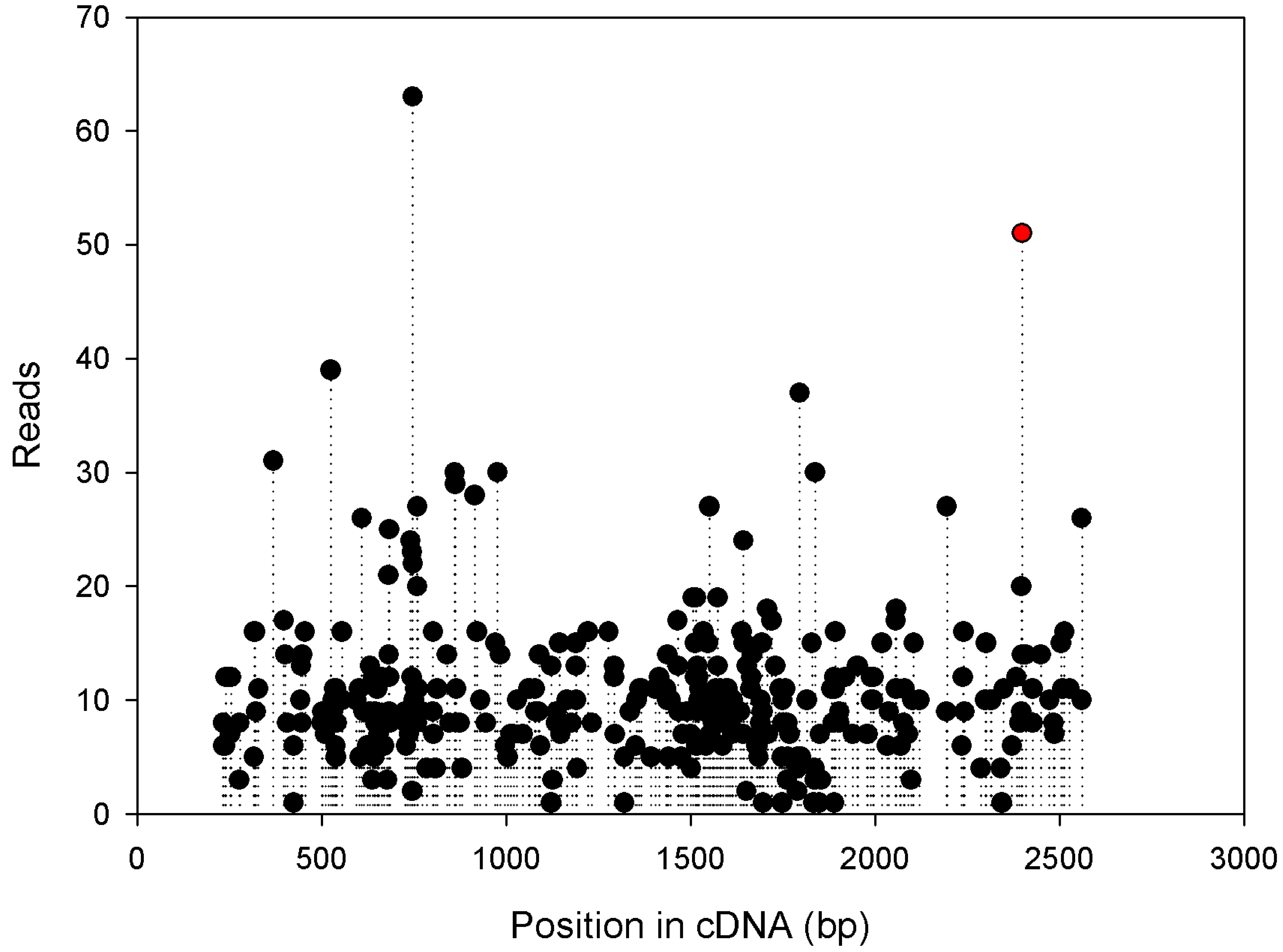


```
5' UGUUCCAUUUUCCAGGA-CUUUGAUUA 3'       Cs3g22270.2
      : : ::: :::::: ::::::
3' ---ACGCAAACGGUCCUAGAAACU--- 5'       Csi-miR482a-3p-Cs5g18480.1-41
```

Csi-miR482a-3p-Cs5g18480.1-77, target=Cs7g19320.2 gene=Cs7g19320
Category:3
Score:4.5
Cleavage site:2948

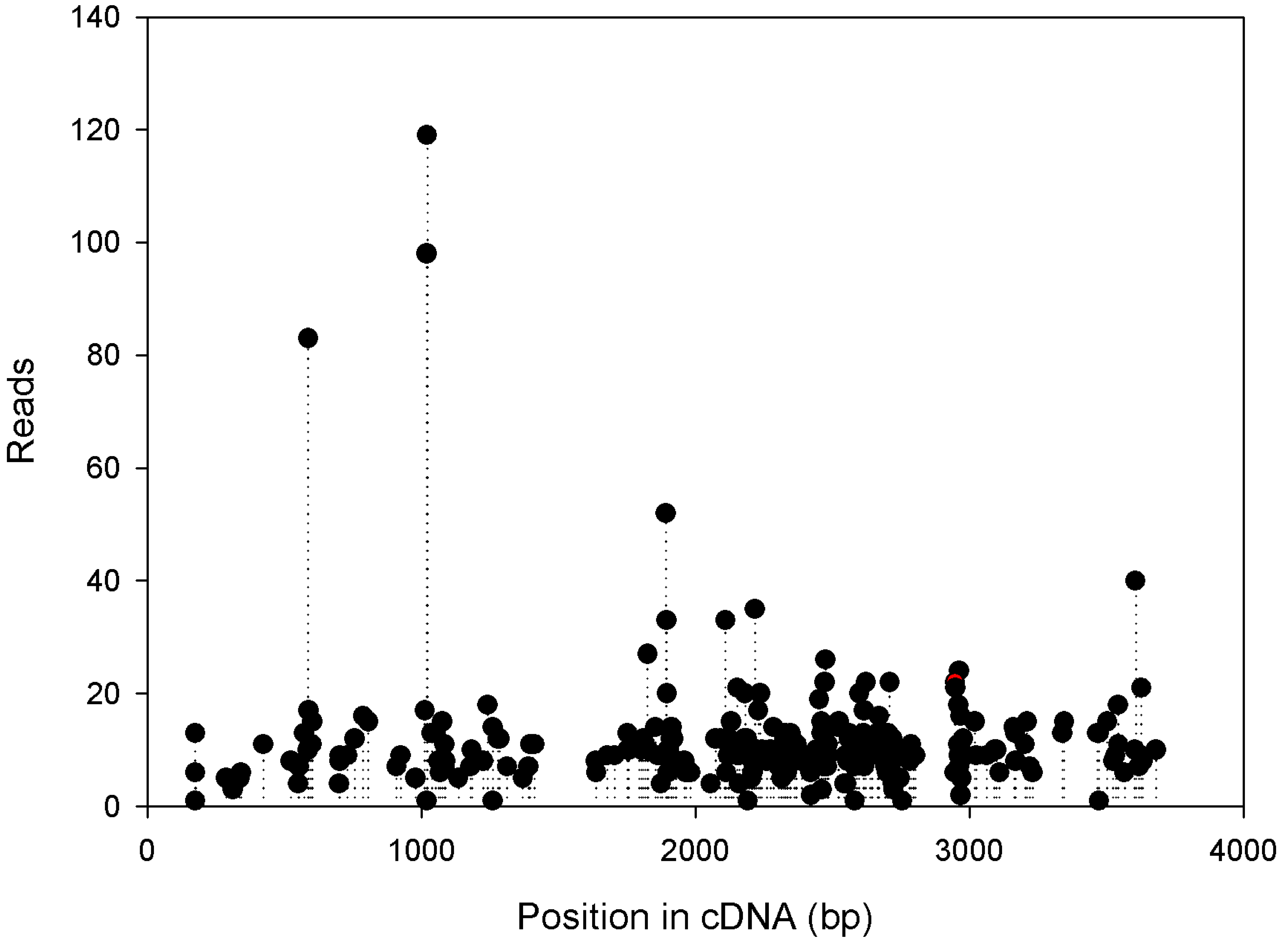


```
5' AAAUGCAGGUGUUUUCUAUUGCGGGG 3'        Cs7g19320.2
     :::  .:: :.::::::::::::
3' --UACCGUCA-AGAAGAUAACGCC-- 5'        Csi-miR482a-3p-Cs5g18480.1-77
```

Csi-miR482a-3p-Cs5g19920.1-36, target=Cs4g03050.1 gene=Cs4g03050
Category:3
Score:4
Cleavage site:1059

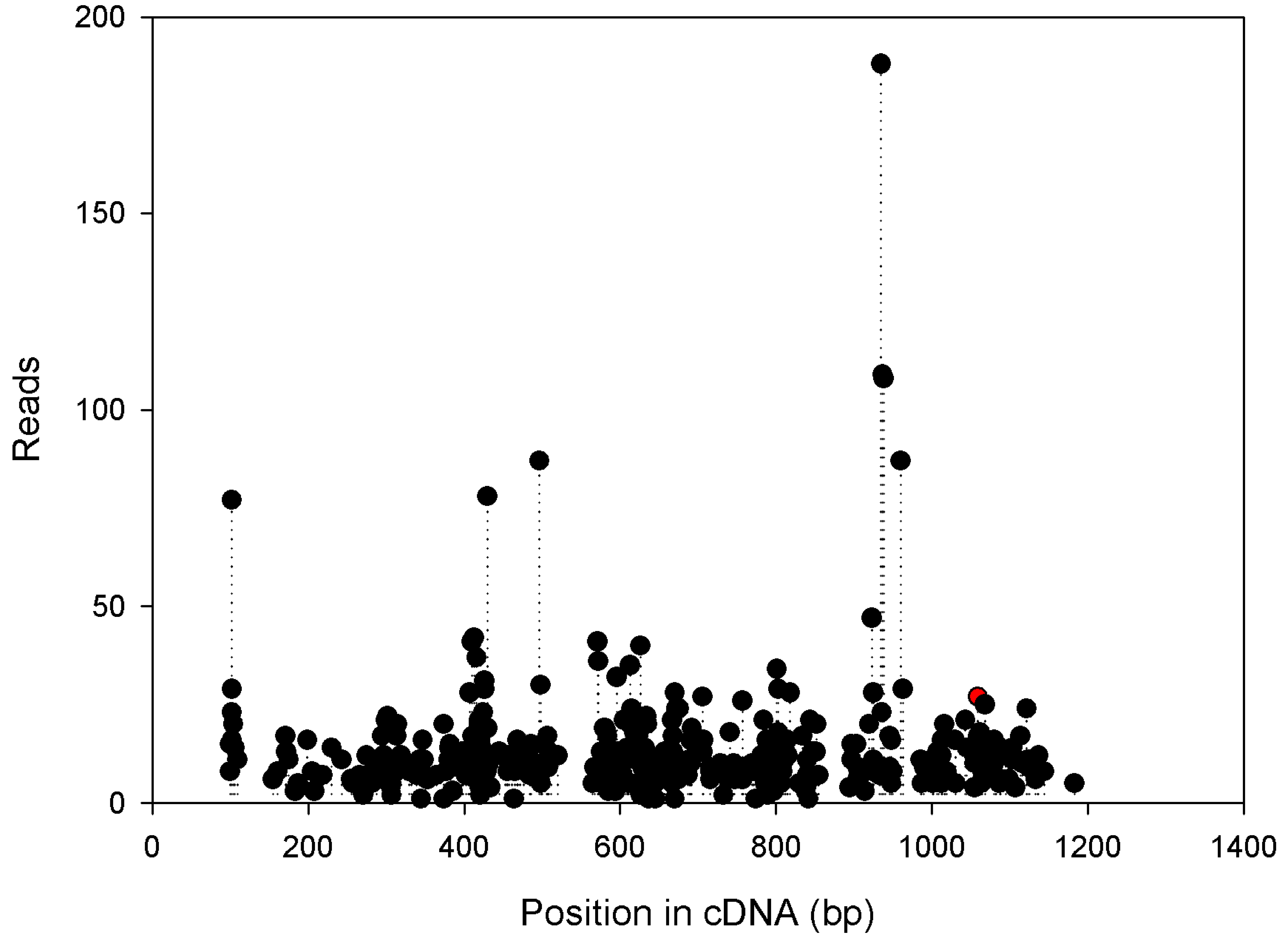


```
5' AAAAUAAUCAACAAGA-CACUUAAAAA 3'       Cs4g03050.1
      : : :::::::::: ::::::
3' --UGAAUAGUUGUUCUAGUGAAU---- 5'       Csi-miR482a-3p-Cs5g19920.1-36
```

Csi-miR482a-3p-Cs5g19920.1-36, target=Cs4g03140.1 gene=Cs4g03140
Category:3
Score:4
Cleavage site:949

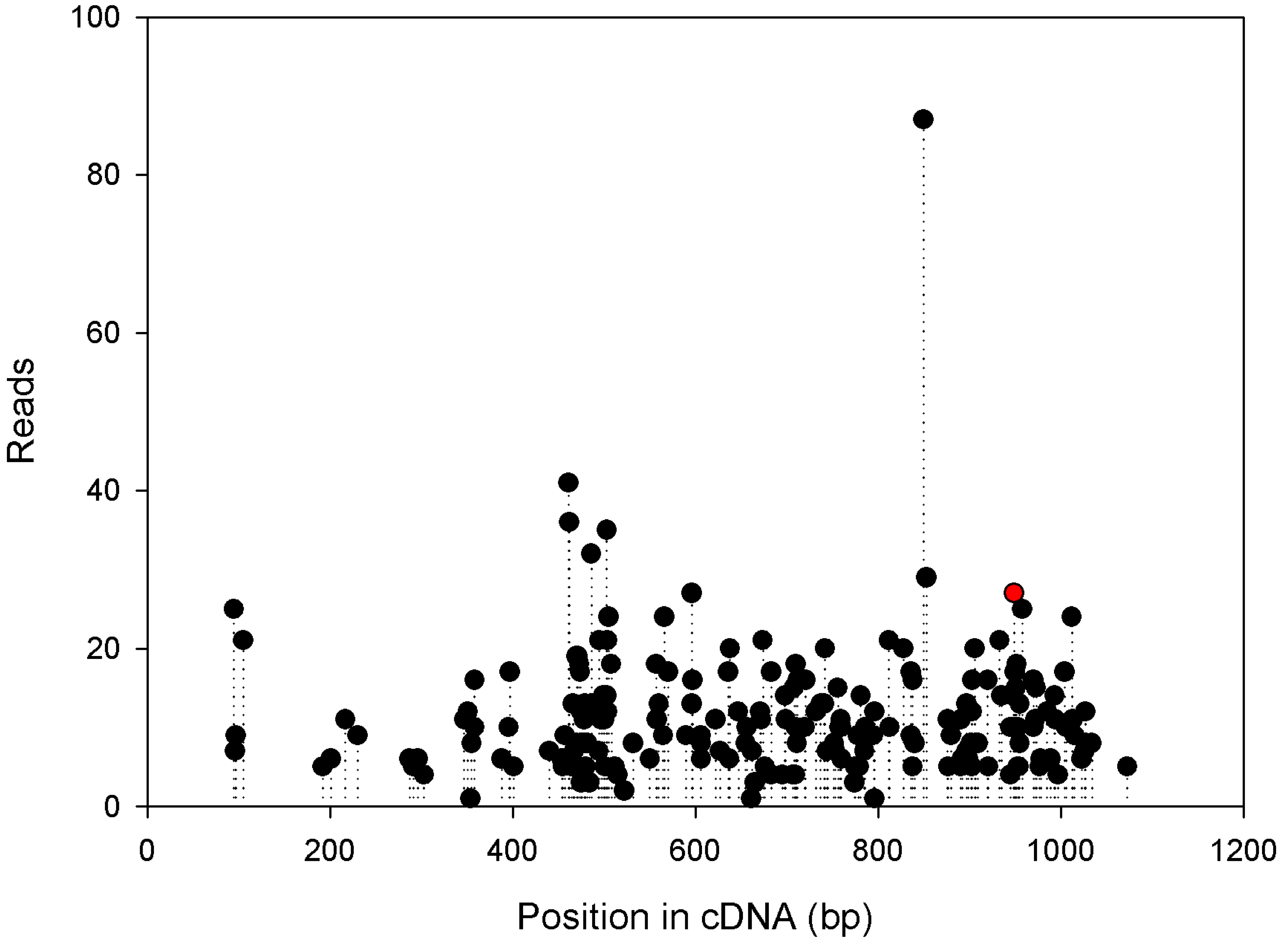


```
5' AAAAUAAUCAACAAGA-CACUUAAAAA 3'       Cs4g03140.1
     : : :::::::::: ::::::
3' --UGAAUAGUUGUUCUAGUGAAU---- 5'       Csi-miR482a-3p-Cs5g19920.1-36
```

Csi-miR482c-Cs9g06846.1-10, target=Cs2g30790.2 gene=Cs2g30790
Category:2
Score:4.5
Cleavage site:1898

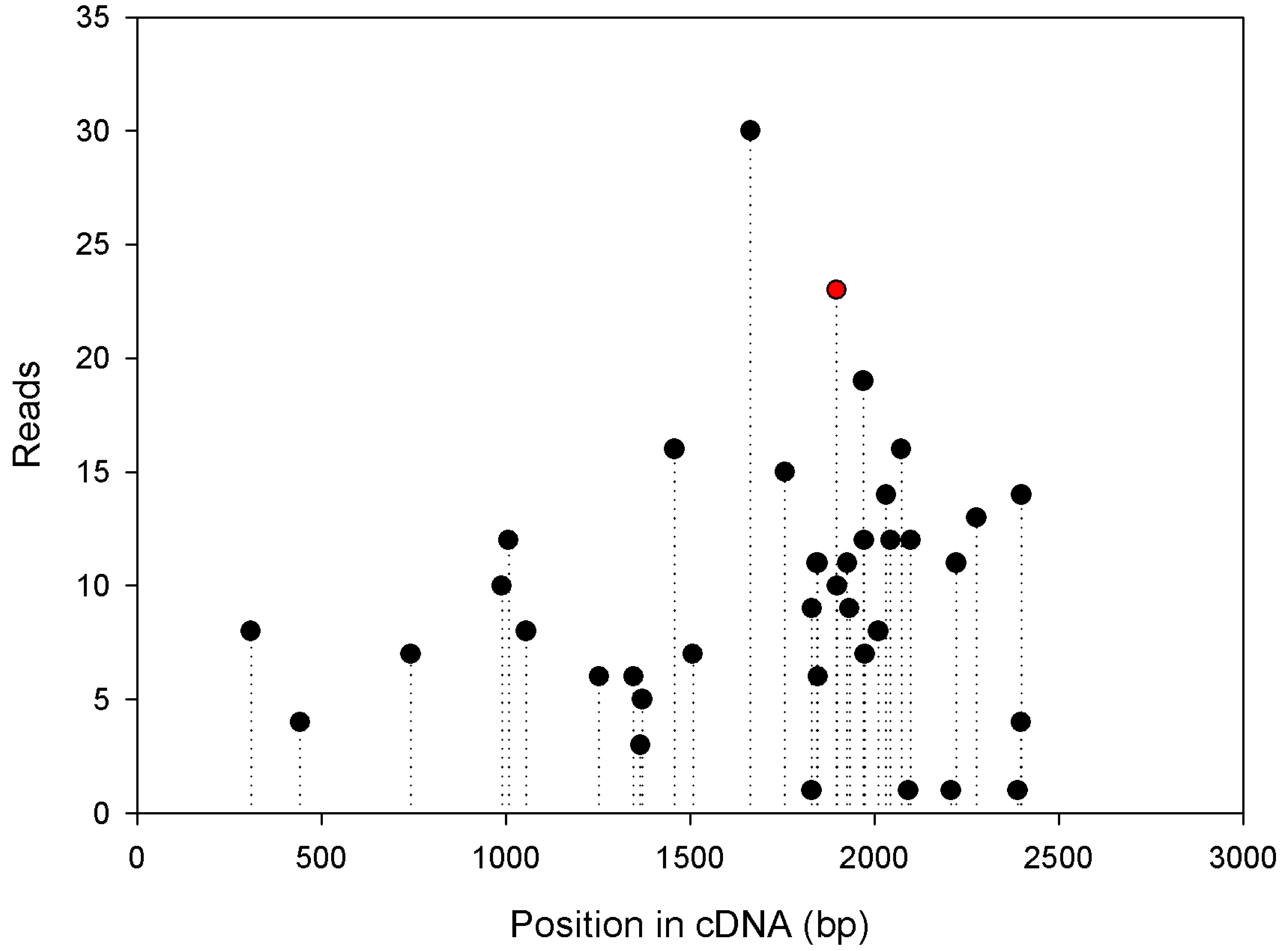


```
5' UGAUAUUCCAUCAUCGUUGAGAAAUU 3'        Cs2g30790.2
       ::. ::: :: ::::::::::
3' ----UAGAGUAUUAACAACUCUUUA- 5'        Csi-miR482c-Cs9g06846.1-10
```

Csi-miR482c-Cs9g06846.1-15, target=Cs1g16450.1 gene=Cs1g16450
Category:2
Score:5
Cleavage site:1954

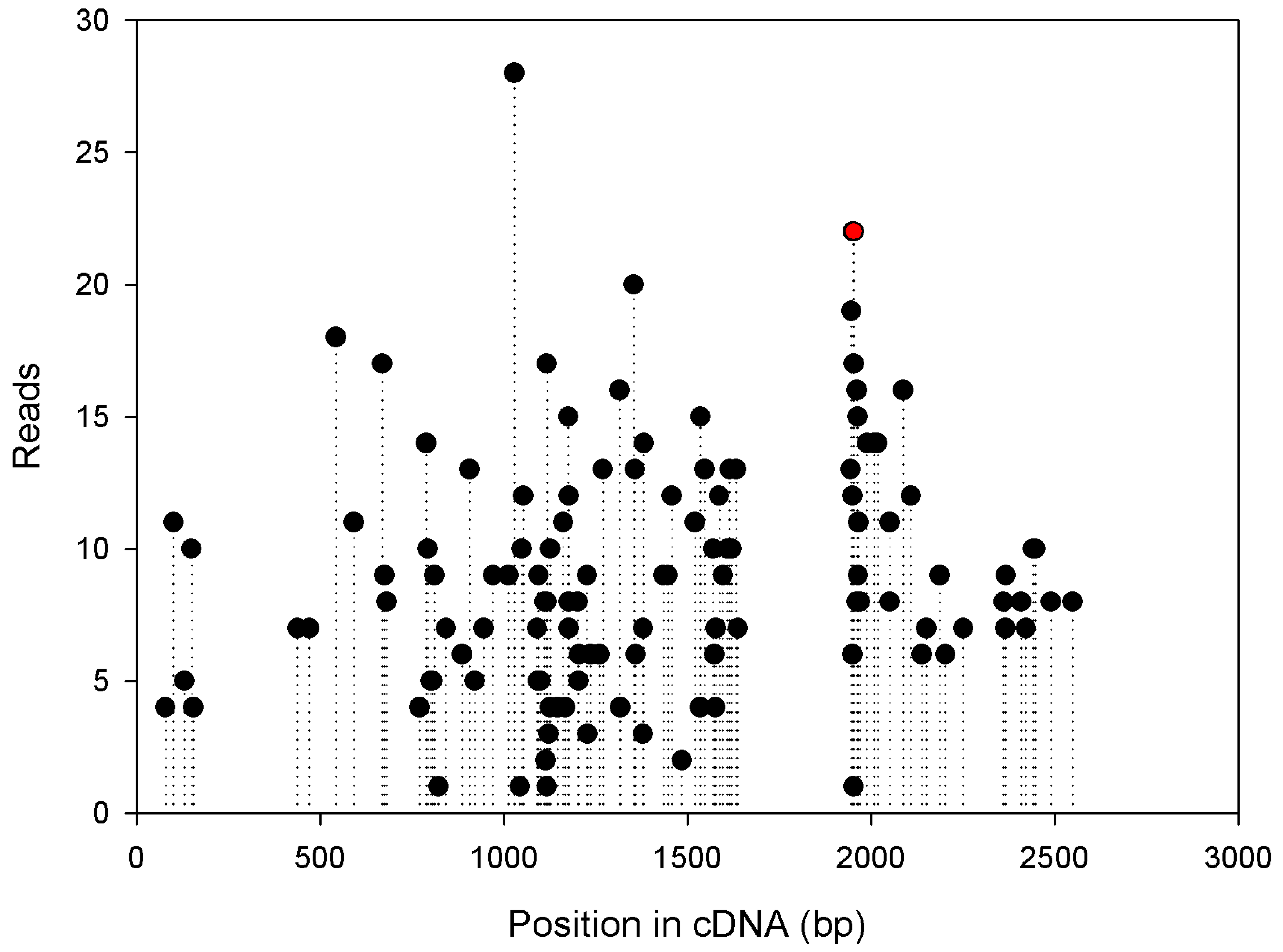


```
5' UACAAUGG-UAUCAUAUUAUGGCACAG 3'        Cs1g16450.1
   ::: :::: ::::::::: ::
3' AUGGUACCUAUAGUAUAAGAC------ 5'        Csi-miR482c-Cs9g06846.1-15
```

Csi-miR482c-Cs9g06846.1-22, target=Cs5g19660.1 gene=Cs5g19660
Category:3
Score:5
Cleavage site:1665

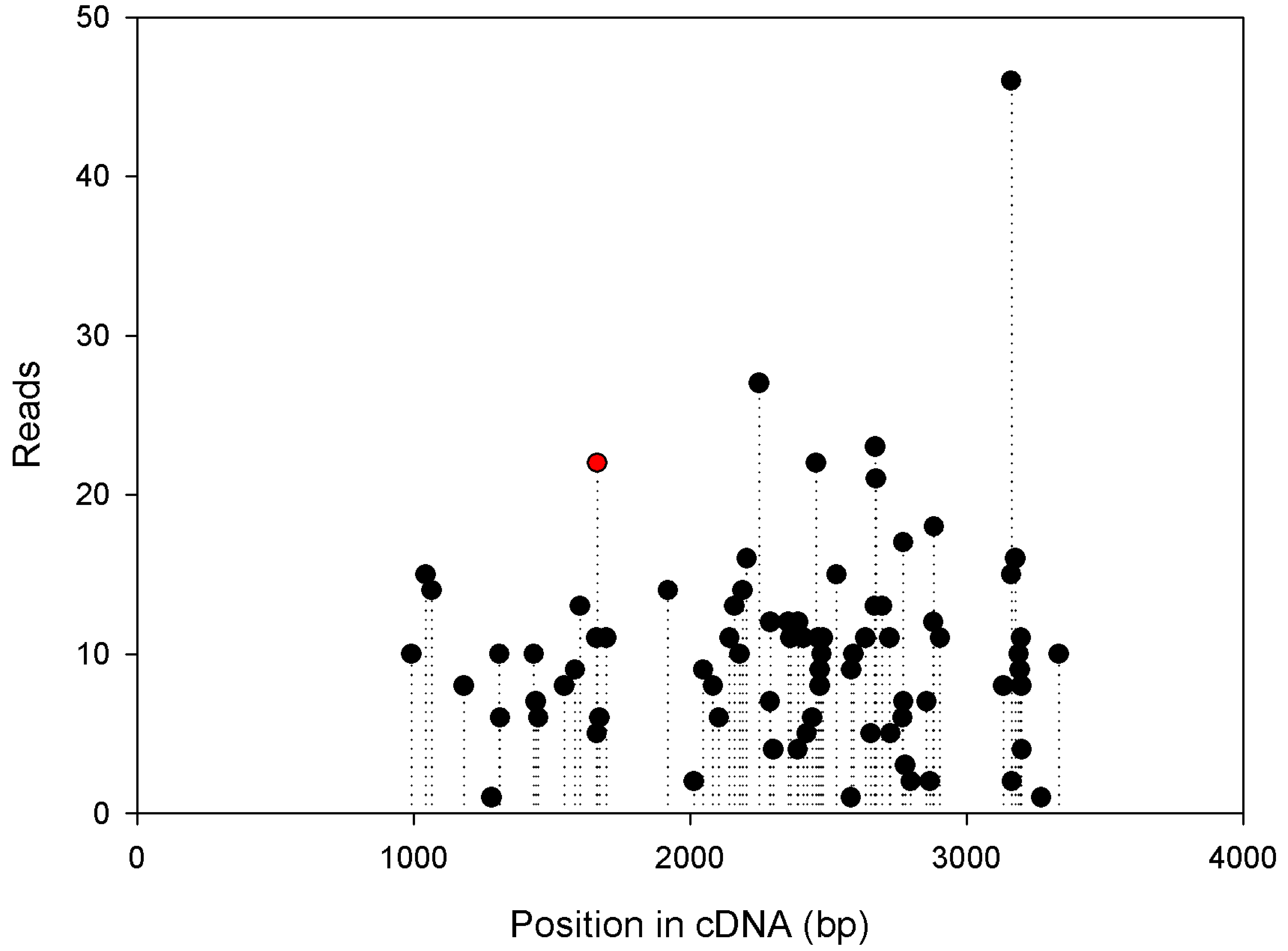


```
5' AGCUUUAU-UAUCUCAAUGCUGGAGGA 3'       Cs5g19660.1
    ::..::: ::::::::: ::
3' -CGGGAUAGAUAGAGUUAGGAA----- 5'       Csi-miR482c-Cs9g06846.1-22
```

Csi-miR482c-Cs9g06846.1-5, target=Cs9g08050.1 gene=Cs9g08050
Category:3
Score:5
Cleavage site:1748

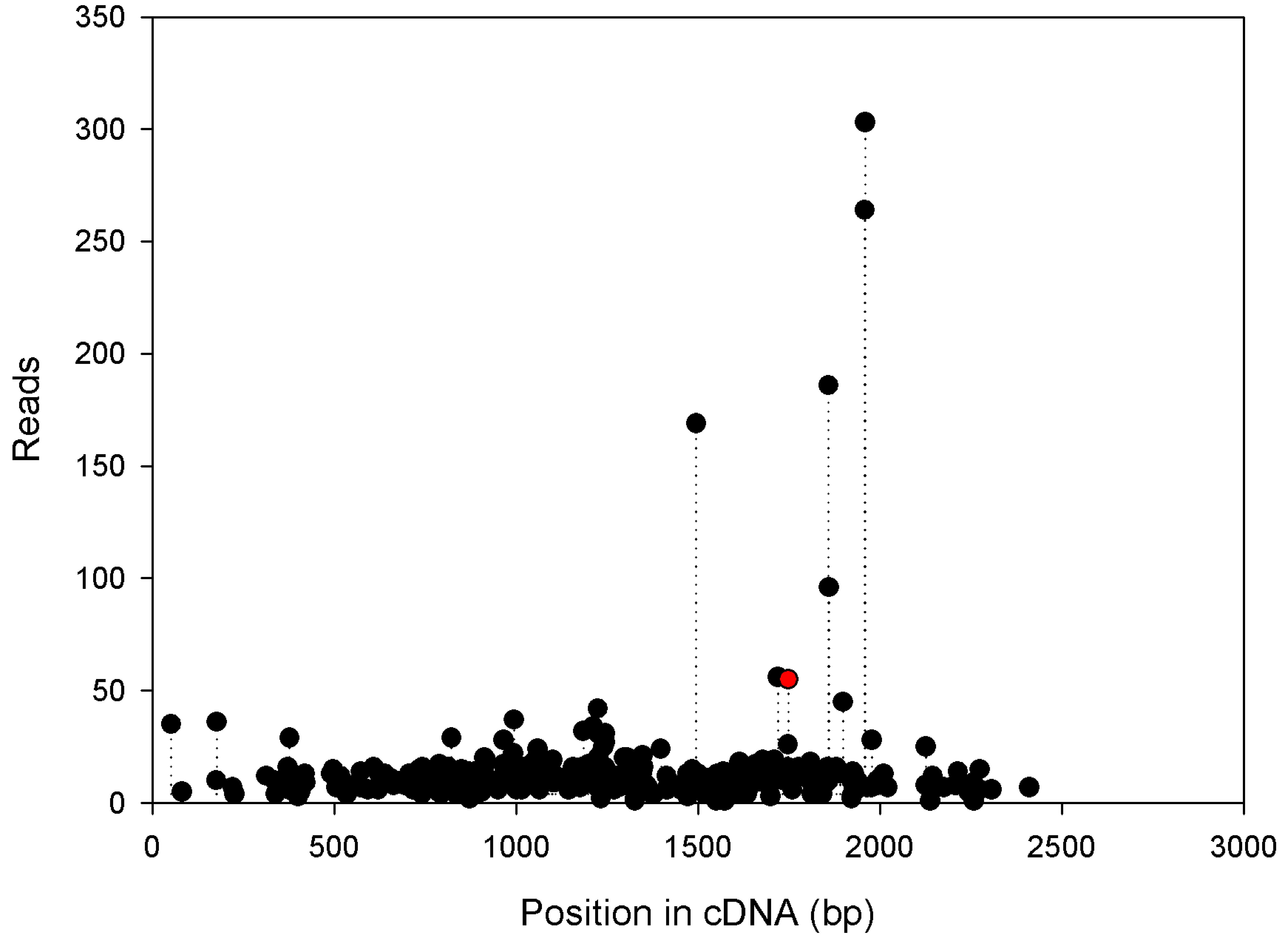


```
5' GAAGGAAUGGAUGGAUGAGAUUCUUU 3'        Cs9g08050.1
     :: :  ::::::.::.:::::
3' -UUACACACCUACUUAUUCUAA---- 5'        Csi-miR482c-Cs9g06846.1-5
```

Csi-miRN20-Cs3g05320.1-23, target=Cs7g19800.1 gene=Cs7g19800
Category:3
Score:4.5
Cleavage site:2805

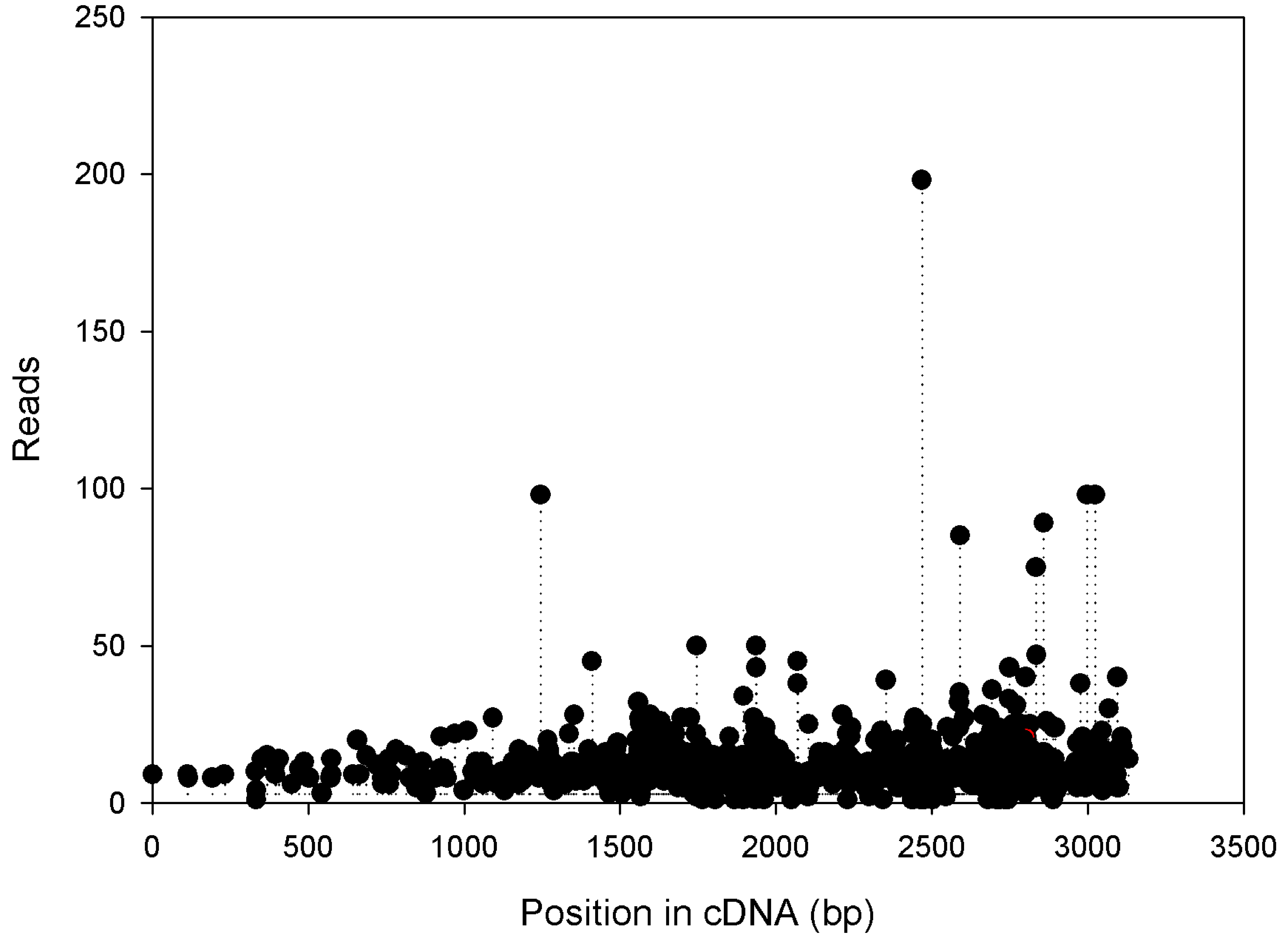


```
5' UGUUGUUUGUACUUCCAUUUUGAUUU 3'        Cs7g19800.1
      :..:. ::::::::::.::::
3' --UAUGAG-AUGAAGGUAAGACUA-- 5'        Csi-miRN20-Cs3g05320.1-23
```

Csi-miRN20-Cs3g05320.1-24, target=Cs1g12760.1 gene=Cs1g12760
Category:1
Score:4.5
Cleavage site:652

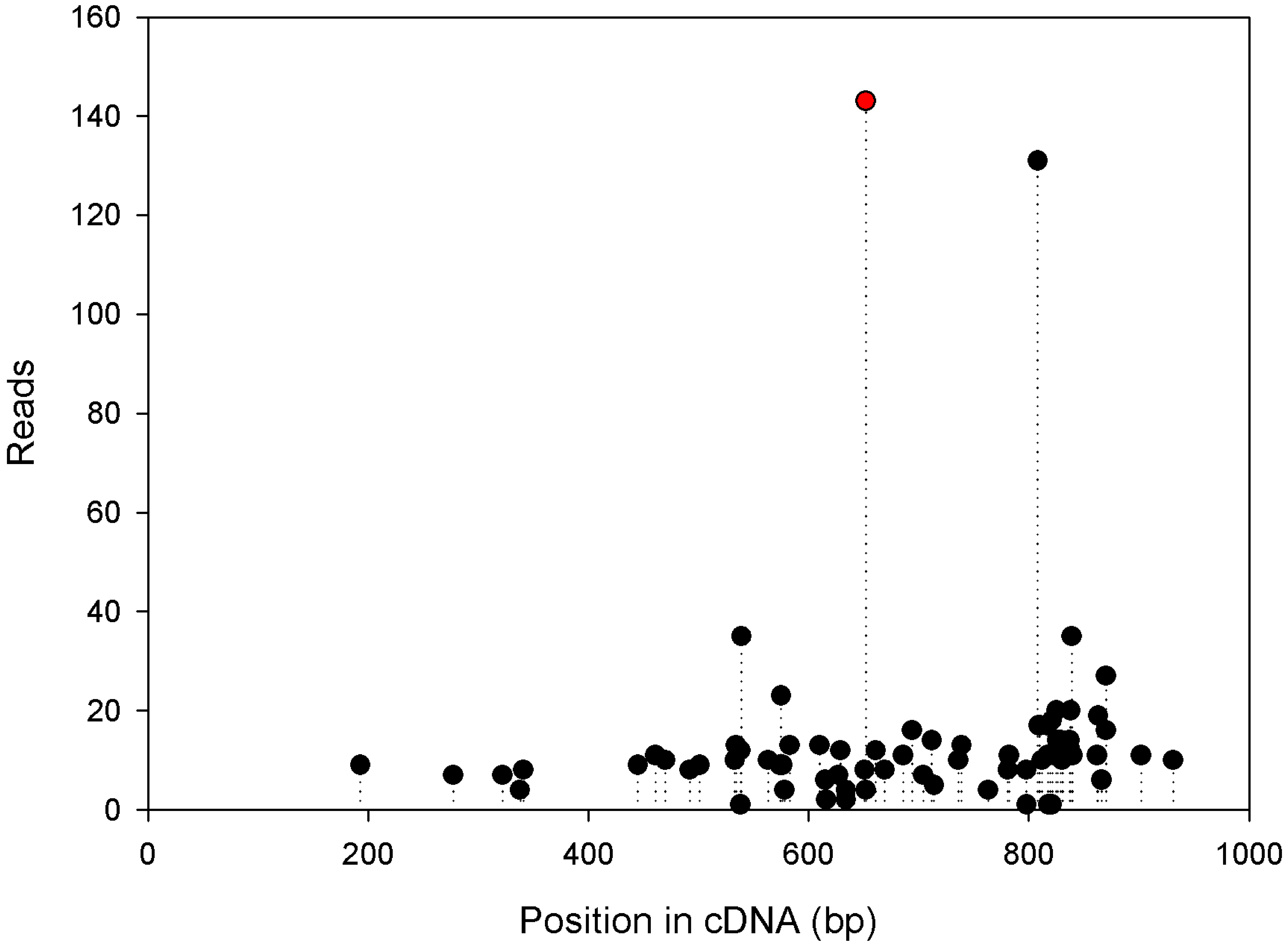


```
5' UAUGUCGGC-UGGGUGACUAUUAUCAU 3'       Cs1g12760.1
         :..: :  .::.:::::::::
3' -----GUUGUAGUCAUUGAUAAUAGU- 5'       Csi-miRN20-Cs3g05320.1-24
```

Csi-miRN20-Cs3g05320.1-26, target=Cs1g15370.1 gene=Cs1g15370
Category:3
Score:4.5
Cleavage site:792

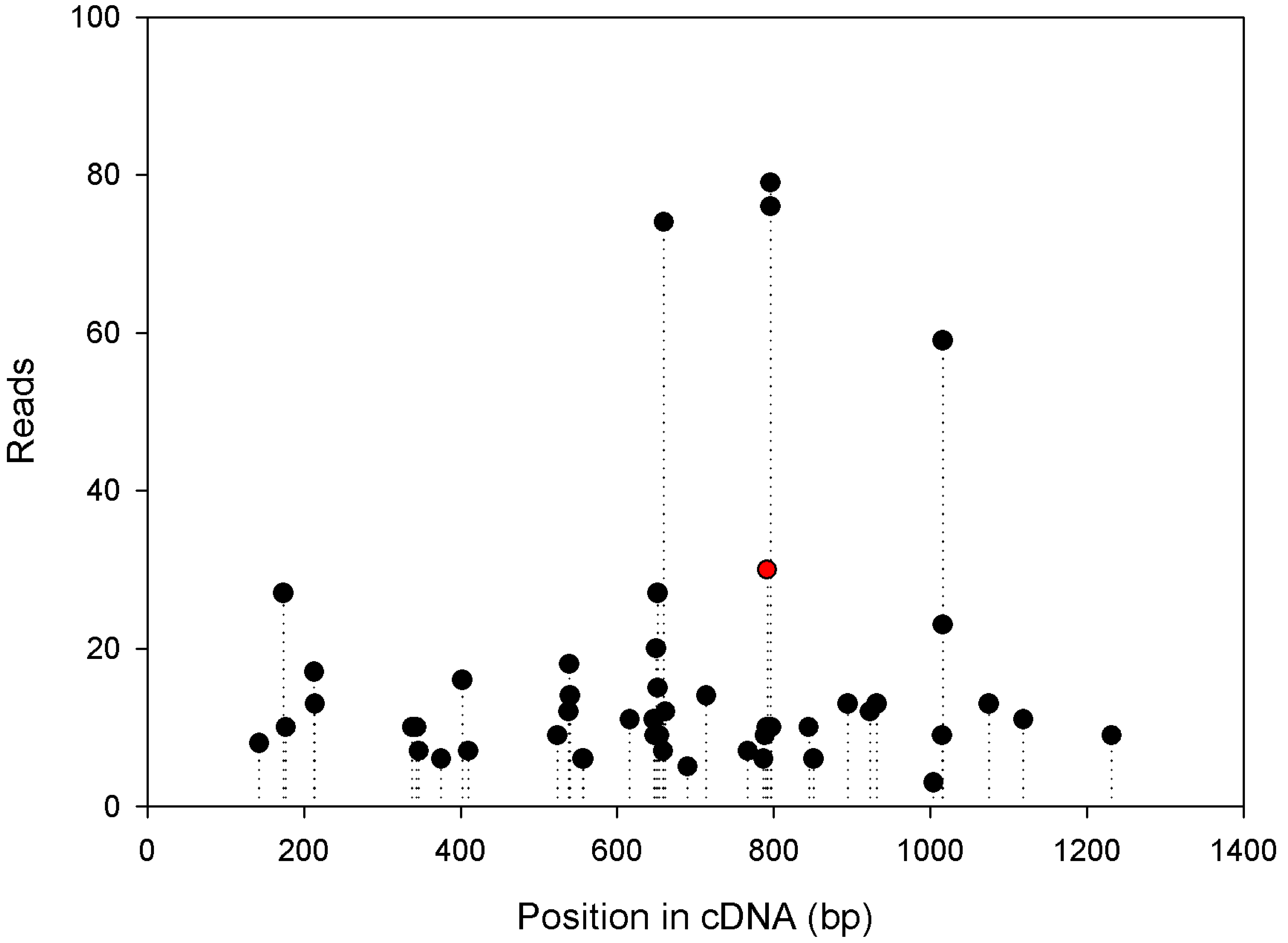


```
5' UACAAUCAU-UCUCUGCUUUCCGCAGA 3'       Cs1g15370.1
     ::::: : :::::: :::::.
3' --GUUAGAAGAGAGACAAAAGGU---- 5'       Csi-miRN20-Cs3g05320.1-26
```

Csi-miRN20-Cs3g05320.1-26, target=Cs3g15060.1 gene=Cs3g15060
Category:3
Score:5
Cleavage site:1378

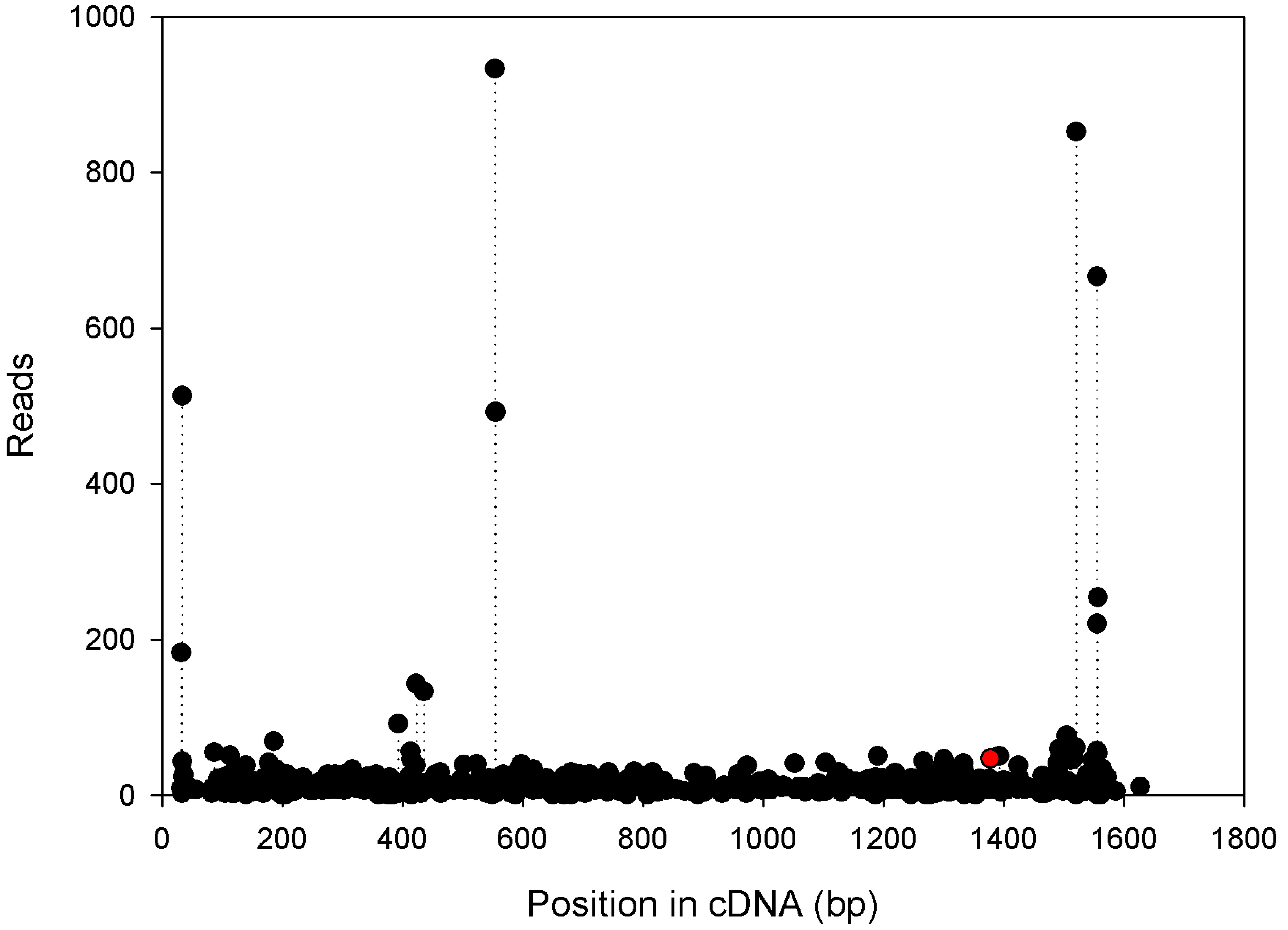


```
5' CAAAAUU-UUUCUGUUUUCUAUUUUGA 3'       Cs3g15060.1
   :::   :: :.:::::::::.:
3' GUUAGAAGAGAGACAAAAGGU------ 5'       Csi-miRN20-Cs3g05320.1-26
```

Csi-miRN20-Cs3g05320.1-26, target=Cs6g18830.1 gene=Cs6g18830
Category:3
Score:5
Cleavage site:676

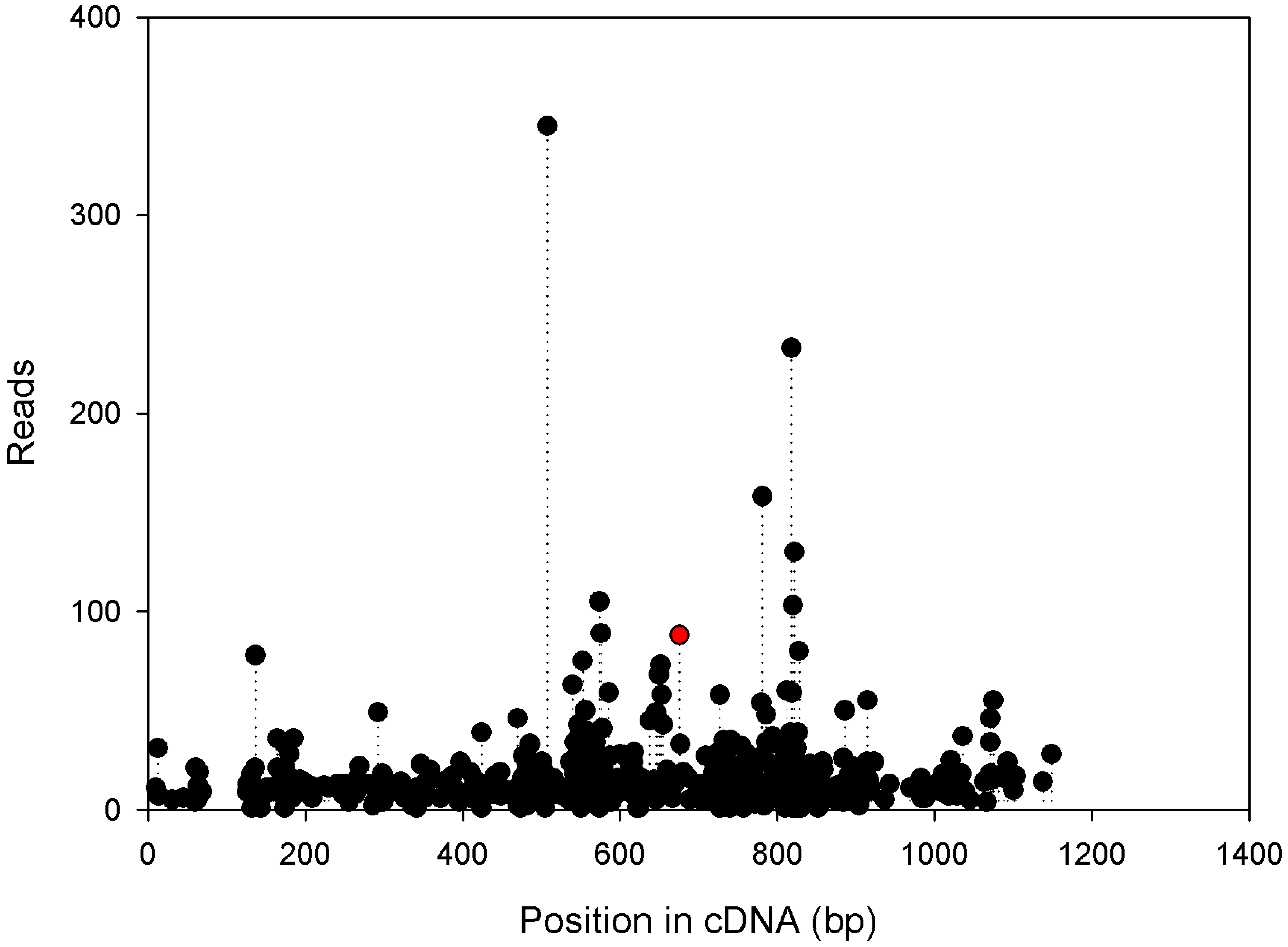


```
5' UGCACUCAACUUCUCUGUUUUCUACU 3'        Cs6g18830.1
     :: ::  : ::::::::::::.:
3' --GUUAGAAG-AGAGACAAAAGGU-- 5'        Csi-miRN20-Cs3g05320.1-26
```

Csi-miRN20-Cs3g05320.1-36, target=Orange1.1t00530.2 gene=Orange1.1t00530
Category:3
Score:5
Cleavage site:1873

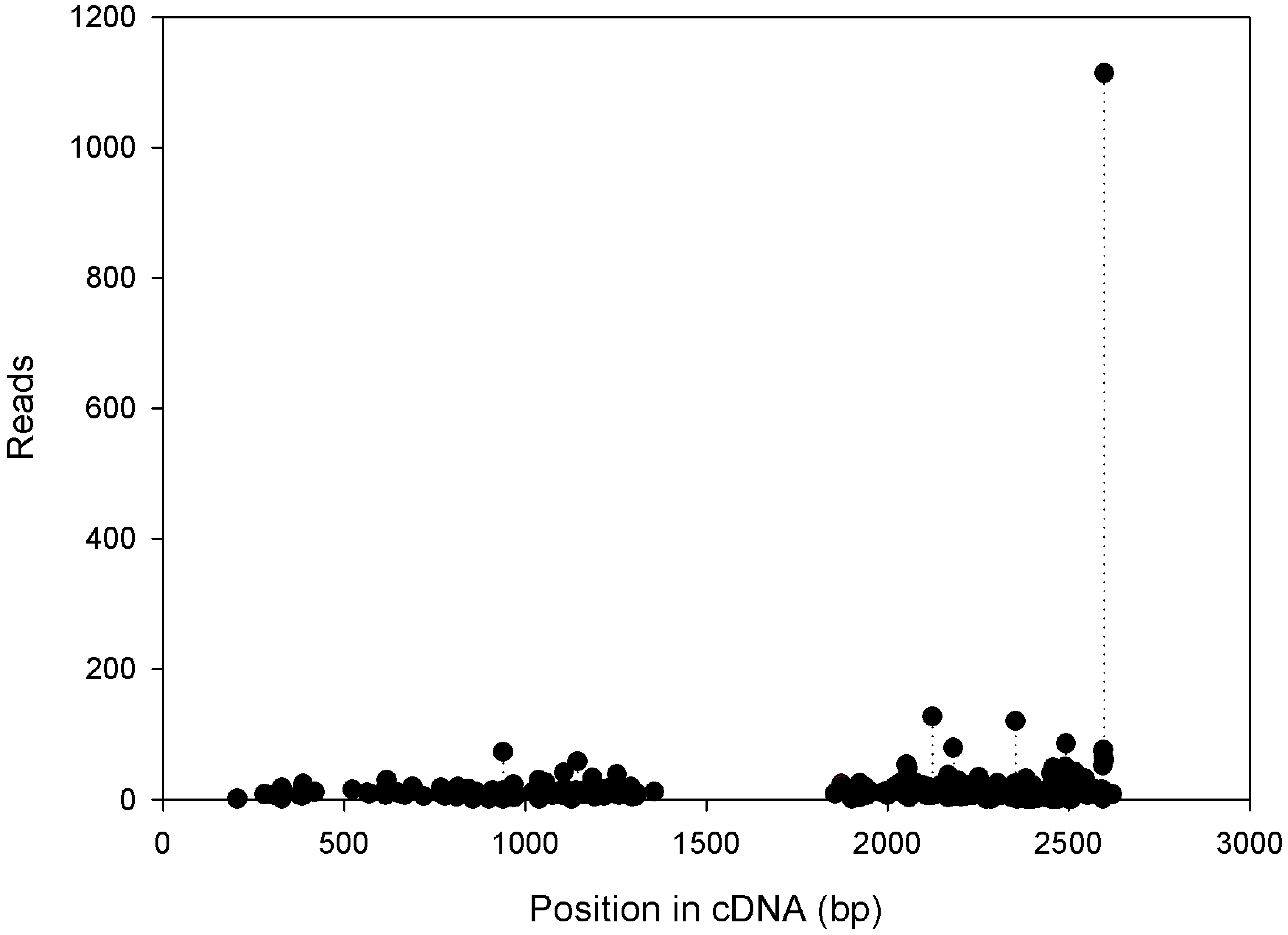


```
5' AUAUCCAAGCA-GCGUGGAAGAUGGAA 3'        Orange1.1t00530.2
         ::::: ::.:::::::..::
3' ------UUCGUACGUACCUUCUGUCUG 5'        Csi-miRN20-Cs3g05320.1-36
```

Csi-miRN20-Cs3g05320.1-4, target=Cs5g18400.1 gene=Cs5g18400
Category:2
Score:5
Cleavage site:726

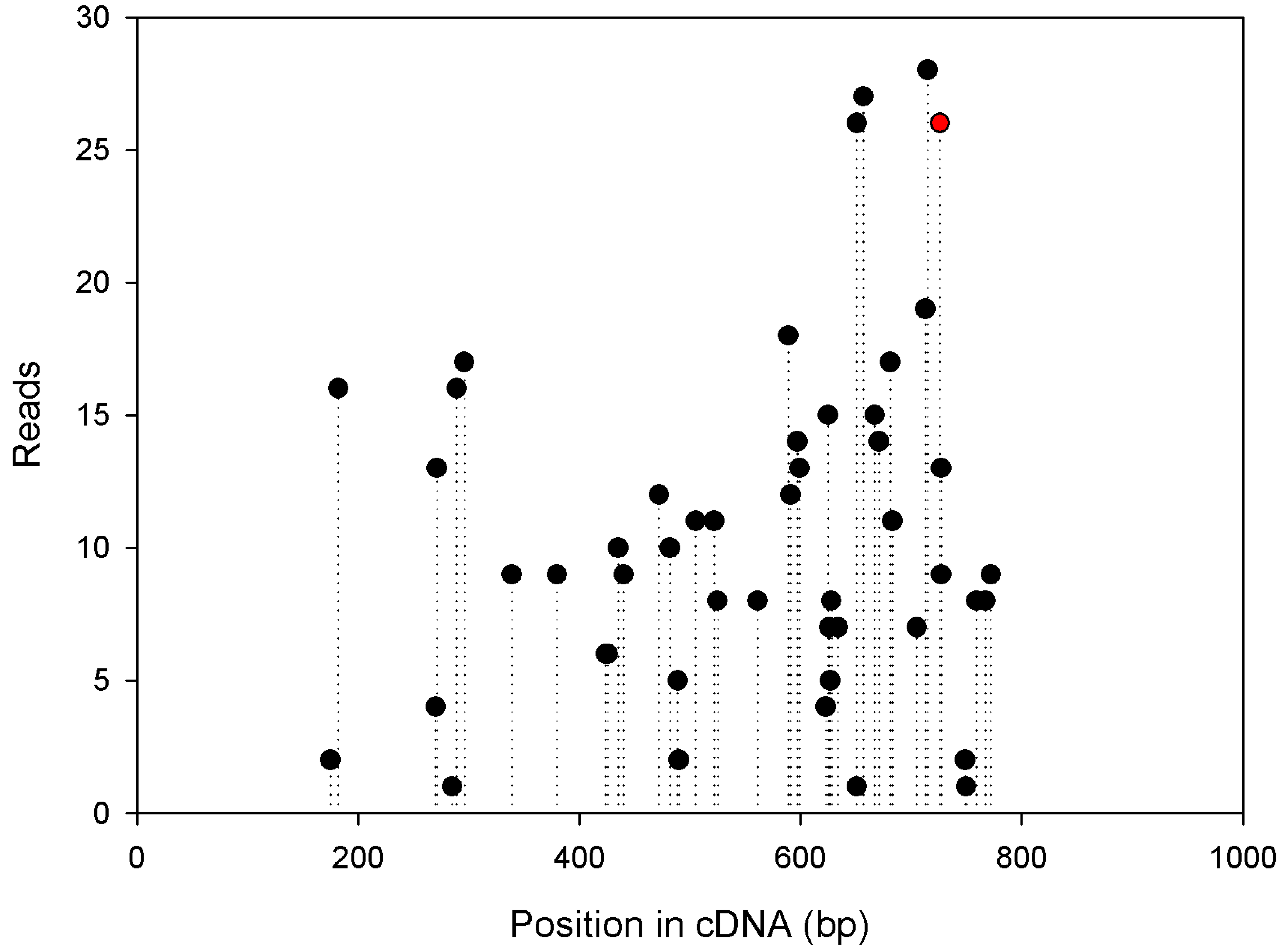


```
5' CUACUGAUAUUAAUGAAUUUUACUGU 3'        Cs5g18400.1
      :.::::.:::: ::::::::
3' ---CAUUAUAGUUAC-UAAAAUGAA- 5'        Csi-miRN20-Cs3g05320.1-4
```

Csi-miRN20-Cs3g05320.1-65, target=Cs3g01450.1 gene=Cs3g01450
Category:3
Score:4.5
Cleavage site:609

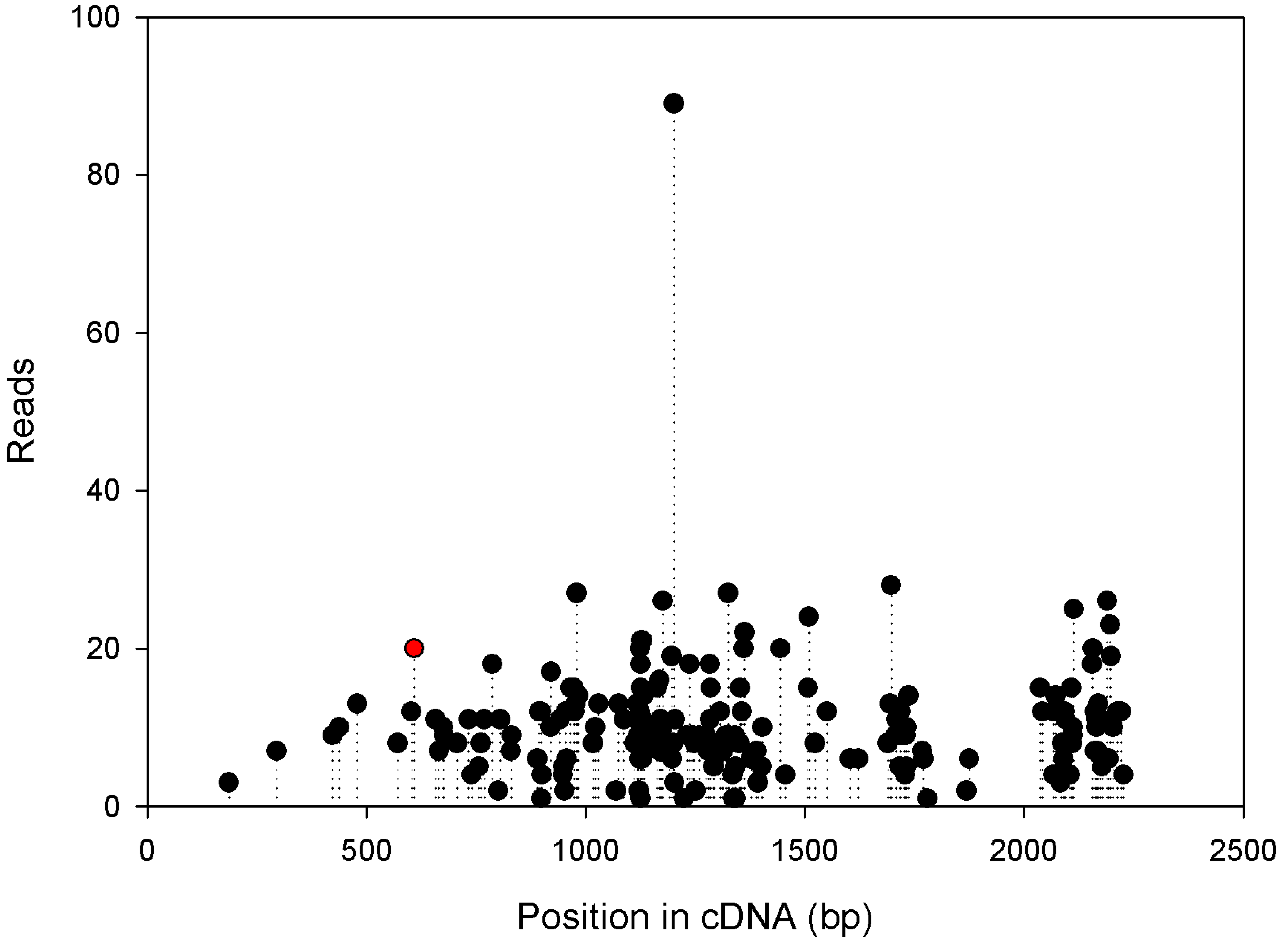


```
5' GACUUCUAU-UUCCAAAUGUACUAUGA 3'       Cs3g01450.1
       :: .:: :::::::::: :::
3' ---AACGUAGAAGGUUUACAGGAU--- 5'       Csi-miRN20-Cs3g05320.1-65
```

Csi-miRN20-Cs3g05320.1-65, target=Orange1.1t01632.1 gene=Orange1.1t01632
Category:3
Score:4.5
Cleavage site:690

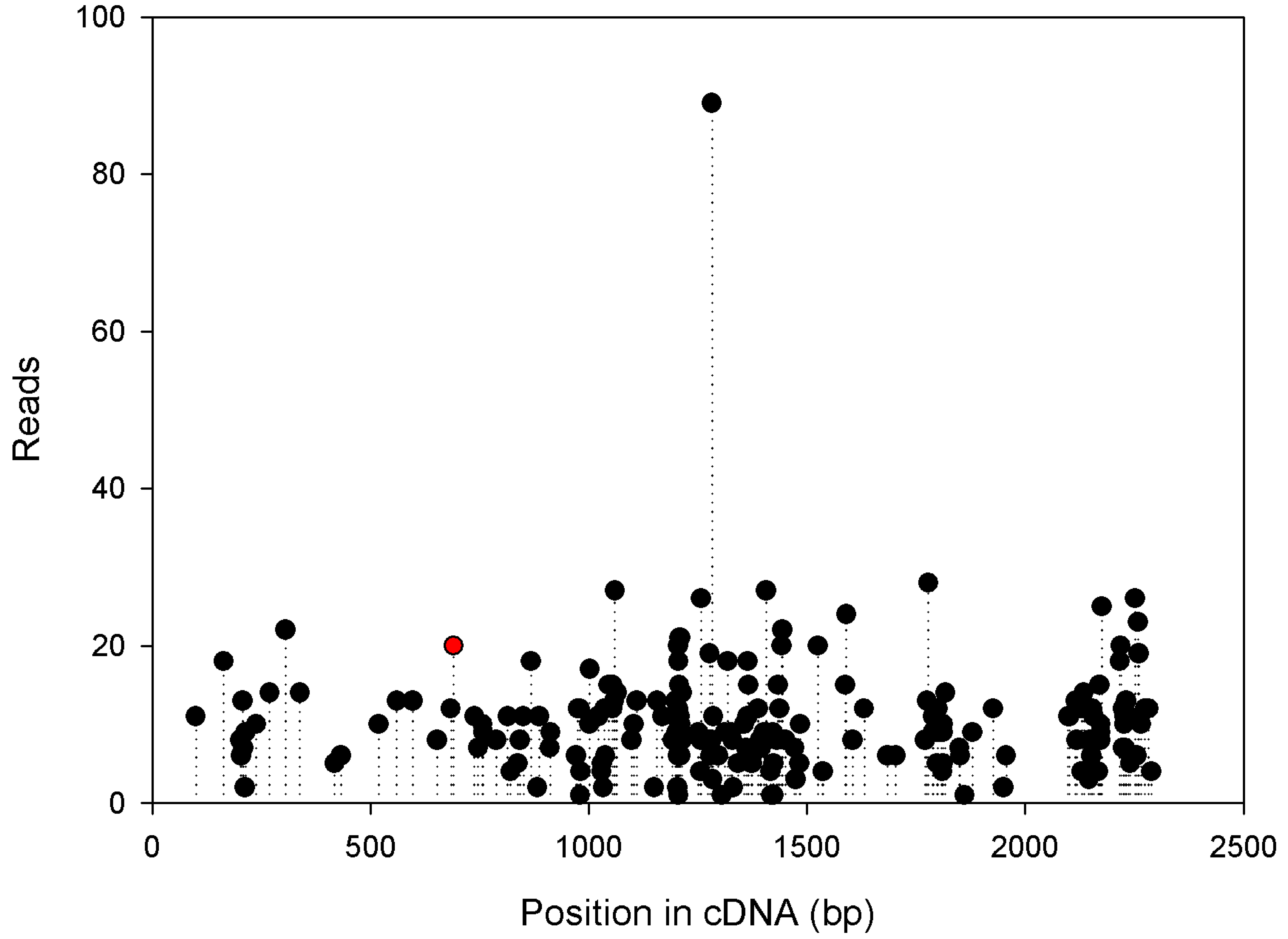


```
5' GACUUCUAU-UUCCAAAUGUACUAUGA 3'       Orange1.1t01632.1
     :: .:: :::::::::: :::
3' ---AACGUAGAAGGUUUACAGGAU--- 5'       Csi-miRN20-Cs3g05320.1-65
```

T-plots for targets of secondary siRNAs in flower

Csi-miR3954a-Cs1g09635.1-5, target=Cs1g01010.1 gene=Cs1g01010
Category:3
Score:5
Cleavage site:1809

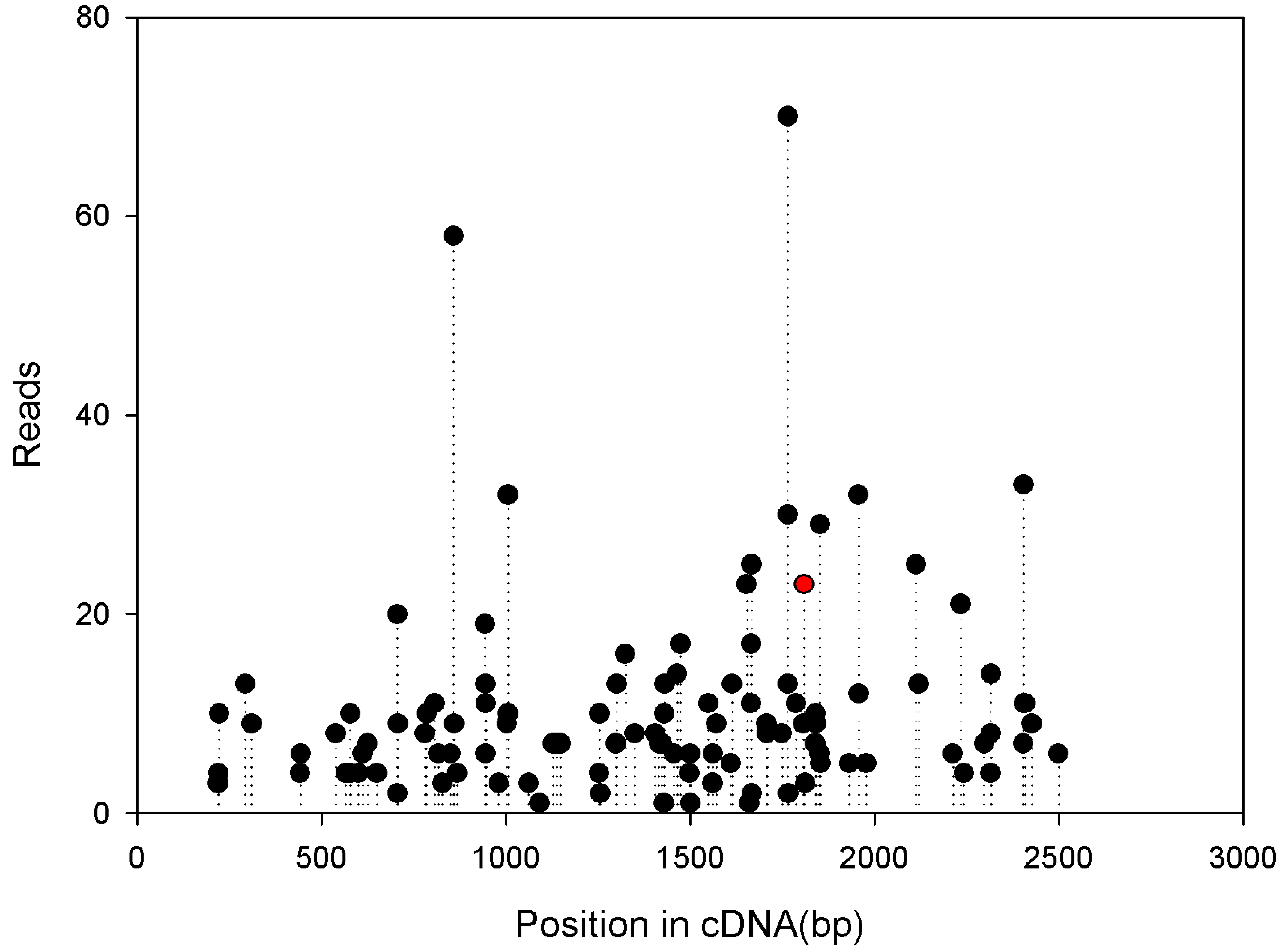


```
5' GAAGAUCUGGGA-UGCAUAUAAUGUGA 3'       Cs1g01010.1
         ::::..: ::::: ::::.::
3' -----AGACUUUAACGUACAUUAUAC- 5'       Csi-miR3954a-Cs1g09635.1-5
```

Csi-miR3954a-Cs1g09635.1-5, target=Cs5g24790.1 gene=Cs5g24790
Category:2
Score:5
Cleaveage site:1682

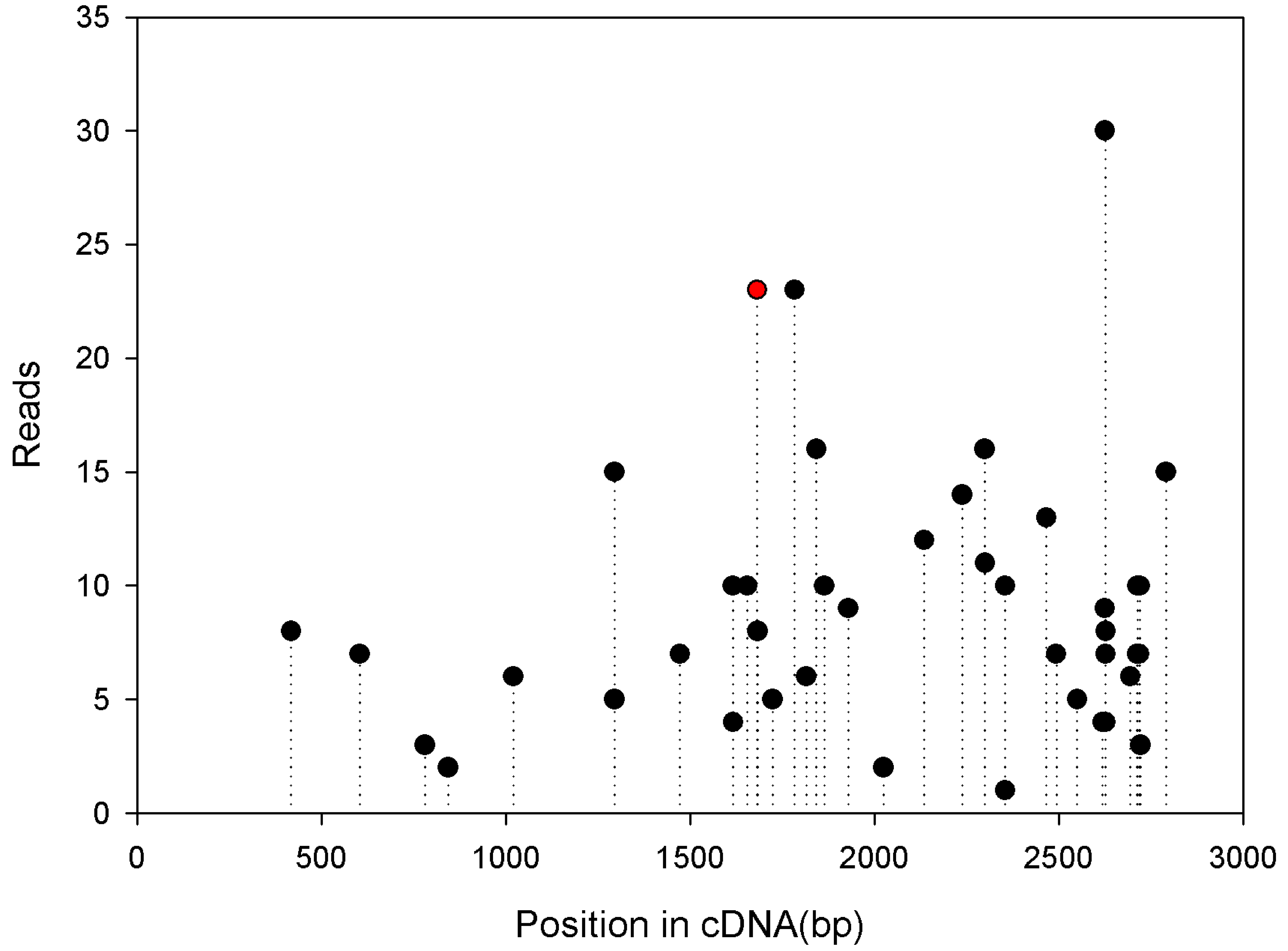


```
5' AGAACACAGAAAAUGCAUG-AAUAUGC 3'       Cs5g24790.1
        : :::: :::::: ::::::
3' -----AGACUUUAACGUACAUUAUAC- 5'       Csi-miR3954a-Cs1g09635.1-5
```

Csi-miR3954a-Cs1g09635.1-5, target=Orange1.1t03220.1 gene=Orange1.1t03220
Category:3
Score:5
Cleavage site:1781

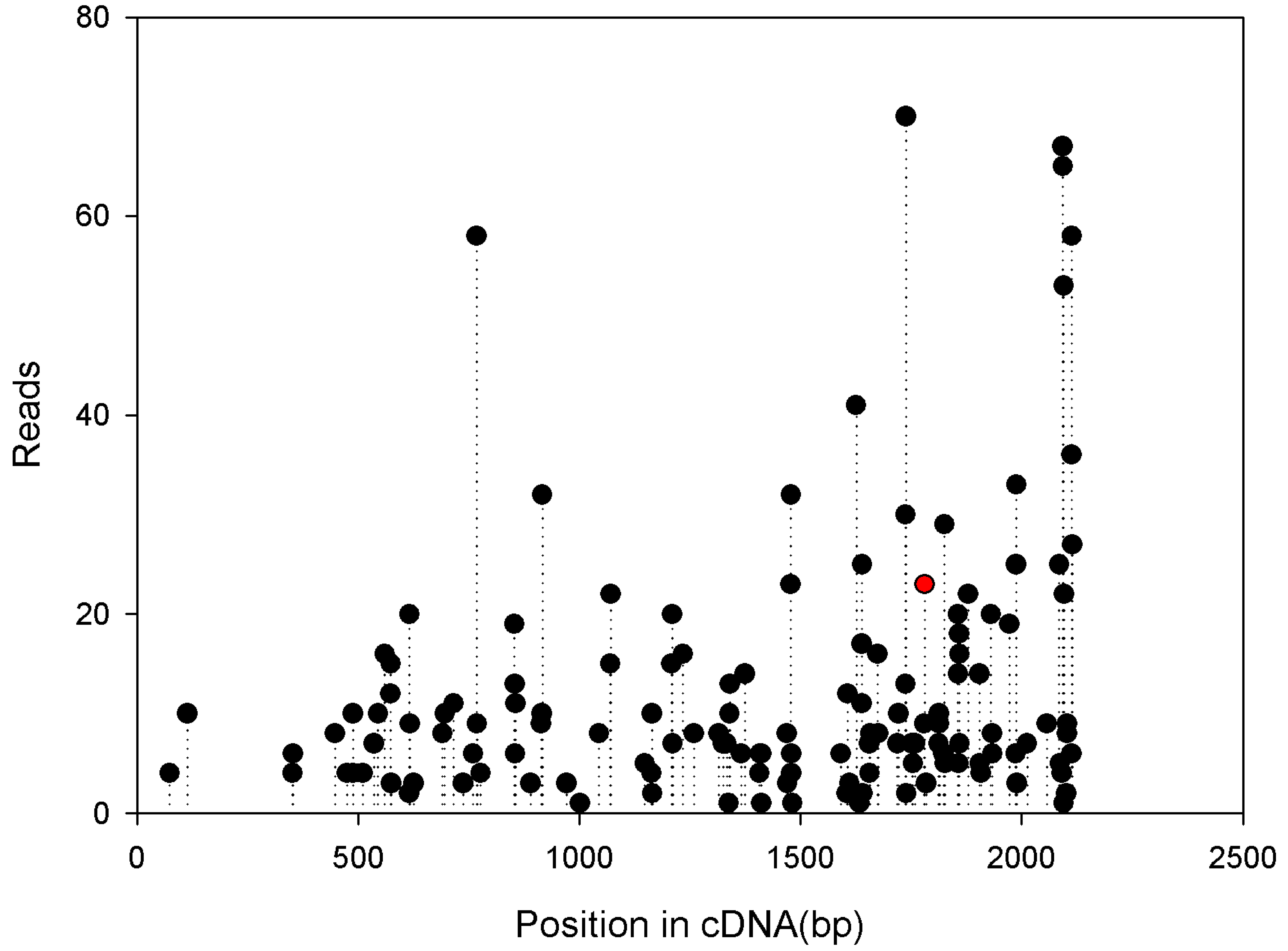


```
5' GAAGAUCUGGGA-UGCAUAUAAUGUGA 3'       Orange1.1t03220.1
         ::::..: ::::: ::::.::
3' -----AGACUUUAACGUACAUUAUAC- 5'       Csi-miR3954a-Cs1g09635.1-5
```

Csi-miR3954a-Cs1g09635.1-7, target=Cs4g11930.1 gene=Cs4g11930
Category:3
Score:3.5
Cleavage site:506

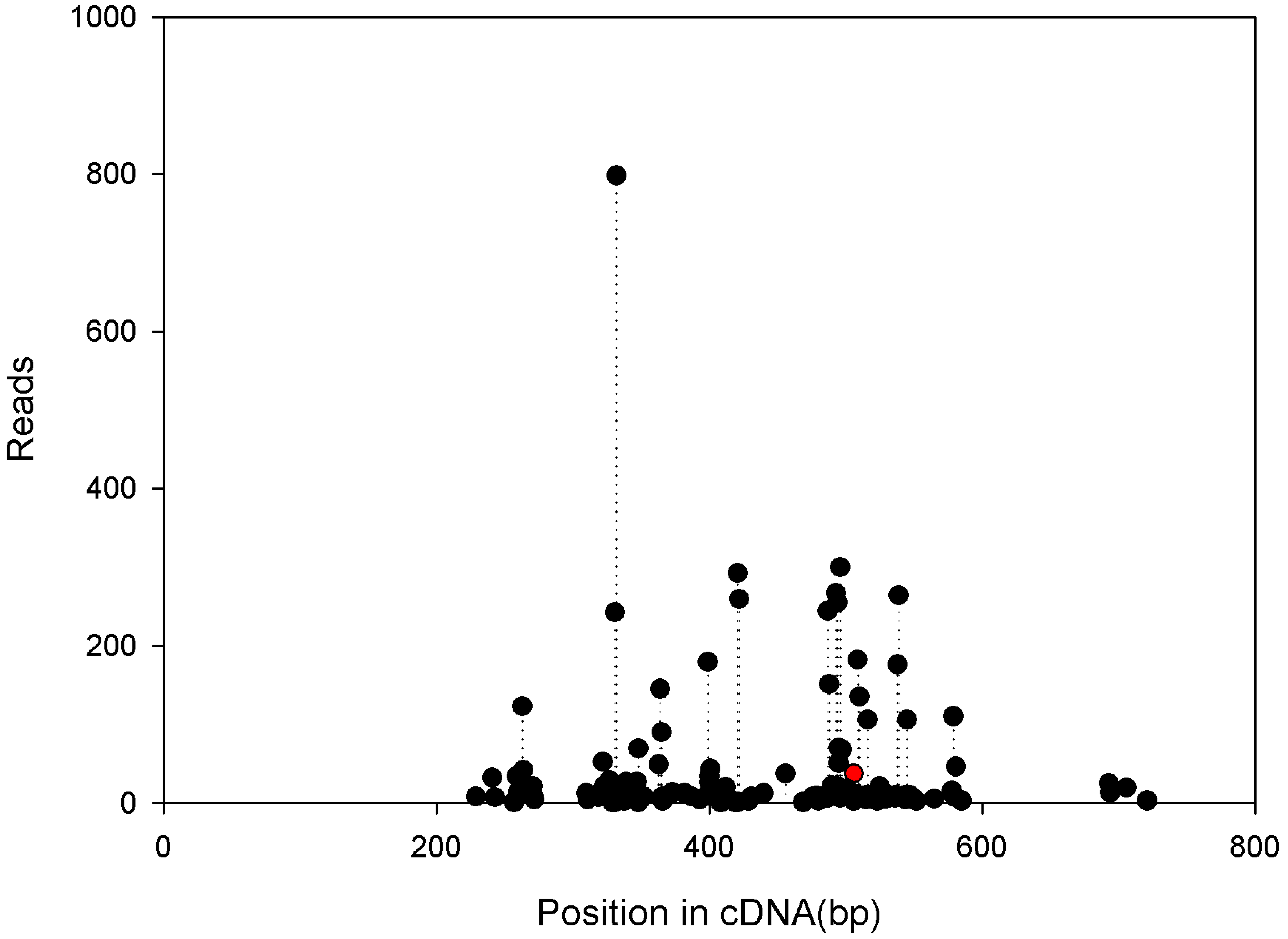


```
5' UCCUGAGGUUUGACCUUUGAGAAUGU 3'        Cs4g11930.1
     :::::::.::::::: ::::
3' --GACUCCAGGCUGGAAAGUCUU--- 5'        Csi-miR3954a-Cs1g09635.1-7
```

Csi-miR3954a-Cs1g09635.1-7, target=Cs9g18330.1 gene=Cs9g18330
Category:2
Score:5
Cleavage site:897

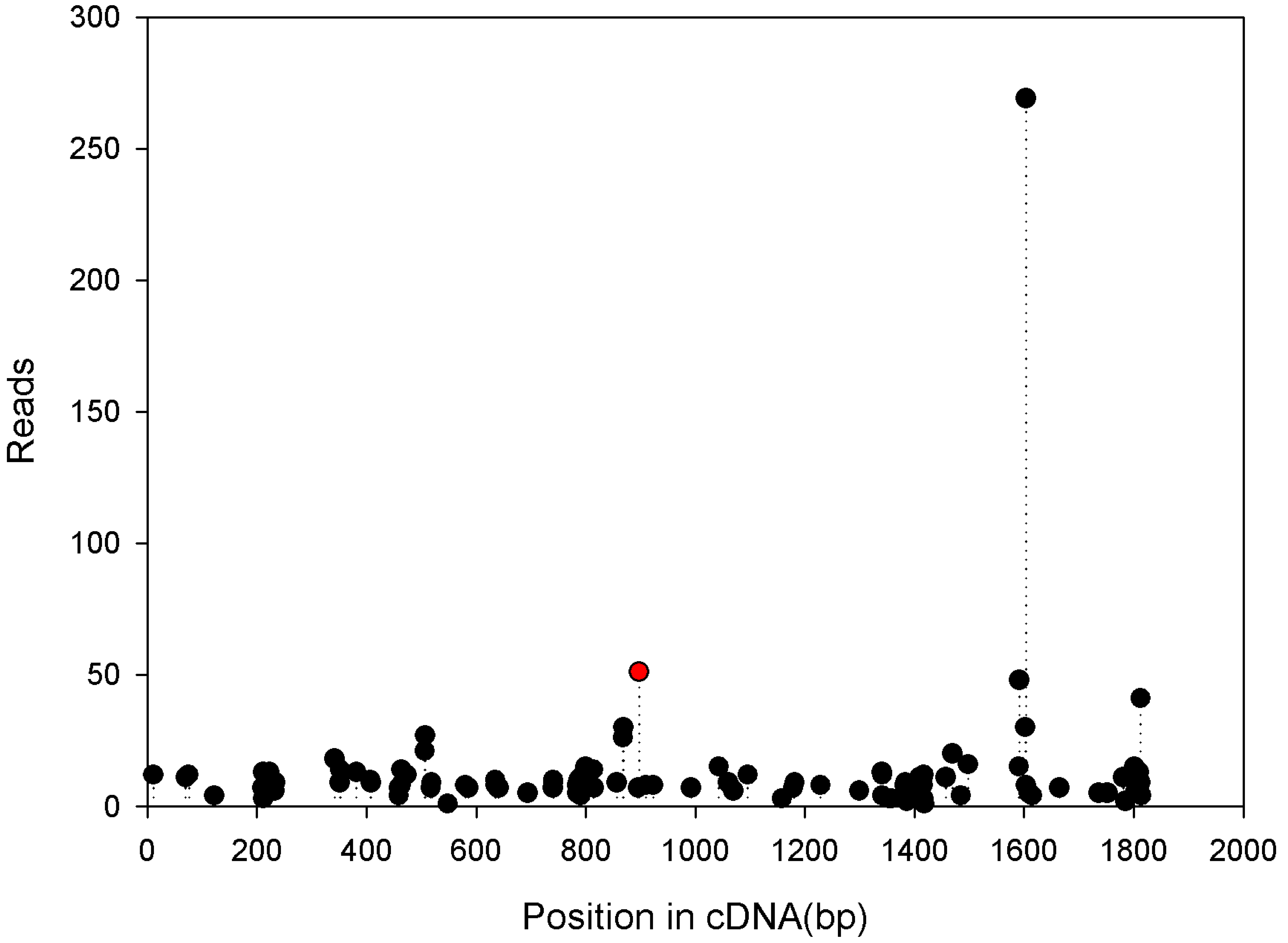


```
5' AGACUGAUGU-CGACUUUUUGGAAUCU 3'       Cs9g18330.1
       :::: :: ::::.:::..:::
3' ---GACUCCAGGCUGGAAAGUCUU--- 5'       Csi-miR3954a-Cs1g09635.1-7
```

Csi-miR3954a-Cs1g09635.1-9, target=Cs2g17570.1 gene=Cs2g17570
Category:3
Score:4
Cleaveage site:3293

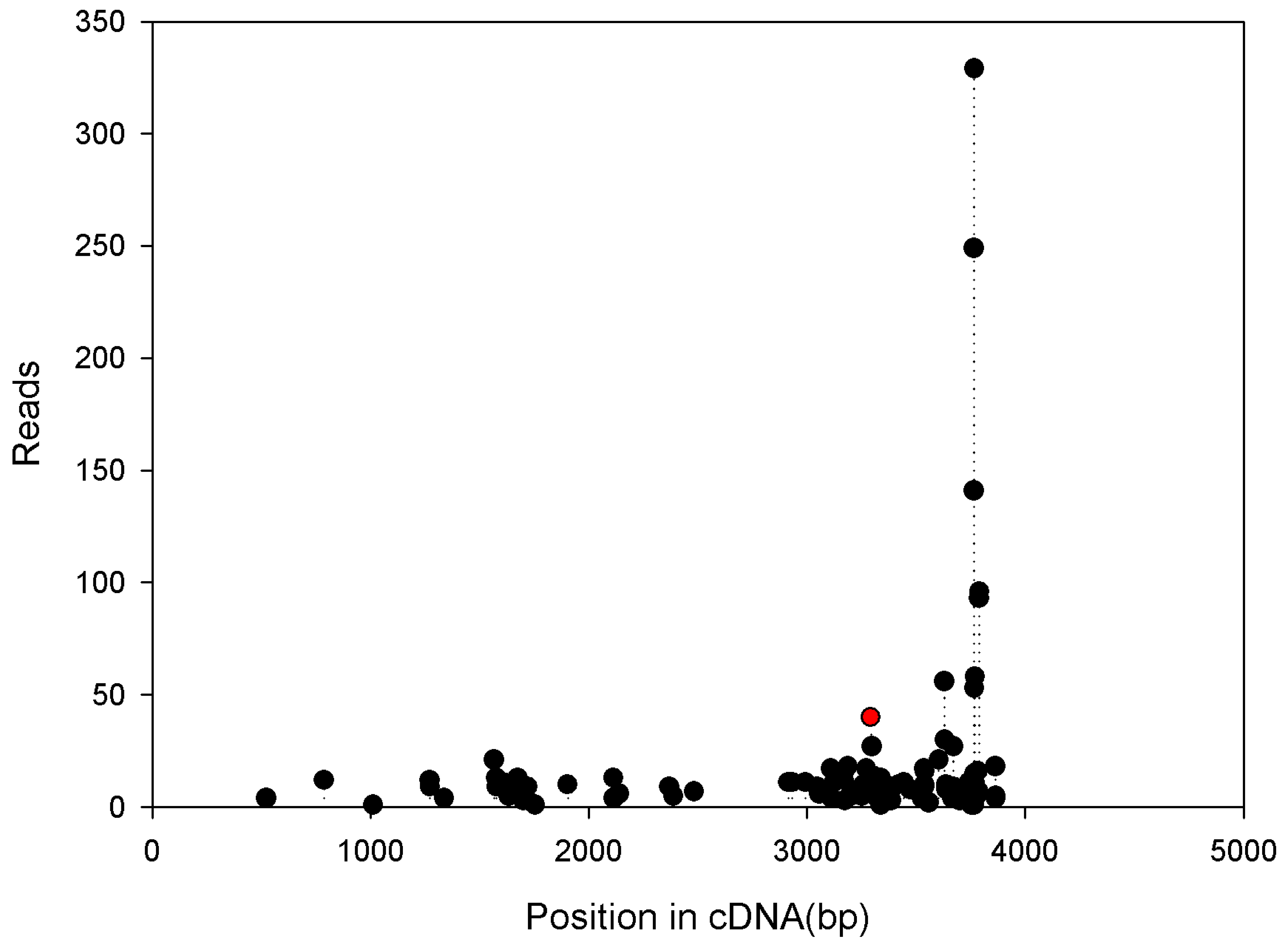


```
5' CGAUGAUGCUAUC-UAUCACAAAGGCA 3'       Cs2g17570.1
        : :::::  ::::::::::::
3' ------AAGAUAGUUUAGUGUUUCCGU 5'       Csi-miR3954a-Cs1g09635.1-9
```

Csi-miR3954a-Cs1g09635.1-9, target=Cs2g17600.1 gene=Cs2g17600
Category:2
Score:4
Cleavage site:1815

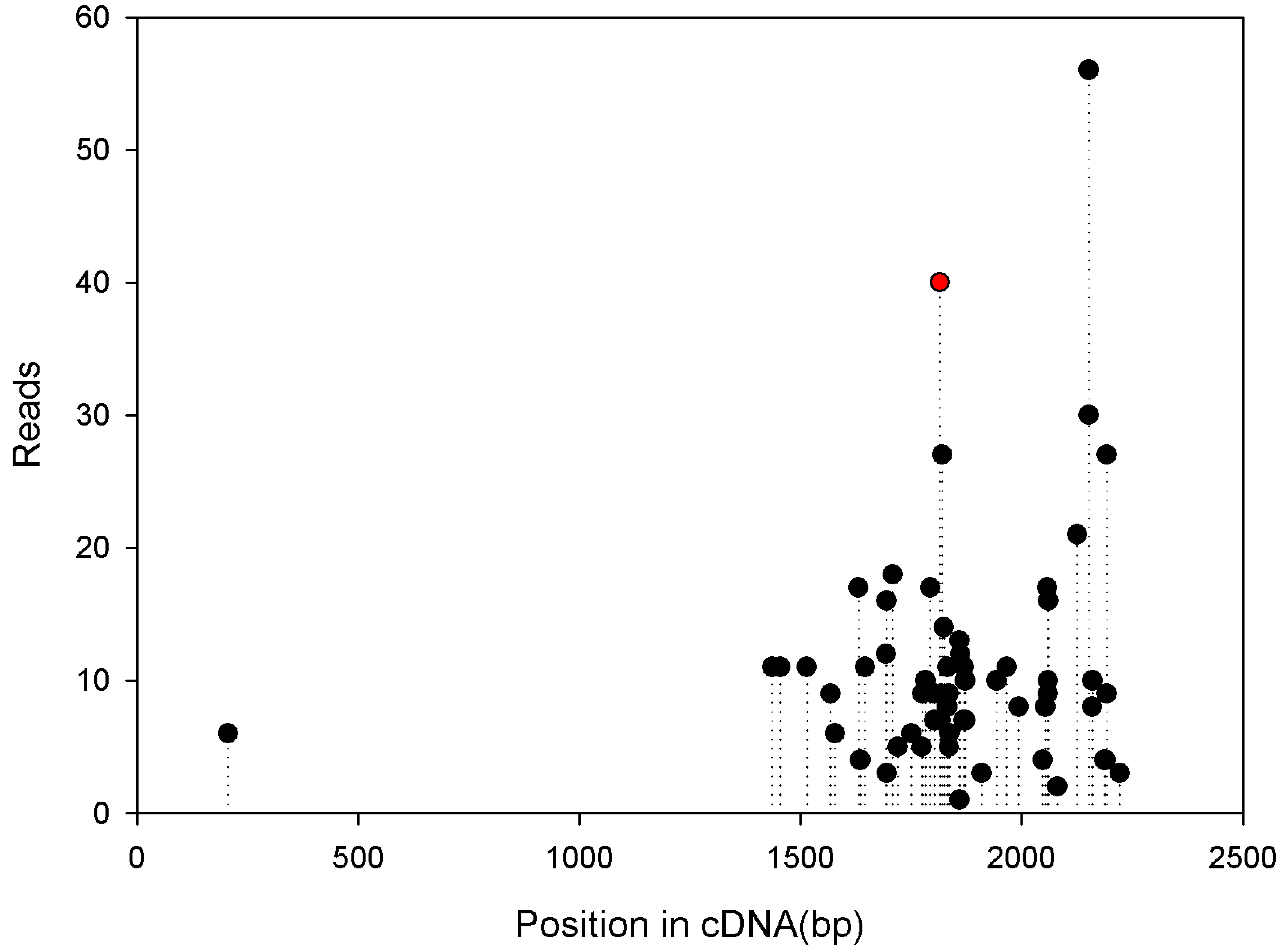


```
5' CGAUGAUGCUAUC-UAUCACAAAGGCA 3'       Cs2g17600.1
          : :::::  ::::::::::::
3' ------AAGAUAGUUUAGUGUUUCCGU 5'       Csi-miR3954a-Cs1g09635.1-9
```

Csi-miR3954a-Cs1g09635.1-9, target=Cs5g11880.1 gene=Cs5g11880
Category:2
Score:5
Cleavage site:406

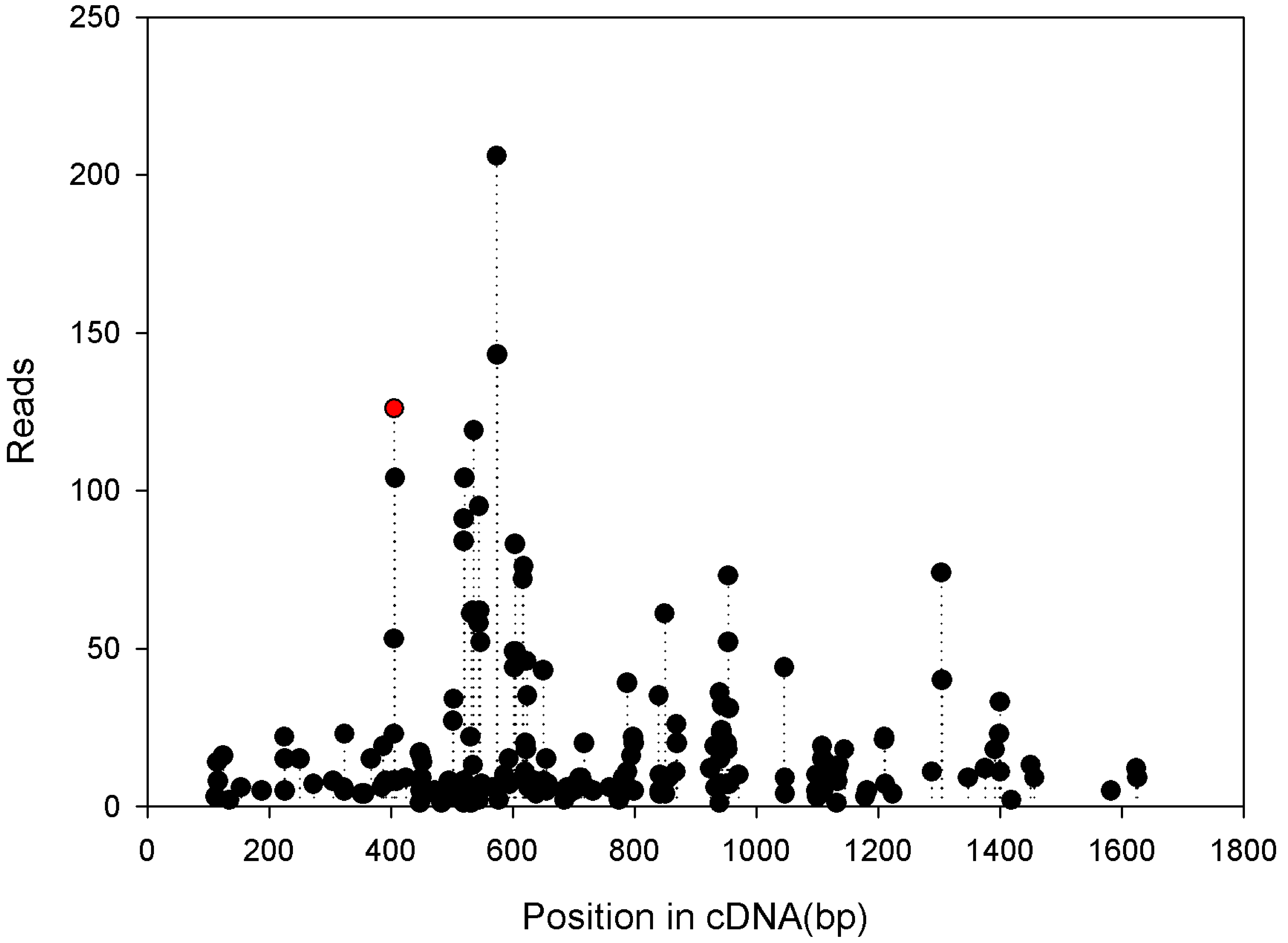


```
5' GAAUAUUC-AGAAAAUUACAAAGGCUA 3'       Cs5g11880.1
        ::: :  ::::.:::::::::
3' -----AAGAUAGUUUAGUGUUUCCGU- 5'       Csi-miR3954a-Cs1g09635.1-9
```

Csi-miR3954a-Cs5g04670.1-21, target=Cs5g09880.1 gene=Cs5g09880
Category:3
Score:3.5
Cleavage site:1356

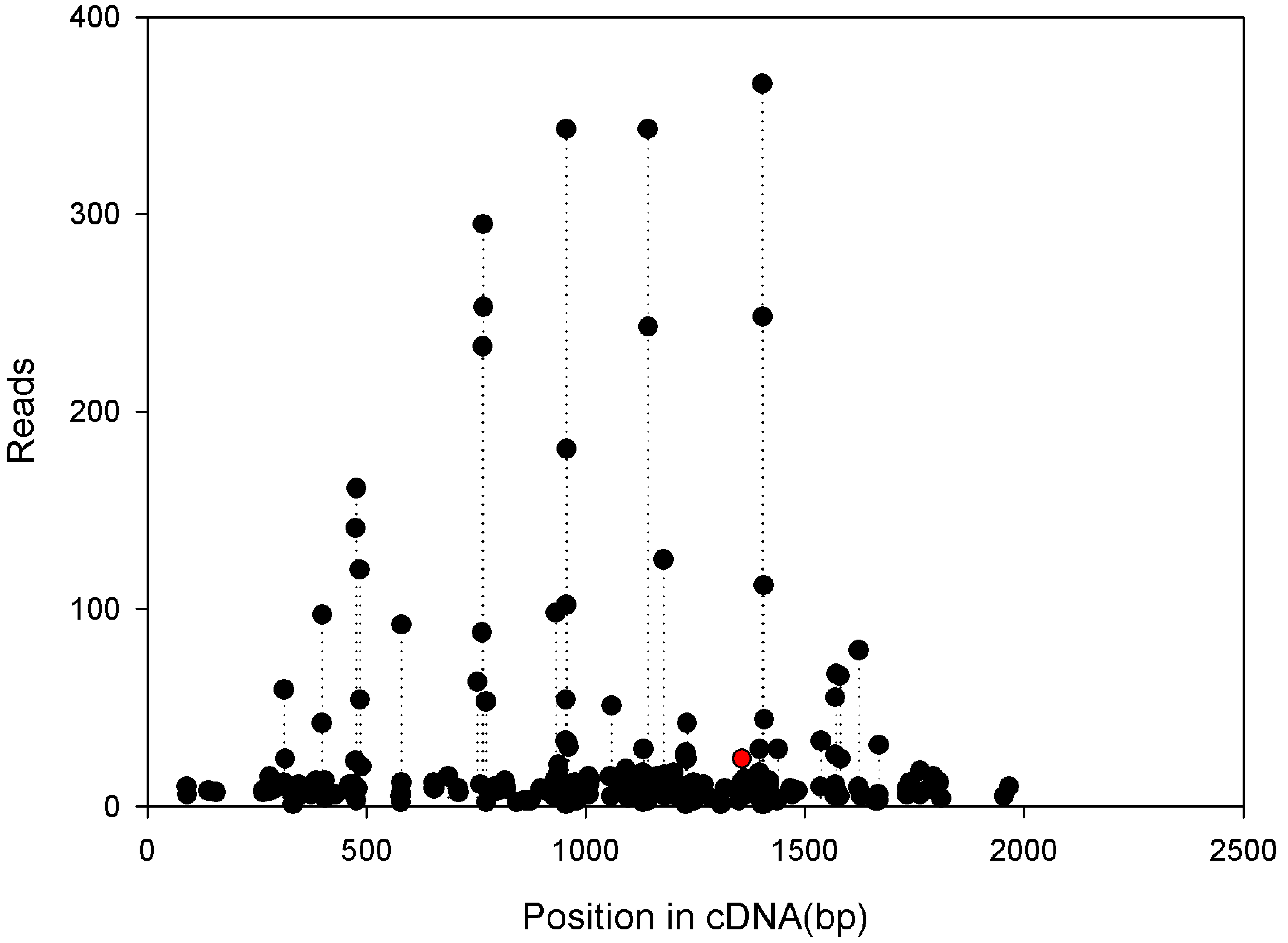


```
5' GAUUCAUGGUGCCAAAUGGUGCACAA 3'        Cs5g09880.1
   :: ::: :.:::::::::::::
3' CUUAGU-CUACGGUUUACCACU---- 5'        Csi-miR3954a-Cs5g04670.1-21
```

Csi-miR482c-Cs9g06846.1-15, target=Cs1g16450.1 gene=Cs1g16450
Category:3
Score:5
Cleavage site:1950

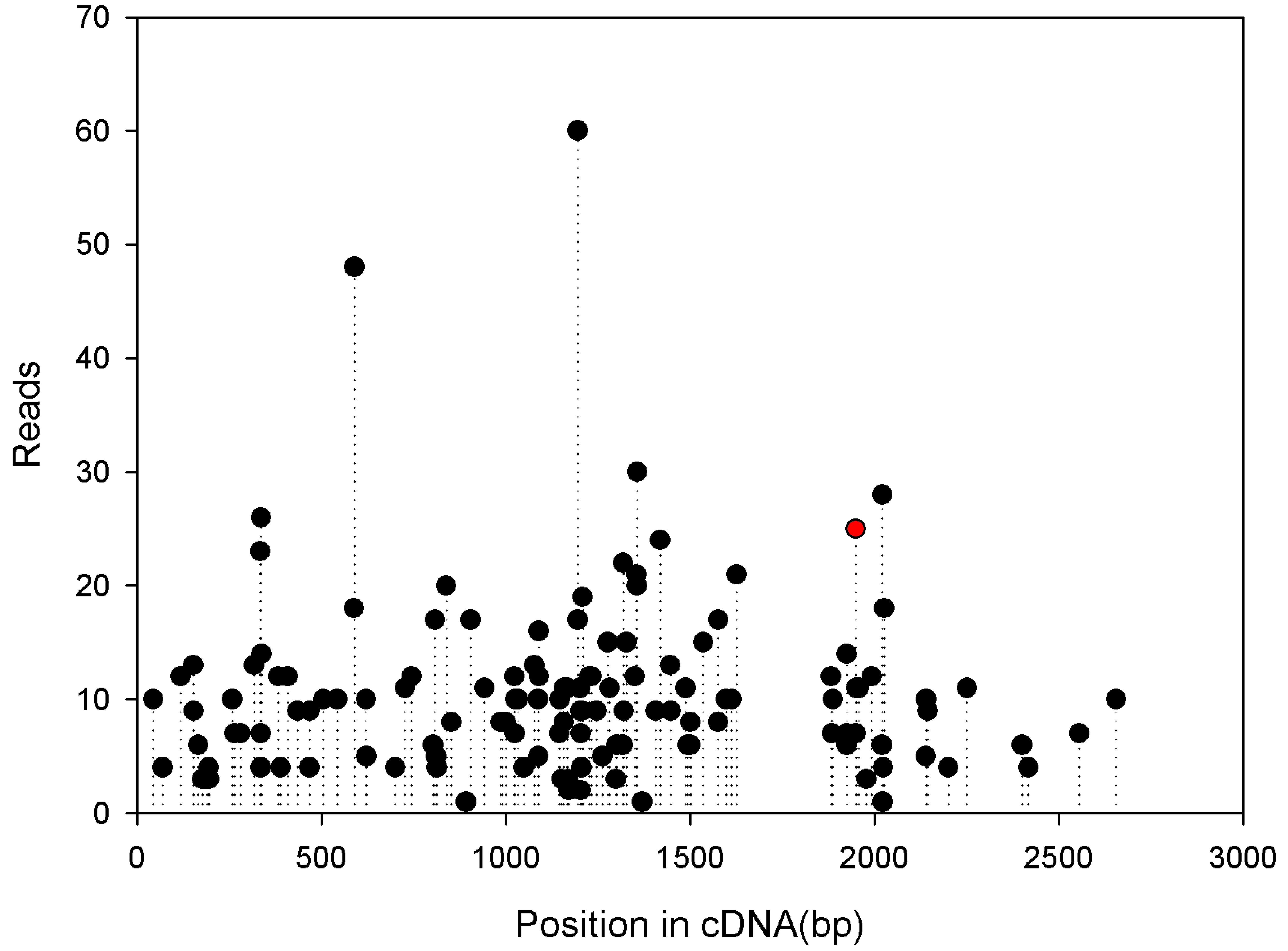


```
5' CAACUACAAUGG-UAUCAUAUUAUGGC 3'       Cs1g16450.1
      ::: :::: ::::::::: ::
3' ----AUGGUACCUAUAGUAUAAGAC-- 5'       Csi-miR482c-Cs9g06846.1-15
```

Csi-miR482c-Cs9g06846.1-5, target=Cs9g08050.1 gene=Cs9g08050
Category:3
Score:5
Cleavage site:1748

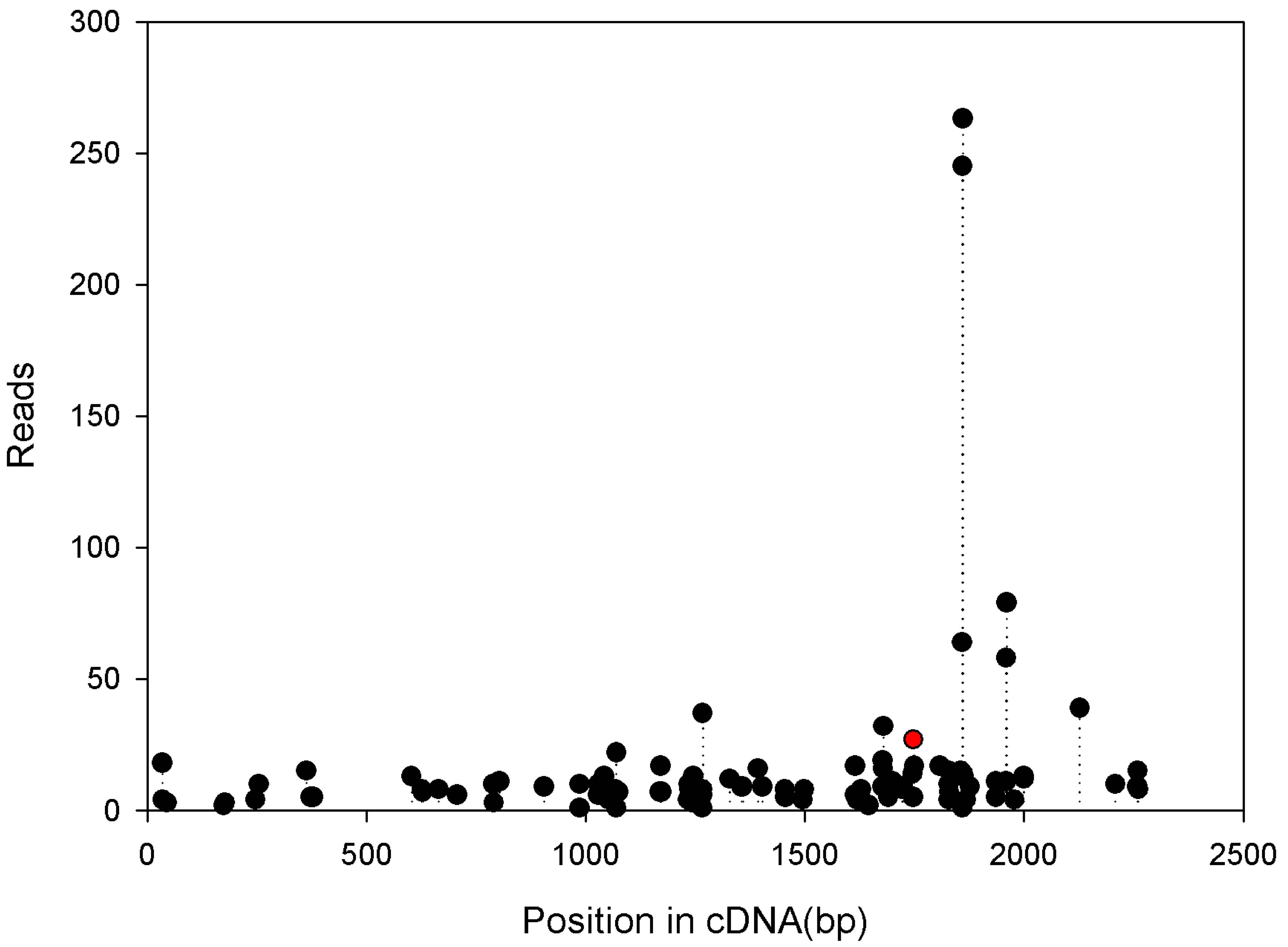


```
5' GAAGGAAUGGAUGGAUGAGAUUCUUU 3'        Cs9g08050.1
    :: :  ::::::.::.:::::
3' -UUACACACCUACUUAUUCUAA---- 5'        Csi-miR482c-Cs9g06846.1-5
```

Csi-miR482c-Cs9g06846.1-6, target=Cs7g31270.1 gene=Cs7g31270
Category:3
Score:5
Cleavage site:897

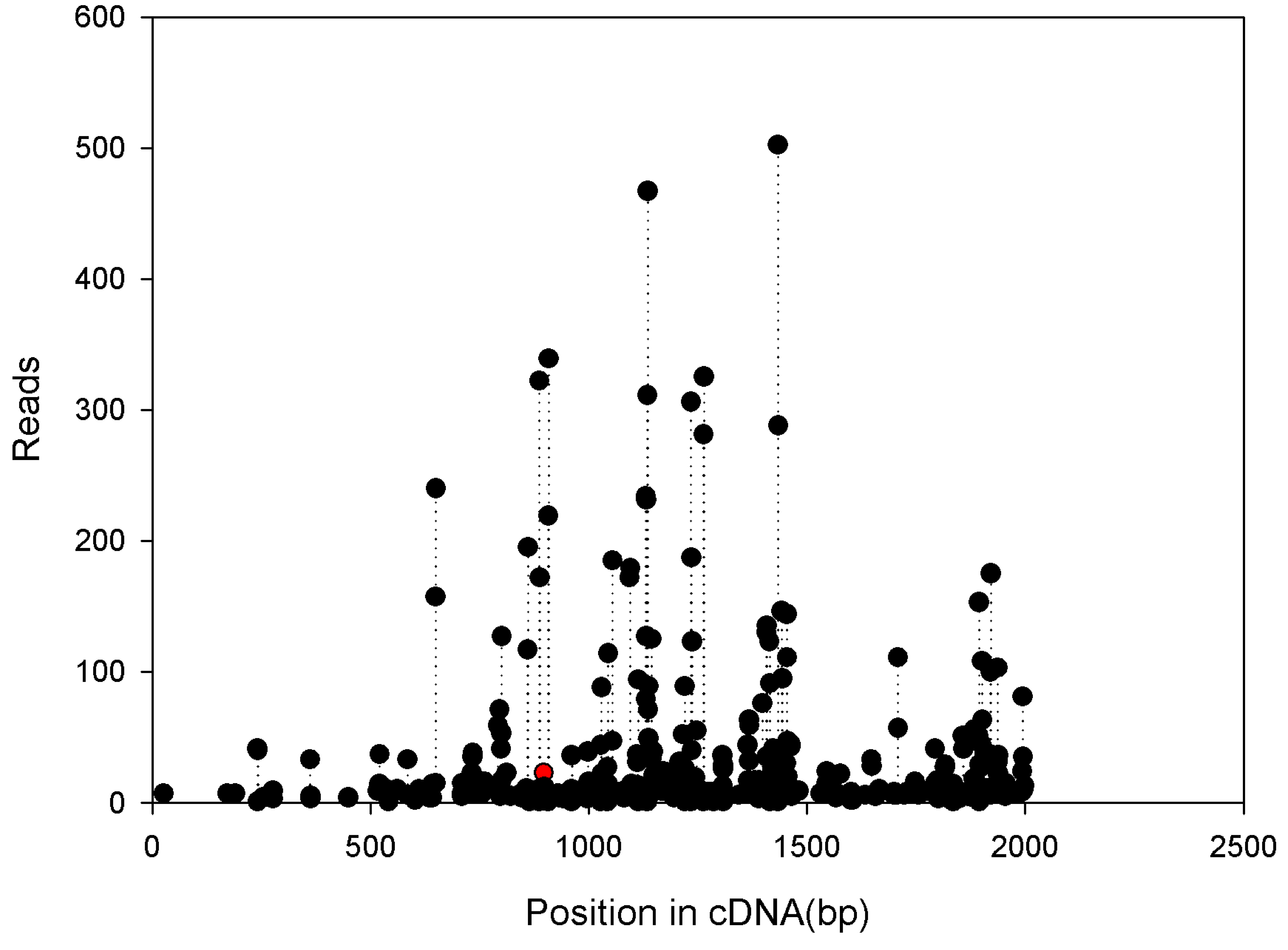


```
5' CAUAUGCUGAGGGAUU-GGAACACUUG 3'       Cs7g31270.1
     ::::::: :::::: : ::::
3' --AUACGACGCCCUAAGCAUUGU---- 5'       Csi-miR482c-Cs9g06846.1-6
```

Csi-miRN20-Cs3g05320.1-14, target=Cs8g19960.1 gene=Cs8g19960
Category:2
Score:4.5
Cleavage site:544

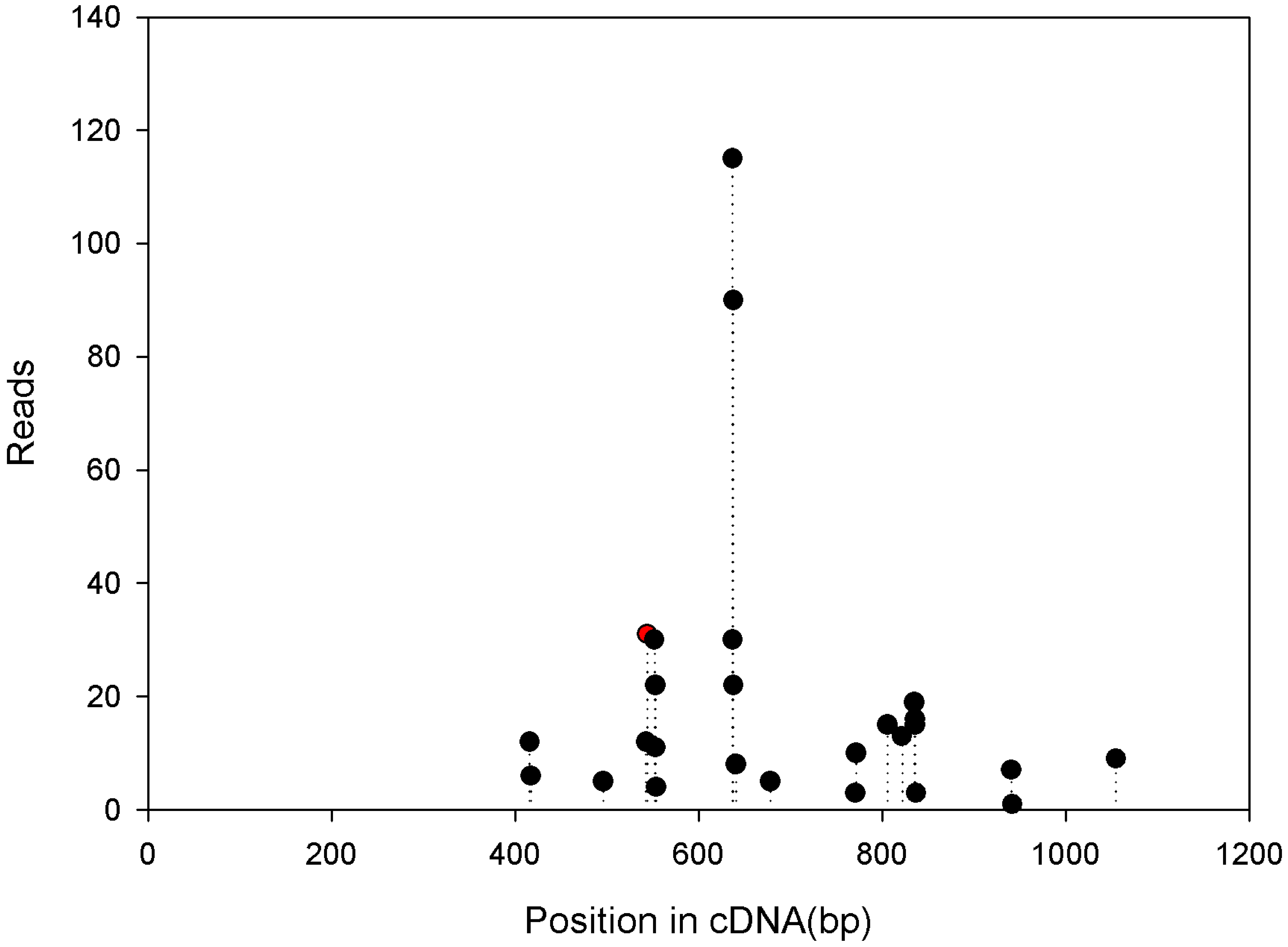


```
5' GAAACAAGAUCAGUUUUAU-GAAACUG 3'       Cs8g19960.1
       ::::.: ::::.:::: :::::
3' ----GUUUUUGUCAGAAUAGCUUUG-- 5'       Csi-miRN20-Cs3g05320.1-14
```

Csi-miRN20-Cs3g05320.1-16, target=Cs9g03400.1 gene=Cs9g03400
Category:3
Score:5
Cleavage site:871

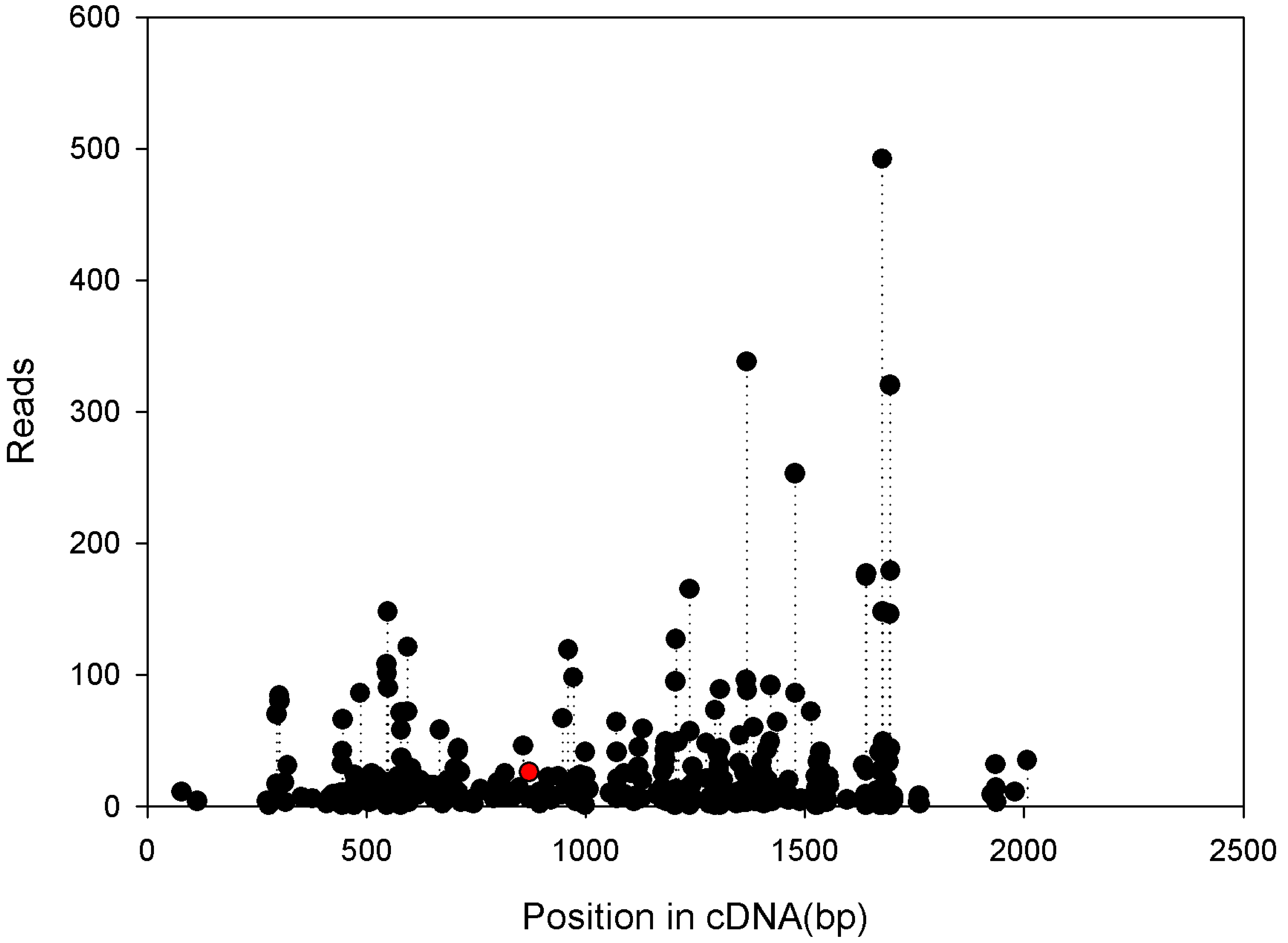


```
5' AAUCCCCAAUCAAA-UUGACAUGAUUG 3'       Cs9g03400.1
         :   ::: ::: ::::::::::
3' ----GUUUUAUUUUGAACUGUACUA-- 5'       Csi-miRN20-Cs3g05320.1-16
```

Csi-miRN20-Cs3g05320.1-19, target=Cs7g05730.1 gene=Cs7g05730
Category:1
Score:5
Cleavage site:2376

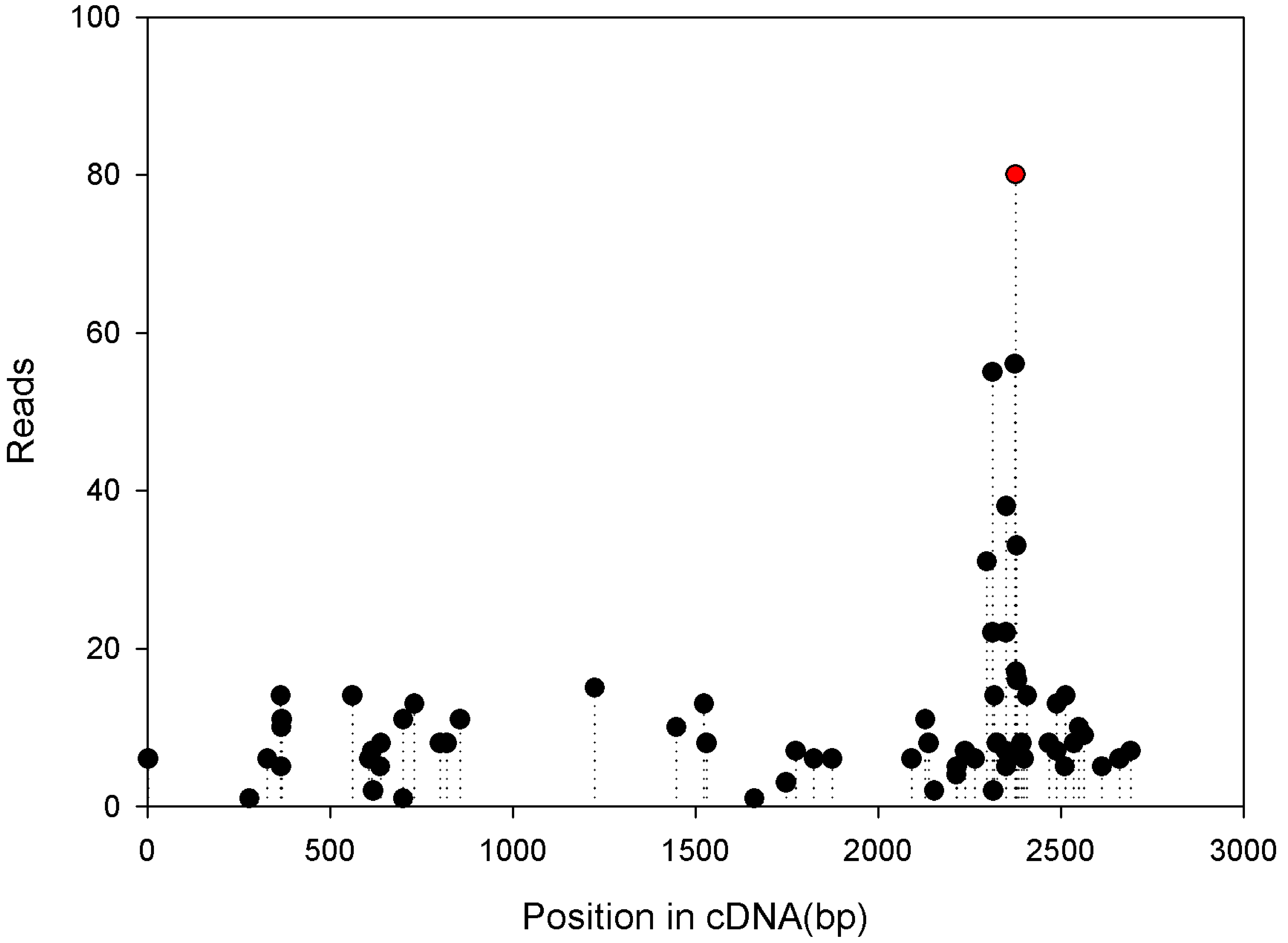


```
5' UGUACAAGAUGAUUUAAUAUCUUGCU 3'        Cs7g05730.1
       ::   :::::..:::::::::
3' ----GUAAUACUAGGUUAUAGAACA- 5'        Csi-miRN20-Cs3g05320.1-19
```

Csi-miRN20-Cs3g05320.1-24, target=Cs1g12760.1 gene=Cs1g12760
Category:2
Score:4.5
Cleavage site:652

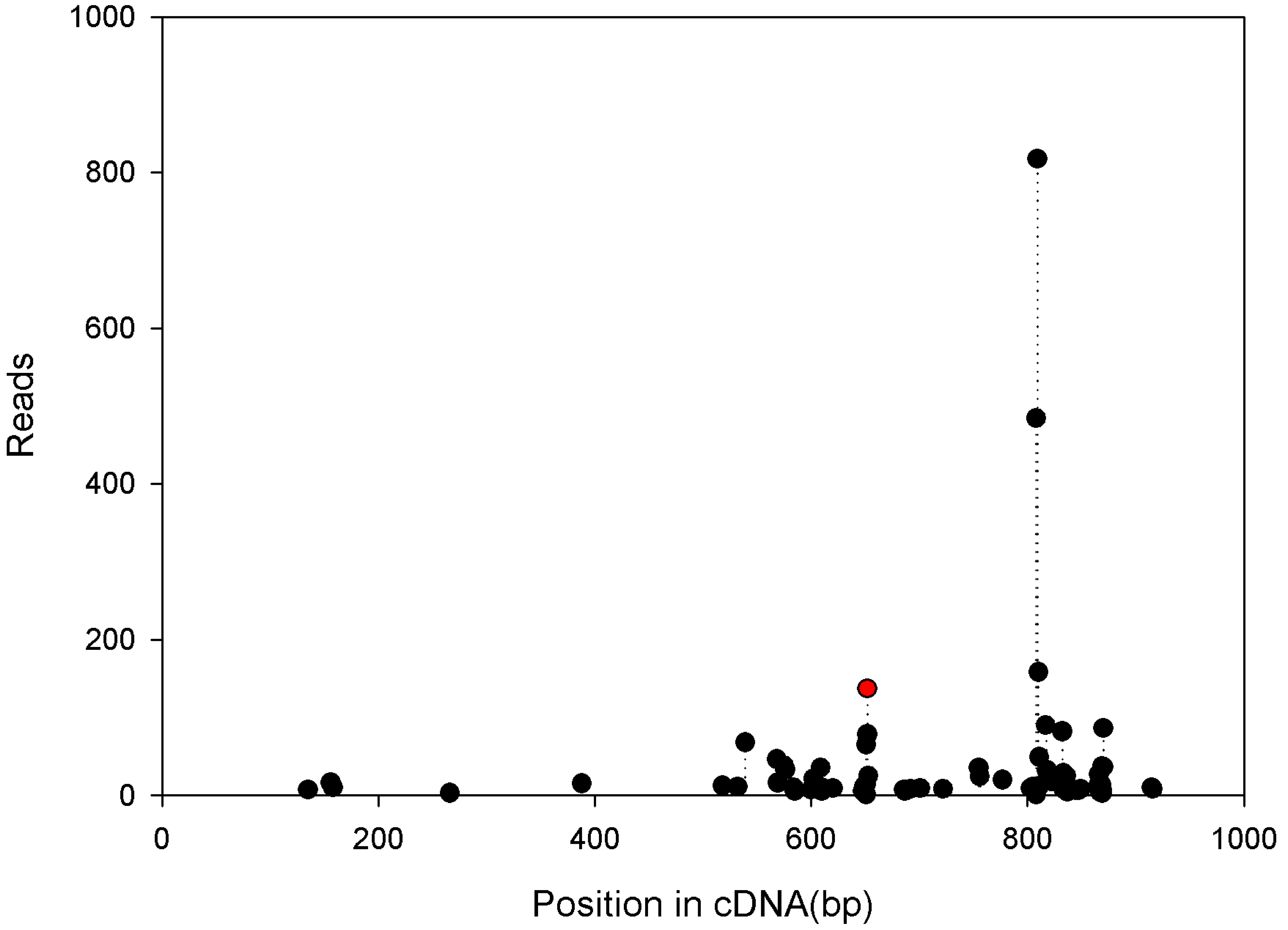


```
5' UAUGUCGGC-UGGGUGACUAUUAUCAU 3'       Cs1g12760.1
        :..: : .::.::::::::::
3' -----GUUGUAGUCAUUGAUAAUAGU- 5'       Csi-miRN20-Cs3g05320.1-24
```

Csi-miRN20-Cs3g05320.1-24, target=Orange1.1t00622.1 gene=Orange1.1t00622
Category:2
Score:5
Cleavage site:1354

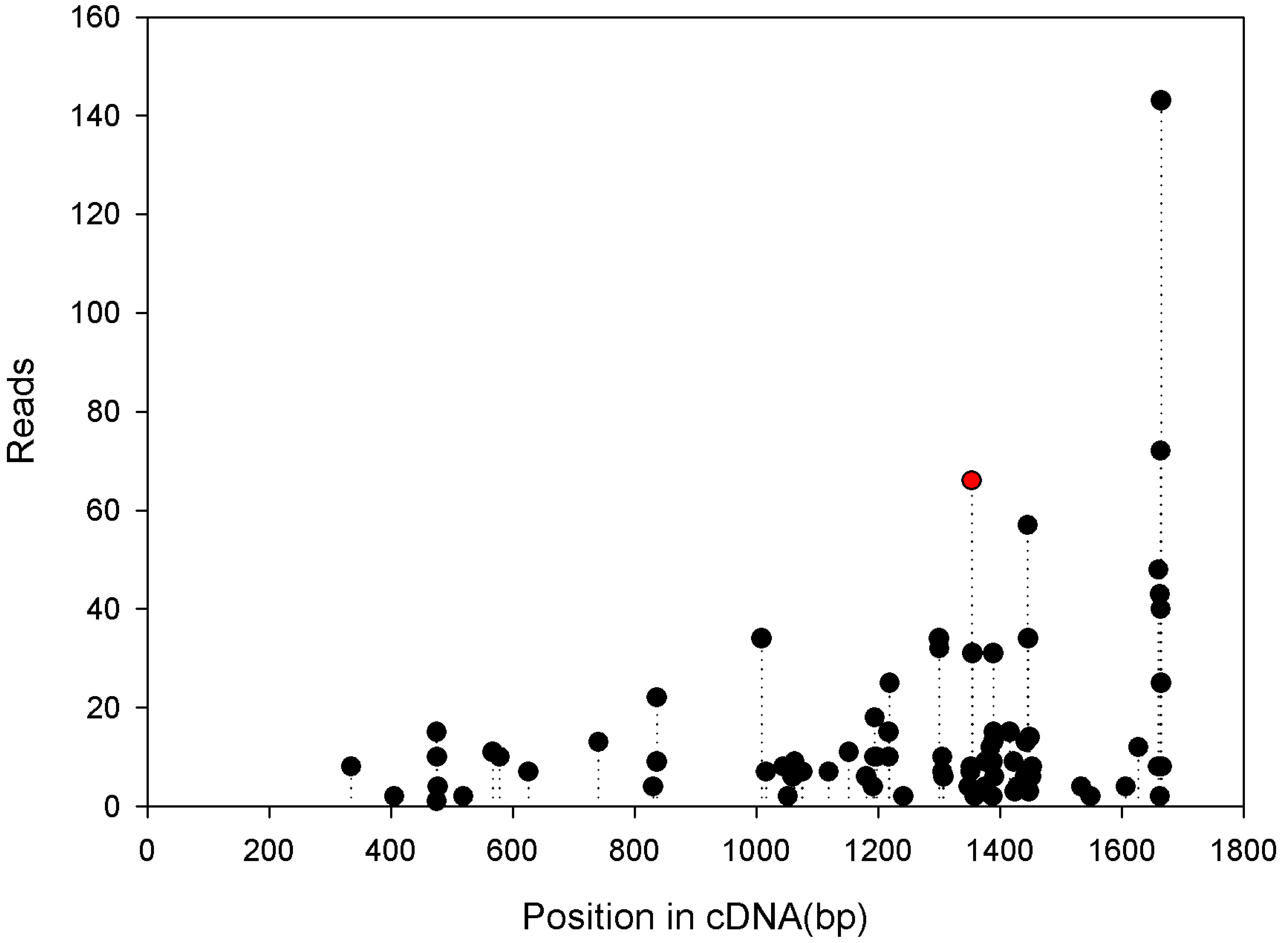


```
5' UGCAAAGUCGGGAACUGUUAUCAUGG 3'        Orange1.1t00622.1
     ::: .::.: ::::.::::::
3' --GUUGUAGUCAUUGAUAAUAGU--- 5'        Csi-miRN20-Cs3g05320.1-24
```

Csi-miRN20-Cs3g05320.1-24, target=Orange1.1t01948.1 gene=Orange1.1t01948
Category:3
Score:4.5
Cleavage site:366

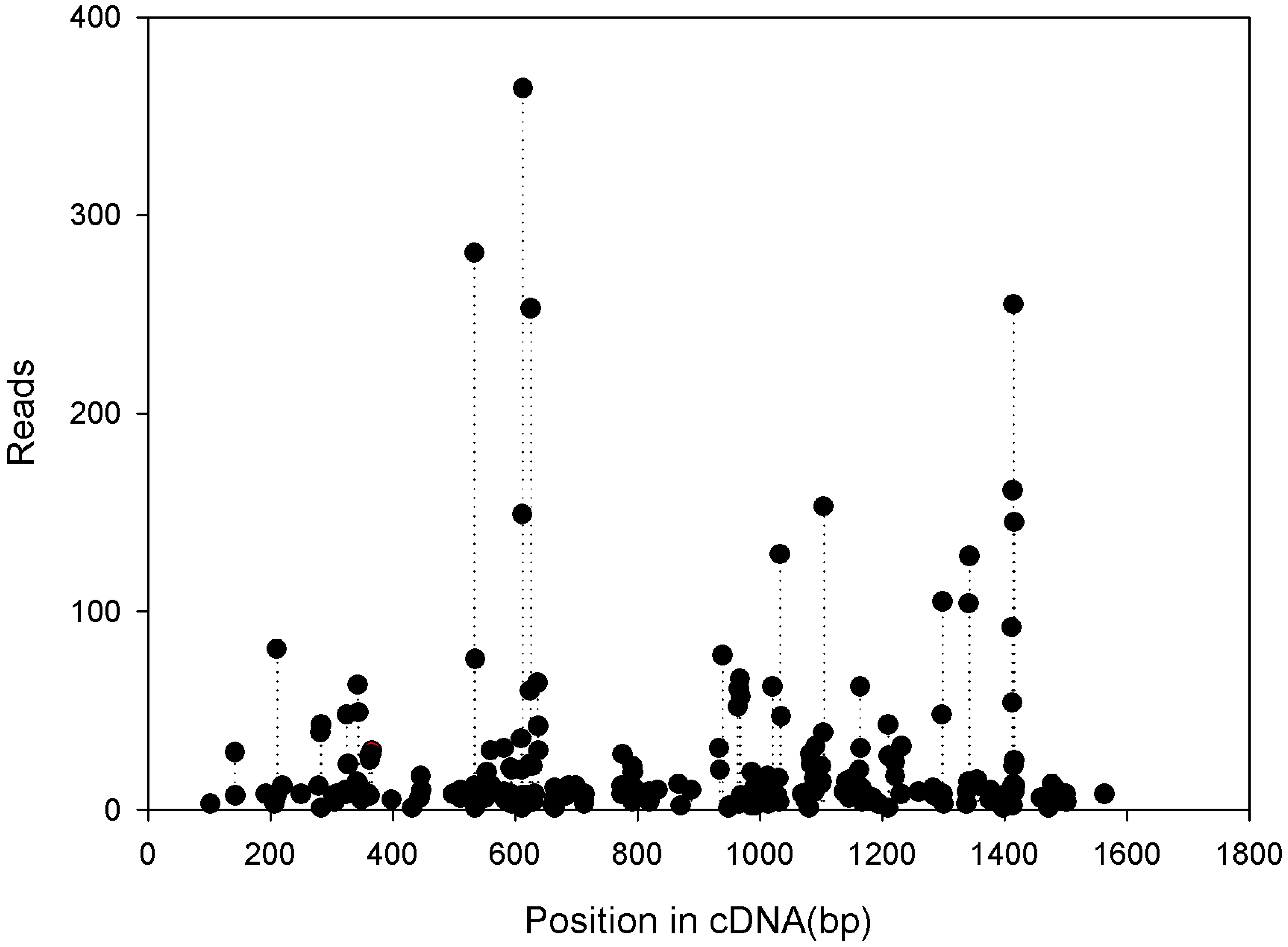


```
5' AUGUGA-AGCAGUAACUAUUAUUGAAA 3'       Orange1.1t01948.1
       ..: : ::::::::::::::..
3' ---GUUGUAGUCAUUGAUAAUAGU--- 5'       Csi-miRN20-Cs3g05320.1-24
```

Csi-miRN20-Cs3g05320.1-26, target=Cs3g11480.1 gene=Cs3g11480
Category:3
Score:4.5
Cleavage site:227

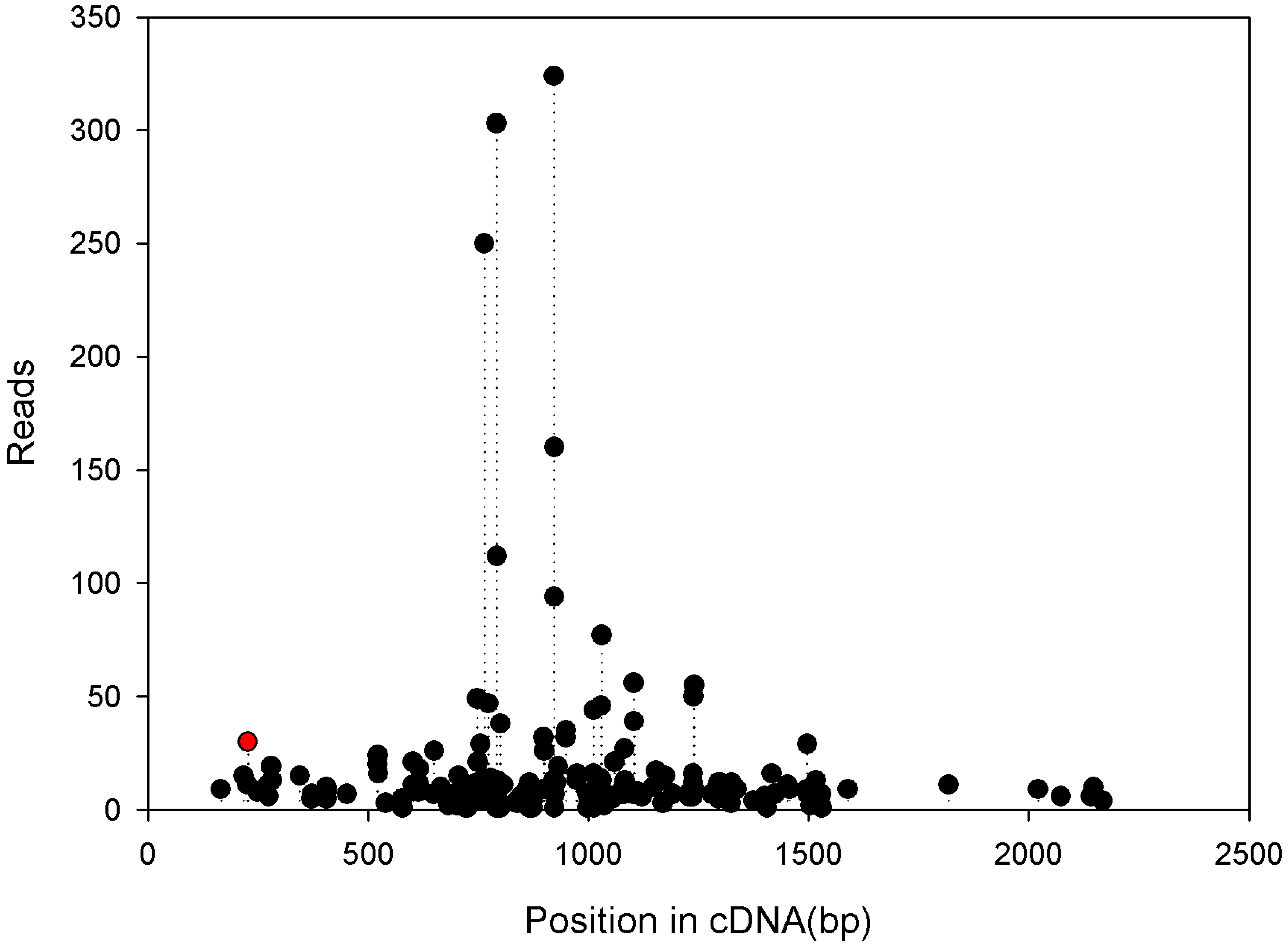


```
5' UCAAUUUUCU-ACUGUUUUCCAGCUGC 3'       Cs3g11480.1
    :::::.::::  ::::::::::
3' -GUUAGAAGAGAGACAAAAGGU----- 5'       Csi-miRN20-Cs3g05320.1-26
```

Csi-miRN20-Cs3g05320.1-26, target=Cs6g18830.1 gene=Cs6g18830
Category:3
Score:5
Cleavage site:676

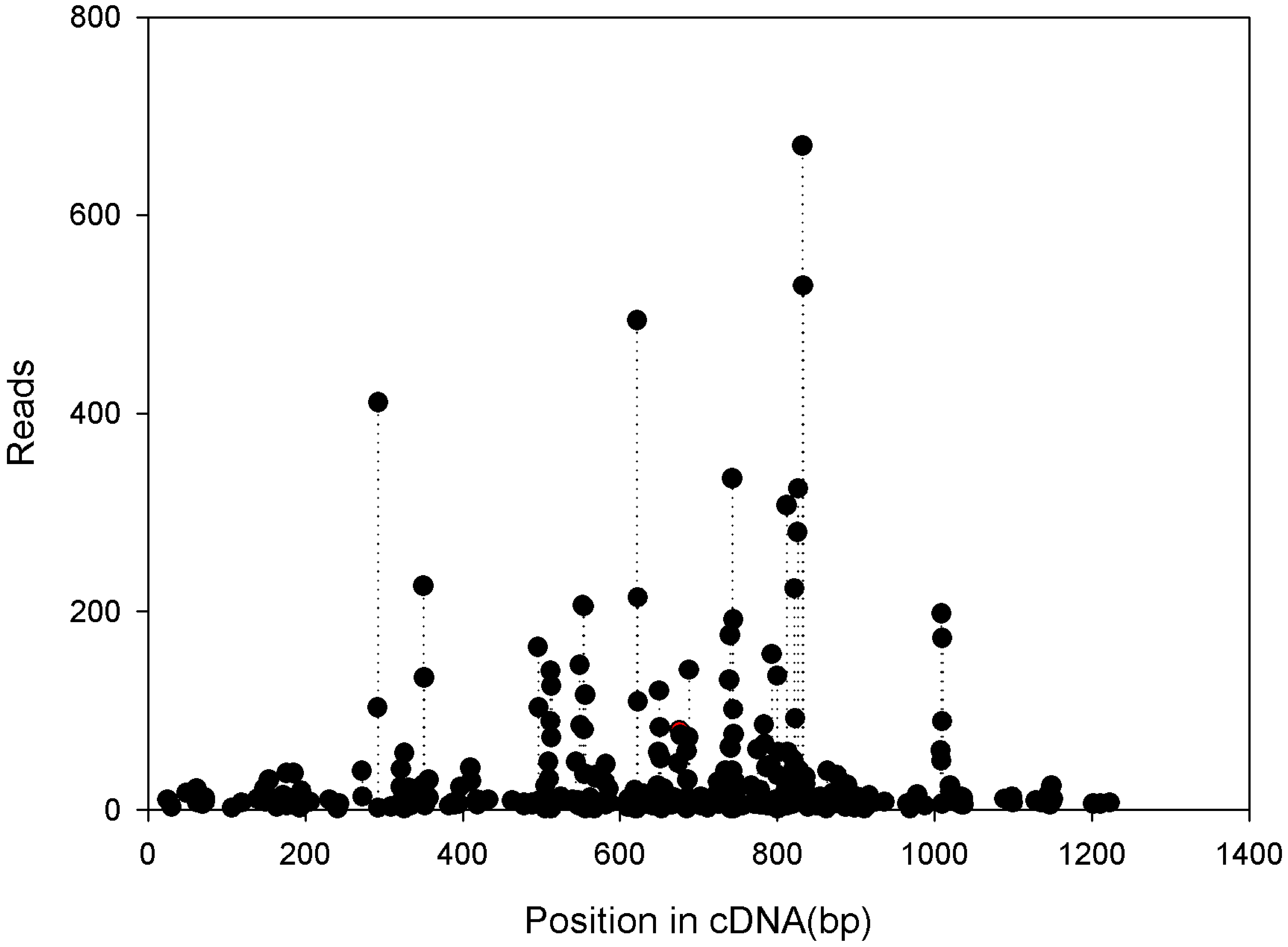


```
5' UGCACUCAACUUCUCUGUUUUCUACU 3'        Cs6g18830.1
     :: ::  : ::::::::::::.:
3' --GUUAGAAG-AGAGACAAAAGGU-- 5'        Csi-miRN20-Cs3g05320.1-26
```

Csi-miRN20-Cs3g05320.1-4, target=Cs5g05630.1 gene=Cs5g05630
Category:2
Score:5
Cleavage site:1096

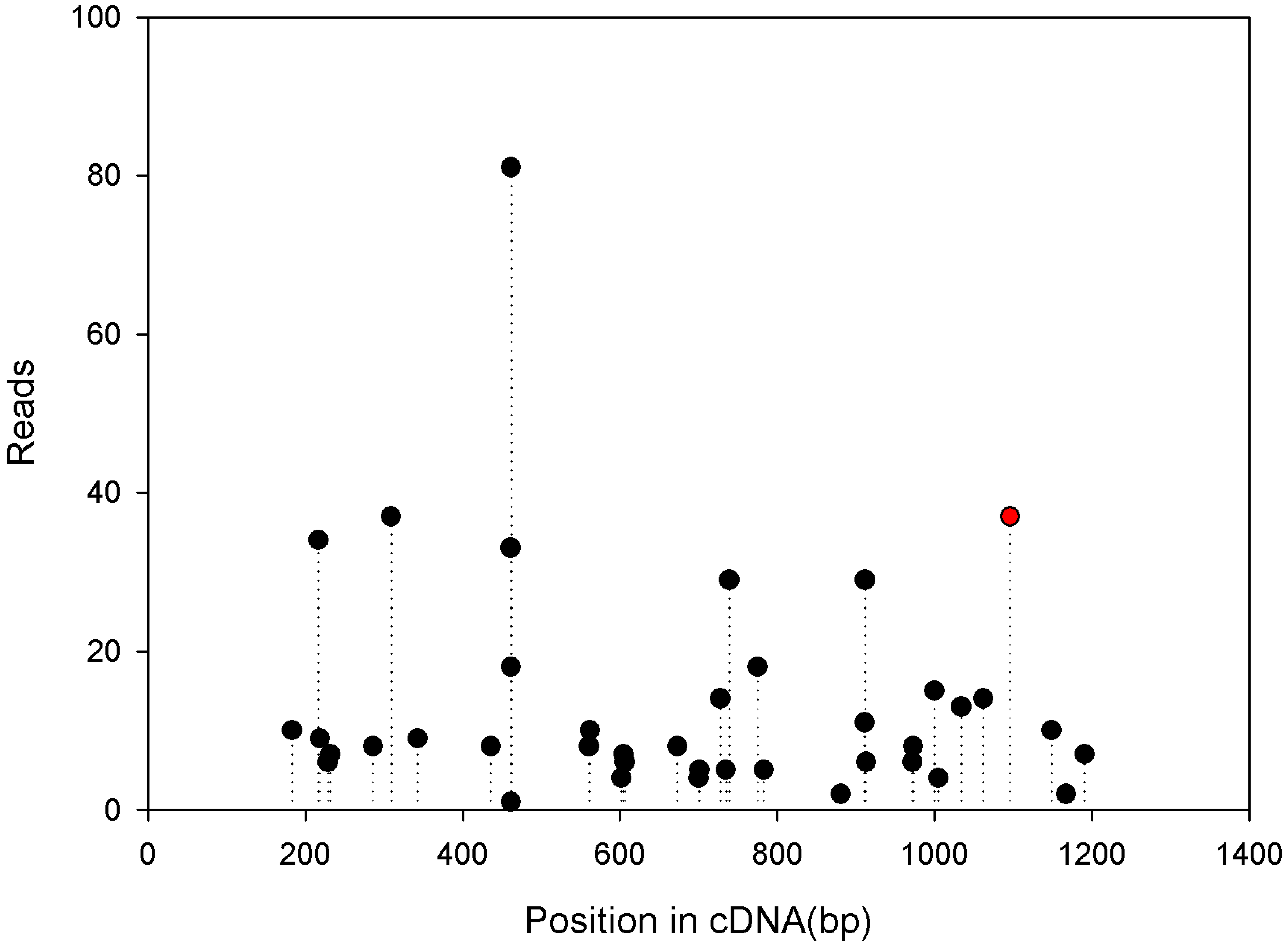


```
5' GAAAUUAAUUAUCUAUGAUUUUGCUU 3'        Cs5g05630.1
         ::: :::: :::::::::.:::
3' ----CAUU-AUAGUUACUAAAAUGAA 5'        Csi-miRN20-Cs3g05320.1-4
```

Csi-miRN20-Cs3g05320.1-4, target=Cs5g18400.1 gene=Cs5g18400
Category:2
Score:5
Cleavage site:729

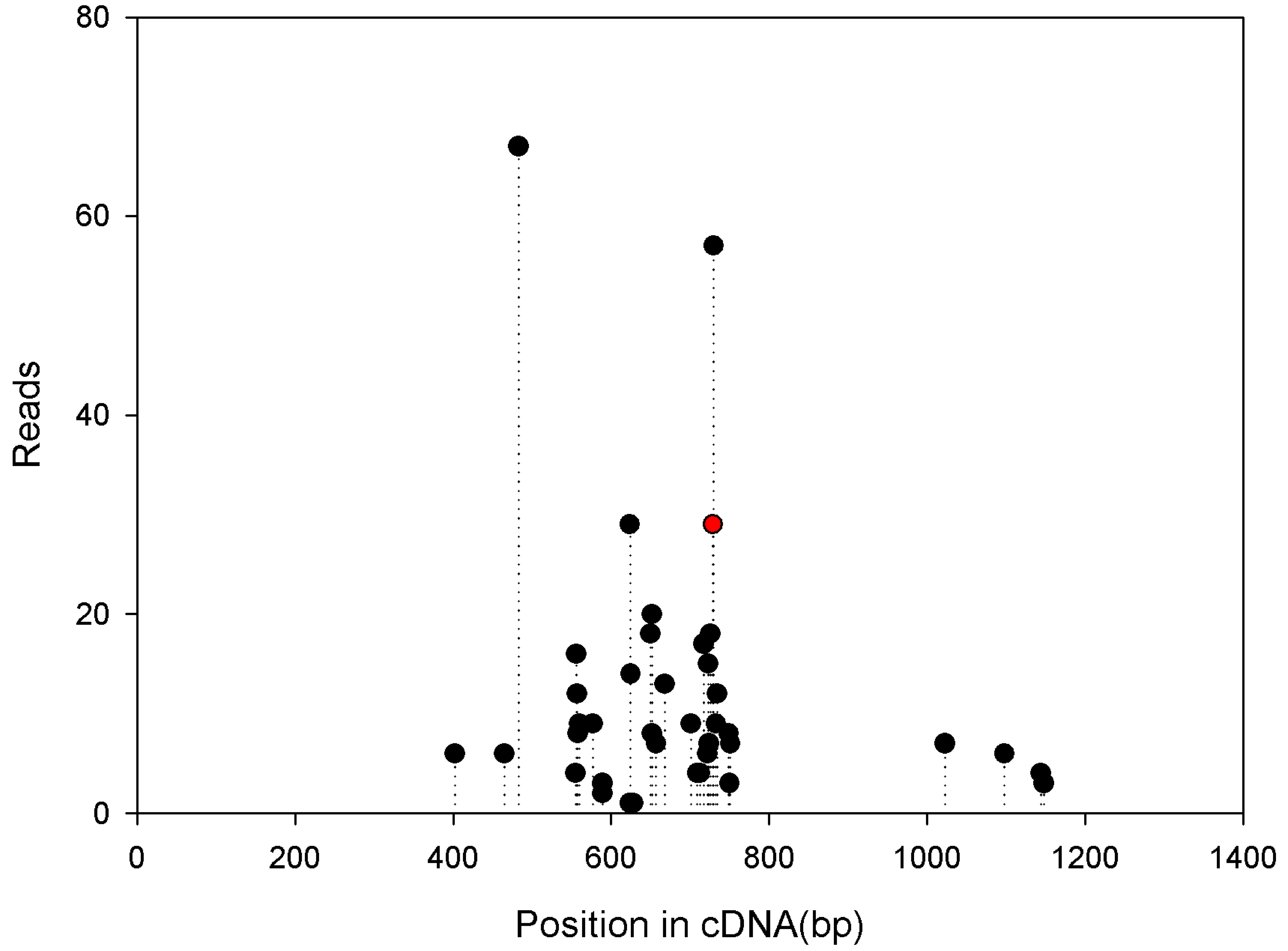


```
5' CUGAUAUUAAUGAAUUUUACUGUGAA 3'        Cs5g18400.1
     :.::::.:::: ::::::::
3' CAUUAUAGUUAC-UAAAAUGAA---- 5'        Csi-miRN20-Cs3g05320.1-4
```

Csi-miRN20-Cs3g05320.1-9, target=Cs3g26640.1 gene=Cs3g26640
Category:3
Score:5
Cleavage site:1670

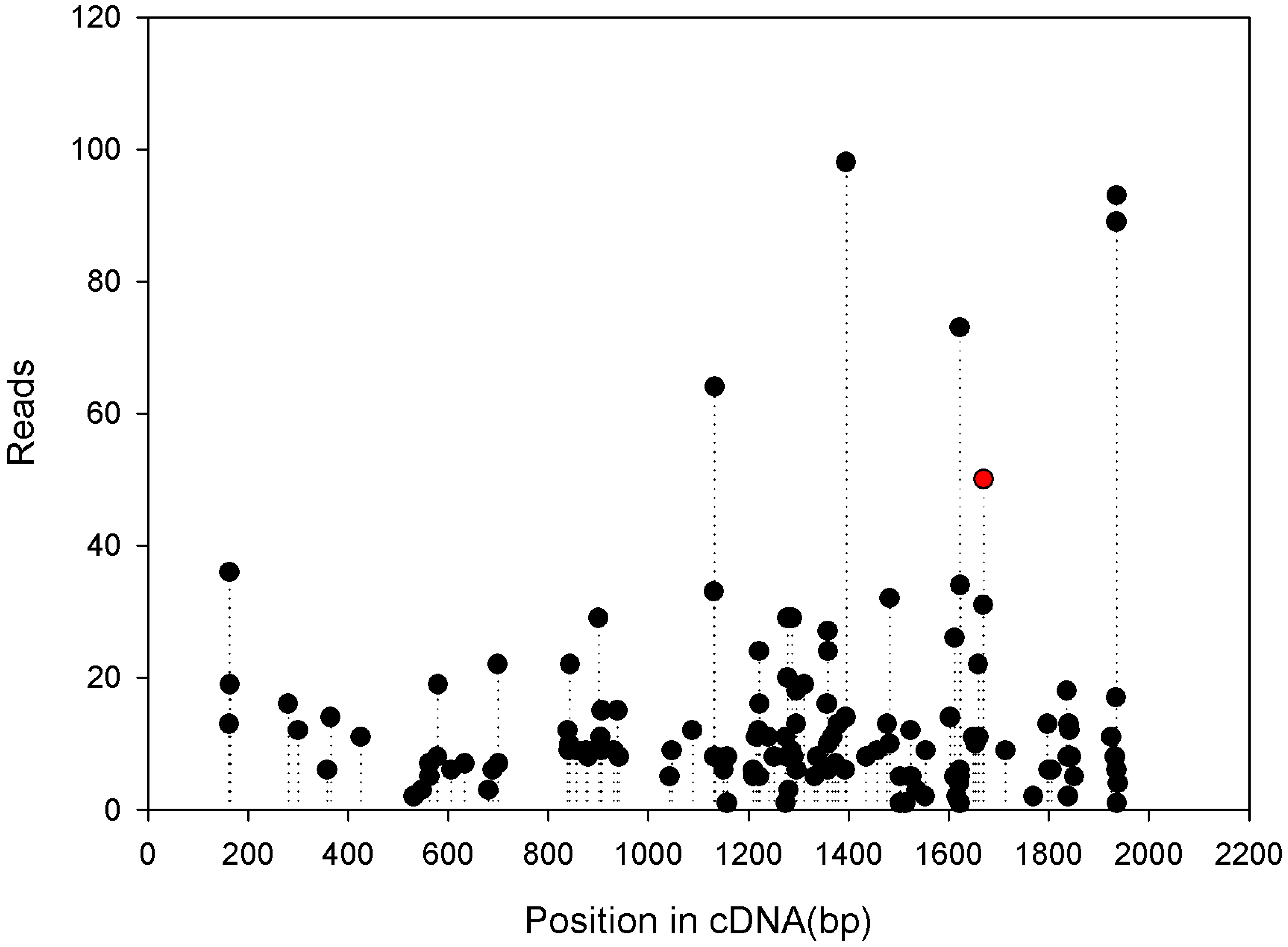


```
5' AUAAAGUAU-AAUAUAUUACUUUCUAG 3'       Cs3g26640.1
    .::: . : ::::::::::::
3' -GUUUGGGACUUAUAUAAUGAA----- 5'       Csi-miRN20-Cs3g05320.1-9
```

T-plots for targets of secondary siRNAs in fruit

Csi-miR482a-3p-Cs1g15550.1-4, target=Cs8g11330.1 gene=Cs8g11330
Category:3
Score:5
Cleavage site:15

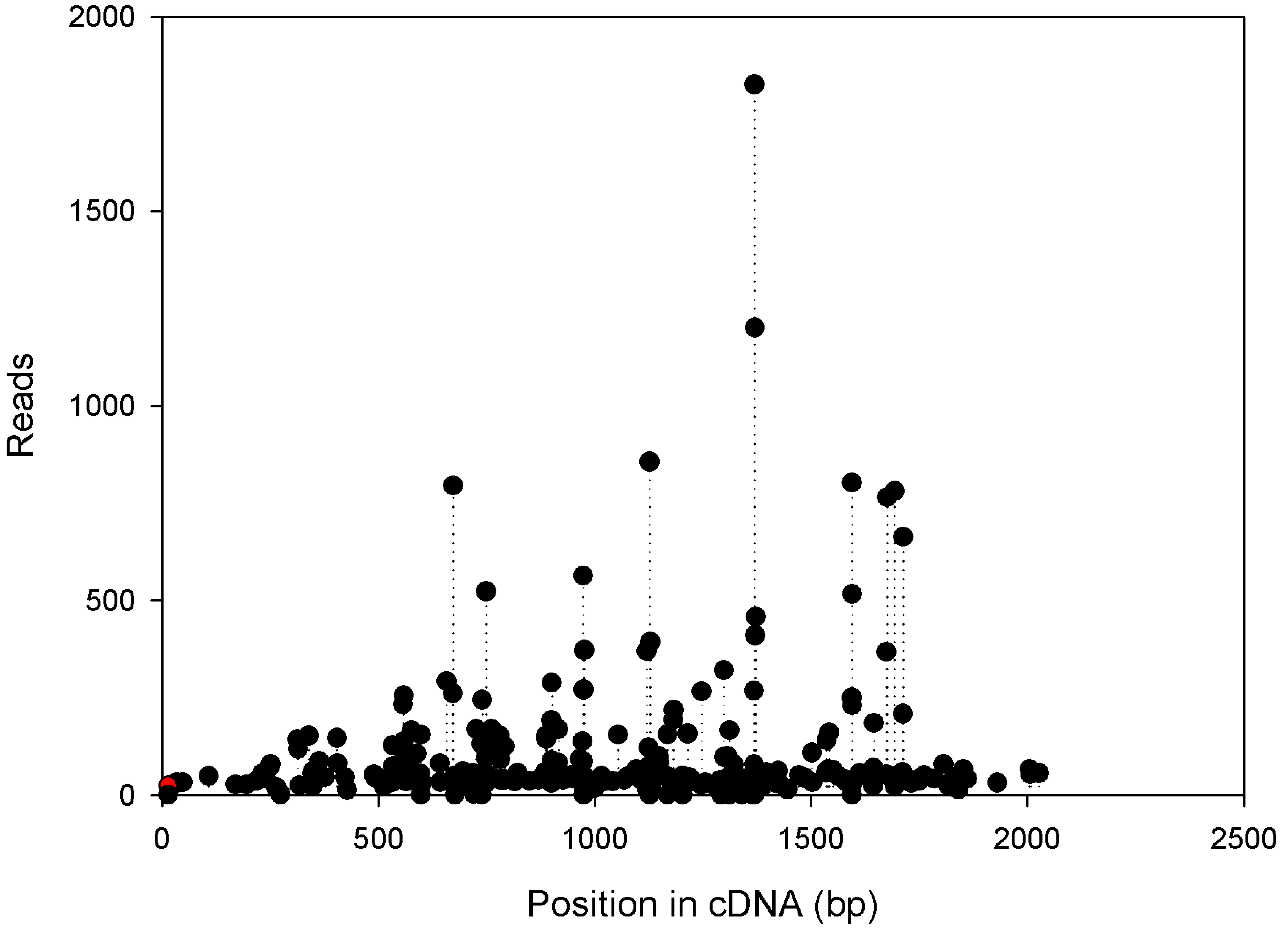


```
5' CAGAAGACACACACACACAUUCUUCU 3'        Cs8g11330.1
      : :::::::: ::::::.:::
3' ----UAUGUGUGUGGGUGUAGGAAU- 5'        Csi-miR482a-3p-Cs1g15550.1-4
```

Csi-miR482a-3p-Cs3g13340.1-20, target=Cs1g07080.1 gene=Cs1g07080
Category:3
Score:5
Cleavage site:930

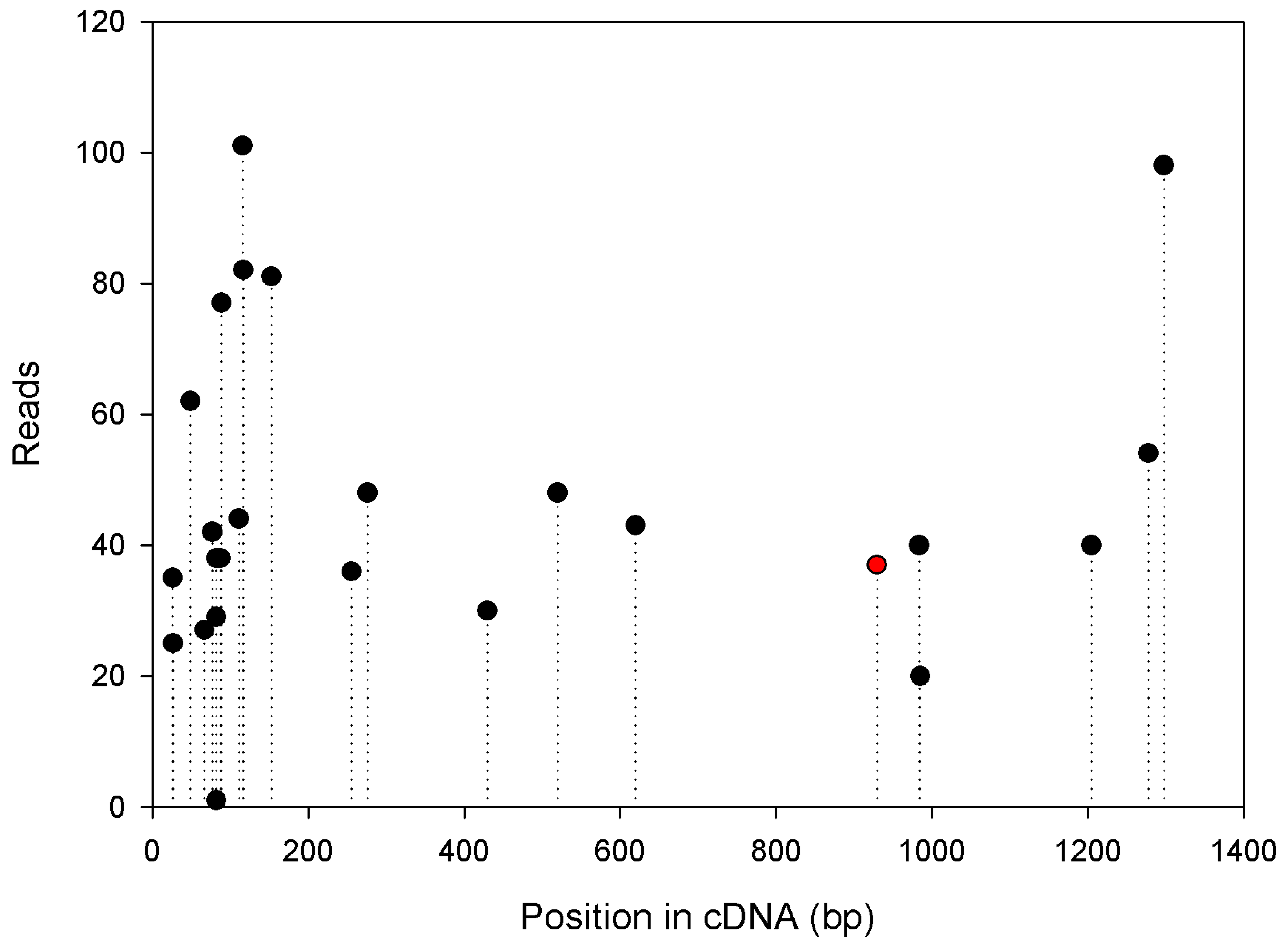


```
5' UGCAGAGUG-GAAUGGCUCAAGCAAUG 3'       Cs1g07080.1
     :.:.::  ::.:::::::.::
3' ---UUUUACGGUUGCCGAGUUUGU--- 5'       Csi-miR482a-3p-Cs3g13340.1-20
```

Csi-miR482a-3p-Cs3g13340.1-20, target=Cs2g11470.1 gene=Cs2g11470
Category:3
Score:3
Cleavage site:1751

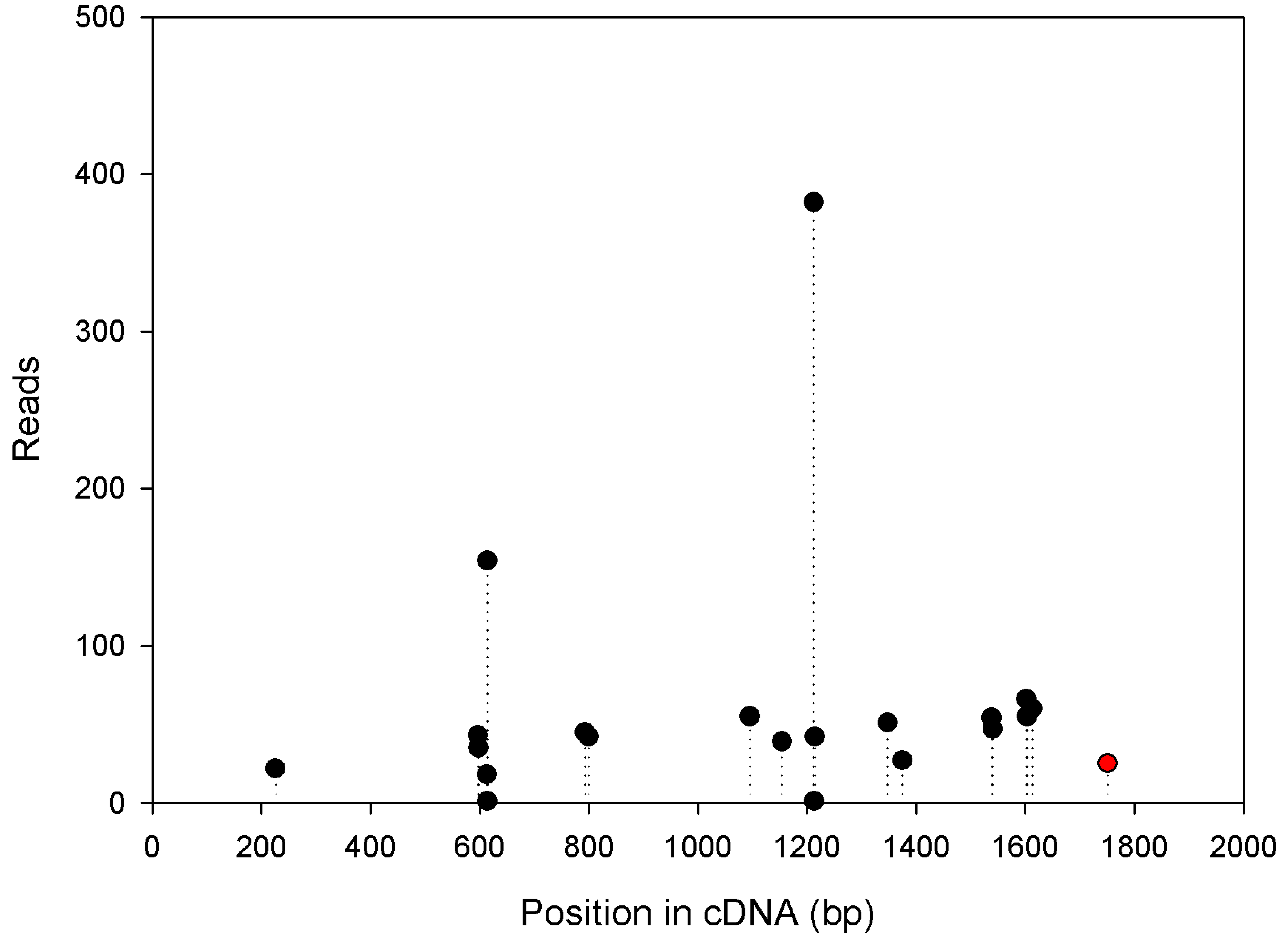


```
5' GUAAAAAAUGCCAAUGGUUCAAACUU 3'        Cs2g11470.1
      ::::::::::.::.::::::
3' ----UUUUACGGUUGCCGAGUUUGU- 5'        Csi-miR482a-3p-Cs3g13340.1-20
```

Csi-miR482a-3p-Cs3g13340.1-35, target=Orange1.1t02629.1 gene=Orange1.1t02629
Category:3
Score:5
Cleavage site:1195

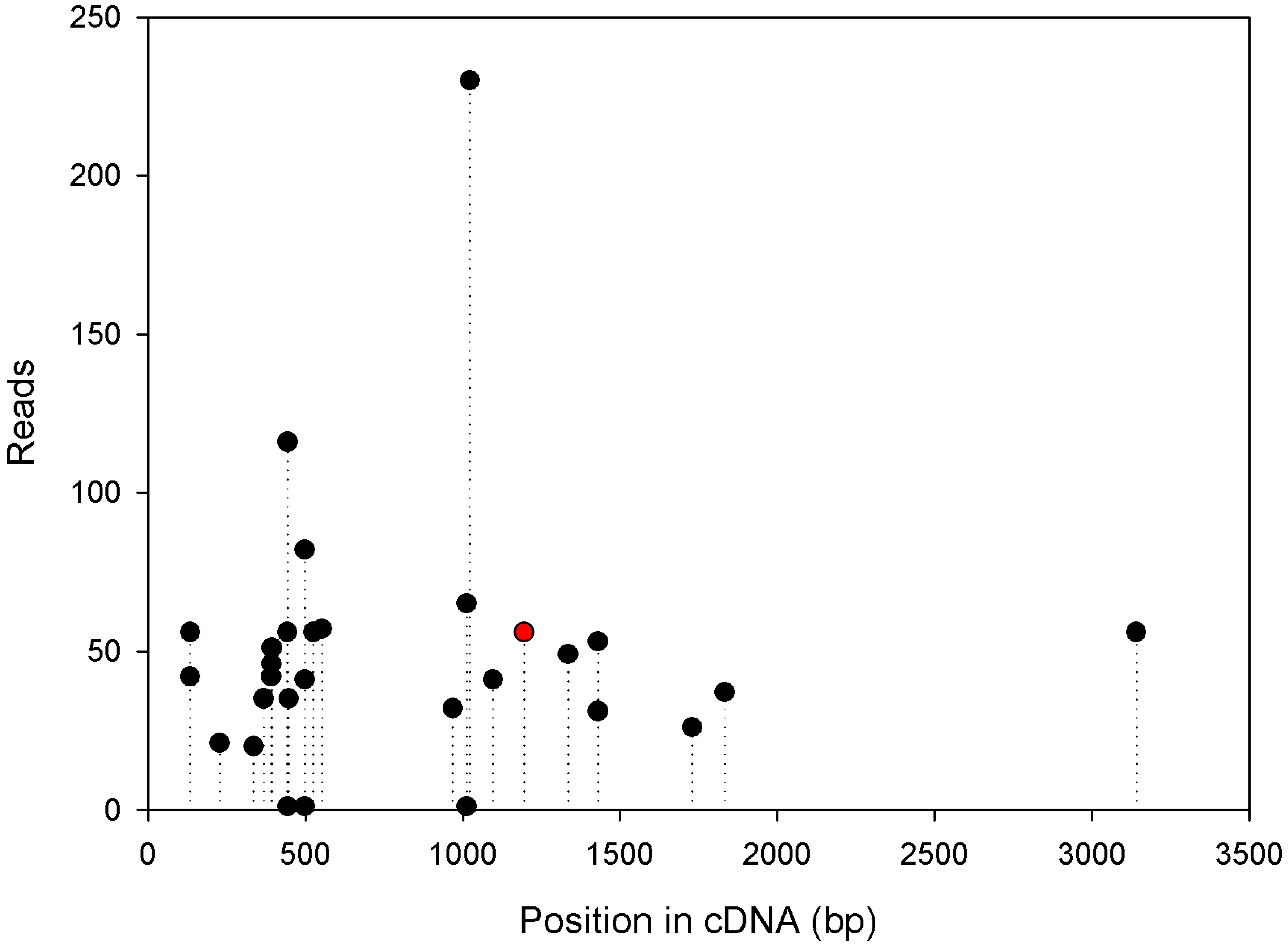


```
5' AGUAUUUUACAUAGUUCAGUUAACUG 3'        Orange1.1t02629.1
    ::  ..  :  ::::::::::.:::
3' -CACGGUAGGUAUCAAGUUAAU---- 5'        Csi-miR482a-3p-Cs3g13340.1-35
```

Csi-miR482a-3p-Orange1.1t01918.1-29, target=Cs2g21880.1 gene=Cs2g21880
Category:3
Score:5
Cleavage site:349

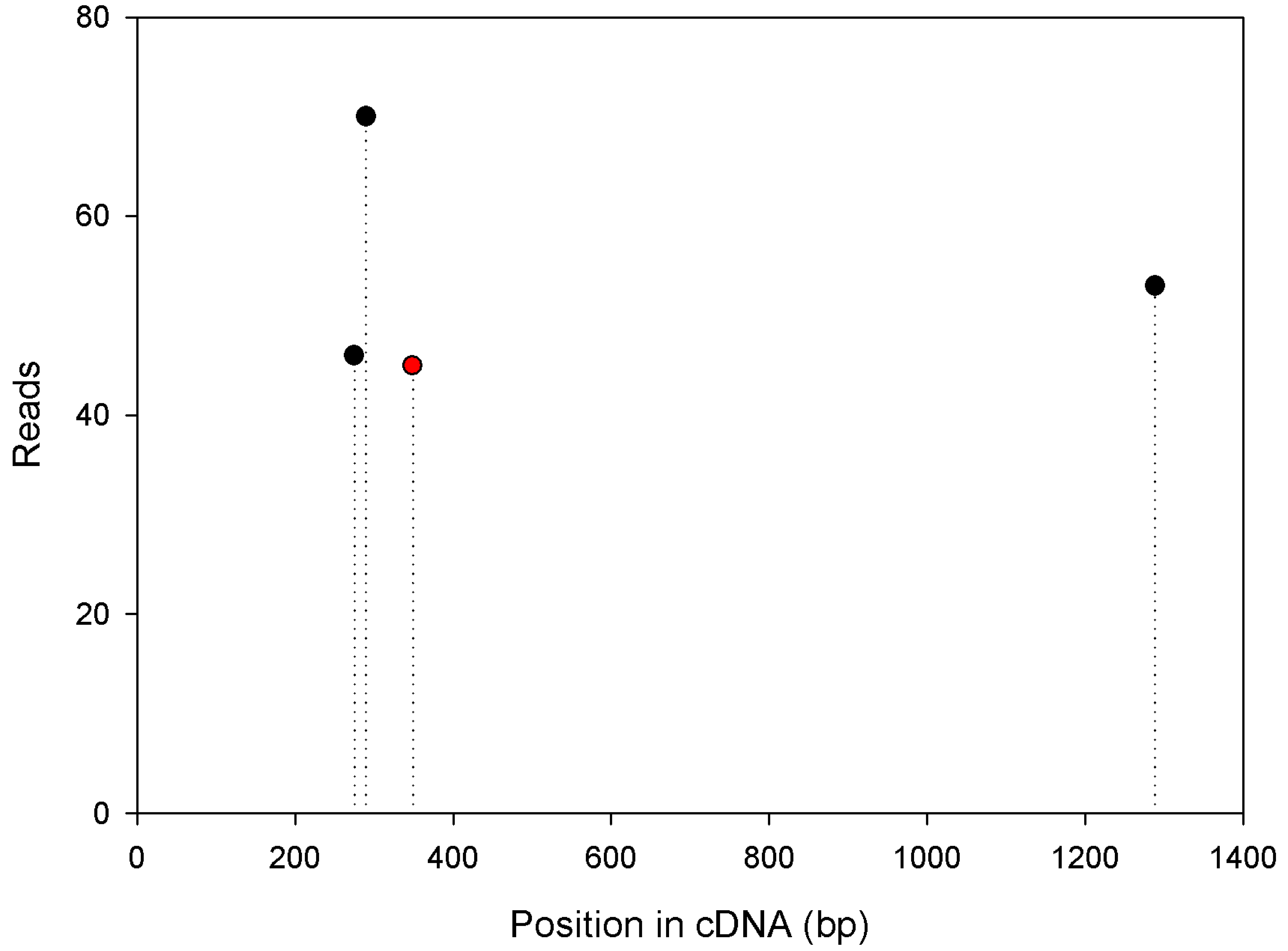


```
5' GAUGUCCAAU-CUCUUCUCGAUCUCUU 3'       Cs2g21880.1
          ::. :. :::::.:::::::
3' ------CUUGUGGAAAGAGUUAGAGAA 5'       Csi-miR482a-3p-Orange1.1t01918.1-29
```

Csi-miR482a-3p-Orange1.1t01918.1-29, target=Cs8g02370.1 gene=Cs8g02370
Category:1
Score:5
Cleavage site:168

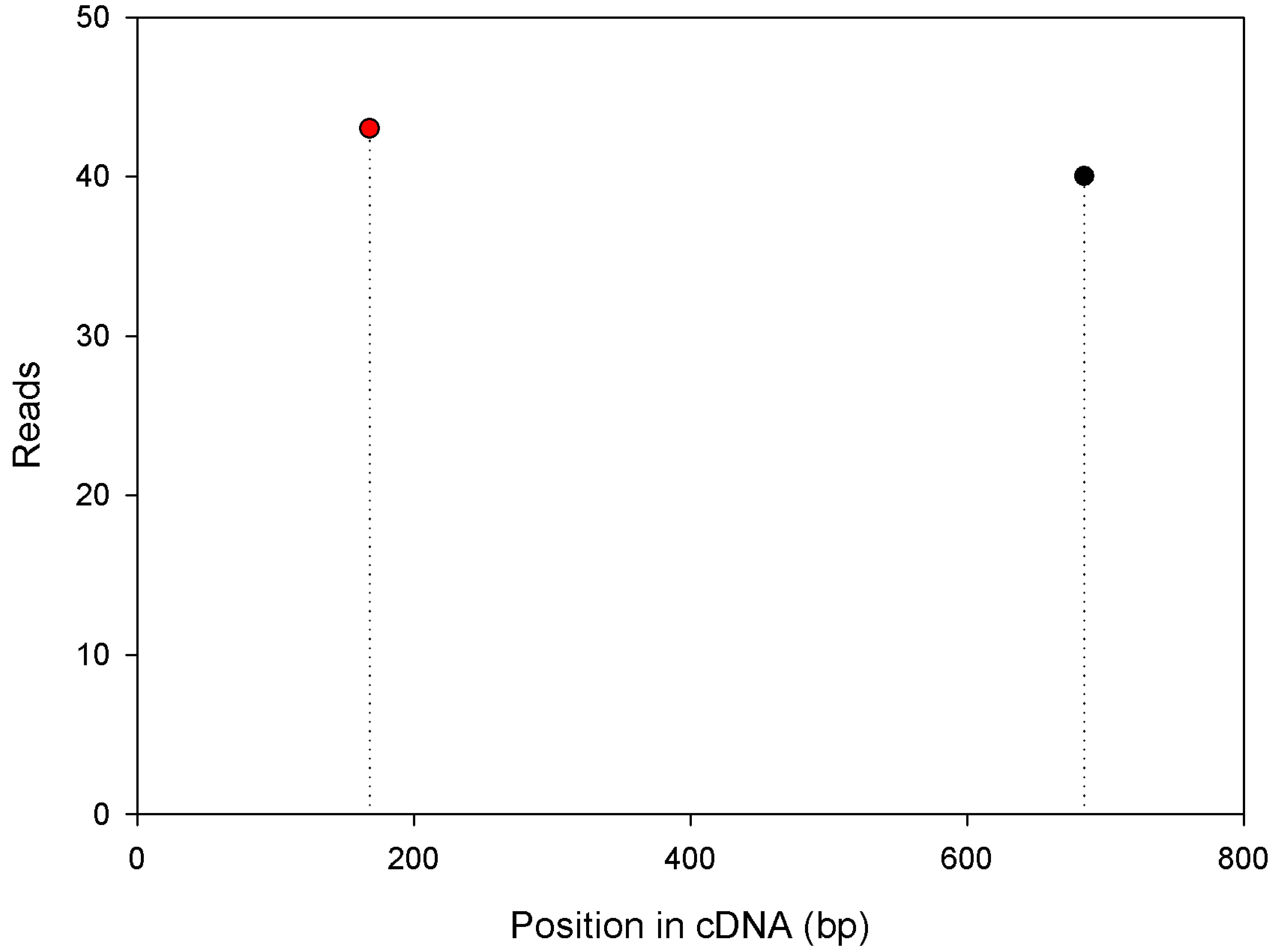


```
5' GGAAAUCCUUUCUGCAAUCUCUAUCU 3'        Cs8g02370.1
    :::  ::::::: ::::::::
3' -CUUGUGGAAAGA-GUUAGAGAA--- 5'        Csi-miR482a-3p-Orange1.1t01918.1-29
```

Csi-miR482a-3p-Orange1.1t01918.1-29, target=Orange1.1t03281.1 gene=Orange1.1t03281
Category:2
Score:5
Cleavage site:45

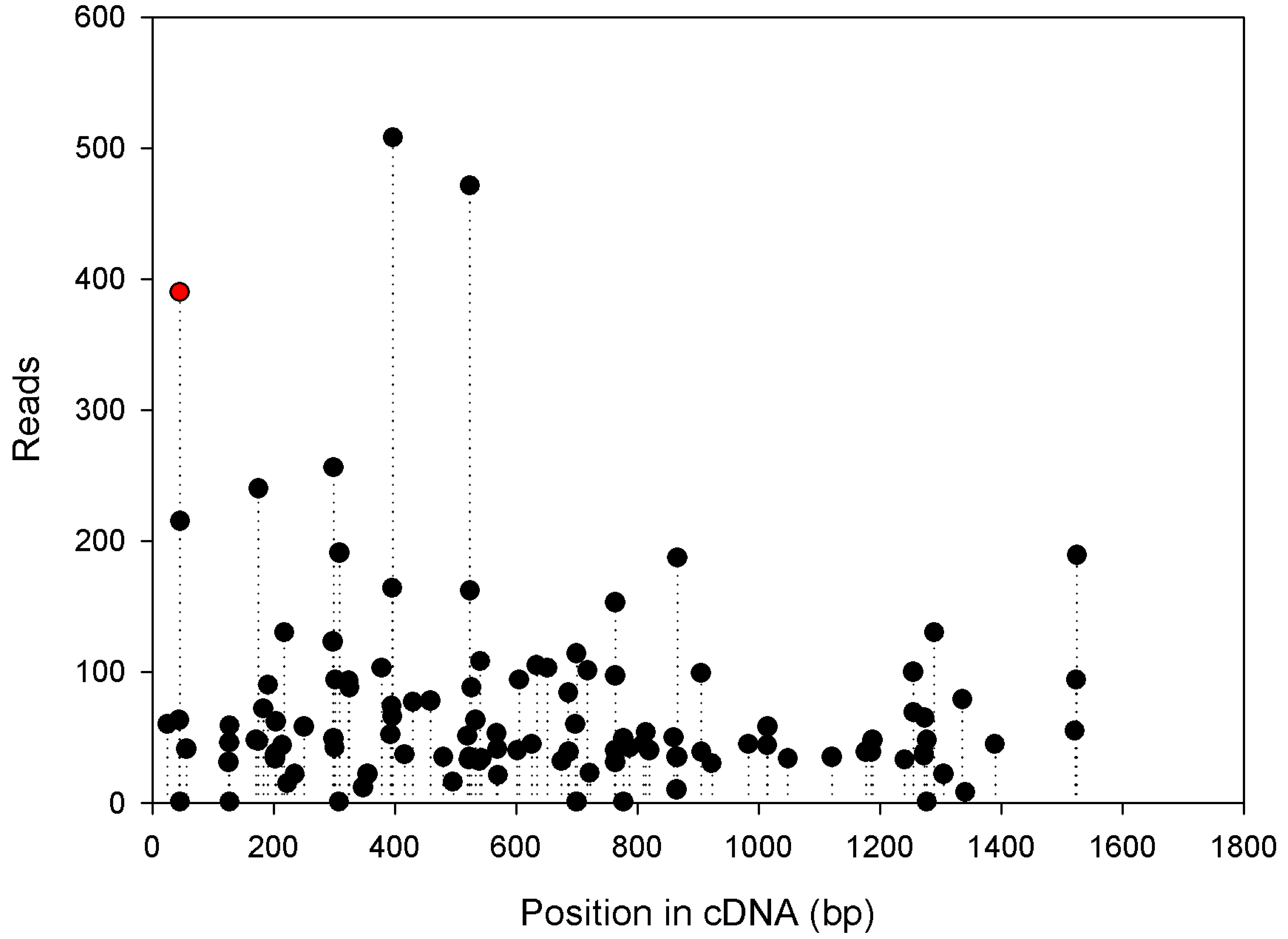


```
5' UAACCAAAAC-CCUUUCUCGAUUUCUC 3'       Orange1.1t03281.1
          ::: :::::::::.::.:::
3' ------CUUGUGGAAAGAGUUAGAGAA 5'       Csi-miR482a-3p-Orange1.1t01918.1-29
```

Csi-miR482a-3p-Orange1.1t01918.1-6, target=Cs6g02560.1 gene=Cs6g02560
Category:3
Score:4
Cleavage site:768

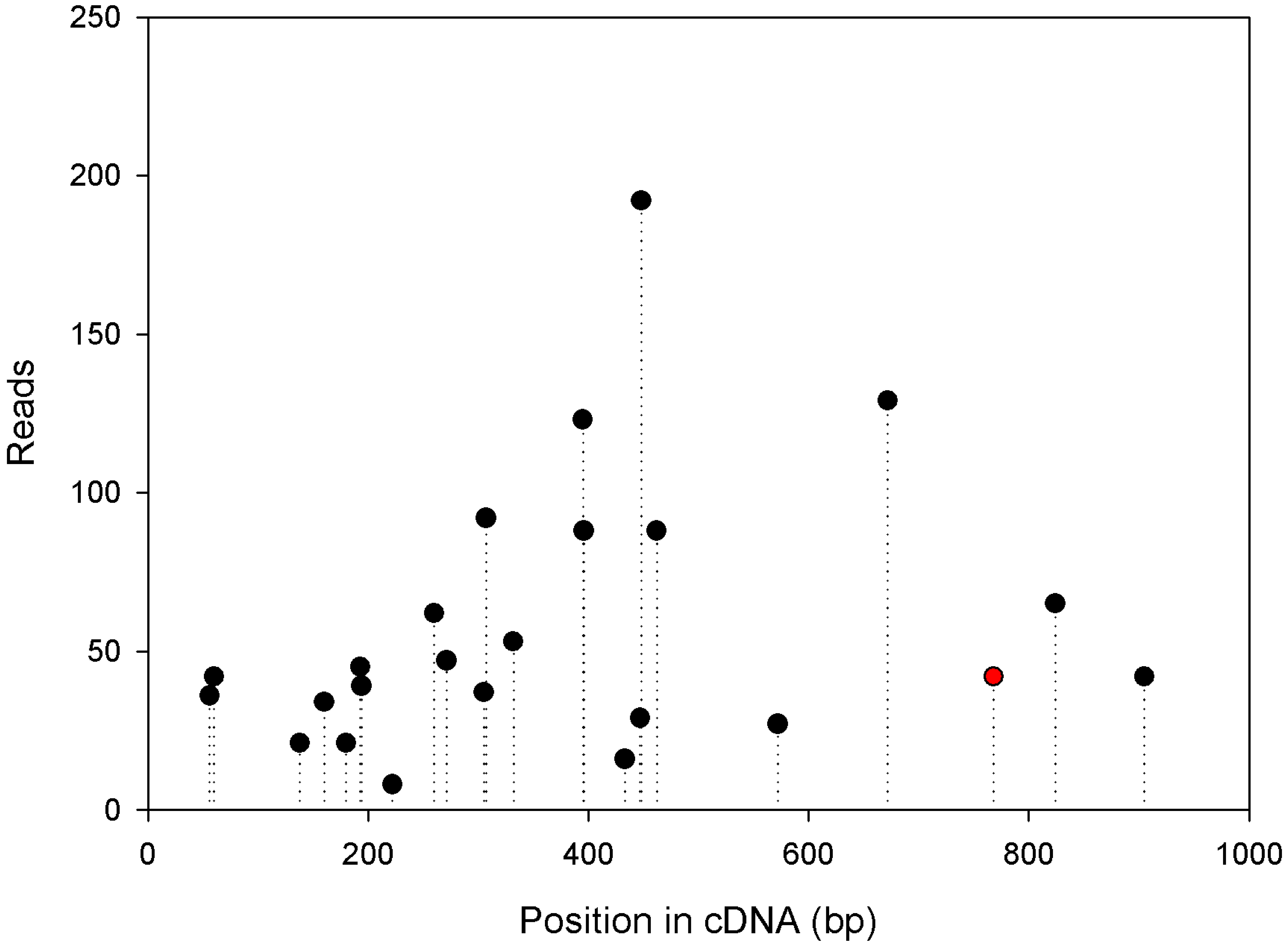


```
5' CCACAAUUGCUUUGGCAACACACAAA 3'        Cs6g02560.1
   : :: :::::::::::::: :
3' GCUGGUAACGAAACCGUUGGG----- 5'        Csi-miR482a-3p-Orange1.1t01918.1-6
```

Csi-miR827.2-Cs5g10180.1-14, target=Cs7g18380.1 gene=Cs7g18380
Category:3
Score:4.5
Cleavage site:894

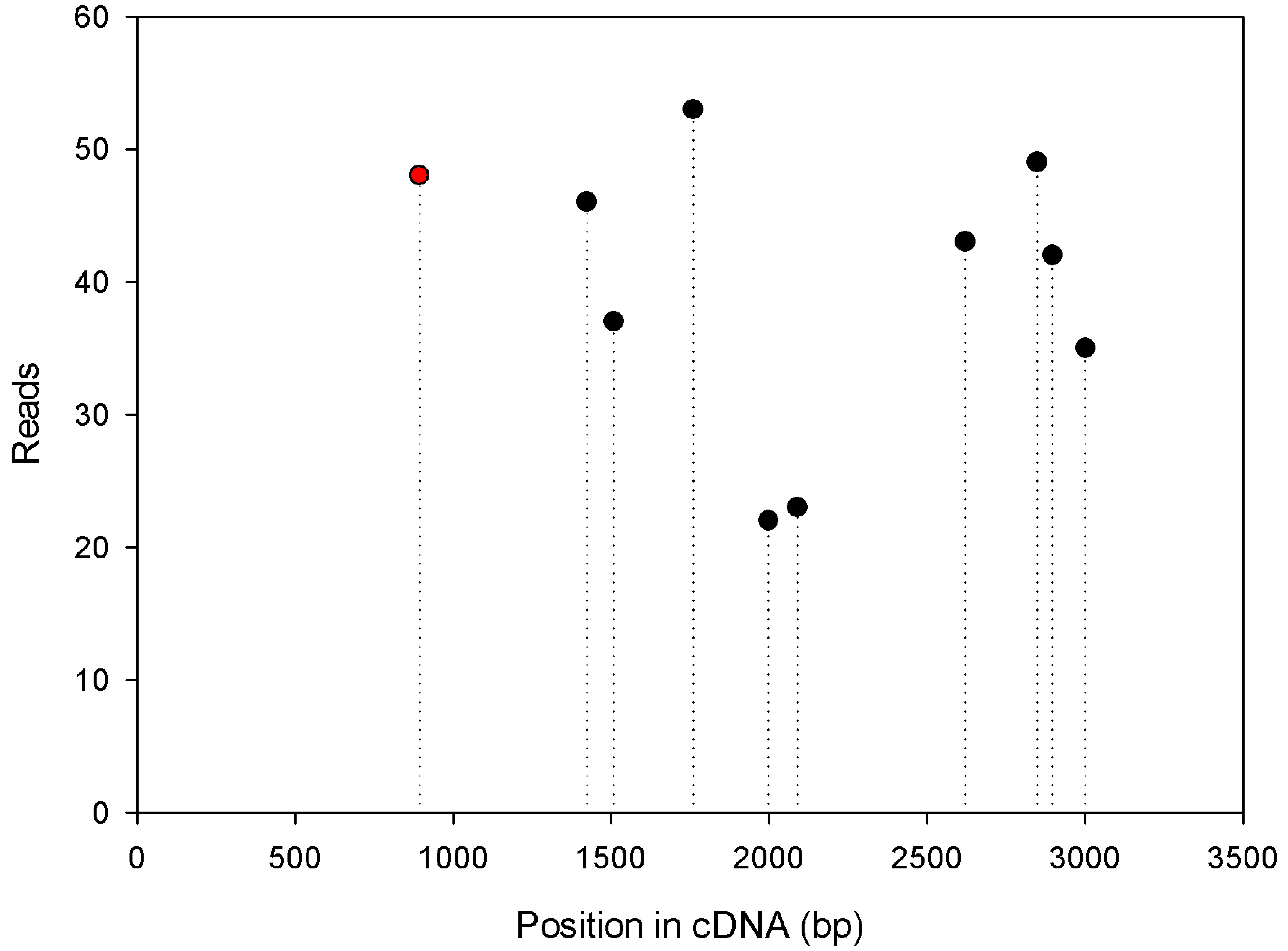


```
5' GAACAAUGCGUCUCAUGCUGUUCUCU 3'        Cs7g18380.1
         :  :  :.  ::::::::::::.::
3' -----UUCUCGUAGUACGACAAGGGA 5'        Csi-miR827.2-Cs5g10180.1-14
```

Csi-miRN20-Cs3g05320.1-12, target=Cs1g17610.1 gene=Cs1g17610
Category:3
Score:5
Cleavage site:655

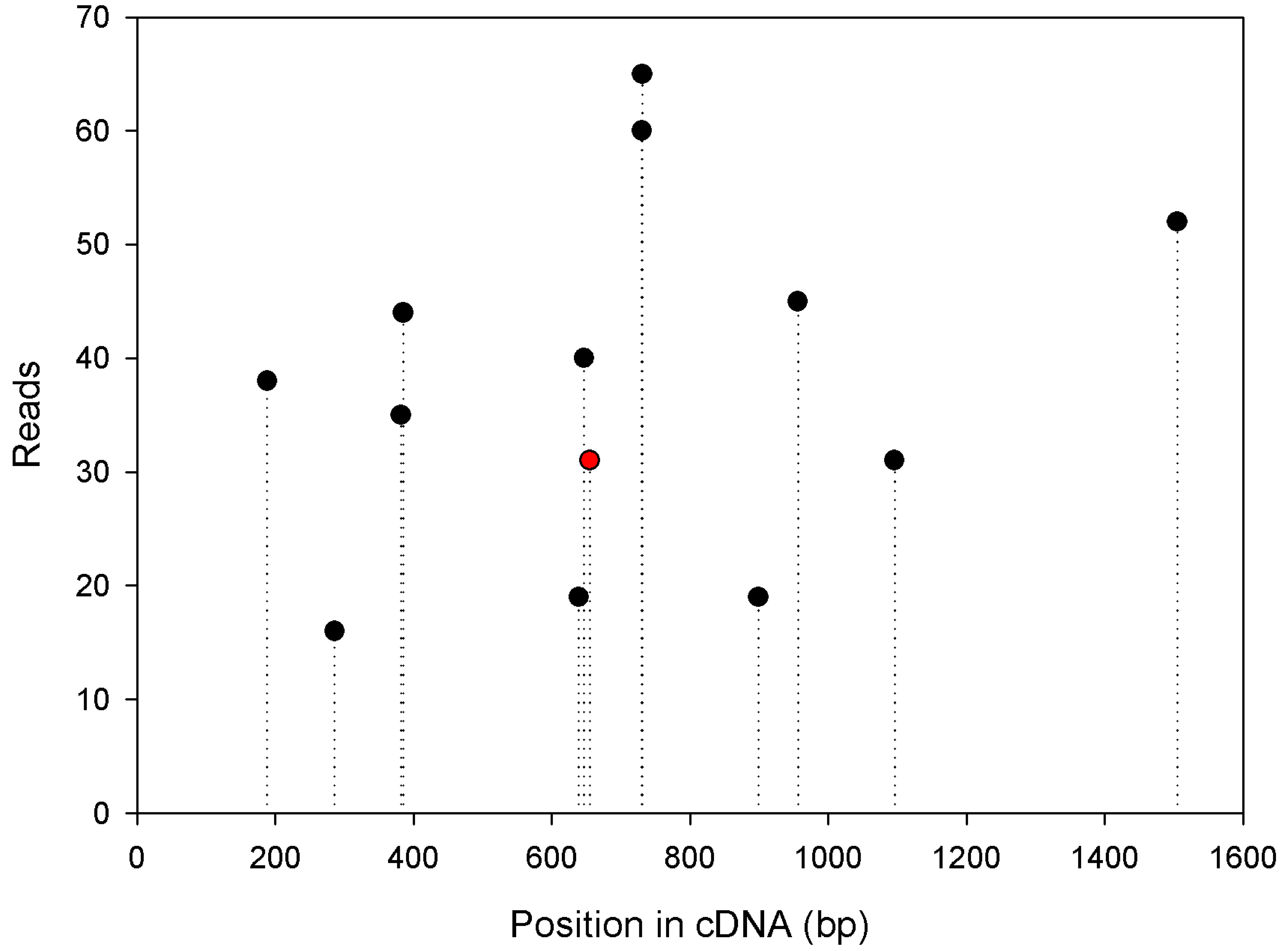


```
5' ACGGCAGAAACCUGGGGUUGUCCCAG 3'        Cs1g17610.1
    : :: :::: :::.:::::::
3' -GACGACUUUCGACUCCAACAG---- 5'        Csi-miRN20-Cs3g05320.1-12
```

Csi-miRN20-Cs3g05320.1-12, target=Cs2g28240.1 gene=Cs2g28240
Category:3
Score:5
Cleavage site:168

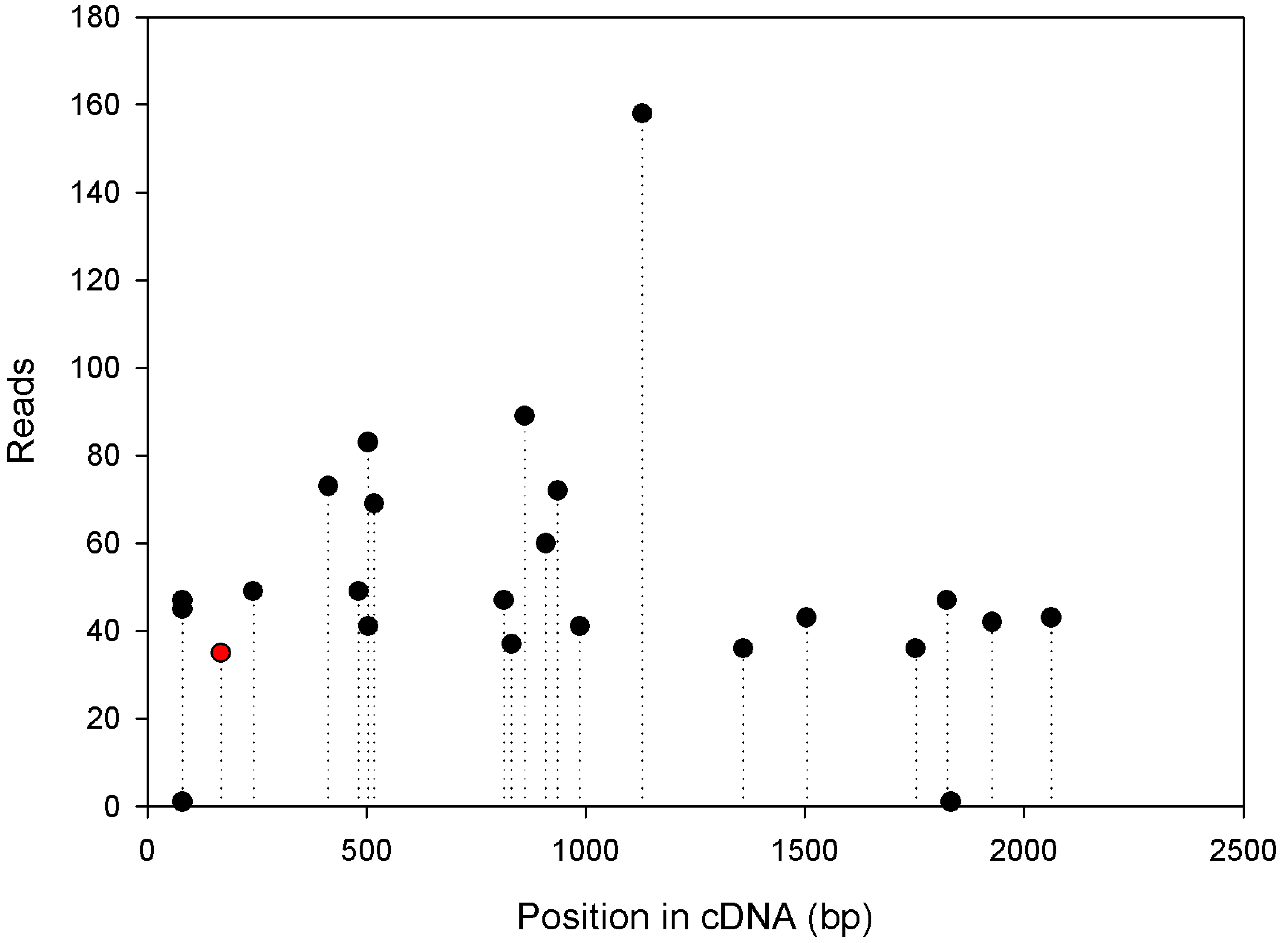


```
5' UUGGUCUGCUG-AAGCUGACGUUGGCG 3'       Cs2g28240.1
         :::::: ::::::: :::: :
3' -----GACGACUUUCGACUCCAACAG- 5'       Csi-miRN20-Cs3g05320.1-12
```

Csi-miRN20-Cs3g05320.1-23, target=Cs7g19680.1 gene=Cs7g19680
Category:3
Score:4.5
Cleavage site:3832

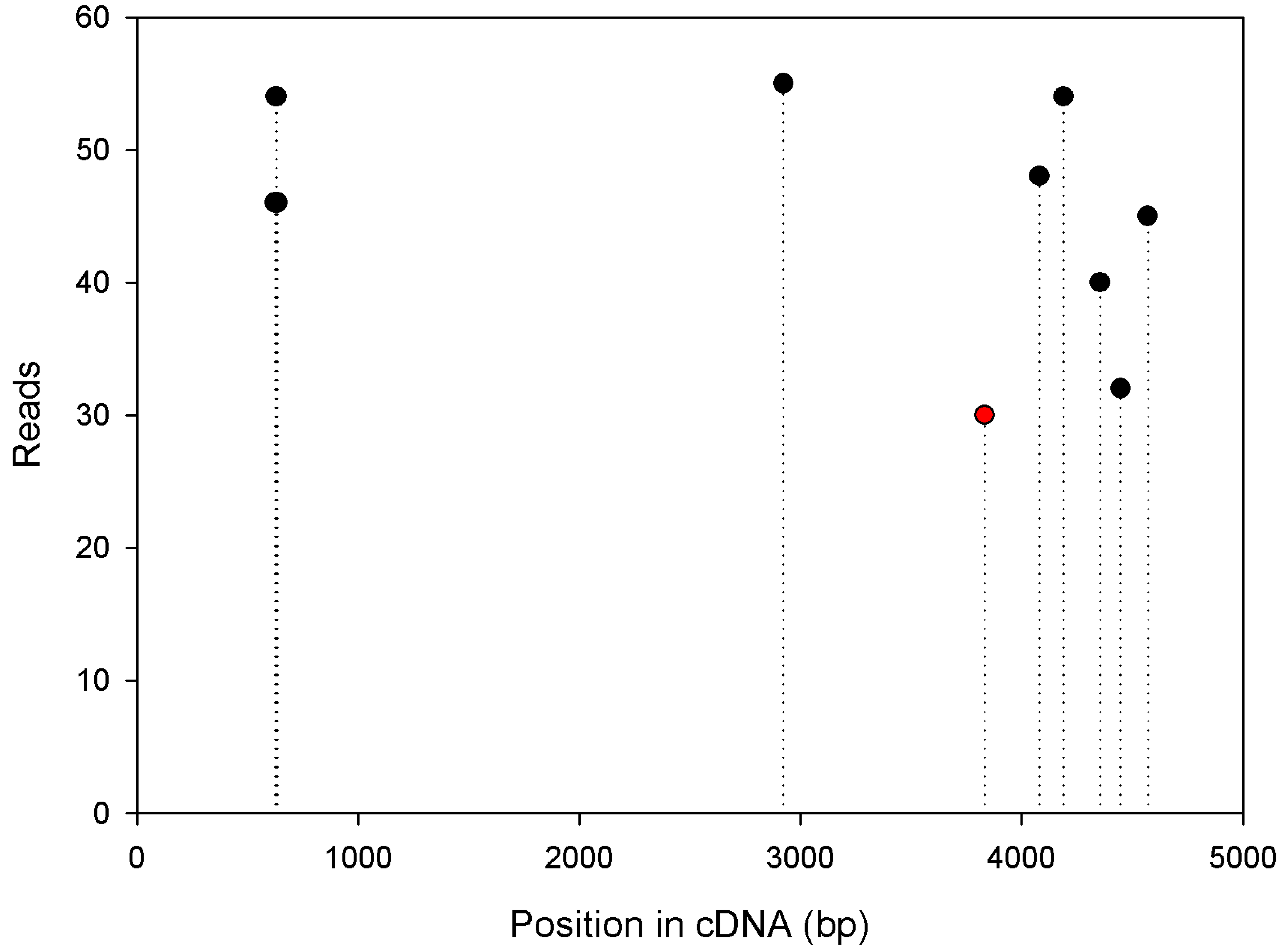


```
5' GCCGUCCUAUUCU-CUUCCAUACUGAU 3'       Cs7g19680.1
         ::.::: ::::::: :::::
3' ------UAUGAGAUGAAGGUAAGACUA 5'       Csi-miRN20-Cs3g05320.1-23
```

Csi-miRN20-Cs3g05320.1-26, target=Cs2g16940.1 gene=Cs2g16940
Category:3
Score:5
Cleavage site:517

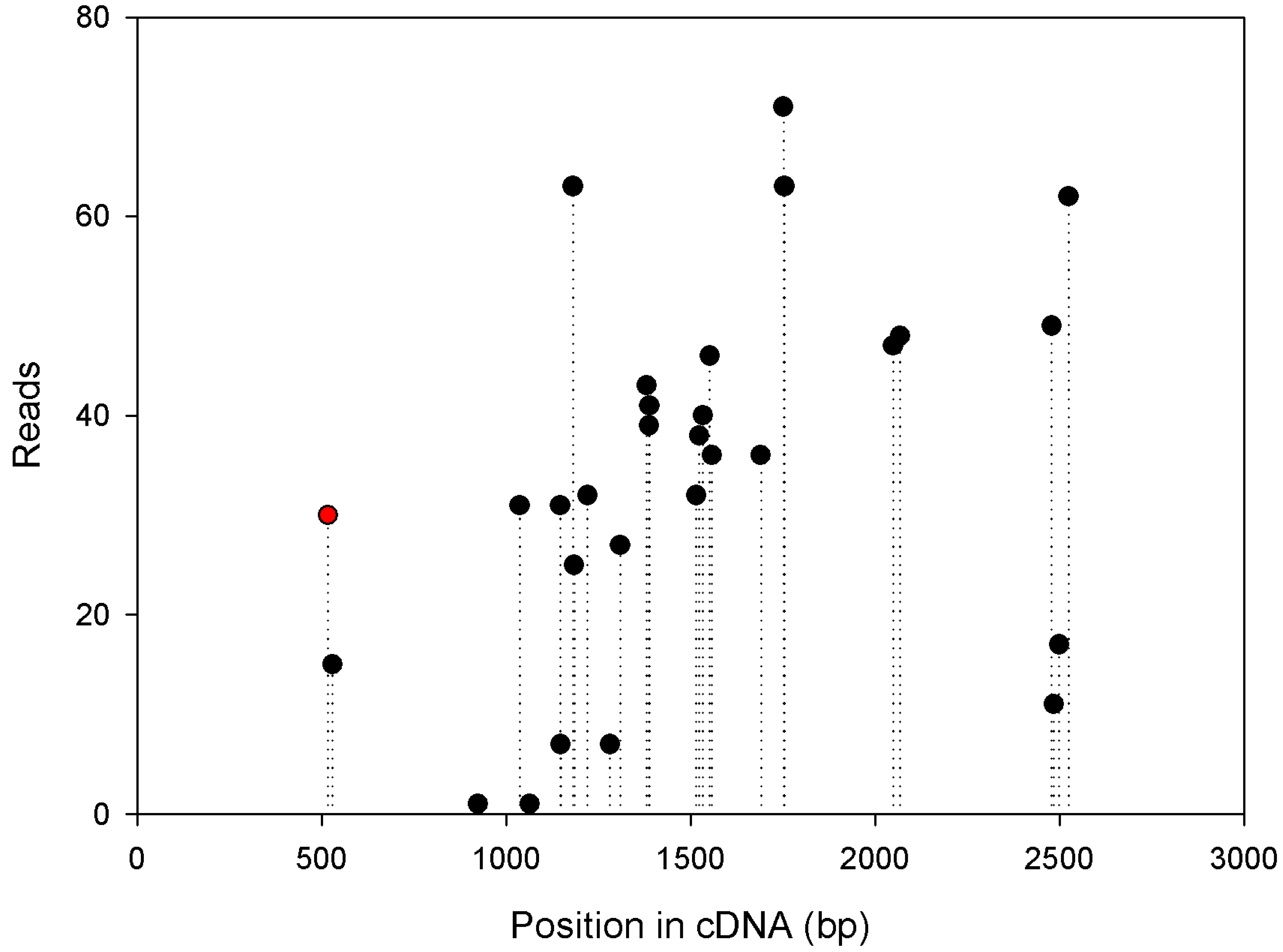


```
5' UAAAGCCUUCUCUUUGUUUUCUGAGC 3'        Cs2g16940.1
     :. ::::::::.::::::::..
3' --GUUAGAAGAGAGACAAAAGGU--- 5'        Csi-miRN20-Cs3g05320.1-26
```

Csi-miRN20-Cs3g05320.1-26, target=Cs6g18830.1 gene=Cs6g18830
Category:3
Score:5
Cleavage site:677

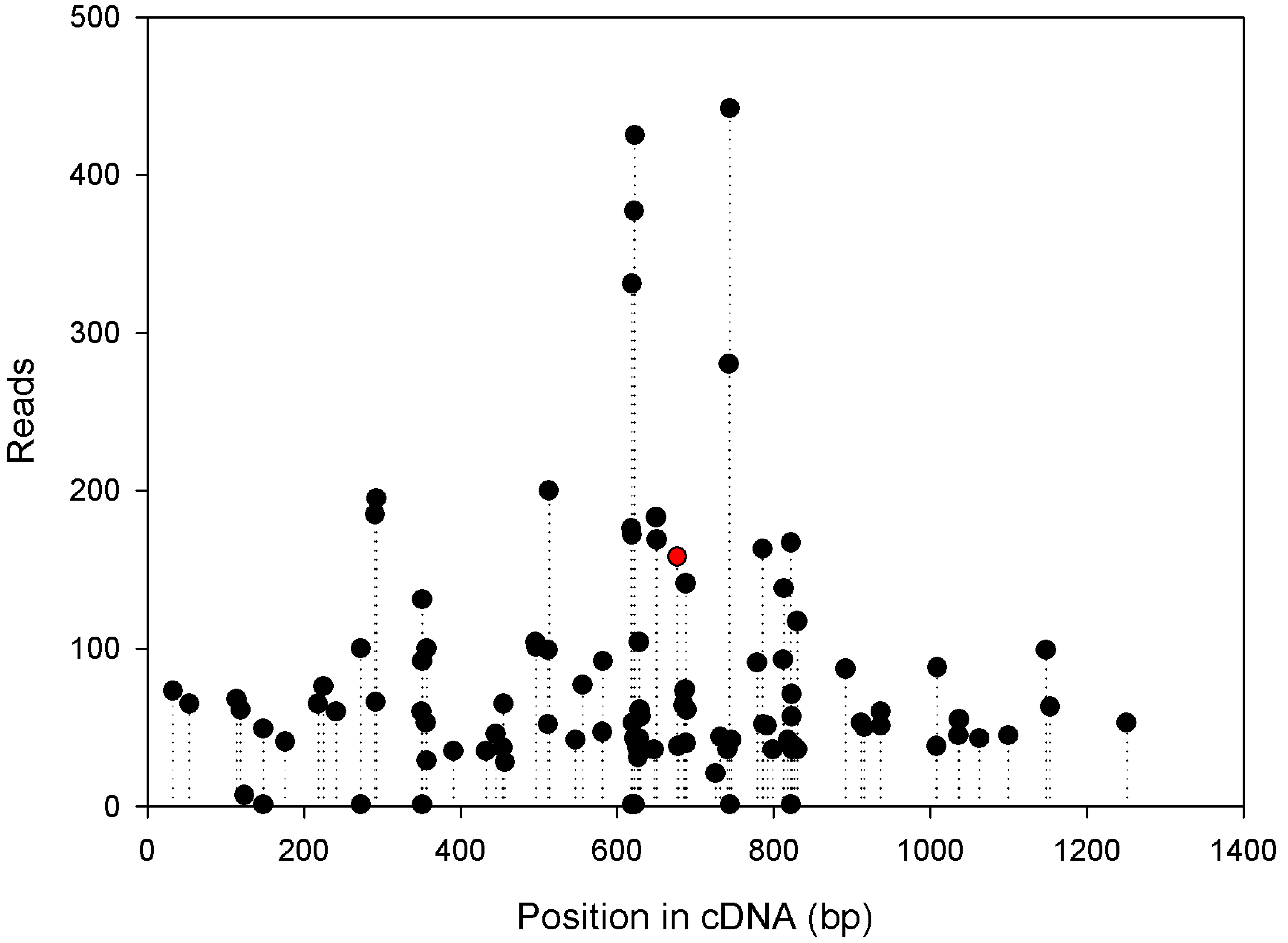


```
5' GCACUCAACUUCUCUGUUUUCUACUA 3'        Cs6g18830.1
    :: ::  : :::::::::::.:
3' -GUUAGAAG-AGAGACAAAAGGU--- 5'        Csi-miRN20-Cs3g05320.1-26
```
