# Supplementary material for: Genome-wide comparison of microRNAs and their targeted transcripts among leaf, flower and fruit of sweet orange
Source: BMC Genomics. 2014 Aug 20;15(1):695. doi: 10.1186/1471-2164-15-695 (PMC4158063; doi:10.1186/1471-2164-15-695)
Supplement: Supplementary file 9 — Additional file 9: Comparative analysis of the presence of SSRs in zma-miR396 and the resulting expression pattern among different tissues in maize. Sequences data were collected from miRBase, the expression data were come from the publication of Zhang et al. (PDF 488 KB) [file 12864_2014_6413_MOESM9_ESM.pdf]

Additional file 9: Comparative analysis of the presence of SSRs in zma-miR396 and the resulting expression pattern among different tissues in maize. Sequences data were collected from miRBase, the expression data were come from the publication of Zhang et al [1].

Sequences of pre-zma-miR396
>zma-MIR396a MI0001801
ACAUGGCCCUCCUUGCCGUCUUCCACAGCUUUCUUGAACUGCAUGCCGCCGGCUGGUGGAUGCUGC
GCGCUUGAAUUCCGGUCGAUCCCAAGAGGCGCAGUUCAAUAAAGCUGUGGGAAACUGCAGAGAGAG
GCCAC
>zma-MIR396b MI0001800
AGAUGGCCUUCUUUGUGAUCUUCCACAGCUUUCUUGAACUGCAUCUCUCAGAGGAGCGGCAGCUUC
AACUCCUCCACCCGCAUCAGCAGGUGCAUGCAGUUCAAUAAAGCUGUGGGAAACUGCAGAGAGAGGC
CAG
>zma-MIR396c MI0010569
CGGGCAUGCUUUCCACAGGCUUUCUUGAACUGUGAACUCGUGGGUGGUGGUGGUAUGUCGGAUGG
AAUGCUUGG
>zma-MIR396d MI0010570
CGGGCAUGCUUUCCACAGGCUUUCUUGAACUGUGAACUCGUGGGCGUGGUGGUGGUGGUGCUGGU
GCUGGGUGU
>zma-MIR396e MI0013225
UUUCCACAGCUUUCUUGAACUUCUUCUUCUUCUCUCUCUUAGAAGGGCCGUAUAGCUUCUUUGAAC
CUCUCUCUCUCUCUCUCUCAAAAGUGUACACGAACACUUUCUUCAUUCUCUGCCUCUCGCUCUCUGA
UCUCCGAAGAAGGUCAAGAAAGCCGUGGGAAGA
>zma-MIR396f MI0013226
UUUCCACAGCUUUCUUGAACUUCUUCUUCUUCUUCUCUCUUGGAACGGCAGCUUUCUUCGAAUCU
GUCUCUCUCUCUCUCAUCUCCGAAGACGAAGGUCAAGAAAGCUGUGGGAAGA
>zma-MIR396g MI0013227
UUCCCACAGCUUUAUUGAACUGCCCUCUUGCUUGCUUGGAUCAAUUGAAACUCGAUCAGGUCCACA
GCACAGCCUGCUGCAUGCAUGAUGCAGUUCAAGAAAGCUGUGGAAGAU

1. Zhang L, Chia J-M, Kumari S, Stein JC, Liu Z, Narechania A, Maher CA, Guill K, McMullen MD, Ware D: **A genome-wide characterization of microRNA genes in maize.** *PLoS Genet* 2009, **5:**e1000716.

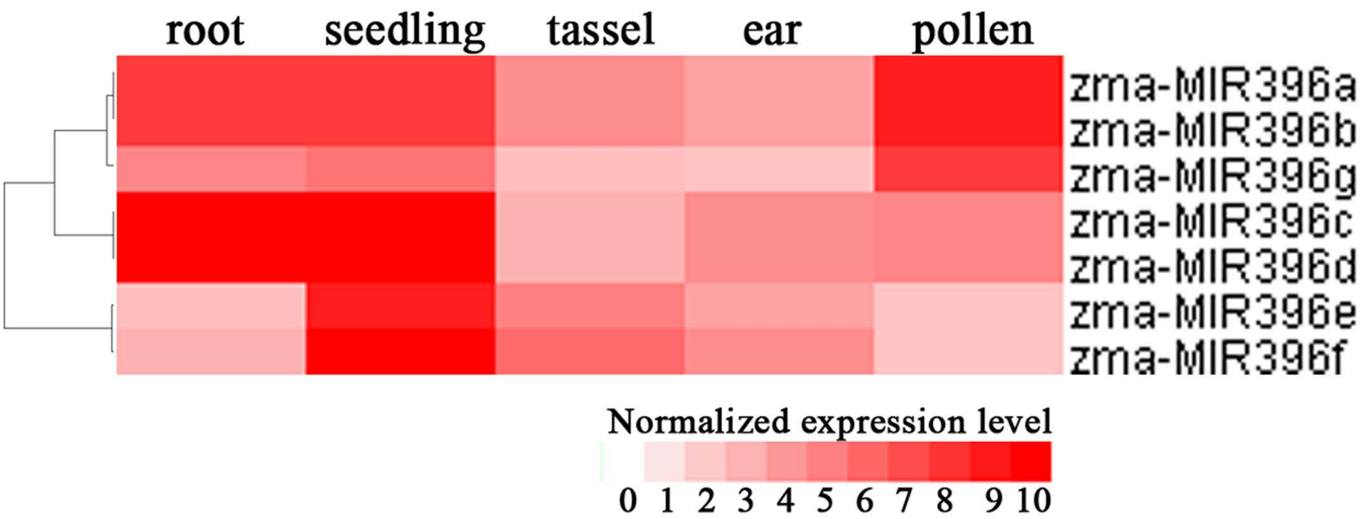
root
seedling
tassel
ear
pollen
zma-MIR396a
zma-MIR396b
zma-MIR396g
zma-MIR396c
zma-MIR396d
zma-MIR396e
zma-MIR396f
Normalized expression level
0 1 2 3 4 5 6 7 8 9 10
