# Supplementary material for: Genome-wide comparison of microRNAs and their targeted transcripts among leaf, flower and fruit of sweet orange
Source: BMC Genomics. 2014 Aug 20;15(1):695. doi: 10.1186/1471-2164-15-695 (PMC4158063; doi:10.1186/1471-2164-15-695)
Supplement: Supplementary file 10 — Additional file 10: Clusters of miRNAs. Two miRNAs located within a single precursor are shown. The mature miRNA sequences are highlighted in yellow and the miRNA* sequences are highlighted in gray. The mismatched nucleotide is highlighted in red. Data in the tables show the digital expression levels (TPM) of miRNAs in different tissues. (a) Csi-miR166j.1 and Csi-miR166j.3. (b) Csi-miR477d.1-3p and Csi-miR477d.2-5p. (PDF 615 KB) [file 12864_2014_6413_MOESM10_ESM.pdf]

Additional file 10. Clusters of miRNAs. Two miRNAs located within a single precursor are shown. The mature miRNA sequences are highlighted in yellow and the miRNA* sequences are highlighted in gray. The mismatched nucleotide is highlighted in red. Data in the tables show the digital expression levels (TPM) of miRNAs in different tissues. (a) Csi-miR166j.1 and Csi-miR166j.3. (b) Csi-miR477d.1-3p and Csi-miR477d.2-5p.

(a)

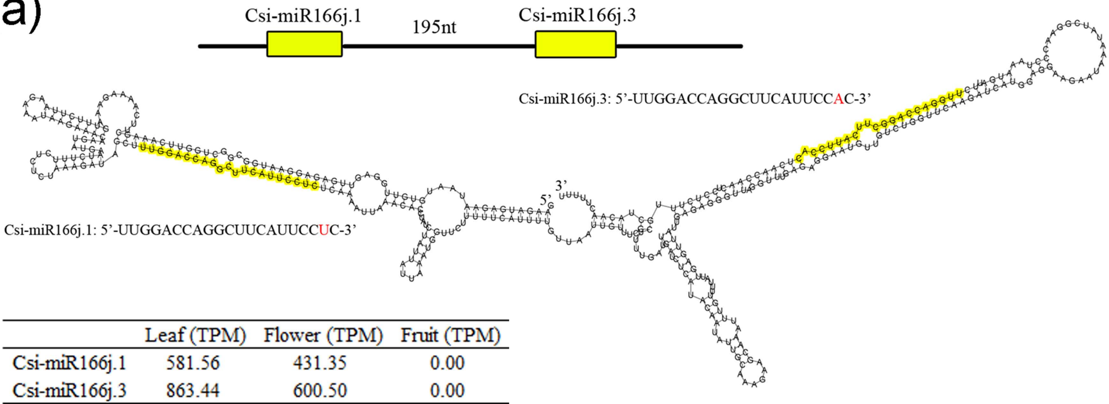

	Leaf (TPM)	Flower (TPM)	Fruit (TPM)
Csi-miR166j.1	581.56	431.35	0.00
Csi-miR166j.3	863.44	600.50	0.00

(b)

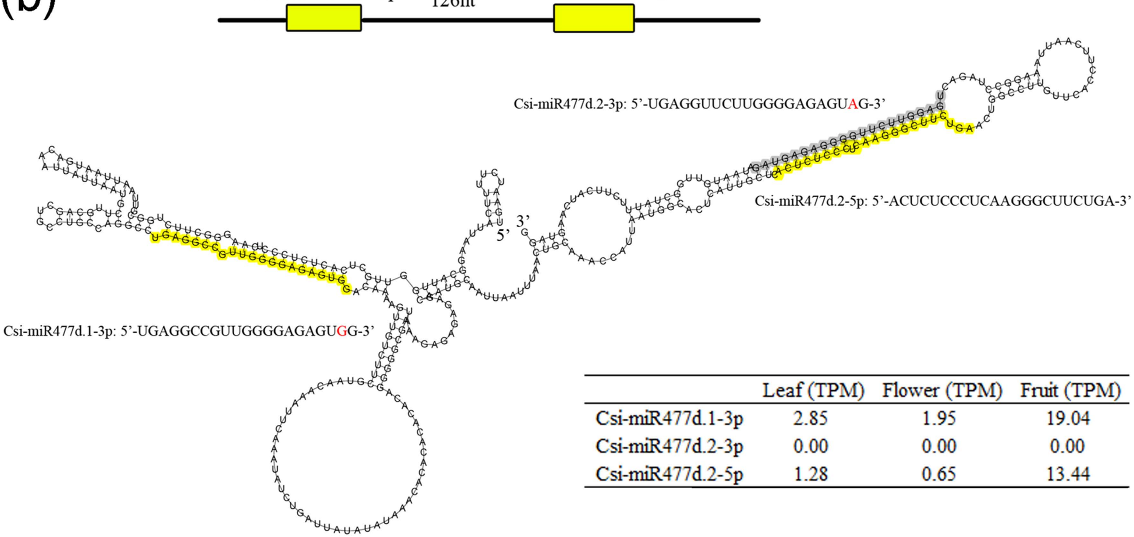

	Leaf (TPM)	Flower (TPM)	Fruit (TPM)
Csi-miR477d.1-3p	2.85	1.95	19.04
Csi-miR477d.2-3p	0.00	0.00	0.00
Csi-miR477d.2-5p	1.28	0.65	13.44
