# Supplementary material for: Genome-wide comparison of microRNAs and their targeted transcripts among leaf, flower and fruit of sweet orange
Source: BMC Genomics. 2014 Aug 20;15(1):695. doi: 10.1186/1471-2164-15-695 (PMC4158063; doi:10.1186/1471-2164-15-695)
Supplement: Supplementary file 11 — Additional file 11: Confirmation of the expression levels of 80 selected miRNAs in different tissues by qRT-PCR. A total of 65 known miRNAs and 15 novel miRNAs were selected according to their differential expression in leaf (L), flower (F) and fruit (Fr), which was derived from high-throughput sequencing. The expression levels of these miRNAs were confirmed using stem-loop qRT-PCR. U4 was used as a loading control gene in qRT-PCR. The data are represented as the mean plus SD of n = 3 biological replicates. (PDF 2 MB) [file 12864_2014_6413_MOESM11_ESM.pdf]

Additional file 11: Confirmation of the expression levels of 80 selected miRNAs in different tissues by qRT-PCR. A total of 65 known miRNAs and 15 novel miRNAs were selected according to their differential expression in leaf (L), flower (F) and fruit (Fr), which was derived from high-throughput sequencing. The expression levels of these miRNAs were confirmed using stem-loop qRT-PCR. U4 was used as a loading control gene in qRT-PCR. The data are represented as the mean plus SD of n = 3 biological replicates.

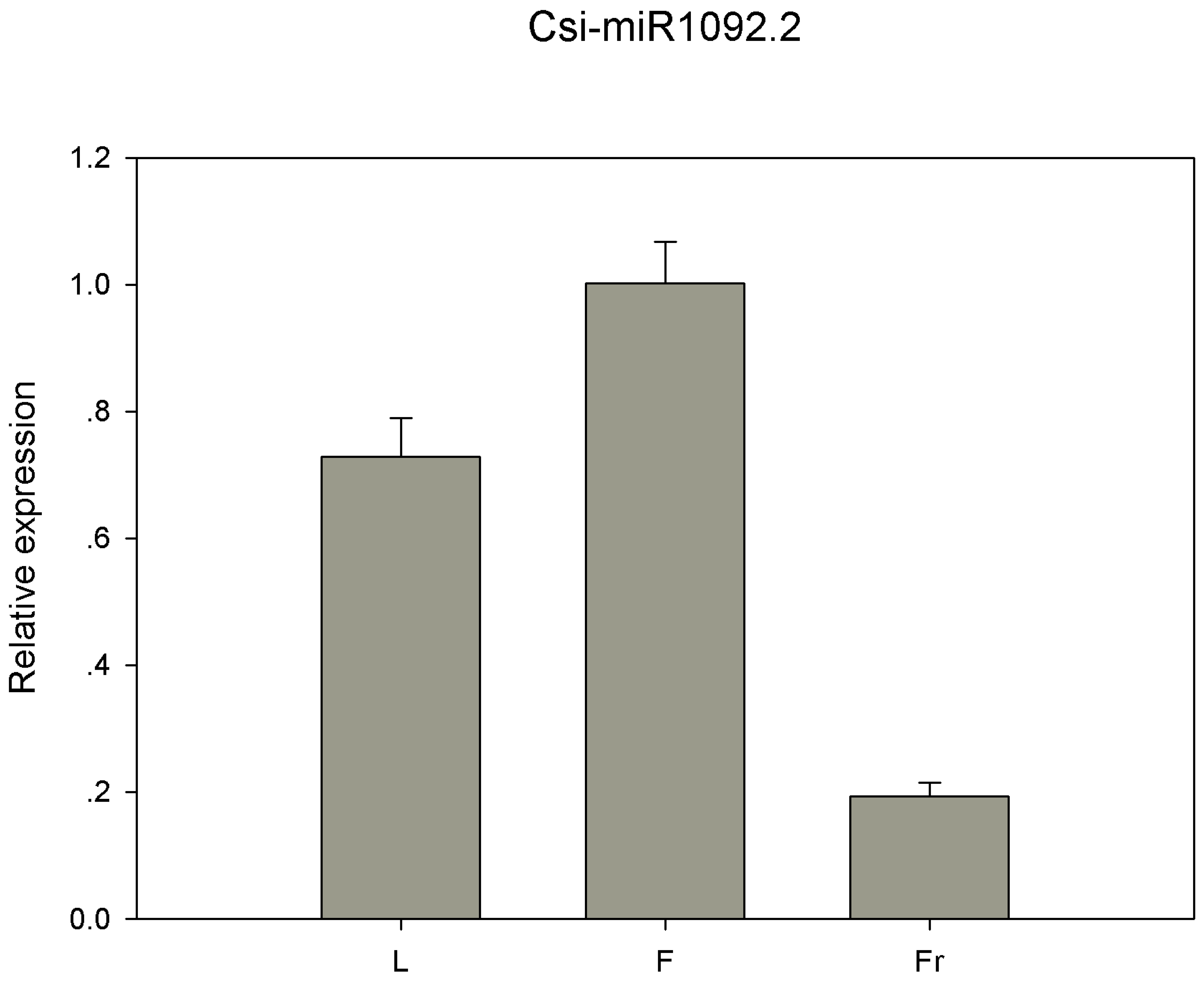
Csi-miR1092.2
Relative expression
1.2
1.0
.8
.6
.4
.2
0.0
L
F
Fr

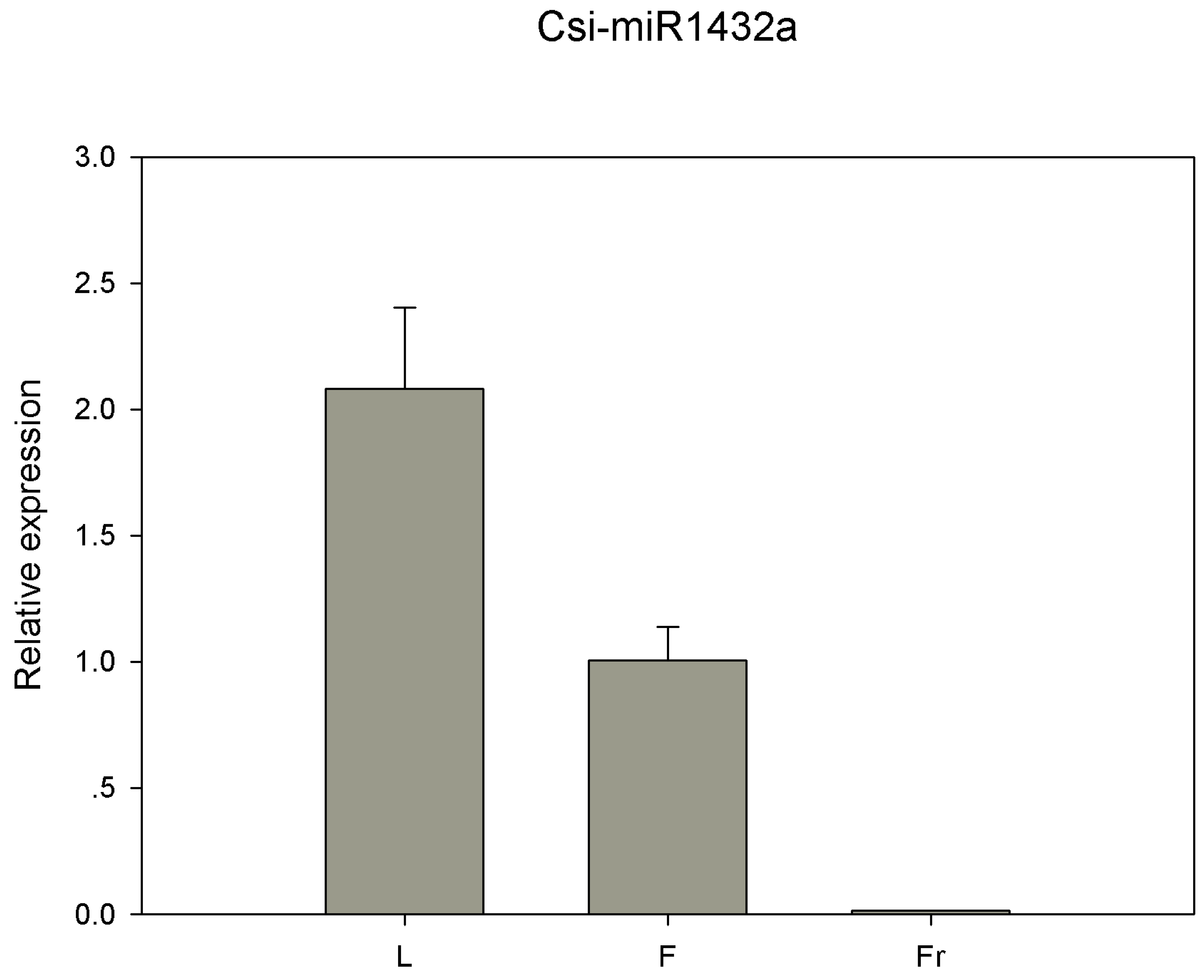

Csi-miR1432a
Relative expression
3.0
2.5
2.0
1.5
1.0
.5
0.0
L
F
Fr

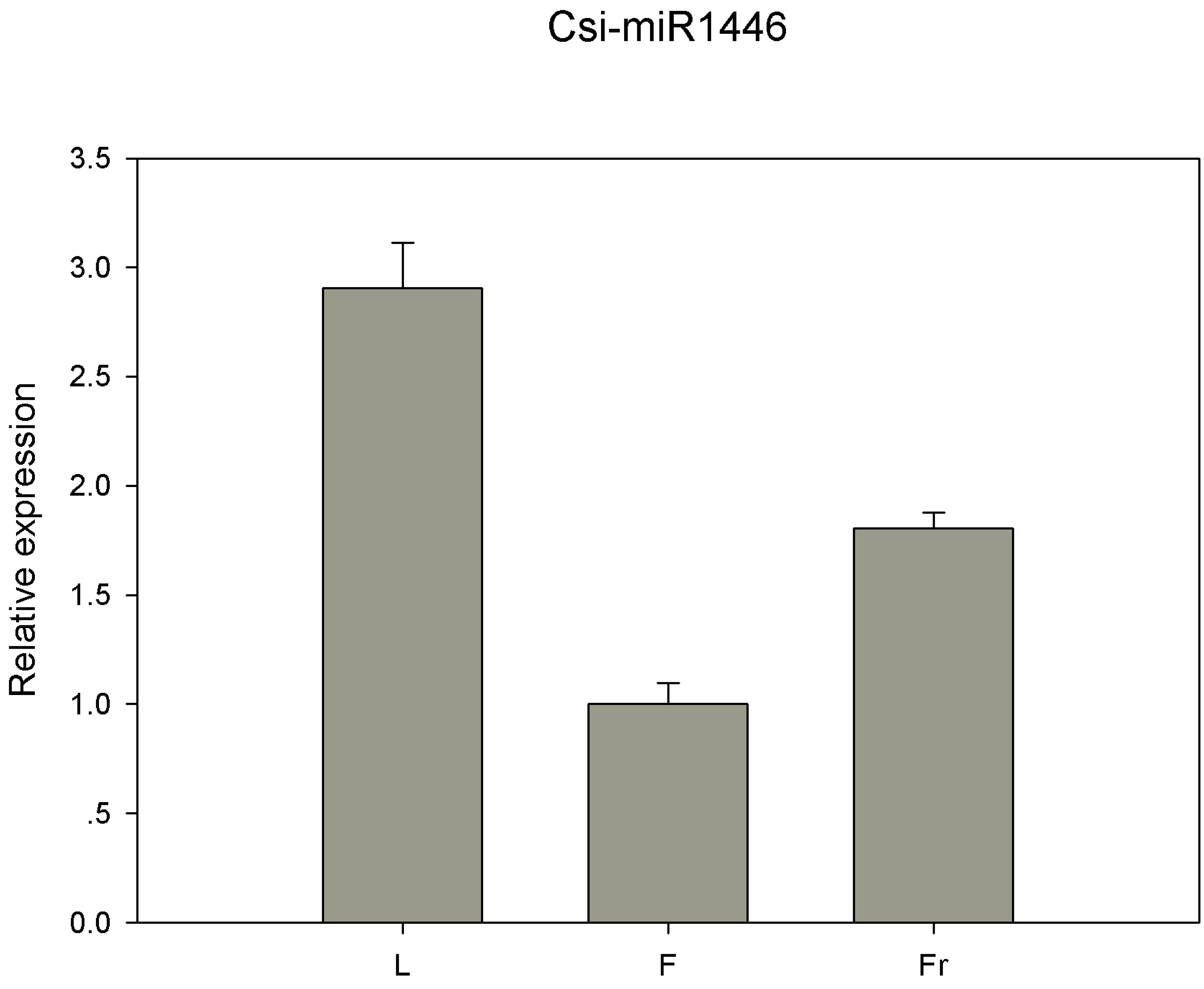

Csi-miR1446
Relative expression
3.5
3.0
2.5
2.0
1.5
1.0
.5
0.0
L
F
Fr

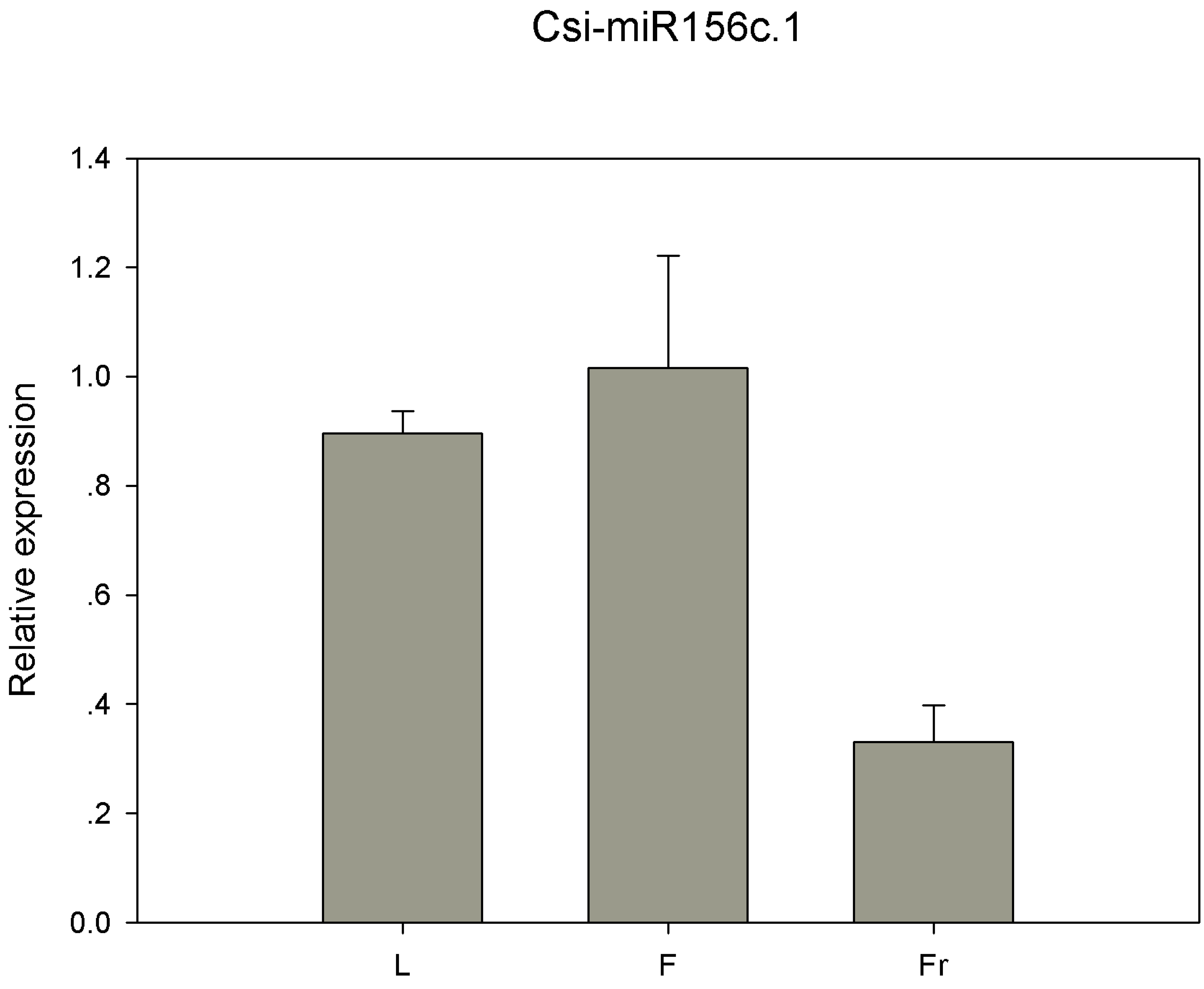
Csi-miR156c.1
Relative expression
1.4
1.2
1.0
.8
.6
.4
.2
0.0
L
F
Fr

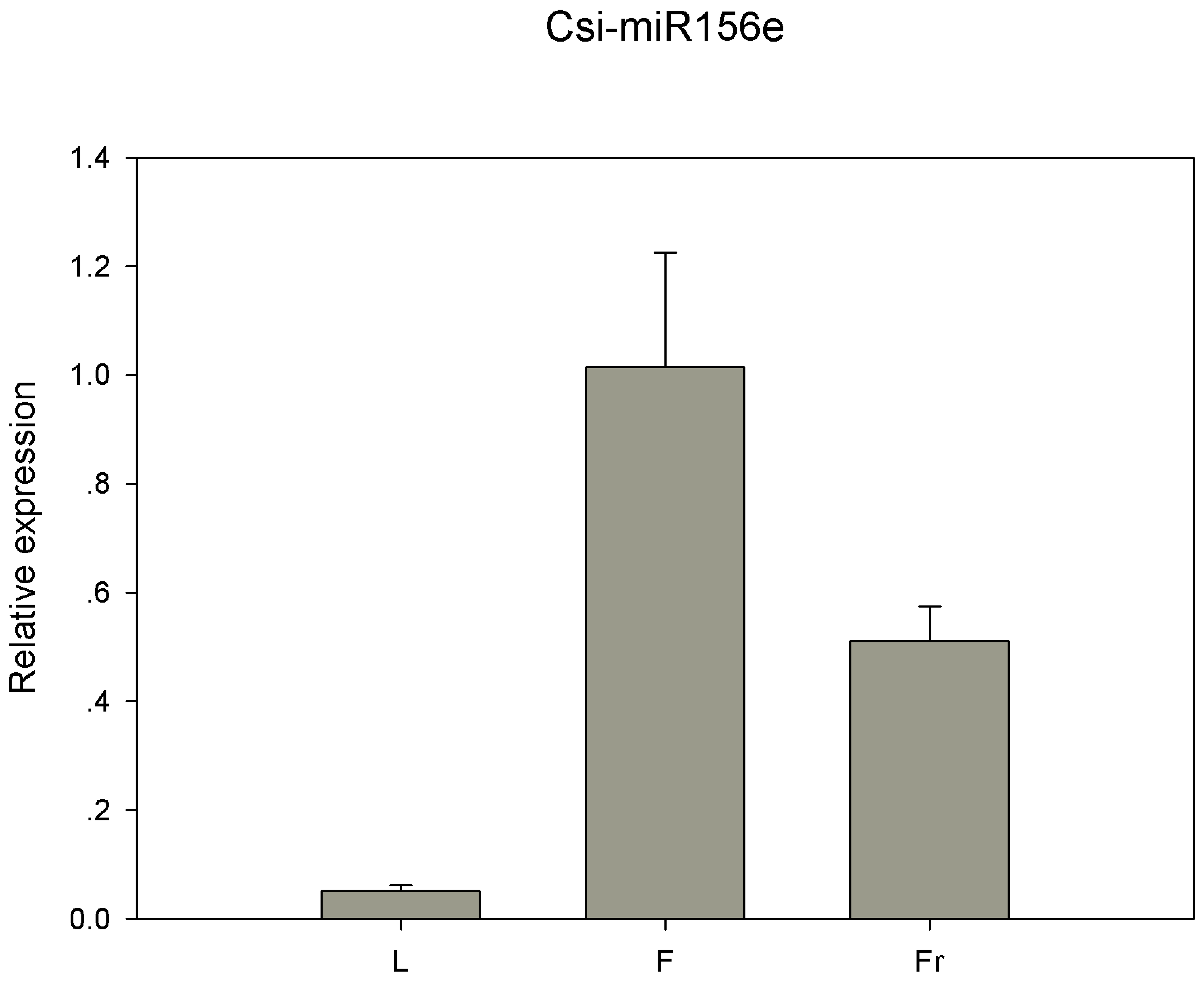
Csi-miR156e
Relative expression
1.4
1.2
1.0
.8
.6
.4
.2
0.0
L
F
Fr

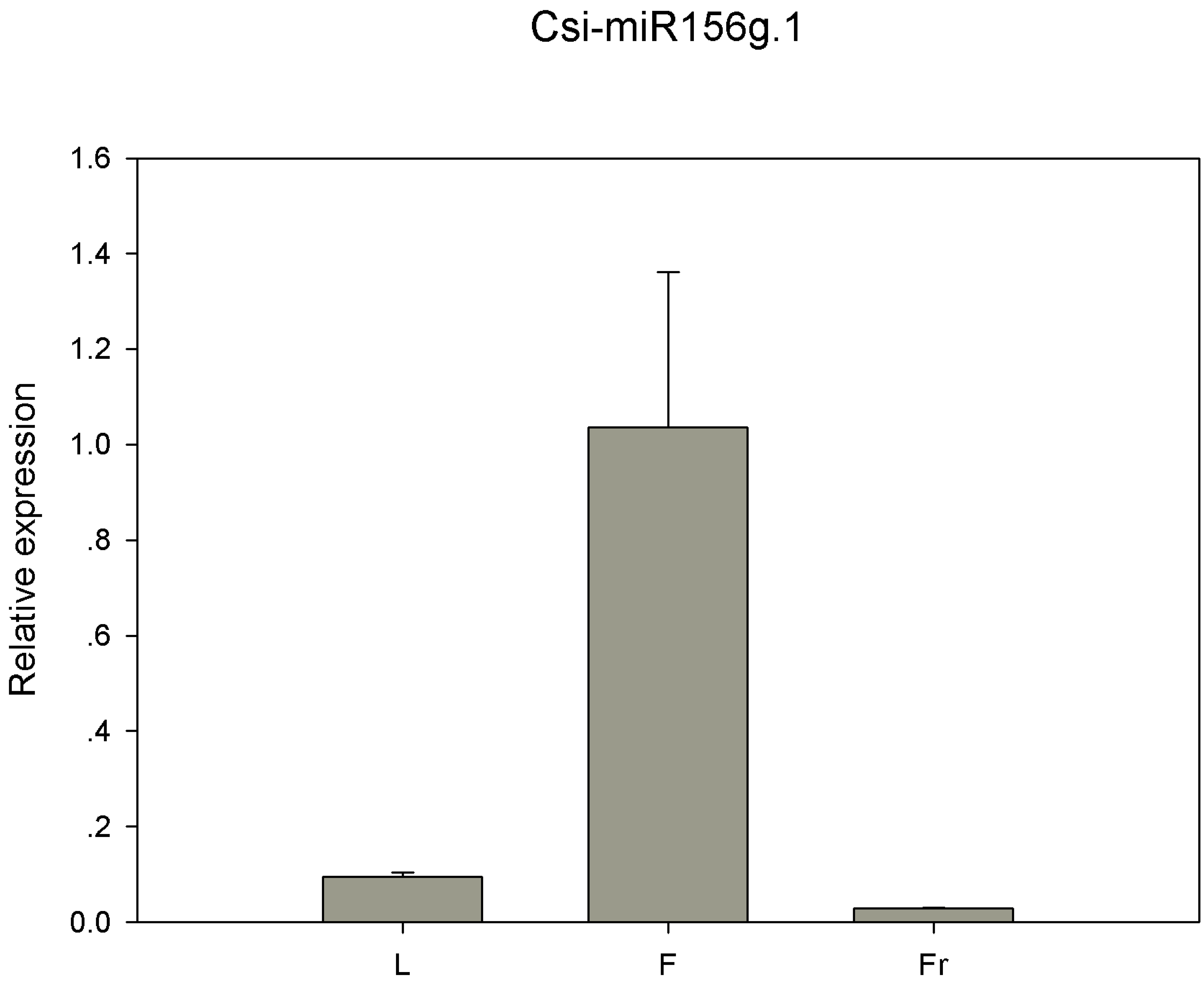

Csi-miR156g.1
Relative expression
1.6
1.4
1.2
1.0
.8
.6
.4
.2
0.0
L
F
Fr

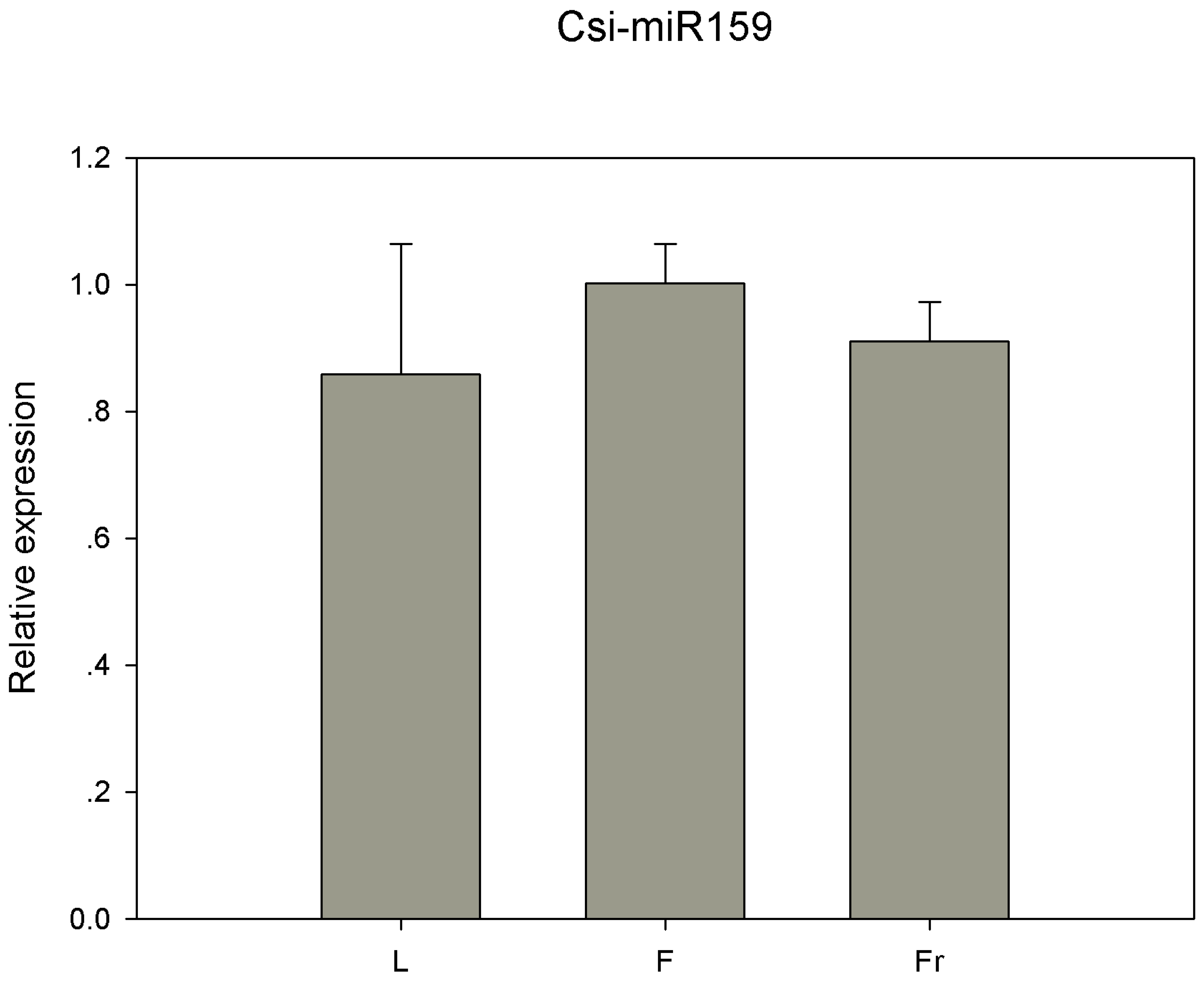

Csi-miR159
Relative expression
1.2
1.0
.8
.6
.4
.2
0.0
L
F
Fr

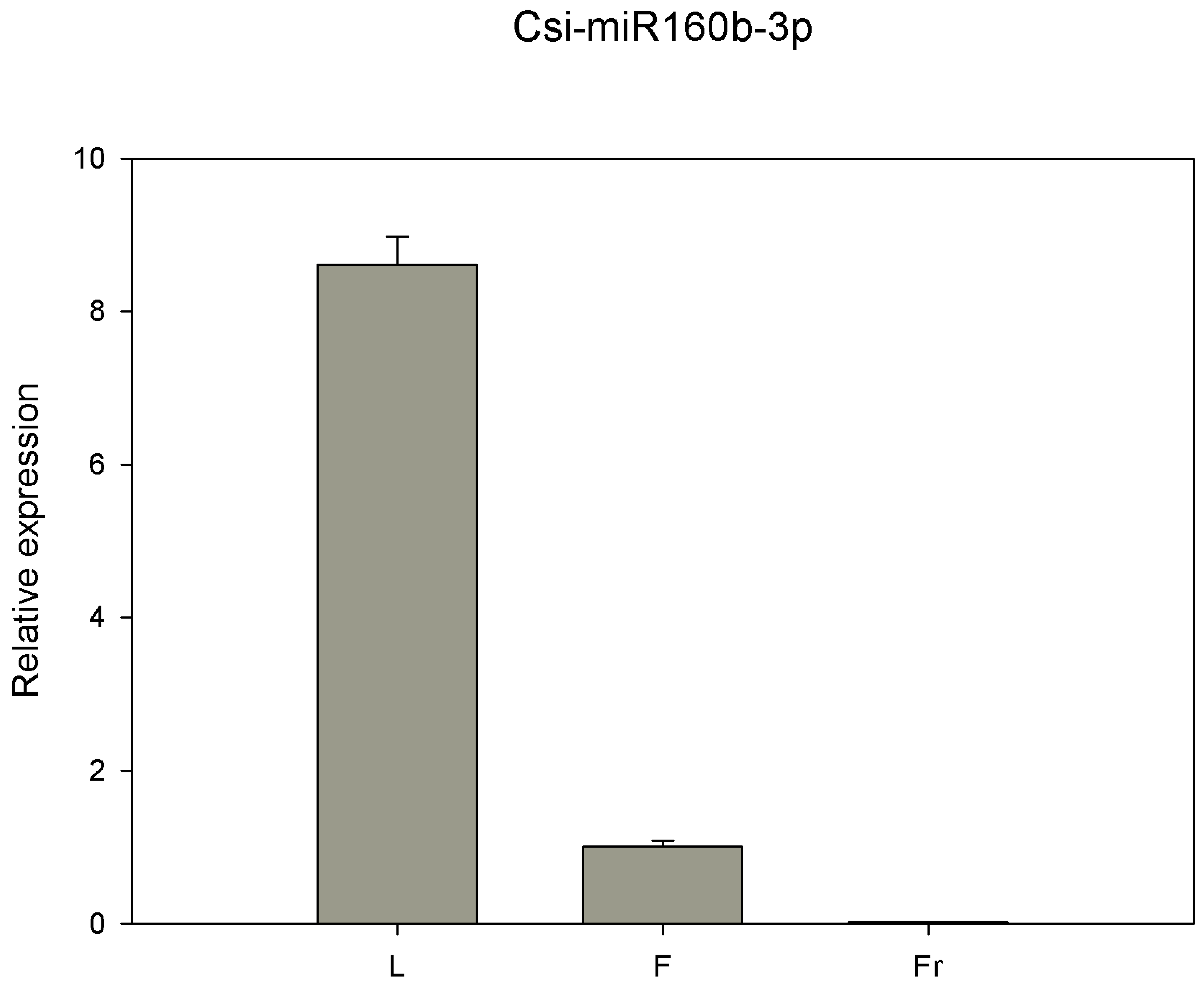
Csi-miR160b-3p
Relative expression
10
8
6
4
2
0
L
F
Fr

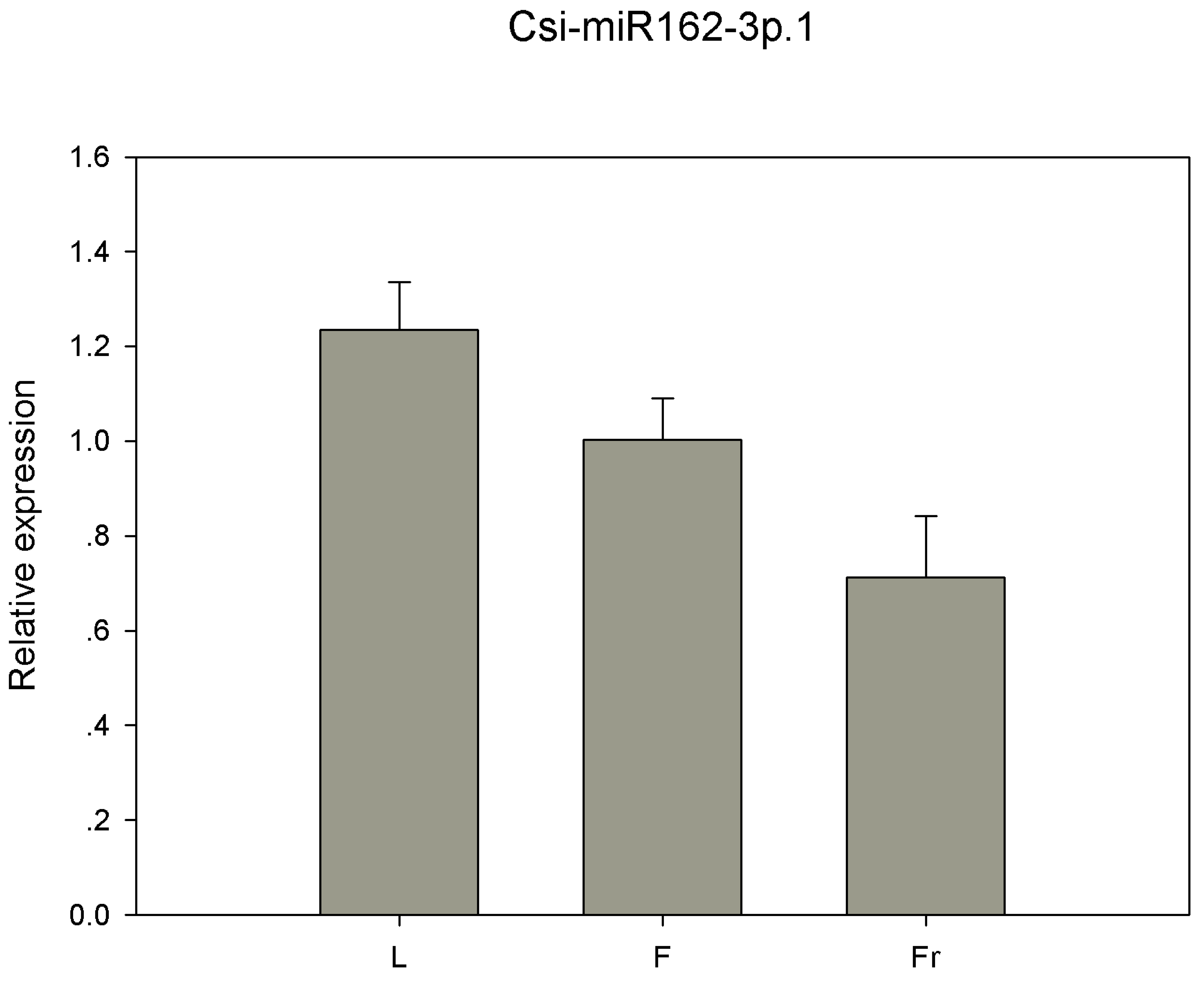
Csi-miR162-3p.1
Relative expression
1.6
1.4
1.2
1.0
.8
.6
.4
.2
0.0
L
F
Fr

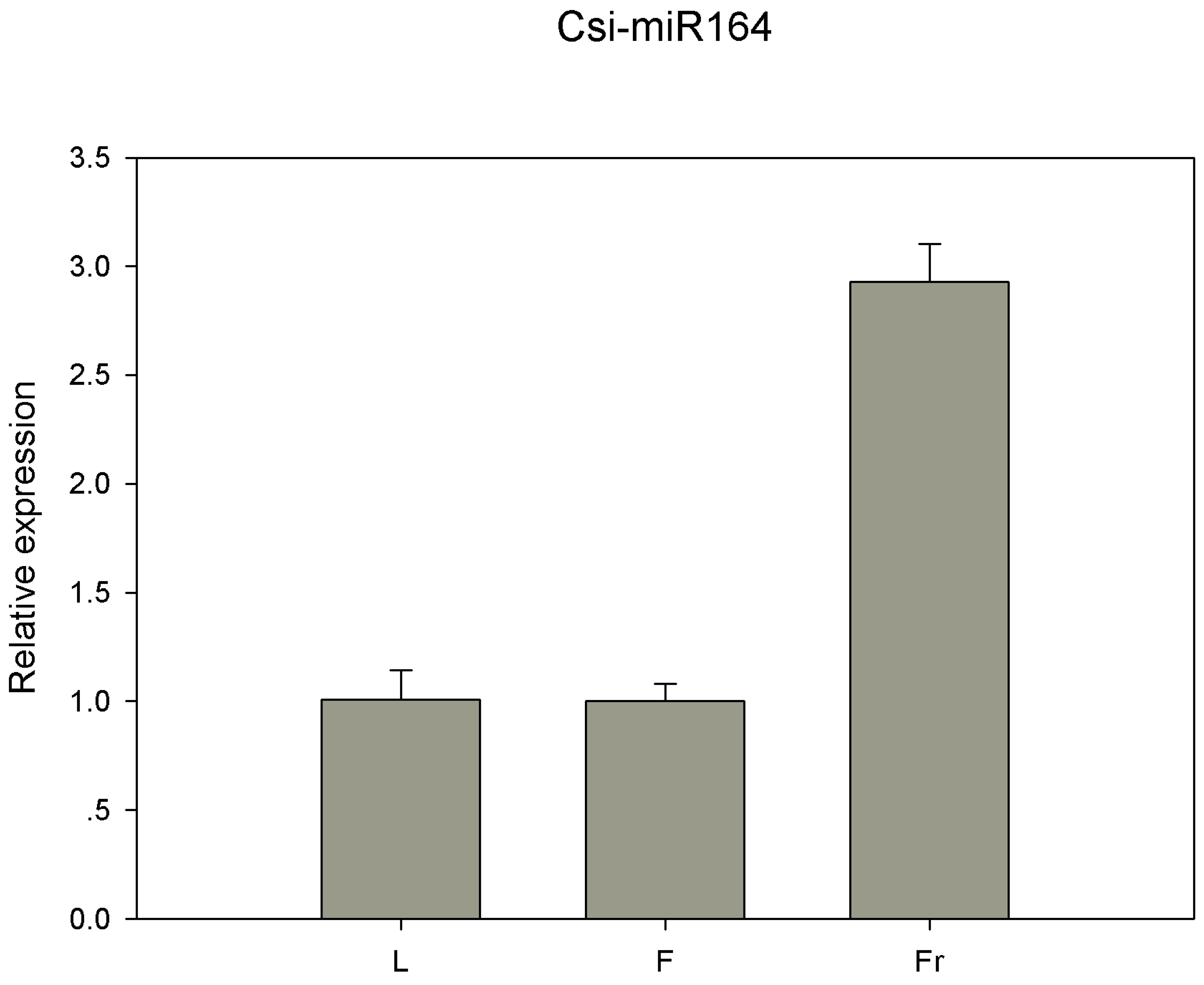

Csi-miR164
Relative expression
3.5
3.0
2.5
2.0
1.5
1.0
.5
0.0
L
F
Fr

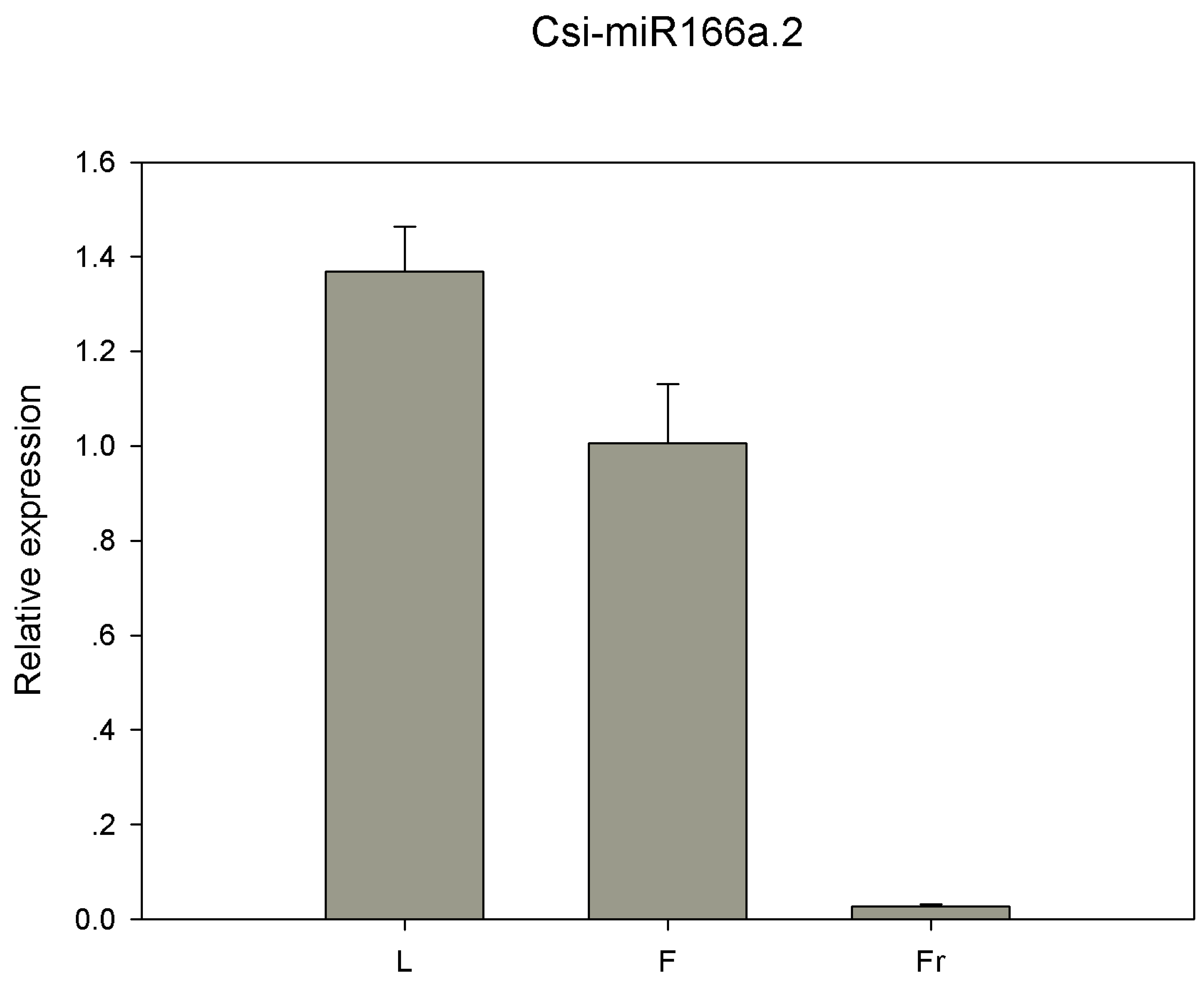

Csi-miR166a.2
Relative expression
0.0
.2
.4
.6
.8
1.0
1.2
1.4
1.6
L
F
Fr

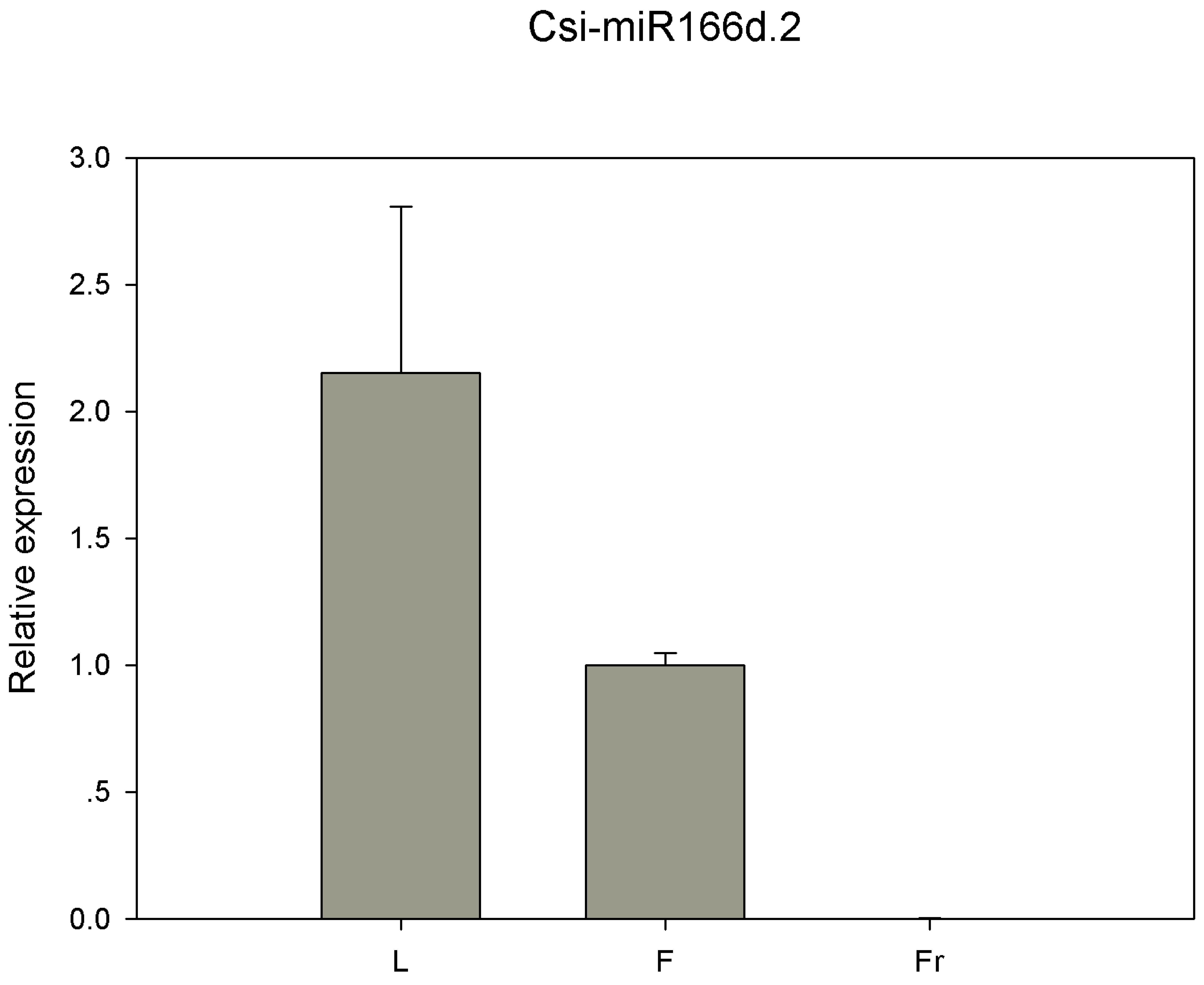

Csi-miR166d.2
Relative expression
3.0
2.5
2.0
1.5
1.0
.5
0.0
L
F
Fr

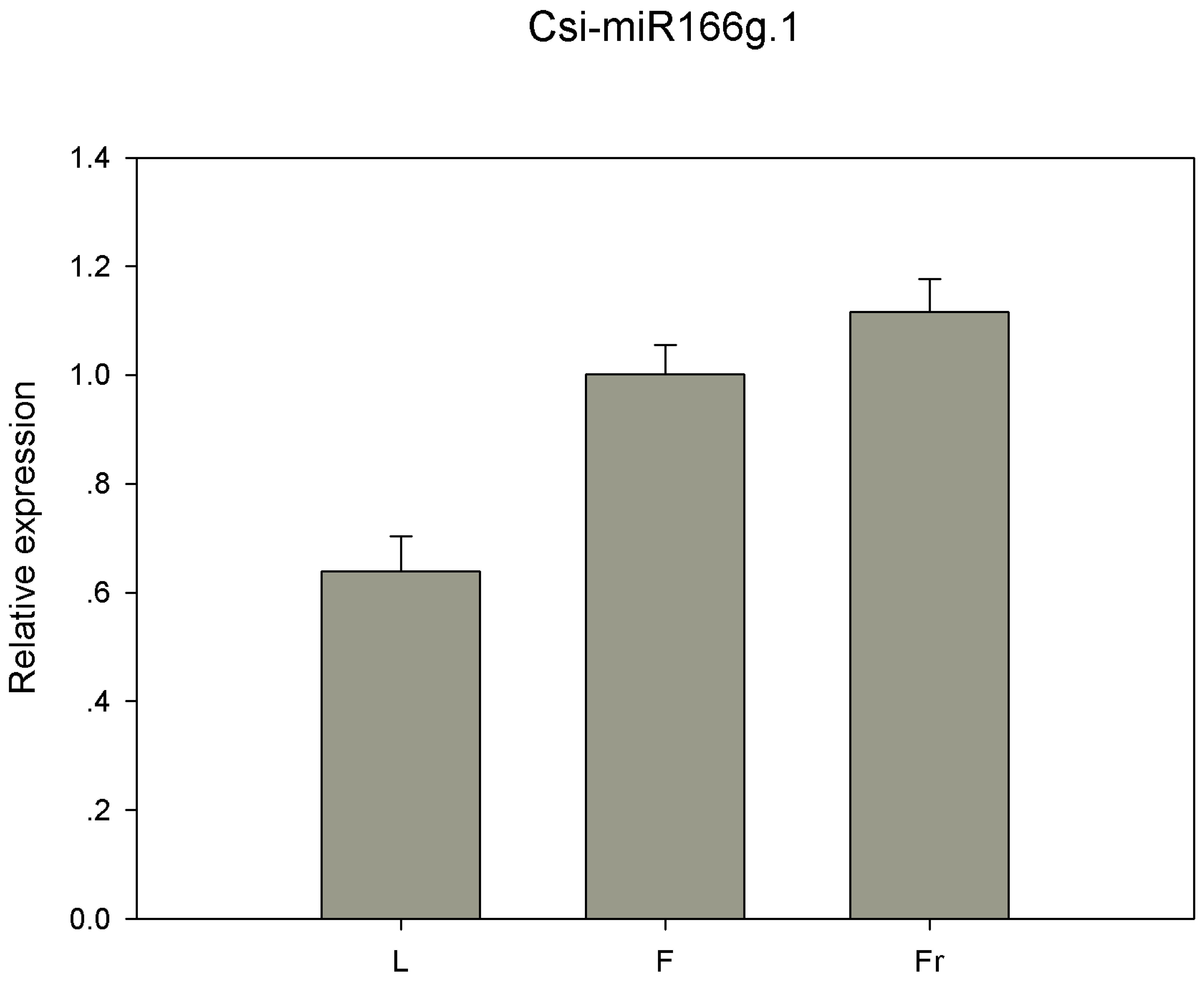

Csi-miR166g.1
Relative expression
0.0
.2
.4
.6
.8
1.0
1.2
1.4
L
F
Fr

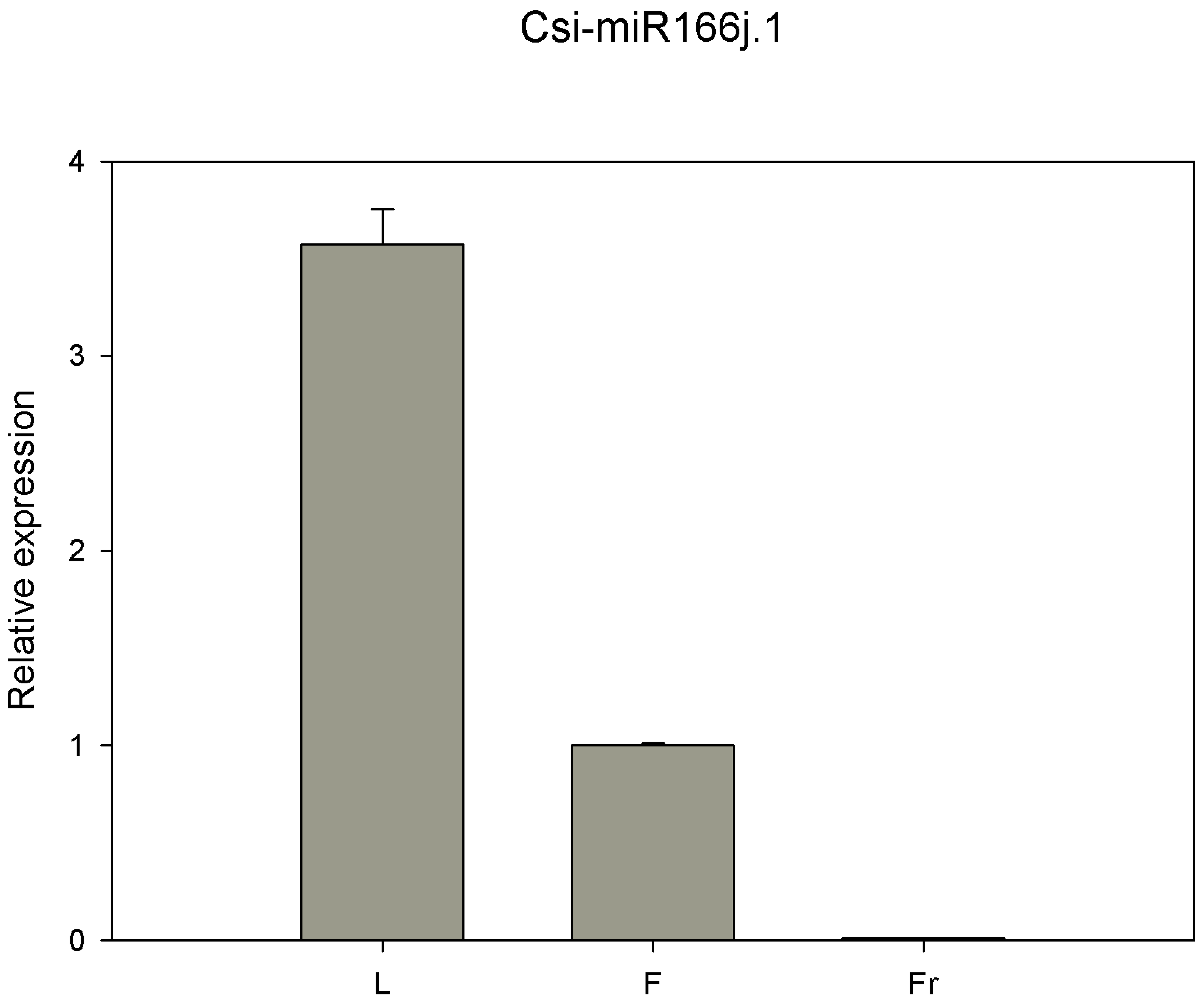

Csi-miR166j.1
Relative expression
0
1
2
3
4
L
F
Fr

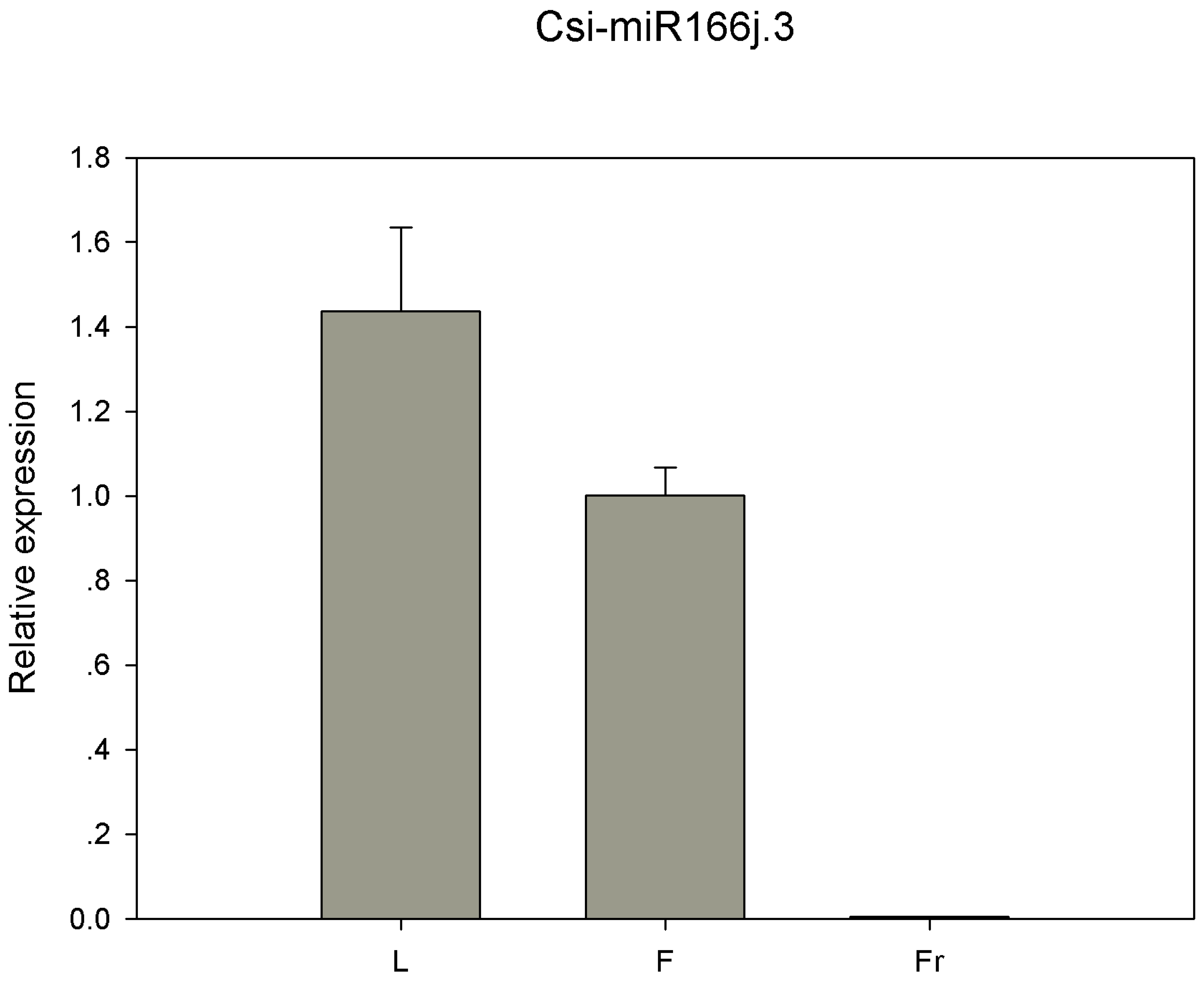
Csi-miR166j.3
Relative expression
0.0
.2
.4
.6
.8
1.0
1.2
1.4
1.6
1.8
L
F
Fr

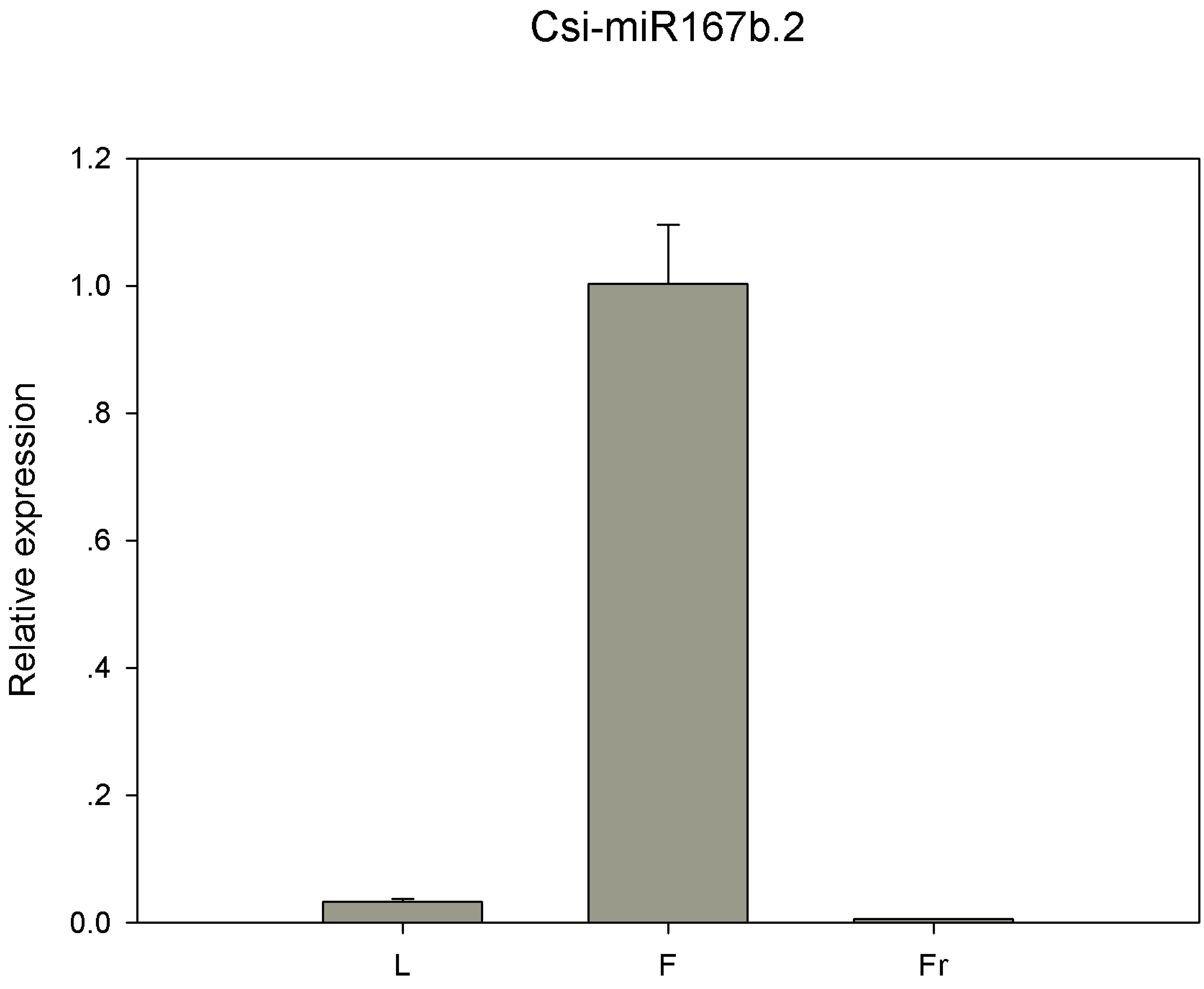

Csi-miR167b.2
Relative expression
1.2
1.0
.8
.6
.4
.2
0.0
L
F
Fr

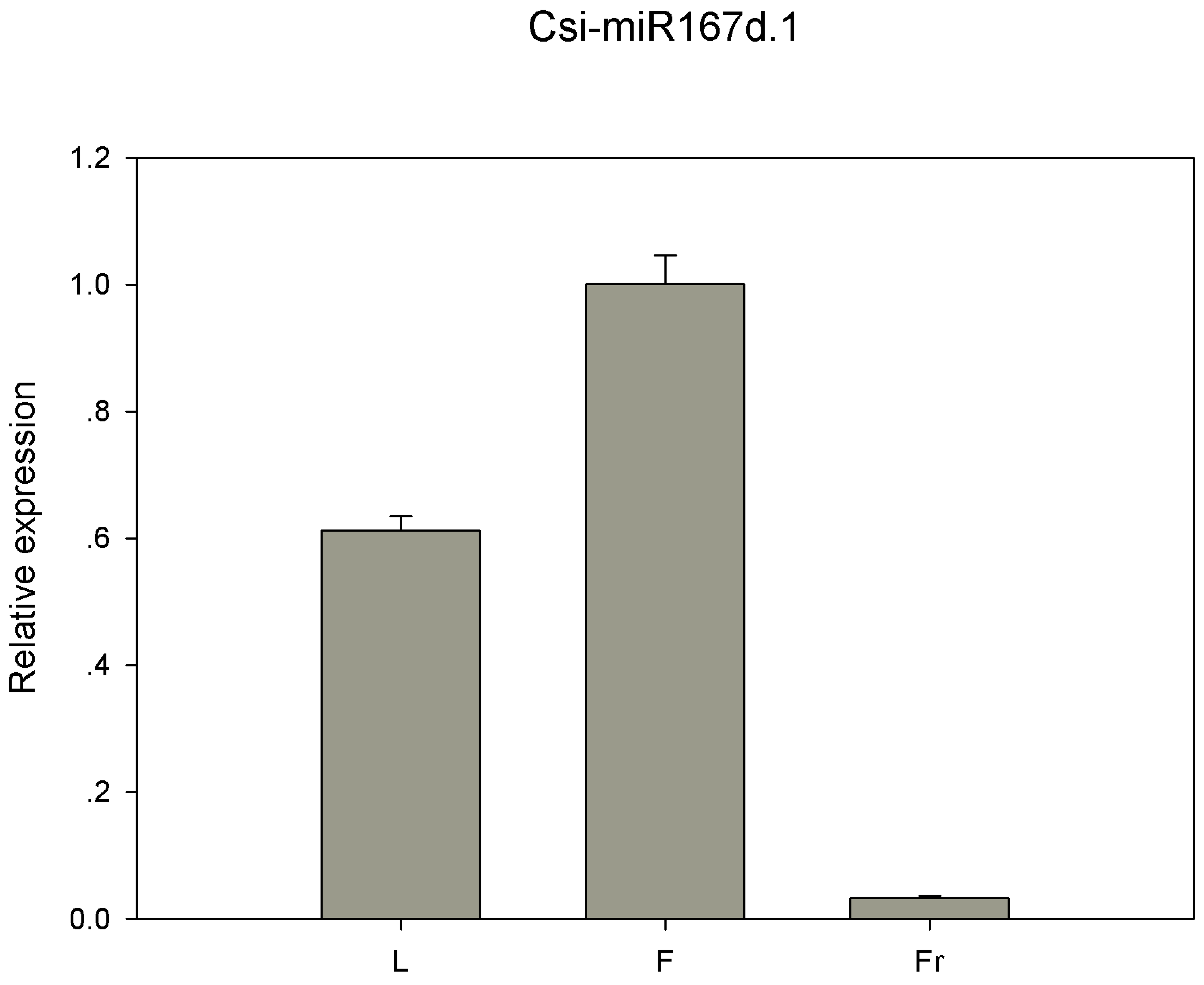

Csi-miR167d.1
Relative expression
1.2
1.0
.8
.6
.4
.2
0.0
L
F
Fr

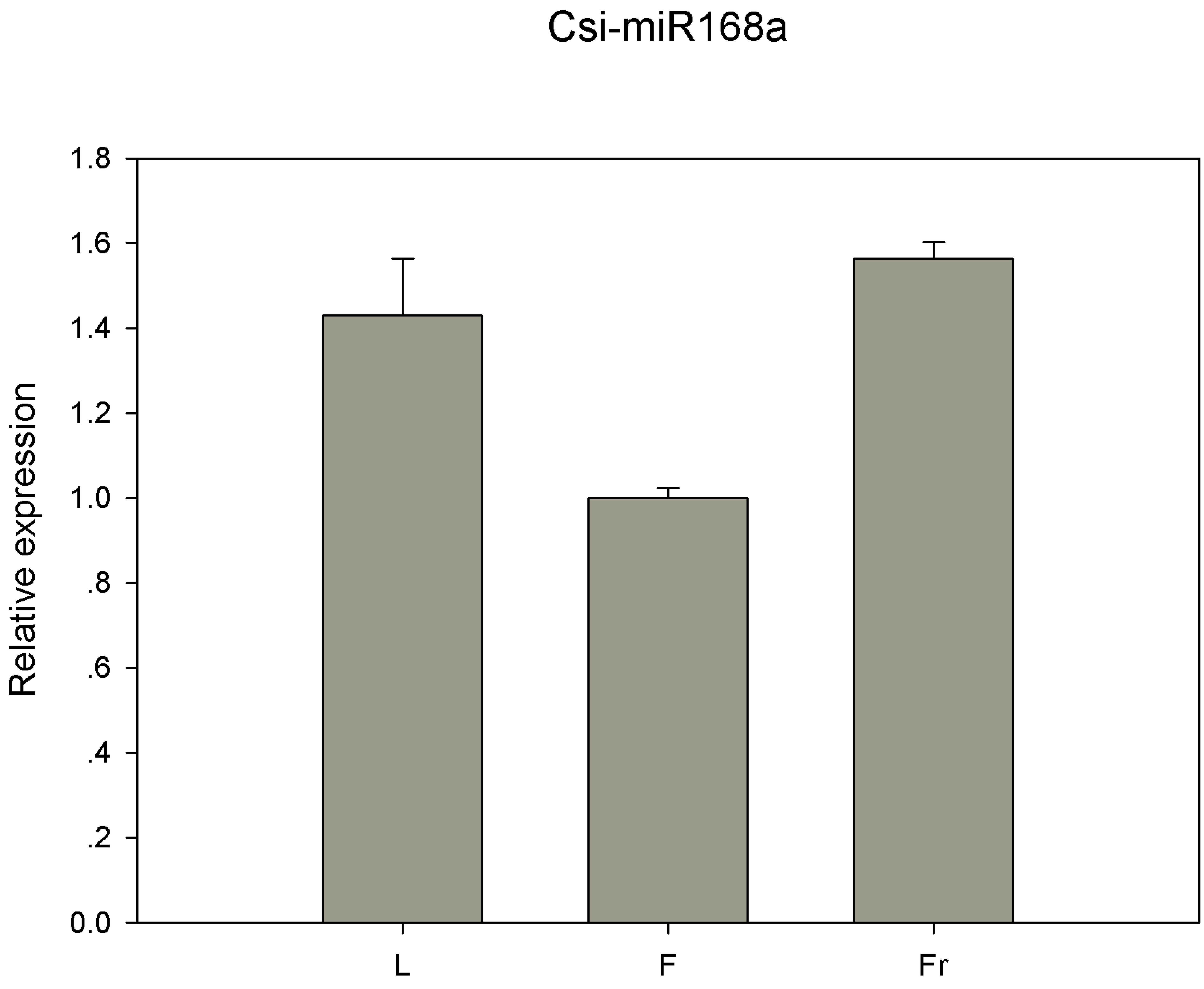
Csi-miR168a
Relative expression
0.0
.2
.4
.6
.8
1.0
1.2
1.4
1.6
1.8
L
F
Fr

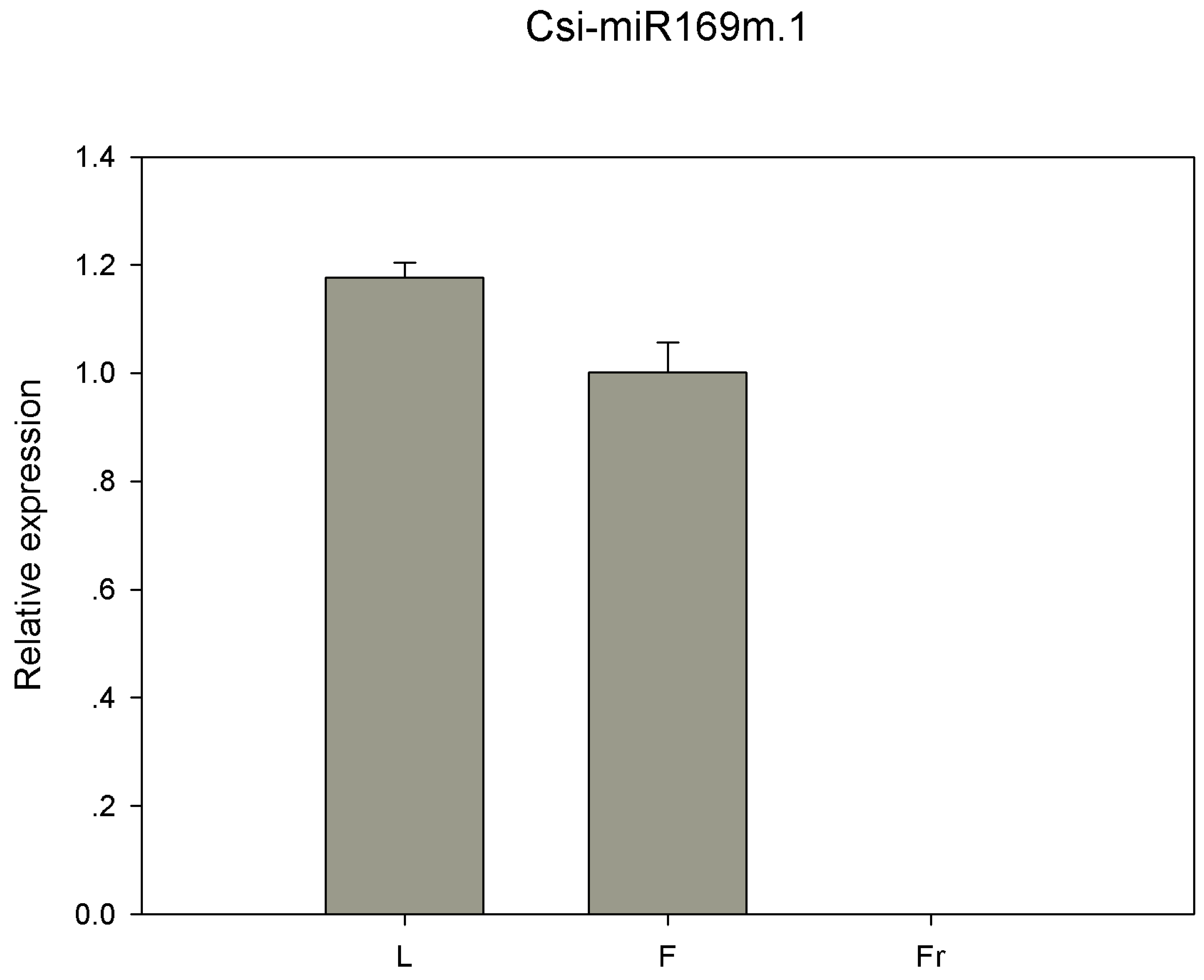
Csi-miR169m.1
Relative expression
1.4
1.2
1.0
.8
.6
.4
.2
0.0
L
F
Fr

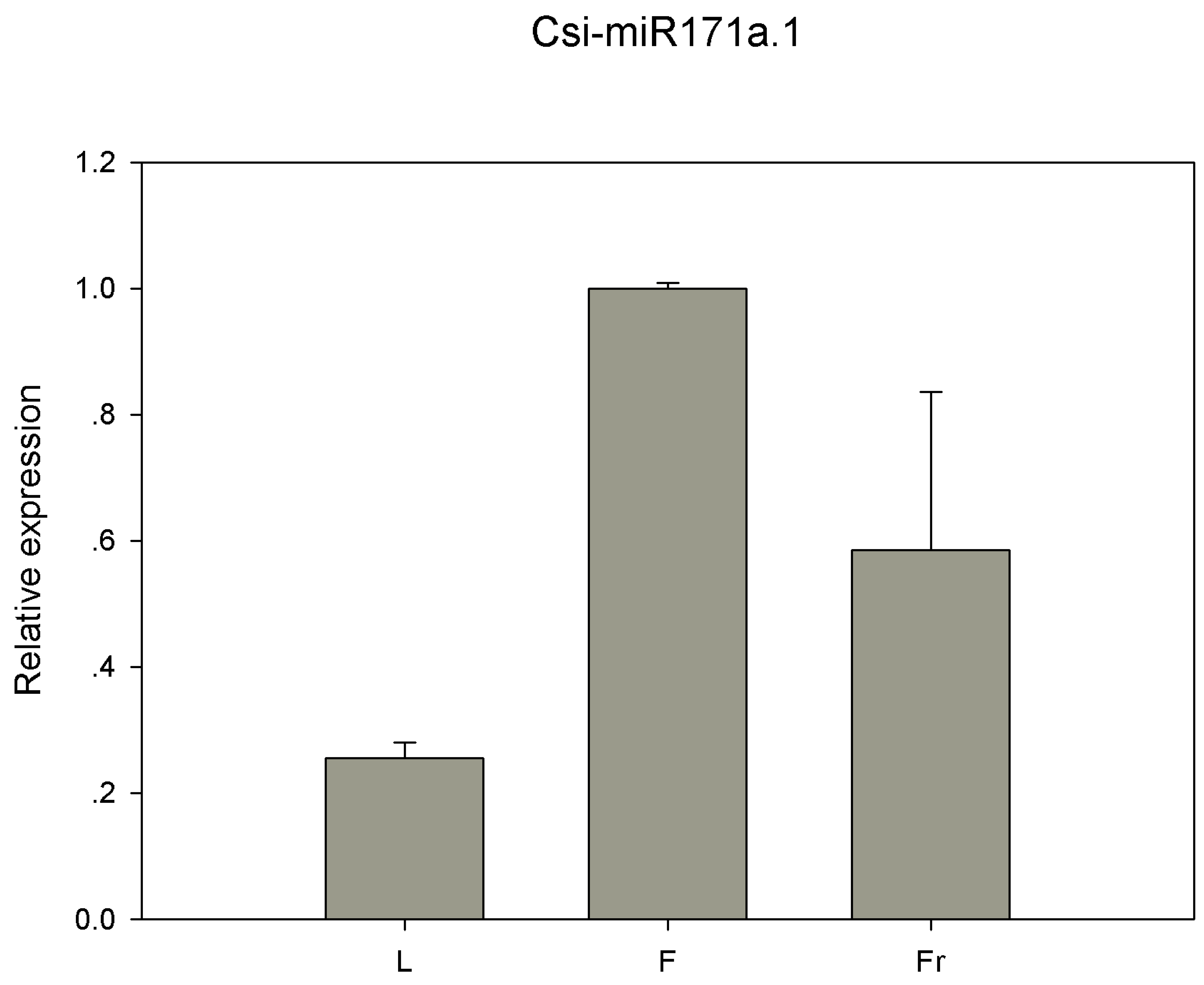
Csi-miR171a.1
Relative expression
1.2
1.0
.8
.6
.4
.2
0.0
L
F
Fr

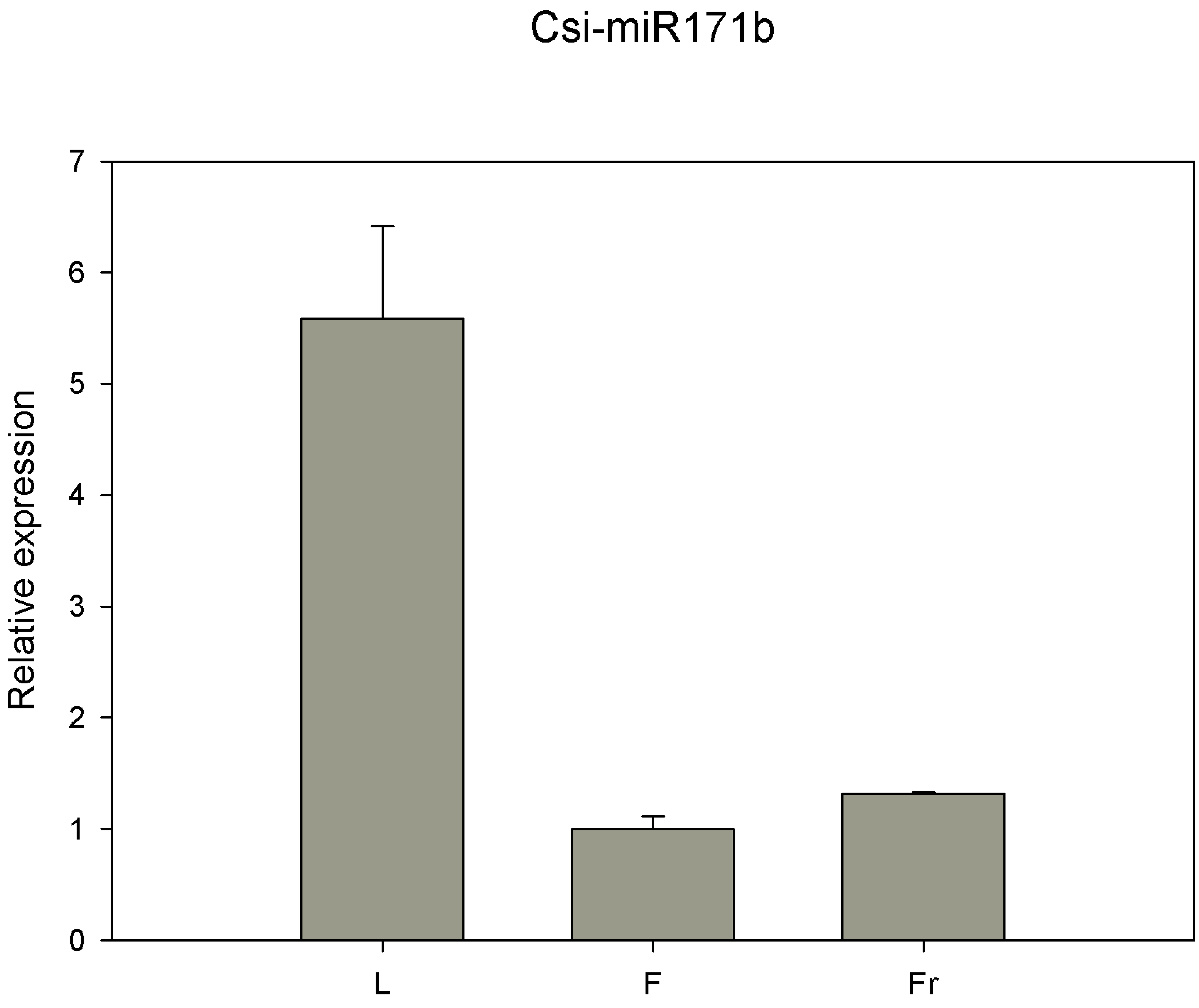

Csi-miR171b
Relative expression
0
1
2
3
4
5
6
7
L
F
Fr

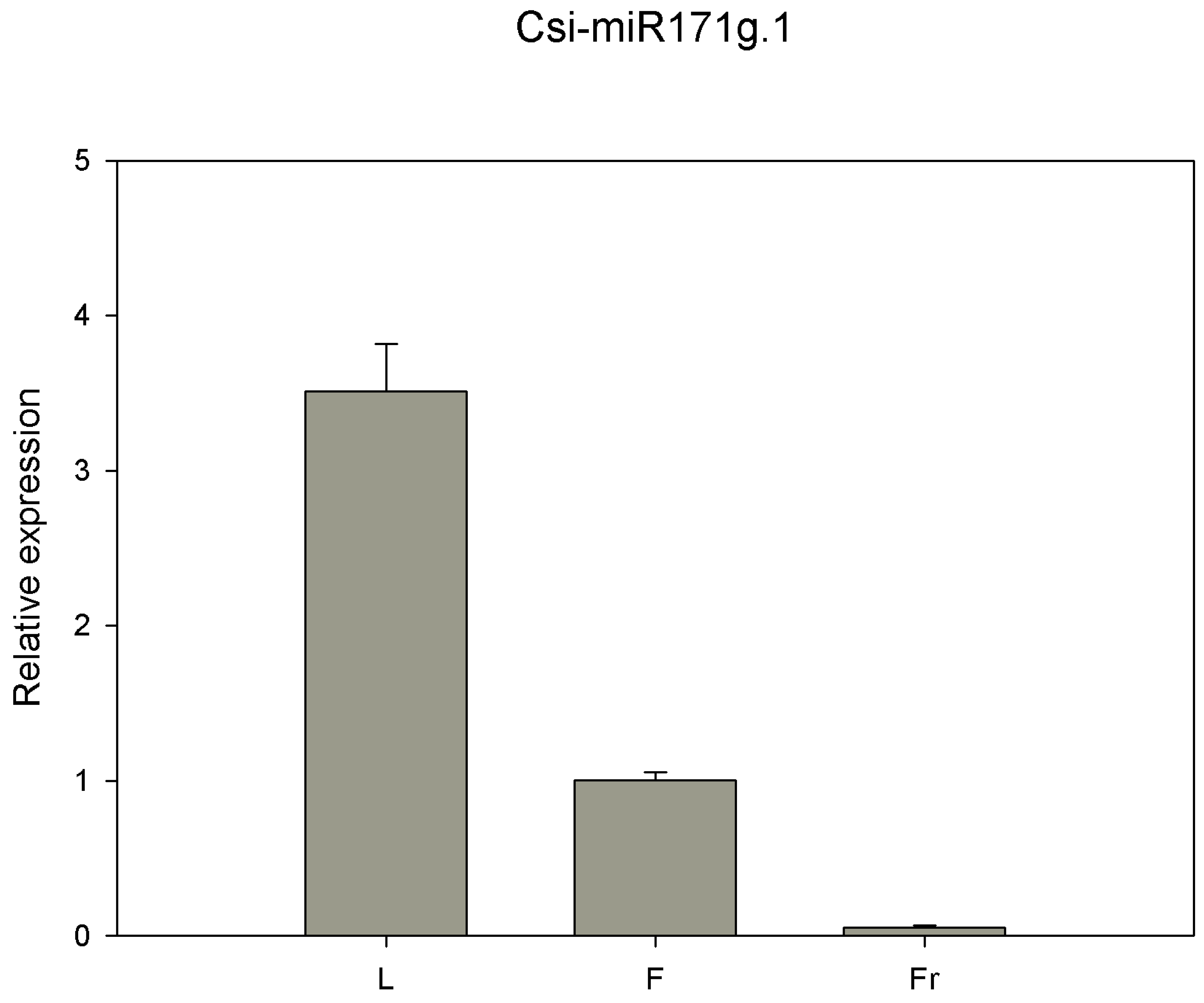
Csi-miR171g.1
Relative expression
0
1
2
3
4
5
L
F
Fr

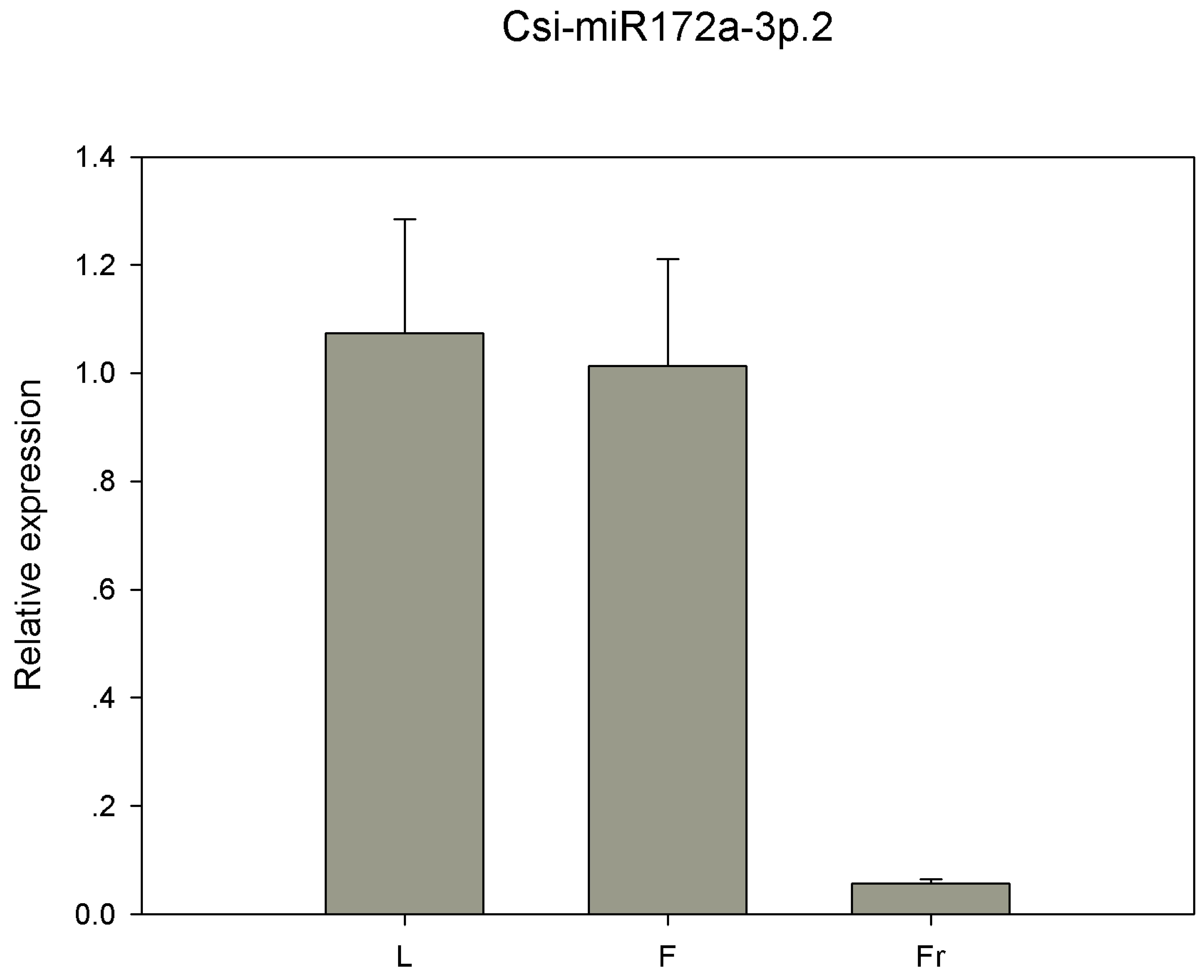
Csi-miR172a-3p.2
Relative expression
0.0
.2
.4
.6
.8
1.0
1.2
1.4
L
F
Fr

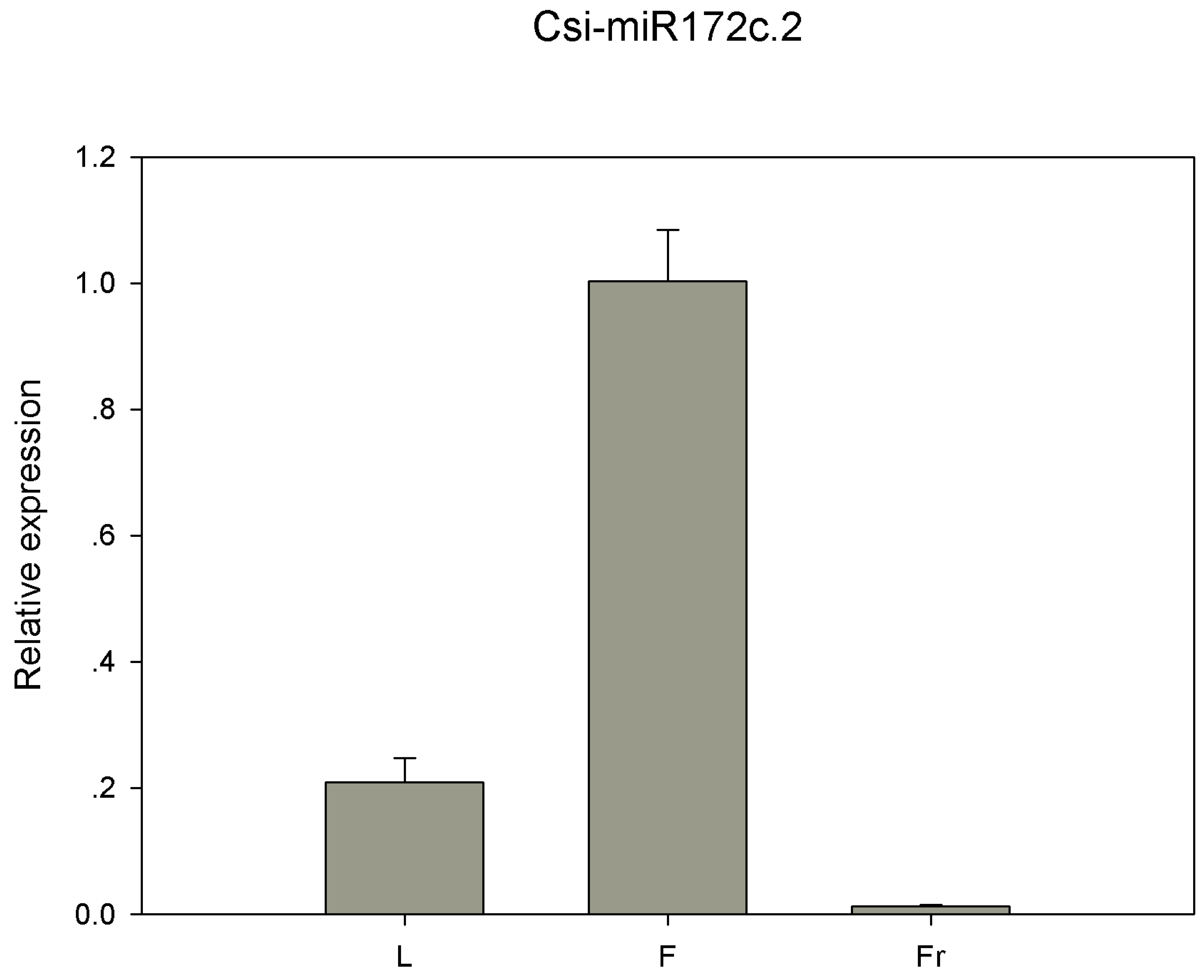
Csi-miR172c.2
Relative expression
1.2
1.0
.8
.6
.4
.2
0.0
L
F
Fr

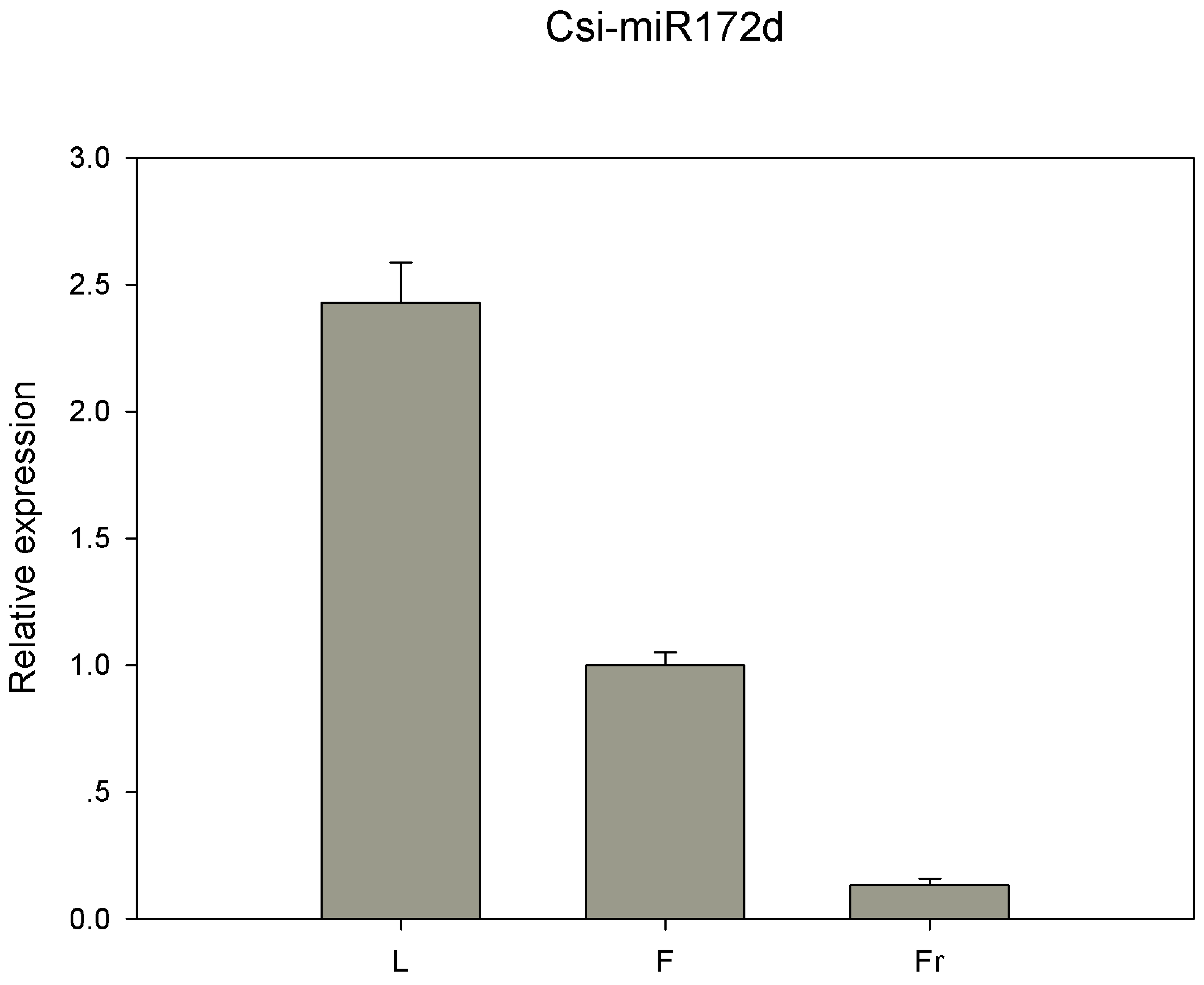
Csi-miR172d
Relative expression
3.0
2.5
2.0
1.5
1.0
.5
0.0
L
F
Fr

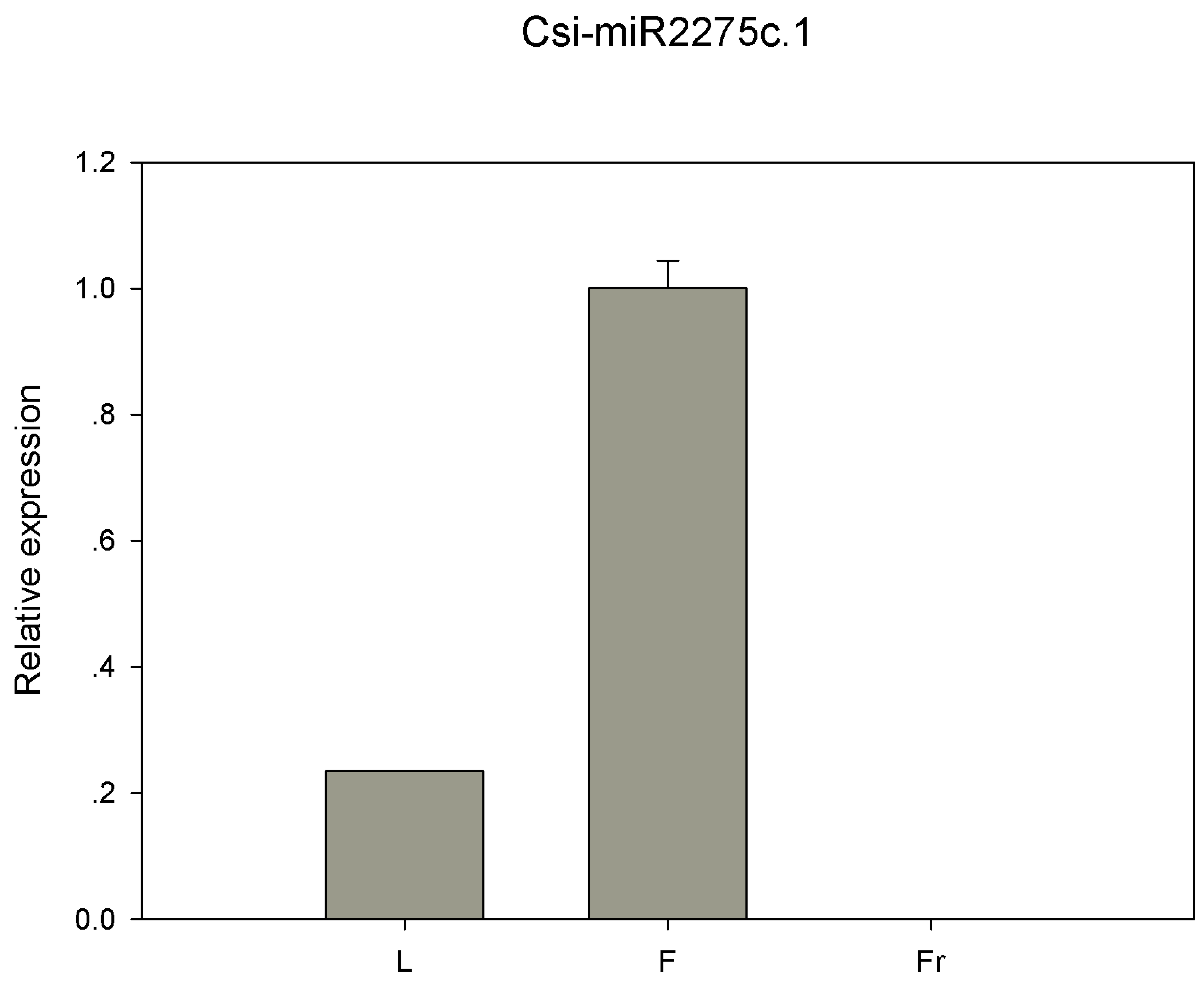
Csi-miR2275c.1
Relative expression
1.2
1.0
.8
.6
.4
.2
0.0
L
F
Fr

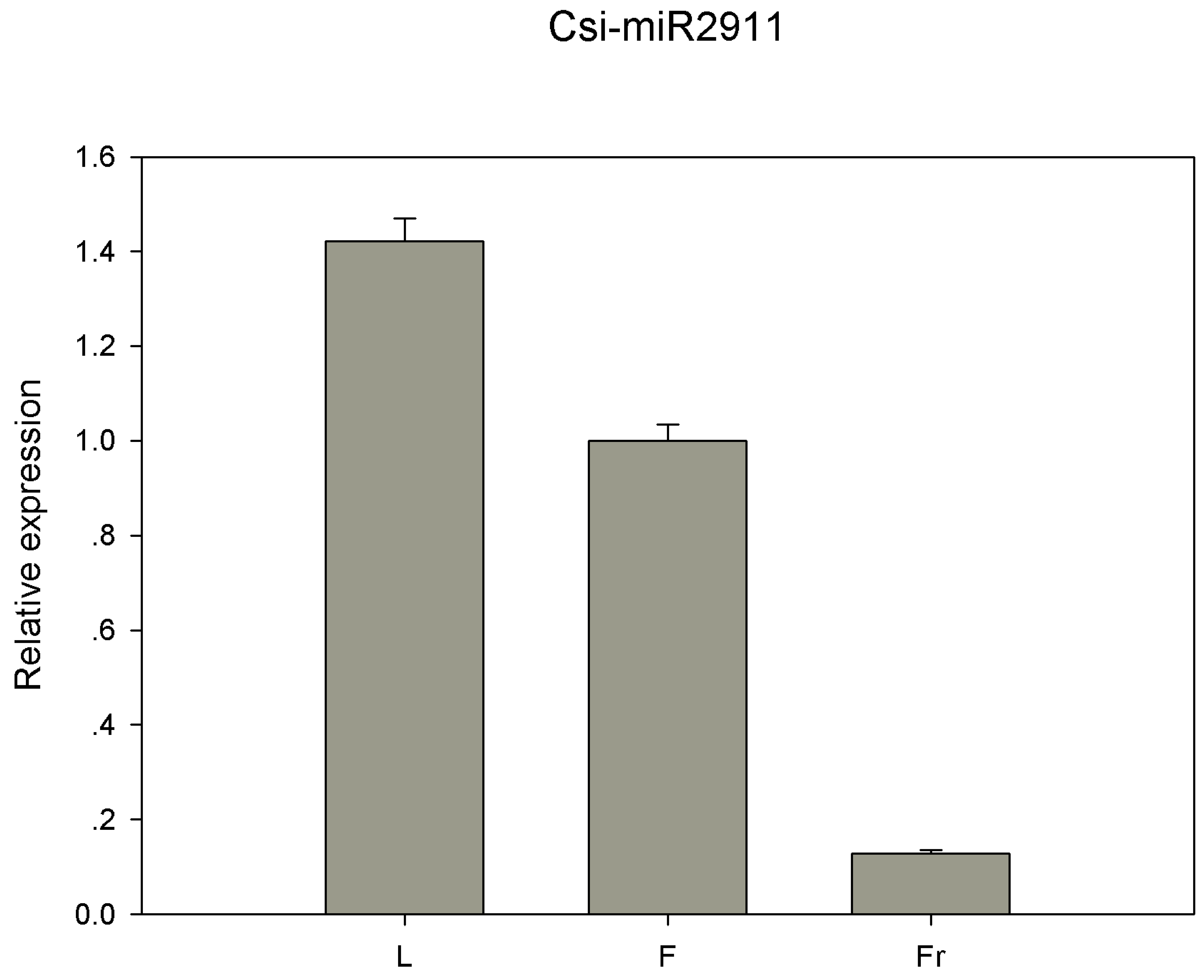

Csi-miR2911
Relative expression
0.0
.2
.4
.6
.8
1.0
1.2
1.4
1.6
L
F
Fr

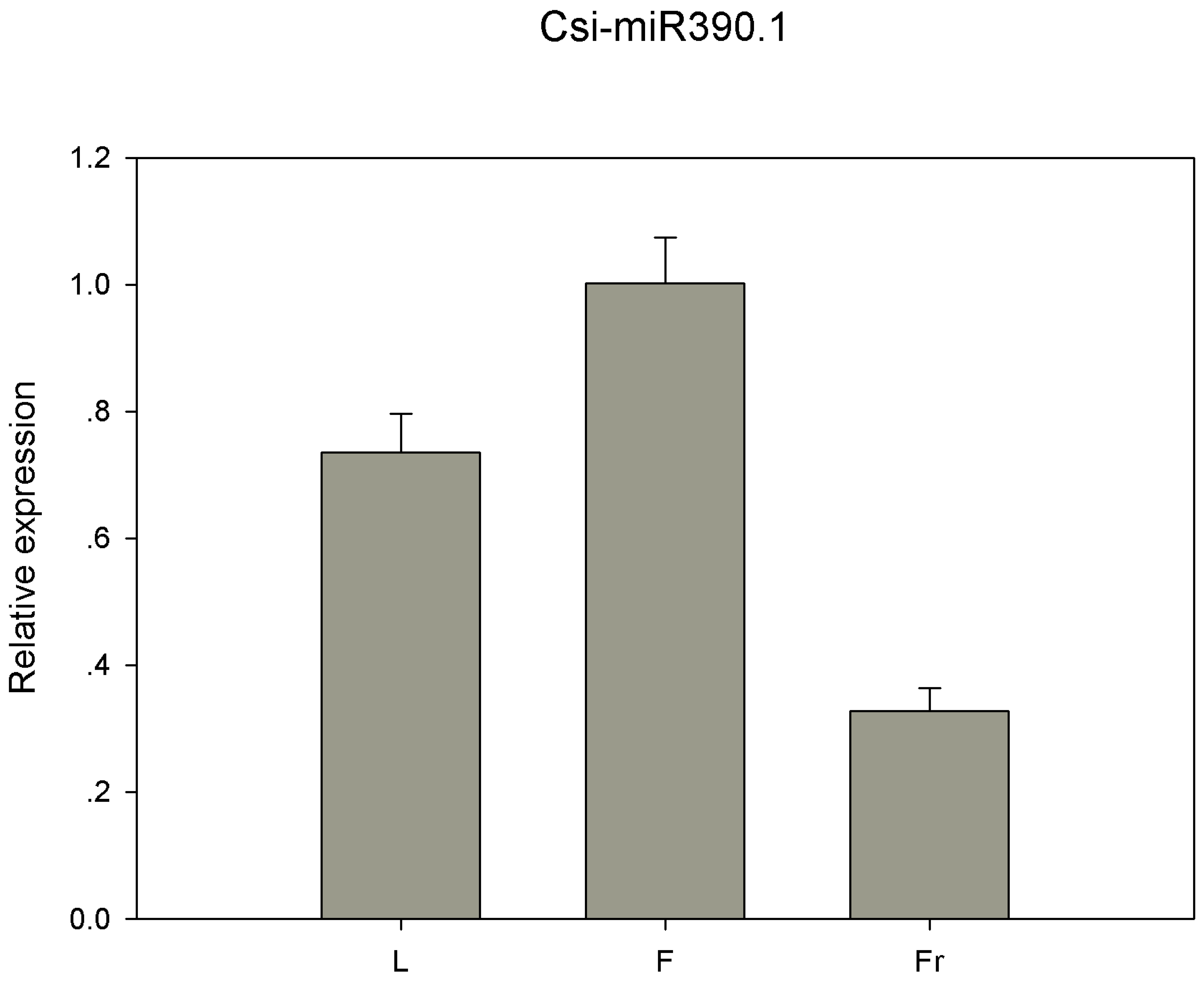

Csi-miR390.1
Relative expression
1.2
1.0
.8
.6
.4
.2
0.0
L
F
Fr

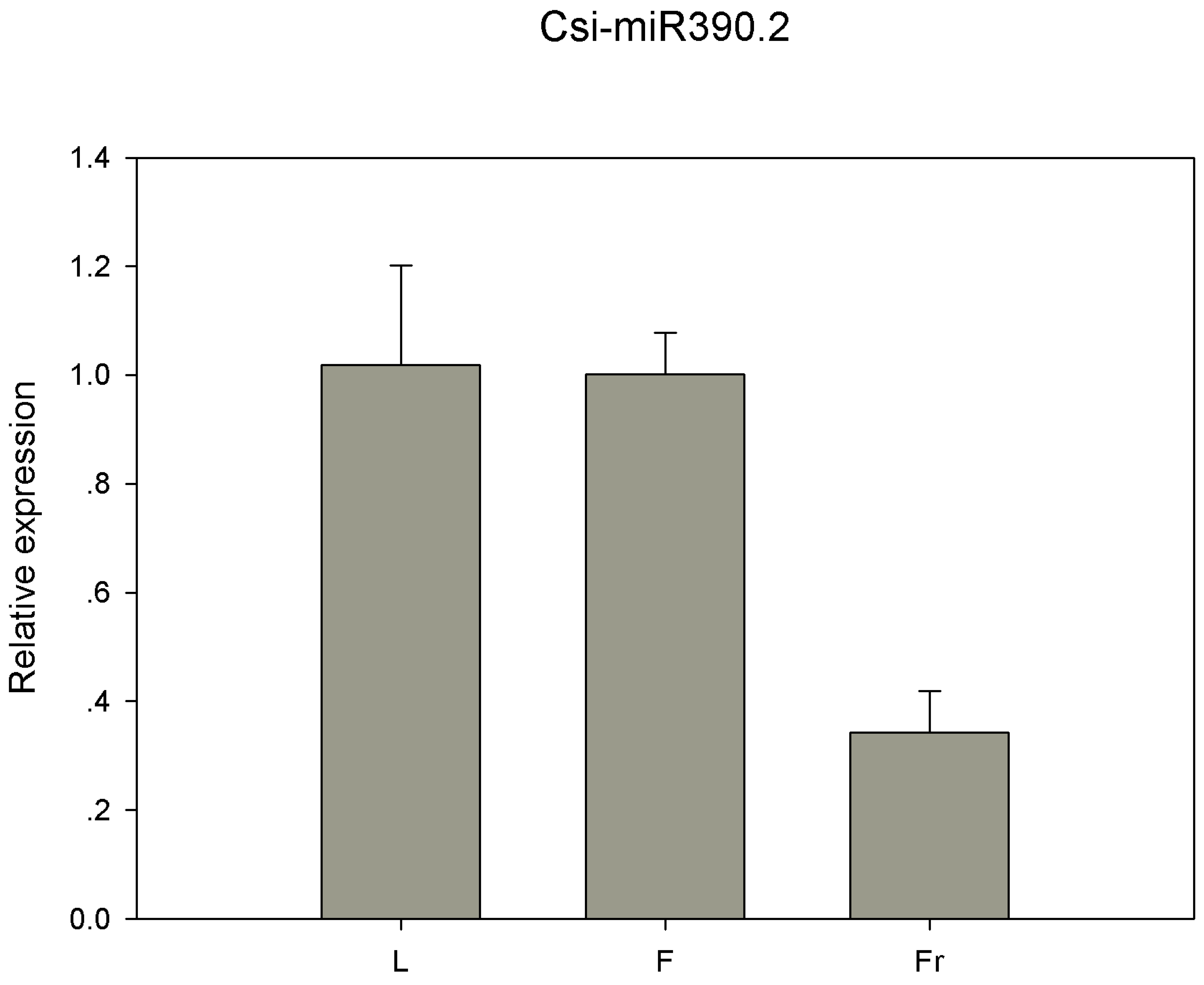

Csi-miR390.2
Relative expression
1.4
1.2
1.0
.8
.6
.4
.2
0.0
L
F
Fr

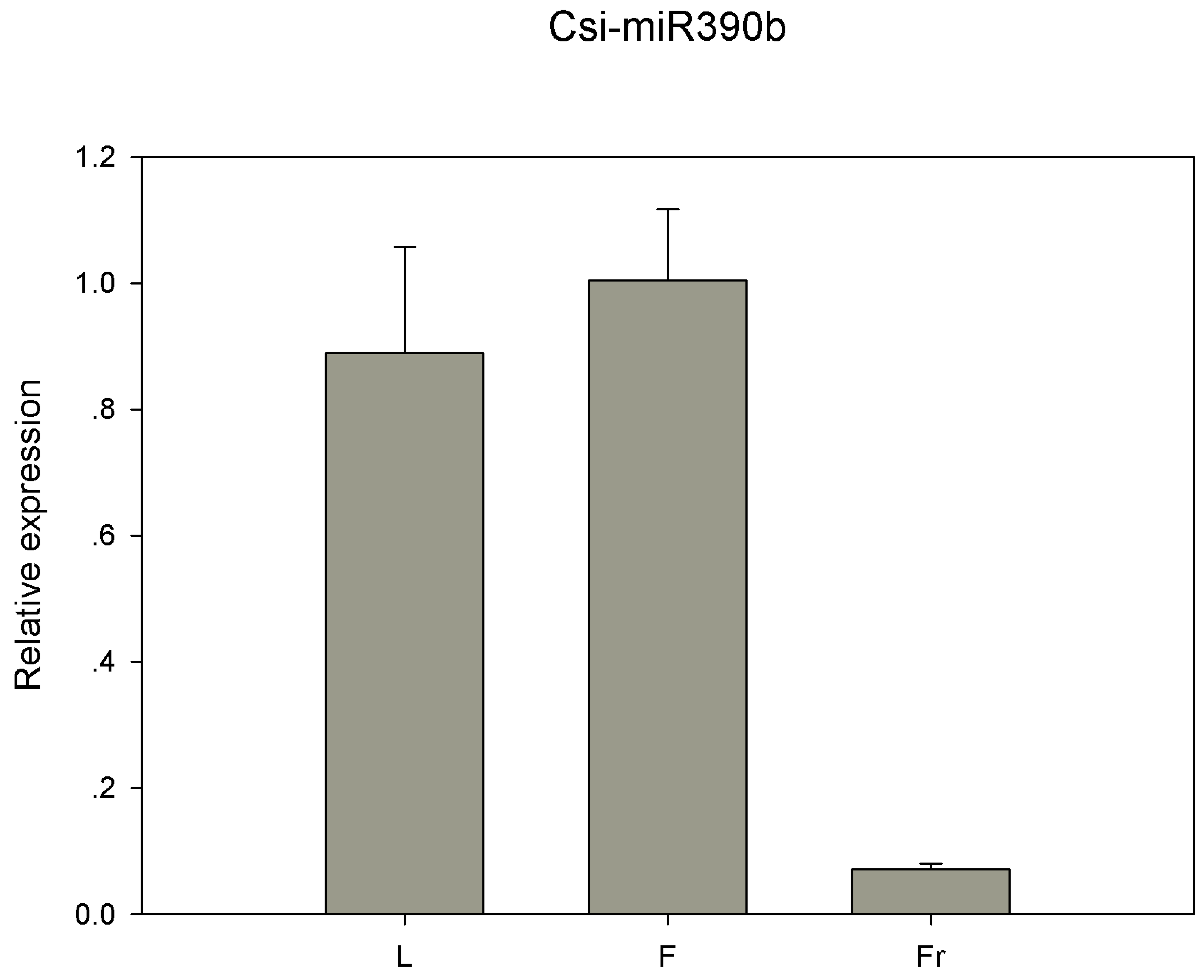
Csi-miR390b
Relative expression
1.2
1.0
.8
.6
.4
.2
0.0
L
F
Fr

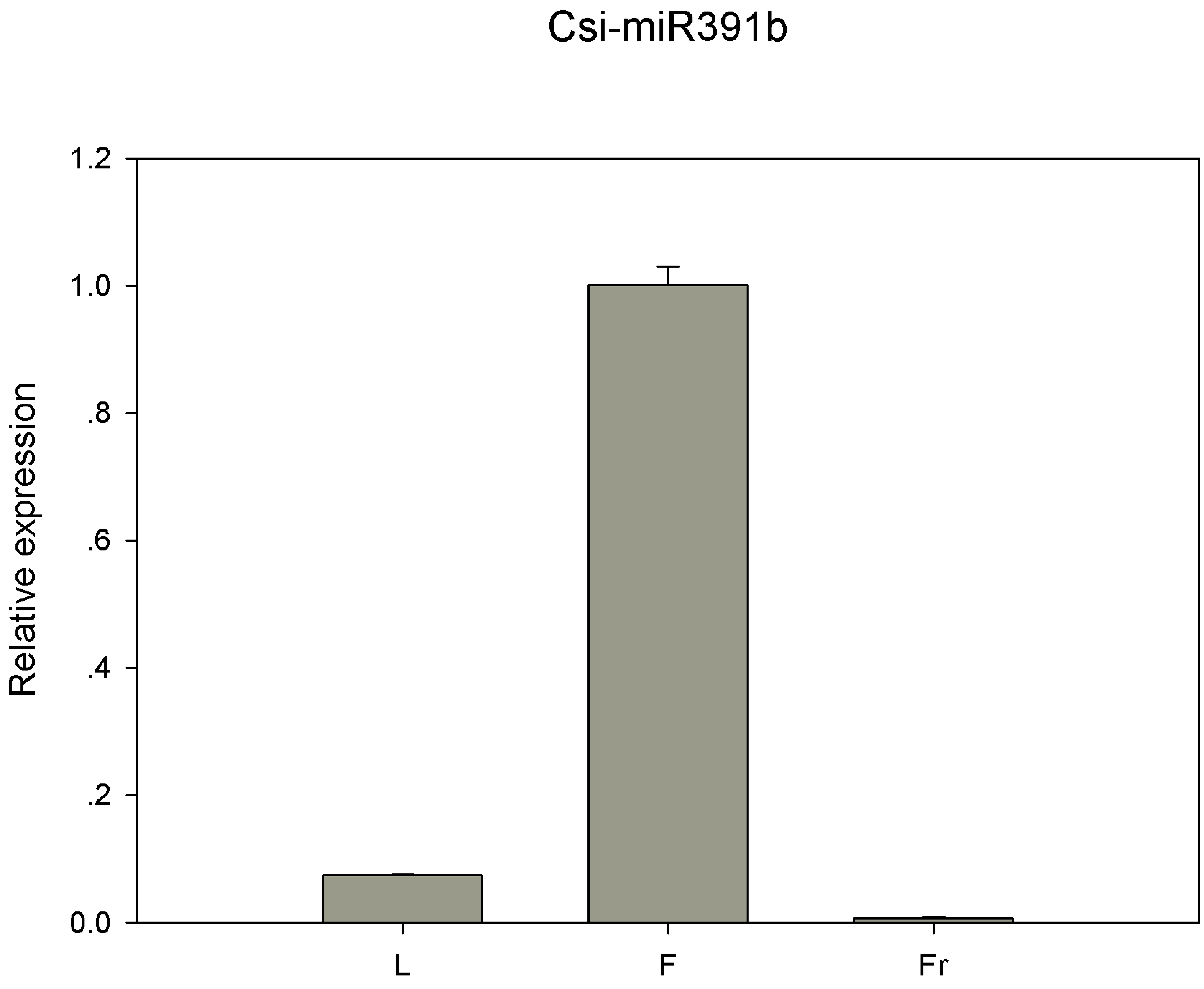

Csi-miR391b
Relative expression
1.2
1.0
.8
.6
.4
.2
0.0
L
F
Fr

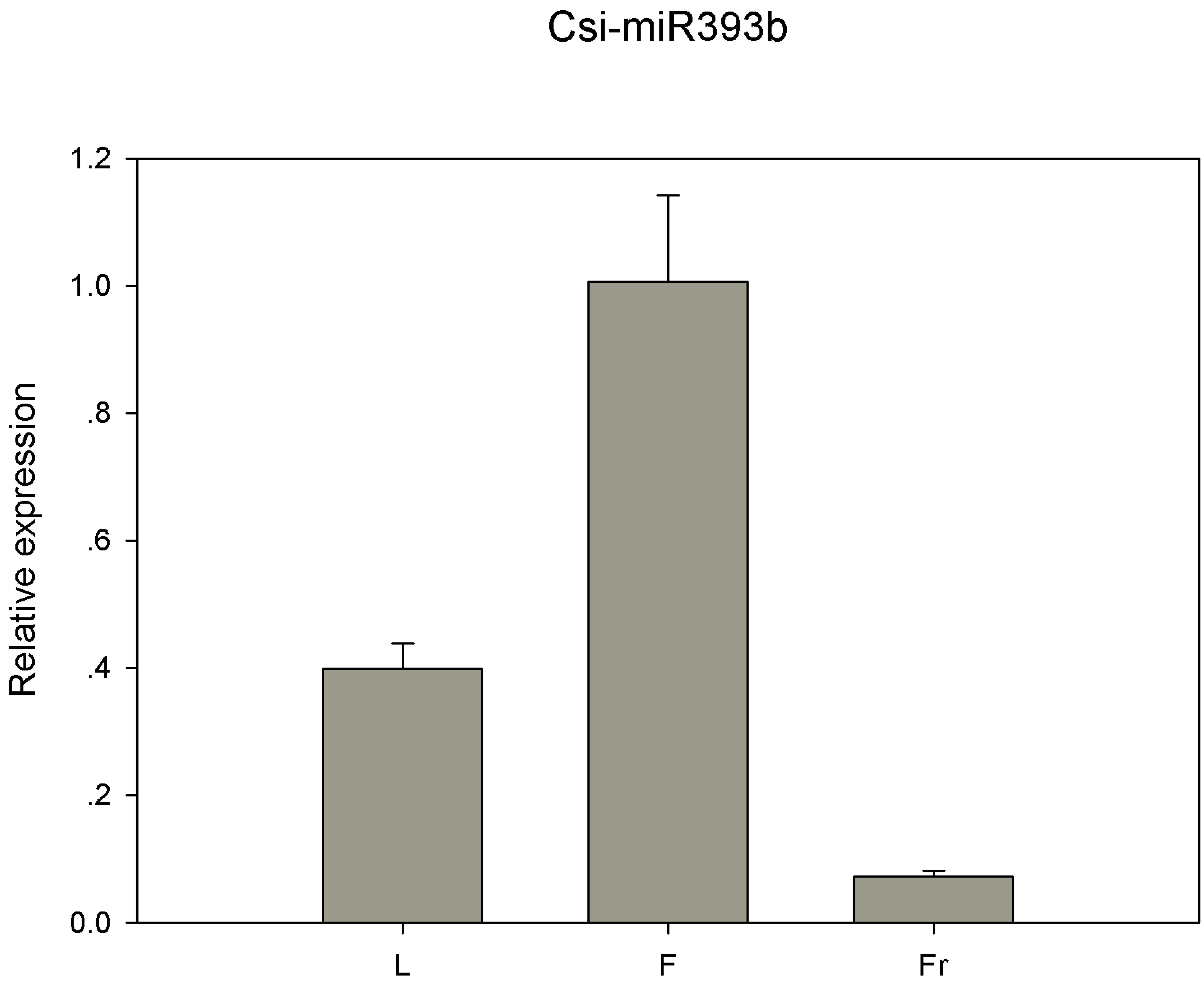

Csi-miR393b
Relative expression
1.2
1.0
.8
.6
.4
.2
0.0
L
F
Fr

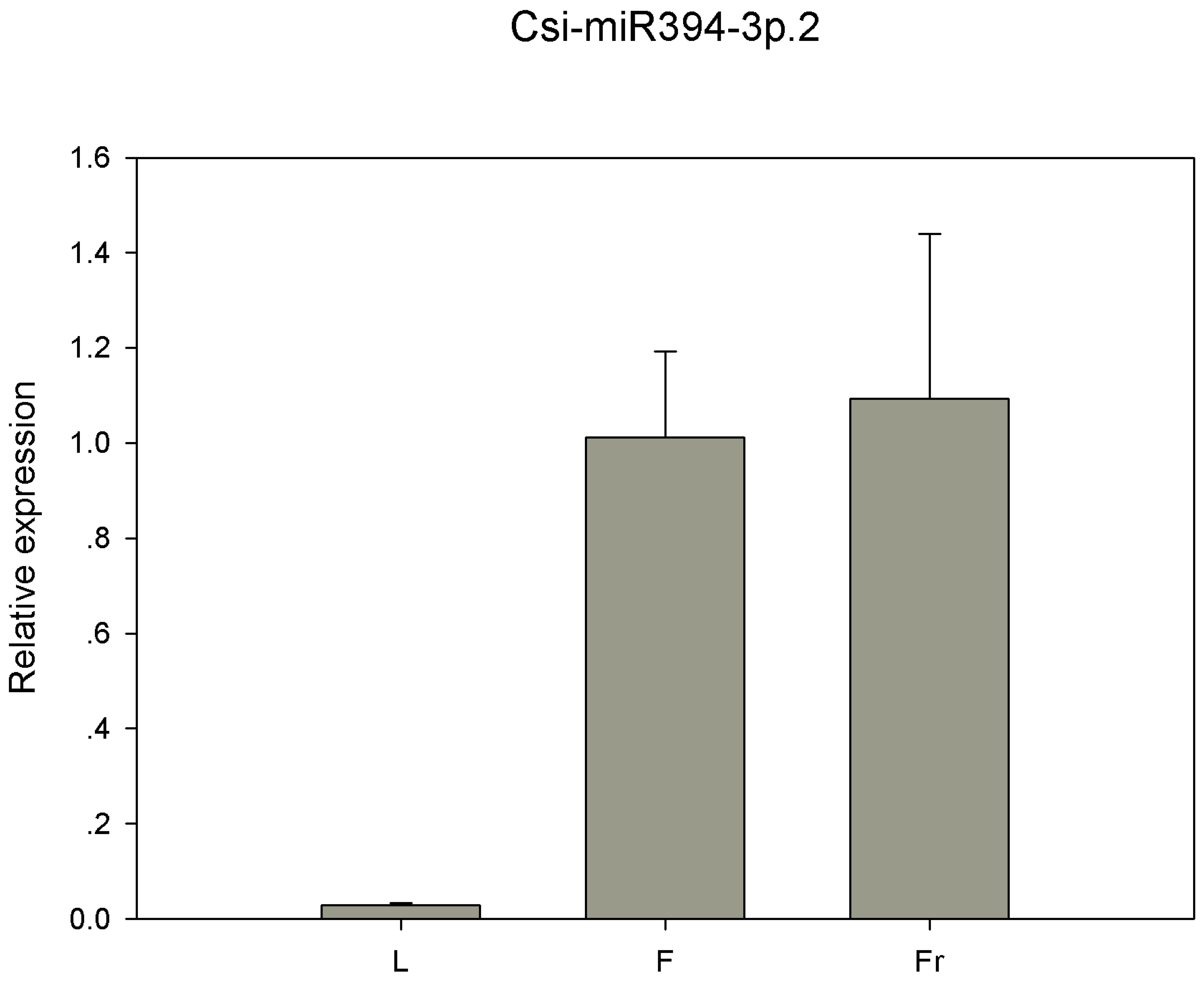

Csi-miR394-3p.2
Relative expression
1.6
1.4
1.2
1.0
.8
.6
.4
.2
0.0
L
F
Fr

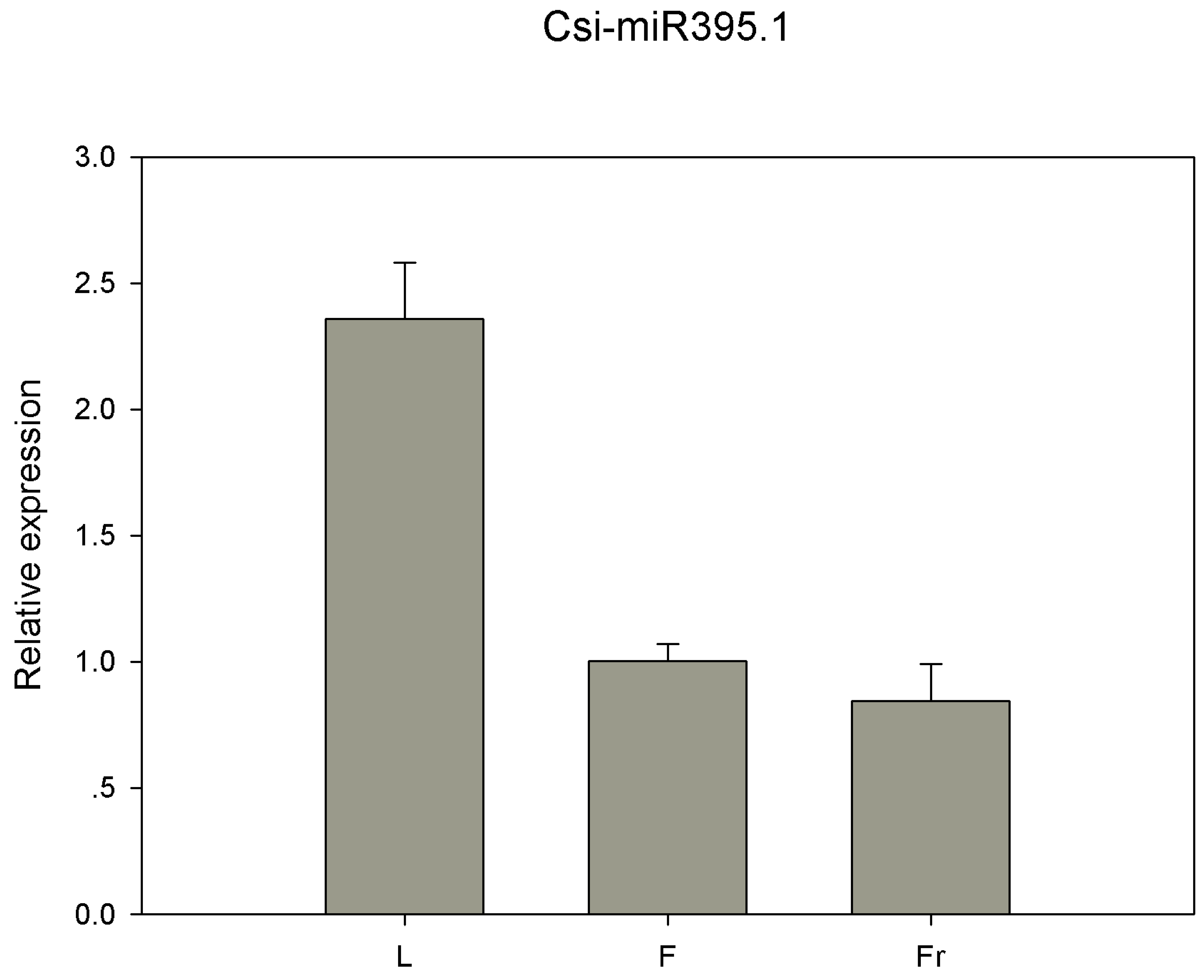

Csi-miR395.1
Relative expression
3.0
2.5
2.0
1.5
1.0
.5
0.0
L
F
Fr

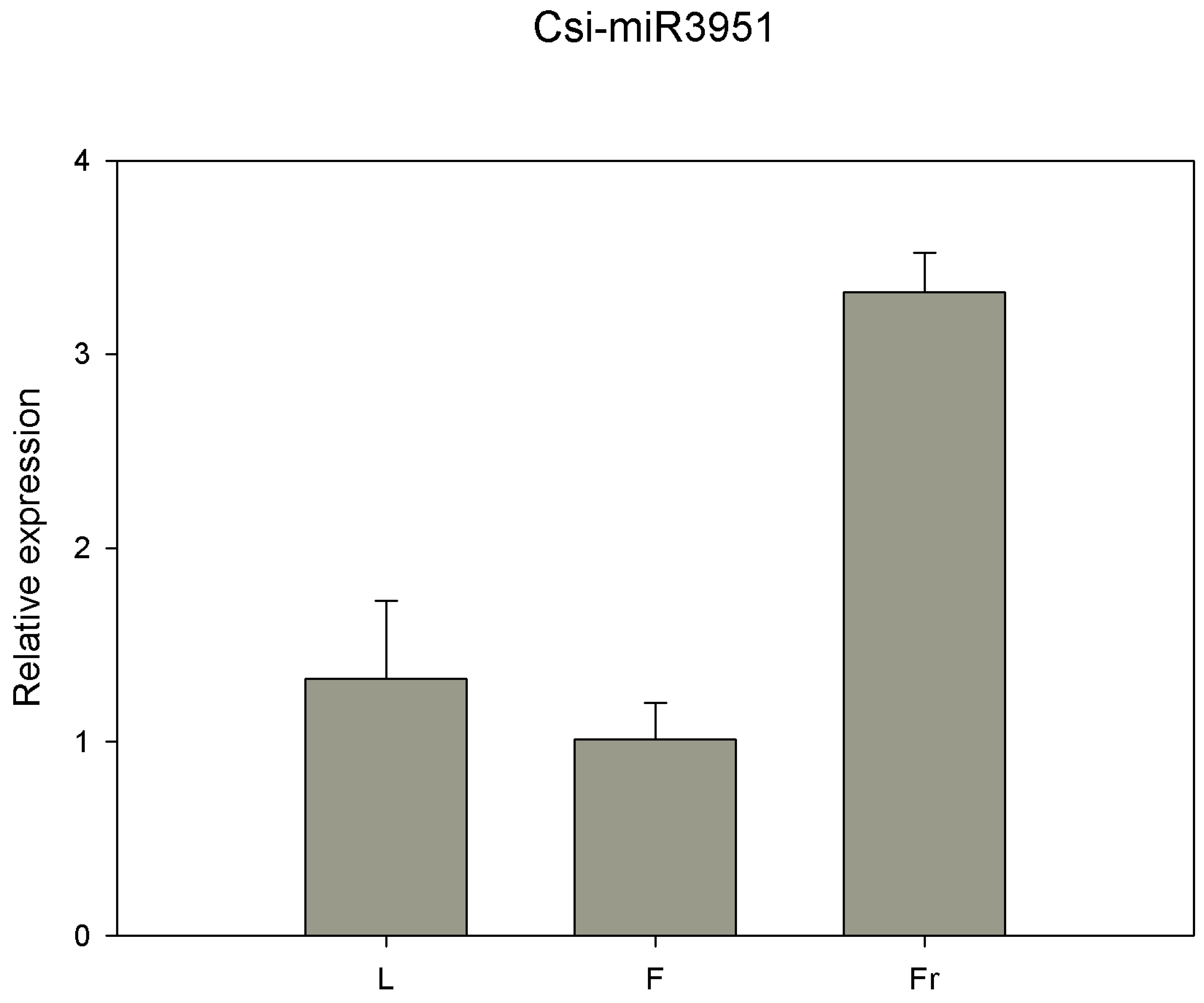
Csi-miR3951
Relative expression
0
1
2
3
4
L
F
Fr

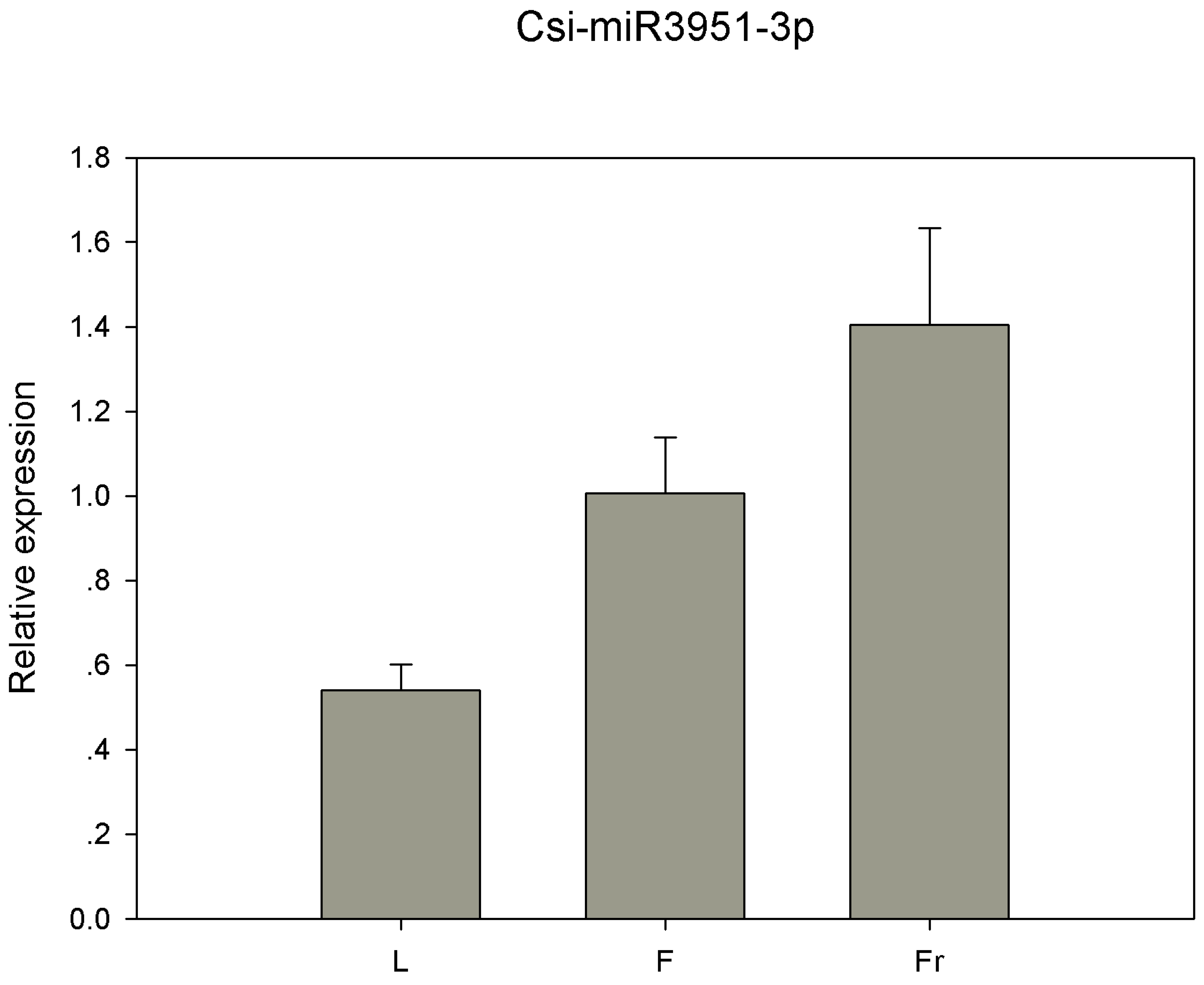

Csi-miR3951-3p
Relative expression
1.8
1.6
1.4
1.2
1.0
.8
.6
.4
.2
0.0
L
F
Fr

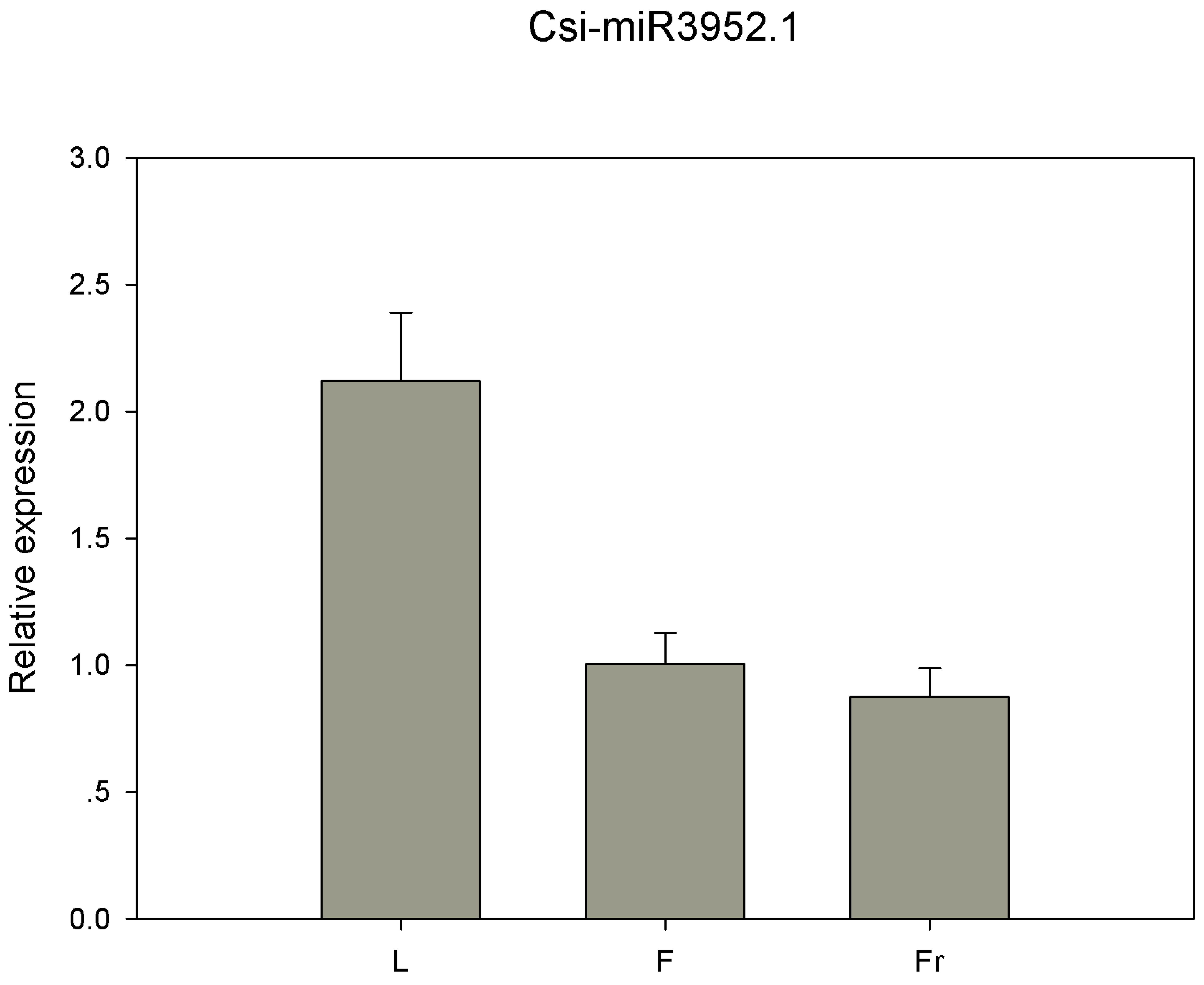
Csi-miR3952.1
Relative expression
3.0
2.5
2.0
1.5
1.0
.5
0.0
L
F
Fr

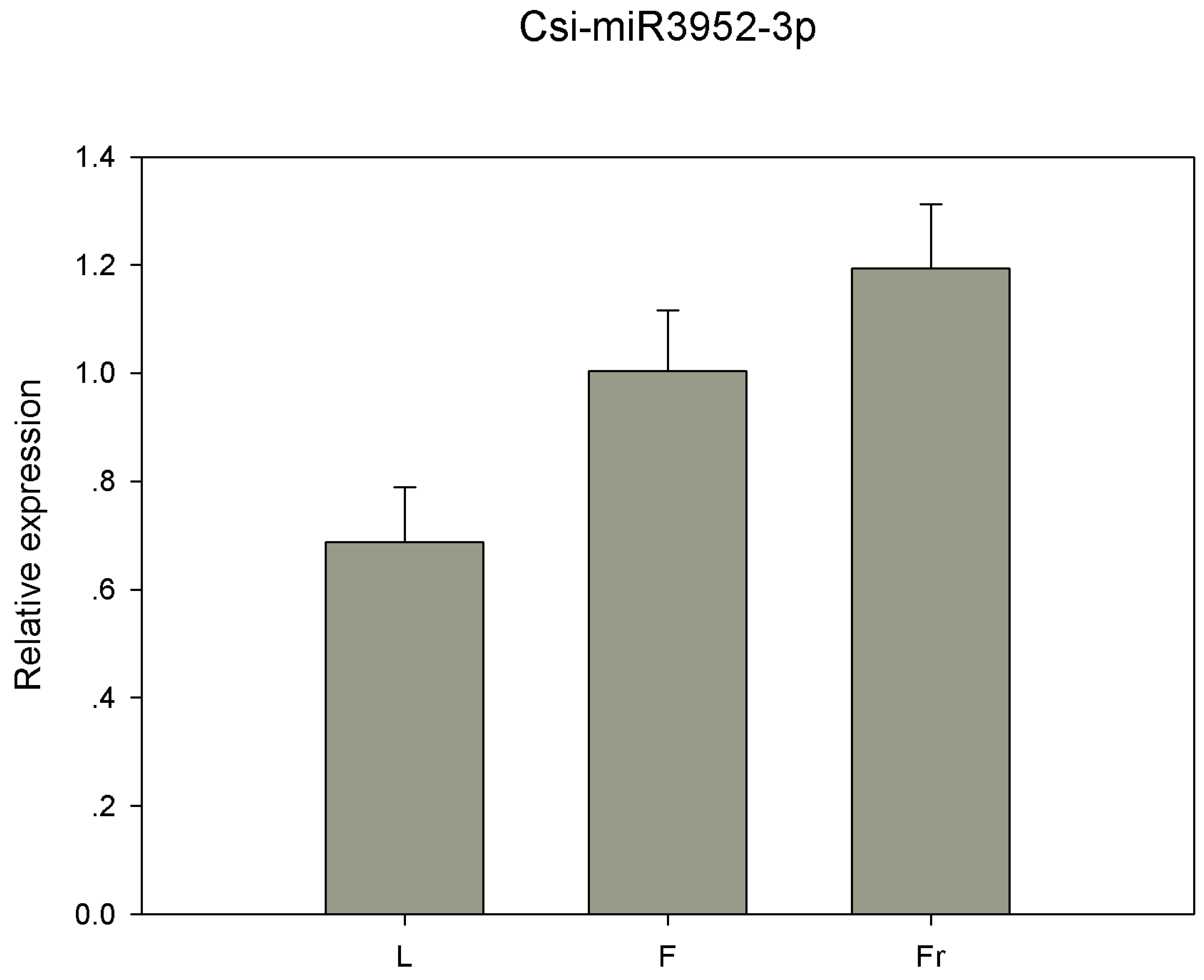
Csi-miR3952-3p
Relative expression
1.4
1.2
1.0
.8
.6
.4
.2
0.0
L
F
Fr

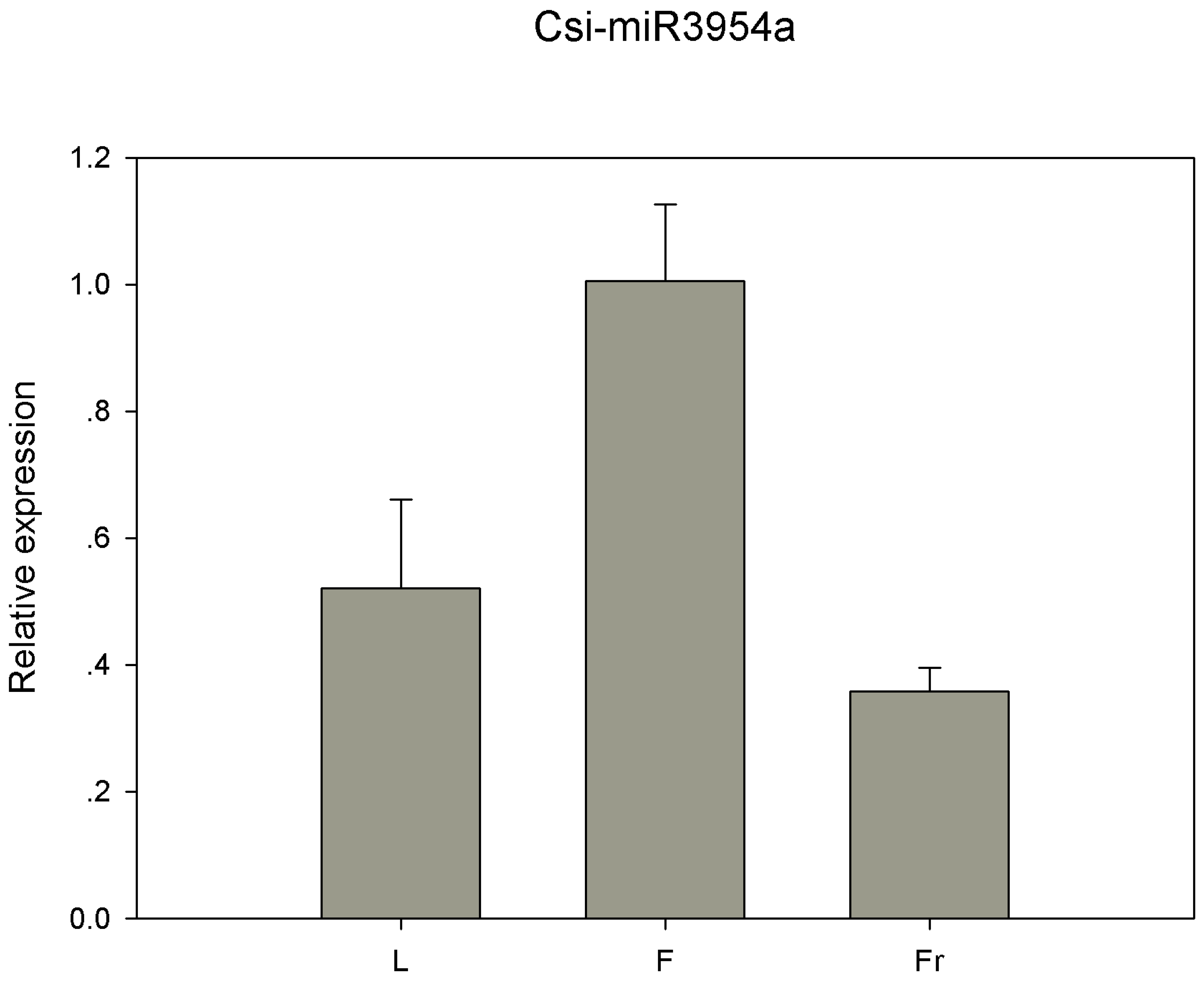
Csi-miR3954a
Relative expression
0.0
.2
.4
.6
.8
1.0
1.2
L
F
Fr

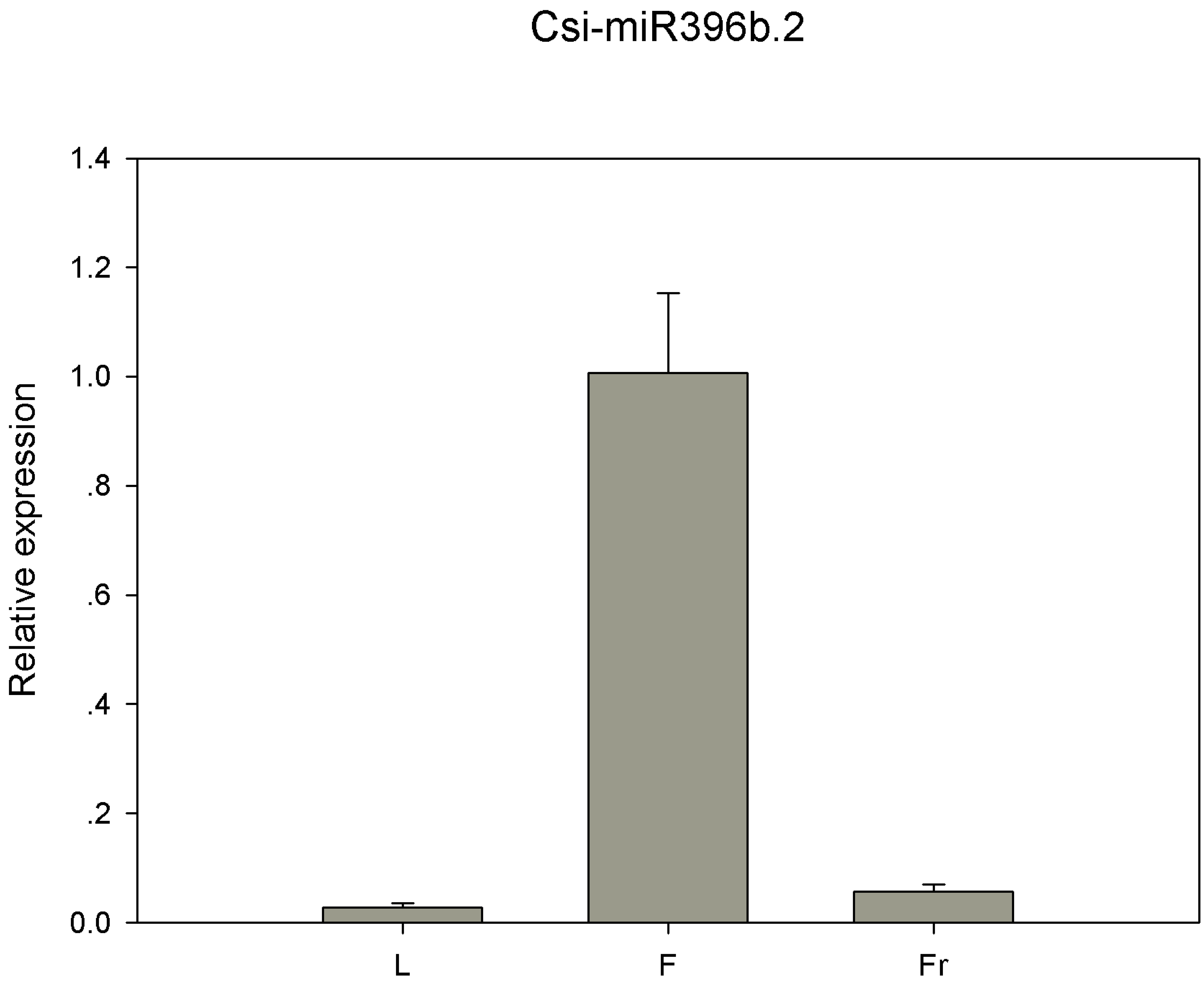

Csi-miR396b.2
Relative expression
1.4
1.2
1.0
.8
.6
.4
.2
0.0
L
F
Fr

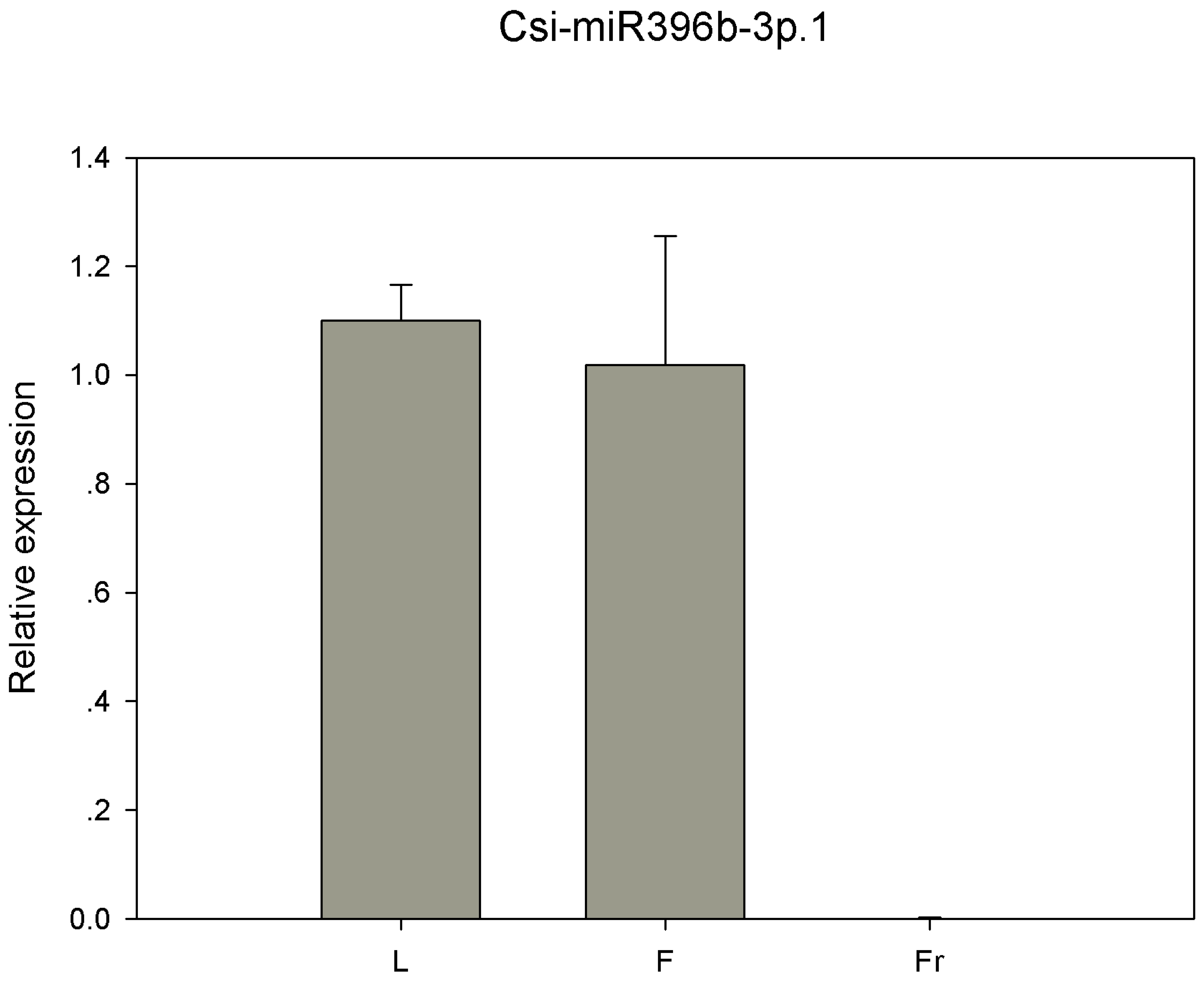

Csi-miR396b-3p.1
Relative expression
1.4
1.2
1.0
.8
.6
.4
.2
0.0
L
F
Fr

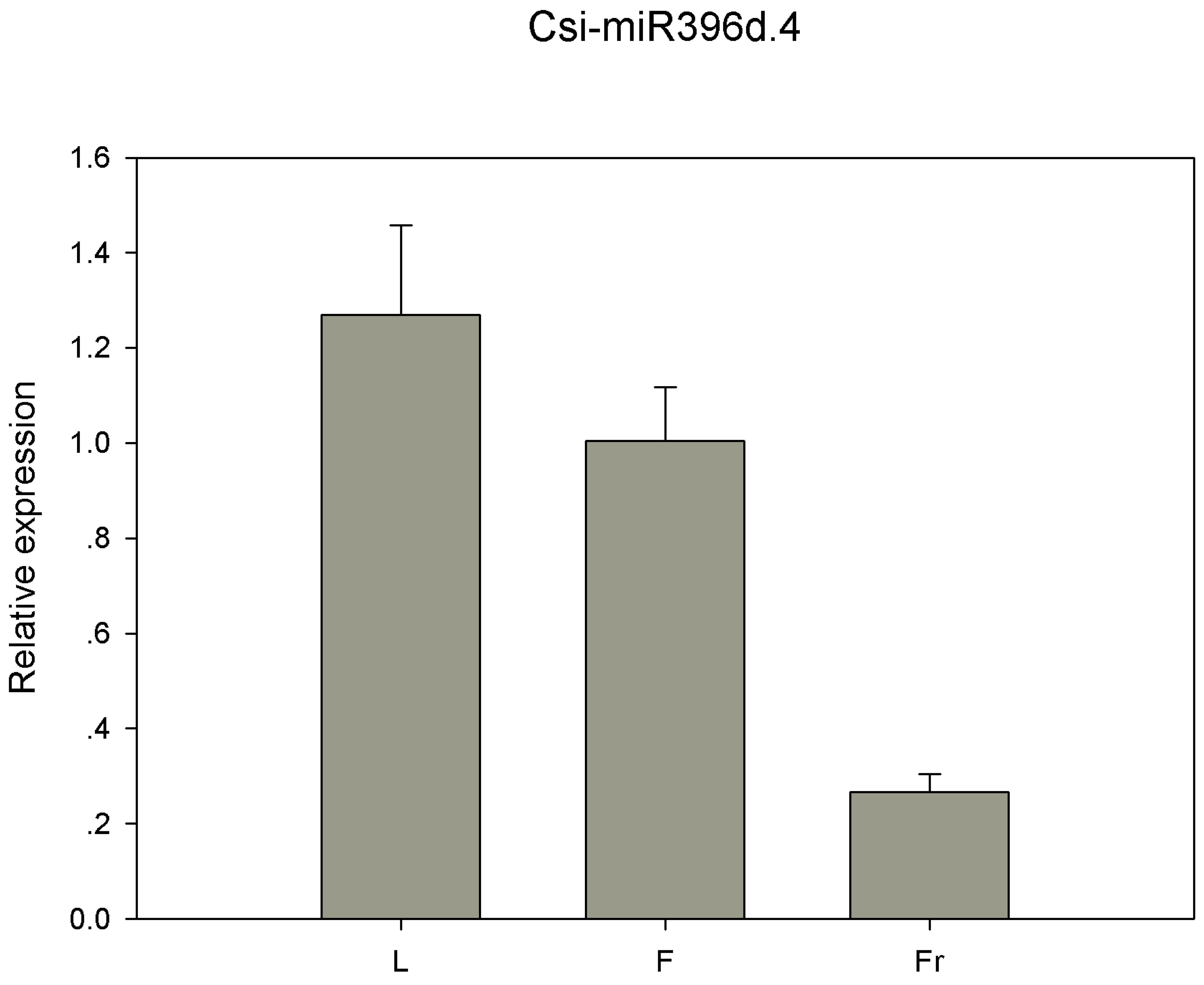

Csi-miR396d.4
Relative expression
1.6
1.4
1.2
1.0
.8
.6
.4
.2
0.0
L
F
Fr

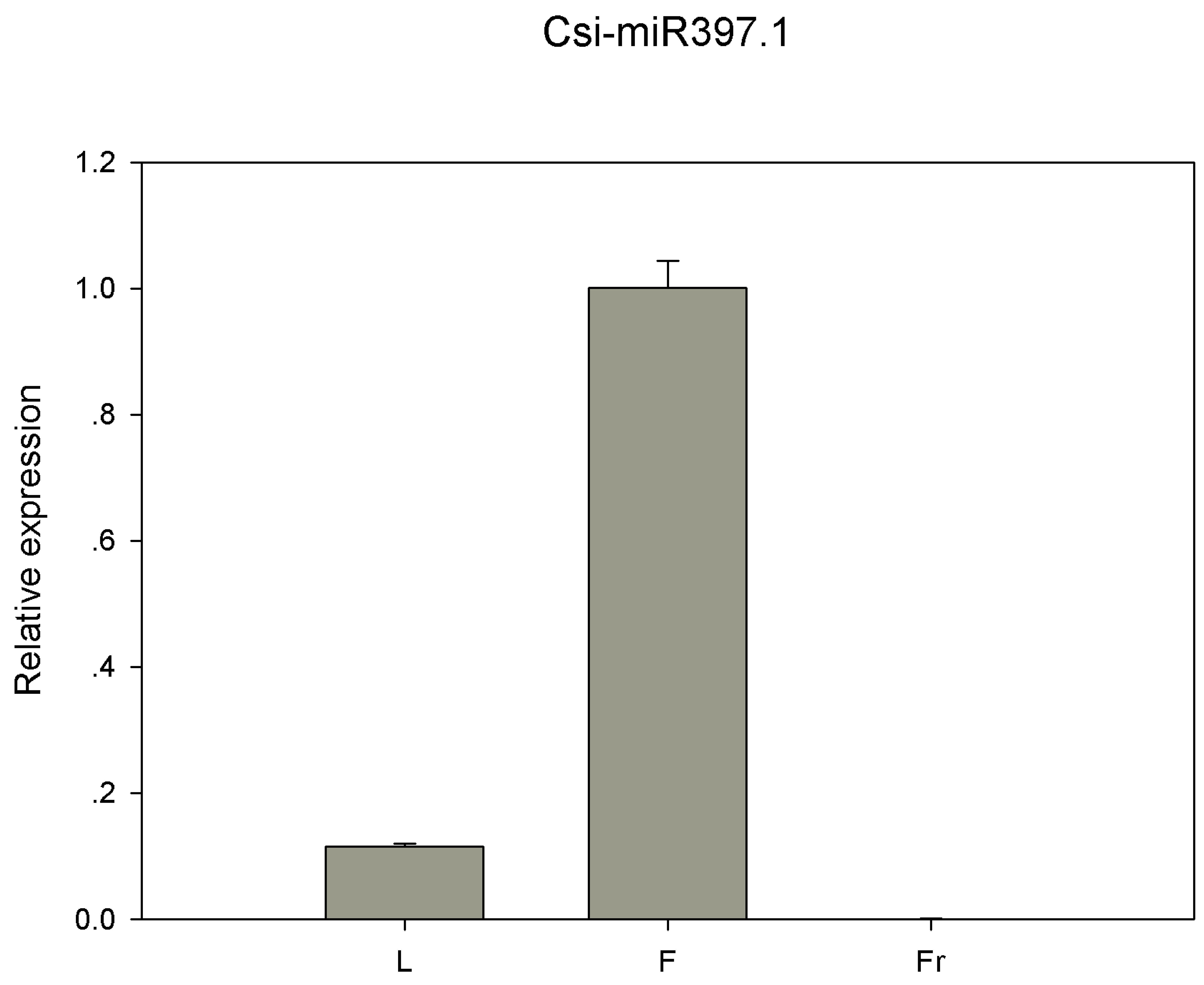
Csi-miR397.1
Relative expression
1.2
1.0
.8
.6
.4
.2
0.0
L
F
Fr

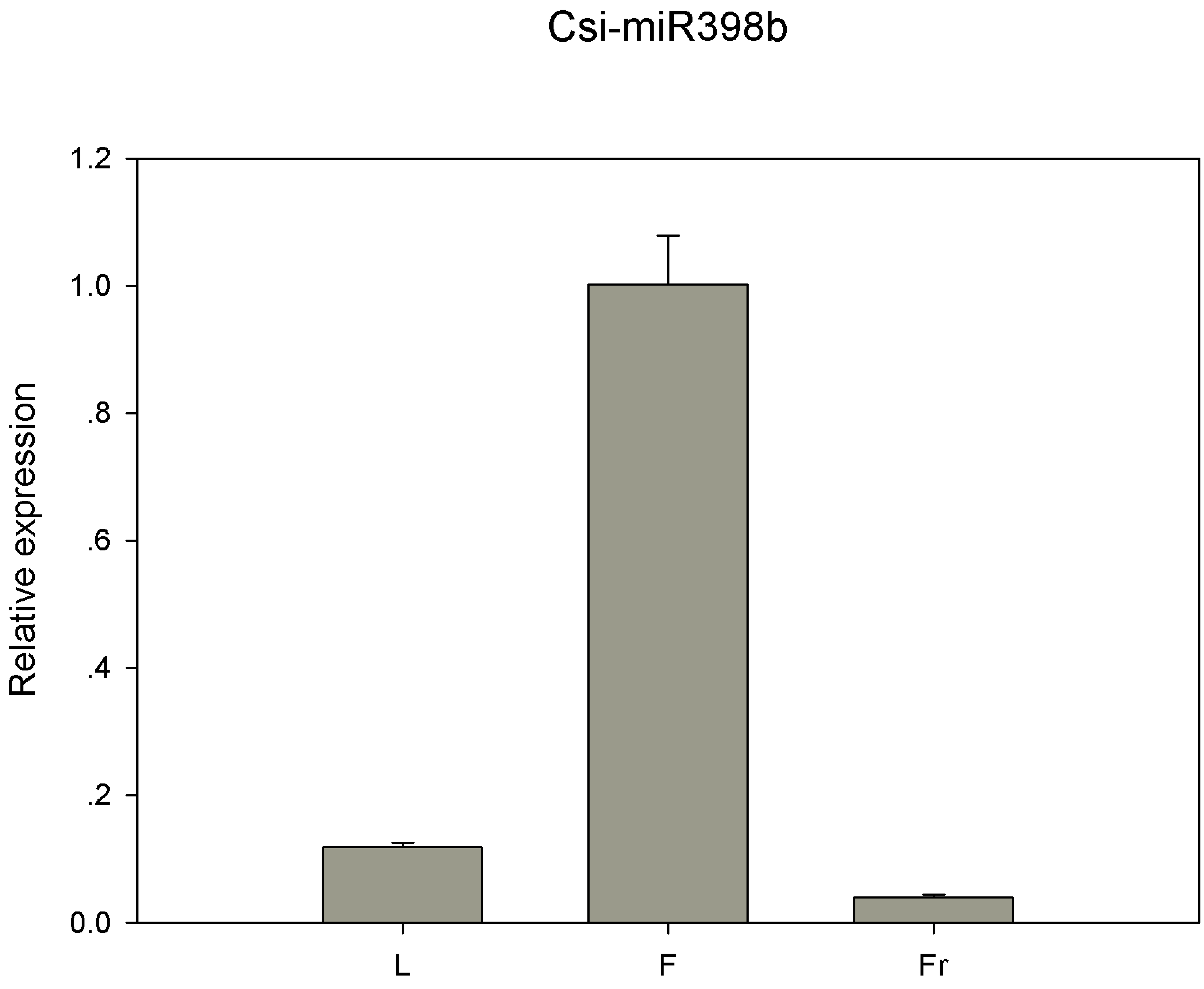

Csi-miR398b
Relative expression
1.2
1.0
.8
.6
.4
.2
0.0
L
F
Fr

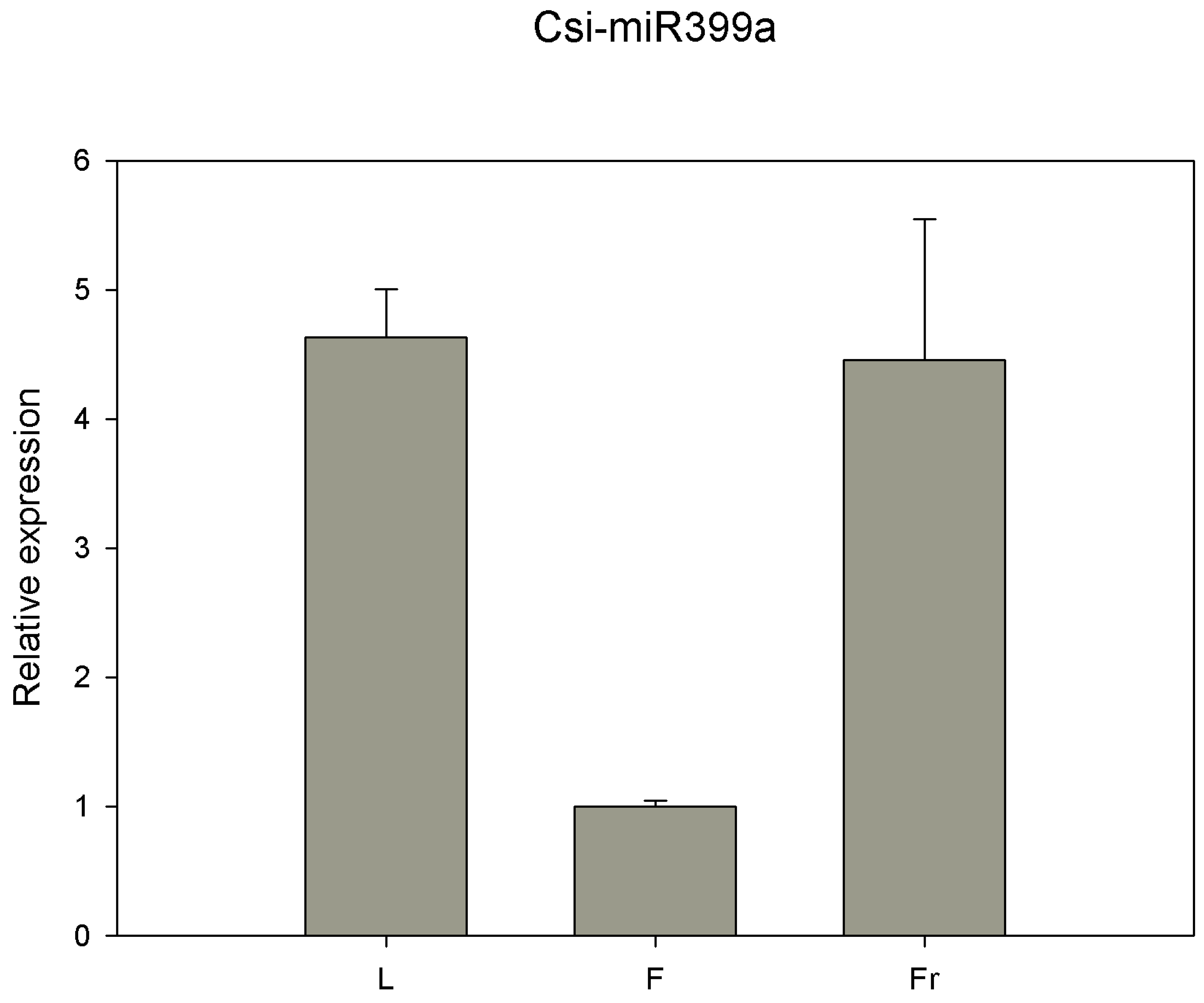
Csi-miR399a
Relative expression
0
1
2
3
4
5
6
L
F
Fr

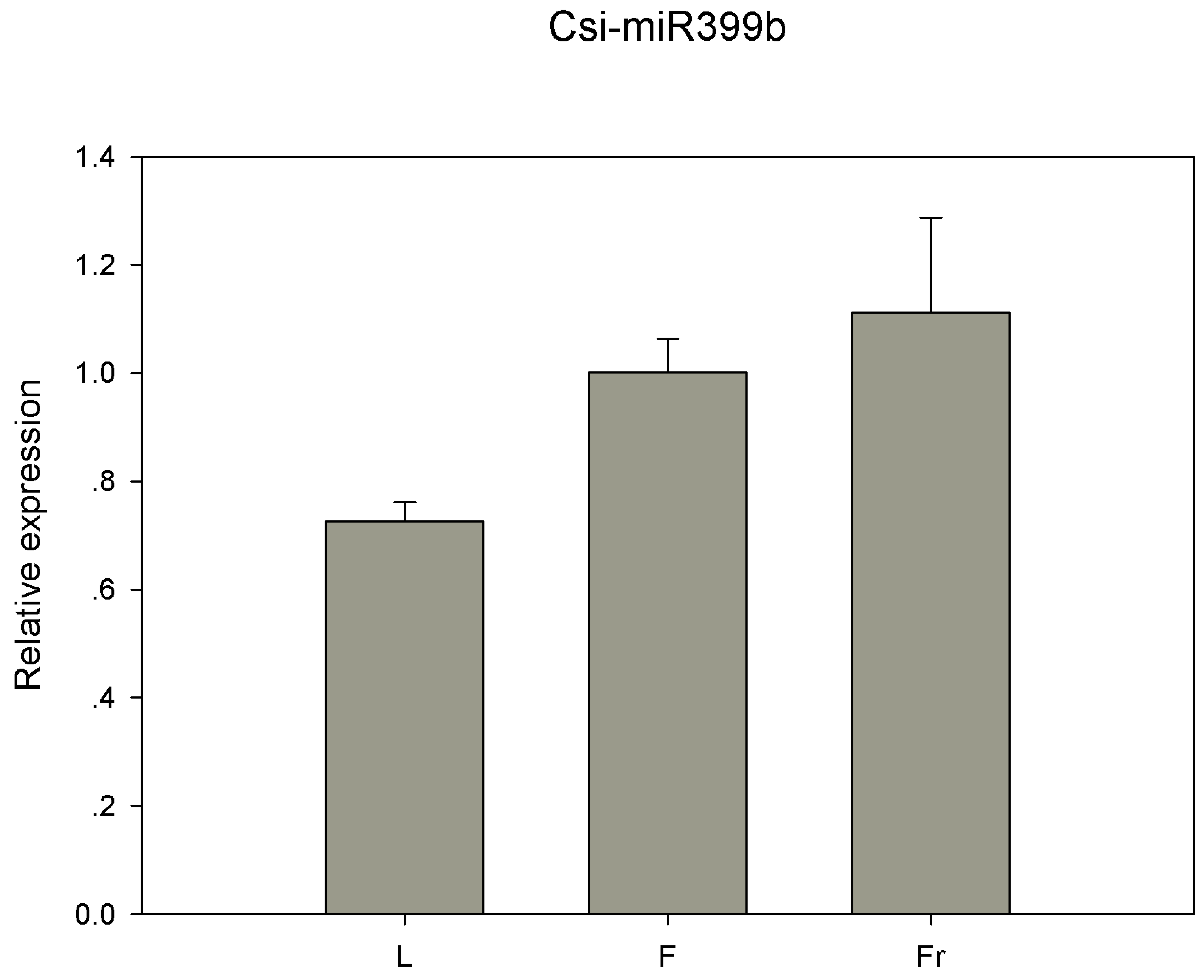

Csi-miR399b
Relative expression
1.4
1.2
1.0
.8
.6
.4
.2
0.0
L
F
Fr

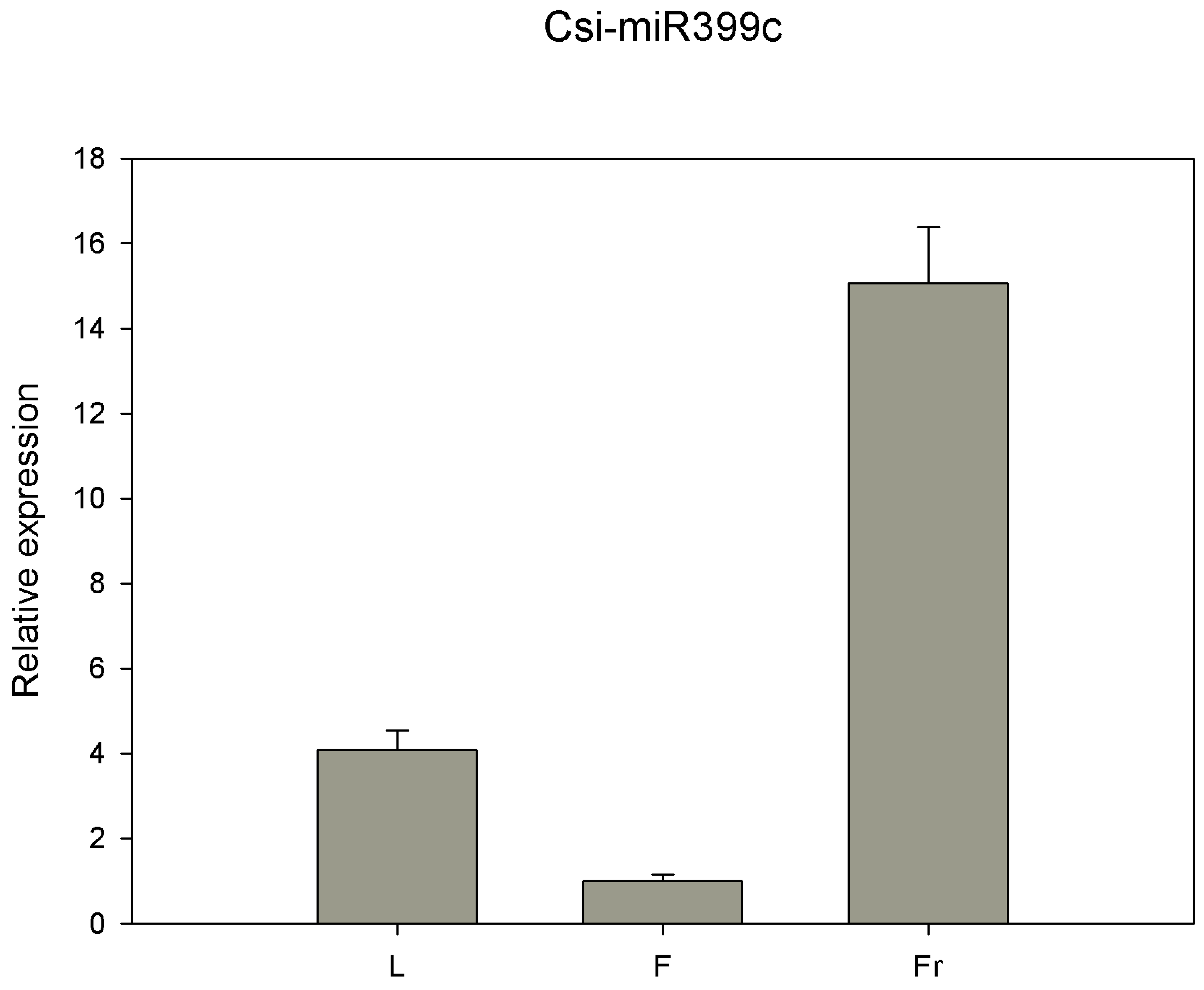

Csi-miR399c
Relative expression
0
2
4
6
8
10
12
14
16
18
L
F
Fr

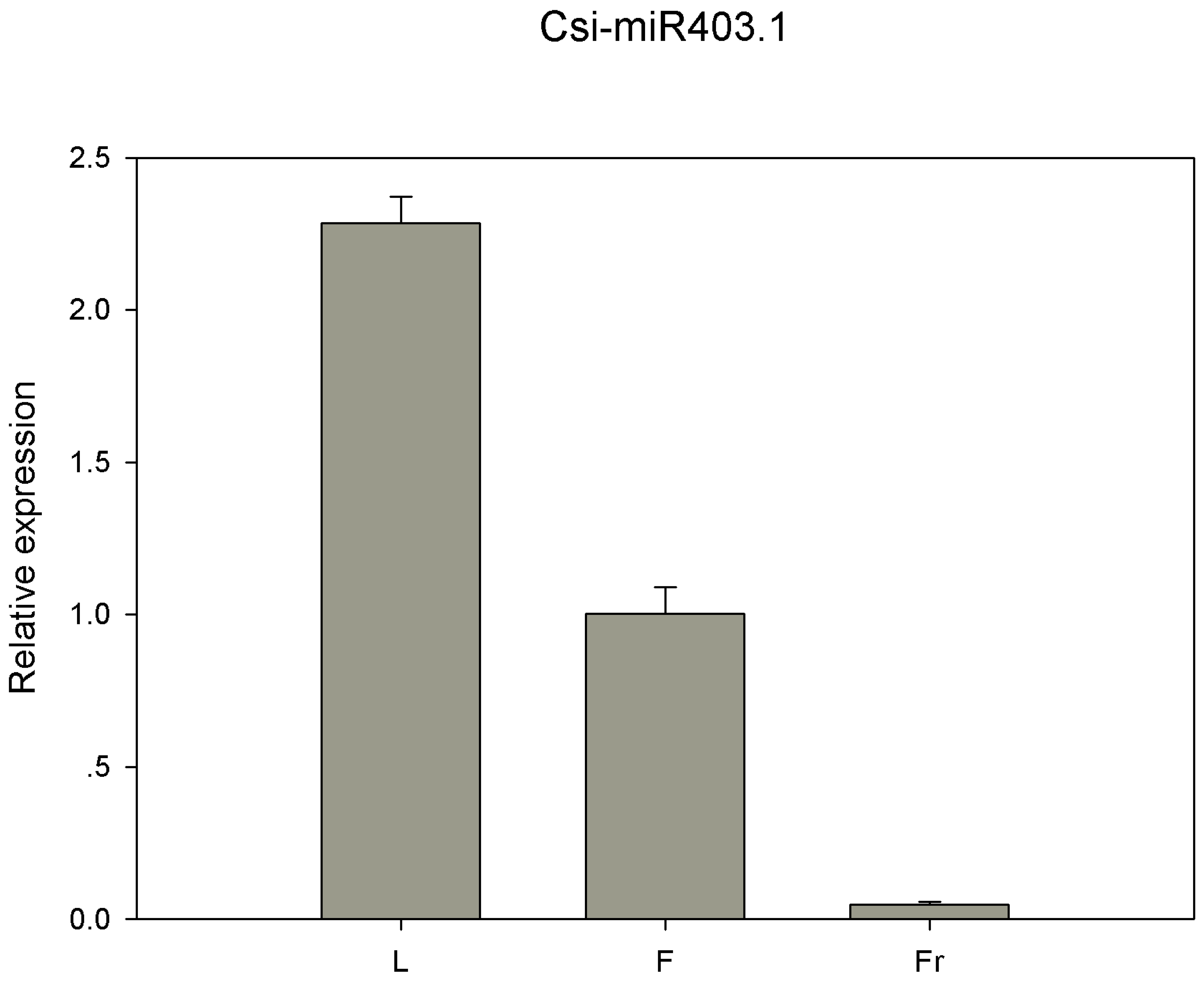
Csi-miR403.1
Relative expression
2.5
2.0
1.5
1.0
.5
0.0
L
F
Fr

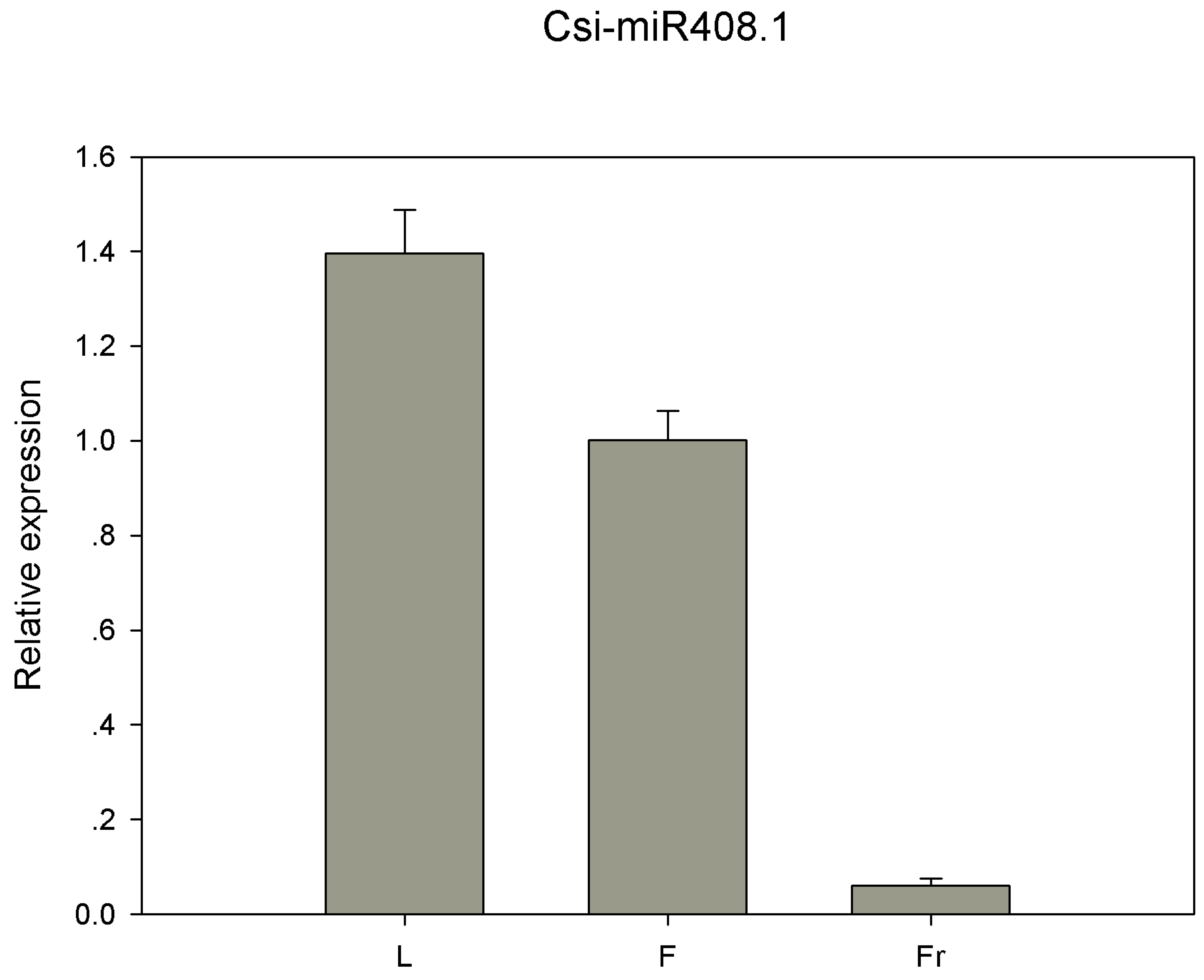

Csi-miR408.1
Relative expression
1.6
1.4
1.2
1.0
.8
.6
.4
.2
0.0
L
F
Fr

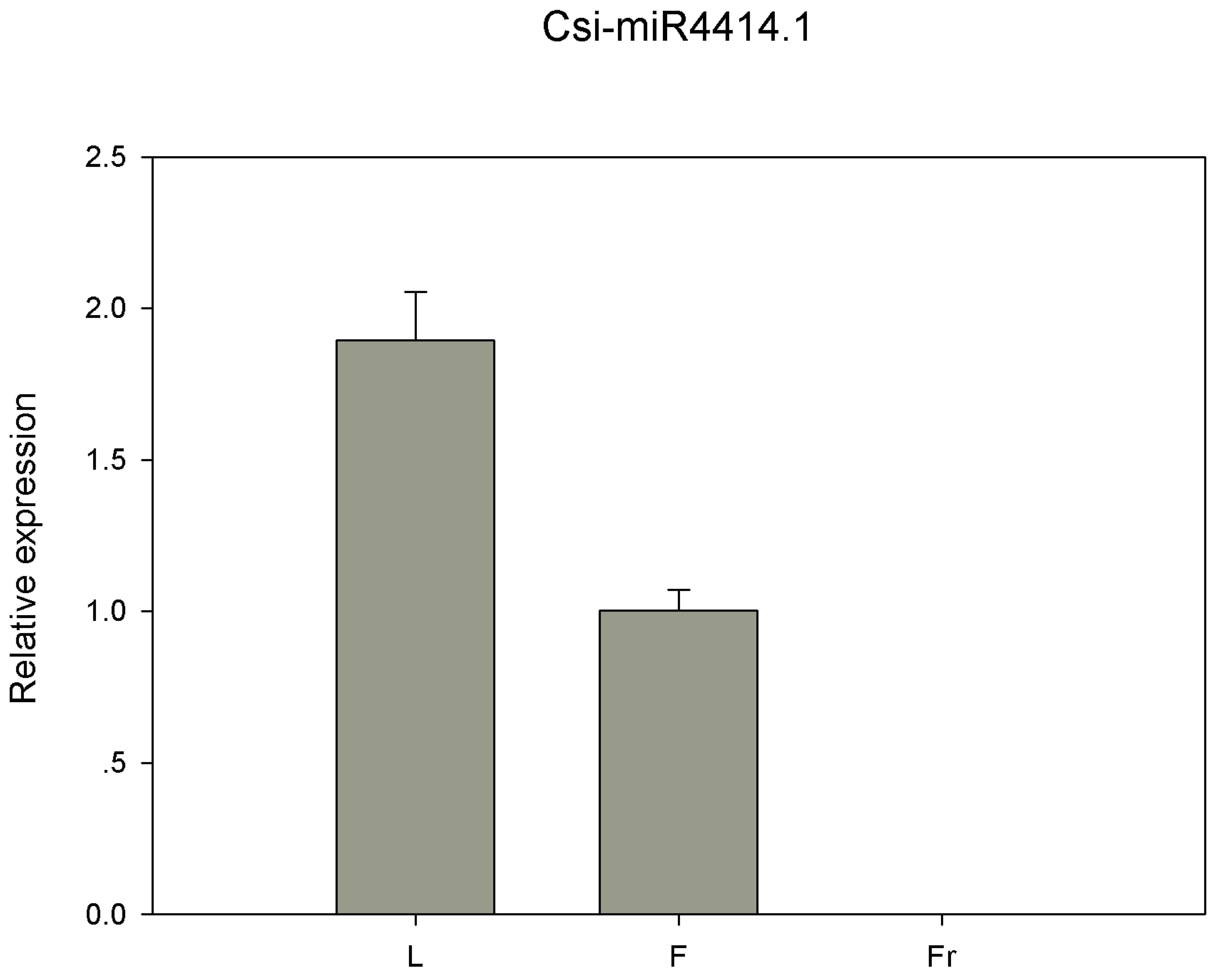
Csi-miR4414.1
Relative expression
2.5
2.0
1.5
1.0
.5
0.0
L
F
Fr

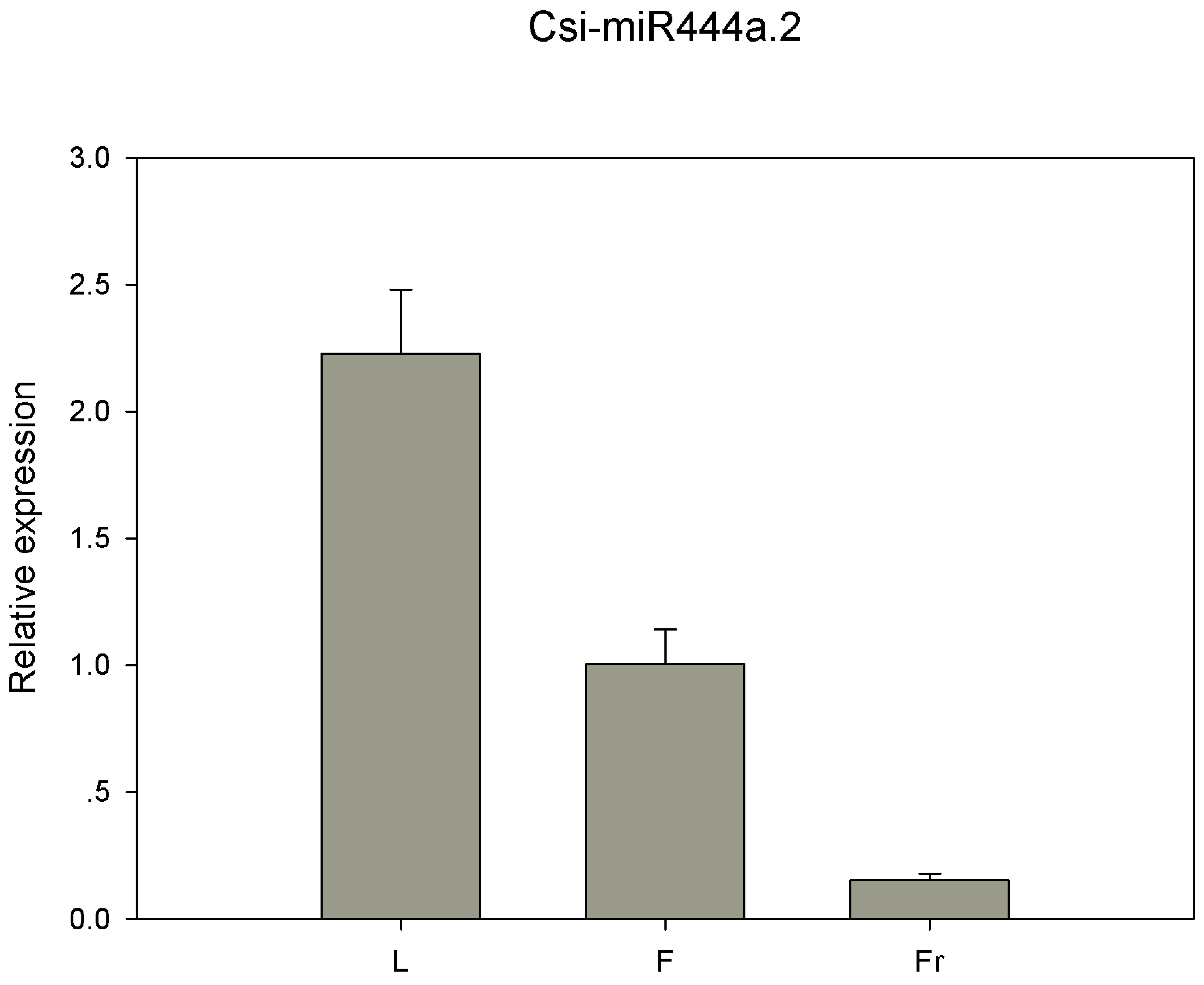
Csi-miR444a.2
Relative expression
3.0
2.5
2.0
1.5
1.0
.5
0.0
L
F
Fr

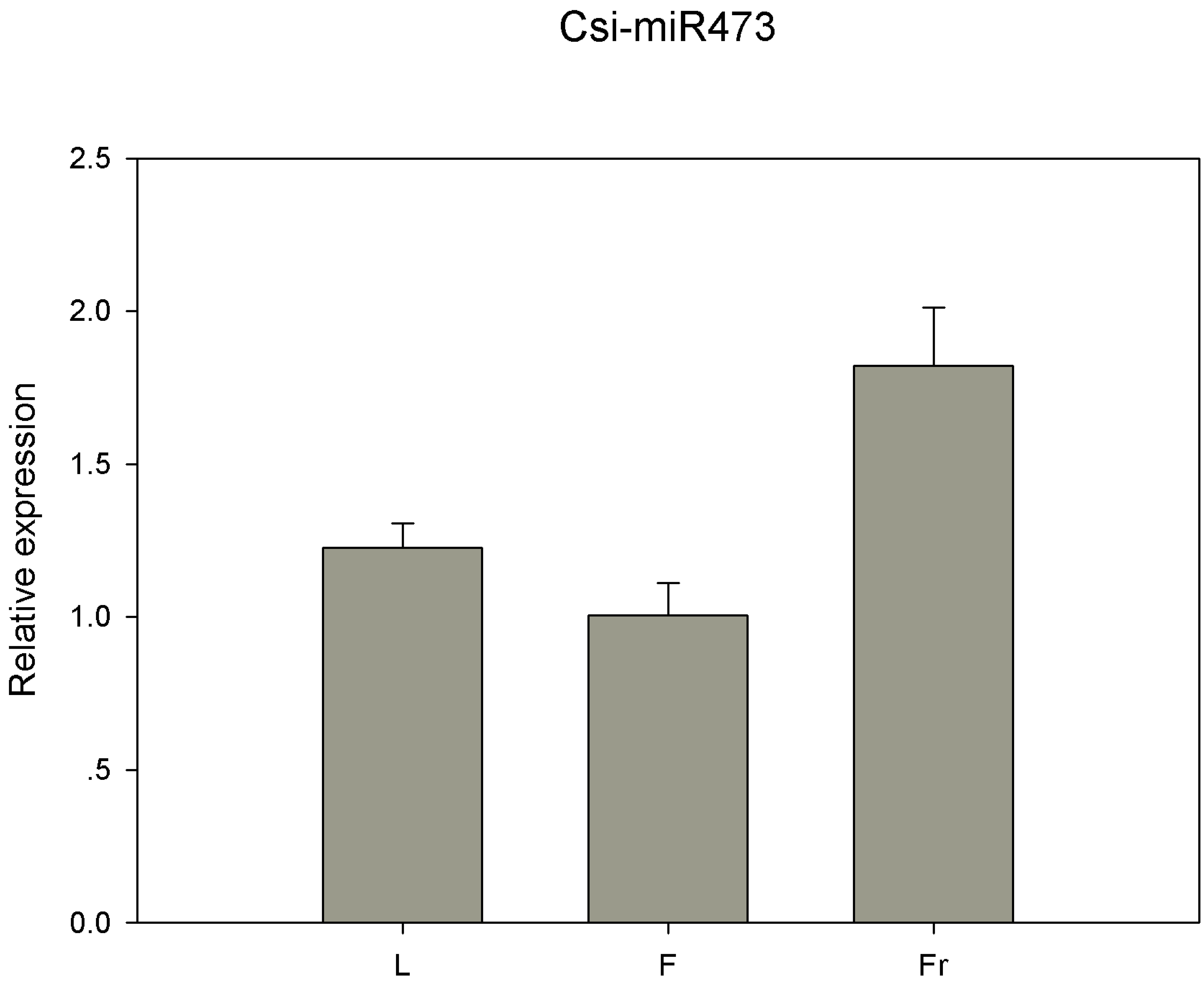

Csi-miR473
Relative expression
2.5
2.0
1.5
1.0
.5
0.0
L
F
Fr

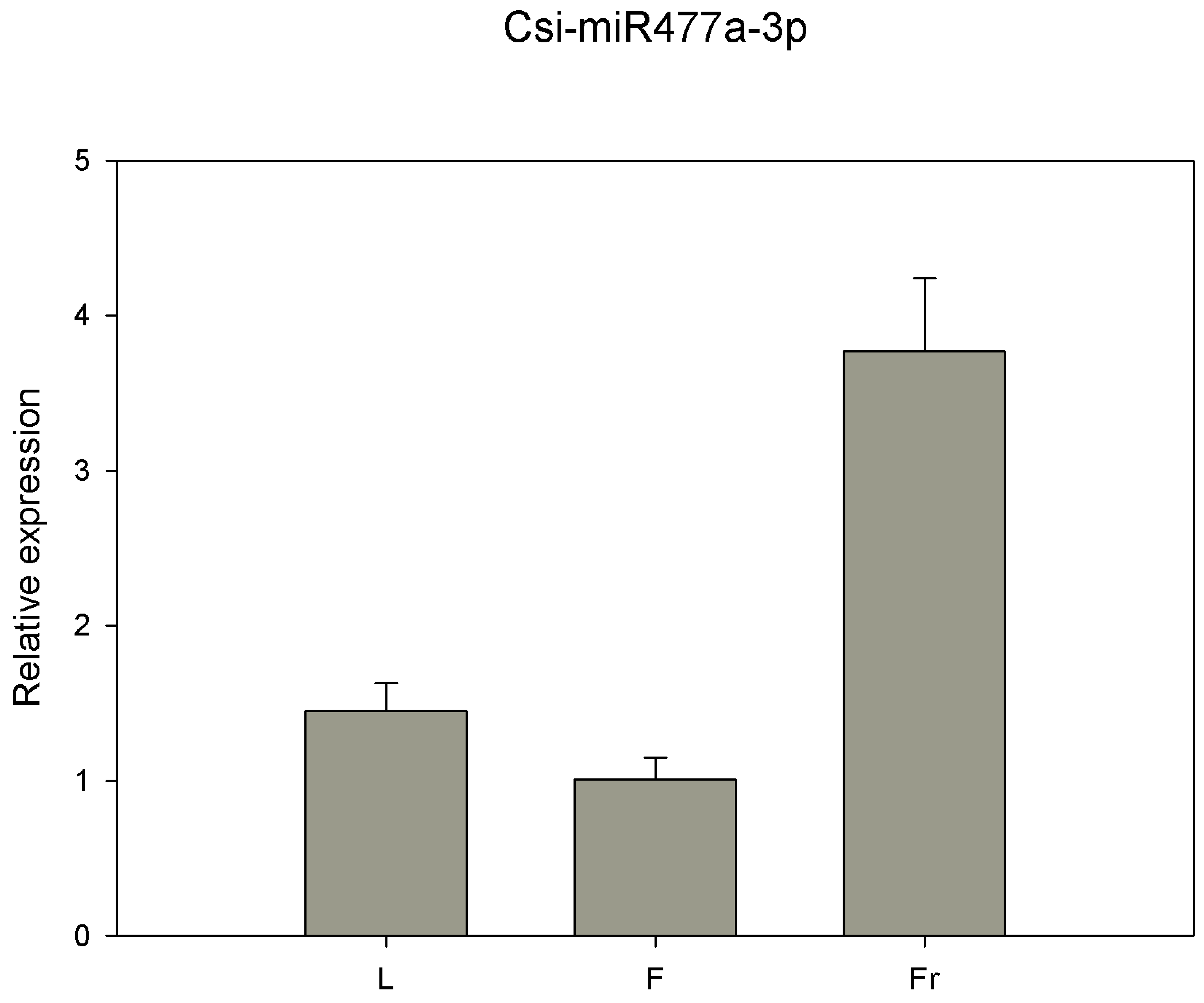
Csi-miR477a-3p
Relative expression
0
1
2
3
4
5
L
F
Fr

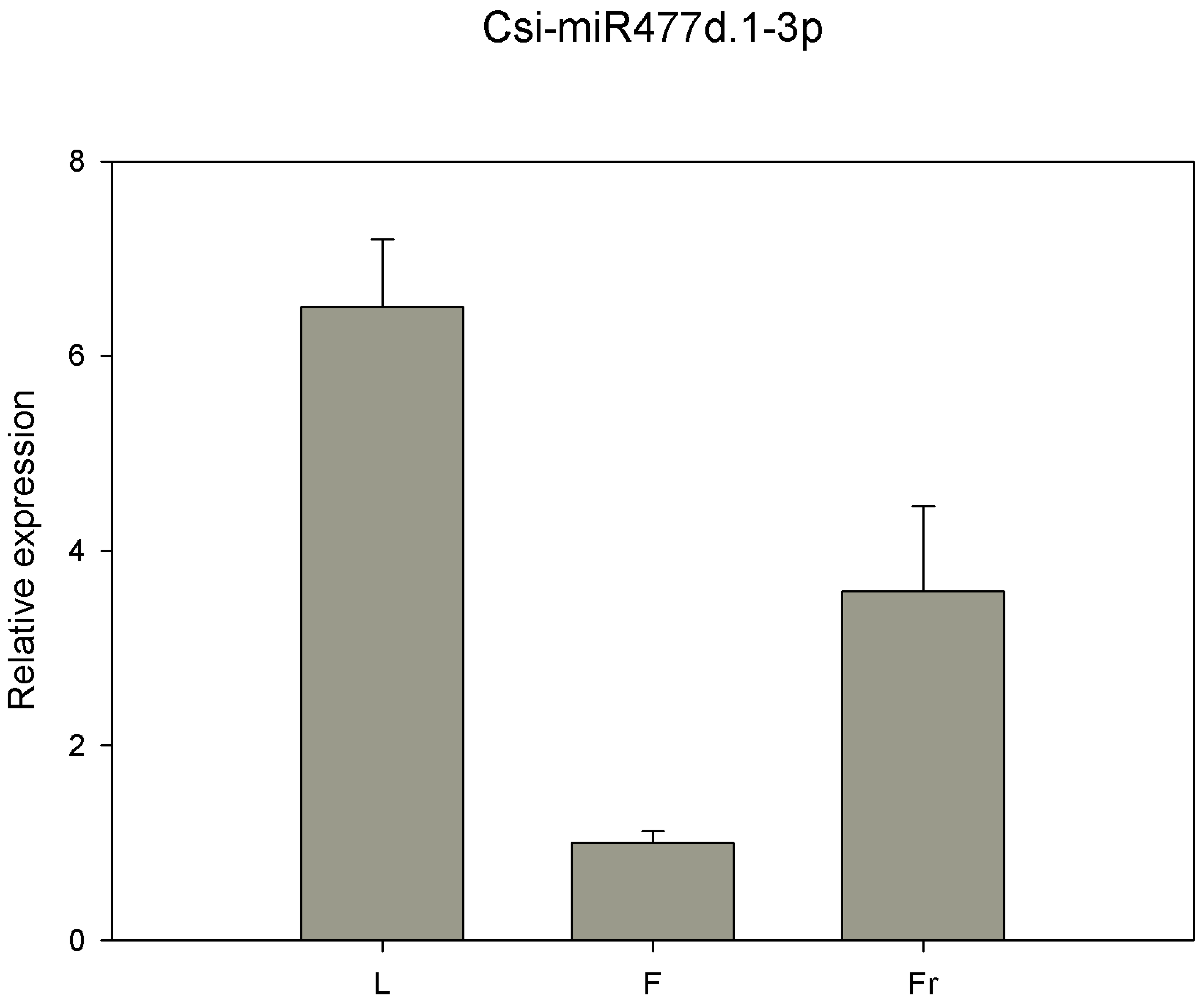
Csi-miR477d.1-3p
8
6
4
2
0
Relative expression
L
F
Fr

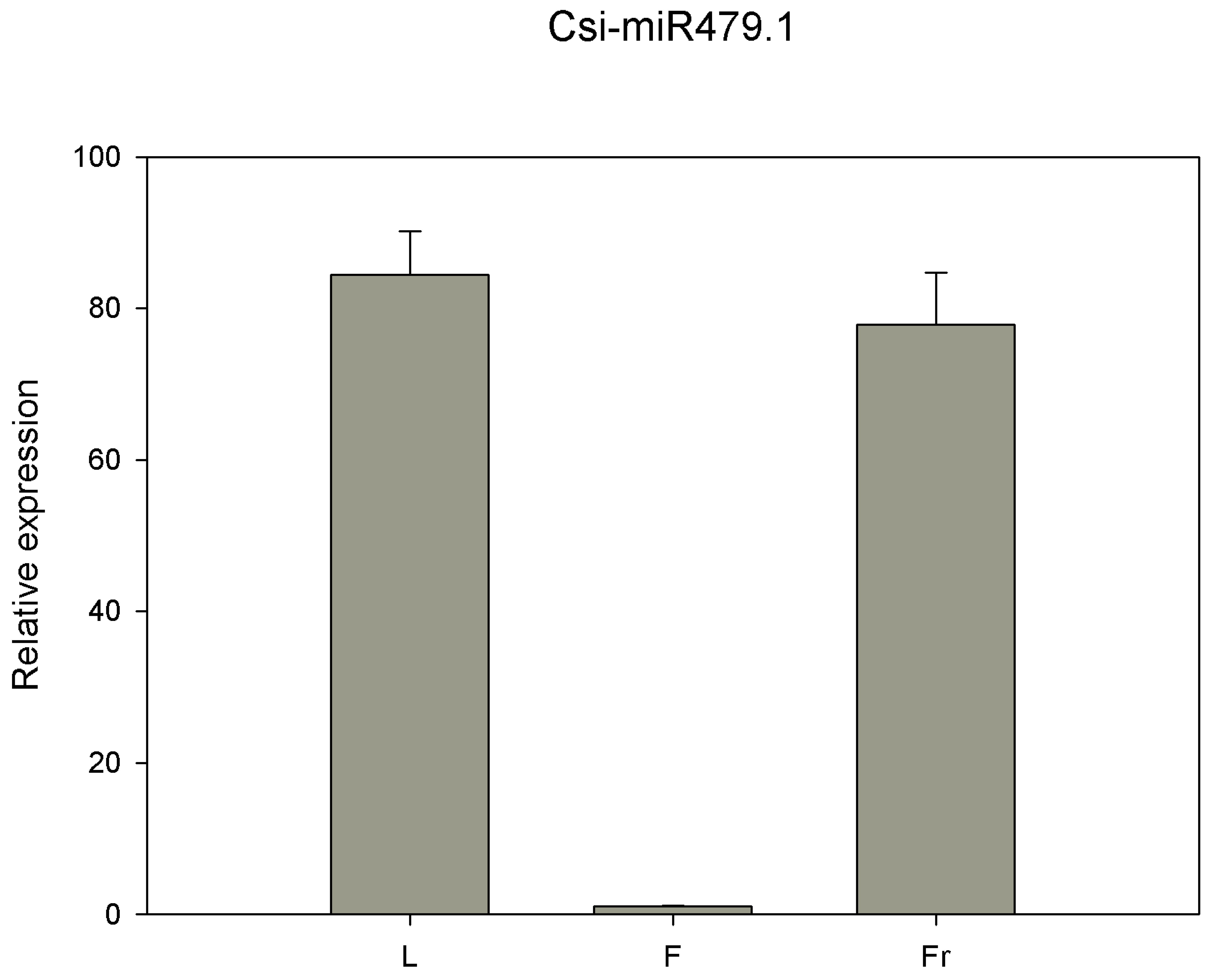

Csi-miR479.1
Relative expression
0
20
40
60
80
100
L
F
Fr

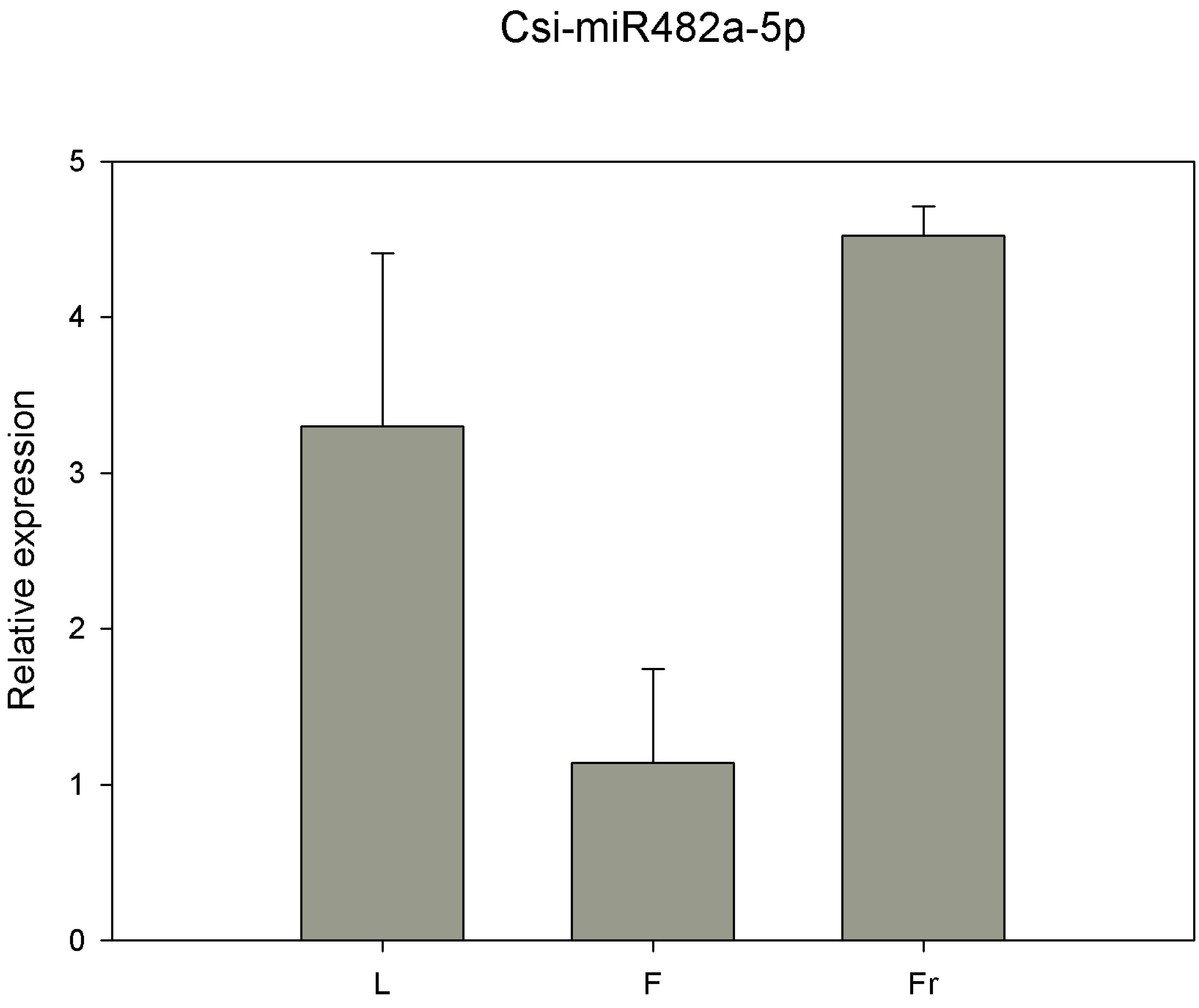

Csi-miR482a-5p
Relative expression
0
1
2
3
4
5
L
F
Fr

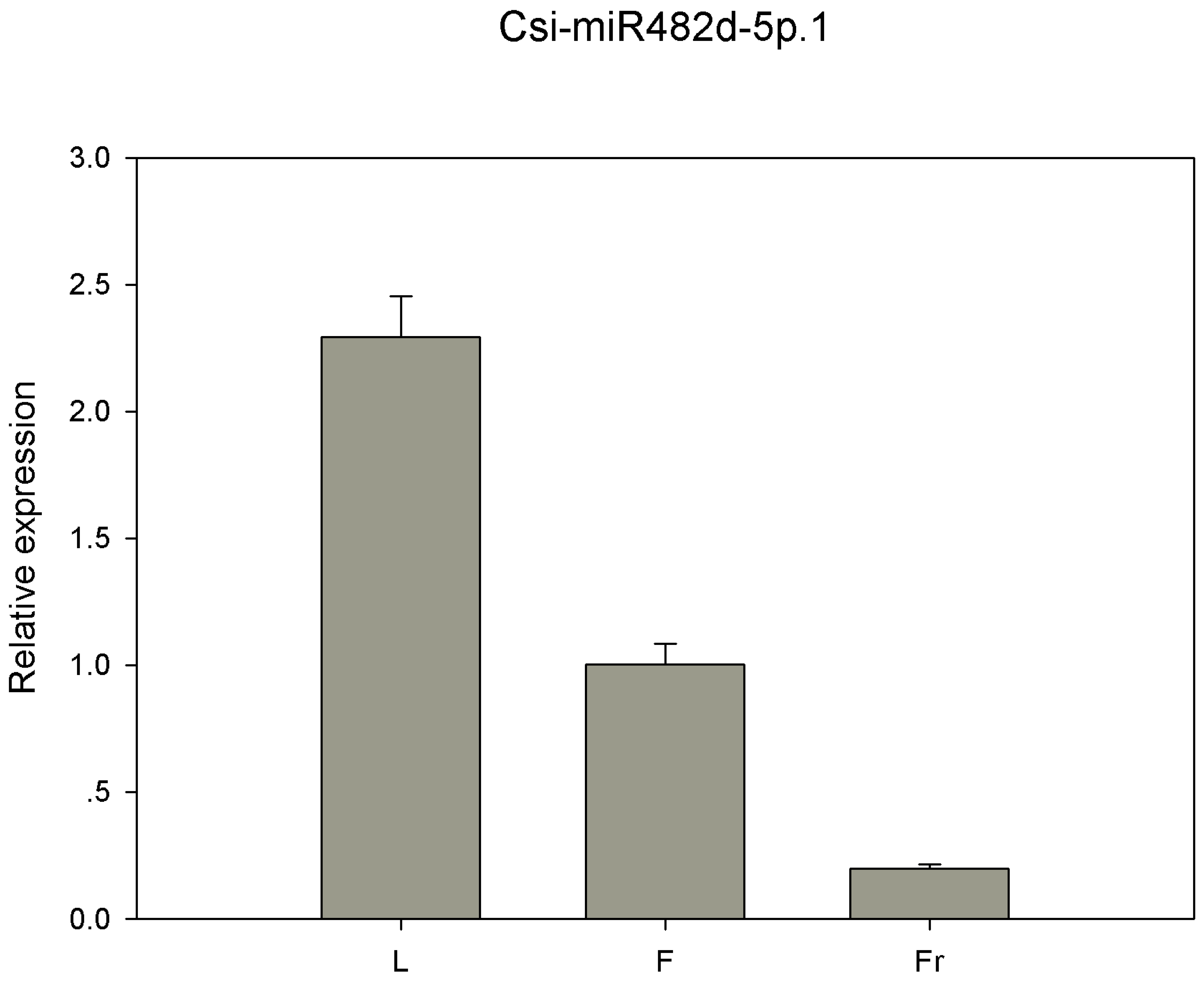
Csi-miR482d-5p.1
Relative expression
3.0
2.5
2.0
1.5
1.0
.5
0.0
L
F
Fr

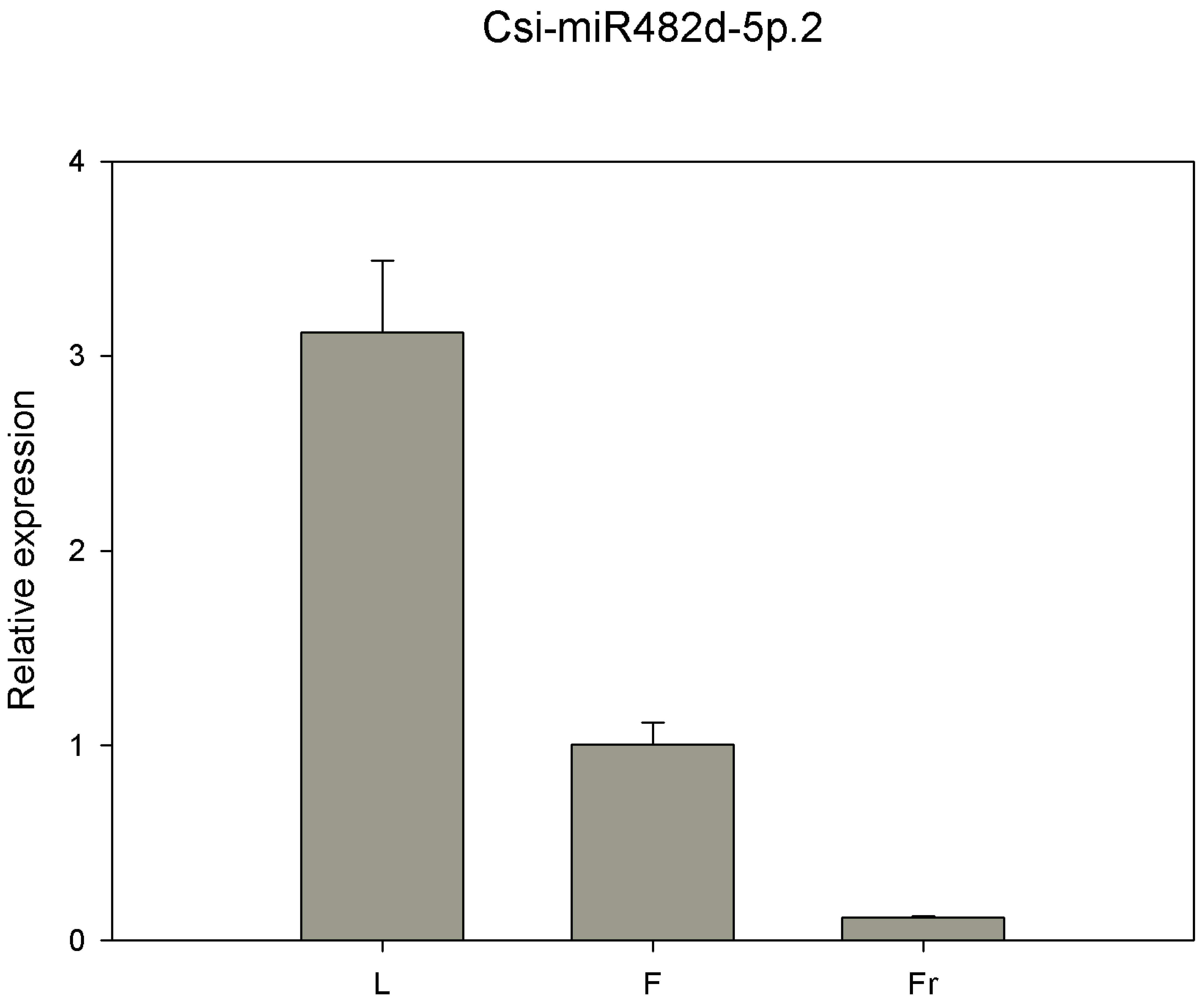
Csi-miR482d-5p.2
Relative expression
0
1
2
3
4
L
F
Fr

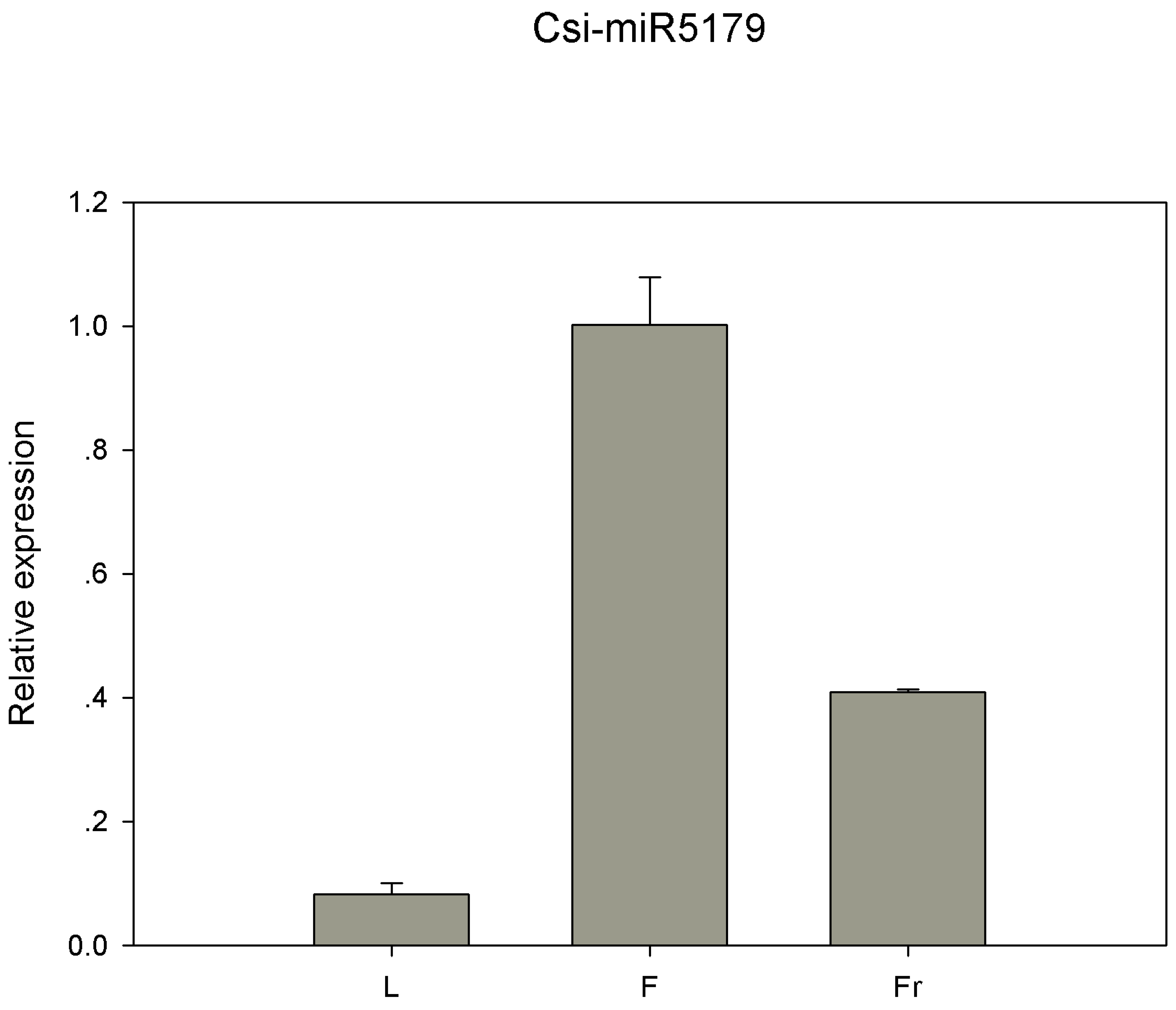

Csi-miR5179
Relative expression
1.2
1.0
.8
.6
.4
.2
0.0
L
F
Fr

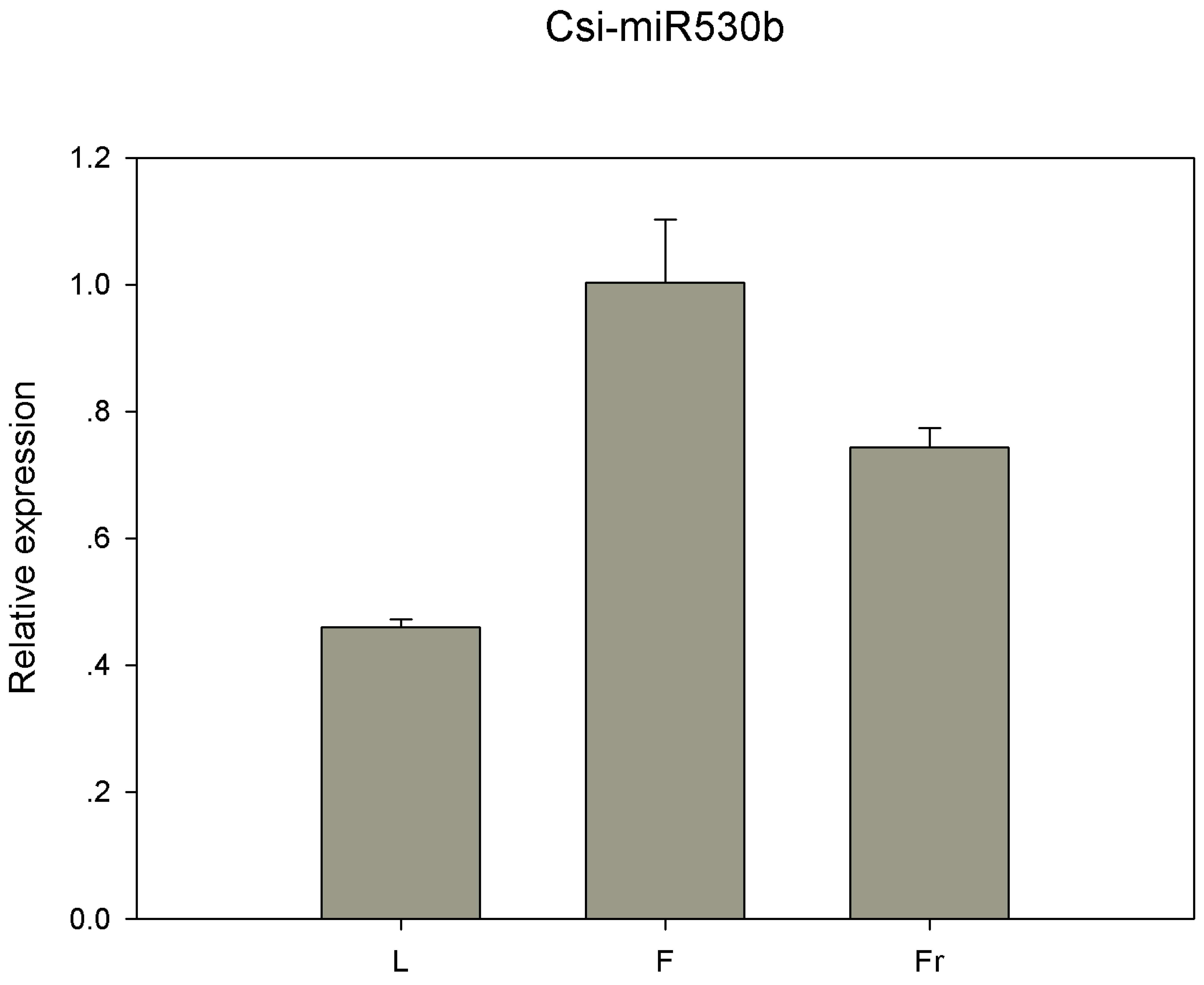

Csi-miR530b
Relative expression
1.2
1.0
.8
.6
.4
.2
0.0
L
F
Fr

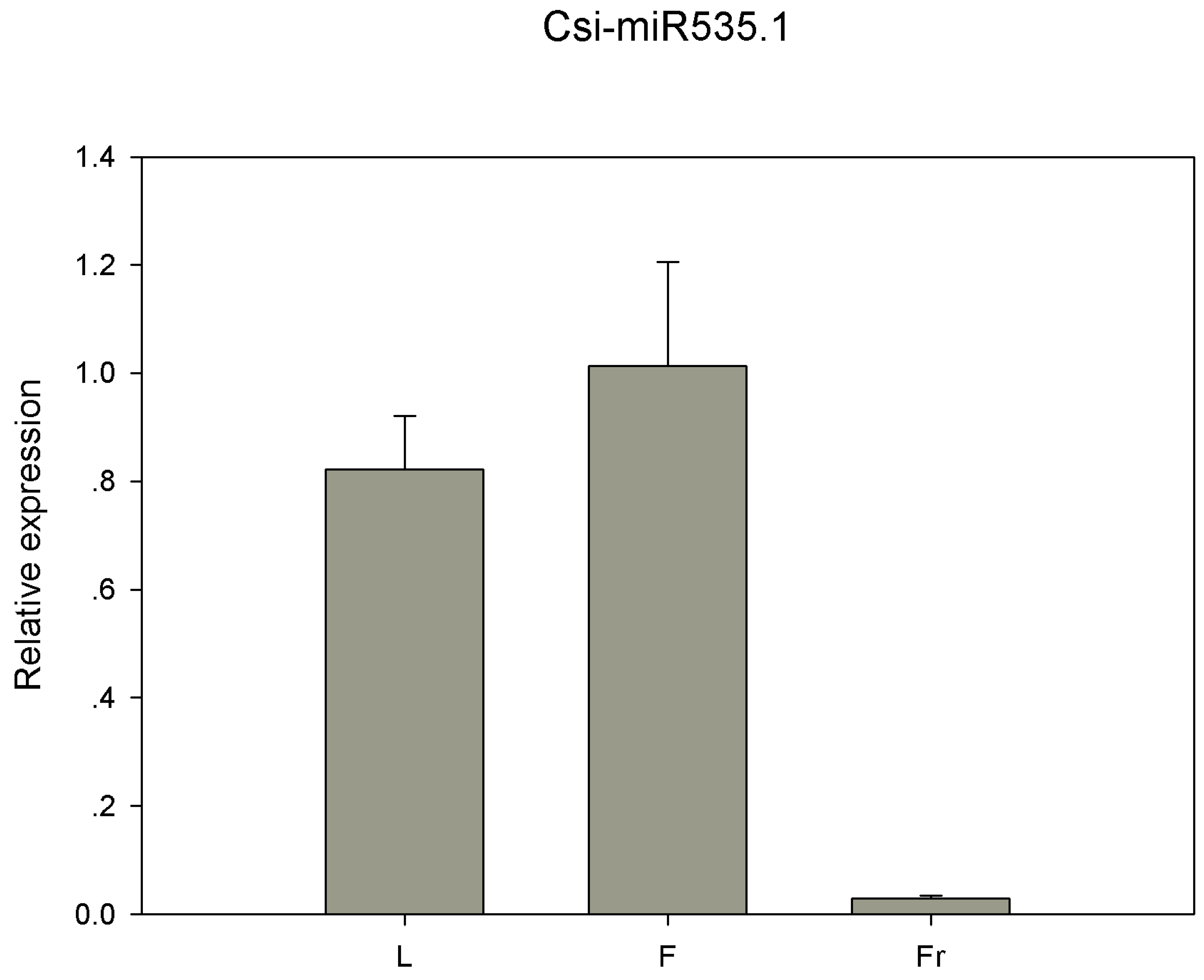
Csi-miR535.1
Relative expression
1.4
1.2
1.0
.8
.6
.4
.2
0.0
L
F
Fr

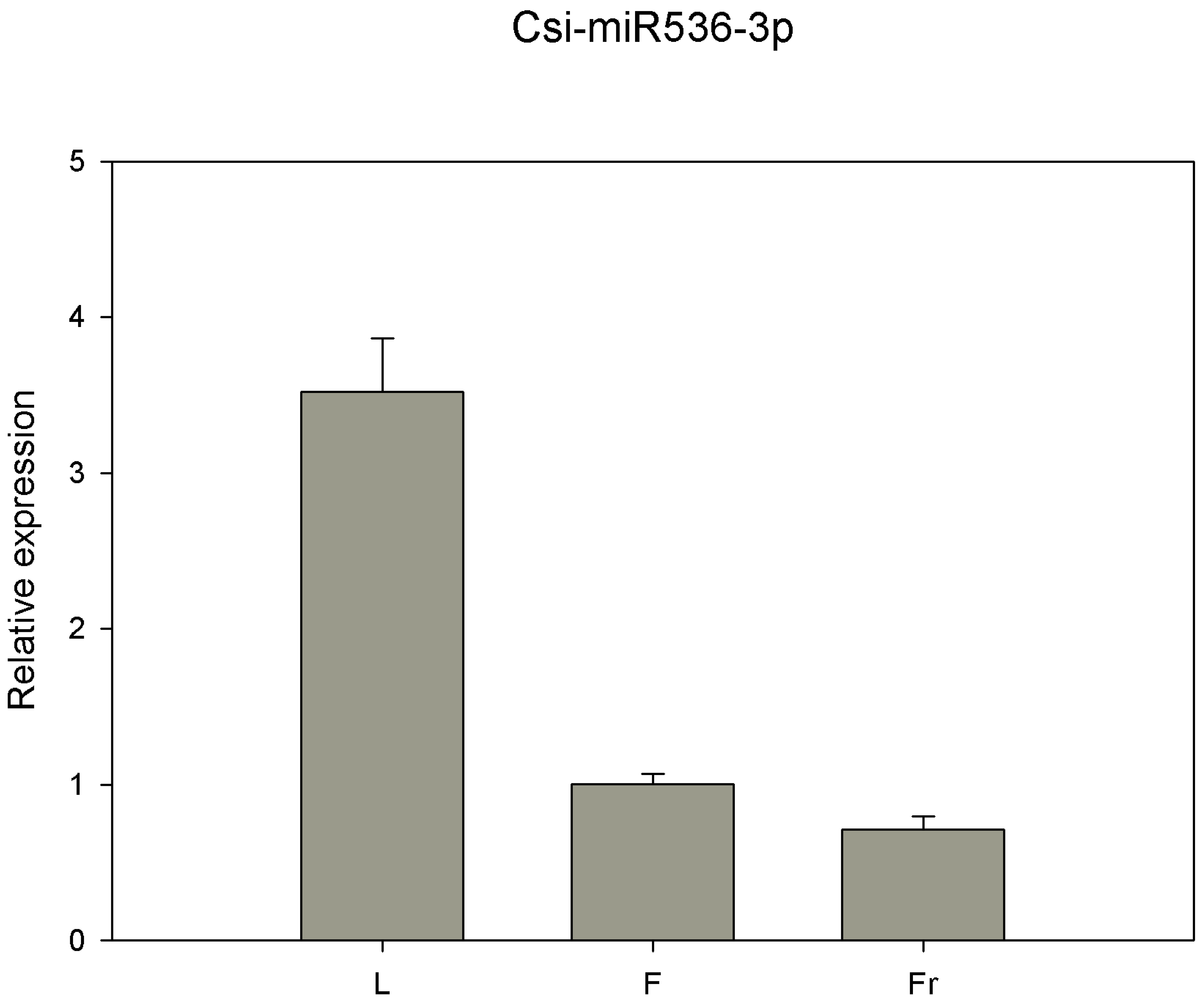
Csi-miR536-3p
Relative expression
0
1
2
3
4
5
L
F
Fr

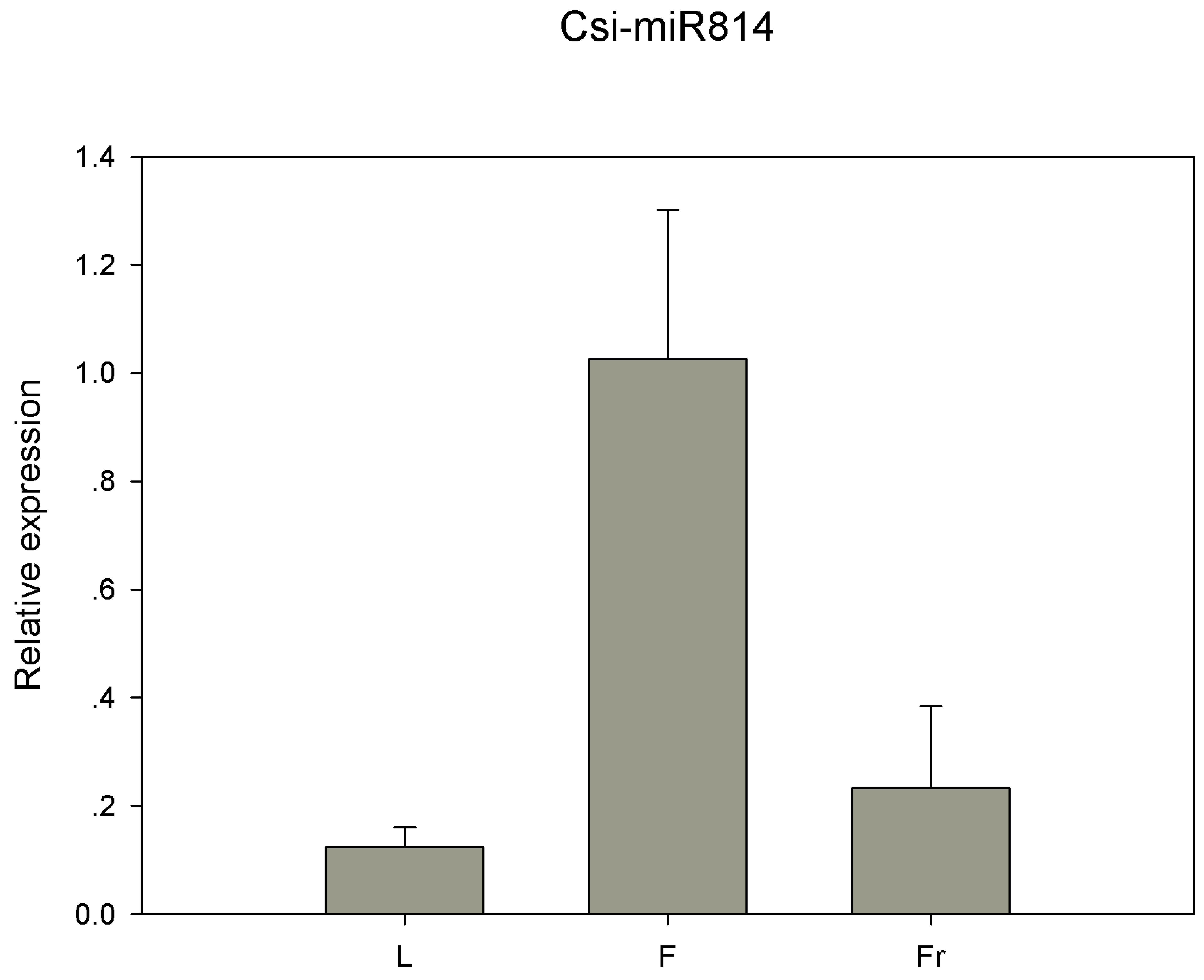
Csi-miR814
Relative expression
1.4
1.2
1.0
.8
.6
.4
.2
0.0
L
F
Fr

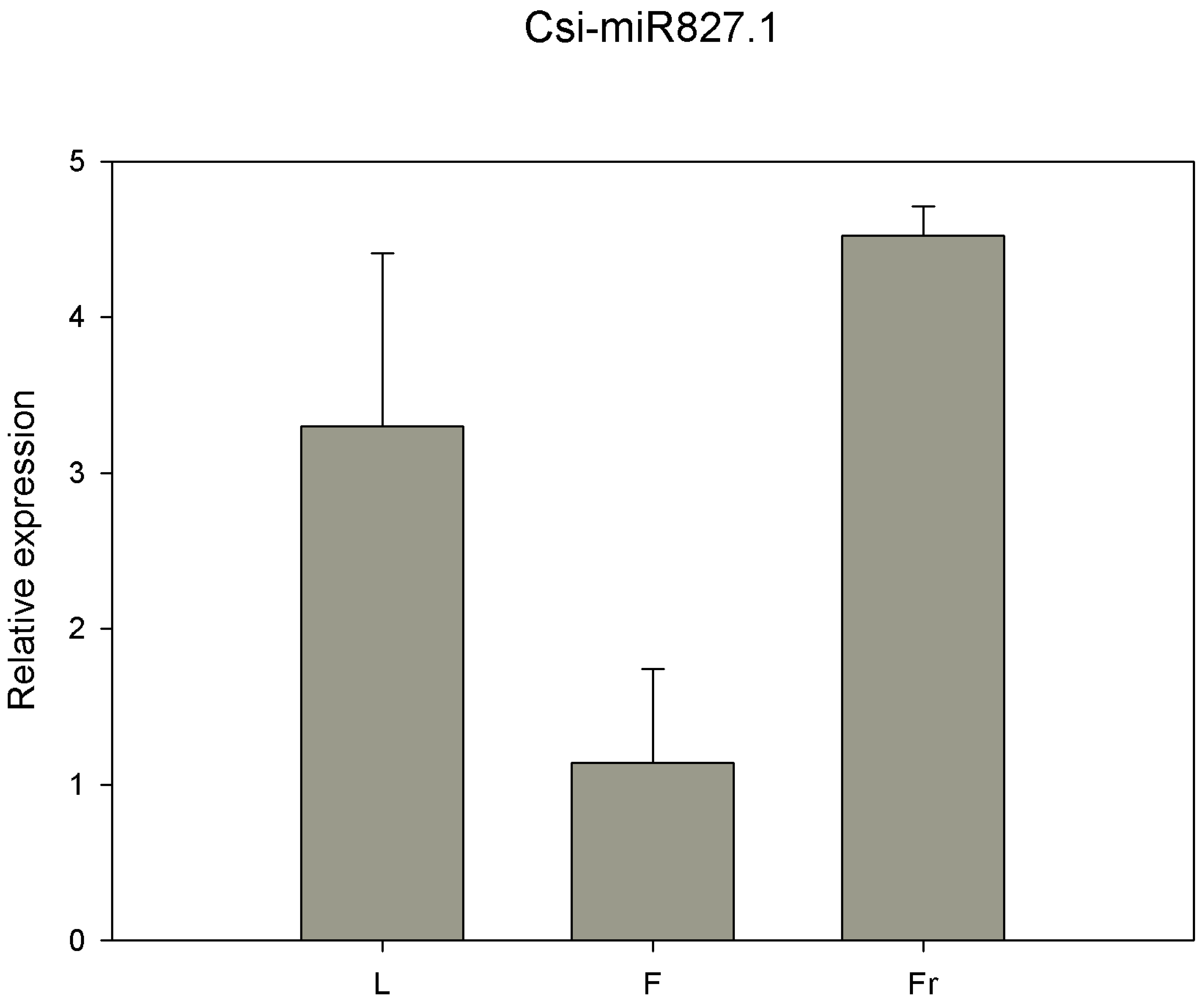
Csi-miR827.1
Relative expression
0
1
2
3
4
5
L
F
Fr

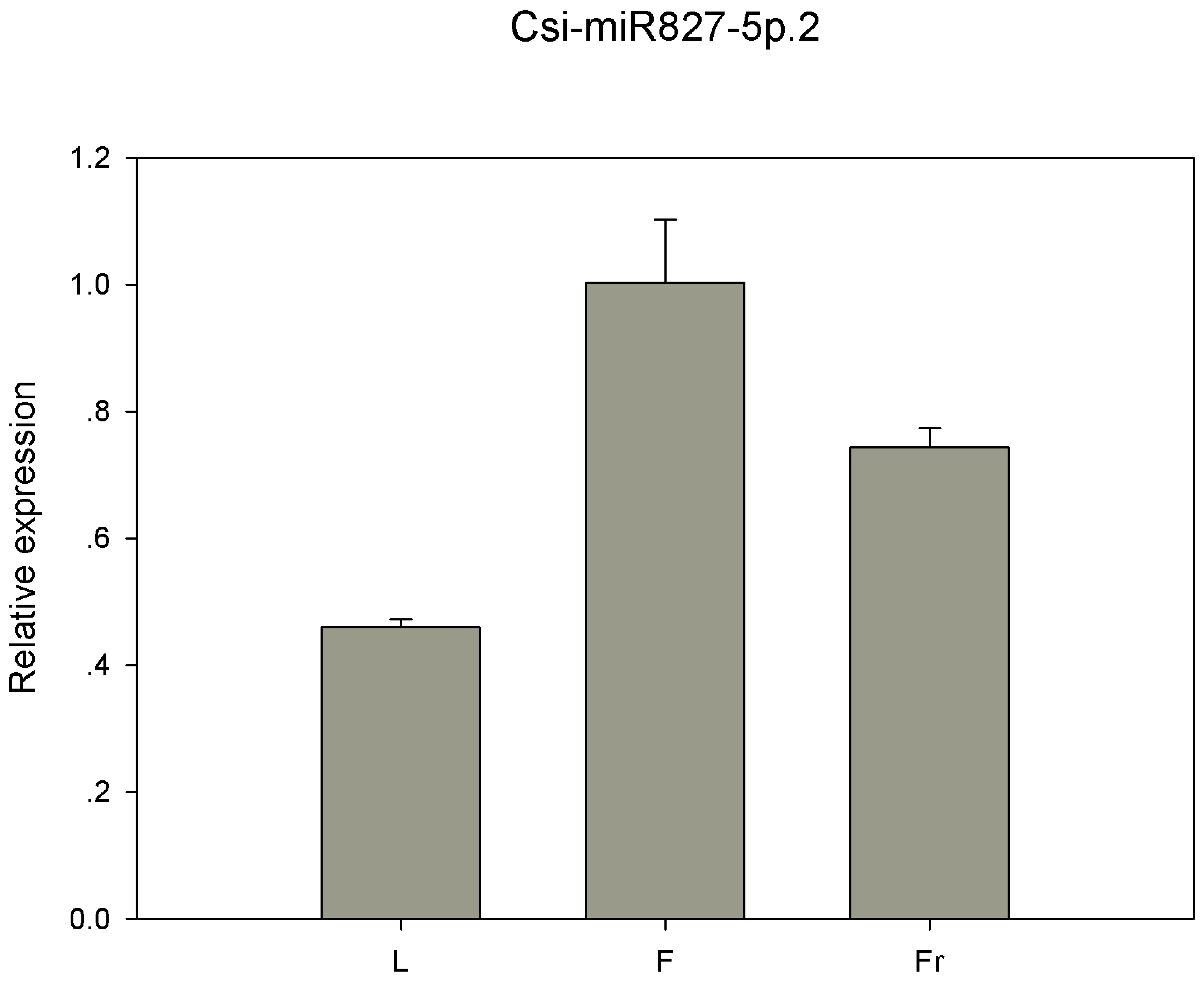

Csi-miR827-5p.2
Relative expression
1.2
1.0
.8
.6
.4
.2
0.0
L
F
Fr

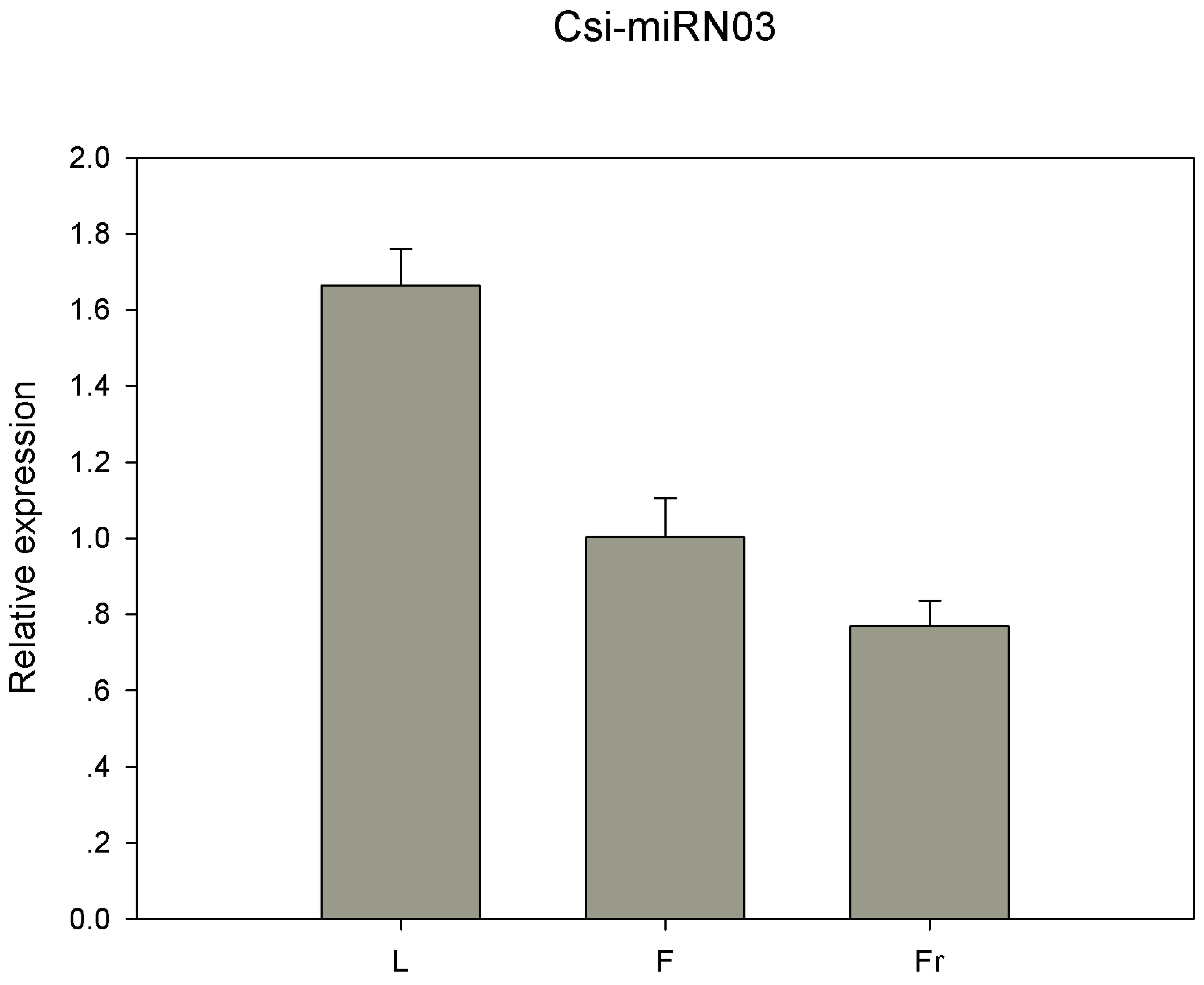
Csi-miRN03
Relative expression
2.0
1.8
1.6
1.4
1.2
1.0
.8
.6
.4
.2
0.0
L
F
Fr

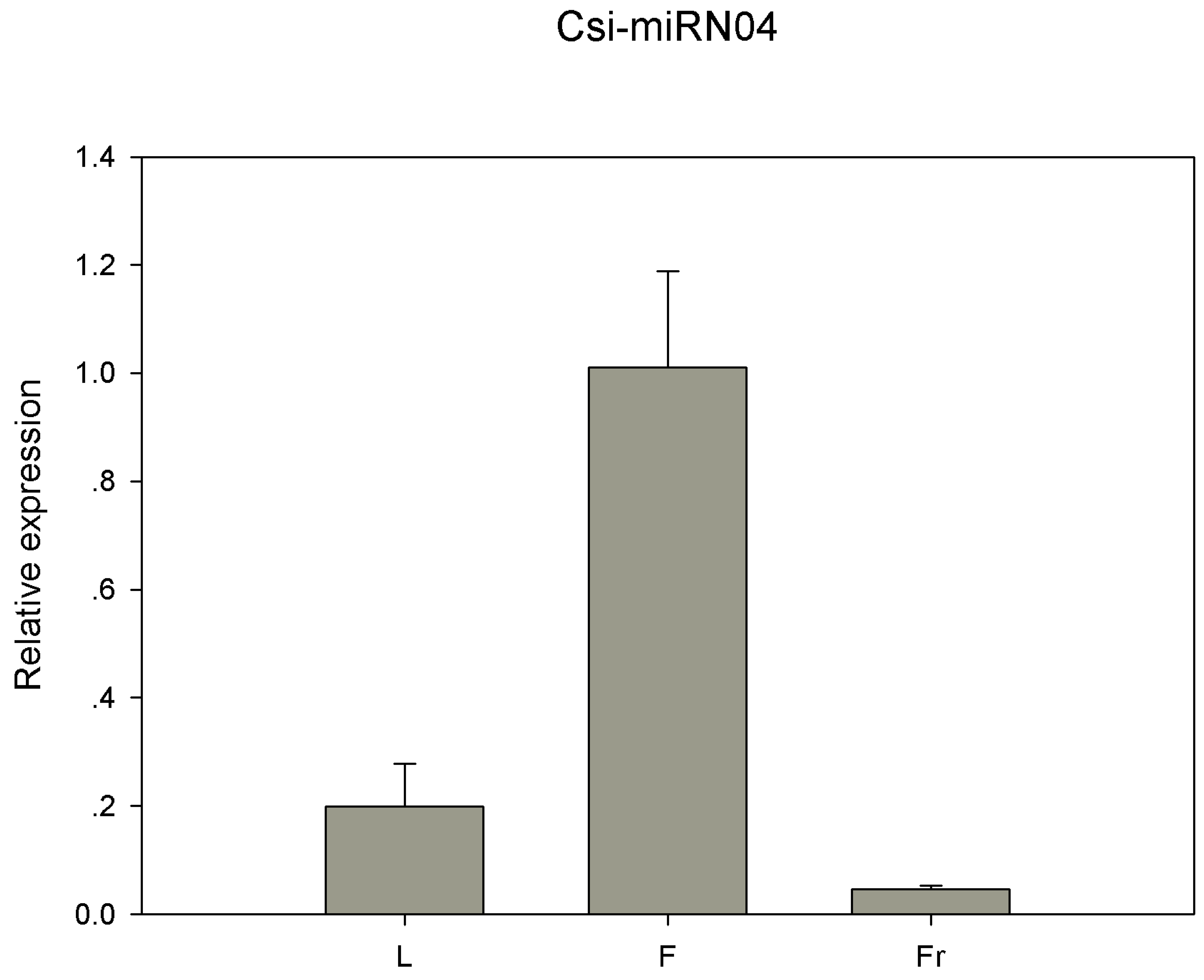
Csi-miRN04
Relative expression
1.4
1.2
1.0
.8
.6
.4
.2
0.0
L
F
Fr

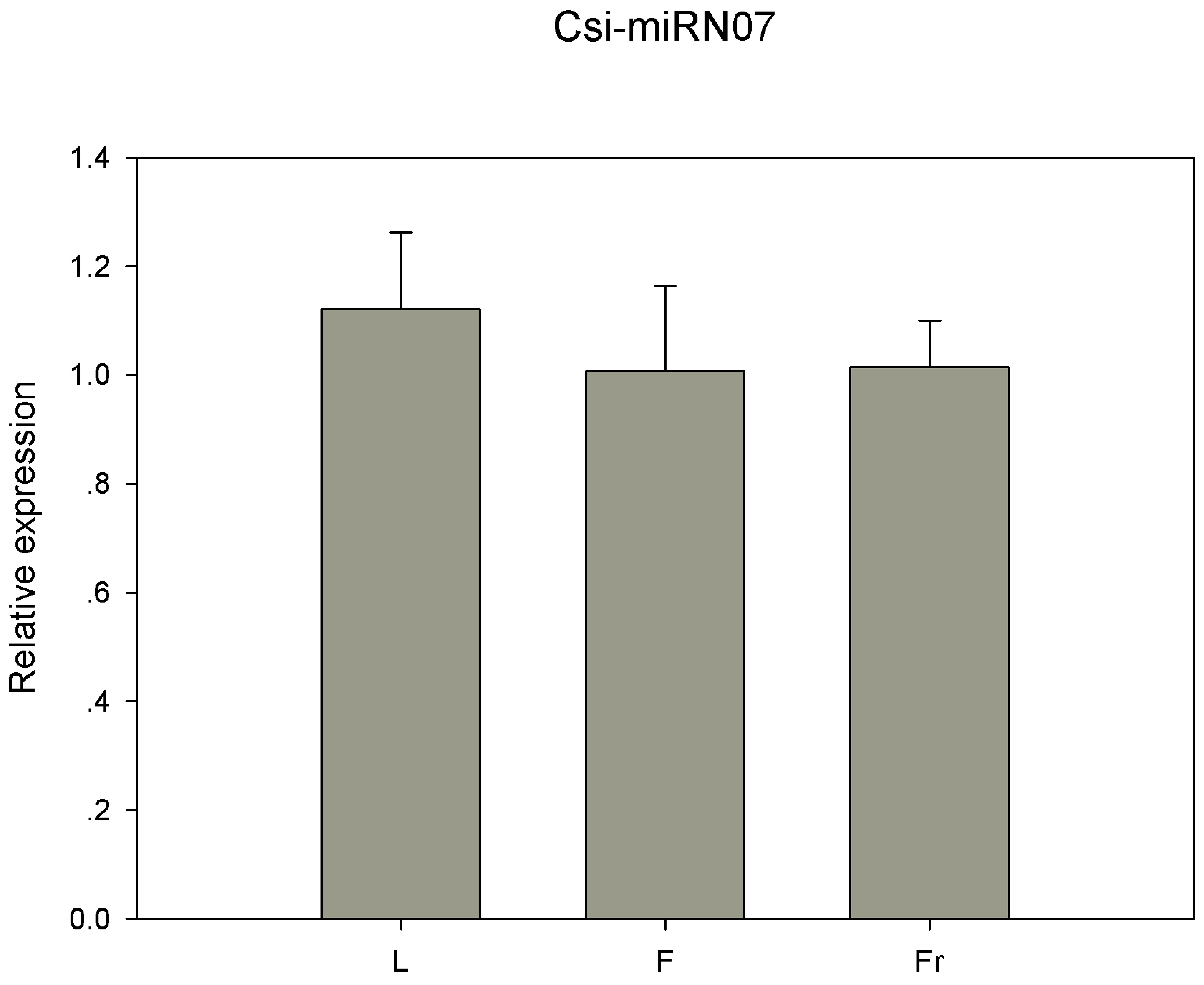

Csi-miRN07
Relative expression
1.4
1.2
1.0
.8
.6
.4
.2
0.0
L
F
Fr

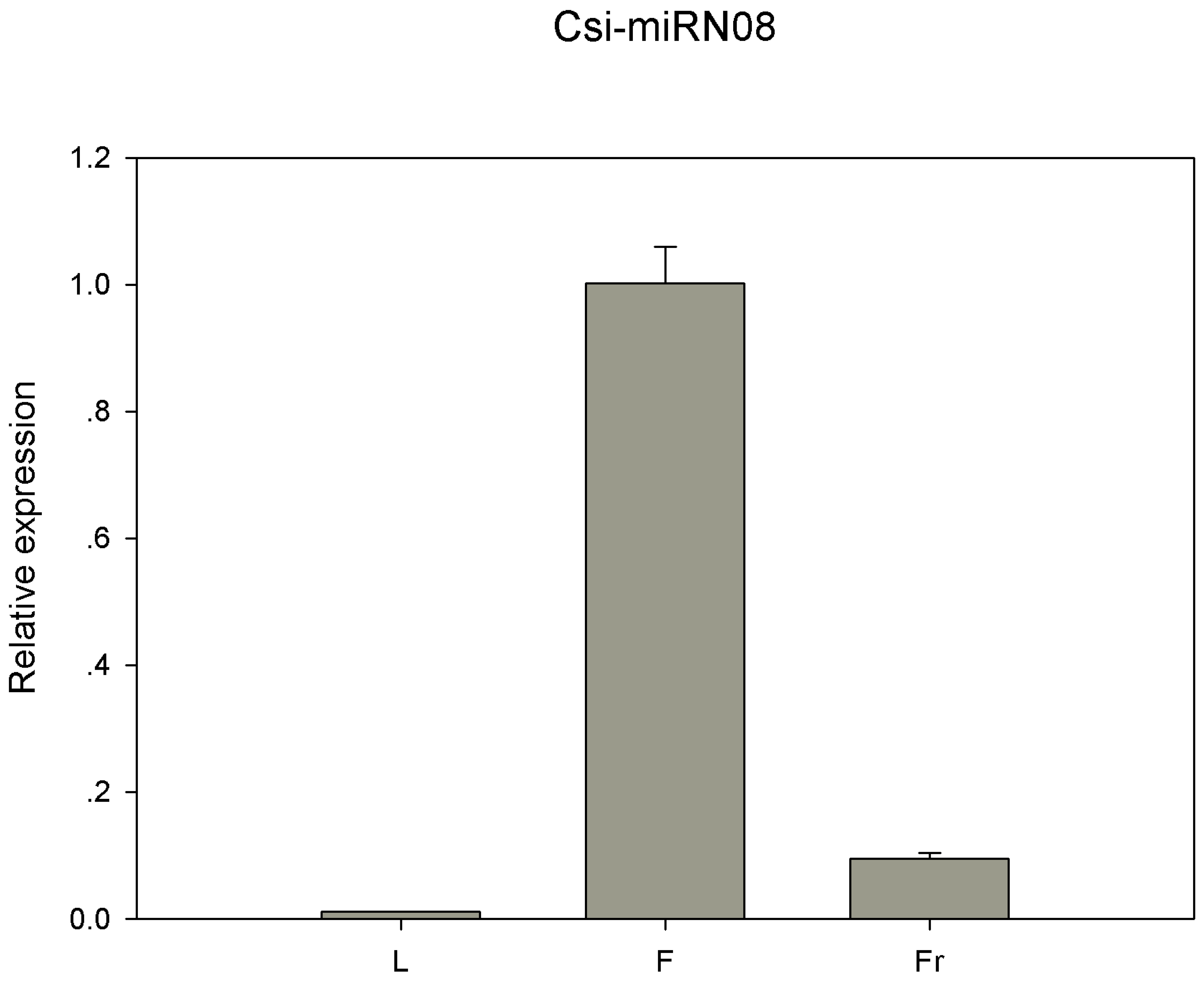
Csi-miRN08
Relative expression
1.2
1.0
.8
.6
.4
.2
0.0
L
F
Fr

Csi-miRN11

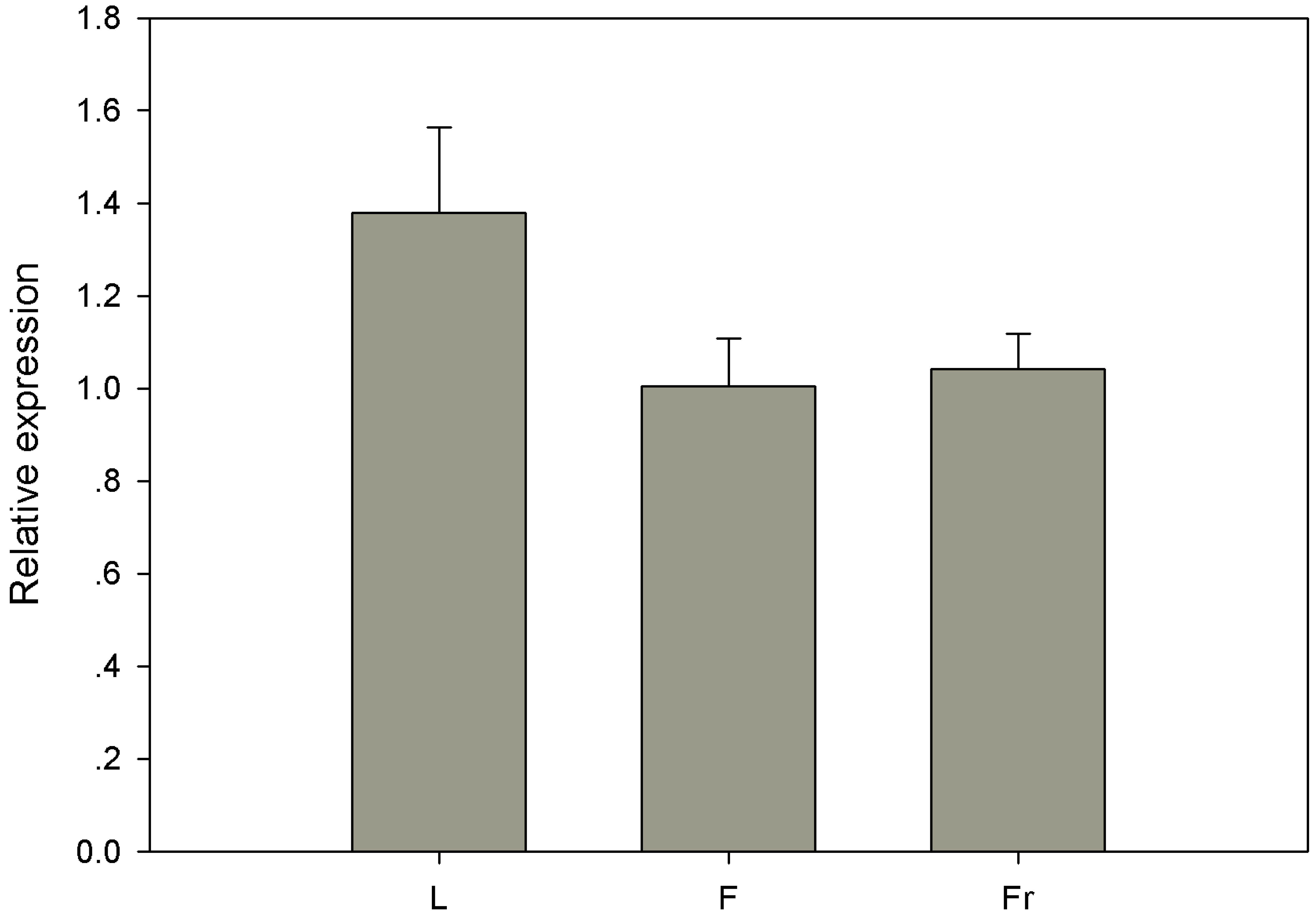

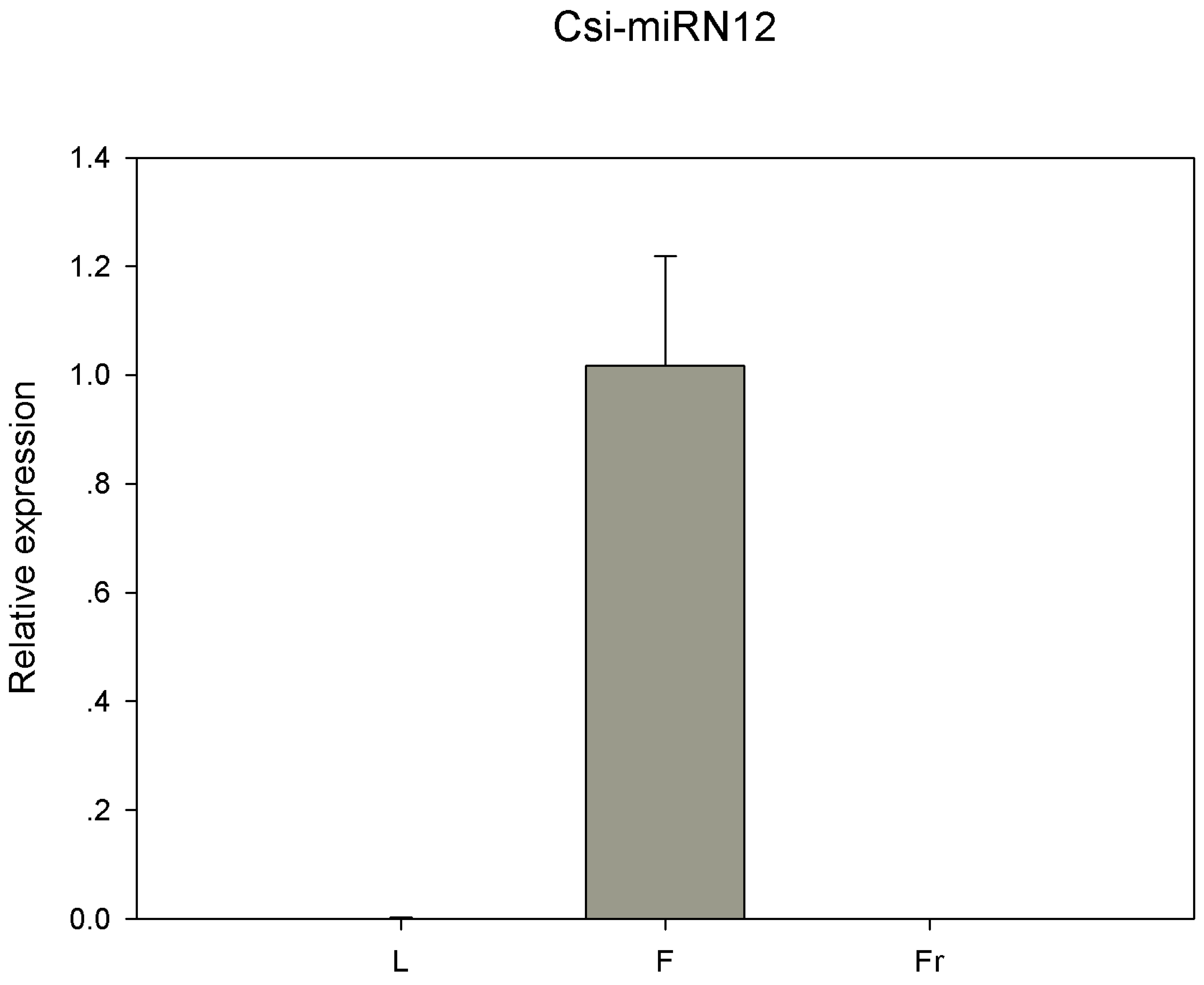

Csi-miRN12
1.4
1.2
1.0
.8
.6
.4
.2
0.0
Relative expression
L
F
Fr

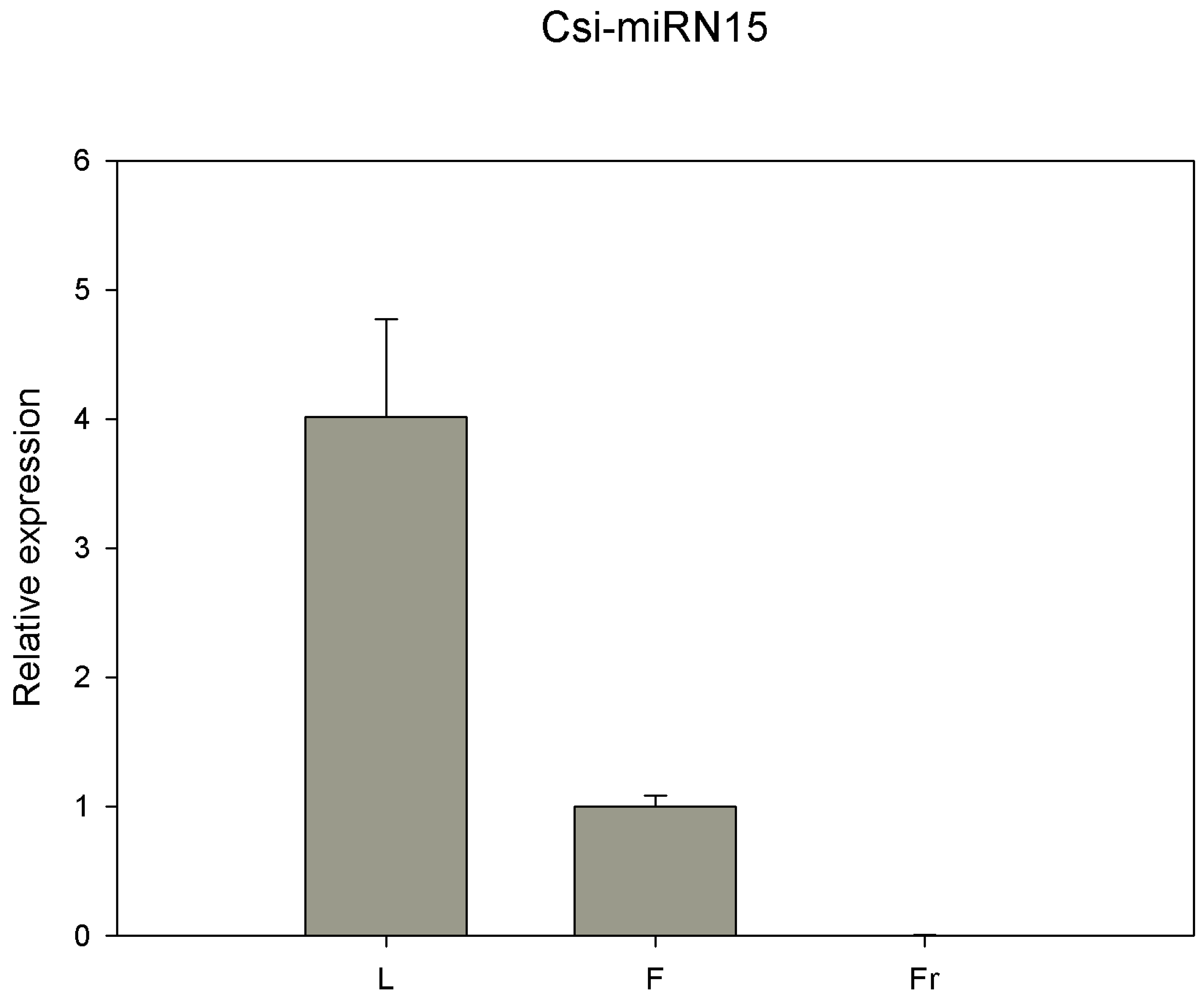

Csi-miRN15
Relative expression
0
1
2
3
4
5
6
L
F
Fr

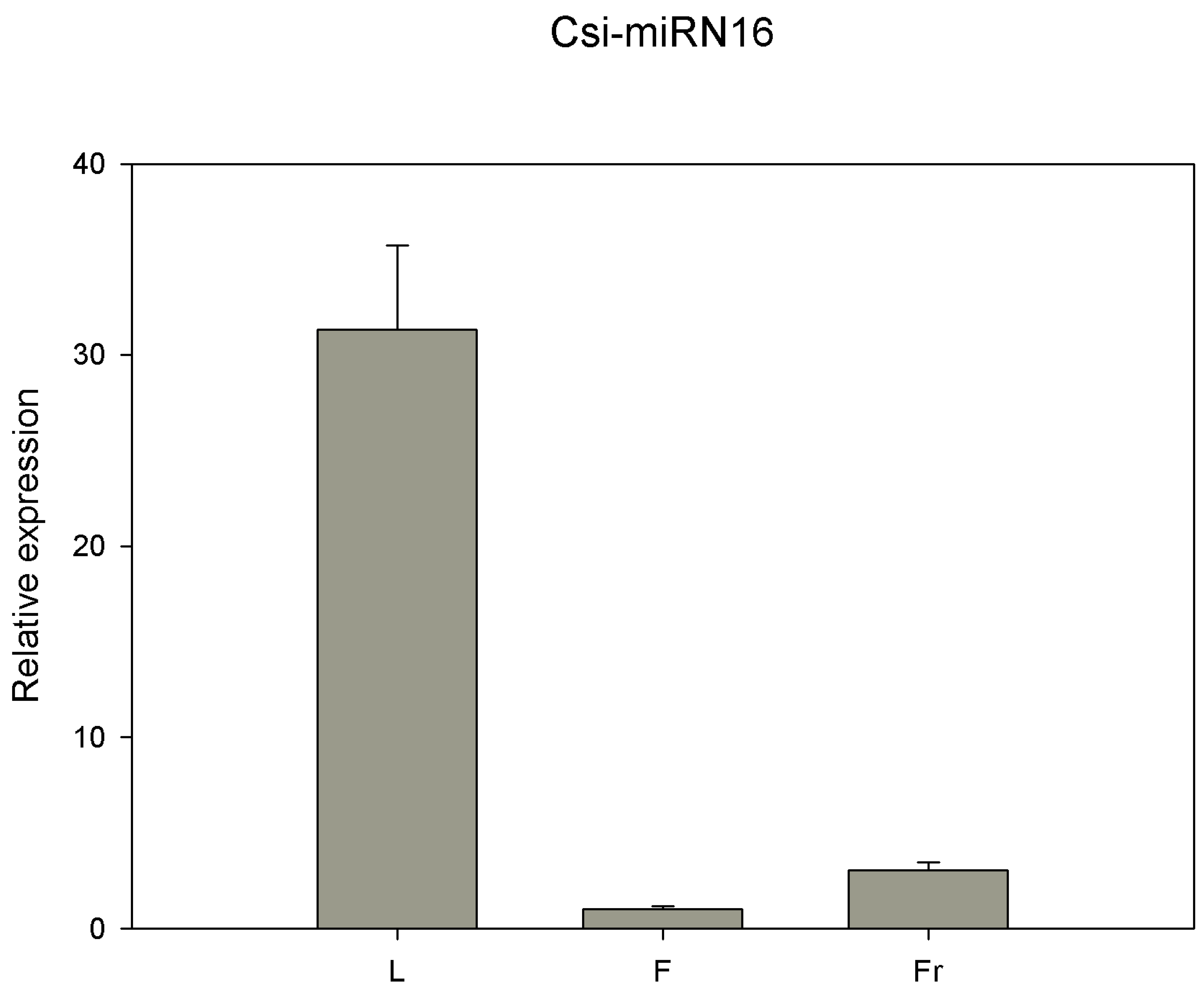
Csi-miRN16
Relative expression
0
10
20
30
40
L
F
Fr

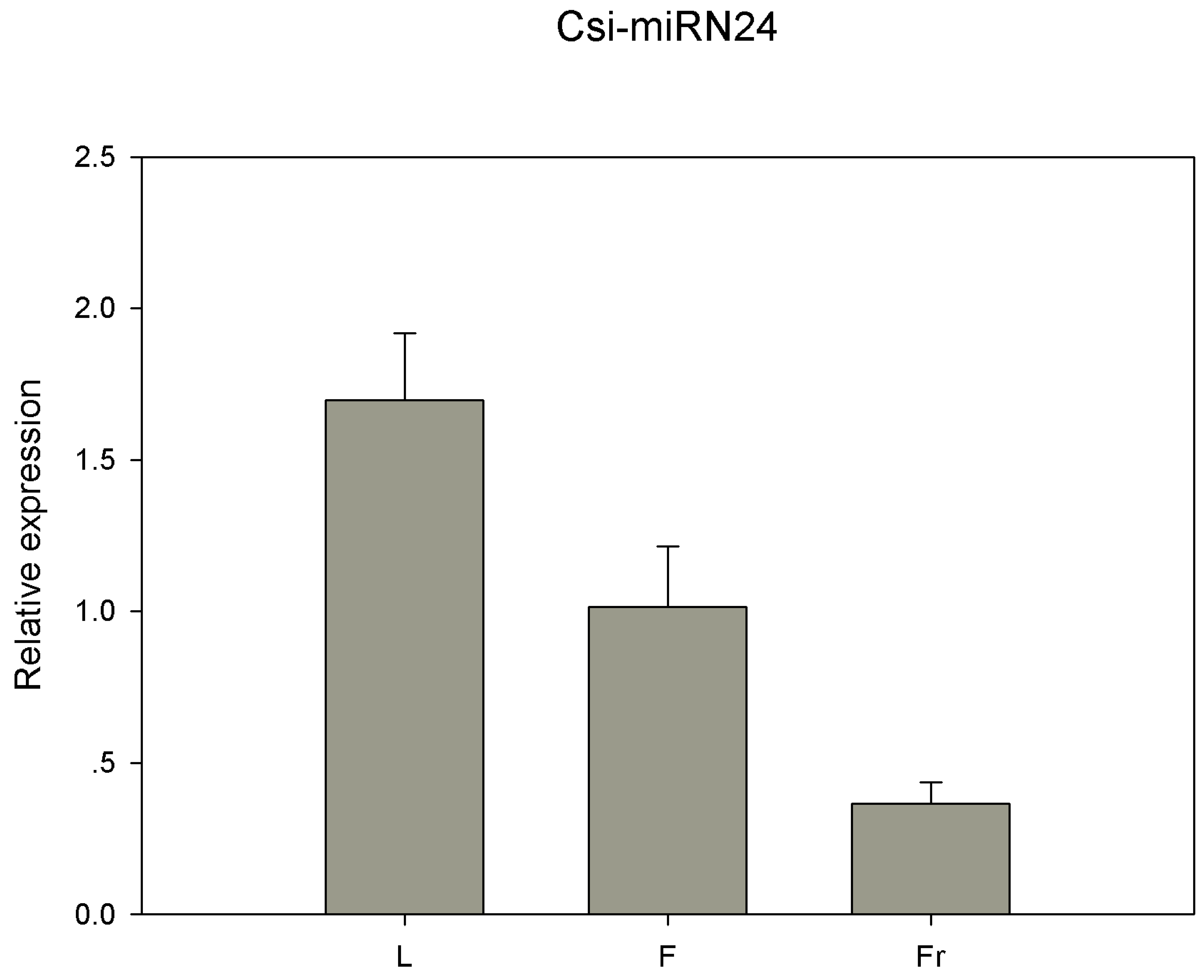

Csi-miRN24
Relative expression
2.5
2.0
1.5
1.0
.5
0.0
L
F
Fr

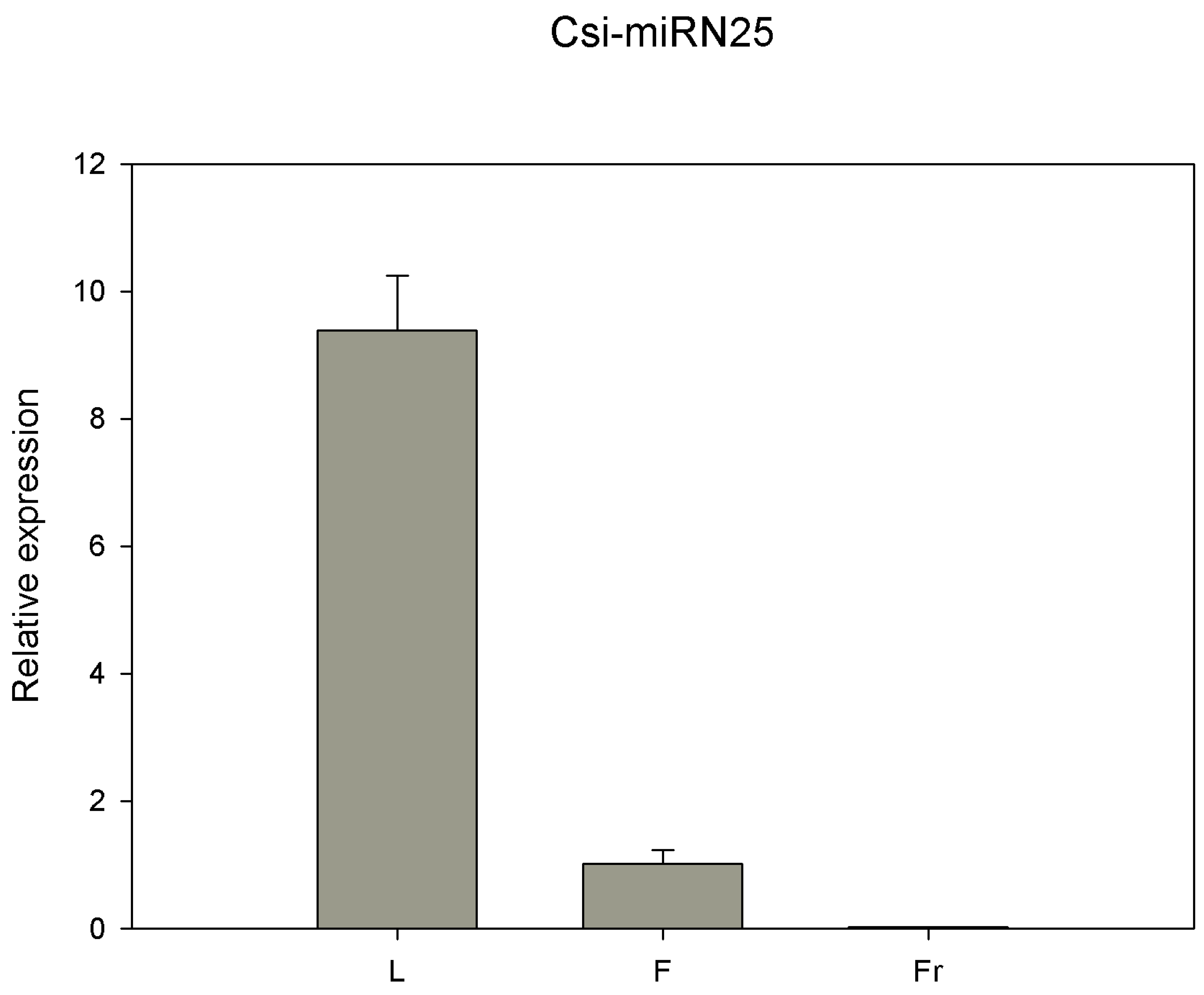
Csi-miRN25
Relative expression
12
10
8
6
4
2
0
L
F
Fr

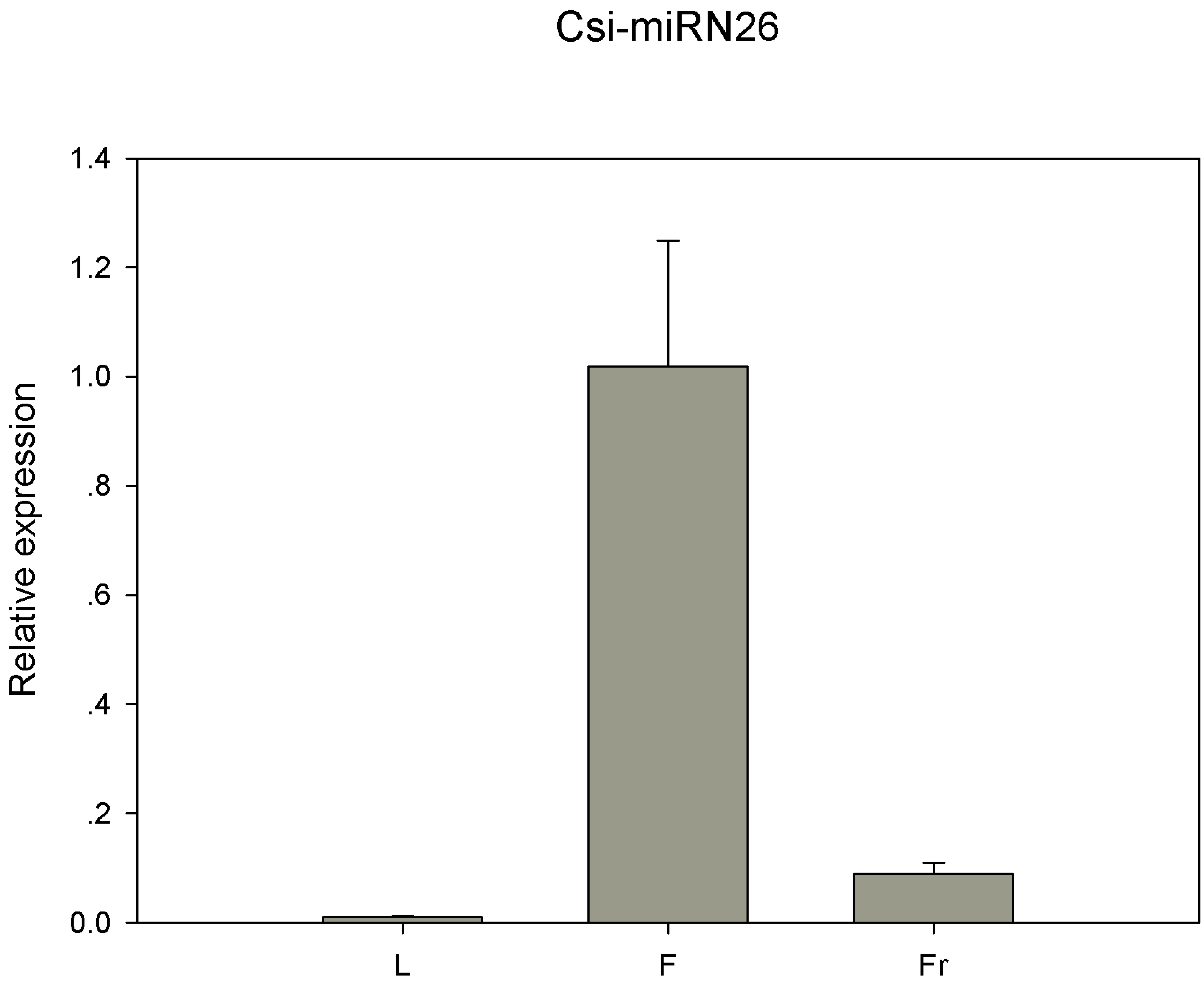
Csi-miRN26
Relative expression
1.4
1.2
1.0
.8
.6
.4
.2
0.0
L
F
Fr

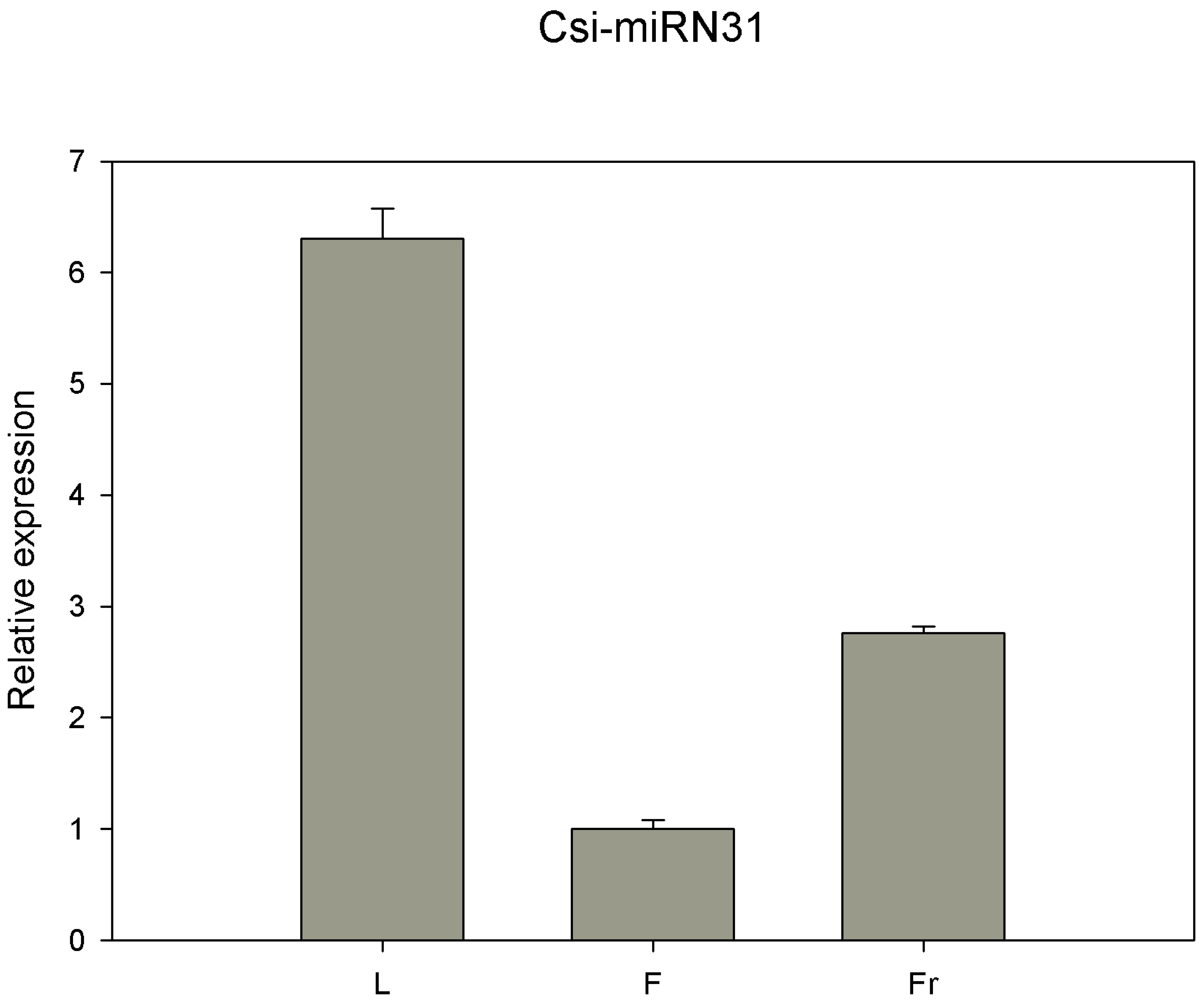

Csi-miRN31
Relative expression
0
1
2
3
4
5
6
7
L
F
Fr

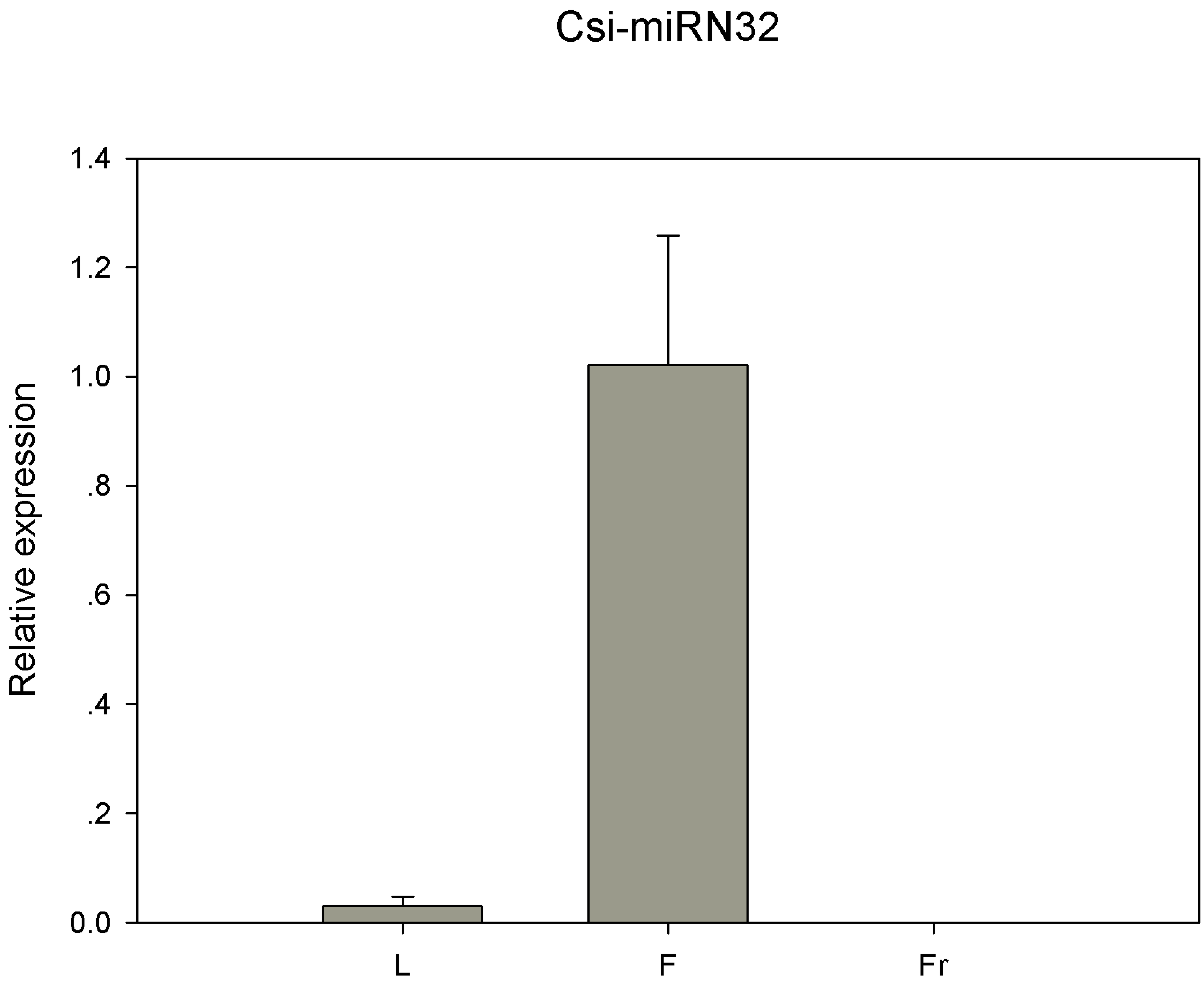
Csi-miRN32
Relative expression
1.4
1.2
1.0
.8
.6
.4
.2
0.0
L
F
Fr

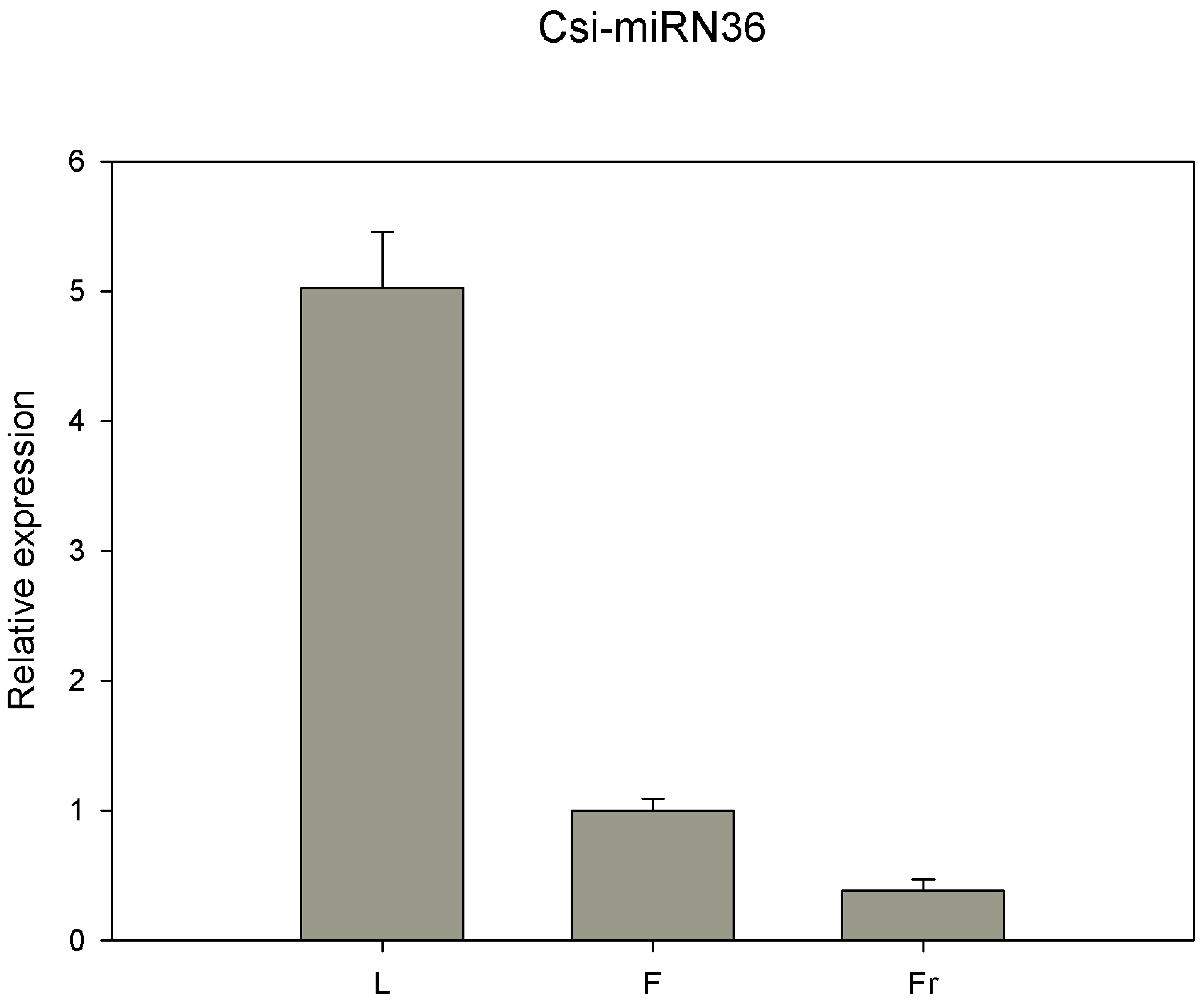

Csi-miRN36
Relative expression
0
1
2
3
4
5
6
L
F
Fr

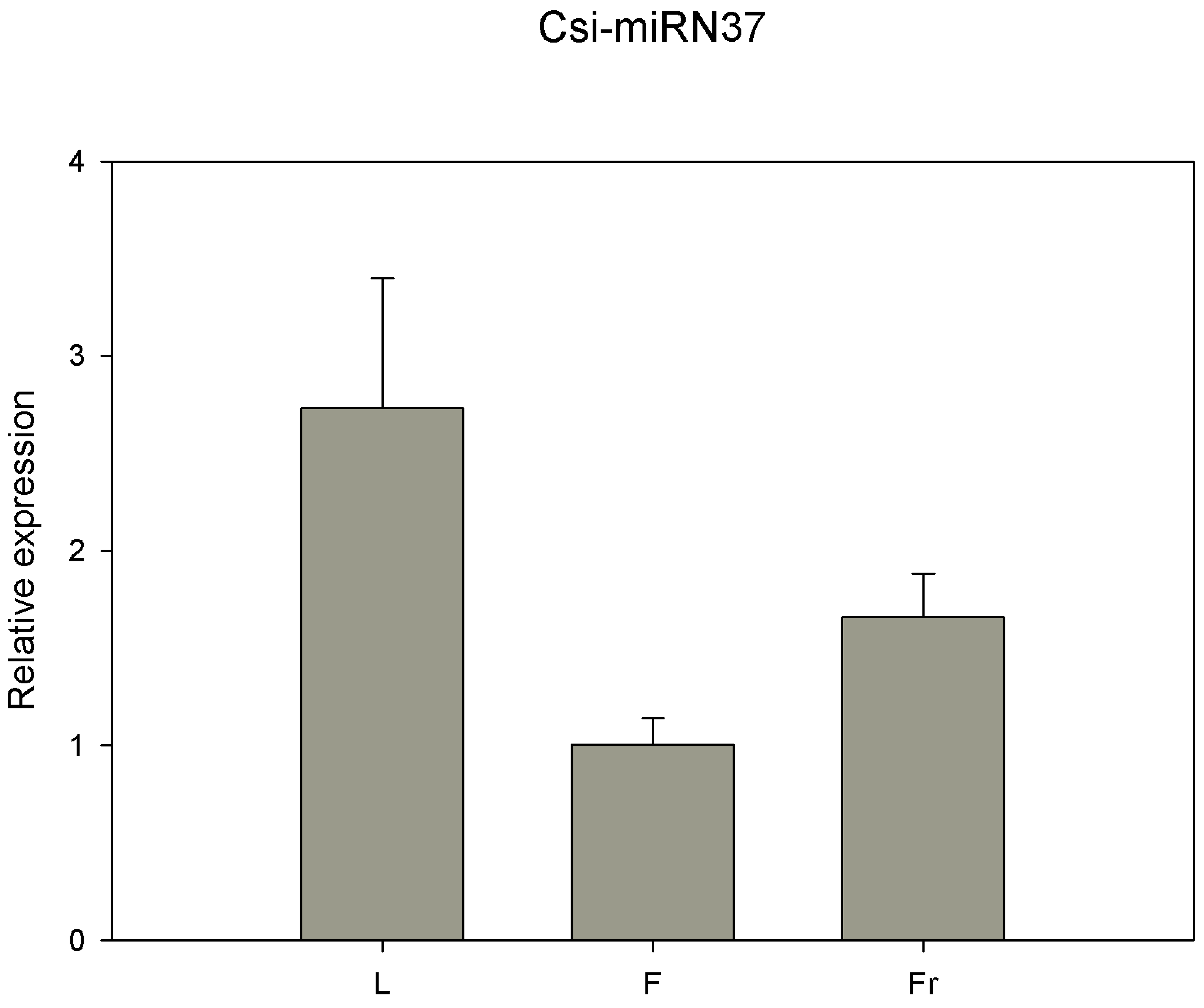
Csi-miRN37
Relative expression
0
1
2
3
4
L
F
Fr
