# Supplementary material for: Genome-wide comparison of microRNAs and their targeted transcripts among leaf, flower and fruit of sweet orange
Source: BMC Genomics. 2014 Aug 20;15(1):695. doi: 10.1186/1471-2164-15-695 (PMC4158063; doi:10.1186/1471-2164-15-695)
Supplement: Supplementary file 13 — Additional file 13: Primers used in miRNA stem-loop RT-PCR. (PDF 38 KB) [file 12864_2014_6413_MOESM13_ESM.pdf]

Additional file 13. Primers used in miRNA stem-loop RT-PCR

	Stem-loop reverse transcription Primer
RT_csi-miR1092.2	GTCGTATCCAGTGCAGGGTCCGAGGTATTCGCACTGGATACGACGGAAAT
RT_csi-miR1432a	GTCGTATCCAGTGCAGGGTCCGAGGTATTCGCACTGGATACGACTGACGG
RT_csi-miR1446	GTCGTATCCAGTGCAGGGTCCGAGGTATTCGCACTGGATACGACGCCGTT
RT_csi-miR156c.1	GTCGTATCCAGTGCAGGGTCCGAGGTATTCGCACTGGATACGACTGTGCT
RT_csi-miR156e	GTCGTATCCAGTGCAGGGTCCGAGGTATTCGCACTGGATACGACGCGCTC
RT_csi-miR156g.1	GTCGTATCCAGTGCAGGGTCCGAGGTATTCGCACTGGATACGACGTGCTC
RT_csi-miR159	GTCGTATCCAGTGCAGGGTCCGAGGTATTCGCACTGGATACGACTAGAGC
RT_csi-miR160b-3p	GTCGTATCCAGTGCAGGGTCCGAGGTATTCGCACTGGATACGACTATGCT
RT_csi-miR162-3p.1	GTCGTATCCAGTGCAGGGTCCGAGGTATTCGCACTGGATACGACCTGGAT
RT_csi-miR164	GTCGTATCCAGTGCAGGGTCCGAGGTATTCGCACTGGATACGACTGCACG
RT_csi-miR166a.2	GTCGTATCCAGTGCAGGGTCCGAGGTATTCGCACTGGATACGACGGGGAA
RT_csi-miR166d.2	GTCGTATCCAGTGCAGGGTCCGAGGTATTCGCACTGGATACGACAGGGAA
RT_csi-miR166g.1	GTCGTATCCAGTGCAGGGTCCGAGGTATTCGCACTGGATACGACGGAATG
RT_csi-miR166j.1	GTCGTATCCAGTGCAGGGTCCGAGGTATTCGCACTGGATACGACGAGGAA
RT_csi-miR166j.3	GTCGTATCCAGTGCAGGGTCCGAGGTATTCGCACTGGATACGACGTGGAA
RT_csi-miR167b.2	GTCGTATCCAGTGCAGGGTCCGAGGTATTCGCACTGGATACGACTAAGAT
RT_csi-miR167d.1	GTCGTATCCAGTGCAGGGTCCGAGGTATTCGCACTGGATACGACTCAGAT
RT_csi-miR168a	GTCGTATCCAGTGCAGGGTCCGAGGTATTCGCACTGGATACGACTTCCCG
RT_csi-miR169m.1	GTCGTATCCAGTGCAGGGTCCGAGGTATTCGCACTGGATACGACCCGGCA
RT_csi-miR171a.1	GTCGTATCCAGTGCAGGGTCCGAGGTATTCGCACTGGATACGACTTTGAT
RT_csi-miR171b	GTCGTATCCAGTGCAGGGTCCGAGGTATTCGCACTGGATACGACGAGTGA
RT_csi-miR171g.1	GTCGTATCCAGTGCAGGGTCCGAGGTATTCGCACTGGATACGACGATATT
RT_csi-miR172a-3p.2	GTCGTATCCAGTGCAGGGTCCGAGGTATTCGCACTGGATACGACTTGCAG
RT_csi-miR172c.2	GTCGTATCCAGTGCAGGGTCCGAGGTATTCGCACTGGATACGACCTGCAG
RT_csi-miR172d	GTCGTATCCAGTGCAGGGTCCGAGGTATTCGCACTGGATACGACATGCAG
RT_csi-miR2275c.1	GTCGTATCCAGTGCAGGGTCCGAGGTATTCGCACTGGATACGACTAAAAT
RT_csi-miR2911	GTCGTATCCAGTGCAGGGTCCGAGGTATTCGCACTGGATACGACTCCCA
RT_csi-miR390.1	GTCGTATCCAGTGCAGGGTCCGAGGTATTCGCACTGGATACGACGGCGCT
RT_csi-miR390.2	GTCGTATCCAGTGCAGGGTCCGAGGTATTCGCACTGGATACGACGGCGCT
RT_csi-miR390b	GTCGTATCCAGTGCAGGGTCCGAGGTATTCGCACTGGATACGACTTGTCT
RT_csi-miR391b	GTCGTATCCAGTGCAGGGTCCGAGGTATTCGCACTGGATACGACGGTGCC
RT_csi-miR393b	GTCGTATCCAGTGCAGGGTCCGAGGTATTCGCACTGGATACGACGATCAA
RT_csi-miR394-3p.1	GTCGTATCCAGTGCAGGGTCCGAGGTATTCGCACTGGATACGACTAGTTT
RT_csi-miR395.1	GTCGTATCCAGTGCAGGGTCCGAGGTATTCGCACTGGATACGACGAGTTC
RT_csi-miR3951	GTCGTATCCAGTGCAGGGTCCGAGGTATTCGCACTGGATACGACTTTTTC
RT_csi-miR3951-3p	GTCGTATCCAGTGCAGGGTCCGAGGTATTCGCACTGGATACGACACAGAT
RT_Csi-miR3952.1	GTCGTATCCAGTGCAGGGTCCGAGGTATTCGCACTGGATACGACTTGAGG
RT_csi-miR3952-3p	GTCGTATCCAGTGCAGGGTCCGAGGTATTCGCACTGGATACGACGTGCTC
RT_csi-miR3954a	GTCGTATCCAGTGCAGGGTCCGAGGTATTCGCACTGGATACGACTGACCG
RT_csi-miR396b.2	GTCGTATCCAGTGCAGGGTCCGAGGTATTCGCACTGGATACGACTTCAAG
RT_csi-miR396b-3p.1	GTCGTATCCAGTGCAGGGTCCGAGGTATTCGCACTGGATACGACTCTCCC
RT_csi-miR396d.4	GTCGTATCCAGTGCAGGGTCCGAGGTATTCGCACTGGATACGACTTCAAG
RT_csi-miR397.1	GTCGTATCCAGTGCAGGGTCCGAGGTATTCGCACTGGATACGACCATCAA
RT_csi-miR398b	GTCGTATCCAGTGCAGGGTCCGAGGTATTCGCACTGGATACGACCAGGGG
RT_csi-miR399a	GTCGTATCCAGTGCAGGGTCCGAGGTATTCGCACTGGATACGACCCGGGC
RT_csi-miR399b	GTCGTATCCAGTGCAGGGTCCGAGGTATTCGCACTGGATACGACTAGGGC
RT_csi-miR399c	GTCGTATCCAGTGCAGGGTCCGAGGTATTCGCACTGGATACGACCAGGGC
RT_csi-miR403.1	GTCGTATCCAGTGCAGGGTCCGAGGTATTCGCACTGGATACGACAGTTTG
RT_Csi-miR408.1	GTCGTATCCAGTGCAGGGTCCGAGGTATTCGCACTGGATACGACCATGCT
RT_csi-miR4414.1	GTCGTATCCAGTGCAGGGTCCGAGGTATTCGCACTGGATACGACTGAACC

RT_Csi-miR444a.2	GTCGTATCCAGTGCAGGGTCCGAGGTATTCGCACTGGATACGACGATGCA
RT_csi-miR473	GTCGTATCCAGTGCAGGGTCCGAGGTATTCGCACTGGATACGACGCGAAG
RT_csi-miR477a-3p	GTCGTATCCAGTGCAGGGTCCGAGGTATTCGCACTGGATACGACCGACCT
RT_csi-miR477d.1-3p	GTCGTATCCAGTGCAGGGTCCGAGGTATTCGCACTGGATACGACCCACTC
RT_csi-miR479.1	GTCGTATCCAGTGCAGGGTCCGAGGTATTCGCACTGGATACGACGATGAG
RT_csi-miR482a-5p	GTCGTATCCAGTGCAGGGTCCGAGGTATTCGCACTGGATACGACCTTCTT
RT_csi-miR482d-5p.1	GTCGTATCCAGTGCAGGGTCCGAGGTATTCGCACTGGATACGACCTTCCC
RT_csi-miR482d-5p.2	GTCGTATCCAGTGCAGGGTCCGAGGTATTCGCACTGGATACGACTATCTT
RT_csi-miR5179	GTCGTATCCAGTGCAGGGTCCGAGGTATTCGCACTGGATACGACATTGCG
RT_csi-miR530b	GTCGTATCCAGTGCAGGGTCCGAGGTATTCGCACTGGATACGACCAAGAT
RT_csi-miR535.1	GTCGTATCCAGTGCAGGGTCCGAGGTATTCGCACTGGATACGACGTGTGC
RT_csi-miR536-3p	GTCGTATCCAGTGCAGGGTCCGAGGTATTCGCACTGGATACGACGACGCA
RT_csi-miR814	GTCGTATCCAGTGCAGGGTCCGAGGTATTCGCACTGGATACGACCTCATC
RT_csi-miR827.1	GTCGTATCCAGTGCAGGGTCCGAGGTATTCGCACTGGATACGACTGTTTG
RT_csi-miR827-5p.1	GTCGTATCCAGTGCAGGGTCCGAGGTATTCGCACTGGATACGACTTAGAT
RT_csi-miRN03	GTCGTATCCAGTGCAGGGTCCGAGGTATTCGCACTGGATACGACAACACC
RT_csi-miRN04	GTCGTATCCAGTGCAGGGTCCGAGGTATTCGCACTGGATACGACTGTAAC
RT_csi-miRN07	GTCGTATCCAGTGCAGGGTCCGAGGTATTCGCACTGGATACGACCACCGT
RT_csi-miRN08	GTCGTATCCAGTGCAGGGTCCGAGGTATTCGCACTGGATACGACGTCCAG
RT_csi-miRN11	GTCGTATCCAGTGCAGGGTCCGAGGTATTCGCACTGGATACGACAACCGT
RT_csi-miRN12	GTCGTATCCAGTGCAGGGTCCGAGGTATTCGCACTGGATACGACTCTTAG
RT_csi-miRN15	GTCGTATCCAGTGCAGGGTCCGAGGTATTCGCACTGGATACGACGTTGGT
RT_csi-miRN16	GTCGTATCCAGTGCAGGGTCCGAGGTATTCGCACTGGATACGACTATCAT
RT_csi-miRN24	GTCGTATCCAGTGCAGGGTCCGAGGTATTCGCACTGGATACGACATTAGC
RT_csi-miRN25	GTCGTATCCAGTGCAGGGTCCGAGGTATTCGCACTGGATACGACAATGCC
RT_csi-miRN26	GTCGTATCCAGTGCAGGGTCCGAGGTATTCGCACTGGATACGACTTTCAT
RT_csi-miRN31	GTCGTATCCAGTGCAGGGTCCGAGGTATTCGCACTGGATACGACCCGAAC
RT_csi-miRN32	GTCGTATCCAGTGCAGGGTCCGAGGTATTCGCACTGGATACGACGCCCTT
RT_csi-miRN36	GTCGTATCCAGTGCAGGGTCCGAGGTATTCGCACTGGATACGACTTTCGC
RT_csi-miRN37	GTCGTATCCAGTGCAGGGTCCGAGGTATTCGCACTGGATACGACCCTTCT

Forward primer for qRT-PCR	
FOR_csi-miR1092.2	ATTCGTTCCACCAAAGCATTC
FOR_csi-miR1432a	ATTCGTGCAGGTGAGATGATA
FOR_csi-miR1446	ATTCGCGAACTCTCTCCCTC
FOR_csi-miR156c.1	ATCCGTGACAGAAGAGAGTG
FOR_csi-miR156e	ACGGCGTGACAGAAGATAGA
FOR_csi-miR156g.1	ACGGCTTGACGGAAGATAGA
FOR_csi-miR159	ACGGCTTTGGATTGAAGGGA
FOR_csi-miR160b-3p	ATAATGCGTACGAGGAGCCA
FOR_csi-miR162-3p.1	ATCCGTCGATAAACCTCTGC
FOR_csi-miR164	ATCATTGGAGAAGCAGGGCA
FOR_csi-miR166a.2	ATCATTCGGACCAGGCTTCA
FOR_csi-miR166d.2	ATCATTCGGACCAGGCTTCA
FOR_csi-miR166g.1	ATCATTCTCGGACCAGGCTT
FOR_csi-miR166j.1	ATTCGTTGGACCAGGCTTCA
FOR_csi-miR166j.3	ATTCGTTGGACCAGGCTTCA
FOR_csi-miR167b.2	ATAATTGAAGCTGCCAGCATG
FOR_csi-miR167d.1	ATAATTGAAGCTGCCAGCATG
FOR_csi-miR168a	ATCATTCGCTTGGTGCAGGT

FOR_csi-miR169m.1	ATTCGCAGCCAAGGATGACT
FOR_csi-miR171a.1	ATTCGTGTTGGAACGGCTCA
FOR_csi-miR171b	ATCCGCGAGCCGAATCAATA
FOR_csi-miR171g.1	ATCATTGATTGAGCCGTGCC
FOR_csi-miR172a-3p.2	CGCCGAGAATCTTGATGATG
FOR_csi-miR172c.2	ATCCGTGGAATCTTGATGATG
FOR_csi-miR172d	CGCCGAGAATCTTGATGATG
FOR_csi-miR2275c.1	CGCCGTTTAATTTCCTCCAAT
FOR_csi-miR2911	CCCGGCCGGGGGACGGA
FOR_csi-miR390.1	ATTCGAAGCTCAGGAGGGAT
FOR_csi-miR390.2	ATTCGAGCTCAGGAGGGAT
FOR_csi-miR390b	ATCATCAGCTCAGGAGGGAT
FOR_csi-miR391b	ATAATTTGTCGCAGGAGCGTT
FOR_csi-miR393b	ATCATTTCCAAAGGGATCGCA
FOR_csi-miR394-3p.1	ATCCGAGCTGGACAAATTGC
FOR_csi-miR395.1	ATTCGCTGAAGTGTTTGGGG
FOR_csi-miR3951	CGGCGTAGATAAAGATGAGA
FOR_csi-miR3951-3p	CGGCGTTTCTCTTATCGTT
FOR_Csi-miR3952.1	ATCCGTGCTGTAGAAAGGCC
FOR_csi-miR3952-3p	ATCCGTGAAGGGCCTTTCTA
FOR_csi-miR3954a	ATCCGTTGGACAGAGAAATCA
FOR_csi-miR396b.2	CGGCGTTCCACAGCTTT
FOR_csi-miR396b-3p.1	ATCCGGCTCAAGAAAGCTGT
FOR_csi-miR396d.4	CGGCGTTCCACGGCTTT
FOR_csi-miR397.1	ATTCGTCATTGAGTGCAGCG
FOR_csi-miR398b	ATTCGTGTGTTCTCAGGTCG
FOR_csi-miR399a	ACGGCTGCCAAAGGAGATTT
FOR_csi-miR399b	ATCCGTGCCAAAGGAGAGTT
FOR_csi-miR399c	ATCCGCGCCAAAGGAGAATT
FOR_csi-miR403.1	CGGCGTTAGATTCACGCA
FOR_Csi-miR408.1	ATTATACGGGGAACAGGCAG
FOR_csi-miR4414.1	ATTCGAGCTGCTGACTCGTT
FOR_Csi-miR444a.2	ATCCGTTTCTTGAGACAAAACA
FOR_csi-miR473	ATCATACTCTCCCTCAAGGG
FOR_csi-miR477a-3p	ATAATGGAAACCCTAGGGGG
FOR_csi-miR477d.1-3p	ATTATTGAGGCCGTTGGGGA
FOR_csi-miR479.1	ATTCGTGTGATATTGGTTCGG
FOR_csi-miR482a-5p	ATTAAGTGGGAGCGTGGGGT
FOR_csi-miR482d-5p.1	ATCGGTGGTATGGGTGAGTA
FOR_csi-miR482d-5p.2	ATTCGTATGGGTGAGTAGGG
FOR_csi-miR5179	ATTCGTCTTGCTCAAGACCG
FOR_csi-miR530b	ATTCGTGCATTTGCACCTGC
FOR_csi-miR535.1	ACGGCTGACAATGAGAGAGA
FOR_csi-miR536-3p	ATTATTGGTGCCACGCTGTG
FOR_csi-miR814	ACCGAAATTAGATTCGTTGTGT
FOR_csi-miR827.1	CGGCGTTAGATGACCATCAA
FOR_csi-miR827-5p.1	ACGGCTGCTTGTTGATTGTC
FOR_csi-miRN03	CGATTTGTTTTGGGTGAAACG
FOR_csi-miRN04	ACGCGTTGTAGAGTGTGTAT
FOR_csi-miRN07	ACACGGGAAGACAAGATCAA
FOR_csi-miRN08	ATTCCCGGGTCACAGCTCC

FOR_csi-miRN11	ATTCGTCGCAGGAGCTTTCT
FOR_csi-miRN12	CGGCATCAATGGTTATGGCA
FOR_csi-miRN15	ATTTCGCAGGGGAGATGGG
FOR_csi-miRN16	ATCTGCTGATGAGAGAGCGA
FOR_csi-miRN24	ATCCGAGCAAGCATCCTGG
FOR_csi-miRN25	ATCCAGGGATTTCATGAAACAT
FOR_csi-miRN26	ATTAACAGGAGGTGGAACAAAT
FOR_csi-miRN31	ATTACCGTTGAGGTAGGGCA
FOR_csi-miRN32	ATTCGATCTATTTGGATGAAGAT
FOR_csi-miRN36	ATTATGGTCATGGGAGGATTG
FOR_csi-miRN37	CGGCATGAGGGAAGAGCTT

Universal reverse primer	
Reverse	GTGCAGGGTCCGAGGT
