# Supplementary material for: Genome-wide comparison of microRNAs and their targeted transcripts among leaf, flower and fruit of sweet orange
Source: BMC Genomics. 2014 Aug 20;15(1):695. doi: 10.1186/1471-2164-15-695 (PMC4158063; doi:10.1186/1471-2164-15-695)
Supplement: Supplementary file 4 — Additional file 4: T-plots of the miRNA targets in different tissues. Densities of the 5’ positions of degradome tags matching each target gene are shown as T-plots. The miRNA-mediated degradome tag is highlighted in red. (PDF 14 MB) [file 12864_2014_6413_MOESM4_ESM.pdf]

Additional file 4: T-plots of the miRNA targets in different tissues.

Densities of the 5’ positions of degradome tags matching each target gene are shown as T-plots. The miRNA-mediated degradome tag is highlighted in red.

T-plots for targets of all miRNAs in leaves

Csi-miR156a.1, target=Cs2g05730.1 gene=Cs2g05730
Category:1
Score=1
Cleavage Site=818

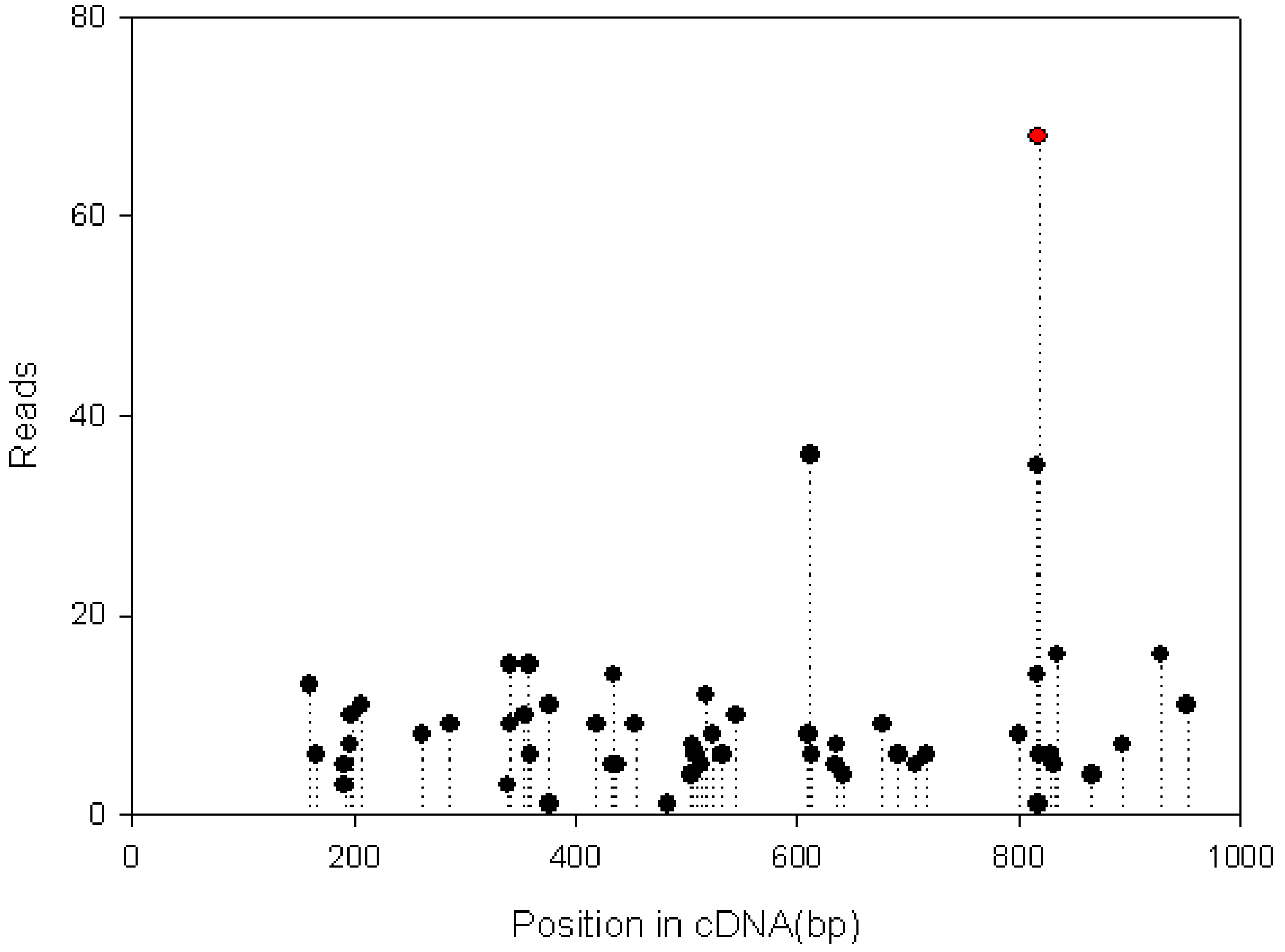


```
5' CGUGCUCUCUCUCUUCUGUCAAUGCU 3'        Cs2g05730.1
    :::::: ::::::::::::::
3' -CACGAGUGAGAGAAGACAGUU---- 5'        Csi-miR156a.1
```

Csi-miR156a.1, target=Cs2g23550.1 gene=Cs2g23550
Category:1
Score=3
Cleavage Site=934

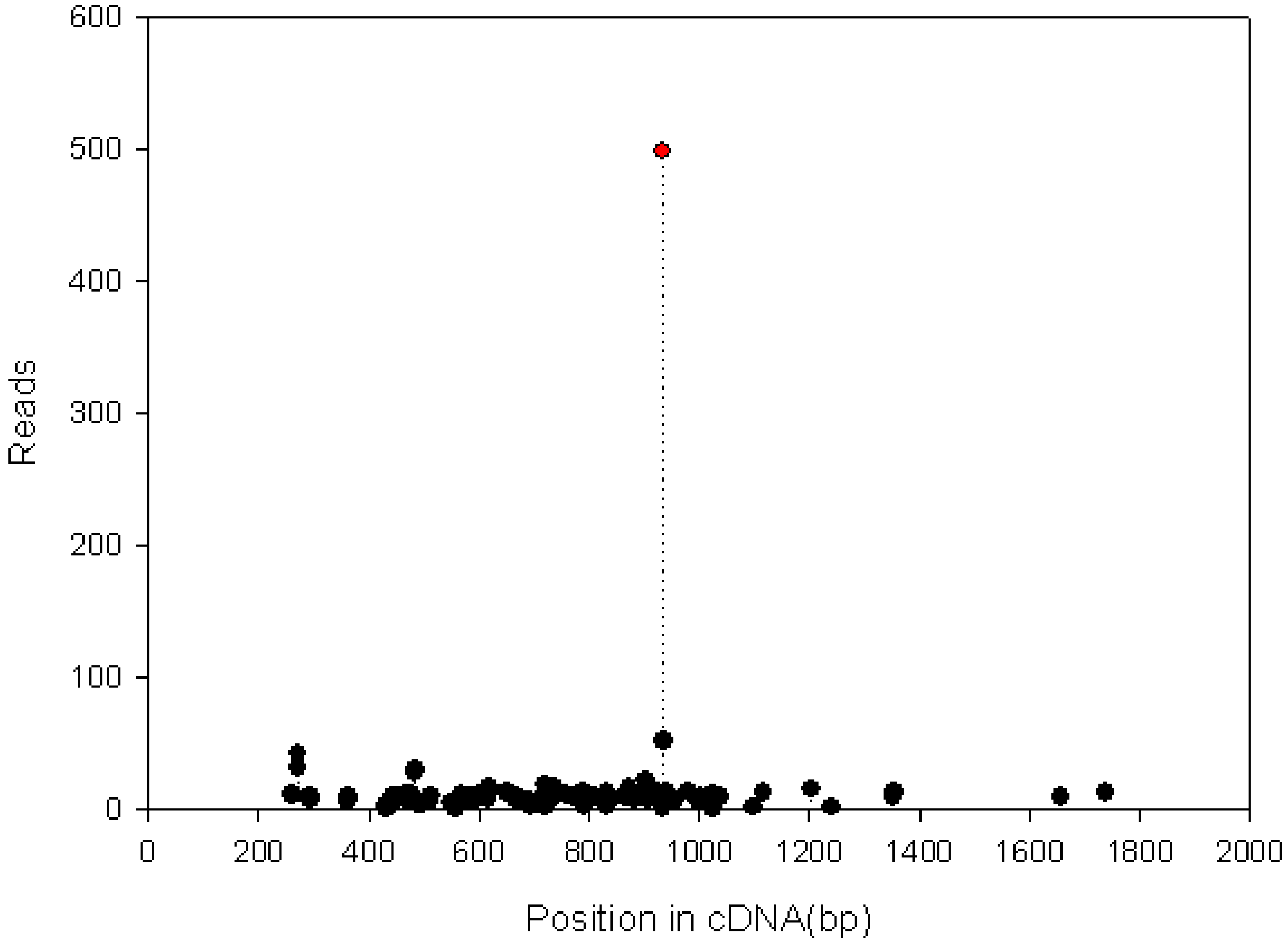


```
5' CAUGCUCUCUCUCUUCUGUCAUCCUA 3'        Cs2g23550.1
      ::::: :::::::::::::
3' -CACGAGUGAGAGAAGACAGUU---- 5'        Csi-miR156a.1
```

Csi-miR156a.1, target=Cs5g11880.1 gene=Cs5g11880
Category:3
Score=5
Cleavage Site=554

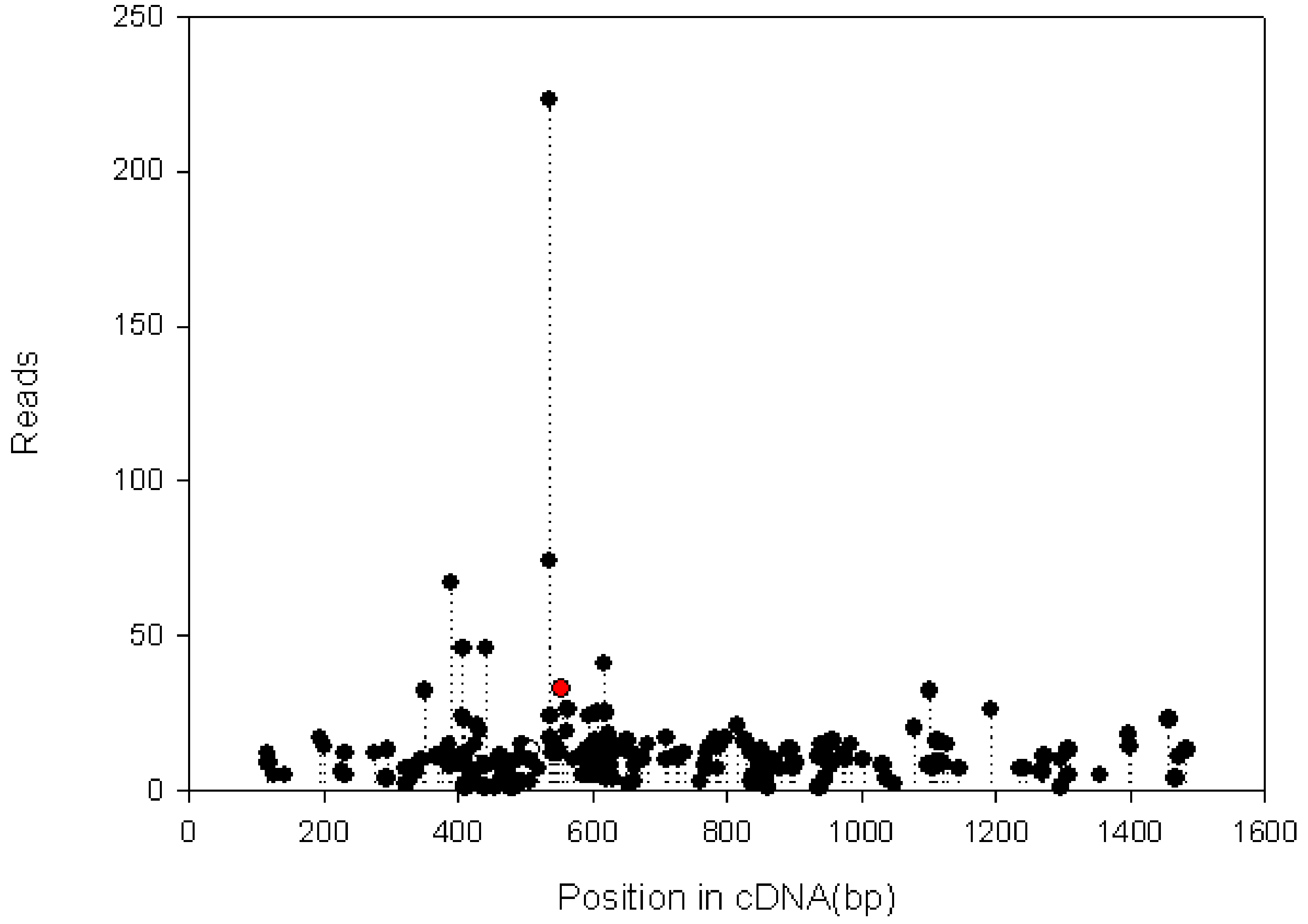


```
5' GUUAUUGUGACCA-UCUCUUCUGACAA 3'        Cs5g11880.1
          :::  :: ::::::::: :::
3' ------CACGAGUGAGAGAAGACAGUU 5'        Csi-miR156a.1
```

Csi-miR156a.1, target=Cs7g10830.1 gene=Cs7g10830
Category:1
Score=1
Cleavage Site=1183

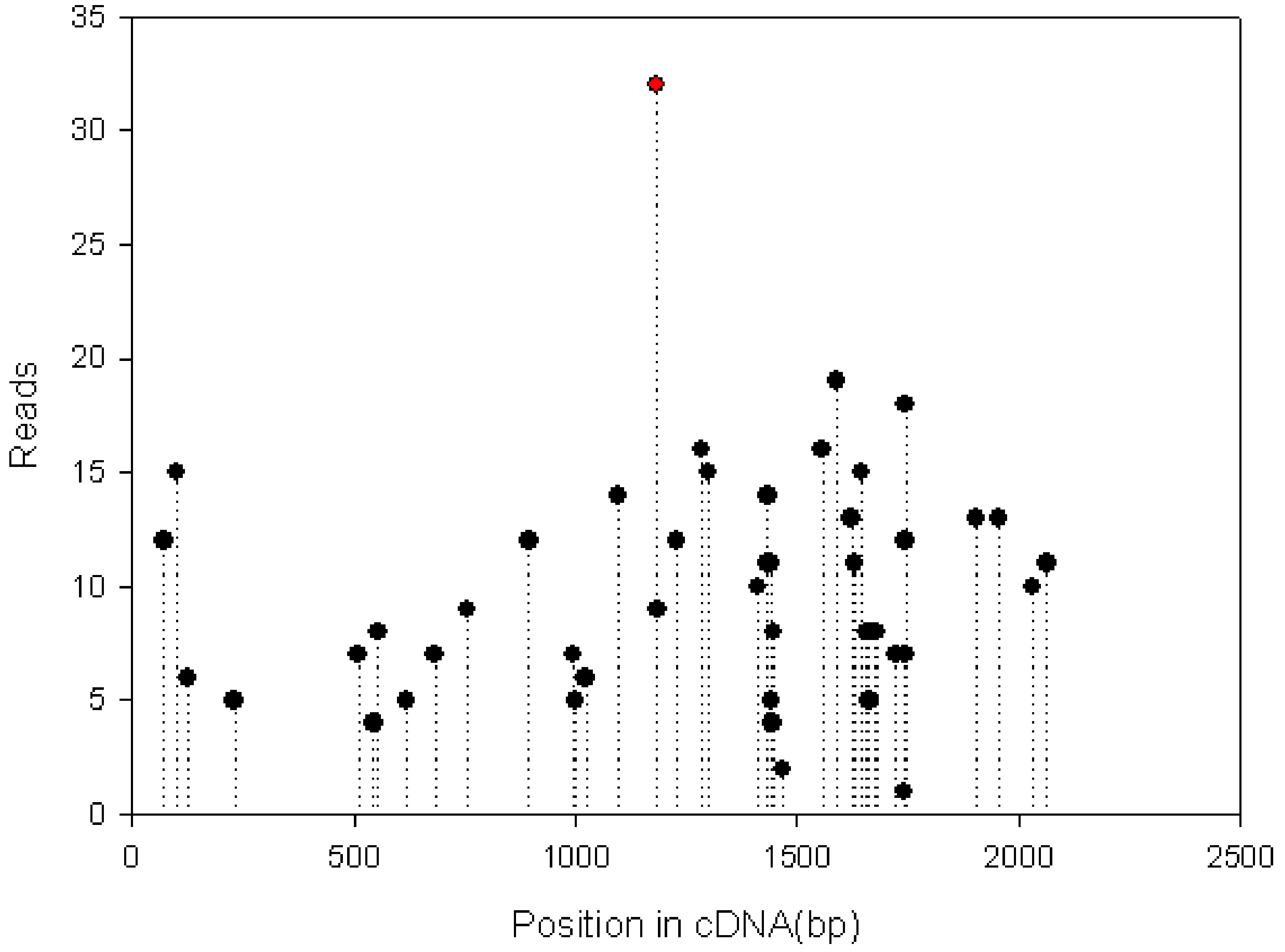


```
5' AGUGCUCUCUCUCUUCUGUCAAAAC 3'         Cs7g10830.1
    :::::: ::::::::::::::
3' -CACGAGUGAGAGAAGACAGUU---- 5'         Csi-miR156a.1
```

Csi-miR156a.1, target=Cs8g17370.1 gene=Cs8g17370
Category:3
Score=5
Cleavage Site=21

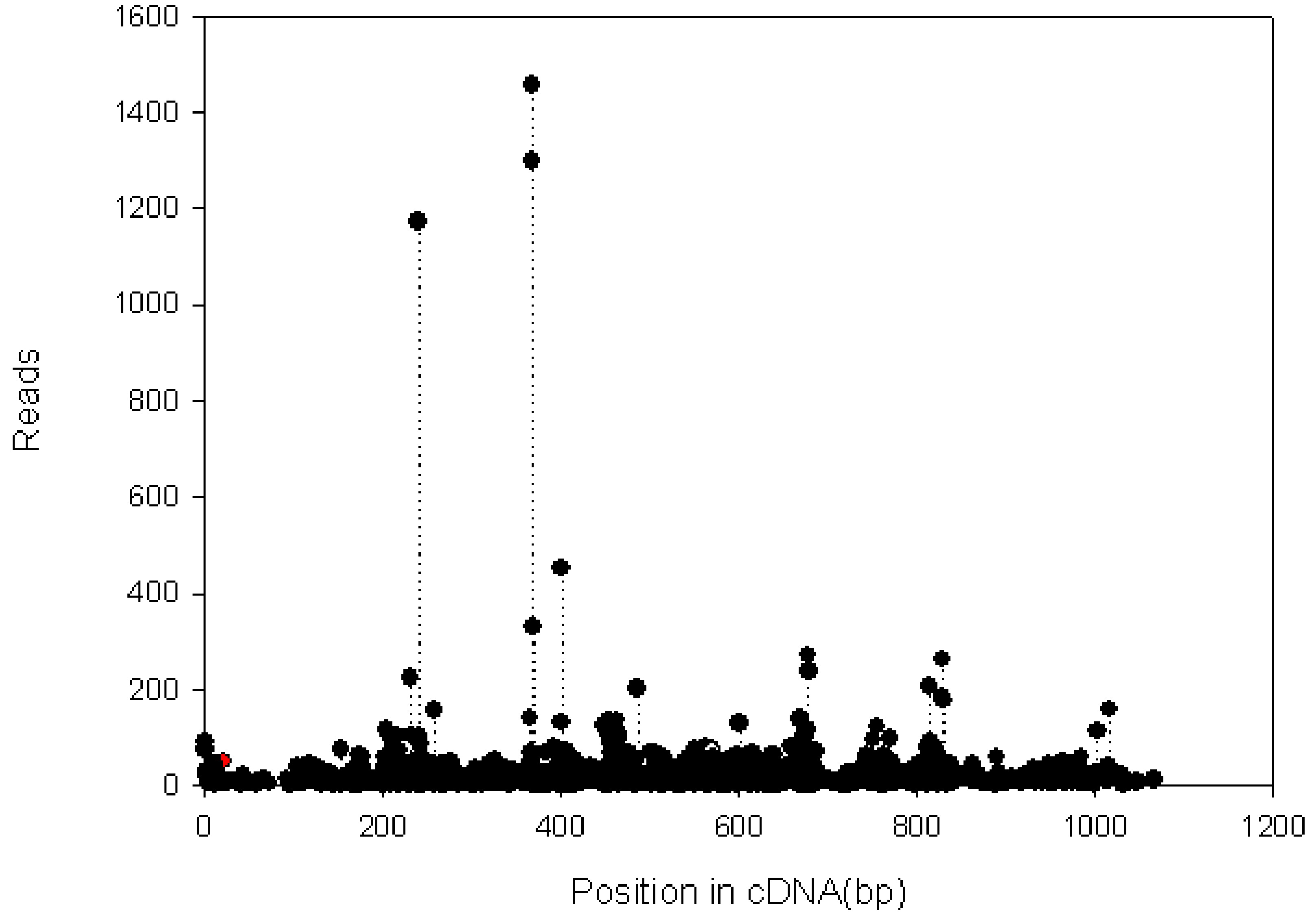


```
5' CUCGUGC-CACUCUCGUUUGUUAAGGA 3'       Cs8g17370.1
     :::: ::::::: :.:::.::
3' ---CACGAGUGAGAGAAGACAGUU--- 5'       Csi-miR156a.1
```

Csi-miR156a.1, target=Orange1.1t02265.1 gene=Orange1.1t02265
Category:1
Score=1
Cleavage Site=982

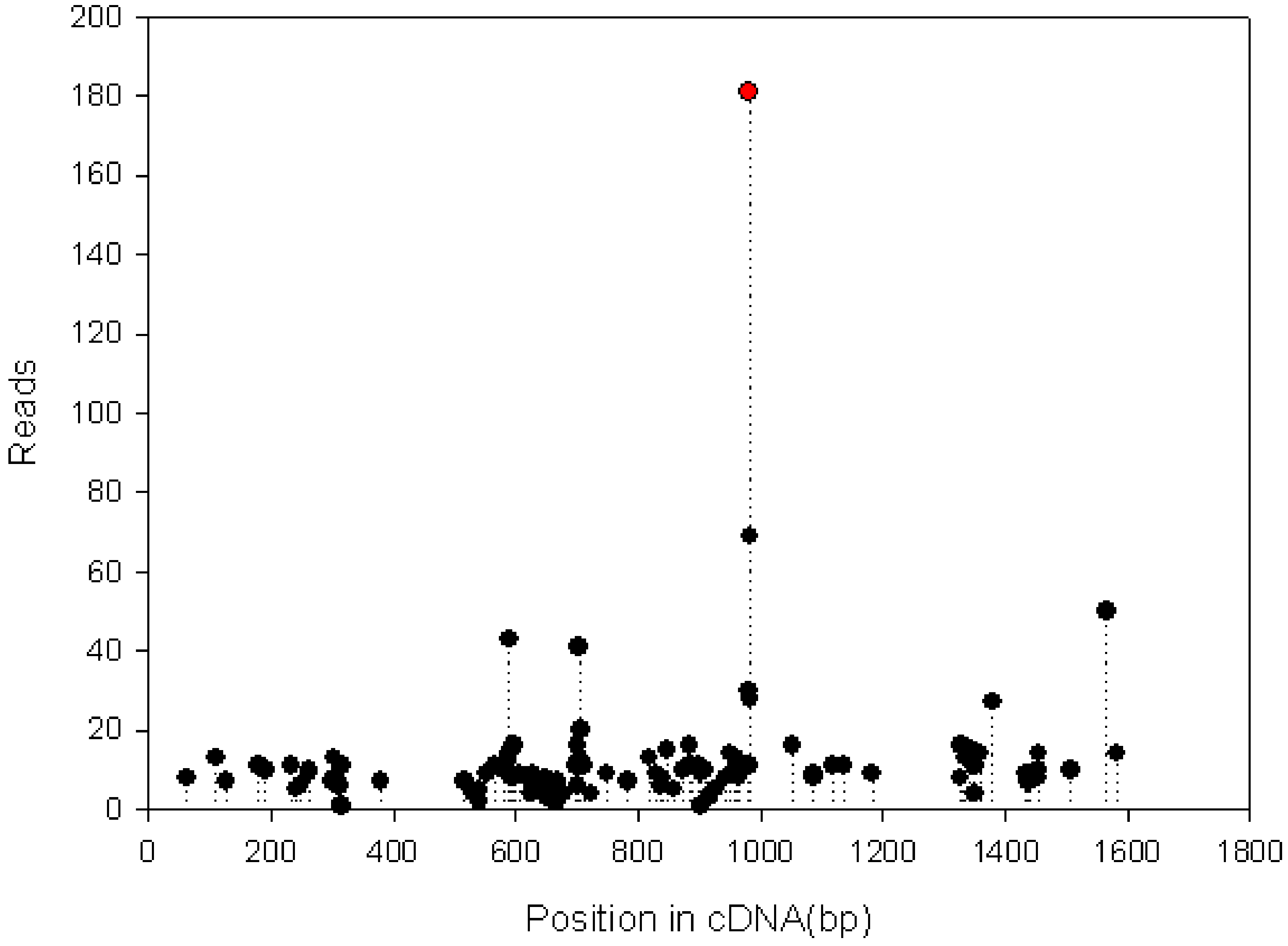


```
5' CUGUGCUCUCUCUCUUCUGUCAAAUC 3'        Orange1.1t02265.1
     :::::: ::::::::::::::
3' --CACGAGUGAGAGAAGACAGUU--- 5'        Csi-miR156a.1
```

Csi-miR156a.2, target=Cs2g05730.1 gene=Cs2g05730
Category:1
Score=1
Cleavage Site=818

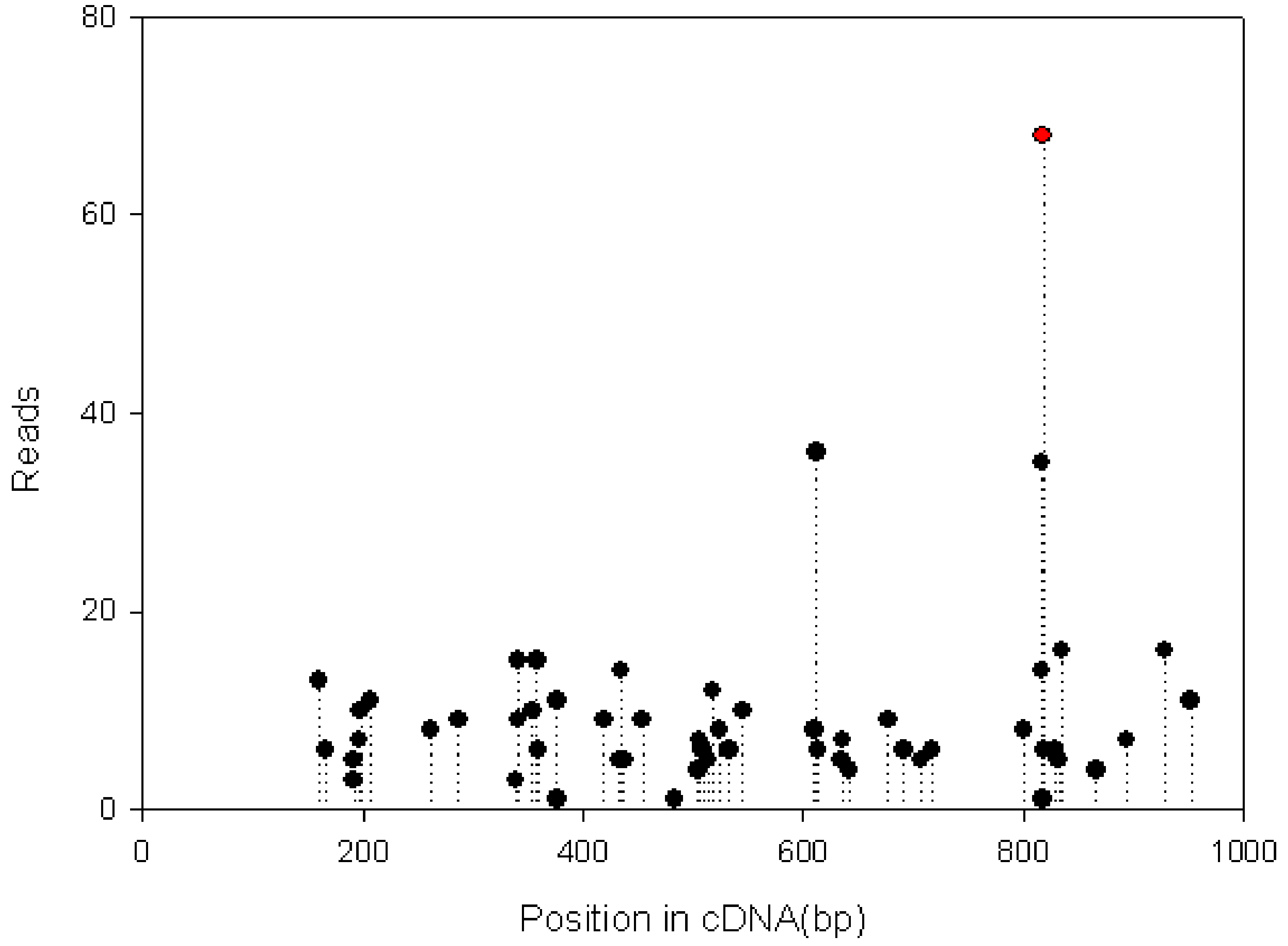


```
5' CGUGCUCUCUCUCUUCUGUCAAUGCU 3'        Cs2g05730.1
   :::::: :::::::::::::
3' -CACGAGUGAGAGAAGACAGU----- 5'        Csi-miR156a.2
```

Csi-miR156a.2, target=Cs2g23550.1 gene=Cs2g23550
Category:1
Score=2
Cleavage Site=934

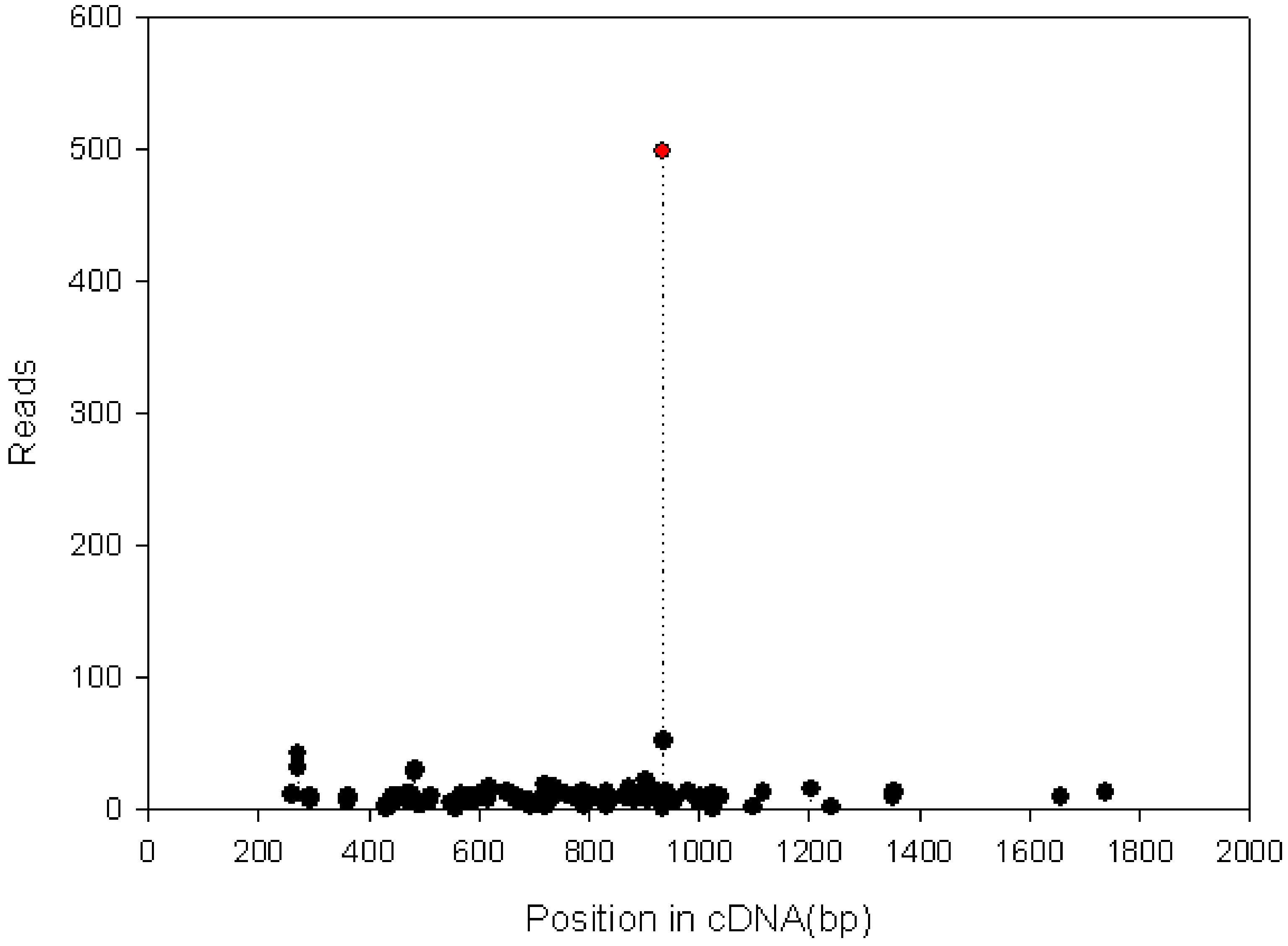


```
5' CAUGCUCUCUCUCUUCUGUCAUCCUA 3'        Cs2g23550.1
     ::::: :::::::::::::
3' -CACGAGUGAGAGAAGACAGU----- 5'        Csi-miR156a.2
```

Csi-miR156a.2, target=Cs7g10830.1 gene=Cs7g10830
Category:1
Score=1
Cleavage Site=1183

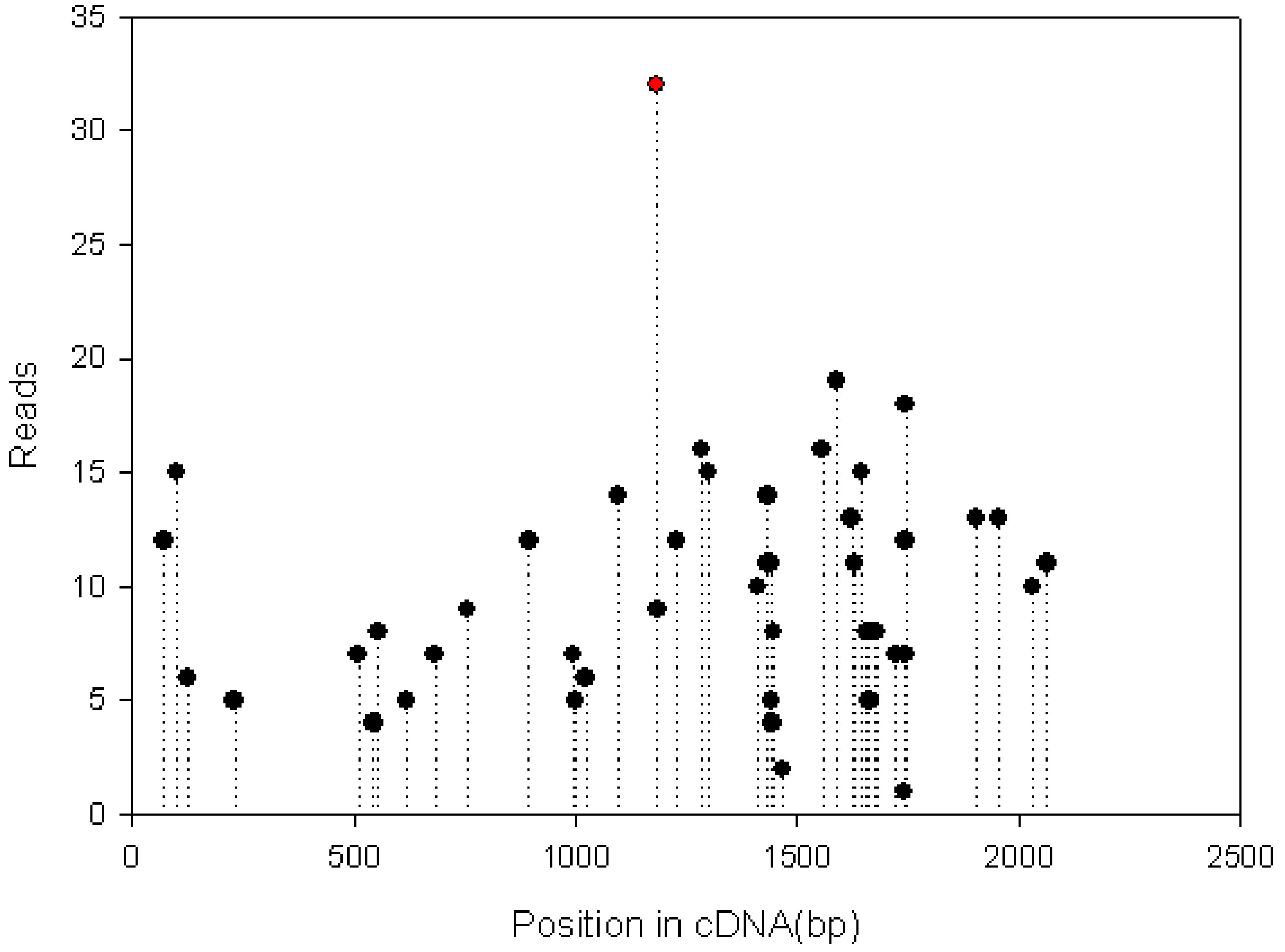


```
5' AGUGCUCUCUCUCUUCUGUCAAAAAC 3'        Cs7g10830.1
    :::::: :::::::::::::
3' -CACGAGUGAGAGAAGACAGU----- 5'        Csi-miR156a.2
```

Csi-miR156a.2, target=Cs8g17370.1 gene=Cs8g17370
Category:3
Score=5
Cleavage Site=21

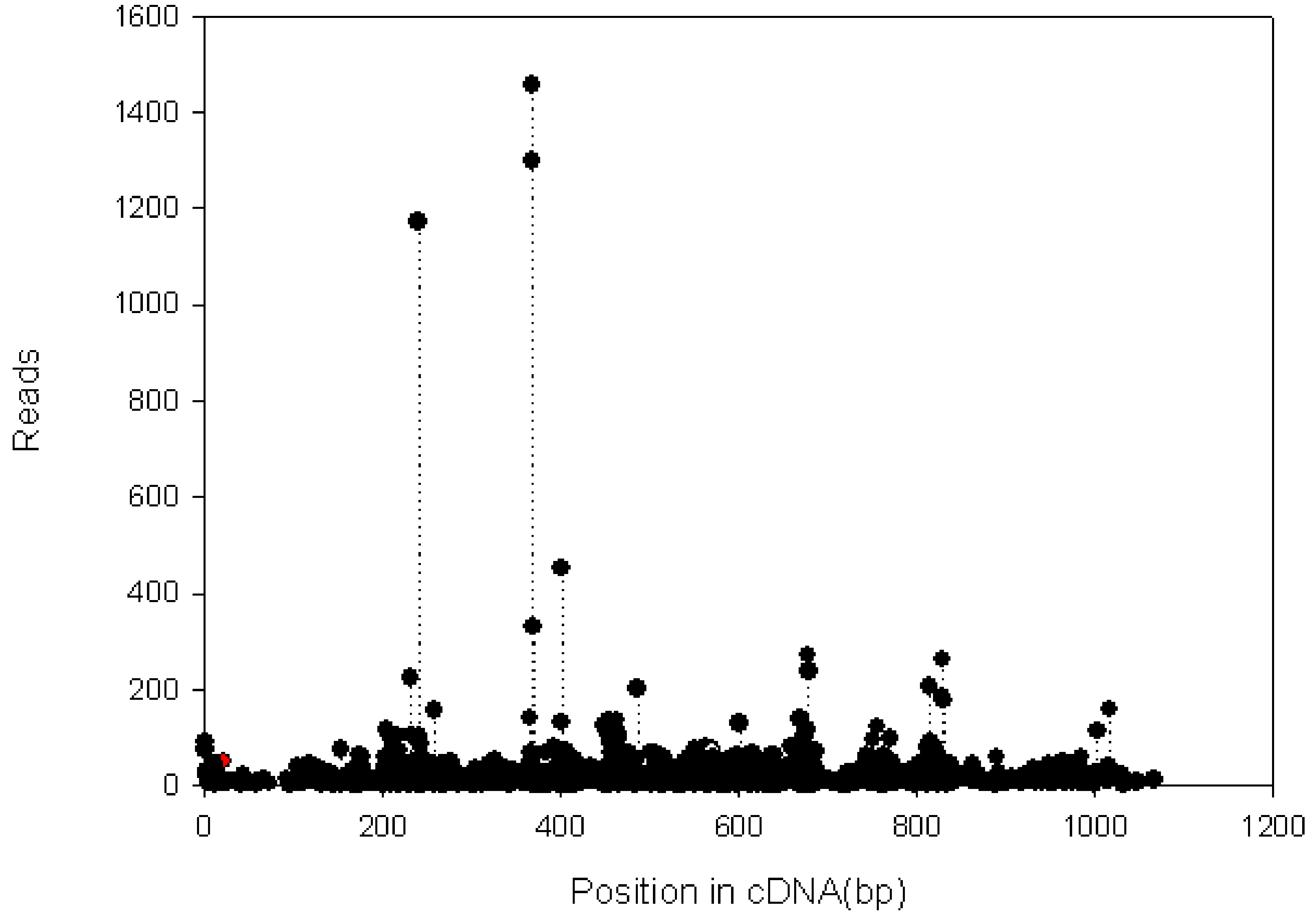


```
5' CUCGUGC-CACUCUCGUUUGUUAAGGA 3'        Cs8g17370.1
       :::: ::::::: :.:::.:
3' ---CACGAGUGAGAGAAGACAGU---- 5'        Csi-miR156a.2
```

Csi-miR156a.2, target=Orange1.1t02265.1 gene=Orange1.1t02265
Category:1
Score=1
Cleavage Site=982

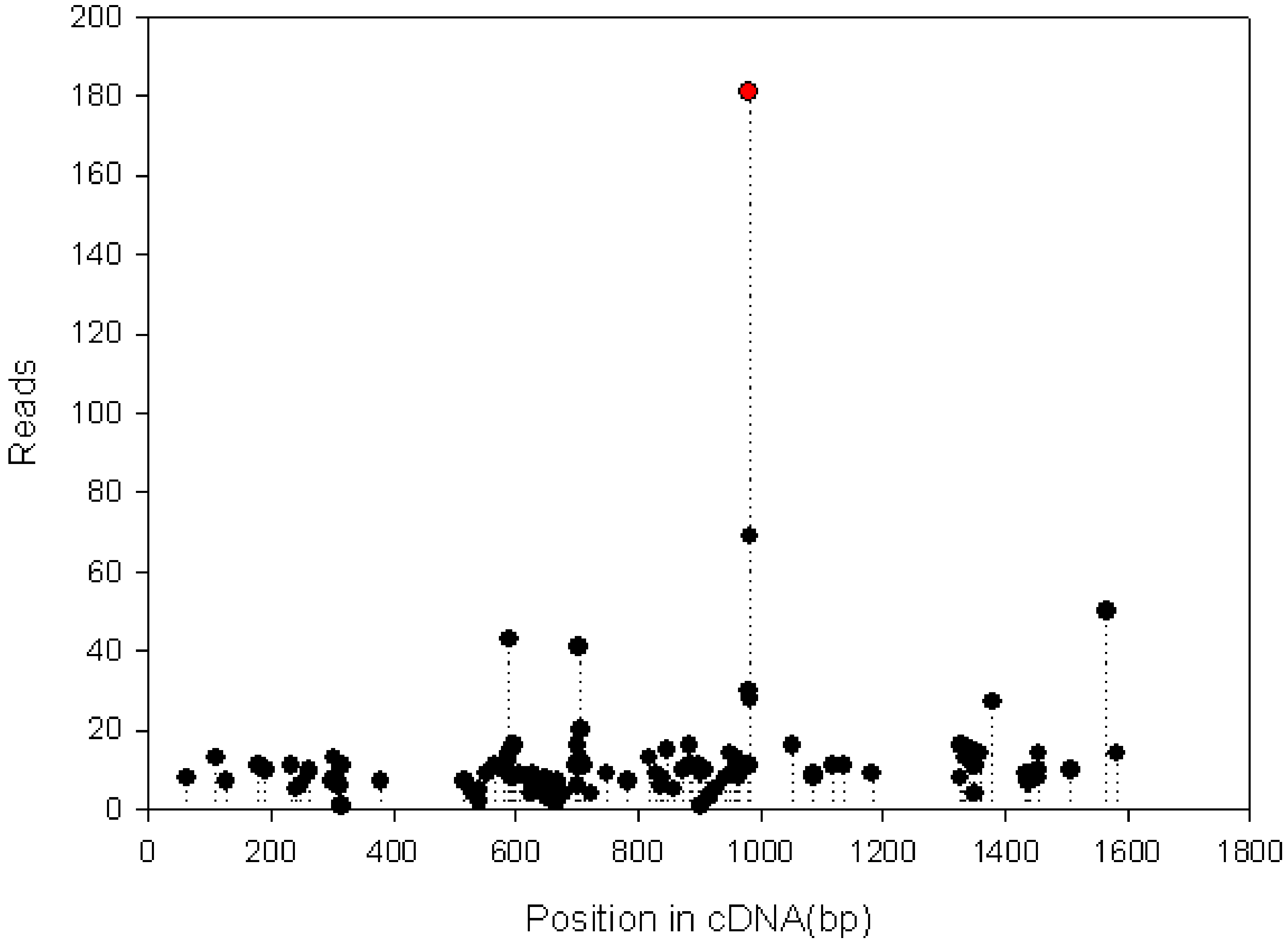


```
5' CUGUGCUCUCUCUCUUCUGUCAAAUC 3'        Orange1.1t02265.1
     :::::: :::::::::::::
3' --CACGAGUGAGAGAAGACAGU---- 5'        Csi-miR156a.2
```

Csi-miR156b.1, target=Cs2g05730.1 gene=Cs2g05730
Category:1
Score=2
Cleavage Site=818

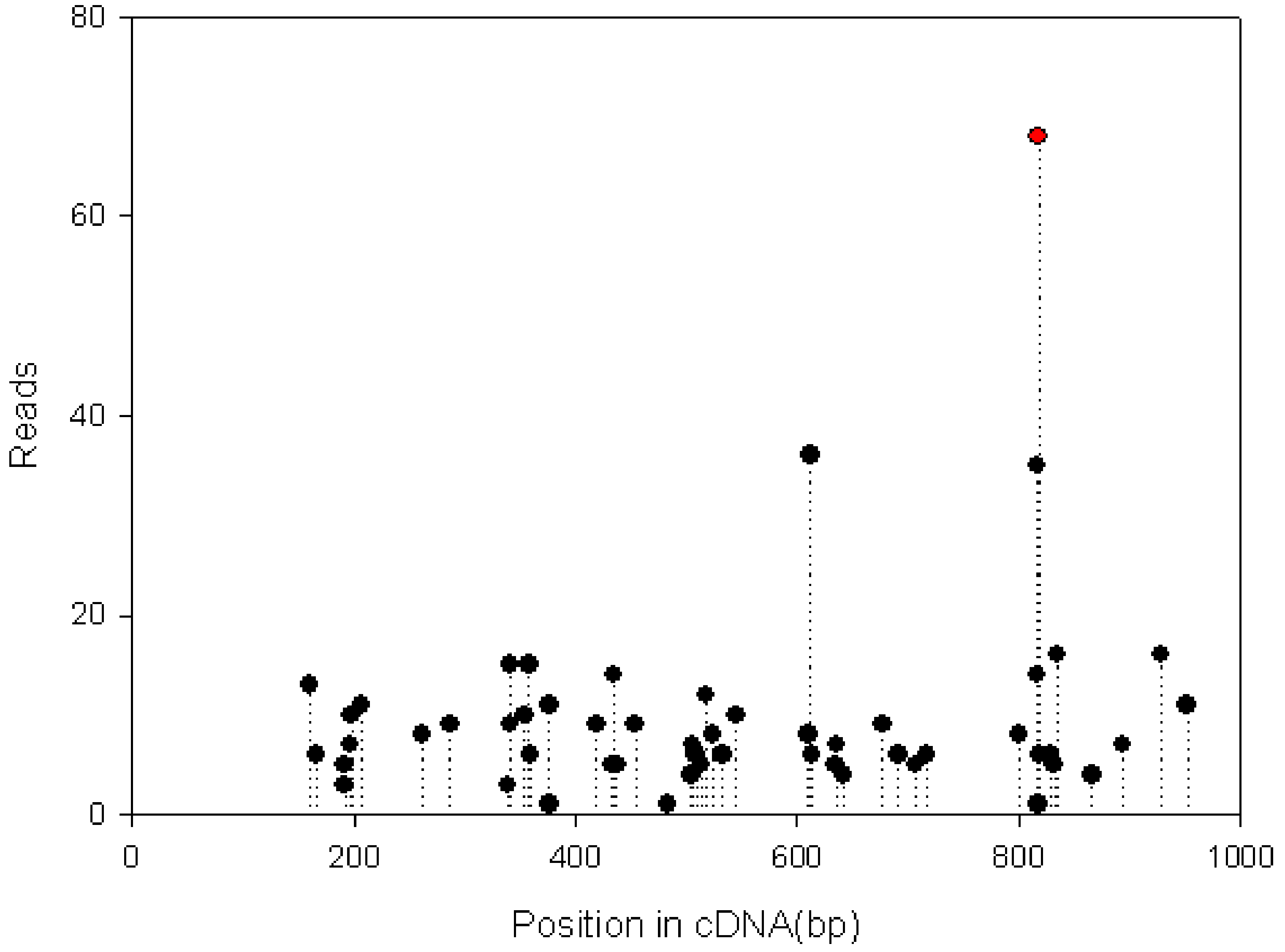


```
5' CGUGCUCUCUCUCUUCUGUCAAUGCU 3'        Cs2g05730.1
    :::::: :::::::::::::
3' -CACGAGUGAGAGAAGACAGUC---- 5'        Csi-miR156b.1
```

Csi-miR156b.1, target=Cs2g23550.1 gene=Cs2g23550
Category:1
Score=3
Cleavage Site=934

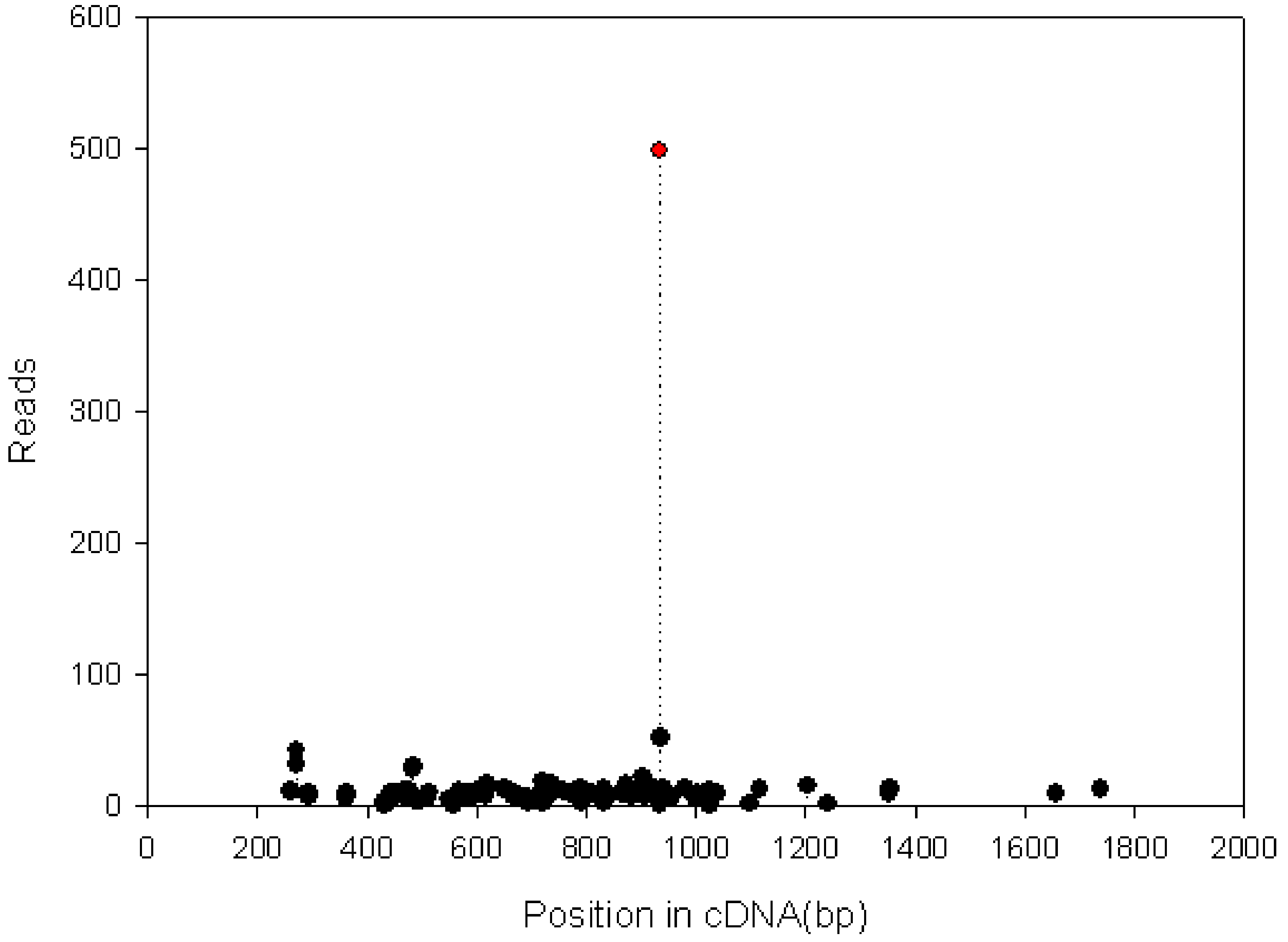


```
5' CAUGCUCUCUCUCUUCUGUCAUCCUA 3'        Cs2g23550.1
      ::::: :::::::::::::
3' -CACGAGUGAGAGAAGACAGUC---- 5'        Csi-miR156b.1
```

Csi-miR156b.1, target=Cs7g10830.1 gene=Cs7g10830
Category:1
Score=2
Cleavage Site=1183

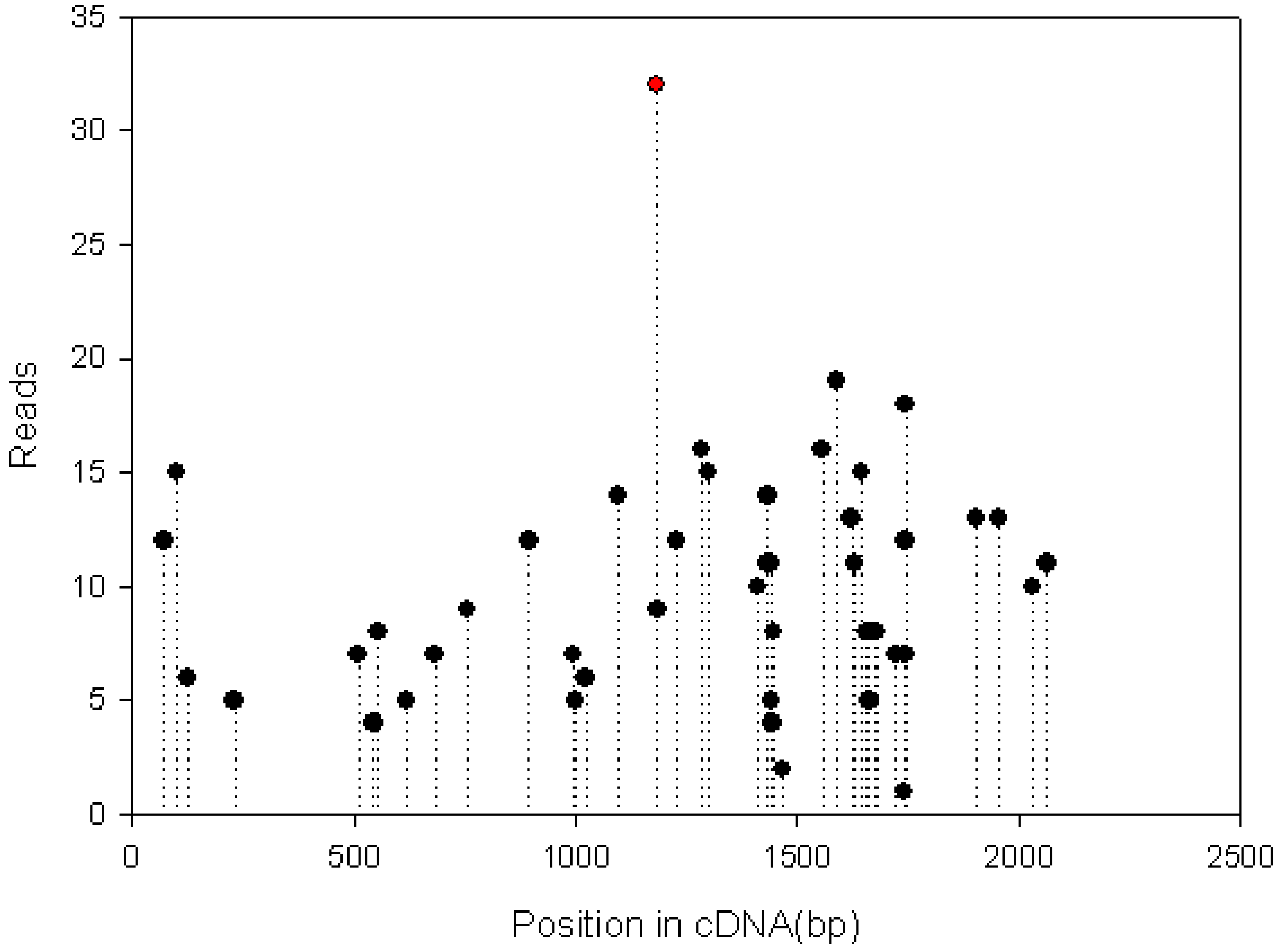


```
5' AGUGCUCUCUCUCUUCUGUCAAAAAC 3'        Cs7g10830.1
    :::::: :::::::::::::
3' -CACGAGUGAGAGAAGACAGUC---- 5'        Csi-miR156b.1
```

Csi-miR156b.1, target=Orange1.1t02265.1 gene=Orange1.1t02265
Category:1
Score=2
Cleavage Site=982

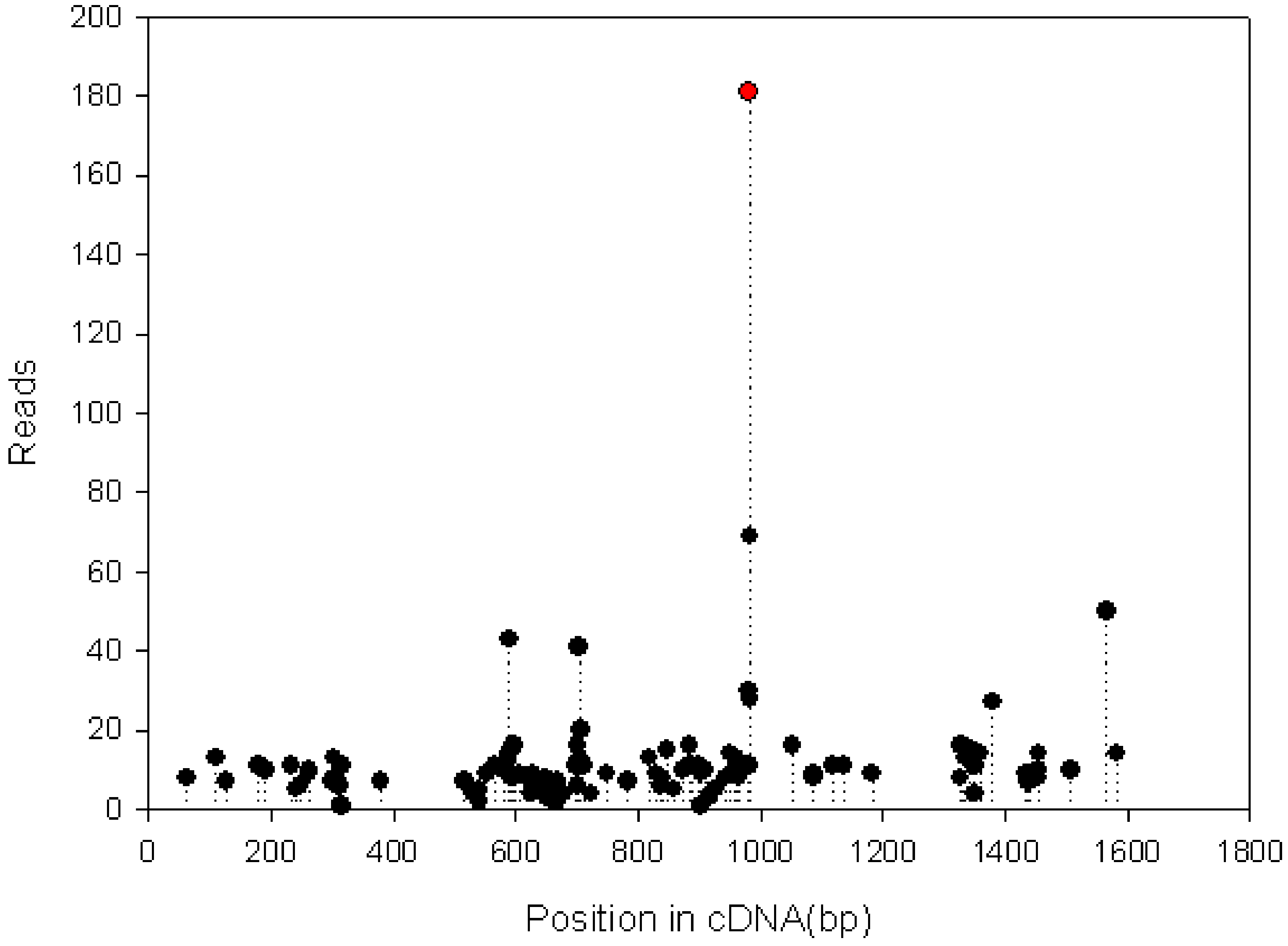


```
5' CUGUGCUCUCUCUCUUCUGUCAAAUC 3'        Orange1.1t02265.1
     :::::: :::::::::::::
3' --CACGAGUGAGAGAAGACAGUC--- 5'        Csi-miR156b.1
```

Csi-miR156c.1, target=Cs2g05730.1 gene=Cs2g05730
Category:1
Score=2
Cleavage Site=818

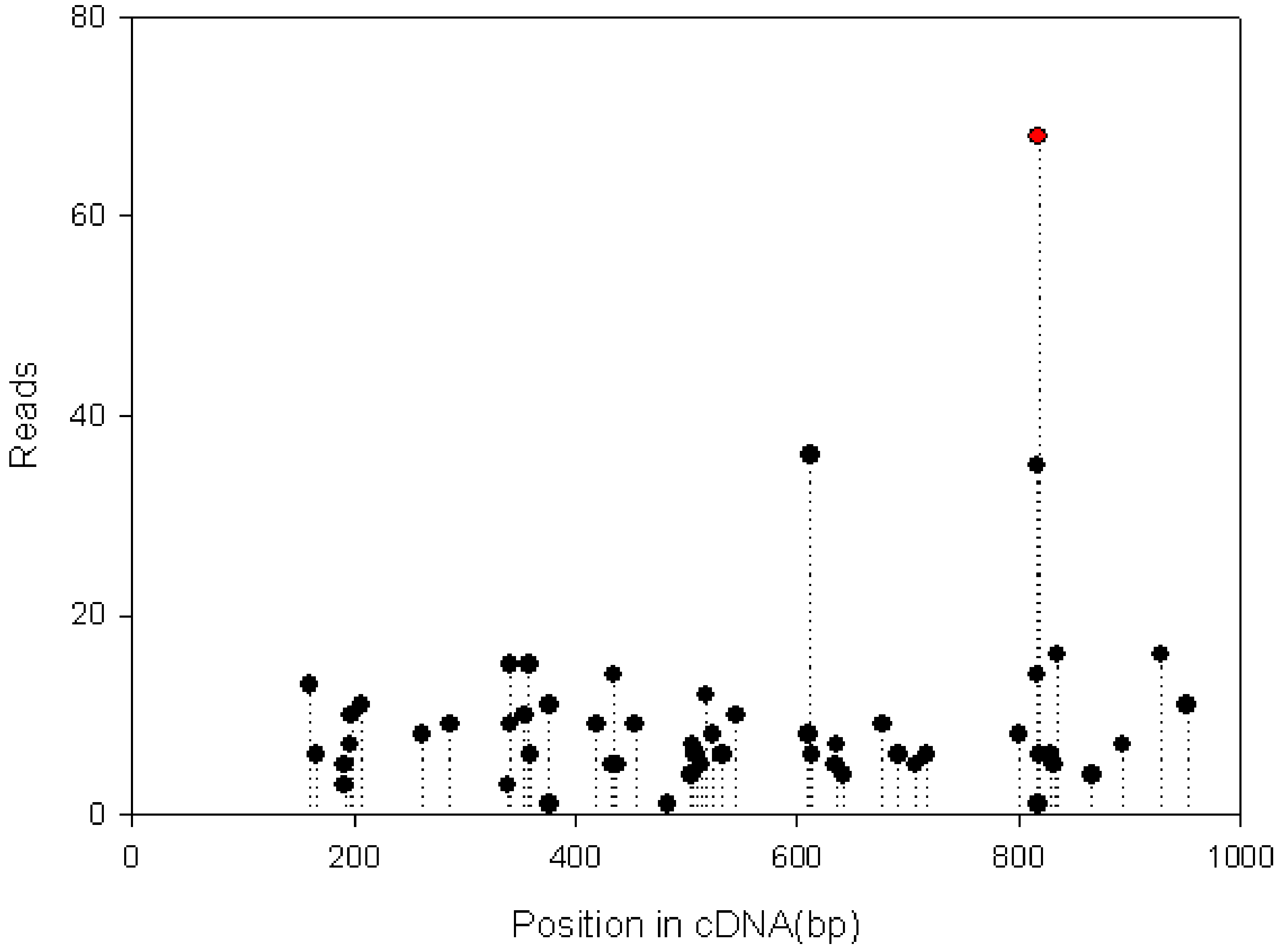


```
5' CGUGCUCUCUCUCUUCUGUCAAUGCU 3'         Cs2g05730.1
    :::::: :::::::::::::
3' ACACGAGUGAGAGAAGACAGU----- 5'         Csi-miR156c.1
```

Csi-miR156c.1, target=Cs2g23550.1 gene=Cs2g23550
Category:1
Score=3
Cleavage Site=934

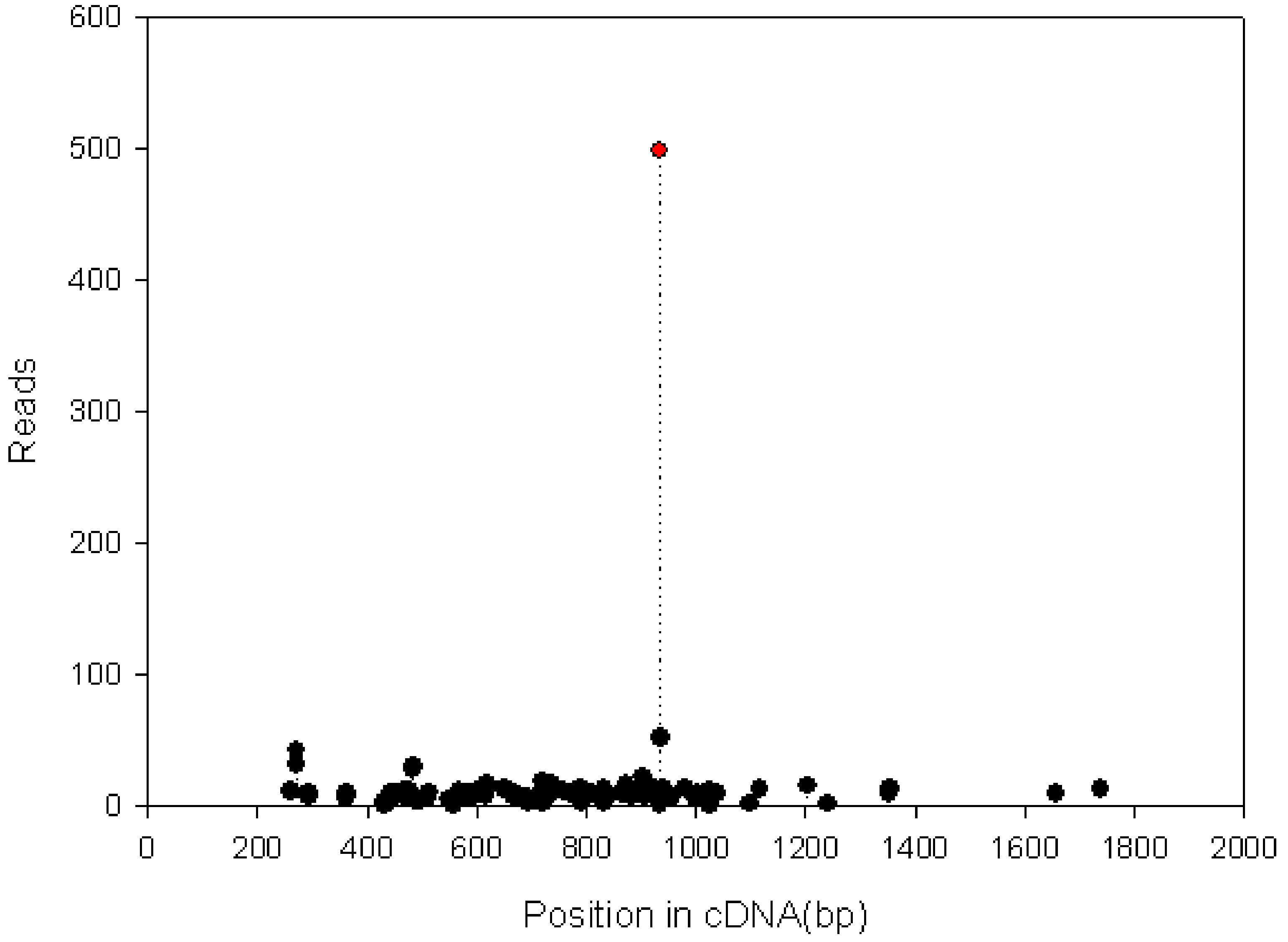


```
5' CAUGCUCUCUCUCUUCUGUCAUCCUA 3'         Cs2g23550.1
     ::::: :::::::::::::
3' ACACGAGUGAGAGAAGACAGU----- 5'         Csi-miR156c.1
```

Csi-miR156c.1, target=Cs7g10830.1 gene=Cs7g10830
Category:1
Score=2
Cleavage Site=1183

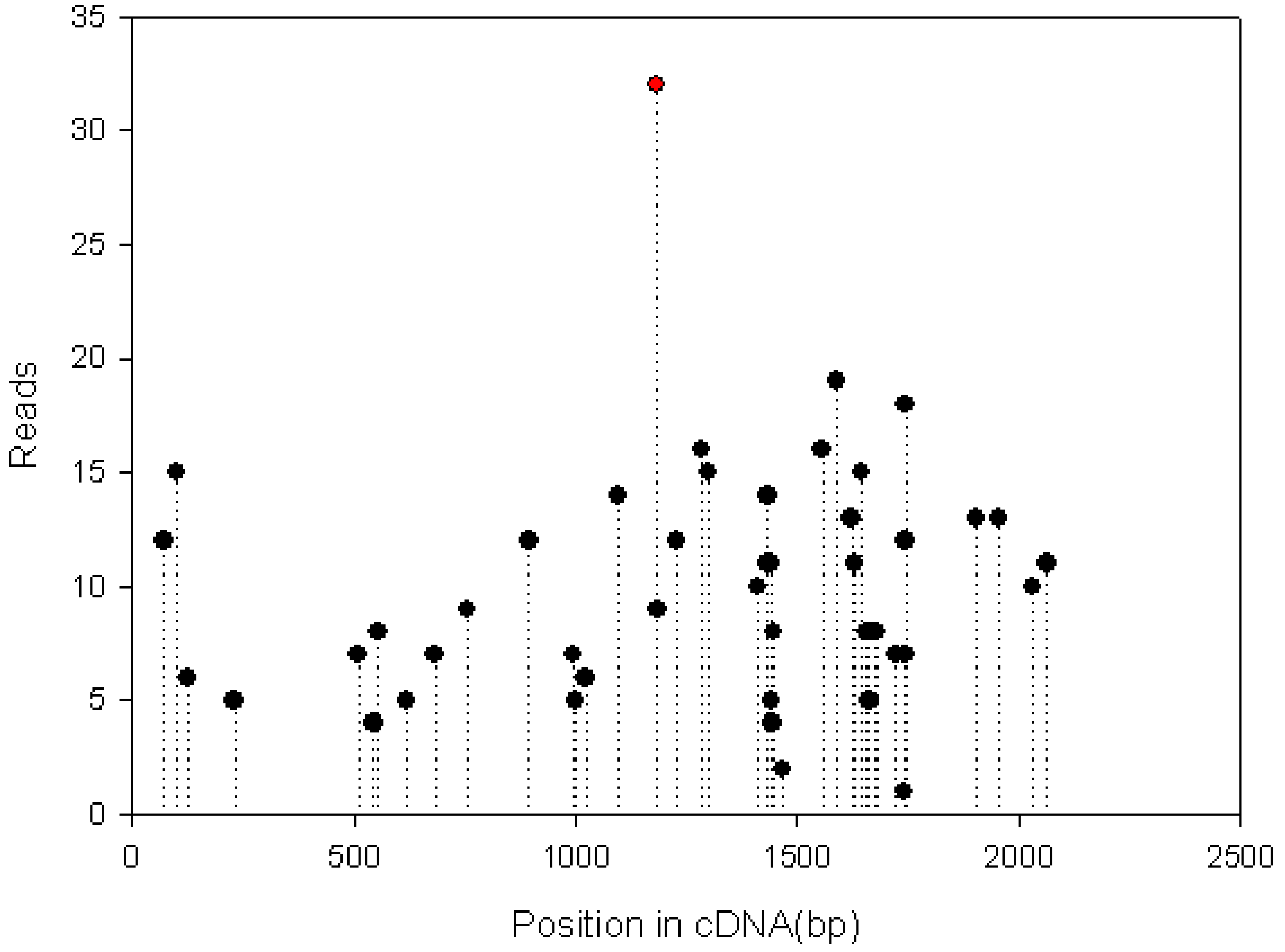


```
5' AGUGCUCUCUCUCUUCUGUCAAAAAC 3'        Cs7g10830.1
    :::::: ::::::::::::::
3' ACACGAGUGAGAGAAGACAGU----- 5'        Csi-miR156c.1
```

Csi-miR156c.1, target=Orange1.1t02265.1 gene=Orange1.1t02265
Category:1
Score=1
Cleavage Site=982

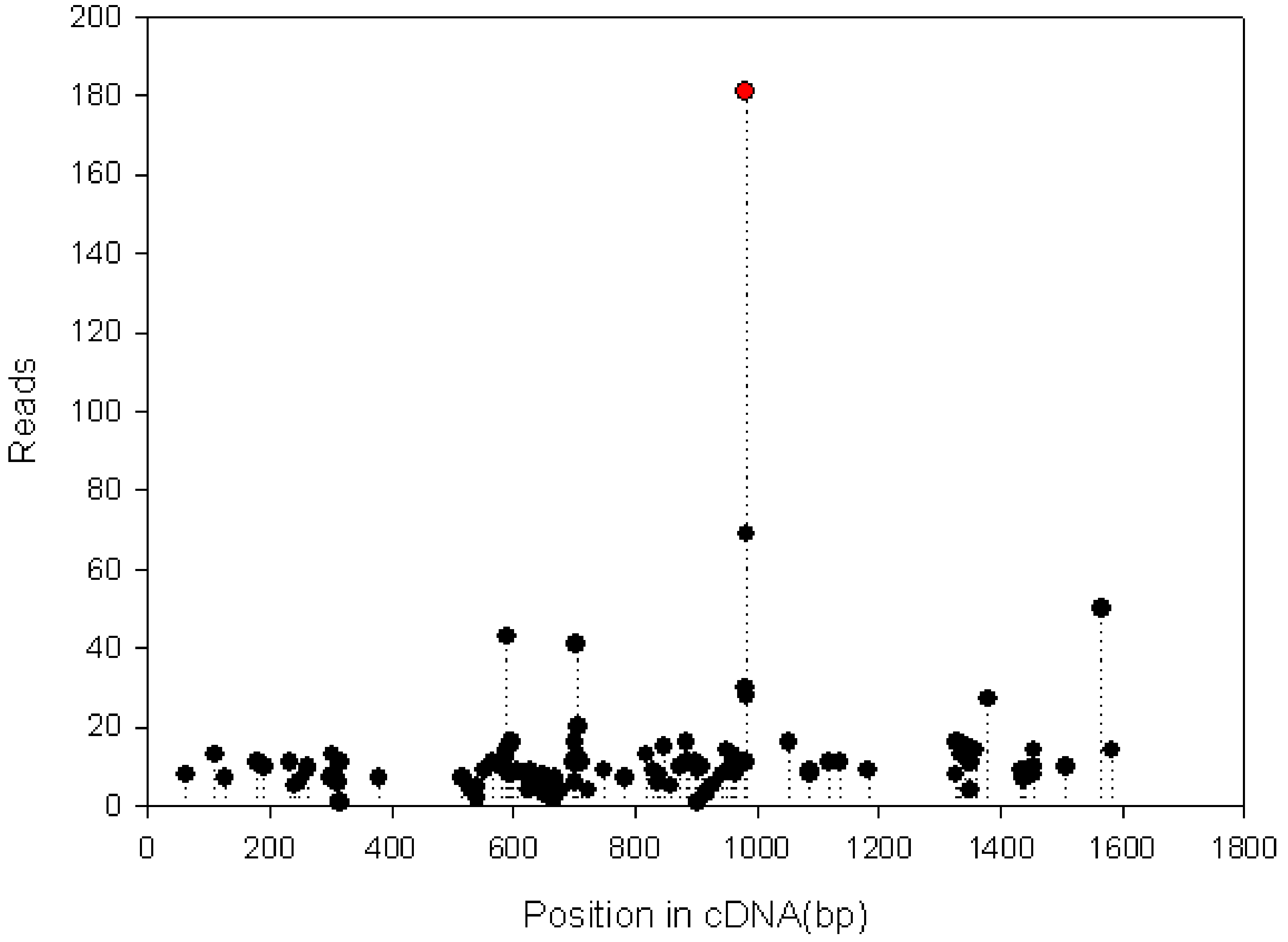


```
5' CUGUGCUCUCUCUCUUCUGUCAAAUC 3'        Orange1.1t02265.1
    ::::::: :::::::::::::
3' -ACACGAGUGAGAGAAGACAGU---- 5'        Csi-miR156c.1
```

Csi-miR156d, target=Cs2g05730.1 gene=Cs2g05730
Category:1
Score=2.5
Cleavage Site=818

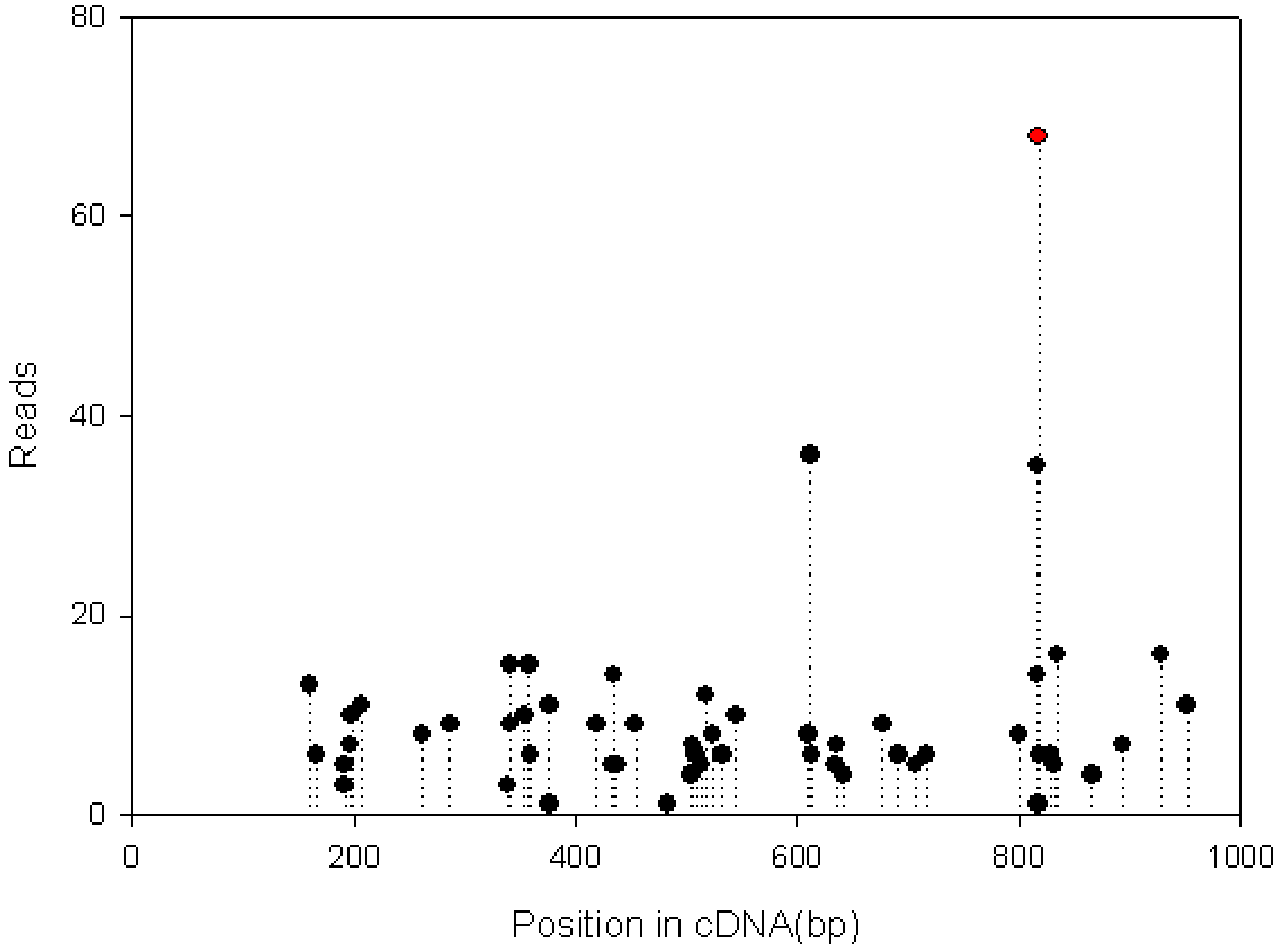


```
5' CGUGCUCUCUCUCUUCUGUCAAUGCU 3'        Cs2g05730.1
    :.::::::: ::::::::::
3' -CGCGAGAGAUAGAAGACAGU----- 5'        Csi-miR156d
```

Csi-miR156d, target=Cs2g23550.1 gene=Cs2g23550
Category:1
Score=5
Cleavage Site=934

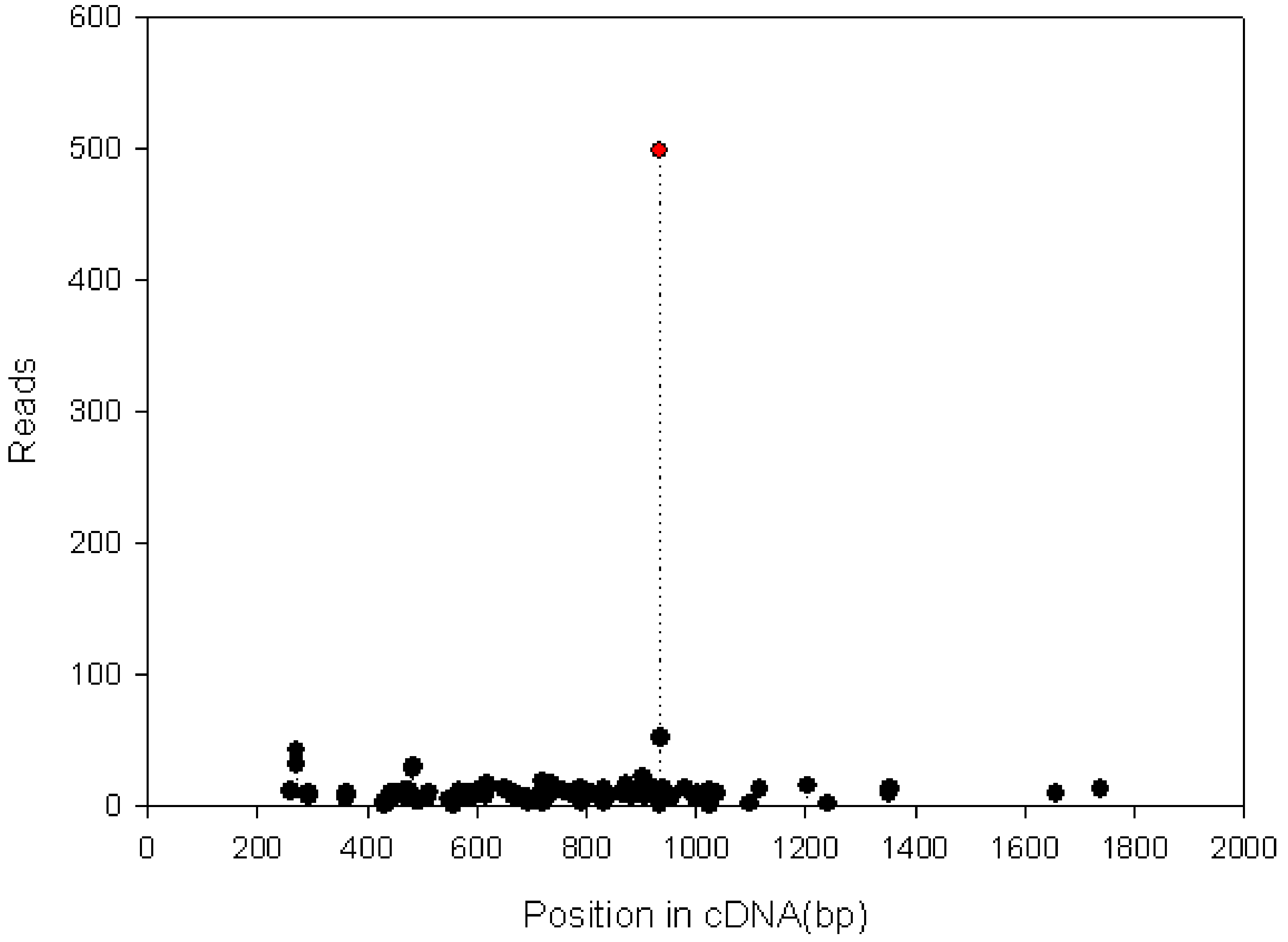


```
5' CAUGCUCUCUCUCUUCUGUCAUCCUA 3'        Cs2g23550.1
     .::::::: :::::::::::
3' -CGCGAGAGAUAGAAGACAGU----- 5'        Csi-miR156d
```

Csi-miR156d, target=Cs7g10830.1 gene=Cs7g10830
Category:1
Score=2.5
Cleavage Site=1183

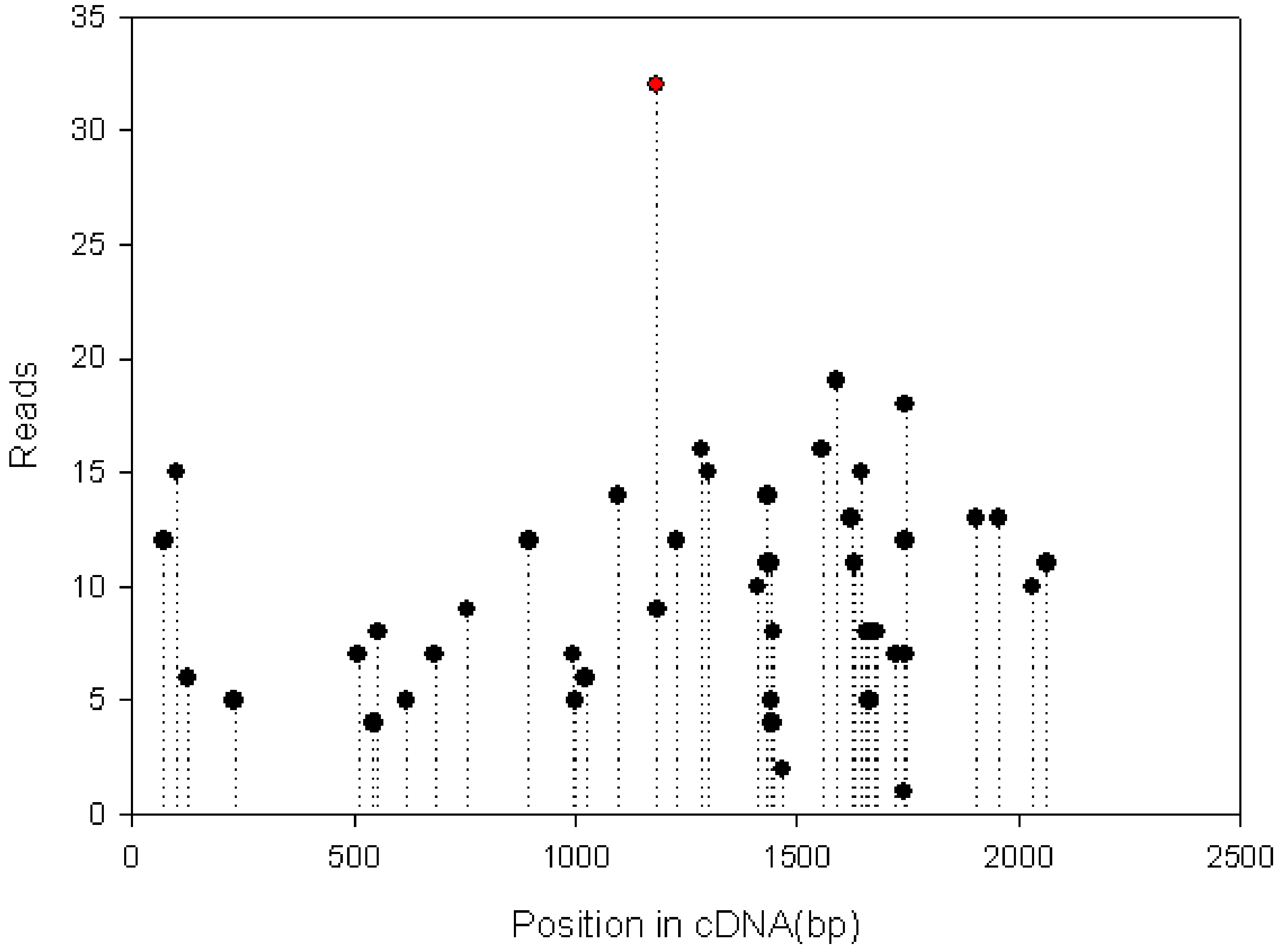


```
5' AGUGCUCUCUCUCUUCUGUCAAAAAC 3'        Cs7g10830.1
    :.:::::::: ::::::::::
3' -CGCGAGAGAUAGAAGACAGU----- 5'        Csi-miR156d
```

Csi-miR156d, target=Orange1.1t02265.1 gene=Orange1.1t02265
Category:1
Score=5
Cleavage Site=982

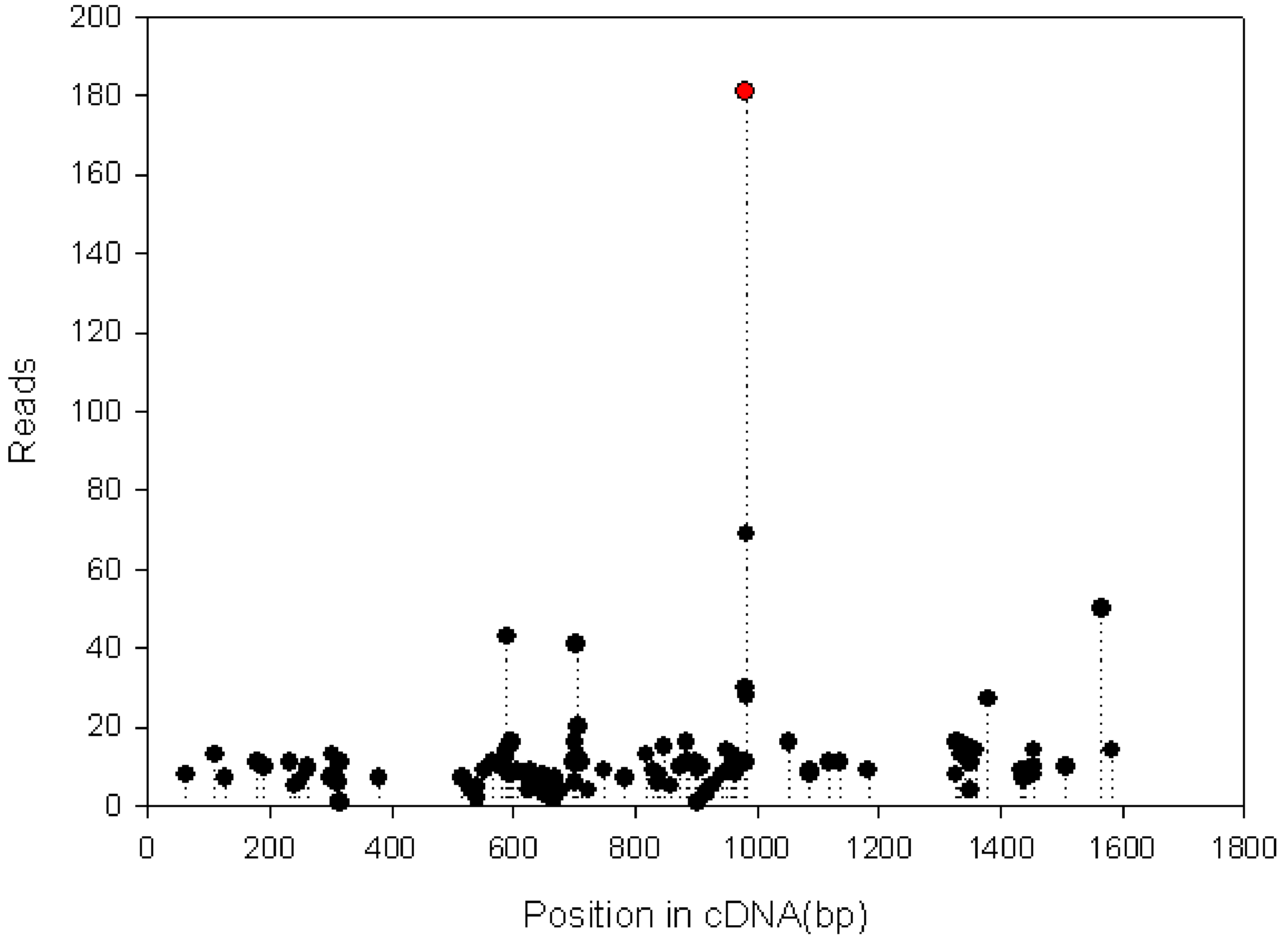


```
5' CUGUGCUCUCUCUCUUCUGUCAAAUC 3'        Orange1.1t02265.1
     :.:::::::: ::::::::::
3' --CGCGAGAGAUAGAAGACAGU---- 5'        Csi-miR156d
```

Csi-miR156e, target=Cs2g05730.1 gene=Cs2g05730
Category:1
Score=2.5
Cleavage Site=818

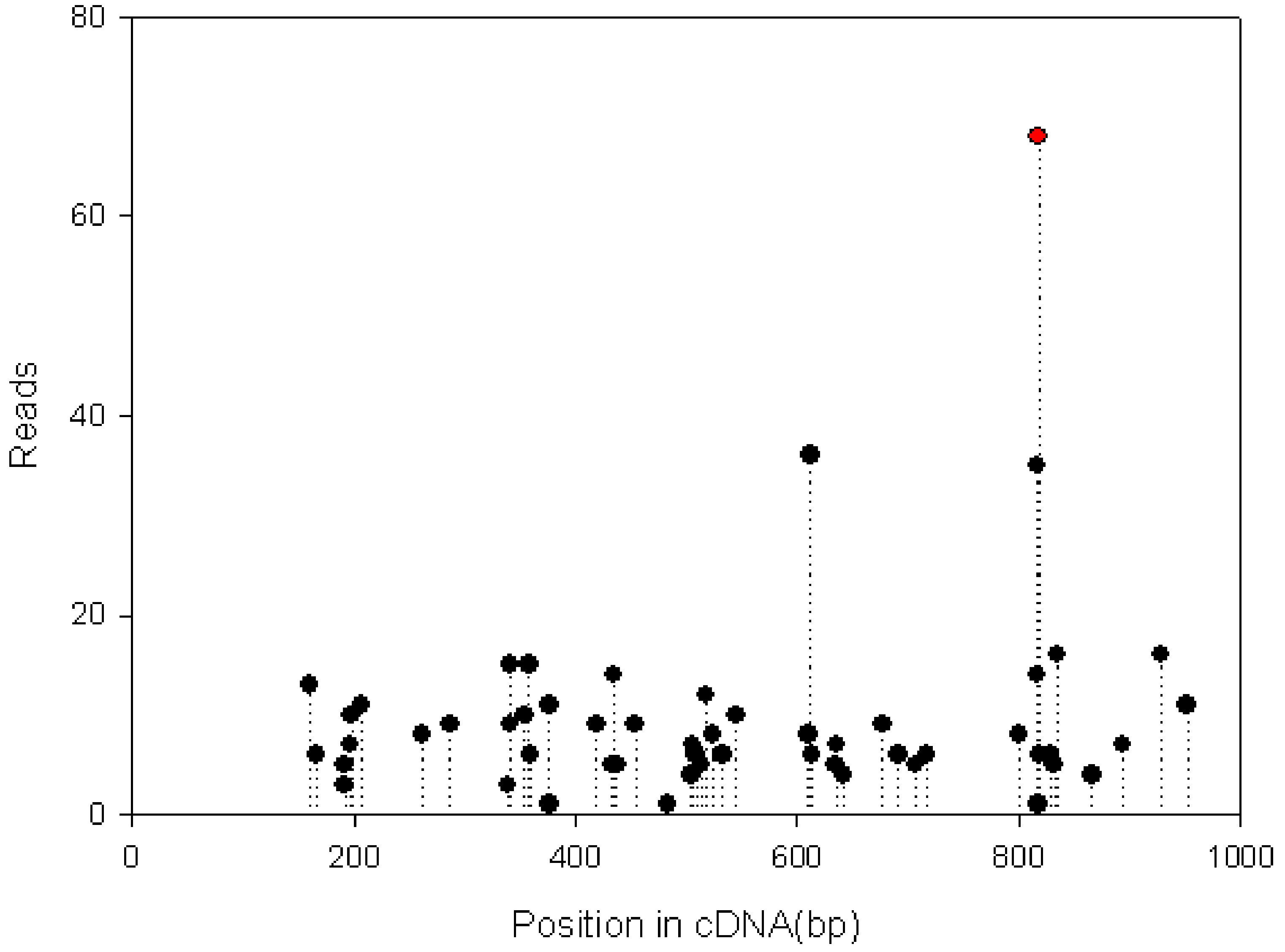


```
5' CGUGCUCUCUCUUCUGUCAAUGCU 3'        Cs2g05730.1
     :.::::::: :::::::::::
3' -CGCGAGAGAUAGAAGACAGUG---- 5'      Csi-miR156e
```

Csi-miR156e, target=Cs2g23550.1 gene=Cs2g23550
Category:1
Score=4
Cleavage Site=934

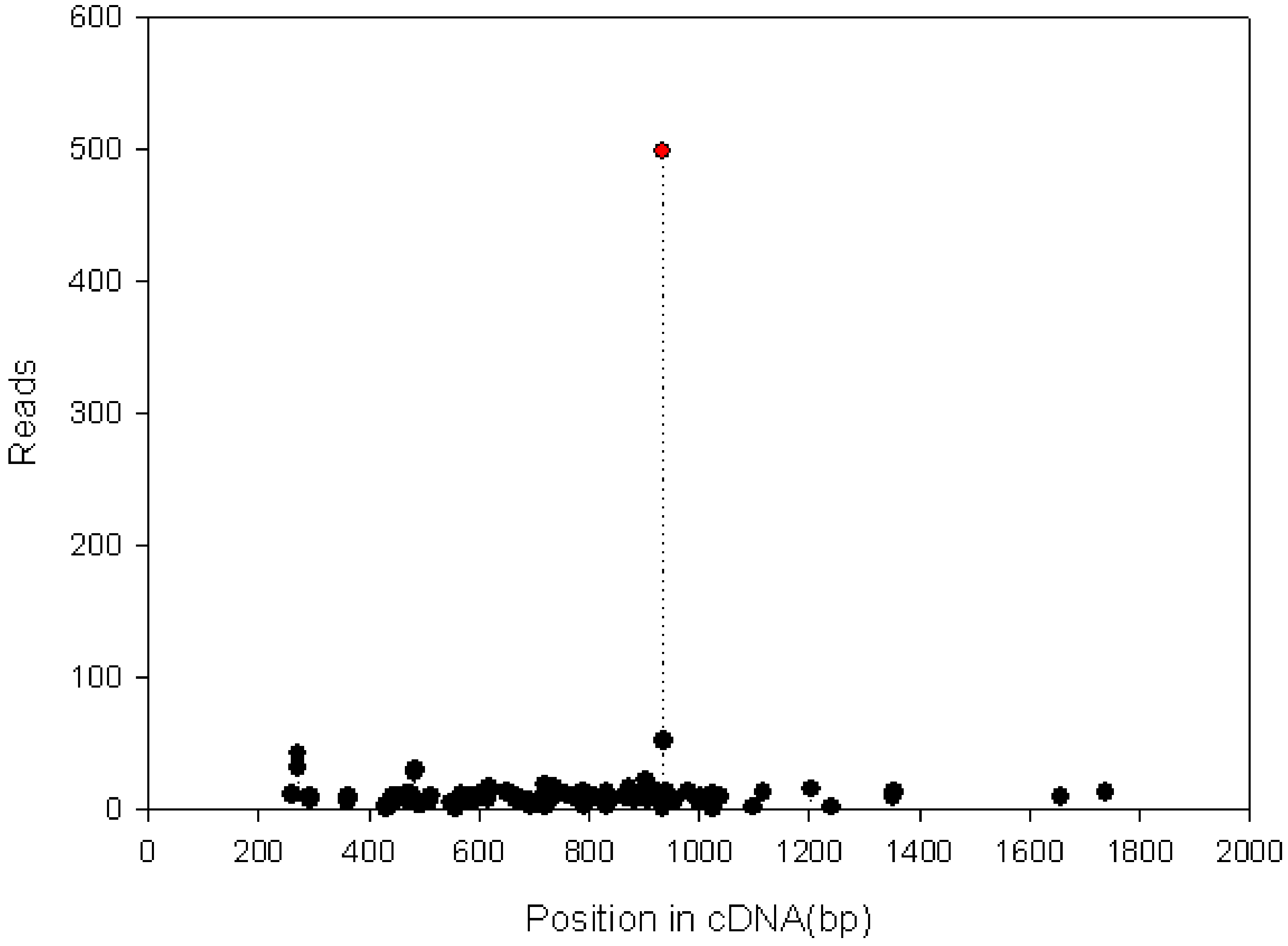


```
5' CAUGCUCUCUCUCUUCUGUCAUCCUA 3'        Cs2g23550.1
     .::::::: ::::::::::.
3' -CGCGAGAGAUAGAAGACAGUG---- 5'        Csi-miR156e
```

Csi-miR156e, target=Cs7g10830.1 gene=Cs7g10830
Category:1
Score=3.5
Cleavage Site=1183

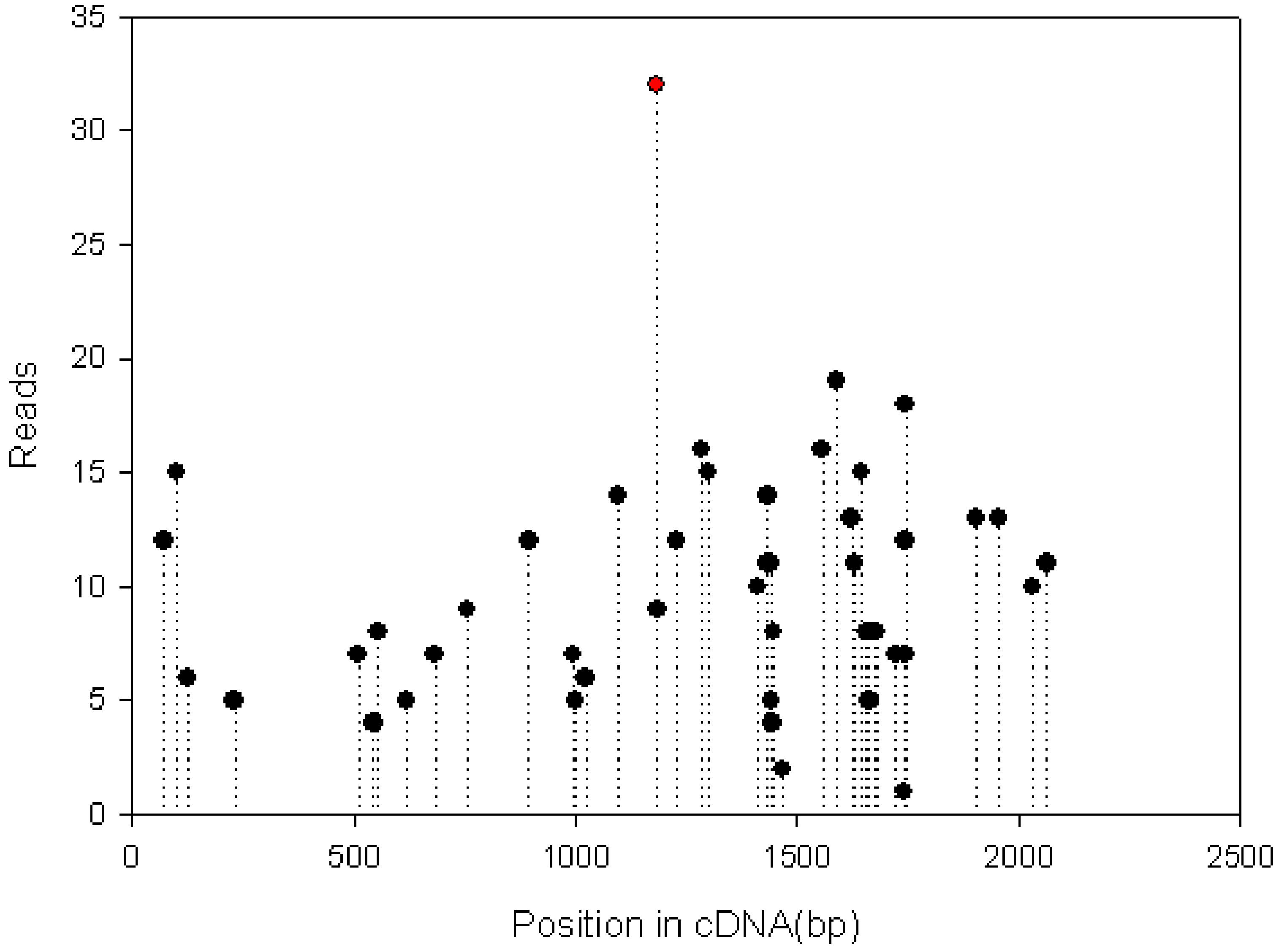


```
5' AGUGCUCUCUCUCUUCUGUCAAAAAC 3'        Cs7g10830.1
    :.::::::: ::::::::::
3' -CGCGAGAGAUAGAAGACAGUG---- 5'        Csi-miR156e
```

Csi-miR156e, target=Orange1.1t02265.1 gene=Orange1.1t02265
Category:1
Score=3.5
Cleavage Site=982

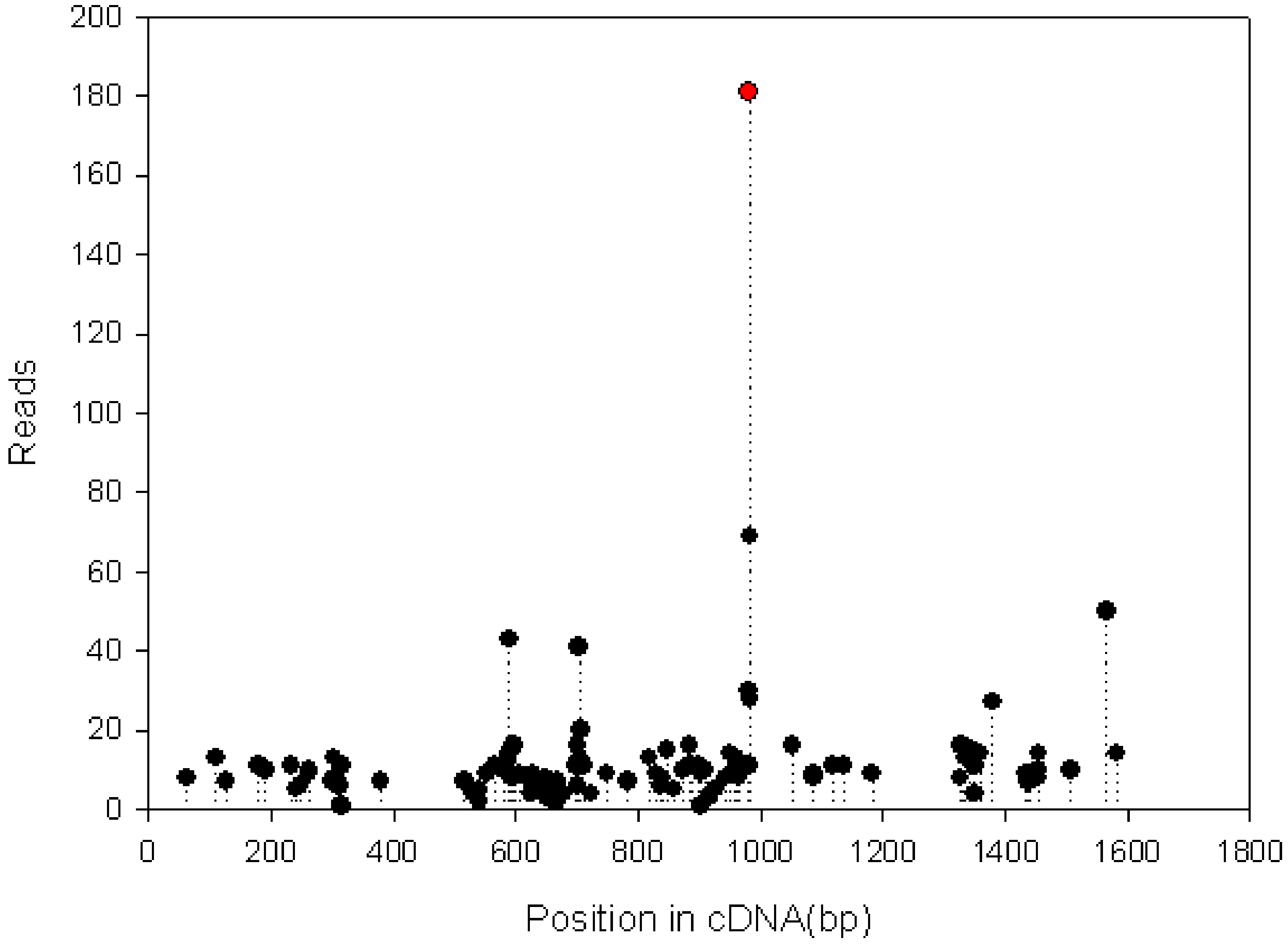


```
5' CUGUGCUCUCUCUCUUCUGUCAAAUC 3'        Orange1.1t02265.1
     :.:::::::: ::::::::::
3' --CGCGAGAGAUAGAAGACAGUG--- 5'        Csi-miR156e
```

Csi-miR156f.2, target=Cs2g05730.1 gene=Cs2g05730
Category:1
Score=1.5
Cleavage Site=818

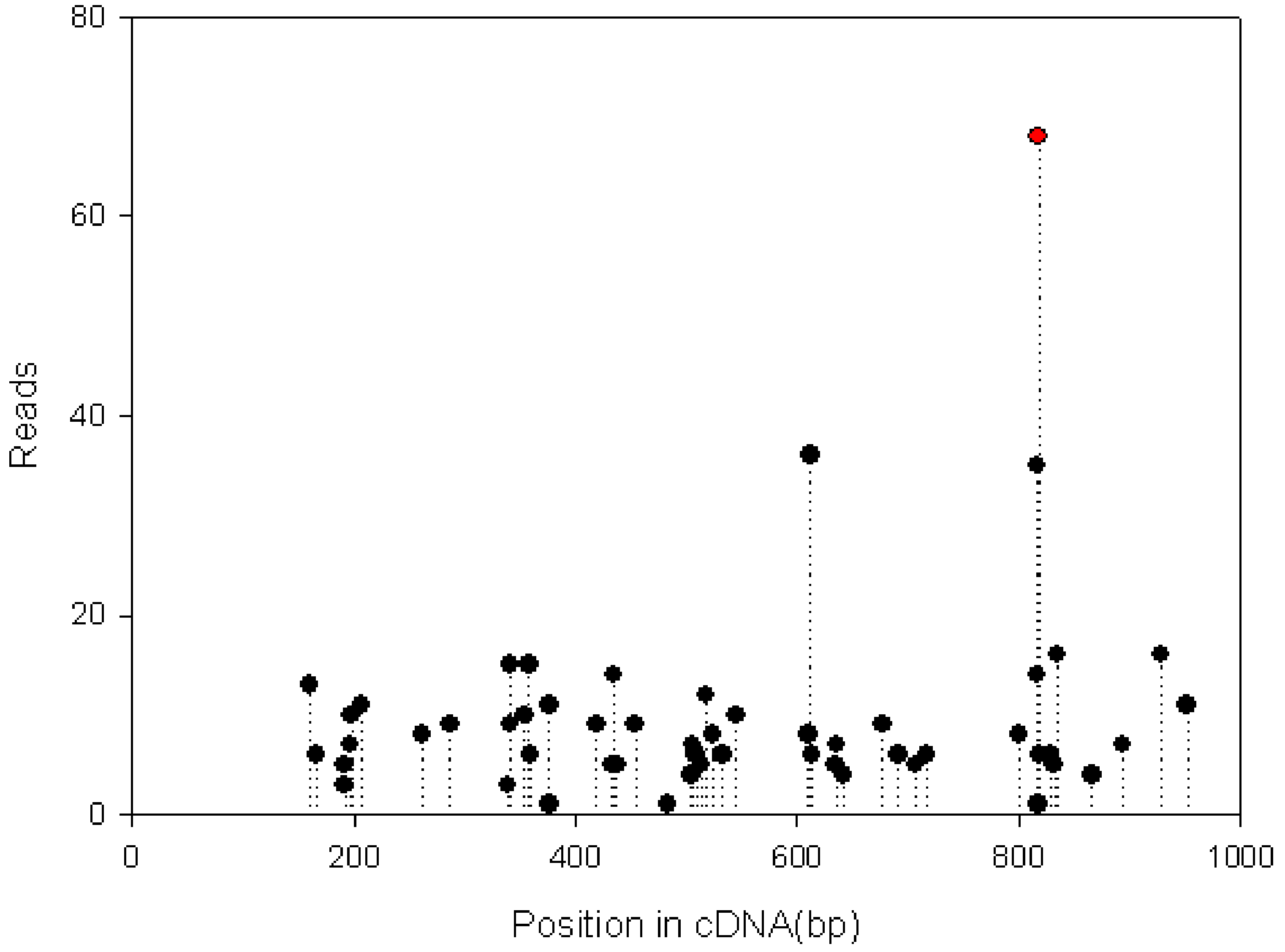


```
5' CGUGCUCUCUCUUCUGUCAAUGCU 3'        Cs2g05730.1
    ::.:::::::::::::::::
3' -CAUGAGAGAGAGAAGACAGUA---- 5'      Csi-miR156f.2
```

Csi-miR156f.2, target=Cs2g23550.1 gene=Cs2g23550
Category:1
Score=1.5
Cleavage Site=934

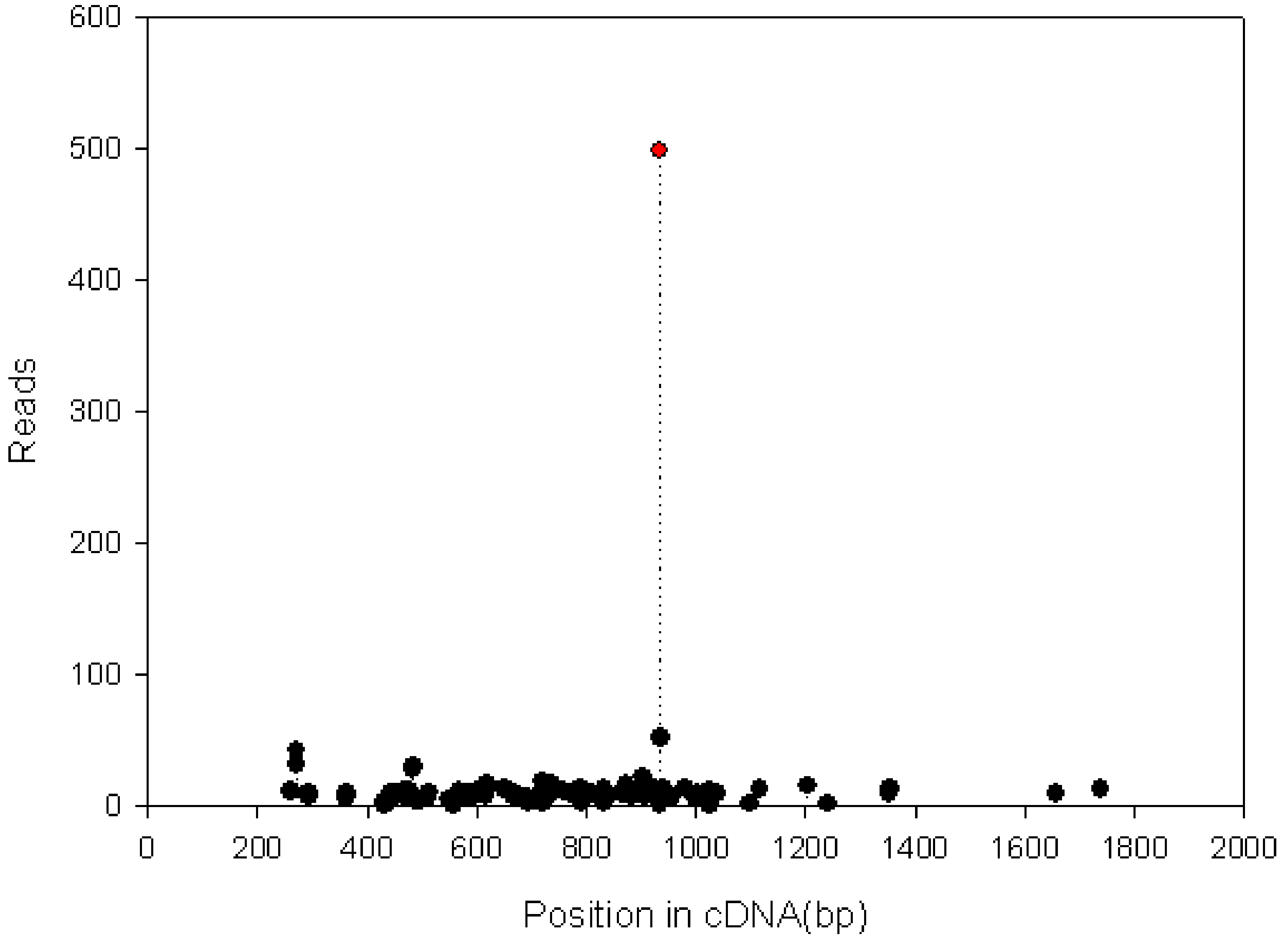


```
5' CAUGCUCUCUCUCUUCUGUCAUCCUA 3'        Cs2g23550.1
     :.::::::::::::::::::::
3' -CAUGAGAGAGAGAAGACAGUA---- 5'        Csi-miR156f.2
```

Csi-miR156f.2, target=Cs7g10830.1 gene=Cs7g10830
Category:1
Score=1.5
Cleavage Site=1183

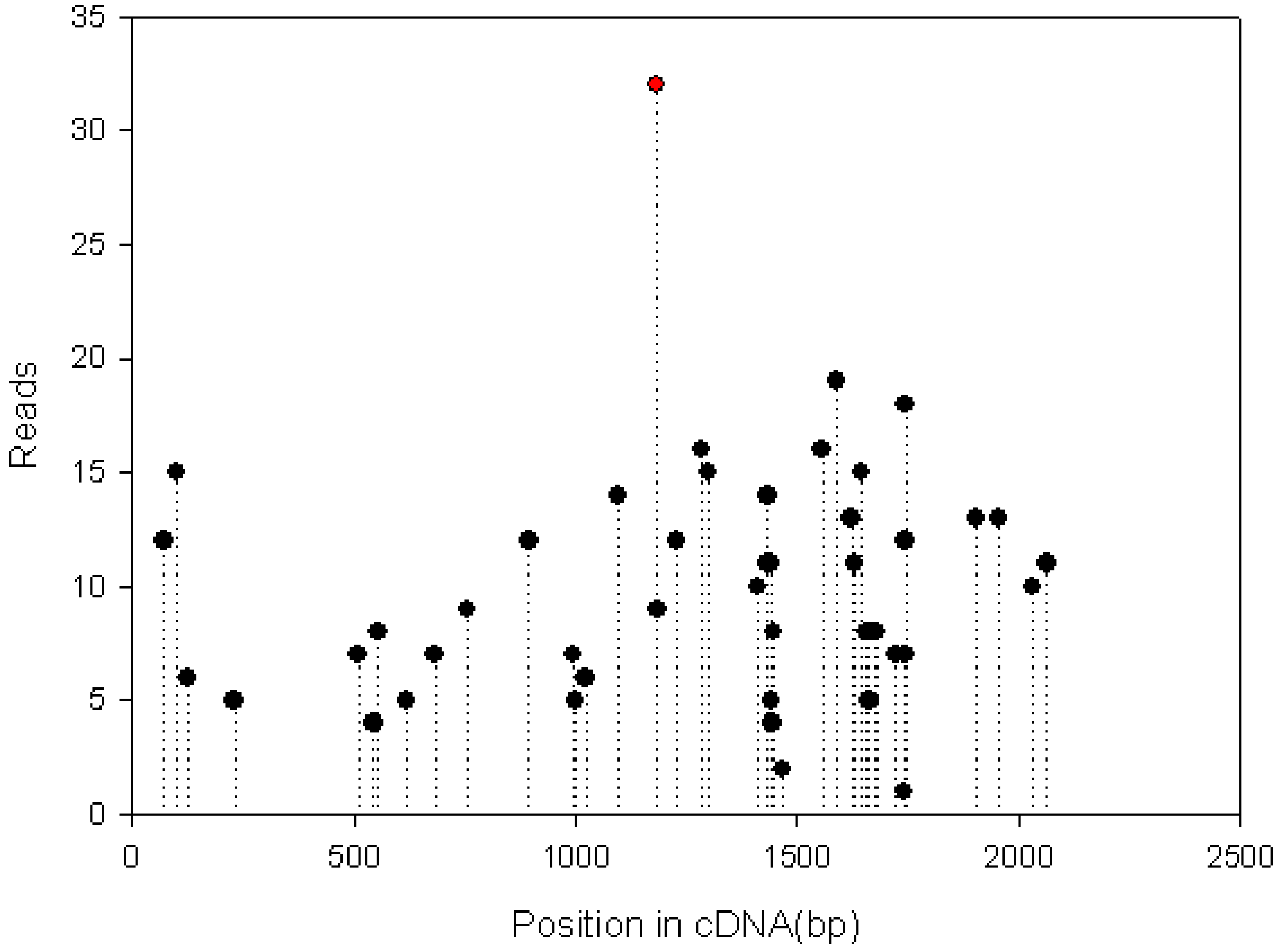


```
5' AGUGCUCUCUCUCUUCUGUCAAAAAC 3'        Cs7g10830.1
    ::.::::::::::::::::::
3' -CAUGAGAGAGAGAAGACAGUA---- 5'        Csi-miR156f.2
```

Csi-miR156f.2, target=Cs8g19900.1 gene=Cs8g19900
Category:2
Score=5
Cleavage Site=2794

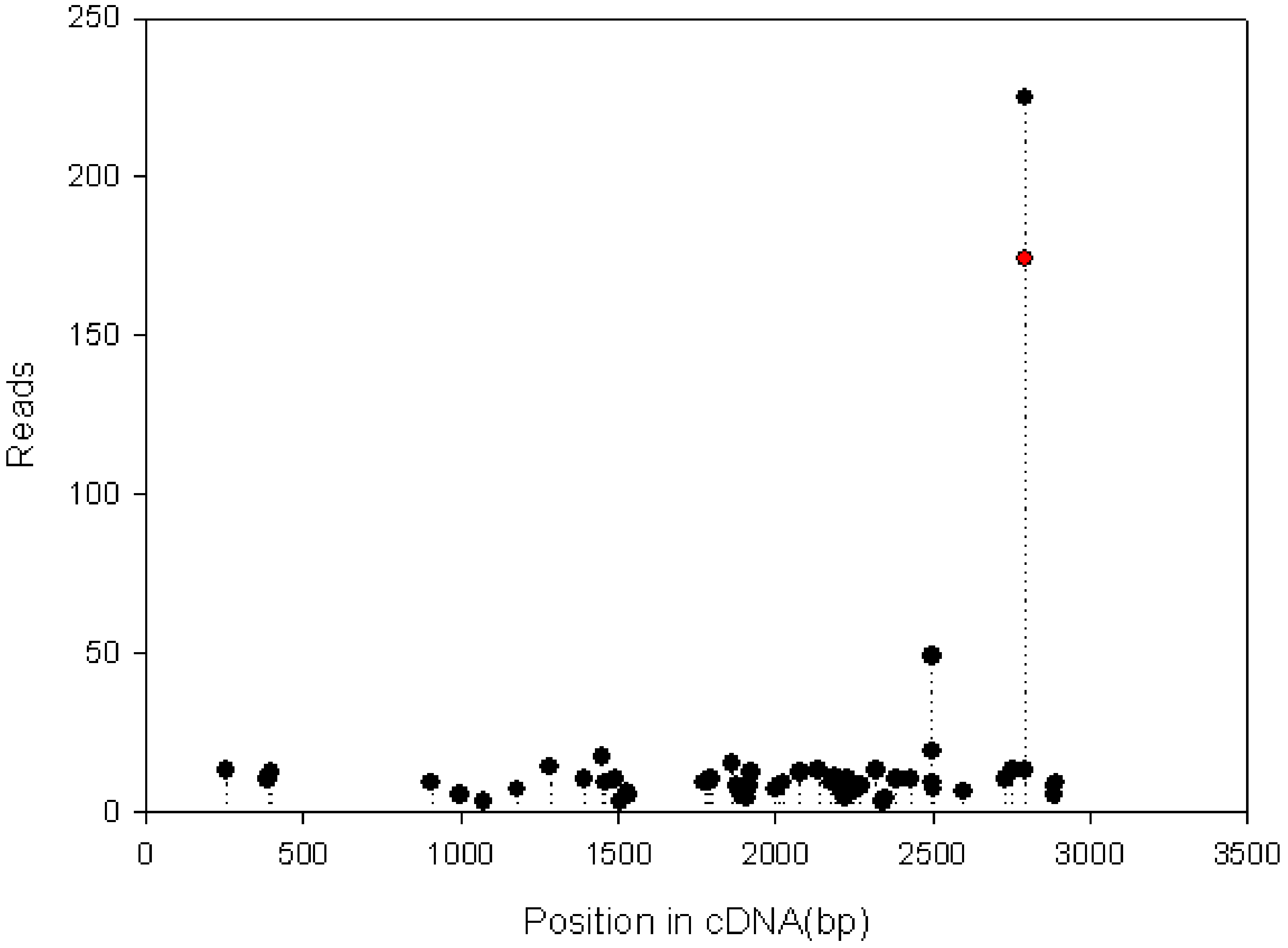


```
5' GUG-UCUUUCUCUUUUGACAUUAUGAG 3'        Cs8g19900.1
   ::. :::.:::::::.:: :::
3' CAUGAGAGAGAGAAGACAGUA------ 5'        Csi-miR156f.2
```

Csi-miR156f.2, target=Orange1.1t02265.1 gene=Orange1.1t02265
Category:1
Score=1.5
Cleavage Site=982

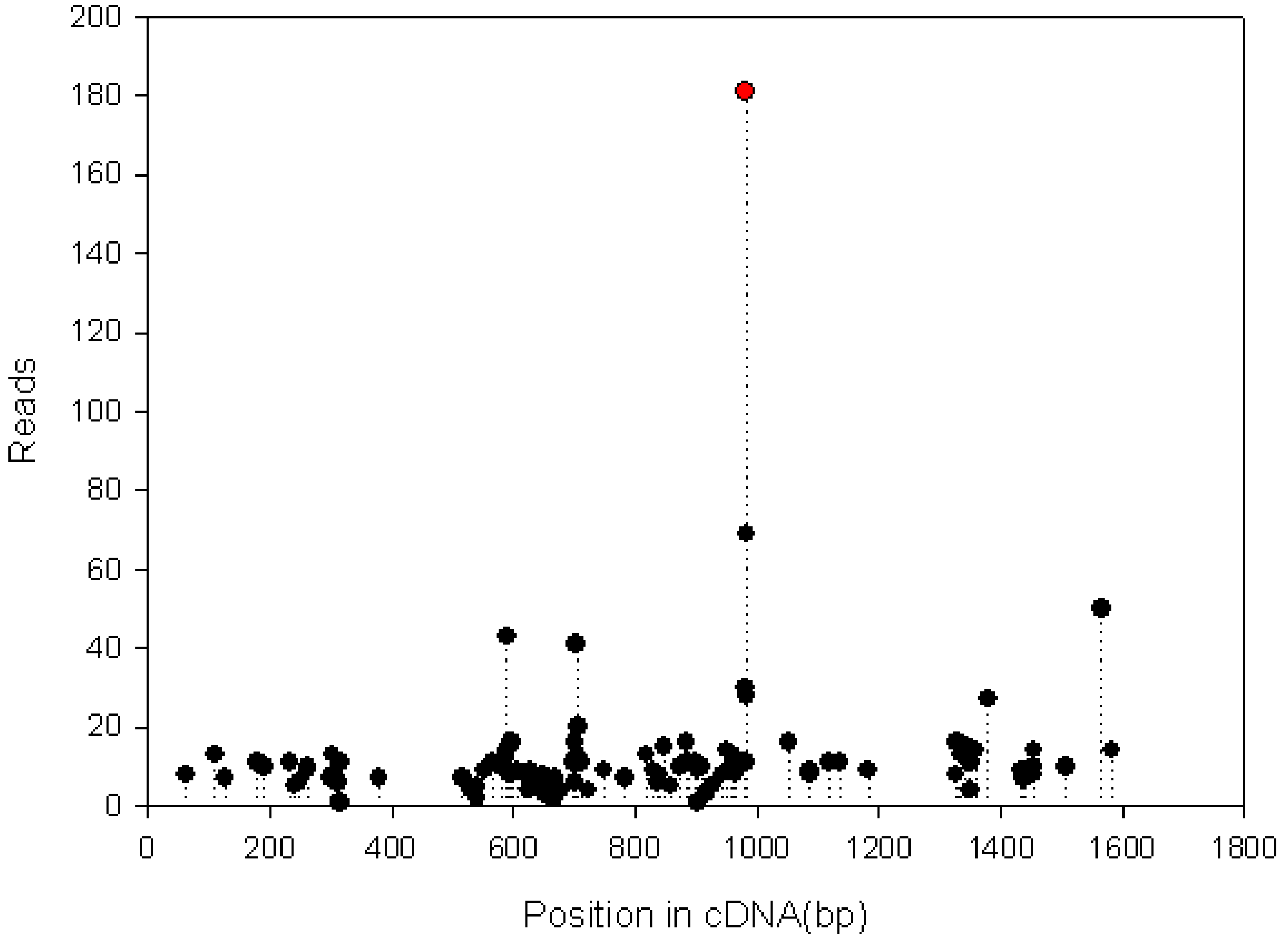


```
5' CUGUGCUCUCUCUCUUCUGUCAAAUC 3'        Orange1.1t02265.1
     ::.:::::::::::::::::::
3' --CAUGAGAGAGAGAAGACAGUA--- 5'        Csi-miR156f.2
```

Csi-miR156g.1, target=Cs2g05730.1 gene=Cs2g05730
Category:1
Score=3
Cleavage Site=818

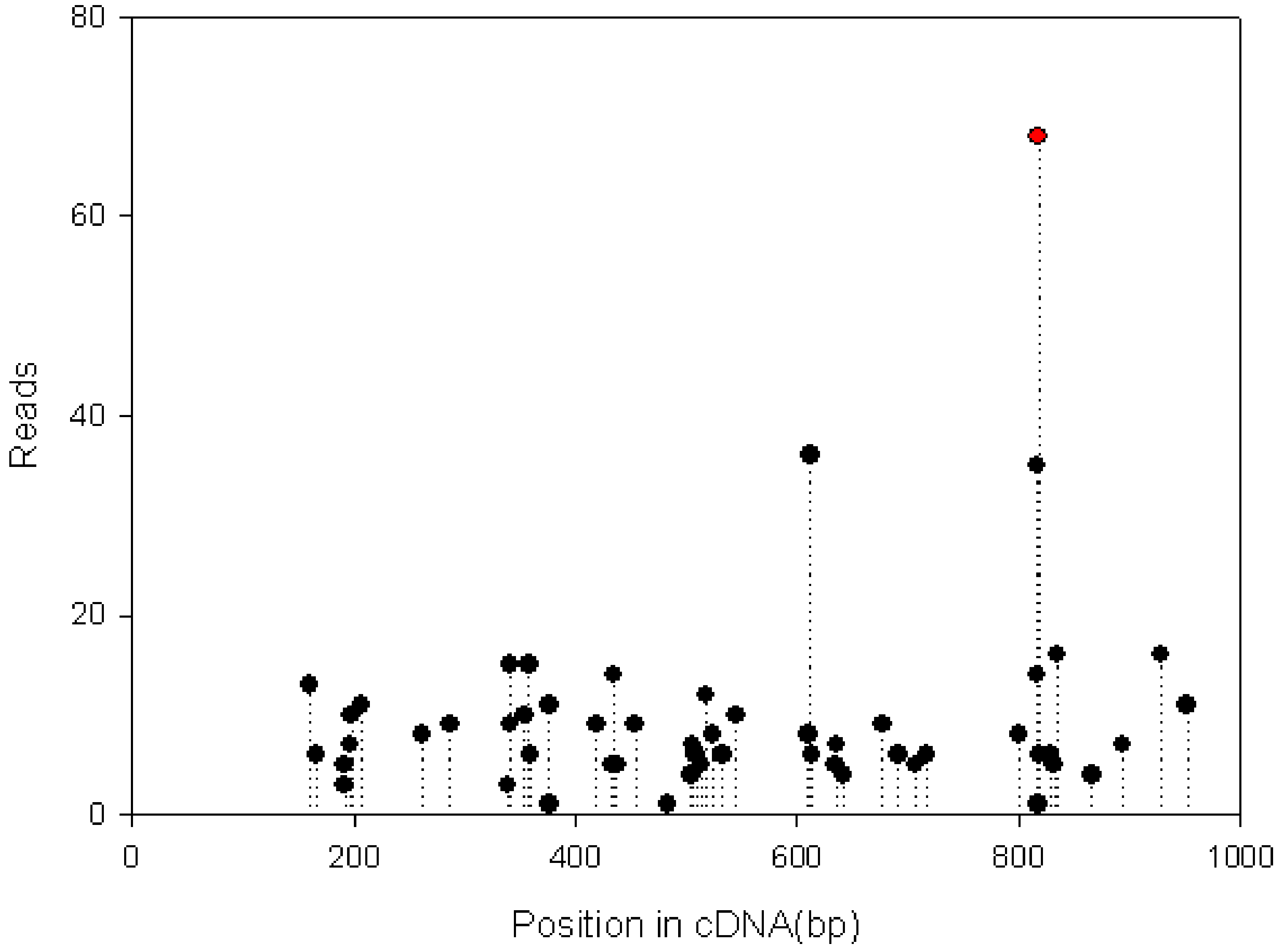


```
5' CGUGCUCUCUCUCUUCUGUCAAUGCU 3'        Cs2g05730.1
    ::::::::: :::::.:::::
3' -CACGAGAGAUAGAAGGCAGUU---- 5'        Csi-miR156g.1
```

Csi-miR156g.1, target=Cs2g23550.1 gene=Cs2g23550
Category:1
Score=5
Cleavage Site=934

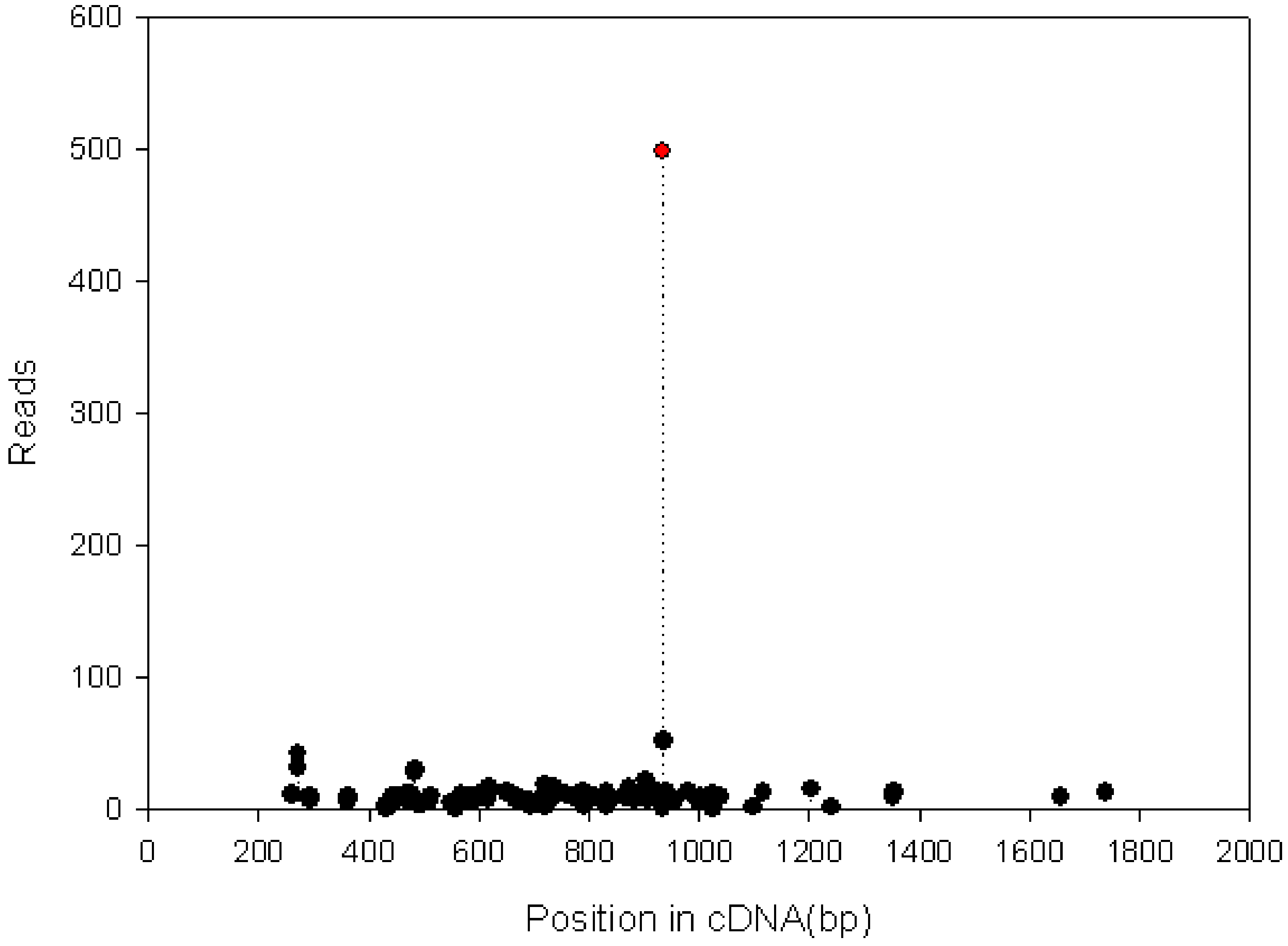


```
5' CAUGCUCUCUCUCUUCUGUCAUCCUA 3'        Cs2g23550.1
     :::::::: ::::::.::::
3' -CACGAGAGAUAGAAGGCAGUU---- 5'        Csi-miR156g.1
```

Csi-miR156g.1, target=Cs7g10830.1 gene=Cs7g10830
Category:1
Score=3
Cleavage Site=1183

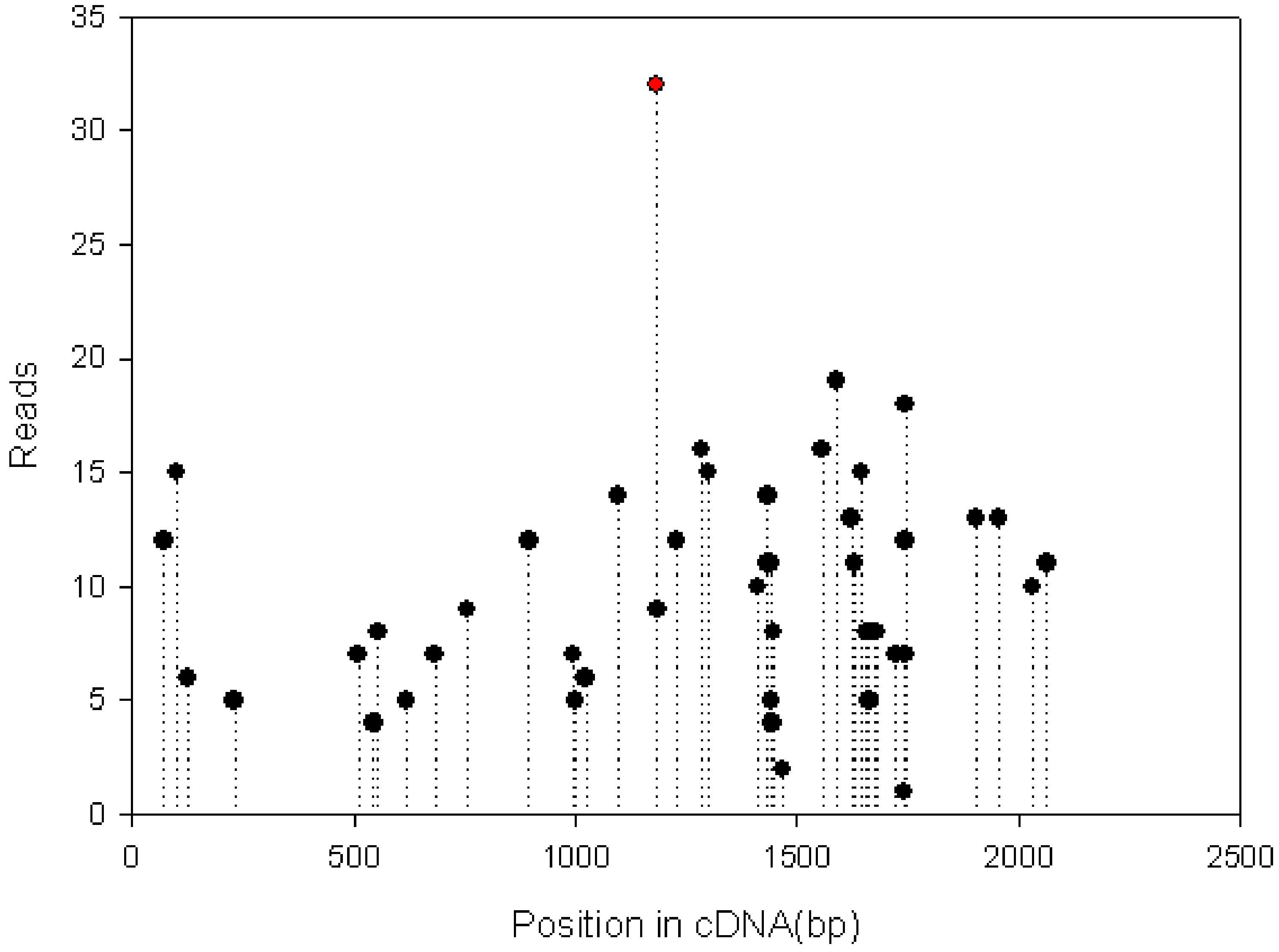


```
5' AGUGCUCUCUCUCUUCUGUCAAAAAC 3'          Cs7g10830.1
    ::::::::: :::::.:::::
3' -CACGAGAGAUAGAAGGCAGUU---- 5'          Csi-miR156g.1
```

Csi-miR156g.1, target=Orange1.1t02265.1 gene=Orange1.1t02265
Category:1
Score=3
Cleavage Site=982

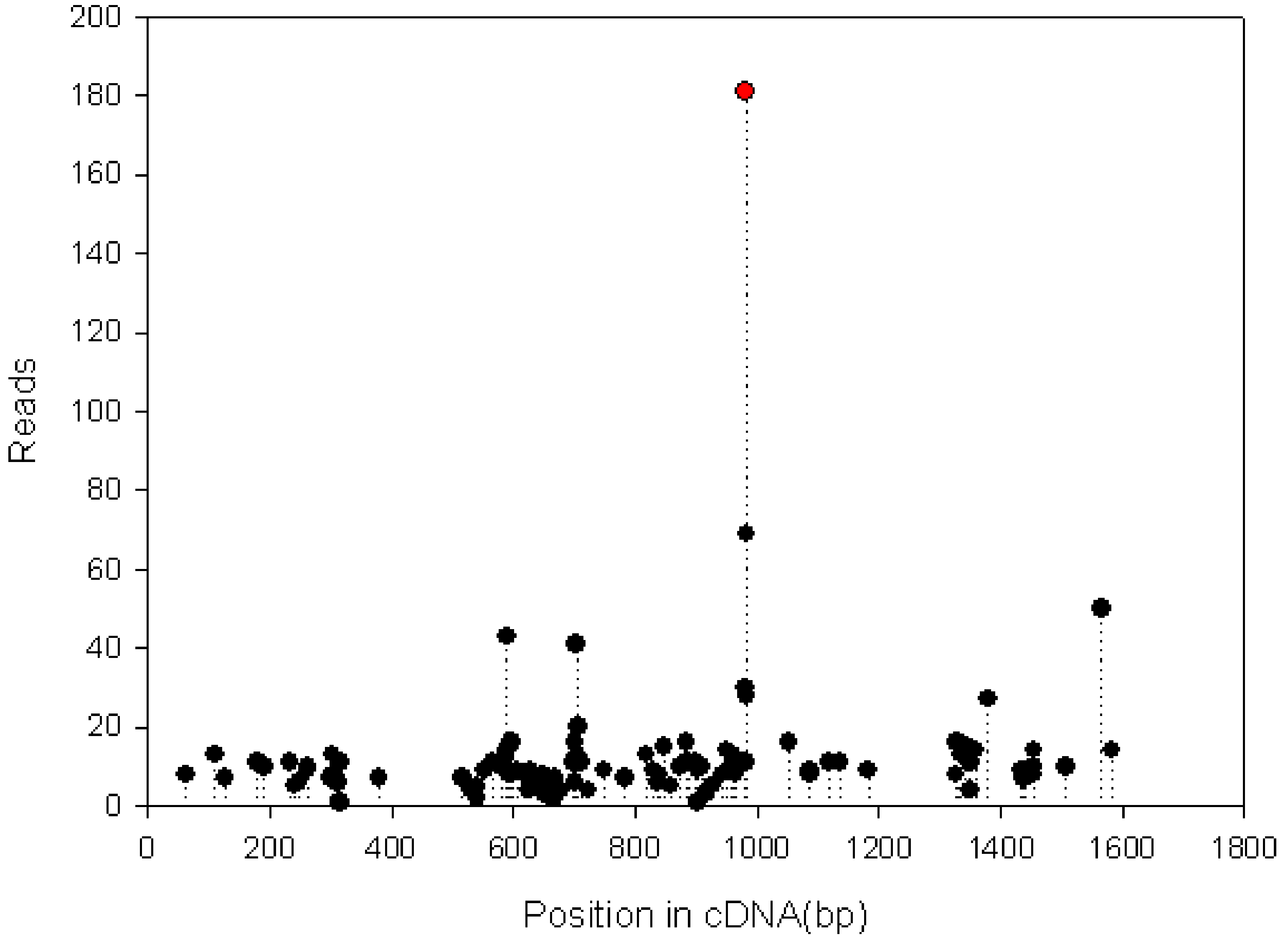


```
5' CUGUGCUCUCUCUUCUGUCAAAUC 3'        Orange1.1t02265.1
     ::::::::: :::::.:::::
3' --CACGAGAGAUAGAAGGCAGUU--- 5'       Csi-miR156g.1
```

Csi-miR156g.2, target=Cs2g05730.1 gene=Cs2g05730
Category:1
Score=3
Cleavage Site=818

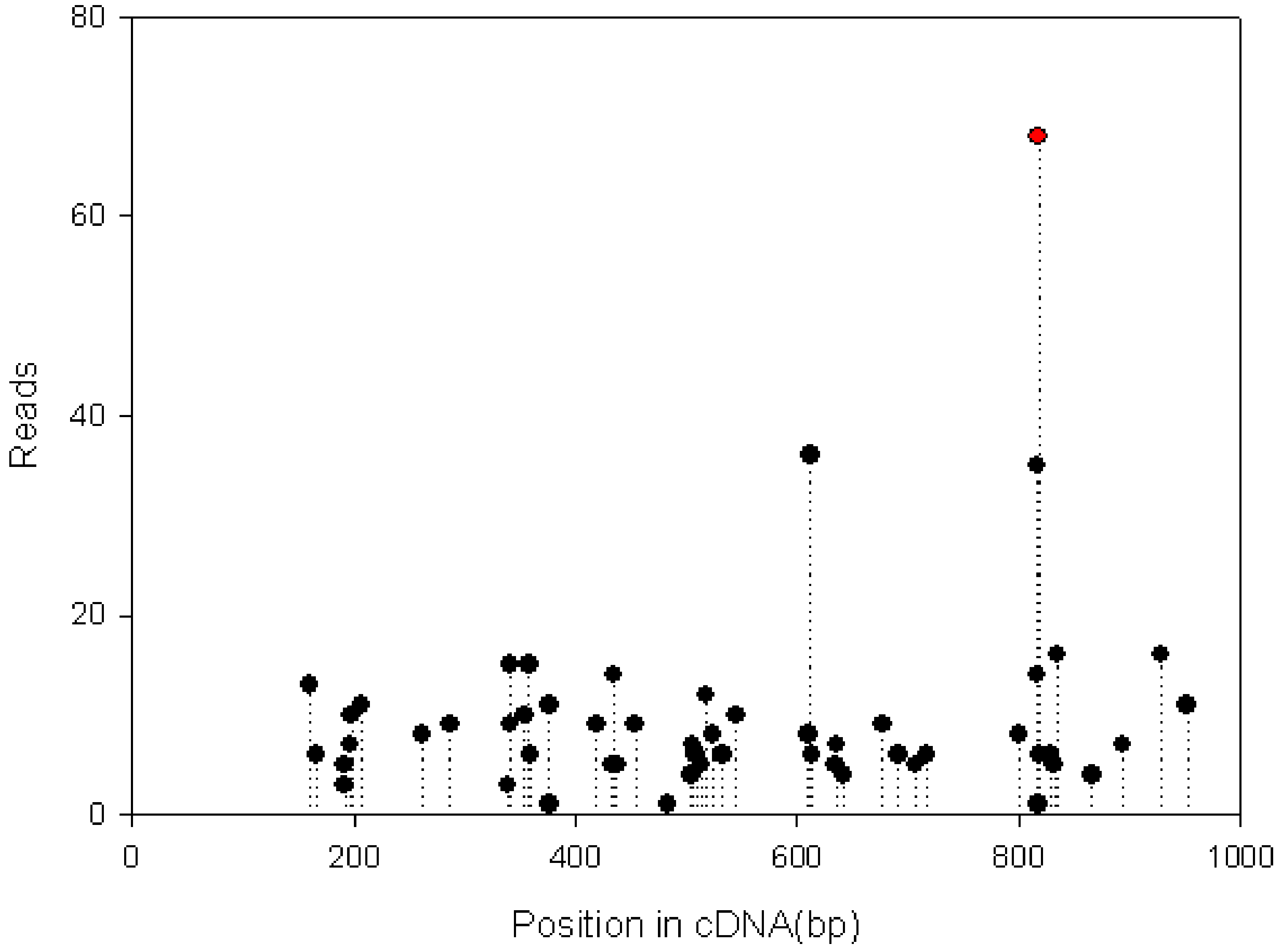


```
5' CGUGCUCUCUCUUCUGUCAAUGCU 3'        Cs2g05730.1
    ::::::::: :::::.::::
3' -CACGAGAGAUAGAAGGCAGU----- 5'      Csi-miR156g.2
```

Csi-miR156g.2, target=Cs2g23550.1 gene=Cs2g23550
Category:1
Score=4
Cleavage Site=934

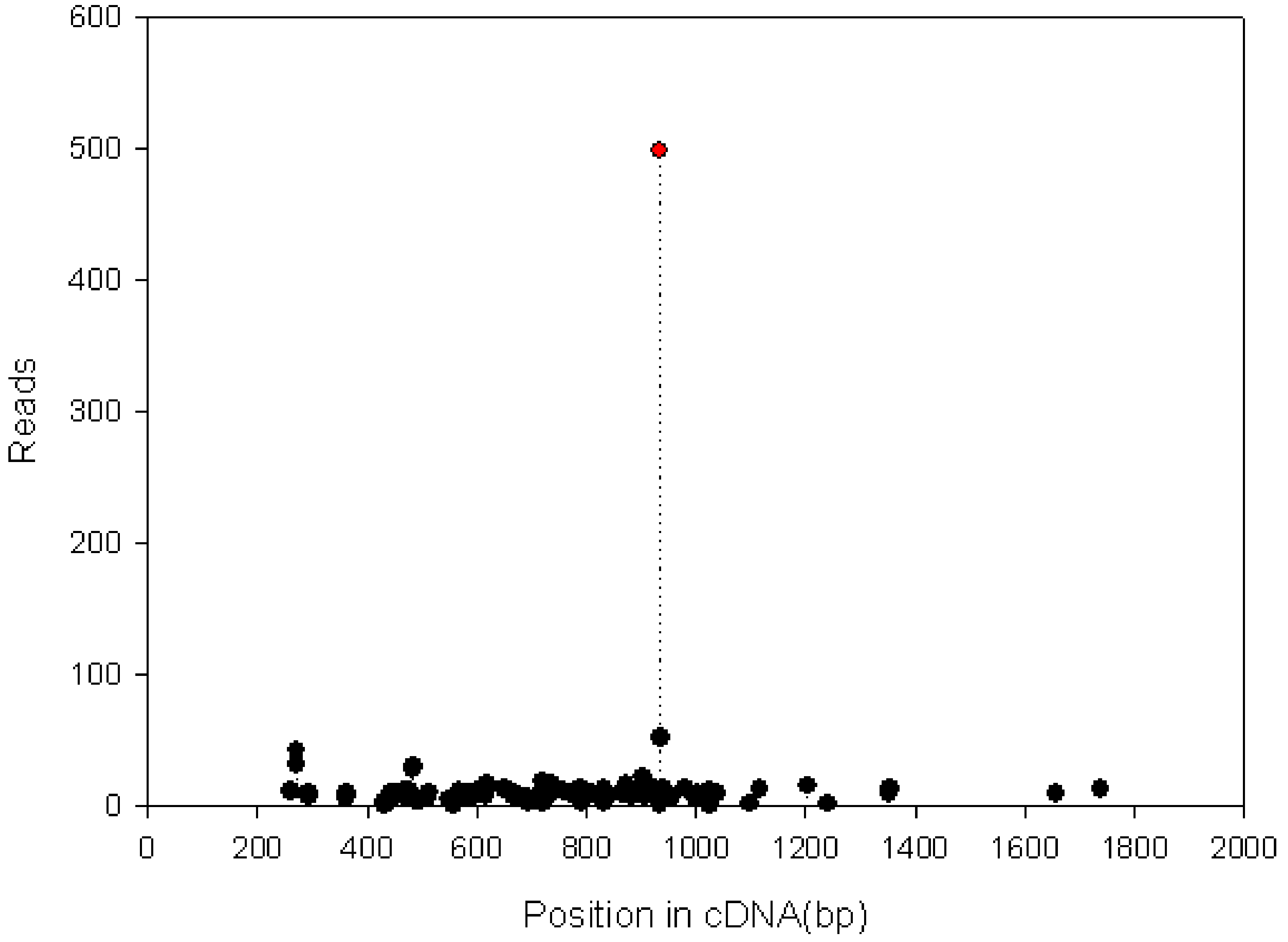


```
5' CAUGCUCUCUCUCUUCUGUCAUCCUA 3'        Cs2g23550.1
     :::::::: ::::::.::::
3' -CACGAGAGAUAGAAGGCAGU----- 5'        Csi-miR156g.2
```

Csi-miR156g.2, target=Cs7g10830.1 gene=Cs7g10830
Category:1
Score=3
Cleavage Site=1183

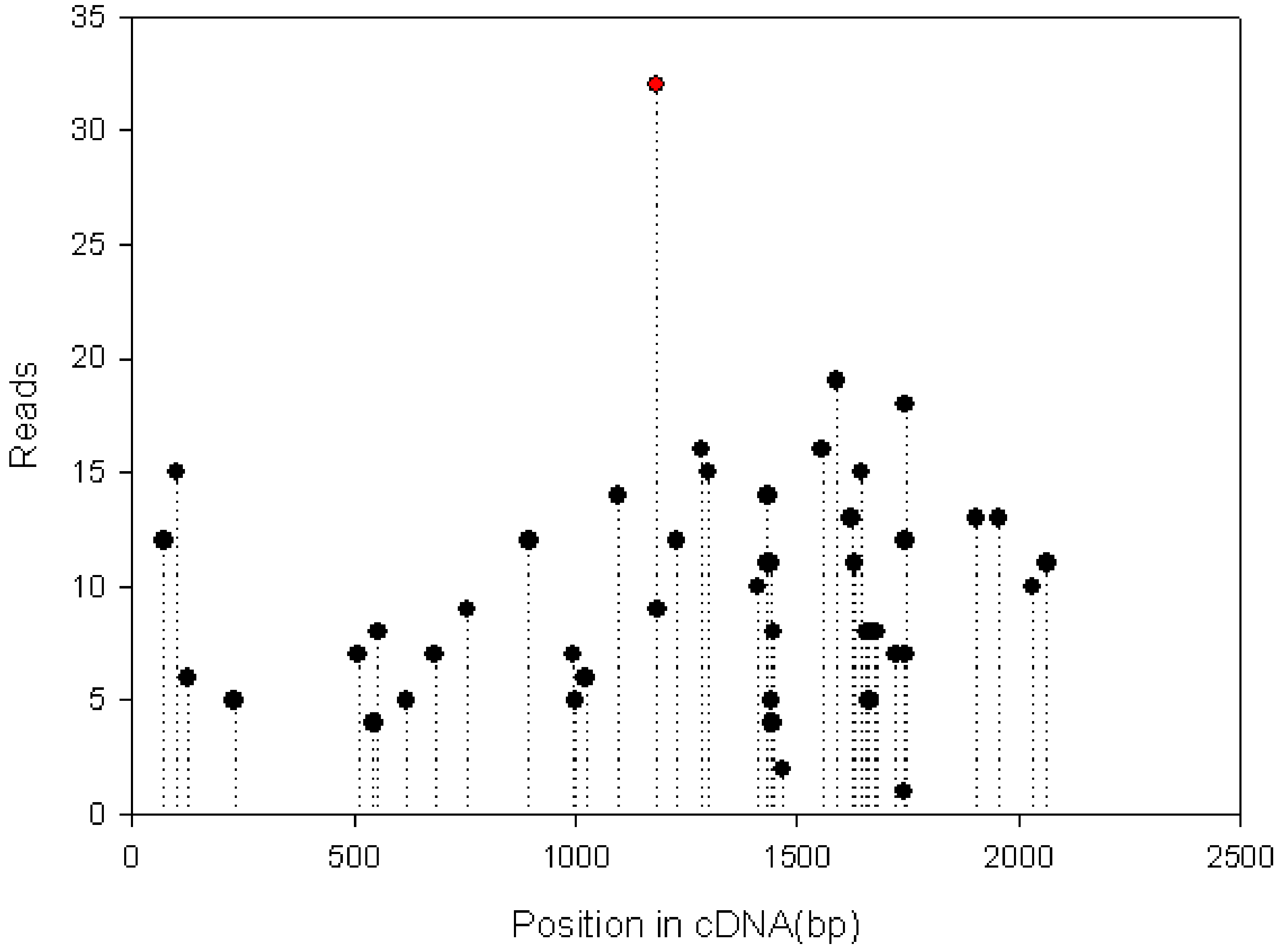


```
5' AGUGCUCUCUCUCUUCUGUCAAAAAC 3'        Cs7g10830.1
    ::::::::: ::::::.::::
3' -CACGAGAGAUAGAAGGCAGU----- 5'        Csi-miR156g.2
```

Csi-miR156g.2, target=Orange1.1t02265.1 gene=Orange1.1t02265
Category:1
Score=3
Cleavage Site=982

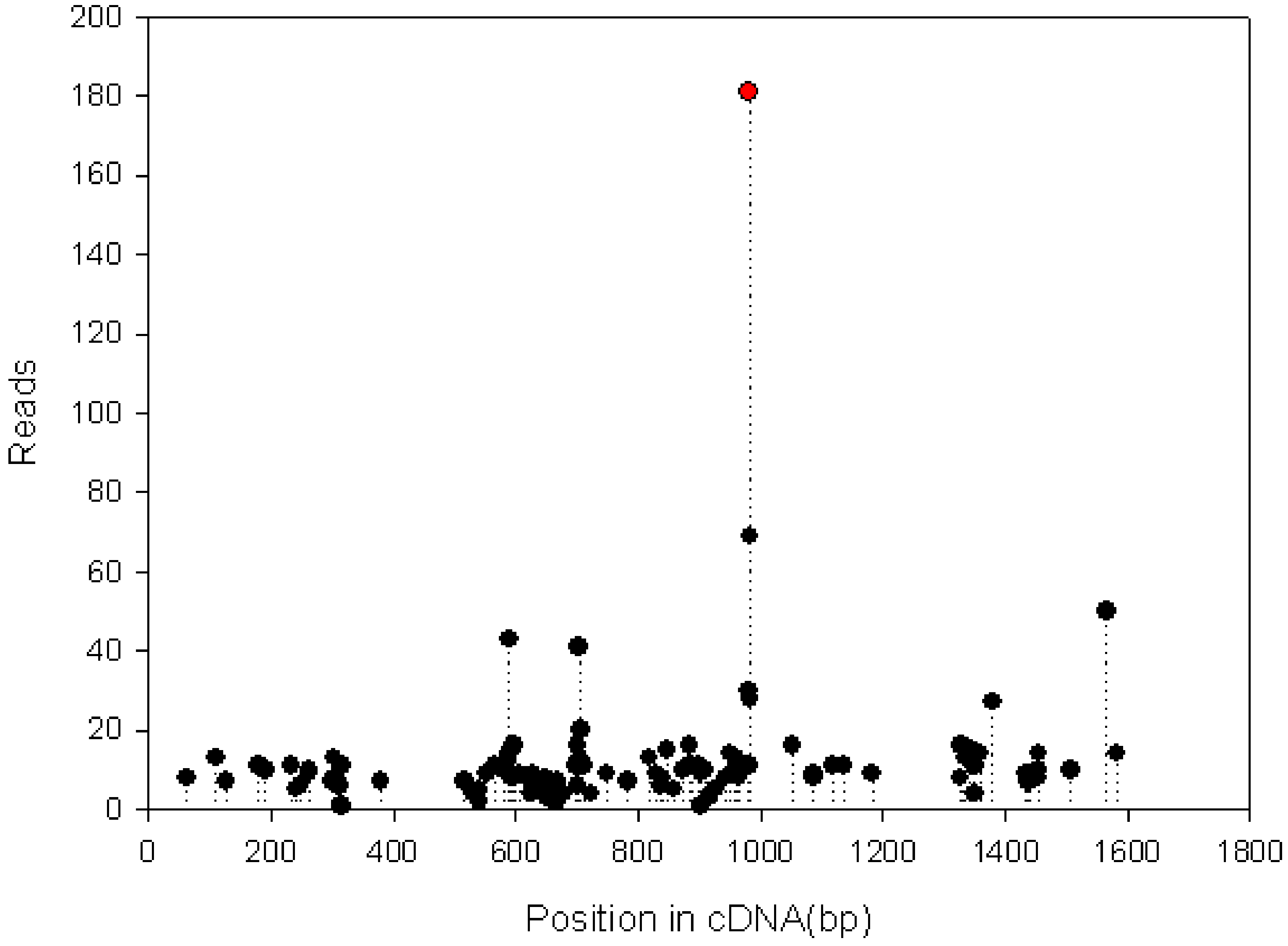


```
5' CUGUGCUCUCUCUCUUCUGUCAAAUC 3'        Orange1.1t02265.1
     ::::::::: :::::.::::
3' --CACGAGAGAUAGAAGGCAGU---- 5'        Csi-miR156g.2
```

Csi-miR156h, target=Cs2g05730.1 gene=Cs2g05730
Category:1
Score=0
Cleavage Site=818

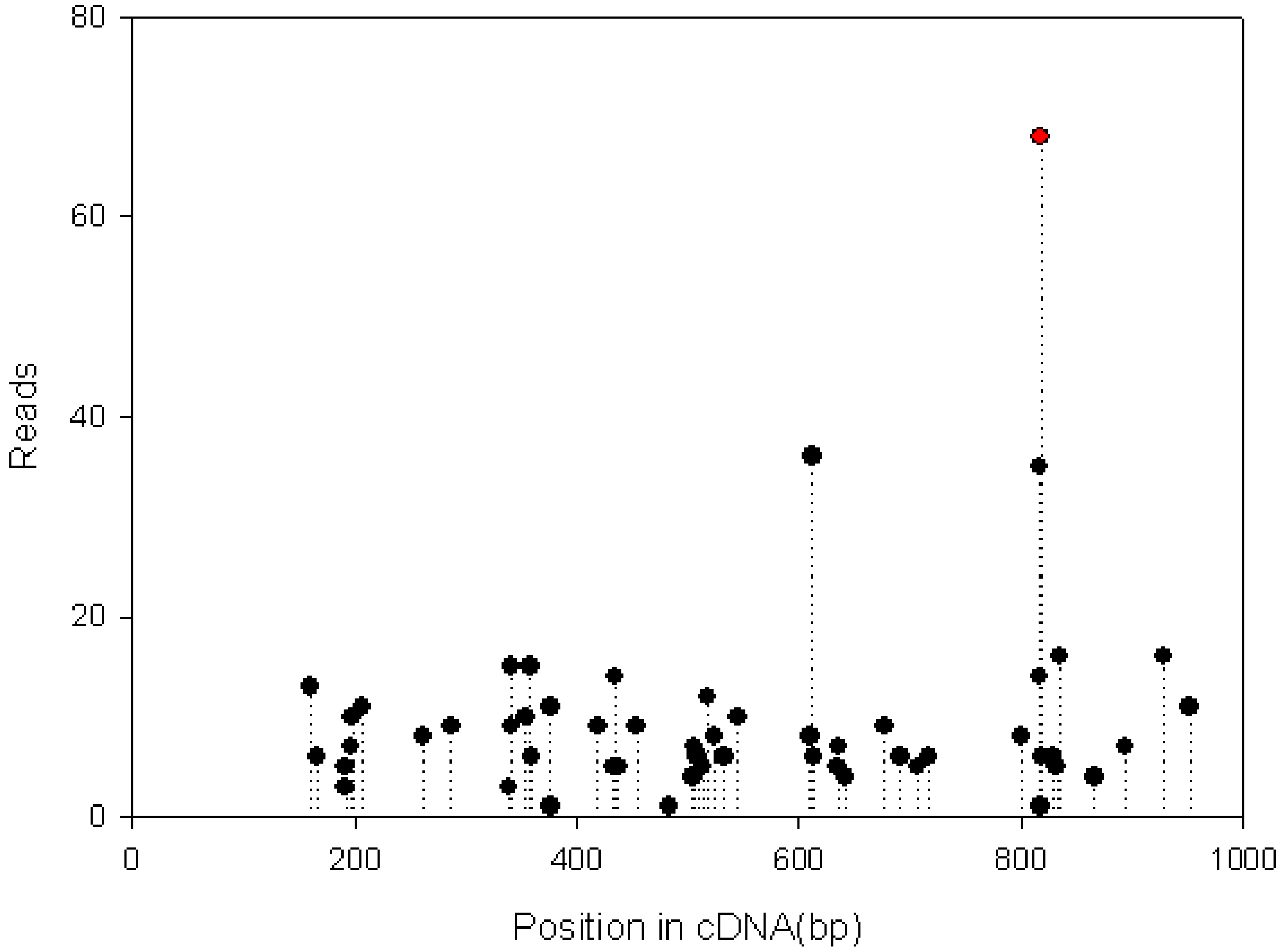


```
5' CGUGCUCUCUCUCUUCUGUCAAUGCU 3'        Cs2g05730.1
    ::::::::::::::::::::::
3' -CACGAGAGAGAGAAGACAGUU---- 5'        Csi-miR156h
```

Csi-miR156h, target=Cs2g23550.1 gene=Cs2g23550
Category:2
Score=2
Cleavage Site=935

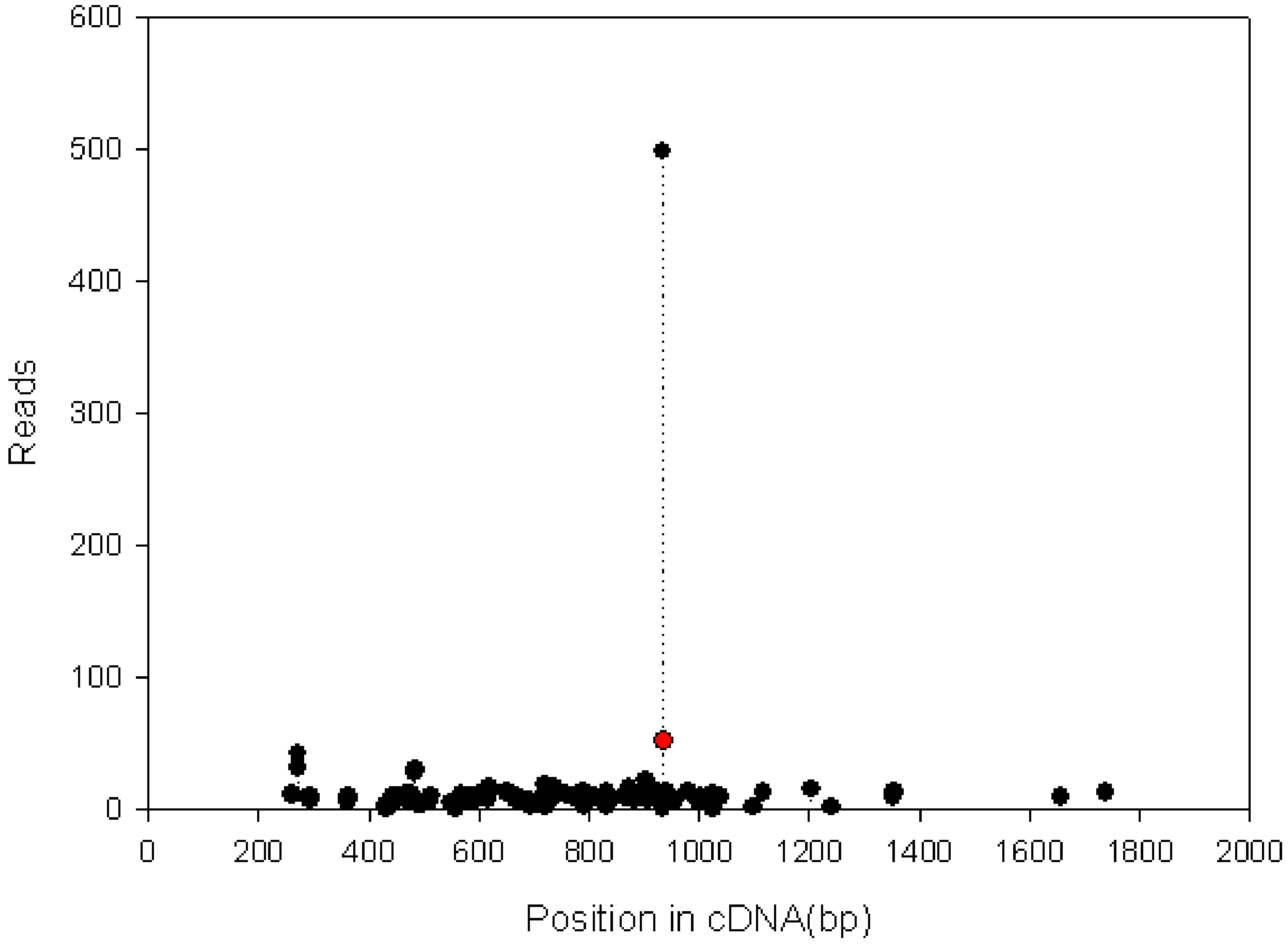


```
5' AUGCUCUCUCUCUUCUGUCAUCCUAG 3'        Cs2g23550.1
    ::::::::::::::::::::
3' CACGAGAGAGAGAAGACAGUU----- 5'        Csi-miR156h
```

Csi-miR156h, target=Cs7g10830.1 gene=Cs7g10830
Category:1
Score=0
Cleavage Site=1183

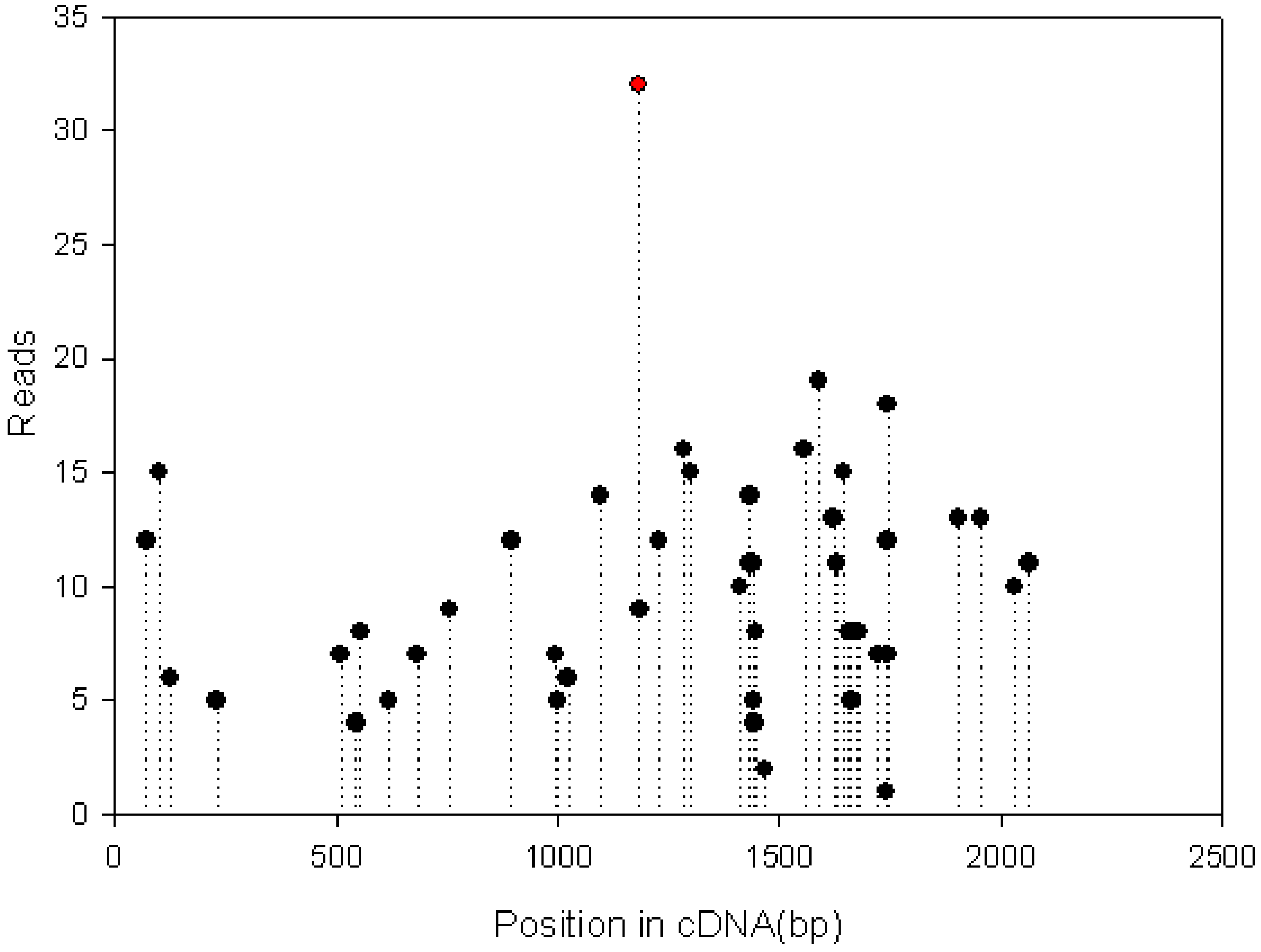


```
5' AGUGCUCUCUCUCUUCUGUCAAAAAC 3'        Cs7g10830.1
    ::::::::::::::::::::::
3' -CACGAGAGAGAGAAGACAGUU---- 5'        Csi-miR156h
```

Csi-miR156h, target=Orange1.1t02265.1 gene=Orange1.1t02265
Category:1
Score=0
Cleavage Site=982

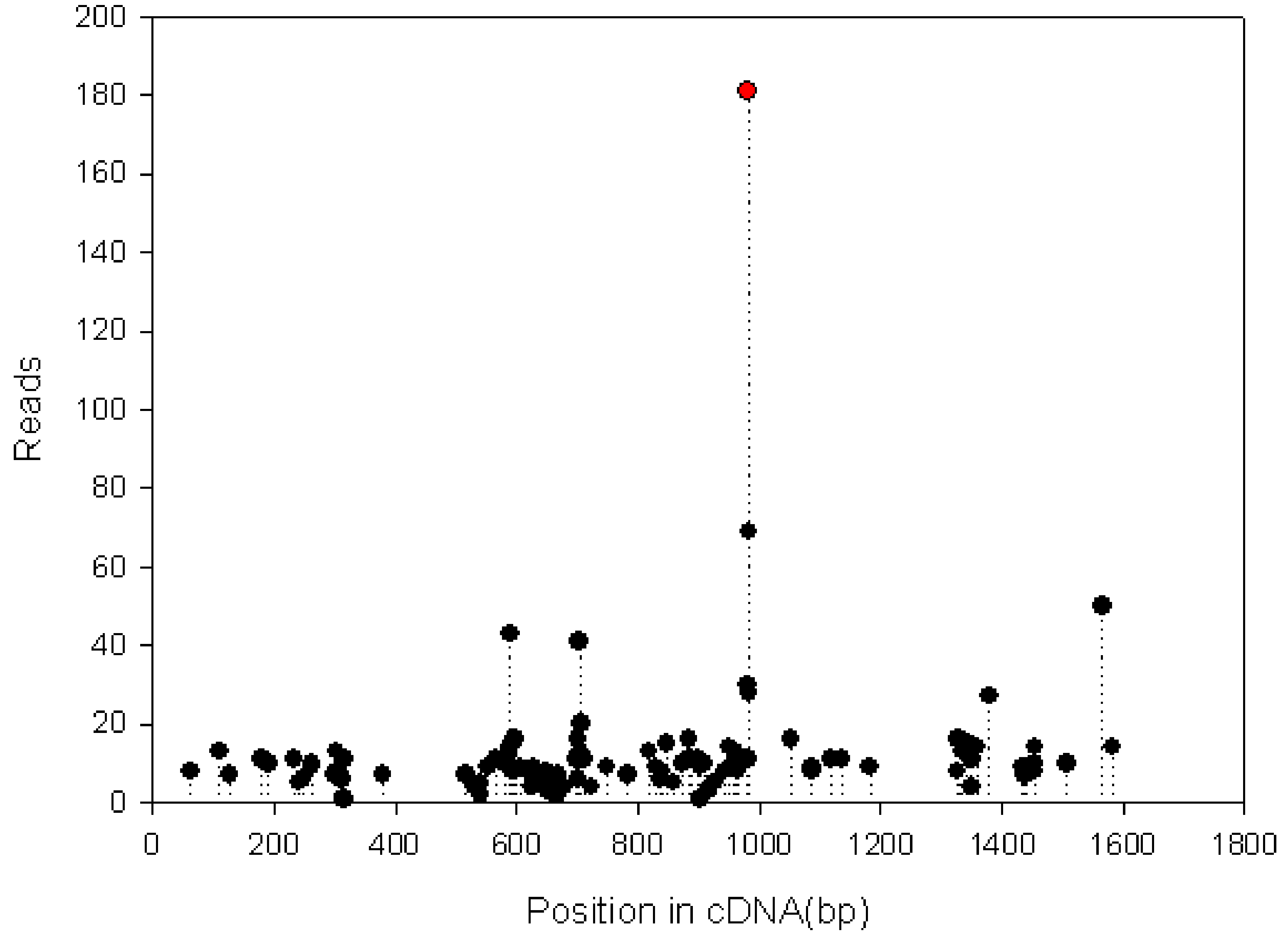


```
5' CUGUGCUCUCUCUCUUCUGUCAAAUC 3'        Orange1.1t02265.1
     ::::::::::::::::::::::
3' --CACGAGAGAGAGAAGACAGUU--- 5'        Csi-miR156h
```

Csi-miR159, target=Cs3g06390.1 gene=Cs3g06390
Category:2
Score=3.5
Cleavage Site=773

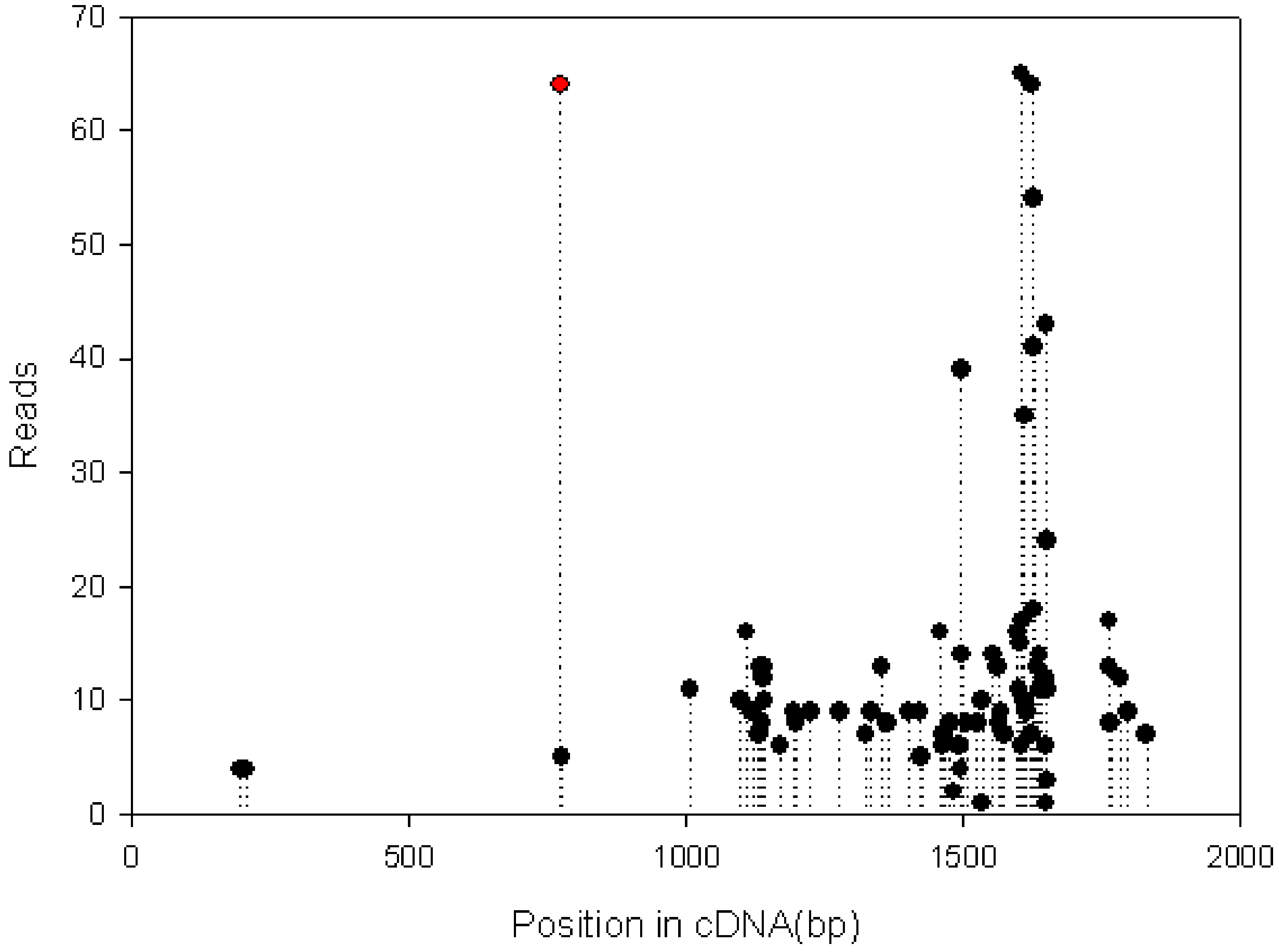


```
5' UUGGAGCUCCCUUCACUCCAAUAUAC 3'        Cs3g06390.1
    :.:::::::::::: :::::
3' -AUCUCGAGGGAAGUUAGGUUU---- 5'        Csi-miR159
```

Csi-miR159, target=Cs6g10950.1 gene=Cs6g10950
Category:1
Score=4
Cleavage Site=5461

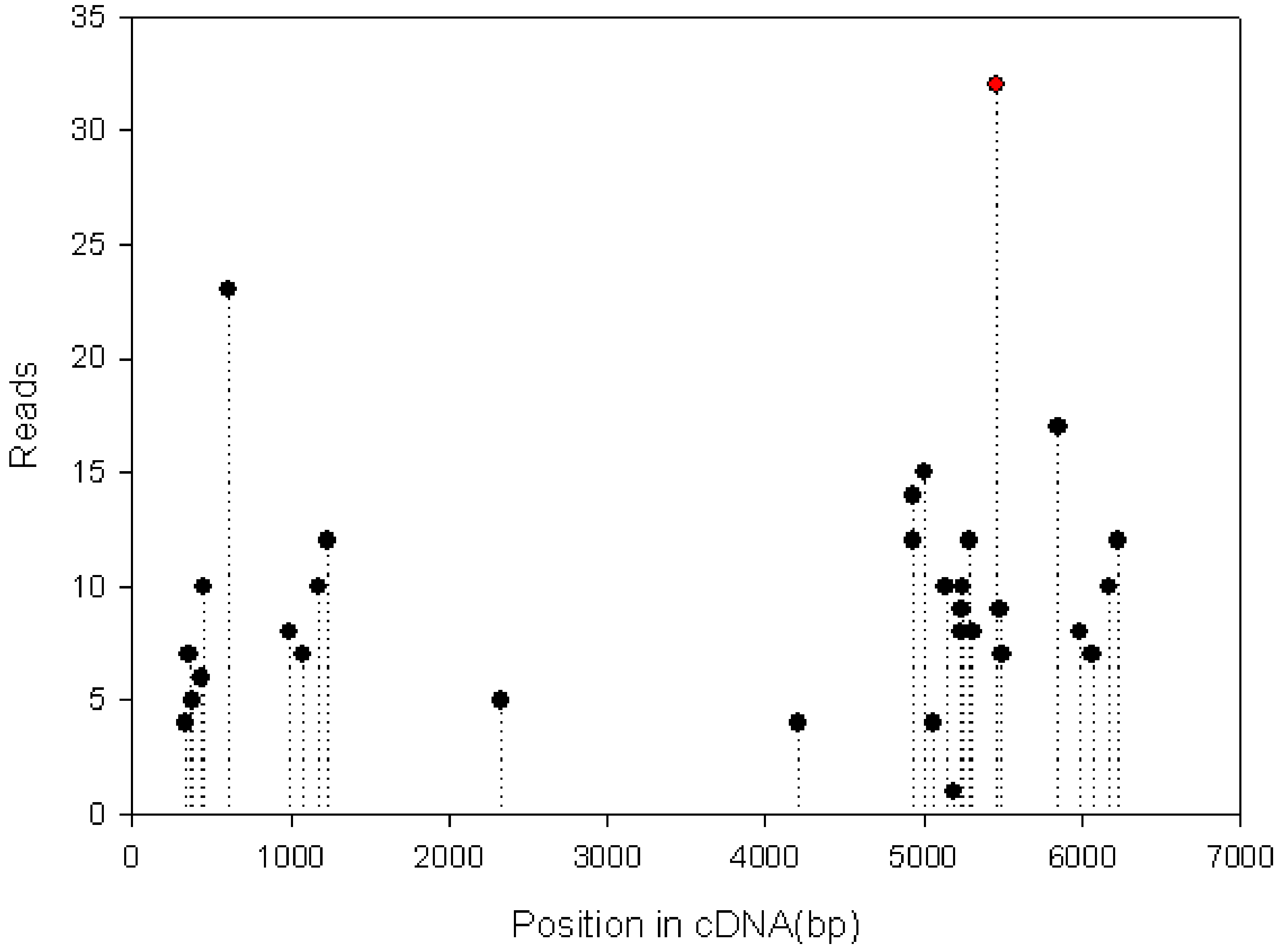


```
5' AAGAGCUCUACUUCAAUCCAAGCUUU 3'        Cs6g10950.1
    ::::::::. ::::::::::::.
3' AUCUCGAGG-GAAGUUAGGUUU---- 5'        Csi-miR159
```

Csi-miR159b, target=Cs3g06390.1 gene=Cs3g06390
Category:2
Score=0
Cleavage Site=773

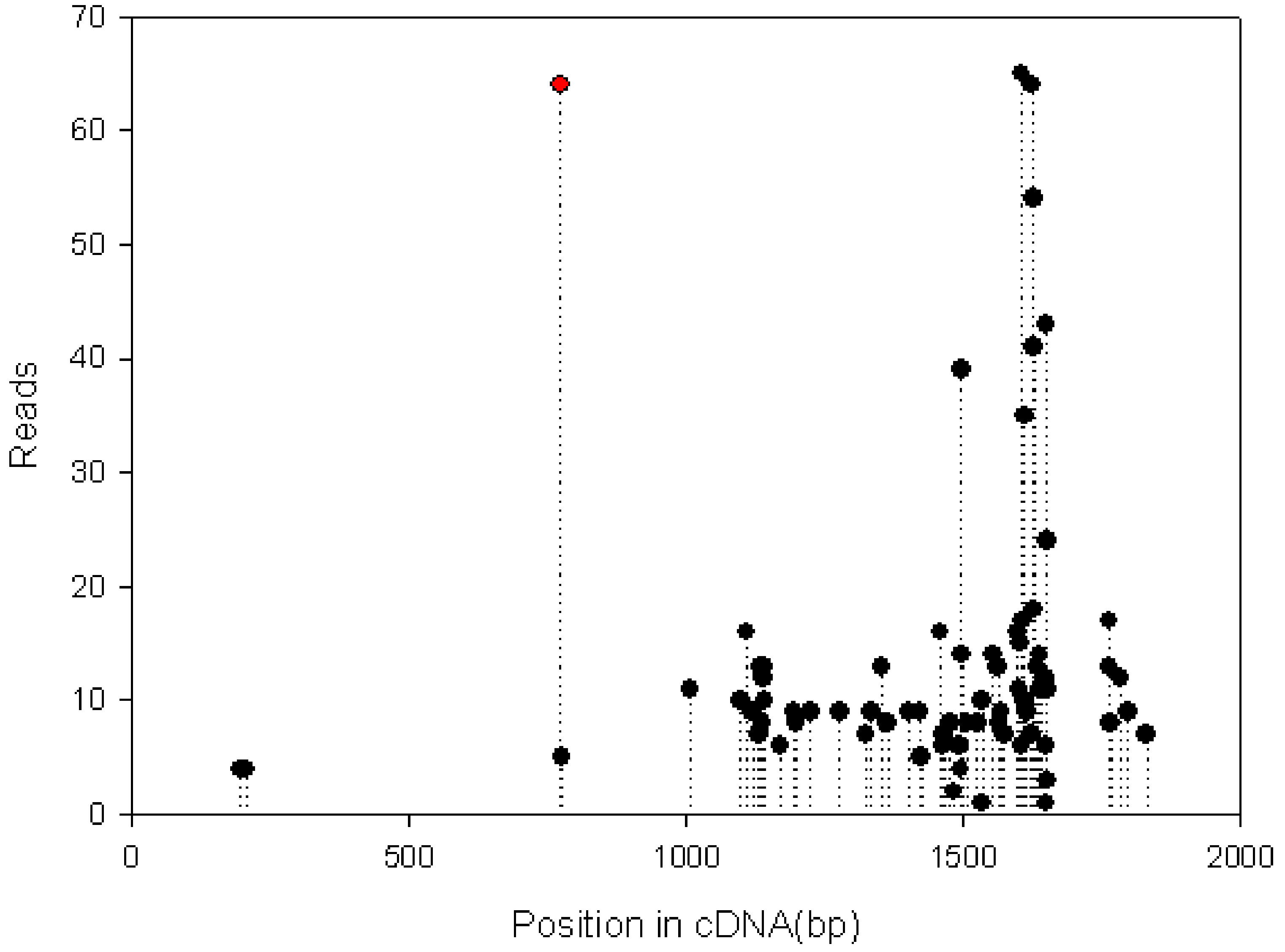


```
5' UUGGAGCUCCCUUCACUCCAAUAUAC 3'        Cs3g06390.1
    :::::::::::::::::::::
3' -ACCUCGAGGGAAGUGAGGUUA---- 5'        Csi-miR159b
```

Csi-miR160a-5p, target=Cs3g18940.1 gene=Cs3g18940
Category:1
Score=0.5
Cleavage Site=1423

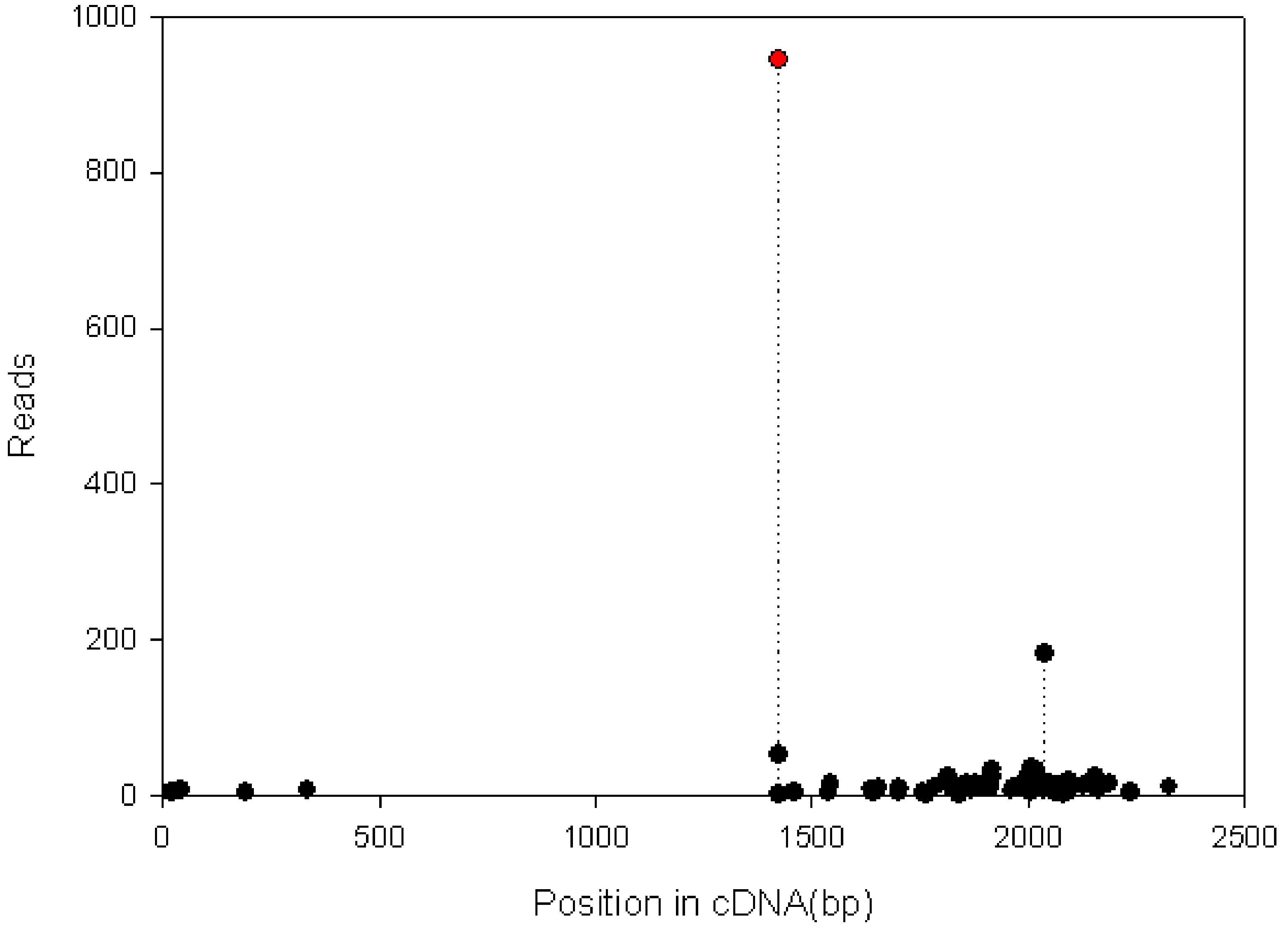


```
5' CUGGCAUGCAGGGAGCCAGGCAAAAU 3'        Cs3g18940.1
    ::::::.:::::::::::::::
3' -ACCGUAUGUCCCUCGGUCCGU---- 5'        Csi-miR160a-5p
```

Csi-miR160a-5p, target=Cs6g11800.1 gene=Cs6g11800
Category:1
Score=0.5
Cleavage Site=2025

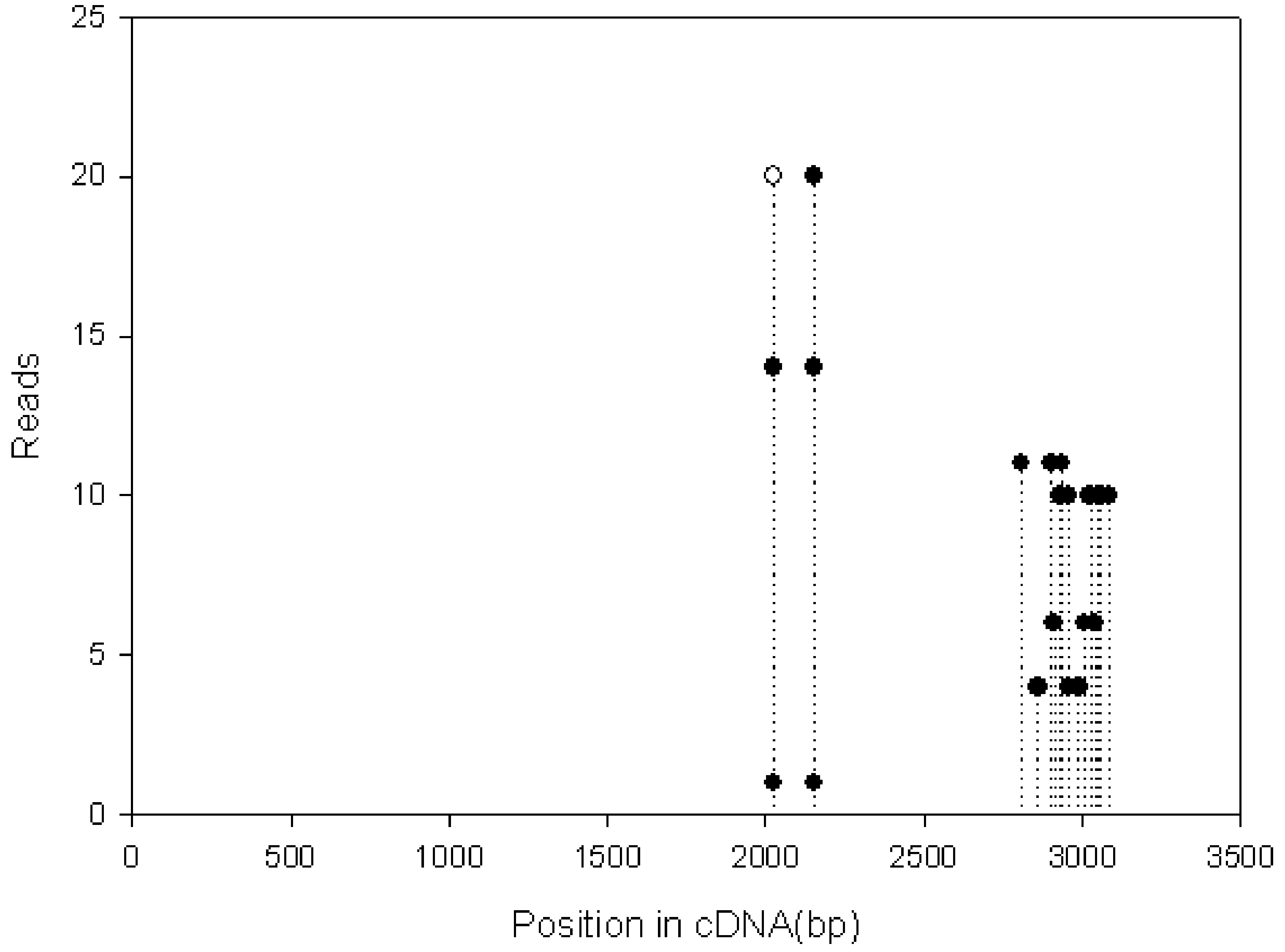


```
5' CUGGCAUGCAGGGAGCCAGGCAUGCU 3'        Cs6g11800.1
    ::::::.:::::::::::::::::
3' -ACCGUAUGUCCCUCGGUCCGU---- 5'        Csi-miR160a-5p
```

Csi-miR160a-5p, target=Cs7g25670.1 gene=Cs7g25670
Category:1
Score=1
Cleavage Site=1693

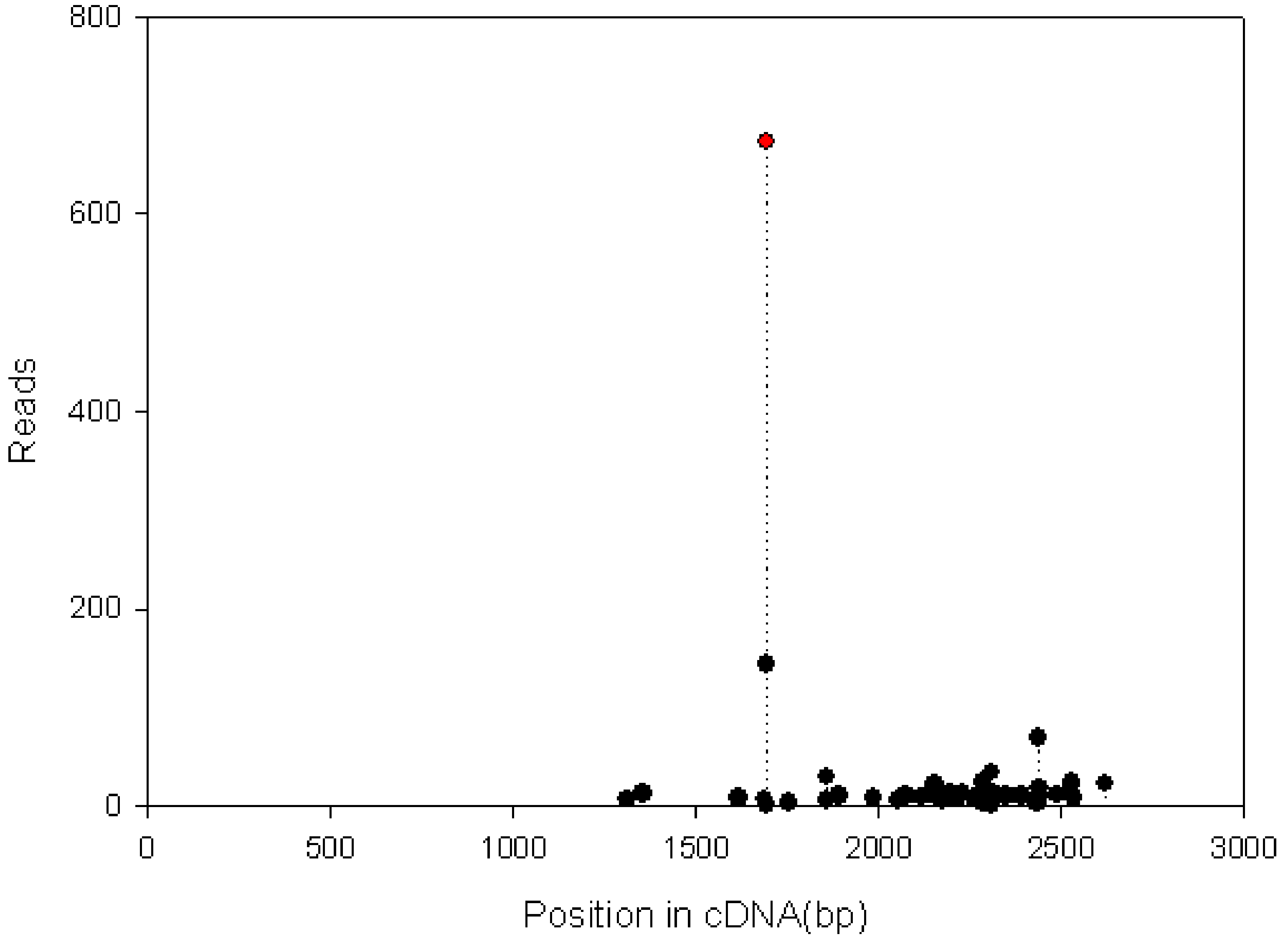


```
5' CAGGCAUACAGGGAGCCAGGCAUGCC 3'        Cs7g25670.1
     ::::::::::::::::::::
3' -ACCGUAUGUCCCUCGGUCCGU---- 5'        Csi-miR160a-5p
```

Csi-miR160a-5p, target=Cs8g16440.1 gene=Cs8g16440
Category:1
Score=5
Cleavage Site=1978

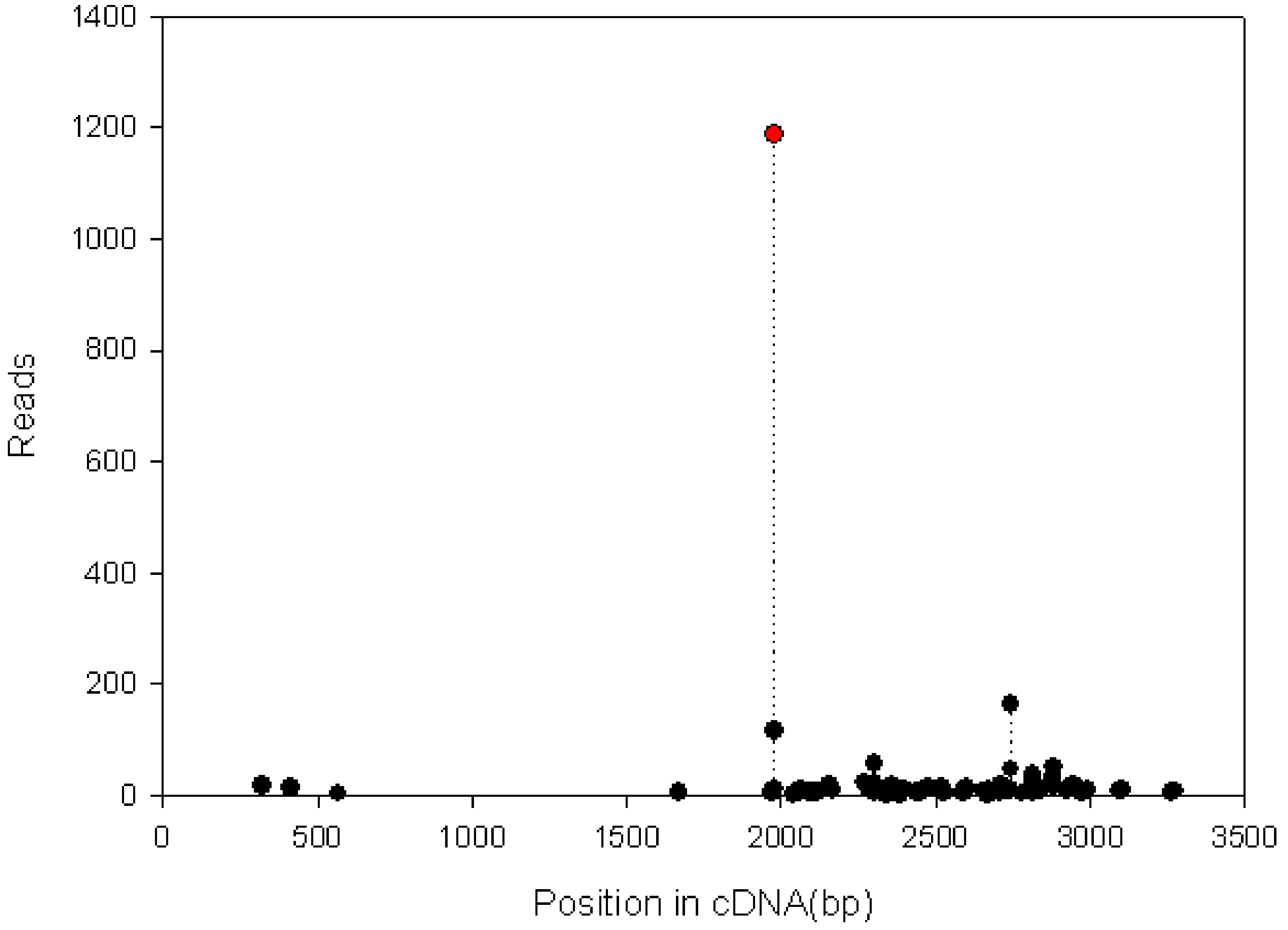


```
5' CAGGCAUACAGGGAGCCAGGCAUGCU 3'        Cs8g16440.1
     ::::::::::::::::::::
3' -ACCGUAUGUCCCUCGGUCCGU---- 5'        Csi-miR160a-5p
```

Csi-miR160b-5p, target=Cs3g18940.1 gene=Cs3g18940
Category:1
Score=1
Cleavage Site=1423

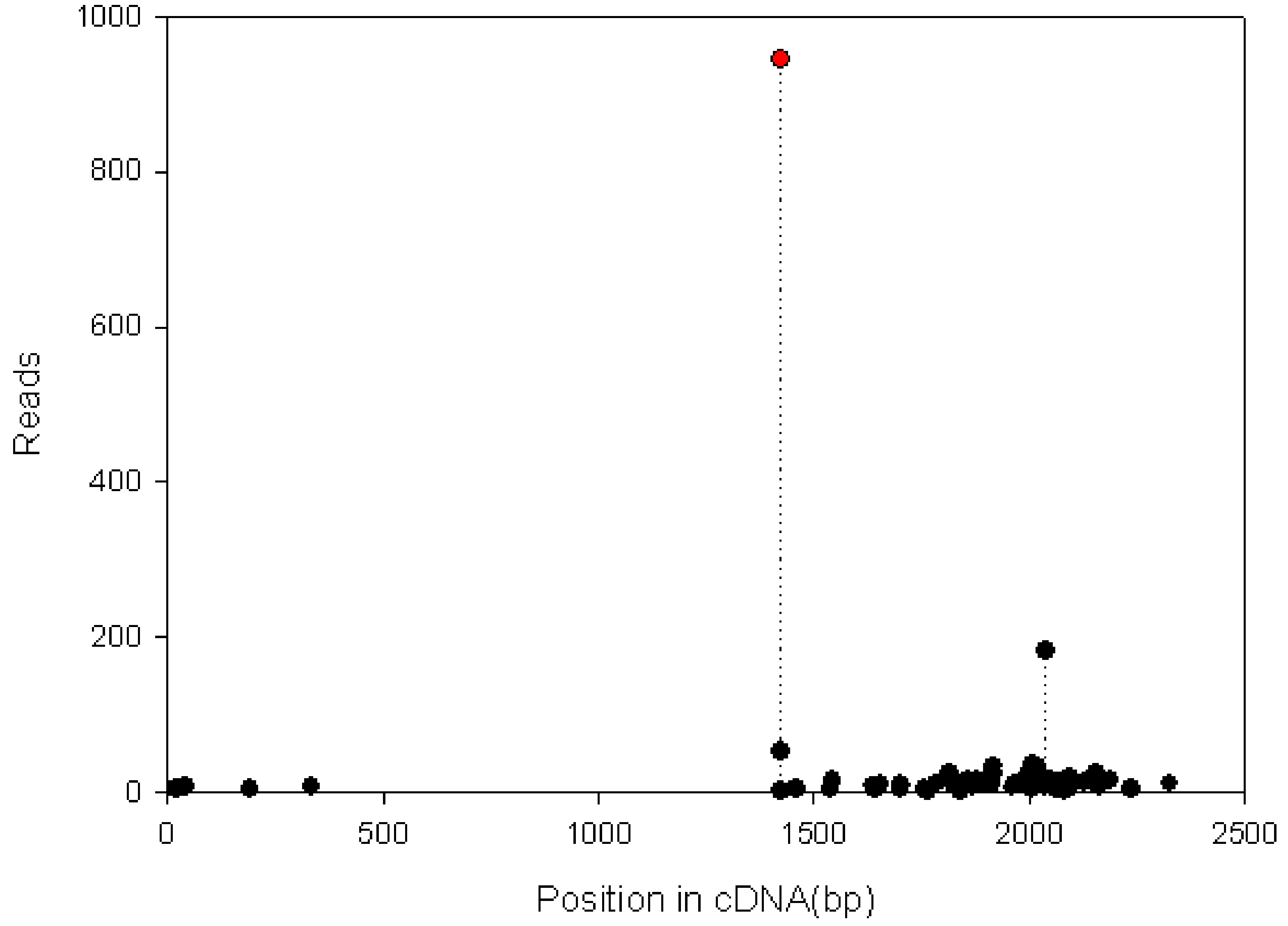


```
5' CUGGCAUGCAGGGAGCCAGGCAAAAU 3'        Cs3g18940.1
    .::::::.::::::::::::::::
3' -GCCGUAUGUCCCUCGGUCCGU---- 5'        Csi-miR160b-5p
```

Csi-miR160b-5p, target=Cs6g11800.1 gene=Cs6g11800
Category:1
Score=1
Cleavage Site=2025

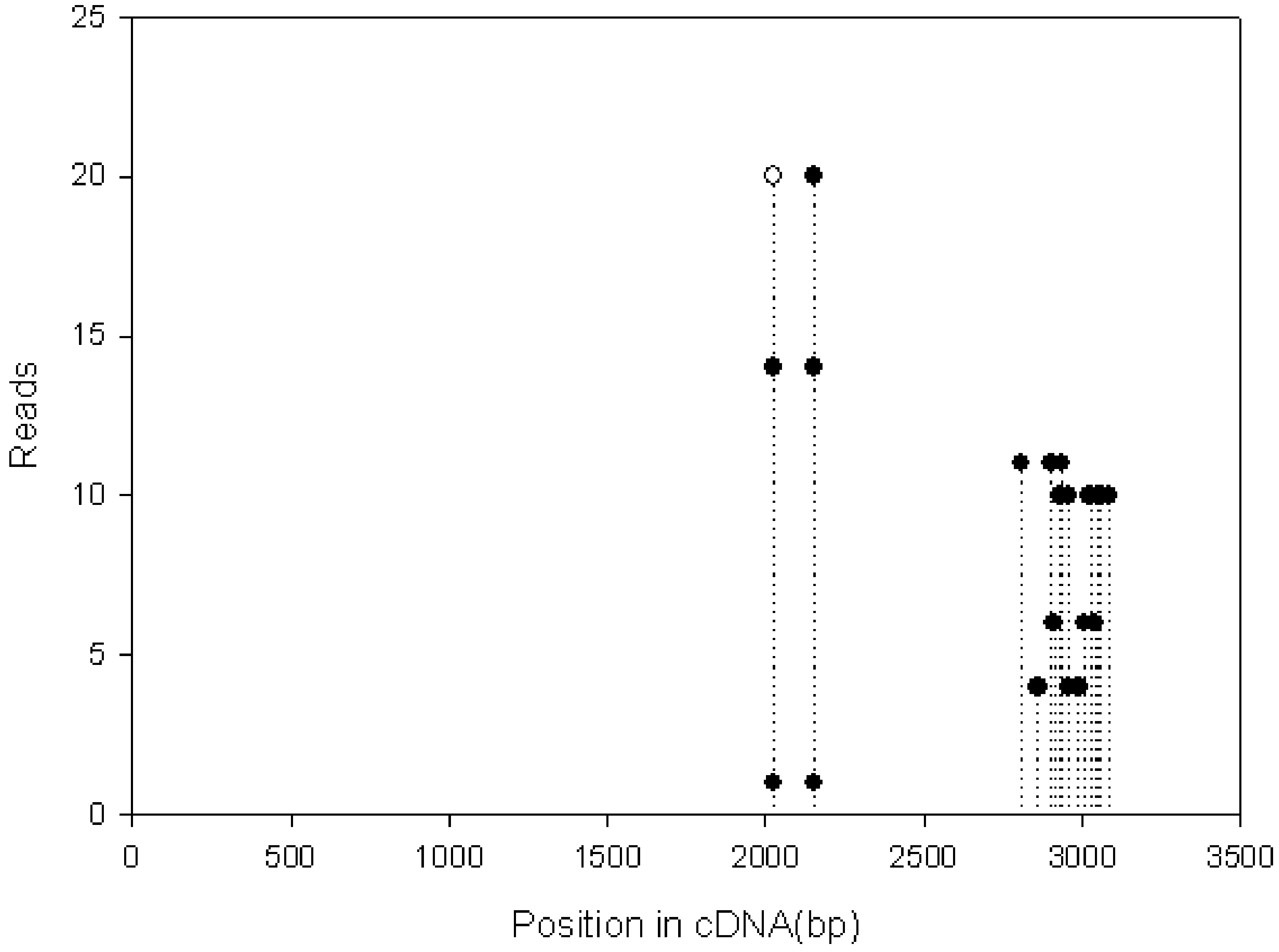


```
5' CUGGCAUGCAGGGAGCCAGGCAUGCU 3'        Cs6g11800.1
    .:::::.:::::::::::::::::
3' -GCCGUAUGUCCCUCGGUCCGU---- 5'        Csi-miR160b-5p
```

Csi-miR160b-5p, target=Cs7g25670.1 gene=Cs7g25670
Category:1
Score=1
Cleavage Site=1693

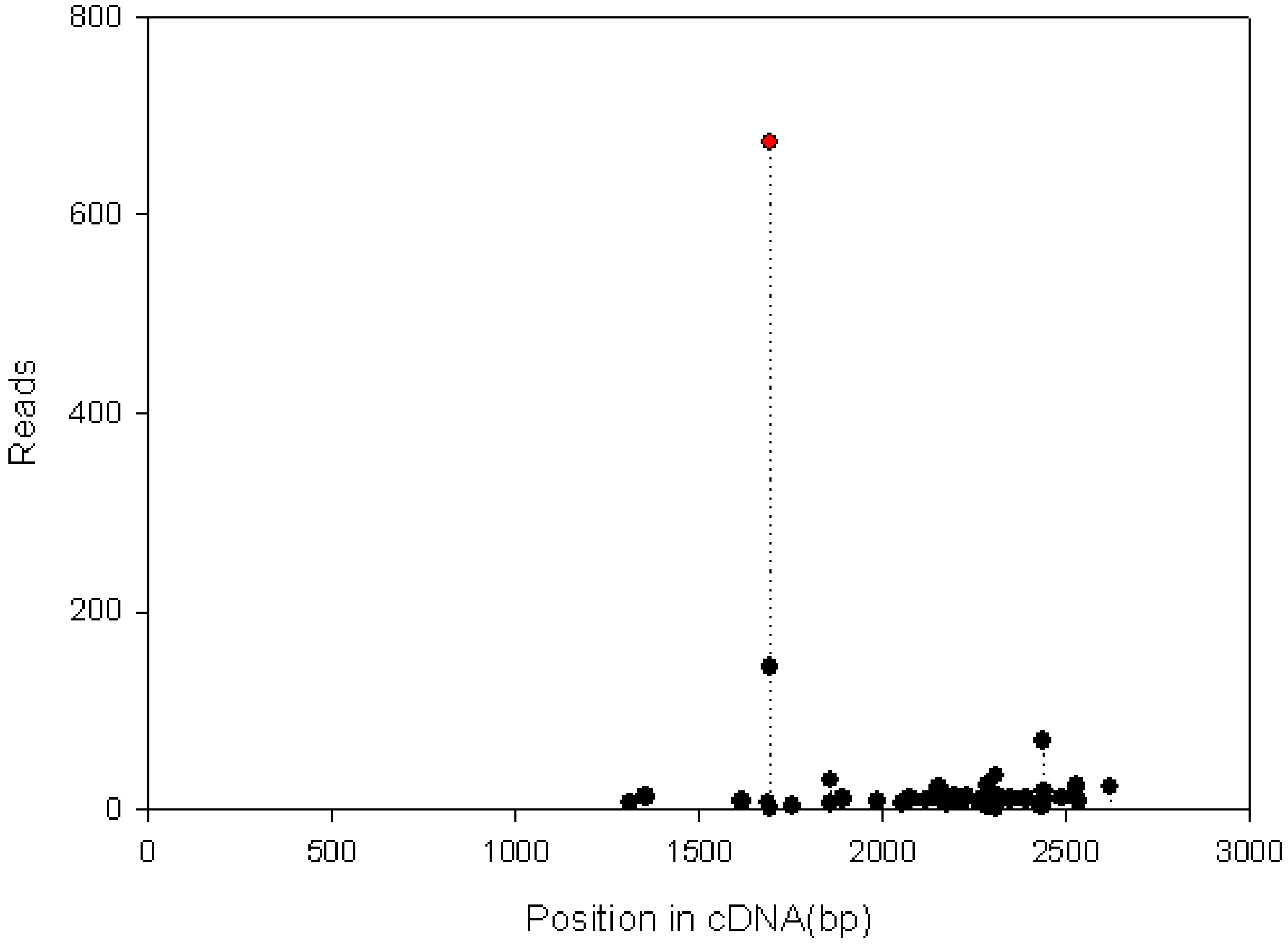


```
5' CAGGCAUACAGGGAGCCAGGCAUGCC 3'        Cs7g25670.1
    ::::::::::::::::::::
3' -GCCGUAUGUCCCUCGGUCCGU---- 5'        Csi-miR160b-5p
```

Csi-miR160b-5p, target=Cs8g16440.1 gene=Cs8g16440
Category:1
Score=5
Cleavage Site=1978

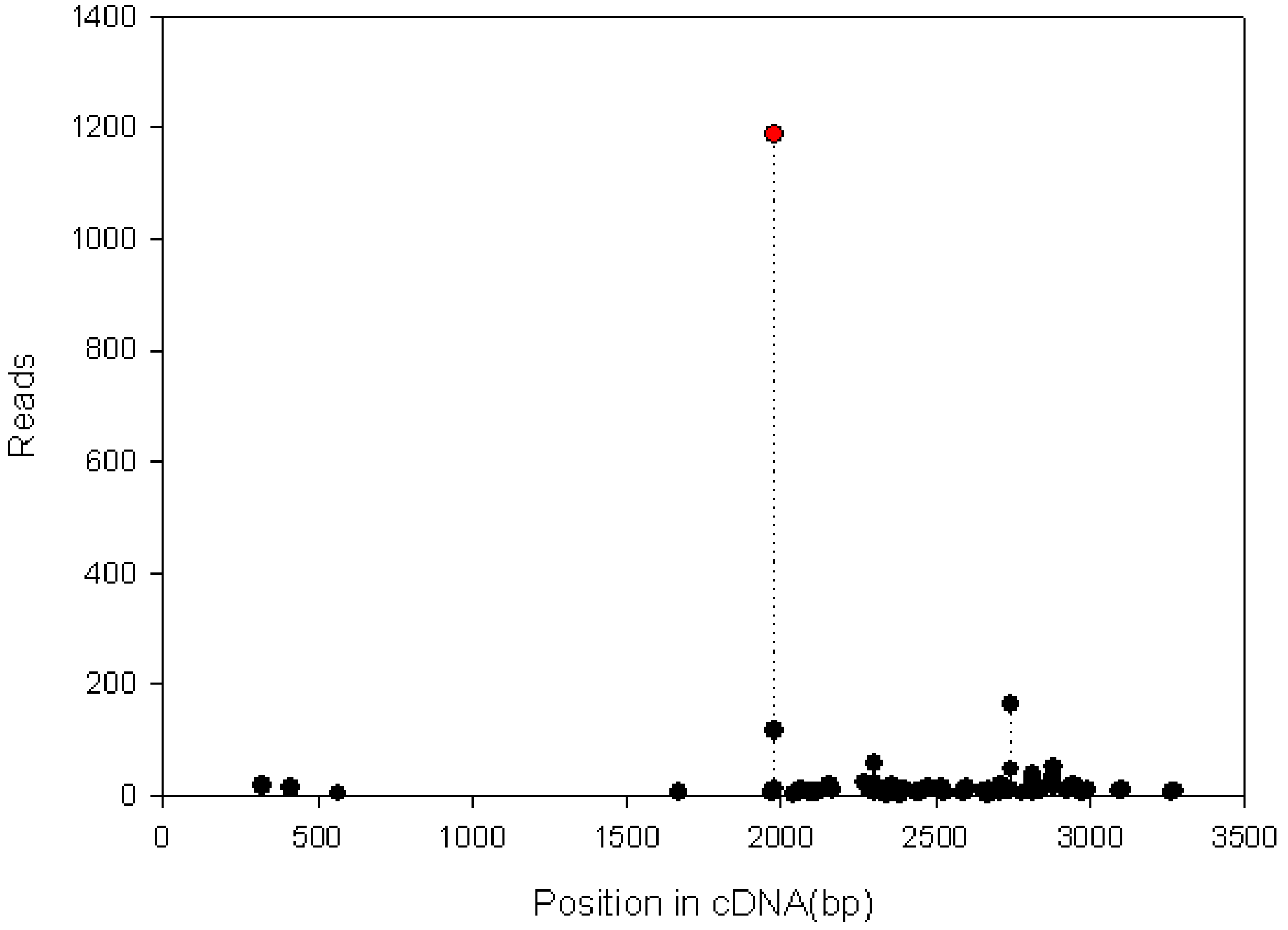


```
5' CAGGCAUACAGGGAGCCAGGCAUGCU 3'        Cs8g16440.1
     ::::::::::::::::::::
3' -GCCGUAUGUCCCUCGGUCCGU---- 5'        Csi-miR160b-5p
```

Csi-miR164, target=Cs5g10870.1 gene=Cs5g10870
Category:1
Score=3.5
Cleavage Site=1062

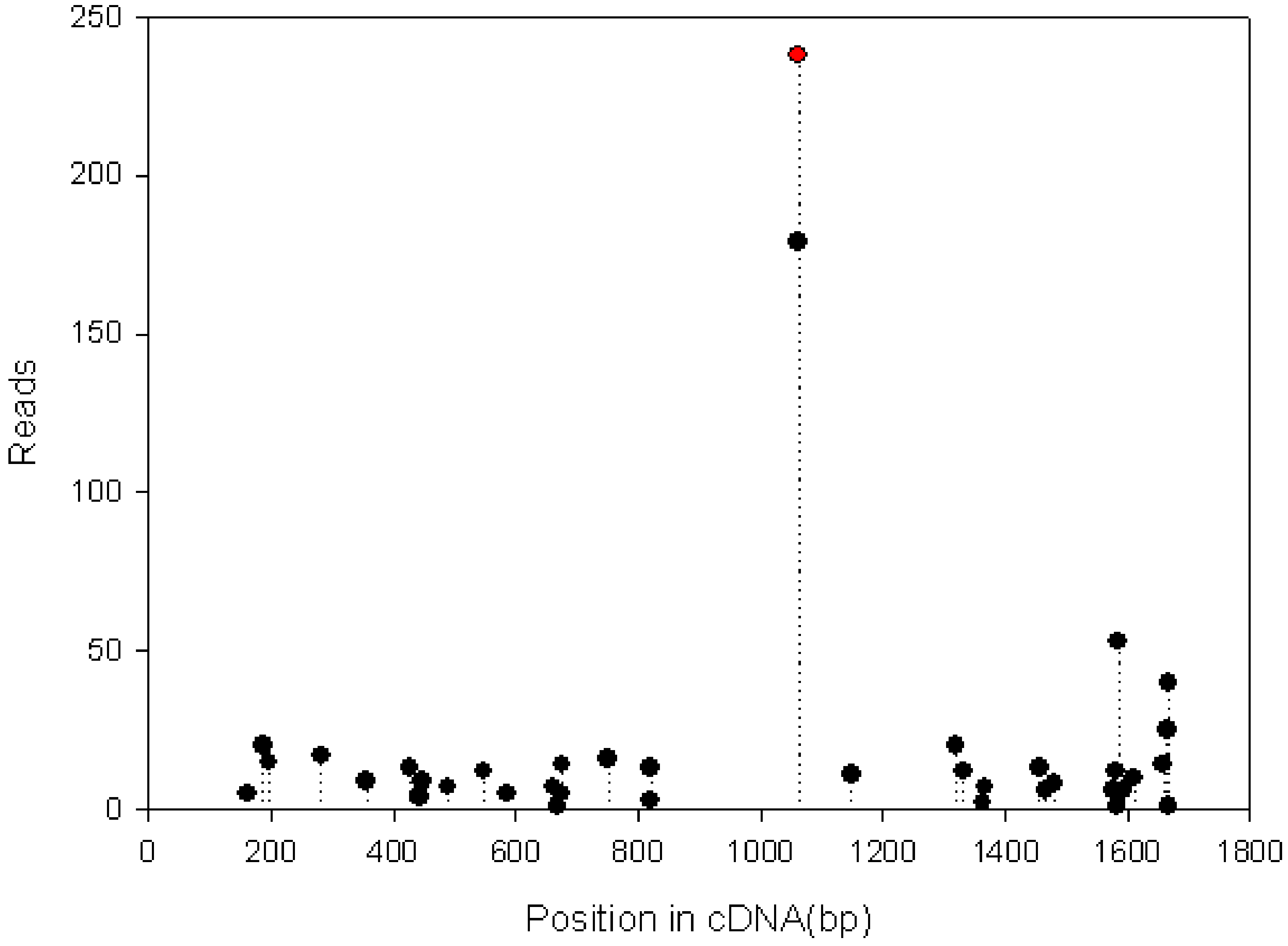


```
5' GCUUACGUGUCCUGCUUCUCCAAUUC 3'        Cs5g10870.1
     .::::::.:::::::::::::
3' -ACGUGCACGGGACGAAGAGGU---- 5'        Csi-miR164
```

Csi-miR164, target=Cs7g01530.1 gene=Cs7g01530
Category:3
Score=5
Cleavage Site=1974

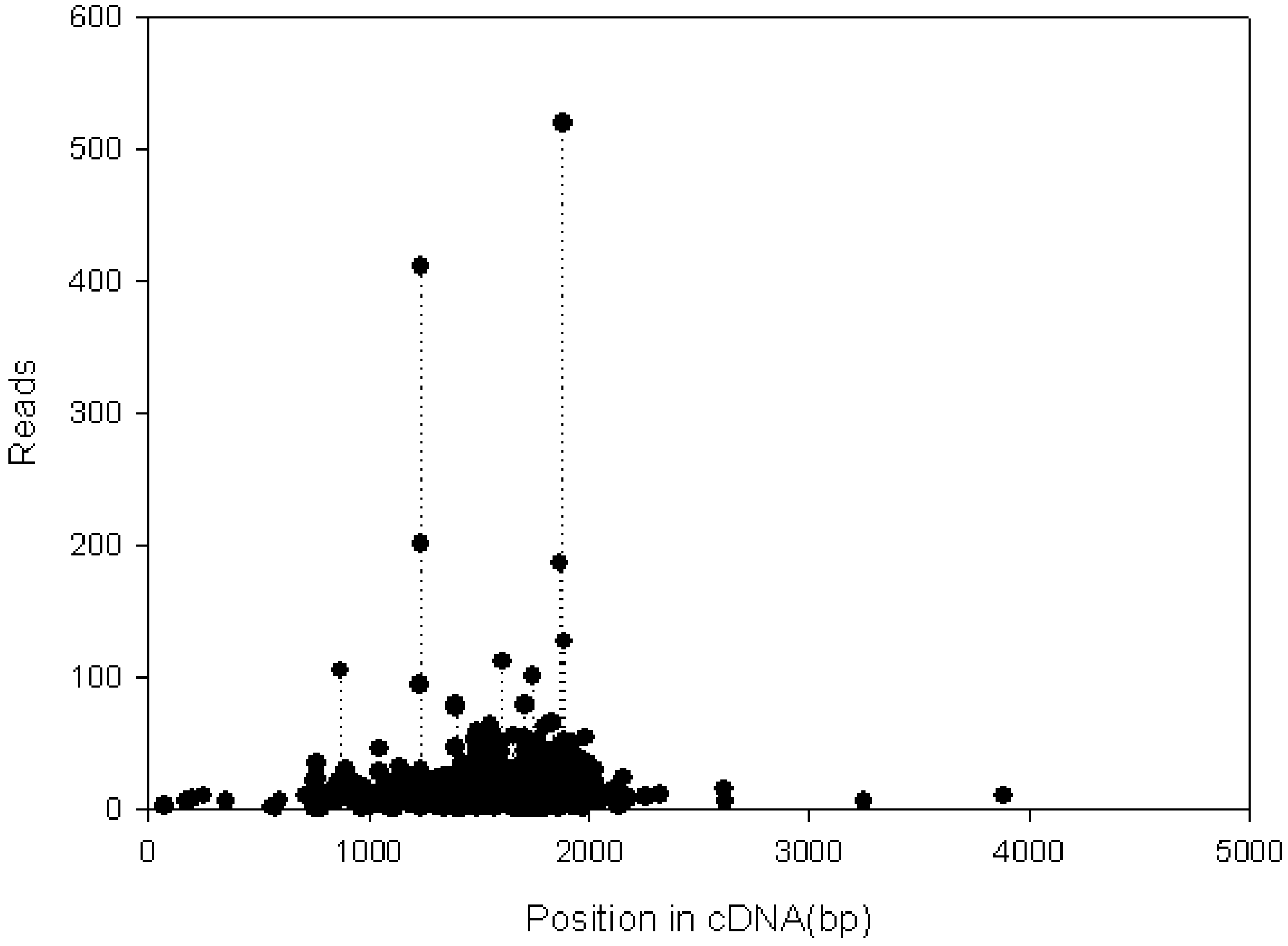


```
5' UAUGAGGCACAUGCUCUGCUUUUCCA 3'        Cs7g01530.1
        :::: :::.:::::::.::::
3' -----ACGUGCACGGGACGAAGAGGU 5'        Csi-miR164
```

Csi-miR166a.1, target=Cs1g15640.1 gene=Cs1g15640
Category:3
Score=3.5
Cleavage Site=1781

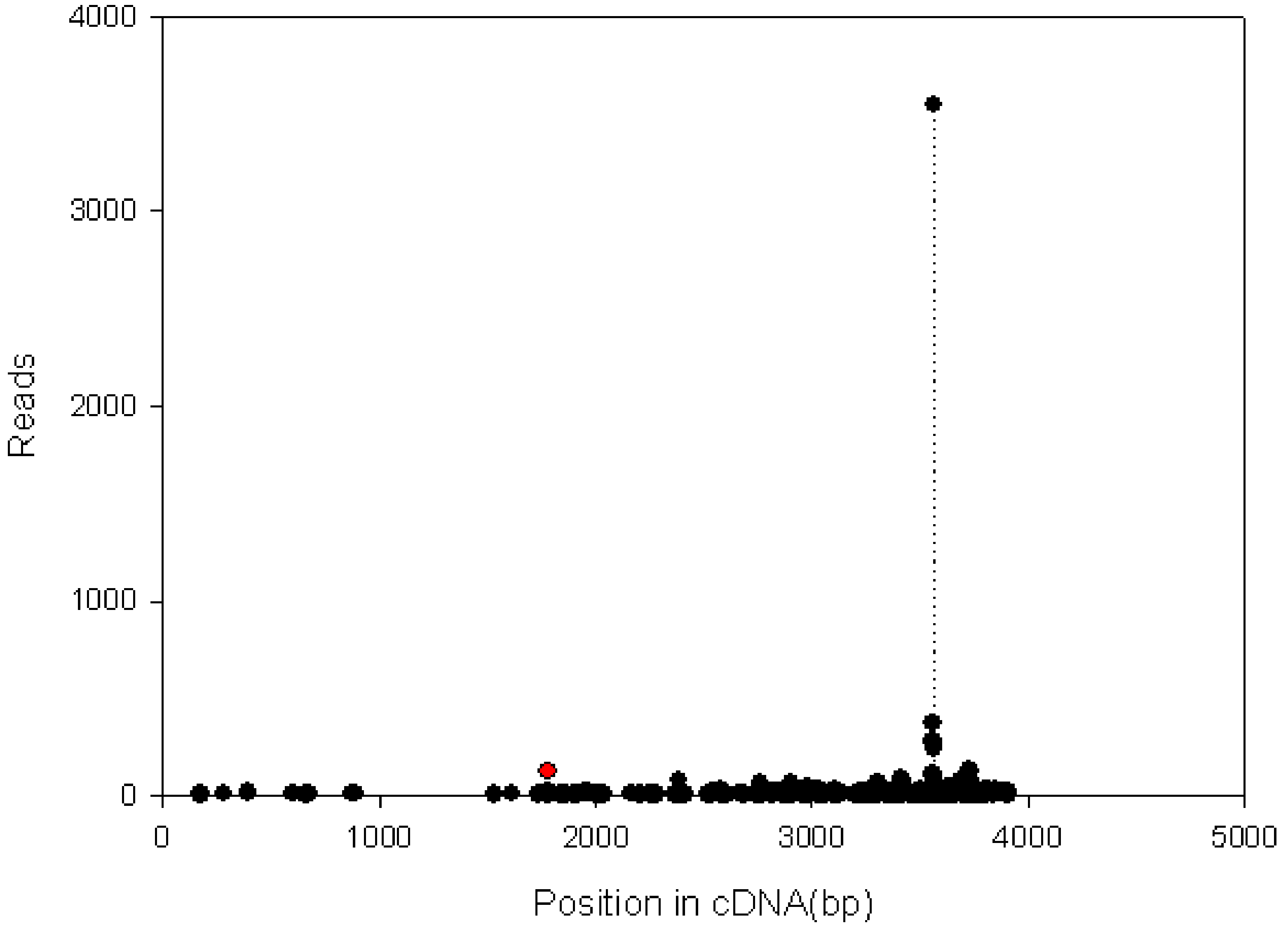


```
5' CCUGGAAUGAAGCCUGGUCCGGAUUC 3'        Cs1g15640.1
      ::::::::::::::::::::.
3' CCCCCUUACUUCGGACCAGGCU---- 5'        Csi-miR166a.1
```

Csi-miR166a.1, target=Cs2g09770.1 gene=Cs2g09770
Category:1
Score=4
Cleavage Site=1058

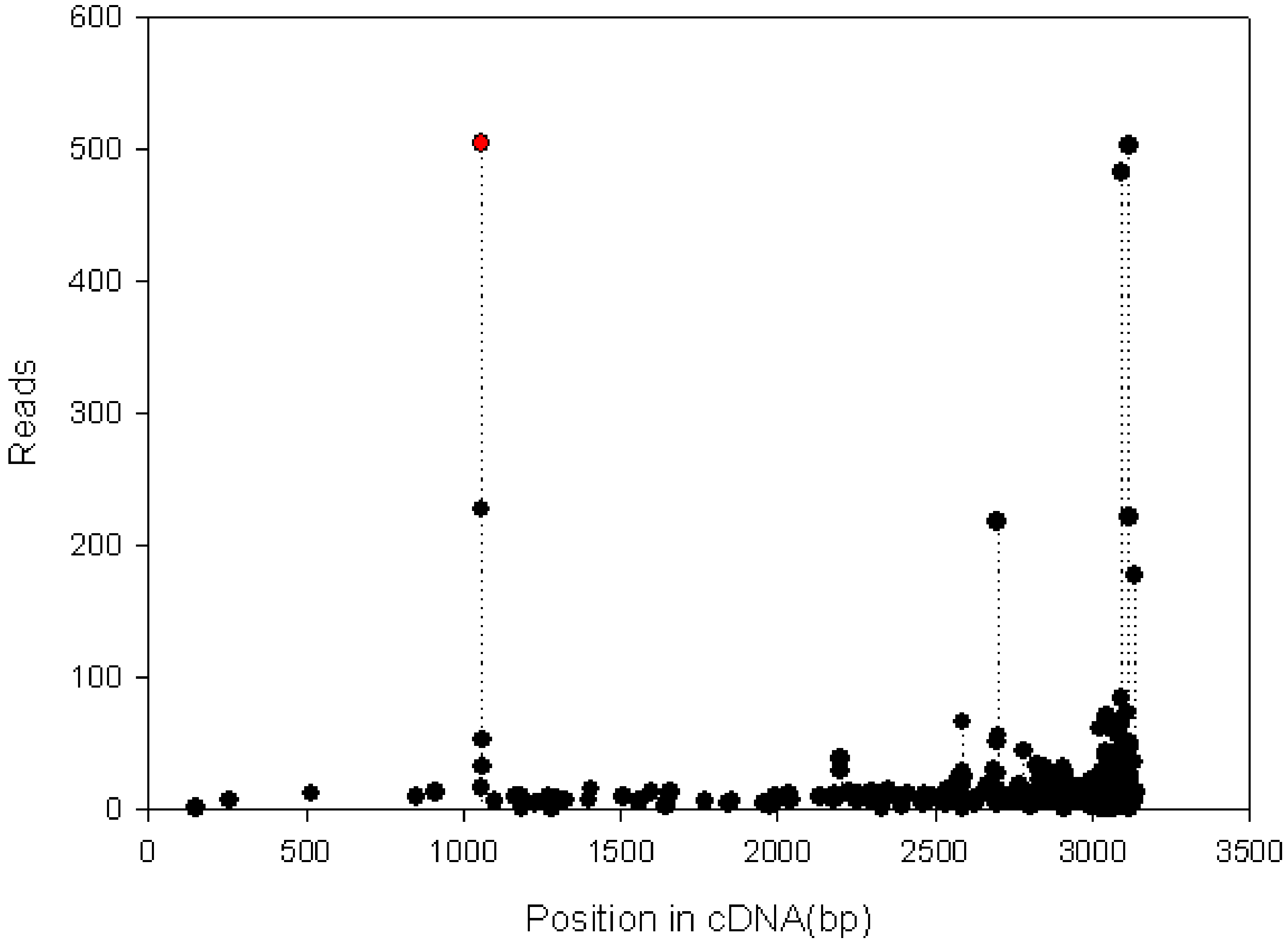


```
5' AUUGGGAUGAAGCCUGGUCCGGAUUC 3'        Cs2g09770.1
     ::.:::::::::::::::::.
3' CCCCCUUACUUCGGACCAGGCU---- 5'        Csi-miR166a.1
```

Csi-miR166a.1, target=Cs4g19310.1 gene=Cs4g19310
Category:1
Score=4
Cleavage Site=677

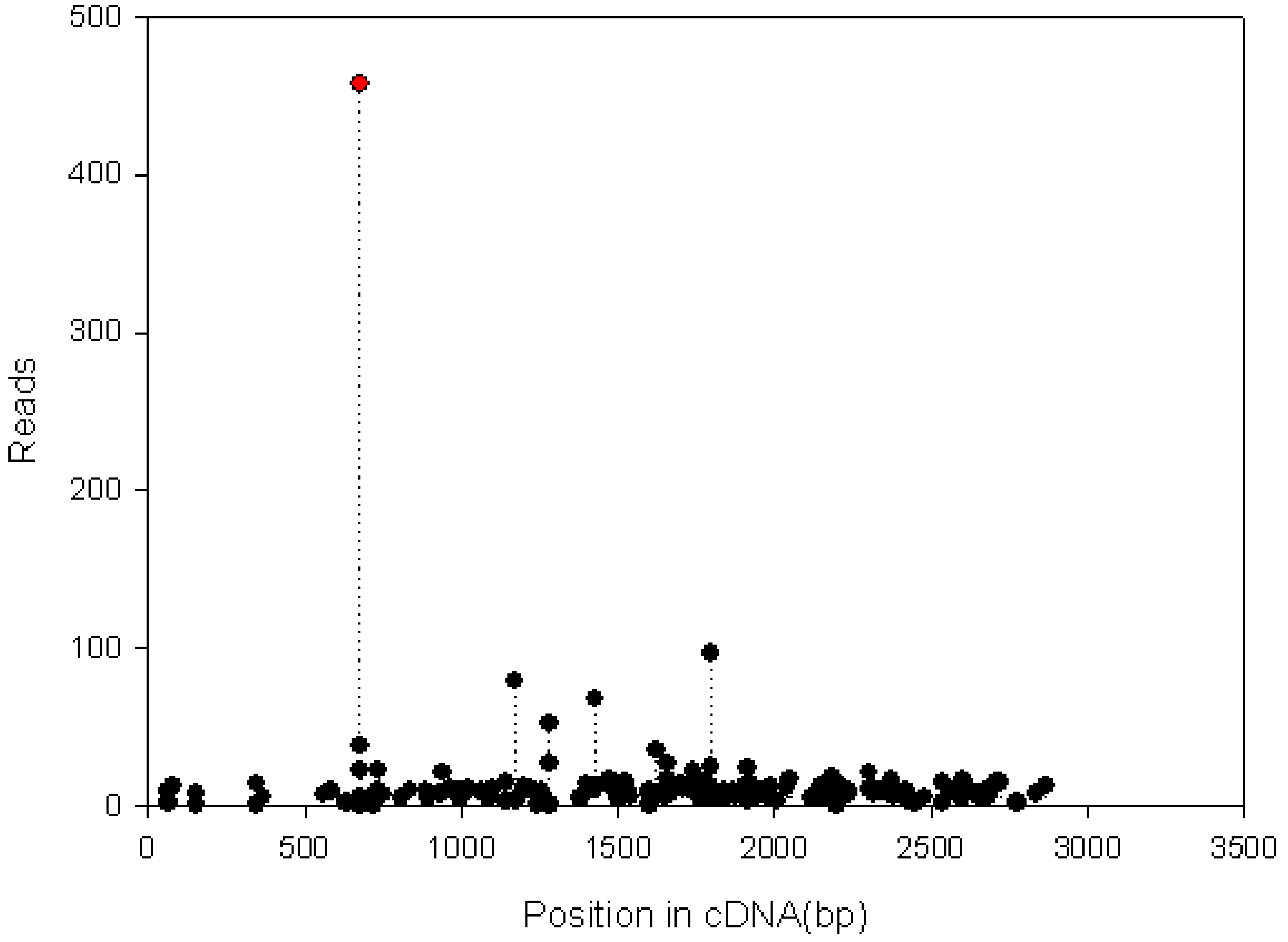


```
5' CCCGGGAUGAAGCCUGGUCCGGAUUC 3'          Cs4g19310.1
      ::.::::::::::::::.
3' CCCCCUUACUUCGGACCAGGCU---- 5'          Csi-miR166a.1
```

Csi-miR166a.1, target=Cs8g16510.1 gene=Cs8g16510
Category:1
Score=4
Cleavage Site=1434

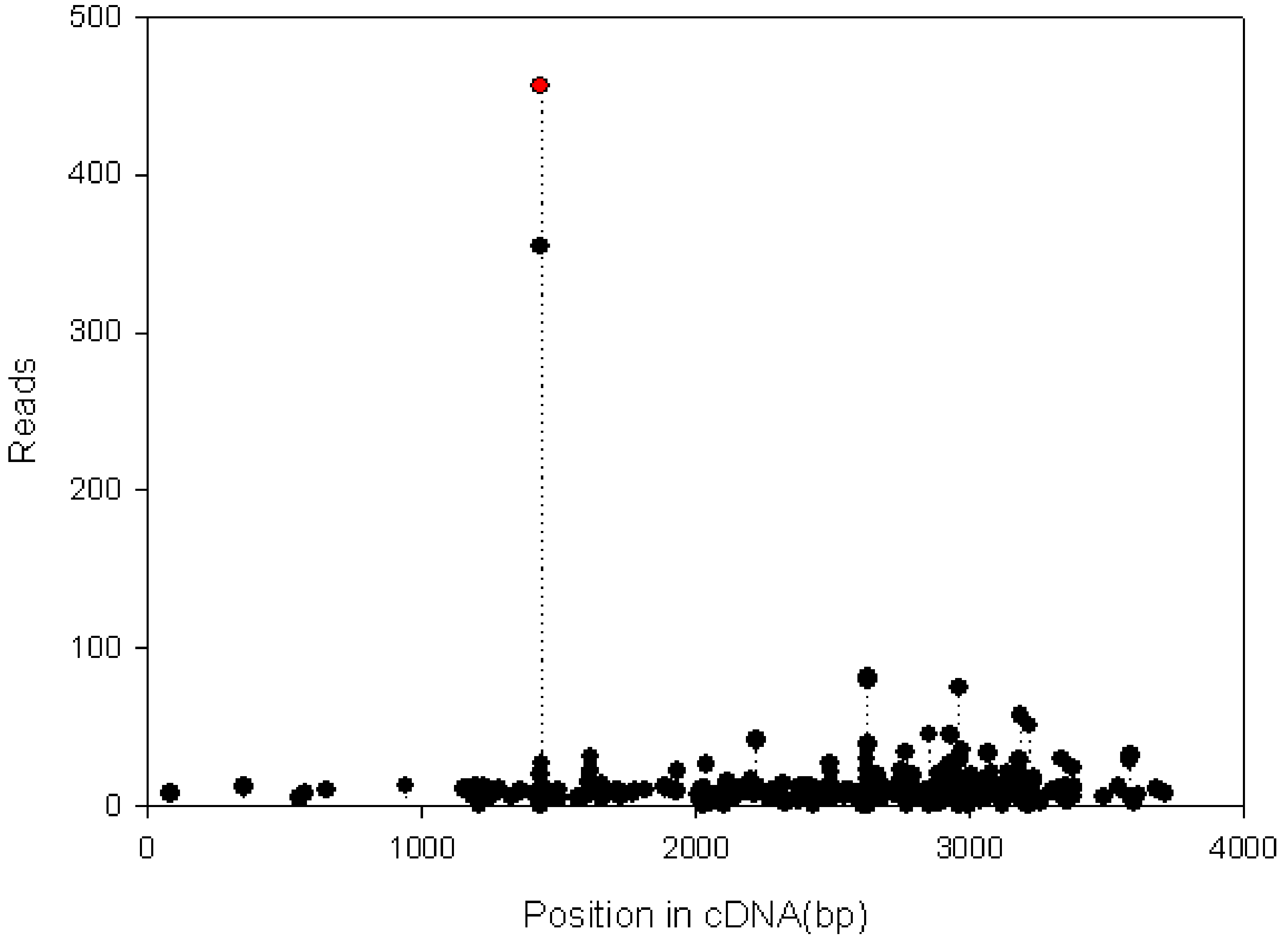


```
5' CCUGGGAUGAAGCCUGGUCCGGAUUC 3'        Cs8g16510.1
       ::.::::::::::::::::.
3' CCCCCUUACUUCGGACCAGGCU---- 5'        Csi-miR166a.1
```

Csi-miR166a.2, target=Cs1g15640.1 gene=Cs1g15640
Category:3
Score=2.5
Cleavage Site=1781

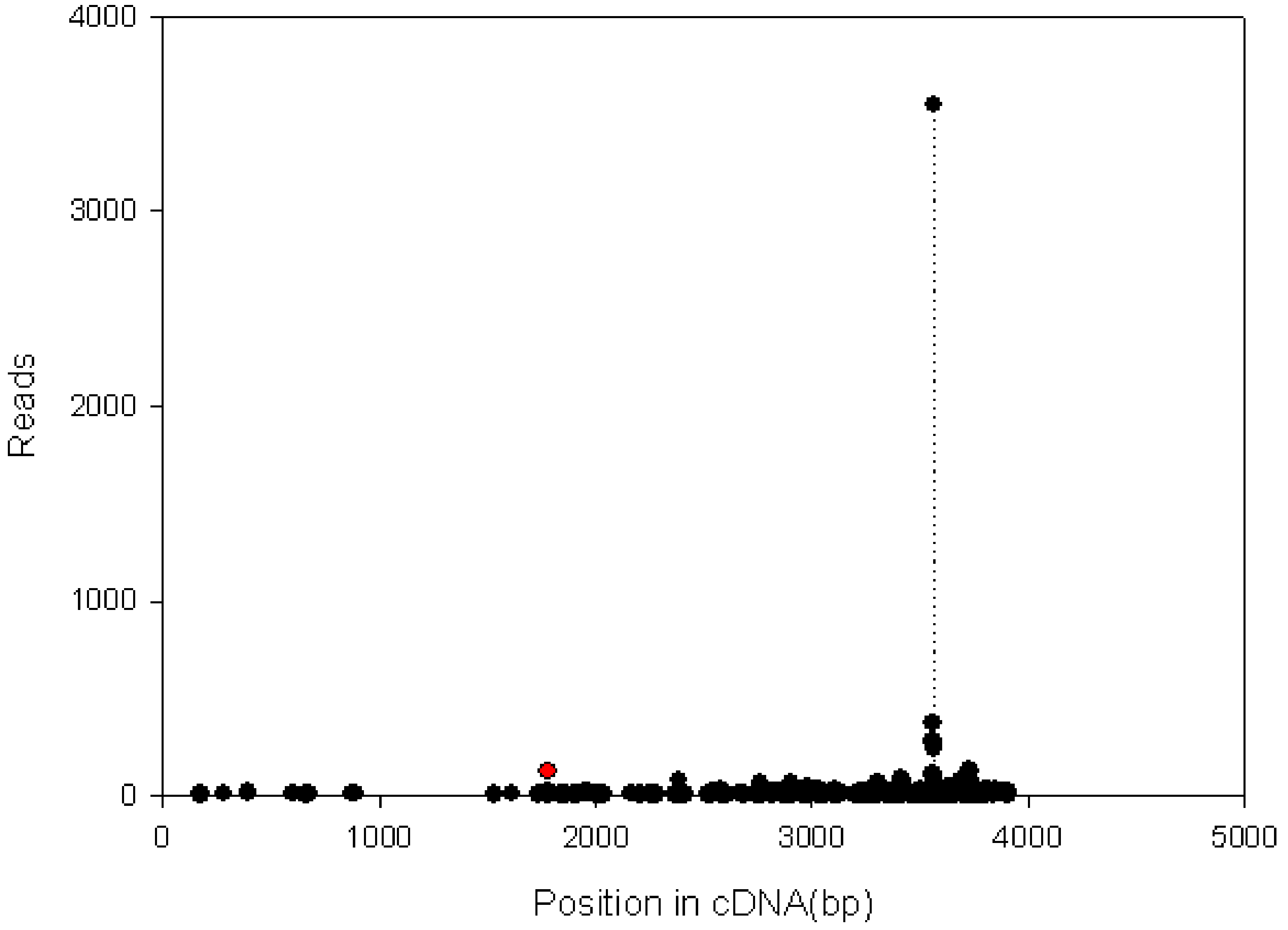


```
5' CCUGGAAUGAAGCCUGGUCCGGAUUC 3'        Cs1g15640.1
     ::::::::::::::::::::.
3' -CCCCUUACUUCGGACCAGGCU---- 5'        Csi-miR166a.2
```

Csi-miR166a.2, target=Cs2g09770.1 gene=Cs2g09770
Category:1
Score=3
Cleavage Site=1058

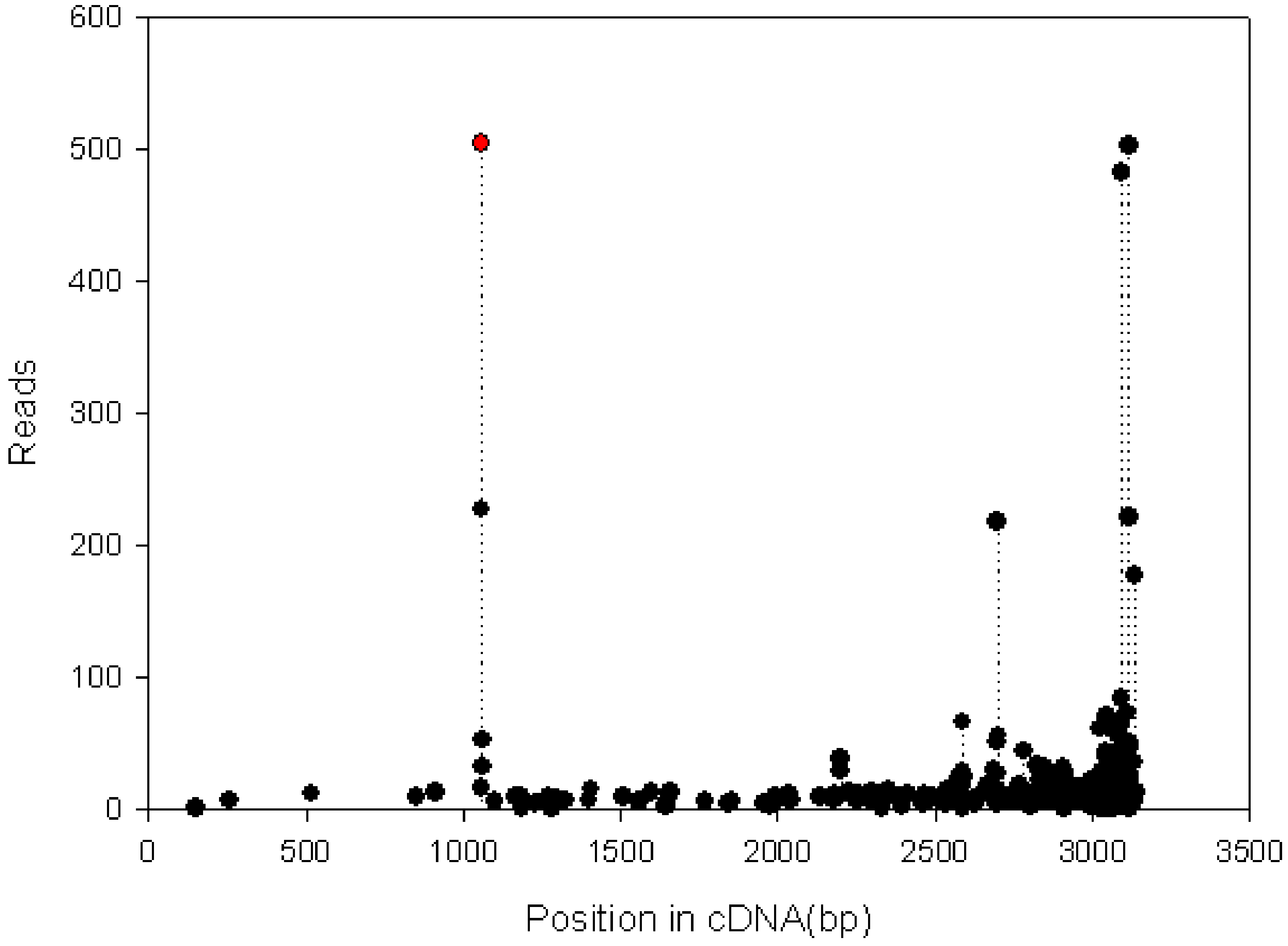


```
5' AUUGGGAUGAAGCCUGGUCCGGAUUC 3'        Cs2g09770.1
     ::.:::::::::::::::::.
3' -CCCCUUACUUCGGACCAGGCU---- 5'        Csi-miR166a.2
```

Csi-miR166a.2, target=Cs4g19310.4 gene=Cs4g19310
Category:1
Score=3
Cleavage Site=677

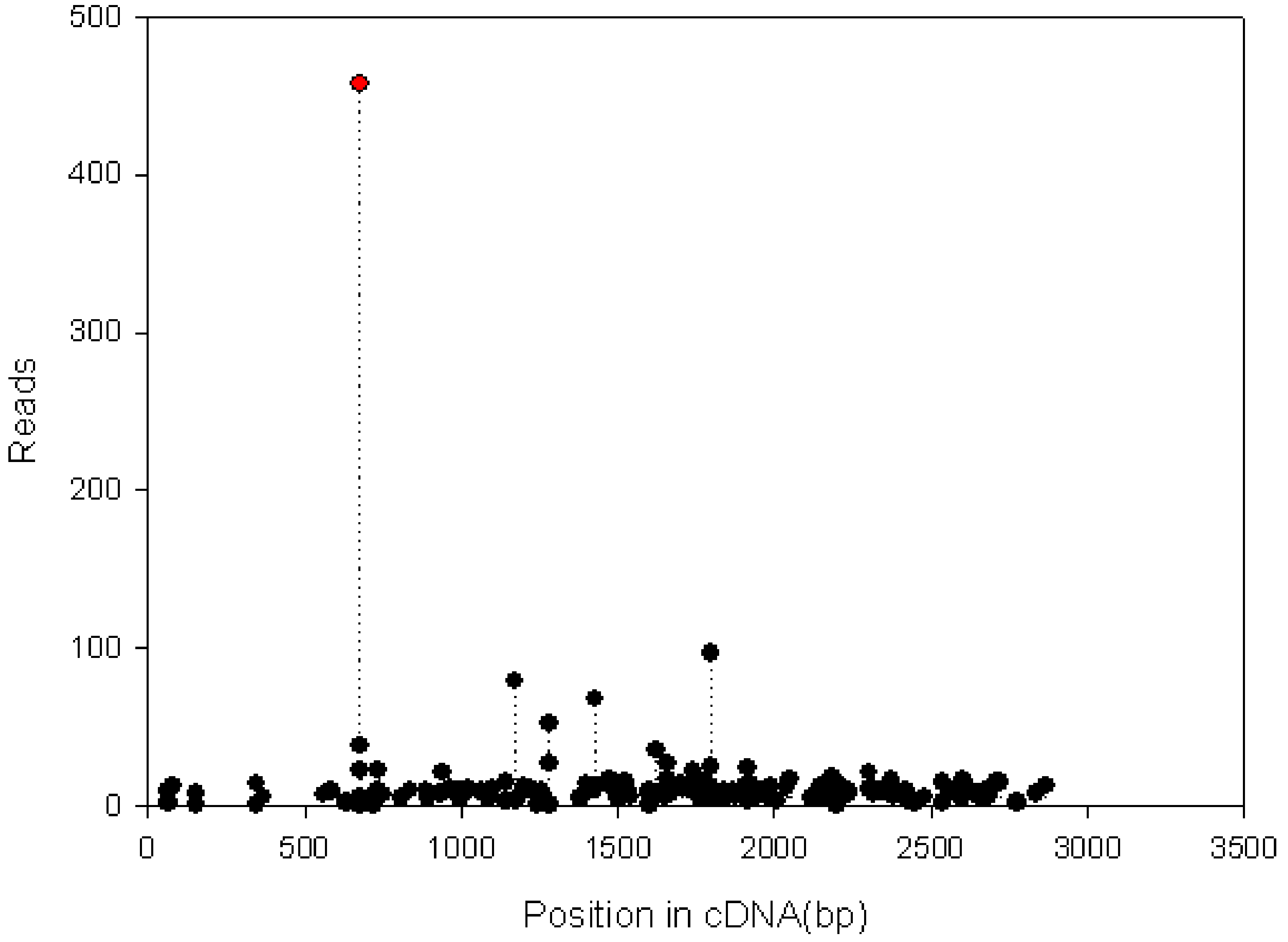


```
5' CCCGGGAUGAAGCCUGGUCCGGAUUC 3'        Cs4g19310.1
      ::.:::::::::::::::::.
3' -CCCCUUACUUCGGACCAGGCU---- 5'        Csi-miR166a.2
```

Csi-miR166a.2, target=Cs8g16510.1 gene=Cs8g16510
Category:1
Score=3
Cleavage Site=1434

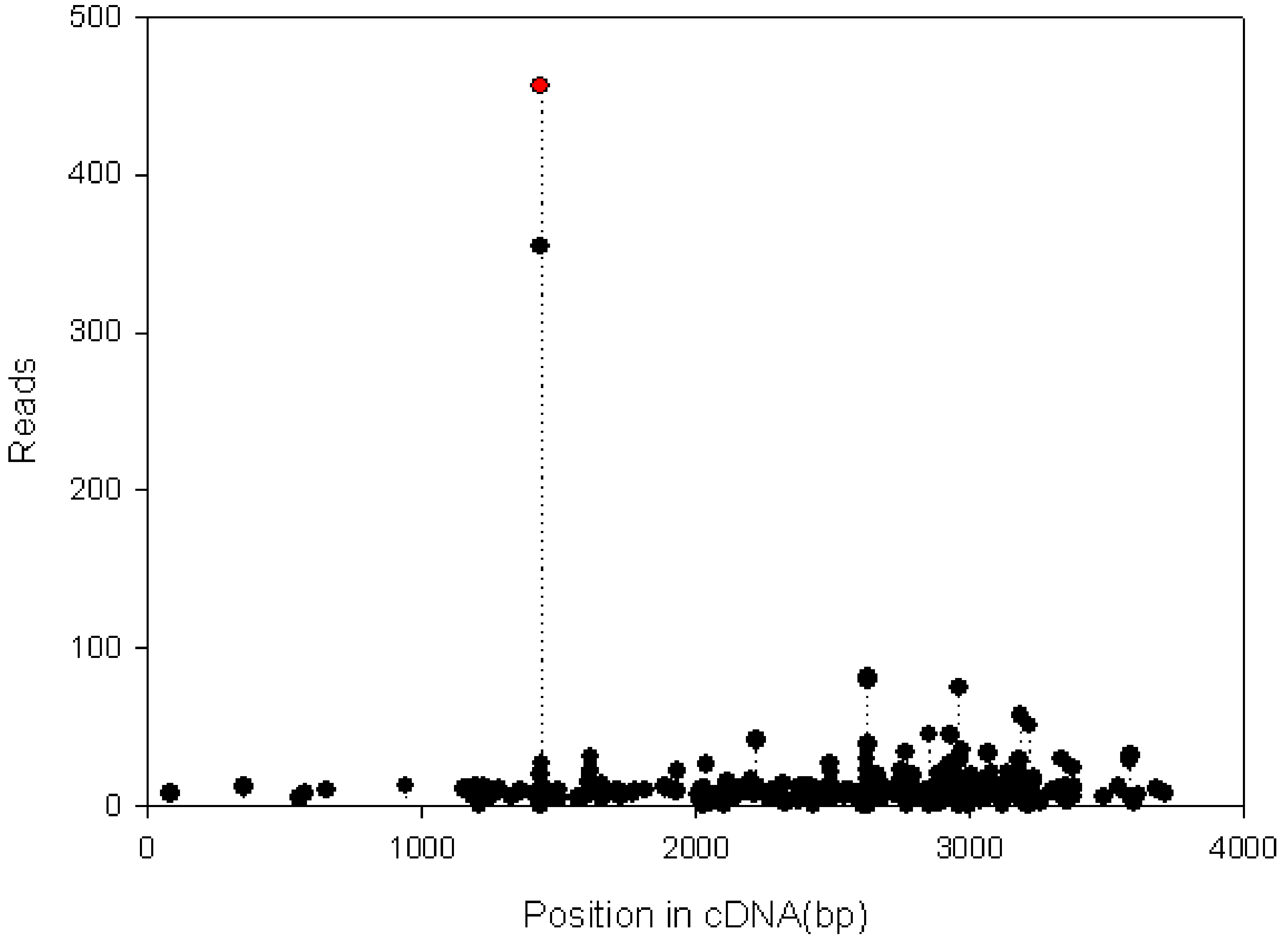


```
5' CCUGGGAUGAAGCCUGGUCCGGAUUC 3'        Cs8g16510.1
      ::.:::::::::::::::::.
3' -CCCCUUACUUCGGACCAGGCU---- 5'        Csi-miR166a.2
```

Csi-miR166b, target=Cs1g15640.1 gene=Cs1g15640
Category:3
Score=2.5
Cleavage Site=1781

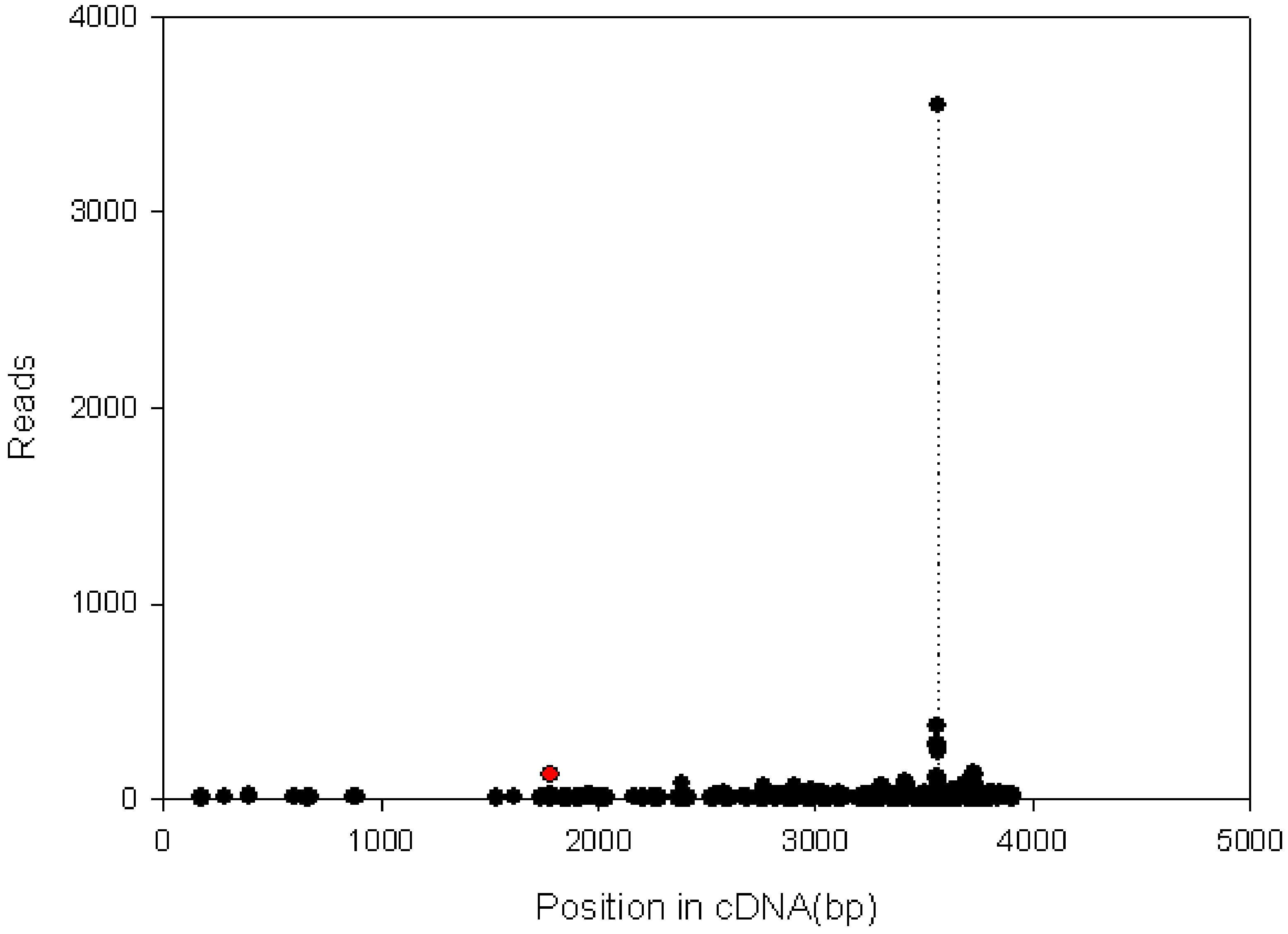


```
5' CCUGGAAUGAAGCCUGGUCCGGAUUC 3'        Cs1g15640.1
    : :::::::::::::::::::.
3' -GCCCUUACUUCGGACCAGGCU---- 5'        Csi-miR166b
```

Csi-miR166b, target=Cs2g09770.1 gene=Cs2g09770
Category:1
Score=3
Cleavage Site=1058

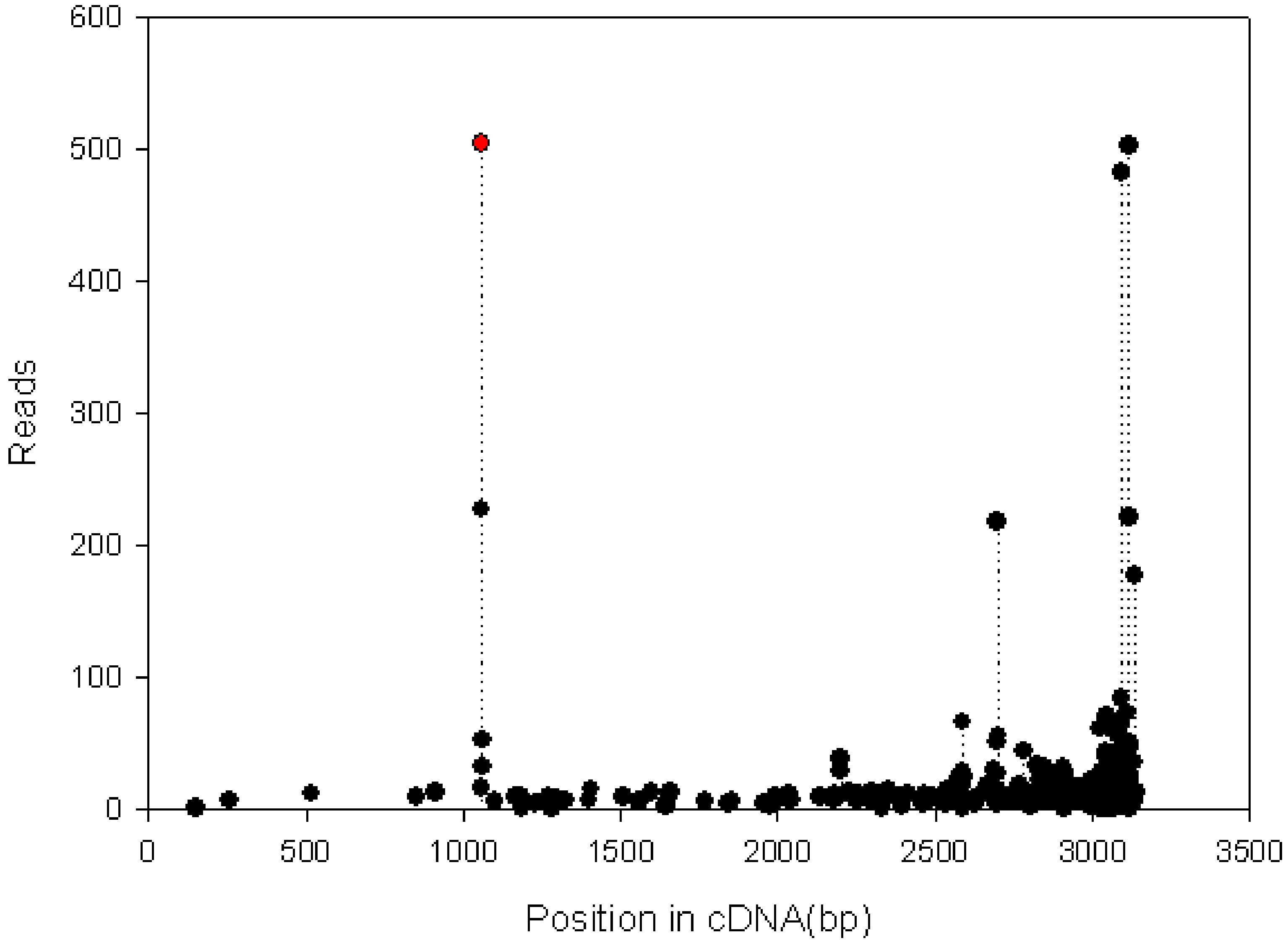


```
5' AUUGGGAUGAAGCCUGGUCCGGAUUC 3'        Cs2g09770.1
    . ::.::::::::::::::.
3' -GCCCUUACUUCGGACCAGGCU---- 5'        Csi-miR166b
```

Csi-miR166b, target=Cs4g19310.1 gene=Cs4g19310
Category:1
Score=3
Cleavage Site=677

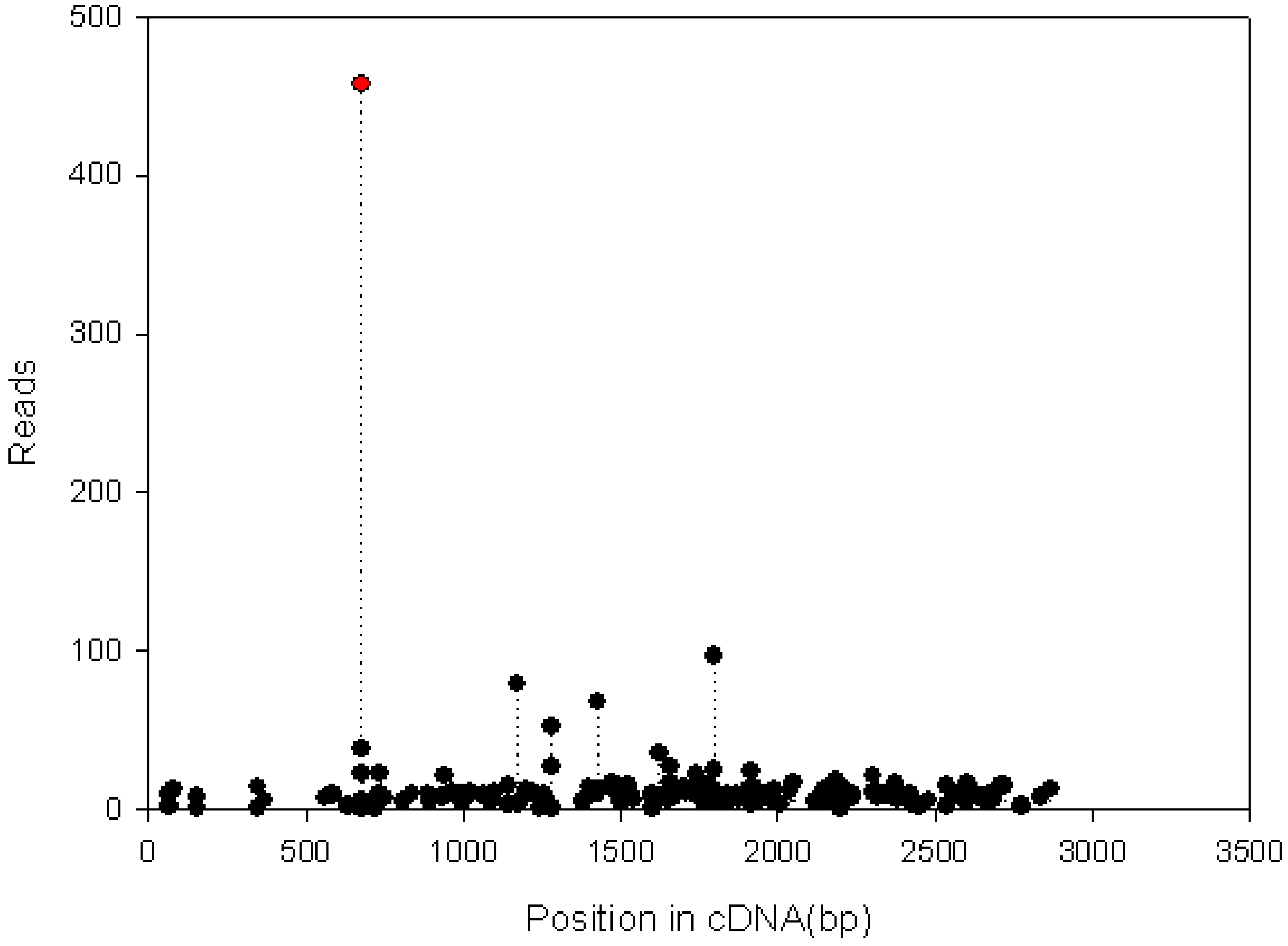


```
5' CCCGGGAUGAAGCCUGGUCCGGAUUC 3'        Cs4g19310.1
    : ::.:::::::::::::::::.
3' -GCCCUUACUUCGGACCAGGCU---- 5'        Csi-miR166b
```

Csi-miR166b, target=Cs8g16510.1 gene=Cs8g16510
Category:1
Score=5
Cleavage Site=1434

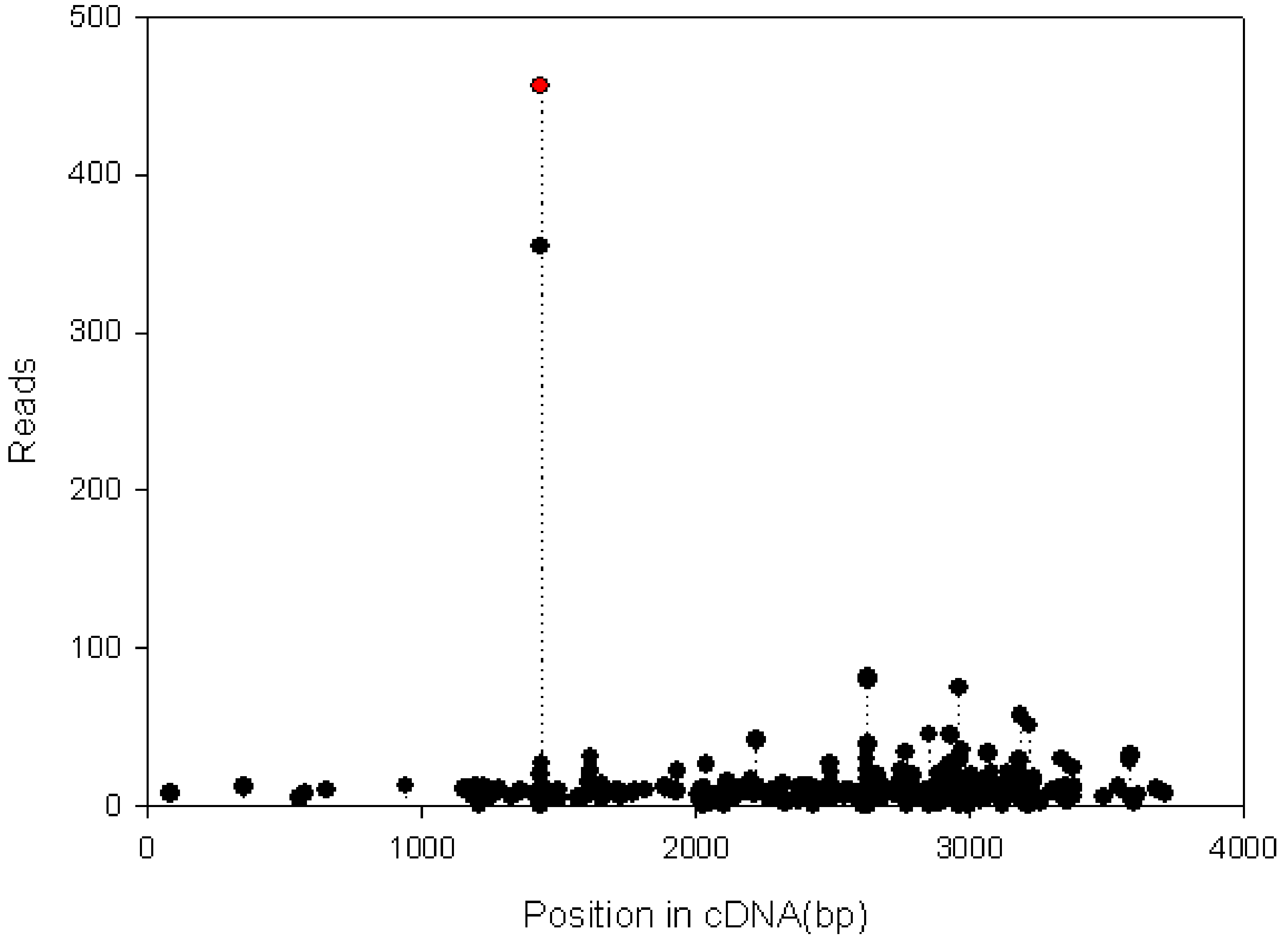


```
5' CCUGGGAUGAAGCCUGGUCCGGAUUC 3'        Cs8g16510.1
    : ::.:::::::::::::::.
3' -GCCCUUACUUCGGACCAGGCU---- 5'        Csi-miR166b
```

Csi-miR166c.1, target=Cs1g15640.1 gene=Cs1g15640
Category:3
Score=0.5
Cleavage Site=1781

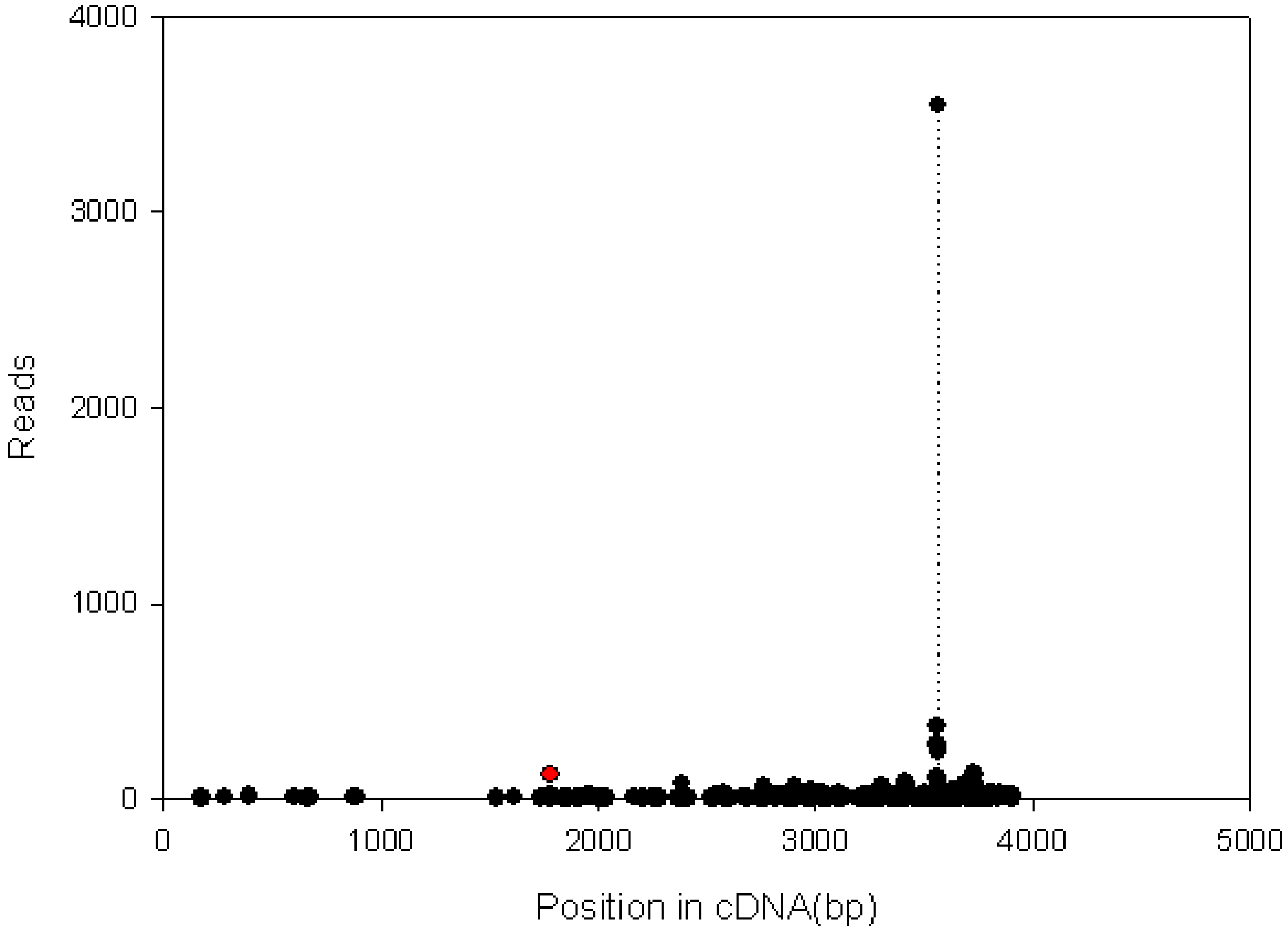


```
5' CCUGGAAUGAAGCCUGGUCCGGAUUC 3'         Cs1g15640.1
       ::::::::::::::::::::.
3' ---CCUUACUUCGGACCAGGCU---- 5'         Csi-miR166c.1
```

Csi-miR166c.1, target=Cs2g09770.1 gene=Cs2g09770
Category:1
Score=1
Cleavage Site=1058

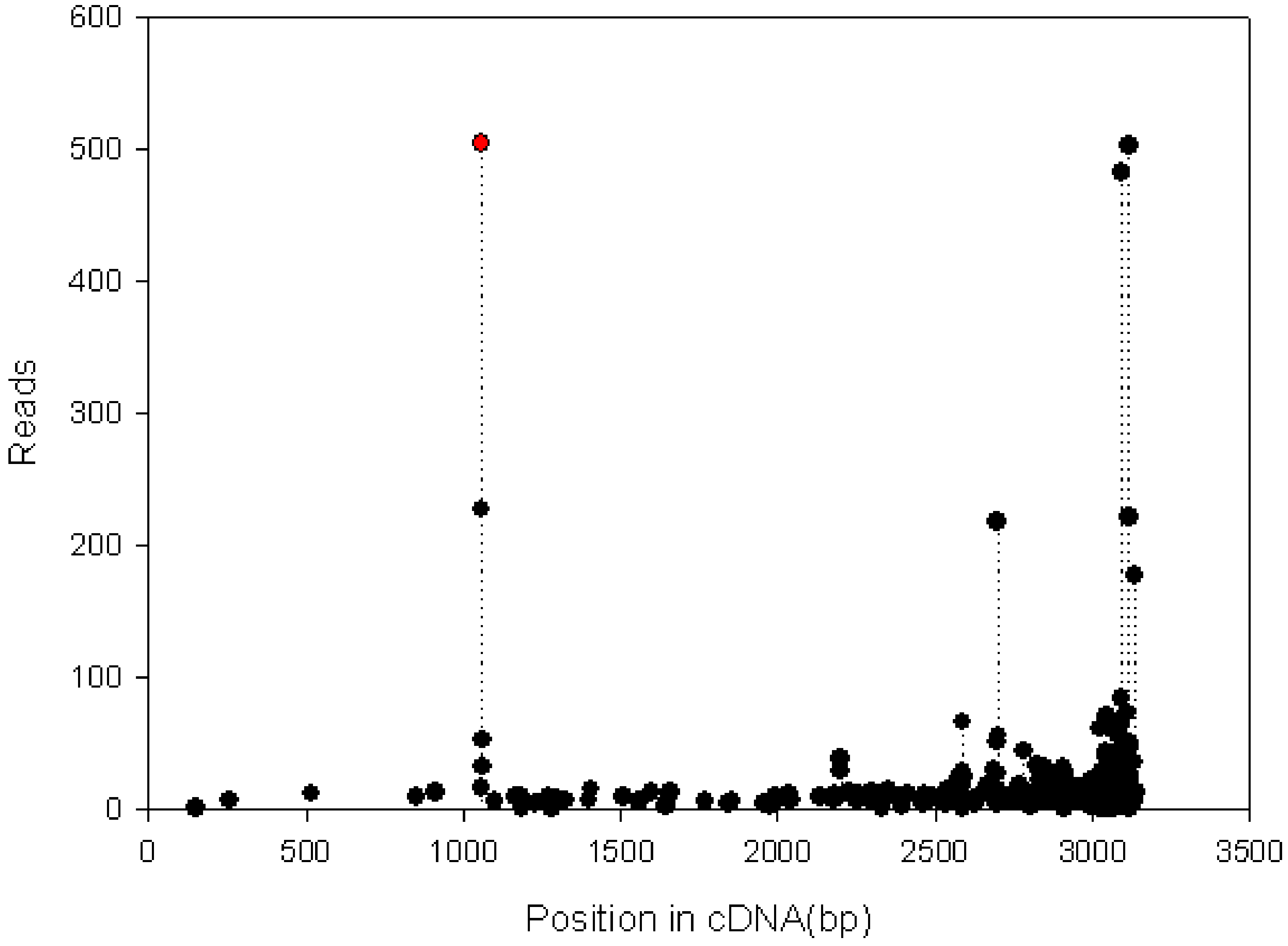


```
5' AUUGGGAUGAAGCCUGGUCCGGAUUC 3'        Cs2g09770.1
      ::.::::::::::::::::::.
3' ---CCUUACUUCGGACCAGGCU---- 5'        Csi-miR166c.1
```

Csi-miR166c.1, target=Cs4g19310.1 gene=Cs4g19310
Category:1
Score=1
Cleavage Site=677

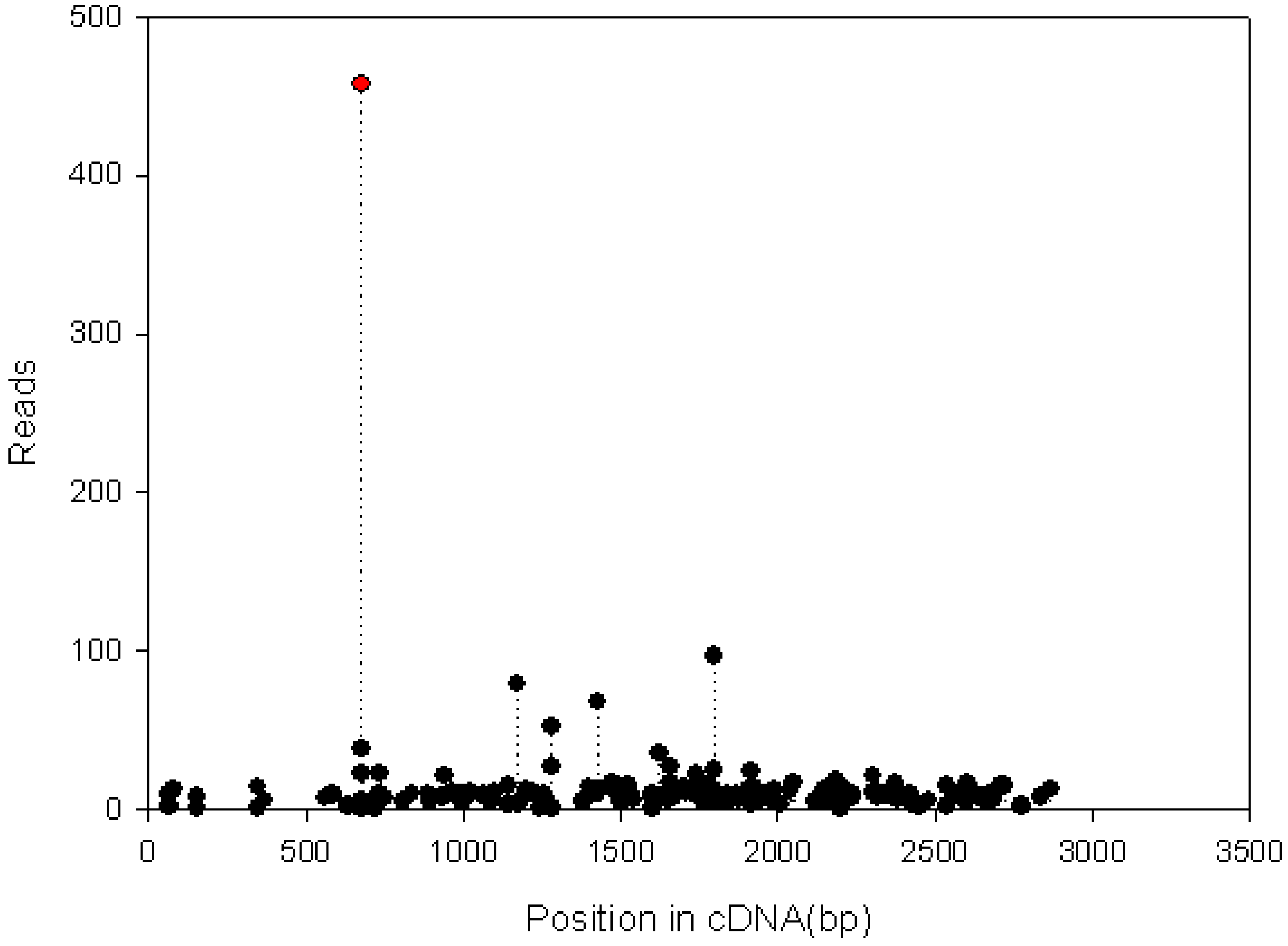


```
5' CCCGGGAUGAAGCCUGGUCCGGAUUC 3'        Cs4g19310.1
      ::.:::::::::::::::.
3' ---CCUUACUUCGGACCAGGCU---- 5'        Csi-miR166c.1
```

Csi-miR166c.1, target=Cs8g16510.1 gene=Cs8g16510
Category:1
Score=1
Cleavage Site=1434

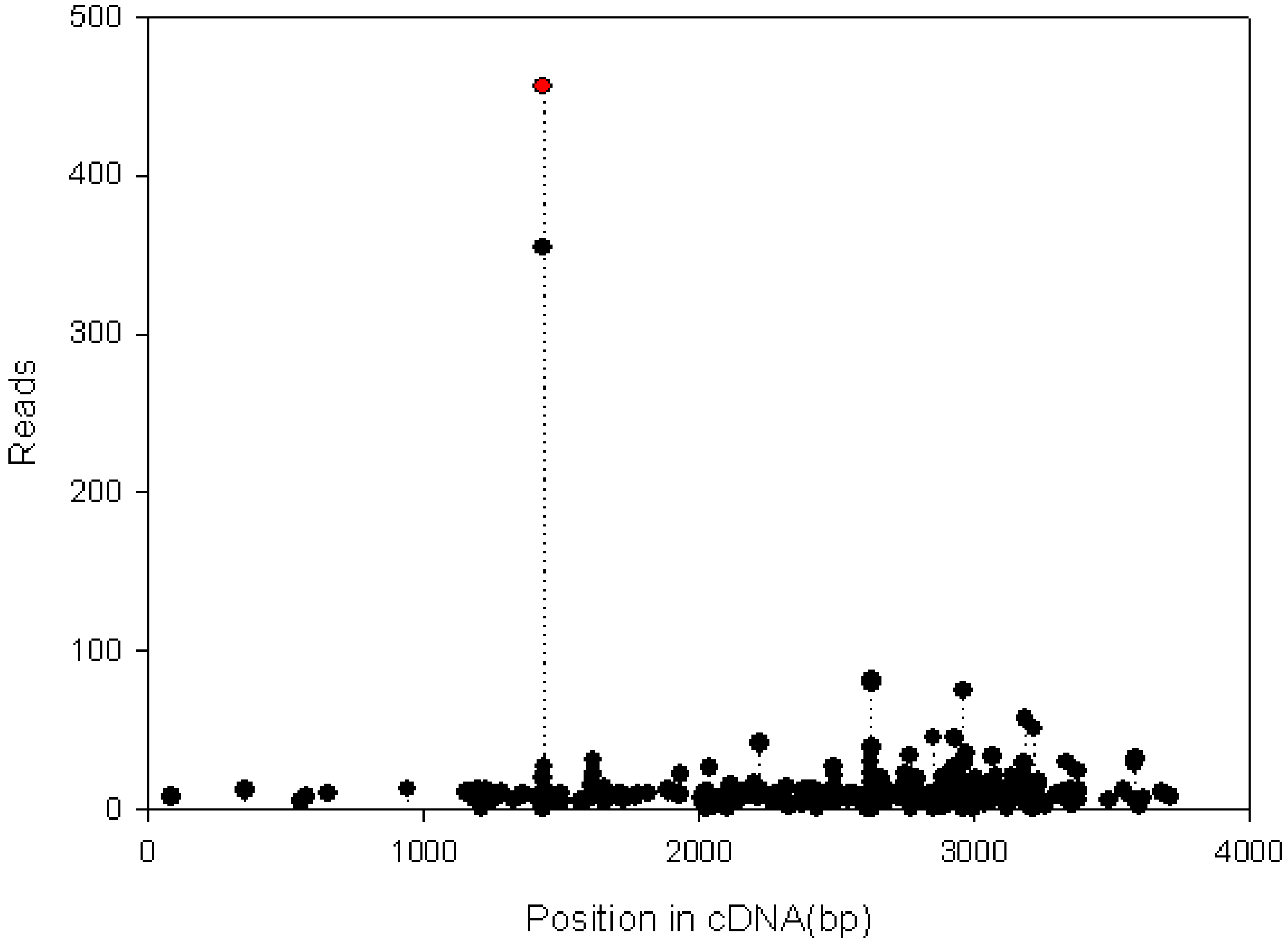


```
5' CCUGGGAUGAAGCCUGGUCCGGAUUC 3'        Cs8g16510.1
       ::.:::::::::::::::::.
3' ---CCUUACUUCGGACCAGGCU---- 5'        Csi-miR166c.1
```

Csi-miR166c.2, target=Cs1g15640.1 gene=Cs1g15640
Category:3
Score=0.5
Cleavage Site=1781

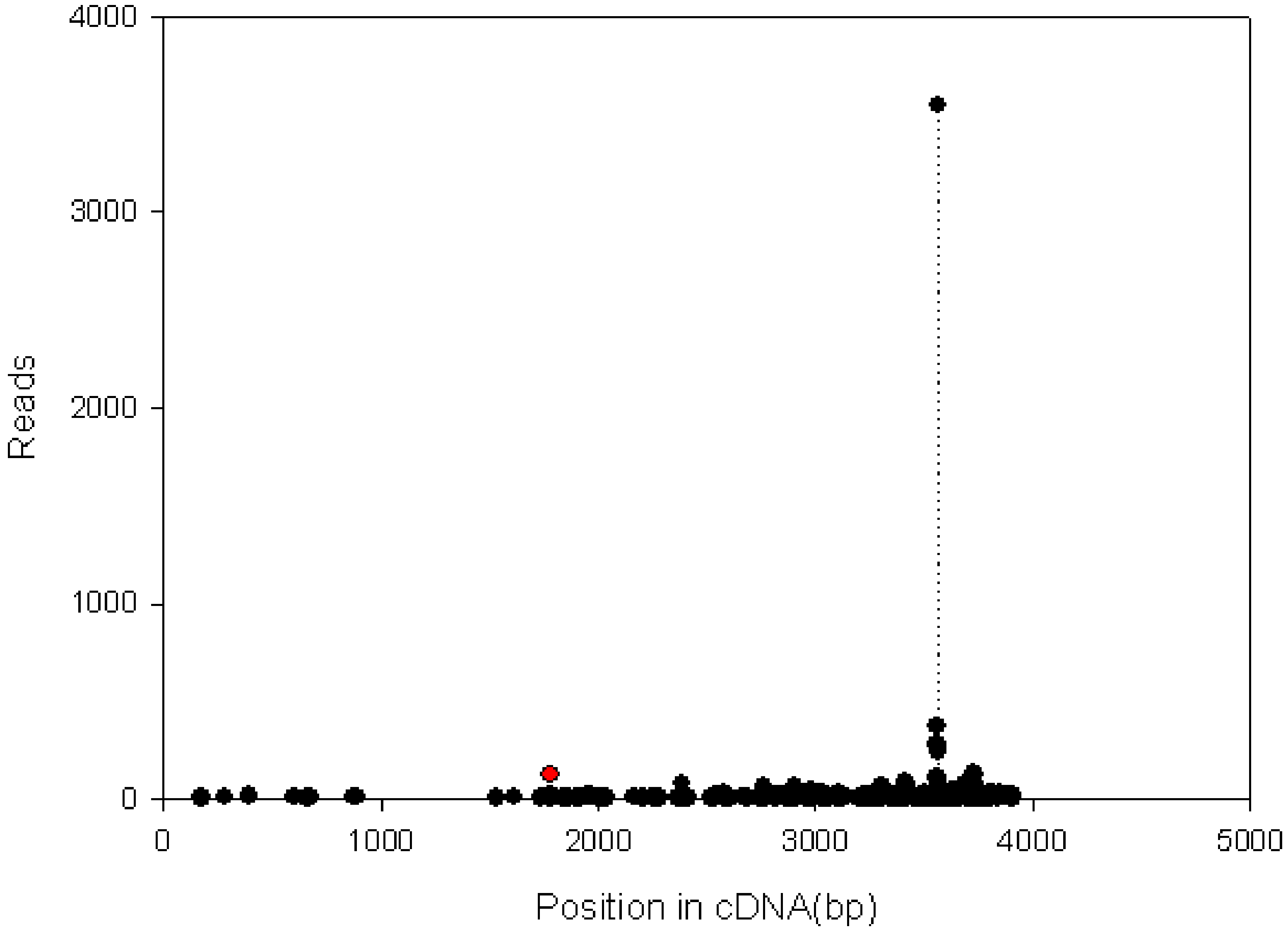


```
5' CCUGGAAUGAAGCCUGGUCCGGAUUC 3'        Cs1g15640.1
       ::::::::::::::::::.
3' ----CUUACUUCGGACCAGGCU---- 5'        Csi-miR166c.2
```

Csi-miR166c.2, target=Cs2g09770.1 gene=Cs2g09770
Category:1
Score=1
Cleavage Site=1058

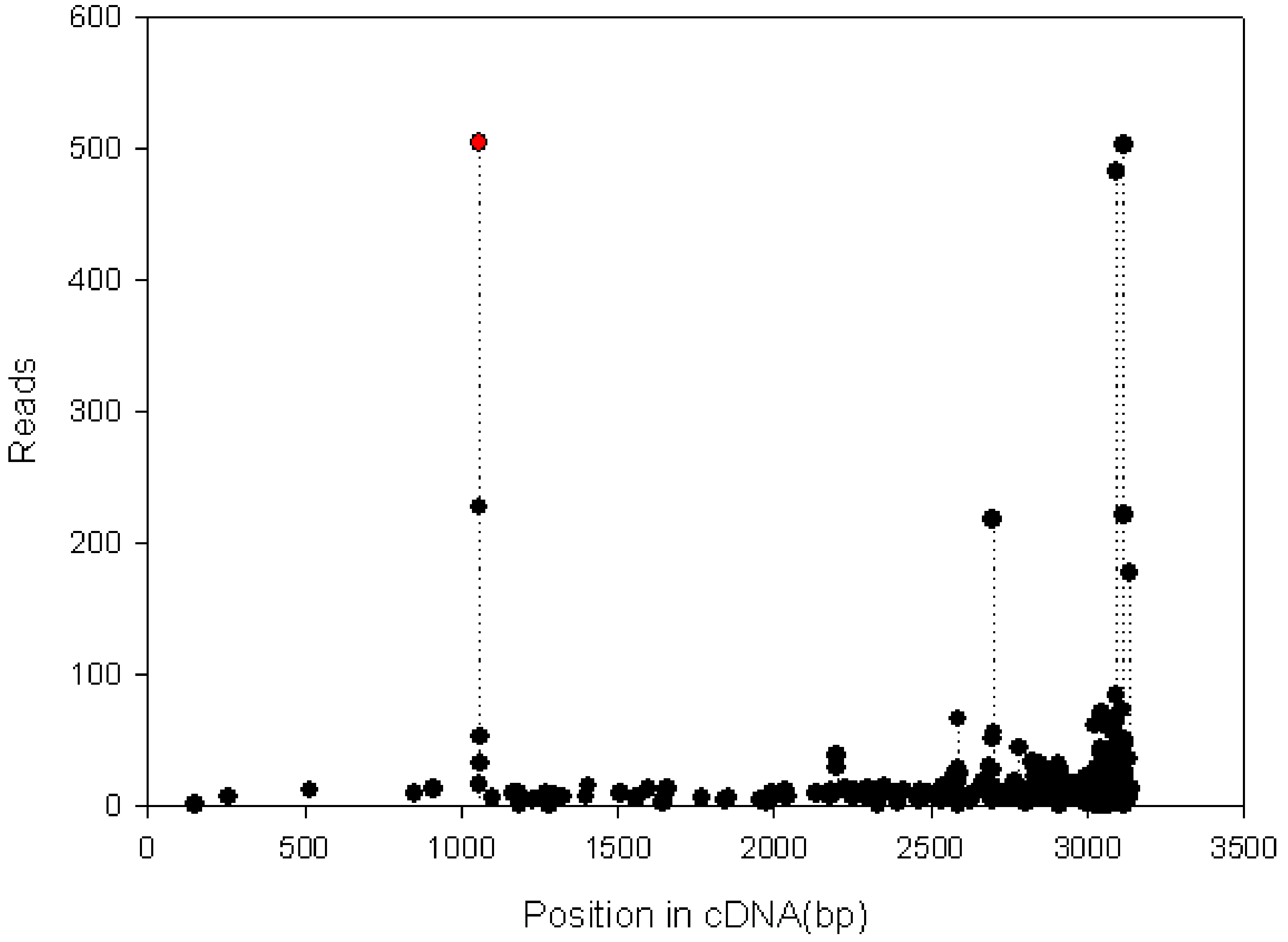


```
5' AUUGGGAUGAAGCCUGGUCCGGAUUC 3'        Cs2g09770.1
       :.:::::::::::::::.
3' ----CUUACUUCGGACCAGGCU---- 5'        Csi-miR166c.2
```

Csi-miR166c.2, target=Cs4g06030.1 gene=Cs4g06030
Category:3
Score=4.5
Cleavage Site=2044

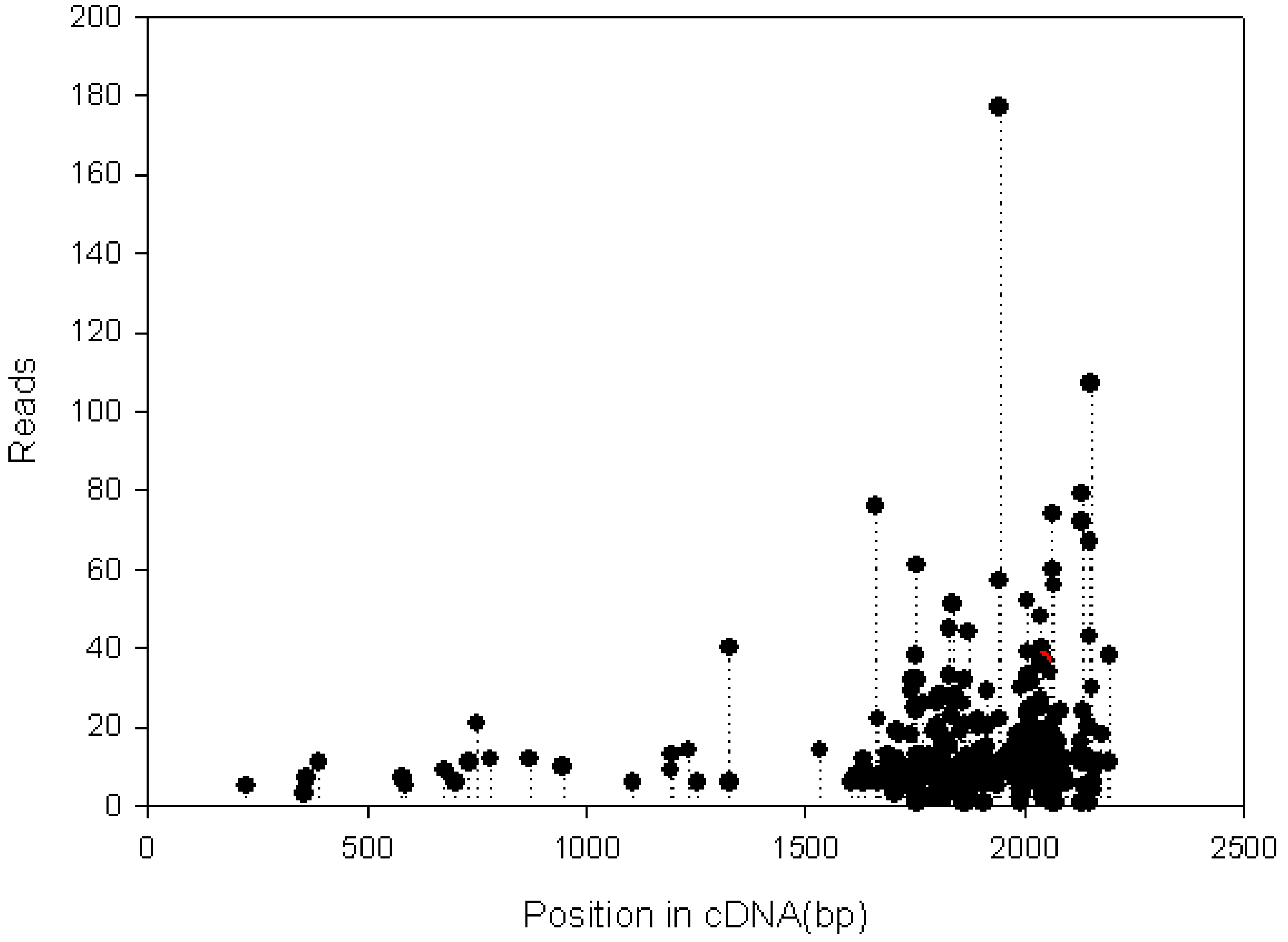


```
5' AGGCUGAAU-AAGCCUGUUCUGGGUUG 3'        Cs4g06030.1
          :::: ::::::: ::.:.
3' -----CUUACUUCGGACCAGGCU---- 5'        Csi-miR166c.2
```

Csi-miR166c.2, target=Cs4g19310.1 gene=Cs4g19310
Category:1
Score=1
Cleavage Site=677

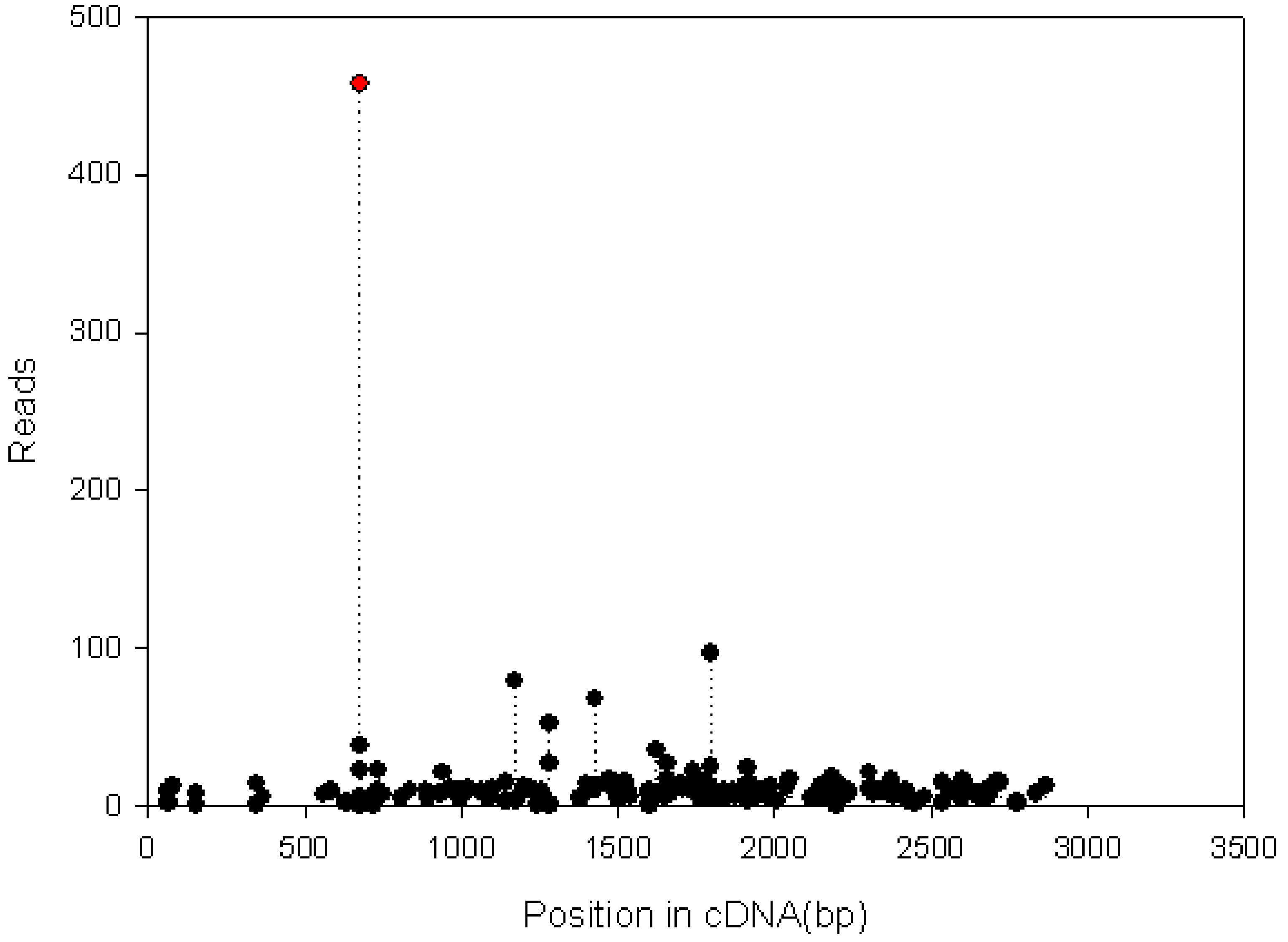


```
5' CCCGGGAUGAAGCCUGGUCCGGAUUC 3'        Cs4g19310.1
        :.:::::::::::::::.
3' ----CUUACUUCGGACCAGGCU---- 5'        Csi-miR166c.2
```

Csi-miR166c.2, target=Cs8g16510.1 gene=Cs8g16510
Category:1
Score=1
Cleavage Site=1434

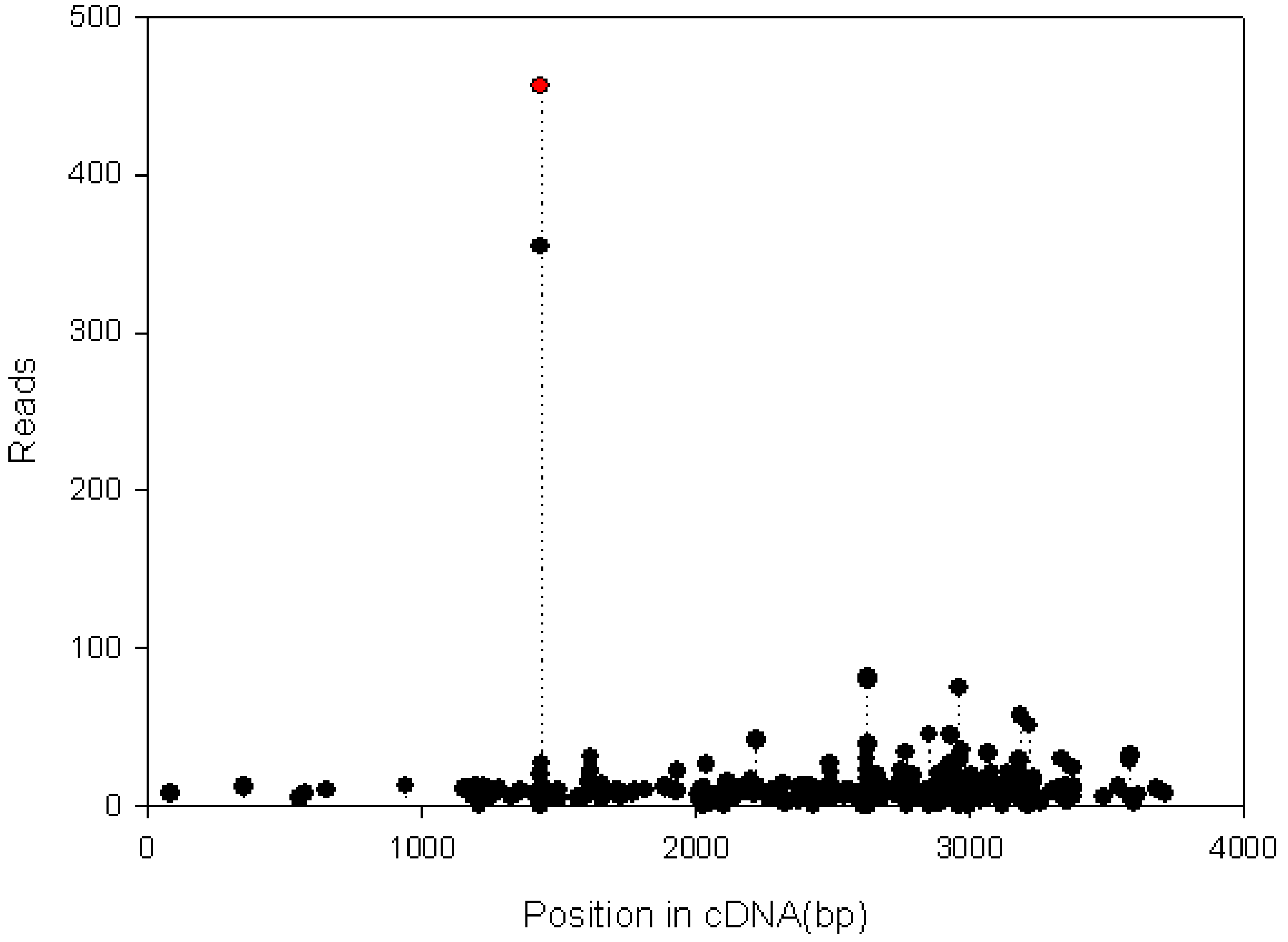


```
5' CCUGGGAUGAAGCCUGGUCCGGAUUC 3'        Cs8g16510.1
         :.:::::::::::::::::.
3' ----CUUACUUCGGACCAGGCU---- 5'        Csi-miR166c.2
```

Csi-miR166c.3, target=Cs1g15640.1 gene=Cs1g15640
Category:3
Score=1.5
Cleavage Site=1781

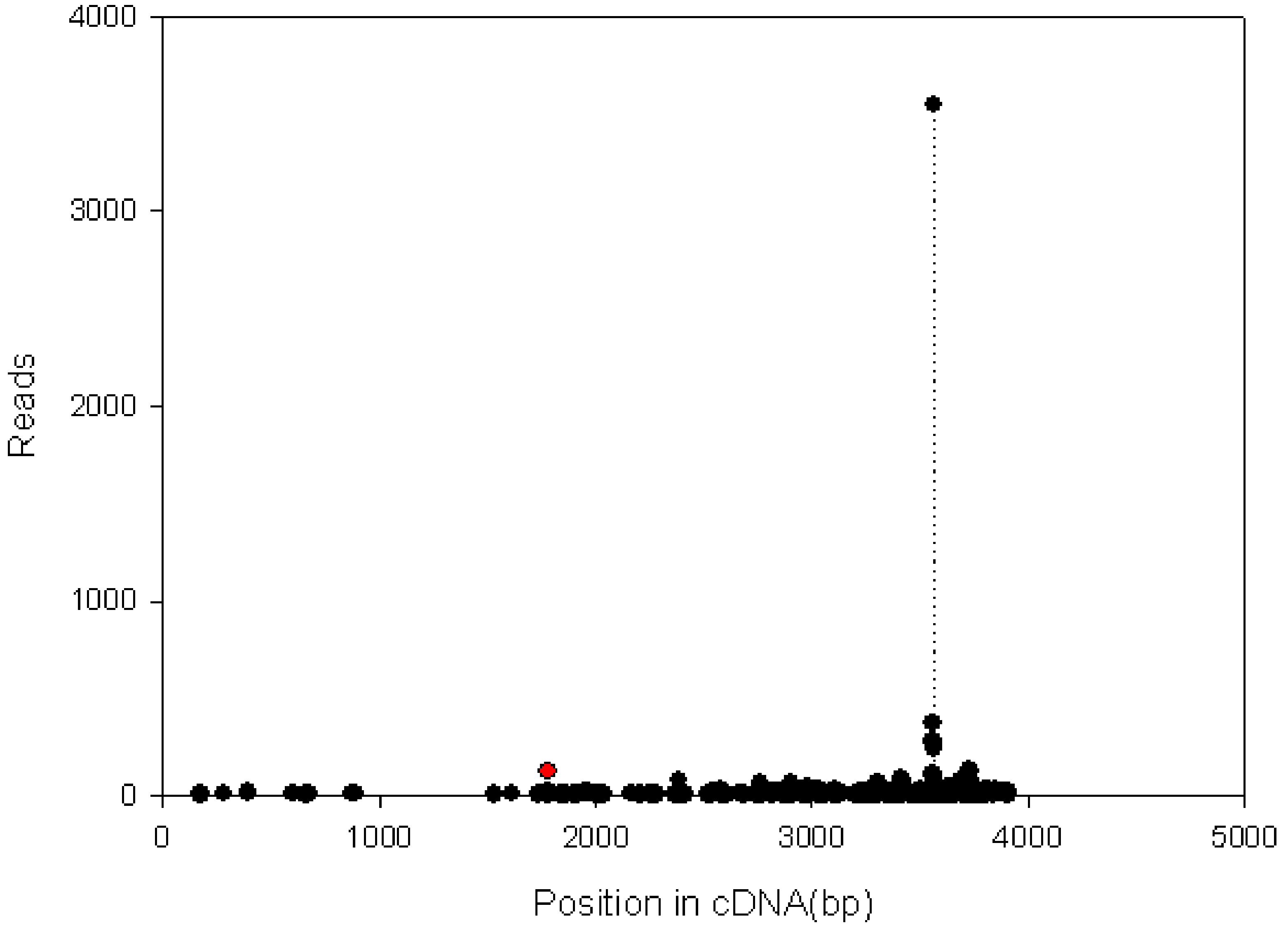


```
5' CCUGGAAUGAAGCCUGGUCCGGAUUC 3'        Cs1g15640.1
     ::::::::::::::::::::.
3' --CCCUUACUUCGGACCAGGCU---- 5'        Csi-miR166c.3
```

Csi-miR166c.3, target=Cs2g09770.1 gene=Cs2g09770
Category:1
Score=2
Cleavage Site=1058

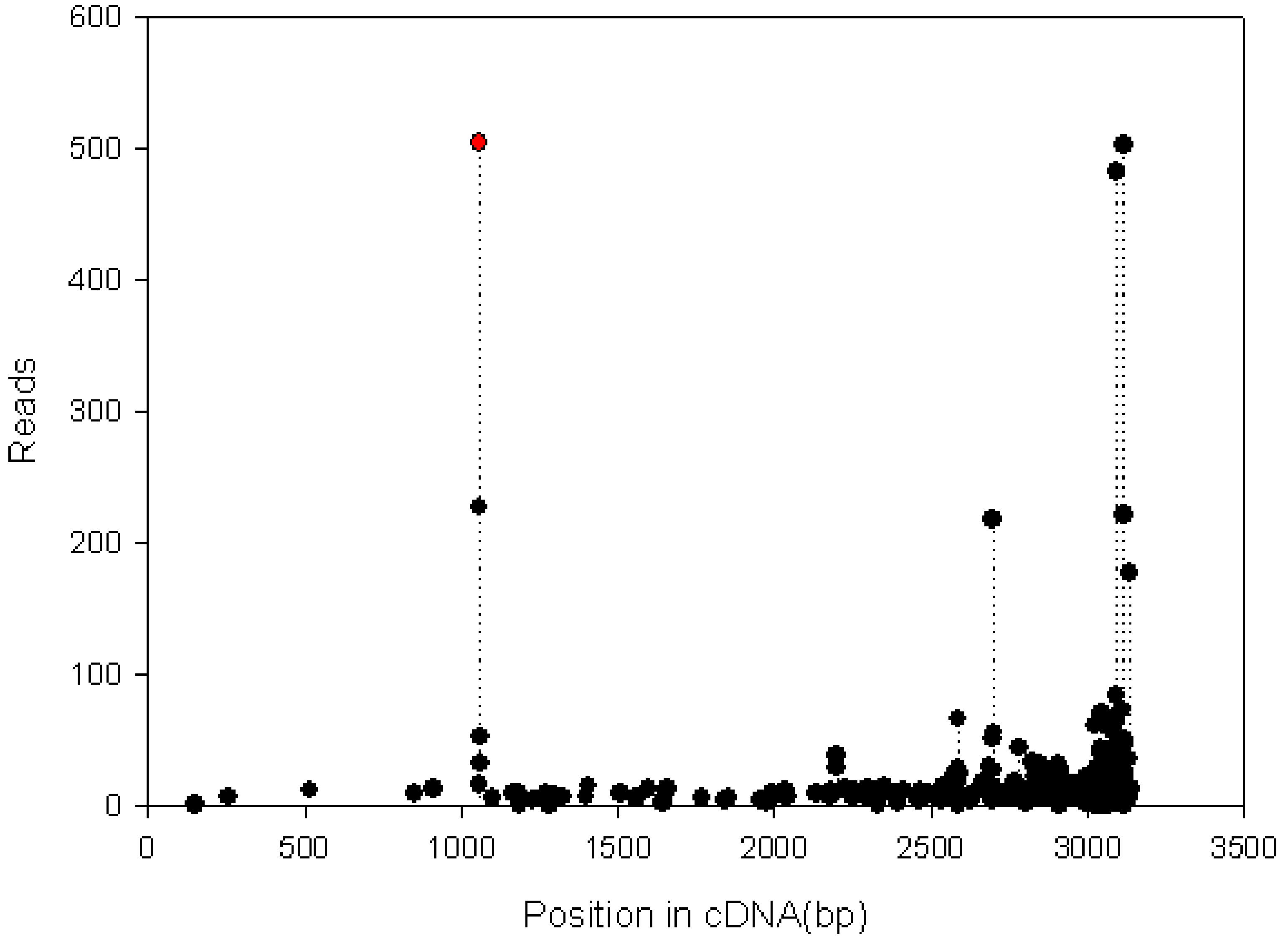


```
5' AUUGGGAUGAAGCCUGGUCCGGAUUC 3'         Cs2g09770.1
     ::.:::::::::::::::::.
3' --CCCUUACUUCGGACCAGGCU---- 5'         Csi-miR166c.3
```

Csi-miR166c.3, target=Cs4g19310.1 gene=Cs4g19310
Category:1
Score=2
Cleavage Site=677

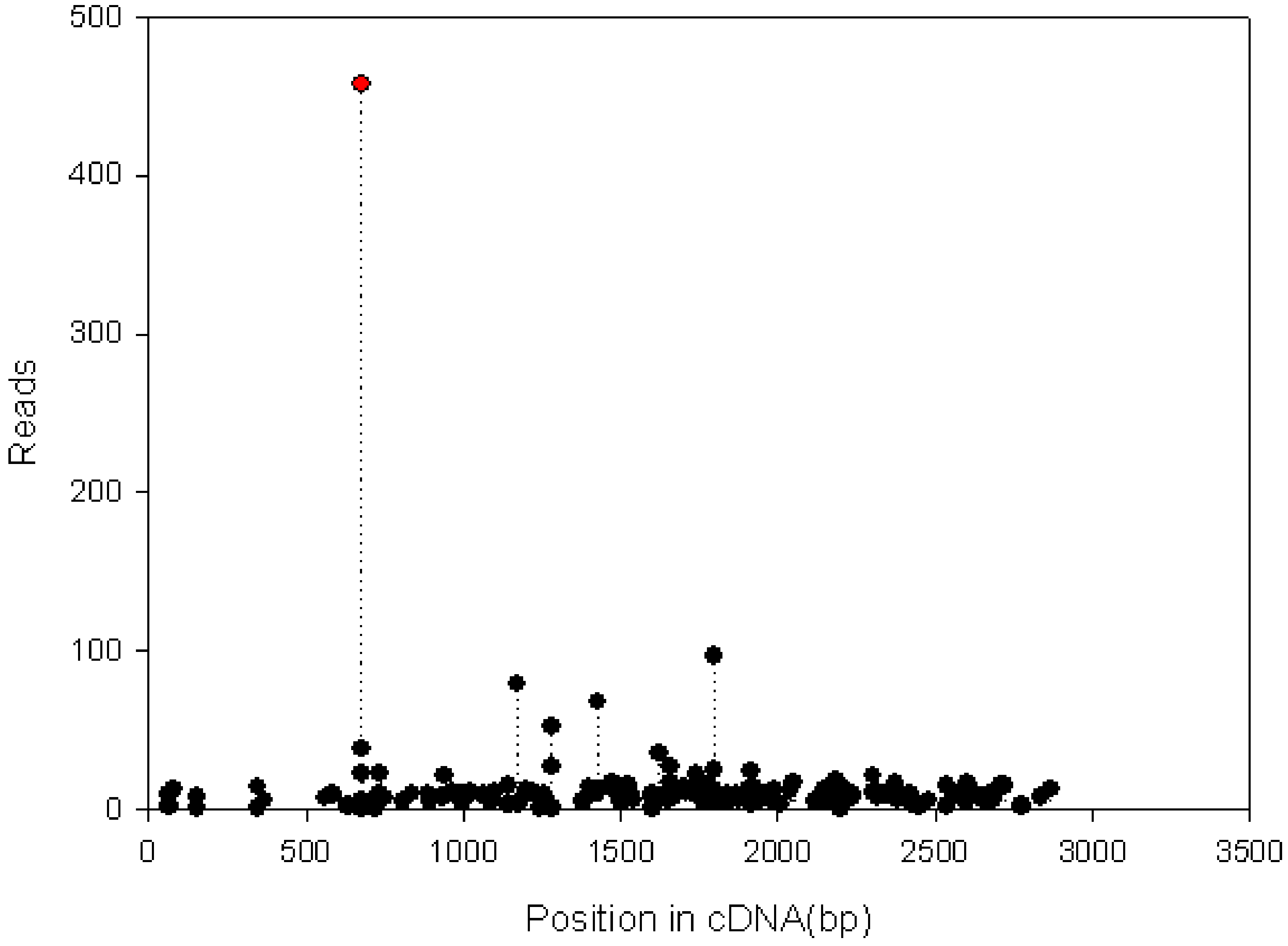


```
5' CCCGGGAUGAAGCCUGGUCCGGAUUC 3'        Cs4g19310.1
     ::.:::::::::::::::::.
3' --CCCUUACUUCGGACCAGGCU---- 5'        Csi-miR166c.3
```

Csi-miR166c.3, target=Cs8g16510.1 gene=Cs8g16510
Category:1
Score=2
Cleavage Site=1434

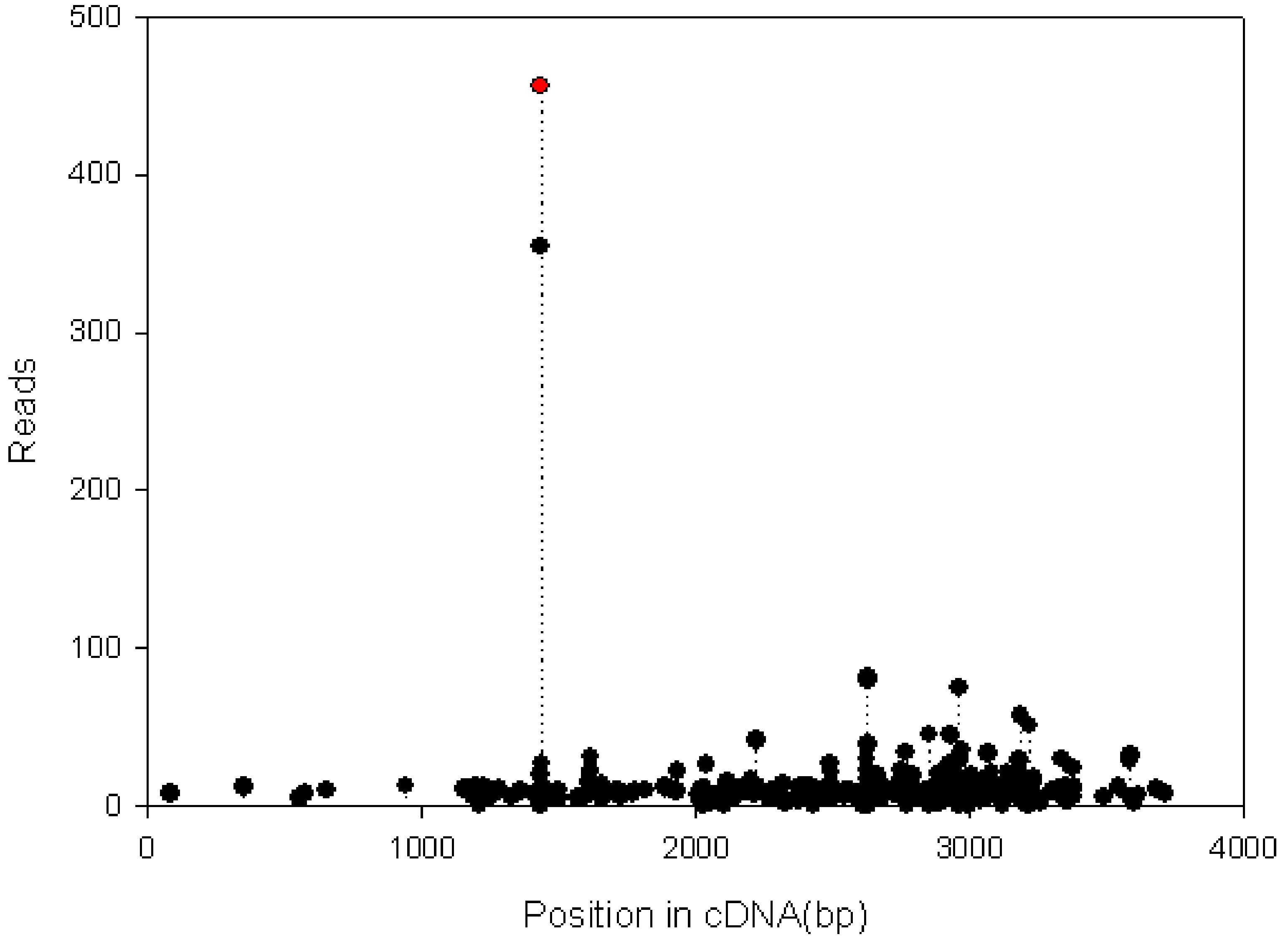


```
5' CCUGGGAUGAAGCCUGGUCCGGAUUC 3'        Cs8g16510.1
      ::.:::::::::::::::::.
3' --CCCUUACUUCGGACCAGGCU---- 5'        Csi-miR166c.3
```

Csi-miR166c.4, target=Cs1g15640.1 gene=Cs1g15640
Category:3
Score=3
Cleavage Site=1781

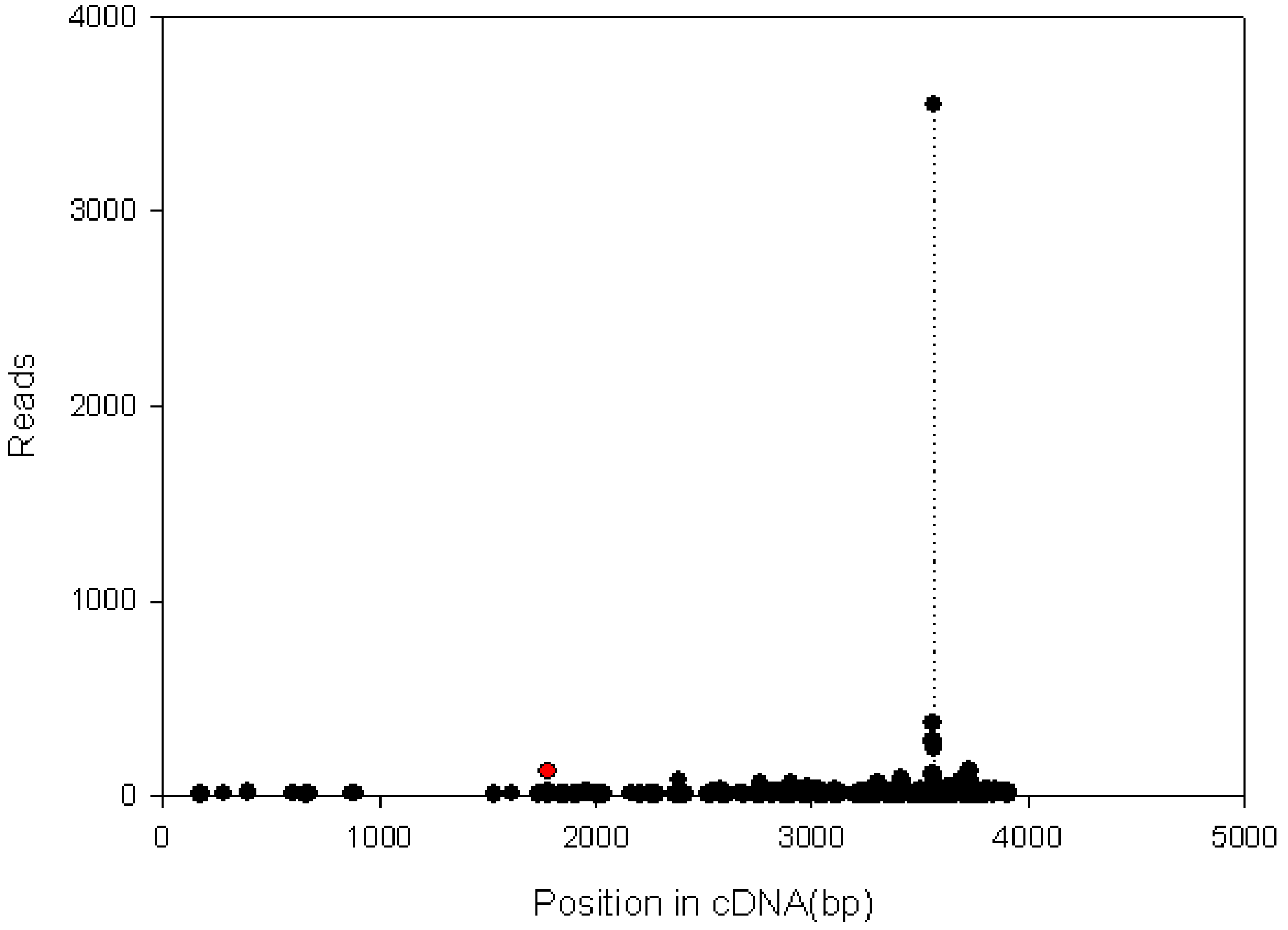


```
5' CCUGGAAUGAAGCCUGGUCCGGAUUC 3'         Cs1g15640.1
      ::::::::::::::::::::.
3' --CCCUUACUUCGGACCAGGCUC--- 5'         Csi-miR166c.4
```

Csi-miR166c.4, target=Cs2g09770.1 gene=Cs2g09770
Category:1
Score=3.5
Cleavage Site=1058

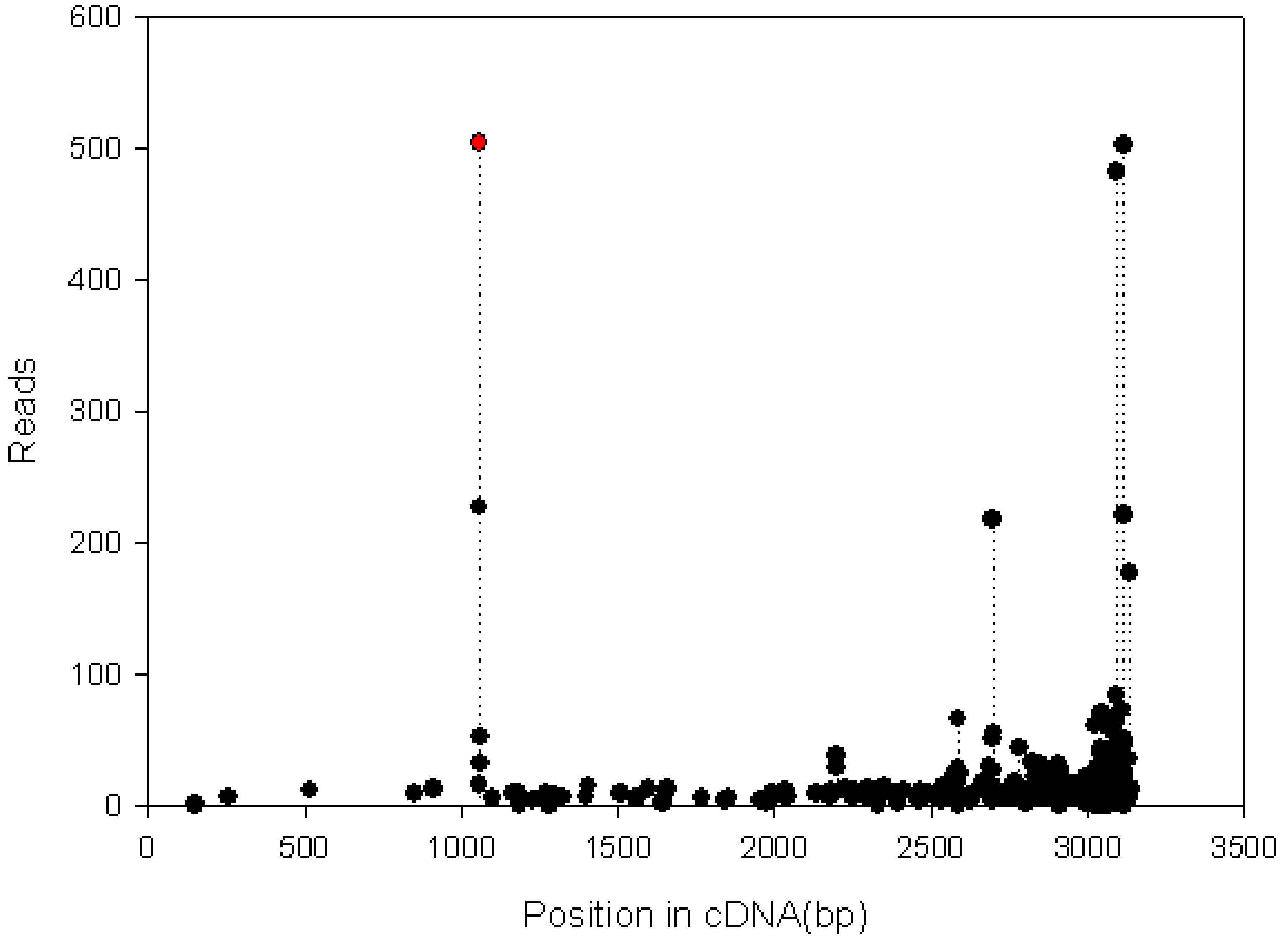


```
5' AUUGGGAUGAAGCCUGGUCCGGAUUC 3'        Cs2g09770.1
     ::.:::::::::::::::::.
3' --CCCUUACUUCGGACCAGGCUC--- 5'        Csi-miR166c.4
```

Csi-miR166c.4, target=Cs4g19310.1 gene=Cs4g19310
Category:1
Score=3.5
Cleavage Site=677

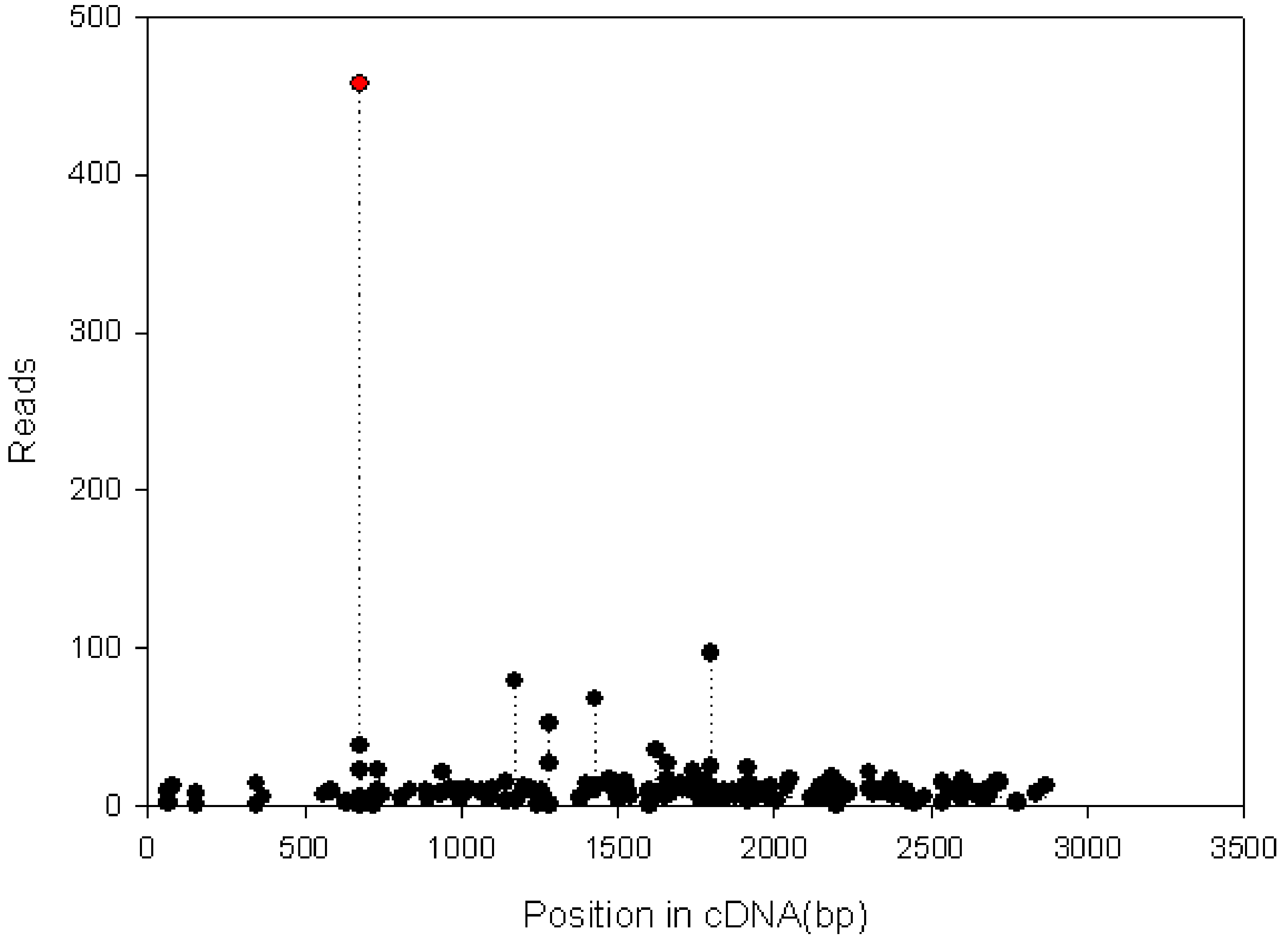


```
5' CCCGGGAUGAAGCCUGGUCCGGAUUC 3'        Cs4g19310.1
       ::.::::::::::::::::::.
3' --CCCUUACUUCGGACCAGGCUC--- 5'        Csi-miR166c.4
```

Csi-miR166c.4, target=Cs8g16510.1 gene=Cs8g16510
Category:1
Score=3.5
Cleavage Site=1434

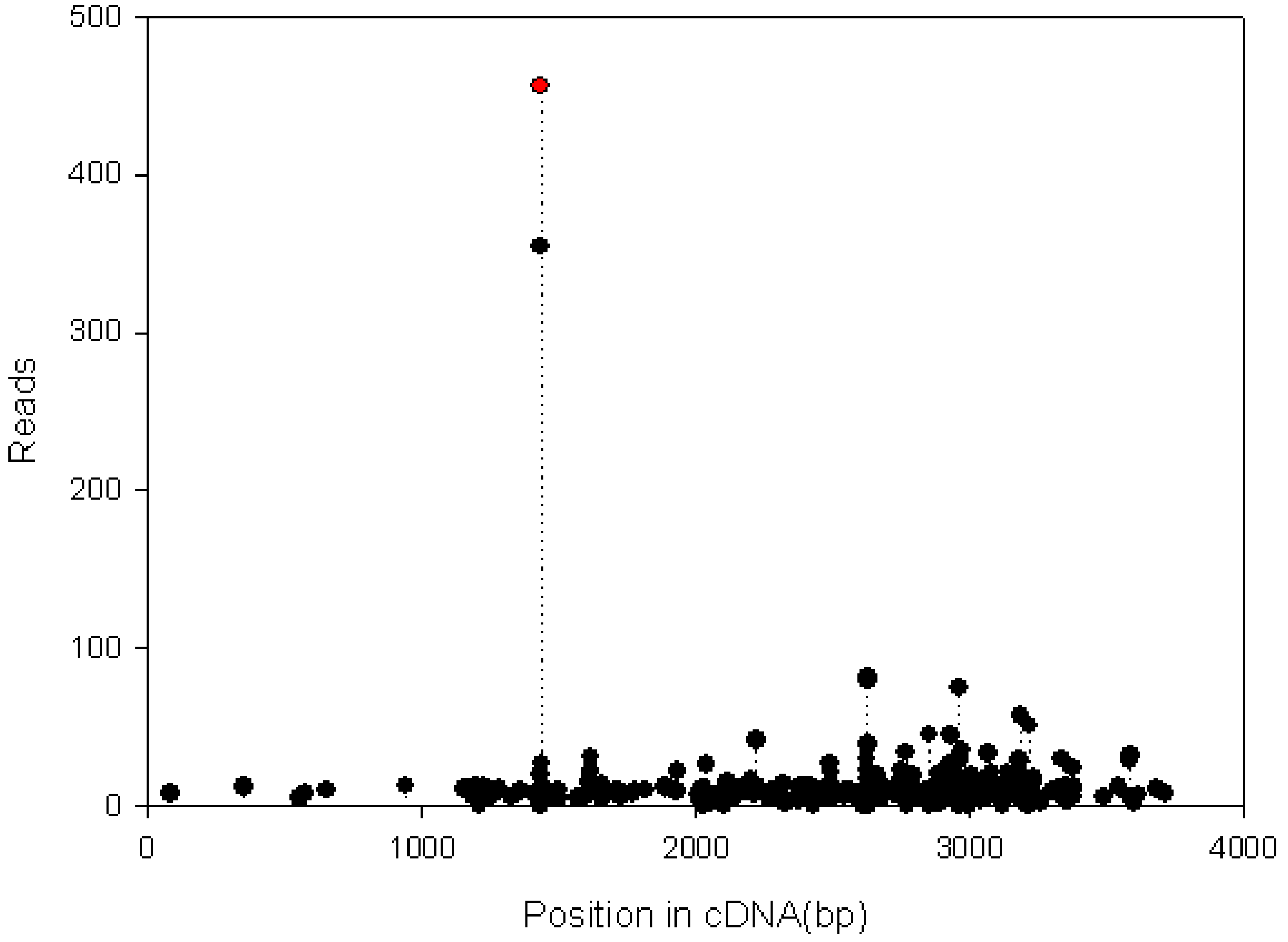


```
5' CCUGGGAUGAAGCCUGGUCCGGAUUC 3'        Cs8g16510.1
       ::.:::::::::::::::.
3' --CCCUUACUUCGGACCAGGCUC--- 5'        Csi-miR166c.4
```

Csi-miR166c.5, target=Cs1g15640.1 gene=Cs1g15640
Category:3
Score=2
Cleavage Site=1781

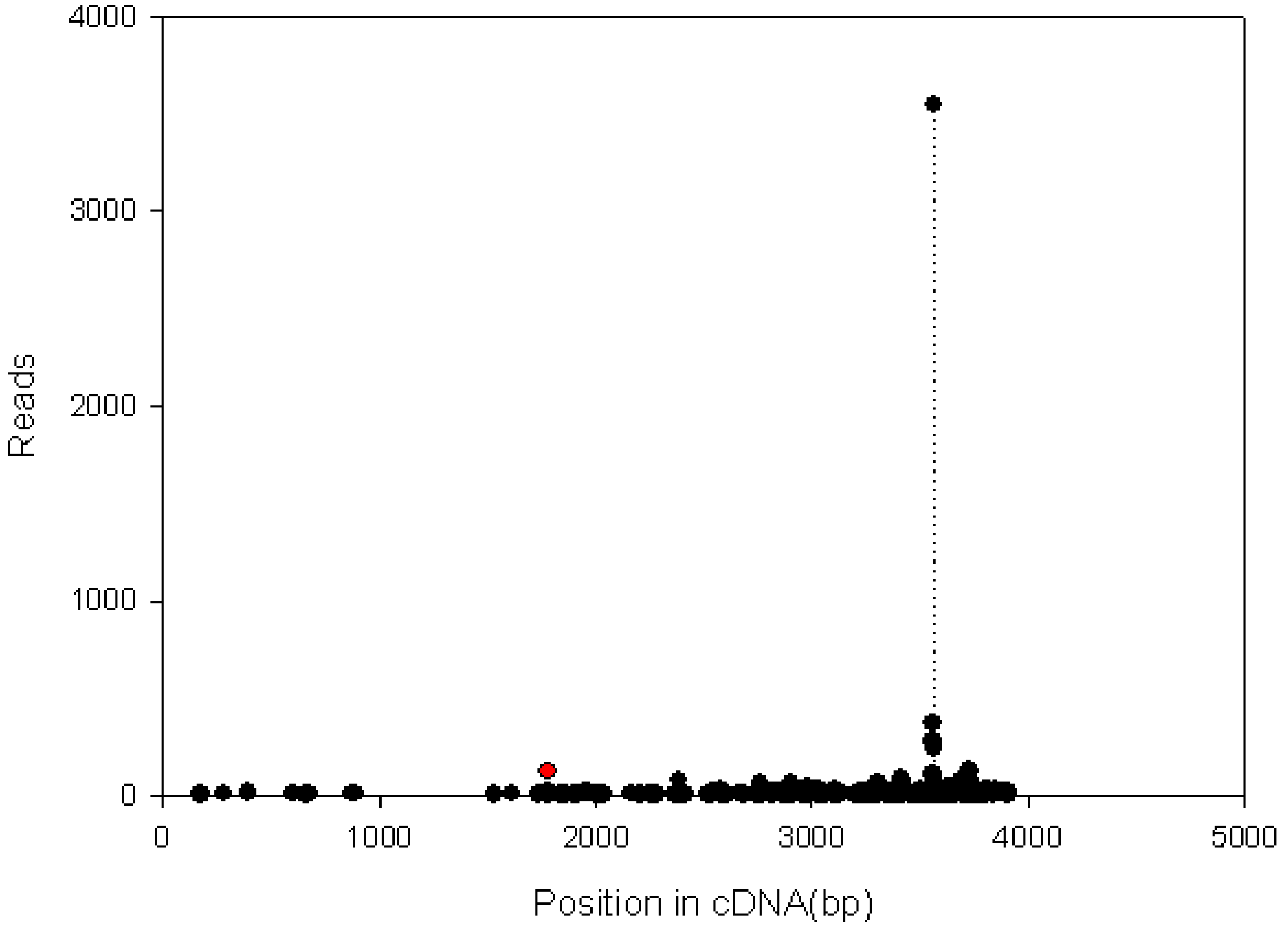


```
5' CCUGGAAUGAAGCCUGGUCCGGAUUC 3'        Cs1g15640.1
       ::::::::::::::::::::.
3' ---CCUUACUUCGGACCAGGCUC--- 5'        Csi-miR166c.5
```

Csi-miR166c.5, target=Cs2g09770.1 gene=Cs2g09770
Category:1
Score=2.5
Cleavage Site=1058

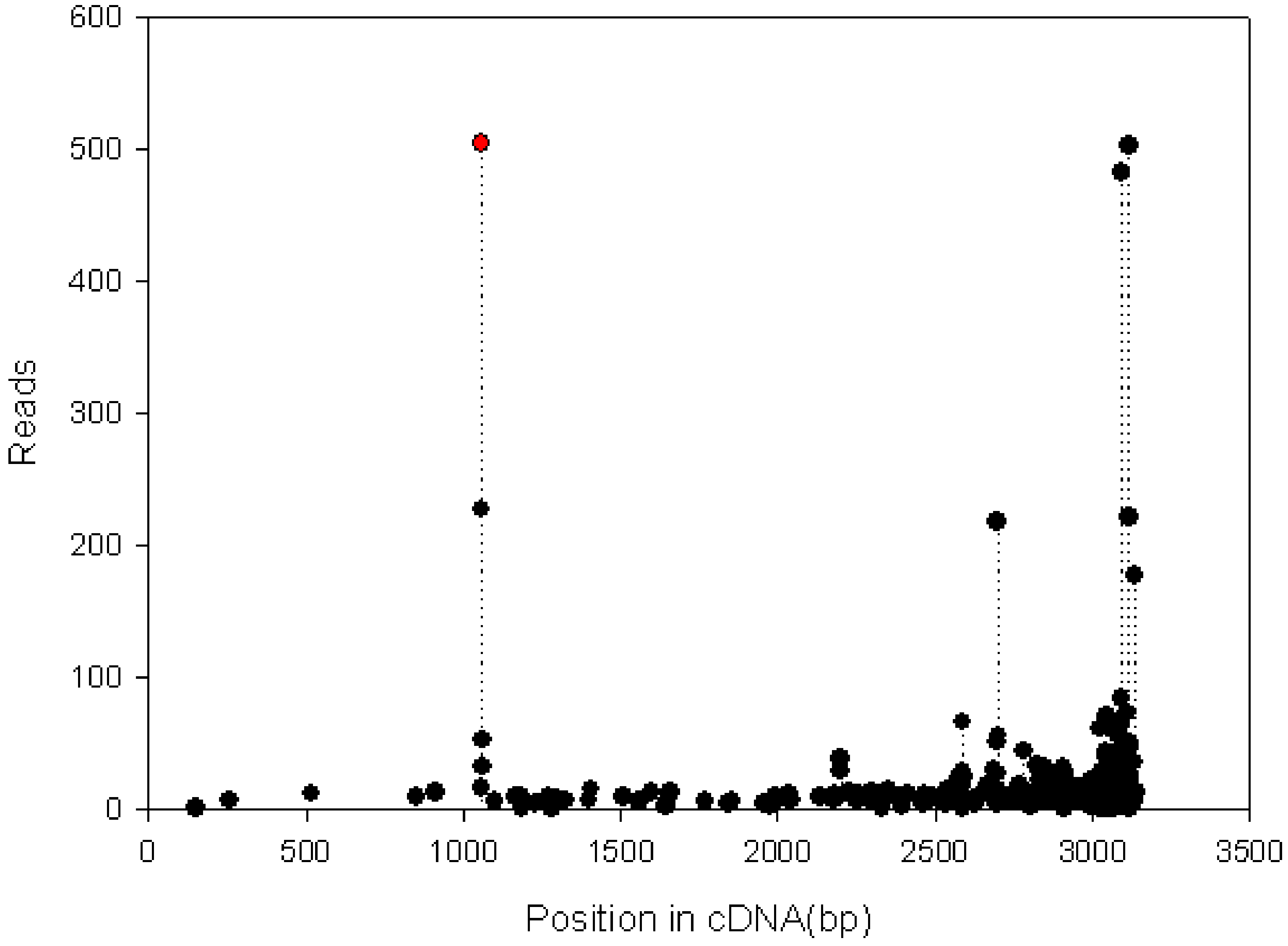


```
5' AUUGGGAUGAAGCCUGGUCCGGAUUC 3'        Cs2g09770.1
      ::.:::::::::::::::::.
3' ---CCUUACUUCGGACCAGGCUC--- 5'        Csi-miR166c.5
```

Csi-miR166c.5, target=Cs4g19310.1 gene=Cs4g19310
Category:1
Score=2.5
Cleavage Site=677

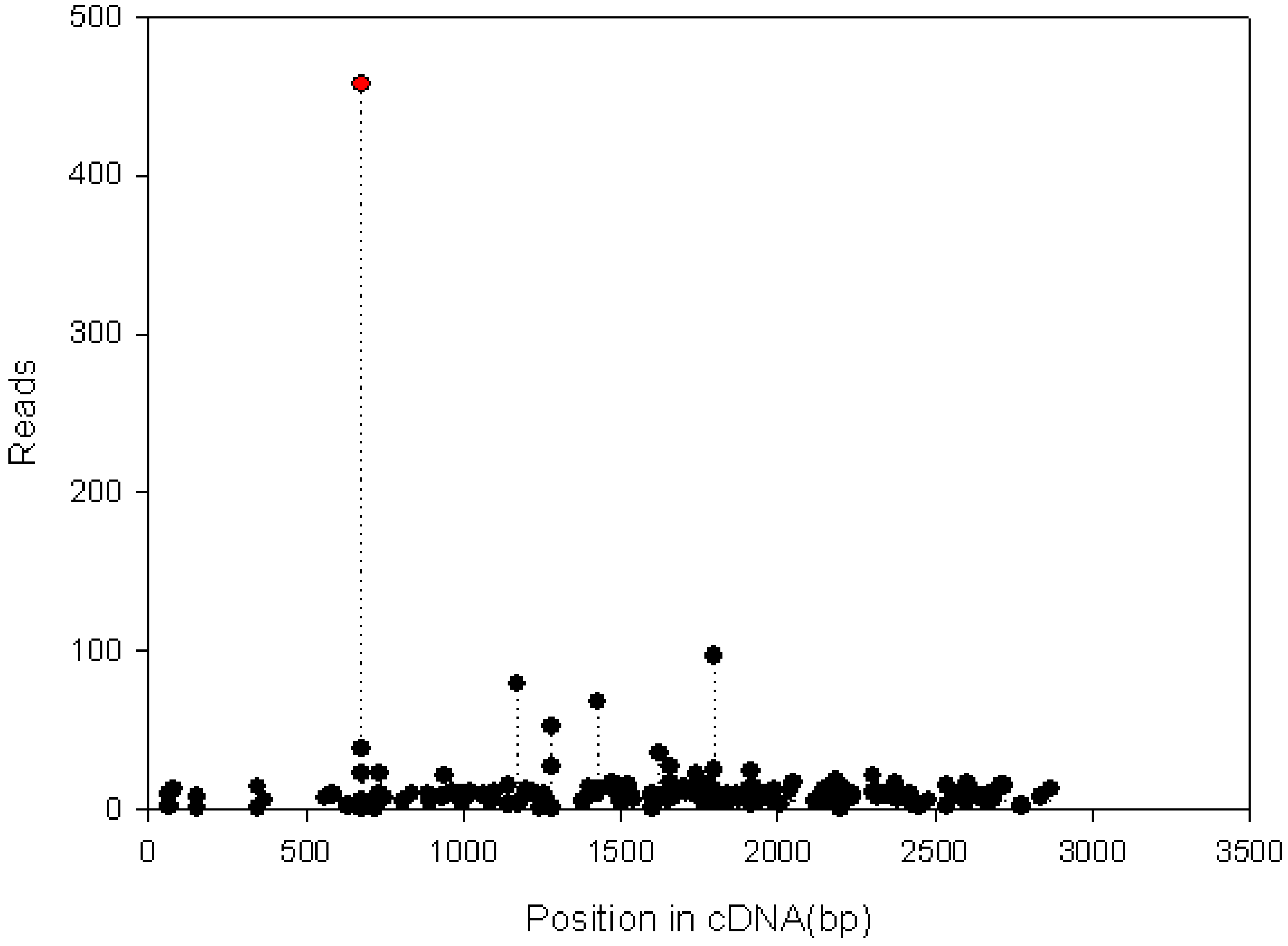


```
5' CCCGGGAUGAAGCCUGGUCCGGAUUC 3'        Cs4g19310.1
      ::.::::::::::::::::.
3' ---CCUUACUUCGGACCAGGCUC--- 5'        Csi-miR166c.5
```

Csi-miR166c.5, target=Cs8g16510.1 gene=Cs8g16510
Category:1
Score=2.5
Cleavage Site=1434

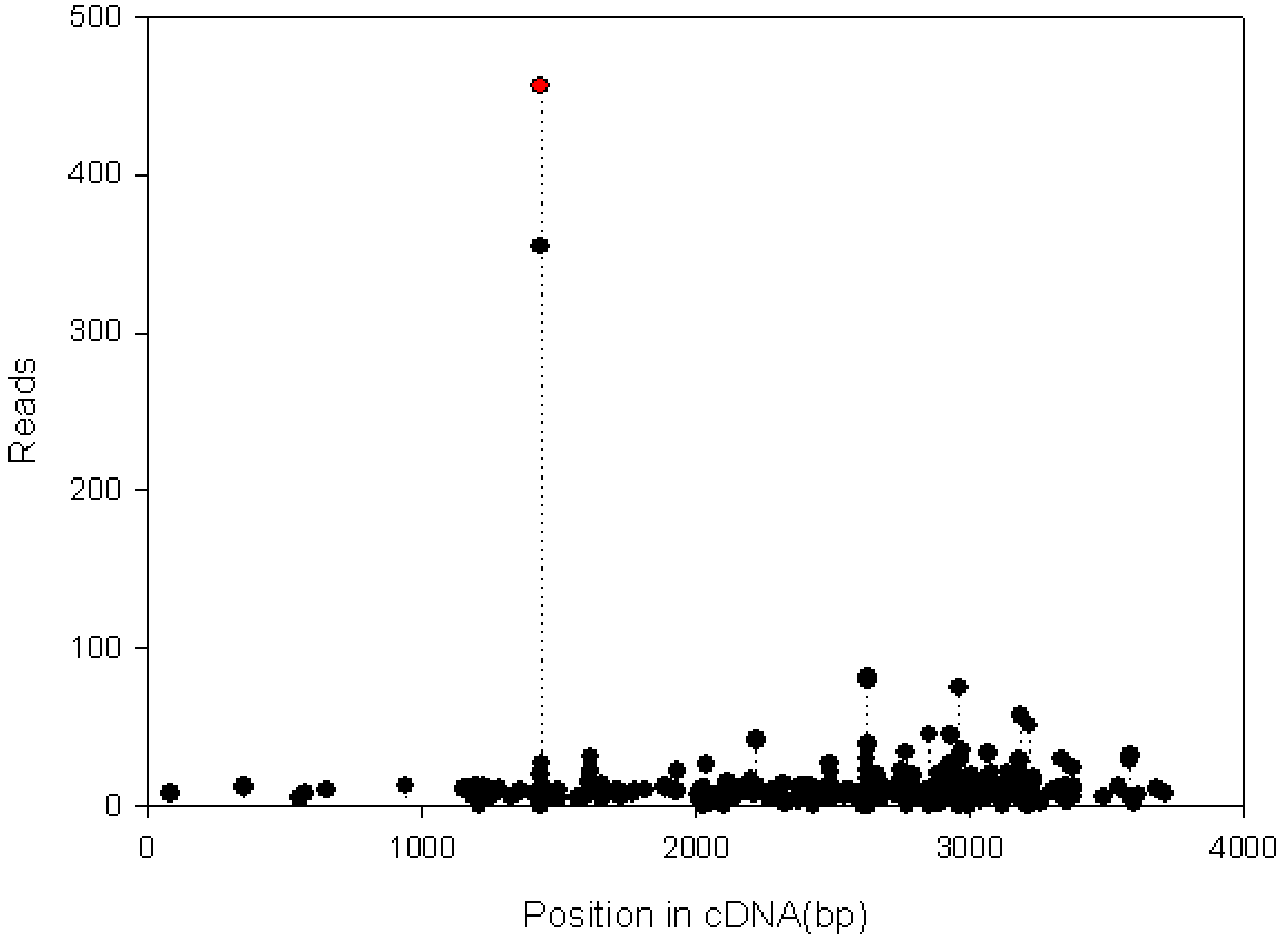


```
5' CCUGGGAUGAAGCCUGGUCCGGAUUC 3'        Cs8g16510.1
      ::.:::::::::::::::::.
3' ---CCUUACUUCGGACCAGGCUC--- 5'        Csi-miR166c.5
```

Csi-miR166d.1, target=Cs1g15640.1 gene=Cs1g15640
Category:3
Score=3.5
Cleavage Site=1781

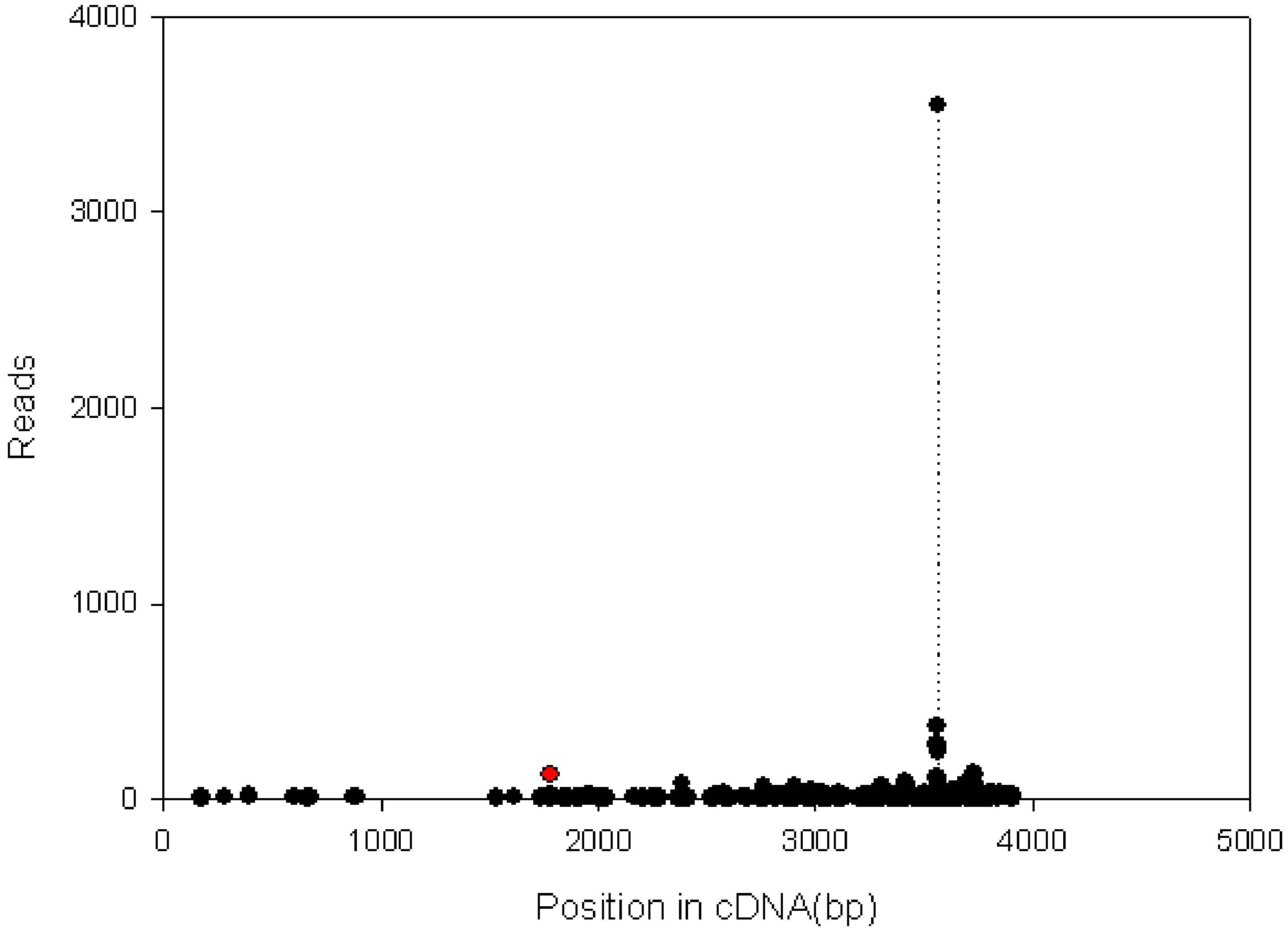


```
5' CCUGGAAUGAAGCCUGGUCCGGAUUC 3'        Cs1g15640.1
       :::::::::::::::::::.
3' UUCCCUUACUUCGGACCAGGCU---- 5'        Csi-miR166d.1
```

Csi-miR166d.1, target=Cs2g09770.1 gene=Cs2g09770
Category:1
Score=3
Cleavage Site=1058

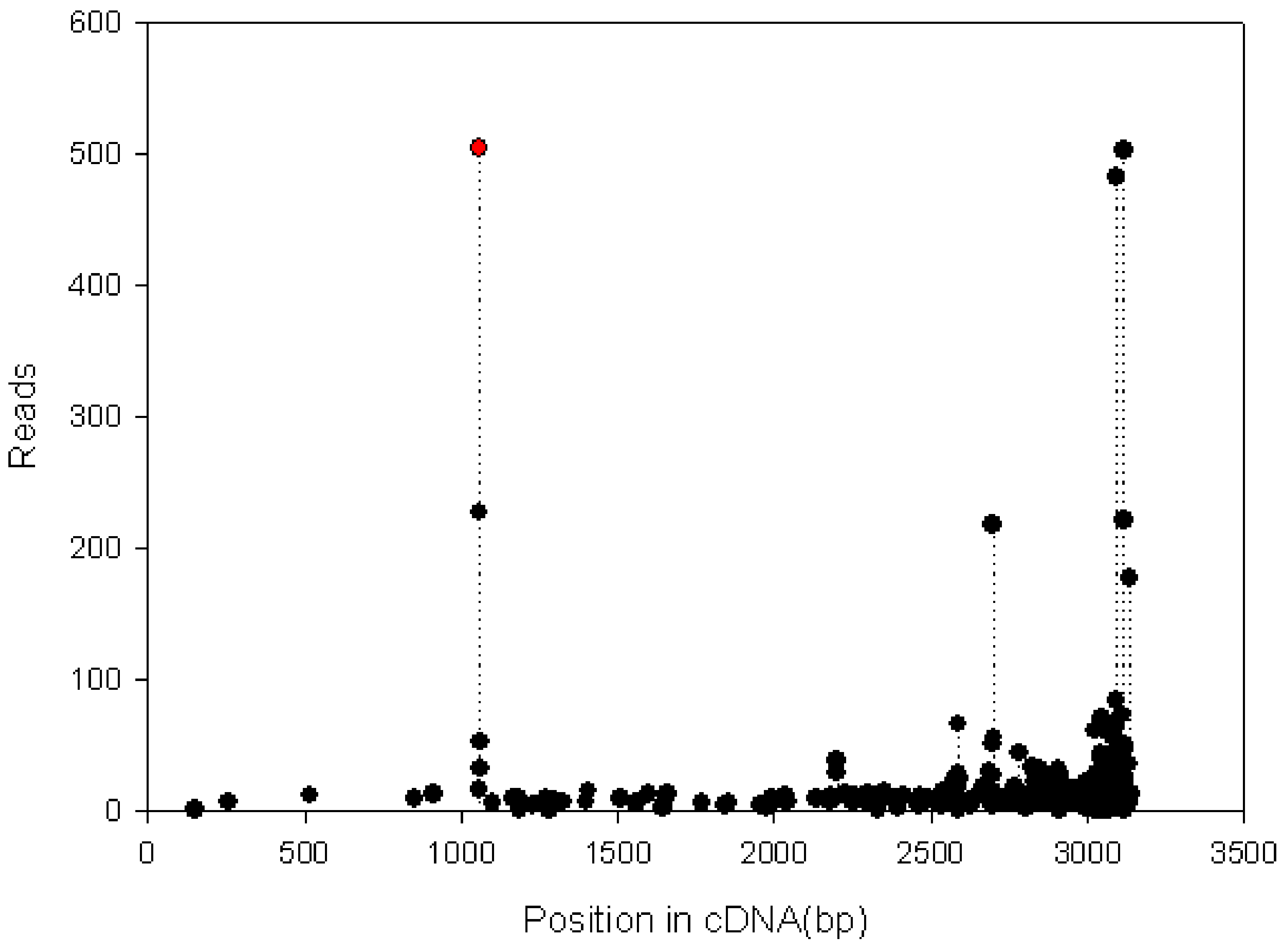


```
5' AUUGGGAUGAAGCCUGGUCCGGAUUC 3'         Cs2g09770.1
   :  ::.:::::::::::::::::.
3' UUCCCUUACUUCGGACCAGGCU---- 5'         Csi-miR166d.1
```

Csi-miR166d.1, target=Cs4g19310.1 gene=Cs4g19310
Category:1
Score=4
Cleavage Site=677

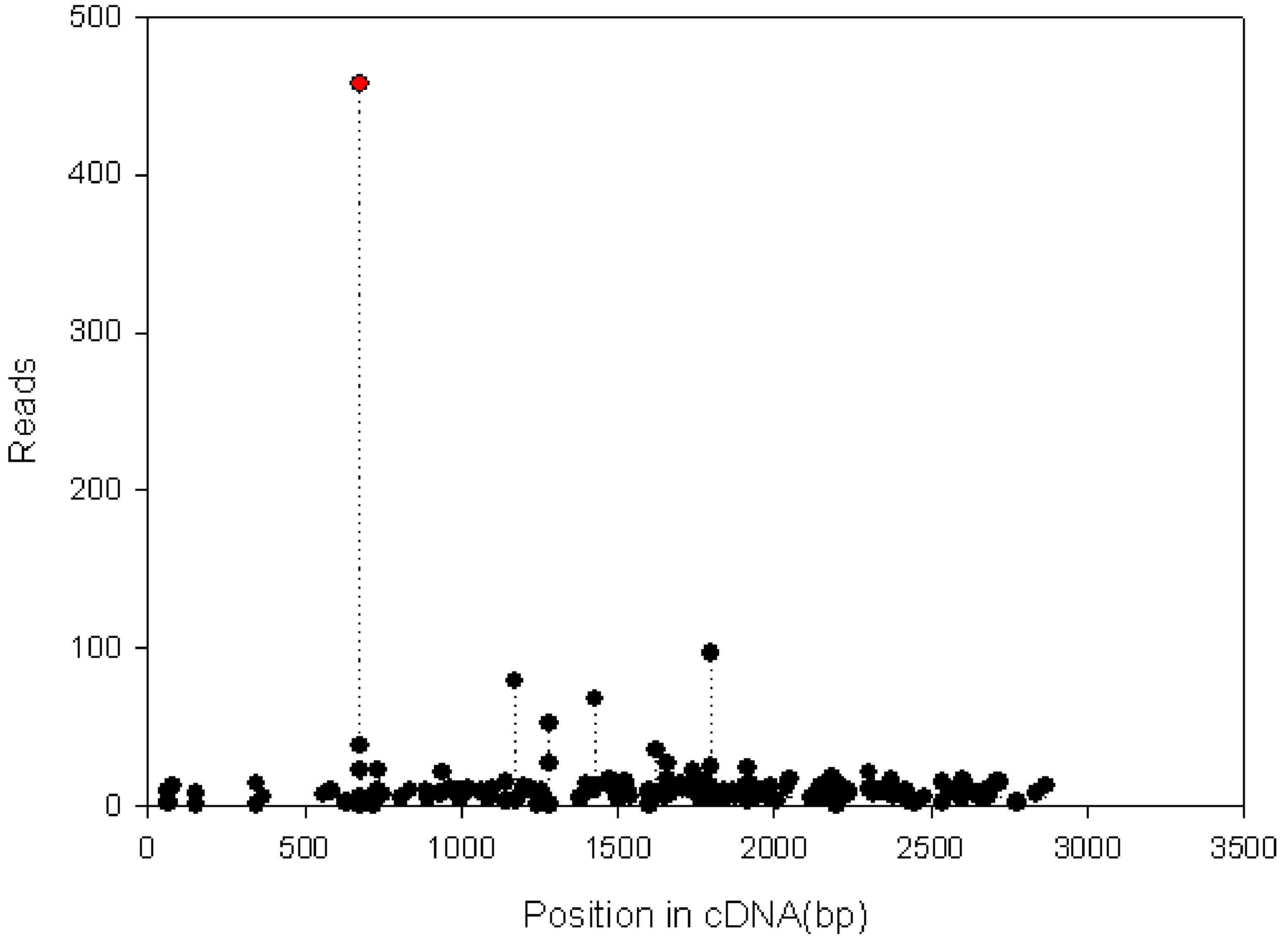


```
5' CCCGGGAUGAAGCCUGGUCCGGAUUC 3'        Cs4g19310.1
      ::.:::::::::::::::::.
3' UUCCCUUACUUCGGACCAGGCU---- 5'        Csi-miR166d.1
```

Csi-miR166d.1, target=Cs8g16510.1 gene=Cs8g16510
Category:1
Score=4
Cleavage Site=1434

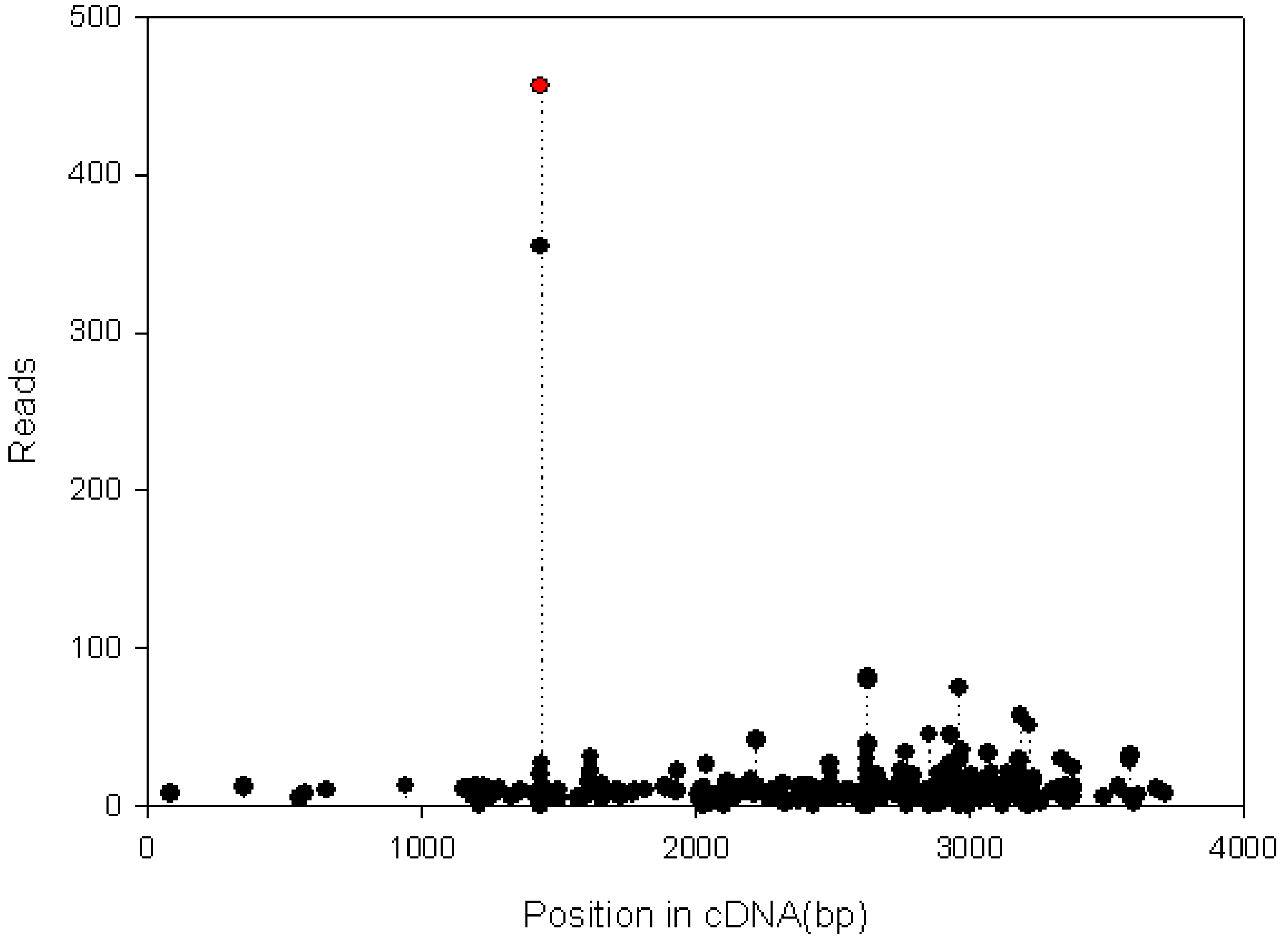


```
5' CCUGGGAUGAAGCCUGGUCCGGAUUC 3'        Cs8g16510.1
       ::.:::::::::::::::::.
3' UUCCCUUACUUCGGACCAGGCU---- 5'        Csi-miR166d.1
```

Csi-miR166d.2, target=Cs1g15640.1 gene=Cs1g15640
Category:3
Score=2.5
Cleavage Site=1781

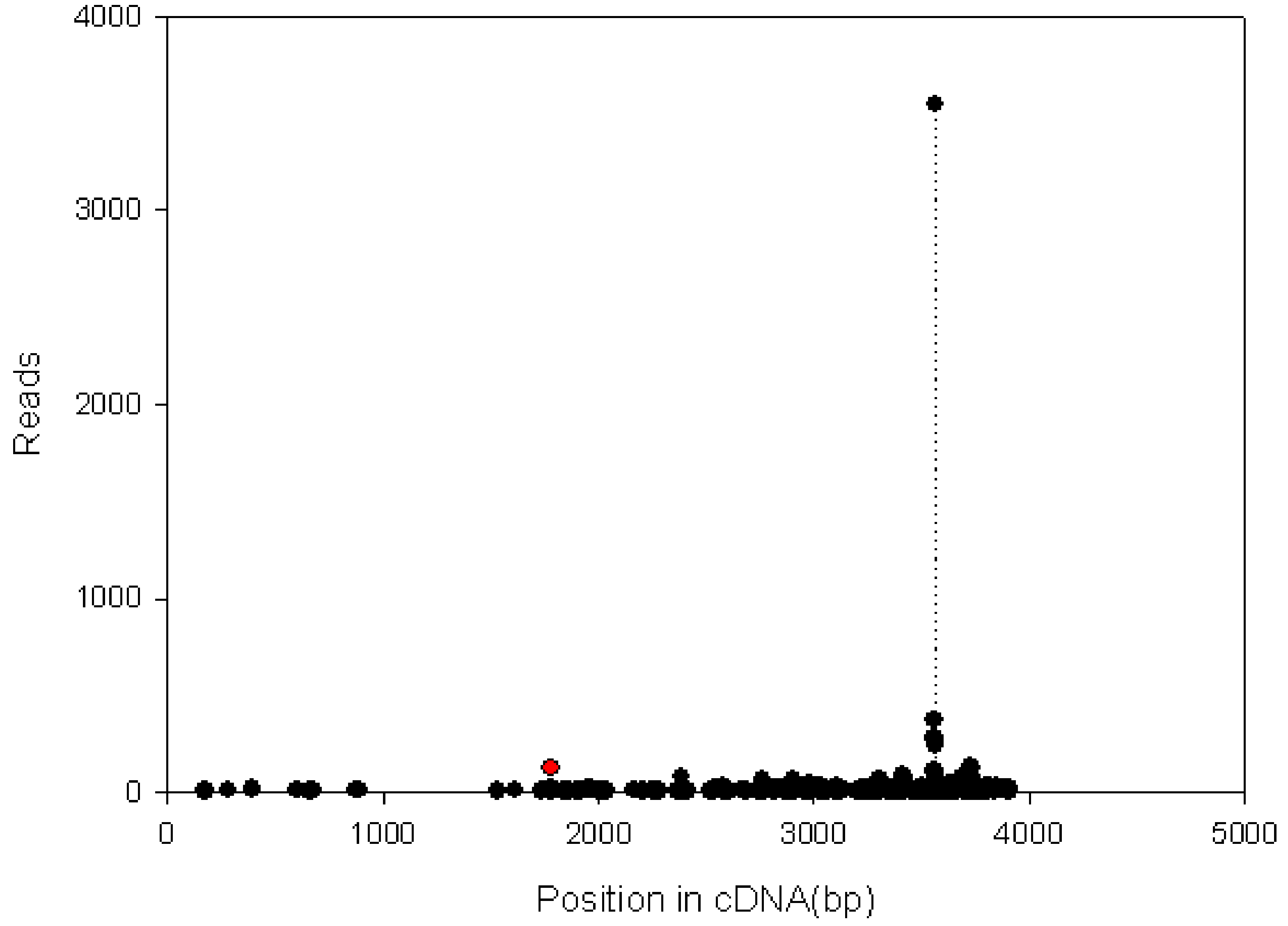


```
5' CCUGGAAUGAAGCCUGGUCCGGAUUC 3'        Cs1g15640.1
       ::::::::::::::::::.
3' -UCCCUUACUUCGGACCAGGCU---- 5'        Csi-miR166d.2
```

Csi-miR166d.2, target=Cs2g09770.1 gene=Cs2g09770
Category:1
Score=3
Cleavage Site=1058

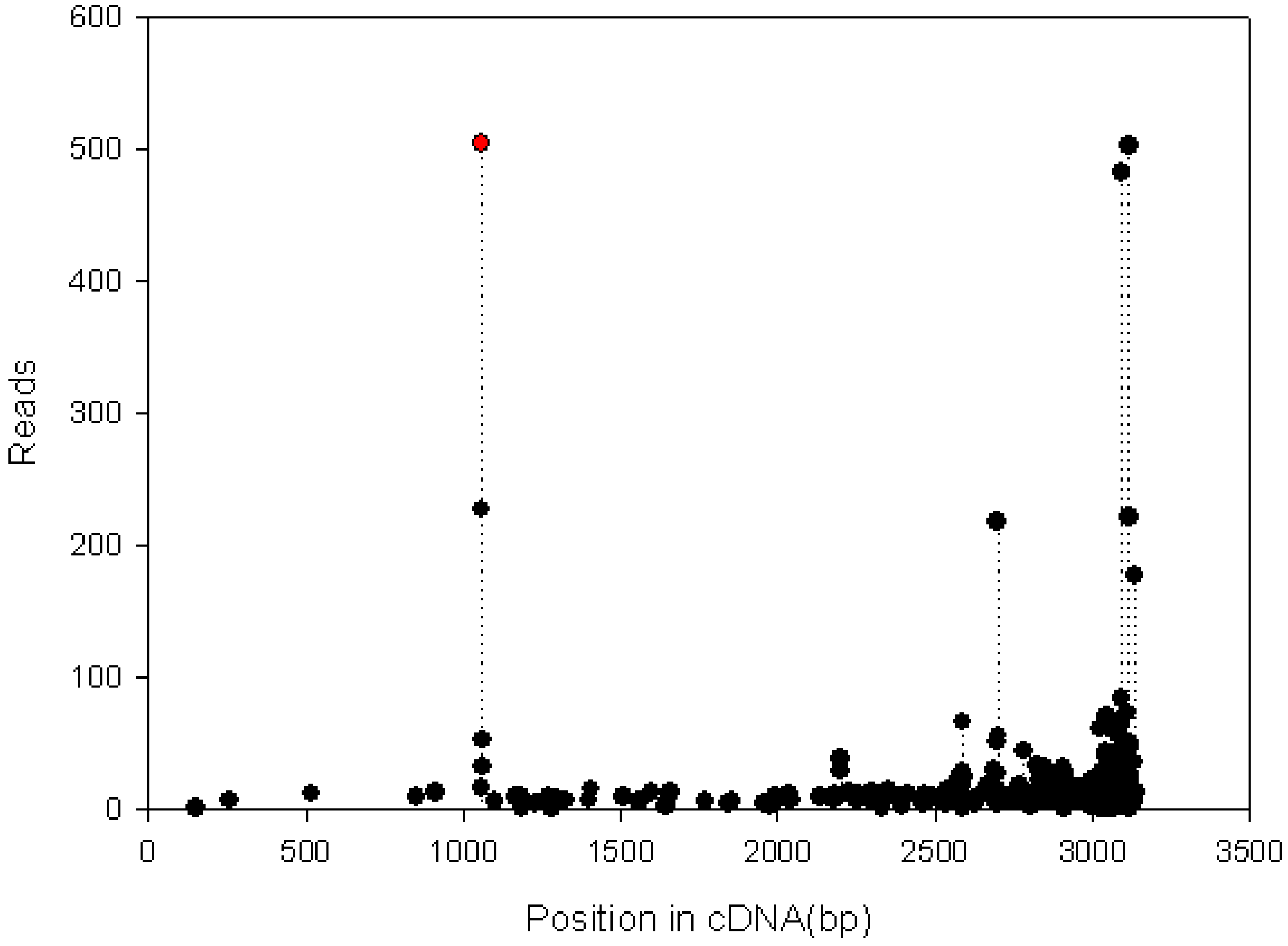


```
5' AUUGGGAUGAAGCCUGGUCCGGAUUC 3'        Cs2g09770.1
     ::.::::::::::::::::.
3' -UCCCUUACUUCGGACCAGGCU---- 5'        Csi-miR166d.2
```

Csi-miR166d.2, target=Cs4g19310.1 gene=Cs4g19310
Category:1
Score=3
Cleavage Site=677

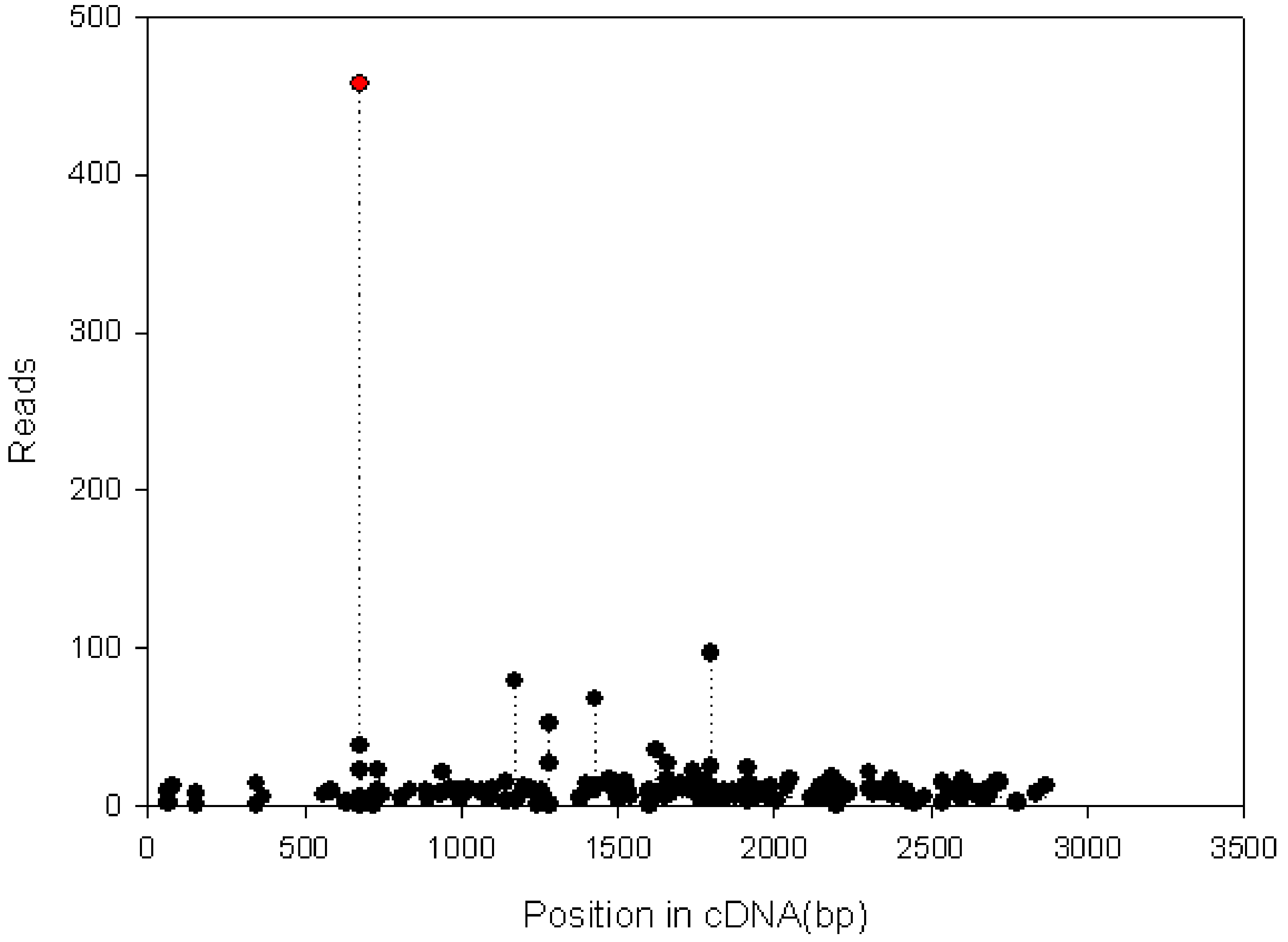


```
5' CCCGGGAUGAAGCCUGGUCCGGAUUC 3'         Cs4g19310.1
     ::.:::::::::::::::::.
3' -UCCCUUACUUCGGACCAGGCU---- 5'         Csi-miR166d.2
```

Csi-miR166d.2, target=Cs8g16510.1 gene=Cs8g16510
Category:1
Score=3
Cleavage Site=1434

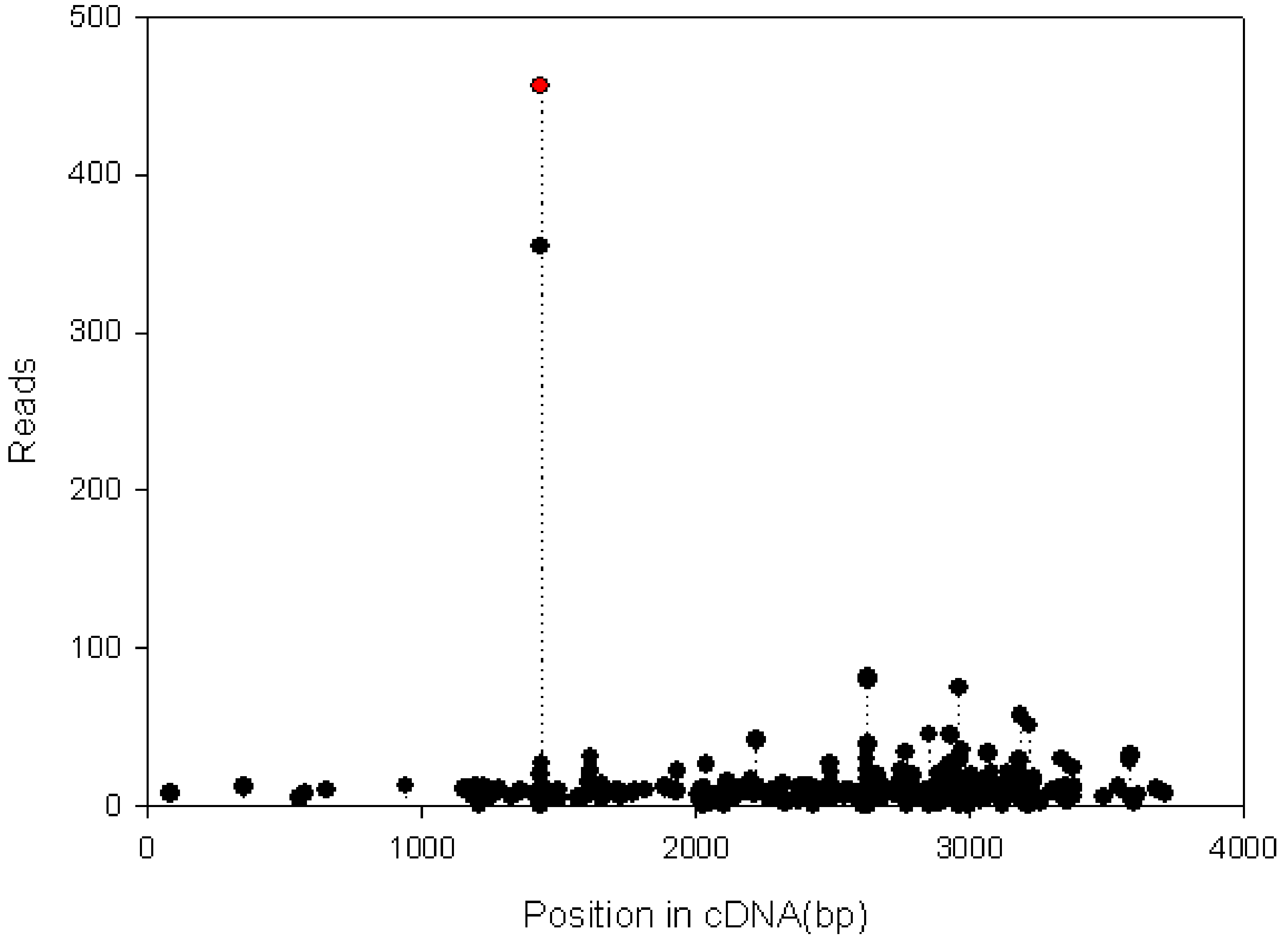


```
5' CCUGGGAUGAAGCCUGGUCCGGAUUC 3'        Cs8g16510.1
       ::.:::::::::::::::.
3' -UCCCUUACUUCGGACCAGGCU---- 5'        Csi-miR166d.2
```

Csi-miR166g.1, target=Cs1g15640.1 gene=Cs1g15640
Category:3
Score=4
Cleavage Site=1781

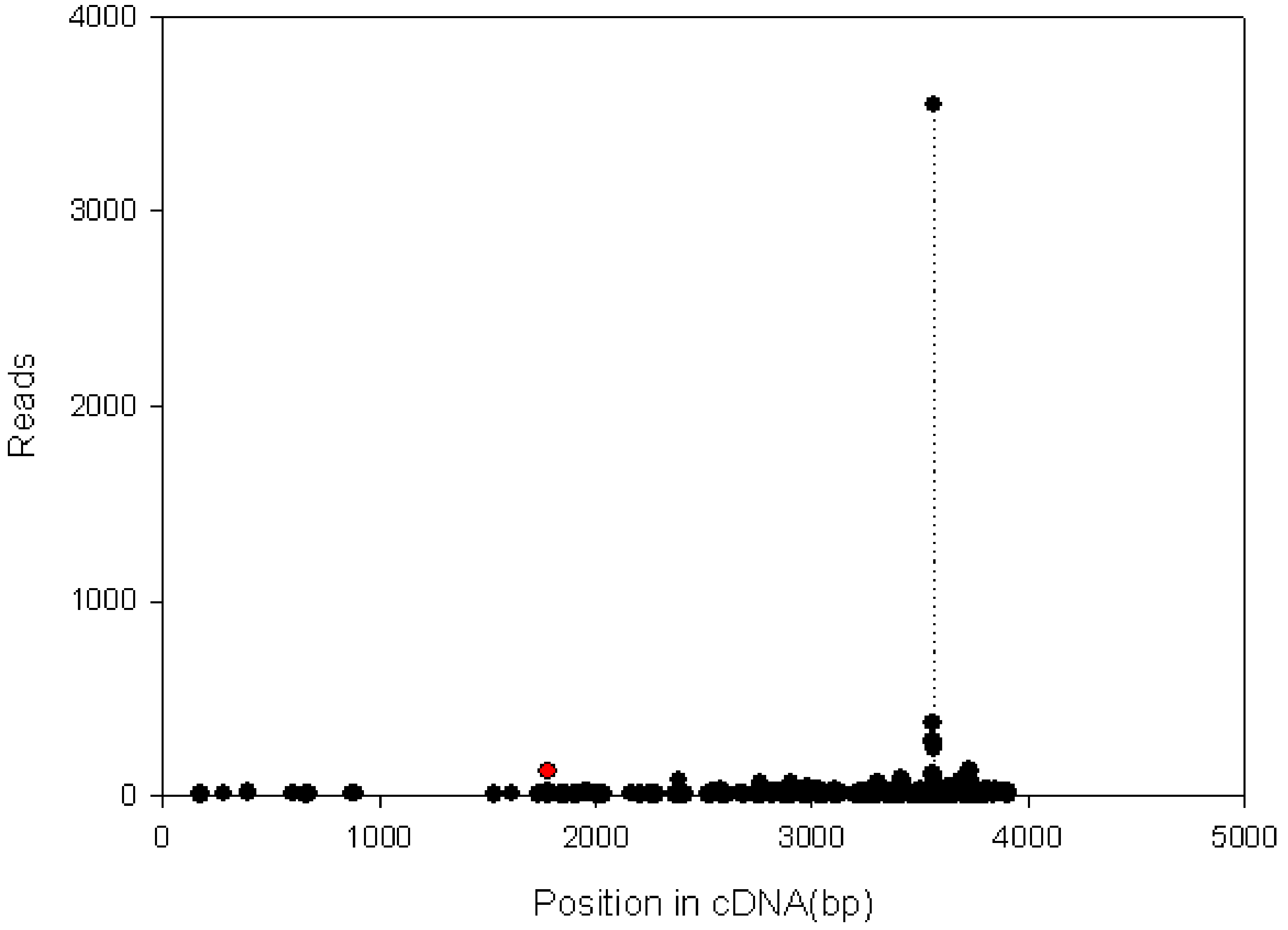


```
5' CCUGGAAUGAAGCCUGGUCCGGAUUC 3'        Cs1g15640.1
      ::::::::::::::::::::.
3' ---CCUUACUUCGGACCAGGCUCU-- 5'        Csi-miR166g.1
```

Csi-miR166g.1, target=Cs2g09770.1 gene=Cs2g09770
Category:1
Score=4.5
Cleavage Site=1058

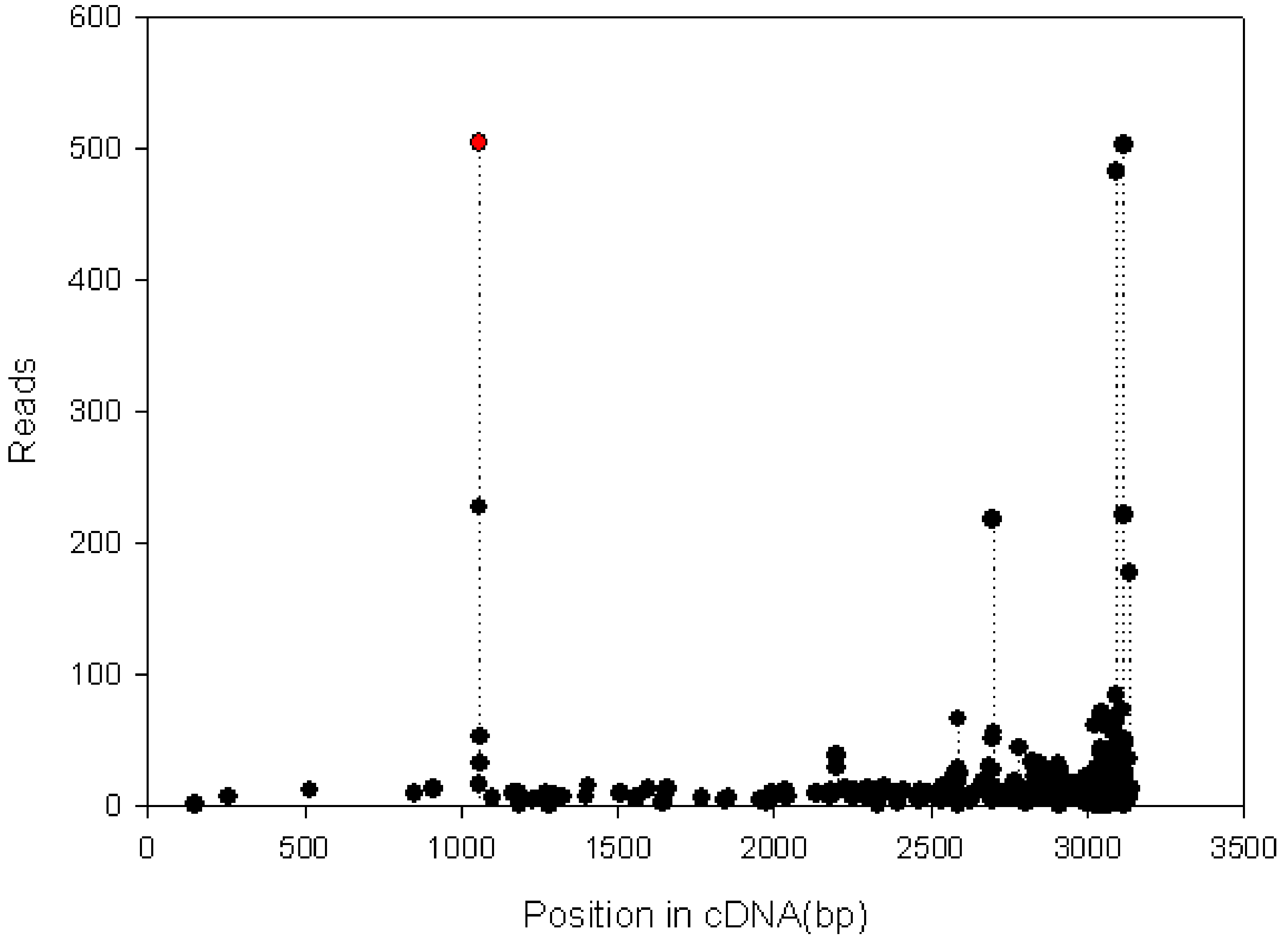


```
5' AUUGGGAUGAAGCCUGGUCCGGAUUC 3'        Cs2g09770.1
      ::.::::::::::::::::.
3' ---CCUUACUUCGGACCAGGCUCU-- 5'        Csi-miR166g.1
```

Csi-miR166g.1, target=Cs4g19310.1 gene=Cs4g19310
Category:1
Score=4.5
Cleavage Site=677

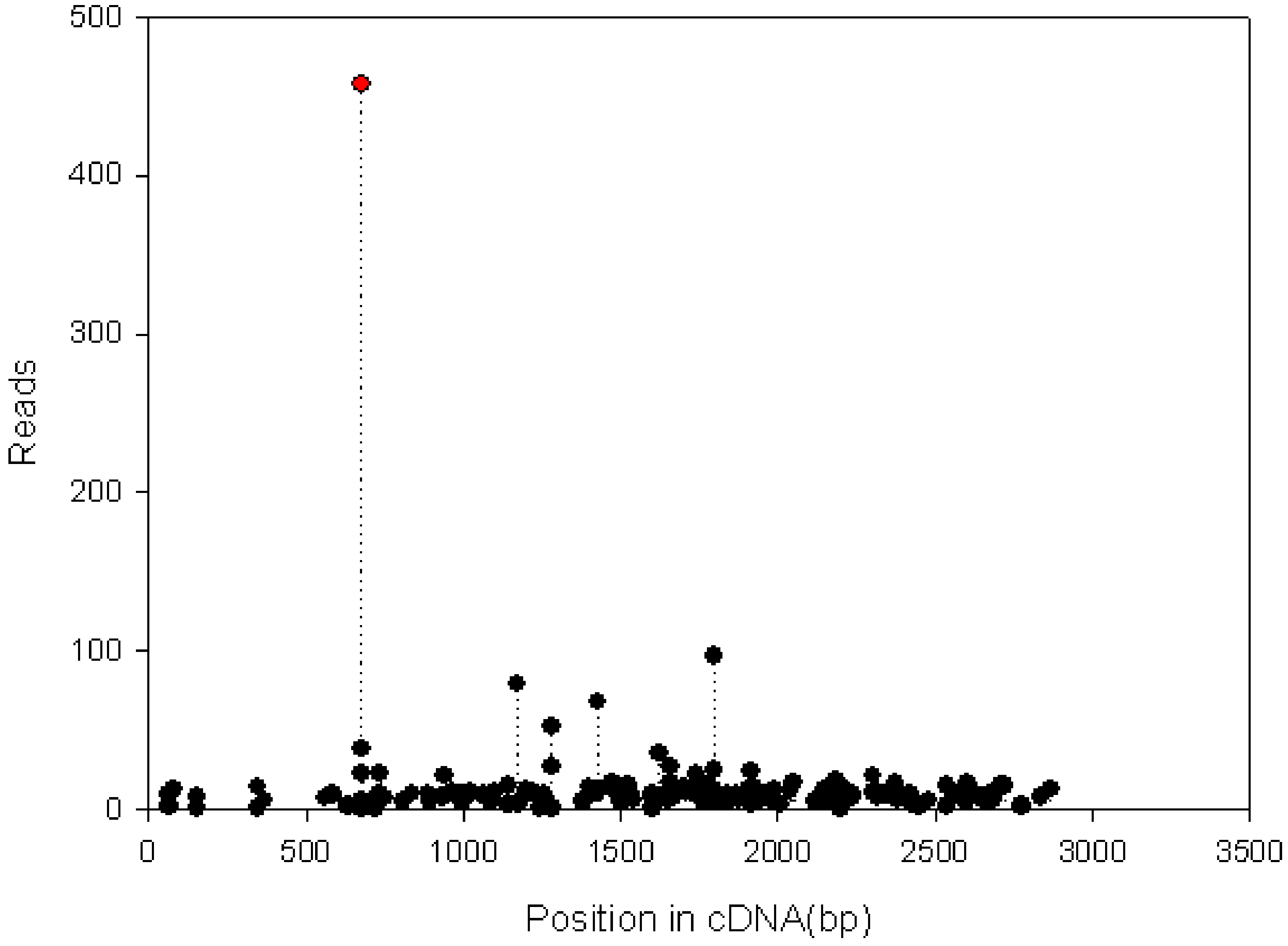


```
5' CCCGGGAUGAAGCCUGGUCCGGAUUC 3'        Cs4g19310.1
      ::.:::::::::::::::::::.
3' ---CCUUACUUCGGACCAGGCUCU-- 5'        Csi-miR166g.1
```

Csi-miR166g.1, target=Cs8g16510.1 gene=Cs8g16510
Category:1
Score=4.5
Cleavage Site=1434

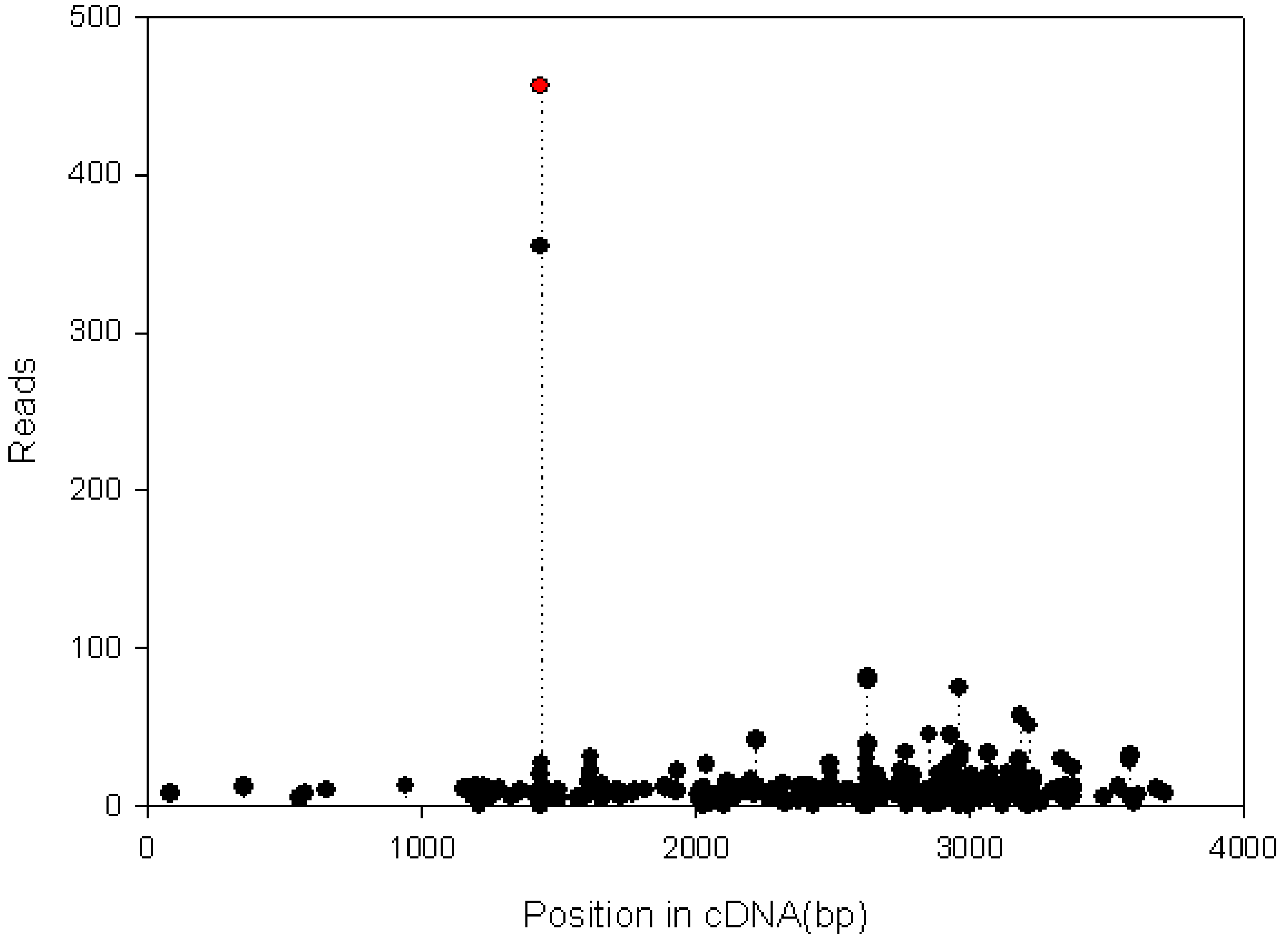


```
5' CCUGGGAUGAAGCCUGGUCCGGAUUC 3'        Cs8g16510.1
       ::.::::::::::::::::::.
3' ---CCUUACUUCGGACCAGGCUCU-- 5'        Csi-miR166g.1
```

Csi-miR166i, target=Cs1g15640.1 gene=Cs1g15640
Category:3
Score=2
Cleavage Site=1781

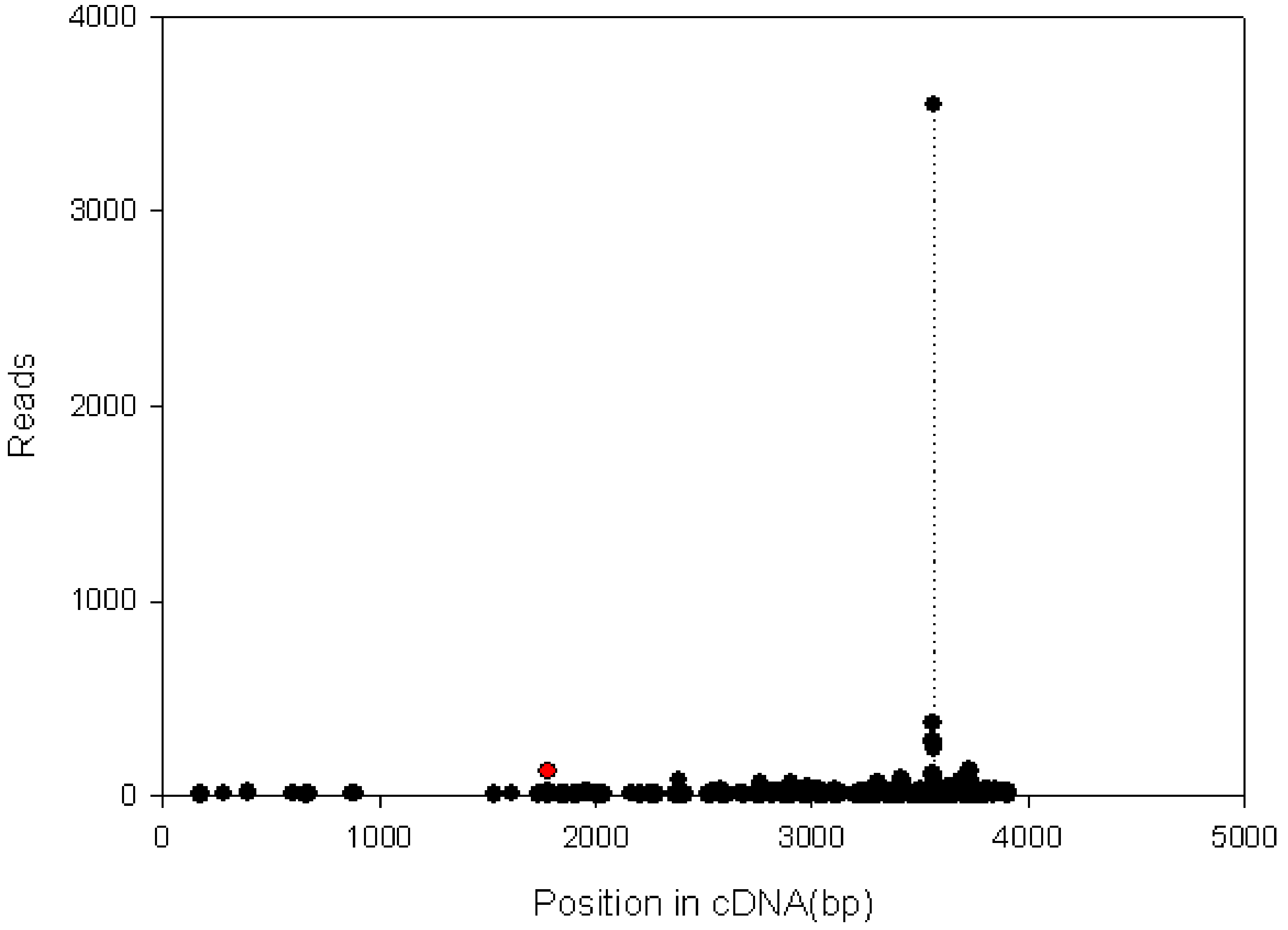


```
5' CCUGGAAUGAAGCCUGGUCCGGAUUC 3'        Cs1g15640.1
       ::::::::::::::::::::.:
3' --CCCUUACUUCGGACCAGGCUU--- 5'        Csi-miR166i
```

Csi-miR166i, target=Cs2g09770.1 gene=Cs2g09770
Category:1
Score=2.5
Cleavage Site=1058

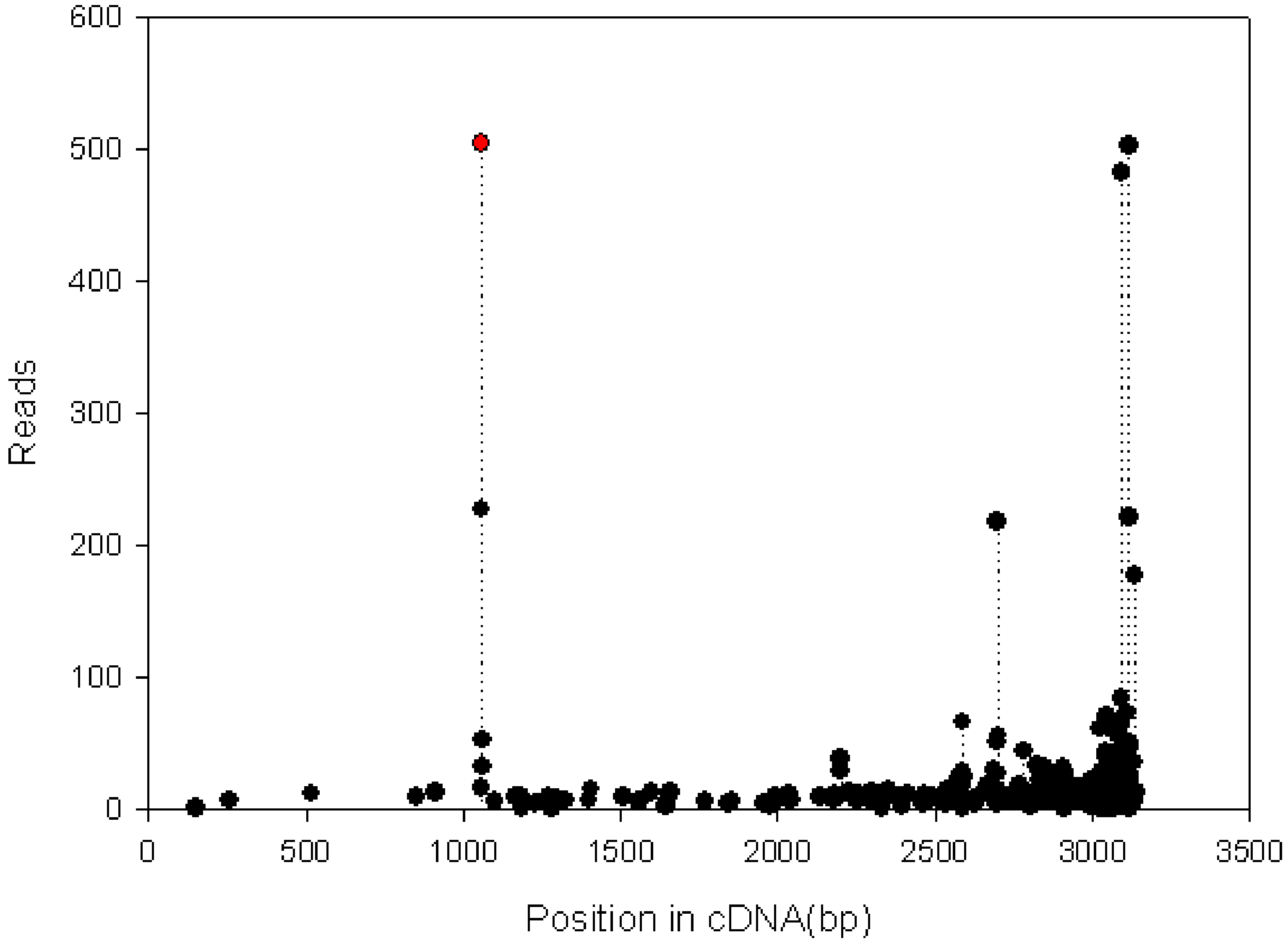


```
5' AUUGGGAUGAAGCCUGGUCCGGAUUC 3'        Cs2g09770.1
     ::.::::::::::::::::.:
3' --CCCUUACUUCGGACCAGGCUU--- 5'        Csi-miR166i
```

Csi-miR166i, target=Cs4g19310.1 gene=Cs4g19310
Category:1
Score=2.5
Cleavage Site=677

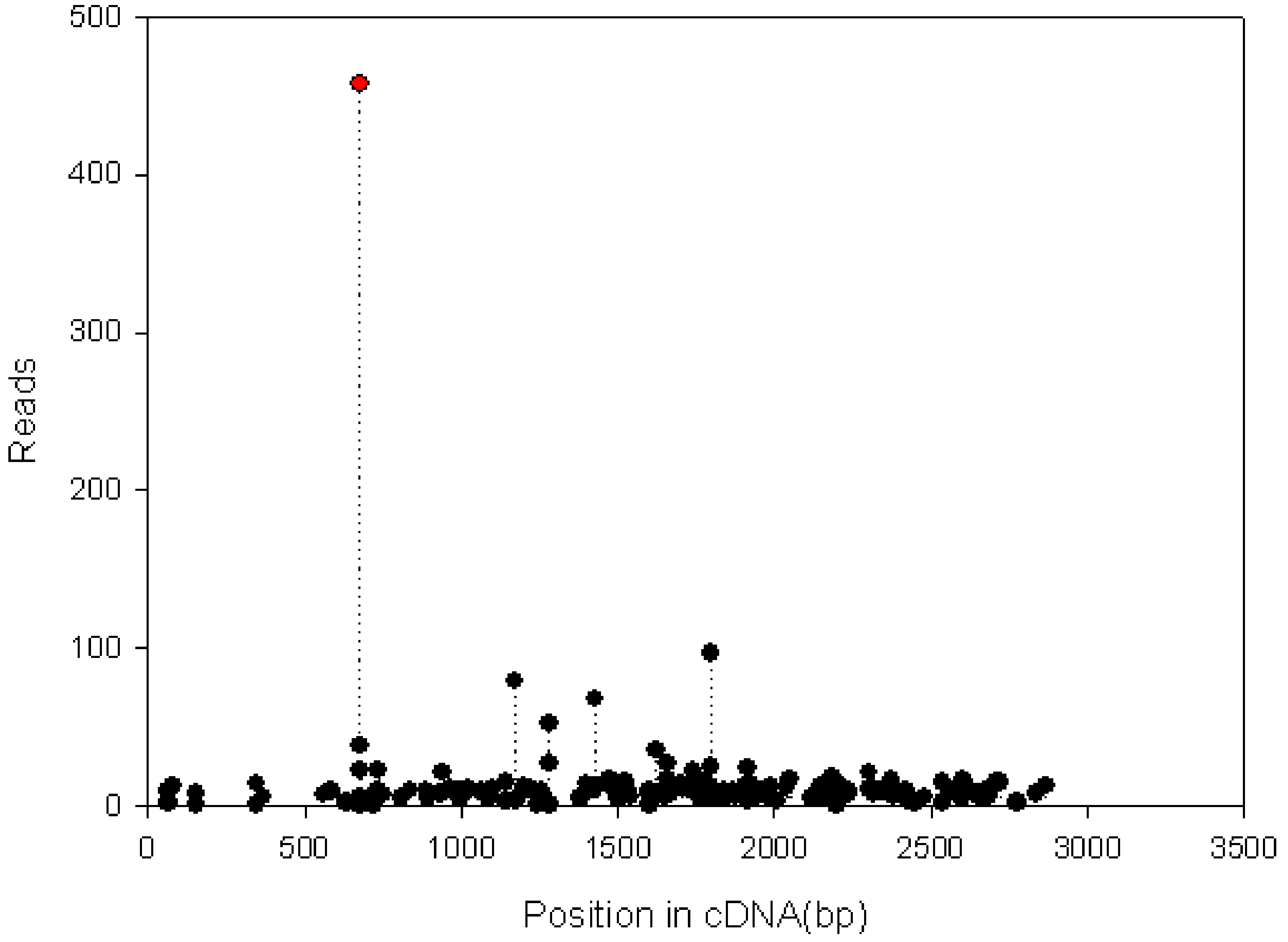


```
5' CCCGGGAUGAAGCCUGGUCCGGAUUC 3'        Cs4g19310.1
     ::.::::::::::::::::.:
3' --CCCUUACUUCGGACCAGGCUU--- 5'        Csi-miR166i
```

Csi-miR166i, target=Cs8g16510.1 gene=Cs8g16510
Category:1
Score=2.5
Cleavage Site=1434

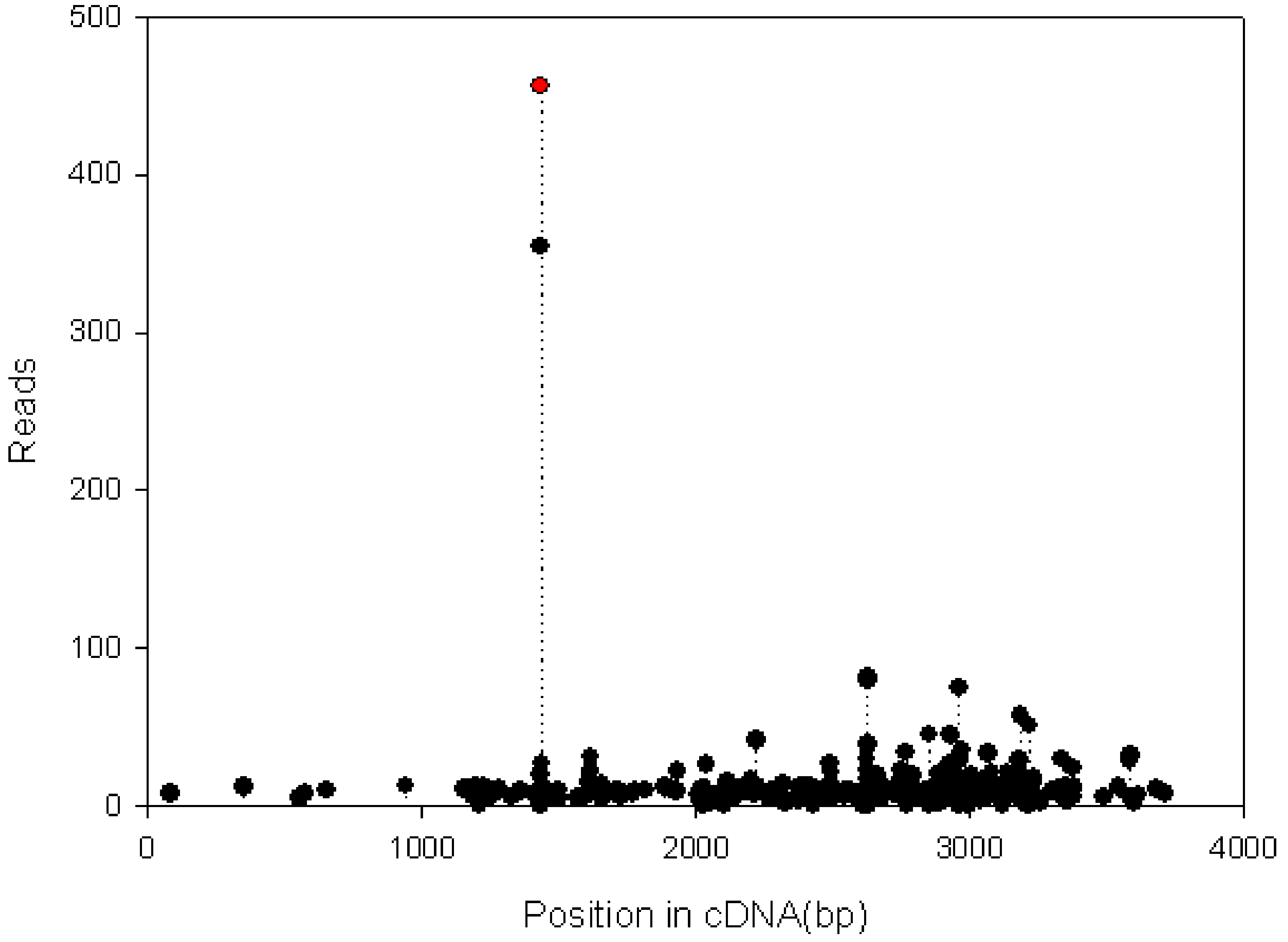


```
5' CCUGGGAUGAAGCCUGGUCCGGAUUC 3'        Cs8g16510.1
      ::.:::::::::::::::::.:
3' --CCCUUACUUCGGACCAGGCUU--- 5'        Csi-miR166i
```

Csi-miR166j.1, target=Cs1g15640.1 gene=Cs1g15640
Category:3
Score=3.5
Cleavage Site=1781

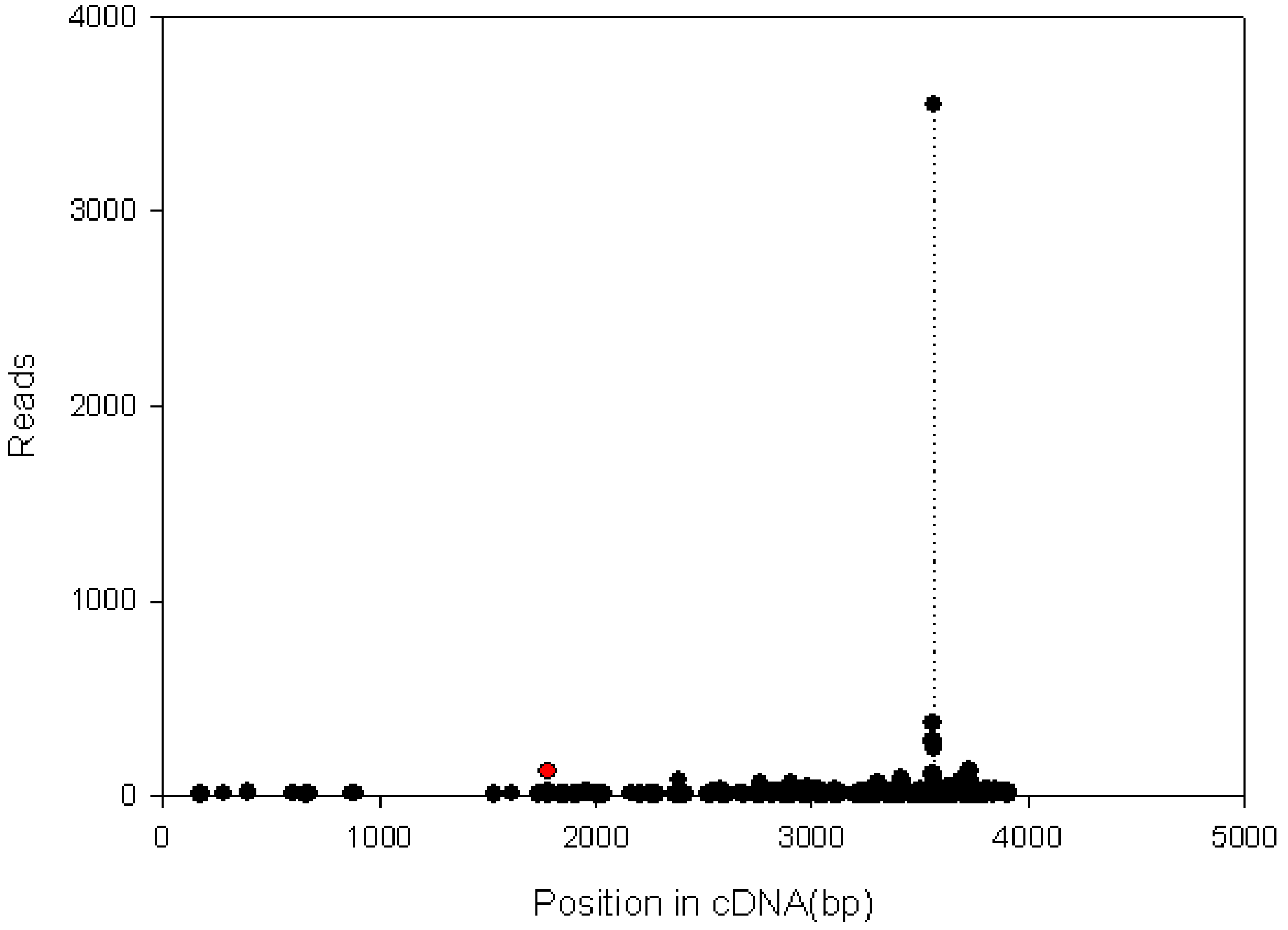


```
5' CCUGGAAUGAAGCCUGGUCCGGAUUC 3'        Cs1g15640.1
     ::::::::::::::::::::..
3' -CUCCUUACUUCGGACCAGGUU---- 5'        Csi-miR166j.1
```

Csi-miR166j.1, target=Cs2g09770.1 gene=Cs2g09770
Category:1
Score=4
Cleavage Site=1058

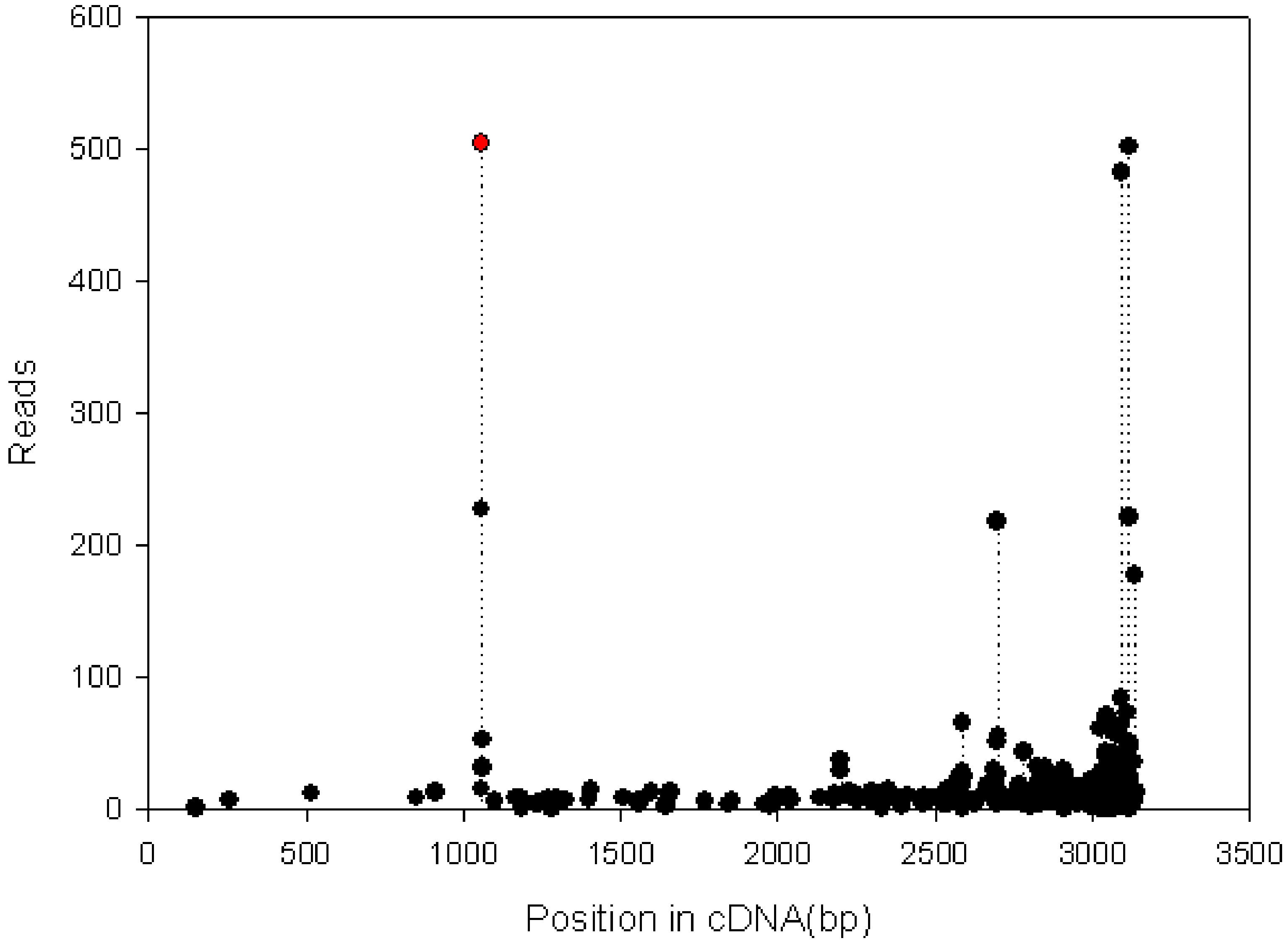


```
5' AUUGGGAUGAAGCCUGGUCCGGAUUC 3'        Cs2g09770.1
     ::.:::::::::::::::::..
3' -CUCCUUACUUCGGACCAGGUU---- 5'        Csi-miR166j.1
```

Csi-miR166j.1, target=Cs4g19310.1 gene=Cs4g19310
Category:1
Score=4
Cleavage Site=677

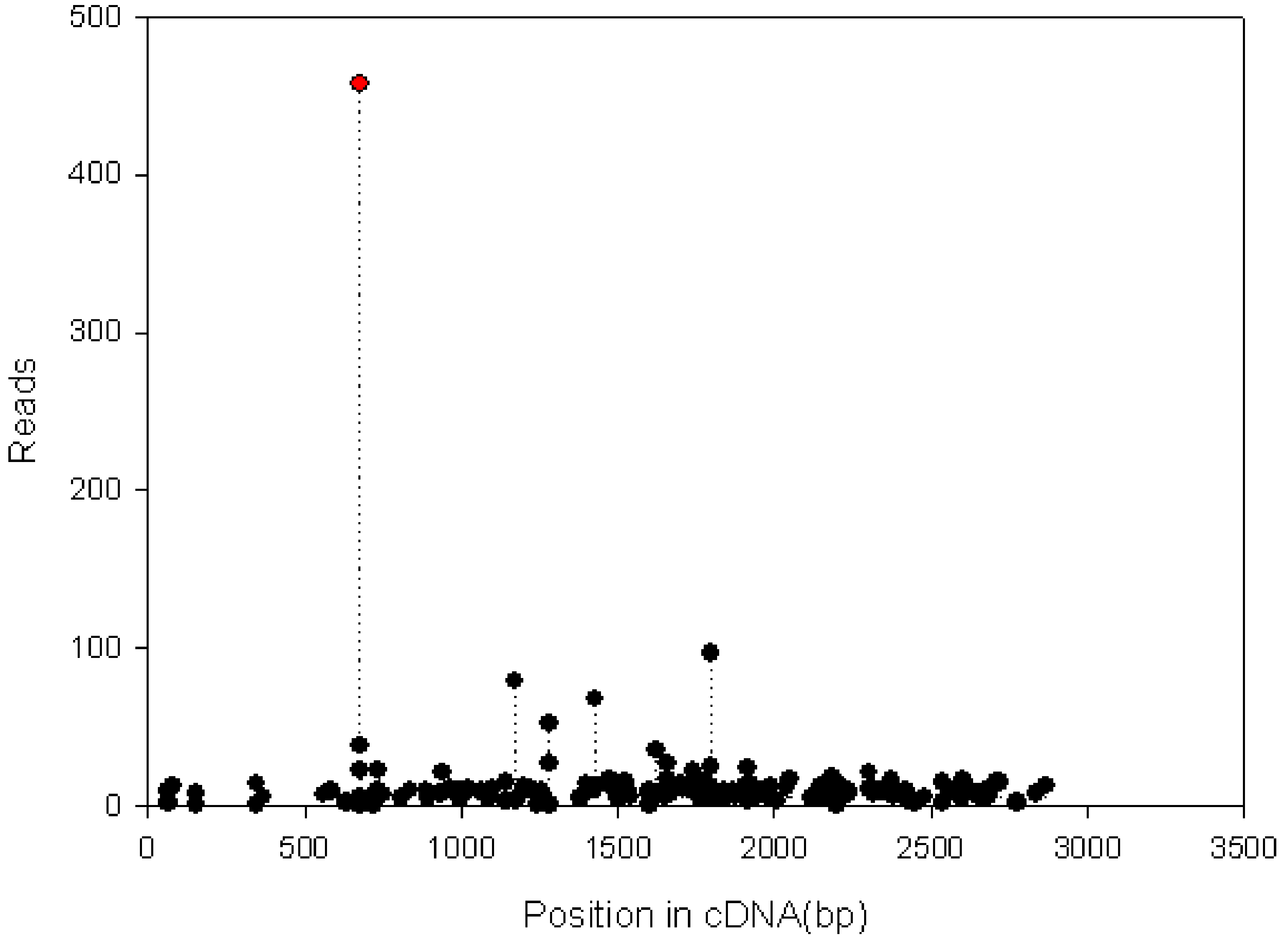


```
5' CCCGGGAUGAAGCCUGGUCCGGAUUC 3'        Cs4g19310.1
       ::.:::::::::::::::..
3' -CUCCUUACUUCGGACCAGGUU---- 5'        Csi-miR166j.1
```

Csi-miR166j.1, target=Cs8g16510.1 gene=Cs8g16510
Category:1
Score=4
Cleavage Site=1434

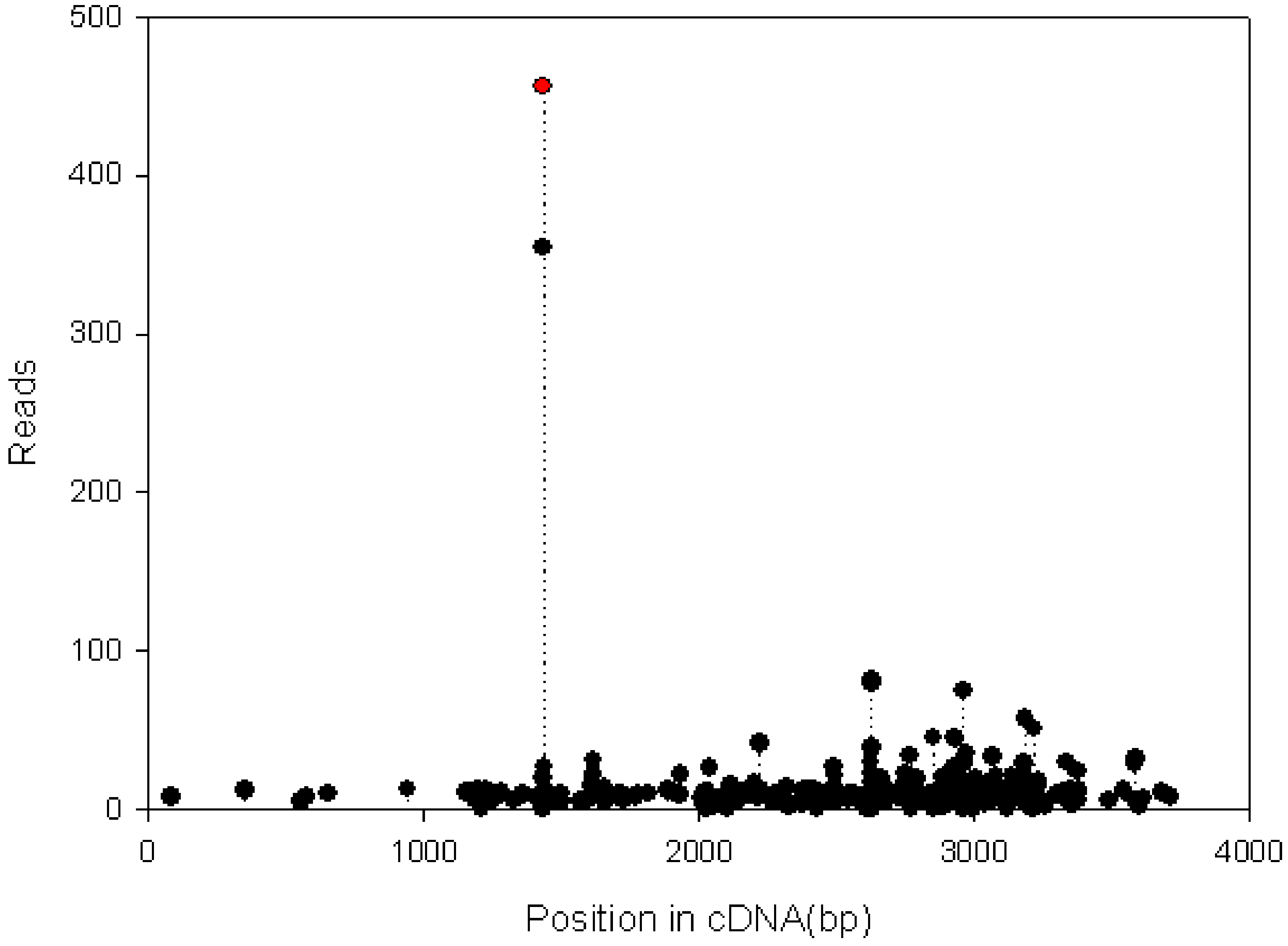


```
5' CCUGGGAUGAAGCCUGGUCCGGAUUC 3'        Cs8g16510.1
      ::.::::::::::::::..
3' -CUCCUUACUUCGGACCAGGUU---- 5'        Csi-miR166j.1
```

Csi-miR166j.2, target=Cs1g15640.1 gene=Cs1g15640
Category:3
Score=5
Cleavage Site=1781

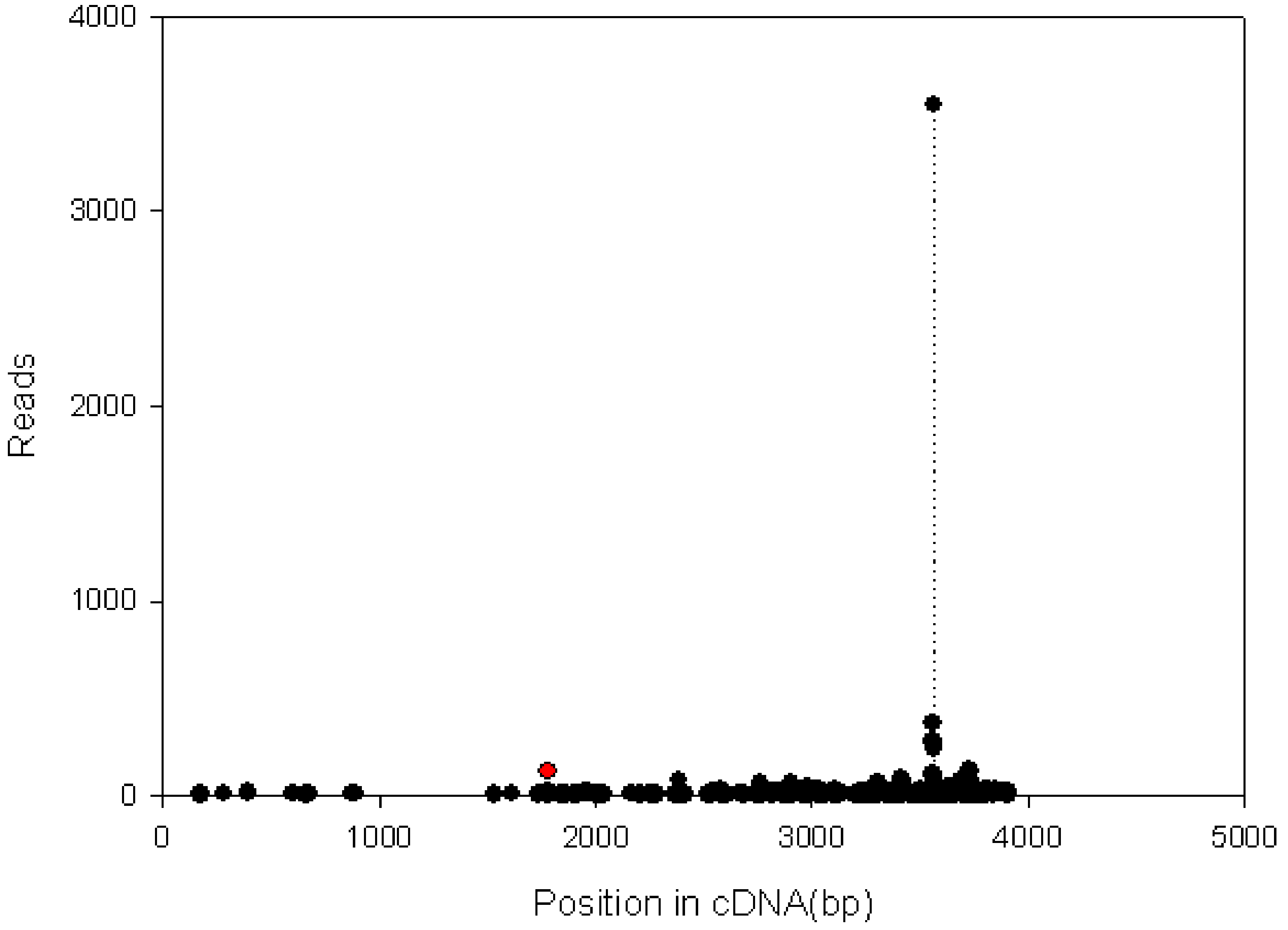


```
5' CCUGGAAUGAAGCCUGGUCCGGAUUC 3'        Cs1g15640.1
      ::::::::::::::::::::..
3' ---CCUUACUUCGGACCAGGUUCU-- 5'        Csi-miR166j.2
```

Csi-miR166j.3, target=Cs1g15640.1 gene=Cs1g15640
Category:3
Score=2.5
Cleavage Site=1781

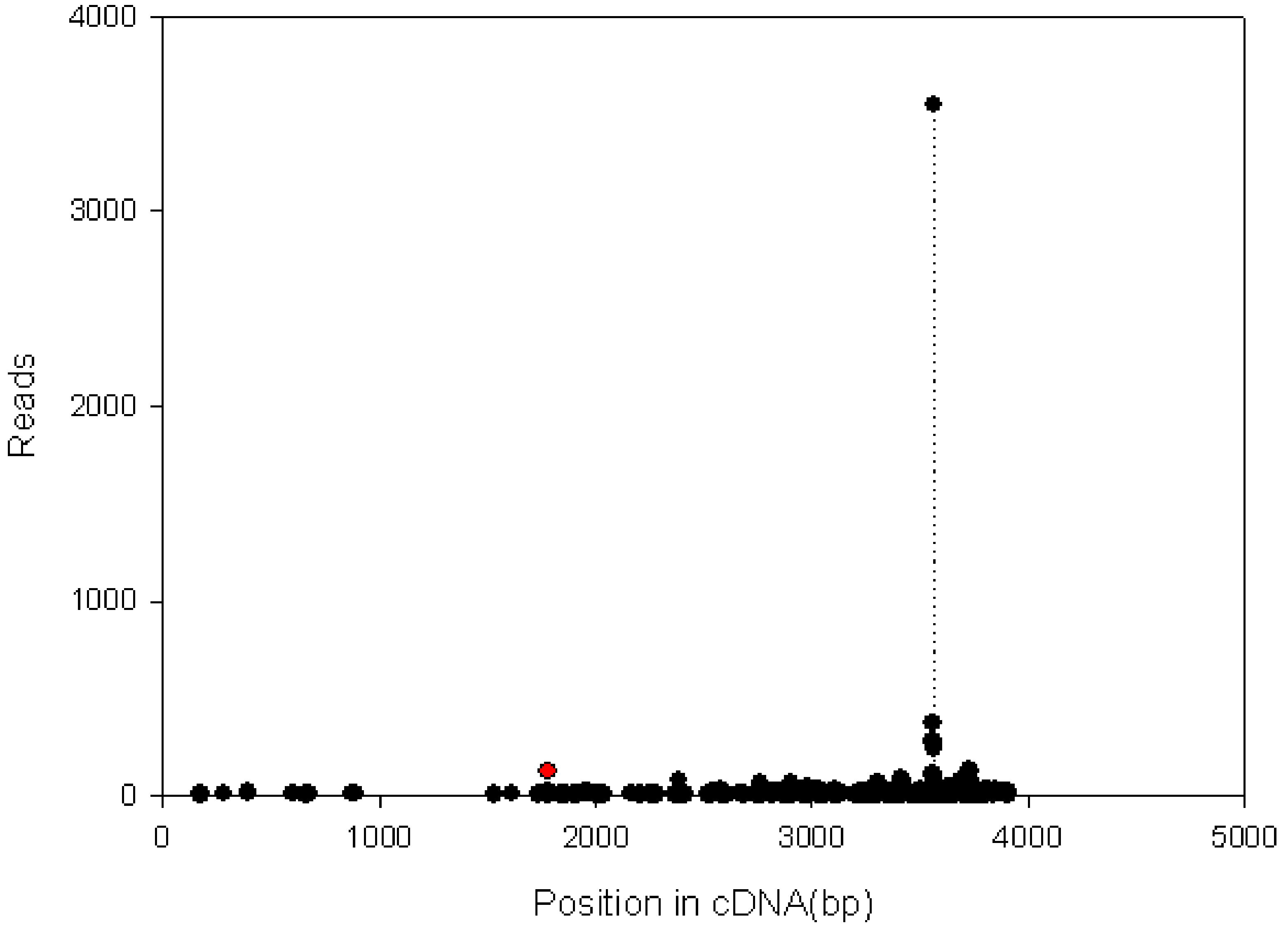


```
5' CCUGGAAUGAAGCCUGGUCCGGAUUC 3'        Cs1g15640.1
    ::::::::::::::::::::..
3' -CACCUUACUUCGGACCAGGUU---- 5'        Csi-miR166j.3
```

Csi-miR166j.3, target=Cs2g09770.1 gene=Cs2g09770
Category:1
Score=3
Cleavage Site=1058

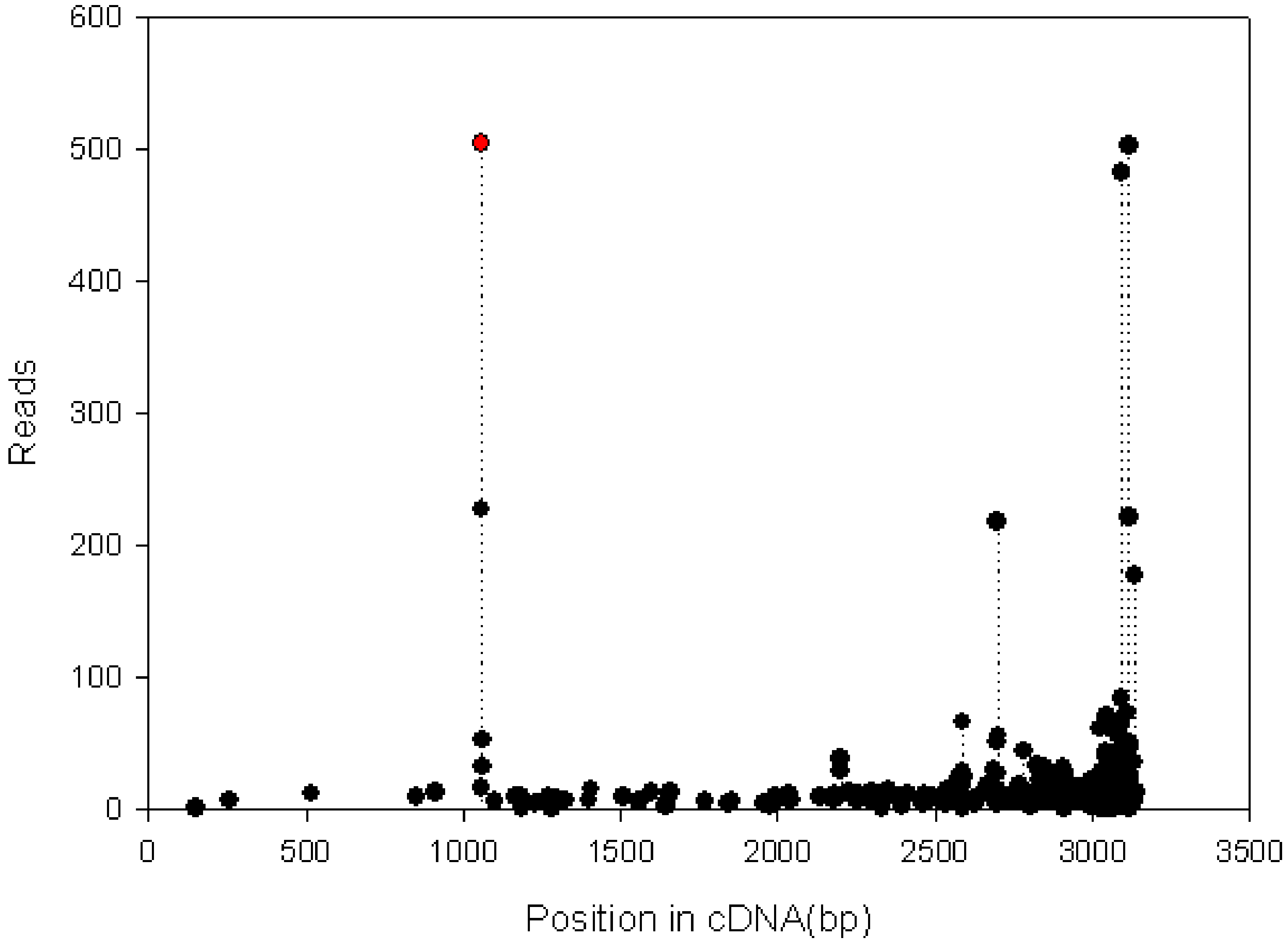


```
5'  AUUGGGAUGAAGCCUGGUCCGGAUUC  3'         Cs2g09770.1
      :::.::::::::::::::..
3'  -CACCUUACUUCGGACCAGGUU----  5'         Csi-miR166j.3
```

Csi-miR166j.3, target=Cs4g19310.1 gene=Cs4g19310
Category:1
Score=4
Cleavage Site=677

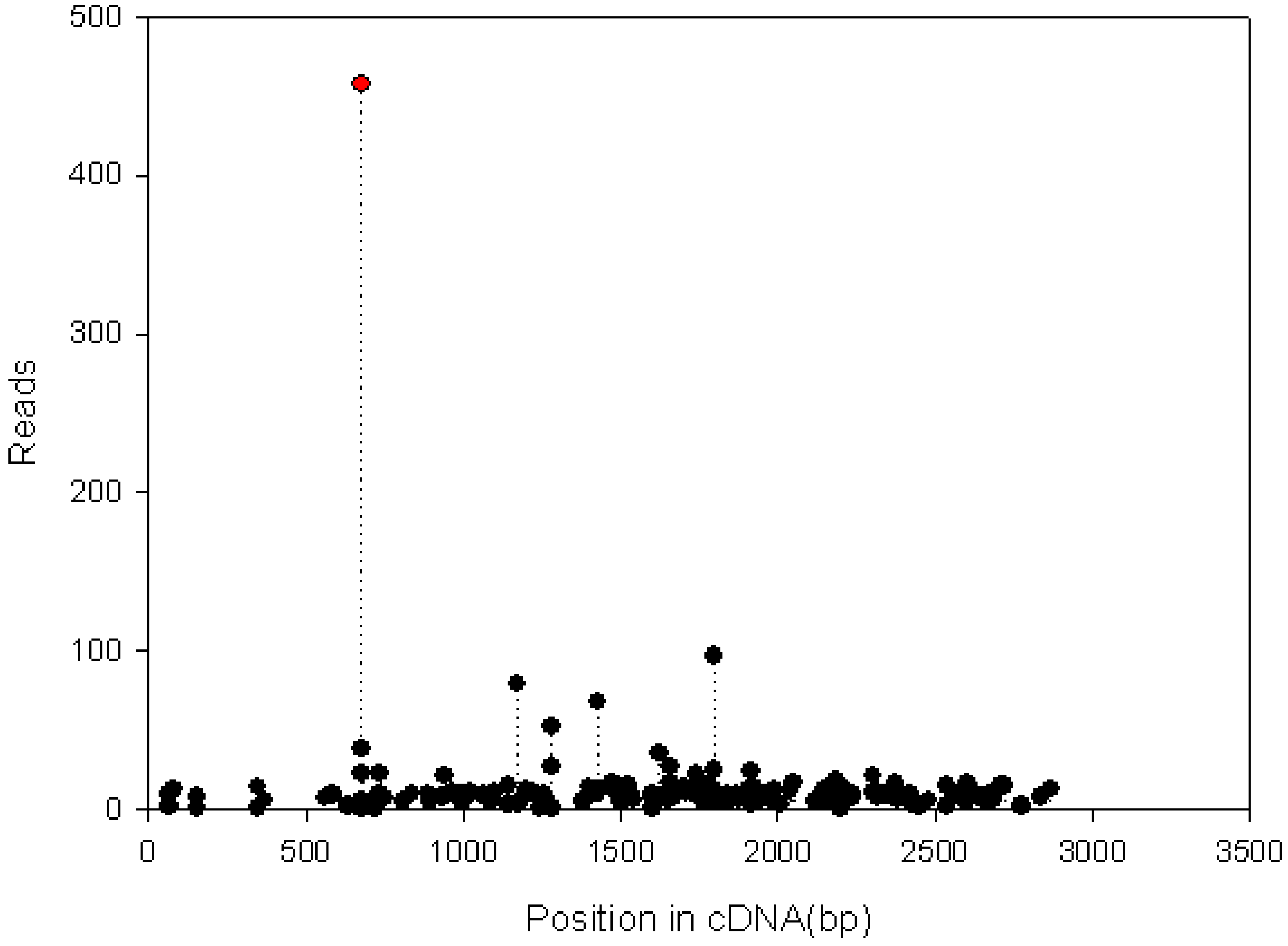


```
5' CCCGGGAUGAAGCCUGGUCCGGAUUC 3'        Cs4g19310.1
     ::.::::::::::::::::..
3' -CACCUUACUUCGGACCAGGUU---- 5'        Csi-miR166j.3
```

Csi-miR166j.3, target=Cs8g16510.1 gene=Cs8g16510
Category:1
Score=3
Cleavage Site=1434

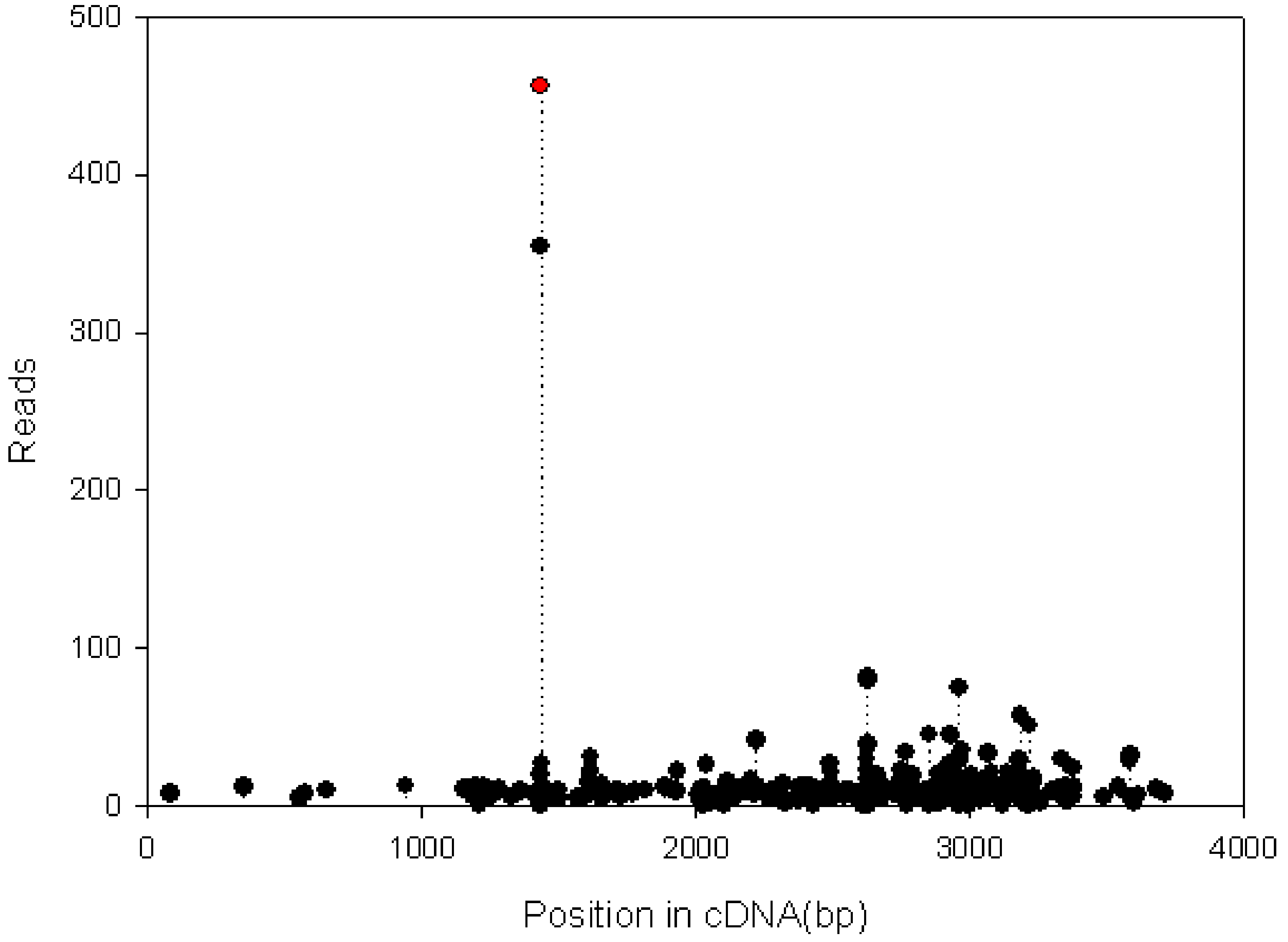


```
5' CCUGGGAUGAAGCCUGGUCCGGAUUC 3'        Cs8g16510.1
     :::.:::::::::::::::::..
3' -CACCUUACUUCGGACCAGGUU---- 5'        Csi-miR166j.3
```

Csi-miR167a.1, target=Cs2g15130.1 gene=Cs2g15130
Category:1
Score=5
Cleavage Site=3444

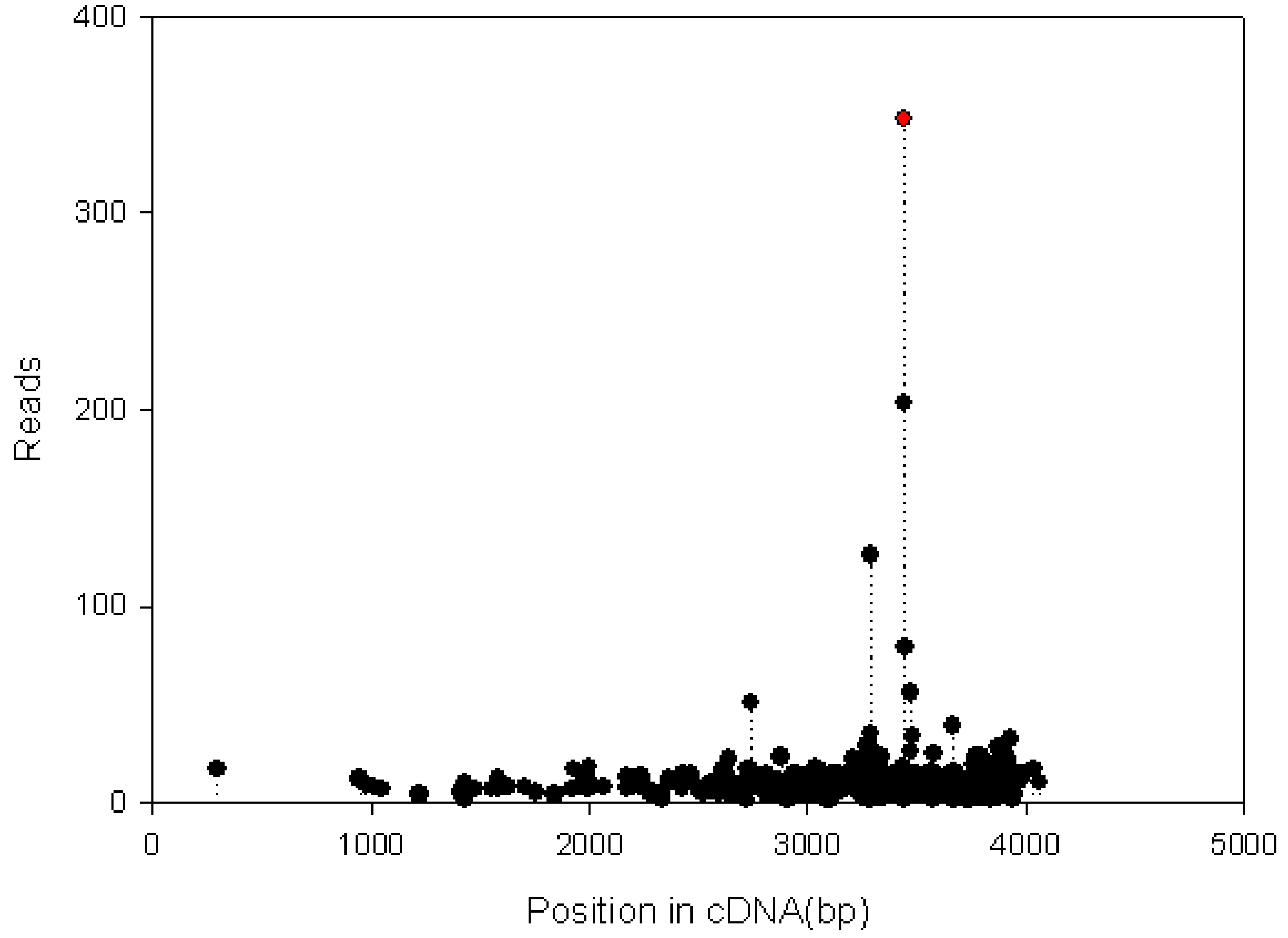


```
5' AGAGAUCAGGCUGGCAGCUUGUAUUU 3'        Cs2g15130.1
     :::::: ::::::::::: .:
3' -AUCUAGUACGACCGUCGAA-GU--- 5'        Csi-miR167a.1
```

Csi-miR167a.1, target=Cs6g16030.1 gene=Cs6g16030
Category:2
Score=5
Cleavage Site=3357

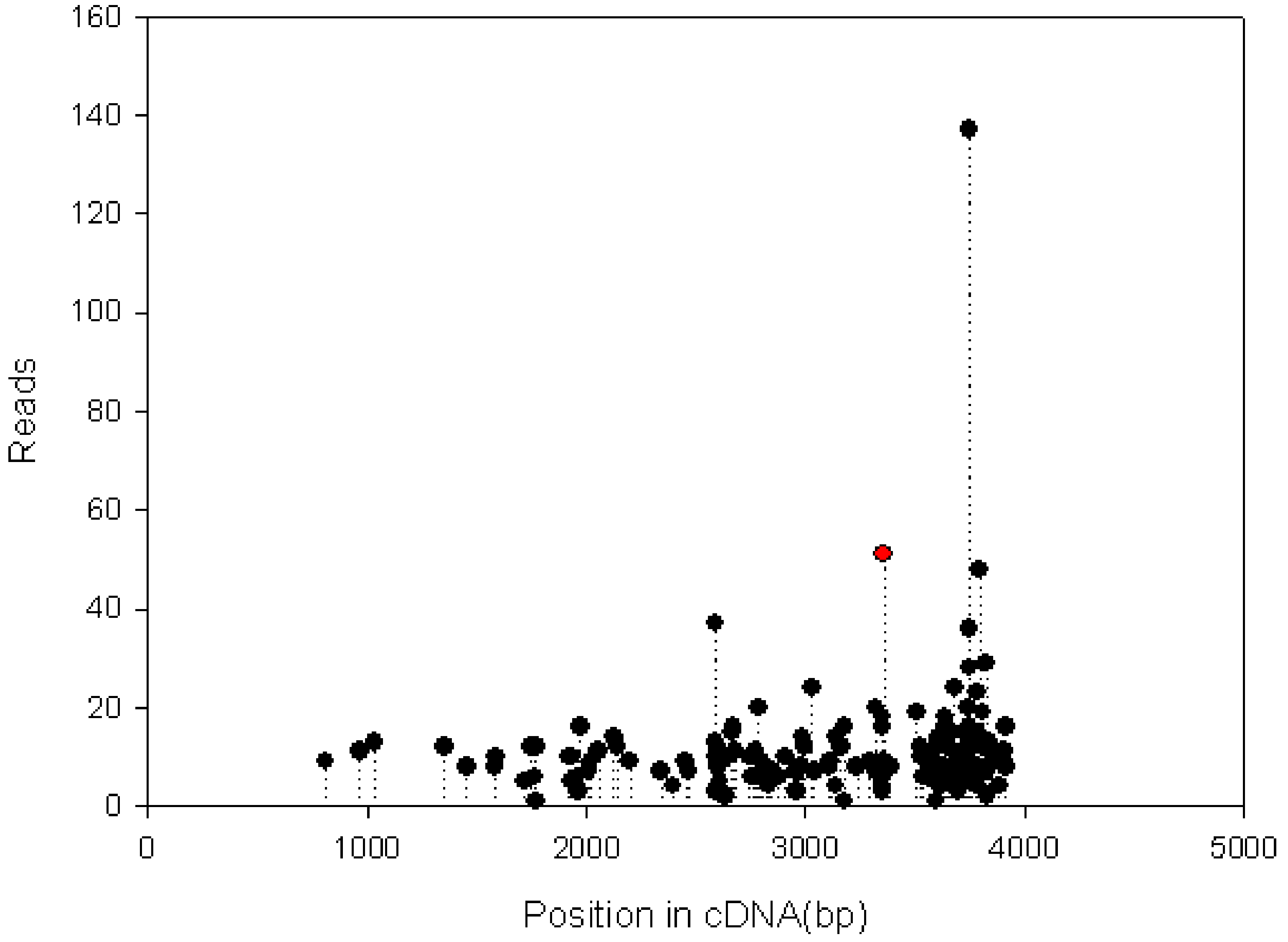


```
5' UUAGAUCAGGCUGGCAGCUUGUAUUC 3'         Cs6g16030.1
    ::::::: ::::::::::: .:
3' -AUCUAGUACGACCGUCGAA-GU--- 5'         Csi-miR167a.1
```

Csi-miR167a.1,target=Cs2g09440.1 gene=Cs2g09440
Category:3
Score=5
Cleavage Site=3257

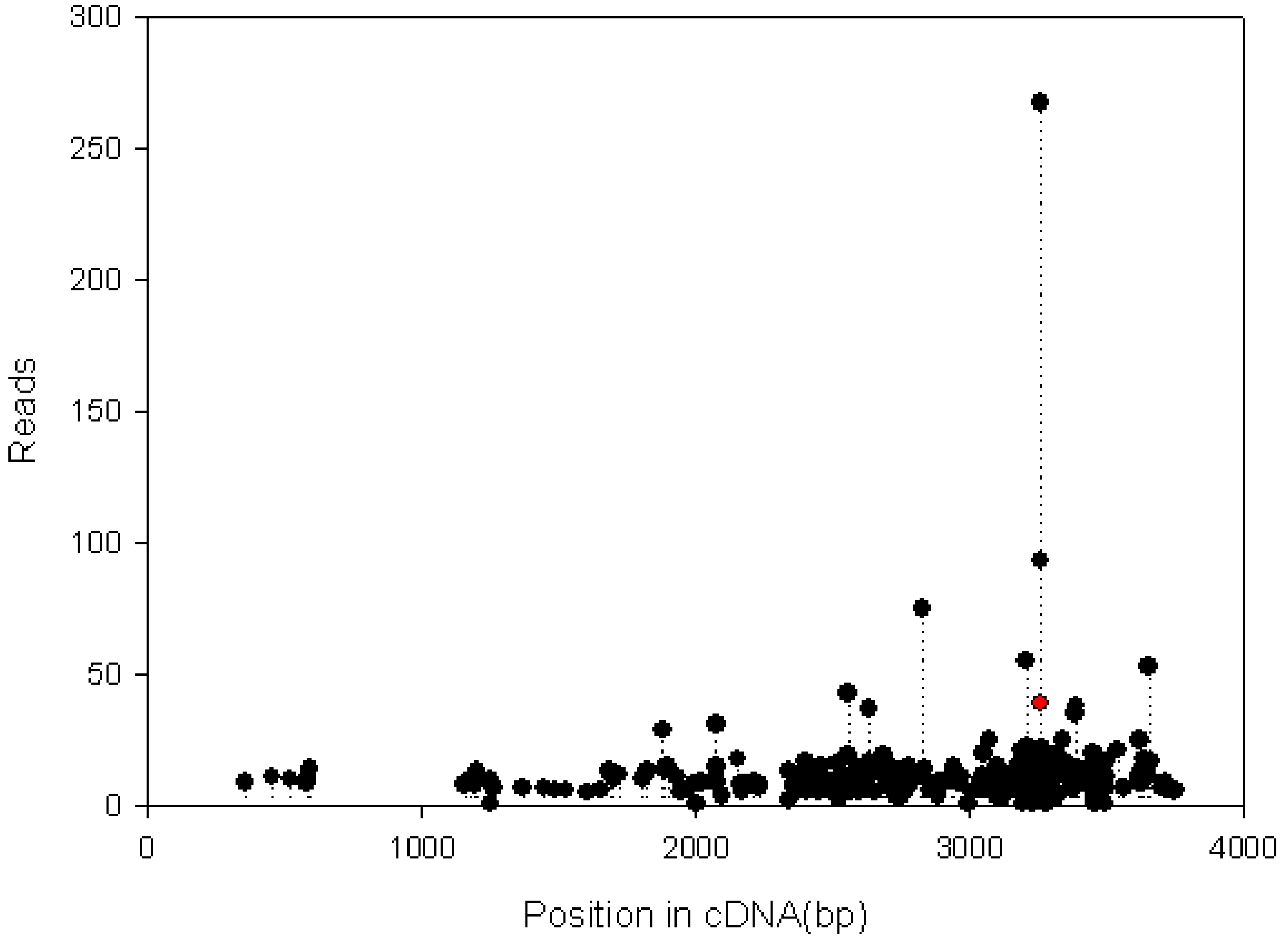


```
5' UGAGAUCAGGCUGGCAGCUUGUAUUC 3'        Cs2g09440.1
    :::::: ::::::::::: .:
3' -AUCUAGUACGACCGUCGAA-GU--- 5'        Csi-miR167a.1
```

Csi-miR167b.1, target=Cs1g09030.1 gene=Cs1g09030
Category:2
Score=4.5
Cleavage Site=1248

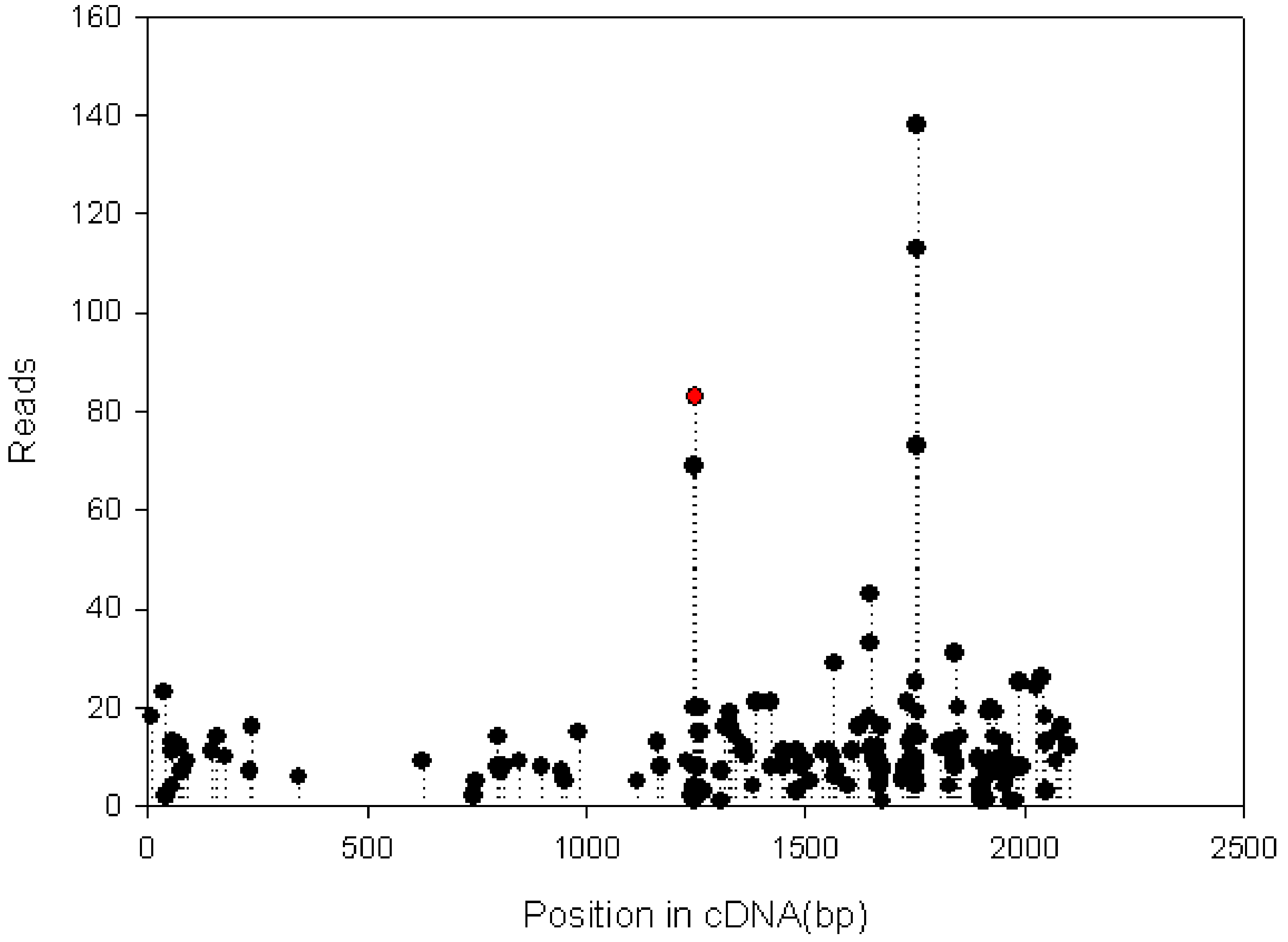


```
5' GUGAAAUCAAGCUGGCAGACUUCGGC 3'         Cs1g09030.1
     : :::: :::::::: ::::.
3' ---UCUAGUACGACCGUC-GAAGU-- 5'         Csi-miR167b.1
```

Csi-miR167b.1, target=Cs2g09440.1 gene=Cs2g09440
Category:2
Score=4
Cleavage Site=3259

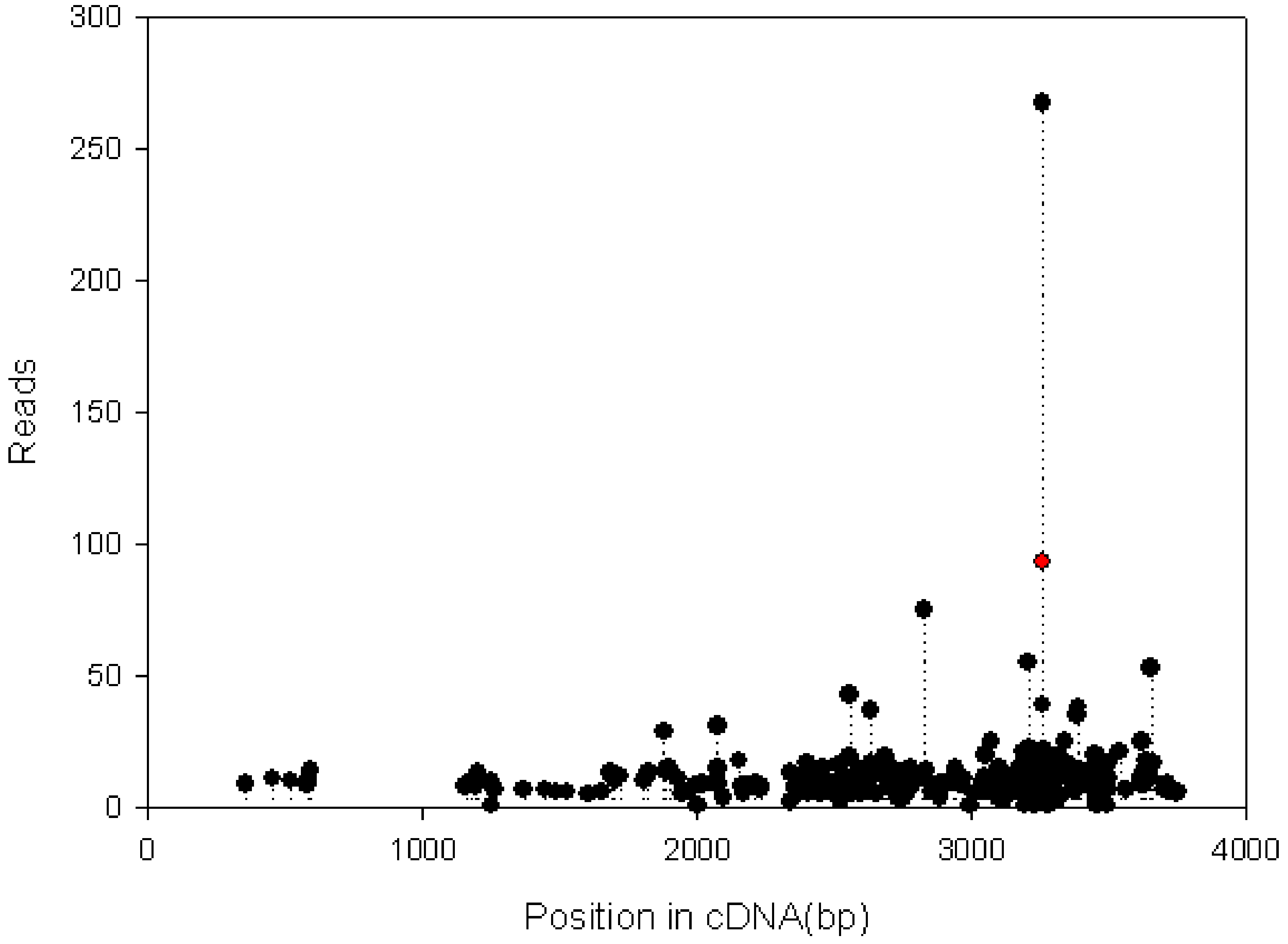


```
5' AGAUCAGGCUGGCAGCUUGUAUUCGU 3'        Cs2g09440.1
   :::::: ::::::::::: .:
3' UCUAGUACGACCGUCGAA-GU----- 5'        Csi-miR167b.1
```

Csi-miR167b.1, target=Cs2g15130.1 gene=Cs2g15130
Category:1
Score=4
Cleavage Site=3444

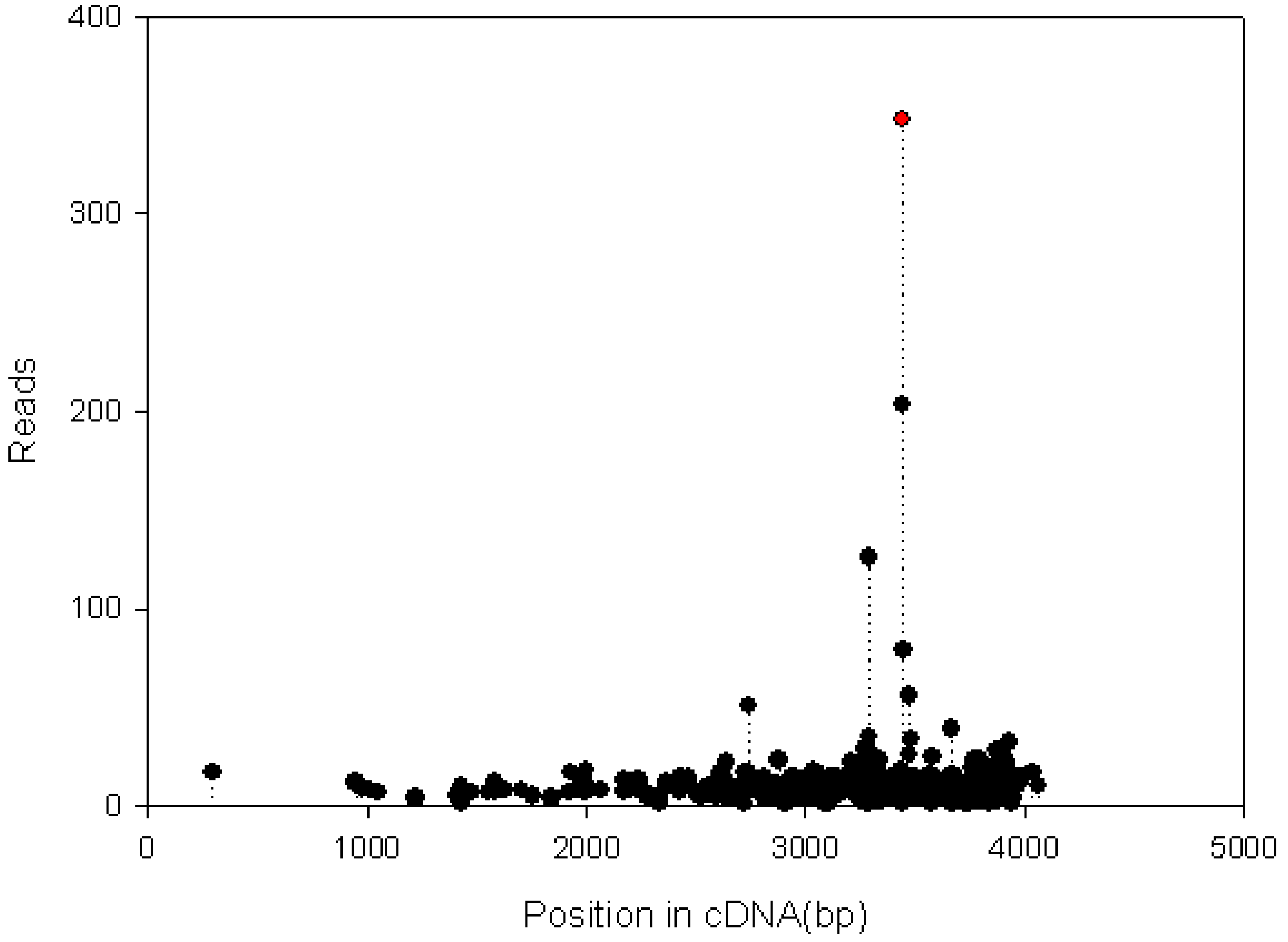


```
5' AGAGAUCAGGCUGGCAGCUUGUAUUU 3'        Cs2g15130.1
     :::::: ::::::::::: .:
3' --UCUAGUACGACCGUCGAA-GU--- 5'        Csi-miR167b.1
```

Csi-miR167b.1, target=Cs6g16030.1 gene=Cs6g16030
Category:2
Score=4
Cleavage Site=3357

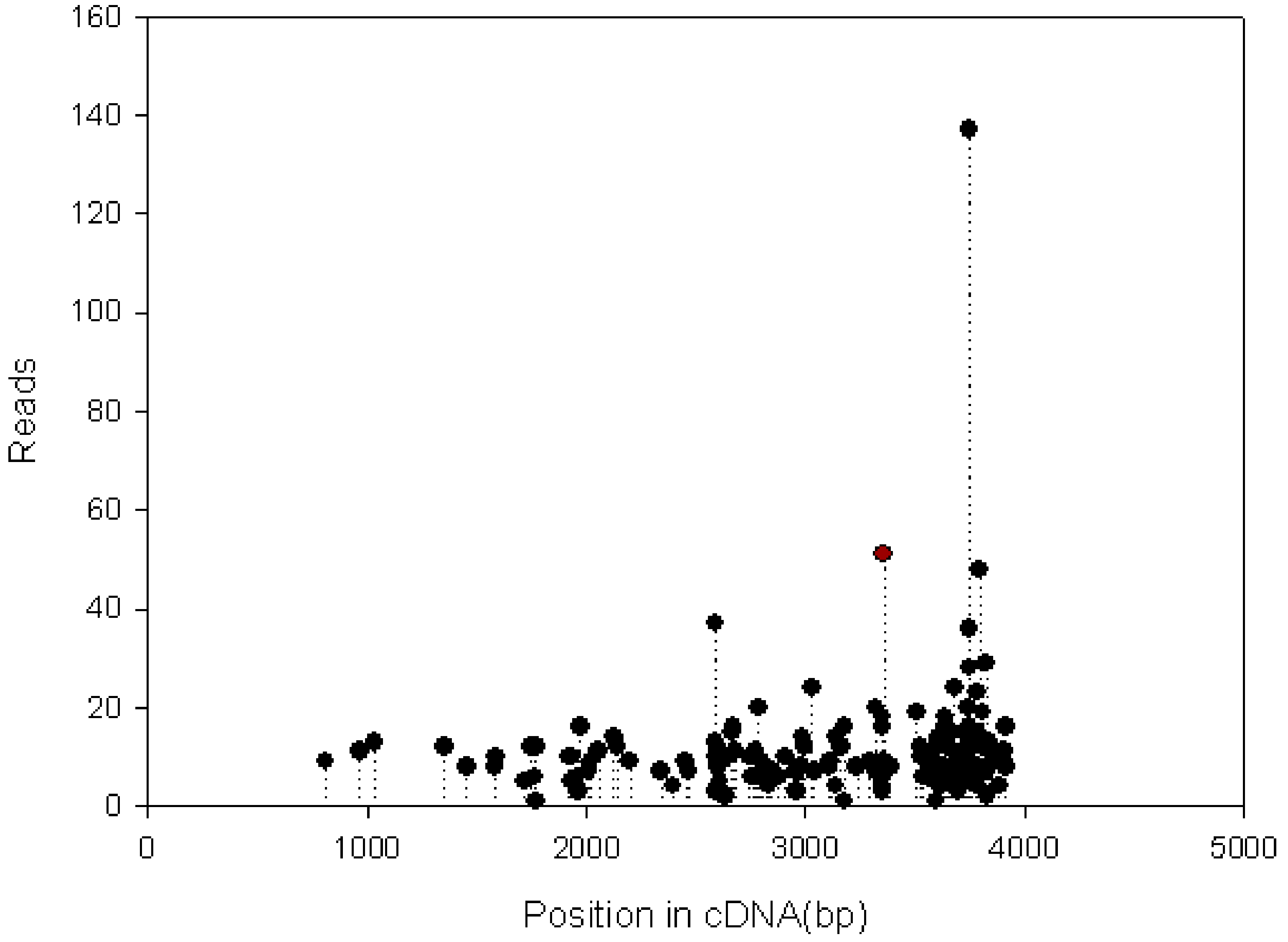


```
5' UUAGAUCAGGCUGGCAGCUUGUAUUC 3'        Cs6g16030.1
     :::::: ::::::::::: .:
3' --UCUAGUACGACCGUCGAA-GU--- 5'        Csi-miR167b.1
```

Csi-miR167b.2, target=Cs1g09030.1 gene=Cs1g09030
Category:2
Score=5
Cleavage Site=1248

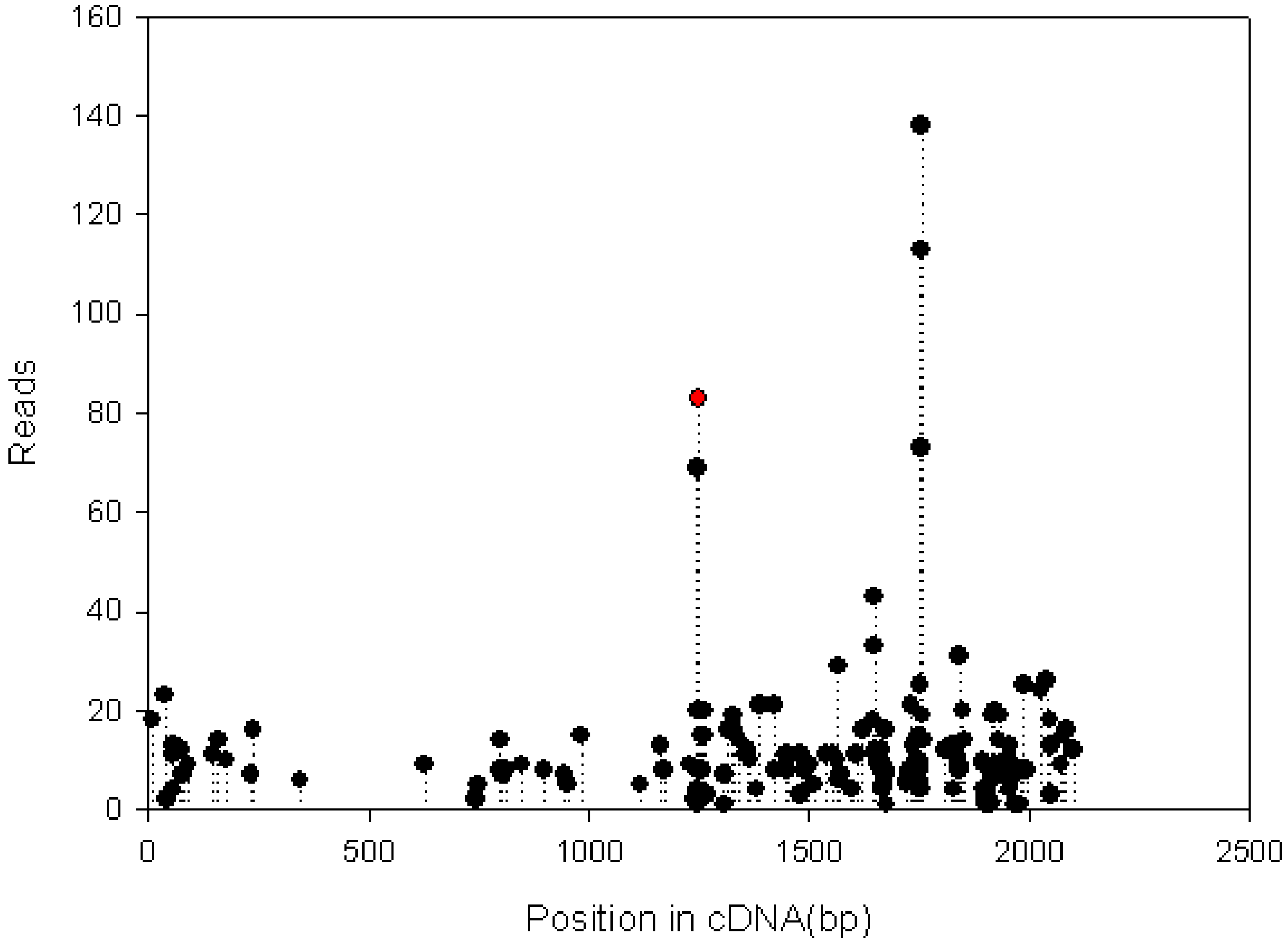


```
5' GUGAAAUCAAGCUGGCAGACUUCGGC 3'        Cs1g09030.1
    :.: :::: :::::::: ::::.
3' -AUUCUAGUACGACCGUC-GAAGU-- 5'        Csi-miR167b.2
```

Csi-miR167b.2, target=Cs2g09440.1 gene=Cs2g09440
Category:3
Score=4.5
Cleavage Site=3257

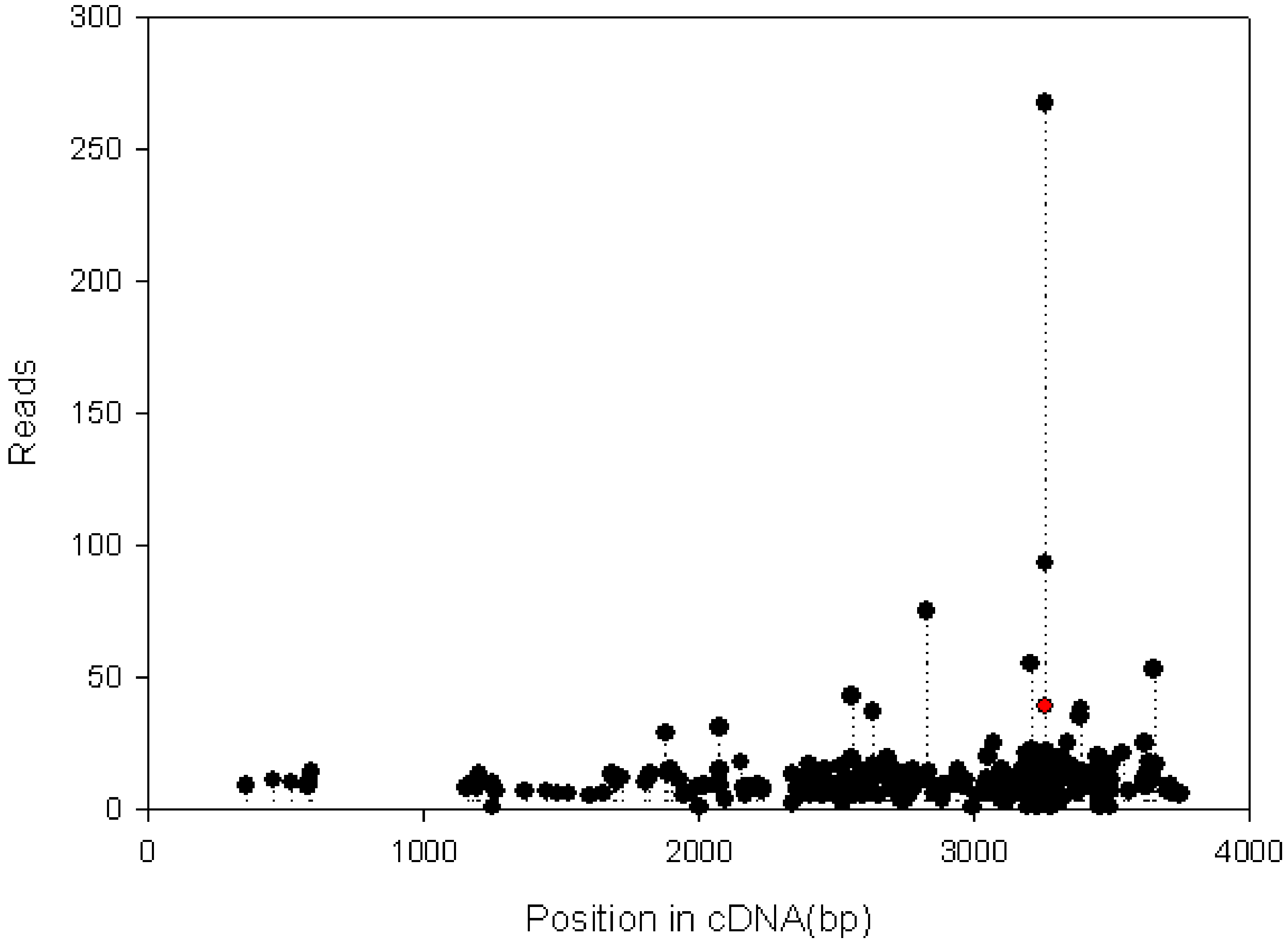


```
5' UGAGAUCAGGCUGGCAGCUUGUAUUC 3'        Cs2g09440.1
   :.:::::: :::::::::: .:
3' AUUCUAGUACGACCGUCGAA-GU--- 5'        Csi-miR167b.2
```

Csi-miR167b.2, target=Cs6g16030.1 gene=Cs6g16030
Category:2
Score=5
Cleavage Site=3357

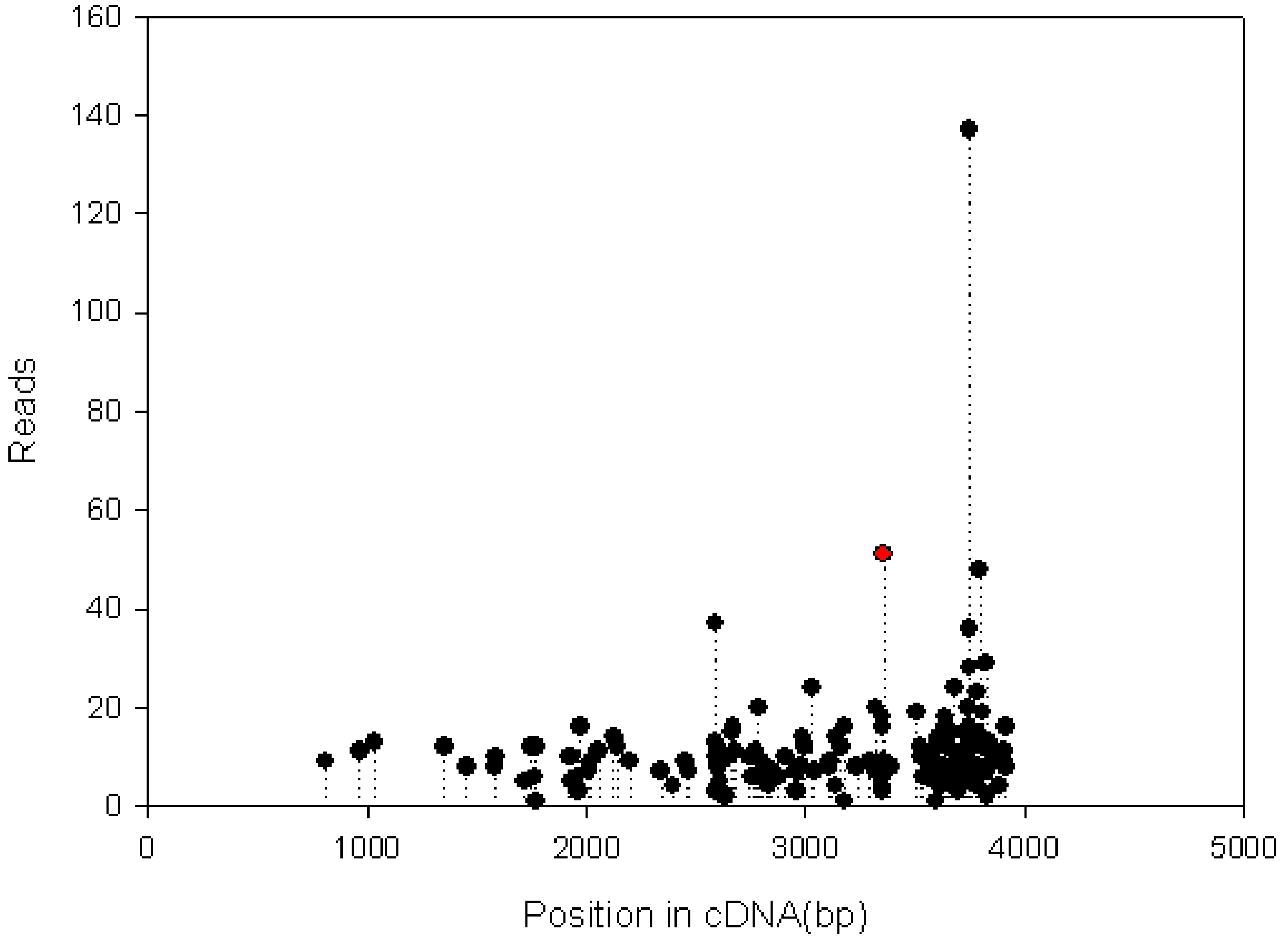


```
5' UUAGAUCAGGCUGGCAGCUUGUAUUC 3'        Cs6g16030.1
   : :::::: ::::::::::: .:
3' AUUCUAGUACGACCGUCGAA-GU--- 5'        Csi-miR167b.2
```

Csi-miR167b.3, target=Cs2g09440.1 gene=Cs2g09440
Category:3
Score=2.5
Cleavage Site=3257

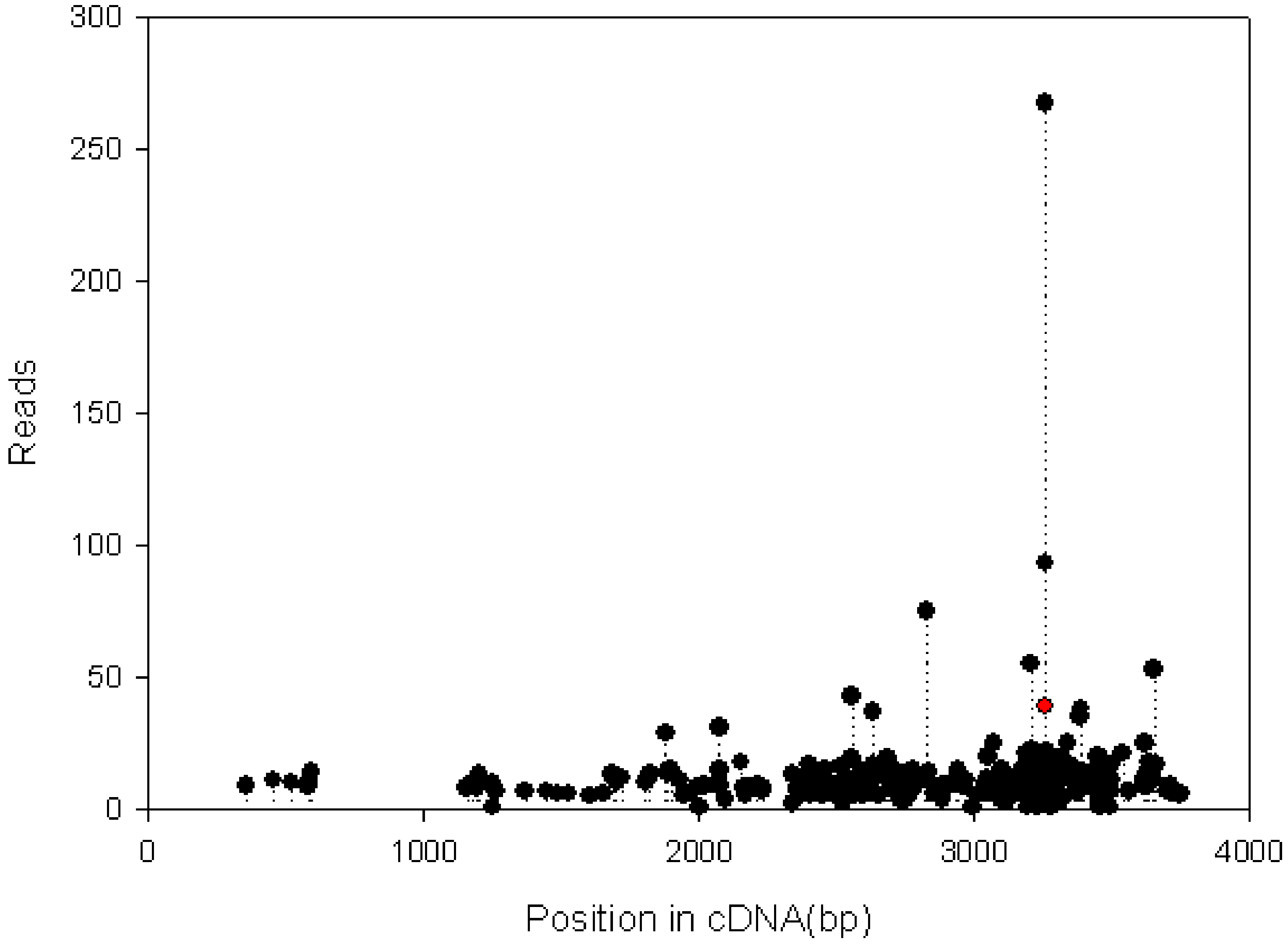


```
5' UGAGAUCAGGCUGGCAGCUUGUAUUC 3'        Cs2g09440.1
   :.:::::: :::::::::::
3' AUUCUAGUACGACCGUCGAA------ 5'        Csi-miR167b.3
```

Csi-miR167b.3, target=Cs2g15130.1 gene=Cs2g15130
Category:1
Score=3.5
Cleavage Site=3444

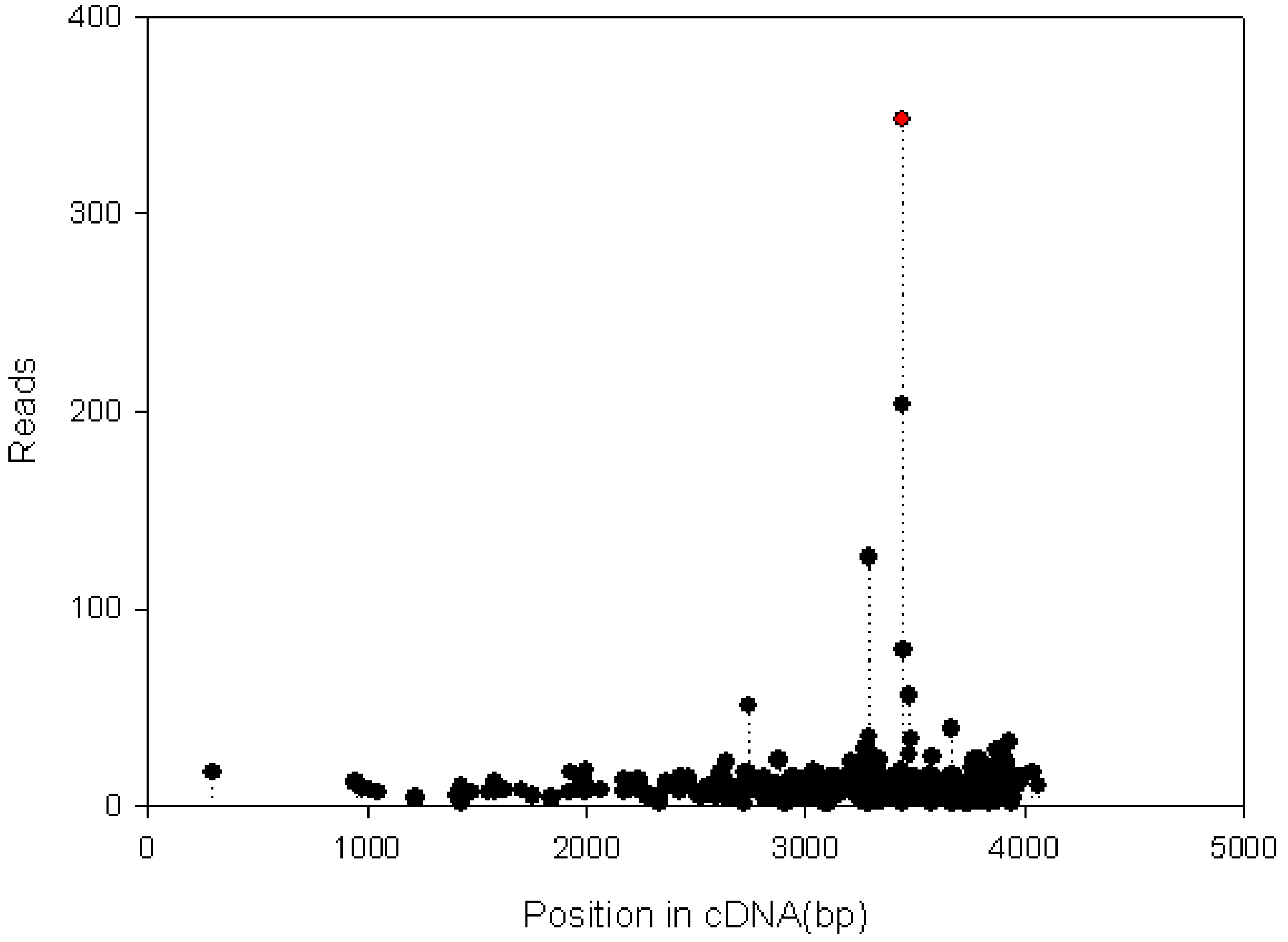


```
5' AGAGAUCAGGCUGGCAGCUUGUAUUU 3'        Cs2g15130.1
    .:::::: :::::::::::
3' AUUCUAGUACGACCGUCGAA------ 5'        Csi-miR167b.3
```

Csi-miR167b.3, target=Cs6g16030.1 gene=Cs6g16030
Category:2
Score=3
Cleavage Site=3357

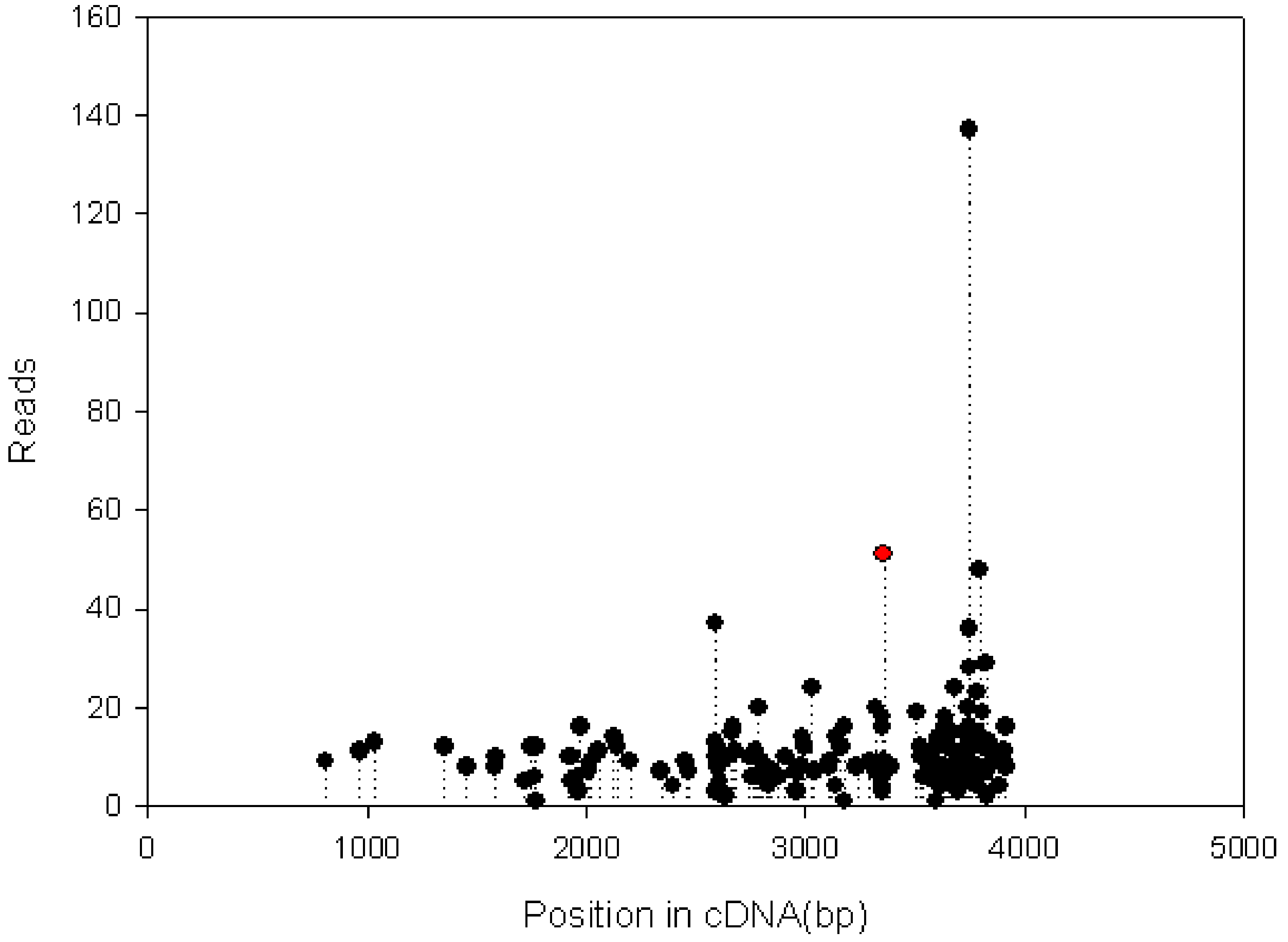


```
5' UUAGAUCAGGCUGGCAGCUUGUAUUC 3'        Cs6g16030.1
   : :::::: ::::::::::
3' AUUCUAGUACGACCGUCGAA------ 5'        Csi-miR167b.3
```

Csi-miR167b.4, target=Cs1g09030.1 gene=Cs1g09030
Category:2
Score=5
Cleavage Site=1248

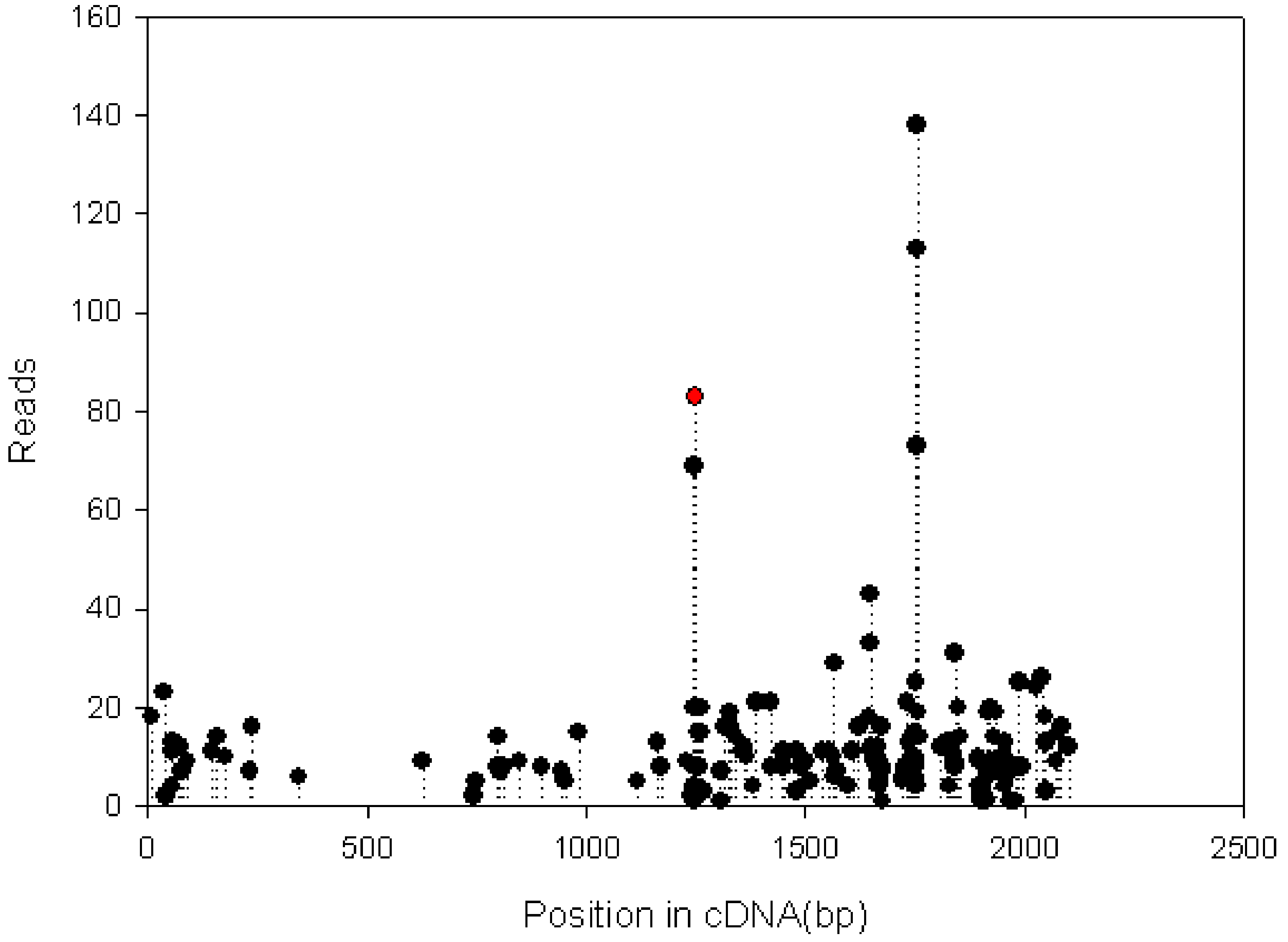


```
5' GUGAAAUCAAGCUGGCAGACUUCGGC 3'        Cs1g09030.1
     .: :::: :::::::: ::::.
3' --UUCUAGUACGACCGUC-GAAGU-- 5'        Csi-miR167b.4
```

Csi-miR167b.4, target=Cs2g09440.1 gene=Cs2g09440
Category:3
Score=4.5
Cleavage Site=3257

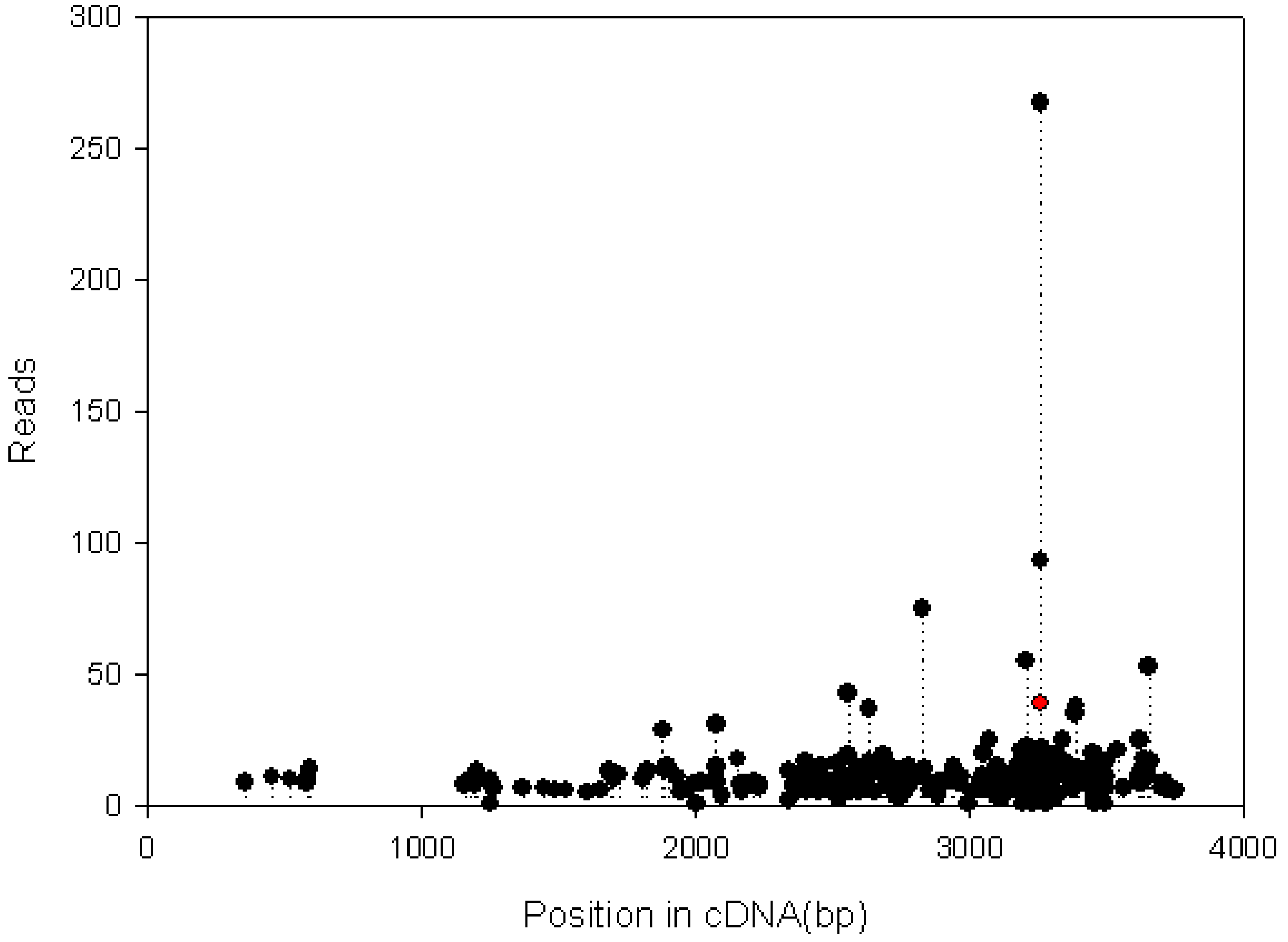


```
5' UGAGAUCAGGCUGGCAGCUUGUAUUC 3'        Cs2g09440.1
    .::::::: :::::::::::  .:
3' -UUCUAGUACGACCGUCGAA-GU--- 5'        Csi-miR167b.4
```

Csi-miR167b.4, target=Cs2g15130.1 gene=Cs2g15130
Category:1
Score=4.5
Cleavage Site=3444

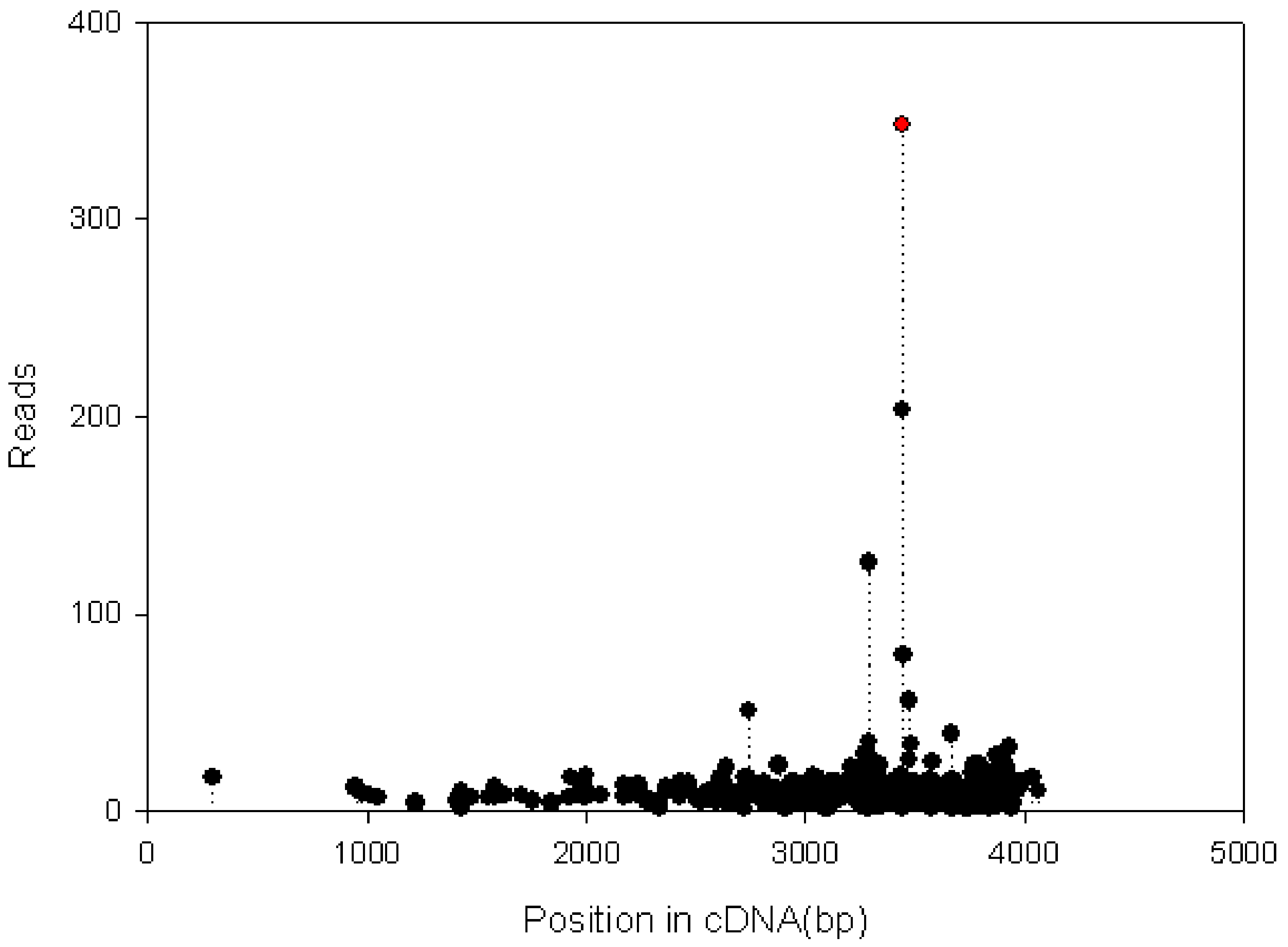


```
5' AGAGAUCAGGCUGGCAGCUUGUAUUU 3'        Cs2g15130.1
    .:::::: ::::::::::: .:
3' -UUCUAGUACGACCGUCGAA-GU--- 5'        Csi-miR167b.4
```

Csi-miR167b.4, target=Cs6g16030.1 gene=Cs6g16030
Category:2
Score=5
Cleavage Site=3357

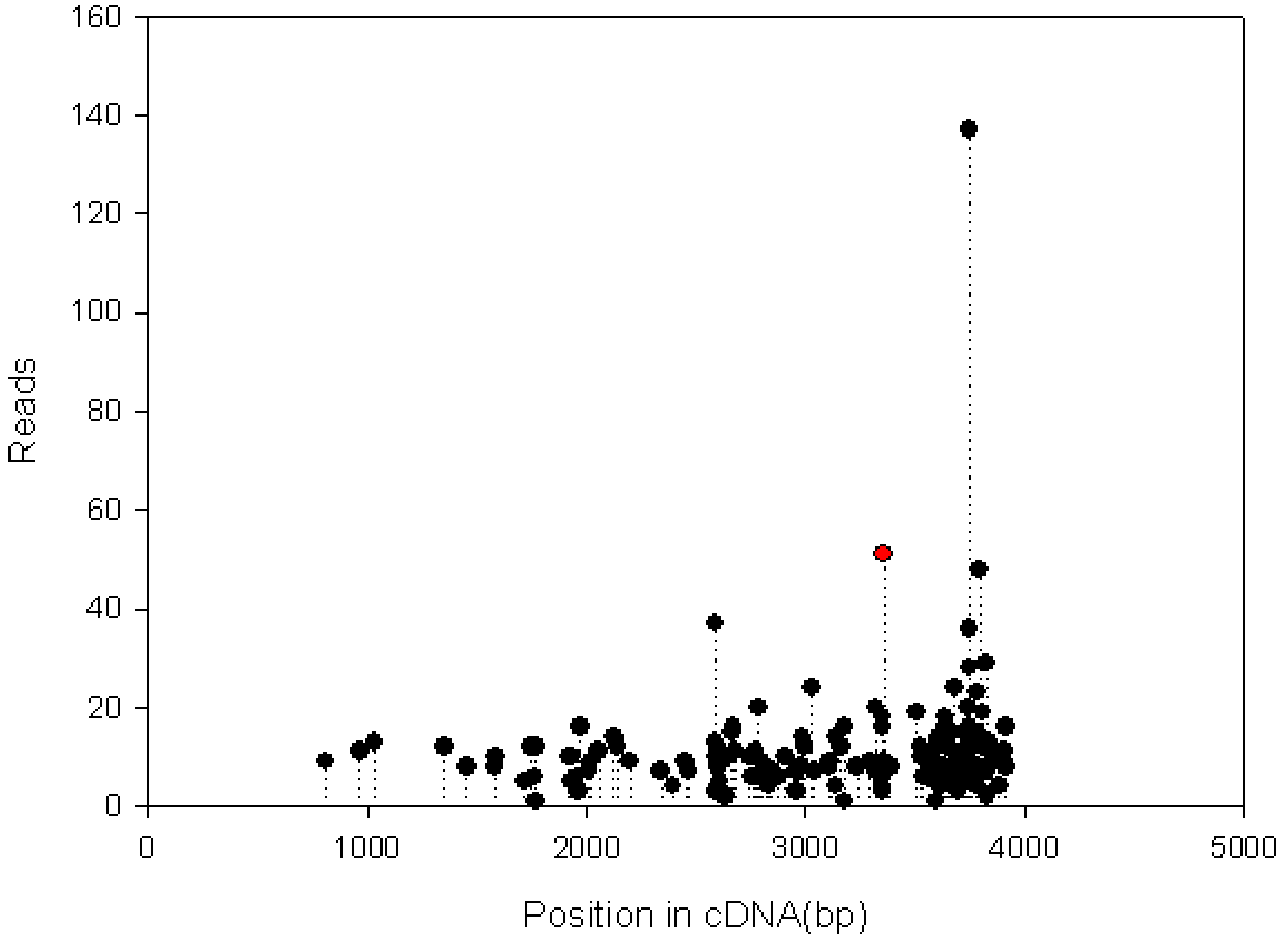


```
5' UUAGAUCAGGCUGGCAGCUUGUAUUC 3'        Cs6g16030.1
    :::::: ::::::::::: .:
3' -UUCUAGUACGACCGUCGAA-GU--- 5'        Csi-miR167b.4
```

Csi-miR167d.1, target=Cs2g09440.1 gene=Cs2g09440
Category:3
Score=5
Cleavage Site=3257

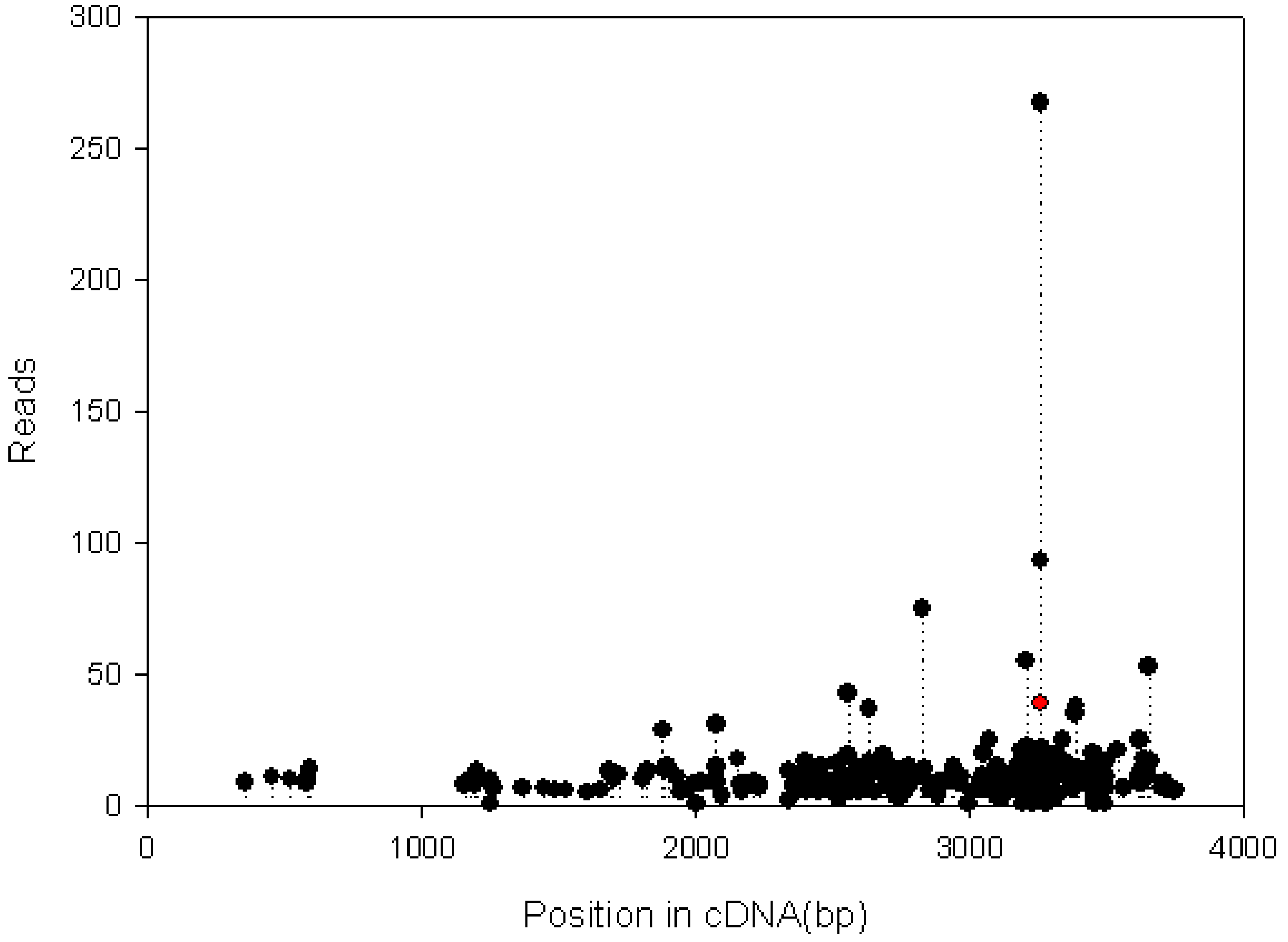


```
5' UGAGAUCAGGCUGGCAGCUUGUAUUC 3'         Cs2g09440.1
   : :::::: :::::::::: .:
3' AGUCUAGUACGACCGUCGAA-GU--- 5'         Csi-miR167d.1
```

Csi-miR167d.1, target=Cs6g16030.1 gene=Cs6g16030
Category:2
Score=4.5
Cleavage Site=3357

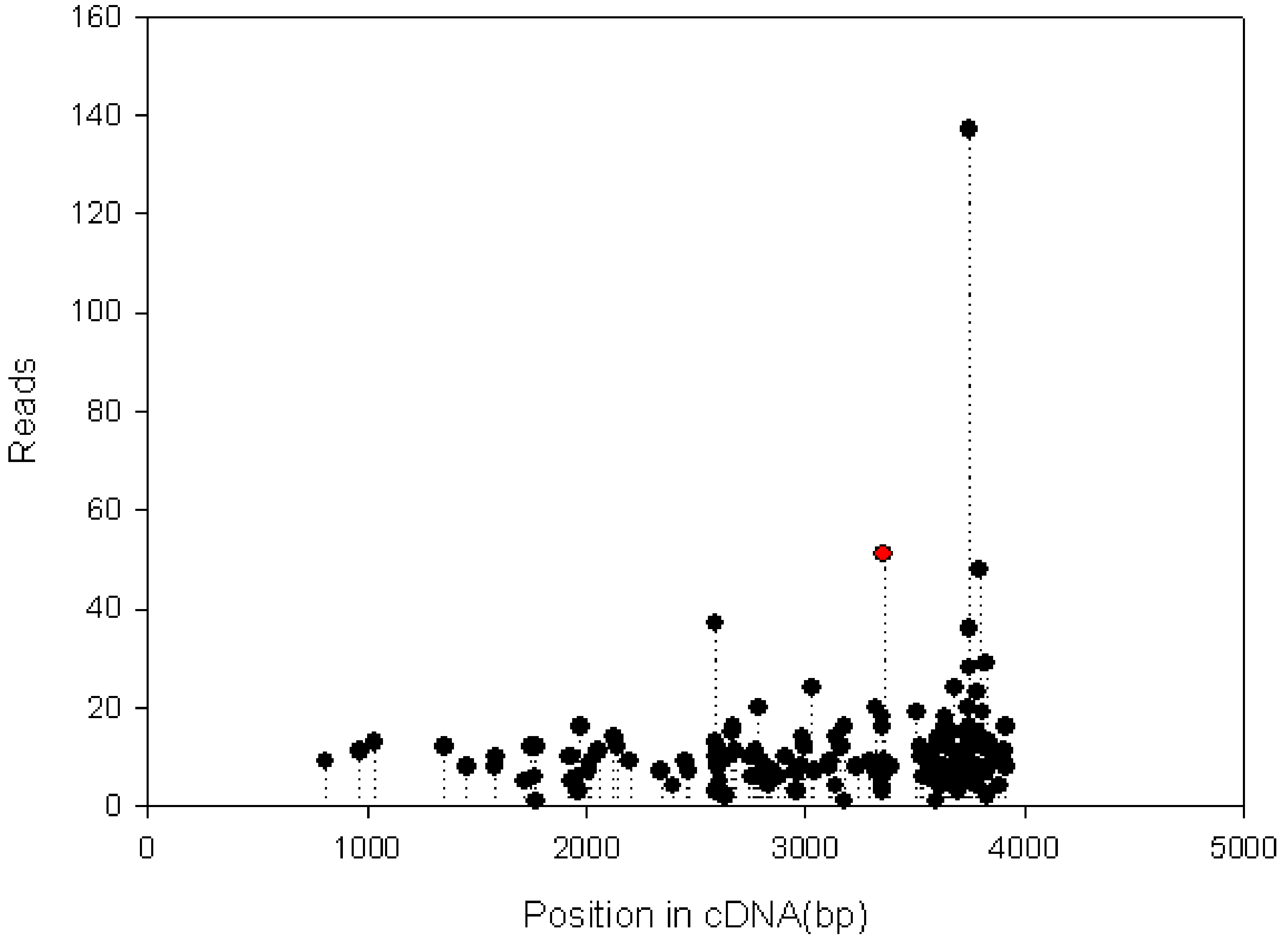


```
5' UUAGAUCAGGCUGGCAGCUUGUAUUC 3'        Cs6g16030.1
   :.:::::: :::::::::: .:
3' AGUCUAGUACGACCGUCGAA-GU--- 5'        Csi-miR167d.1
```

Csi-miR167d.2, target=Cs2g09440.1 gene=Cs2g09440
Category:3
Score=5
Cleavage Site=3257

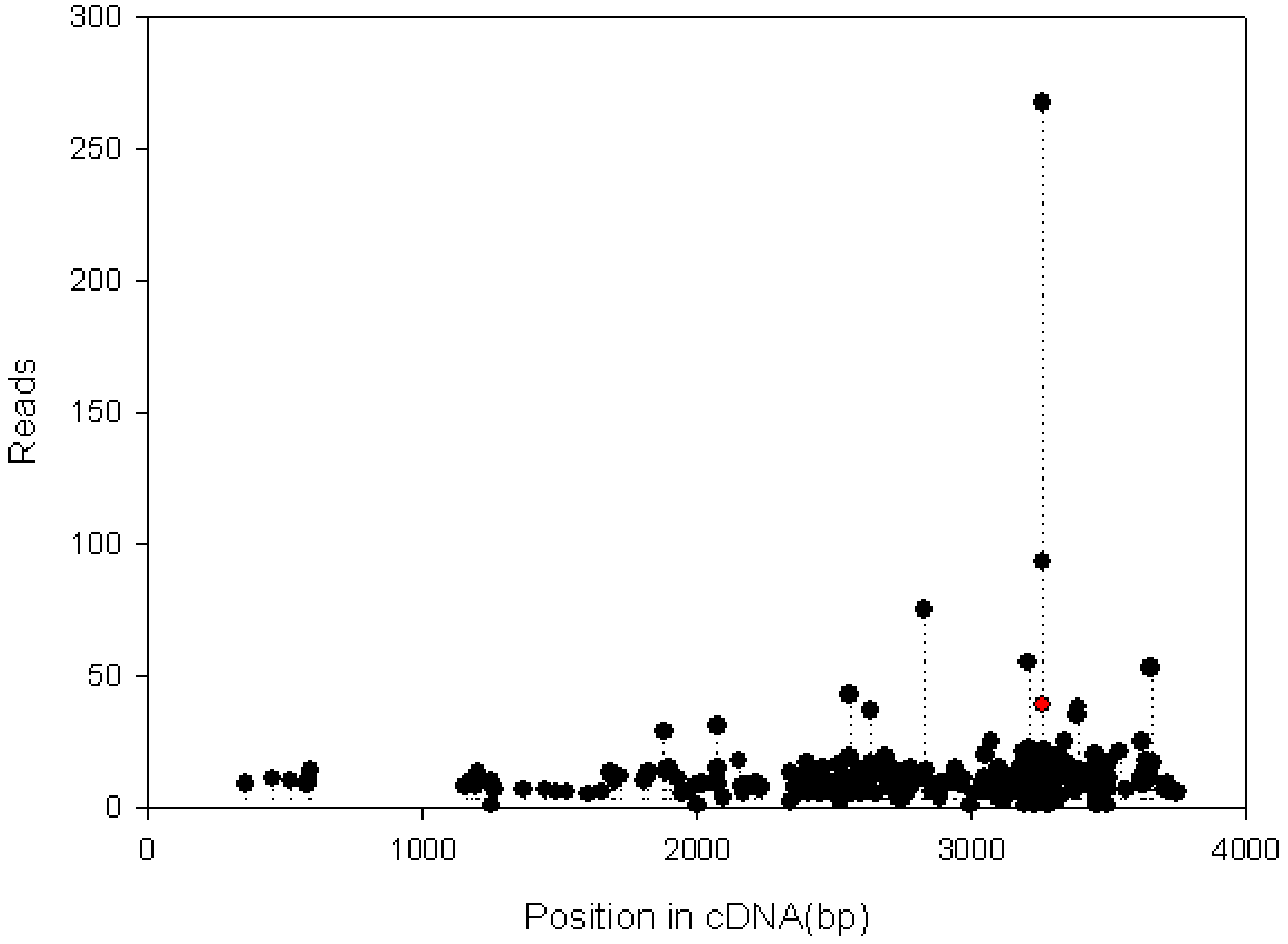


```
5' UGAGAUCAGGCUGGCAGCUUGUAUUC 3'        Cs2g09440.1
    :::::: :::::::::::: .:
3' -GUCUAGUACGACCGUCGAA-GU--- 5'        Csi-miR167d.2
```

Csi-miR167d.2, target=Cs2g15130.1 gene=Cs2g15130
Category:1
Score=5
Cleavage Site=3444

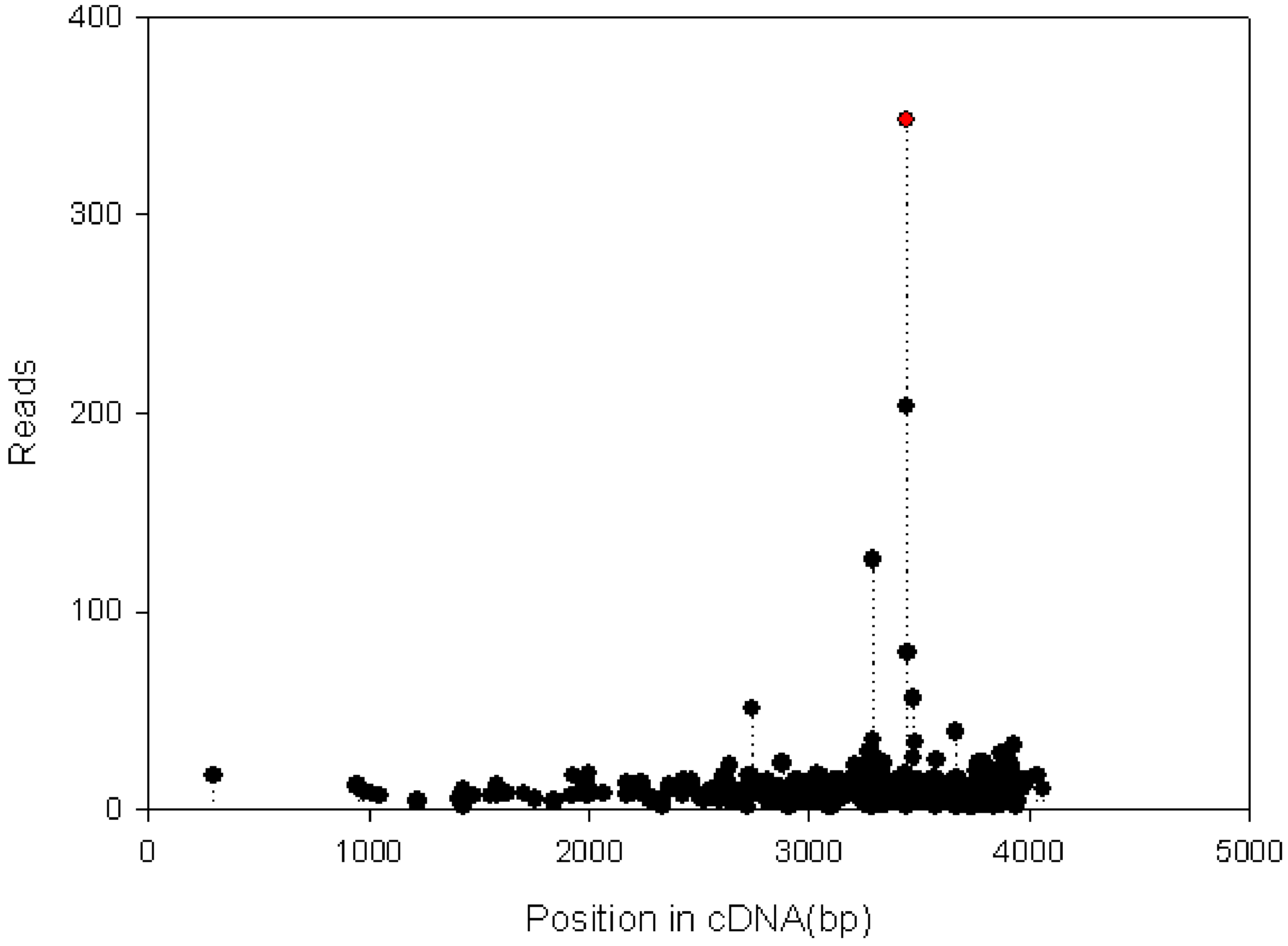


```
5' AGAGAUCAGGCUGGCAGCUUGUAUUU 3'        Cs2g15130.1
     :::::: :::::::::::: .:
3' -GUCUAGUACGACCGUCGAA-GU--- 5'        Csi-miR167d.2
```

Csi-miR167d.2, target=Cs6g16030.1 gene=Cs6g16030
Category:2
Score=4.5
Cleavage Site=3357

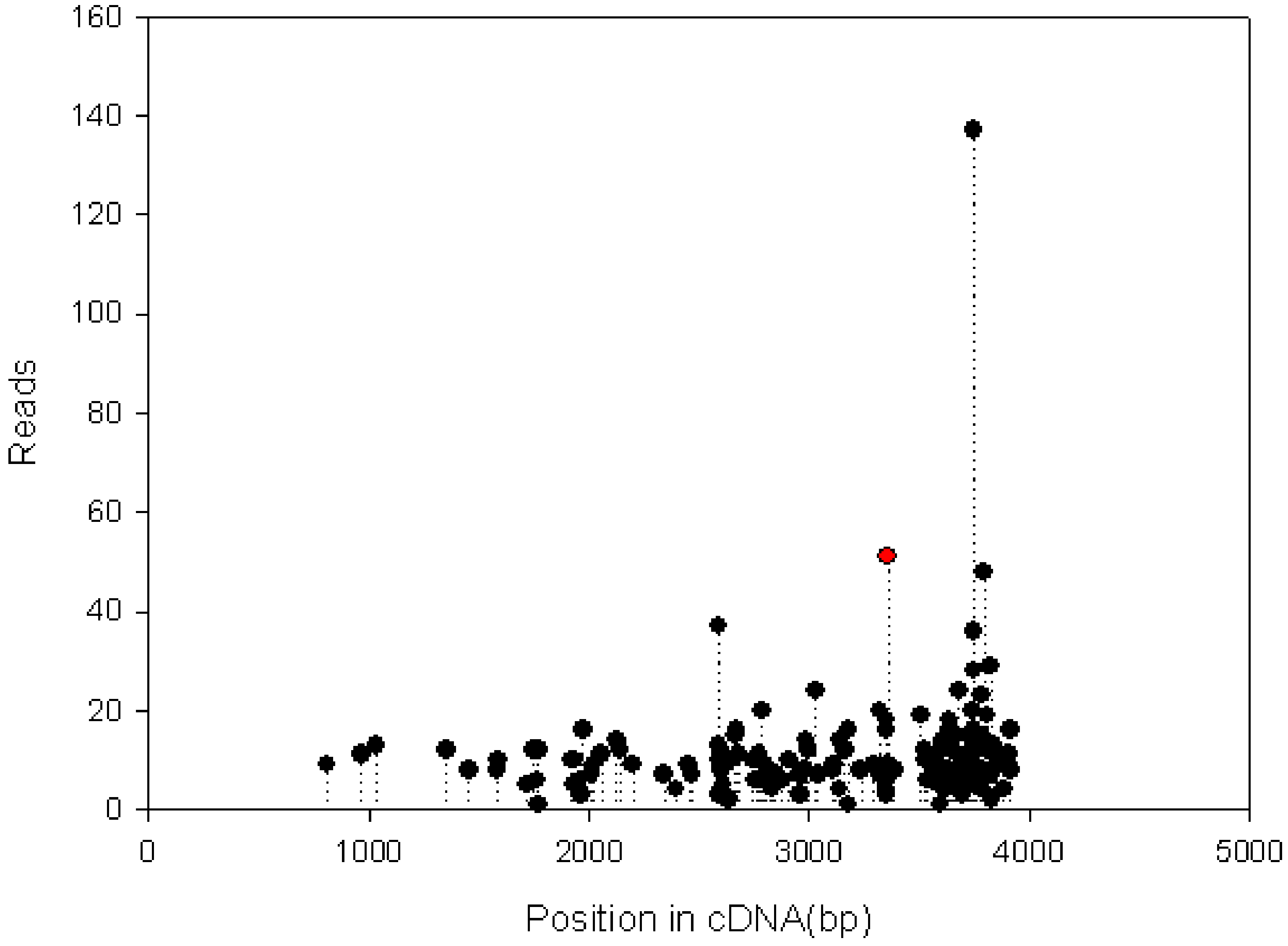


```
5' UUAGAUCAGGCUGGCAGCUUGUAUUC 3'        Cs6g16030.1
    .:::::: :::::::::: .:
3' -GUCUAGUACGACCGUCGAA-GU--- 5'        Csi-miR167d.2
```

Csi-miR168a, target=Cs5g16710.1 gene=Cs5g16710
Category:3
Score=5
Cleavage Site=797

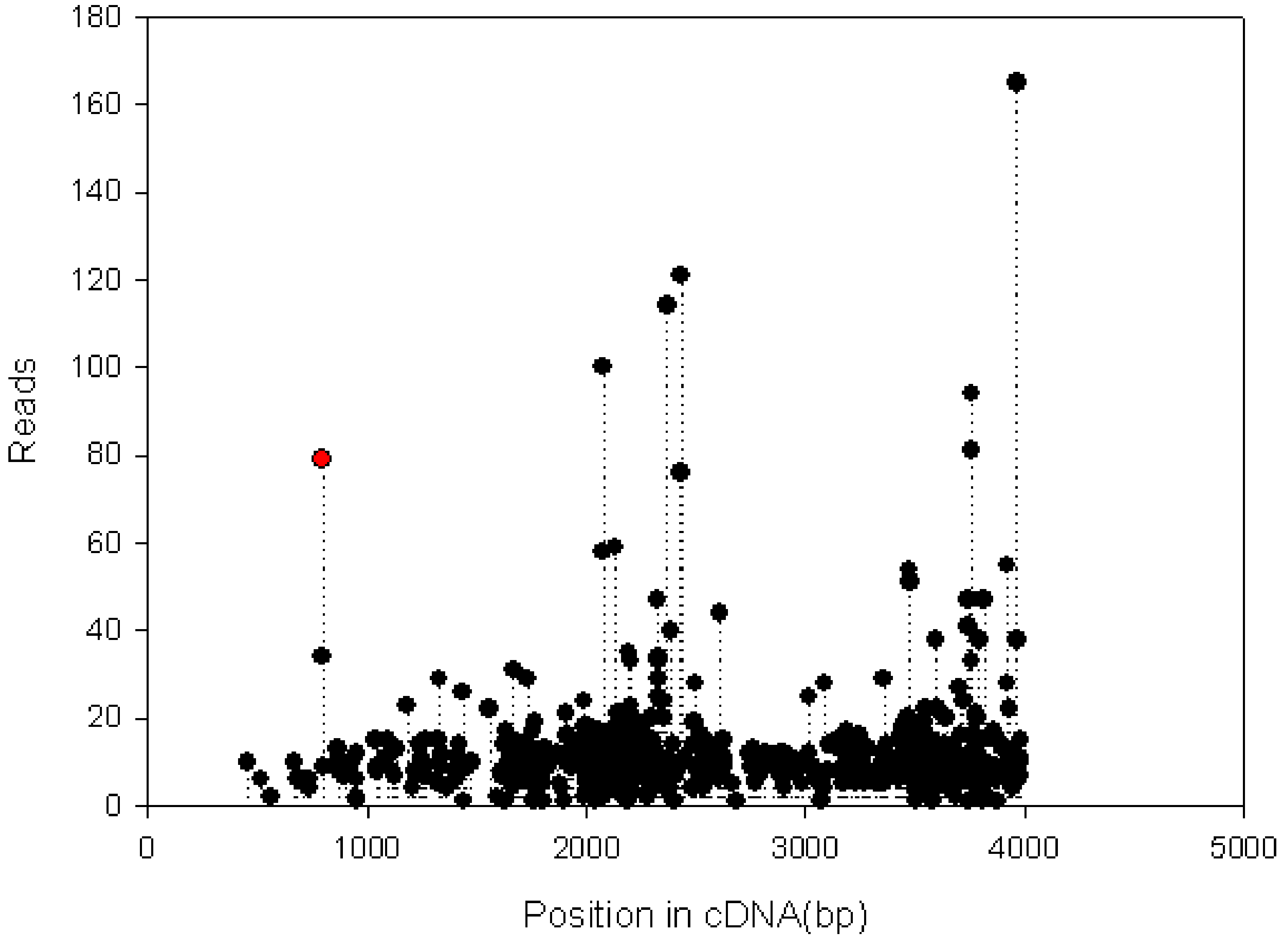


```
5' AUUCCCGAGCUACACCAAGCAACCCC 3'        Cs5g16710.1
    ::::::: :: :::::::: :
3' -AAGGGCUGGACGUGGUUCGCU---- 5'        Csi-miR168a
```

Csi-miR168a, target=Orange1.1t00591.1 gene=Orange1.1t00591
Category:1
Score=3.5
Cleavage Site=500

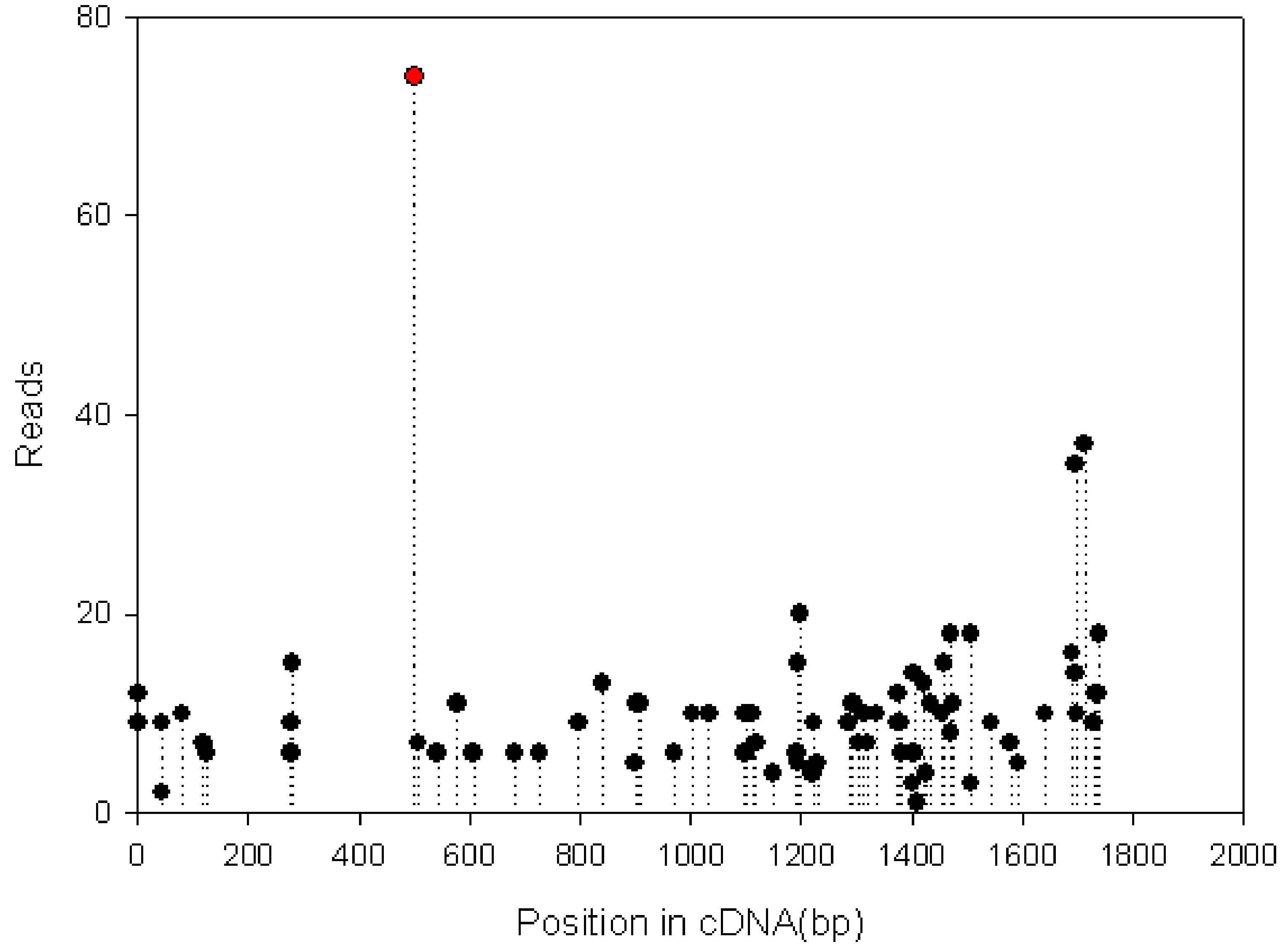


```
5' AUACCCGAGCUGCAUCAAGCGGACAU 3'      Orange1.1t00591.1
    : ::::: :::::.::::::.
3' -AAGGGCUGGACGUGGUUCGCU---- 5'      Csi-miR168a
```

Csi-miR169b.1, target=Cs1g17780.1 gene=Cs1g17780
Category:1
Score=3
Cleavage Site=1200

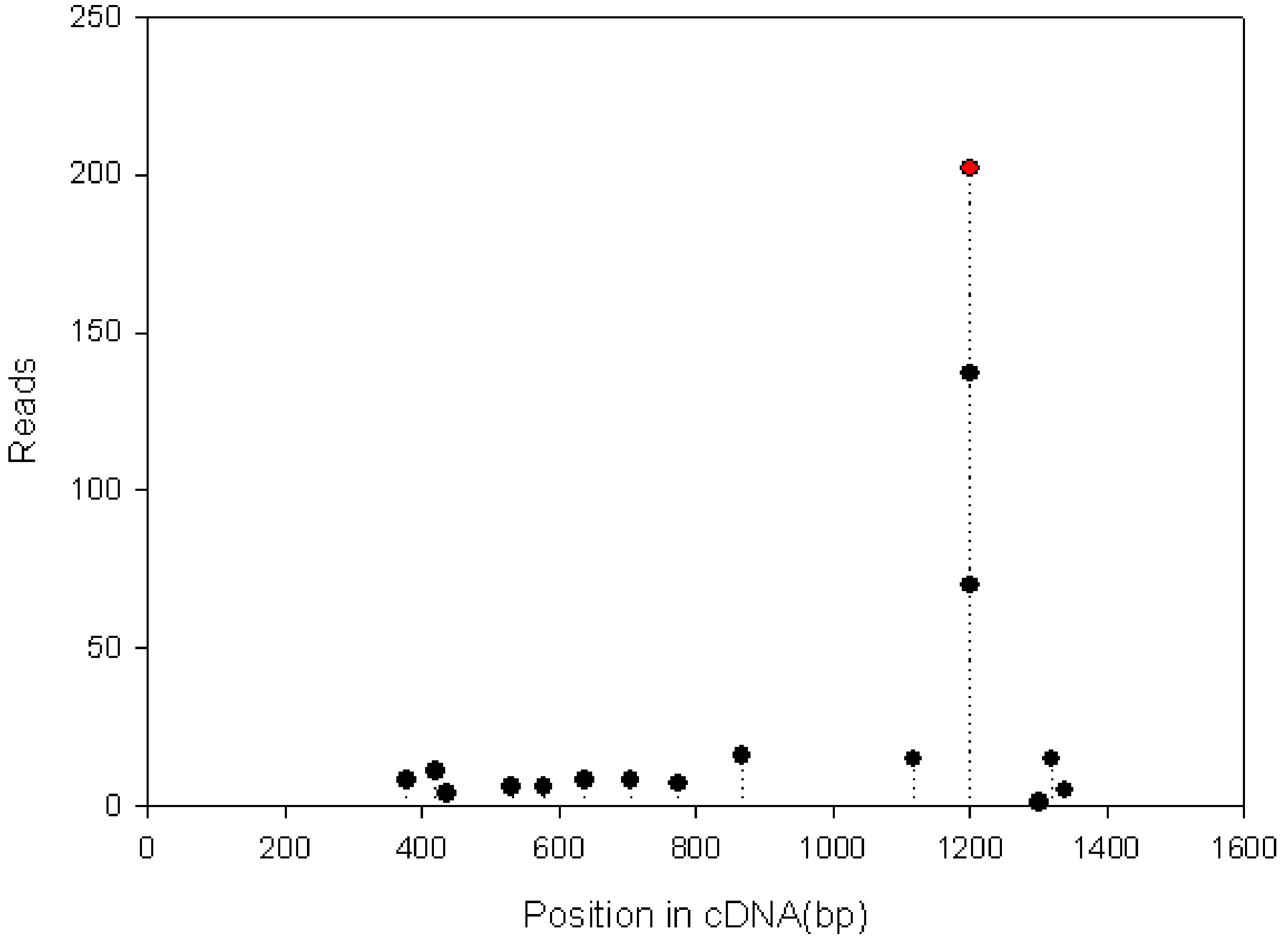


```
5' CUUGGG-AAGUCAUCCUUGGCUCUUUA 3'        Cs1g17780.1
     ..:: :::::::::::::::
3' --GUCCGUUCAGUAGGAACCGAU---- 5'        Csi-miR169b.1
```

Csi-miR169b.1, target=Cs2g30350.1 gene=Cs2g30350
Category:1
Score=4
Cleavage Site=1229

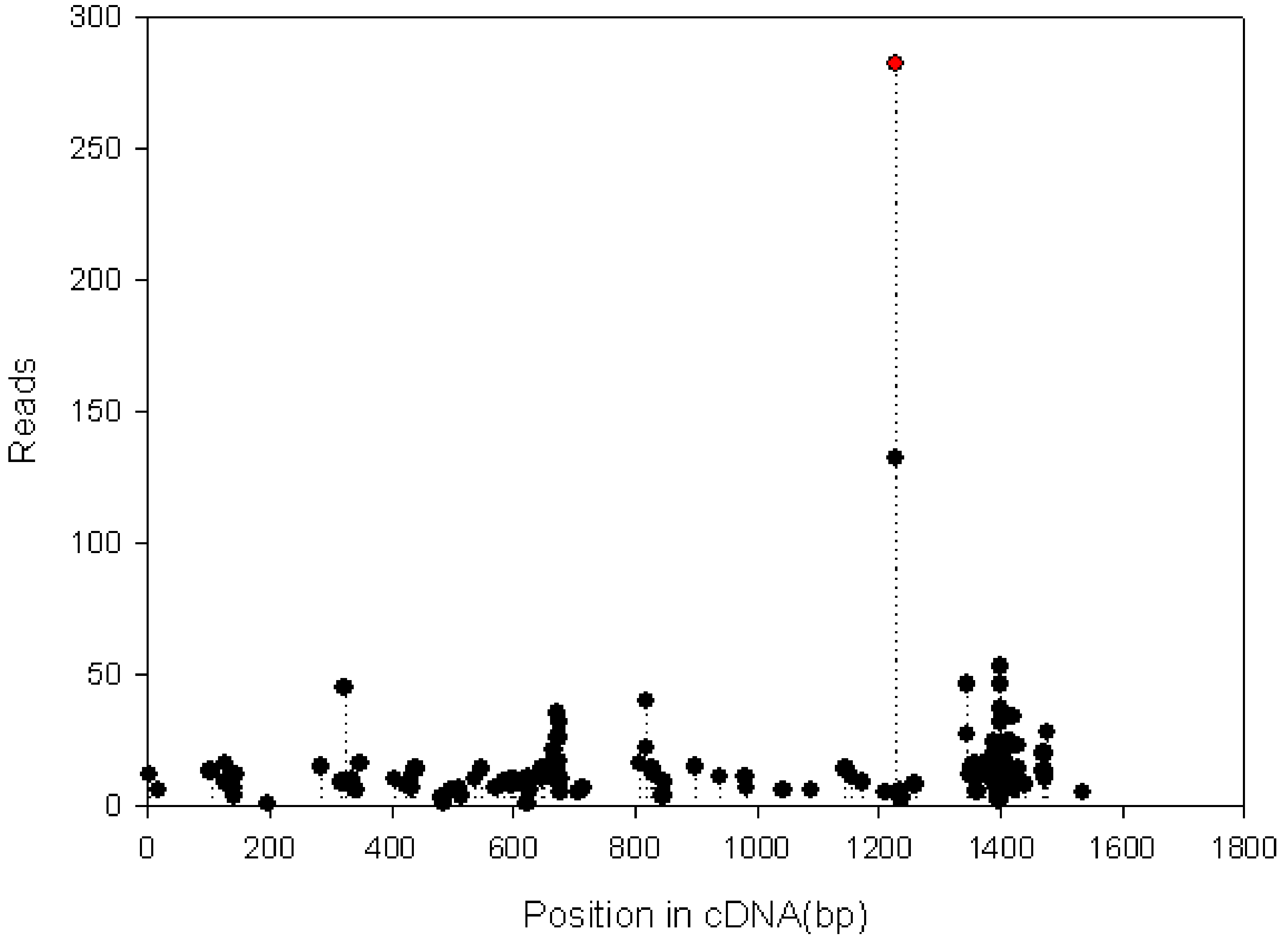


```
5' GCCGGCAAAUCAUUCUUGGCUUCGUU 3'        Cs2g30350.1
    : ::::: ::::.:::::::
3' -GUCCGUUCAGUAGGAACCGAU---- 5'        Csi-miR169b.1
```

Csi-miR169b.1, target=Cs6g13560.1 gene=Cs6g13560
Category:1
Score=3.5
Cleavage Site=1307

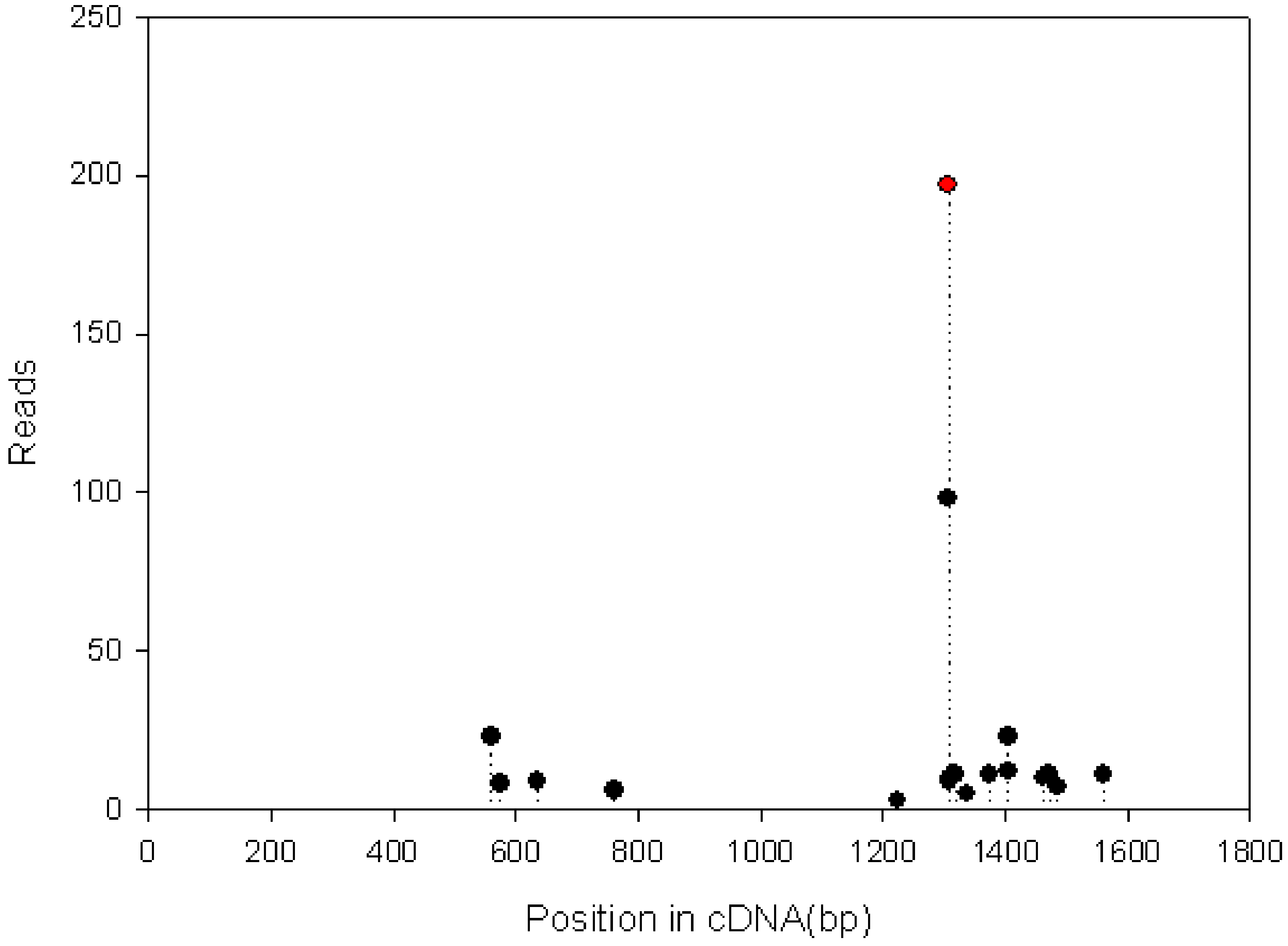


```
5' UUAGGCAAAUCAUUCUUGGCUCAUCU 3'        Cs6g13560.1
    .:::::: ::::.::::::::
3' -GUCCGUUCAGUAGGAACCGAU---- 5'        Csi-miR169b.1
```

Csi-miR169b.1, target=Cs7g01720.1 gene=Cs7g01720
Category:1
Score=3
Cleavage Site=1331

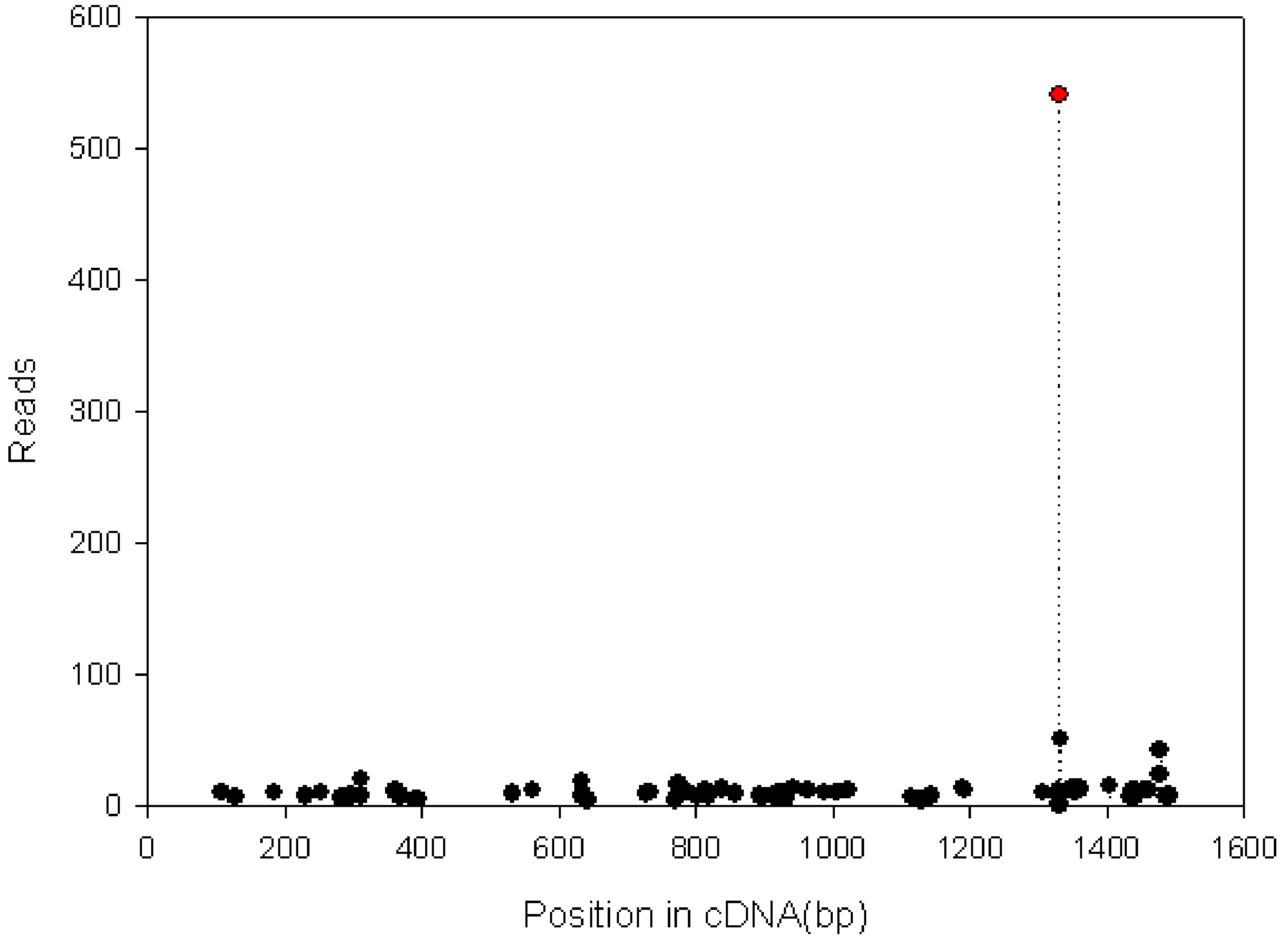


```
5' UCAGGCAAUUCAUUCUUGGCUUUGCA 3'        Cs7g01720.1
    ::::::: ::::.::::::::
3' -GUCCGUUCAGUAGGAACCGAU---- 5'        Csi-miR169b.1
```

Csi-miR169i.1, target=Cs1g17780.1 gene=Cs1g17780
Category:1
Score=3
Cleavage Site=1200

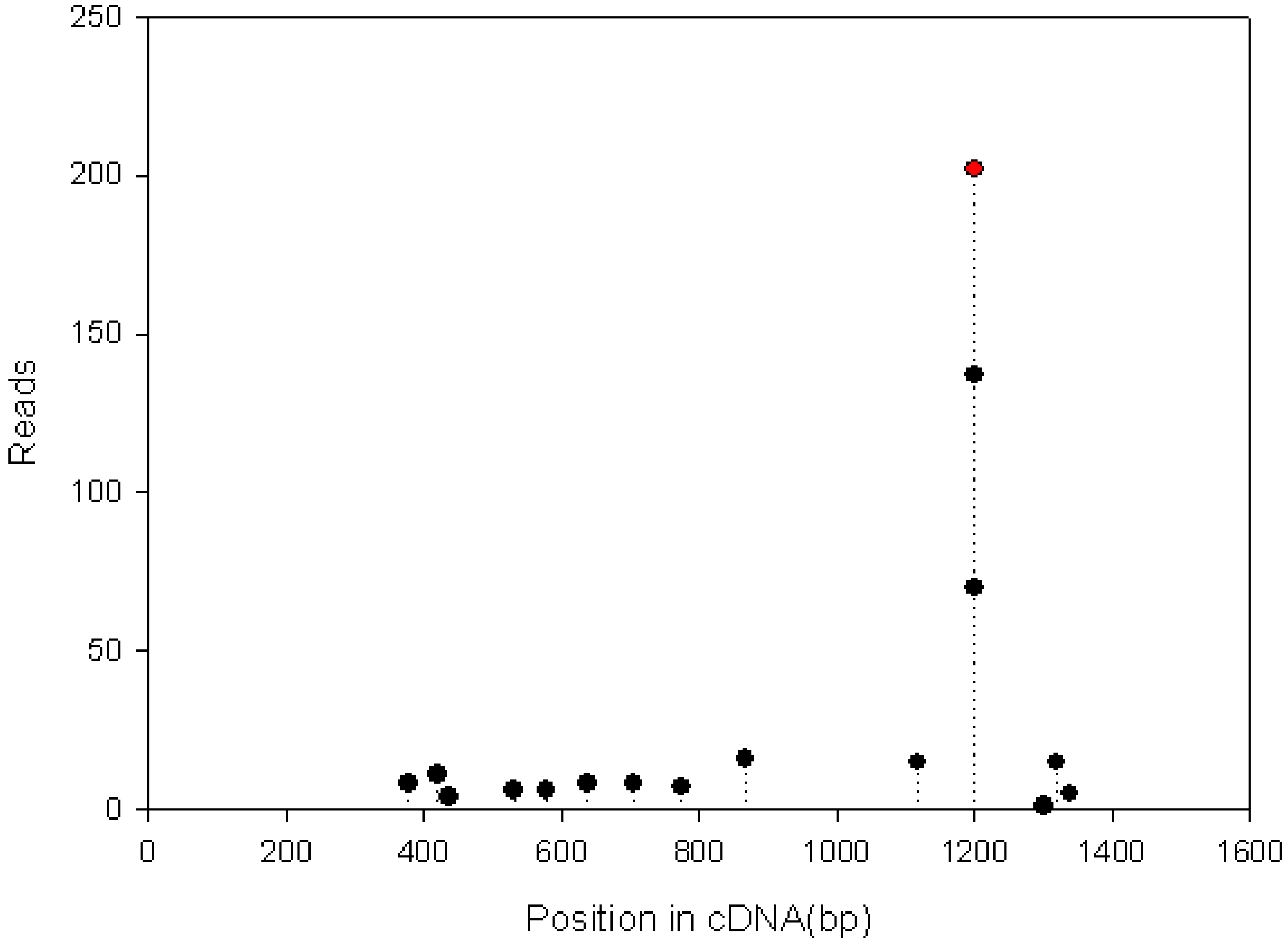


```
5' CUUGGG-AAGUCAUCCUUGGCUCUUUA 3'       Cs1g17780.1
    :..:: ::::::::::::::::
3' -AGUCCGUUCAGUAGGAACCGAU---- 5'       Csi-miR169i.1
```

Csi-miR169i.1, target=Cs2g30350.1 gene=Cs2g30350
Category:1
Score=5
Cleavage Site=1229

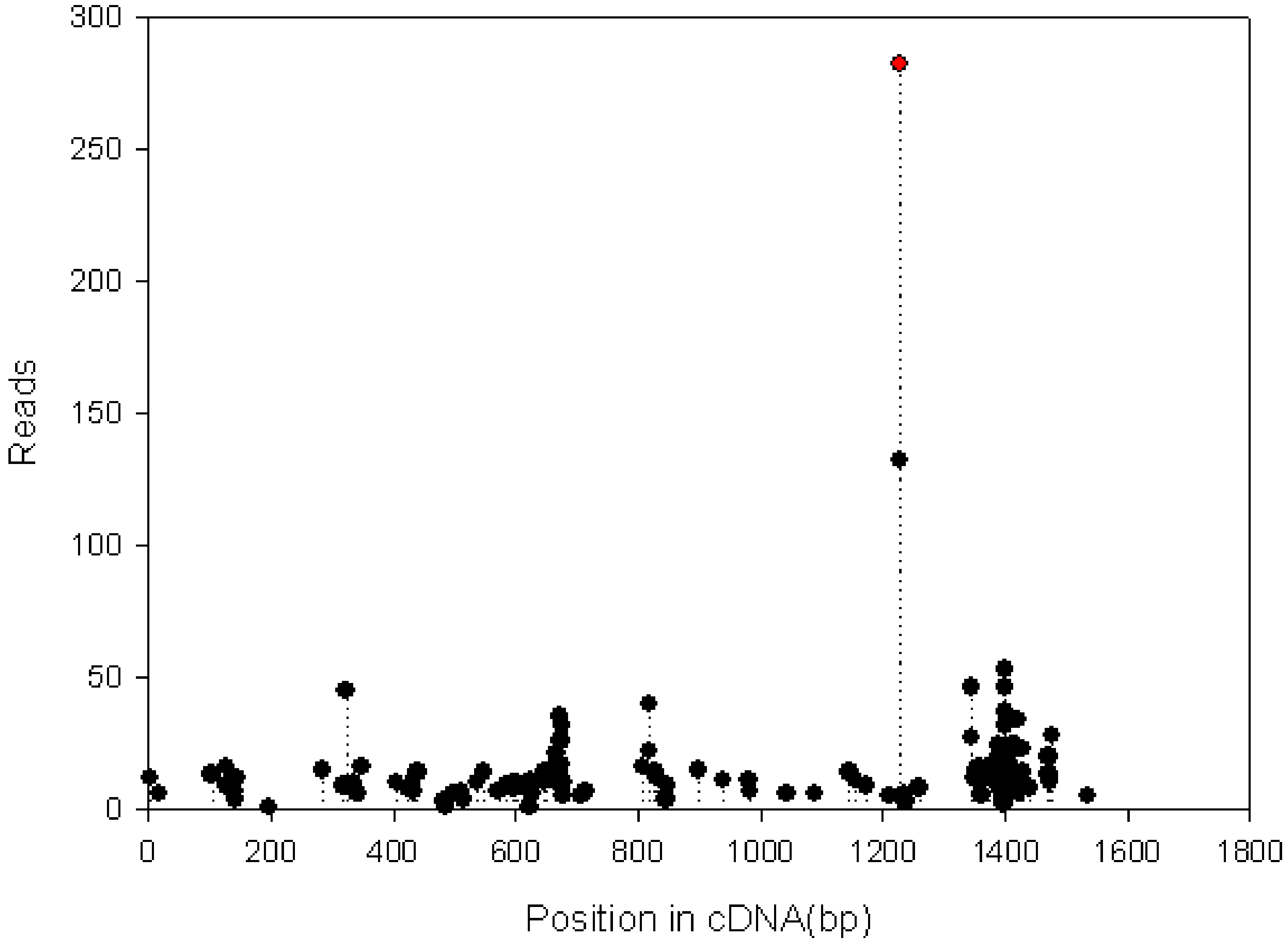


```
5' GCCGGCAAAUCAUUCUUGGCUUCGUU 3'        Cs2g30350.1
    : ::::: ::::.::::::::
3' AGUCCGUUCAGUAGGAACCGAU---- 5'        Csi-miR169i.1
```

Csi-miR169i.1, target=Cs6g13560.1 gene=Cs6g13560
Category:1
Score=3.5
Cleavage Site=1307

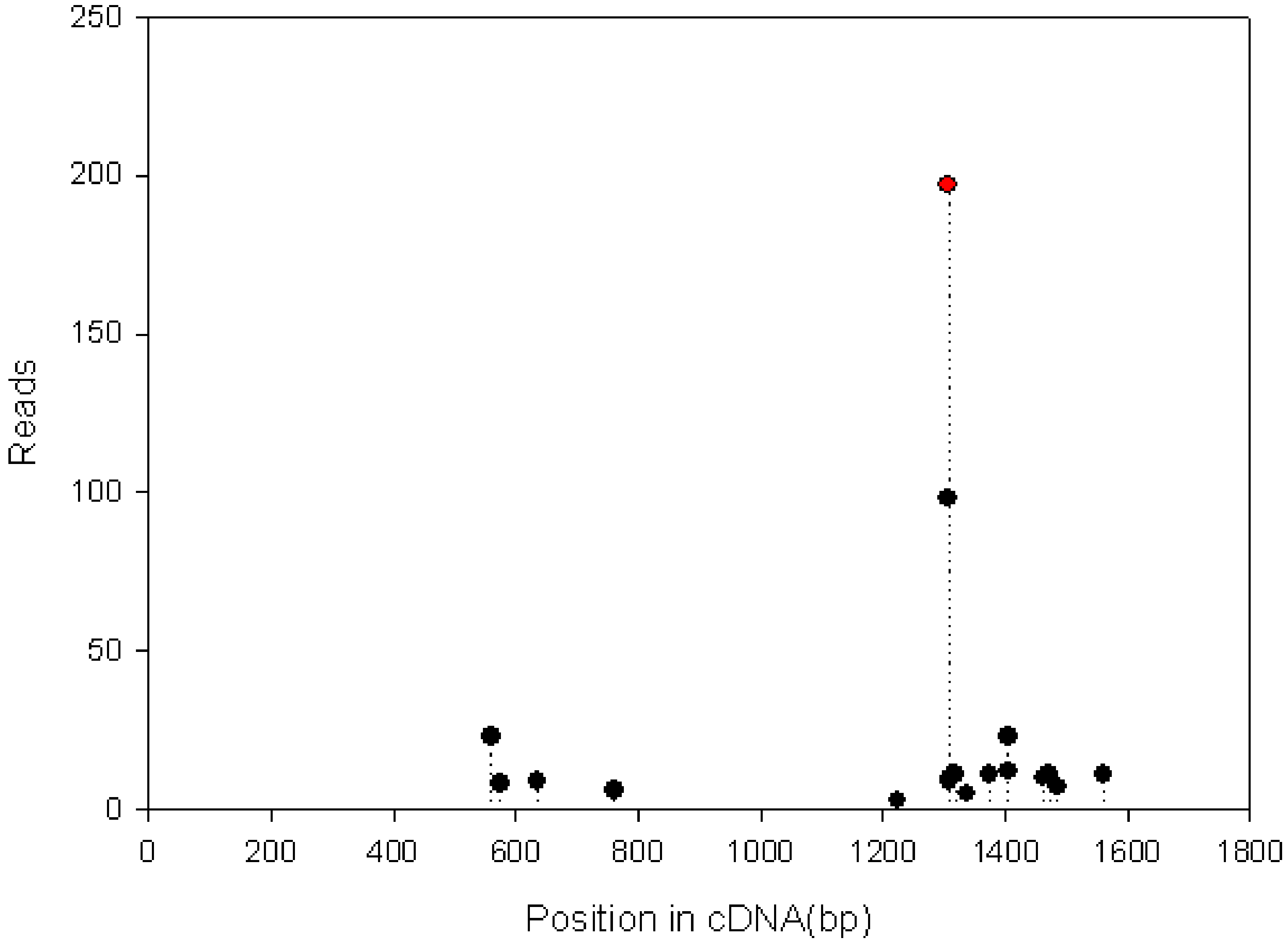


```
5' UUAGGCAAAUCAUUCUUGGCUCAUCU 3'        Cs6g13560.1
   :.:::::: ::::.::::::::
3' AGUCCGUUCAGUAGGAACCGAU---- 5'        Csi-miR169i.1
```

Csi-miR169i.1, target=Cs7g01720.1 gene=Cs7g01720
Category:1
Score=3
Cleavage Site=1331

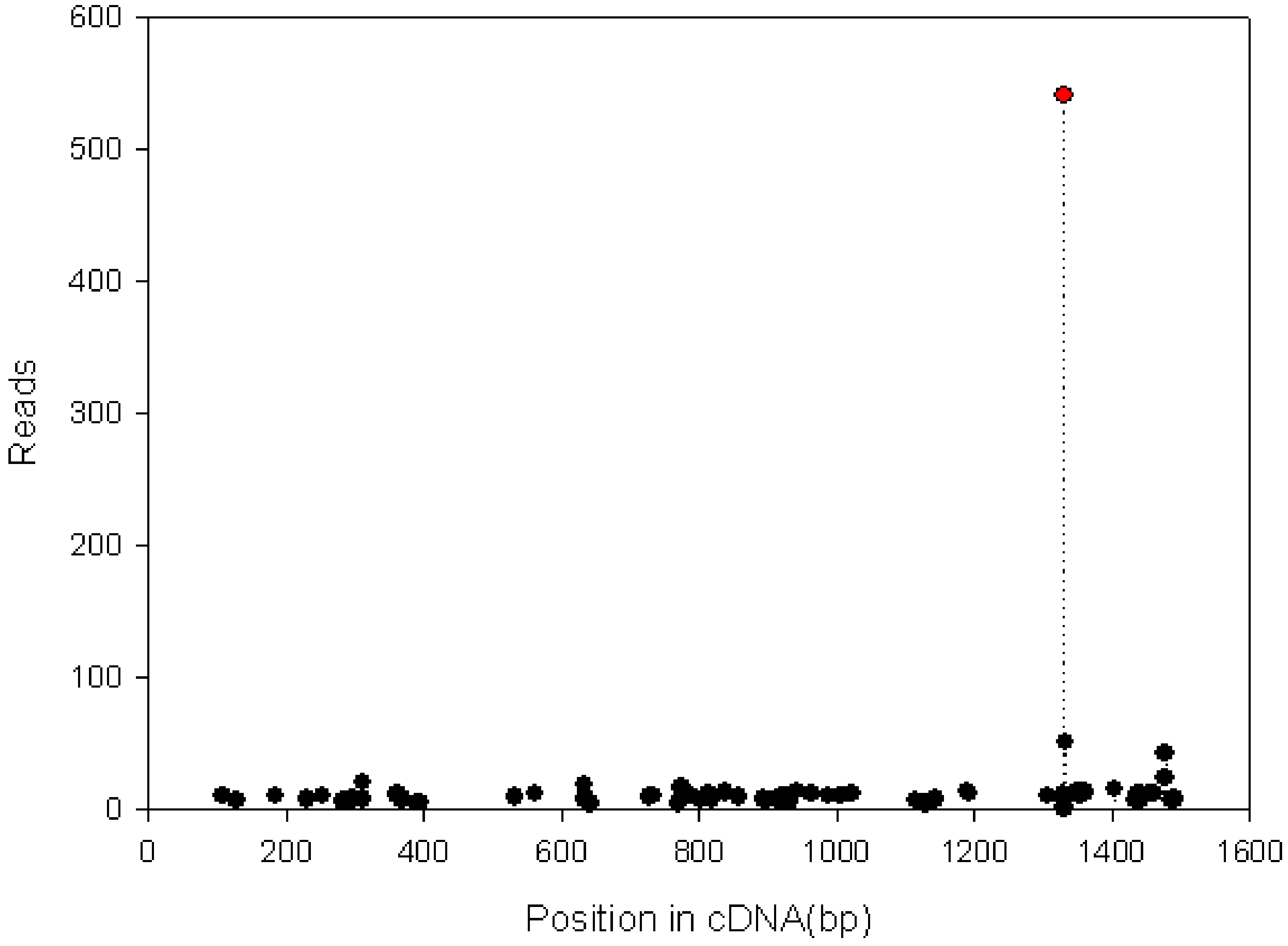


```
5' UCAGGCAAUUCAUUCUUGGCUUUGCA 3'        Cs7g01720.1
   :::::::: :::::.:::::::
3' AGUCCGUUCAGUAGGAACCGAU---- 5'        Csi-miR169i.1
```

Csi-miR169m.1, target=Cs1g17780.1 gene=Cs1g17780
Category:1
Score=3
Cleavage Site=1200

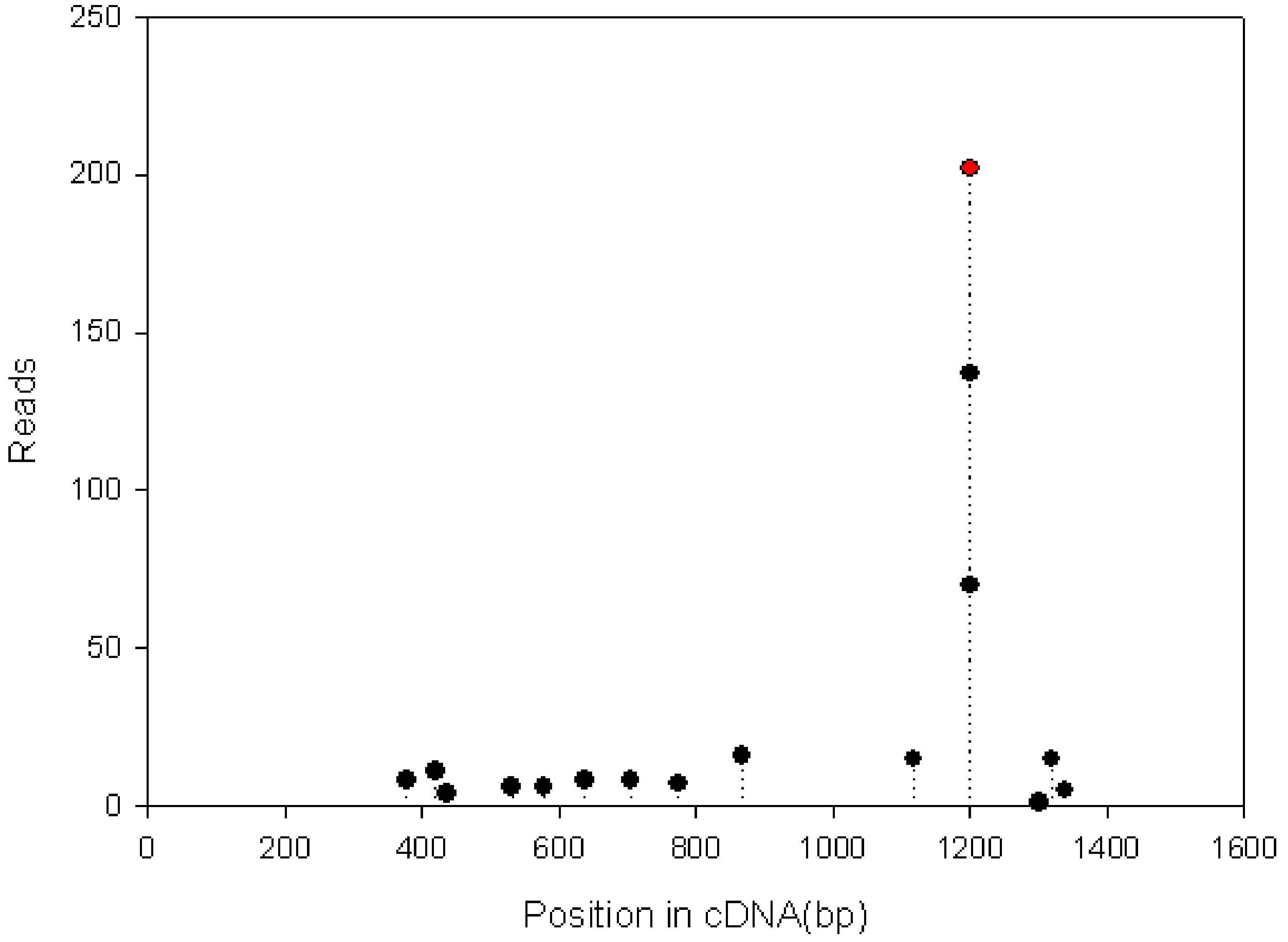


```
5' CUUGGGAAGUCAUCCUUGGCUCUUUA 3'        Cs1g17780.1
    ..:: :::::::::::::::
3' -GGCCGUUCAGUAGGAACCGAC---- 5'        Csi-miR169m.1
```

Csi-miR169m.1, target=Cs2g30350.1 gene=Cs2g30350
Category:1
Score=3
Cleavage Site=1229

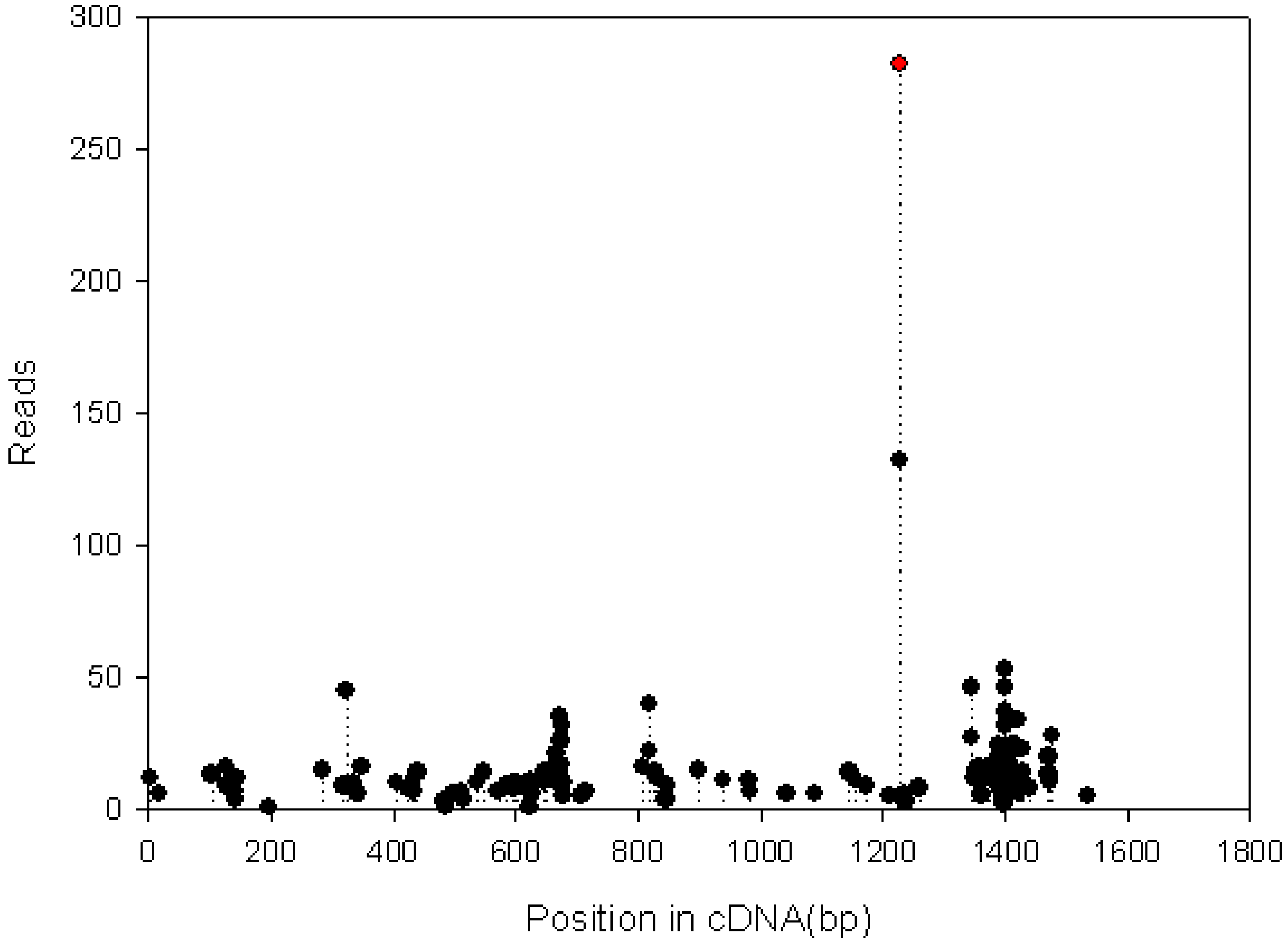


```
5' GCCGGCAAAUCAUUCUUGGCUUCGUU 3'        Cs2g30350.1
    ::::::: ::::.:::::::
3' -GGCCGUUCAGUAGGAACCGAC---- 5'        Csi-miR169m.1
```

Csi-miR169m.1, target=Cs6g13560.1 gene=Cs6g13560
Category:1
Score=4.5
Cleavage Site=1307

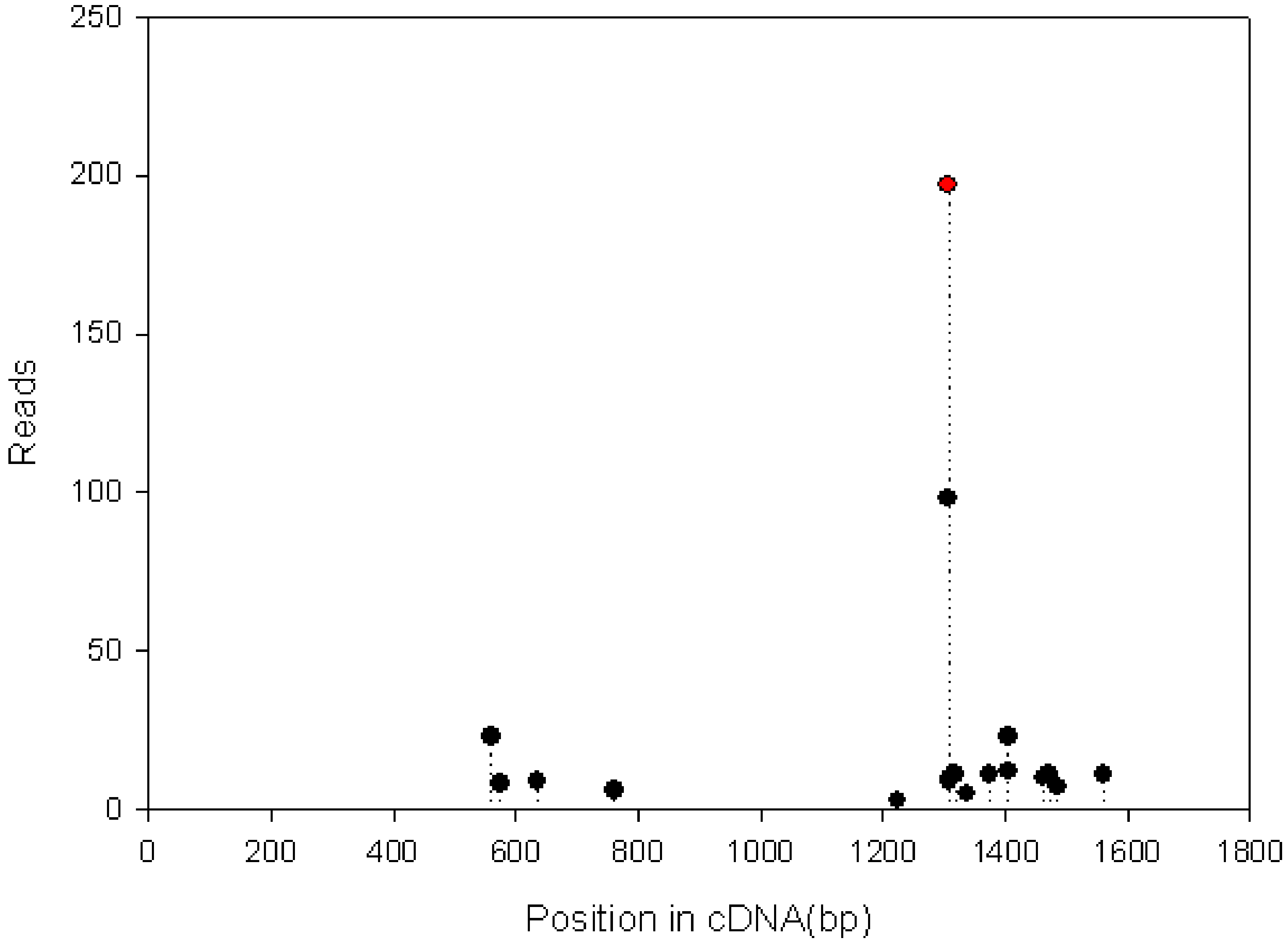


```
5' UUAGGCAAAUCAUUCUUGGCUCAUCU 3'        Cs6g13560.1
    .  :::::  ::::.::::::::
3' -GGCCGUUCAGUAGGAACCGAC---- 5'        Csi-miR169m.1
```

Csi-miR169m.1, target=Cs7g01720.1 gene=Cs7g01720
Category:1
Score=4
Cleavage Site=1331

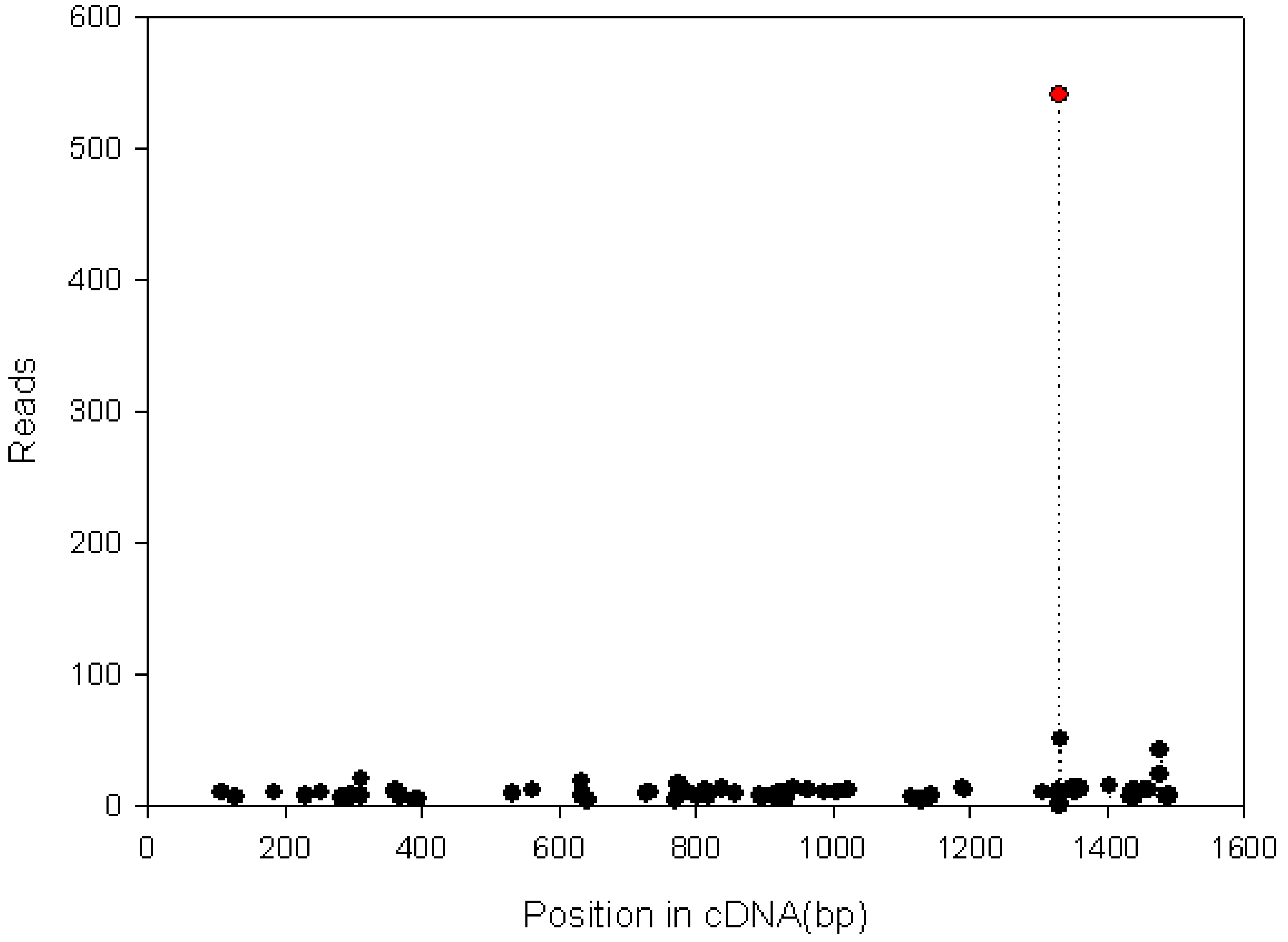


```
5' UCAGGCAAUUCAUUCUUGGCUUUGCA 3'        Cs7g01720.1
    : ::::: ::::.::::::::
3' -GGCCGUUCAGUAGGAACCGAC---- 5'        Csi-miR169m.1
```

Csi-miR169m.2, target=Cs1g17780.1 gene=Cs1g17780
Category:1
Score=3
Cleavage Site=1200

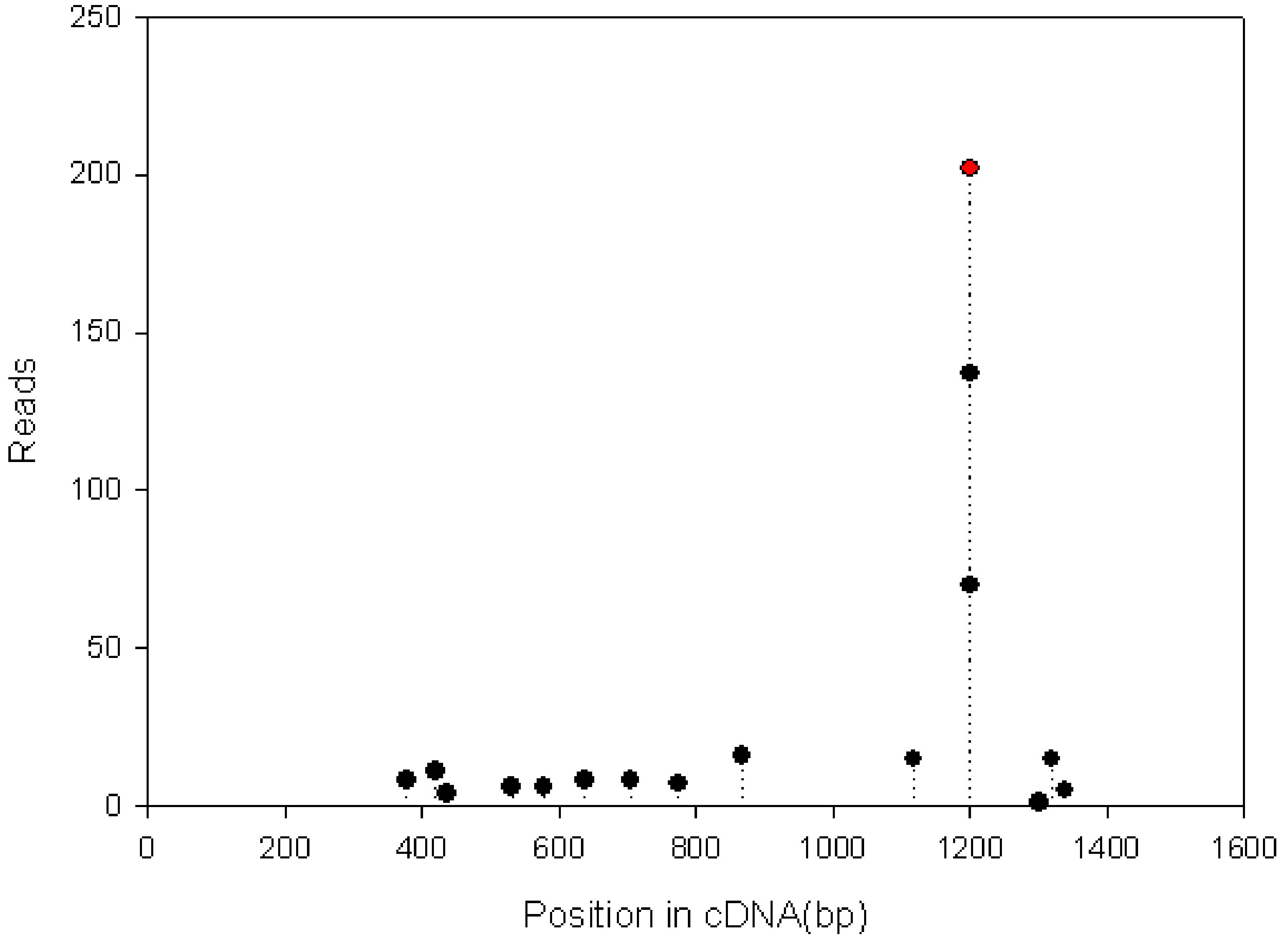


```
5' CUUGGGAAGUCAUCCUUGGCUCUUUA 3'        Cs1g17780.1
     ..:: ::::::::::::::::
3' CGGCCGUUCAGUAGGAACCGA----- 5'        Csi-miR169m.2
```

Csi-miR169m.2, target=Cs2g30350.1 gene=Cs2g30350
Category:1
Score=3
Cleavage Site=1229

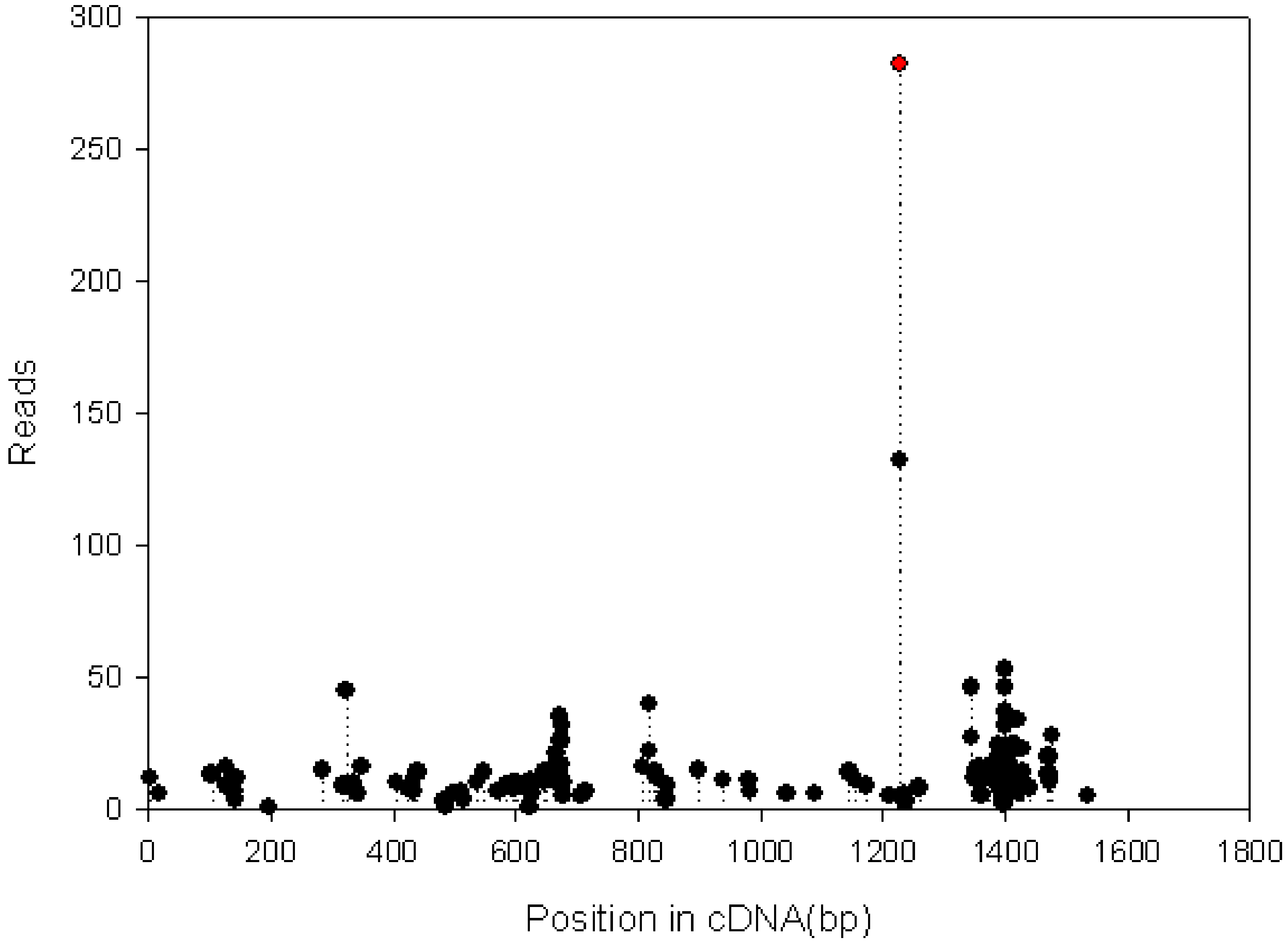


```
5' GCCGGCAAAUCAUUCUUGGCUUCGUU 3'        Cs2g30350.1
   :::::::: ::::.::::::::
3' CGGCCGUUCAGUAGGAACCGA----- 5'        Csi-miR169m.2
```

Csi-miR169m.2, target=Cs7g01720.1 gene=Cs7g01720
Category:1
Score=5
Cleavage Site=1331

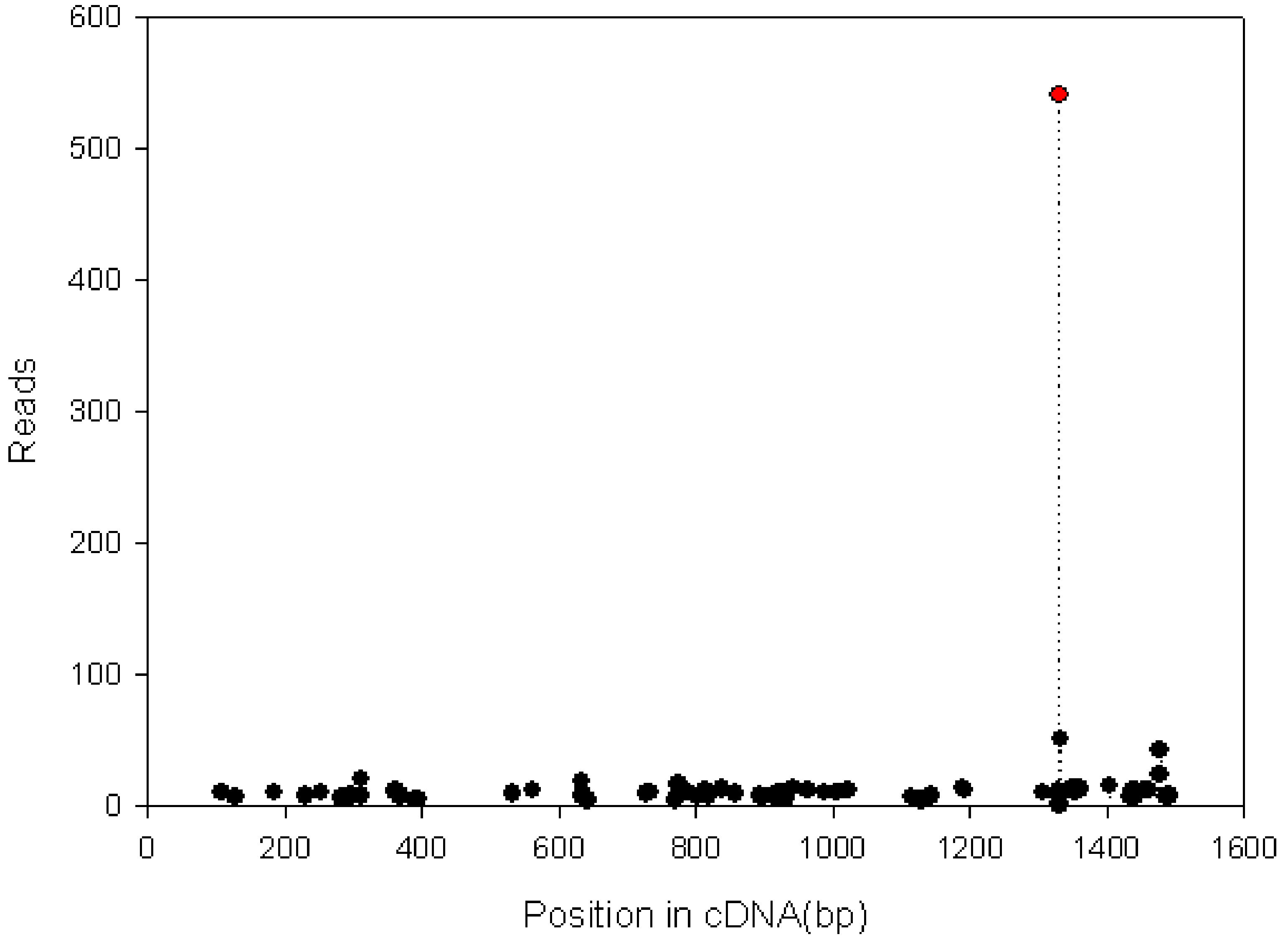


```
5' UCAGGCAAUUCAUUCUUGGCUUUGCA 3'        Cs7g01720.1
    :  :::::  :::::.::::::::
3' CGGCCGUUCAGUAGGAACCGA----- 5'        Csi-miR169m.2
```

Csi-miR169m.3, target=Cs1g17780.1 gene=Cs1g17780
Category:1
Score=4
Cleavage Site=1200

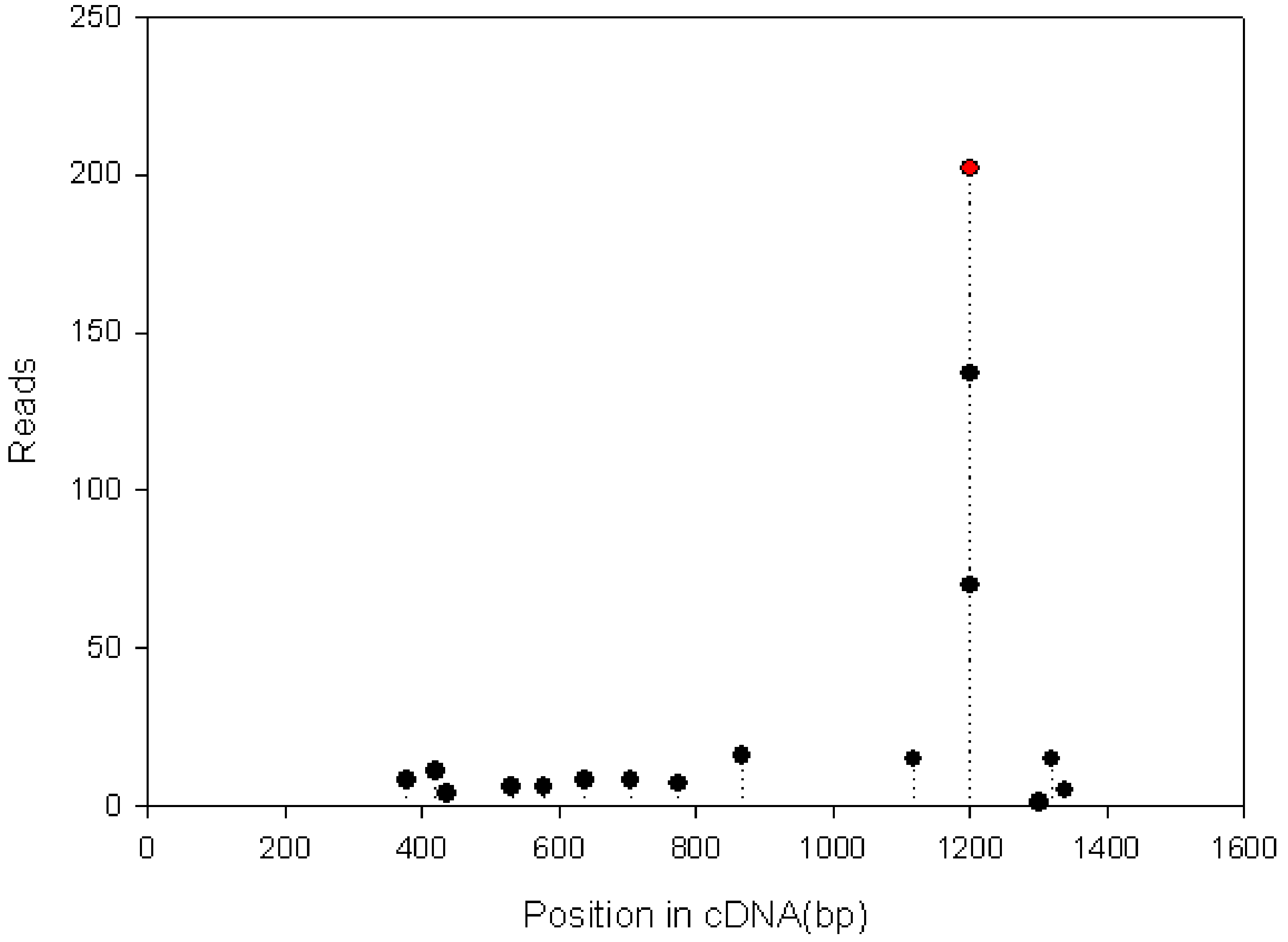


```
5' CUUGGGAAGUCAUCCUUGGCUCUUUA 3'        Cs1g17780.1
     .:: ::::::::::::::::: .
3' --GCCGUUCAGUAGGAACCGACG--- 5'        Csi-miR169m.3
```

Csi-miR169m.3, target=Cs2g30350.1 gene=Cs2g30350
Category:1
Score=4
Cleavage Site=1229

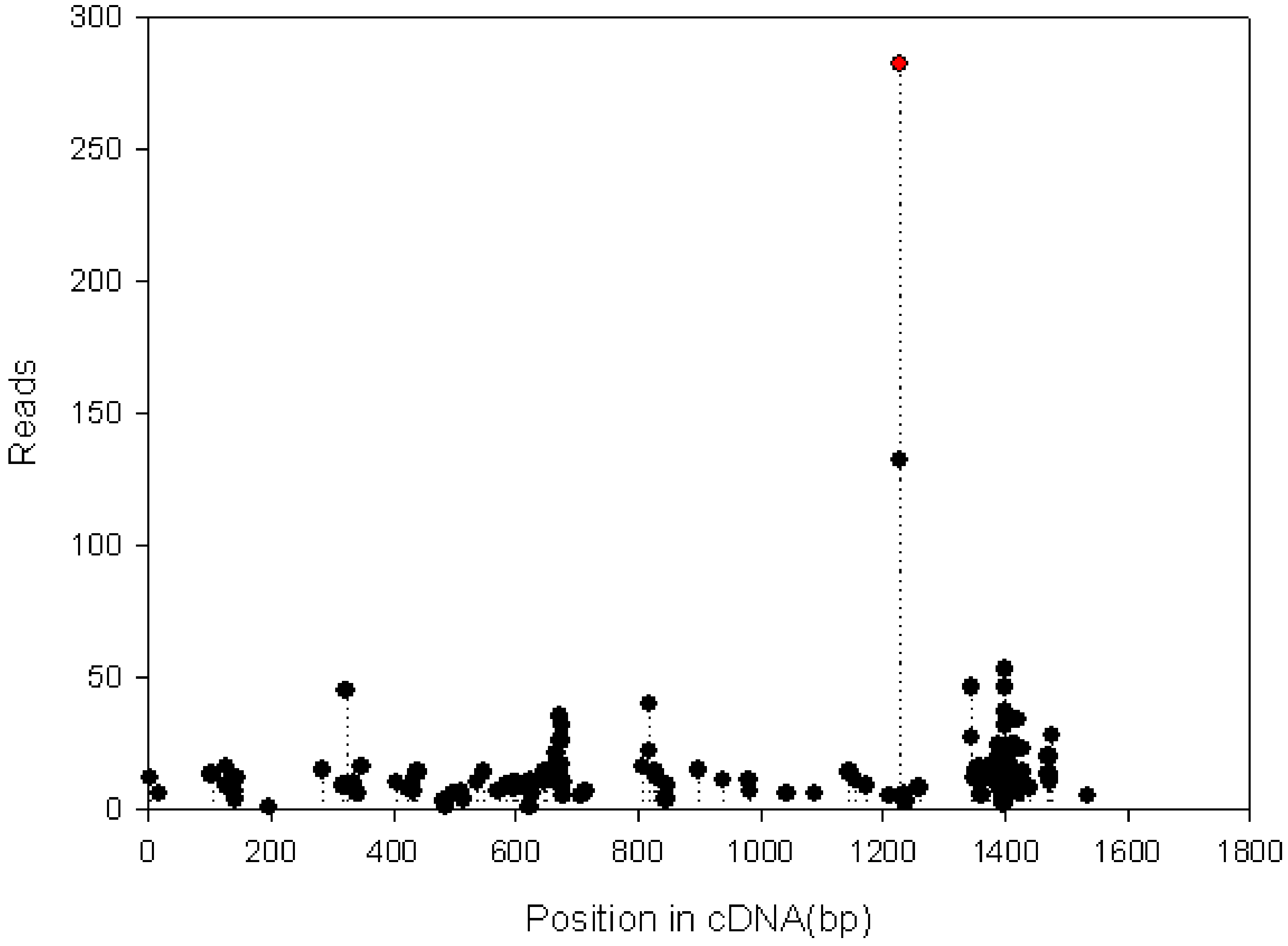


```
5' GCCGGCAAAUCAUUCUUGGCUUCGUU 3'        Cs2g30350.1
     :::::: ::::.:::::::: :
3' --GCCGUUCAGUAGGAACCGACG--- 5'        Csi-miR169m.3
```

Csi-miR171d, target=Cs5g08980.1 gene=Cs5g08980
Category:1
Score=2.5
Cleavage Site=1697

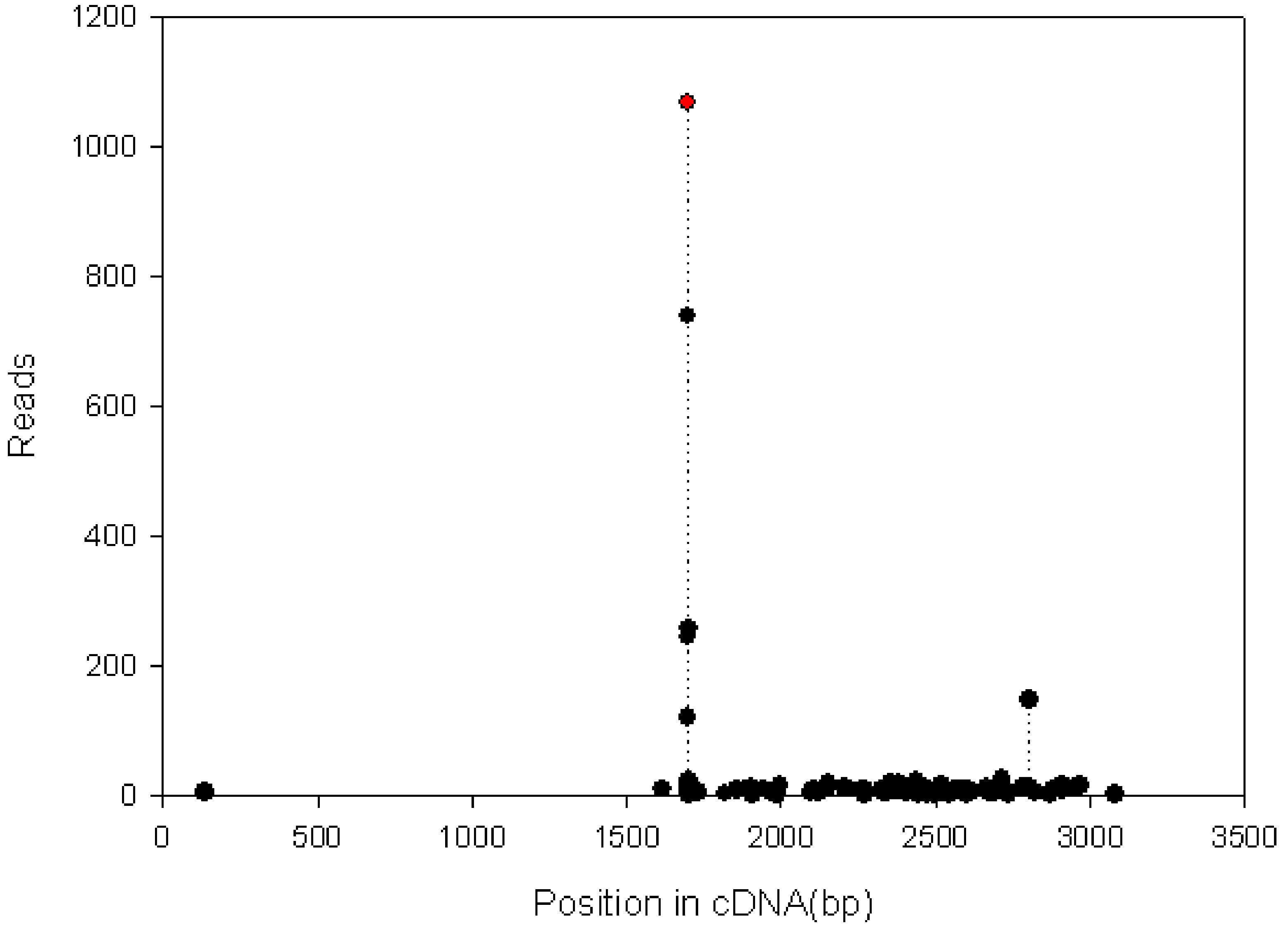


```
5' AAGGGAUAUUGGCGCGGCUCAAUCAC 3'        Cs5g08980.1
     :.:::::::.:::::::::::
3' -CCUCUAUAACUGCGCCGAGUU---- 5'        Csi-miR171d
```

Csi-miR171d, target=Orange1.1t00199.1 gene=Orange1.1t00199
Category:1
Score=2.5
Cleavage Site=1612

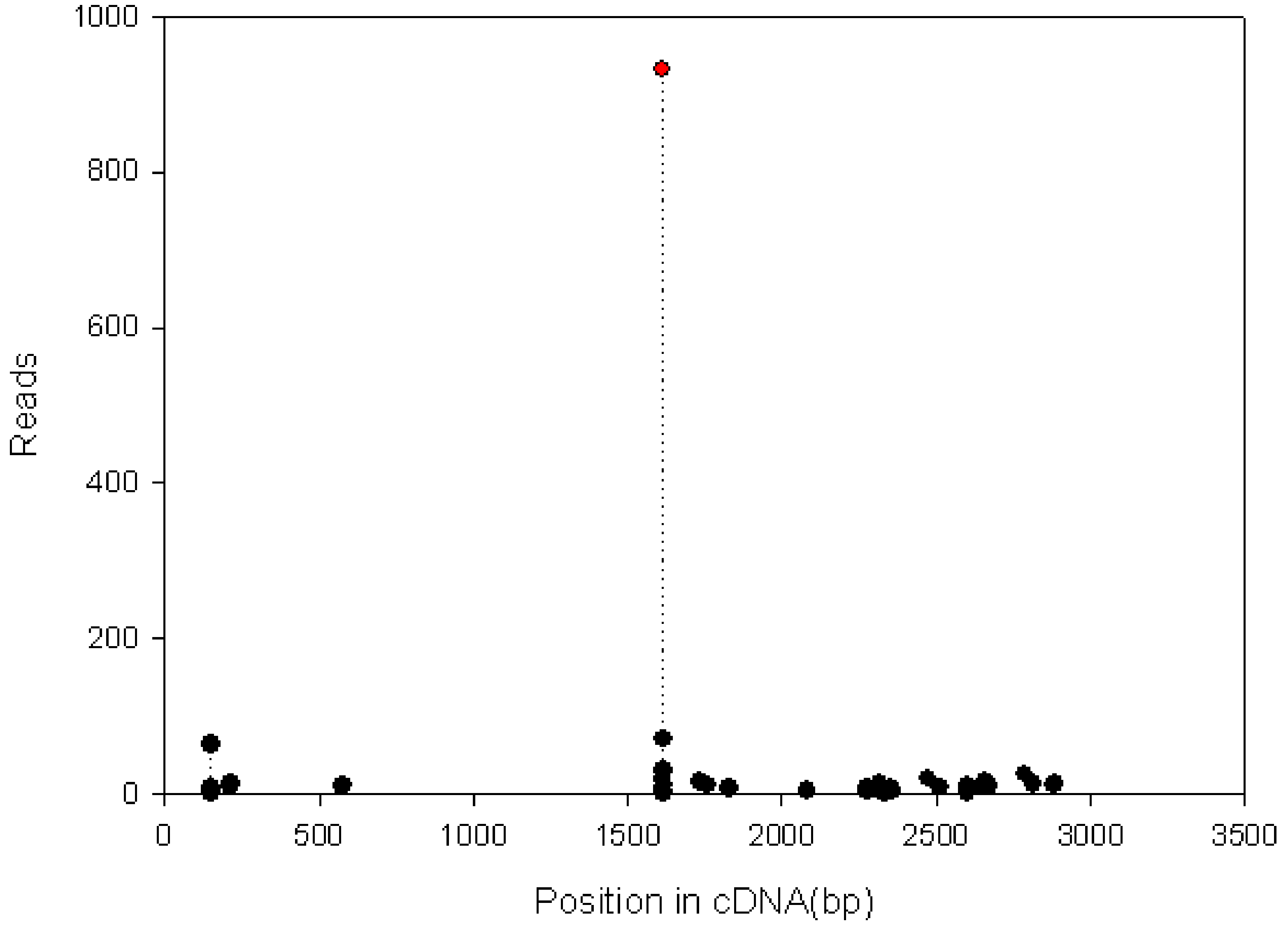


```
5' AAGGGAUAUUGGCGCGGCUCAAUCAC 3'        Orange1.1t00199.1
     :.:::::::.::::::::::
3' -CCUCUAUAACUGCGCCGAGUU---- 5'        Csi-miR171d
```

Csi-miR171d, target=Orange1.1t00200.1 gene=Orange1.1t00200
Category:1
Score=2.5
Cleavage Site=1511

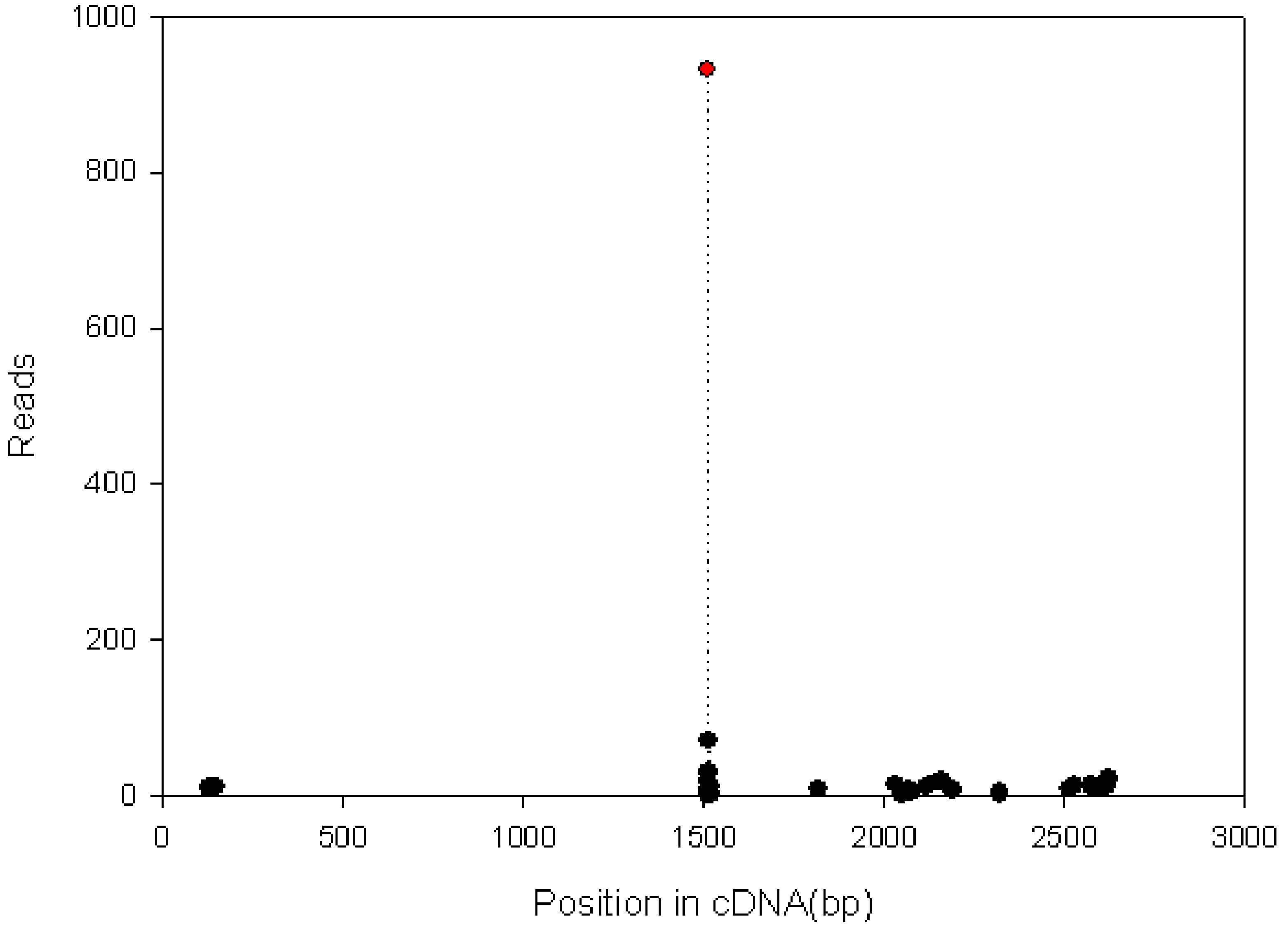


```
5' AAGGGAUAUUGGCGCGGCUCAAUCAC 3'        Orange1.1t00200.1
     :.:::::::.::::::::::
3' -CCUCUAUAACUGCGCCGAGUU---- 5'        Csi-miR171d
```

Csi-miR171g.1, target=Cs5g08980.1 gene=Cs5g08980
Category:1
Score=1
Cleavage Site=1697

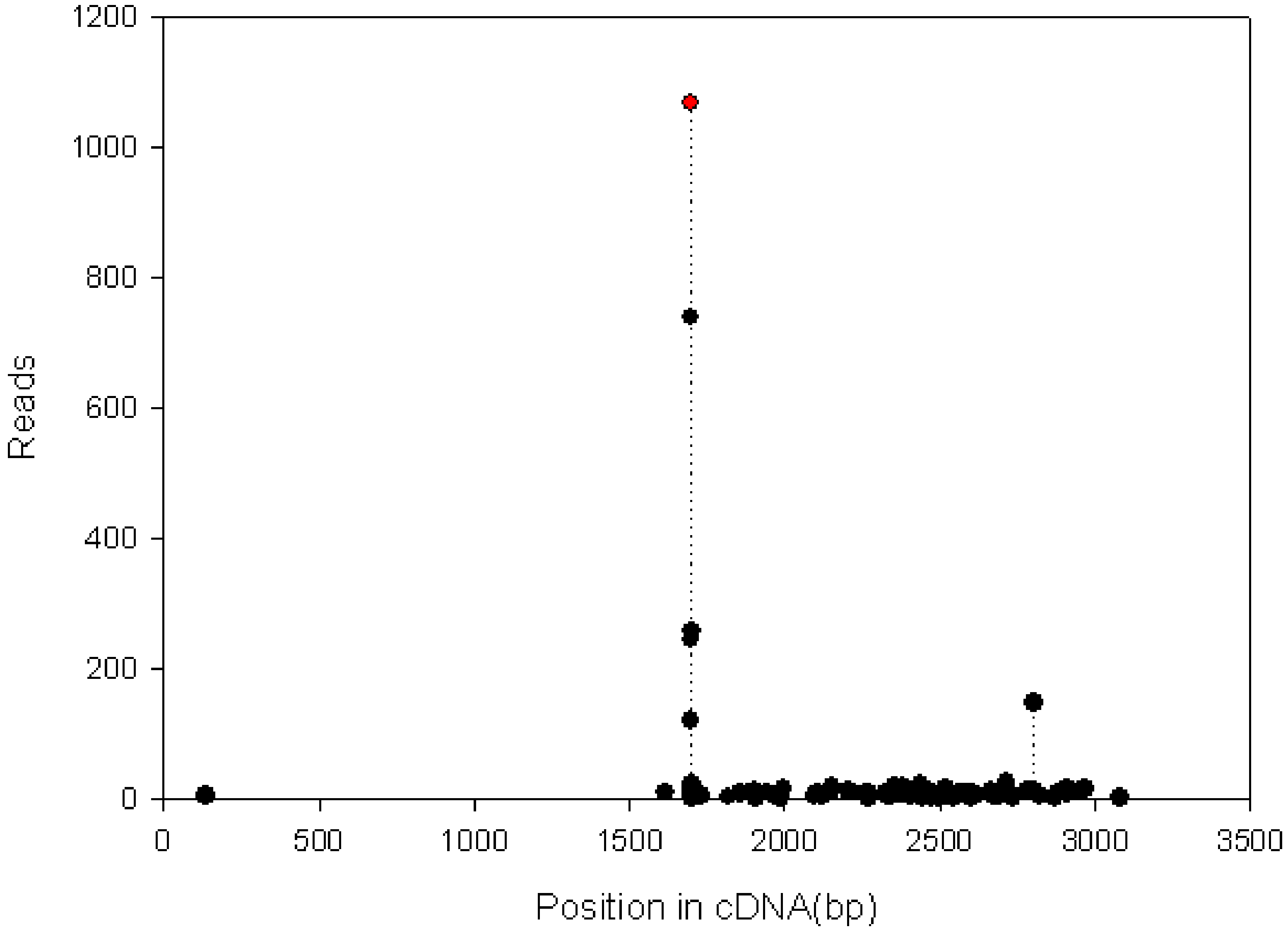


```
5' AAGGGAUAUUGGCGCGGCUCAAUCAC 3'        Cs5g08980.1
       :::::::::.:::::::::::
3' ----CUAUAACCGUGCCGAGUUAGU- 5'        Csi-miR171g.1
```

Csi-miR171g.1, target=Orange1.1t00199.1 gene=Orange1.1t00199
Category:1
Score=1
Cleavage Site=1612

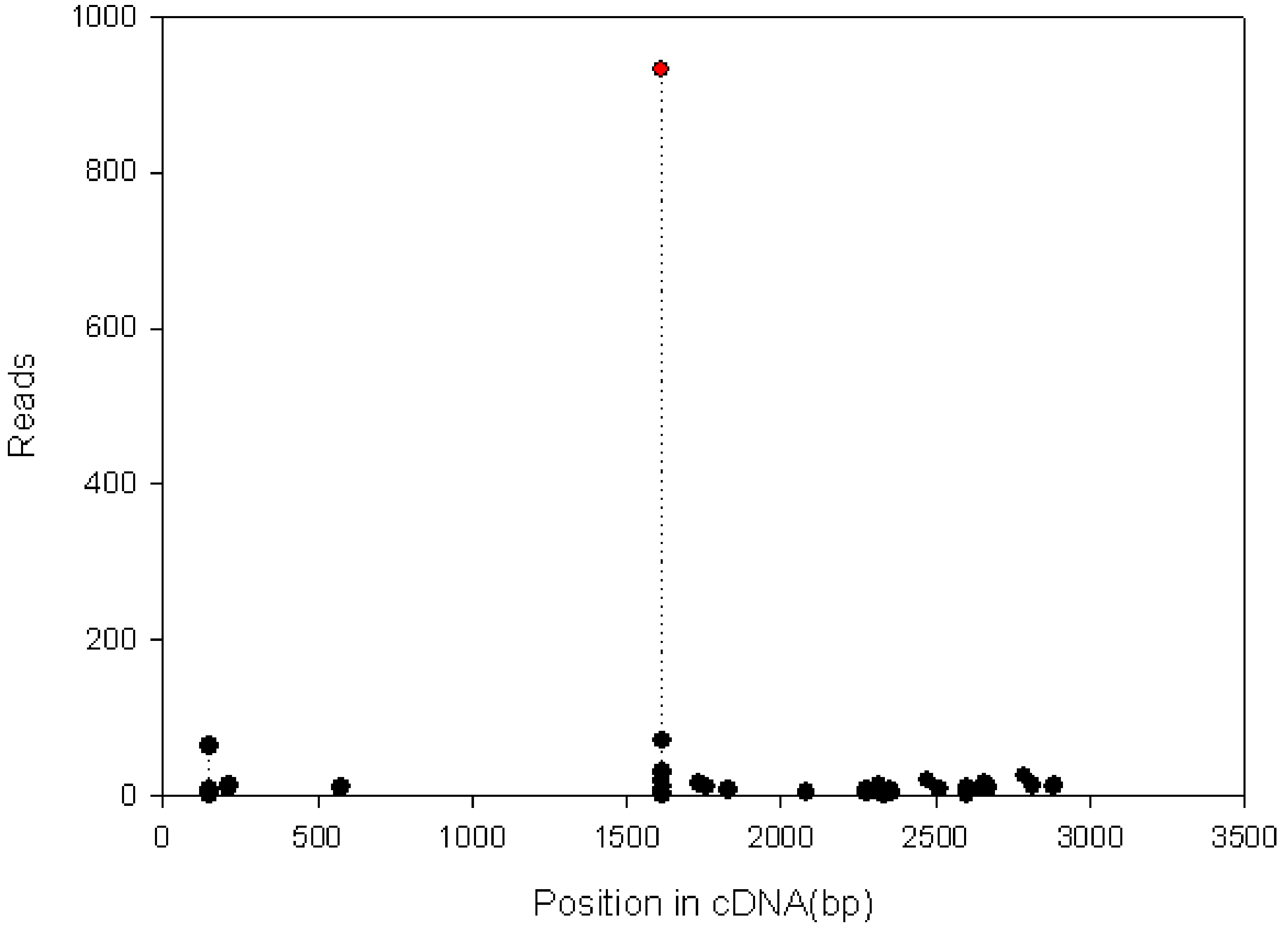


```
5' AAGGGAUAUUGGCGCGGCUCAAUCAC 3'        Orange1.1t00199.1
         :::::::::.:::::::::::
3' ----CUAUAACCGUGCCGAGUUAGU- 5'        Csi-miR171g.1
```

Csi-miR171g.1, target=Orange1.1t00200.1 gene=Orange1.1t00200
Category:1
Score=1
Cleavage Site=1511

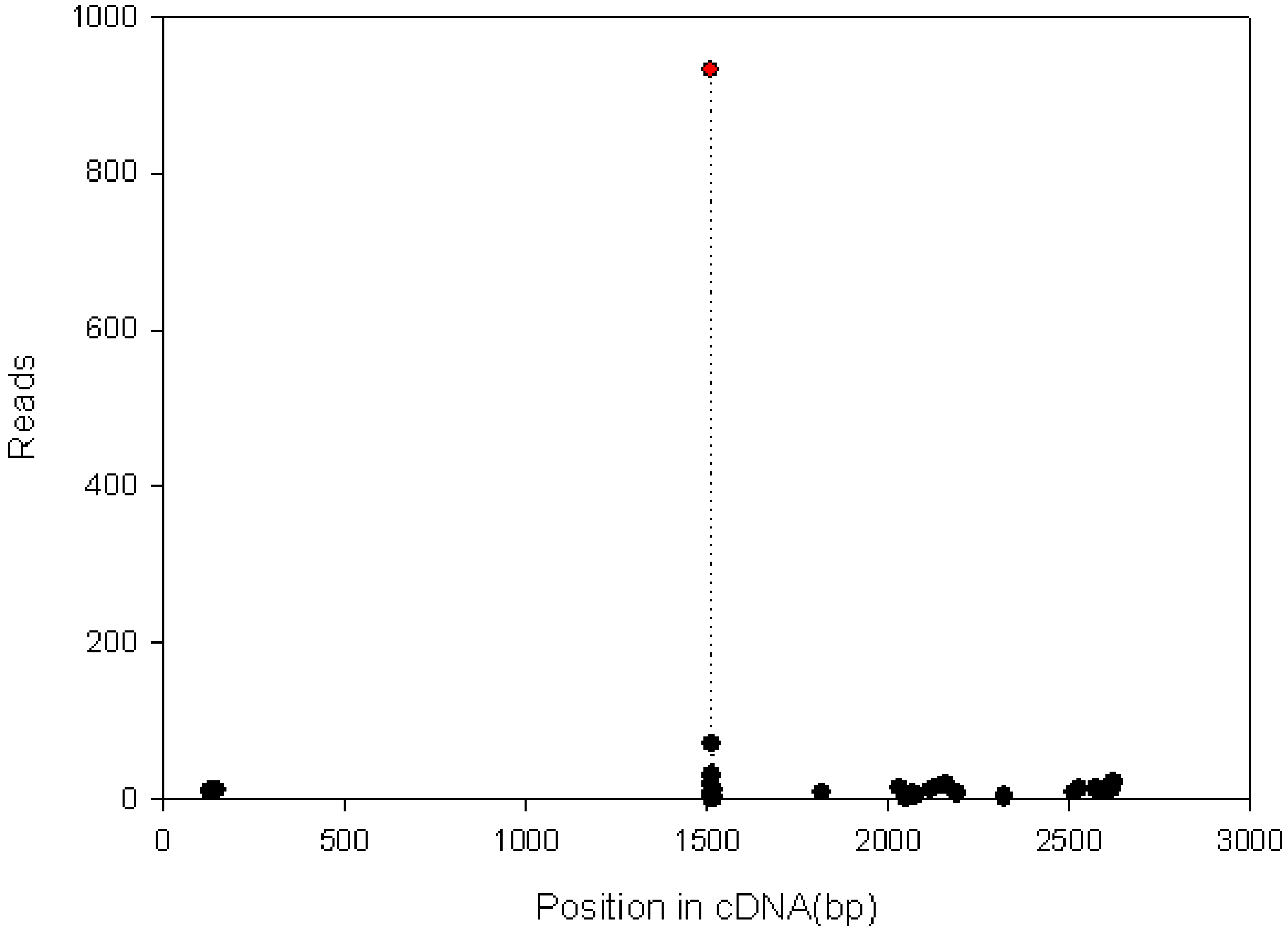


```
5' AAGGGAUAUUGGCGCGGCUCAAUCAC 3'        Orange1.1t00200.1
       :::::::::.:::::::::::
3' ----CUAUAACCGUGCCGAGUUAGU- 5'        Csi-miR171g.1
```

Csi-miR172a-3p.1, target=Cs6g11940.1 gene=Cs6g11940
Category:3
Score=5
Cleavage Site=2040

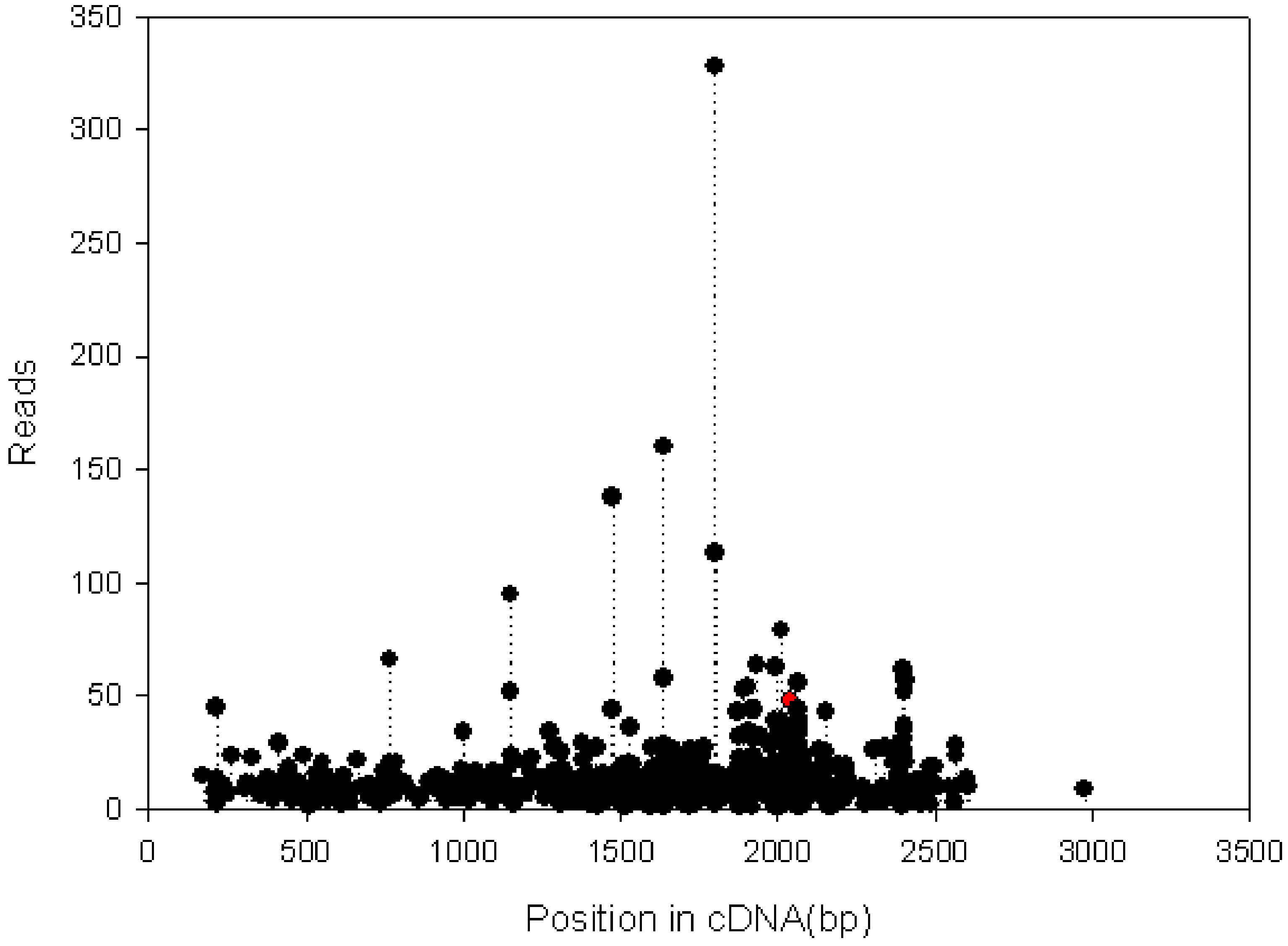


```
5' AGAGCUUCAUCCAUCAAGAUUCUGUG 3'        Cs6g11940.1
      ::  ::: ::::::::::::
3' --ACGUCGUA-GUAGUUCUAAGA--- 5'        Csi-miR172a-3p.1
```

Csi-miR172a-3p.1, target=Cs7g27790.1 gene=Cs7g27790
Category:1
Score=1
Cleavage Site=1614

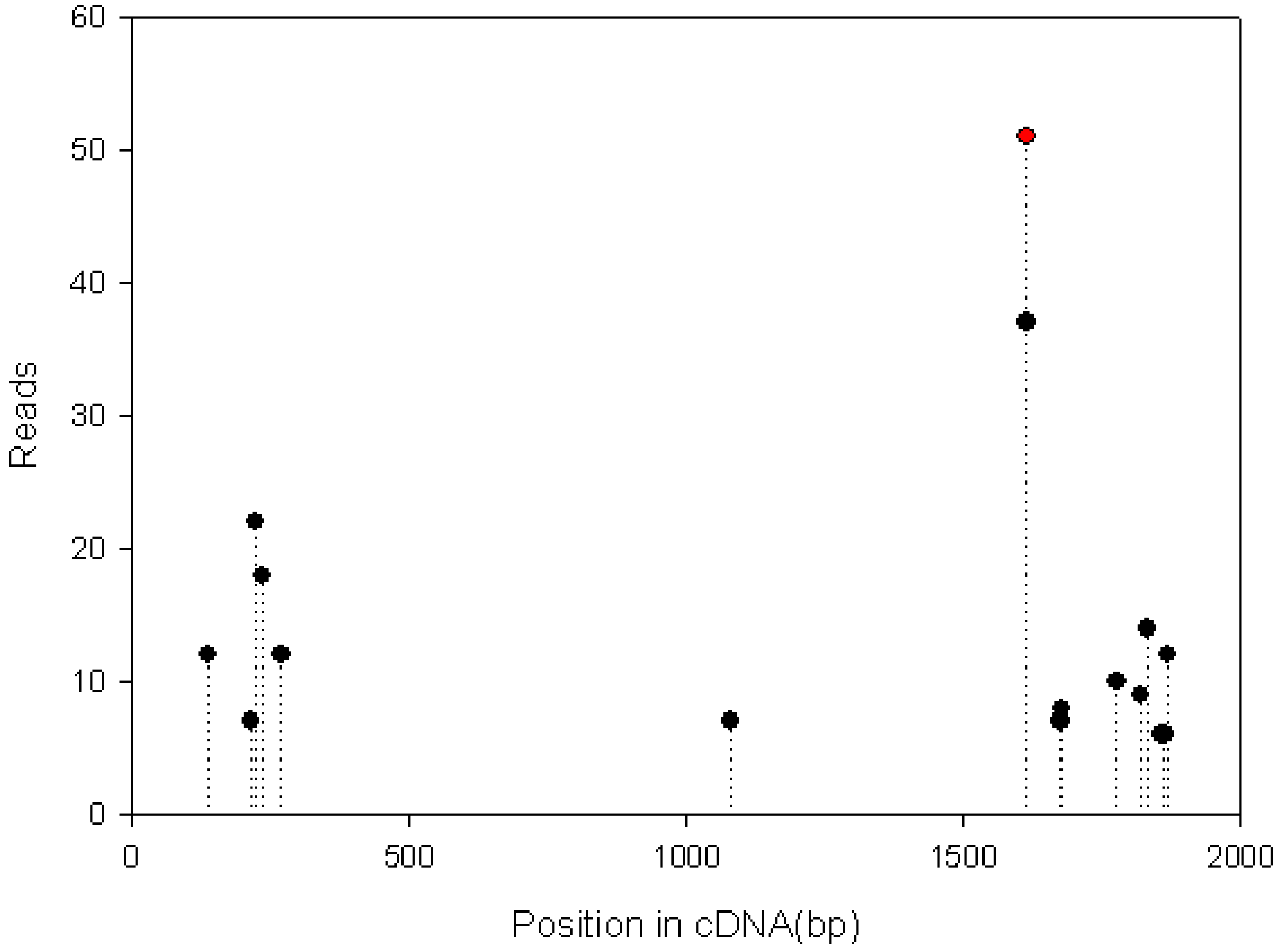


```
5' AGUGCAGCAUCAUCAGGAUUCUCUUC 3'        Cs7g27790.1
     :::::::::::::.::::::
3' --ACGUCGUAGUAGUUCUAAGA---- 5'        Csi-miR172a-3p.1
```

Csi-miR172a-3p.1, target=Cs8g17390.1 gene=Cs8g17390
Category:1
Score=2
Cleavage Site=1865

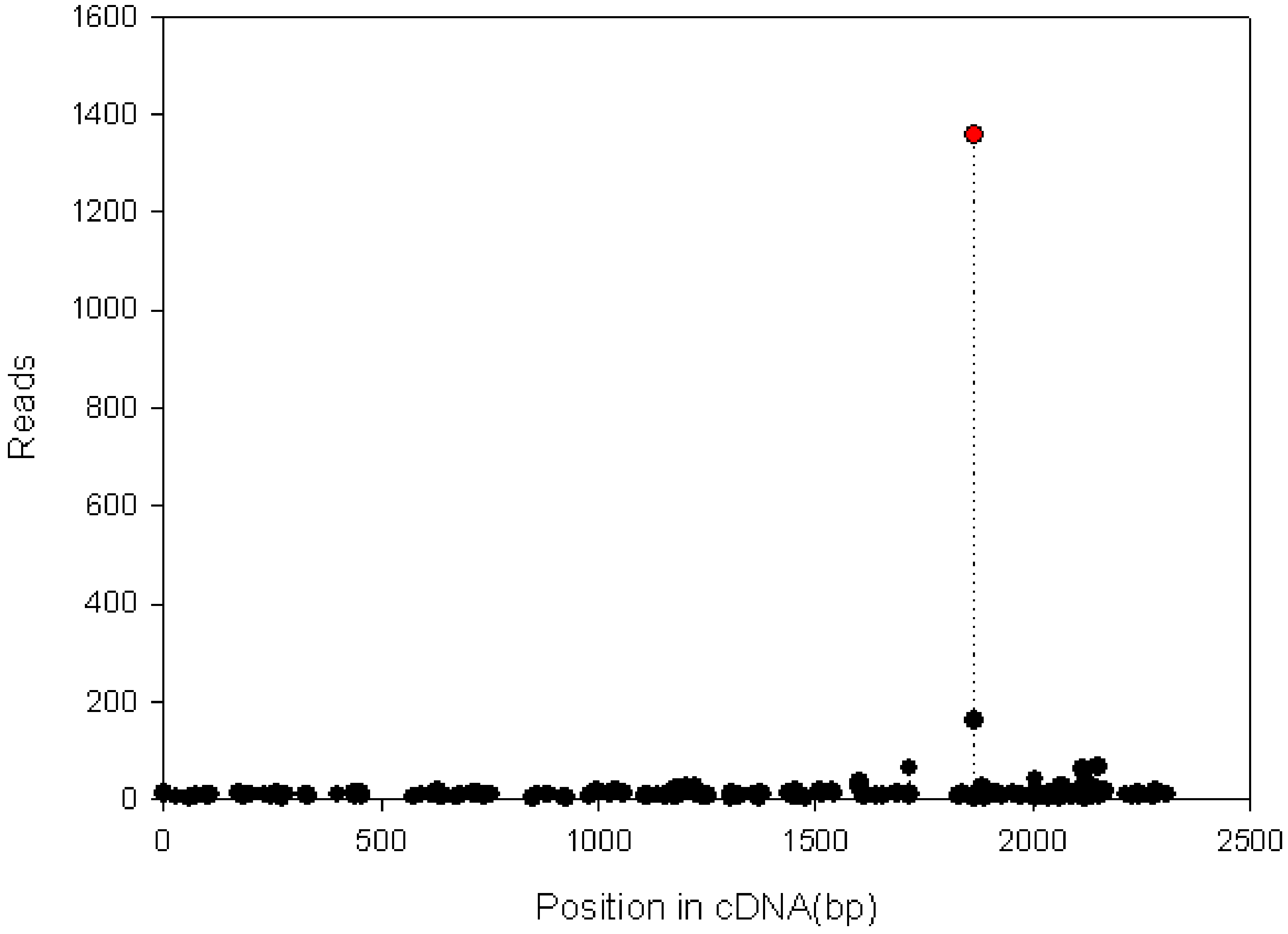


```
5' AGUGCAGCAUCAUCAGGAUUCCCAUU 3'        Cs8g17390.1
     ::::::::::::::.:::::
3' --ACGUCGUAGUAGUUCUAAGA---- 5'        Csi-miR172a-3p.1
```

Csi-miR172a-3p.1, target=Orange1.1t04055.1 gene=Orange1.1t04055
Category:1
Score=2
Cleavage Site=2049

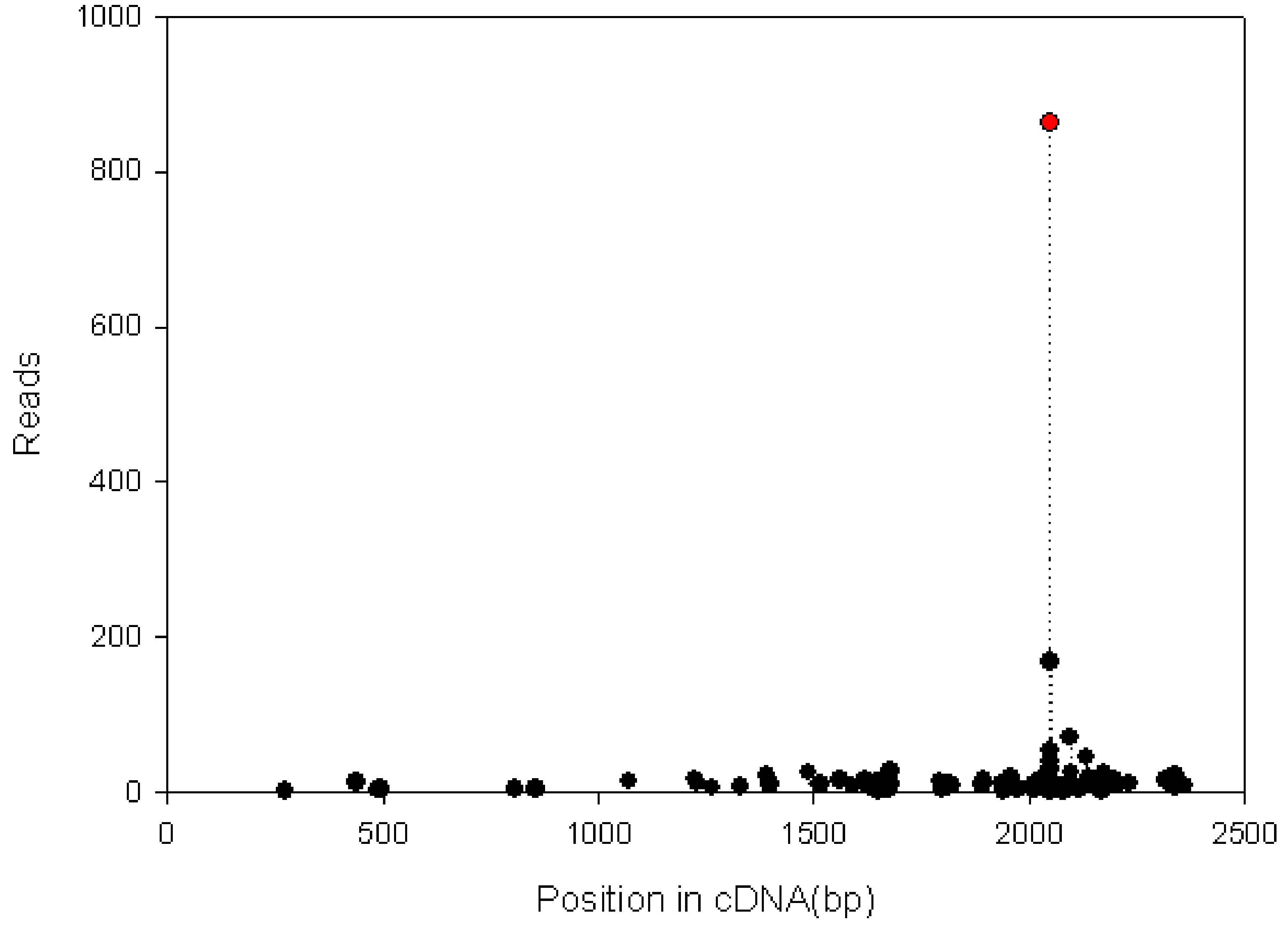


```
5' GCUGCAGCAUCAUCAGGAUUCCCUCA 3'        Orange1.1t04055.1
     ::::::::::::::::.:::::
3' --ACGUCGUAGUAGUUCUAAGA---- 5'        Csi-miR172a-3p.1
```

Csi-miR172a-3p.2, target=Cs7g27790.1 gene=Cs7g27790
Category:1
Score=2
Cleavage Site=1614

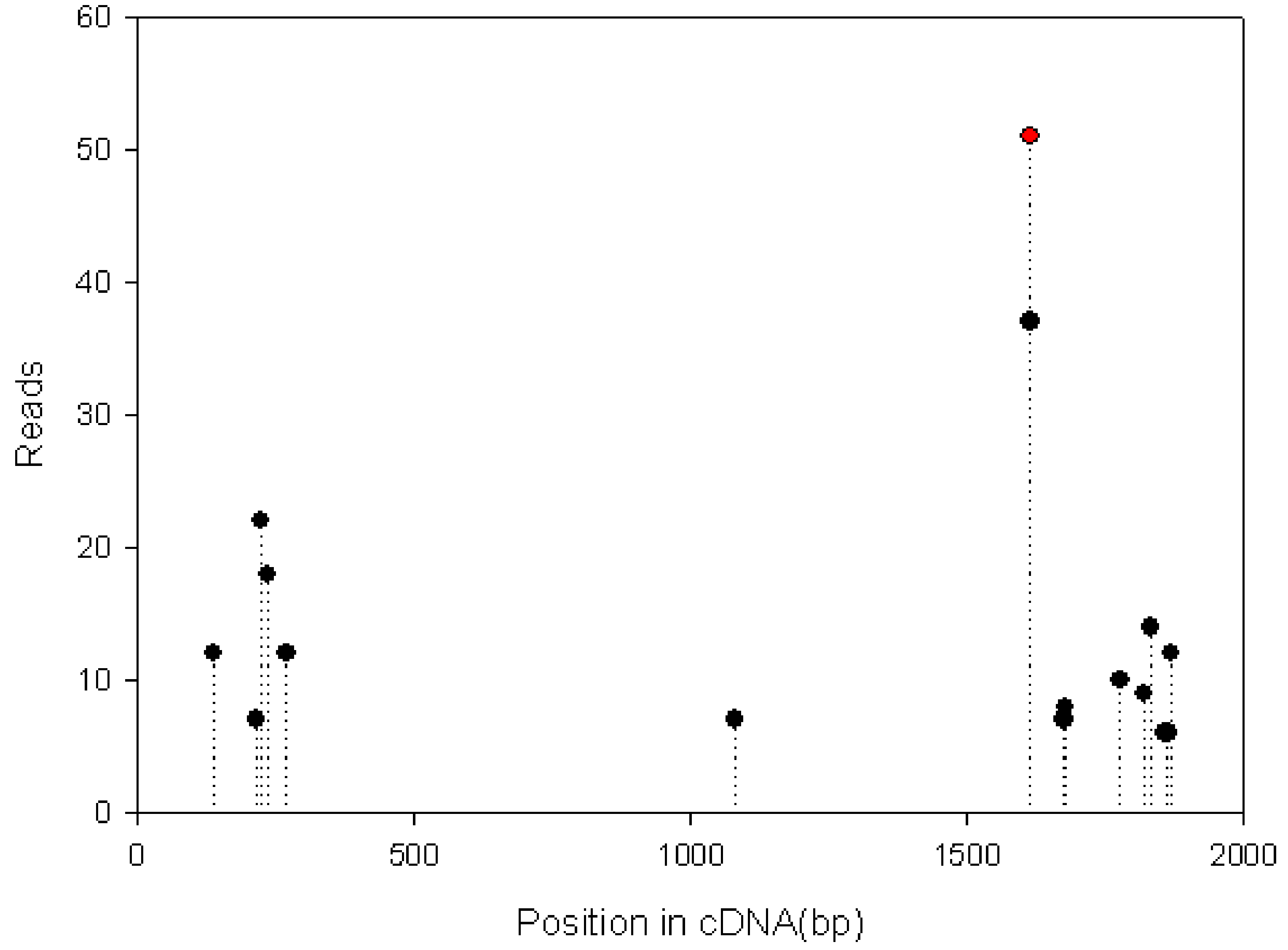


```
5' AGUGCAGCAUCAUCAGGAUUCUCUUC 3'        Cs7g27790.1
     ::::::::::::::.::::::
3' -AACGUCGUAGUAGUUCUAAGA---- 5'        Csi-miR172a-3p.2
```

Csi-miR172a-3p.2, target=Cs8g17390.1 gene=Cs8g17390
Category:1
Score=3
Cleavage Site=1865

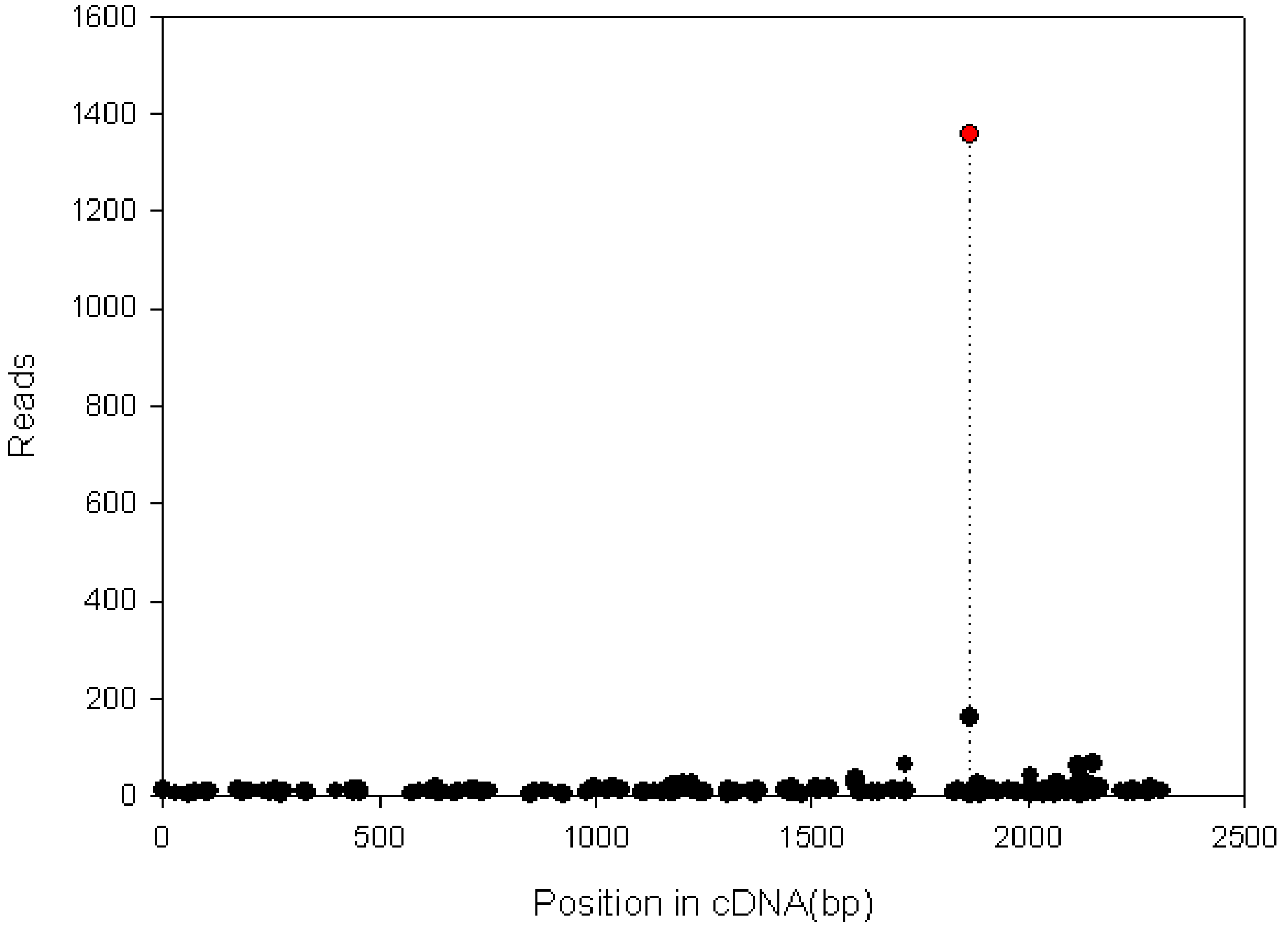


```
5' AGUGCAGCAUCAUCAGGAUUCCCAUU 3'        Cs8g17390.1
     :::::::::::::.:::::
3' -AACGUCGUAGUAGUUCUAAGA---- 5'        Csi-miR172a-3p.2
```

Csi-miR172a-3p.2, target=Orange1.1t04055.1 gene=Orange1.1t04055
Category:1
Score=3
Cleavage Site=2049

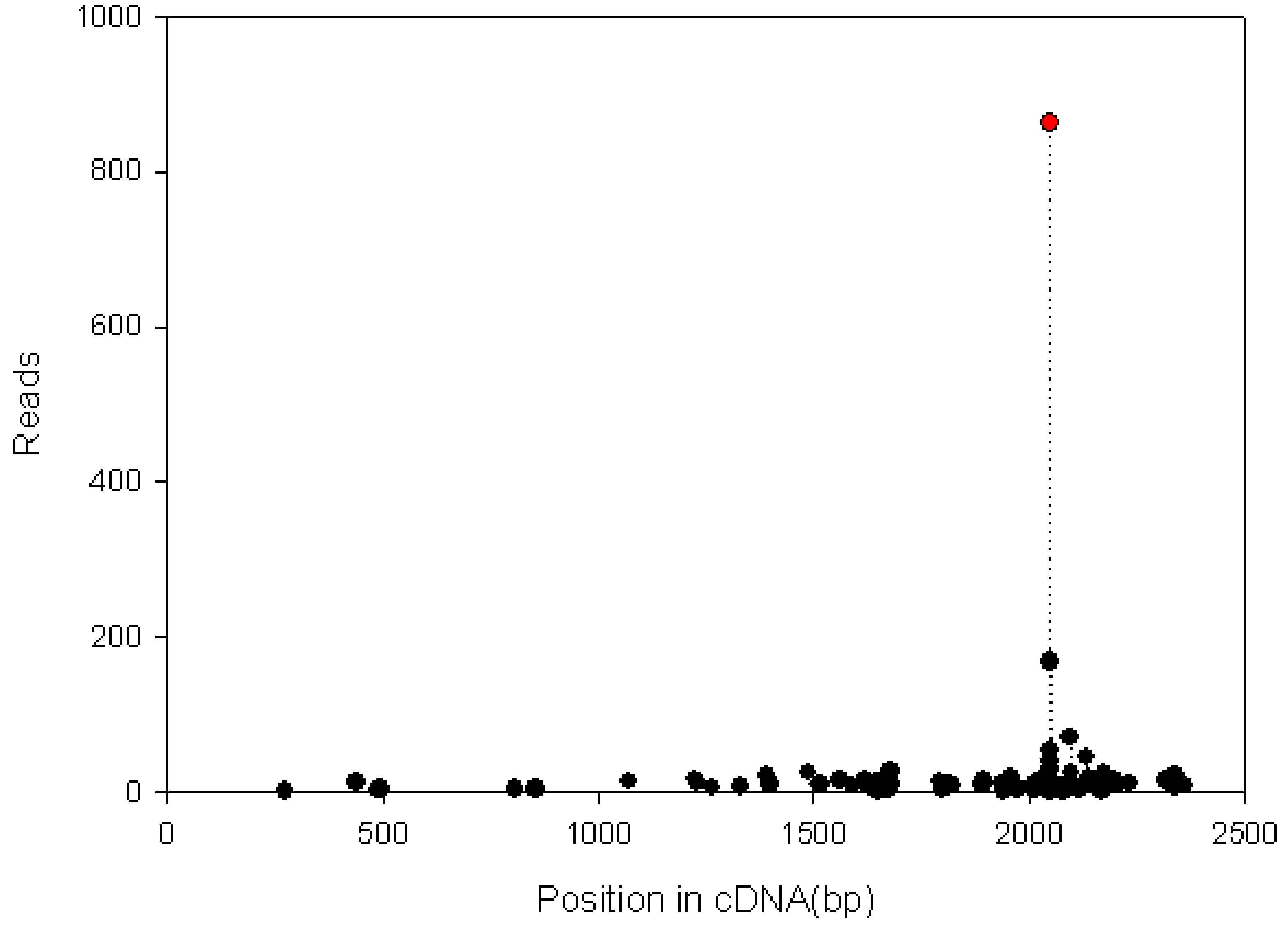


```
5' GCUGCAGCAUCAUCAGGAUUCCCUCA 3'        Orange1.1t04055.1
     ::::::::::::::.:::::
3' -AACGUCGUAGUAGUUCUAAGA---- 5'        Csi-miR172a-3p.2
```

Csi-miR172a-3p.3, target=Cs2g21180.1 gene=Cs2g21180
Category:3
Score=5
Cleavage Site=437

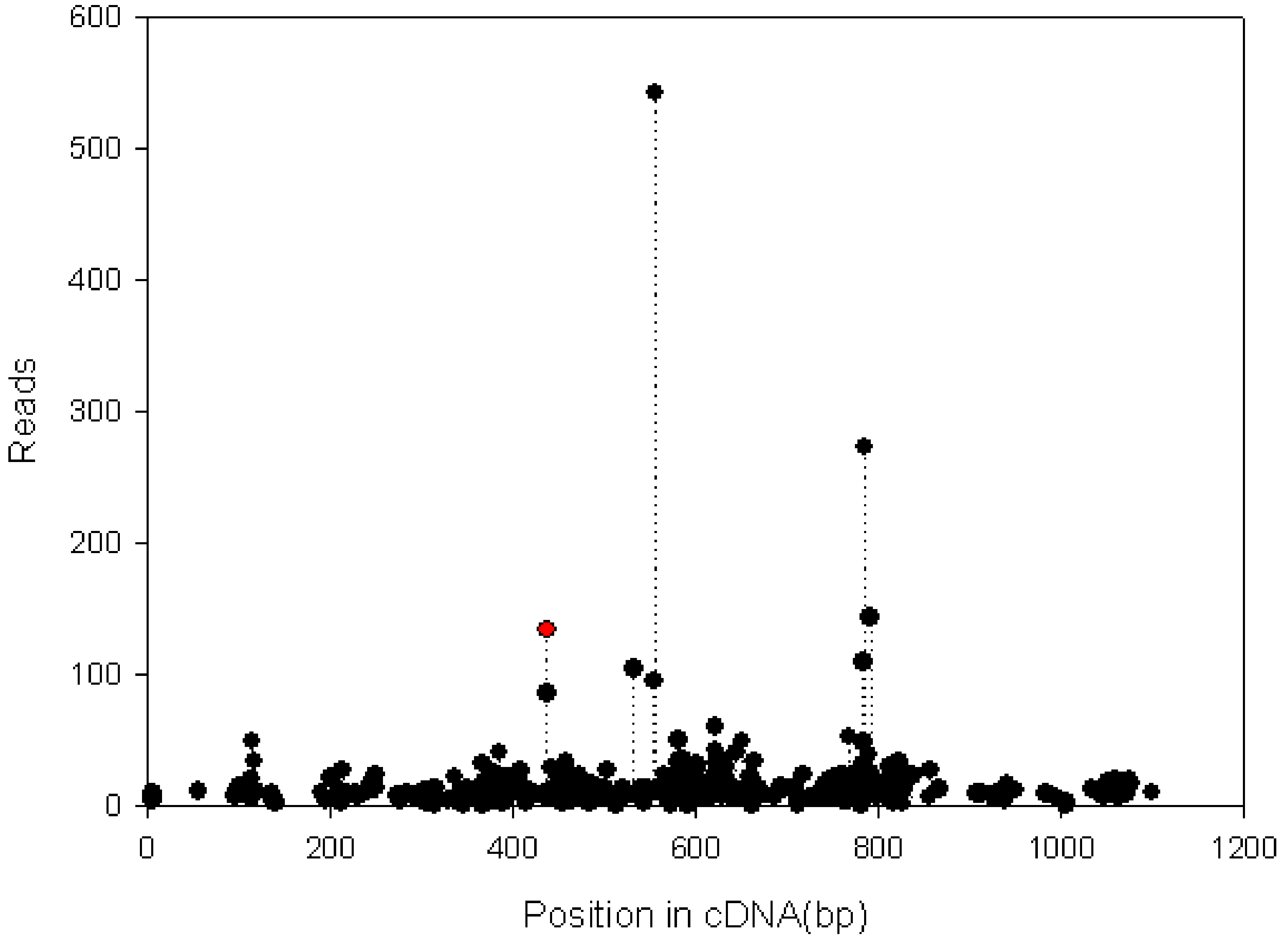


```
5' UUUACAGCAUCCUCAAGAUCCAGCAA 3'        Cs2g21180.1
    :: ::::::: ::::::: :
3' -AACGUCGUAGUAGUUCUAAG----- 5'        Csi-miR172a-3p.3
```

Csi-miR172a-3p.3, target=Cs7g27790.1 gene=Cs7g27790
Category:1
Score=2
Cleavage Site=1614

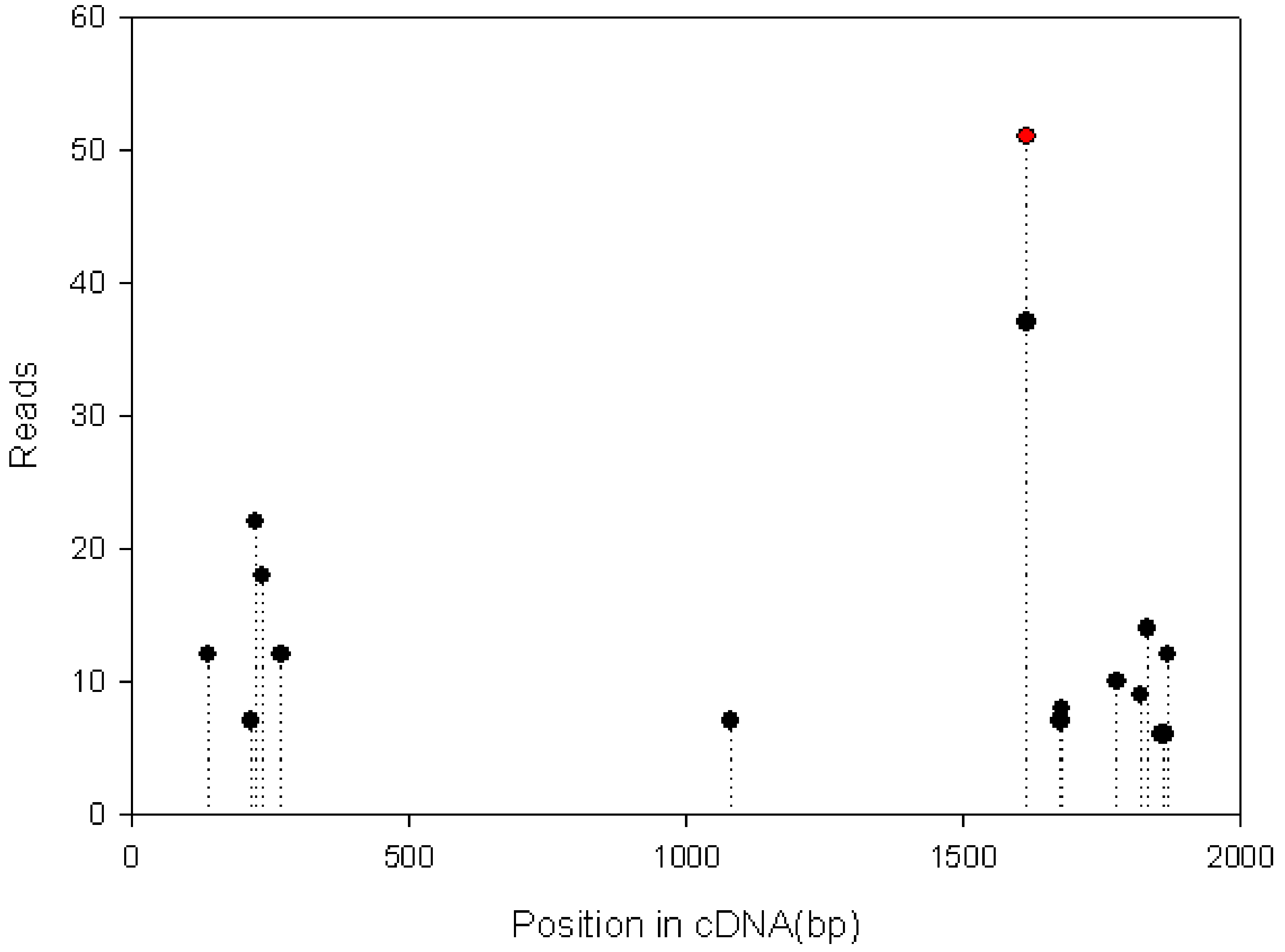


```
5' AGUGCAGCAUCAUCAGGAUUCUCUUC 3'        Cs7g27790.1
    :::::::::::::.:::::
3' -AACGUCGUAGUAGUUCUAAG----- 5'        Csi-miR172a-3p.3
```

Csi-miR172a-3p.3, target=Cs8g17390.1 gene=Cs8g17390
Category:1
Score=2
Cleavage Site=1865

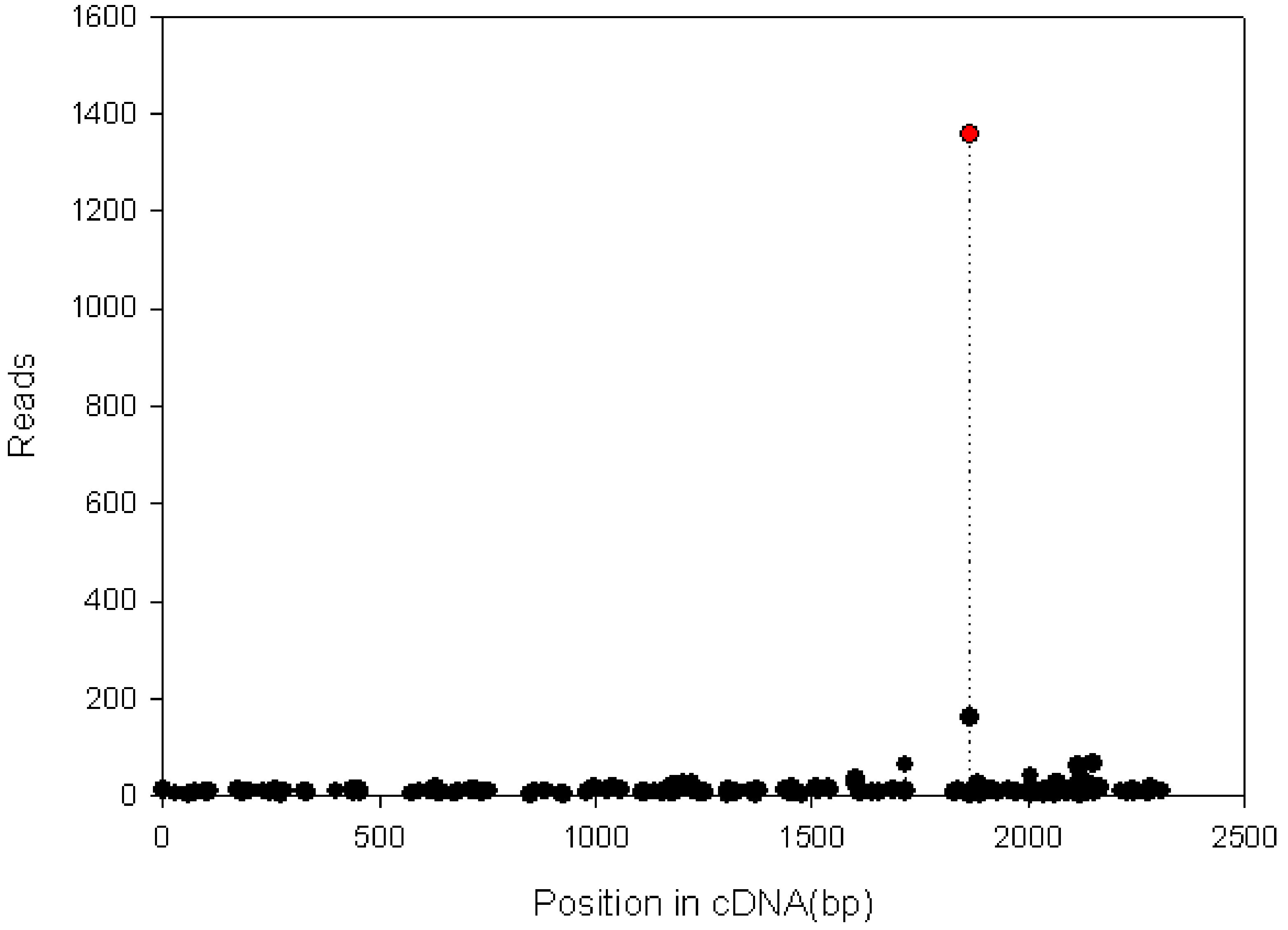


```
5' AGUGCAGCAUCAUCAGGAUUCCCAUU 3'        Cs8g17390.1
    :::::::::::::.:::::
3' -AACGUCGUAGUAGUUCUAAG----- 5'        Csi-miR172a-3p.3
```

Csi-miR172a-3p.3, target=Orange1.1t04055.1 gene=Orange1.1t04055
Category:1
Score=2
Cleavage Site=2049

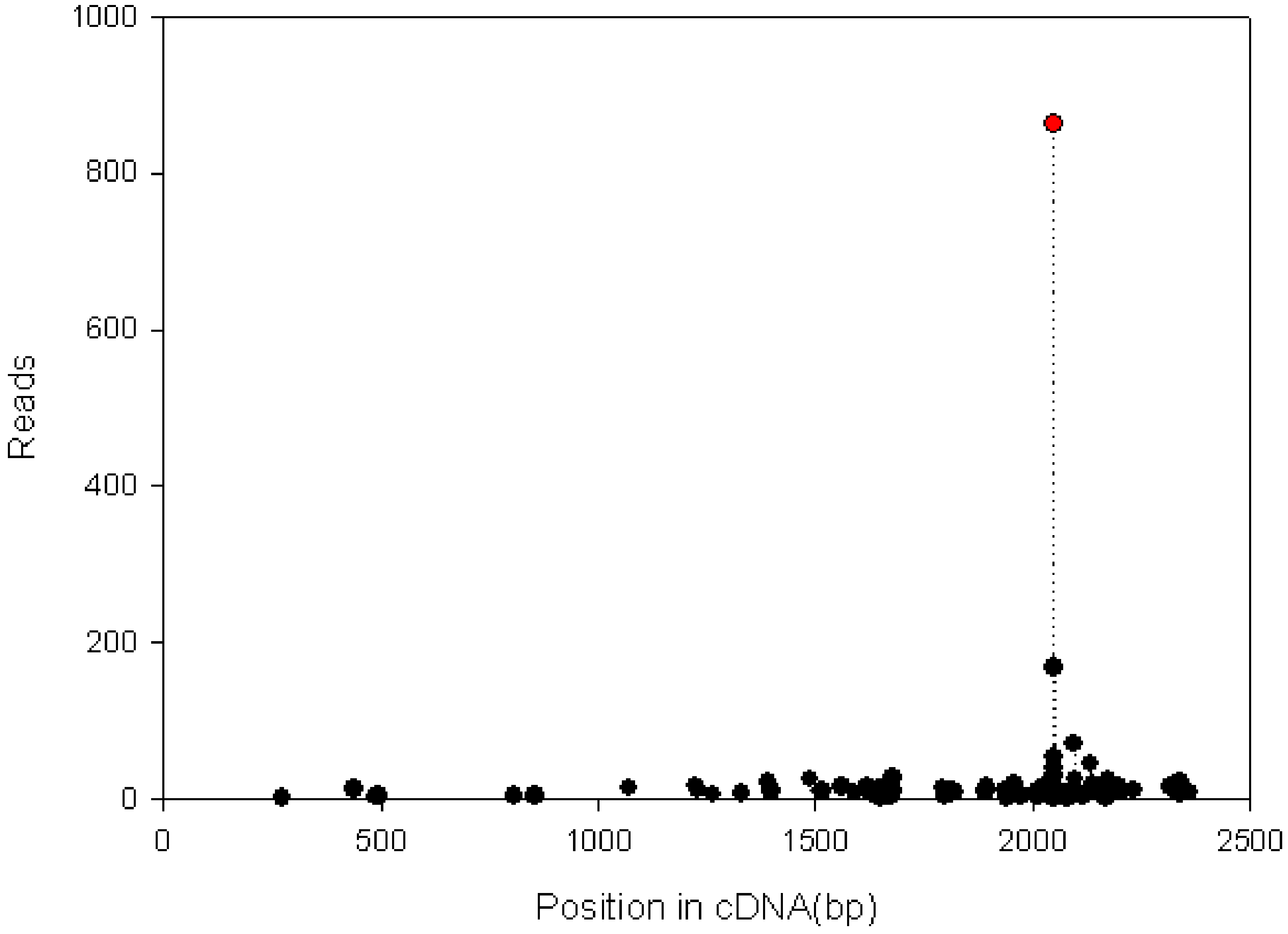


```
5' GCUGCAGCAUCAUCAGGAUUCCCUCA 3'        Orange1.1t04055.1
     ::::::::::::::::.:::::
3' -AACGUCGUAGUAGUUCUAAG----- 5'        Csi-miR172a-3p.3
```

Csi-miR172c.1, target=Cs2g21180.1 gene=Cs2g21180
Category:3
Score=4
Cleavage Site=437

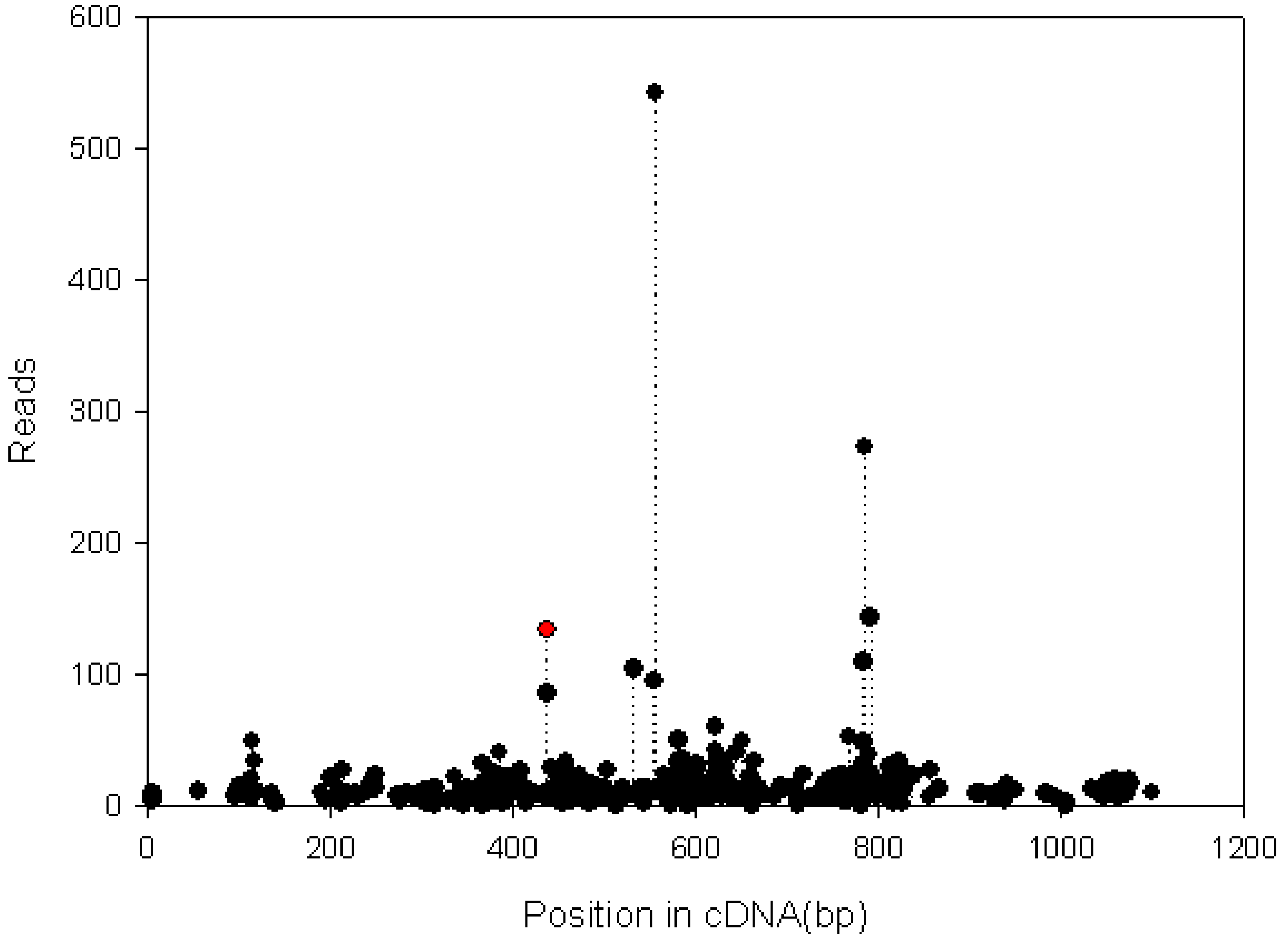


```
5' UUUACAGCAUCCUCAAGA-UCCAGCAA 3'        Cs2g21180.1
        :::::: :::::: ::::
3' -----UCGUAGUAGUUCUAAGGU---- 5'        Csi-miR172c.1
```

Csi-miR172c.1, target=Cs3g06140.1 gene=Cs3g06140
Category:1
Score=4.5
Cleavage Site=480

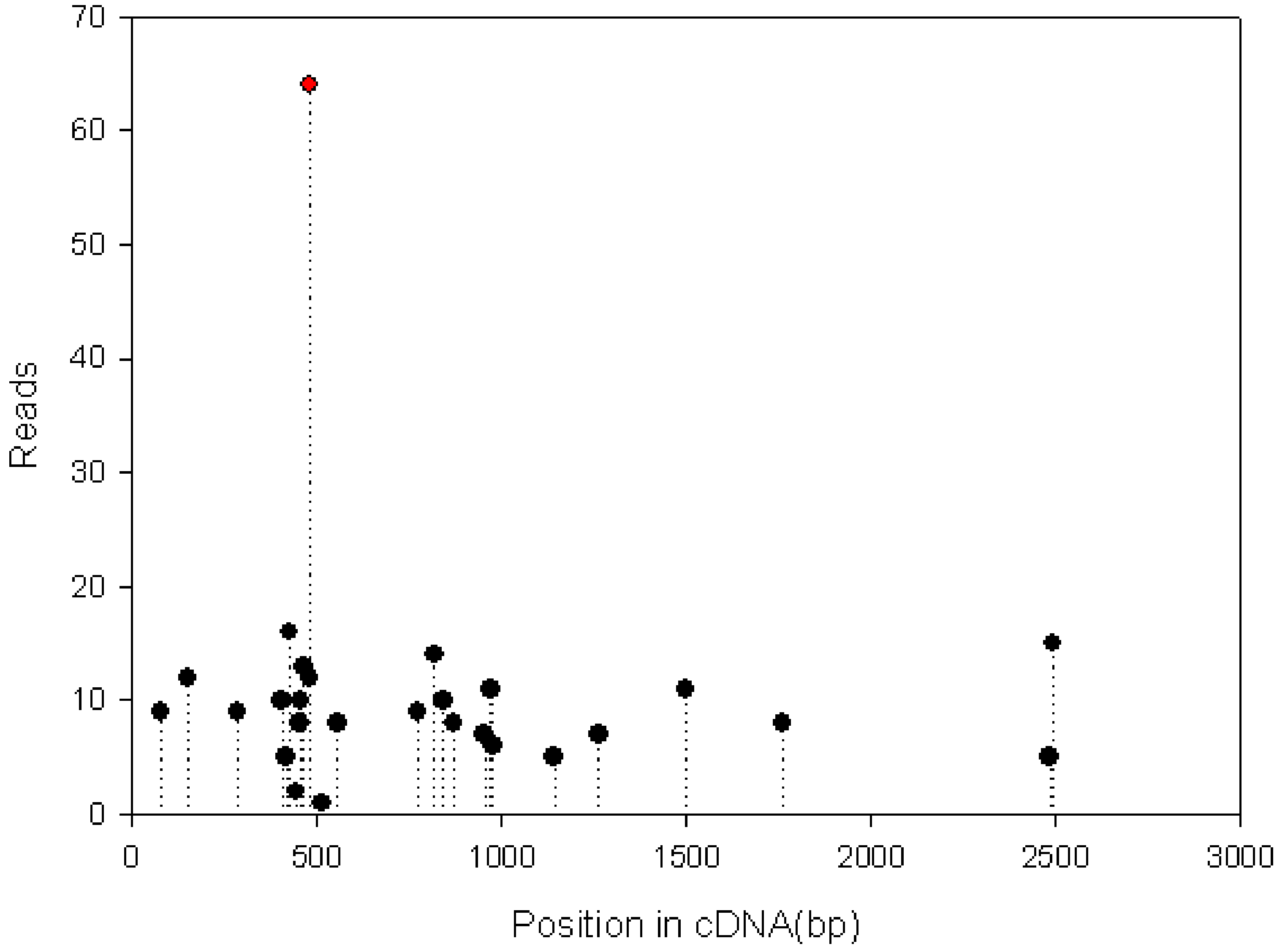


```
5' UGAGUCUGCCACCAUUAAGAUUCCUG 3'        Cs3g06140.1
             . :: :::.:::::::::
3' -------UCGUAGUAGUUCUAAGGU- 5'        Csi-miR172c.1
```

Csi-miR172c.1, target=Cs6g11940.1 gene=Cs6g11940
Category:3
Score=4.5
Cleavage Site=2040

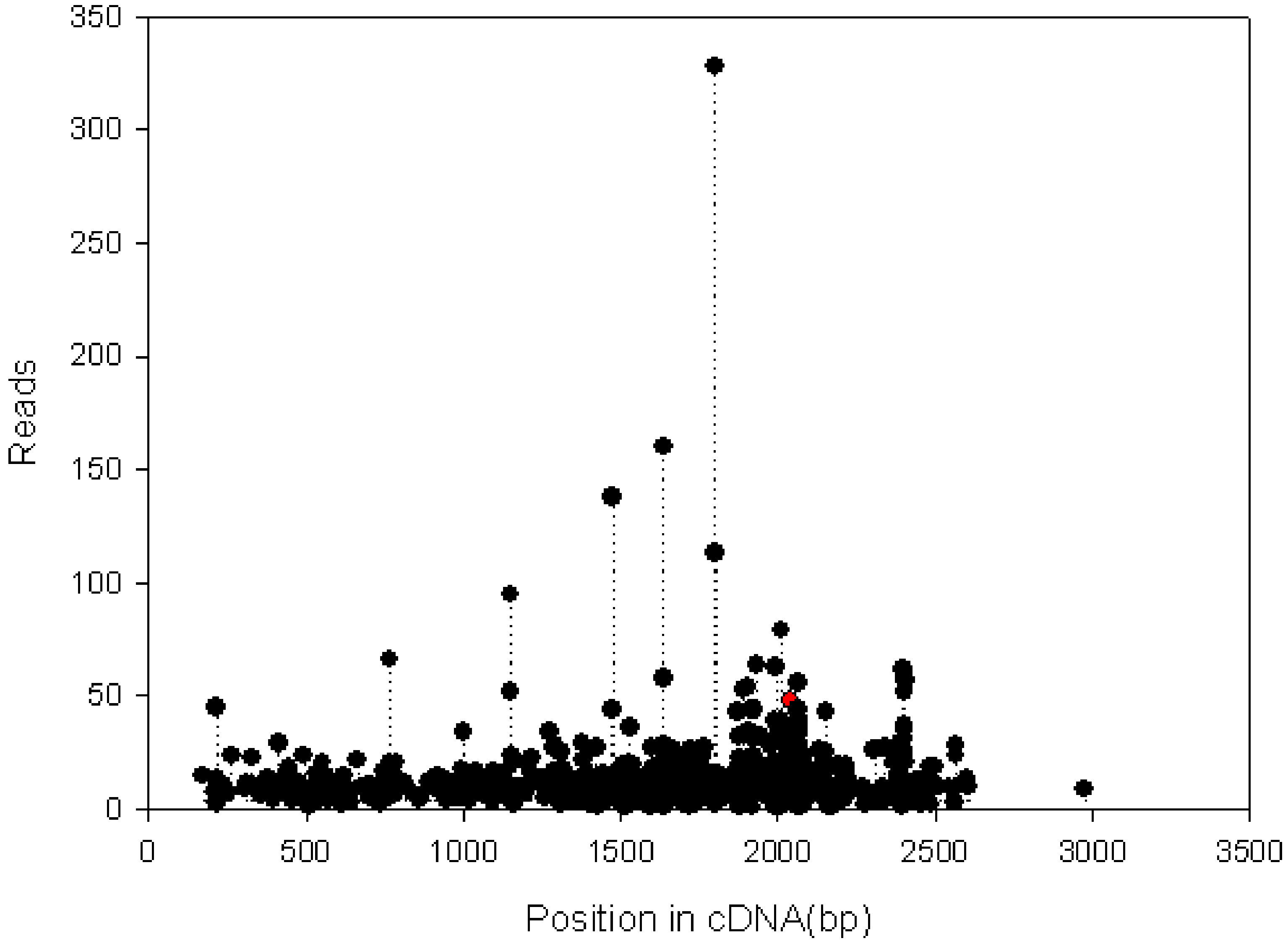


```
5' AGAGCUUCAUCCAUCAAGAUUCUGUG 3'        Cs6g11940.1
         ::: ::::::::::::..
3' -----UCGUA-GUAGUUCUAAGGU-- 5'        Csi-miR172c.1
```

Csi-miR172c.1, target=Cs7g23640.1 gene=Cs7g23640
Category:2
Score=5
Cleavage Site=3757

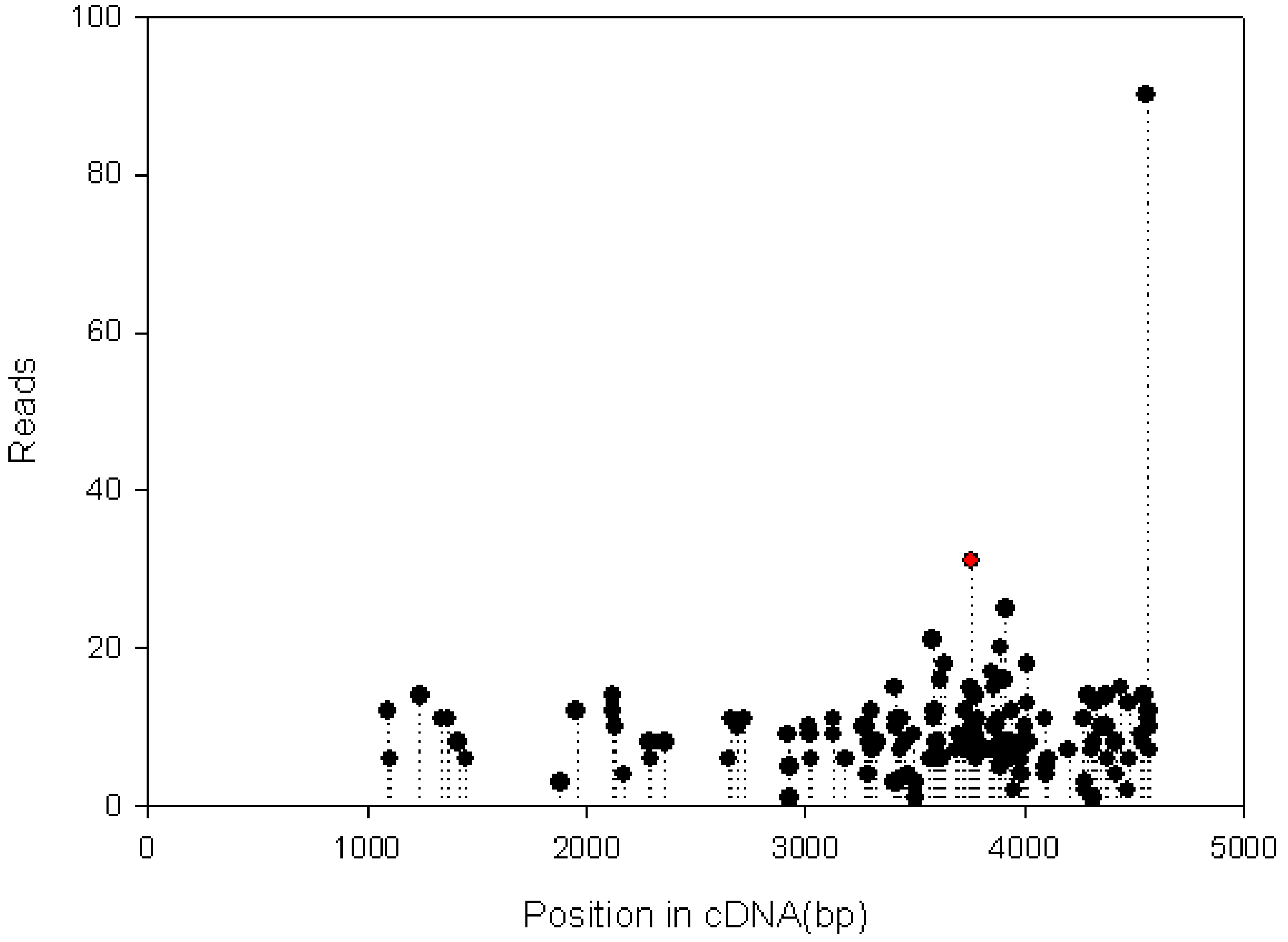


```
5' CCAAUGCCUCGUCAAG-UUCCAUGUUC 3'        Cs7g23640.1
         :: ::.::::: :::::
3' ----UCGUAGUAGUUCUAAGGU----- 5'        Csi-miR172c.1
```

Csi-miR172c.1, target=Cs7g27790.1 gene=Cs7g27790
Category:1
Score=3
Cleavage Site=1614

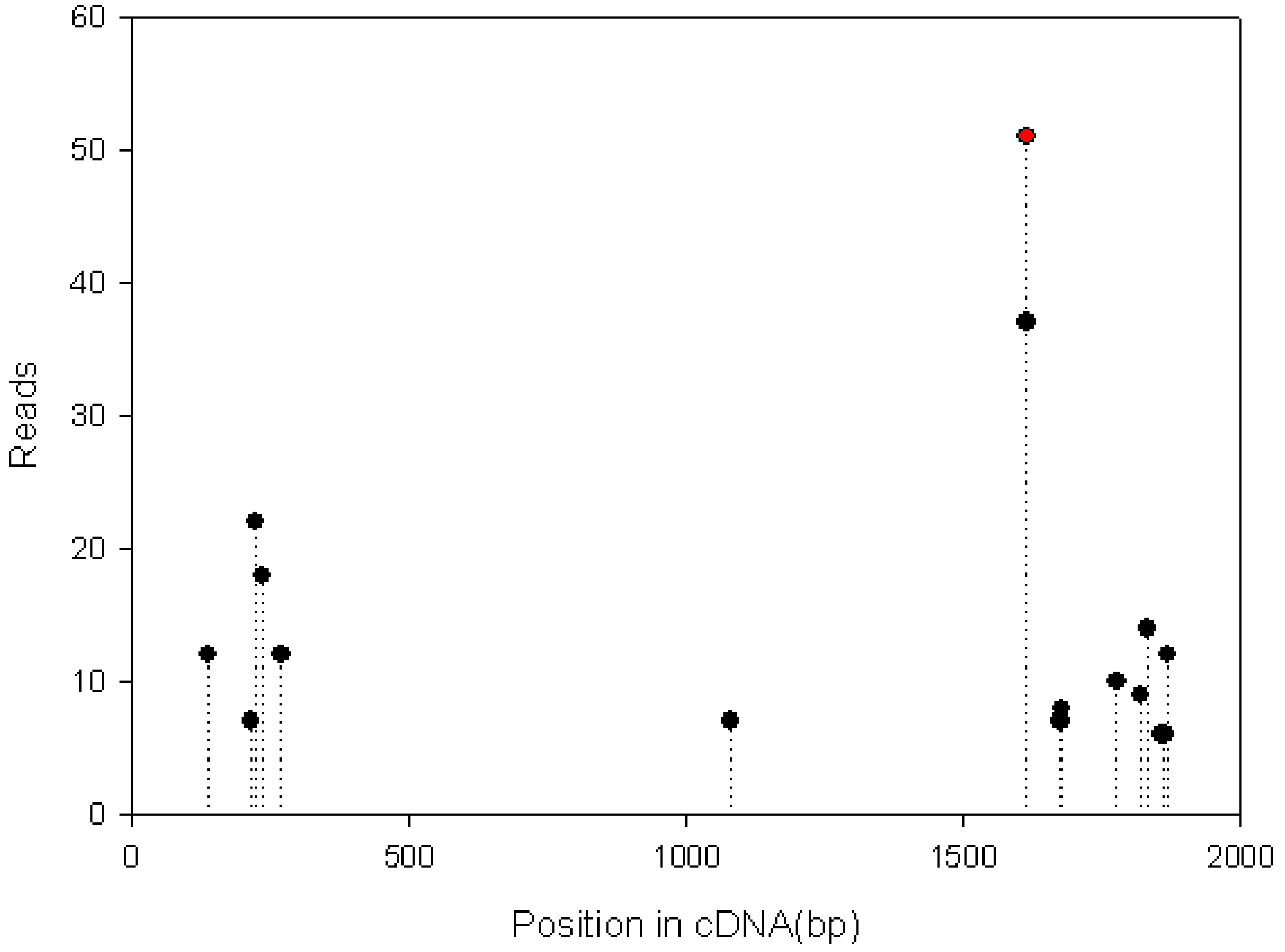


```
5' AGUGCAGCAUCAUCAGGAUUCUCUUC 3'        Cs7g27790.1
         ::::::::::::.:::::.
3' -----UCGUAGUAGUUCUAAGGU--- 5'        Csi-miR172c.1
```

Csi-miR172c.1, target=Cs7g31800.1 gene=Cs7g31800
Category:3
Score=5
Cleavage Site=762

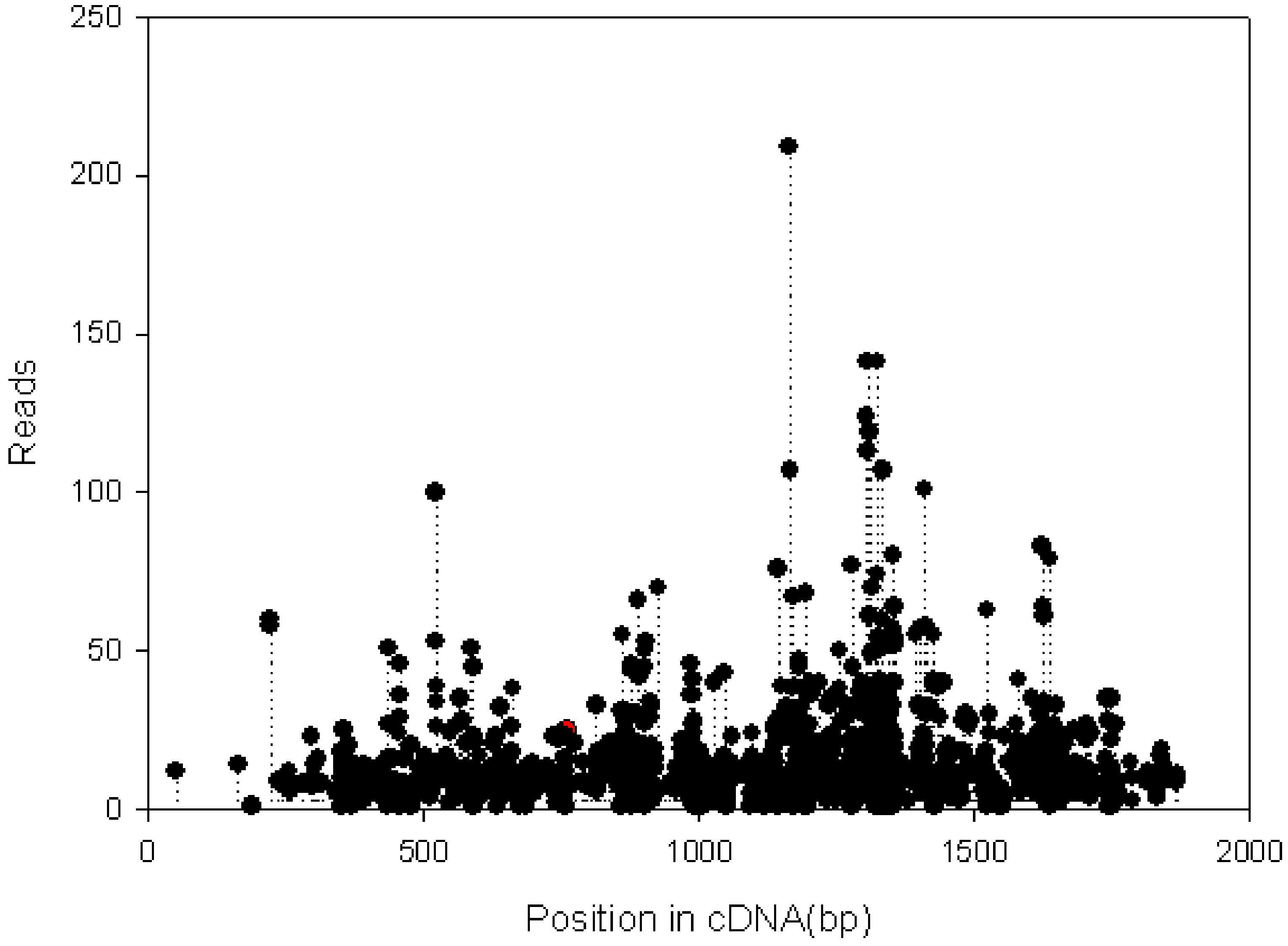


```
5' CUGCCCAACAUCAAGGUUCCUCUCAU 3'        Cs7g31800.1
          :: :::::::::.::::
3' ---UCGUAGUAGUUCUAAGGU----- 5'        Csi-miR172c.1
```

Csi-miR172c.1, target=Cs8g17390.1 gene=Cs8g17390
Category:1
Score=2
Cleavage Site=1865

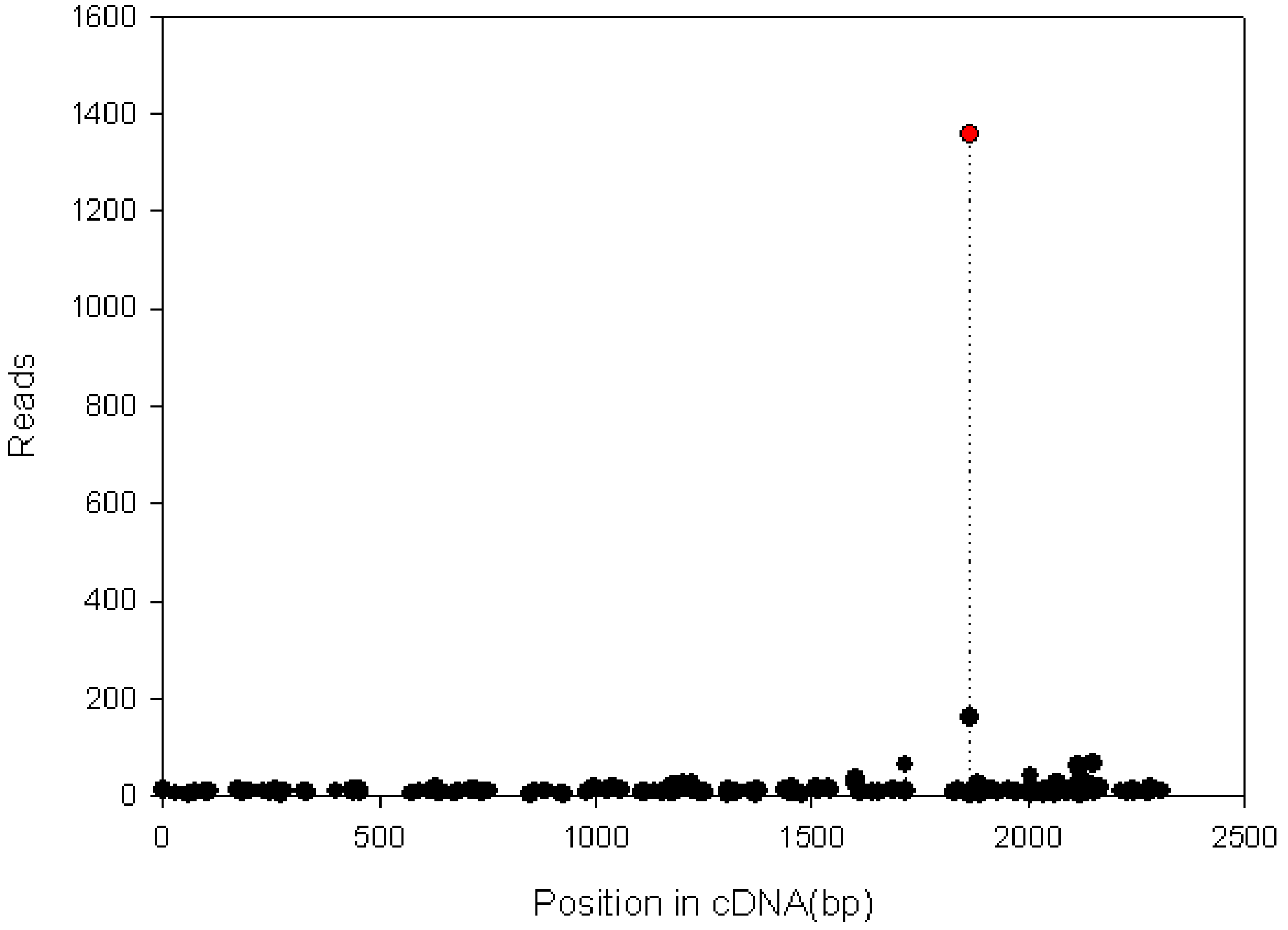


```
5' AGUGCAGCAUCAUCAGGAUUCCCAUU 3'          Cs8g17390.1
         :::::::::::.::::::
3' -----UCGUAGUAGUUCUAAGGU--- 5'          Csi-miR172c.1
```

Csi-miR172c.1, target=Orange1.1t04055.1 gene=Orange1.1t04055
Category:1
Score=2
Cleavage Site=2049

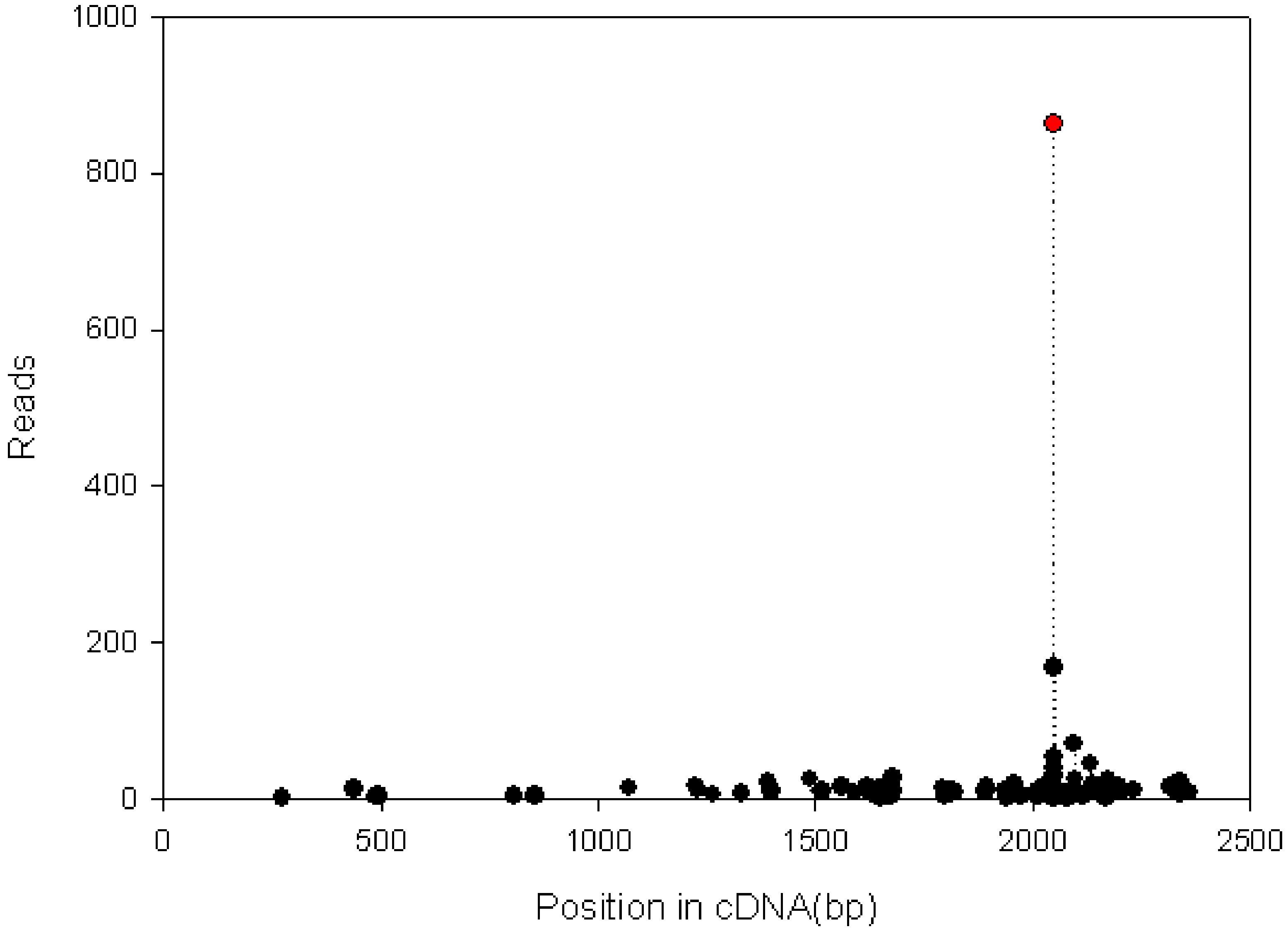


```
5' GCUGCAGCAUCAUCAGGAUUCCCUCA 3'        Orange1.1t04055.1
         :::::::::::.::::::
3' -----UCGUAGUAGUUCUAAGGU--- 5'        Csi-miR172c.1
```

Csi-miR172c.2, target=Cs7g27790.1 gene=Cs7g27790
Category:1
Score=4
Cleavage Site=1614

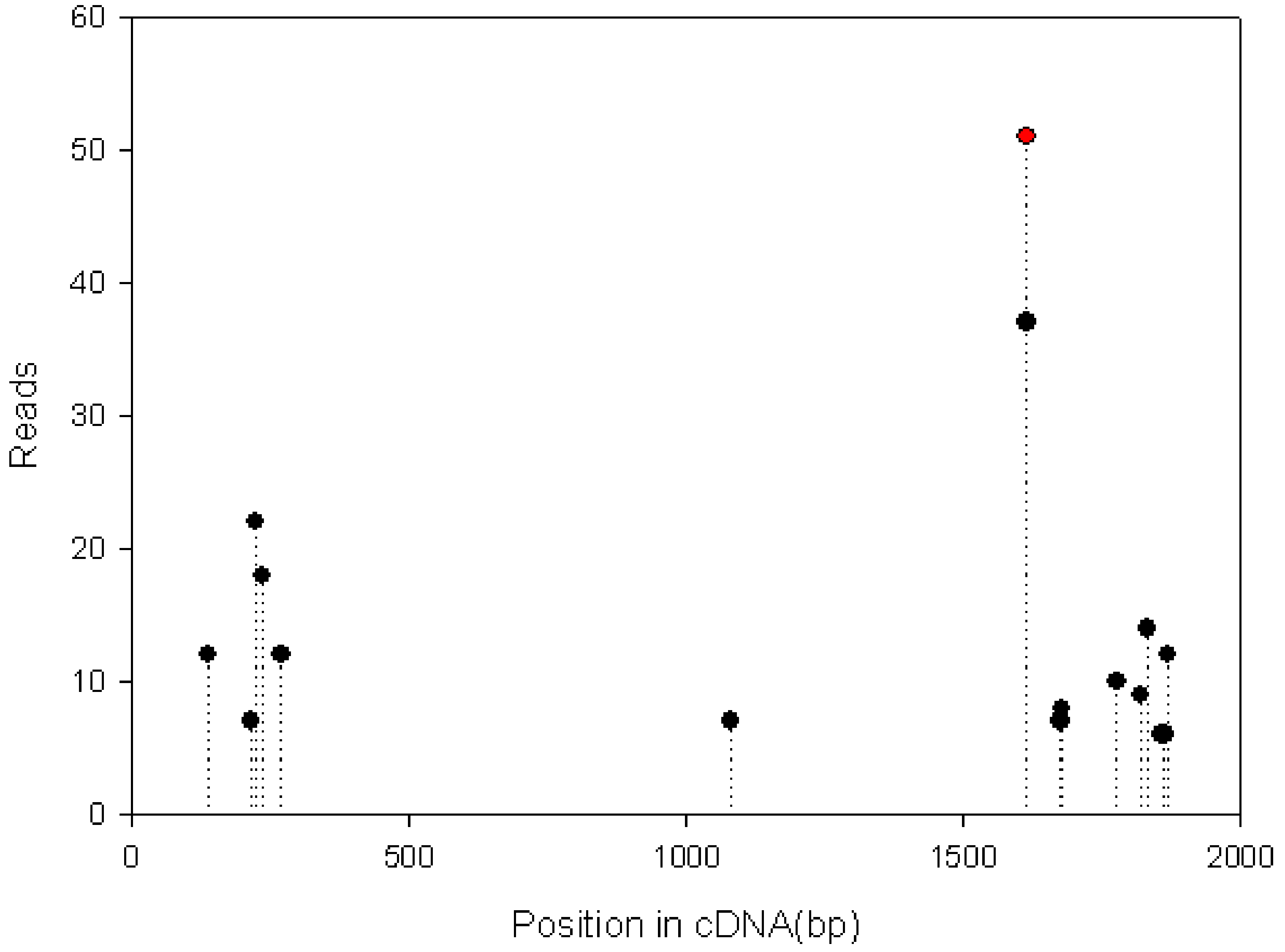


```
5' AGUGCAGCAUCAUCAGGAUUCUCUUC 3'        Cs7g27790.1
    ::::::::::::::::.:::::.
3' -GACGUCGUAGUAGUUCUAAGGU--- 5'        Csi-miR172c.2
```

Csi-miR172c.2, target=Cs7g31800.1 gene=Cs7g31800
Category:3
Score=5
Cleavage Site=762

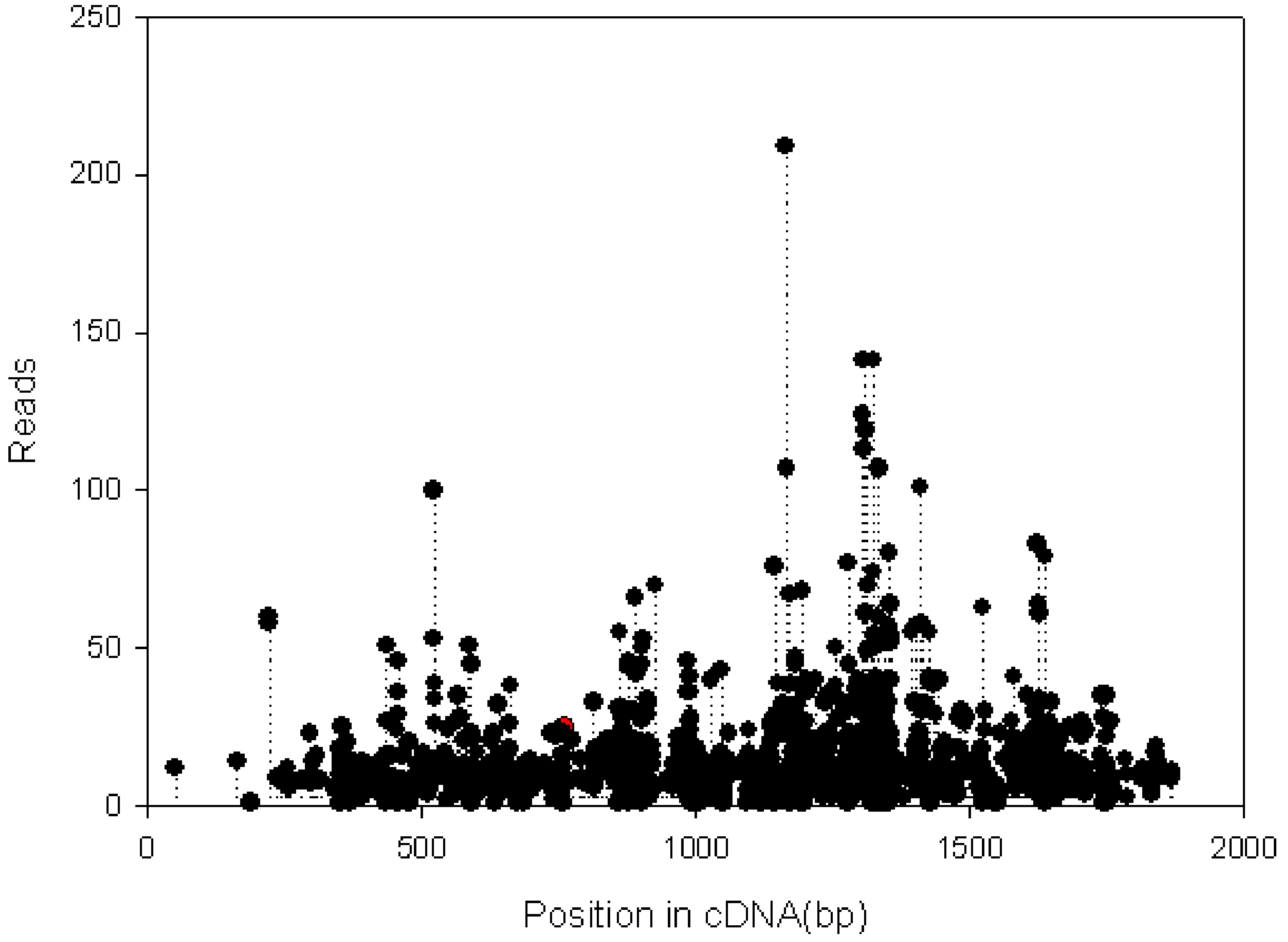


```
5' CUGC-CCAACAUCAAGGUUCCUCUCAU 3'       Cs7g31800.1
   ::::  :: :::::::.::::
3' GACGUCGUAGUAGUUCUAAGGU----- 5'       Csi-miR172c.2
```

Csi-miR172c.2, target=Cs8g17390.1 gene=Cs8g17390
Category:1
Score=3
Cleavage Site=1865

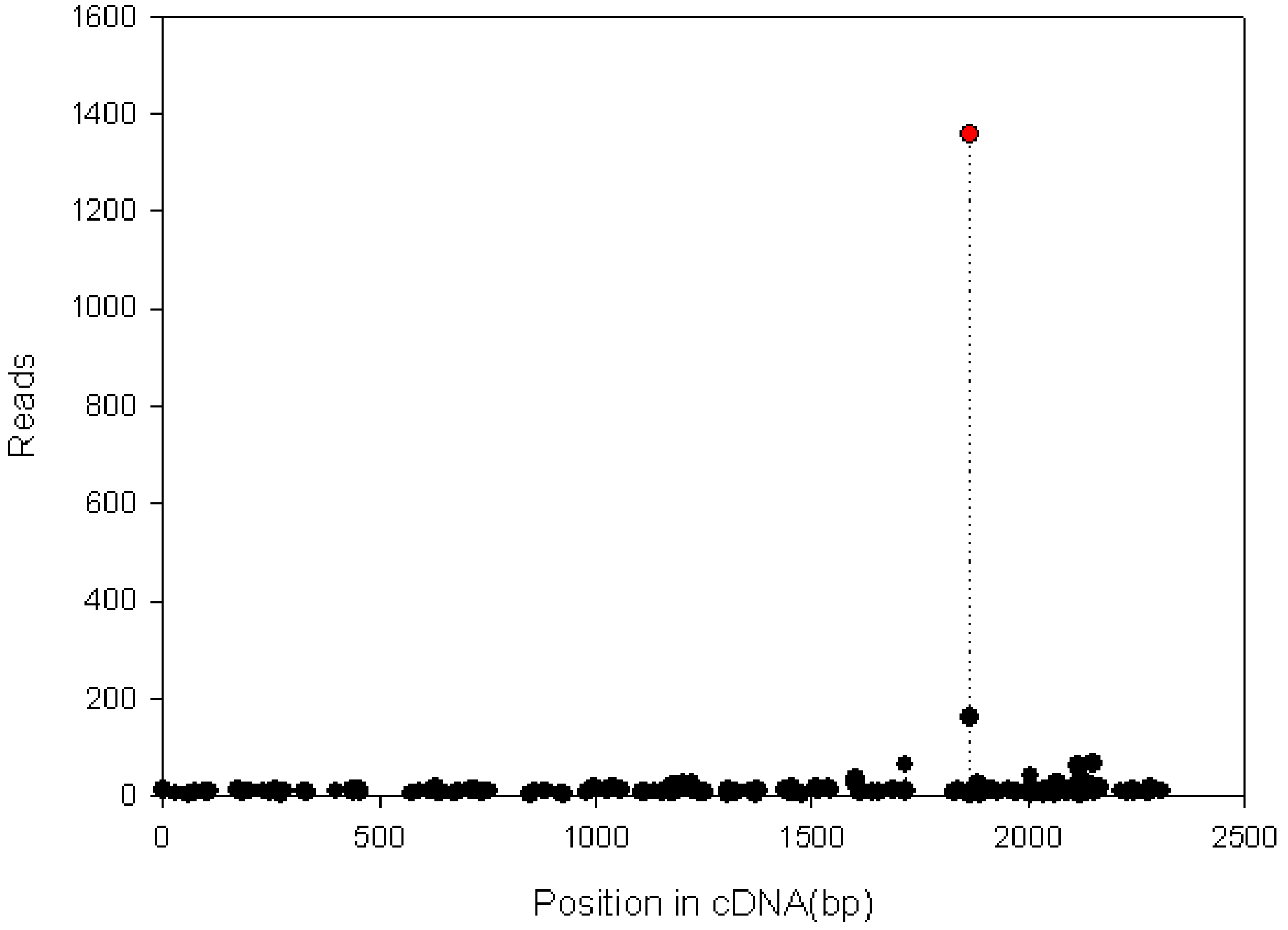


```
5' AGUGCAGCAUCAUCAGGAUUCCCAUU 3'        Cs8g17390.1
    ::::::::::::::.:::::
3' -GACGUCGUAGUAGUUCUAAGGU--- 5'        Csi-miR172c.2
```

Csi-miR172c.2, target=Orange1.1t04055.1 gene=Orange1.1t04055
Category:1
Score=2
Cleavage Site=2049

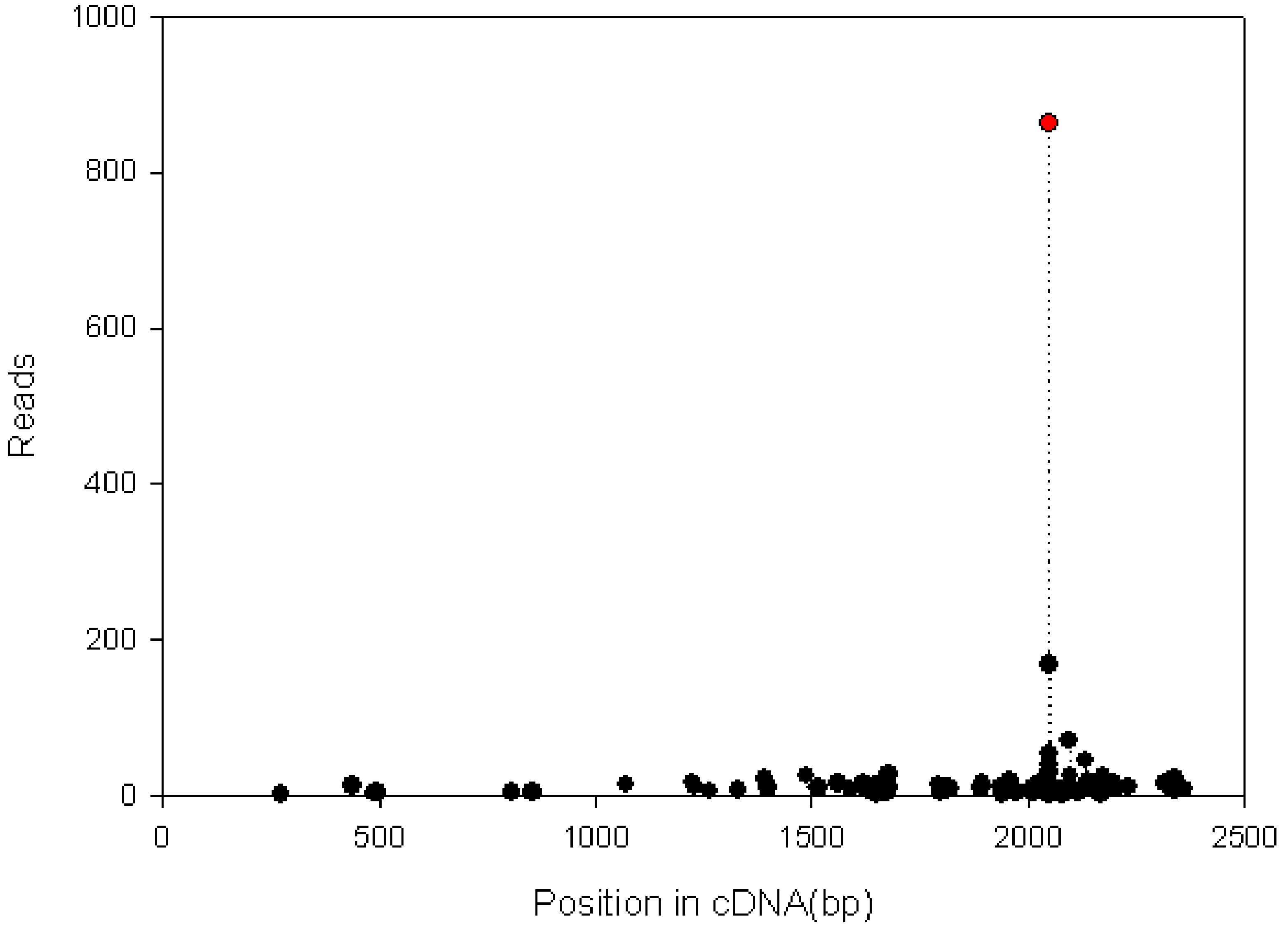


```
5' GCUGCAGCAUCAUCAGGAUUCCCUCA 3'        Orange1.1t04055.1
    ::::::::::::::::::.::::::
3' -GACGUCGUAGUAGUUCUAAGGU--- 5'        Csi-miR172c.2
```

Csi-miR172d, target=Cs7g27790.1 gene=Cs7g27790
Category:1
Score=1.5
Cleavage Site=1614

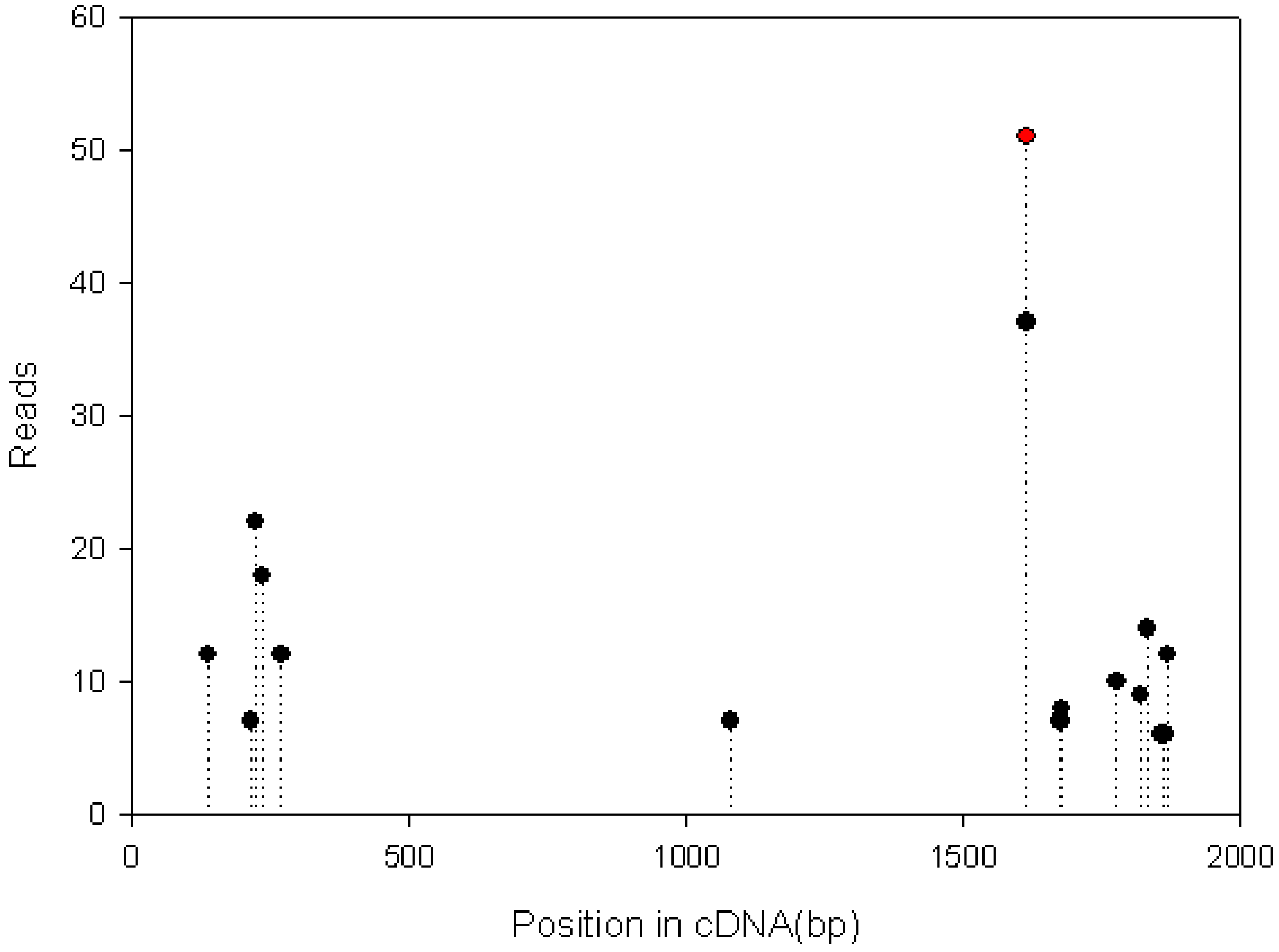


```
5' AGUGCAGCAUCAUCAGGAUUCUCUUC 3'        Cs7g27790.1
    .::::::::::::::::.::::::
3' -UACGUCGUAGUAGUUCUAAGA---- 5'        Csi-miR172d
```

Csi-miR172d, target=Cs8g17390.1 gene=Cs8g17390
Category:1
Score=2.5
Cleavage Site=1865

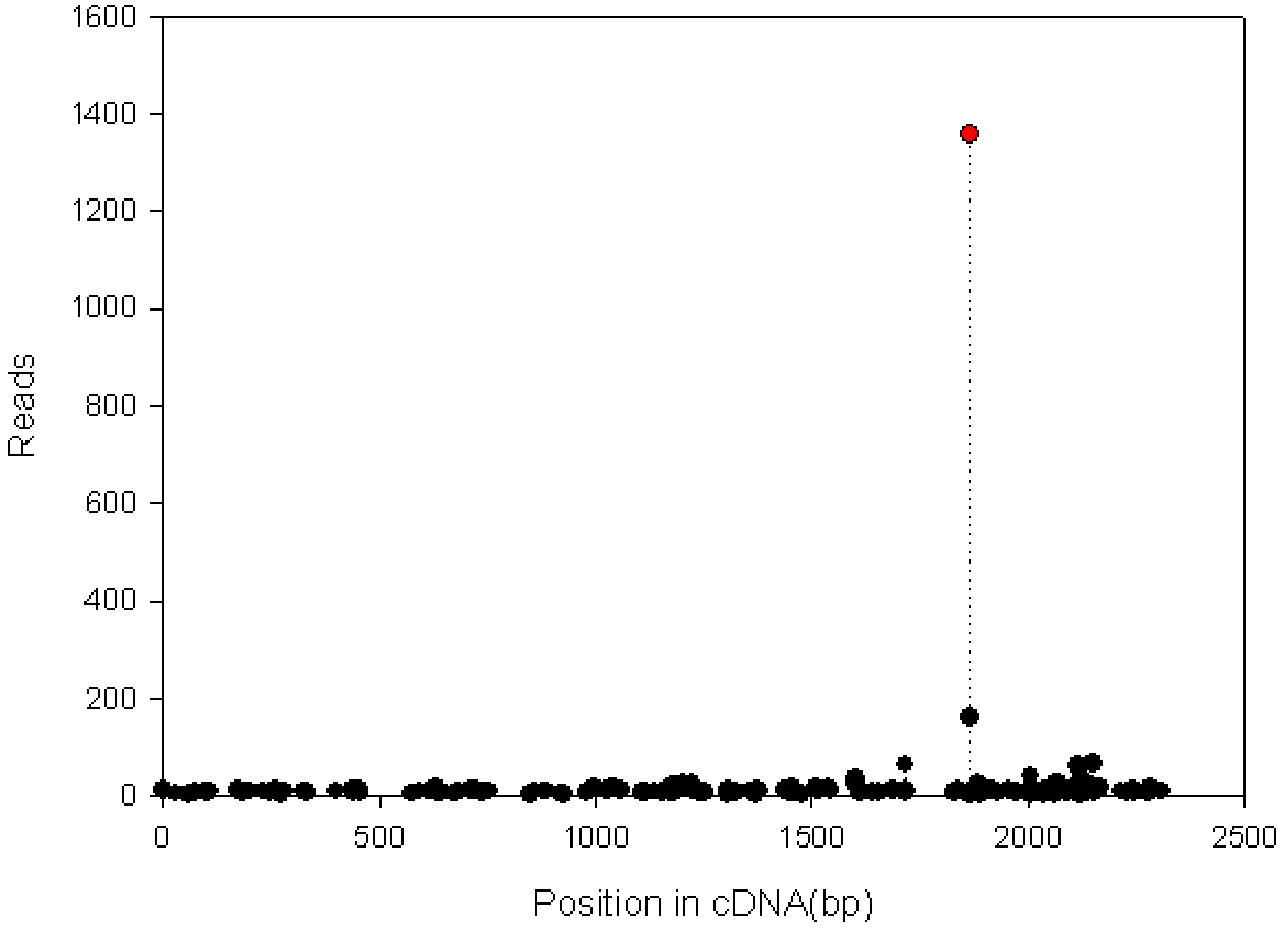


```
5' AGUGCAGCAUCAUCAGGAUUCCCAUU 3'        Cs8g17390.1
    .:::::::::::::::::.:::::
3' -UACGUCGUAGUAGUUCUAAGA---- 5'        Csi-miR172d
```

Csi-miR172d, target=Orange1.1t04055.1 gene=Orange1.1t04055
Category:1
Score=3
Cleavage Site=2049

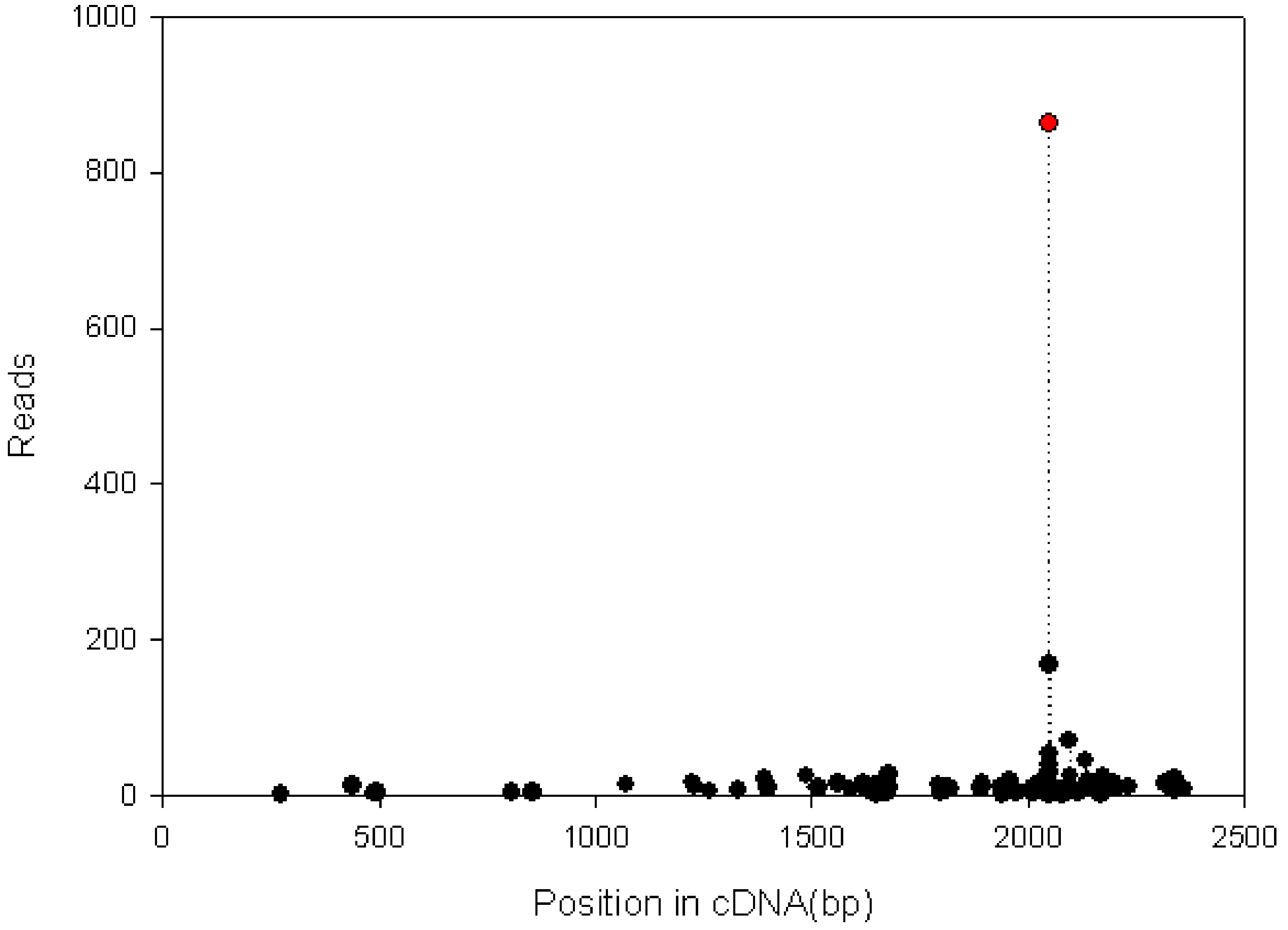


```
5' GCUGCAGCAUCAUCAGGAUUCCCUCA 3'        Orange1.1t04055.1
     ::::::::::::::.:::::
3' -UACGUCGUAGUAGUUCUAAGA---- 5'        Csi-miR172d
```

Csi-miR172d-3p, target=Cs5g21000.1 gene=Cs5g21000
Category:3
Score=5
Cleavage Site=748

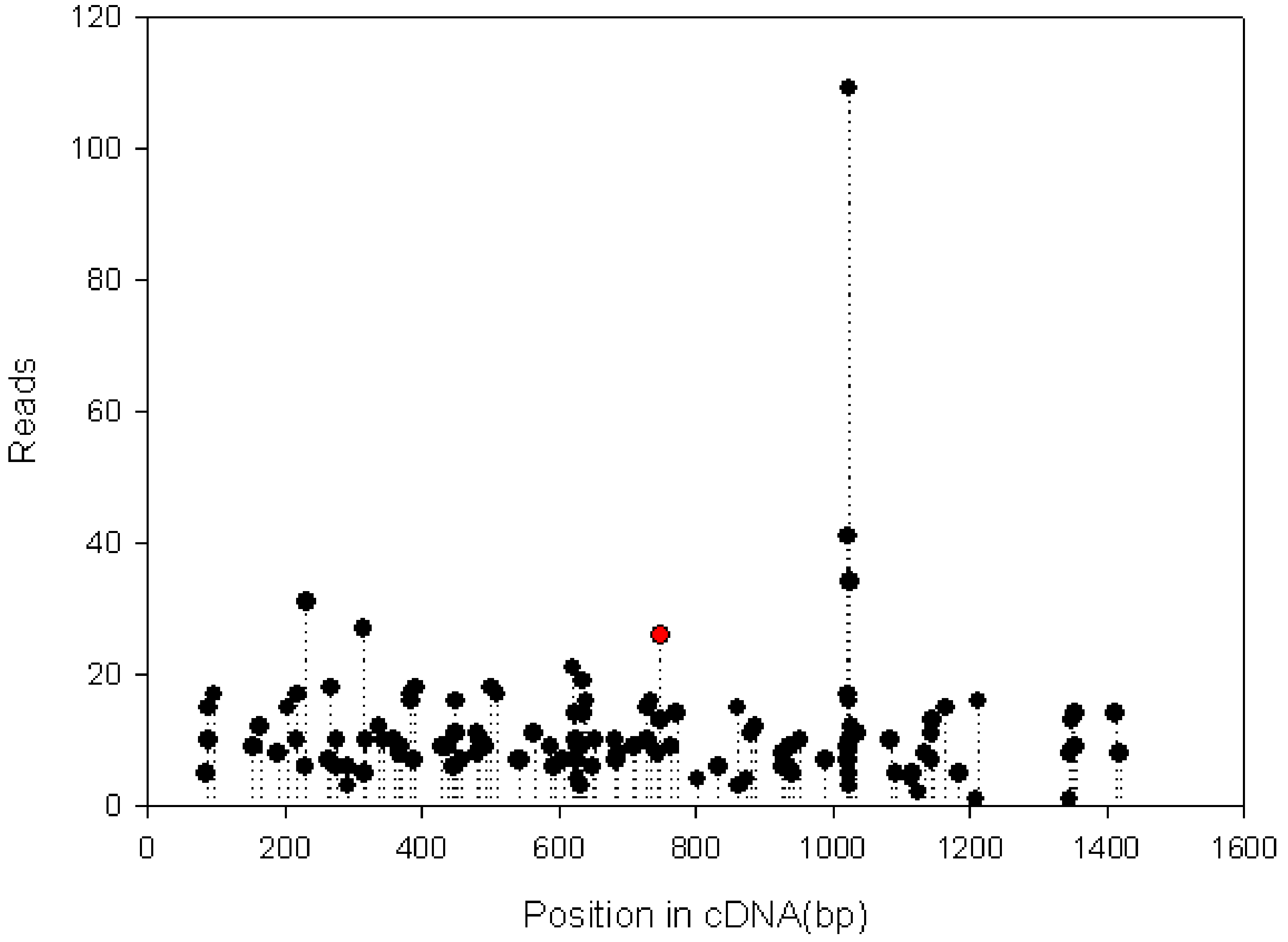


```
5' AACAAGUUUGGAU-UUGAUGAUGCUUU 3'        Cs5g21000.1
          : ::.:: :::::::::: .
3' ------ACACUUAGAACUACUACGACG 5'        Csi-miR172d-3p
```

Csi-miR172d-3p, target=Cs9g03090.1 gene=Cs9g03090
Category:3
Score=4.5
Cleavage Site=2766

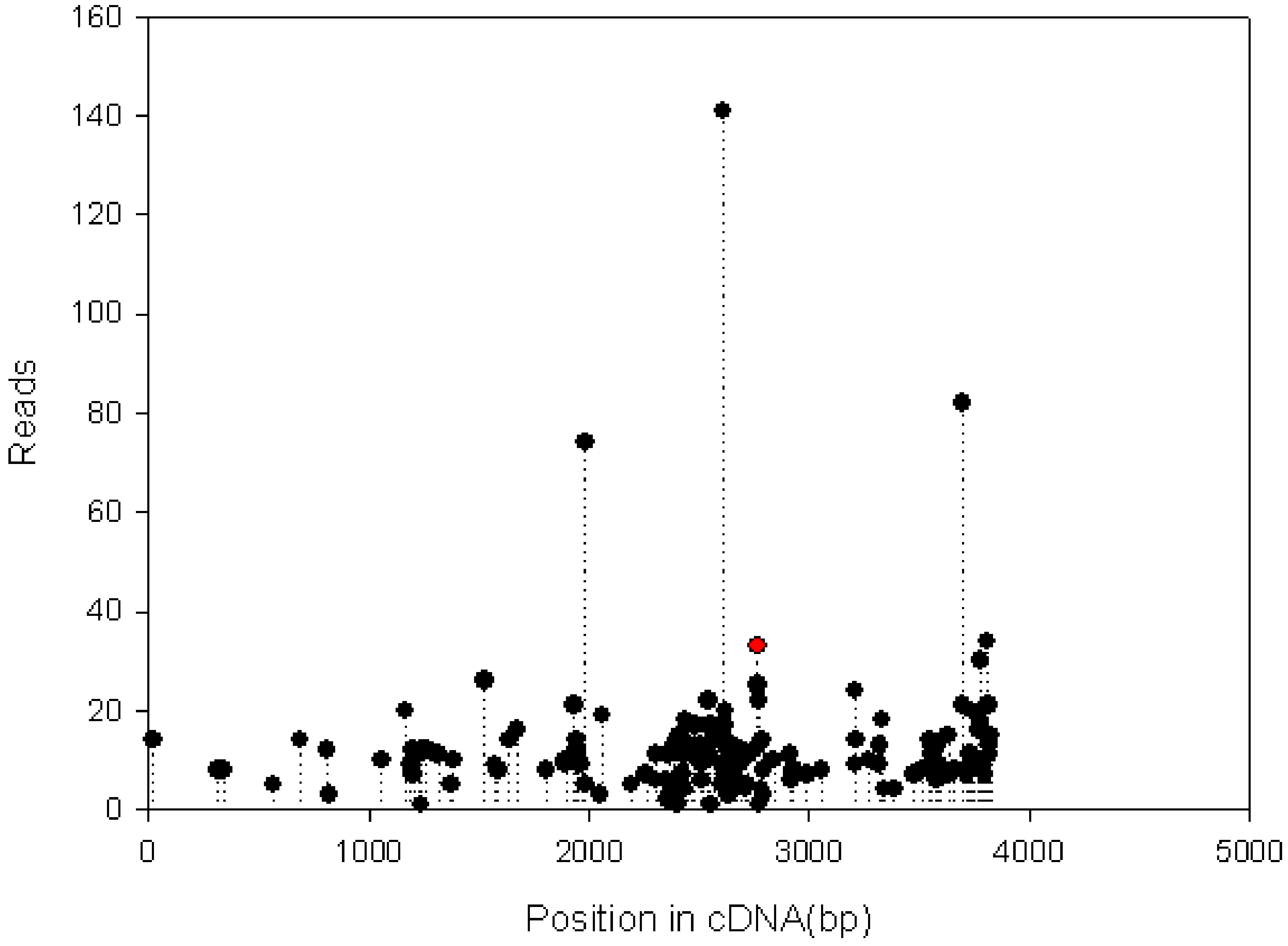


```
5' CAAUCUGCAUUUUUGAUGAUGUUGCU 3'        Cs9g03090.1
      :  ::  :  :.:::::::::.:::
3' ---ACAC-UUAGAACUACUACGACG- 5'        Csi-miR172d-3p
```

Csi-miR172e.2, target=Cs7g27790.1 gene=Cs7g27790
Category:1
Score=1.5
Cleavage Site=1614

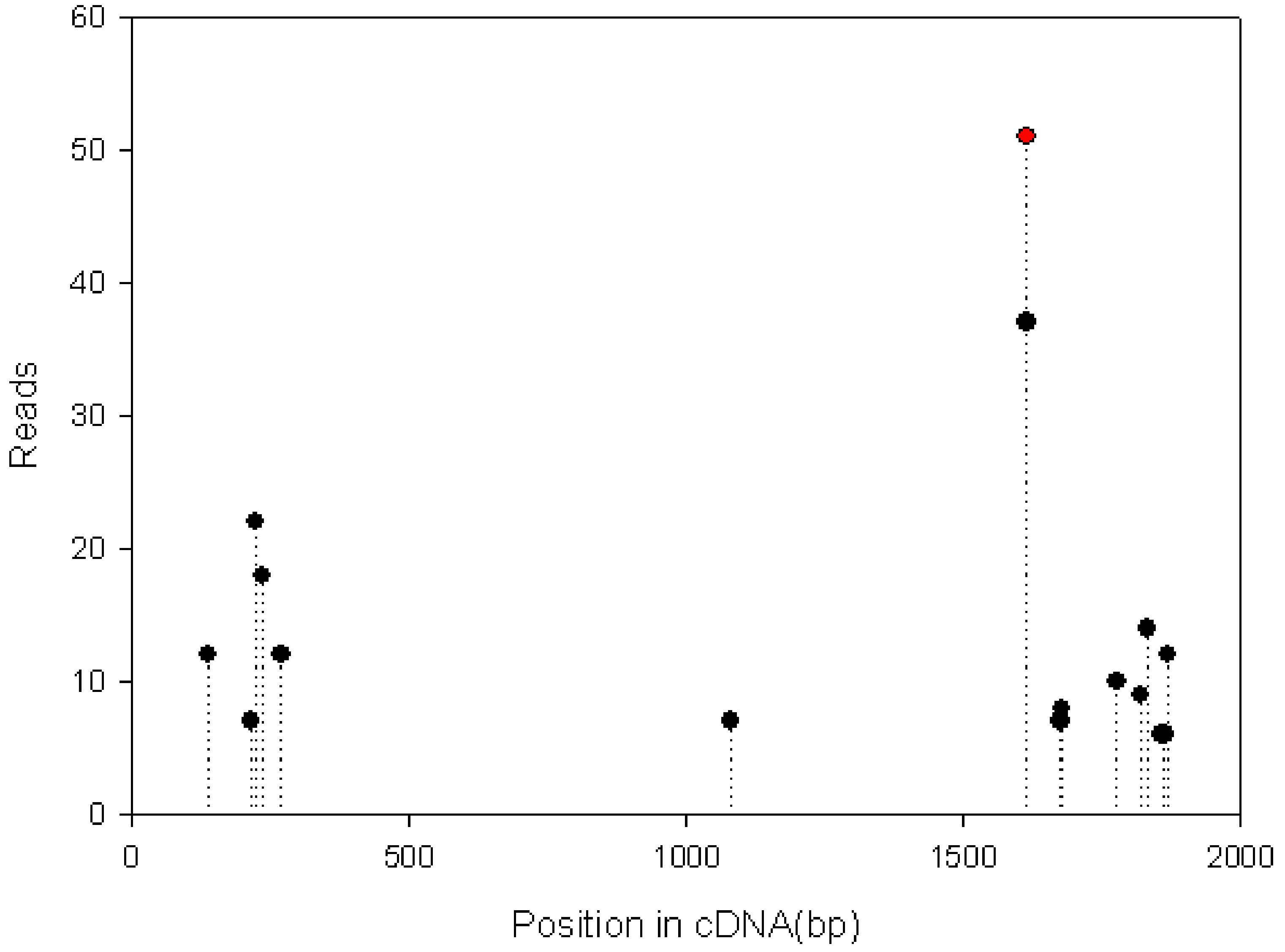


```
5' AGUGCAGCAUCAUCAGGAUUCUCUUC 3'        Cs7g27790.1
    .:::::::::::::::.:::::
3' -UACGUCGUAGUAGUUCUAAG----- 5'        Csi-miR172e.2
```

Csi-miR172e.2, target=Cs8g17390.1 gene=Cs8g17390
Category:1
Score=1.5
Cleavage Site=1865

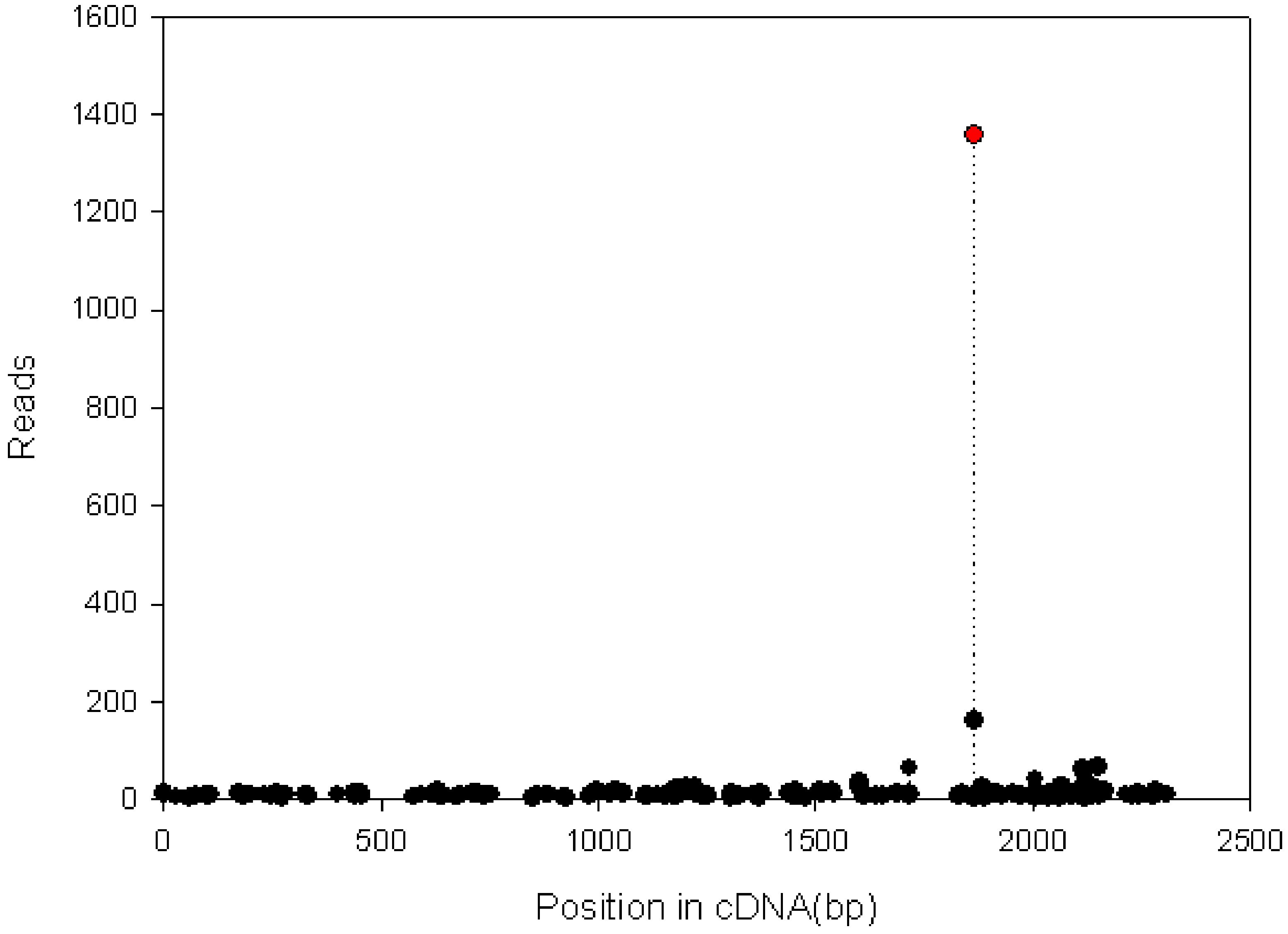


```
5' AGUGCAGCAUCAUCAGGAUUCCCAUU 3'        Cs8g17390.1
    .::::::::::::::.:::::
3' -UACGUCGUAGUAGUUCUAAG----- 5'        Csi-miR172e.2
```

Csi-miR172e.2, target=Cs9g07740.1 gene=Cs9g07740
Category:3
Score=5
Cleavage Site=3319

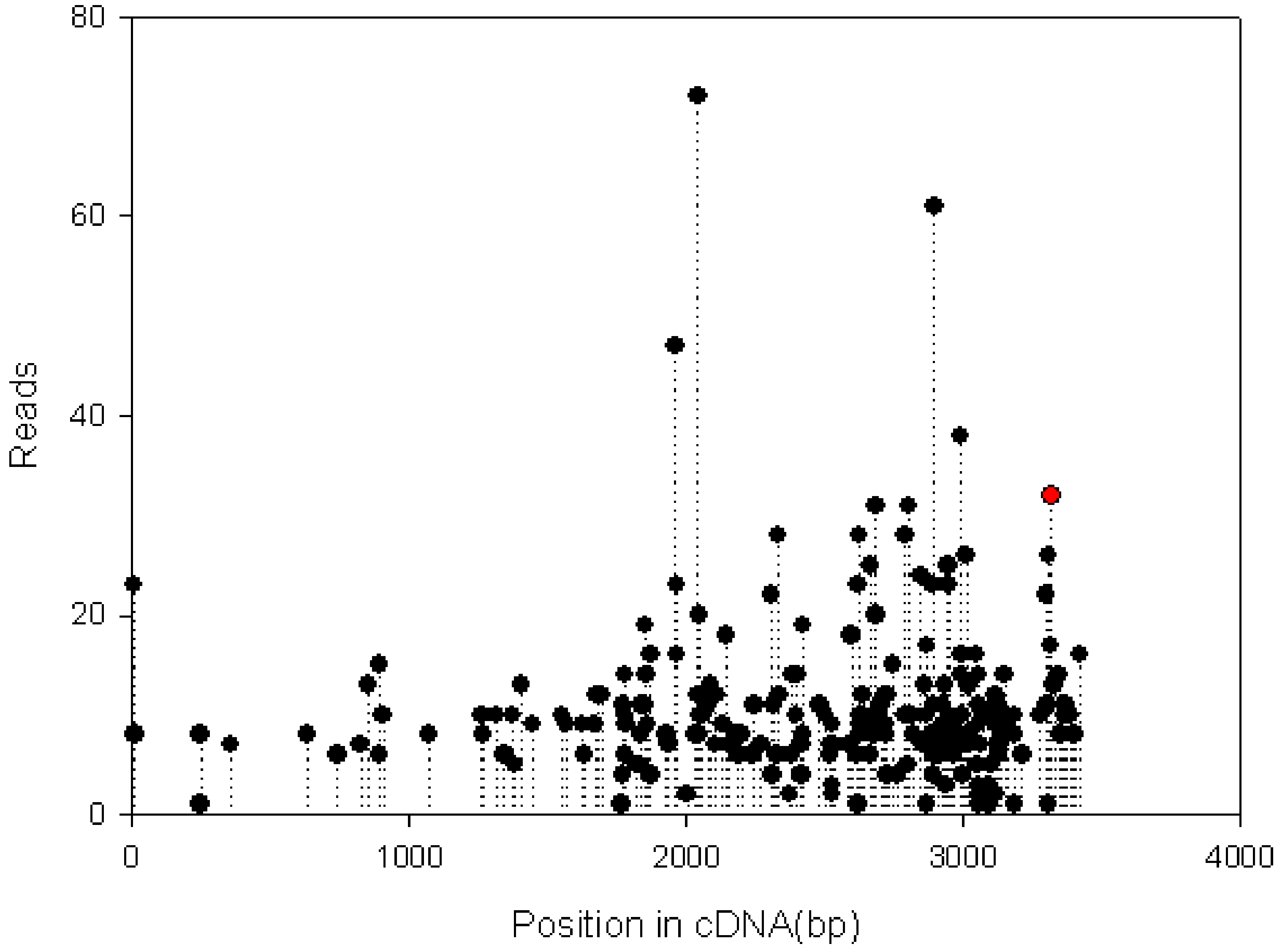


```
5' AUGUAUGAUCAUGAAGAUUUGGCAUU 3'        Cs9g07740.1
   ::::.:  ::::: :::::::.
3' UACGUCGUAGUAGUUCUAAG------ 5'        Csi-miR172e.2
```

Csi-miR172e.2, target=Orange1.1t04055.1 gene=Orange1.1t04055
Category:1
Score=2
Cleavage Site=2049

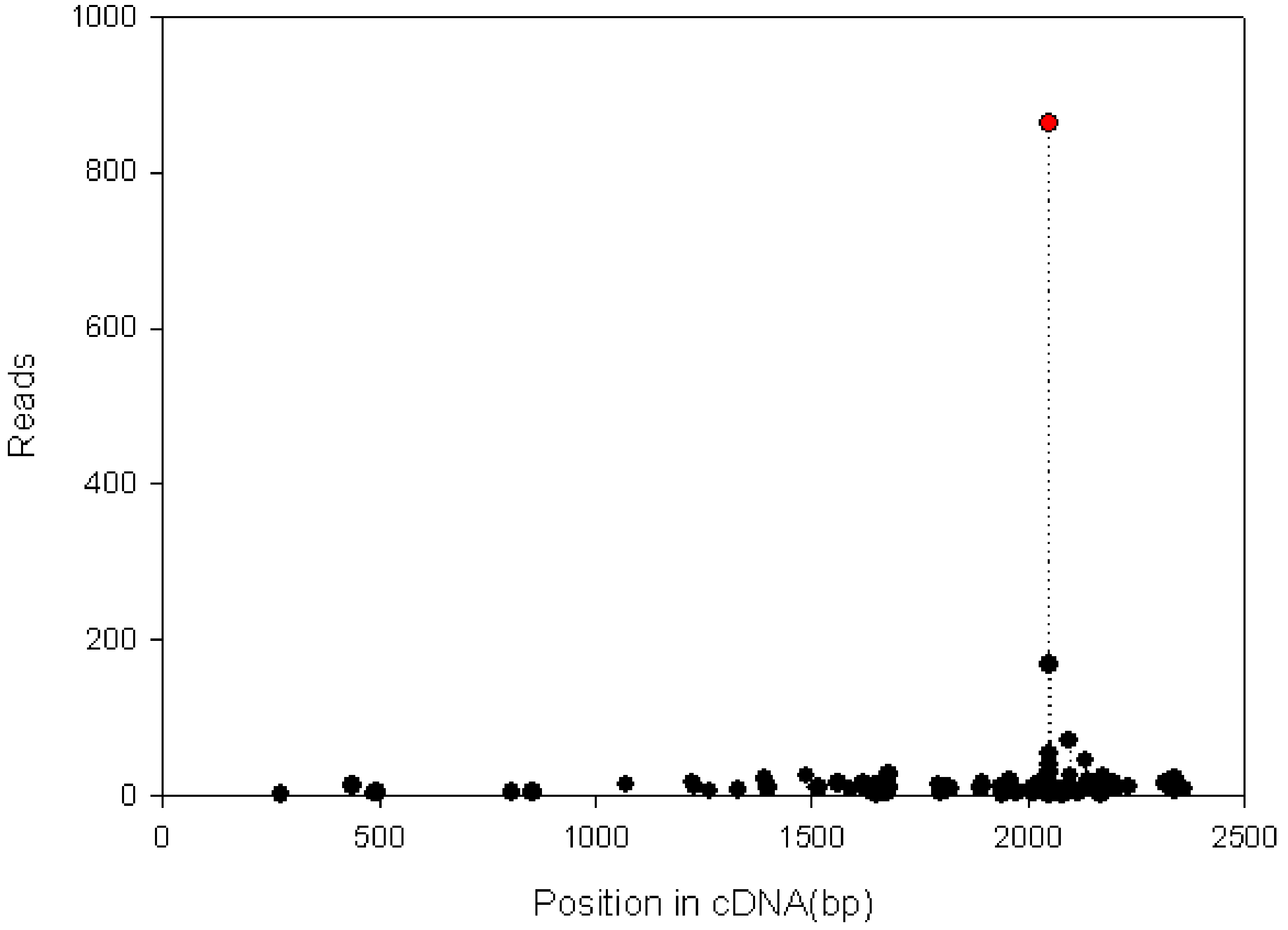


```
5' GCUGCAGCAUCAUCAGGAUUCCCUCA 3'        Orange1.1t04055.1
      ::::::::::::::.:::::
3' -UACGUCGUAGUAGUUCUAAG----- 5'        Csi-miR172e.2
```

Csi-miR172k, target=Cs7g27790.1 gene=Cs7g27790
Category:1
Score=2.5
Cleavage Site=1614

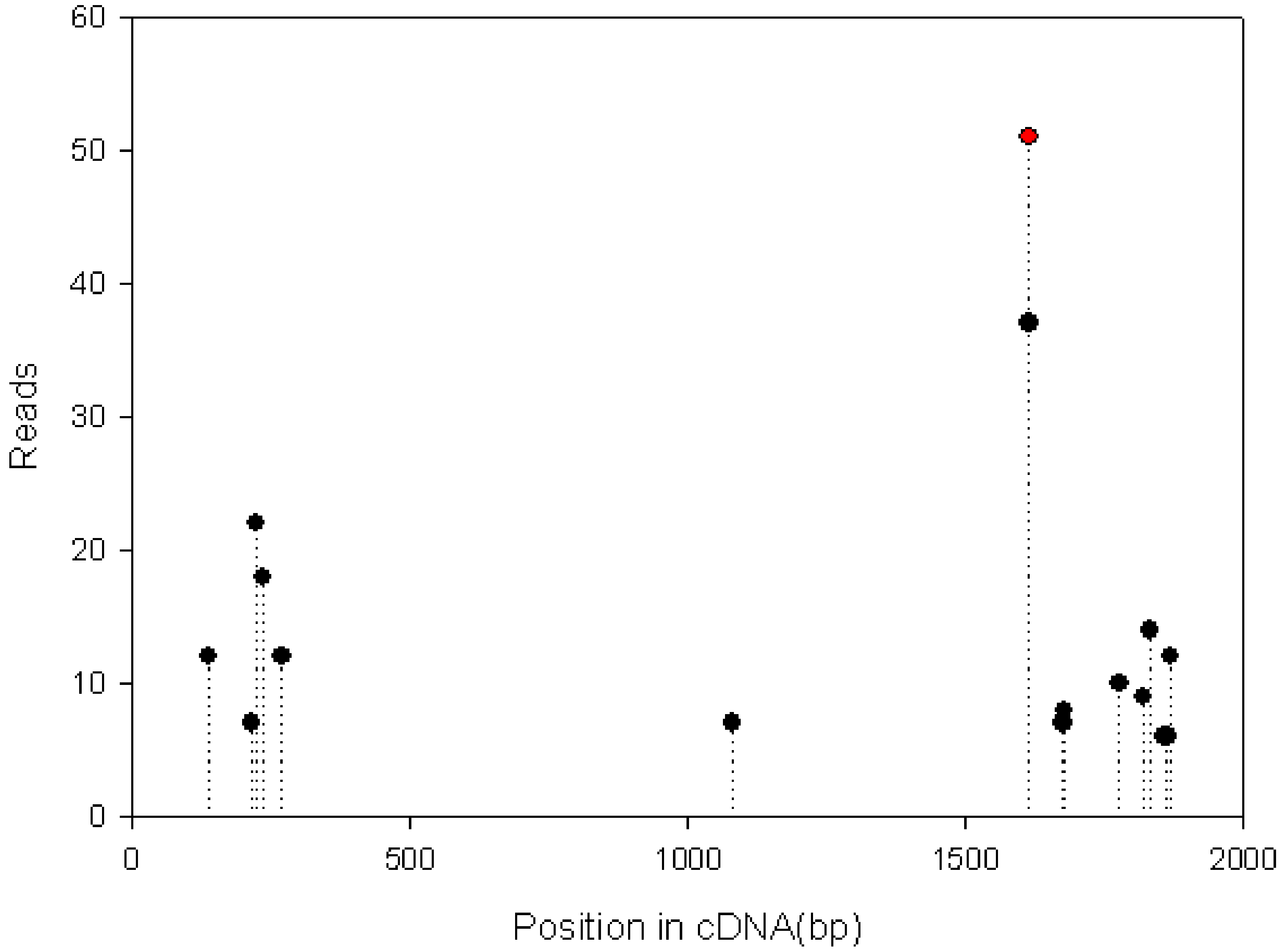


```
5' AGUGCAGCAUCAUCAGGAUUCUCUUC 3'        Cs7g27790.1
     .::::::::::::::::.:::::
3' -UACGUCGUAGUAGUUCUAAGU---- 5'        Csi-miR172k
```

Csi-miR172k, target=Cs8g17390.1 gene=Cs8g17390
Category:1
Score=2.5
Cleavage Site=1865

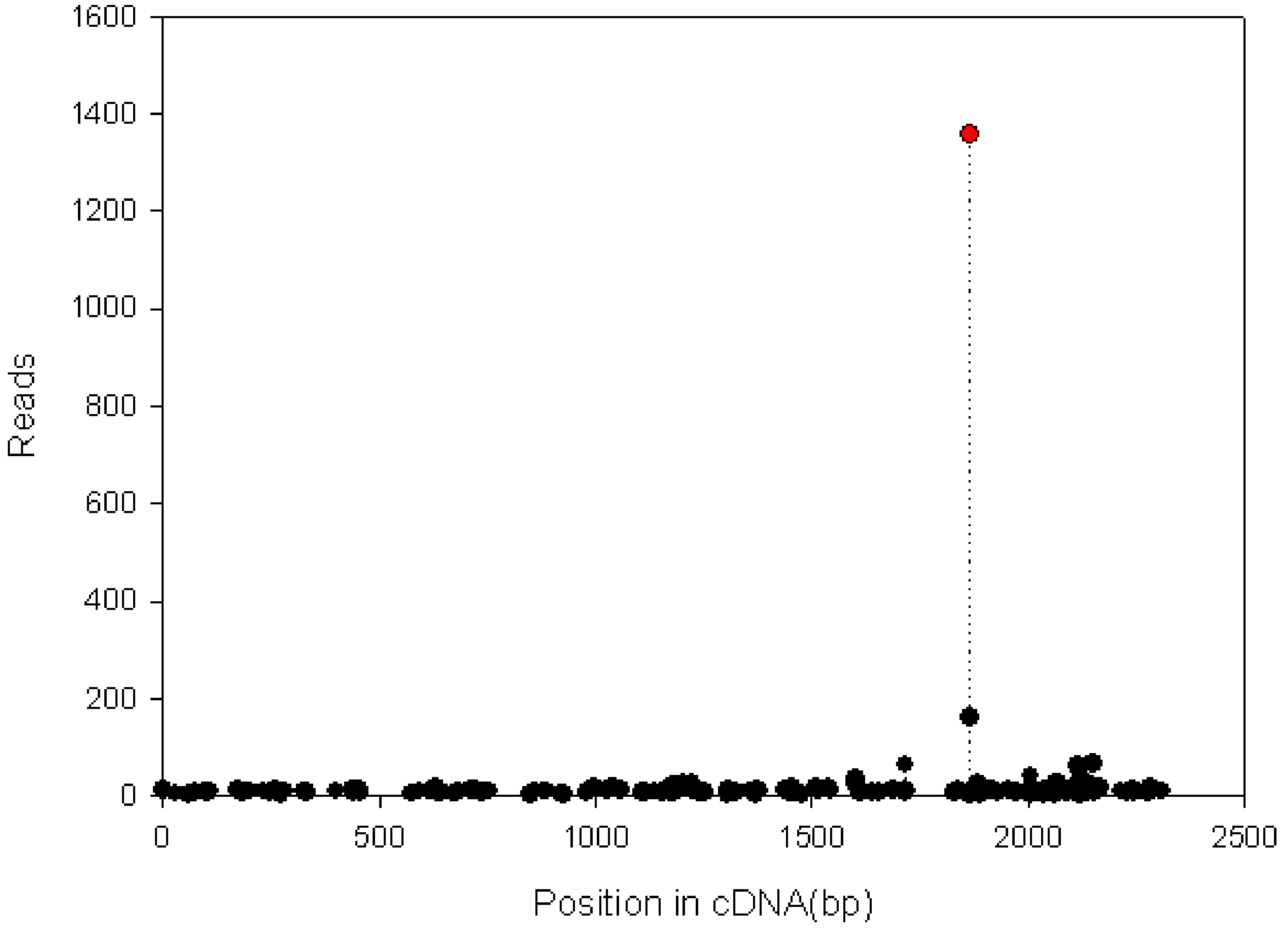


```
5' AGUGCAGCAUCAUCAGGAUUCCCAUU 3'        Cs8g17390.1
    .:::::::::::::::.:::::
3' -UACGUCGUAGUAGUUCUAAGU---- 5'        Csi-miR172k
```

Csi-miR172k, target=Orange1.1t04055.1 gene=Orange1.1t04055
Category:1
Score=5
Cleavage Site=2049

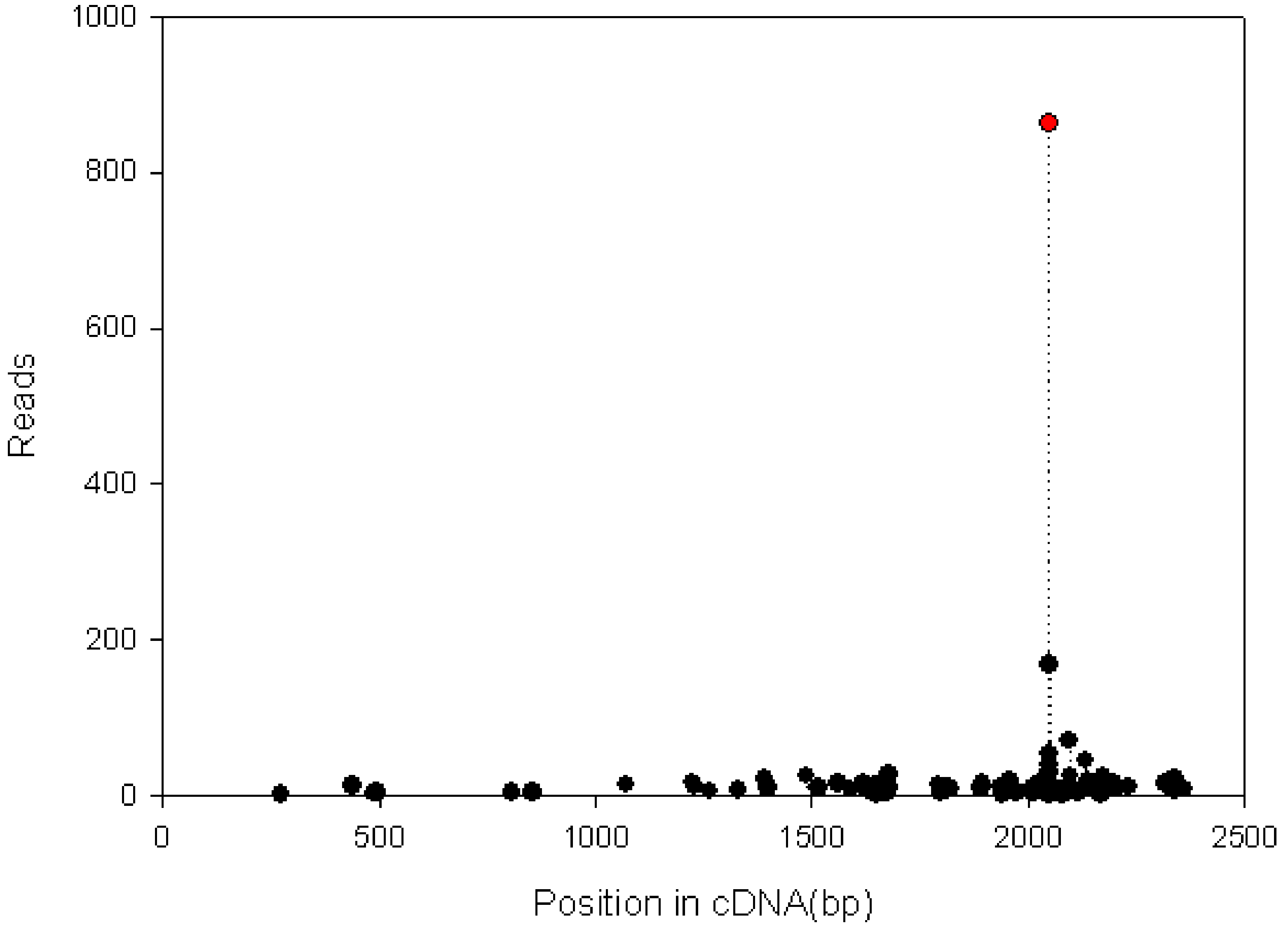


```
5' GCUGCAGCAUCAUCAGGAUUCCCUCA 3'        Orange1.1t04055.1
     ::::::::::::::::.:::::
3' -UACGUCGUAGUAGUUCUAAGU---- 5'        Csi-miR172k
```

Csi-miR2118.1, target=Orange1.1t00149.1 gene=Orange1.1t00149
Category:2
Score=4.5
Cleavage Site=98

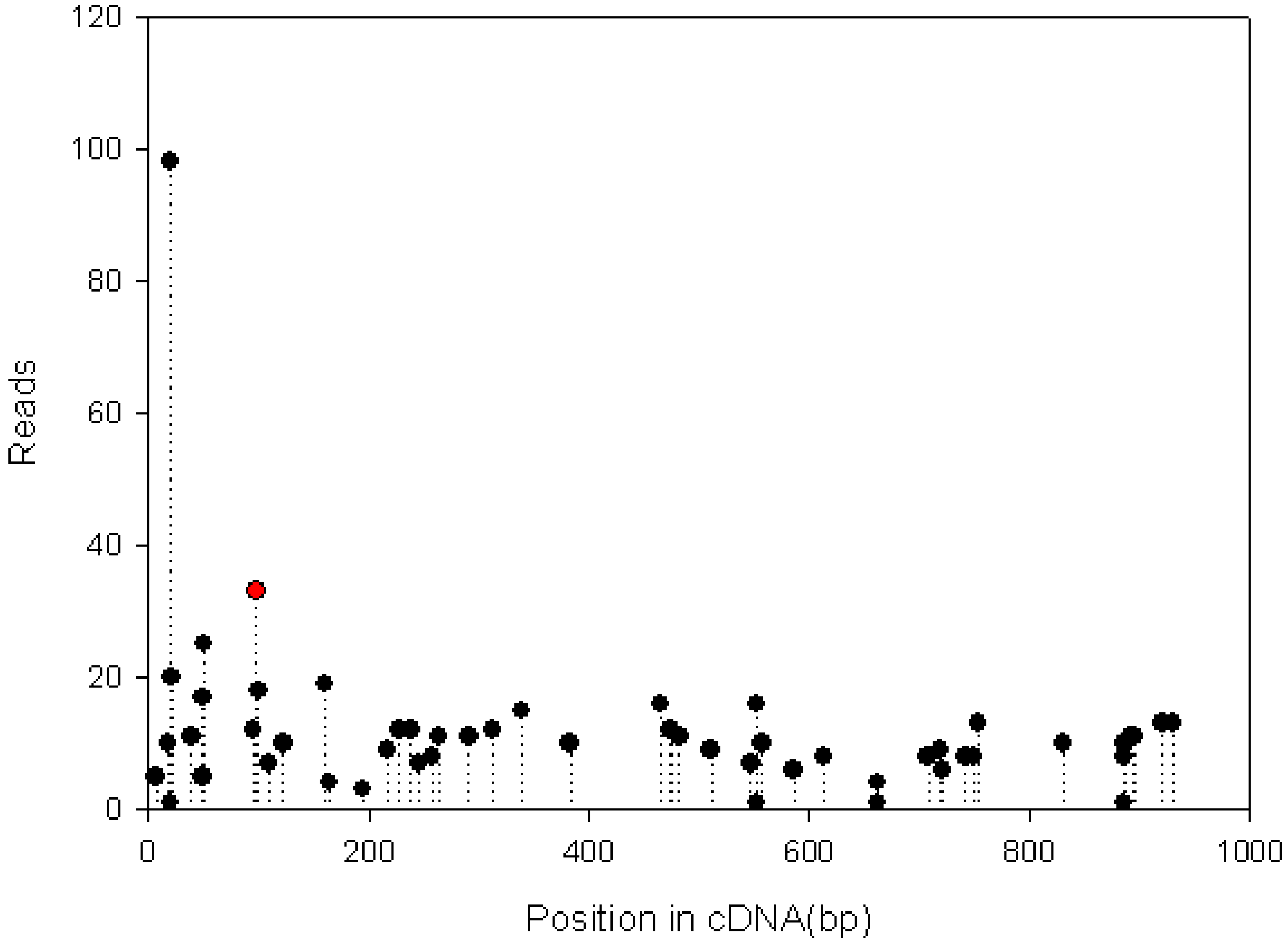


```
5' ACGGAGUGGGUGGCAUA-GUGAUGGCC 3'        Orange1.1t00149.1
   : :::.::::::::::: ::.:
3' UUCCUUACCCACCGUAUCCAUU----- 5'        Csi-miR2118.1
```

Csi-miR2118.1, target=Orange1.1t00557.1 gene=Orange1.1t00557
Category:1
Score=4
Cleavage Site=131

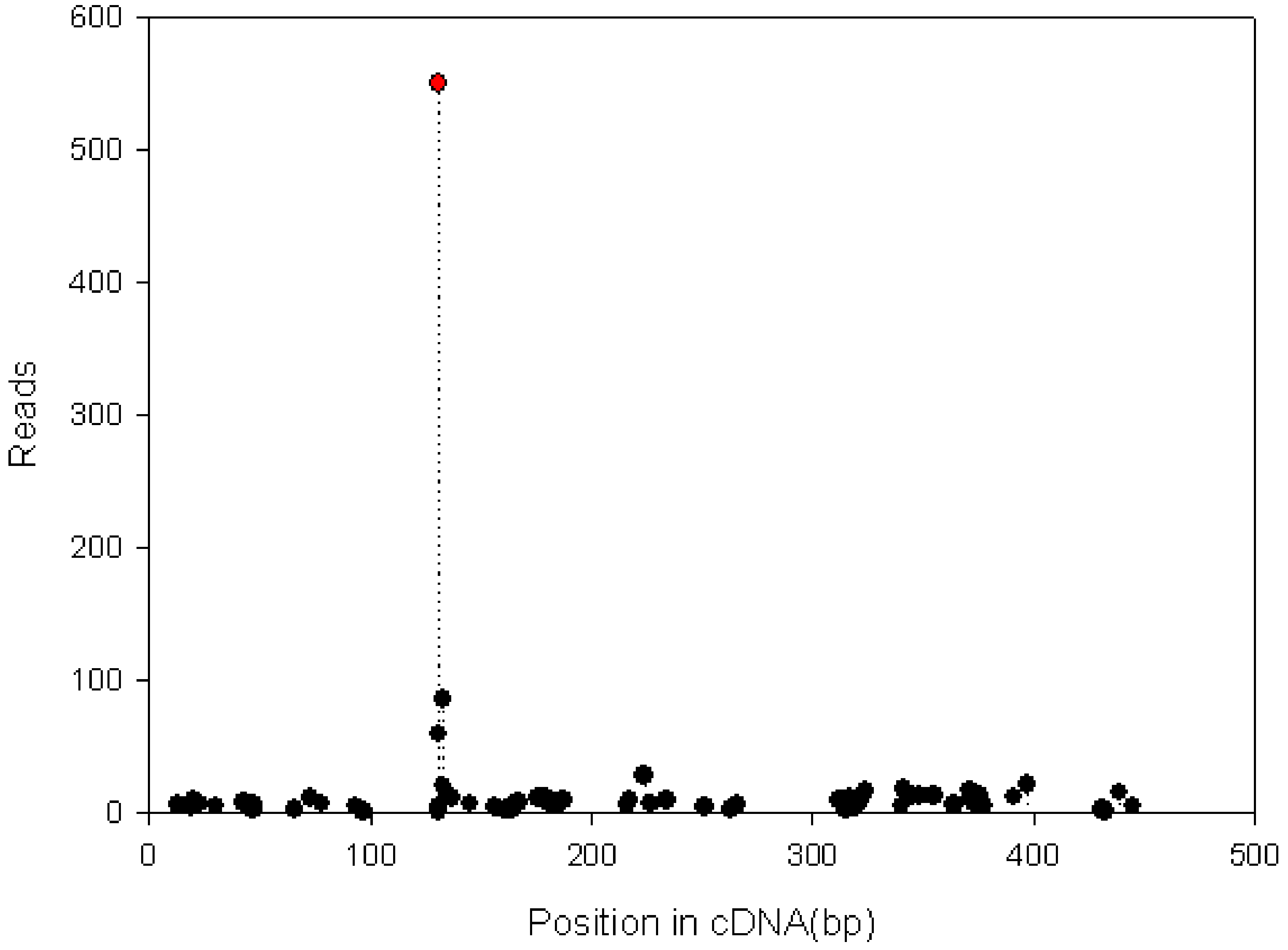


```
5' AAGACAUGGGUGGCAUAGGUCACAUC 3'        Orange1.1t00557.1
   :::  ::::::::::::::: :
3' UUCCUUACCCACCGUAUCCAUU---- 5'        Csi-miR2118.1
```

Csi-miR2118.1, target=Orange1.1t02171.1 gene=Orange1.1t02171
Category:3
Score=5
Cleavage Site=662

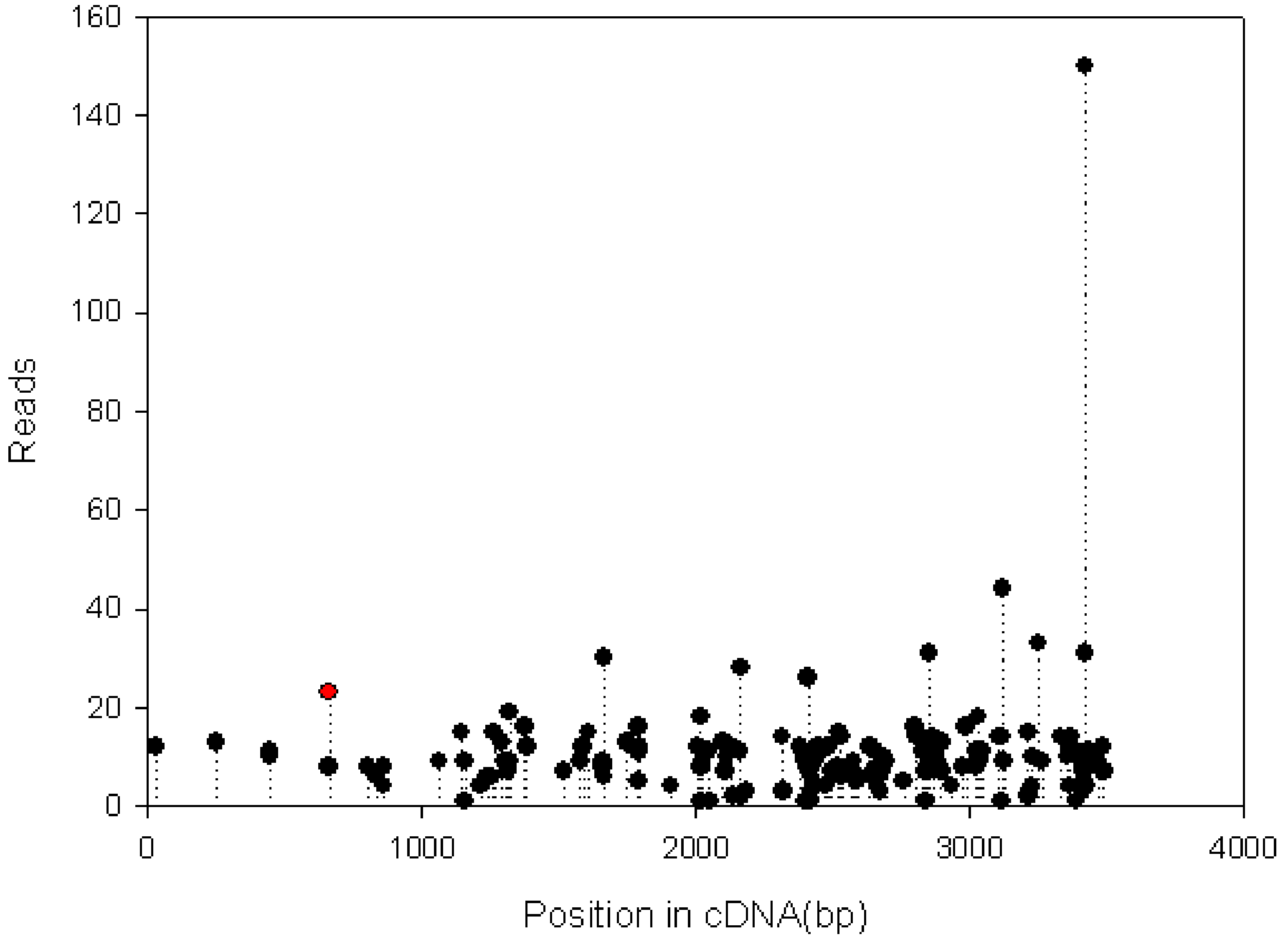


```
5' GGGGCAUGGGUGGUAUAGGCAAAACC 3'        Orange1.1t02171.1
   ..:: :::::::::.::::: ::
3' UUCCUUACCCACCGUAUCCAUU---- 5'        Csi-miR2118.1
```

Csi-miR2118.1, target=Orange1.1t02175.1 gene=Orange1.1t02175
Category:3
Score=5
Cleavage Site=735

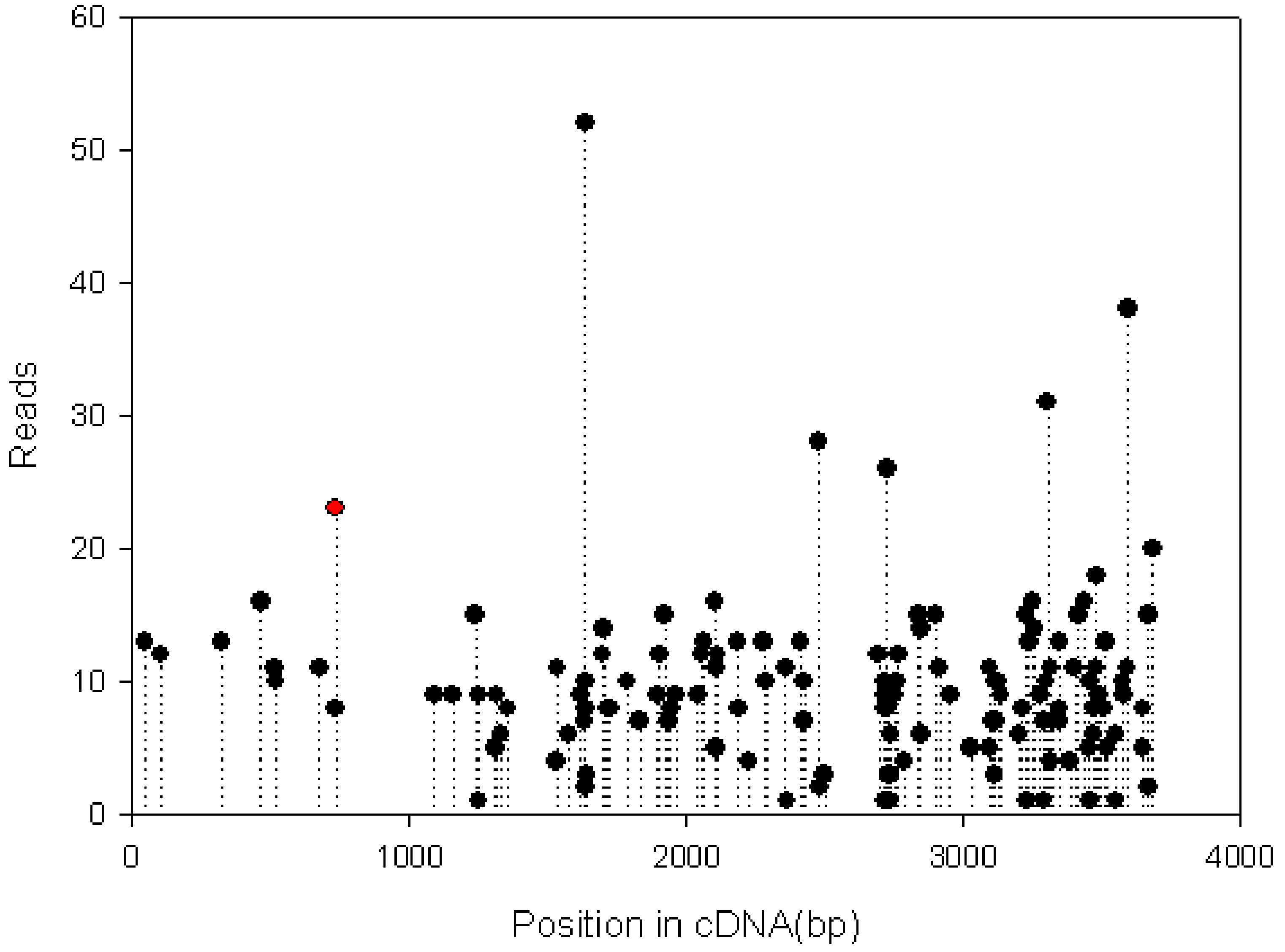


```
5' GGGGCAUGGGUGGUAUAGGCAAAACC 3'        Orange1.1t02175.1
   ..:: :::::::::.::::: ::
3' UUCCUUACCCACCGUAUCCAUU---- 5'        Csi-miR2118.1
```

Csi-miR2118.2, target=Cs2g09590.1 gene=Cs2g09590
Category:1
Score=4
Cleavage Site=769

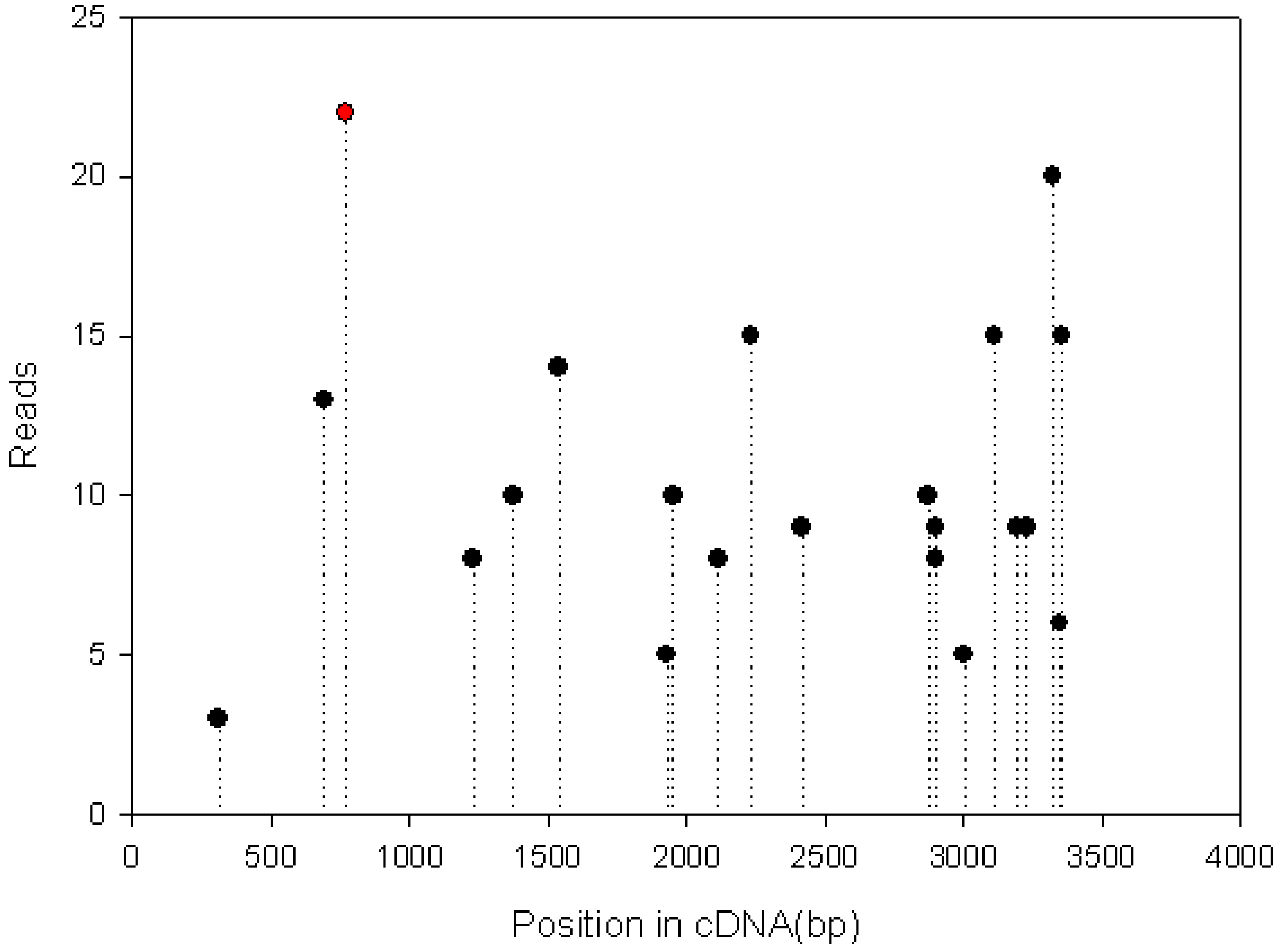


```
5' GGUAUGGGUGGCAUAGGGGACUCAUU 3'        Cs2g09590.1
   :: ::::::::::::::: .:
3' CCUUACCCACCGUAUCCAUU------ 5'        Csi-miR2118.2
```

Csi-miR2118.2, target=Cs2g30960.1 gene=Cs2g30960
Category:1
Score=5
Cleavage Site=694

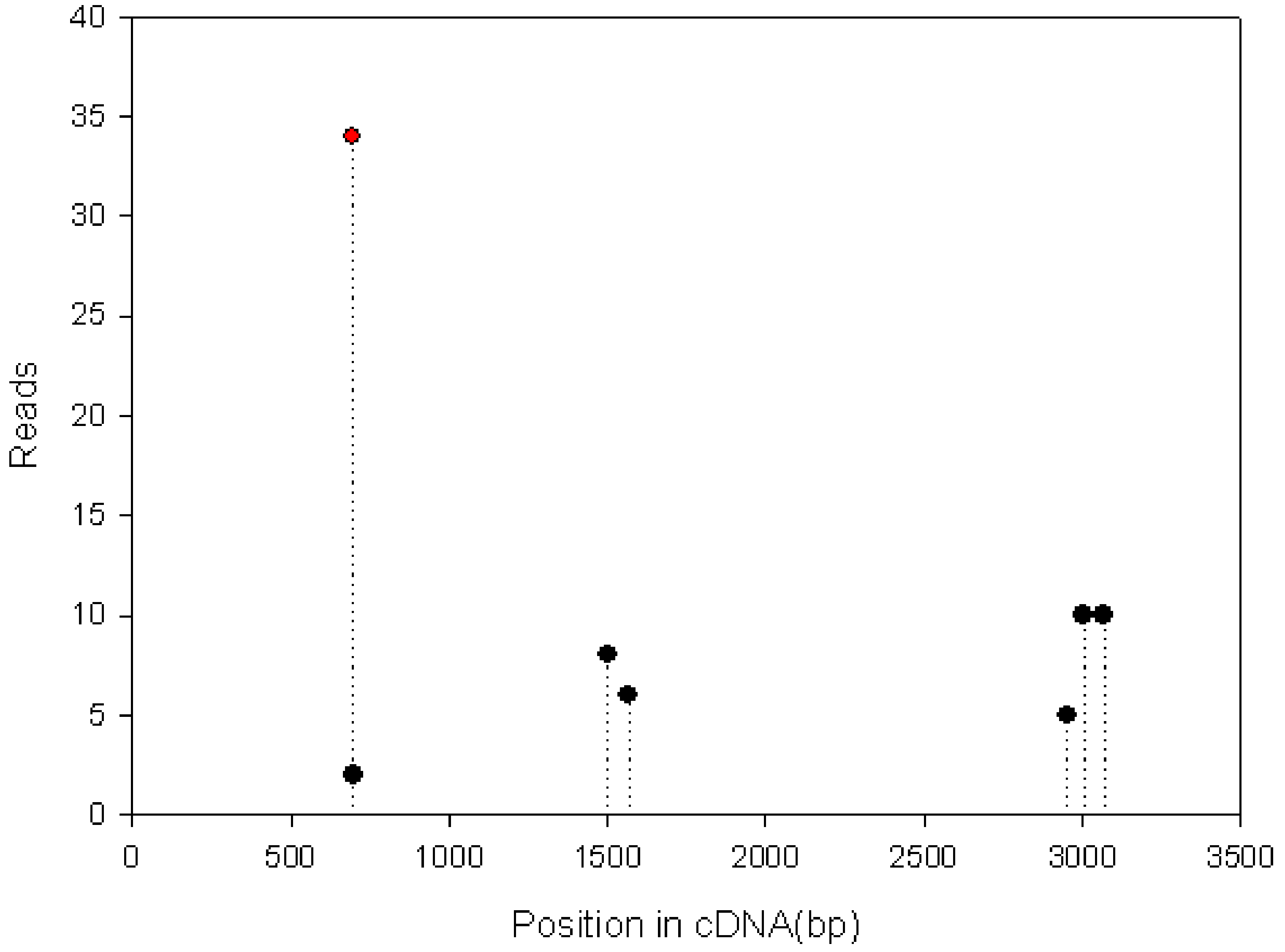


```
5' GGCAUUGGUGGUAUAGGCAAGACAAC 3'        Cs2g30960.1
   :: :: :::::.::::: ::
3' CCUUACCCACCGUAUCCAUU------ 5'        Csi-miR2118.2
```

Csi-miR2118.2, target=Cs2g30970.1 gene=Cs2g30970
Category:1
Score=5
Cleavage Site=673

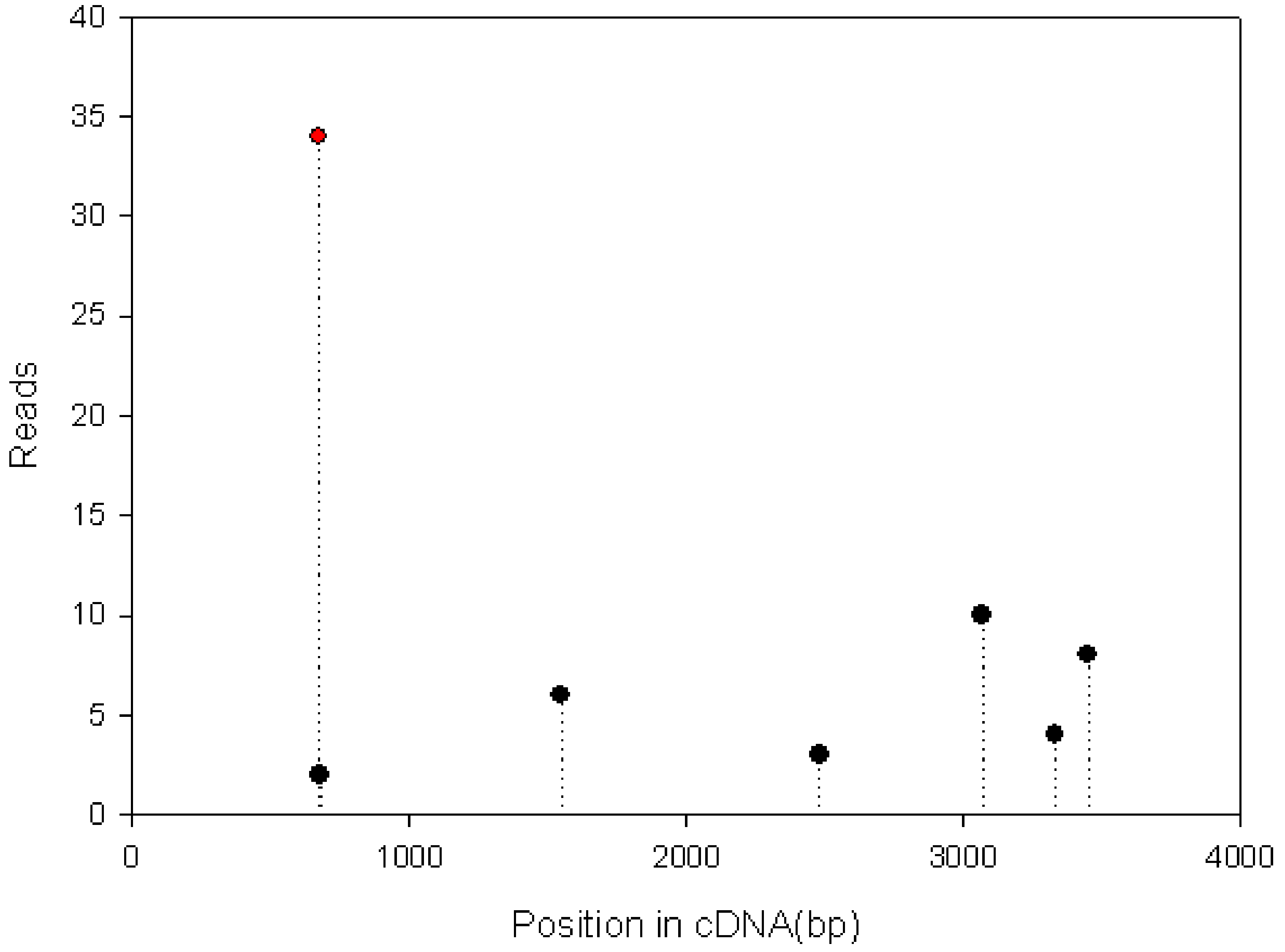


```
5'  GGCAUUGGUGGUAUAGGCAAGACAAC 3'        Cs2g30970.1
    :: :: :::::.::::: ::
3'  CCUUACCCACCGUAUCCAUU------ 5'        Csi-miR2118.2
```

Csi-miR2118.2, target=Cs3g12720.1 gene=Cs3g12720
Category:1
Score=5
Cleavage Site=547

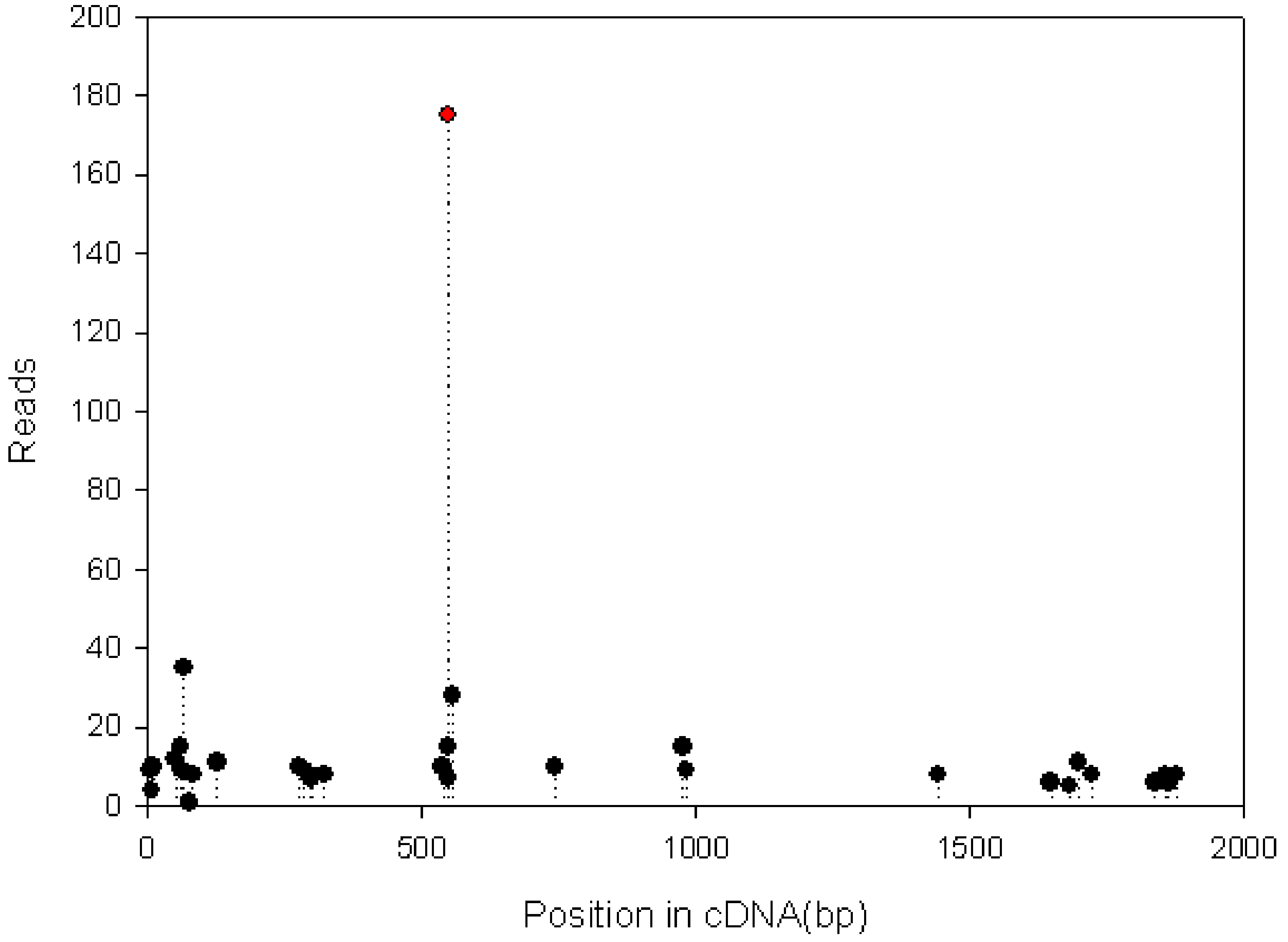


```
5' GGCAUGGGUGGCAUUGGAAAGACAAC 3'        Cs3g12720.1
   :: :::::::::: :: ::
3' CCUUACCCACCGUAUCCAUU------ 5'        Csi-miR2118.2
```

Csi-miR2118.2, target=Cs3g12760.1 gene=Cs3g12760
Category:1
Score=4.5
Cleavage Site=1037

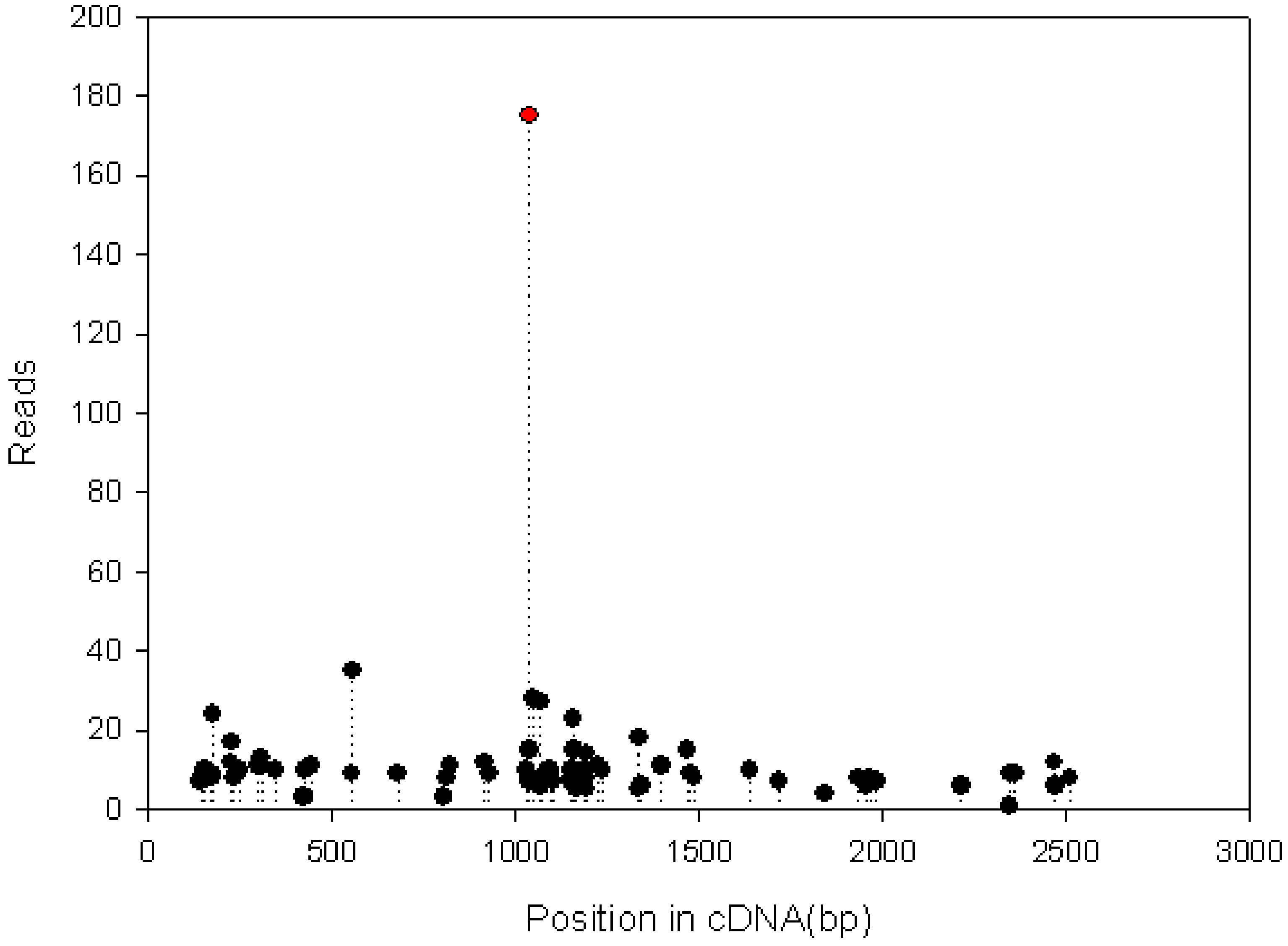


```
5' GGGAUGGGUGGCAUUGGAAAGACAAC 3'          Cs3g12760.1
   ::.:::::::::::: :: ::
3' CCUUACCCACCGUAUCCAUU------ 5'          Csi-miR2118.2
```

Csi-miR2118.2, target=Cs3g12850.1 gene=Cs3g12850
Category:1
Score=4.5
Cleavage Site=547

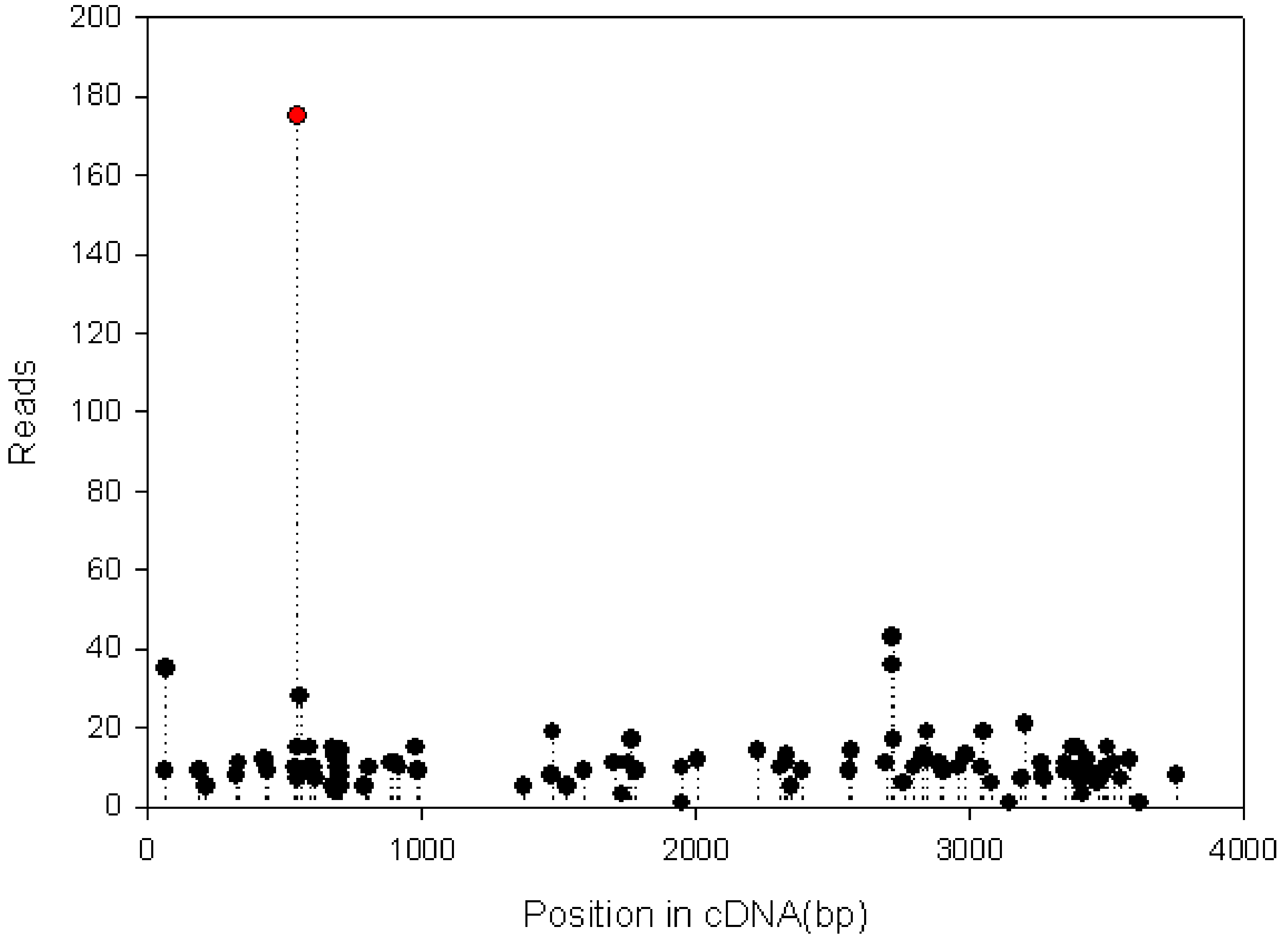


```
5' GGGAUGGGUGGCAUUGGAAAGACAAC 3'        Cs3g12850.1
   ::.:::::::::::: :: ::
3' CCUUACCCACCGUAUCCAUU------ 5'        Csi-miR2118.2
```

Csi-miR2118.2, target=Cs5g18480.1 gene=Cs5g18480
Category:1
Score=4
Cleavage Site=700

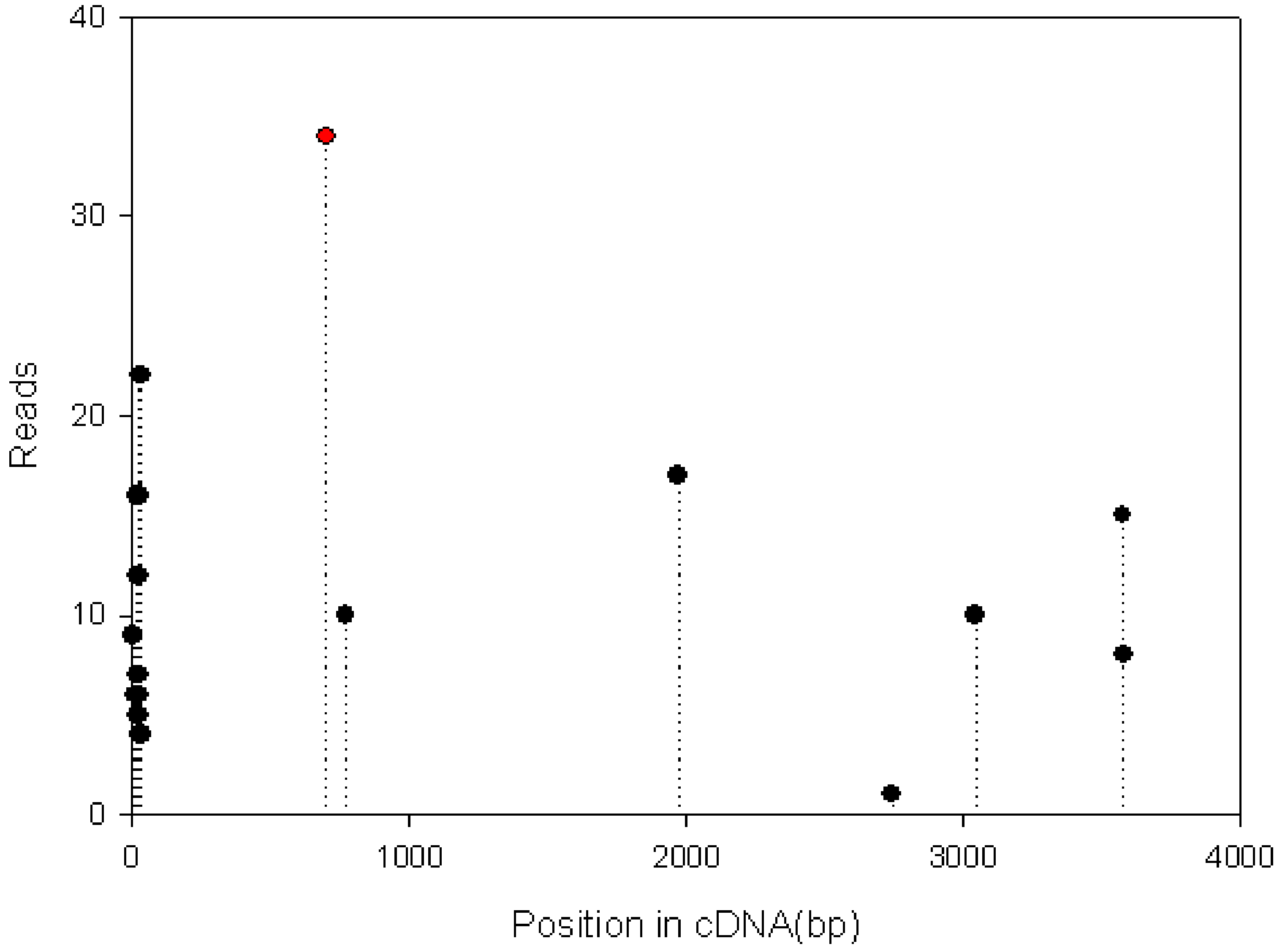


```
5' GGCAUGGGUGGUAUAGGCAAGACAAC 3'        Cs5g18480.1
   :: :::::::::.::::: ::
3' CCUUACCCACCGUAUCCAUU------ 5'        Csi-miR2118.2
```

Csi-miR2118.2, target=Cs5g19310.1 gene=Cs5g19310
Category:1
Score=5
Cleavage Site=859

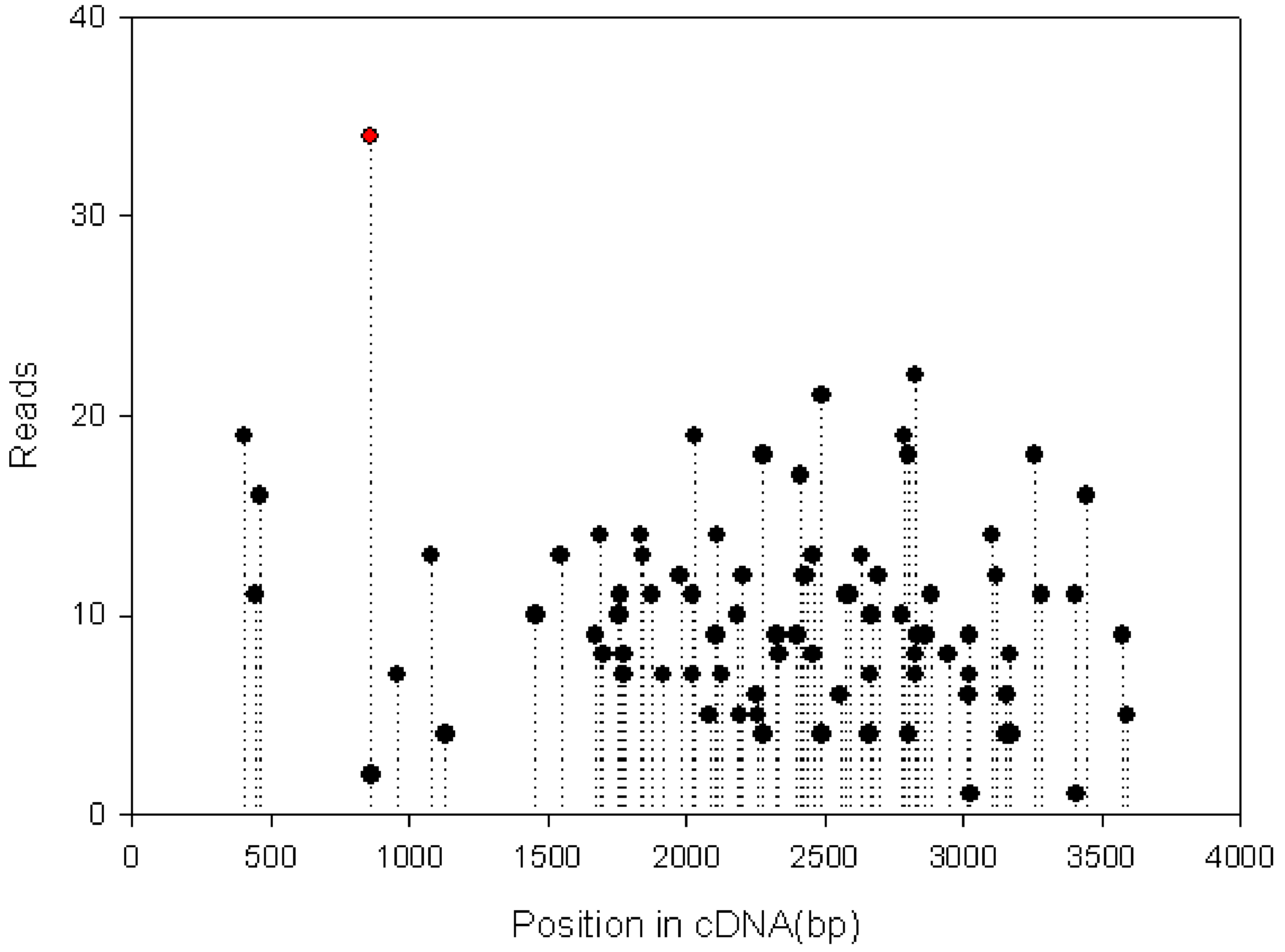


```
5' GGCAUCGGUGGUAUAGGCAAGACAAC 3'        Cs5g19310.1
   :: :: :::::.:::::  ::
3' CCUUACCCACCGUAUCCAUU------ 5'        Csi-miR2118.2
```

Csi-miR2118.2, target=Cs5g19850.1 gene=Cs5g19850
Category:1
Score=5
Cleavage Site=646

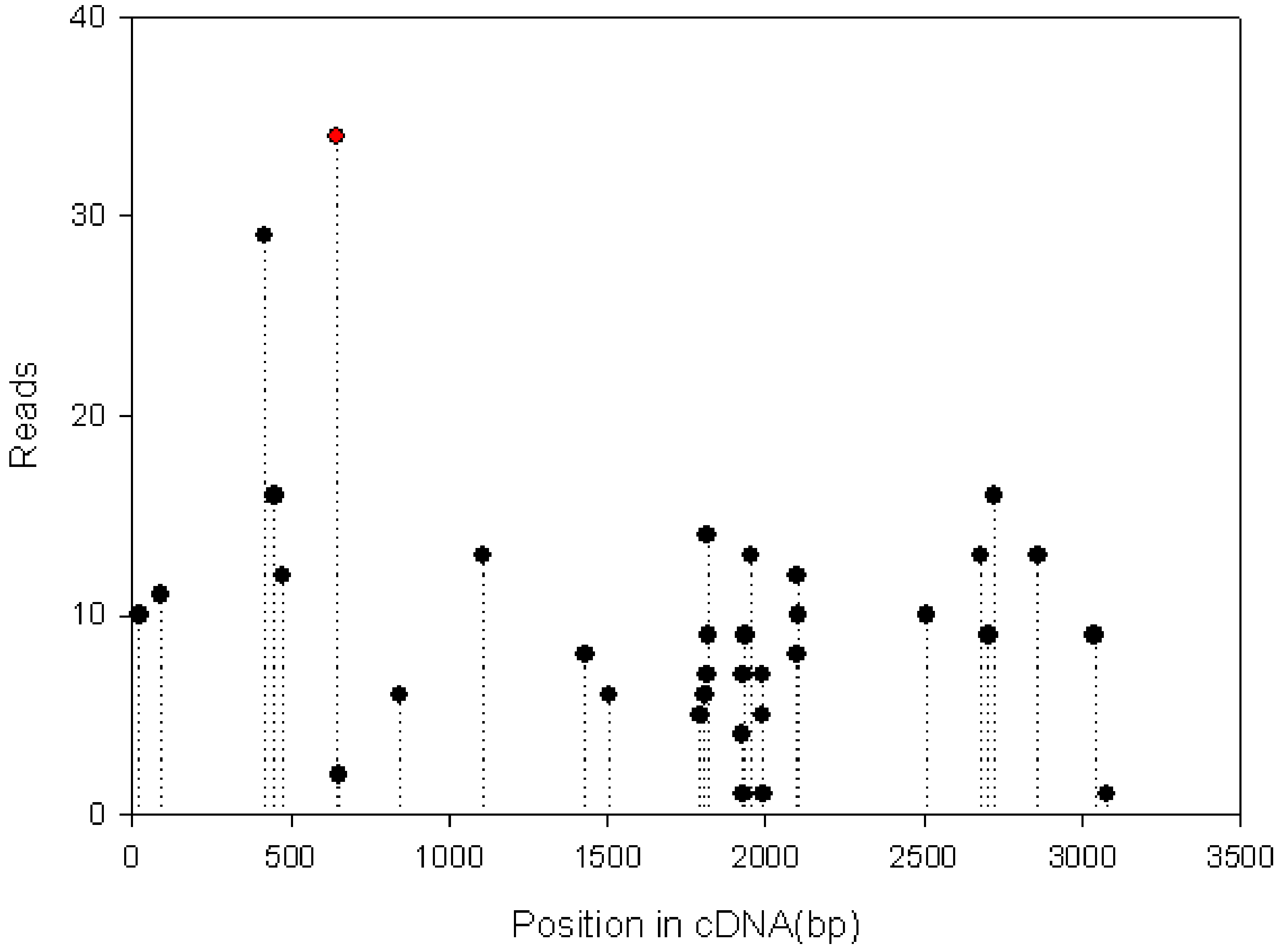


```
5' GGCAUUGGUGGUAUAGGCAAGACAAC 3'        Cs5g19850.1
   :: :: :::::.::::: ::
3' CCUUACCCACCGUAUCCAUU------ 5'        Csi-miR2118.2
```

Csi-miR2118.2, target=Cs5g19920.1 gene=Cs5g19920
Category:1
Score=4
Cleavage Site=715

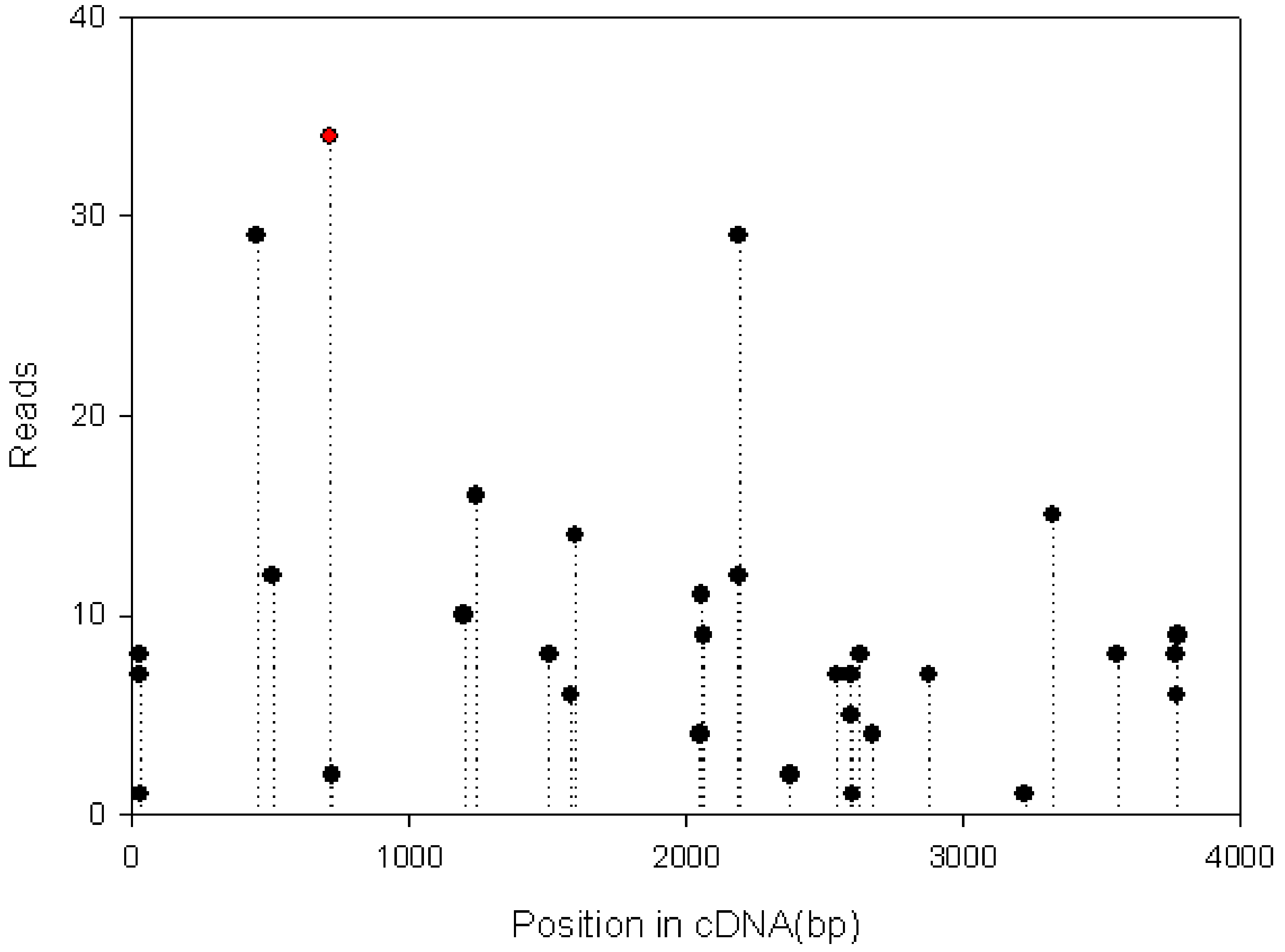


```
5' GGCAUGGGUGGUAUAGGCAAGACAAC 3'        Cs5g19920.1
   :: ::::::::.::::: ::
3' CCUUACCCACCGUAUCCAUU------ 5'        Csi-miR2118.2
```

Csi-miR2118.2, target=Cs8g10560.1 gene=Cs8g10560
Category:2
Score=5
Cleavage Site=652

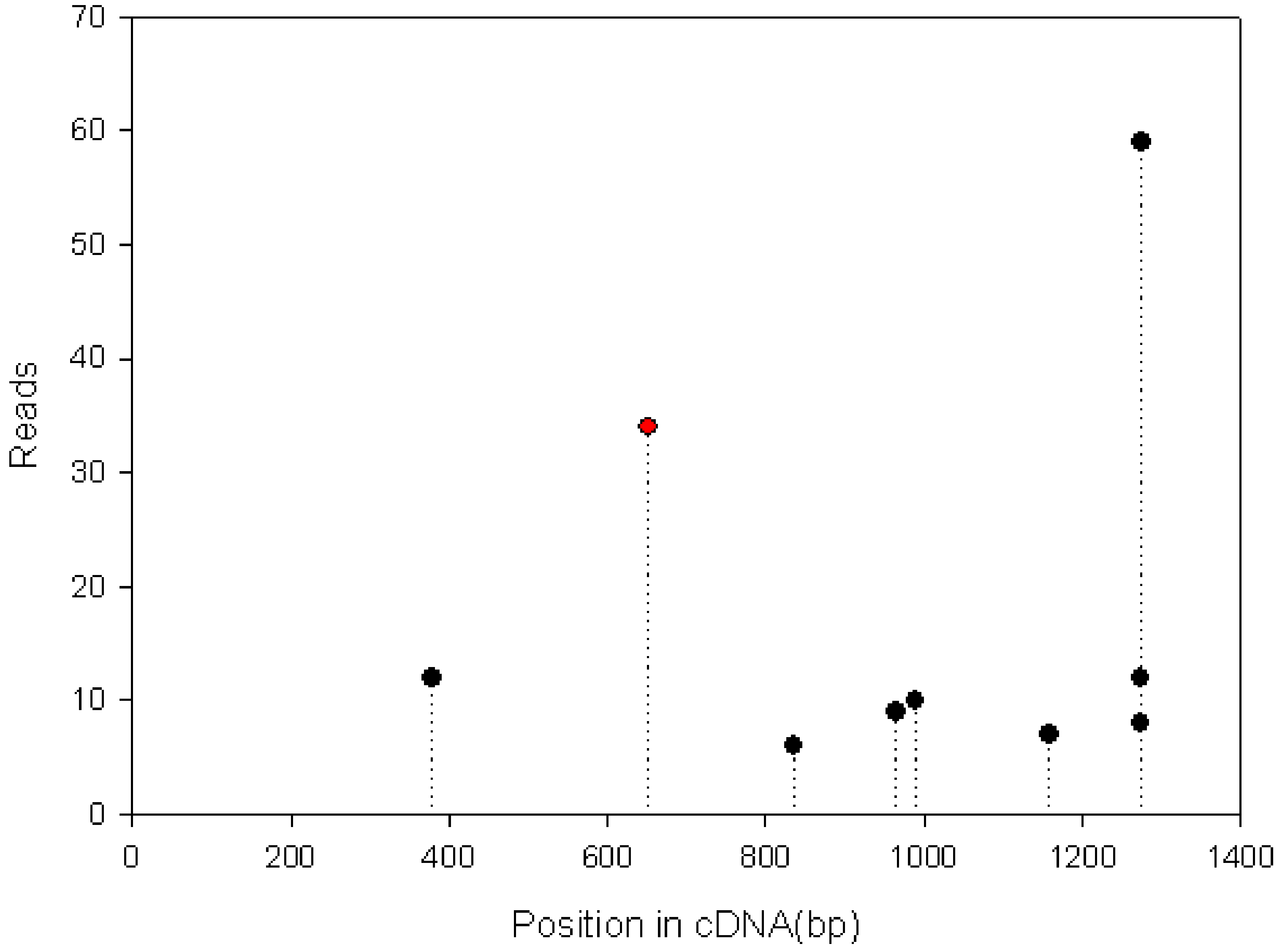


```
5' GGCAUCGGUGGUAUAGGCAAGACAAC 3'        Cs8g10560.1
   :: :: :::::.::::: ::
3' CCUUACCCACCGUAUCCAUU------ 5'        Csi-miR2118.2
```

Csi-miR2118.2, target=Orange1.1t00149.1 gene=Orange1.1t00149
Category:2
Score=3.5
Cleavage Site=98

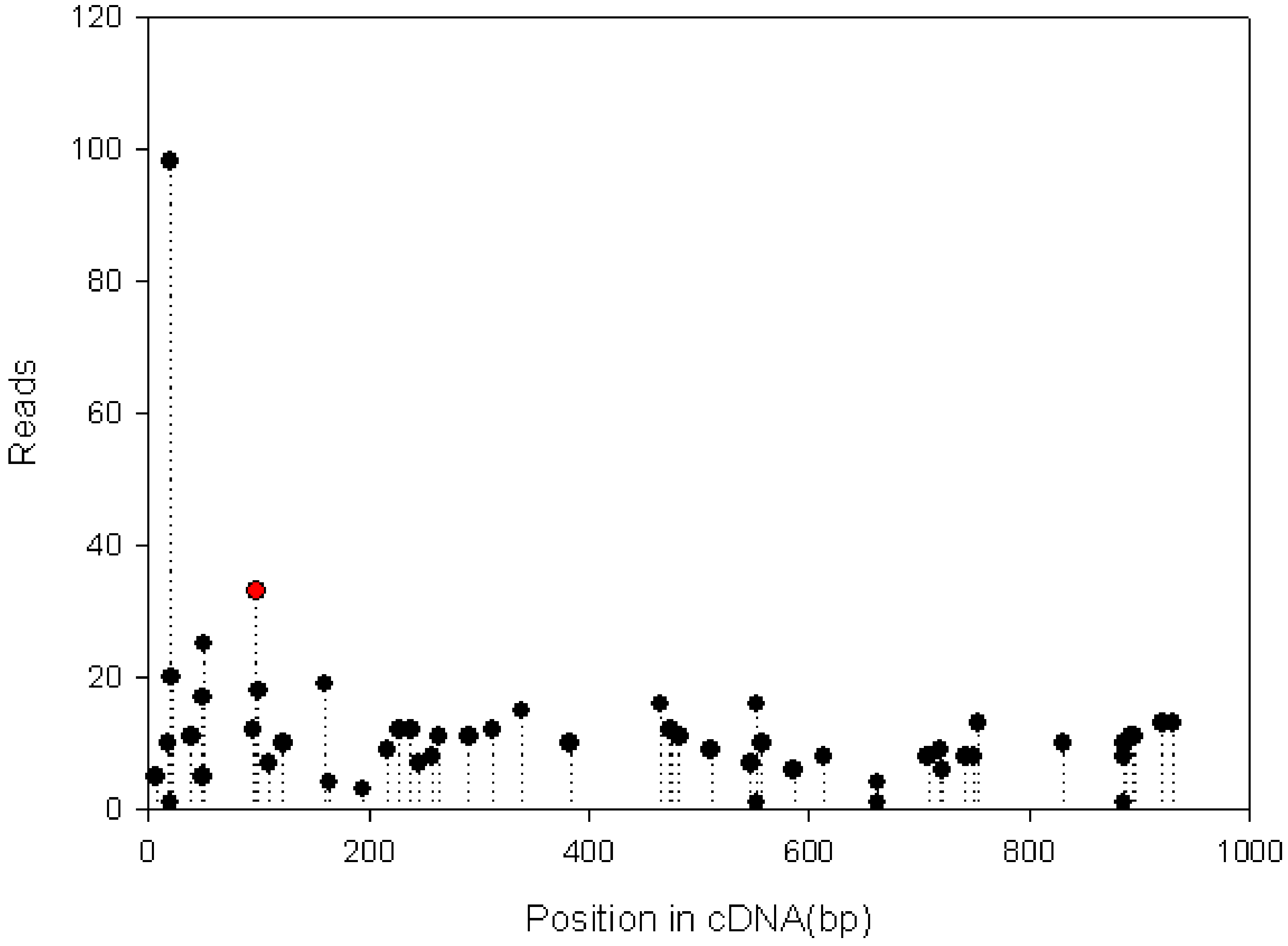


```
5' ACGGAGUGGGUGGCAUA-GUGAUGGCC 3'        Orange1.1t00149.1
    :::.:::::::::::: ::.:
3' --CCUUACCCACCGUAUCCAUU----- 5'        Csi-miR2118.2
```

Csi-miR2118.2, target=Orange1.1t00557.1 gene=Orange1.1t00557
Category:1
Score=4
Cleavage Site=131

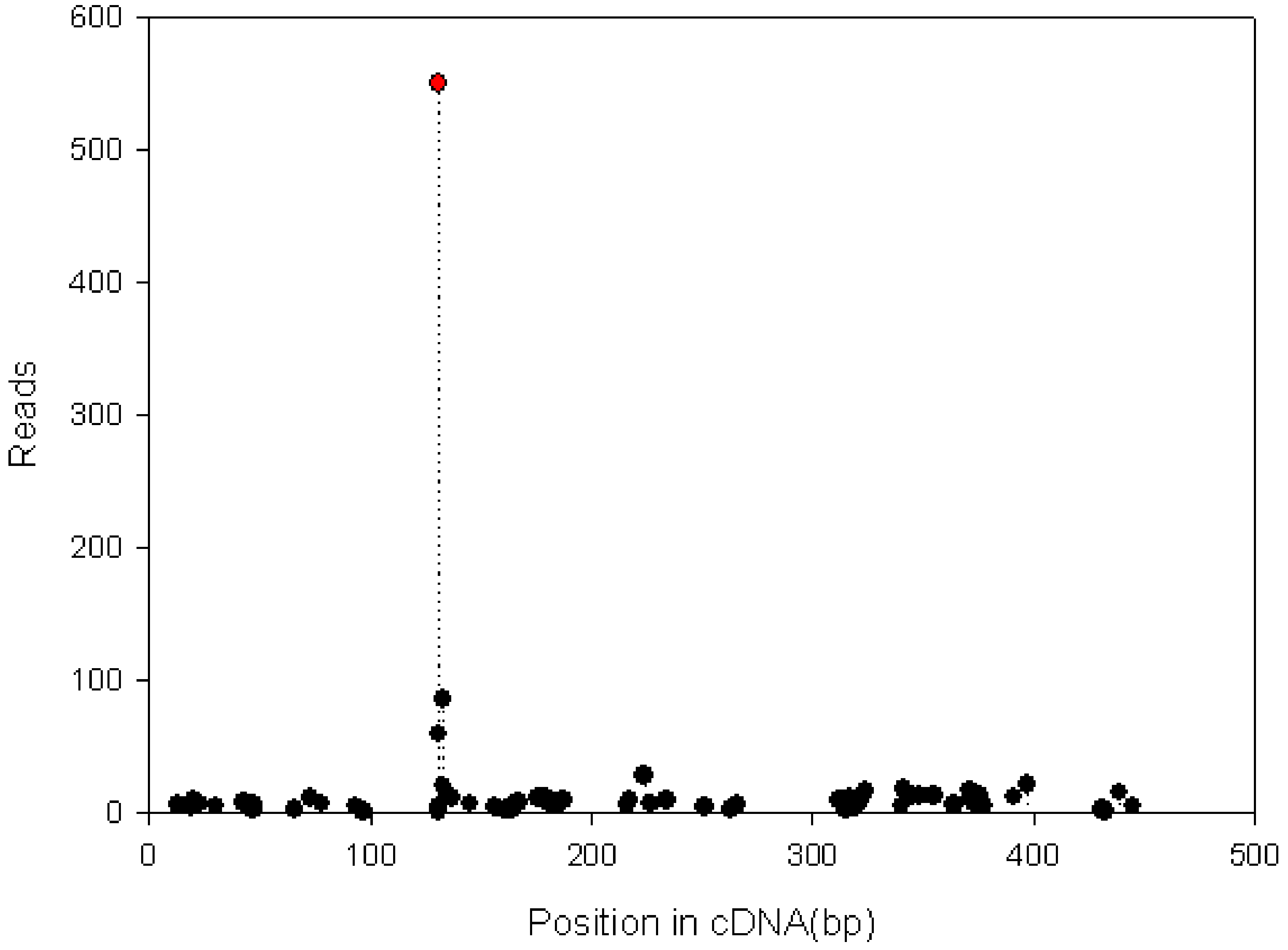


```
5' AAGACAUGGGUGGCAUAGGUCACAUC 3'        Orange1.1t00557.1
      :: ::::::::::::::: :
3' -CCU-UACCCACCGUAUCCAUU---- 5'        Csi-miR2118.2
```

Csi-miR2118.2, target=Orange1.1t02171.1 gene=Orange1.1t02171
Category:3
Score=4
Cleavage Site=662

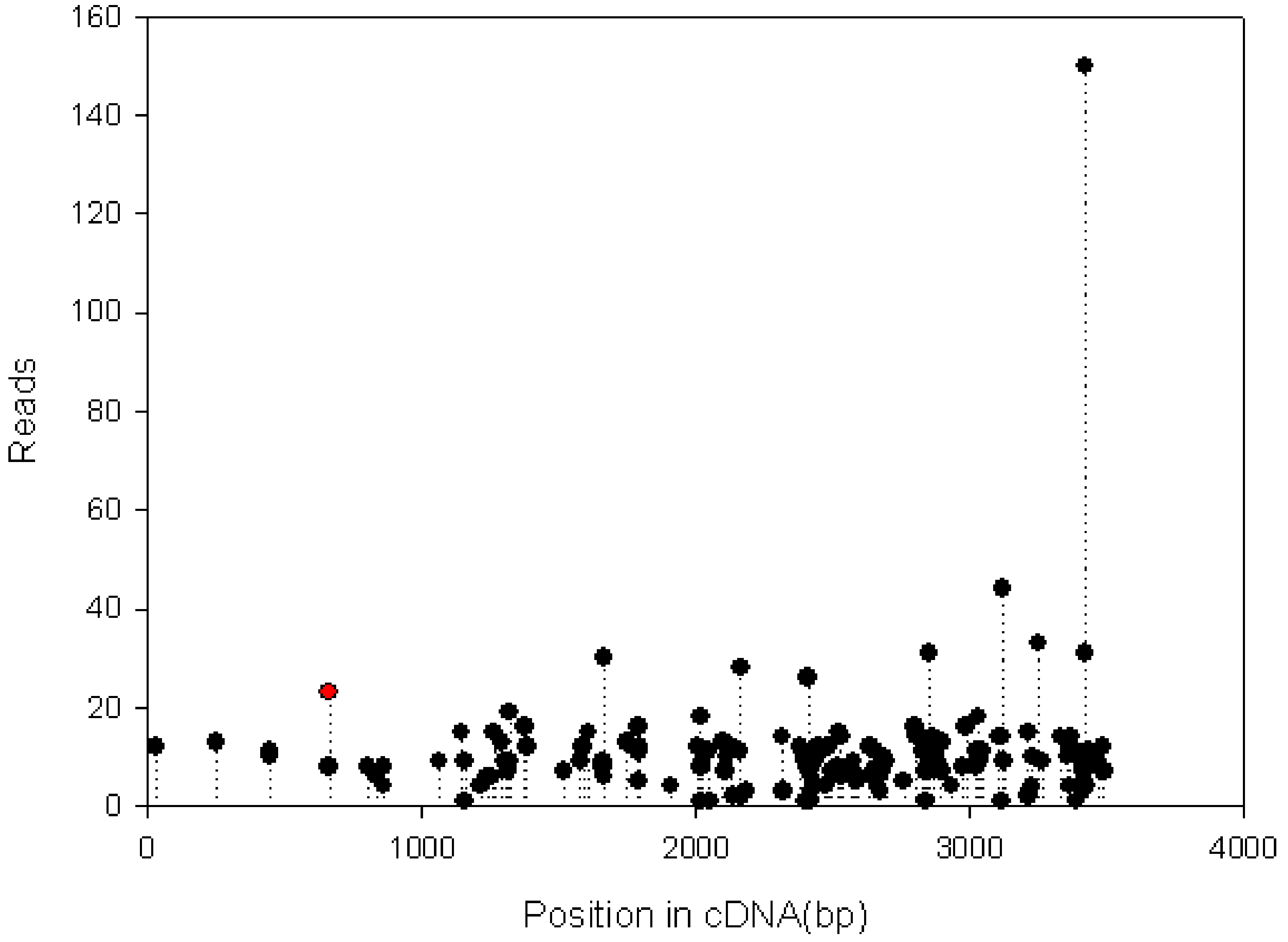


```
5' GGGGCAUGGGUGGUAUAGGCAAAACC 3'        Orange1.1t02171.1
     :: ::::::::.::::: ::
3' --CCUUACCCACCGUAUCCAUU---- 5'        Csi-miR2118.2
```

Csi-miR2118.2, target=Orange1.1t02175.1 gene=Orange1.1t02175
Category:3
Score=4
Cleavage Site=735

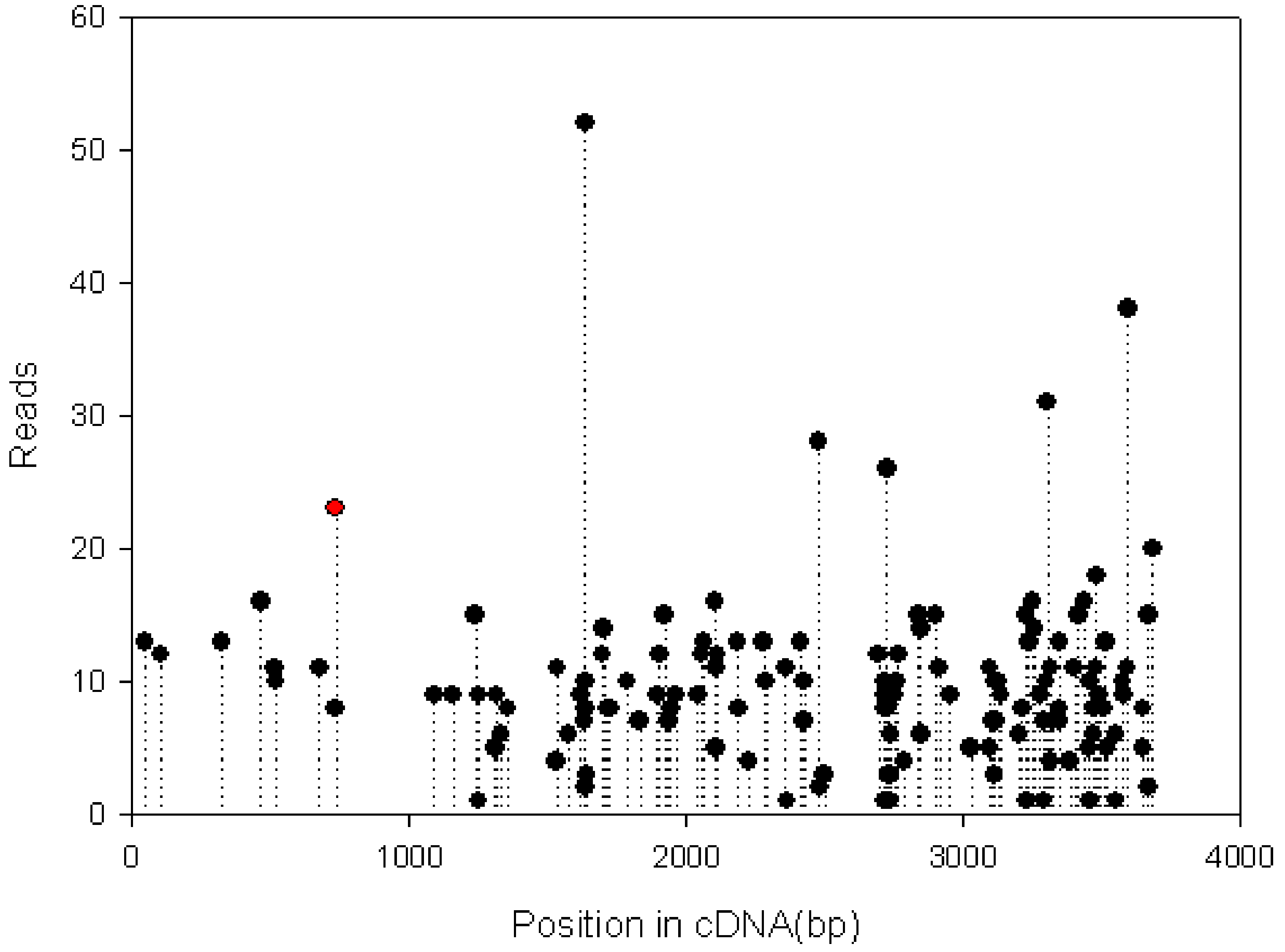


```
5' GGGGCAUGGGUGGUAUAGGCAAAACC 3'        Orange1.1t02175.1
     :: ::::::::.::::: ::
3' --CCUUACCCACCGUAUCCAUU---- 5'        Csi-miR2118.2
```

Csi-miR2275d, target=Cs5g35220.1 gene=Cs5g35220
Category:1
Score=5
Cleavage Site=605

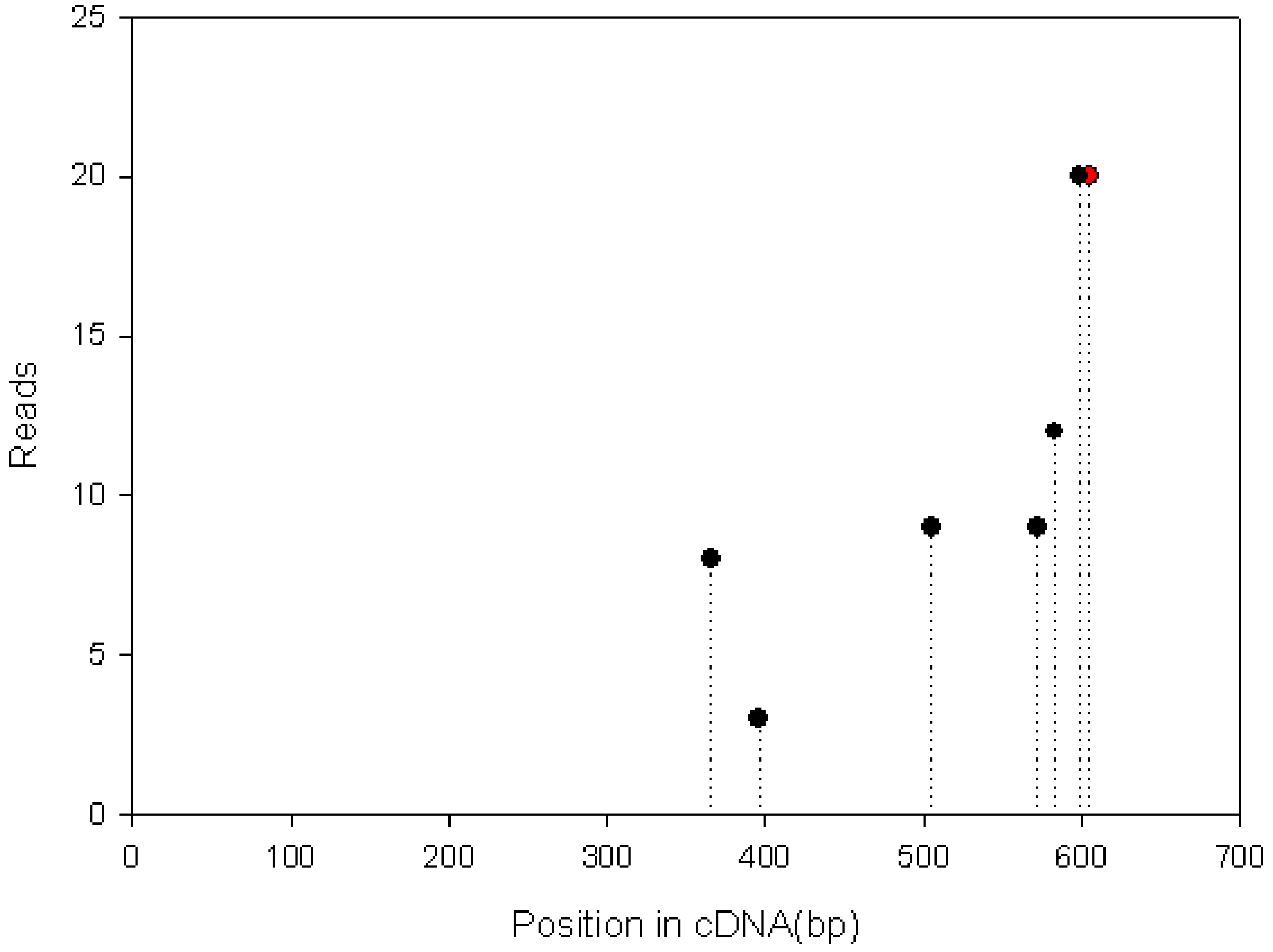


```
5' UUAAGUUU-GUUCCAGCUAAUUCUGGU 3'        Cs5g35220.1
       :::: :::::: :.::::::
3' ---ACAAAUCAAGGUAGGUUAAGA--- 5'        Csi-miR2275d
```

Csi-miR2275d, target=Cs8g03105.1 gene=Cs8g03105
Category:1
Score=5
Cleavage Site=297

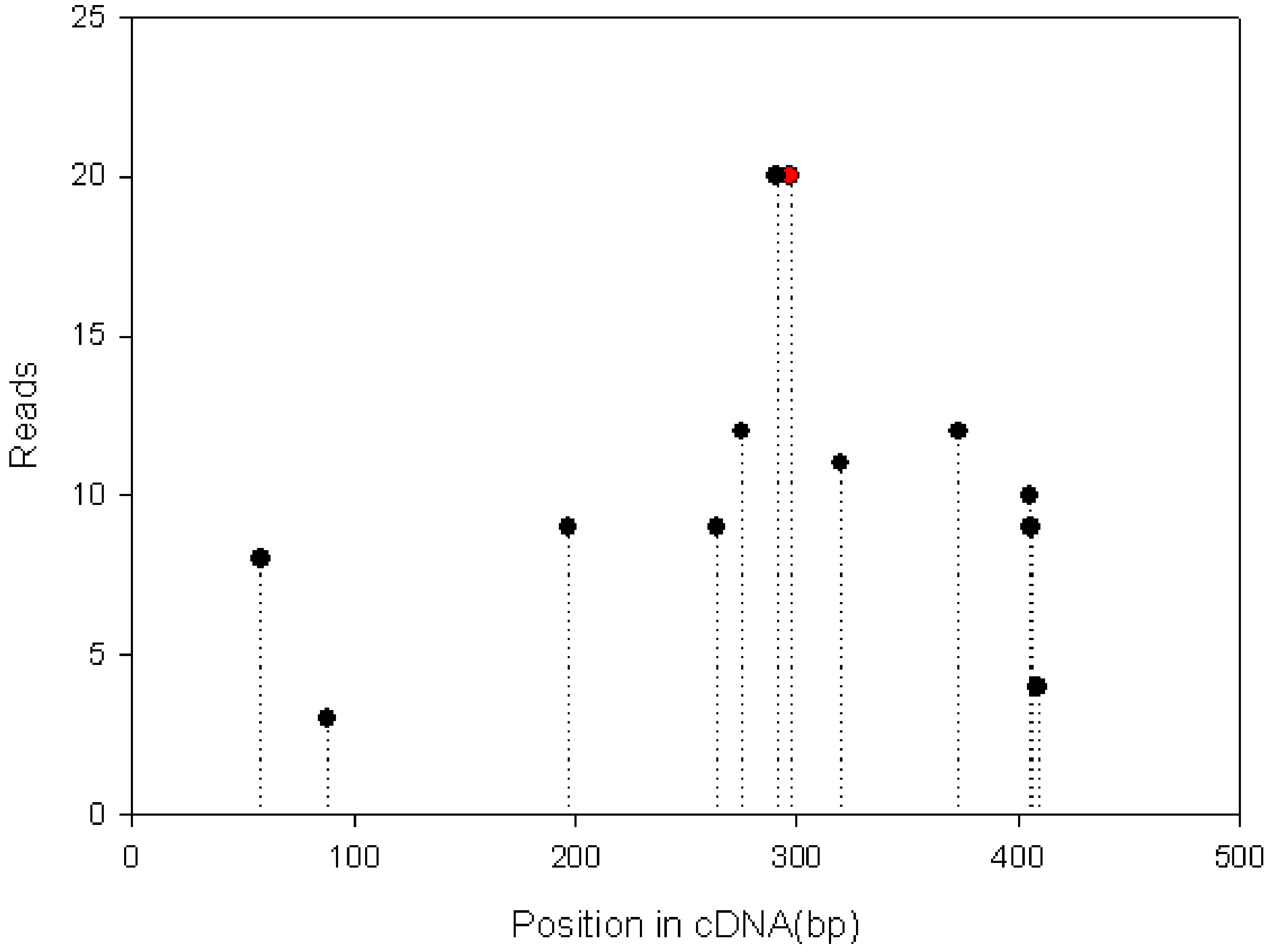


```
5' UUAAGUUU-GUUCCAGCUAAUUCUGGU 3'        Cs8g03105.1
        :::: :::::: :.::::::
3' ---ACAAAUCAAGGUAGGUUAAGA--- 5'        Csi-miR2275d
```

Csi-miR319, target=Cs3g06390.1 gene=Cs3g06390
Category:2
Score=4
Cleavage Site=773

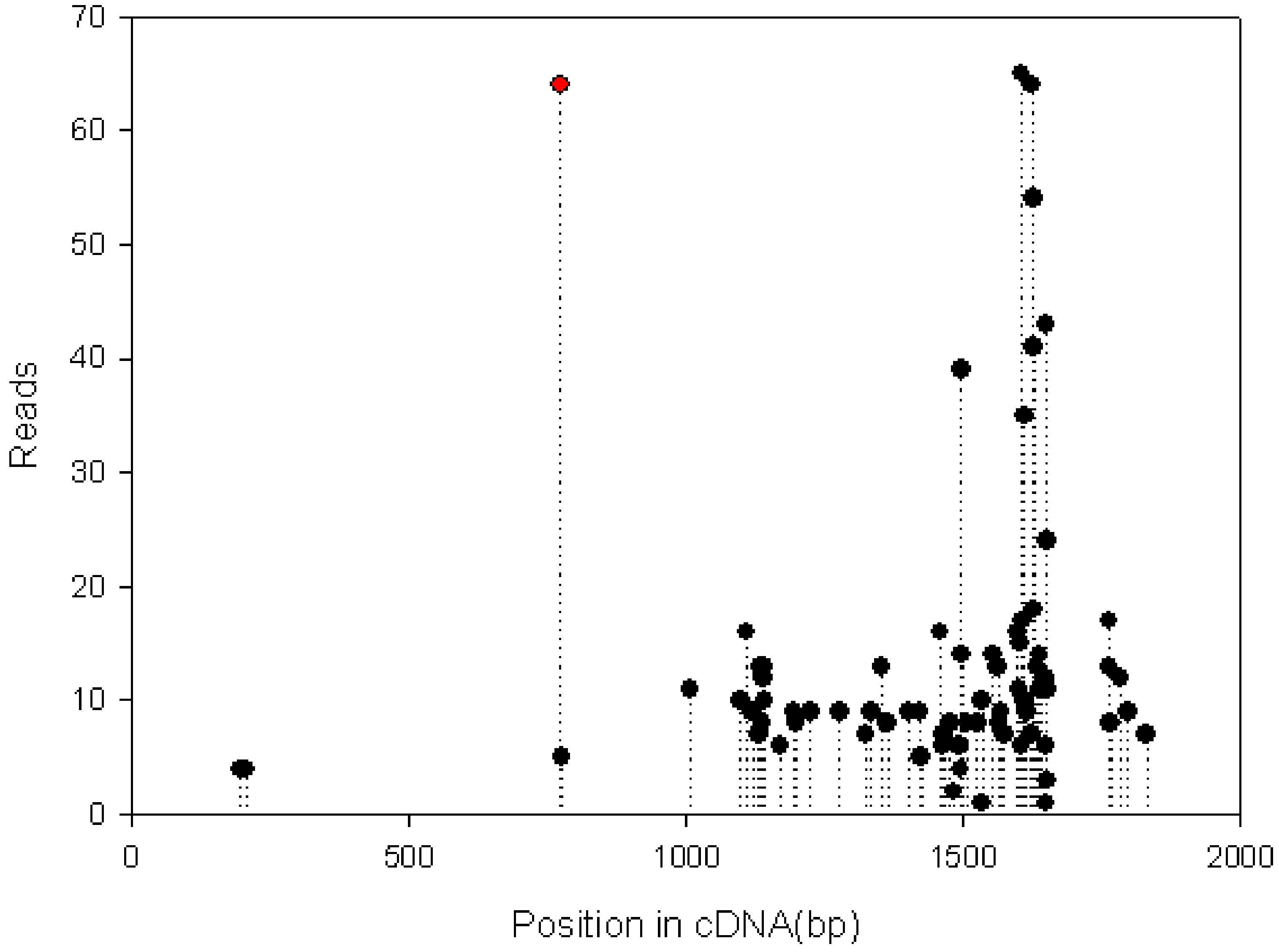


```
5' UUGGAGCUCCCUUCACUCCAAUAUAC 3'        Cs3g06390.1
     ::::::::::::: :::::
3' -UCCUCGAGGGAAGUCAGGUUU---- 5'        Csi-miR319
```

Csi-miR319b, target=Cs3g06390.1 gene=Cs3g06390
Category:2
Score=3
Cleavage Site=773

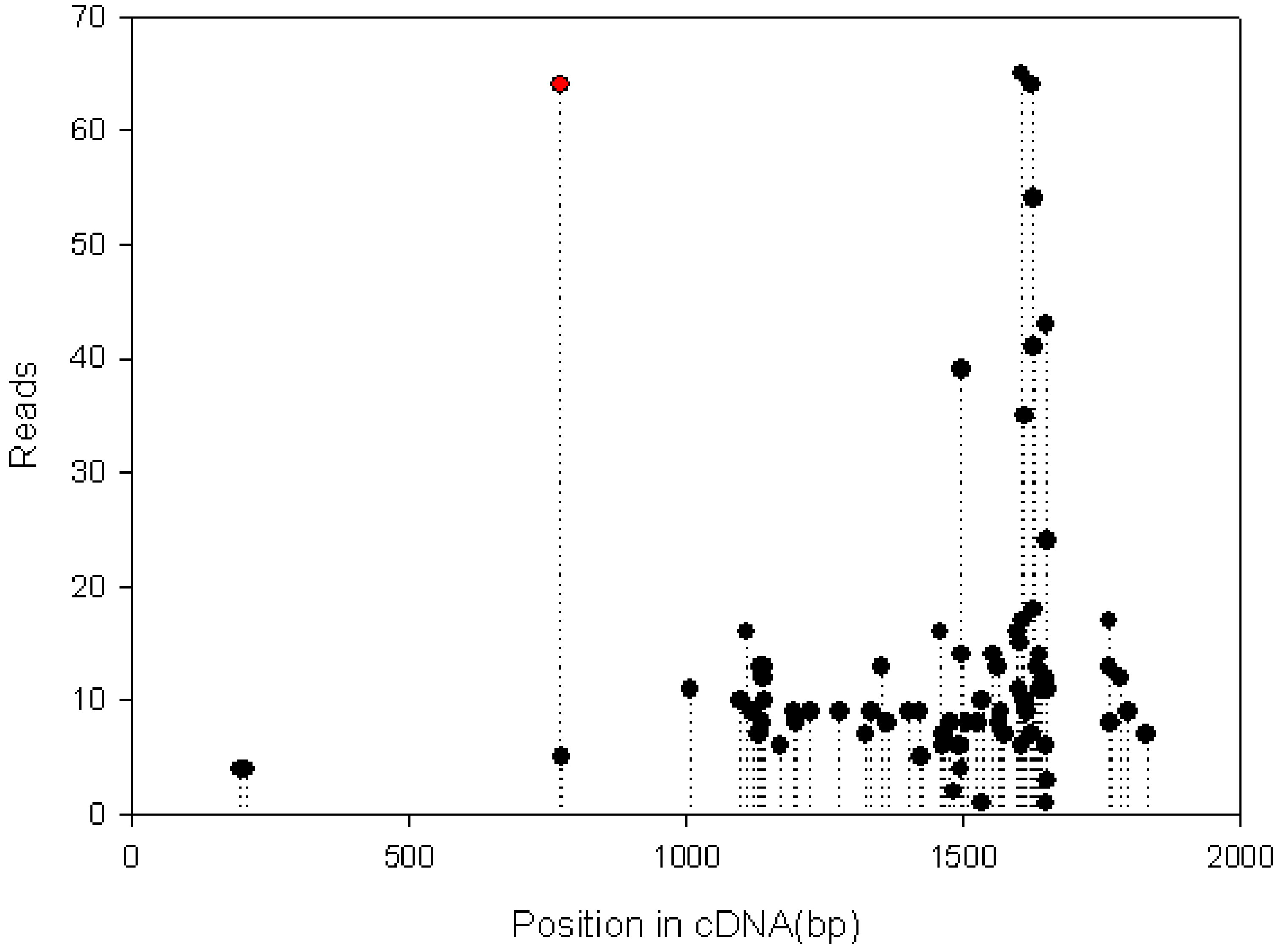


```
5' UUGGAGCUCCCUUCACUCCAAUAUAC 3'        Cs3g06390.1
     ::::::::::::: :::::
3' -CCCUCGAGGGAAGUCAGGUU----- 5'        Csi-miR319b
```

Csi-miR390.1, target=Cs9g01780.1 gene=Cs9g01780
Category:2
Score=4.5
Cleavage Site=495

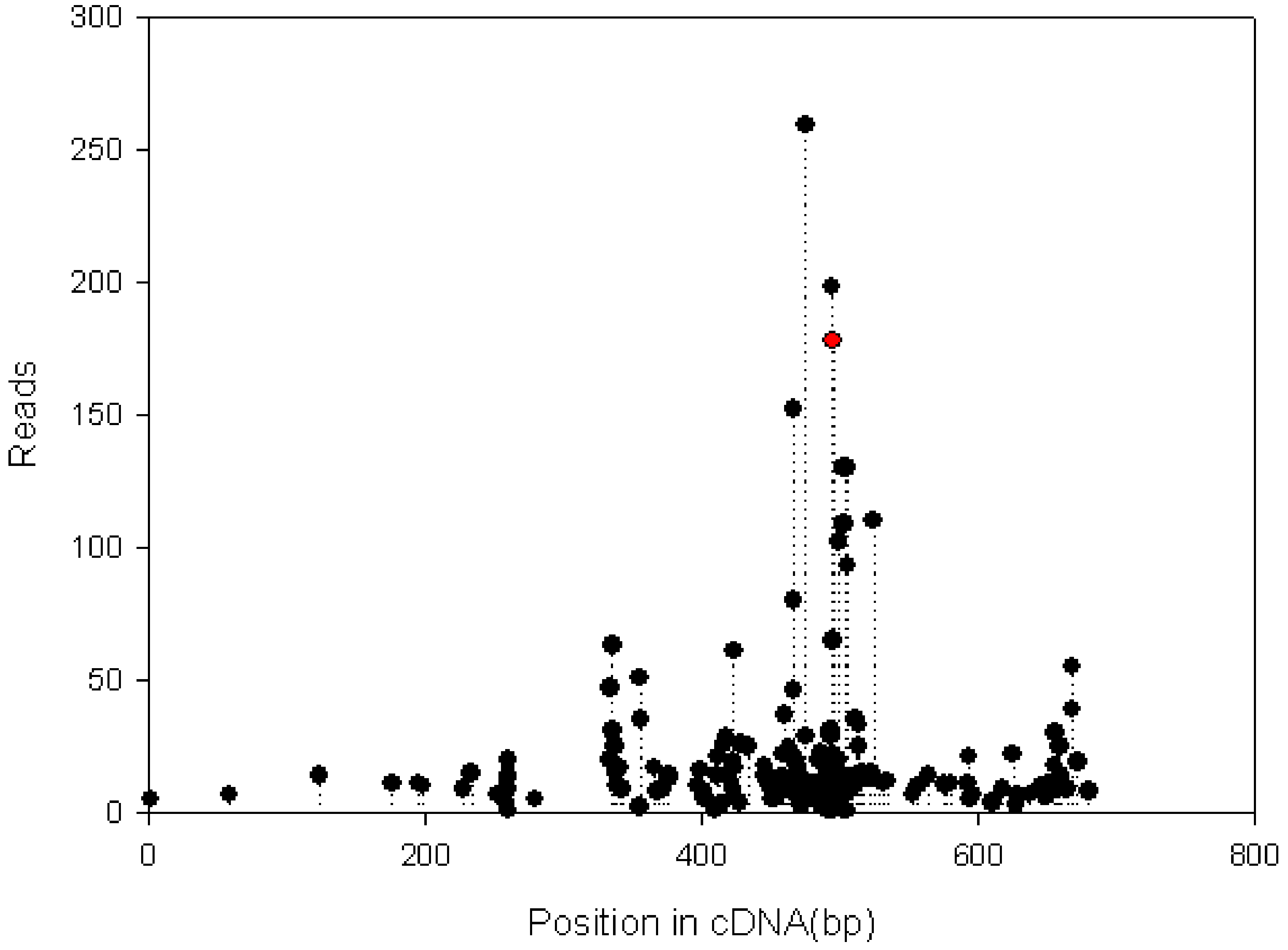


```
5' CUUCCUUGUCUAUCCCUCCUGAGCUG 3'        Cs9g01780.1
         .: :::::::::::::::::
3' ----CCGC-GAUAGGGAGGACUCGAA 5'        Csi-miR390.1
```

Csi-miR390.2, target=Cs9g01780.1 gene=Cs9g01780
Category:2
Score=3.5
Cleavage Site=494

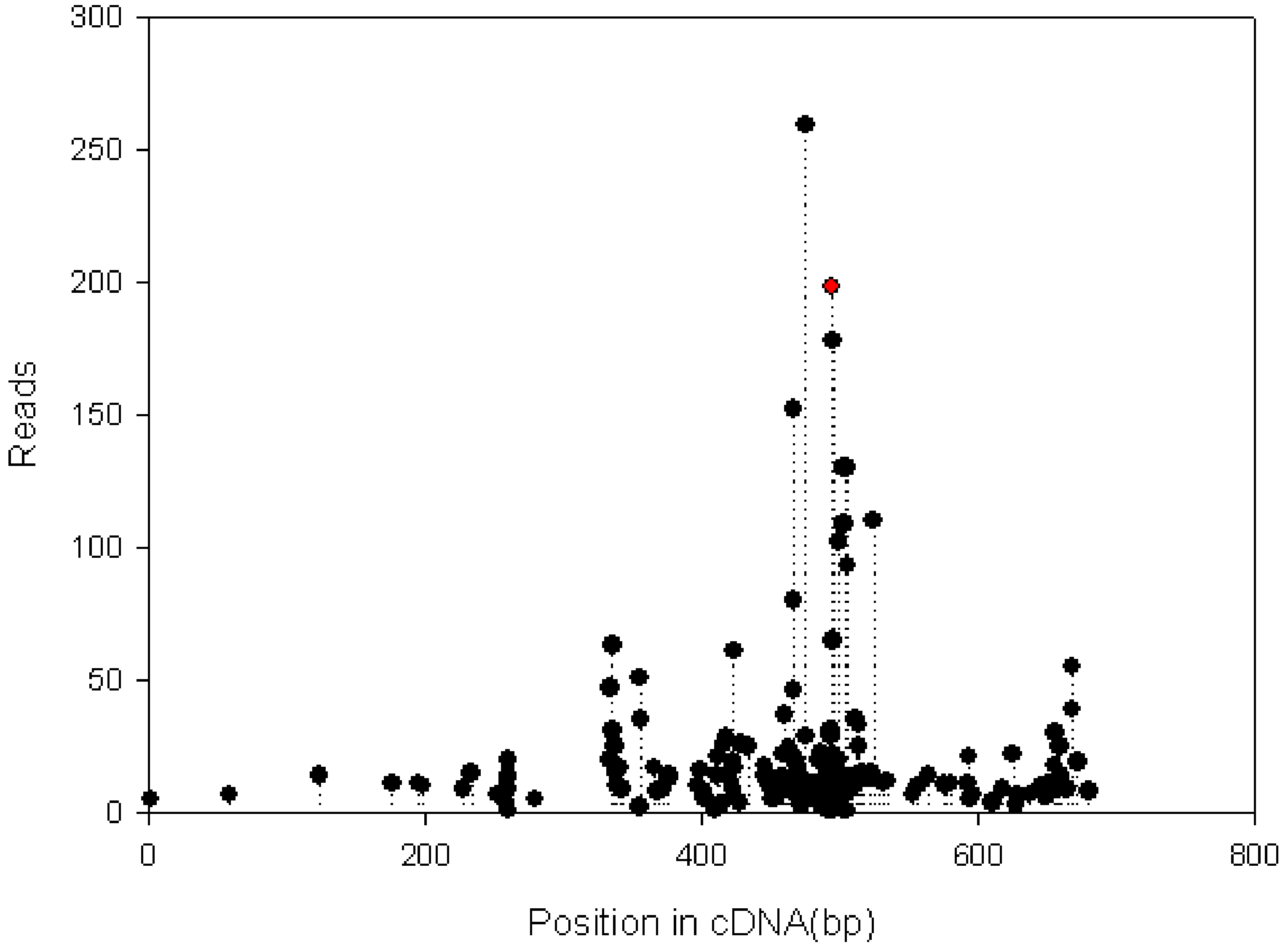


```
5' CCUUCCUUGUCUAUCCCUCCUGAGCU 3'        Cs9g01780.1
         .: ::::::::::::::::
3' -----CCGC-GAUAGGGAGGACUCGA 5'        Csi-miR390.2
```

Csi-miR390b, target=Cs9g01780.1 gene=Cs9g01780
Category:2
Score=0
Cleavage Site=495

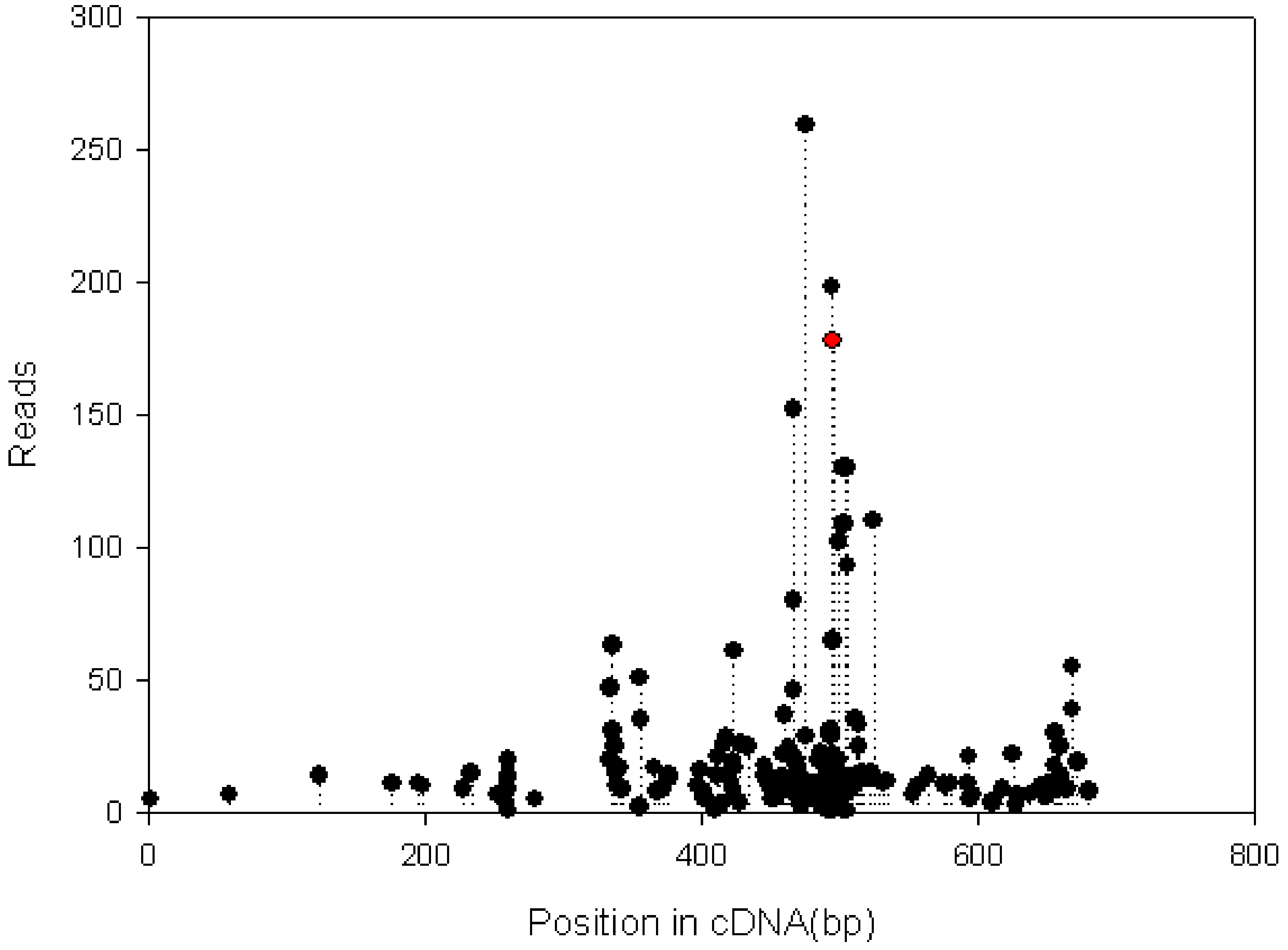


```
5' CUUCCUUGUCUAUCCCUCCUGAGCUG 3'        Cs9g01780.1
         :::::::::::::::::::::
3' -----AACAGAUAGGGAGGACUCGAC 5'        Csi-miR390b
```

Csi-miR393a.1, target=Cs2g14270.1 gene=Cs2g14270
Category:1
Score=1.5
Cleavage Site=2491

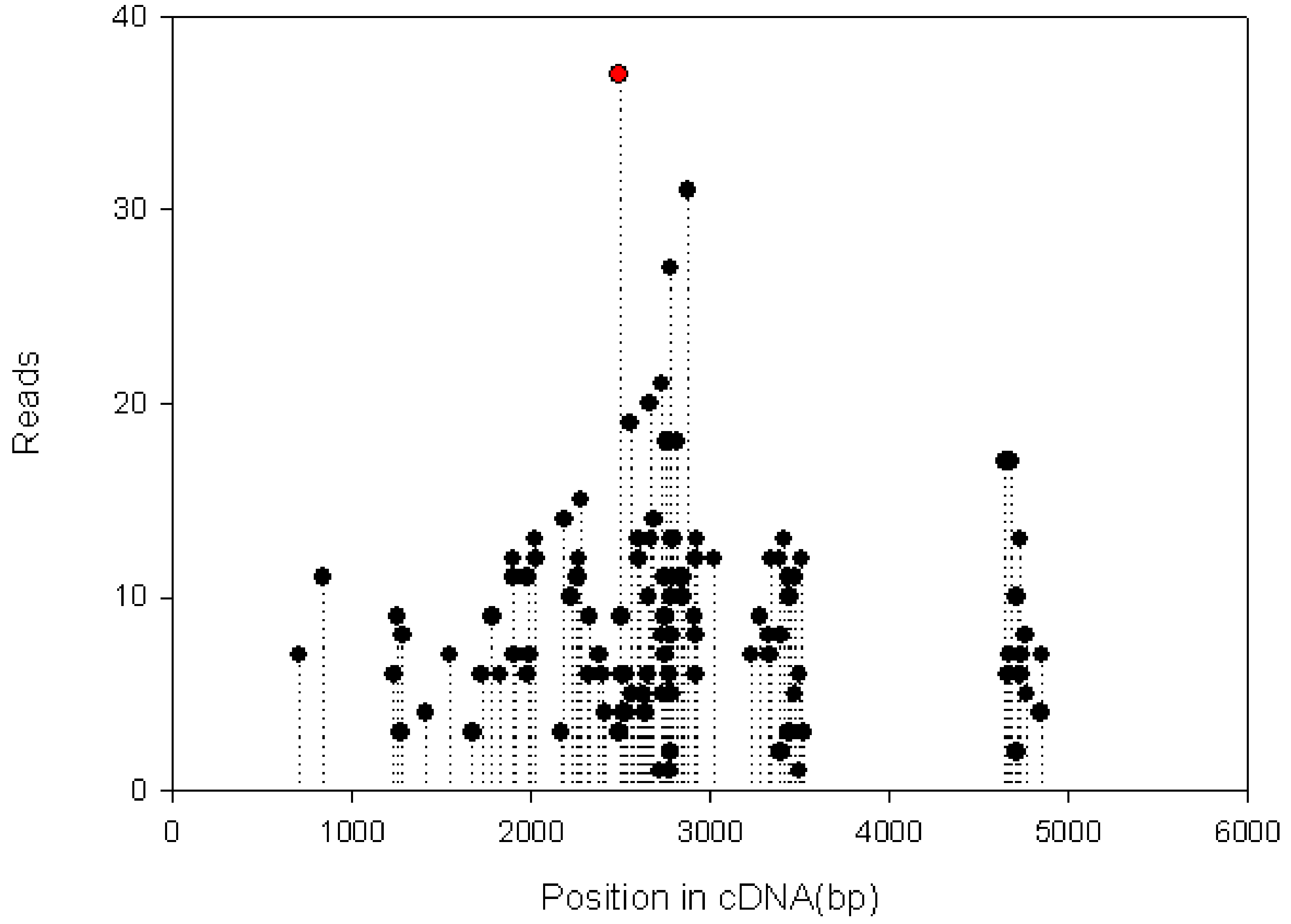


```
5' GAGACAAUGCGAUCCCUUUGGAUGUC 3'        Cs2g14270.1
      . ::::::::::::::::::
3' --UAGUUACGCUAGGGAAACCU---- 5'        Csi-miR393a.1
```

Csi-miR393a.1, target=Cs7g31800.1 gene=Cs7g31800
Category:2
Score=5
Cleavage Site=1323

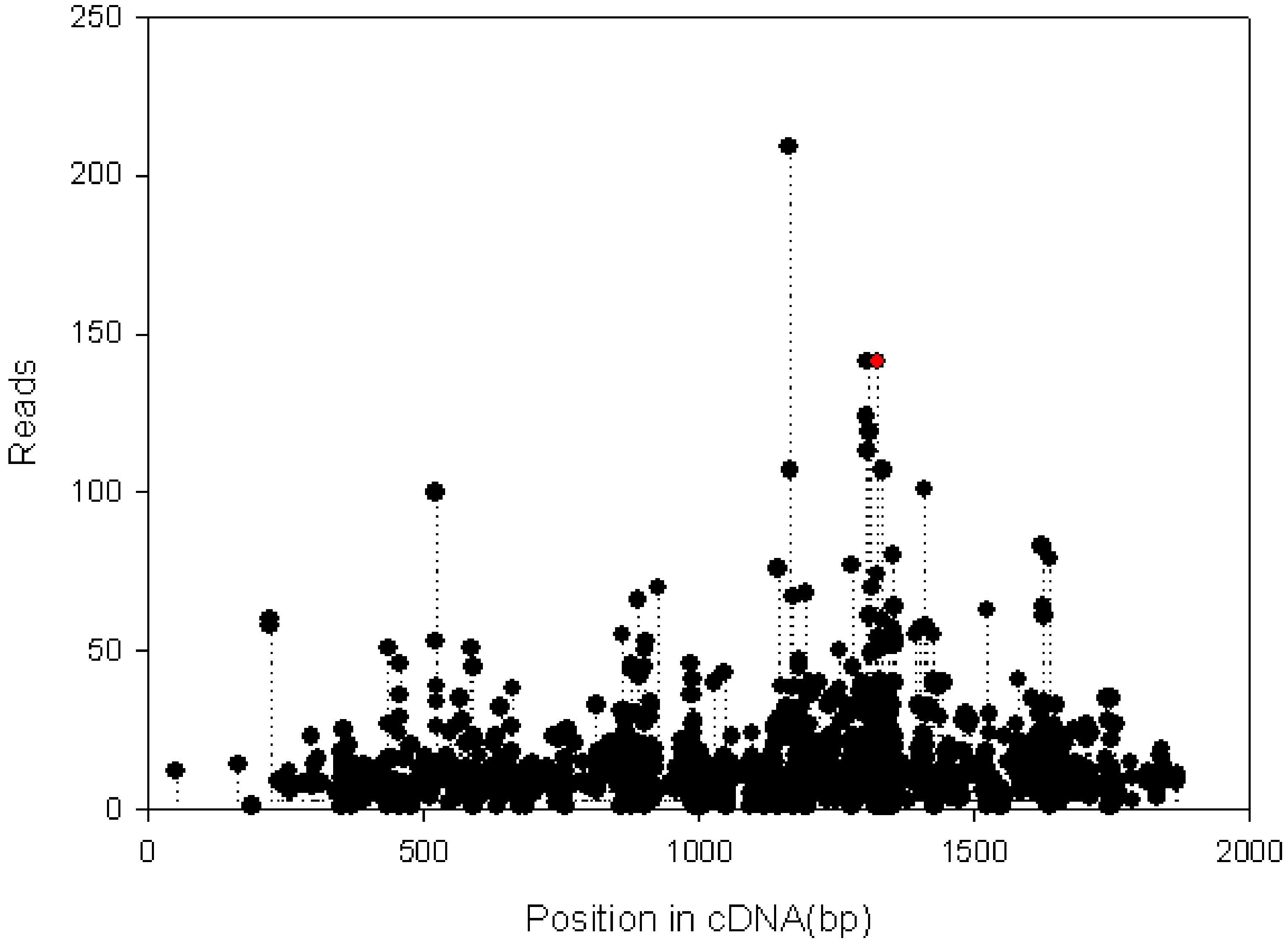


```
5' GGCCAAUCCAU-UGAUUUCUUUGGAGC 3'        Cs7g31800.1
         ::: :: .:::..:::::::
3' -----UAGUUACGCUAGGGAAACCU-- 5'        Csi-miR393a.1
```

Csi-miR393a.2, target=Cs2g14270.1 gene=Cs2g14270
Category:1
Score=1
Cleavage Site=2491

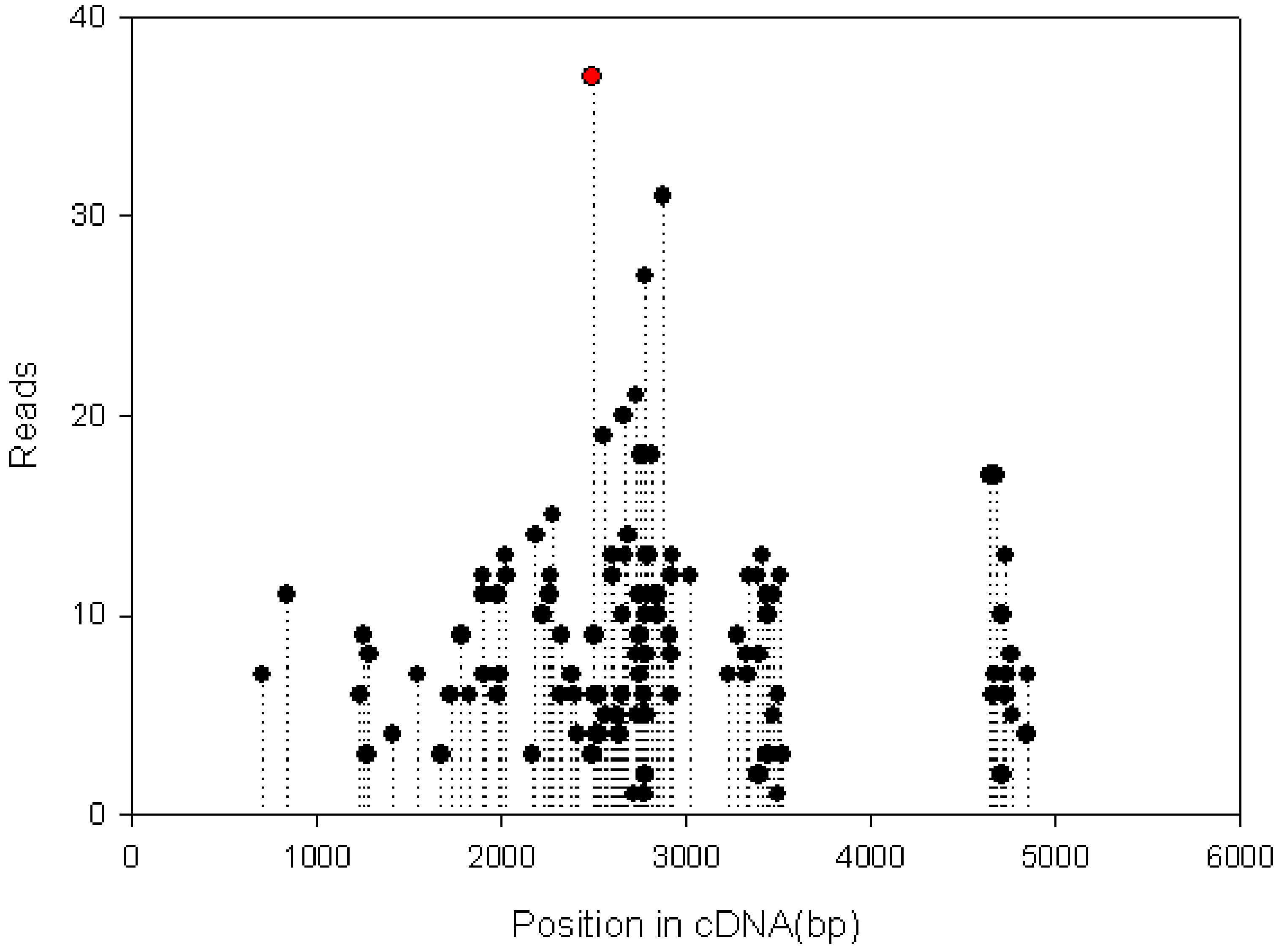


```
5' GAGA-CAAUGCGAUCCCUUUGGAUGUC 3'        Cs2g14270.1
     :: :::::::::::::::::::
3' --CUAGUUACGCUAGGGAAACCU---- 5'        Csi-miR393a.2
```

Csi-miR393a.3, target=Cs2g14270.1 gene=Cs2g14270
Category:1
Score=2.5
Cleavage Site=2491

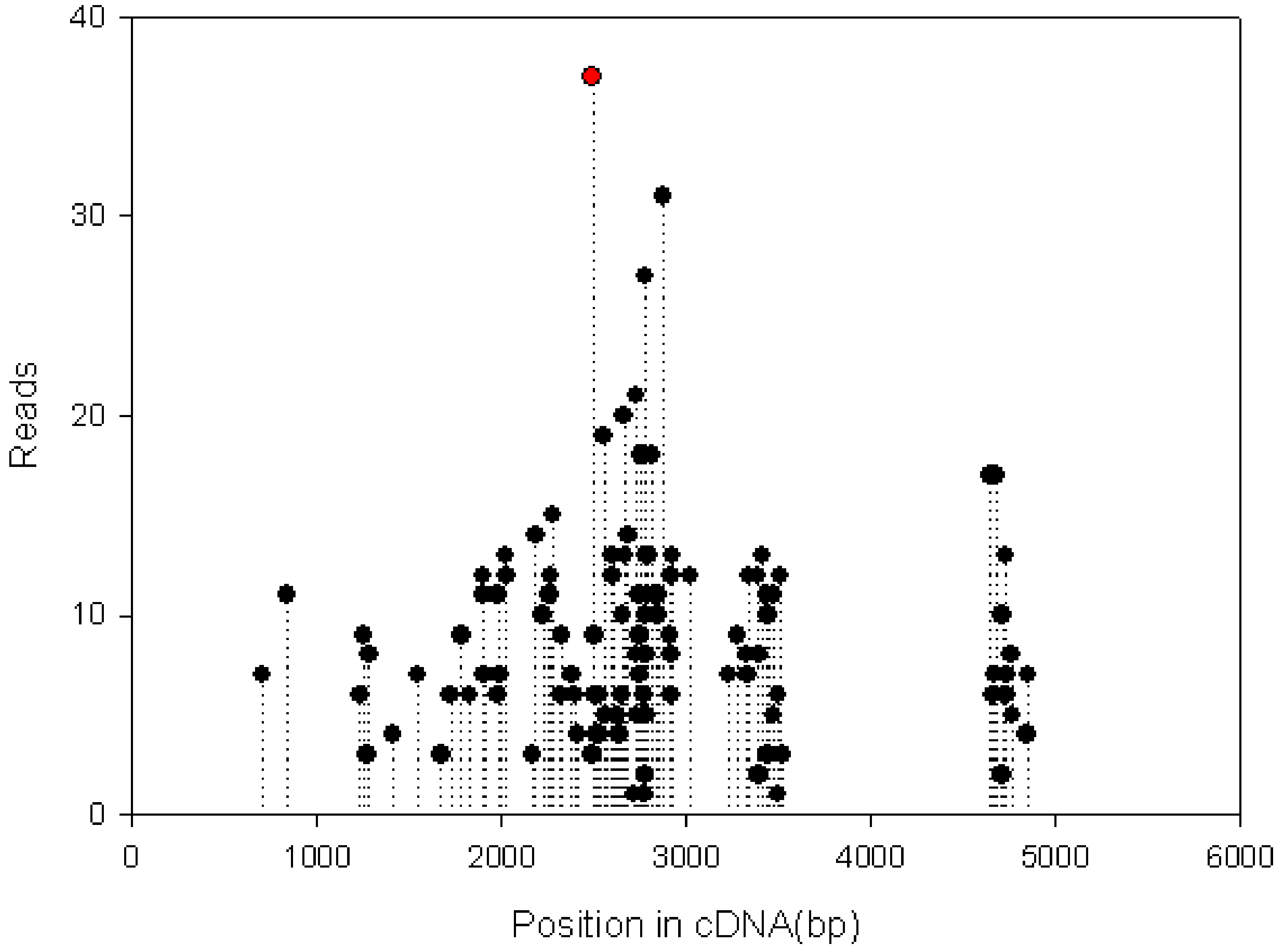


```
5' GAGACAAUGCGAUCCCUUUGGAUGUC 3'        Cs2g14270.1
   : . ::::::::::::::::::::
3' CCUAGUUACGCUAGGGAAACCU---- 5'        Csi-miR393a.3
```

Csi-miR393b, target=Cs2g14270.1 gene=Cs2g14270
Category:1
Score=2
Cleavage Site=2491

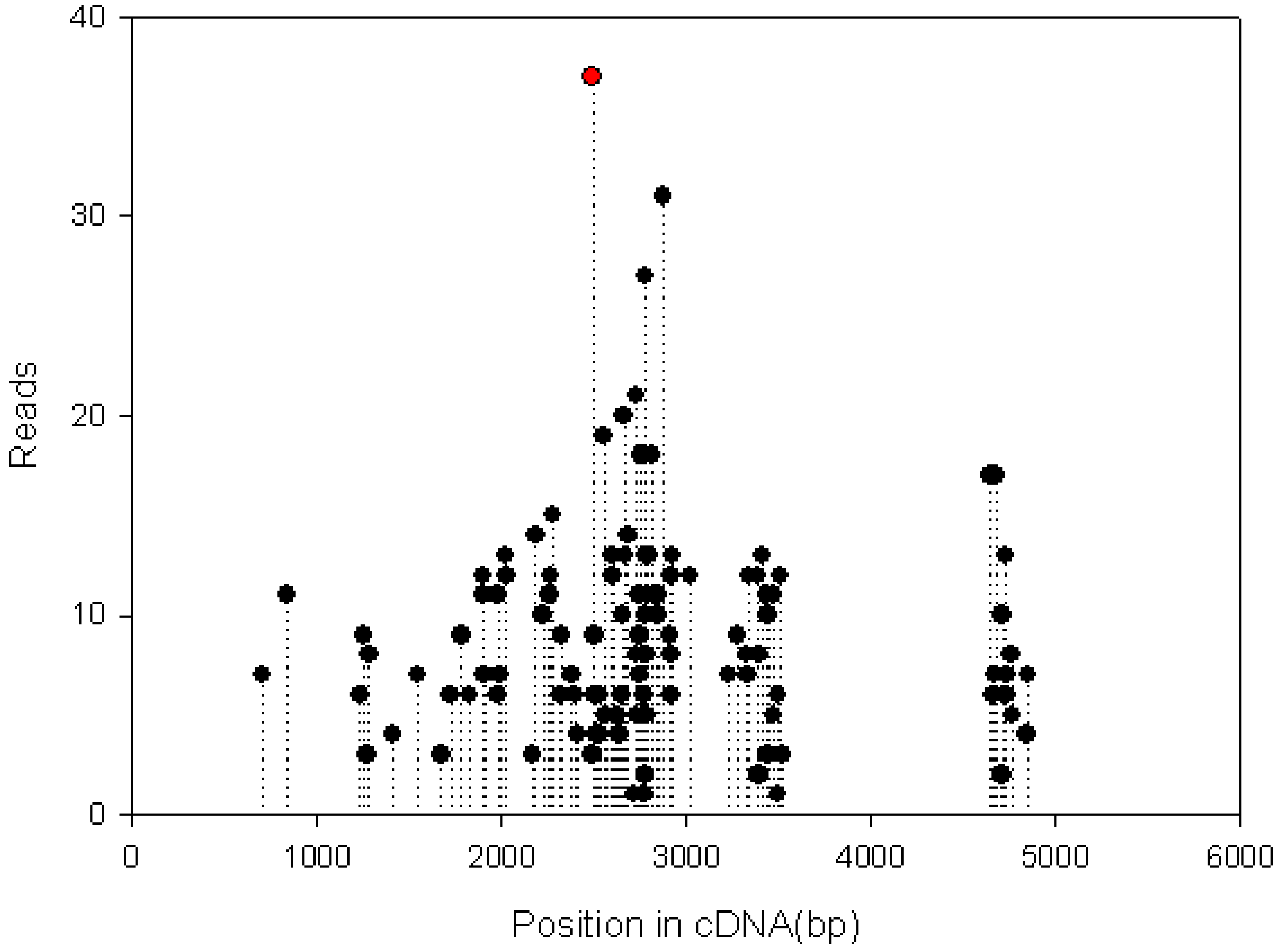


```
5' GAGA-CAAUGCGAUCCCUUUGGAUGUC 3'        Cs2g14270.1
     :: ::::::::::::::::::::
3' --CUAGUUACGCUAGGGAAACCUU--- 5'        Csi-miR393b
```

Csi-miR394, target=Cs2g02980.1 gene=Cs2g02980
Category:3
Score=5
Cleavage Site=1005

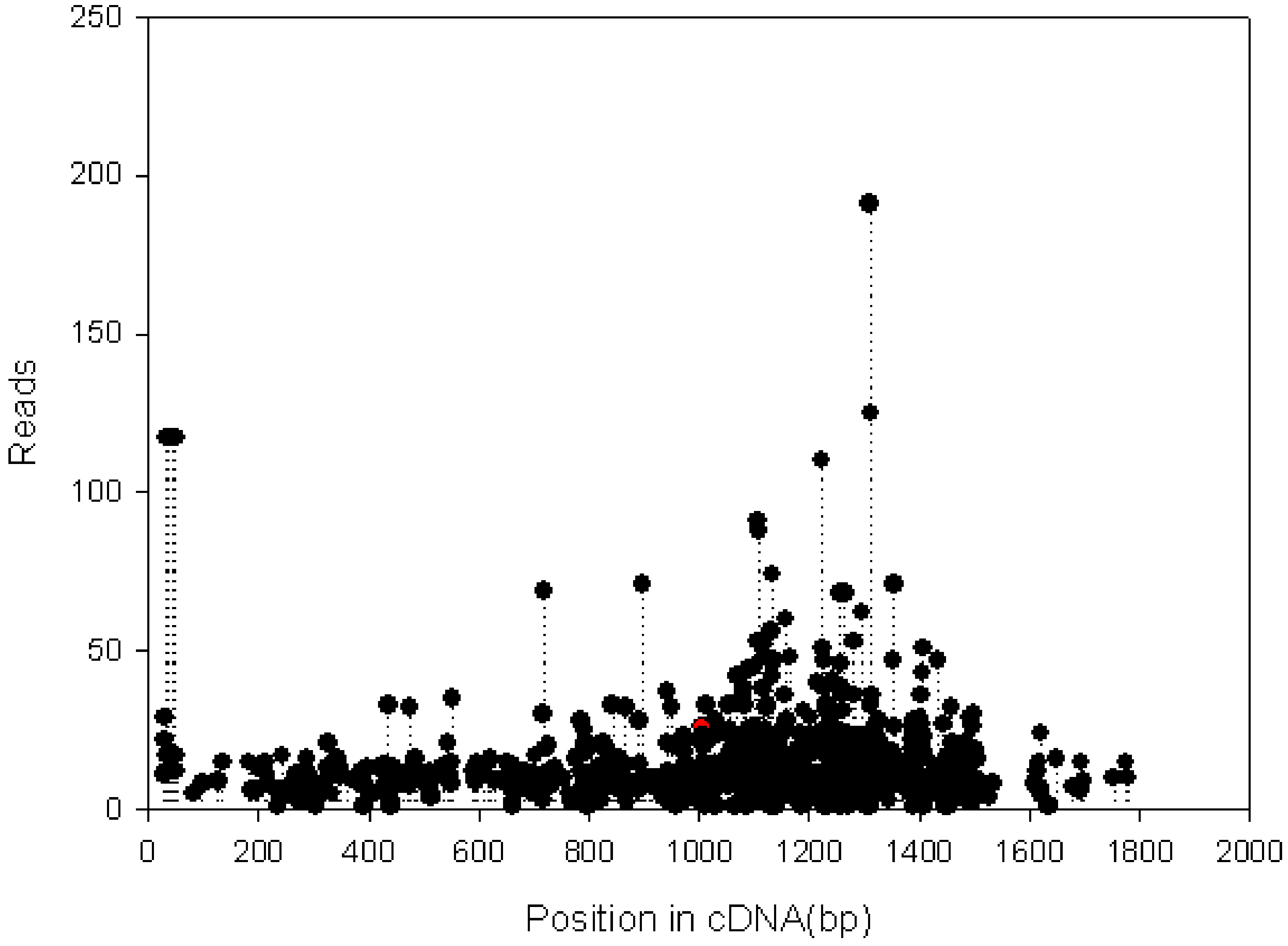


```
5' GAAGGUGGUGGUGGG-AGGAUGCCAAG 3'       Cs2g02980.1
         :: :::::. ::.:::::::
3' ------CCUCCACCUGUCUUACGGUU- 5'       Csi-miR394
```

Csi-miR394, target=Cs7g10850.1 gene=Cs7g10850
Category:2
Score=1
Cleavage Site=1168

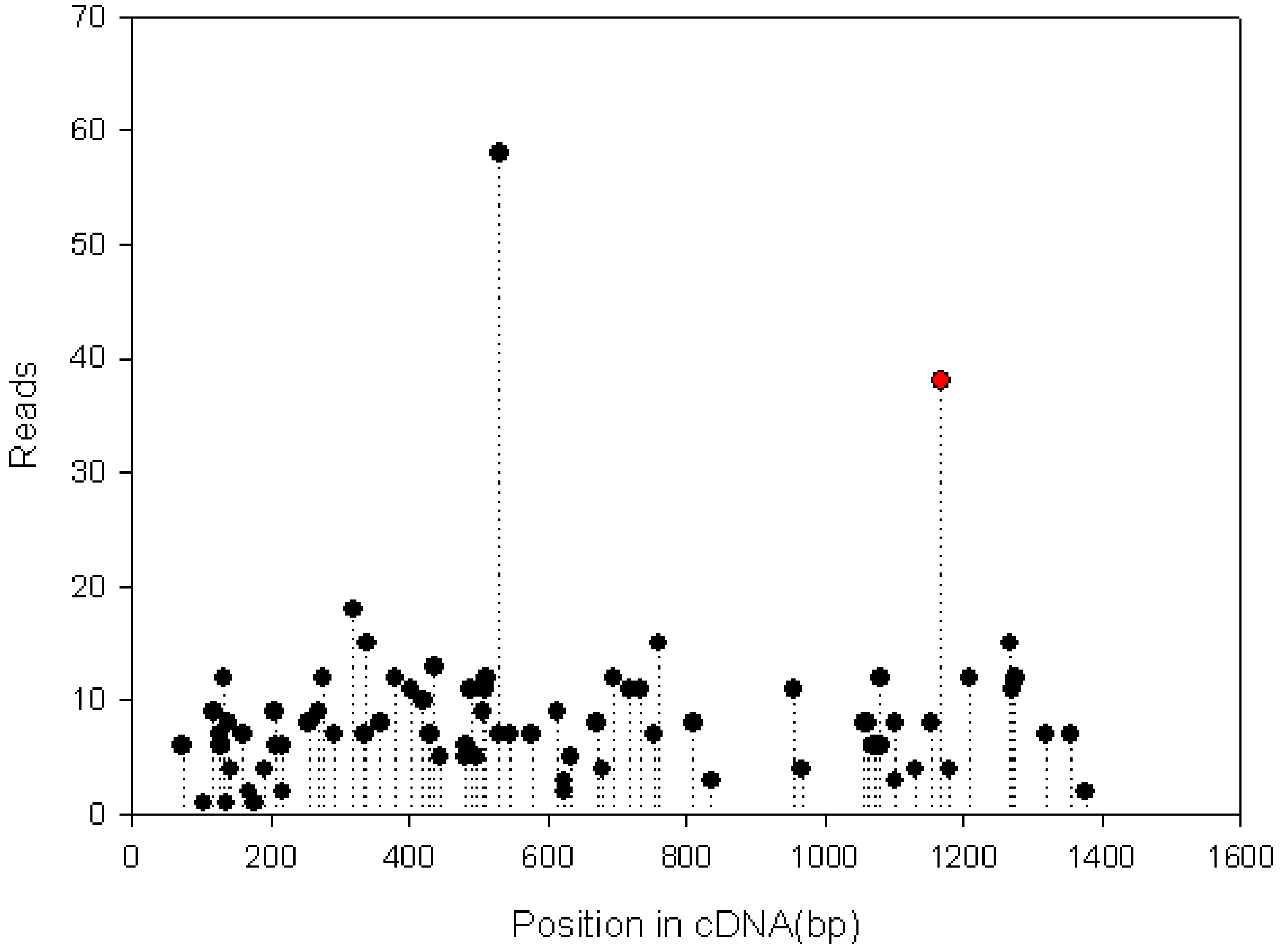


```
5' AAGGAGGUUGACAGAAUGCCAAAUAU 3'        Cs7g10850.1
     :::::: :::::::::::::
3' --CCUCCACCUGUCUUACGGUU---- 5'        Csi-miR394
```

Csi-miR395.2, target=Cs5g34330.1 gene=Cs5g34330
Category:1
Score=5
Cleavage Site=1443

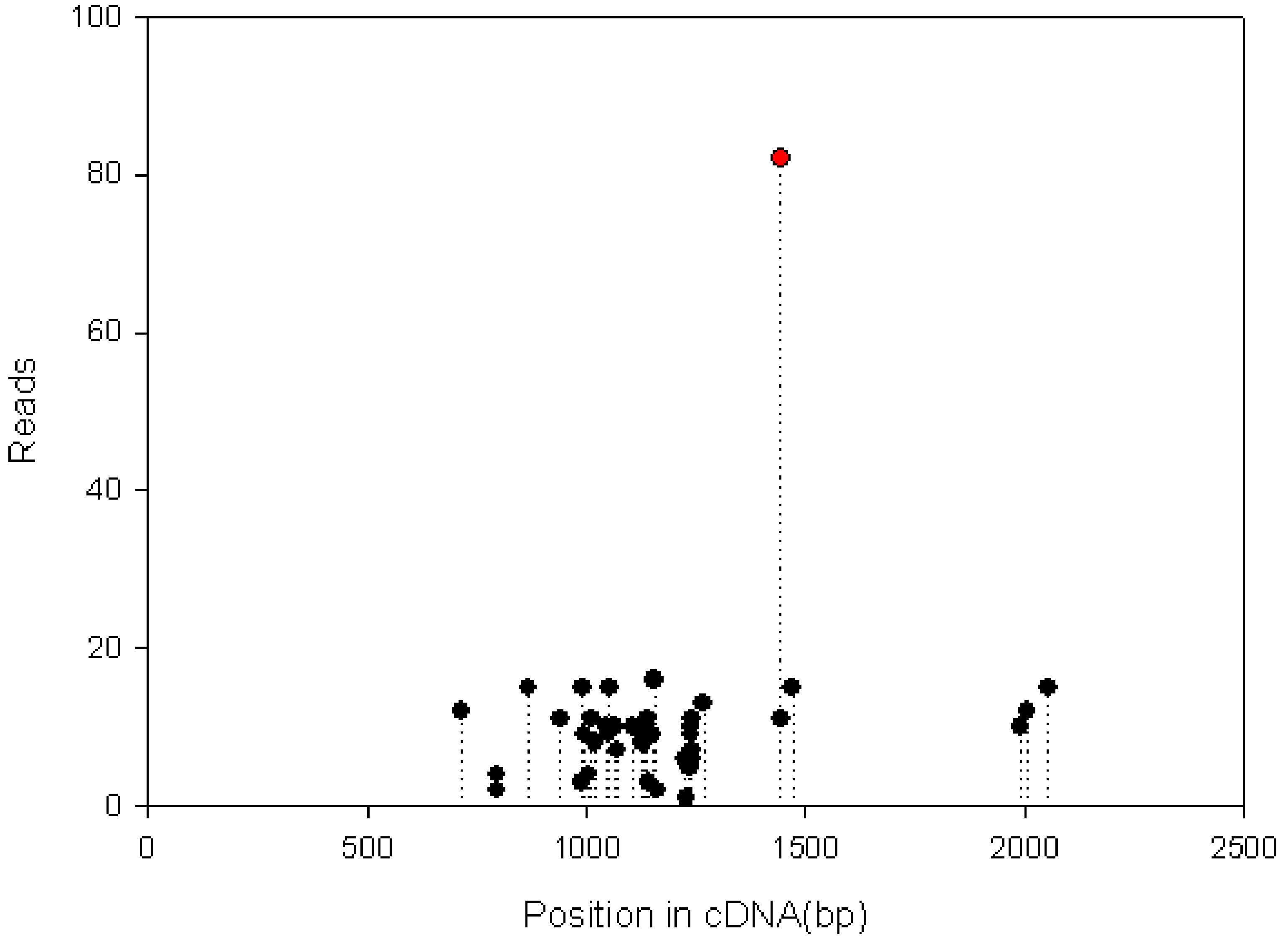


```
5' UUUUUAAGUU-UCCCAGACACUUUGGC 3'        Cs5g34330.1
         :::: .::::.::::::..
3' -----CUCAAGGGGGUUUGUGAAGU-- 5'        Csi-miR395.2
```

Csi-miR3951, target=Cs1g06060.1 gene=Cs1g06060
Category:1
Score=2.5
Cleavage Site=174

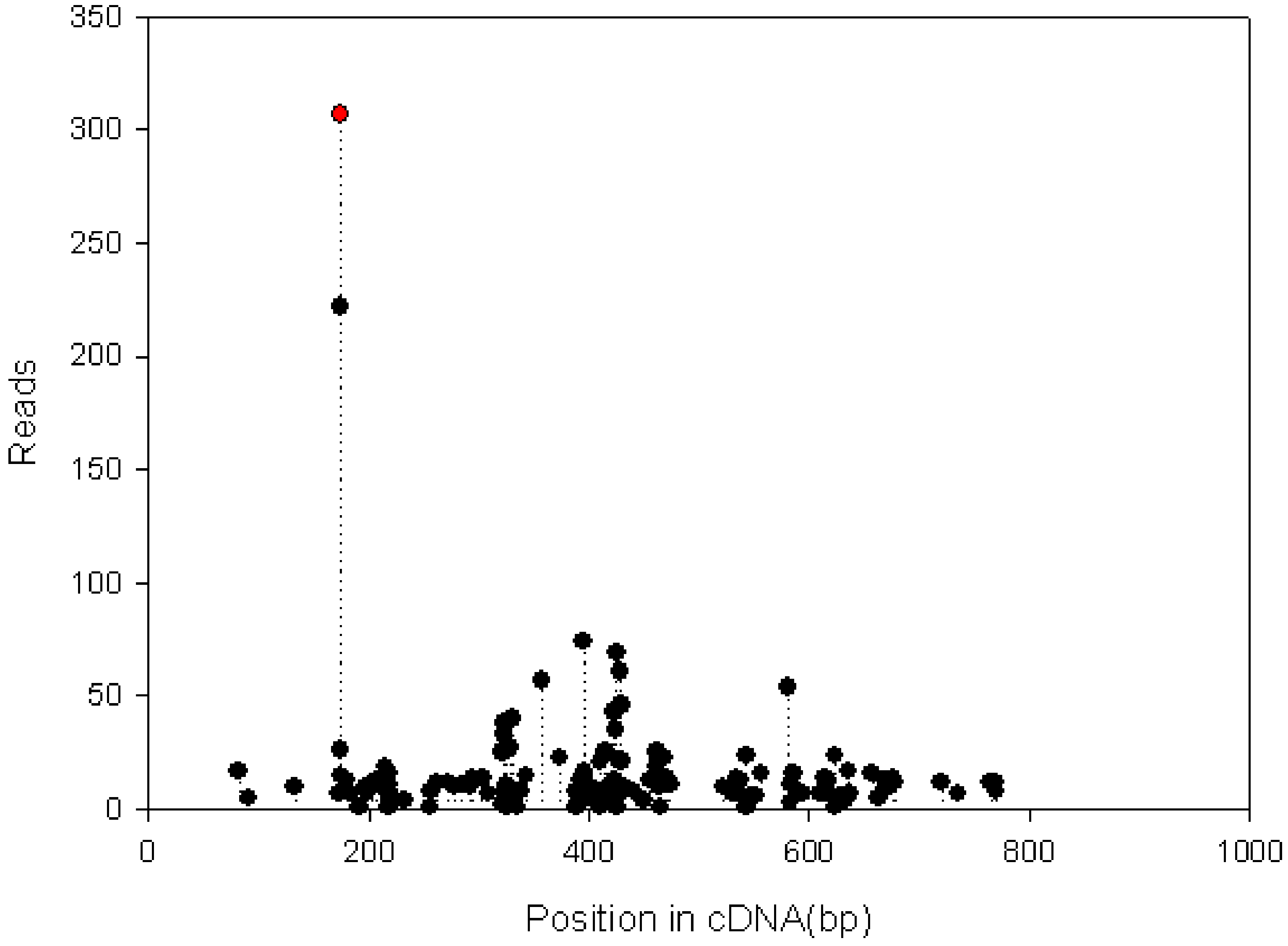


```
5' UUUUUUUCUCUC-UCUUUAUCUGUGGG 3'        Cs1g06060.1
     :::::::::: :::::::::.
3' --AAAAAGAGAGUAGAAAUAGAU---- 5'        Csi-miR3951
```

Csi-miR3951, target=Orange1.1t05622.1 gene=Orange1.1t05622
Category:1
Score=2.5
Cleavage Site=113

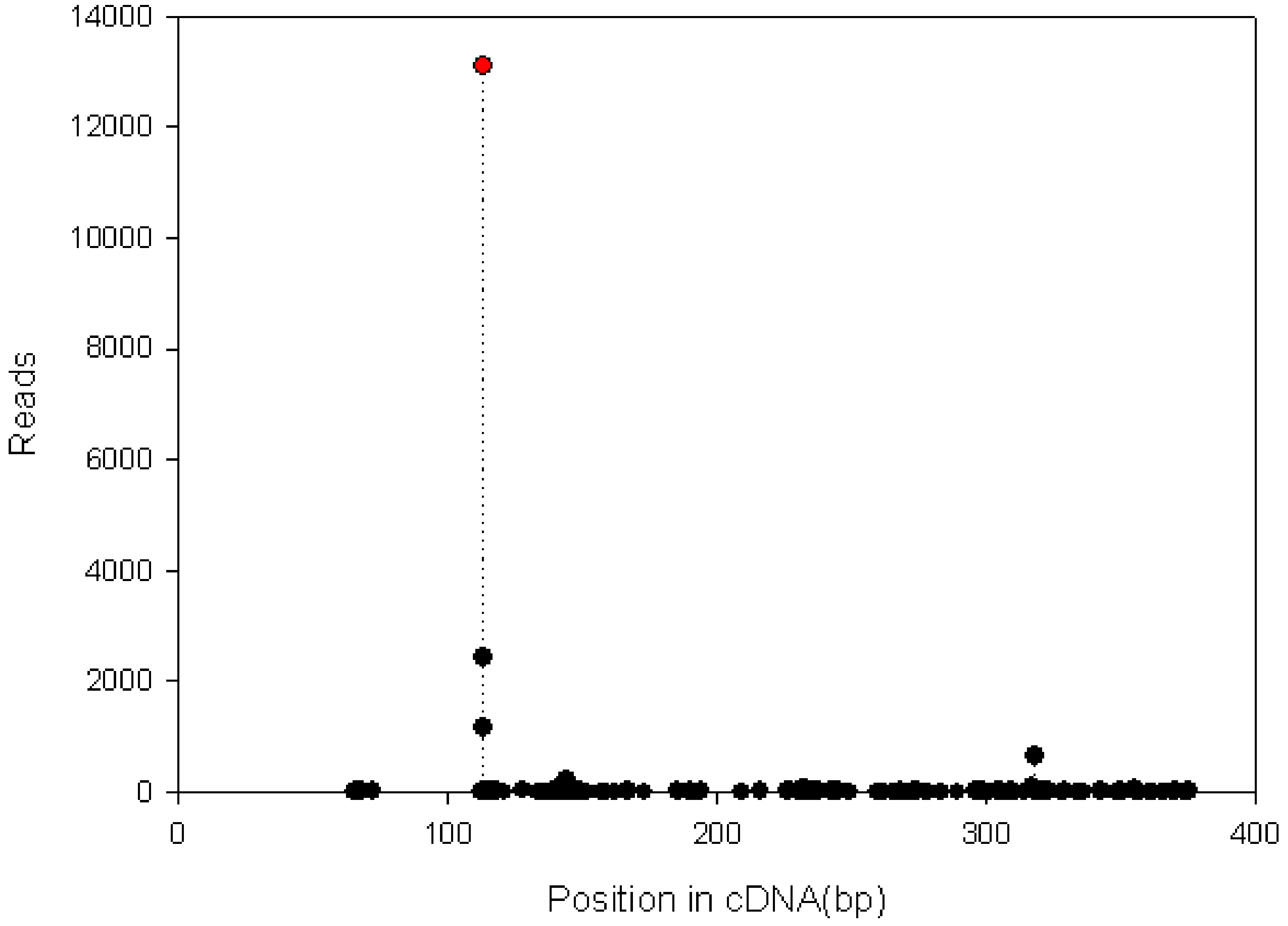


```
5' UUUUUUUCUCUC-UCUUUAUCUGUGGG 3'       Orange1.1t05622.1
     :::::::::: :::::::::.
3' --AAAAAGAGAGUAGAAAUAGAU---- 5'       Csi-miR3951
```

Csi-miR3952-3p, target=Orange1.1t02252.1 gene=Orange1.1t02252
Category:2
Score=3.5
Cleavage Site=3970

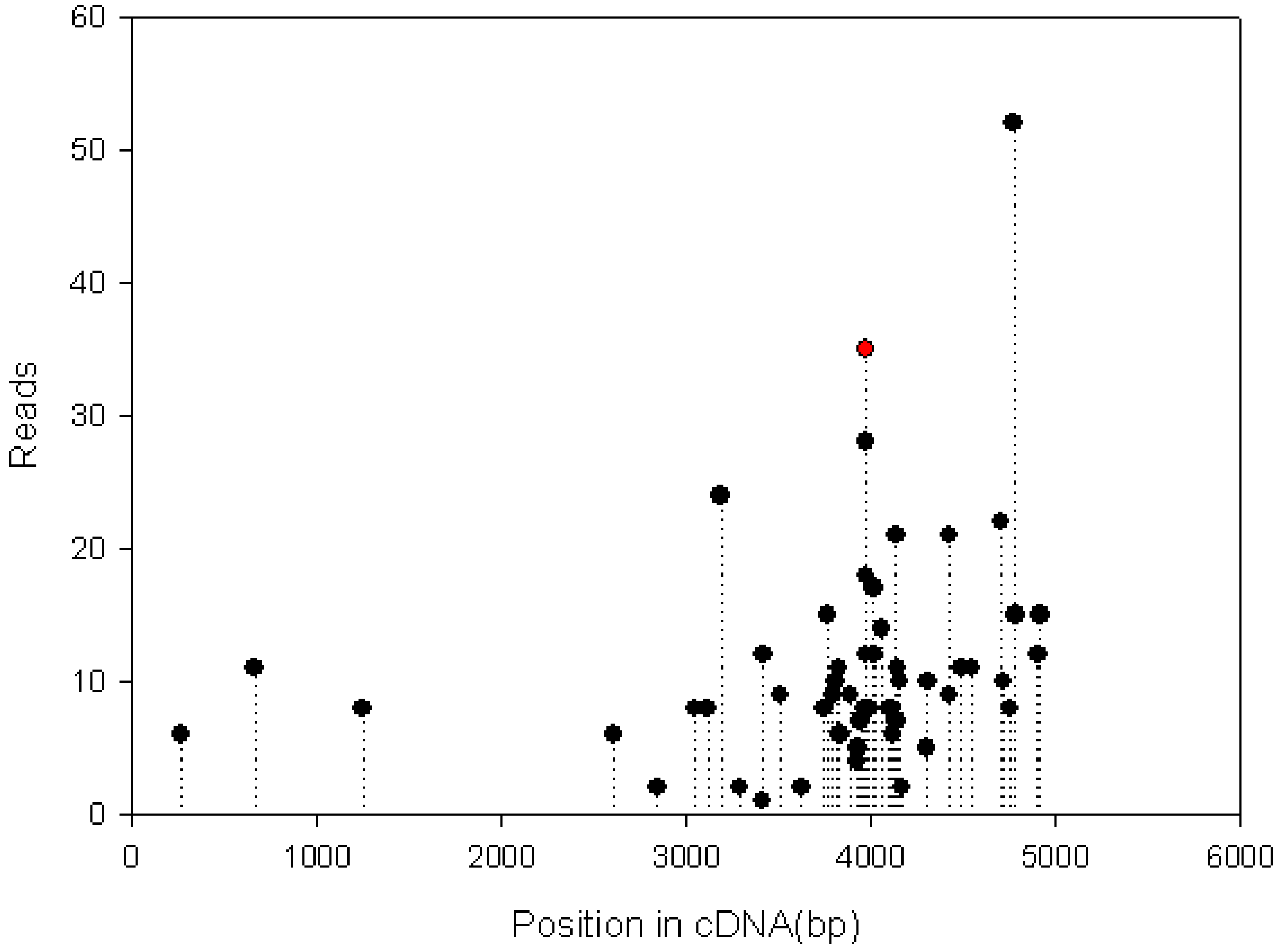


```
5' AAUGUGGGCUCUGGAAAGGCUCUUCU 3'        Orange1.1t02252.1
         :  :::::.::::::::.::::
3' -----CACGAGAUCUUUCCGGGAAGU 5'        Csi-miR3952-3p
```

Csi-miR3954a, target=Cs1g09600.1 gene=Cs1g09600
Category:2
Score=1
Cleavage Site=416

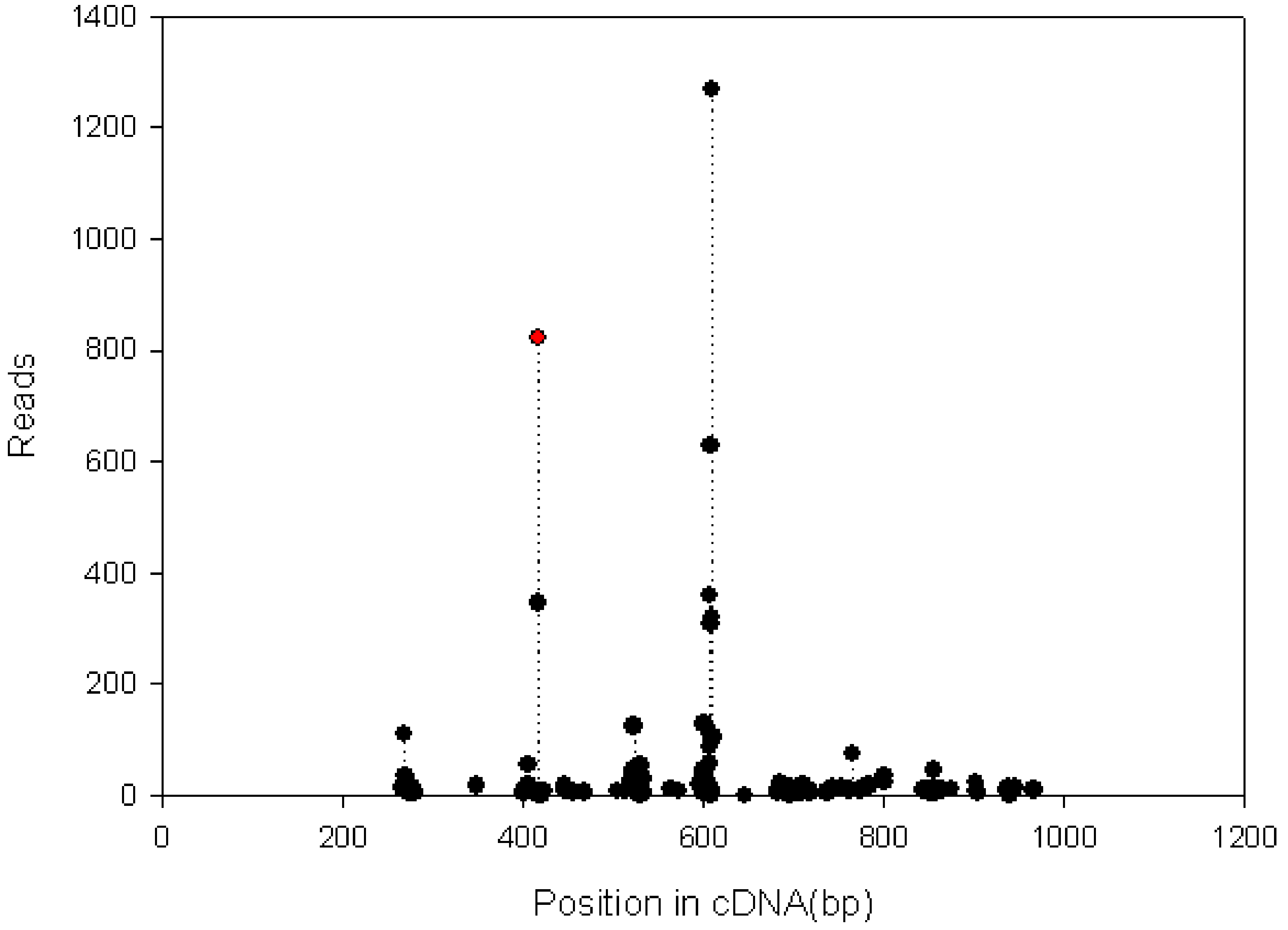


```
5' UGACCCUGAUUUCUCUGUCCAAGCAA 3'        Cs1g09600.1
   ::::: ::::::::::::::::
3' ACUGGCACUAAAGAGACAGGUU---- 5'        Csi-miR3954a
```

Csi-miR3954a, target=Cs1g09635.1 gene=Cs1g09635
Category:2
Score=1
Cleavage Site=351

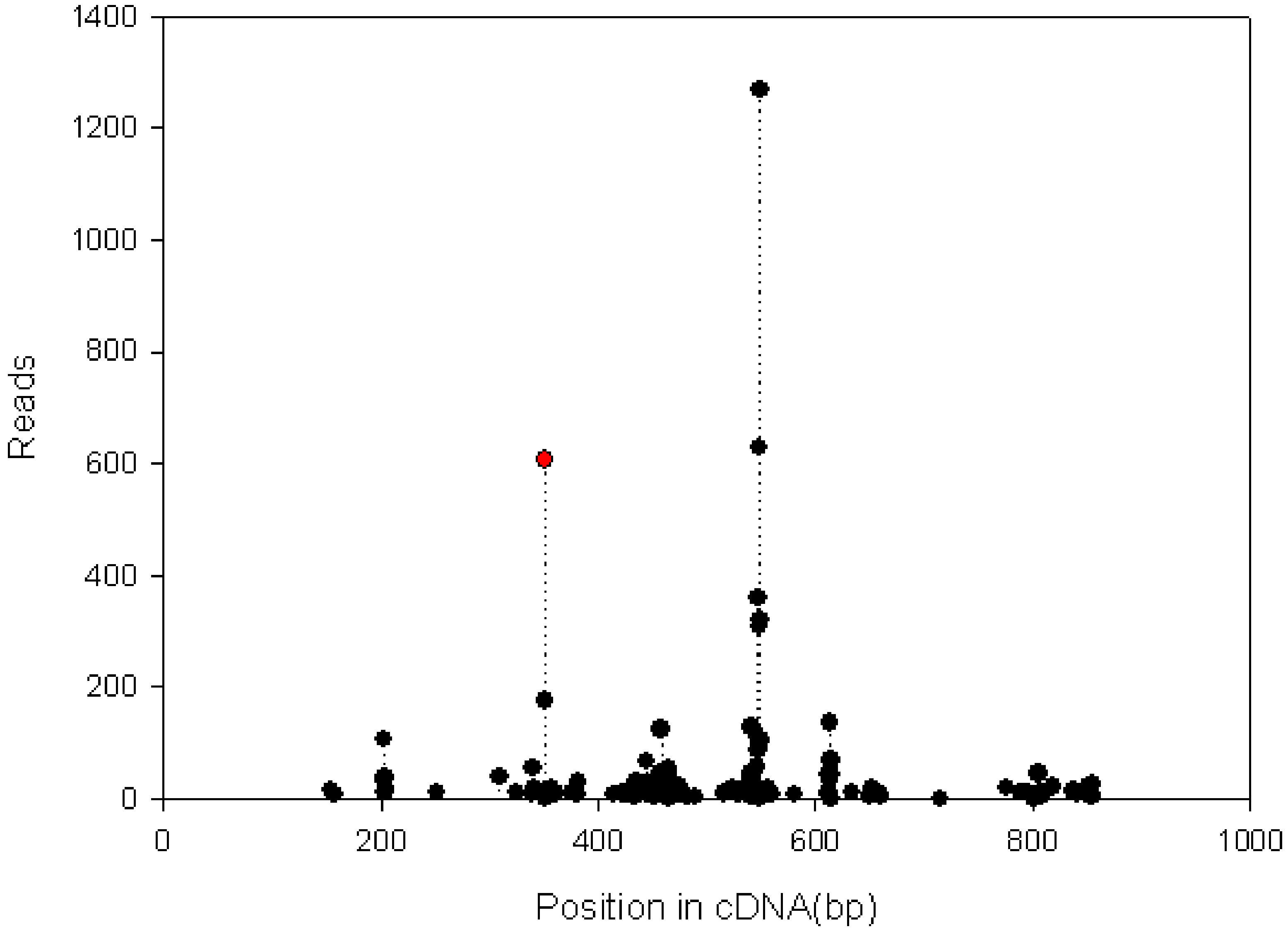


```
5' UGACCCUGAUUUCUCUGUCCAAACAG 3'        Cs1g09635.1
   ::::: ::::::::::::::::
3' ACUGGCACUAAAGAGACAGGUU---- 5'        Csi-miR3954a
```

Csi-miR3954a, target=Cs5g04670.1 gene=Cs5g04670
Category:3
Score=1
Cleavage Site=142

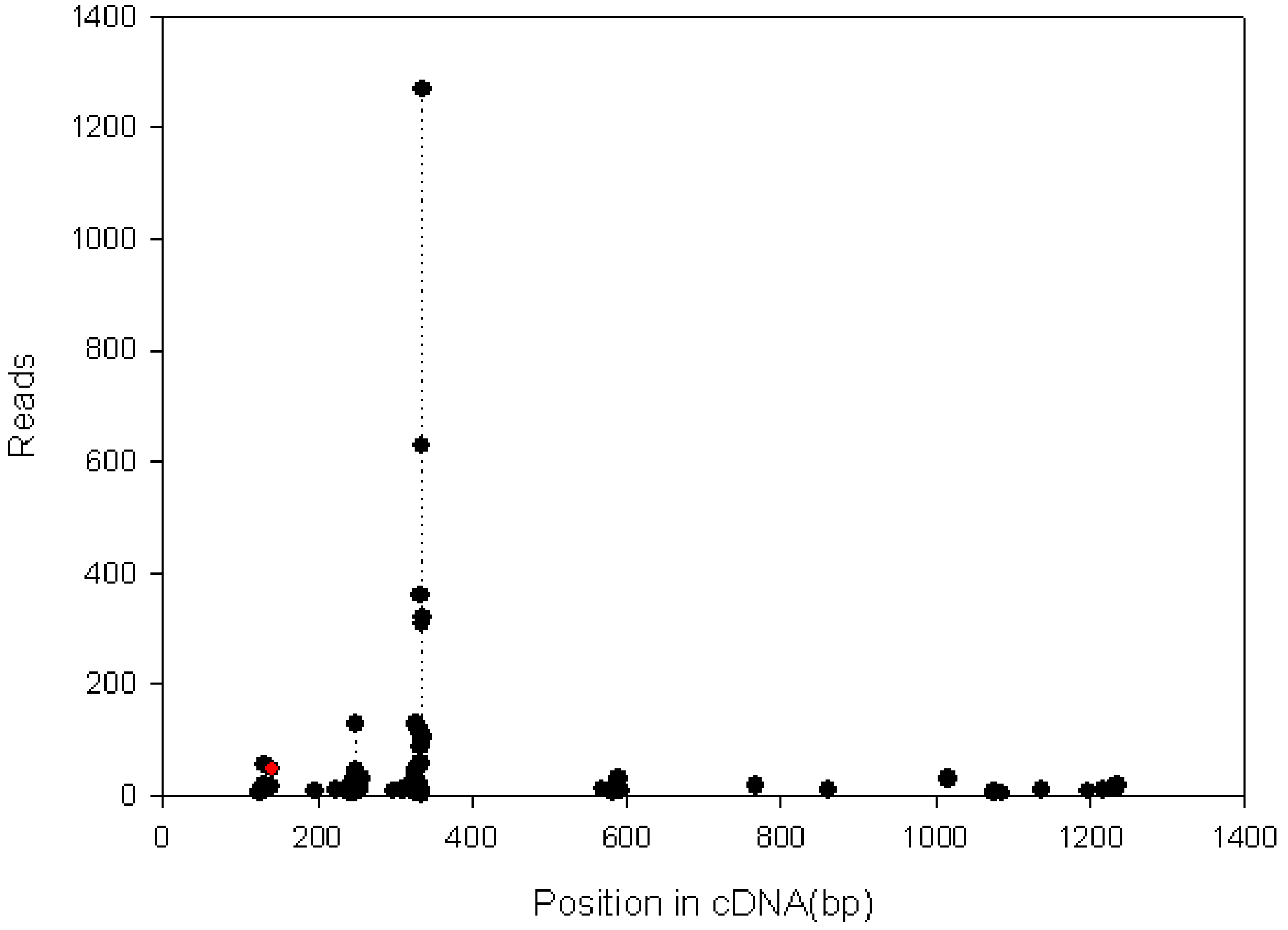


```
5' UGACCCUGAUUUCUCUGUCCAAACAA 3'        Cs5g04670.1
   ::::: ::::::::::::::::
3' ACUGGCACUAAAGAGACAGGUU---- 5'        Csi-miR3954a
```

Csi-miR3954a, target=Cs7g22460.1 gene=Cs7g22460
Category:2
Score=0.5
Cleavage Site=214

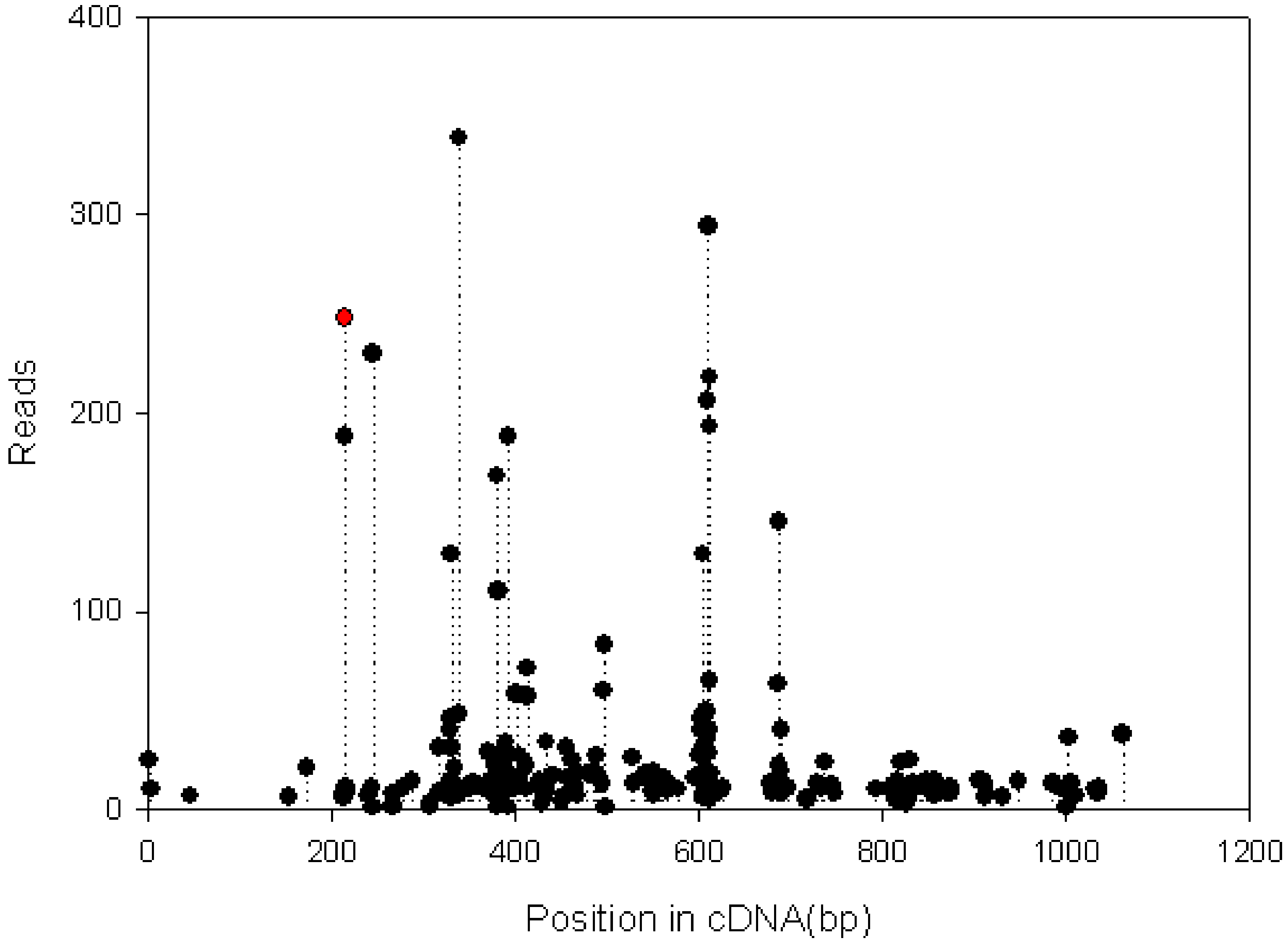


```
5' UGACCGUGAUUUCUCUGUCCAGCCAA 3'        Cs7g22460.1
   ::::::::::::::::::::::.
3' ACUGGCACUAAAGAGACAGGUU---- 5'        Csi-miR3954a
```

Csi-miR3954b, target=Cs1g09600.1 gene=Cs1g09600
Category:2
Score=1
Cleavage Site=416

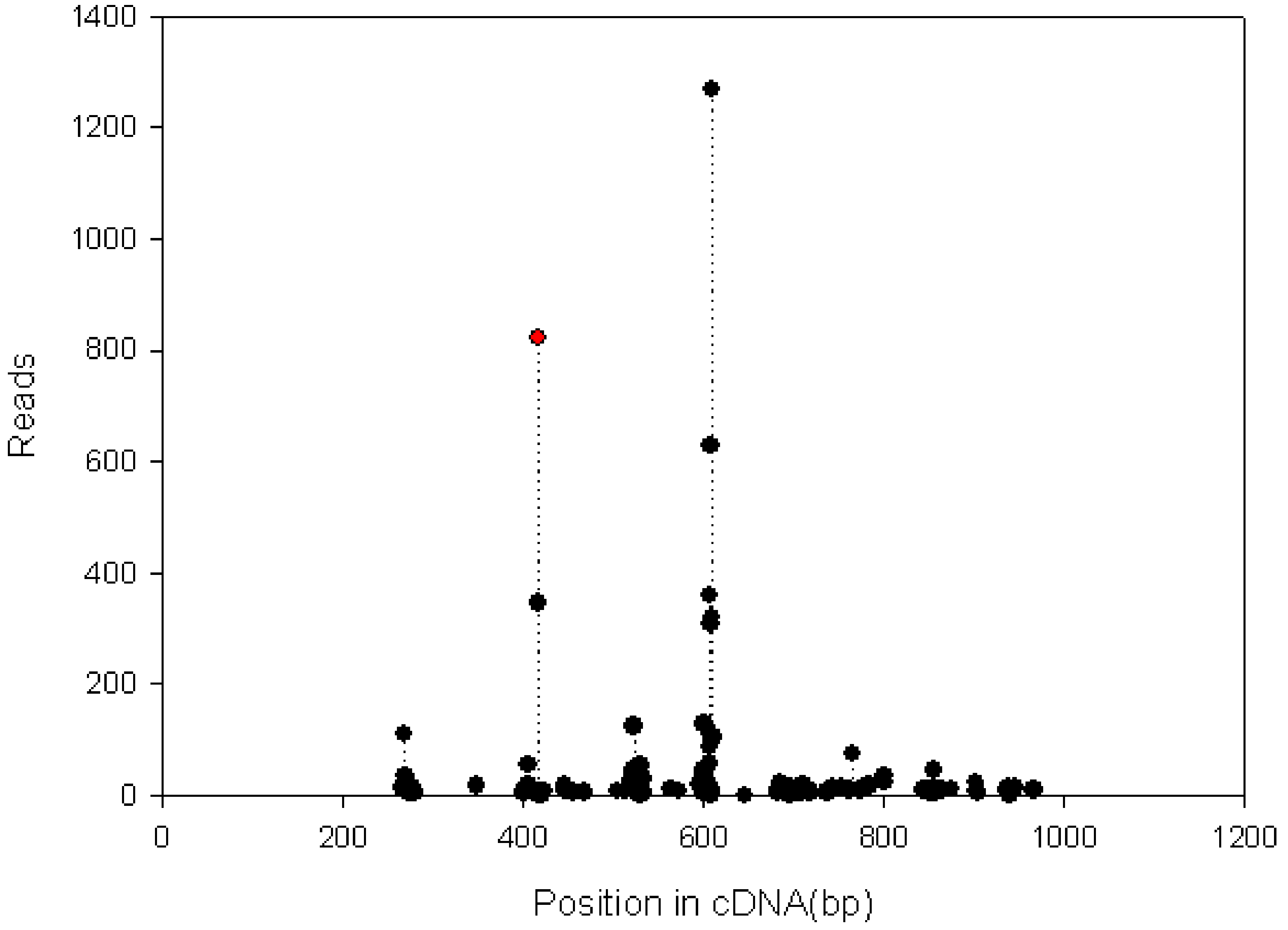


```
5' UGACCCUGAUUUCUCUGUCCAAGCAA 3'        Cs1g09600.1
   ::::: ::::::::::::::
3' ACUGGCACUAAAGAGACAGG------ 5'        Csi-miR3954b
```

Csi-miR3954b, target=Cs1g09635.1 gene=Cs1g09635
Category:2
Score=1
Cleavage Site=351

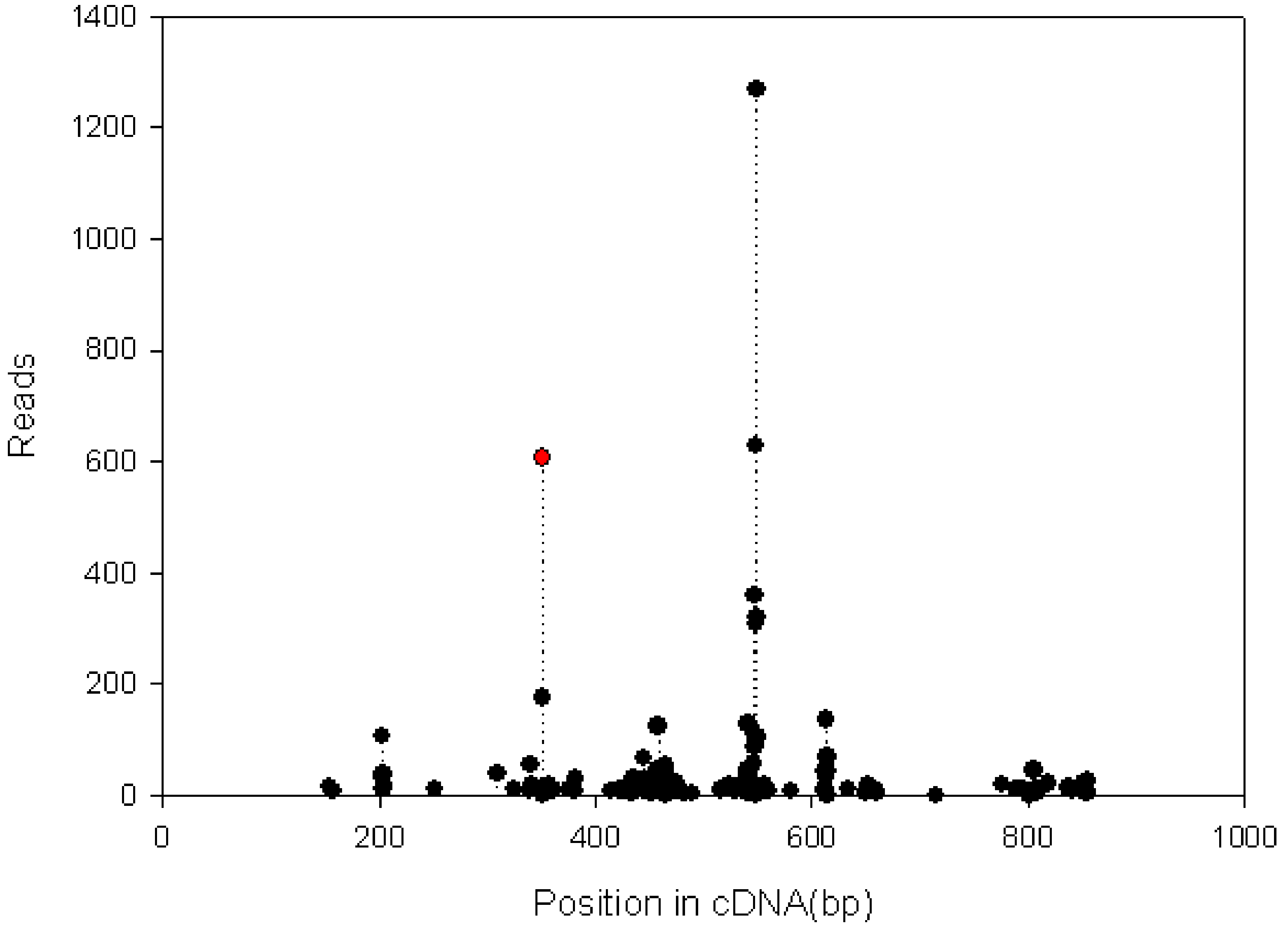


```
5' UGACCCUGAUUUCUCUGUCCAAACAG 3'        Cs1g09635.1
   ::::: ::::::::::::::
3' ACUGGCACUAAAGAGACAGG------ 5'        Csi-miR3954b
```

Csi-miR3954b, target=Cs1g09665.1 gene=Cs1g09665
Category:3
Score=0
Cleavage Site=416

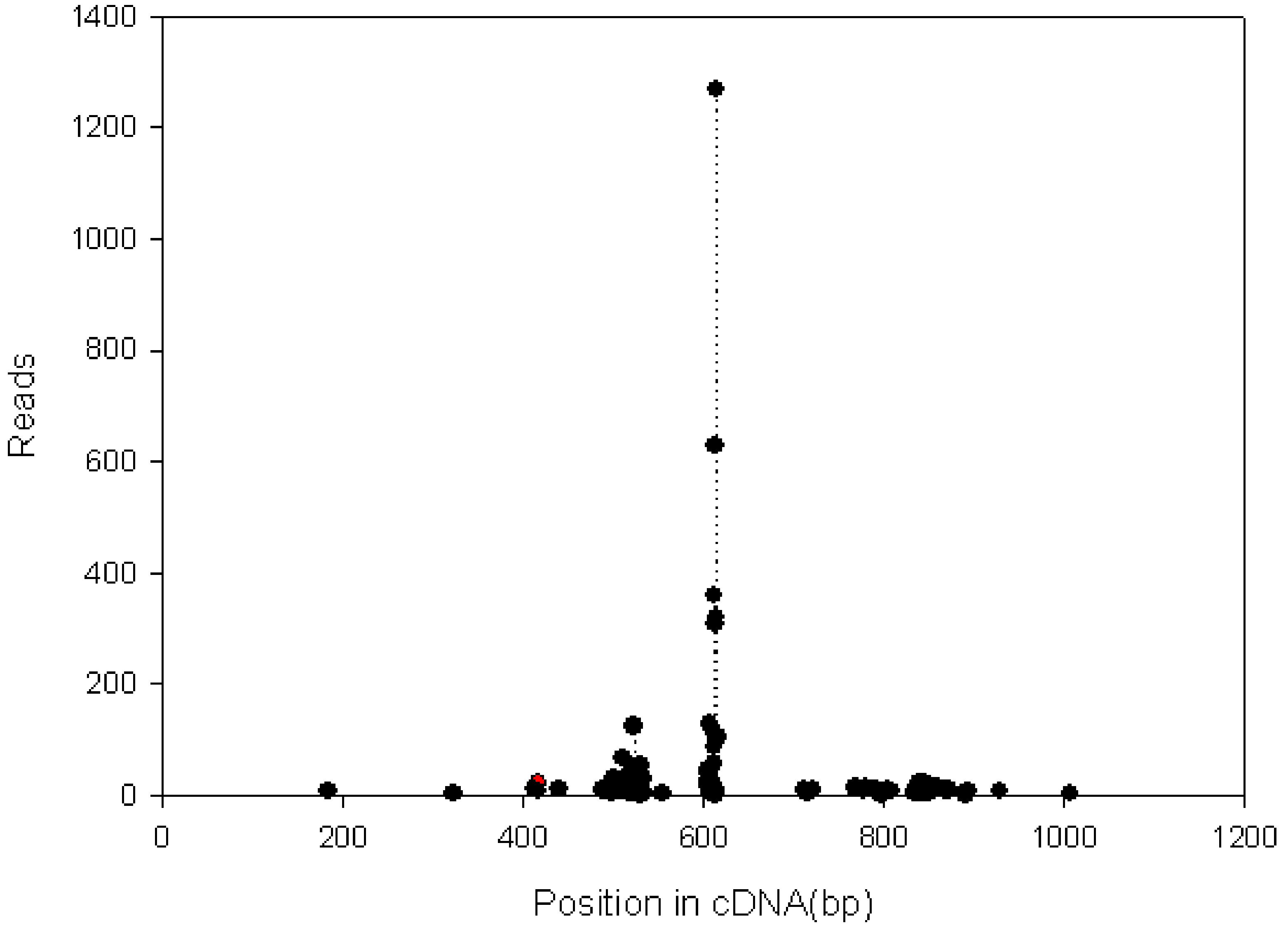


```
5' UGACCGUGAUUUCUCUGUCCAAGCAG 3'        Cs1g09665.1
   ::::::::::::::::::::
3' ACUGGCACUAAAGAGACAGG------ 5'        Csi-miR3954b
```

Csi-miR3954b, target=Cs2g29120.1 gene=Cs2g29120
Category:1
Score=4.5
Cleavage Site=941

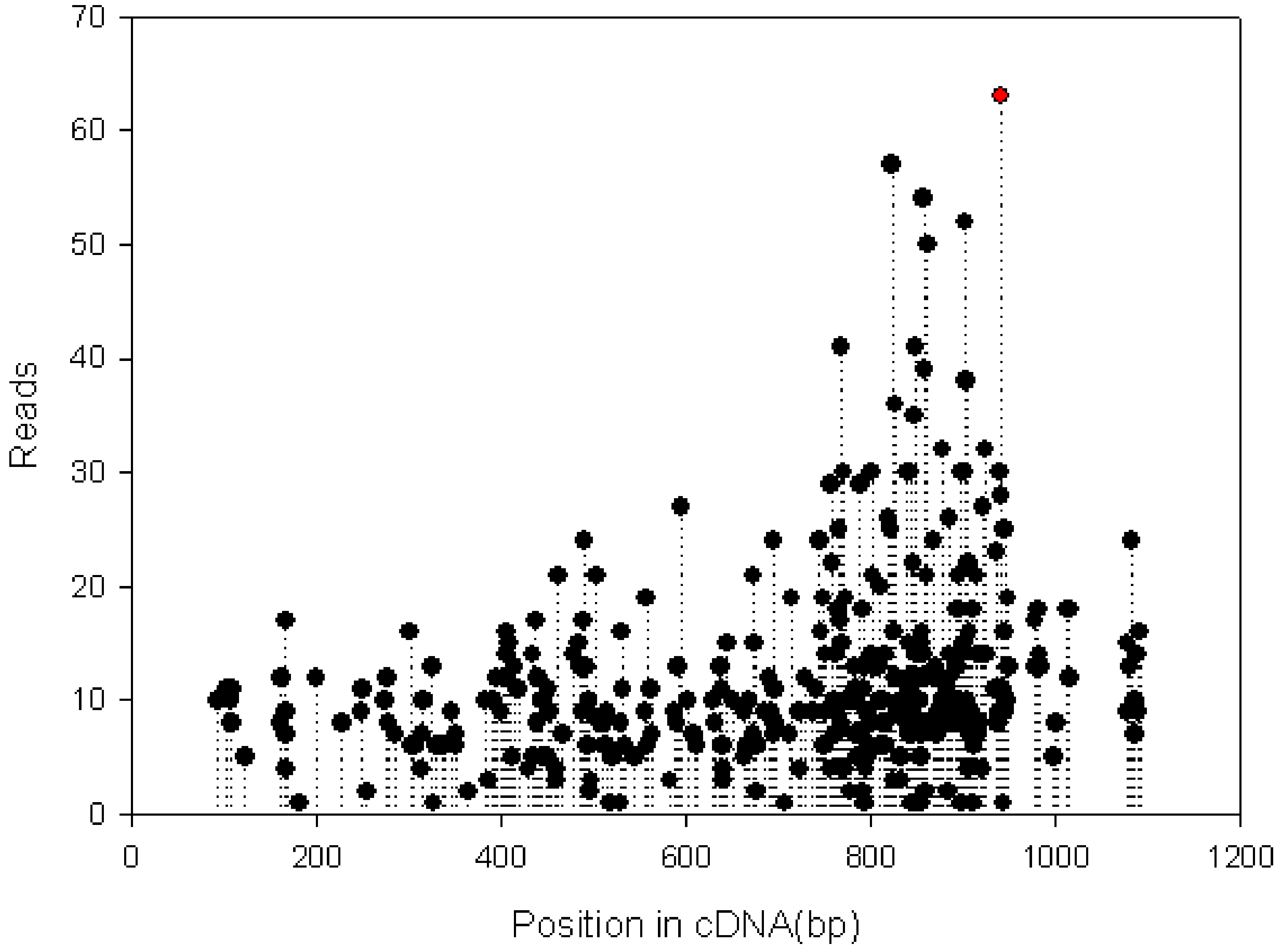


```
5' CUAAGGACUGUGAUUACUCUGUCAAA 3'        Cs2g29120.1
        :::.:::::: :::::::
3' ----ACUGGCACUAAAGAGACAGG-- 5'        Csi-miR3954b
```

Csi-miR3954b, target=Cs5g04670.1 gene=Cs5g04670
Category:3
Score=1
Cleavage Site=142

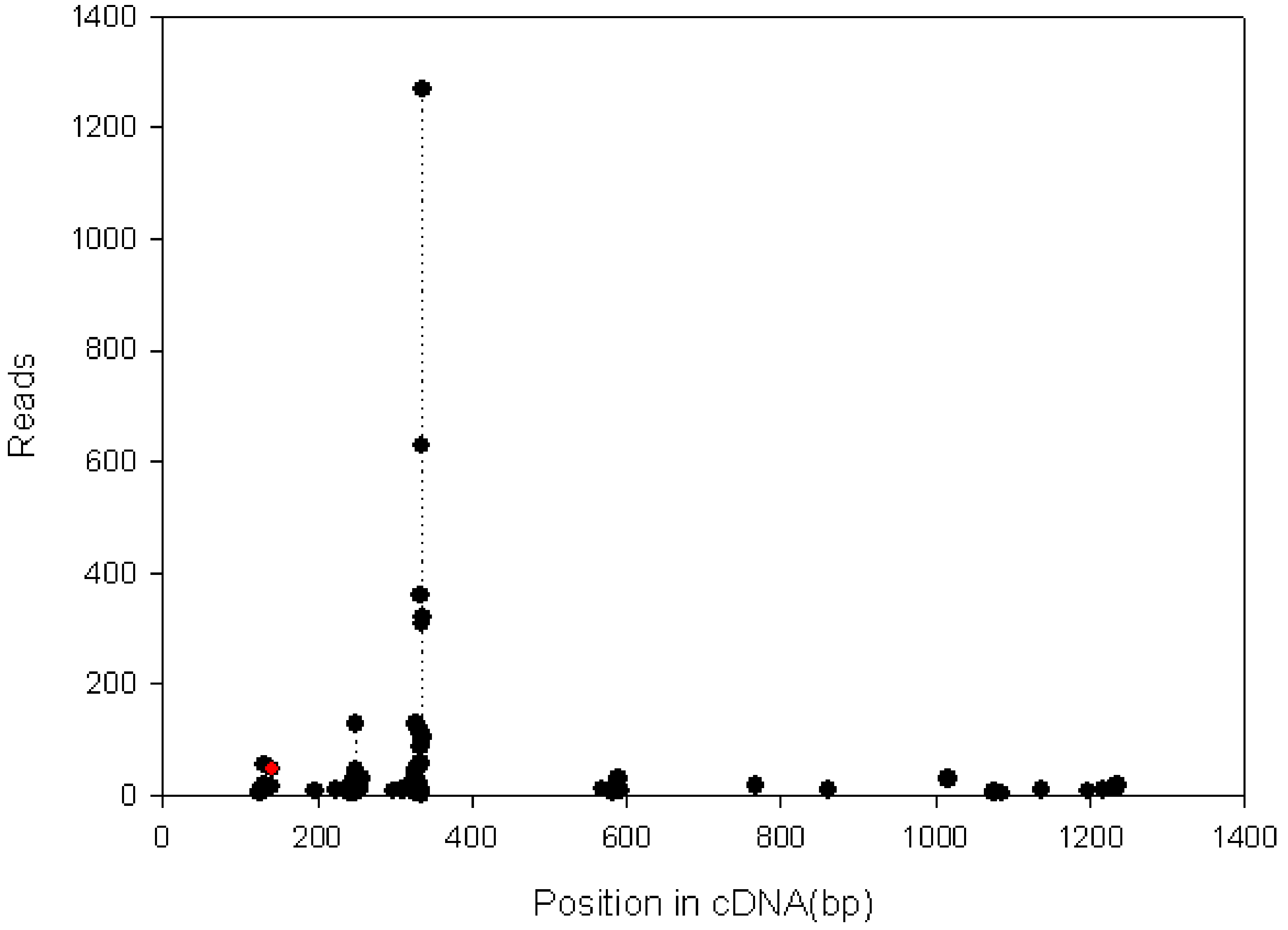


```
5' UGACCCUGAUUUCUCUGUCCAAACAA 3'        Cs5g04670.1
   ::::: ::::::::::::::
3' ACUGGCACUAAAGAGACAGG------ 5'        Csi-miR3954b
```

Csi-miR3954b, target=Cs7g22460.1 gene=Cs7g22460
Category:2
Score=0
Cleavage Site=214

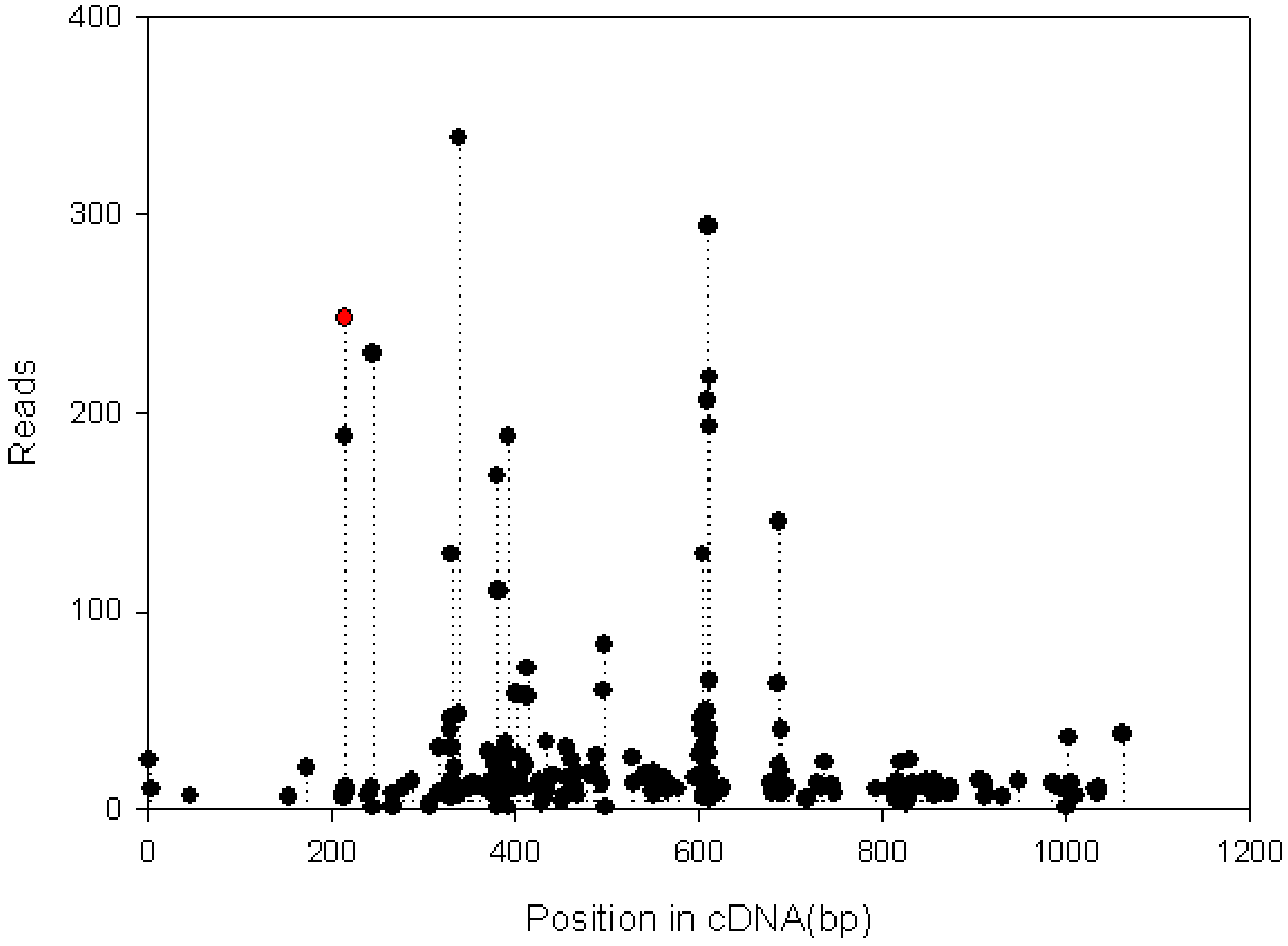


```
5' UGACCGUGAUUUCUCUGUCCAGCCAA 3'        Cs7g22460.1
   :::::::::::::::::::::
3' ACUGGCACUAAAGAGACAGG------ 5'        Csi-miR3954b
```

Csi-miR396a, target=Cs1g21350.1 gene=Cs1g21350
Category:1
Score=3
Cleavage Site=1164

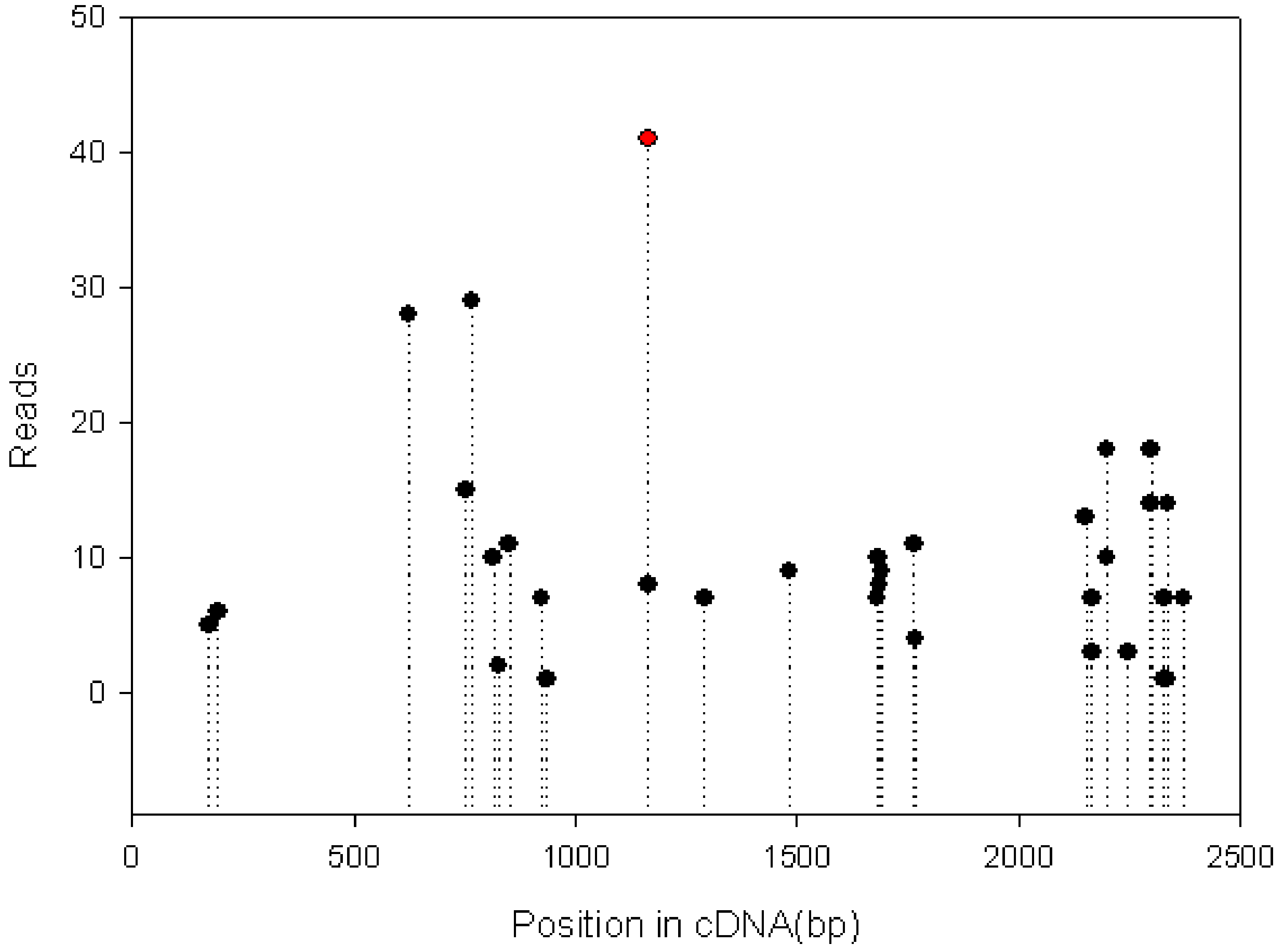


```
5' ACCGUUCAAGAAAGCCUGUGGAAGGC 3'        Cs1g21350.1
    : :::::::::::: ::::::::
3' -GUCAAGUUCUUUC-GACACCUU--- 5'        Csi-miR396a
```

Csi-miR396a, target=Cs1g22520.1 gene=Cs1g22520
Category:1
Score=5
Cleavage Site=226

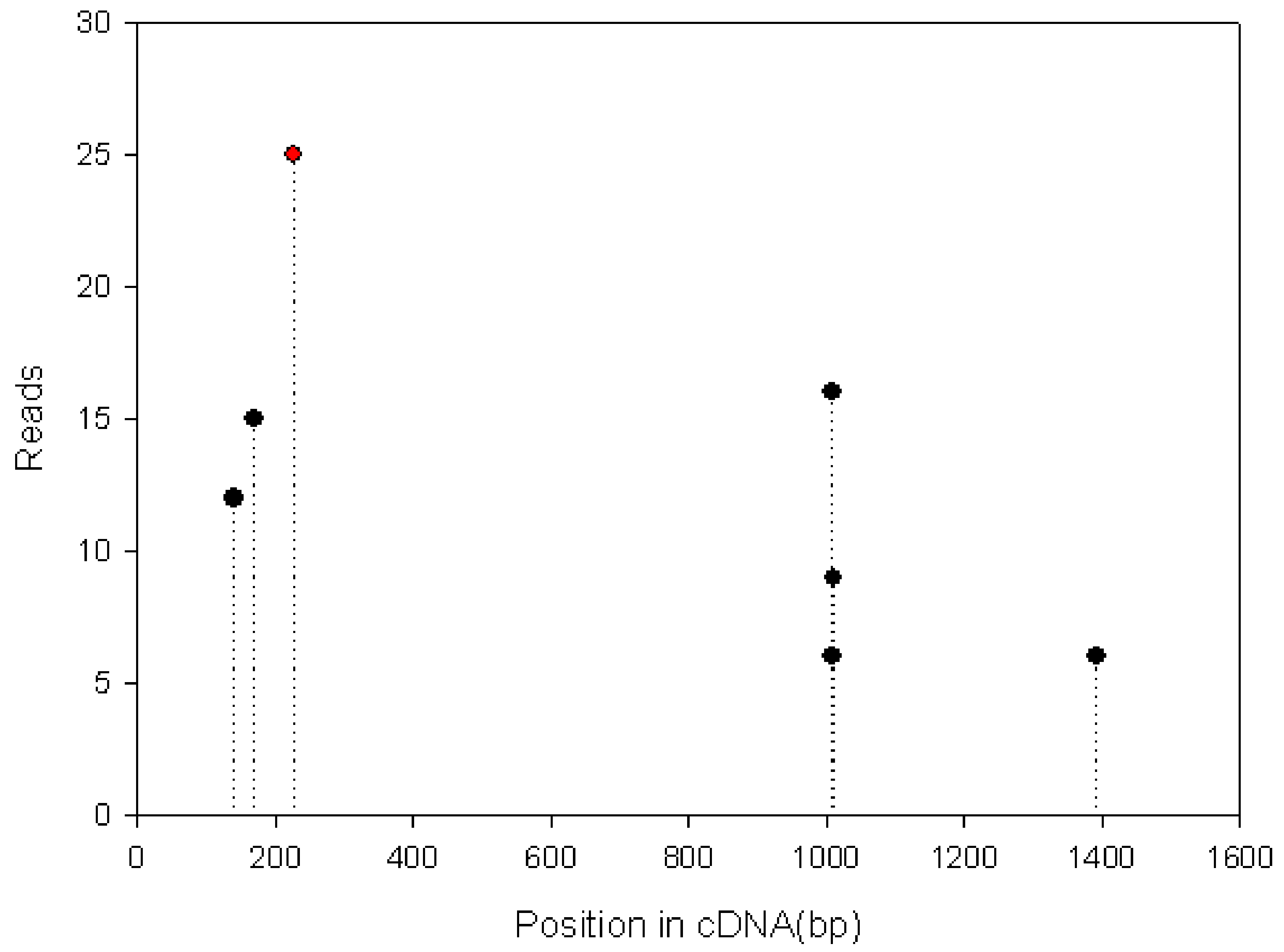


```
5' GAAGUUCAAGAAAUCUGUGGGAAGAA 3'        Cs1g22520.1
     ::::::::::: :::::::.:
3' -GUCAAGUUCUUUCGACACCUU---- 5'        Csi-miR396a
```

Csi-miR396a, target=Cs3g23180.1 gene=Cs3g23180
Category:1
Score=4
Cleavage Site=625

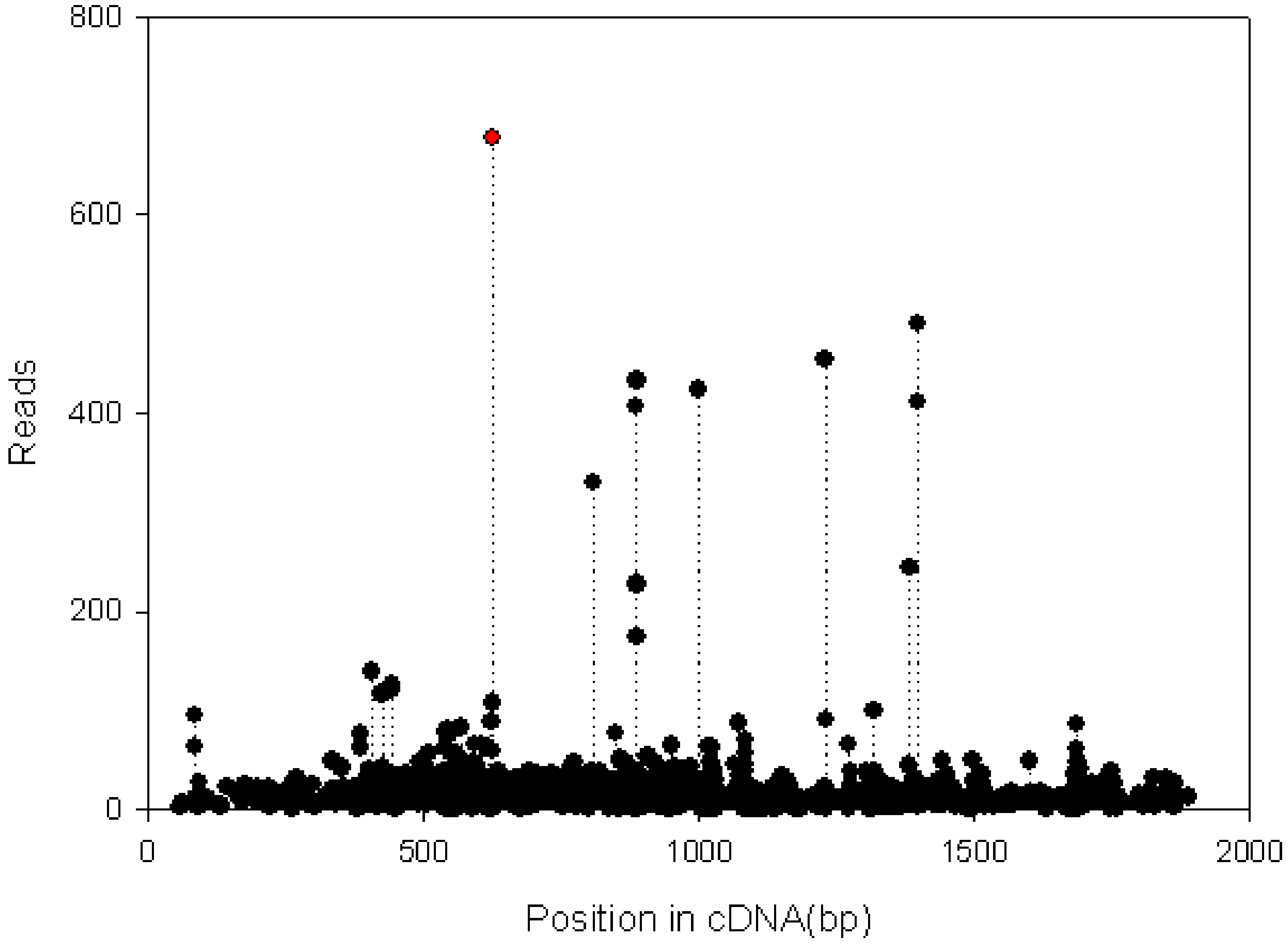


```
5' AAAGAUCAAGGAAGCUGUGGGAGUUG 3'        Cs3g23180.1
     :: :::::.::::::::::.:
3' -GUCAAGUUCUUUCGACACCUU---- 5'        Csi-miR396a
```

Csi-miR396a, target=Cs4g05000.1 gene=Cs4g05000
Category:3
Score=4
Cleavage Site=1249

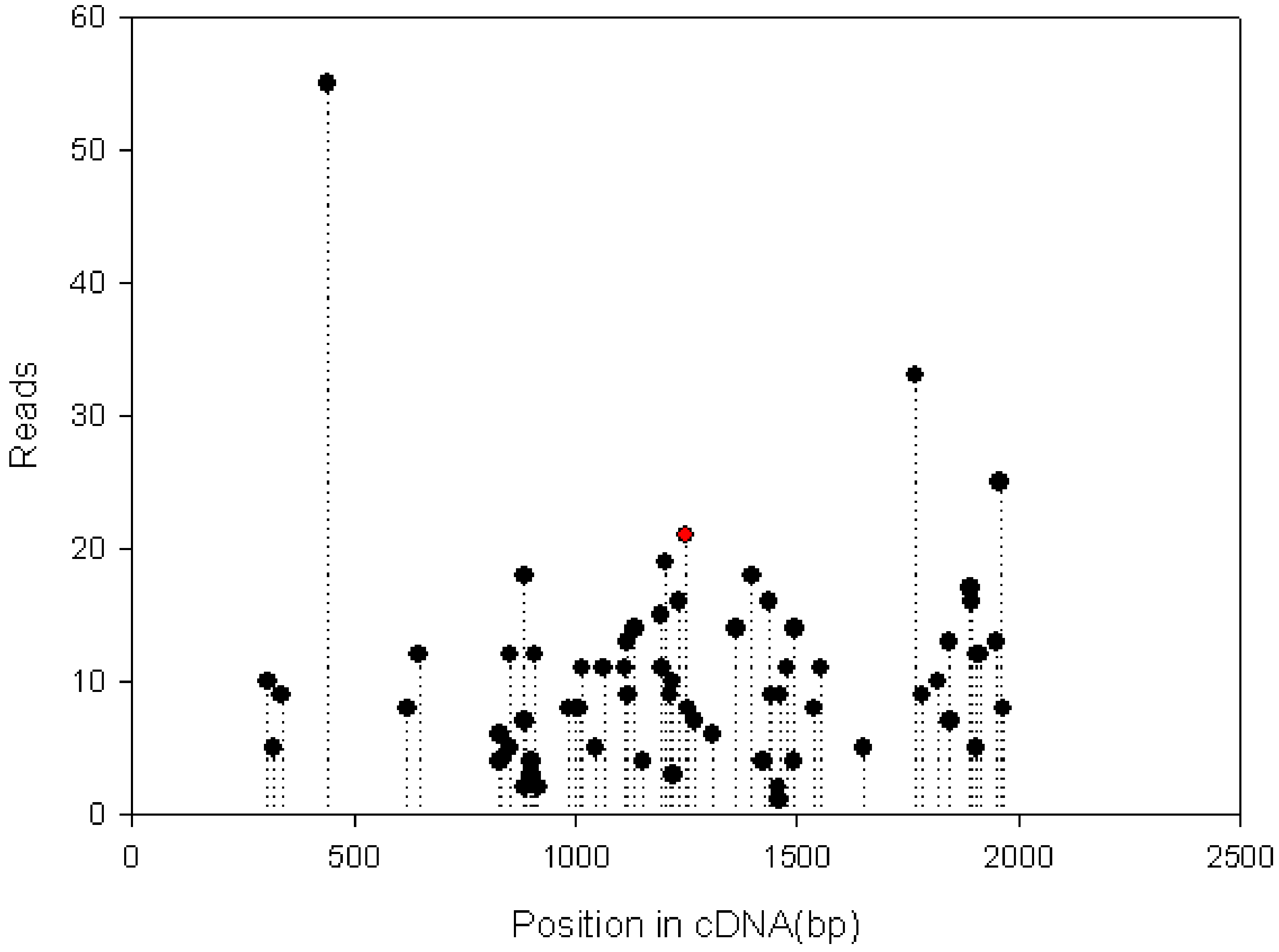


```
5' ACCCUUGAAGAAAGCUGUGGAUAUAG 3'        Cs4g05000.1
    :   :: ::::::::::::::
3' -GUCAAGUUCUUUCGACACCUU---- 5'        Csi-miR396a
```

Csi-miR396a, target=Cs5g01380.1 gene=Cs5g01380
Category:1
Score=3
Cleavage Site=1181

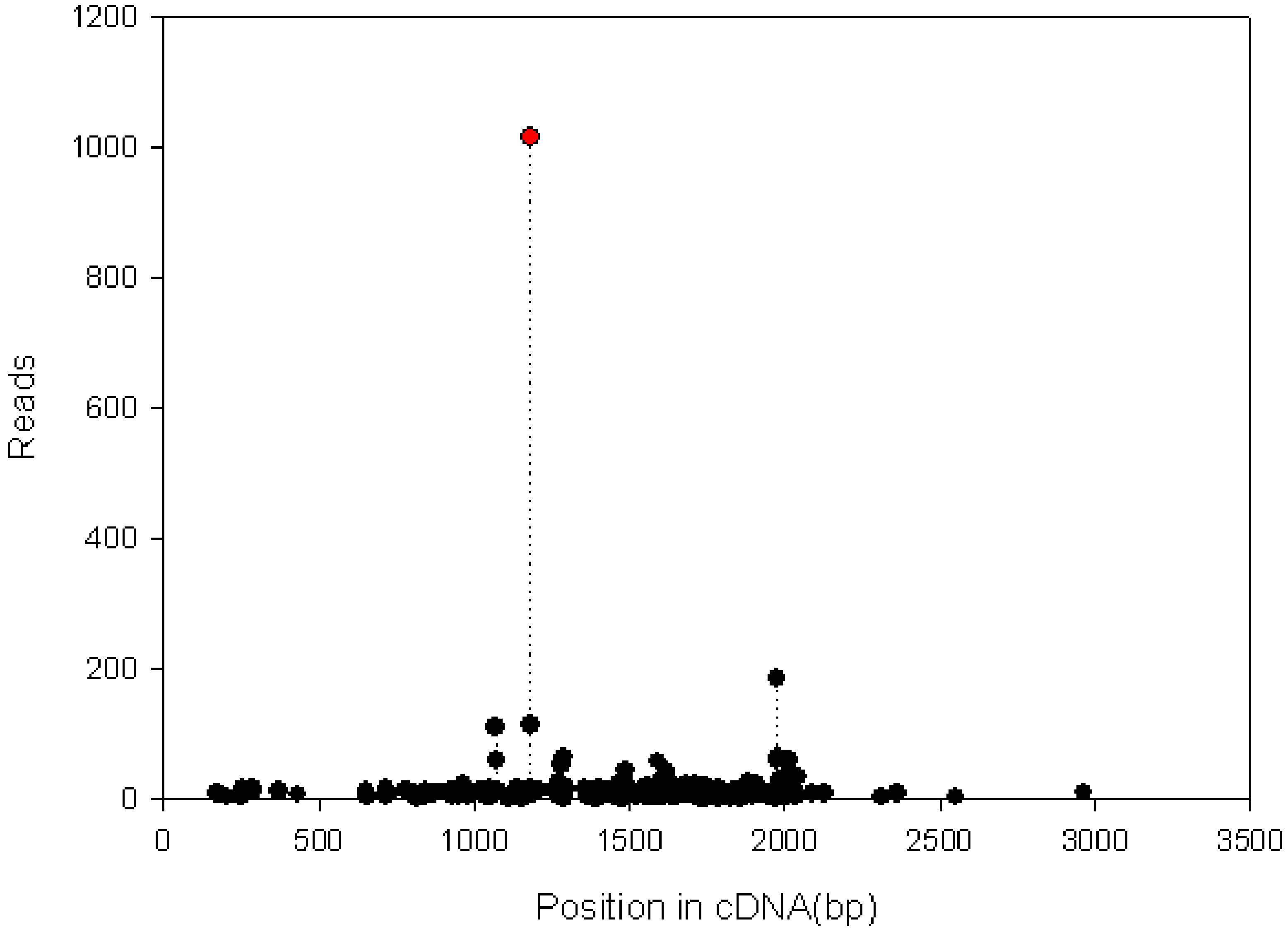


```
5' CCCGUUCAAGAAAGCCUGUGGAAUUA 3'        Cs5g01380.1
    : :::::::::: ::::::::
3' -GUCAAGUUCUUUC-GACACCUU--- 5'        Csi-miR396a
```

Csi-miR396a, target=Cs5g09850.1 gene=Cs5g09850
Category:3
Score=4
Cleavage Site=767

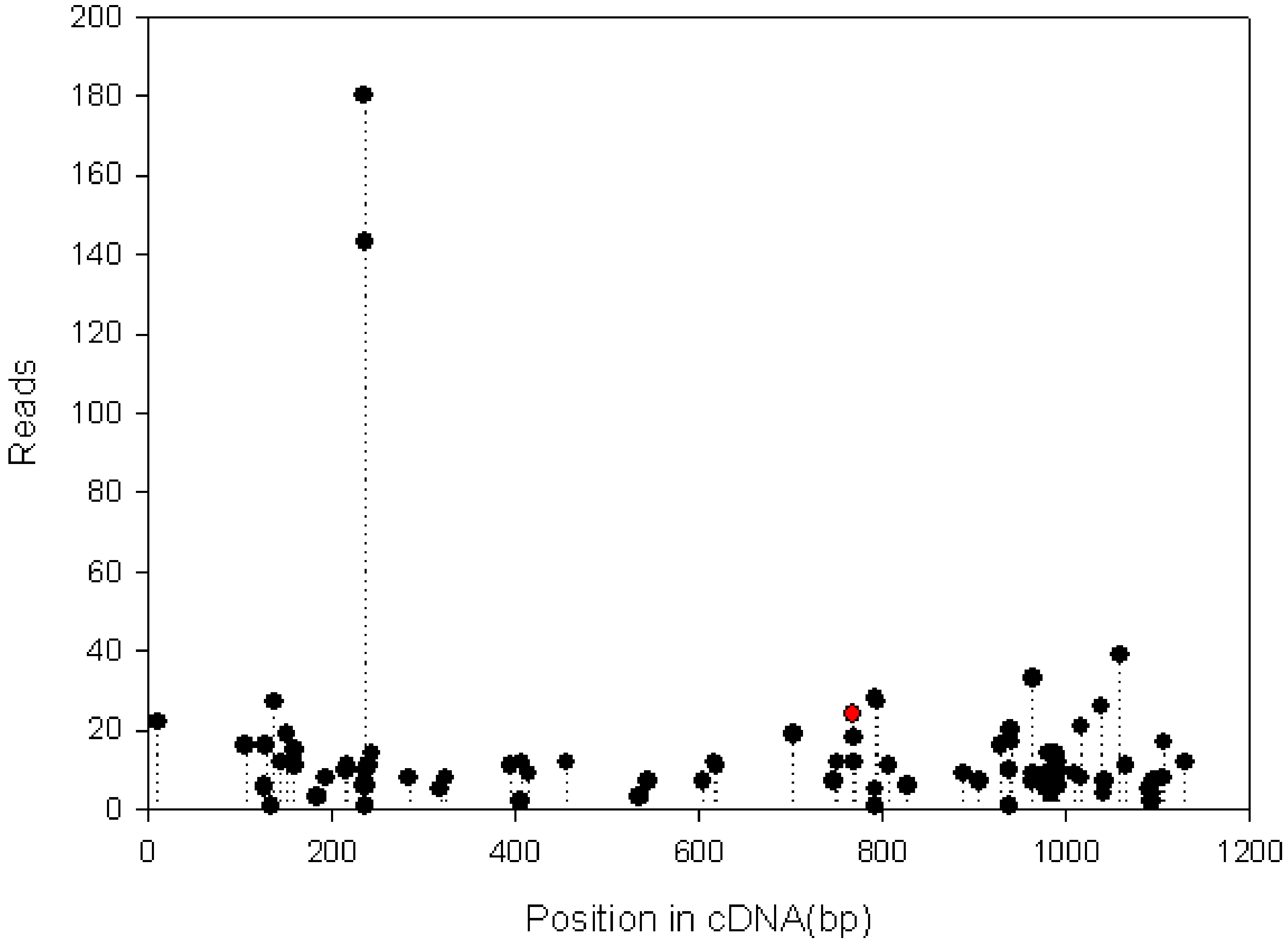


```
5' UGCGUUCAAGAAAGCUUGUGGAAGCU 3'        Cs5g09850.1
     ::::::::::::: :::::::
3' -GUCAAGUUCUUUCG-ACACCUU--- 5'        Csi-miR396a
```

Csi-miR396a, target=Cs7g15220.1 gene=Cs7g15220
Category:1
Score=3
Cleavage Site=377

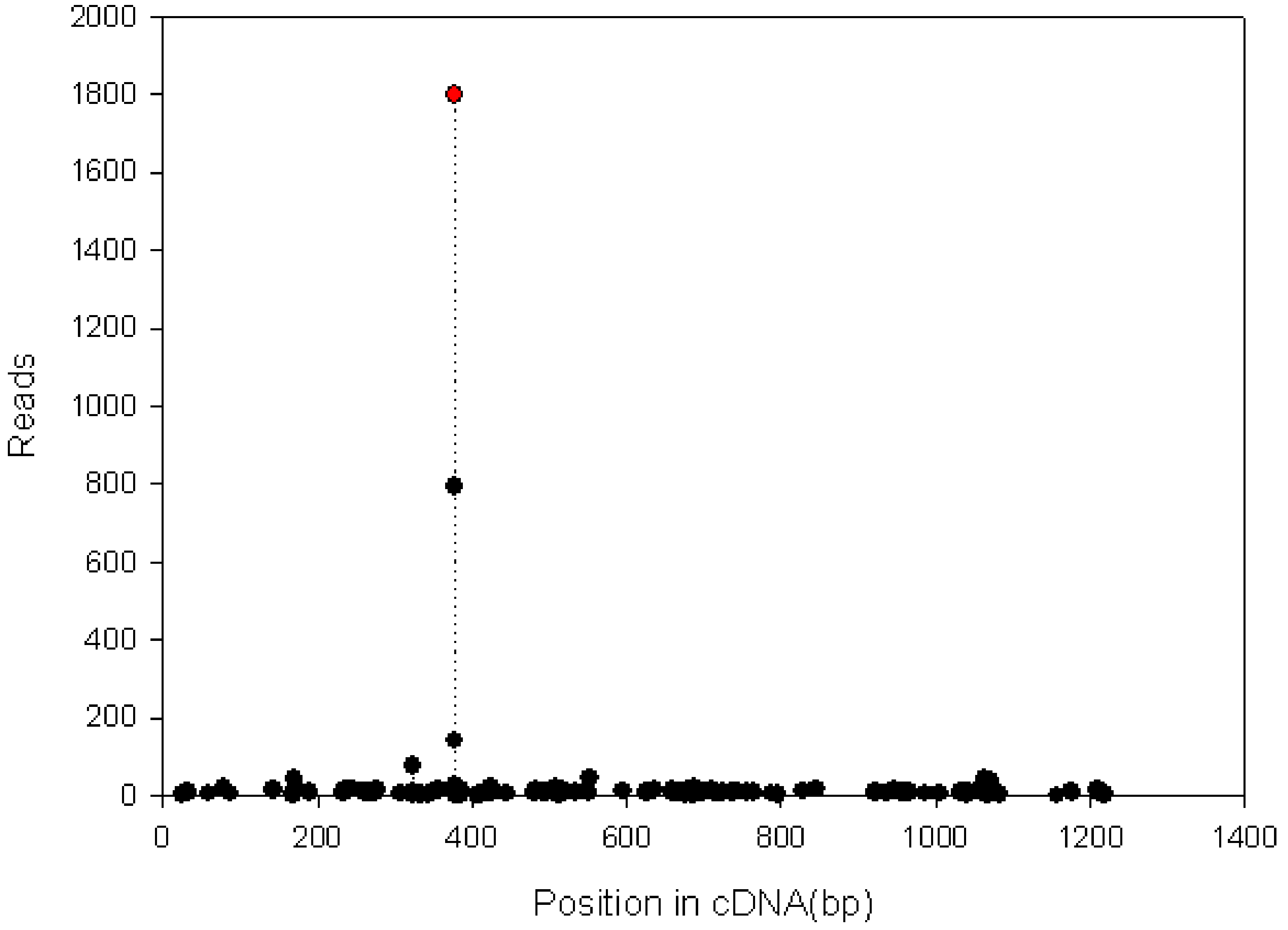


```
5' ACCGUUCAAGAAAGCCUGUGGAAUCA 3'        Cs7g15220.1
    : ::::::::::: ::::::::
3' -GUCAAGUUCUUUC-GACACCUU--- 5'        Csi-miR396a
```

Csi-miR396a, target=Cs7g27670.1 gene=Cs7g27670
Category:1
Score=5
Cleavage Site=653

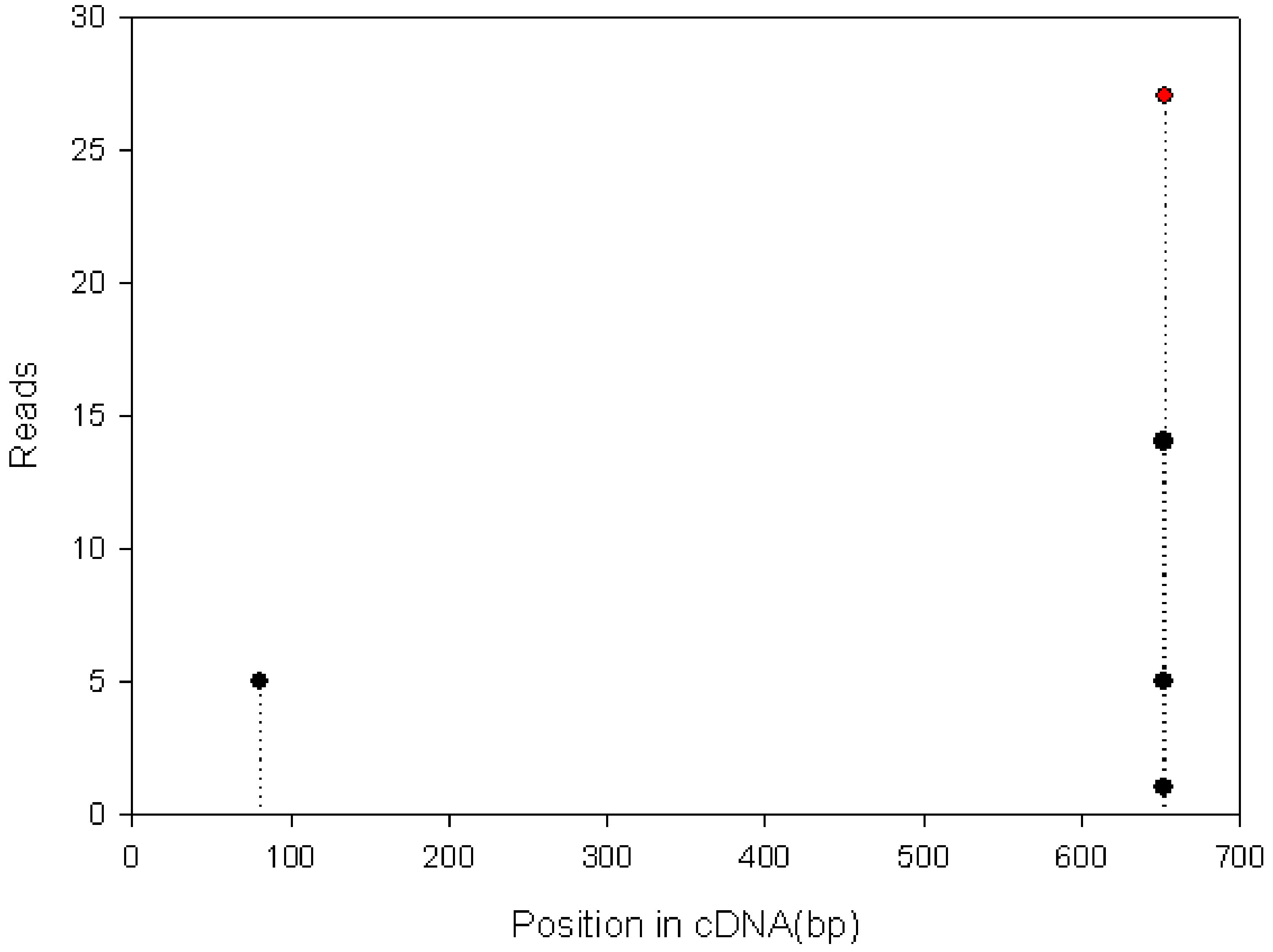


```
5' UCA-UCCAAGAAGGCUAUGGAAUGGAC 3'      Cs7g27670.1
    :: : ::::::.::: :::::
3' -GUCAAGUUCUUUCGACACCUU----- 5'      Csi-miR396a
```

Csi-miR396a, target=Cs7g27680.1 gene=Cs7g27680
Category:1
Score=5
Cleavage Site=713

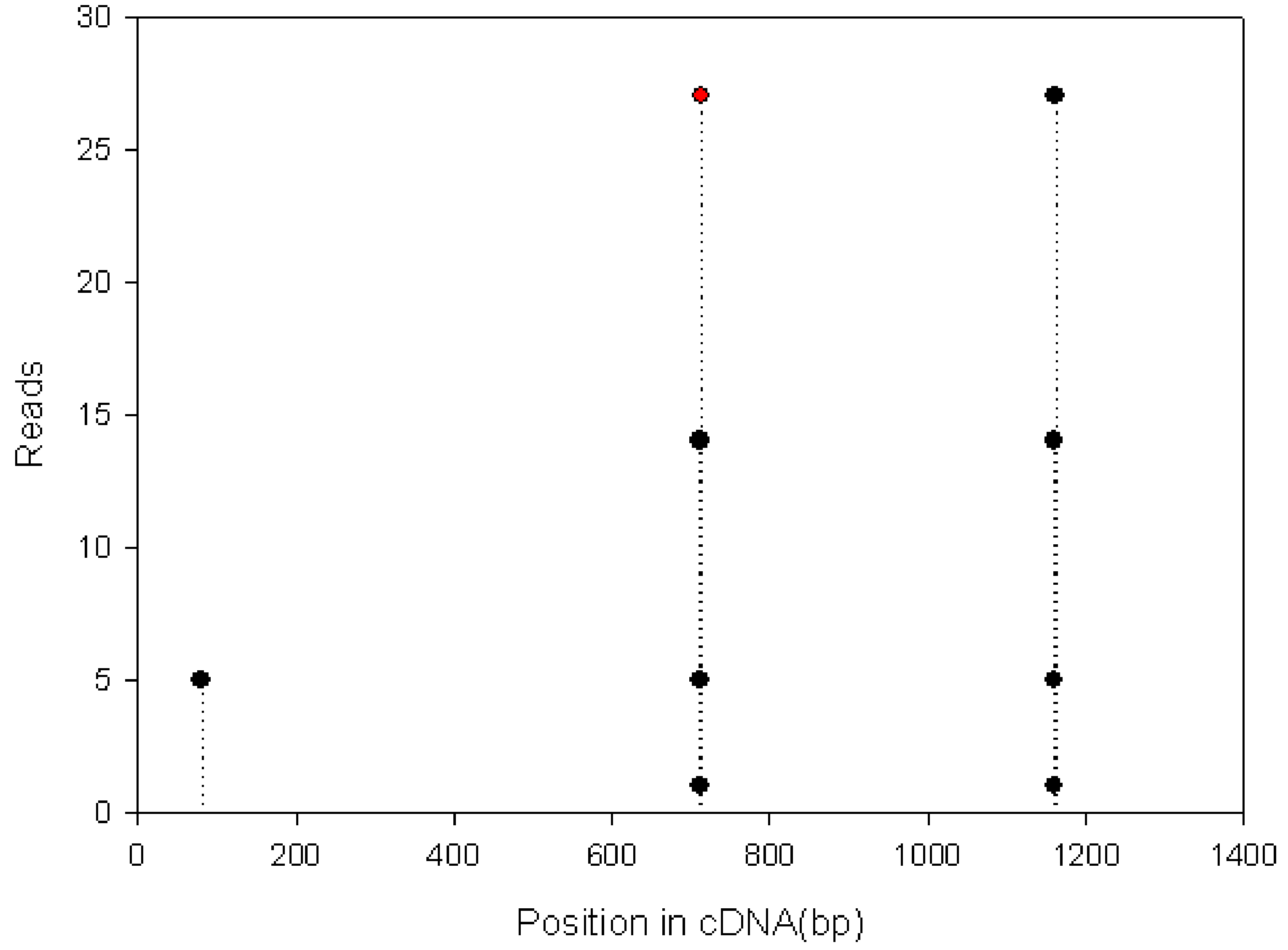


```
5' UCA-UCCAAGAAGGCUAUGGAAUGGAC 3'       Cs7g27680.1
    :: : ::::::.::: :::::
3' -GUCAAGUUCUUUCGACACCUU----- 5'       Csi-miR396a
```

Csi-miR396a, target=Orange1.1t00172.1 gene=Orange1.1t00172
Category:1
Score=3
Cleavage Site=656

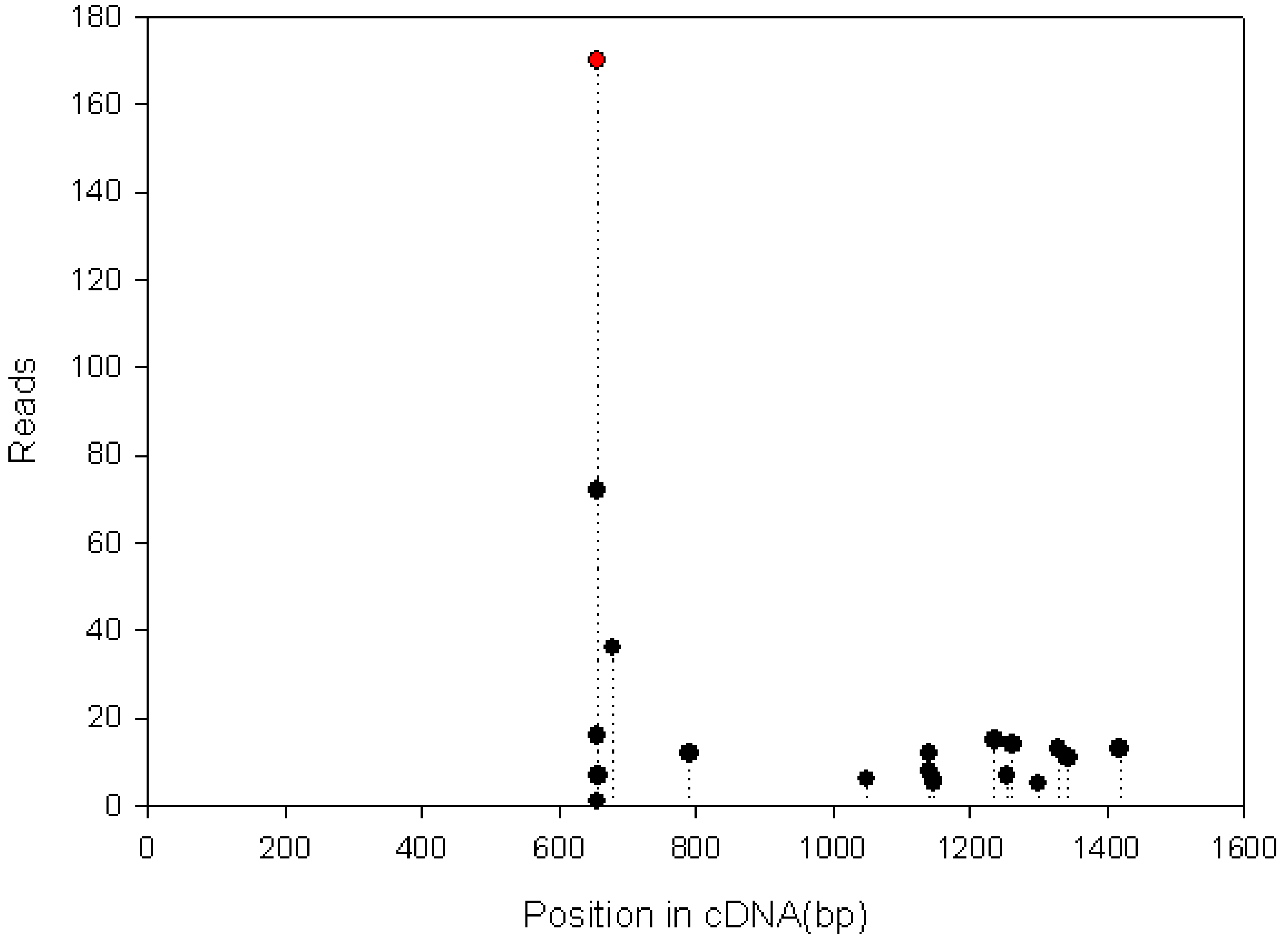


```
5' ACCGUUCAAGAAAGCCUGUGGAACUU 3'        Orange1.1t00172.1
    : ::::::::::: ::::::::
3' -GUCAAGUUCUUUC-GACACCUU--- 5'        Csi-miR396a
```

Csi-miR396a, target=Orange1.1t03122.1 gene=Orange1.1t03122
Category:1
Score=3
Cleavage Site=583

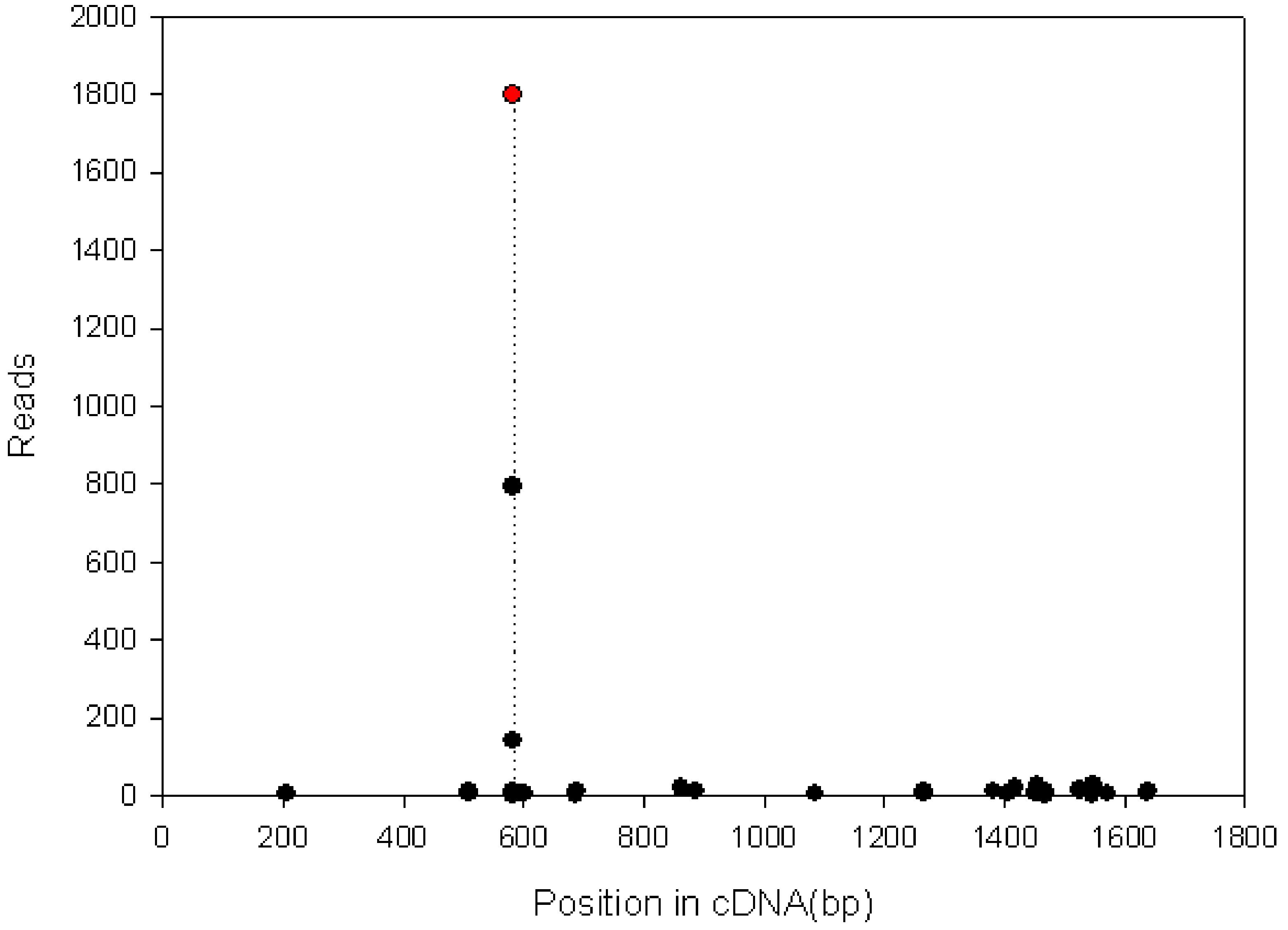


```
5' ACCGUUCAAGAAAGCCUGUGGAAUCA 3'        Orange1.1t03122.1
    : :::::::::: ::::::::
3' -GUCAAGUUCUUUC-GACACCUU--- 5'        Csi-miR396a
```

Csi-miR396b.1, target=Cs1g21350.1 gene=Cs1g21350
Category:1
Score=2
Cleavage Site=1164

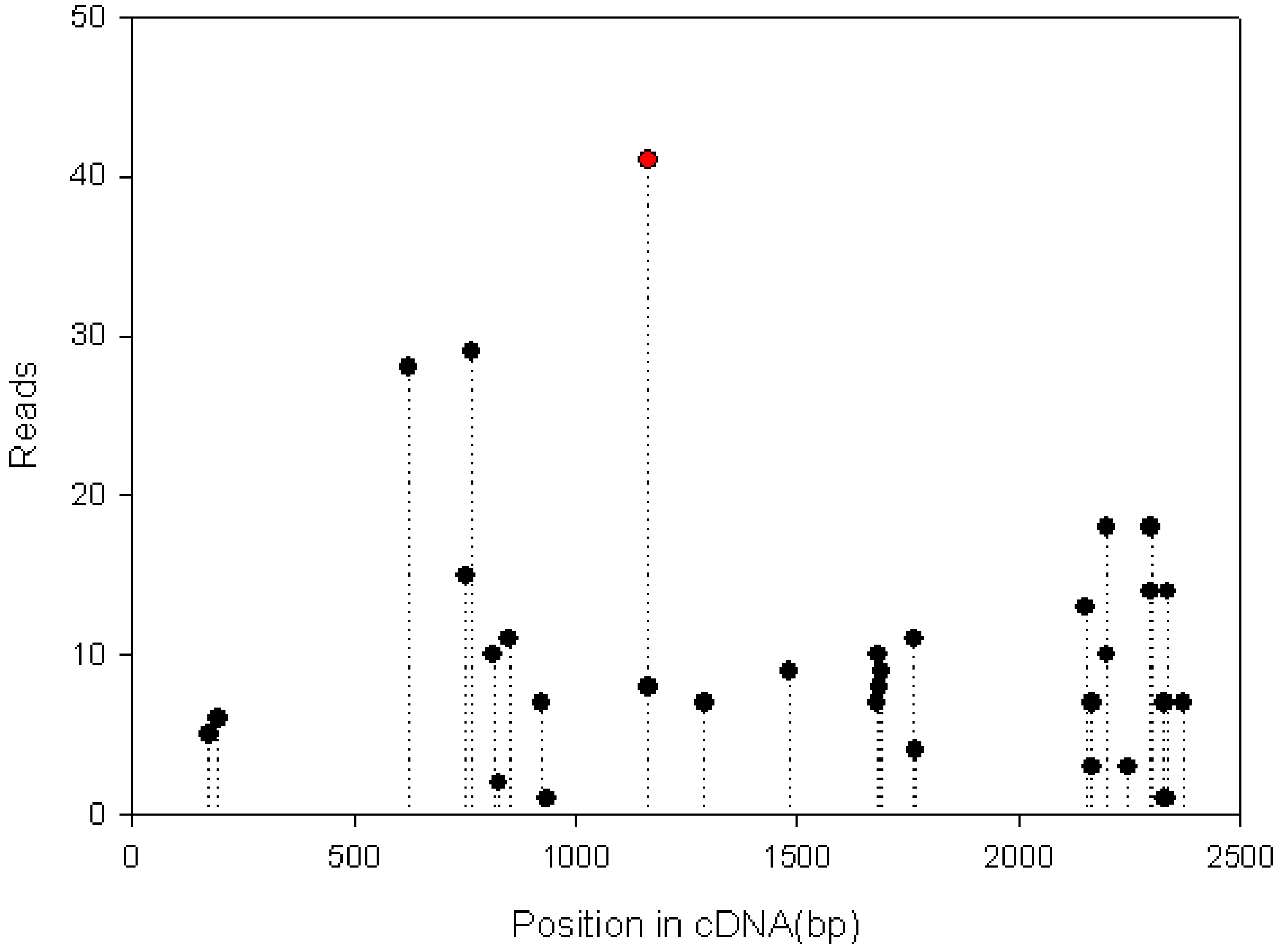


```
5' ACCGUUCAAGAAAGCCUGUGGAAGGC 3'        Cs1g21350.1
     ::::::::::: ::::::::
3' ---CAAGUUCUUUC-GACACCUU--- 5'        Csi-miR396b.1
```

Csi-miR396b.1, target=Cs1g22520.1 gene=Cs1g22520
Category:1
Score=3
Cleavage Site=226

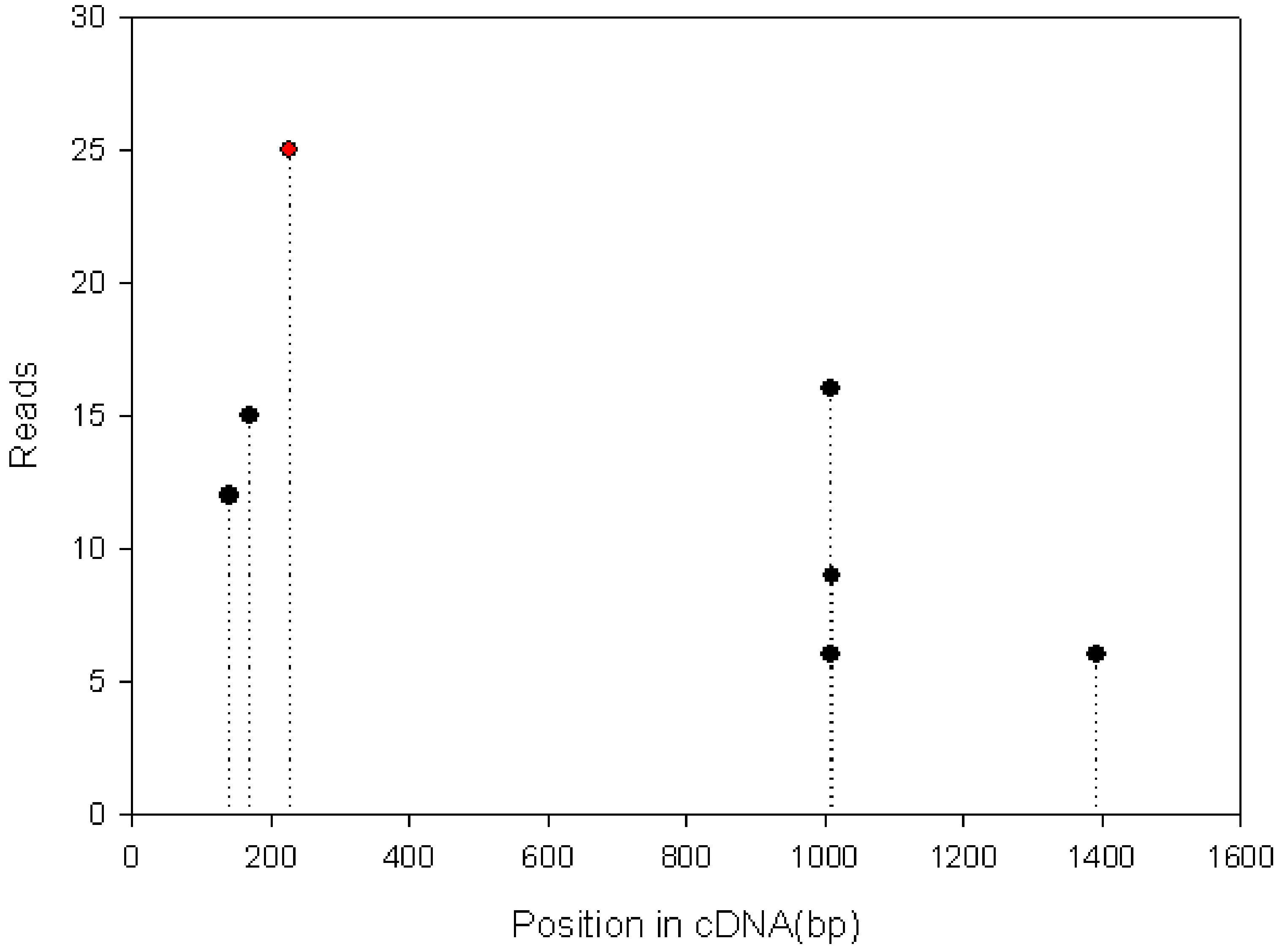


```
5' GAAGUUCAAGAAAUCUGUGGGAAGAA 3'        Cs1g22520.1
     :::::::::: :::::::.:
3' ---CAAGUUCUUUCGACACCUU---- 5'        Csi-miR396b.1
```

Csi-miR396b.1, target=Cs3g23180.1 gene=Cs3g23180
Category:1
Score=3
Cleavage Site=625

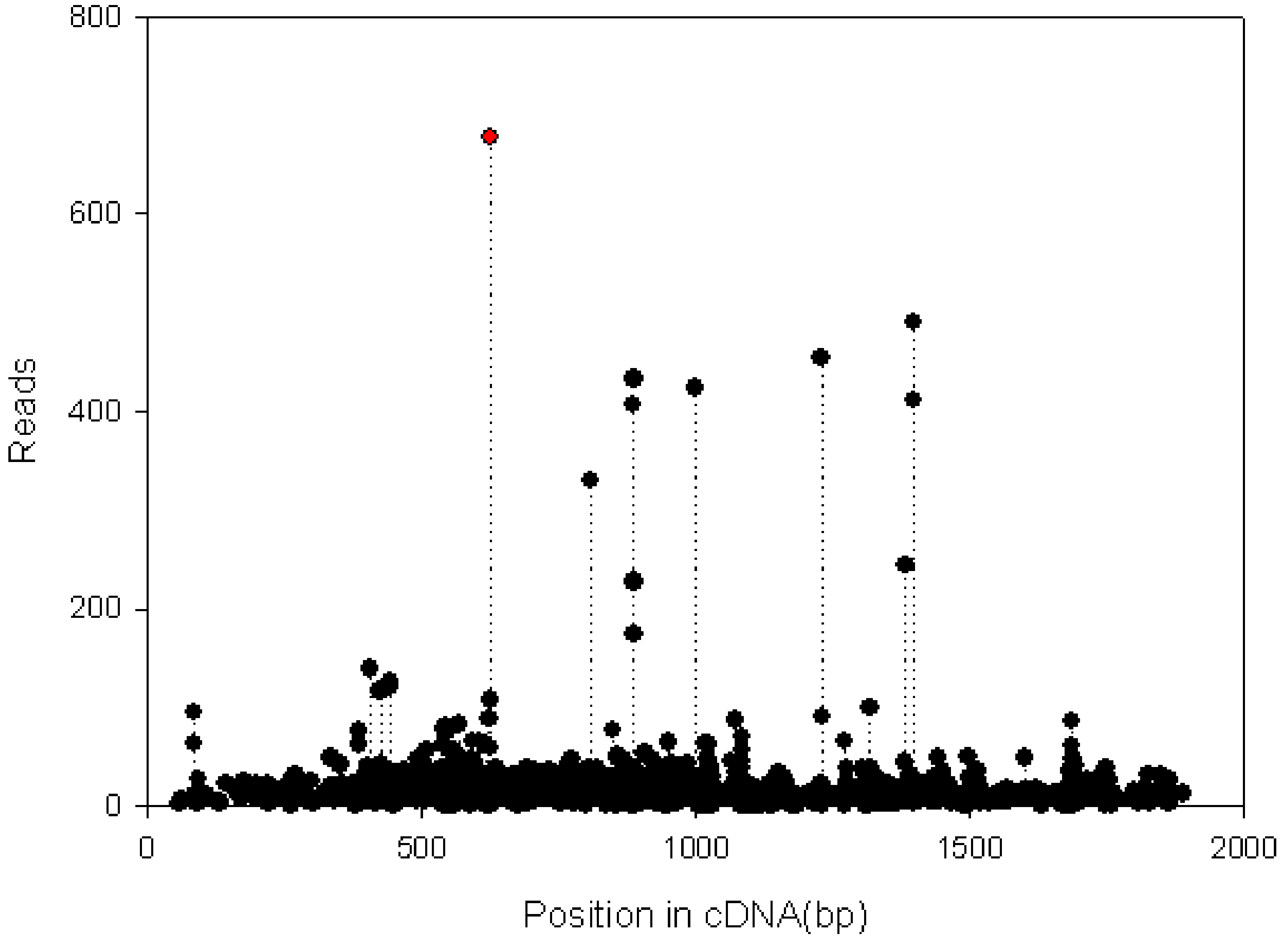


```
5' AAAGAUCAAGGAAGCUGUGGGAGUUG 3'         Cs3g23180.1
      :  :::::.::::::::::.:
3' ---CAAGUUCUUUCGACACCUU---- 5'         Csi-miR396b.1
```

Csi-miR396b.1, target=Cs4g05000.1 gene=Cs4g05000
Category:3
Score=3
Cleavage Site=1249

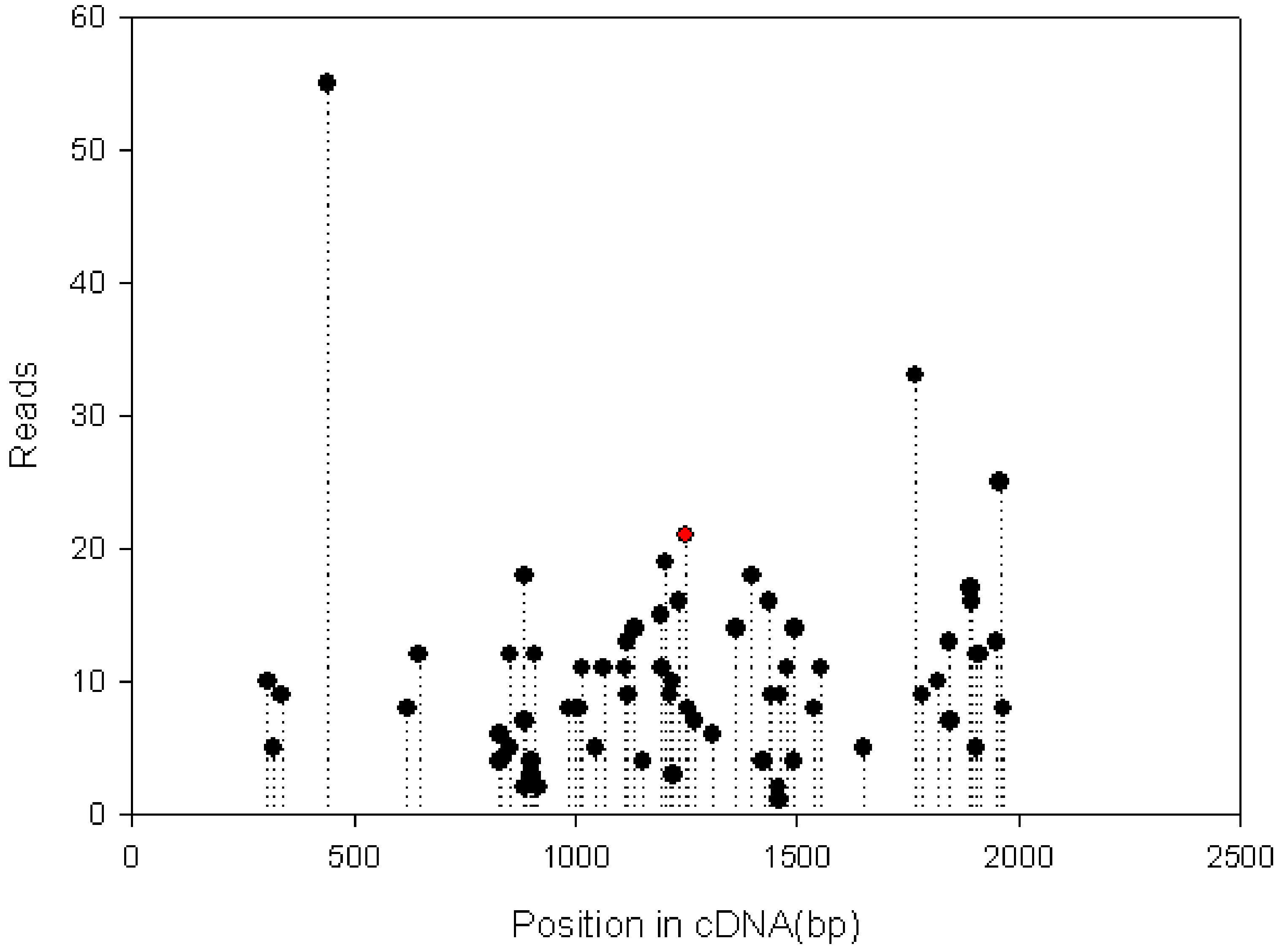


```
5' ACCCUUGAAGAAAGCUGUGGAUAUAG 3'        Cs4g05000.1
      :: ::::::::::::::
3' ---CAAGUUCUUUCGACACCUU---- 5'        Csi-miR396b.1
```

Csi-miR396b.1, target=Cs5g01380.1 gene=Cs5g01380
Category:1
Score=2
Cleavage Site=1181

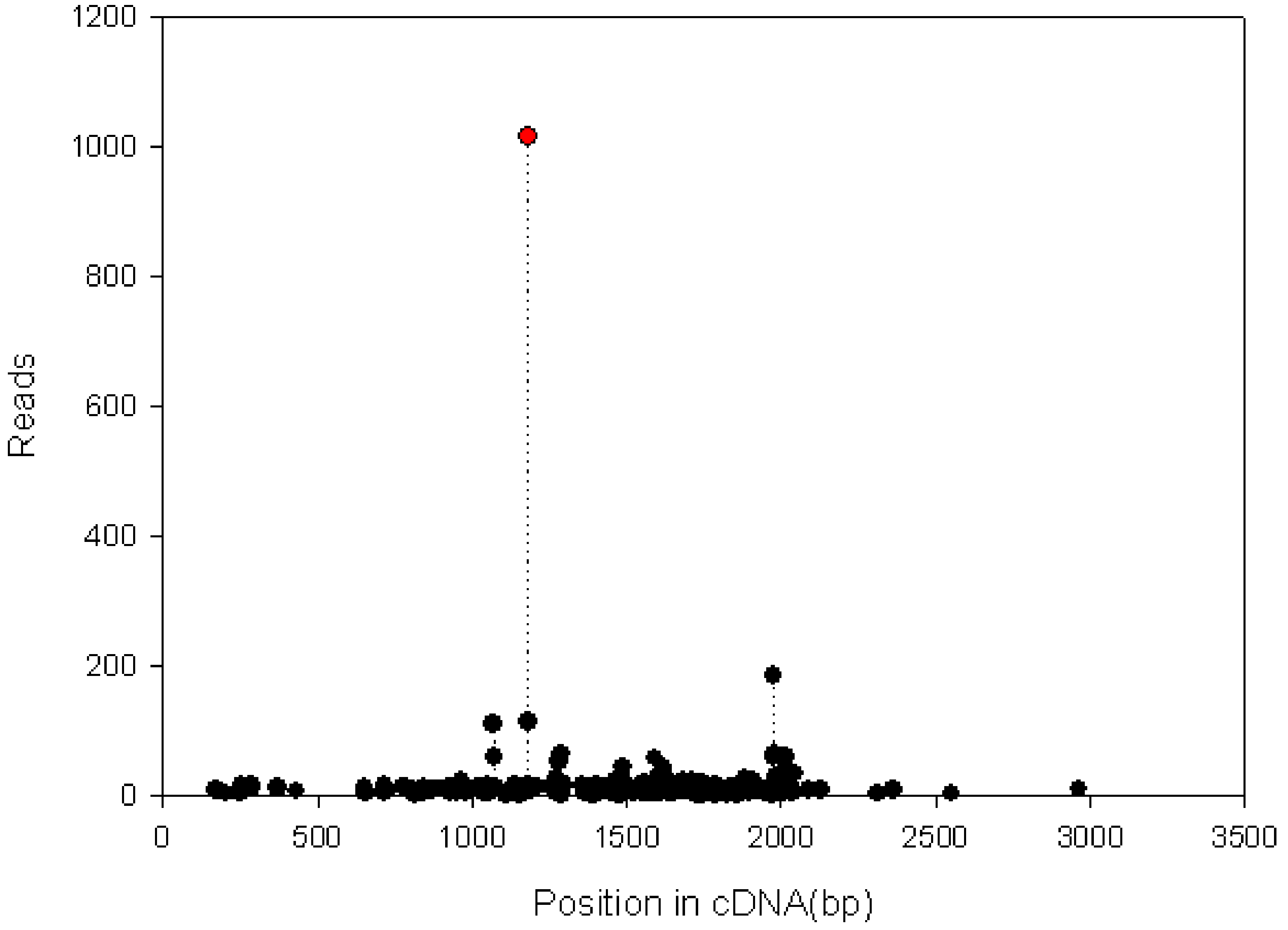


```
5'  CCCGUUCAAGAAAGCCUGUGGAAUUA 3'        Cs5g01380.1
       ::::::::::: ::::::::
3'  ---CAAGUUCUUUC-GACACCUU--- 5'        Csi-miR396b.1
```

Csi-miR396b.1, target=Cs5g06510.1 gene=Cs5g06510
Category:3
Score=5
Cleavage Site=1185

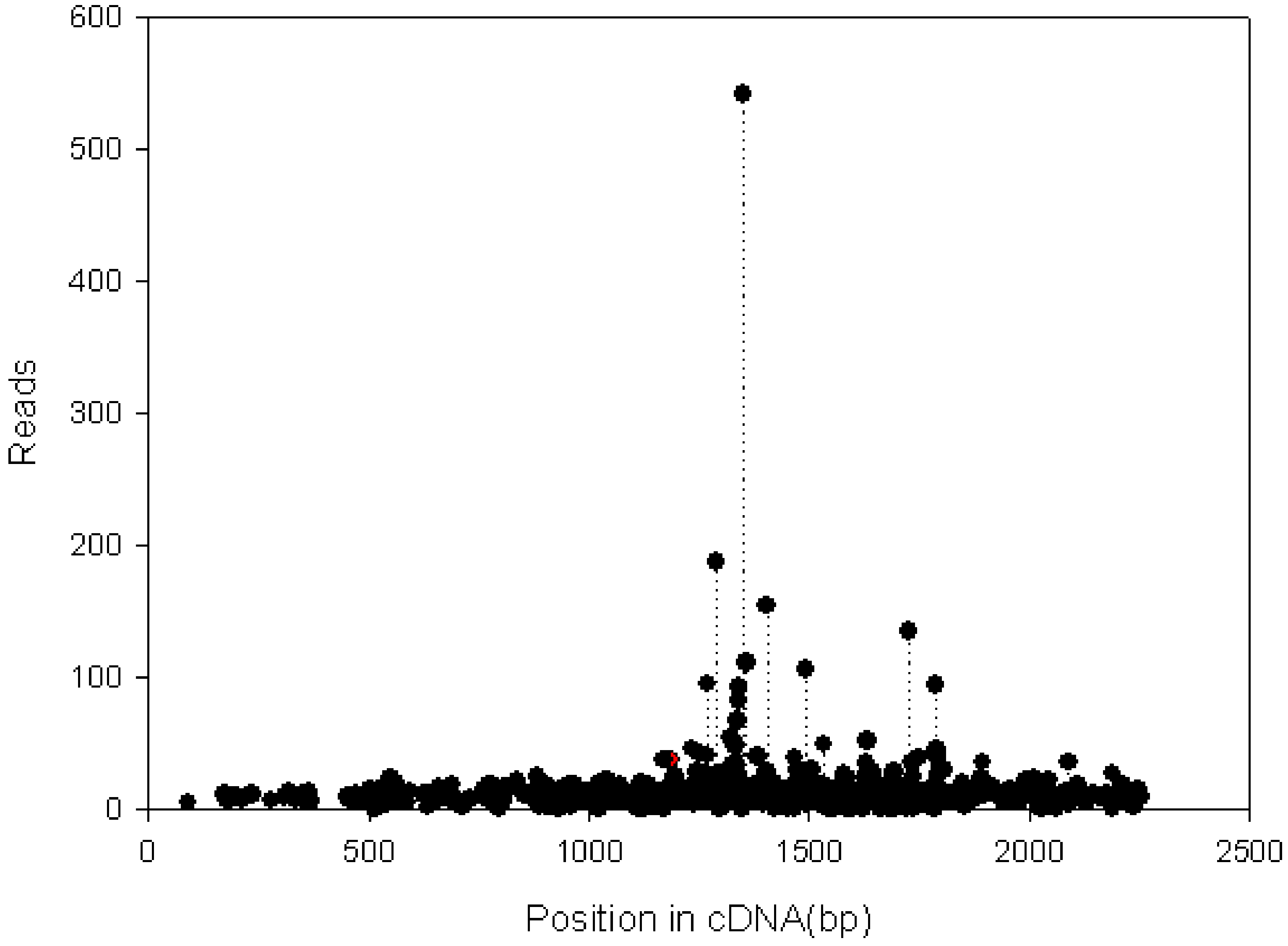


```
5' UAAUCAAGAAAGCUGAGGACUUAGCU 3'        Cs5g06510.1
     :::::::::::: :::
3' -CAAGUUCUUUCGACACCUU------ 5'        Csi-miR396b.1
```

Csi-miR396b.1, target=Cs5g09850.1 gene=Cs5g09850
Category:3
Score=2
Cleavage Site=767

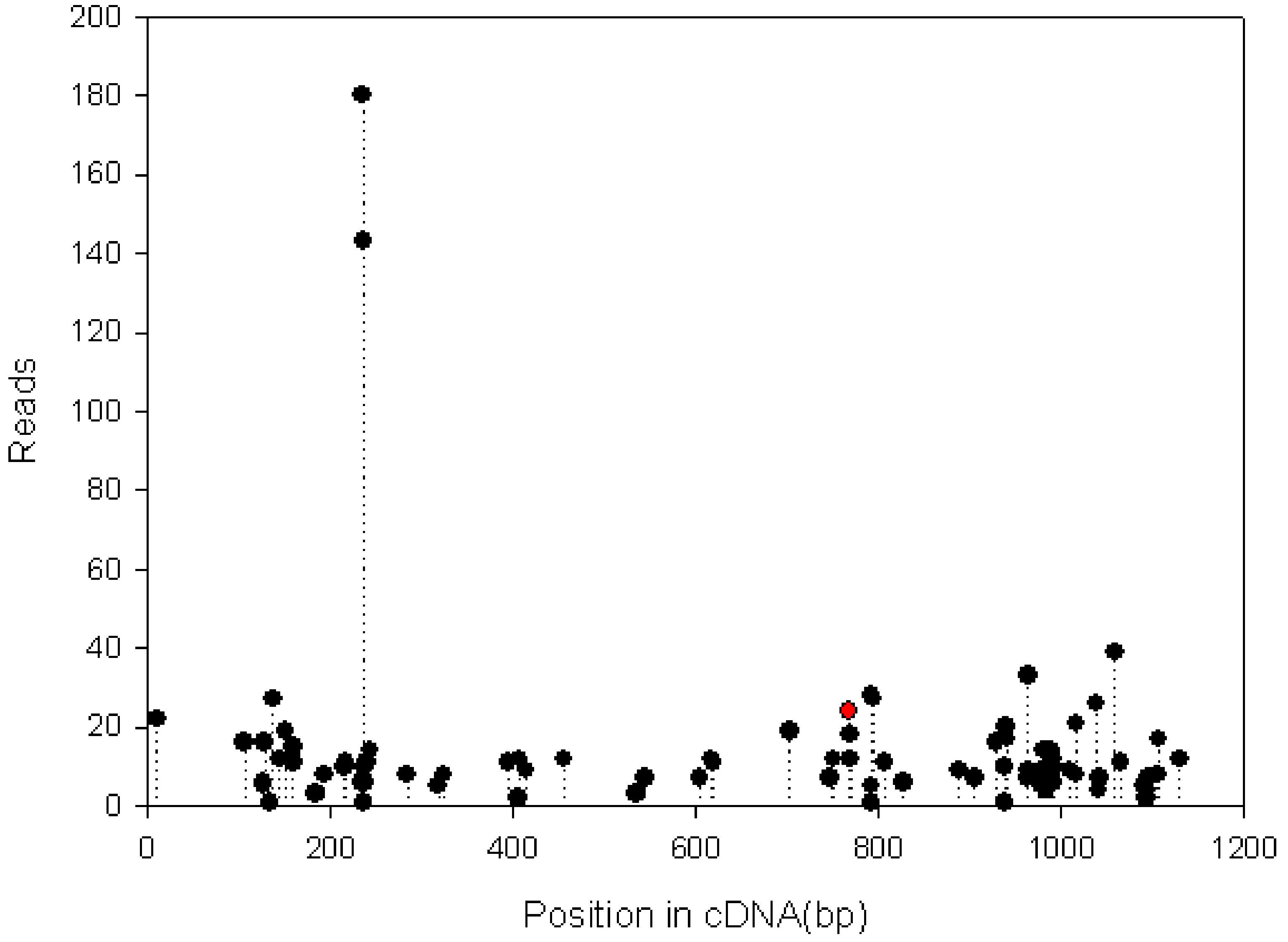


```
5' UGCGUUCAAGAAAGCUUGUGGAAGCU 3'        Cs5g09850.1
     ::::::::::::: :::::::
3' ---CAAGUUCUUUCG-ACACCUU--- 5'        Csi-miR396b.1
```

Csi-miR396b.1, target=Cs5g24150.1 gene=Cs5g24150
Category:3
Score=5
Cleavage Site=3251

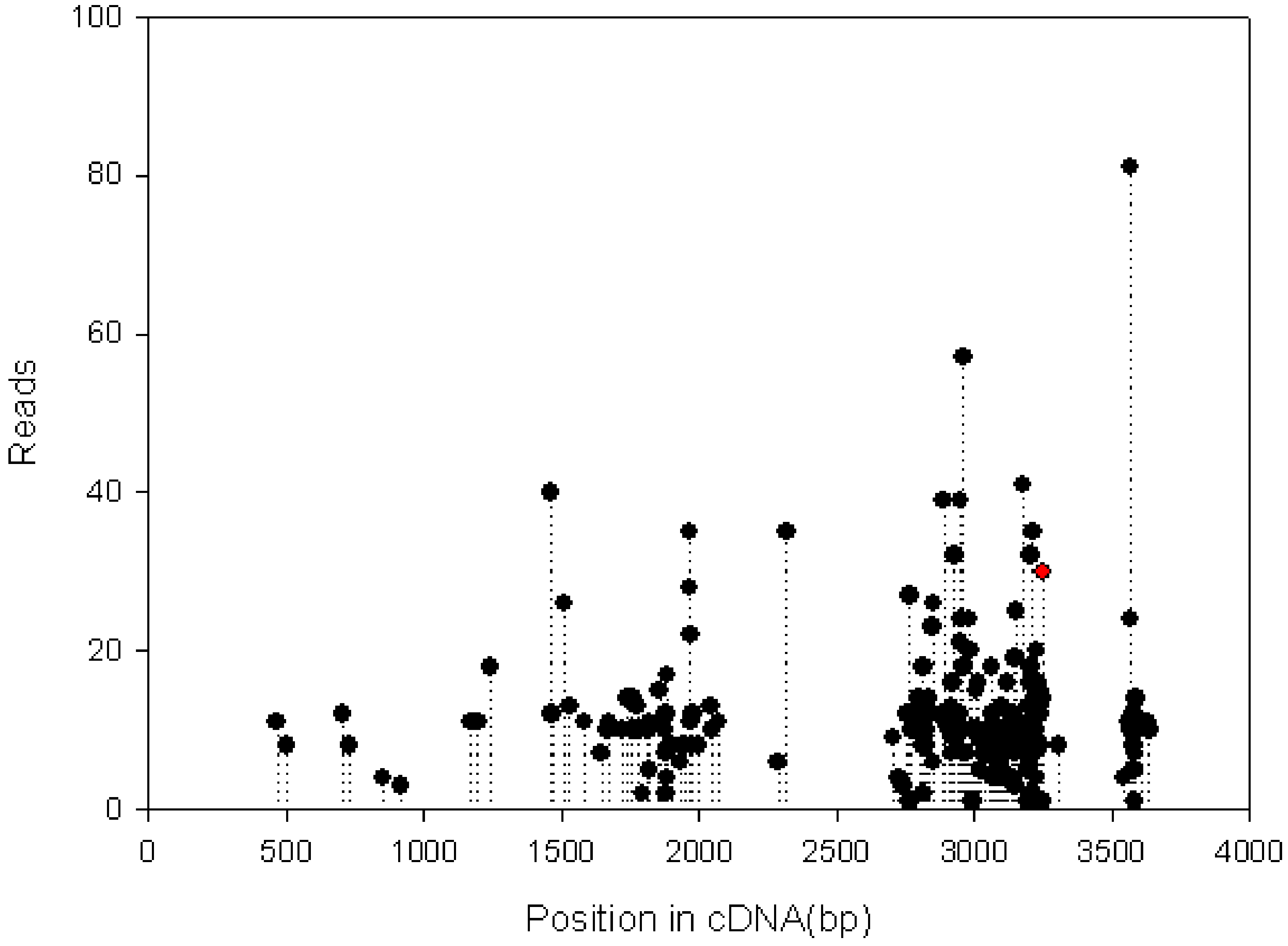


```
5' CGAUCAA-AAAGCUGUAGAAAAUCACA 3'       Cs5g24150.1
    : :::: :::::::: :::
3' -CAAGUUCUUUCGACACCUU------- 5'       Csi-miR396b.1
```

Csi-miR396b.1, target=Cs6g19380.1 gene=Cs6g19380
Category:2
Score=5
Cleavage Site=1152

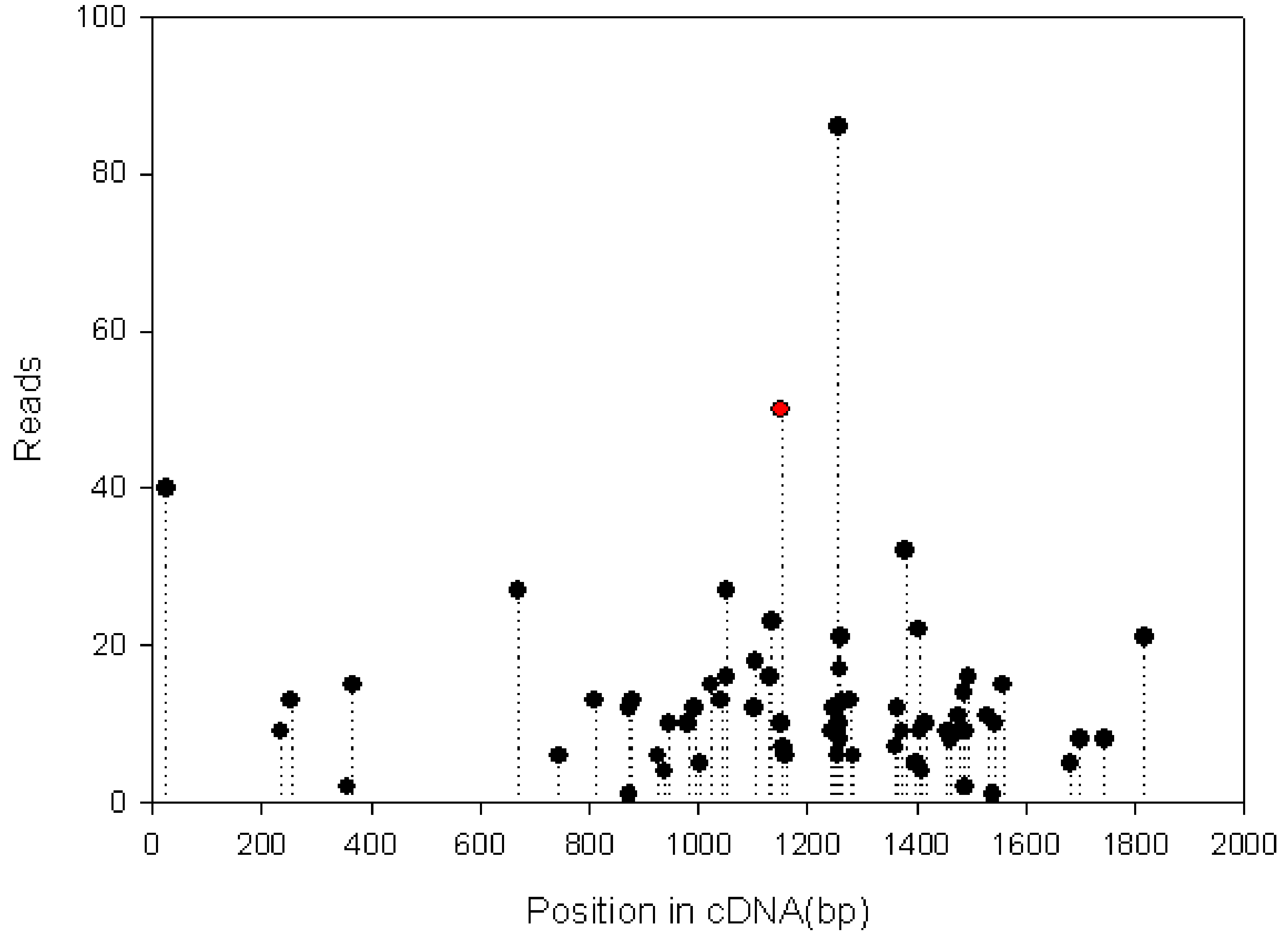


```
5' CUUUUACUUUUAGAGAAGCUGUGGAA 3'        Cs6g19380.1
           ::.:.  .::::::::::
3' -------CAAGUUCUUUCGACACCUU 5'        Csi-miR396b.1
```

Csi-miR396b.1, target=Cs7g15220.1 gene=Cs7g15220
Category:1
score=2
Cleavage Site=377

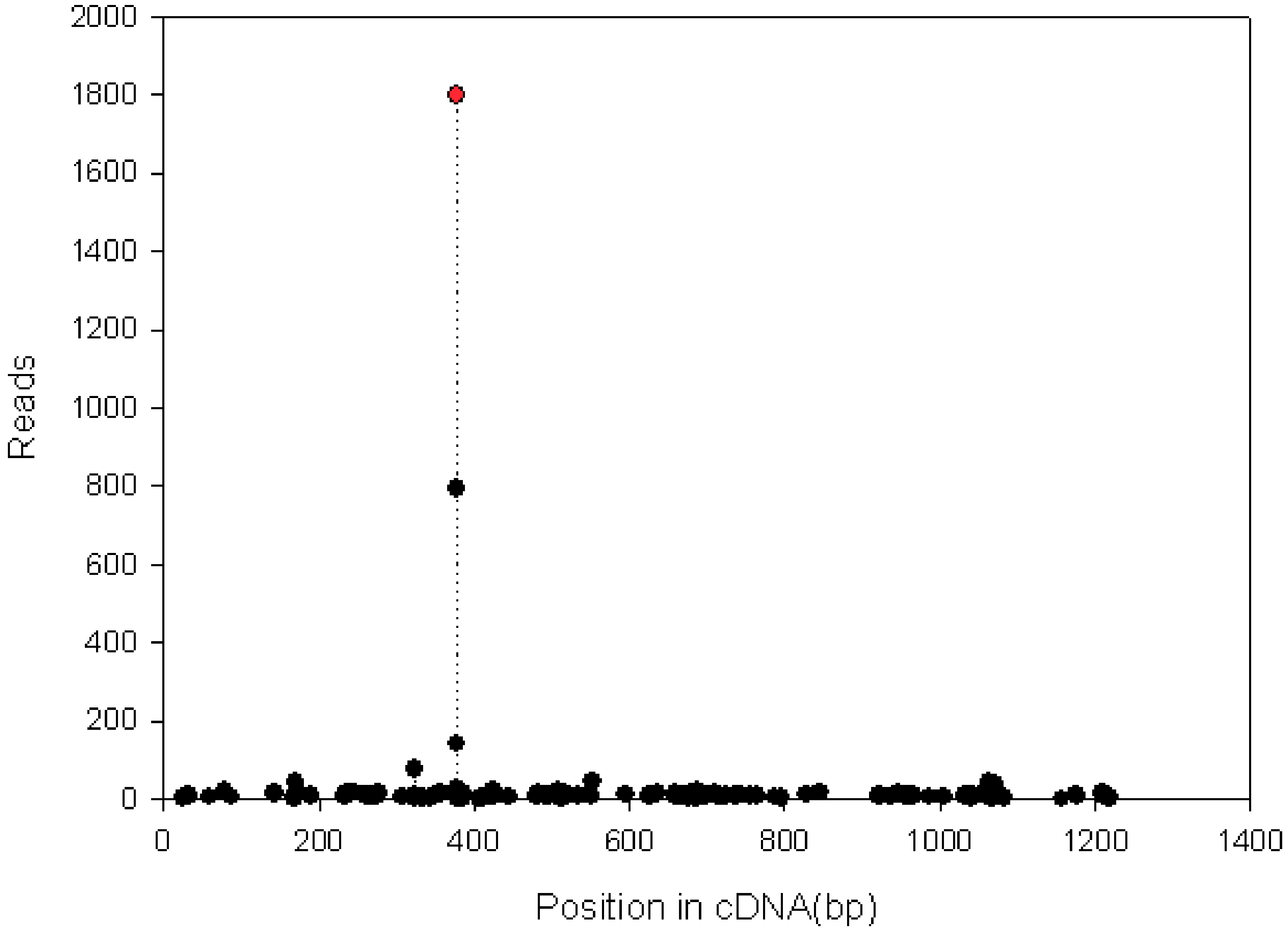


```
5' ACCGUUCAAGAAAGCCUGUGGAAUCA 3'        Cs7g15220.1
      ::::::::::: ::::::::
3' ---CAAGUUCUUUC-GACACCUU--- 5'        Csi-miR396b.1
```

Csi-miR396b.1, target=Cs7g27670.1 gene=Cs7g27670
Category:1
Score=5
Cleavage Site=653

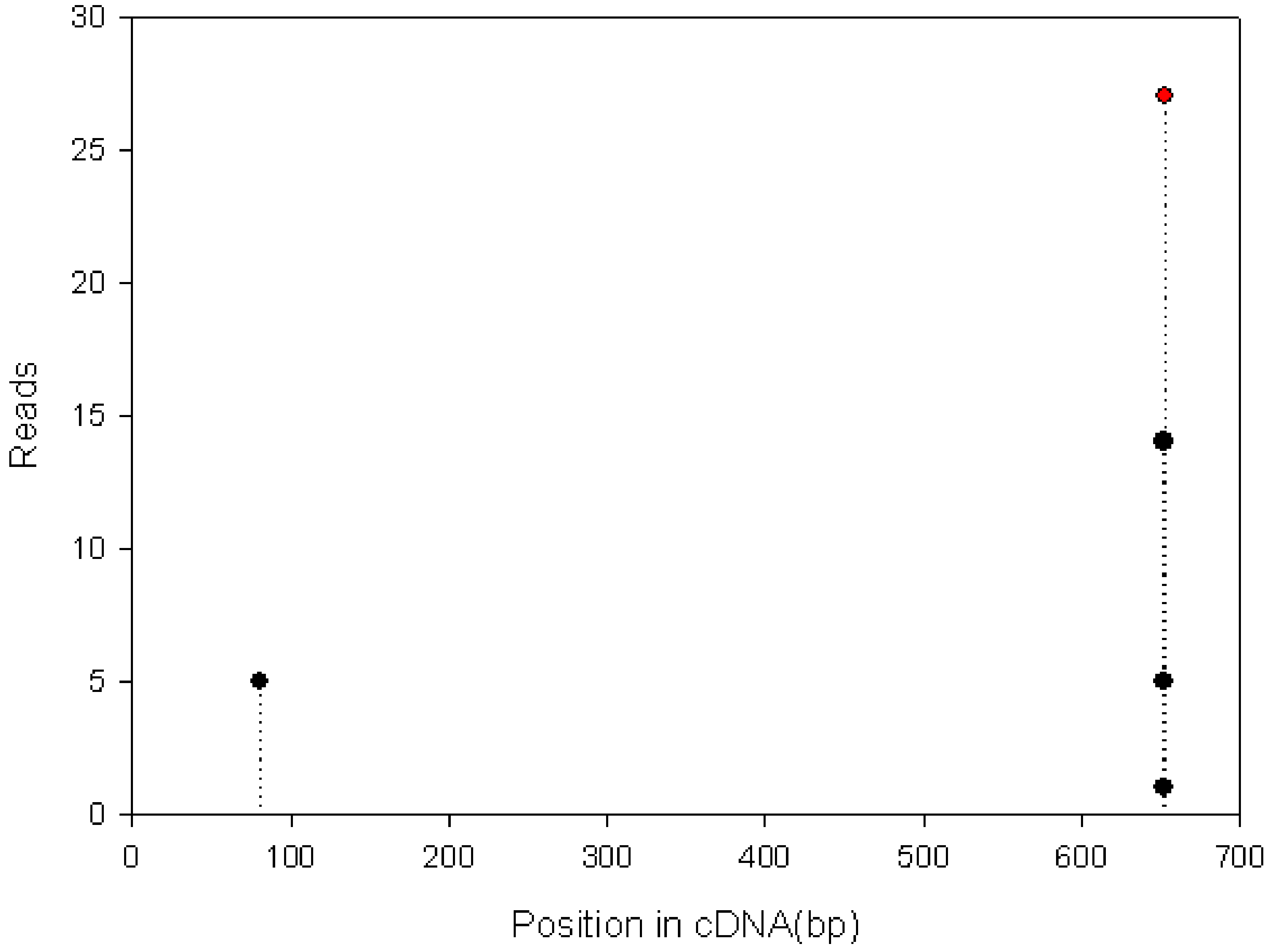


```
5' UCAUCCAAGAAGGCUAUGGAAUGGAC 3'        Cs7g27670.1
       : :::::::.::: :::::
3' --CAAGUUCUUUCGACACCUU----- 5'        Csi-miR396b.1
```

Csi-miR396b.1, target=Cs7g27680.1 gene=Cs7g27680
Category:1
Score=5
Cleavage Site=713

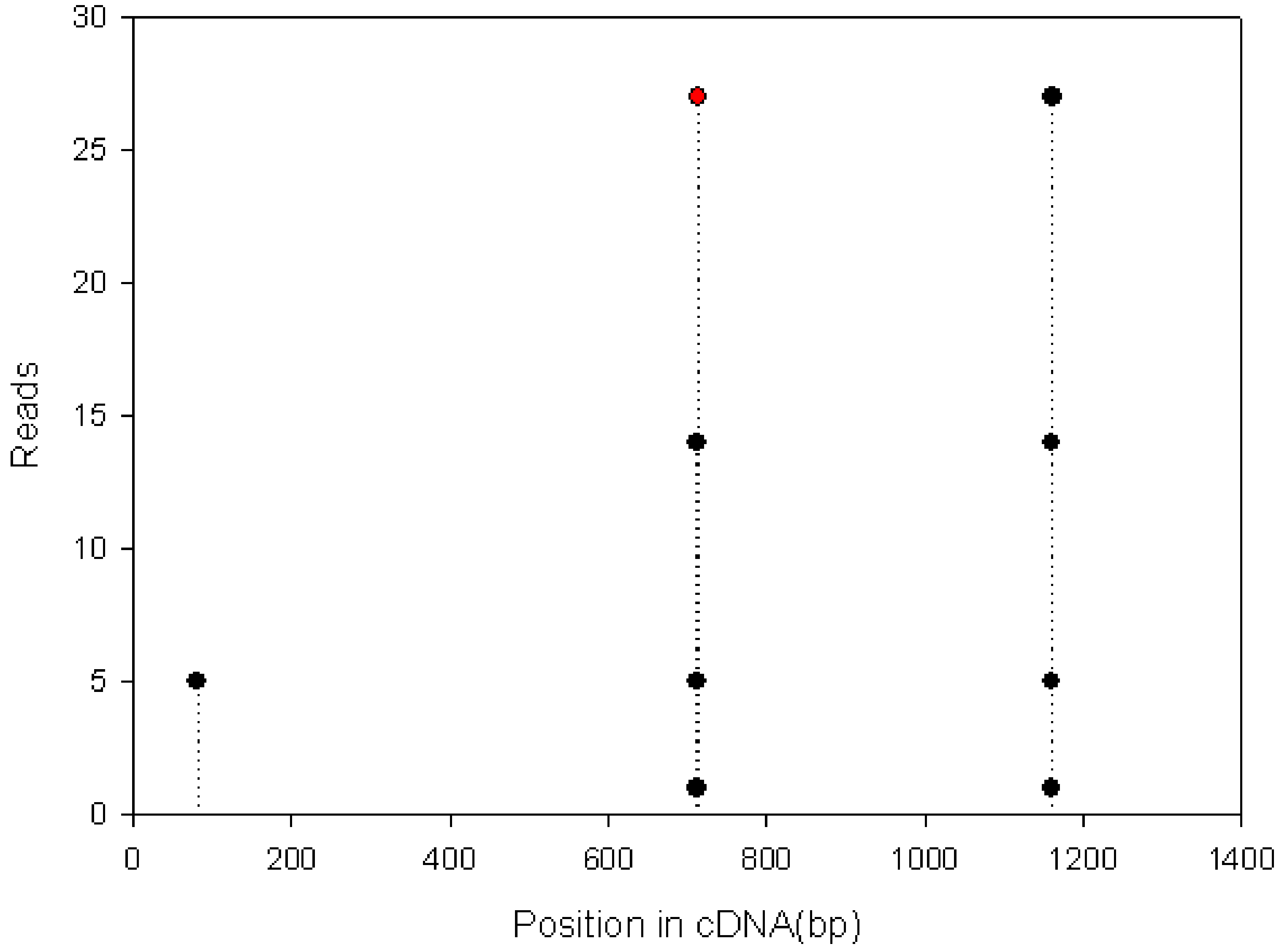


```
5' UCAUCCAAGAAGGCUAUGGAAUGGAC 3'        Cs7g27680.1
      : :::::::.::: :::::
3' --CAAGUUCUUUCGACACCUU----- 5'        Csi-miR396b.1
```

Csi-miR396b.1, target=Orange1.1t00172.1 gene=Orange1.1t00172
Category:1
Score=2
Cleavage Site=656

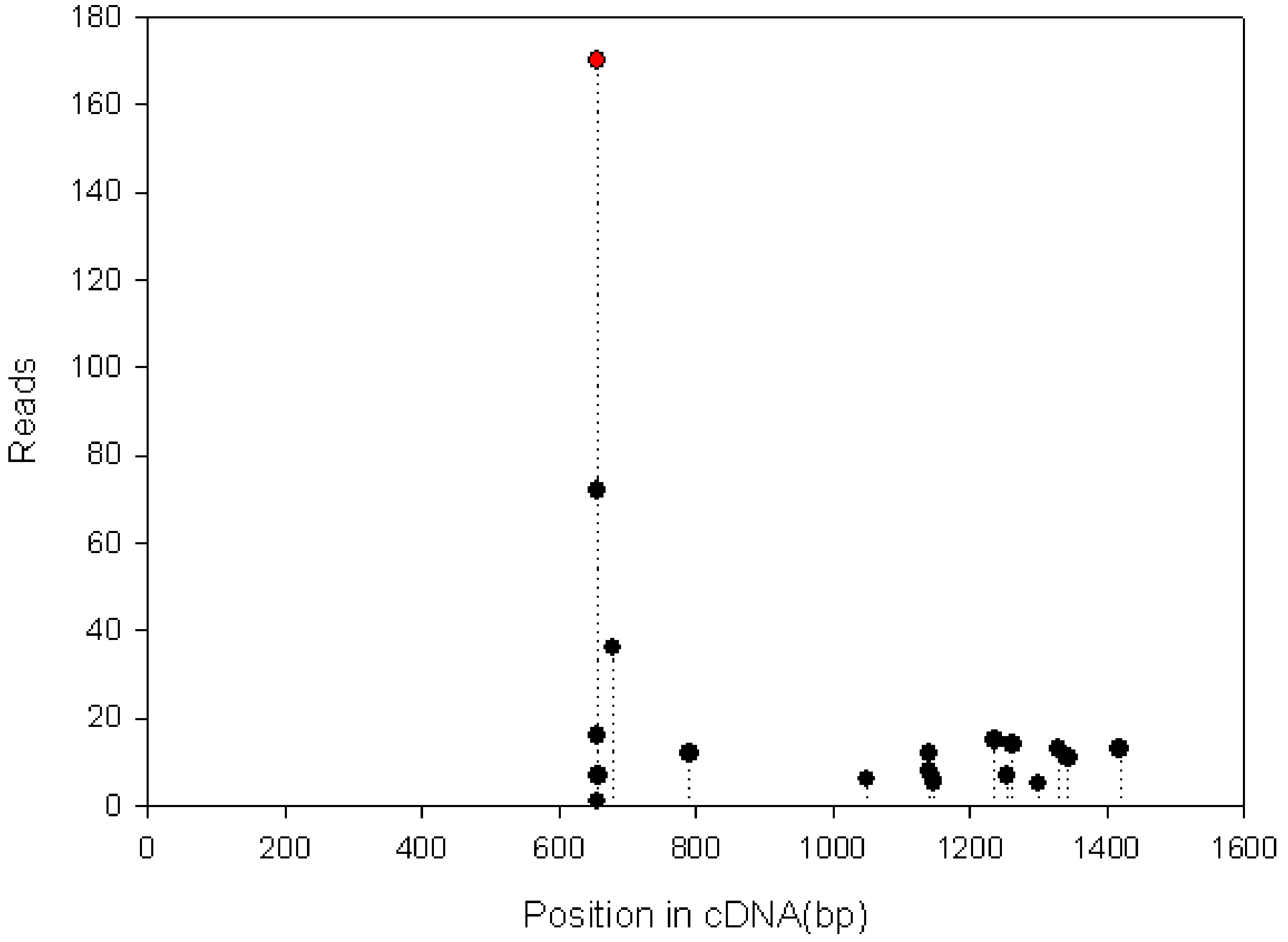


```
5' ACCGUUCAAGAAAGCCUGUGGAACUU 3'        Orange1.1t00172.1
      :::::::::: ::::::::
3' ---CAAGUUCUUUC-GACACCUU--- 5'        Csi-miR396b.1
```

Csi-miR396b.1, target=Orange1.1t02555.1 gene=Orange1.1t02555
Category:1
Score=2
Cleavage Site=920

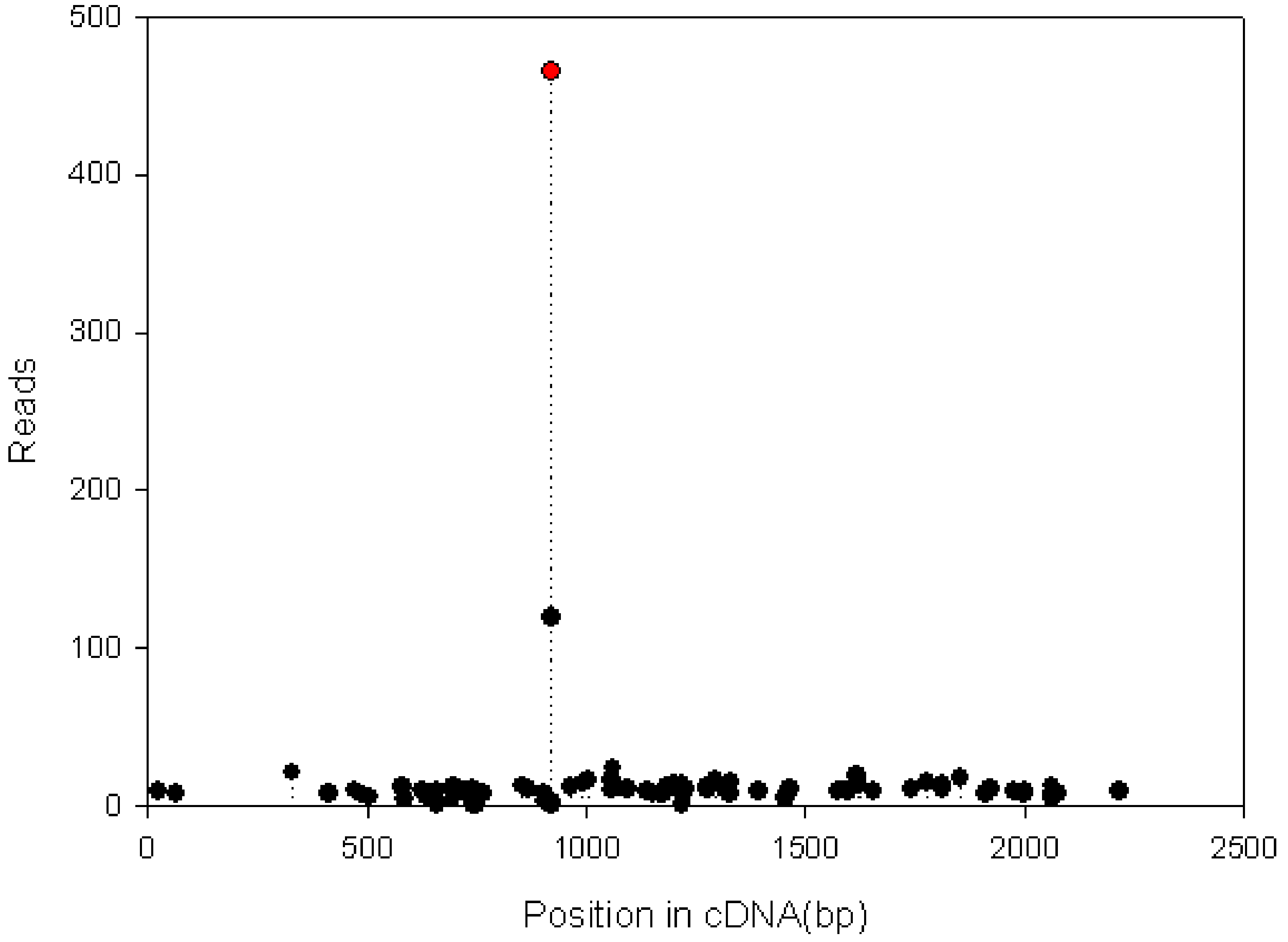


```
5' CACGUUCAAGAAAGCCUGUGGAACUU 3'        Orange1.1t02555.1
      ::::::::::: ::::::::
3' ---CAAGUUCUUUC-GACACCUU--- 5'        Csi-miR396b.1
```

Csi-miR396b.1, target=Orange1.1t03122.1 gene=Orange1.1t03122
Category:1
Score=2
Cleavage Site=583

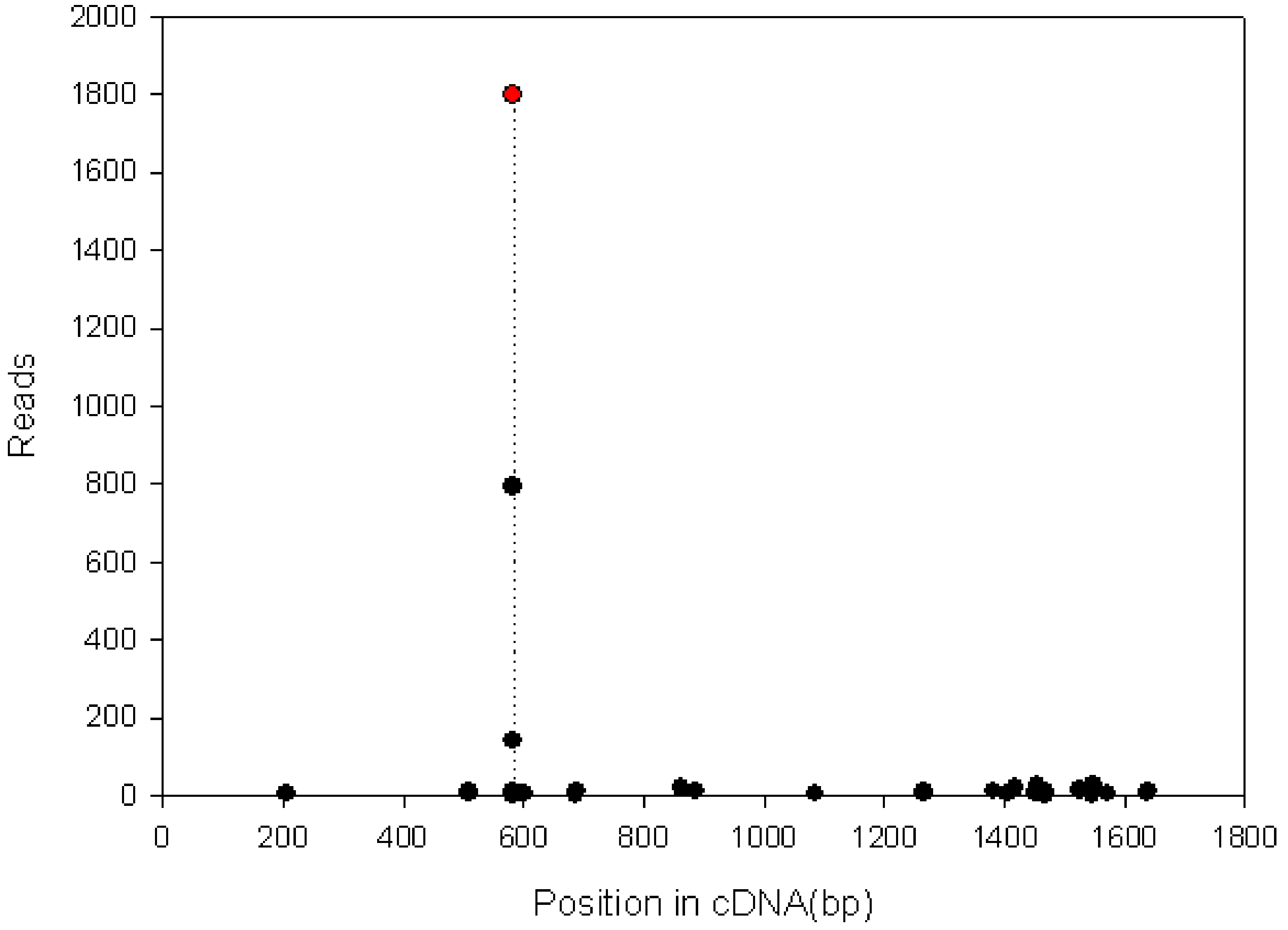


```
5' ACCGUUCAAGAAAGCCUGUGGAAUCA 3'        Orange1.1t03122.1
      ::::::::::: ::::::::
3' ---CAAGUUCUUUC-GACACCUU--- 5'        Csi-miR396b.1
```

Csi-miR396b.2, target=Cs1g21350.1 gene=Cs1g21350
Category:1
Score=2
Cleavage Site=1164

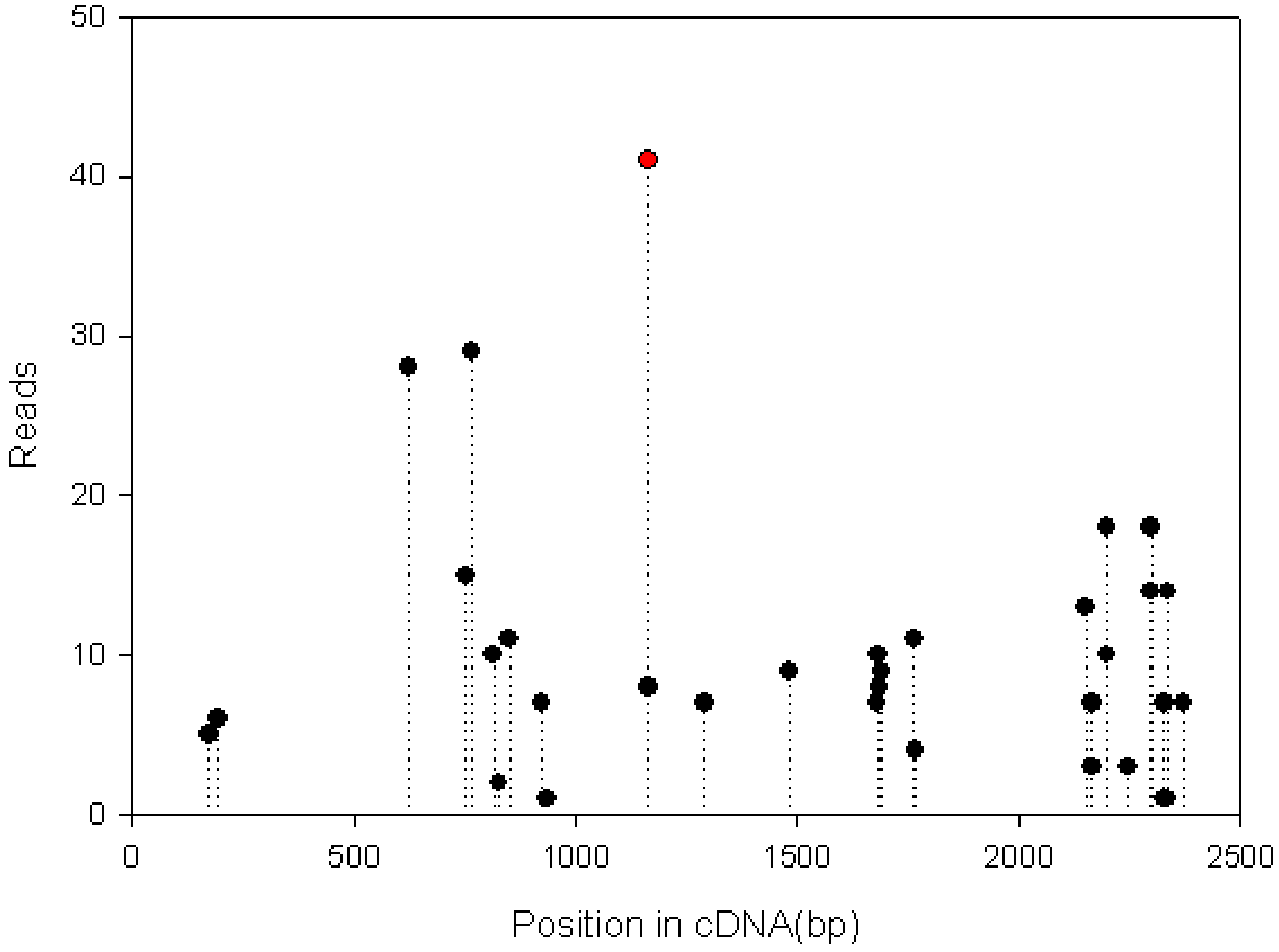


```
5' ACCGUUCAAGAAAGCCUGUGGAAGGC 3'        Cs1g21350.1
      :::::::::: ::::::::
3' ----AAGUUCUUUC-GACACCUU--- 5'        Csi-miR396b.2
```

Csi-miR396b.2, target=Cs3g23180.1 gene=Cs3g23180
Category:1
Score=3
Cleavage Site=625

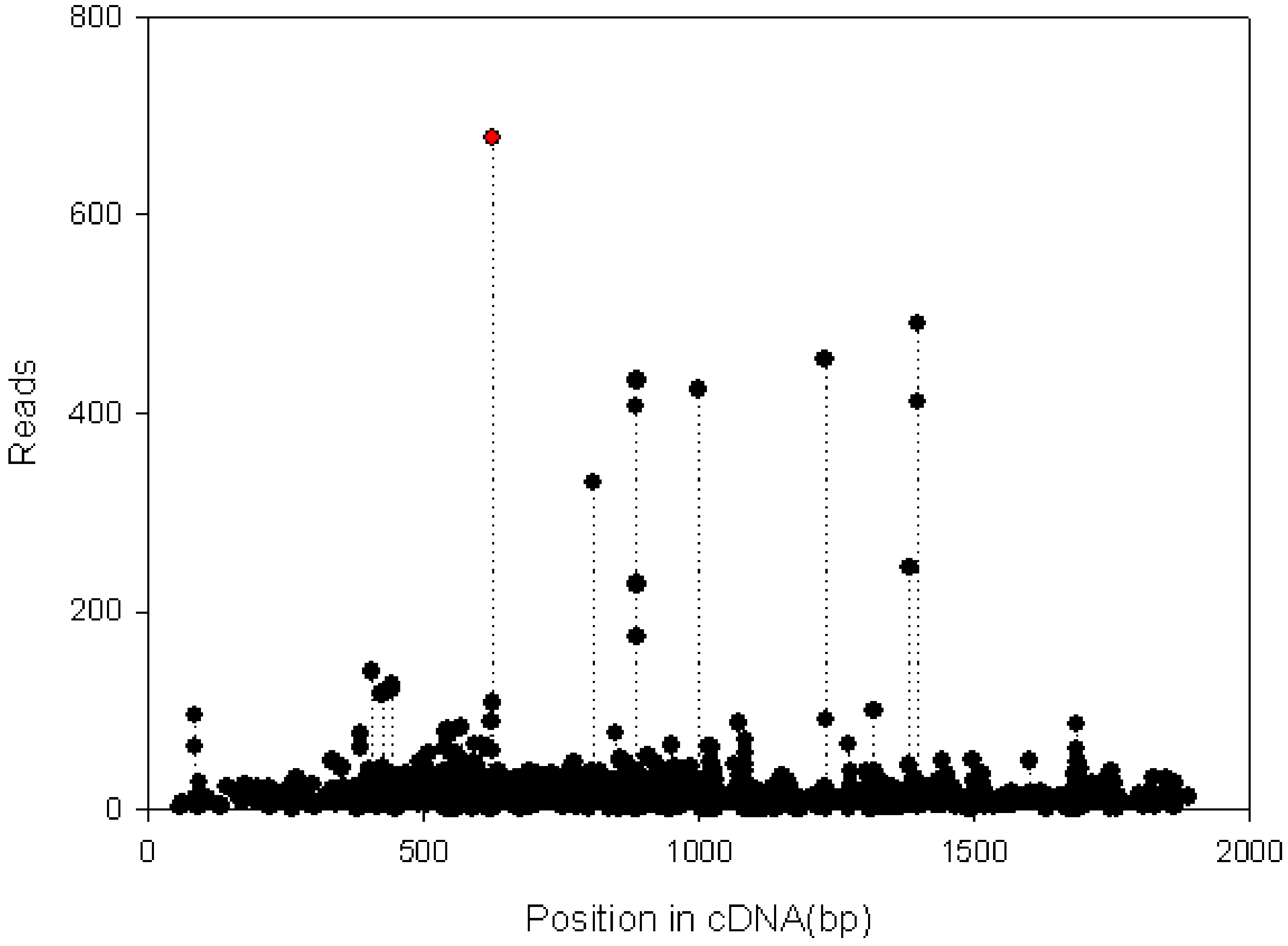


```
5' AAAGAUCAAGGAAGCUGUGGAGUUG 3'        Cs3g23180.1
       :::::.::::::::::.:
3' ----AAGUUCUUUCGACACCUU---- 5'        Csi-miR396b.2
```

Csi-miR396b.2, target=Cs4g05000.1 gene=Cs4g05000
Category:3
Score=2
Cleavage Site=1249

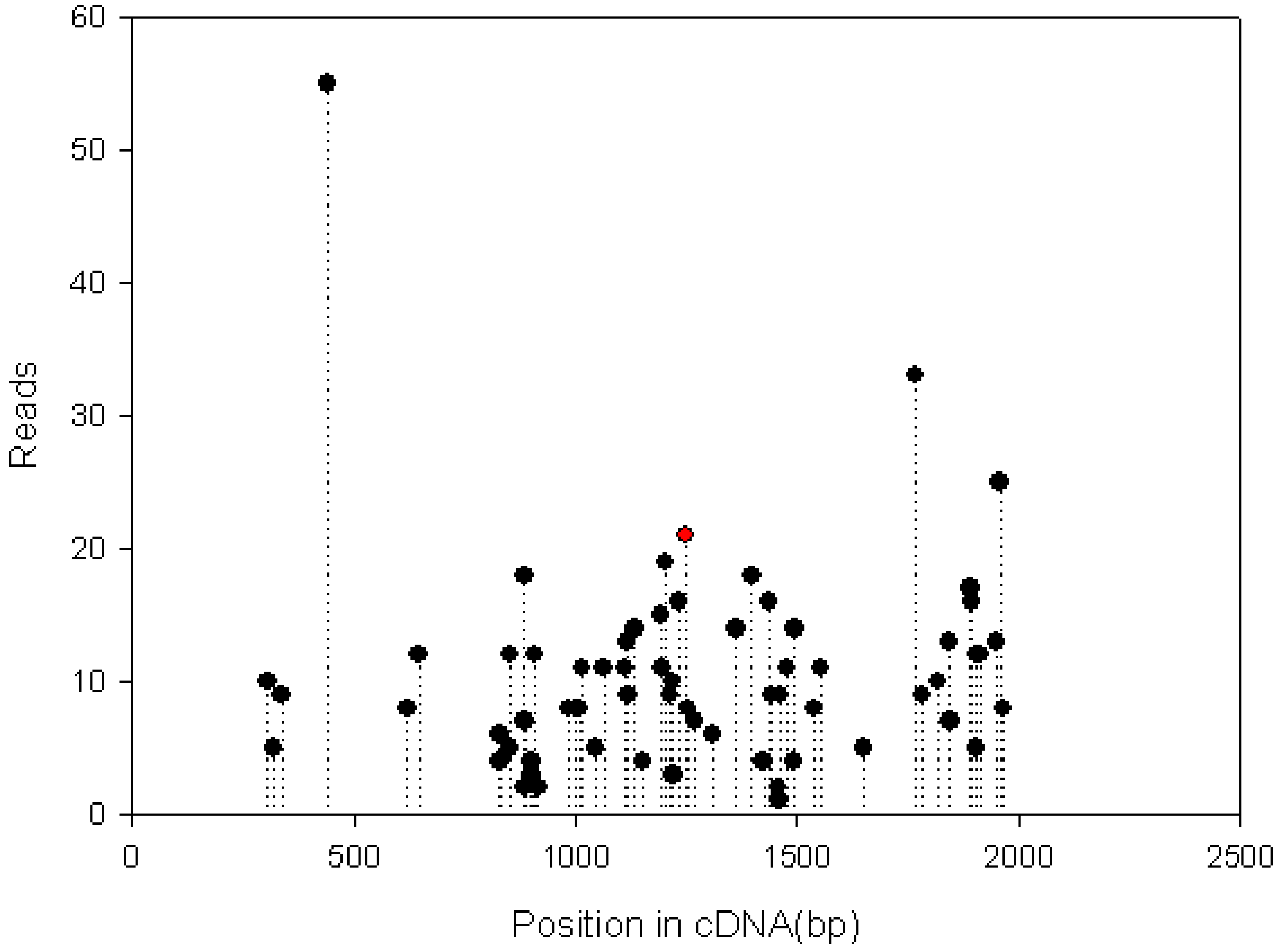


```
5' ACCCUUGAAGAAAGCUGUGGAUAUAG 3'        Cs4g05000.1
       :: ::::::::::::::
3' ----AAGUUCUUUCGACACCUU---- 5'        Csi-miR396b.2
```

Csi-miR396b.2, target=Cs4g06230.1 gene=Cs4g06230
Category:3
Score=5
Cleavage Site=3187

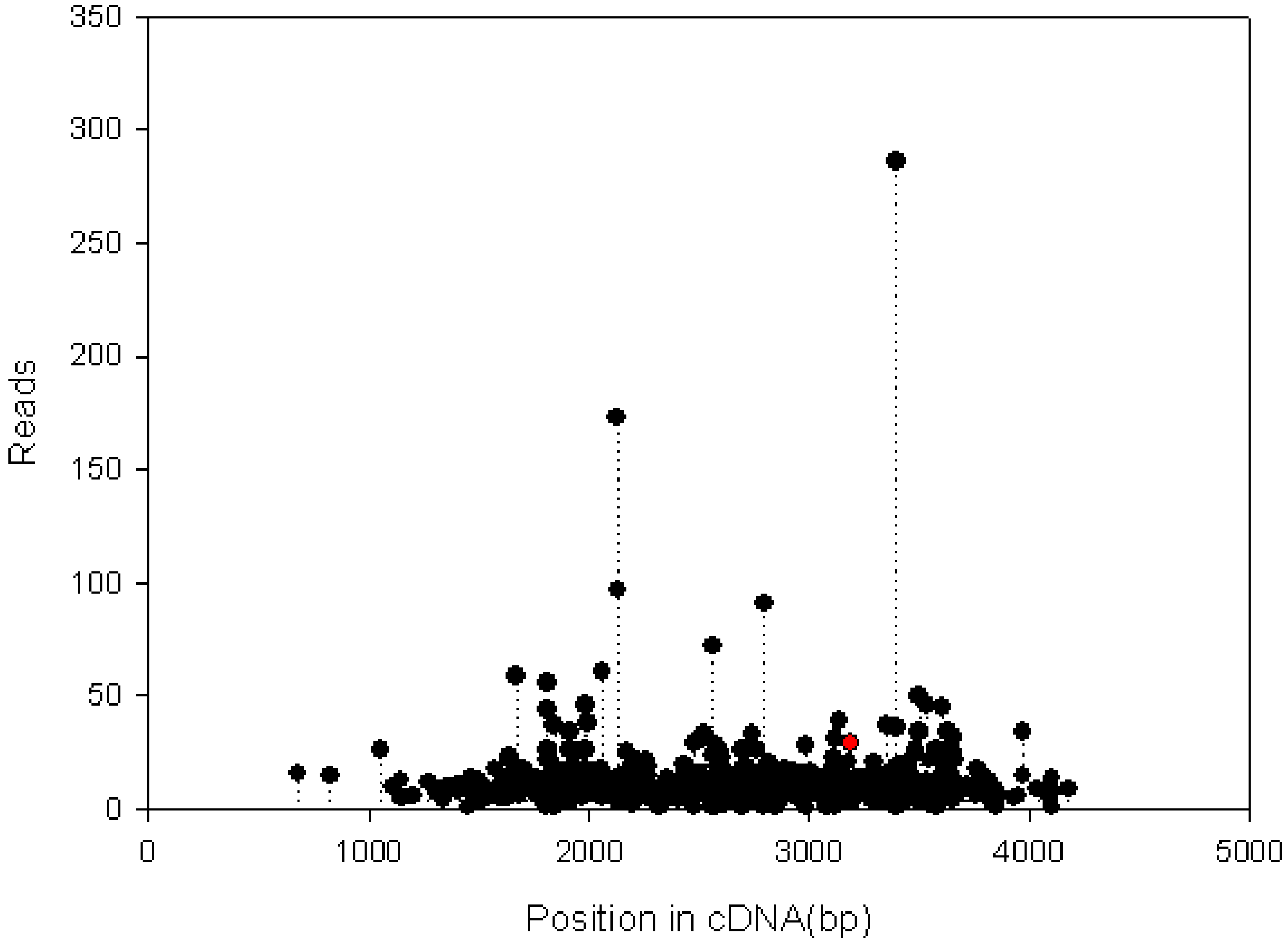


```
5' UACUGGAAGAAAAGCUGUGGAUAACG 3'        Cs4g06230.1
        :   :::  :::::::::::
3' ---AAGUUC-UUUCGACACCUU---- 5'        Csi-miR396b.2
```

Csi-miR396b.2, target=Cs5g01380.1 gene=Cs5g01380
Category:1
Score=2
Cleavage Site=1181

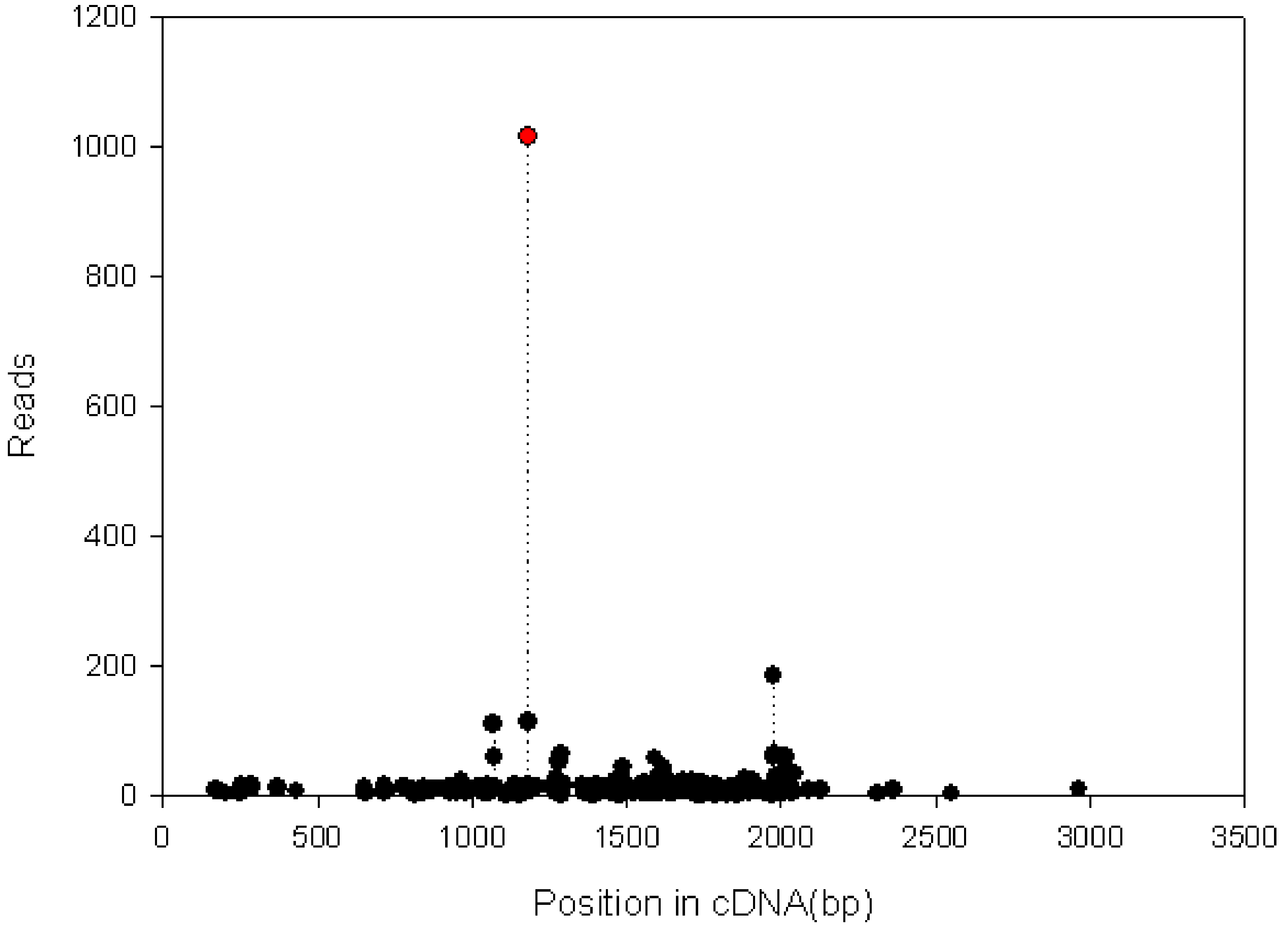


```
5' CCCGUUCAAGAAAGCCUGUGGAAUUA 3'        Cs5g01380.1
       :::::::::: ::::::::
3' ----AAGUUCUUUC-GACACCUU--- 5'        Csi-miR396b.2
```

Csi-miR396b.2, target=Cs5g05620.1 gene=Cs5g05620
Category:3
Score=5
Cleavage Site=403

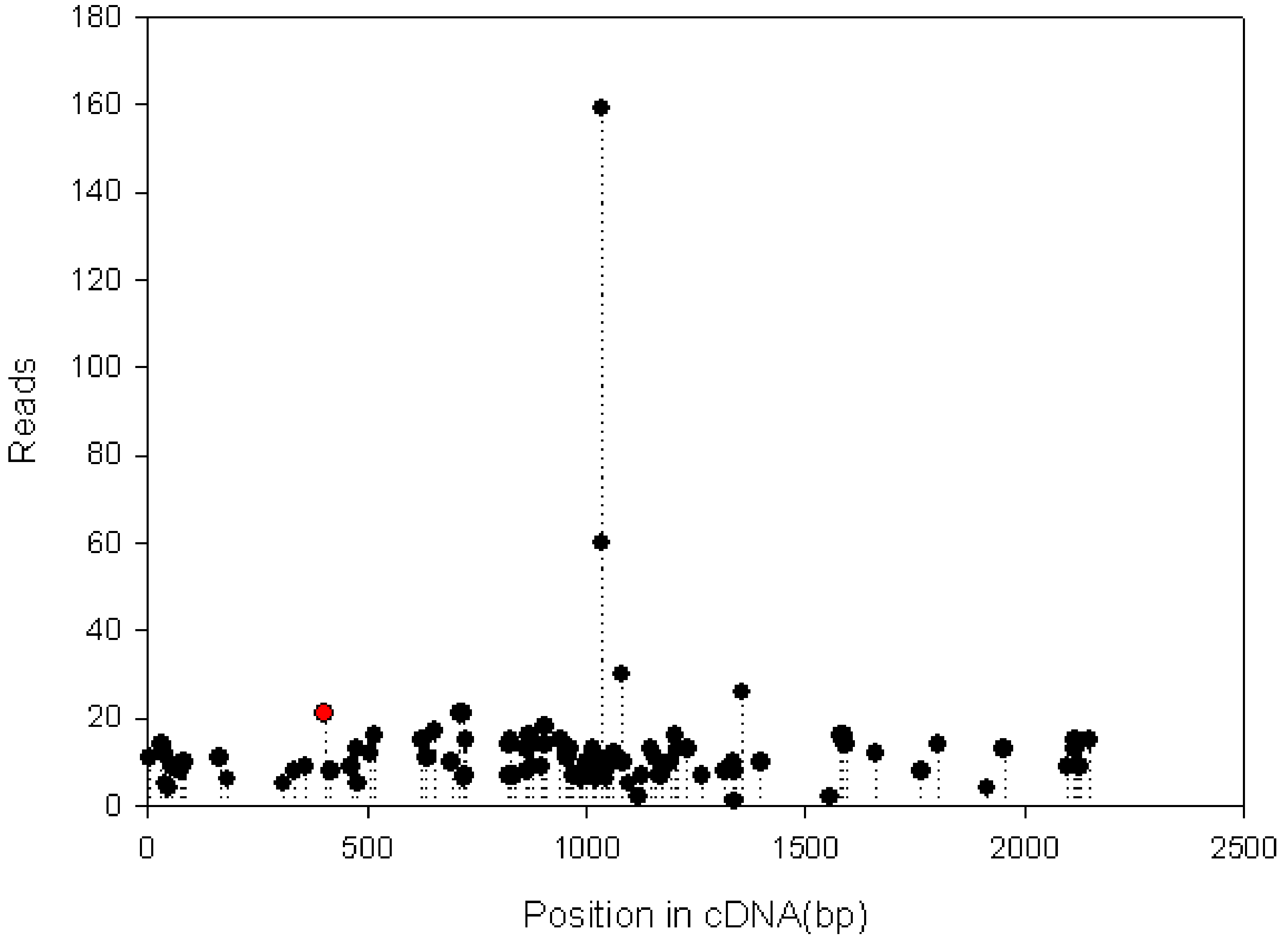


```
5' GAAGGUGAGAAGAAGGUUGUGGAAAG 3'        Cs5g05620.1
              :::::.:.:::::::
3' ------AAGUUCUUUCGACACCUU-- 5'        Csi-miR396b.2
```

Csi-miR396b.2, target=Cs5g06510.1 gene=Cs5g06510
Category:3
Score=4
Cleavage Site=1185

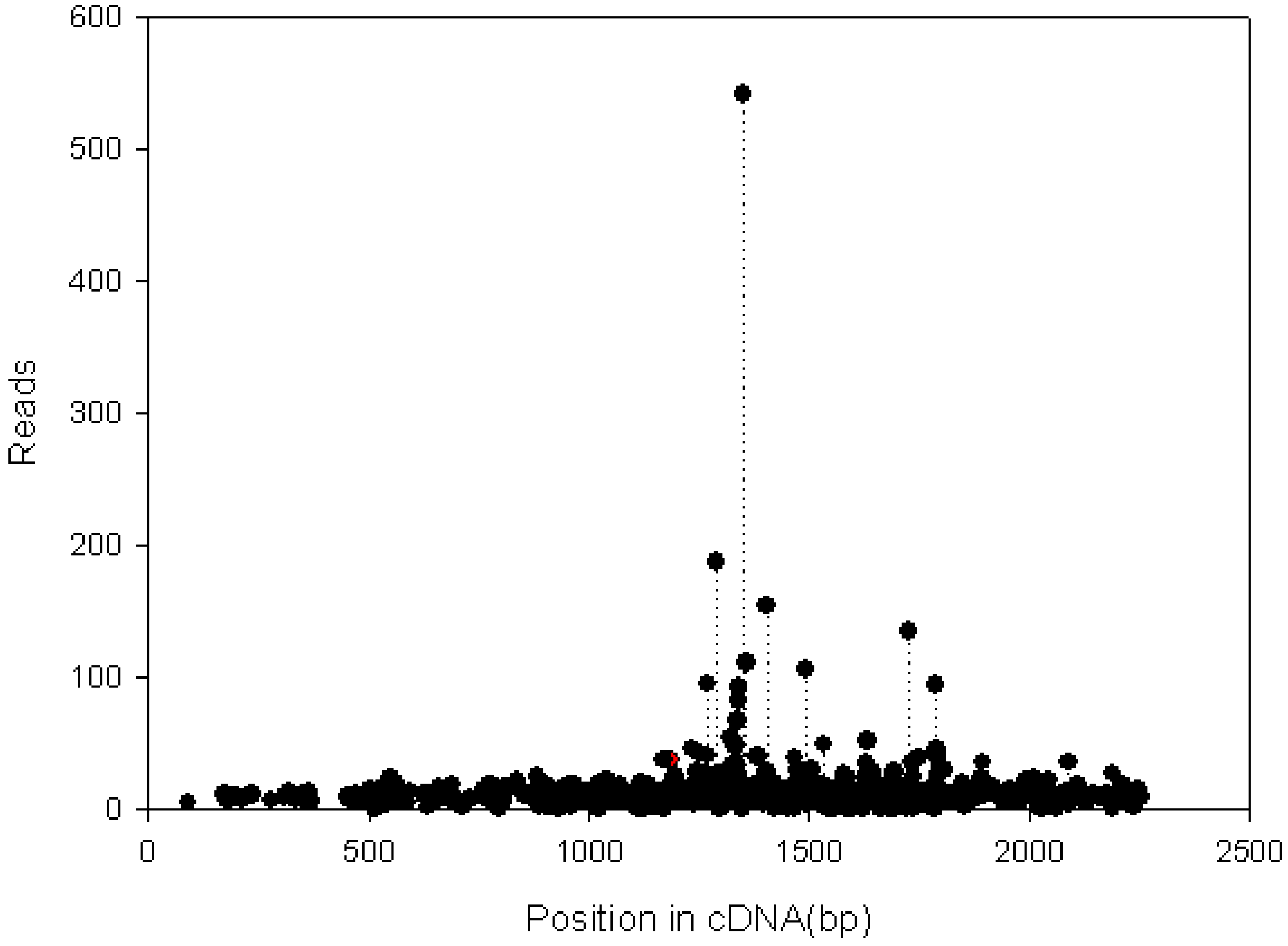


```
5' UAAUCAAGAAAGCUGAGGACUUAGCU 3'        Cs5g06510.1
     :::::::::::: :::
3' --AAGUUCUUUCGACACCUU------ 5'        Csi-miR396b.2
```

Csi-miR396b.2, target=Cs5g09850.1 gene=Cs5g09850
Category:3
Score=2
Cleavage Site=767

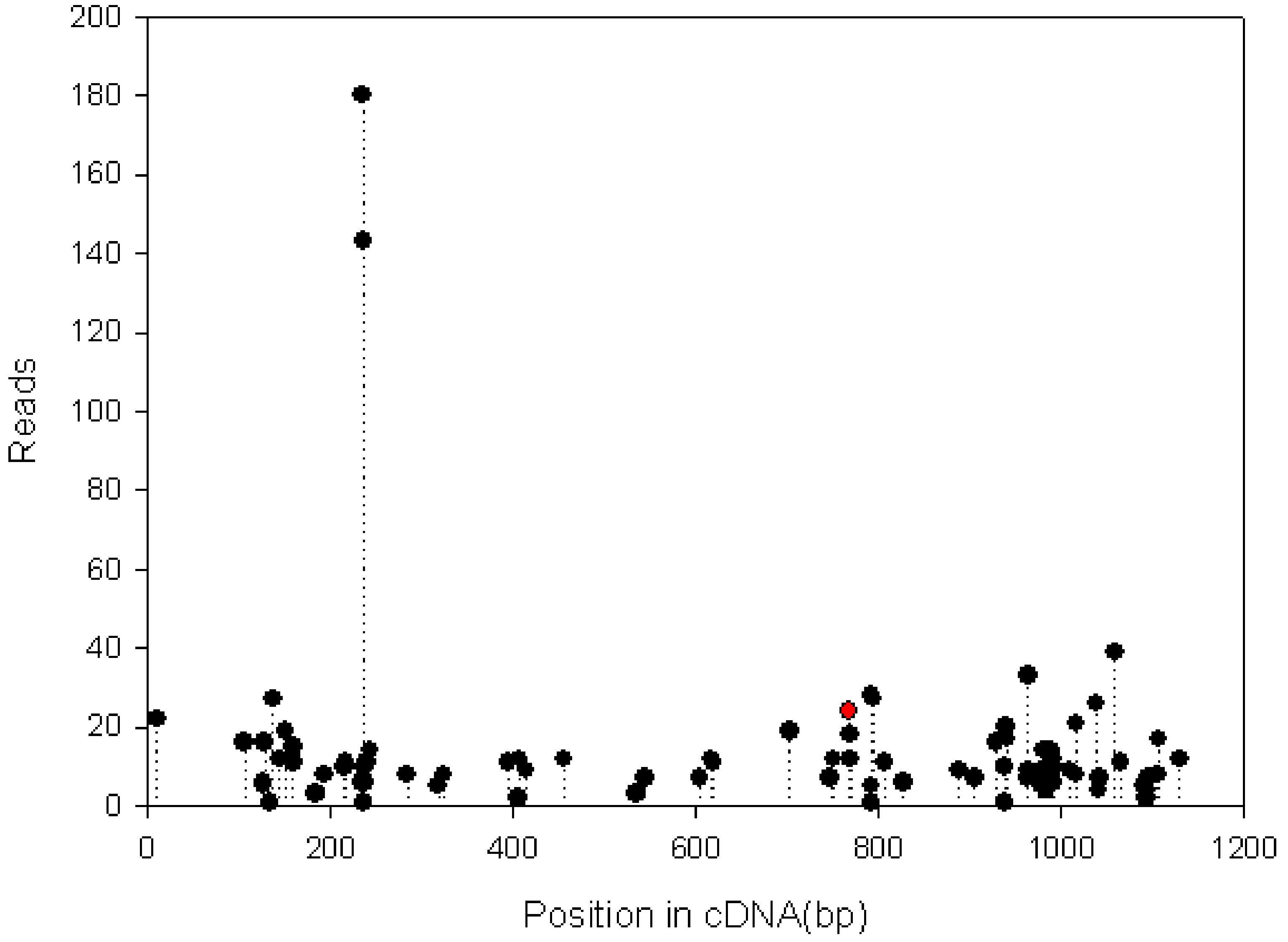


```
5' UGCGUUCAAGAAAGCUUGUGGAAGCU 3'        Cs5g09850.1
        ::::::::::: :::::::
3' ----AAGUUCUUUCG-ACACCUU--- 5'        Csi-miR396b.2
```

Csi-miR396b.2, target=Cs5g24150.1 gene=Cs5g24150
Category:1
Score=5
Cleavage Site=3251

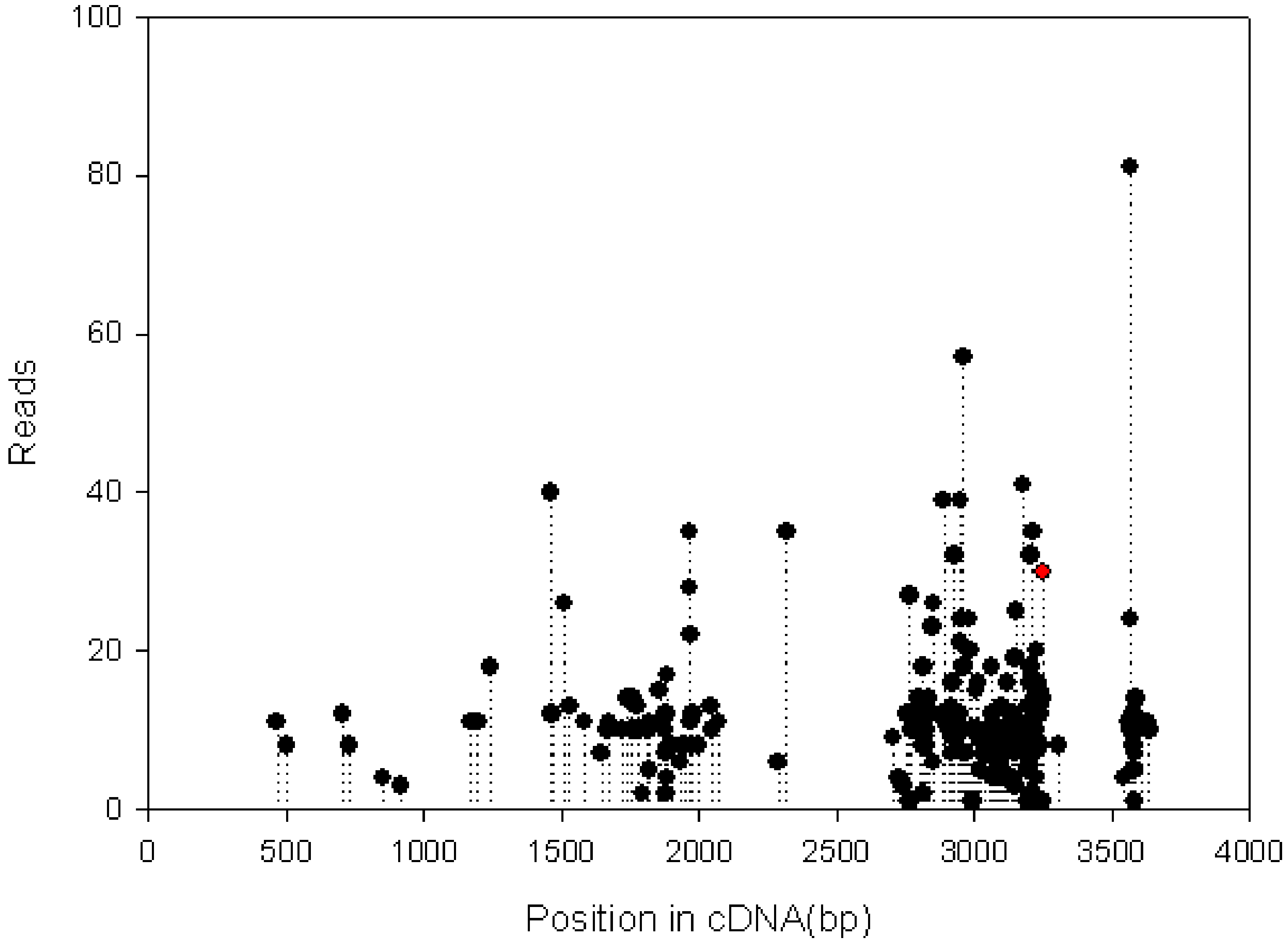


```
5' CGAUCAA-AAAGCUGUAGAAAAUCACA 3'       Cs5g24150.1
       :::: :::::::: :::
3' --AAGUUCUUUCGACACCUU------- 5'       Csi-miR396b.2
```

Csi-miR396b.2, target=Cs6g19000.1 gene=Cs6g19000
Category:3
Score=5
Cleavage Site=1901

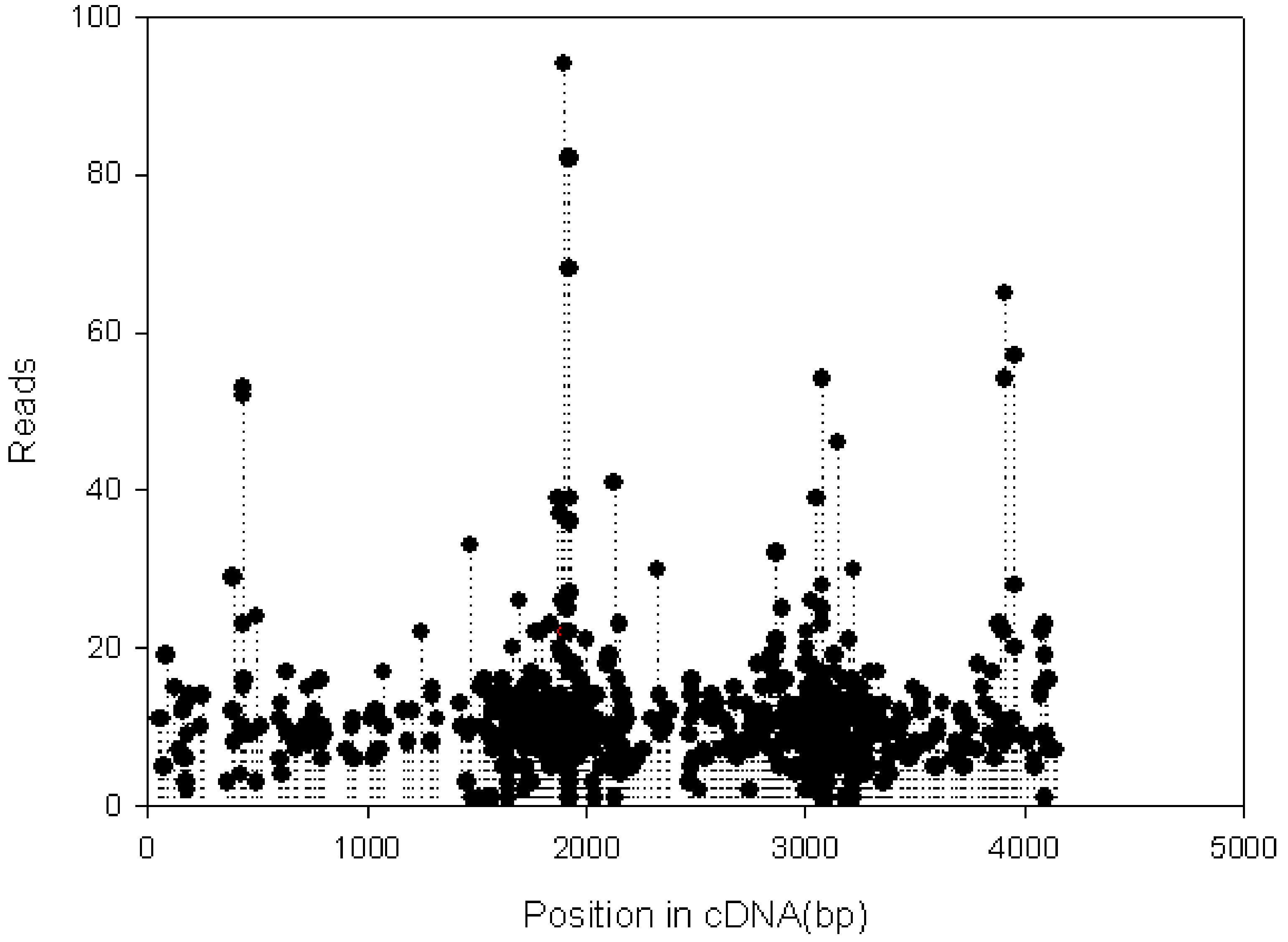


```
5' AAAUGCUUUUGAAGGAAGCUGAGGAU 3'        Cs6g19000.1
            :: :::.:::::: :::
3' --------AAGUUCUUUCGACACCUU 5'        Csi-miR396b.2
```

Csi-miR396b.2, target=Cs6g19380.1 gene=Cs6g19380
Category:2
Score=4
Cleavage Site=1152

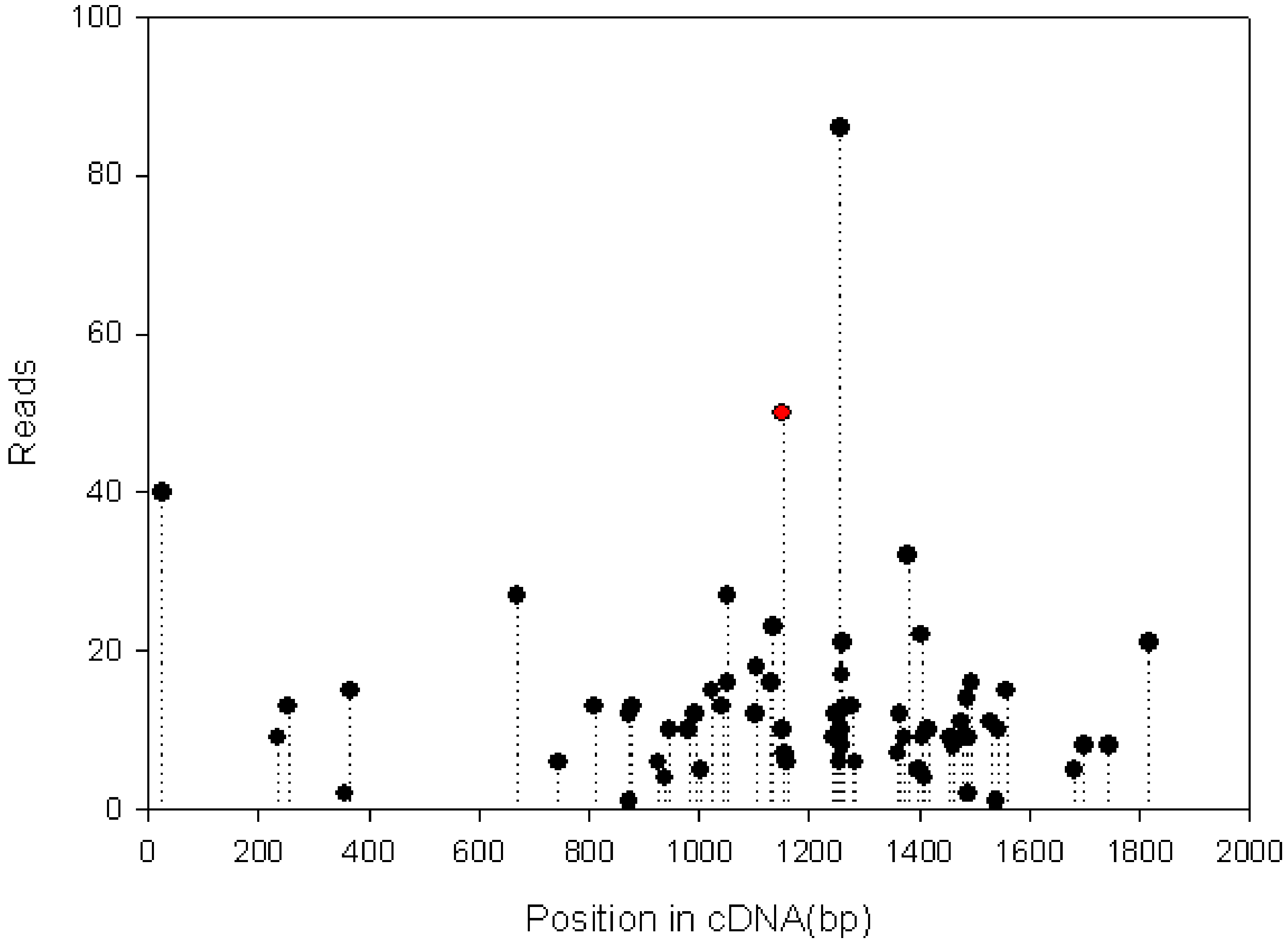


```
5' CUUUUACUUUUAGAGAAGCUGUGGAA 3'        Cs6g19380.1
           ::.:. .:::::::::::
3' --------AAGUUCUUUCGACACCUU 5'        Csi-miR396b.2
```

Csi-miR396b.2, target=Cs7g15220.1 gene=Cs7g15220
Category:1
Score=2
Cleavage Site=377

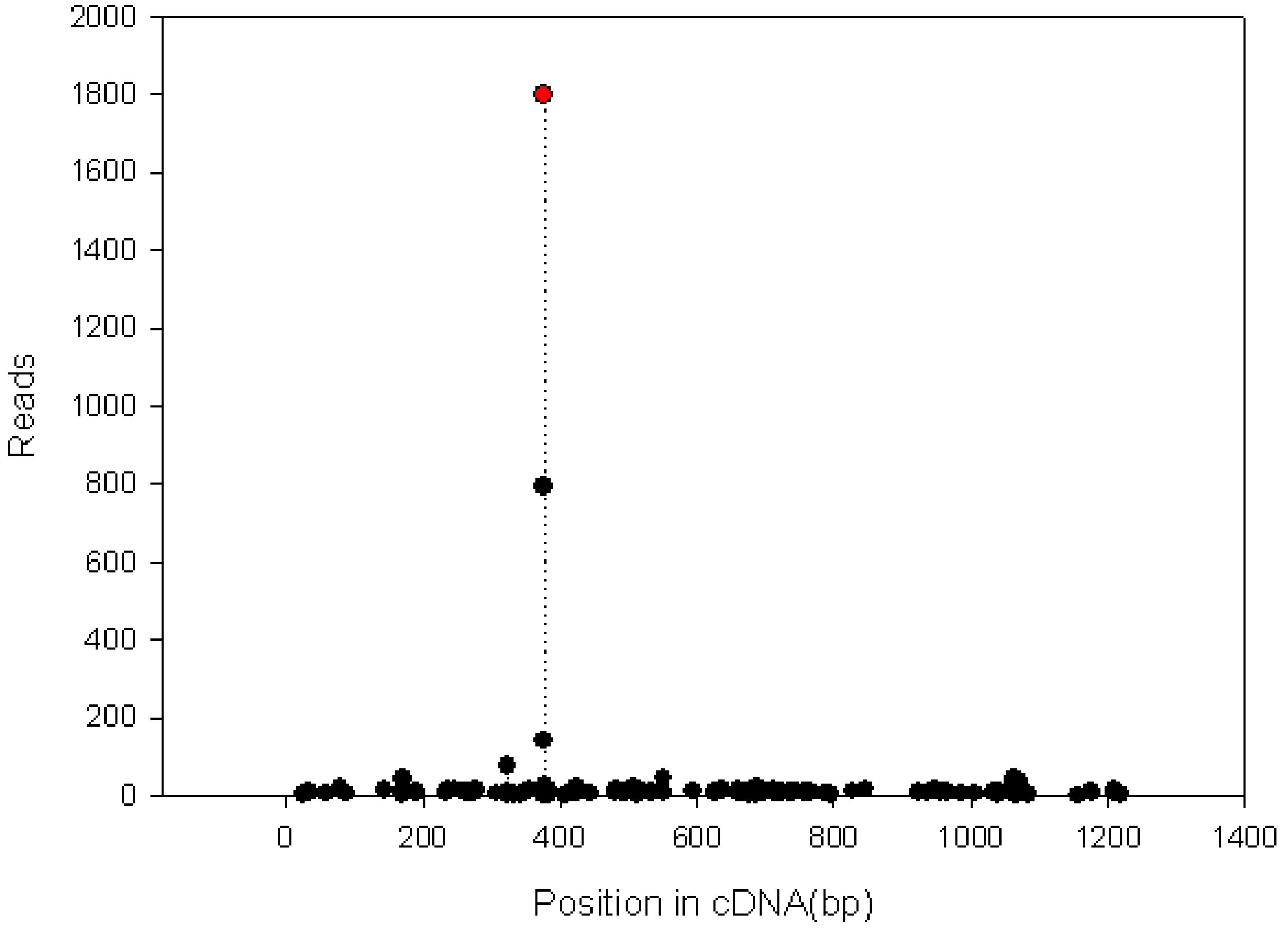


```
5' ACCGUUCAAGAAAGCCUGUGGAAUCA 3'        Cs7g15220.1
        :::::::::: ::::::::
3' ----AAGUUCUUUC-GACACCUU--- 5'        Csi-miR396b.2
```

Csi-miR396b.2, target=Cs7g27670.1 gene=Cs7g27670
Category:1
Score=4
Cleavage Site=653

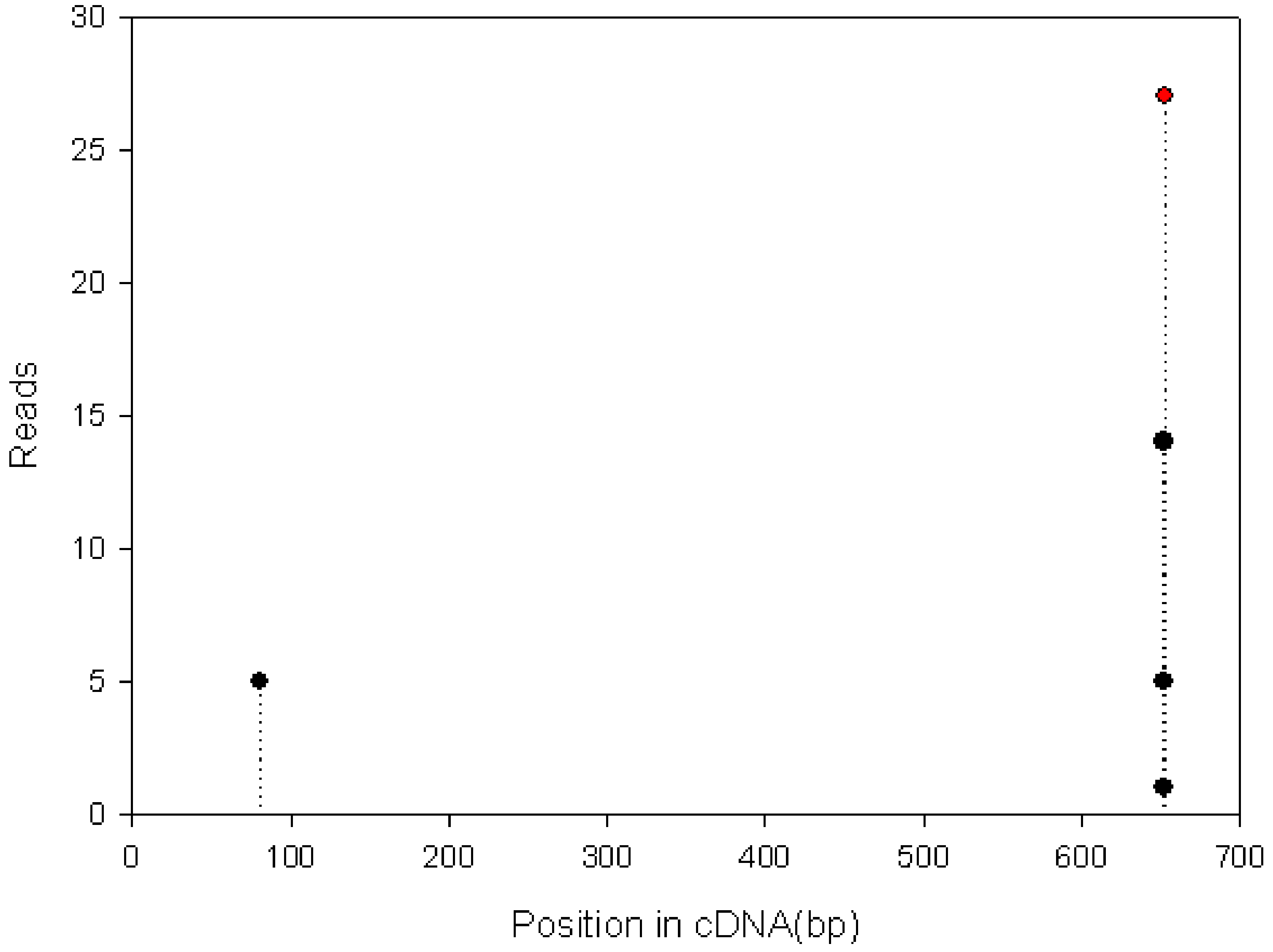


```
5' UCAUCCAAGAAGGCUAUGGAAUGGAC 3'        Cs7g27670.1
      : :::::::.::: :::::
3' ---AAGUUCUUUCGACACCUU----- 5'        Csi-miR396b.2
```

Csi-miR396b.2, target=Cs7g27680.1 gene=Cs7g27680
Category:1
Score=4
Cleavage Site=713

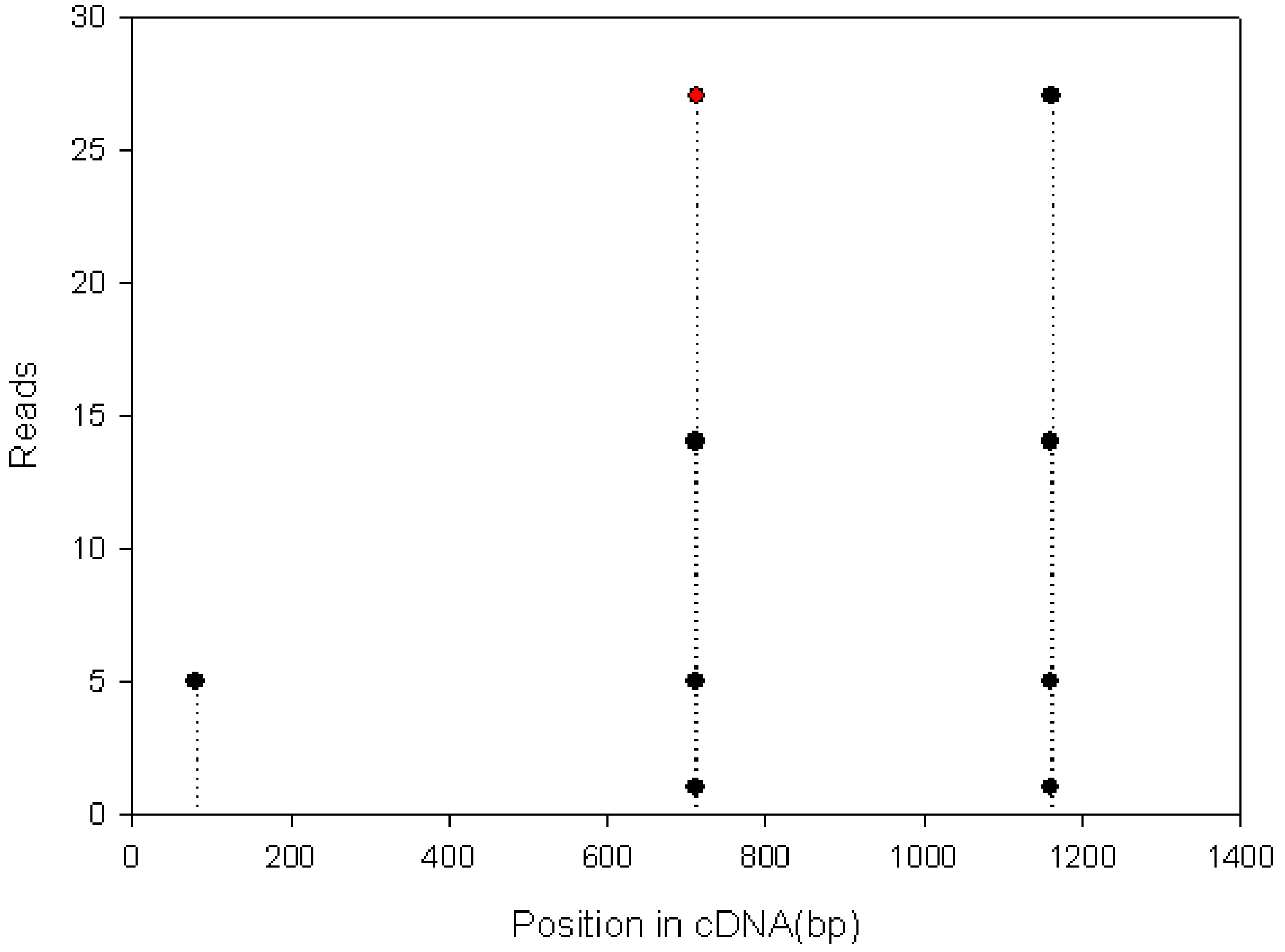


```
5' UCAUCCAAGAAGGCUAUGGAAUGGAC 3'        Cs7g27680.1
       : ::::::.::: :::::
3' ---AAGUUCUUUCGACACCUU----- 5'        Csi-miR396b.2
```

Csi-miR396b.2, target=Cs8g03020.2 gene=Cs8g03020
Category:3
Score=5
Cleavage Site=5475

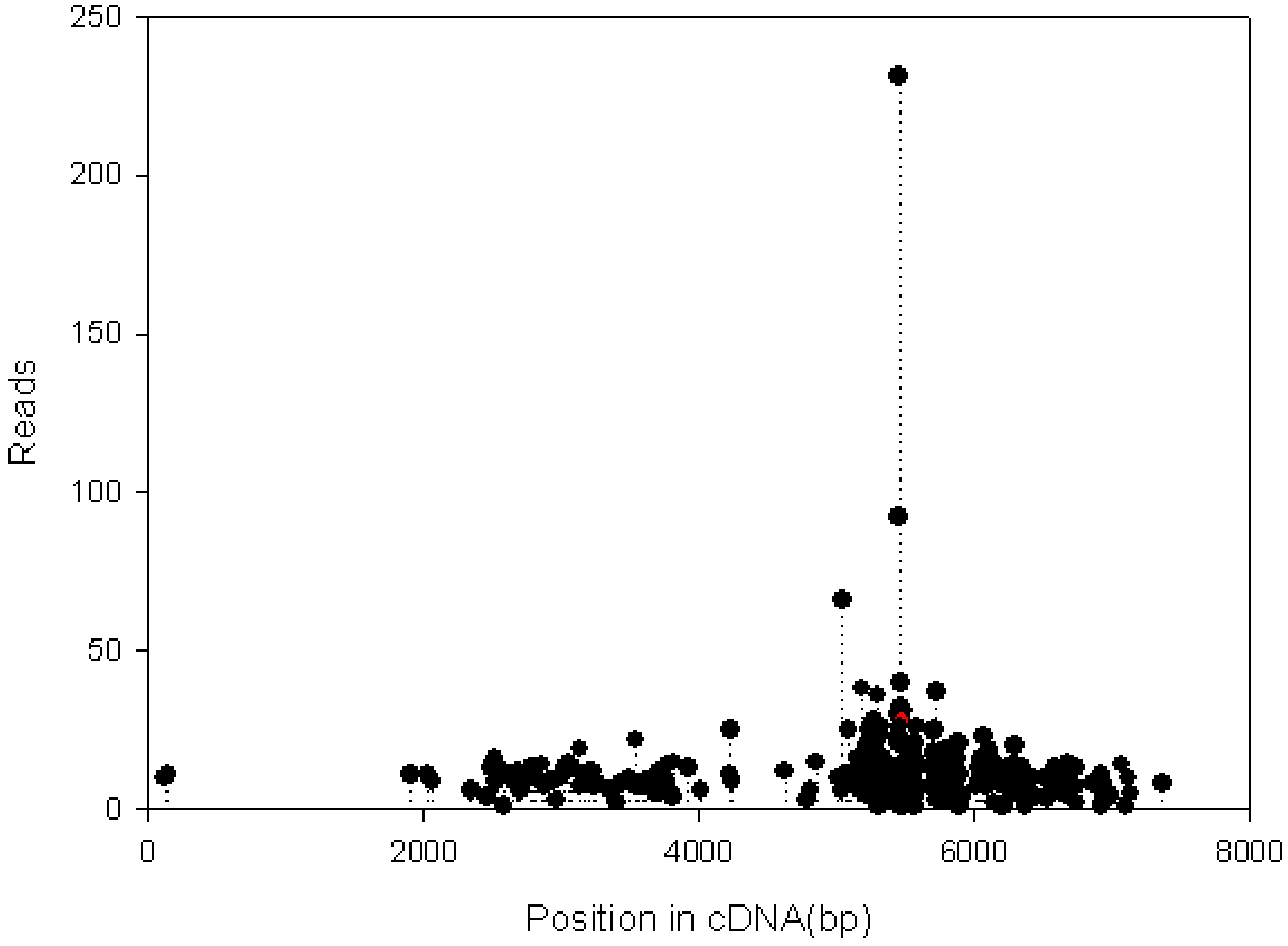


```
5' CCUGCACGAAAGCUUUGGACCAAUUC 3'        Cs8g03020.2
     : :: ::::::: ::::
3' --AAGUUCUUUCGACACCUU------ 5'        Csi-miR396b.2
```

Csi-miR396b.2, target=Cs8g17370.1 gene=Cs8g17370
Category:3
Score=4.5
Cleavage Site=140

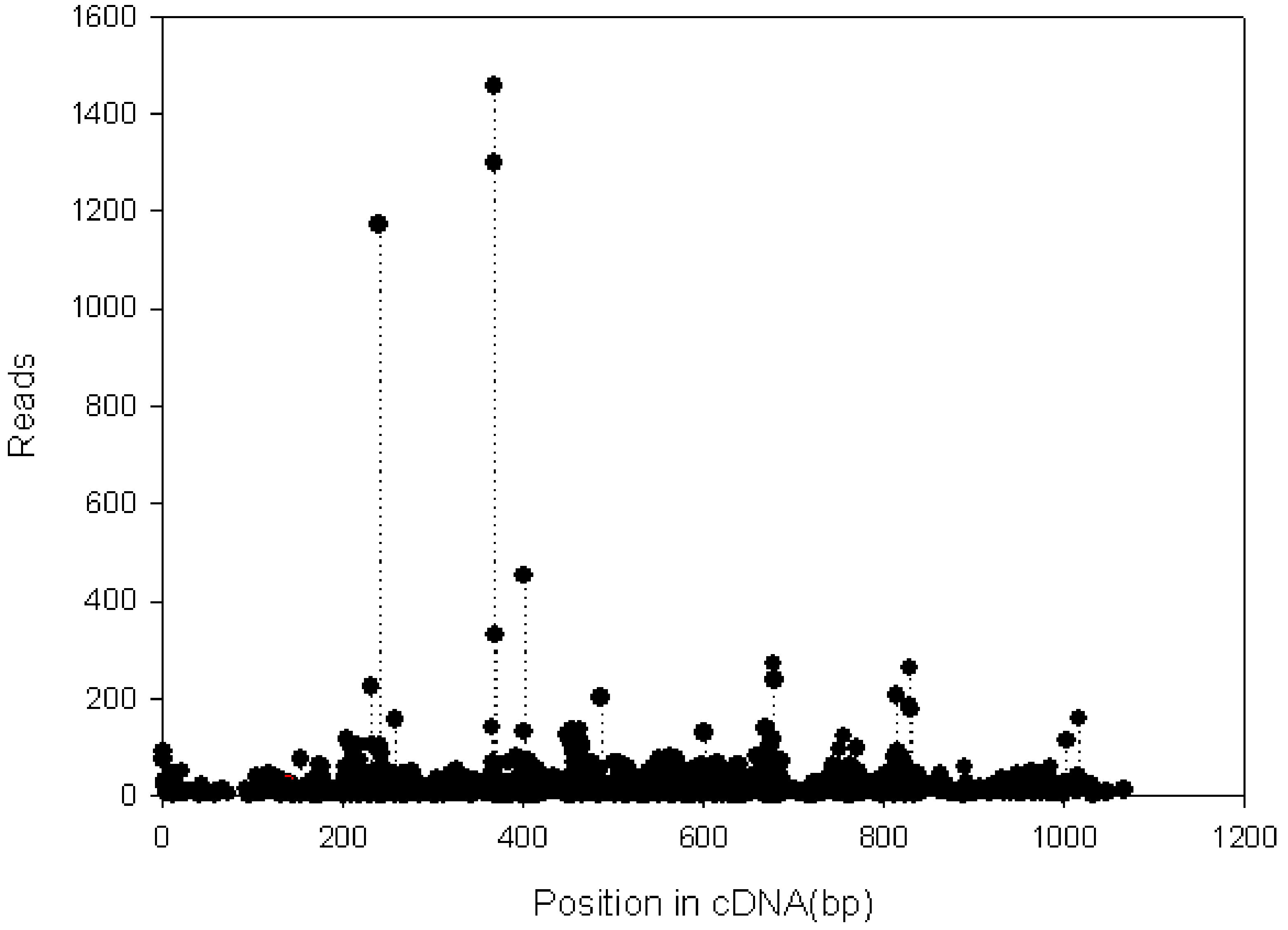


```
5' AGGAUUACAAGAAGGCCGUGGAGAAA 3'        Cs8g17370.1
        : :::::::.:: :::::.
3' -----AAGUUCUUUCGACACCUU--- 5'        Csi-miR396b.2
```

Csi-miR396b.2, target=Orange1.1t00172.1 gene=Orange1.1t00172
Category:1
Score=2
Cleavage Site=656

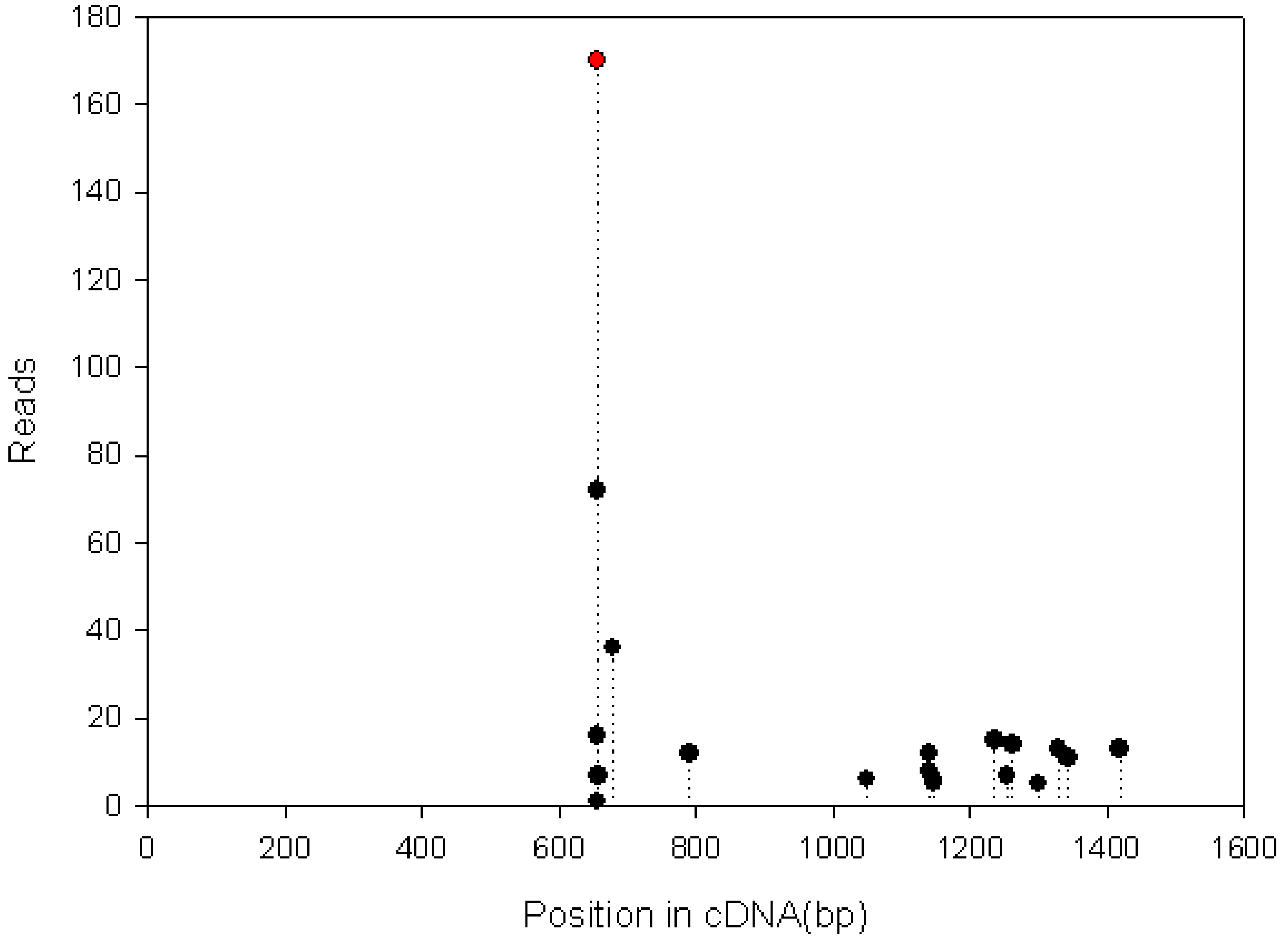


```
5' ACCGUUCAAGAAAGCCUGUGGAACUU 3'        Orange1.1t00172.1
      :::::::::: ::::::::
3' ----AAGUUCUUUC-GACACCUU--- 5'        Csi-miR396b.2
```

Csi-miR396b.2, target=Orange1.1t02555.1 gene=Orange1.1t02555
Category:1
Score=2
Cleavage Site=920

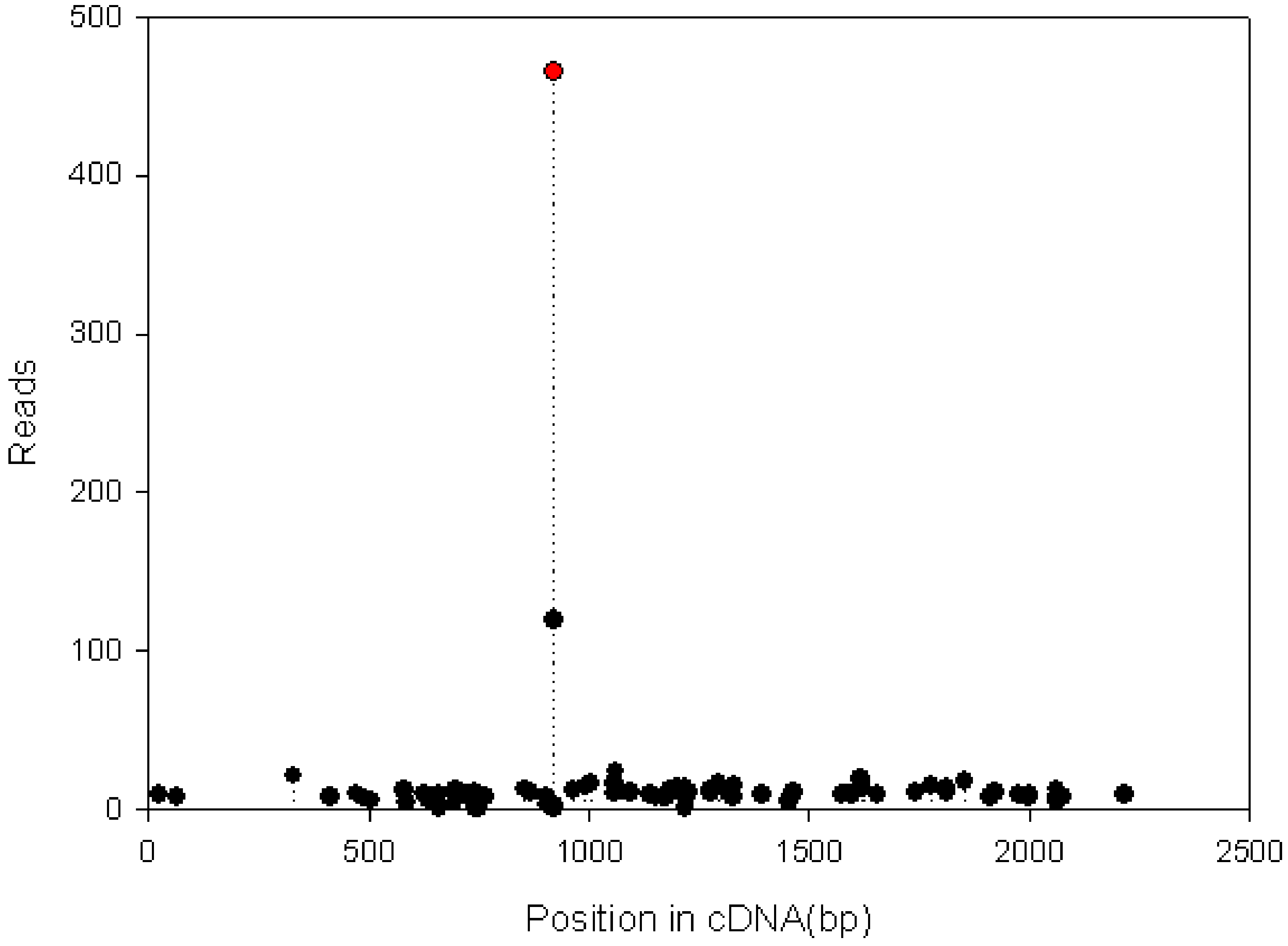


```
5' CACGUUCAAGAAAGCCUGUGGAACUU 3'        Orange1.1t02555.1
         :::::::::: ::::::::
3' ----AAGUUCUUUC-GACACCUU--- 5'        Csi-miR396b.2
```

Csi-miR396b.2, target=Orange1.1t03122.1 gene=Orange1.1t03122
Category:1
Score=2
Cleavage Site=583

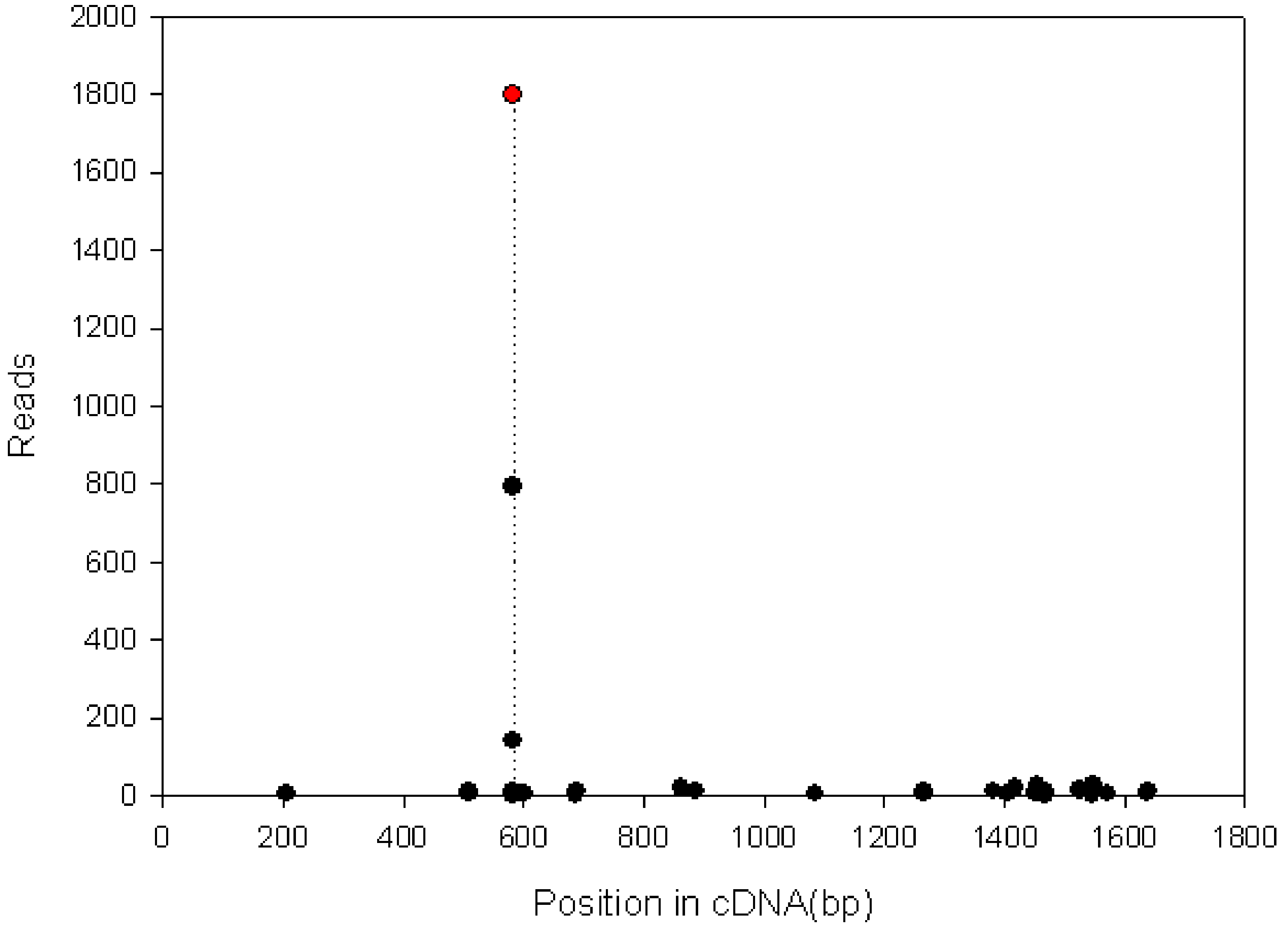


```
5' ACCGUUCAAGAAAGCCUGUGGAAUCA 3'        Orange1.1t03122.1
       :::::::::: ::::::::
3' ----AAGUUCUUUC-GACACCUU--- 5'        Csi-miR396b.2
```

Csi-miR396b.3, target=Cs1g21350.1 gene=Cs1g21350
Category:1
Score=4
Cleavage Site=1164

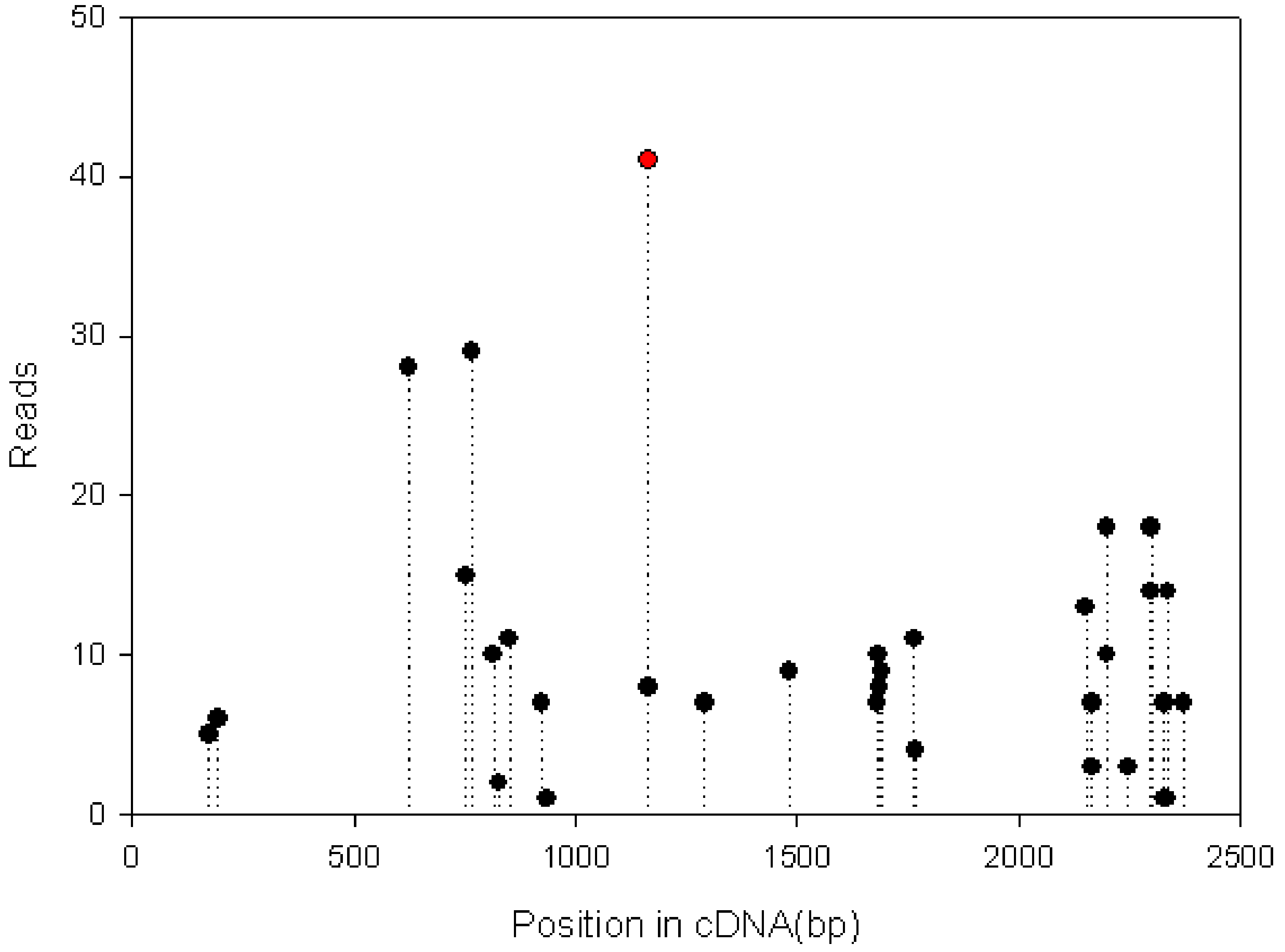


```
5' ACCGUUCAAGAAAGCCUGUGGAAGGC 3'        Cs1g21350.1
      ::::::::::: ::::::::
3' -UUCAAGUUCUUUC-GACACCUU--- 5'        Csi-miR396b.3
```

Csi-miR396b.3, target=Cs1g22520.1 gene=Cs1g22520
Category:1
Score=3
Cleavage Site=226

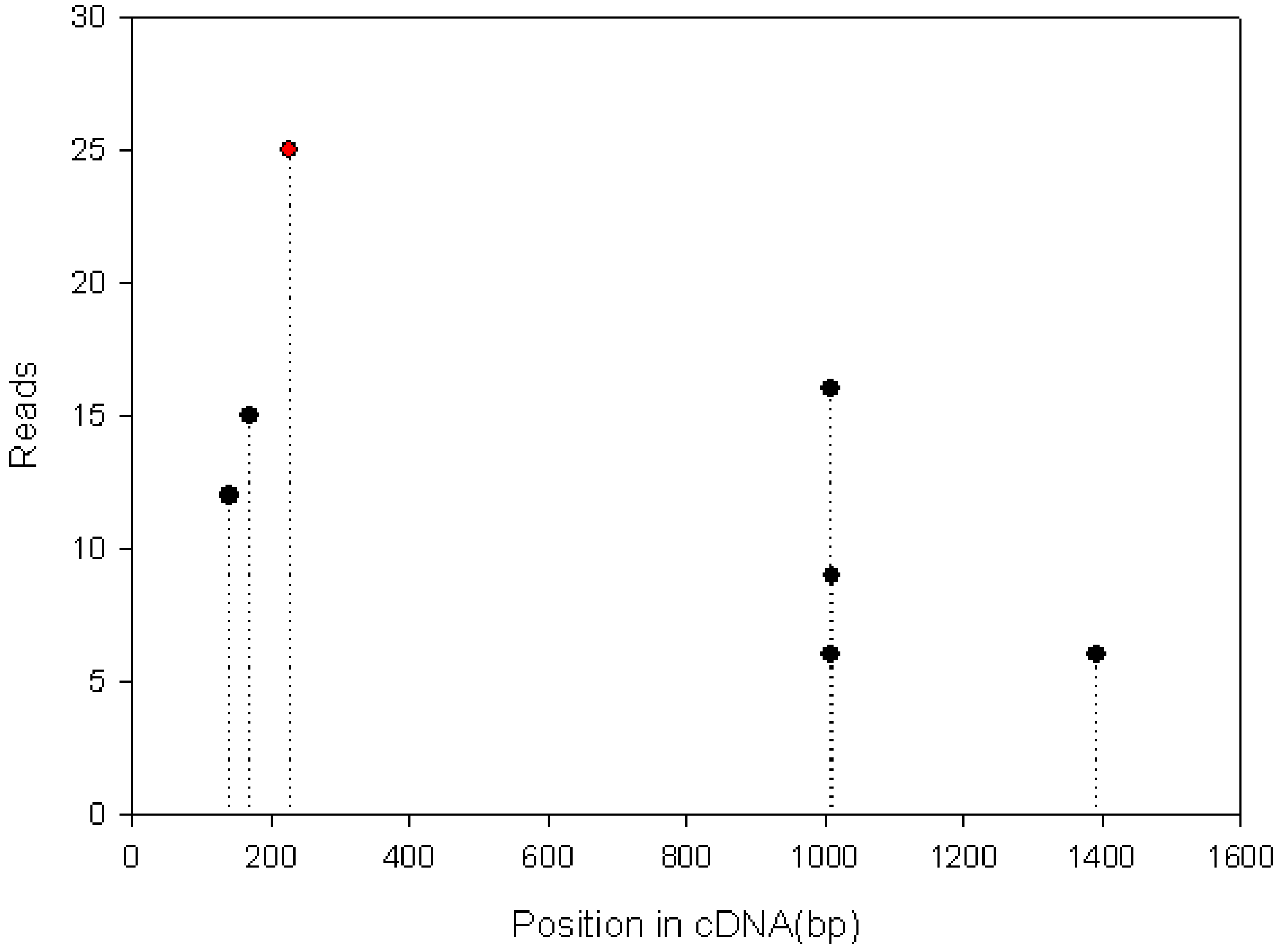


```
5' GAAGUUCAAGAAAUCUGUGGGAAGAA 3'        Cs1g22520.1
    ::::::::::::: :::::::.:
3' -UUCAAGUUCUUUCGACACCUU---- 5'        Csi-miR396b.3
```

Csi-miR396b.3, target=Cs3g23180.1 gene=Cs3g23180
Category:1
Score=3
Cleavage Site=625

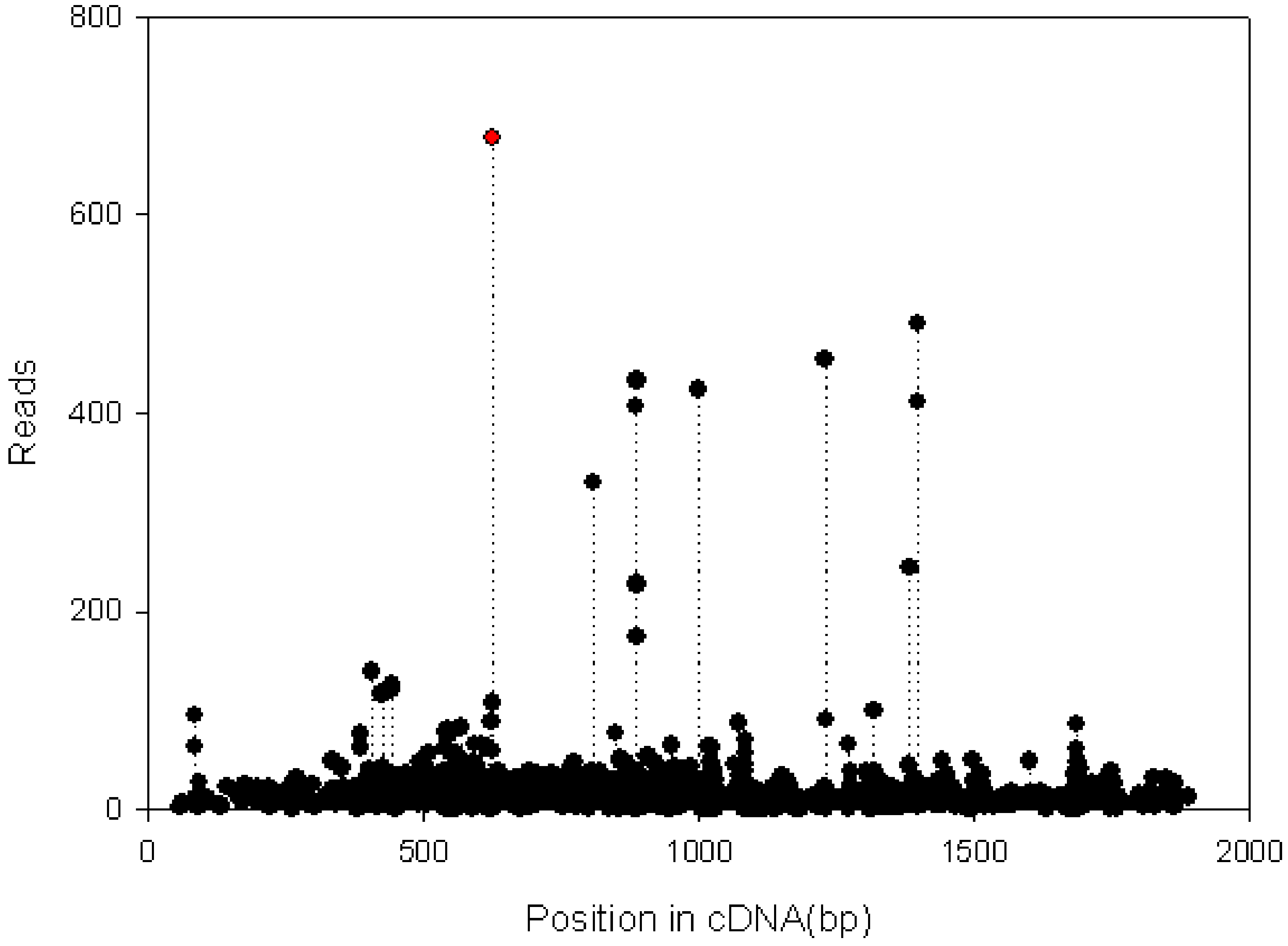


```
5' AAAGAUCAAGGAAGCUGUGGGAGUUG 3'        Cs3g23180.1
    ::: ::::::.:::::::::.:
3' -UUCAAGUUCUUUCGACACCUU---- 5'        Csi-miR396b.3
```

Csi-miR396b.3, target=Cs5g01380.1 gene=Cs5g01380
Category:1
Score=4
Cleavage Site=1181

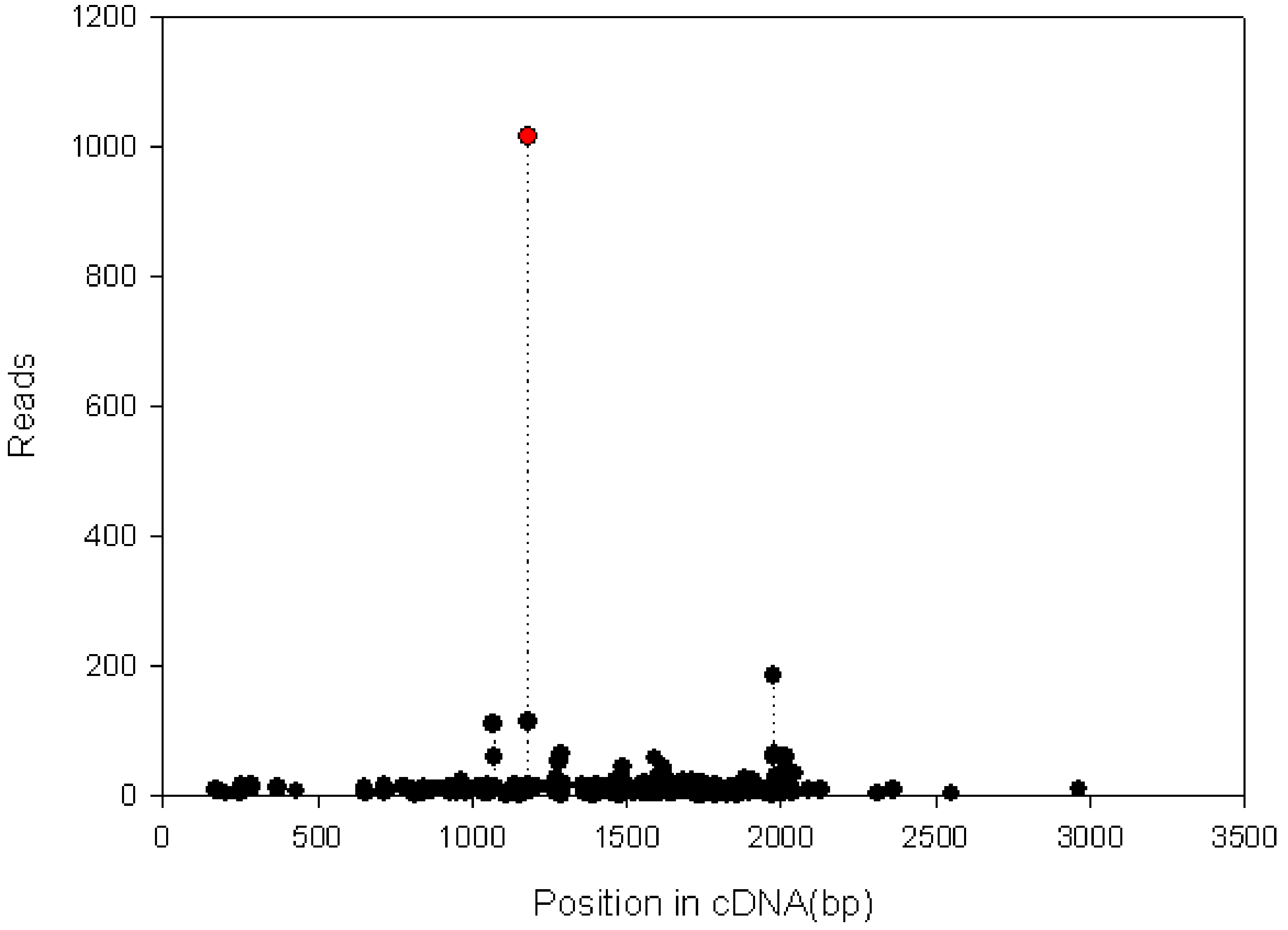


```
5' CCCGUUCAAGAAAGCCUGUGGAAUUA 3'        Cs5g01380.1
      ::::::::::: ::::::::
3' -UUCAAGUUCUUUC-GACACCUU--- 5'        Csi-miR396b.3
```

Csi-miR396b.3, target=Cs5g09850.1 gene=Cs5g09850
Category:3
Score=3.5
Cleavage Site=767

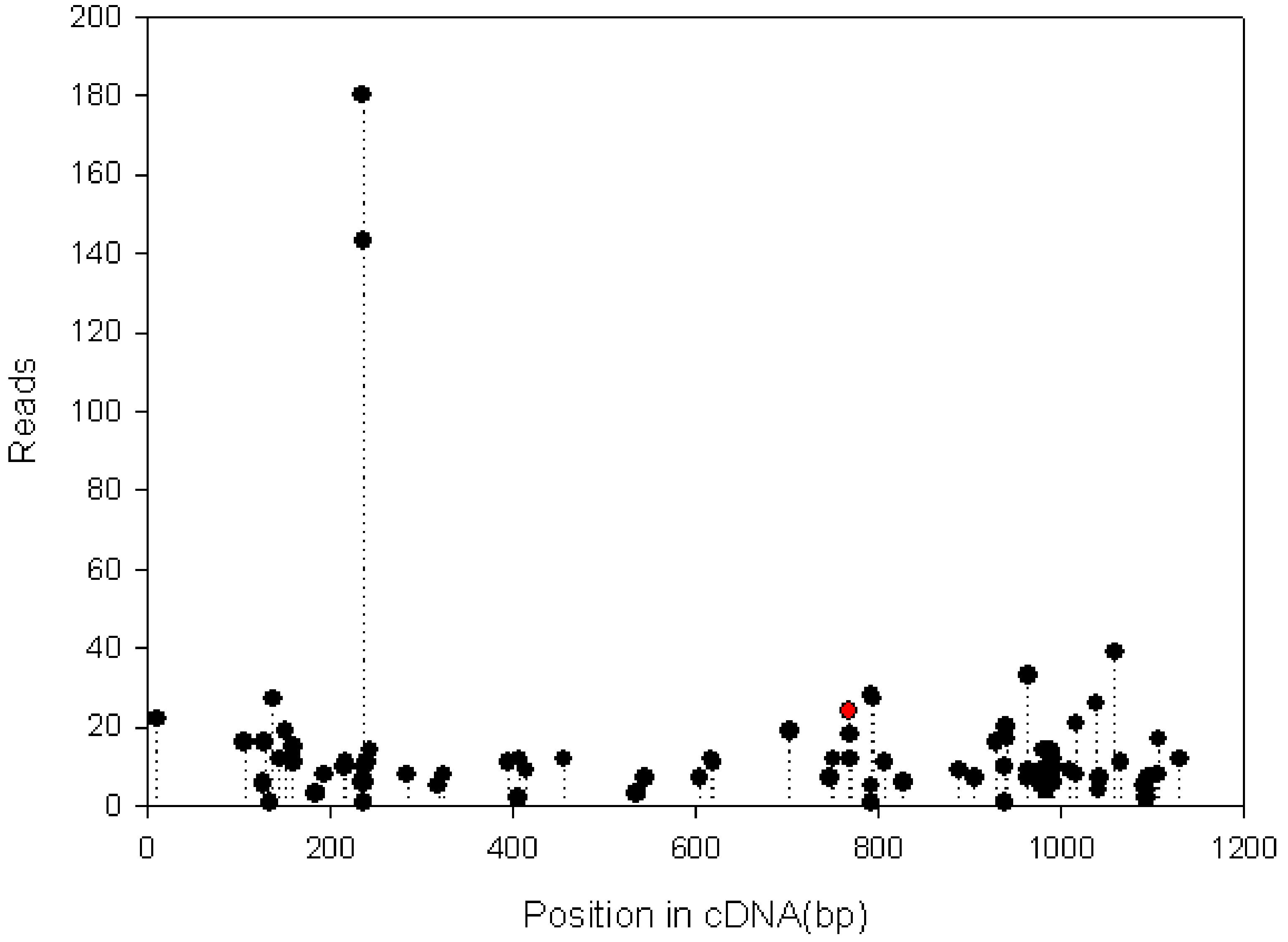


```
5' UGCGUUCAAGAAAGCUUGUGGAAGCU 3'        Cs5g09850.1
    . ::::::::::::: :::::::
3' -UUCAAGUUCUUUCG-ACACCUU--- 5'        Csi-miR396b.3
```

Csi-miR396b.3, target=Cs7g15220.1 gene=Cs7g15220
Category:1
Score=4
Cleavage Site=377

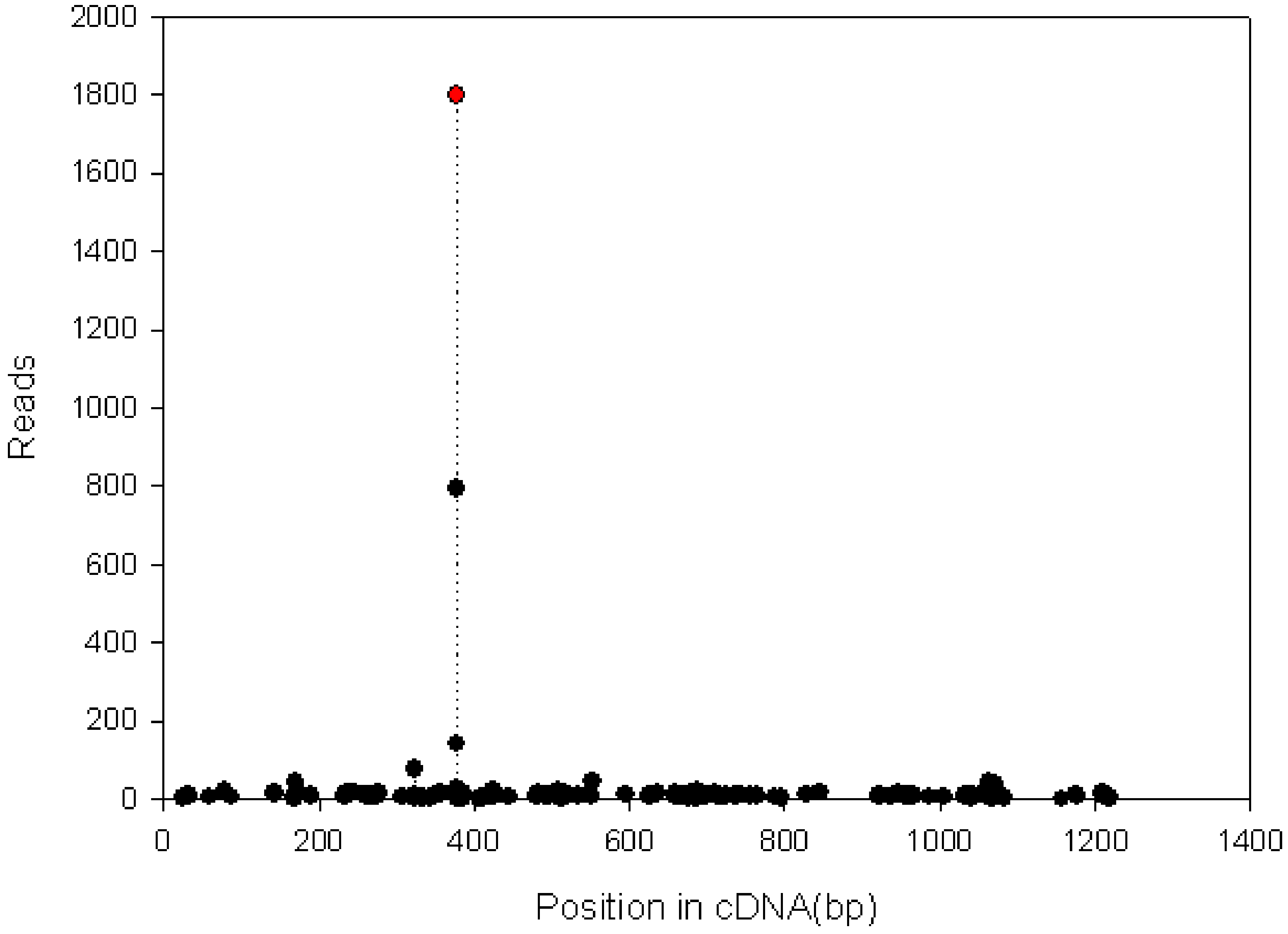


```
5' ACCGUUCAAGAAAGCCUGUGGAAUCA 3'        Cs7g15220.1
     :::::::::::: ::::::::
3' -UUCAAGUUCUUUC-GACACCUU--- 5'        Csi-miR396b.3
```

Csi-miR396b.3, target=Orange1.1t00172.1 gene=Orange1.1t00172
Category:1
Score=4
Cleavage Site=656

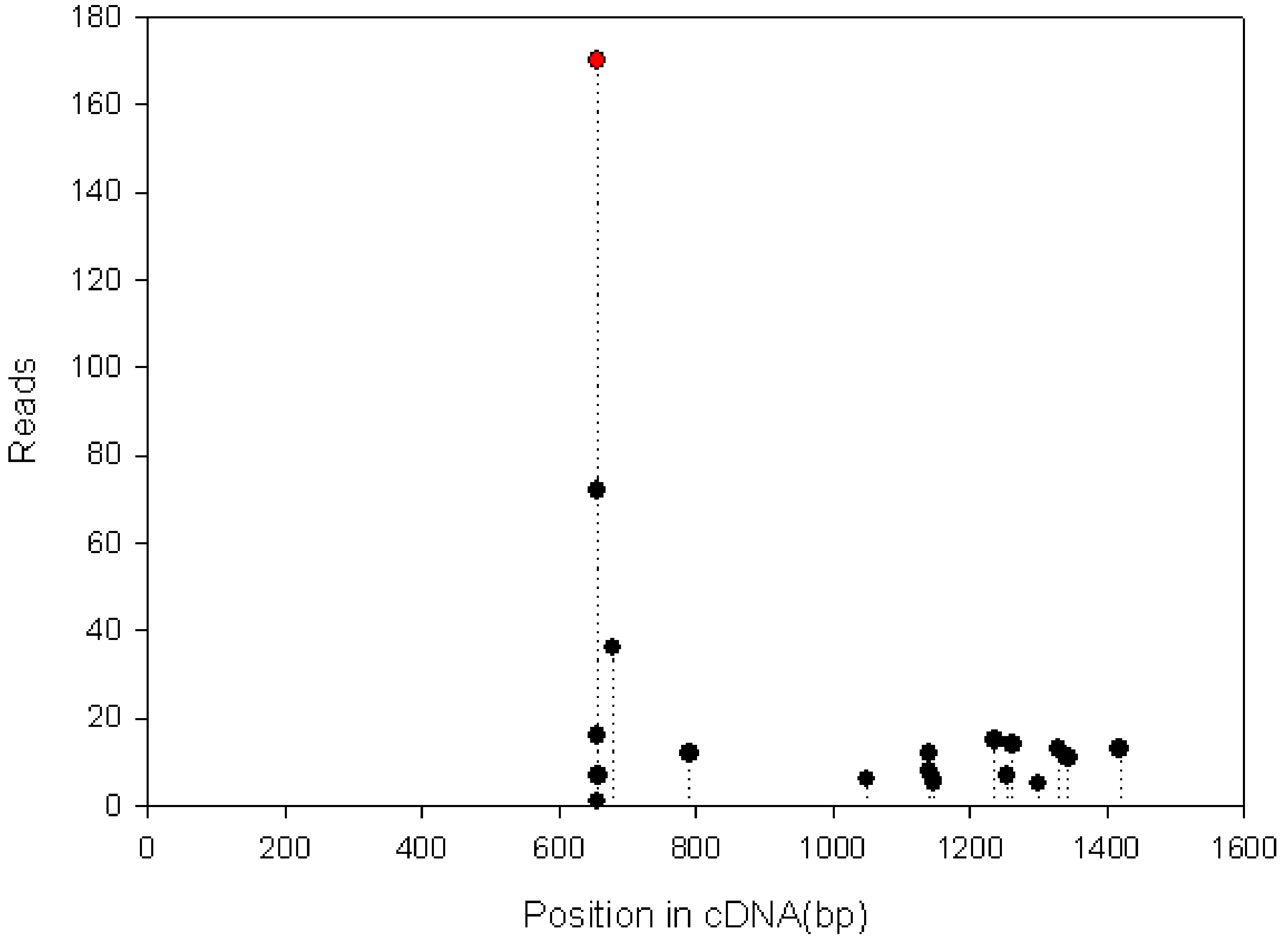


```
5' ACCGUUCAAGAAAGCCUGUGGAACUU 3'        Orange1.1t00172.1
     ::::::::::: ::::::::
3' -UUCAAGUUCUUUC-GACACCUU--- 5'        Csi-miR396b.3
```

Csi-miR396b.3, target=Orange1.1t02555.1 gene=Orange1.1t02555
Category:1
Score=3
Cleavage Site=920

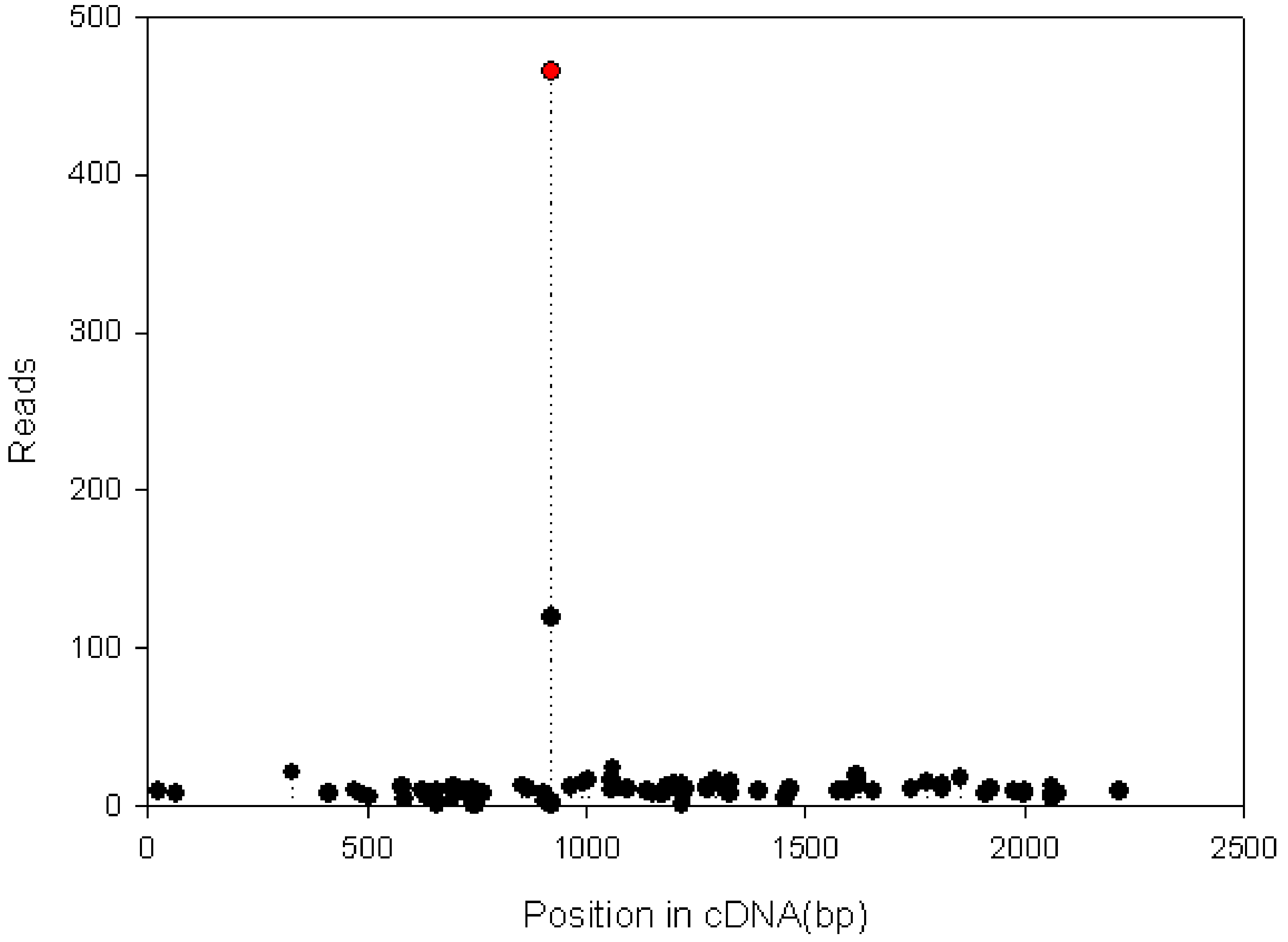


```
5' CACGUUCAAGAAAGCCUGUGGAACUU 3'        Orange1.1t02555.1
    : ::::::::::: ::::::::
3' -UUCAAGUUCUUUC-GACACCUU--- 5'        Csi-miR396b.3
```

Csi-miR396b.3, target=Orange1.1t03122.1 gene=Orange1.1t03122
Category:1
Score=4
Cleavage Site=583

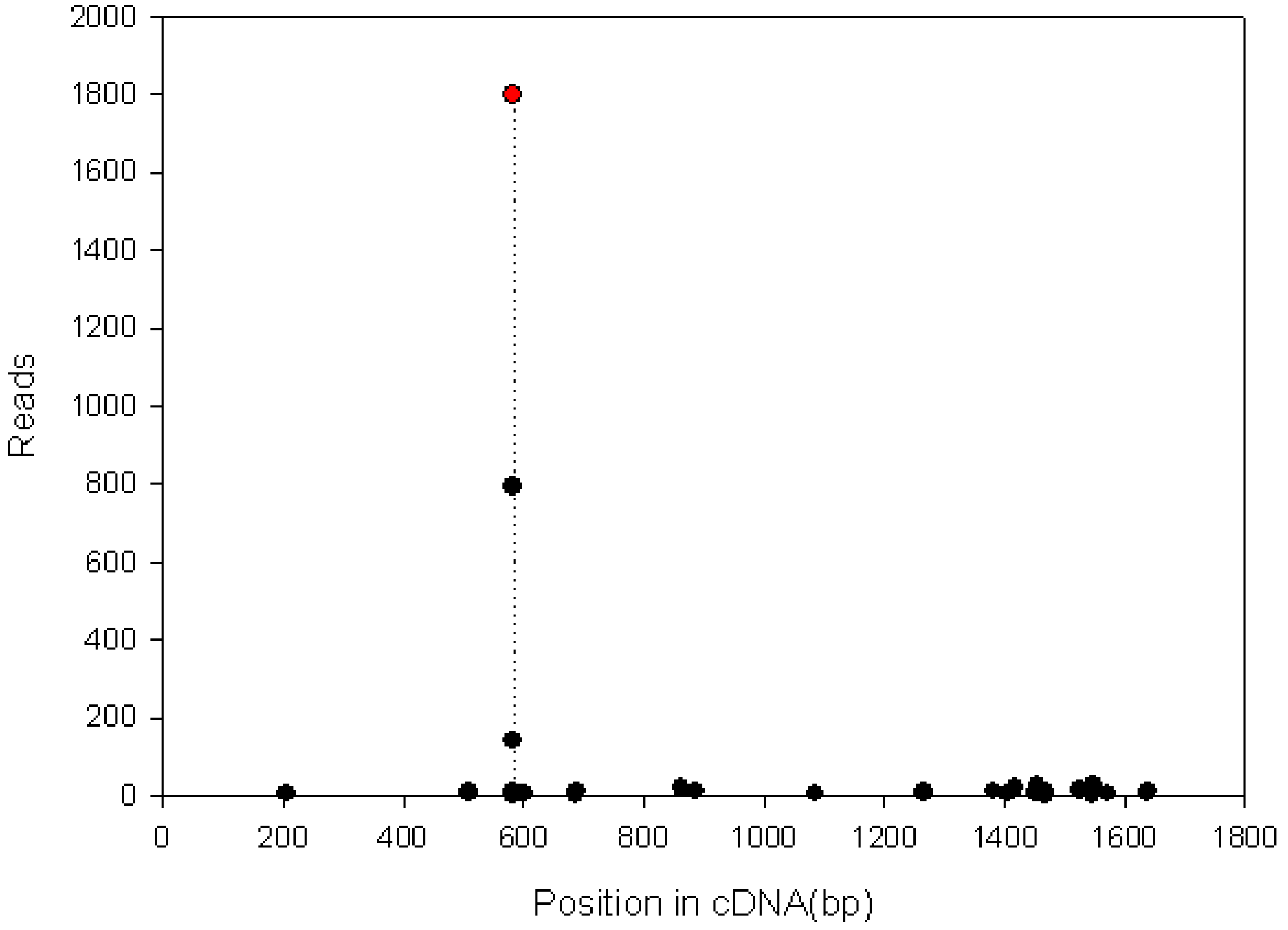


```
5' ACCGUUCAAGAAAGCCUGUGGAAUCA 3'        Orange1.1t03122.1
     ::::::::::: ::::::::
3' -UUCAAGUUCUUUC-GACACCUU--- 5'        Csi-miR396b.3
```

Csi-miR396b-3p.2, target=Cs2g01990.1 gene=Cs2g01990
Category:3
Score=5
Cleavage Site=1431

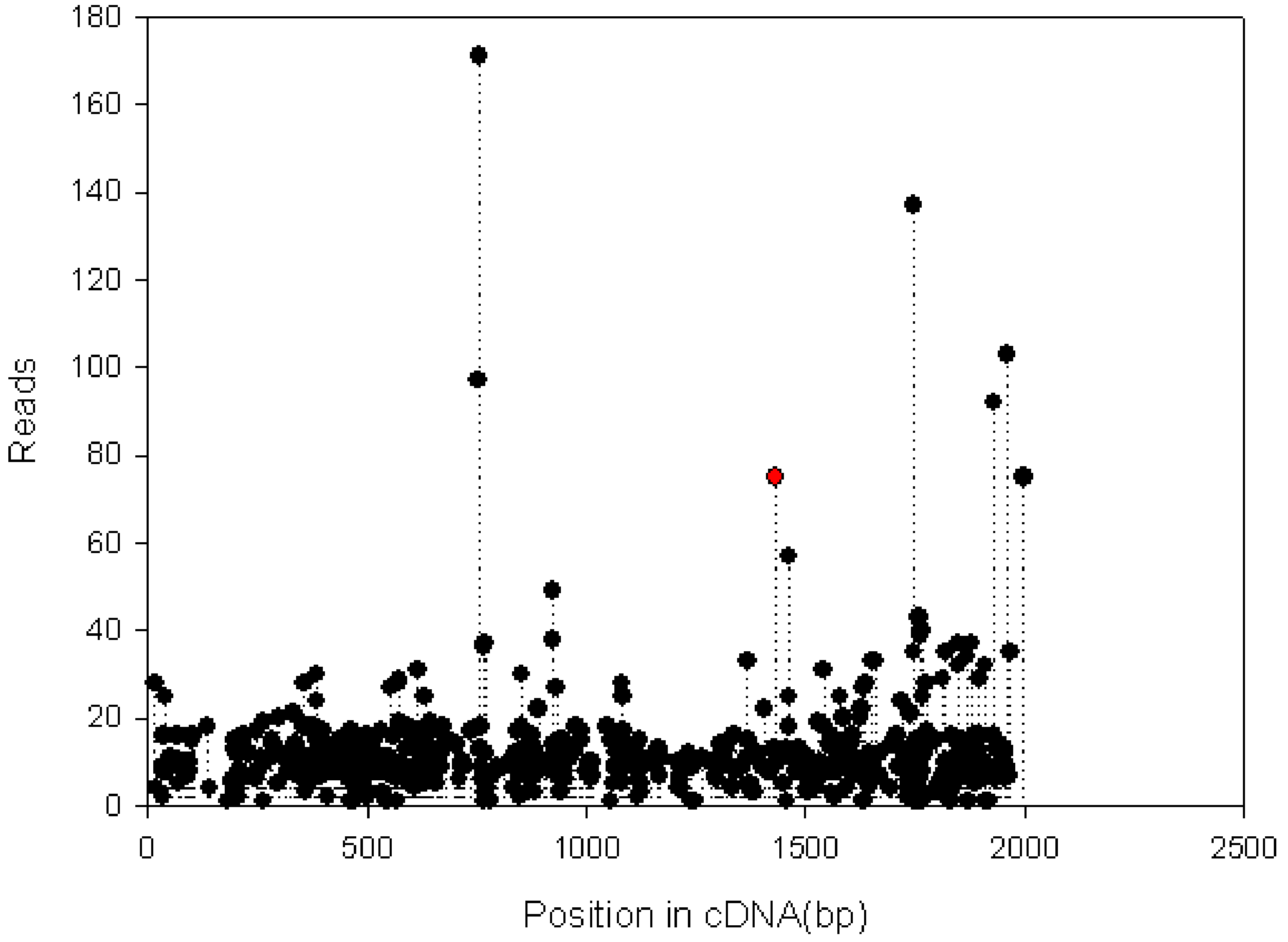


```
5' AUUCUUUCACAGGUUCCUUGAGACAC 3'        Cs2g01990.1
     :::..::::: :: ::::::
3' --AGAGGGUGUCGAAAGAACUC---- 5'        Csi-miR396b-3p.2
```

Csi-miR396d.1, target=Cs1g21350.1 gene=Cs1g21350
Category:1
Score=3.5
Cleavage Site=1164

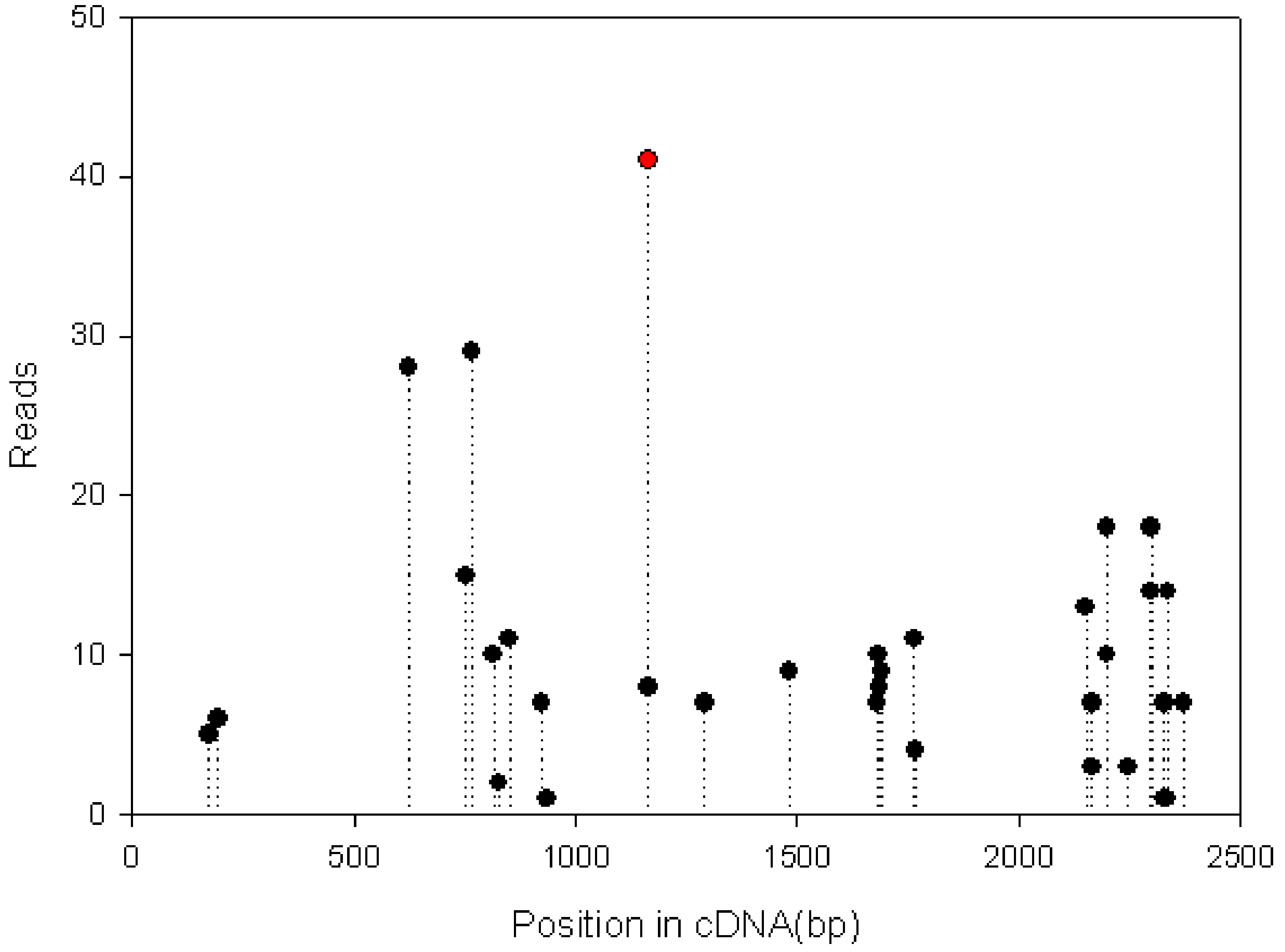


```
5' ACCGUUCAAGAAAGCCUGUGGAAGGC 3'        Cs1g21350.1
     ::::::::::::: :::::::.
3' --UCAAGUUCUUUCGG-CACCUUU-- 5'        Csi-miR396d.1
```

Csi-miR396d.1, target=Cs1g22520.1 gene=Cs1g22520
Category:1
Score=4
Cleavage Site=226

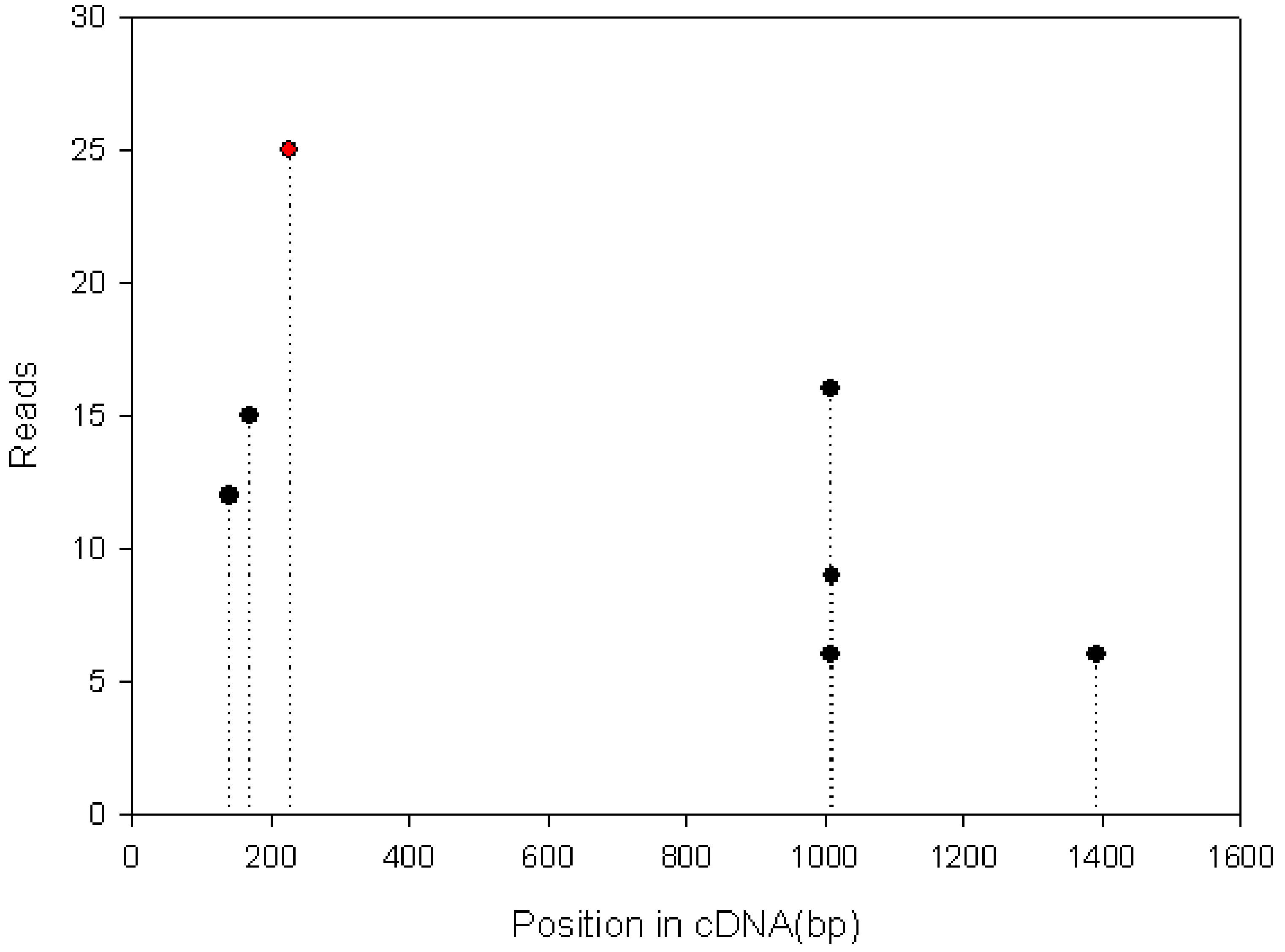


```
5' GAAGUUCAAGAAAUCUGUGGGAAGAA 3'        Cs1g22520.1
     ::::::::::: :.::::.::
3' --UCAAGUUCUUUCGGCACCUUU--- 5'        Csi-miR396d.1
```

Csi-miR396d.1, target=Cs3g23180.1 gene=Cs3g23180
Category:1
Score=4.5
Cleavage Site=625

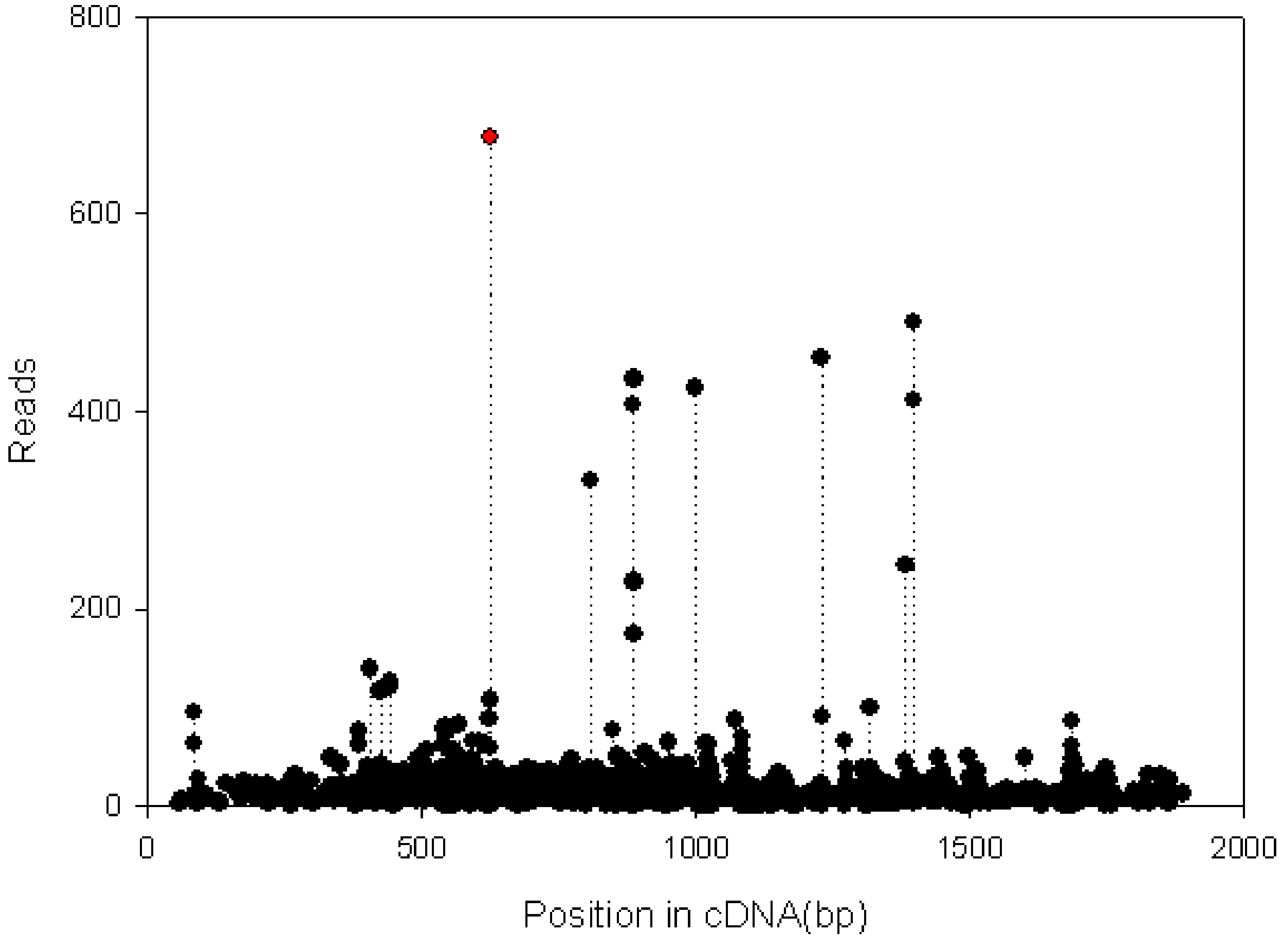


```
5' AAAGAUCAAGGAAGCUGUGGGAGUUG 3'        Cs3g23180.1
     :: :::::.::::.::::.:.
3' --UCAAGUUCUUUCGGCACCUUU--- 5'        Csi-miR396d.1
```

Csi-miR396d.1, target=Cs4g12200.1 gene=Cs4g12200
Category:3
Score=4.5
Cleavage Site=228

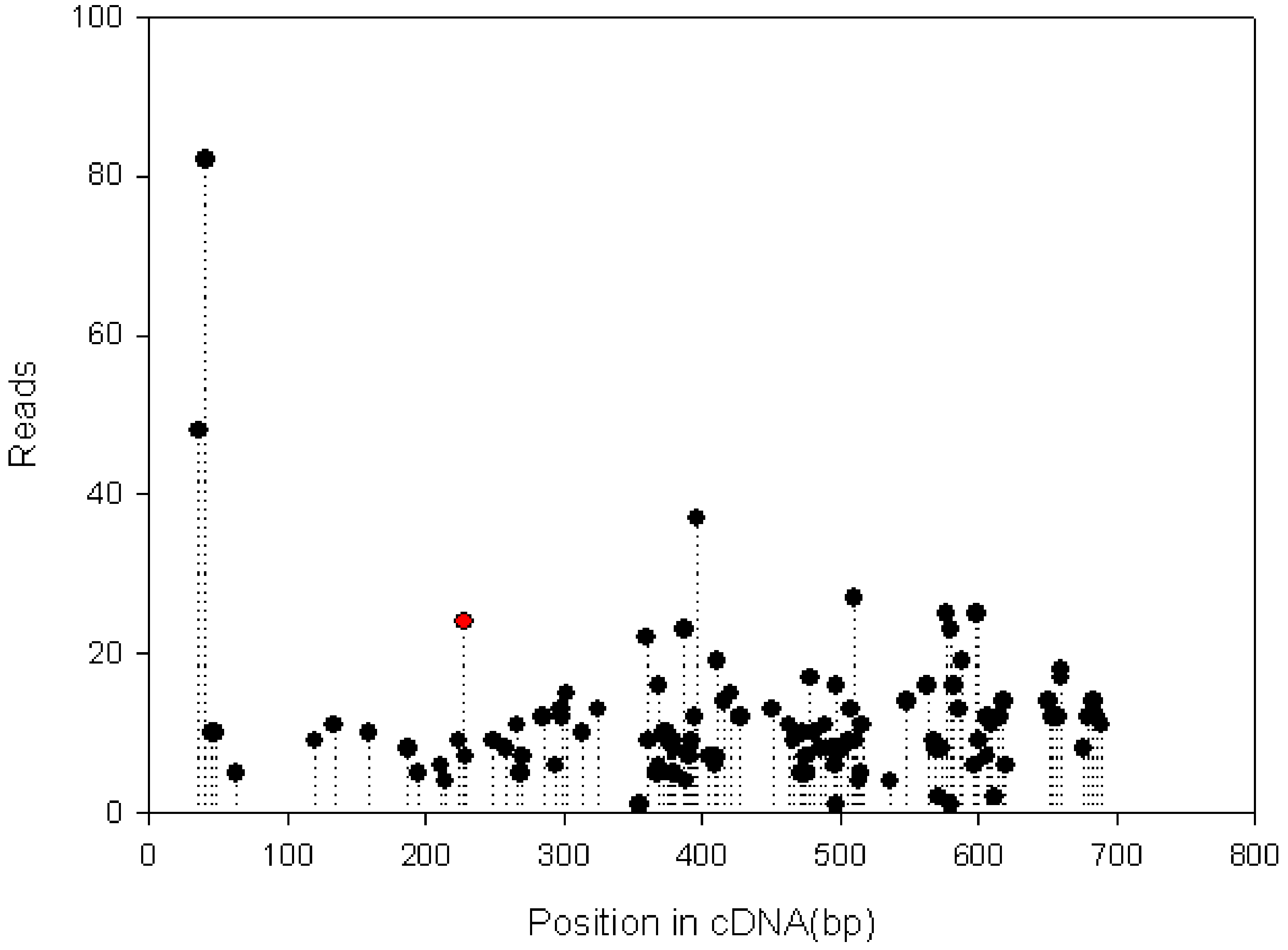


```
5' GGUUCCUGAAAGCCG-GGAAAUAUGCU 3'        Cs4g12200.1
   .::::  :::::::: :::::
3' UCAAGUUCUUUCGGCACCUUU------ 5'        Csi-miR396d.1
```

Csi-miR396d.1, target=Cs5g01380.1 gene=Cs5g01380
Category:1
Score=4
Cleavage Site=1181

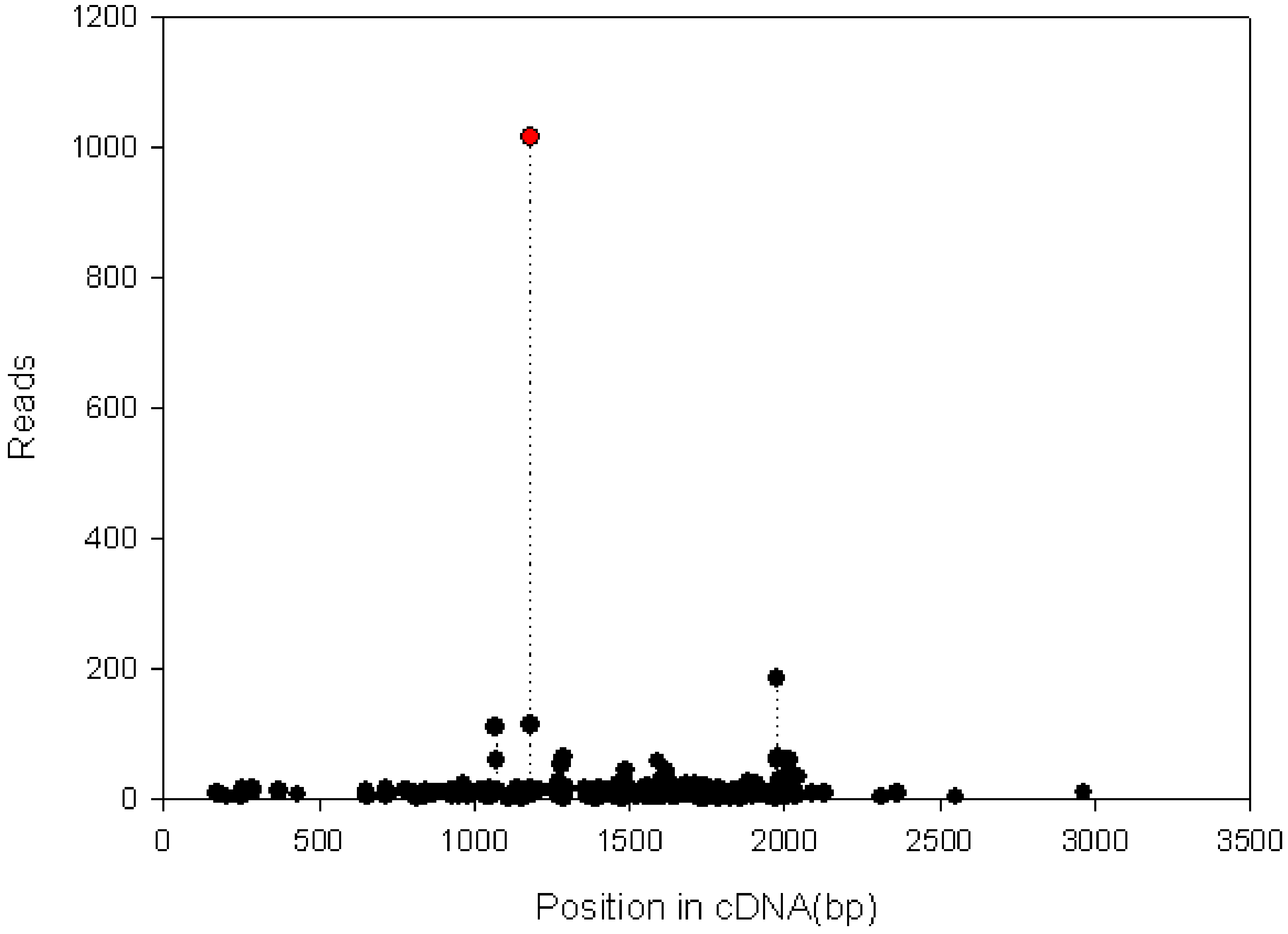


```
5' CCCGUUCAAGAAAGCCUGUGGAAUUA 3'        Cs5g01380.1
     ::::::::::::: ::::::
3' --UCAAGUUCUUUCGG-CACCUUU-- 5'        Csi-miR396d.1
```

Csi-miR396d.1, target=Cs5g09850.1 gene=Cs5g09850
Category:3
Score=4.5
Cleavage Site=767

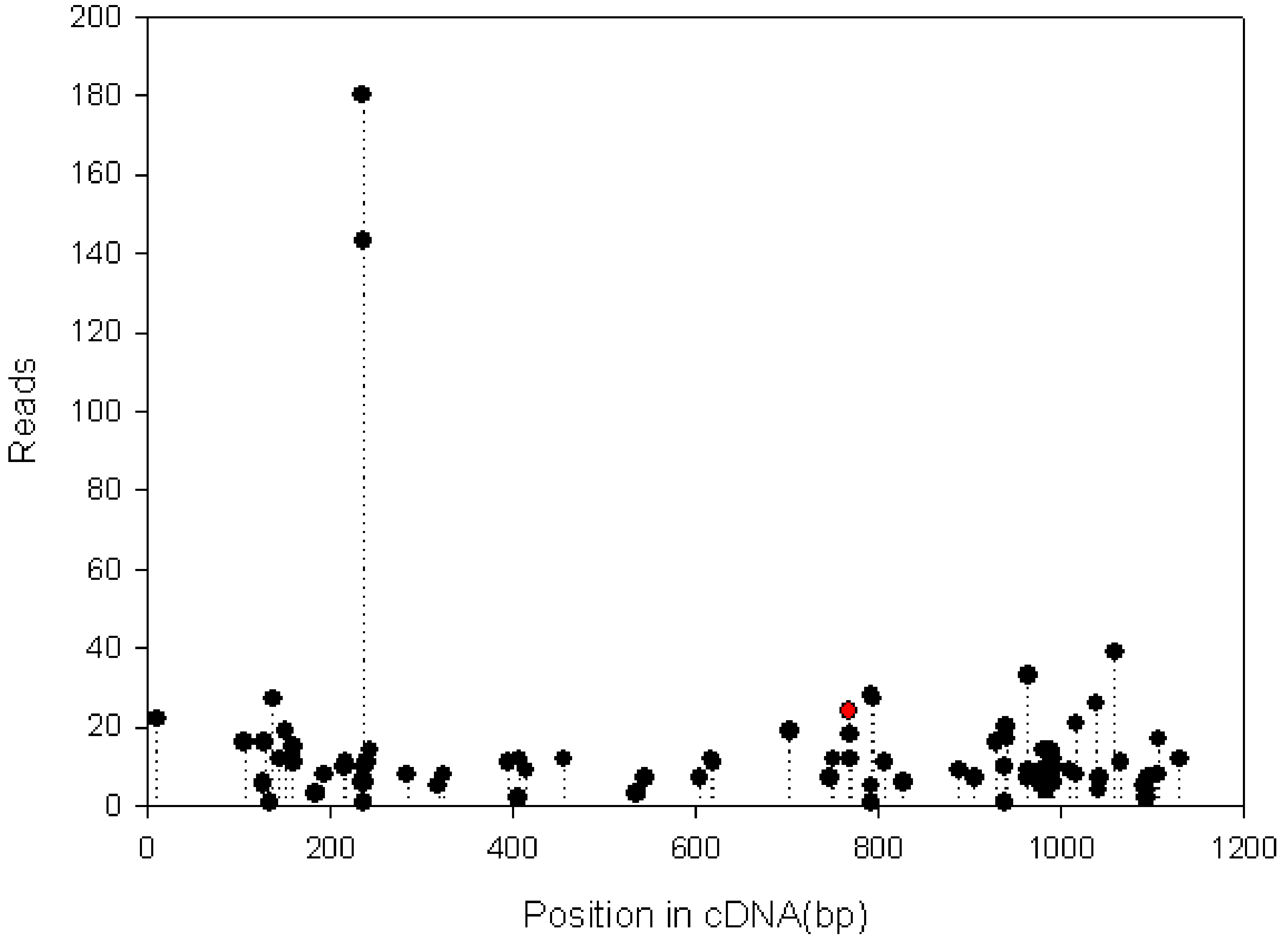


```
5' UGCGUUCAAGAAAGCUUGUGGAAGCU 3'        Cs5g09850.1
     ::::::::::::: .:::::::.
3' --UCAAGUUCUUUCG-GCACCUUU-- 5'        Csi-miR396d.1
```

Csi-miR396d.1, target=Cs7g15220.1 gene=Cs7g15220
Category:1
Score=4
Cleavage Site=377

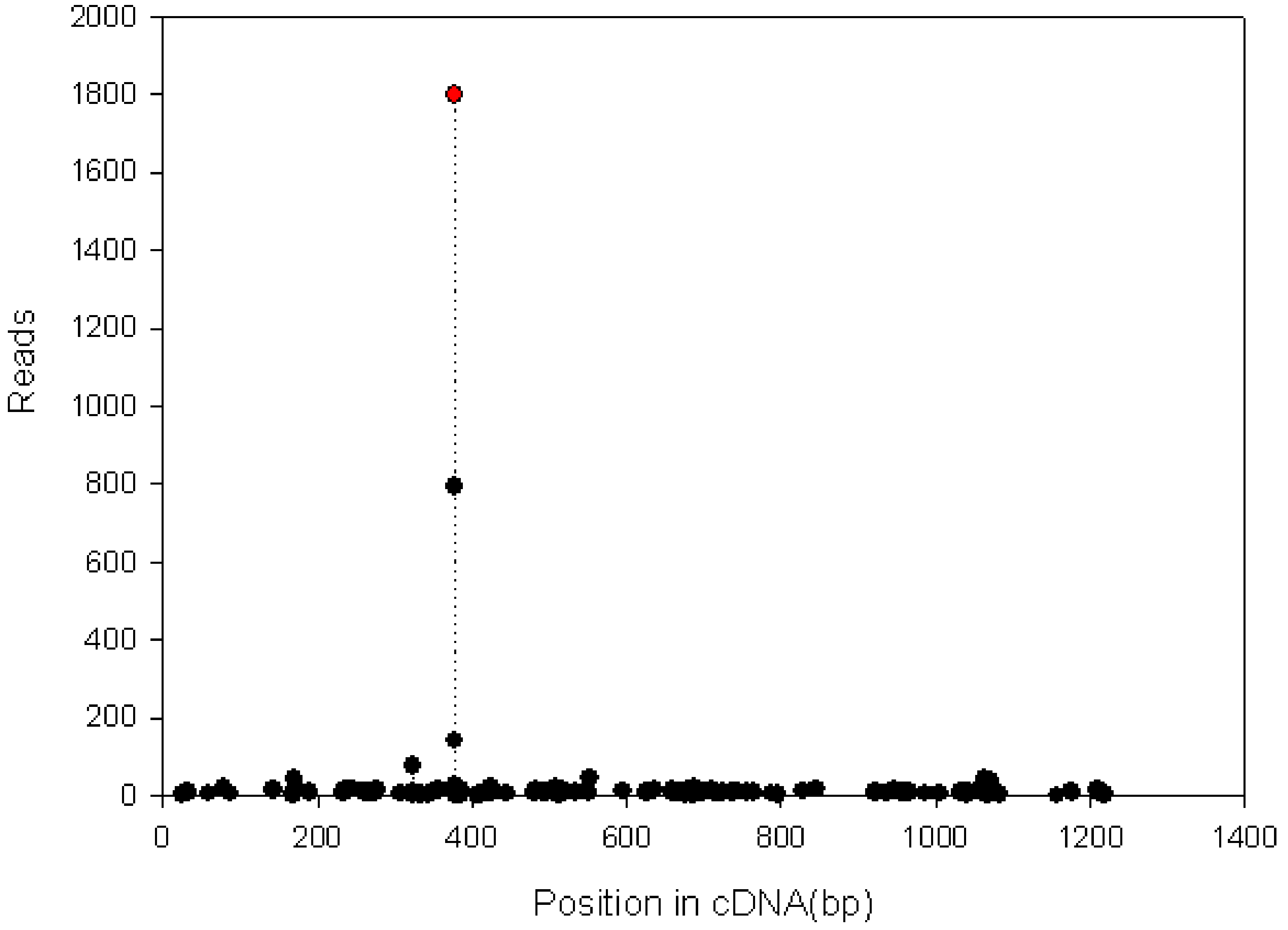


```
5' ACCGUUCAAGAAAGCCUGUGGAAUCA 3'        Cs7g15220.1
     ::::::::::::: ::::::
3' --UCAAGUUCUUUCGG-CACCUUU-- 5'        Csi-miR396d.1
```

Csi-miR396d.1, target=Cs8g17370.1 gene=Cs8g17370
Category:3
Score=4
Cleavage Site=140

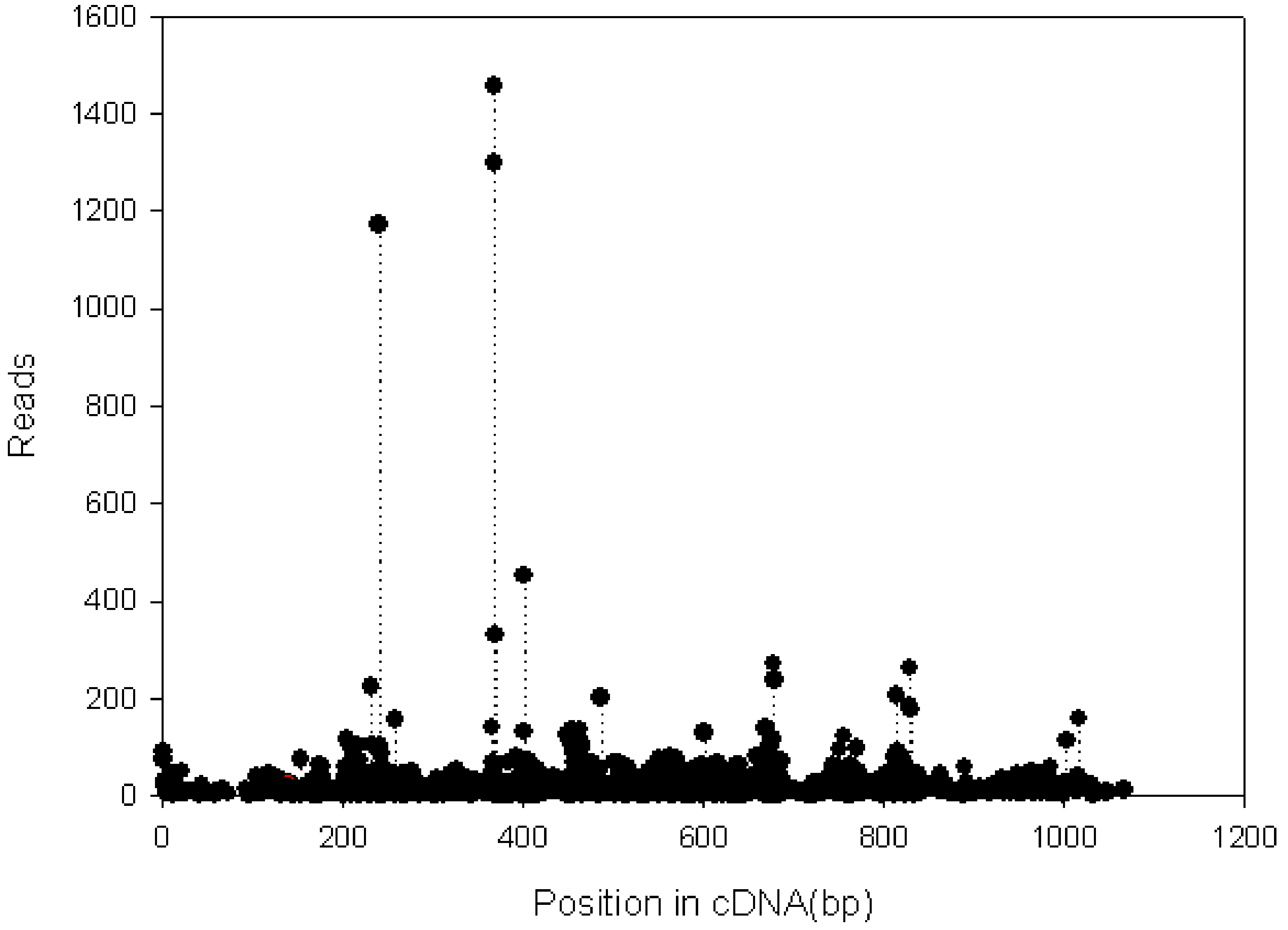


```
5' AGGAUUACAAGAAGGCCGUGGAGAAA 3'        Cs8g17370.1
     :  :  :::::::.::::::::.:
3' ---UCAAGUUCUUUCGGCACCUUU-- 5'        Csi-miR396d.1
```

Csi-miR396d.1, target=Orange1.1t00172.1 gene=Orange1.1t00172
Category:1
Score=4
Cleavage Site=656

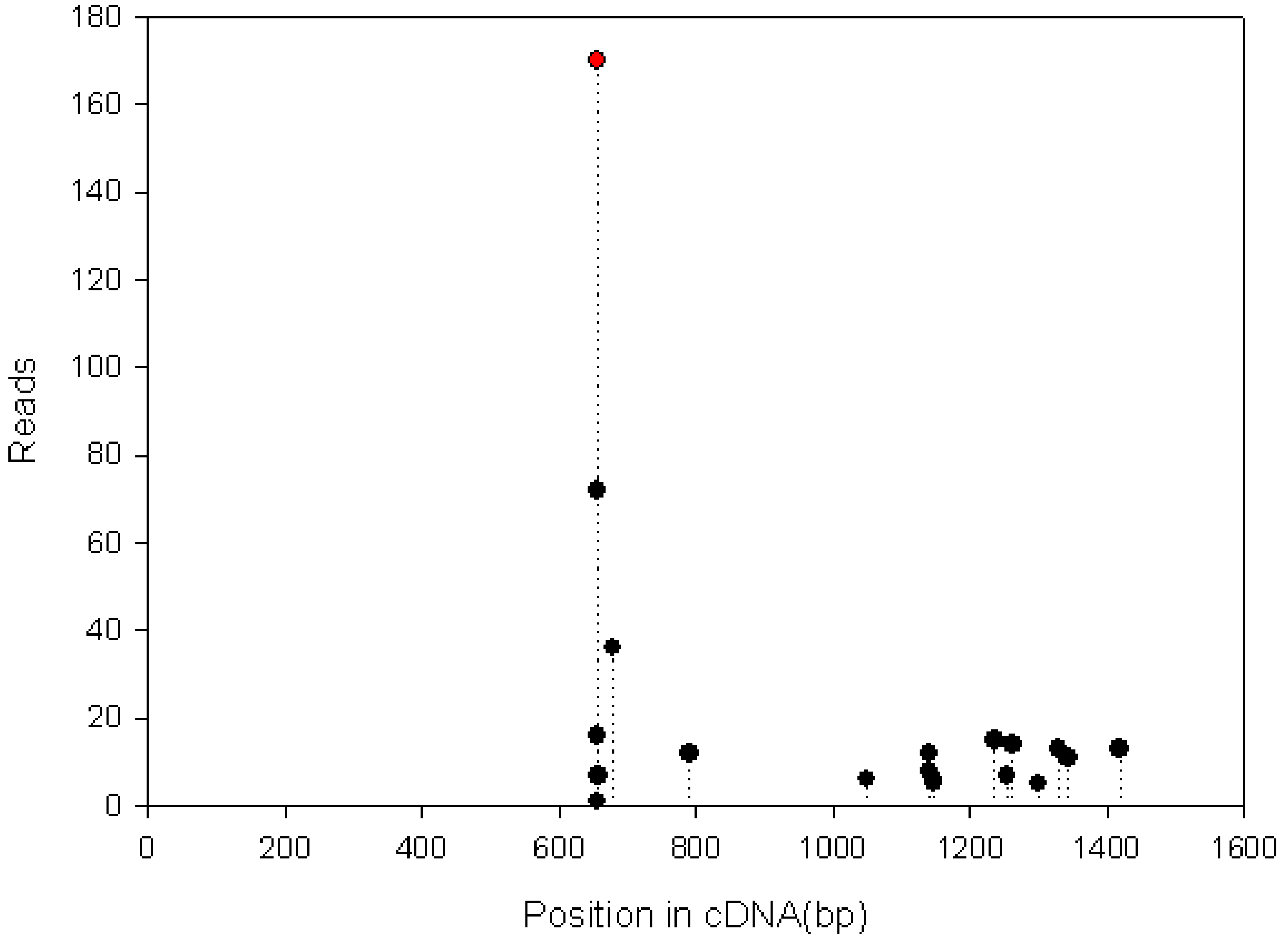


```
5' ACCGUUCAAGAAAGCCUGUGGAACUU 3'        Orange1.1t00172.1
     ::::::::::::: ::::::
3' --UCAAGUUCUUUCGG-CACCUUU-- 5'        Csi-miR396d.1
```

Csi-miR396d.1, target=Orange1.1t02555.1 gene=Orange1.1t02555
Category:1
Score=4
Cleavage Site=920

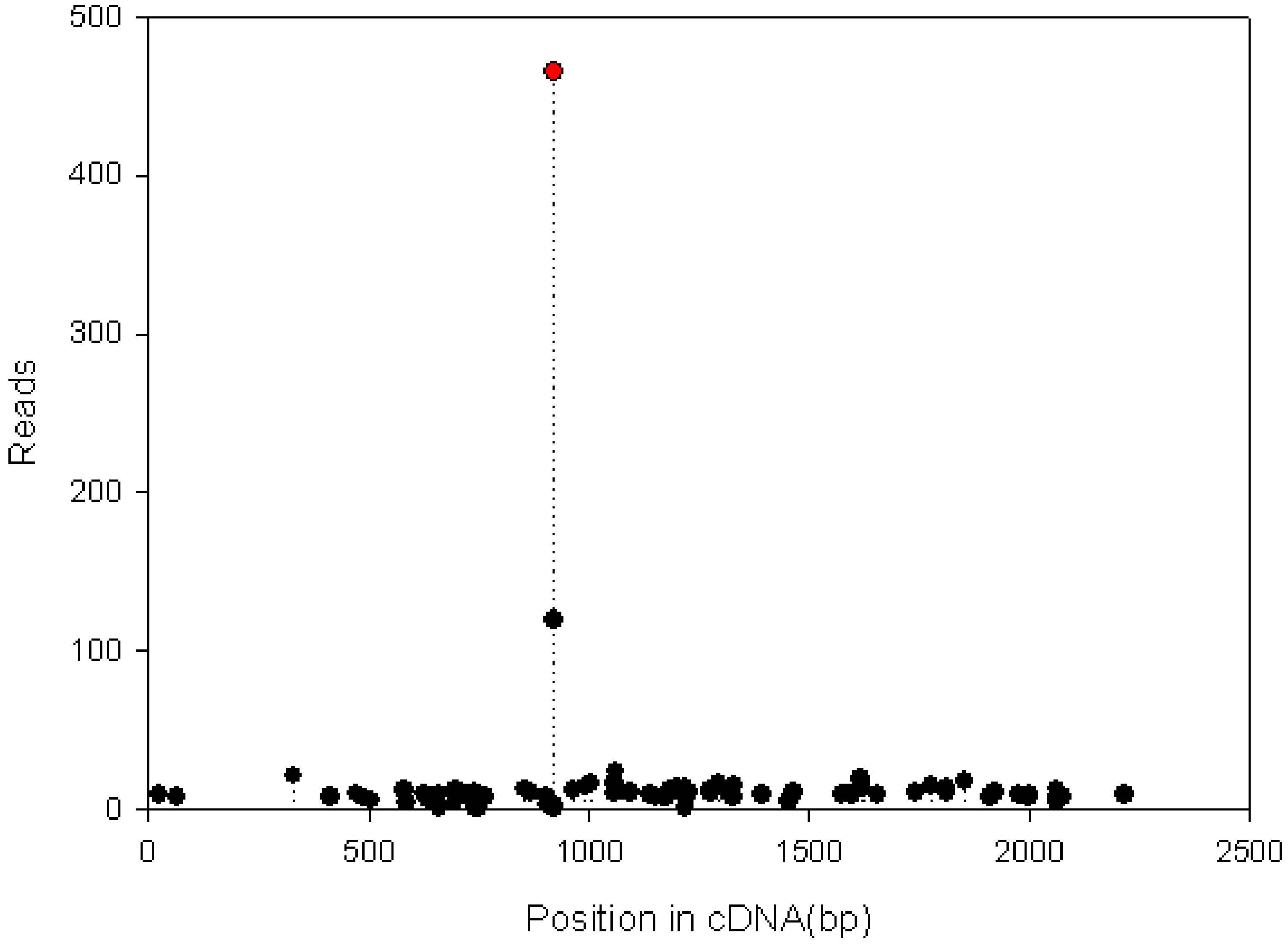


```
5' CACGUUCAAGAAAGCCUGUGGAACUU 3'        Orange1.1t02555.1
     ::::::::::::: ::::::
3' --UCAAGUUCUUUCGG-CACCUUU-- 5'        Csi-miR396d.1
```

Csi-miR396d.1, target=Orange1.1t03122.1 gene=Orange1.1t03122
Category:1
Score=4
Cleavage Site=583

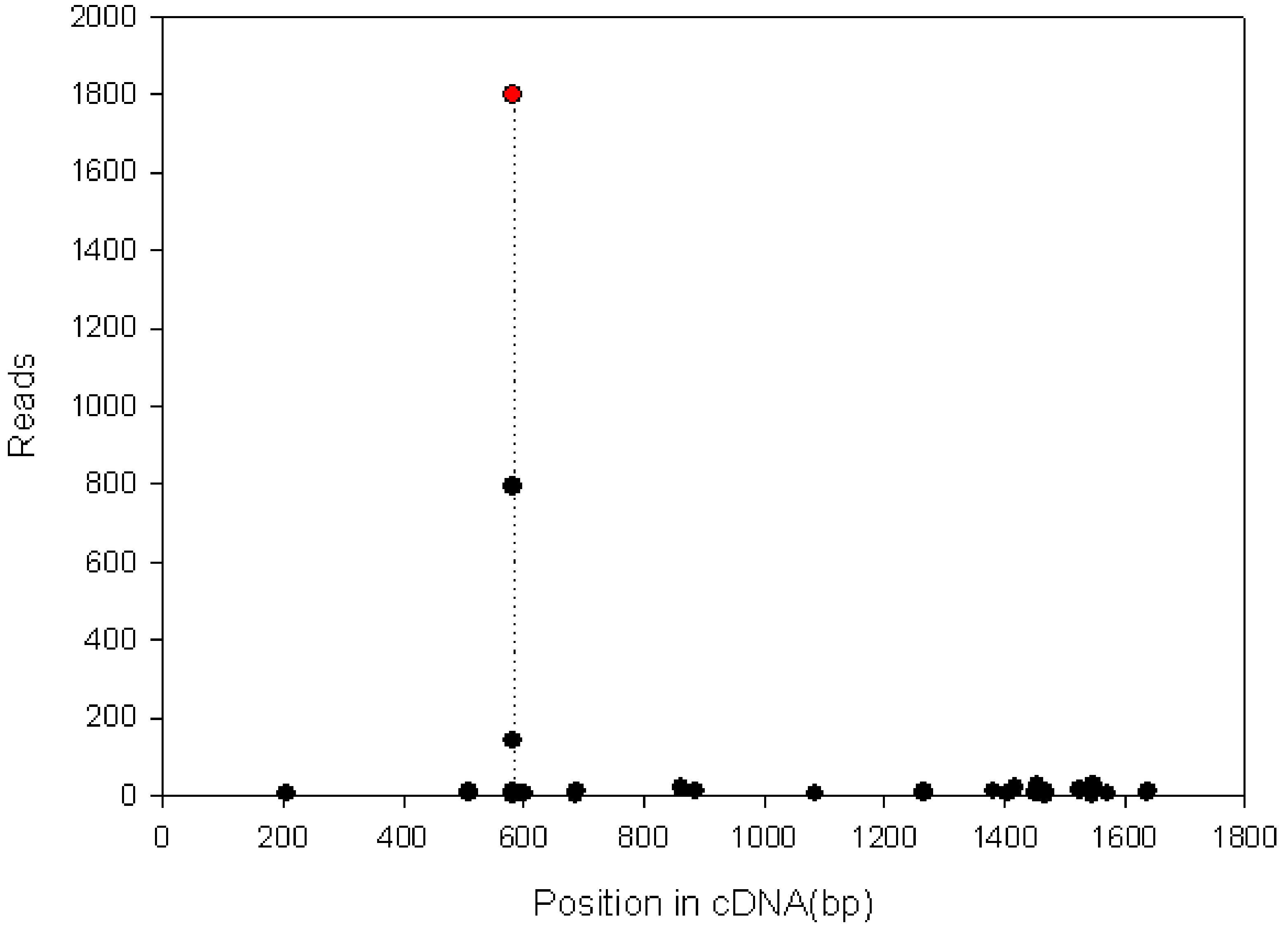


```
5' ACCGUUCAAGAAAGCCUGUGGAAUCA 3'        Orange1.1t03122.1
     ::::::::::::: ::::::
3' --UCAAGUUCUUUCGG-CACCUUU-- 5'        Csi-miR396d.1
```

Csi-miR396d.2, target=Cs1g21350.1 gene=Cs1g21350
Category:1
Score=4
Cleavage Site=1164

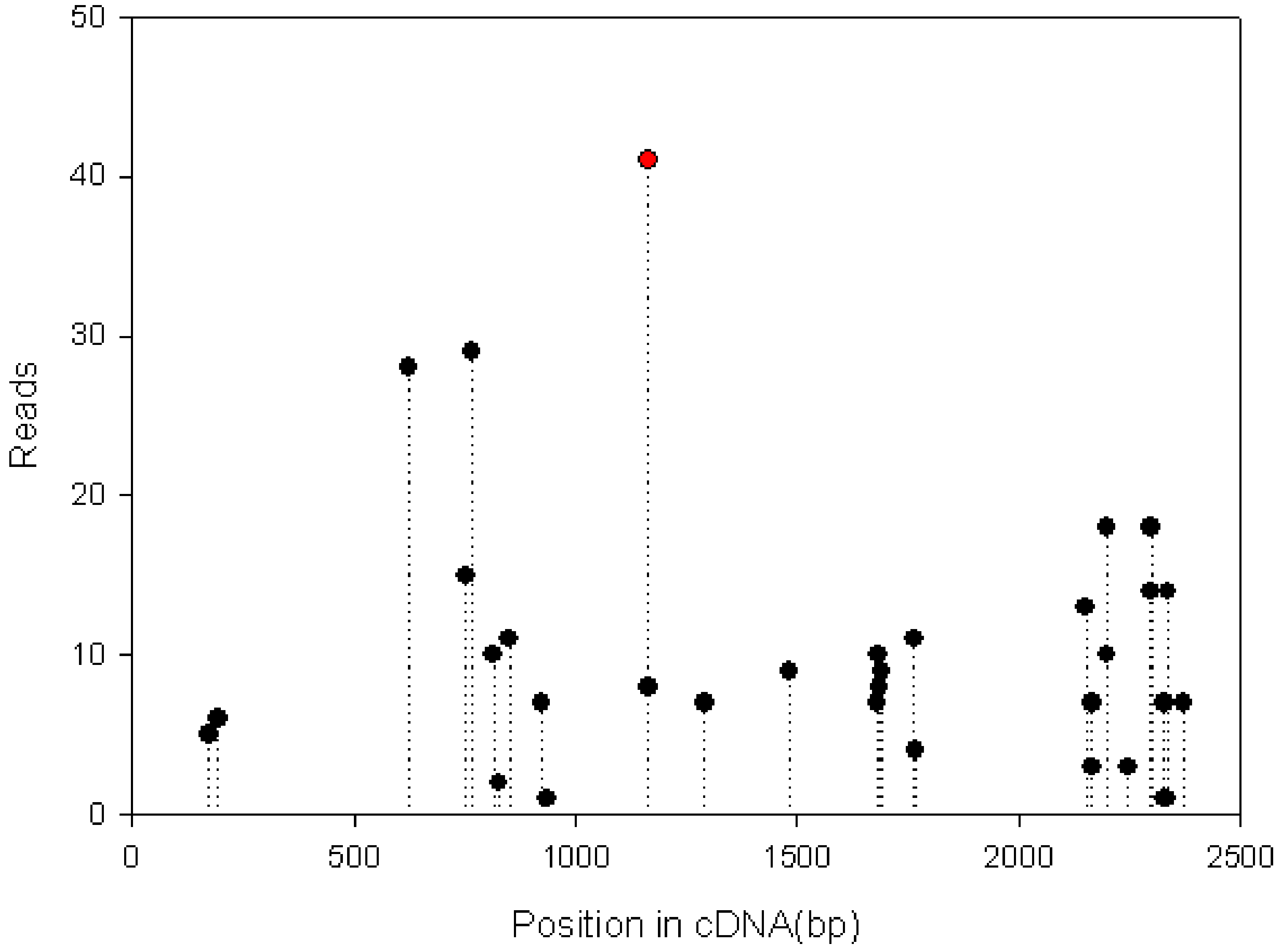


```
5' ACCGUUCAAGAAAGCCUGUGGAAGGC 3'        Cs1g21350.1
      ::::::::::::: ::::::
3' -UUCAAGUUCUUUCGG-CACCUU--- 5'        Csi-miR396d.2
```

Csi-miR396d.2, target=Cs1g22520.1 gene=Cs1g22520
Category:1
Score=4
Cleavage Site=226

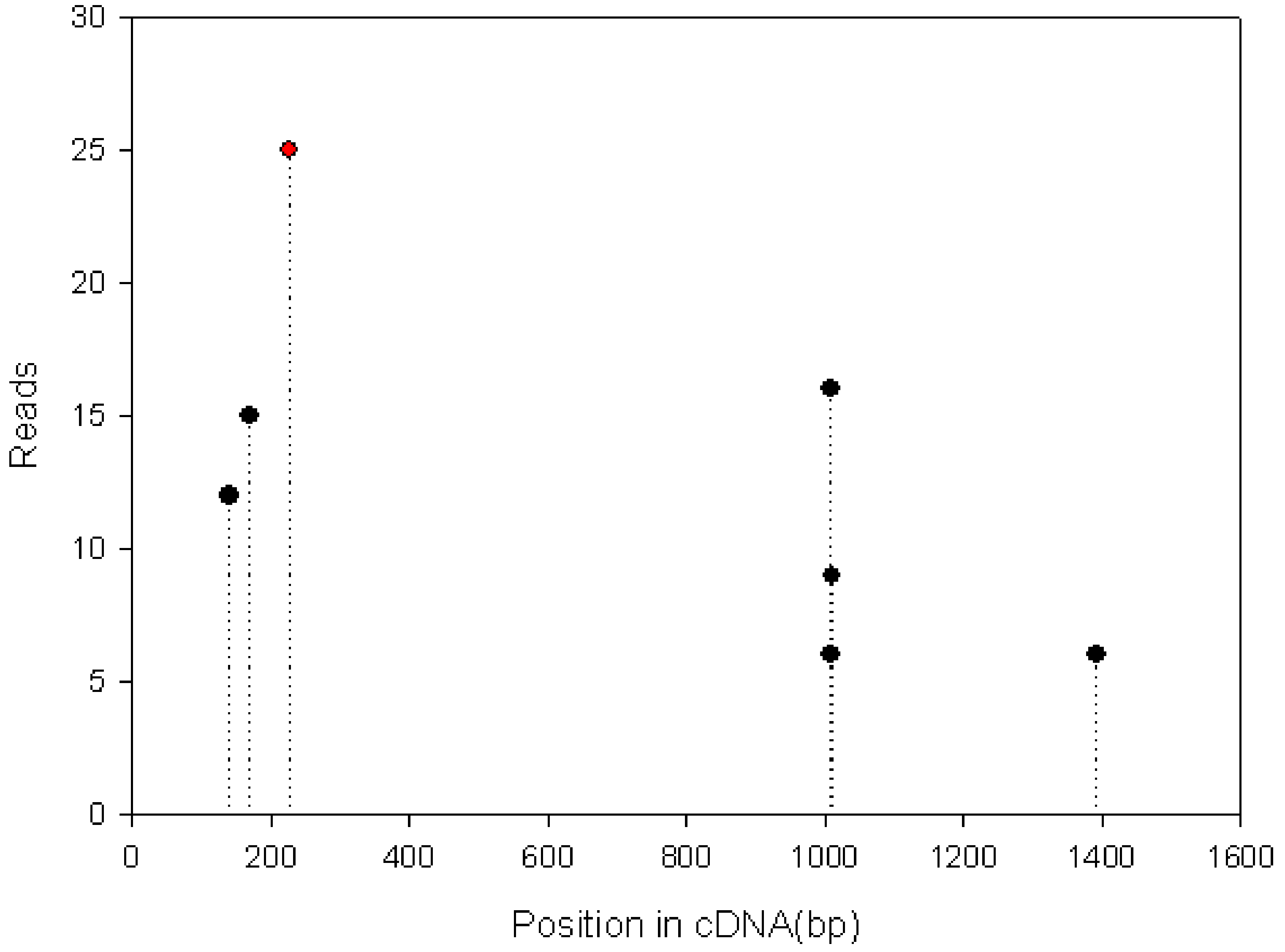


```
5' GAAGUUCAAGAAAUCUGUGGGAAGAA 3'        Cs1g22520.1
     :::::::::::: :.::::.:
3' -UUCAAGUUCUUUCGGCACCUU---- 5'        Csi-miR396d.2
```

Csi-miR396d.2, target=Cs2g09620.1 gene=Cs2g09620
Category:3
Score=5
Cleavage Site=3513

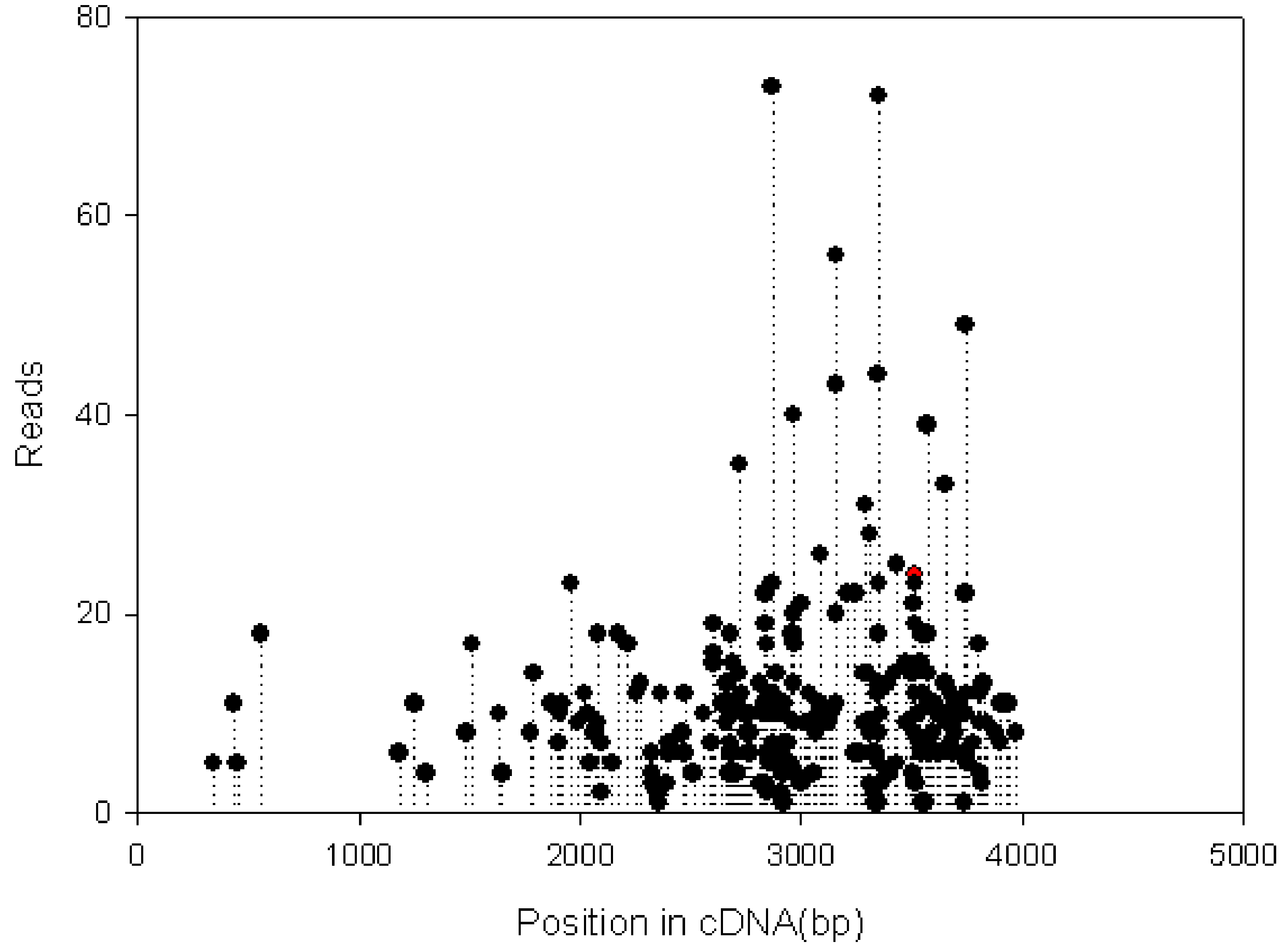


```
5' AAGUAC-UGAAAUCCGUGGAACUUUUG 3'        Cs2g09620.1
   :::: :  :::: ::::::::
3' UUCAAGUUCUUUCGGCACCUU------ 5'        Csi-miR396d.2
```

Csi-miR396d.2, target=Cs3g23180.1 gene=Cs3g23180
Category:1
Score=4
Cleavage Site=625

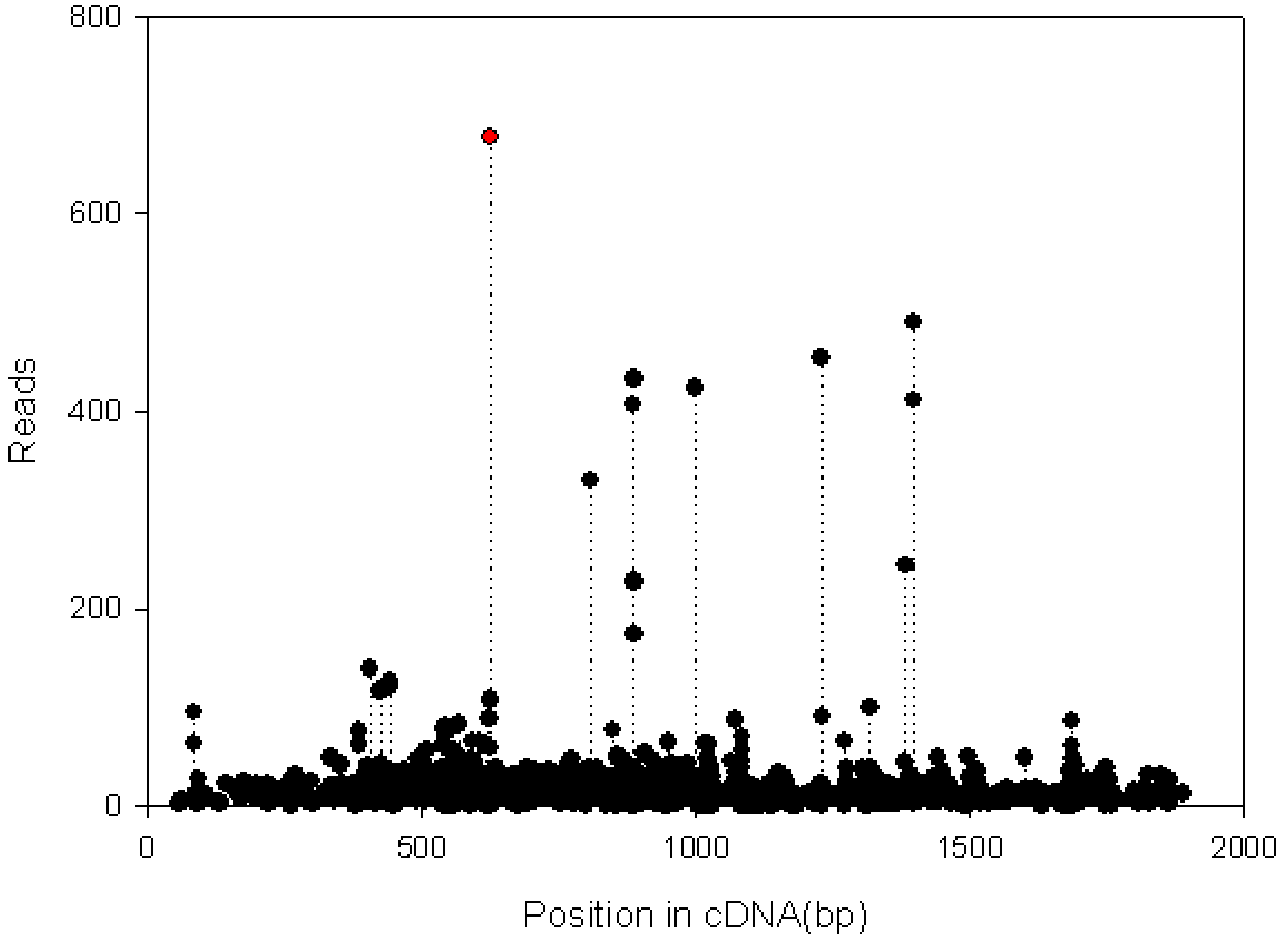


```
5' AAAGAUCAAGGAAGCUGUGGGAGUUG 3'        Cs3g23180.1
    ::: :::::.::::.::::.:
3' -UUCAAGUUCUUUCGGCACCUU---- 5'        Csi-miR396d.2
```

Csi-miR396d.2, target=Cs5g01380.1 gene=Cs5g01380
Category:1
Score=4
Cleavage Site=1181

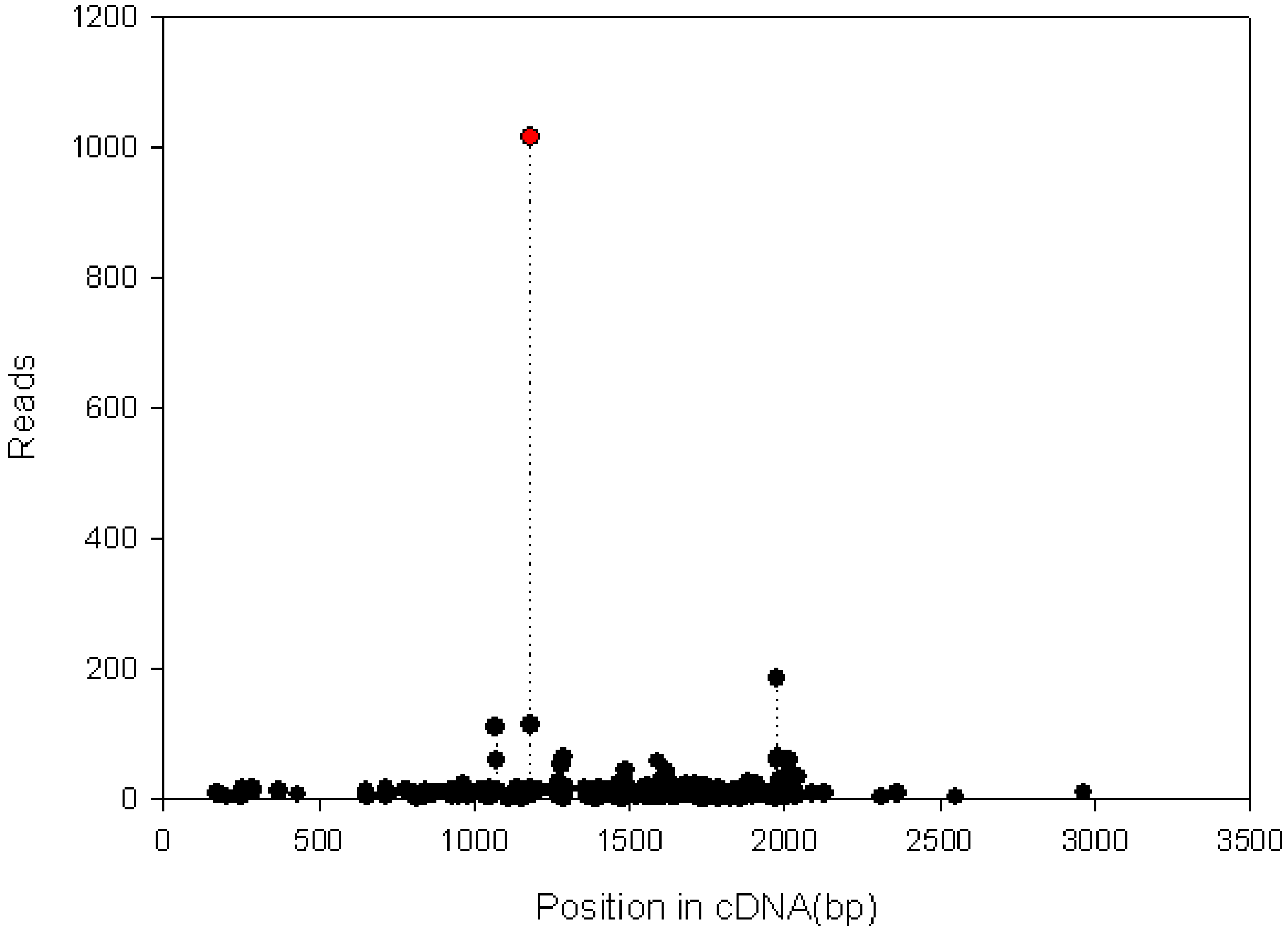


```
5' CCCGUUCAAGAAAGCCUGUGGAAUUA 3'        Cs5g01380.1
     ::::::::::::: ::::::
3' -UUCAAGUUCUUUCGG-CACCUU--- 5'        Csi-miR396d.2
```

Csi-miR396d.2, target=Cs5g09850.1 gene=Cs5g09850
Category:3
Score=4.5
Cleavage Site=767

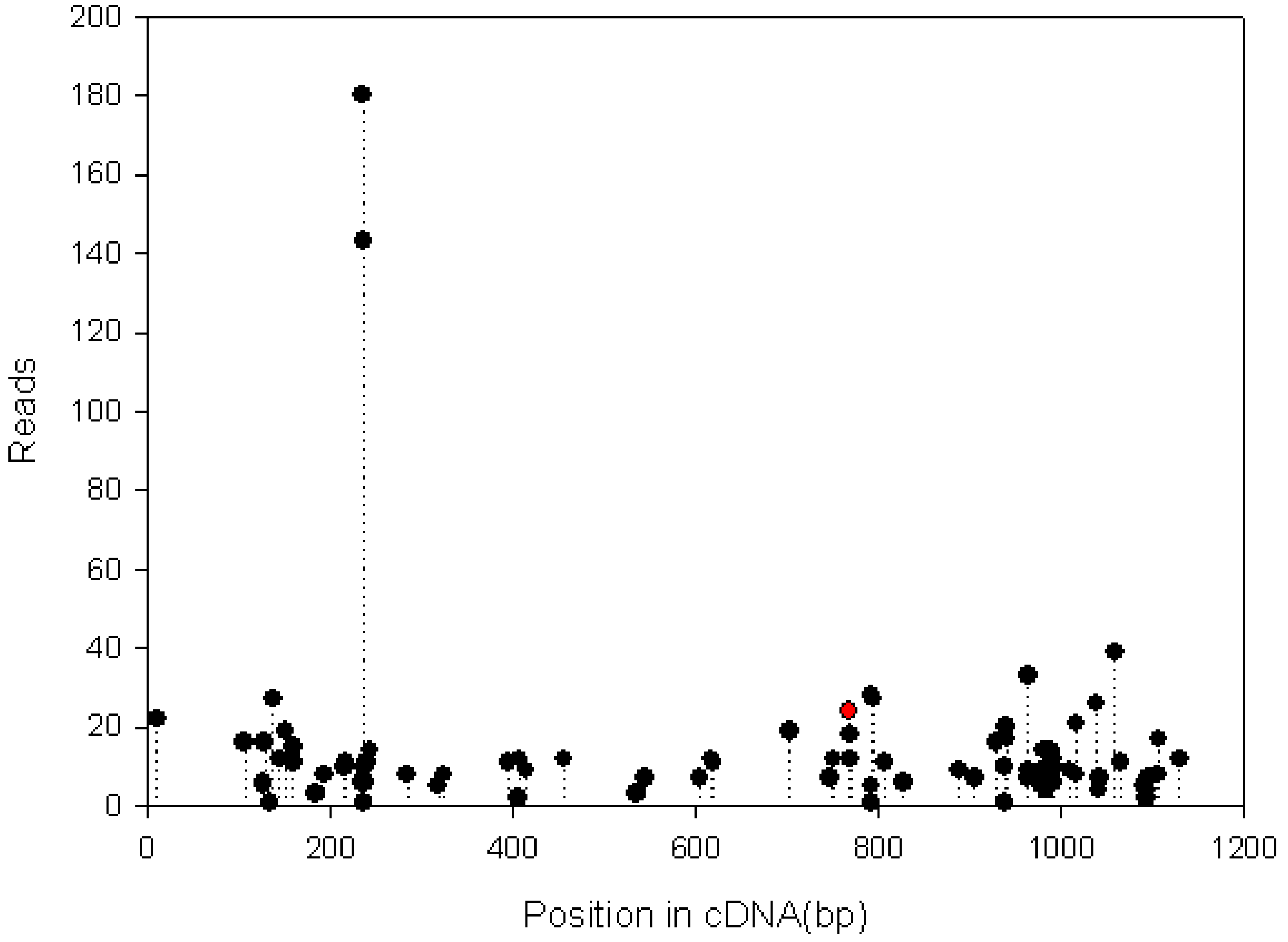


```
5' UGCGUUCAAGAAAGCUUGUGGAAGCU 3'        Cs5g09850.1
    . :::::::::::: .::::::
3' -UUCAAGUUCUUUCG-GCACCUU--- 5'        Csi-miR396d.2
```

Csi-miR396d.2, target=Cs7g15220.1 gene=Cs7g15220
Category:1
Score=4
Cleavage Site=377

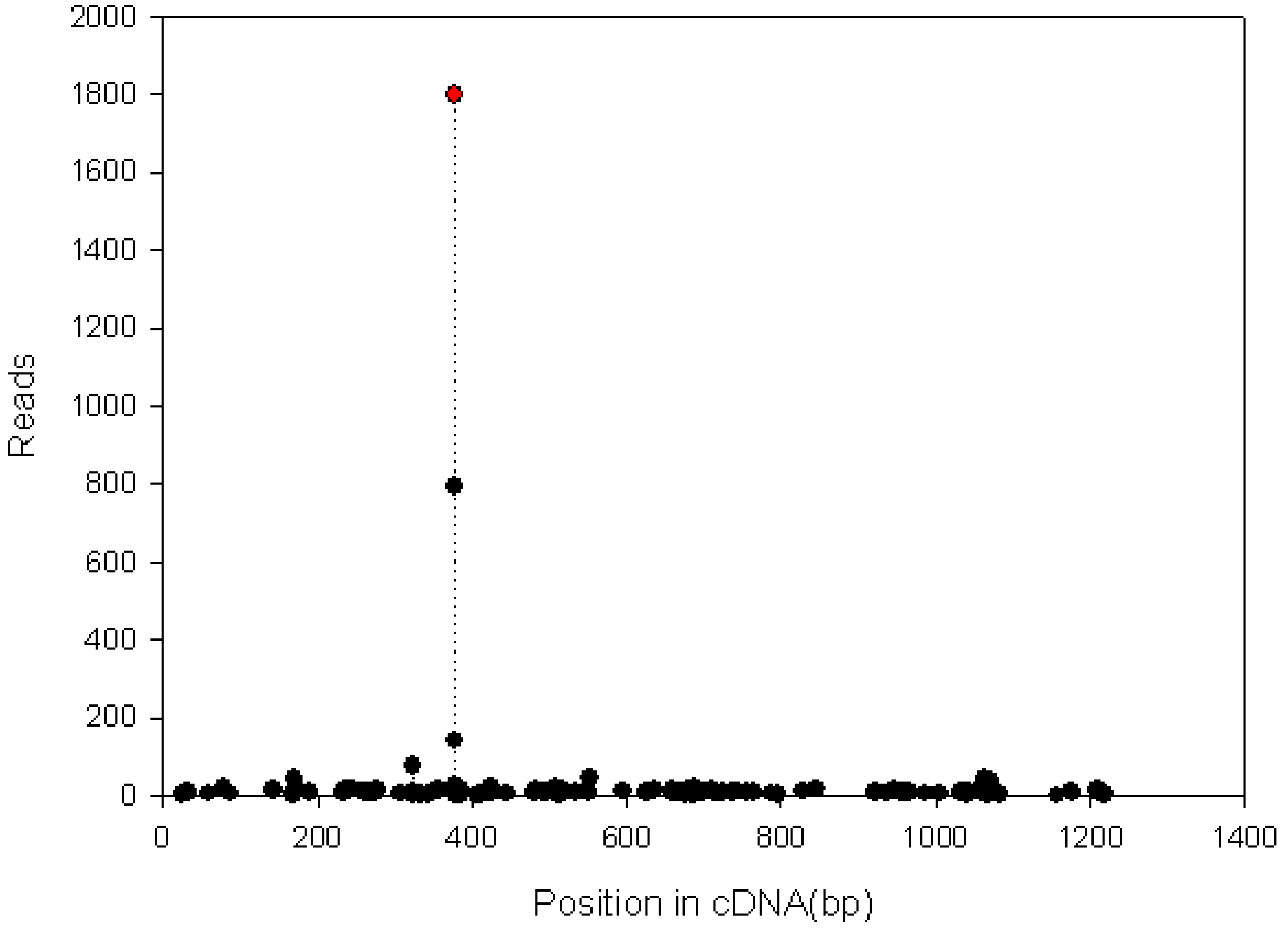


```
5' ACCGUUCAAGAAAGCCUGUGGAAUCA 3'        Cs7g15220.1
      ::::::::::::: ::::::
3' -UUCAAGUUCUUUCGG-CACCUU--- 5'        Csi-miR396d.2
```

Csi-miR396d.2, target=Cs8g17370.1 gene=Cs8g17370
Category:3
Score=4
Cleavage Site=140

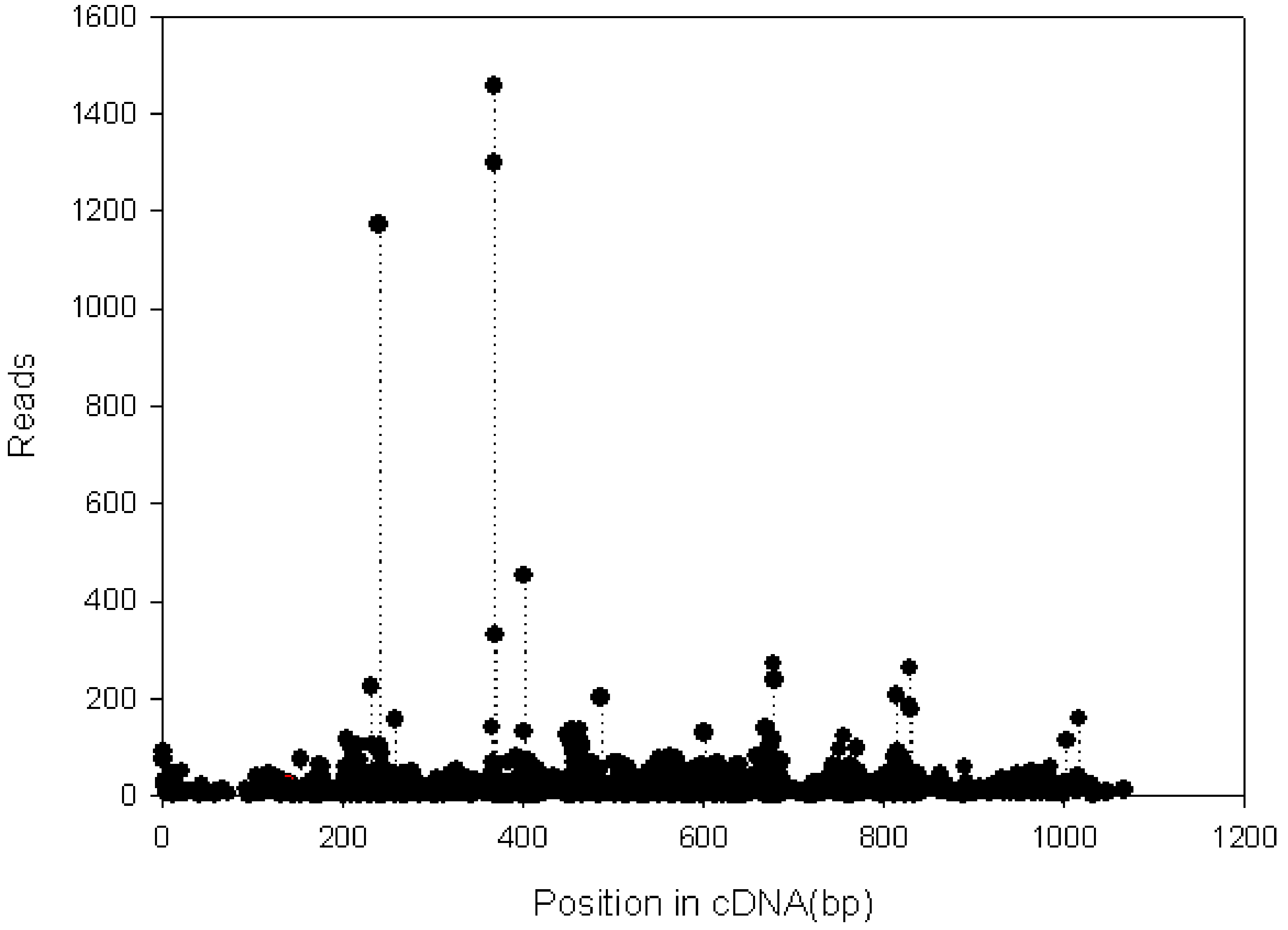


```
5' AGGAUUACAAGAAGGCCGUGGAGAAA 3'        Cs8g17370.1
     .: : ::::::.:::::::::.
3' --UUCAAGUUCUUUCGGCACCUU--- 5'        Csi-miR396d.2
```

Csi-miR396d.2, target=Orange1.1t00172.1 gene=Orange1.1t00172
Category:1
Score=4
Cleavage Site=656

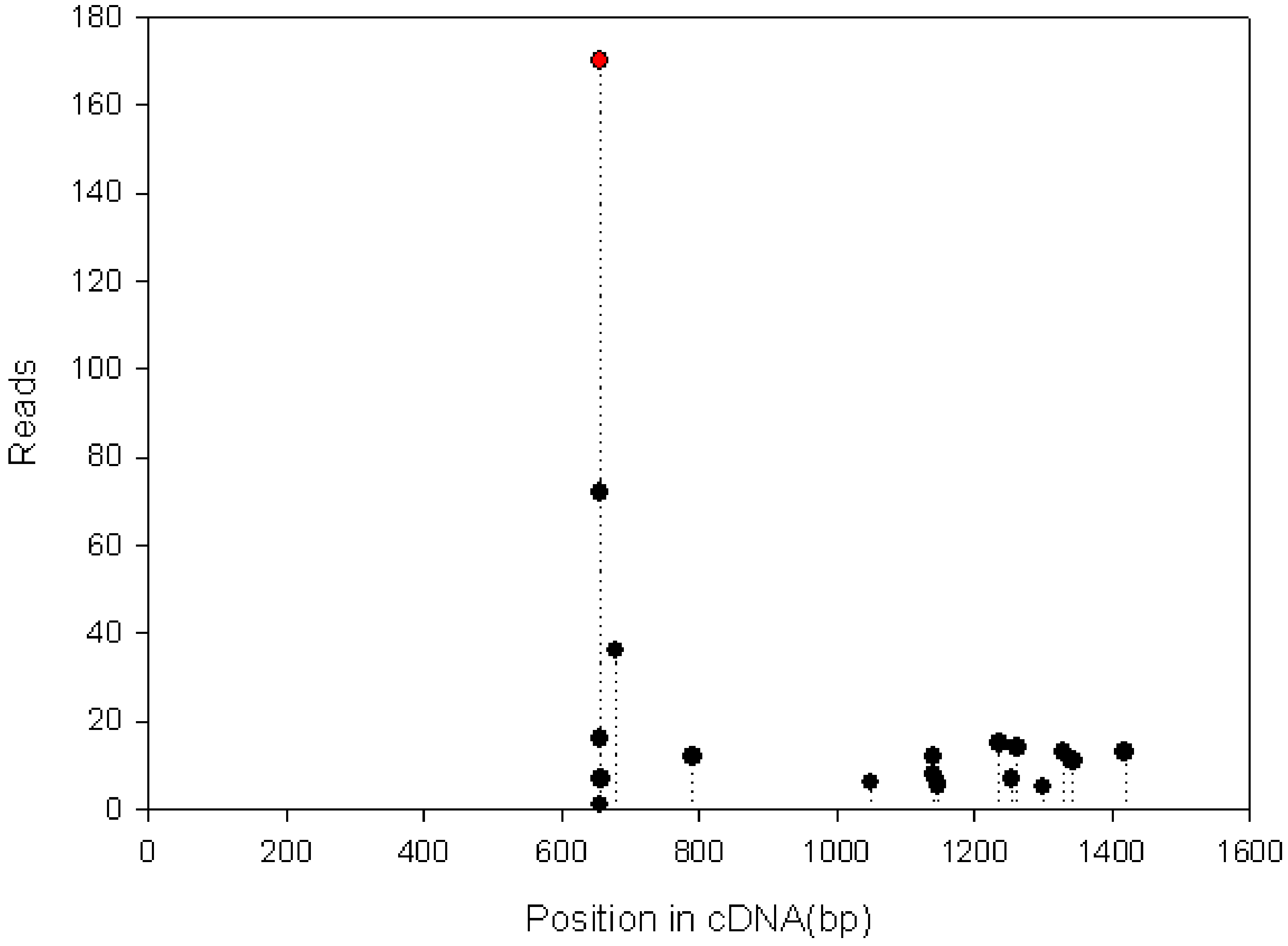


```
5' ACCGUUCAAGAAAGCCUGUGGAACUU 3'        Orange1.1t00172.1
      ::::::::::::: ::::::
3' -UUCAAGUUCUUUCGG-CACCUU--- 5'        Csi-miR396d.2
```

Csi-miR396d.2, target=Orange1.1t02555.1 gene=Orange1.1t02555
Category:1
Score=3
Cleavage Site=920

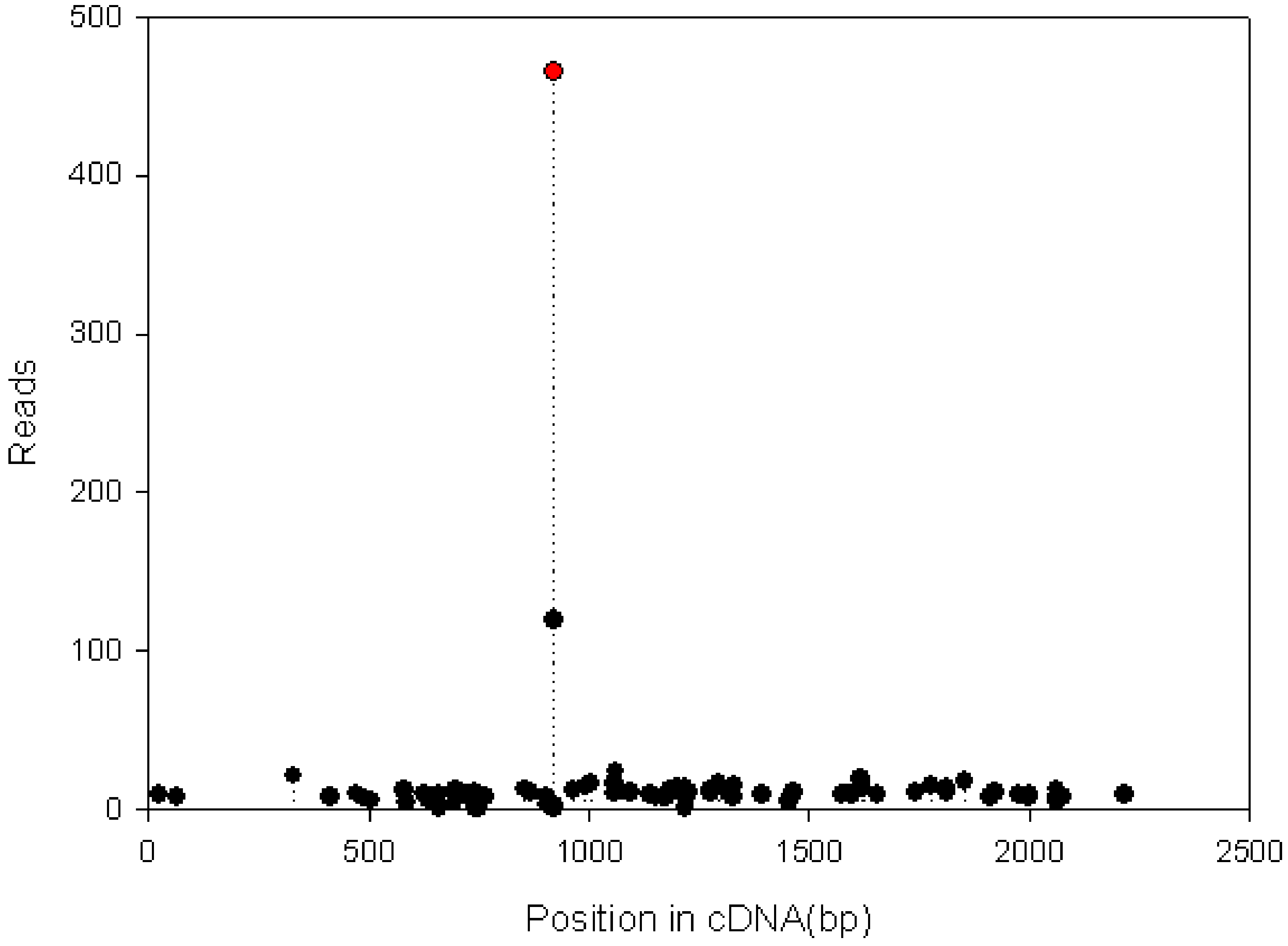


```
5' CACGUUCAAGAAAGCCUGUGGAACUU 3'        Orange1.1t02555.1
    : ::::::::::::: ::::::
3' -UUCAAGUUCUUUCGG-CACCUU--- 5'        Csi-miR396d.2
```

Csi-miR396d.2, target=Orange1.1t03122.1 gene=Orange1.1t03122
Category:1
Score=4
Cleavage Site=583

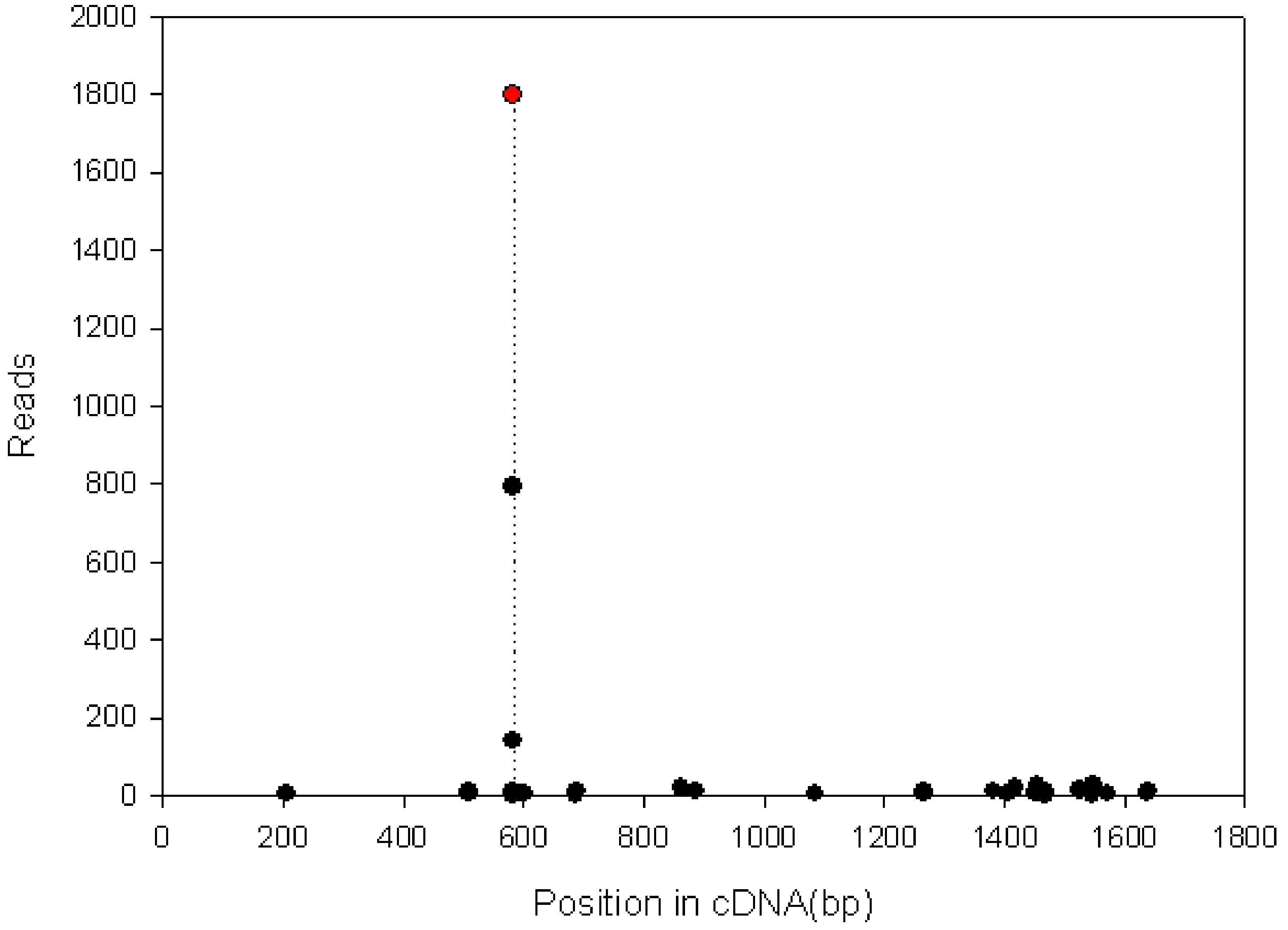


```
5' ACCGUUCAAGAAAGCCUGUGGAAUCA 3'        Orange1.1t03122.1
     ::::::::::::: ::::::
3' -UUCAAGUUCUUUCGG-CACCUU--- 5'        Csi-miR396d.2
```

Csi-miR396d.3, target=Cs1g15720.1 gene=Cs1g15720
Category:3
Score=5
Cleavage Site=1610

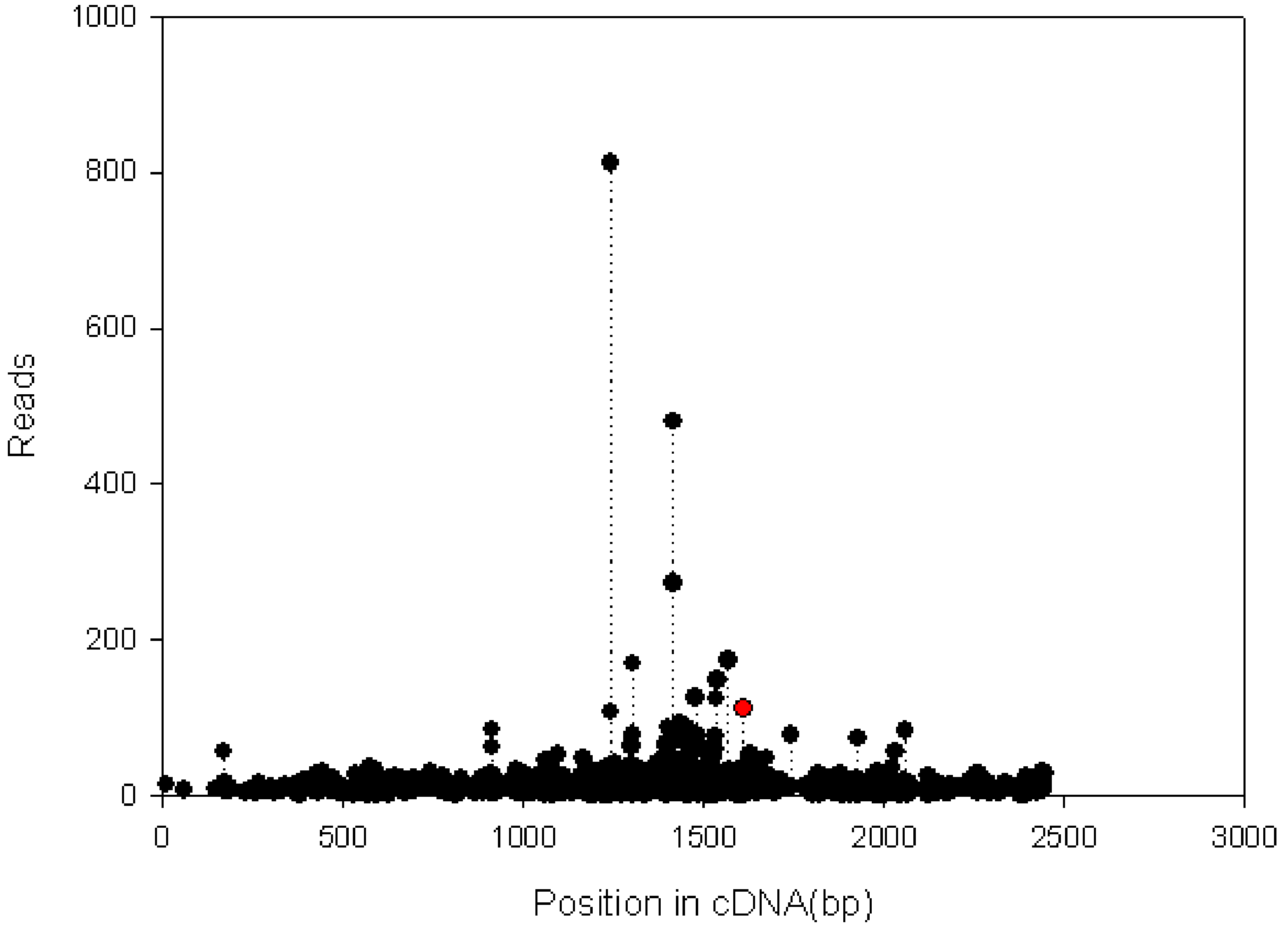


```
5' CAACAUUUCAA-AAAGCCGUGGGCUCA 3'        Cs1g15720.1
          ::::: ::::::::::.
3' -----CAAGUUCUUUCGGCACCUU--- 5'        Csi-miR396d.3
```

Csi-miR396d.3, target=Cs1g21350.1 gene=Cs1g21350
Category:1
Score=2
Cleavage Site=1164

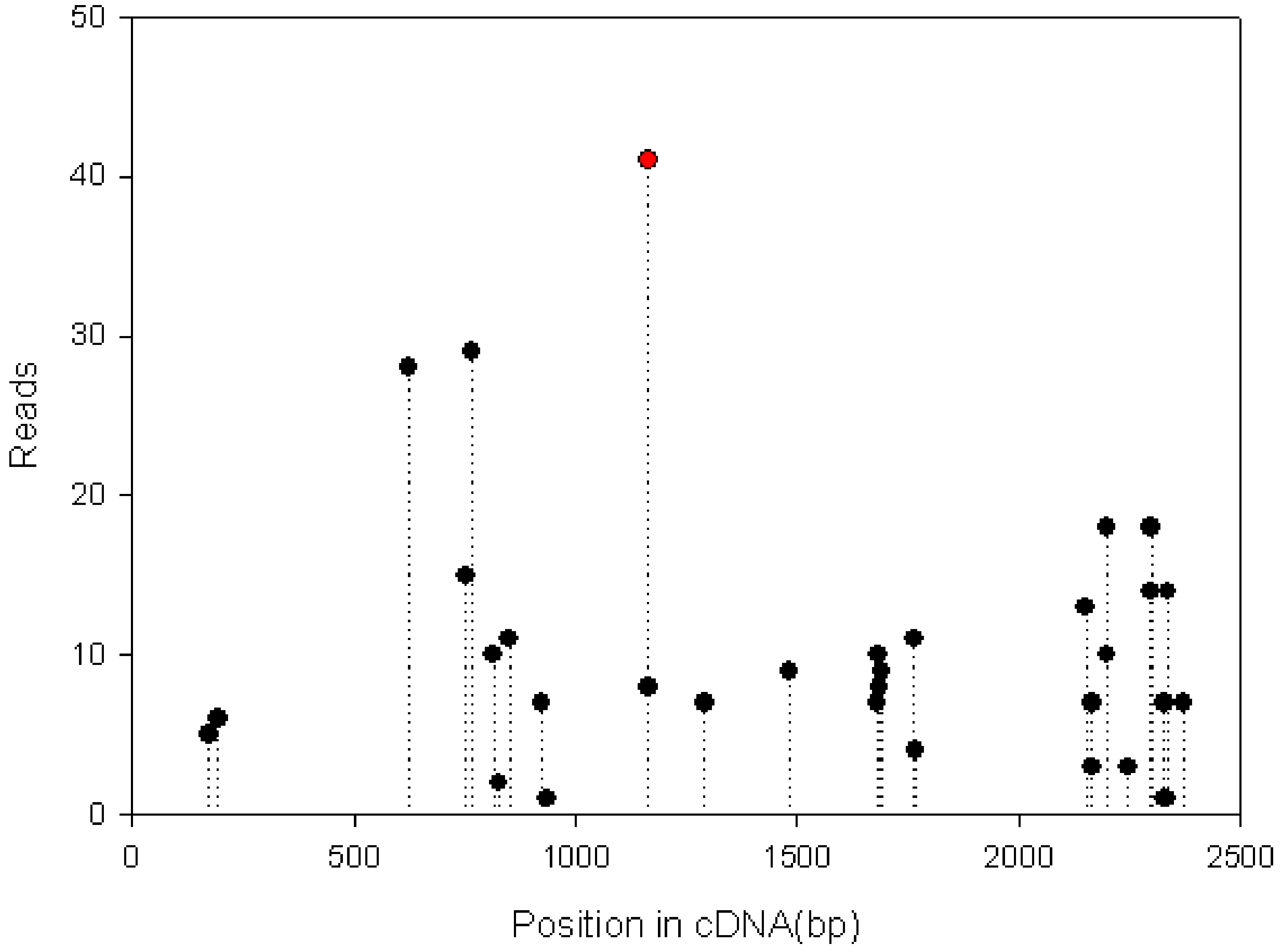


```
5' ACCGUUCAAGAAAGCCUGUGGAAGGC 3'        Cs1g21350.1
      ::::::::::::: ::::::
3' ---CAAGUUCUUUCGG-CACCUU--- 5'        Csi-miR396d.3
```

Csi-miR396d.3, target=Cs1g22520.1 gene=Cs1g22520
Category:1
Score=4
Cleavage Site=226

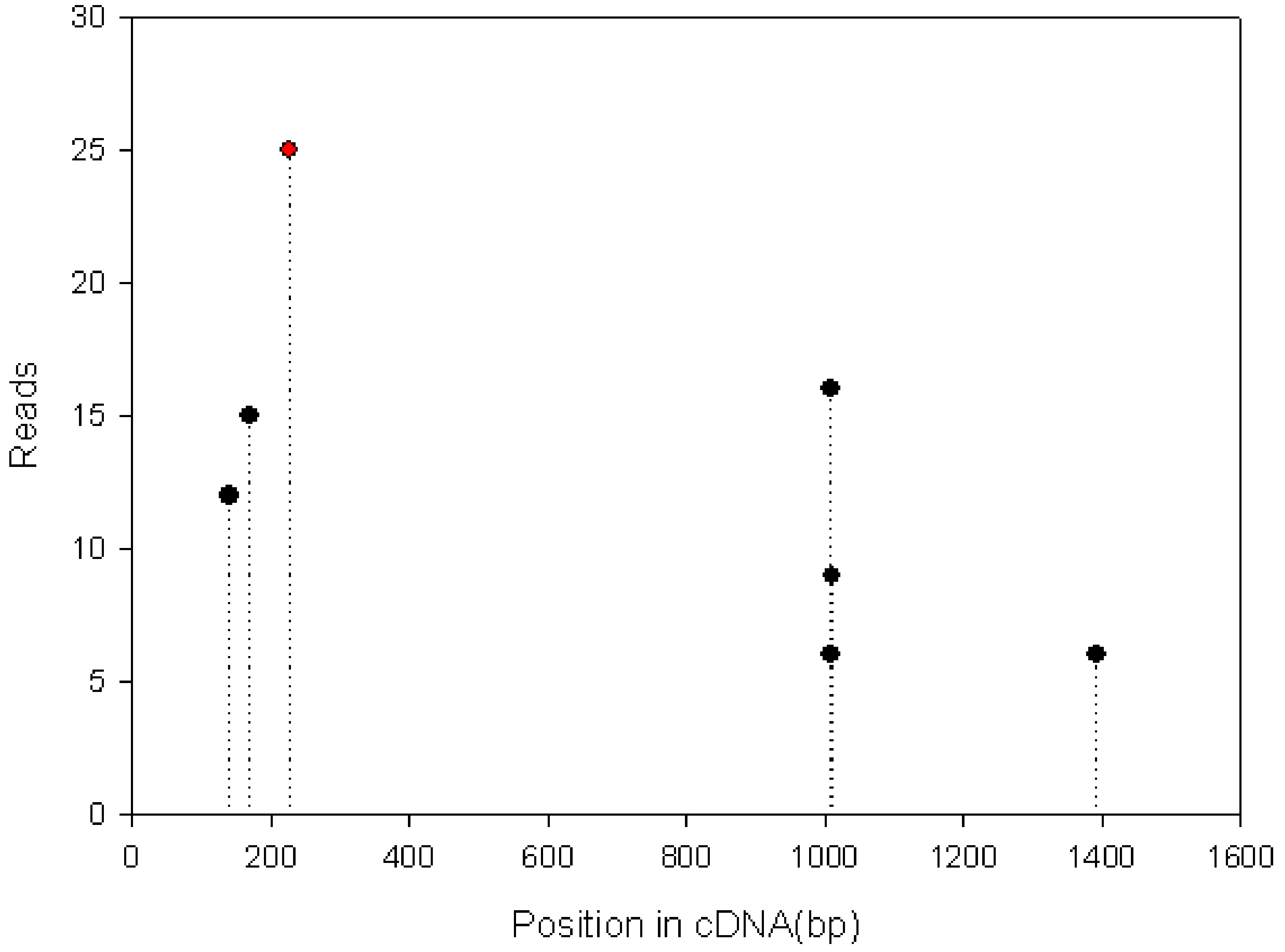


```
5' GAAGUUCAAGAAAUCUGUGGGAAGAA 3'        Cs1g22520.1
       :::::::::: :.::::.:
3' ---CAAGUUCUUUCGGCACCUU---- 5'        Csi-miR396d.3
```

Csi-miR396d.3, target=Cs2g21180.1 gene=Cs2g21180
Category:3
Score=4.5
Cleavage Site=600

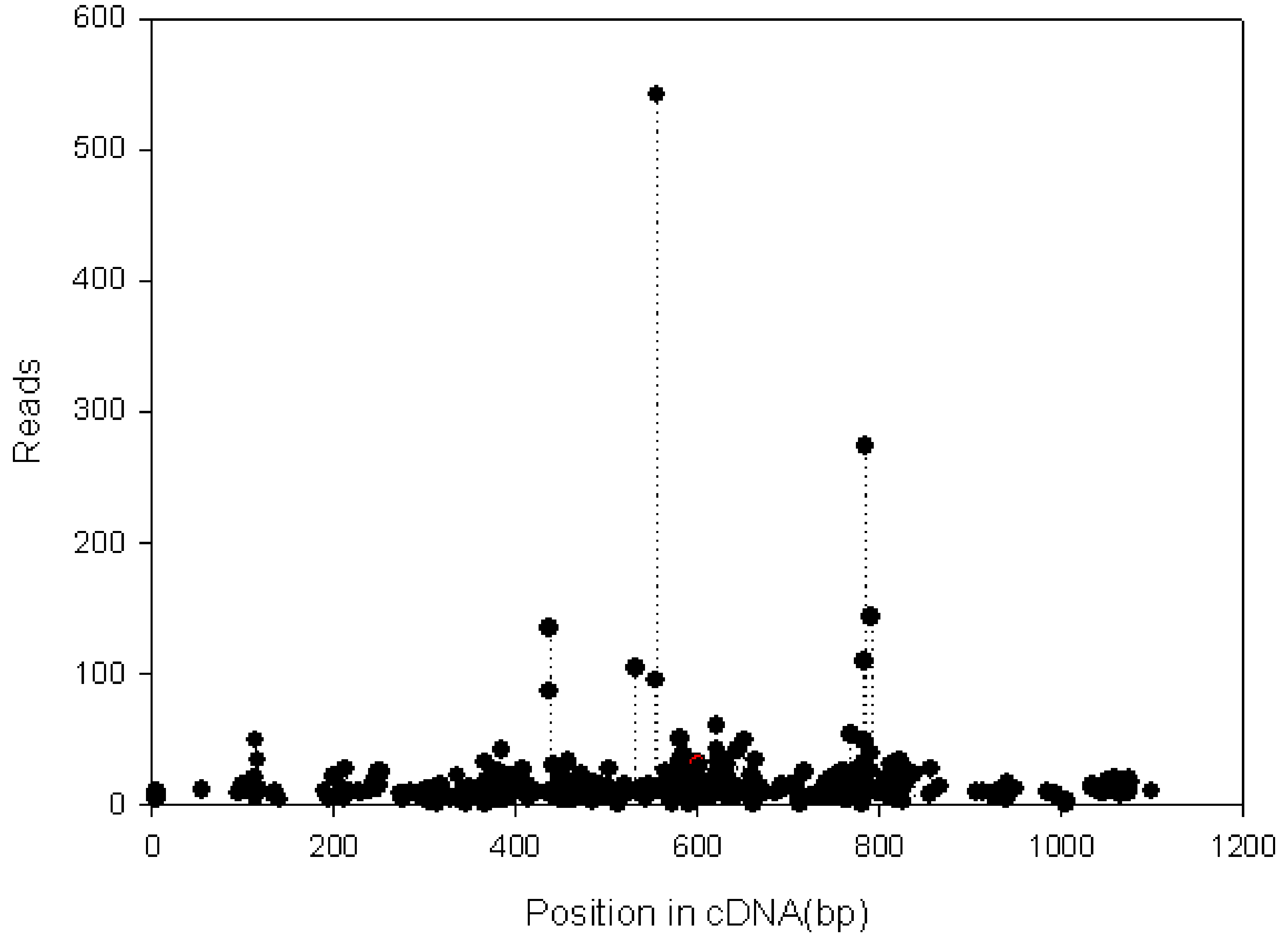


```
5' UCGAUUGCUCAGGAAGGCCAUGGAAG 3'        Cs2g21180.1
          : :::.:::.::: :::::
3' ------CAAGUUCUUUCGGCACCUU- 5'        Csi-miR396d.3
```

Csi-miR396d.3, target=Cs3g23180.1 gene=Cs3g23180
Category:1
Score=4
Cleavage Site=625

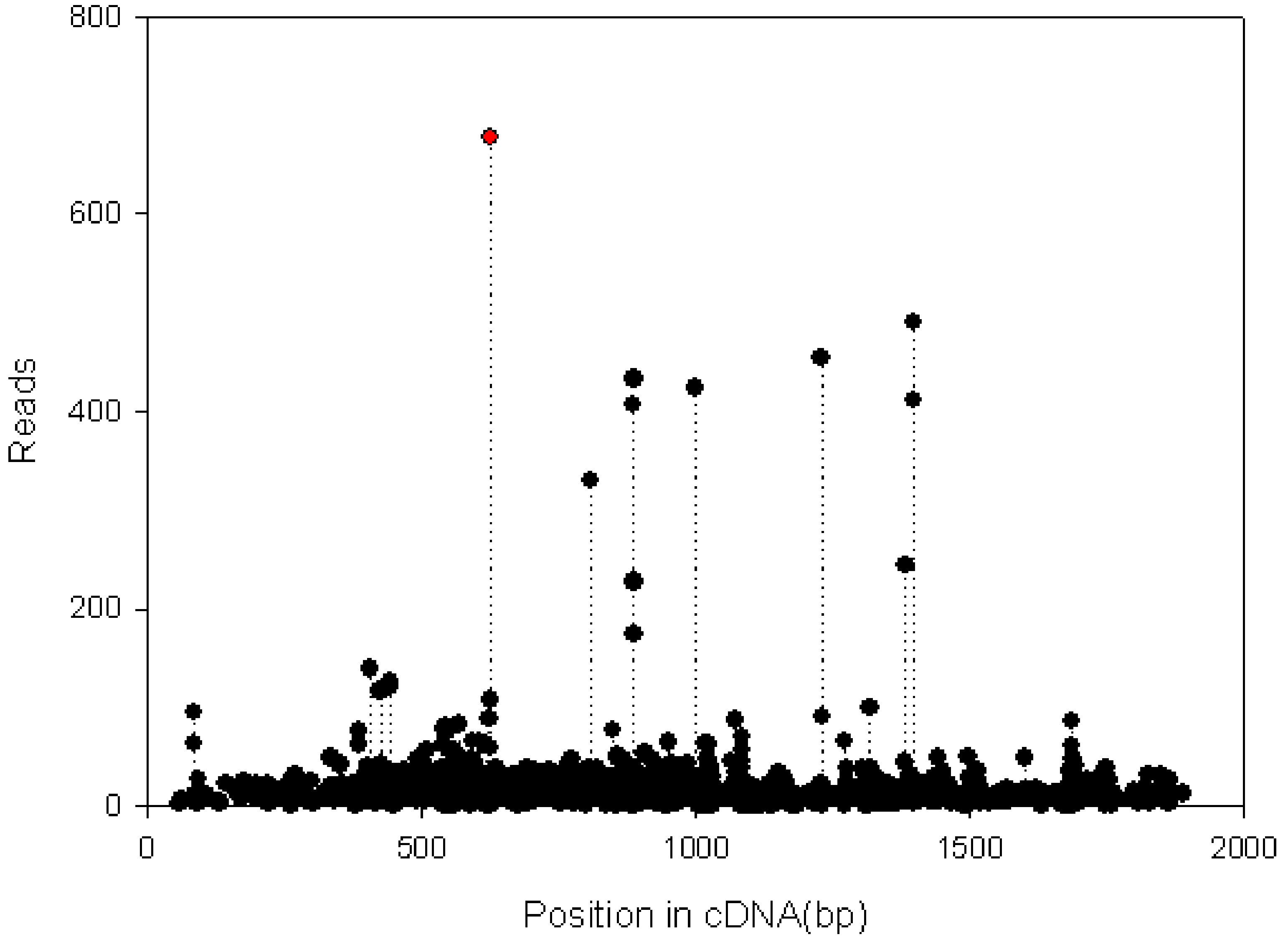


```
5' AAAGAUCAAGGAAGCUGUGGGAGUUG 3'        Cs3g23180.1
      : :::::.::::.::::.:
3' ---CAAGUUCUUUCGGCACCUU---- 5'        Csi-miR396d.3
```

Csi-miR396d.3, target=Cs4g05000.1 gene=Cs4g05000
Category:3
Score=4
Cleavage Site=1249

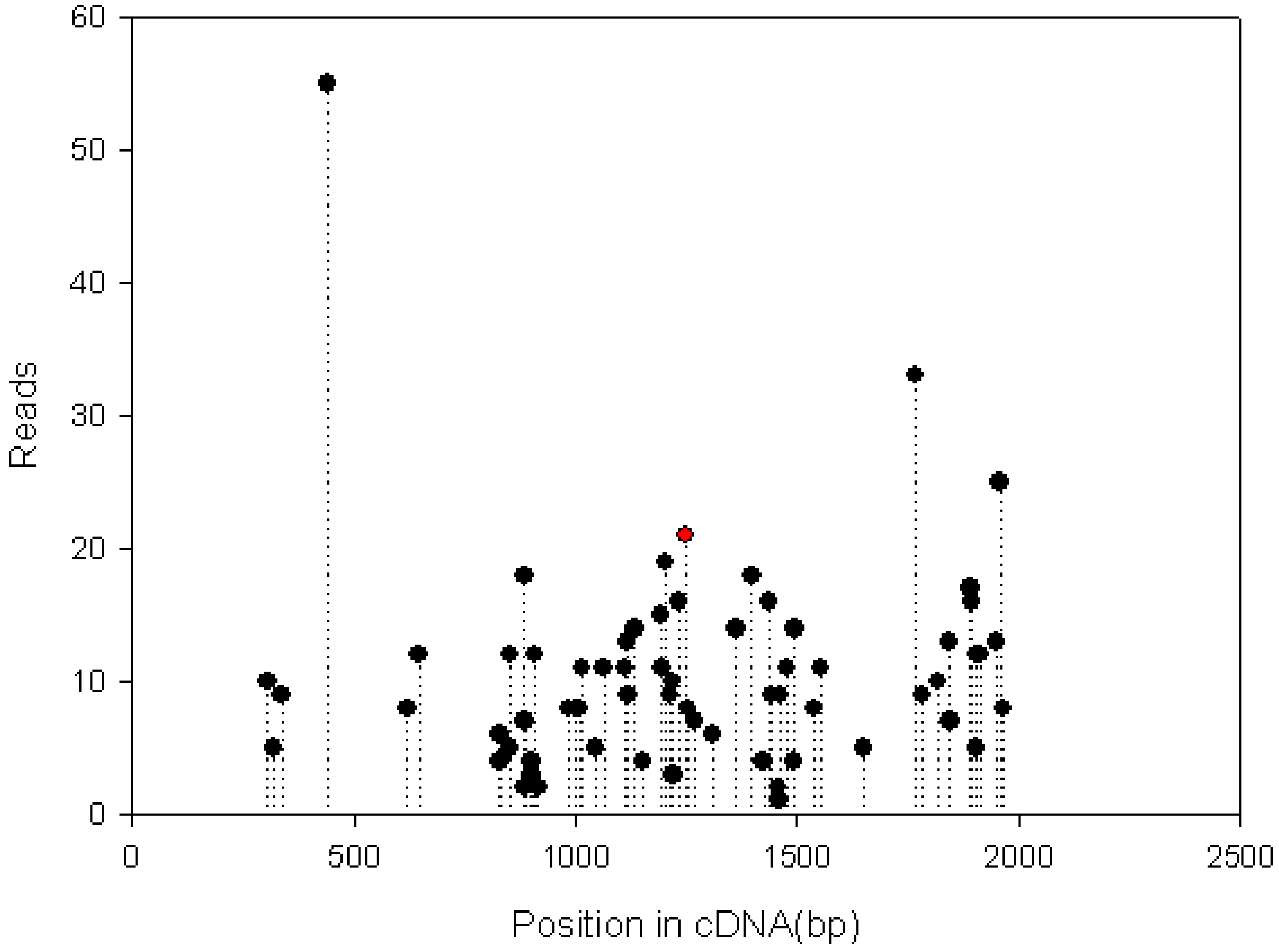


```
5' ACCCUUGAAGAAAGCUGUGGAUAUAG 3'        Cs4g05000.1
      :: ::::::::.:::::
3' ---CAAGUUCUUUCGGCACCUU---- 5'        Csi-miR396d.3
```

Csi-miR396d.3, target=Cs4g12200.1 gene=Cs4g12200
Category:3
Score=4
Cleavage Site=228

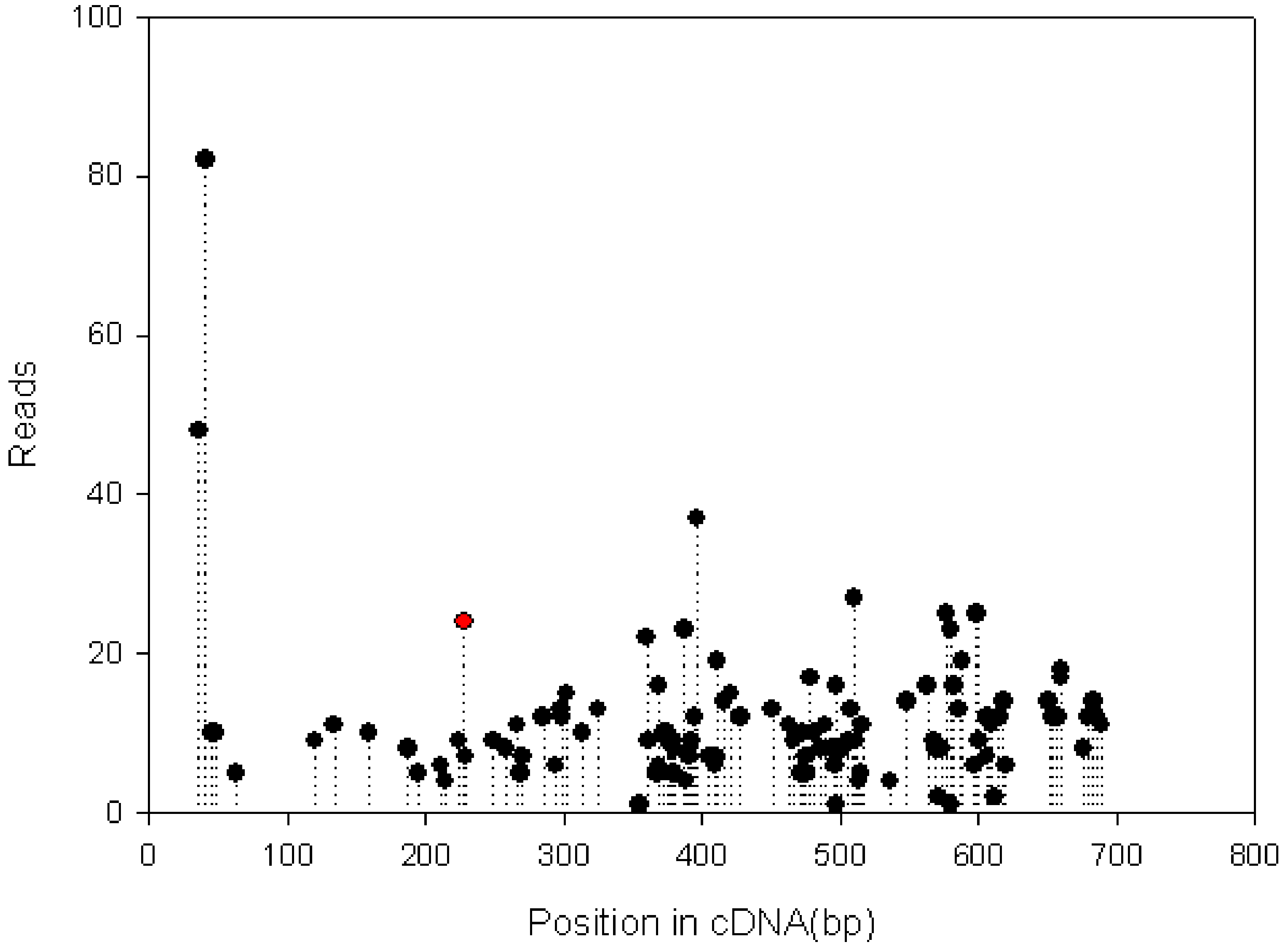


```
5' GGUUCCUGAAAGCCG-GGAAAUAUGCU 3'        Cs4g12200.1
    ::::  :::::::: ::::
3' -CAAGUUCUUUCGGCACCUU------- 5'        Csi-miR396d.3
```

Csi-miR396d.3, target=Cs5g01380.1 gene=Cs5g01380
Category:1
Score=2
Cleavage Site=1181

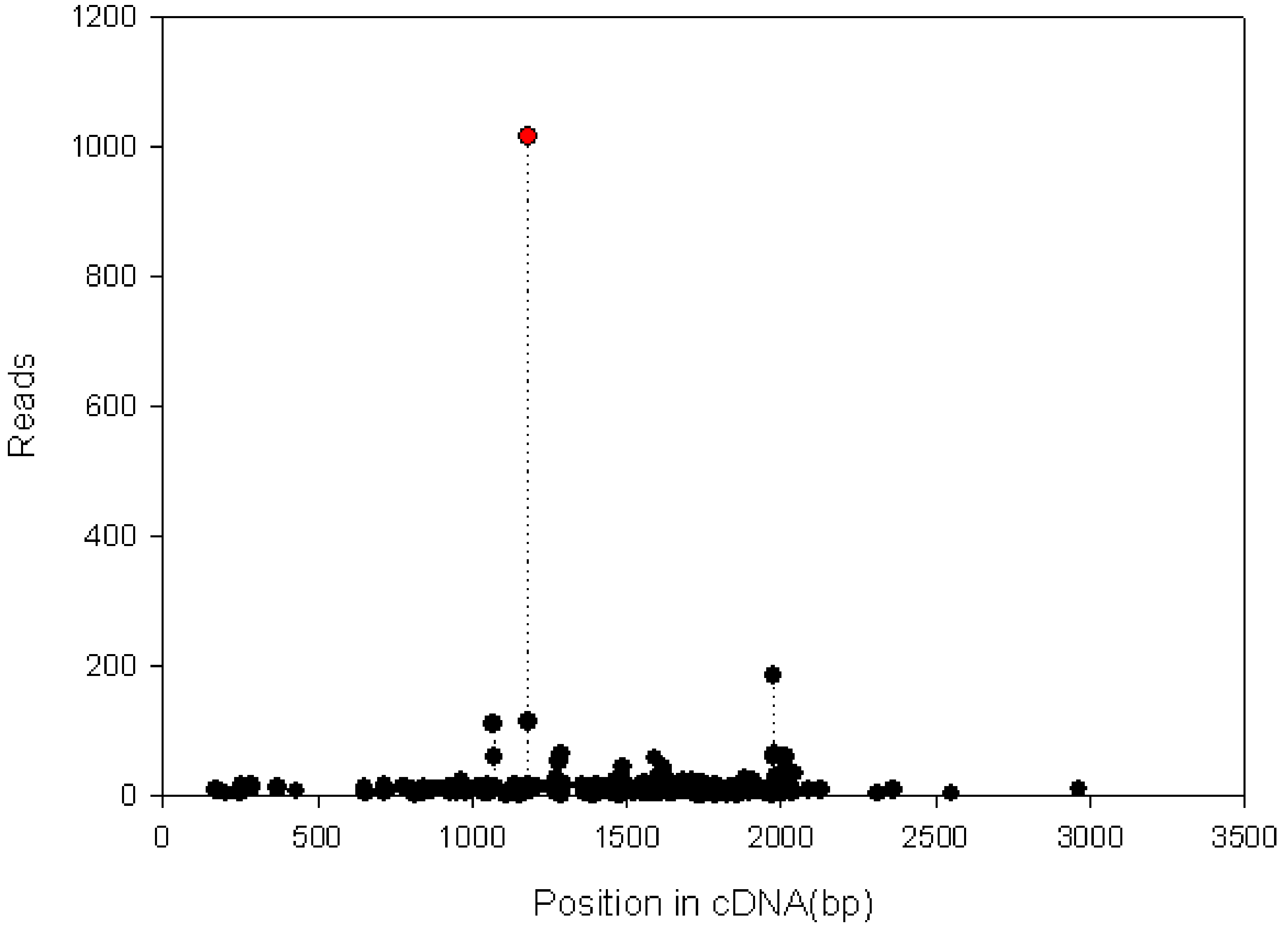


```
5' CCCGUUCAAGAAAGCCUGUGGAAUUA 3'        Cs5g01380.1
      ::::::::::::: ::::::
3' ---CAAGUUCUUUCGG-CACCUU--- 5'        Csi-miR396d.3
```

Csi-miR396d.3, target=Cs5g09850.1 gene=Cs5g09850
Category:3
Score=3
Cleavage Site=767

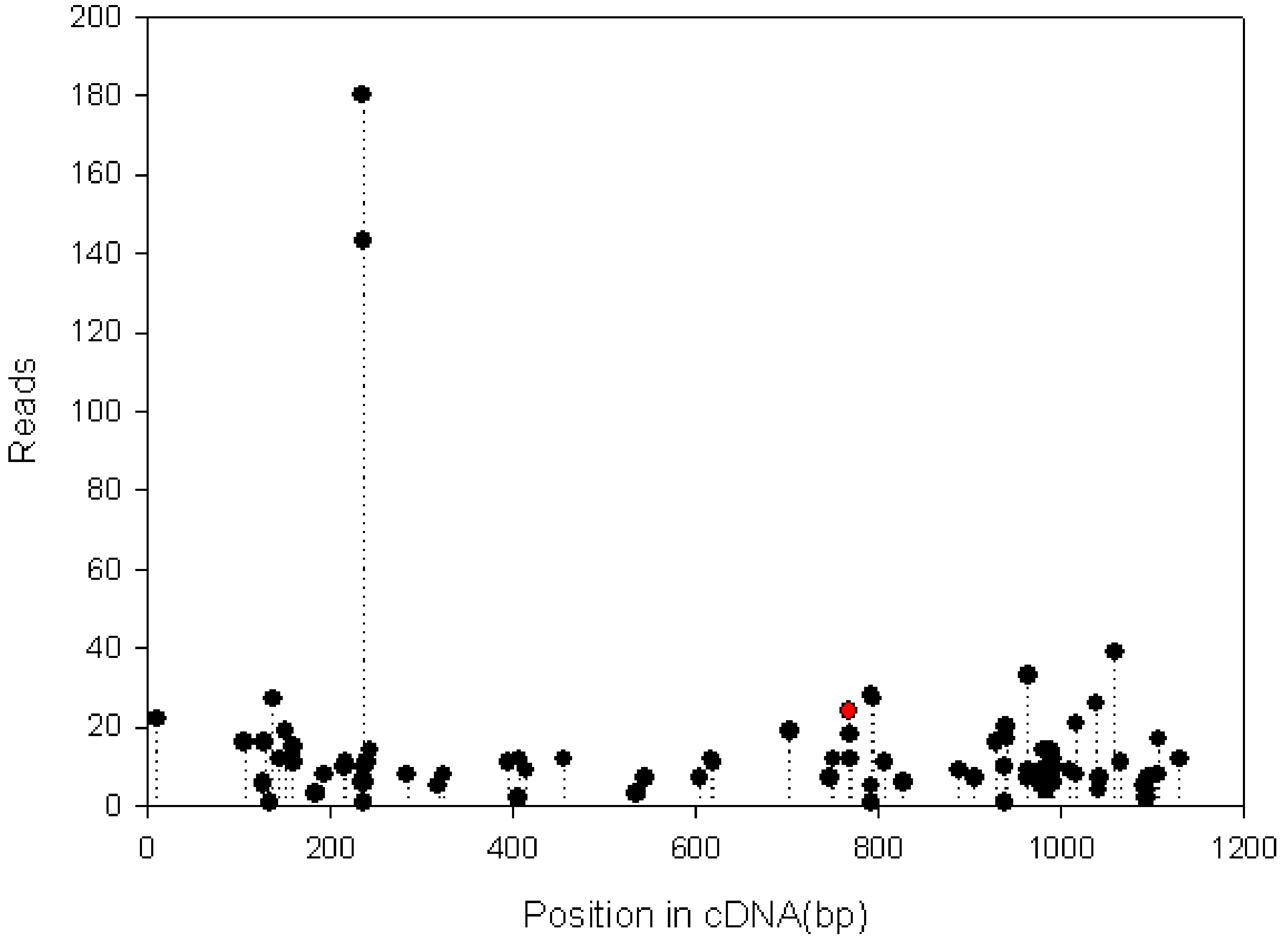


```
5' UGCGUUCAAGAAAGCUUGUGGAAGCU 3'        Cs5g09850.1
      :::::::::::: .::::::
3' ---CAAGUUCUUUCG-GCACCUU--- 5'        Csi-miR396d.3
```

Csi-miR396d.3, target=Cs7g15220.1 gene=Cs7g15220
Category:1
Score=2
Cleavage Site=377

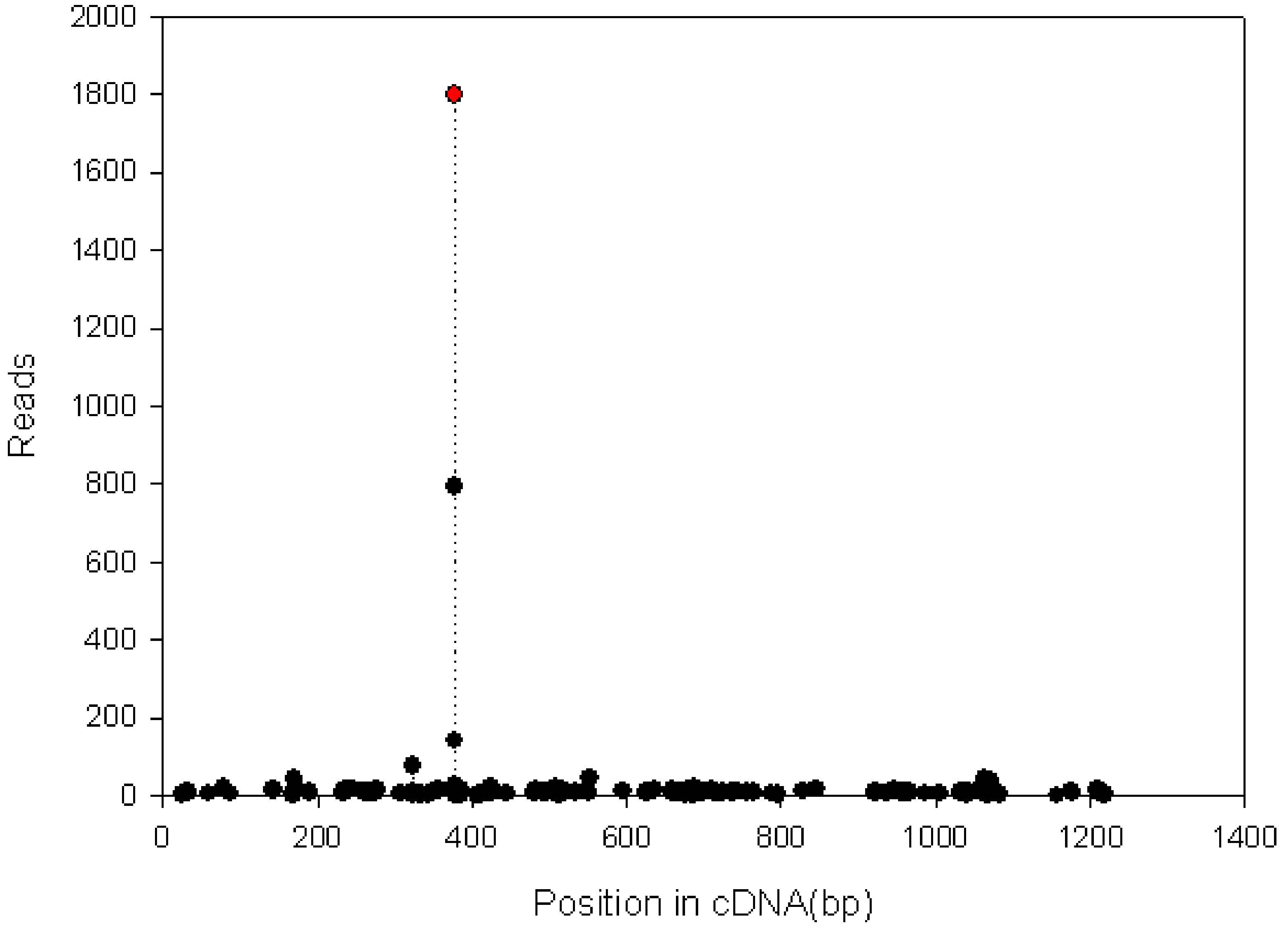


```
5' ACCGUUCAAGAAAGCCUGUGGAAUCA 3'        Cs7g15220.1
      ::::::::::::: ::::::
3' ---CAAGUUCUUUCGG-CACCUU--- 5'        Csi-miR396d.3
```

Csi-miR396d.3, target=Cs8g17370.1 gene=Cs8g17370
Category:3
Score=3.5
Cleavage Site=140

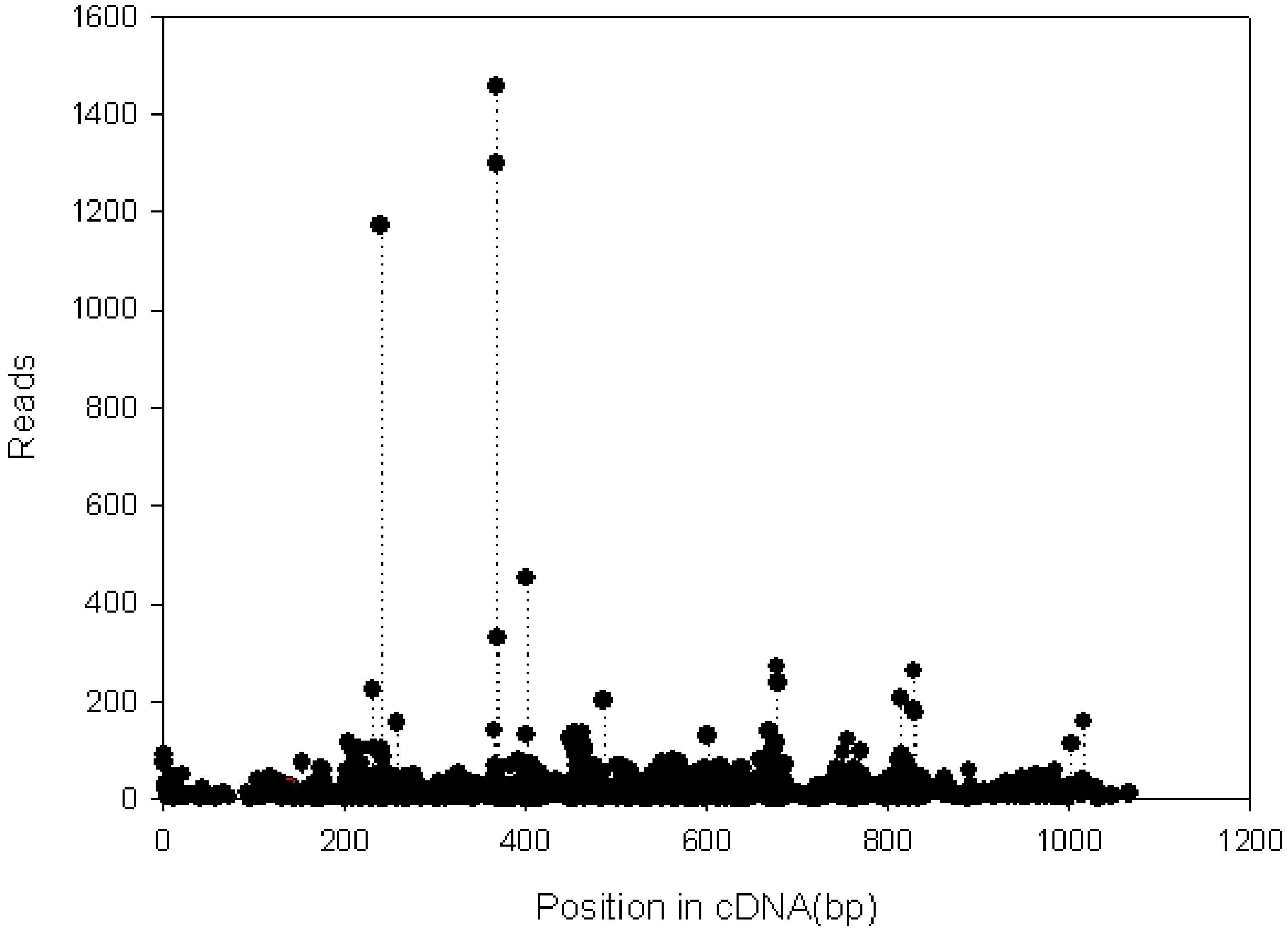


```
5' AGGAUUACAAGAAGGCCGUGGAGAAA 3'        Cs8g17370.1
         : ::::::.::::::::.
3' ----CAAGUUCUUUCGGCACCUU--- 5'        Csi-miR396d.3
```

Csi-miR396d.3, target=Orange1.1t00172.1 gene=Orange1.1t00172
Category:1
Score=2
Cleavage Site=656

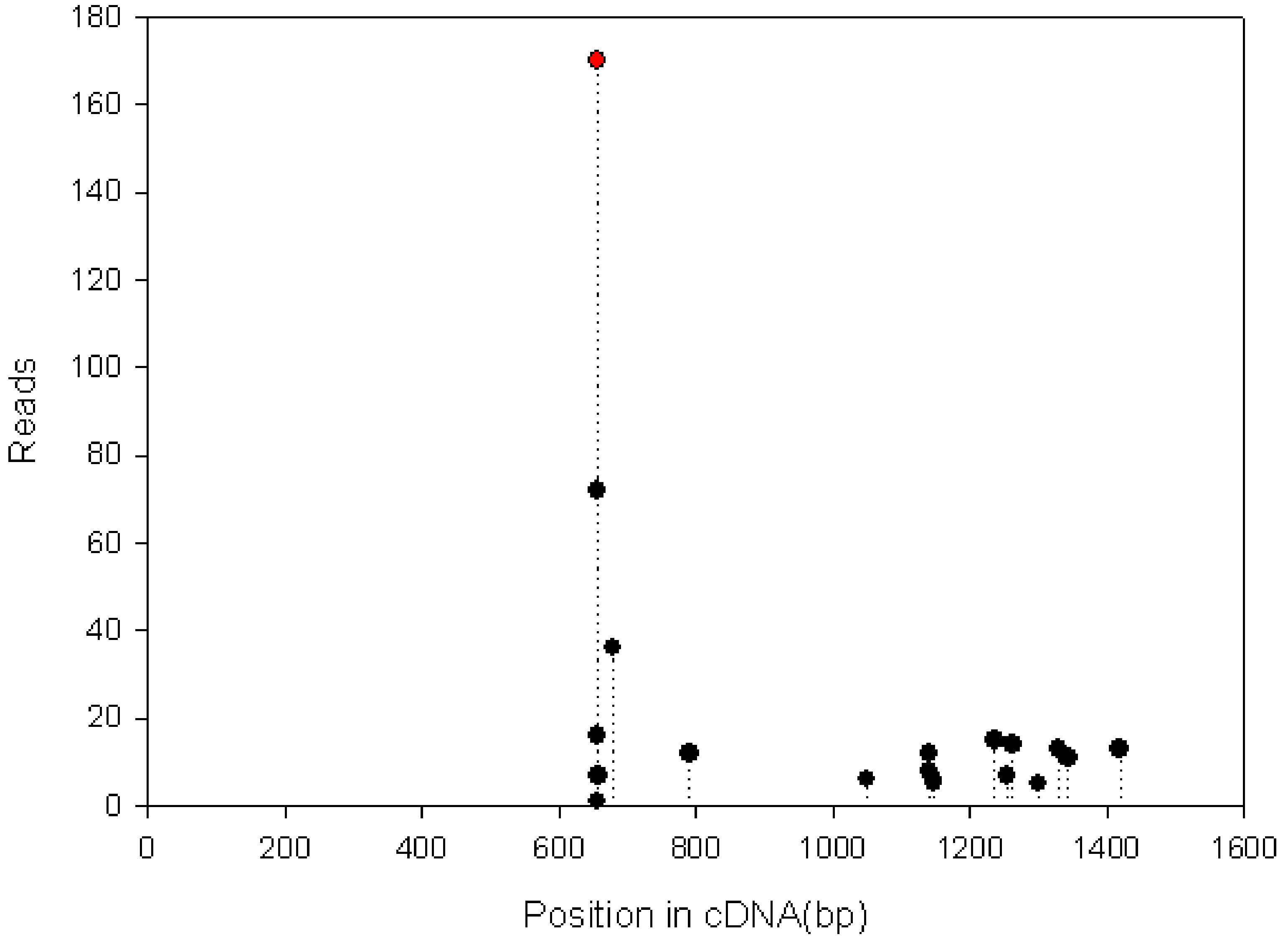


```
5' ACCGUUCAAGAAAGCCUGUGGAACUU 3'        Orange1.1t00172.1
     ::::::::::::: ::::::
3' ---CAAGUUCUUUCGG-CACCUU--- 5'        Csi-miR396d.3
```

Csi-miR396d.3, target=Orange1.1t02555.1 gene=Orange1.1t02555
Category:1
Score=2
Cleavage Site=920

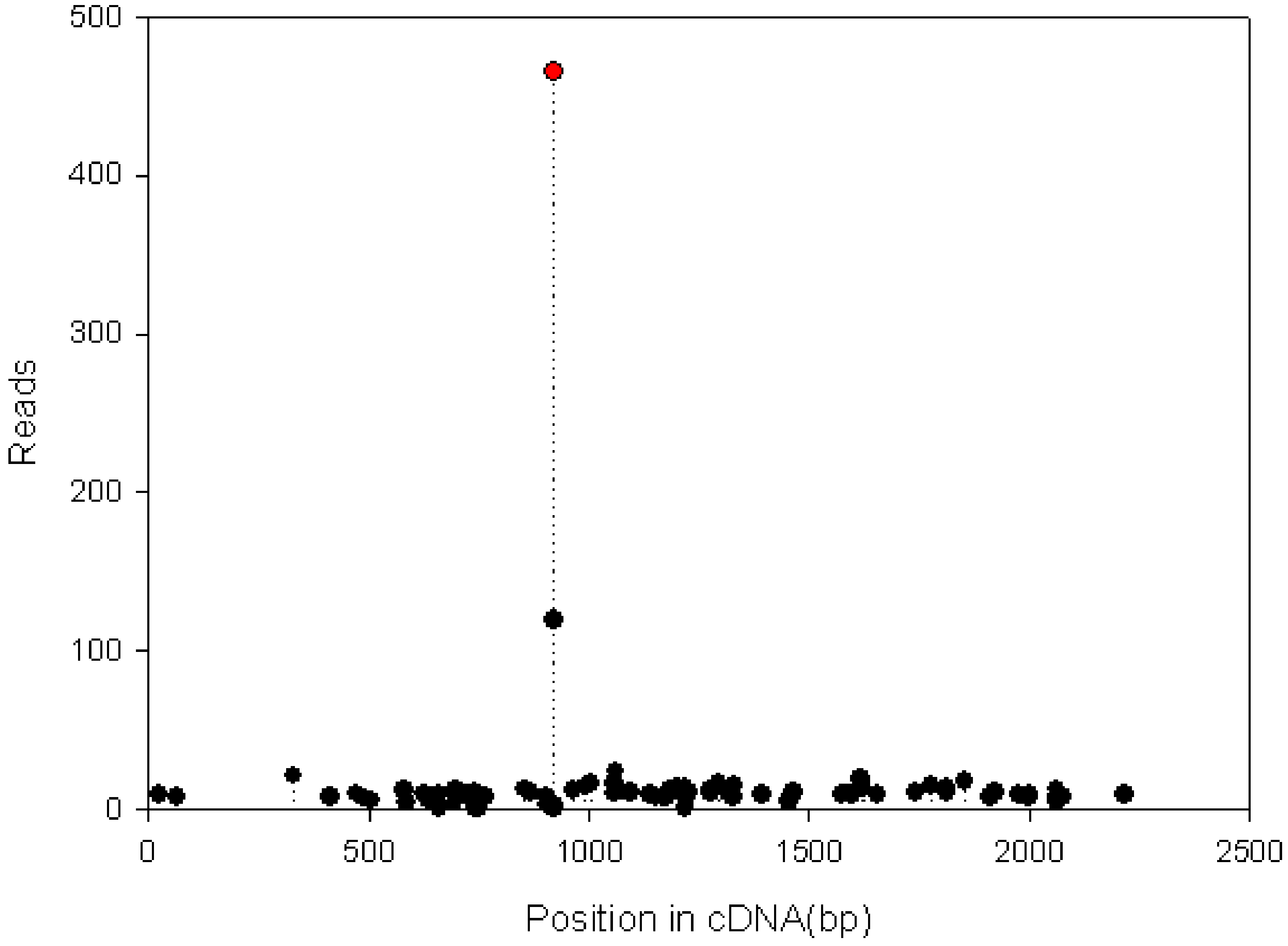


```
5' CACGUUCAAGAAAGCCUGUGGAACUU 3'        Orange1.1t02555.1
     ::::::::::::: ::::::
3' ---CAAGUUCUUUCGG-CACCUU--- 5'        Csi-miR396d.3
```

Csi-miR396d.3, target=Orange1.1t03122.1 gene=Orange1.1t03122
Category:1
Score=2
Cleavage Site=583

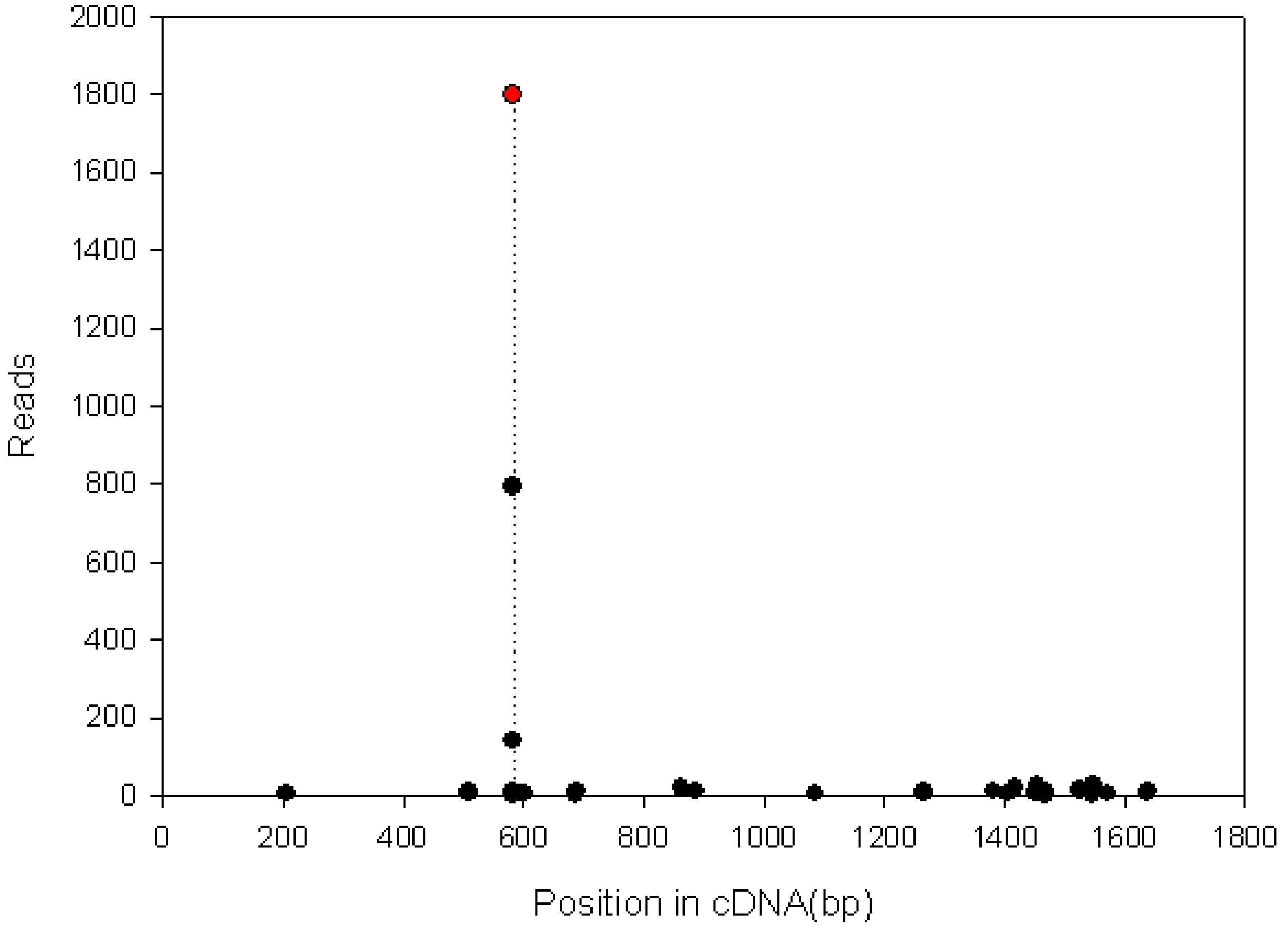


```
5' ACCGUUCAAGAAAGCCUGUGGAAUCA 3'        Orange1.1t03122.1
     ::::::::::::: ::::::
3' ---CAAGUUCUUUCGG-CACCUU--- 5'        Csi-miR396d.3
```

Csi-miR396d.4, target=Cs1g15720.1 gene=Cs1g15720
Category:3
Score=4
Cleavage Site=1610

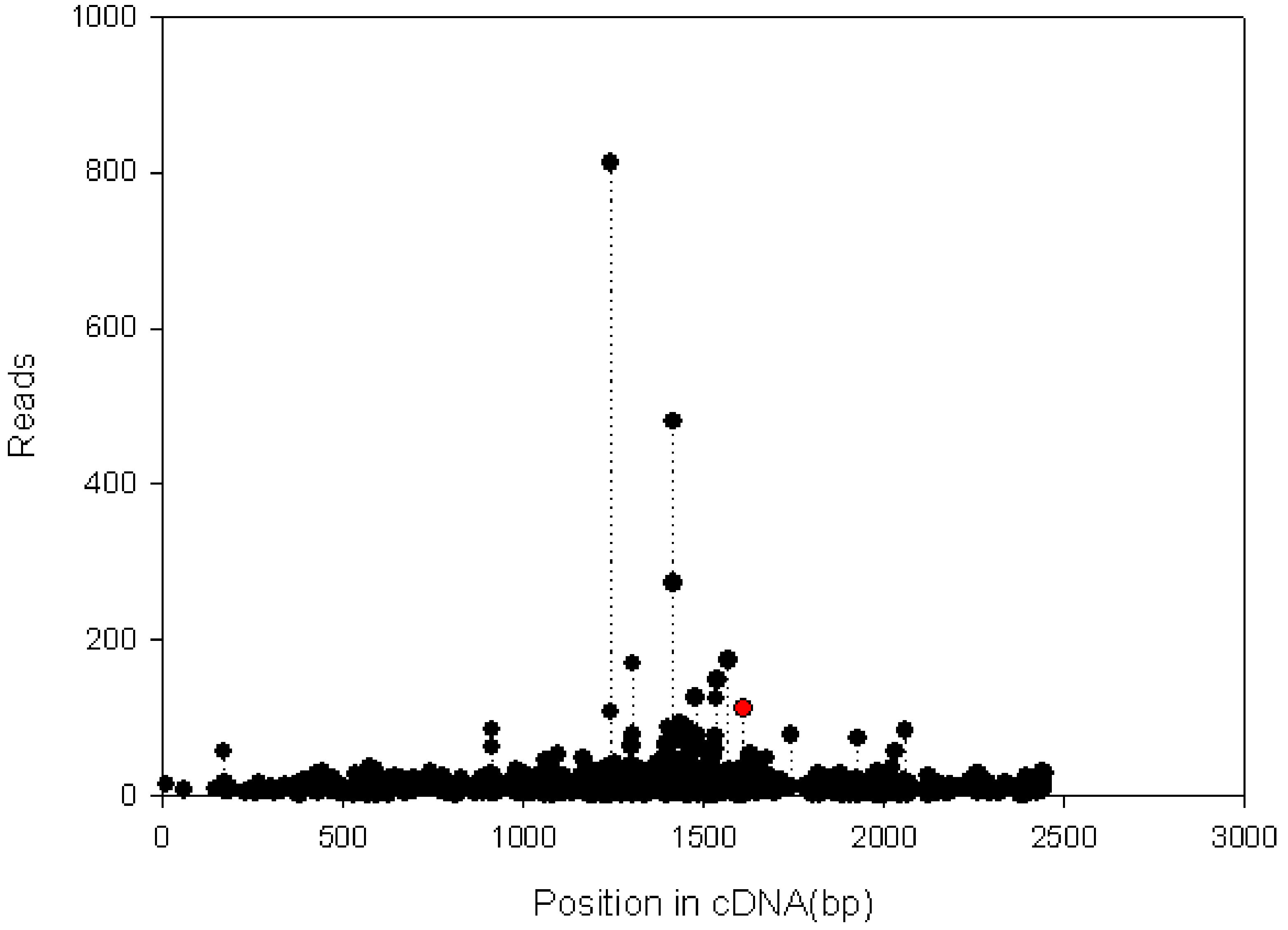


```
5' CAACAUUUCAA-AAAGCCGUGGGCUCA 3'       Cs1g15720.1
         ::::: ::::::::::.
3' ------AAGUUCUUUCGGCACCUU--- 5'       Csi-miR396d.4
```

Csi-miR396d.4, target=Cs1g21350.1 gene=Cs1g21350
Category:1
Score=2
Cleavage Site=1164

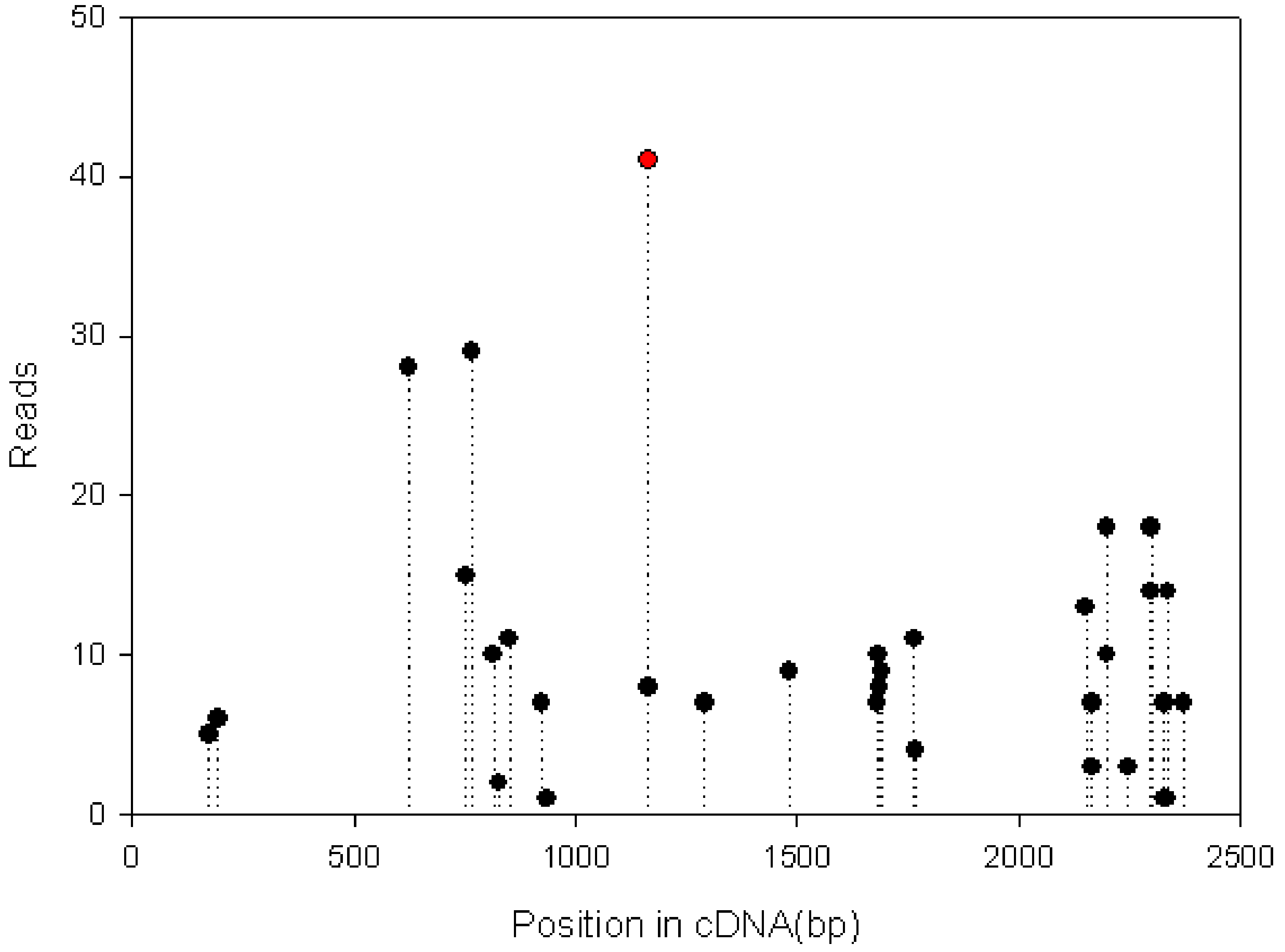


```
5' ACCGUUCAAGAAAGCCUGUGGAAGGC 3'        Cs1g21350.1
       :::::::::::: ::::::
3' ----AAGUUCUUUCGG-CACCUU--- 5'        Csi-miR396d.4
```

Csi-miR396d.4, target=Cs1g22520.1 gene=Cs1g22520
Category:1
Score=4
Cleavage Site=226

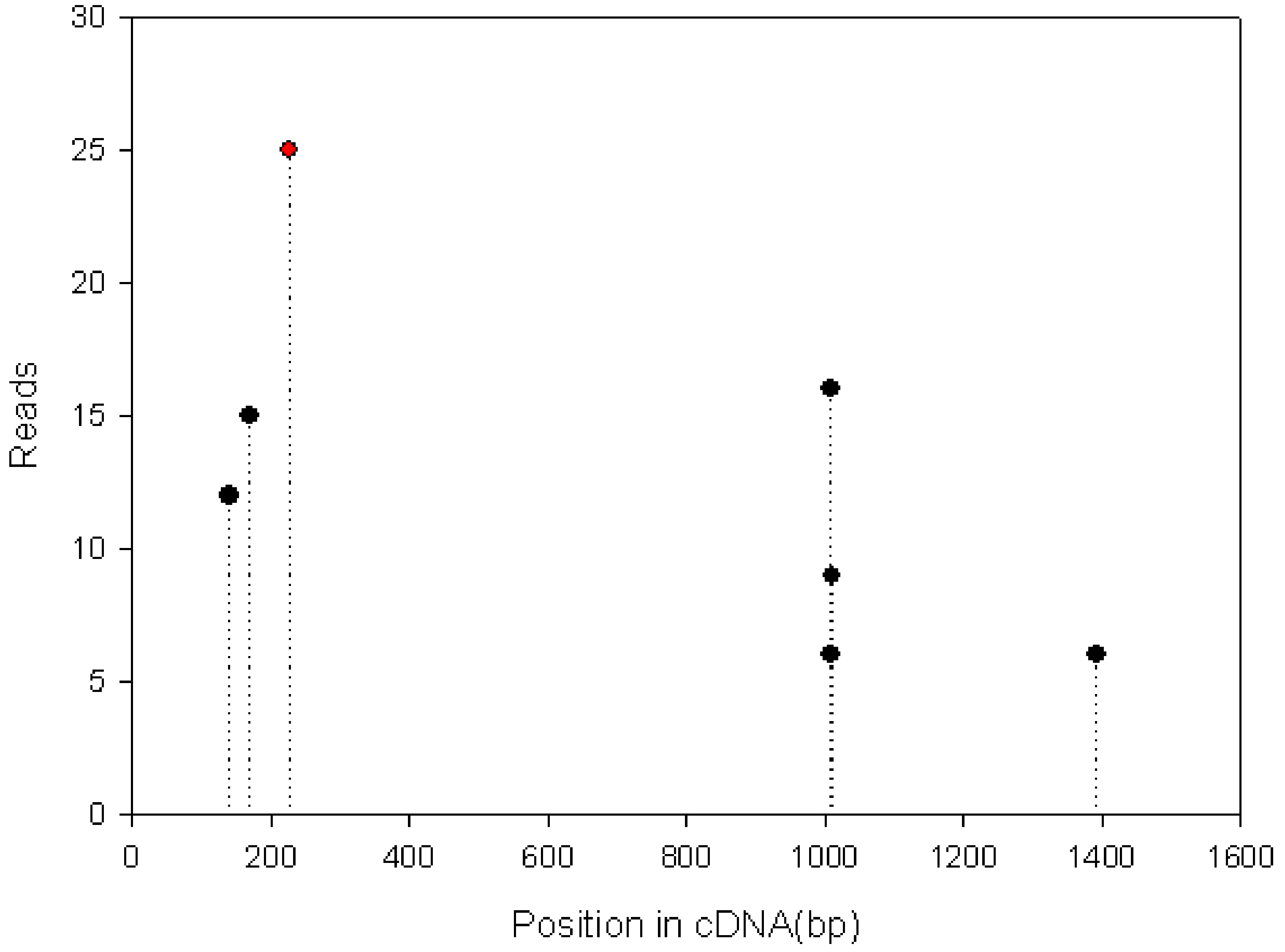


```
5' GAAGUUCAAGAAAUCUGUGGGAAGAA 3'        Cs1g22520.1
         ::::::::: :.::::.:
3' ----AAGUUCUUUCGGCACCUU---- 5'        Csi-miR396d.4
```

Csi-miR396d.4, target=Cs2g21180.1 gene=Cs2g21180
Category:3
Score=4.5
Cleavage Site=600

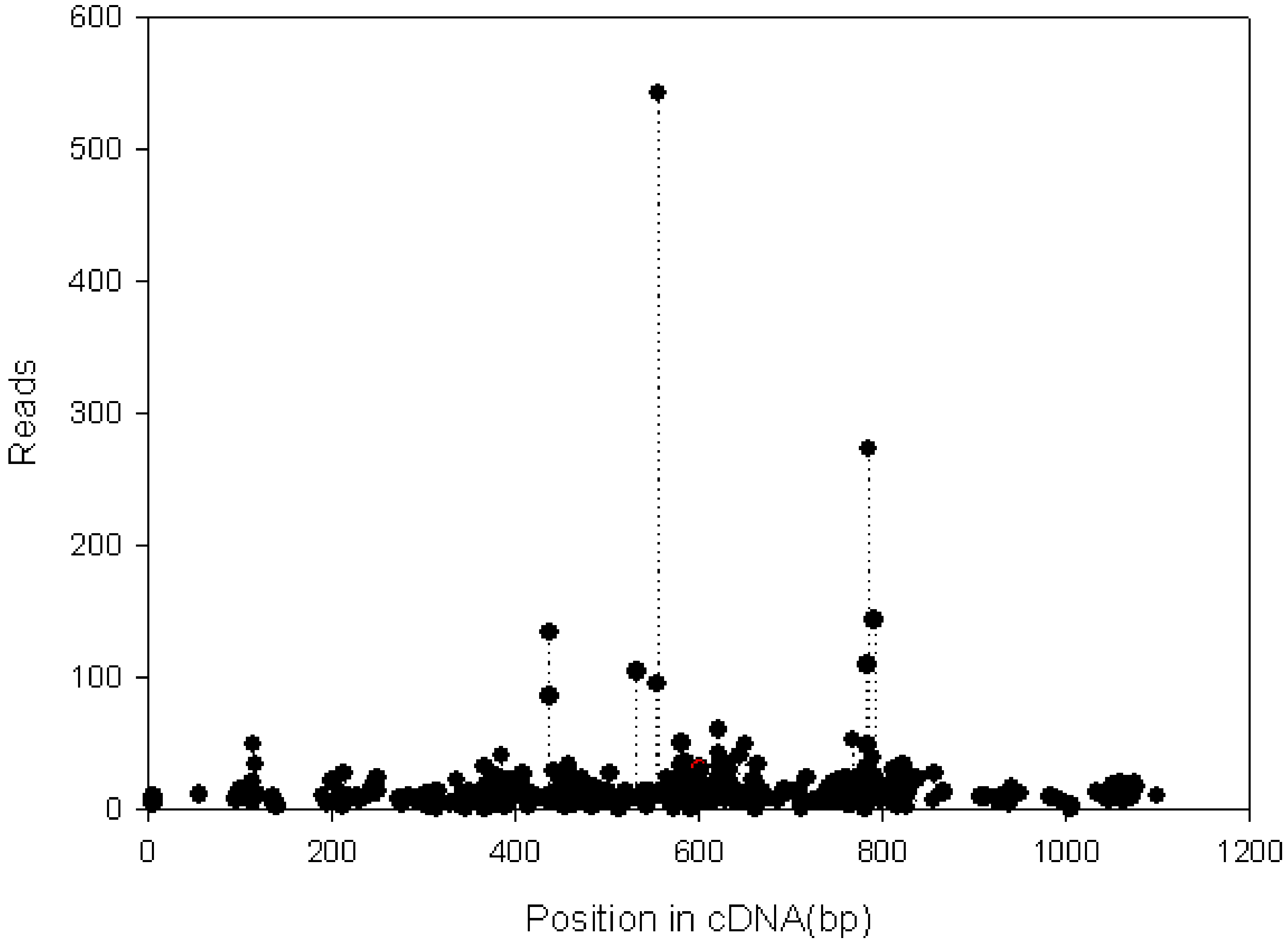


```
5' UCGAUUGCUCAGGAAGGCCAUGGAAG 3'        Cs2g21180.1
           :::.:::.::: :::::
3' -------AAGUUCUUUCGGCACCUU- 5'        Csi-miR396d.4
```

Csi-miR396d.4, target=Cs3g23180.1 gene=Cs3g23180
Category:1
Score=4
Cleavage Site=625

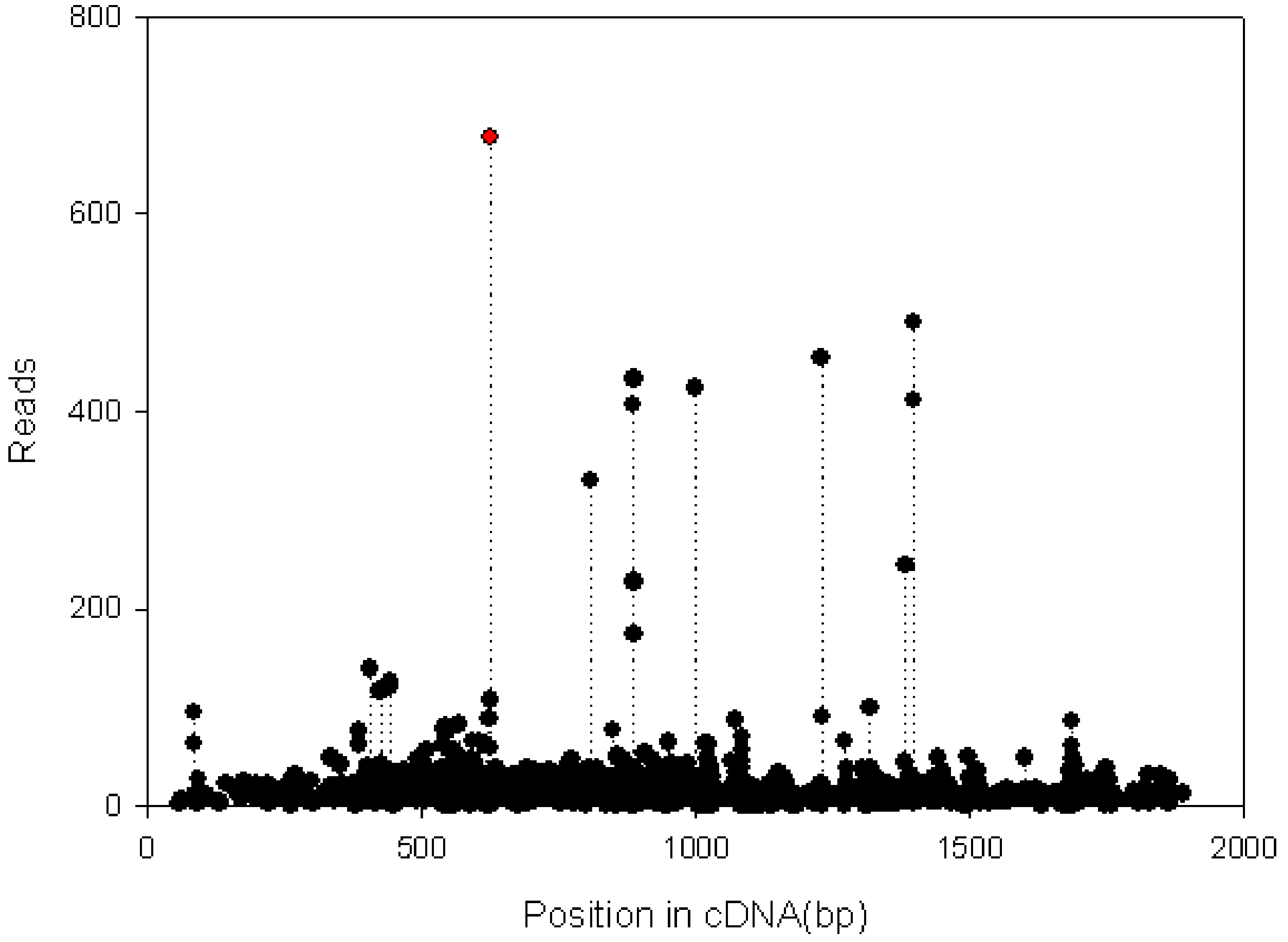


```
5' AAAGAUCAAGGAAGCUGUGGGAGUUG 3'        Cs3g23180.1
         :::::.::::.::::.:
3' ----AAGUUCUUUCGGCACCUU---- 5'        Csi-miR396d.4
```

Csi-miR396d.4, target=Cs4g05000.1 gene=Cs4g05000
Category:3
Score=5
Cleavage Site=1249

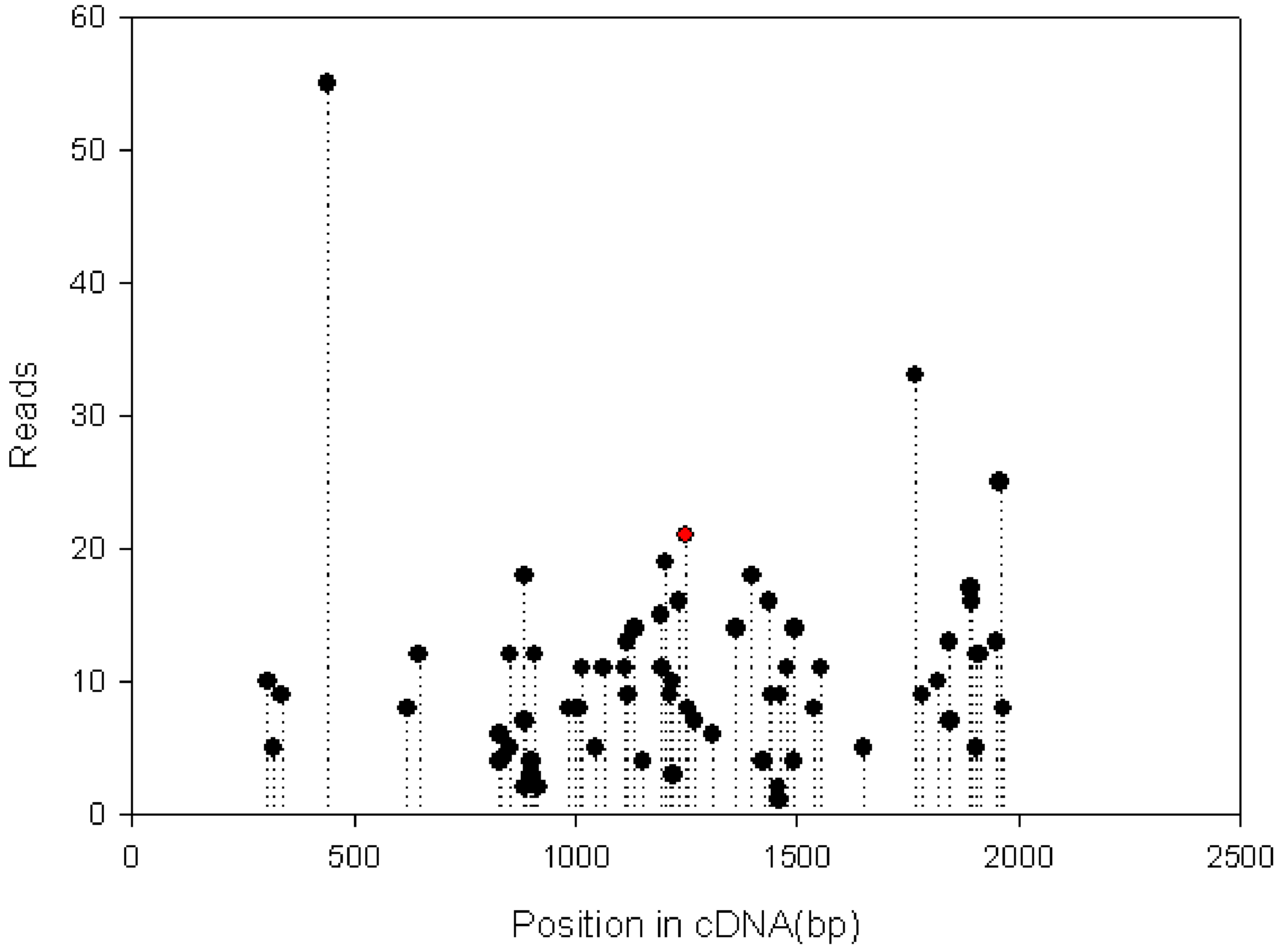


```
5' ACCCUUGAAGAAAGCUGUGGAUAUAG 3'        Cs4g05000.1
       :: ::::::::.:::::
3' ----AAGUUCUUUCGGCACCUU---- 5'        Csi-miR396d.4
```

Csi-miR396d.4, target=Cs4g10880.1 gene=Cs4g10880
Category:3
Score=5
Cleavage Site=803

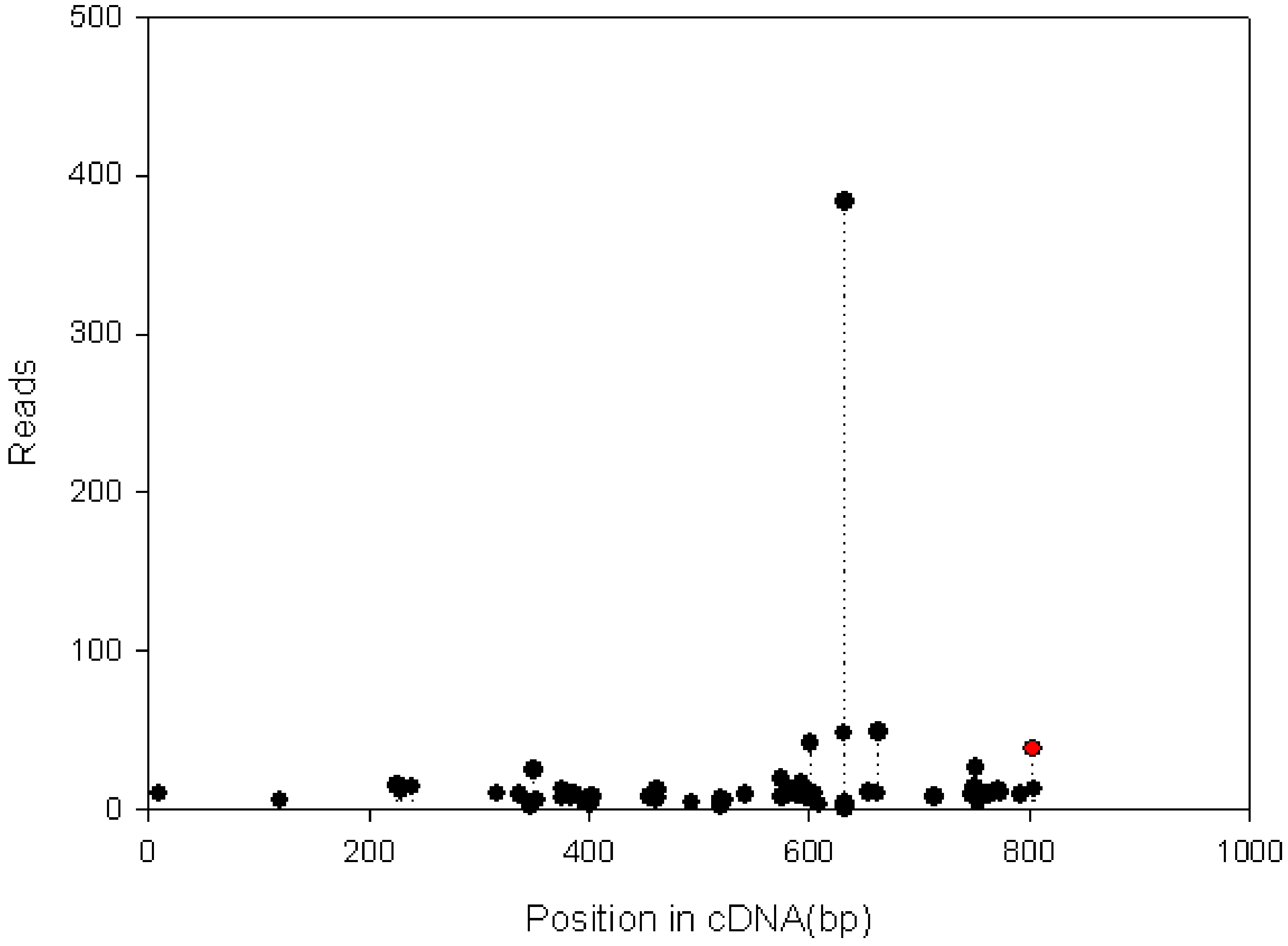


```
5' UUUUGAGGGAGCC-UGGAACUGAUUUC 3'        Cs4g10880.1
    ::..::..:::: :::::
3' -AAGUUCUUUCGGCACCUU-------- 5'        Csi-miR396d.4
```

Csi-miR396d.4, target=Cs4g12200.1 gene=Cs4g12200
Category:3
Score=4
Cleavage Site=228

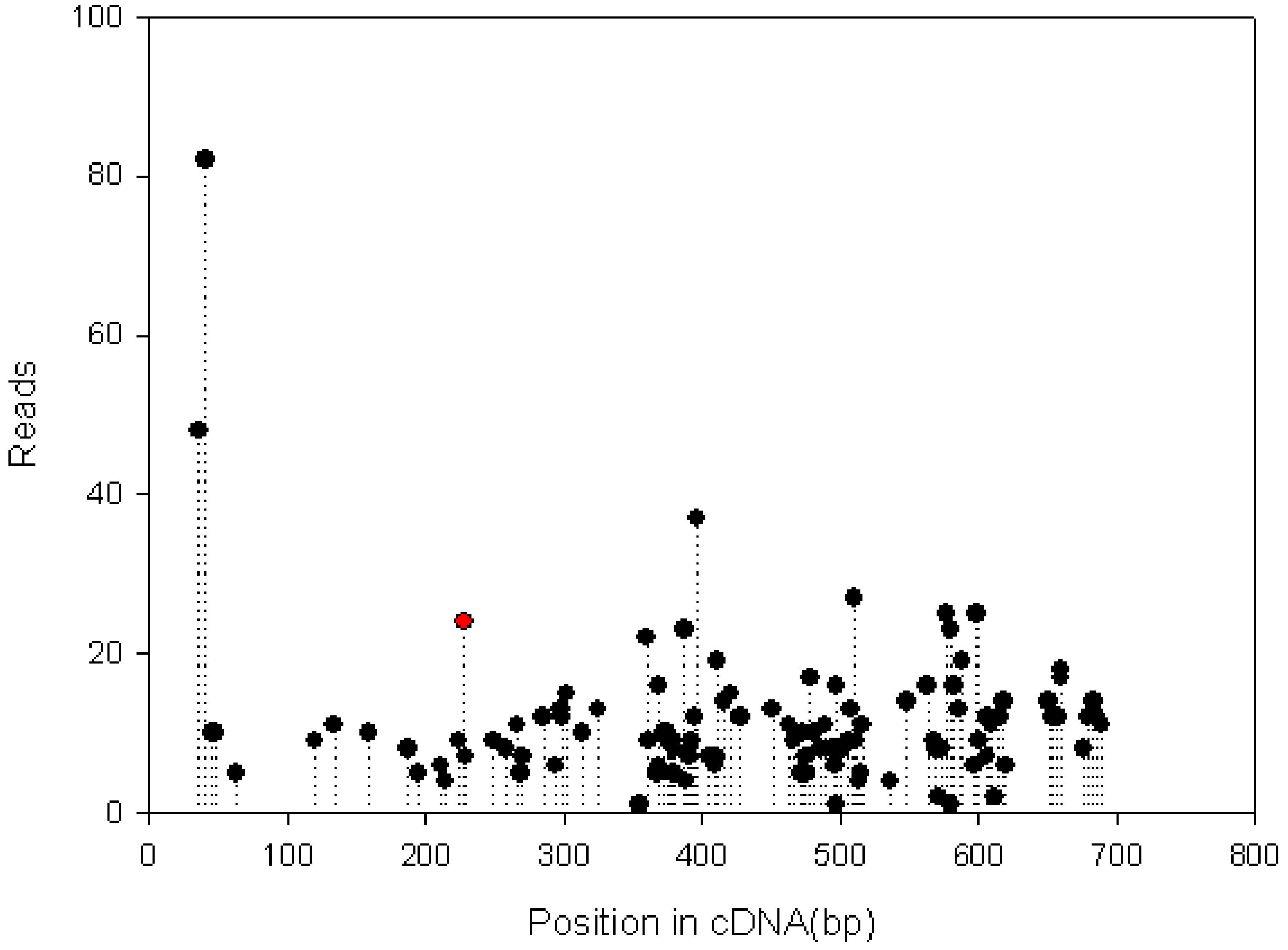


```
5' GGUUCCUGAAAGCCG-GGAAAUAUGCU 3'        Cs4g12200.1
     :::  :::::::: ::::
3' --AAGUUCUUUCGGCACCUU------- 5'        Csi-miR396d.4
```

Csi-miR396d.4, target=Cs5g01380.1 gene=Cs5g01380
Category:1
Score=2
Cleavage Site=1181

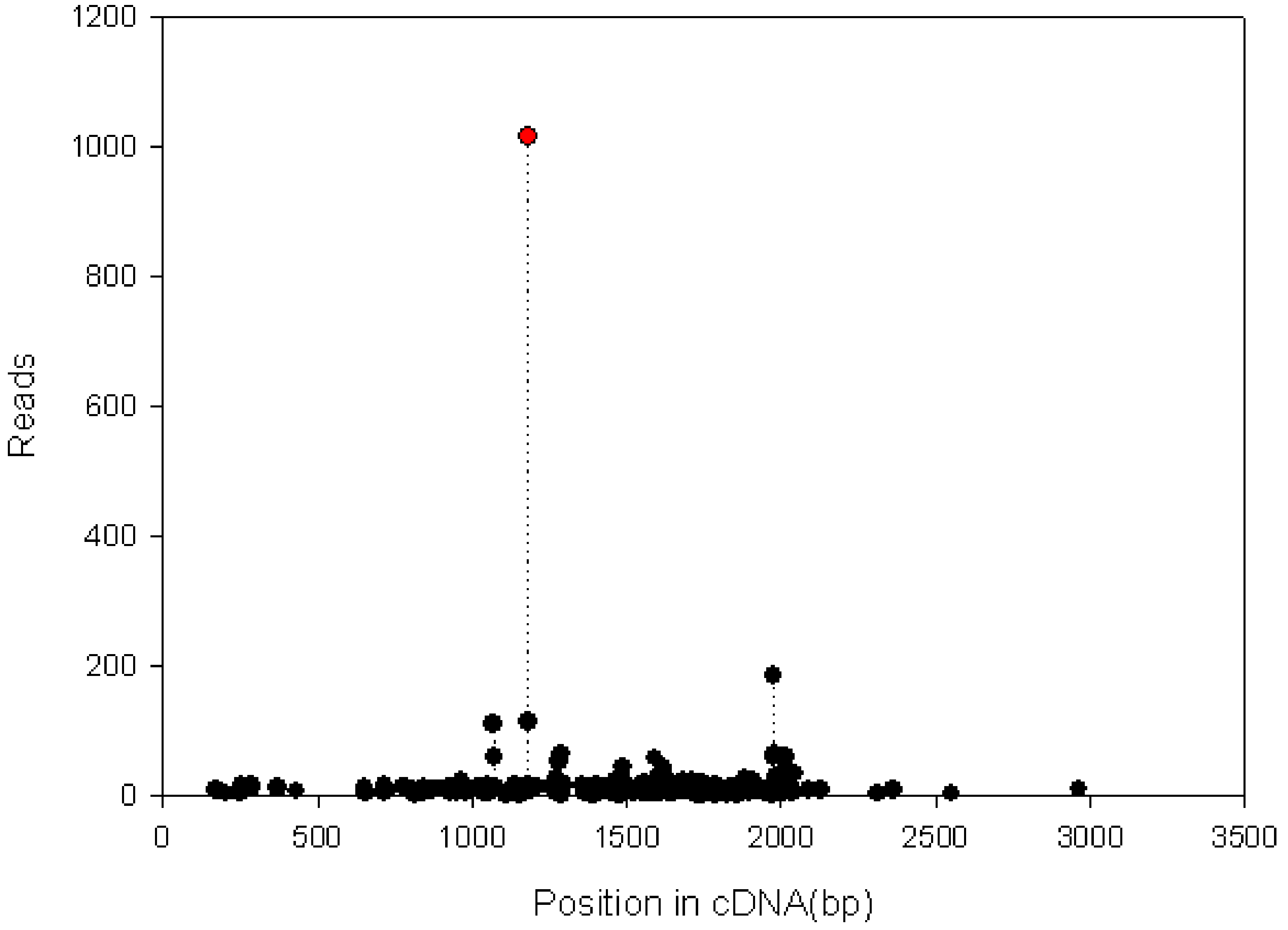


```
5' CCCGUUCAAGAAAGCCUGUGGAAUUA 3'        Cs5g01380.1
       :::::::::::: ::::::
3' ----AAGUUCUUUCGG-CACCUU--- 5'        Csi-miR396d.4
```

Csi-miR396d.4, target=Cs5g01840.1 gene=Cs5g01840
Category:3
Score=5
Cleavage Site=1251

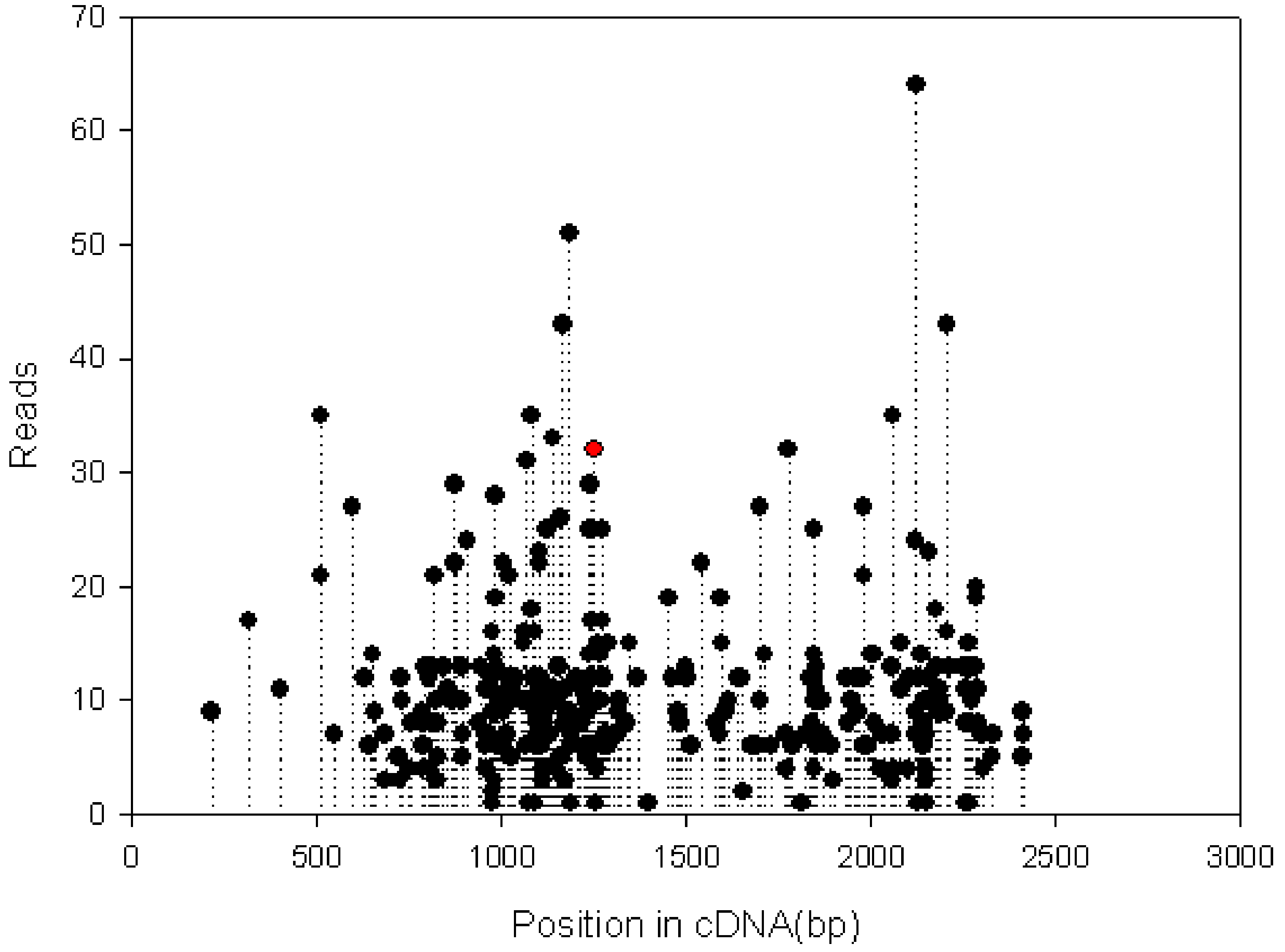


```
5' CCUGUGAAGAAGGCCAUGGAAGAUGC 3'        Cs5g01840.1
        : ::::::.::: :::::
3' ---AAGUUCUUUCGGCACCUU----- 5'        Csi-miR396d.4
```

Csi-miR396d.4, target=Cs5g06510.1 gene=Cs5g06510
Category:3
Score=5
Cleavage Site=1185

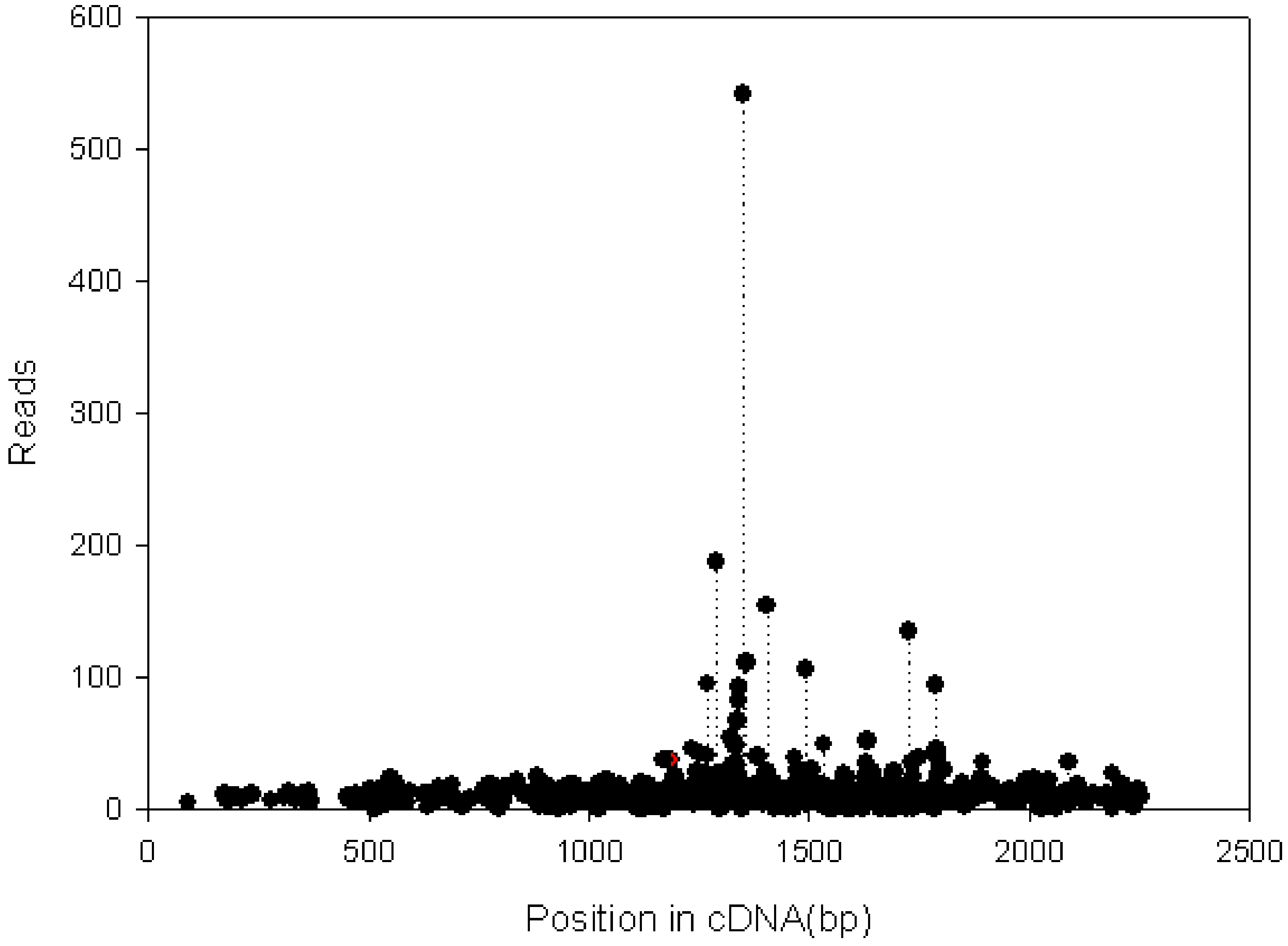


```
5' UAAUCAAGAAAGCUGAGGACUUAGCU 3'        Cs5g06510.1
     ::::::::::.: :::
3' --AAGUUCUUUCGGCACCUU------ 5'        Csi-miR396d.4
```

Csi-miR396d.4, target=Cs5g09850.1 gene=Cs5g09850
Category:3
Score=3
Cleavage Site=767

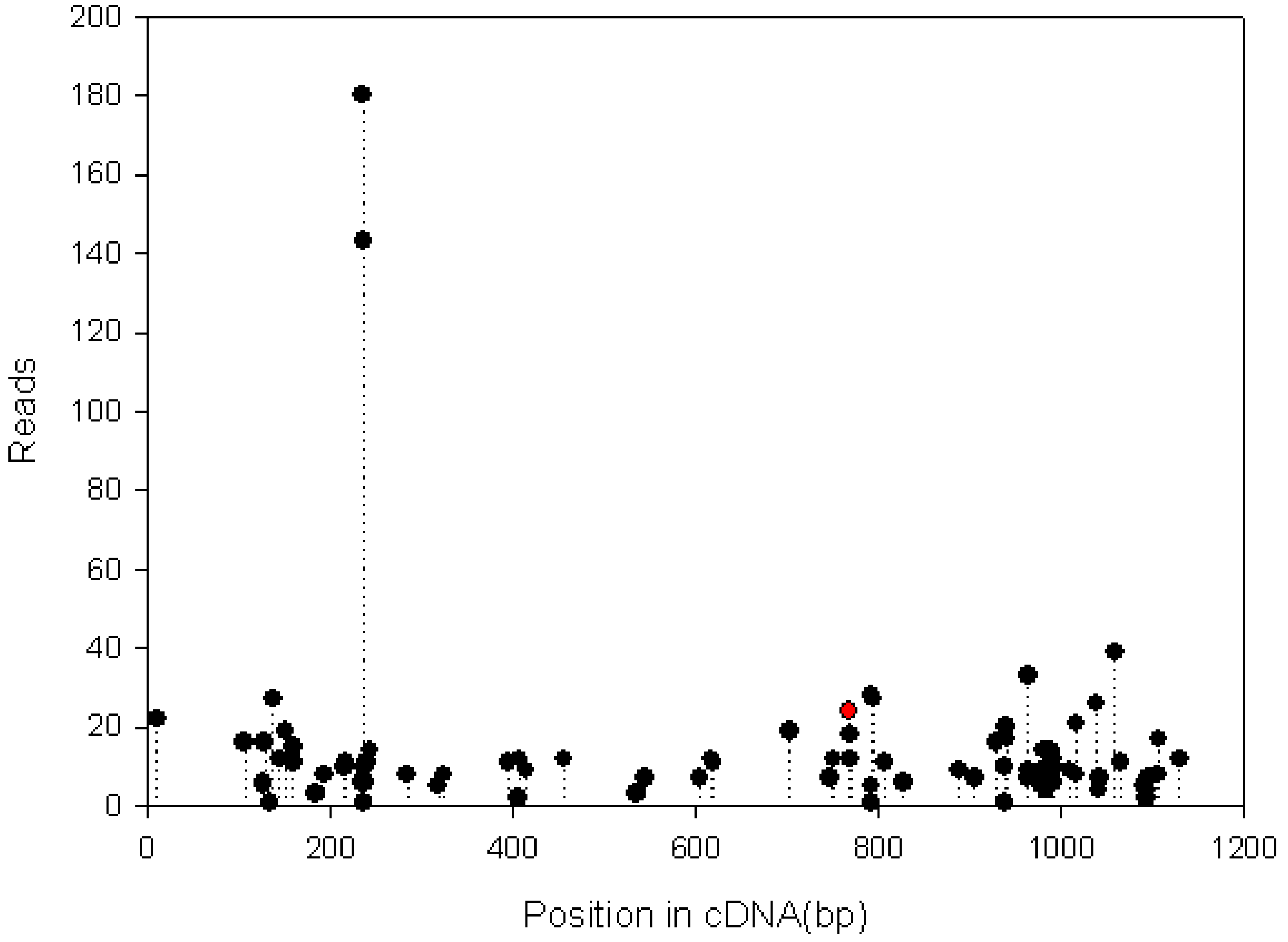


```
5' UGCGUUCAAGAAAGCUUGUGGAAGCU 3'        Cs5g09850.1
         :::::::::: .::::::
3' ----AAGUUCUUUCG-GCACCUU--- 5'        Csi-miR396d.4
```

Csi-miR396d.4, target=Cs6g17090.1 gene=Cs6g17090
Category:3
Score=4.5
Cleavage Site=1398

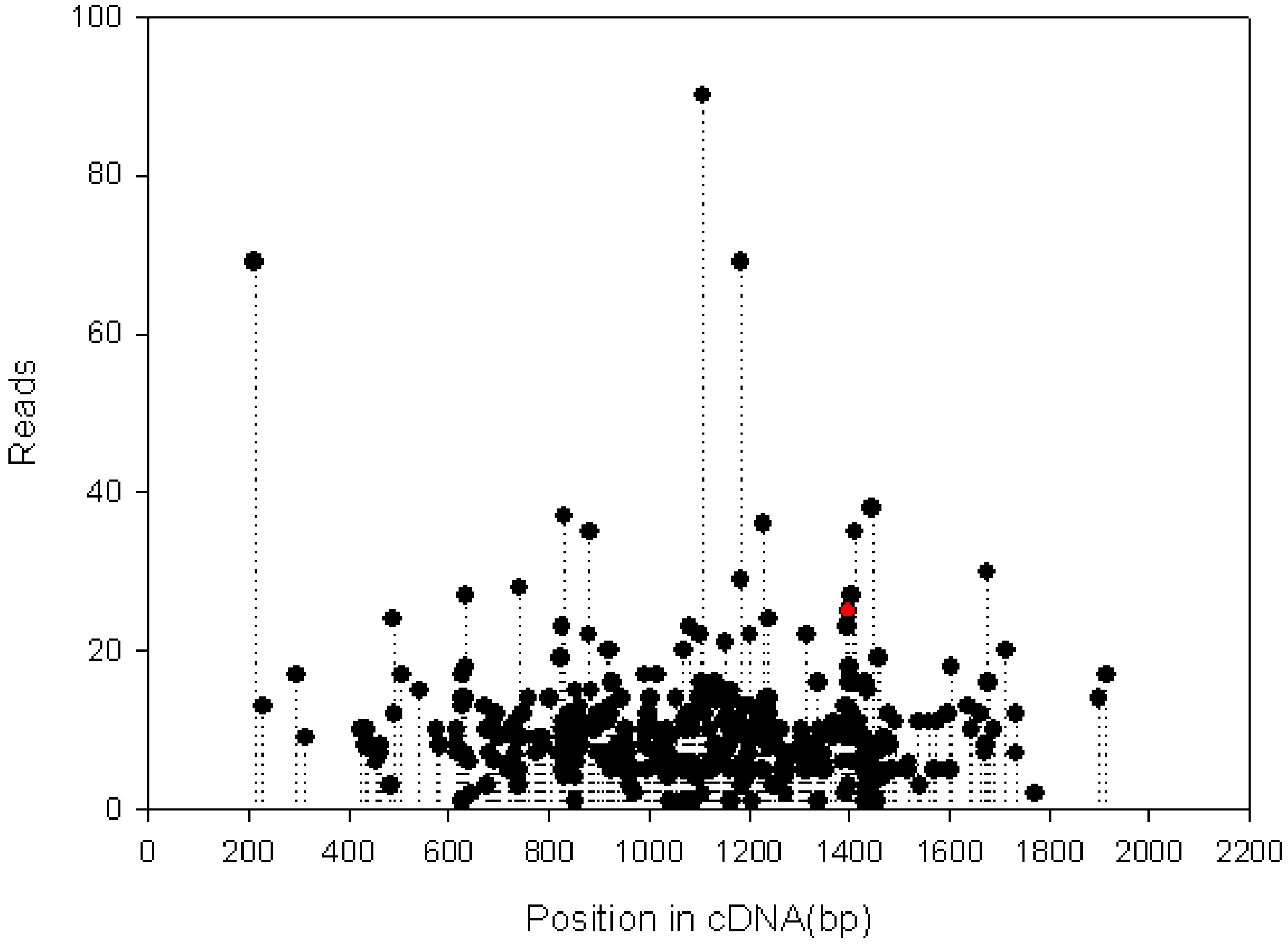


```
5' AAUUUGAGAGAGGCGUGGAGAUUCUG 3'        Cs6g17090.1
     ::..:::.:: :::::.
3' --AAGUUCUUUCGGCACCUU------ 5'        Csi-miR396d.4
```

Csi-miR396d.4, target=Cs6g19380.1 gene=Cs6g19380
Category:2
Score=5
Cleavage Site=1152

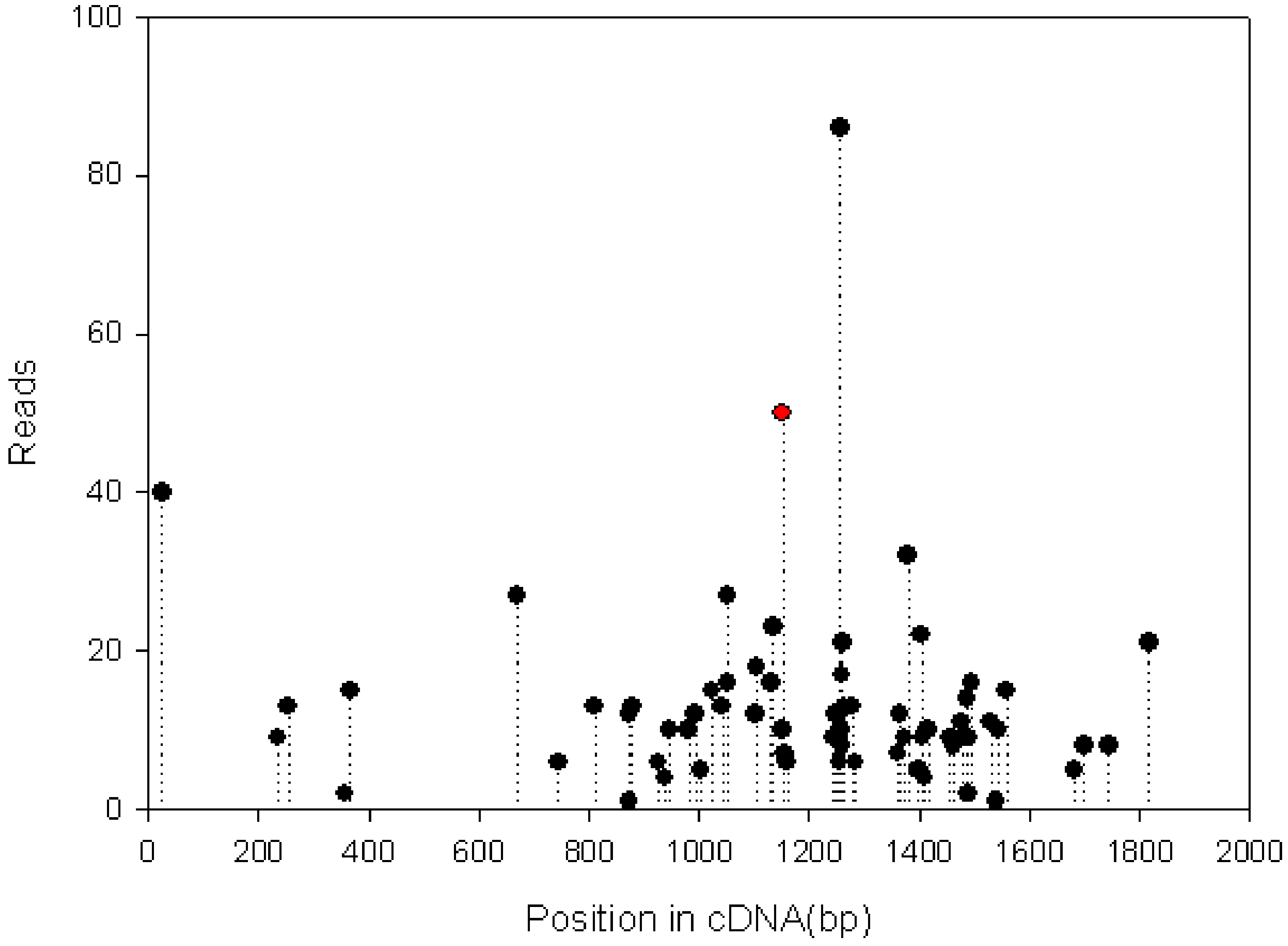


```
5' CUUUUACUUUUAGAGAAGCUGUGGAA 3'        Cs6g19380.1
           ::.:. .::::.::::::
3' --------AAGUUCUUUCGGCACCUU 5'        Csi-miR396d.4
```

Csi-miR396d.4, target=Cs7g15220.1 gene=Cs7g15220
Category:1
Score=2
Cleavage Site=377

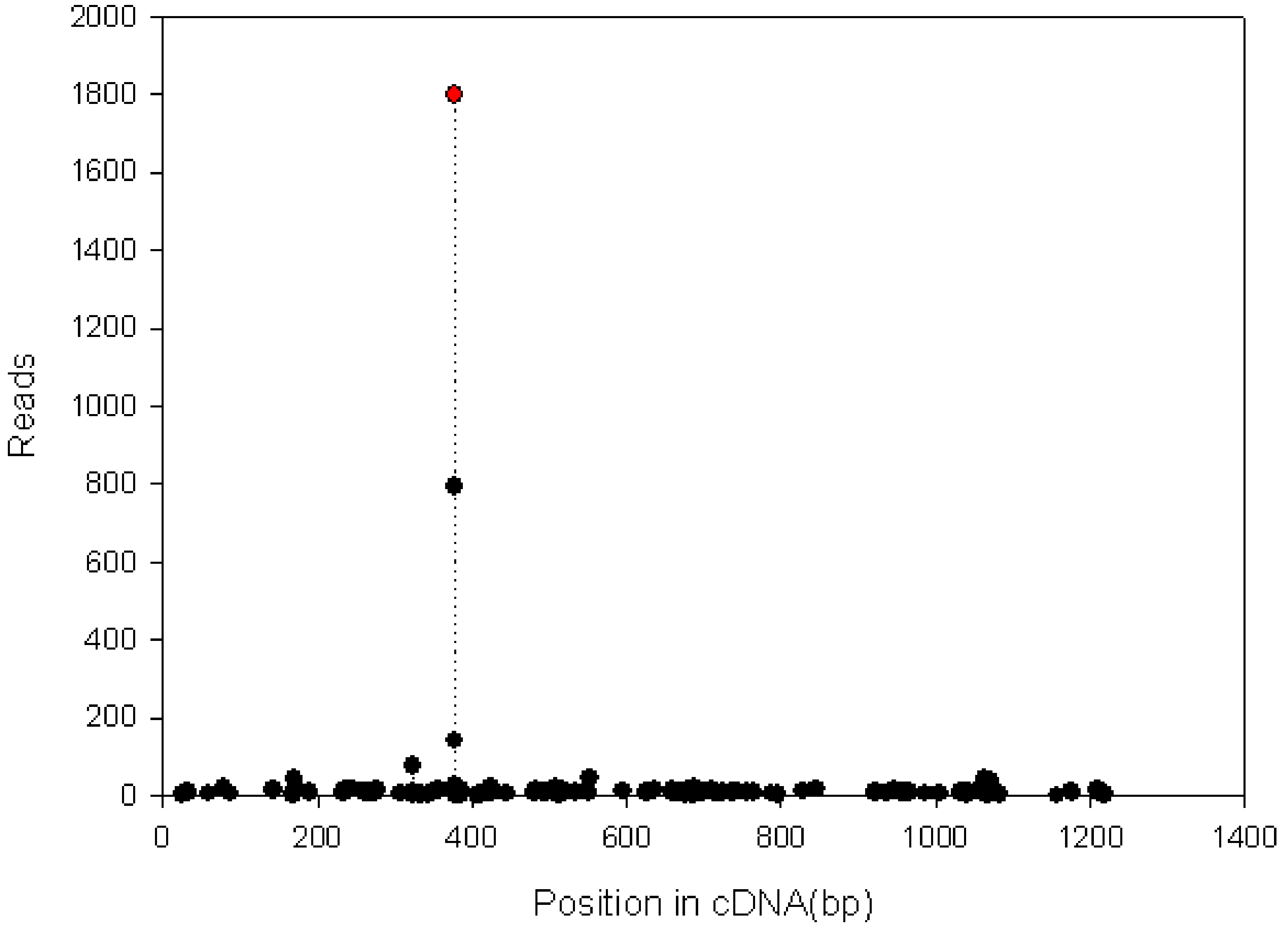


```
5' ACCGUUCAAGAAAGCCUGUGGAAUCA 3'        Cs7g15220.1
         :::::::::::: ::::::
3' ----AAGUUCUUUCGG-CACCUU--- 5'        Csi-miR396d.4
```

Csi-miR396d.4, target=Cs7g27670.1 gene=Cs7g27670
Category:1
Score=5
Cleavage Site=653

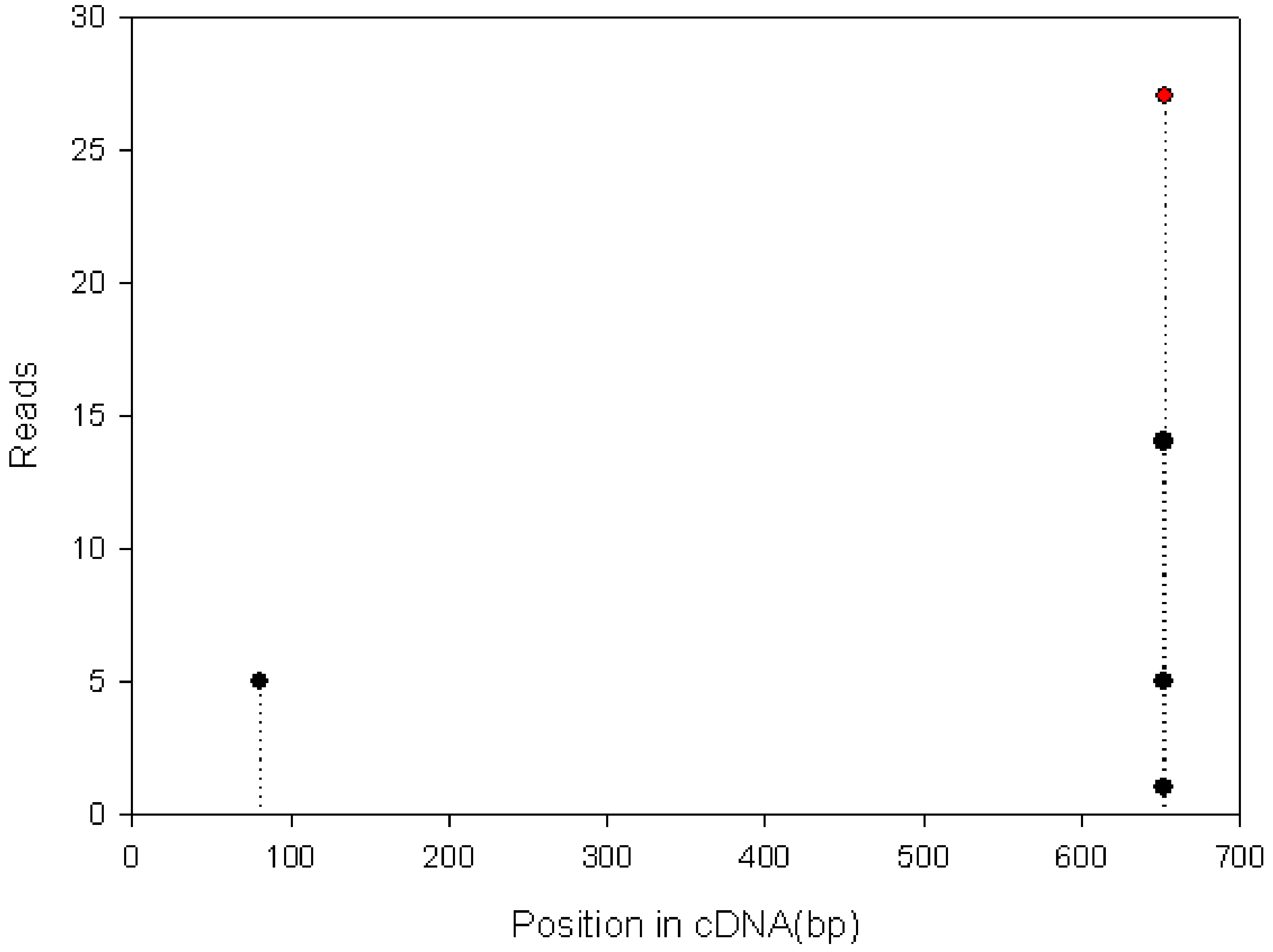


```
5' UCAUCCAAGAAGGCUAUGGAAUGGAC 3'        Cs7g27670.1
      : :::::::.::. :::::
3' ---AAGUUCUUUCGGCACCUU----- 5'        Csi-miR396d.4
```

Csi-miR396d.4, target=Cs7g27680.1 gene=Cs7g27680
Category:1
Score=5
Cleavage Site=713

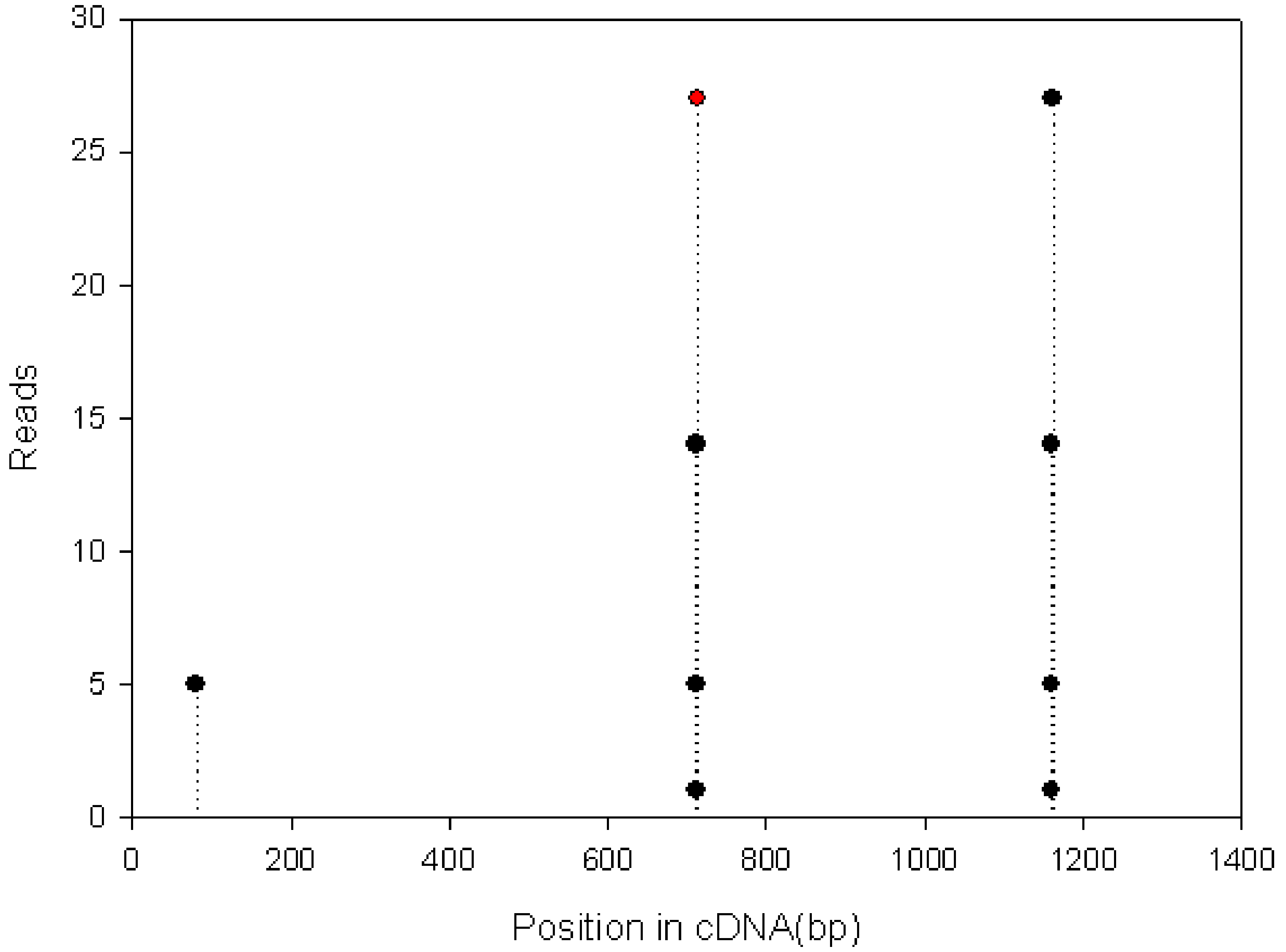


```
5' UCAUCCAAGAAGGCUAUGGAAUGGAC 3'        Cs7g27680.1
      : ::::::.::. :::::
3' ---AAGUUCUUUCGGCACCUU----- 5'        Csi-miR396d.4
```

Csi-miR396d.4, target=Cs8g03230.1 gene=Cs8g03230
Category:3
Score=4.5
Cleavage Site=666

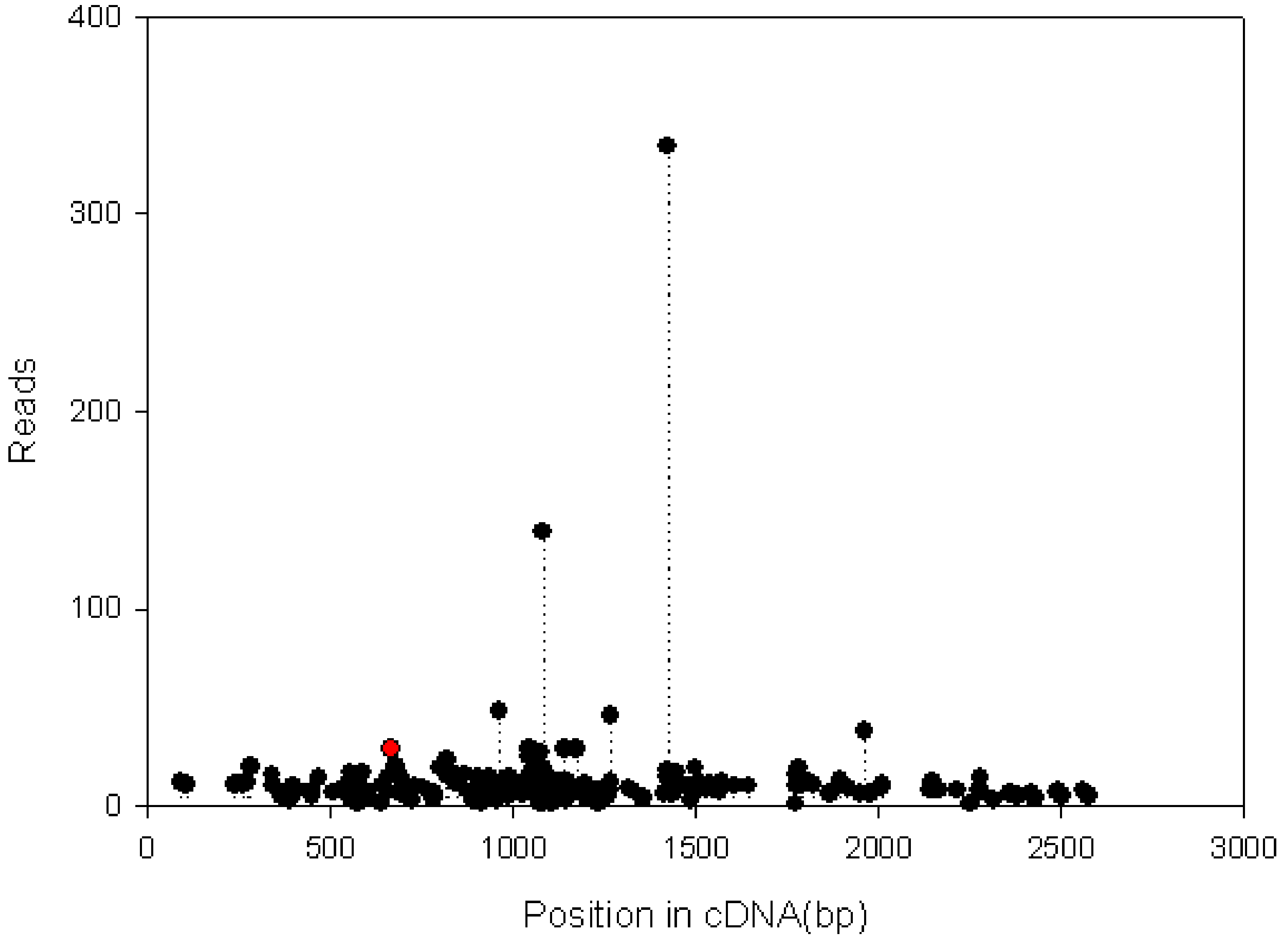


```
5' GCCGGCGUUCCGAGGAAGCC-UGGAAU 3'        Cs8g03230.1
           :  :.::.:::::  :::::
3' --------AAGUUCUUUCGGCACCUU- 5'        Csi-miR396d.4
```

Csi-miR396d.4, target=Cs8g17370.1 gene=Cs8g17370
Category:3
Score=2.5
Cleavage Site=140

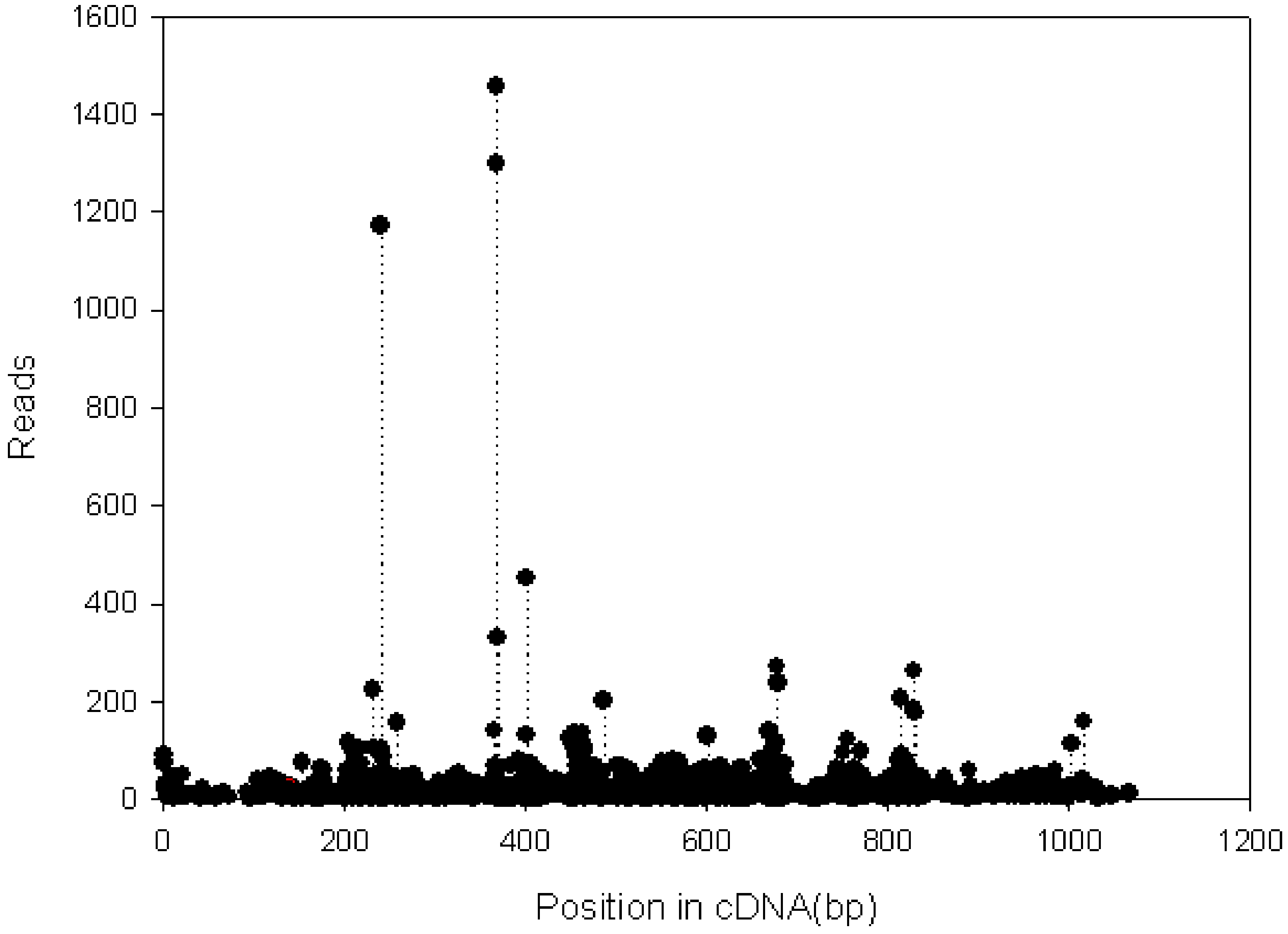


```
5' AGGAUUACAAGAAGGCCGUGGAGAAA 3'        Cs8g17370.1
         : ::::::.::::::::.
3' -----AAGUUCUUUCGGCACCUU--- 5'        Csi-miR396d.4
```

Csi-miR396d.4, target=Orange1.1t00172.1 gene=Orange1.1t00172
Category:1
Score=2
Cleavage Site=656

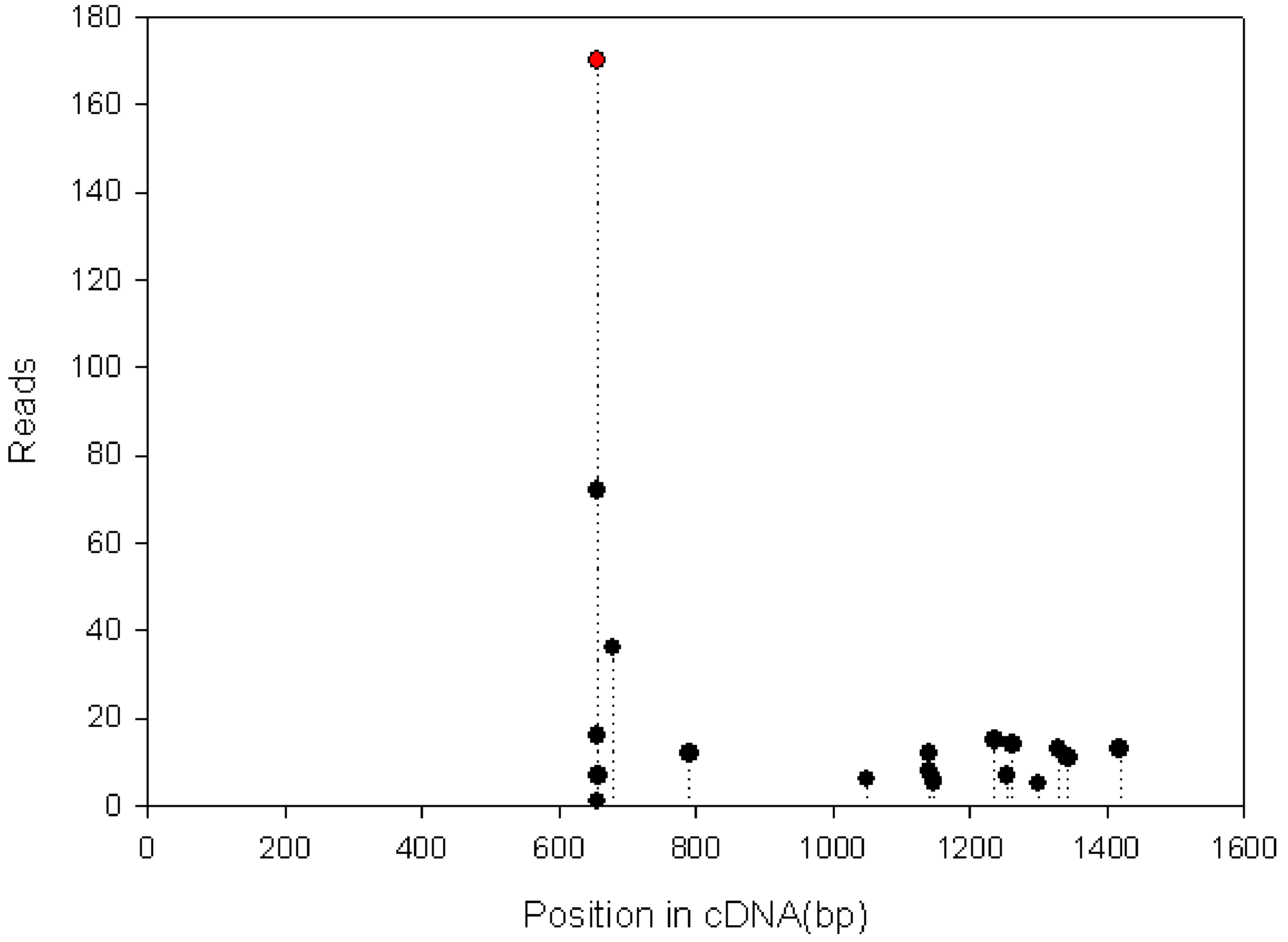


```
5' ACCGUUCAAGAAAGCCUGUGGAACUU 3'        Orange1.1t00172.1
       :::::::::::: ::::::
3' ----AAGUUCUUUCGG-CACCUU--- 5'        Csi-miR396d.4
```

Csi-miR396d.4, target=Orange1.1t02555.1 gene=Orange1.1t02555
Category:1
Score=2
Cleavage Site=920

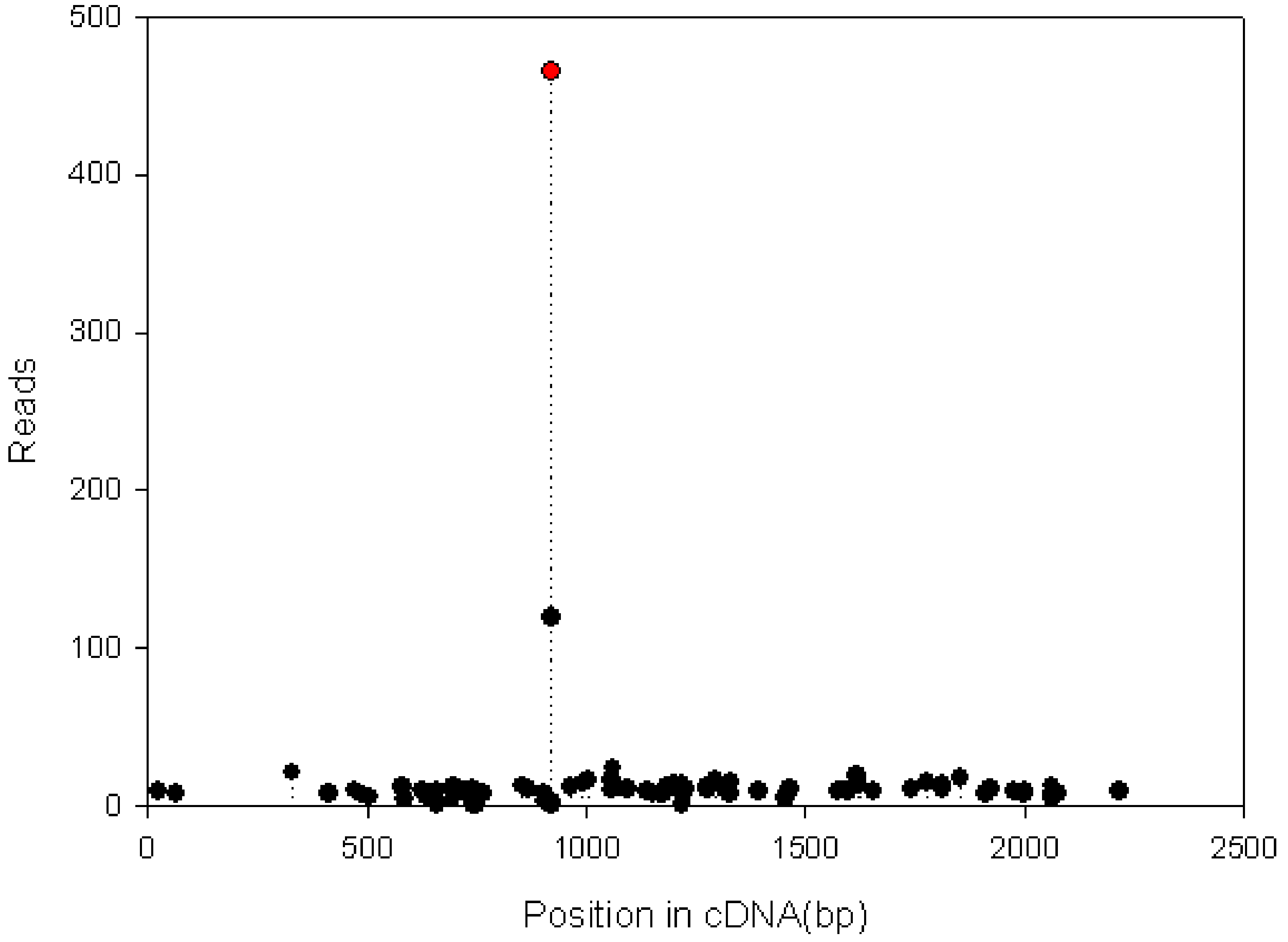


```
5' CACGUUCAAGAAAGCCUGUGGAACUU 3'        Orange1.1t02555.1
       :::::::::::: ::::::
3' ----AAGUUCUUUCGG-CACCUU--- 5'        Csi-miR396d.4
```

Csi-miR396d.4, target=Orange1.1t03122.1 gene=Orange1.1t03122
Category:1
Score=2
Cleavage Site=583

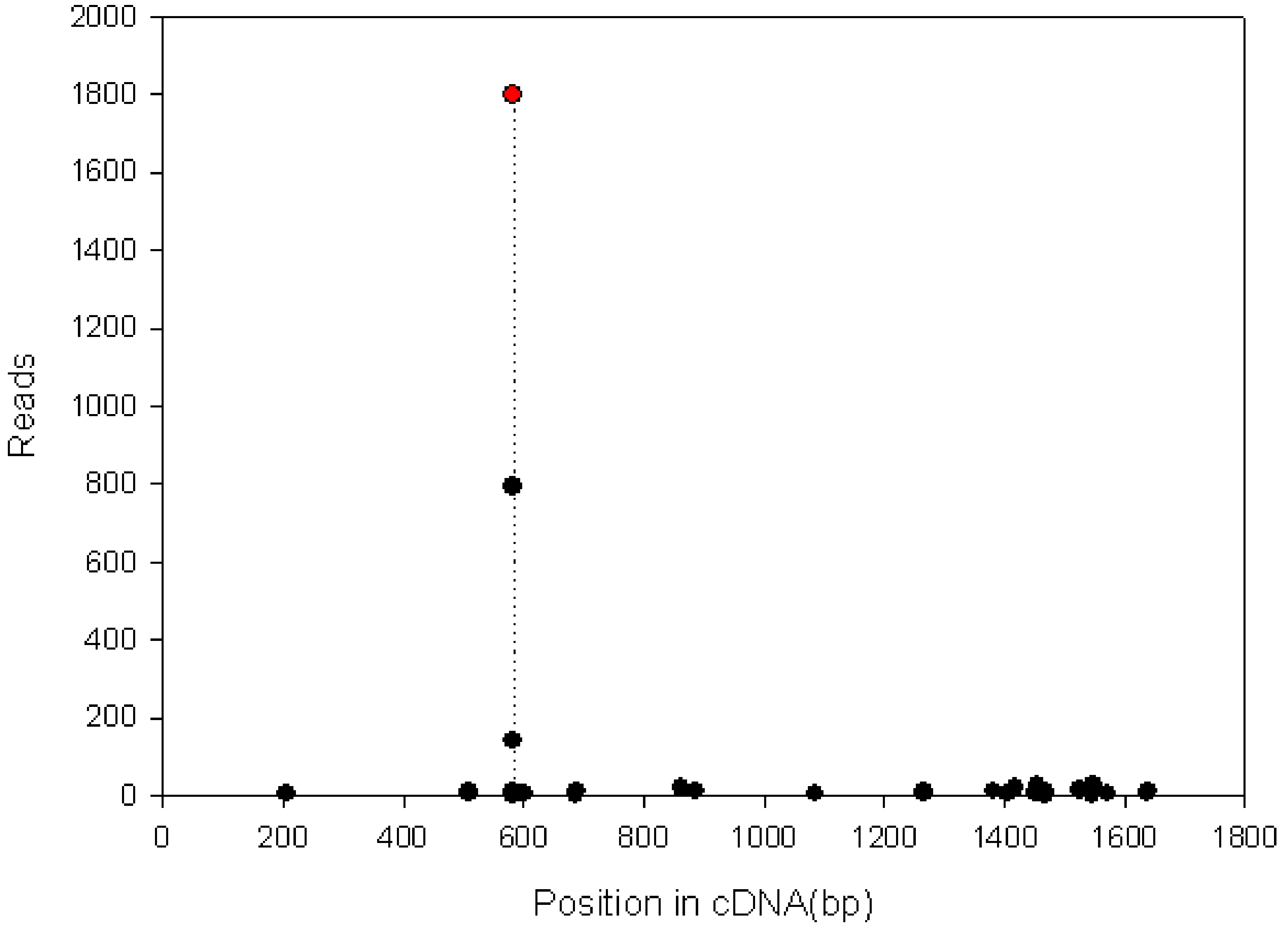


```
5' ACCGUUCAAGAAAGCCUGUGGAAUCA 3'        Orange1.1t03122.1
       :::::::::::: ::::::
3' ----AAGUUCUUUCGG-CACCUU--- 5'        Csi-miR396d.4
```

Csi-miR397.1, target=Orange1.1t03059.1 gene=Orange1.1t03059
Category:3
Score=3.5
Cleavage Site=1926

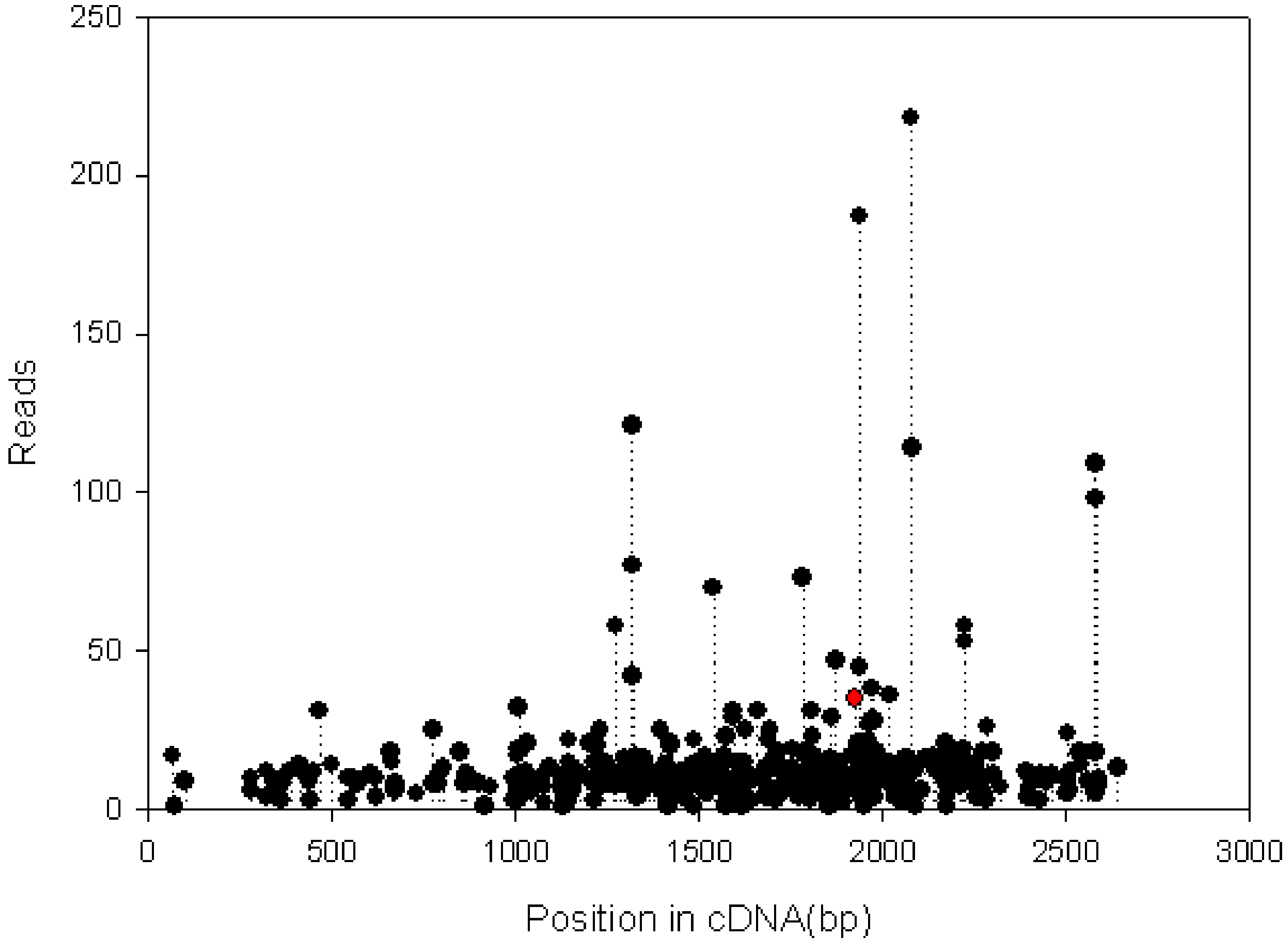


```
5' UAGCAUGCAAUGGUGCACUCAAUGAG 3'        Orange1.1t03059.1
      ::: :::.: ::::::::::::
3' ---GUA-GUUGCGACGUGAGUUACU- 5'        Csi-miR397.1
```

Csi-miR397.2, target=Orange1.1t03059.1 gene=Orange1.1t03059
Category:3
Score=4.5
Cleavage Site=1926

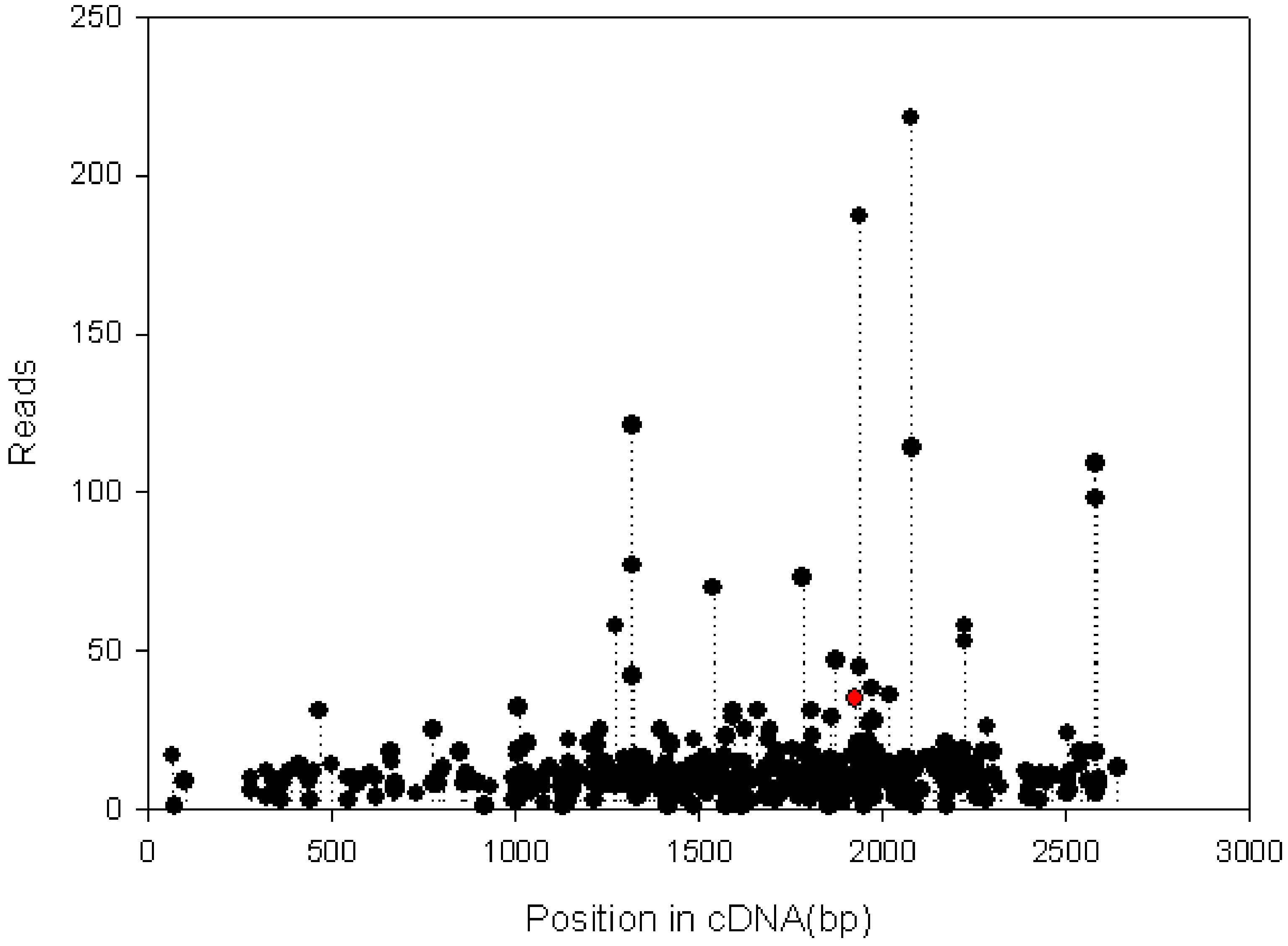


```
5' UAGCAUGCAAUGGUGCACUCAAUGAG 3'        Orange1.1t03059.1
     ::: :::.: :::::::::::
3' --AGUA-GUUGCGACGUGAGUUAC-- 5'        Csi-miR397.2
```

Csi-miR403.1, target=Cs2g10760.1 gene=Cs2g10760
Category:1
Score=0
Cleavage Site=3364

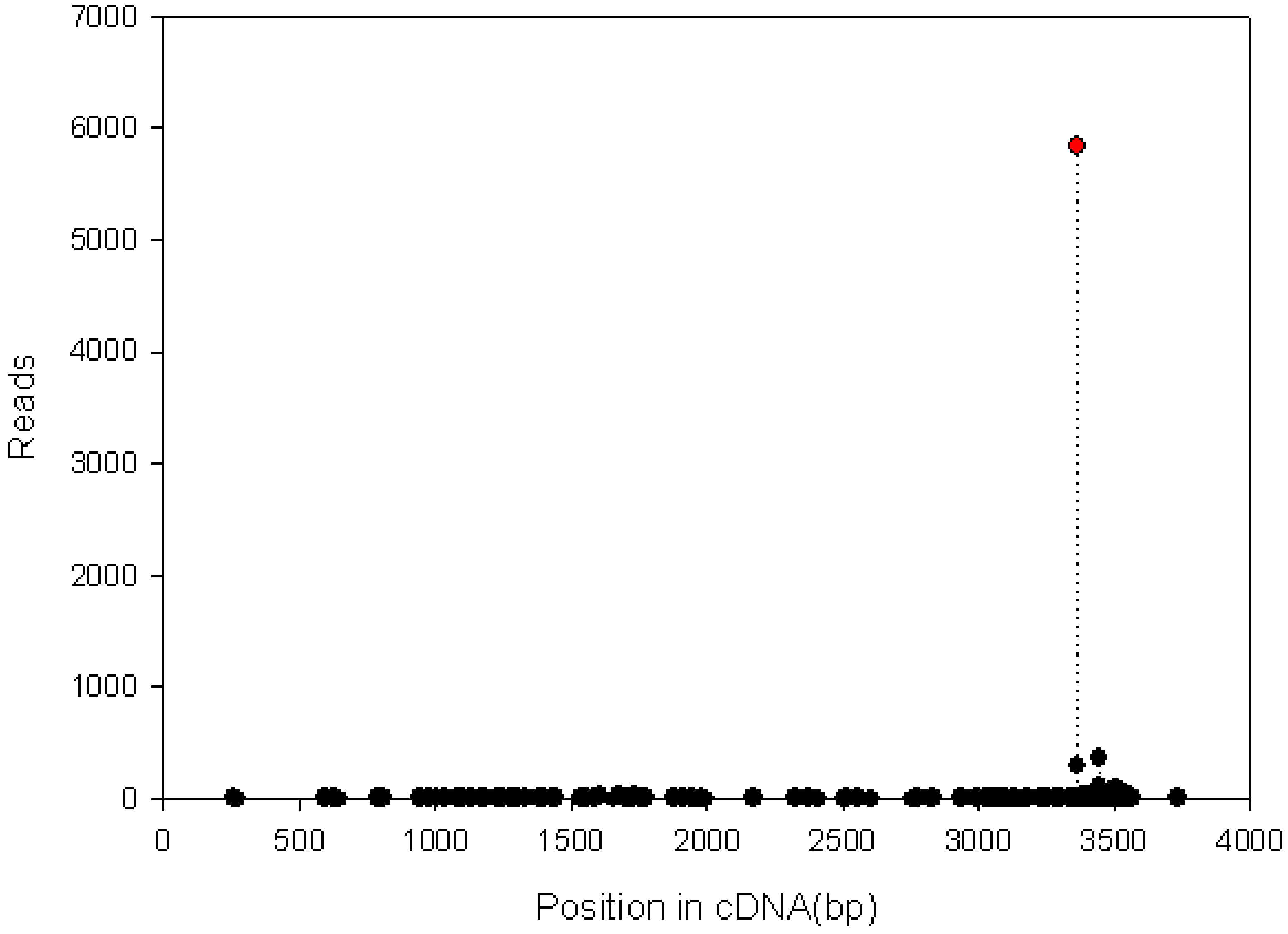


```
5' AGGAGUUUGUGCGUGAAUCUAAUGAG 3'        Cs2g10760.1
      ::::::::::::::::::::
3' ---UCAAACACGCACUUAGAUU---- 5'        Csi-miR403.1
```

Csi-miR403.1, target=Cs2g10770.1 gene=Cs2g10770
Category:3
Score=1
Cleavage Site=3478

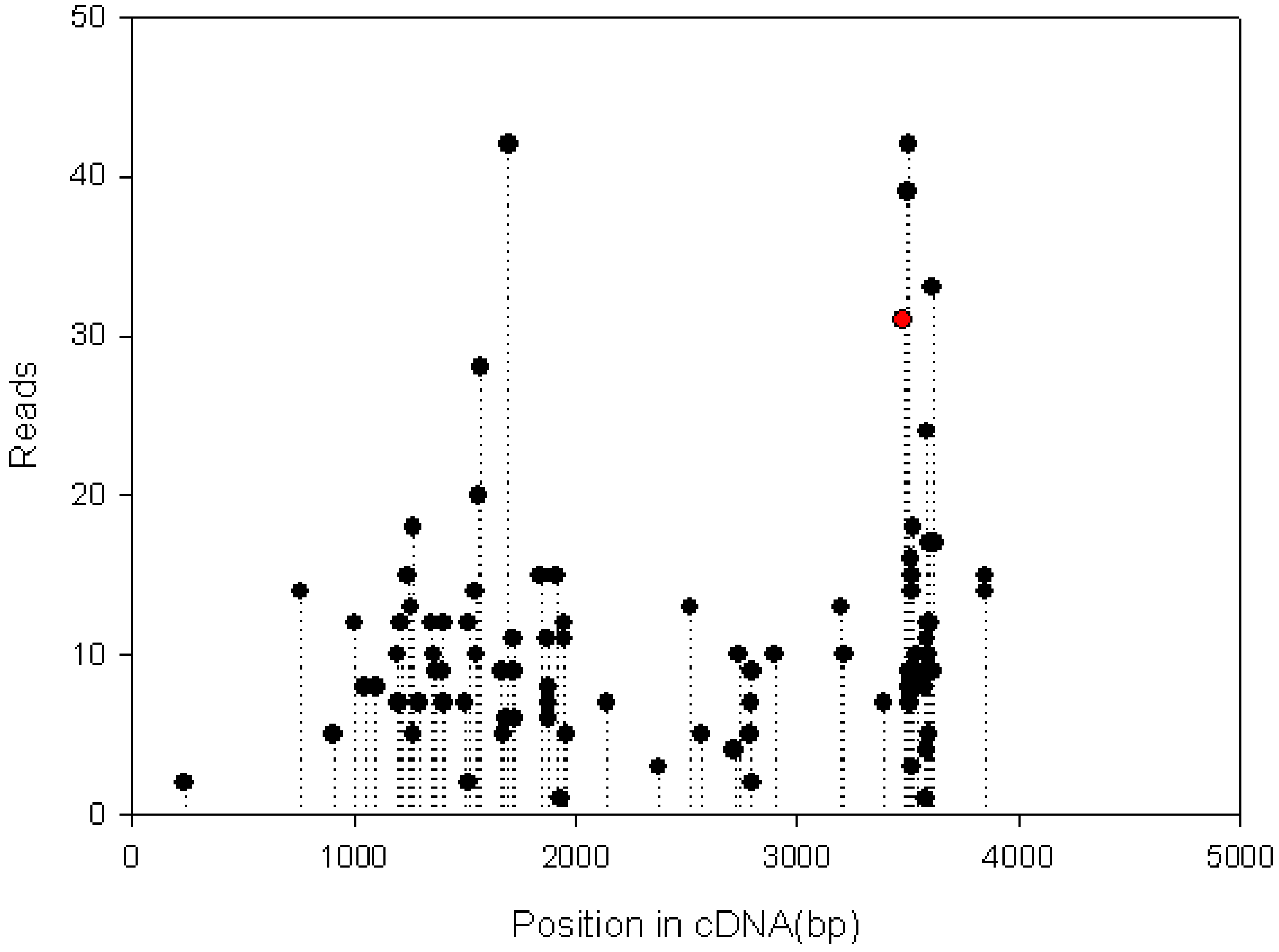


```
5' AGGAGUUCGUGCGUGAAUCUAAUGCG 3'        Cs2g10770.1
     :::: ::::::::::::::
3' ---UCAAACACGCACUUAGAUU---- 5'        Csi-miR403.1
```

Csi-miR403.1, target=Cs3g01650.1 gene=Cs3g01650
Category:2
Score=5
Cleavage Site=2428

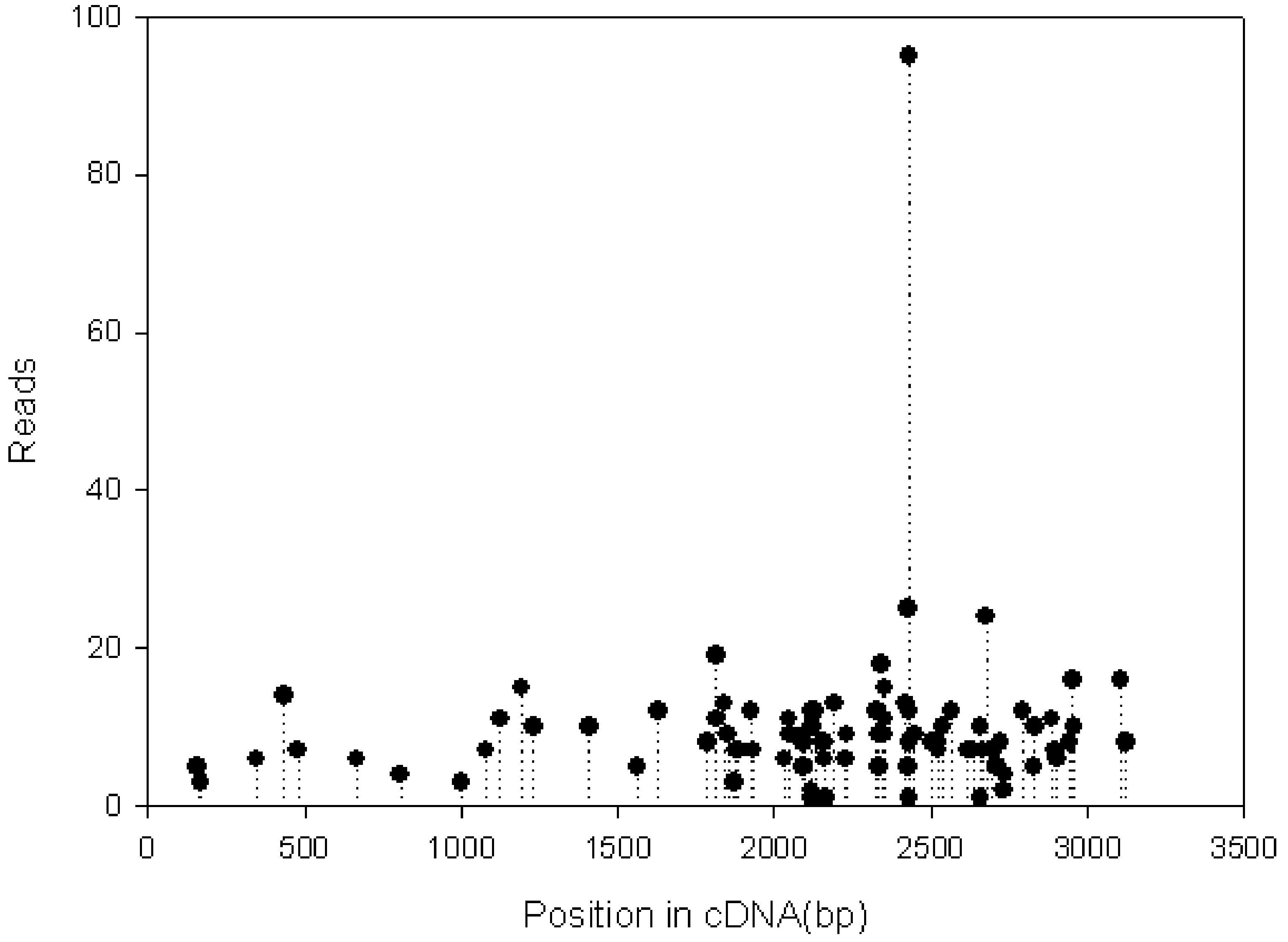


```
5' AGUUU-UGUGAGAAUUUAAGUGGAGCU 3'        Cs3g01650.1
   ::::: ::.: ::::.:::
3' UCAAACACGCACUUAGAUU-------- 5'        Csi-miR403.1
```

Csi-miR403.1, target=Cs4g01460.1 gene=Cs4g01460
Category:1
Score=5
Cleavage Site=1693

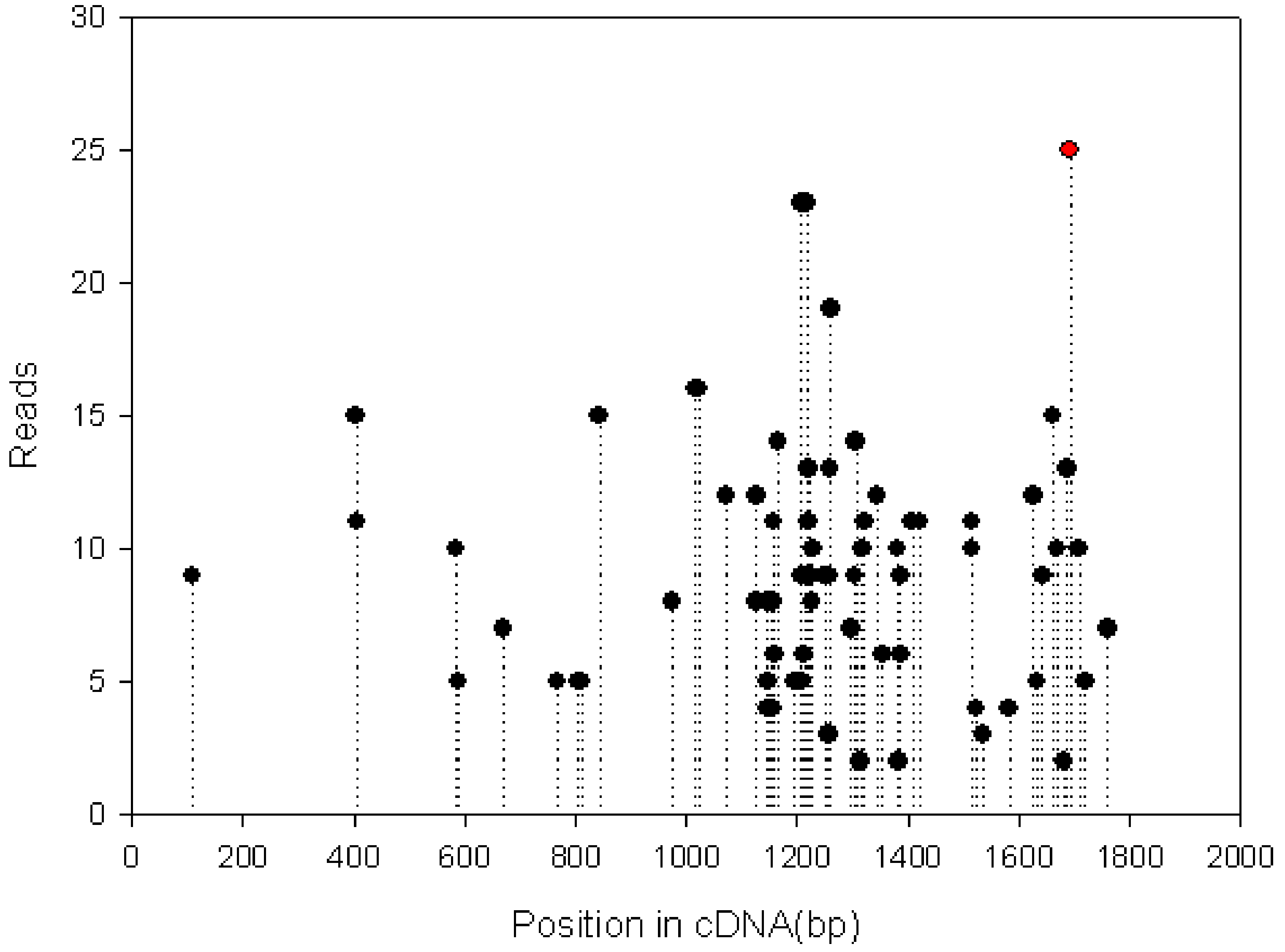


```
5' UGUGUAGACUUUGUGACUGAAUCUAA 3'        Cs4g01460.1
         : ::::::  :::::::::
3' -------UCAAACACGCACUUAGAUU 5'        Csi-miR403.1
```

Csi-miR403.1, target=Cs4g19605.1 gene=Cs4g19605
Category:1
Score=5
Cleavage Site=792

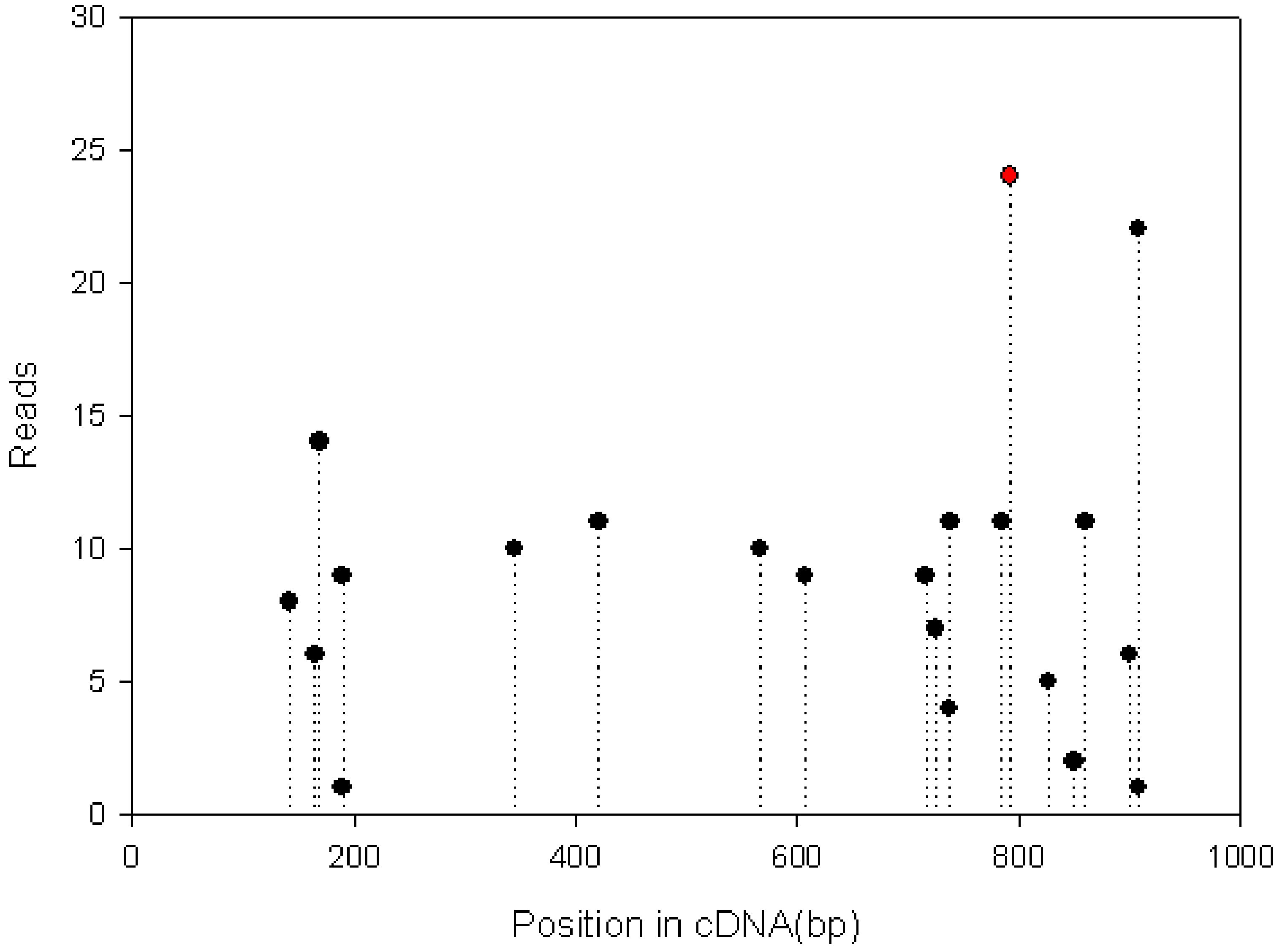


```
5' AGGUAUUUGUGUGUGGAUCUAUAUAU 3'        Cs4g19605.1
         ::::::.:::.:::::
3' ---UCAAACACGCACUUAGAUU---- 5'        Csi-miR403.1
```

Csi-miR403.2, target=Cs2g10760.1 gene=Cs2g10760
Category:1
Score=1
Cleavage Site=3364

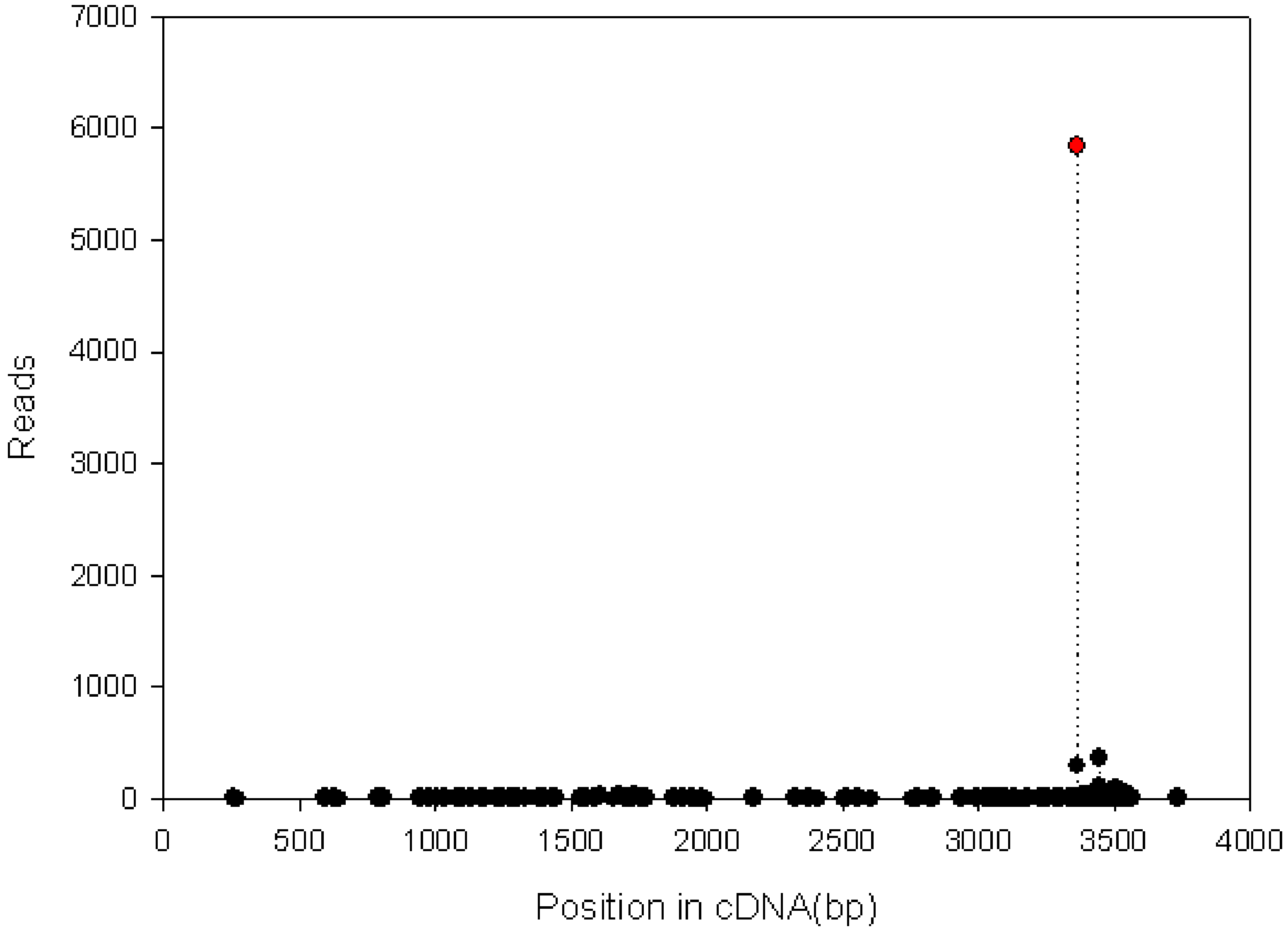


```
5' AGGAGUUUGUGCGUGAAUCUAAUGAG 3'        Cs2g10760.1
    ::::::::::::::::::::
3' -GCUCAAACACGCACUUAGAUU---- 5'        Csi-miR403.2
```

Csi-miR403.2, target=Cs2g10770.1 gene=Cs2g10770
Category:3
Score=2
Cleavage Site=3478

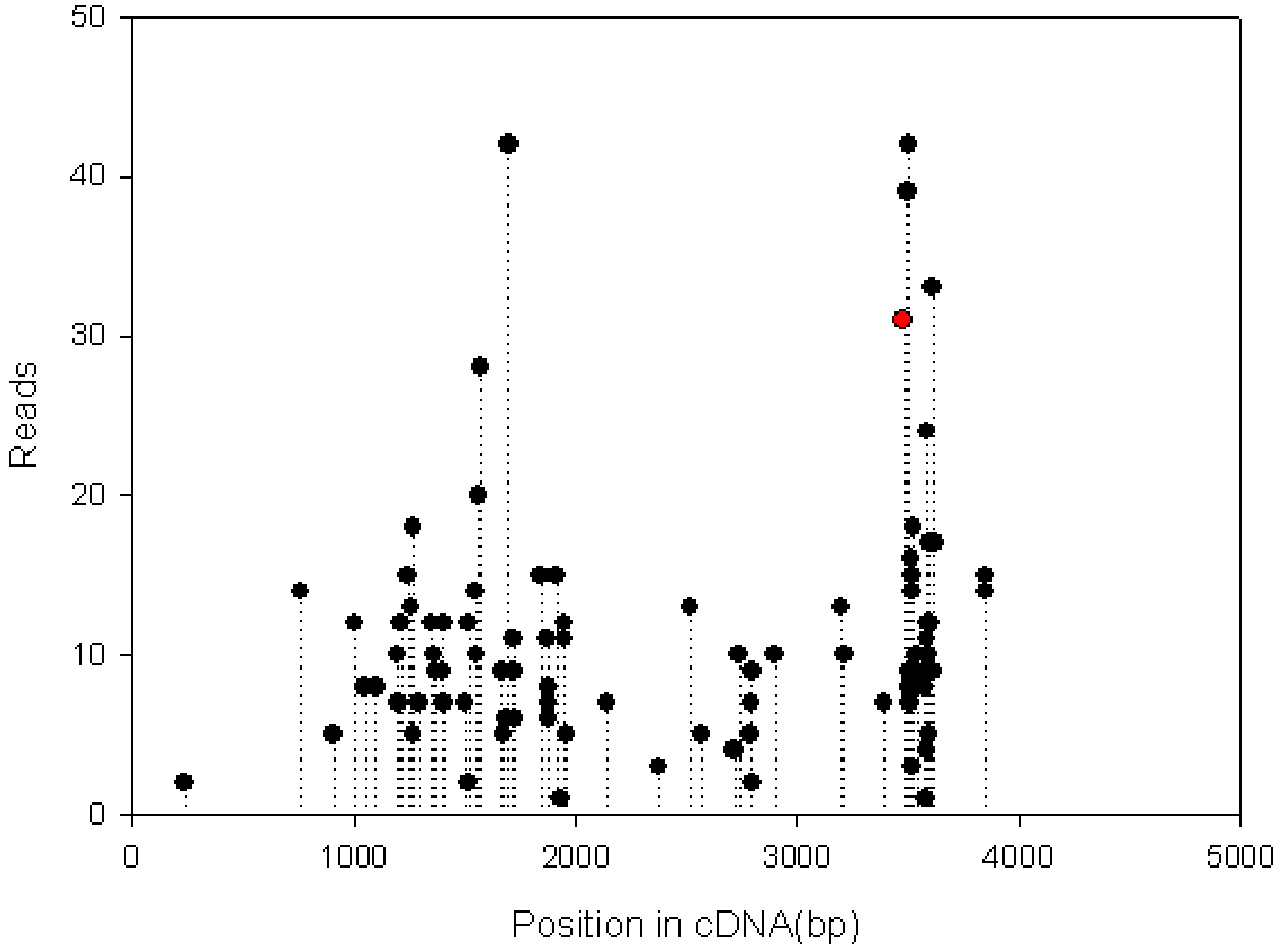


```
5' AGGAGUUCGUGCGUGAAUCUAAUGCG 3'        Cs2g10770.1
     ::::: ::::::::::::::
3' -GCUCAAACACGCACUUAGAUU---- 5'        Csi-miR403.2
```

Csi-miR403.3, target=Cs2g10760.1 gene=Cs2g10760
Category:1
Score=0
Cleavage Site=3364

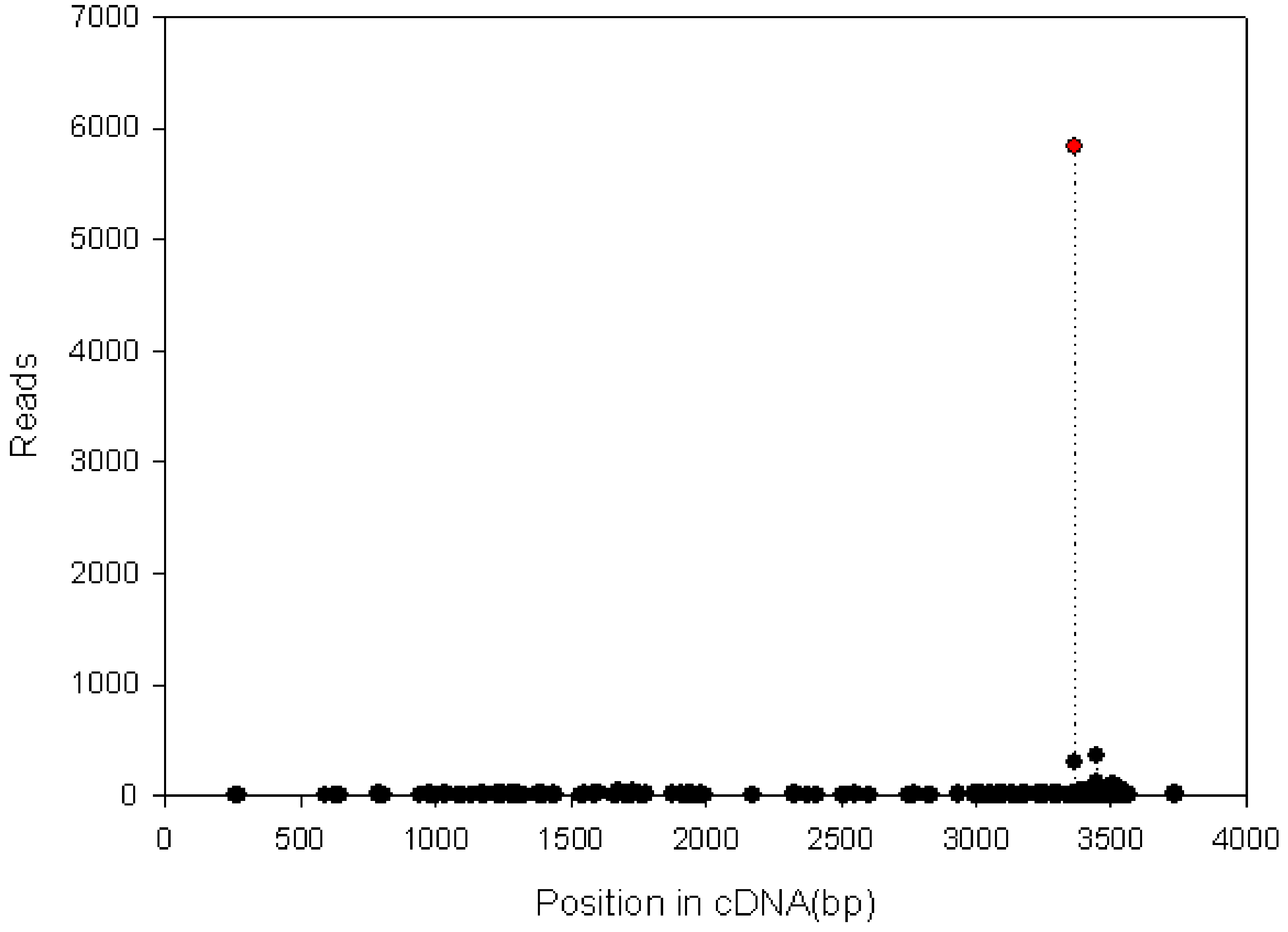


```
5' AGGAGUUUGUGCGUGAAUCUAAUGAG 3'        Cs2g10760.1
       ::::::::::::::::::
3' ----CAAACACGCACUUAGAUU---- 5'        Csi-miR403.3
```

Csi-miR403.3, target=Cs2g10770.1 gene=Cs2g10770
Category:3
Score=1
Cleavage Site=3478

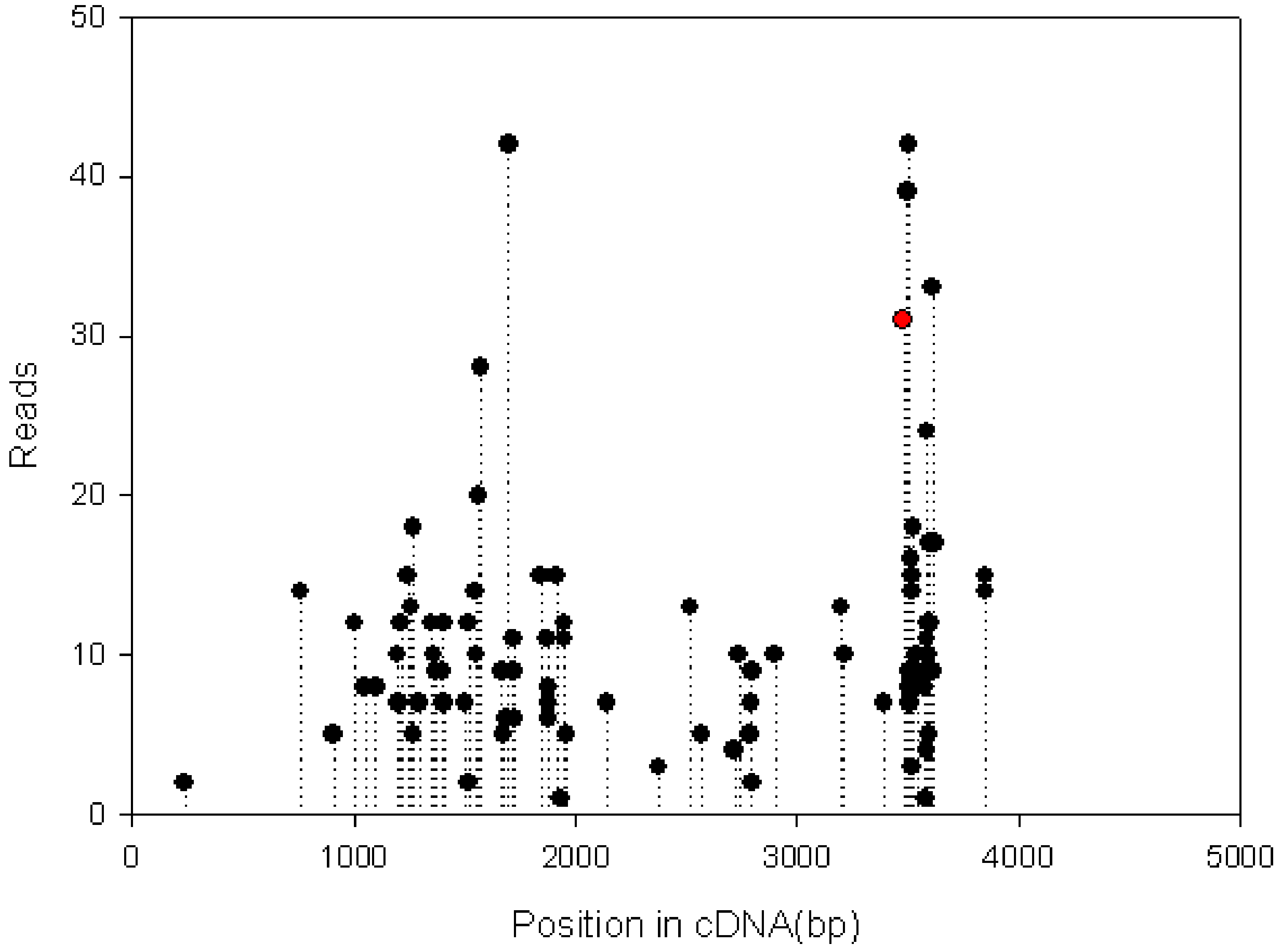


```
5' AGGAGUUCGUGCGUGAAUCUAAUGCG 3'        Cs2g10770.1
       ::: ::::::::::::::
3' ----CAAACACGCACUUAGAUU---- 5'        Csi-miR403.3
```

Csi-miR403.3, target=Cs3g01650.1 gene=Cs3g01650
Category:1
Score=5
Cleavage Site=2429

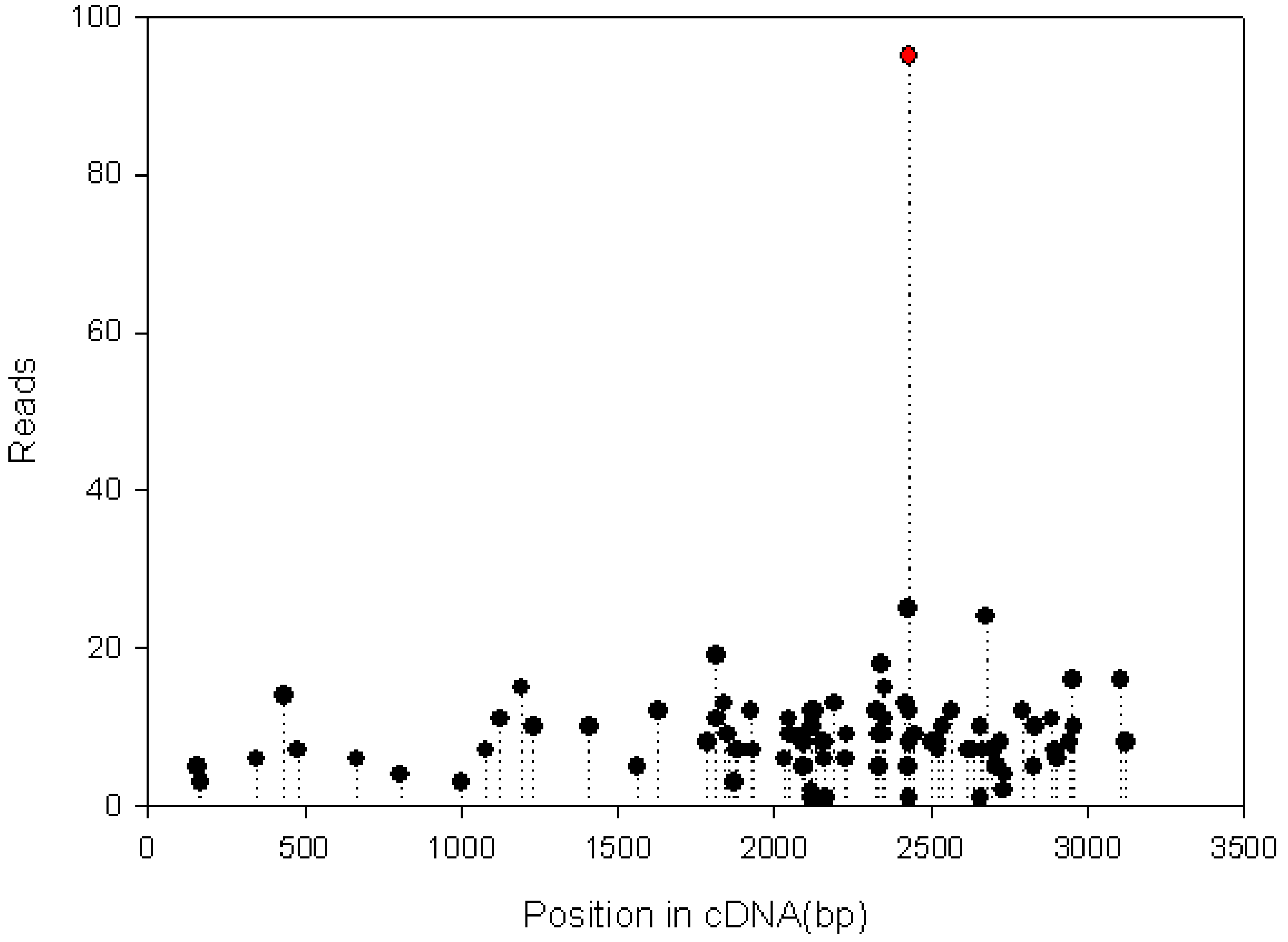


```
5' GUUU-UGUGAGAAUUUAAGUGGAGCUG 3'        Cs3g01650.1
   :::: ::.: ::::.:::
3' CAAACACGCACUUAGAUU--------- 5'        Csi-miR403.3
```

Csi-miR403.3, target=Cs4g01460.1 gene=Cs4g01460
Category:1
Score=5
Cleavage Site=1693

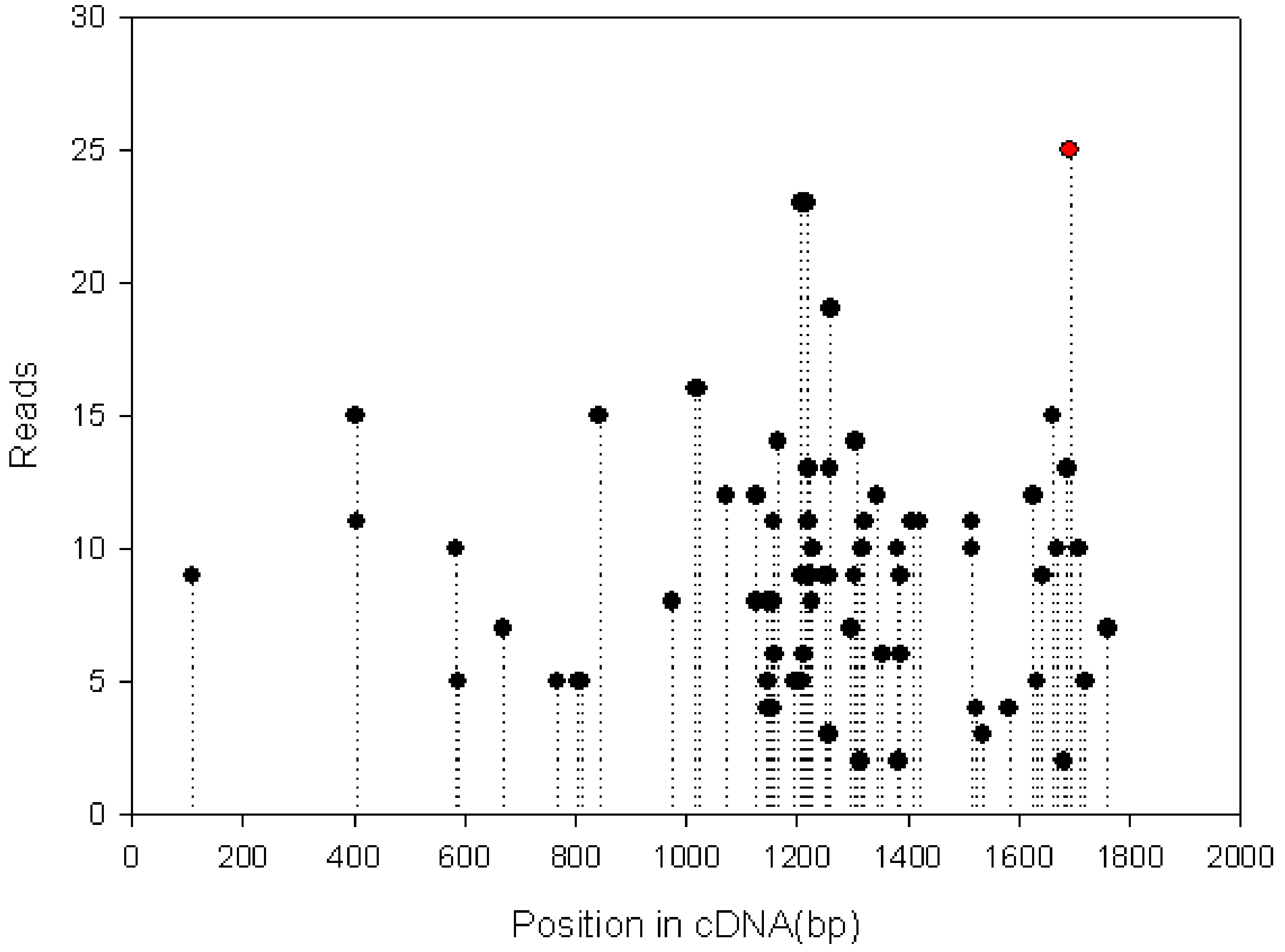


```
5' UGUGUAGACUUUGUGACUGAAUCUAA 3'        Cs4g01460.1
                 ::::::  :::::::::
3' --------CAAACACGCACUUAGAUU 5'        Csi-miR403.3
```

Csi-miR403.3, target=Cs4g19605.1 gene=Cs4g19605
Category:1
Score=4
Cleavage Site=792

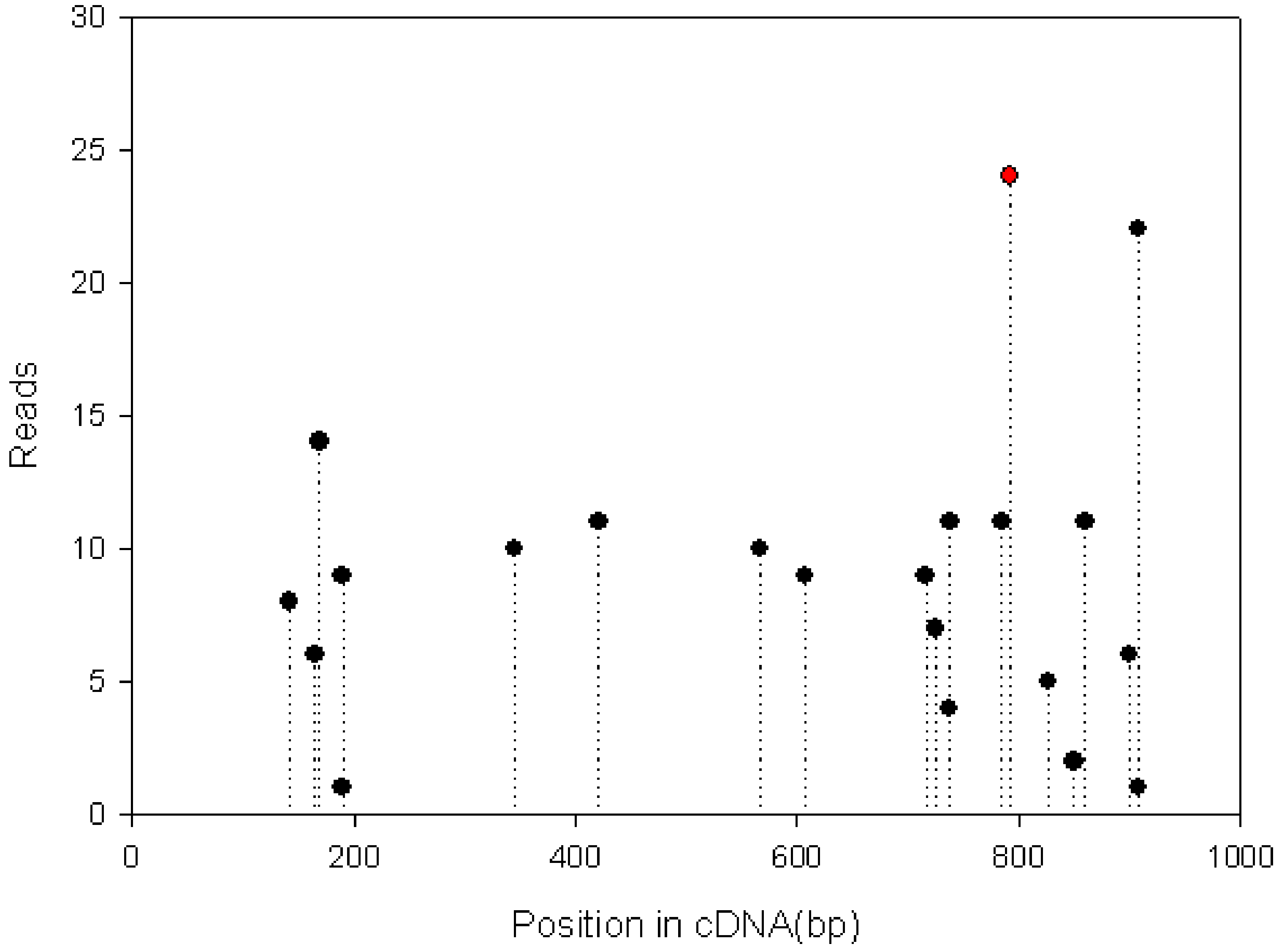


```
5' AGGUAUUUGUGUGUGGAUCUAUAUAU 3'        Cs4g19605.1
         ::::::.:::.:::::
3' ----CAAACACGCACUUAGAUU---- 5'        Csi-miR403.3
```

Csi-miR444a.1, target=Cs9g07680.1 gene=Cs9g07680
Category:3
Score=3
Cleavage Site=121

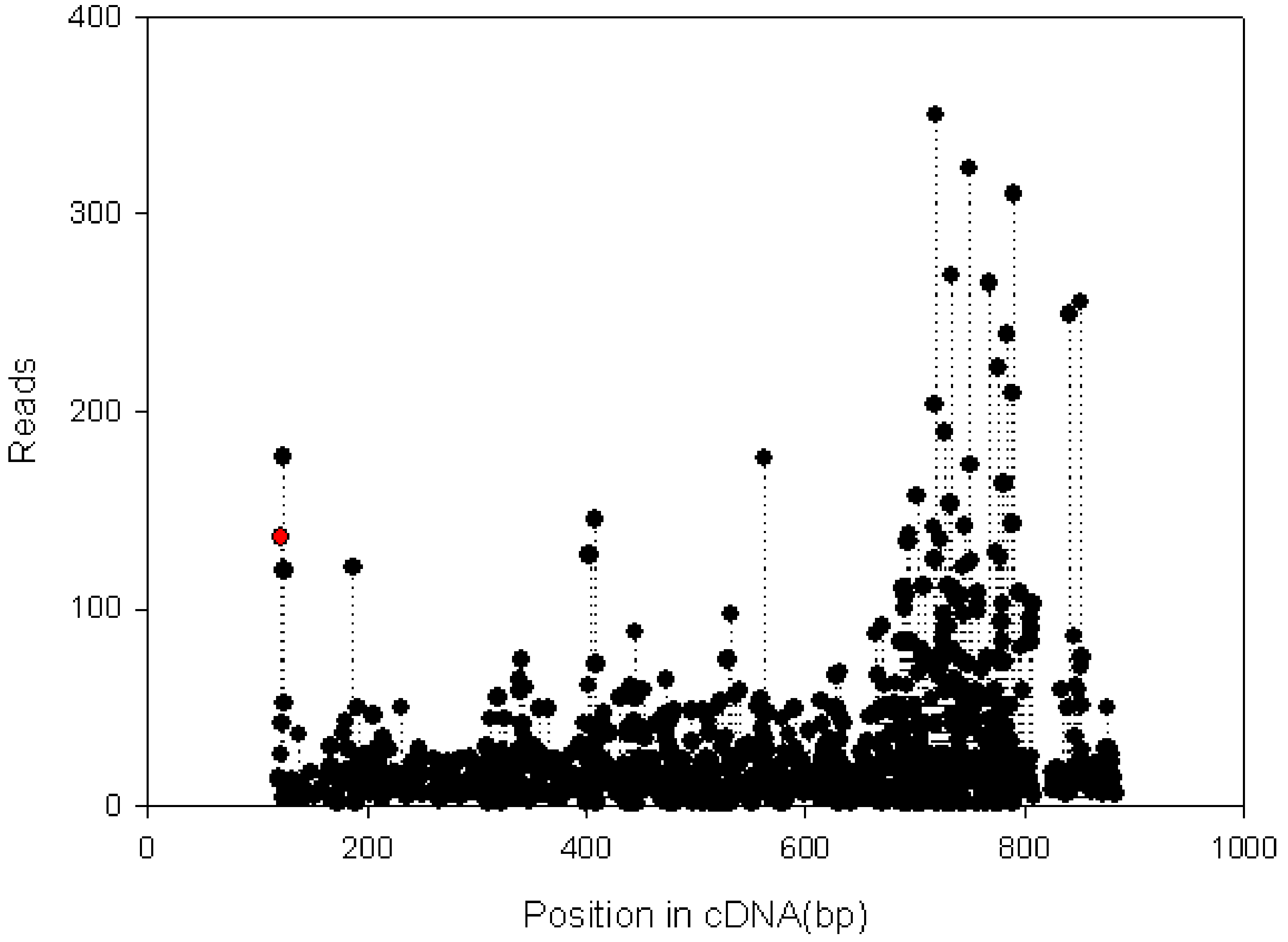


```
5' UUGC-UCUCUUGUCUCAAGAAAUUGCA 3'       Cs9g07680.1
    ::: : : :::::::::::::
3' -ACGUACAAAACAGAGUUCUUU----- 5'       Csi-miR444a.1
```

Csi-miR444a.1, target=Orange1.1t02003.1 gene=Orange1.1t02003
Category:3
Score=5
Cleavage Site=544

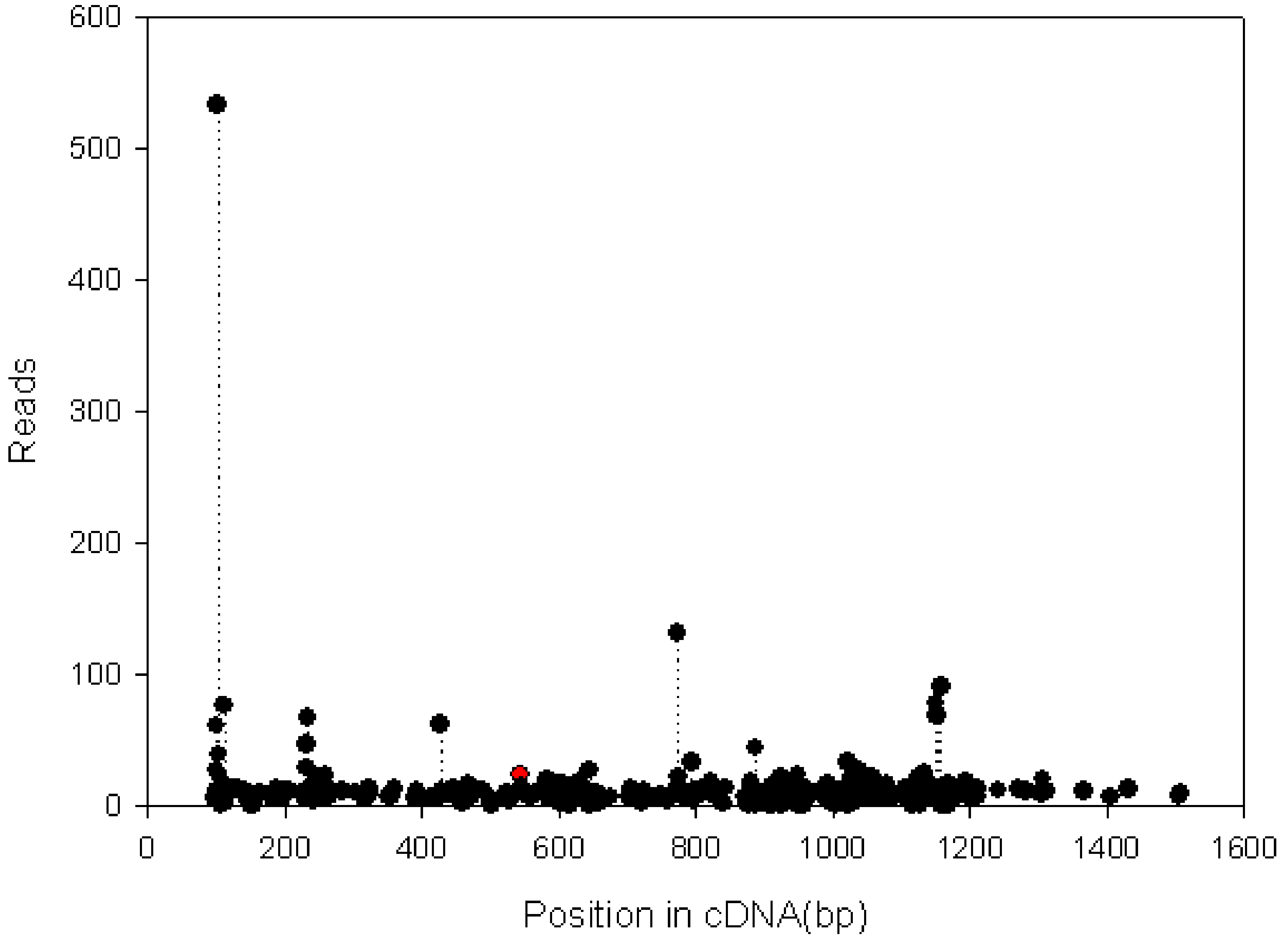


```
5' UUUGCUUGCUUUGUC-CAAGAAAGUAA 3'        Orange1.1t02003.1
     ::: :: :::::: :::::::
3' --ACGUACAAAACAGAGUUCUUU---- 5'        Csi-miR444a.1
```

Csi-miR472, target=Cs2g30590.1 gene=Cs2g30590
Category:1
Score=5
Cleavage Site=889

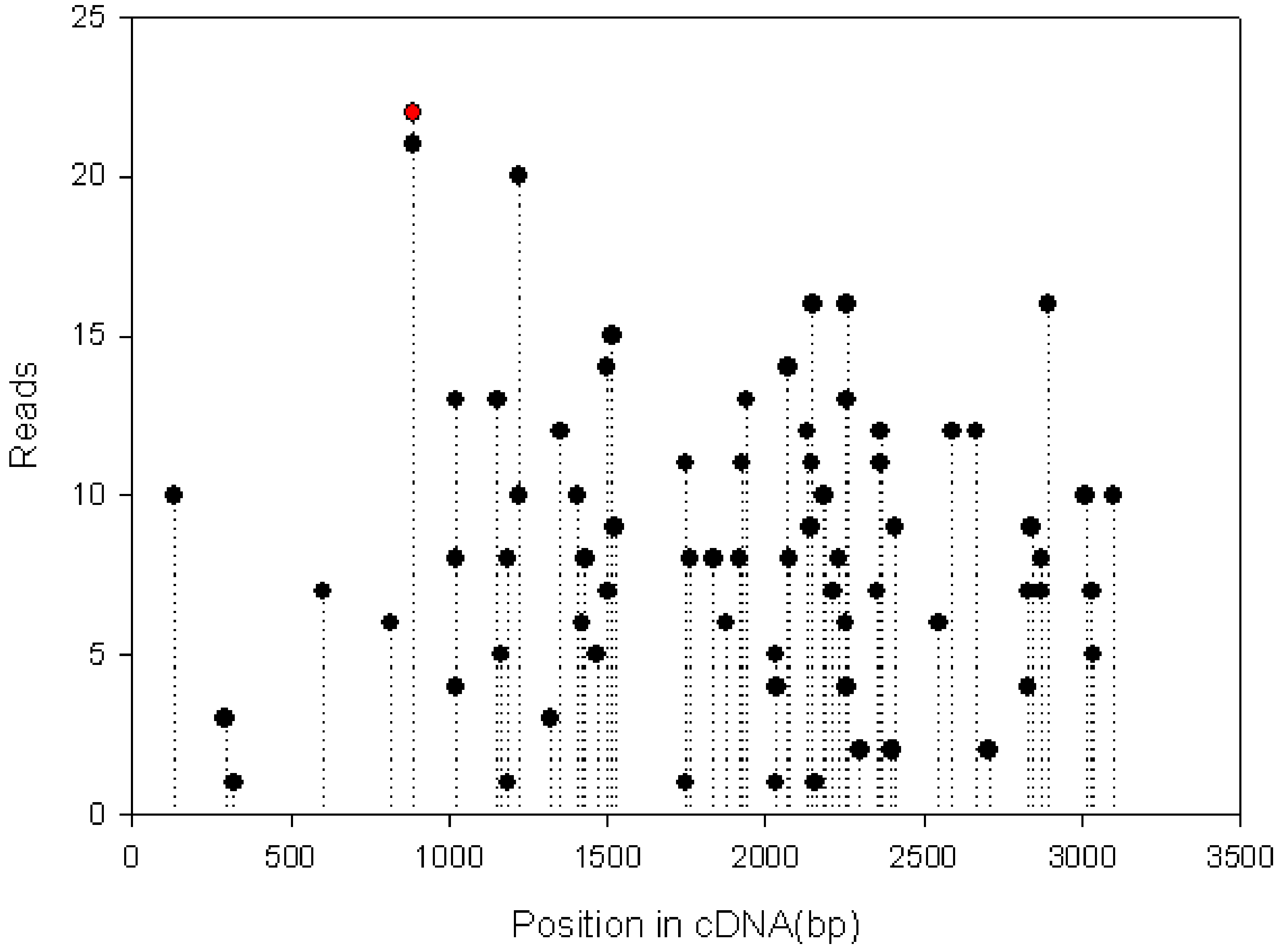


```
5' GGUAUGGGAGGUUUGGGCAAAACAAC 3'        Cs2g30590.1
   :: :::::::::: :::: :::
3' CCCUACCCUCCACACCCUUUU----- 5'        Csi-miR472
```

Csi-miR472, target=Cs7g26730.1 gene=Cs7g26730
Category:1
Score=3
Cleavage Site=1411

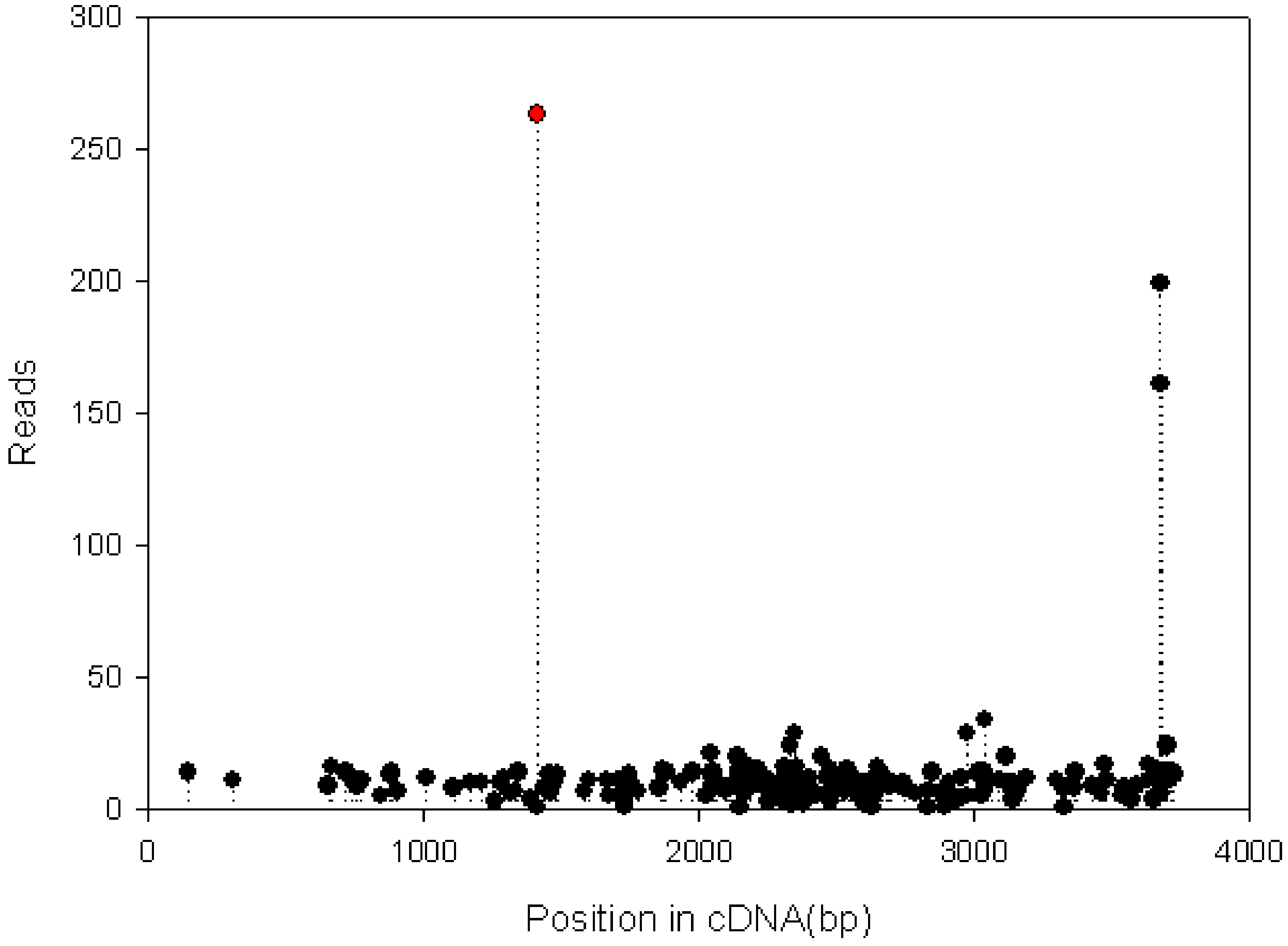


```
5' GAGAAGAGAGGUGUGGGAAAAUGCAA 3'          Cs7g26730.1
   : :: : ::::::::::::::
3' CCCUACCCUCCACACCCUUUU----- 5'          Csi-miR472
```

Csi-miR473-3p, target=Cs1g08770.1 gene=Cs1g08770
Category:3
Score=4
Cleavage Site=1281

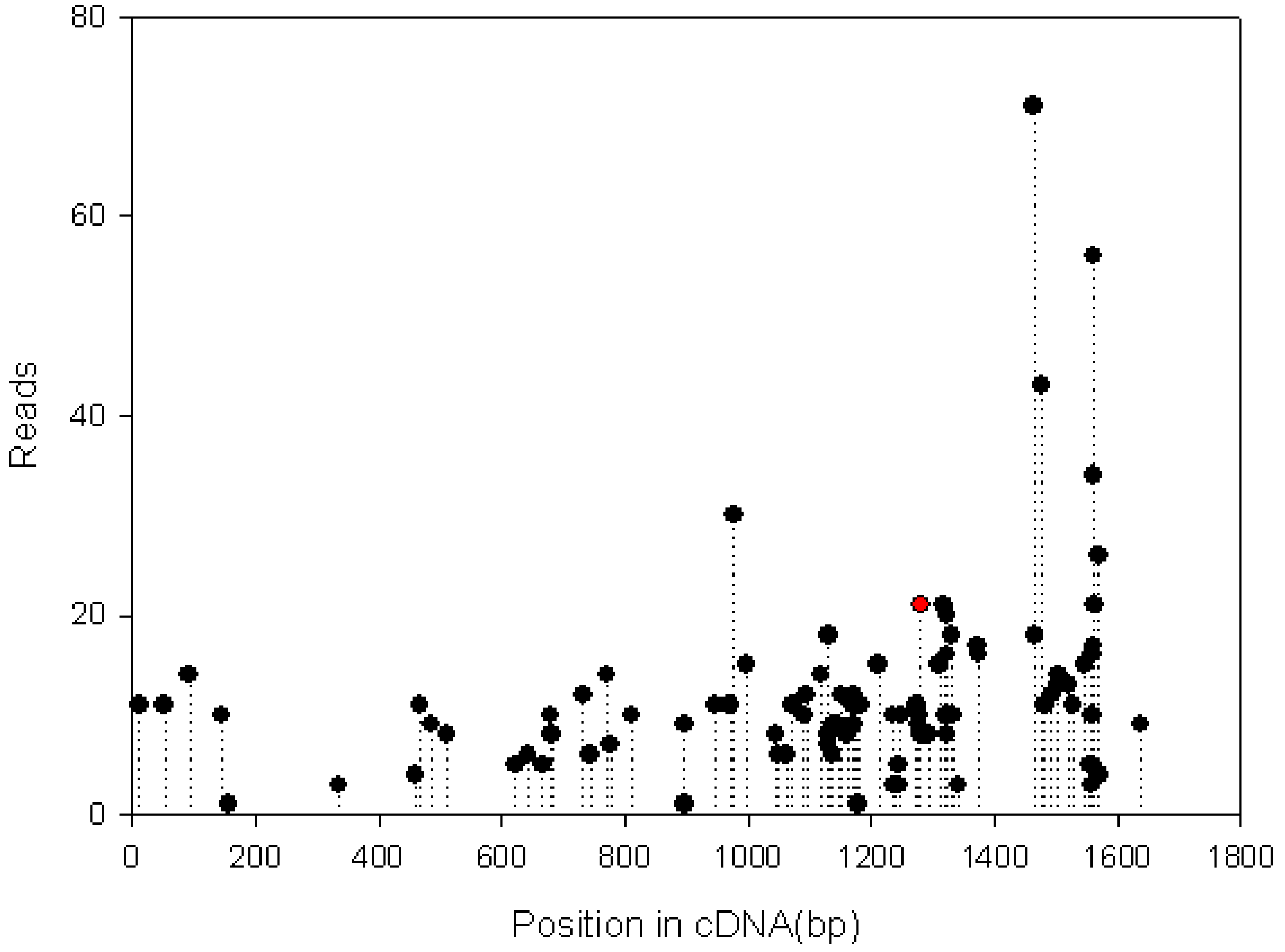


```
5' UGUCAUAC-UCCCCAAGGACUUUGAAA 3'        Cs1g08770.1
    ::::. :  :::::::::::::.
3' --AGUGAGUUGGGGUUCCUGAAG---- 5'        Csi-miR473-3p
```

Csi-miR477a-3p, target=Cs7g09520.1 gene=Cs7g09520
Category:2
Score=5
Cleavage Site=461

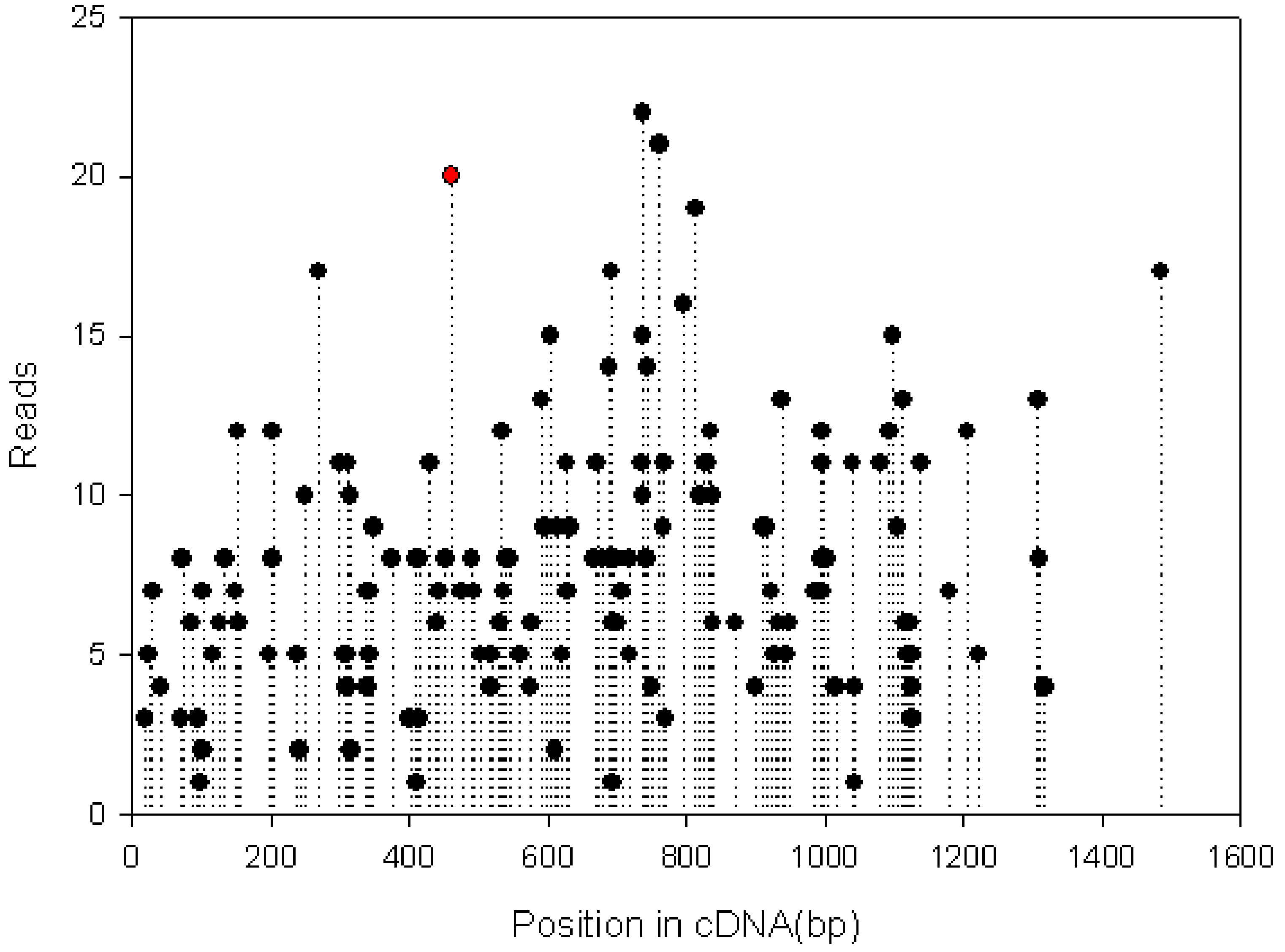


```
5' GGGGUCCACCUCCUAGGGUUUCGGAU 3'        Cs7g09520.1
      : :: ::.::::::::::
3' --GCUGGAGGGGGAUCCCAAAGG--- 5'        Csi-miR477a-3p
```

Csi-miR482a-3p, target=Cs5g18480.1 gene=Cs5g18480
Category:1
Score=5
Cleavage Site=700

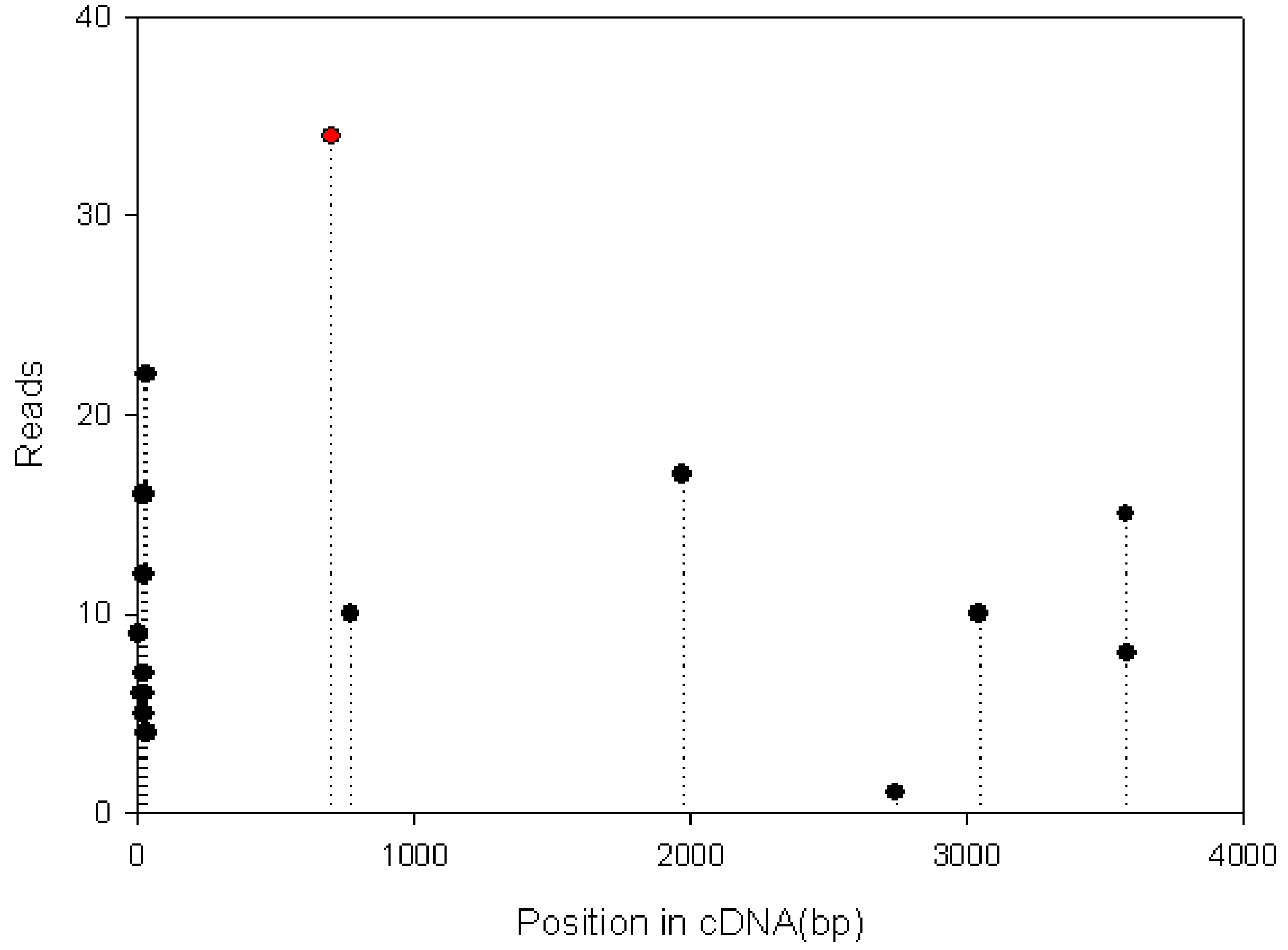


```
5' GGCAUGGGUGGUAUAGGCAAGACAAC 3'        Cs5g18480.1
   :: ::::: ::.::::: ::::
3' CCUUACCCUCCGUAUCCCUUCU---- 5'        Csi-miR482a-3p
```

Csi-miR482a-3p, target=Cs5g19920.1 gene=Cs5g19920
Category:1
Score=5
Cleavage Site=715

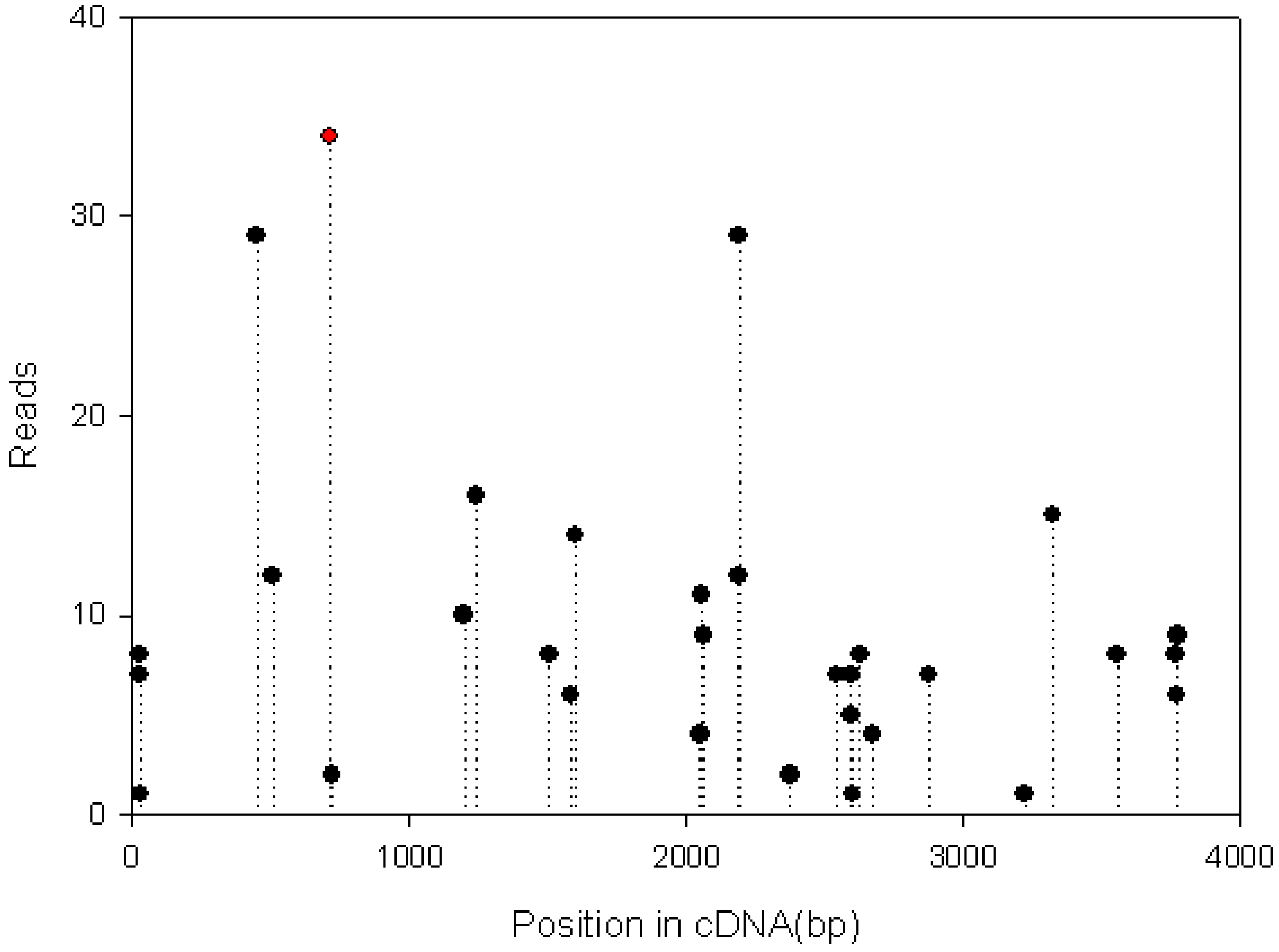


```
5' GGCAUGGGUGGUAUAGGCAAGACAAC 3'        Cs5g19920.1
   :: ::::: ::.::::: ::::
3' CCUUACCCUCCGUAUCCCUUCU---- 5'        Csi-miR482a-3p
```

Csi-miR482b, target=Cs1g11740.1 gene=Cs1g11740
Category:1
Score=4.5
Cleavage Site=2101

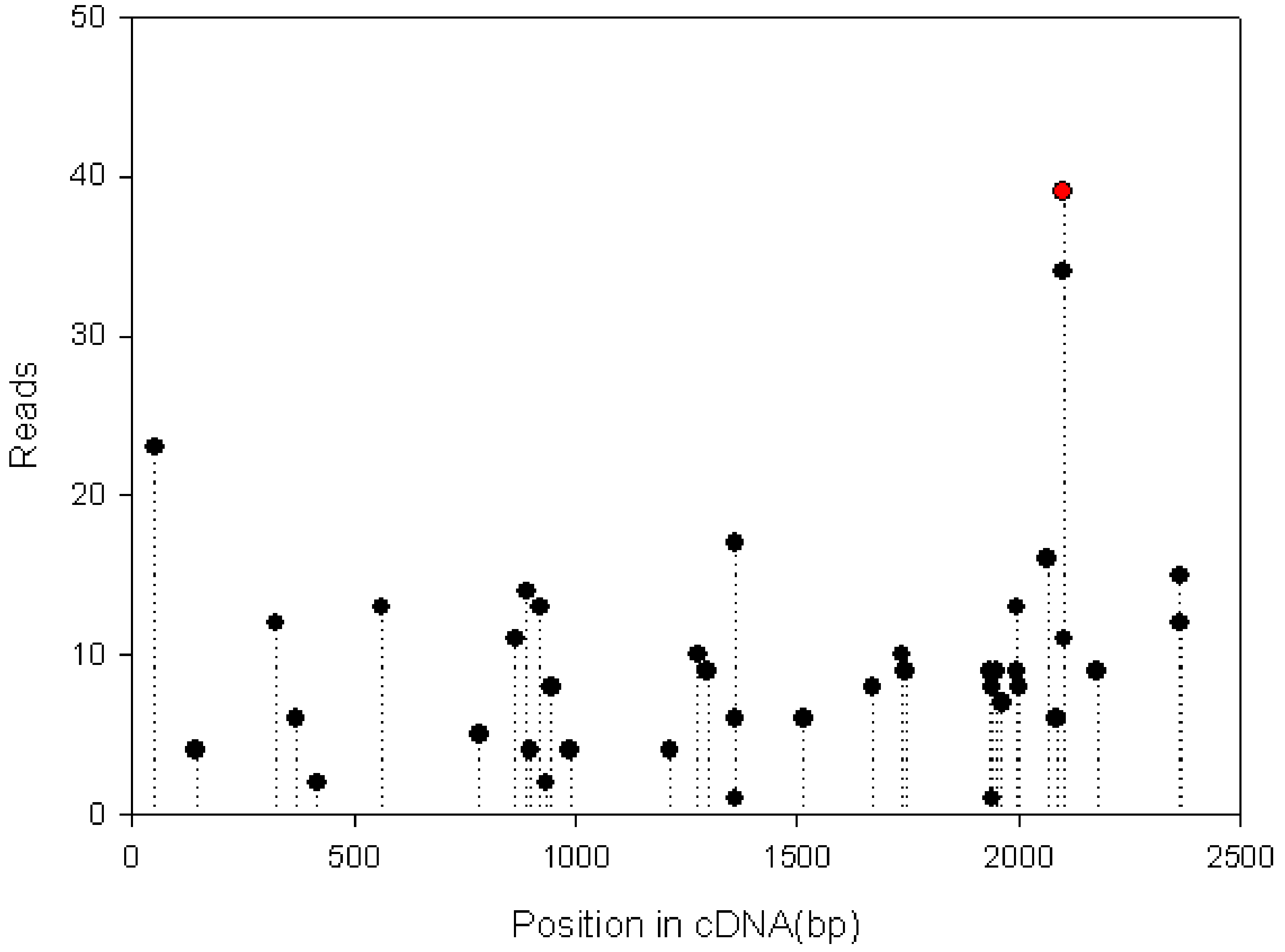


```
5' GCGGCGUGGGGGAGUGGGCAAGGUU 3'        Cs1g11740.1
     :: .::::.:: :::::::::.
3' --CCUUACCCUCCCCACCCGUUCU-- 5'        Csi-miR482b
```

Csi-miR482c, target=Cs9g06846.1 gene=Cs9g06846
Category:1
Score=2.5
Cleavage Site=158

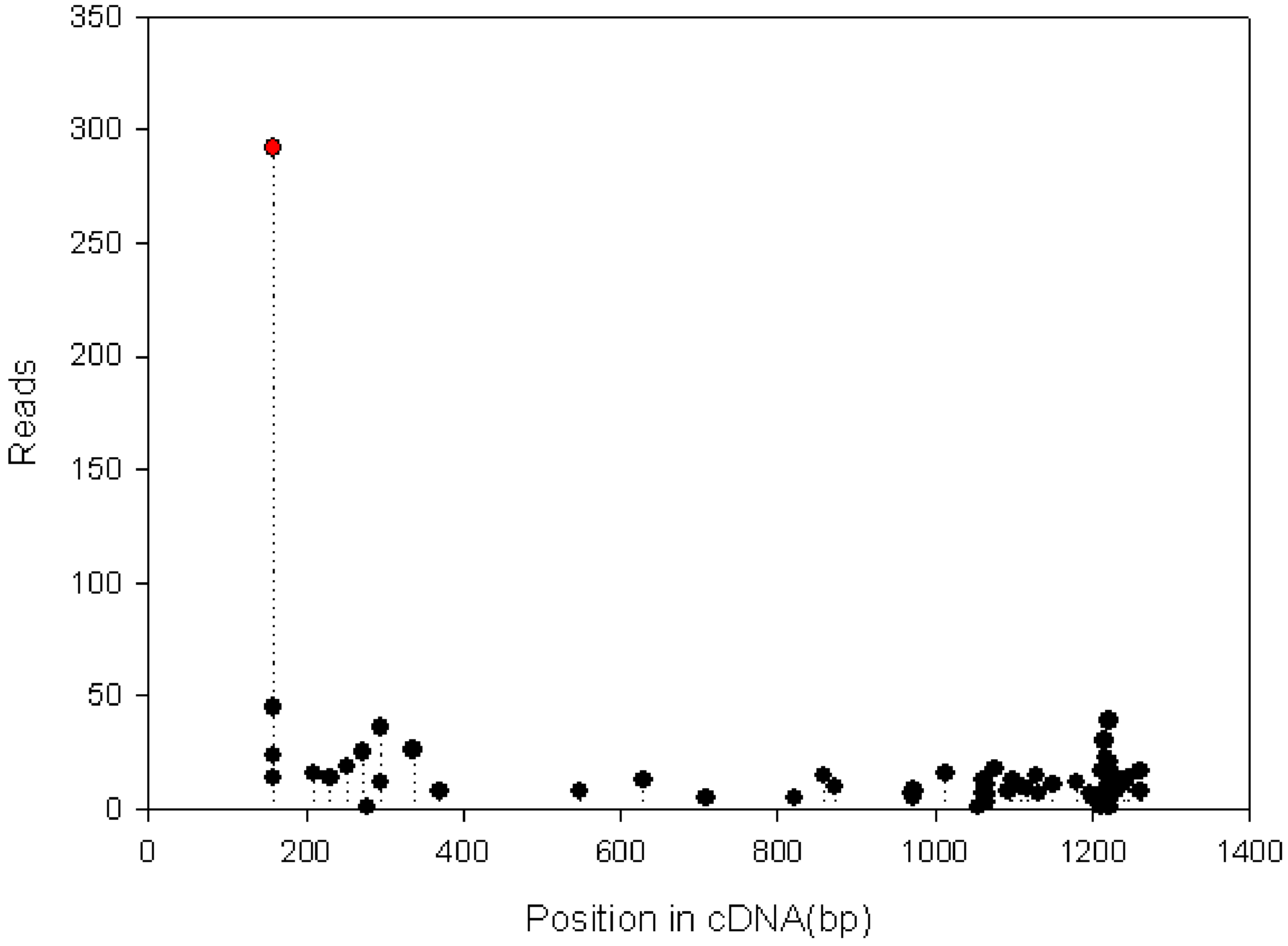


```
5' UGGGAAUAGGUGGACUAGGAAAAUU 3'        Cs9g06846.1
   :.::::::::: :::::::::::
3' AUCCUUAUCCCCCUGAUCCCUU---- 5'        Csi-miR482c
```

Csi-miR482d, target=Cs1g11740.1 gene=Cs1g11740
Category:1
Score=5
Cleavage Site=2101

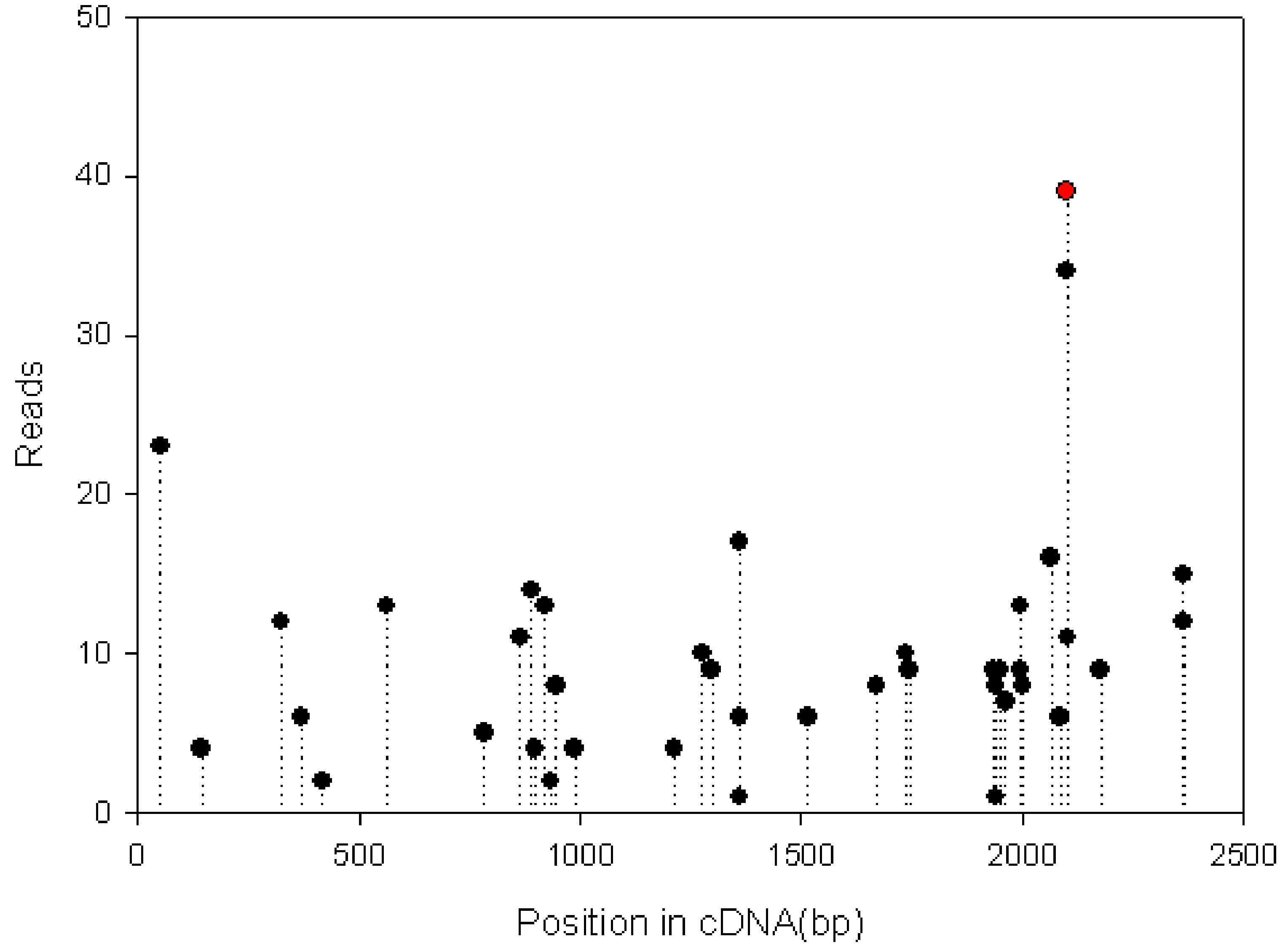


```
5' GCGGCGUGGGGGAGUGGGCAAGGUU 3'        Cs1g11740.1
     :::.:::: ::::::.:: ::::.
3' --CCGUACCCACCUCAUCCCUUCU-- 5'       Csi-miR482d
```

Csi-miR530b, target=Cs6g11650.1 gene=Cs6g11650
Category:3
Score=5
Cleavage Site=260

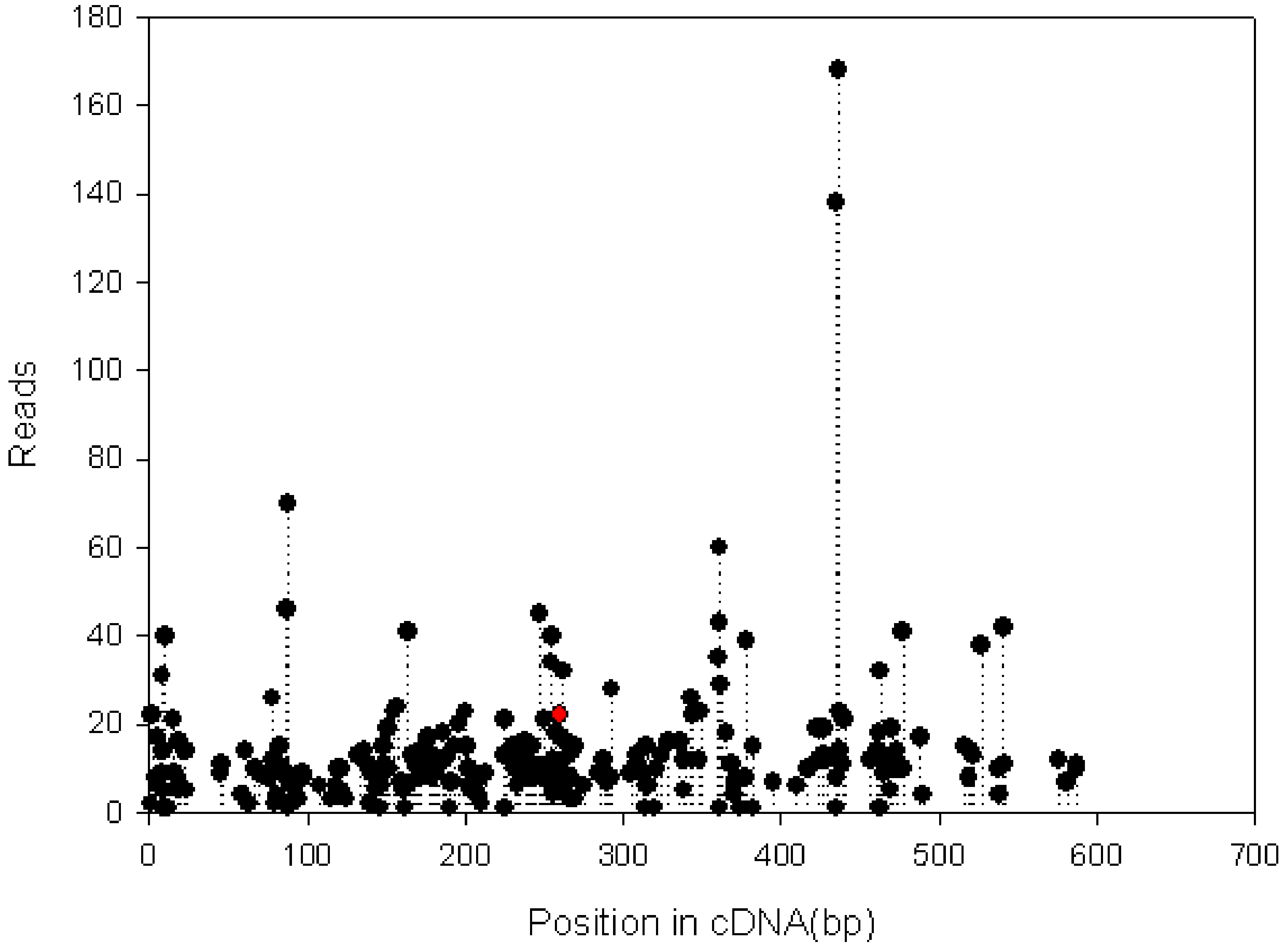


```
5' UGGAGAUUCAGGUGCGAAUGUGAAGA 3'        Cs6g11650.1
     .:::: ::::::::.::::..
3' -GUUCUACGUCCACGUUUACGU---- 5'        Csi-miR530b
```

Csi-miR535.1, target=Cs2g05730.1 gene=Cs2g05730
Category:2
Score=5
Cleavage Site=817

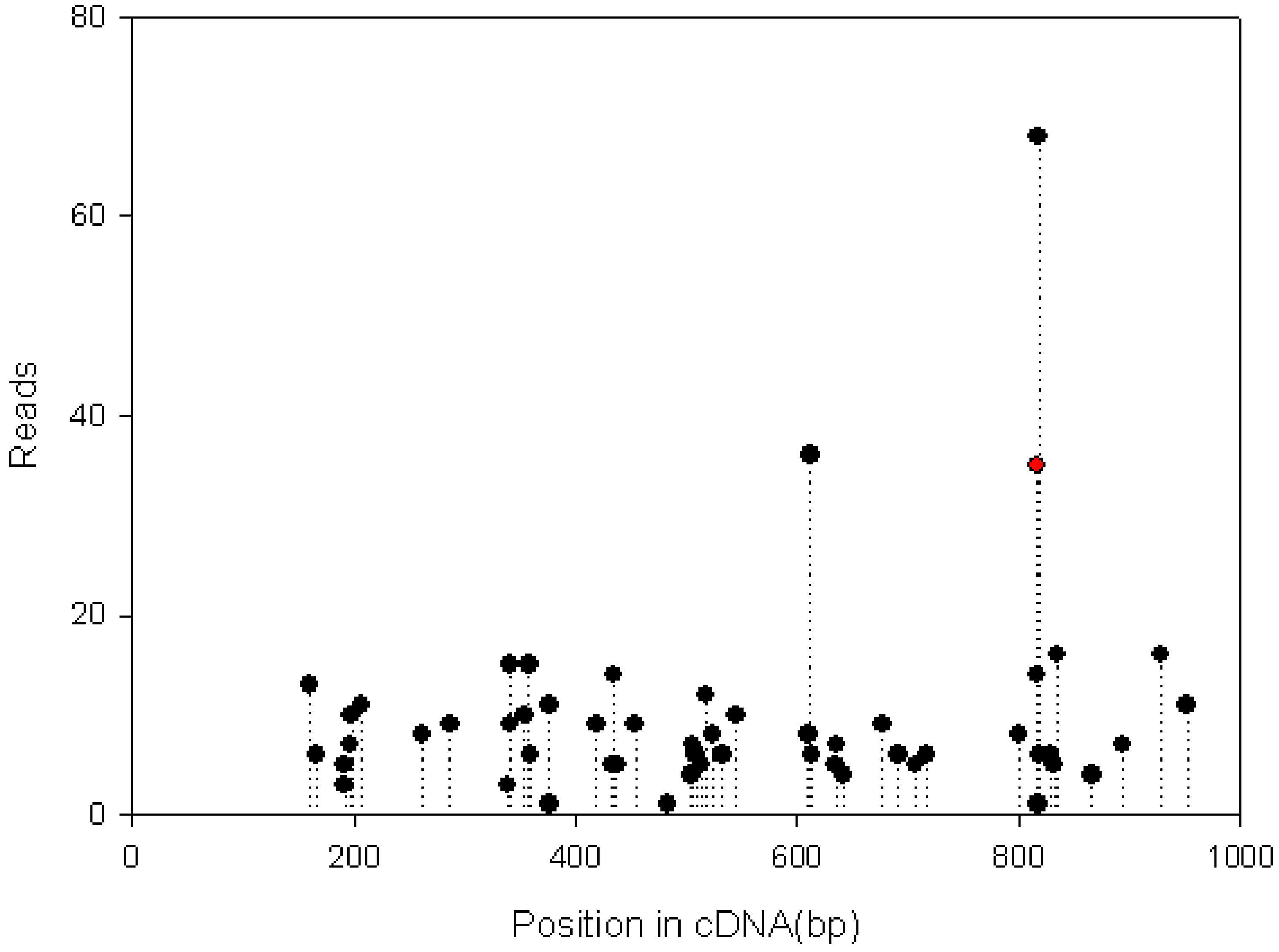


```
5' GCGUGCUCUCUCUCUUCUGUCAAUGC 3'        Cs2g05730.1
   : ::::::::::::: : :::::
3' CACACGAGAGAGAGUA-ACAGU---- 5'        Csi-miR535.1
```

Csi-miR535.1, target=Cs4g05470.1 gene=Cs4g05470
Category:3
Score=4
Cleavage Site=1460

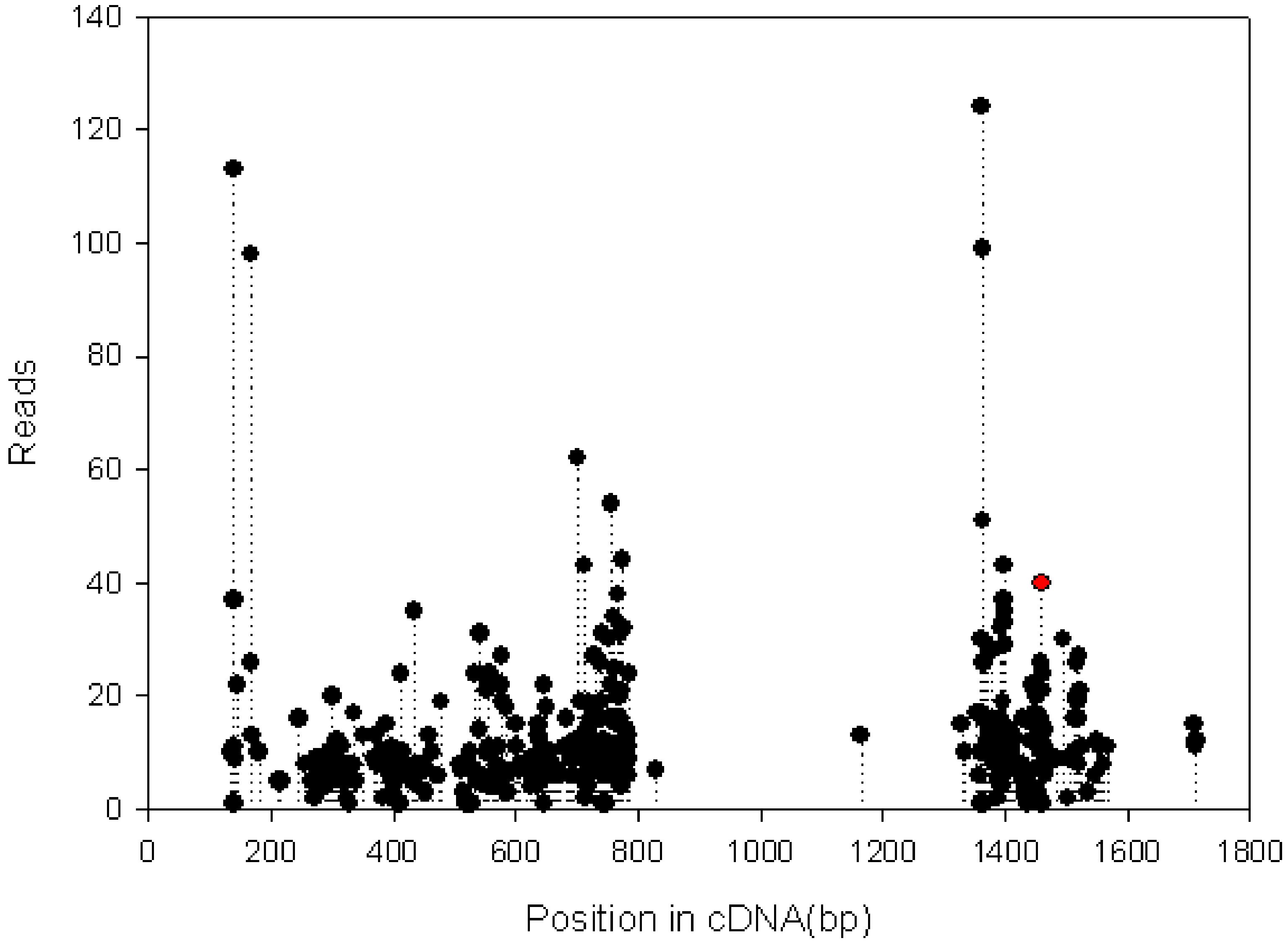


```
5' CUCUGUG-UUUCUCUCAUUGUUGGGAU 3'        Cs4g05470.1
     :::: :.:::::::::::::..
3' --CACACGAGAGAGAGUAACAGU---- 5'        Csi-miR535.1
```

Csi-miR535.1, target=Orange1.1t02265.1 gene=Orange1.1t02265
Category:1
Score=5
Cleavage Site=982

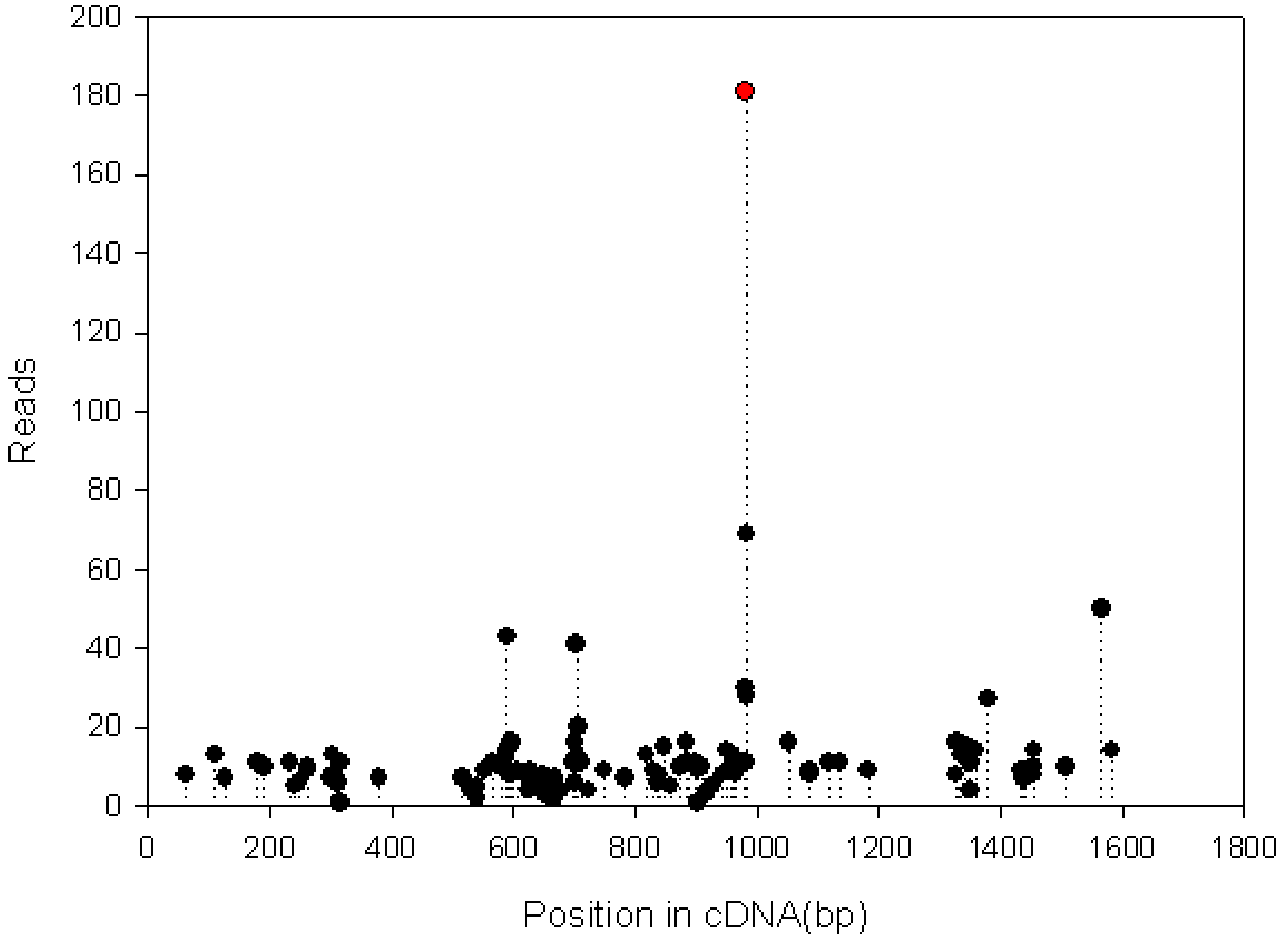


```
5' CUGUGCUCUCUCUCUUCUGUCAAAUC 3'        Orange1.1t02265.1
    ::::::::::::: : :::::
3' CACACGAGAGAGAGUA-ACAGU---- 5'        Csi-miR535.1
```

Csi-miR535.2, target=Cs2g05730.1 gene=Cs2g05730
Category:1
Score=5
Cleavage Site=818

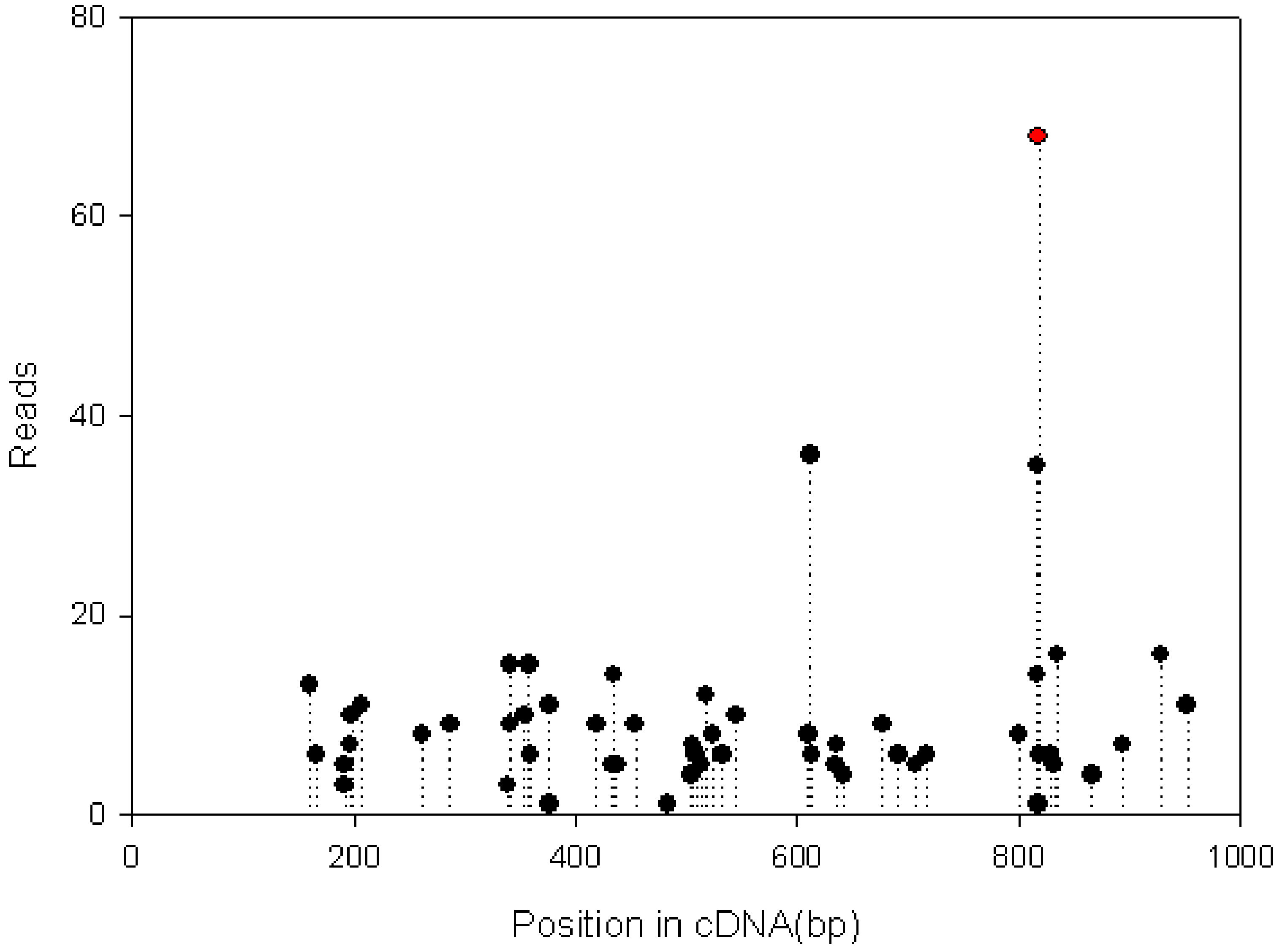


```
5' CGUGCUCUCUCUCUUCUGUCAAUGCU 3'        Cs2g05730.1
    :::::::::::: : :::::
3' ACACGAGAGAGAGUA-ACAGU----- 5'        Csi-miR535.2
```

Csi-miR535.2, target=Cs4g05470.1 gene=Cs4g05470
Category:3
Score=3
Cleavage Site=1460

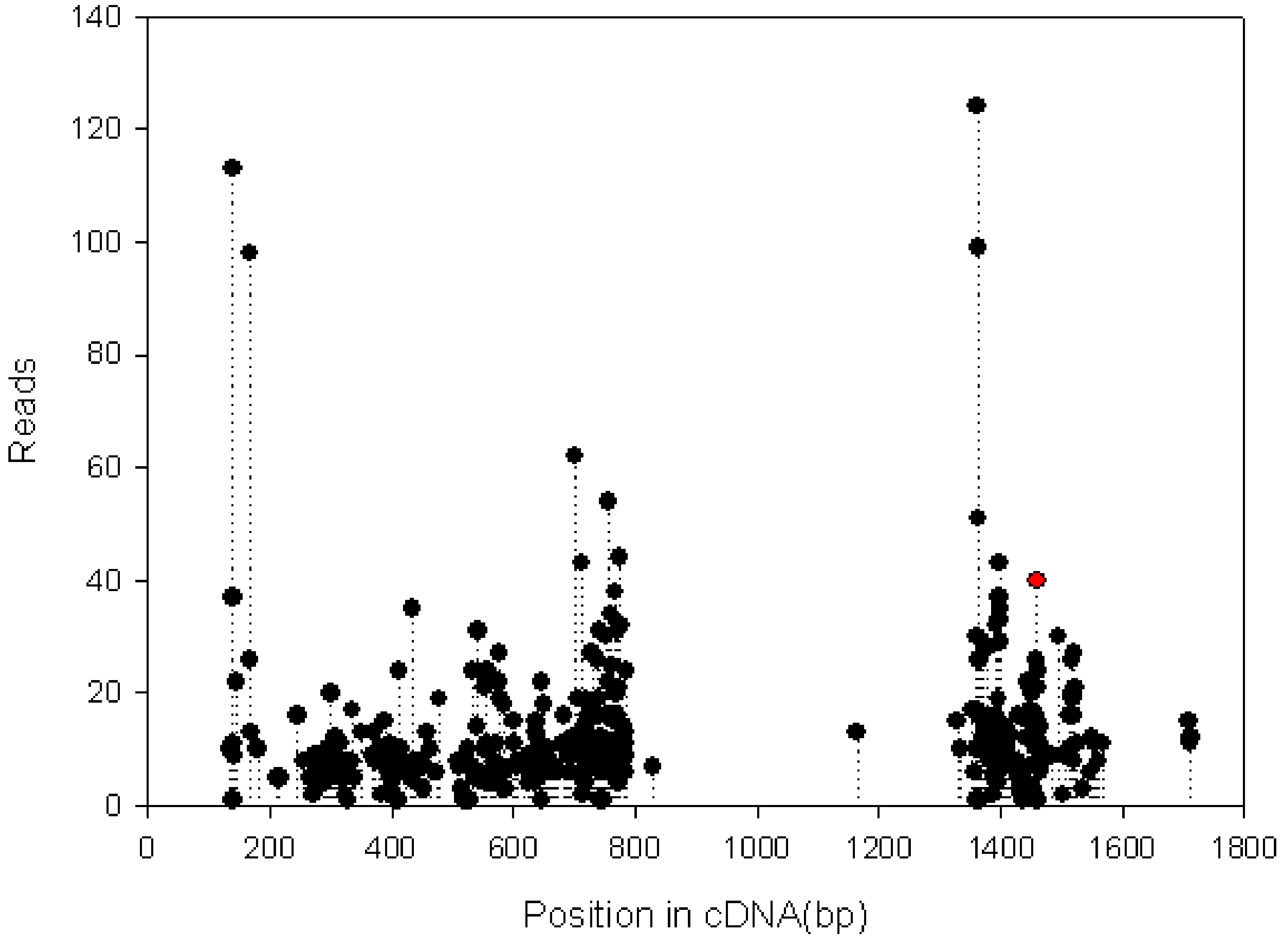


```
5' CUCUGUG-UUUCUCUCAUUGUUGGGAU 3'        Cs4g05470.1
       :::: :.:::::::::::..
3' ---ACACGAGAGAGAGUAACAGU---- 5'        Csi-miR535.2
```

Csi-miR535.2, target=Cs7g10830.1 gene=Cs7g10830
Category:1
Score=5
Cleavage Site=1183

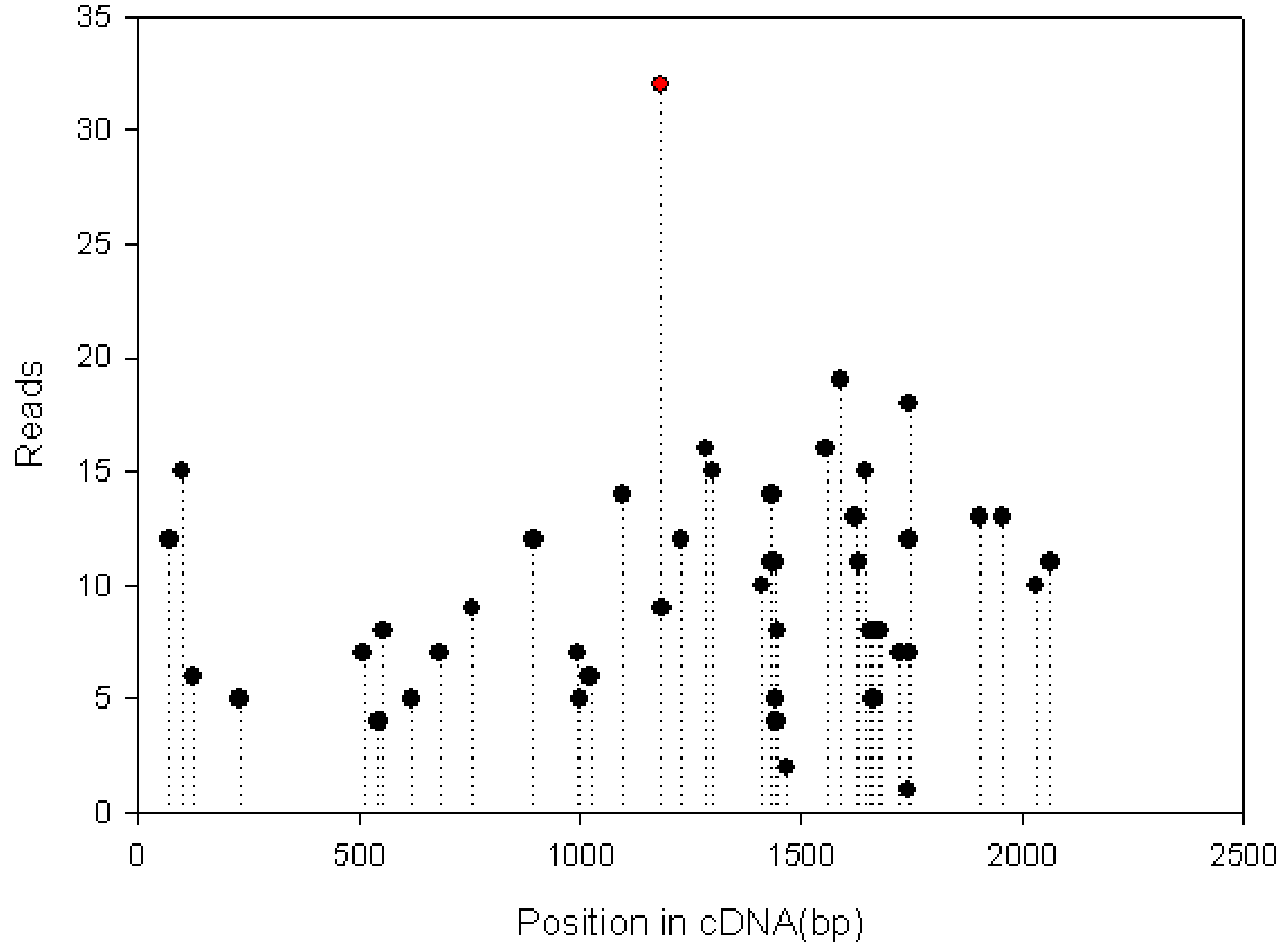


```
5' AGUGCUCUCUCUCUUCUGUCAAAAAC 3'         Cs7g10830.1
    ::::::::::::: : :::::
3' ACACGAGAGAGAGUA-ACAGU----- 5'         Csi-miR535.2
```

Csi-miR535.2, target=Orange1.1t02265.1 gene=Orange1.1t02265
Category:1
Score=4
Cleavage Site=982

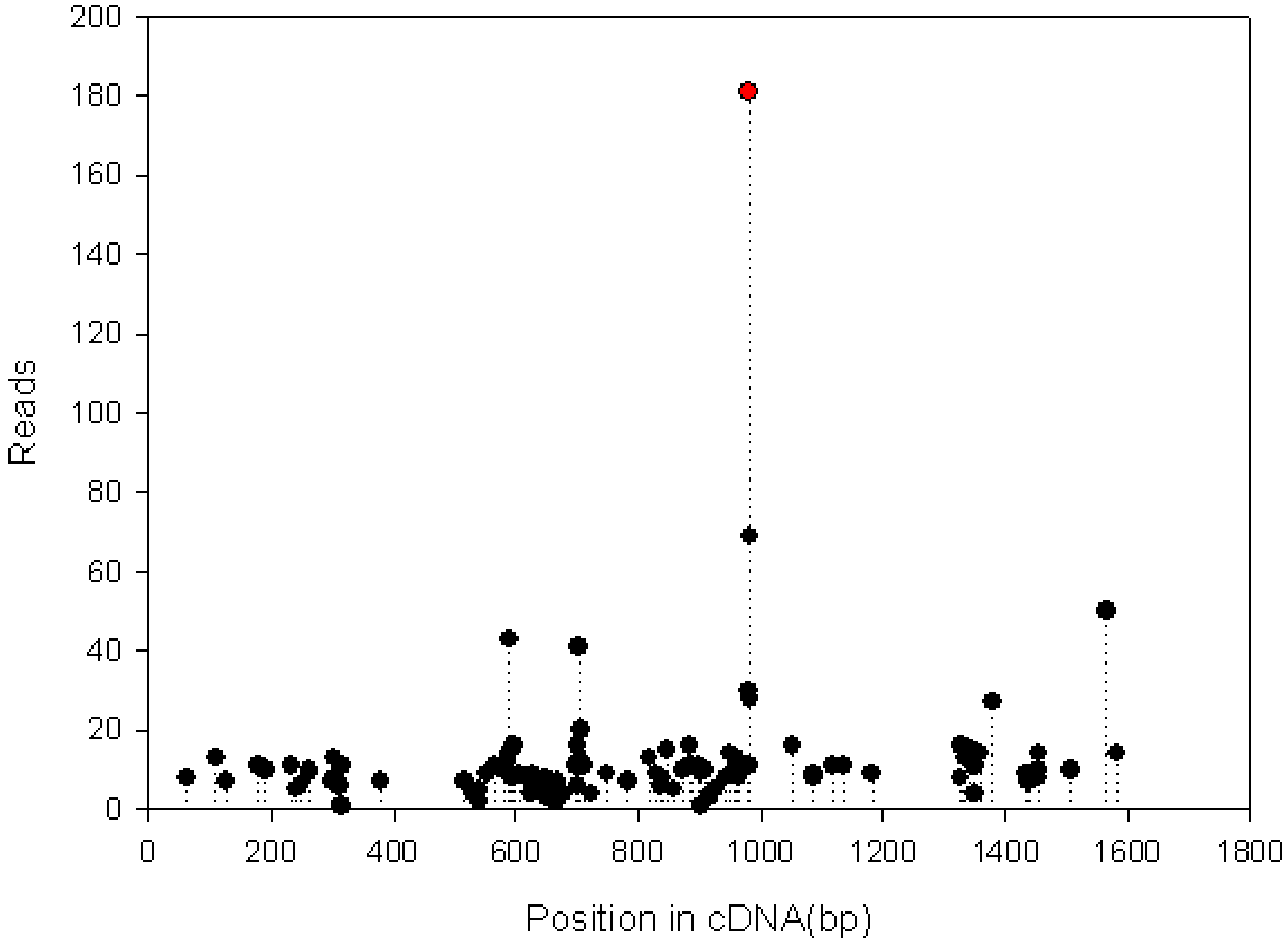


```
5' CUGUGCUCUCUCUCUUCUGUCAAAUC 3'        Orange1.1t02265.1
    ::::::::::::: : :::::
3' -ACACGAGAGAGAGUA-ACAGU---- 5'        Csi-miR535.2
```

Csi-miR536-3p, target=Orange1.1t01470.1 gene=Orange1.1t01470
Category:1
Score=1.5
Cleavage Site=195

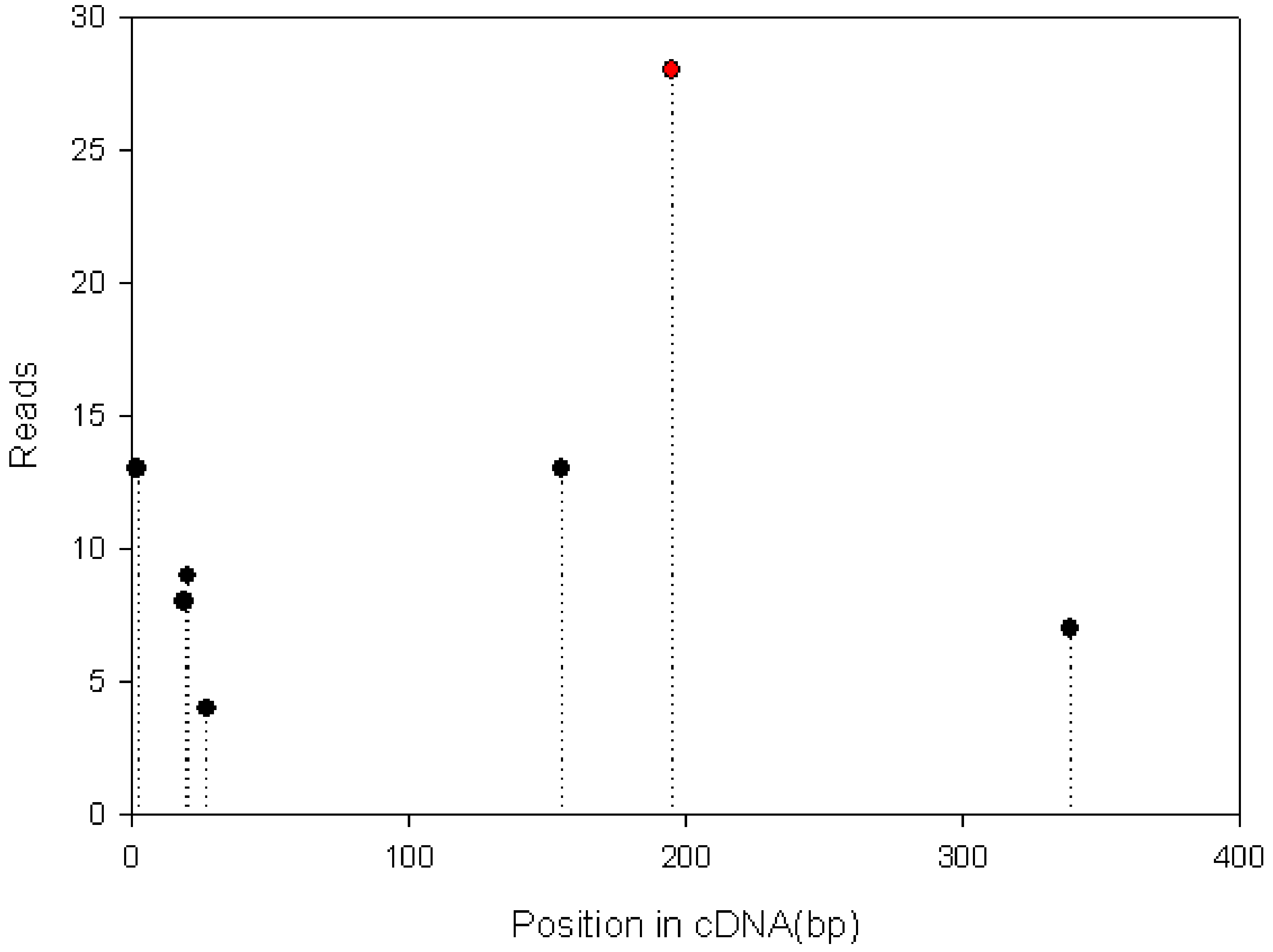


```
5' CGACGCACGCAGCGUGGCACUAUCUC 3'        Orange1.1t01470.1
    :::::::.:::::::::::::.:
3' -CUGCGUGUGUCGCACCGUGGU---- 5'        Csi-miR536-3p
```

Csi-miR536-3p, target=Orange1.1t04955.1 gene=Orange1.1t04955
Category:1
Score=2
Cleavage Site=195

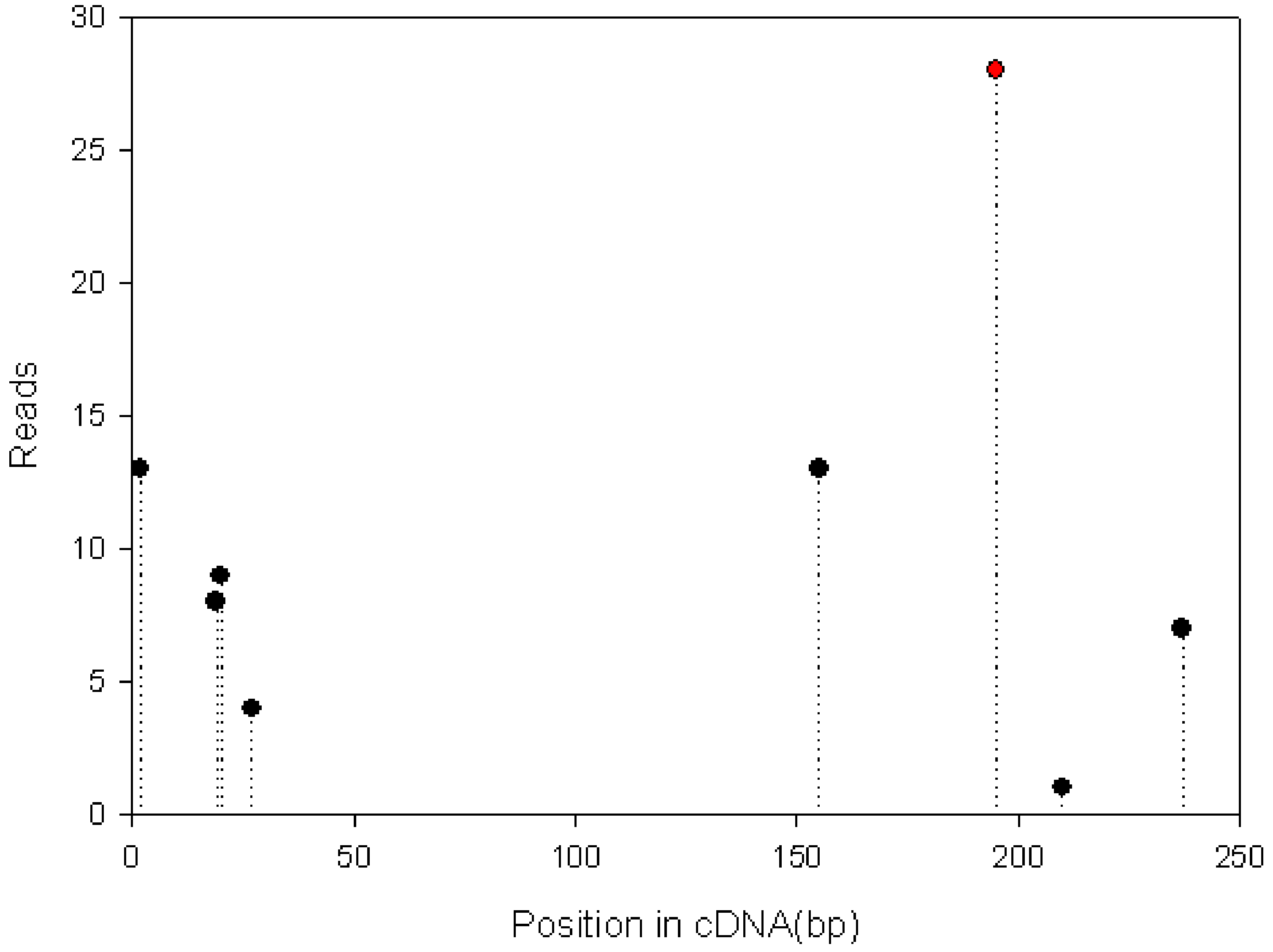


```
5' CGAUGCACGCAGCGUGGCACUAUCUC 3'        Orange1.1t04955.1
    ::.::::.::::::::::::.:
3' -CUGCGUGUGUCGCACCGUGGU---- 5'        Csi-miR536-3p
```

Csi-miR536-3p, target=Orange1.1t05081.1 gene=Orange1.1t05081
Category:1
Score=2
Cleavage Site=405

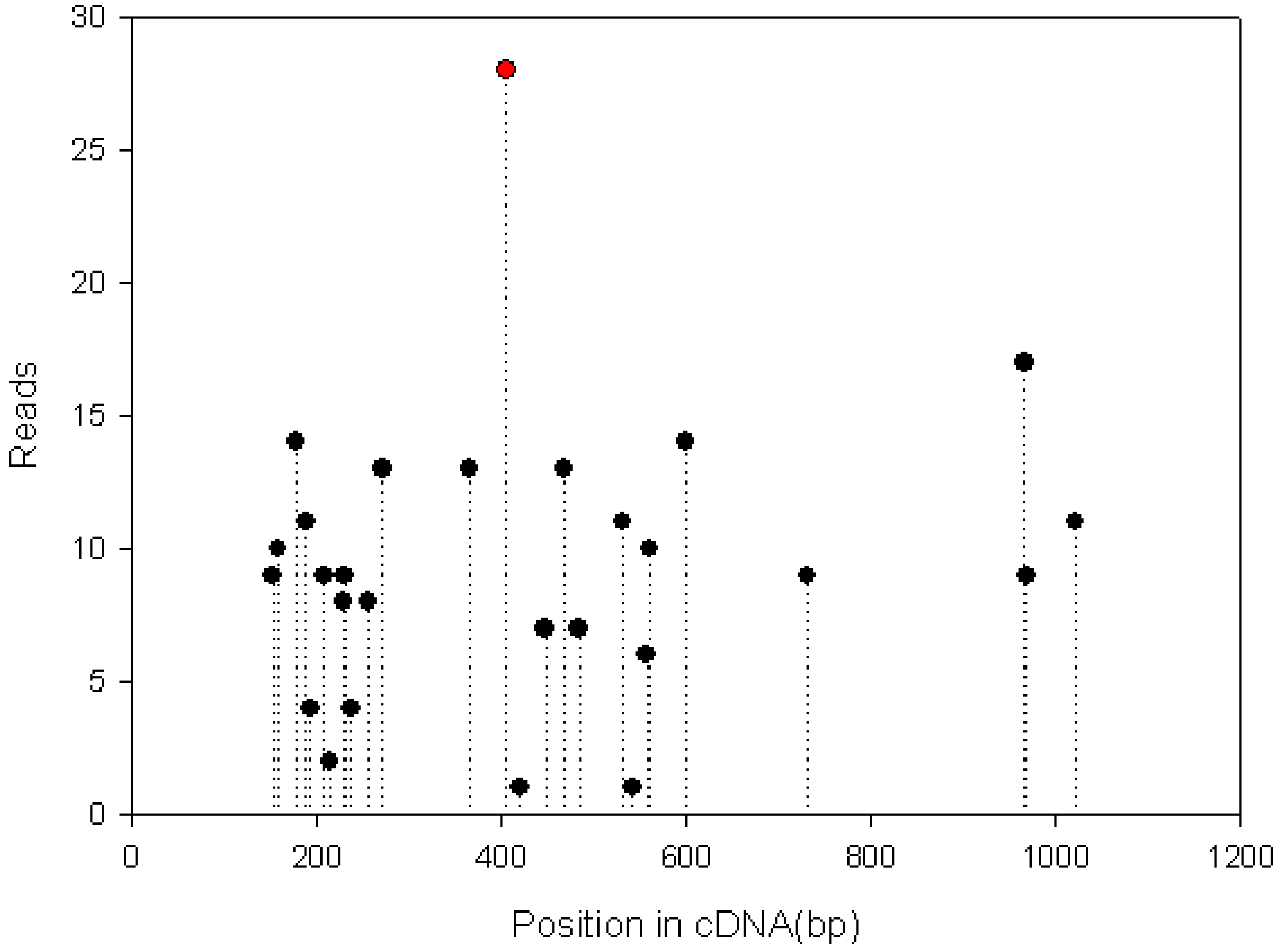


```
5' CGAUGCACGCAGCGUGGCACUAUCUC 3'        Orange1.1t05081.1
    ::.::::.:::::::::::::.:
3' -CUGCGUGUGUCGCACCGUGGU---- 5'        Csi-miR536-3p
```

Csi-miR827-5p.2, target=Cs4g14890.1 gene=Cs4g14890
Category:3
Score=4.5
Cleavage Site=492

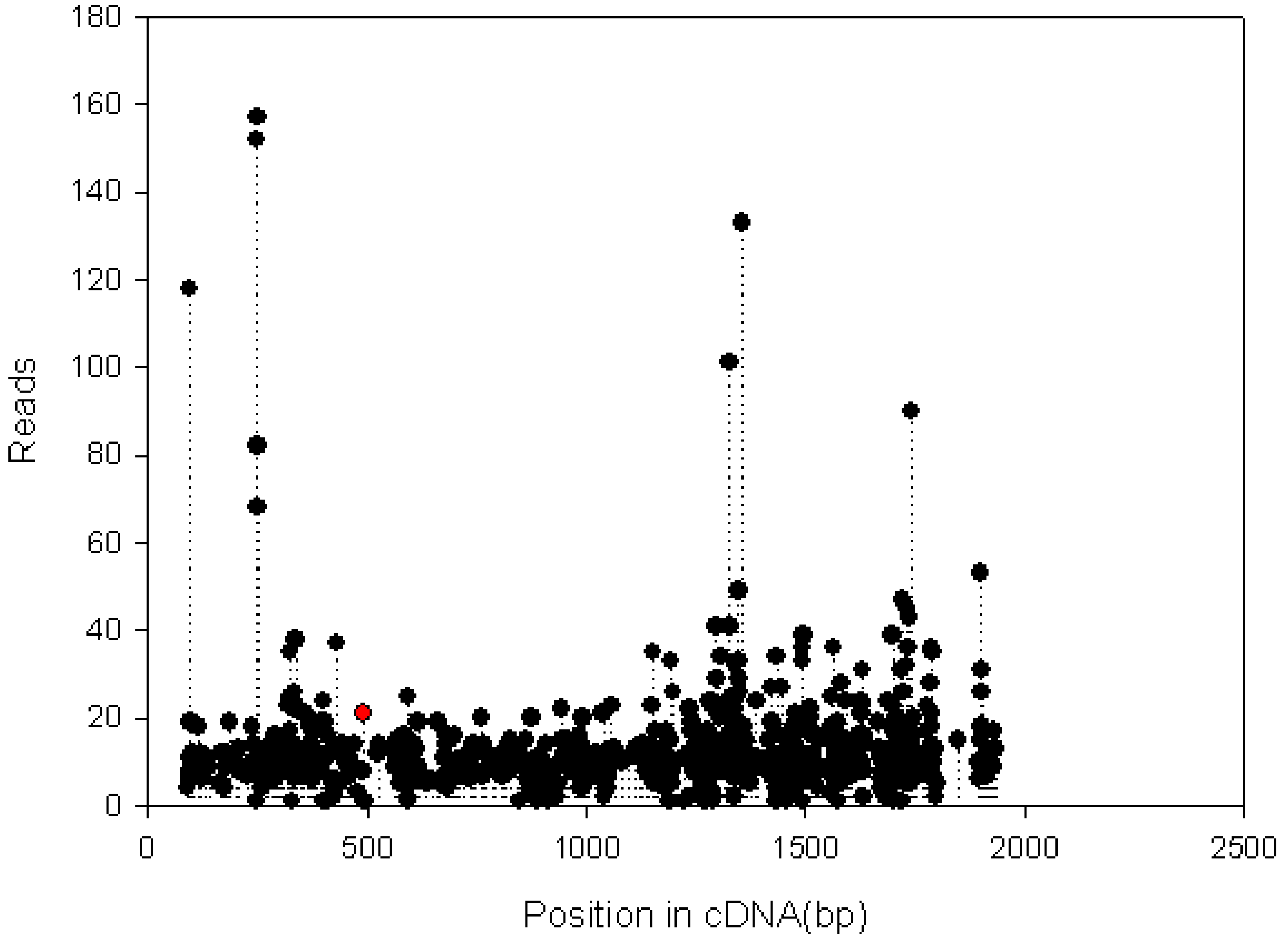


```
5' GUCGAUGACAAUC-ACAAGCGAAAGGA 3'        Cs4g14890.1
    : :::::::::: ::::::.
3' AAUCUACUGUUAGUUGUUCGU------ 5'        Csi-miR827-5p.2
```

Csi-miR833.1, target=Cs8g03110.1 gene=Cs8g03110
Category:3
Score=4.5
Cleavage Site=1324

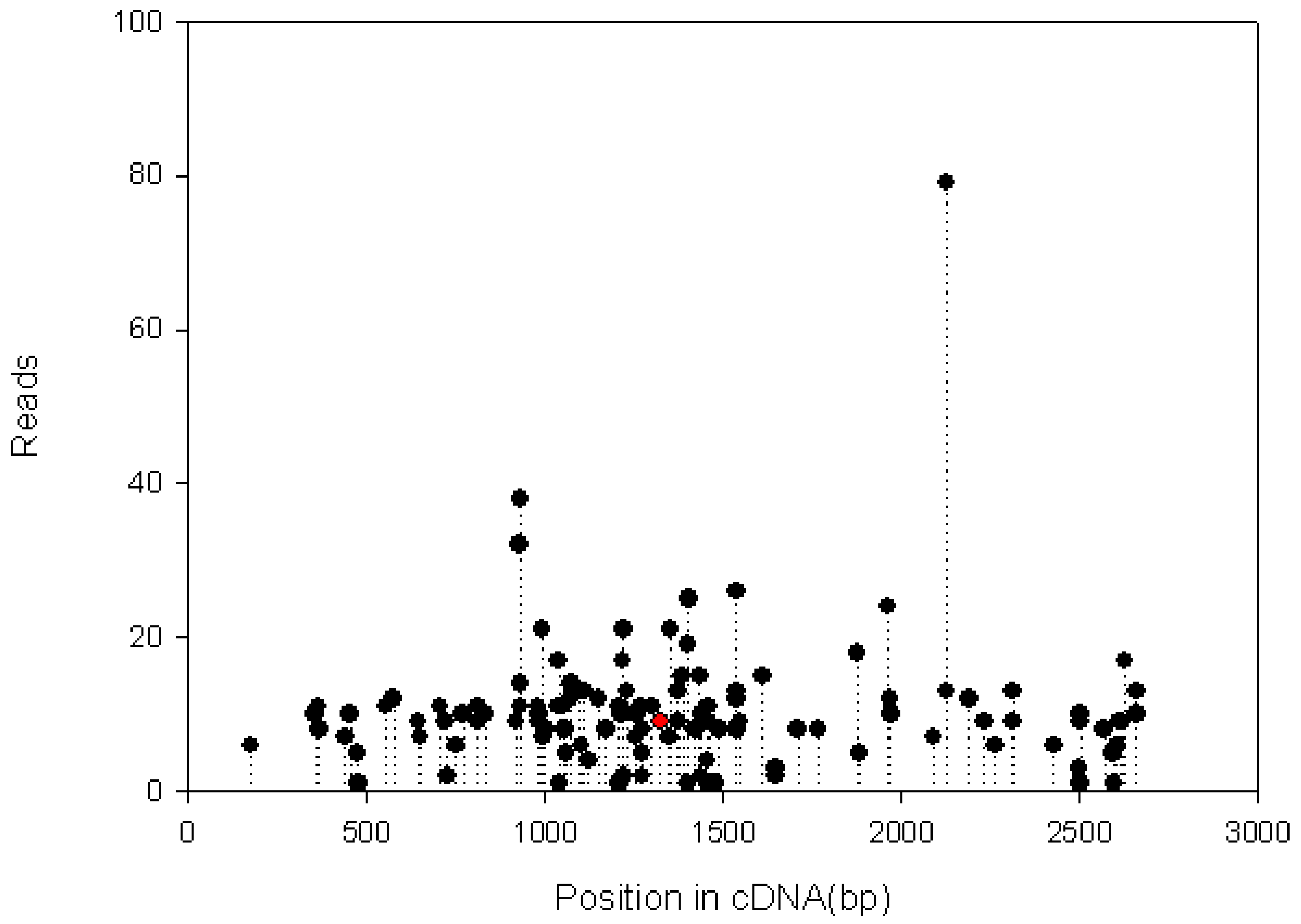


```
5' CUGA-UCAUAUCAGCAAUAGGUCCCCG 3'        Cs8g03110.1
   ::: .:::::::.::::::..:
3' -ACUGGGUAUAGUUGUUAUUUA----- 5'        Csi-miR833.1
```

Csi-miRN02, target=Cs1g08400.1 gene=Cs1g08400
Category:1
Score=2
Cleavage Site=333

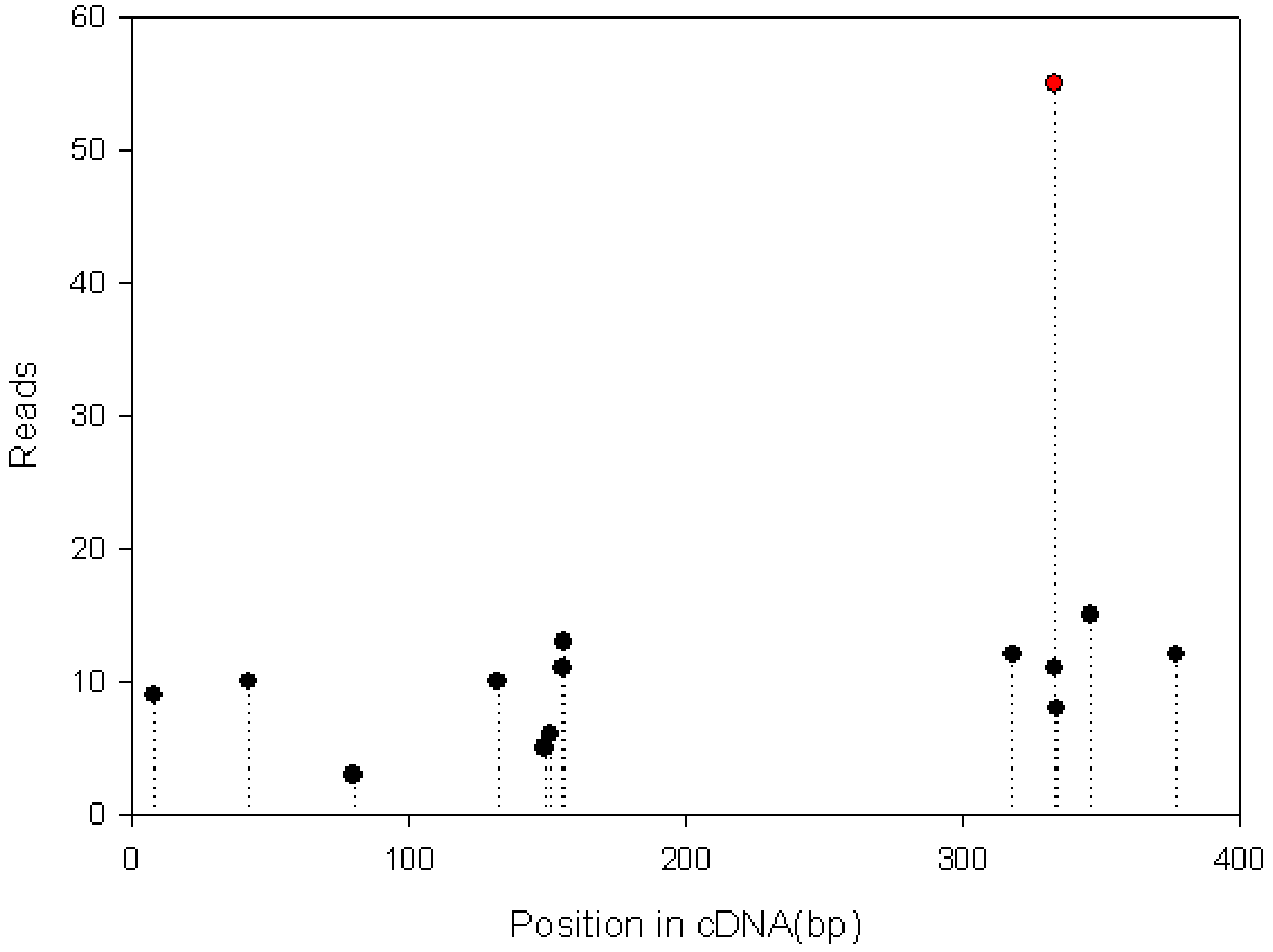


```
5' AACUGUGACACGGUACUAUAGUUGCA 3'        Cs1g08400.1
     ::::: ::::::::::.::::
3' --GACACCGUGCCAUGAUGUCAA--- 5'        Csi-miRN02
```

Csi-miRN02, target=Cs3g18790.1 gene=Cs3g18790
Category:1
Score=2
Cleavage Site=1819

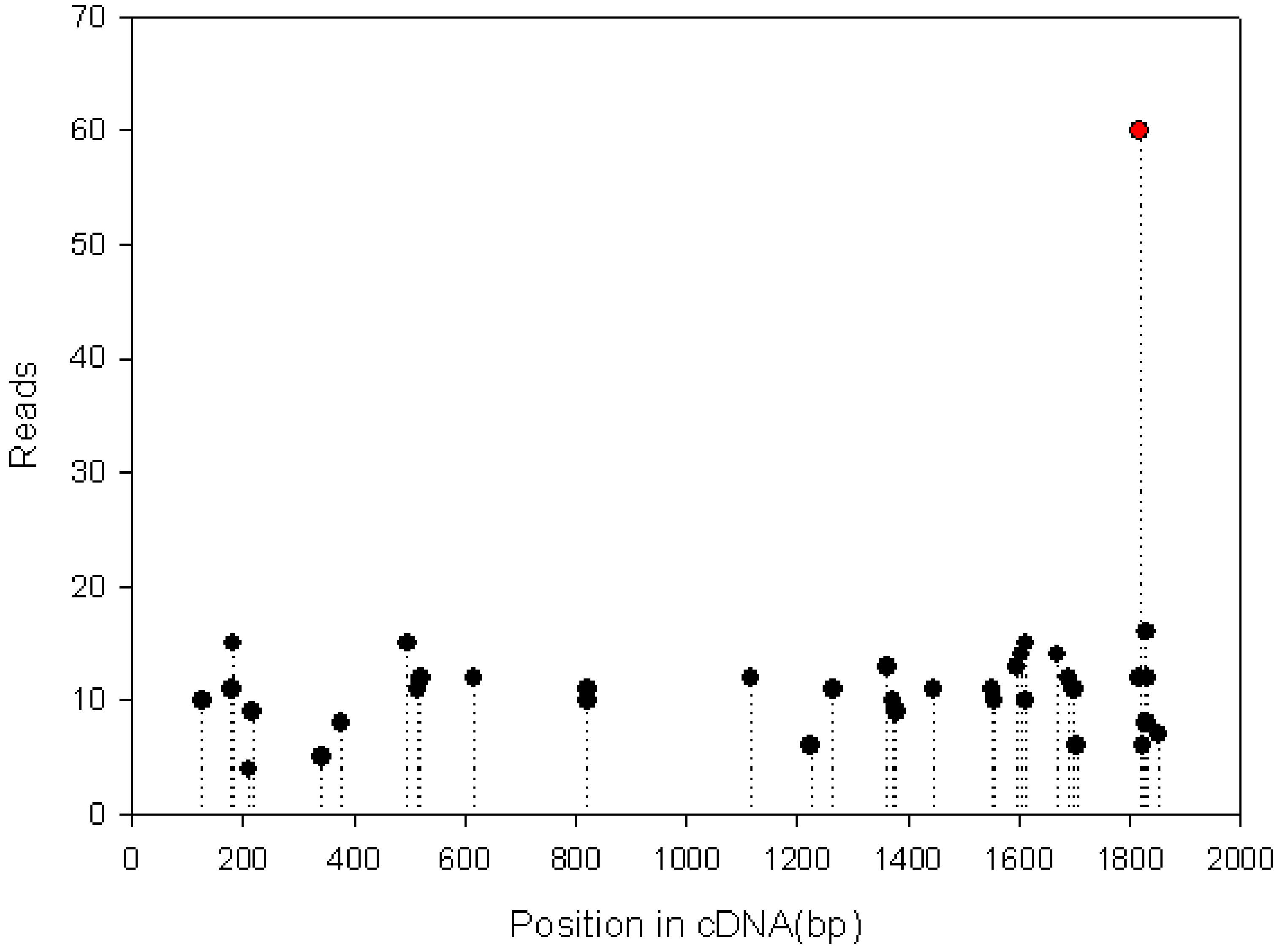


```
5'  GCAACUGUGGCACGGUACCACAGUUG 3'        Cs3g18790.1
        :::::::::::::: ::::::
3'  ----GACACCGUGCCAUGAUGUCAA- 5'        Csi-miRN02
```

Csi-miRN02, target=Cs8g12570.1 gene=Cs8g12570
Category:1
Score=4.5
Cleavage Site=3176

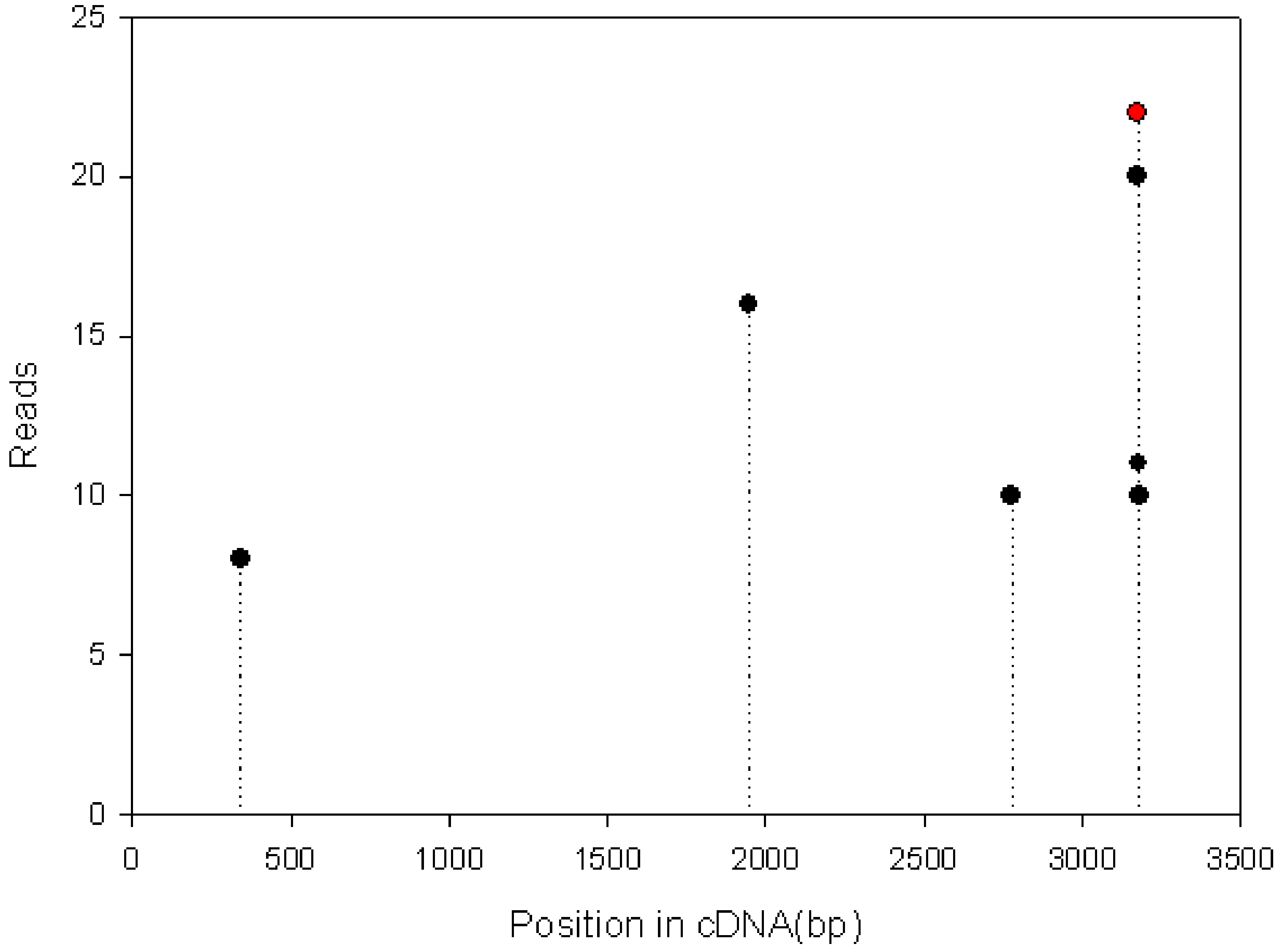


```
5' AACAGUGGUAUGGUACCACAGUUGCA 3'        Cs8g12570.1
     : ::::.:.::::: ::::::
3' --GACACCGUGCCAUGAUGUCAA--- 5'        Csi-miRN02
```

Csi-miRN02, target=Orange1.1t01714.1 gene=Orange1.1t01714
Category:3
Score=3.5
Cleavage Site=2127

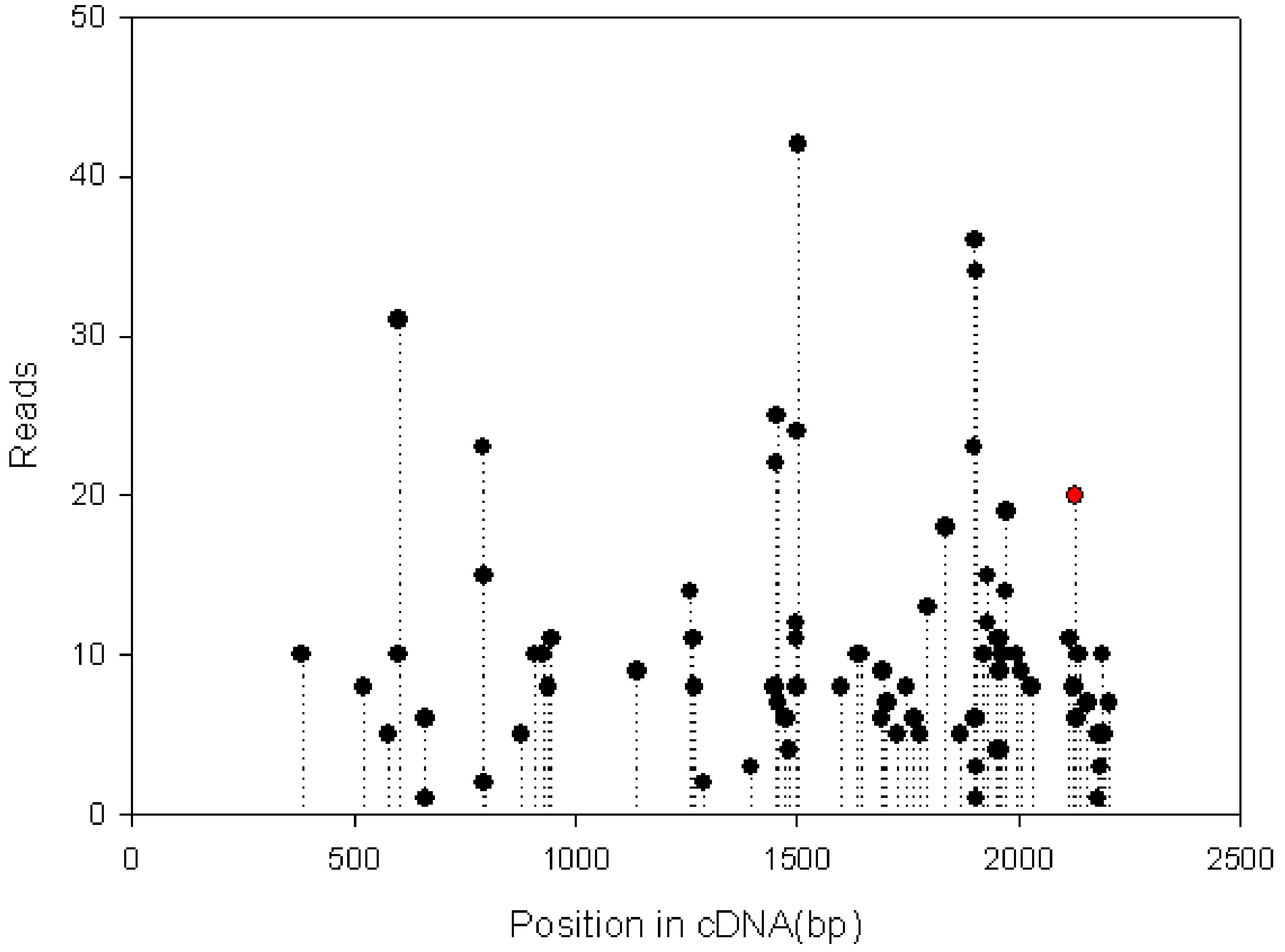


```
5' AAUUGUGGCACGGUGCCACAGUUGCA 3'        Orange1.1t01714.1
     .:::::::::::::.: ::::::
3' --GACACCGUGCCAUGAUGUCAA--- 5'        Csi-miRN02
```

Csi-miRN05, target=Cs4g19940.1 gene=Cs4g19940
Category:2
Score=3.5
Cleavage Site=3042

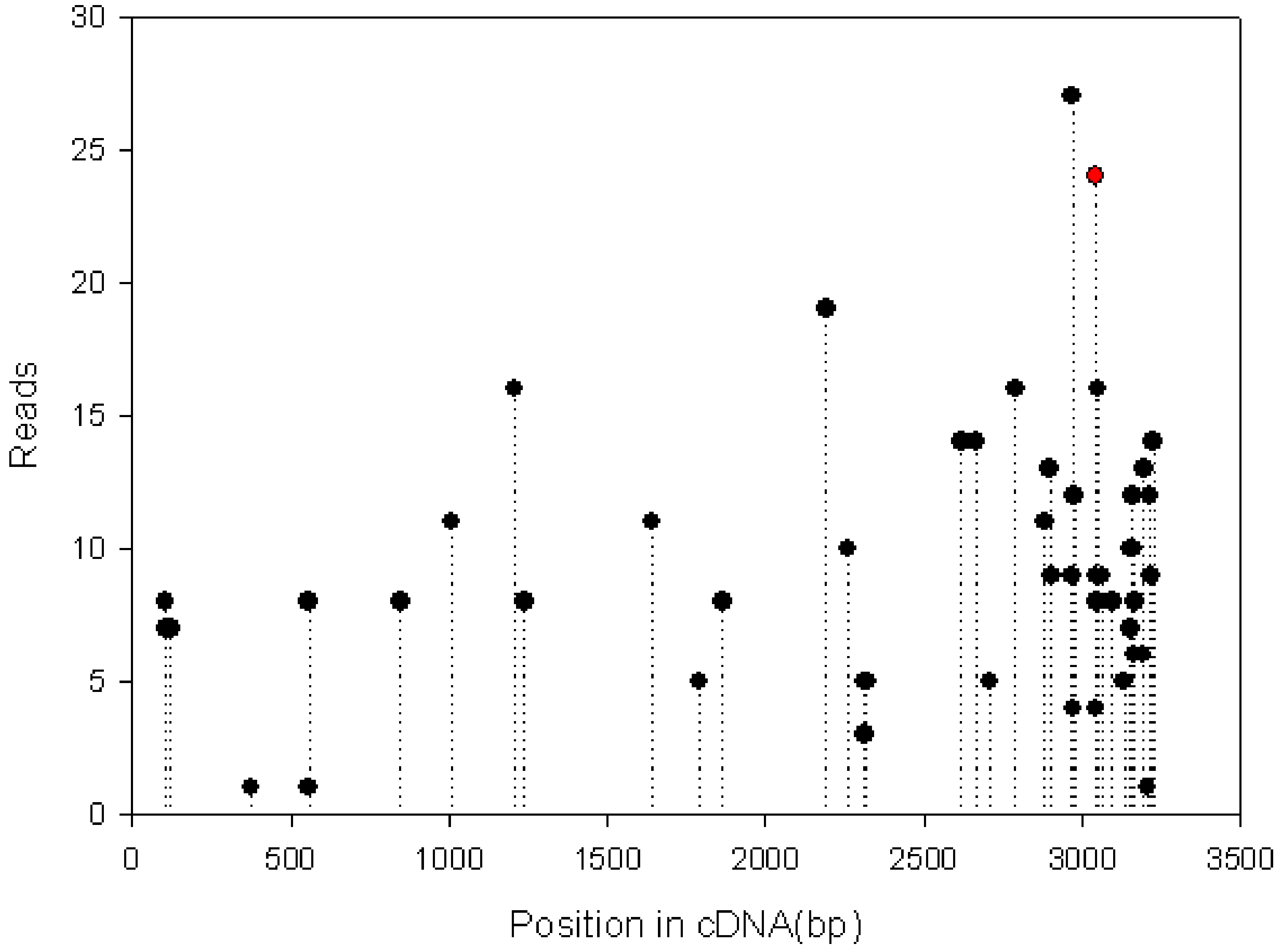


```
5' AGCUAGGAAUGUAUCUUACAUUAUAU 3'        Cs4g19940.1
     :   ::.:::::::::::::::
3' ACUCUCUUUACAUAGAAUGUA----- 5'        Csi-miRN05
```

Csi-miRN07, target=Cs8g09620.1 gene=Cs8g09620
Category:3
Score=4
Cleavage Site=353

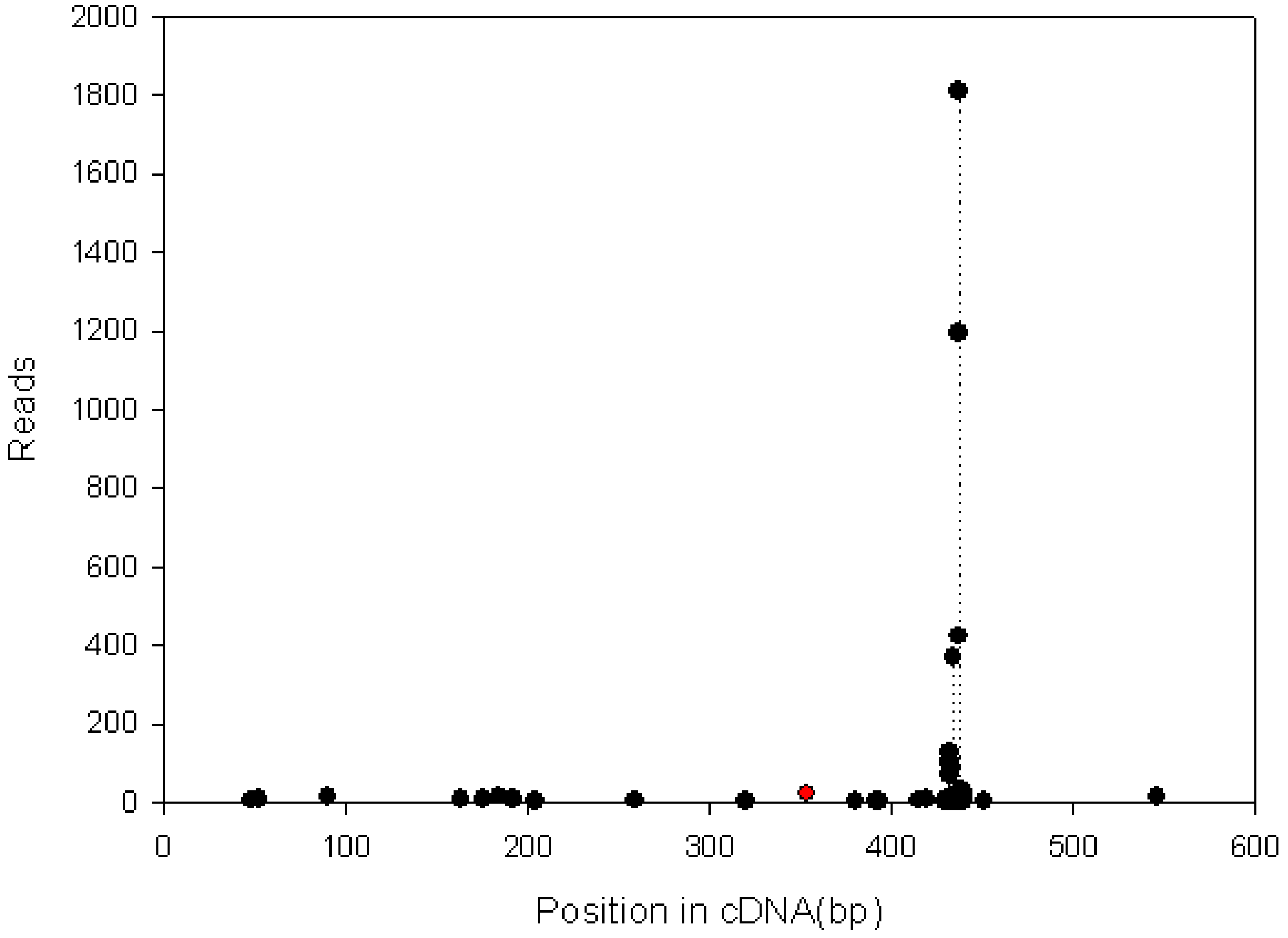


```
5' UCCGACACCGUUUCAUCUUGUCCUCC 3'        Cs8g09620.1
         :::::::: :::::::: :::
3' -----GUGGCAAACUAGAACAGAAGG 5'        Csi-miRN07
```

Csi-miRN11, target=Cs8g13560.1 gene=Cs8g13560
Category:1
score=2
Cleavage Site=302

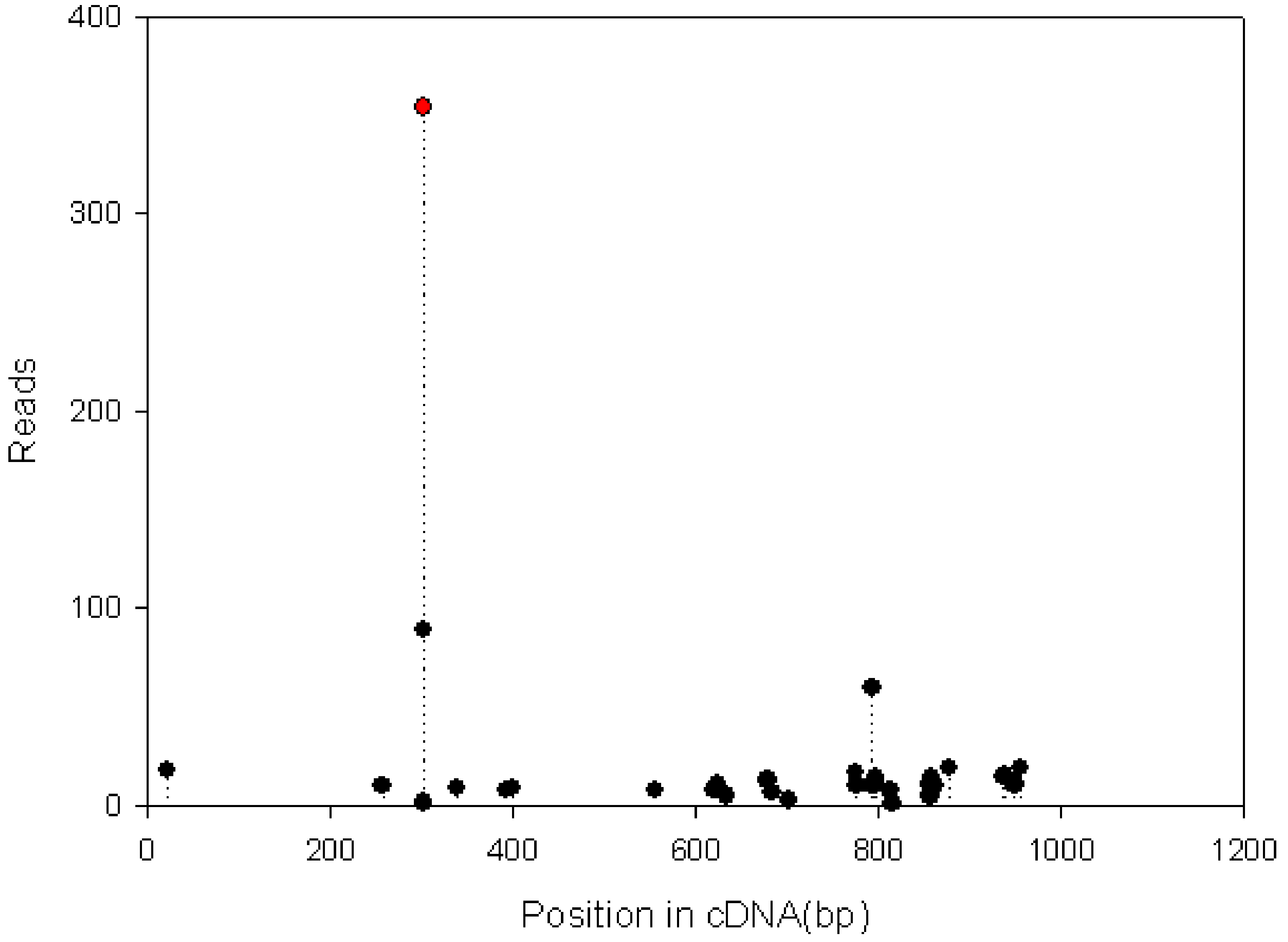


```
5' GUACCGUAAAAAGCUCCUGCGACAUG 3'        Cs8g13560.1
    :::::: :::::::::::::
3' -UUGGCAUCUUUCGAGGACGCU---- 5'        Csi-miRN11
```

Csi-miRN12, target=Cs4g06030.1 gene=Cs4g06030
Category:3
Score=5
Cleavage Site=2072

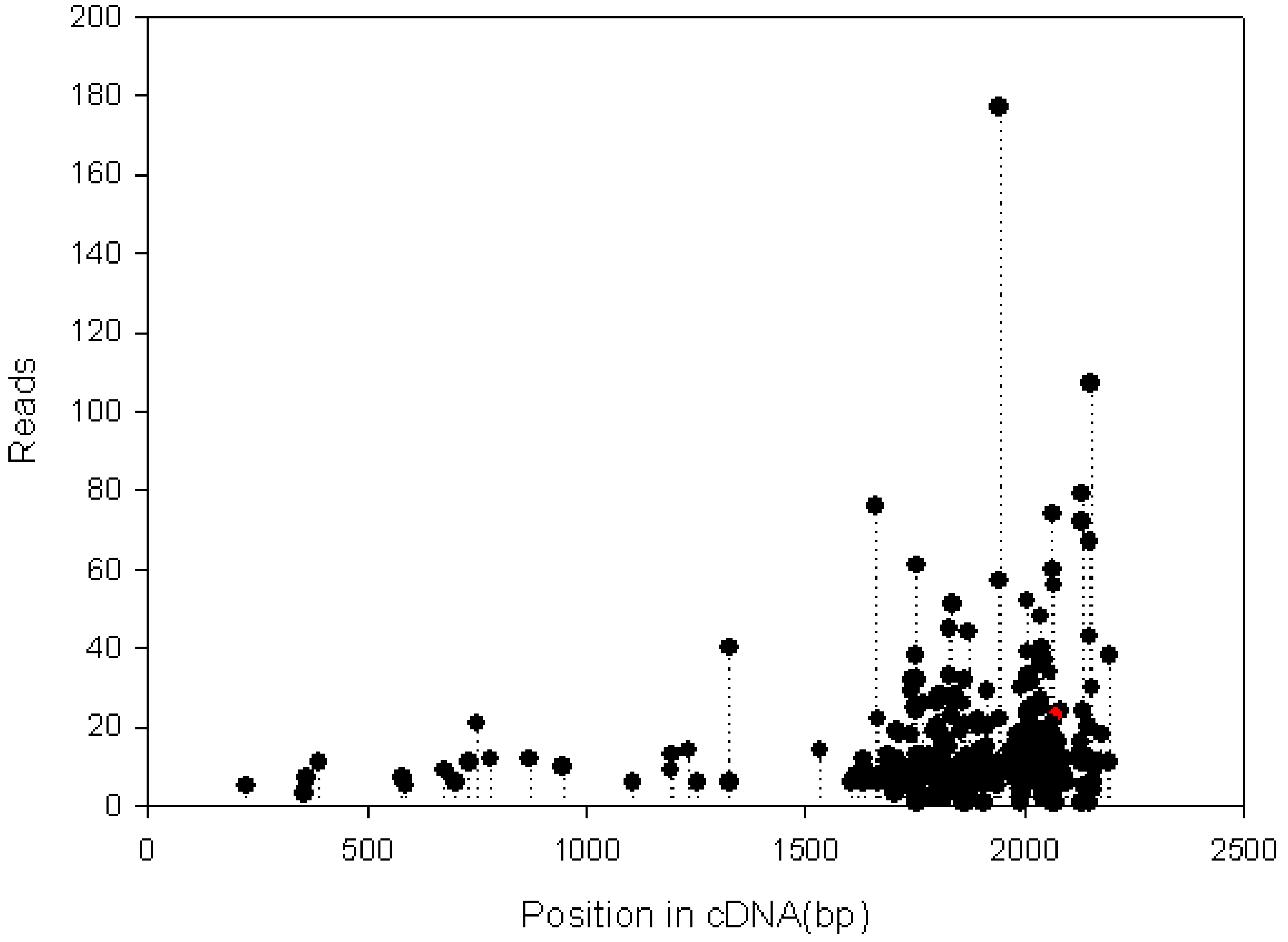


```
5' GAGCUCAGUGGCAUAGCCAUUGAAAC 3'        Cs4g06030.1
     :: :::: ::::.:::::::
3' --AGAAUCACGGUAUUGGUAACU--- 5'        Csi-miRN12
```

Csi-miRN20, target=Cs3g05320.1 gene=Cs3g05320
Category:1
Score=0
Cleavage Site=451

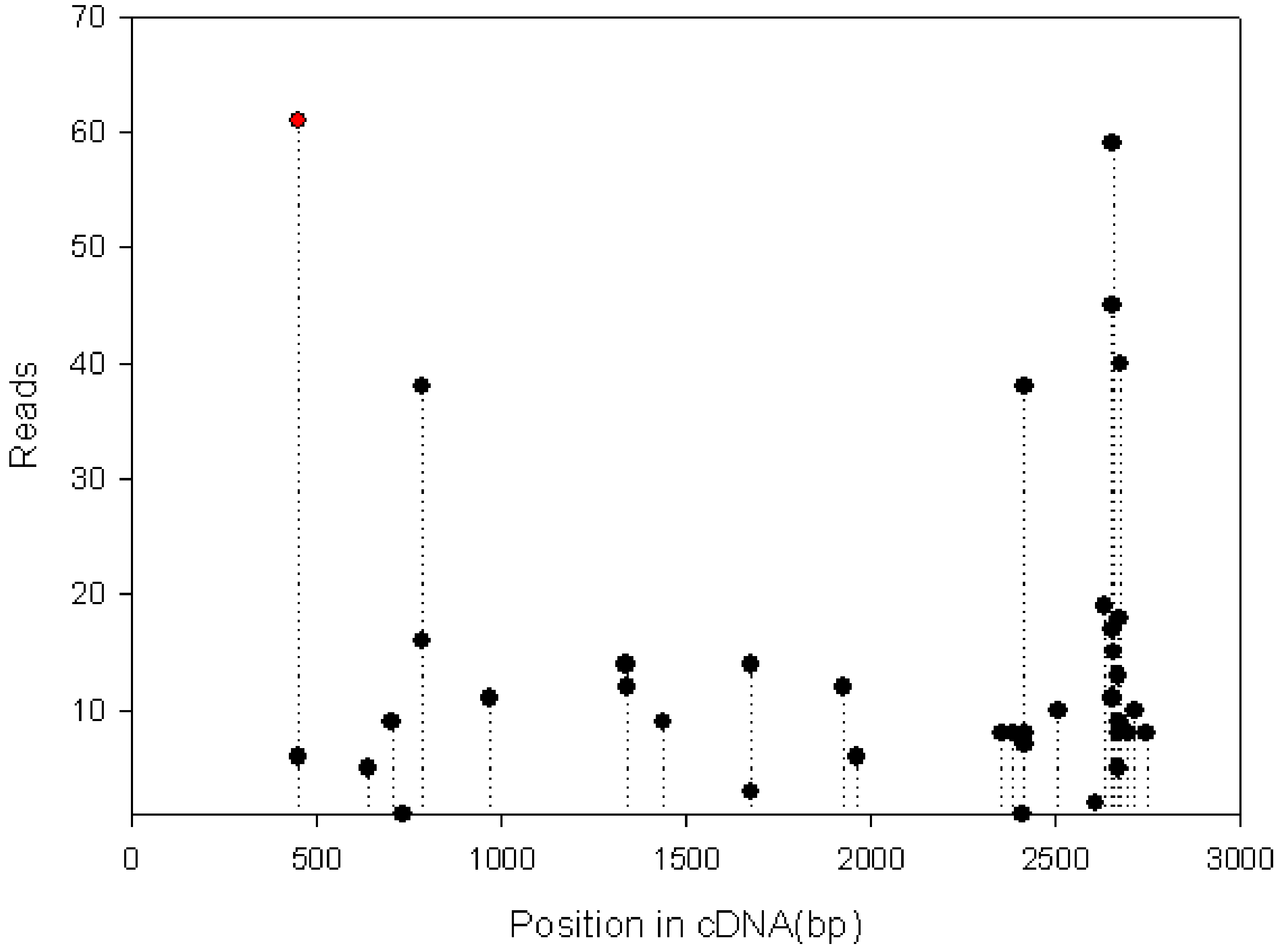


```
5' UUAUCAGCAUCAUGCAACAAAAUAGU 3'        Cs3g05320.1
   ::::::::::::::::::::::
3' AAUAGUCGUAGUACGUUGUUUU---- 5'        Csi-miRN20
```

Csi-miRN37, target=Orange1.1t02400.1 gene=Orange1.1t02400
Category:2
Score=5
Cleavage Site=772

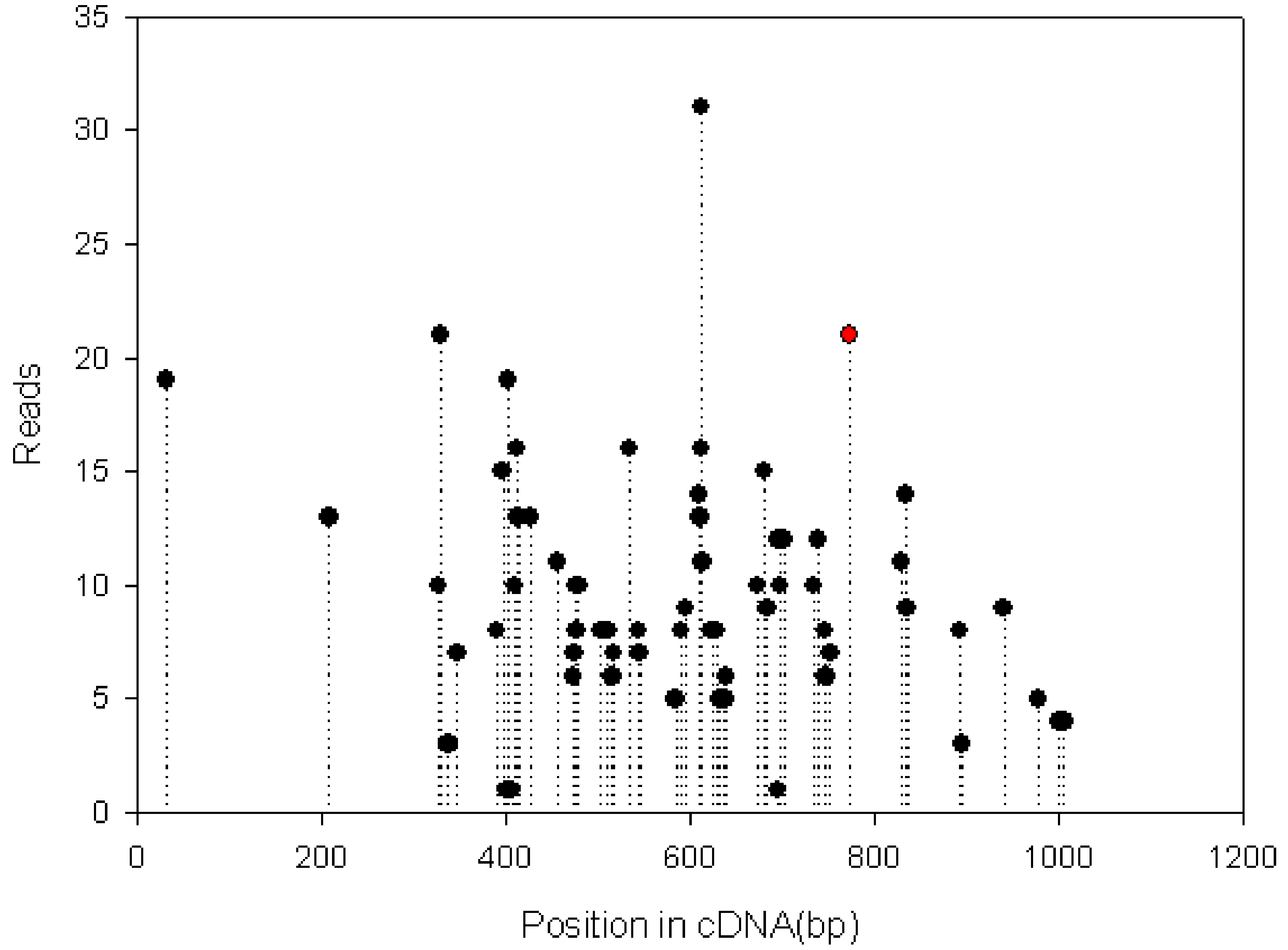


```
5' GAGUACAACU-AGCUUUUCCCUCAAGA 3'        Orange1.1t02400.1
         :   ::  ::::.:::::::::
3' ----GGAAGAUUCGAGAAGGGAGU--- 5'        Csi-miRN37
```

T-plots for targets of all miRNAs in flower

Csi-miR1092.2, target=Cs8g11330.1 gene=Cs8g11330
Category=3
Score=5
Cleavage Site=1168

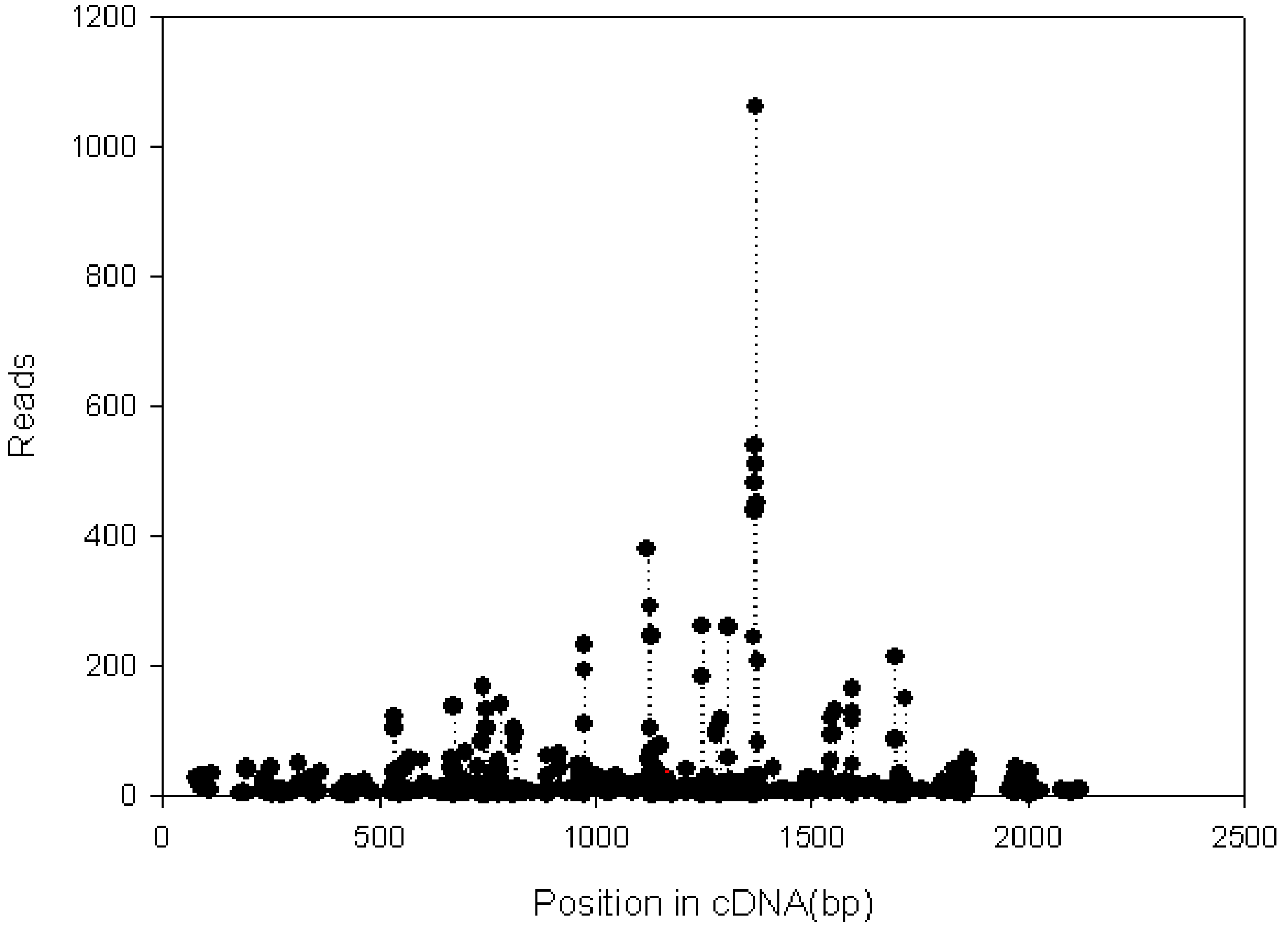


```
5' GGAAAUGUAUGCCAUUGGUGGAAGUG 3'        Cs8g11330.1
   ::::::: ::: : :::::::::
3' CCUUUACUUAC-GAAACCACCUU--- 5'        csi-miR1092.2
```

Csi-miR1432a, target=Cs1g21570.1 gene=Cs1g21570
Category=3
Score=4
Cleavage Site=702

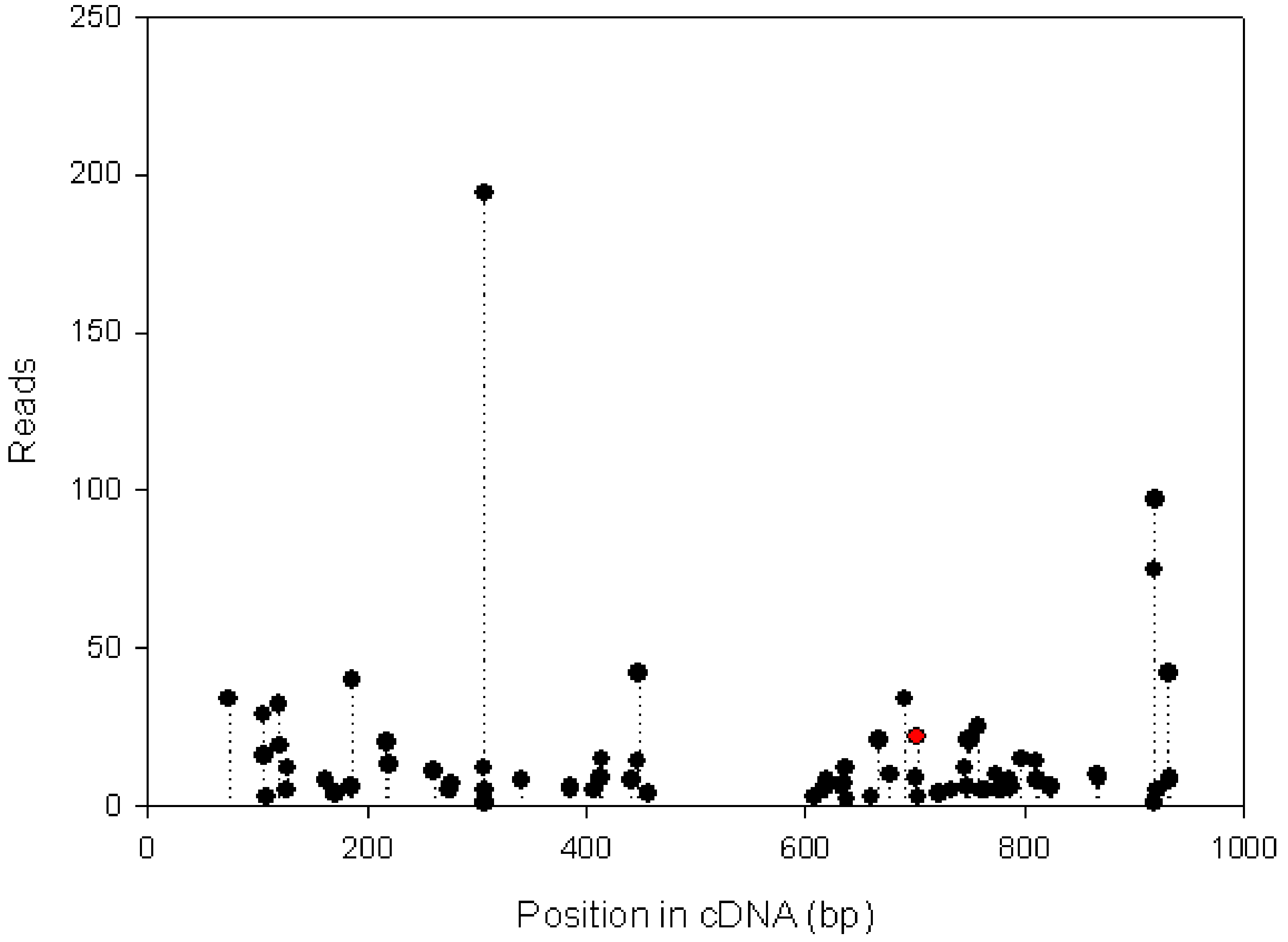


```
5' AAACUGAUUGUGUUGUCUCACCUGCA 3'        Cs1g21570.1
       ::::. ::.:..::::::::::
3' ----ACUGCCAUAGUAGAGUGGACGU 5'        Csi-miR1432a
```

Csi-miR1446, target=Cs7g23010.1 gene=Cs7g23010
Category=1
Score=4
Cleavage Site=3881

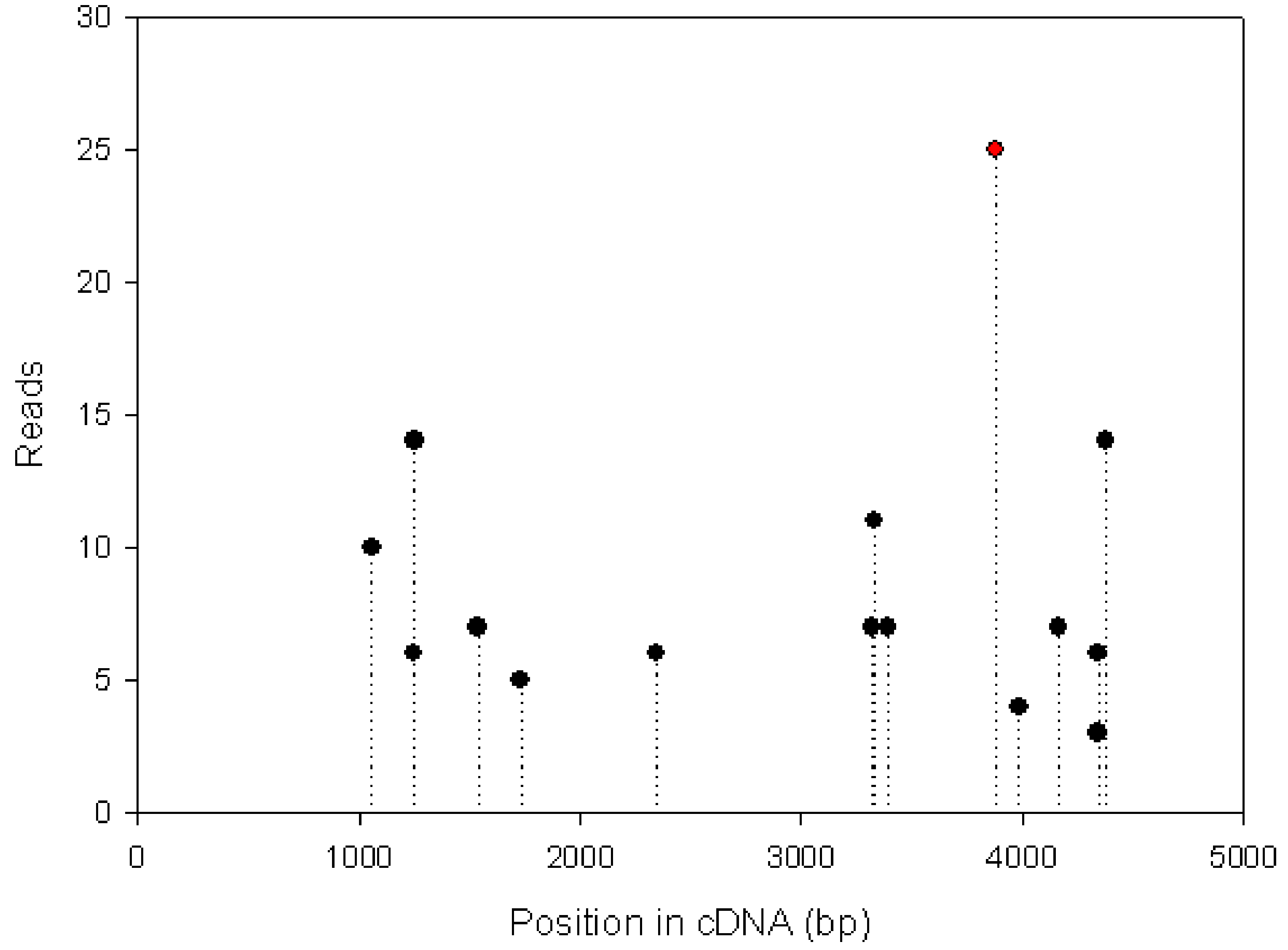


```
5' AAAGACGUUGAAGGAGAGAGUUUGAA 3'        Cs7g23010.1
     : :::::: :::::::::::.:
3' ---CGGCAACUCCCUCUCUCAAGC-- 5'        Csi-miR1446
```

Csi-miR156a.1, target=Cs2g05730.1 gene=Cs2g05730
Category:1
Score=1
Cleavage Site=818

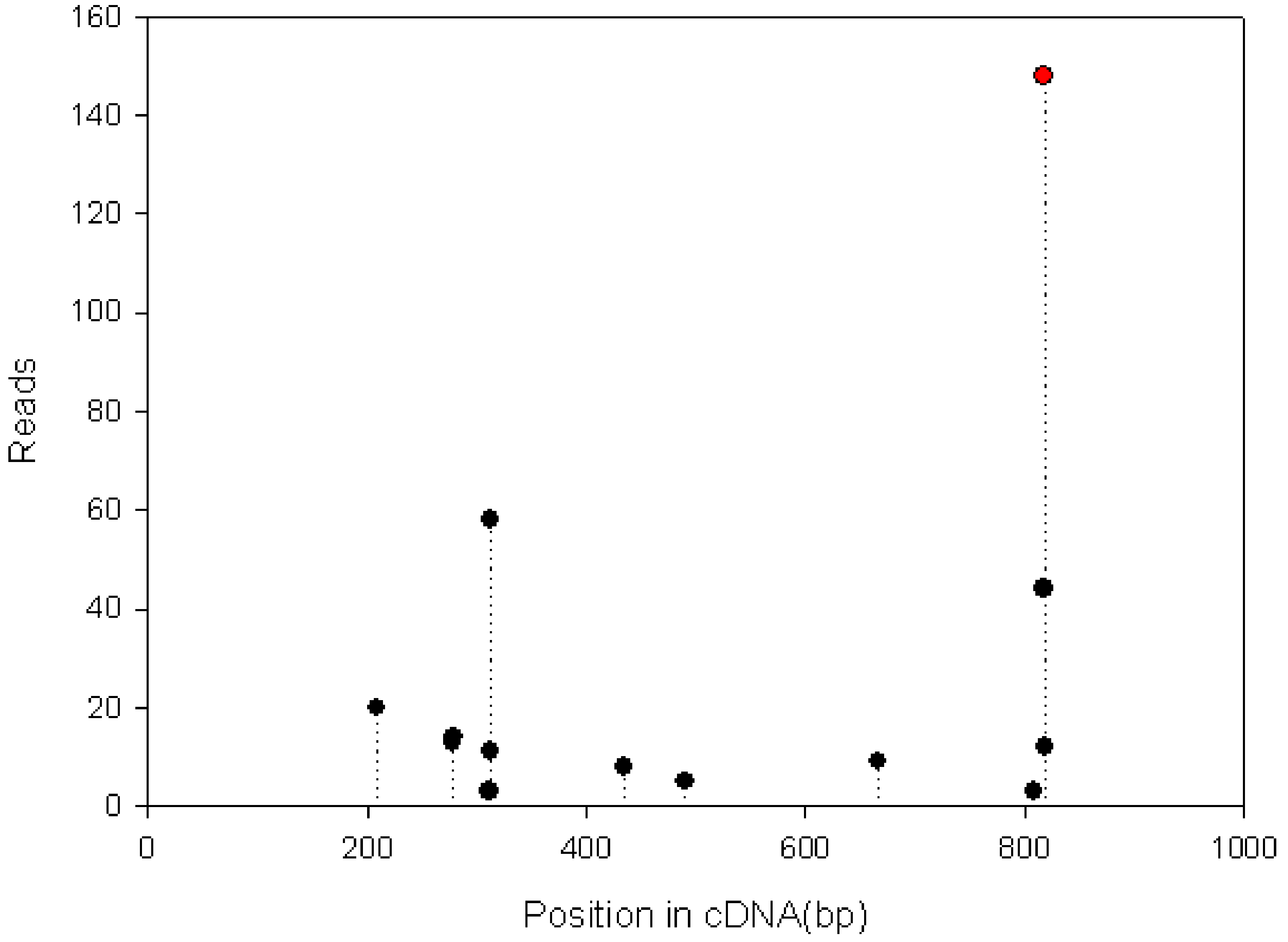


```
5' CGUGCUCUCUCUCUUCUGUCAAUGCU 3'        Cs2g05730.1
     :::::: ::::::::::::::
3' -CACGAGUGAGAGAAGACAGUU---- 5'        Csi-miR156a.1
```

Csi-miR156a.1, target=Cs2g23550.1 gene=Cs2g23550
Category=1
Score=3
Cleavage Site=934

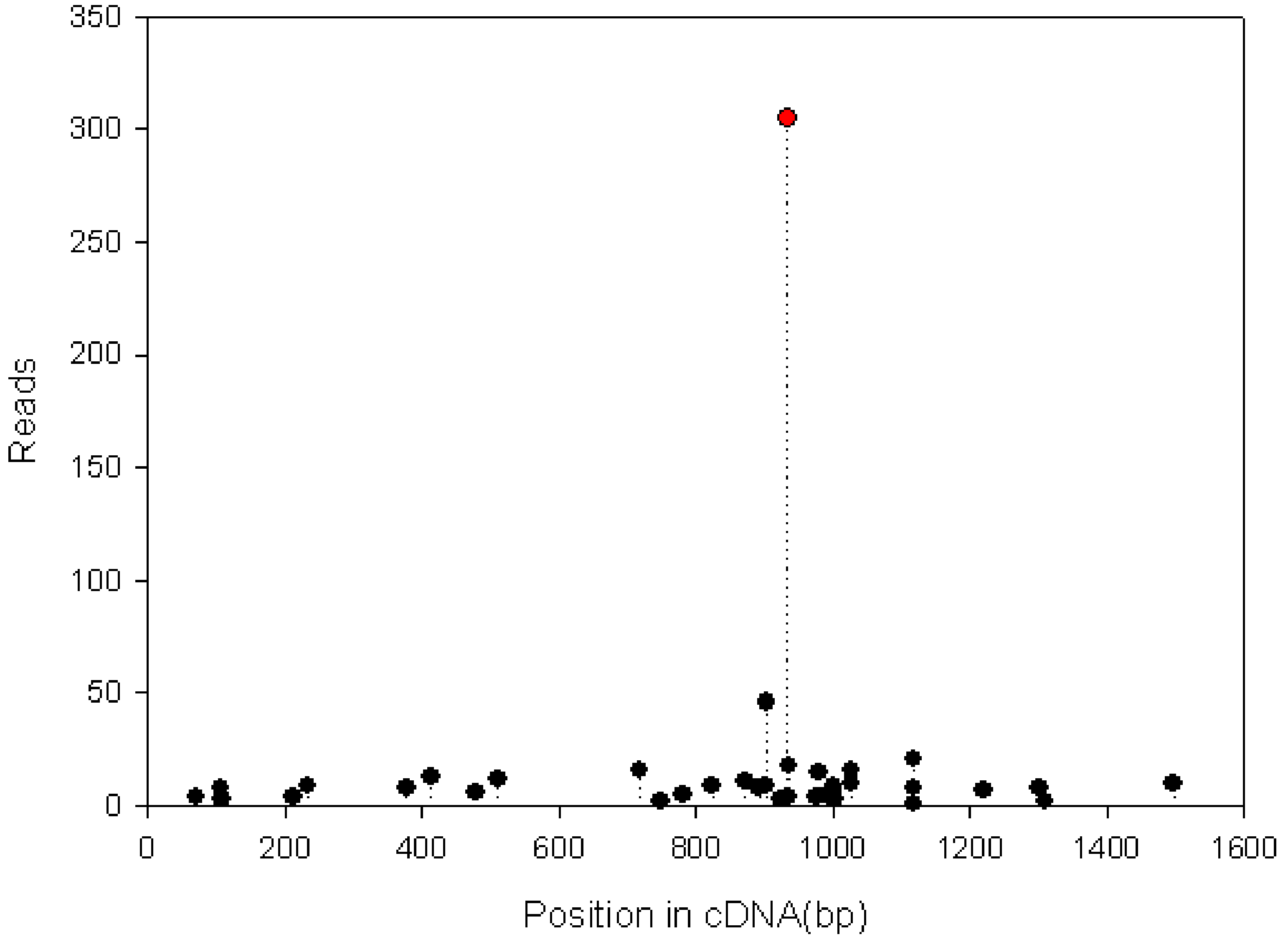


```
5' CAUGCUCUCUCUCUUCUGUCAUCCUA 3'        Cs2g23550.1
     ::::: :::::::::::::
3' -CACGAGUGAGAGAAGACAGUU---- 5'        Csi-miR156a.1
```

Csi-miR156a.2, target=Cs2g05730.1 gene=Cs2g05730
Category:1
Score=1
Cleavage Site=818

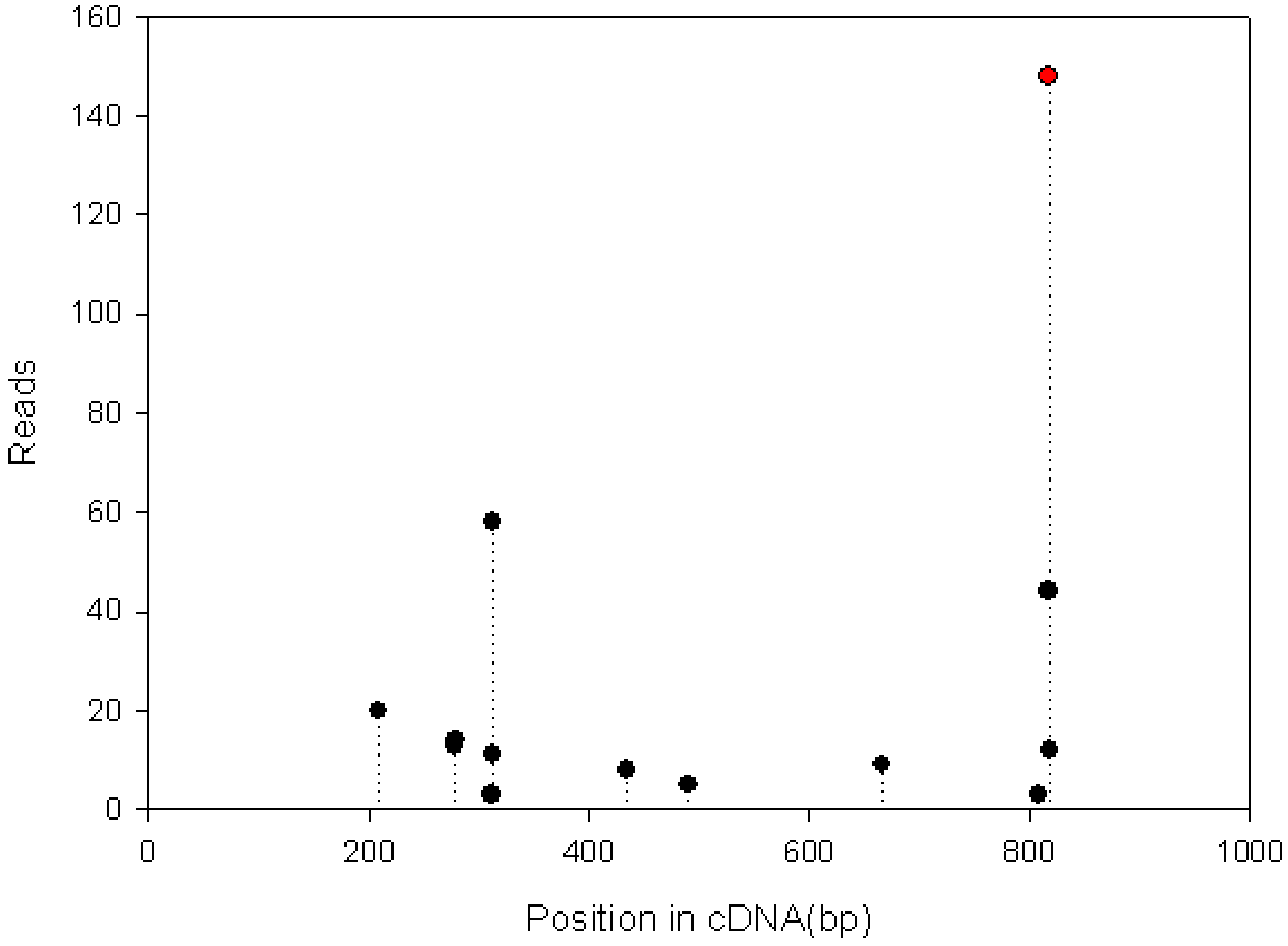


```
5' CGUGCUCUCUCUCUUCUGUCAAUGCU 3'        Cs2g05730.1
    :::::: :::::::::::::
3' -CACGAGUGAGAGAAGACAGU----- 5'        Csi-miR156a.2
```

Csi-miR156a.2, target=Cs2g23550.1 gene=Cs2g23550
Category=1
Score=2
Cleavage Site=934

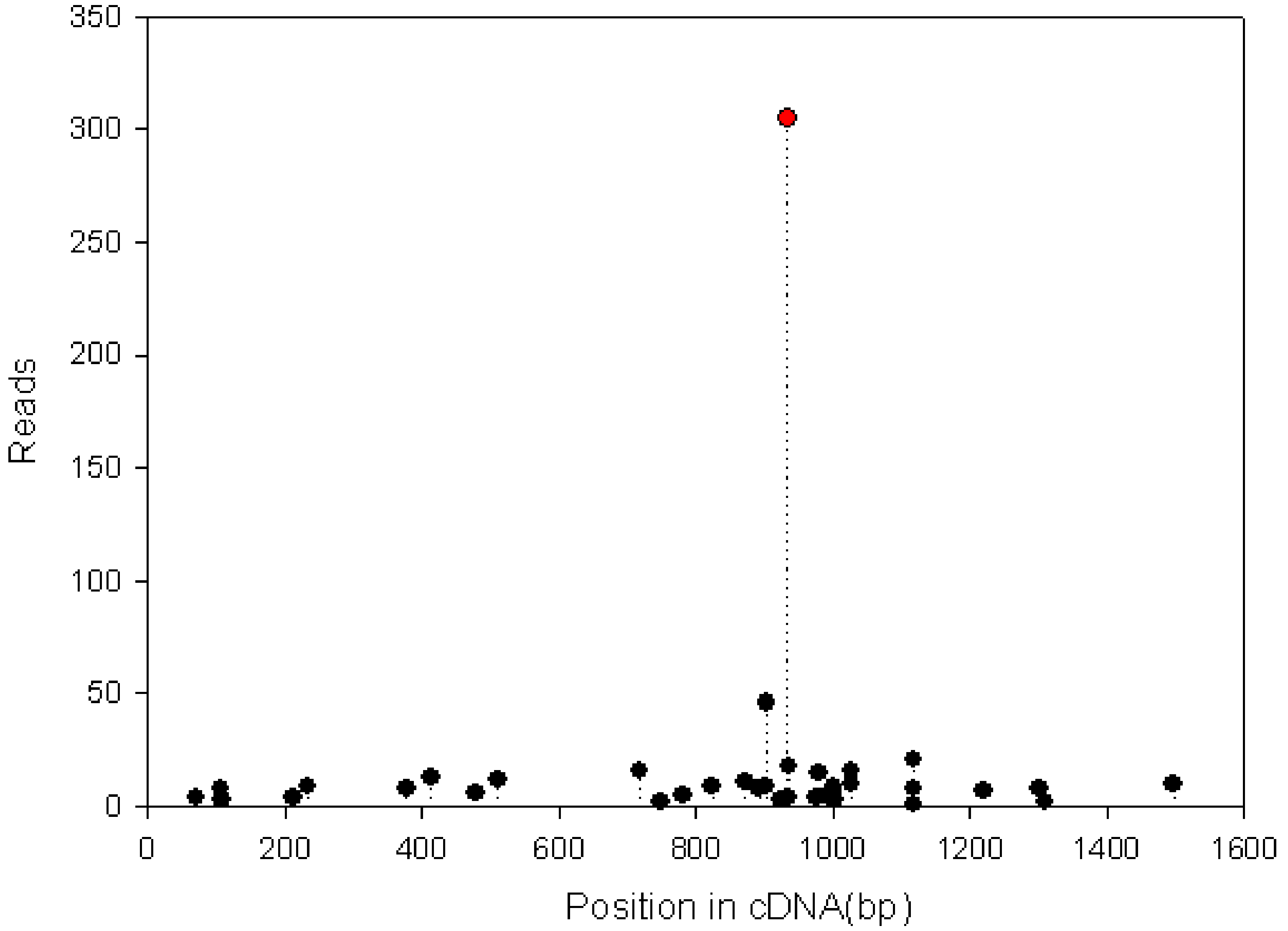


```
5' CAUGCUCUCUCUUCUGUCAUCCUA 3'        Cs2g23550.1
     ::::: ::::::::::::
3' -CACGAGUGAGAGAAGACAGU----- 5'       Csi-miR156a.2
```

Csi-miR156b.1, target=Cs2g05730.1 gene=Cs2g05730
Category:1
Score=2
Cleavage Site=818

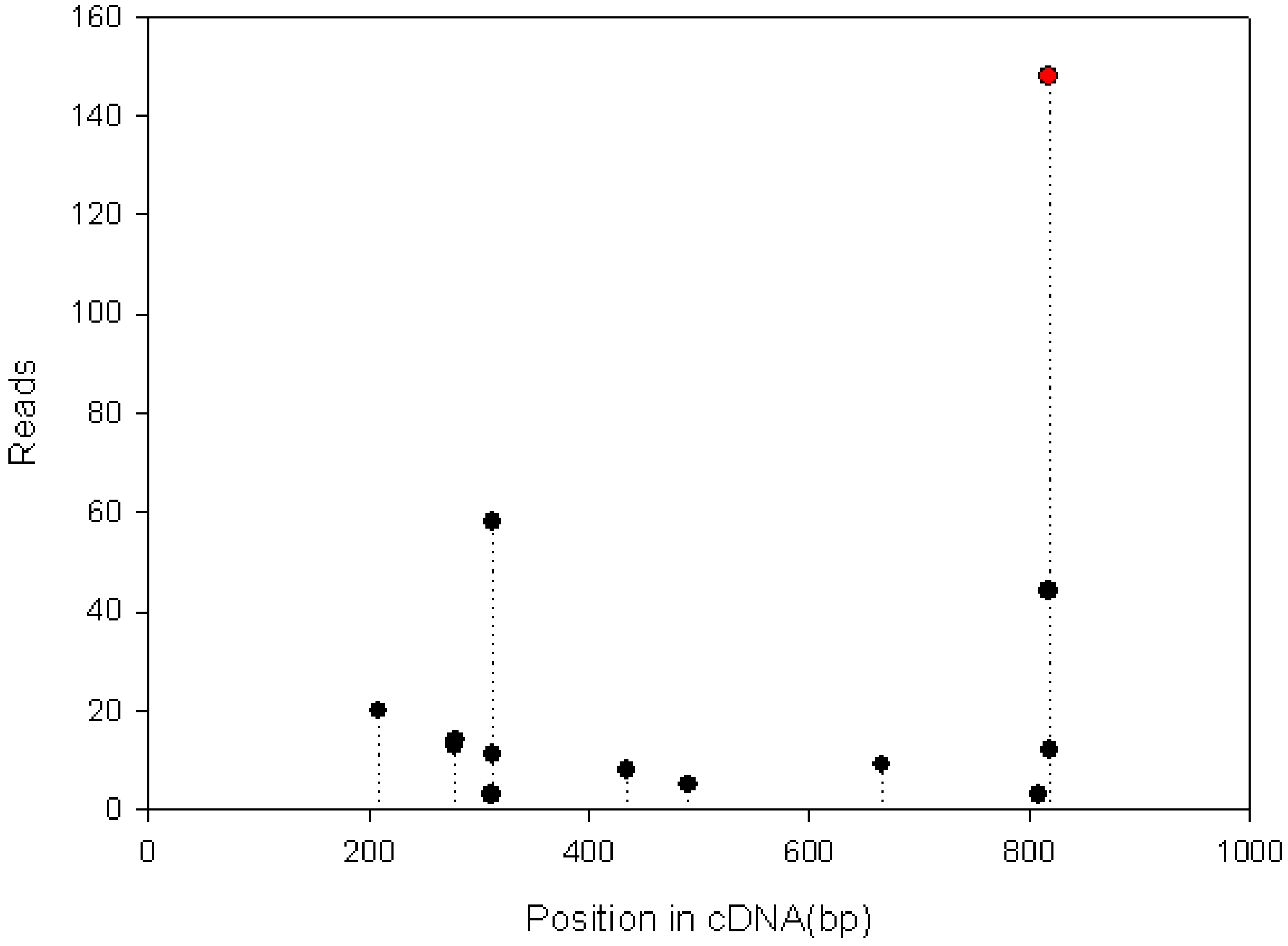


```
5' CGUGCUCUCUCUCUUCUGUCAAUGCU 3'        Cs2g05730.1
    :::::: :::::::::::::
3' -CACGAGUGAGAGAAGACAGUC---- 5'        Csi-miR156b.1
```

Csi-miR156b.1, target=Cs2g23550.1 gene=Cs2g23550
Category=1
Score=3
Cleavage Site=934

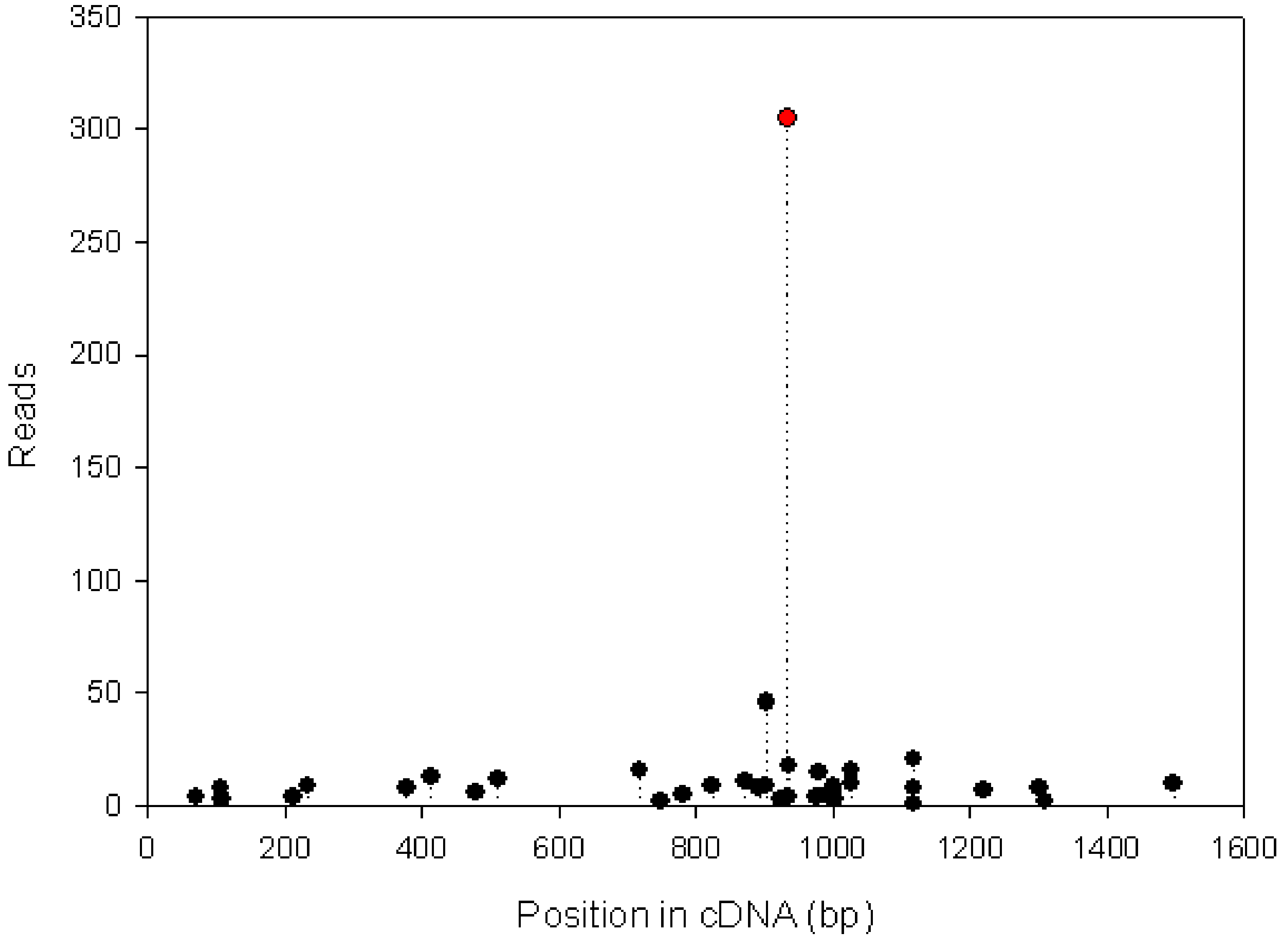


```
5' CAUGCUCUCUCUCUUCUGUCAUCCUA 3'        Cs2g23550.1
     ::::: :::::::::::::
3' -CACGAGUGAGAGAAGACAGUC---- 5'        Csi-miR156b.1
```

Csi-miR156c.1, target=Cs2g05730.1 gene=Cs2g05730
Category:1
Score=2
Cleavage Site=818

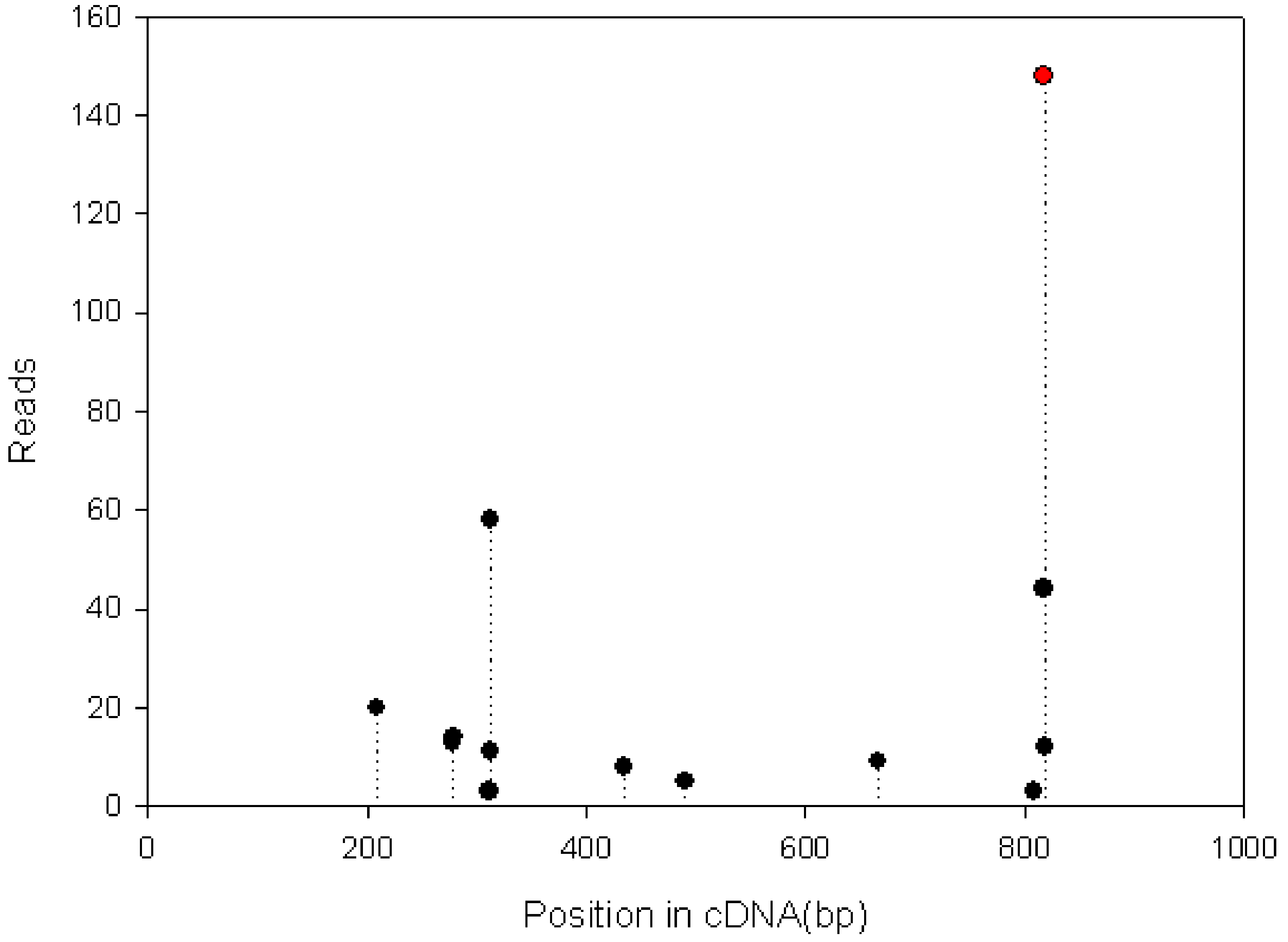


```
5' CGUGCUCUCUCUCUUCUGUCAAUGCU 3'        Cs2g05730.1
     :::::: :::::::::::::
3' ACACGAGUGAGAGAAGACAGU----- 5'        Csi-miR156c.1
```

Csi-miR156c.1, target=Cs2g23550.1 gene=Cs2g23550
Category=1
Score=3
Cleavage Site=934

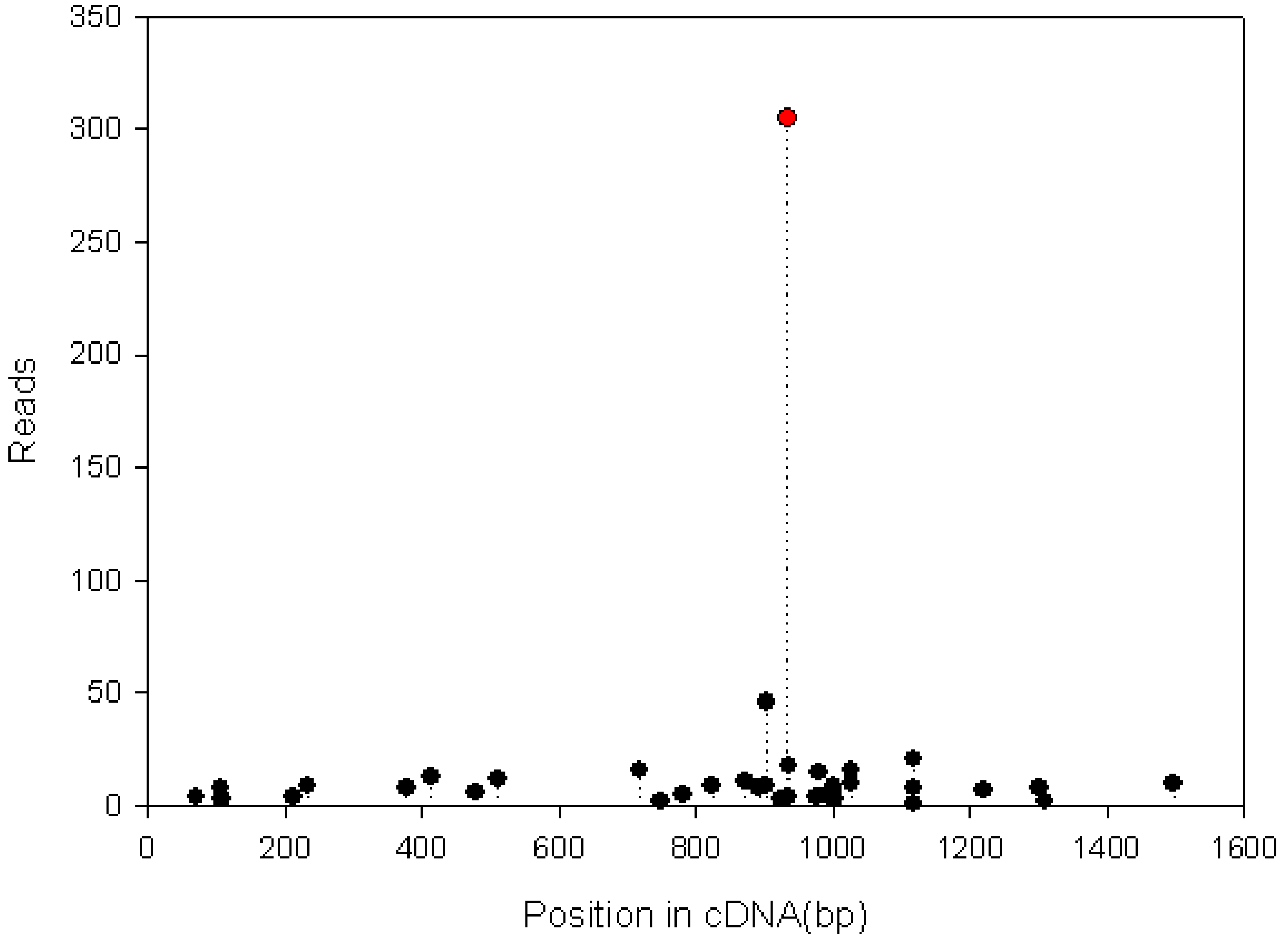


```
5' CAUGCUCUCUCUCUUCUGUCAUCCUA 3'        Cs2g23550.1
     ::::: :::::::::::::
3' ACACGAGUGAGAGAAGACAGU----- 5'        Csi-miR156c.1
```

Csi-miR156d, target=Cs2g05730.1 gene=Cs2g05730
Category:1
Score=2.5
Cleavage Site=818

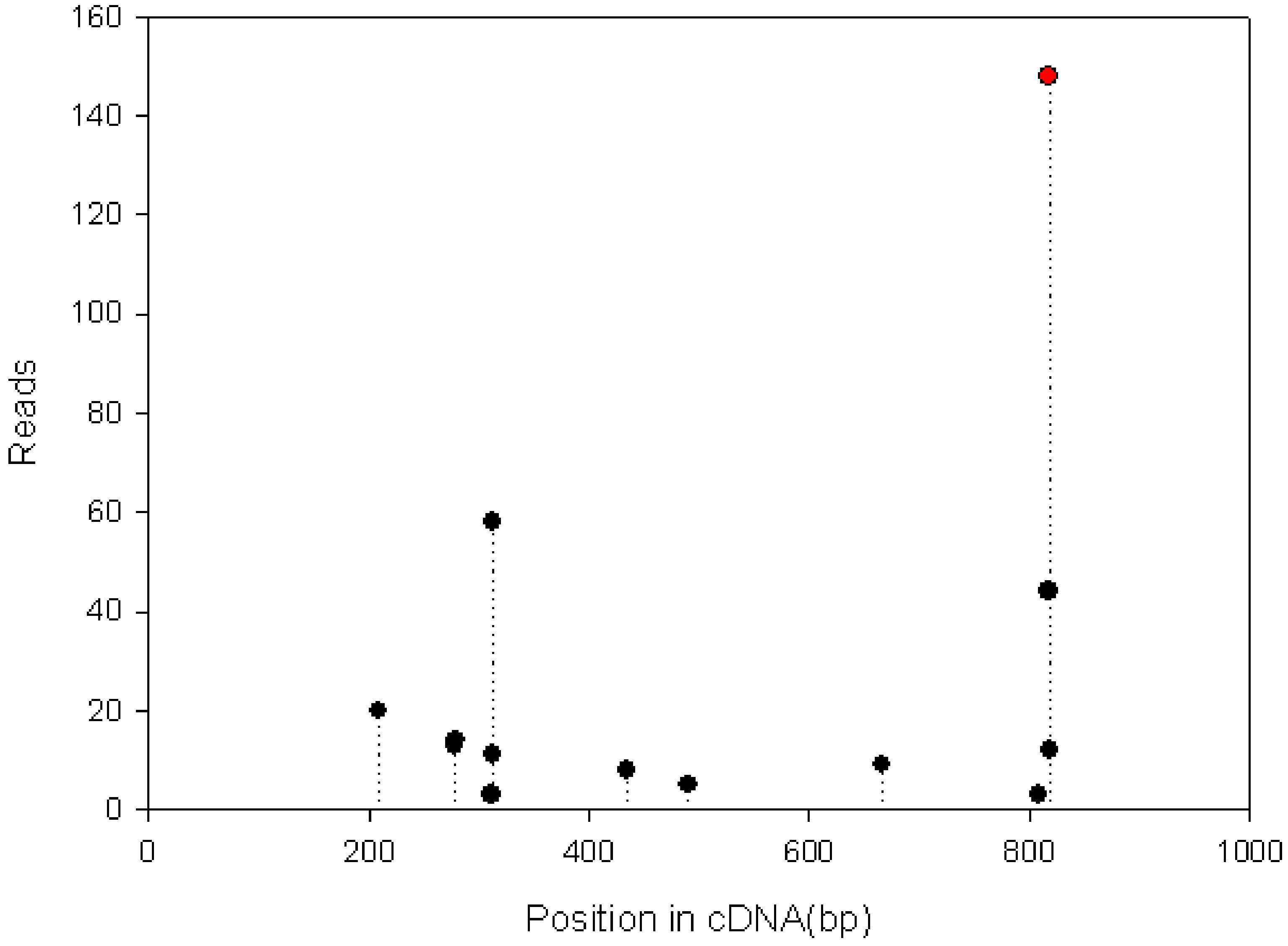


```
5' CGUGCUCUCUCUCUUCUGUCAAUGCU 3'        Cs2g05730.1
    :.:::::::: ::::::::::
3' -CGCGAGAGAUAGAAGACAGU----- 5'        Csi-miR156d
```

Csi-miR156d, target=Cs2g23550.1 gene=Cs2g23550
Category=1
Score=3.5
Cleavage Site=934

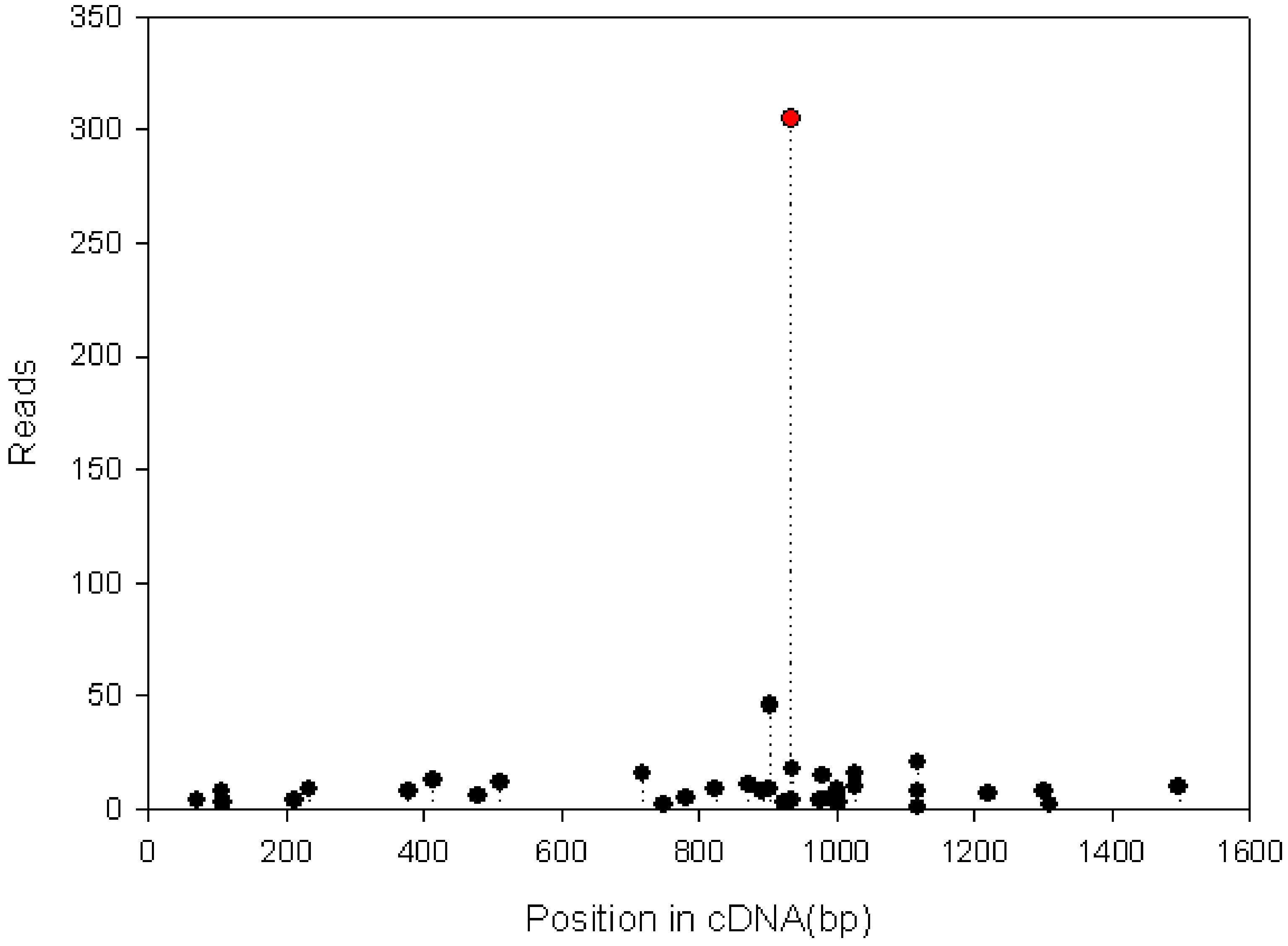


```
5' CAUGCUCUCUCUCUUCUGUCAUCCUA 3'        Cs2g23550.1
     .:::::::: :::::::::::
3' -CGCGAGAGAUAGAAGACAGU----- 5'        Csi-miR156d
```

Csi-miR156e, target=Cs2g05730.1 gene=Cs2g05730
Category:1
Score=3.5
Cleavage Site=818

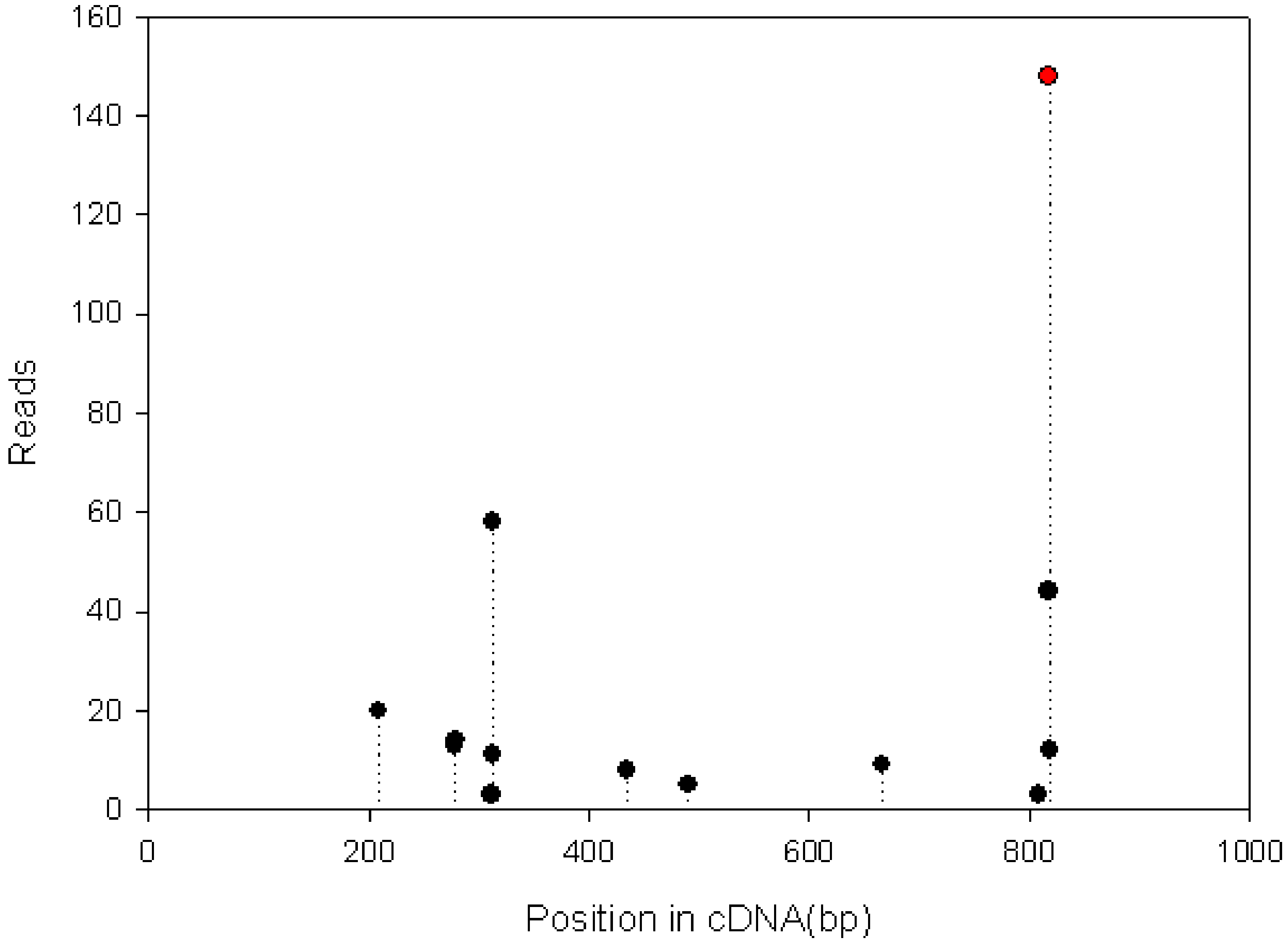


```
5' CGUGCUCUCUCUCUUCUGUCAAUGCU 3'        Cs2g05730.1
    :.:::::::: ::::::::::
3' -CGCGAGAGAUAGAAGACAGUG---- 5'        Csi-miR156e
```

Csi-miR156e, target=Cs2g23550.1 gene=Cs2g23550
Category=1
Score=4
Cleavage Site=934

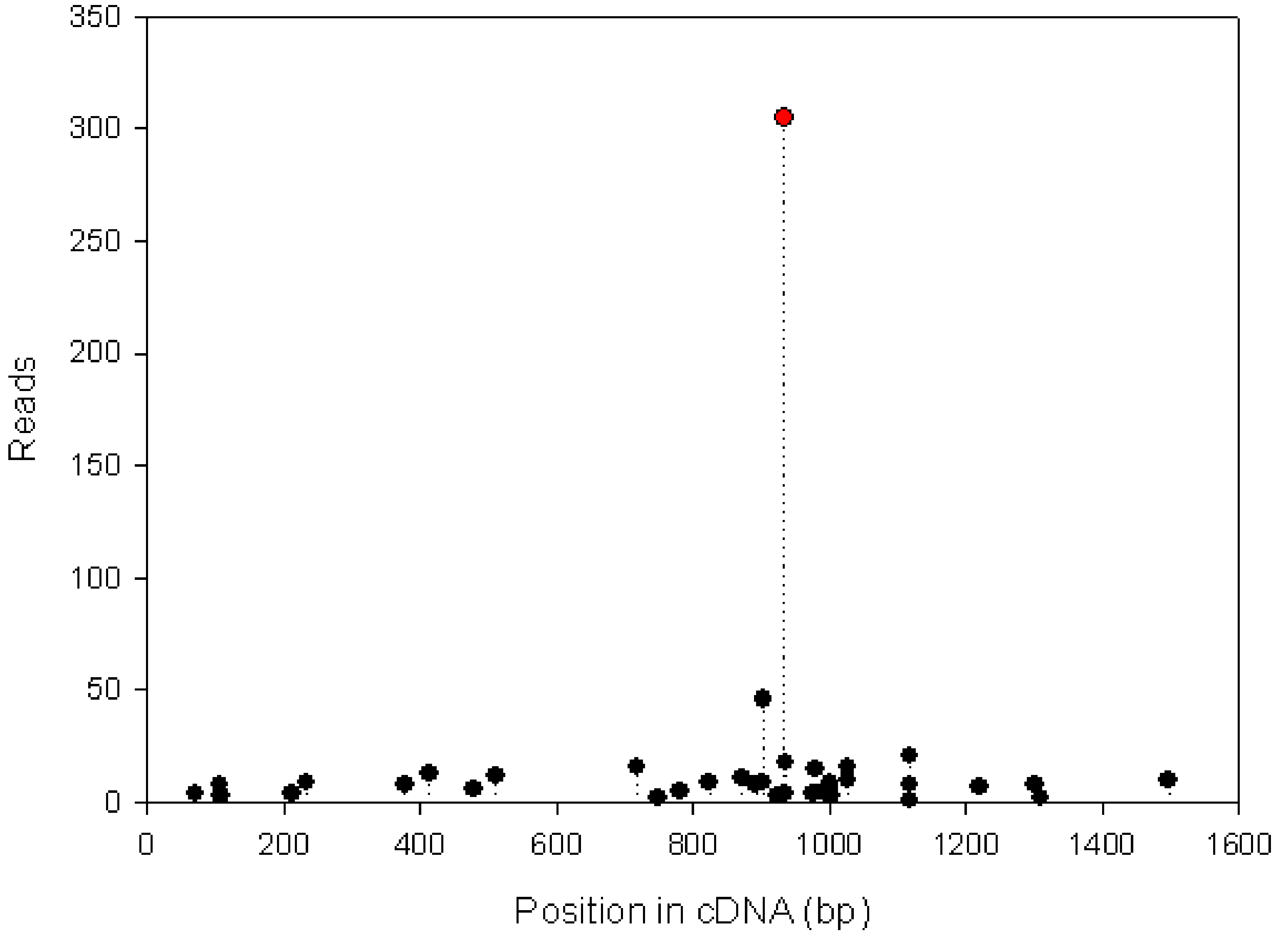


```
5' CAUGCUCUCUCUCUUCUGUCAUCCUA 3'        Cs2g23550.1
     .::::::: ::::::::::.
3' -CGCGAGAGAUAGAAGACAGUG---- 5'        Csi-miR156e
```

Csi-miR156f.2, target=Cs2g05730.1 gene=Cs2g05730
Category:1
Score=1.5
Cleavage Site=818

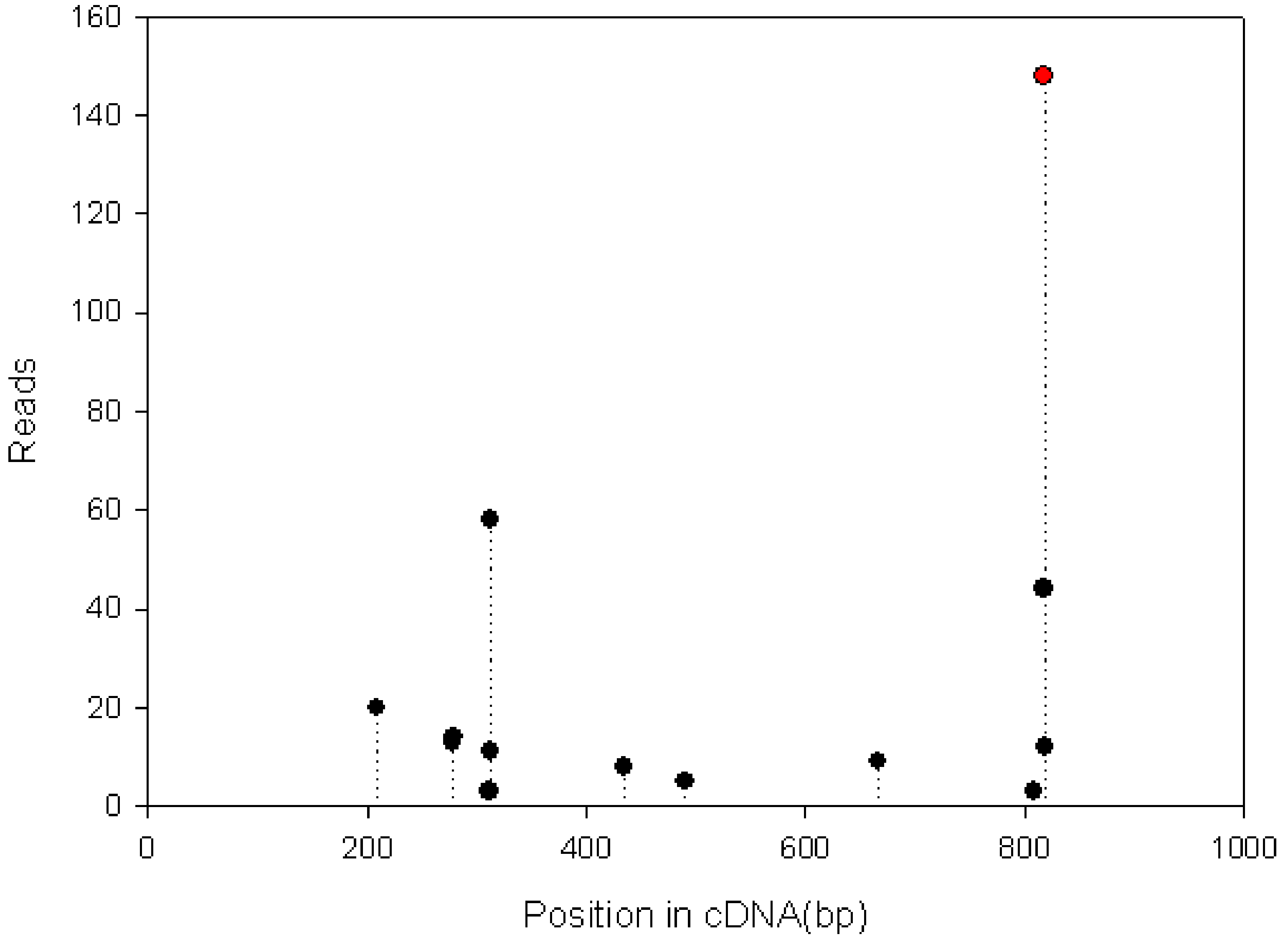


```
5' CGUGCUCUCUCUCUUCUGUCAAUGCU 3'          Cs2g05730.1
   ::.:::::::::::::::::::
3' -CAUGAGAGAGAGAAGACAGUA---- 5'          Csi-miR156f.2
```

Csi-miR156f.2, target=Cs2g23550.1 gene=Cs2g23550
Category=1
Score=1.5
Cleavage Site=934

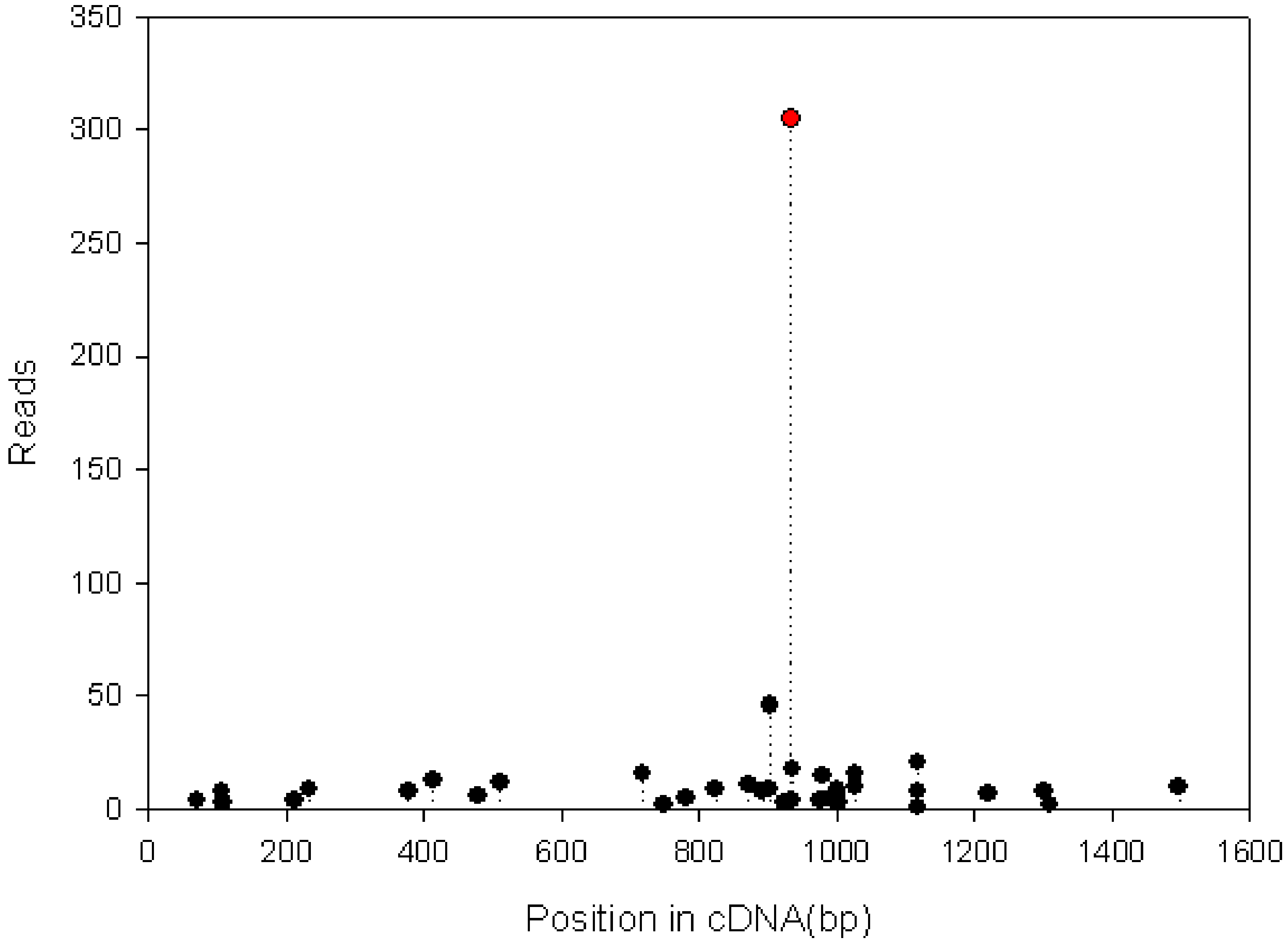


```
5' CAUGCUCUCUCUCUUCUGUCAUCCUA 3'          Cs2g23550.1
     :.:::::::::::::::::::
3' -CAUGAGAGAGAGAAGACAGUA---- 5'          Csi-miR156f.2
```

Csi-miR156f.2, target=Cs8g19900.1 gene=Cs8g19900
Category=2
Score=5
Cleavage Site=2794

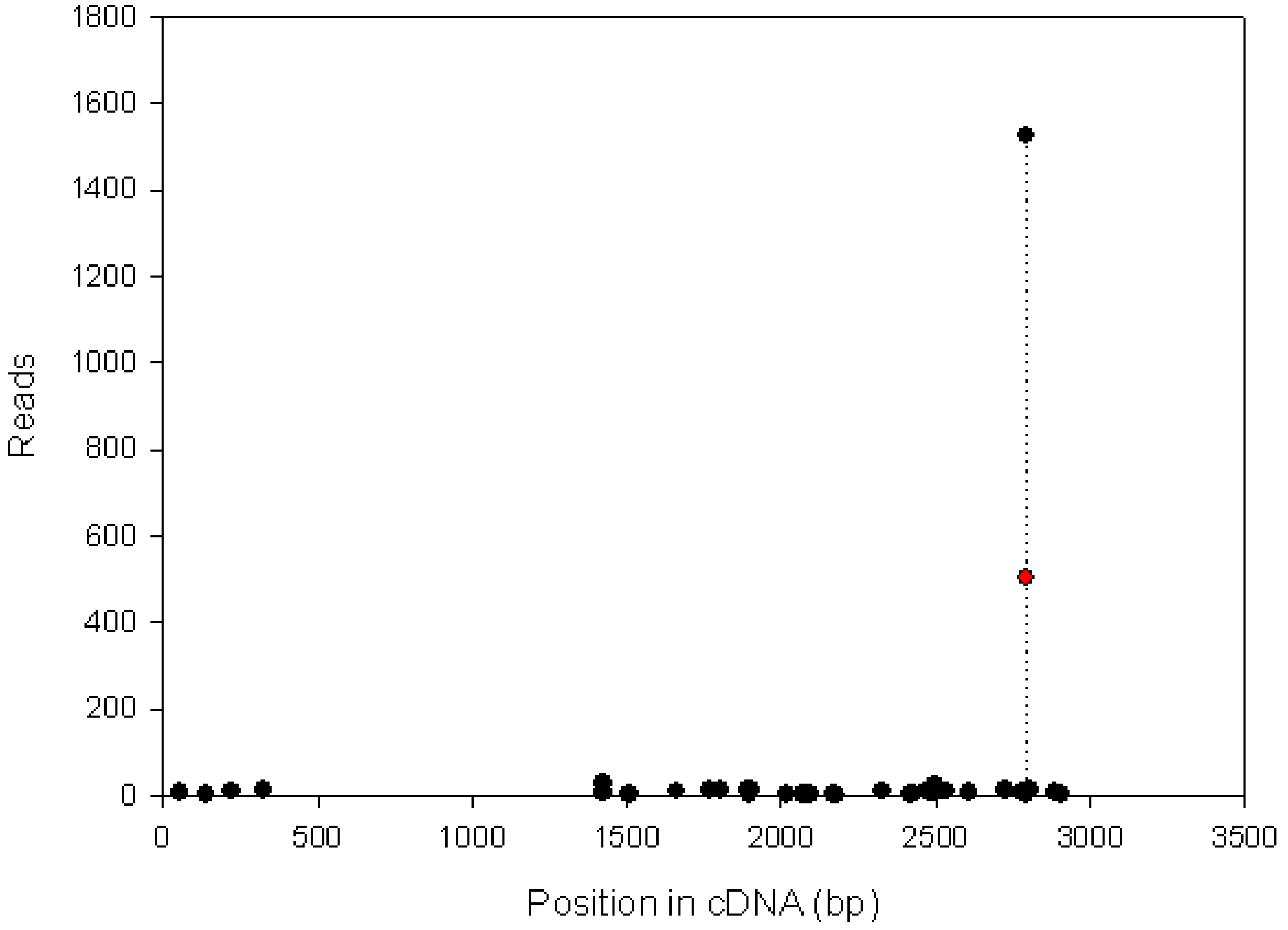


```
5' GUG-UCUUUCUCUUUUGACAUUAUGAG 3'        Cs8g19900.1
   ::. :::.:::::::.:: :::
3' CAUGAGAGAGAGAAGACAGUA------ 5'        Csi-miR156f.2
```

Csi-miR156g.1, target=Cs2g05730.1 gene=Cs2g05730
Category:1
Score=3
Cleavage Site=818

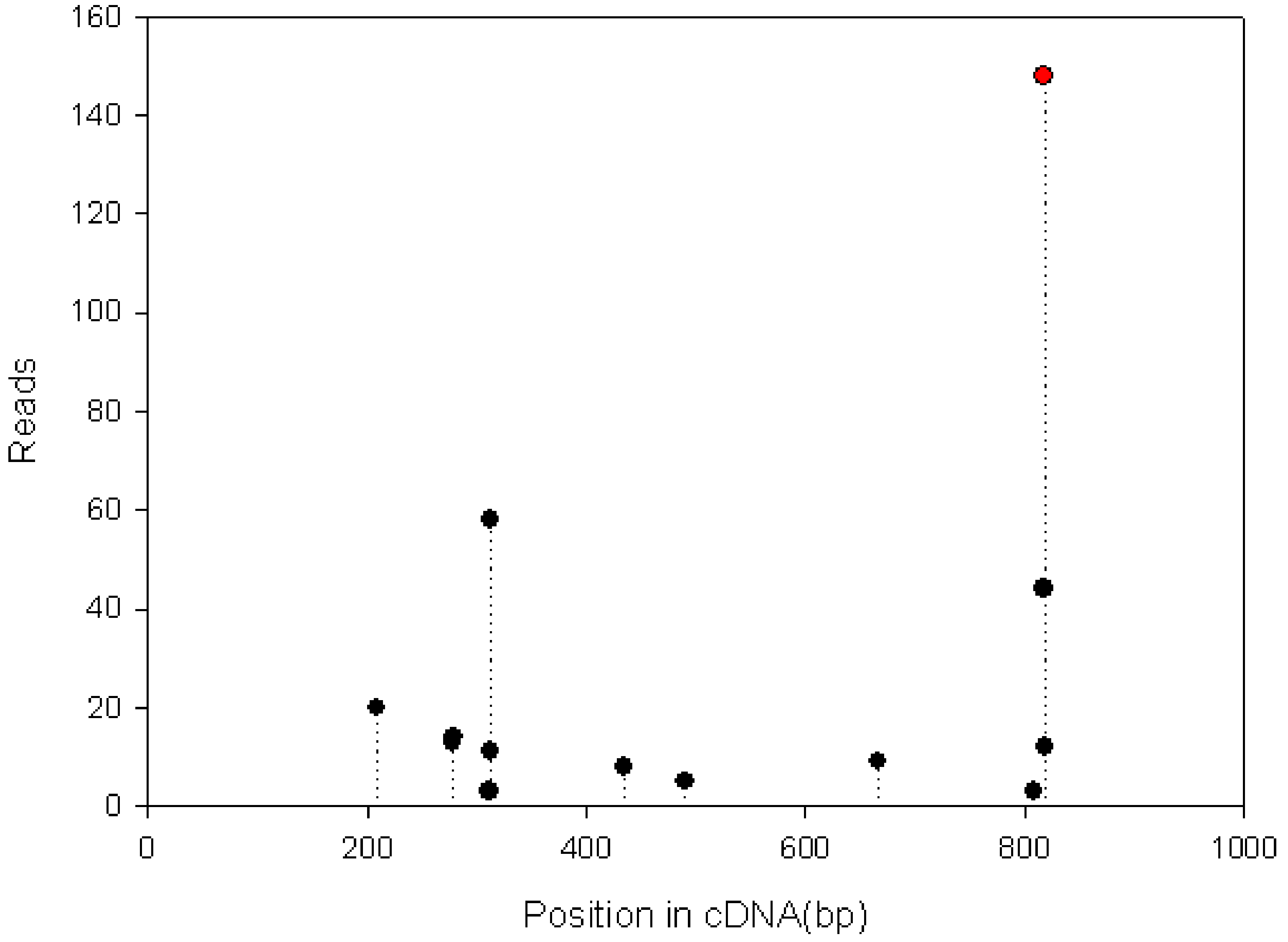


```
5' CGUGCUCUCUCUCUUCUGUCAAUGCU 3'        Cs2g05730.1
    ::::::::: :::::.:::::
3' -CACGAGAGAUAGAAGGCAGUU---- 5'        Csi-miR156g.1
```

Csi-miR156g.1, target=Cs2g23550.1 gene=Cs2g23550
Category=1
Score=5
Cleavage Site=934

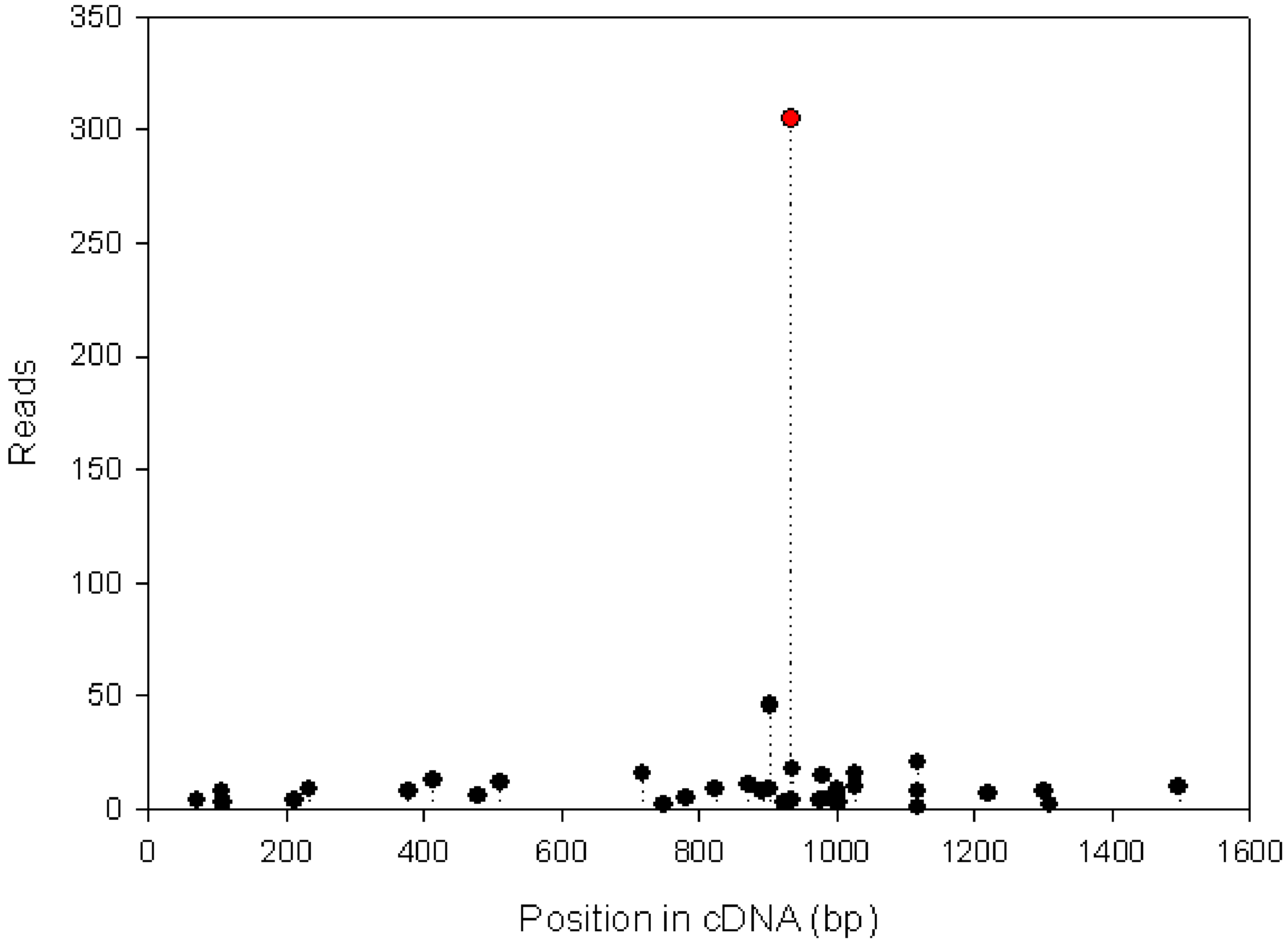


```
5' CAUGCUCUCUCUUCUGUCAUCCUA 3'        Cs2g23550.1
     :::::::: :::::.::::
3' -CACGAGAGAUAGAAGGCAGUU---- 5'      Csi-miR156g.1
```

Csi-miR156g.2, target=Cs2g05730.1 gene=Cs2g05730
Category:1
Score=3
Cleavage Site=818

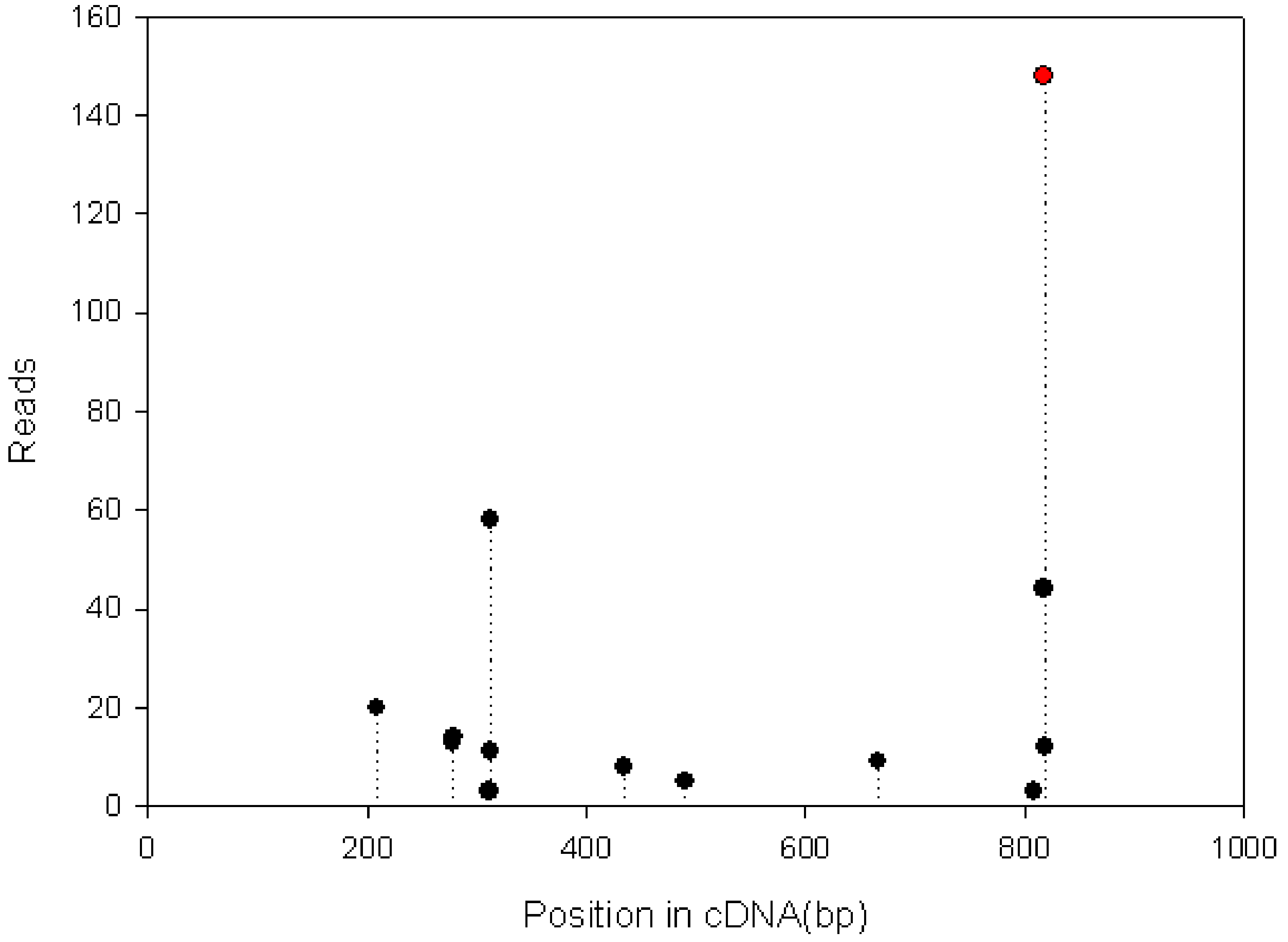


```
5' CGUGCUCUCUCUCUUCUGUCAAUGCU 3'        Cs2g05730.1
    ::::::::: :::::.::::
3' -CACGAGAGAUAGAAGGCAGU----- 5'        Csi-miR156g.2
```

Csi-miR156g.2, target=Cs2g23550.1 gene=Cs2g23550
Category=1
Score=4
Cleavage Site=934

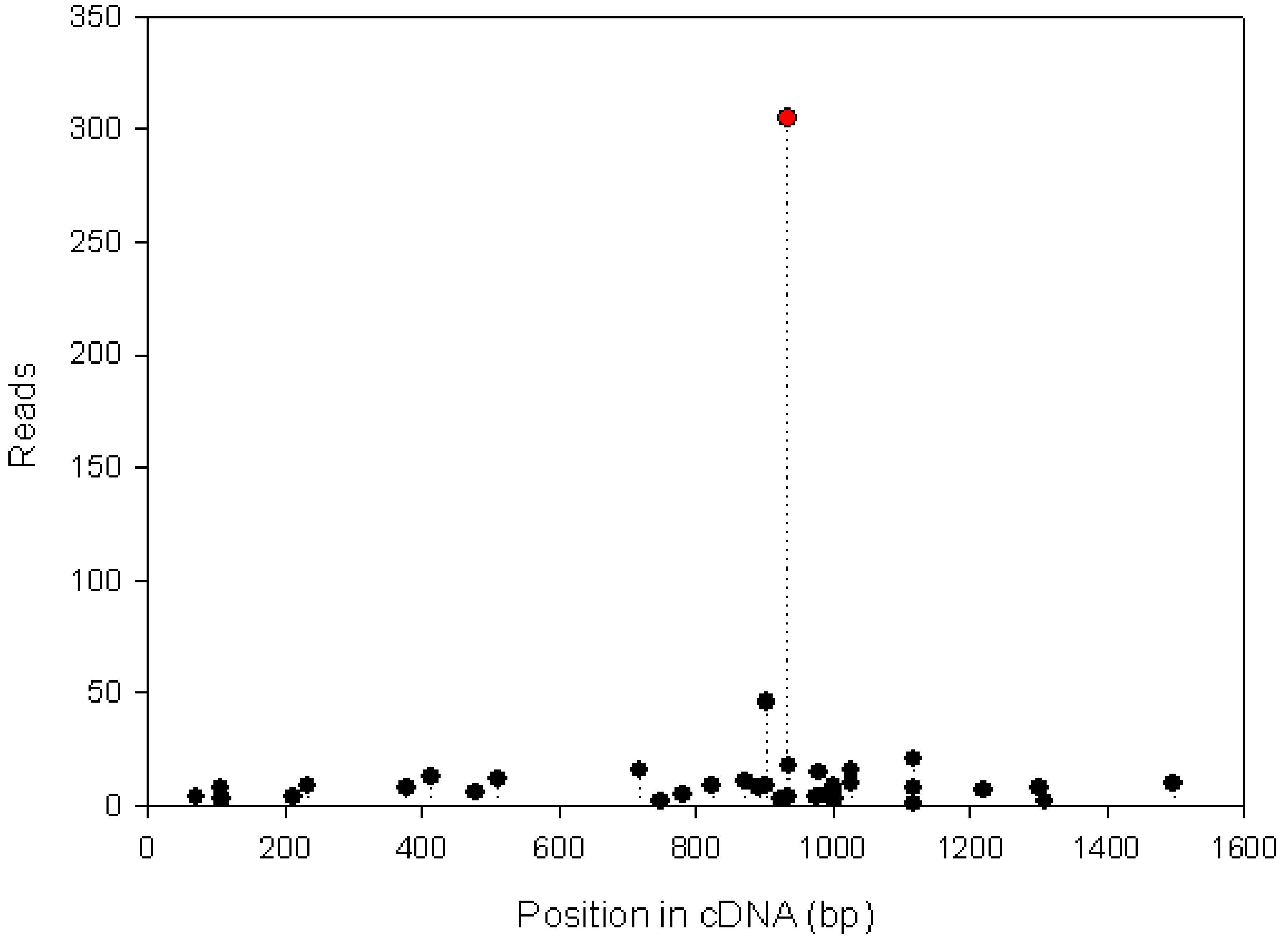


```
5' CAUGCUCUCUCUCUUCUGUCAUCCUA 3'        Cs2g23550.1
     :::::::: ::::::.::::
3' -CACGAGAGAUAGAAGGCAGU----- 5'        Csi-miR156g.2
```

Csi-miR156h, target=Cs2g05730.1 gene=Cs2g05730
Category:1
Score=0
Cleavage Site=818

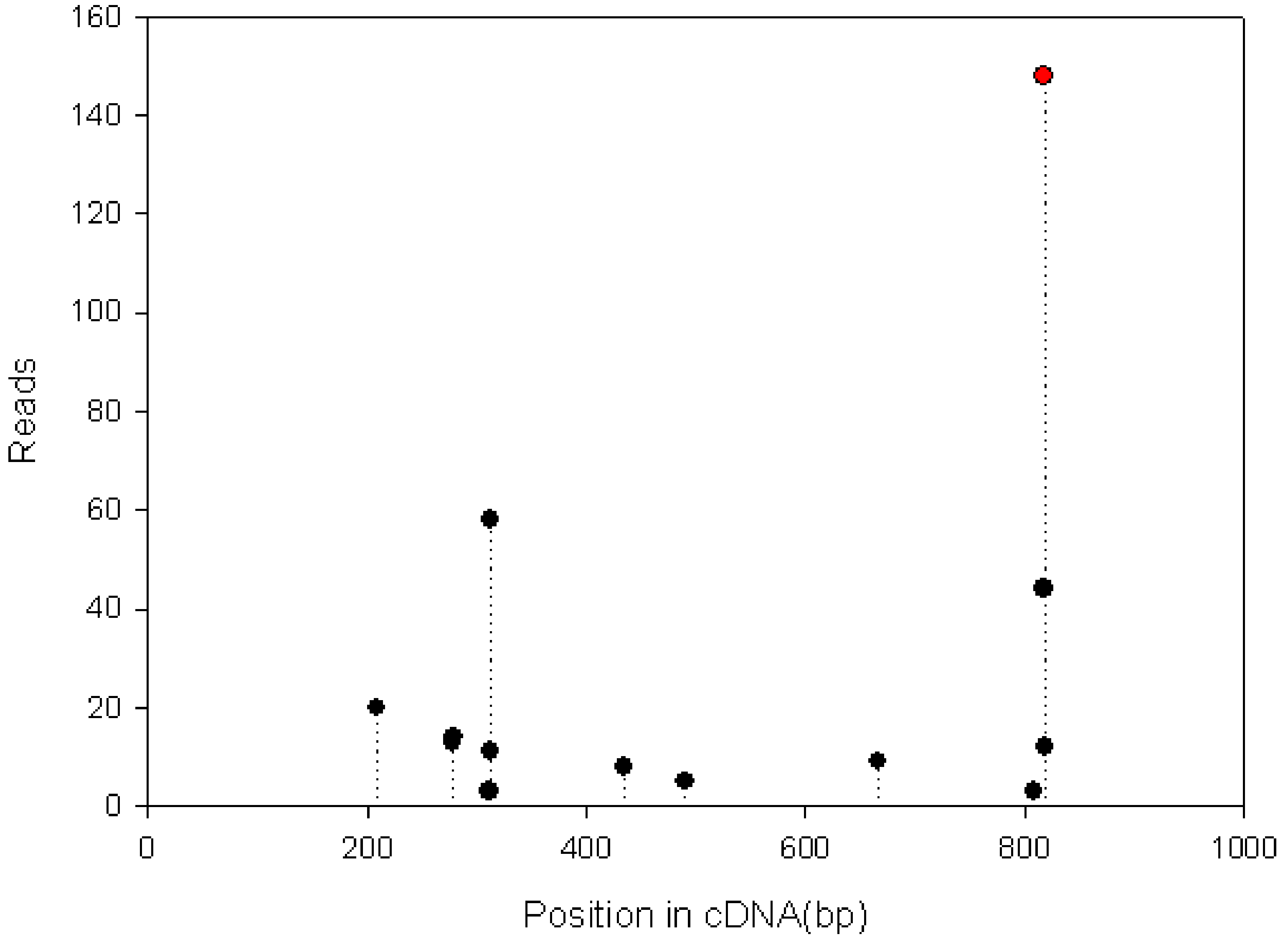


```
5' CGUGCUCUCUCUCUUCUGUCAAUGCU 3'        Cs2g05730.1
    :::::::::::::::::::::
3' -CACGAGAGAGAGAAGACAGUU---- 5'        Csi-miR156h
```

Csi-miR156h, target=Cs2g23550.1 gene=Cs2g23550
Category=1
Score=2
Cleavage Site=934

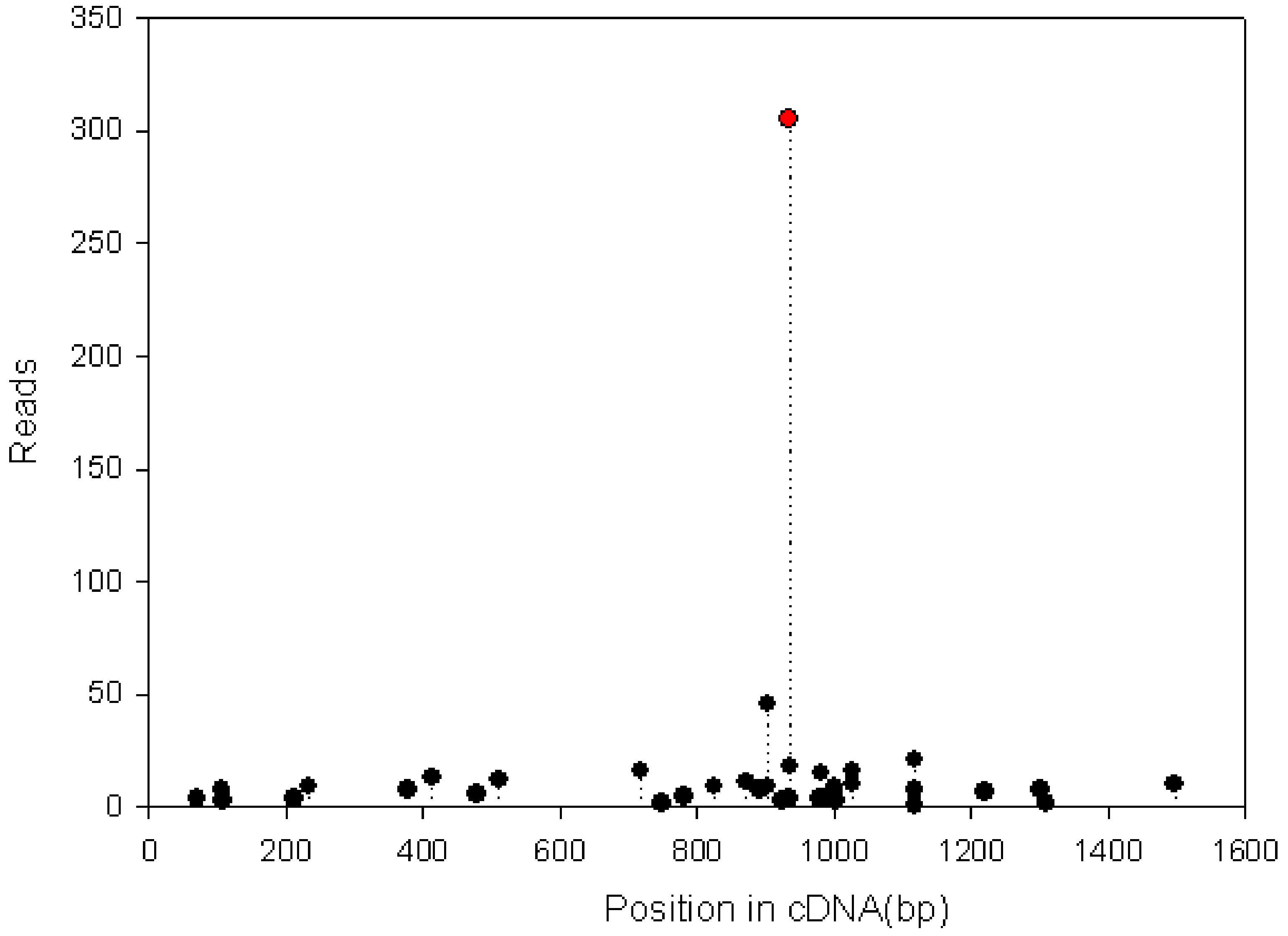


```
5' CAUGCUCUCUCUCUUCUGUCAUCCUA 3'        Cs2g23550.1
    ::::::::::::::::::::
3' -CACGAGAGAGAGAAGACAGUU---- 5'        Csi-miR156h
```

Csi-miR159, target=Cs3g06390.1 gene=Cs3g06390
Category=1
Score=3.5
Cleavage Site=773

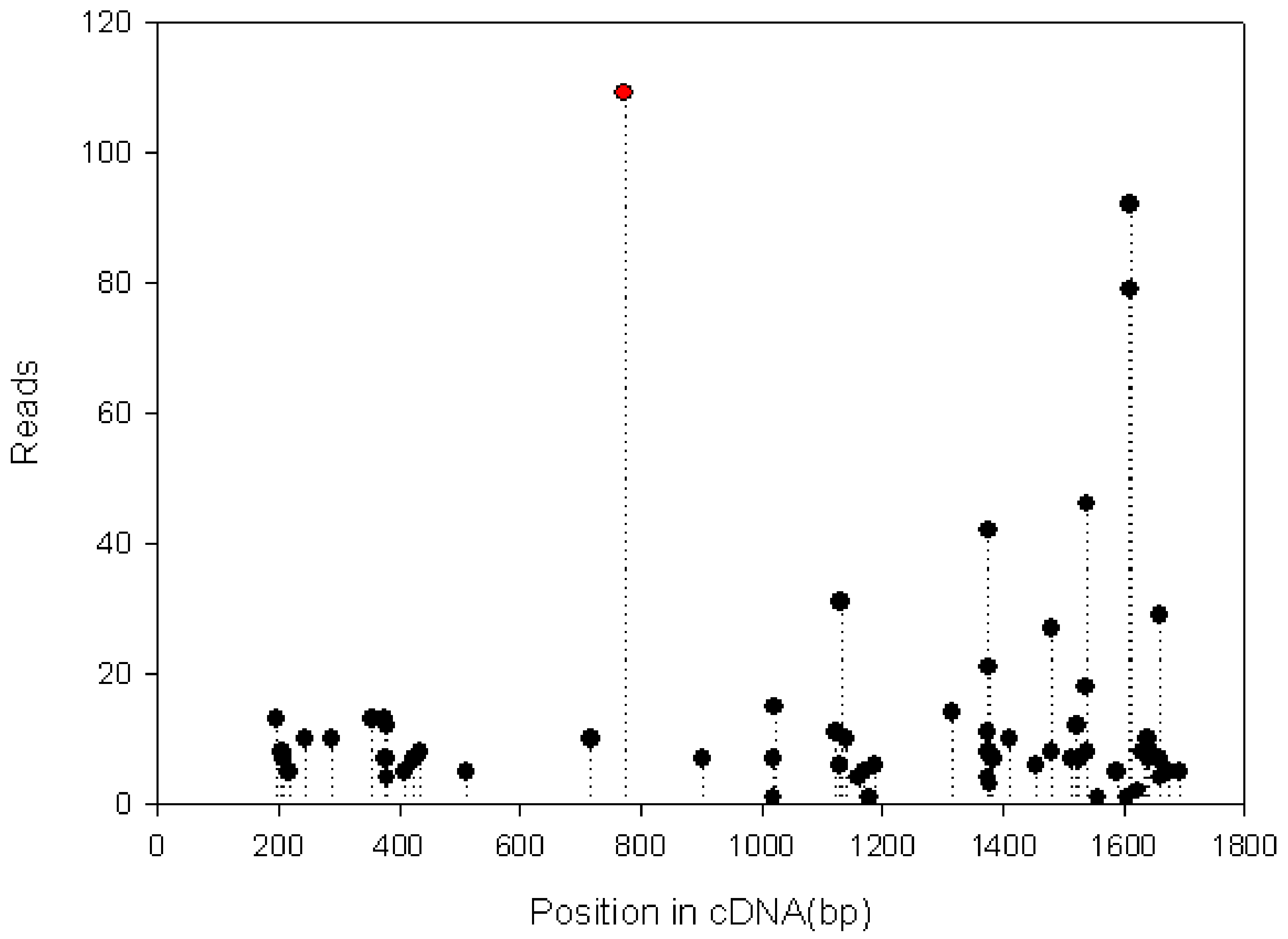


```
5' UUGGAGCUCCCUUCACUCCAAUAUAC 3'        Cs3g06390.1
    :.:::::::::::: :::::
3' -AUCUCGAGGGAAGUUAGGUUU---- 5'        Csi-miR159
```

Csi-miR159b, target=Cs3g06390.1 gene=Cs3g06390
Category=1
Score=0
Cleavage Site=773

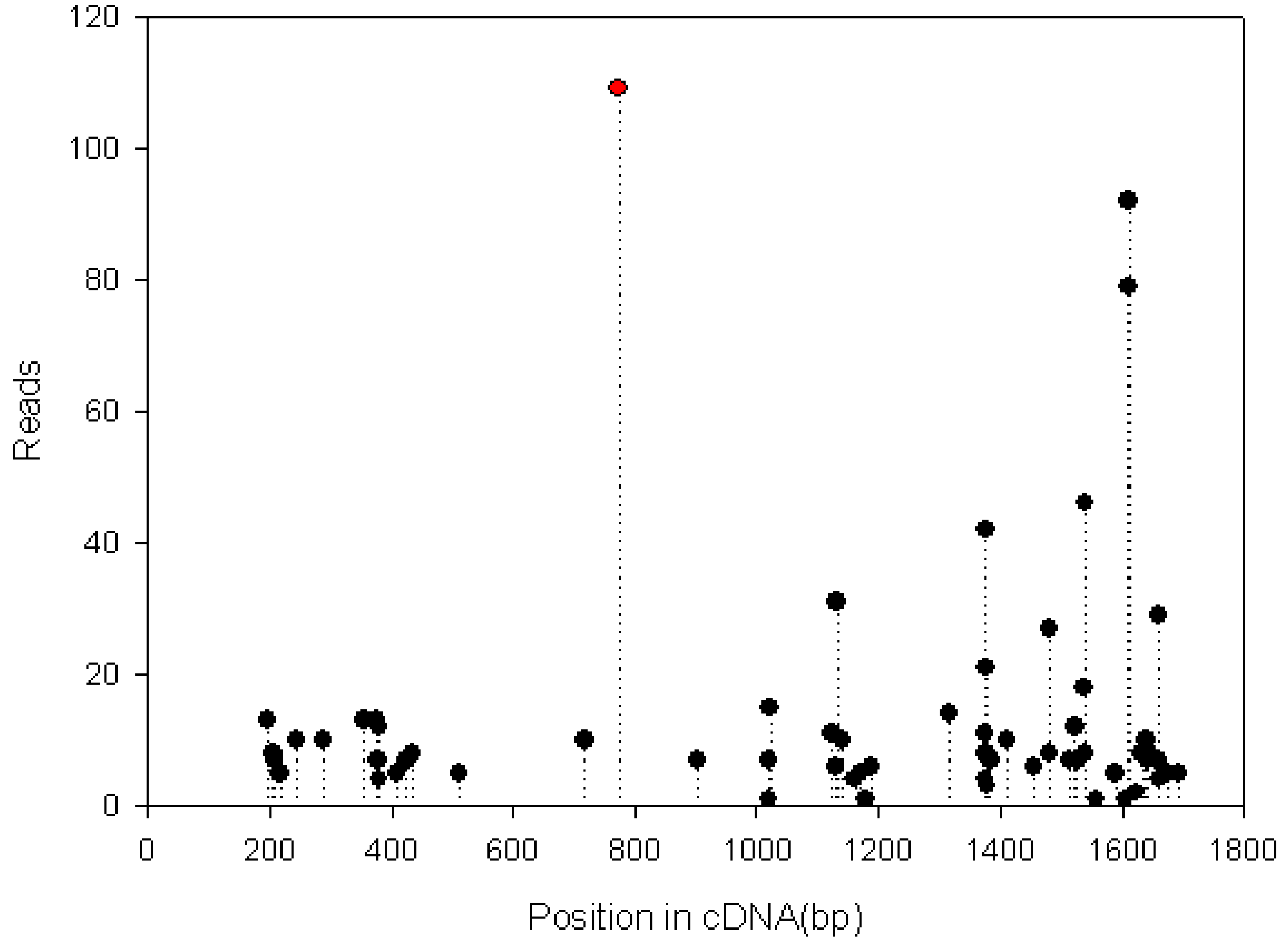


```
5' UUGGAGCUCCCUUCACUCCAAUAUAC 3'        Cs3g06390.1
    :::::::::::::::::::::
3' -ACCUCGAGGGAAGUGAGGUUA---- 5'        Csi-miR159b
```

Csi-miR160a-3p, target=Cs9g06430.1 gene=Cs9g06430
Category=3
Score=5
Cleavage Site=5175

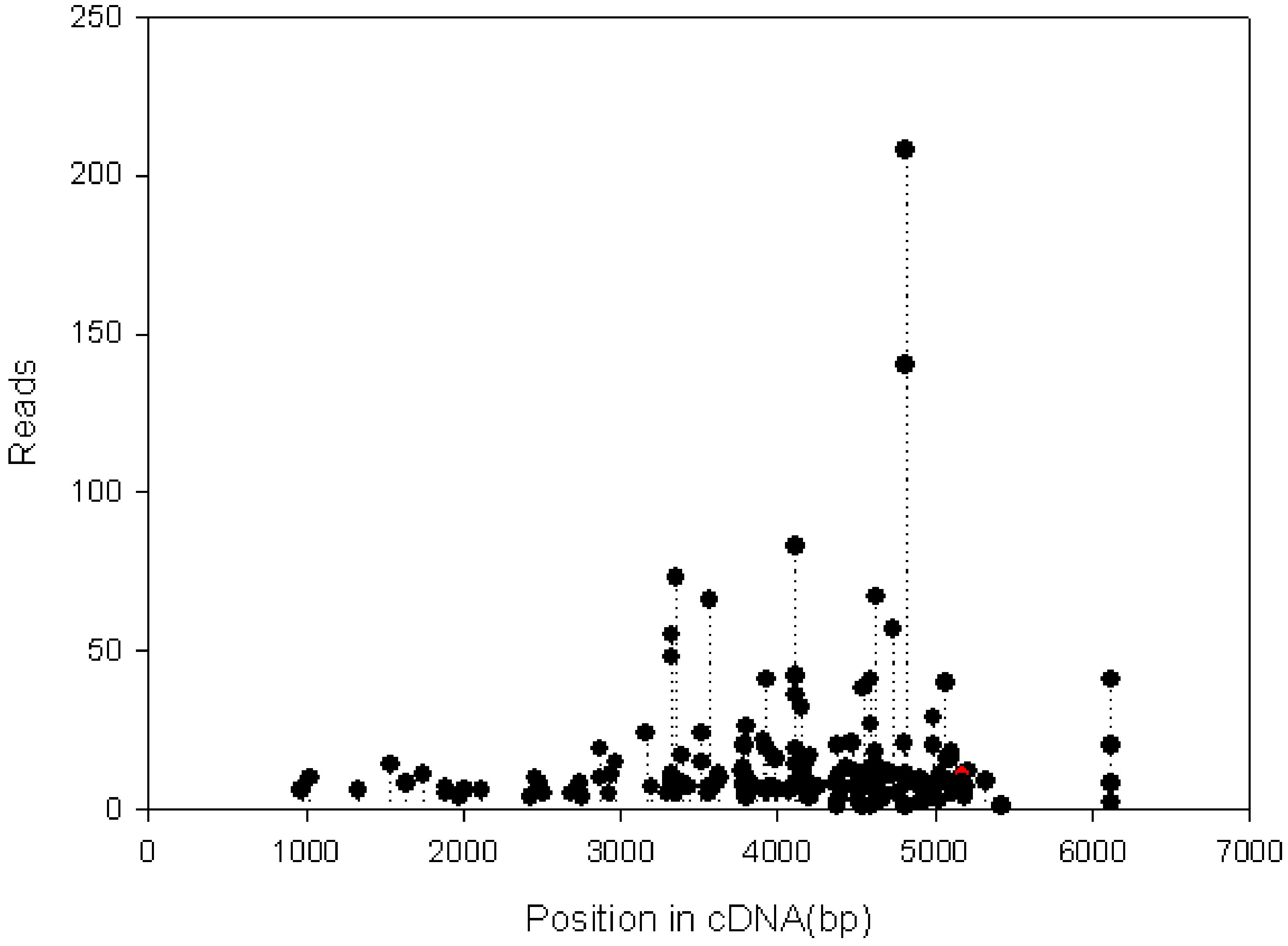


```
5' UGAG-AUUGUUCCUCAUACGUUCAACA 3'        Cs9g06430.1
   :. : :: :.:::::::::::.
3' AUACGUACCGAGGAGUAUGCG------ 5'        Csi-miR160a-3p
```

Csi-miR162-3p.1, target=Orange1.1t00584.1 gene=Orange1.1t00584
Category=2
Score=2
Cleavage Site=3231

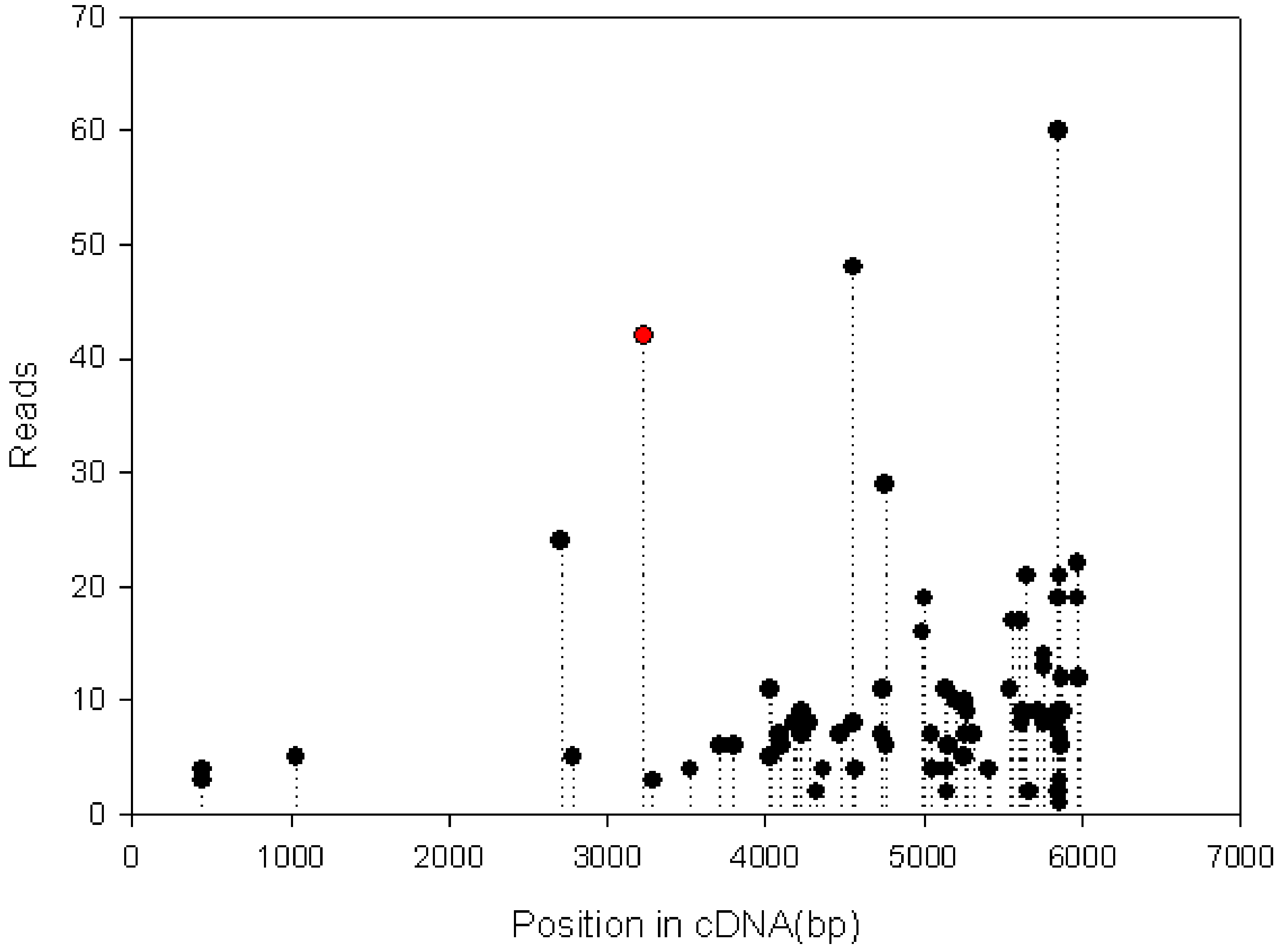


```
5' GCUGGAUGCAGAGGUGUUAUCGAUGU 3'        Orange1.1t00584.1
    :::::::::::::: :::::::
3' -GACCUACGUCUCCA-AAUAGCU--- 5'        Csi-miR162-3p.1
```

Csi-miR162-3p.2, target=Orange1.1t00584.1 gene=Orange1.1t00584
Category=2
Score=2
Cleavage Site=3231

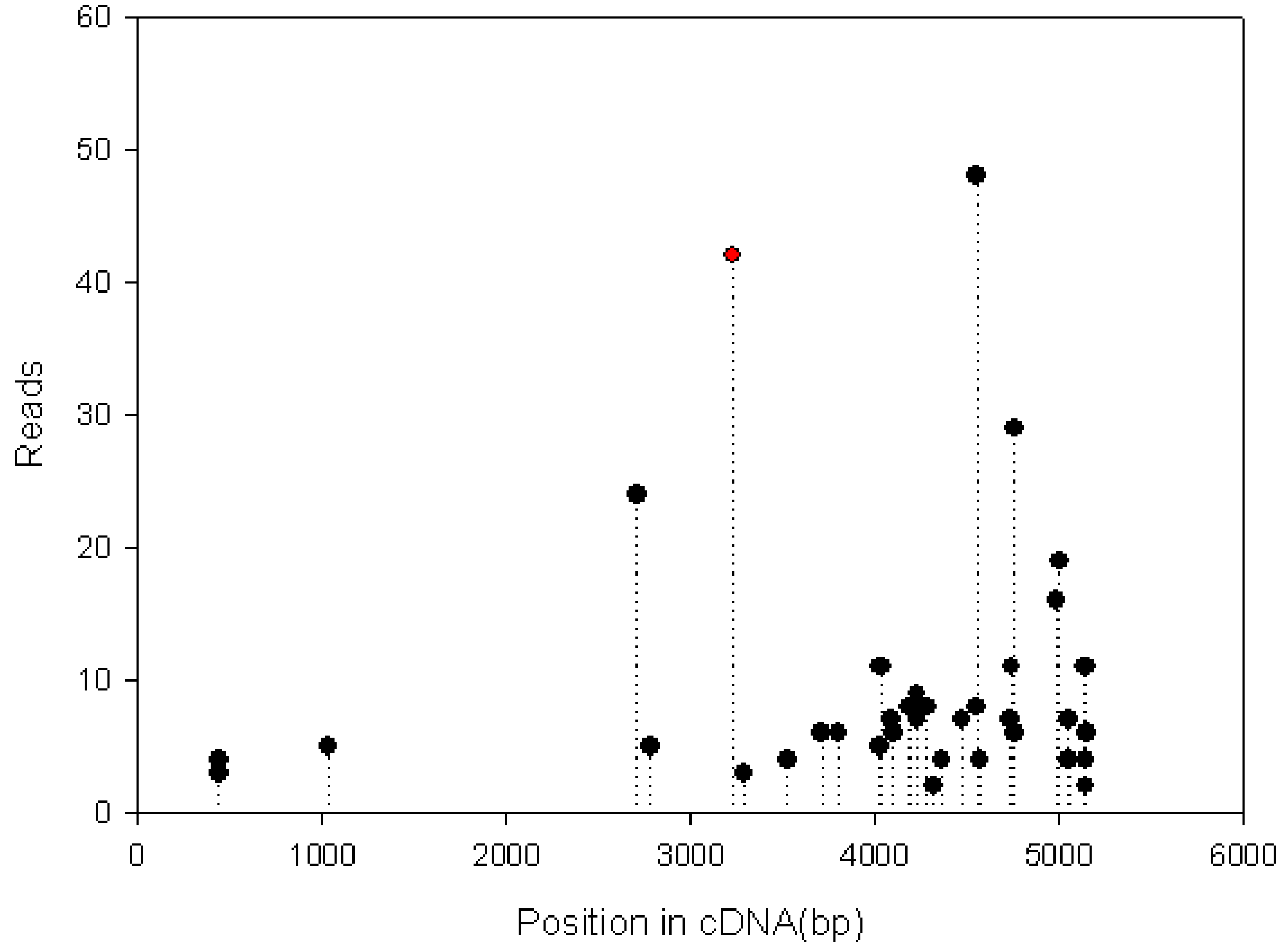


```
5' GCUGGAUGCAGAGGUGUUAUCGAUGU 3'       Orange1.1t00584.1
    ::::::::::::: :::::
3' -GACCUACGUCUCCA-AAUAG----- 5'       Csi-miR162-3p.2
```

Csi-miR164, target=Cs5g10870.1 gene=Cs5g10870
Category=1
Score=3.5
Cleavage Site=1062

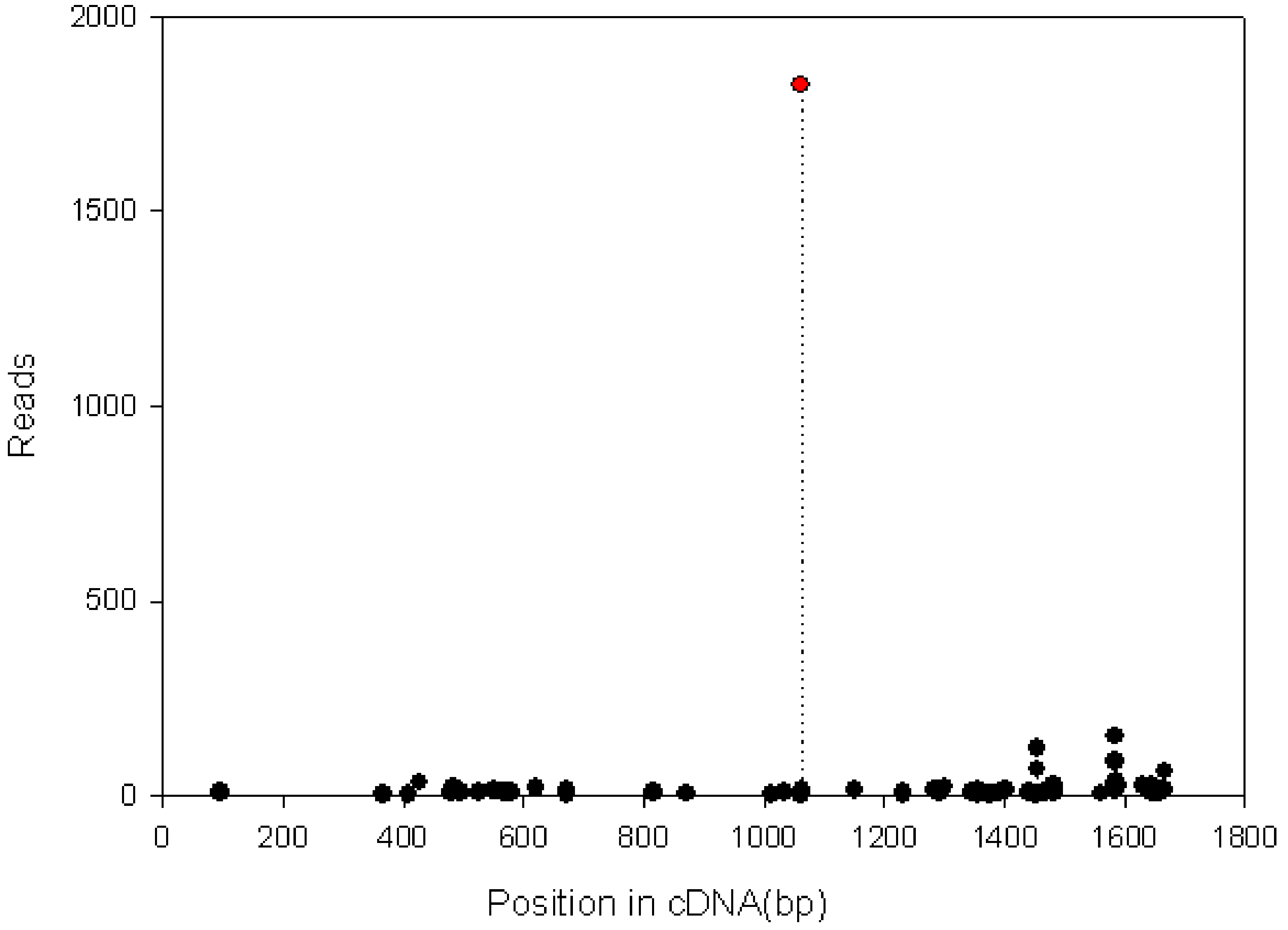


```
5' GCUUACGUGUCCUGCUUCUCCAAUUC 3'        Cs5g10870.1
      .::::::.::::::::::::
3' -ACGUGCACGGGACGAAGAGGU---- 5'        Csi-miR164
```

Csi-miR166a.1, target=Cs2g09770.1 gene=Cs2g09770
Category:3
Score=4
Cleavage Site=1058

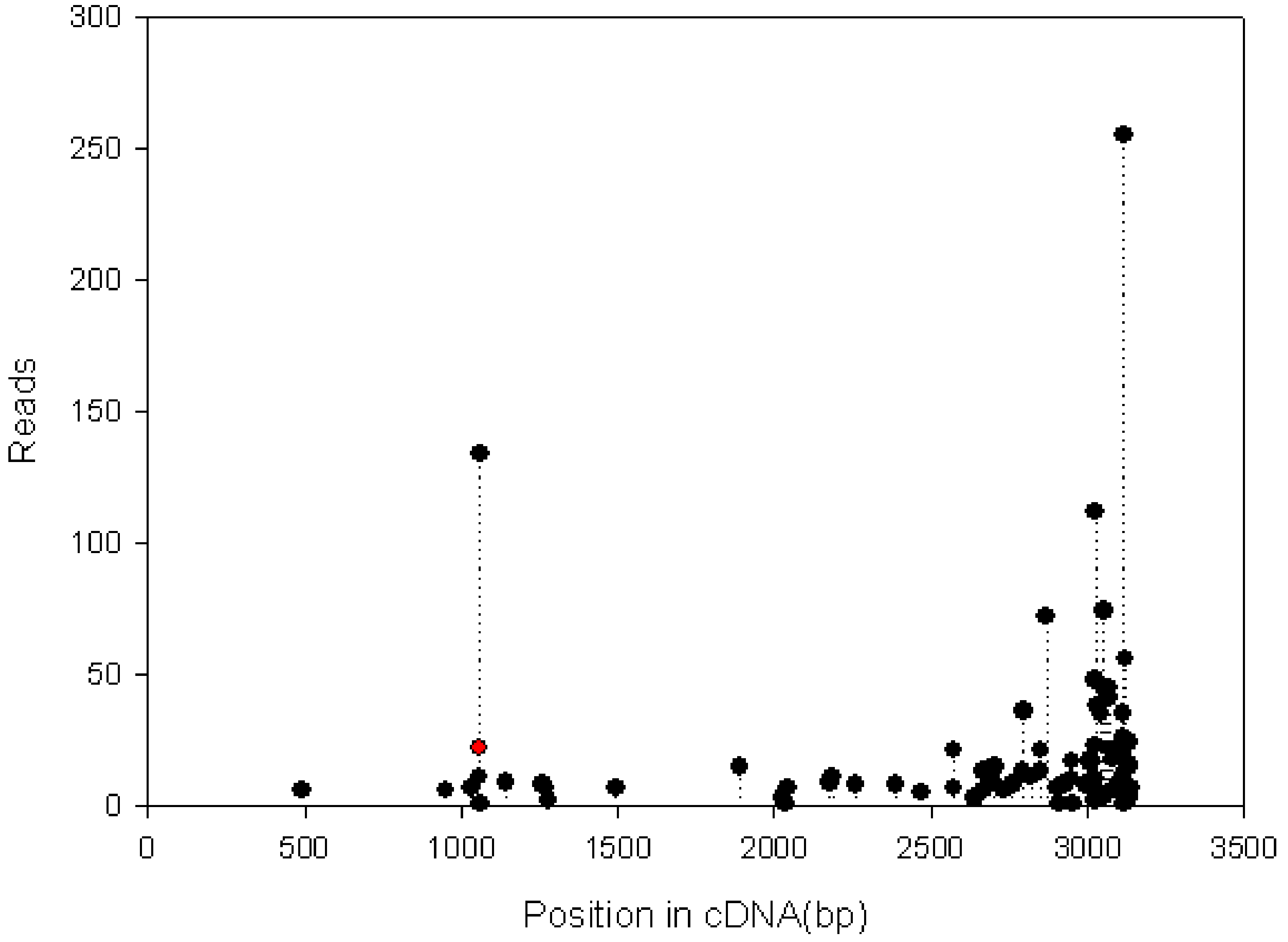


```
5' AUUGGGAUGAAGCCUGGUCCGGAUUC 3'        Cs2g09770.1
     ::.:::::::::::::::::.
3' CCCCCUUACUUCGGACCAGGCU---- 5'        Csi-miR166a.1
```

Csi-miR166a.1, target=Cs8g16510.1 gene=Cs8g16510
Category=1
Score=4
Cleavage Site=1434

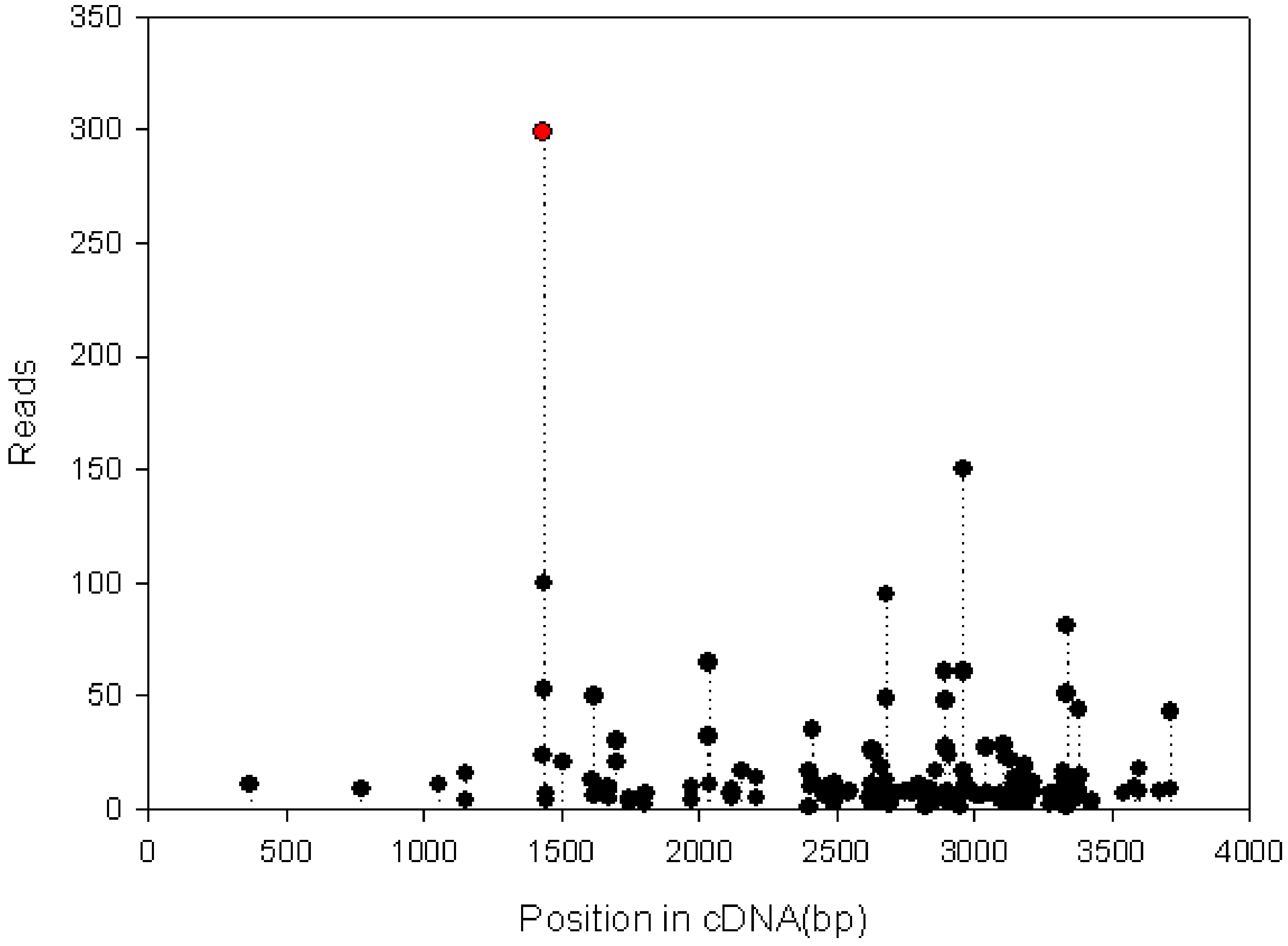


```
5' CCUGGGAUGAAGCCUGGUCCGGAUUC 3'        Cs8g16510.1
       ::.::::::::::::::::.
3' CCCCCUUACUUCGGACCAGGCU---- 5'        Csi-miR166a.1
```

Csi-miR166a.2, target=Cs2g09770.1 gene=Cs2g09770
Category:3
Score=3
Cleavage Site=1058

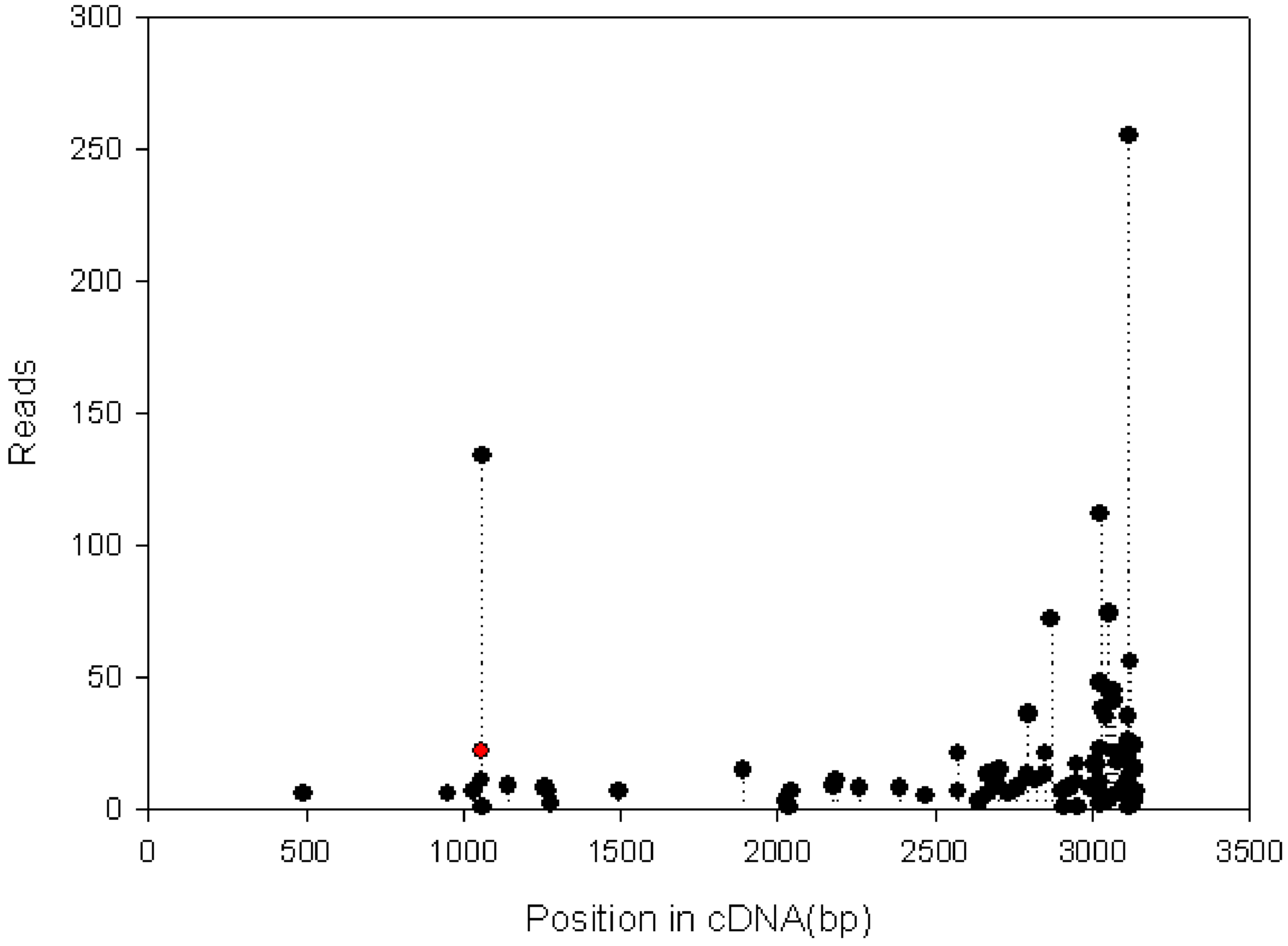


```
5' AUUGGGAUGAAGCCUGGUCCGGAUUC 3'        Cs2g09770.1
     ::.:::::::::::::::::.
3' -CCCCUUACUUCGGACCAGGCU---- 5'        Csi-miR166a.2
```

Csi-miR166a.2, target=Cs8g16510.1 gene=Cs8g16510
Category=1
Score=3
Cleavage Site=1434

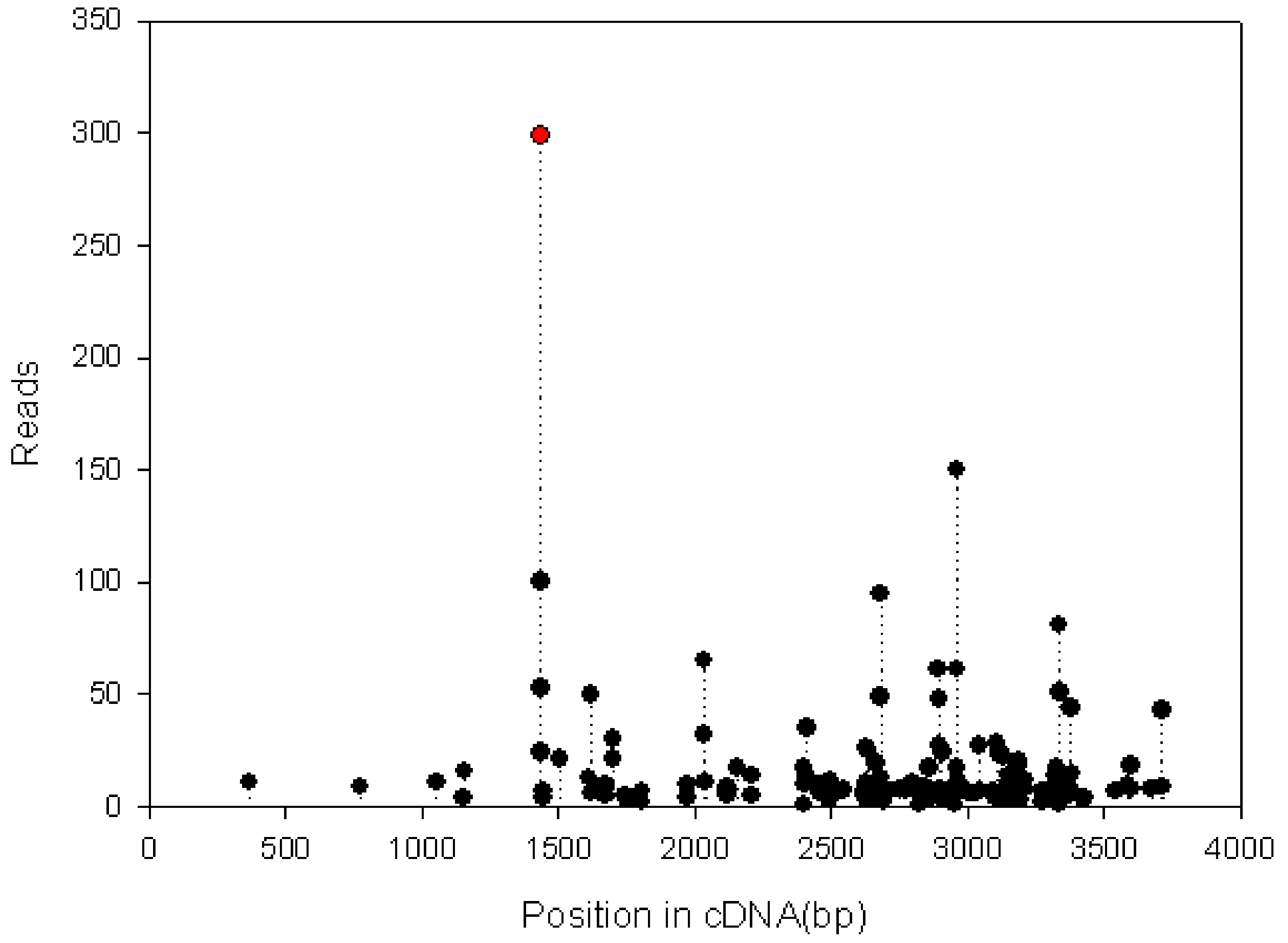


```
5' CCUGGGAUGAAGCCUGGUCCGGAUUC 3'        Cs8g16510.1
      ::.::::::::::::::::.
3' -CCCCUUACUUCGGACCAGGCU---- 5'        Csi-miR166a.2
```

Csi-miR166b, target=Cs2g09770.1 gene=Cs2g09770
Category:3
Score=2.5
Cleavage Site=1058

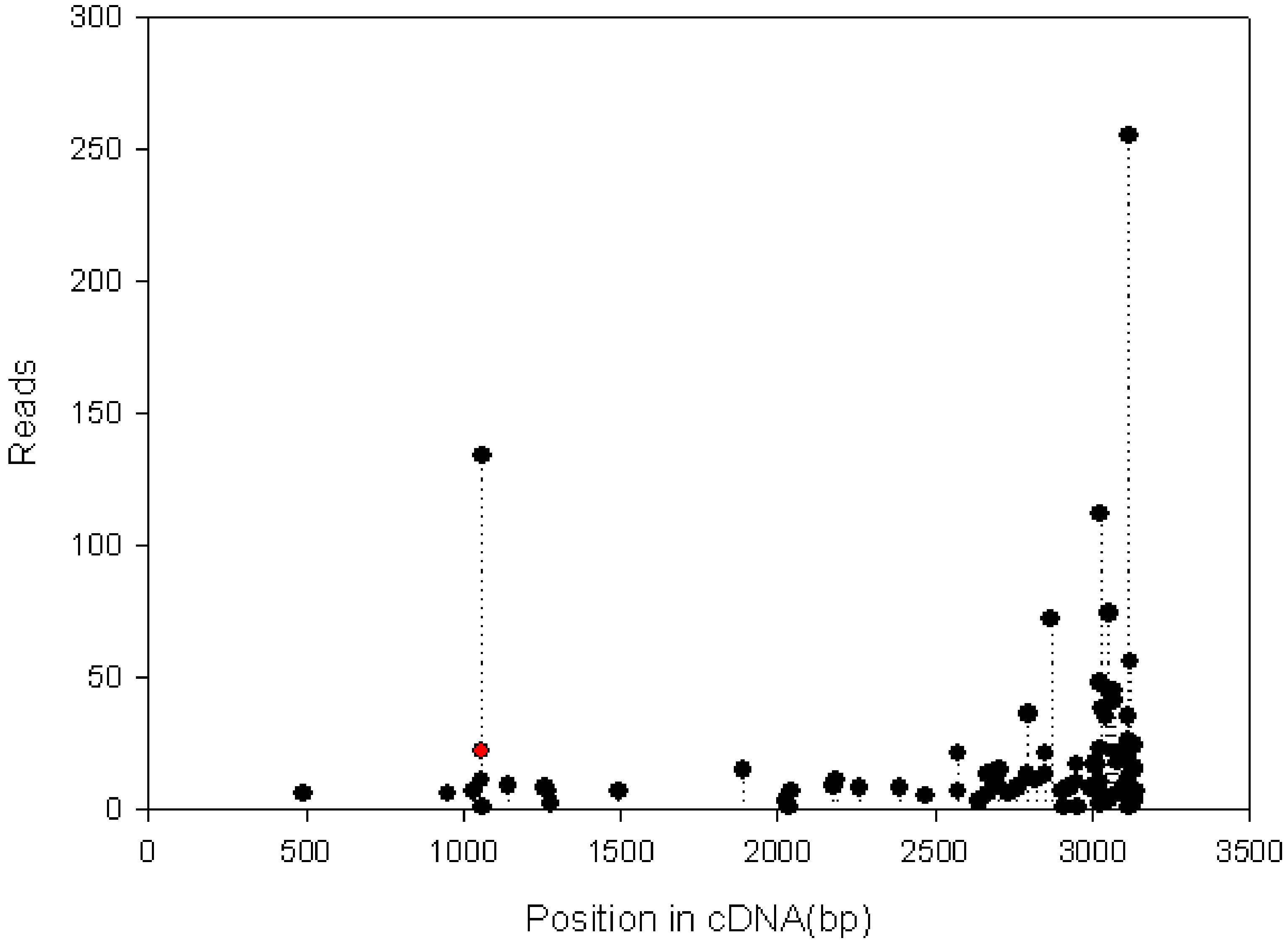


```
5' AUUGGGAUGAAGCCUGGUCCGGAUUC 3'        Cs2g09770.1
    . ::.::::::::::::::::.
3' -GCCCUUACUUCGGACCAGGCU---- 5'        Csi-miR166b
```

Csi-miR166b, target=Cs8g16510.1 gene=Cs8g16510
Category=1
Score=2
Cleavage Site=1434

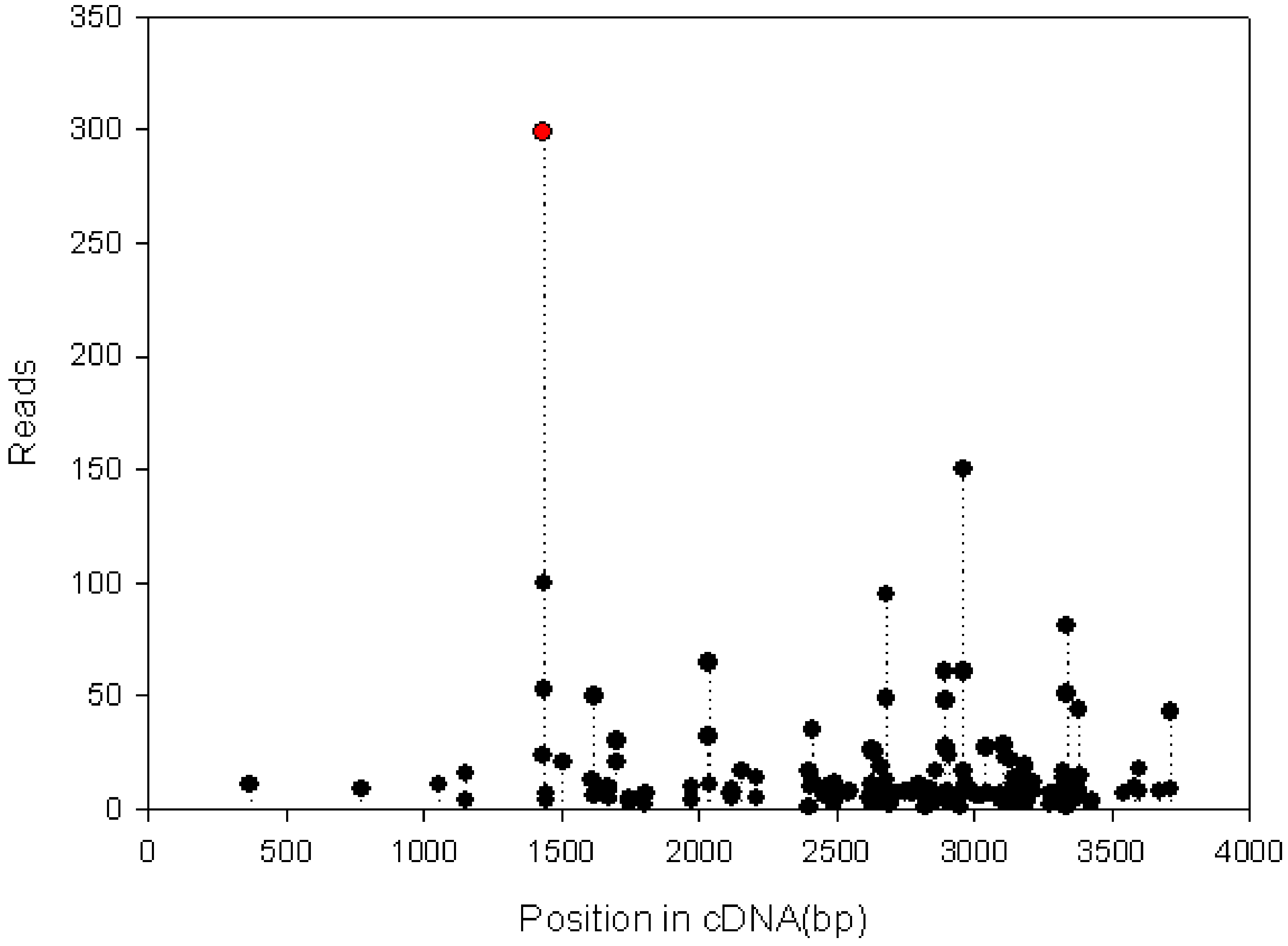


```
5' CCUGGGAUGAAGCCUGGUCCGGAUUC 3'        Cs8g16510.1
    : ::.:::::::::::::::::.
3' -GCCCUUACUUCGGACCAGGCU---- 5'        Csi-miR166b
```

Csi-miR166c.1, target=Cs2g09770.1 gene=Cs2g09770
Category:2
Score=1
Cleavage Site=1060

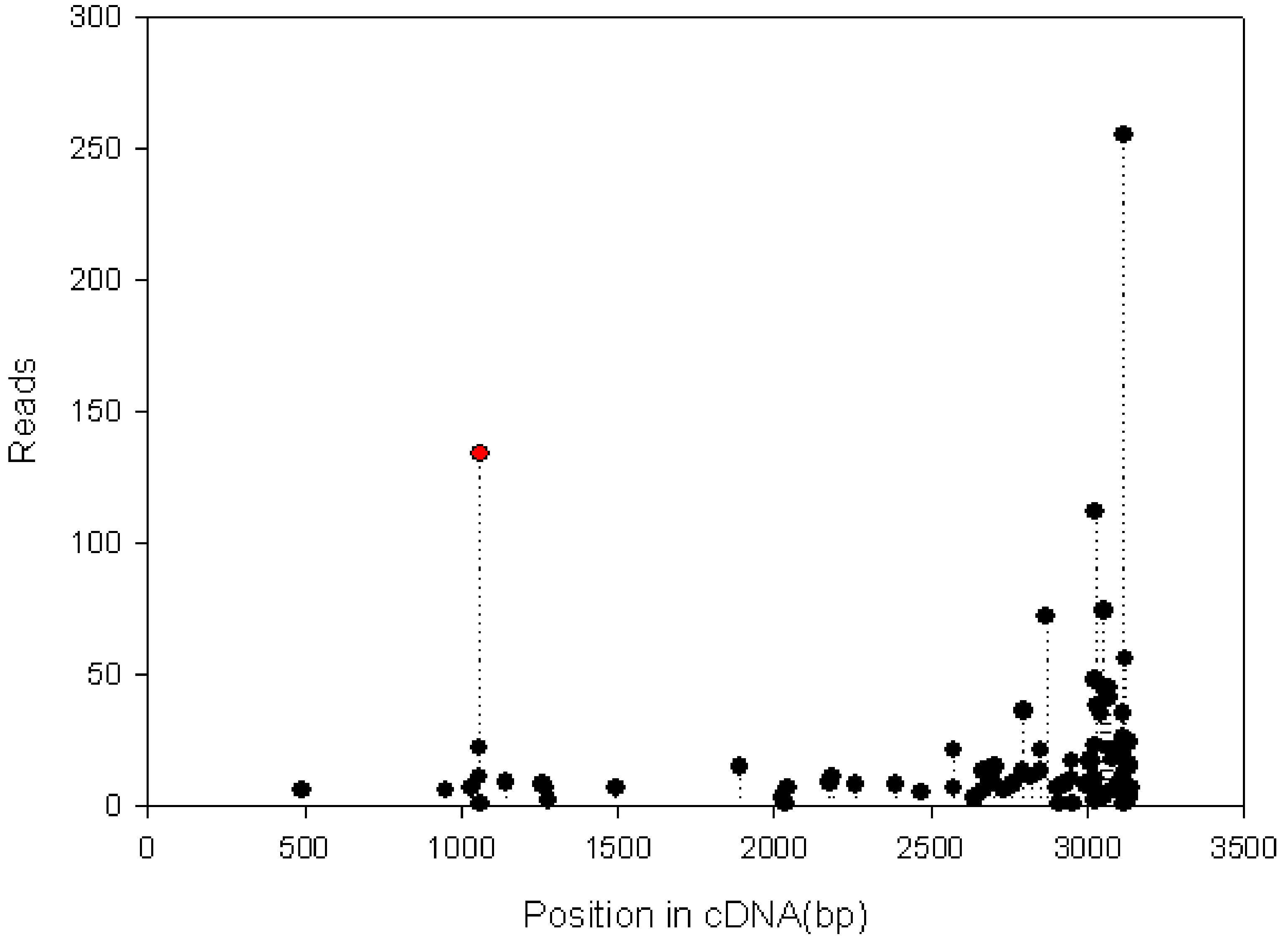


```
5' UGGGAUGAAGCCUGGUCCGGAUUCUA 3'        Cs2g09770.1
    ::.:::::::::::::::::.
3' -CCUUACUUCGGACCAGGCU------ 5'        Csi-miR166c.1
```

Csi-miR166c.1, target=Cs8g16510.1 gene=Cs8g16510
Category=1
Score=1
Cleavage Site=1434

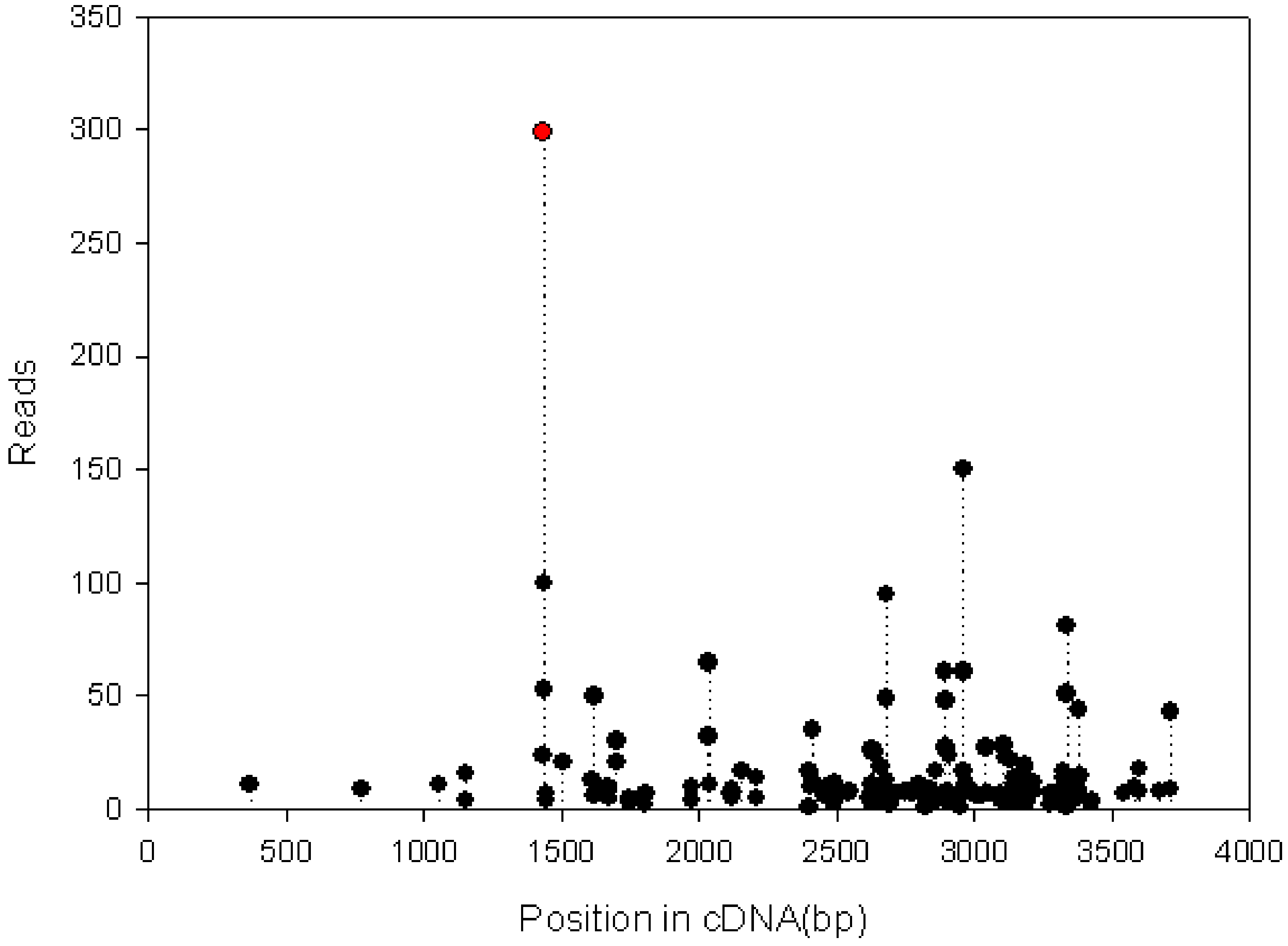


```
5' CCUGGGAUGAAGCCUGGUCCGGAUUC 3'        Cs8g16510.1
       ::.:::::::::::::::::.
3' ---CCUUACUUCGGACCAGGCU---- 5'        Csi-miR166c.1
```

Csi-miR166c.2, target=Cs2g09770.1 gene=Cs2g09770
Category:2
Score=1
Cleavage Site=1060

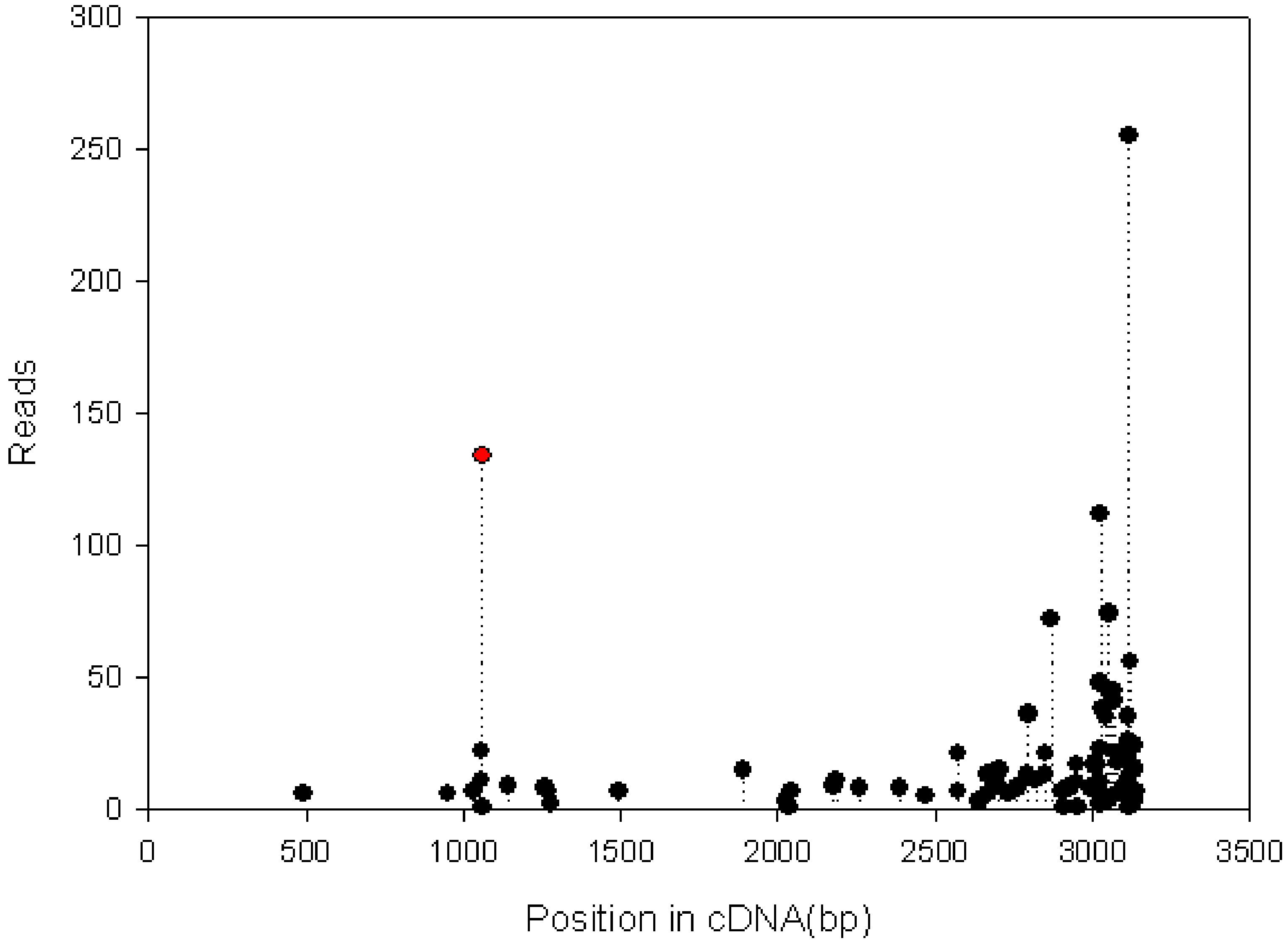


```
5' UGGGAUGAAGCCUGGUCCGGAUUCUA 3'        Cs2g09770.1
     :.:::::::::::::::::.
3' --CUUACUUCGGACCAGGCU------ 5'        Csi-miR166c.2
```

Csi-miR166c.2, target=Cs8g16510.1 gene=Cs8g16510
Category=1
Score=1
Cleavage Site=1434

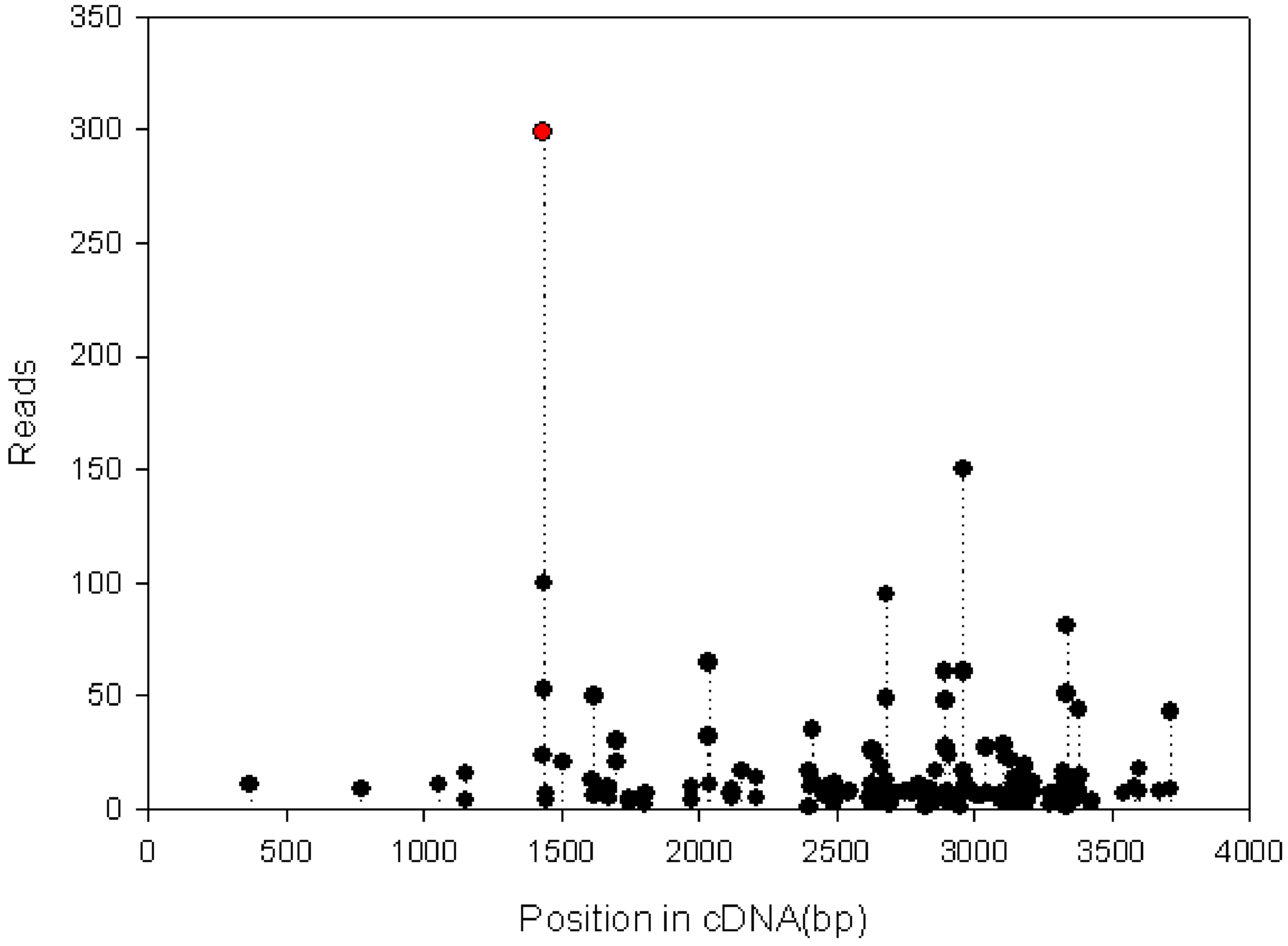


```
5' CCUGGGAUGAAGCCUGGUCCGGAUUC 3'        Cs8g16510.1
         :.::::::::::::::::.
3' ----CUUACUUCGGACCAGGCU---- 5'        Csi-miR166c.2
```

Csi-miR166c.3, target=Cs2g09770.1 gene=Cs2g09770
Category:2
Score=2
Cleavage Site=1060

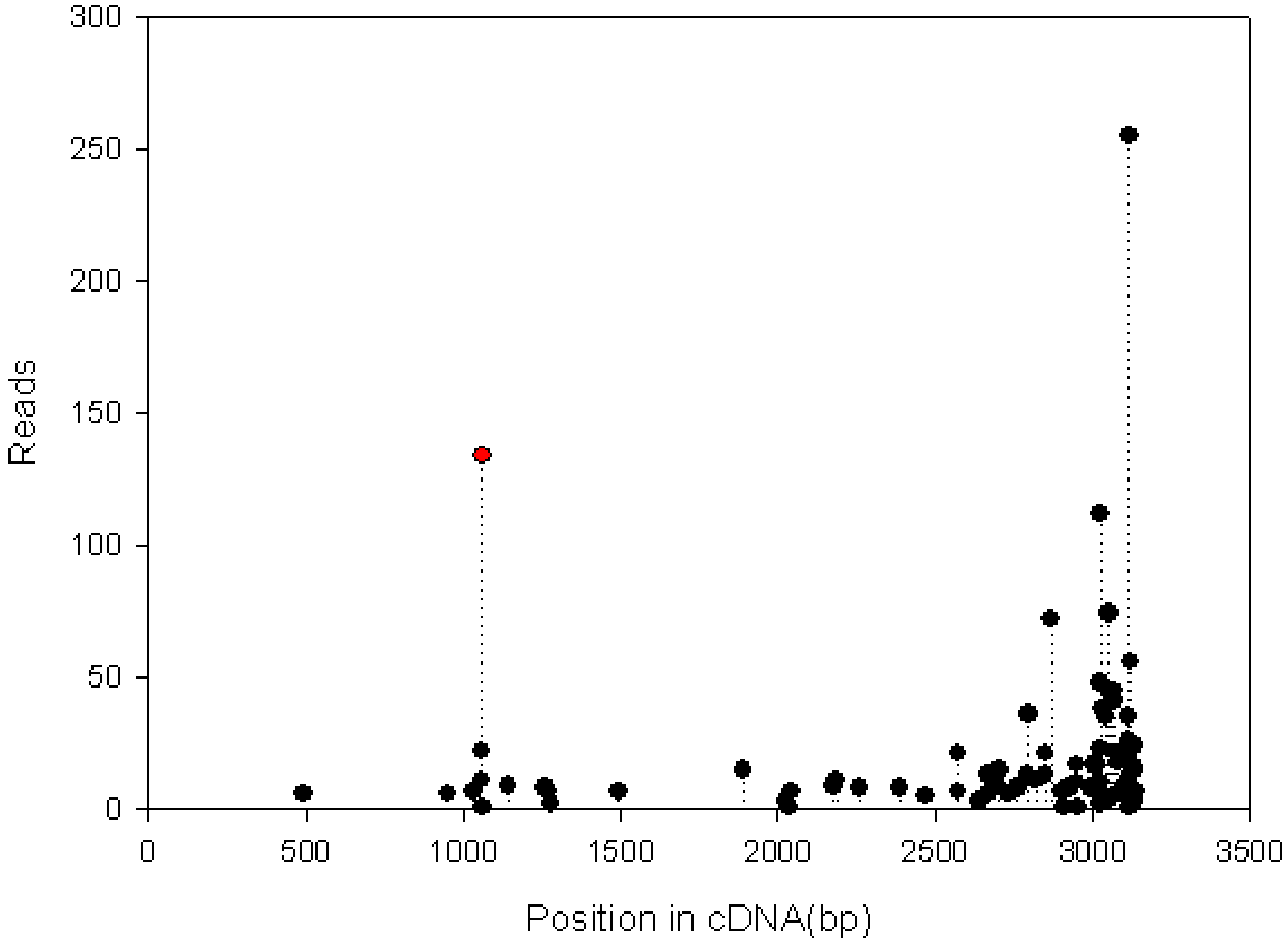


```
5' UGGGAUGAAGCCUGGUCCGGAUUCUA 3'        Cs2g09770.1
    ::.:::::::::::::::::.
3' CCCUUACUUCGGACCAGGCU------ 5'        Csi-miR166c.3
```

Csi-miR166c.3, target=Cs8g16510.1 gene=Cs8g16510
Category=1
Score=2
Cleavage Site=1434

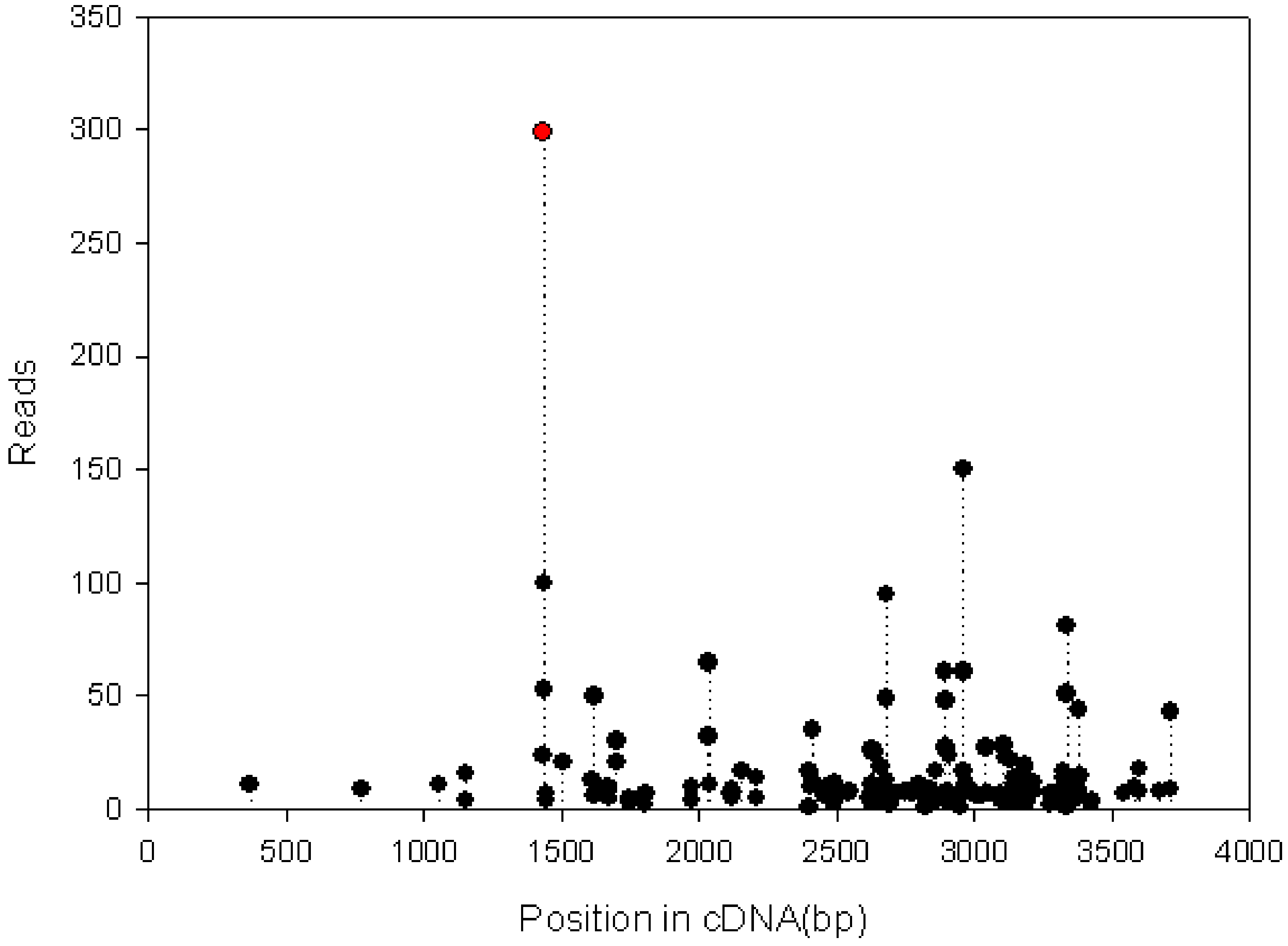


```
5' CCUGGGAUGAAGCCUGGUCCGGAUUC 3'        Cs8g16510.1
     ::.::::::::::::::::.
3' --CCCUUACUUCGGACCAGGCU---- 5'        Csi-miR166c.3
```

Csi-miR166c.4, target=Cs2g09770.1 gene=Cs2g09770
Category:2
Score=3.5
Cleavage Site=1060

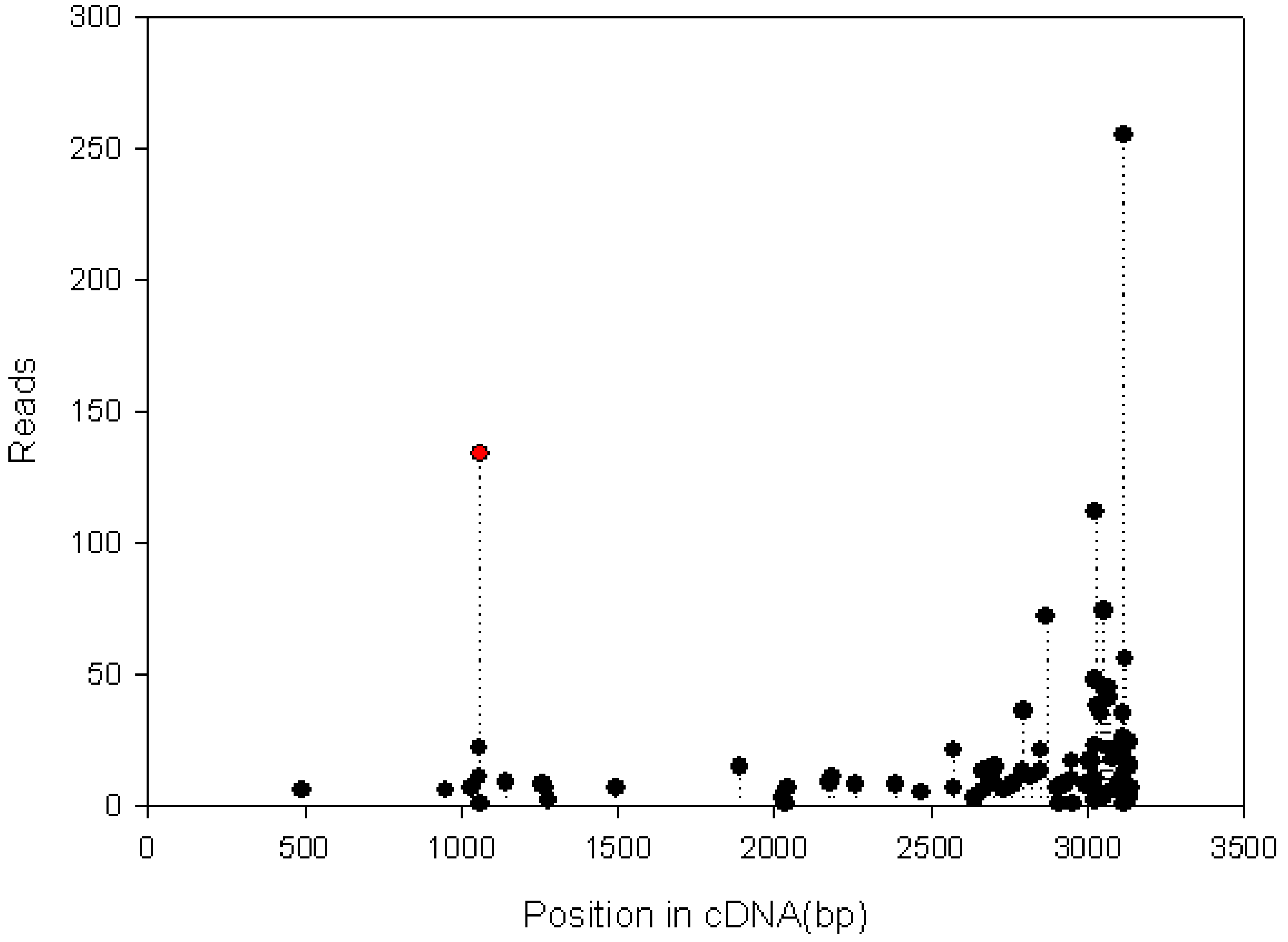


```
5' UGGGAUGAAGCCUGGUCCGGAUUCUA 3'        Cs2g09770.1
   ::.:::::::::::::::::.
3' CCCUUACUUCGGACCAGGCUC----- 5'        Csi-miR166c.4
```

Csi-miR166c.4, target=Cs8g16510.1 gene=Cs8g16510
Category=1
Score=3.5
Cleavage Site=1434

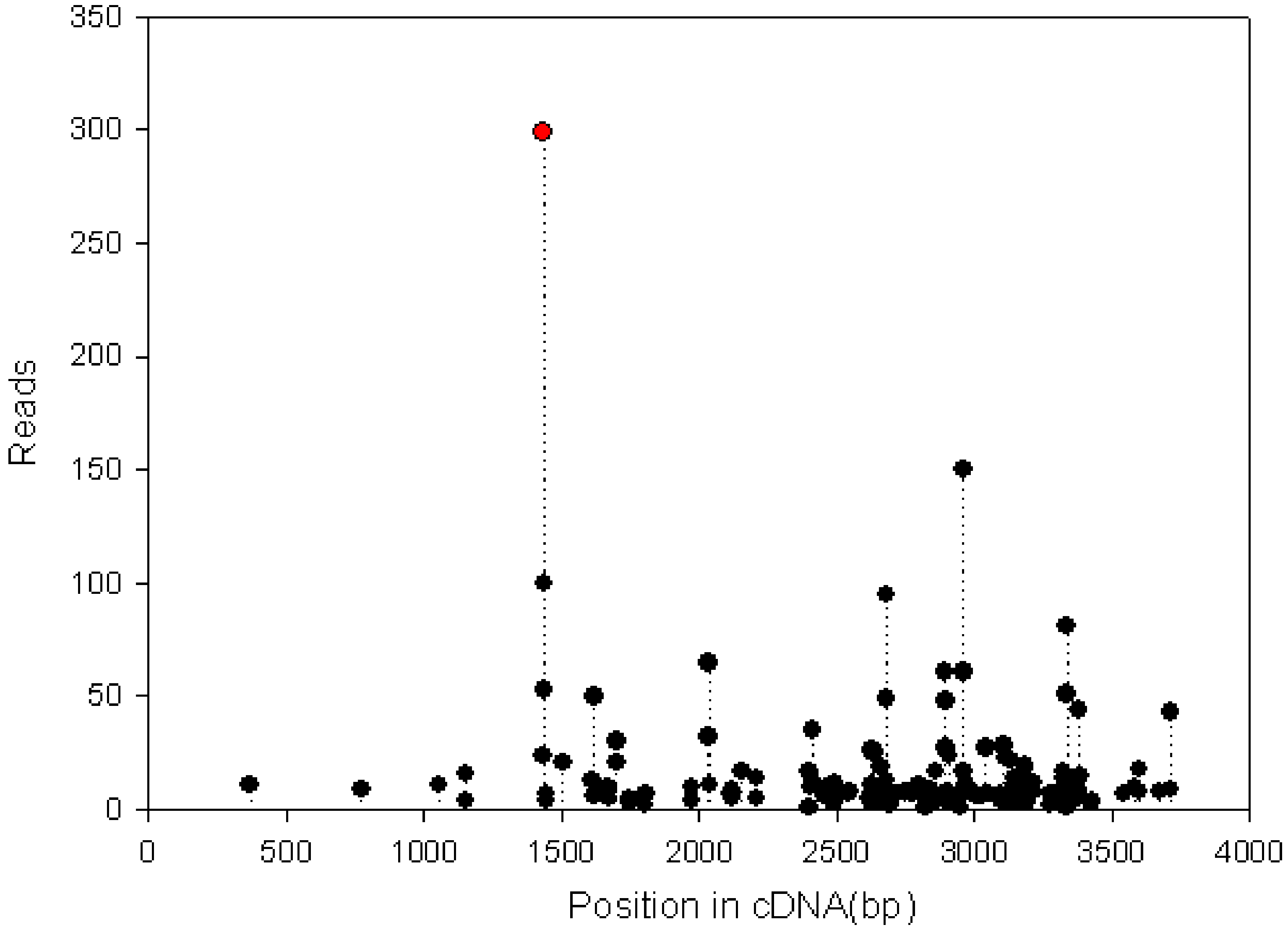


```
5' CCUGGGAUGAAGCCUGGUCCGGAUUC 3'         Cs8g16510.1
     ::.:::::::::::::::::.
3' --CCCUUACUUCGGACCAGGCUC--- 5'         Csi-miR166c.4
```

Csi-miR166c.5, target=Cs2g09770.1 gene=Cs2g09770
Category:2
Score=2.5
Cleavage Site=1060

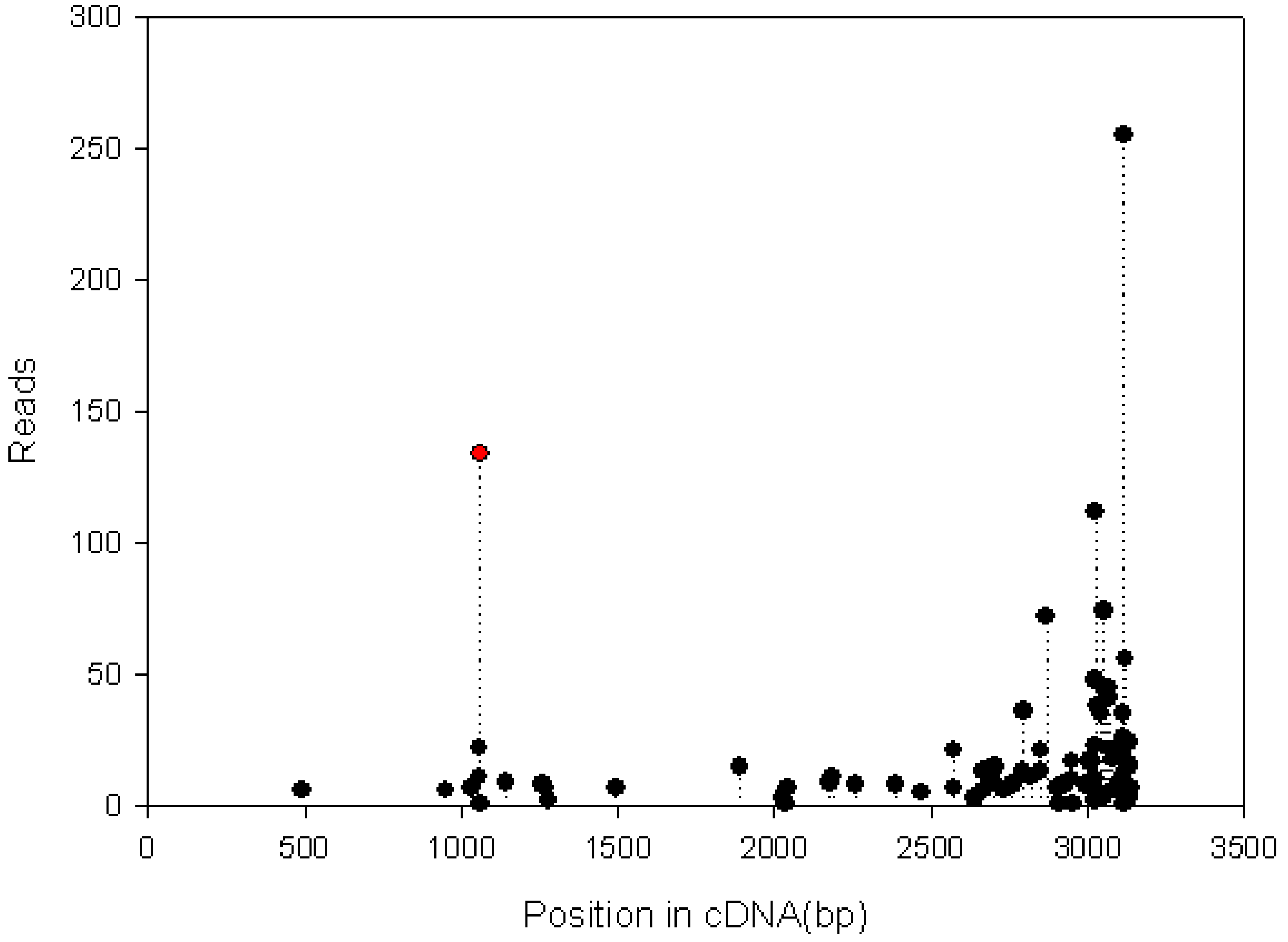


```
5' UGGGAUGAAGCCUGGUCCGGAUUCUA 3'        Cs2g09770.1
    ::.:::::::::::::::::.
3' -CCUUACUUCGGACCAGGCUC----- 5'        Csi-miR166c.5
```

Csi-miR166c.5, target=Cs8g16510.1 gene=Cs8g16510
Category=1
Score=2.5
Cleavage Site=1434

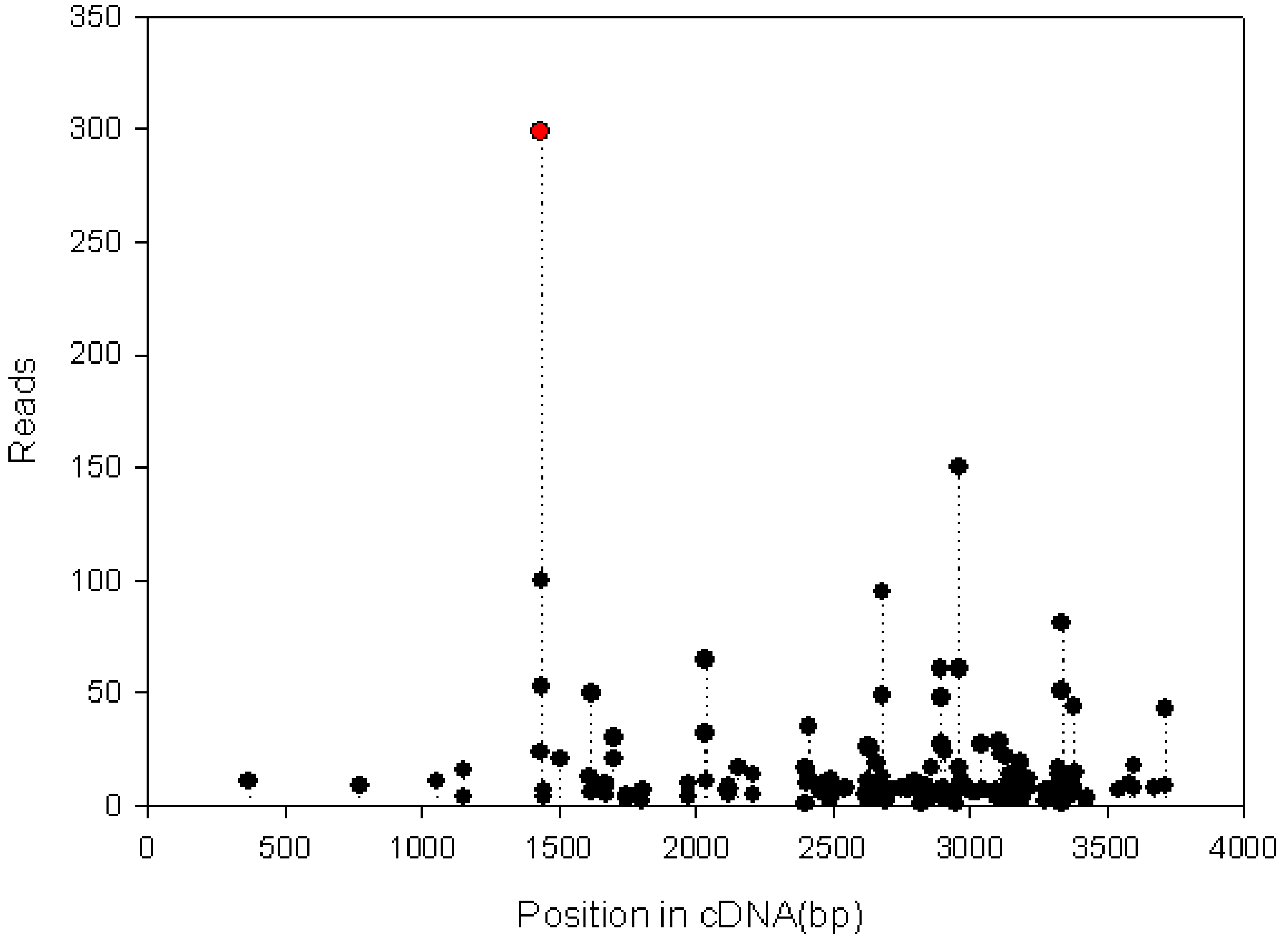


```
5' CCUGGGAUGAAGCCUGGUCCGGAUUC 3'        Cs8g16510.1
      ::.:::::::::::::::::.
3' ---CCUUACUUCGGACCAGGCUC--- 5'        Csi-miR166c.5
```

Csi-miR166d.1, target=Cs2g09770.1 gene=Cs2g09770
Category:3
Score=3
Cleavage Site=1058

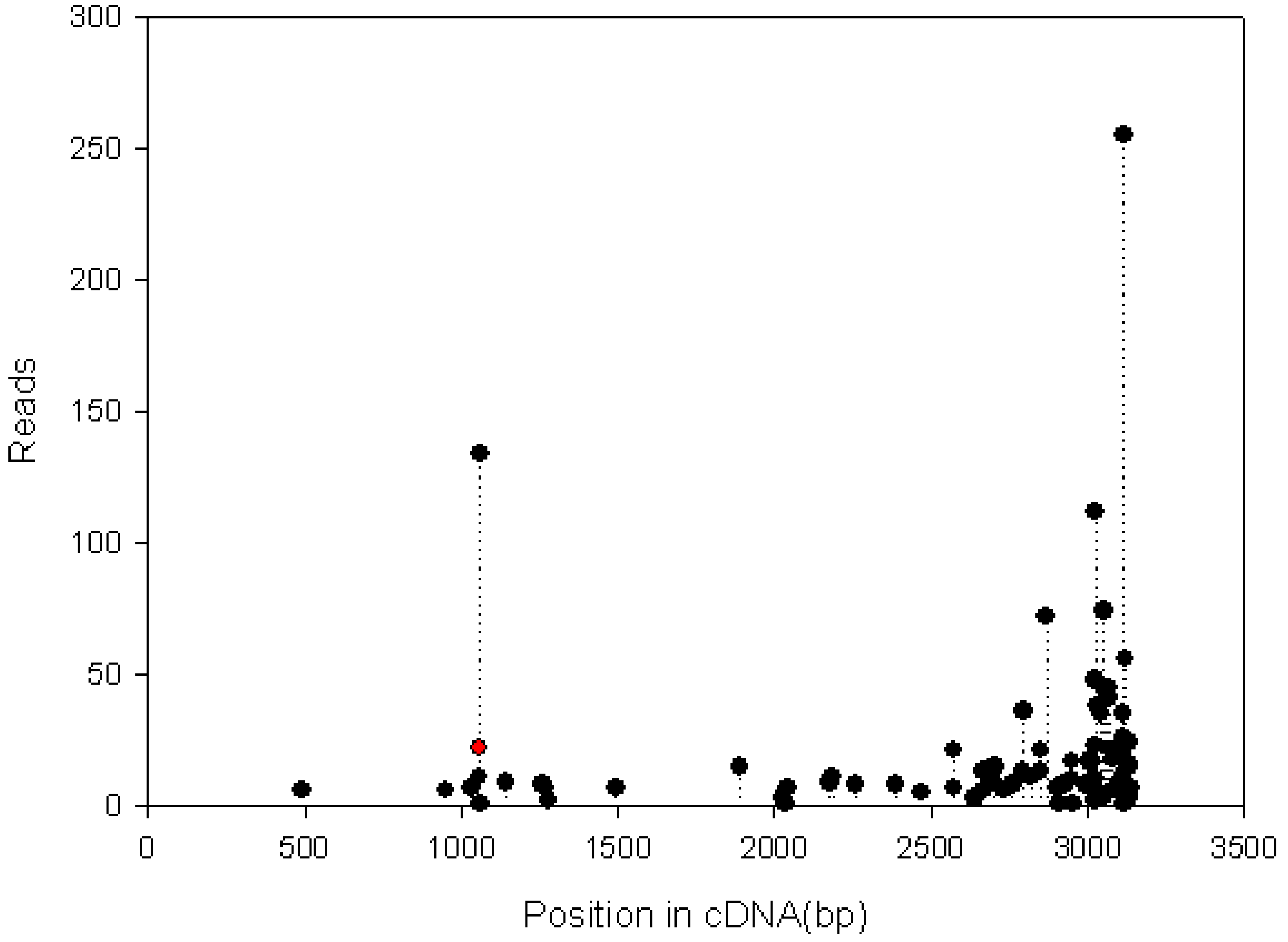


```
5' AUUGGGAUGAAGCCUGGUCCGGAUUC 3'        Cs2g09770.1
   :  ::.:::::::::::::::::.
3' UUCCCUUACUUCGGACCAGGCU---- 5'        Csi-miR166d.1
```

Csi-miR166d.1, target=Cs8g16510.1 gene=Cs8g16510
Category=1
Score=4
Cleavage Site=1434

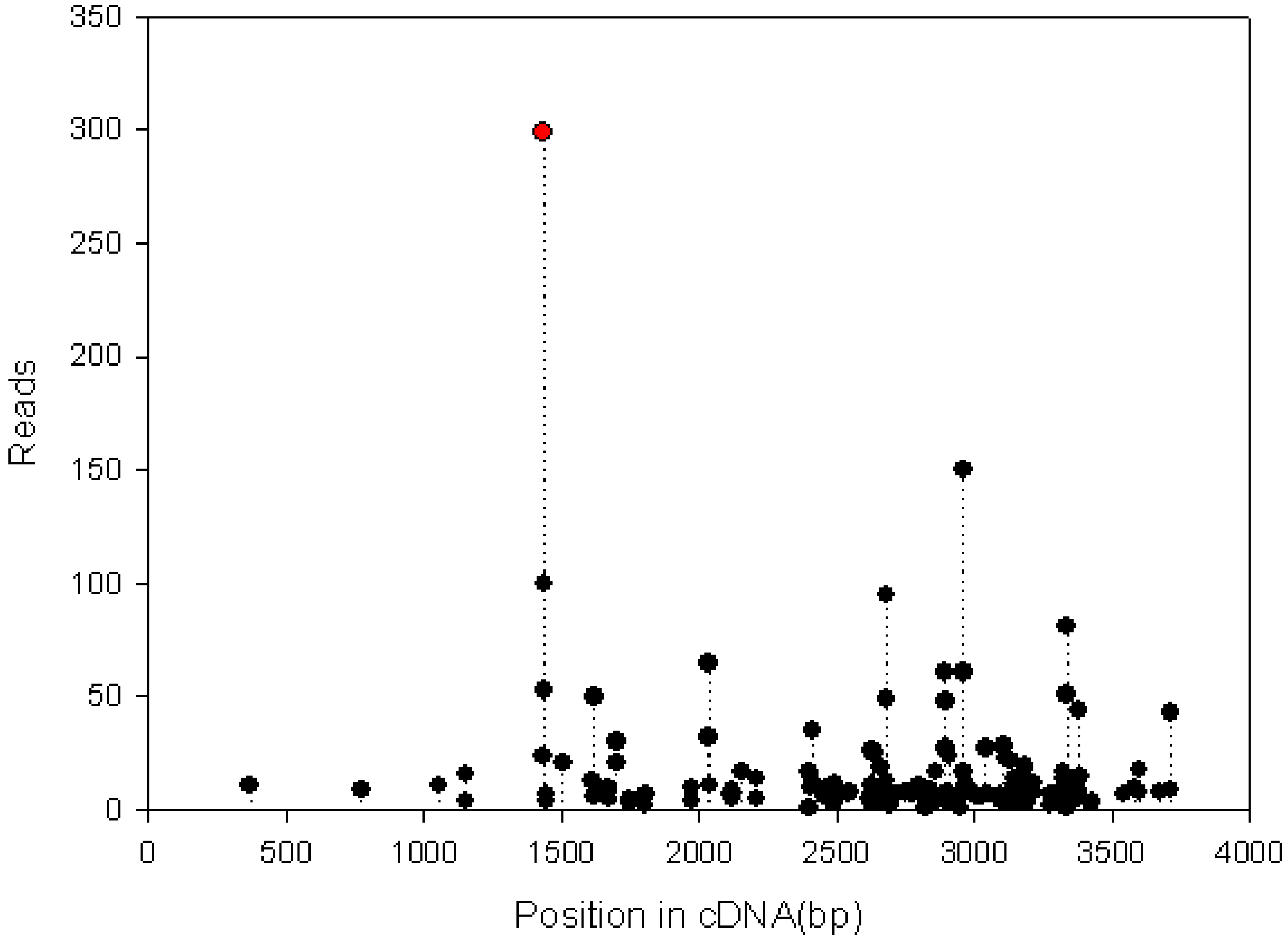


```
5' CCUGGGAUGAAGCCUGGUCCGGAUUC 3'        Cs8g16510.1
       ::.:::::::::::::::::.
3' UUCCCUUACUUCGGACCAGGCU---- 5'        Csi-miR166d.1
```

Csi-miR166d.2, target=Cs2g09770.1 gene=Cs2g09770
Category:3
Score=3
Cleavage Site=1058

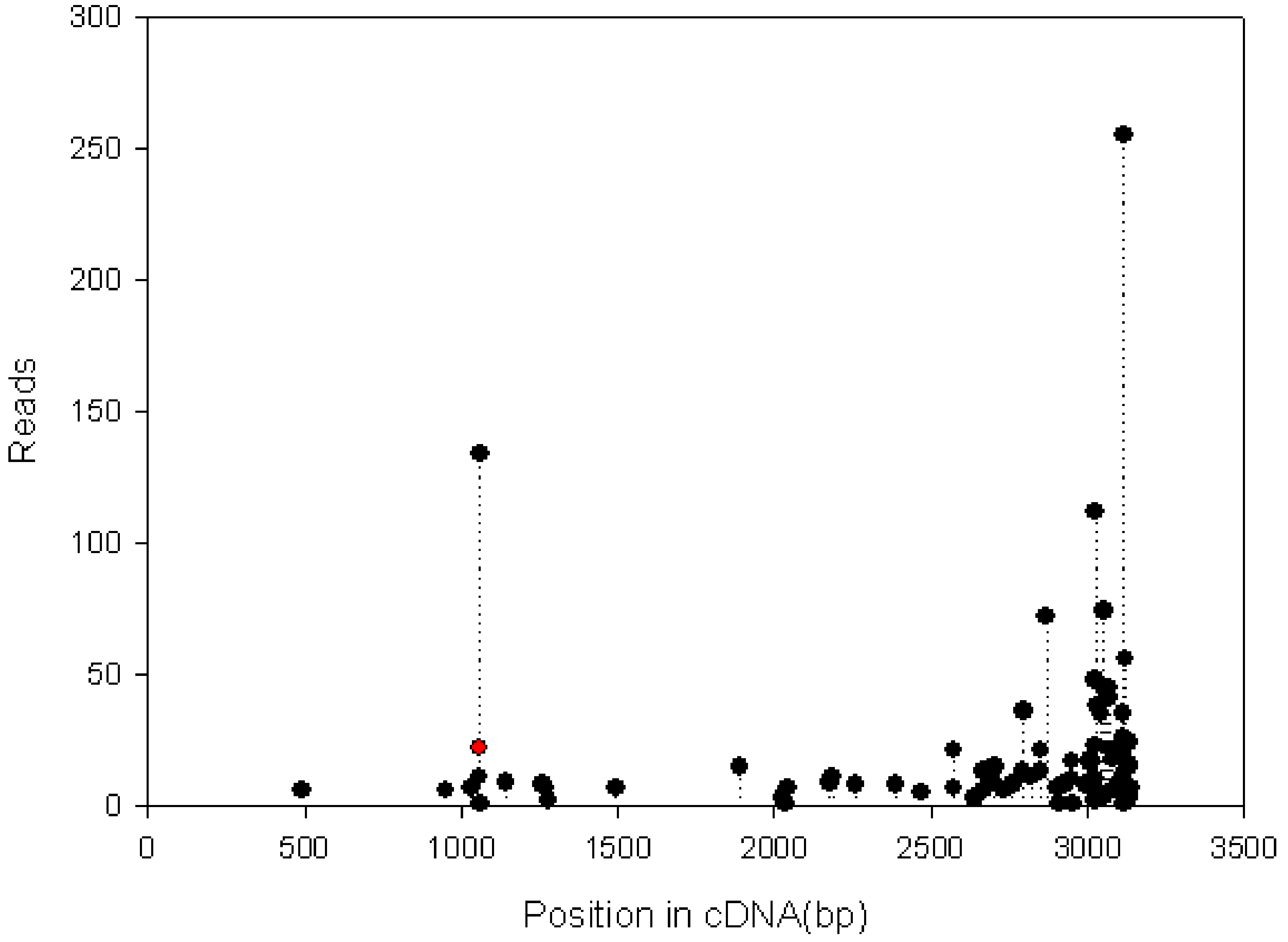


```
5' AUUGGGAUGAAGCCUGGUCCGGAUUC 3'         Cs2g09770.1
     ::.:::::::::::::::::.
3' -UCCCUUACUUCGGACCAGGCU---- 5'         Csi-miR166d.2
```

Csi-miR166d.2, target=Cs8g16510.1 gene=Cs8g16510
Category=1
Score=3
Cleavage Site=1434

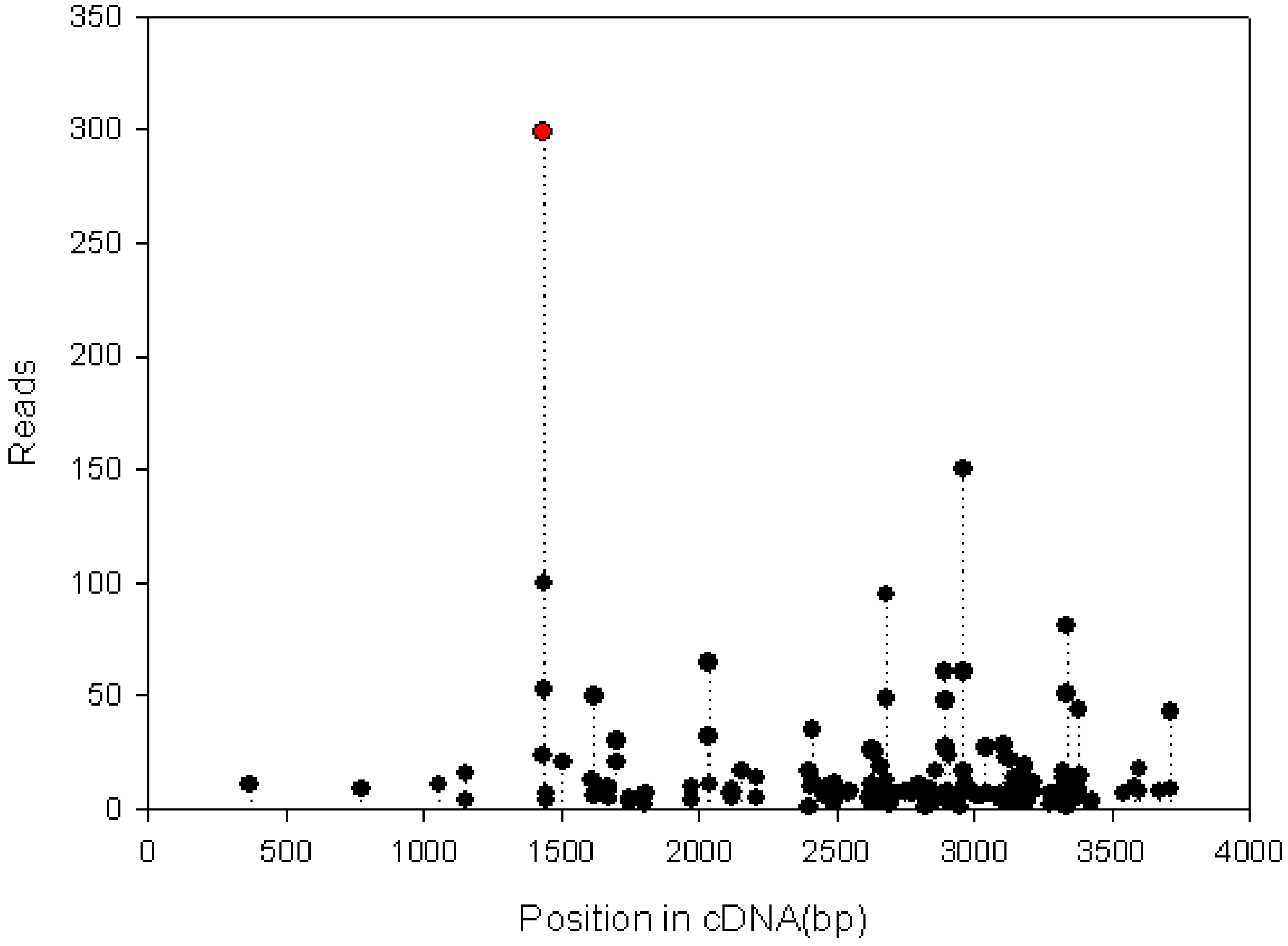


```
5' CCUGGGAUGAAGCCUGGUCCGGAUUC 3'        Cs8g16510.1
       ::.:::::::::::::::::.
3' -UCCCUUACUUCGGACCAGGCU---- 5'        Csi-miR166d.2
```

Csi-miR166g.1, target=Cs2g09770.1 gene=Cs2g09770
Category:2
Score=4.5
Cleavage Site=1060

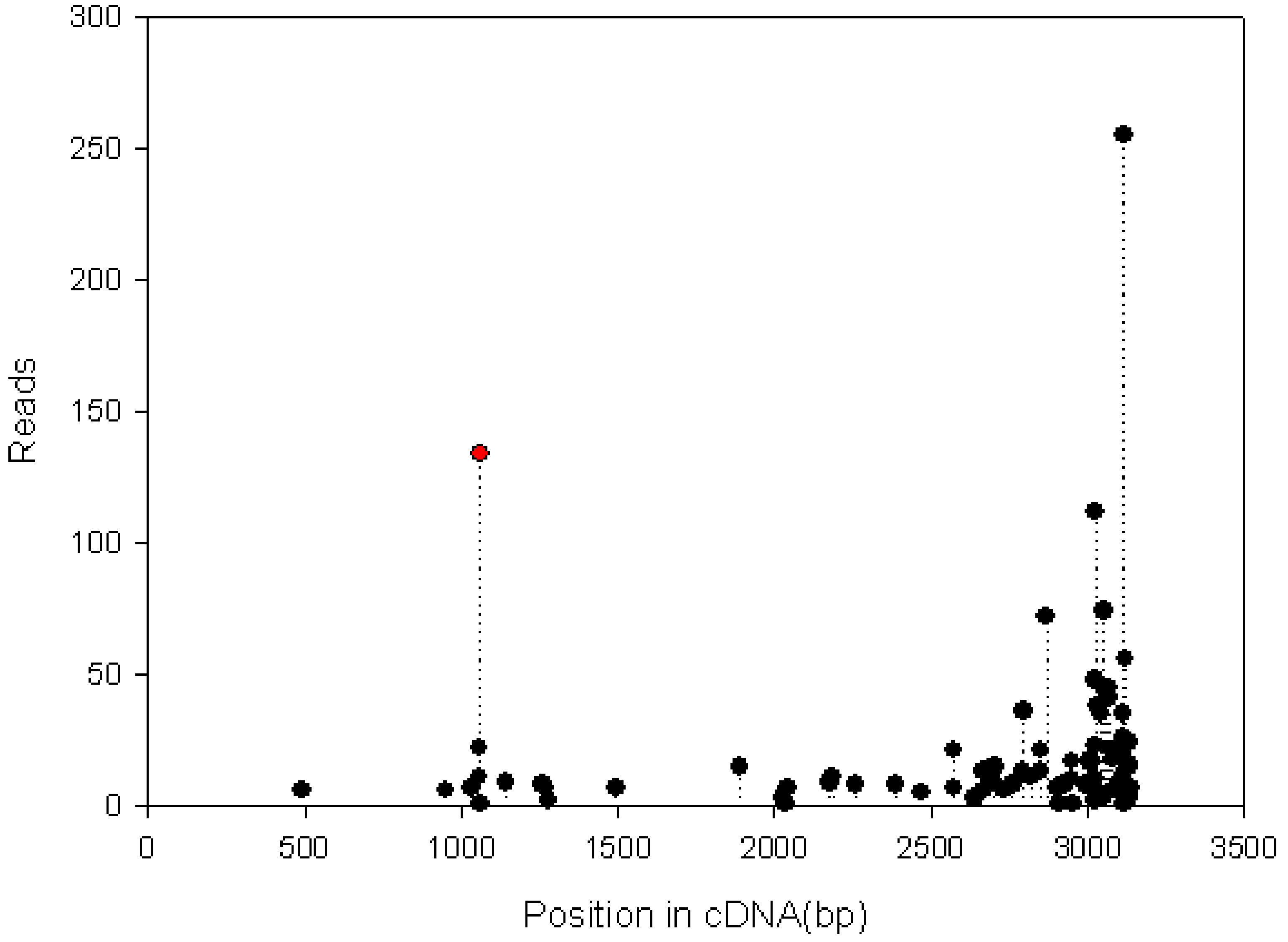


```
5' UGGGAUGAAGCCUGGUCCGGAUUCUA 3'        Cs2g09770.1
    ::.::::::::::::::::.
3' -CCUUACUUCGGACCAGGCUCU---- 5'        Csi-miR166g.1
```

Csi-miR166g.1, target=Cs8g16510.1 gene=Cs8g16510
Category=1
Score=4.5
Cleavage Site=1434

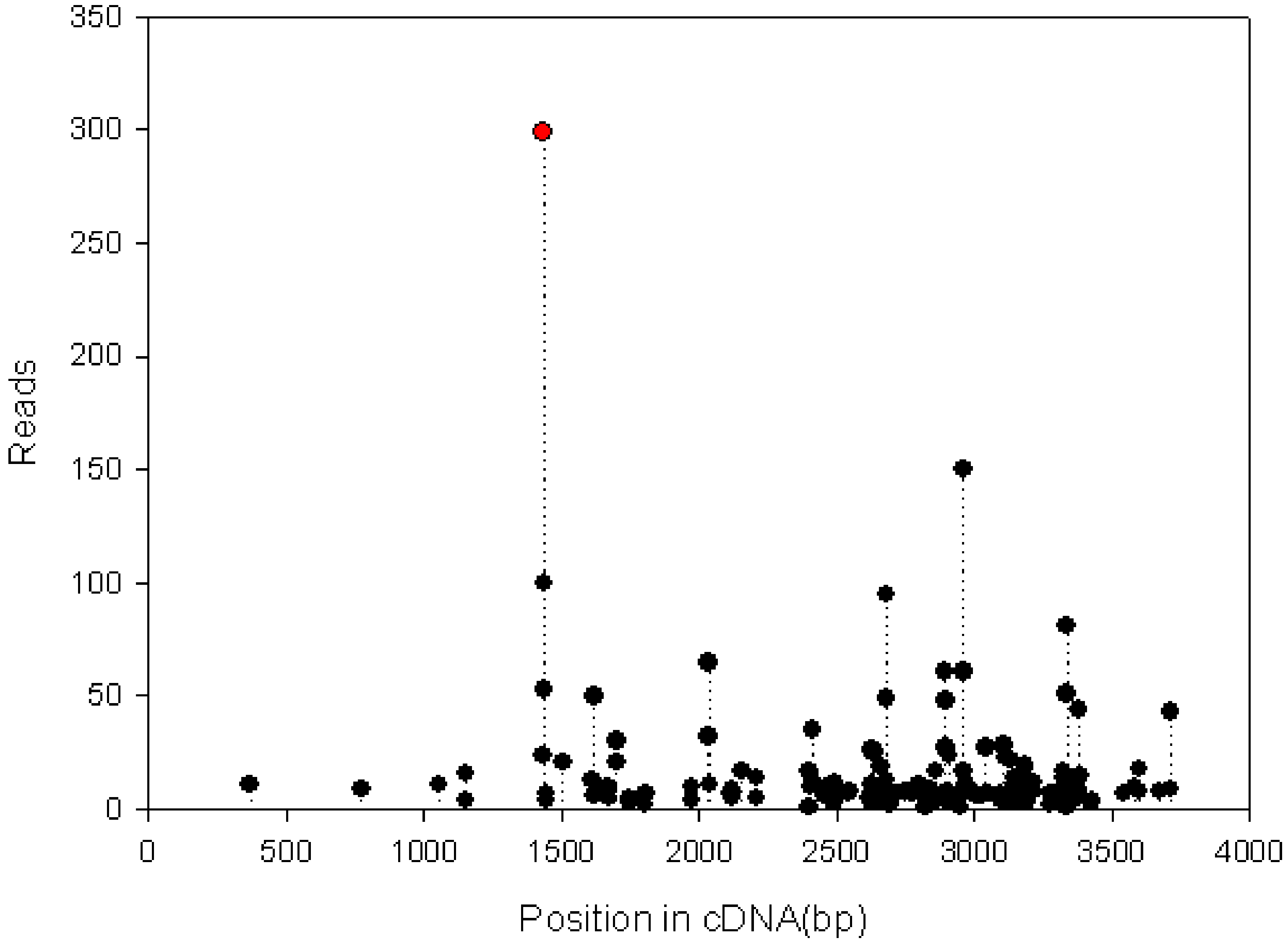


```
5' CCUGGGAUGAAGCCUGGUCCGGAUUC 3'        Cs8g16510.1
     ::.:::::::::::::::::.
3' ---CCUUACUUCGGACCAGGCUCU-- 5'        Csi-miR166g.1
```

Csi-miR166i, target=Cs2g09770.1 gene=Cs2g09770
Category:2
Score=2.5
Cleavage Site=1060

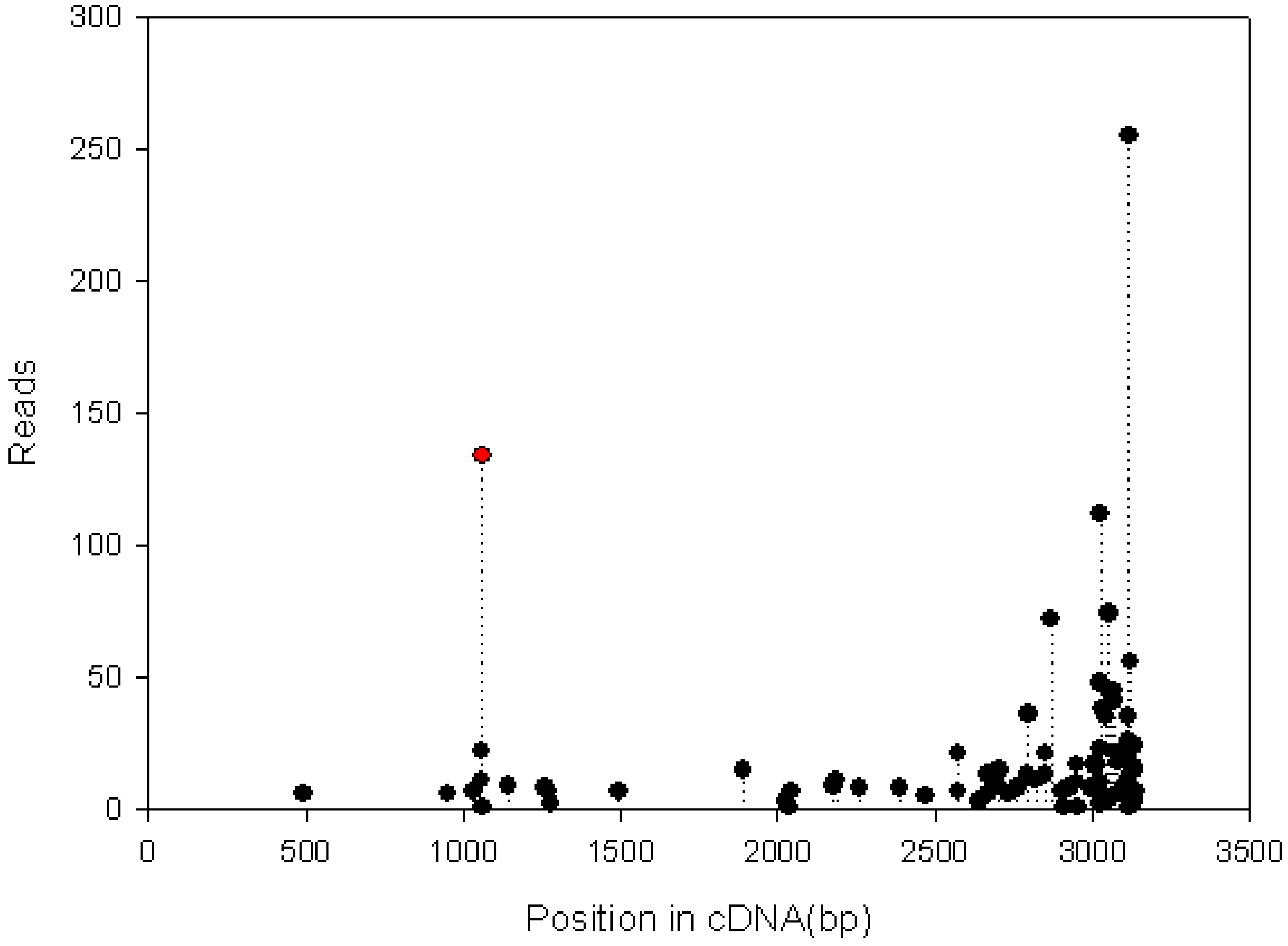


```
5' UGGGAUGAAGCCUGGUCCGGAUUCUA 3'        Cs2g09770.1
     ::.:::::::::::::::::.:
3' CCCUUACUUCGGACCAGGCUU----- 5'        Csi-miR166i
```

Csi-miR166i, target=Cs8g16510.1 gene=Cs8g16510
Category=1
Score=2.5
Cleavage Site=1434

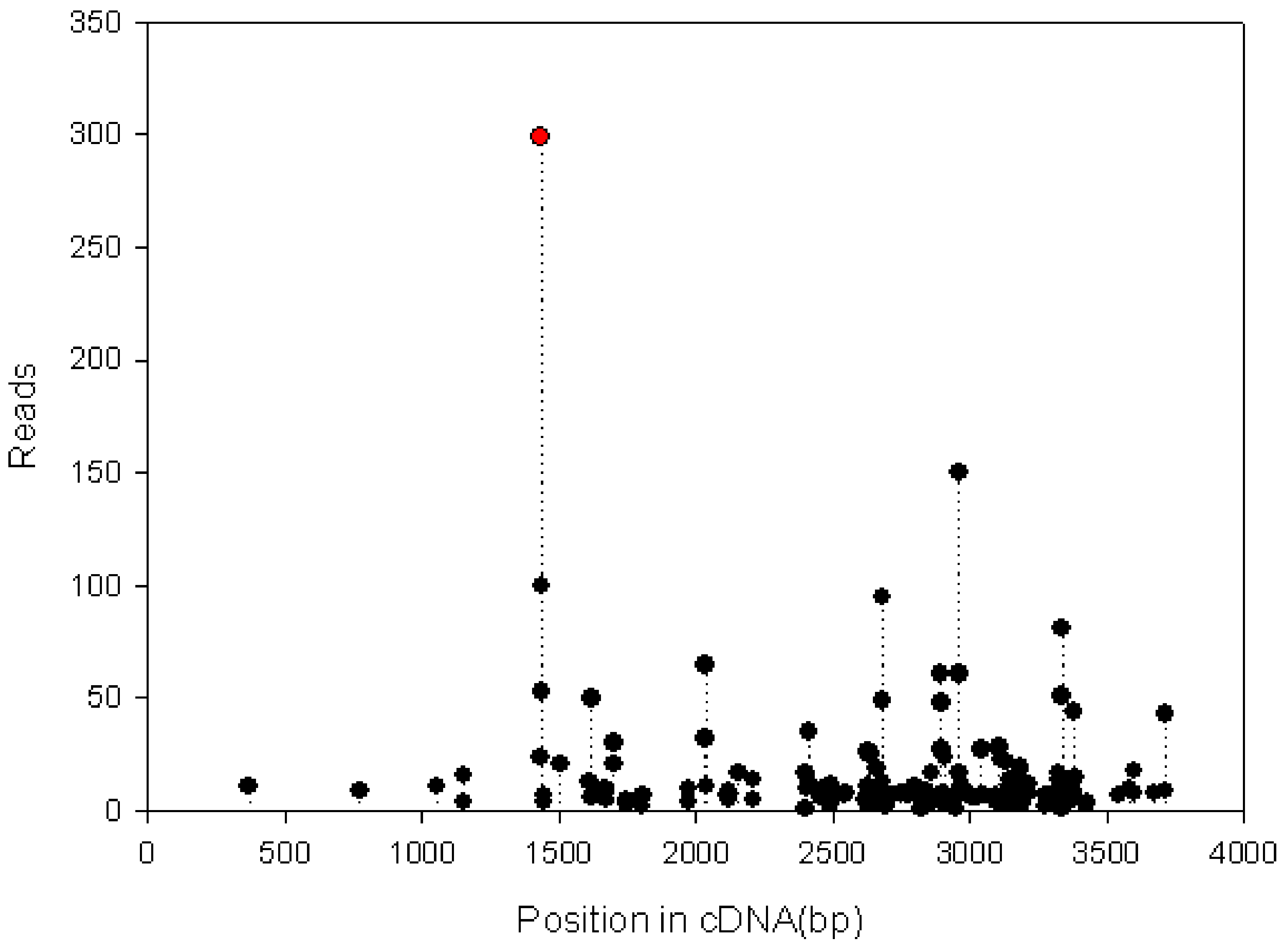


```
5' CCUGGGAUGAAGCCUGGUCCGGAUUC 3'        Cs8g16510.1
       ::.:::::::::::::::::.:
3' --CCCUUACUUCGGACCAGGCUU--- 5'        Csi-miR166i
```

Csi-miR166j.1, target=Cs2g09770.1 gene=Cs2g09770
Category:3
Score=4
Cleavage Site=1058

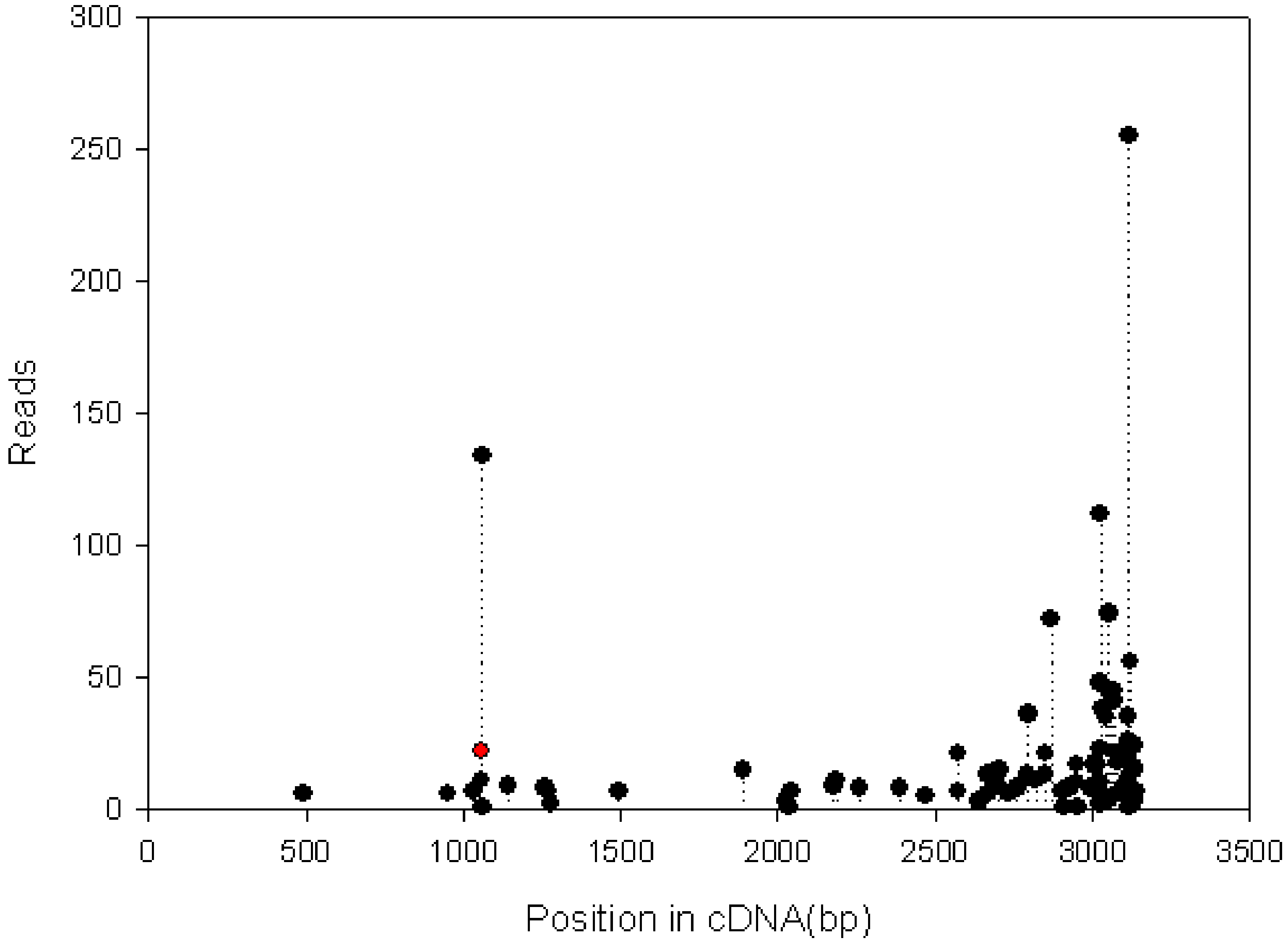


```
5' AUUGGGAUGAAGCCUGGUCCGGAUUC 3'        Cs2g09770.1
     ::.:::::::::::::::..
3' -CUCCUUACUUCGGACCAGGUU---- 5'        Csi-miR166j.1
```

Csi-miR166j.1, target=Cs8g16510.1 gene=Cs8g16510
Category=1
Score=4
Cleavage Site=1434

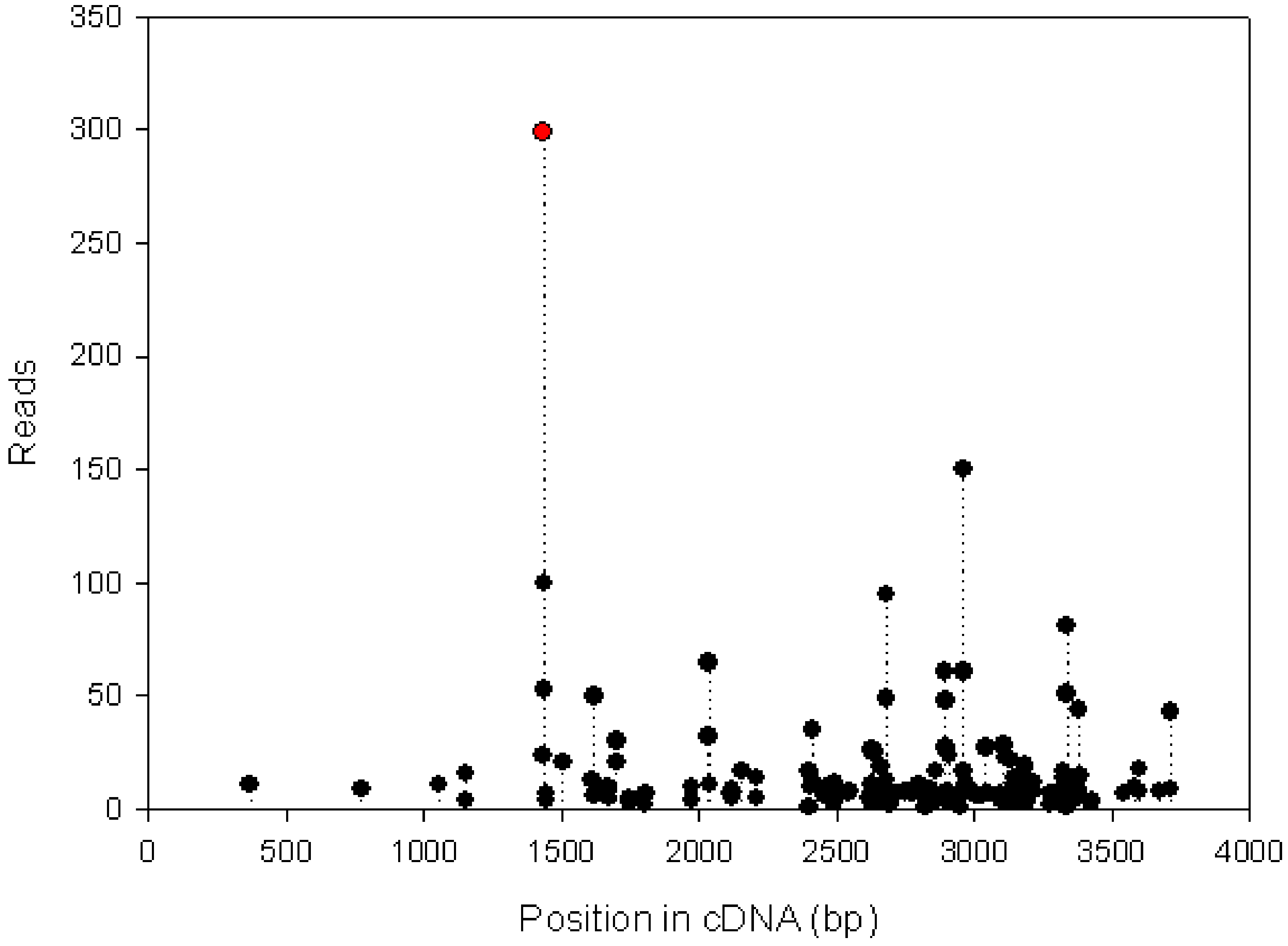


```
5' CCUGGGAUGAAGCCUGGUCCGGAUUC 3'        Cs8g16510.1
      ::.:::::::::::::::::..
3' -CUCCUUACUUCGGACCAGGUU---- 5'        Csi-miR166j.1
```

Csi-miR166j.2, target=Cs4g14480.1 gene=Cs4g14480
Category=3
Score=3
Cleavage Site=1338

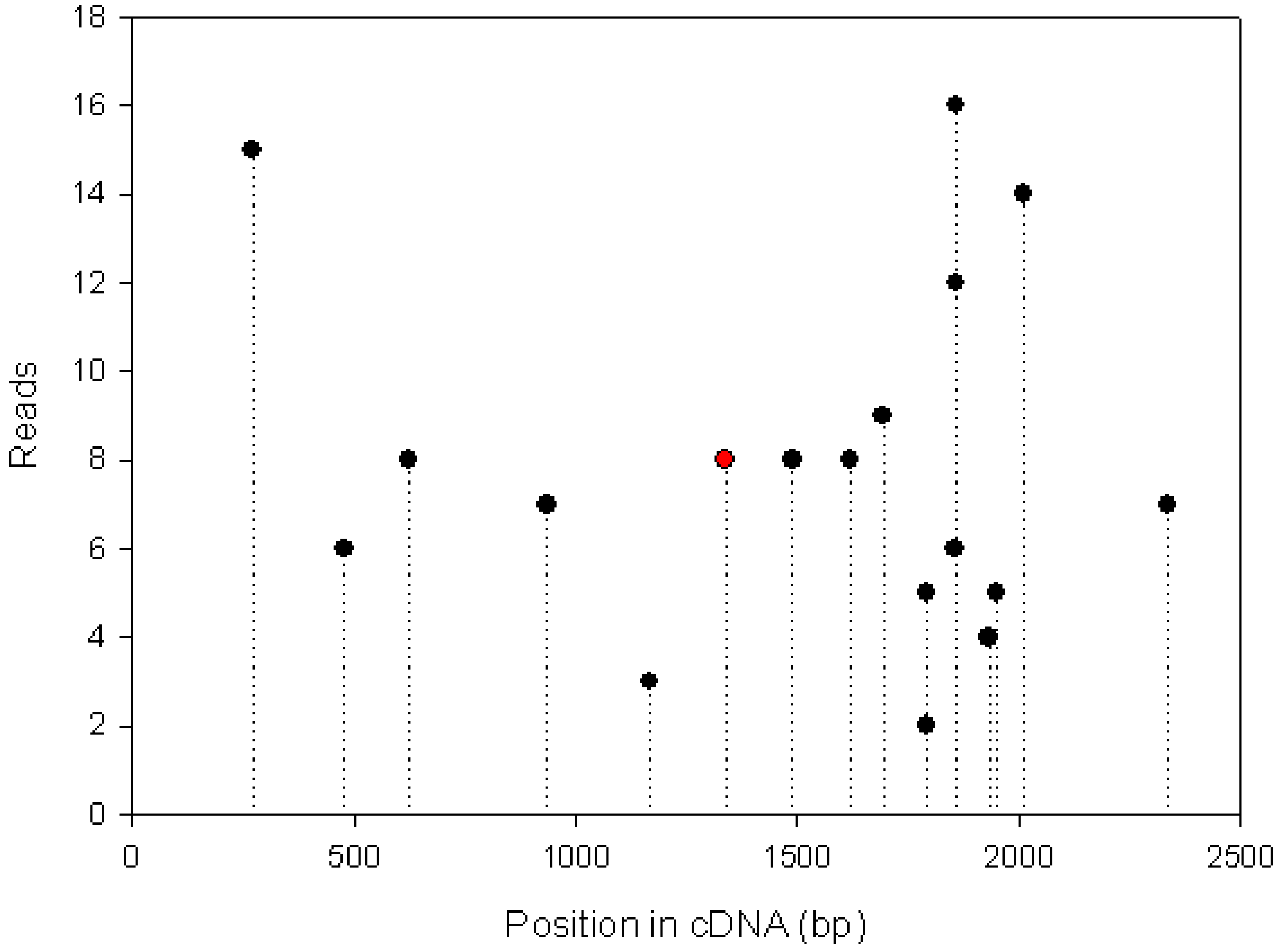


```
5' UGGUAUAAAGCCUGGUCUAAGAGACU 3'        Cs4g14480.1
    :: :: :::::::::::.::::
3' -CCUUACUUCGGACCAGGUUCU---- 5'        Csi-miR166j.2
```

Csi-miR166j.3, target=Cs2g09770.1 gene=Cs2g09770
Category:3
Score=3
Cleavage Site=1058

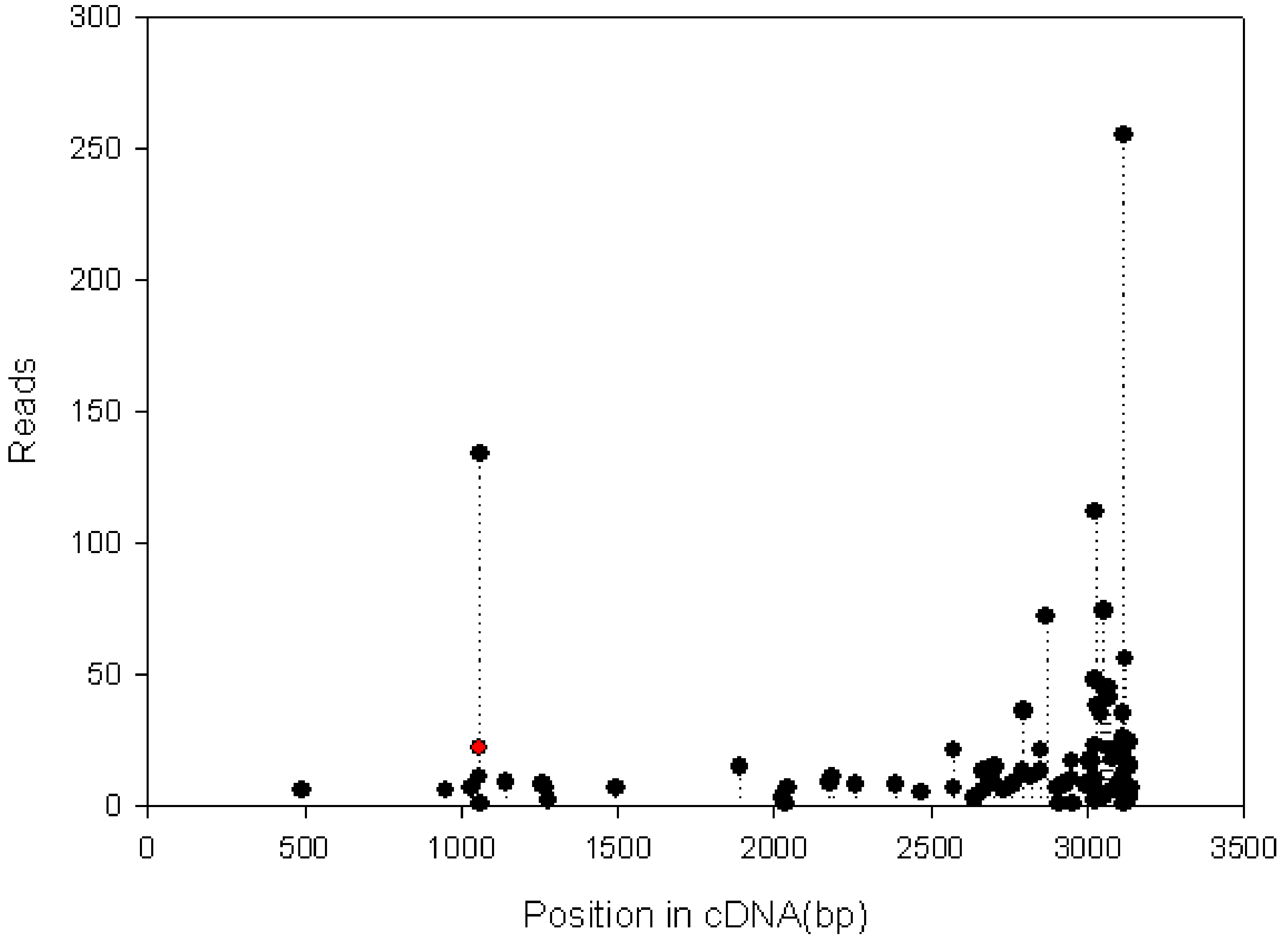


```
5' AUUGGGAUGAAGCCUGGUCCGGAUUC 3'        Cs2g09770.1
     :::.:::::::::::::::::..
3' -CACCUUACUUCGGACCAGGUU---- 5'        Csi-miR166j.3
```

Csi-miR166j.3, target=Cs8g16510.1 gene=Cs8g16510
Category=1
Score=3
Cleavage Site=1434

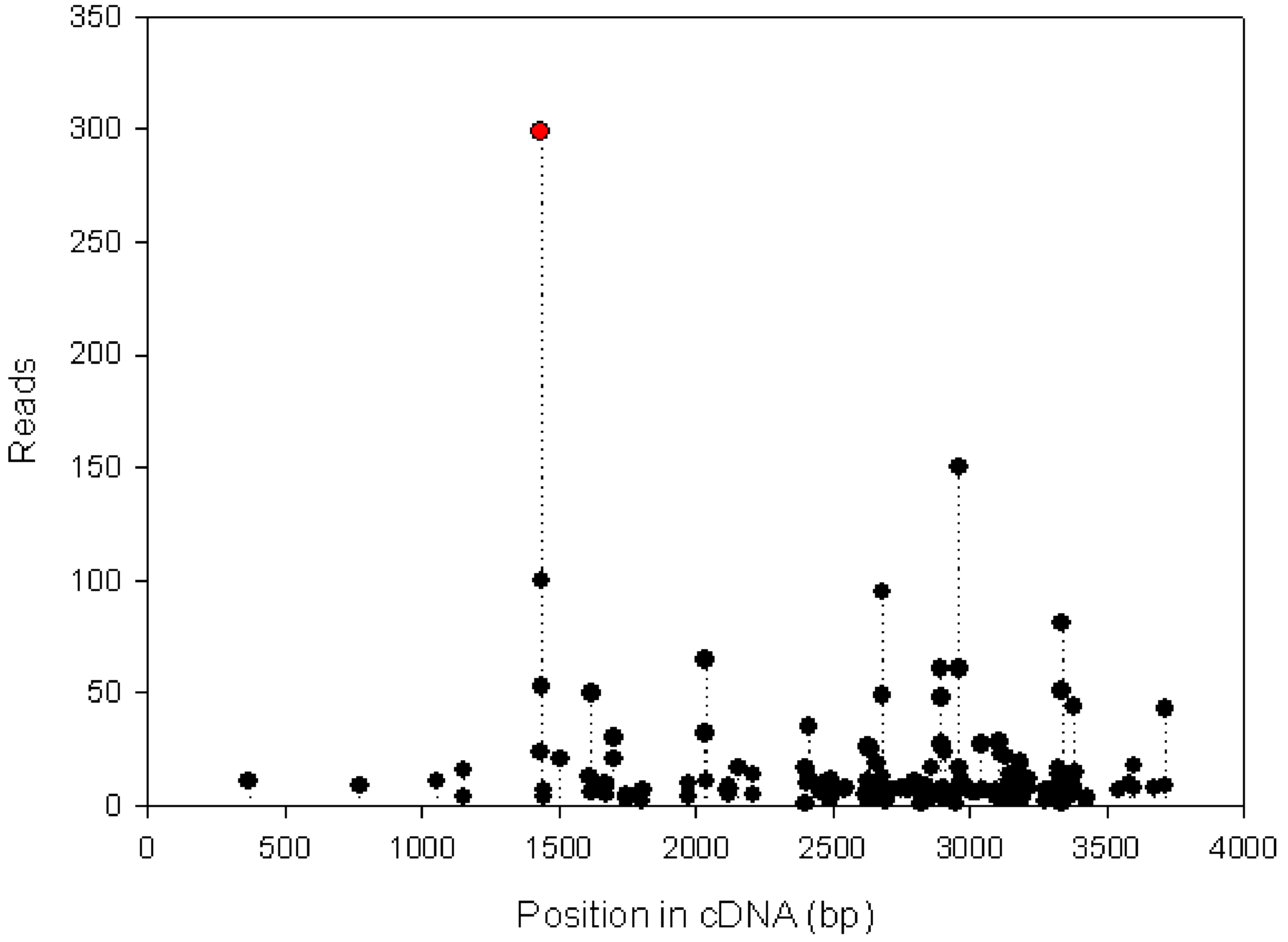


```
5' CCUGGGAUGAAGCCUGGUCCGGAUUC 3'        Cs8g16510.1
     :::.::::::::::::::..
3' -CACCUUACUUCGGACCAGGUU---- 5'        Csi-miR166j.3
```

Csi-miR167a.1, target=Cs2g15130.1 gene=Cs2g15130
Category=1
Score=5
Cleavage Site=3444

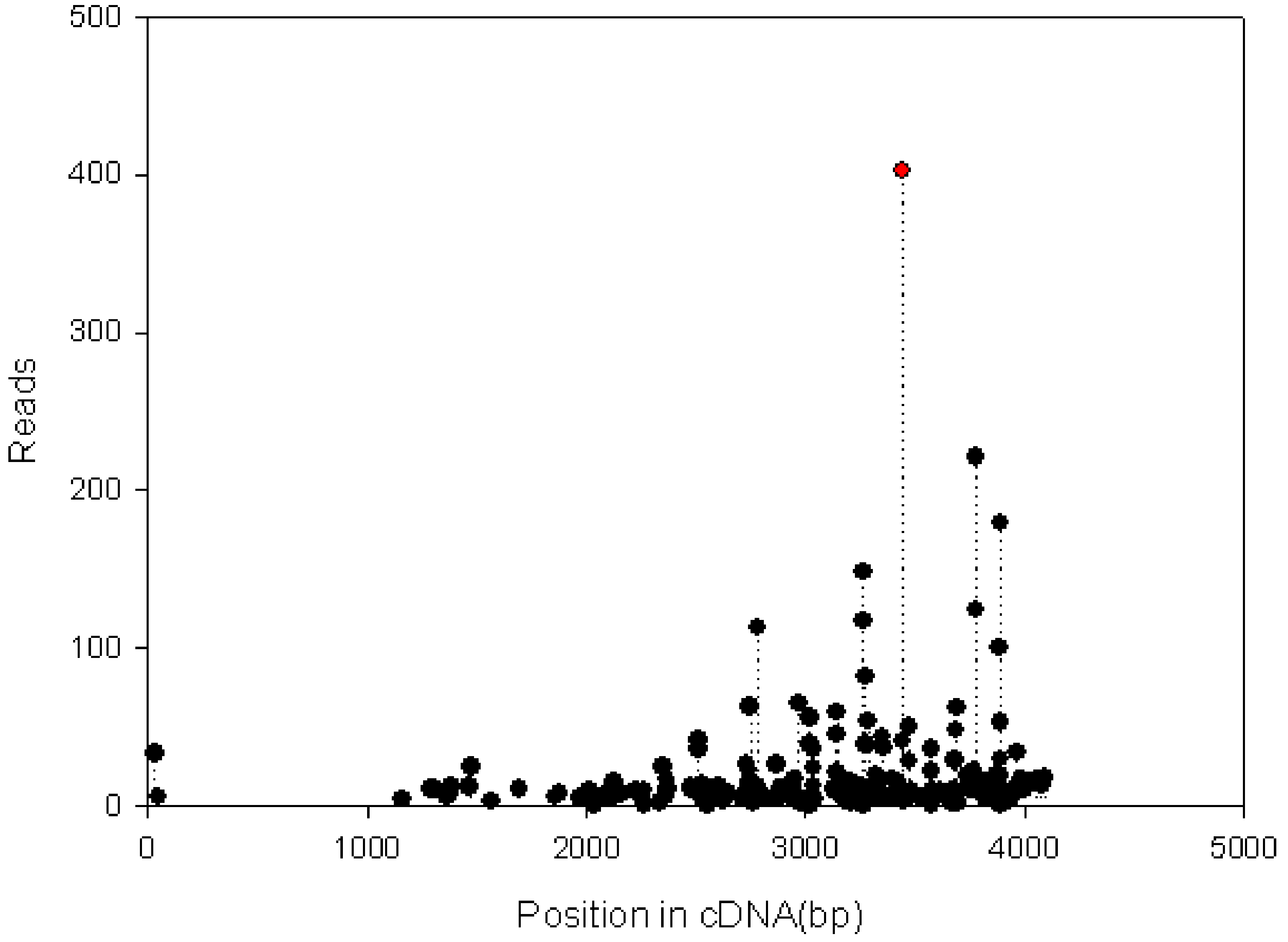


```
5' AGAGAUCAGGCUGGCAGCUUGUAUUU 3'        Cs2g15130.1
    :::::: ::::::::::: .:
3' -AUCUAGUACGACCGUCGAA-GU--- 5'        Csi-miR167a.1
```

Csi-miR167a.1, target=Cs8g08320.1 gene=Cs8g08320
Category:3
Score=5
Cleavage Site=584

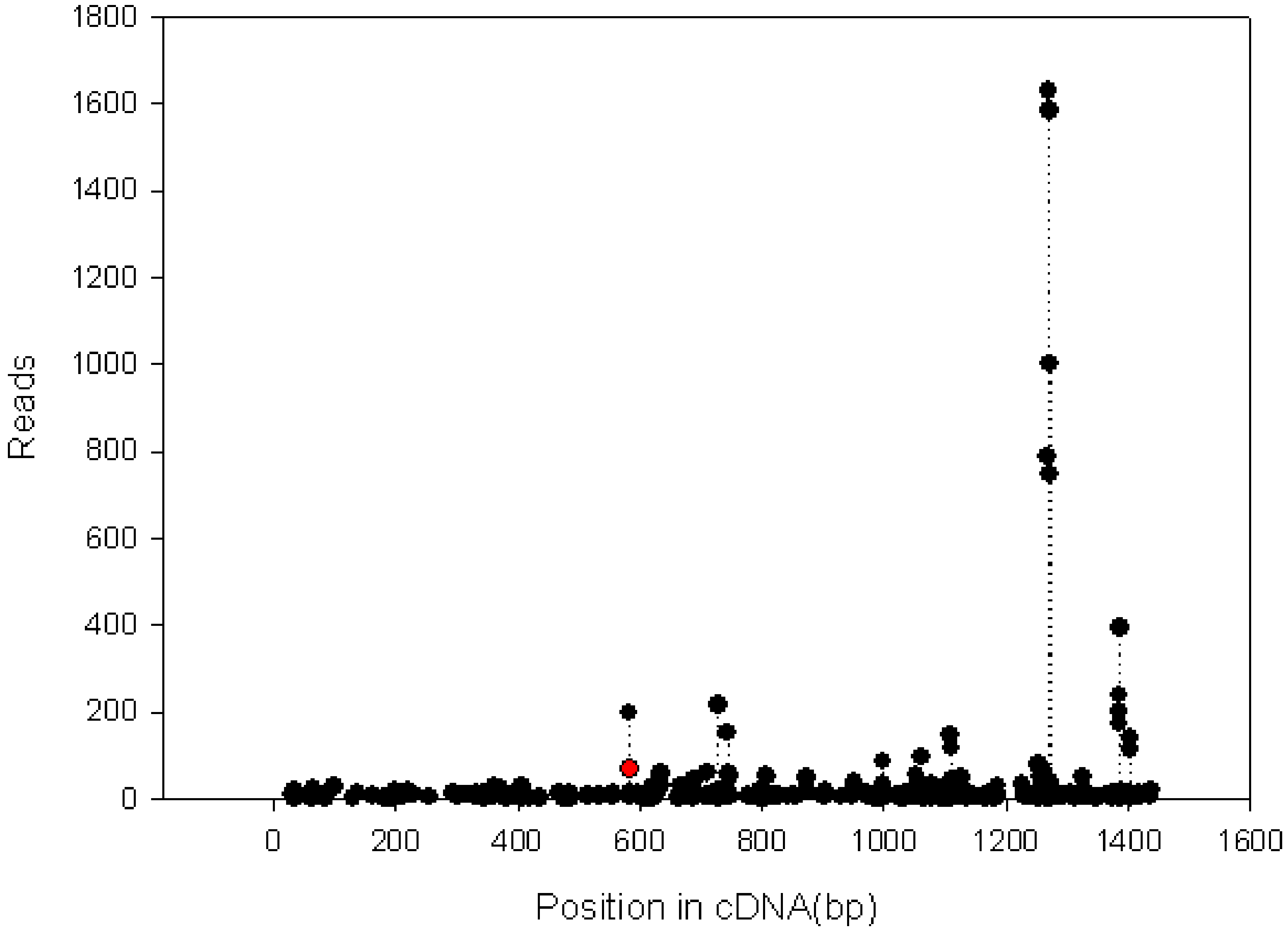


```
5' UUCAUGCGGAUCAUGCUGG-AGUUUCA 3'        Cs8g08320.1
         .:::::::::::: ::.::::
3' ------AUCUAGUACGACCGUCGAAGU 5'        Csi-miR167a.1
```

Csi-miR167b.1, target=Cs2g15130.1 gene=Cs2g15130
Category=1
Score=4
Cleavage Site=3444

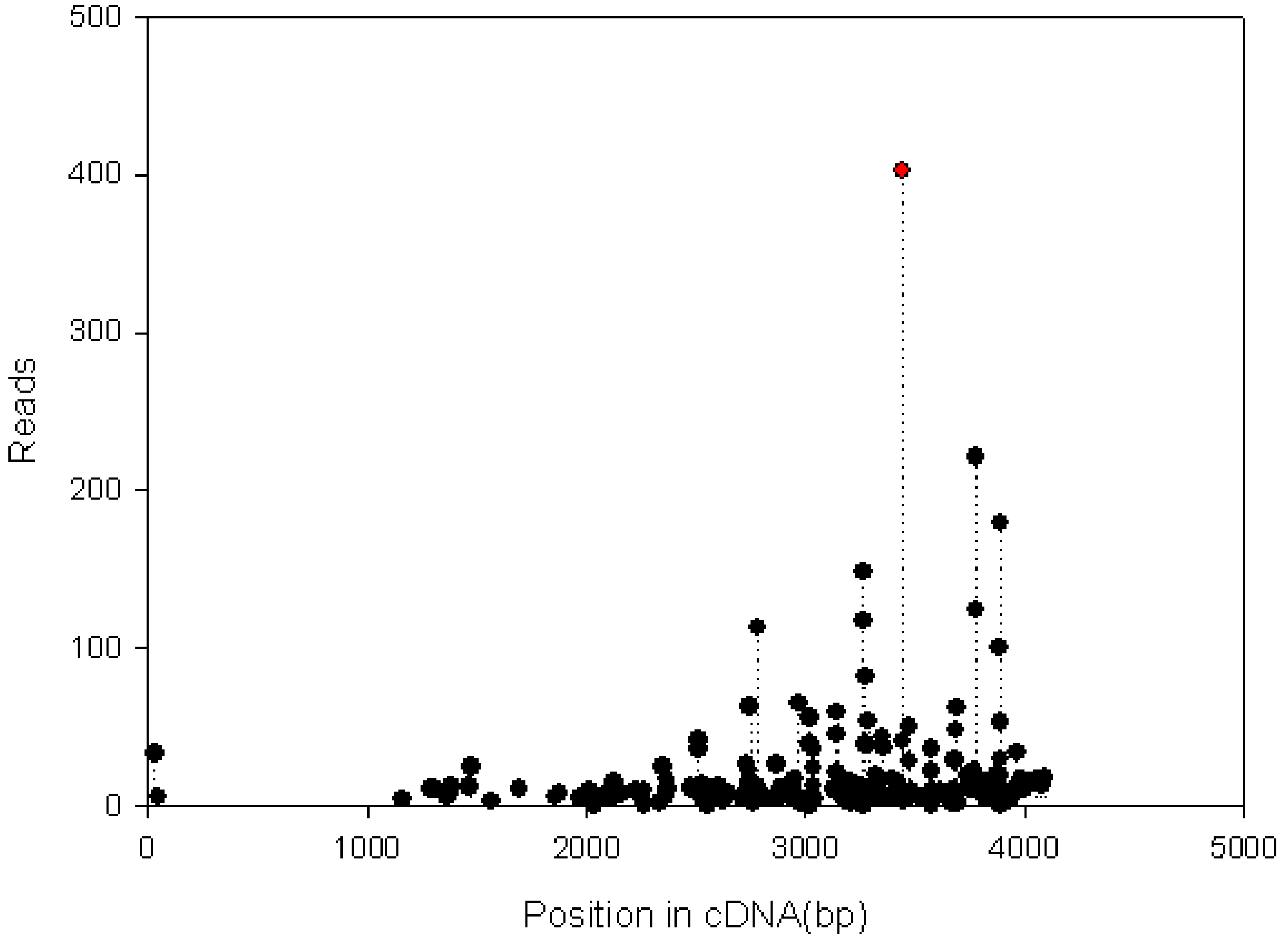


```
5' AGAGAUCAGGCUGGCAGCUUGUAUUU 3'        Cs2g15130.1
     :::::: ::::::::::: .:
3' --UCUAGUACGACCGUCGAA-GU--- 5'        Csi-miR167b.1
```

Csi-miR167b.1, target=Cs8g08320.1 gene=Cs8g08320
Category:3
Score=3.5
Cleavage Site=584

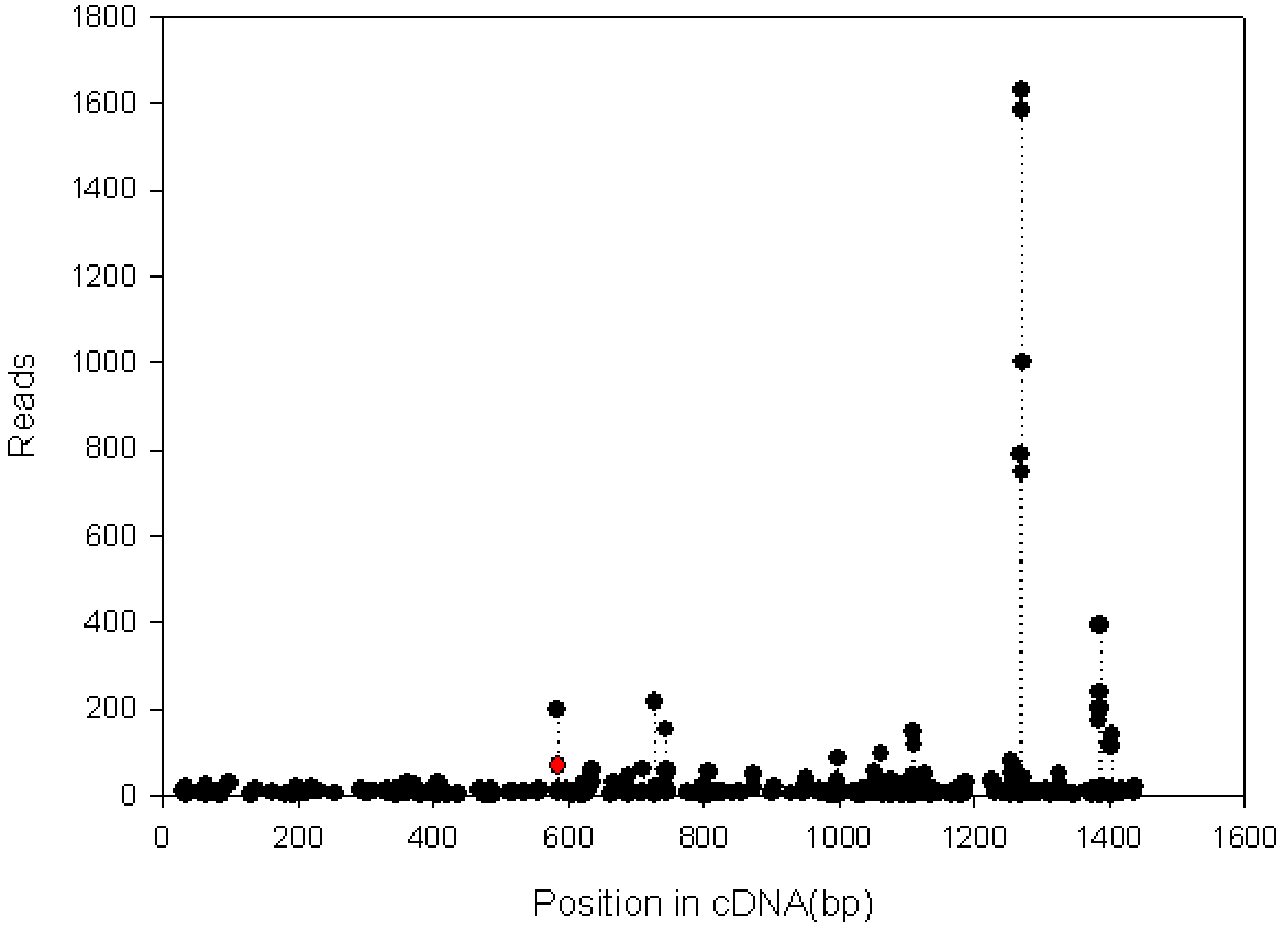


```
5' UUCAUGCGGAUCAUGCUGG-AGUUUCA 3'        Cs8g08320.1
          .:::::::::::: ::.::::
3' -------UCUAGUACGACCGUCGAAGU 5'        Csi-miR167b.1
```

Csi-miR167b.2, target=Cs1g09030.1 gene=Cs1g09030
Category=3
Score=5
Cleavage Site=1246

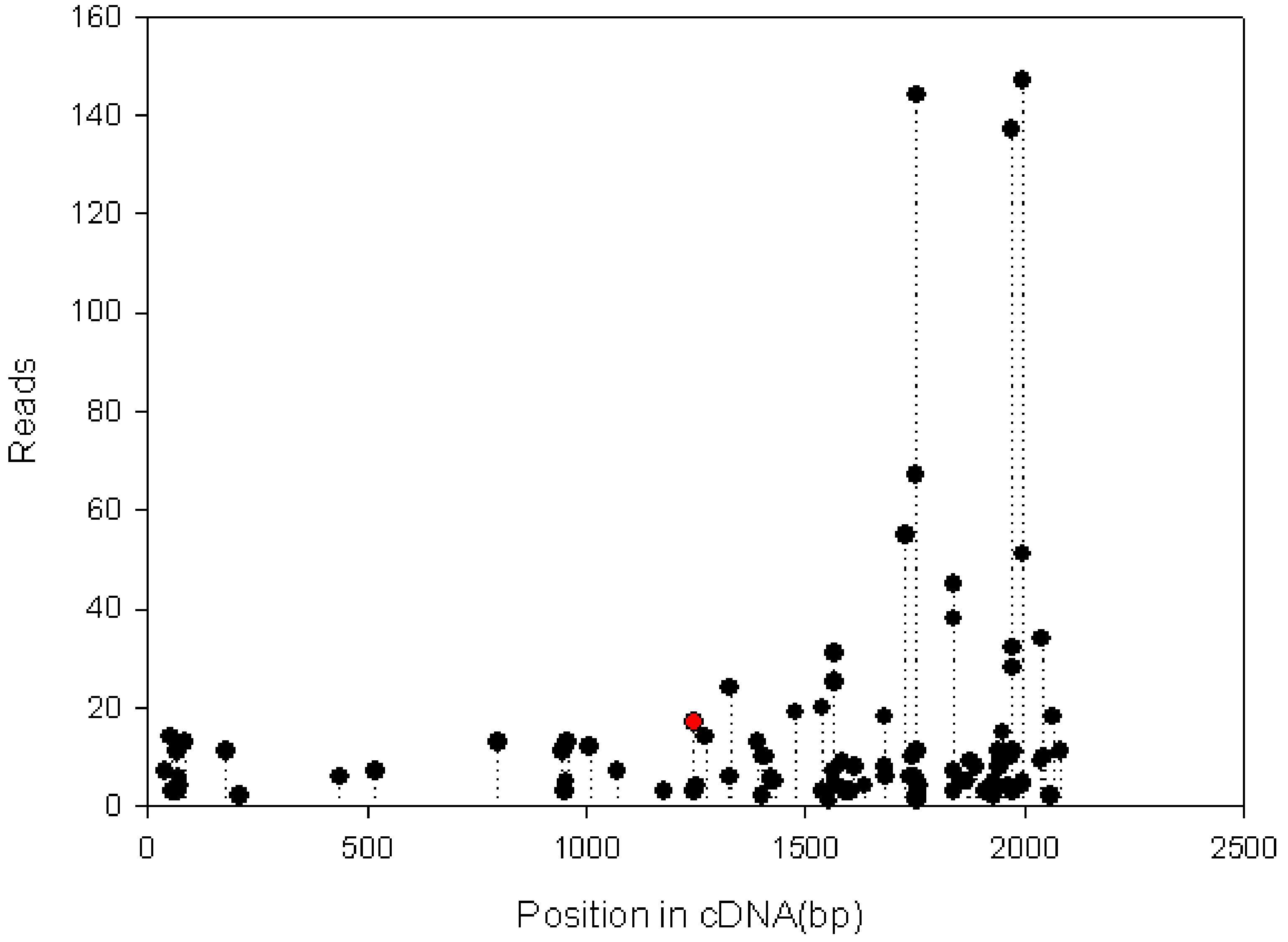


```
5' UGGUGAAAUCAAGCUGGCAGACUUCG 3'        Cs1g09030.1
      :.: :::: :::::::: ::::.
3' ---AUUCUAGUACGACCGUC-GAAGU 5'        Csi-miR167b.2
```

Csi-miR167b.3, target=Cs2g15130.1 gene=Cs2g15130
Category=1
Score=3.5
Cleavage Site=3444

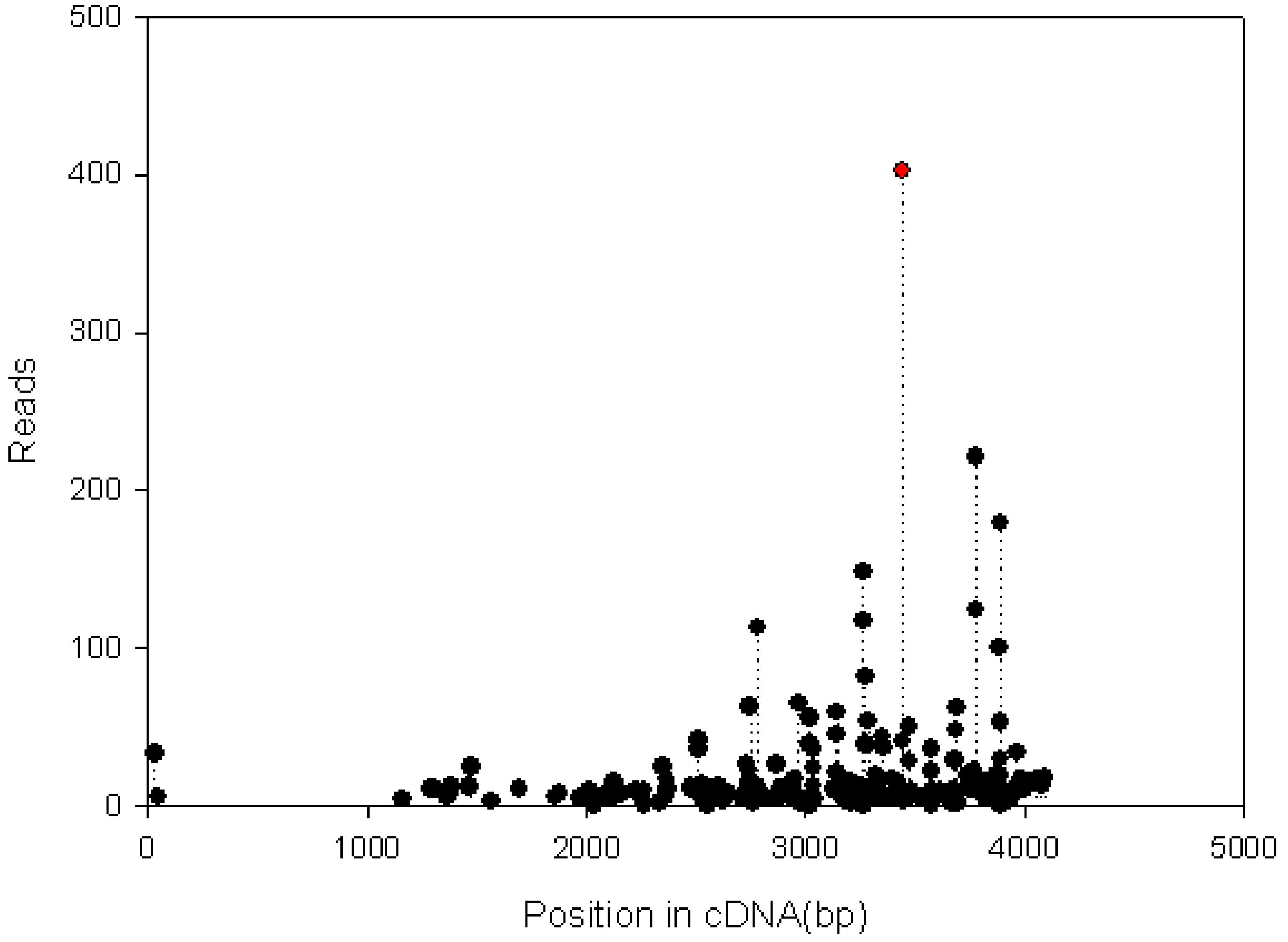


```
5' AGAGAUCAGGCUGGCAGCUUGUAUUU 3'        Cs2g15130.1
    .:::::: :::::::::::
3' AUUCUAGUACGACCGUCGAA------ 5'        Csi-miR167b.3
```

Csi-miR167b.4, target=Cs2g15130.1 gene=Cs2g15130
Category=1
Score=4.5
Cleavage Site=3444

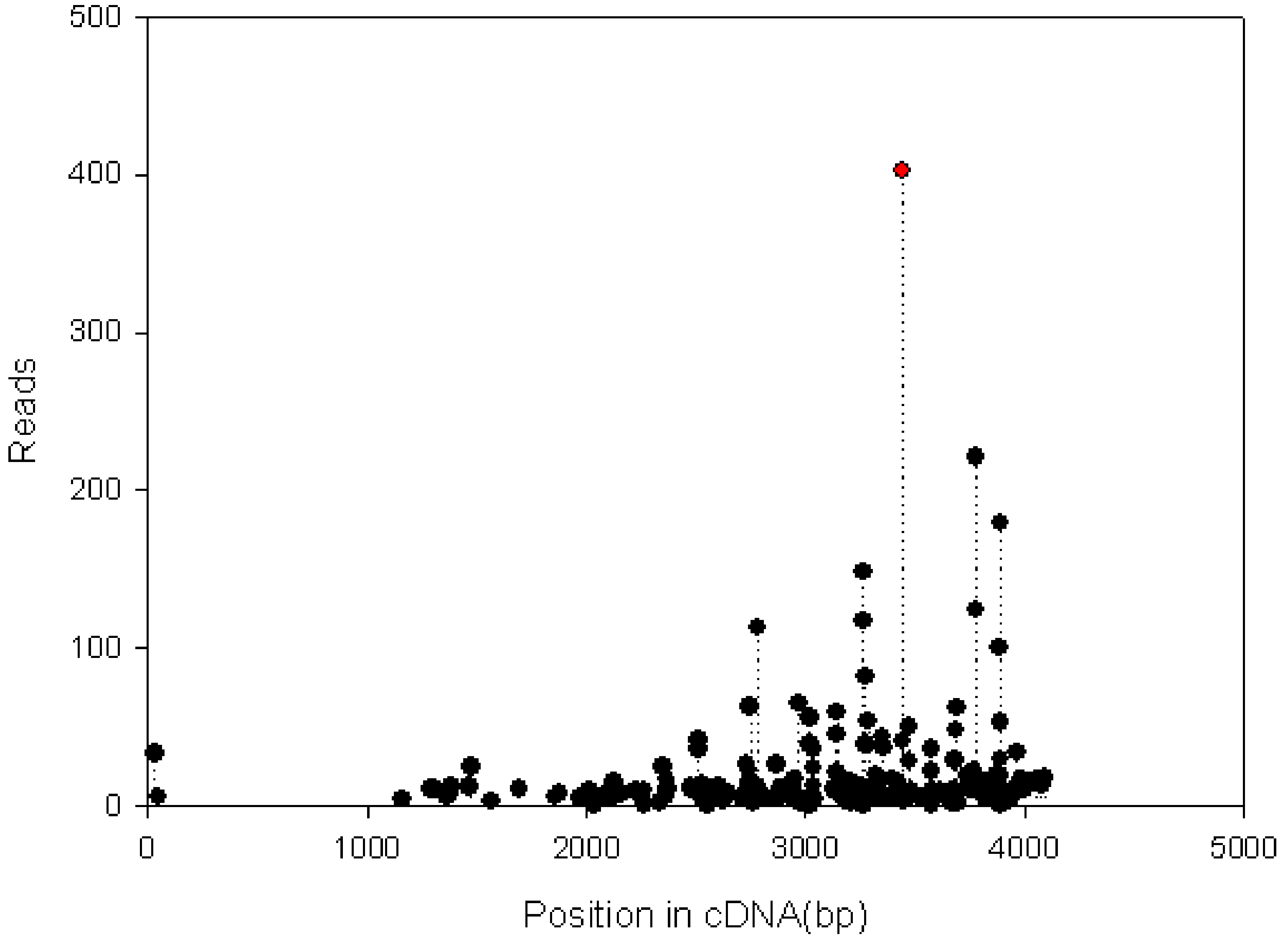


```
5' AGAGAUCAGGCUGGCAGCUUGUAUUU 3'        Cs2g15130.1
    .:::::: :::::::::::: .:
3' -UUCUAGUACGACCGUCGAA-GU--- 5'        Csi-miR167b.4
```

Csi-miR167b.4, target=Cs8g08320.1 gene=Cs8g08320
Category:3
Score=4.5
Cleavage Site=584

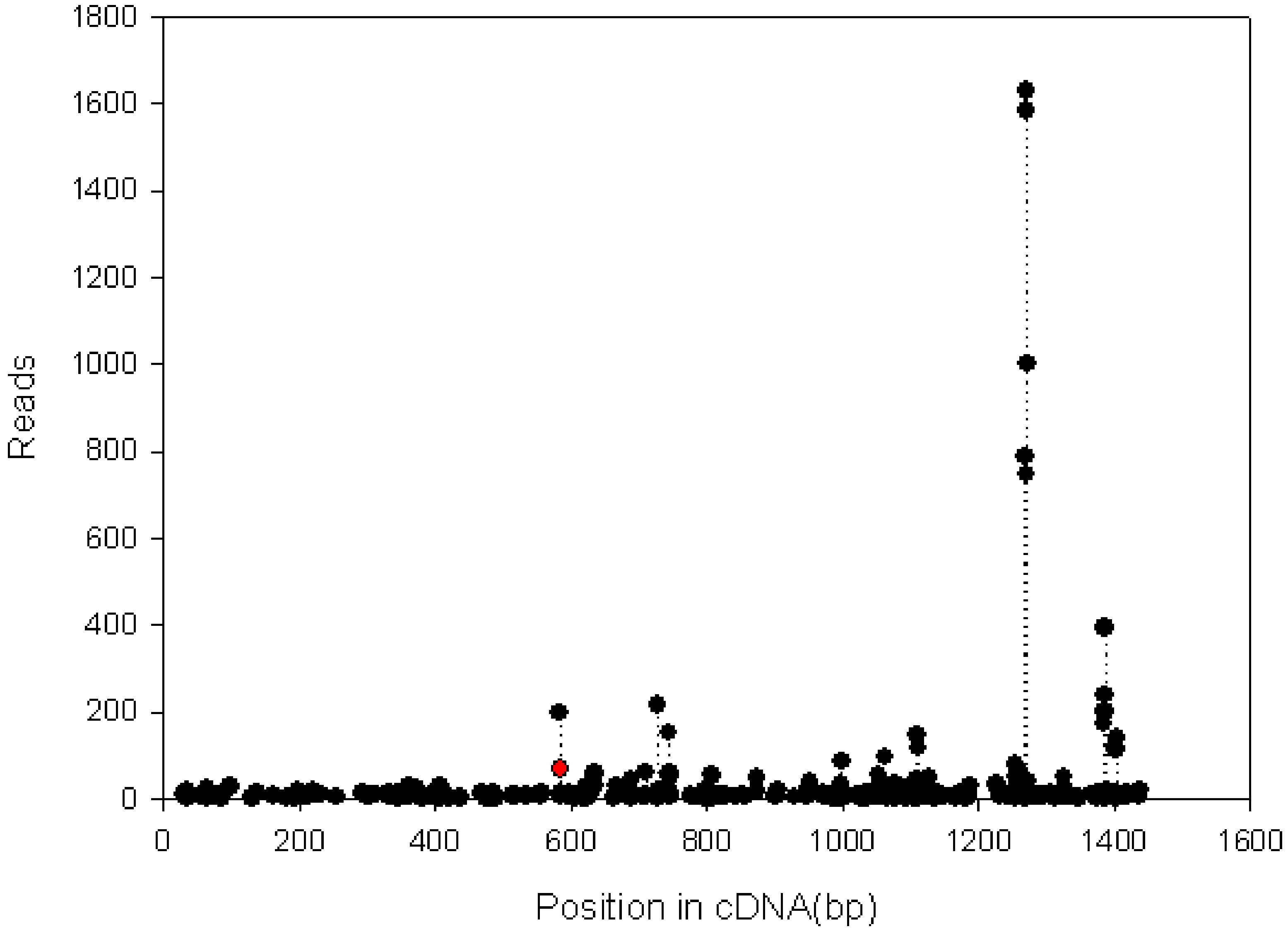


```
5' UUCAUGCGGAUCAUGCUGG-AGUUUCA 3'       Cs8g08320.1
          .:::::::::::: ::.::::
3' ------UUCUAGUACGACCGUCGAAGU 5'       Csi-miR167b.4
```

Csi-miR167d.1, target=Cs8g08320.1 gene=Cs8g08320
Category=3
Score=4.5
Cleavage Site=584

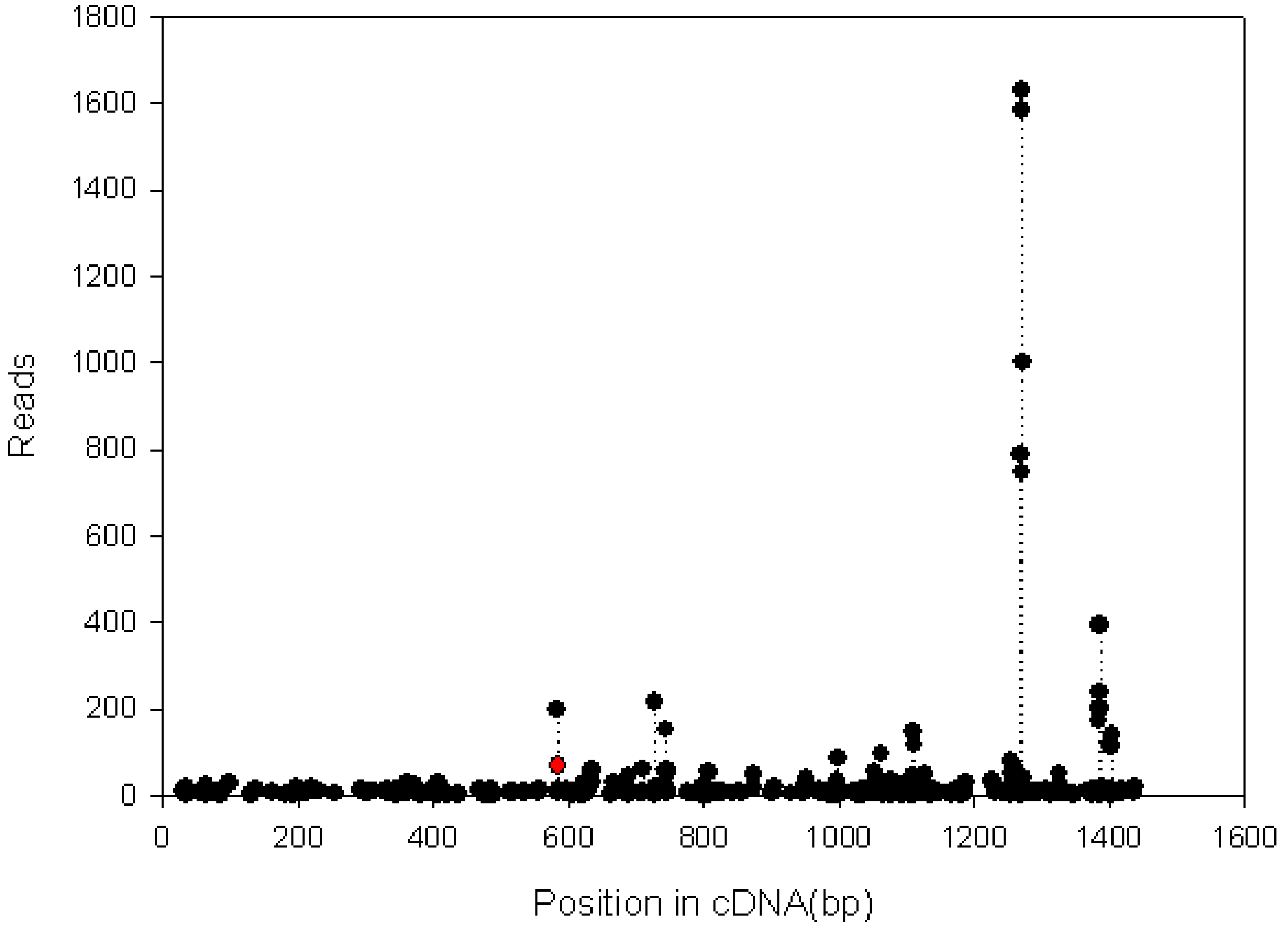


```
5' UUCAUGCGGAUCAUGCUGG-AGUUUCA 3'        Cs8g08320.1
         :.:::::::::: ::.::::
3' -----AGUCUAGUACGACCGUCGAAGU 5'        Csi-miR167d.1
```

Csi-miR167d.2, target=Cs2g15130.1 gene=Cs2g15130
Category=1
Score=5
Cleavage Site=3444

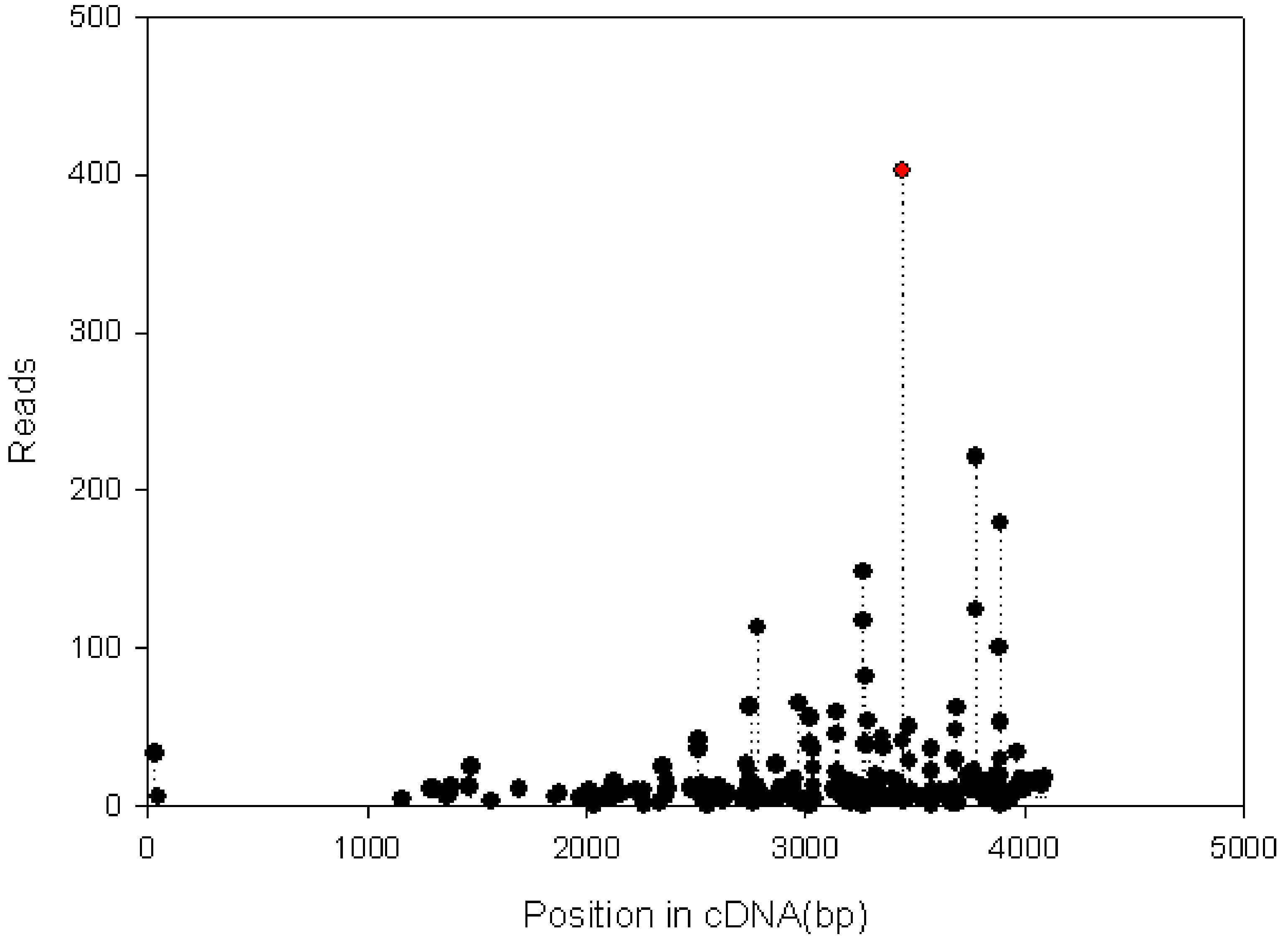


```
5' AGAGAUCAGGCUGGCAGCUUGUAUUU 3'        Cs2g15130.1
     :::::: ::::::::::: .:
3' -GUCUAGUACGACCGUCGAA-GU--- 5'        Csi-miR167d.2
```

Csi-miR167d.2, target=Cs8g08320.1 gene=Cs8g08320
Category:3
Score=3.5
Cleavage Site=584

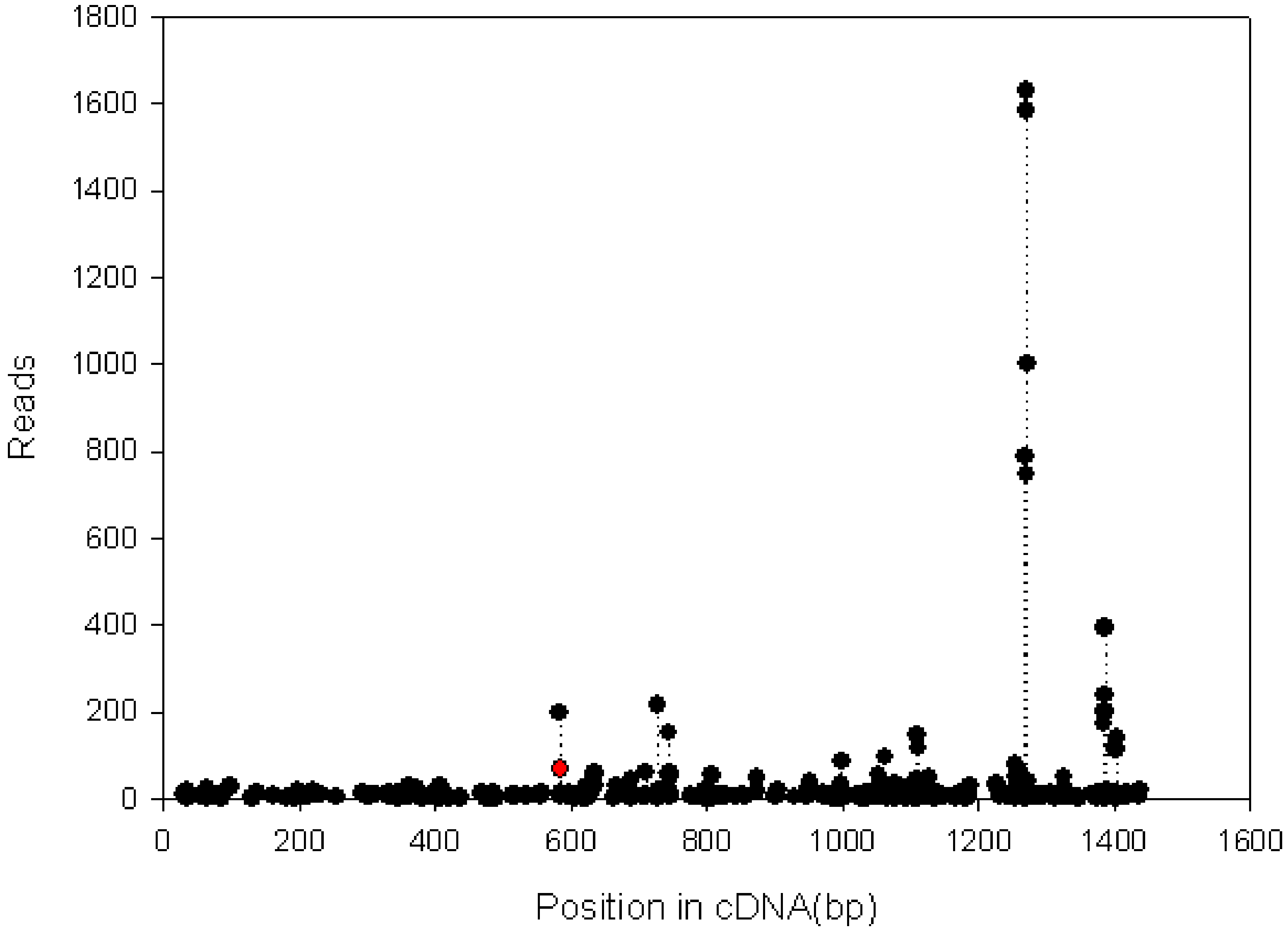


```
5' UUCAUGCGGAUCAUGCUGG-AGUUUCA 3'        Cs8g08320.1
          :.:::::::::::: ::.::::
3' ------GUCUAGUACGACCGUCGAAGU 5'        Csi-miR167d.2
```

Csi-miR168a, target=Cs5g16710.1 gene=Cs5g16710
Category=3
Score=5
Cleavage Site=797

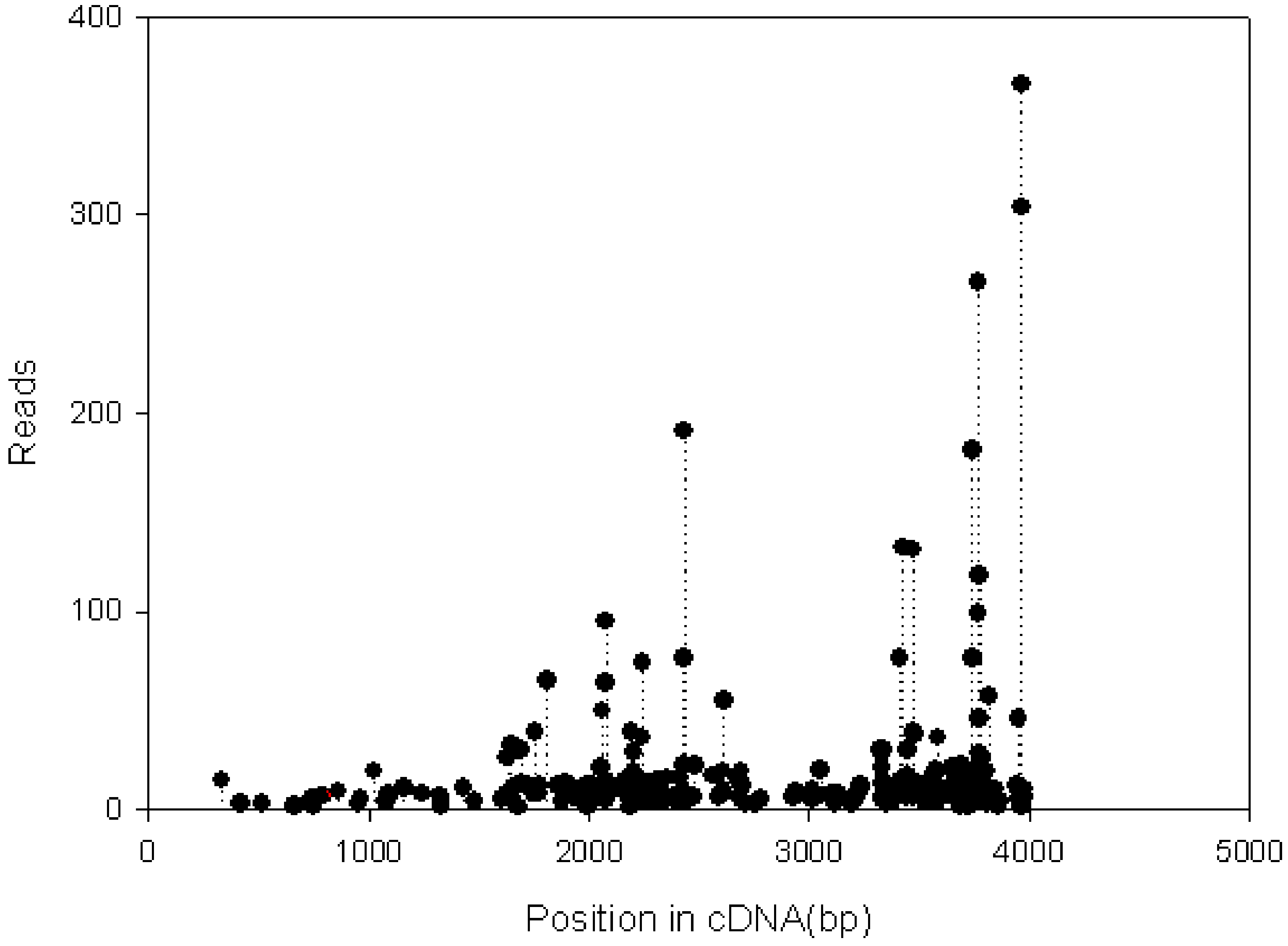


```
5' AUUCCCGAGCUACACCAAGCAACCCC 3'        Cs5g16710.1
    ::::::: :: :::::::: :
3' -AAGGGCUGGACGUGGUUCGCU---- 5'        Csi-miR168a
```

Csi-miR169b.1, target=Cs6g13560.1 gene=Cs6g13560
Category=1
Score=3.5
Cleavage Site=1307

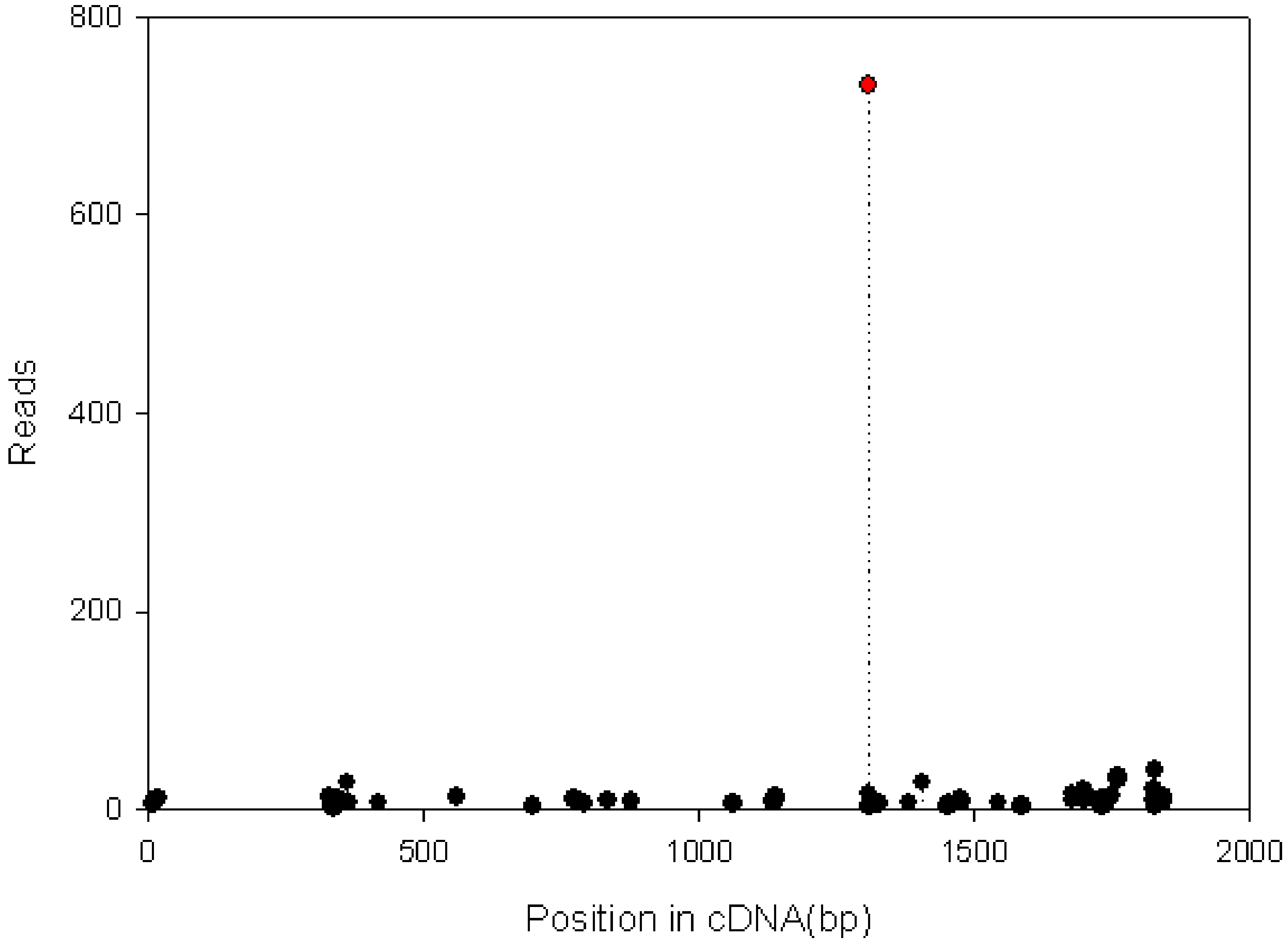


```
5' UUAGGCAAAUCAUUCUUGGCUCAUCU 3'        Cs6g13560.1
    .:::::: ::::.:::::::
3' -GUCCGUUCAGUAGGAACCGAU---- 5'        Csi-miR169b.1
```

Csi-miR169b.1-3p, target=Cs2g28180.1 gene=Cs2g28180
Category:3
Score=5
Cleavage Site=546

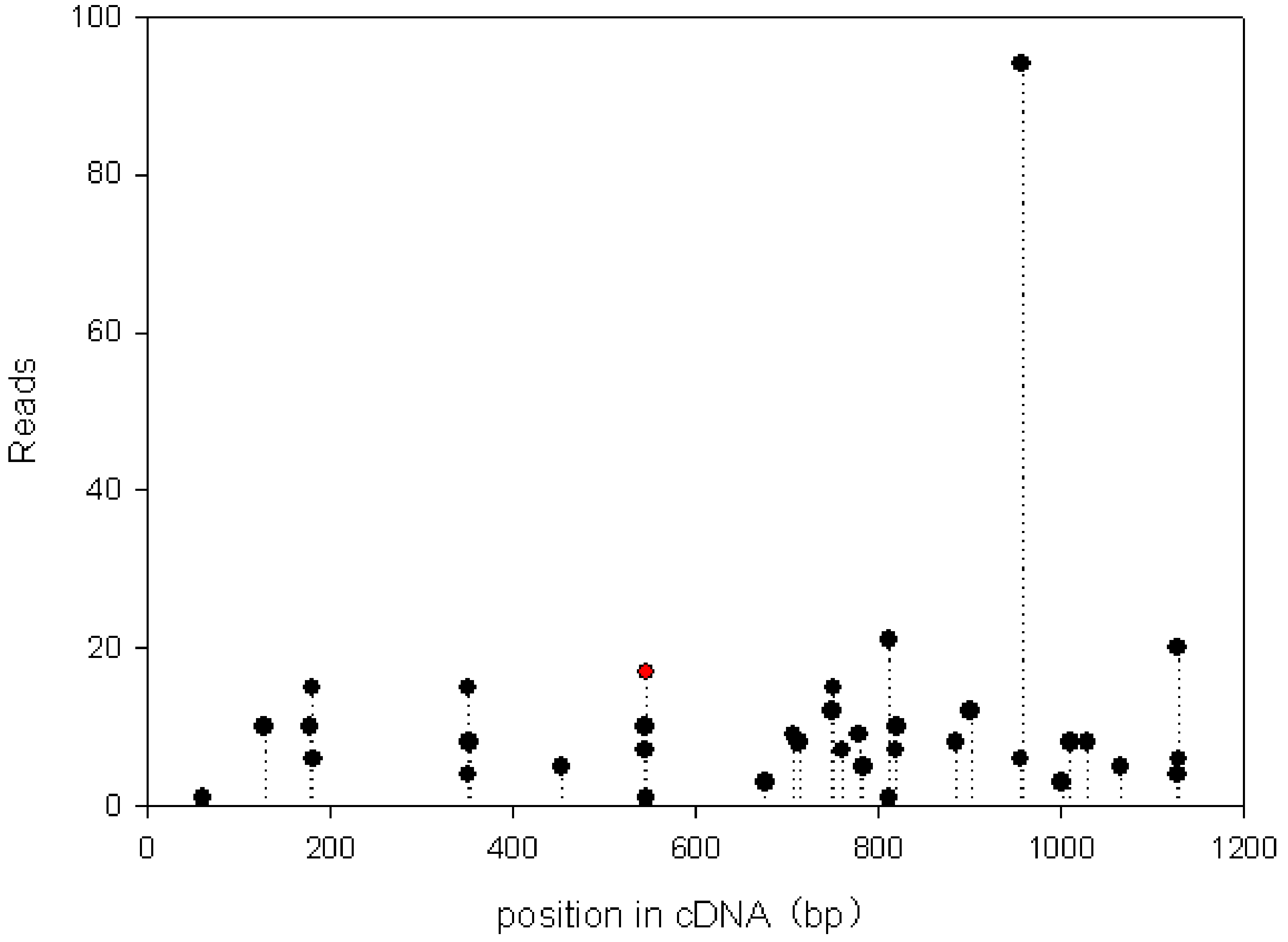


```
5' UUCCCCUU-GCCACUGAGACUGCCUAU 3'        Cs2g28180.1
        ::: ::::  ::::::::::
3' -----GAAUCGGUUCCUCUGACGGA-- 5'        Csi-miR169b.1-3p
```

Csi-miR169b.1-3p, target=Cs9g16380.1 gene=Cs9g16380
Category=3
Score=5
Cleavage Site=619

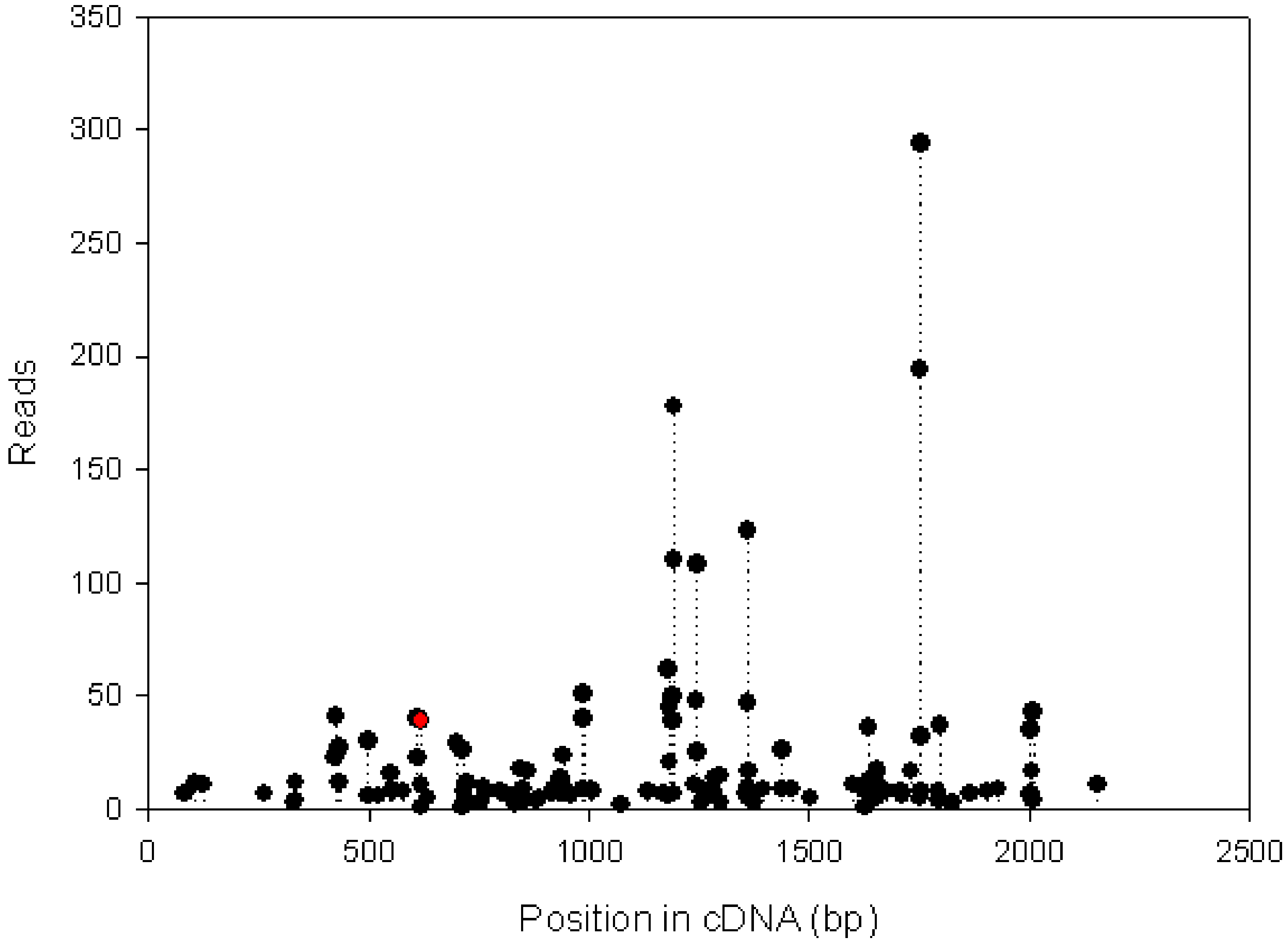


```
5' GACAUCGCCAAGGGGAUUGCUUACCU 3'        Cs9g16380.1
     : : :::::::.::.::::.:
3' --GAAUCGGUUCCUCUGACGGA---- 5'        Csi-miR169b.1-3p
```

Csi-miR169i.1, target=Cs6g13560.1 gene=Cs6g13560
Category=1
Score=3.5
Cleavage Site=1307

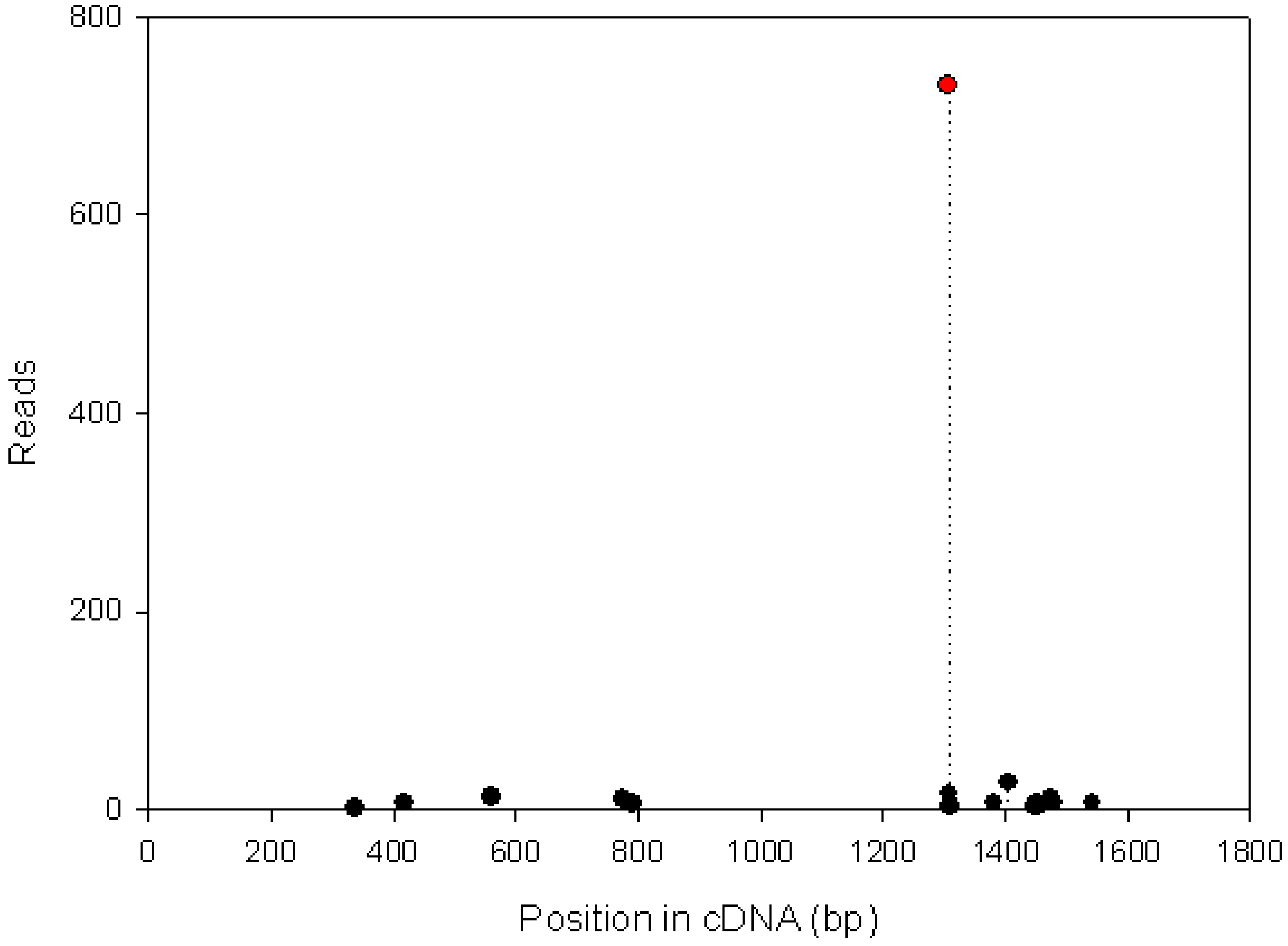


```
5' UUAGGCAAAUCAUUCUUGGCUCAUCU 3'        Cs6g13560.1
   :.:::::: :::::.:::::::
3' AGUCCGUUCAGUAGGAACCGAU---- 5'        Csi-miR169i.1
```

Csi-miR169i.1-3p, target=Orange1.1t00423.1 gene=Orange1.1t00423
Category=3
Score=4.5
Cleavage Site=1434

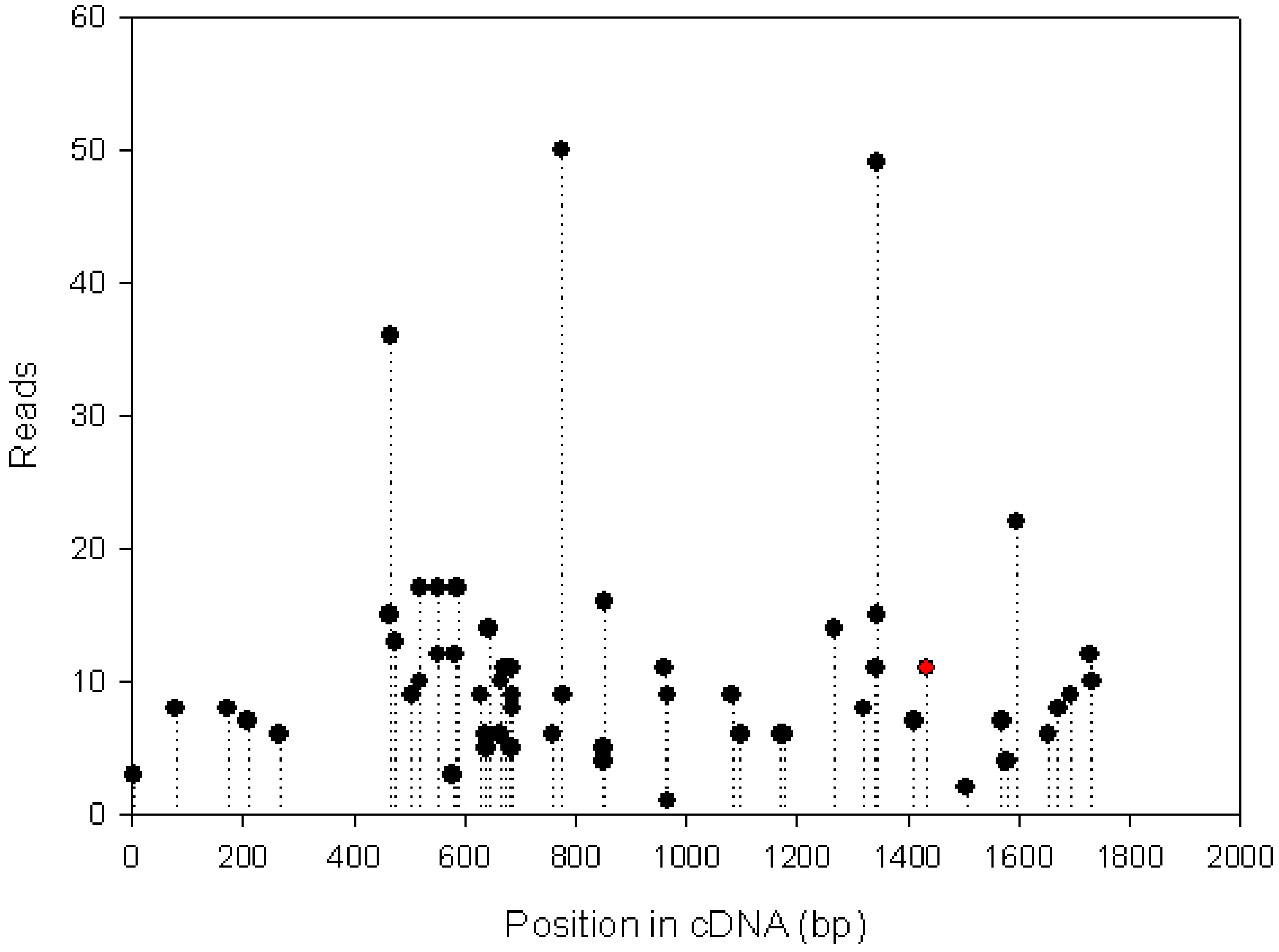


```
5' AGGUGGCCAUGGAGACUGCAUCUCAA 3'        Orange1.1t00423.1
    : :.:::: :::::::::
3' -CAAUCGGUUCCUCUGACGG------ 5'        Csi-miR169i.1-3p
```

Csi-miR169m.1, target=Cs3g24090.1 gene=Cs3g24090
Category=1
Score=5
Cleavage Site=1205

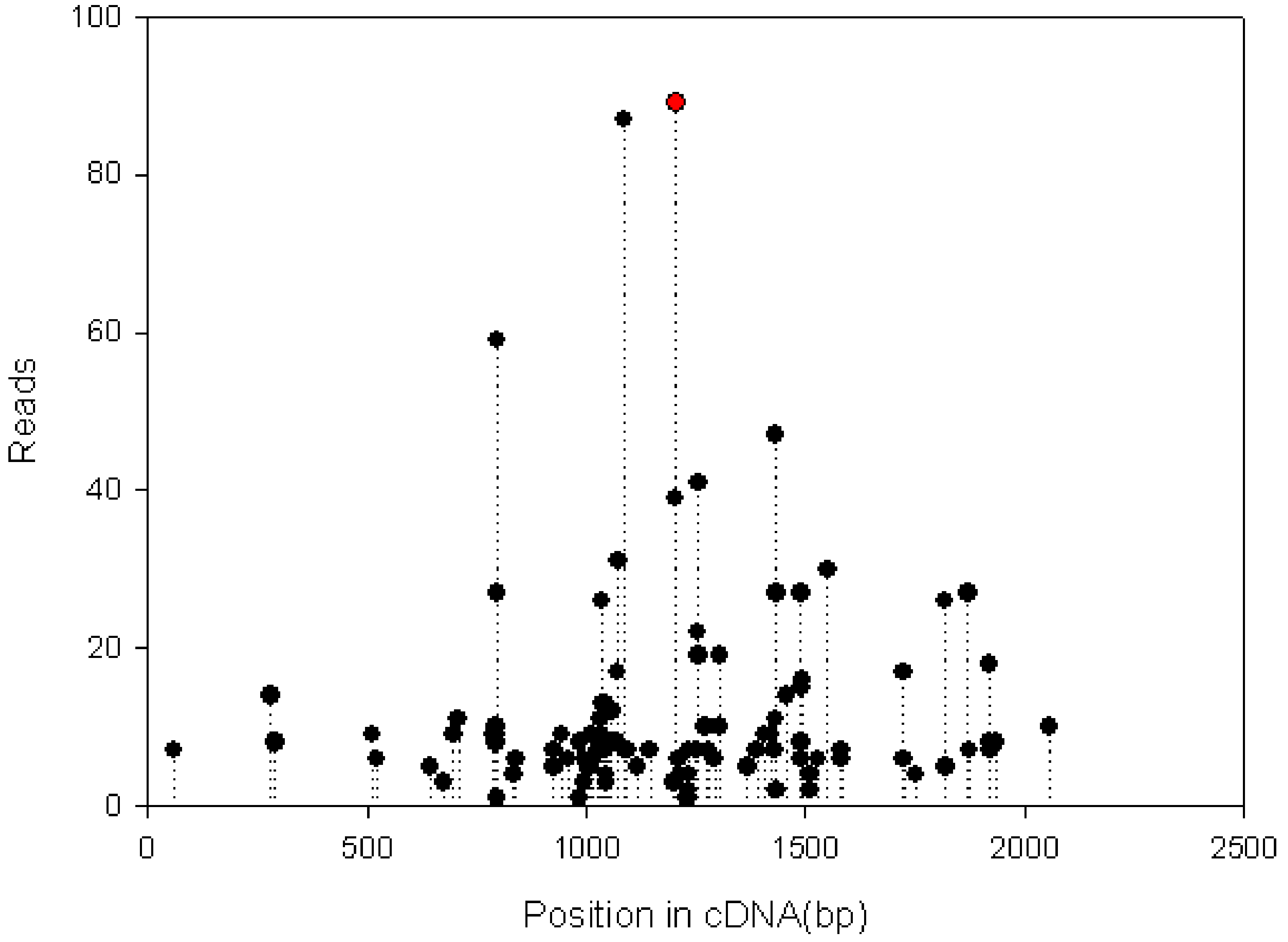


```
5' UUUGUUGUUCAAGUCGUUCUUGGCUG 3'        Cs3g24090.1
       ..:   ::::::.:.::::::::
3' ----GGC-CGUUCAGUAGGAACCGAC 5'        Csi-miR169m.1
```

Csi-miR169m.1, target=Cs6g13560.1 gene=Cs6g13560
Category=1
Score=4.5
Cleavage Site=1307

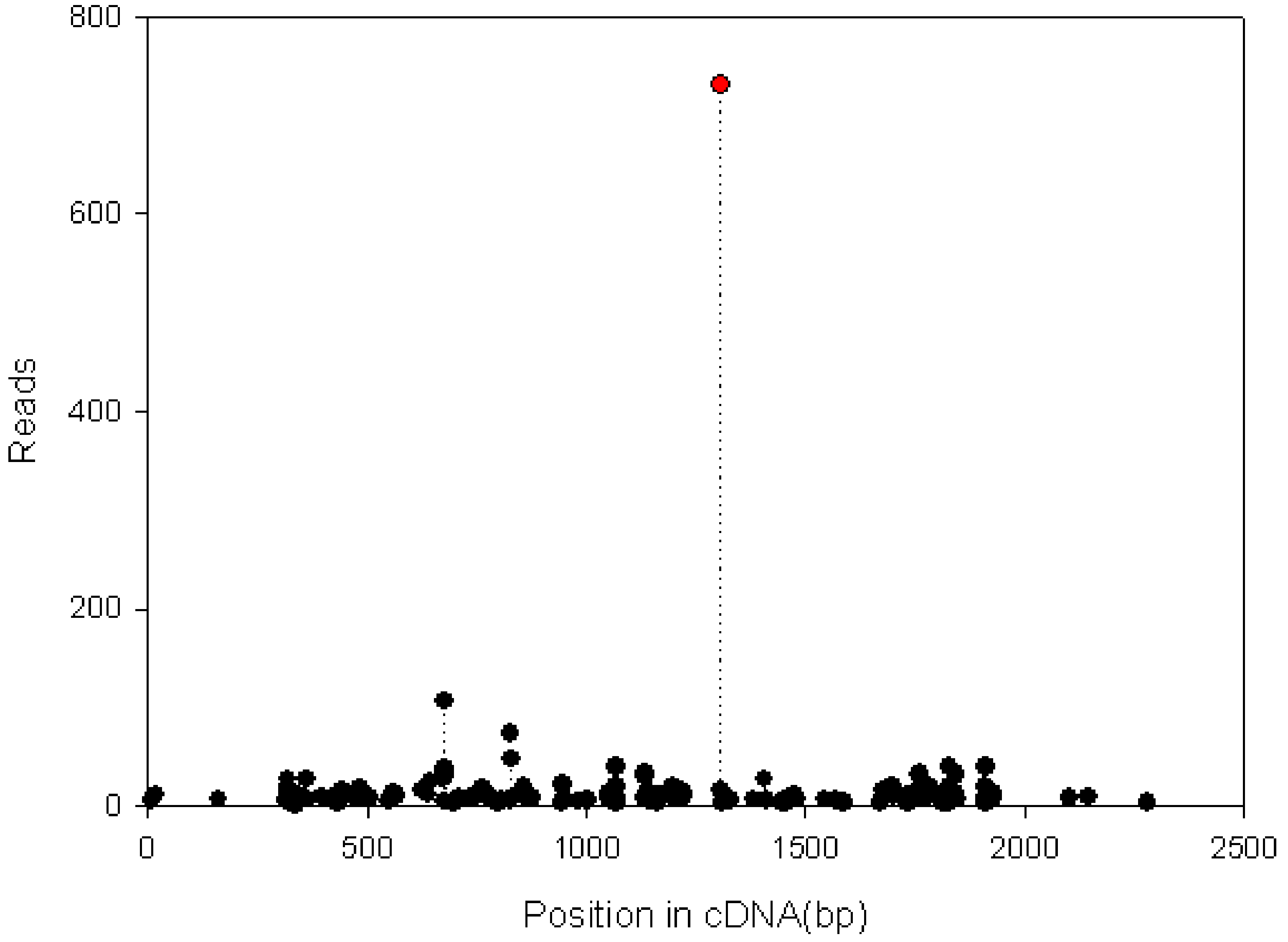


```
5' UUAGGCAAAUCAUUCUUGGCUCAUCU 3'        Cs6g13560.1
    . ::::: ::::.::::::::
3' -GGCCGUUCAGUAGGAACCGAC---- 5'        Csi-miR169m.1
```

Csi-miR169m.2, target=Cs3g24090.1 gene=Cs3g24090
Category=1
Score=5
Cleavage Site=1205

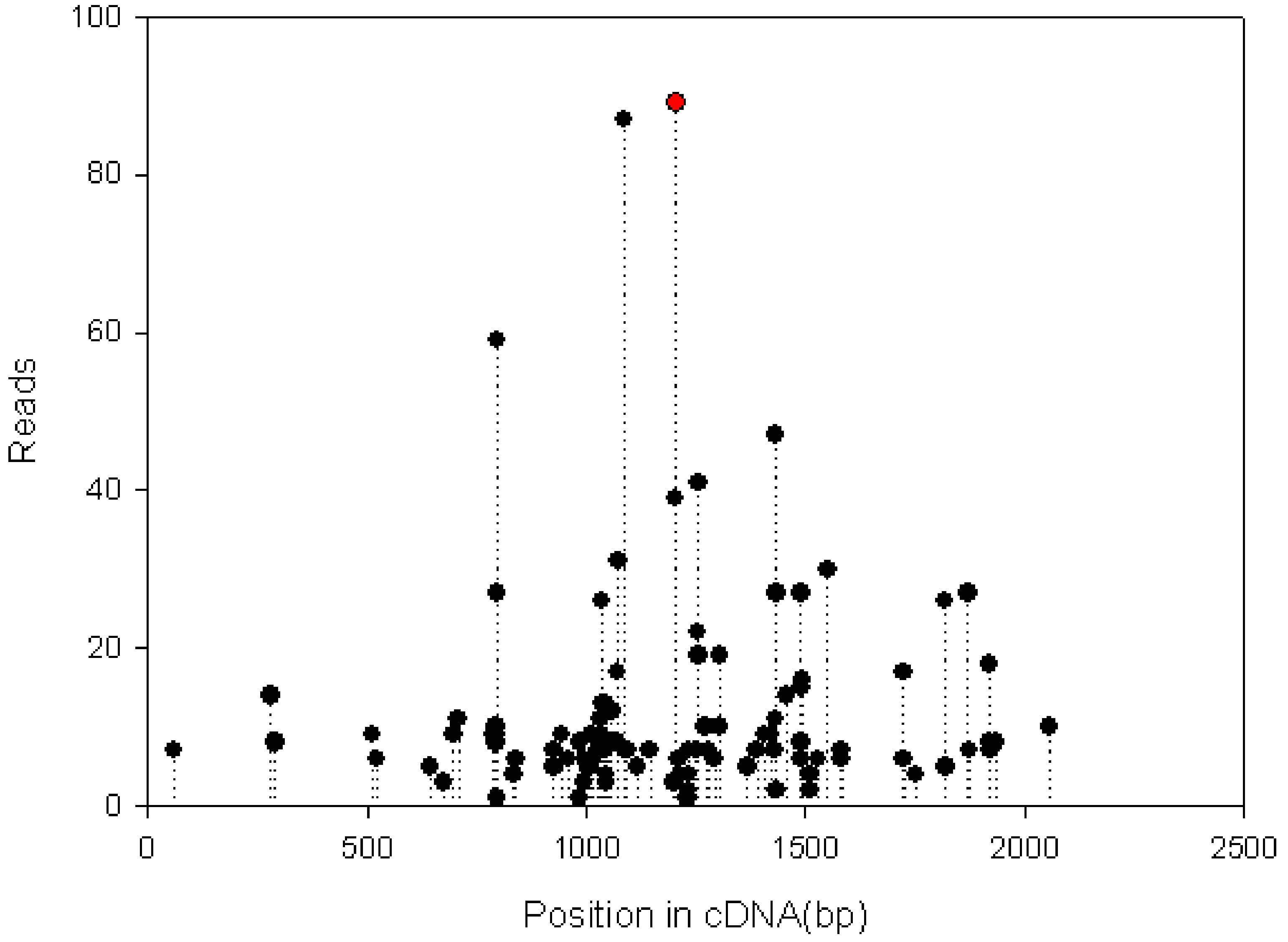


```
5' UUUGUUGUUCAAGUCGUUCUUGGCUG 3'        Cs3g24090.1
       :..:  ::::::.:.:::::::
3' ---CGGC-CGUUCAGUAGGAACCGA- 5'        Csi-miR169m.2
```

Csi-miR169m.3, target=Cs7g06000.1 gene=Cs7g06000
Category=3
Score=5
Cleavage Site=2907

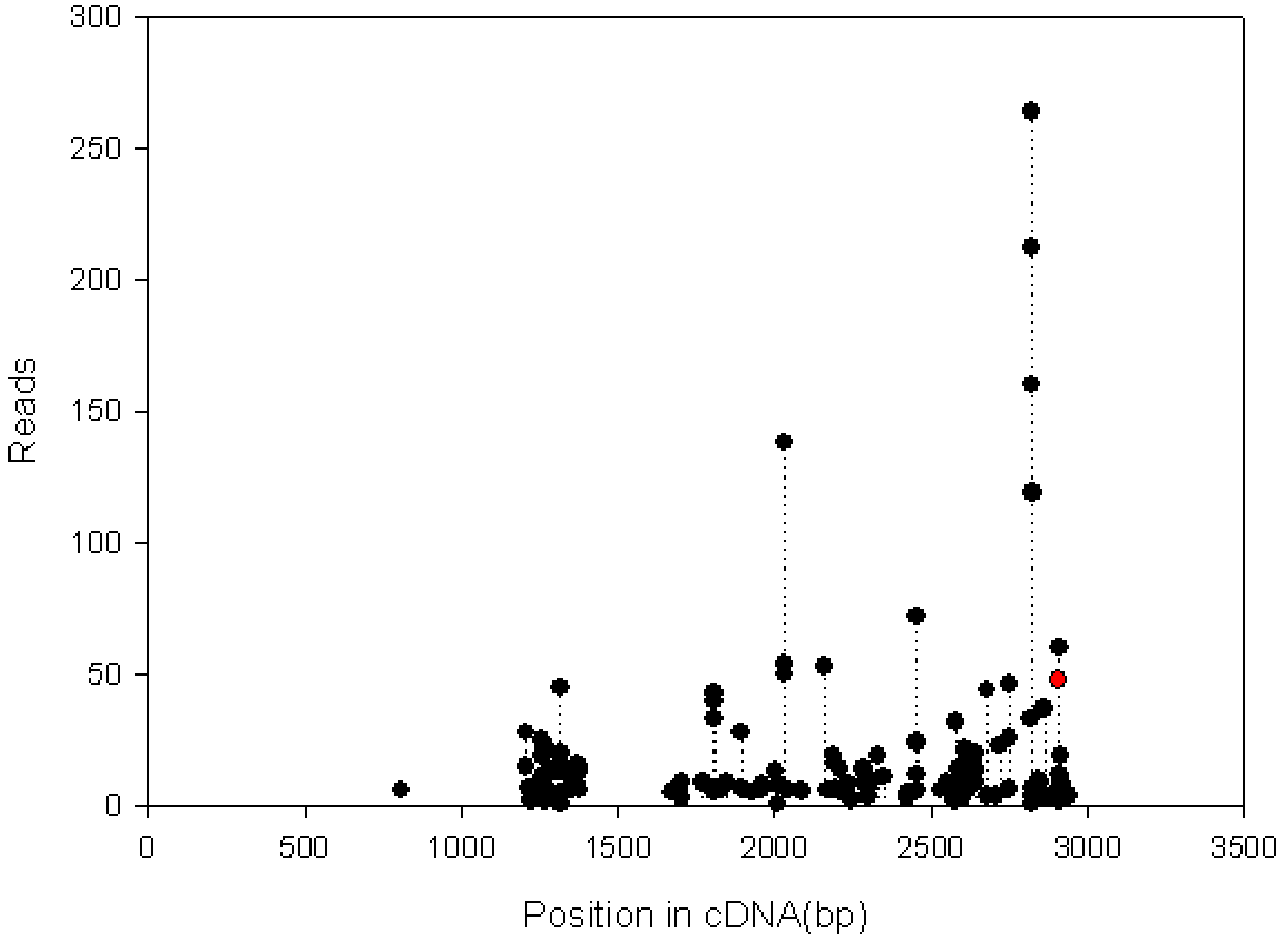


```
5' UGGGAGGAUAUCCUUGGCUGGAUUGU 3'        Cs7g06000.1
   .:: :.: .:::::::::::
3' GCCGUUCAGUAGGAACCGACG----- 5'        Csi-miR169m.3
```

Csi-miR171a.1, target=Cs2g24890.1 gene=Cs2g24890
Category=3
Score=5
Cleavage Site=778

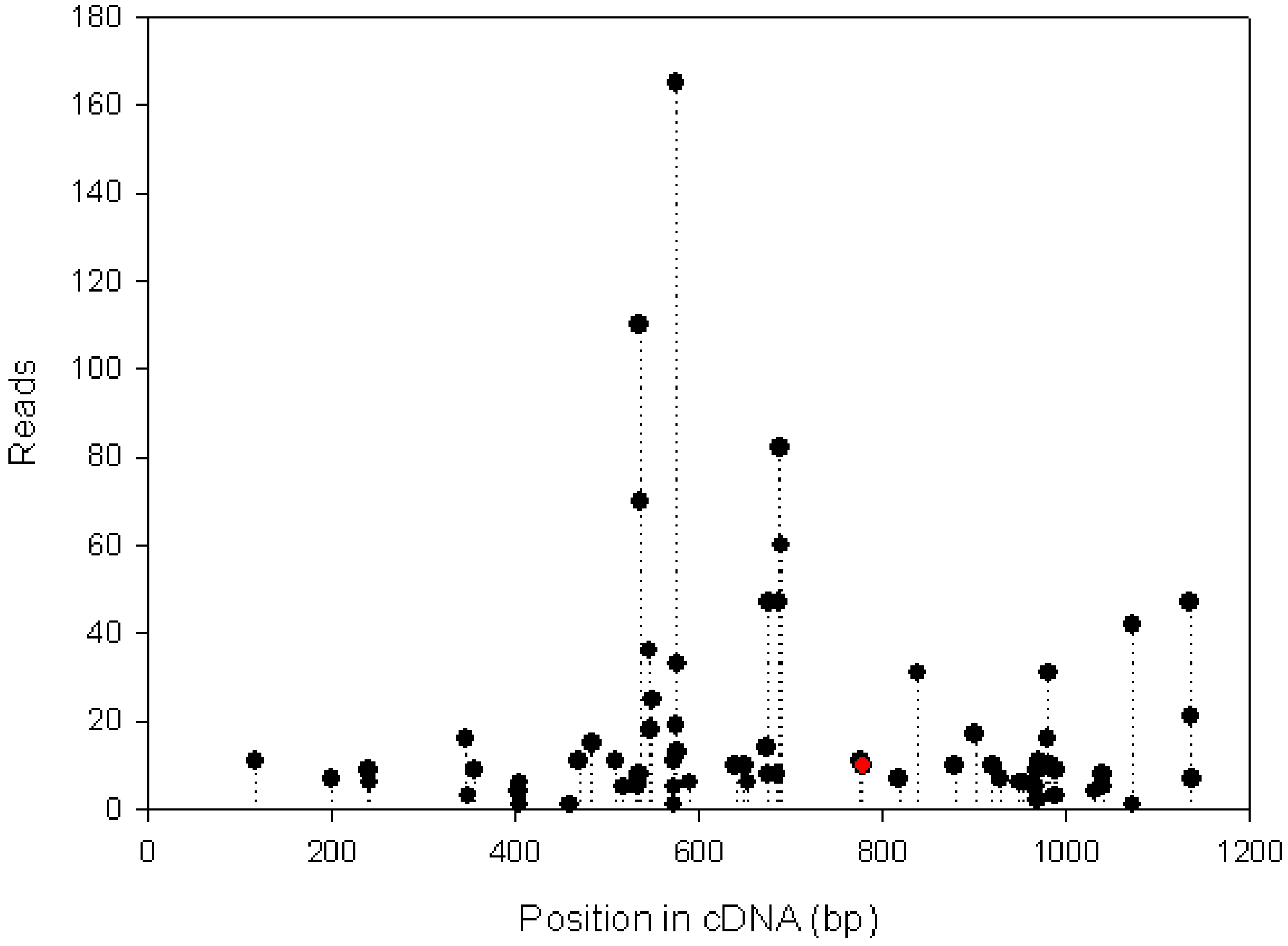


```
5' ACGUUUGAUUGCAGCUAUUCCAGCAU 3'        Cs2g24890.1
     :::::::: :::. :::::.::
3' ---AAACUAAC-UCGGCAAGGUUGU- 5'        Csi-miR171a.1
```

Csi-miR171b, target=Cs4g09320.1 gene=Cs4g09320
Category=3
Score=4
Cleavage Site=527

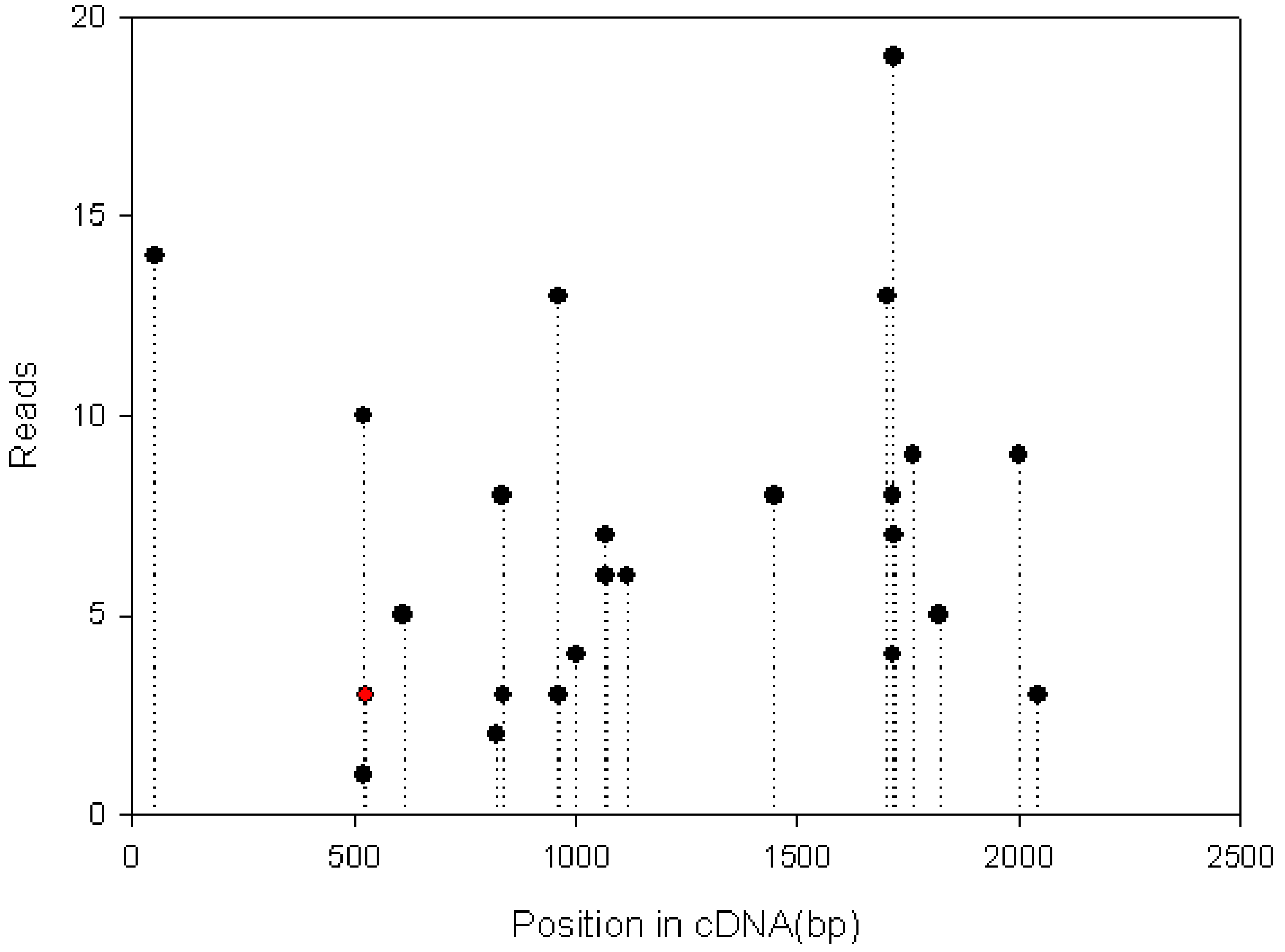


```
5' AAGAGCGAGAAUUGAUUCGGCUUGAG 3'        Cs4g09320.1
     ::: ::  ::::::::::::.:
3' --CUCACU-AUAACUAAGCCGAGC-- 5'        Csi-miR171b
```

Csi-miR171d, target=Cs9g19410.1 gene=Cs9g19410
Category=3
Score=4.5
Cleavage Site=1275

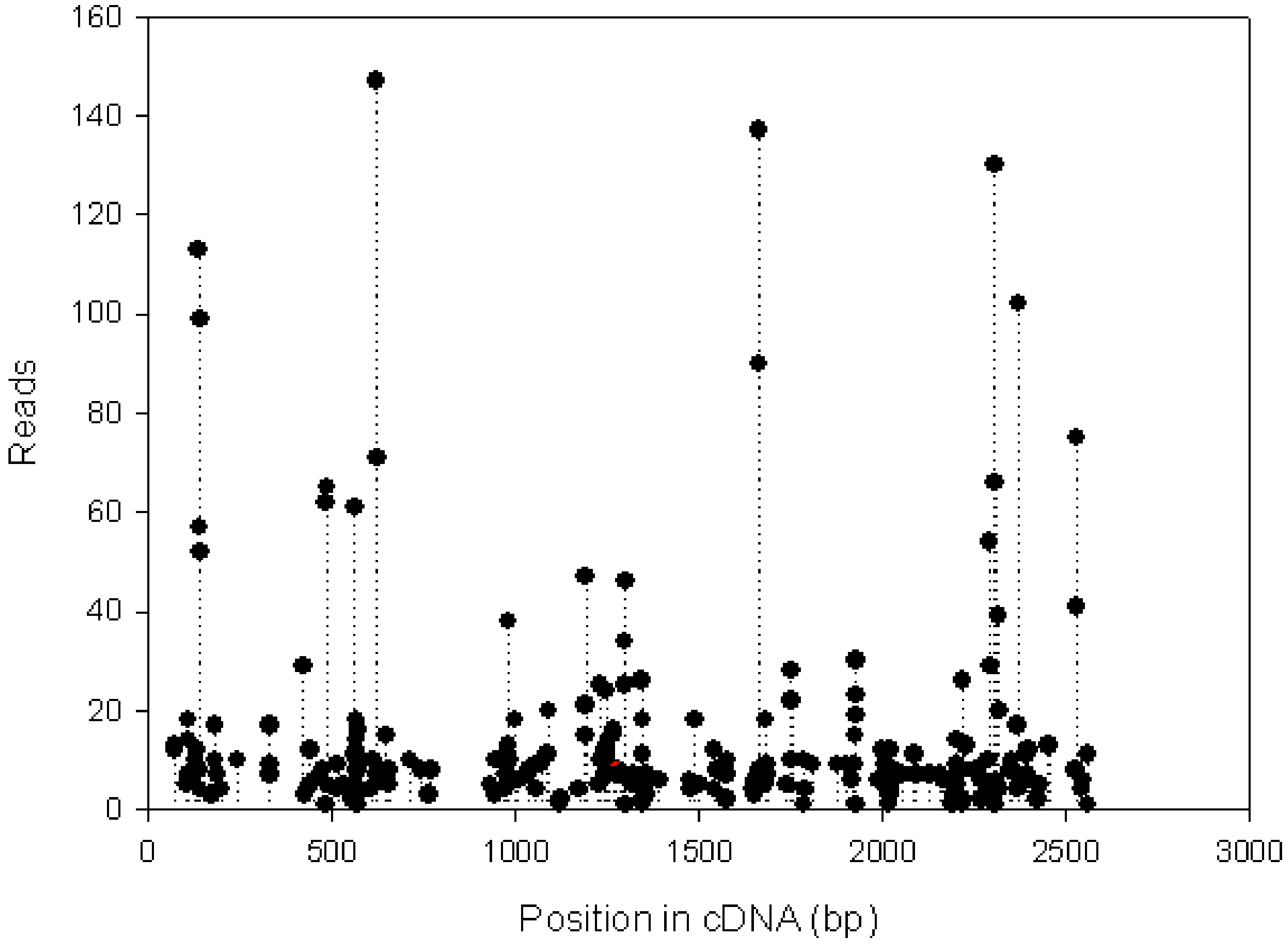


```
5' CGAGAUCUUGACGCGGCUGAGCCAGG 3'        Cs9g19410.1
    ::::: :::::::::::: :.
3' CCUCUAUAACUGCGCCGAGUU----- 5'        Csi-miR171d
```

Csi-miR171g.1, target=Orange1.1t00199.1 gene=Orange1.1t00199
Category=1
Score=1
Cleavage Site=1615

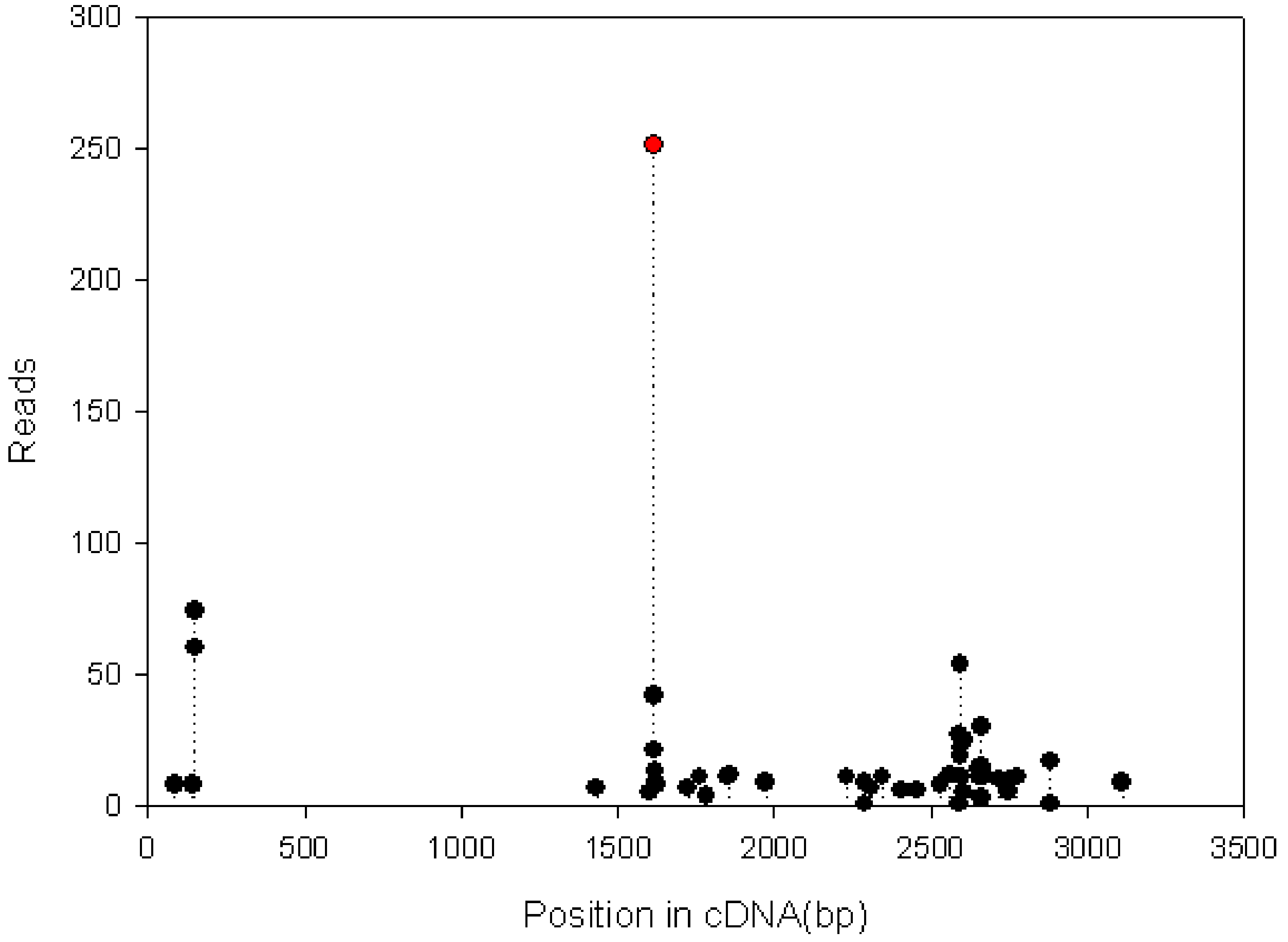


```
5' GGAUAUUGGCGCGGCUCAAUCACCAG 3'        Orange1.1t00199.1
    ::::::::::.:::::::::::
3' -CUAUAACCGUGCCGAGUUAGU---- 5'        Csi-miR171g.1
```

Csi-miR171g.1, target=Orange1.1t00200.1 gene=Orange1.1t00200
Category:1
Score=1
Cleavage Site=1514

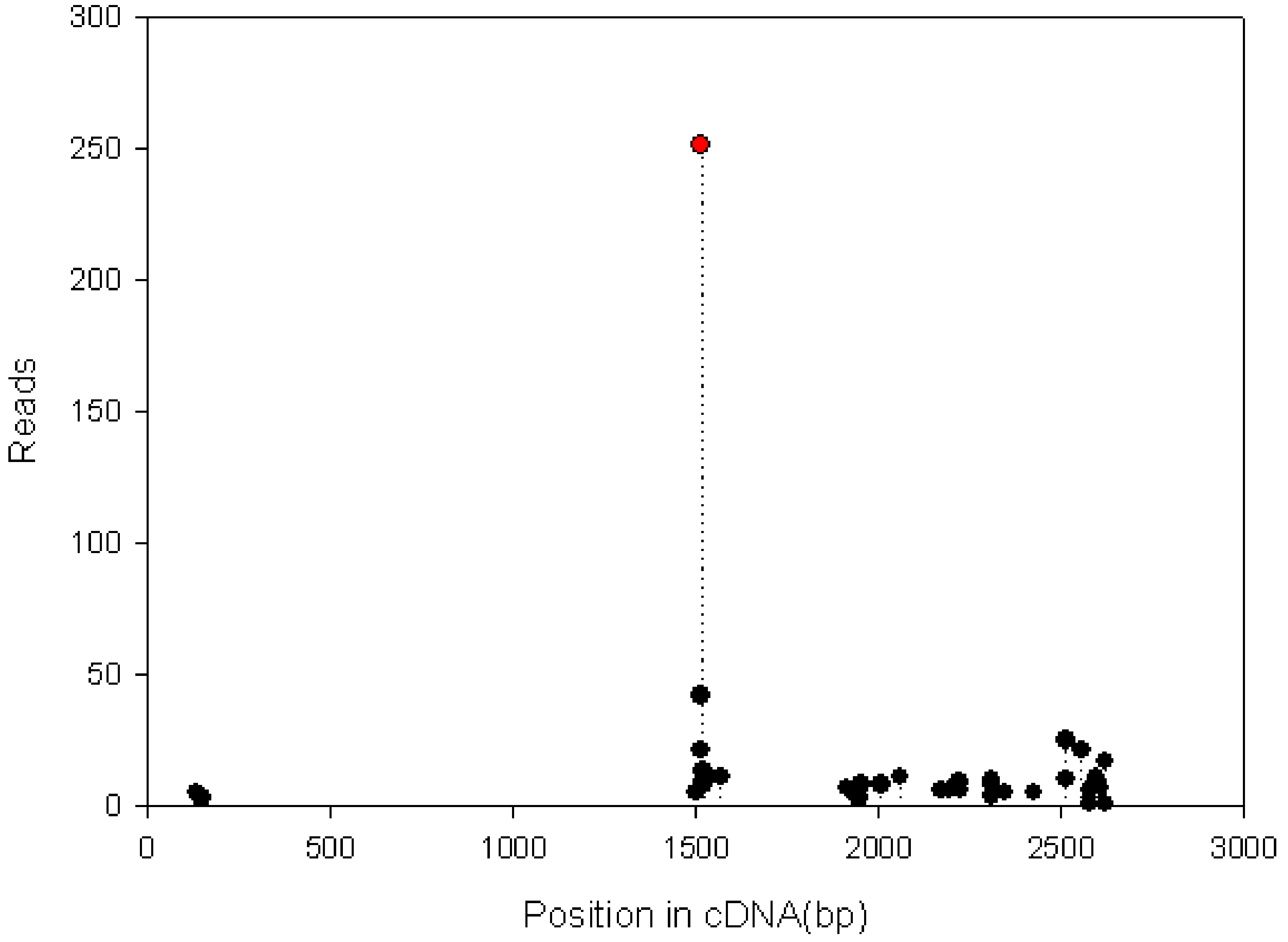


```
5' GGAUAUUGGCGCGGCUCAAUCACCAG 3'        Orange1.1t00200.1
    ::::::::::.:::::::::::
3' -CUAUAACCGUGCCGAGUUAGU---- 5'        Csi-miR171g.1
```

Csi-miR172a-3p.1, target=Cs8g17390.1 gene=Cs8g17390
Category:3
Score=2
Cleavage Site=1865

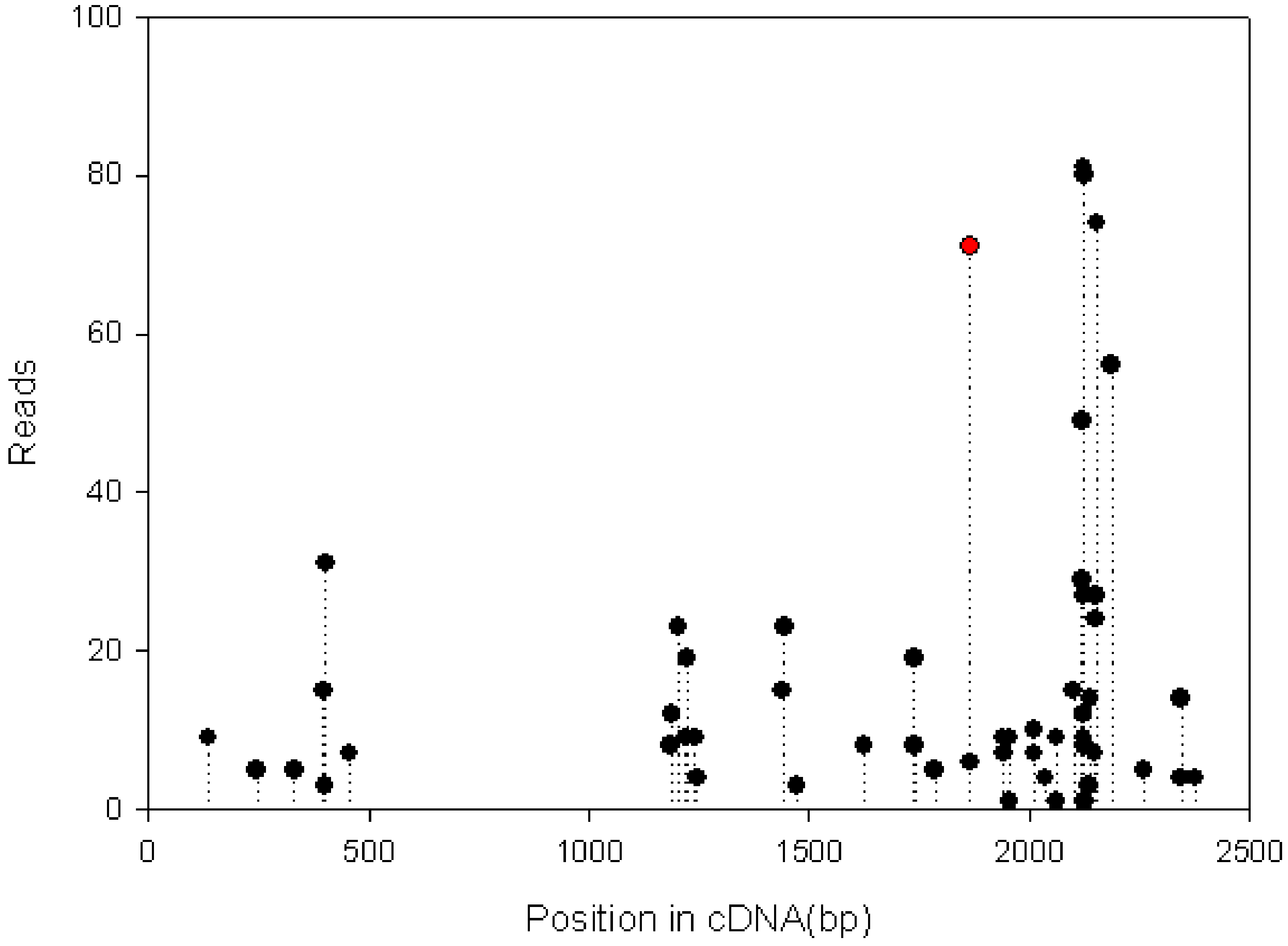


```
5' AGUGCAGCAUCAUCAGGAUUCCCAUU 3'        Cs8g17390.1
     ::::::::::::::::.:::::
3' --ACGUCGUAGUAGUUCUAAGA---- 5'        Csi-miR172a-3p.1
```

Csi-miR172a-3p.1, target=Orange1.1t04055.1 gene=Orange1.1t04055
Category=3
Score=2
Cleavage Site=2049

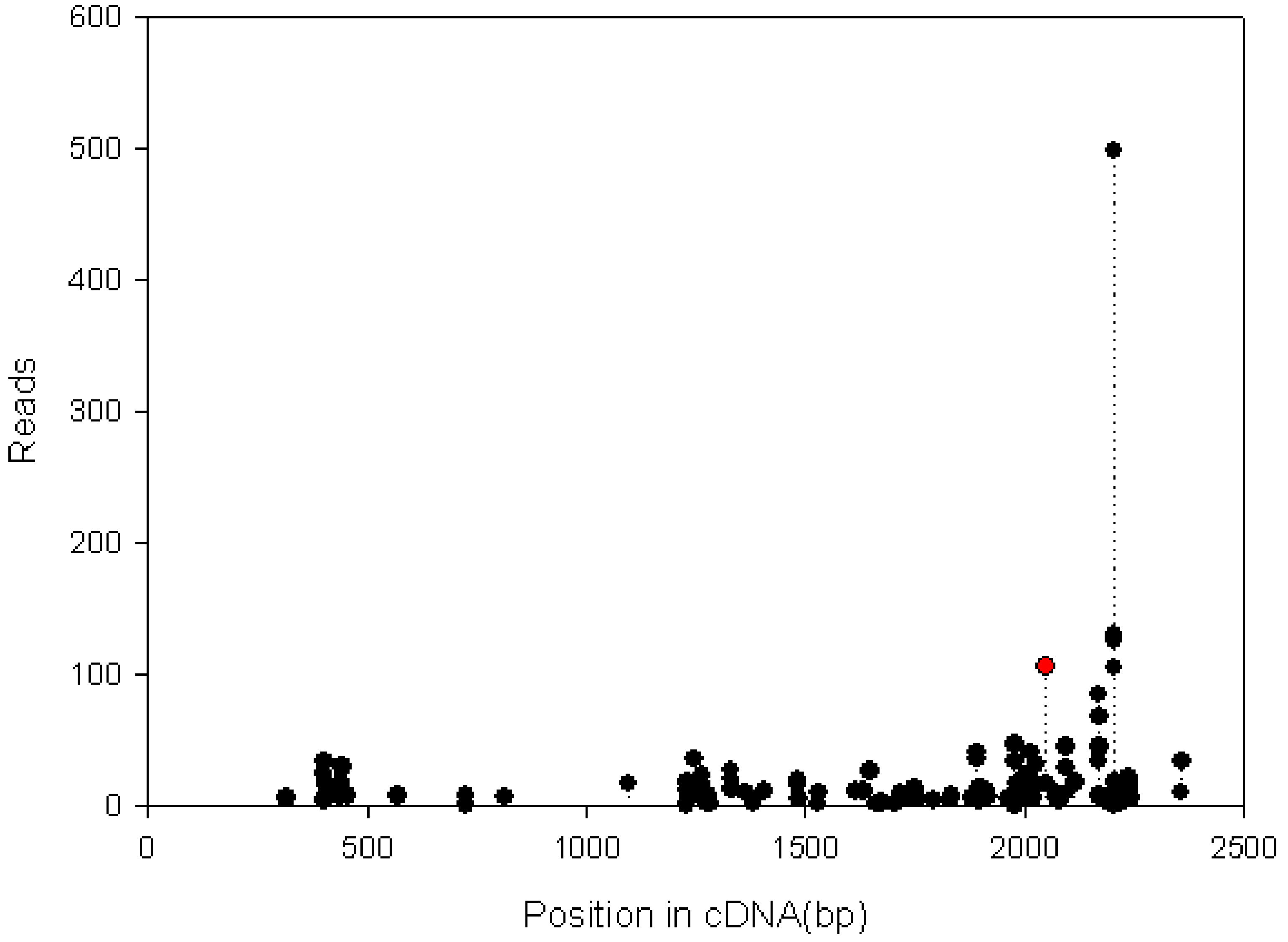


```
5' GCUGCAGCAUCAUCAGGAUUCCCUCA 3'        Orange1.1t04055.1
     ::::::::::::::::.:::::
3' --ACGUCGUAGUAGUUCUAAGA---- 5'        Csi-miR172a-3p.1
```

Csi-miR172a-3p.2, target=Cs8g17390.1 gene=Cs8g17390
Category:3
Score=3
Cleavage Site=1865

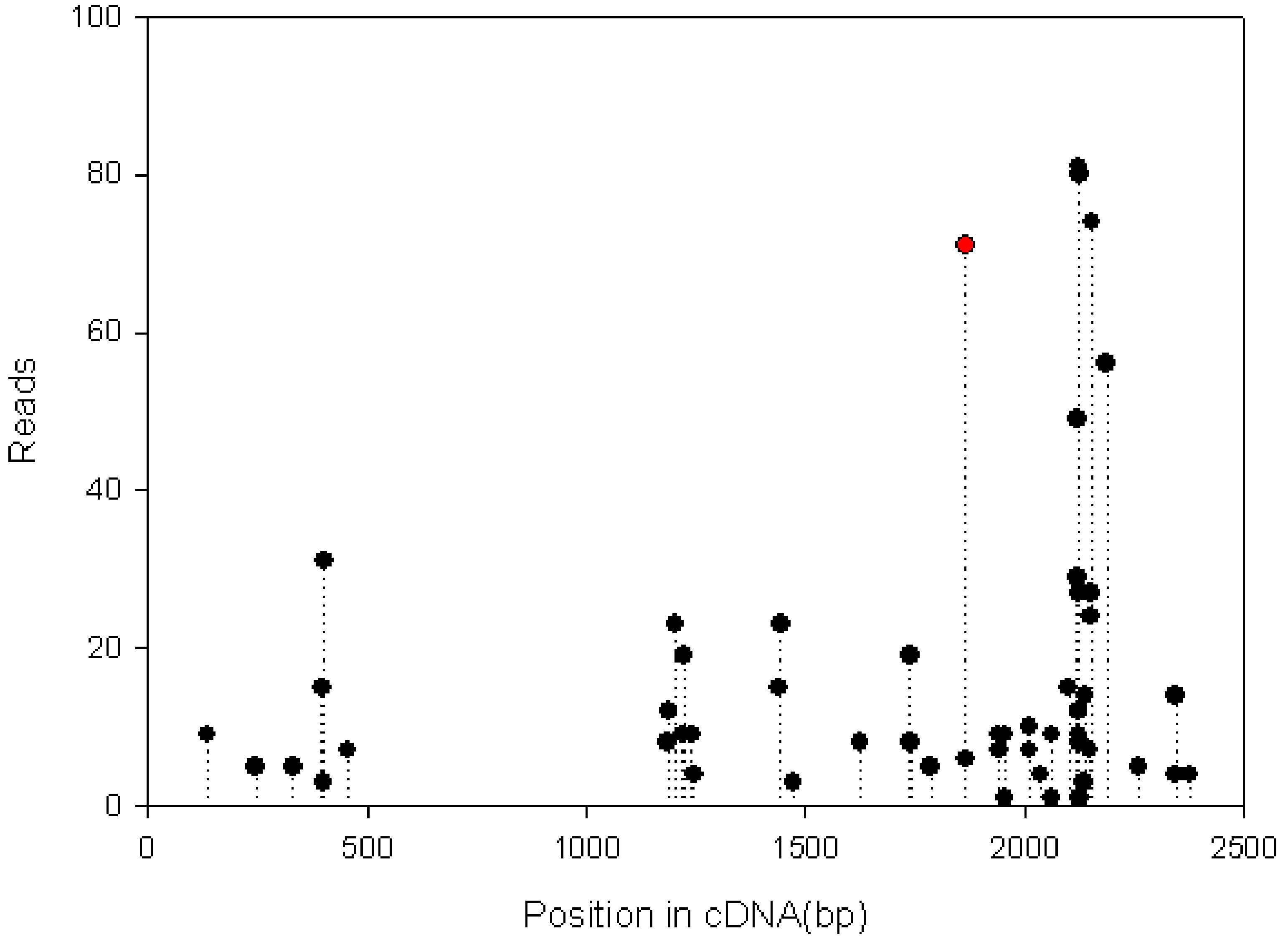


```
5' AGUGCAGCAUCAUCAGGAUUCCCAUU 3'        Cs8g17390.1
     ::::::::::::::.:::::
3' -AACGUCGUAGUAGUUCUAAGA---- 5'        Csi-miR172a-3p.2
```

Csi-miR172a-3p.2, target=Orange1.1t04055.1 gene=Orange1.1t04055
Category=3
Score=3
Cleavage Site=2049

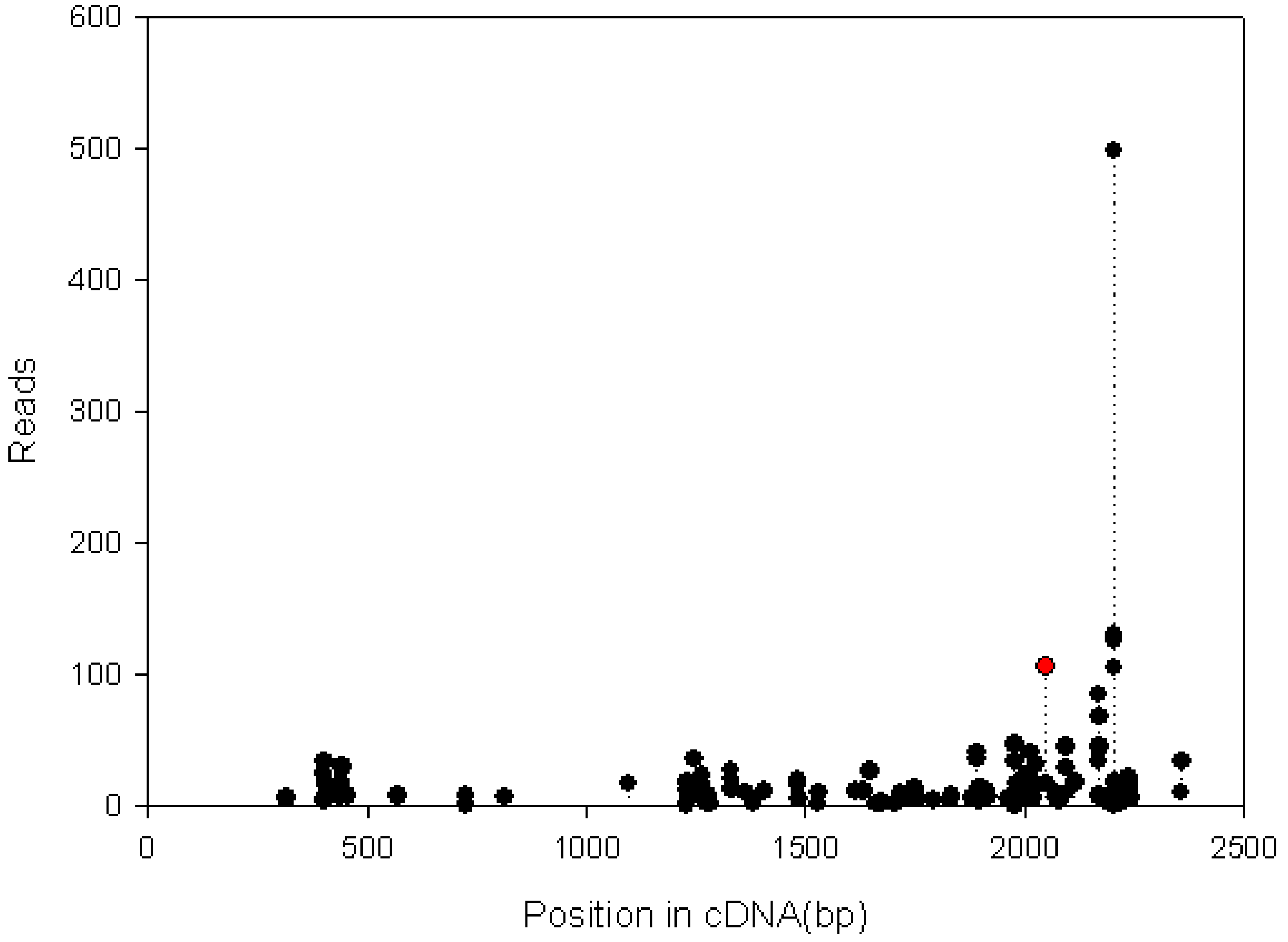


```
5' GCUGCAGCAUCAUCAGGAUUCCCUCA 3'        Orange1.1t04055.1
    :::::::::::::.:::::
3' -AACGUCGUAGUAGUUCUAAGA---- 5'        Csi-miR172a-3p.2
```

Csi-miR172a-3p.3, target=Cs8g17390.1 gene=Cs8g17390
Category:3
Score=2
Cleavage Site=1865

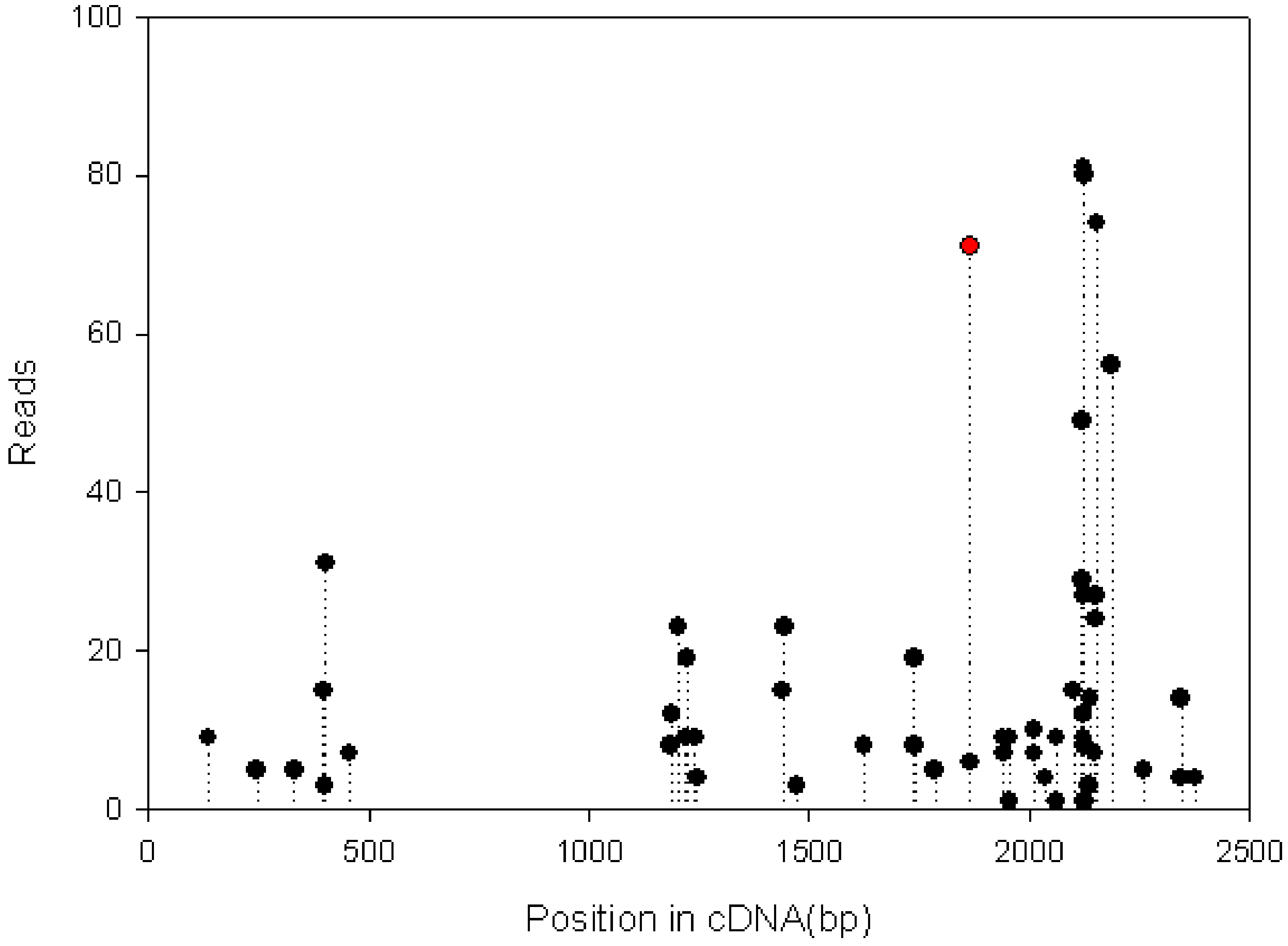


```
5' AGUGCAGCAUCAUCAGGAUUCCCAUU 3'        Cs8g17390.1
     ::::::::::::::.:::::
3' -AACGUCGUAGUAGUUCUAAG----- 5'        Csi-miR172a-3p.3
```

Csi-miR172a-3p.3, target=Orange1.1t04055.1 gene=Orange1.1t04055
Category=3
Score=2
Cleavage Site=2049

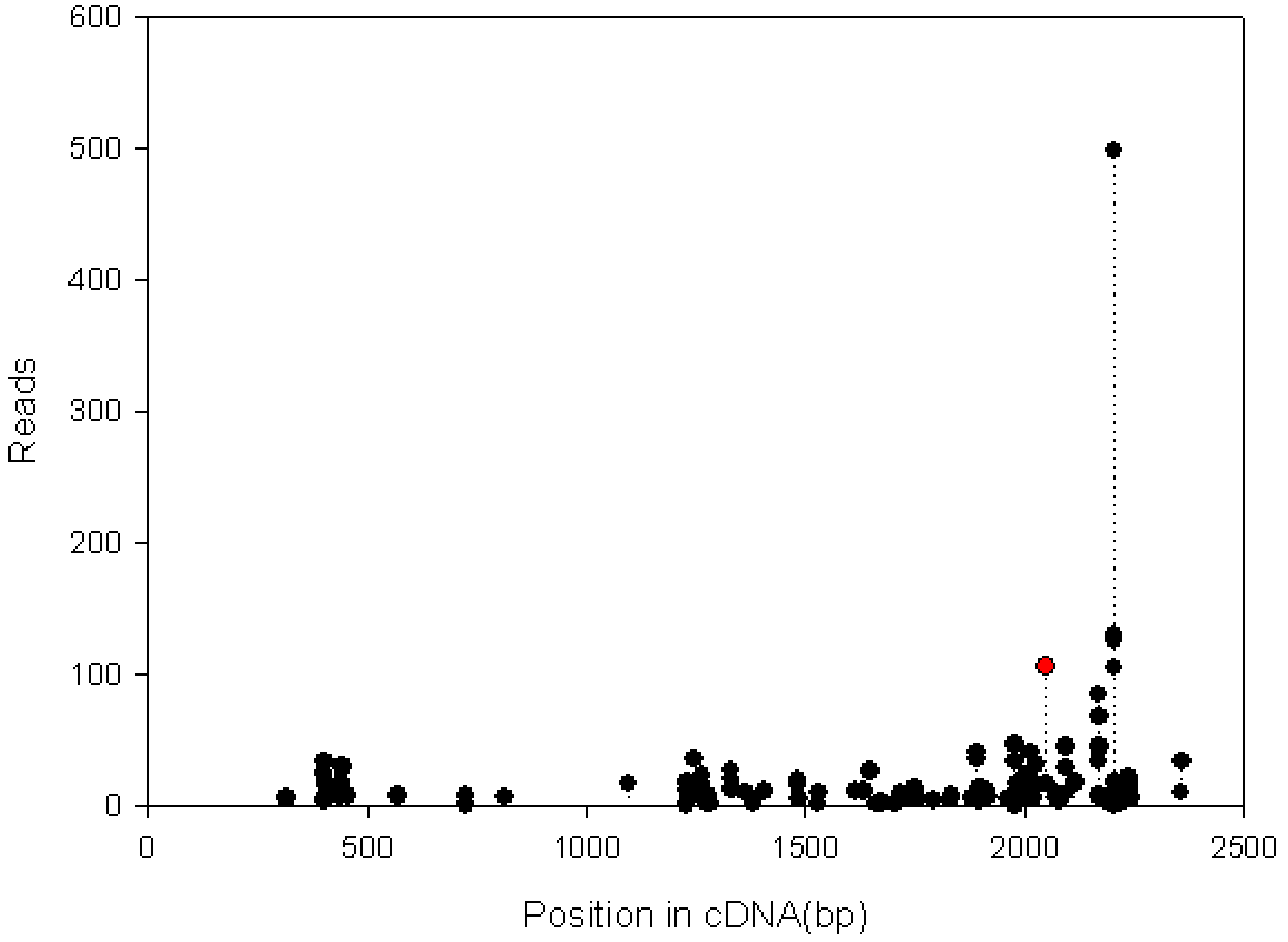


```
5' GCUGCAGCAUCAUCAGGAUUCCCUCA 3'        Orange1.1t04055.1
     ::::::::::::::.:::::
3' -AACGUCGUAGUAGUUCUAAG----- 5'        Csi-miR172a-3p.3
```

Csi-miR172c.1, target=Cs1g21180.1 gene=Cs1g21180
Category:3
Score=4
Cleavage Site=3253

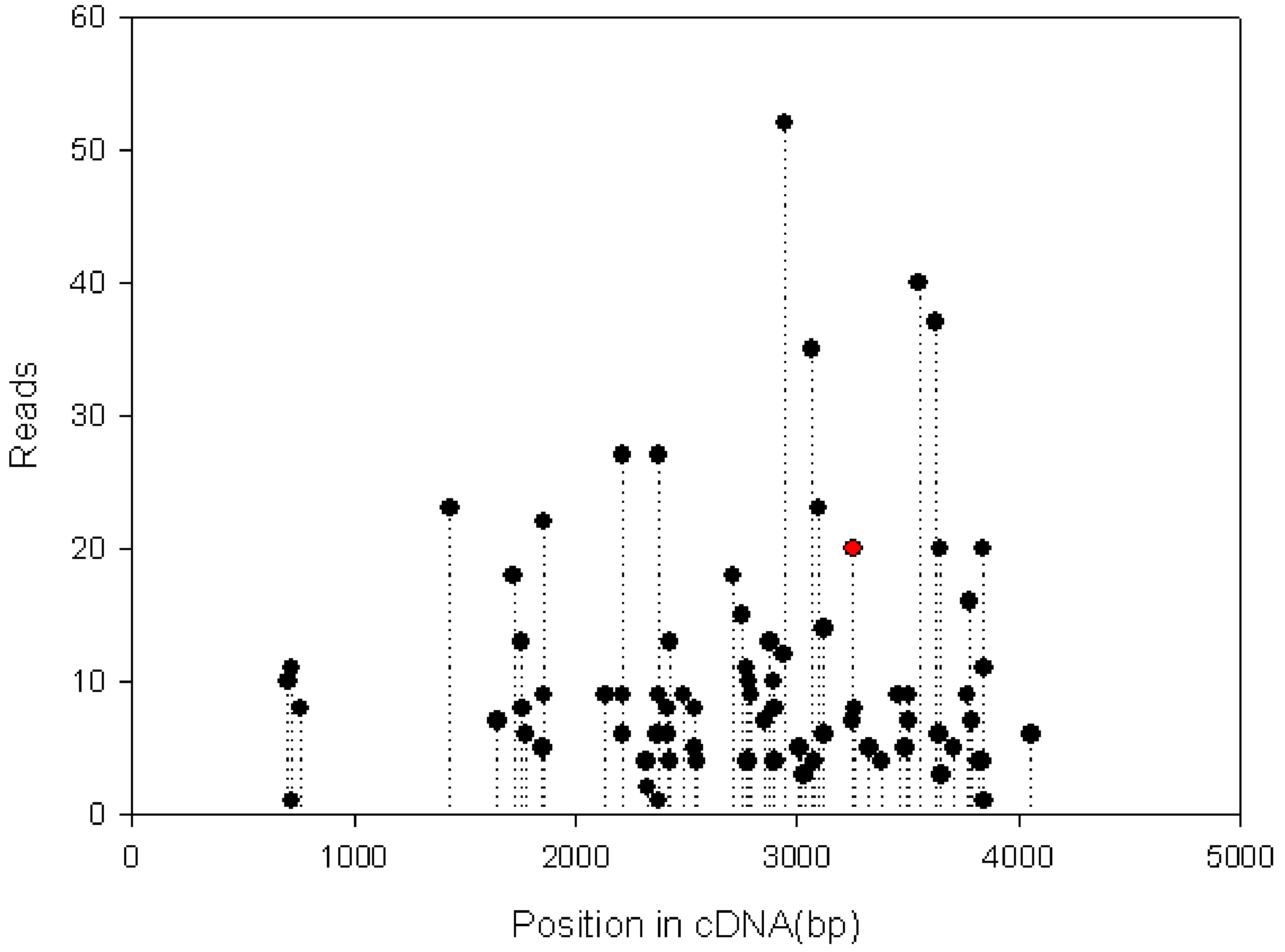


```
5' GUAGAUAAG-AUCAUUAAGAUUUCUGA 3'       Cs1g21180.1
          :: :::::.::::::.:
3' -------UCGUAGUAGUUCUAAGGU-- 5'       Csi-miR172c.1
```

Csi-miR172c.1, target=Cs3g06140.1 gene=Cs3g06140
Category:1
Score=4.5
Cleavage Site=480

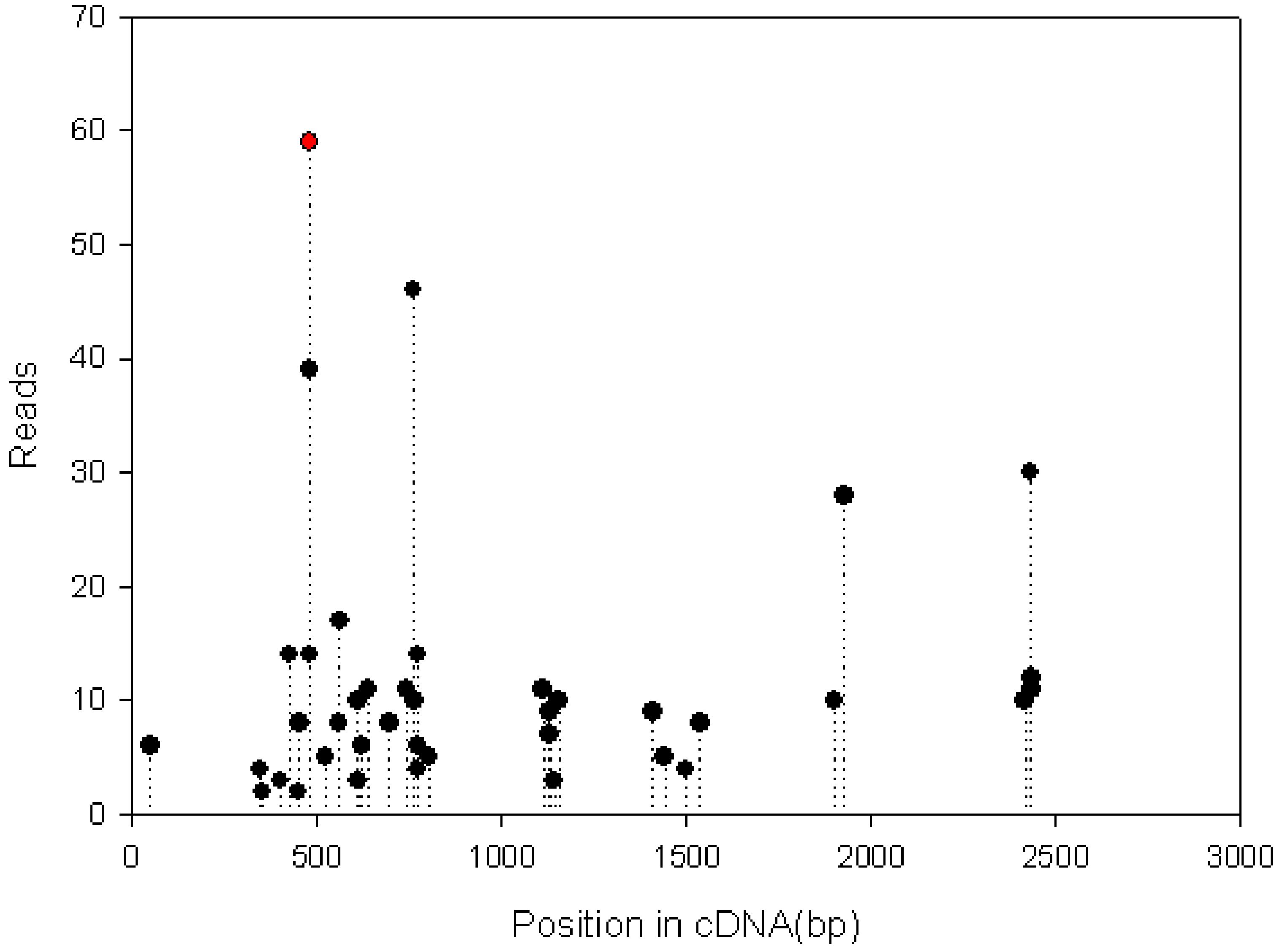


```
5' UGAGUCUGCCACCAUUAAGAUUCCUG 3'        Cs3g06140.1
           .  ::  :::.:::::::::
3' -------UCGUAGUAGUUCUAAGGU- 5'        Csi-miR172c.1
```

Csi-miR172c.1, target=Cs8g17390.1 gene=Cs8g17390
Category:3
Score=2
Cleavage Site=1865

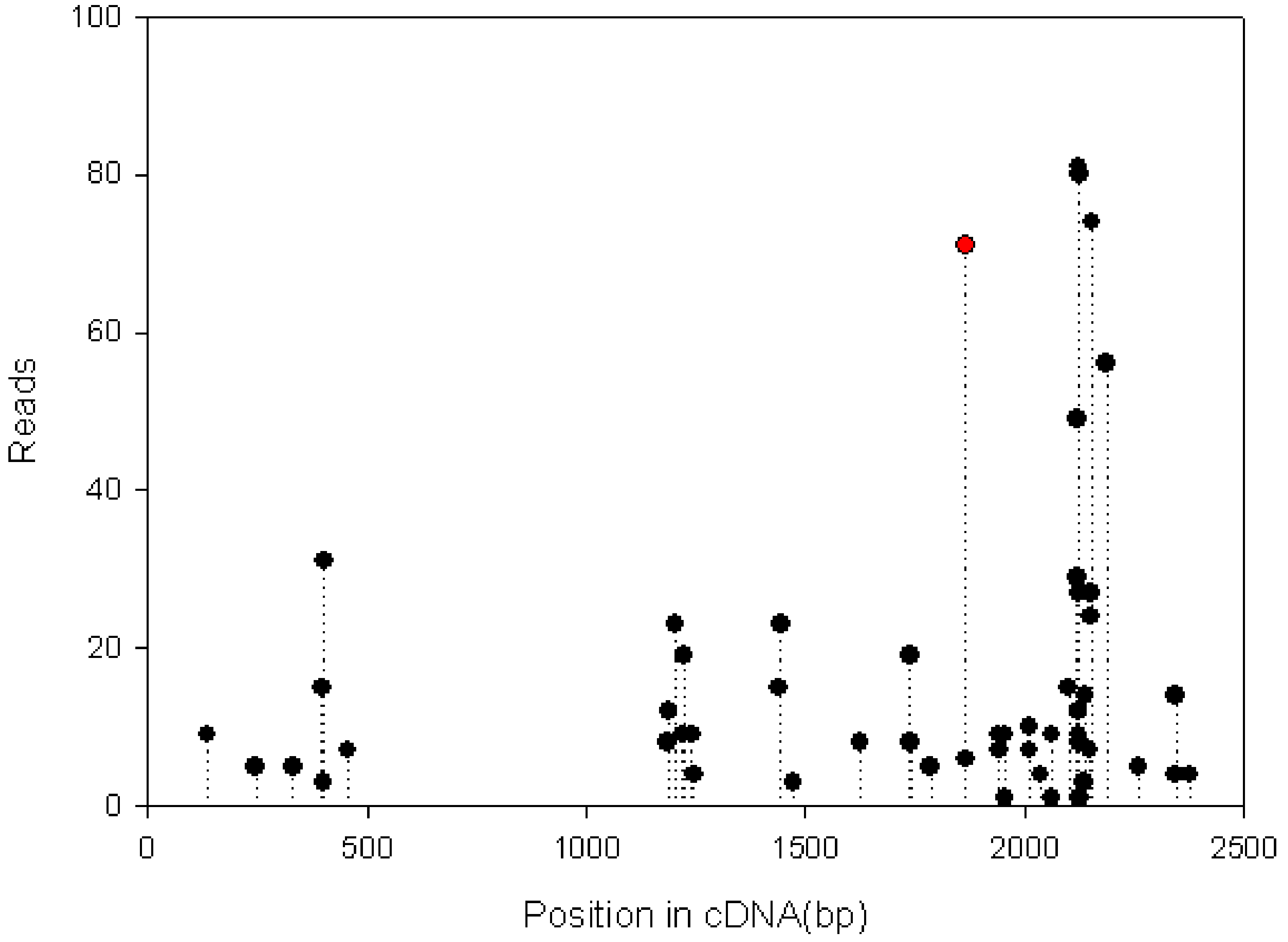


```
5' AGUGCAGCAUCAUCAGGAUUCCCAUU 3'        Cs8g17390.1
         :::::::::::.::::::
3' -----UCGUAGUAGUUCUAAGGU--- 5'        Csi-miR172c.1
```

Csi-miR172c.1, target=Orange1.1t04055.1 gene=Orange1.1t04055
Category=3
Score=2
Cleavage Site=2049

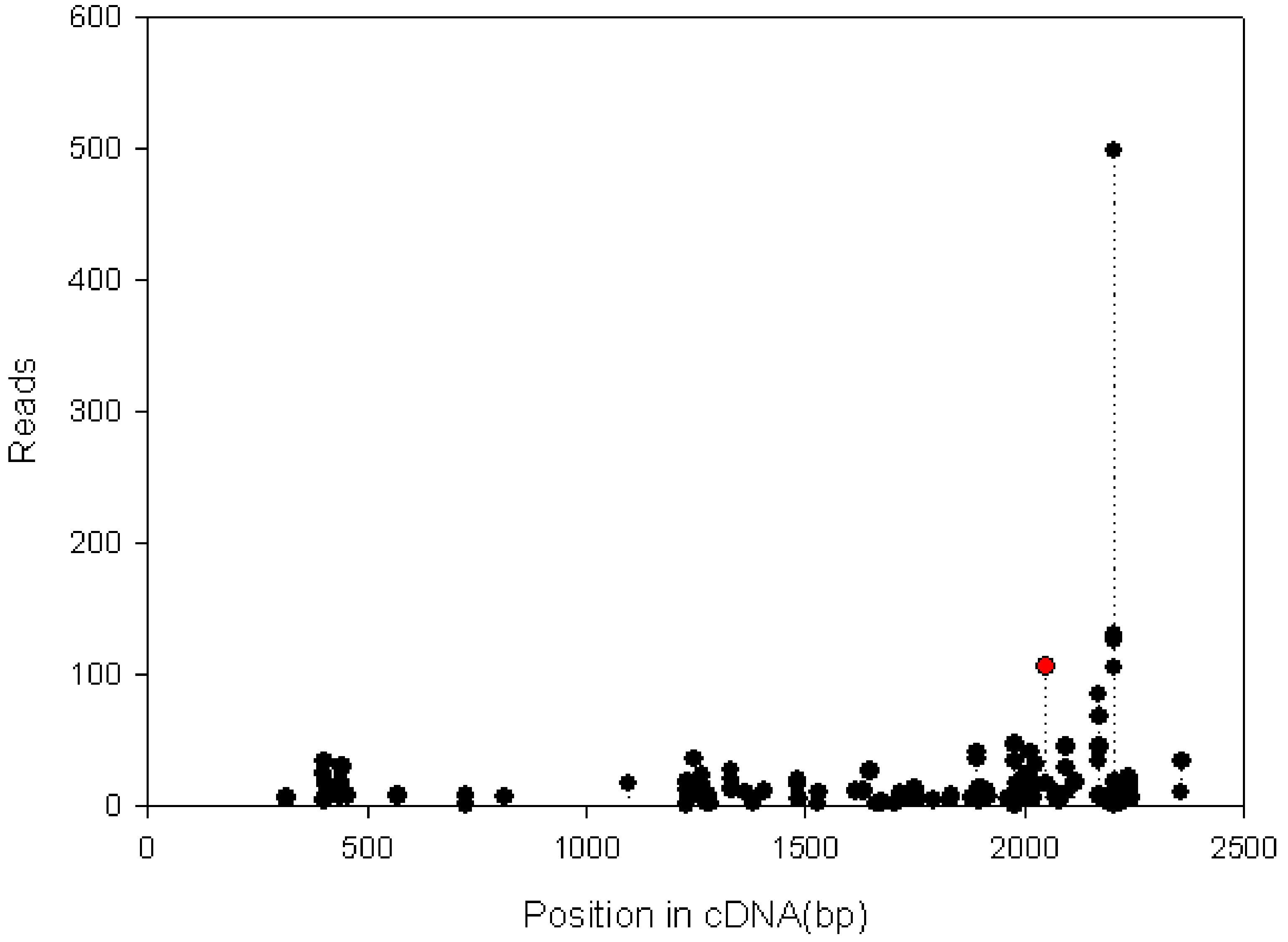


```
5' GCUGCAGCAUCAUCAGGAUUCCCUCA 3'        Orange1.1t04055.1
         ::::::::::.::::::
3' -----UCGUAGUAGUUCUAAGGU--- 5'        Csi-miR172c.1
```

Csi-miR172c.2, target=Cs8g17390.1 gene=Cs8g17390
Category:3
Score=3
Cleavage Site=1865

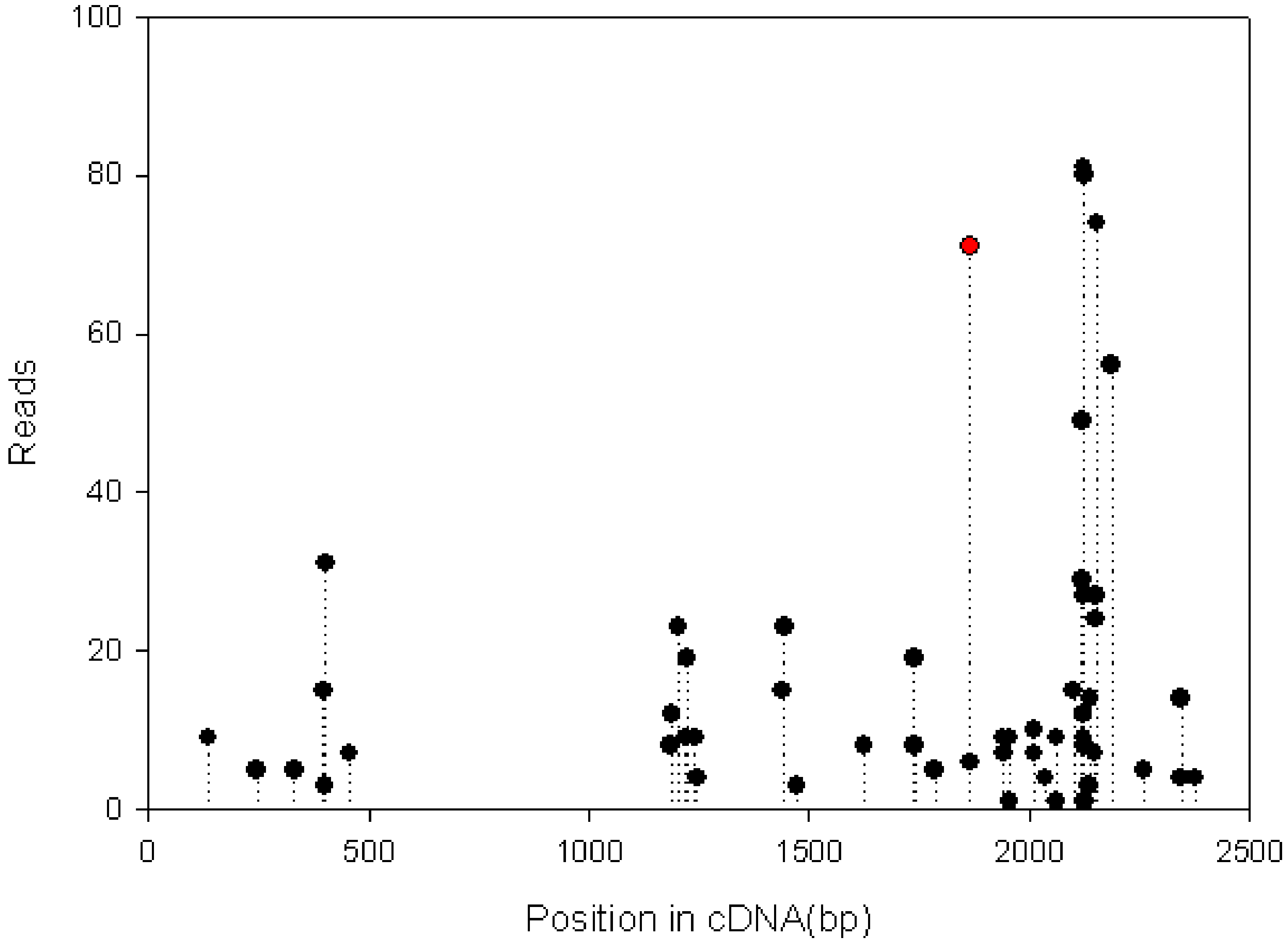


```
5' AGUGCAGCAUCAUCAGGAUUCCCAUU 3'        Cs8g17390.1
      :::::::::::::.::::::
3' -GACGUCGUAGUAGUUCUAAGGU--- 5'        Csi-miR172c.2
```

Csi-miR172c.2, target=Orange1.1t04055.1 gene=Orange1.1t04055
Category=3
Score=2
Cleavage Site=2049

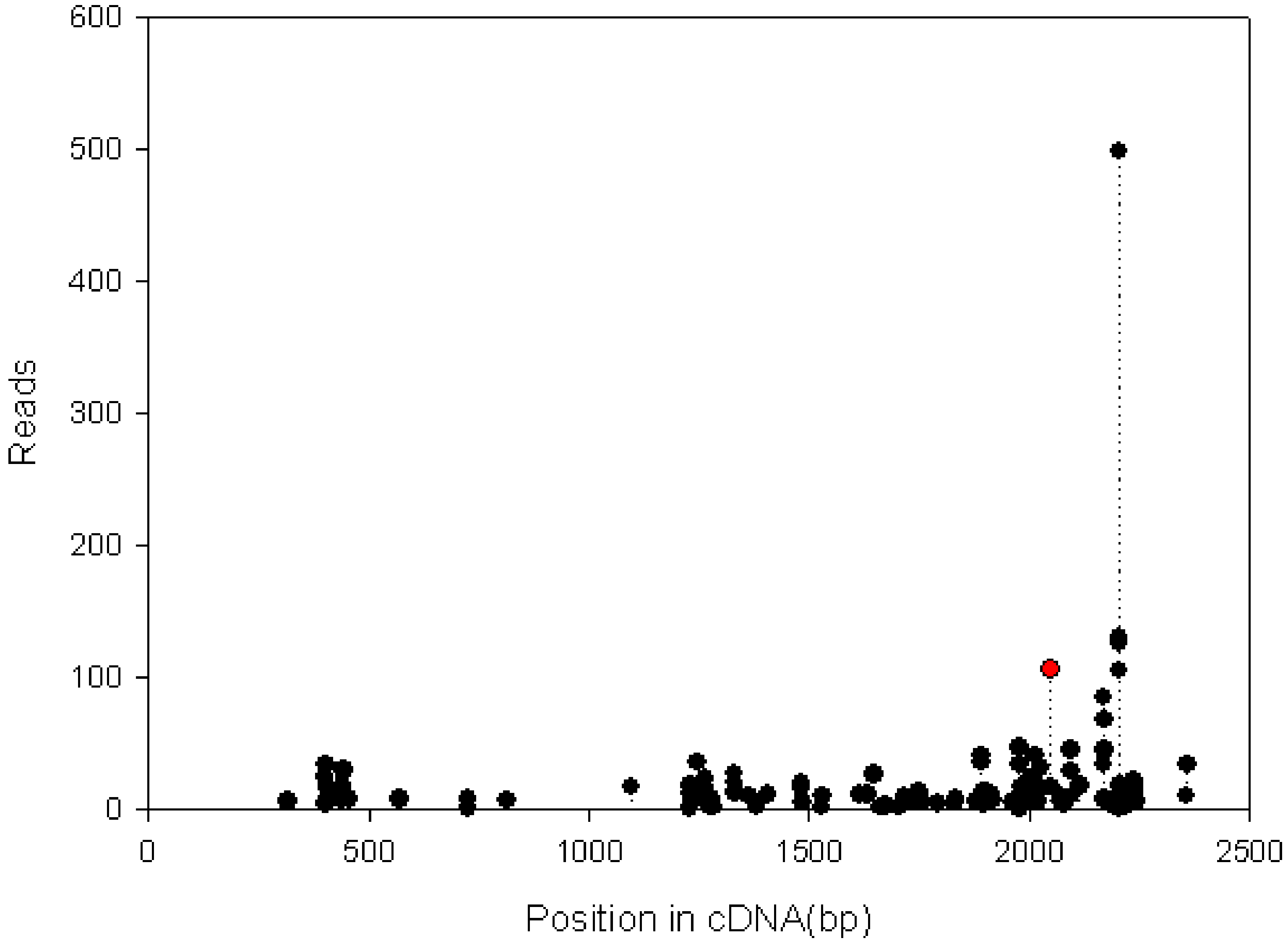


```
5' GCUGCAGCAUCAUCAGGAUUCCCUCA 3'        Orange1.1t04055.1
    ::::::::::::::.:::::::
3' -GACGUCGUAGUAGUUCUAAGGU--- 5'        Csi-miR172c.2
```

Csi-miR172d, target=Cs8g17390.1 gene=Cs8g17390
Category:3
Score=2.5
Cleavage Site=1865

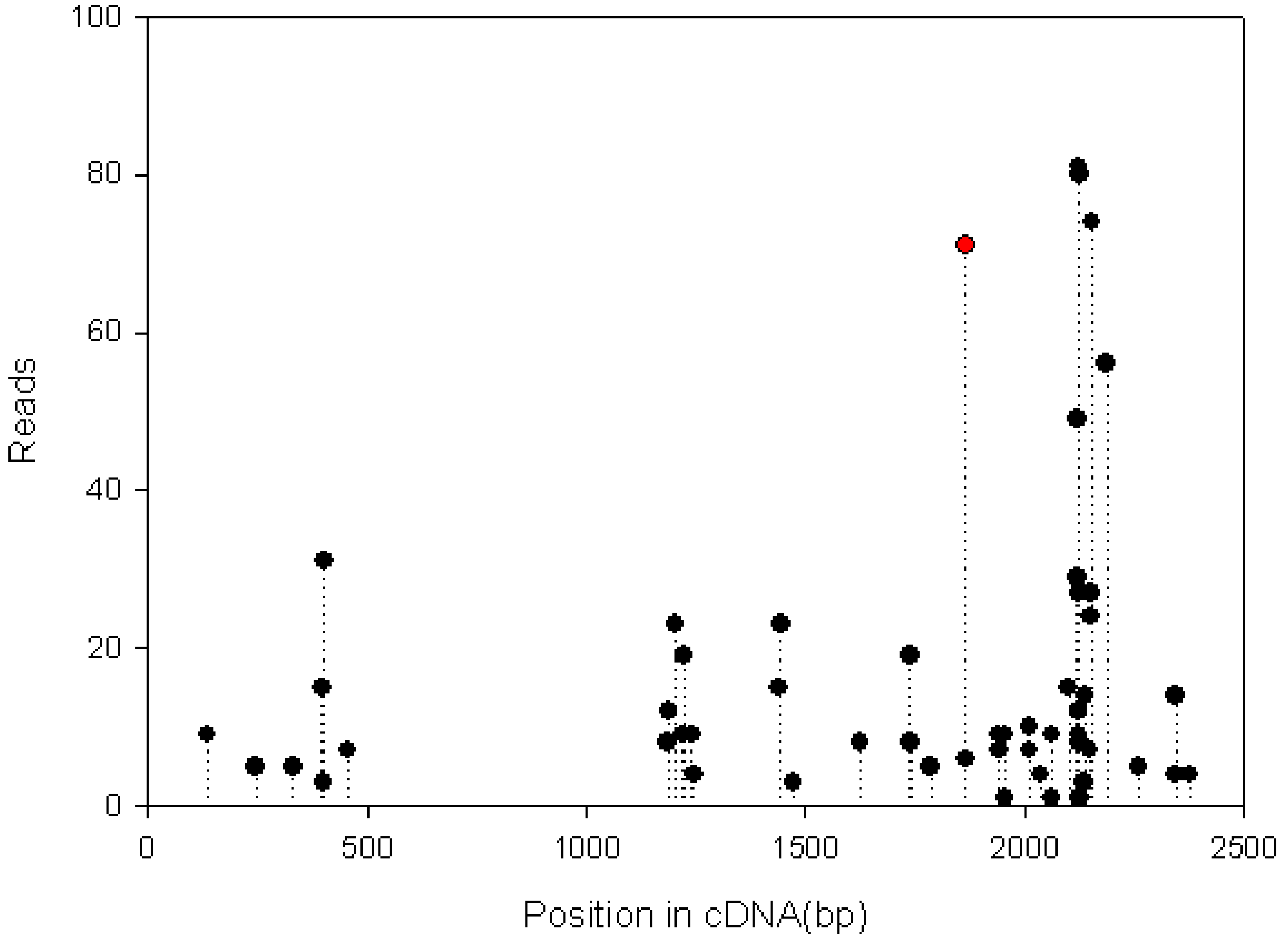


```
5' AGUGCAGCAUCAUCAGGAUUCCCAUU 3'        Cs8g17390.1
    .::::::::::::::::.:::::
3' -UACGUCGUAGUAGUUCUAAGA---- 5'        Csi-miR172d
```

Csi-miR172d, target=Orange1.1t04055.1 gene=Orange1.1t04055
Category=3
Score=3
Cleavage Site=2049

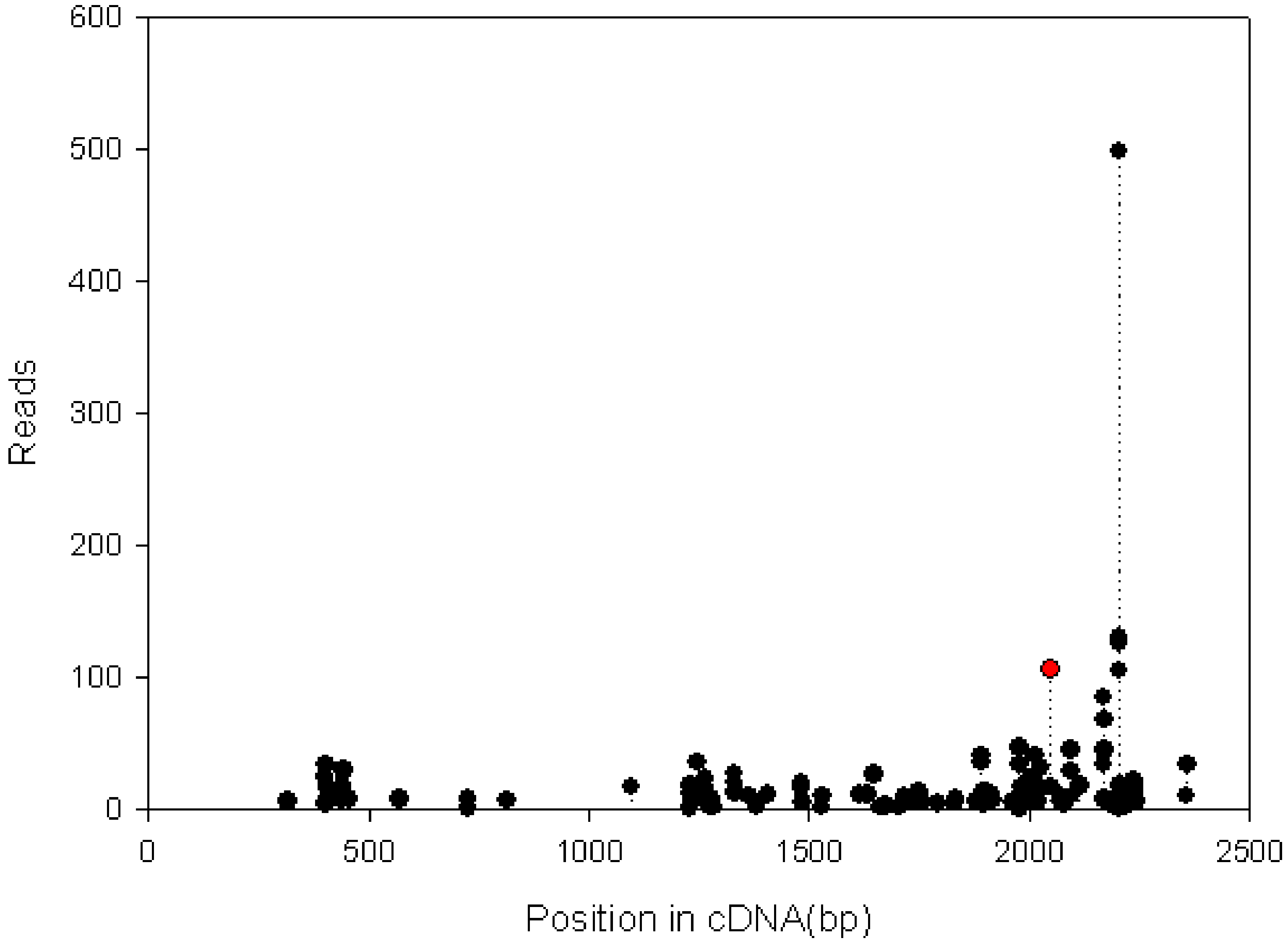


```
5' GCUGCAGCAUCAUCAGGAUUCCCUCA 3'        Orange1.1t04055.1
     ::::::::::::::.:::::
3' -UACGUCGUAGUAGUUCUAAGA---- 5'        Csi-miR172d
```

Csi-miR172d-3p, target=Cs9g03090.1 gene=Cs9g03090
Category=1
Score=4.5
Cleavage Site=2765

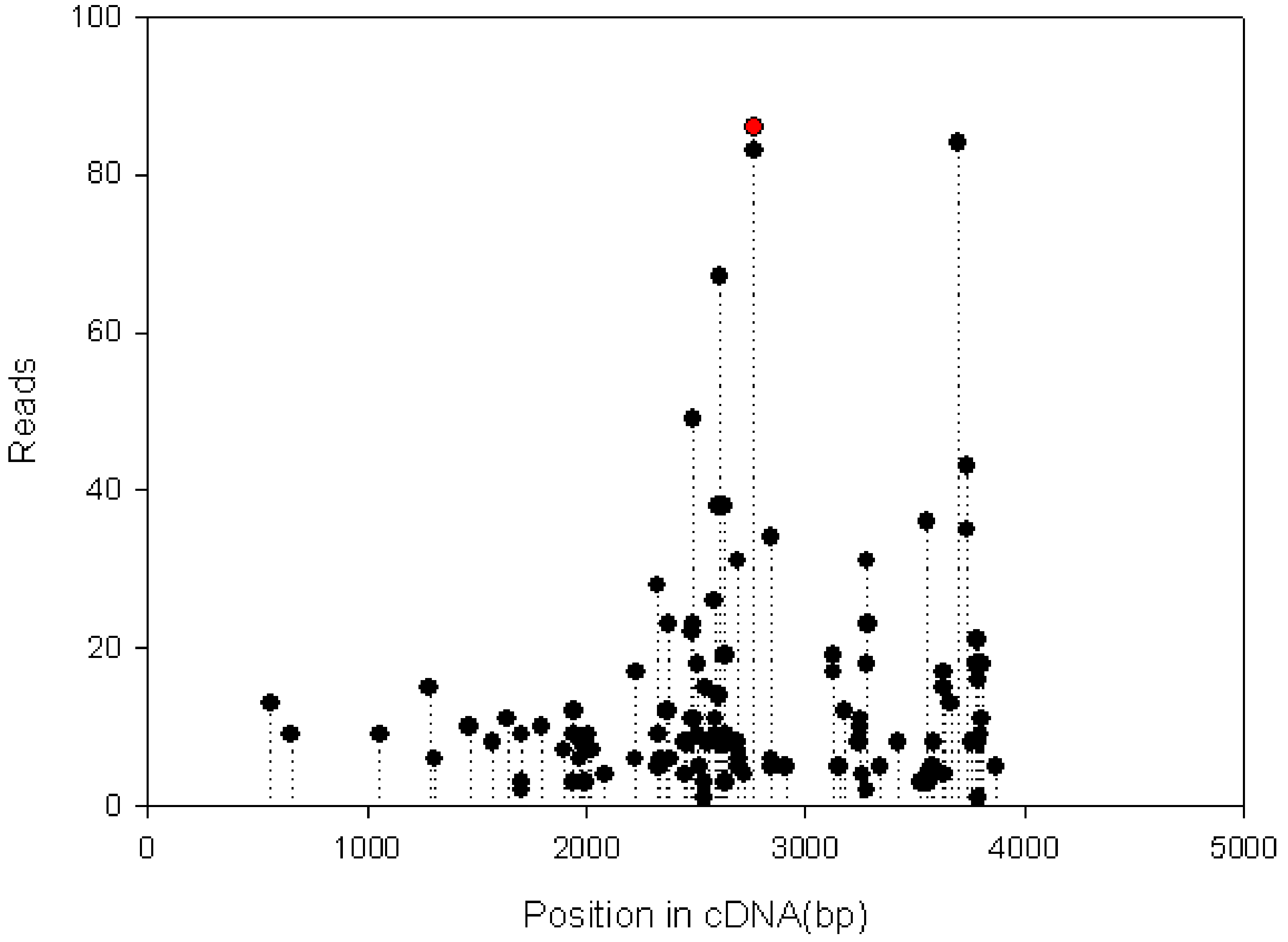


```
5' GCAAUCUGCAUUUUUGAUGAUGUUGC 3'        Cs9g03090.1
       :  :: :  :.:::::::::.:::
3' ----ACAC-UUAGAACUACUACGACG 5'        Csi-miR172d-3p
```

Csi-miR172e.2, target=Cs8g17390.1 gene=Cs8g17390
Category:3
Score=1.5
Cleavage Site=1865

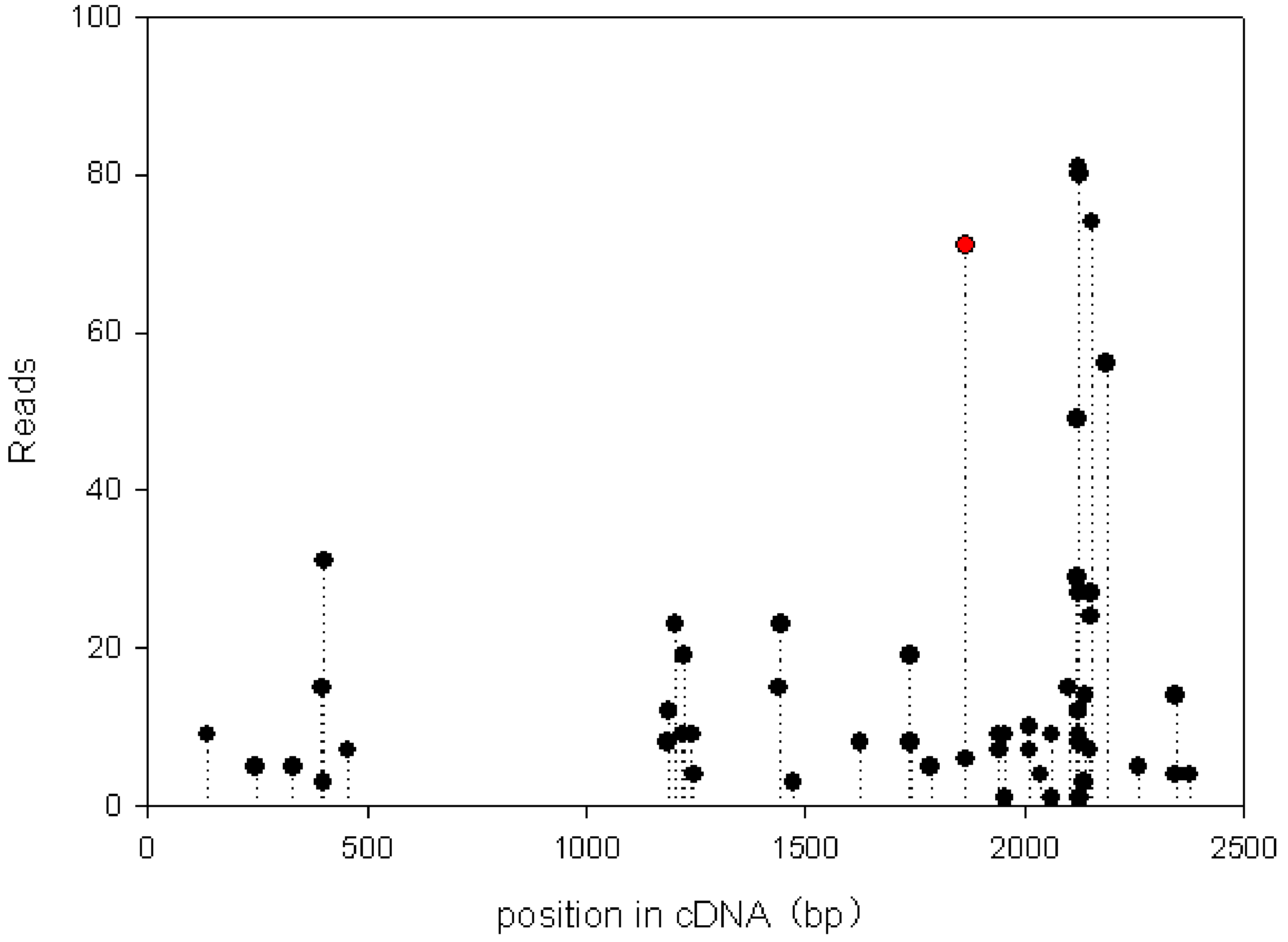


```
5' AGUGCAGCAUCAUCAGGAUUCCCAUU 3'        Cs8g17390.1
    .:::::::::::::::.:::::
3' -UACGUCGUAGUAGUUCUAAG----- 5'        Csi-miR172e.2
```

Csi-miR172e.2, target=Orange1.1t04055.1 gene=Orange1.1t04055
Category=2
Score=2
Cleavage Site=2049

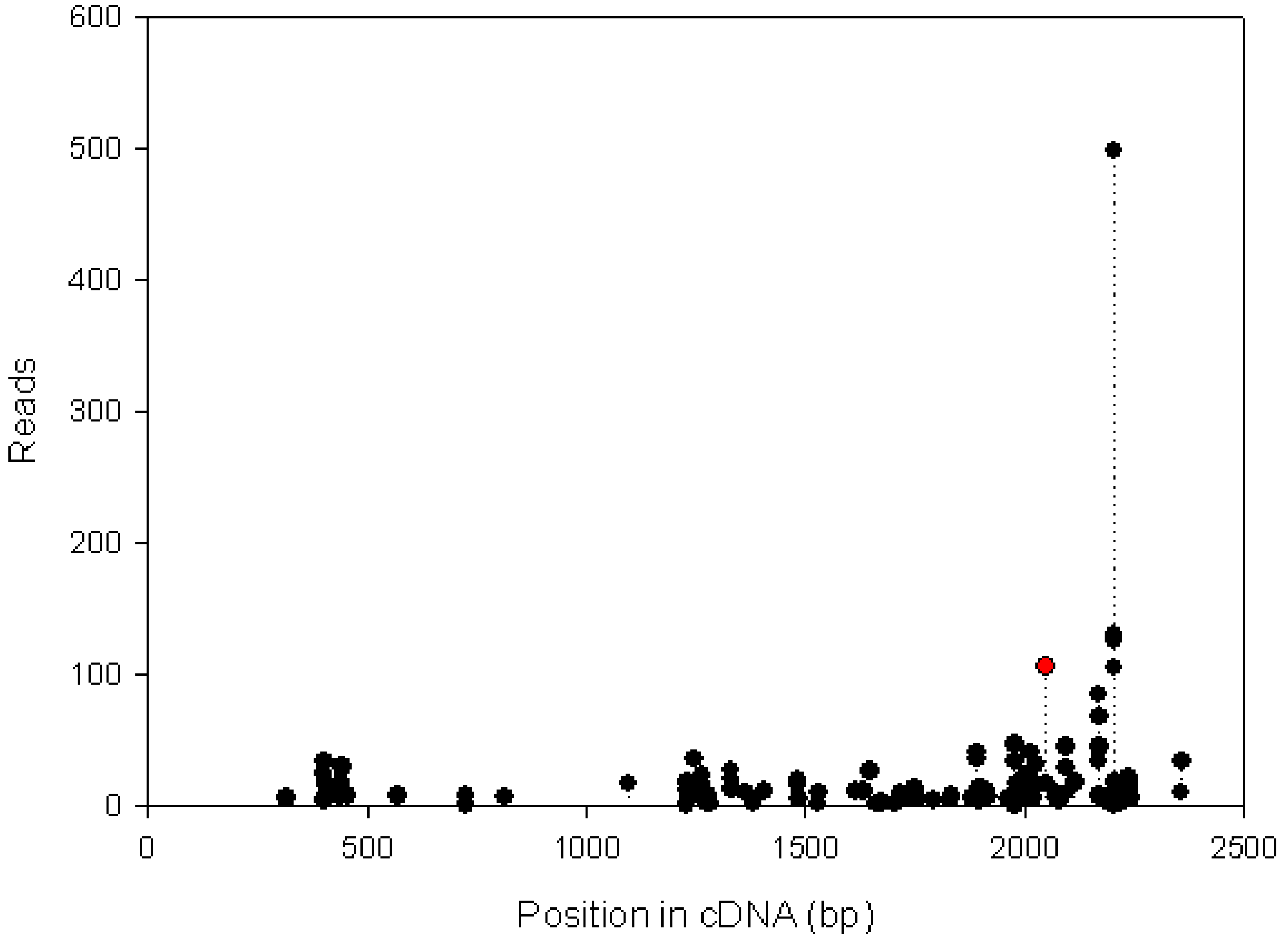


```
5' GCUGCAGCAUCAUCAGGAUUCCCUCA 3'        Orange1.1t04055.1
    ::::::::::::::.:::::
3' -UACGUCGUAGUAGUUCUAAG----- 5'        Csi-miR172e.2
```

Csi-miR172k, target=Cs8g17390.1 gene=Cs8g17390
Category:3
Score=2.5
Cleavage Site=1865

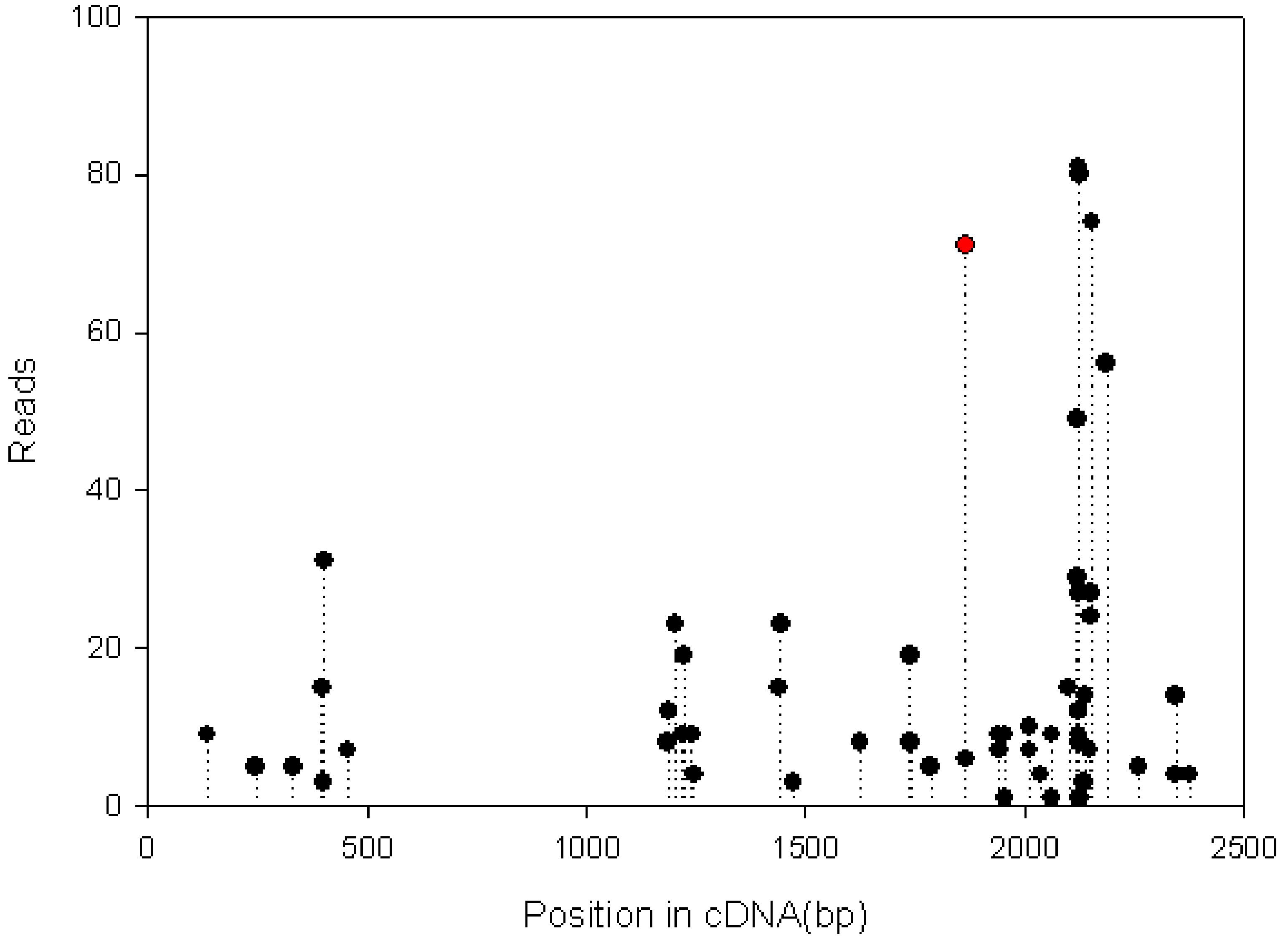


```
5' AGUGCAGCAUCAUCAGGAUUCCCAUU 3'        Cs8g17390.1
    .:::::::::::::::.:::::
3' -UACGUCGUAGUAGUUCUAAGU---- 5'        Csi-miR172k
```

Csi-miR172k, target=Orange1.1t04055.1 gene=Orange1.1t04055
Category=3
Score=3
Cleavage Site=2049

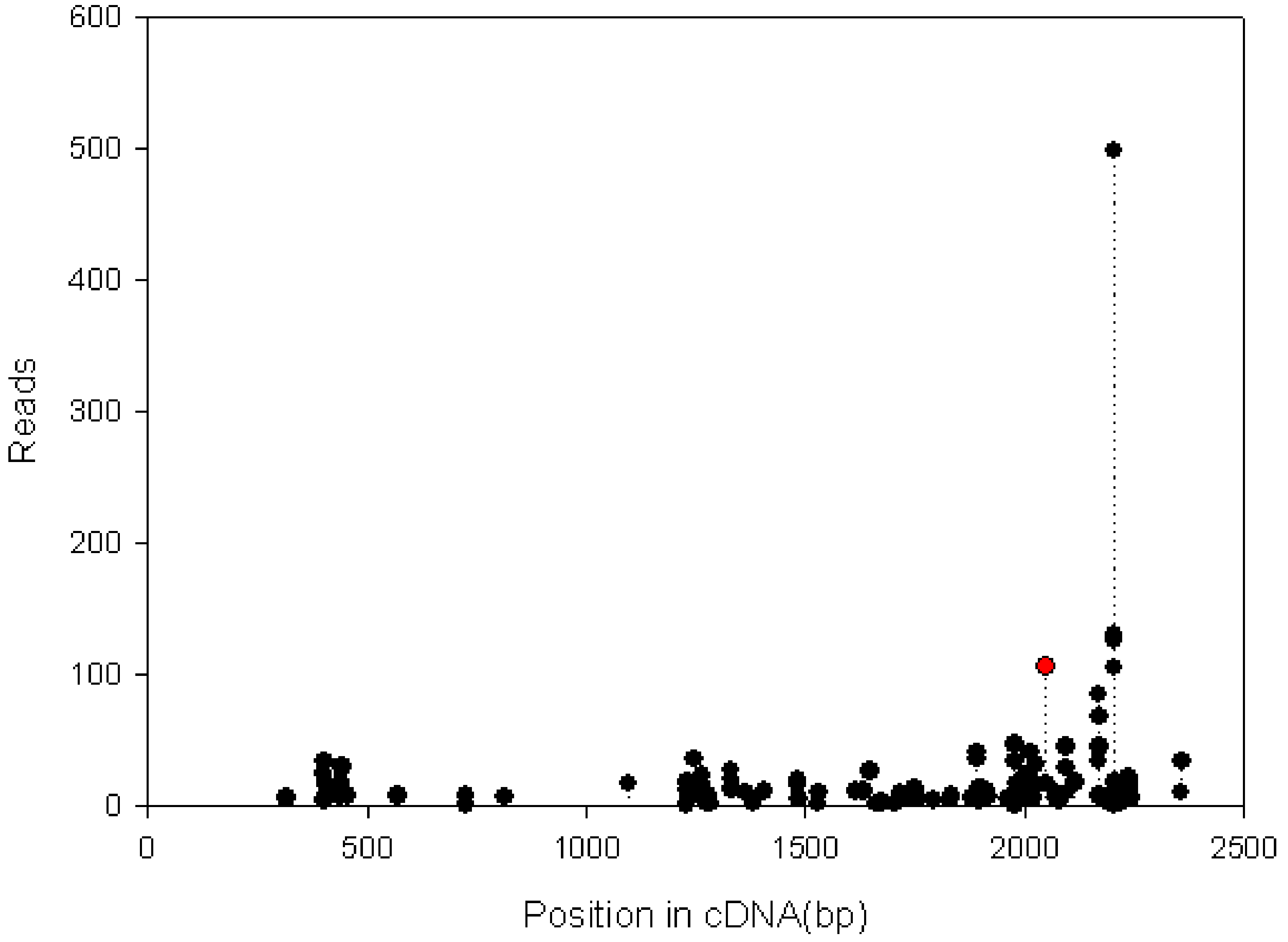


```
5' GCUGCAGCAUCAUCAGGAUUCCCUCA 3'        Orange1.1t04055.1
     :::::::::::::::.:::::
3' -UACGUCGUAGUAGUUCUAAGU---- 5'        Csi-miR172k
```

Csi-miR2111, target=Cs5g29630.1 gene=Cs5g29630
Category=3
Score=5
Cleavage Site=1384

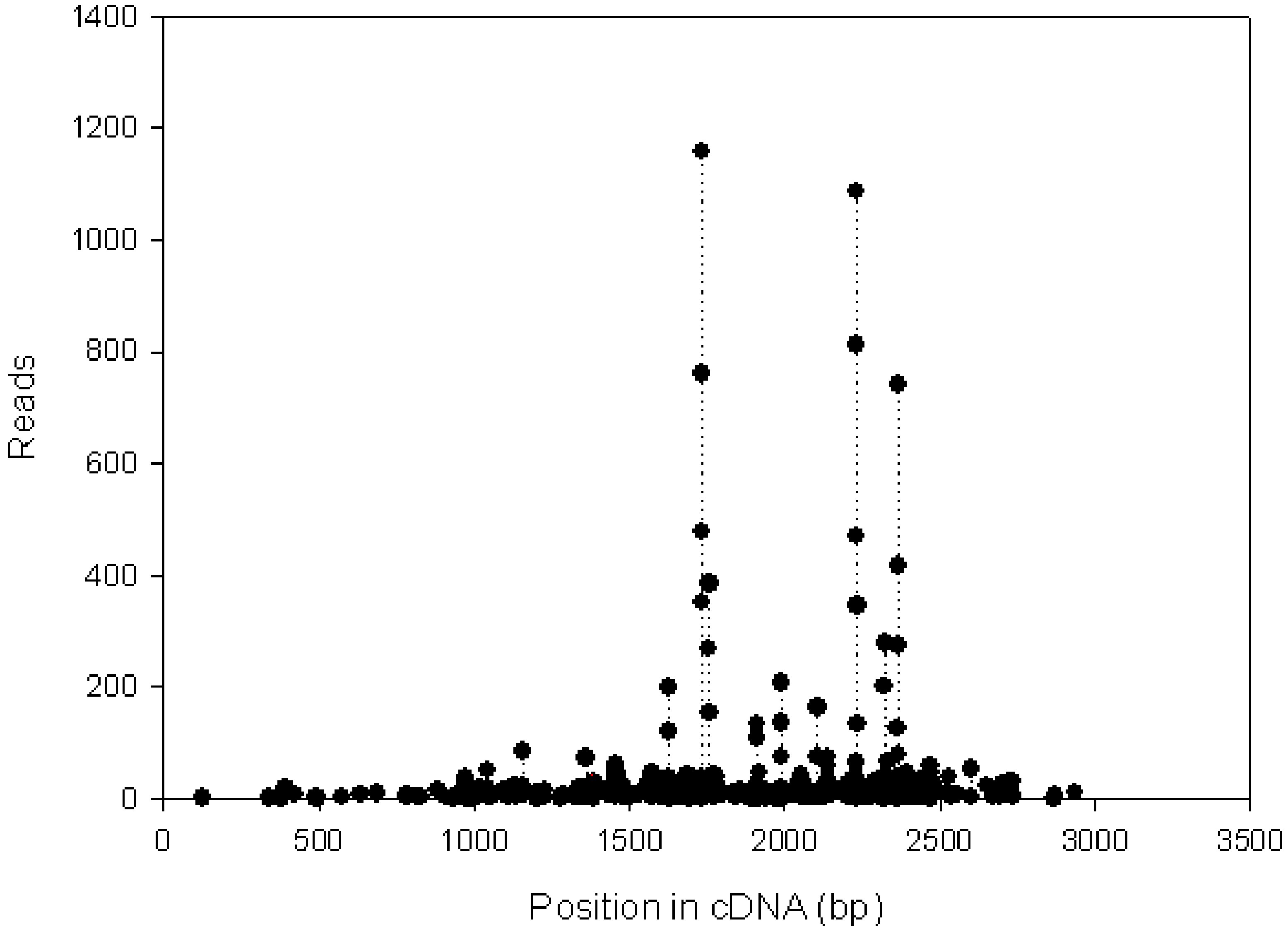


```
5' UUUGACCCCCAGGAUGCAGUUUAUUU 3'        Cs5g29630.1
     ..: :: ::::::::::: :::
3' --GUUUGGAGUCCUACGUCUAAU--- 5'        Csi-miR2111
```

Csi-miR2118.1,target=Cs6g12050.1 gene=Cs6g12050
Category=3
Score=5
Cleavage Site=655

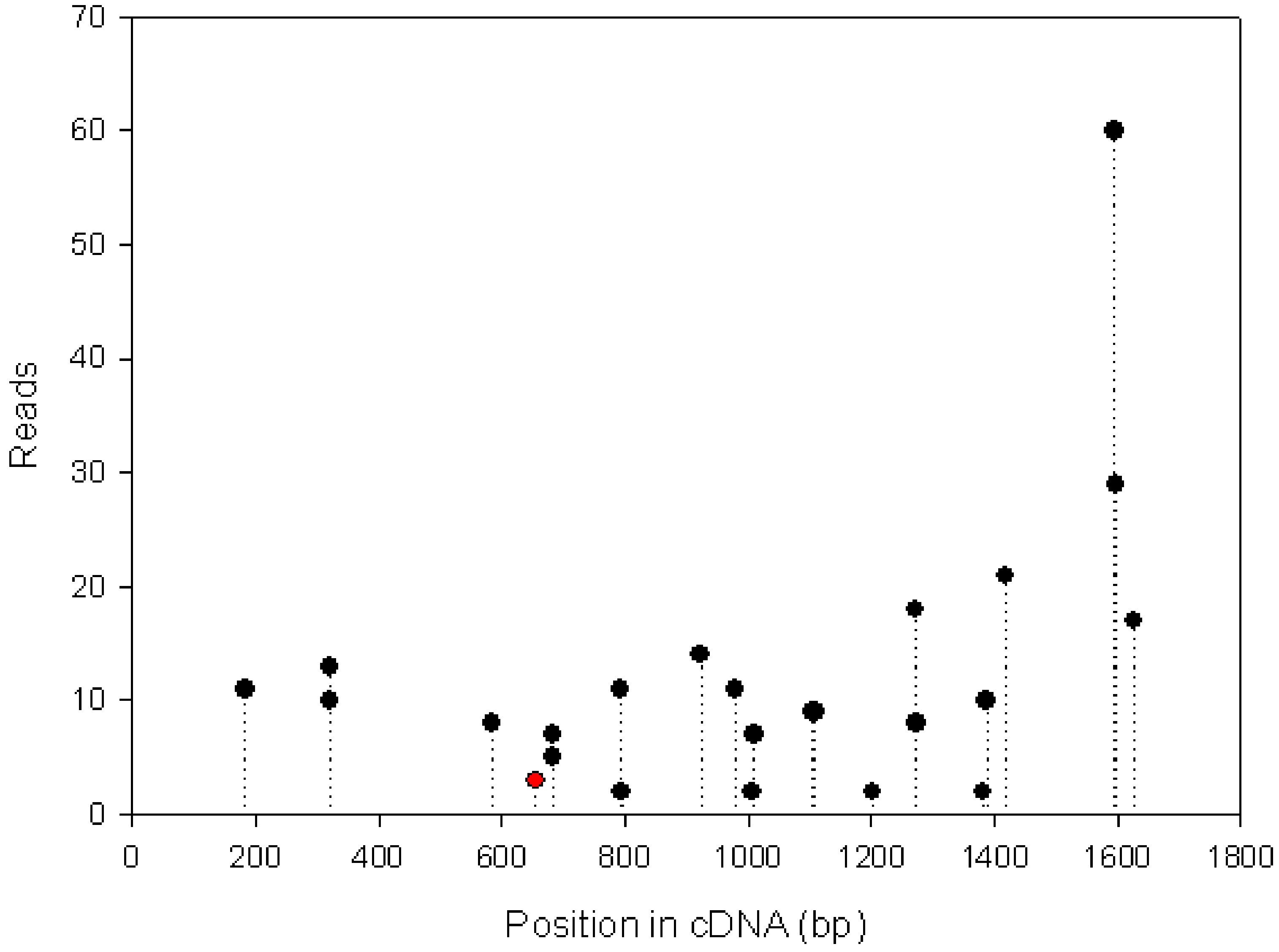


```
5' AAGGAAUGGGUGACAGAGGUGAAUUU 3'        Cs6g12050.1
   :::::::::::: :: ::::.:
3' UUCCUUACCCACCGUAUCCAUU---- 5'        Csi-miR2118.1
```

Csi-miR2118.2, target=Cs3g12720.1 gene=Cs3g12720
Category=1
Score=5
Cleavage Site=547

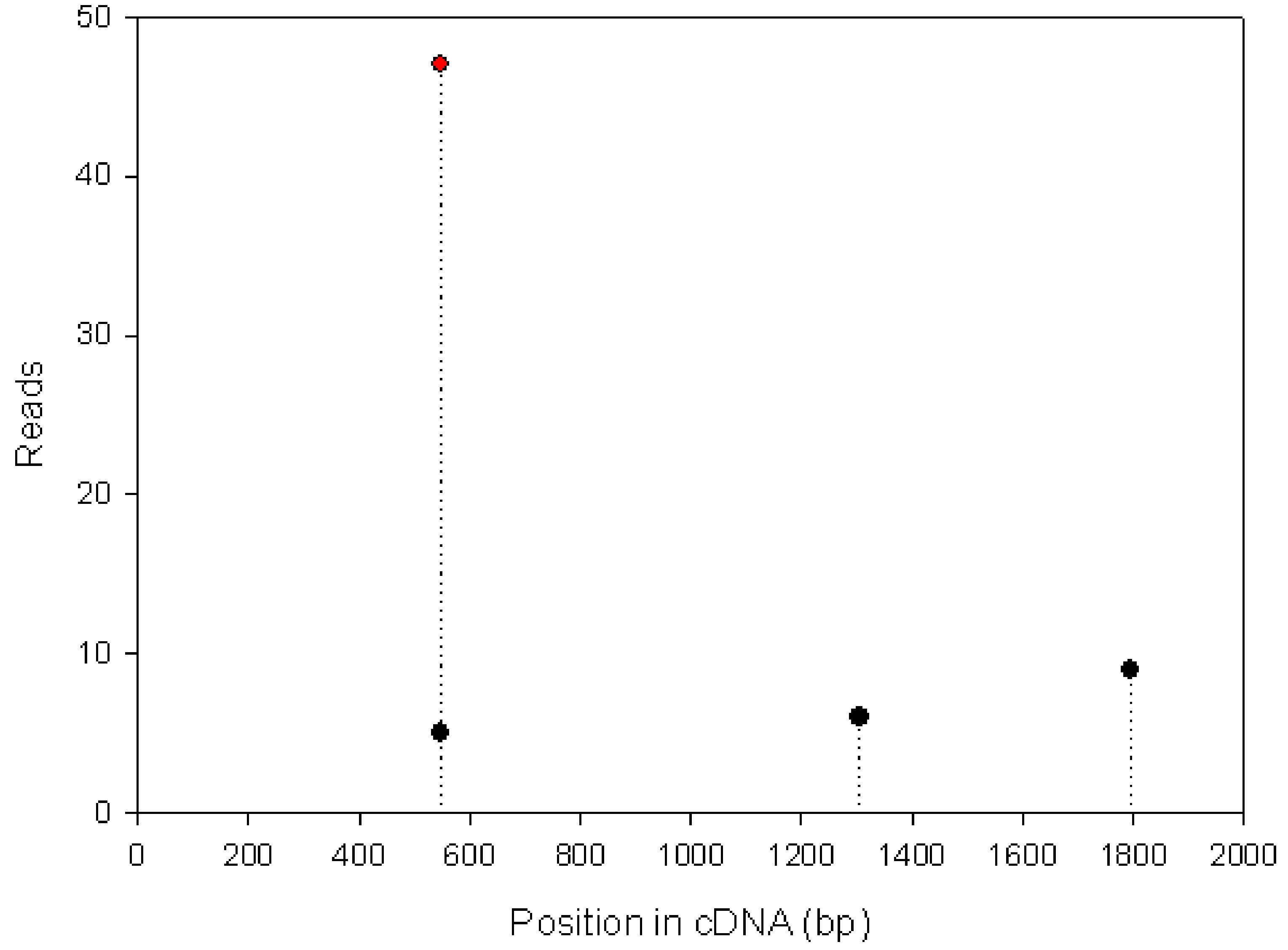


```
5' GGCAUGGGUGGCAUUGGAAAGACAAC 3'        Cs3g12720.1
   :: :::::::::::: :: ::
3' CCUUACCCACCGUAUCCAUU------ 5'        Csi-miR2118.2
```

Csi-miR2118.2, target=Cs3g12850.1 gene=Cs3g12850
Category=1
Score=4.5
Cleavage Site=547

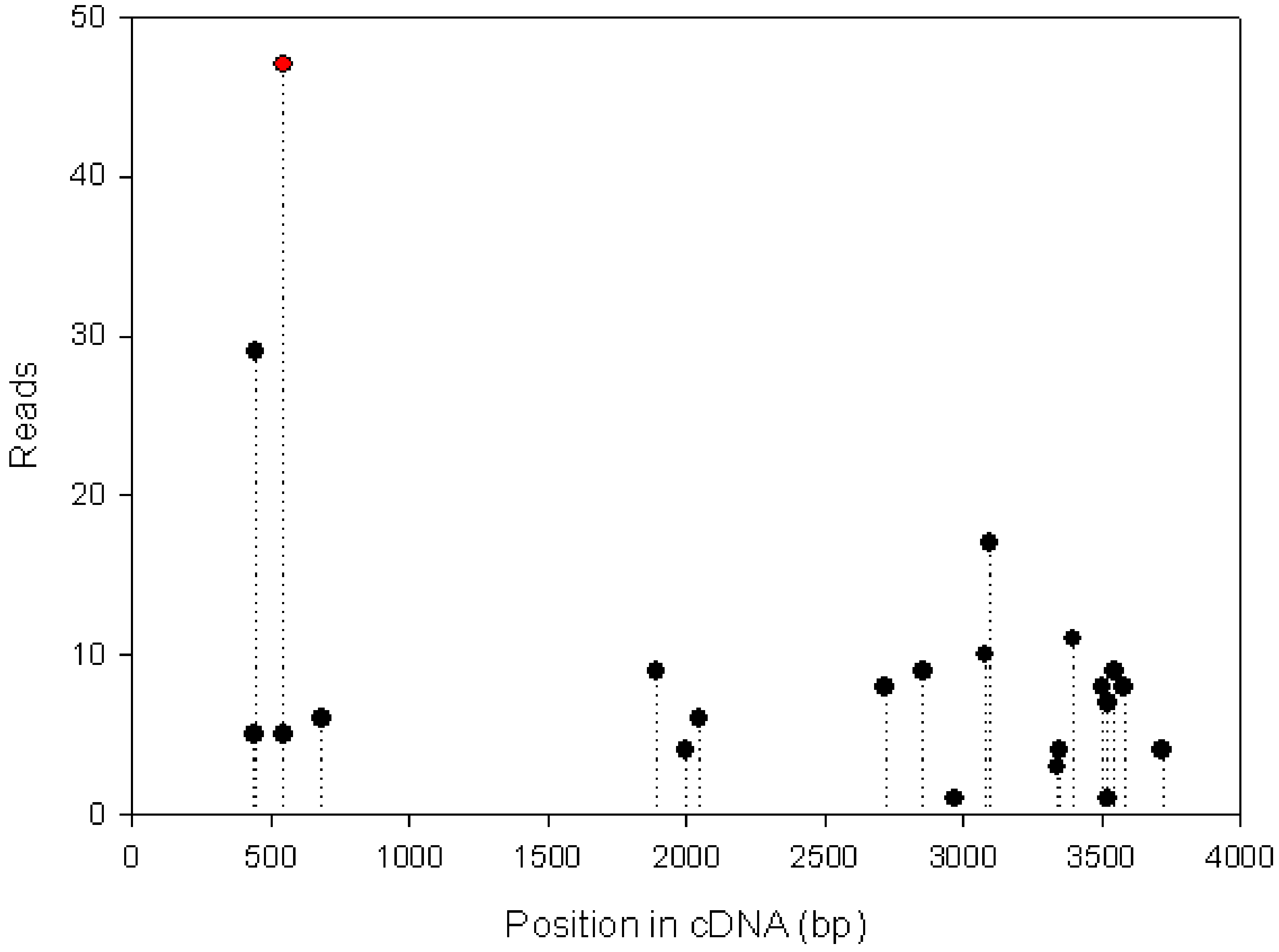


```
5' GGGAUGGGUGGCAUUGGAAAGACAAC 3'        Cs3g12850.1
   ::.:::::::::::: :: ::
3' CCUUACCCACCGUAUCCAUU------ 5'        Csi-miR2118.2
```

Csi-miR2118.2, target=Orange1.1t00149.1 gene=Orange1.1t00149
Category:2
Score=3.5
Cleavage Site=100

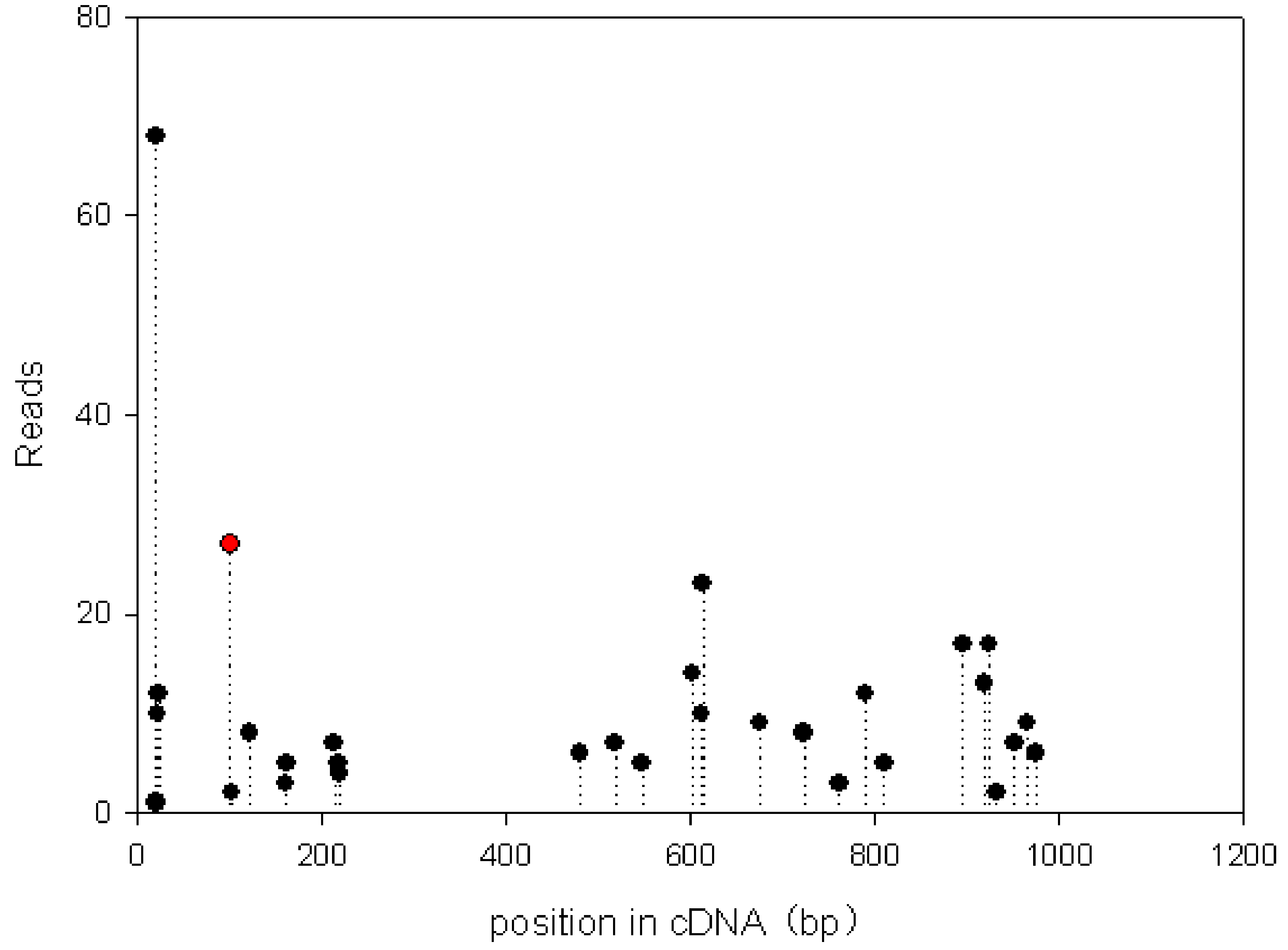


```
5' GGAGUGGGUGGCAUA-GUGAUGGCCGG 3'       Orange1.1t00149.1
   ::::.:::::::::::: :::.:
3' CCUUACCCACCGUAUCCAUU------- 5'       Csi-miR2118.2
```

Csi-miR2118.2arget=Cs3g12760.1 gene=Cs3g12760
Category=1
Score=4.5
Cleavage Site=1037

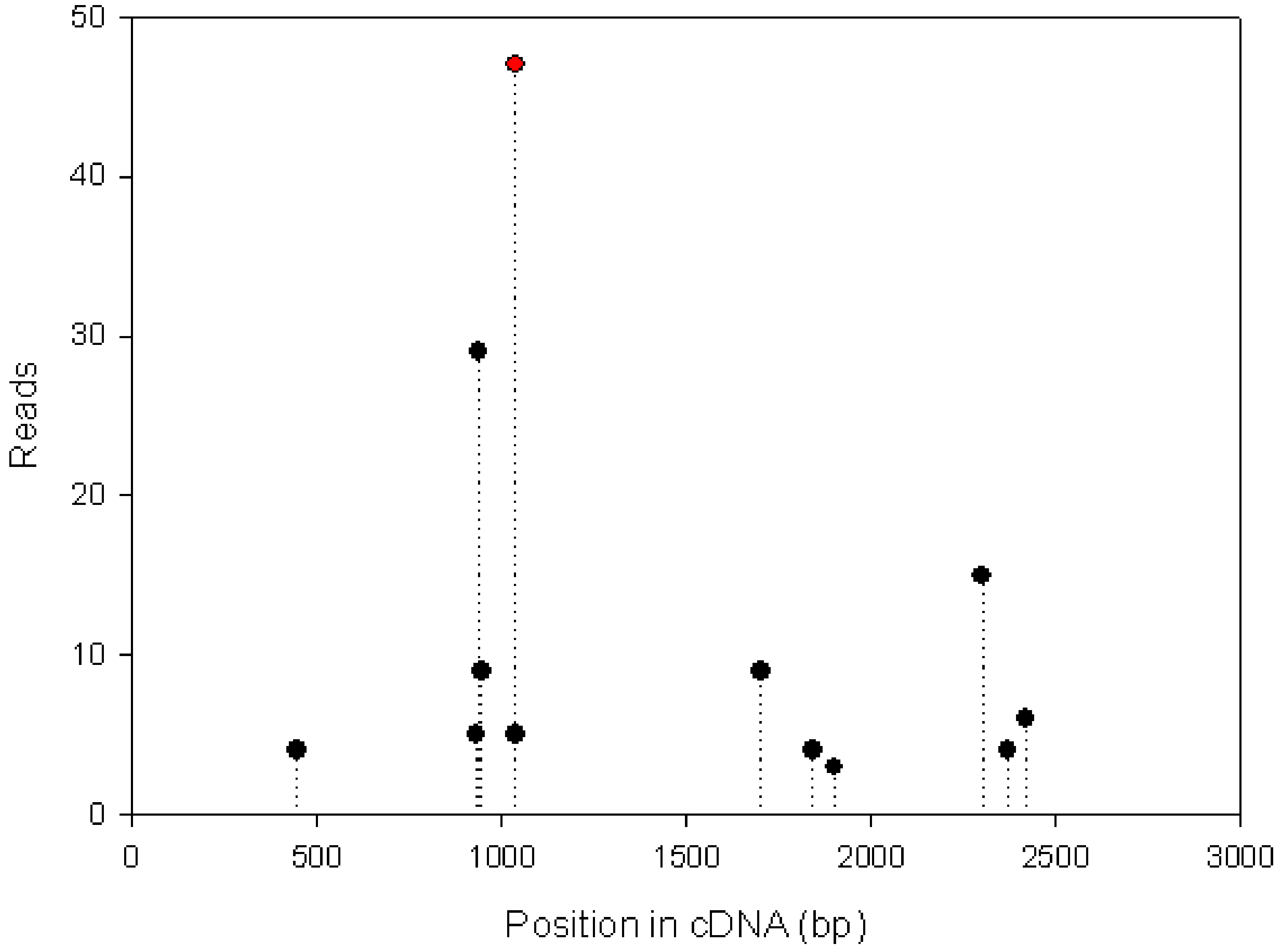


```
5' GGGAUGGGUGGCAUUGGAAAGACAAC 3'        Cs3g12760.1
   ::.:::::::::::: :: ::
3' CCUUACCCACCGUAUCCAUU------ 5'        Csi-miR2118.2
```

Csi-miR2275a, target=Orange1.1t02313.1 gene=Orange1.1t02313
Category=3
Score=4.5
Cleavage Site=2627

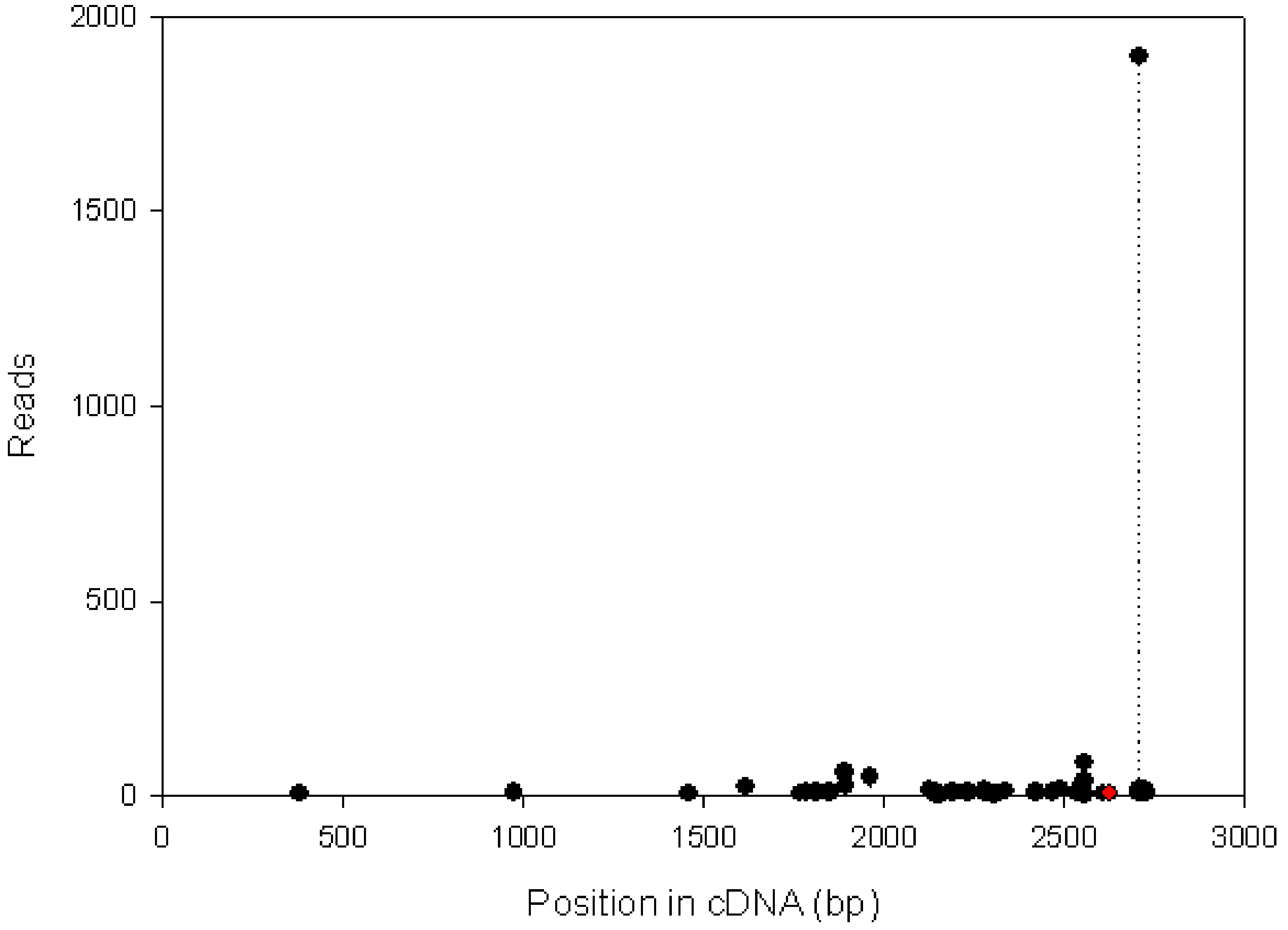


```
5' CCUGAGA-AUUGGAGGGAAUAAAAGUU 3'       Orange1.1t02313.1
      :.::: :::::::::.::: :::
3' --AUUCUAUAACCUCCUUUAAUUU--- 5'       Csi-miR2275a
```

Csi-miR2275b, target=Cs2g10790.1 gene=Cs2g10790
Category=3
Score=4.5
Cleavage Site=1371

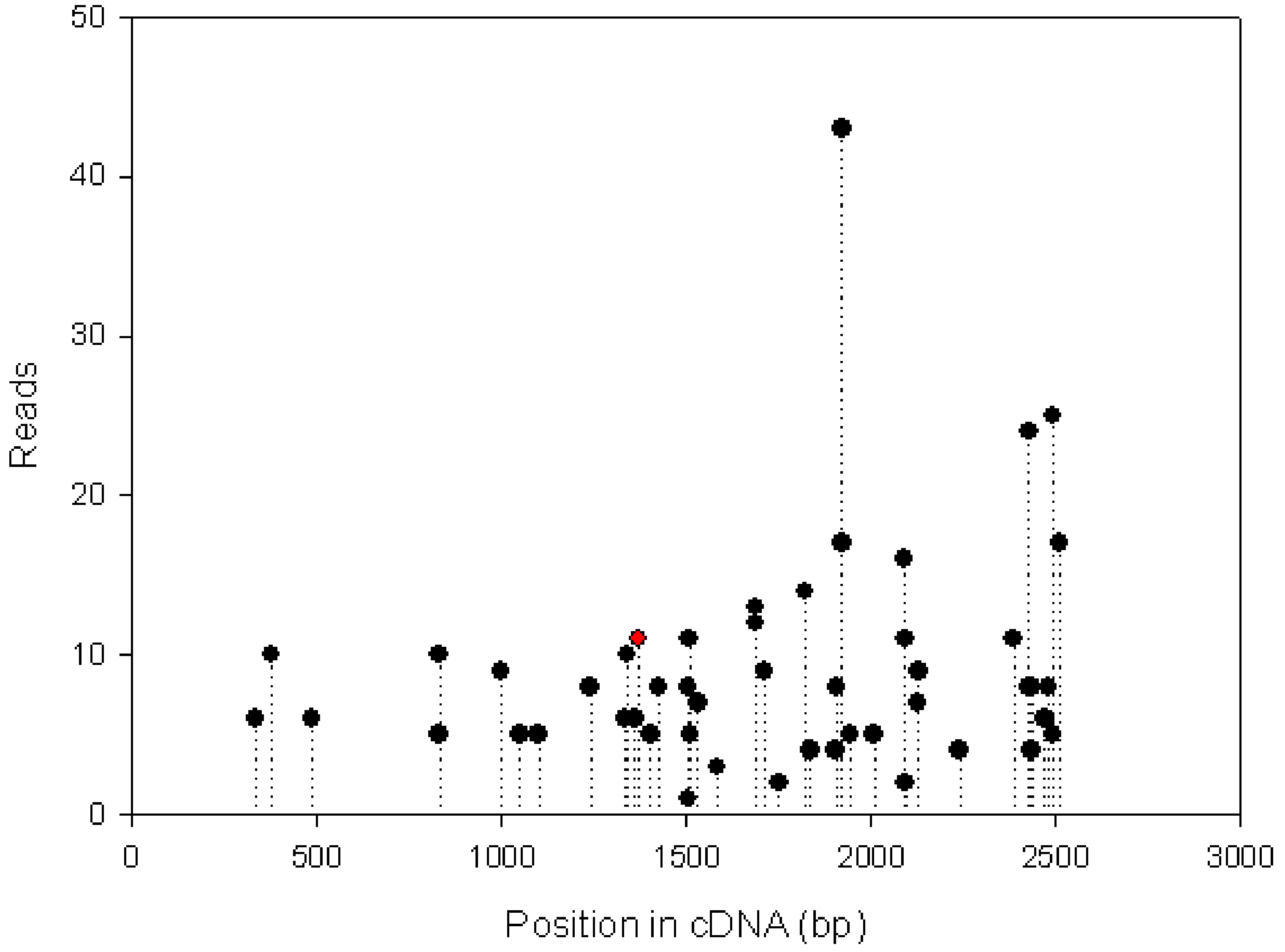


```
5' UGAAGUUGCGGAGGAAAUUAGAAUGC 3'        Cs2g10790.1
   ::::.:   ::::::::::::.:
3' ACUUUAUAACCUCCUUUAAUUU---- 5'        Csi-miR2275b
```

Csi-miR2275c.1, target=Cs2g10790.1 gene=Cs2g10790
Category=3
Score=5
Cleavage Site=1371

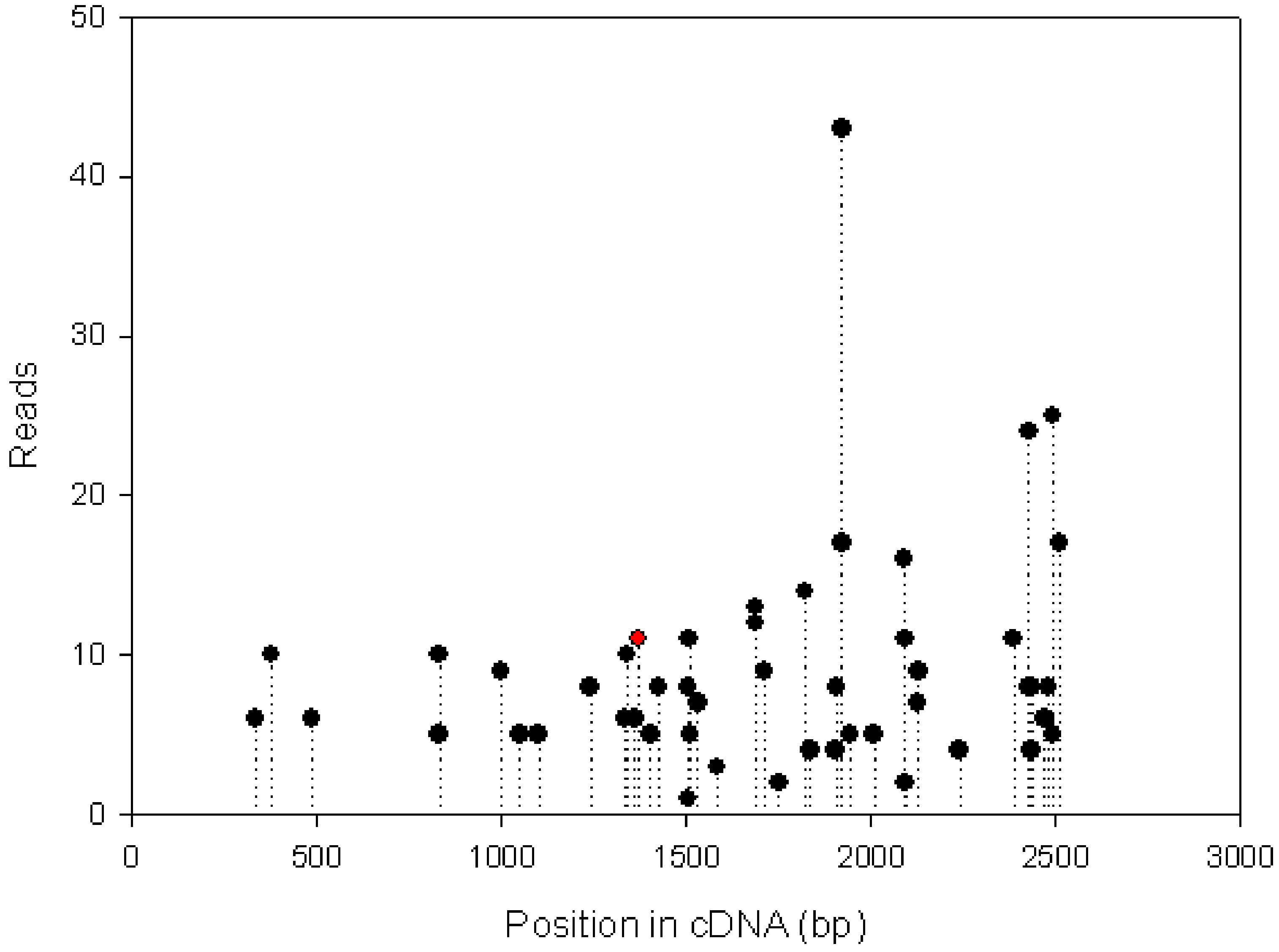


```
5' UGAAGUUGCGGAGGAAAUUAGAAUGC 3'        Cs2g10790.1
   :.::.:   :::::::::::.:
3' AUUUUAUAACCUCCUUUAAUUU---- 5'        Csi-miR2275c.1
```

Csi-miR2275d, target=Cs2g06980.1 gene=Cs2g06980
Category: 3
Score=5
Cleavage Site=3782

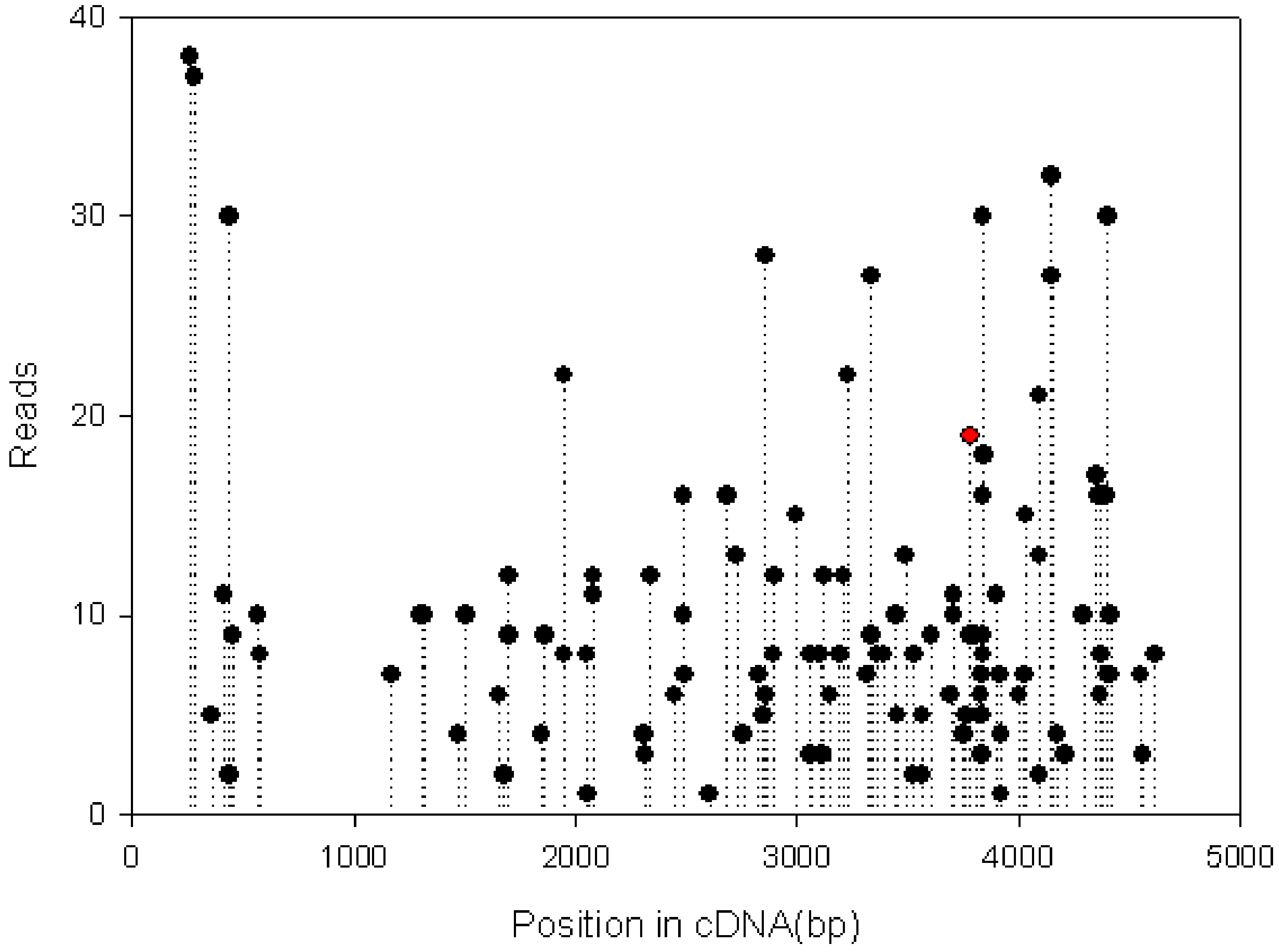


```
5' AGAUCAUGUAGUU-CAUCCAAUUCUUG 3'        Cs2g06980.1
         :  ::::: :::::::::::
3' ----ACAAAUCAAGGUAGGUUAAGA-- 5'        Csi-miR2275d
```

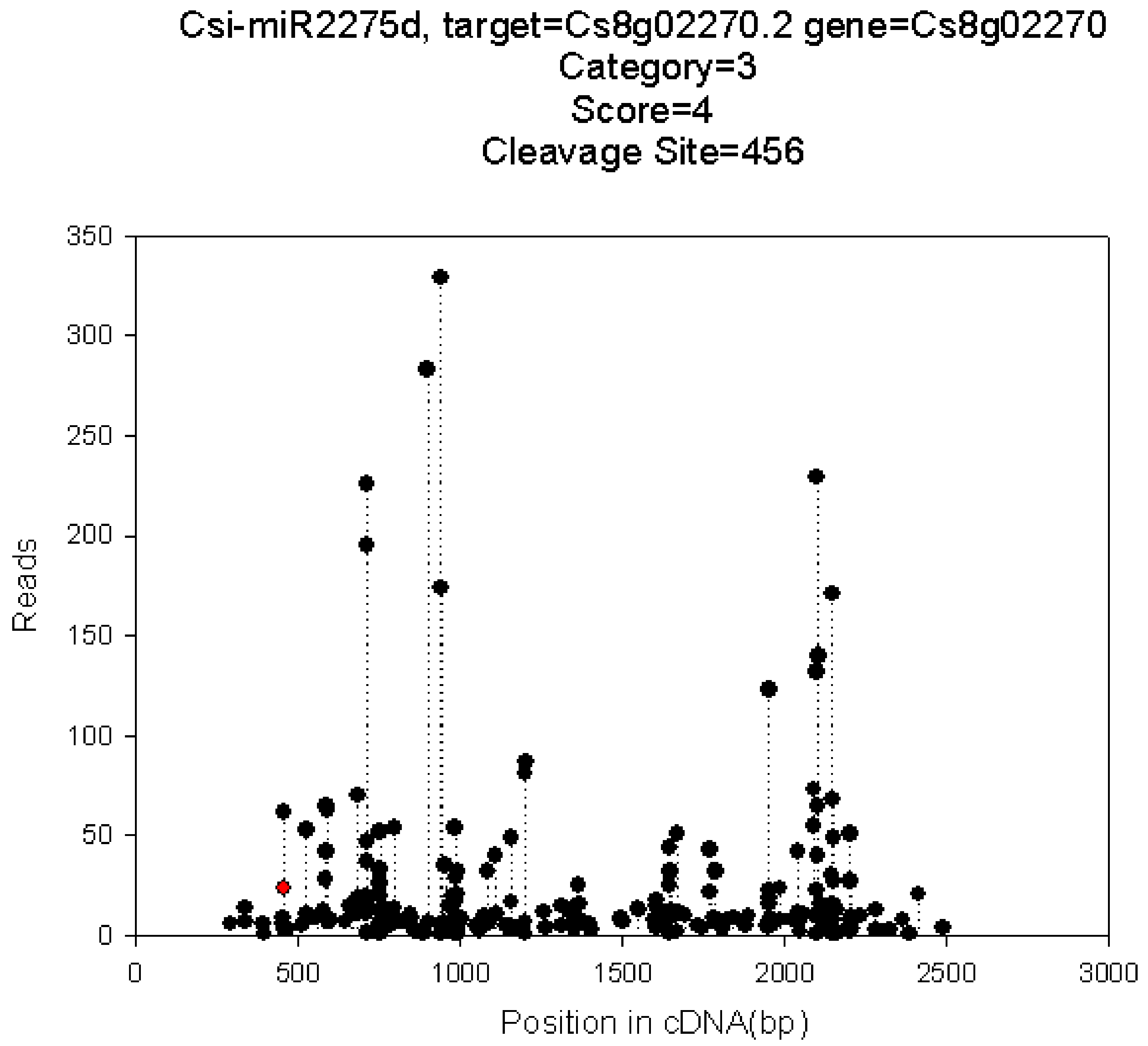


```
5' AGUUUUCAUUUGUUUCAUCCAAUUCU 3'        Cs8g02270.2
          :   ::  :::.:::::::::::
3' -----ACAAAUCAAGGUAGGUUAAGA 5'        Csi-miR2275d
```

Csi-miR2275f, target=Cs4g12820.1 gene=Cs4g12820
Category=3
Score=5
Cleavage Site=522

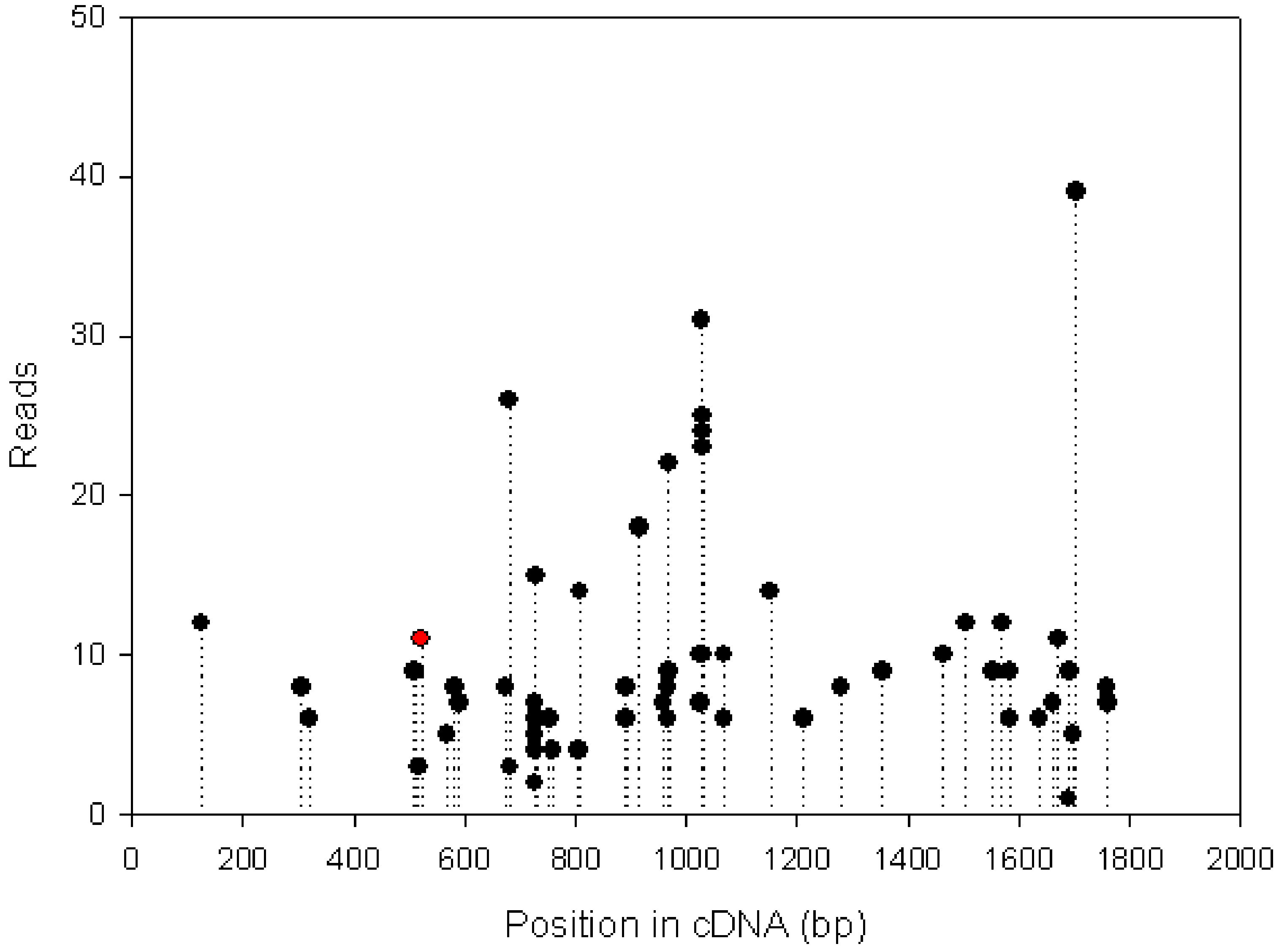


```
5' UUUAGAAAUUGCAGAAAACCAACGGA 3'        Cs4g12820.1
    : ::: :::: ::::::::::
3' -ACUCUAUAACCUCUUUUGGUUU--- 5'        Csi-miR2275f
```

Csi-miR319, target=Cs3g06390.1 gene=Cs3g06390
Category=1
Score=4
Cleavage Site=773

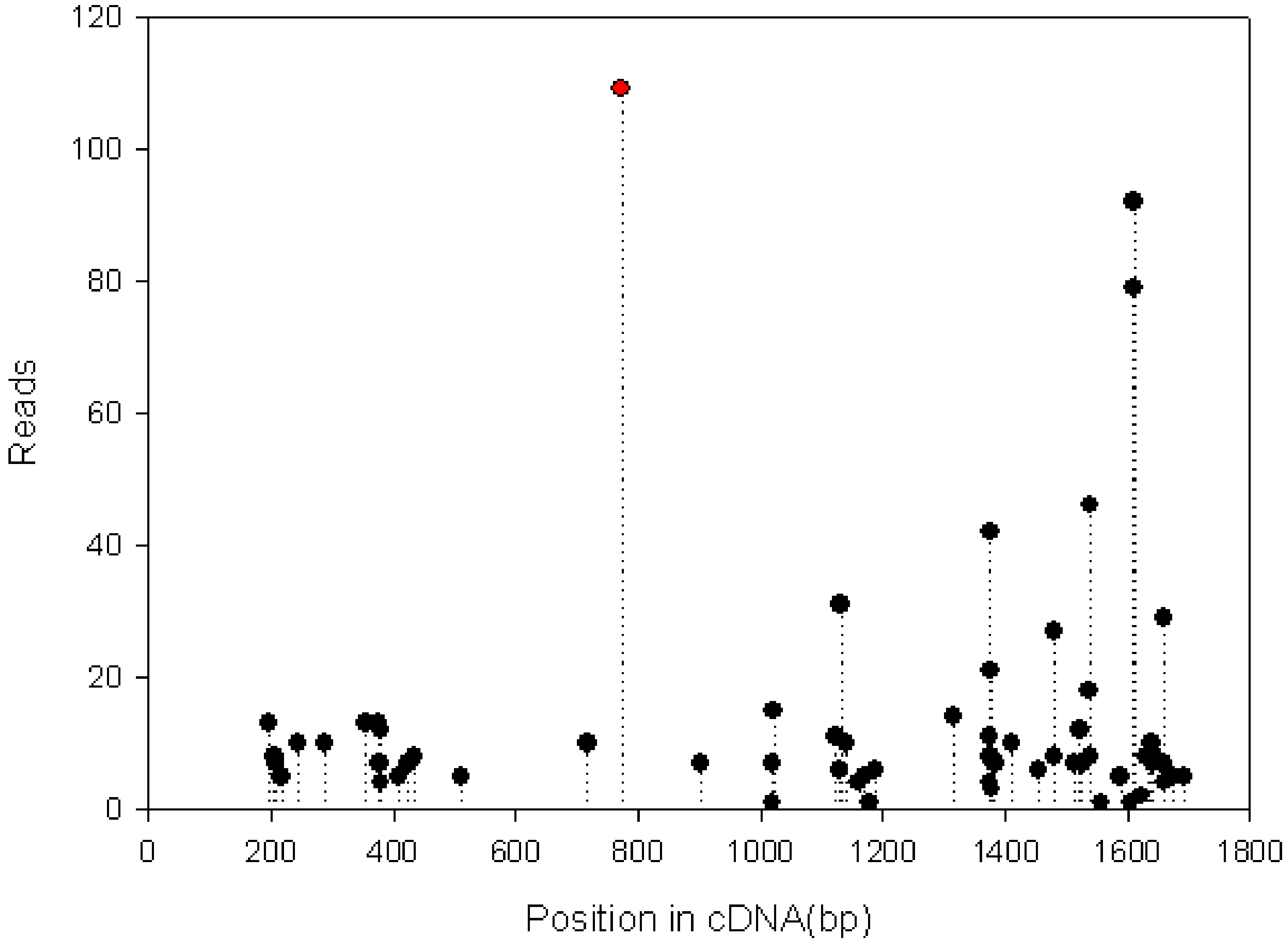


```
5' UUGGAGCUCCCUUCACUCCAAUAUAC 3'        Cs3g06390.1
    ::::::::::::: :::::
3' -UCCUCGAGGGAAGUCAGGUUU---- 5'        Csi-miR319
```

Csi-miR319a, target=Cs1g06290.1 gene=Cs1g06290
Category=3
Score=3
Cleavage Site=1242

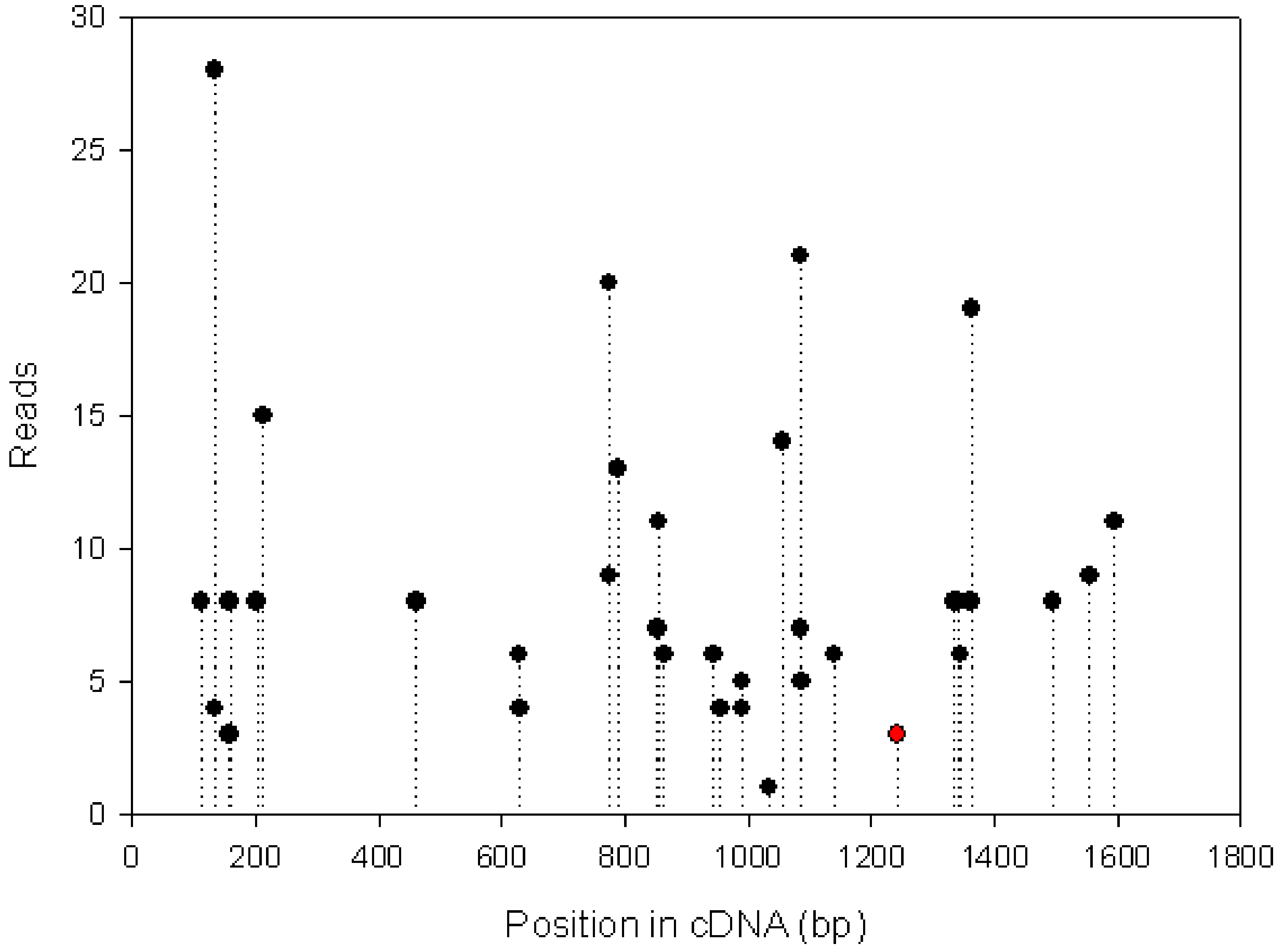


```
5' GUGCGGAUGAAUGAGUUGGUAGUUCA 3'        Cs1g06290.1
       :.::::::::::.::.::.:
3' ---ACUUACUUACUCAGCCGUCGA-- 5'        Csi-miR319a
```

Csi-miR319b, target=Cs3g06390.1 gene=Cs3g06390
Category=1
Score=3
Cleavage Site=773

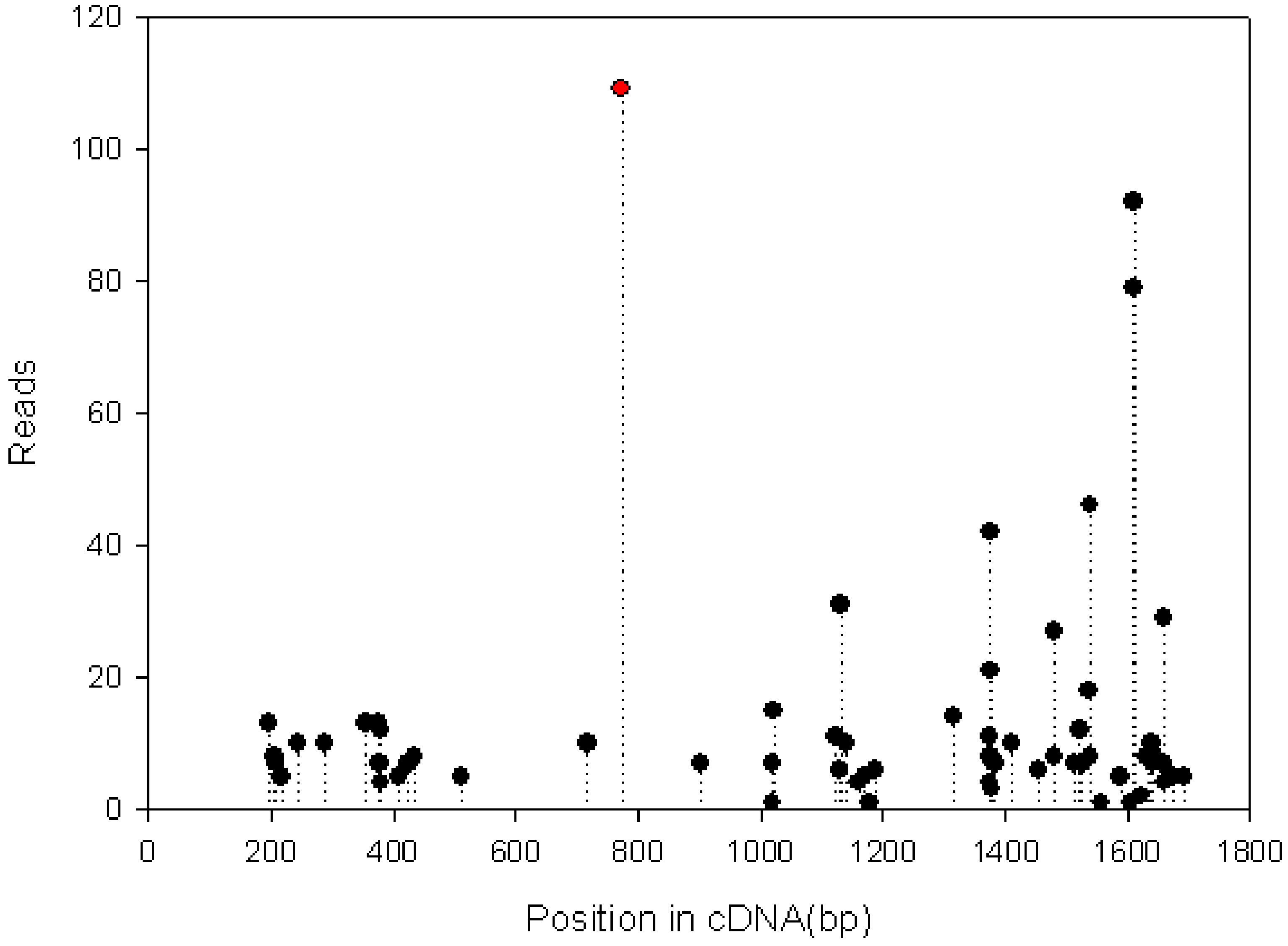


```
5' UUGGAGCUCCCUUCACUCCAAUAUAC 3'        Cs3g06390.1
     ::::::::::::: :::::
3' -CCCUCGAGGGAAGUCAGGUU----- 5'        Csi-miR319b
```

Csi-miR390.1, target=Cs9g01780.1 gene=Cs9g01780
Category=1
Score=4.5
Cleavage Site=495

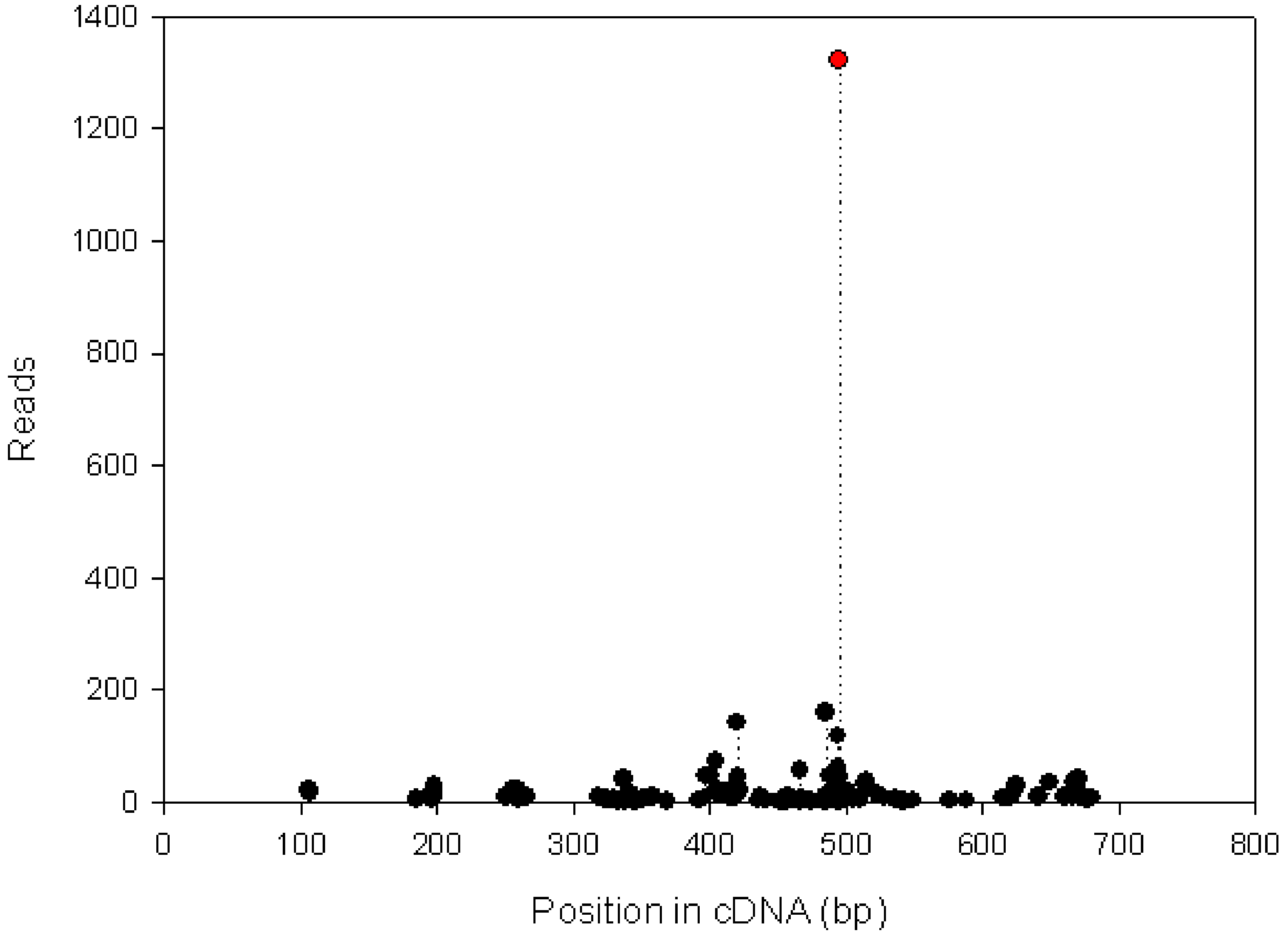


```
5' CUUCCUUGUCUAUCCCUCCUGAGCUG 3'        Cs9g01780.1
         .: ::::::::::::::::
3' ----CCGC-GAUAGGGAGGACUCGAA 5'        Csi-miR390.1
```

Csi-miR390.2, target=Cs9g01780.1 gene=Cs9g01780
Category=1
Score=3.5
Cleavage Site=495

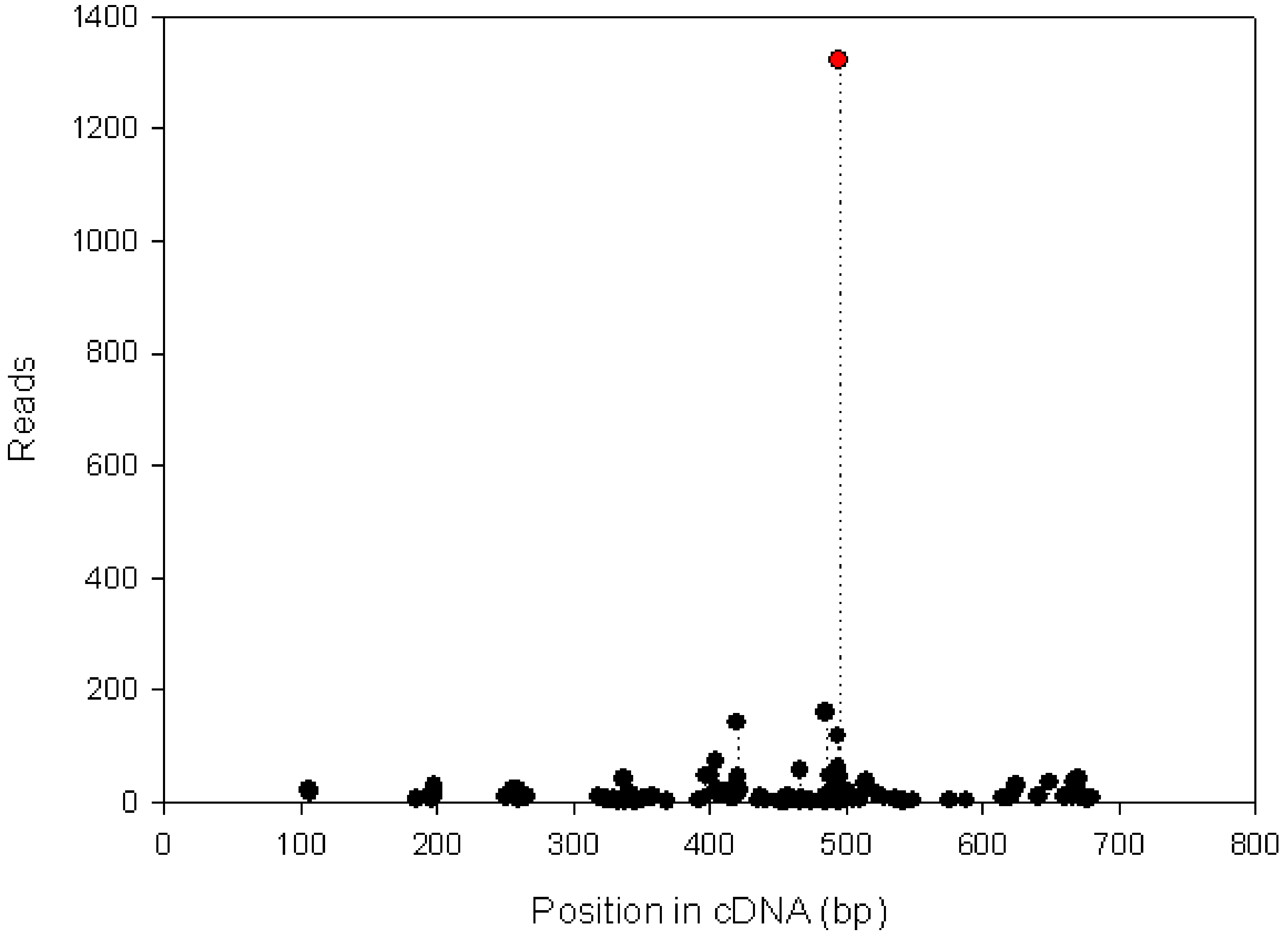


```
5' CUUCCUUGUCUAUCCCUCCUGAGCUG 3'        Cs9g01780.1
         .: ::::::::::::::::
3' ----CCGC-GAUAGGGAGGACUCGA- 5'        Csi-miR390.2
```

Csi-miR390-3p.2, target=Cs8g04530.1 gene=Cs8g04530
Category=3
Score=5
Cleavage Site=871

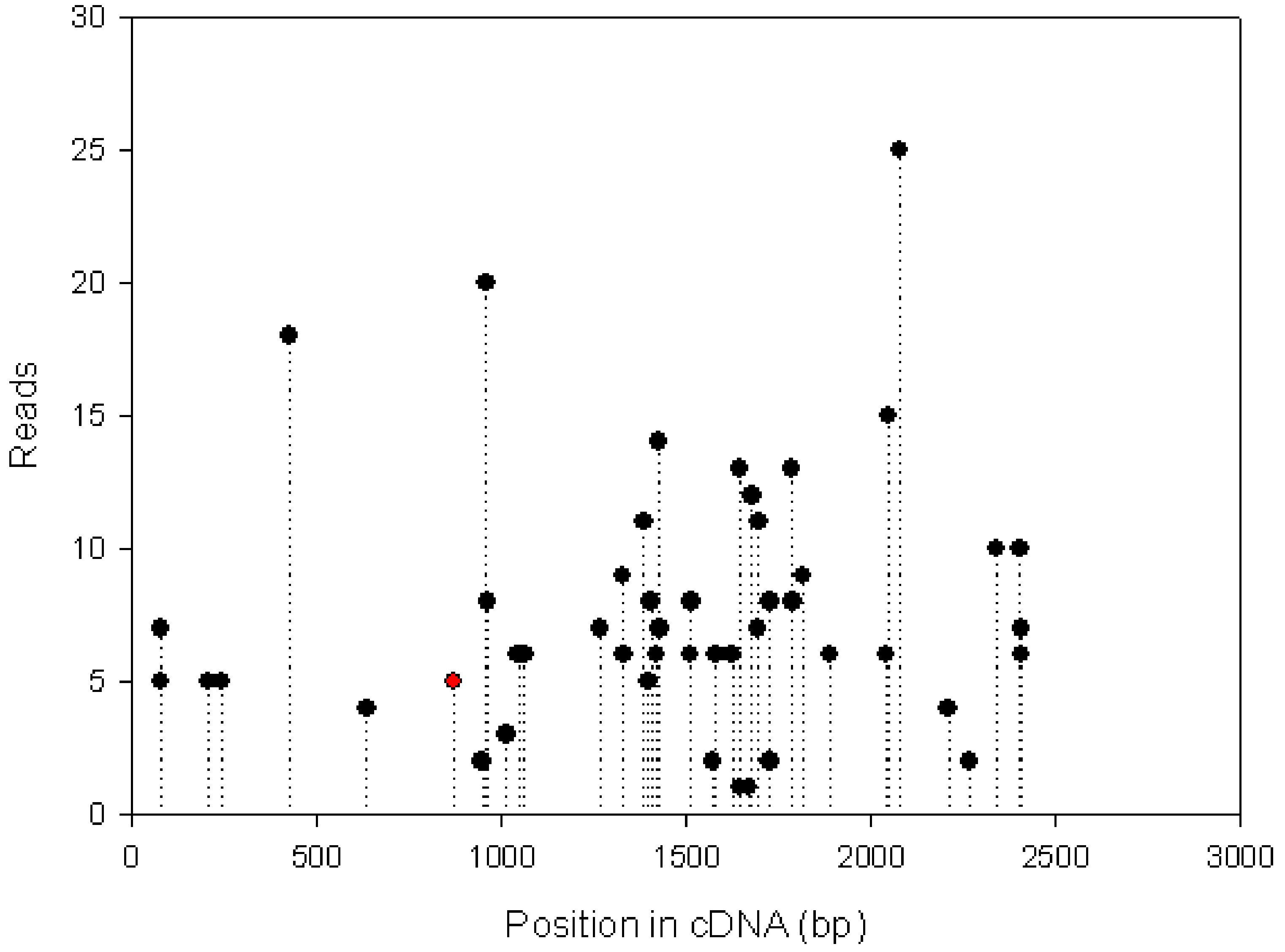


```
5' CAAUGGGGAAGGUUAGGAUGG-UAGCC 3'        Cs8g04530.1
           :::. :.:::::::: ::::
3' -------CUUUGAGUCCUACCUAUCGC 5'        Csi-miR390-3p.2
```

Csi-miR390b, target=Cs9g01780.1 gene=Cs9g01780
Category=1
Score=0
Cleavage Site=495

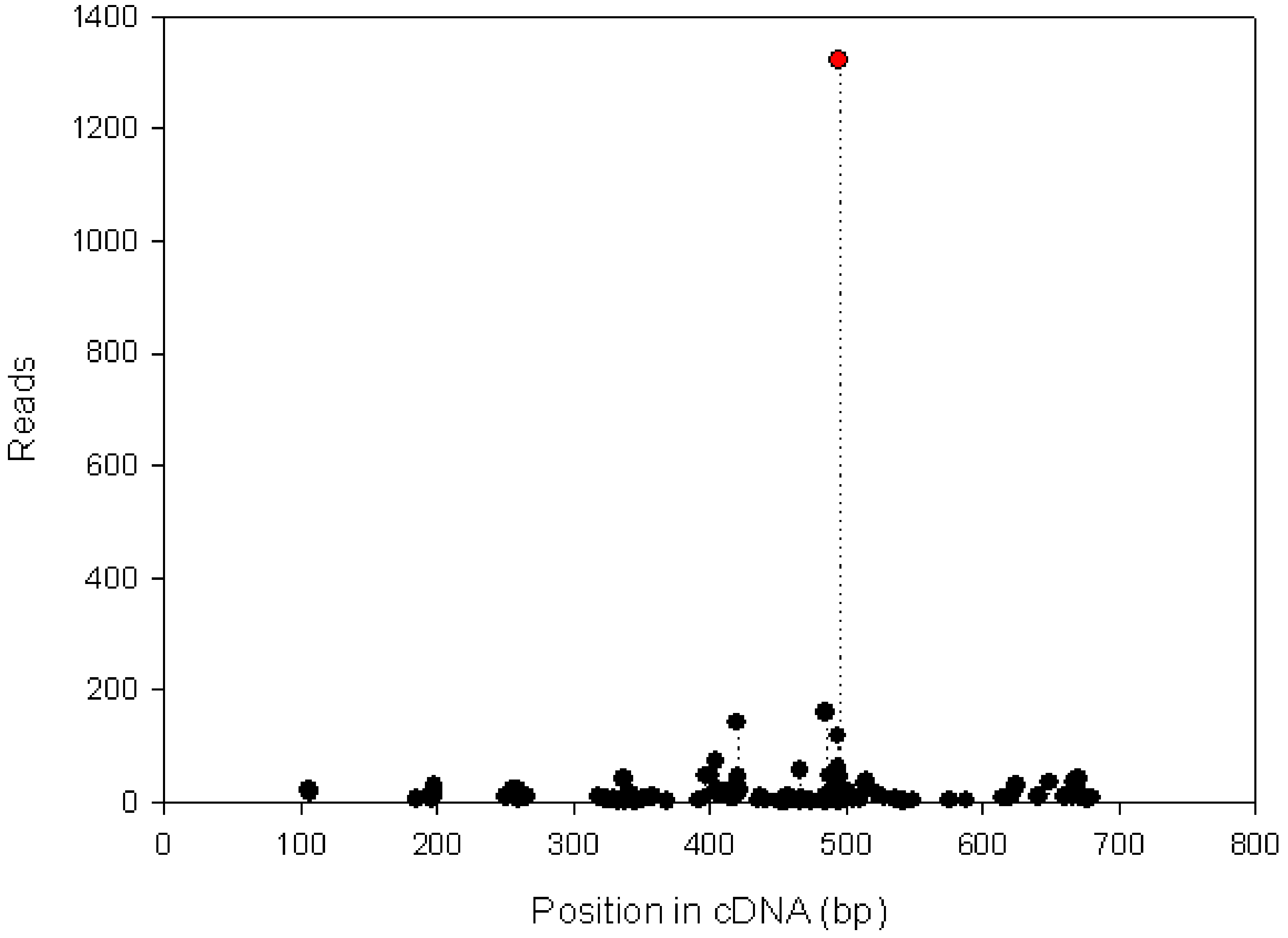


```
5' CUUCCUUGUCUAUCCCUCCUGAGCUG 3'         Cs9g01780.1
        :::::::::::::::::::::
3' -----AACAGAUAGGGAGGACUCGAC 5'         Csi-miR390b
```

Csi-miR391a.1, target=Cs5g17560.1 gene=Cs5g17560
Category=3
Score=4.5
Cleavage Site=2319

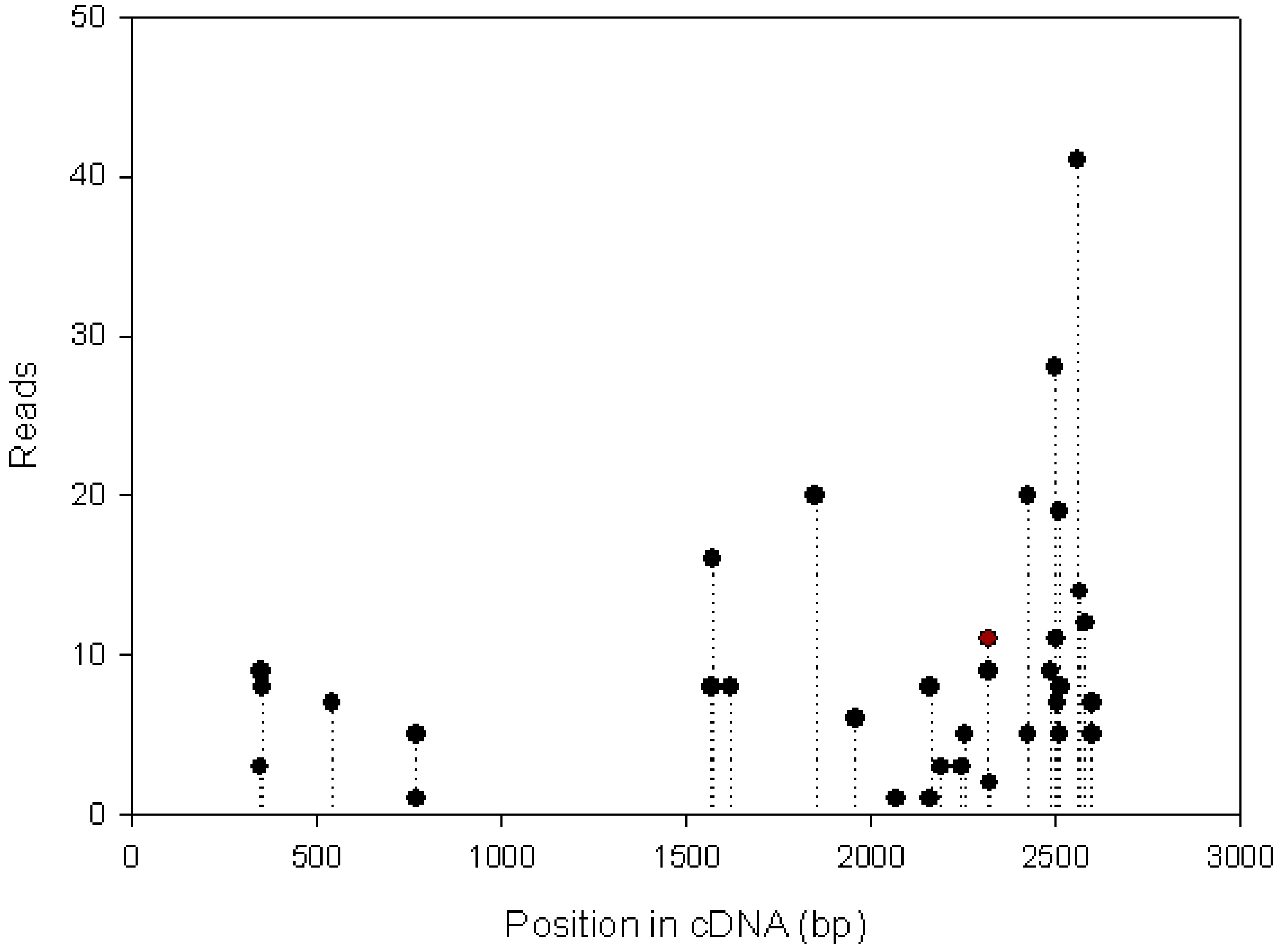


```
5' UUGC-AUUUUUCUGGUGACAAUCUCAA 3'        Cs5g17560.1
    ::: ::.:.::.::.:::::
3' -ACGAUAGAGAGGCCGCUGUU------ 5'        Csi-miR391a.1
```

Csi-miR393a.1, target=Cs7g31800.1 gene=Cs7g31800
Category=3
Score=5
Cleavage Site=1321

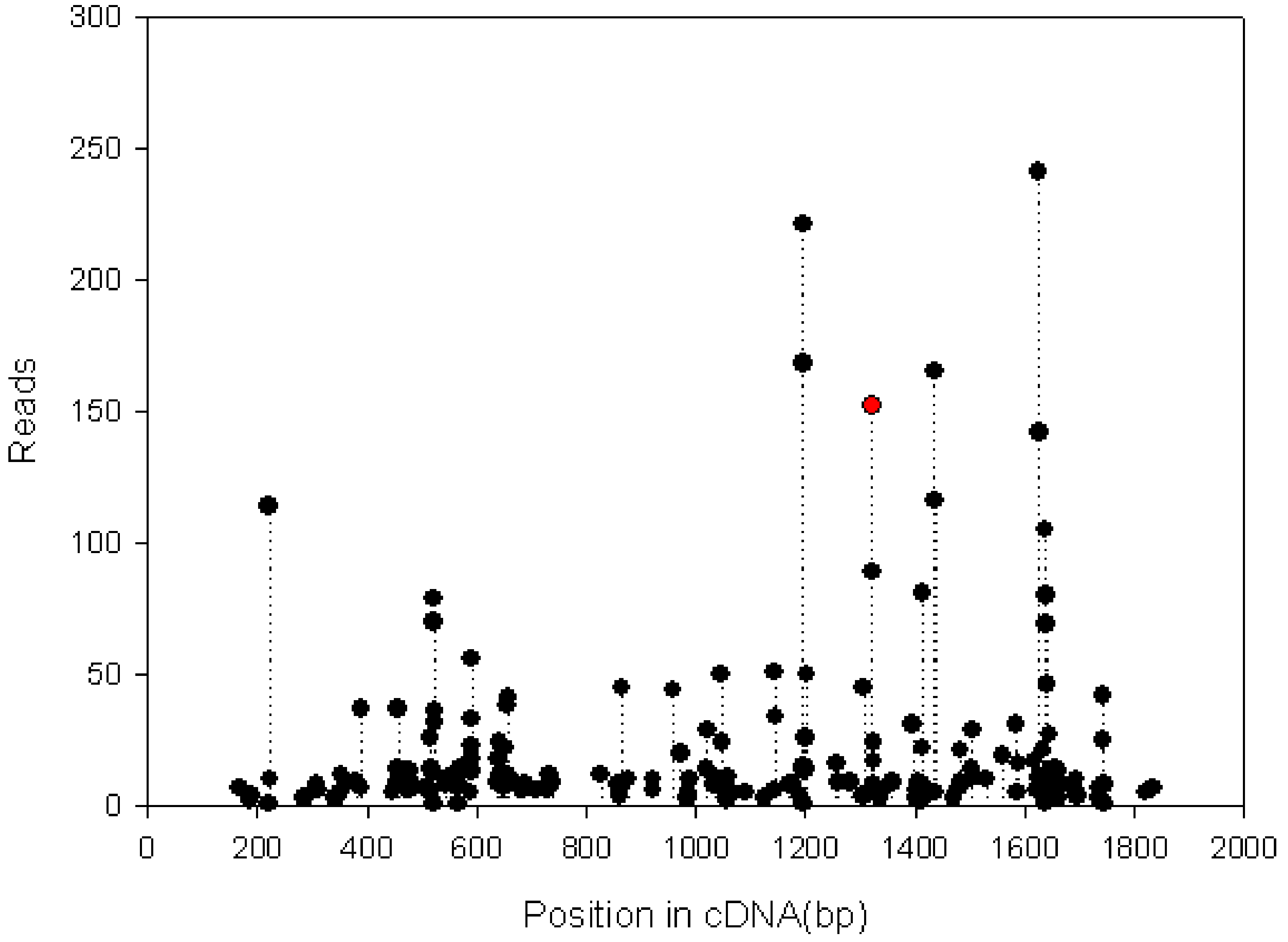


```
5' CCGGCCAAUCCAU-UGAUUUCUUUGGA 3'        Cs7g31800.1
          ::: :: .:::..:::::::
3' -------UAGUUACGCUAGGGAAACCU 5'        Csi-miR393a.1
```

Csi-miR393a.2, target=Orange1.1t02367.1 gene=Orange1.1t02367
Category=3
Score=3.5
Cleavage Site=424

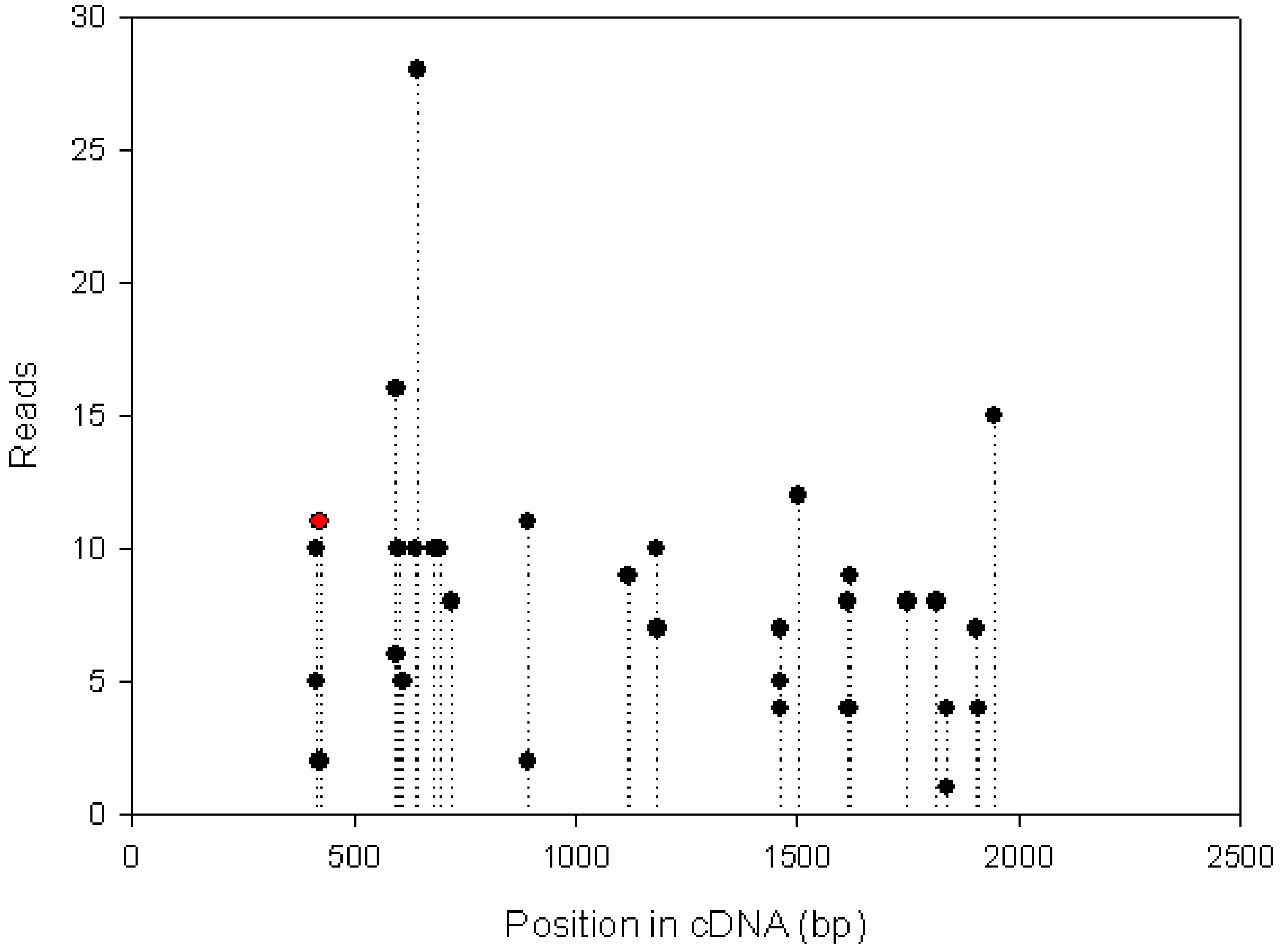


```
5' GGAUCAGAGCGAUCCCUUUGAAUCAG 3'        Orange1.1t02367.1
    :::::. :::::::::::: :
3' -CUAGUUACGCUAGGGAAACCU---- 5'        Csi-miR393a.2
```

Csi-miR393a.2-3p, target=Cs9g05070.1 gene=Cs9g05070
Category=3
Score=4
Cleavage Site=1601

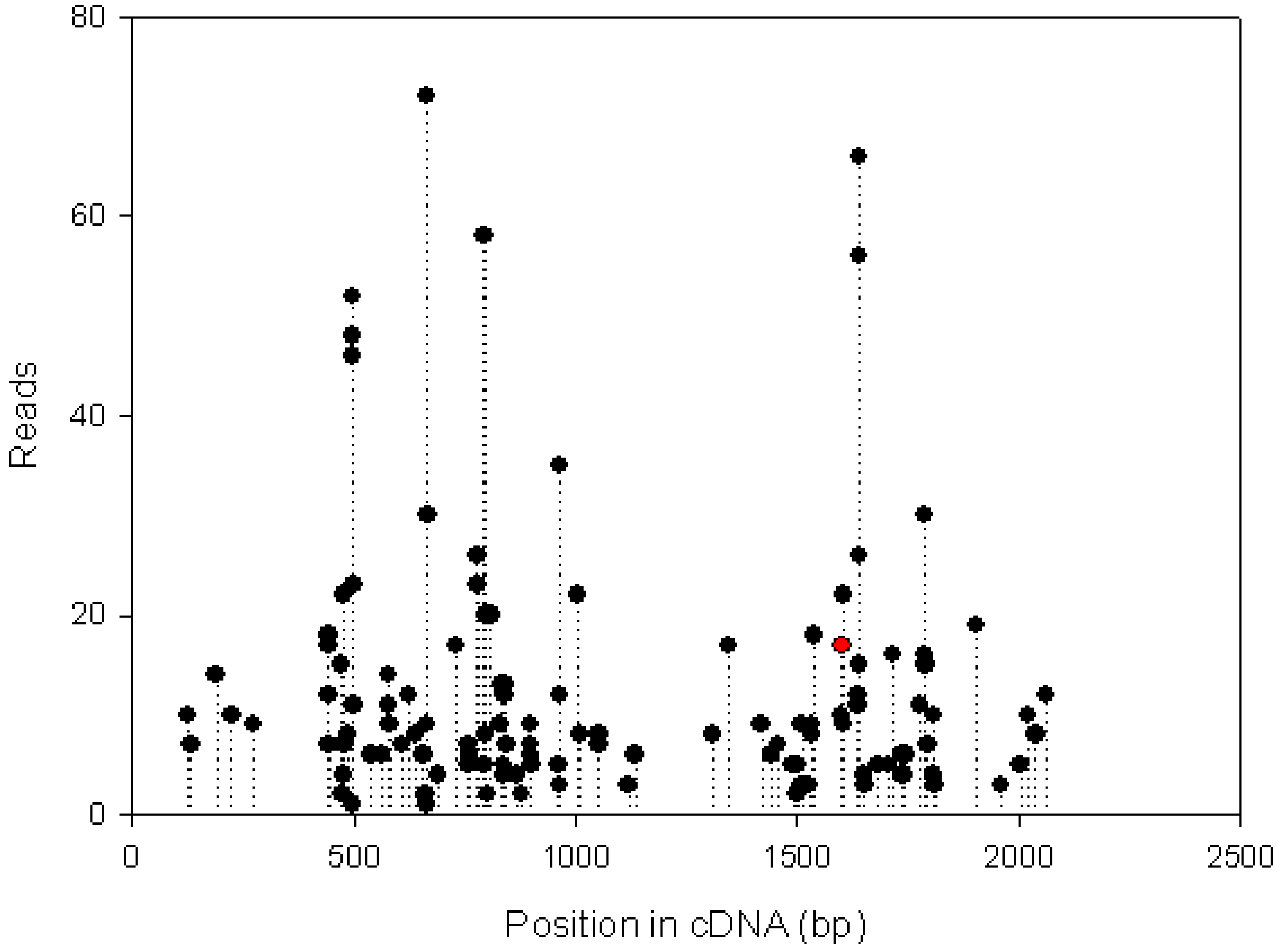


```
5' UAAUCCGGA-GGAUGGCAUGAUUCUUG 3'       Cs9g05070.1
    ::::::..: ::::.::::::::
3' -UUAGGUUUCCCUAUCGUACUA----- 5'       Csi-miR393a.2-3p
```

Csi-miR393a.3, target=Orange1.1t02367.1 gene=Orange1.1t02367
Category=3
Score=3.5
Cleavage Site=424

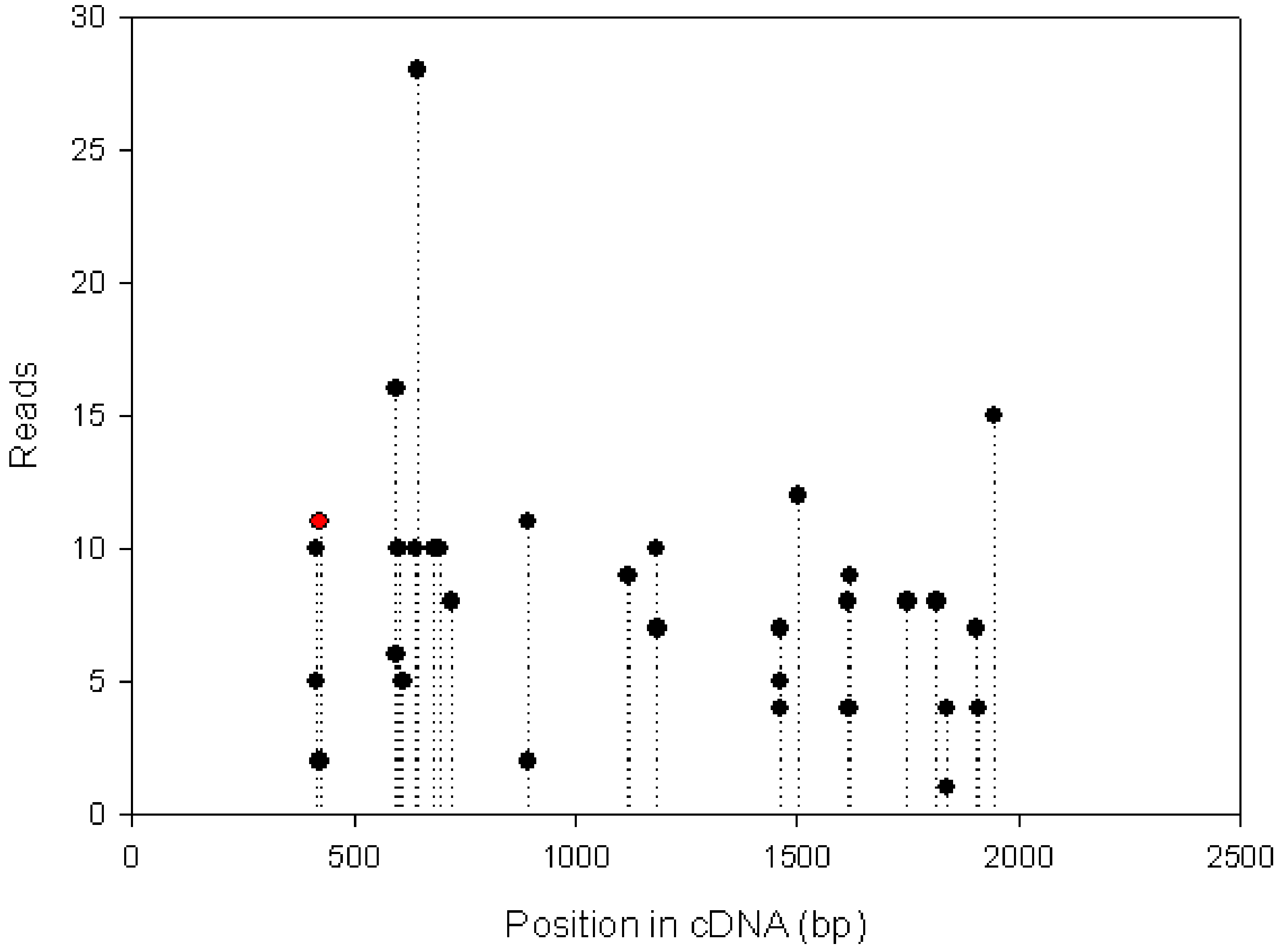


```
5' GGAUCAGAGCGAUCCCUUUGAAUCAG 3'        Orange1.1t02367.1
   :::::::. :::::::::::: :
3' CCUAGUUACGCUAGGGAAACCU---- 5'        Csi-miR393a.3
```

Csi-miR393b, target=Orange1.1t02367.1 gene=Orange1.1t02367
Category=3
Score=4.5
Cleavage Site=424

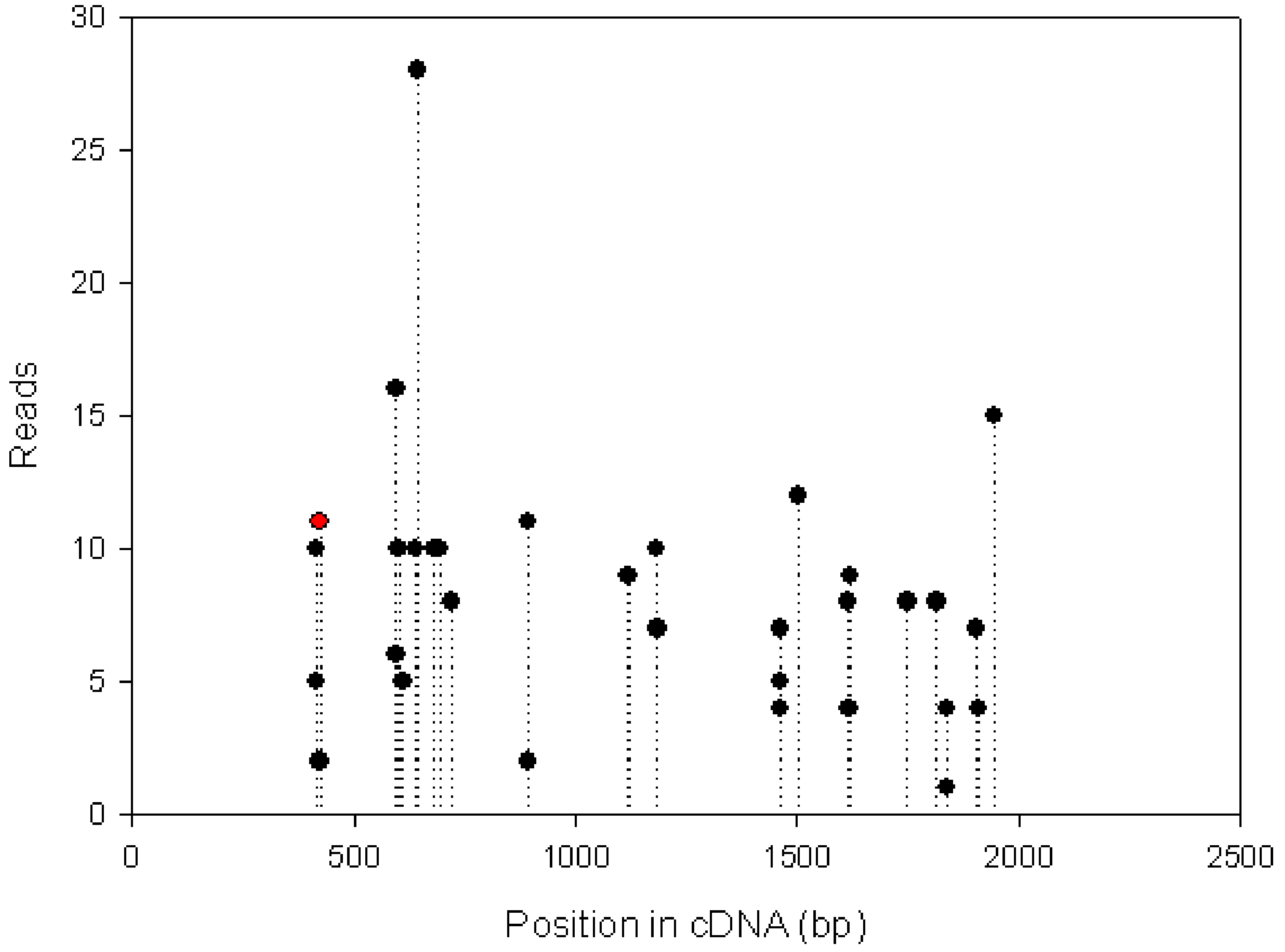


```
5' GGAUCAGAGCGAUCCCUUUGAAUCAG 3'        Orange1.1t02367.1
    :::::. ::::::::::::: :
3' -CUAGUUACGCUAGGGAAACCUU--- 5'        Csi-miR393b
```

Csi-miR393b-3p,target=Cs5g31220.1 gene=Cs5g31220
Category=3
Score=5
Cleavage Site=483

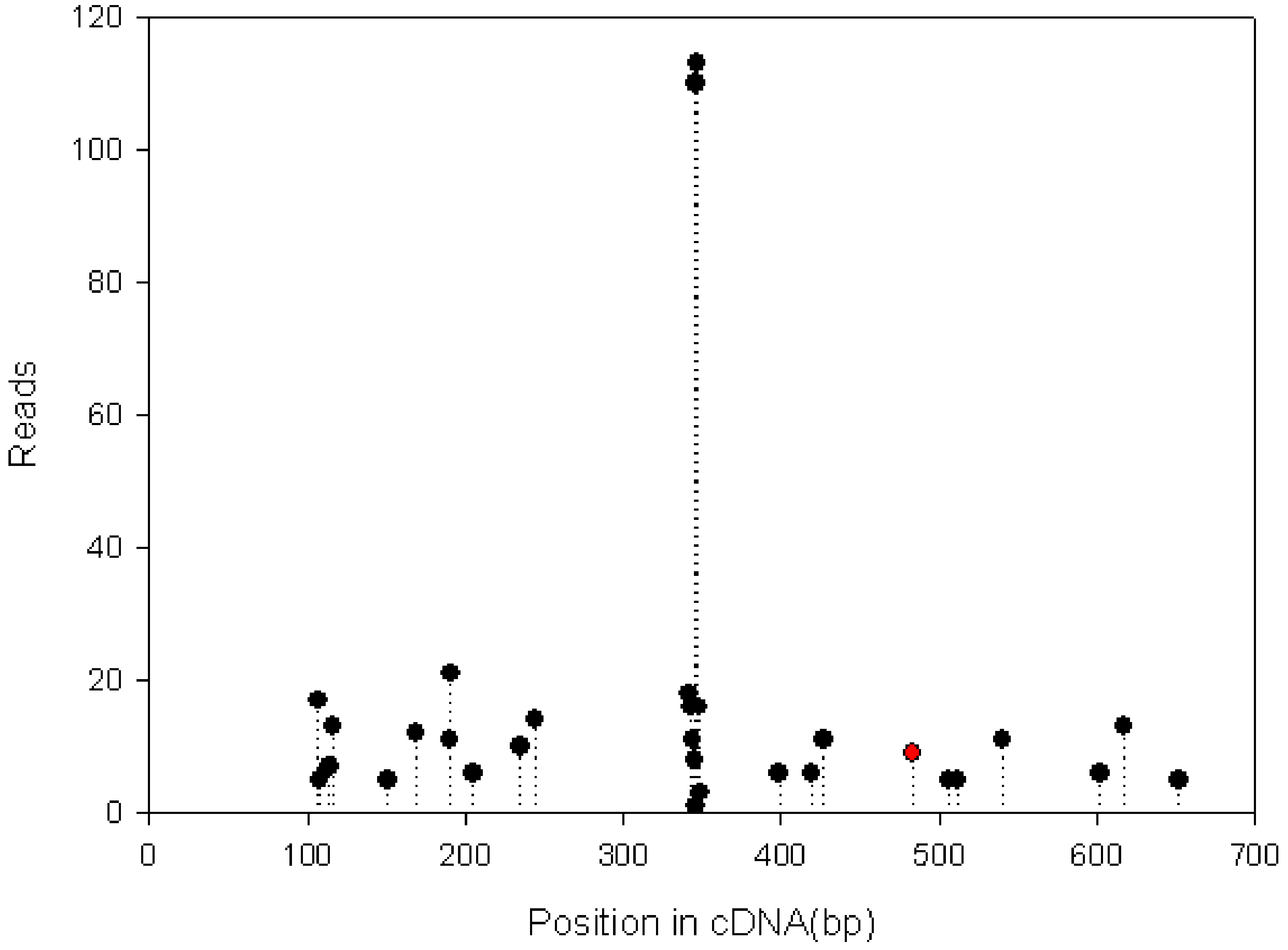


```
5' AAUUCCUAAGGGAUC-UGUGAUUGAGC 3'        Cs5g31220.1
   :::::: :::::::: ..:::
3' UUAAGGCUUCCCUAGCGUACU------ 5'        Csi-miR393b-3p
```

Csi-miR394, target=Cs5g08980.1 gene=Cs5g08980
Category=3
Score=3.5
Cleavage Site=2526

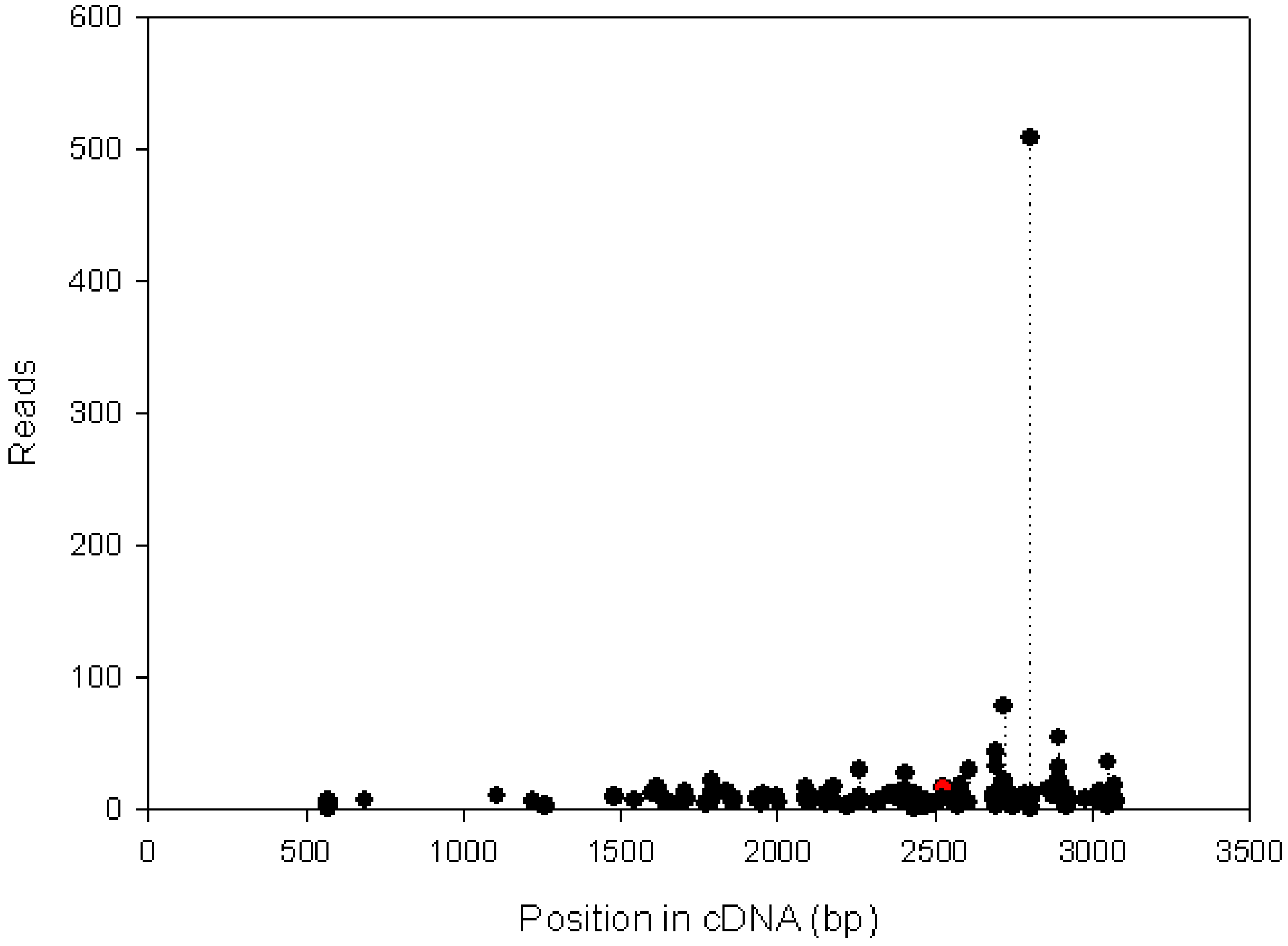


```
5' GGGCCCGGACAGAAUGCCAAUGUGGA 3'        Cs5g08980.1
   ::.    ::::::::::::::
3' CCUCCACCUGUCUUACGGUU------ 5'        Csi-miR394
```

Csi-miR394-3p.1, target=Cs3g09210.1 gene=Cs3g09210
Category=3
Score=5
Cleavage Site=1866

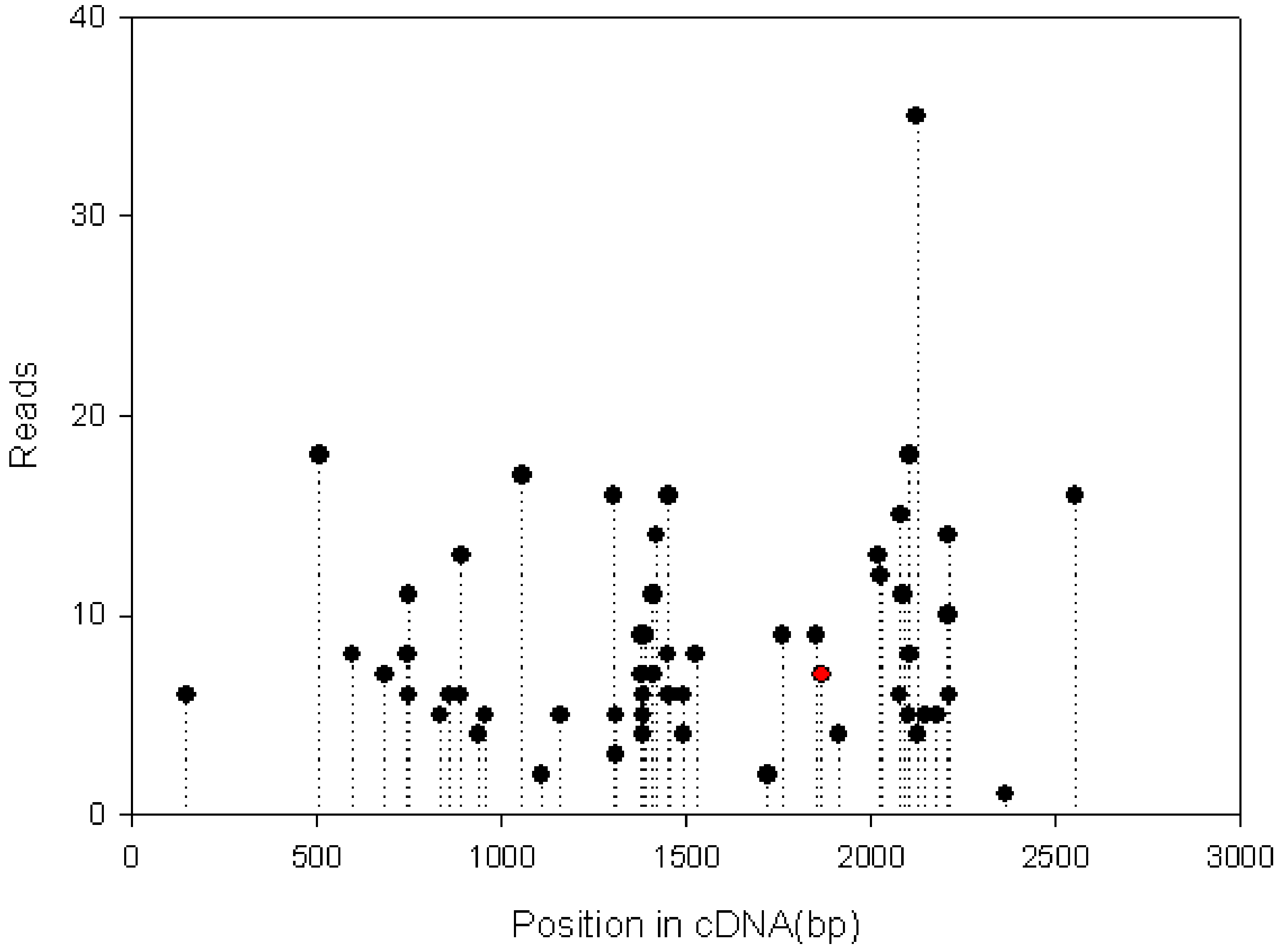


```
5' GUUUGUUUGGAAUUU-UCUAGCUGAUA 3'        Cs3g09210.1
     : ::::: ::::: ::.::::
3' --AUCAAACGUUAAACAGGUCGA---- 5'        Csi-miR394-3p.1
```

Csi-miR394-3p.2, target=Cs8g14600.1 gene=Cs8g14600
Category=3
Score=5
Cleavage Site=996

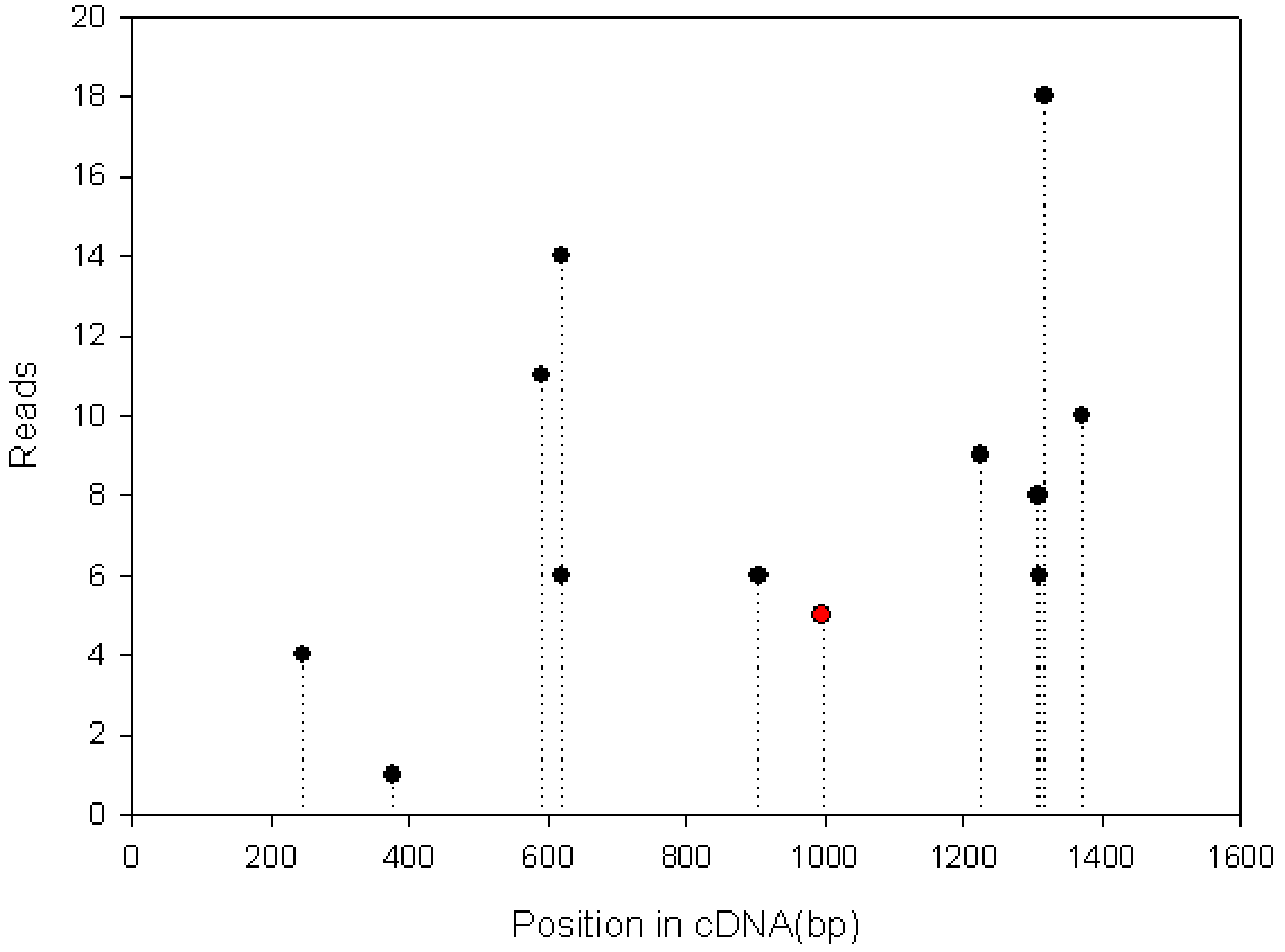


```
5' UGAAG-CUGCAGUUUGUCCAUCCAAAC 3'        Cs8g14600.1
   :: ::  ::::.::::::::
3' ACAUCAAACGUUAAACAGGUC------ 5'        Csi-miR394-3p.2
```

Csi-miR395.1, target=Orange1.1t01708.1 gene=Orange1.1t01708
Category=3
Score=4.5
Cleavage Site=1338

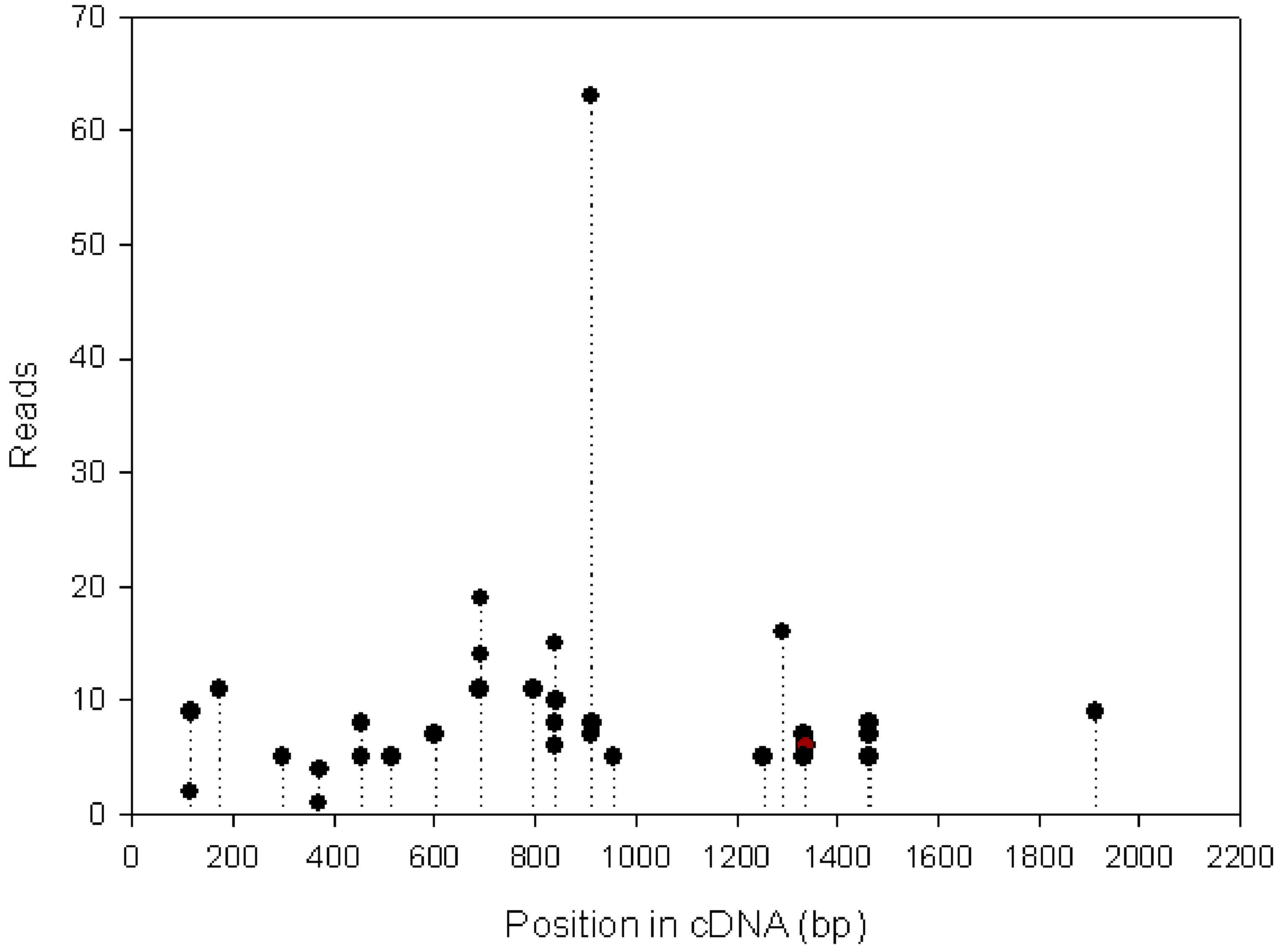


```
5' AACCGGGCU-CACCAAAUACUUCAGAU 3'      Orange1.1t01708.1
       :.:  :  :  :::::.:::::::
3' ----CUCAAGGGGGUUUGUGAAGUC-- 5'      Csi-miR395.1
```

Csi-miR395.2, target=Cs5g34330.1 gene=Cs5g34330
Category=3
Score=5
Cleavage Site=1443

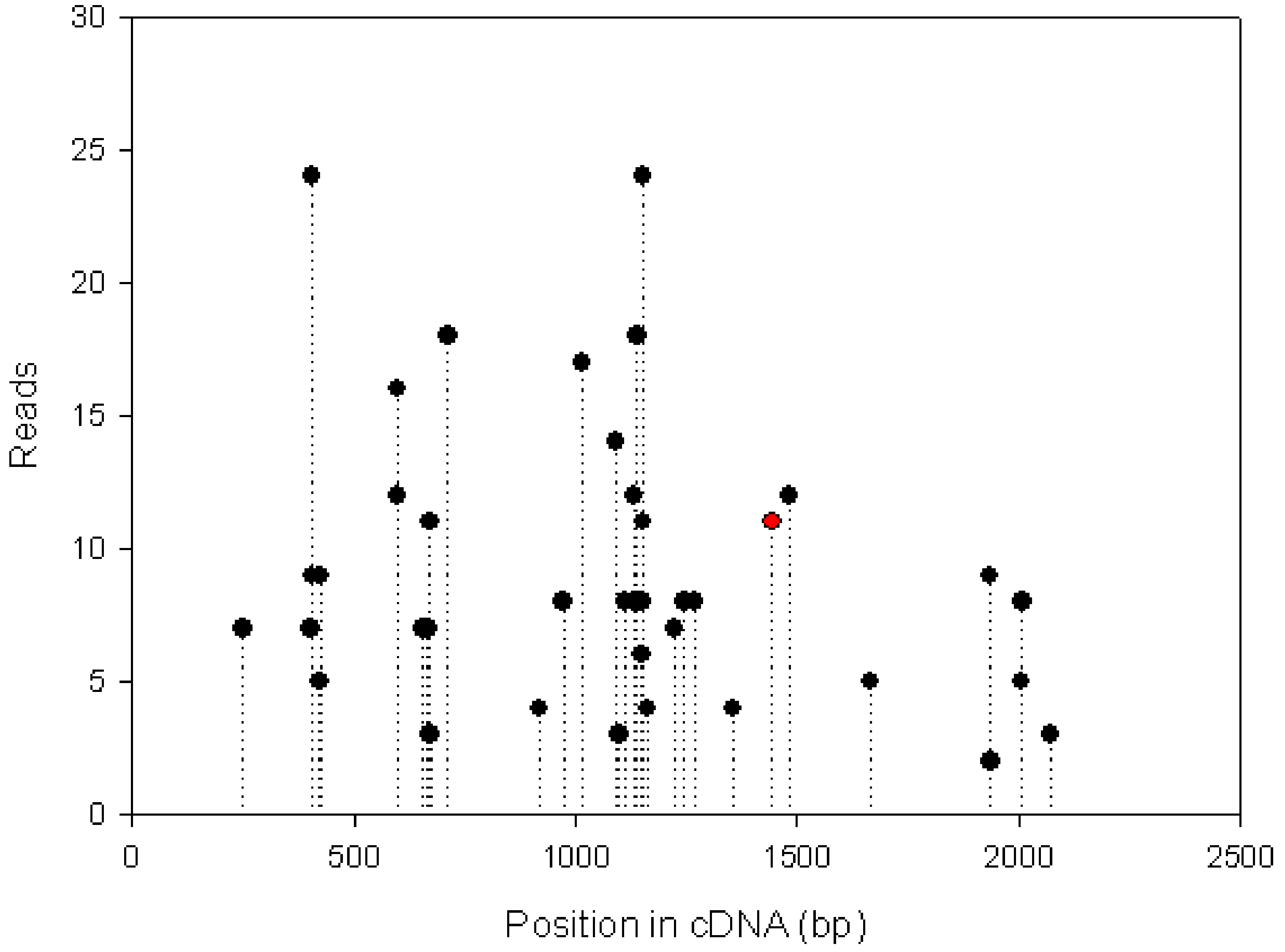


```
5' UUUUUAAGUU-UCCCAGACACUUUGGC 3'        Cs5g34330.1
          :::: .::::.:::::..
3' -----CUCAAGGGGGUUUGUGAAGU-- 5'        Csi-miR395.2
```

Csi-miR3951, target=Orange1.1t05622.1 gene=Orange1.1t05622
Category=1
Score=2.5
Cleavage Site=113

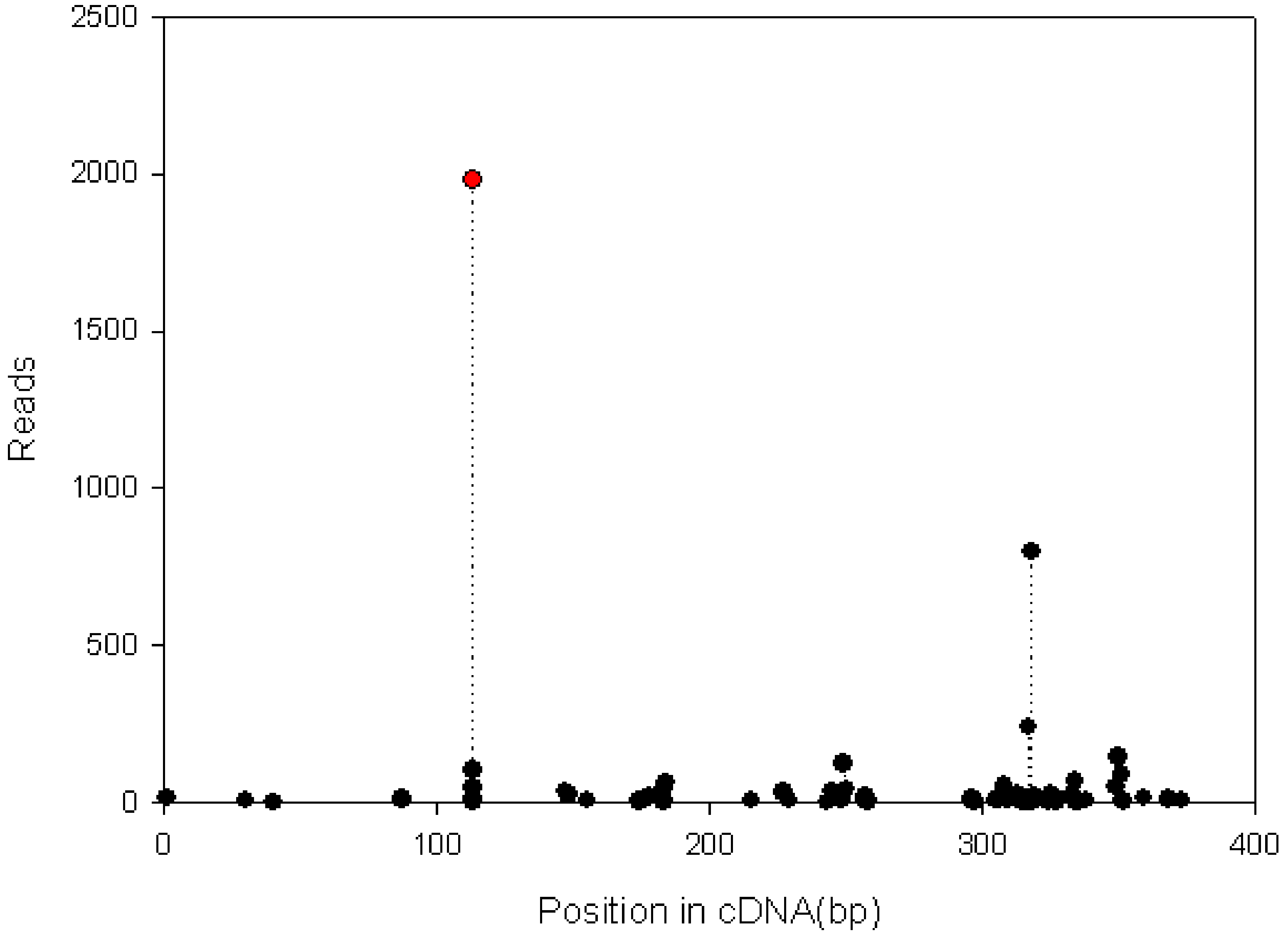


```
5' UUUUUUUCUCUC-UCUUUAUCUGUGGG 3'        Orange1.1t05622.1
     :::::::::: :::::::::.
3' --AAAAAGAGAGUAGAAAUAGAU---- 5'        Csi-miR3951
```

Csi-miR3951-3p, target=Cs2g03130.1 gene=Cs2g03130
Category=3
Score=5
Cleavage Site=1813

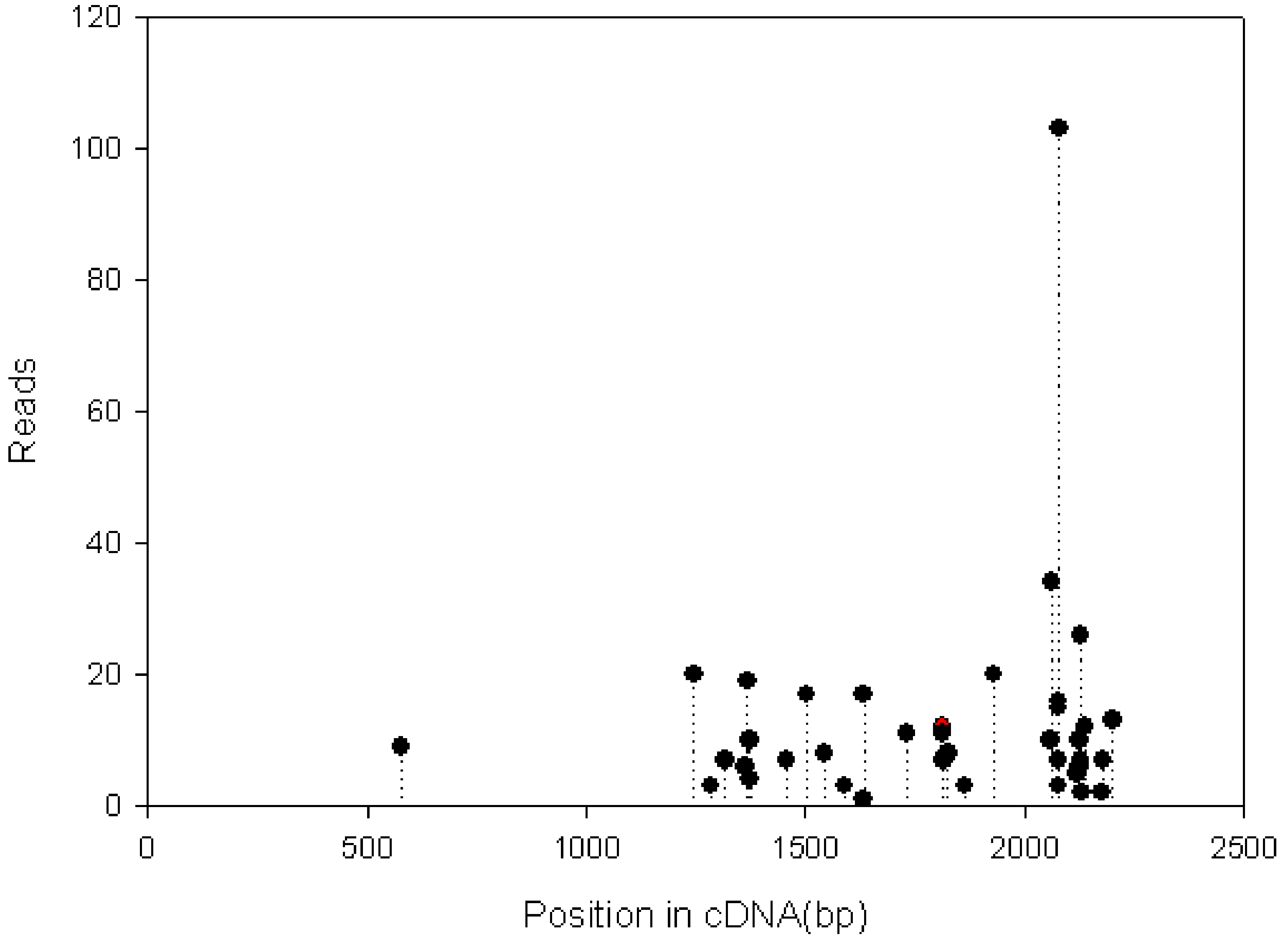


```
5' AUACAGAUAAUGAUGGGAGGAUCAAC 3'        Cs2g03130.1
     :::::::.:::..::::.:
3' --UGUCUAUUGCUAUUCUCUUU---- 5'        Csi-miR3951-3p
```

Csi-miR3952.1, target=Cs8g13286.1 gene=Cs8g13286
Category=2
Score=3
Cleavage Site=304

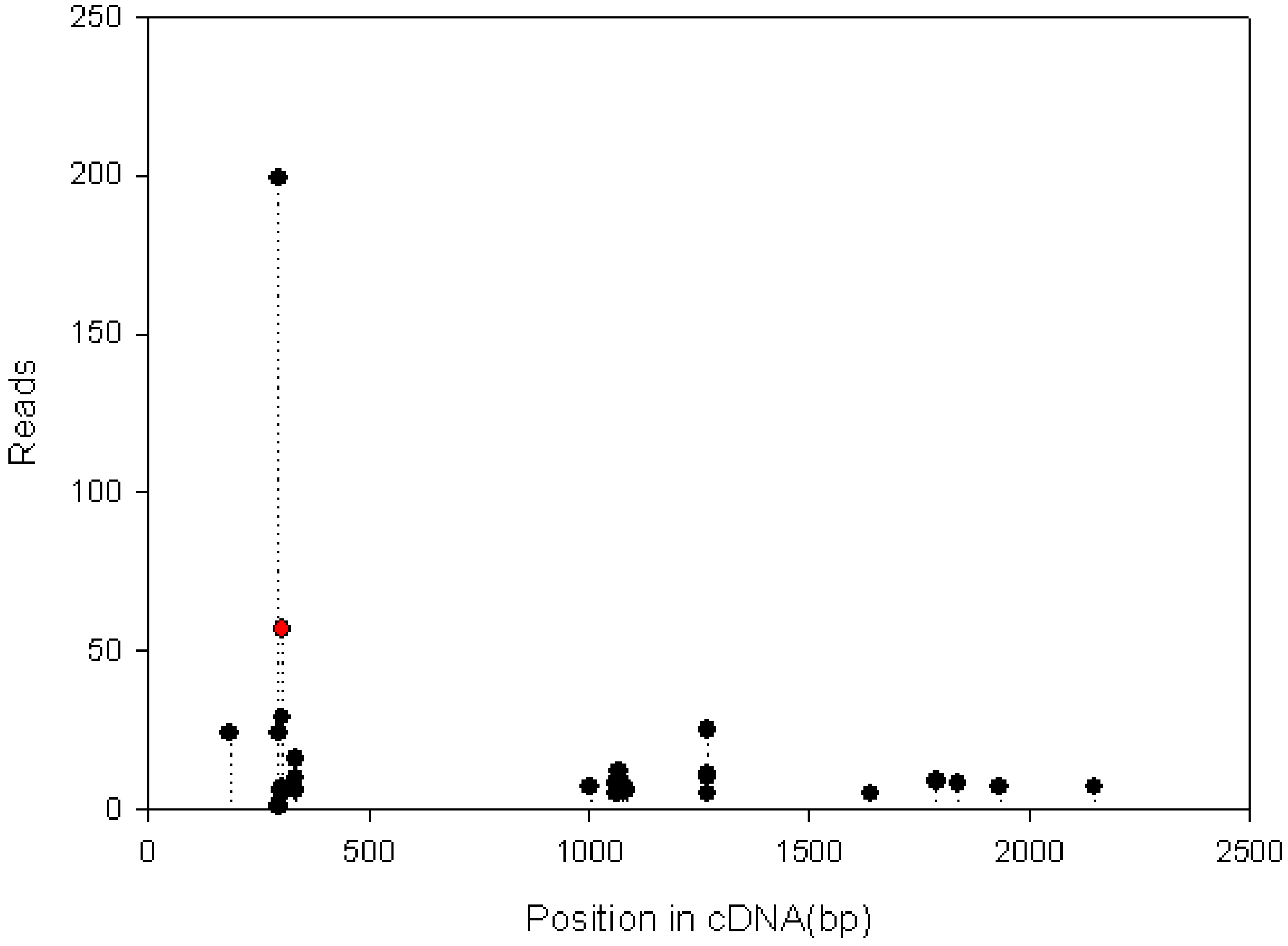


```
5' AGUUGAAGGGCCUUUCUAGAGCACUG 3'        Cs8g13286.1
    :::: ::::::::::: ::::
3' --AACUCCCCGGAAAGAUGUCGU--- 5'        Csi-miR3952.1
```

Csi-miR3952.2, target=Cs8g13286.1 gene=Cs8g13286
Category=2
Score=3
Cleavage Site=304

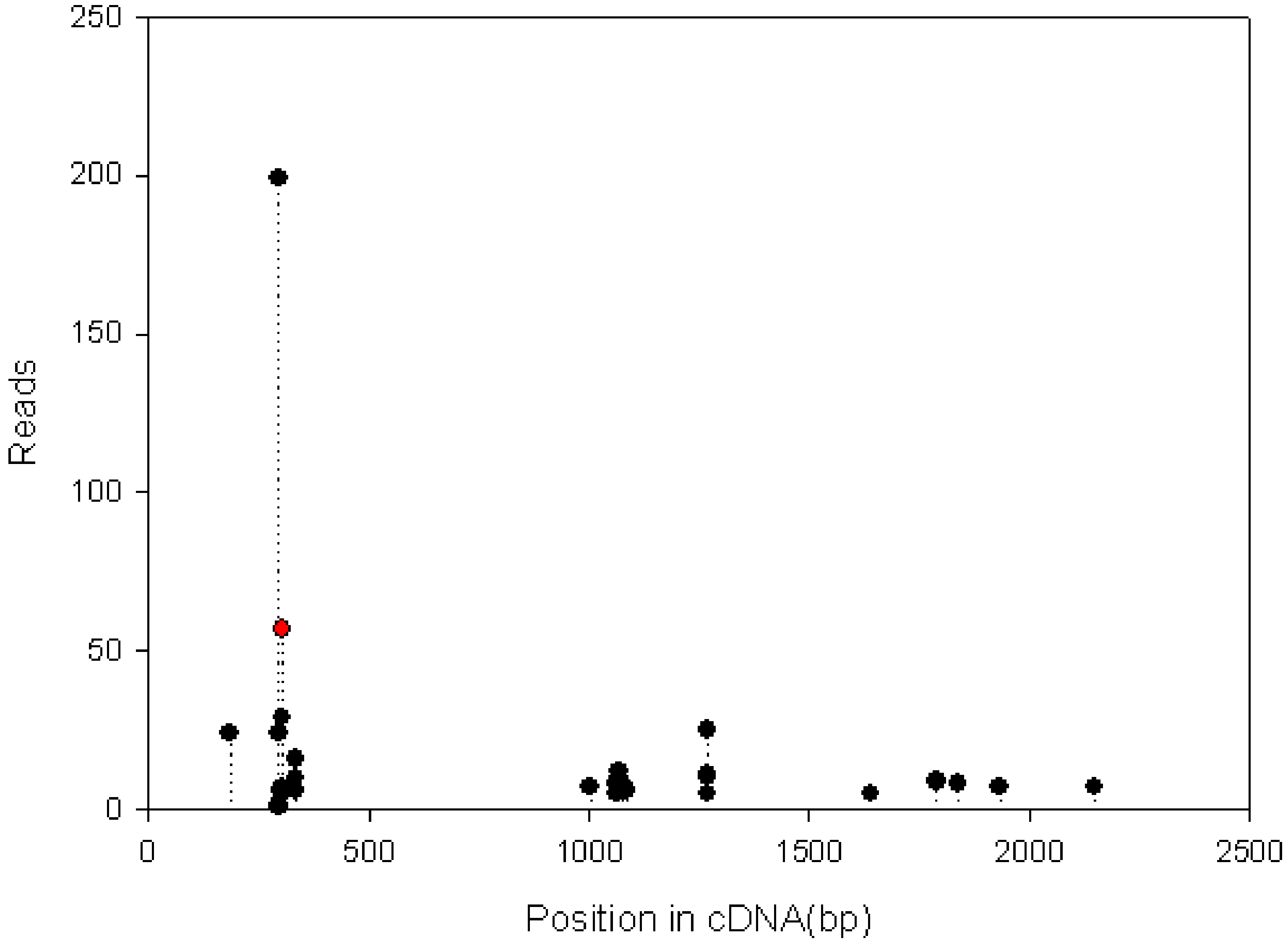


```
5' AGUUGAAGGGCCUUUCUAGAGCACUG 3'        Cs8g13286.1
    ::::: ::::::::::: :::
3' -CAACUCCCCGGAAAGAUGUCG---- 5'        Csi-miR3952.2
```

Csi-miR3952-3p, target=Cs8g13286.1 gene=Cs8g13286
Category=3
Score=3
Cleavage Site=185

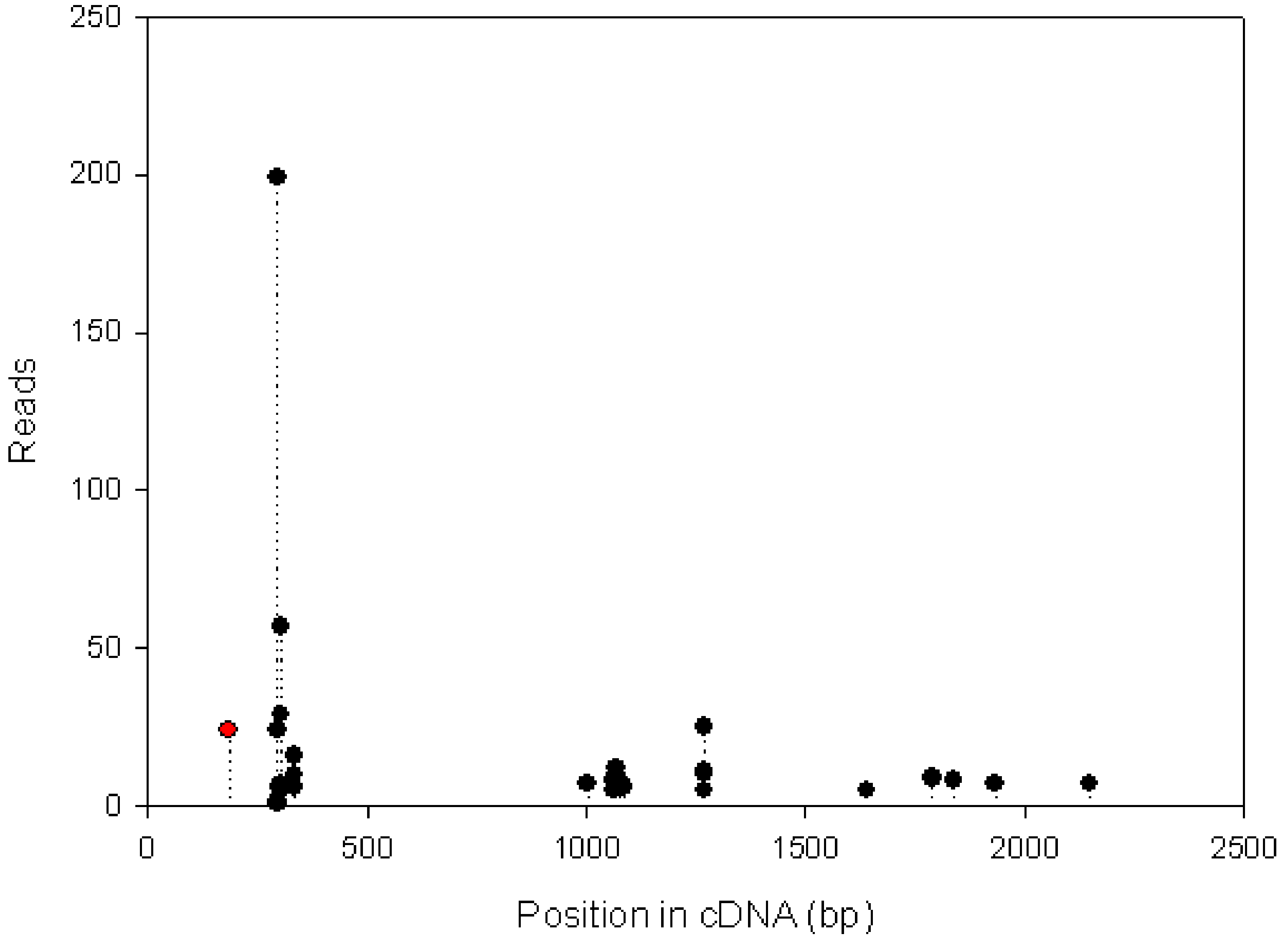


```
5' UGUGCUGUAGAAAGGCCCCUCAACCU 3'        Cs8g13286.1
    ::::: ::::::::::: :::
3' -CACGAGAUCUUUCCGGGAAGU---- 5'        Csi-miR3952-3p
```

Csi-miR3954a, target=Cs1g09600.1 gene=Cs1g09600
Category=1
Score=1
Cleavage Site=416

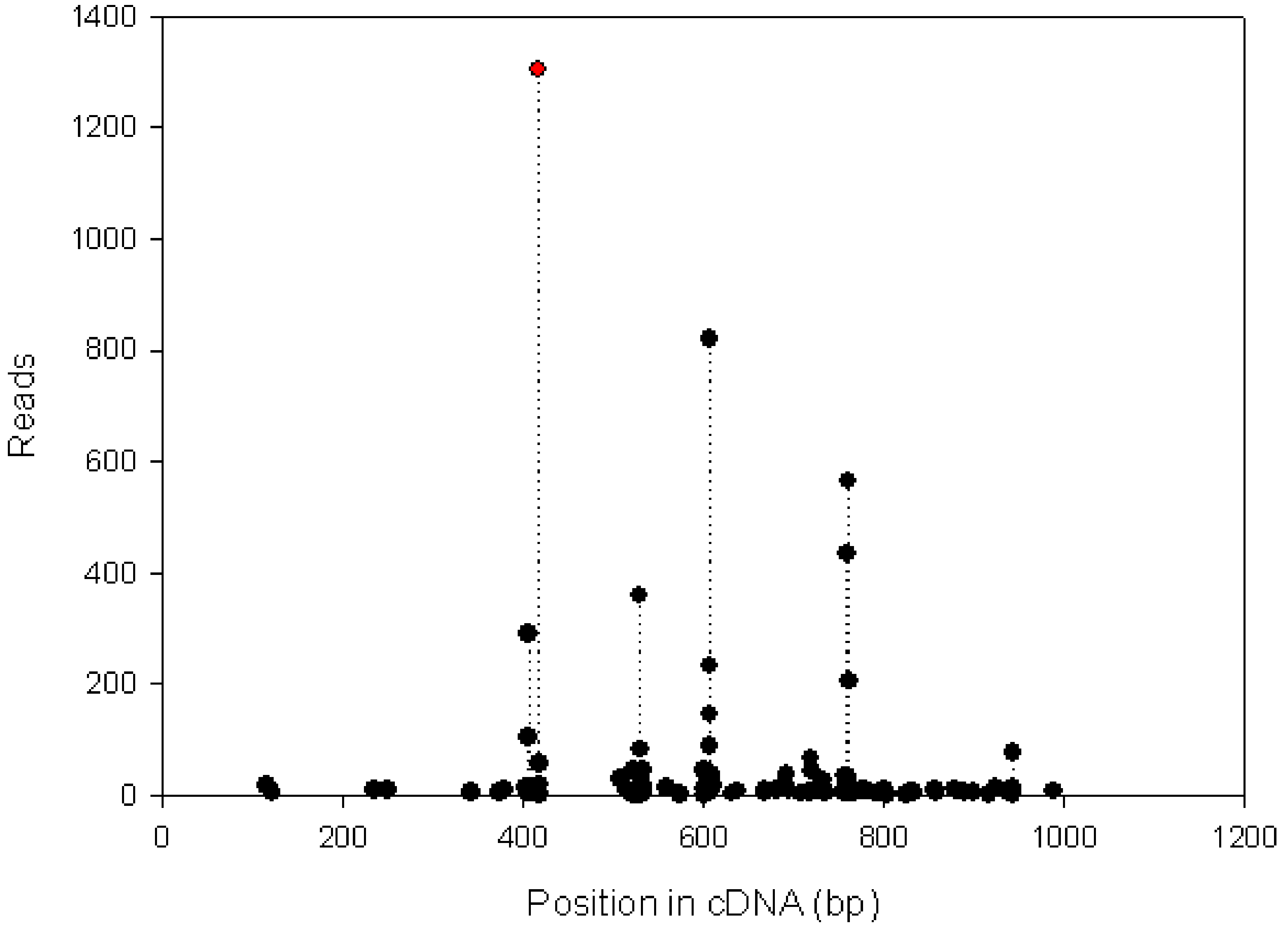


```
5' UGACCCUGAUUUCUCUGUCCAAGCAA 3'        Cs1g09600.1
   ::::: ::::::::::::::::
3' ACUGGCACUAAAGAGACAGGUU---- 5'        Csi-miR3954a
```

Csi-miR3954a, target=Cs1g09635.1 gene=Cs1g09635
Category:3
Score=1
Cleavage Site=351

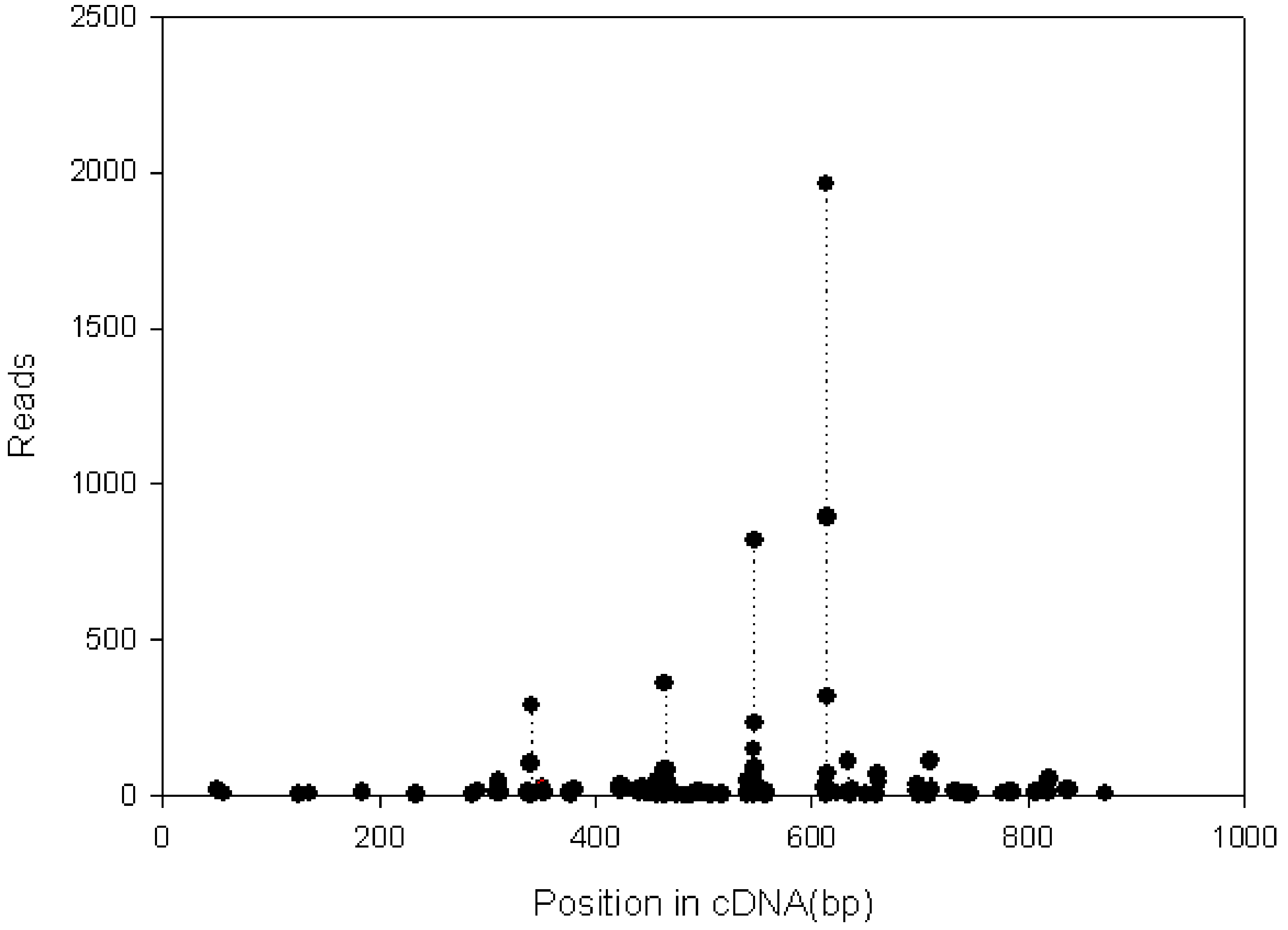


```
5' UGACCCUGAUUUCUCUGUCCAAACAG 3'        Cs1g09635.1
   ::::: ::::::::::::::::
3' ACUGGCACUAAAGAGACAGGUU---- 5'        Csi-miR3954a
```

Csi-miR3954a, target=Cs5g04670.1 gene=Cs5g04670
Category:3
Score=1
Cleavage Site=142

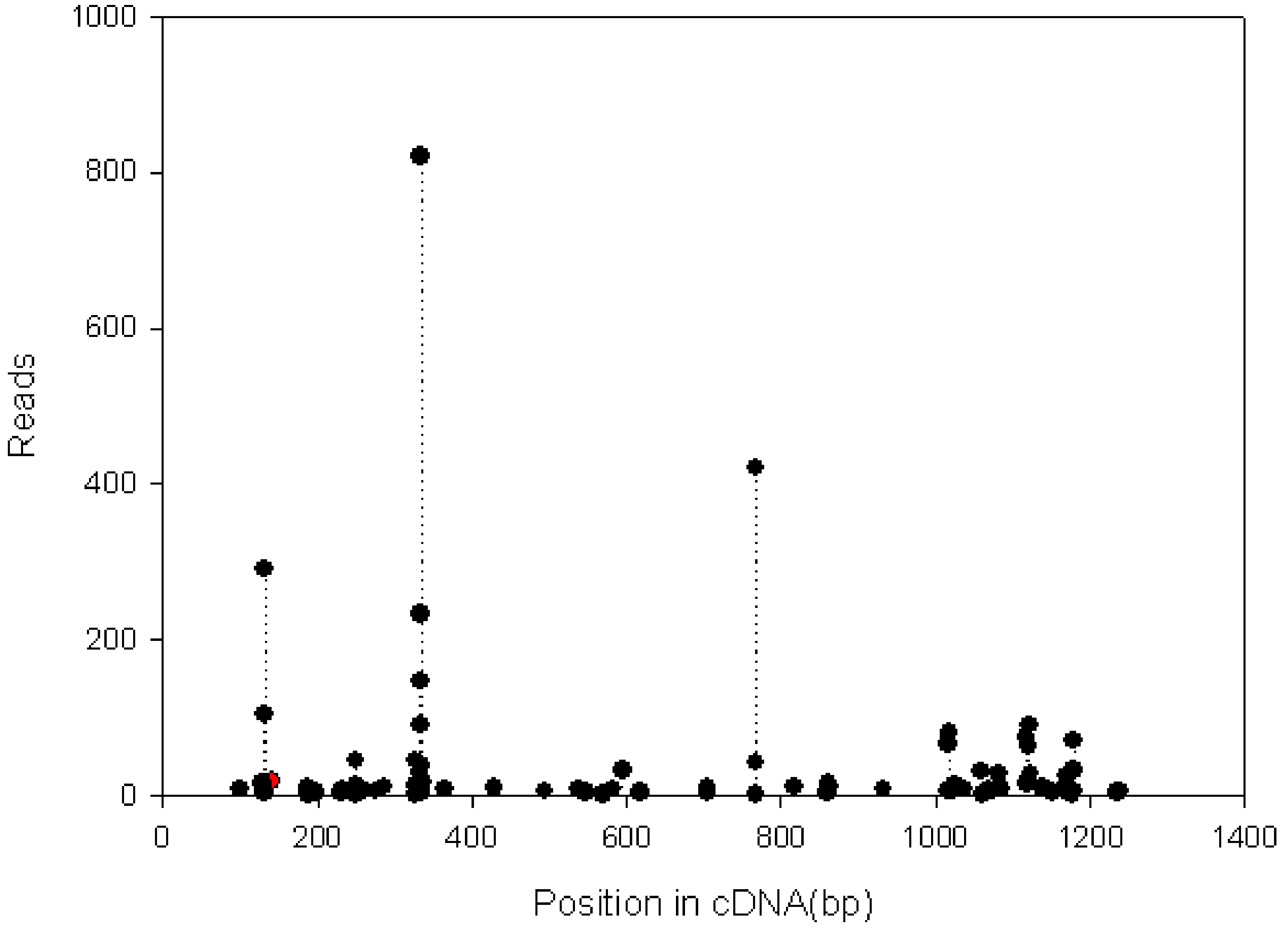


```
5' UGACCCUGAUUUCUCUGUCCAAACAA 3'        Cs5g04670.1
   ::::: ::::::::::::::::
3' ACUGGCACUAAAGAGACAGGUU---- 5'        Csi-miR3954a
```

Csi-miR3954b, target=Cs1g09600.1 gene=Cs1g09600
Category=1
Score=1
Cleavage Site=416

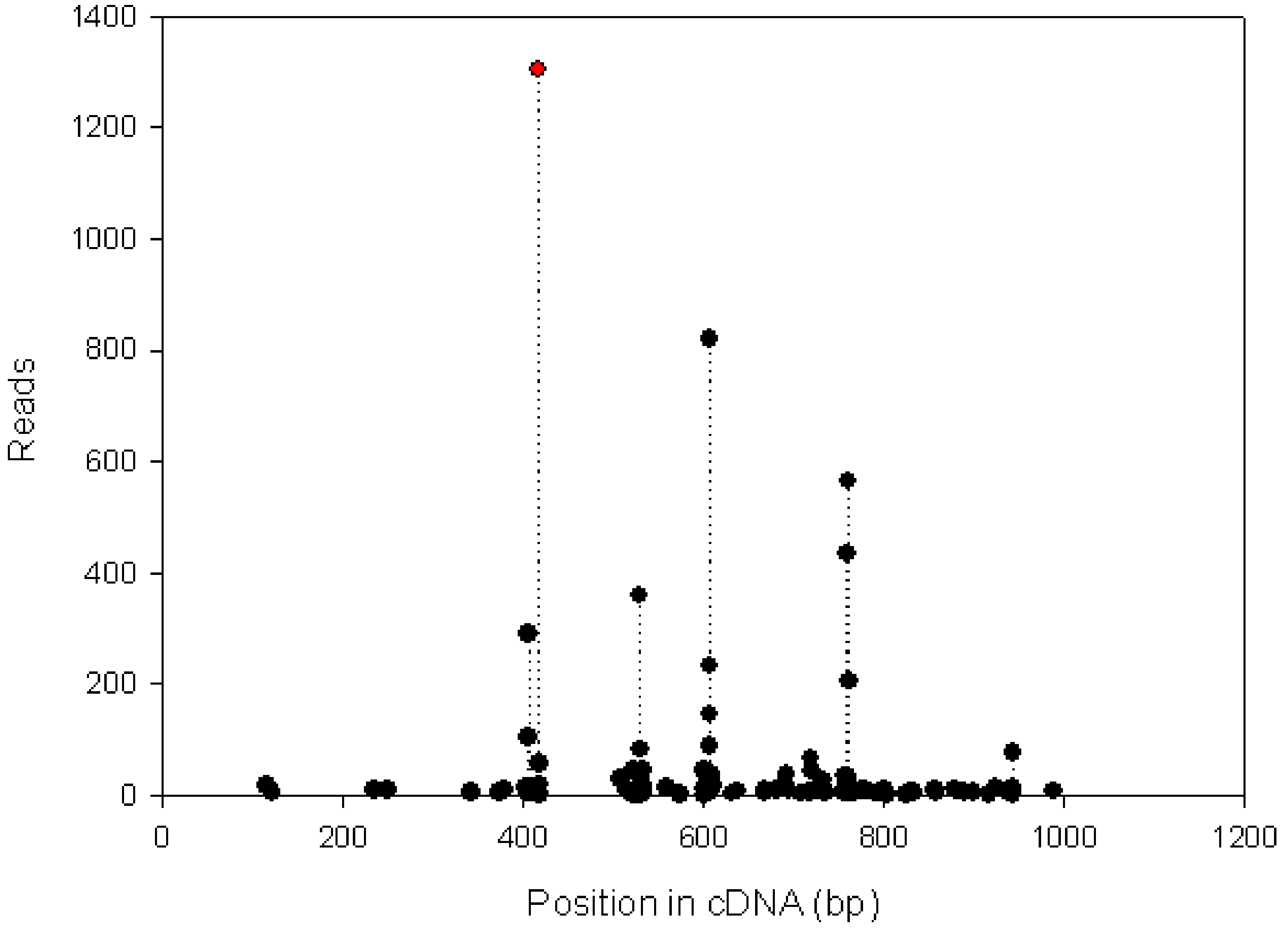


```
5' UGACCCUGAUUUCUCUGUCCAAGCAA 3'        Cs1g09600.1
   ::::: ::::::::::::::
3' ACUGGCACUAAAGAGACAGG------ 5'        Csi-miR3954b
```

Csi-miR3954b, target=Cs1g09635.1 gene=Cs1g09635
Category:3
Score=1
Cleavage Site=351

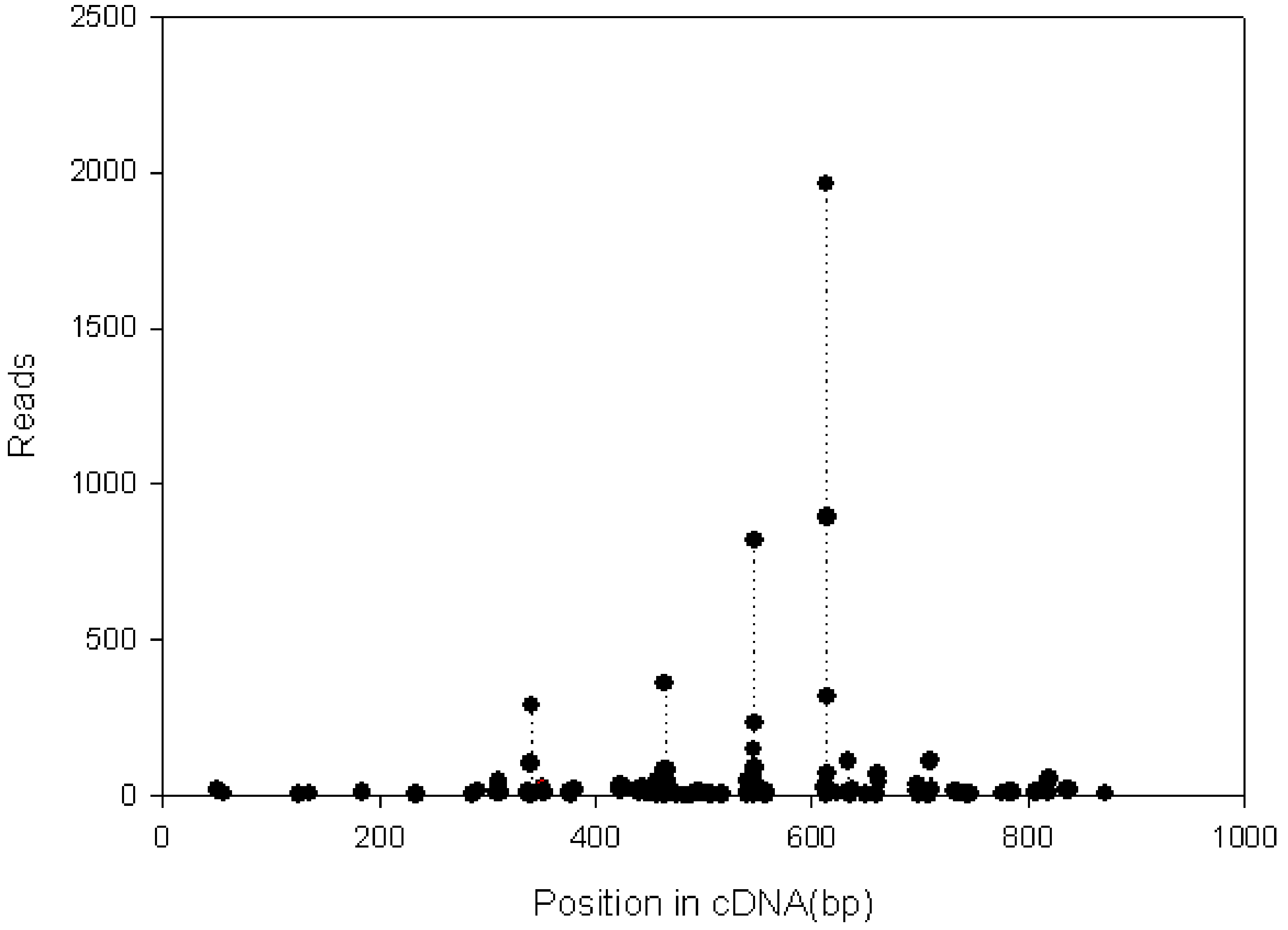


```
5' UGACCCUGAUUUCUCUGUCCAAACAG 3'        Cs1g09635.1
   ::::: ::::::::::::::
3' ACUGGCACUAAAGAGACAGG------ 5'        Csi-miR3954b
```

Csi-miR3954b, target=Cs2g29120.1 gene=Cs2g29120
Category:1
Score=4.5
Cleavage Site=942

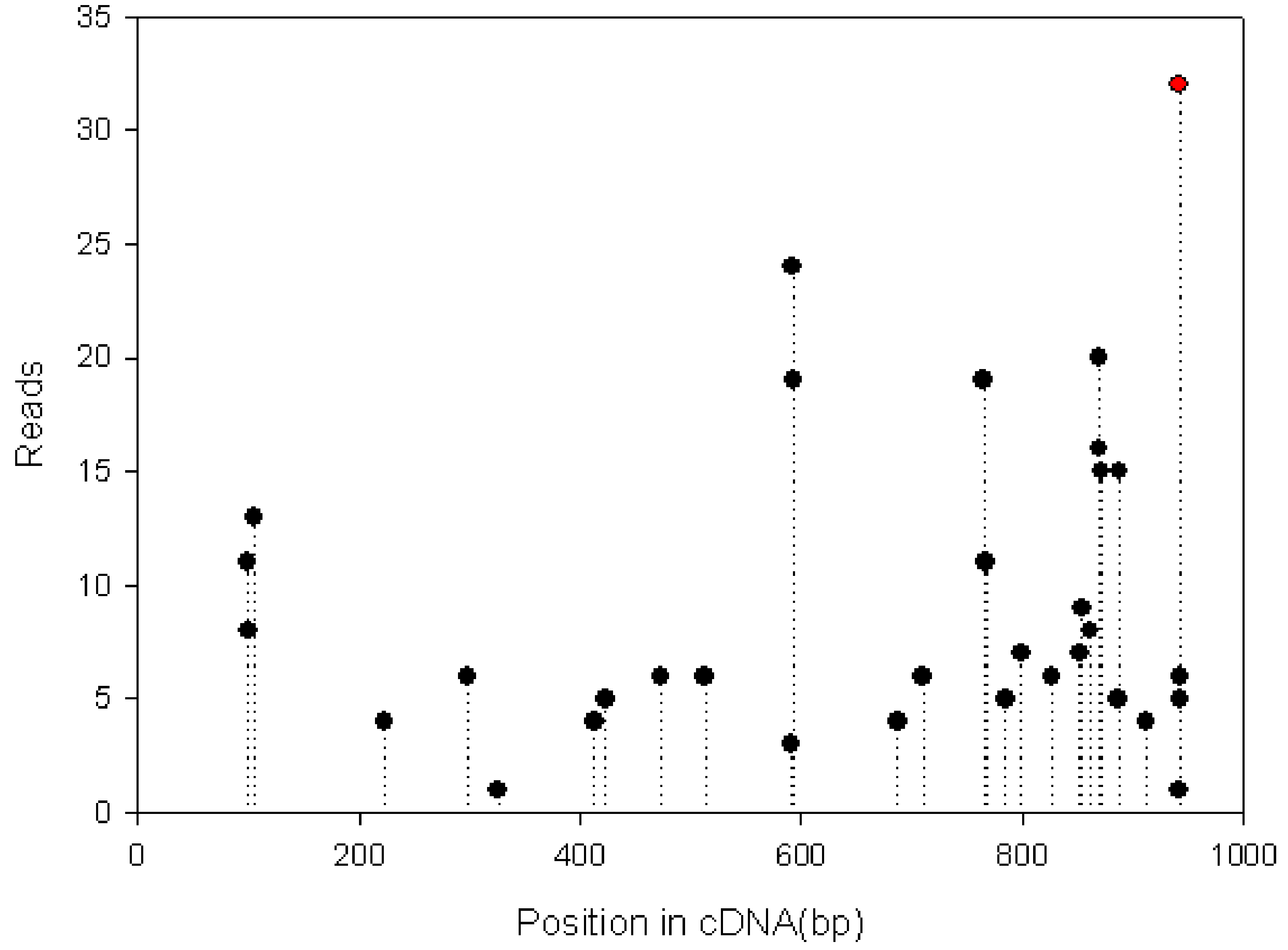


```
5' UAAGGACUGUGAUUACUCUGUCAAAC 3'        Cs2g29120.1
        :::.:::::: :::::::
3' ---ACUGGCACUAAAGAGACAGG--- 5'        Csi-miR3954b
```

Csi-miR3954b, target=Cs5g04670.1 gene=Cs5g04670
Category:3
Score=1
Cleavage Site=142

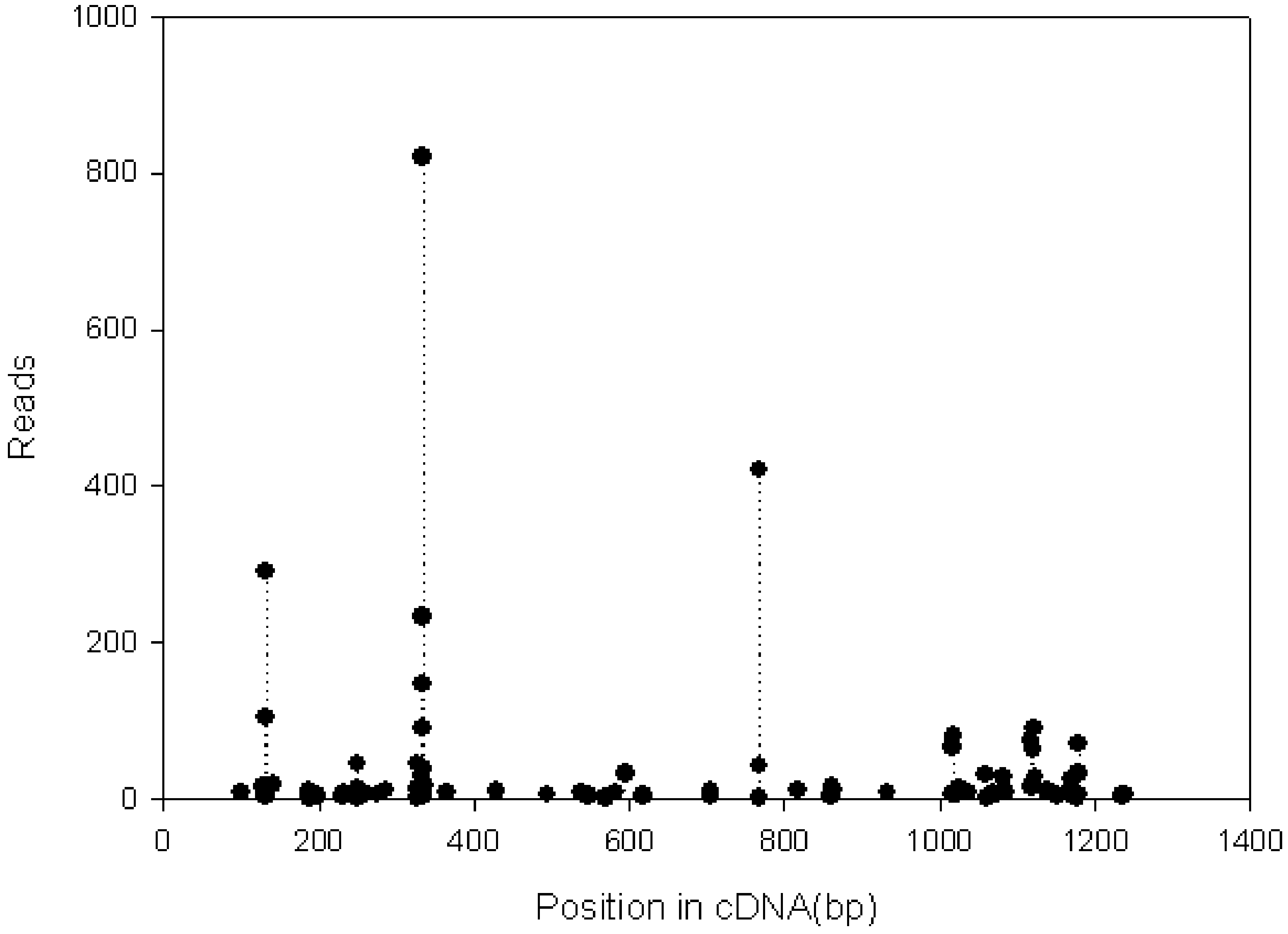


```
5' UGACCCUGAUUUCUCUGUCCAAACAA 3'        Cs5g04670.1
   ::::: ::::::::::::::
3' ACUGGCACUAAAGAGACAGG------ 5'        Csi-miR3954b
```

Csi-miR396a, target=Cs1g22520.1 gene=Cs1g22520
Category:1
Score=4
Cleavage Site=226

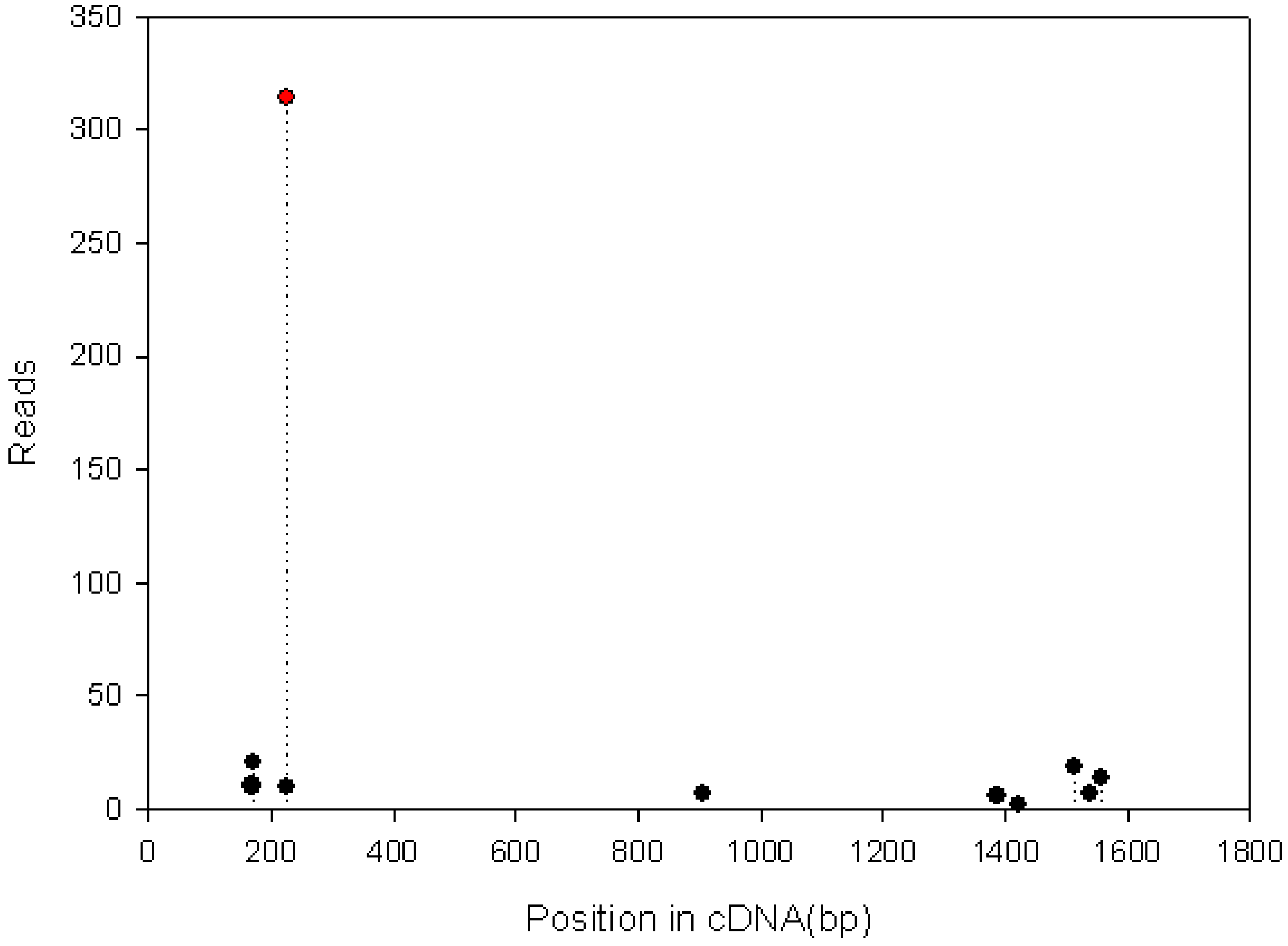


```
5' GAAGUUCAAGAAAUCUGUGGGAAGAA 3'        Cs1g22520.1
    ::::::::::: :::::::.:
3' -GUCAAGUUCUUUCGACACCUU---- 5'        Csi-miR396a
```

Csi-miR396a, target=Cs3g23180.1 gene=Cs3g23180
Category:3
Score=4
Cleavage Site=625

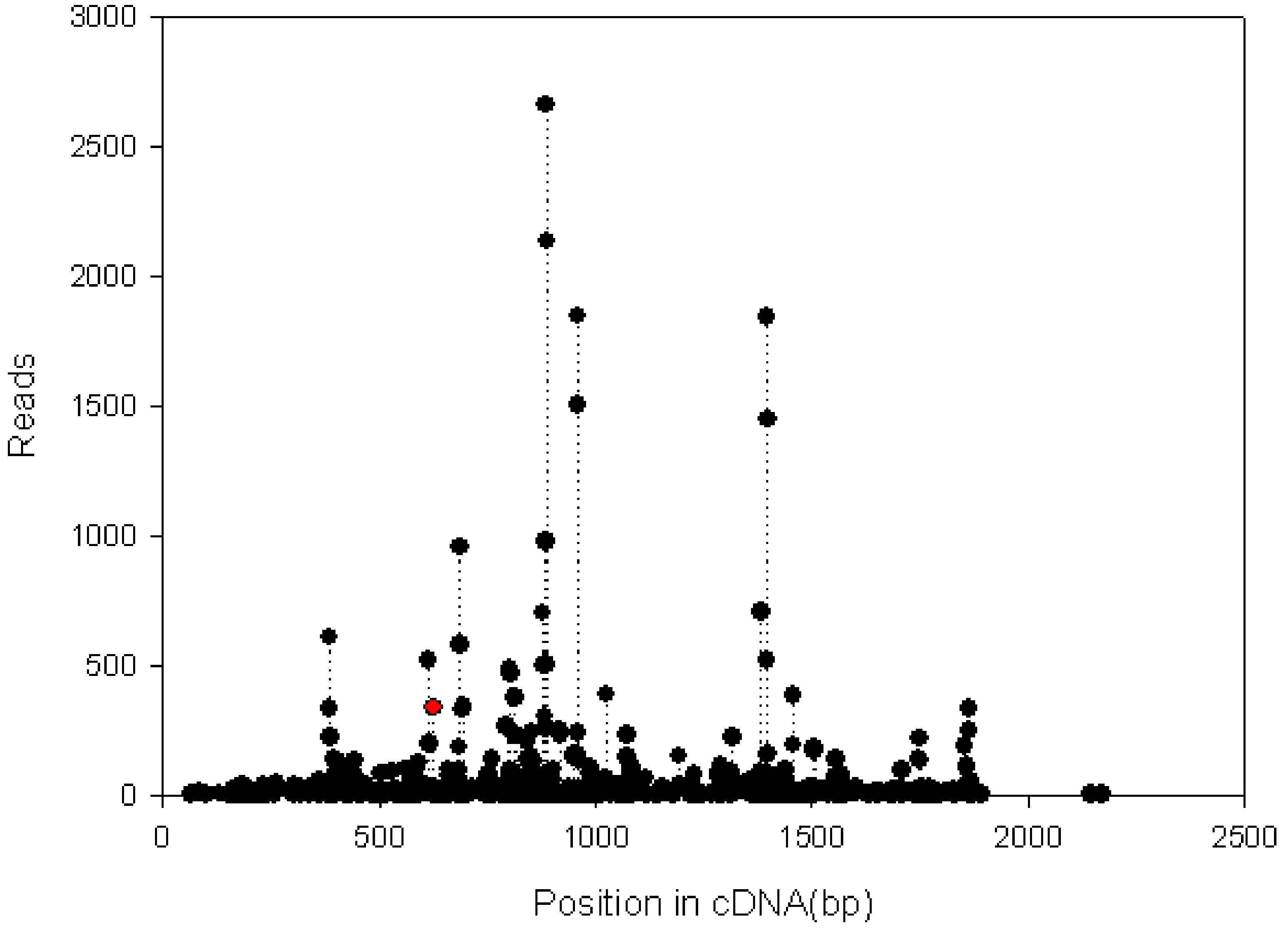


```
5' AAAGAUCAAGGAAGCUGUGGGAGUUG 3'        Cs3g23180.1
     :: :::::.::::::::::.:
3' -GUCAAGUUCUUUCGACACCUU---- 5'        Csi-miR396a
```

Csi-miR396a, target=Cs5g09850.1 gene=Cs5g09850
Category:2
Score=4
Cleavage Site=767

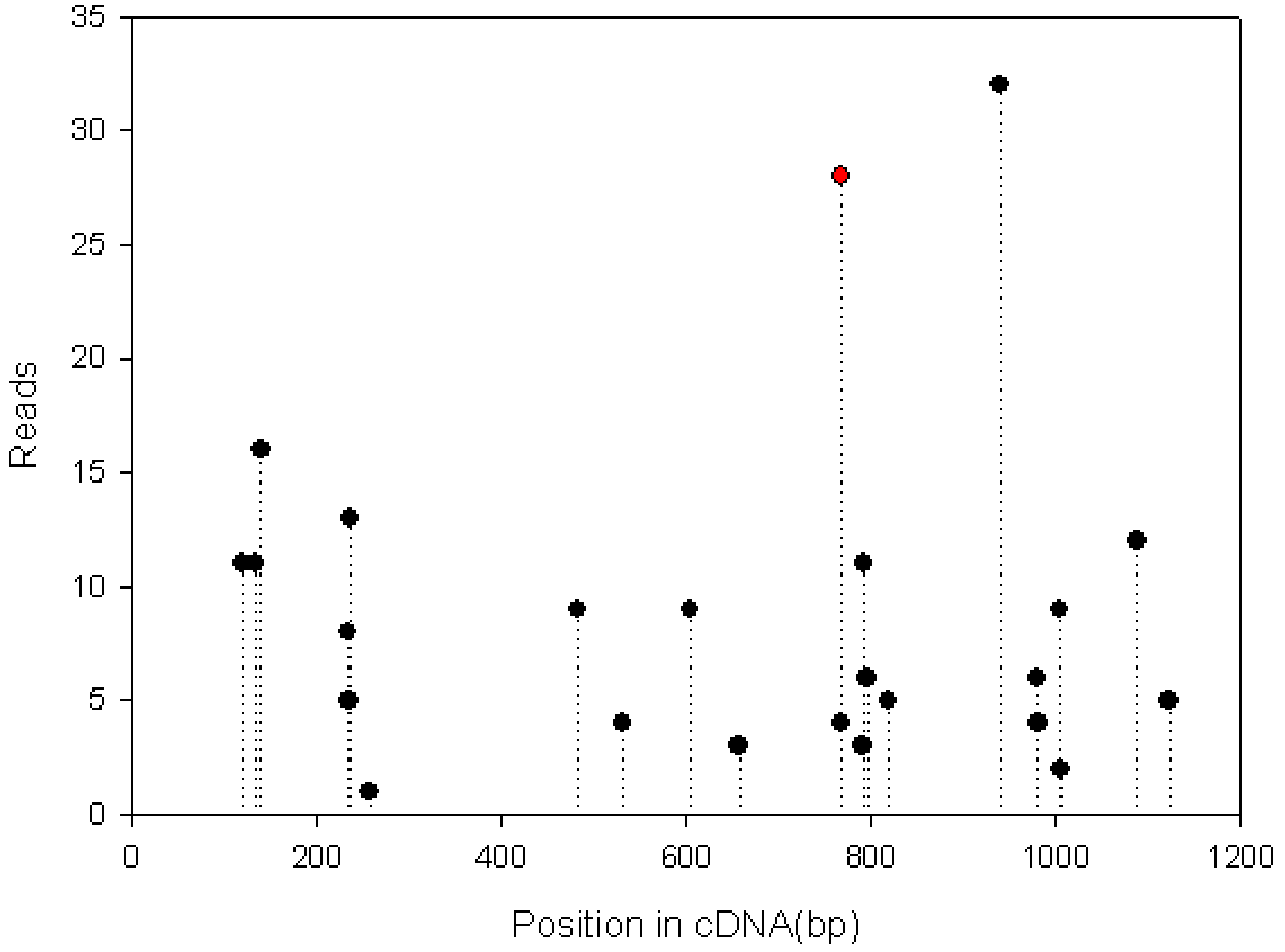


```
5' UGCGUUCAAGAAAGCUUGUGGAAGCU 3'        Cs5g09850.1
     :::::::::::: :::::::
3' -GUCAAGUUCUUUCG-ACACCUU--- 5'        Csi-miR396a
```

Csi-miR396a, target=Cs6g15330.1 gene=Cs6g15330
Category: 1
Score=3
Cleavage Site=449

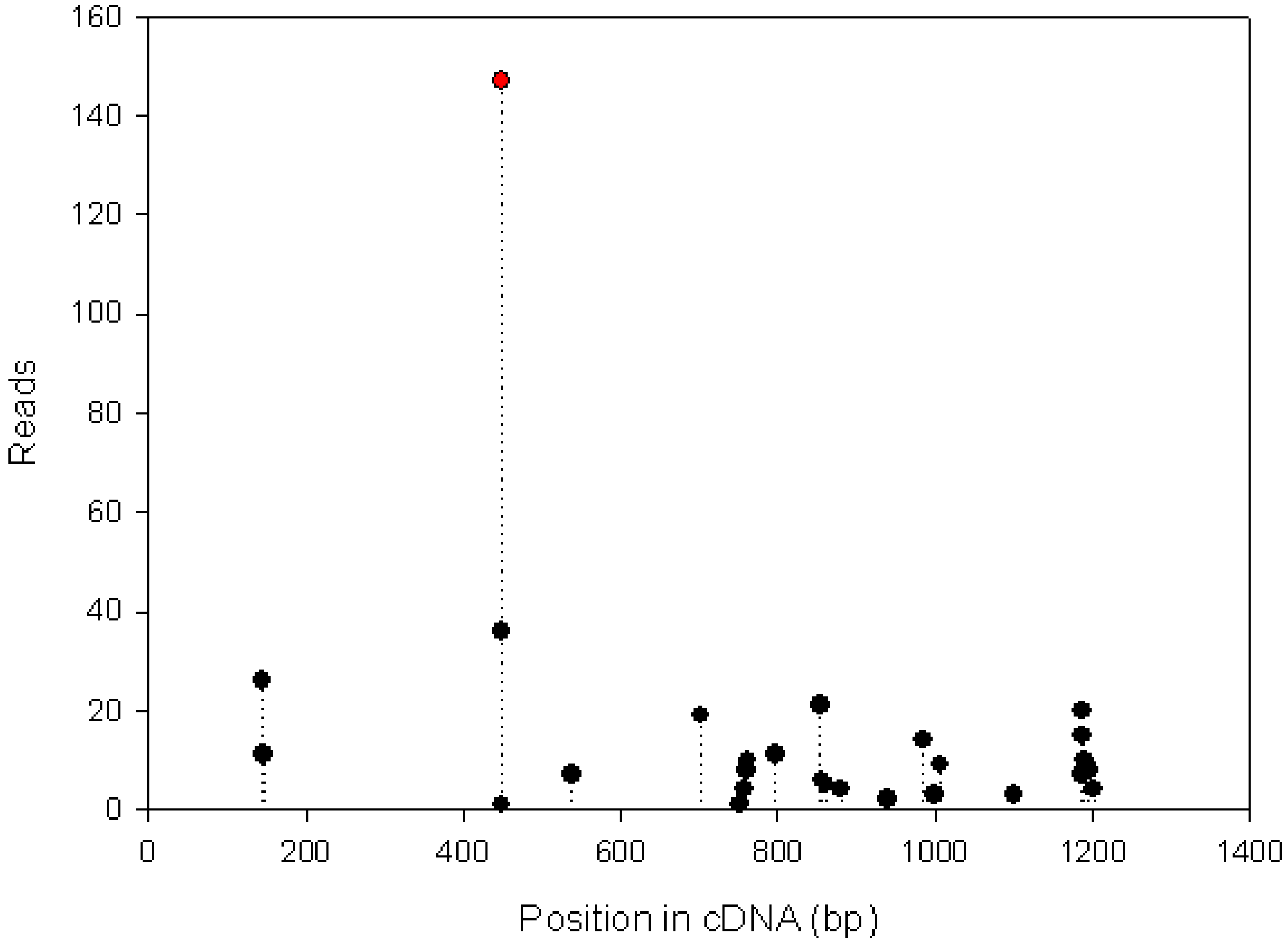


```
5' ACCGUUCAAGAAAGCAUGUGGAAAUC 3'         Cs6g15330.1
    : :::::::::::: :::::::
3' -GUCAAGUUCUUUCG-ACACCUU--- 5'         Csi-miR396a
```

Csi-miR396a, target=Cs7g15220.1 gene=Cs7g15220
Category=1
Score=3
Cleavage Site=377

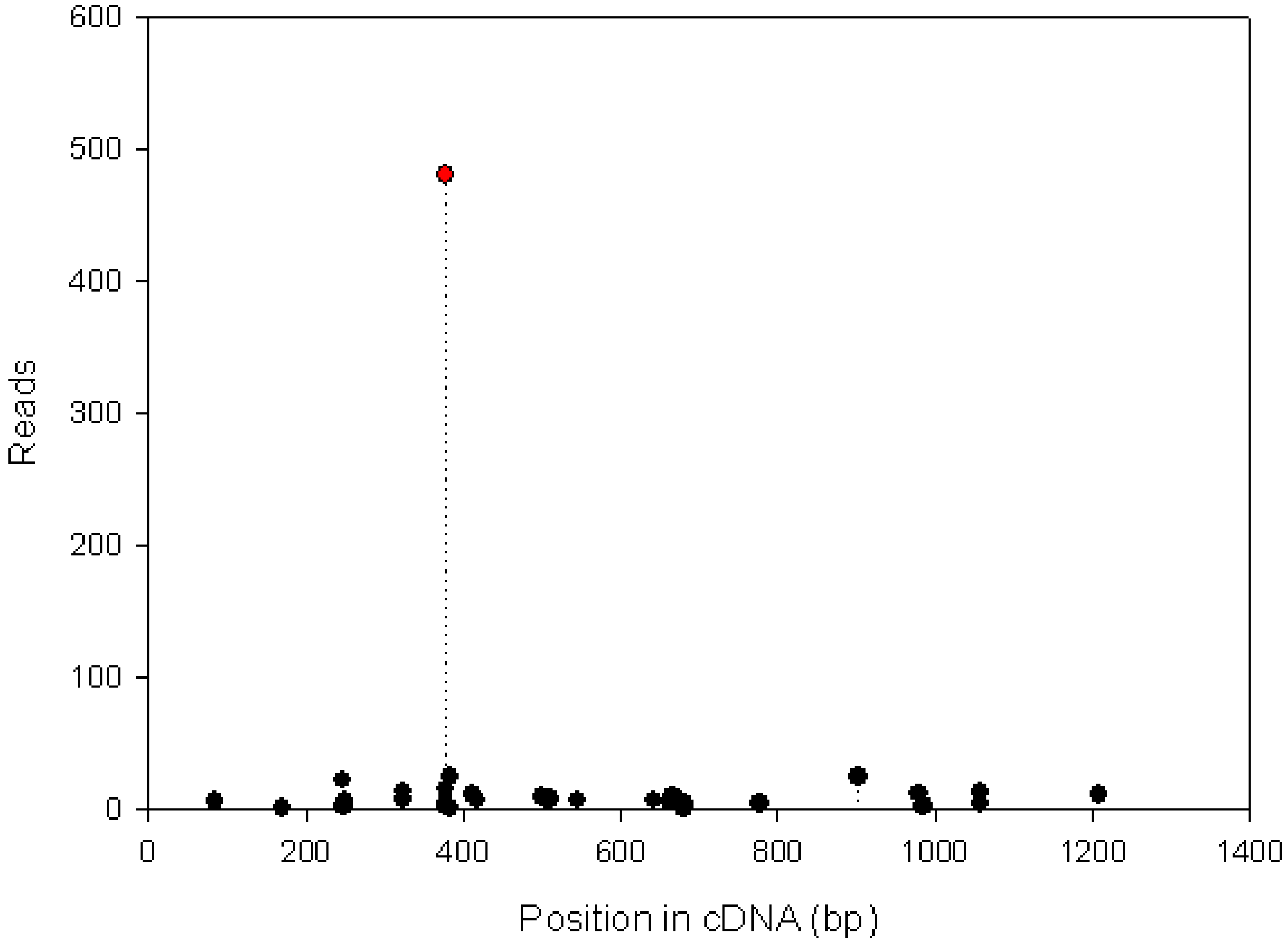


```
5' ACCGUUCAAGAAAGCCUGUGGAAUCA 3'        Cs7g15220.1
    : :::::::::::: ::::::::
3' -GUCAAGUUCUUUC-GACACCUU--- 5'        Csi-miR396a
```

Csi-miR396a, target=Orange1.1t03122.1 gene=Orange1.1t03122
Category=1
Score=3
Cleavage Site=583

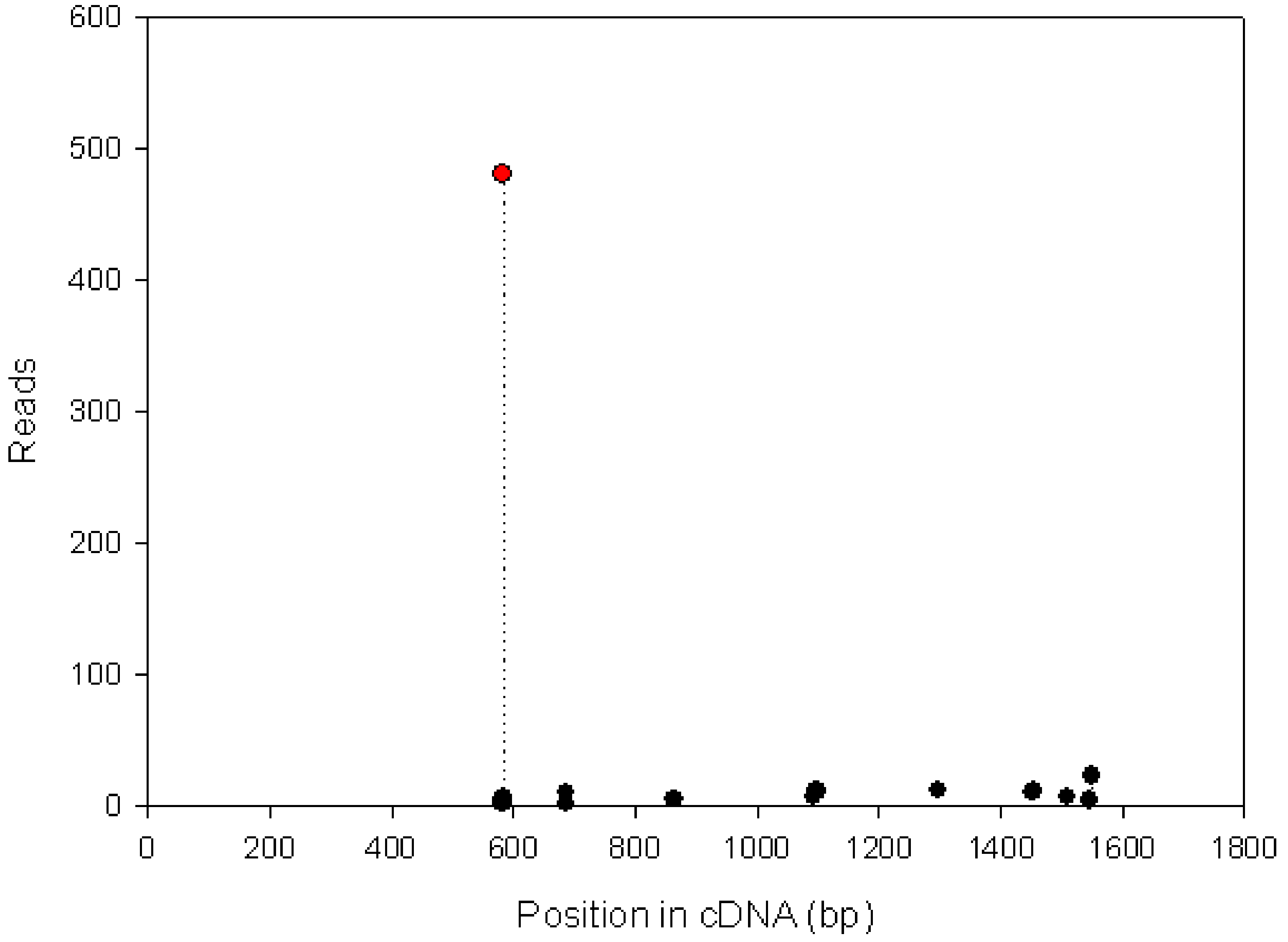


```
5' ACCGUUCAAGAAAGCCUGUGGAAUCA 3'        Orange1.1t03122.1
     : ::::::::::: ::::::::
3' -GUCAAGUUCUUUC-GACACCUU--- 5'        Csi-miR396a
```

Csi-miR396b.1, target=Cs1g22520.1 gene=Cs1g22520
Category:1
Score=3
Cleavage Site=226

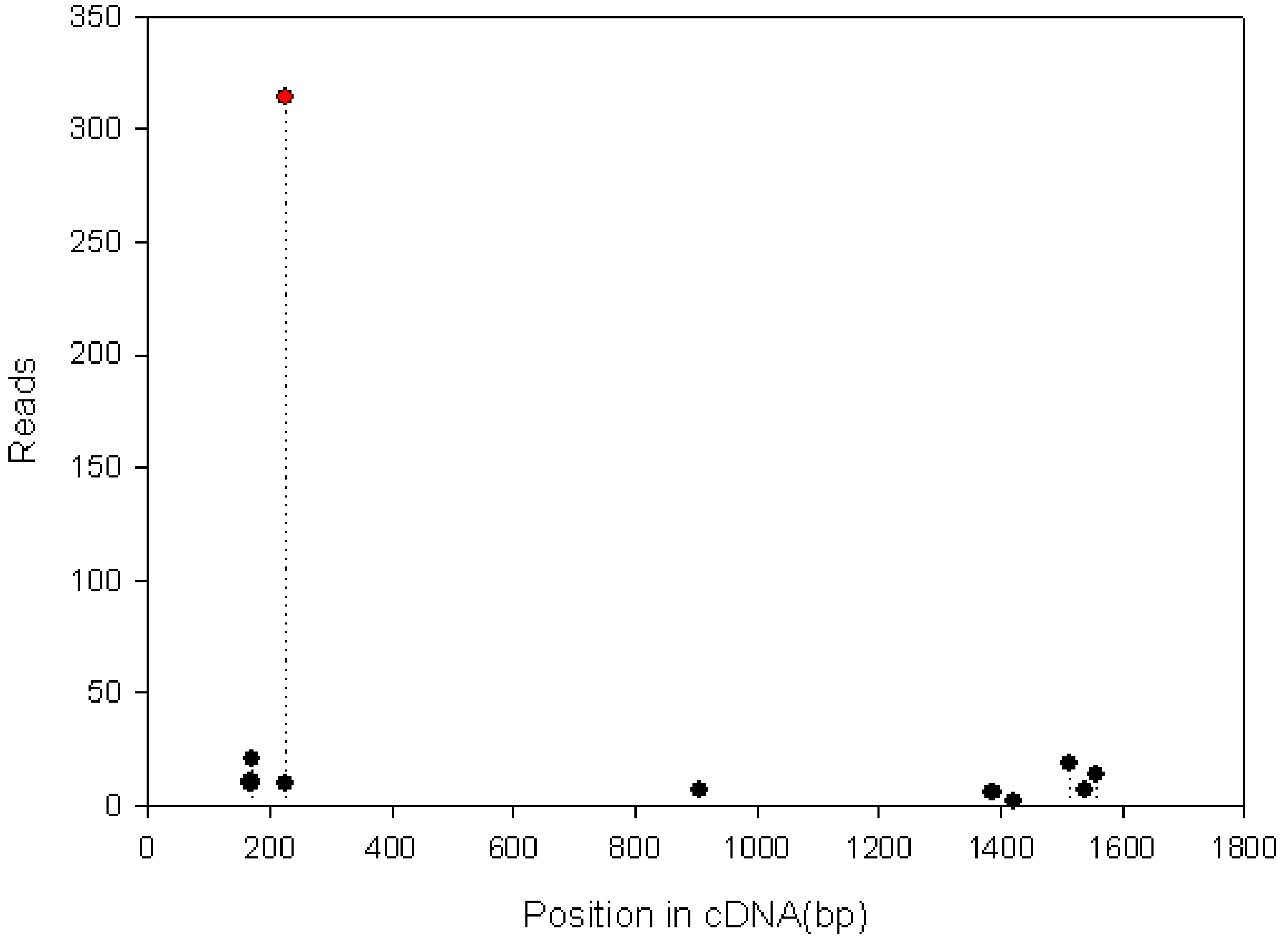


```
5' GAAGUUCAAGAAAUCUGUGGGAAGAA 3'        Cs1g22520.1
      :::::::::: :::::::.:
3' ---CAAGUUCUUUCGACACCUU---- 5'        Csi-miR396b.1
```

Csi-miR396b.1, target=Cs3g23180.1 gene=Cs3g23180
Category:3
Score=3
Cleavage Site=625

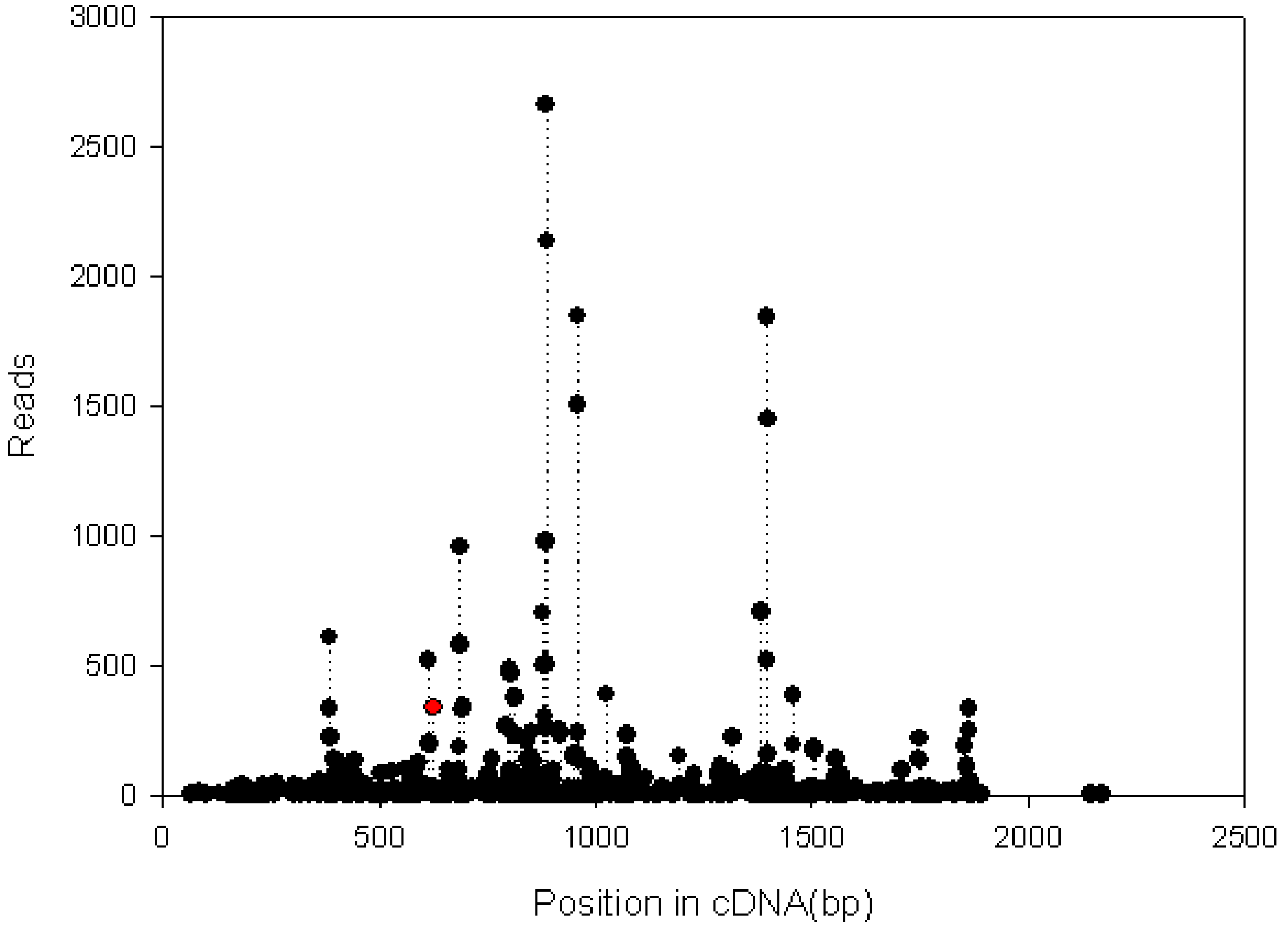


```
5' AAAGAUCAAGGAAGCUGUGGGAGUUG 3'        Cs3g23180.1
      : :::::.:::::::::.:
3' ---CAAGUUCUUUCGACACCUU---- 5'        Csi-miR396b.1
```

Csi-miR396b.1, target=Cs5g09850.1 gene=Cs5g09850
Category:2
Score=2
Cleavage Site=767

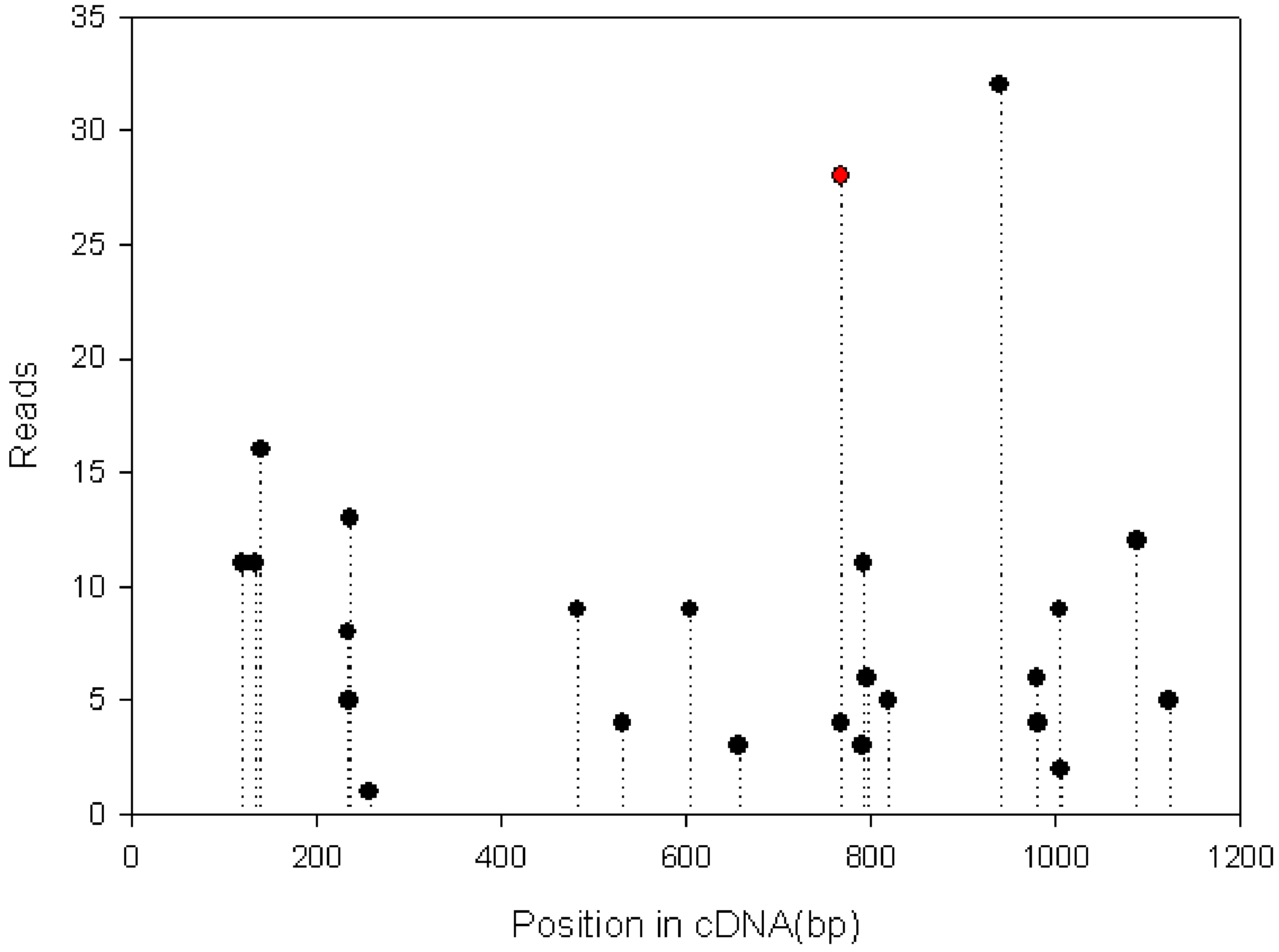


```
5' UGCGUUCAAGAAAGCUUGUGGAAGCU 3'        Cs5g09850.1
      :::::::::::: :::::::
3' ---CAAGUUCUUUCG-ACACCUU--- 5'        Csi-miR396b.1
```

Csi-miR396b.1, target=Cs6g15330.1 gene=Cs6g15330
Category: 1
Score=2
Cleavage Site=449

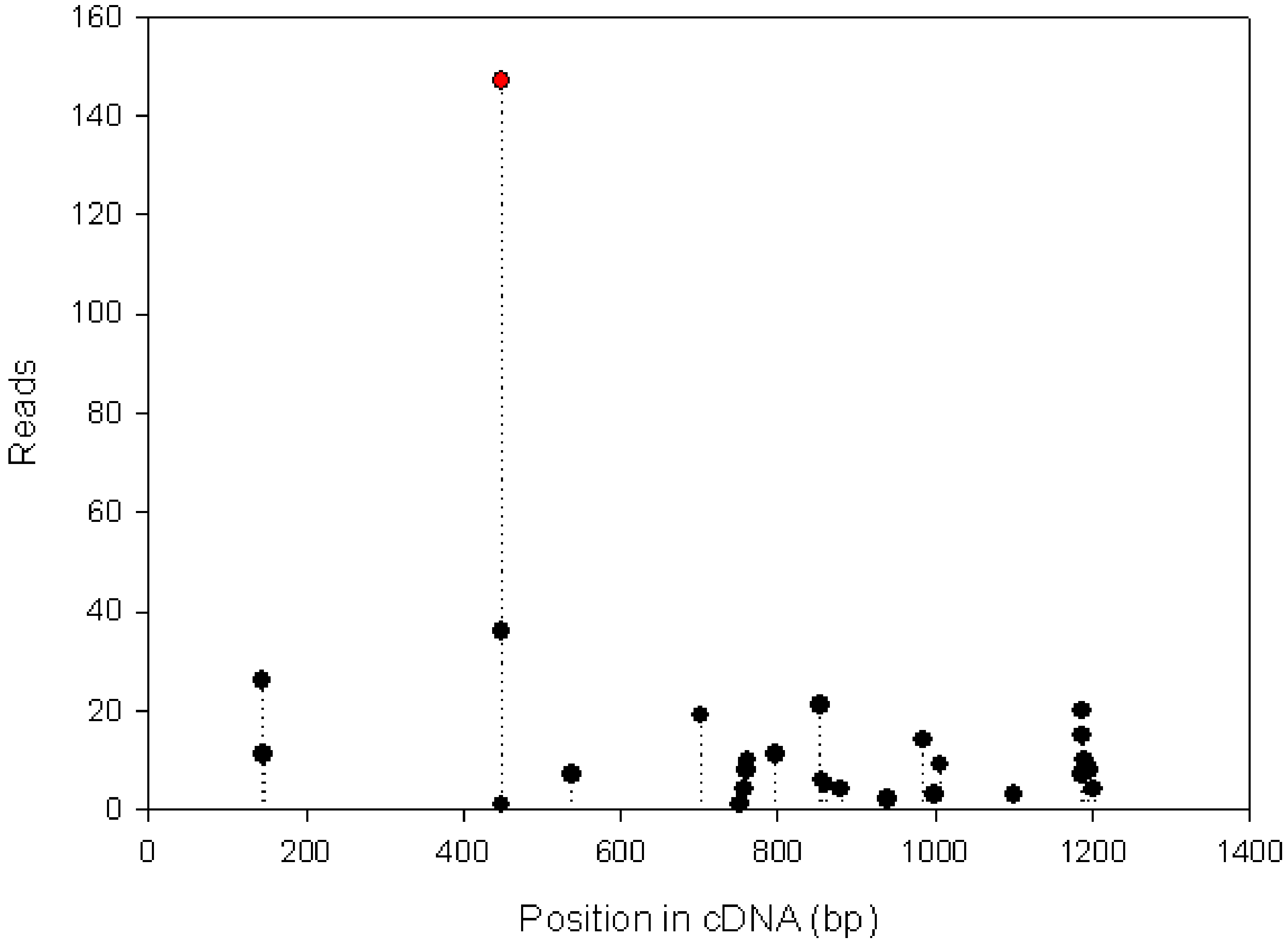


```
5' ACCGUUCAAGAAAGCAUGUGGAAAUC 3'        Cs6g15330.1
     :::::::::::: :::::::
3' ---CAAGUUCUUUCG-ACACCUU--- 5'        Csi-miR396b.1
```

Csi-miR396b.1, target=Cs7g15220.1 gene=Cs7g15220
Category=1
Score=2
Cleavage Site=377

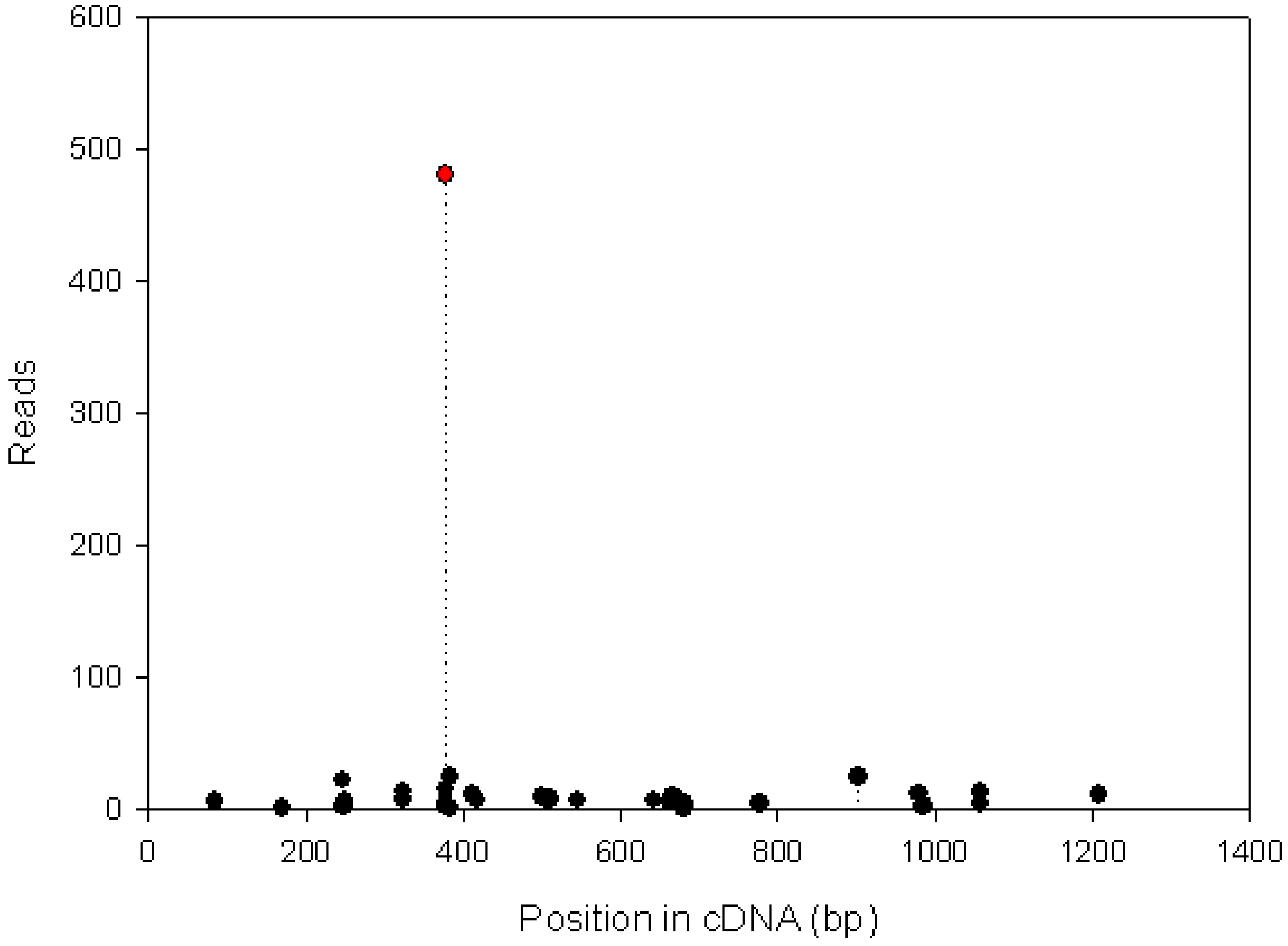


```
5' ACCGUUCAAGAAAGCCUGUGGAAUCA 3'        Cs7g15220.1
      ::::::::::: ::::::::
3' ---CAAGUUCUUUC-GACACCUU--- 5'        Csi-miR396b.1
```

Csi-miR396b.1, target=Orange1.1t03122.1 gene=Orange1.1t03122
Category=1
Score=2
Cleavage Site=583

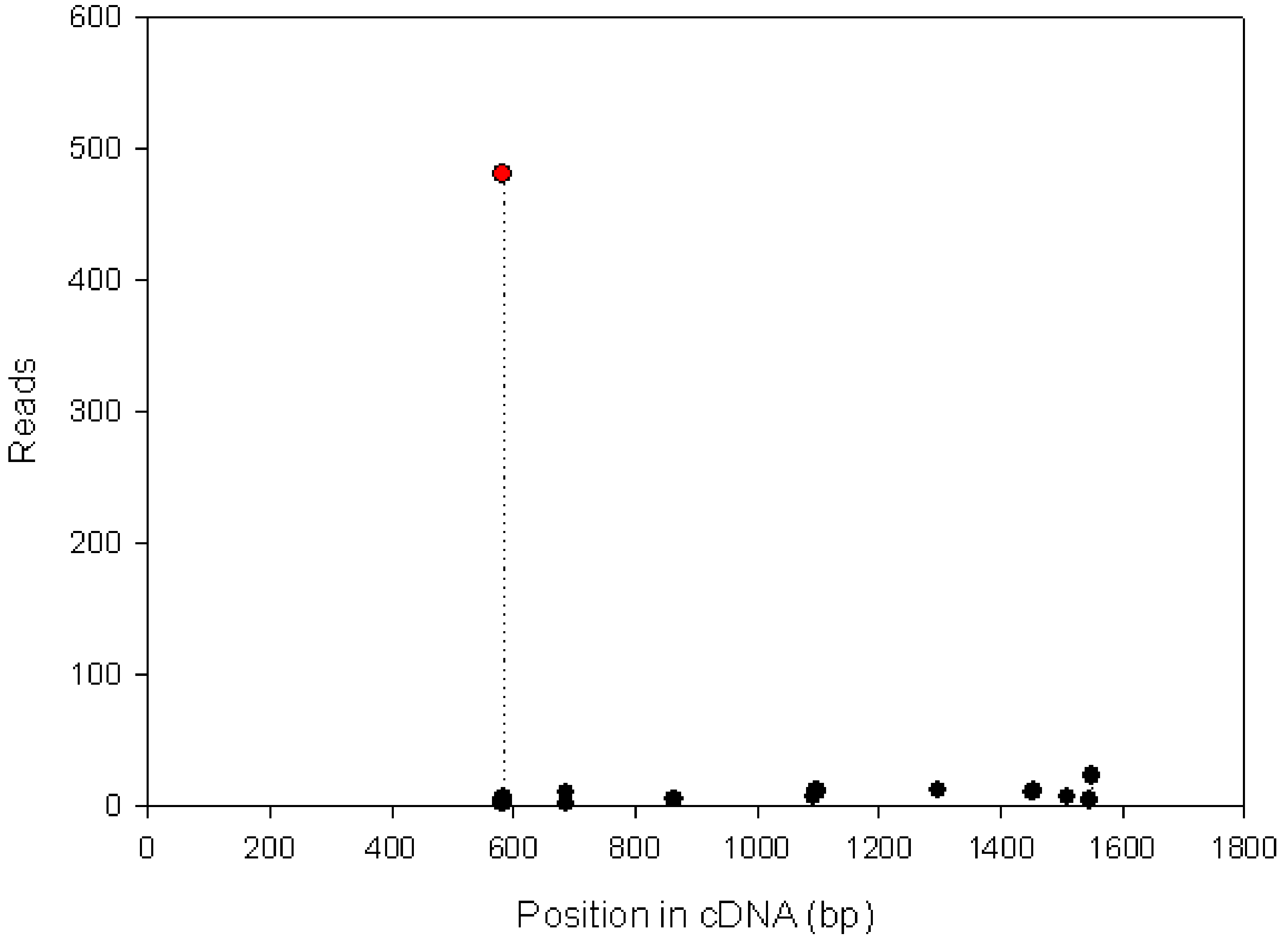


```
5' ACCGUUCAAGAAAGCCUGUGGAAUCA 3'        Orange1.1t03122.1
      ::::::::::: ::::::::
3' ---CAAGUUCUUUC-GACACCUU--- 5'        Csi-miR396b.1
```

Csi-miR396b.2, target=Cs1g22520.1 gene=Cs1g22520
Category:1
Score=3
Cleavage Site=226

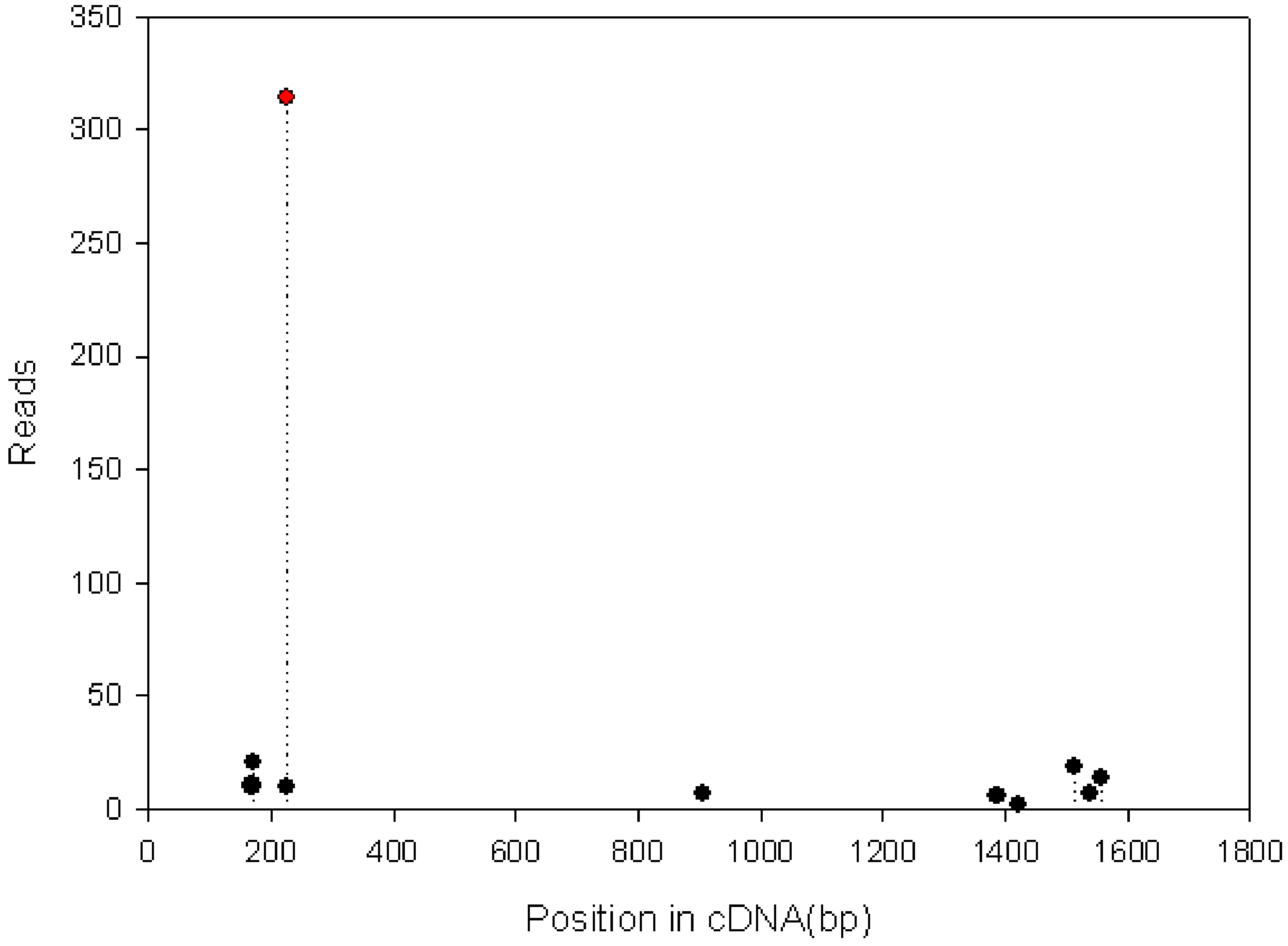


```
5' GAAGUUCAAGAAAUCUGUGGGAAGAA 3'        Cs1g22520.1
         ::::::::: :::::::.:
3' ----AAGUUCUUUCGACACCUU---- 5'        Csi-miR396b.2
```

Csi-miR396b.2, target=Cs3g23180.1 gene=Cs3g23180
Category:3
Score=3
Cleavage Site=625

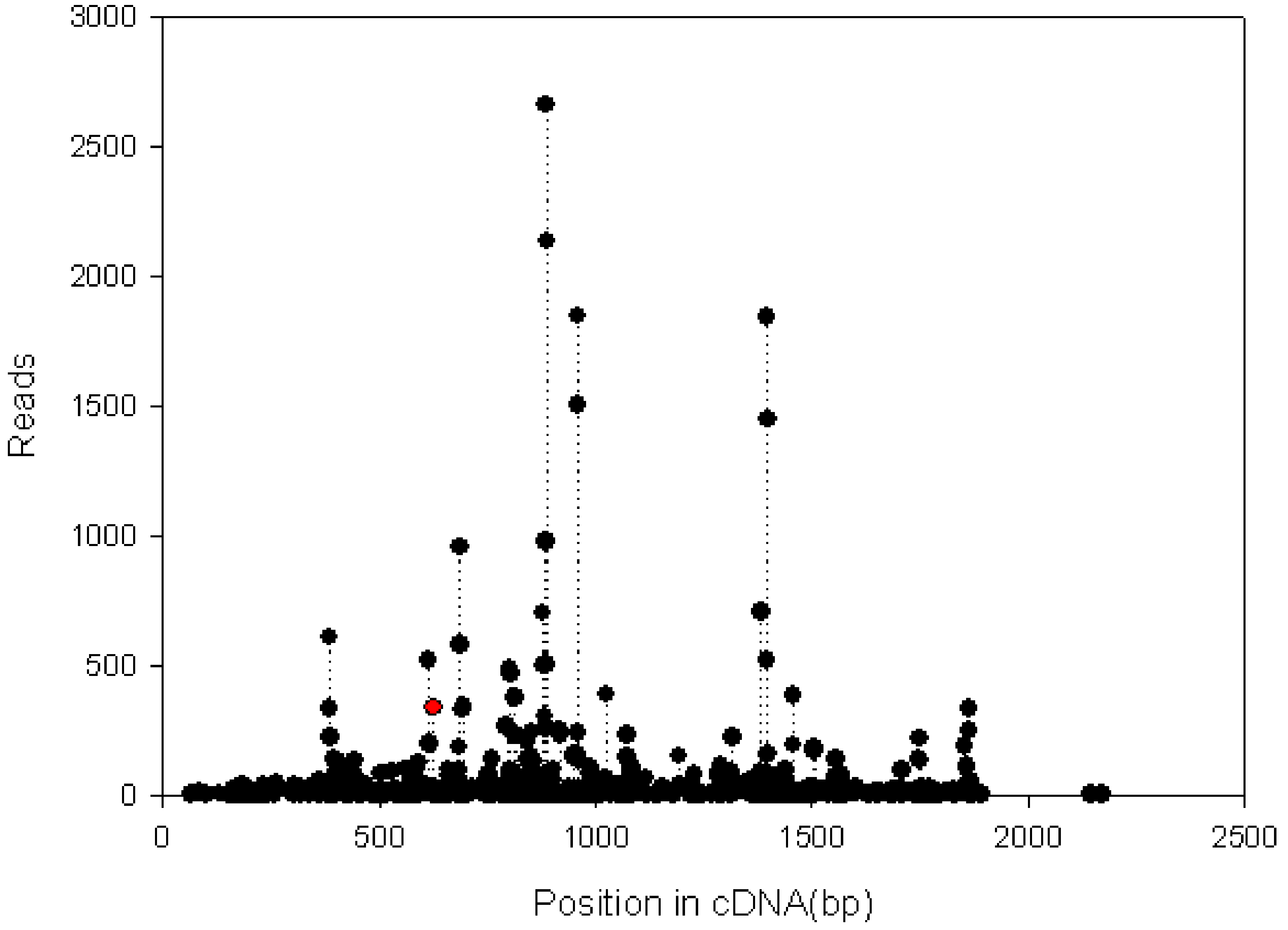


```
5' AAAGAUCAAGGAAGCUGUGGGAGUUG 3'        Cs3g23180.1
       :::::.:::::::::.:
3' ----AAGUUCUUUCGACACCUU---- 5'        Csi-miR396b.2
```

Csi-miR396b.2, target=Cs5g09850.1 gene=Cs5g09850
Category:2
Score=2
Cleavage Site=767

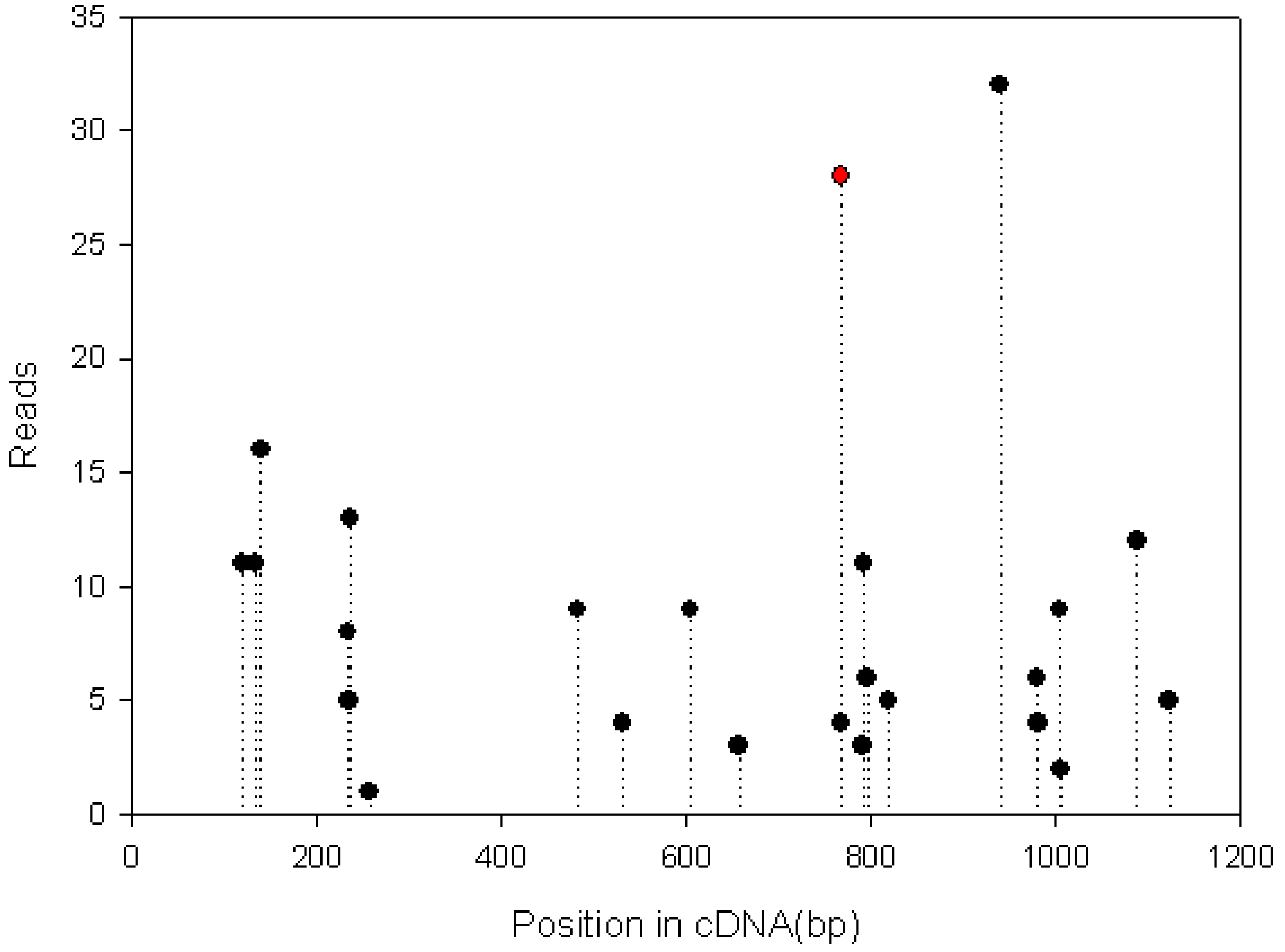


```
5' UGCGUUCAAGAAAGCUUGUGGAAGCU 3'        Cs5g09850.1
       ::::::::::: :::::::
3' ----AAGUUCUUUCG-ACACCUU--- 5'        Csi-miR396b.2
```

Csi-miR396b.2, target=Cs6g15330.1 gene=Cs6g15330
Category: 1
Score=2
Cleavage Site=449

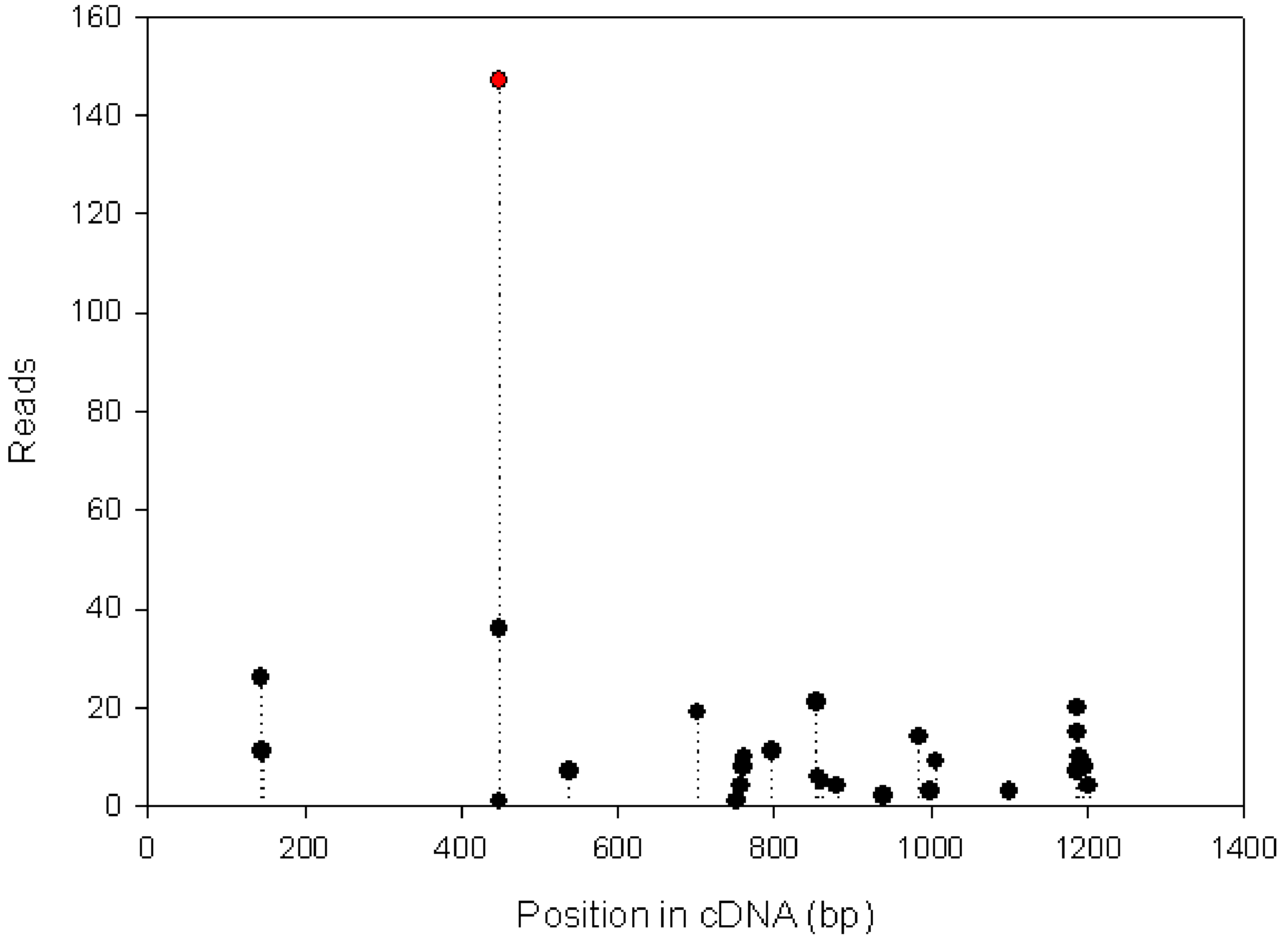


```
5' ACCGUUCAAGAAAGCAUGUGGAAAUC 3'        Cs6g15330.1
       ::::::::::: :::::::
3' ----AAGUUCUUUCG-ACACCUU--- 5'        Csi-miR396b.2
```

Csi-miR396b.2, target=Cs7g15220.1 gene=Cs7g15220
Category=1
Score=2
Cleavage Site=377

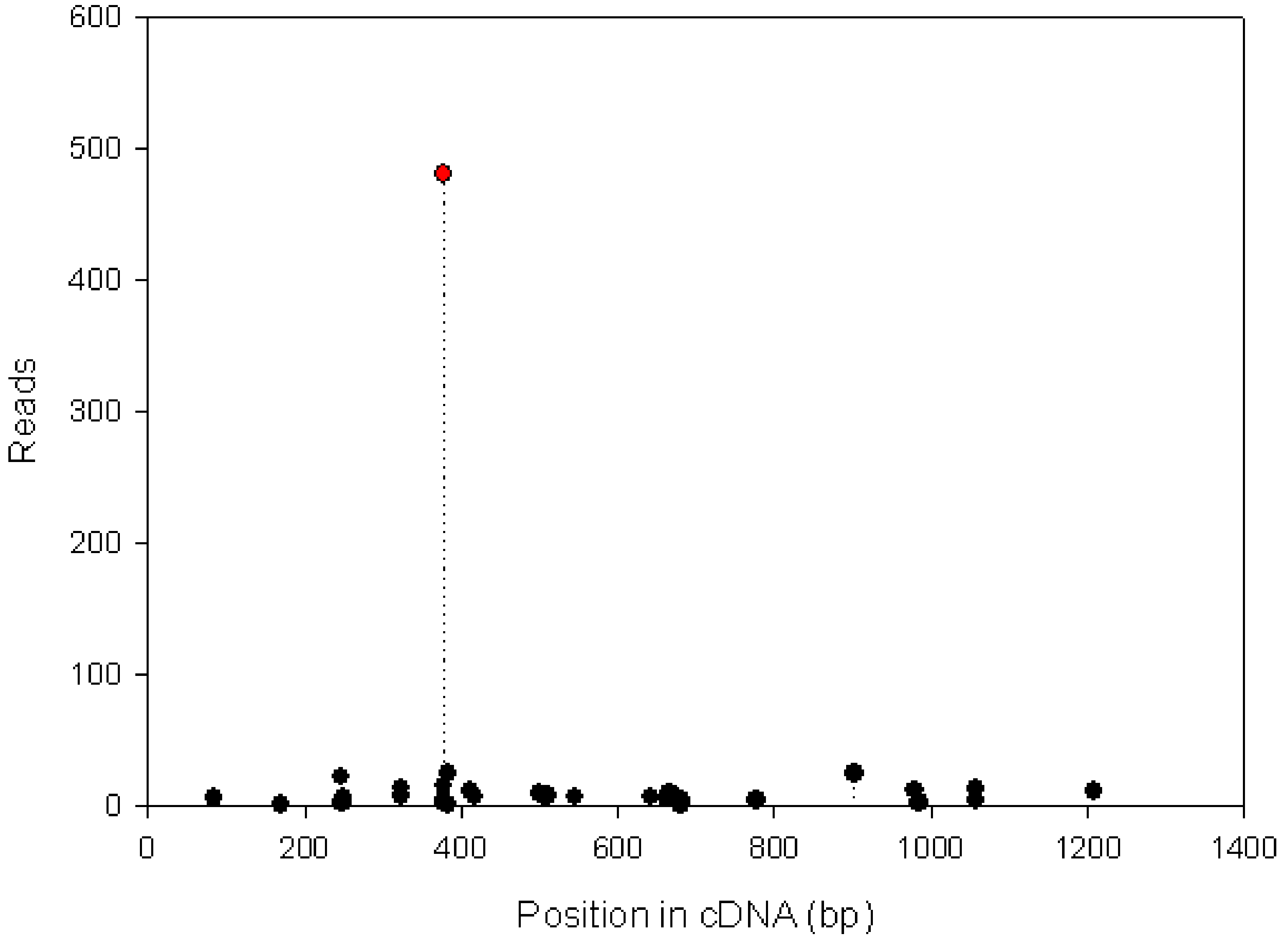


```
5' ACCGUUCAAGAAAGCCUGUGGAAUCA 3'        Cs7g15220.1
       :::::::::: ::::::::
3' ----AAGUUCUUUC-GACACCUU--- 5'        Csi-miR396b.2
```

Csi-miR396b.2, target=Cs8g17370.1 gene=Cs8g17370
Category:3
Score=4.5
Cleavage Site=138

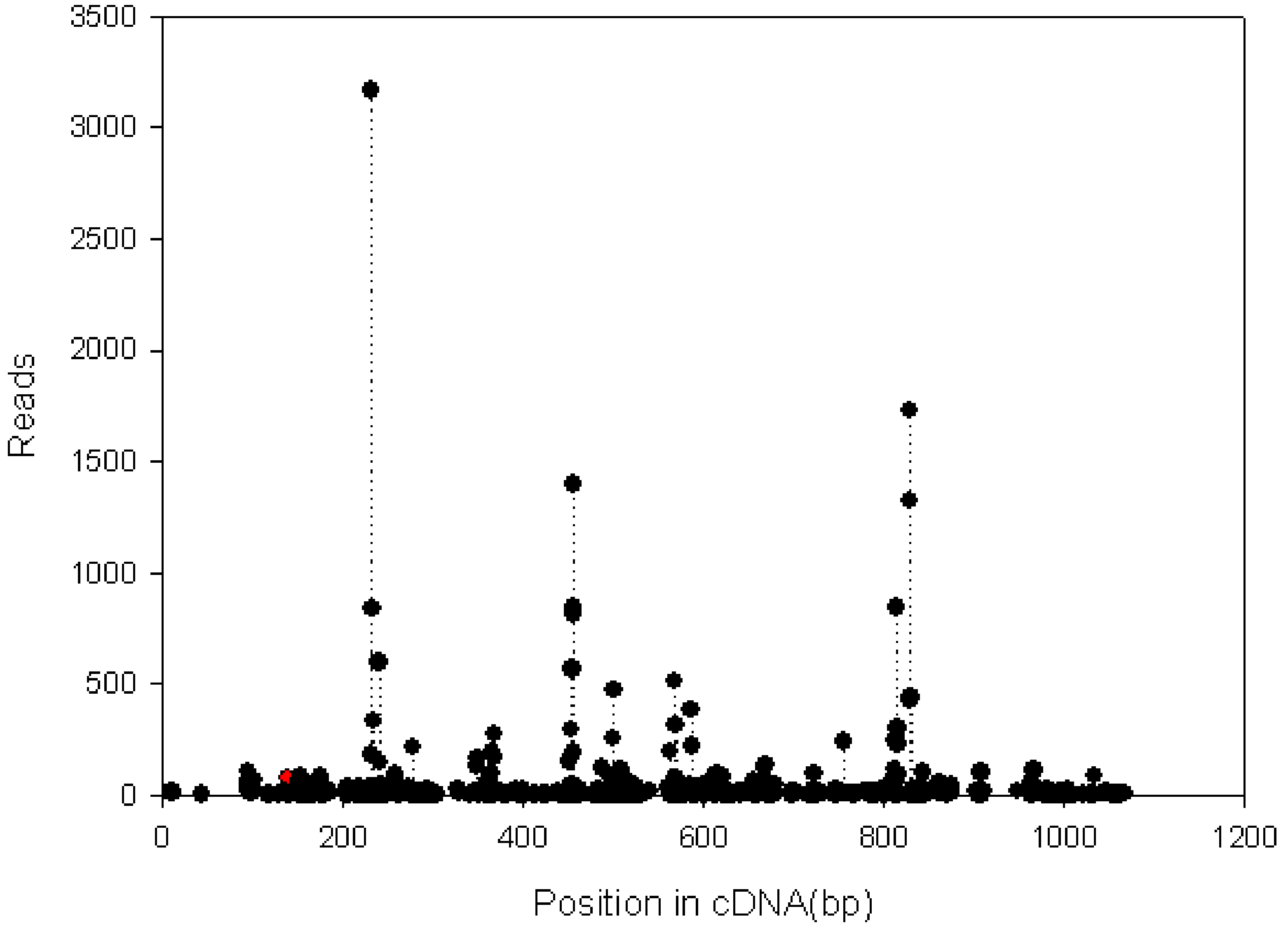


```
5' CGAGGAUUACAAGAAGGCCGUGGAGA 3'        Cs8g17370.1
          : ::::::.:: :::::.
3' -------AAGUUCUUUCGACACCUU- 5'        Csi-miR396b.2
```

Csi-miR396b.2, target=Orange1.1t02555.1 gene=Orange1.1t02555
Category:1
Score=2
Cleavage Site=920

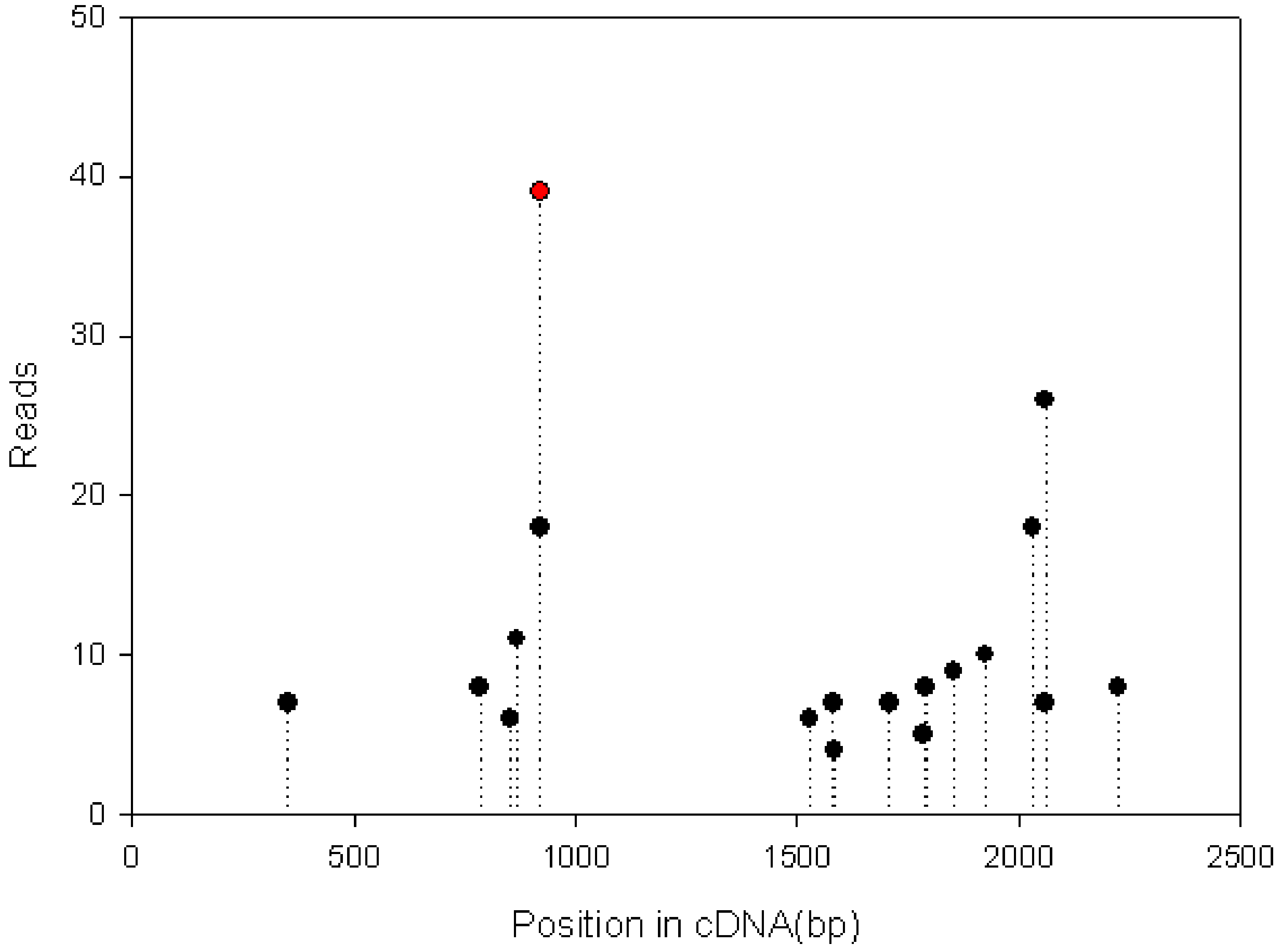


```
5' CACGUUCAAGAAAGCCUGUGGAACUU 3'        Orange1.1t02555.1
       :::::::::: ::::::::
3' ----AAGUUCUUUC-GACACCUU--- 5'        Csi-miR396b.2
```

Csi-miR396b.2, target=Orange1.1t03122.1 gene=Orange1.1t03122
Category=1
Score=2
Cleavage Site=583

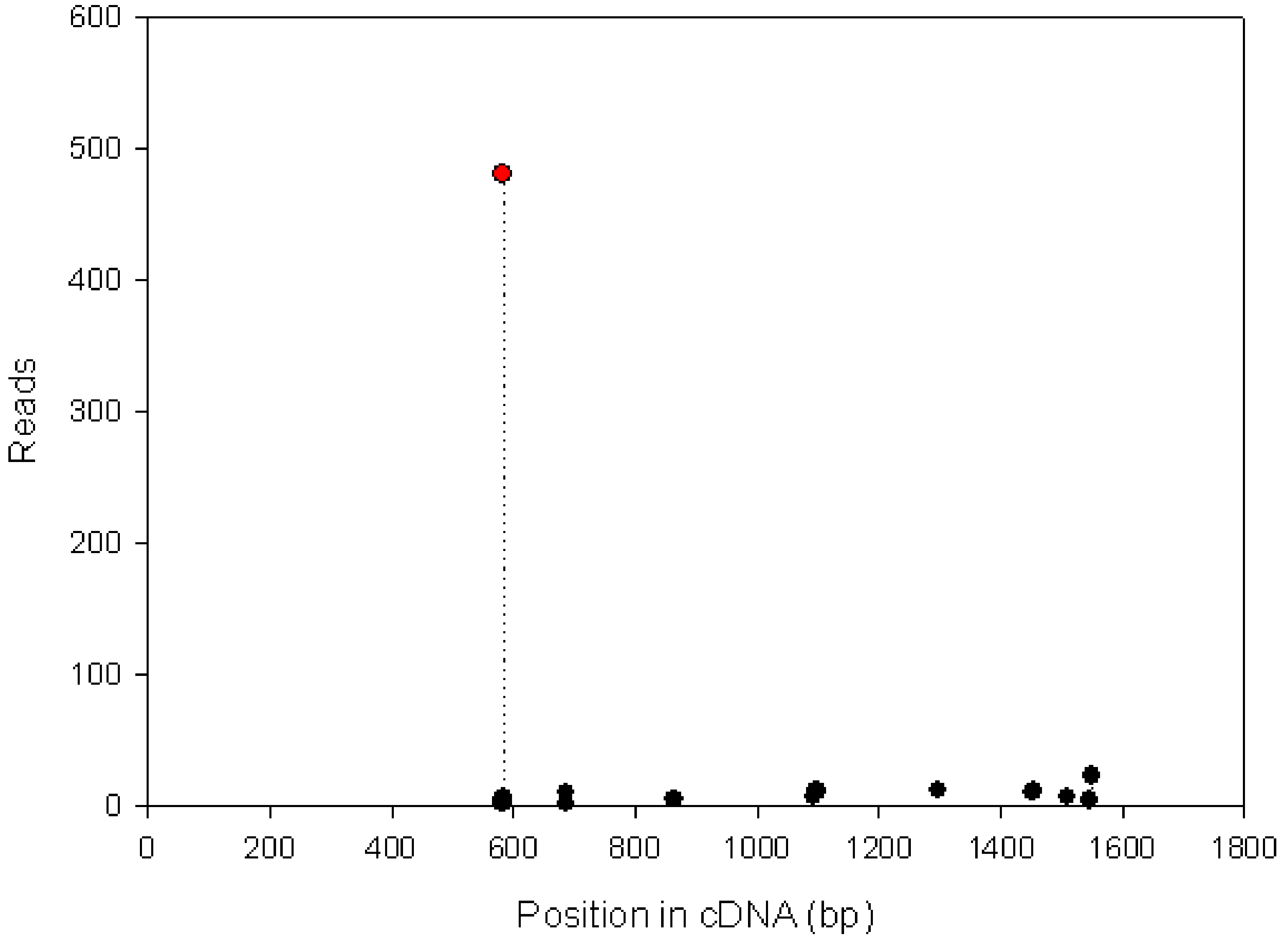


```
5' ACCGUUCAAGAAAGCCUGUGGAAUCA 3'        Orange1.1t03122.1
       :::::::::: ::::::::
3' ----AAGUUCUUUC-GACACCUU--- 5'        Csi-miR396b.2
```

Csi-miR396b.3, target=Cs1g22520.1 gene=Cs1g22520
Category:1
Score=3
Cleavage Site=226

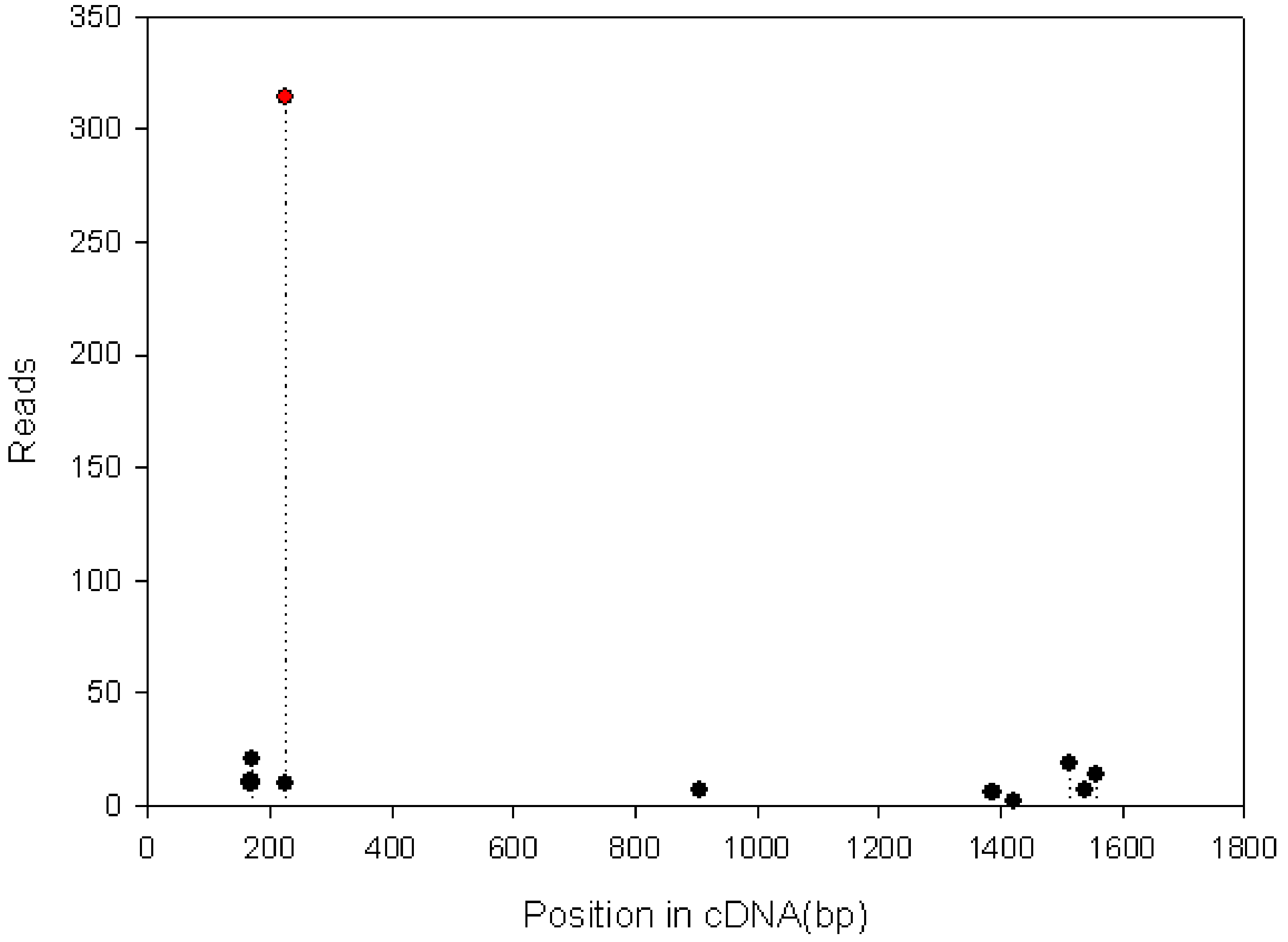


```
5' GAAGUUCAAGAAAUCUGUGGGAAGAA 3'        Cs1g22520.1
    :::::::::::: :::::::.:
3' -UUCAAGUUCUUUCGACACCUU---- 5'        Csi-miR396b.3
```

Csi-miR396b.3, target=Cs3g23180.1 gene=Cs3g23180
Category:3
Score=3
Cleavage Site=625

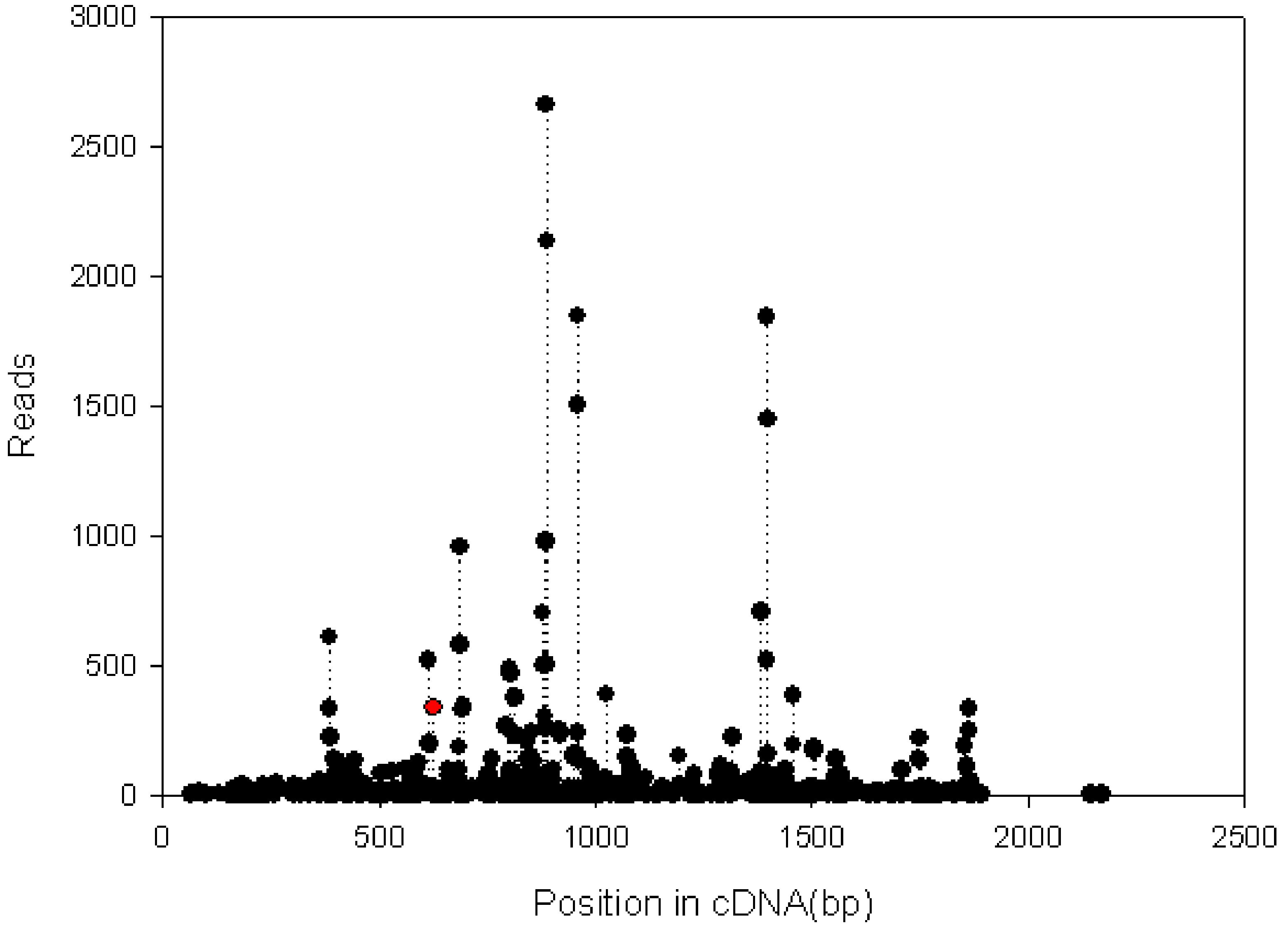


```
5' AAAGAUCAAGGAAGCUGUGGGAGUUG 3'        Cs3g23180.1
    ::: :::::.:::::::::::.:
3' -UUCAAGUUCUUUCGACACCUU---- 5'        Csi-miR396b.3
```

Csi-miR396b.3, target=Cs5g09850.1 gene=Cs5g09850
Category:2
Score=3.5
Cleavage Site=767

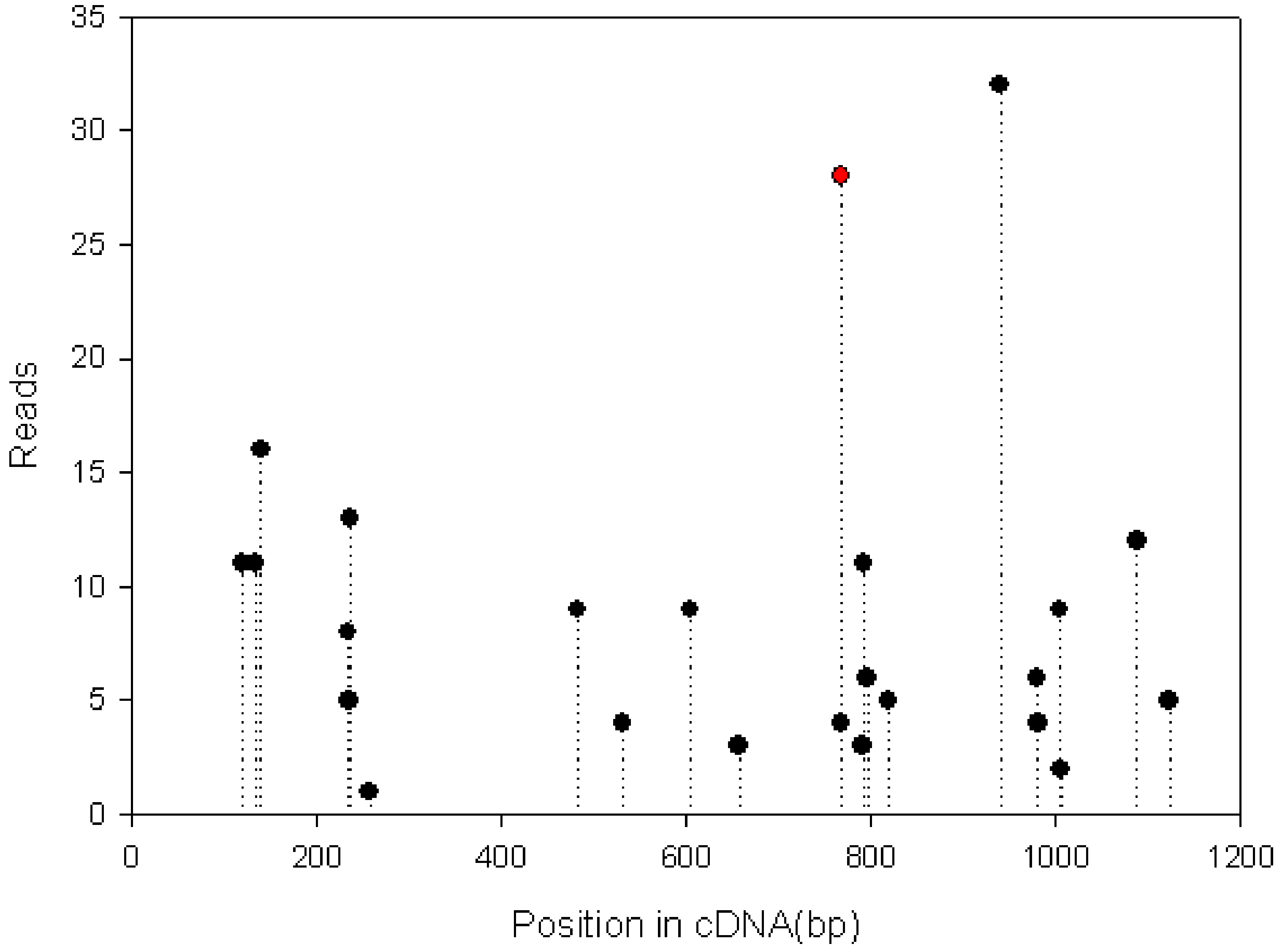


```
5' UGCGUUCAAGAAAGCUUGUGGAAGCU 3'        Cs5g09850.1
    . ::::::::::::: :::::::
3' -UUCAAGUUCUUUCG-ACACCUU--- 5'        Csi-miR396b.3
```

Csi-miR396b.3, target=Cs6g15330.1 gene=Cs6g15330
Category: 1
Score=4
Cleavage Site=449

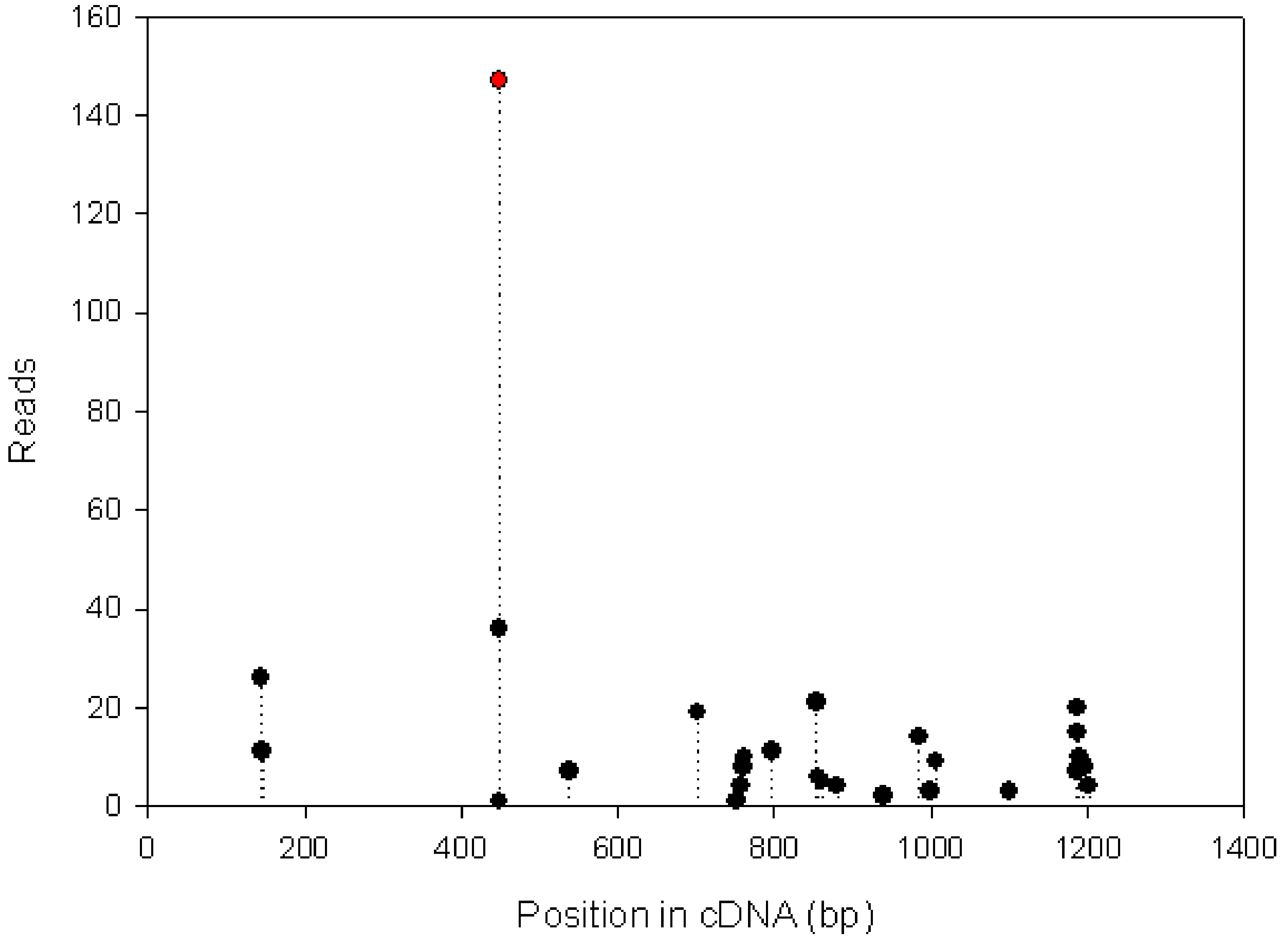


```
5' ACCGUUCAAGAAAGCAUGUGGAAAUC 3'        Cs6g15330.1
     :::::::::::: :::::::
3' -UUCAAGUUCUUUCG-ACACCUU--- 5'        Csi-miR396b.3
```

Csi-miR396b.3, target=Cs7g15220.1 gene=Cs7g15220
Category=1
Score=4
Cleavage Site=377

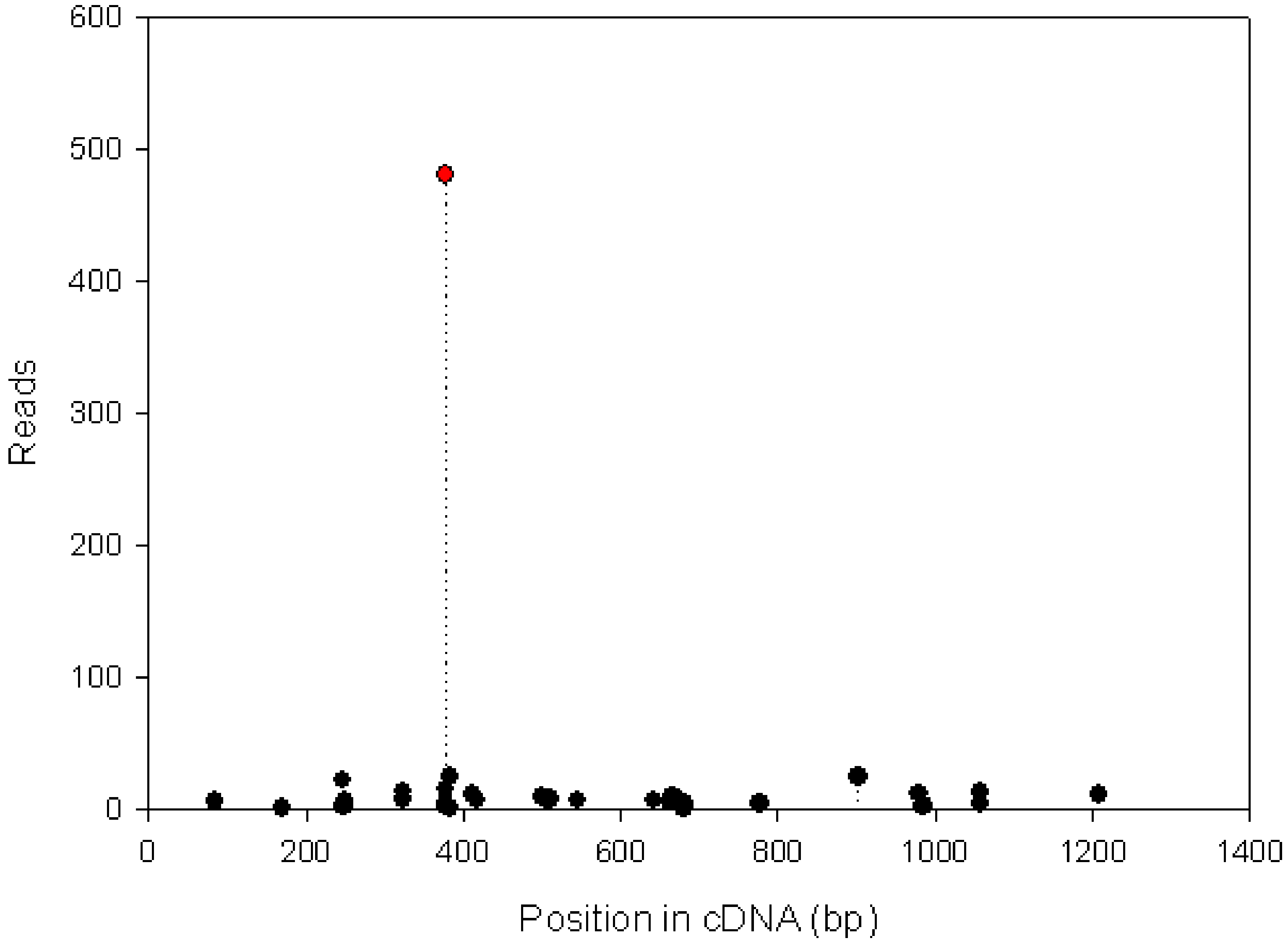


```
5' ACCGUUCAAGAAAGCCUGUGGAAUCA 3'        Cs7g15220.1
      ::::::::::: ::::::::
3' -UUCAAGUUCUUUC-GACACCUU--- 5'        Csi-miR396b.3
```

Csi-miR396b.3, target=Orange1.1t03122.1 gene=Orange1.1t03122
Category=1
Score=4
Cleavage Site=583

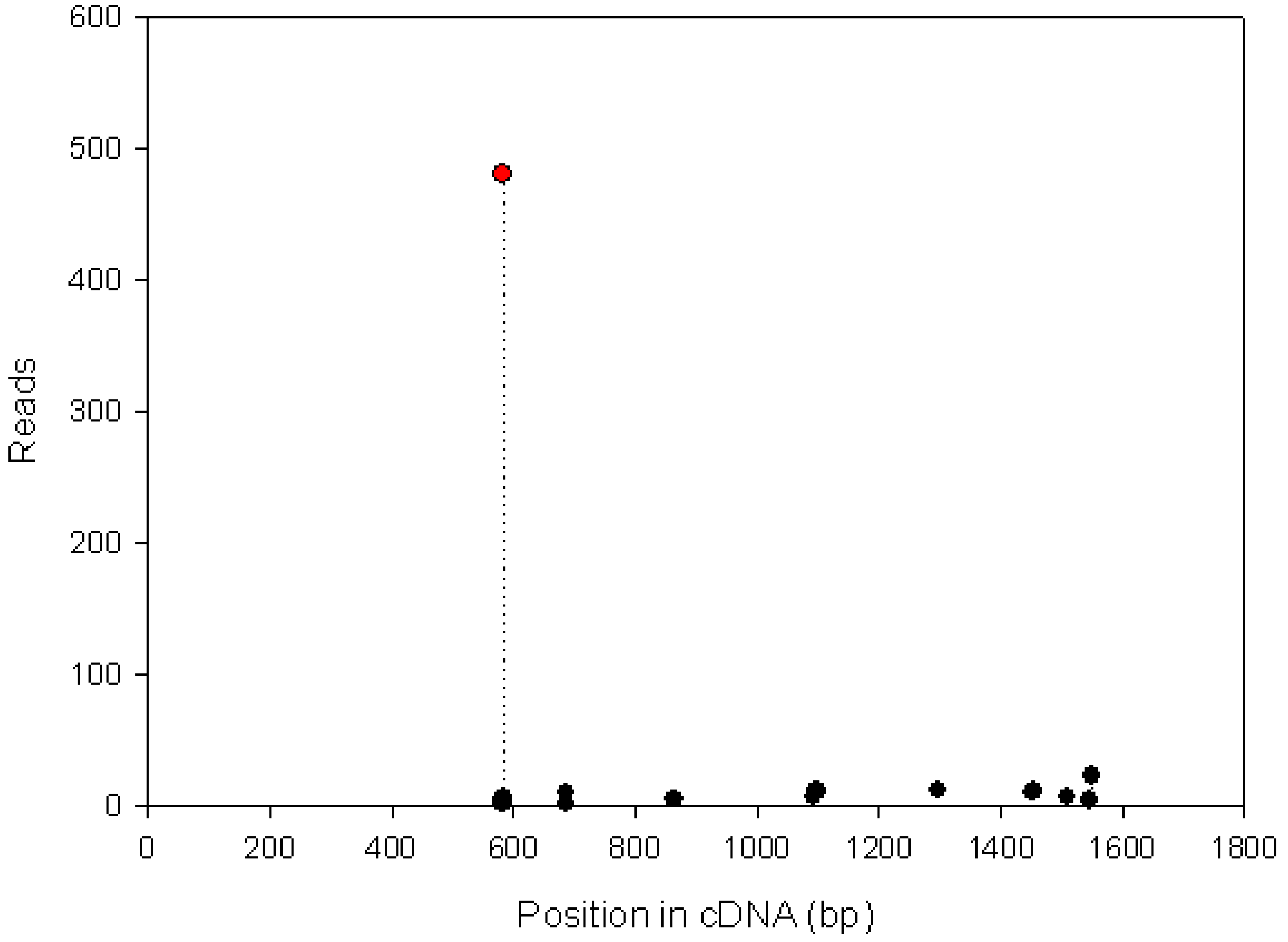


```
5' ACCGUUCAAGAAAGCCUGUGGAAUCA 3'        Orange1.1t03122.1
      ::::::::::: ::::::::
3' -UUCAAGUUCUUUC-GACACCUU--- 5'        Csi-miR396b.3
```

Csi-miR396b-3p.1, target=Cs9g04080.1 gene=Cs9g04080
Category=3
Score=5
Cleavage Site=311

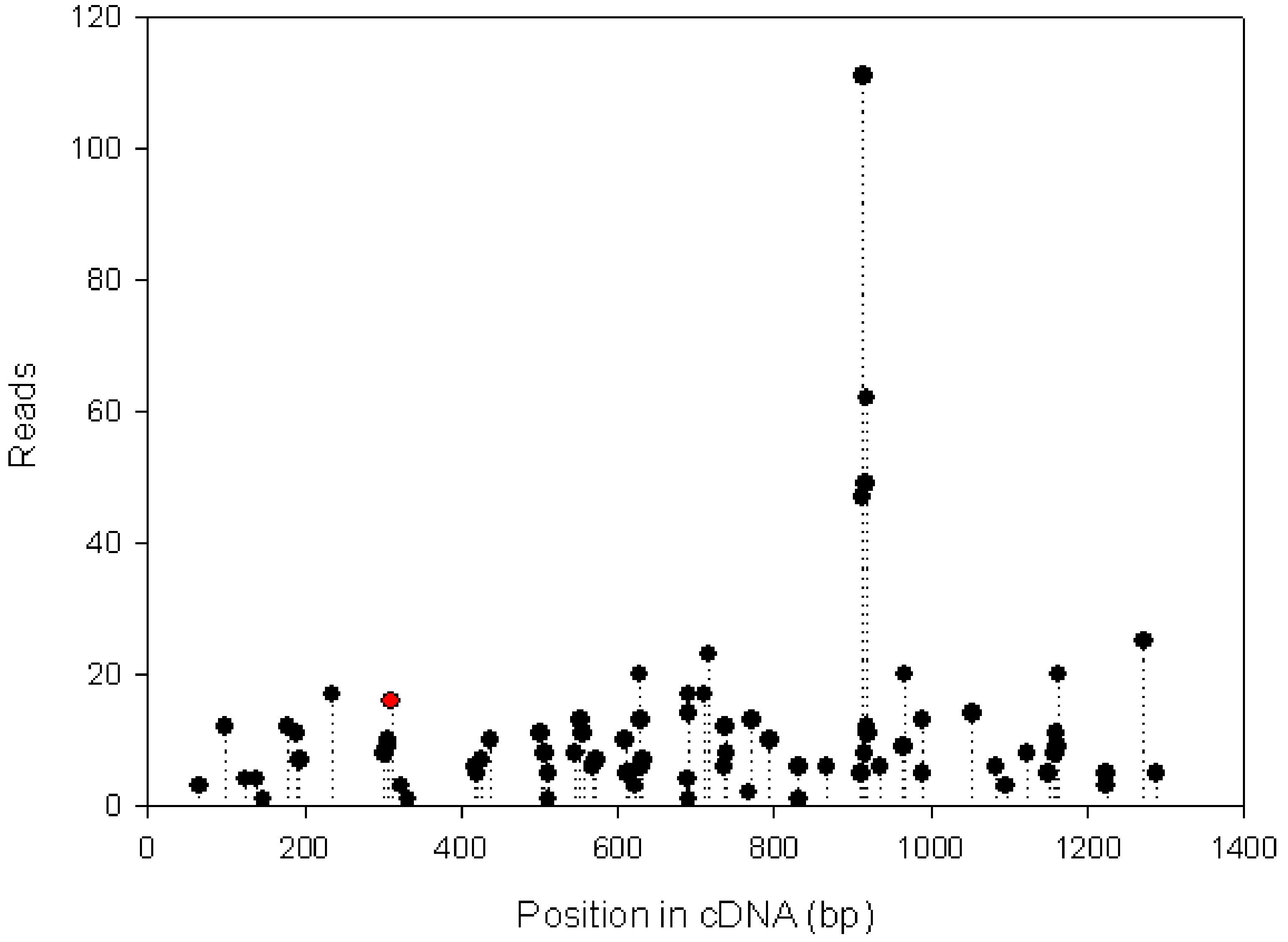


```
5' UCUCUUCCUUCACGGCUUUCCUGAGC 3'        Cs9g04080.1
        :: ..:::.:::::: :::::
3' -----AGAGGGUGUCGAAAGAACUCG 5'        Csi-miR396b-3p.1
```

Csi-miR396b-3p.2, target=Cs9g04080.1 gene=Cs9g04080
Category=3
Score=5
Cleavage Site=311

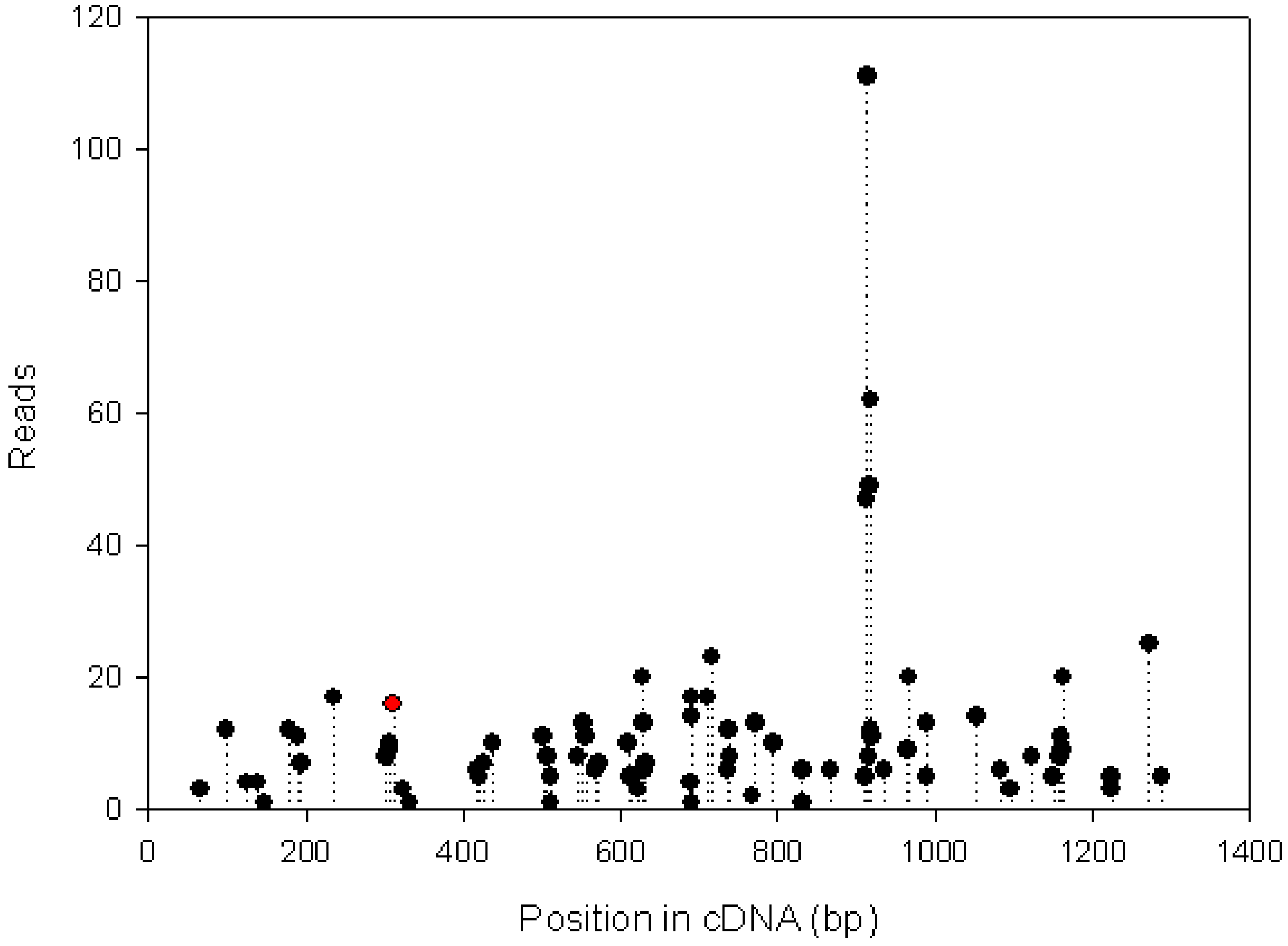


```
5' UCUCUUCCUUCACGGCUUUCCUGAGC 3'        Cs9g04080.1
           ::  ..:::.::::::  ::::
3' -----AGAGGGUGUCGAAAGAACUC- 5'        Csi-miR396b-3p.2
```

Csi-miR396c, target=Cs2g09460.1 gene=Cs2g09460
Category=3
Score=5
Cleavage Site=812

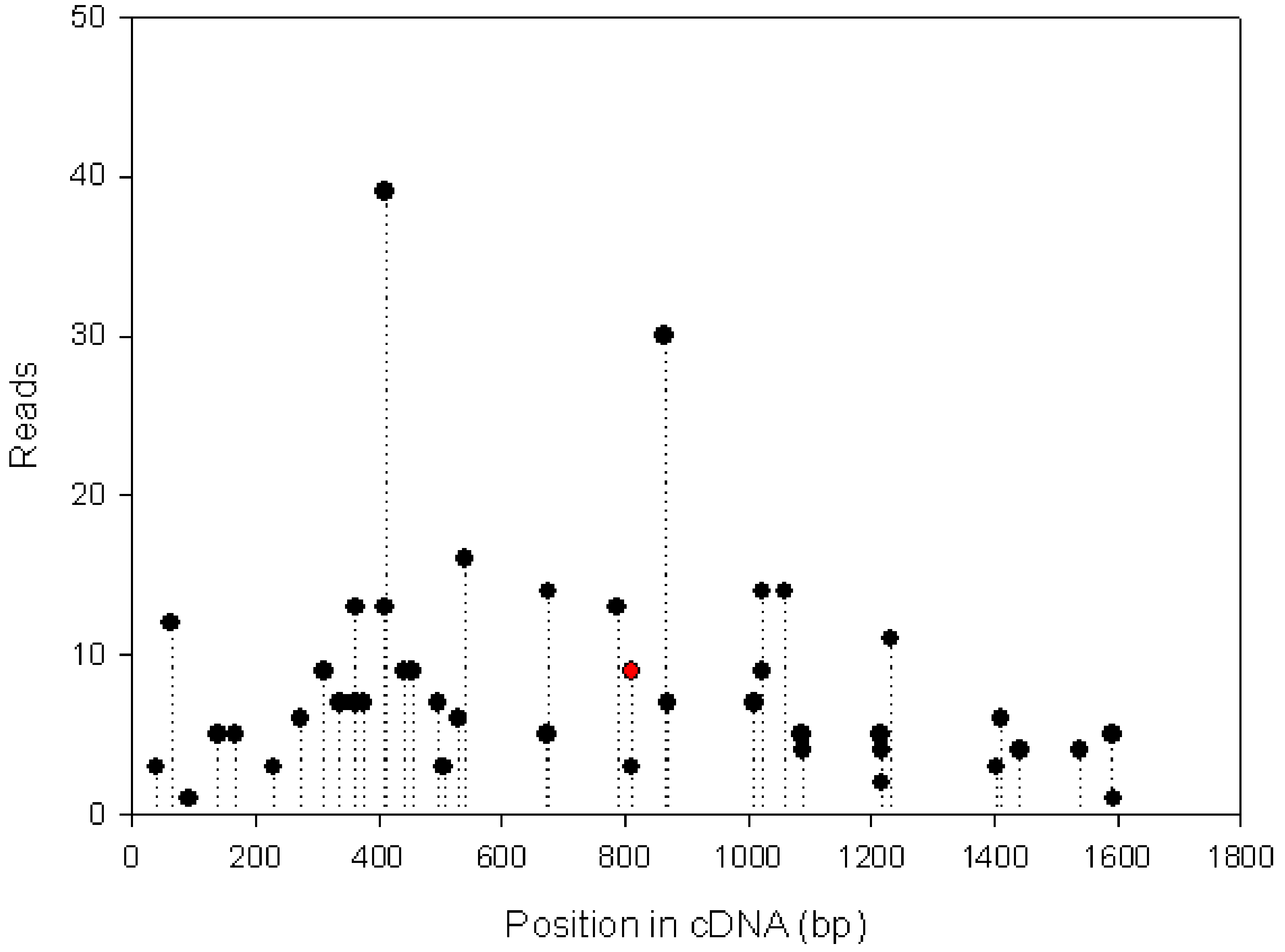


```
5' UCUGCUUCCCUCAGA-CUCUUGAAGCU 3'        Cs2g09460.1
       :::::: ::::  :::::::
3' ----GAAGGGUGUCUAAAGAACUU--- 5'        Csi-miR396c
```

Csi-miR396d.1, target=Cs1g22520.1 gene=Cs1g22520
Category:1
Score=4
Cleavage Site=226

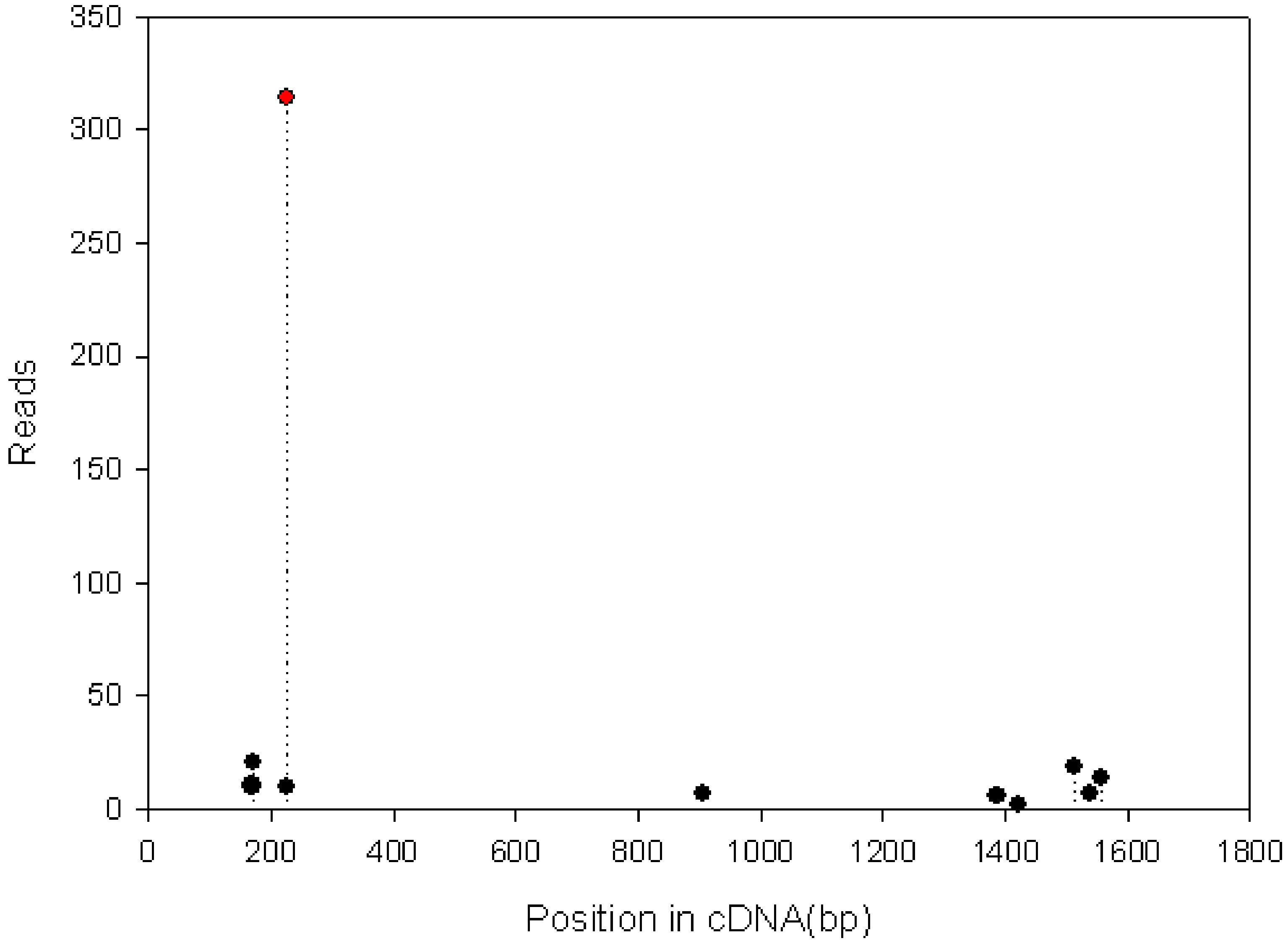


```
5' GAAGUUCAAGAAAUCUGUGGGAAGAA 3'        Cs1g22520.1
     ::::::::::: :.::::.::
3' --UCAAGUUCUUUCGGCACCUUU--- 5'        Csi-miR396d.1
```

Csi-miR396d.1, target=Cs3g23180.1 gene=Cs3g23180
Category:3
Score=4.5
Cleavage Site=625

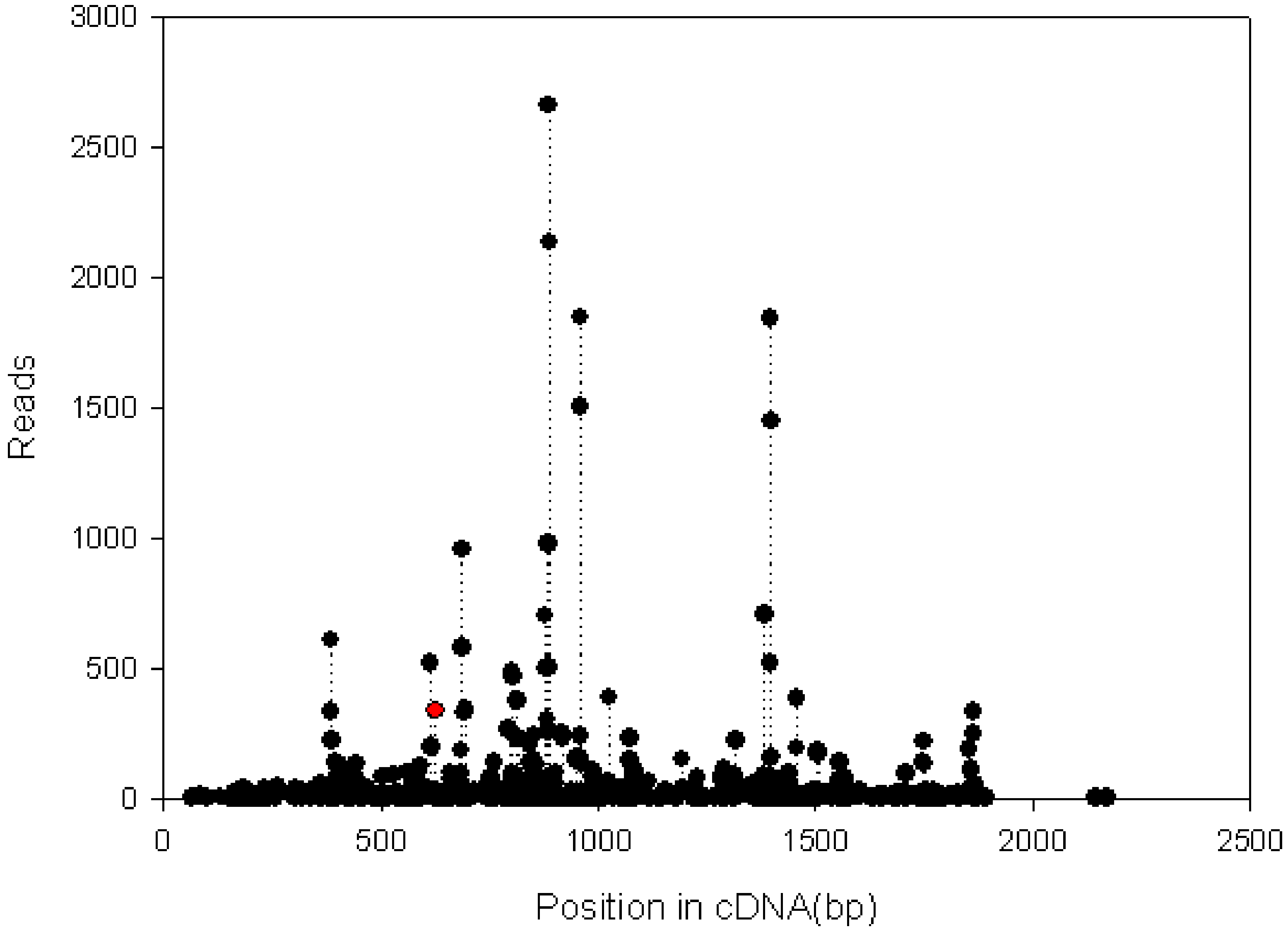


```
5' AAAGAUCAAGGAAGCUGUGGGAGUUG 3'        Cs3g23180.1
     :: :::::.::::.::::.:.
3' --UCAAGUUCUUUCGGCACCUUU--- 5'        Csi-miR396d.1
```

Csi-miR396d.1, target=Cs5g09850.1 gene=Cs5g09850
Category:2
Score=4.5
Cleavage Site=767

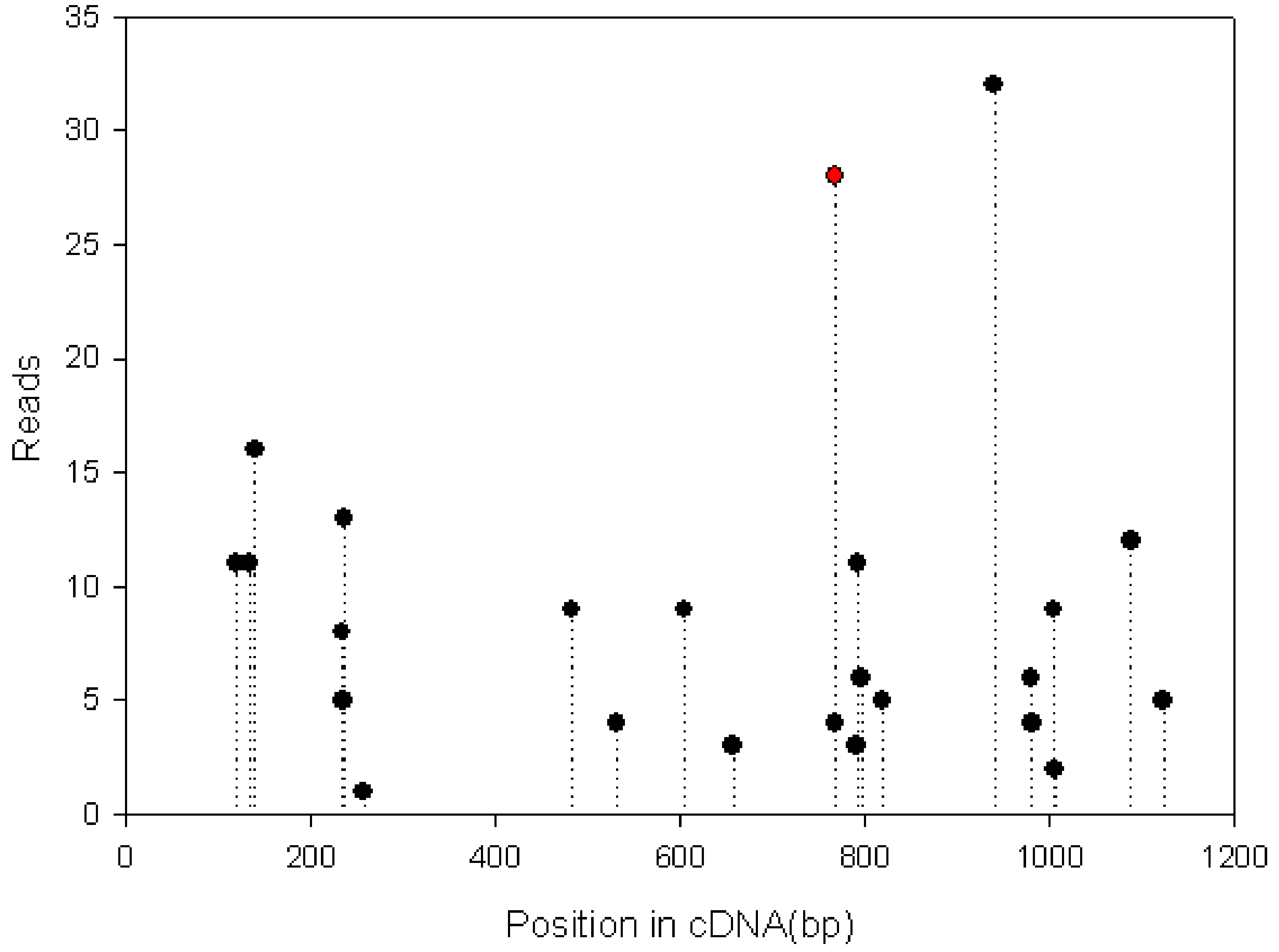


```
5' UGCGUUCAAGAAAGCUUGUGGAAGCU 3'        Cs5g09850.1
     :::::::::::: .::::::.
3' --UCAAGUUCUUUCG-GCACCUUU-- 5'        Csi-miR396d.1
```

Csi-miR396d.1, target=Cs6g15330.1 gene=Cs6g15330
Category: 1
Score=4
Cleavage Site=449

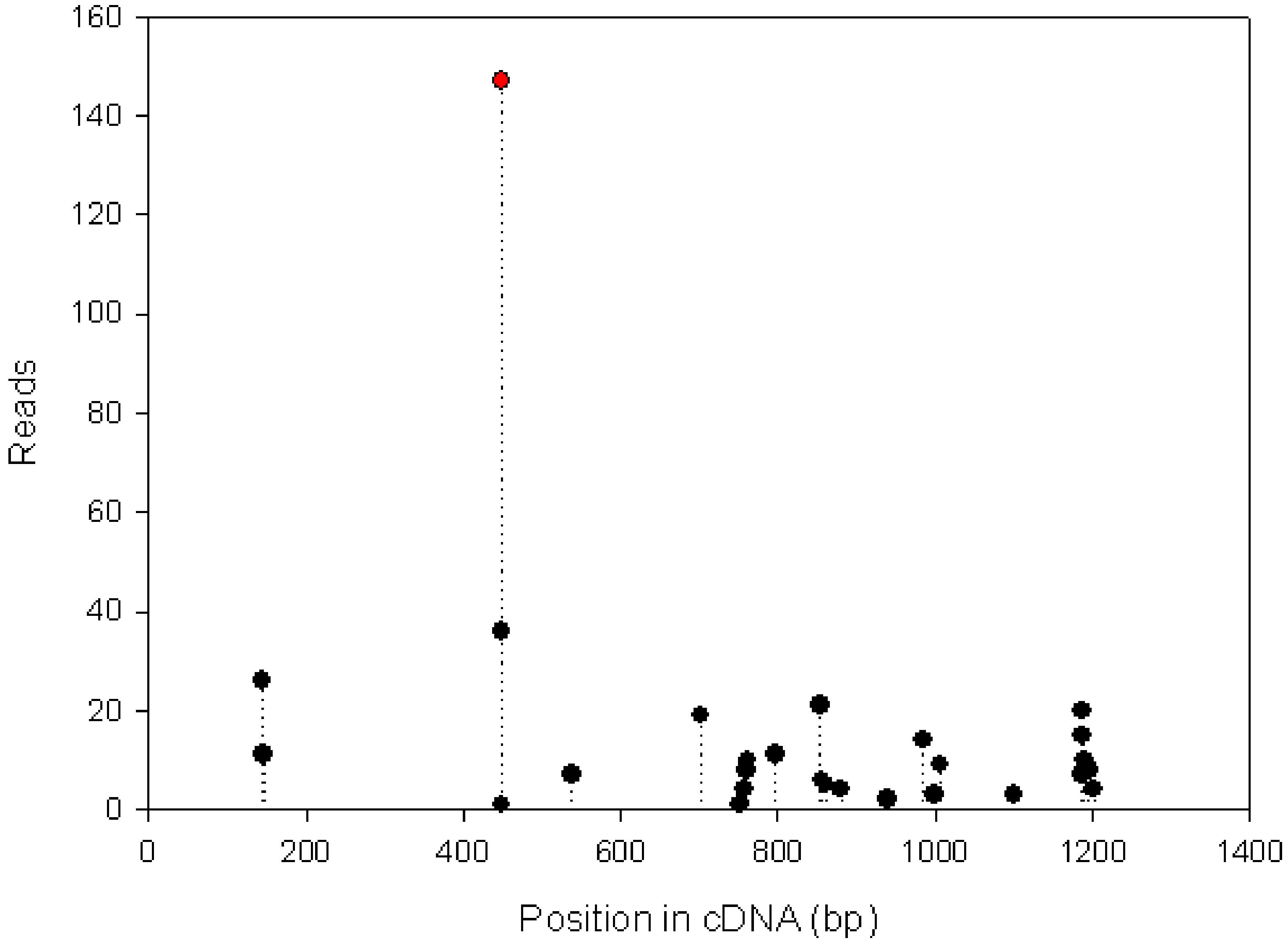


```
5' ACCGUUCAAGAAAGCAUGUGGAAAUC 3'        Cs6g15330.1
     :::::::::::: ::::::::
3' --UCAAGUUCUUUCG-GCACCUUU-- 5'        Csi-miR396d.1
```

Csi-miR396d.1, target=Cs7g15220.1 gene=Cs7g15220
Category=1
Score=4
Cleavage Site=377

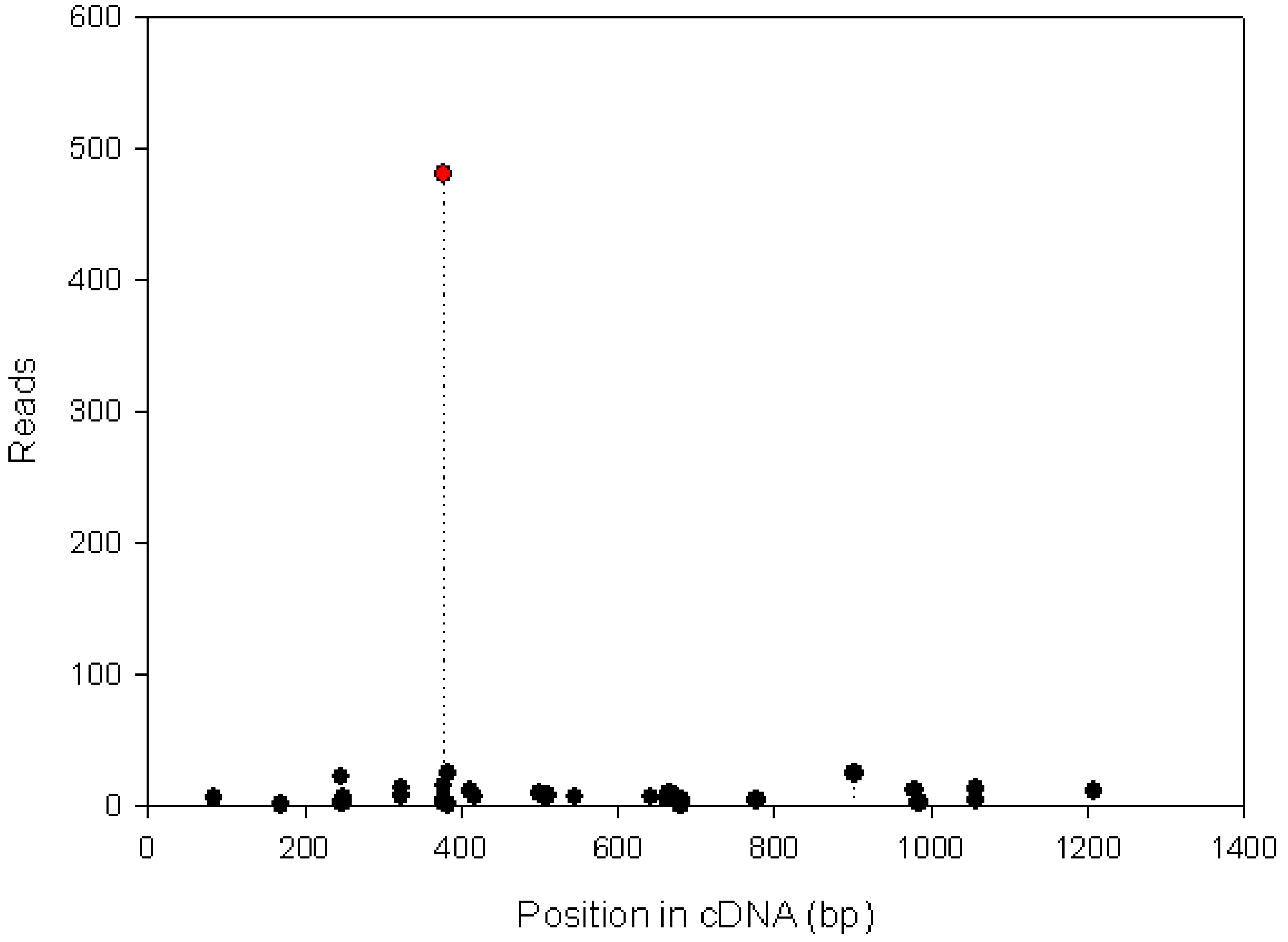


```
5' ACCGUUCAAGAAAGCCUGUGGAAUCA 3'        Cs7g15220.1
     ::::::::::::: ::::::
3' --UCAAGUUCUUUCGG-CACCUUU-- 5'        Csi-miR396d.1
```

Csi-miR396d.1, target=Orange1.1t03122.1 gene=Orange1.1t03122
Category=1
Score=4
Cleavage Site=583

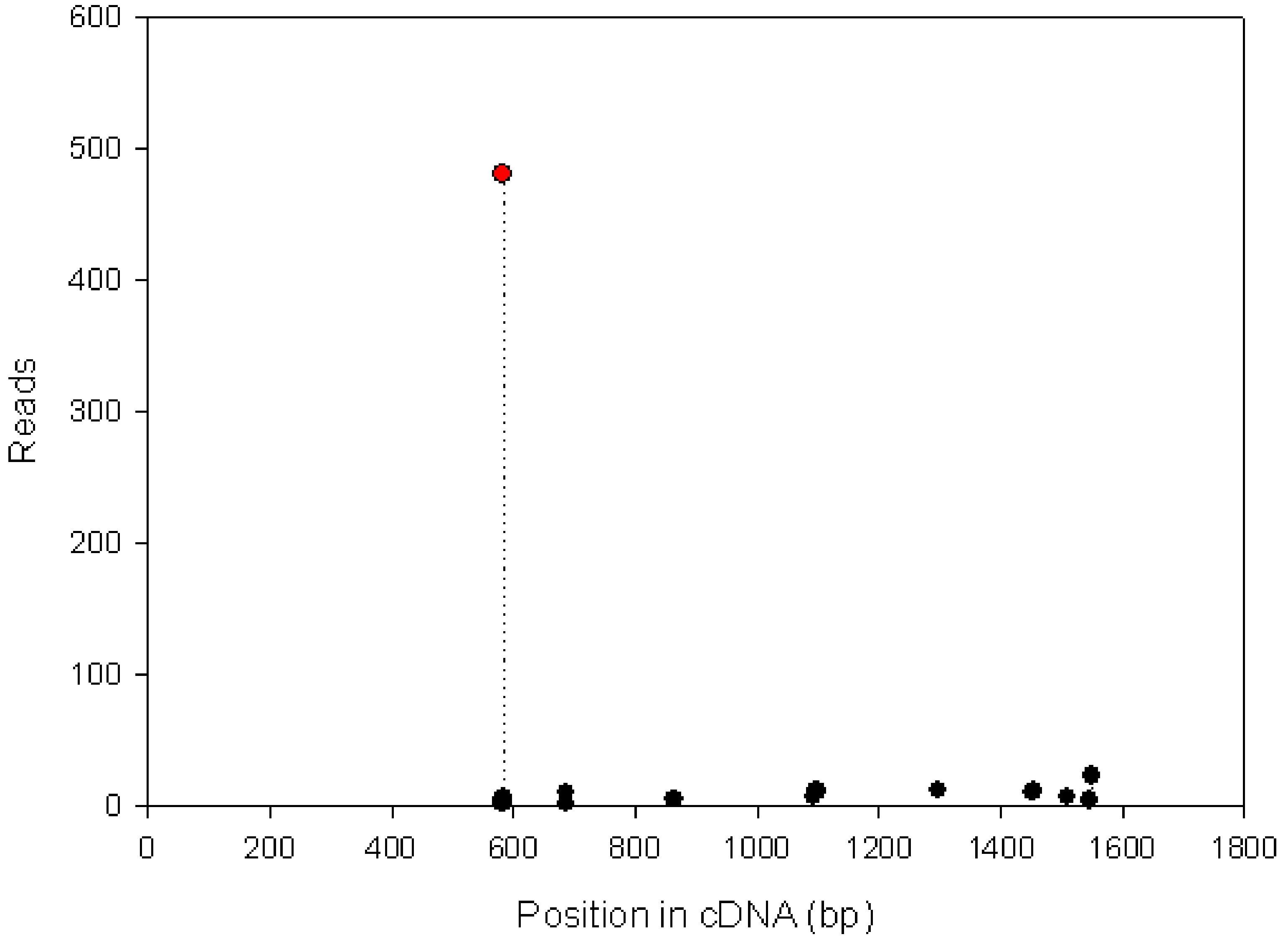


```
5' ACCGUUCAAGAAAGCCUGUGGAAUCA 3'        Orange1.1t03122.1
     ::::::::::::: ::::::
3' --UCAAGUUCUUUCGG-CACCUUU-- 5'        Csi-miR396d.1
```

Csi-miR396d.2, target=Cs1g22520.1 gene=Cs1g22520
Category:1
Score=4
Cleavage Site=226

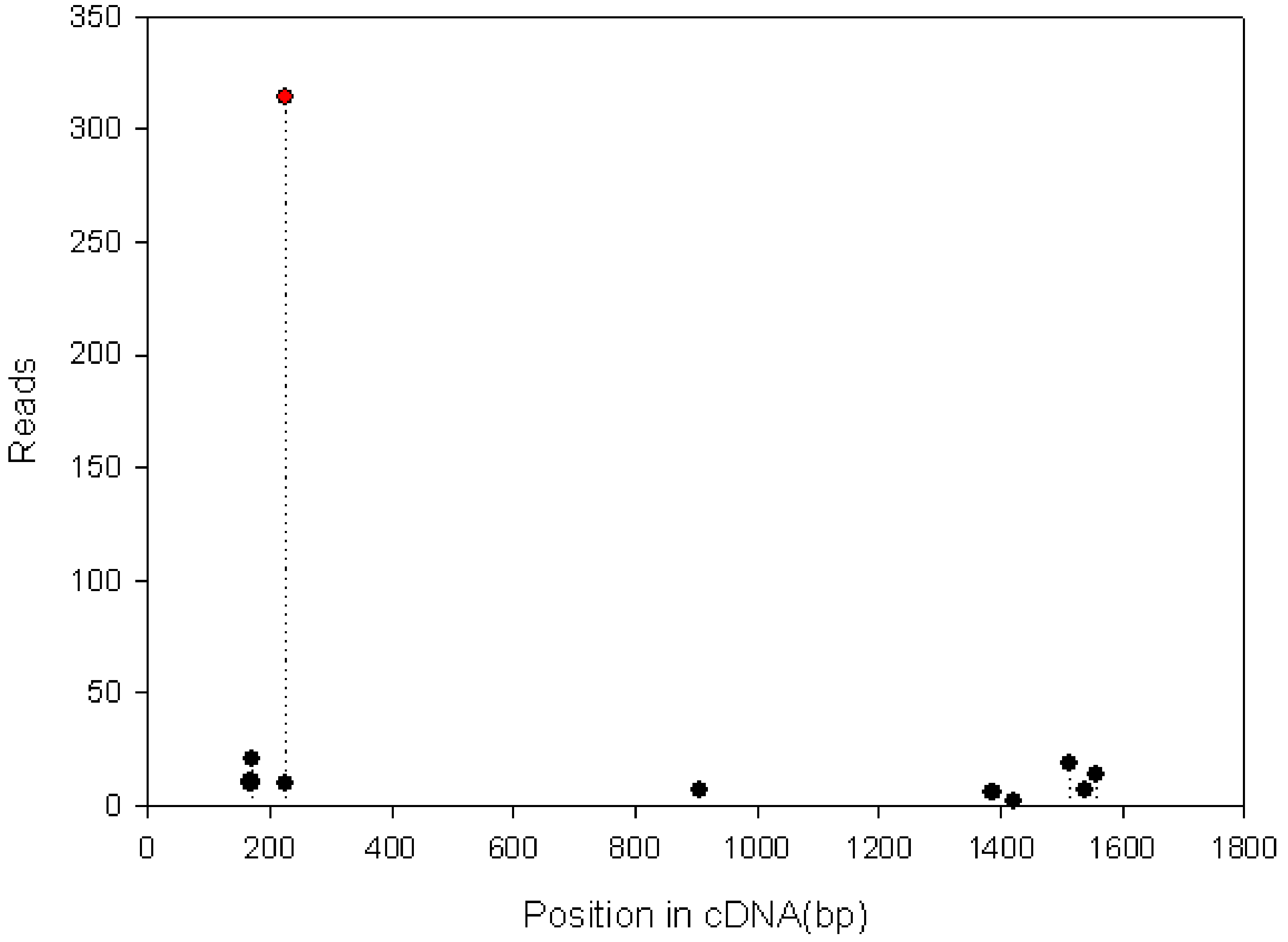


```
5' GAAGUUCAAGAAAUCUGUGGGAAGAA 3'        Cs1g22520.1
    :::::::::::: :.::::.:
3' -UUCAAGUUCUUUCGGCACCUU---- 5'        Csi-miR396d.2
```

Csi-miR396d.2, target=Cs3g23180.1 gene=Cs3g23180
Category:3
Score=4
Cleavage Site=625

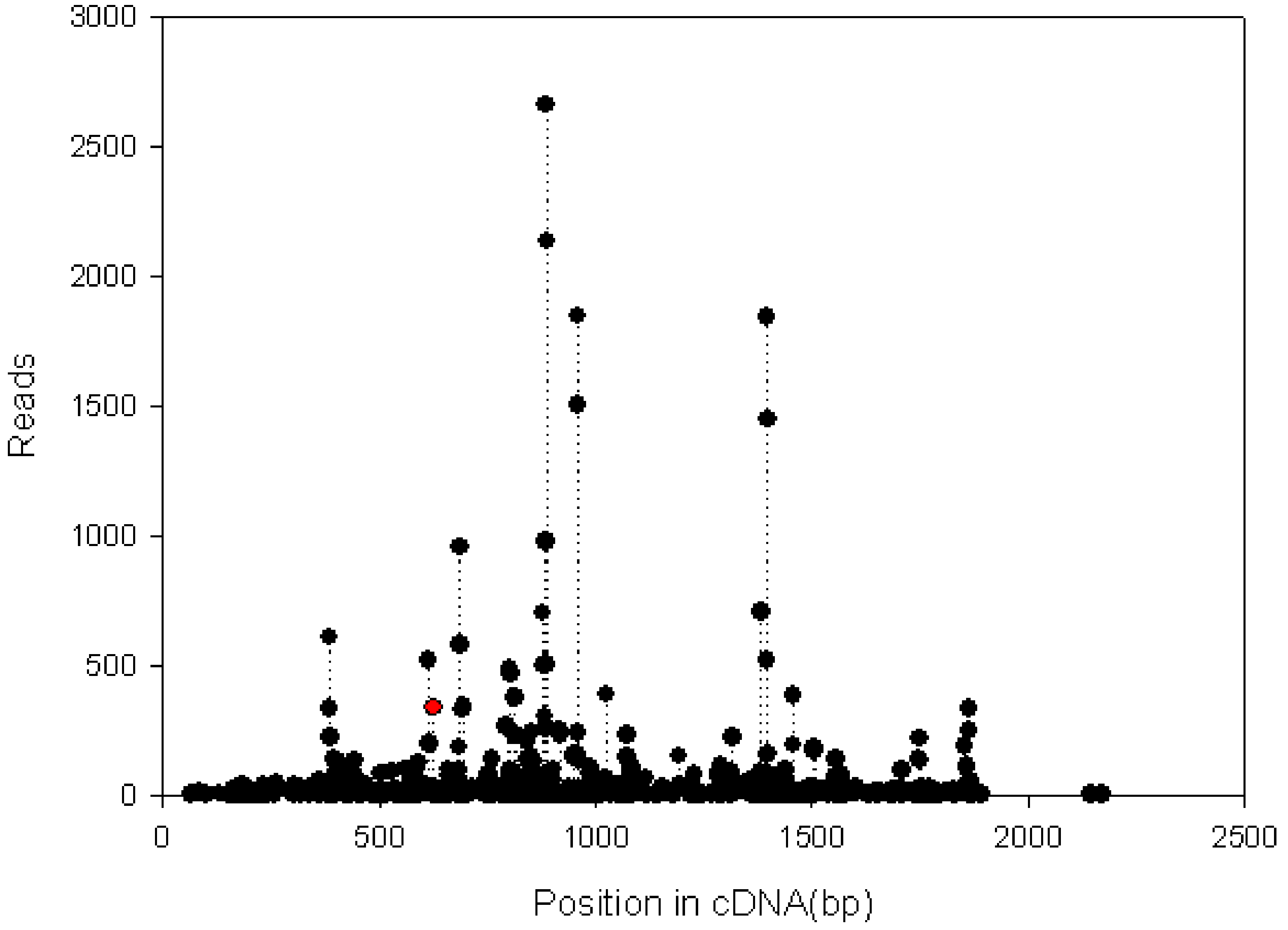


```
5' AAAGAUCAAGGAAGCUGUGGGAGUUG 3'        Cs3g23180.1
    ::: ::::::.::::.::::.:
3' -UUCAAGUUCUUUCGGCACCUU---- 5'        Csi-miR396d.2
```

Csi-miR396d.2, target=Cs5g09850.1 gene=Cs5g09850
Category:2
Score=4.5
Cleavage Site=767

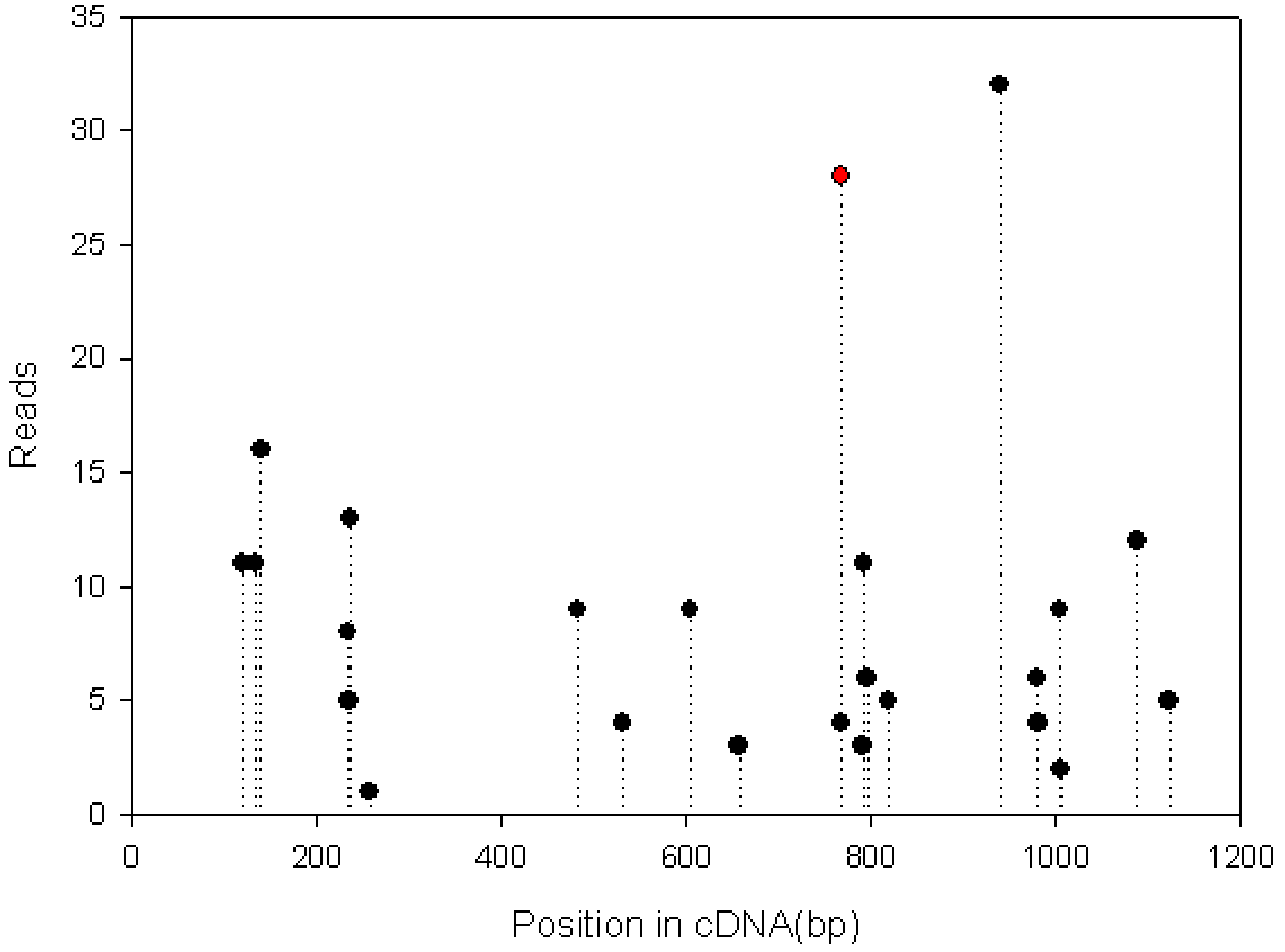


```
5' UGCGUUCAAGAAAGCUUGUGGAAGCU 3'        Cs5g09850.1
    . ::::::::::::  .::::::
3' -UUCAAGUUCUUUCG-GCACCUU--- 5'        Csi-miR396d.2
```

Csi-miR396d.2, target=Cs6g15330.1 gene=Cs6g15330
Category: 1
Score=5
Cleavage Site=449

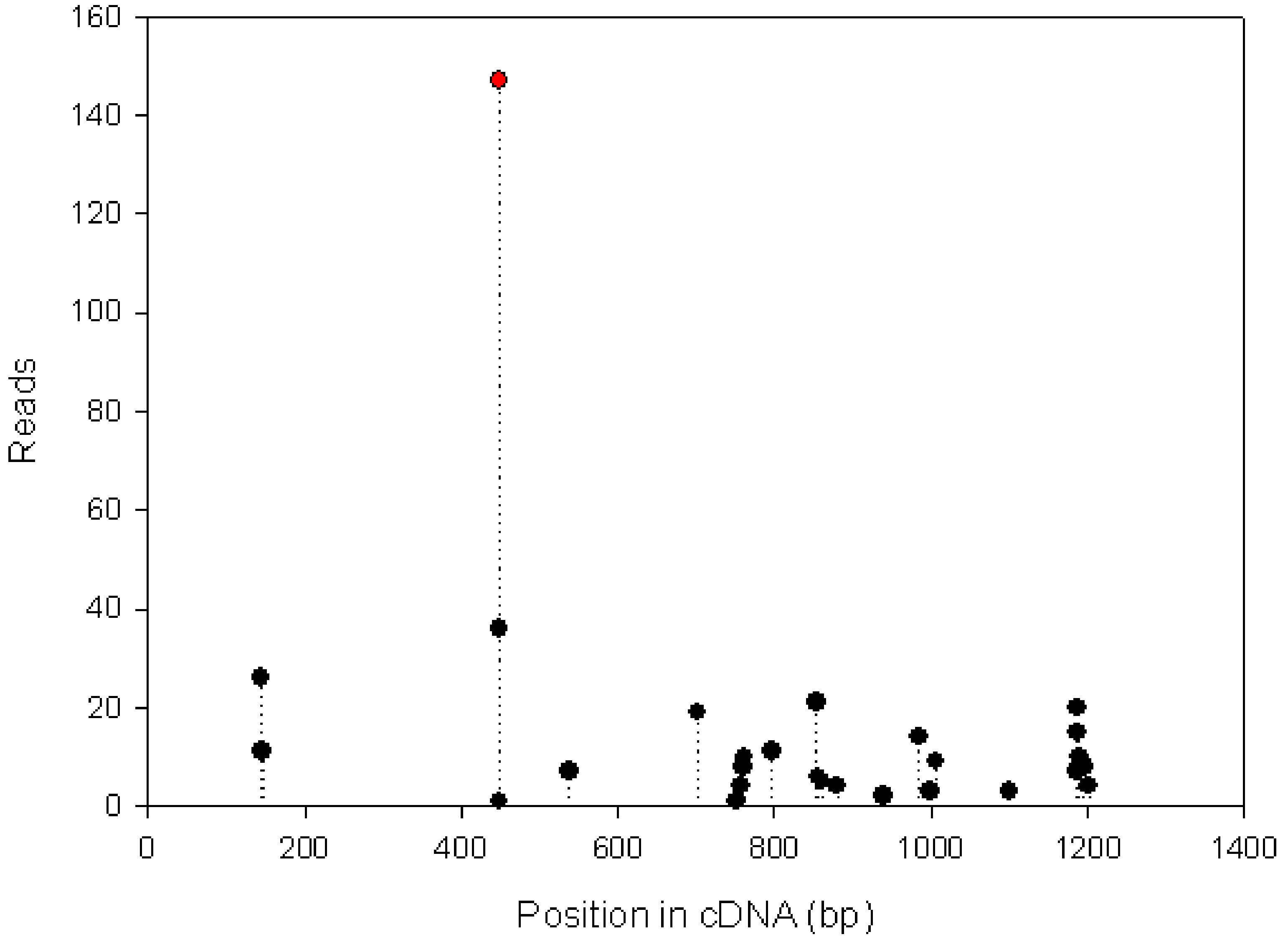


```
5' ACCGUUCAAGAAAGCAUGUGGAAAUC 3'        Cs6g15330.1
     :::::::::::: .::::::
3' -UUCAAGUUCUUUCG-GCACCUU--- 5'        Csi-miR396d.2
```

Csi-miR396d.2, target=Cs7g15220.1 gene=Cs7g15220
Category=1
Score=4
Cleavage Site=377

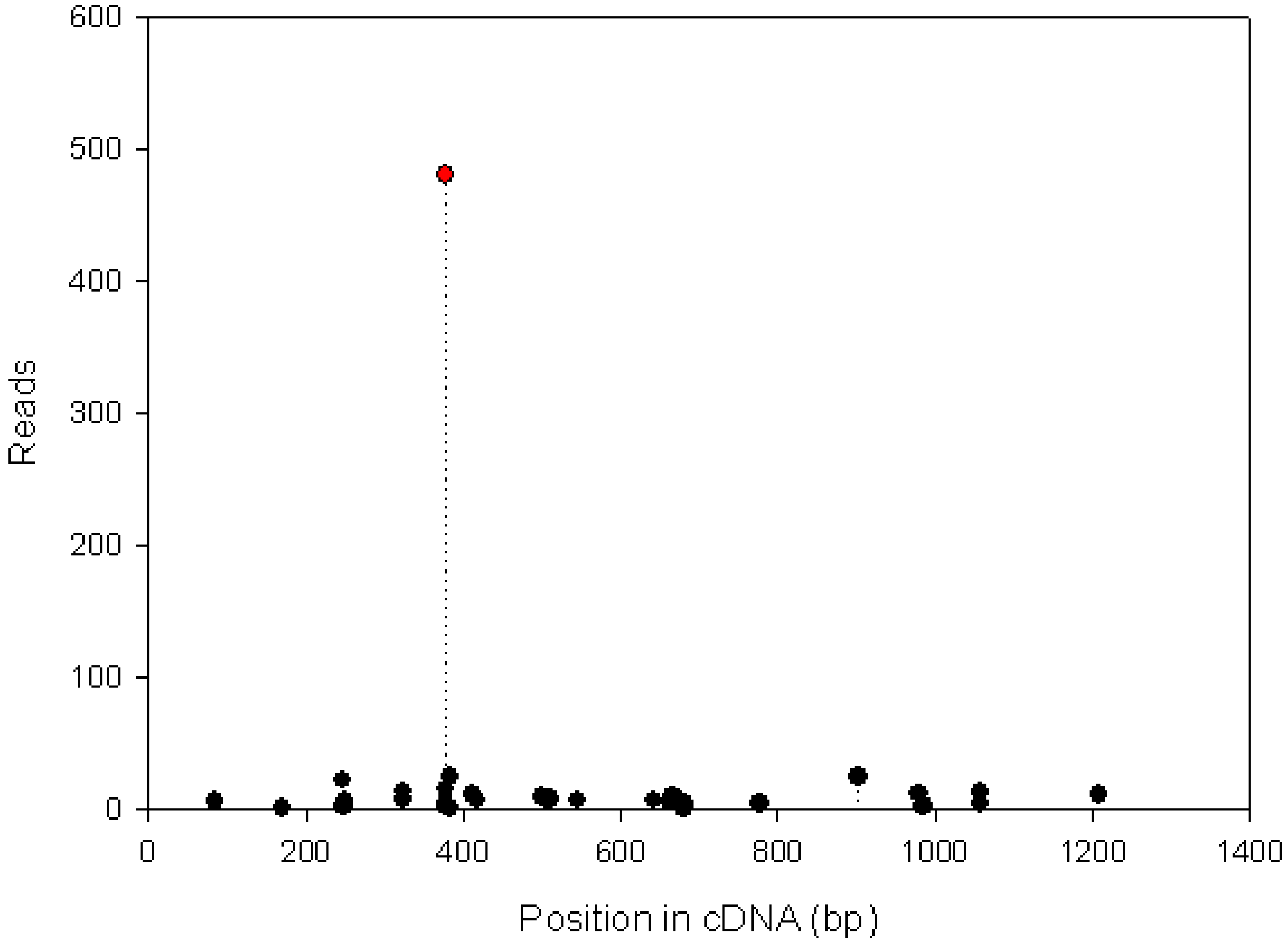


```
5' ACCGUUCAAGAAAGCCUGUGGAAUCA 3'        Cs7g15220.1
     ::::::::::::: ::::::
3' -UUCAAGUUCUUUCGG-CACCUU--- 5'        Csi-miR396d.2
```

Csi-miR396d.2, target=Orange1.1t03122.1 gene=Orange1.1t03122
Category=1
Score=4
Cleavage Site=583

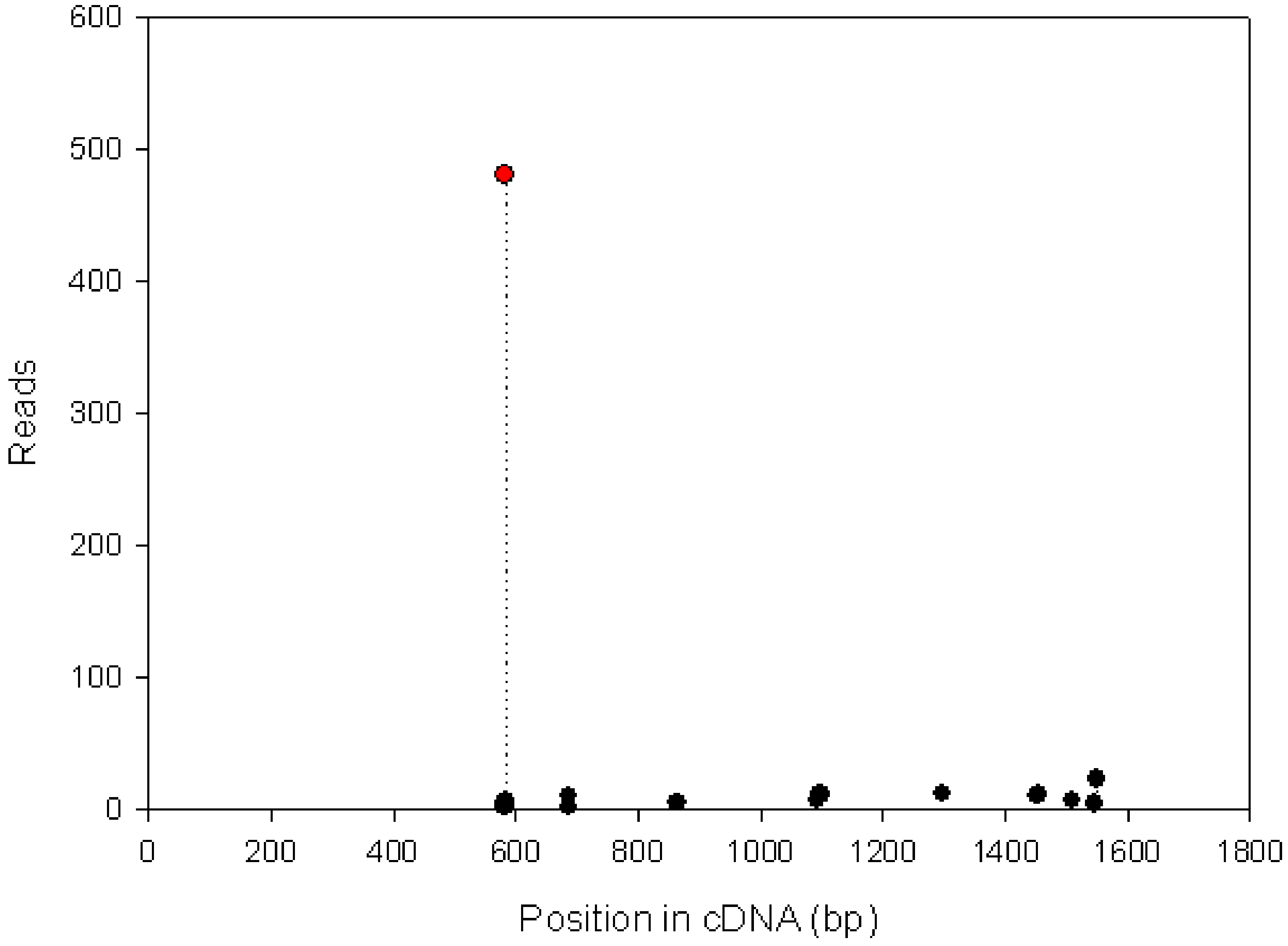


```
5' ACCGUUCAAGAAAGCCUGUGGAAUCA 3'        Orange1.1t03122.1
     ::::::::::::: ::::::
3' -UUCAAGUUCUUUCGG-CACCUU--- 5'        Csi-miR396d.2
```

Csi-miR396d.3, target=Cs1g22520.1 gene=Cs1g22520
Category:1
Score=4
Cleavage Site=226

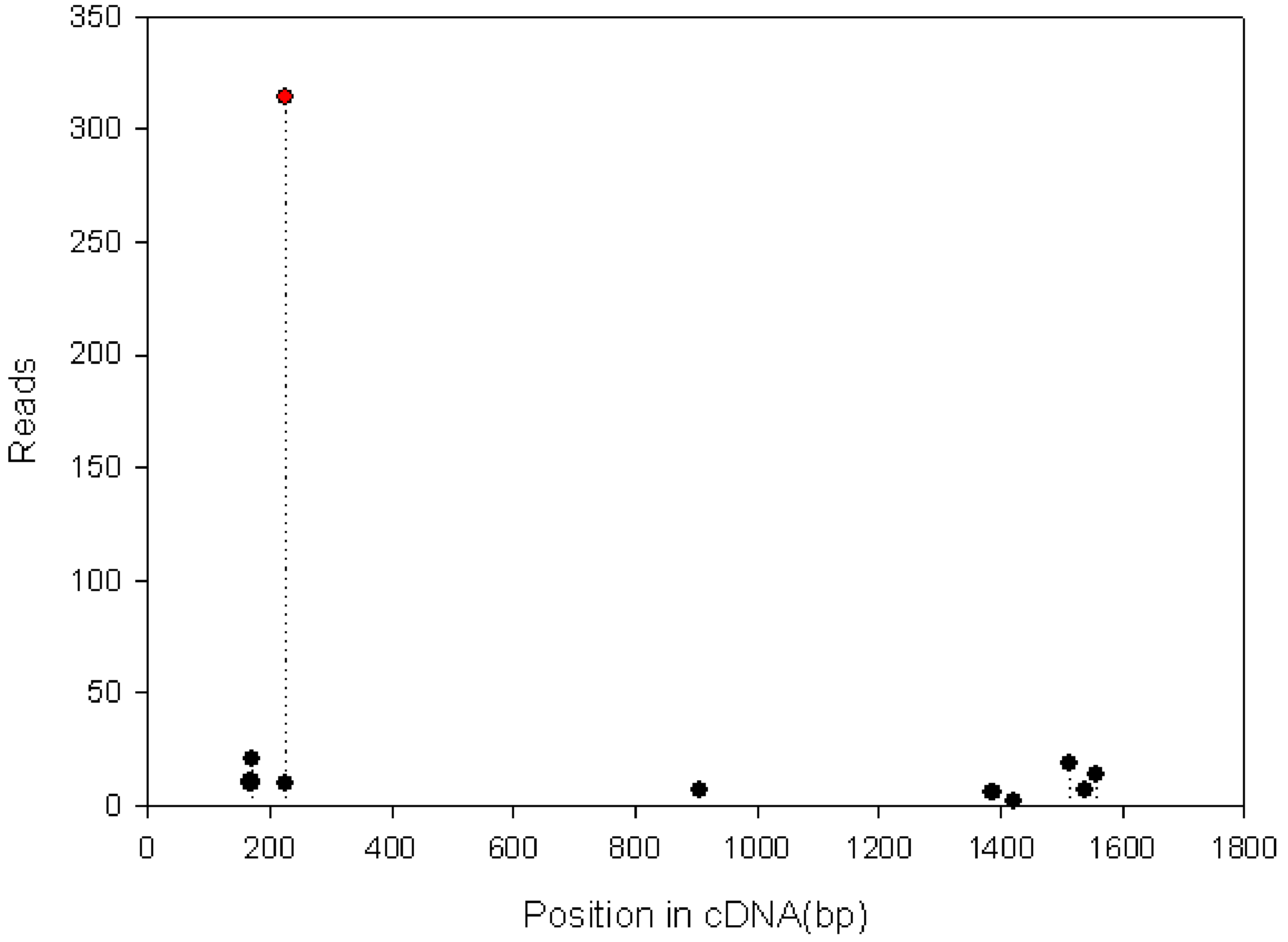


```
5' GAAGUUCAAGAAAUCUGUGGGAAGAA 3'        Cs1g22520.1
      :::::::::: :.::::.:
3' ---CAAGUUCUUUCGGCACCUU---- 5'        Csi-miR396d.3
```

Csi-miR396d.3, target=Cs3g23180.1 gene=Cs3g23180
Category:3
Score=4
Cleavage Site=625

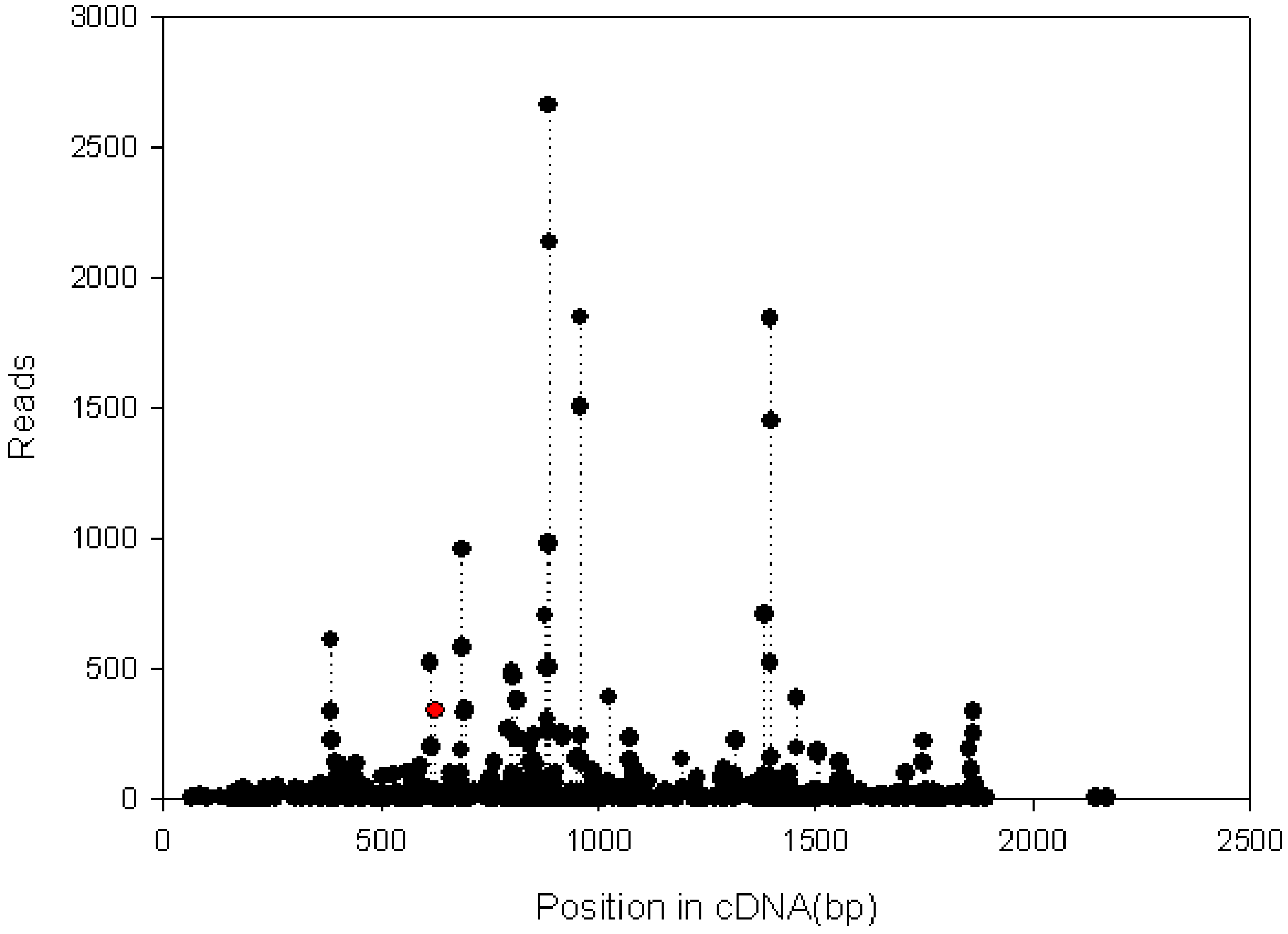


```
5' AAAGAUCAAGGAAGCUGUGGGAGUUG 3'        Cs3g23180.1
       : :::::.::::.::::.:
3' ---CAAGUUCUUUCGGCACCUU---- 5'        Csi-miR396d.3
```

Csi-miR396d.3, target=Cs5g09850.1 gene=Cs5g09850
Category:2
Score=3
Cleavage Site=767

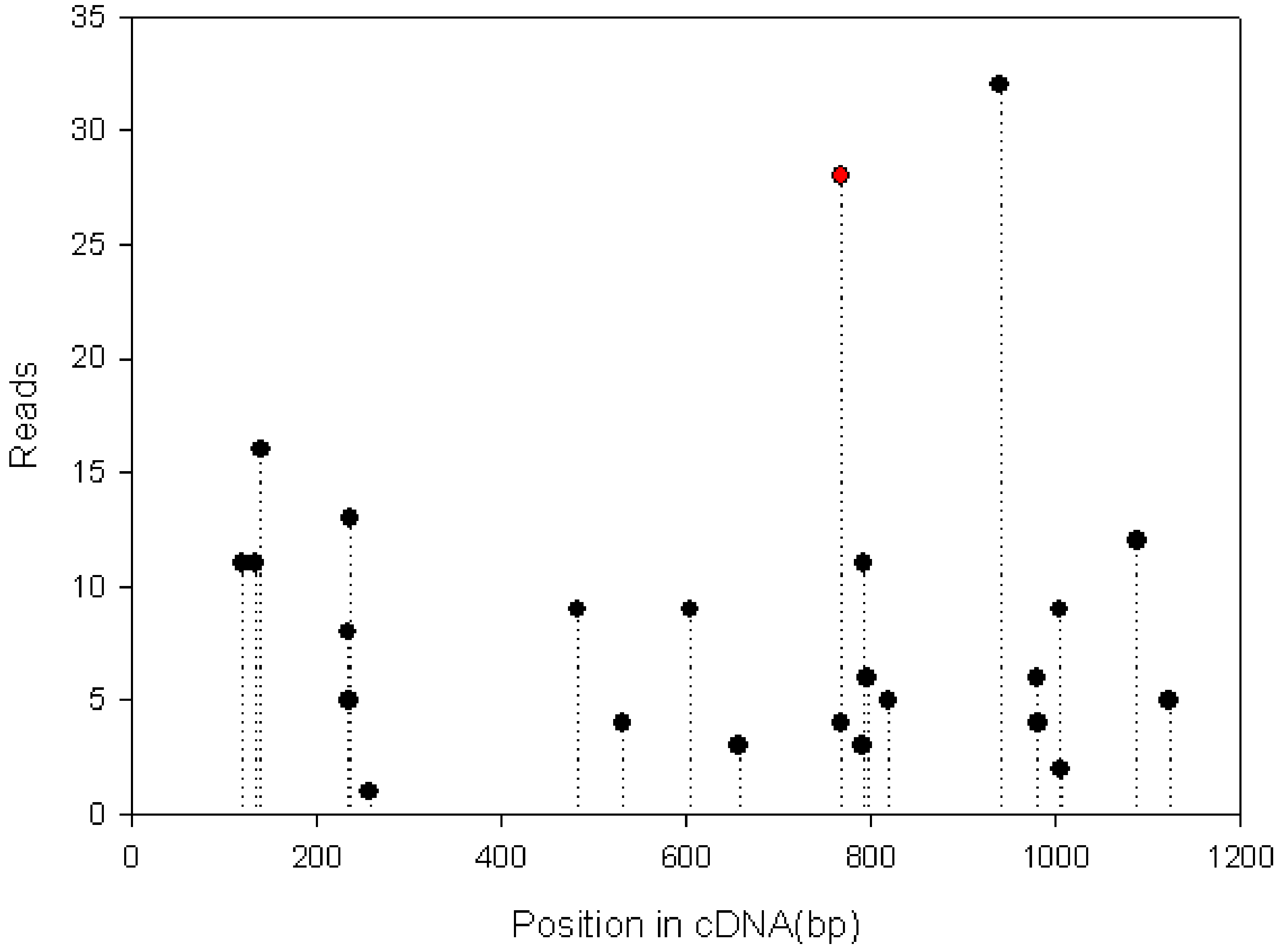


```
5' UGCGUUCAAGAAAGCUUGUGGAAGCU 3'        Cs5g09850.1
     :::::::::::: .::::::
3' ---CAAGUUCUUUCG-GCACCUU--- 5'        Csi-miR396d.3
```

Csi-miR396d.3, target=Cs6g15330.1 gene=Cs6g15330
Category: 1
Score=3
Cleavage Site=449

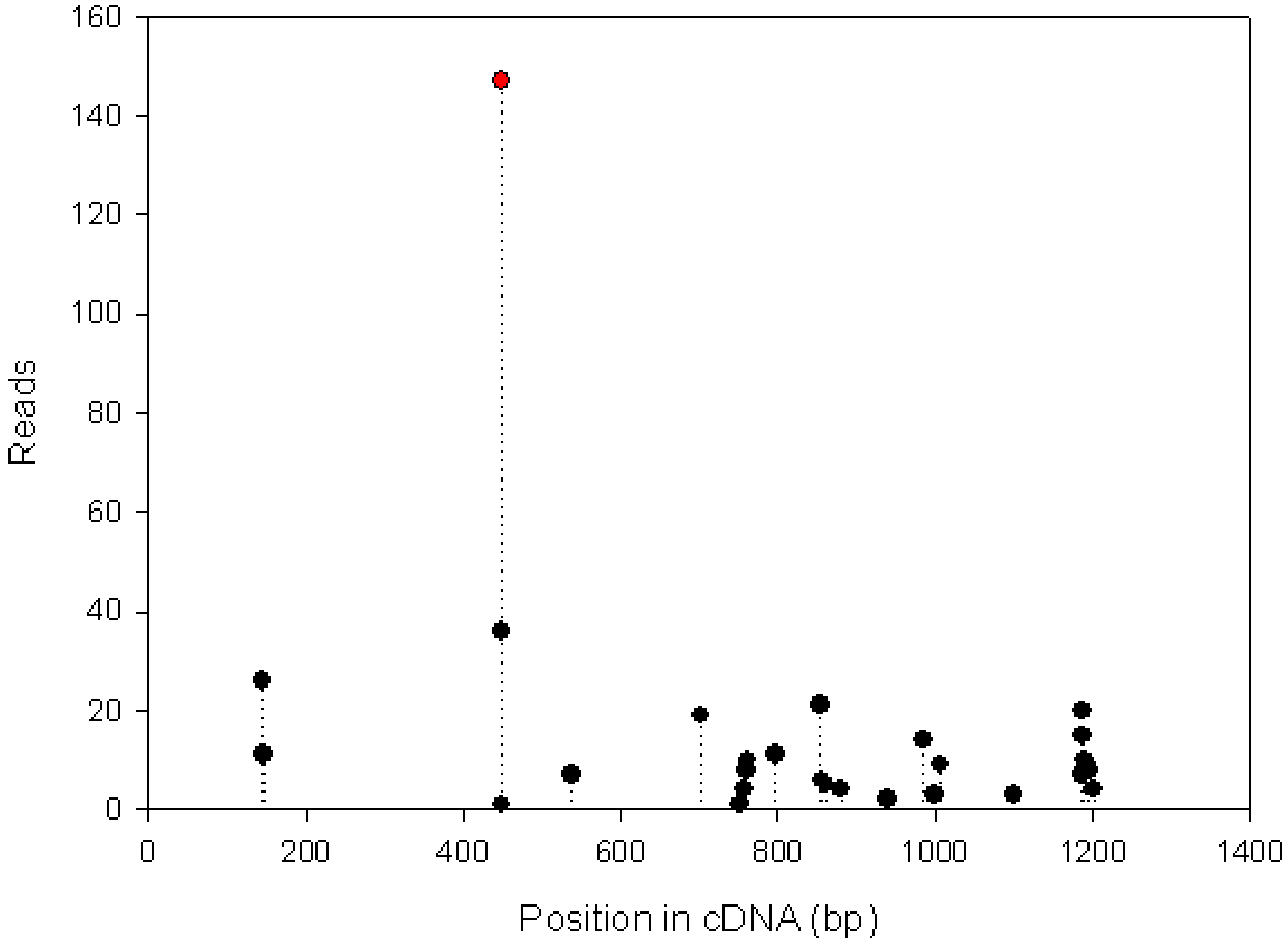


```
5' ACCGUUCAAGAAAGCAUGUGGAAAUC 3'         Cs6g15330.1
       :::::::::::: .::::::
3' ---CAAGUUCUUUCG-GCACCUU--- 5'         Csi-miR396d.3
```

Csi-miR396d.3, target=Cs7g15220.1 gene=Cs7g15220
Category=1
Score=2
Cleavage Site=377

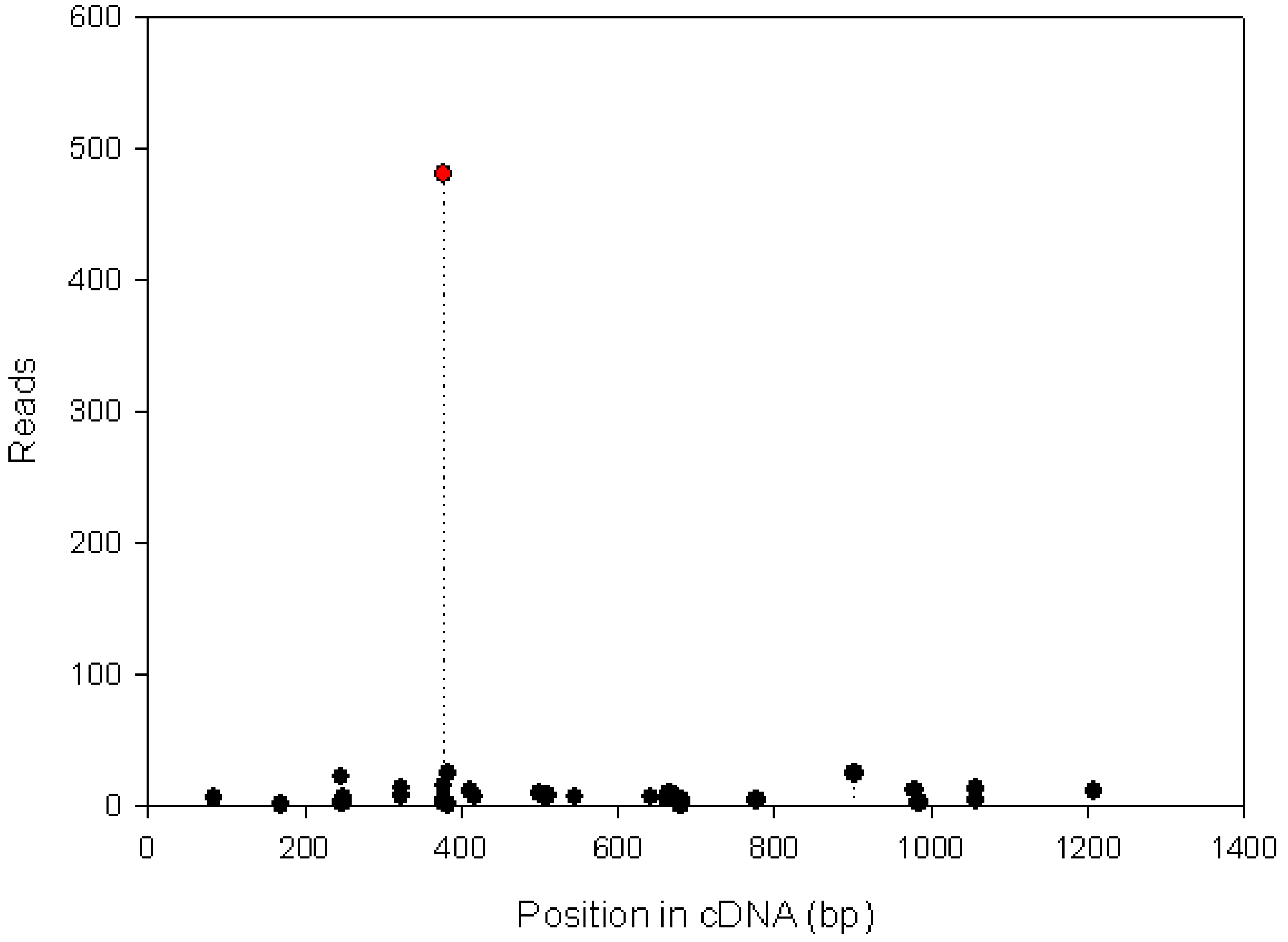


```
5' ACCGUUCAAGAAAGCCUGUGGAAUCA 3'        Cs7g15220.1
      ::::::::::::: ::::::
3' ---CAAGUUCUUUCGG-CACCUU--- 5'        Csi-miR396d.3
```

Csi-miR396d.3, target=Orange1.1t03122.1 gene=Orange1.1t03122
Category=1
Score=2
Cleavage Site=583

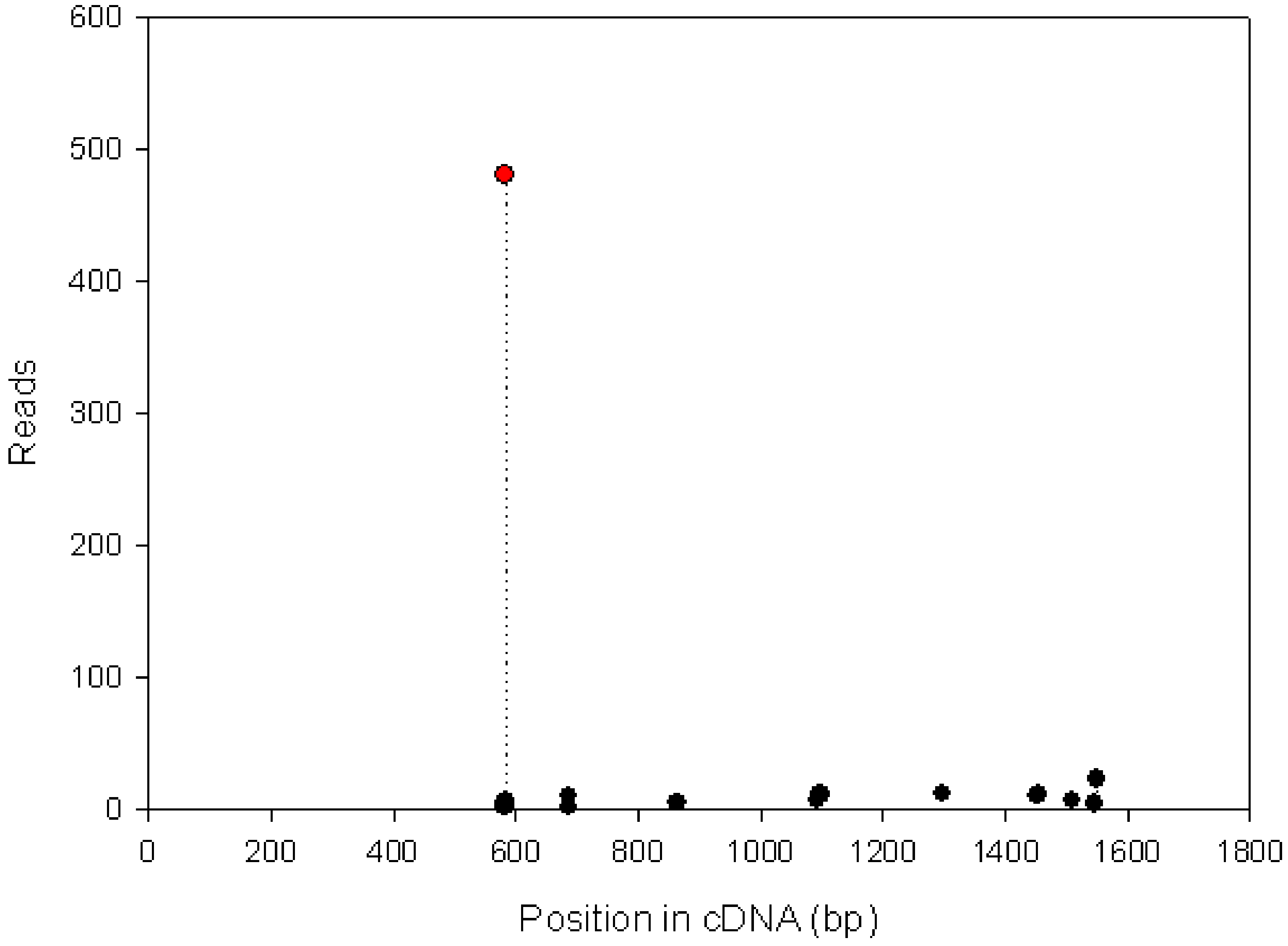


```
5' ACCGUUCAAGAAAGCCUGUGGAAUCA 3'        Orange1.1t03122.1
      ::::::::::::: ::::::
3' ---CAAGUUCUUUCGG-CACCUU--- 5'        Csi-miR396d.3
```

Csi-miR396d.4, target=Cs1g22520.1 gene=Cs1g22520
Category:1
Score=4
Cleavage Site=226

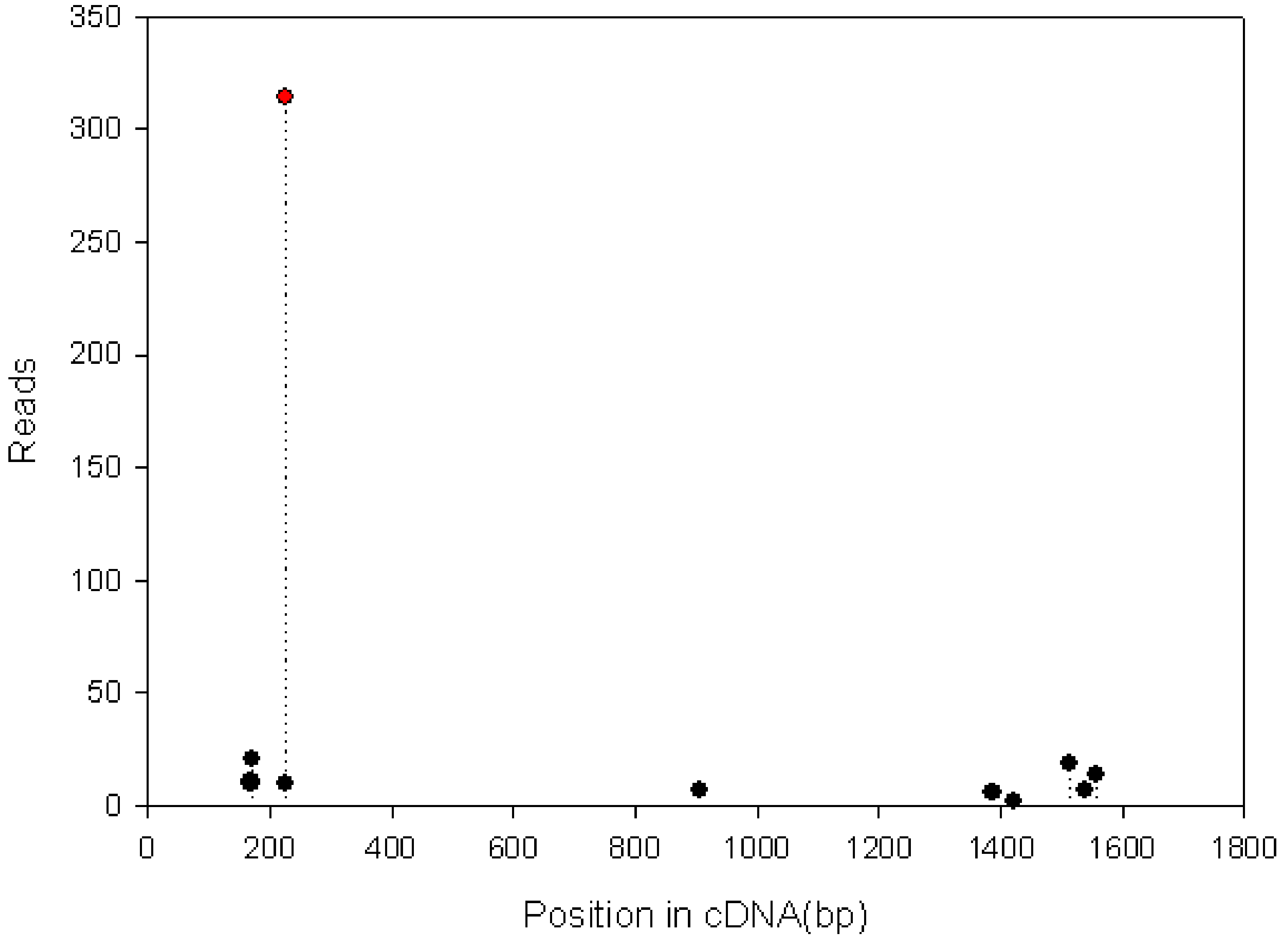


```
5' GAAGUUCAAGAAAUCUGUGGGAAGAA 3'        Cs1g22520.1
       ::::::::: :.::::.:
3' ----AAGUUCUUUCGGCACCUU---- 5'        Csi-miR396d.4
```

Csi-miR396d.4, target=Cs3g23180.1 gene=Cs3g23180
Category:3
Score=4
Cleavage Site=625

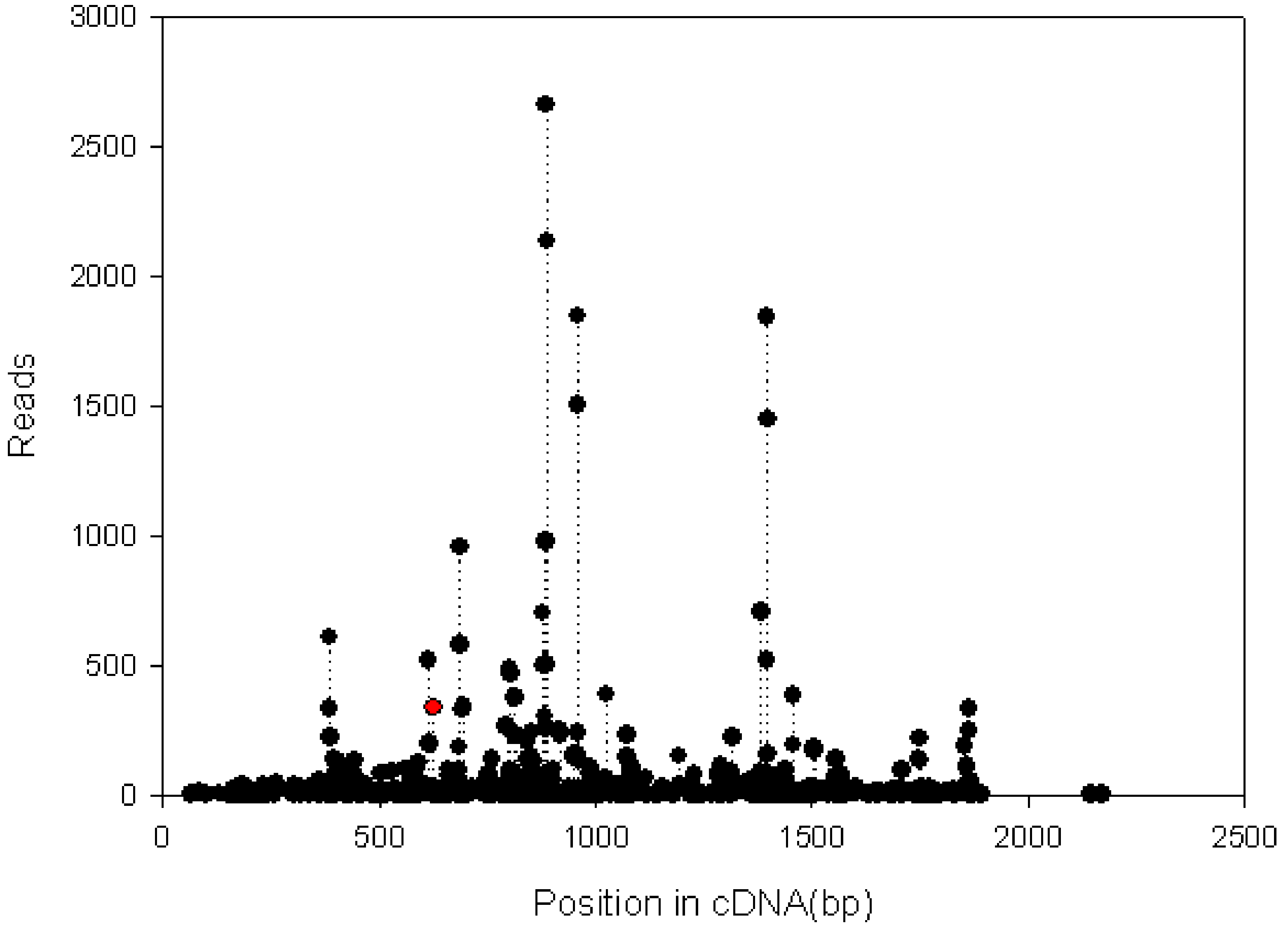


```
5' AAAGAUCAAGGAAGCUGUGGGAGUUG 3'        Cs3g23180.1
          ::::::.::::.:::::.:
3' ----AAGUUCUUUCGGCACCUU---- 5'        Csi-miR396d.4
```

Csi-miR396d.4, target=Cs5g09850.1 gene=Cs5g09850
Category:2
Score=3
Cleavage Site=767

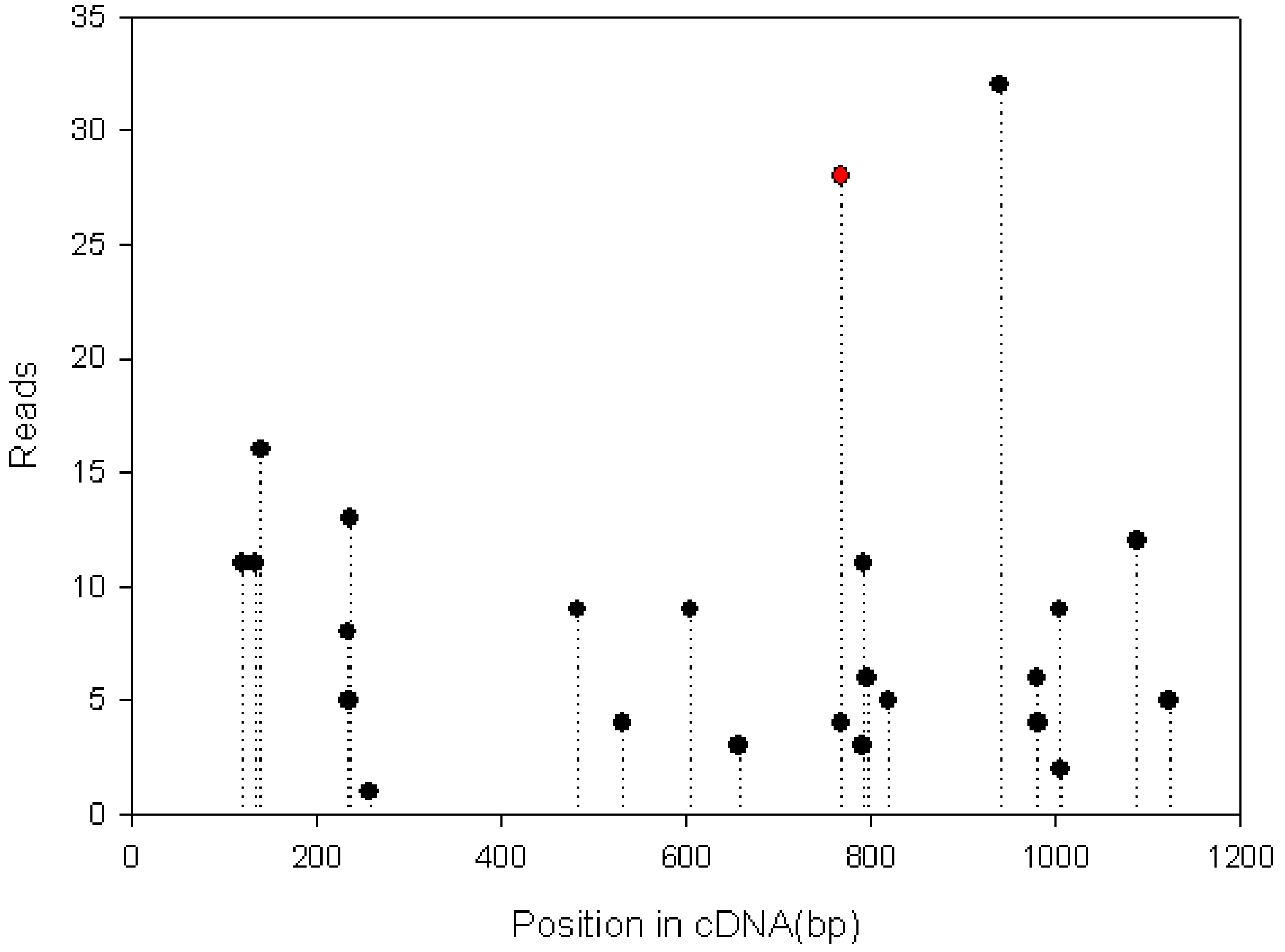


```
5' UGCGUUCAAGAAAGCUUGUGGAAGCU 3'        Cs5g09850.1
       ::::::::::: .::::::
3' ----AAGUUCUUUCG-GCACCUU--- 5'        Csi-miR396d.4
```

Csi-miR396d.4, target=Cs6g15330.1 gene=Cs6g15330
Category: 1
Score=3
Cleavage Site=449

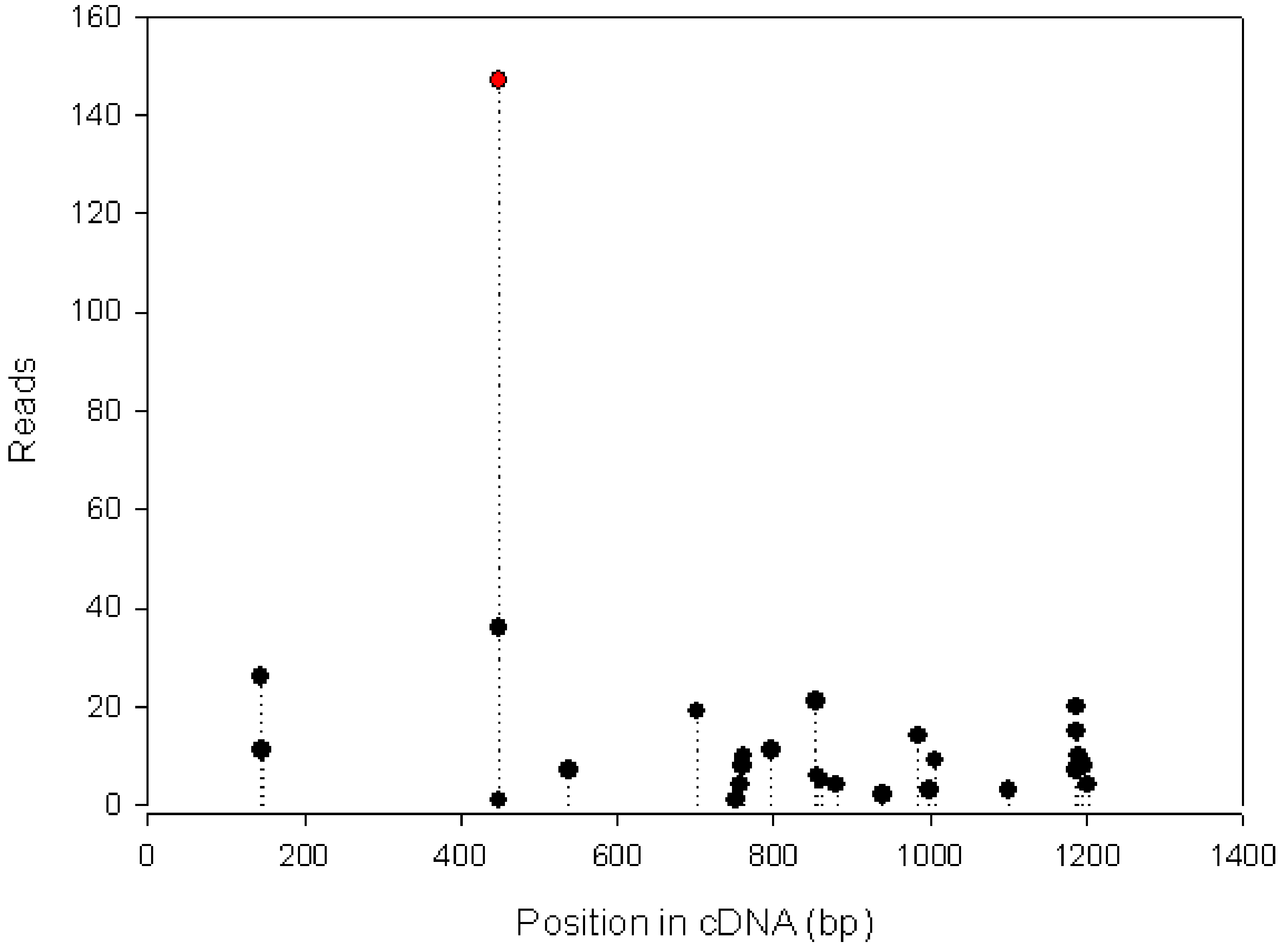


```
5' ACCGUUCAAGAAAGCAUGUGGAAAUC 3'        Cs6g15330.1
       ::::::::::: .::::::
3' ----AAGUUCUUUCG-GCACCUU--- 5'        Csi-miR396d.4
```

Csi-miR396d.4, target=Cs7g15220.1 gene=Cs7g15220
Category=1
Score=2
Cleavage Site=377

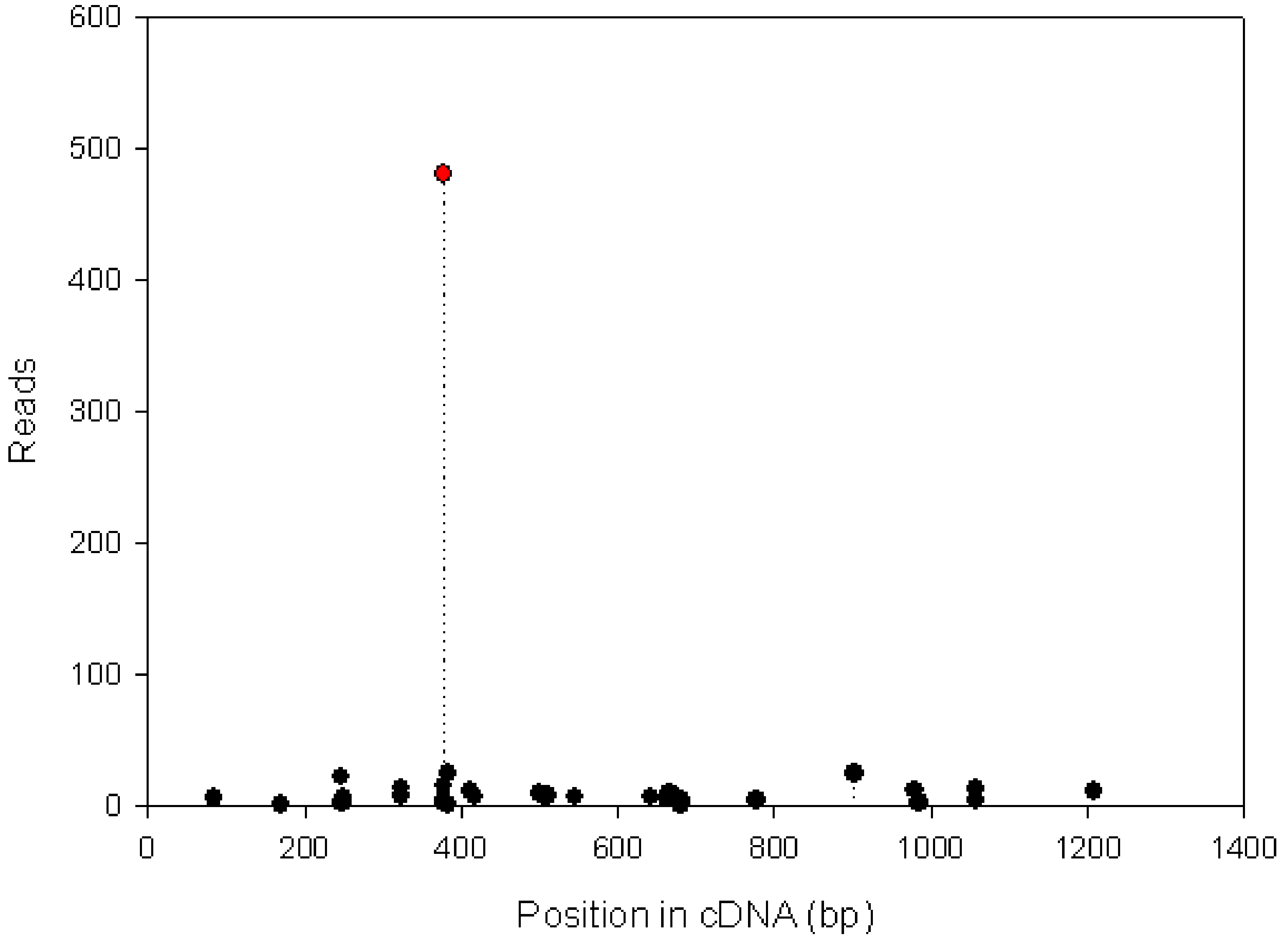


```
5' ACCGUUCAAGAAAGCCUGUGGAAUCA 3'        Cs7g15220.1
       :::::::::::: ::::::
3' ----AAGUUCUUUCGG-CACCUU--- 5'        Csi-miR396d.4
```

Csi-miR396d.4, target=Cs8g17370.1 gene=Cs8g17370
Category:3
Score=2.5
Cleavage Site=138

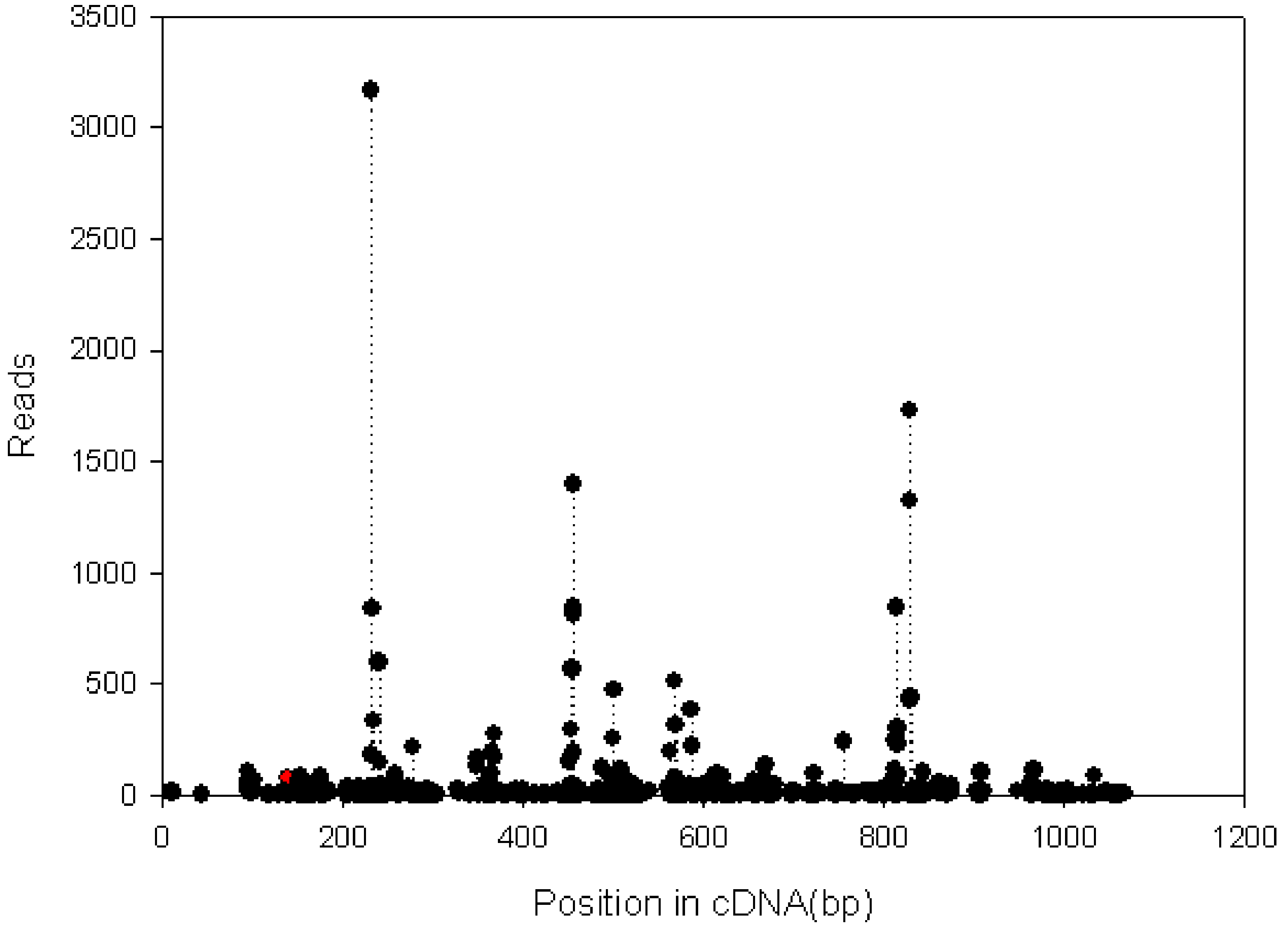


```
5' CGAGGAUUACAAGAAGGCCGUGGAGA 3'        Cs8g17370.1
          : ::::::.::::::::.
3' -------AAGUUCUUUCGGCACCUU- 5'        Csi-miR396d.4
```

Csi-miR396d.4, target=Orange1.1t02555.1 gene=Orange1.1t02555
Category:1
Score=2
Cleavage Site=920

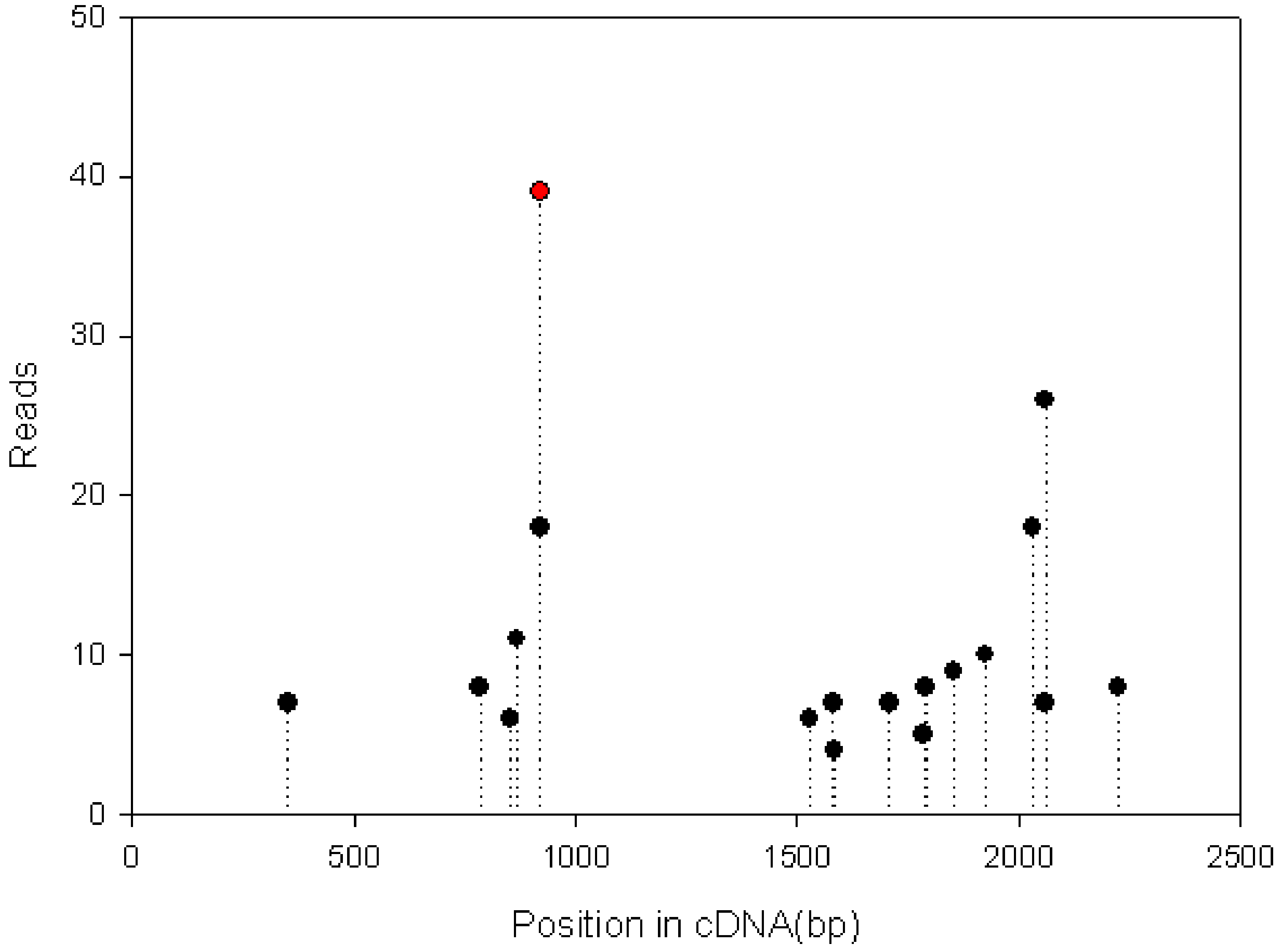


```
5' CACGUUCAAGAAAGCCUGUGGAACUU 3'        Orange1.1t02555.1
       :::::::::::: ::::::
3' ----AAGUUCUUUCGG-CACCUU--- 5'        Csi-miR396d.4
```

Csi-miR396d.4, target=Orange1.1t03122.1 gene=Orange1.1t03122
Category=1
Score=2
Cleavage Site=583

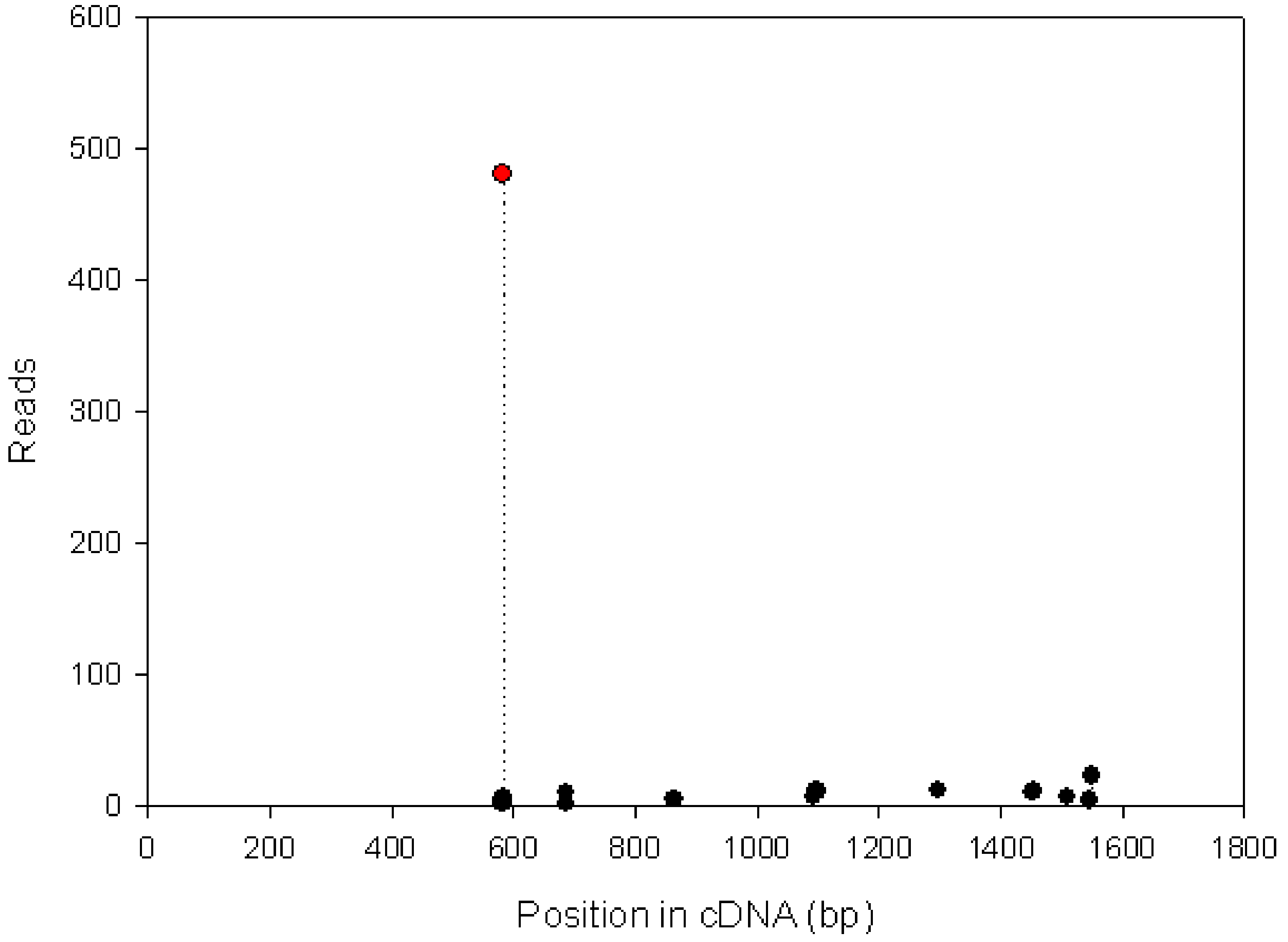


```
5' ACCGUUCAAGAAAGCCUGUGGAAUCA 3'        Orange1.1t03122.1
       :::::::::::: ::::::
3' ----AAGUUCUUUCGG-CACCUU--- 5'        Csi-miR396d.4
```

Csi-miR396d-3p, target=Cs8g05290.1 gene=Cs8g05290
Category=3
Score=5
Cleavage Site=160

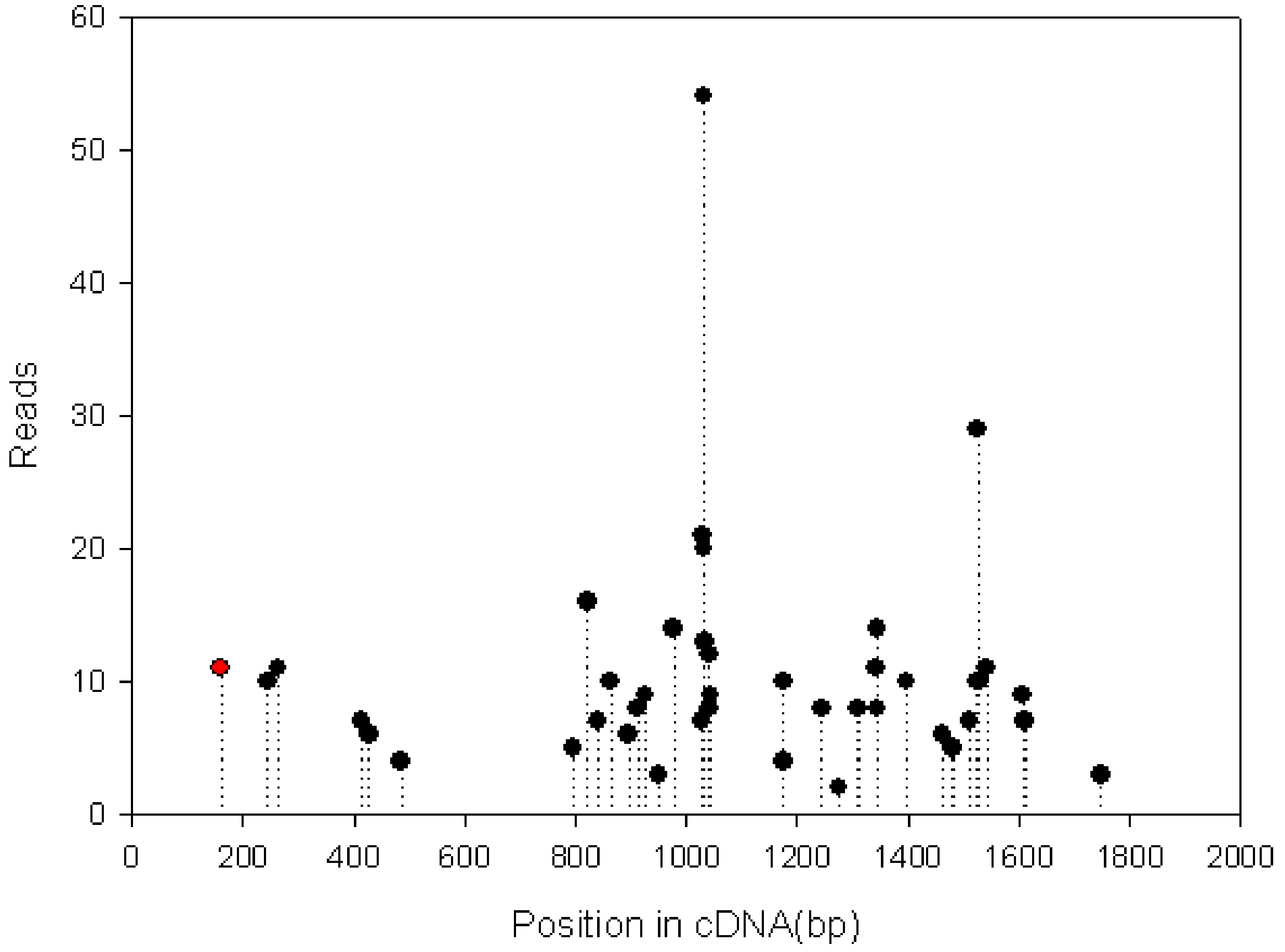


```
5' UAUAUUUUACGGCUUUCUUGAGUCCA 3'        Cs8g05290.1
     : :...::::: ::::::::.
3' --AAAGGGUGCCGUAAGAACUCG--- 5'        Csi-miR396d-3p
```

Csi-miR397.1, target=Cs6g07450.1 gene=Cs6g07450
Category=2
Score=2.5
Cleavage Site=889

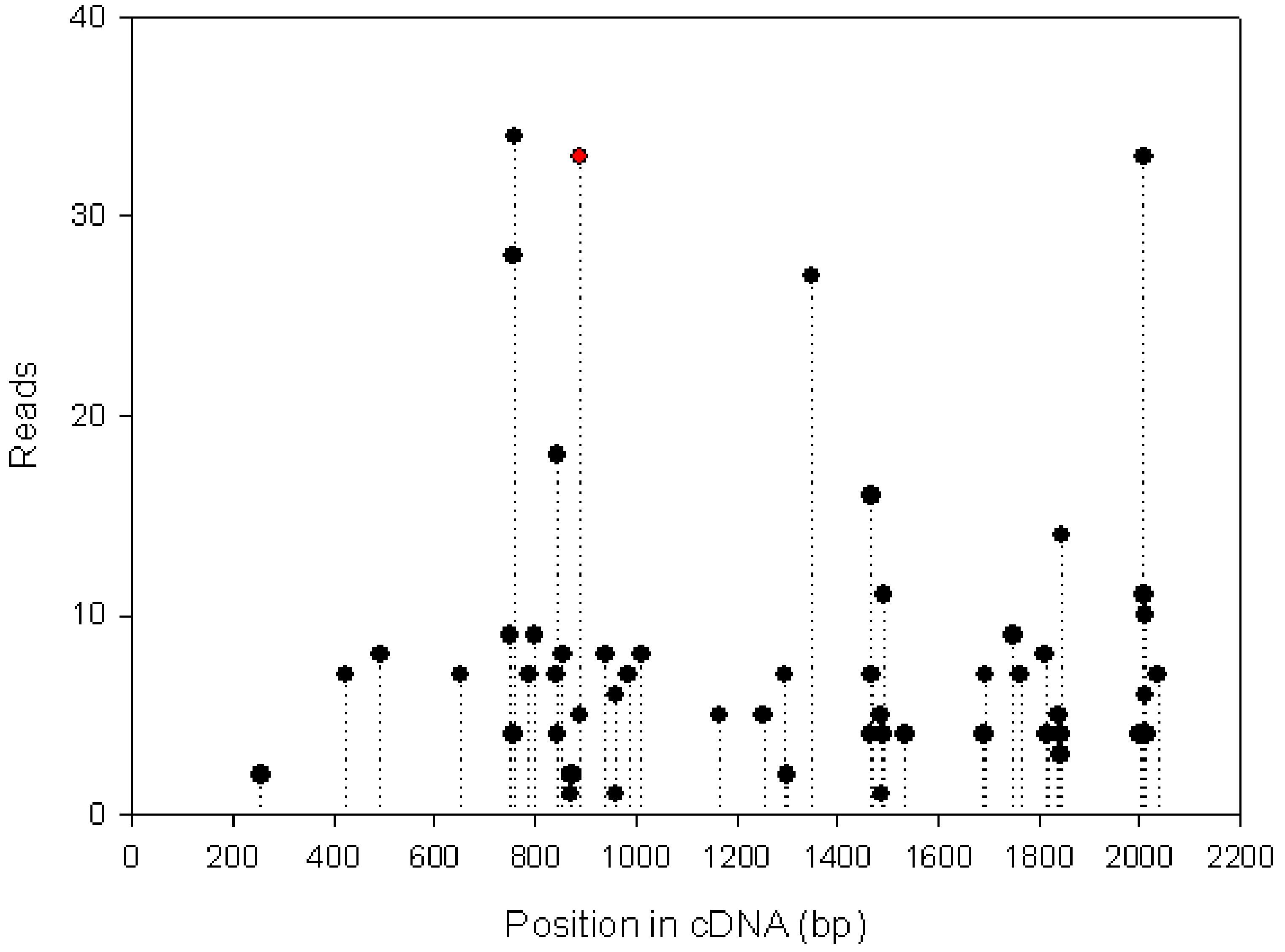


```
5' UCAUCAAUGCUGCACUCAAUAACCAG 3'          Cs6g07450.1
    ::::::.::::::::::::: :
3' -GUAGUUGCGACGUGAGUUACU---- 5'          Csi-miR397.1
```

Csi-miR397.2, target=Cs6g07410.1 gene=Cs6g07410
Category=1
Score=1
Cleavage Site=775

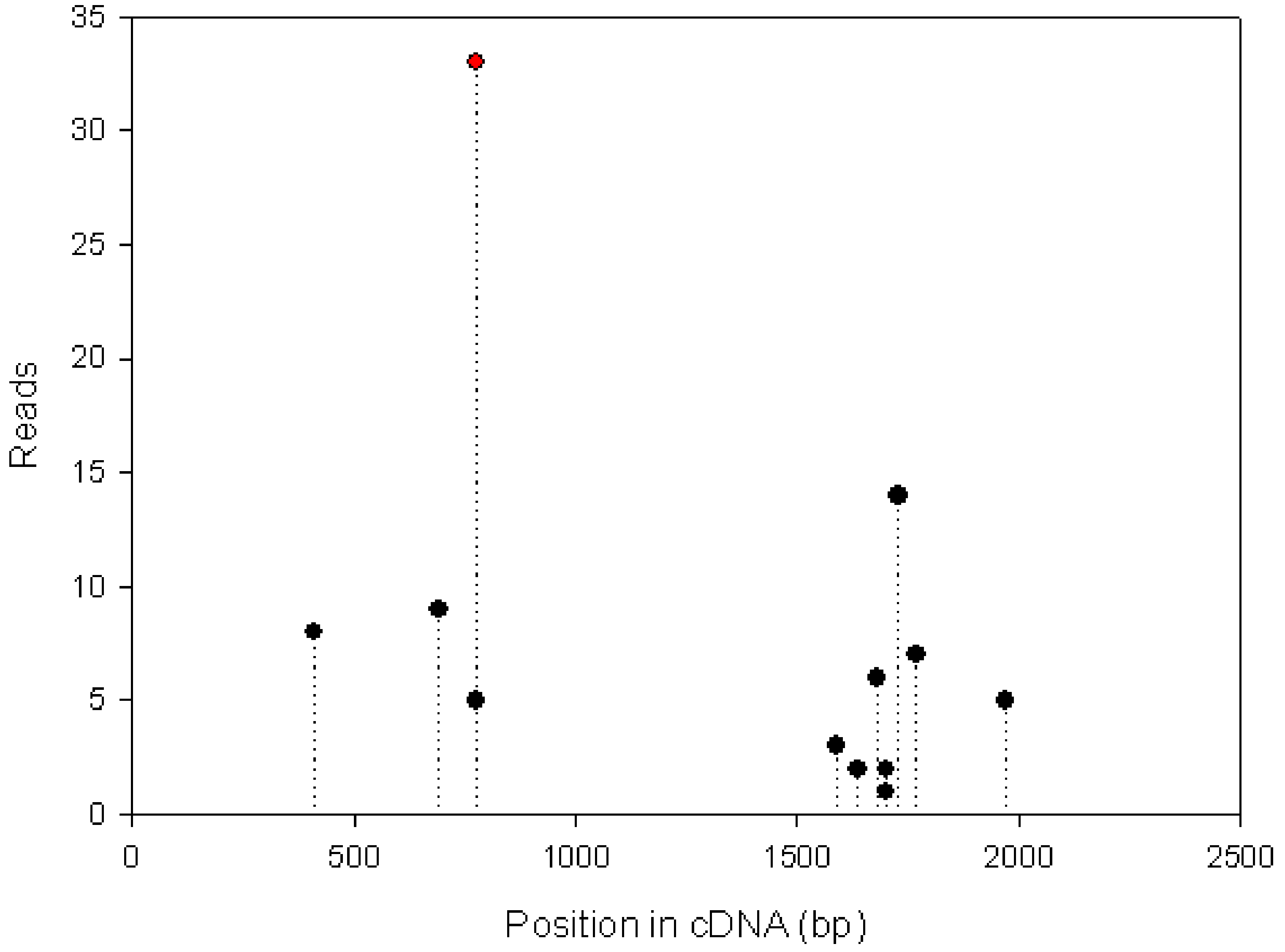


```
5' UCAUCAACGCUGCACUCAAUAACCAG 3'        Cs6g07410.1
   ::::::::::::::::::::
3' AGUAGUUGCGACGUGAGUUAC----- 5'        Csi-miR397.2
```

Csi-miR398b, target=Cs1g13240.1 gene=Cs1g13240
Category=1
Score=4
Cleavage Site=273

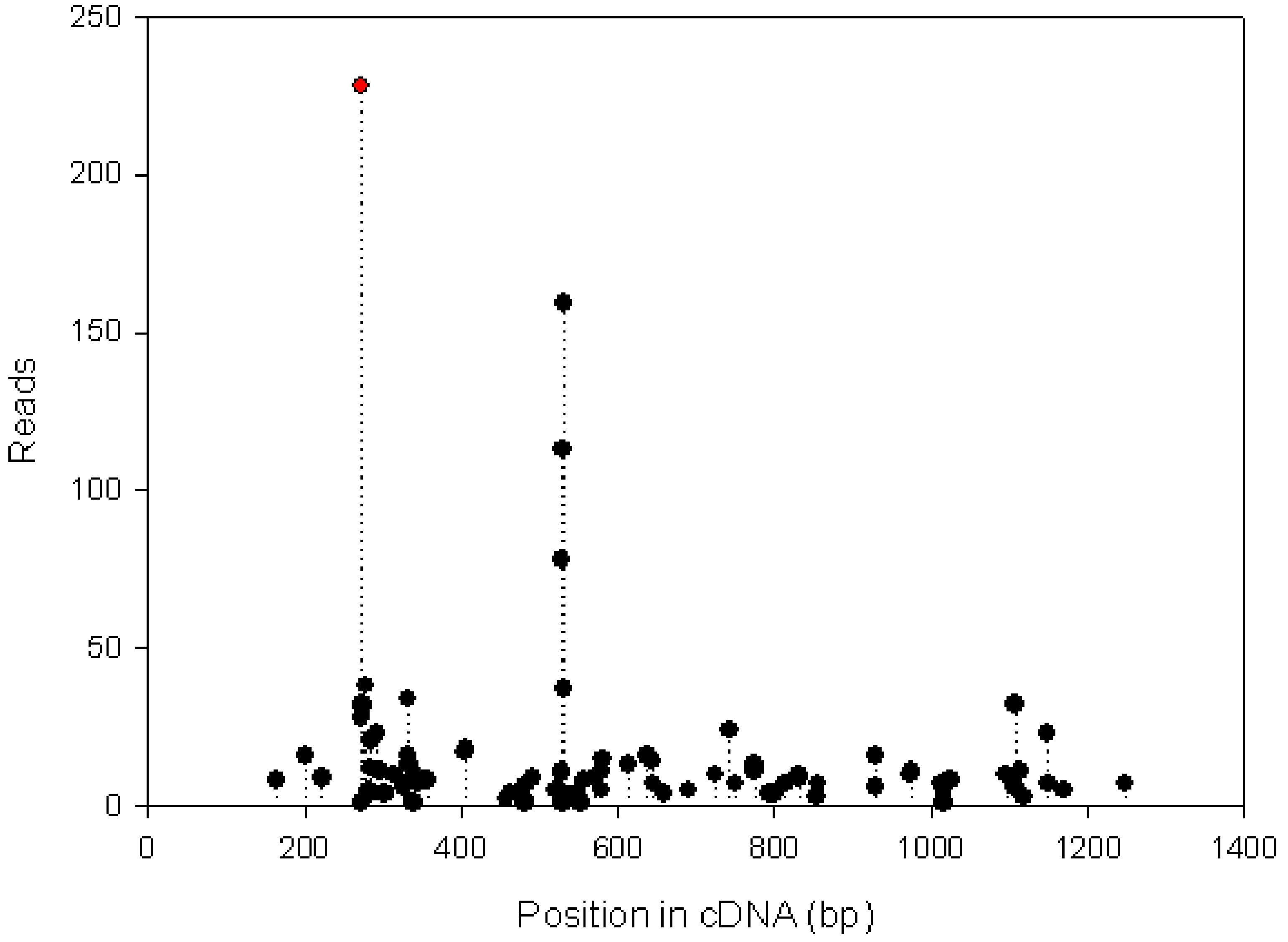


```
5' GAAAAAAAGGGGCGACCUGAGAAU-CA 3'        Cs1g13240.1
         ::::::::::::::::. ::
3' ------GUCCCCGCUGGACUCUUGUGU 5'        Csi-miR398b
```

Csi-miR399a, target=Cs3g22510.1 gene=Cs3g22510
Category=3
Score=5
Cleavage Site=115

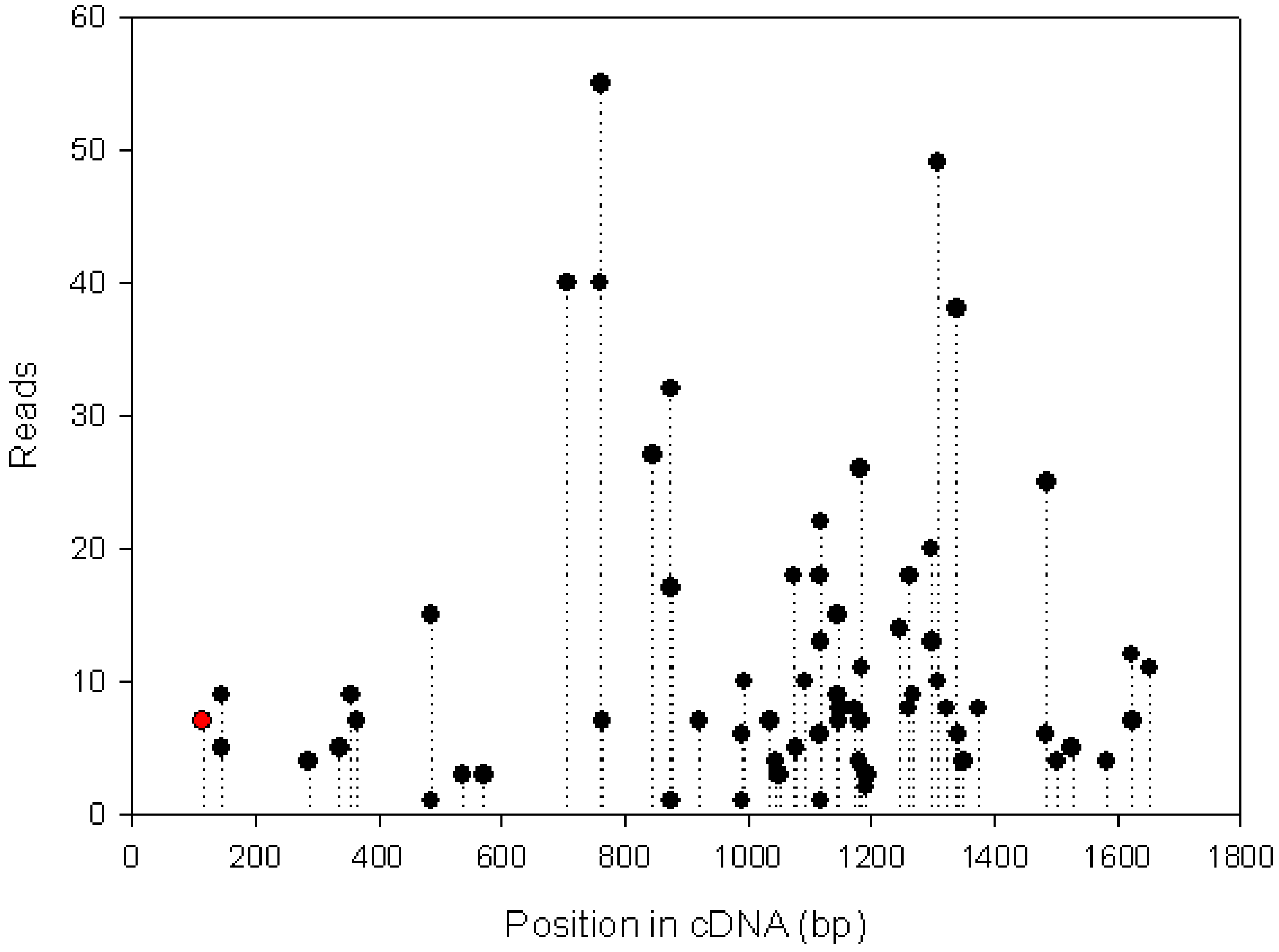


```
5' ACUUGGGCGAAGUCUUCUUUGGCUGA 3'        Cs3g22510.1
     ..:::: ::.:::.::::::::
3' --GGCCCG-UUUAGAGGAAACCGU-- 5'        Csi-miR399a
```

Csi-miR399b, target=Orange1.1t01536.1 gene=Orange1.1t01536
Category=3
Score=5
Cleavage Site=1918

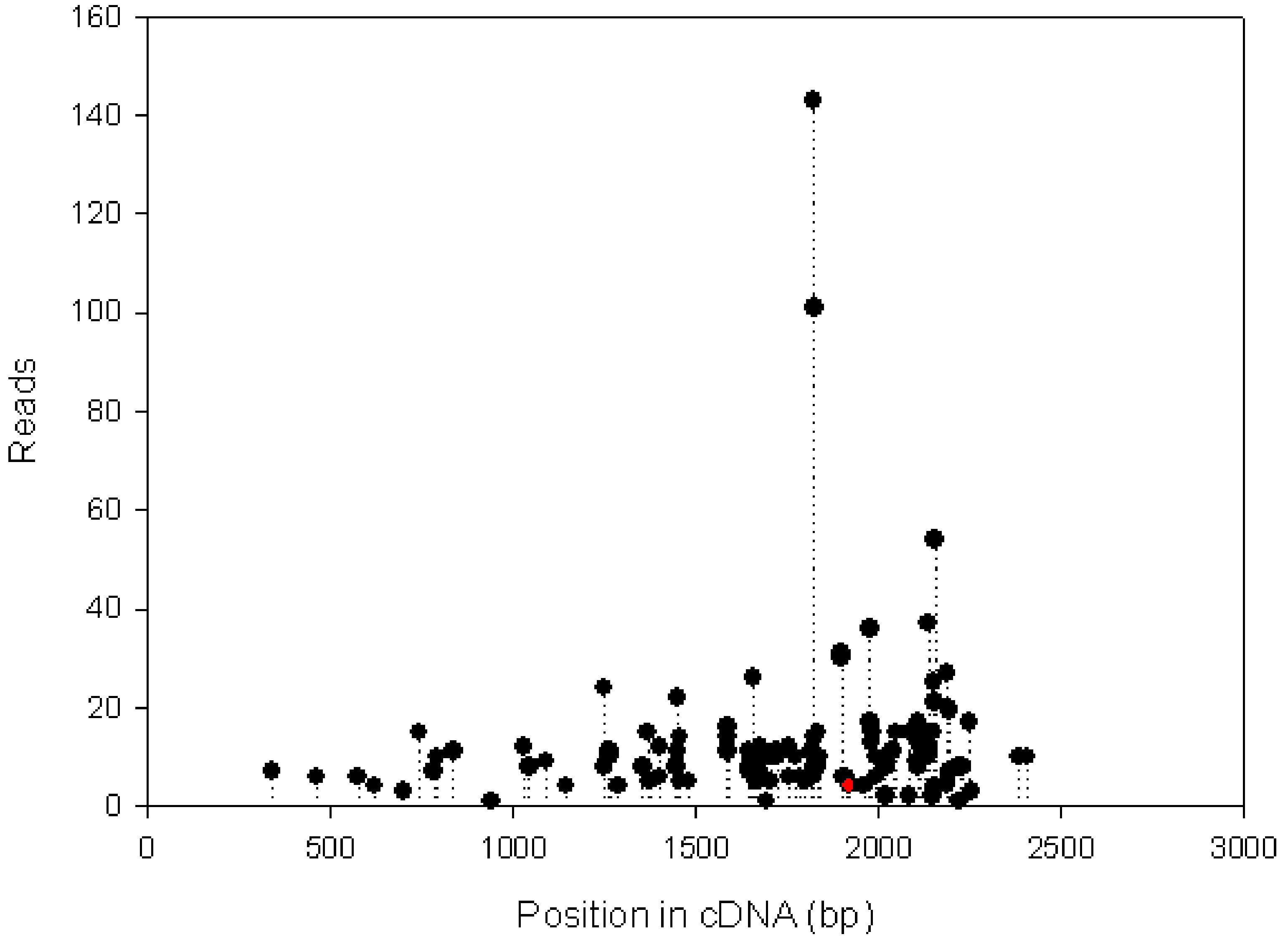


```
5' GGUUCCUGGCCAACUCUCCUCUGGCA 3'        Orange1.1t01536.1
         :: :::::::::: :::::
3' -----AUCCCGUUGAGAGGAAACCGU 5'        Csi-miR399b
```

Csi-miR399d, target=Cs2g30260.1 gene=Cs2g30260
Category=3
Score=4.5
Cleavage Site=1438

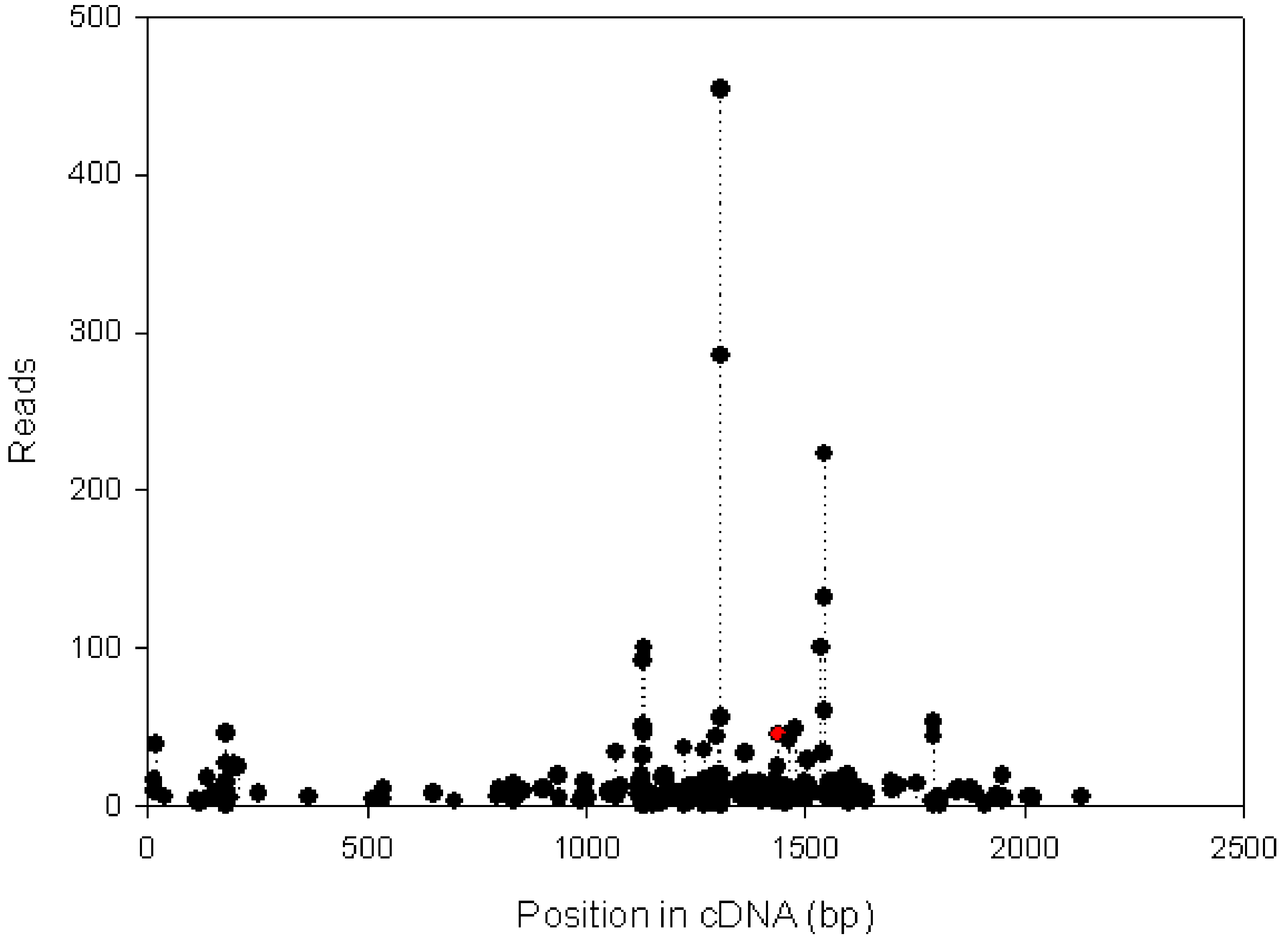


```
5' UCUUUCAUGGCAGCUAUCUUUUGGCA 3'        Cs2g30260.1
         :: ::::.:: ::.:::::::
3' -----GUCCCGUUGAGAGGAAACCGU 5'        Csi-miR399d
```

Csi-miR399e-5p, target=Cs1g02320.1 gene=Cs1g02320
Category=3
Score=5
Cleavage Site=1630

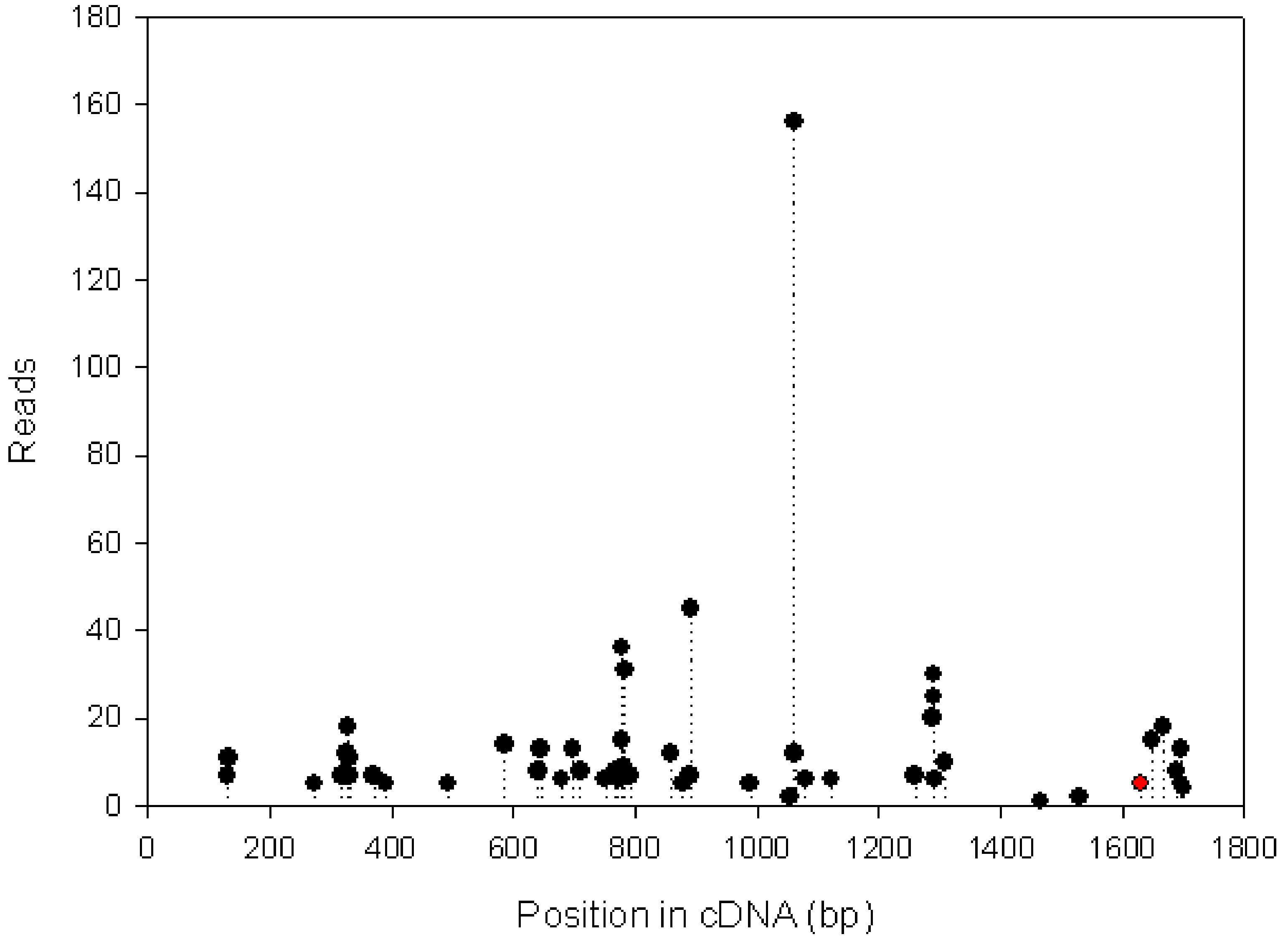


```
5' UUCUCAUUGGCAAAGGAGGACUACAU 3'        Cs1g02320.1
        :: .: :::::::::::: ::.
3' ----GU-GCGGUUUCCUCCUGACGUG 5'        Csi-miR399e-5p
```

Csi-miR403.1, target=Cs2g10760.1 gene=Cs2g10760
Category=1
Score=0
Cleavage Site=3364

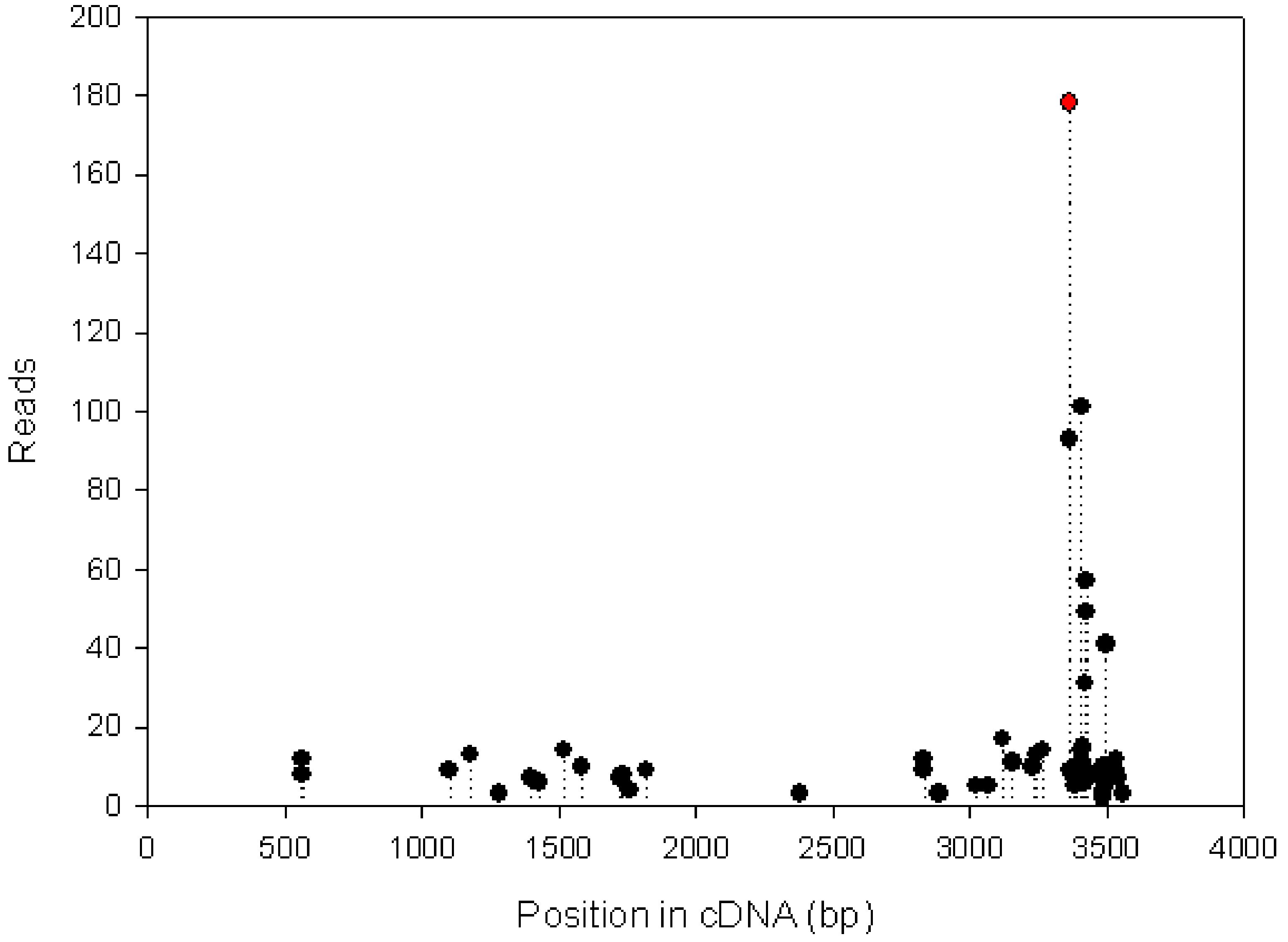


```
5' AGGAGUUUGUGCGUGAAUCUAAUGAG 3'        Cs2g10760.1
      :::::::::::::::::::
3' ---UCAAACACGCACUUAGAUU---- 5'        Csi-miR403.1
```

Csi-miR403.2, target=Cs2g10760.1 gene=Cs2g10760
Category=1
Score=1
Cleavage Site=3364

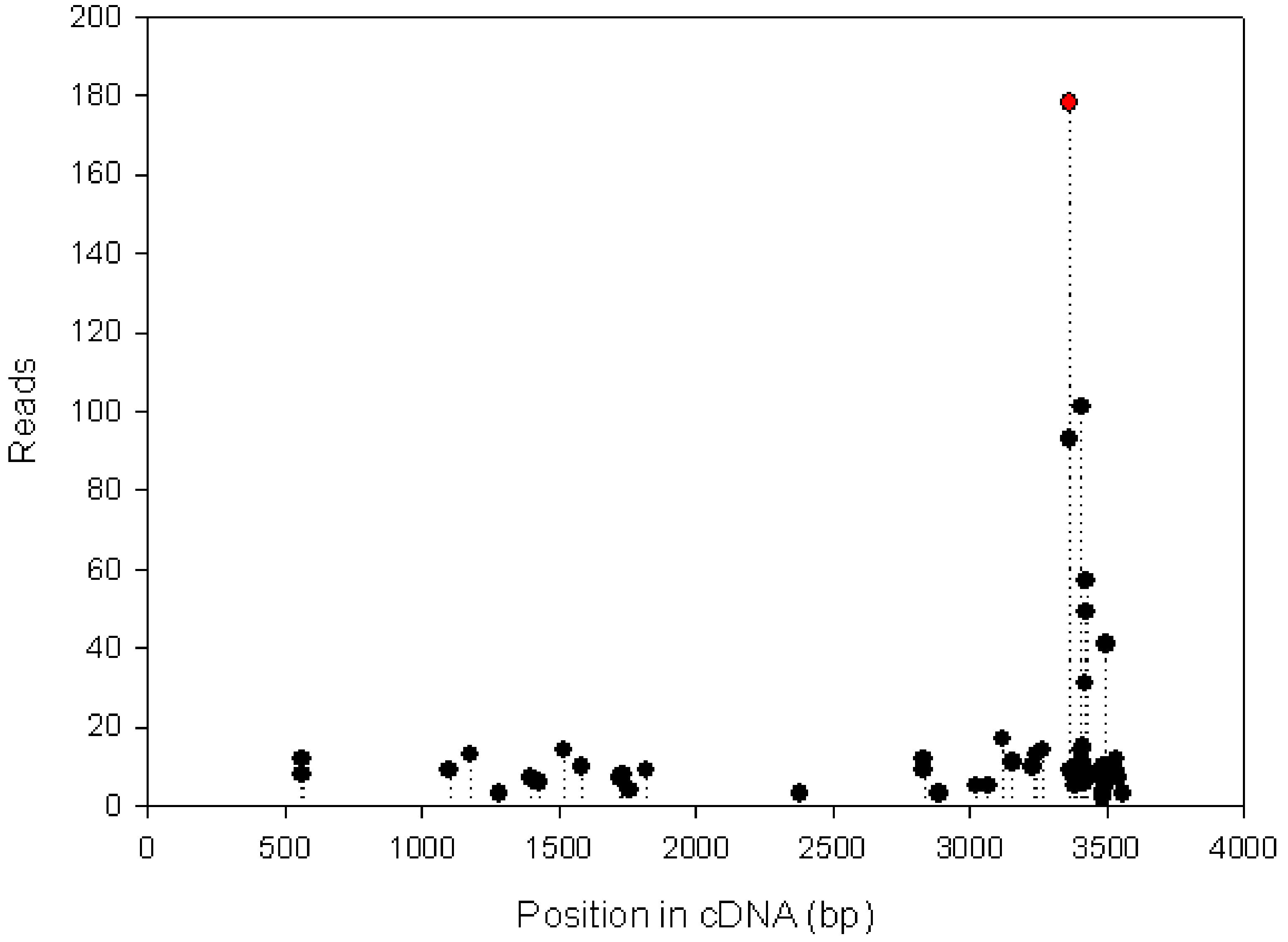


```
5' AGGAGUUUGUGCGUGAAUCUAAUGAG 3'        Cs2g10760.1
    ::::::::::::::::::::
3' -GCUCAAACACGCACUUAGAUU---- 5'        Csi-miR403.2
```

Csi-miR403.3, target=Cs2g10760.1 gene=Cs2g10760
Category=1
Score=0
Cleavage Site=3364

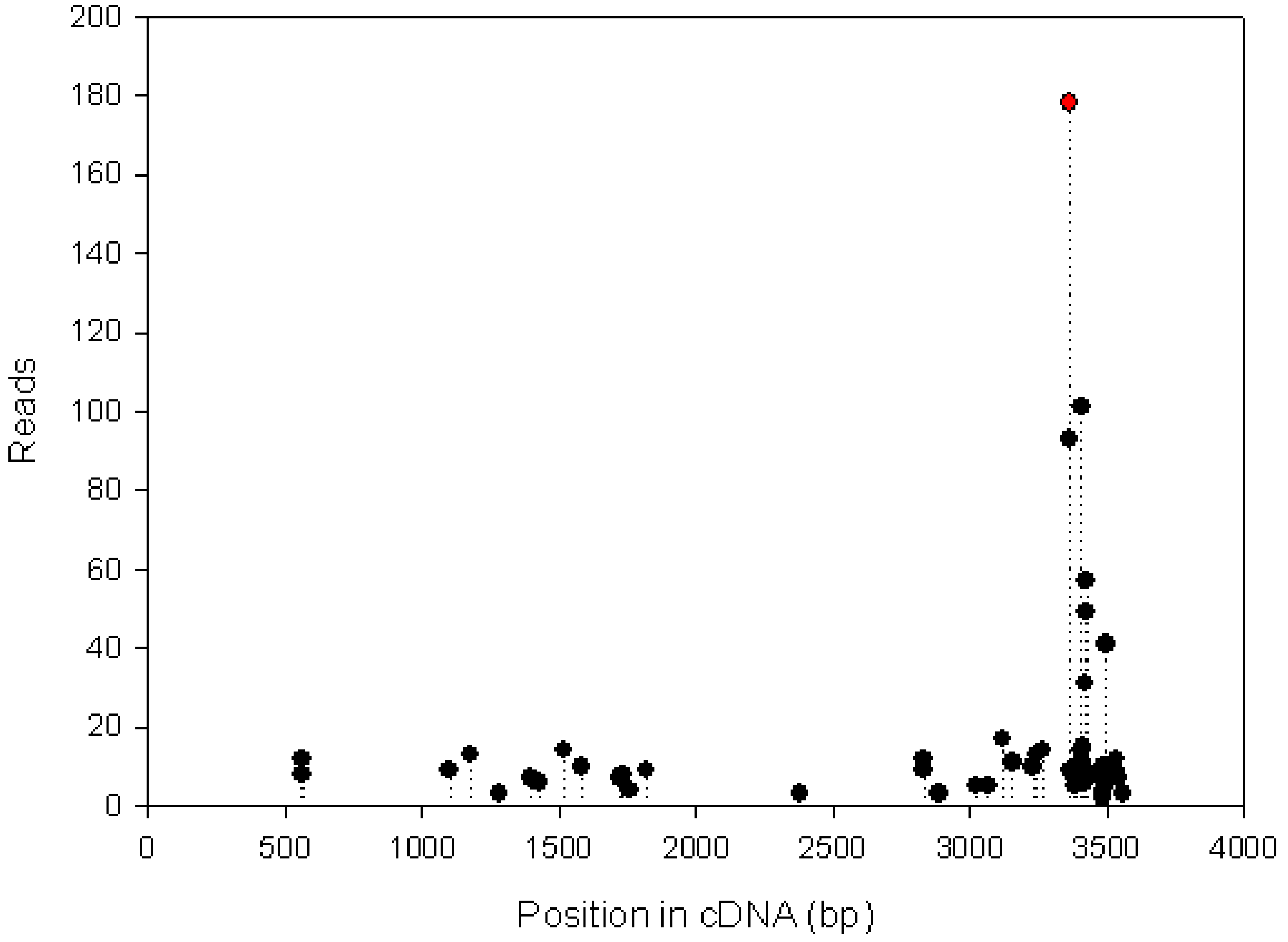


```
5' AGGAGUUUGUGCGUGAAUCUAAUGAG 3'        Cs2g10760.1
        ::::::::::::::::::::
3' ----CAAACACGCACUUAGAUU---- 5'        Csi-miR403.3
```

Csi-miR403.3, target=Cs8g07240.1 gene=Cs8g07240
Category:3
Score=5
Cleavage Site=532

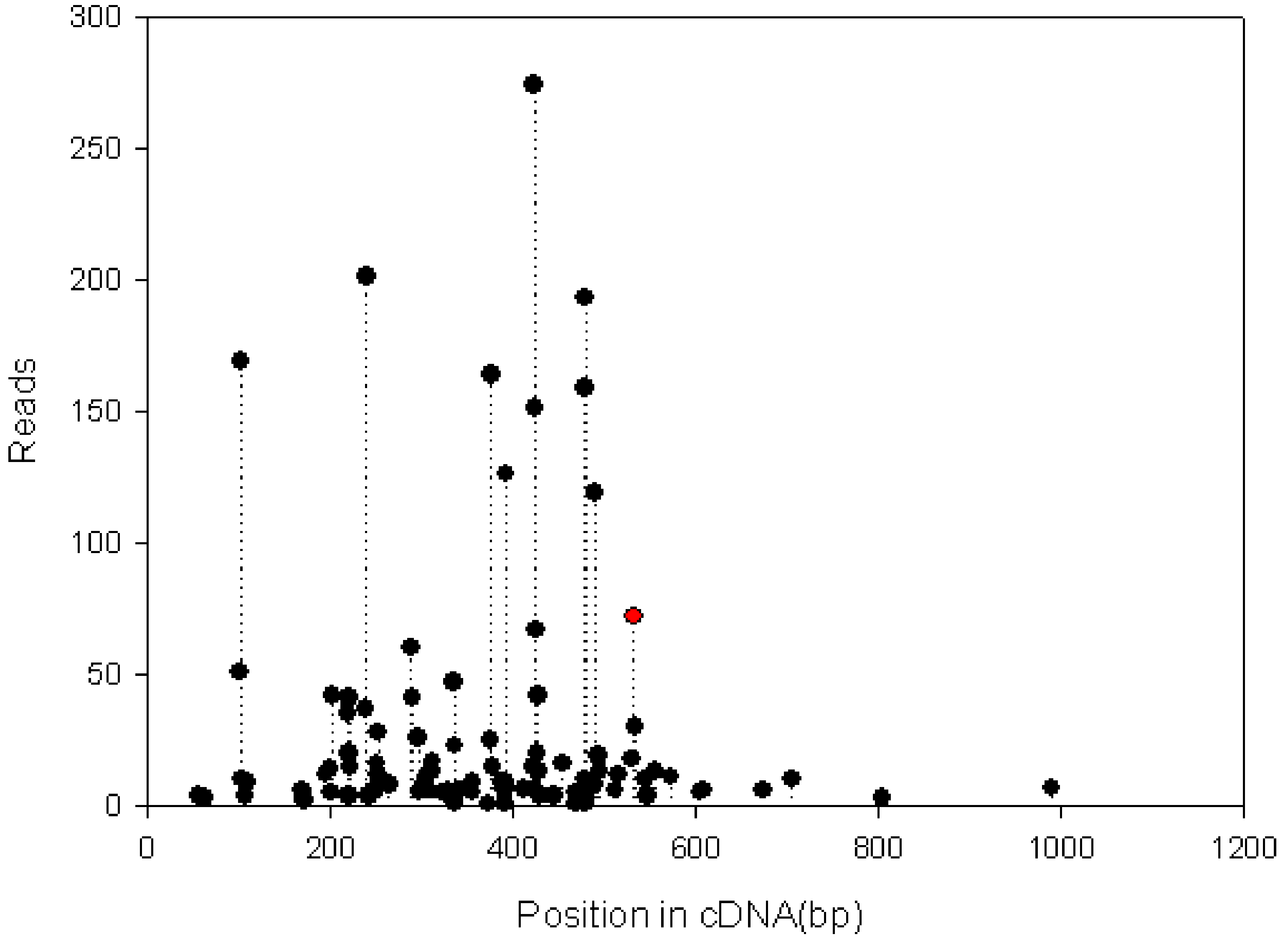


```
5' UUGUUUCU-UGUGAAUCUAUUUGAGAG 3'        Cs8g07240.1
     :::: : .:::::::::
3' --CAAACACGCACUUAGAUU------- 5'        Csi-miR403.3
```

Csi-miR408.1, target=Cs2g13550.1 gene=Cs2g13550
Category=3
Score=5
Cleavage Site=1964

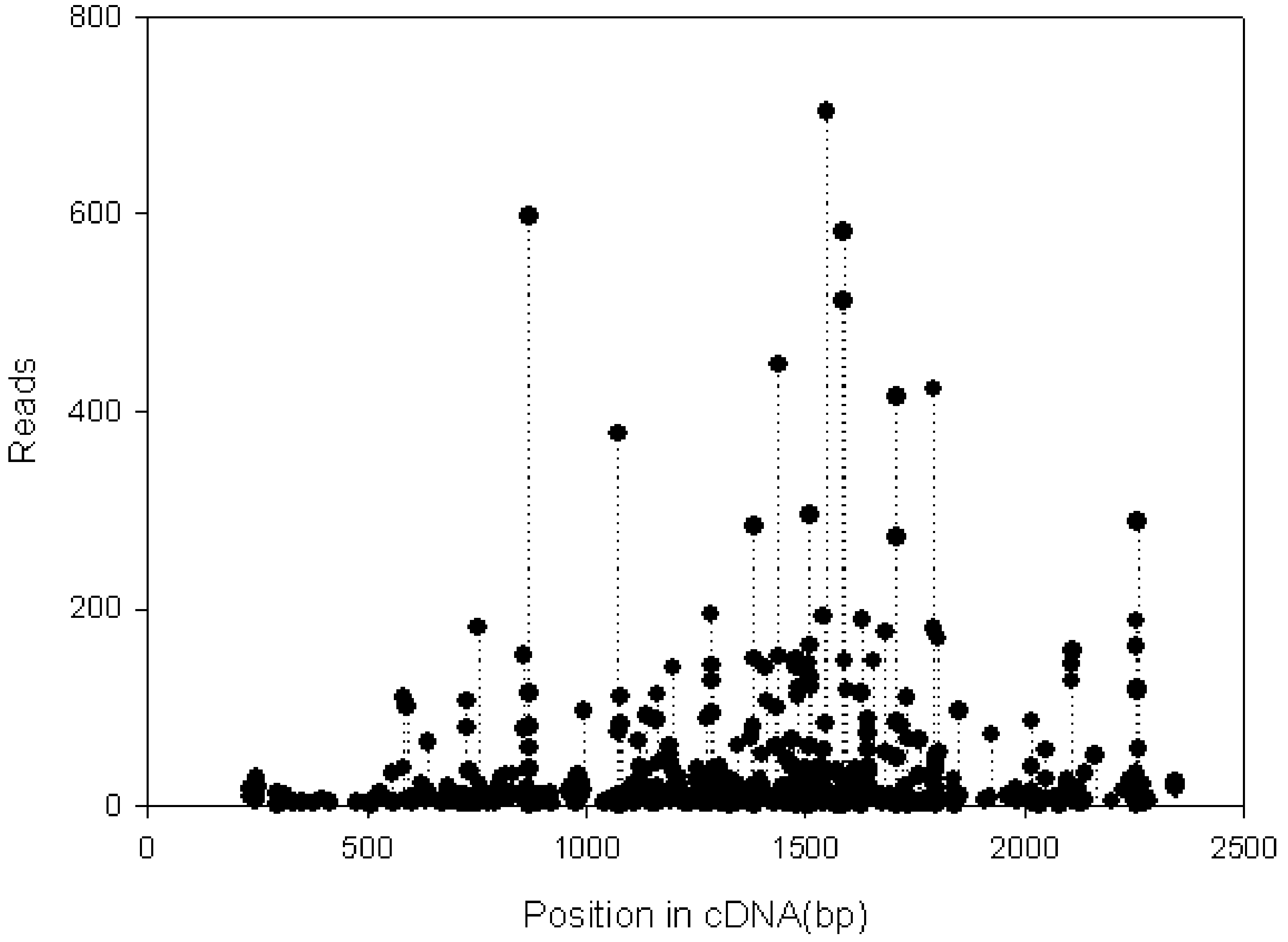


```
5' CUCAUGCUC-GUCUCUUCCCUGUUCCU 3'       Cs2g13550.1
     ::::::: :.:: ::::::.::
3' --GUACGAGACGGACAAGGGGCA---- 5'       Csi-miR408.1
```

Csi-miR408.2, target=Cs6g02740.1 gene=Cs6g02740
Category=3
Score=5
Cleavage Site=1532

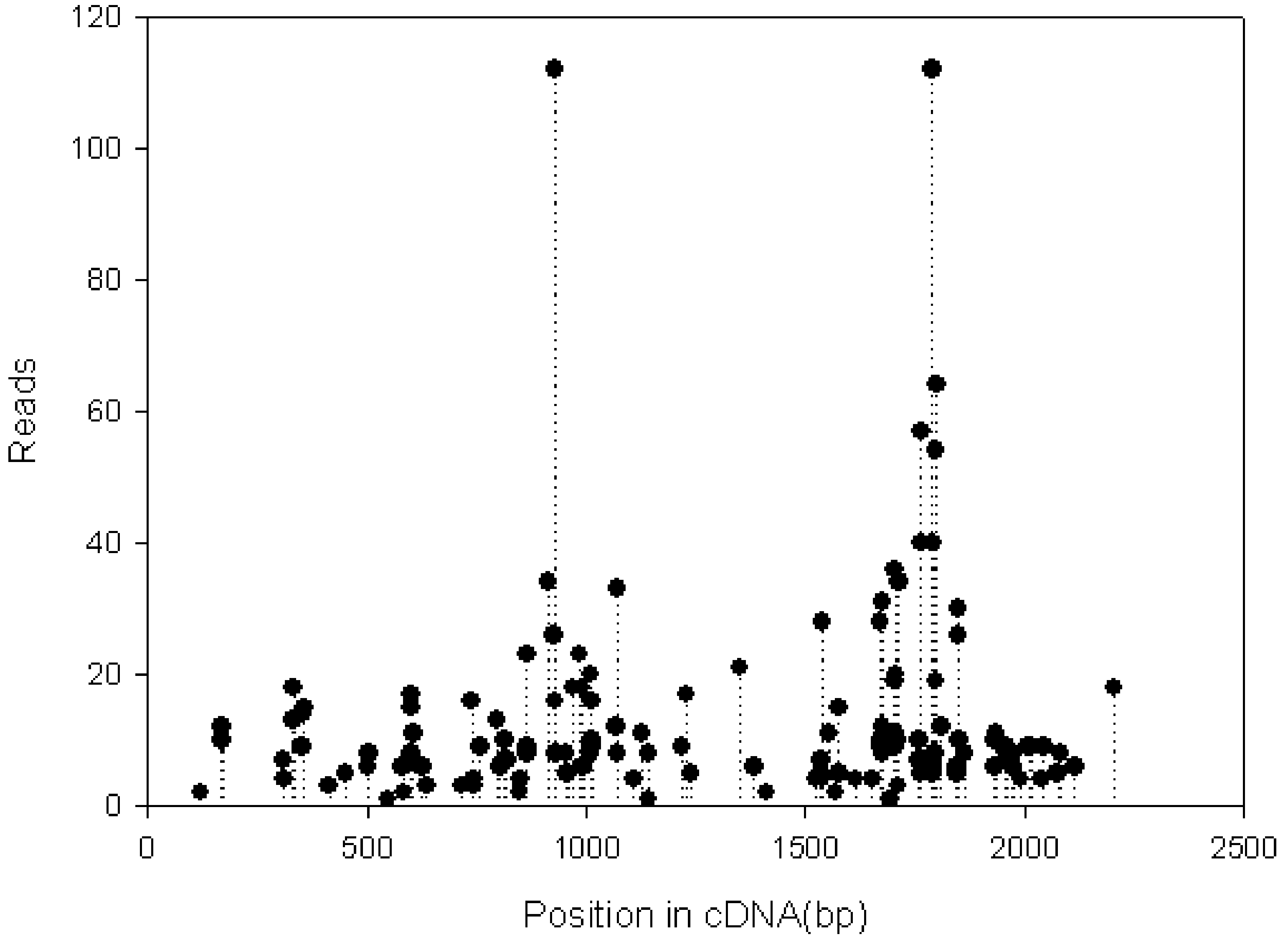


```
5' GCUGUGGUAUGCCUGUUCCUUGACUC 3'        Cs6g02740.1
   :..:: : :::::::::..:
3' -GGUACGAGACGGACAAGGGGC---- 5'        Csi-miR408.2
```

Csi-miR4414.2, target=Cs3g26540.1 gene=Cs3g26540
Category=1
Score=5
Cleavage Site=736

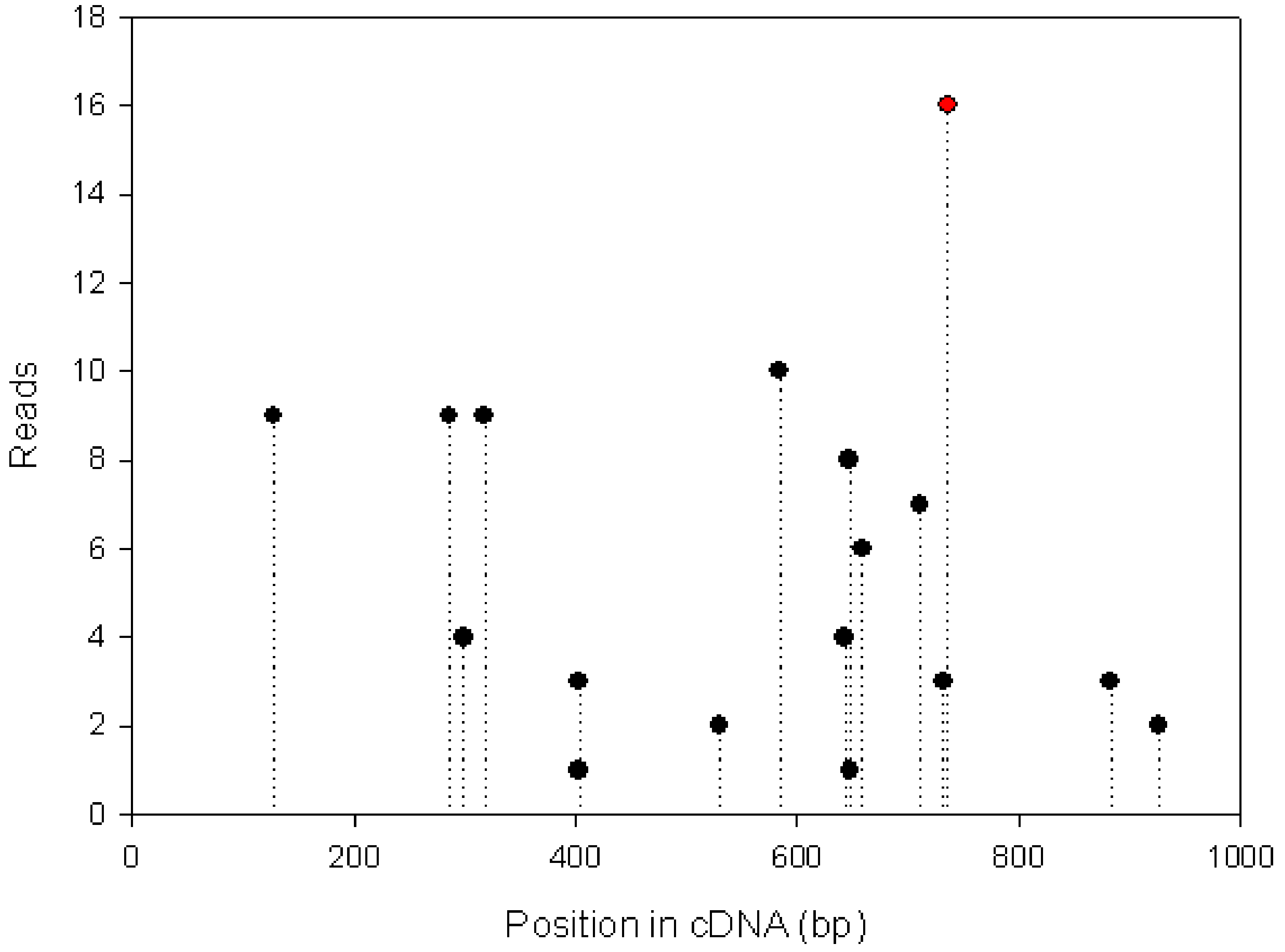


```
5' AGAAUCGA-GAGUCAGCCGCUCGCUCC 3'        Cs3g26540.1
    ::::.:.: :::::::: :::
3' -CUUGGUUGCUCAGUCGUCGA------ 5'        Csi-miR4414.2
```

Csi-miR4414.2, target=Orange1.1t02428.1 gene=Orange1.1t02428
Category:1
Score=4.5
Cleavage Site=58

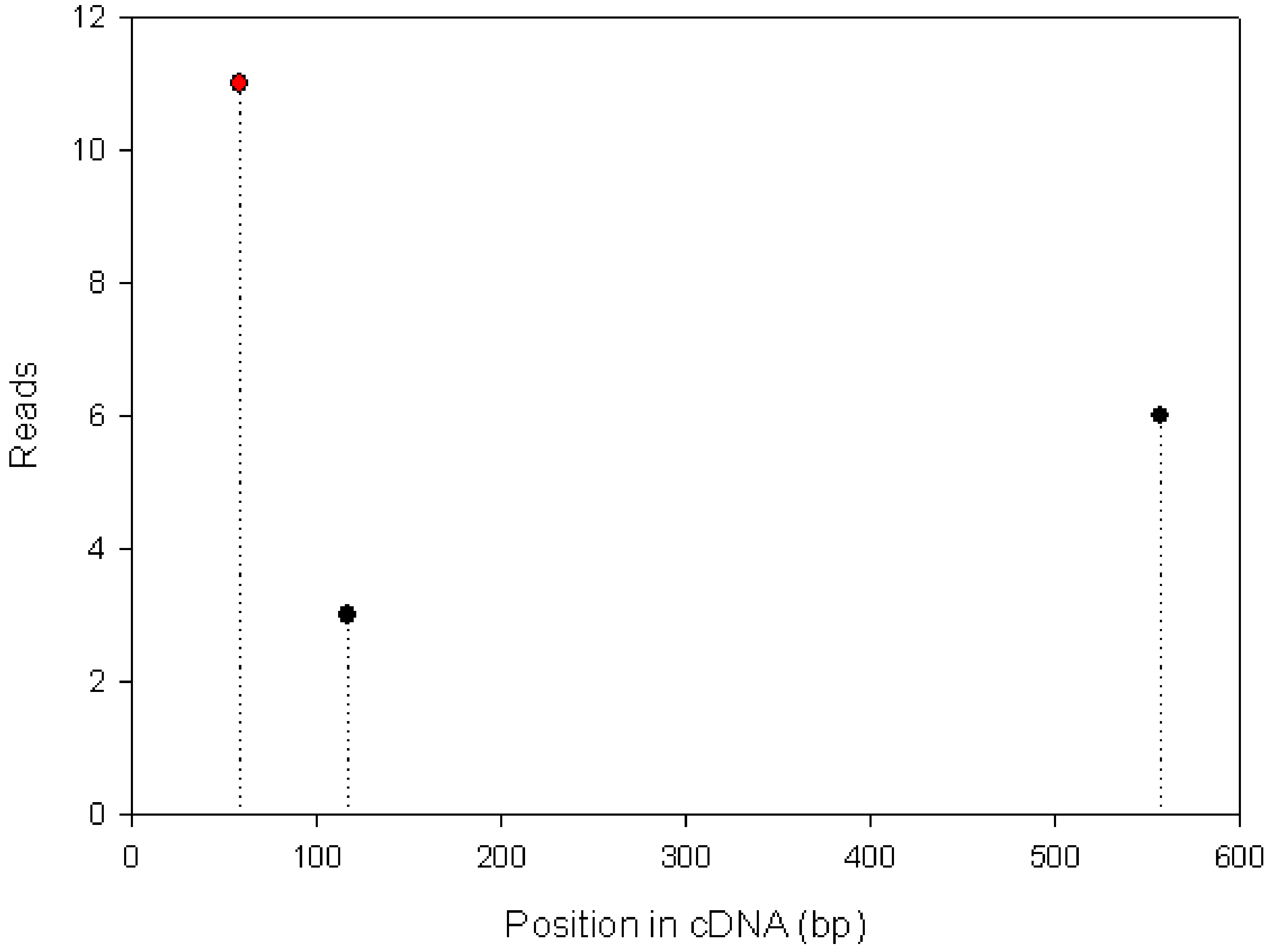


```
5' CCUACAACUACUGAGUCAGCAGCCAA 3'        Orange1.1t02428.1
         ::::.: .:::::::::::
3' ----CUUGGUUGCUCAGUCGUCGA-- 5'        Csi-miR4414.2
```

Csi-miR444a.1, target=Orange1.1t02003.1 gene=Orange1.1t02003
Category=2
Score=4
Cleavage Site=545

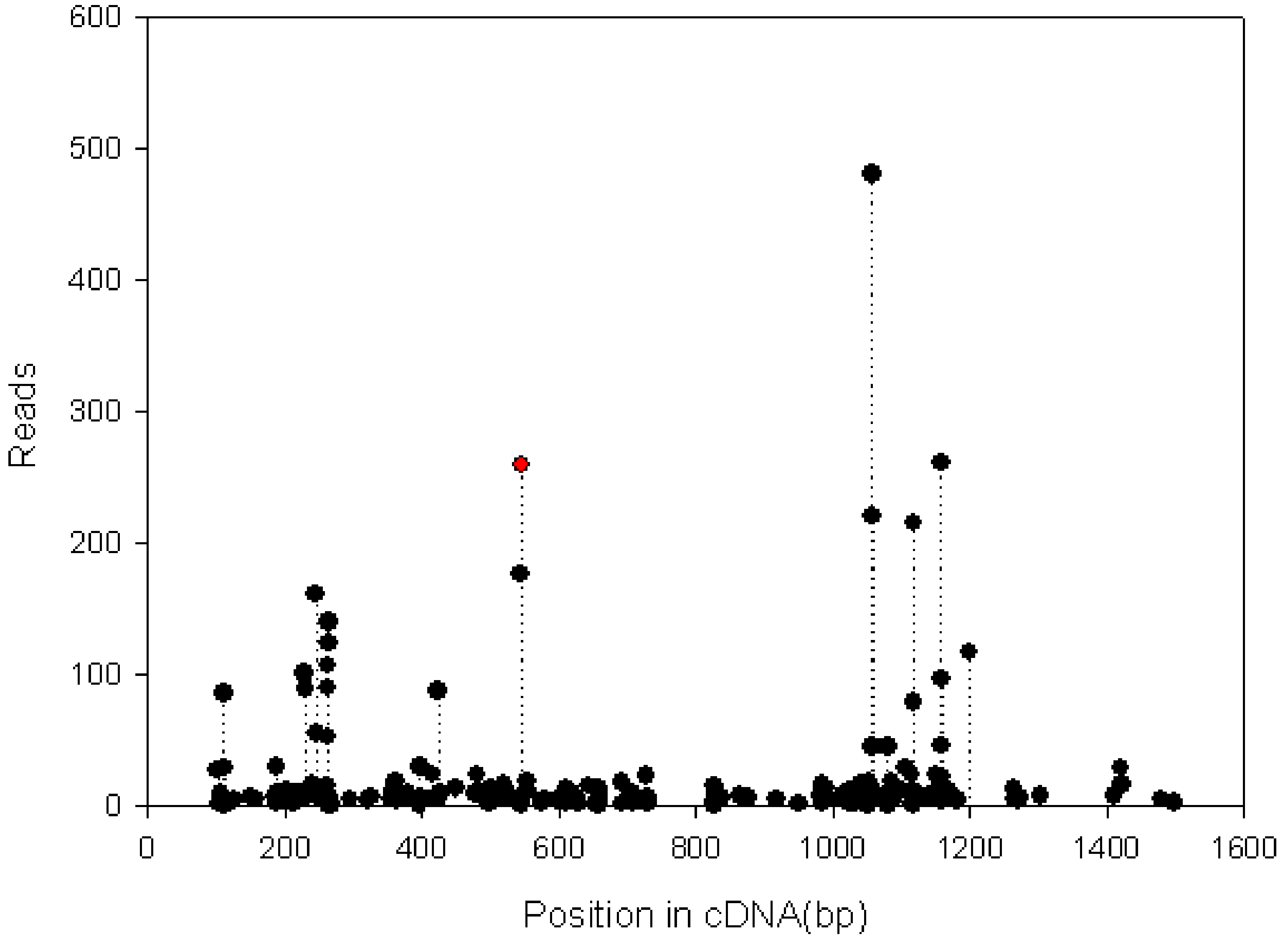


```
5' UUGCUUGCUUUGUC-CAAGAAAGUAAC 3'        Orange1.1t02003.1
    ::: :: :::::: :::::::
3' -ACGUACAAAACAGAGUUCUUU----- 5'        Csi-miR444a.1
```

Csi-miR472, target=Cs7g26730.1 gene=Cs7g26730
Category=3
Score=3
Cleavage Site=1411

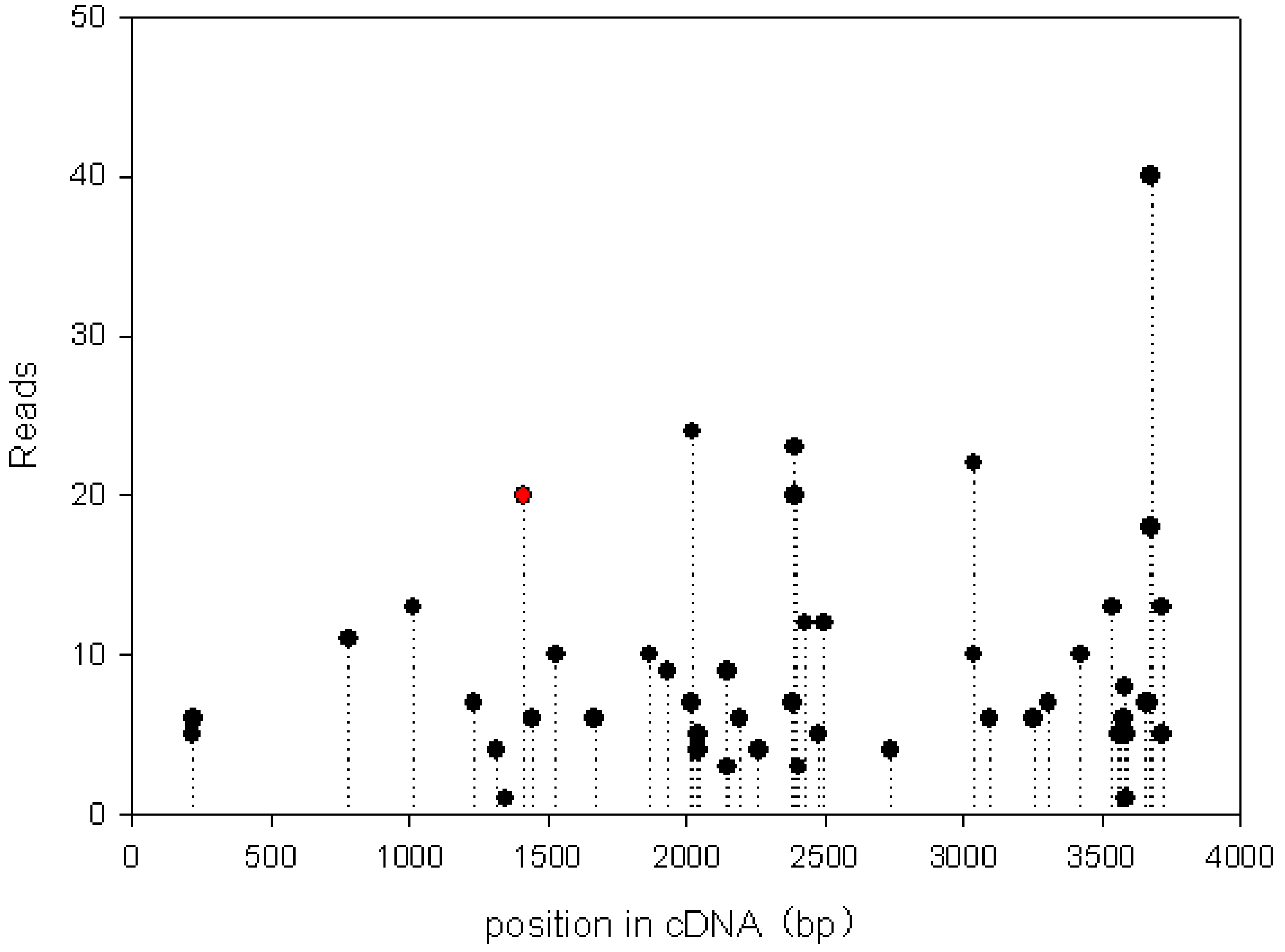


```
5' GAGAAGAGAGGUGUGGGAAAAUGCAA 3'        Cs7g26730.1
    : :: :  ::::::::::::::
3' CCCUACCCUCCACACCCUUUU----- 5'        Csi-miR472
```

Csi-miR473-3p, target=Orange1.1t02213.1 gene=Orange1.1t02213
Category=3
Score=4
Cleavage Site=534

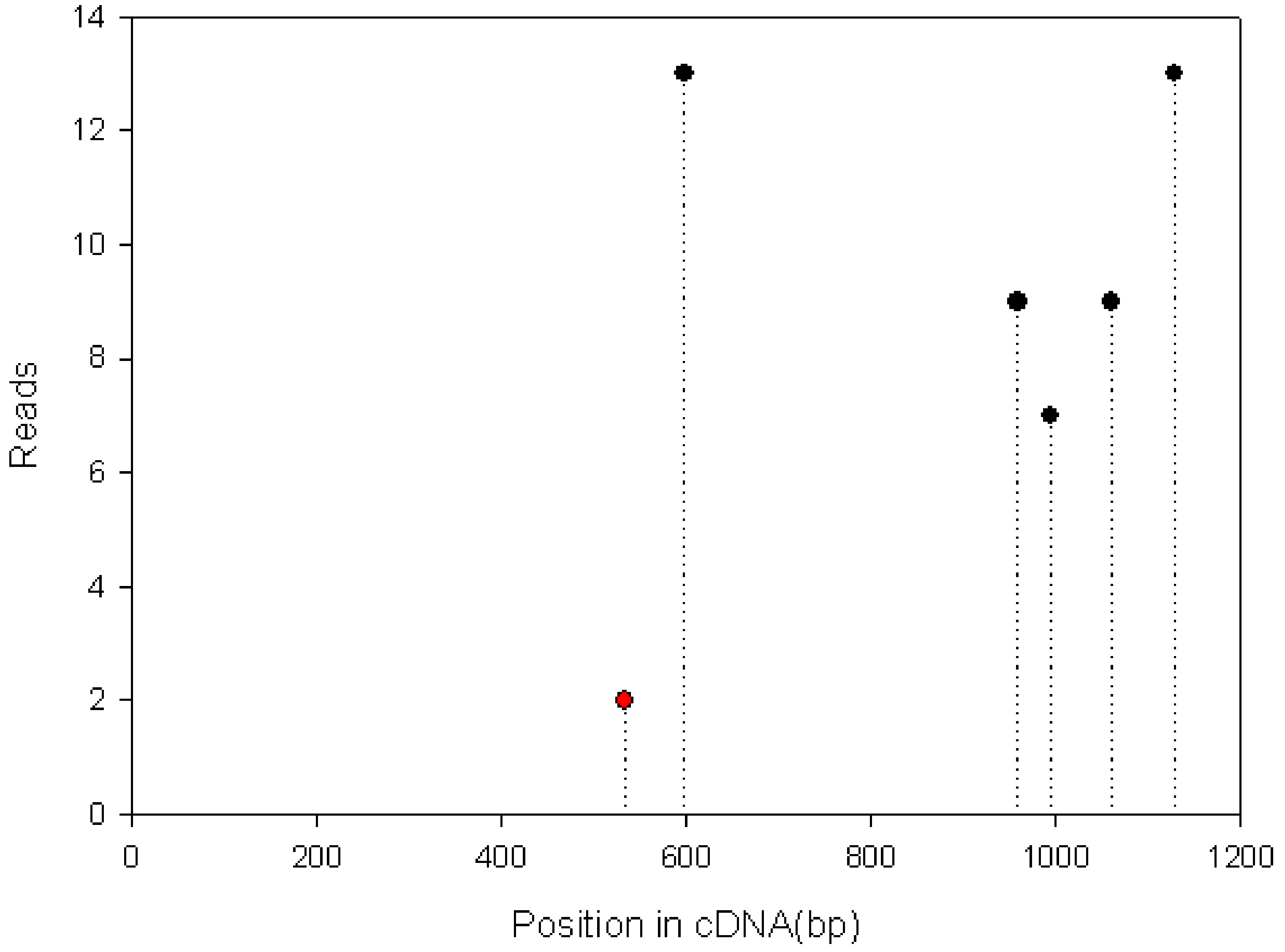


```
5' GUCUGUCA-UCAACUUCGAGGACUUCA 3'       Orange1.1t02213.1
         ::: :::::..:.::::::::
3' -----AGUGAGUUGGGGUUCCUGAAG- 5'       csi-miR473-3p
```

Csi-miR477a-3p, target=Cs7g09520.1 gene=Cs7g09520
Category=2
Score=5
Cleavage Site=463

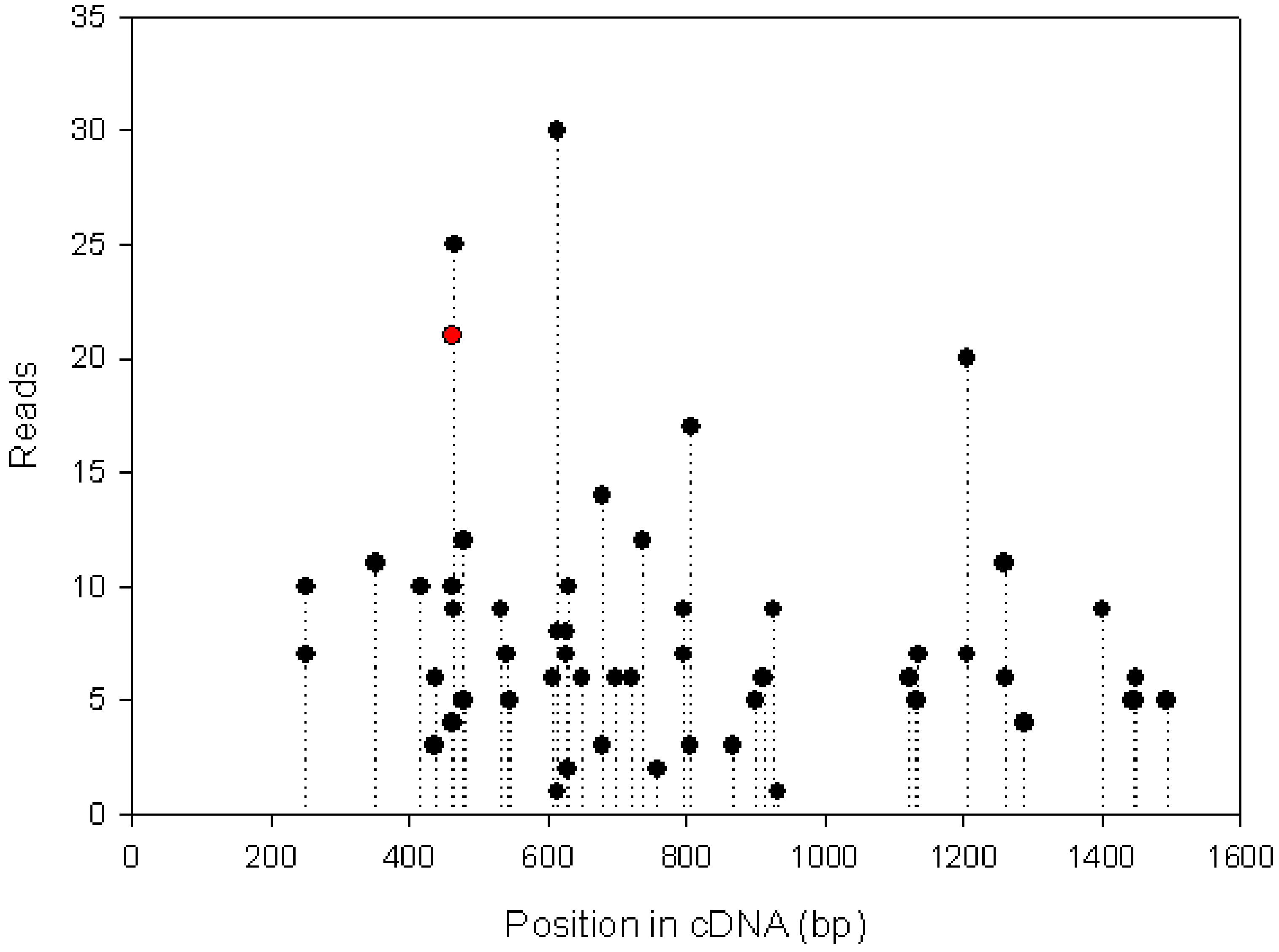


```
5'  GGUCCACCUCCUAGGGUUUCGGAUUU 3'          Cs7g09520.1
      :  ::  ::.:::::::::::
3'  GCUGGAGGGGAUCCCAAAGG----- 5'           Csi-miR477a-3p
```

Csi-miR477b.2, target=Cs8g17030.1 gene=Cs8g17030
Category=3
Score=5
Cleavage Site=373

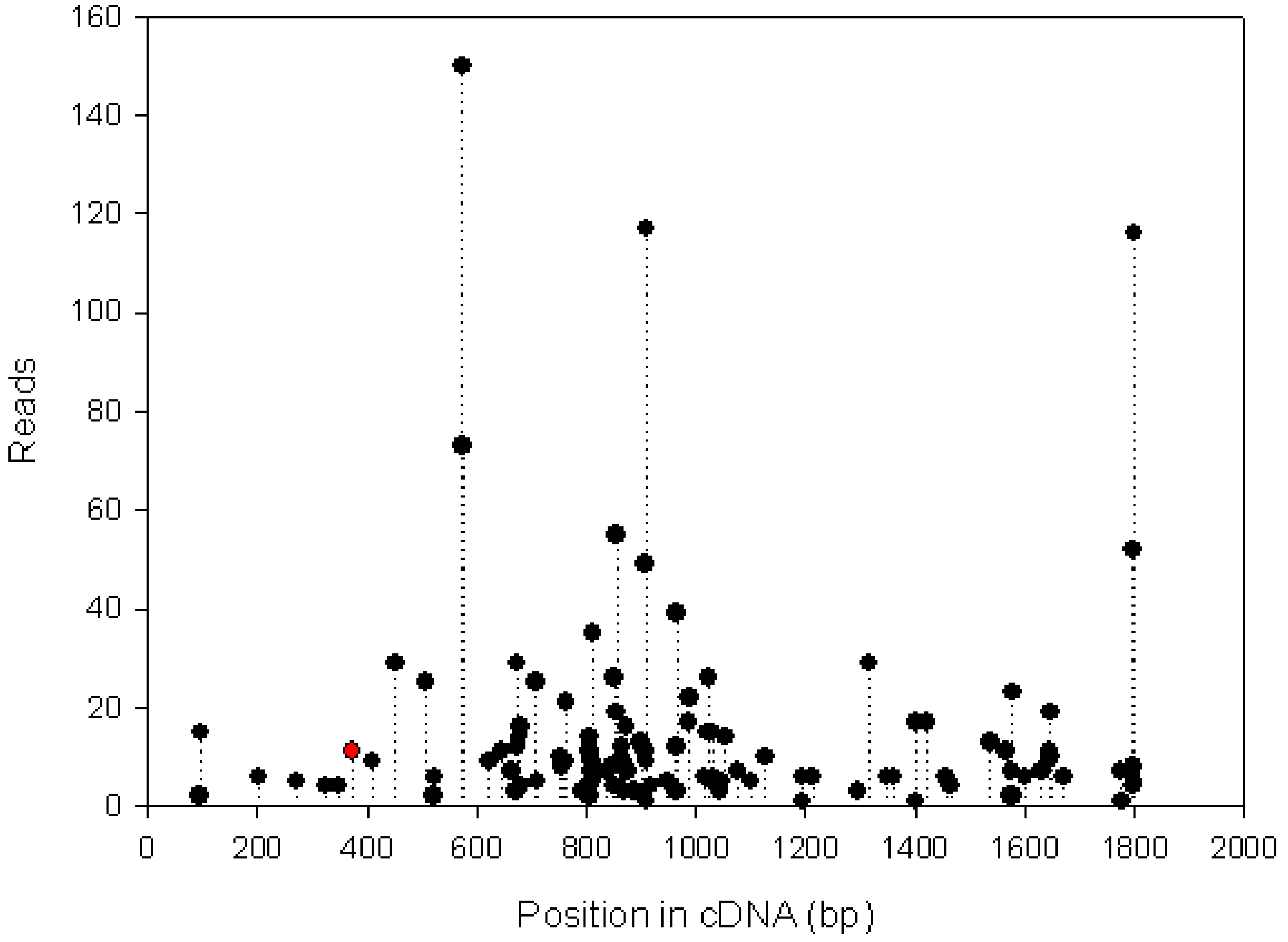


```
5' UGUGAGGAGUCCUGGAGGGAGAAUUU 3'        Cs8g17030.1
     :::.::.:::  :::::::: :
3' ---CUCUUCGGGAACUCCCUCUCA-- 5'        Csi-miR477b.2
```

Csi-miR477d.1-3p, target=Cs2g02350.1 gene=Cs2g02350
Category=3
Score=5
Cleavage Site=67

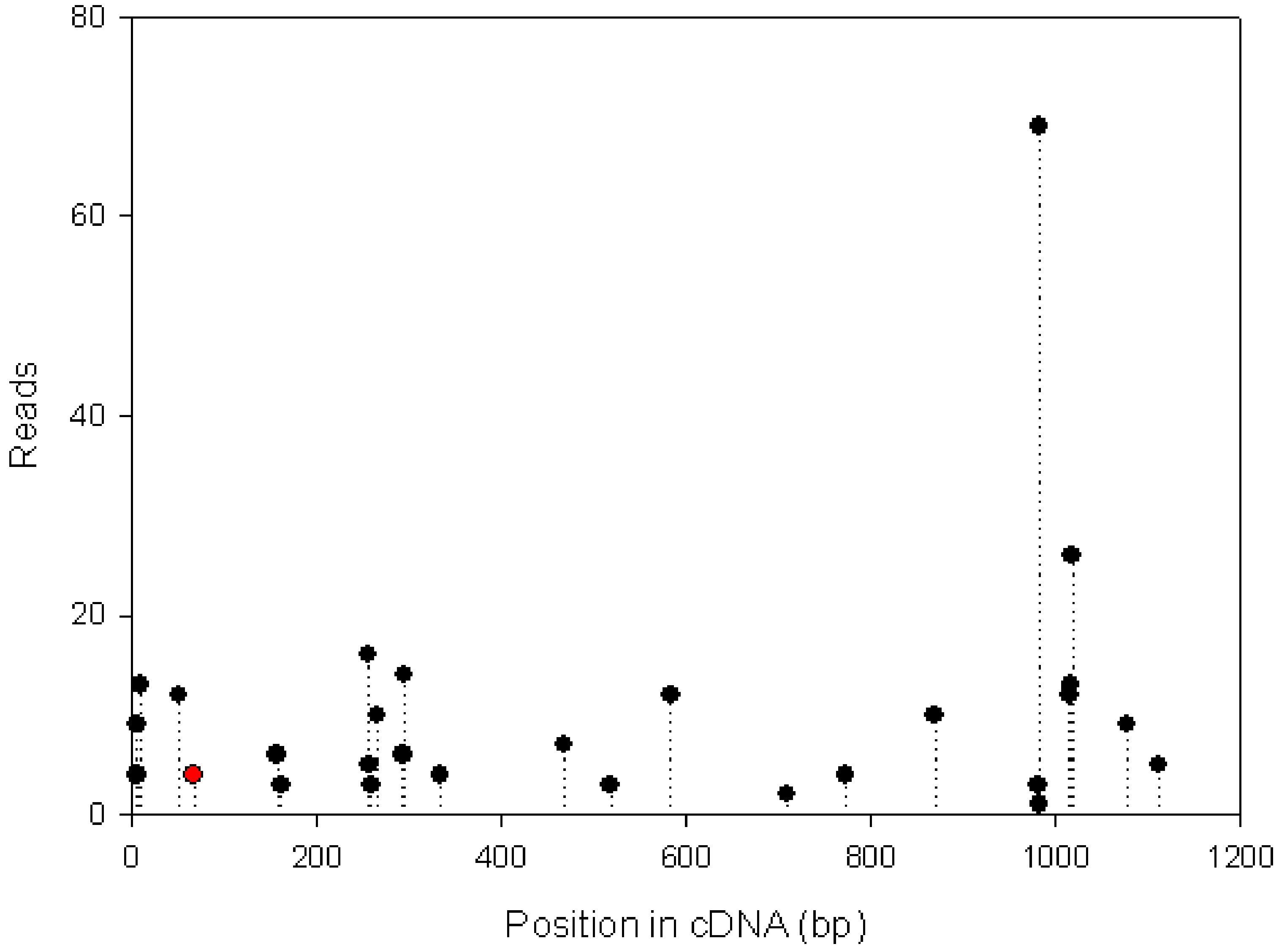


```
5' AACCACUUCACUUCAACGGCUUCAAA 3'        Cs2g02350.1
     :::: :: :..:::::::.:::
3' --GGUG-AGAGGGGUUGCCGGAGU-- 5'        Csi-miR477d.1-3p
```

Csi-miR477d.2-5p, target=Cs3g10900.1 gene=Cs3g10900
Category=3
Score=3.5
Cleavage Site=1314

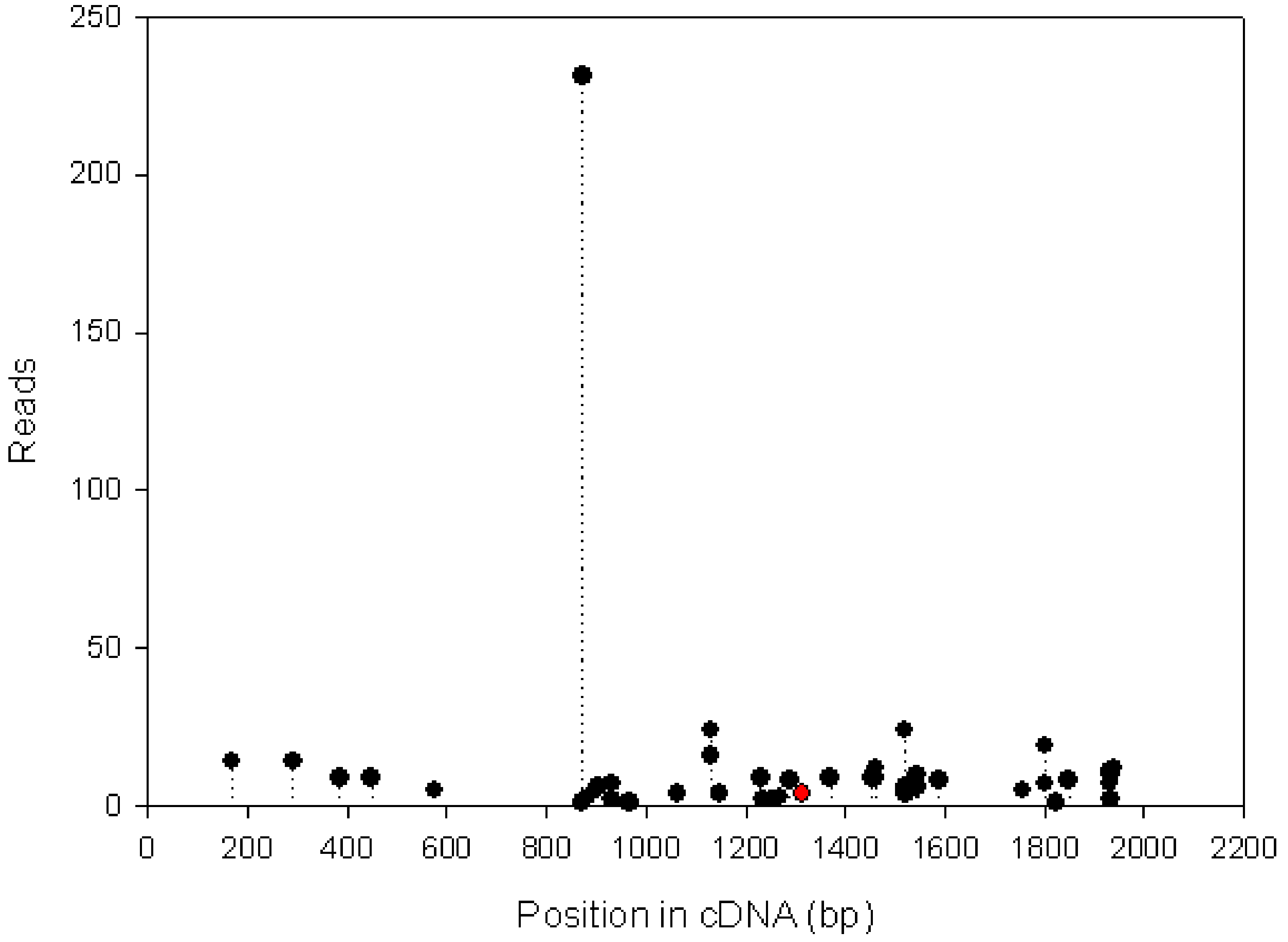


```
5' UCUGUCAUCAGCUCUUGAGGGAGAGA 3'        Cs3g10900.1
      :::  :::.:::::::::::::
3' ----AGUCUUCGGGAACUCCCUCUCA 5'        Csi-miR477d.2-5p
```

Csi-miR479.1, target=Cs5g24810.1 gene=Cs5g24810
Category=3
Score=5
Cleavage Site=4314

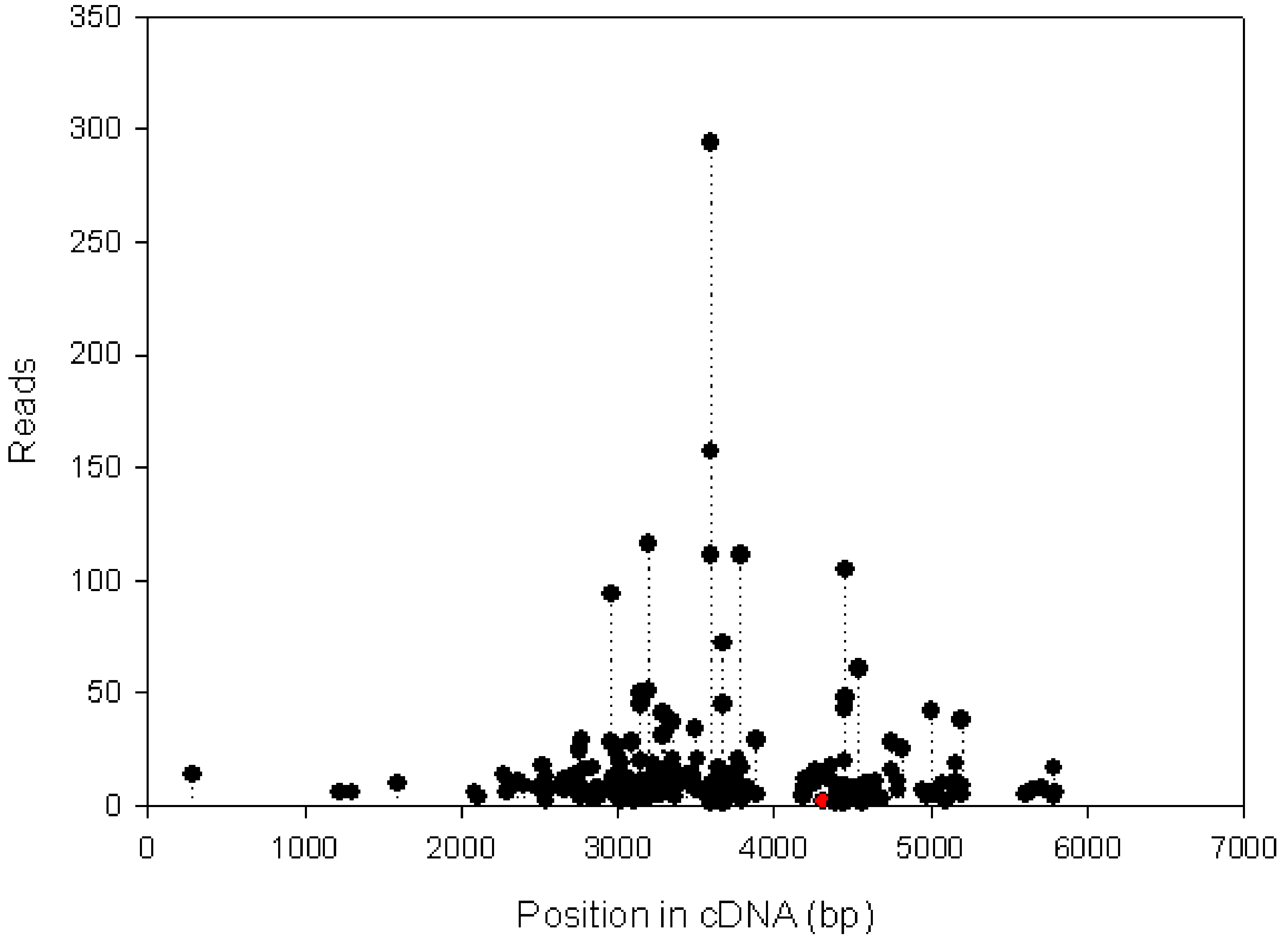


```
5' GA-GCGCCGGAUCAAUAUUACAUAUGG 3'        Cs5g24810.1
   :: : ::::.:.:::::::.:::
3' CUACUCGGCUUGGUUAUAGUGU----- 5'        Csi-miR479.1
```

Csi-miR479.2, target=Cs6g21570.1 gene=Cs6g21570
category=3
score=5
Cleavage Site=205

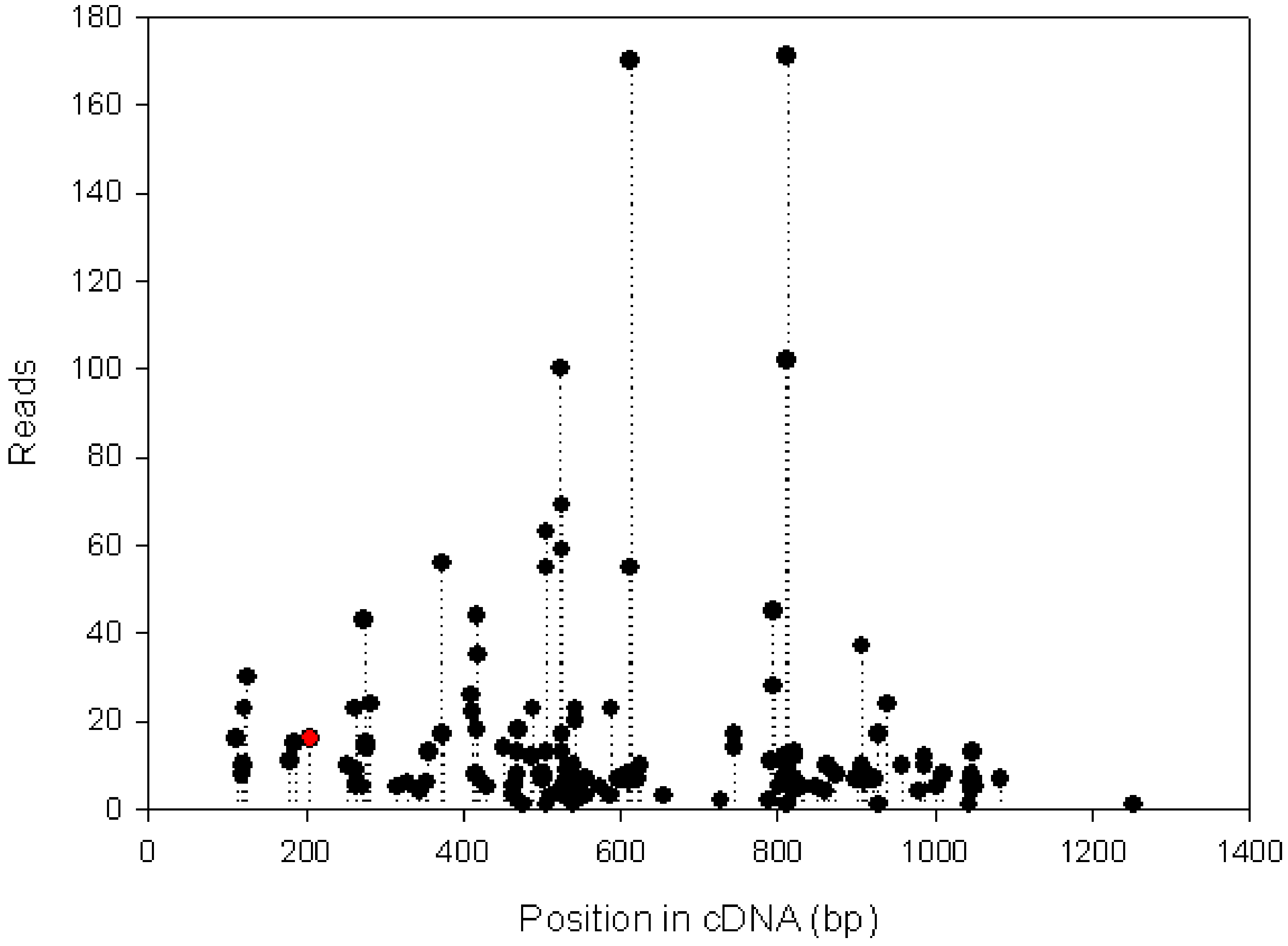


```
5' AUGA-CCGUACCAAUAUCAAAUCUUGU 3'        Cs6g21570.1
    ::: ::: :::::::::: :
3' -ACUCGGCUUGGUUAUAGUGU------ 5'        Csi-miR479.2
```

Csi-miR482b, target=Cs1g13430.1 gene=Cs1g13430
Category=3
Score=4
Cleavage Site=700

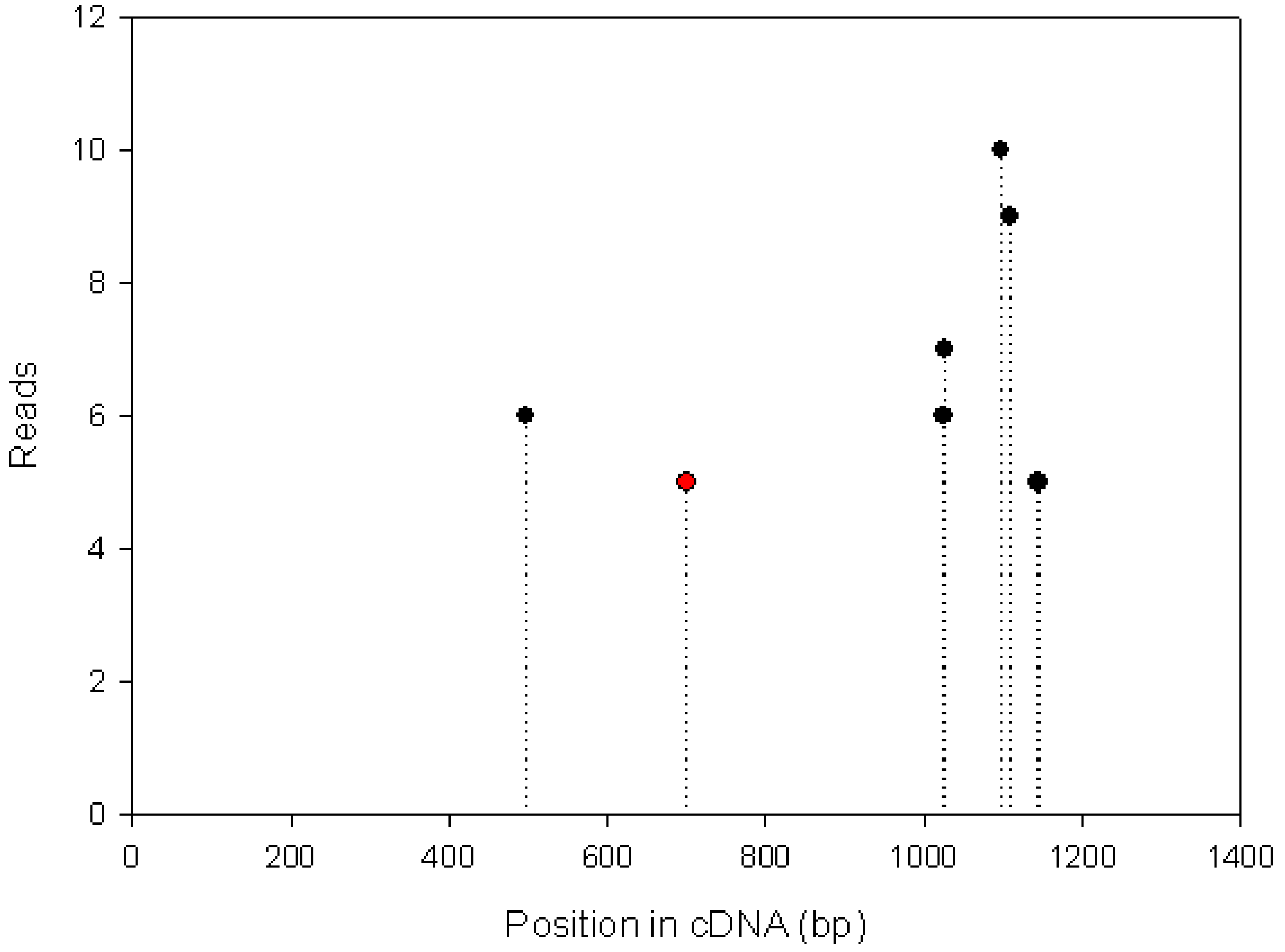


```
5' GGCAUGGGAGGGGUGGGUAAAACUAC 3'        Cs1g13430.1
   :: :::::::::::::::.:: :
3' CCUUACCCUCCCCACCCGUUCU---- 5'        Csi-miR482b
```

Csi-miR482c, target=Cs9g06846.1 gene=Cs9g06846
Category=3
Score=3
Cleavage Site=158

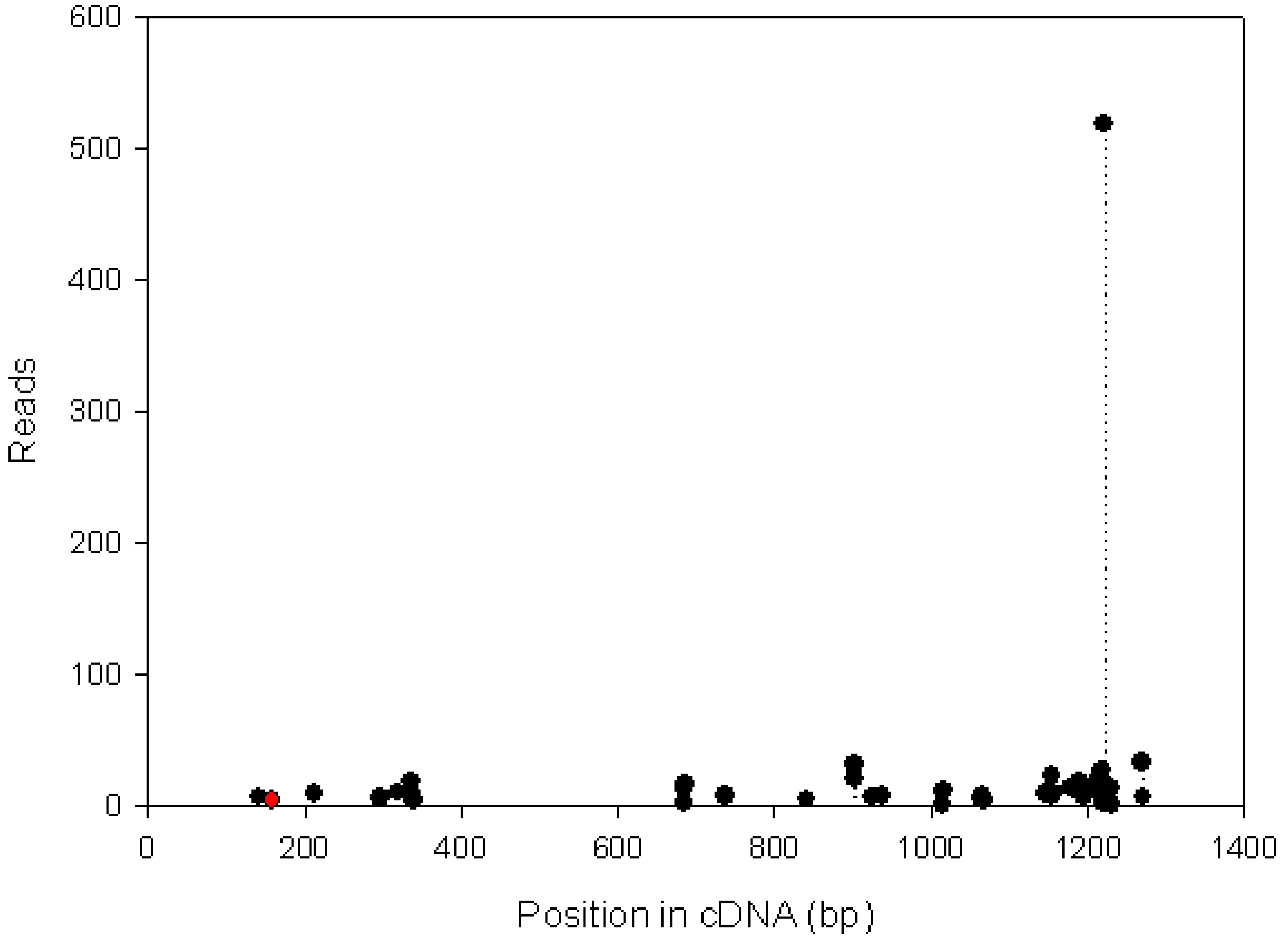


```
5' UGGGAAUAGGUGGACUAGGGAAAAUU 3'        Cs9g06846.1
   :.:::::::: :::::::::::
3' AUCCUUAUCCCCCUGAUCCCUU---- 5'        Csi-miR482c
```

Csi-miR482d, target=Cs6g10170.1 gene=Cs6g10170
Category=3
Score=5
Cleavage Site=1389

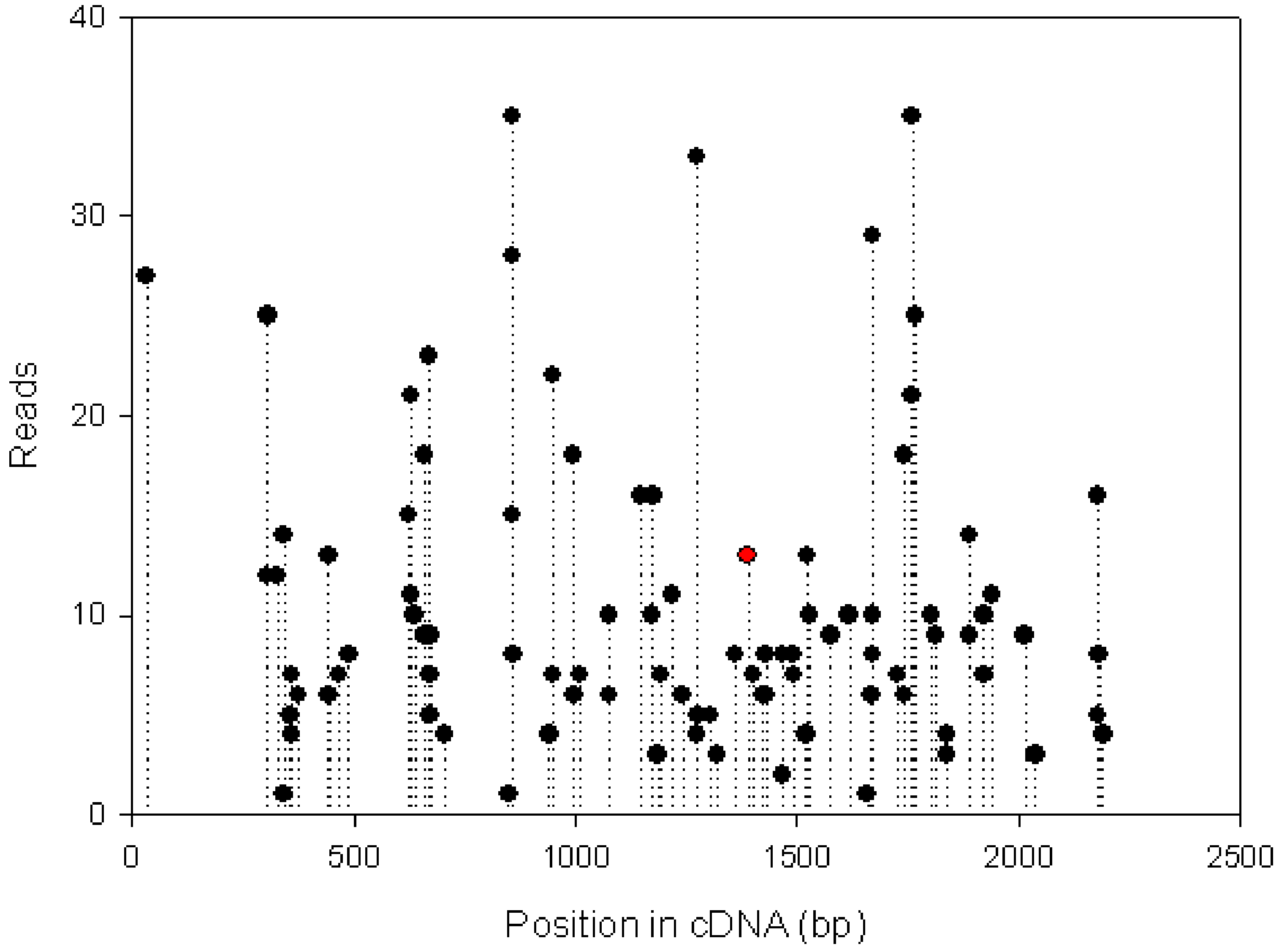


```
5' AGACAAGGGAGGAG-AGGGAAGAGAGG 3'        Cs6g10170.1
    : :: ::: :::: ::::::::
3' -CCGUACCCACCUCAUCCCUUCU---- 5'        Csi-miR482d
```

Csi-miR5054.2, target=Cs7g07710.1 gene=Cs7g07710
Category=3
Score=4.5
Cleavage Site=3045

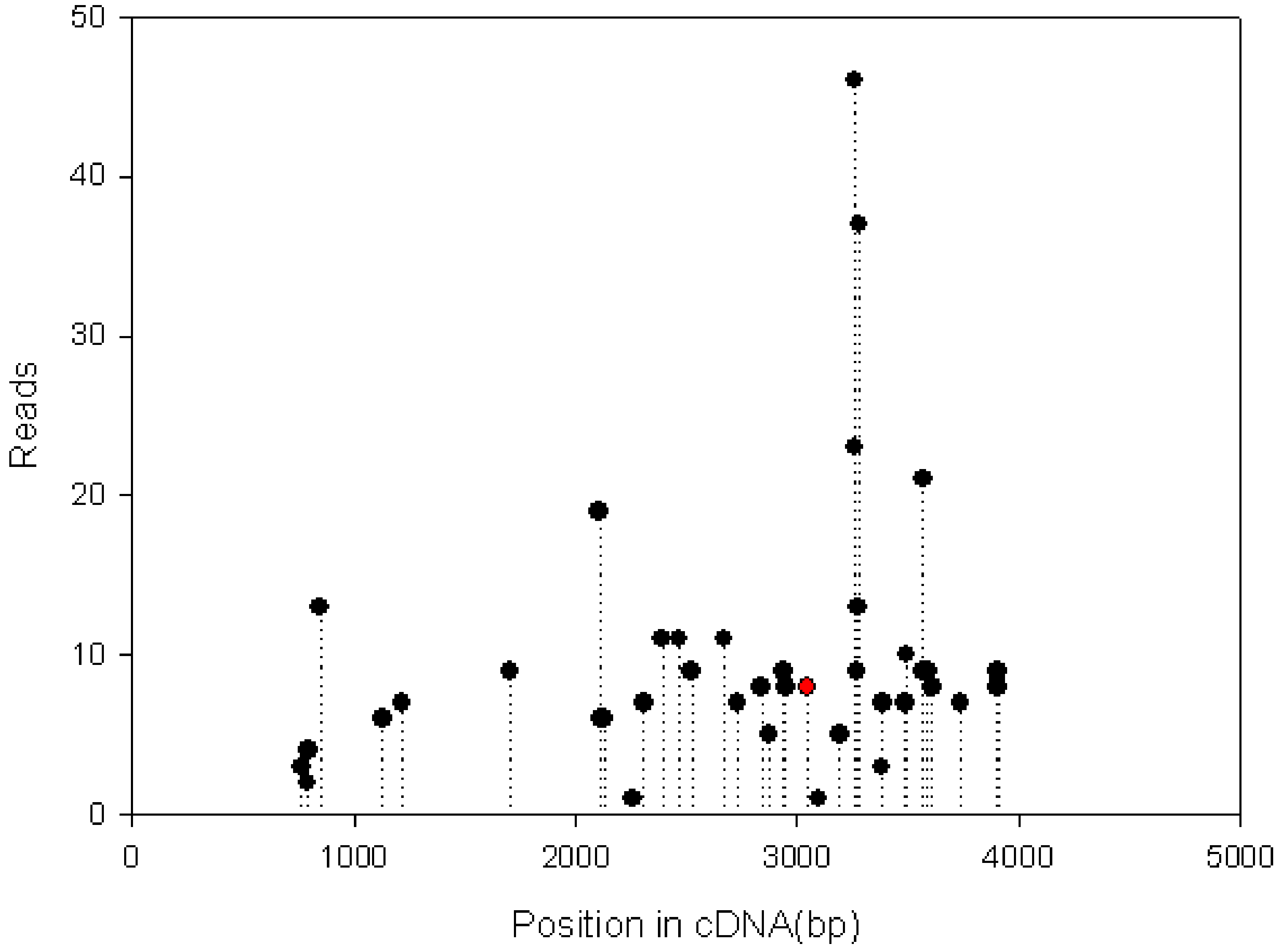


```
5' GGAGUUGUCCAGUCUGUGGGGAAGGC 3'        Cs7g07710.1
       : :. : ::::::::::::
3' ----ACCGCGGCAGACACCCCUUG-- 5'        Csi-miR5054.2
```

Csi-miR5179, target=Cs1g24860.1 gene=Cs1g24860
Category=3
Score=3.5
Cleavage Site=820

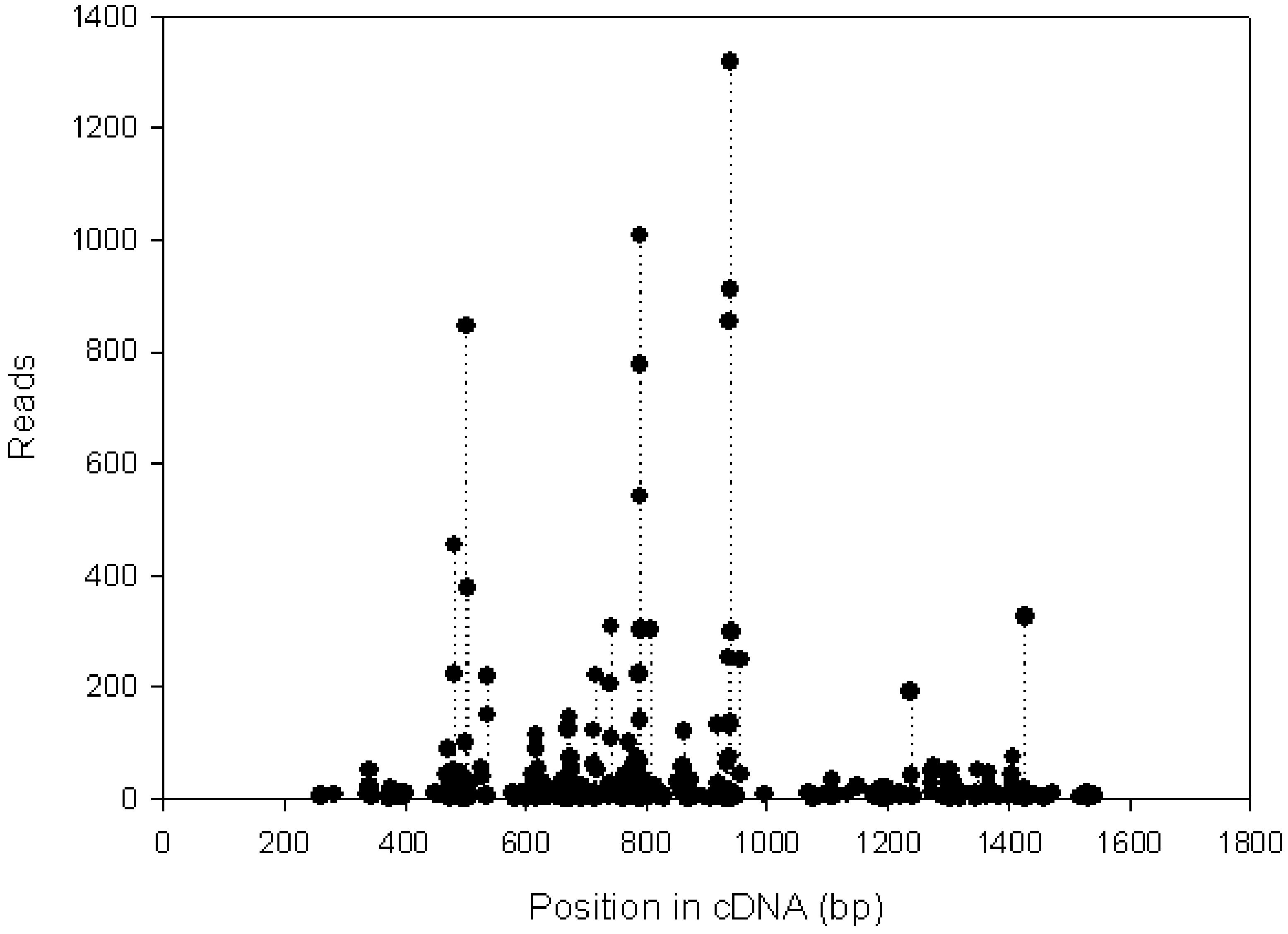


```
5' AACUGCGUGGUCUUGAGCAAAACAUG 3'        Cs1g24860.1
    :  ::::.:::::::::::::  :
3' -UAACGCGCCAGAACUCGUUCU---- 5'        Csi-miR5179
```

Csi-miR5291a, target=Cs1g02730.1 gene=Cs1g02730
Category=3
Score=4.5
Cleavage Site=665

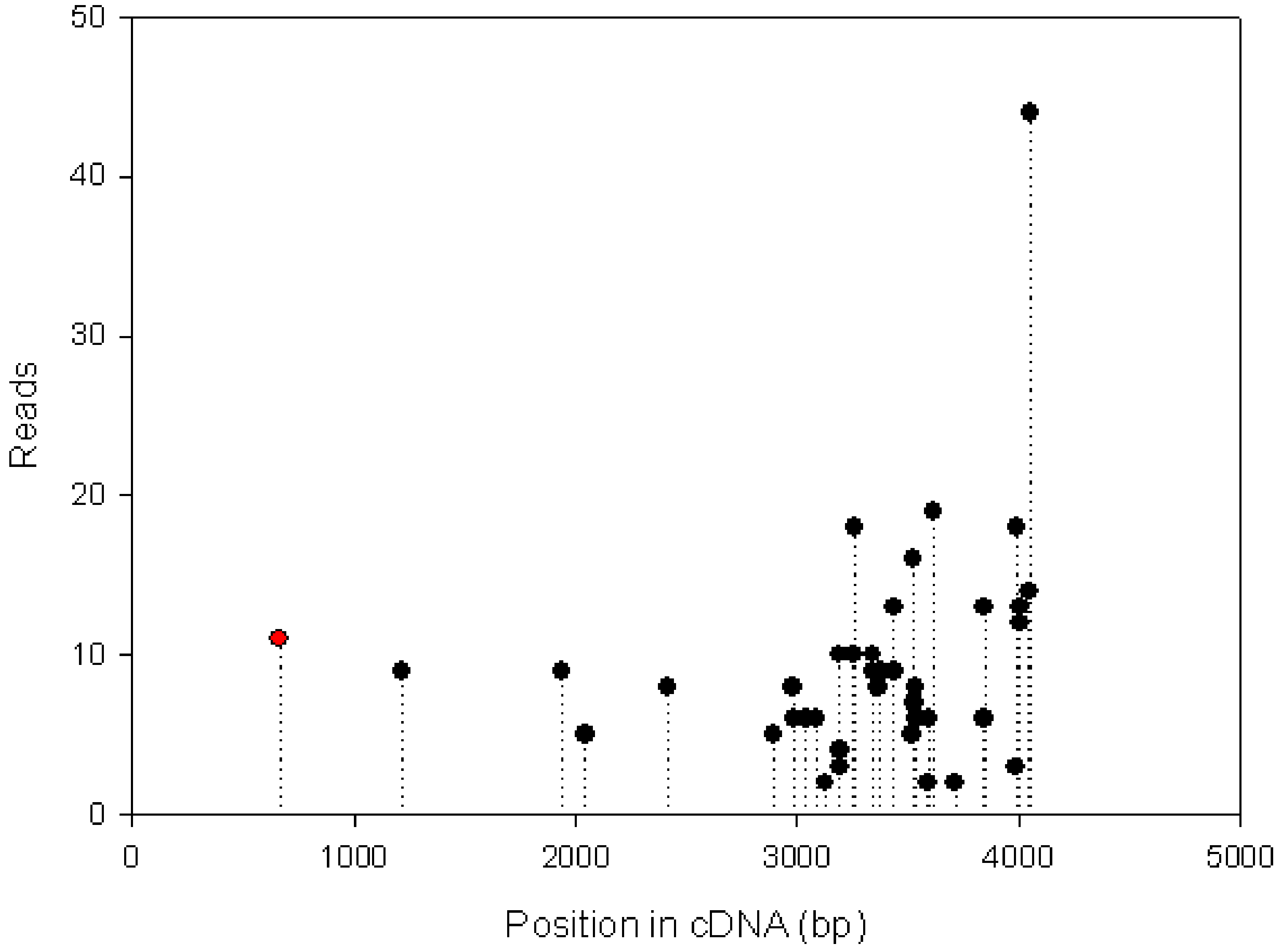


```
5' GACUCAACUCUUUGACCAUUCAUCAA 3'        Cs1g02730.1
       ::: ::.: : :::::::::::
3' ----GUUUAGGAUC-GGUAAGUAGUU 5'        Csi-miR5291a
```

Csi-miR530a, target=Cs3g18550.1 gene=Cs3g18550
Category=3
Score=5
Cleavage Site=332

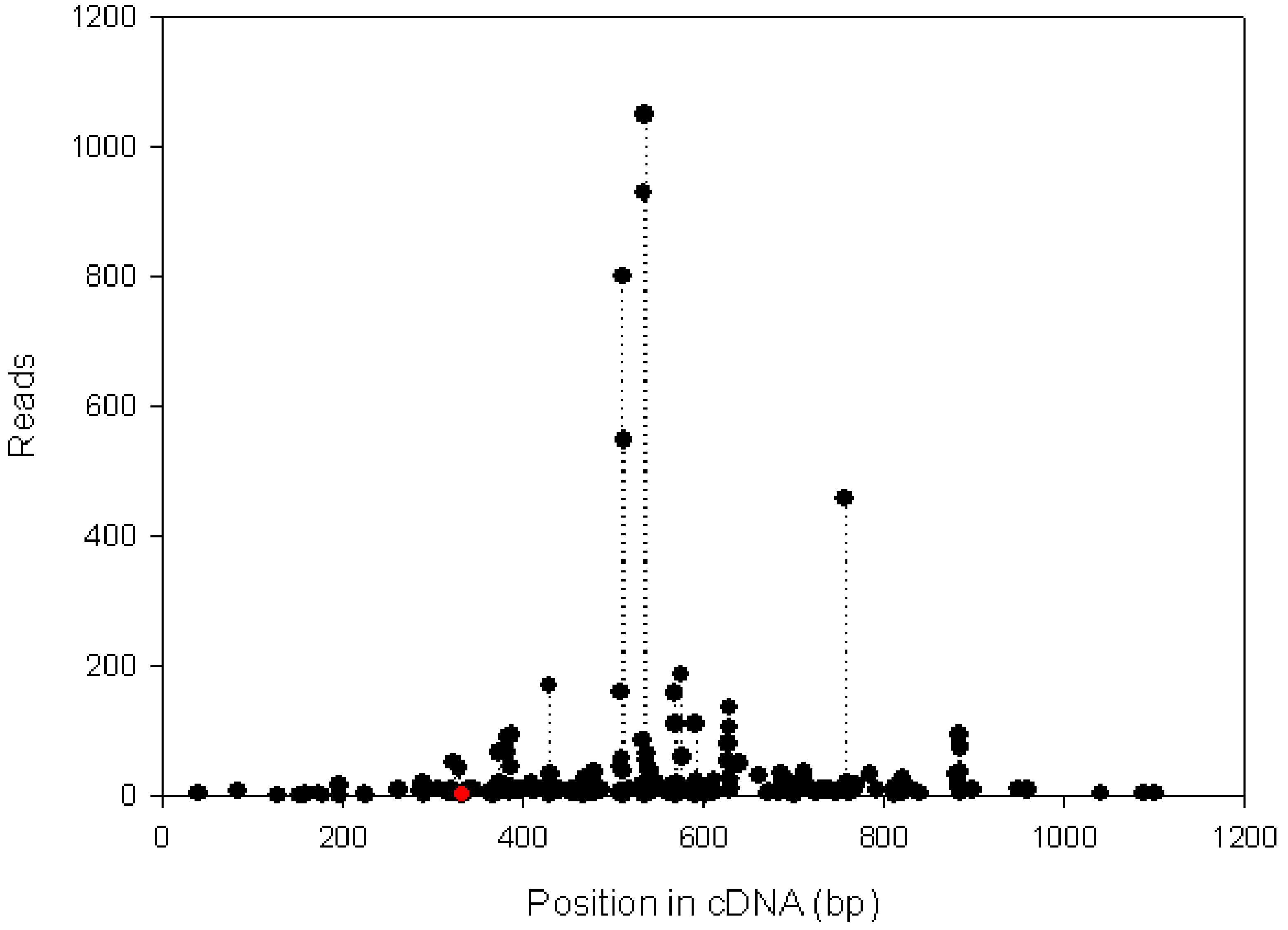


```
5' GUGCCAAGGUGAAGGU-UGAAUGCAAA 3'        Cs3g18550.1
       ::::::: :::: ..::::::
3' ----GUUCCACGUCCACGUUUACGU-- 5'        Csi-miR530a
```

Csi-miR530b, target=Cs4g05330.1 gene=Cs4g05330
Category=3
Score=5
Cleavage Site=2322

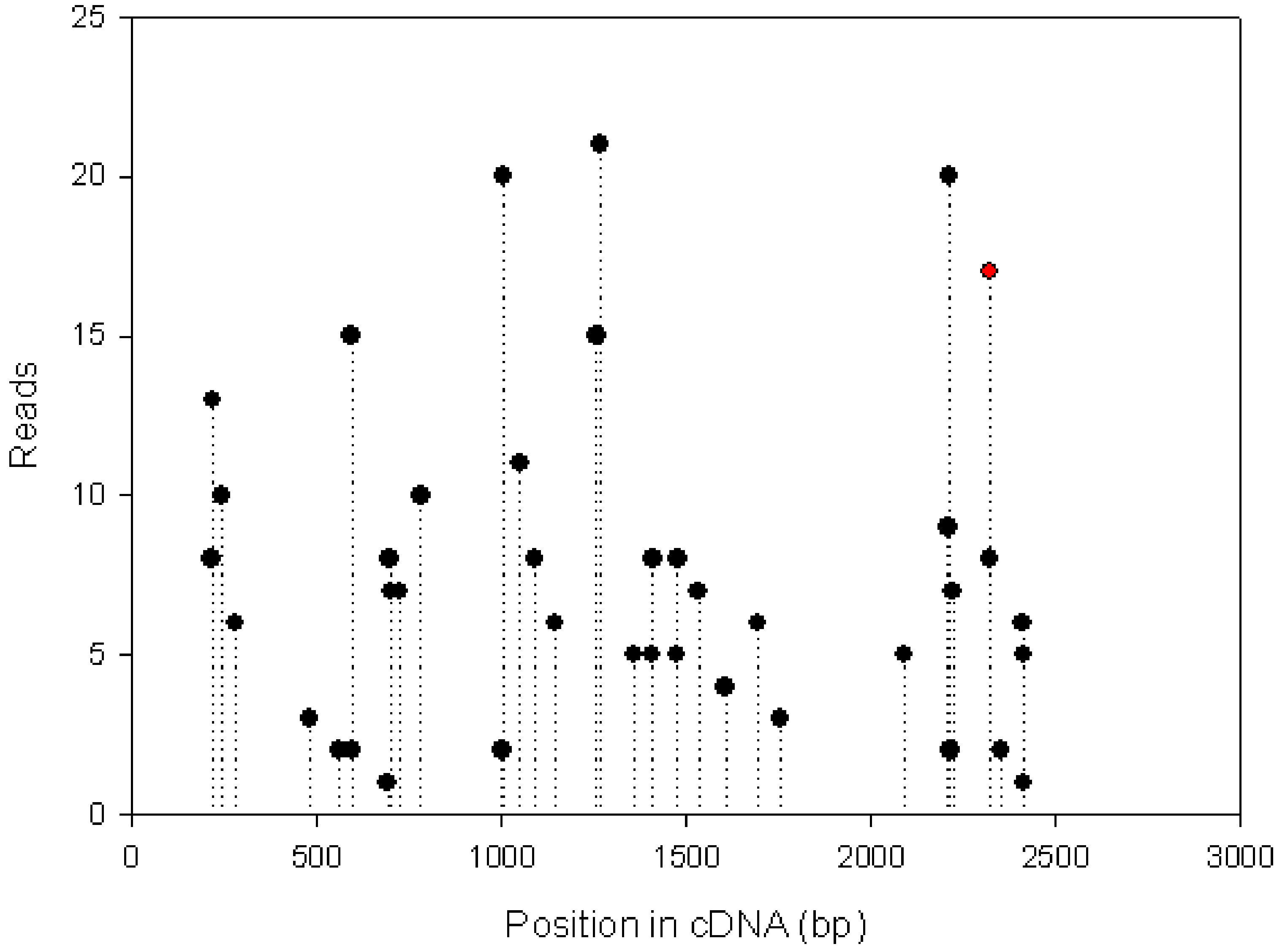


```
5' AAUGGGGACGCAGGUGCUAAUGCAUG 3'        Cs4g05330.1
         ..:: :::::::: ::::::
3' ---GUUCUACGUCCACGUUUACGU-- 5'        Csi-miR530b
```

Csi-miR530b, target=Cs6g11650.1 gene=Cs6g11650
Category:3
Score=5
Cleavage Site=261

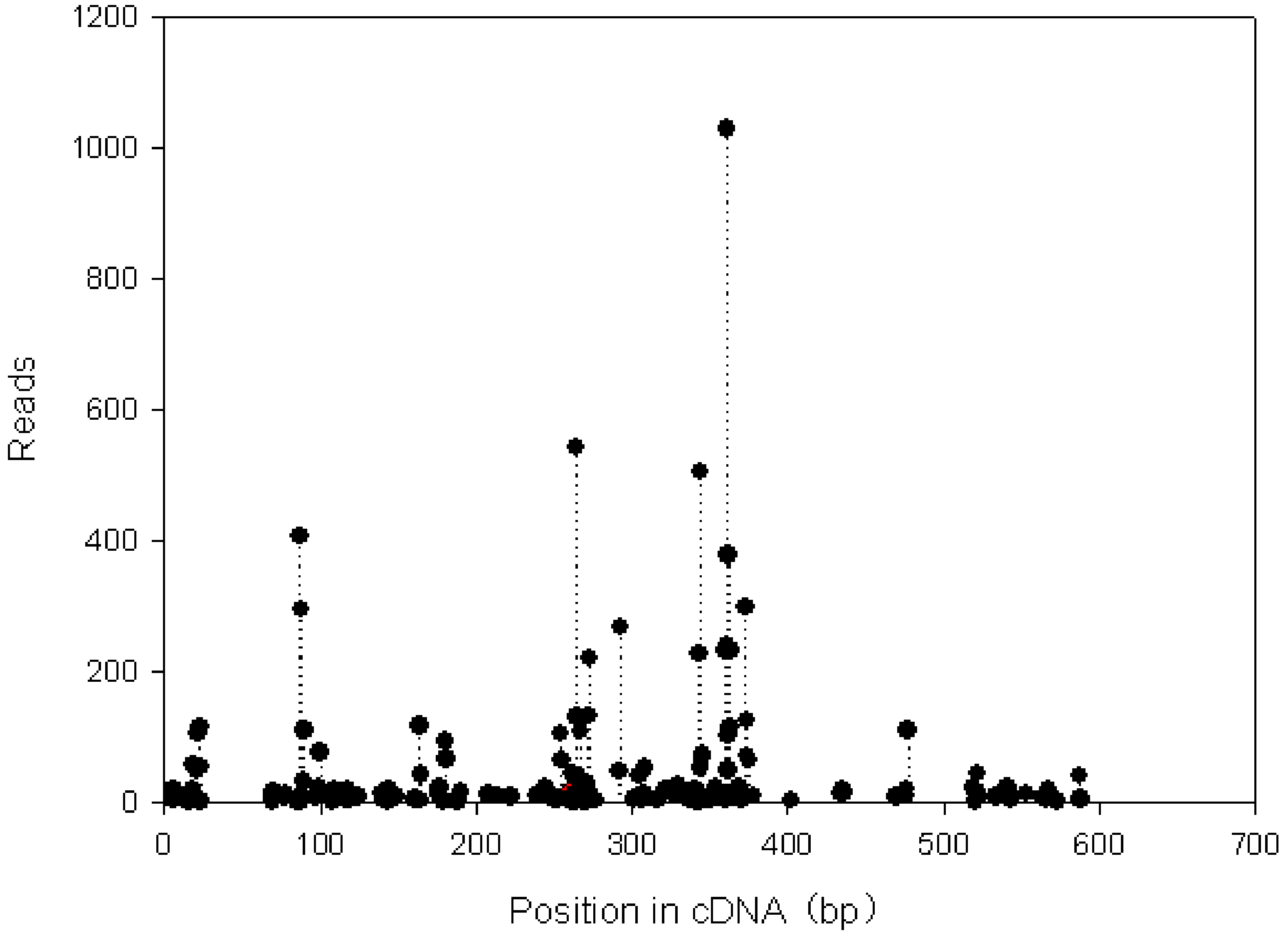


```
5' GGAGAUUCAGGUGCGAAUGUGAAGAG 3'        Cs6g11650.1
     .:::: ::::::::.::::..
3' GUUCUACGUCCACGUUUACGU----- 5'        Csi-miR530b
```

Csi-miR535.1, target=Cs4g05470.1 gene=Cs4g05470
Category=3
Score=4
Cleavage Site=1460

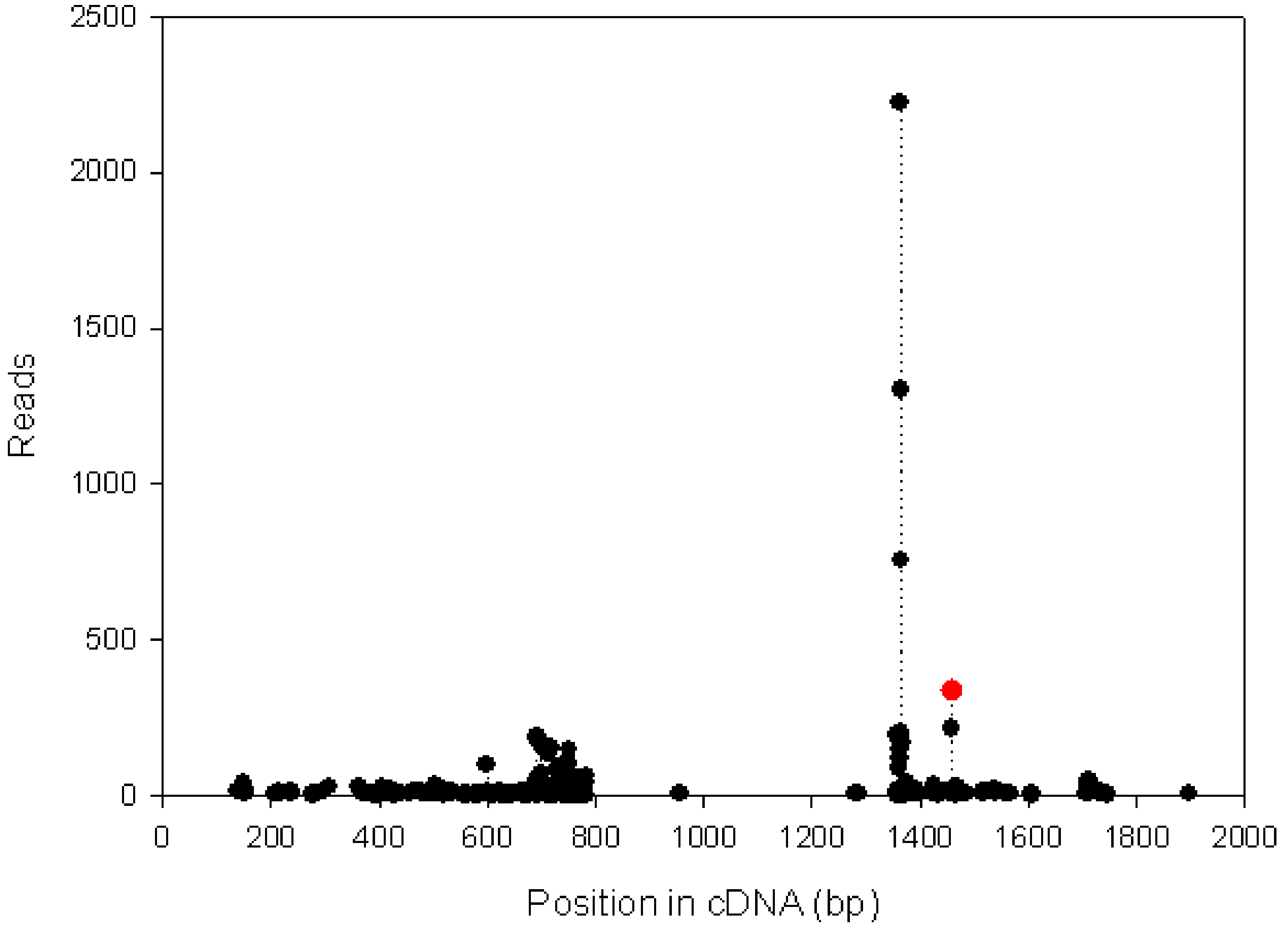


```
5' CUCUGUG-UUUCUCUCAUUGUUGGGAU 3'        Cs4g05470.1
       :::: :.:::::::::::..
3' --CACACGAGAGAGAGUAACAGU---- 5'        Csi-miR535.1
```

Csi-miR535.2, target=Cs2g05730.1 gene=Cs2g05730
Category:1
Score=5
Cleavage Site=818

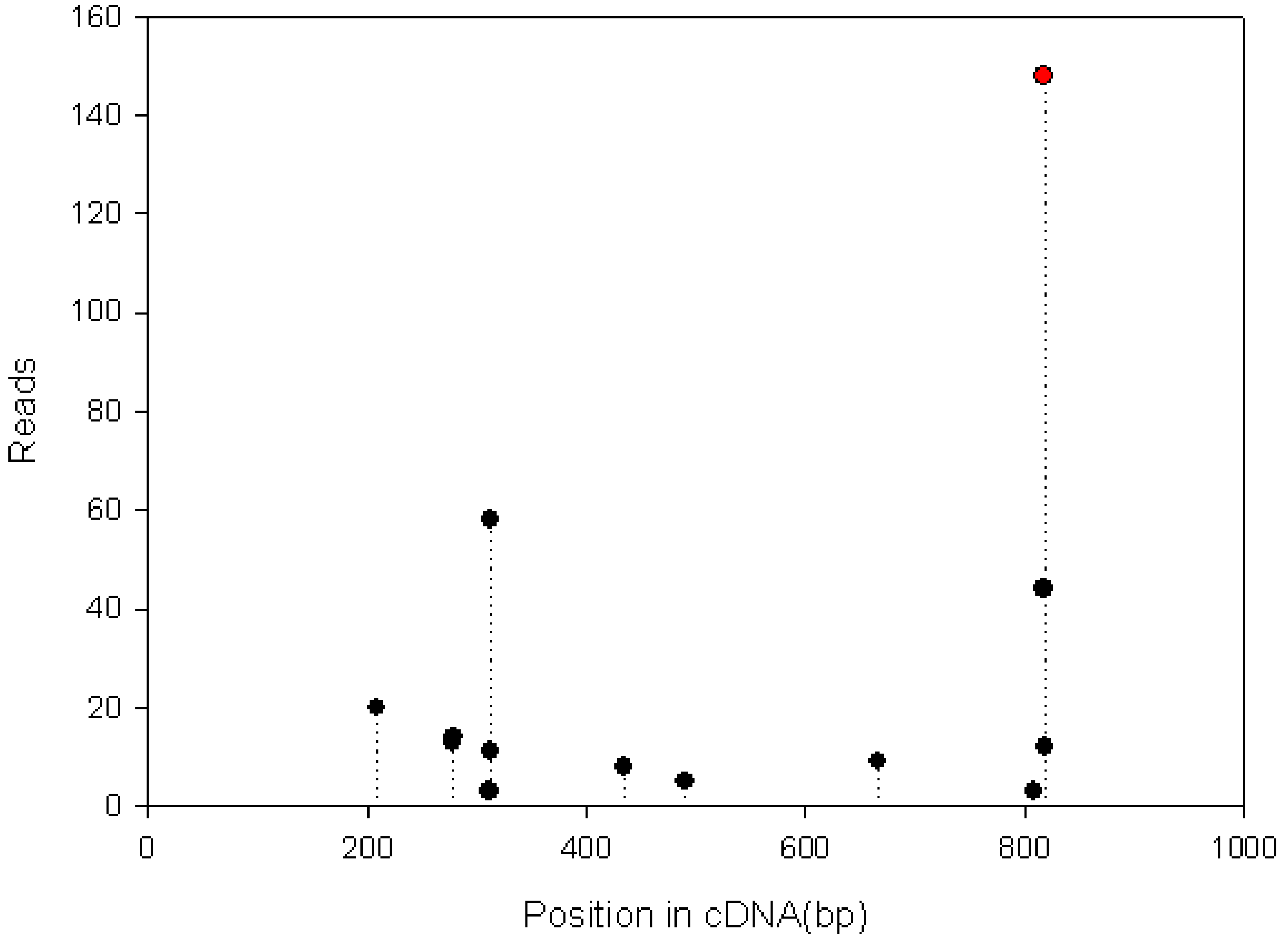


```
5' CGUGCUCUCUCUCUUCUGUCAAUGCU 3'        Cs2g05730.1
    :::::::::::: : :::::
3' ACACGAGAGAGAGUA-ACAGU----- 5'        Csi-miR535.2
```

Csi-miR535.2, target=Cs4g05470.1 gene=Cs4g05470
Category=3
Score=3
Cleavage Site=1459

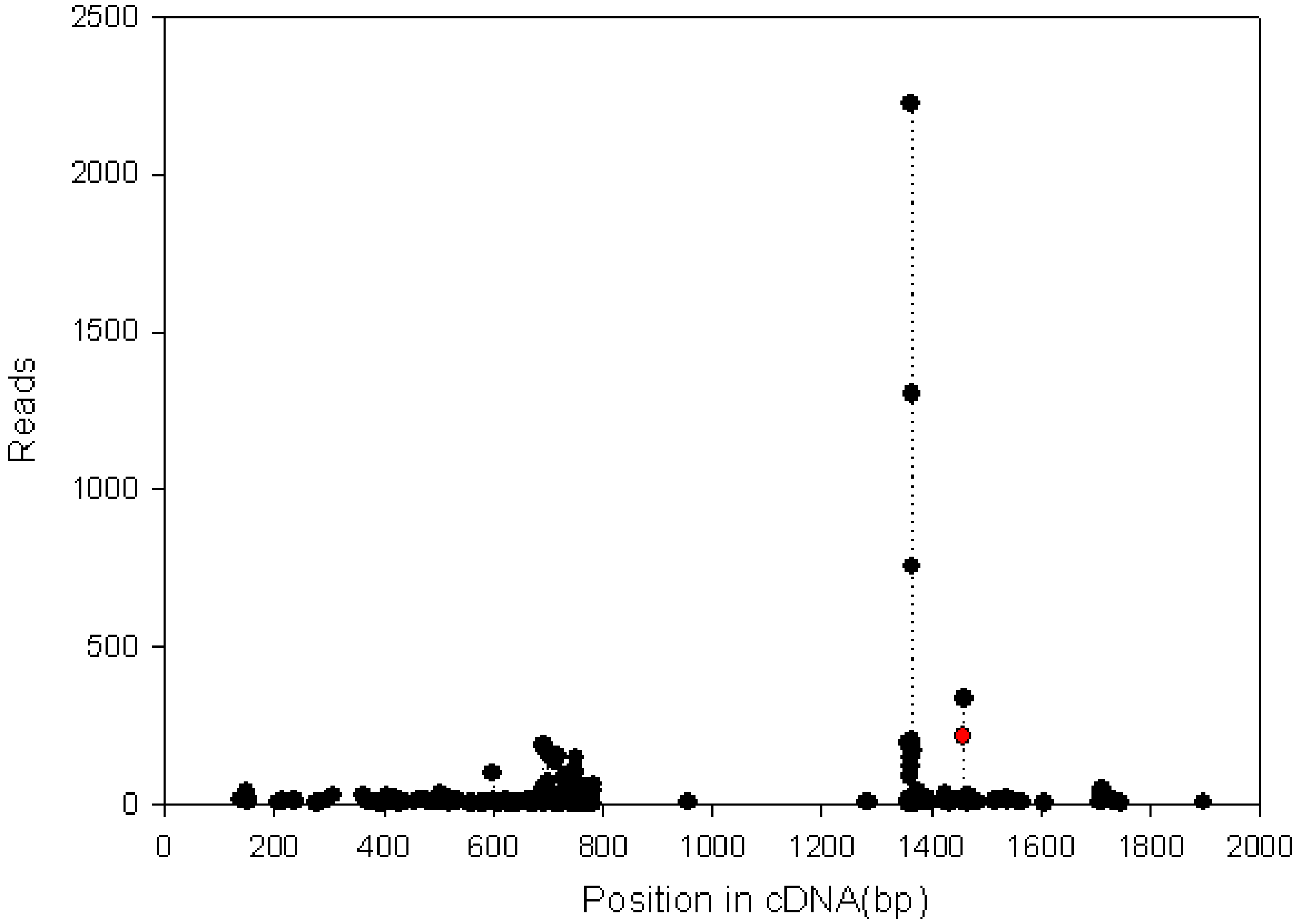


```
5' UCUCUGUG-UUUCUCUCAUUGUUGGGA 3'        Cs4g05470.1
       :::: :.:::::::::::..
3' ----ACACGAGAGAGAGUAACAGU--- 5'        Csi-miR535.2
```

Csi-miR536-5p, target=Cs1g15460.1 gene=Cs1g15460
Category=3
Score=5
Cleavage Site=2245

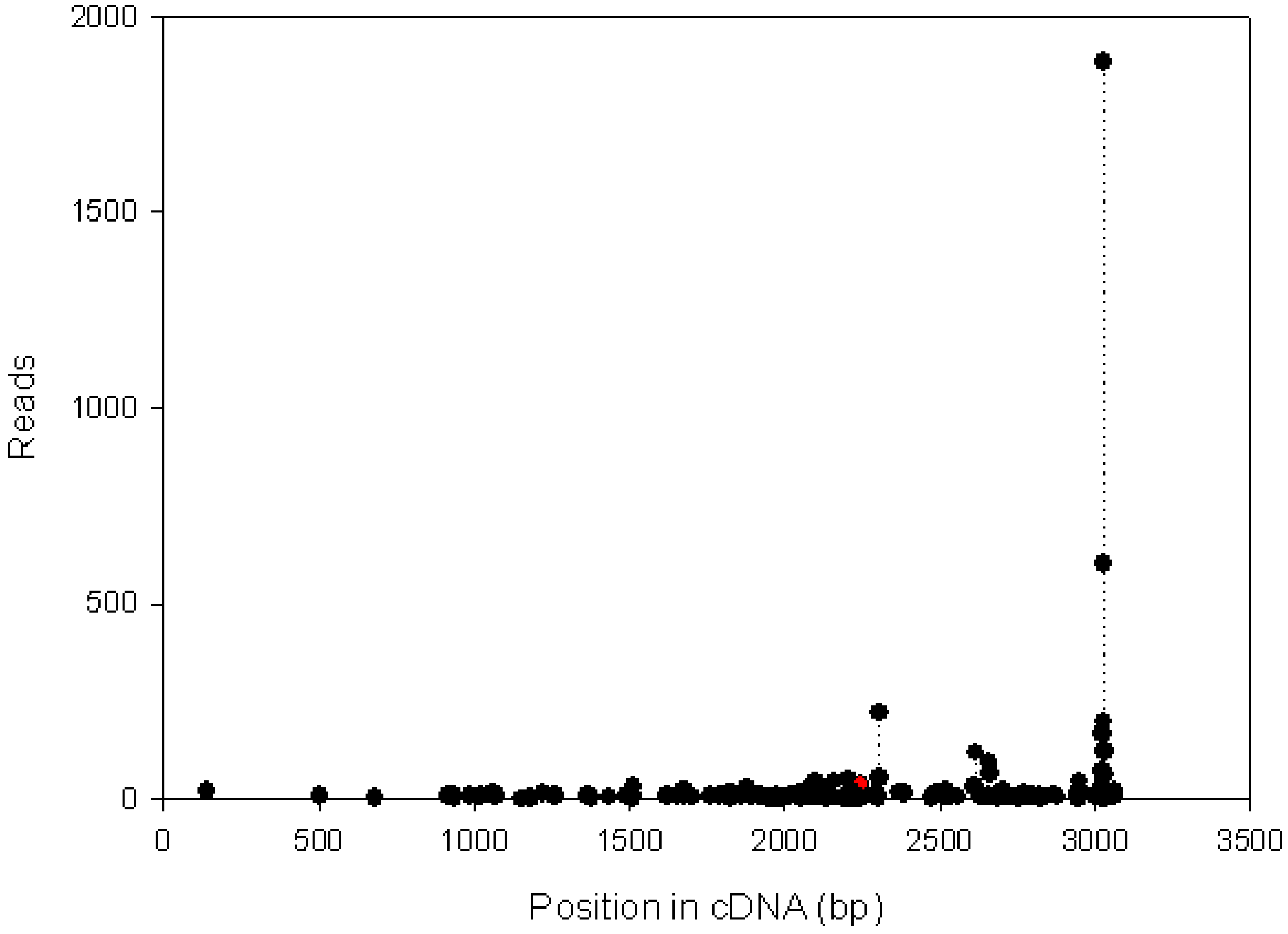


```
5' AGAAGGUUCCUCUGCUGGGGUGCCAU 3'        Cs1g15460.1
    :: :::::: : ::::::::::
3' -CUACCAAGGUG-CGACCCCACGC-- 5'        Csi-miR536-5p
```

Csi-miR827-5p.1, target=Cs1g24800.1 gene=Cs1g24800
Category=3
Score=3.5
Cleavage Site=2095

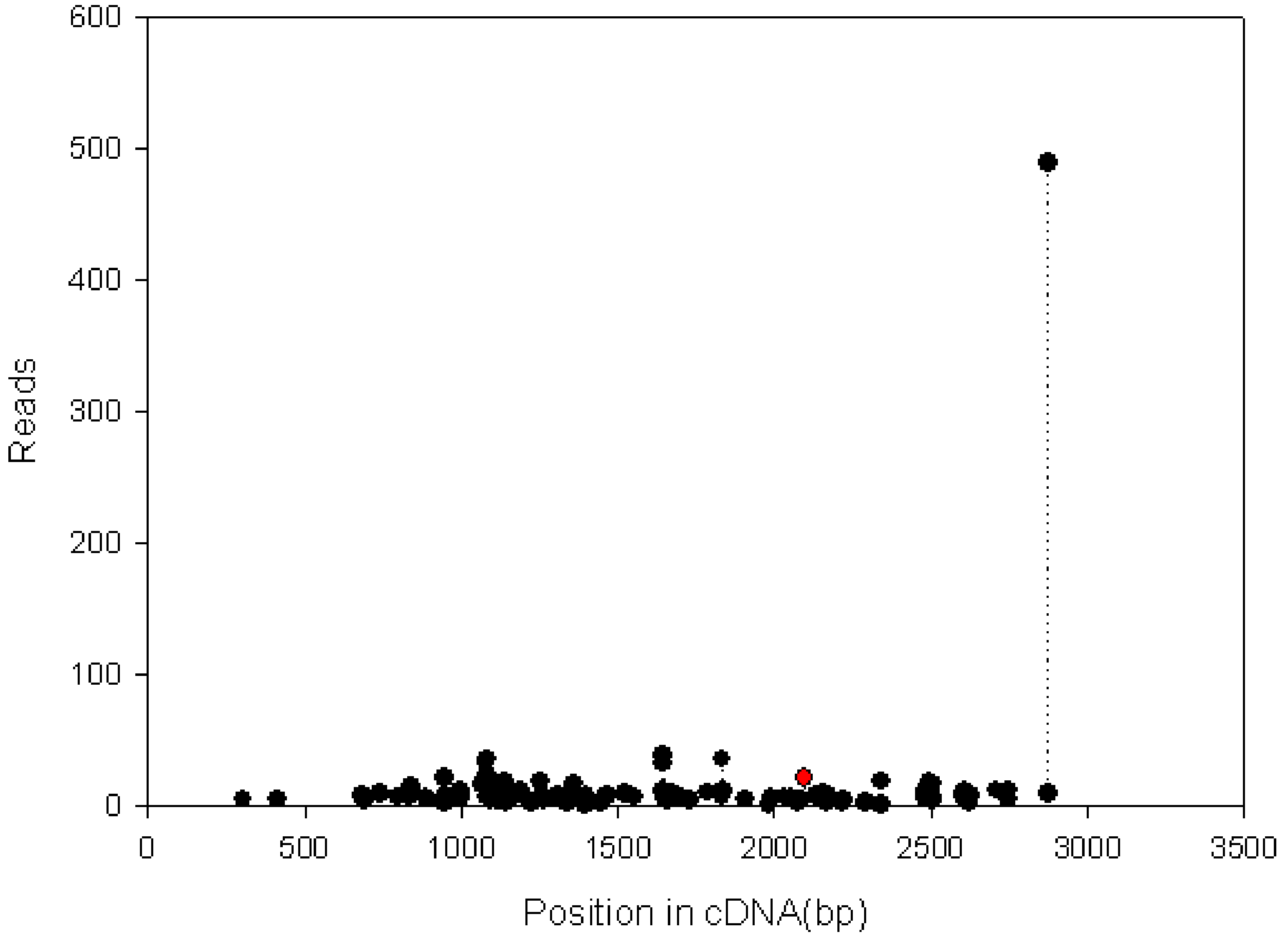


```
5' UGCGAGUGCAUGACAAUCAACAAAUG 3'        Cs1g24800.1
      :: :. ::::::::::::::
3' ---CUAAUCUACUGUUAGUUGUUC-- 5'        Csi-miR827-5p.1
```

Csi-miR827-5p.2, target=Cs9g10630.1 gene=Cs9g10630
Category=3
Score=4.5
Cleavage Site=194

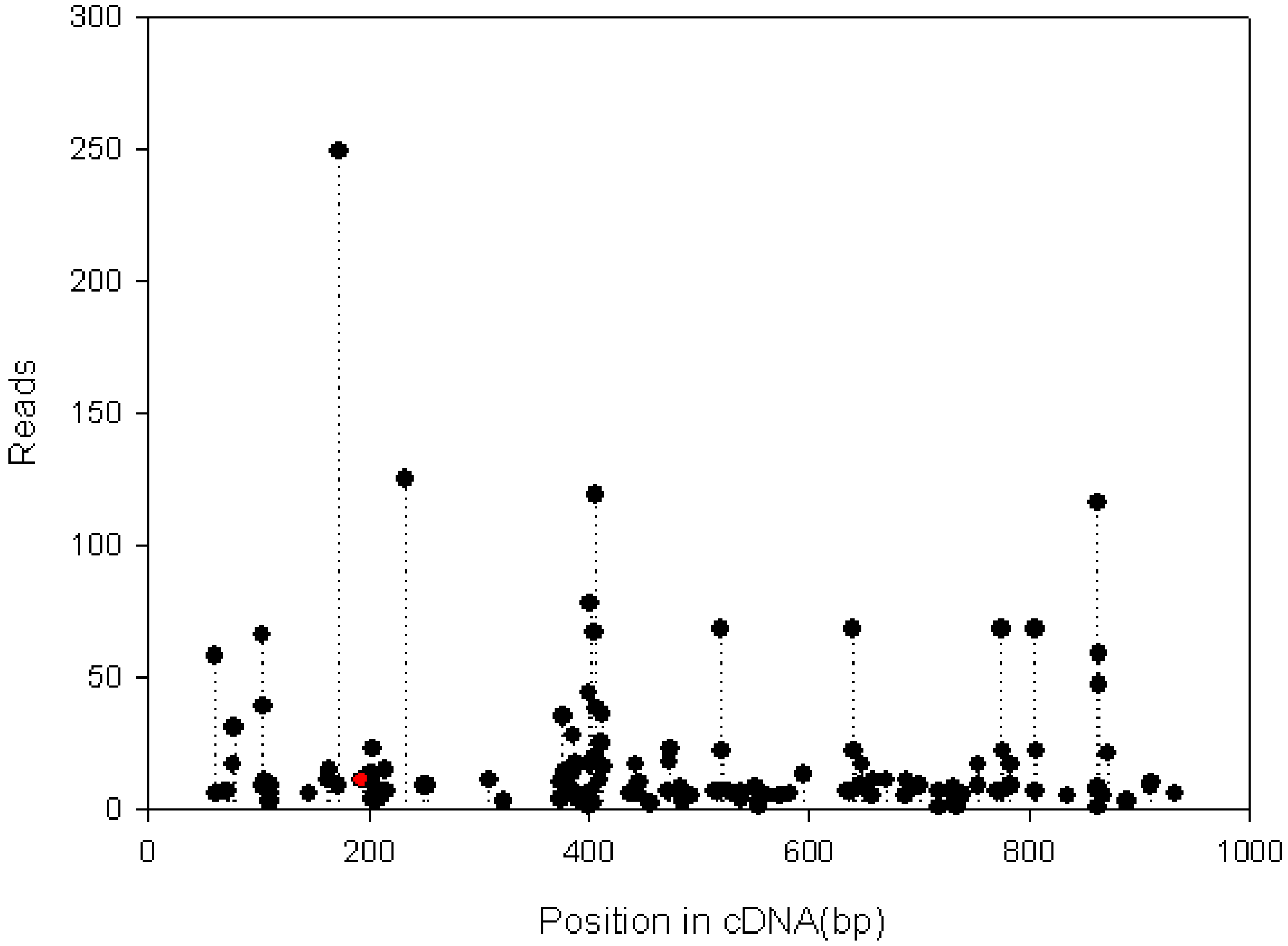


```
5' GCCUACAAG-CAAUCAACAAGCGAAUG 3'        Cs9g10630.1
     :: : : ::::::::::::.
3' --AAUCUACUGUUAGUUGUUCGU---- 5'        Csi-miR827-5p.2
```

Csi-miR828, target=Orange1.1t00180.1 gene=Orange1.1t00180
Category=3
Score=5
Cleavage Site=652

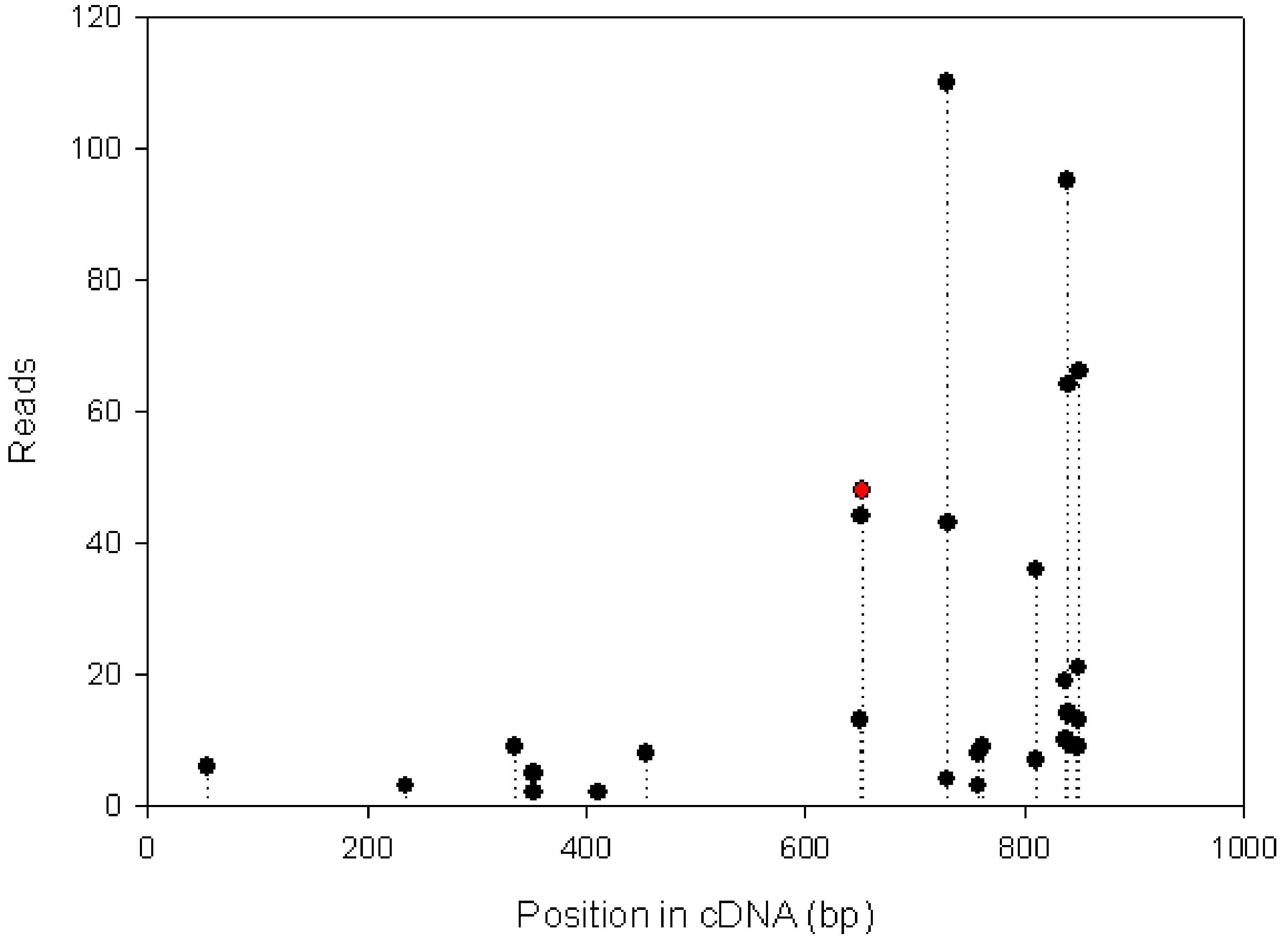


```
5' AGCUACUUG-CUAAAUGGGUAUGCUCU 3'        Orange1.1t00180.1
     :   :::: ::::::::.::::
3' --GCAGAACGGAUUUACUCAUAG---- 5'        Csi-miR828
```

Csi-miR833.1, target=Cs9g02420.1 gene=Cs9g02420
Category=2
Score=4.5
Cleavage Site=936

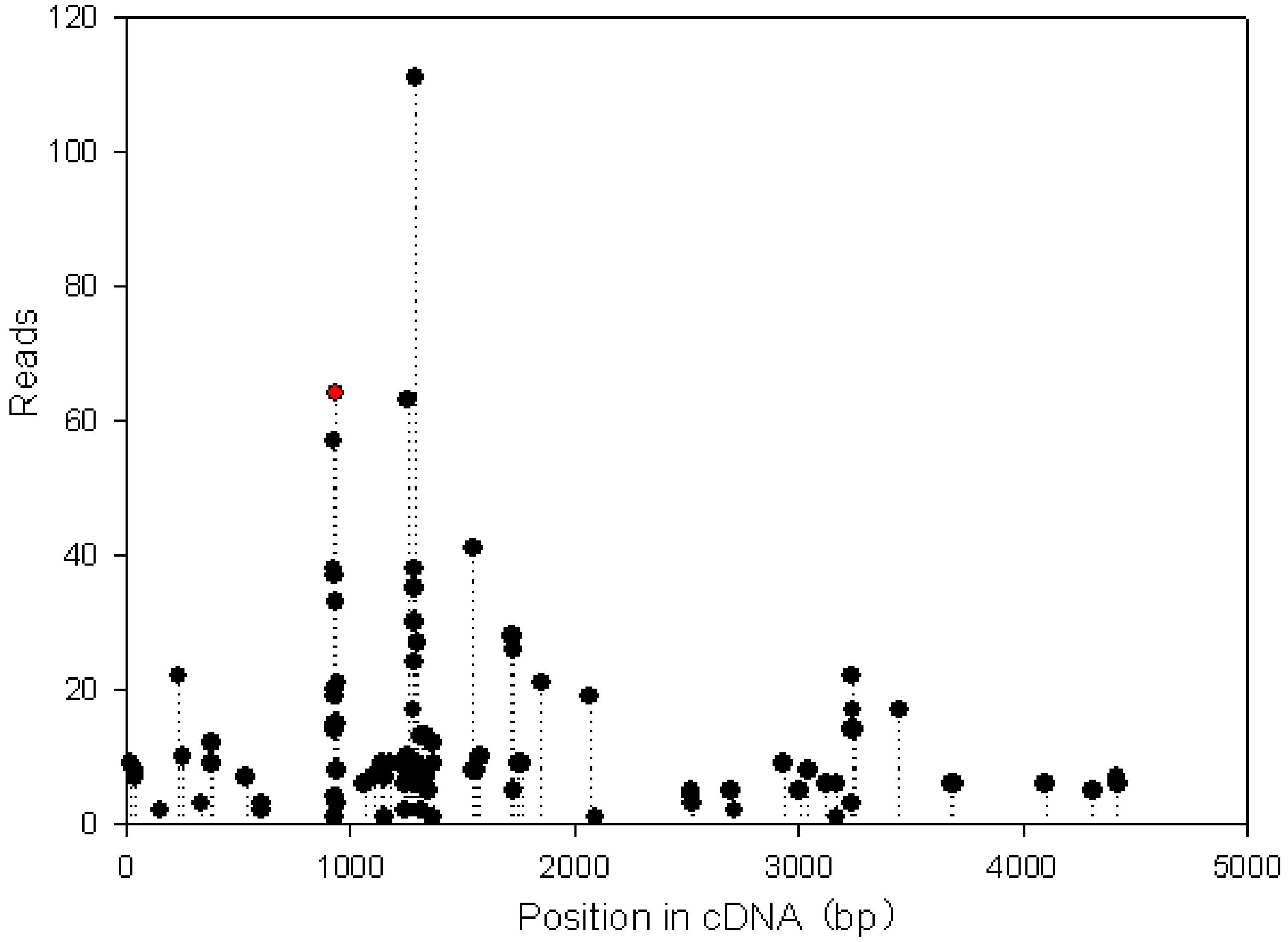


```
5' CUGUUCUGUA-CAACAAUAAAUUUUCC 3'       Cs9g02420.1
    :: .:..:: :::::::::::
3' -ACUGGGUAUAGUUGUUAUUUA----- 5'       Csi-miR833.1
```

Csi-miRN01, target=Cs5g31745.1 gene=Cs5g31745
Category=3
Score=5
Cleavage Site=148

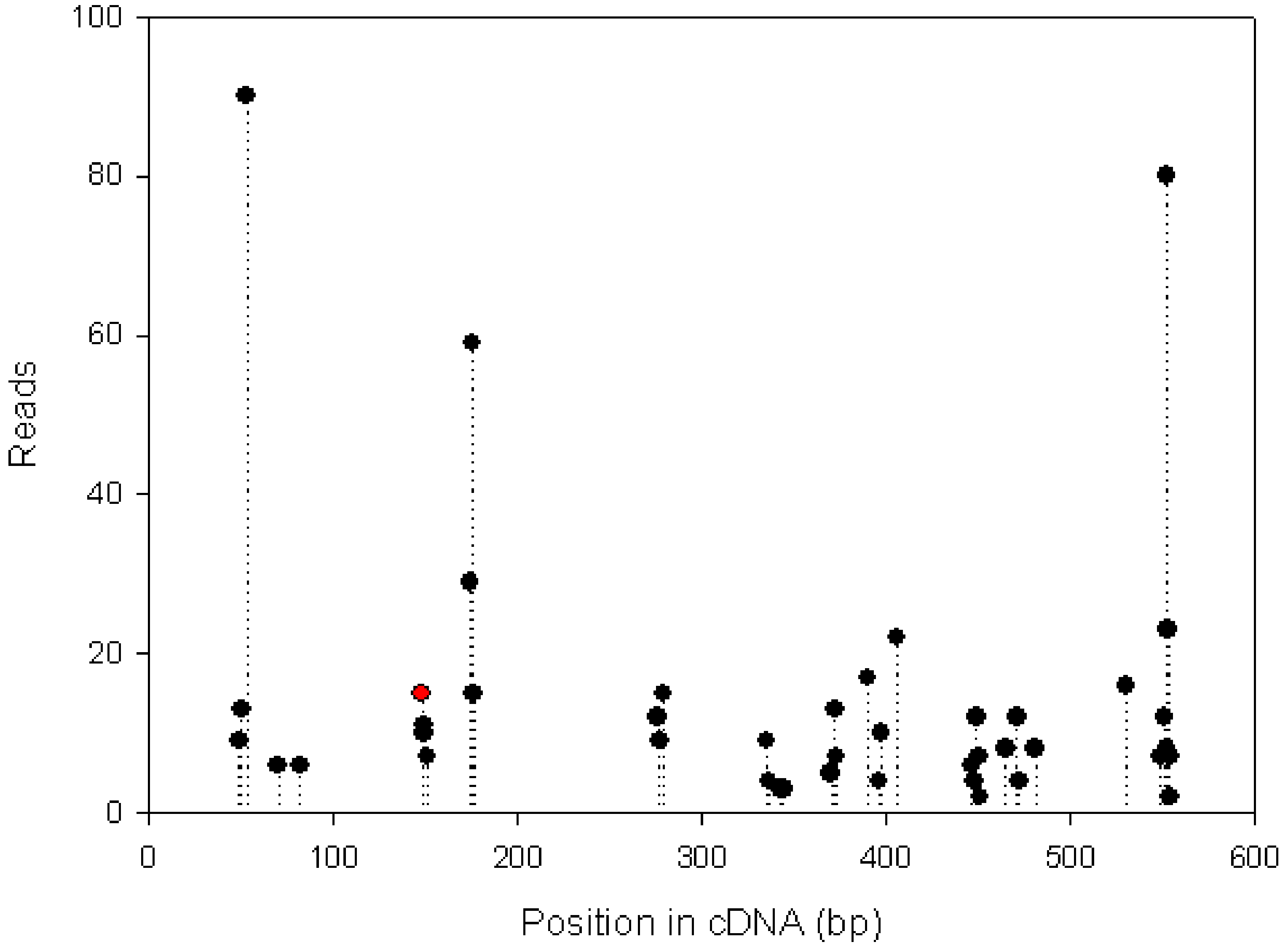


```
5' UGGUGGGCAGAGAAGUGACU-GUUGAG 3'       Cs5g31745.1
      : ..::::.:::::::: :::
3' ---AAUUGUCUUUUCACUGAGCAA--- 5'       Csi-miRN01
```

Csi-miRN02, target=Cs3g18790.1 gene=Cs3g18790
Category=2
Score=2
Cleavage Site=1819

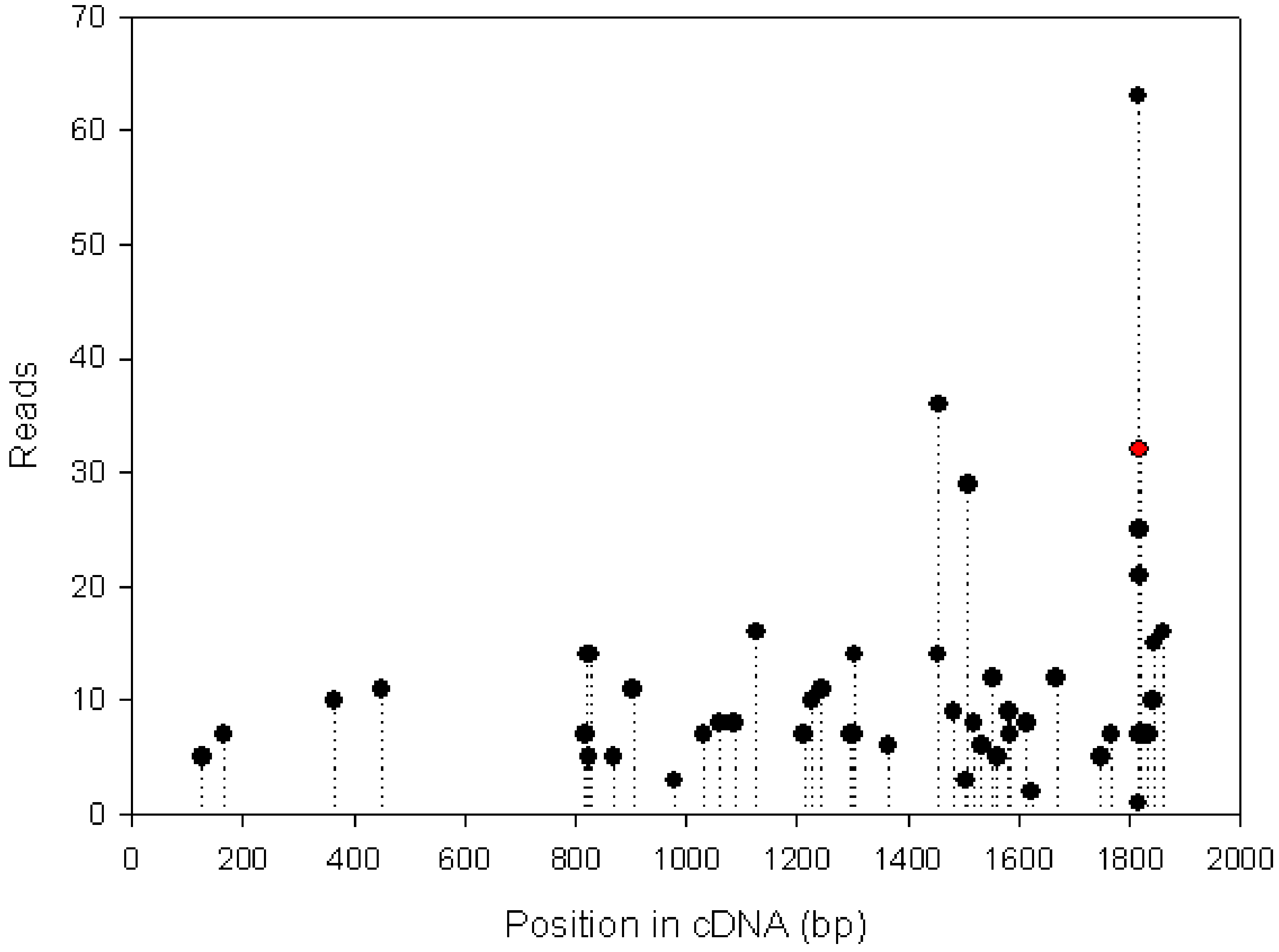


```
5' GCAACUGUGGCACGGUACCACAGUUG 3'        Cs3g18790.1
       ::::::::::::: ::::::
3' ----GACACCGUGCCAUGAUGUCAA- 5'        Csi-miRN02
```

Csi-miRN03, target=Cs5g26760.1 gene=Cs5g26760
Category=3
Score=5
Cleavage Site=457

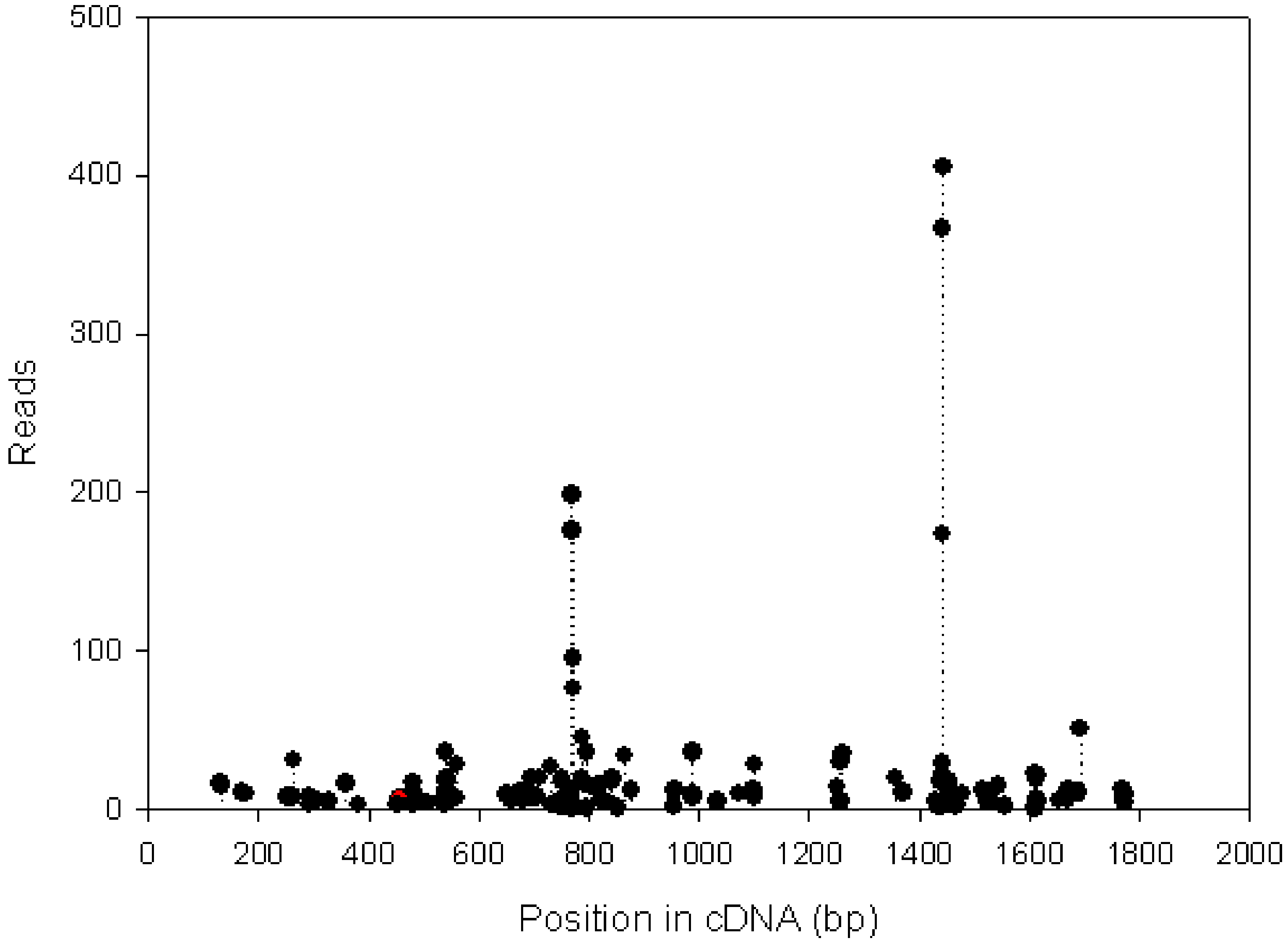


```
5' UAUAGACAACUUUUUCACCCAGAGCA 3'        Cs5g26760.1
       .::: :. :::::::::.:.::
3' ----UUGUGGGCAAAGUGGGUUUUGU 5'        Csi-miRN03
```

Csi-miRN04, target=Orange1.1t00471.1 gene=Orange1.1t00471
Category=4
Score=4
Cleavage Site=2386

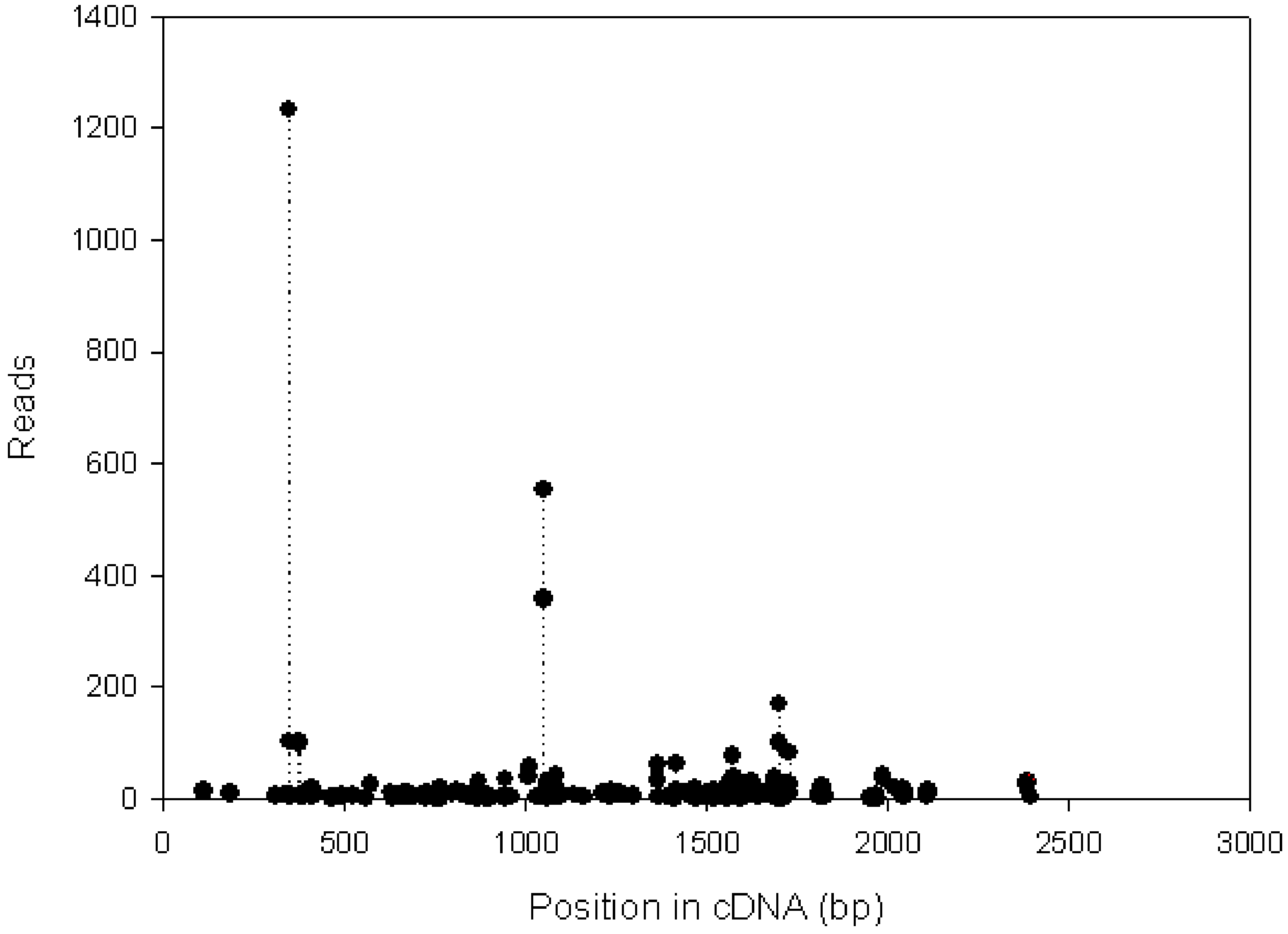


```
5' CAUGUAACAUACAUAUUCCACAACAA 3'        Orange1.1t00471.1
     :::::::::::.:.:: ::::
3' --ACAUUGUAUGUGUGAGAUGUU--- 5'        Csi-miRN04
```

Csi-miRN05, target=Cs4g19940.1 gene=Cs4g19940
Category=3
Score=3.5
Cleavage Site=3041

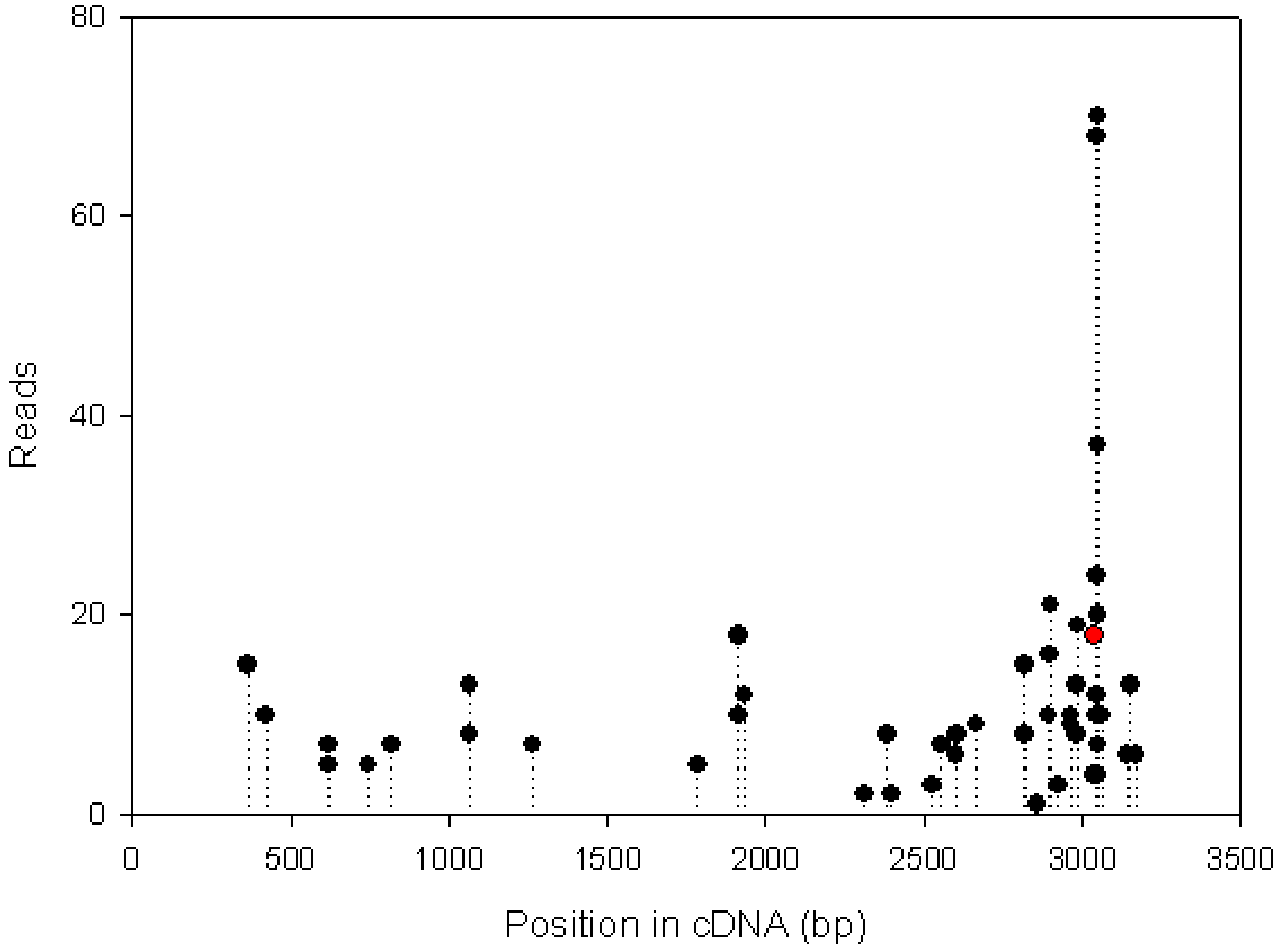


```
5' AAGCUAGGAAUGUAUCUUACAUUAUA 3'        Cs4g19940.1
     :  ::.:::::::::::::::
3' -ACUCUCUUUACAUAGAAUGUA---- 5'        Csi-miRN05
```

Csi-miRN07, target=Cs8g09620.1 gene=Cs8g09620
Category=1
Score=4
Cleavage Site=357

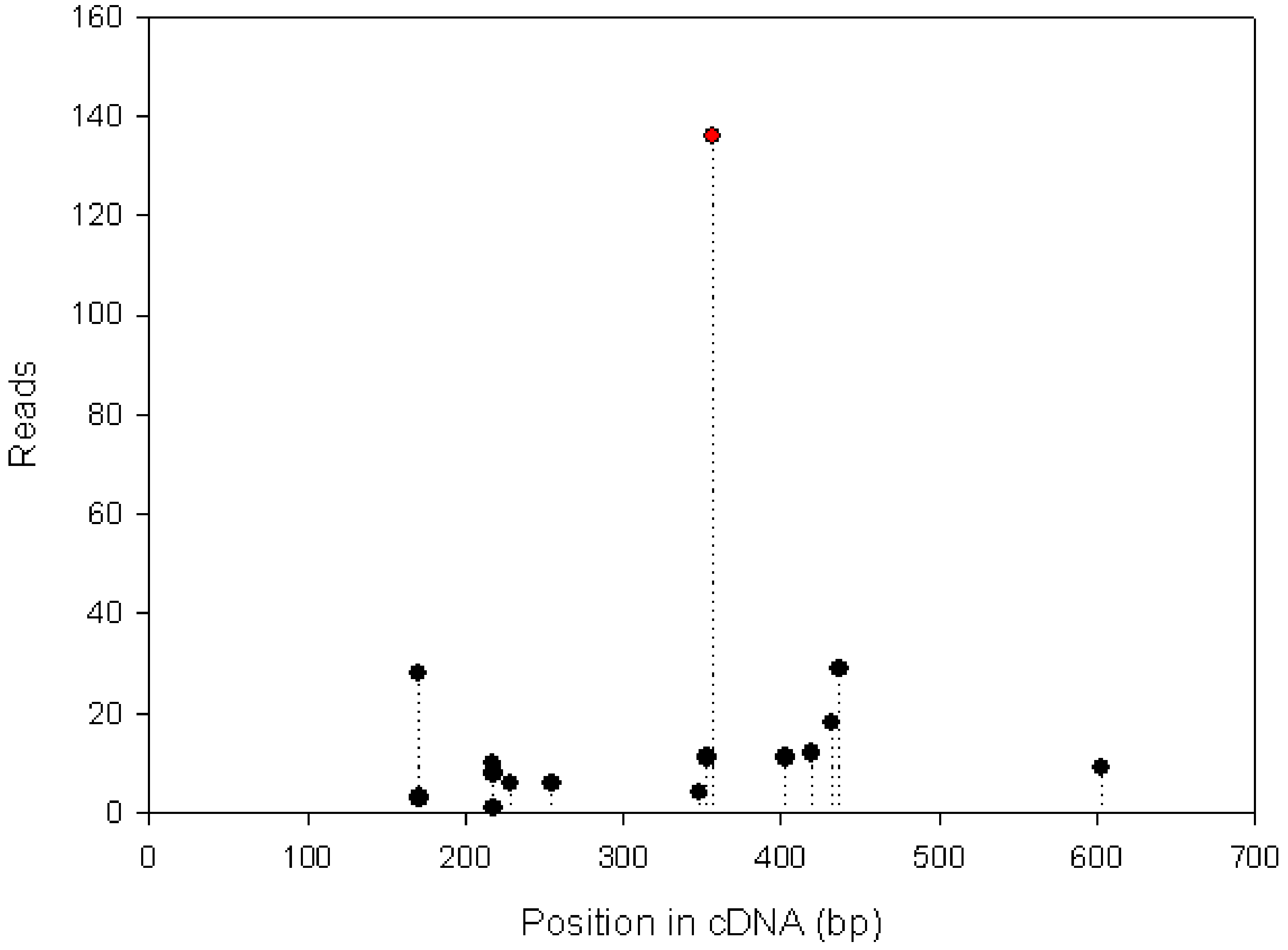


```
5' ACACCGUUUCAUCUUGUCCUCCAGAA 3'        Cs8g09620.1
    :::::::: :::::::: :::
3' -GUGGCAAACUAGAACAGAAGG---- 5'        Csi-miRN07
```

Csi-miRN10, target=Cs9g04330.1 gene=Cs9g04330
Category=3
Score=5
Cleavage Site=1822

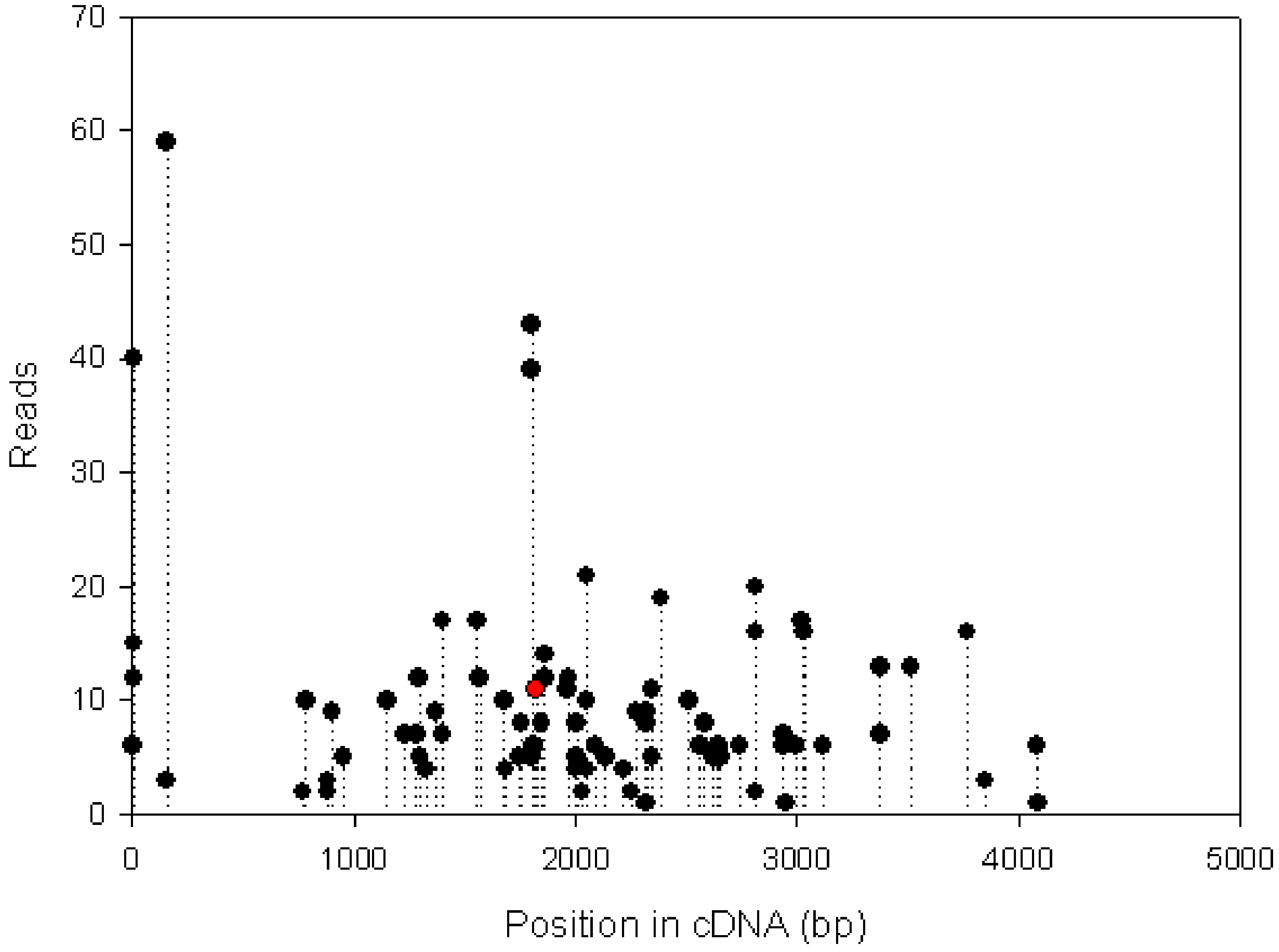


```
5' AUCAUGUGA-GCAUACACUCAAUACUU 3'        Cs9g04330.1
        ::::.:  .  :.::::::::::
3' ----ACAUUGUAUGUGUGAGUUAUU-- 5'        Csi-miRN10
```

Csi-miRN11, target=Cs8g13560.1 gene=Cs8g13560
Category=3
Score=2
Cleavage Site=302

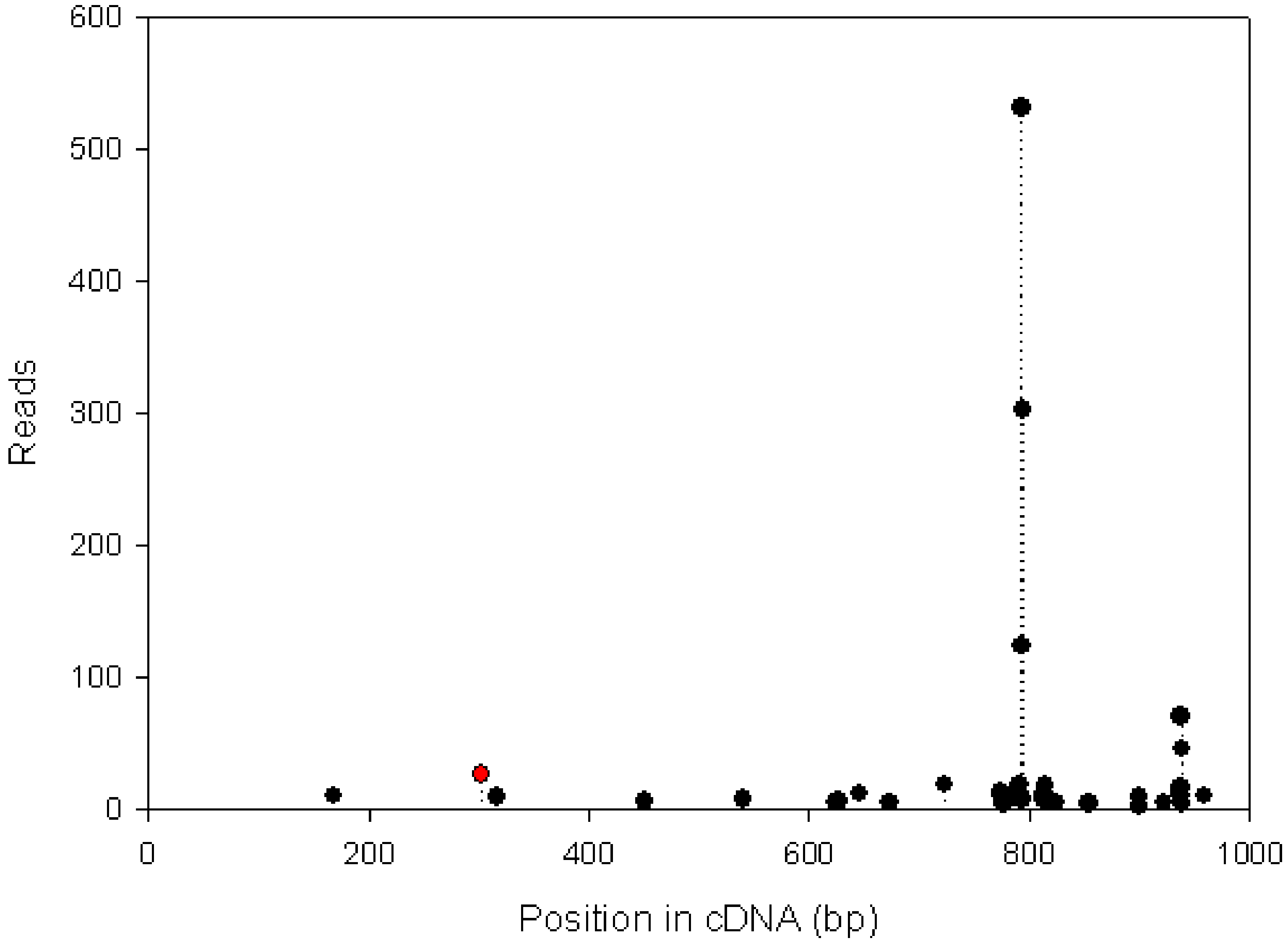


```
5' GUACCGUAAAAAGCUCCUGCGACAUG 3'        Cs8g13560.1
    :::::: :::::::::::::
3' -UUGGCAUCUUUCGAGGACGCU---- 5'        Csi-miRN11
```

Csi-miRN12, target=Cs4g06030.1 gene=Cs4g06030
Category=2
Score=5
Cleavage Site=2071

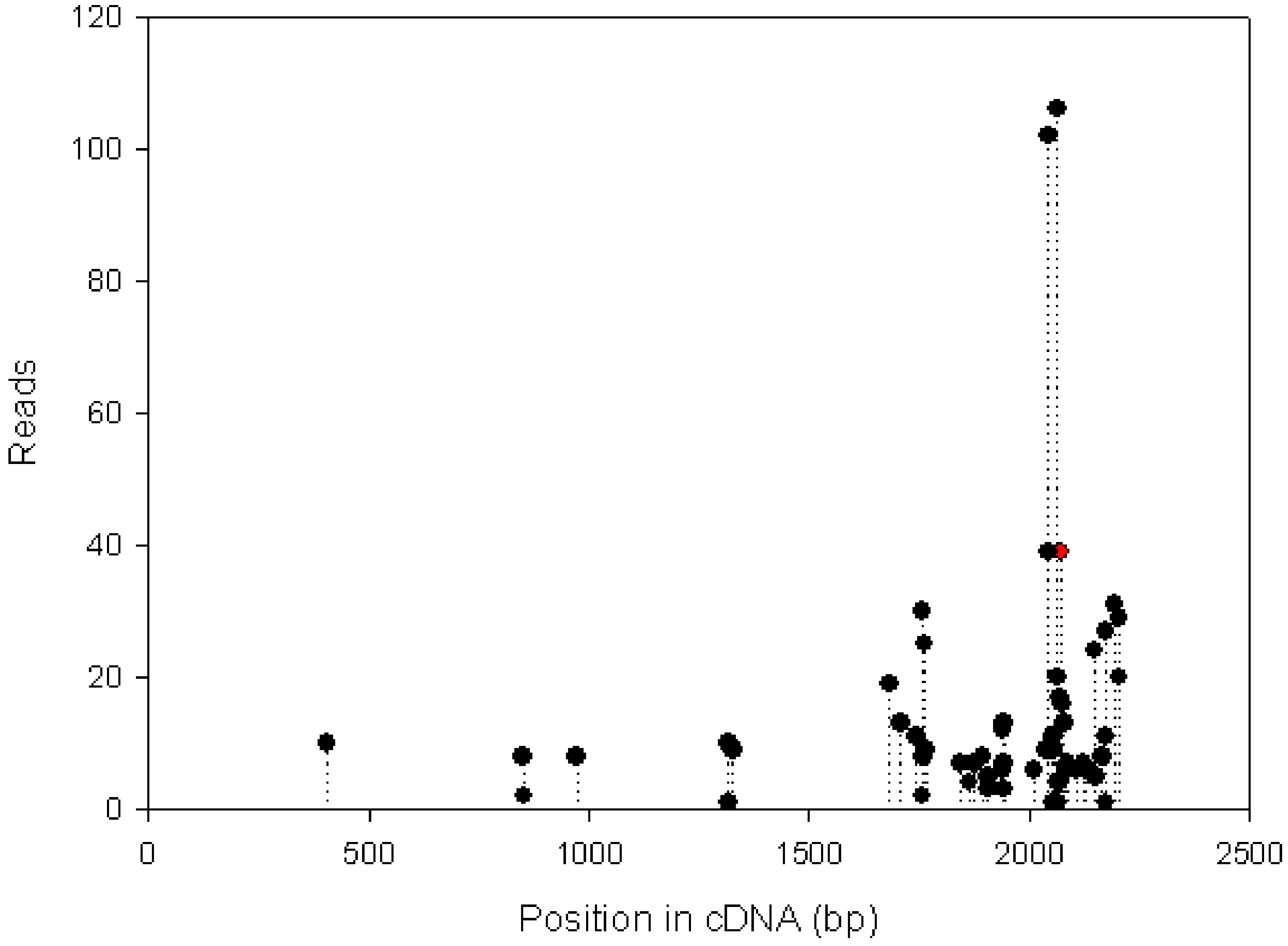


```
5' UGAGCUCAGUGGCAUAGCCAUUGAAA 3'        Cs4g06030.1
      :: :::: ::::.::::::::
3' ---AGAAUCACGGUAUUGGUAACU-- 5'        Csi-miRN12
```

Csi-miRN14, target=Cs7g14990.1 gene=Cs7g14990
Category=3
Score=4.5
Cleavage Site=2345

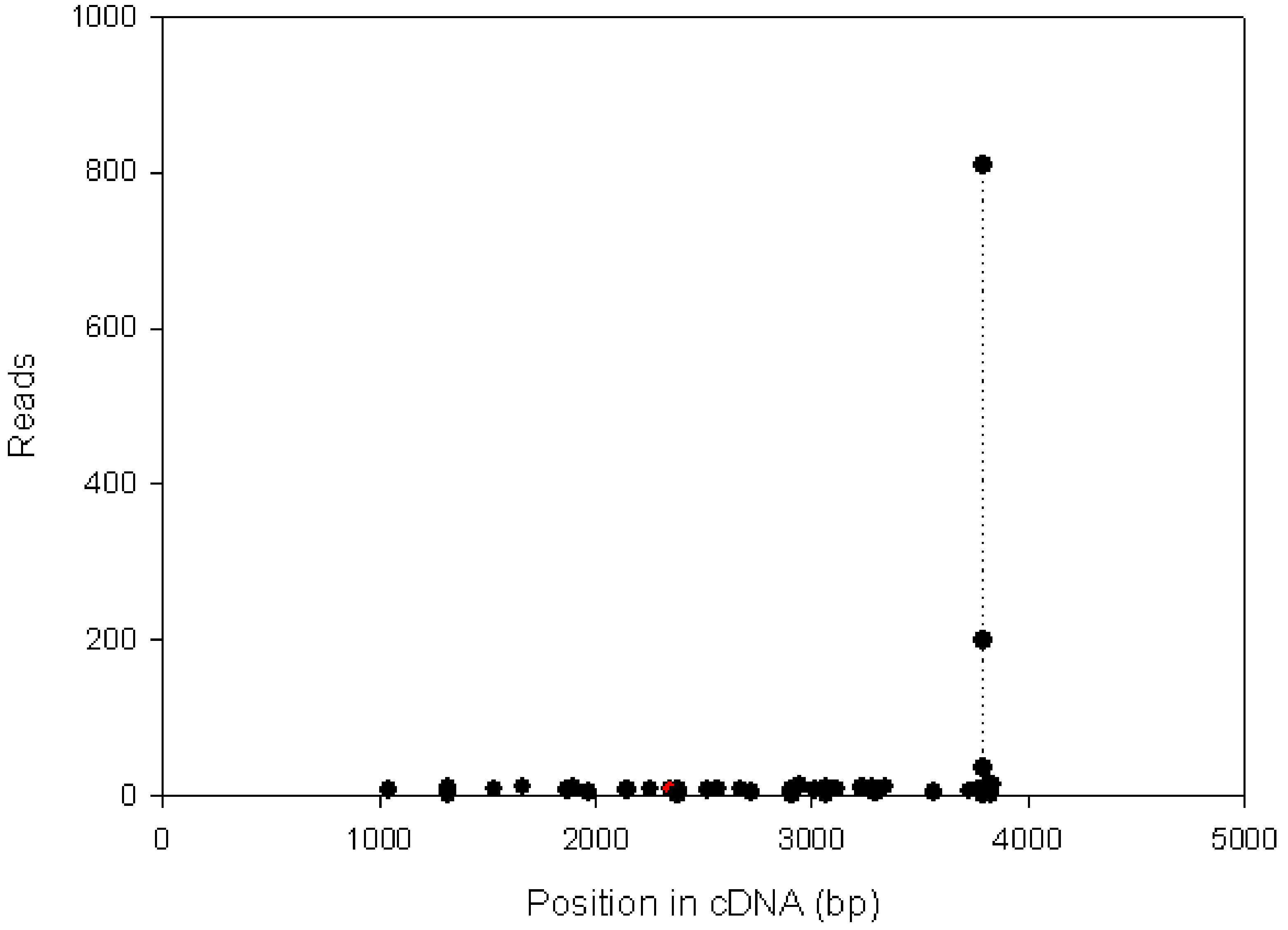


```
5' GAUGAAUAUUU-UUAUCAGGACUAUGA 3'       Cs7g14990.1
     :::: .:: :::::::.:::::::
3' ---CUUAAGAAUAAUAGUUCUGAUACG 5'       Csi-miRN14
```

Csi-miRN16, target=Cs7g06555.1 gene=Cs7g06555
Category=3
Score=5
Cleavage Site=132

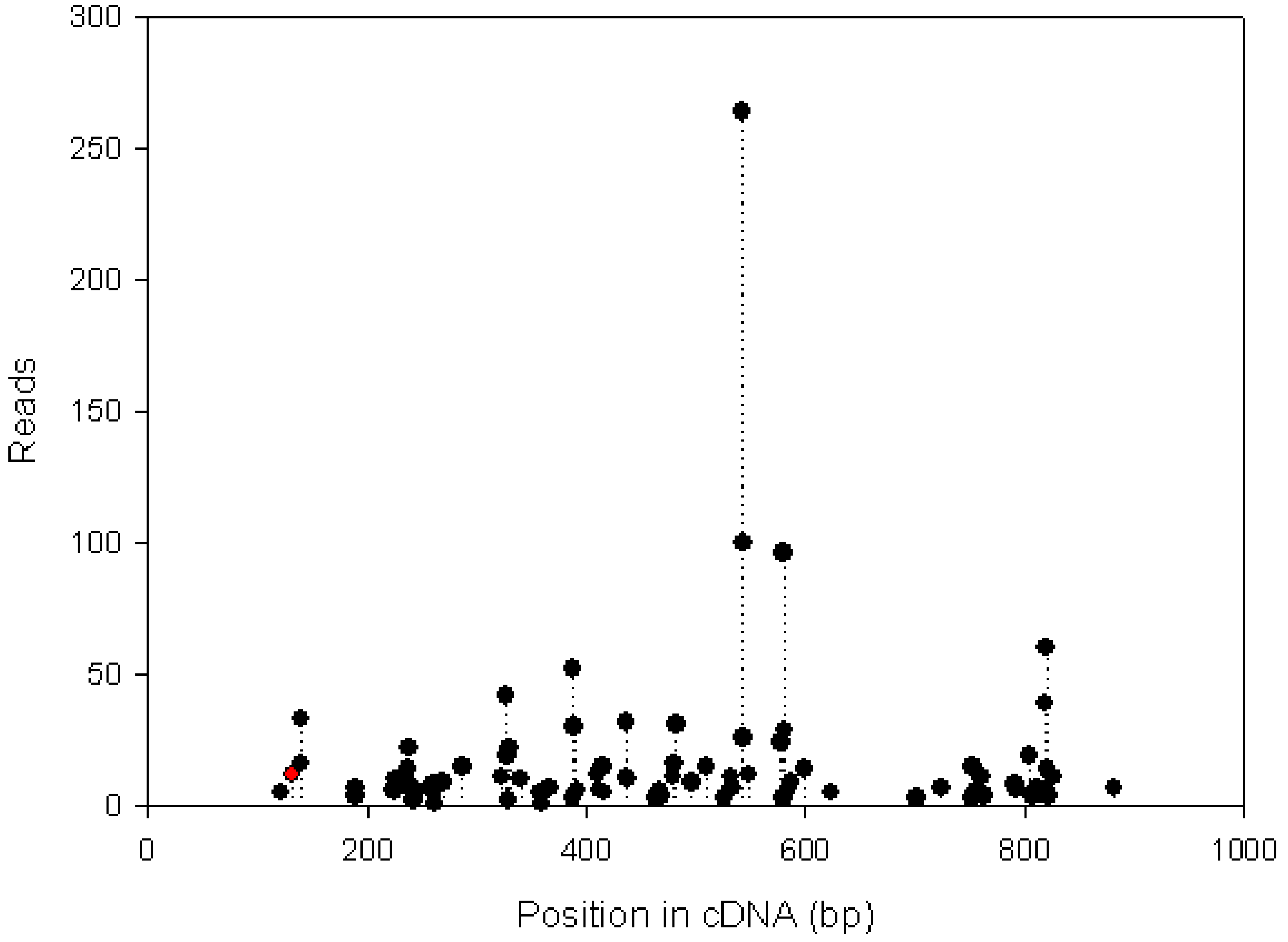


```
5' CCUUCAUUUUUUGUUCUCUUAUCAGC 3'        Cs7g06555.1
         ::. ::.:.:::::.:::::
3' ----AUAGUAAGCGAGAGAGUAGUC- 5'        Csi-miRN16
```

Csi-miRN17, target=Orange1.1t00471.1 gene=Orange1.1t00471
Category=3
Score=2
Cleavage Site=2386

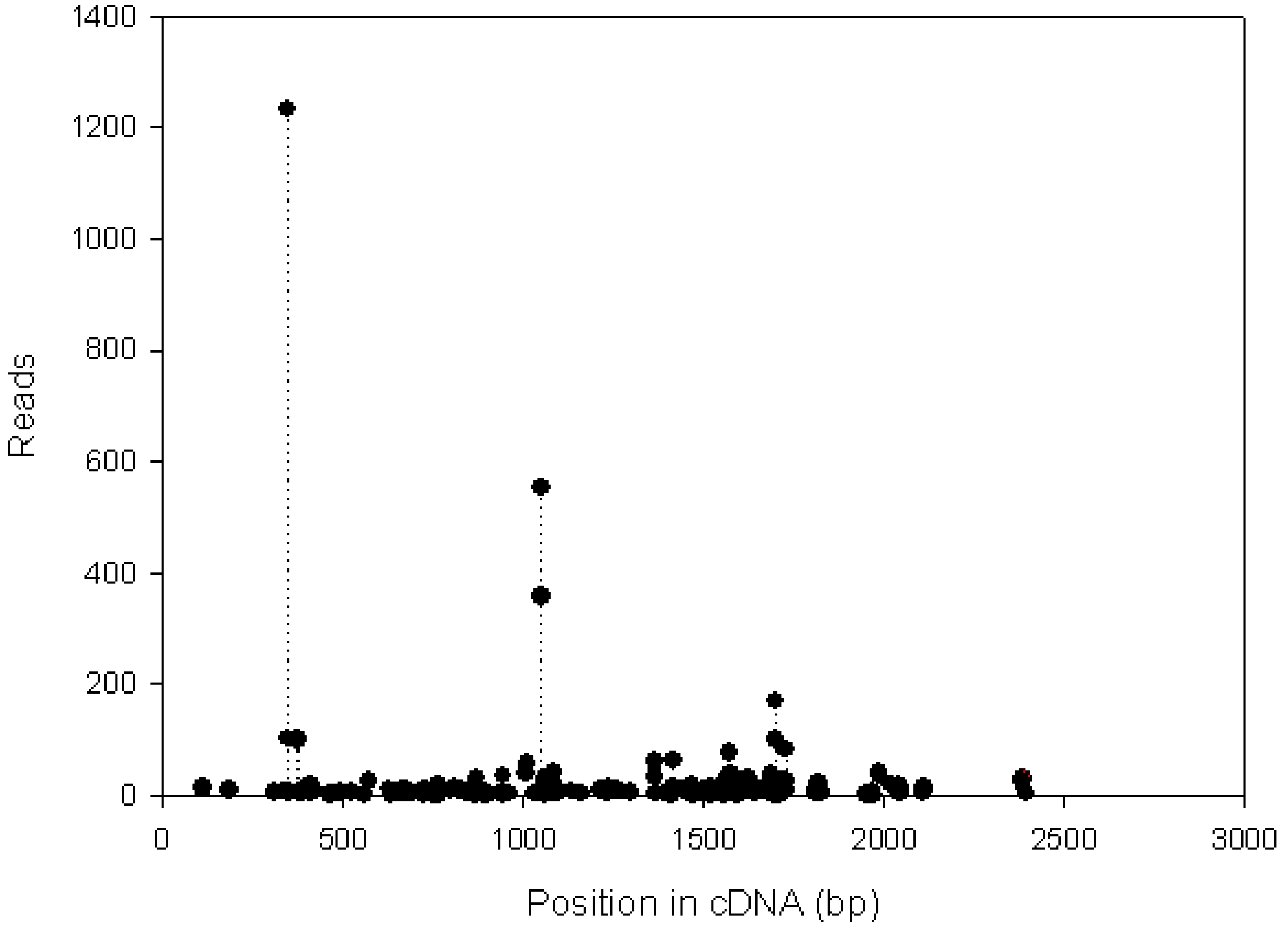


```
5' CAUGUAACAUACAUAUUCCACAACAA 3'        Orange1.1t00471.1
     ::::::::::.:.:::::::
3' --ACAUUGUAUGUGUGAGGUGUU--- 5'        Csi-miRN17
```

Csi-miRN18, target=Cs7g24200.1 gene=Cs7g24200
Category=3
Score=5
Cleavage Site=1104

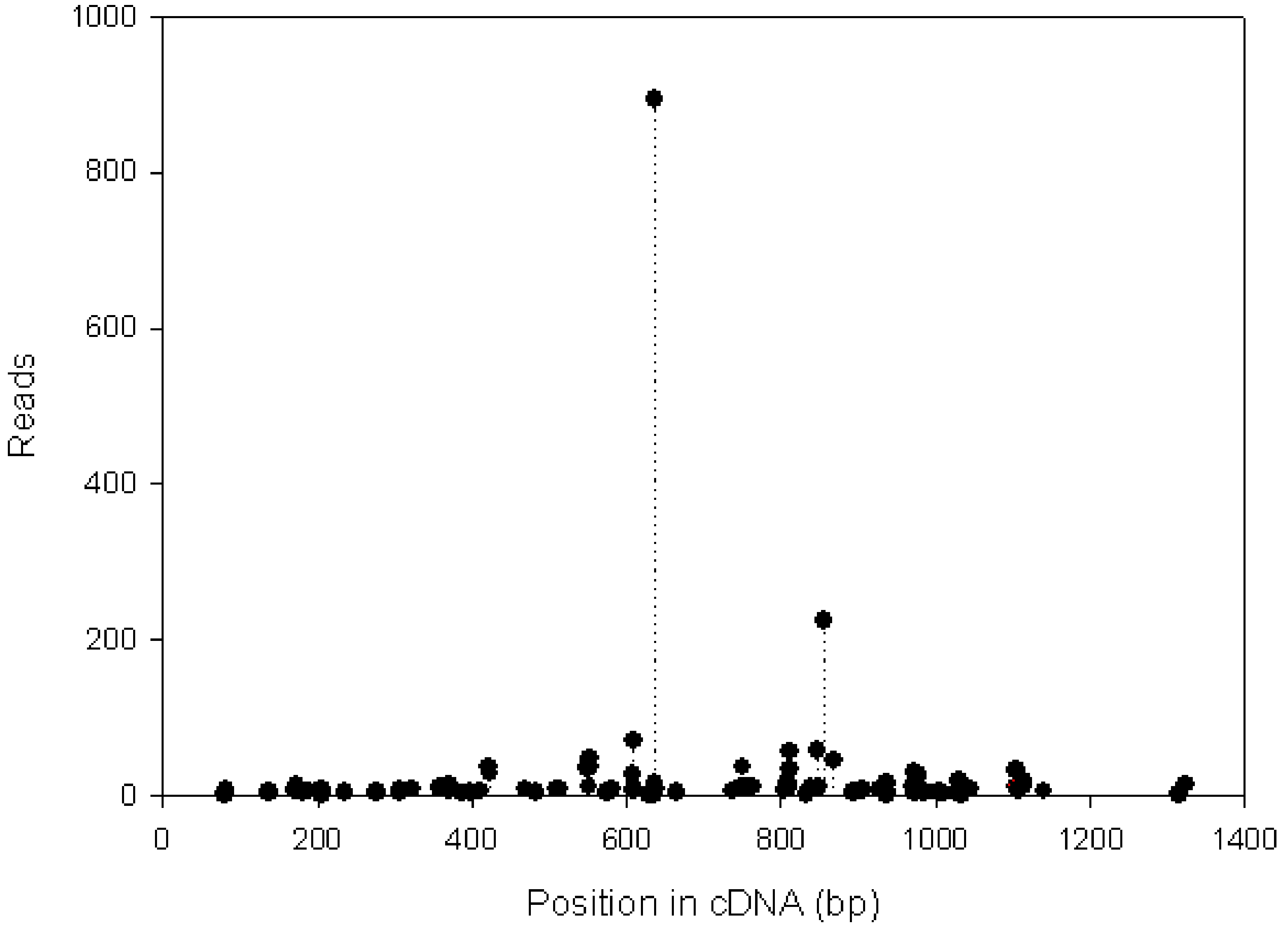


```
5'  CAUAAUAGGGUUGCAUCCAGUUAGGA 3'          Cs7g24200.1
    :  ::  .:  .:::::::::::.
3'  GCAUGGUGUCAACGUAGGUCG----- 5'          Csi-miRN18
```

Csi-miRN19, target=Cs5g32800.1 gene=Cs5g32800
Category=3
Score=5
Cleavage Site=1085

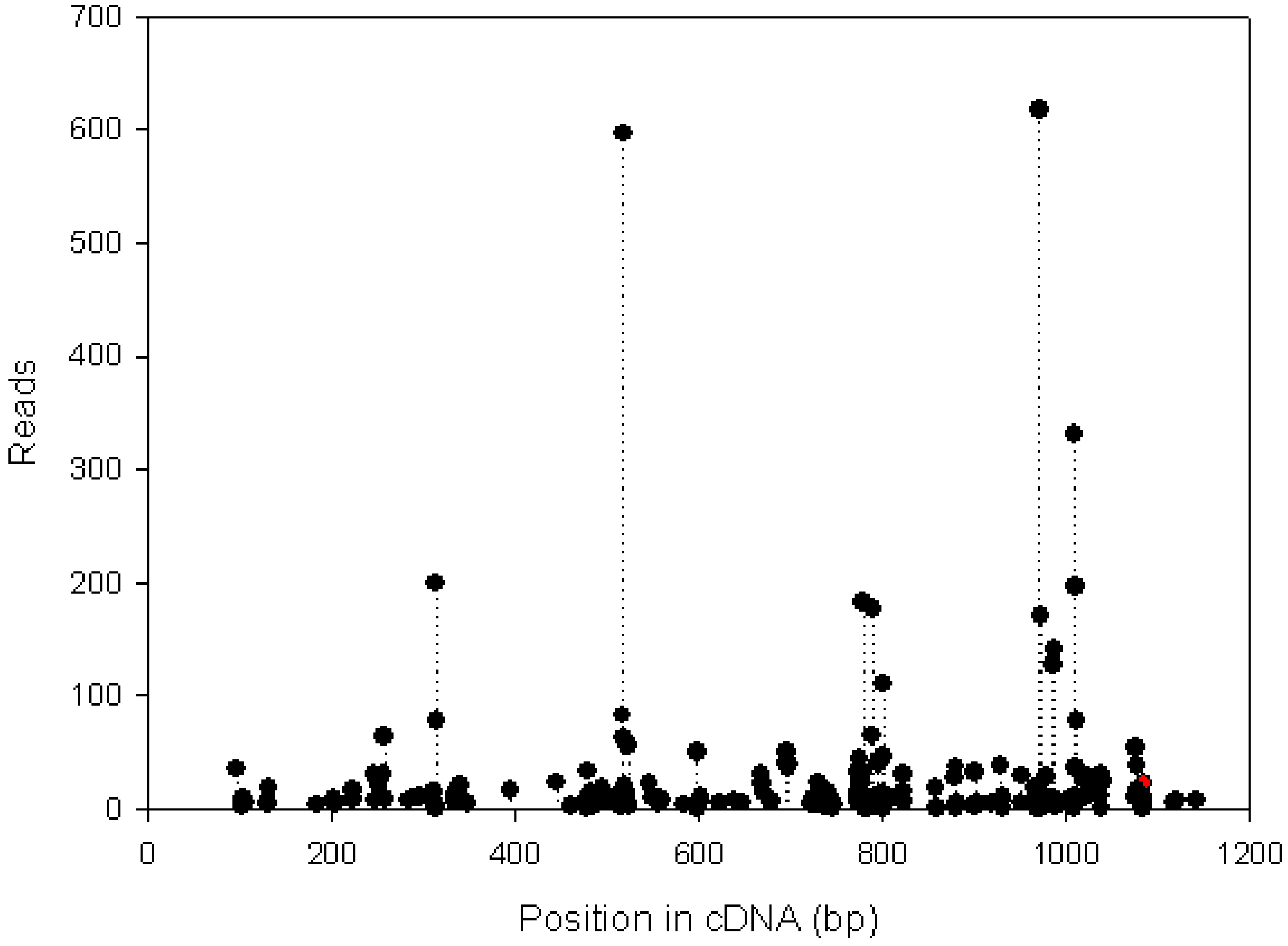


```
5' UAAAAUUUAG-UAUUCAAUGAACGACU 3'        Cs5g32800.1
        :::::    :::::.::::::
3' -----AAAUCUCAAAGUUGCUUGCUU- 5'        Csi-miRN19
```

Csi-miRN20, target=Cs3g05320.1 gene=Cs3g05320
Category=3
Score=0
Cleavage Site=451

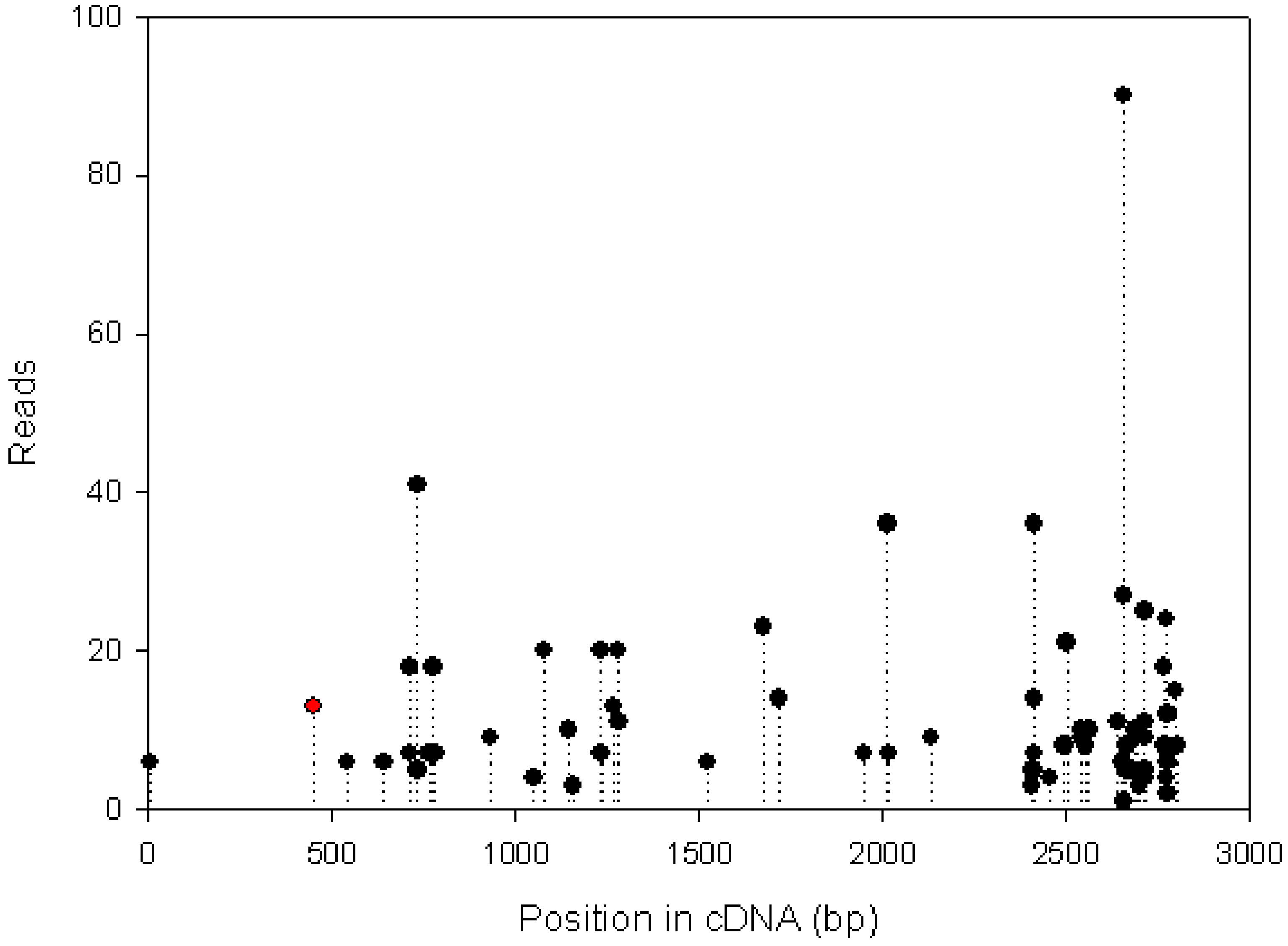


```
5' UUAUCAGCAUCAUGCAACAAAAUAGU 3'         Cs3g05320.1
   ::::::::::::::::::::::
3' AAUAGUCGUAGUACGUUGUUUU---- 5'         Csi-miRN20
```

Csi-miRN23, target=Cs1g07330.1 gene=Cs1g07330
Category=3
Score=5
Cleavage Site=130

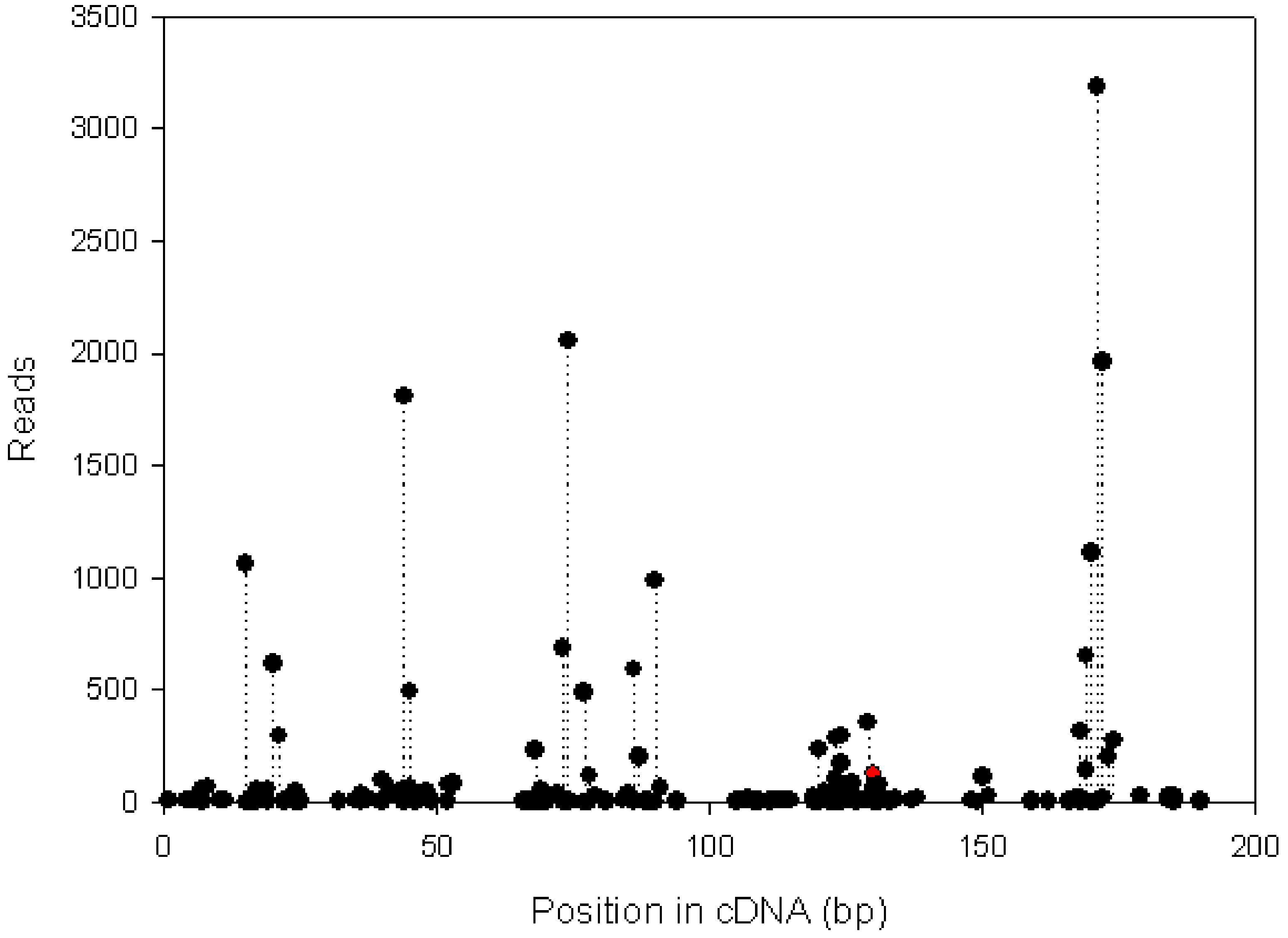


```
5' GUCAUGGACGUU-CAGGCUGCUGAGAC 3'        Cs1g07330.1
          :::::..  :::::::::::.:
3' ------CUGCGGCCUCCGACGACUUUC 5'        Csi-miRN23
```

Csi-miRN24, target=Cs4g01970.1 gene=Cs4g01970
Category=3
Score=4.5
Cleavage Site=2471

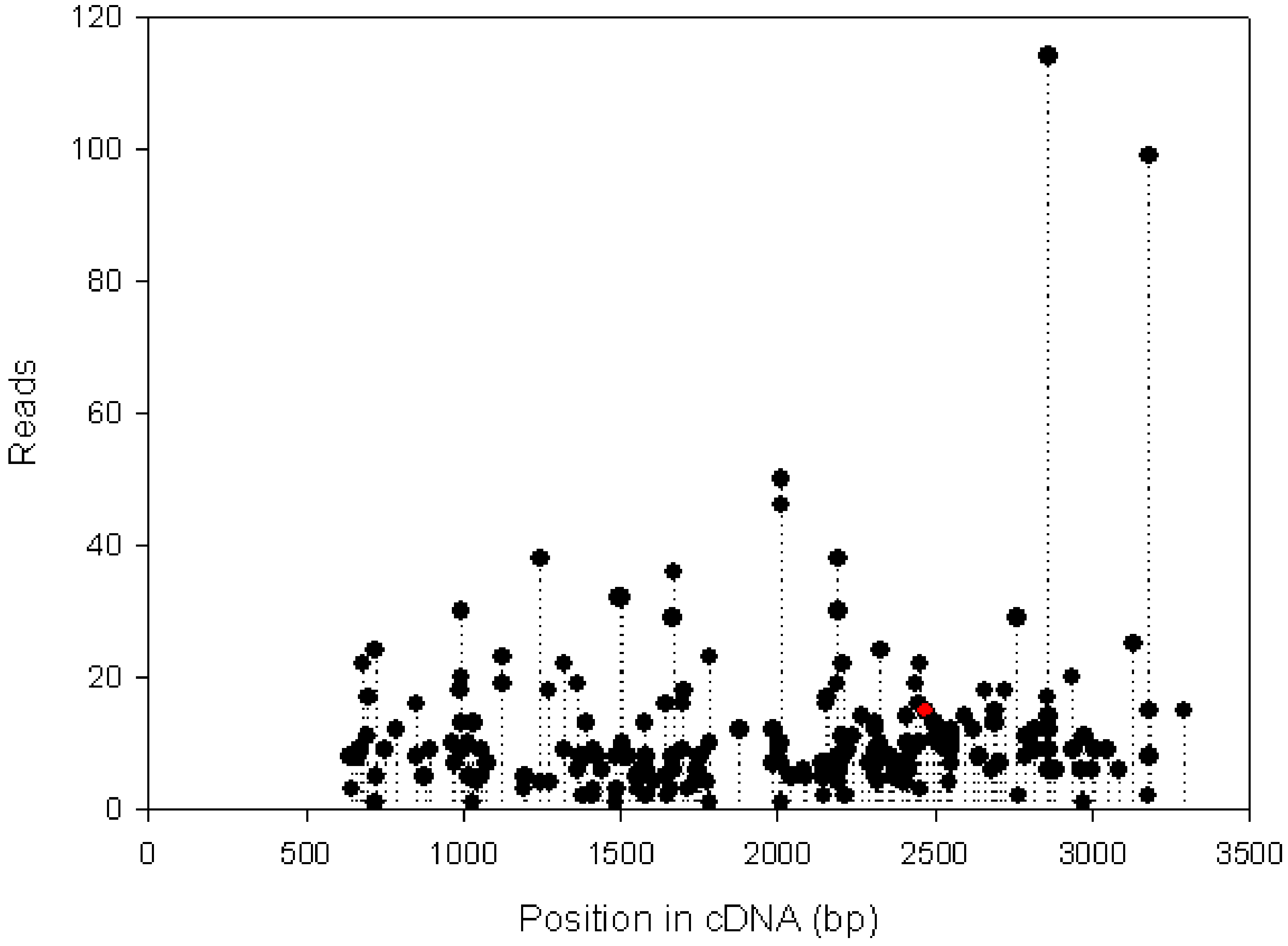


```
5' GAGACAUUGGGACGGGAUGUUUGCUC 3'          Cs4g01970.1
         ::::.:   :.:::::.:::::
3' -----UAAUCGGGUCCUACGAACGA- 5'          Csi-miRN24
```

Csi-miRN26, target=Cs9g18000.1 gene=Cs9g18000
Category=3
Score=5
Cleavage Site=3291

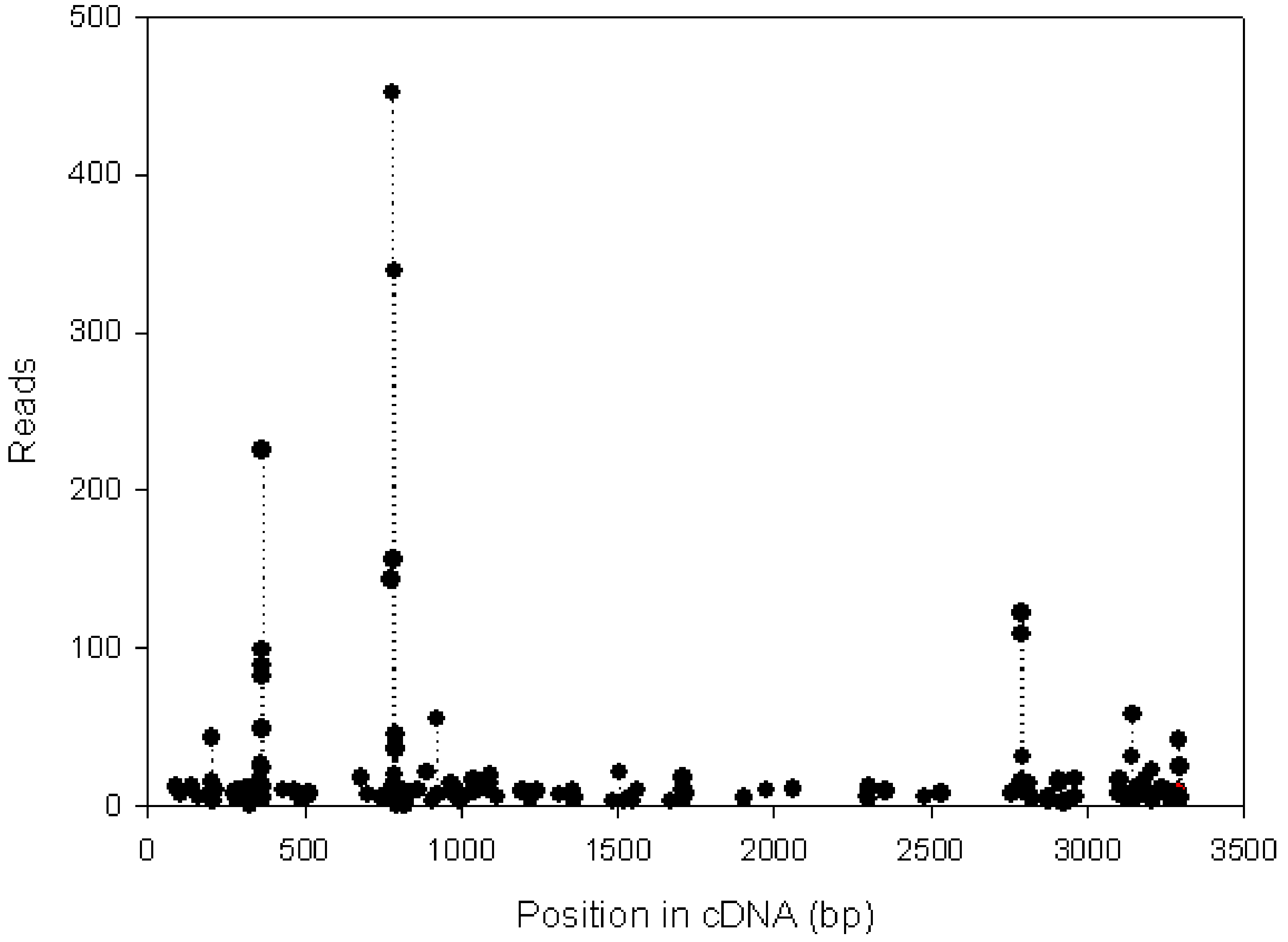


```
5' GUUCA-ACUUGUUCUGCCUCCUGUAAA 3'        Cs9g18000.1
    :::: : ::::::..::::::::
3' AAAGUAUAAACAAGGUGGAGGACA--- 5'        Csi-miRN26
```

Csi-miRN28, target=Cs3g17940.1 gene=Cs3g17940
Category=3
Score=5
Cleavage Site=610

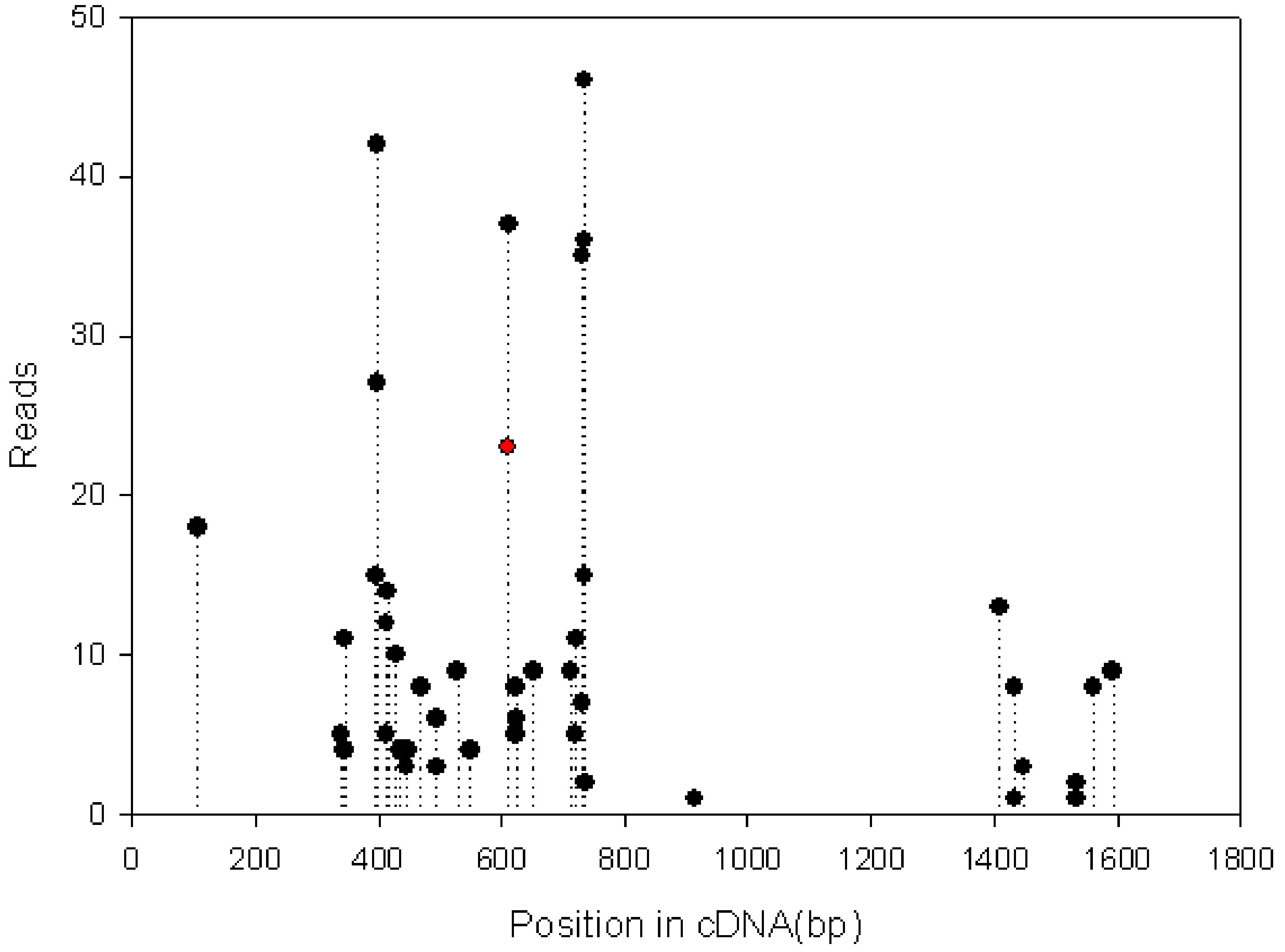


```
5' ACUGGAAGGCUACUCCCCAUAACUCU 3'          Cs3g17940.1
   ::.:    .:::::::::::::
3' UGGCGCGUCGAUGAGGGGUAC----- 5'          Csi-miRN28
```

Csi-miRN33, target=Cs4g07210.1 gene=Cs4g07210
Category=3
Score=5
Cleavage Site=1803

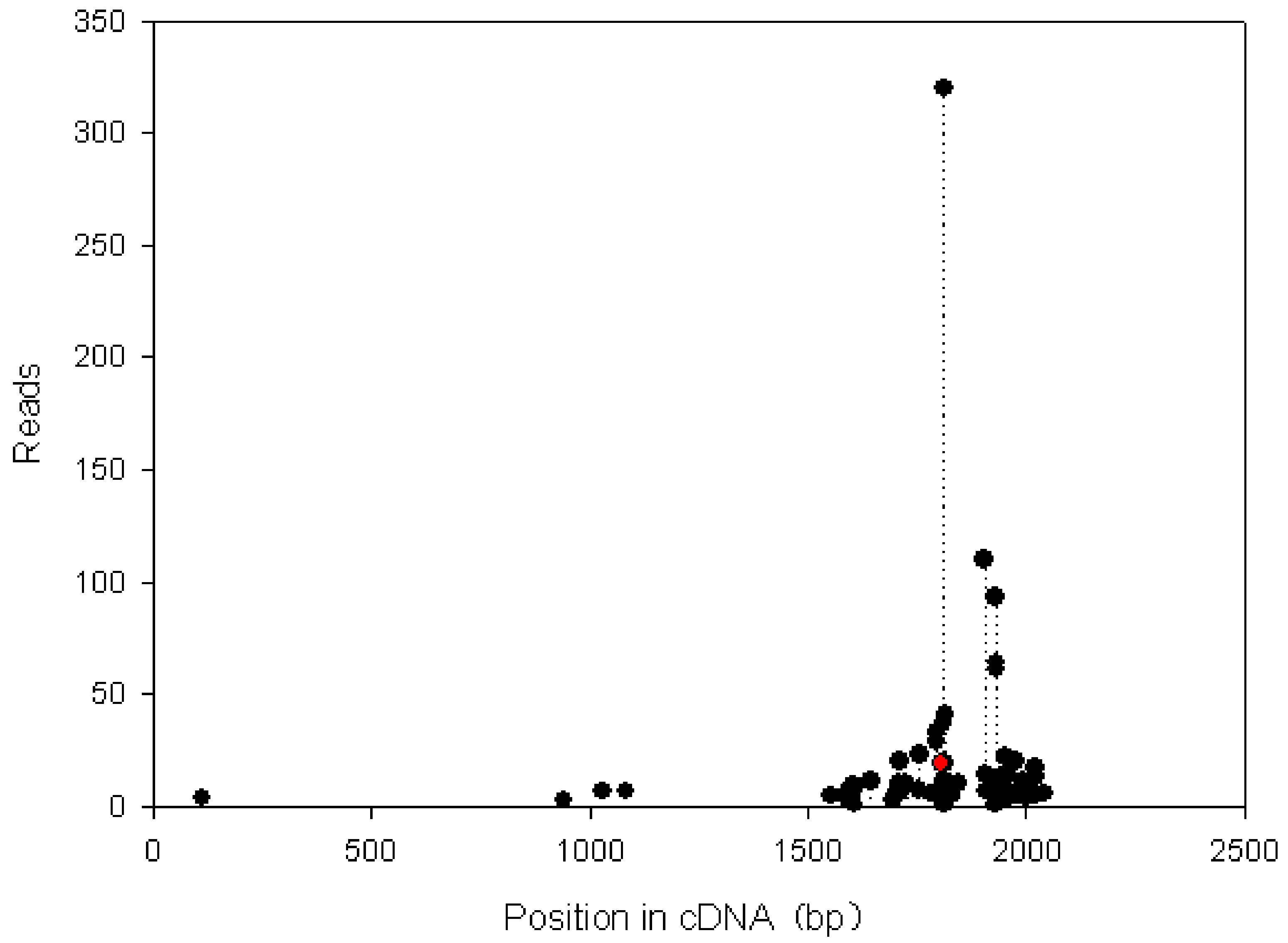


```
5' UUGGUCAUUUAGCUUGUUACAACACU 3'        Cs4g07210.1
   :::::: ::::::::::. :
3' AACCAGAAAAUCGAACAGGGA----- 5'        Csi-miRN33
```

Csi-miRN37, target=Orange1.1t02400.1 gene=Orange1.1t02400
Category=1
Score=5
Cleavage Site=775

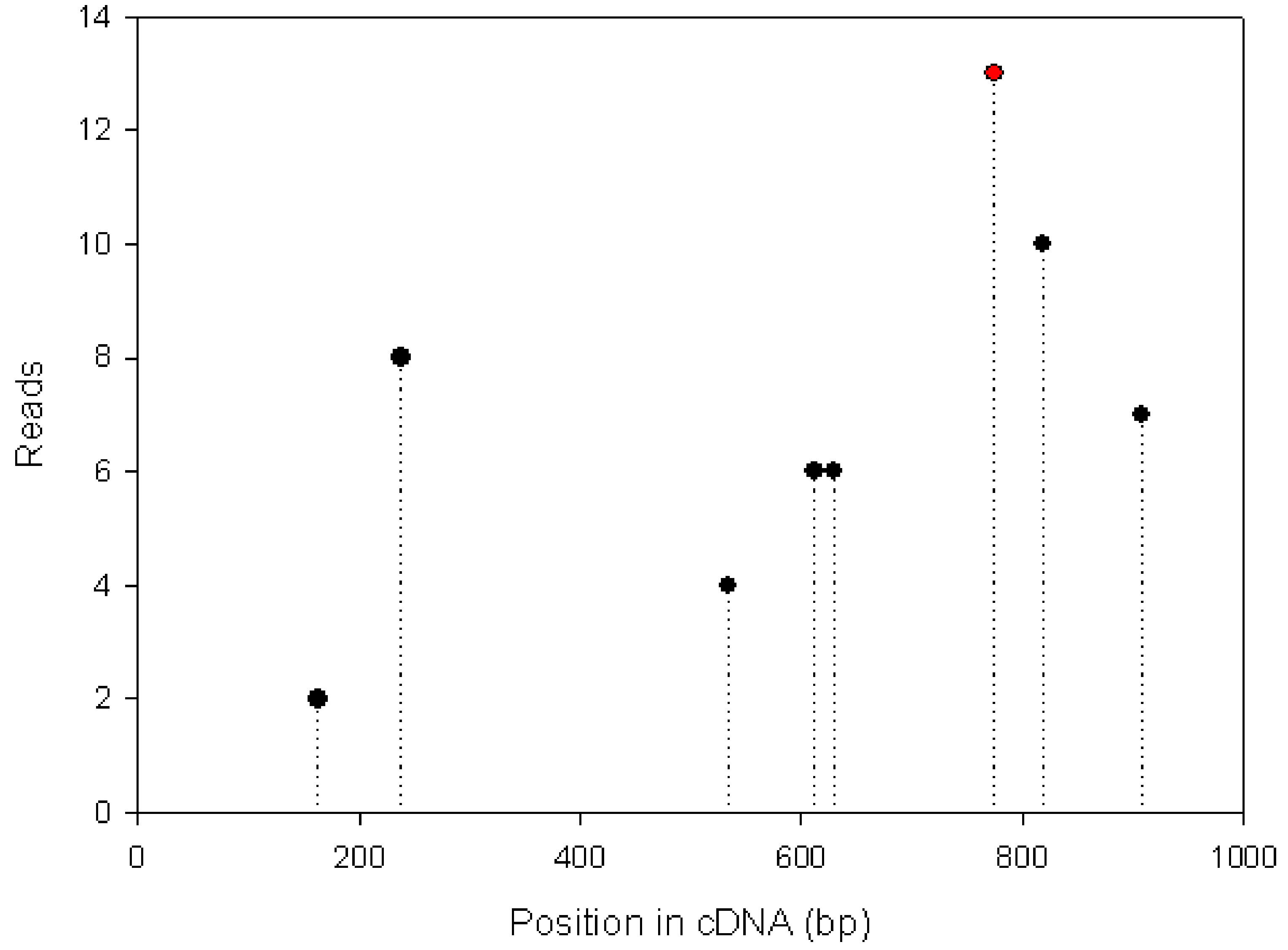


```
5' UACAACU-AGCUUUUCCCUCAAGAAAG 3'        Orange1.1t02400.1
     :  :: ::::.::::::::
3' -GGAAGAUUCGAGAAGGGAGU------ 5'        Csi-miRN37
```

T-plots for targets of all miRNAs in fruit

Csi-miR1092.2, target=Cs8g11330.1 gene=Cs8g11330
Category:3
Score=5
Cleavage Site=1168

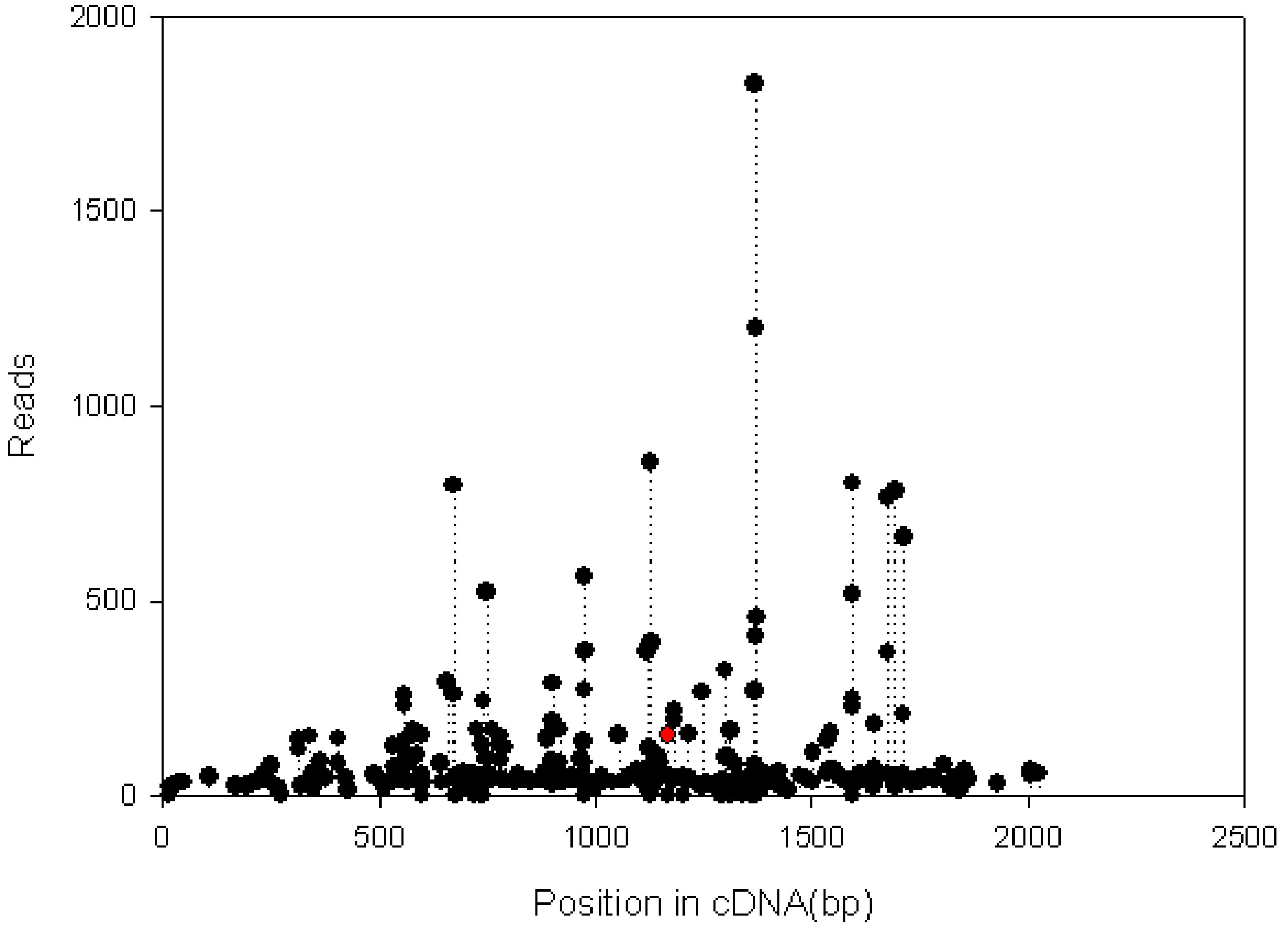


```
5' GGAAAUGUAUGCCAUUGGUGGAAGUG 3'        Cs8g11330.1
   ::::::: ::: : ::::::::::
3' CCUUUACUUAC-GAAACCACCUU--- 5'        Csi-miR1092.2
```

Csi-miR1446, target=Cs2g01340.1 gene=Cs2g01340
Category:3
Score=5
Cleavage Site=353

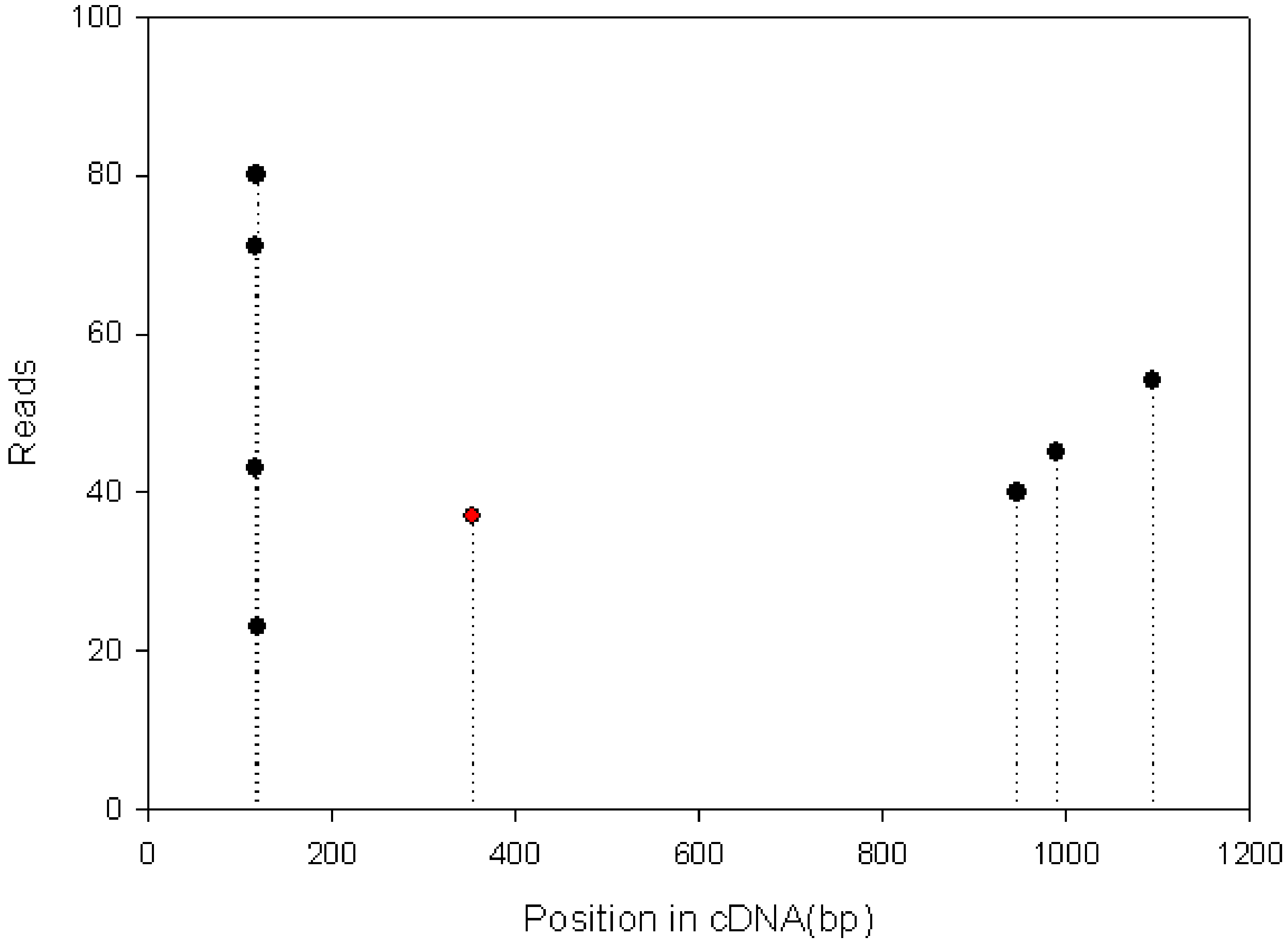


```
5' UUUGUUUAGCGAGAGAGUUCGGAUUU 3'        Cs2g01340.1
    ..::: :: :::::::::::
3' CGGCAACUCCCUCUCUCAAGC----- 5'        Csi-miR1446
```

Csi-miR156a.1, target=Cs2g23550.1 gene=Cs2g23550
Category:1
Score=3
Cleavage Site=934

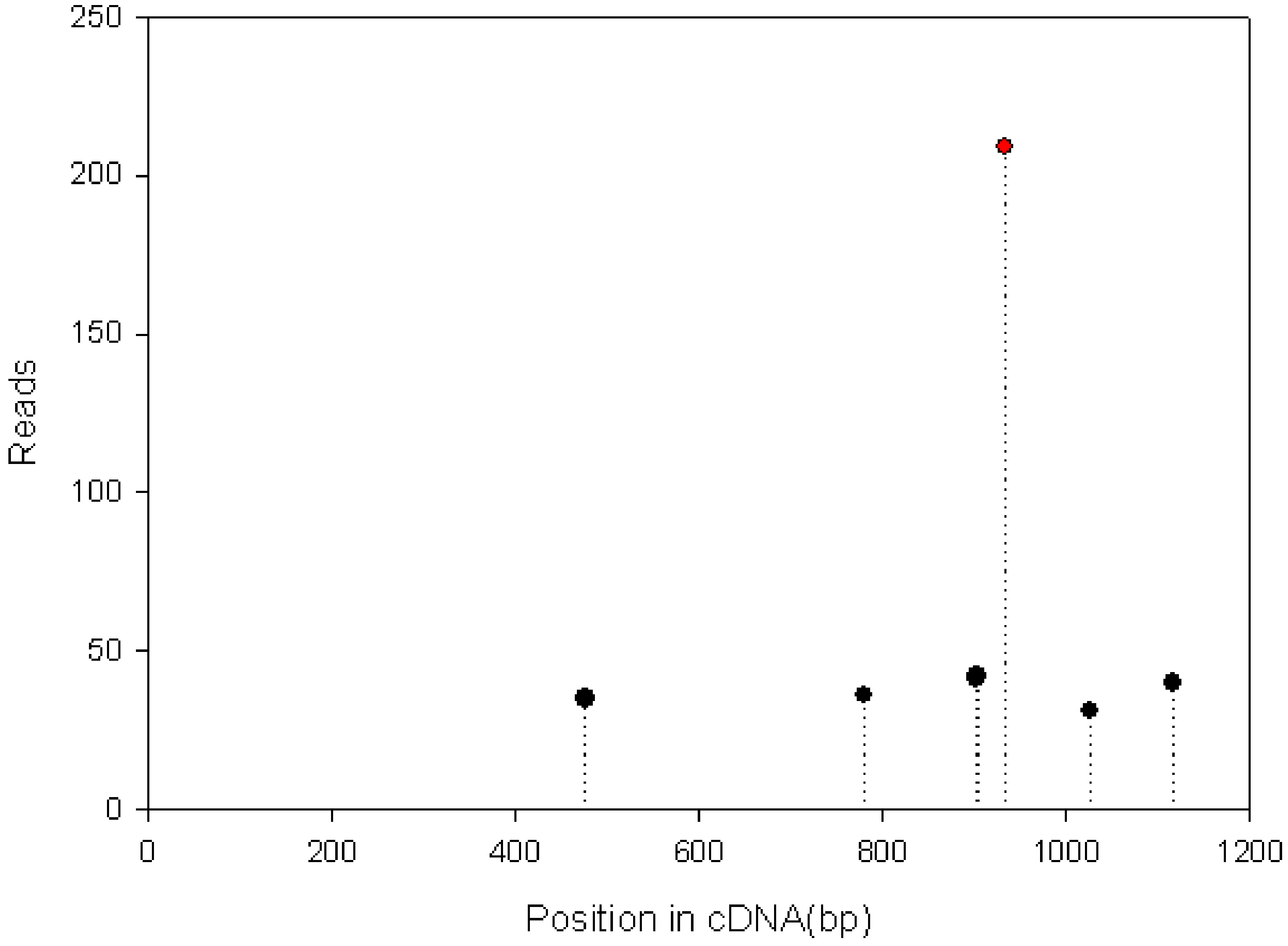


```
5' CAUGCUCUCUCUCUUCUGUCAUCCUA 3'        Cs2g23550.1
     ::::: :::::::::::::
3' -CACGAGUGAGAGAAGACAGUU---- 5'        Csi-miR156a.1
```

Csi-miR156a.1, target=Cs3g10870.1 gene=Cs3g10870
Category:1
Score=3.5
Cleavage Site=244

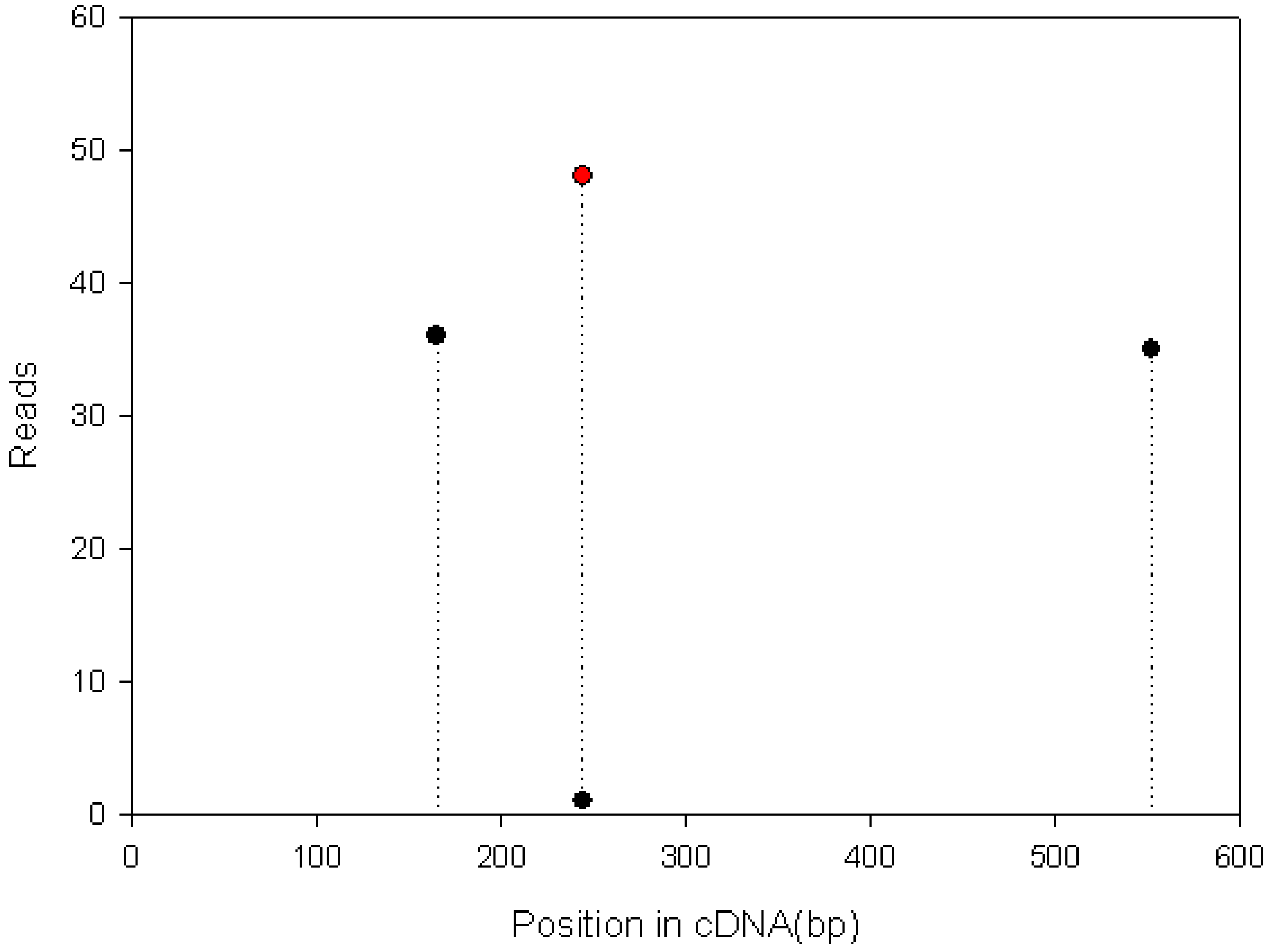


```
5' CUUCGUCCUC-UUCUUUUCUGUCAACA 3'       Cs3g10870.1
       :: ::: .:::.:::::::::
3' ----CACGAGUGAGAGAAGACAGUU-- 5'       Csi-miR156a.1
```

Csi-miR156a.1, target=Cs5g12260.1 gene=Cs5g12260
Category:1
Score=4
Cleavage Site=1693

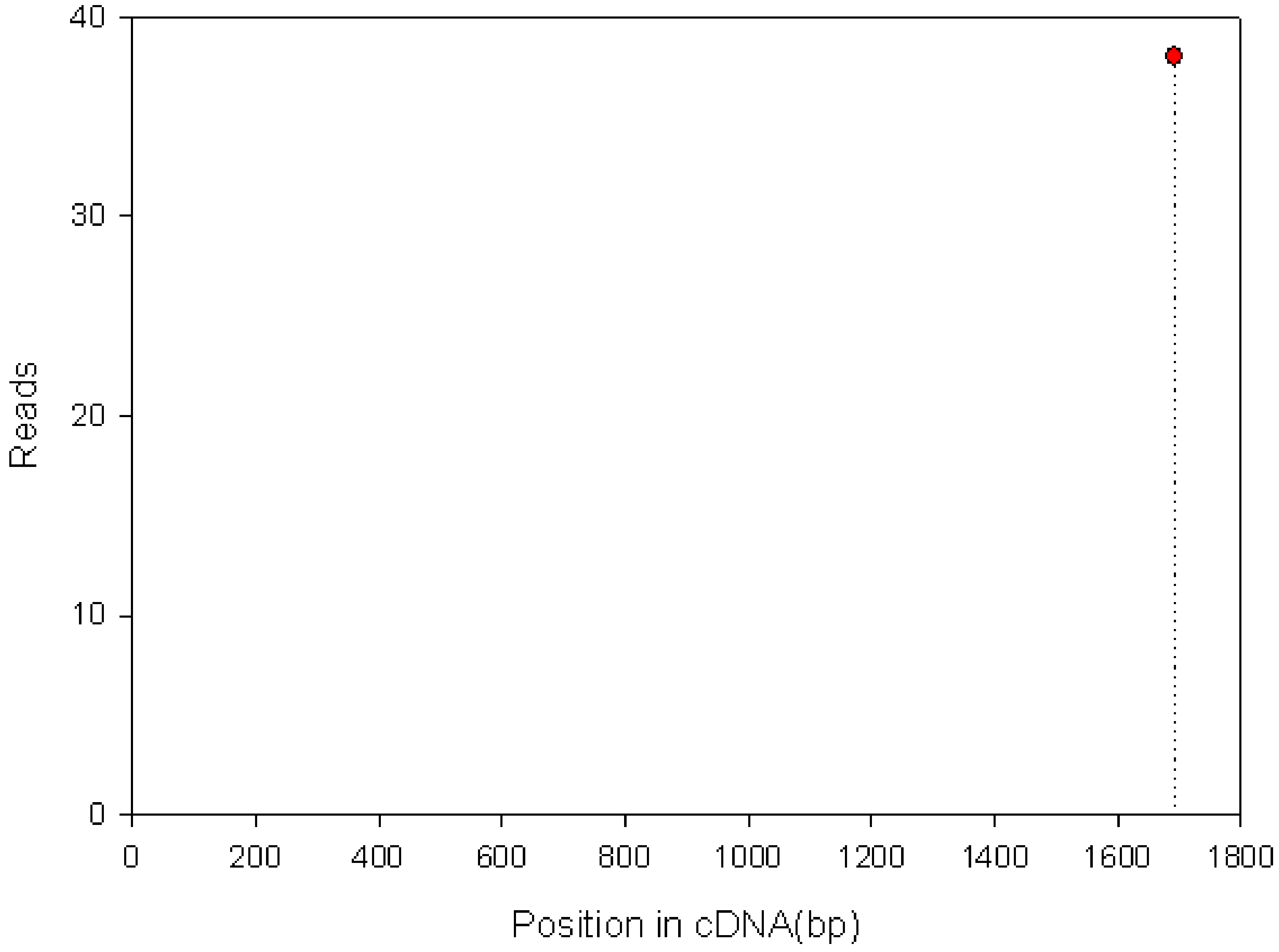


```
5' CUAUGCUCACCCUCUUCUGUCAUCUA 3'        Cs5g12260.1
      ::::::: ::::::::::
3' --CACGAGUGAGAGAAGACAGUU--- 5'        Csi-miR156a.1
```

Csi-miR156a.1, target=Cs7g11770.1 gene=Cs7g11770
Category:1
Score=1.5
Cleavage Site=1487

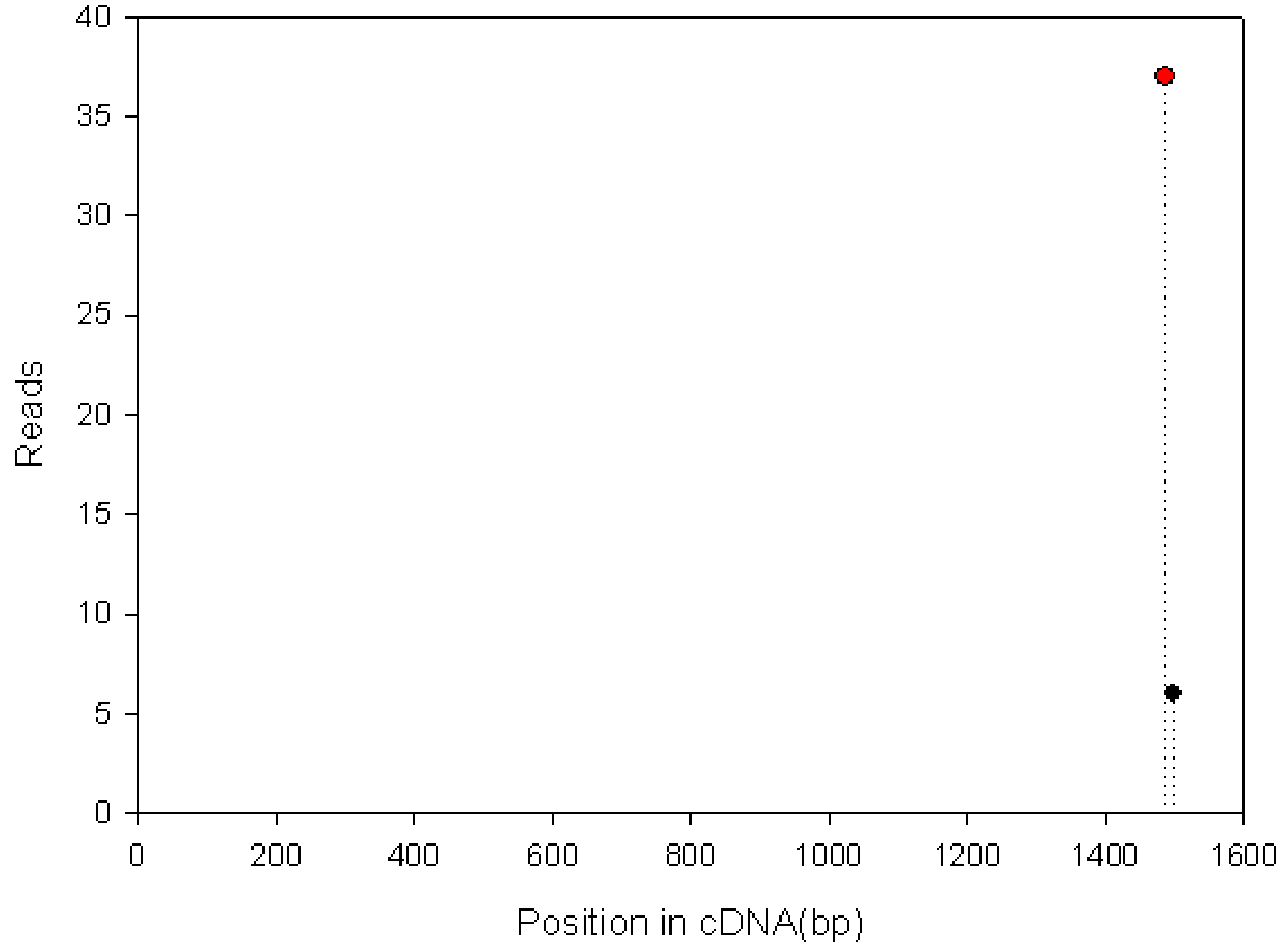


```
5' UGGUGCUCUCUCUUCUGUCAGCUC 3'        Cs7g11770.1
     :::::: :::::::::::::.
3' --CACGAGUGAGAGAAGACAGUU--- 5'        Csi-miR156a.1
```

Csi-miR156a.1, target=Orange1.1t01983.1 gene=Orange1.1t01983
Category:3
Score=5
Cleavage Site=693

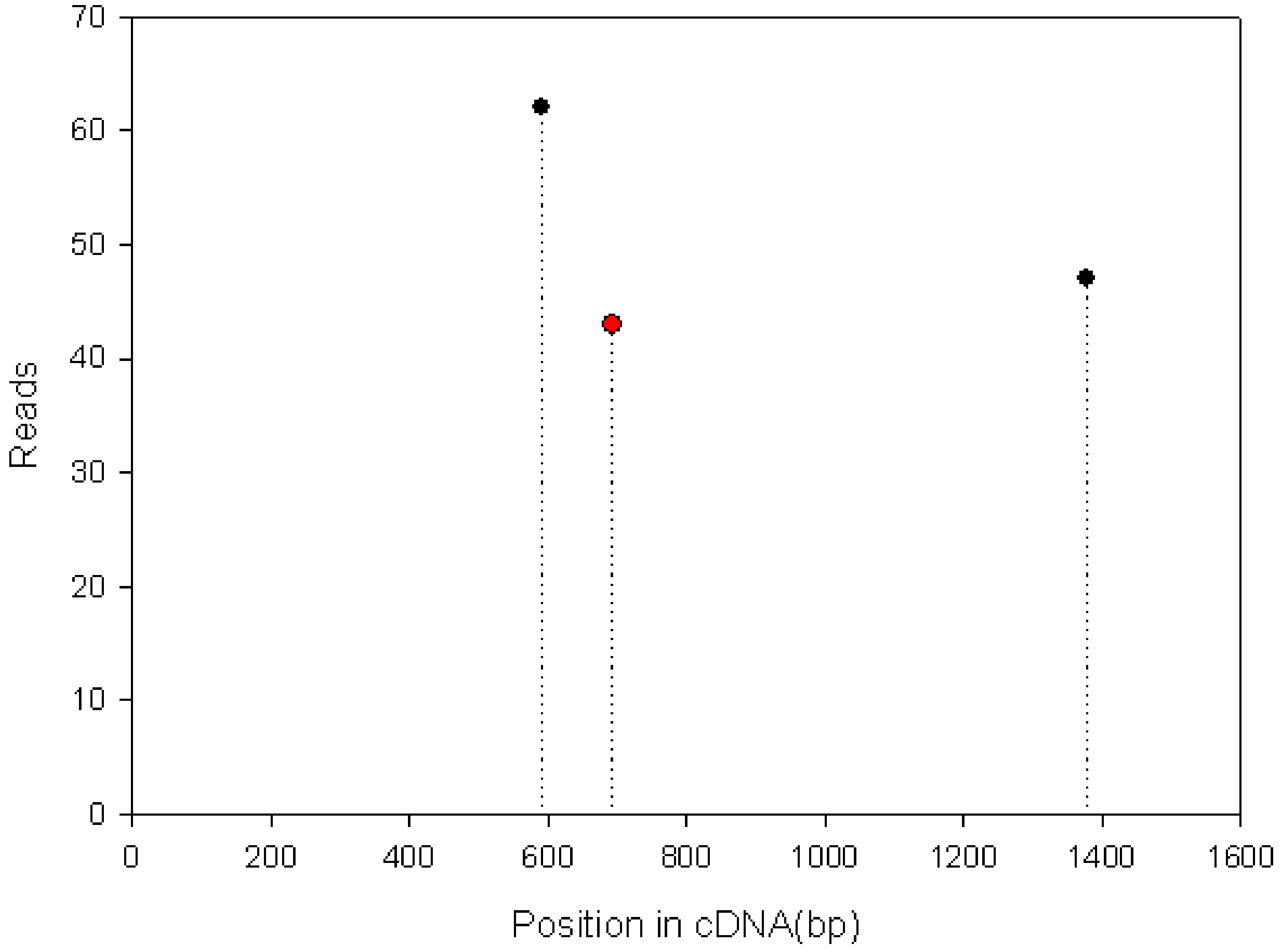


```
5' CUUGCUUCUGUCUCUUCUGUCAUCAC 3'        Orange1.1t01983.1
     ::::. . ::::::::::::
3' -CACGAGUG-AGAGAAGACAGUU--- 5'        Csi-miR156a.1
```

Csi-miR156a.2, target=Cs2g17270.1 gene=Cs2g17270
Category:3
Score=4.5
Cleavage Site=274

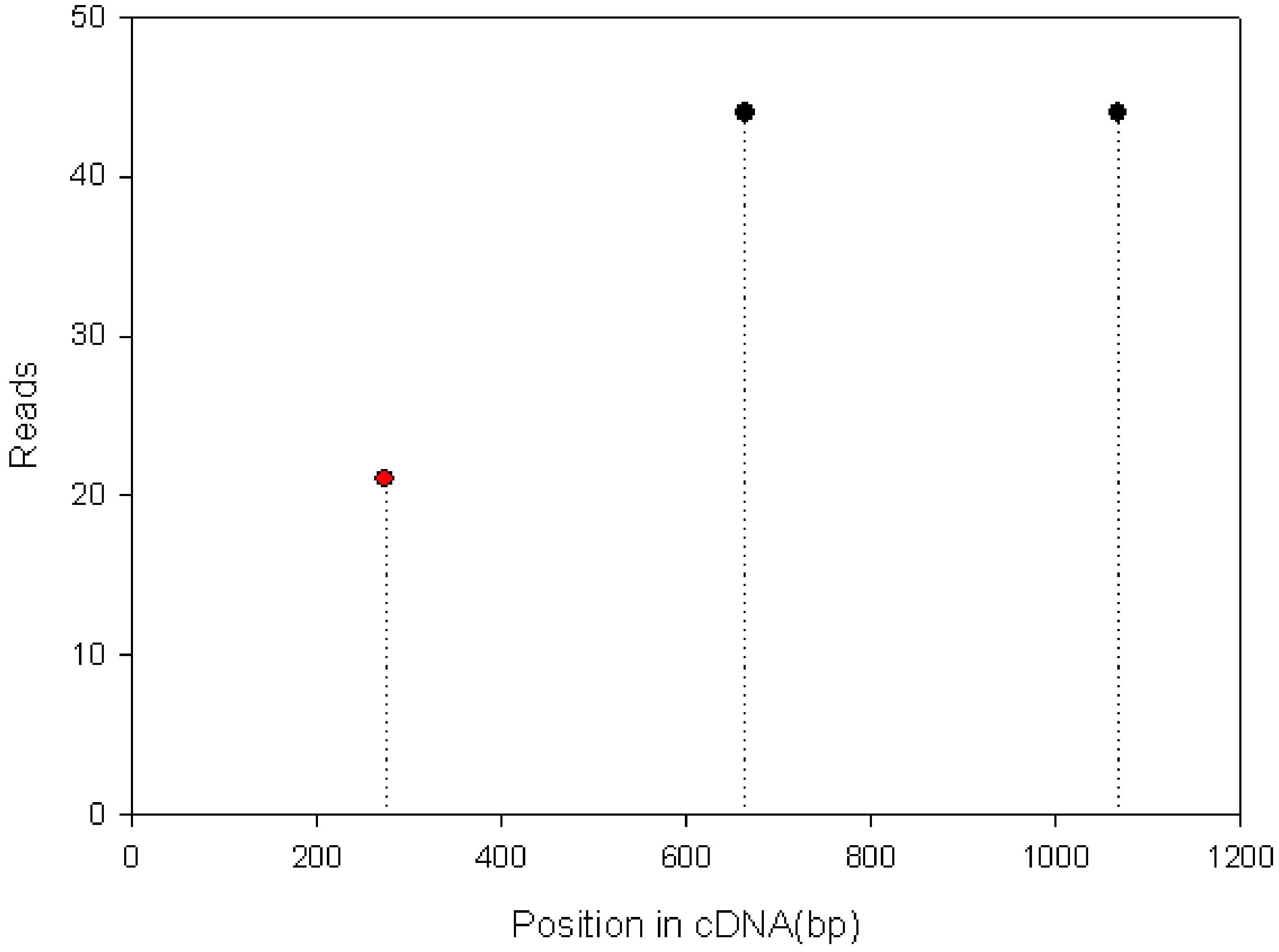


```
5' CUCUCUGCACGCU-UCUUCUGUCAUGG 3'       Cs2g17270.1
       ::: :.:: ::::::::::
3' ----CACGAGUGAGAGAAGACAGU--- 5'       Csi-miR156a.2
```

Csi-miR156a.2, target=Cs2g23550.1 gene=Cs2g23550
Category:1
Score=2
Cleavage Site=934

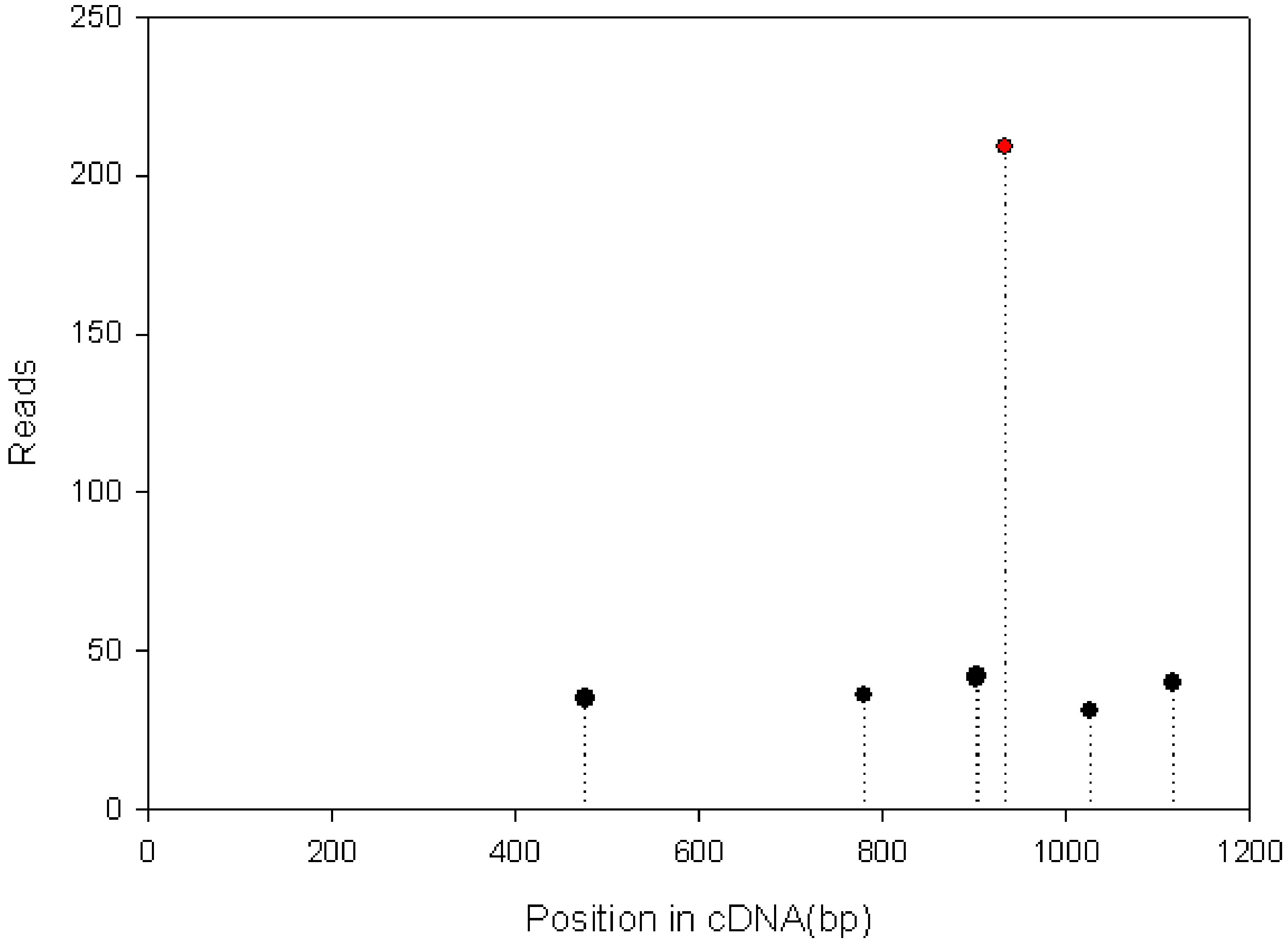


```
5' CAUGCUCUCUCUCUUCUGUCAUCCUA 3'        Cs2g23550.1
     ::::: :::::::::::::
3' -CACGAGUGAGAGAAGACAGU----- 5'        Csi-miR156a.2
```

Csi-miR156a.2, target=Cs3g10870.1 gene=Cs3g10870
Category:1
Score=4
Cleavage Site=244

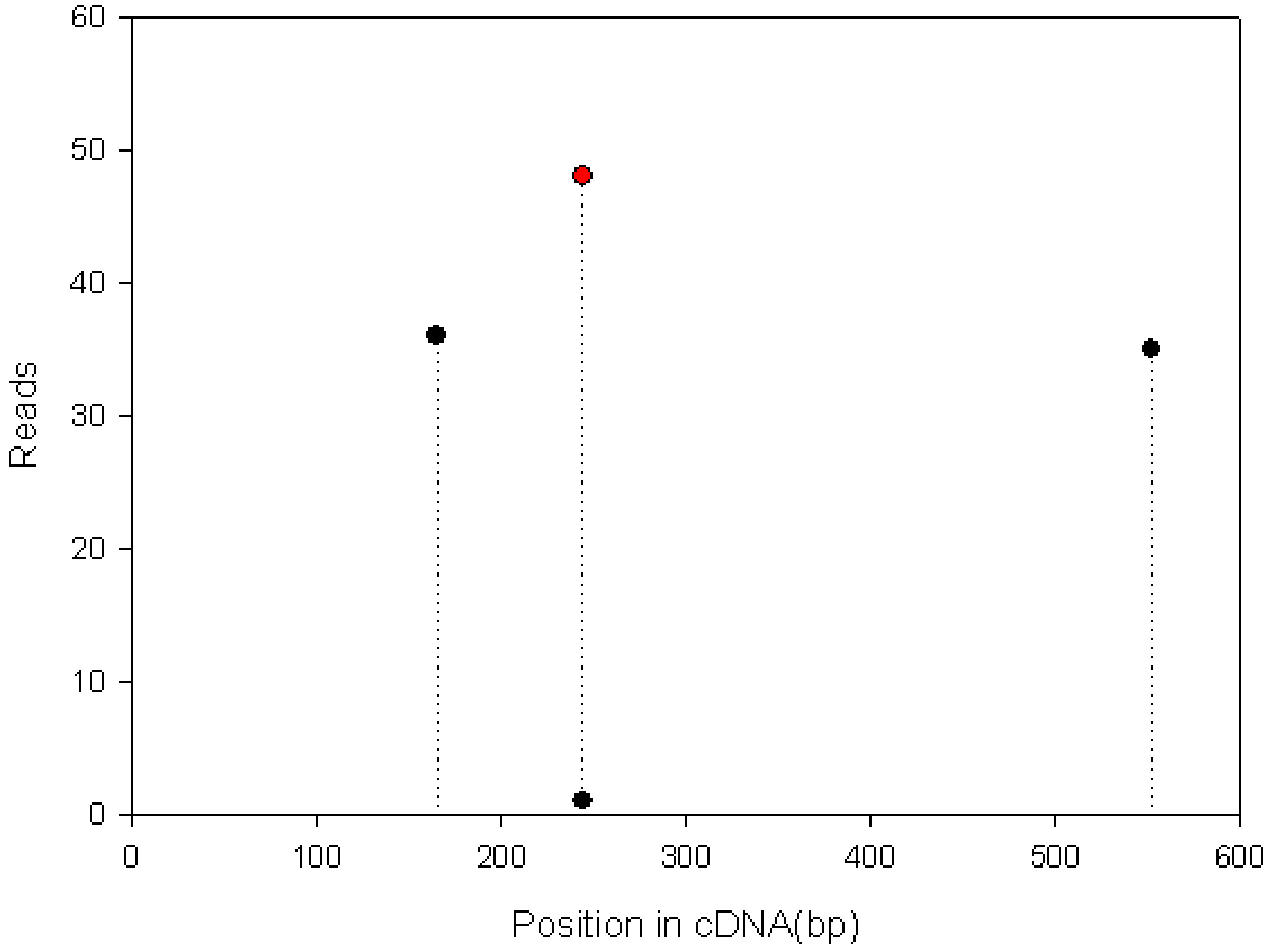


```
5' CUUCGUCCUC-UUCUUUUCUGUCAACA 3'        Cs3g10870.1
       :: :::  .:::.::::::::
3' ----CACGAGUGAGAGAAGACAGU--- 5'        Csi-miR156a.2
```

Csi-miR156a.2, target=Cs5g12260.1 gene=Cs5g12260
Category:1
Score=3
Cleavage Site=1693

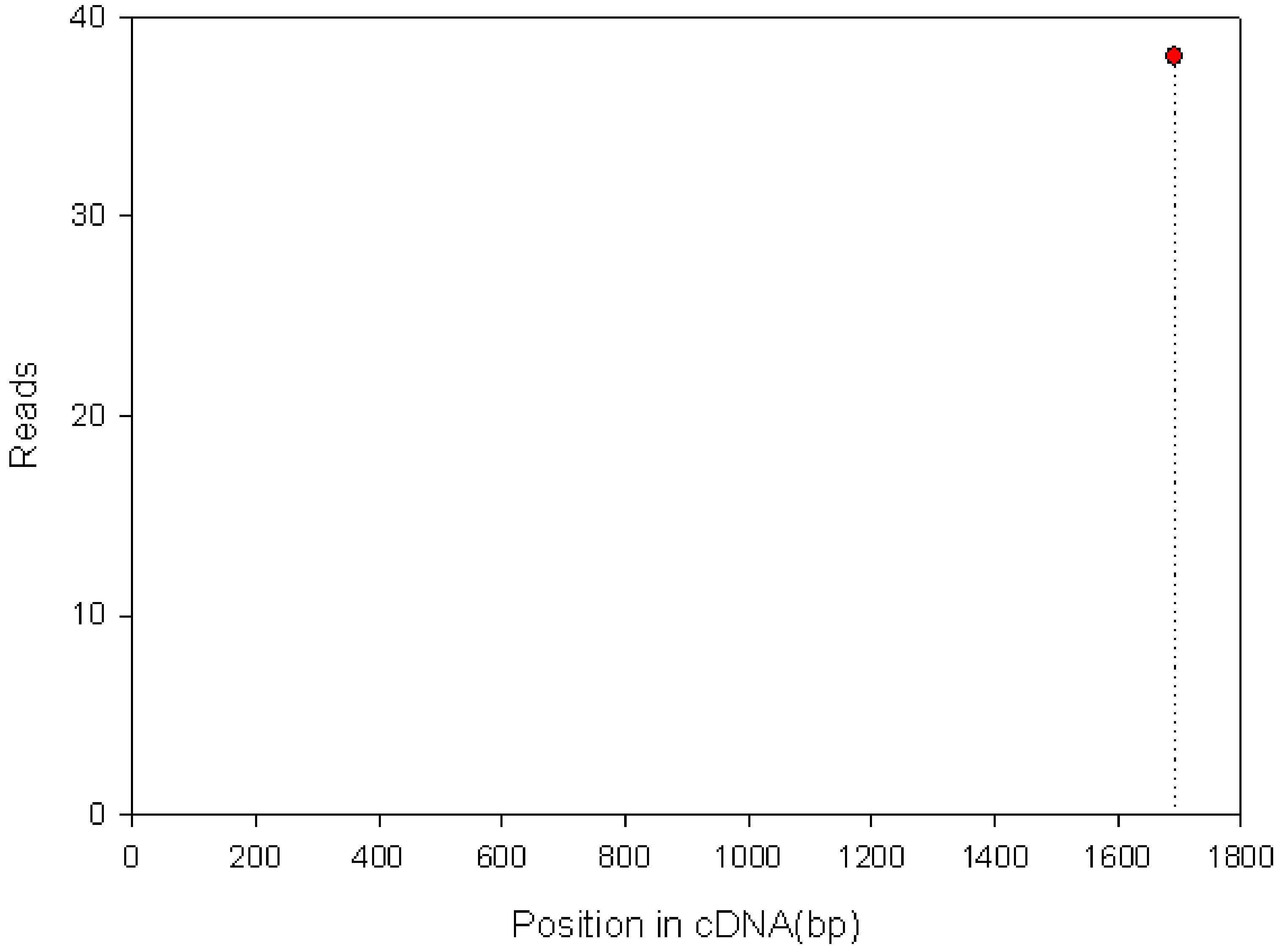


```
5' CUAUGCUCACCCUCUUCUGUCAUCUA 3'        Cs5g12260.1
      ::::::: :::::::::::
3' --CACGAGUGAGAGAAGACAGU---- 5'        Csi-miR156a.2
```

Csi-miR156a.2, target=Cs7g11770.1 gene=Cs7g11770
Category:1
Score=1
Cleavage Site=1487

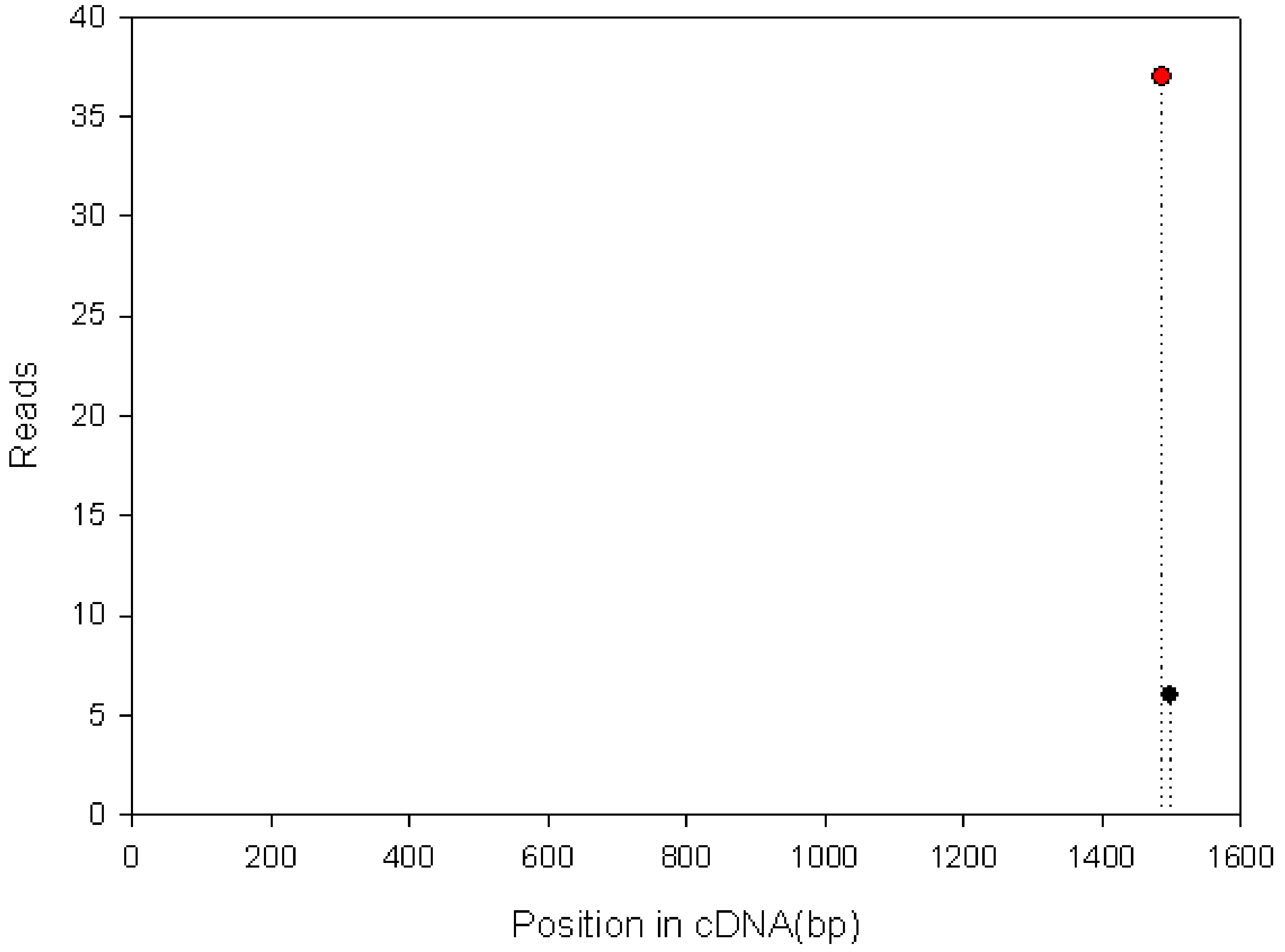


```
5' UGGUGCUCUCUCUCUUCUGUCAGCUC 3'        Cs7g11770.1
    :::::: :::::::::::::
3' --CACGAGUGAGAGAAGACAGU---- 5'        Csi-miR156a.2
```

Csi-miR156a.2, target=Orange1.1t01983.1 gene=Orange1.1t01983
Category:3
Score=5
Cleavage Site=693

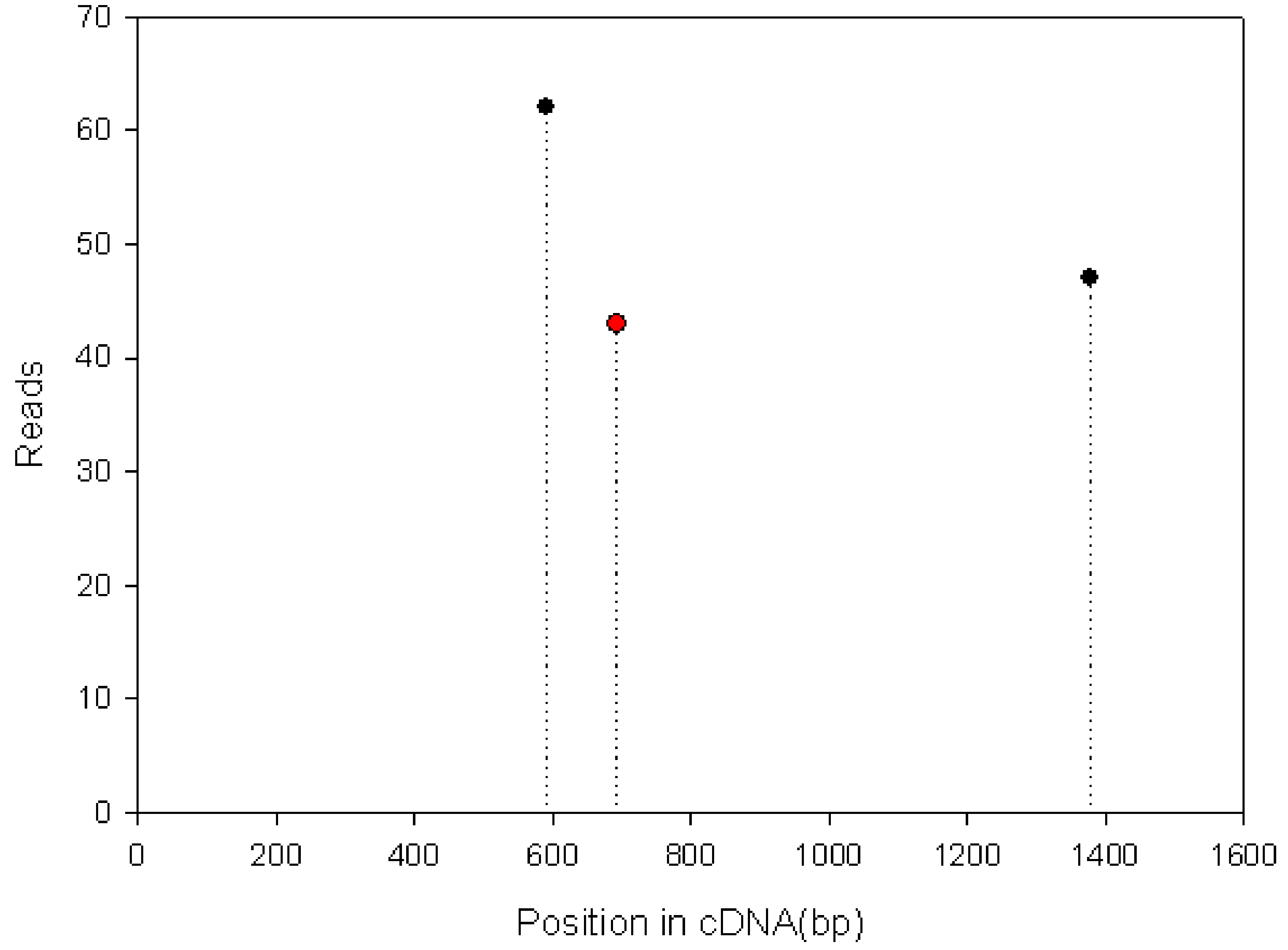


```
5' CUUGCUUCUGUCUCUUCUGUCAUCAC 3'        Orange1.1t01983.1
    ::::. . :::::::::::
3' -CACGAGUG-AGAGAAGACAGU---- 5'        Csi-miR156a.2
```

Csi-miR156b.1, target=Cs2g23550.1 gene=Cs2g23550
Category:1
Score=3
Cleavage Site=934

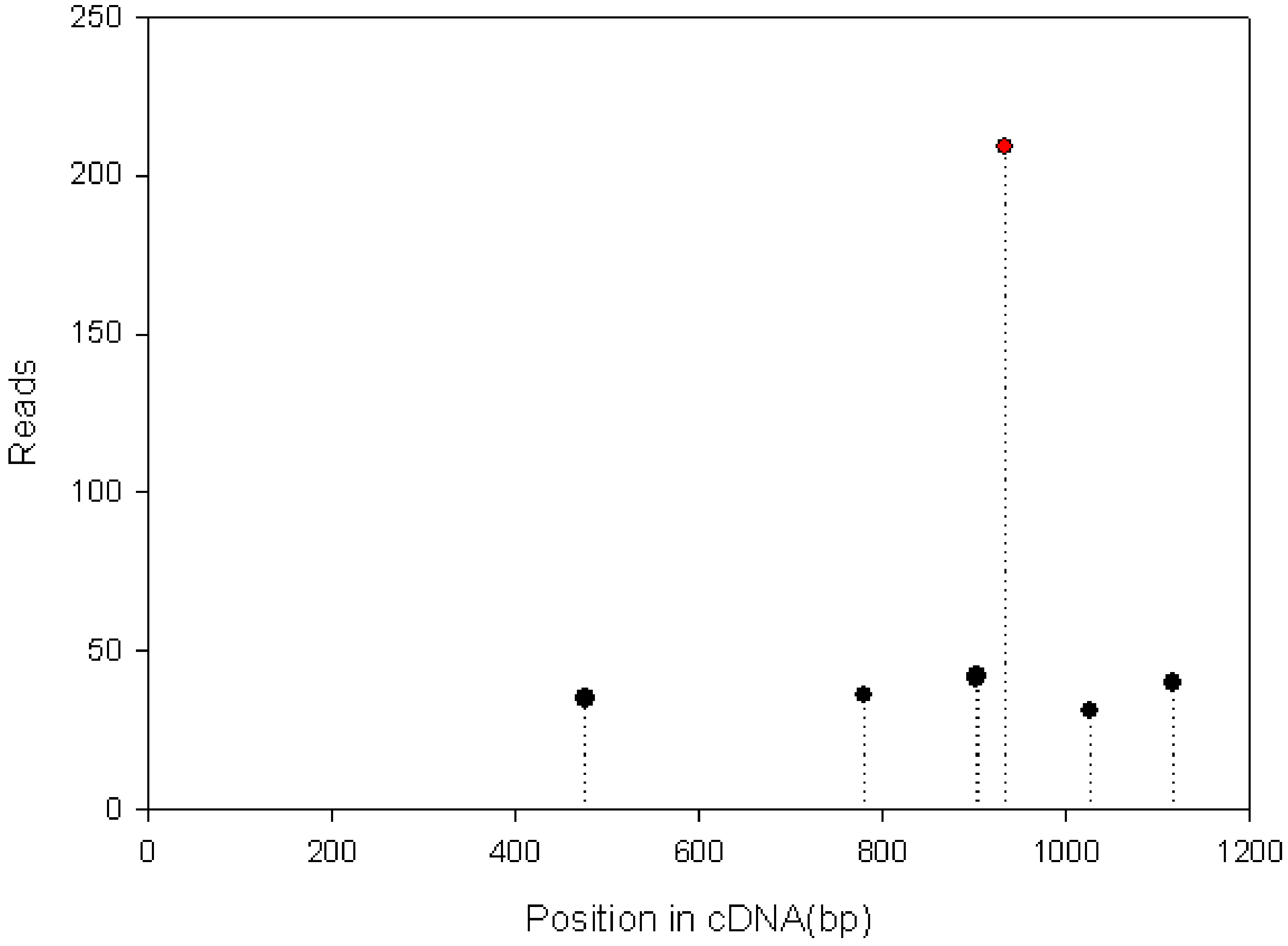


```
5' CAUGCUCUCUCUCUUCUGUCAUCCUA 3'        Cs2g23550.1
     ::::: ::::::::::::::
3' -CACGAGUGAGAGAAGACAGUC---- 5'        Csi-miR156b.1
```

Csi-miR156b.1, target=Cs3g10870.1 gene=Cs3g10870
Category:1
Score=4.5
Cleavage Site=244

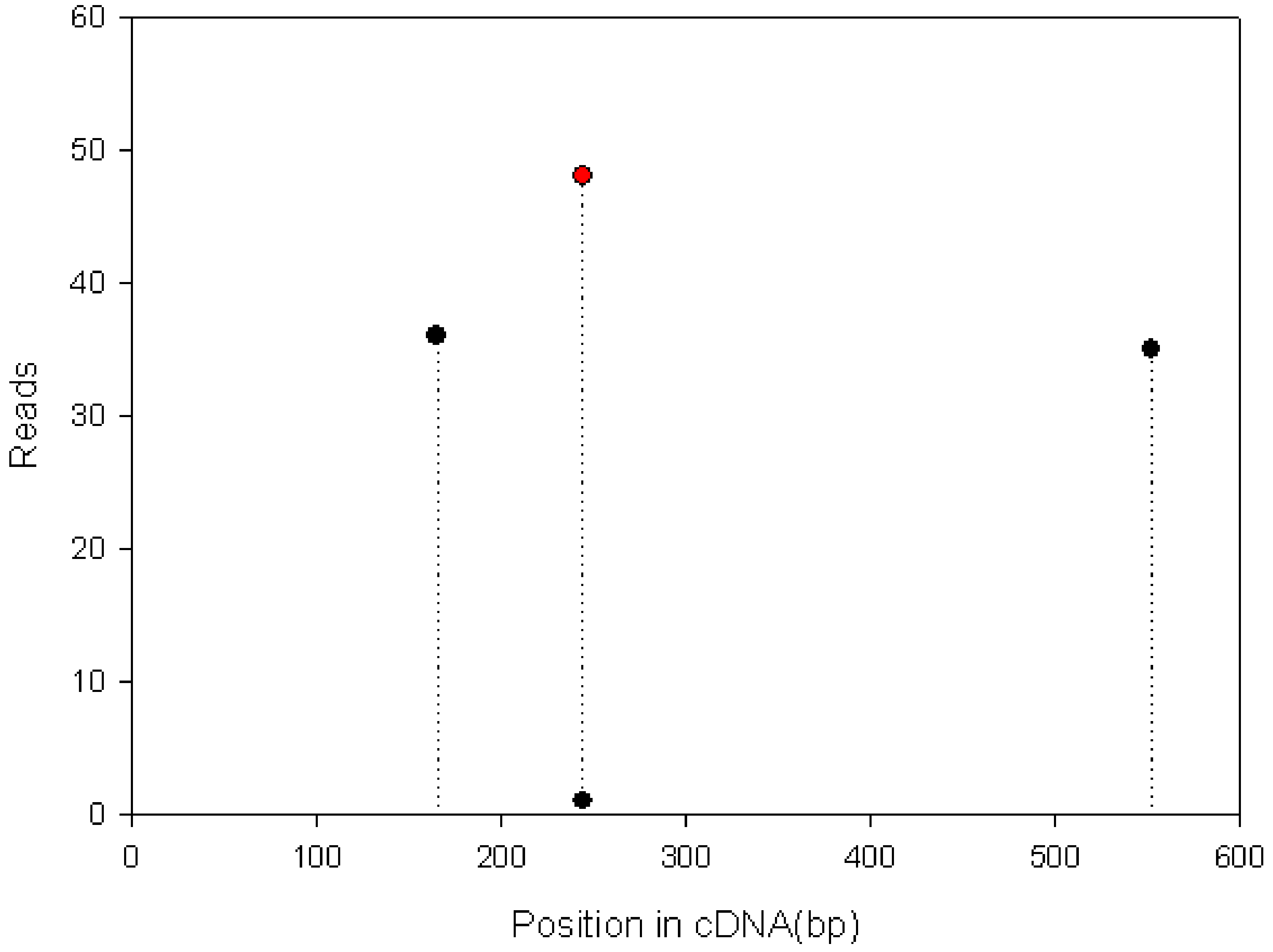


```
5' CUUCGUCCUC-UUCUUUUCUGUCAACA 3'       Cs3g10870.1
       :: ::: .:::.::::::::
3' ----CACGAGUGAGAGAAGACAGUC-- 5'       Csi-miR156b.1
```

Csi-miR156b.1, target=Cs5g12260.1 gene=Cs5g12260
Category:1
Score=4
Cleavage Site=1693

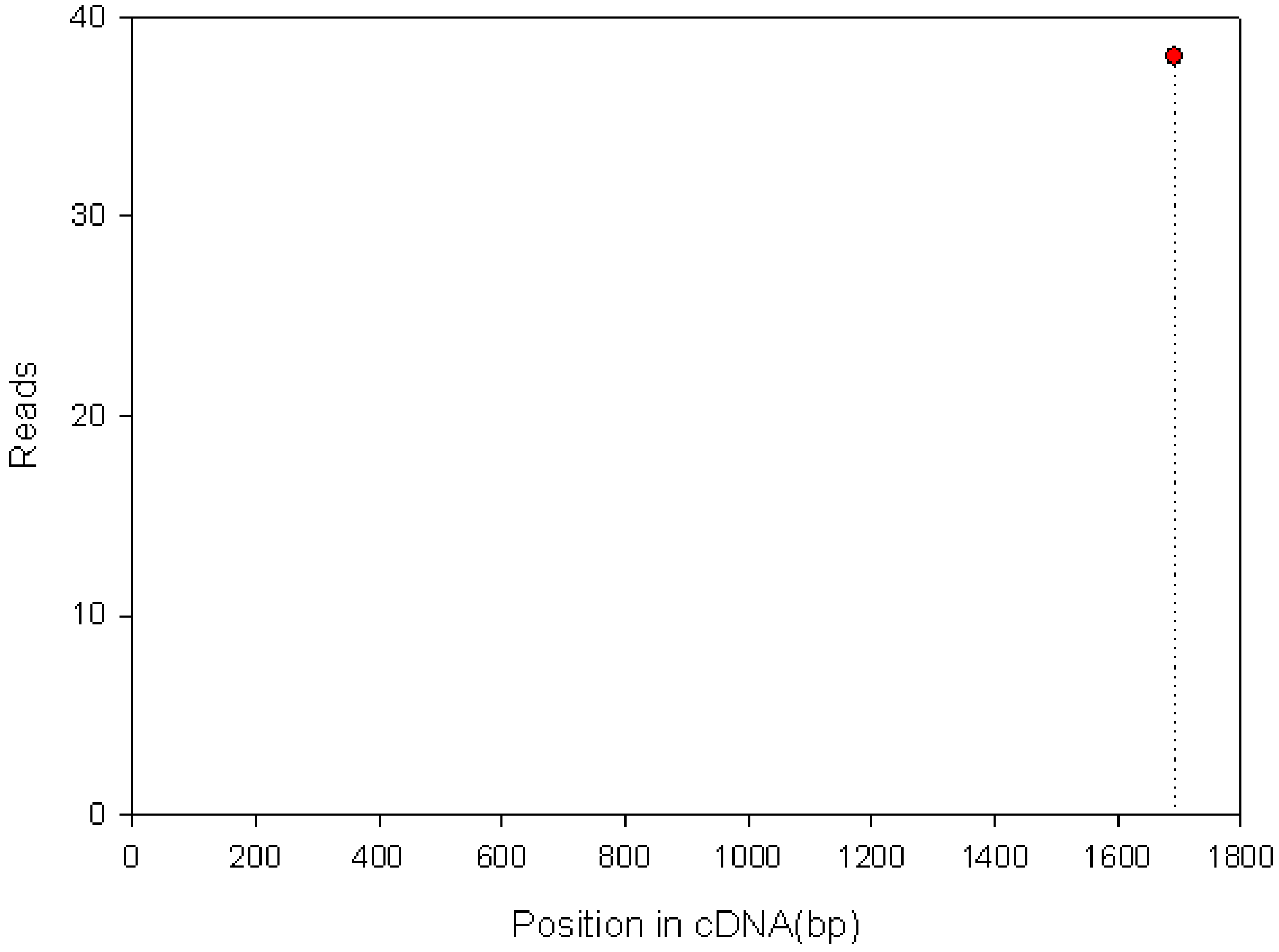


```
5' CUAUGCUCACCCUCUUCUGUCAUCUA 3'        Cs5g12260.1
      ::::::: :::::::::::
3' --CACGAGUGAGAGAAGACAGUC--- 5'        Csi-miR156b.1
```

Csi-miR156b.1, target=Cs7g11770.1 gene=Cs7g11770
Category:1
Score=1
Cleavage Site=1487

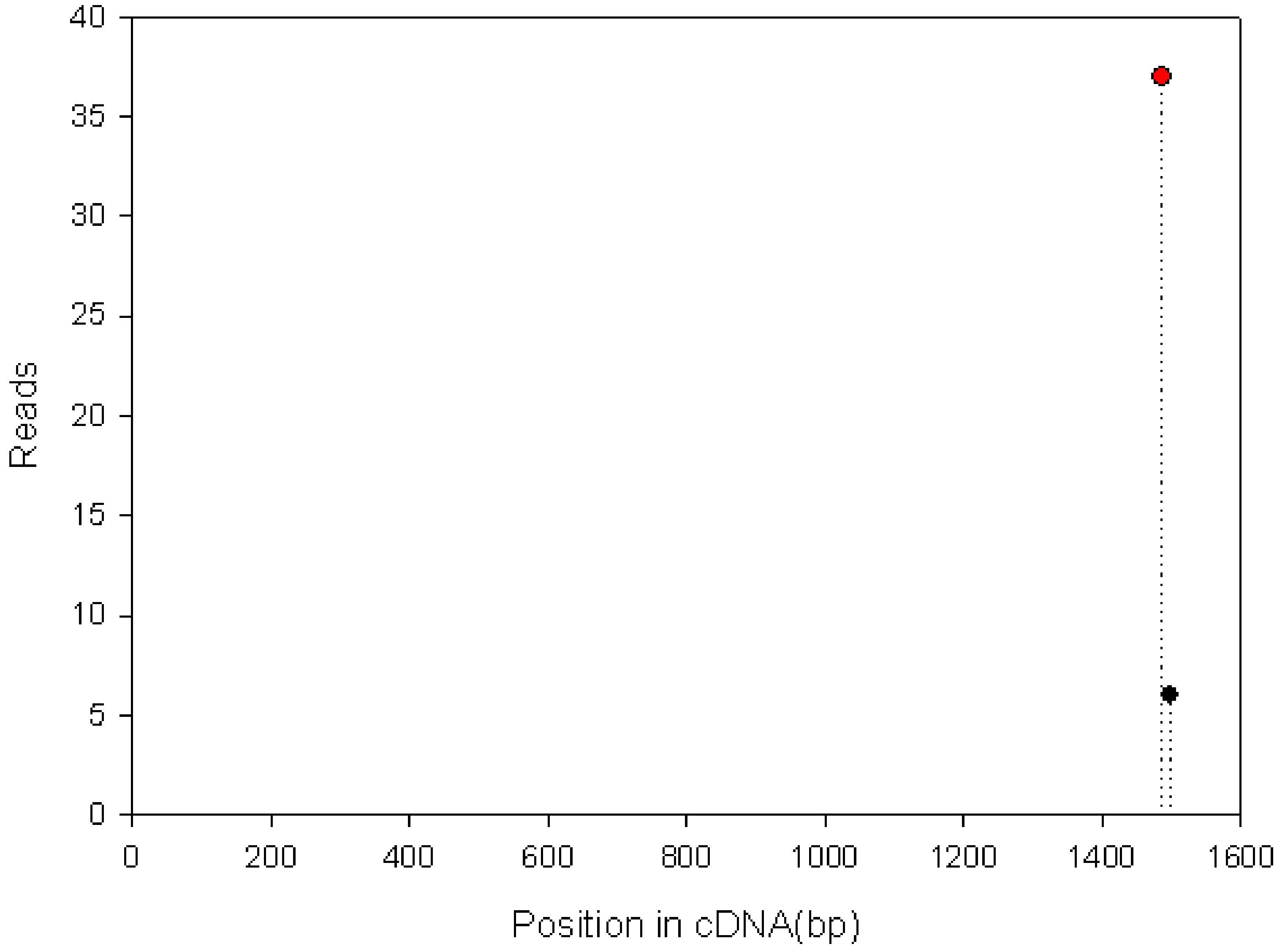


```
5' UGGUGCUCUCUCUUCUGUCAGCUC 3'        Cs7g11770.1
     :::::: :::::::::::::
3' --CACGAGUGAGAGAAGACAGUC--- 5'      Csi-miR156b.1
```

Csi-miR156b.1, target=Orange1.1t01983.1 gene=Orange1.1t01983
Category:3
Score=5
Cleavage Site=693

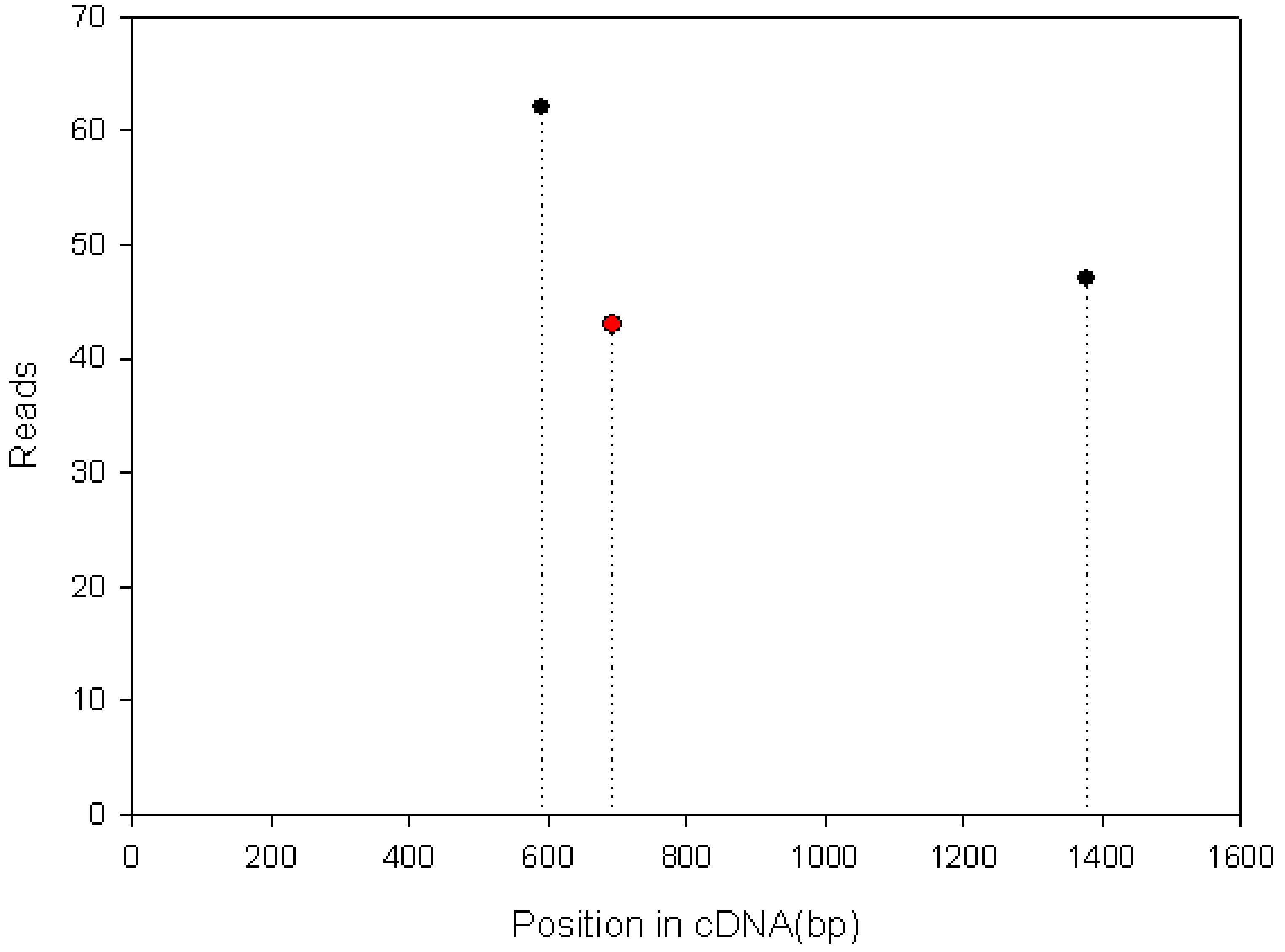


```
5' CUUGCUUCUGUCUCUUCUGUCAUCAC 3'        Orange1.1t01983.1
    ::::. . ::::::::::::
3' -CACGAGUG-AGAGAAGACAGUC--- 5'        Csi-miR156b.1
```

Csi-miR156c.1, target=Cs2g17270.1 gene=Cs2g17270
Category:3
Score=4.5
Cleavage Site=274

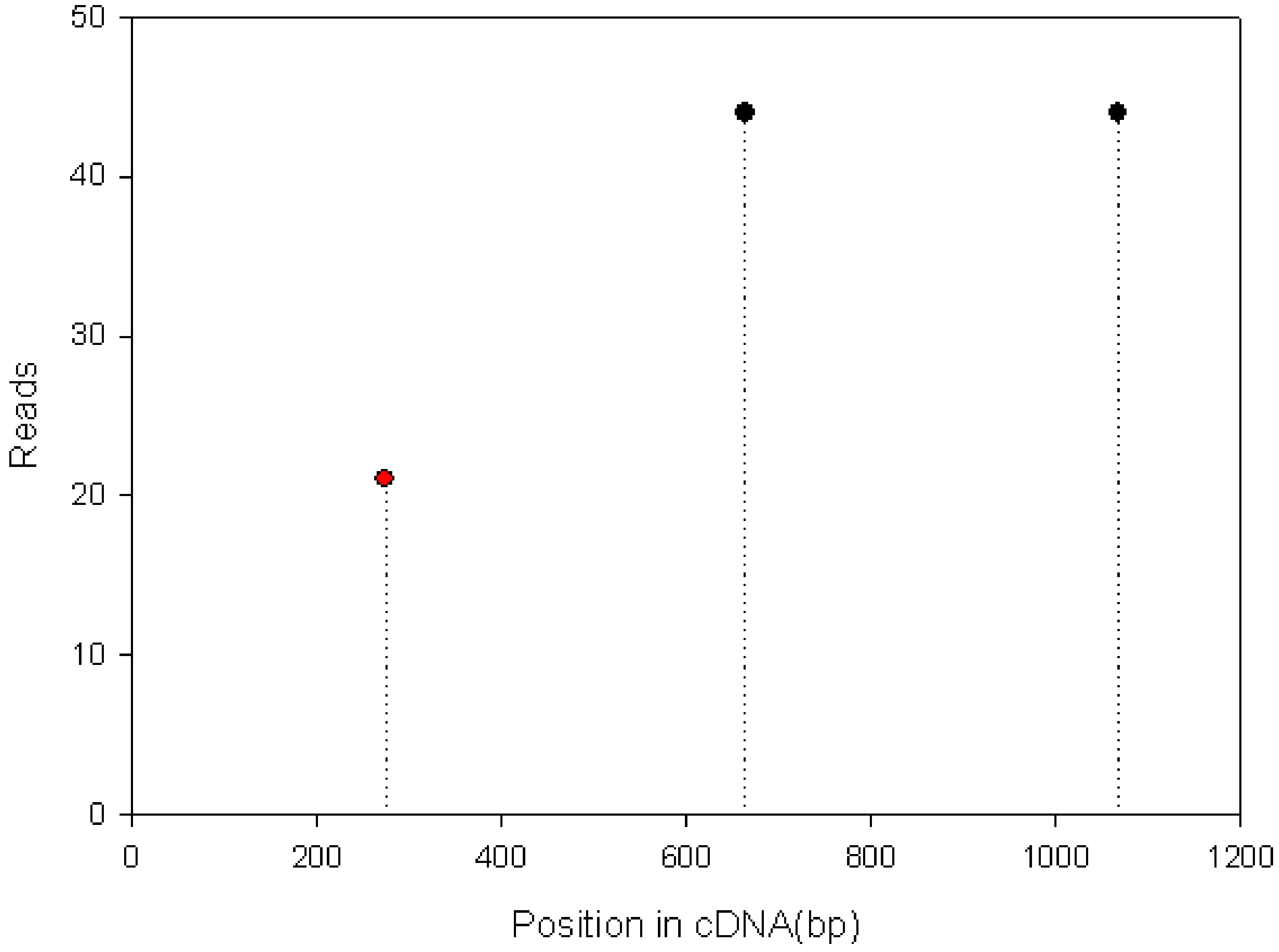


```
5' CUCUCUGCACGCU-UCUUCUGUCAUGG 3'        Cs2g17270.1
       :  :::  :.::  ::::::::::
3' ---ACACGAGUGAGAGAAGACAGU--- 5'        Csi-miR156c.1
```

Csi-miR156c.1, target=Cs2g23550.1 gene=Cs2g23550
Category:1
Score=3
Cleavage Site=934

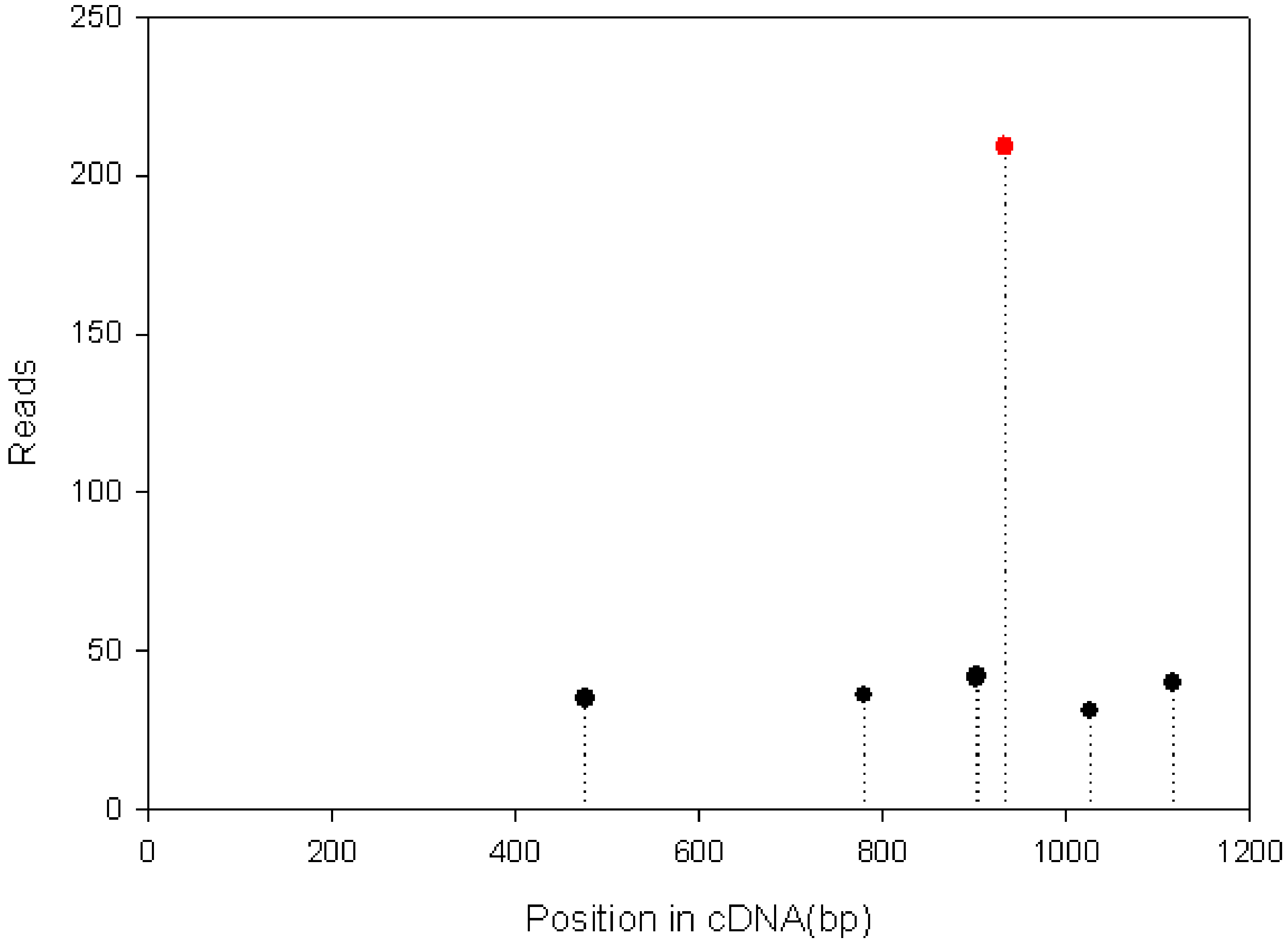


```
5' CAUGCUCUCUCUCUUCUGUCAUCCUA 3'        Cs2g23550.1
     ::::: :::::::::::::
3' ACACGAGUGAGAGAAGACAGU----- 5'        Csi-miR156c.1
```

Csi-miR156c.1, target=Cs3g10870.1 gene=Cs3g10870
Category:1
Score=5
Cleavage Site=244

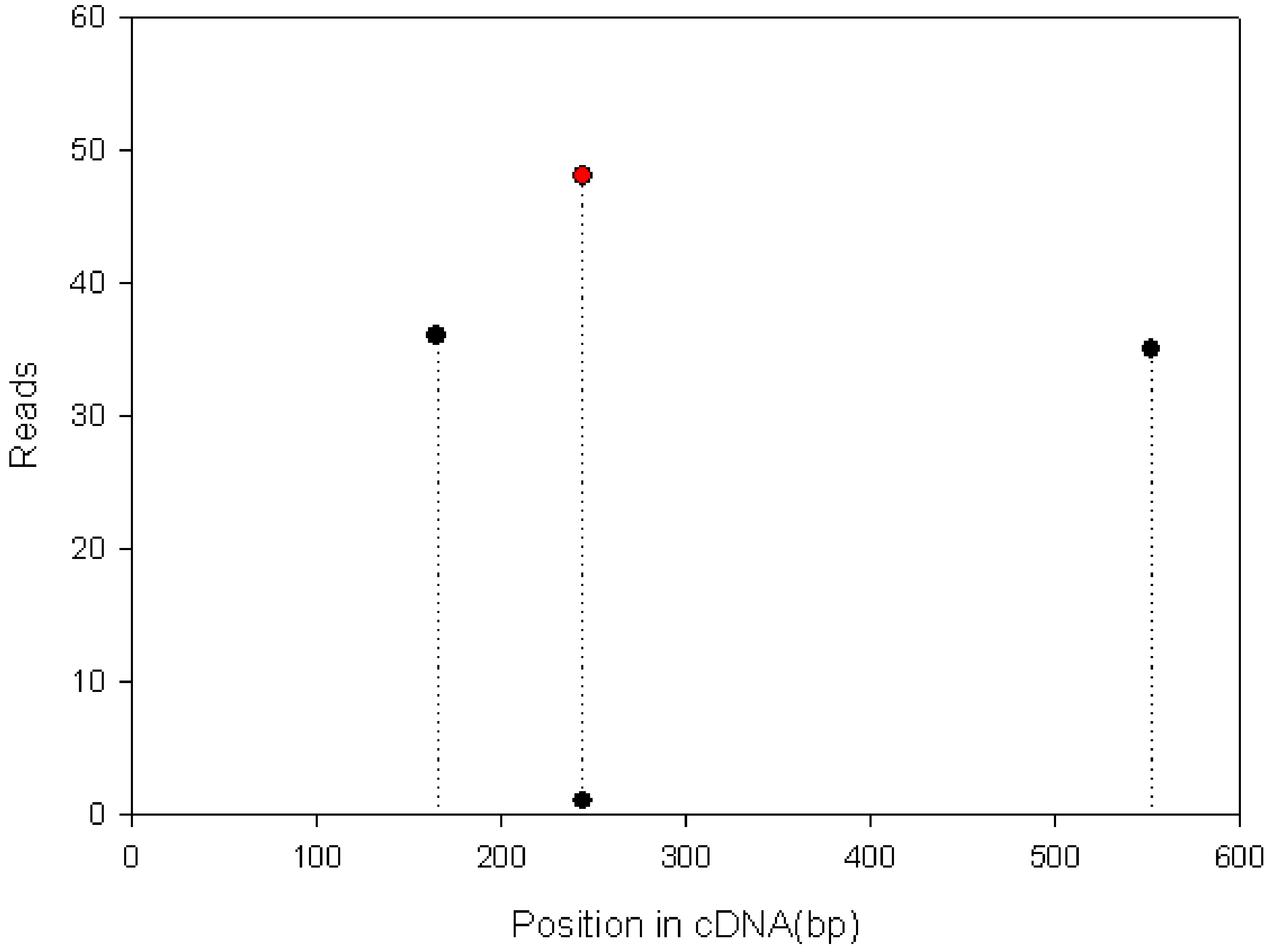


```
5' CUUCGUCCUC-UUCUUUUCUGUCAACA 3'        Cs3g10870.1
      :: ::: .:::.::::::::
3' ---ACACGAGUGAGAGAAGACAGU--- 5'        Csi-miR156c.1
```

Csi-miR156c.1, target=Cs5g12260.1 gene=Cs5g12260
Category:1
Score=3
Cleavage Site=1693

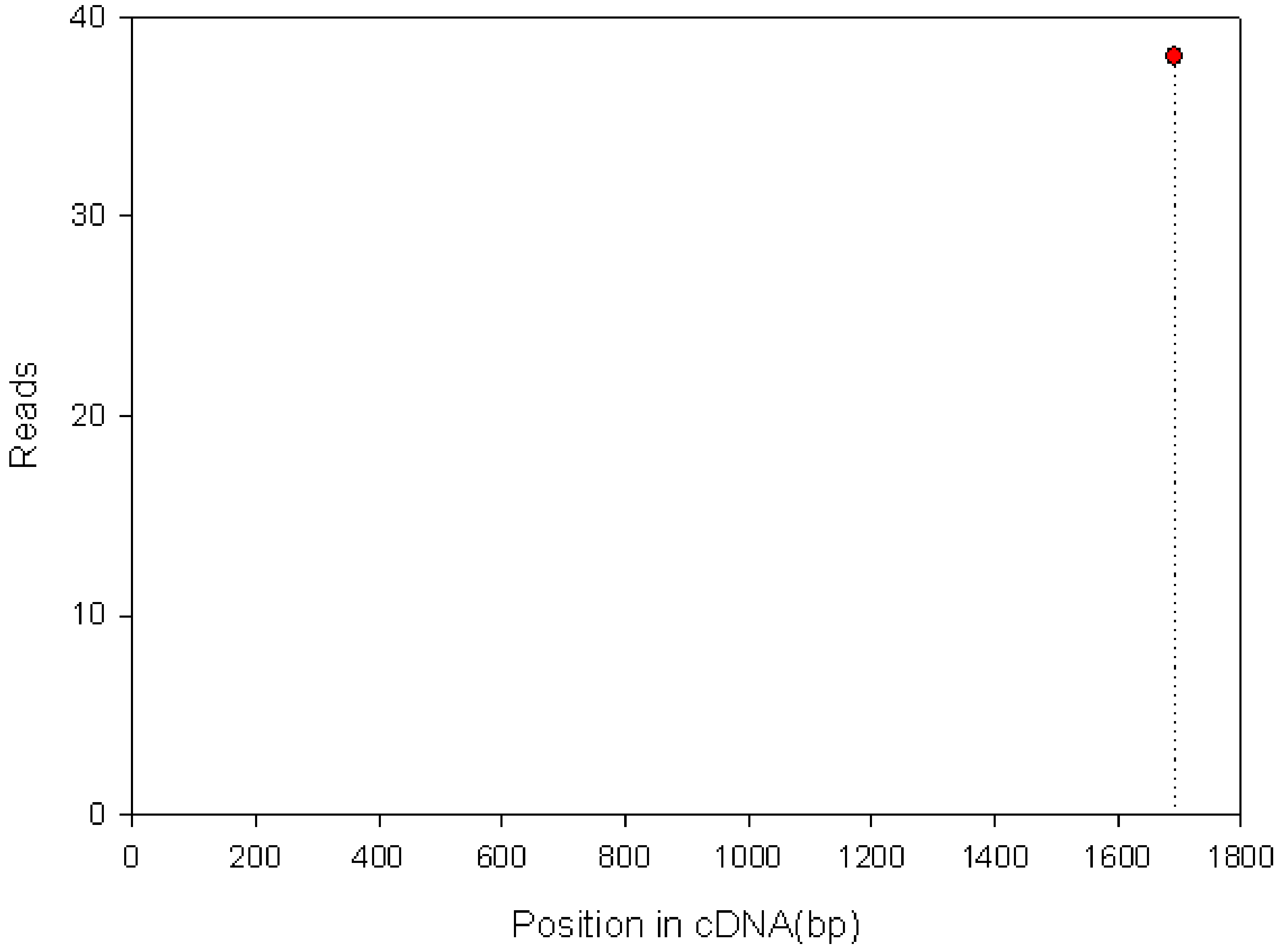


```
5' CUAUGCUCACCCUCUUCUGUCAUCUA 3'        Cs5g12260.1
    : ::::::: :::::::::::
3' -ACACGAGUGAGAGAAGACAGU---- 5'        Csi-miR156c.1
```

Csi-miR156c.1, target=Cs7g11770.1 gene=Cs7g11770
Category:1
Score=2
Cleavage Site=1487

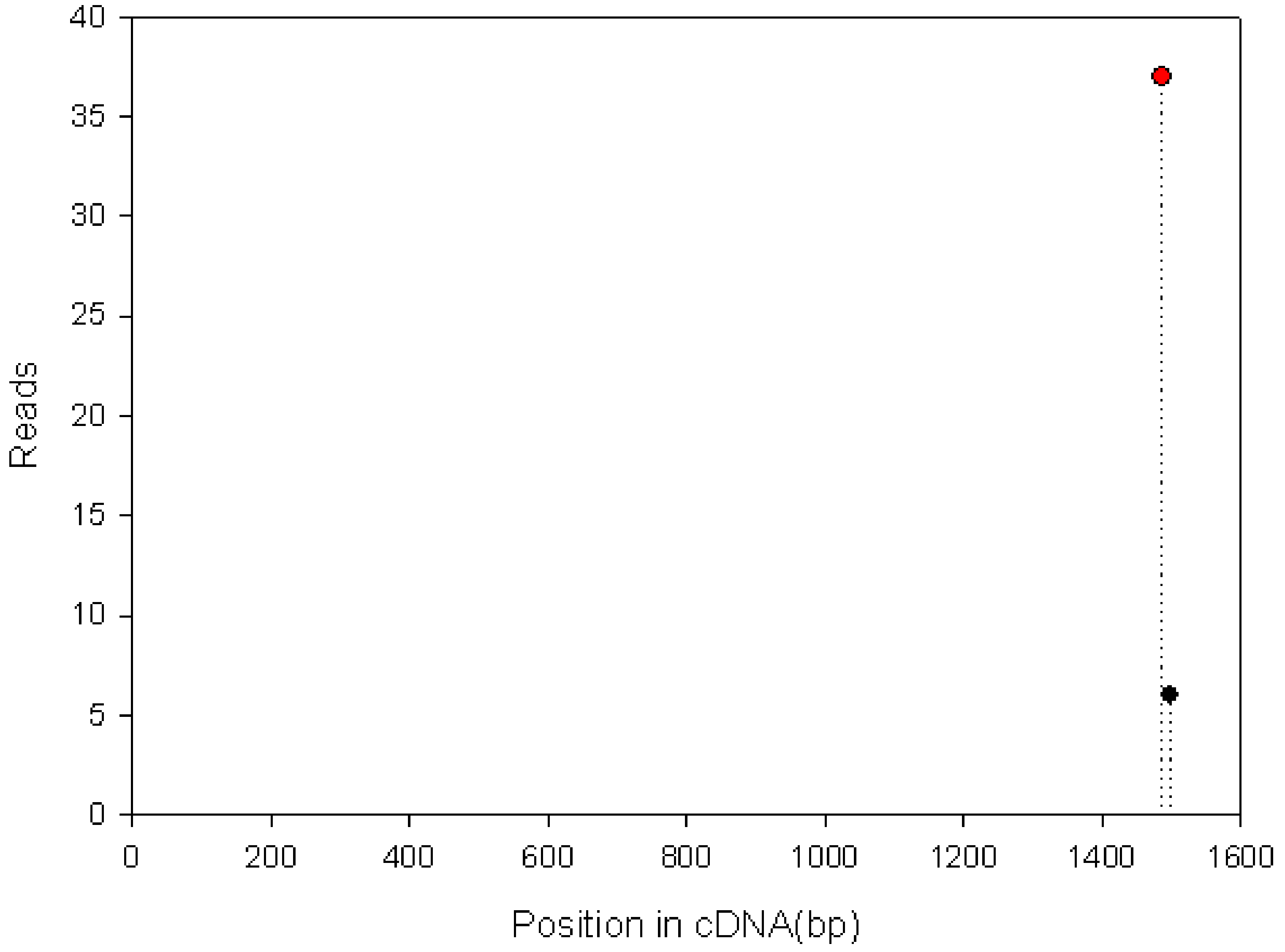


```
5' UGGUGCUCUCUCUCUUCUGUCAGCUC 3'        Cs7g11770.1
    :::::: :::::::::::::
3' -ACACGAGUGAGAGAAGACAGU---- 5'        Csi-miR156c.1
```

Csi-miR156d, target=Cs2g23550.1 gene=Cs2g23550
Category:1
Score=3.5
Cleavage Site=934

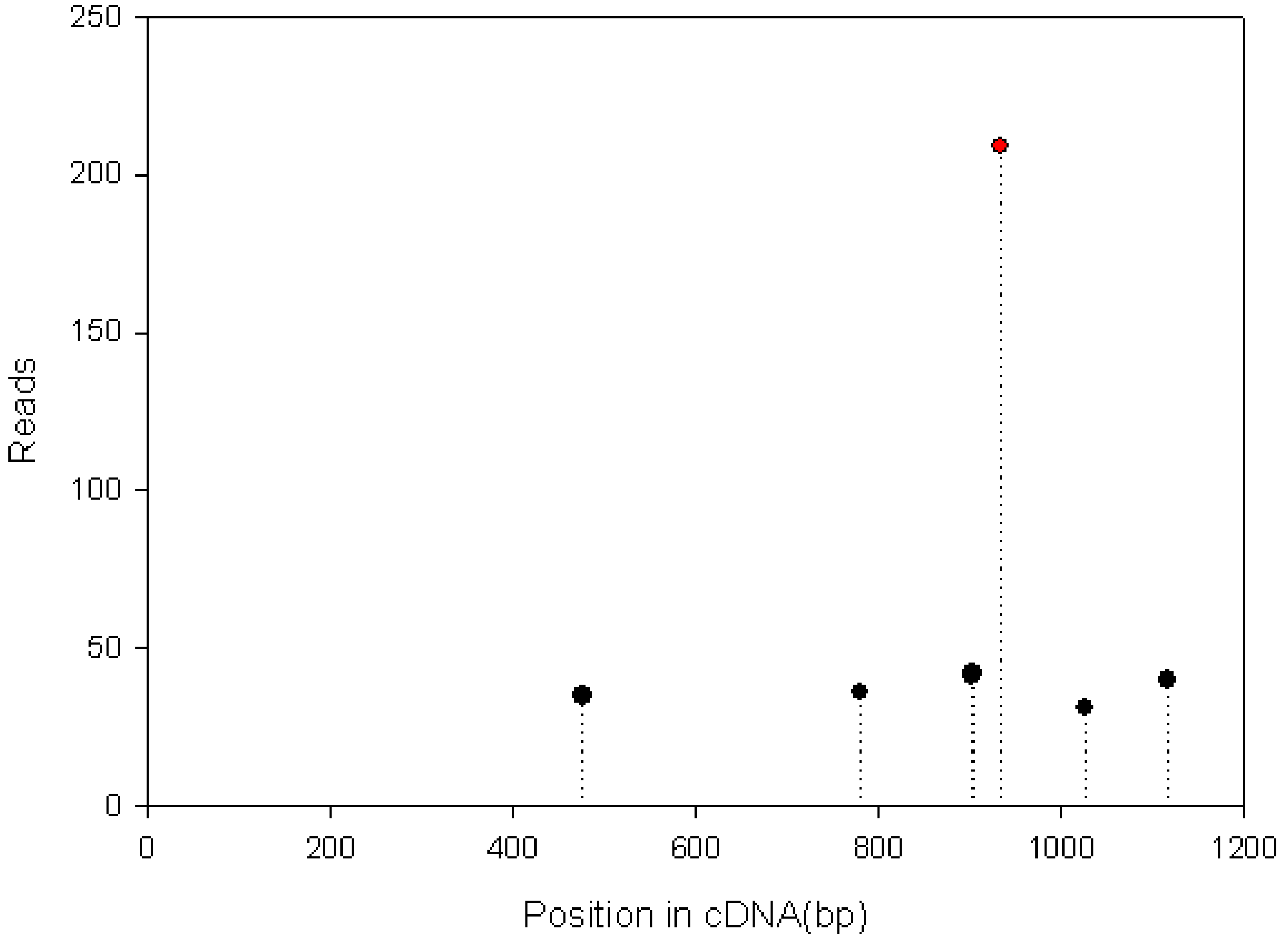


```
5' CAUGCUCUCUCUCUUCUGUCAUCCUA 3'      Cs2g23550.1
     .::::::: ::::::::::
3' -CGCGAGAGAUAGAAGACAGU----- 5'      Csi-miR156d
```

Csi-miR156d, target=Cs7g11770.1 gene=Cs7g11770
Category:1
Score=2.5
Cleavage Site=1487

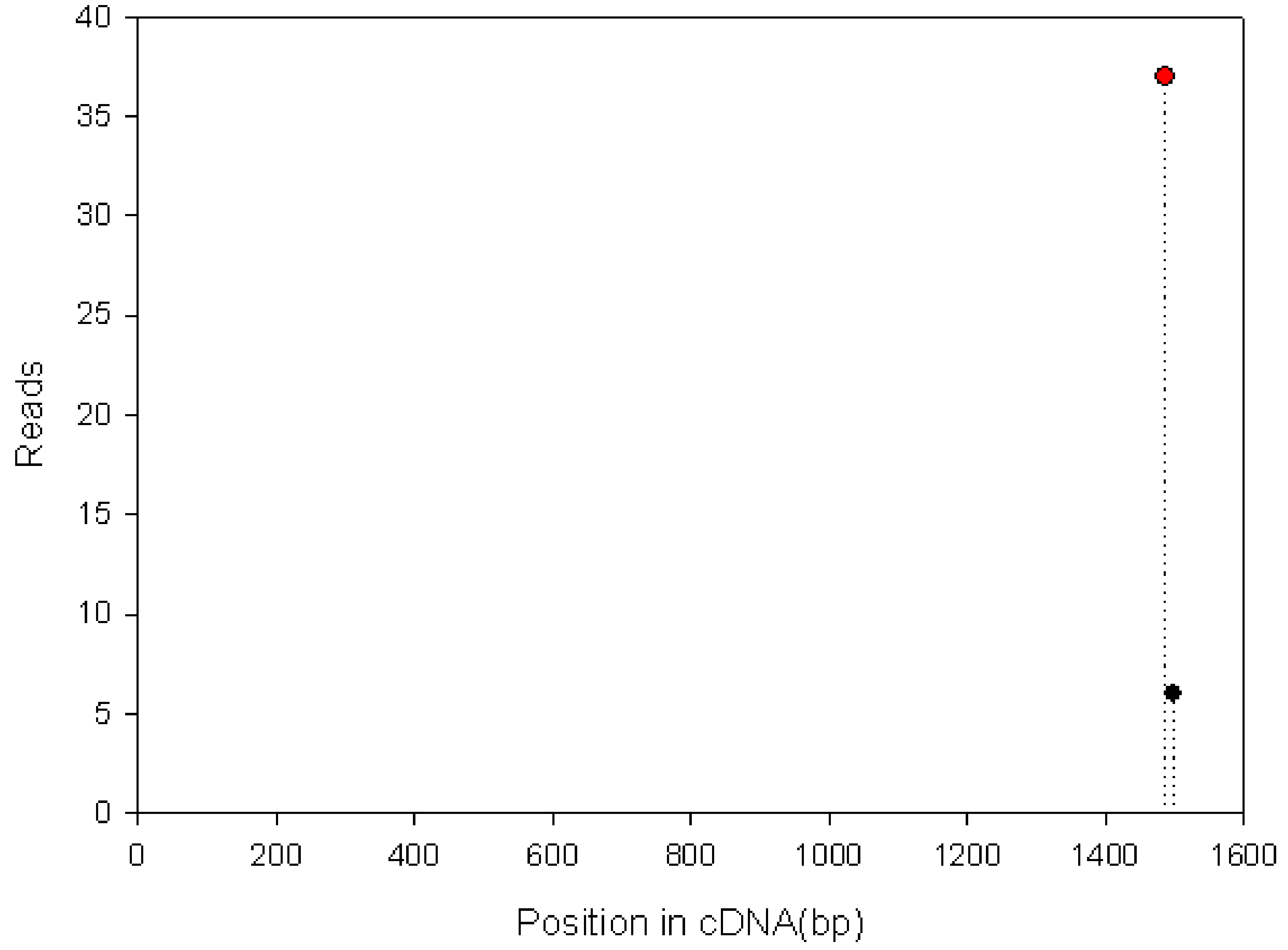


```
5' UGGUGCUCUCUCUCUUCUGUCAGCUC 3'        Cs7g11770.1
     :.::::::: ::::::::::
3' --CGCGAGAGAUAGAAGACAGU---- 5'        Csi-miR156d
```

Csi-miR156e, target=Cs2g23550.1 gene=Cs2g23550
Category:1
Score=4
Cleavage Site=934

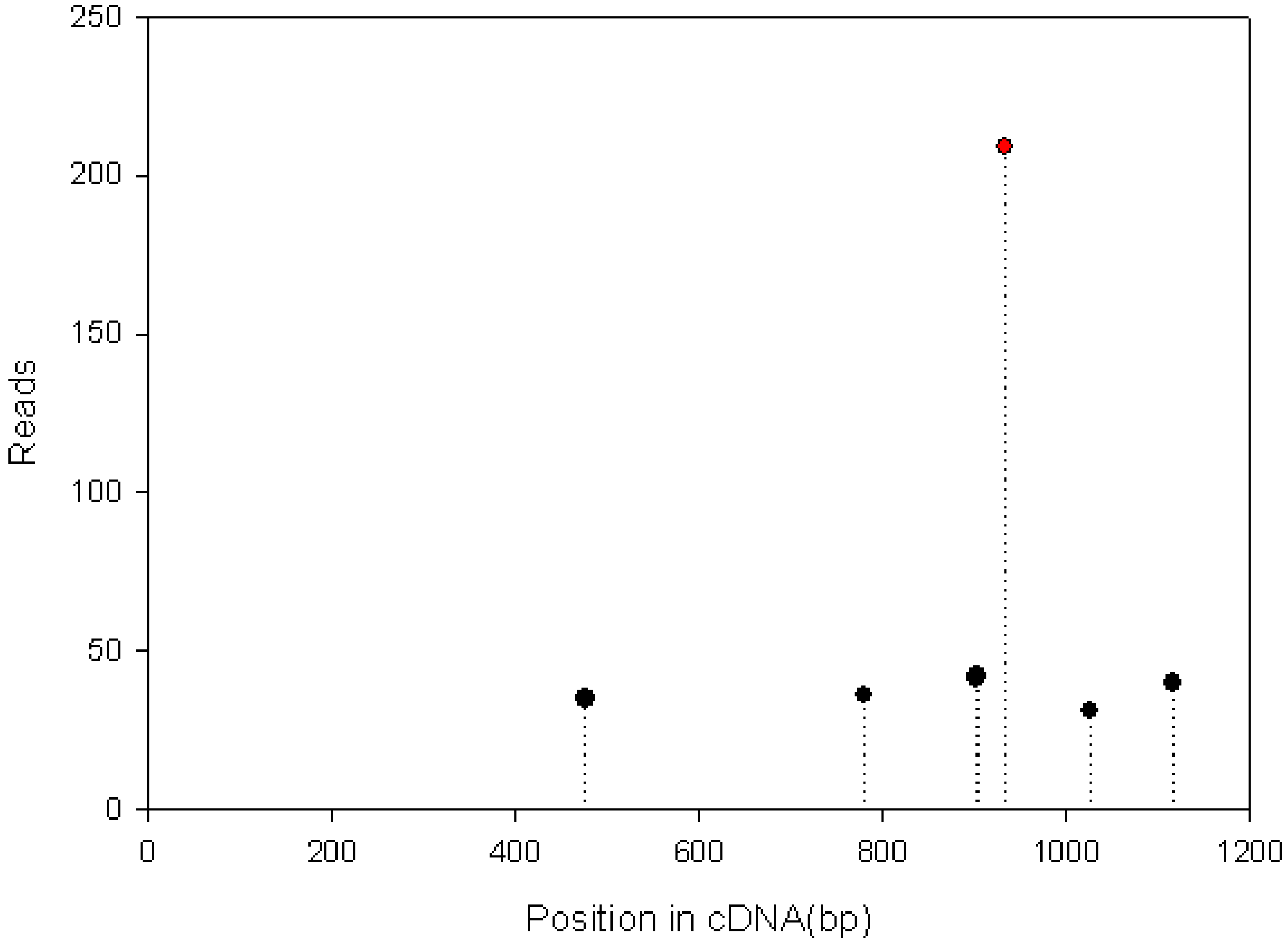


```
5' CAUGCUCUCUCUCUUCUGUCAUCCUA 3'        Cs2g23550.1
    .::::::: :::::::::::.
3' -CGCGAGAGAUAGAAGACAGUG---- 5'        Csi-miR156e
```

Csi-miR156e, target=Cs4g07790.1 gene=Cs4g07790
Category:3
Score=5
Cleavage Site=424

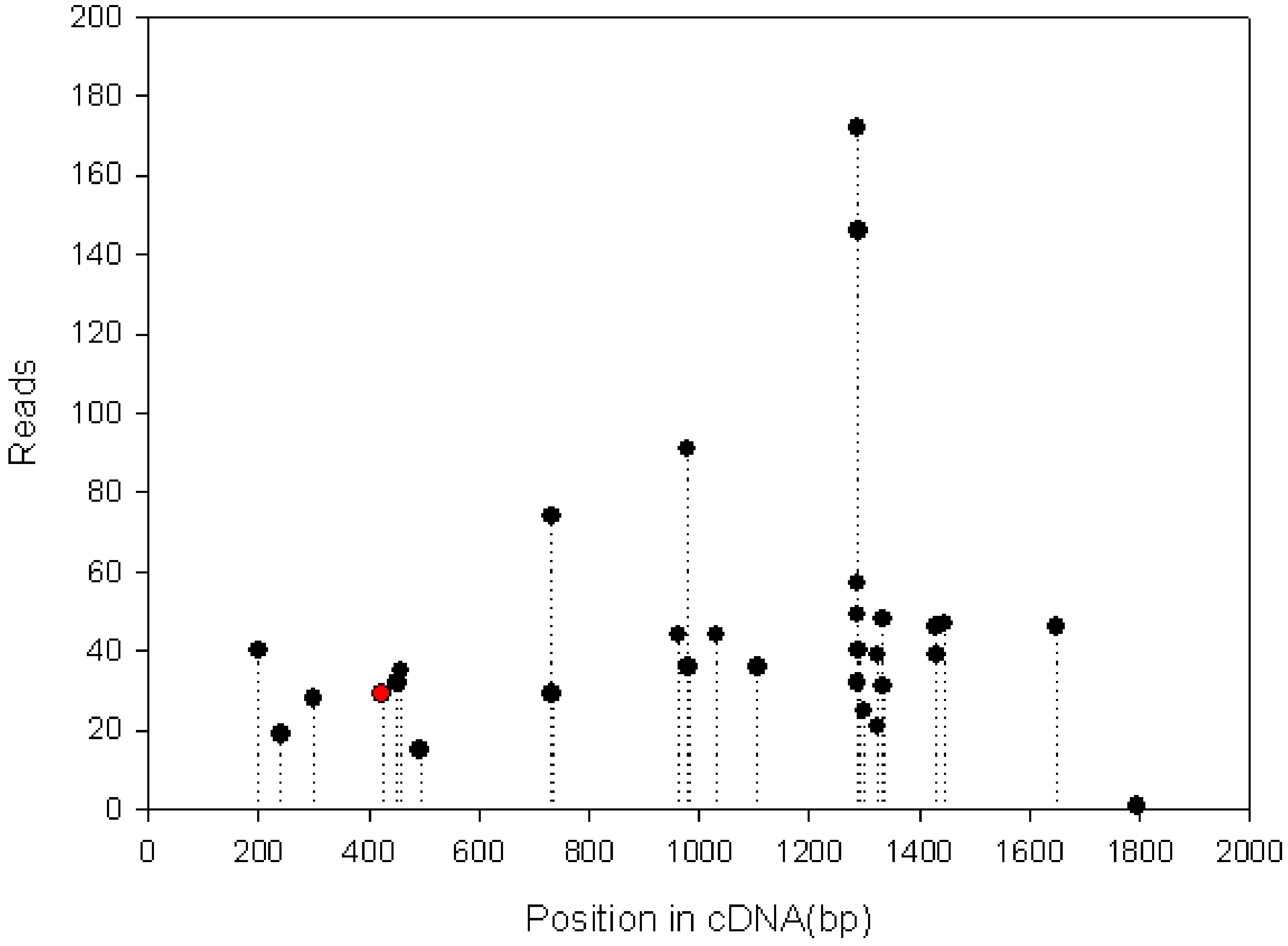


```
5' GCAAUGGUGAUCUUUG-CUUCUGUCAC 3'        Cs4g07790.1
         :.: :::.:. ::::::::::
3' ------CGCGAGAGAUAGAAGACAGUG 5'        Csi-miR156e
```

Csi-miR156e, target=Cs7g11770.1 gene=Cs7g11770
Category:1
Score=3.5
Cleavage Site=1487

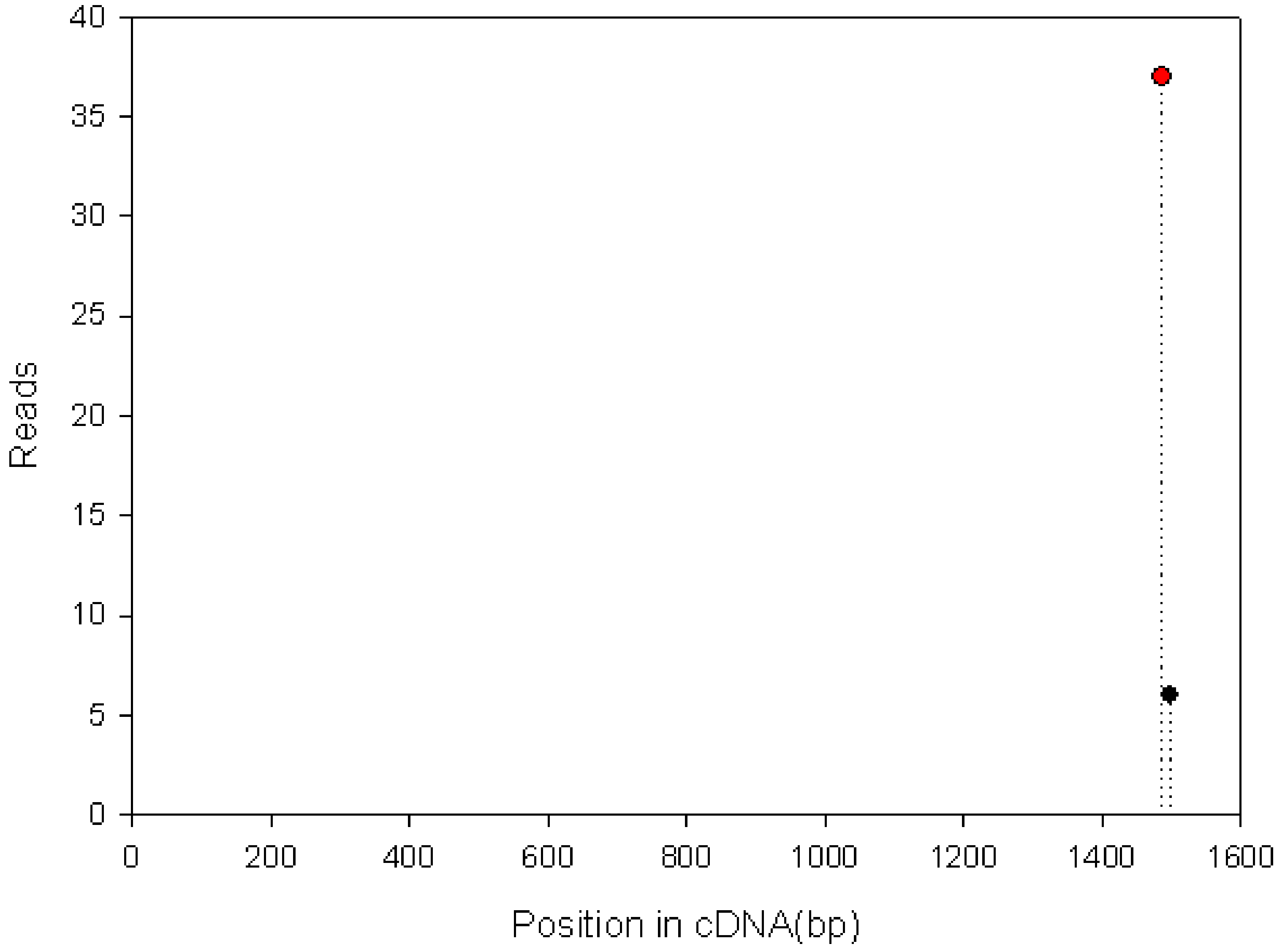


```
5' UGGUGCUCUCUCUCUUCUGUCAGCUC 3'        Cs7g11770.1
     :.::::::: ::::::::::
3' --CGCGAGAGAUAGAAGACAGUG--- 5'        Csi-miR156e
```

Csi-miR156f.2, taget=Cs2g23550.1 gene=Cs2g23550
Category:1
Score=1.5
Cleavage Site=934

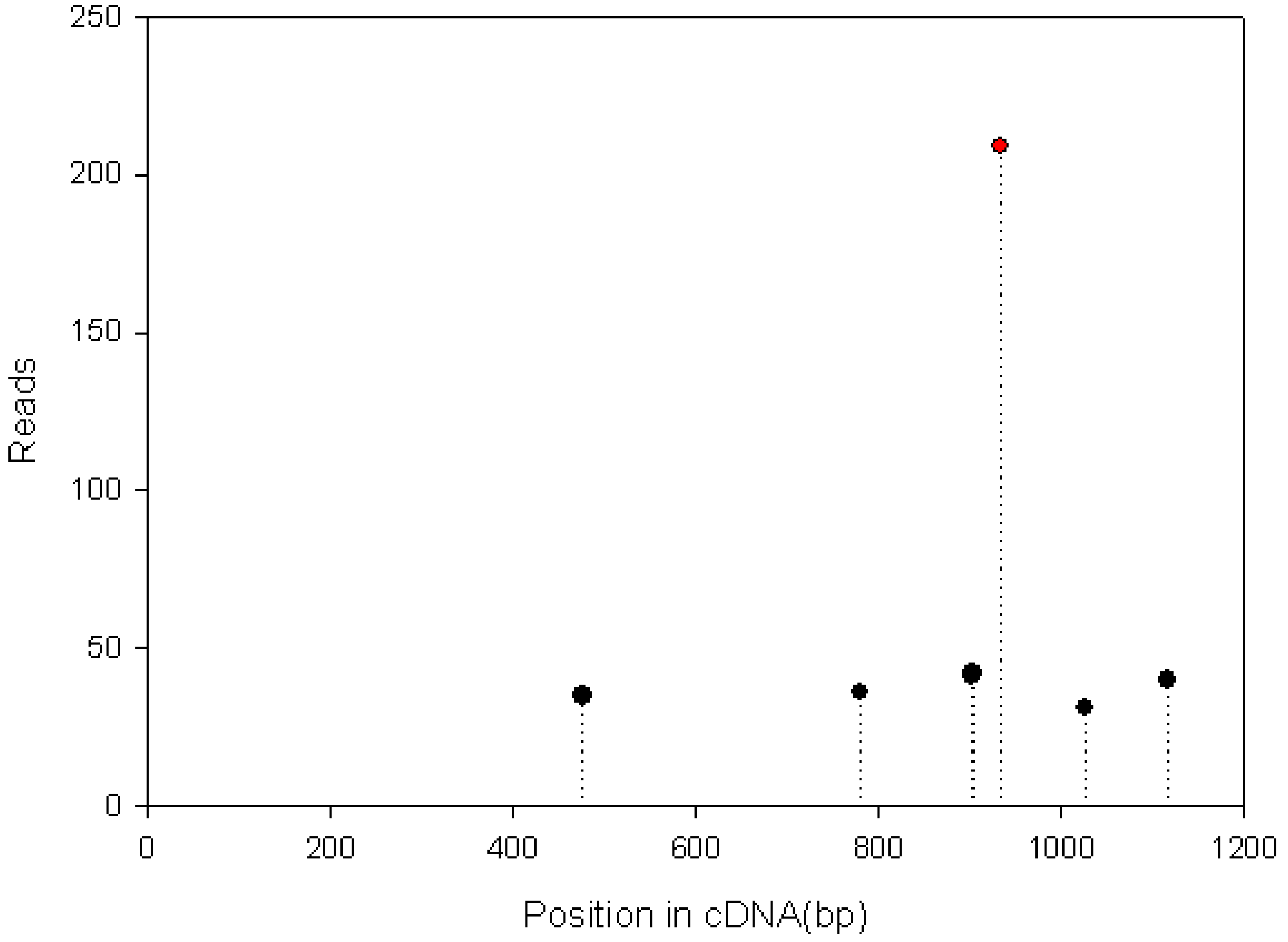


```
5' CAUGCUCUCUCUCUUCUGUCAUCCUA 3'        Cs2g23550.1
     :.:::::::::::::::::::
3' -CAUGAGAGAGAGAAGACAGUA---- 5'        Csi-miR156f.2
```

Csi-miR156f.2, target=Cs3g10870.1 gene=Cs3g10870
Category:1
Score=4
Cleavage Site=244

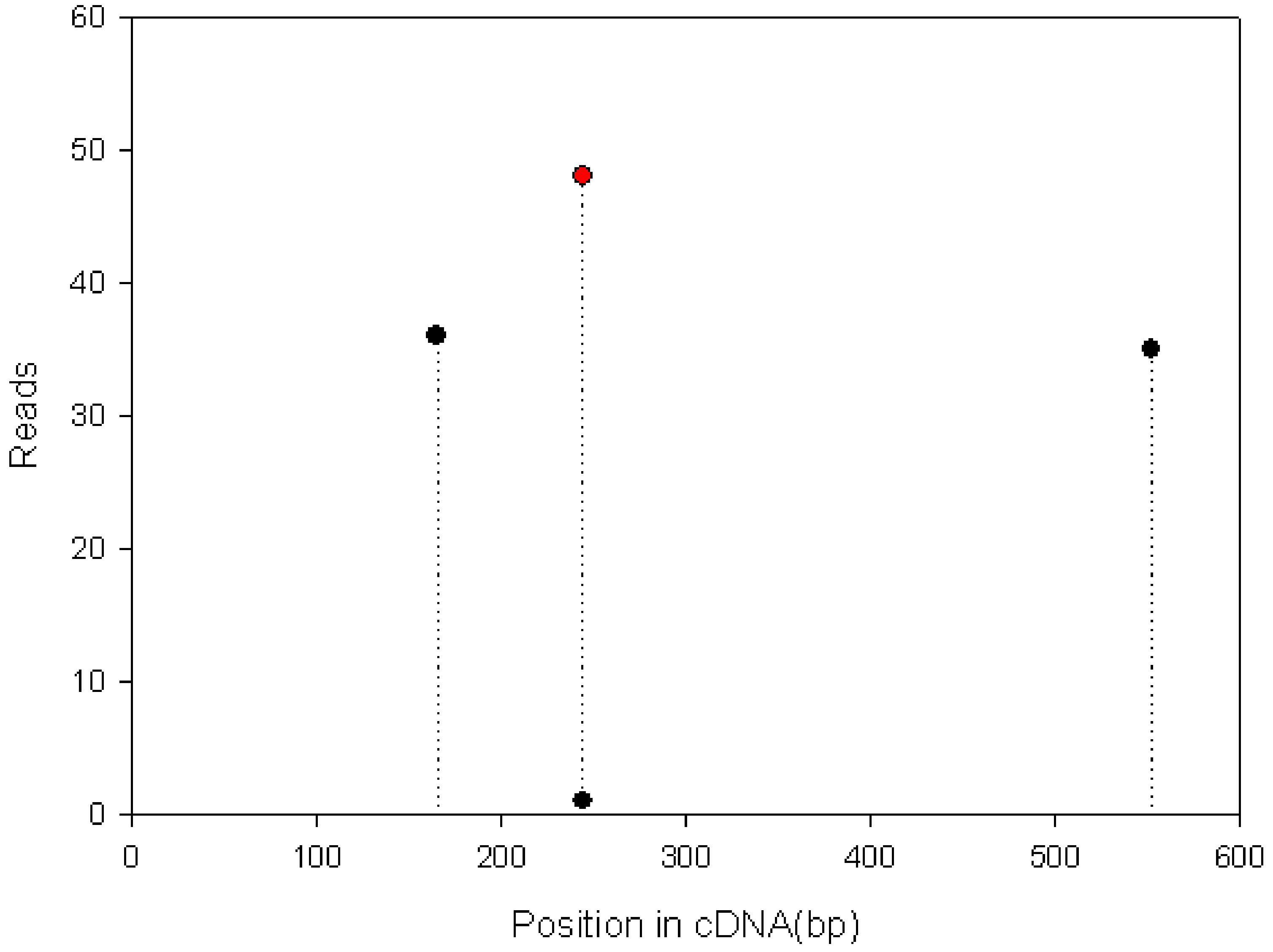


```
5' CUUCGUCCUCU-UCUUUUCUGUCAACA 3'        Cs3g10870.1
      :: :::: :::.:::::::::
3' ----CAUGAGAGAGAGAAGACAGUA-- 5'        Csi-miR156f.2
```

Csi-miR156f.2, target=Cs5g12260.1 gene=Cs5g12260
Category:1
Score=4.5
Cleavage Site=1693

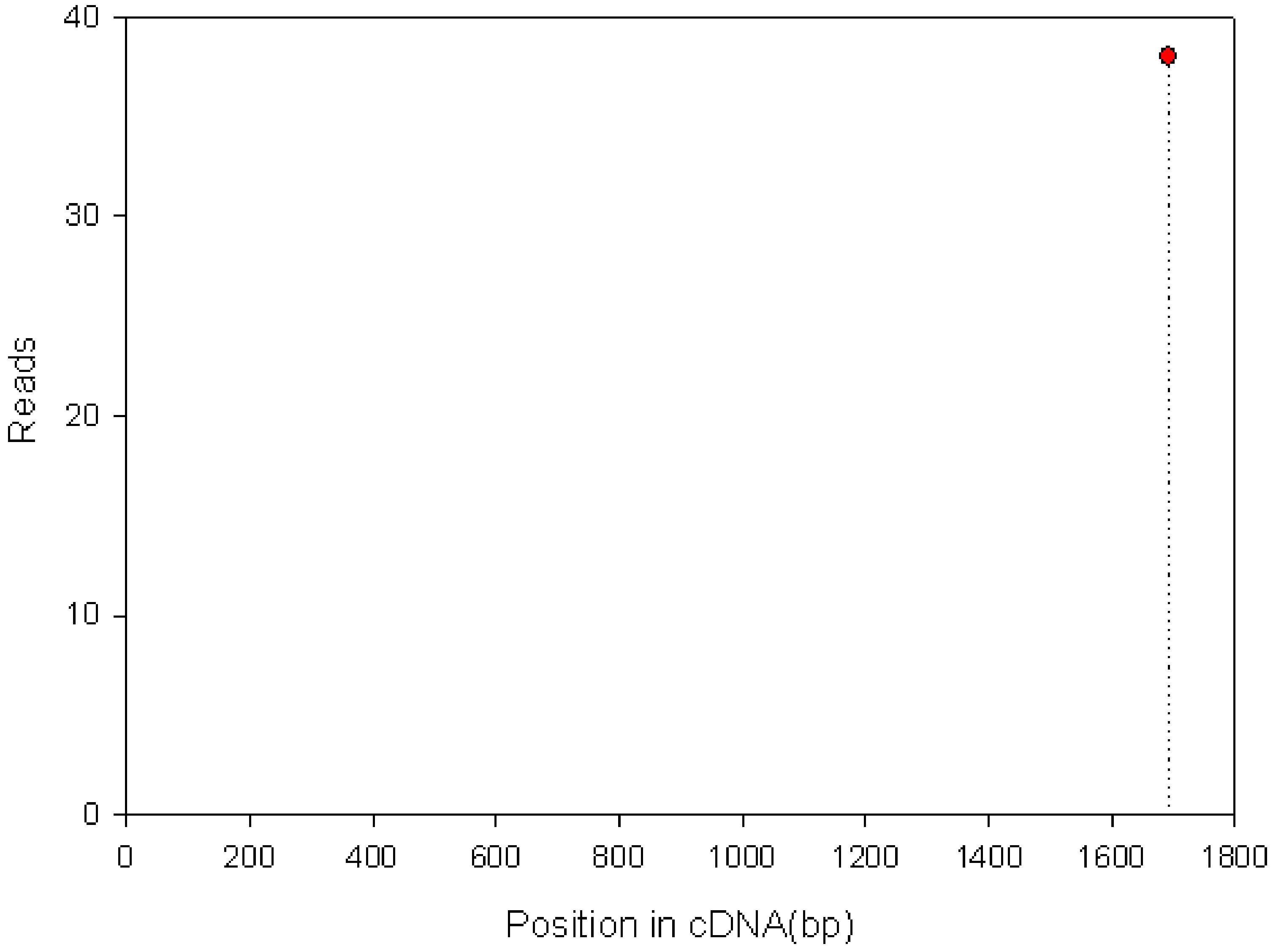


```
5' CUAUGCUCACCCUCUUCUGUCAUCUA 3'        Cs5g12260.1
      :.::: : ::::::::::::
3' --CAUGAGAGAGAGAAGACAGUA--- 5'        Csi-miR156f.2
```

Csi-miR156f.2, target=Cs7g11770.1 gene=Cs7g11770
Category:1
Score=1.5
Cleavage Site=1487

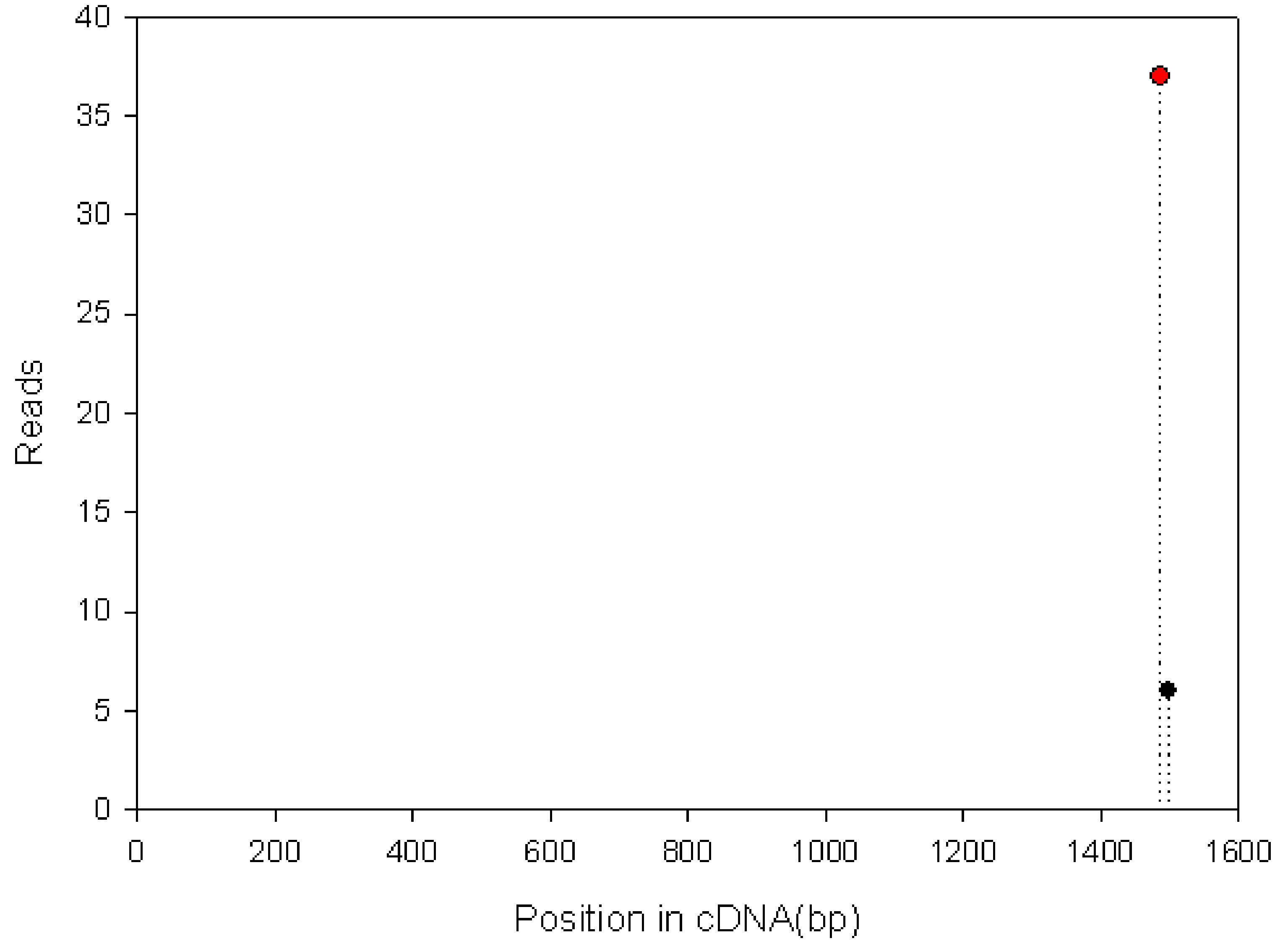


```
5' UGGUGCUCUCUCUCUUCUGUCAGCUC 3'        Cs7g11770.1
     ::.::::::::::::::::::
3' --CAUGAGAGAGAGAAGACAGUA--- 5'        Csi-miR156f.2
```

Csi-miR156f.2, target=Cs8g19900.1 gene=Cs8g19900
Category:2
Score=5
Cleavage Site=2794

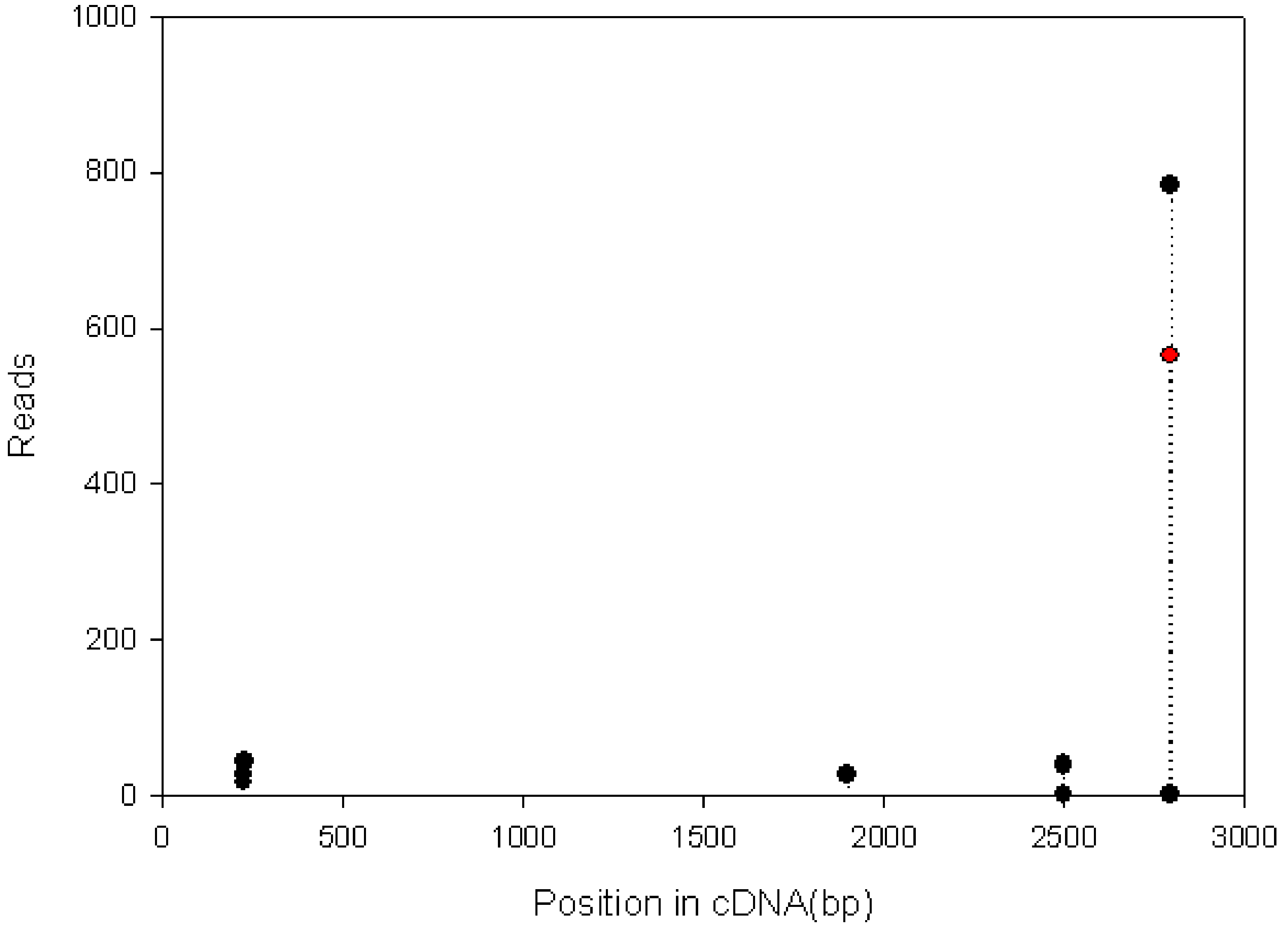


```
5' GUG-UCUUUCUCUUUUGACAUUAUGAG 3'       Cs8g19900.1
   ::. :::.:::::::.:: :::
3' CAUGAGAGAGAGAAGACAGUA------ 5'       Csi-miR156f.2
```

Csi-miR156f.2, target=Orange1.1t01983.1 gene=Orange1.1t01983
Category:3
Score=3.5
Cleavage Site=693

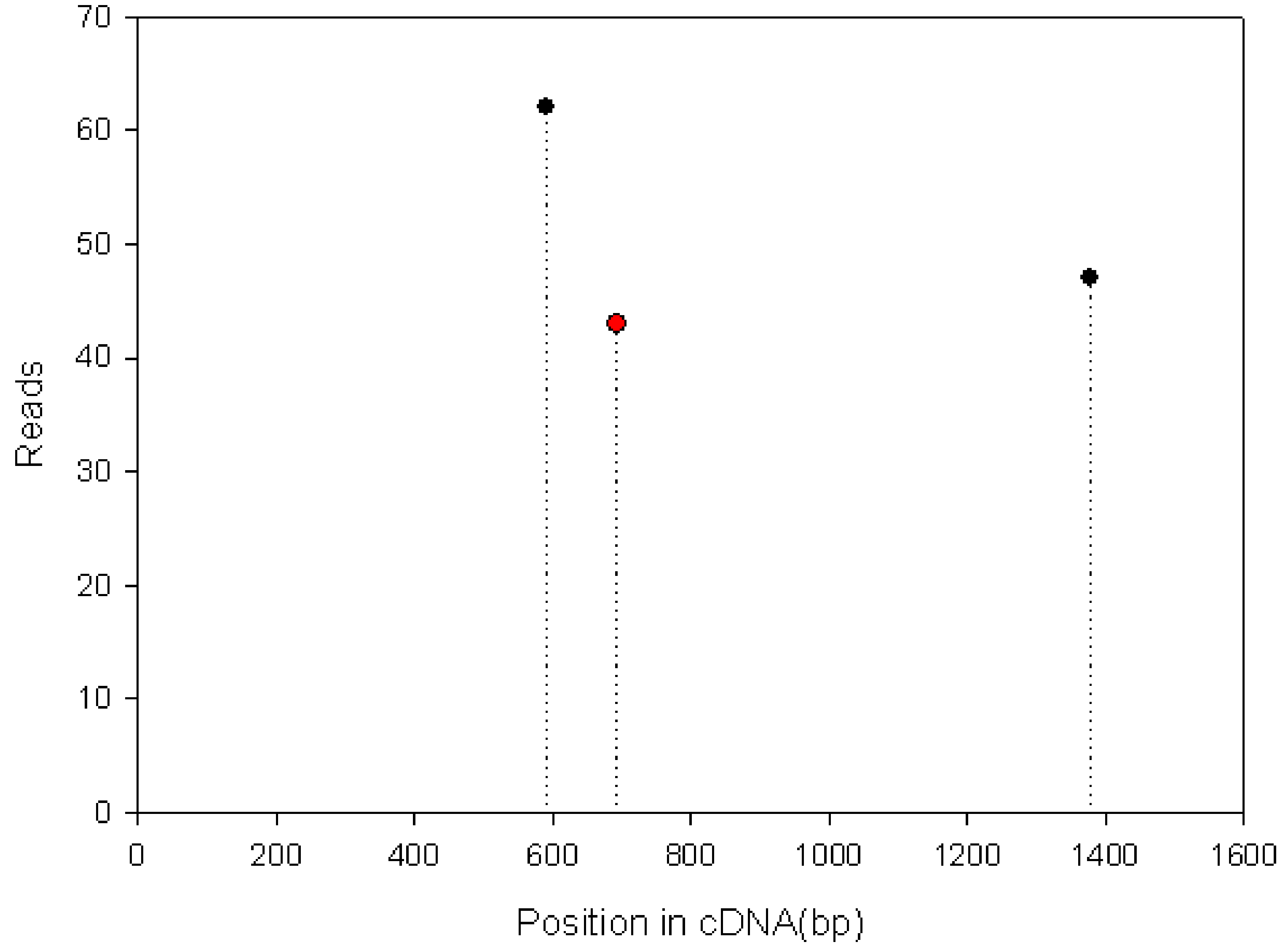


```
5' CUUGCUUCUGUCUCUUCUGUCAUCAC 3'        Orange1.1t01983.1
     :.: ::: :::::::::::::
3' -CAUG-AGAGAGAGAAGACAGUA--- 5'        Csi-miR156f.2
```

Csi-miR156g.1, target=Cs2g23550.1 gene=Cs2g23550
Category:1
Score=5
Cleavage Site=934

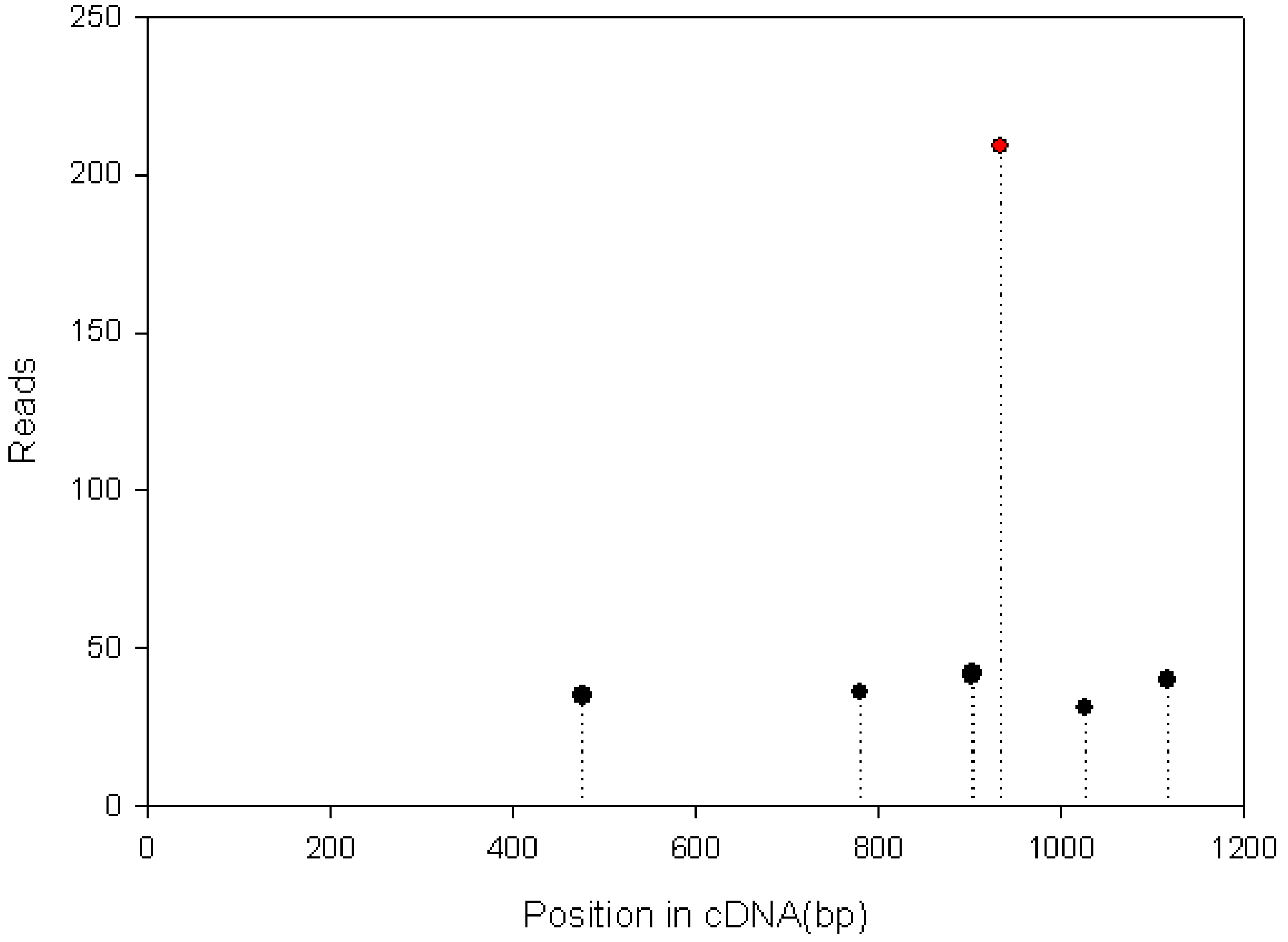


```
5' CAUGCUCUCUCUCUUCUGUCAUCCUA 3'        Cs2g23550.1
     :::::::: ::::::.::::
3' -CACGAGAGAUAGAAGGCAGUU---- 5'        Csi-miR156g.1
```

Csi-miR156g.1, target=Cs7g11770.1 gene=Cs7g11770
Category:1
Score=3.5
Cleavage Site=1487

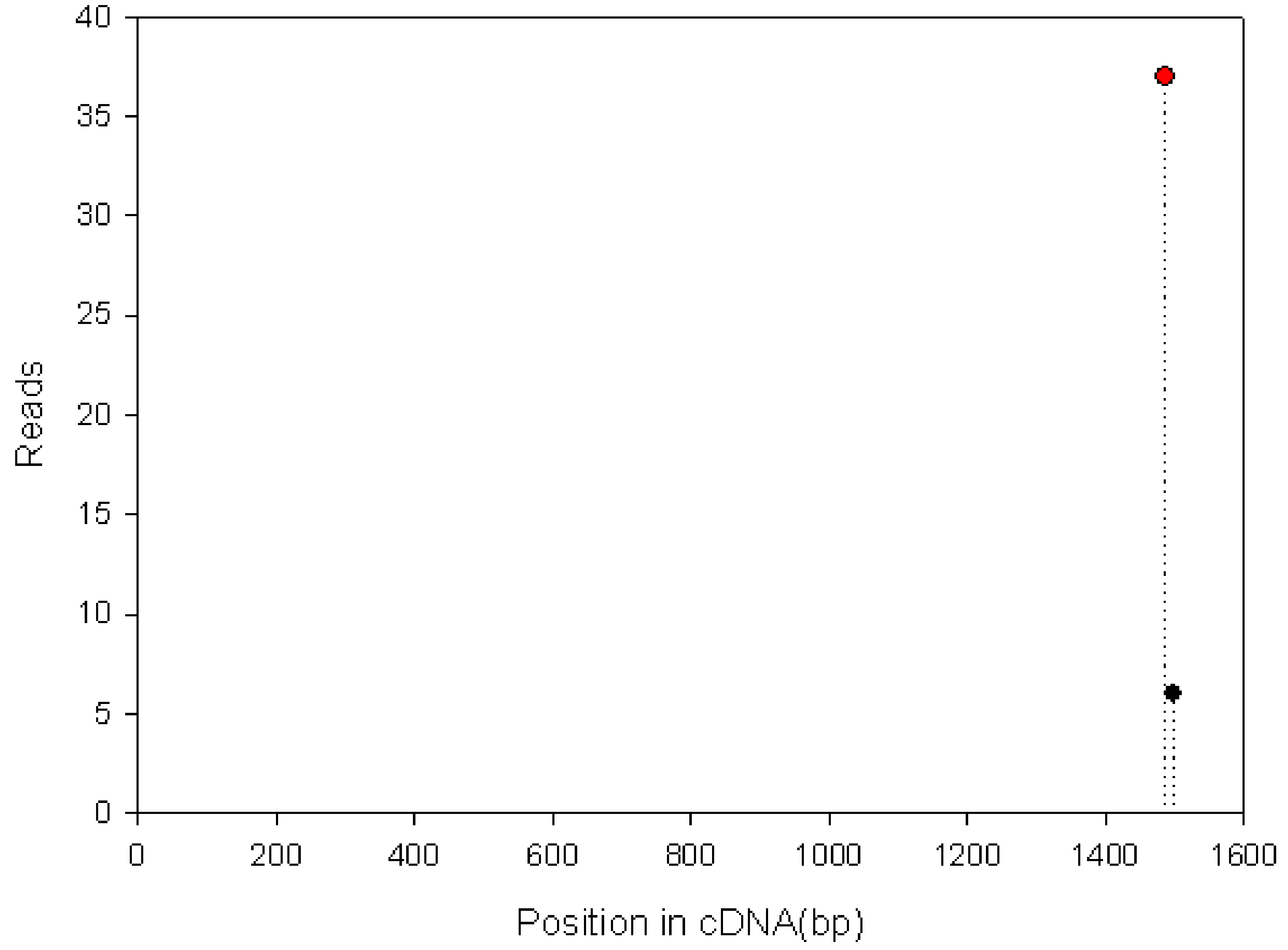


```
5' UGGUGCUCUCUCUCUUCUGUCAGCUC 3'        Cs7g11770.1
     ::::::::: :::::.::::.
3' --CACGAGAGAUAGAAGGCAGUU--- 5'        Csi-miR156g.1
```

Csi-miR156g.2, target=Cs2g23550.1 gene=Cs2g23550
Category:1
Score=4
Cleavage Site=934

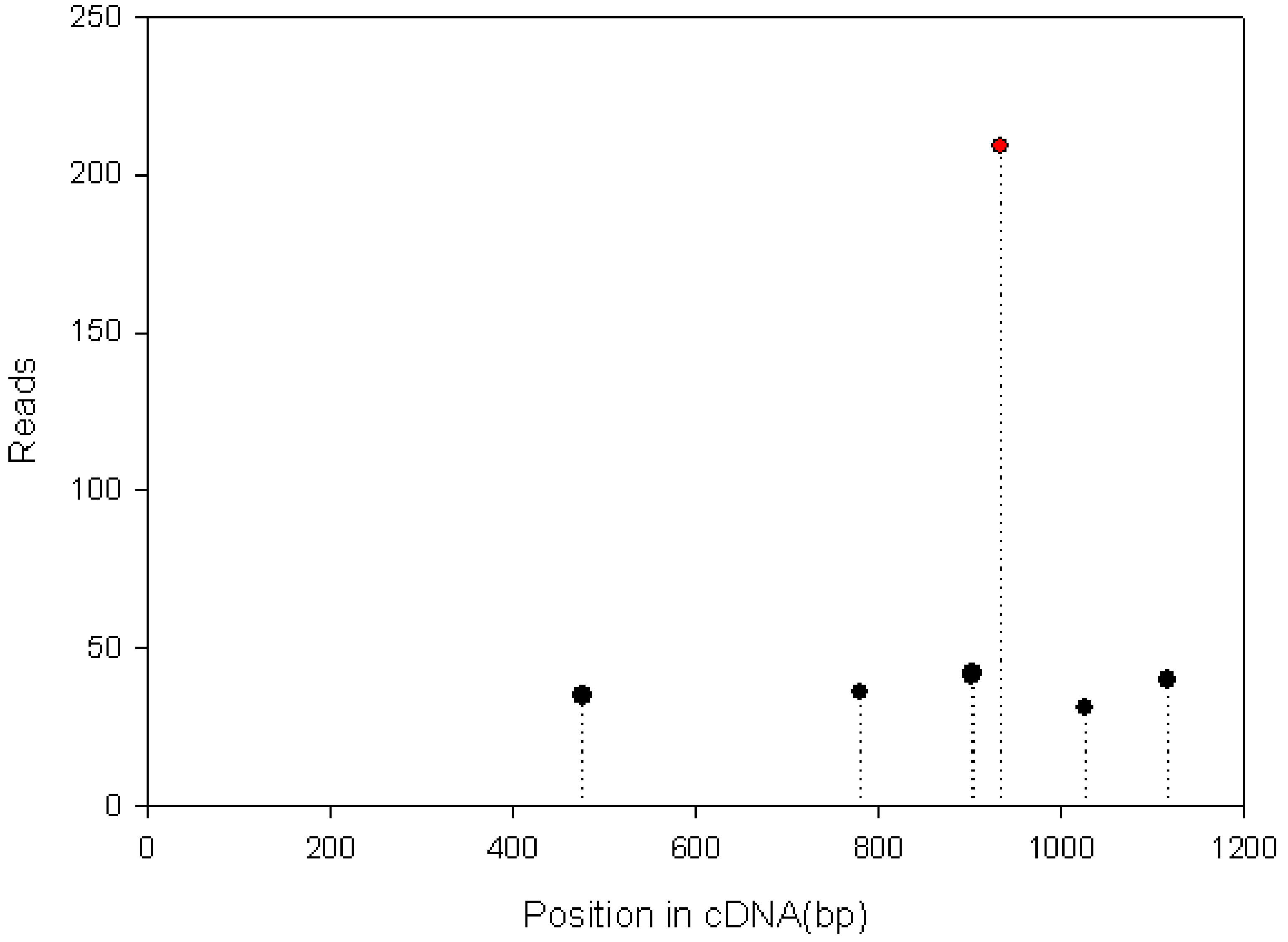


```
5' CAUGCUCUCUCUUCUGUCAUCCUA 3'        Cs2g23550.1
     :::::::: ::::::.::::
3' -CACGAGAGAUAGAAGGCAGU----- 5'       Csi-miR156g.2
```

Csi-miR156g.2, target=Cs7g11770.1 gene=Cs7g11770
Category:1
Score=3
Cleavage Site=1487

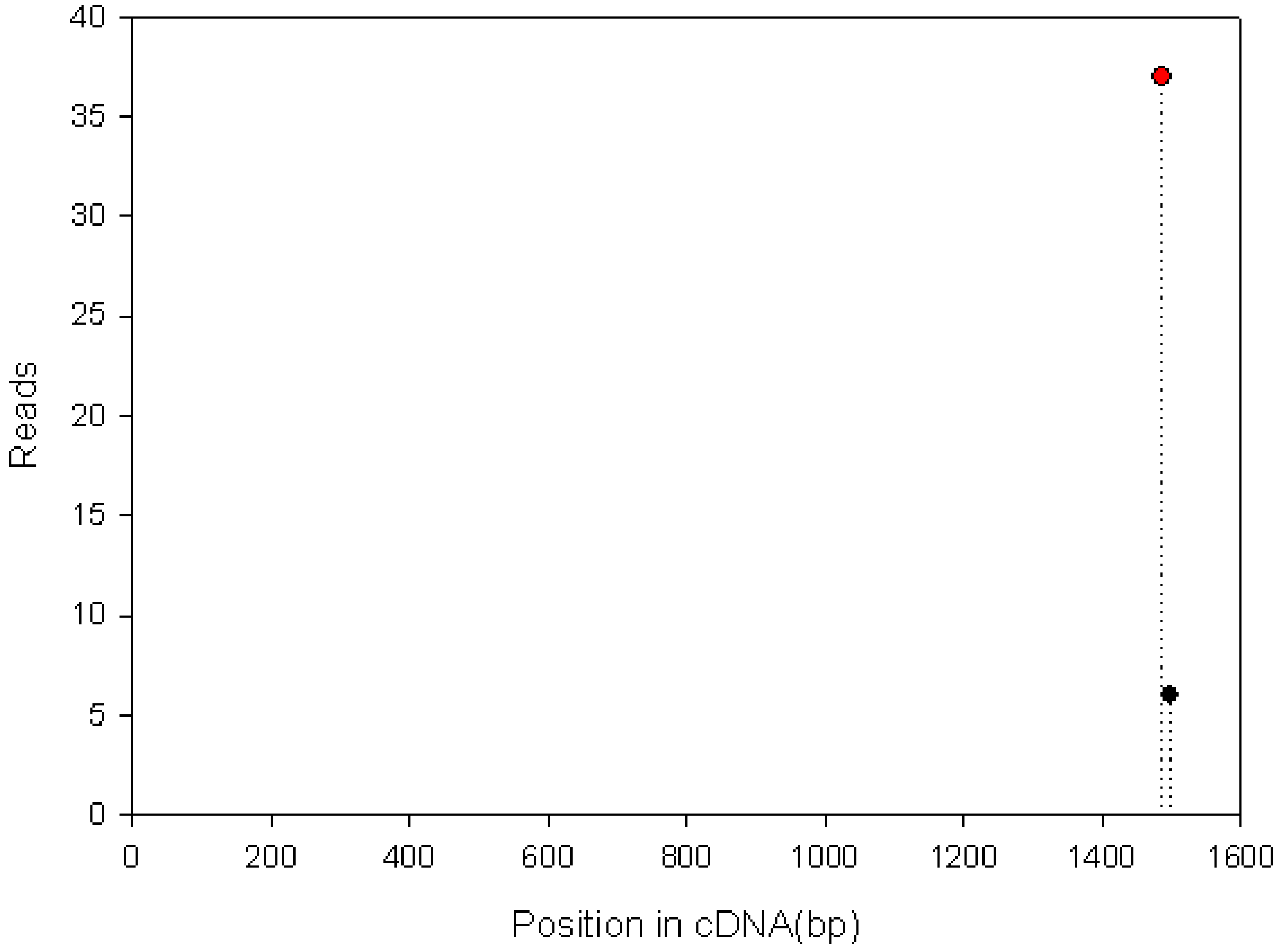


```
5' UGGUGCUCUCUCUCUUCUGUCAGCUC 3'        Cs7g11770.1
     ::::::::: :::::.::::
3' --CACGAGAGAUAGAAGGCAGU---- 5'        Csi-miR156g.2
```

Csi-miR156h, target=Cs1g26030.1 gene=Cs1g26030
Category:1
Score=5
Cleavage Site=1018

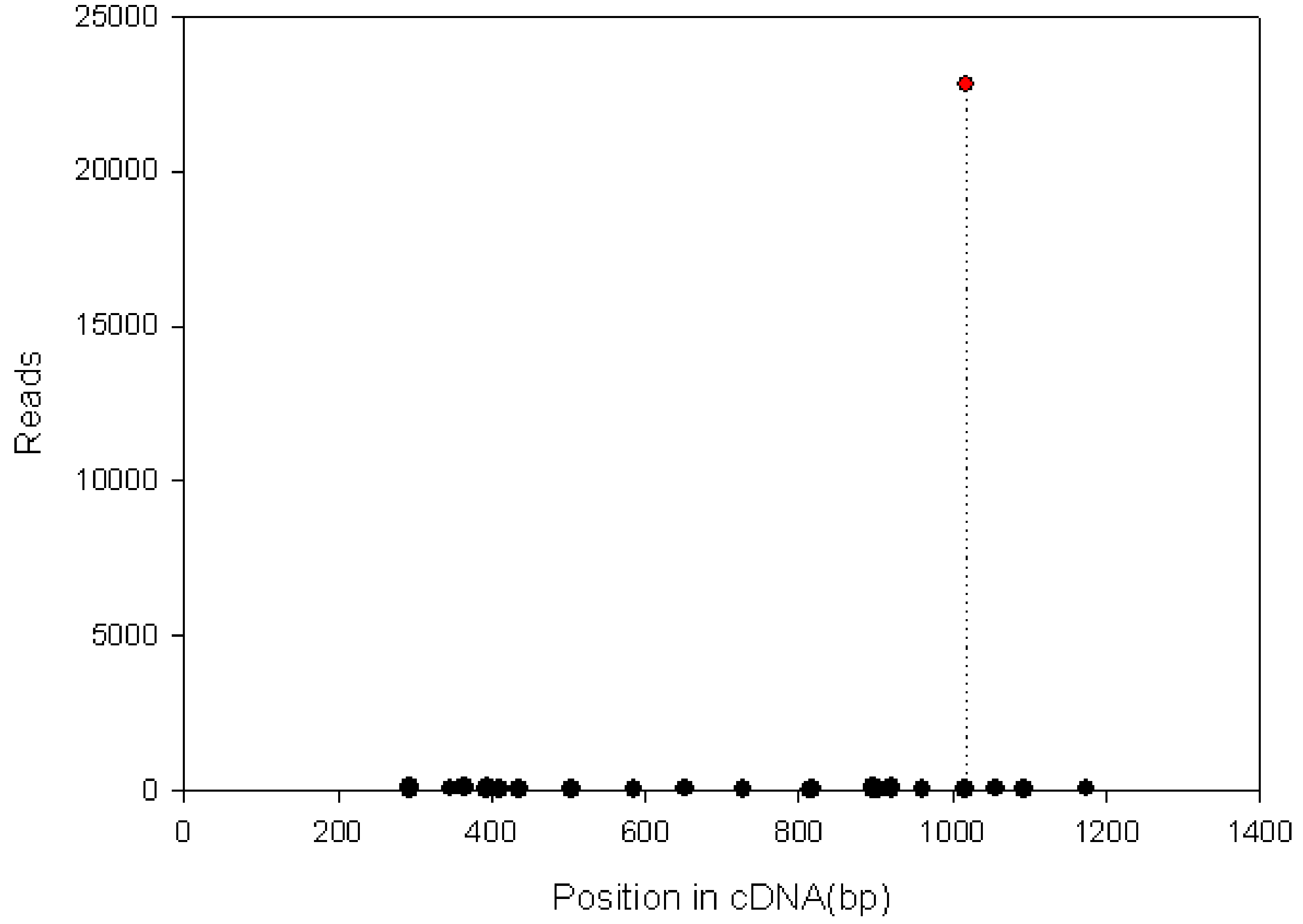


```
5' GUGCAUUUUCUUUUCUGUAAAUAACU 3'        Cs1g26030.1
   ::::  .:.:::.:::::: ::
3' CACGAGAGAGAGAAGACAGUU----- 5'        Csi-miR156h
```

Csi-miR156h, target=Cs2g23550.1 gene=Cs2g23550
Category:1
Score=2
Cleavage Site=934

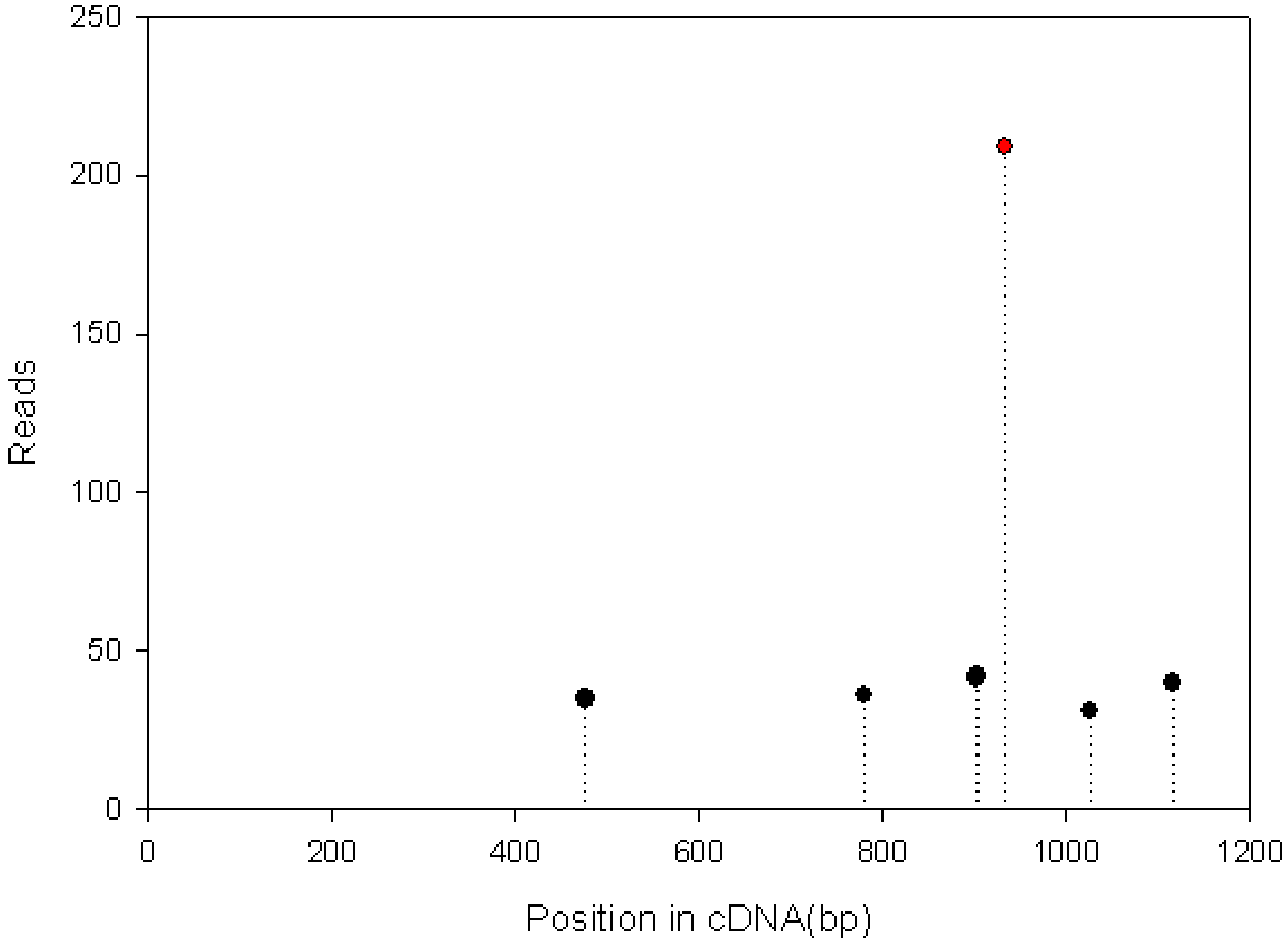


```
5' CAUGCUCUCUCUUCUGUCAUCCUA 3'        Cs2g23550.1
     ::::::::::::::::::::
3' -CACGAGAGAGAGAAGACAGUU---- 5'        Csi-miR156h
```

Csi-miR156h, target=Cs3g10870.1 gene=Cs3g10870
Category:1
Score=3
Cleavage Site=244

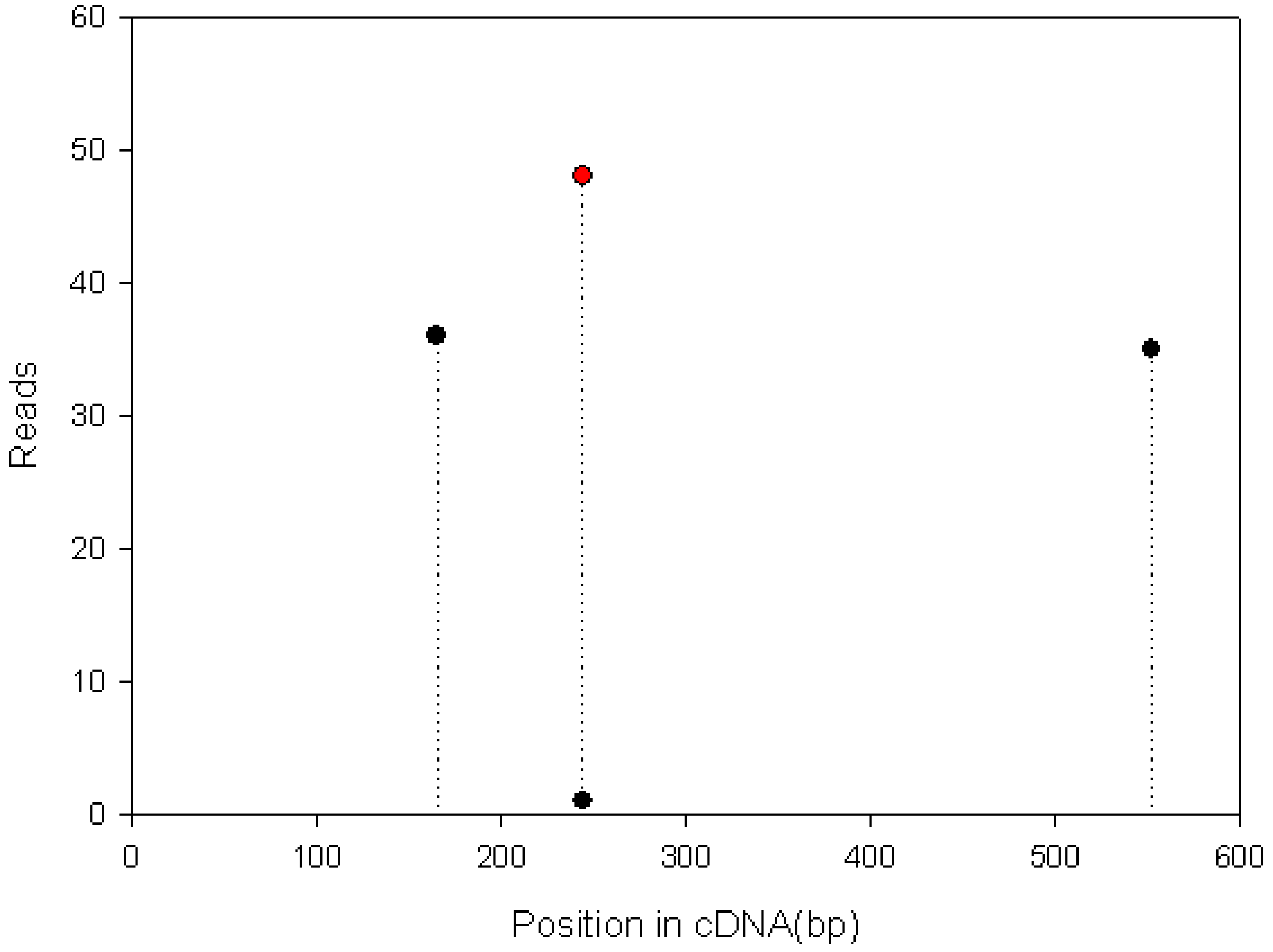


```
5' CUUCGUCCUCU-UCUUUUCUGUCAACA 3'        Cs3g10870.1
     :: :::: :::.::::::::::
3' ----CACGAGAGAGAGAAGACAGUU-- 5'        Csi-miR156h
```

Csi-miR156h, target=Cs5g12260.1 gene=Cs5g12260
Category:1
Score=5
Cleavage Site=1693

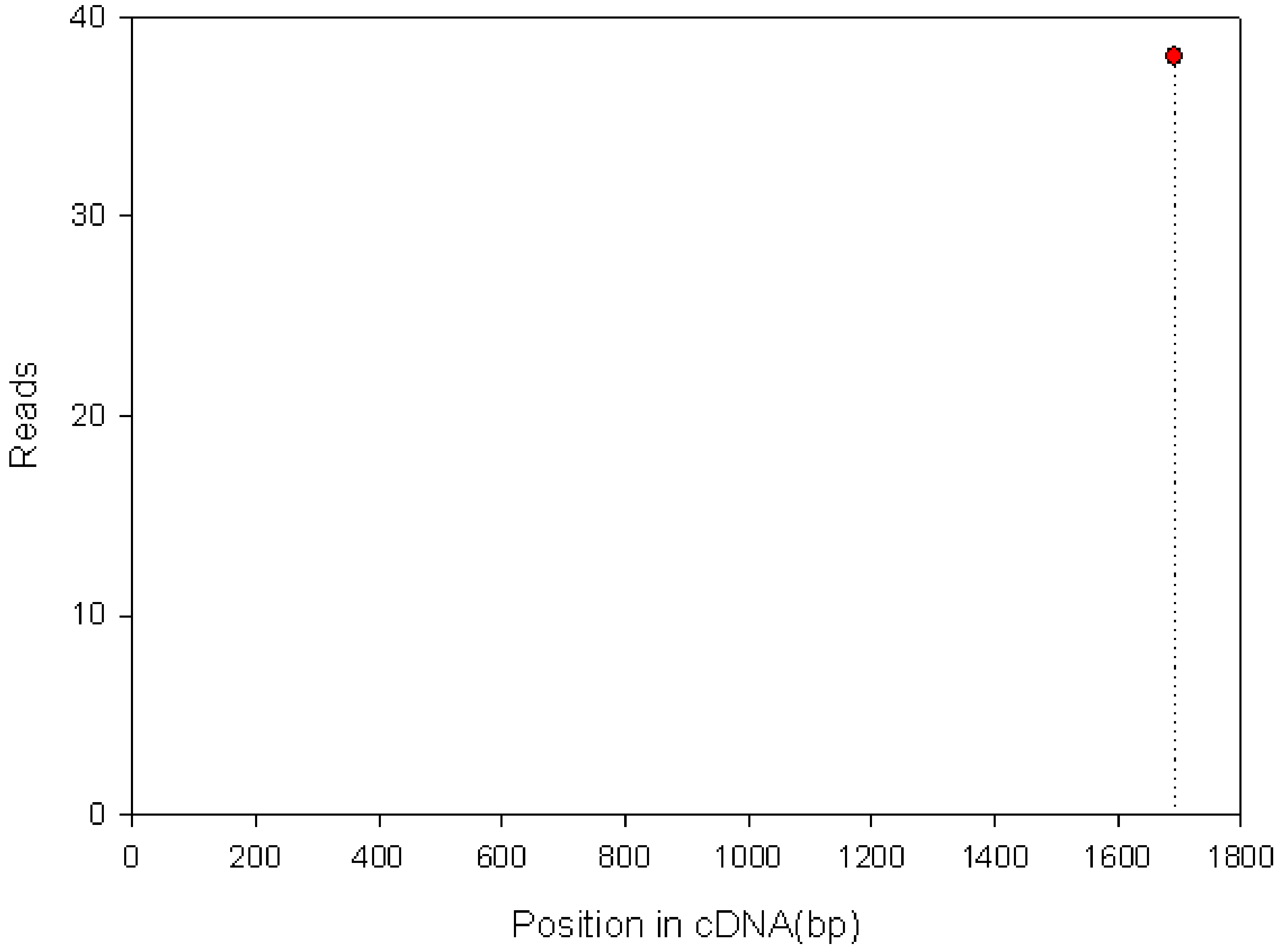


```
5' CUAUGCUCACCCUCUUCUGUCAUCUA 3'        Cs5g12260.1
       ::::: : :::::::::::
3' --CACGAGAGAGAGAAGACAGUU--- 5'        Csi-miR156h
```

Csi-miR156h, target=Cs7g11770.1 gene=Cs7g11770
Category:1
Score=0.5
Cleavage Site=1487

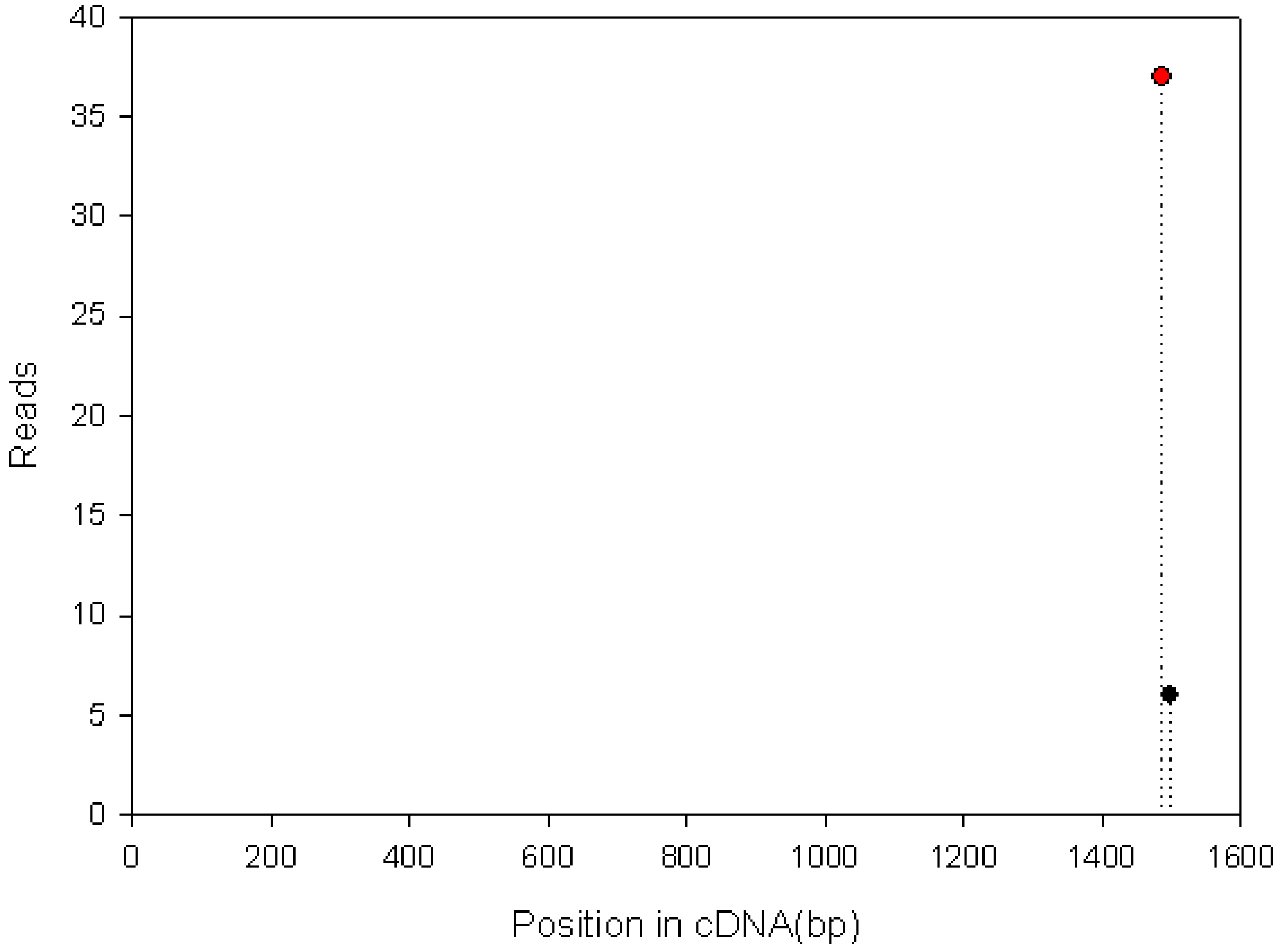


```
5' UGGUGCUCUCUCUCUUCUGUCAGCUC 3'        Cs7g11770.1
     ::::::::::::::::::::.
3' --CACGAGAGAGAGAAGACAGUU--- 5'        Csi-miR156h
```

Csi-miR156h, target=Orange1.1t01983.1 gene=Orange1.1t01983
Category:3
Score=4
Cleavage Site=693

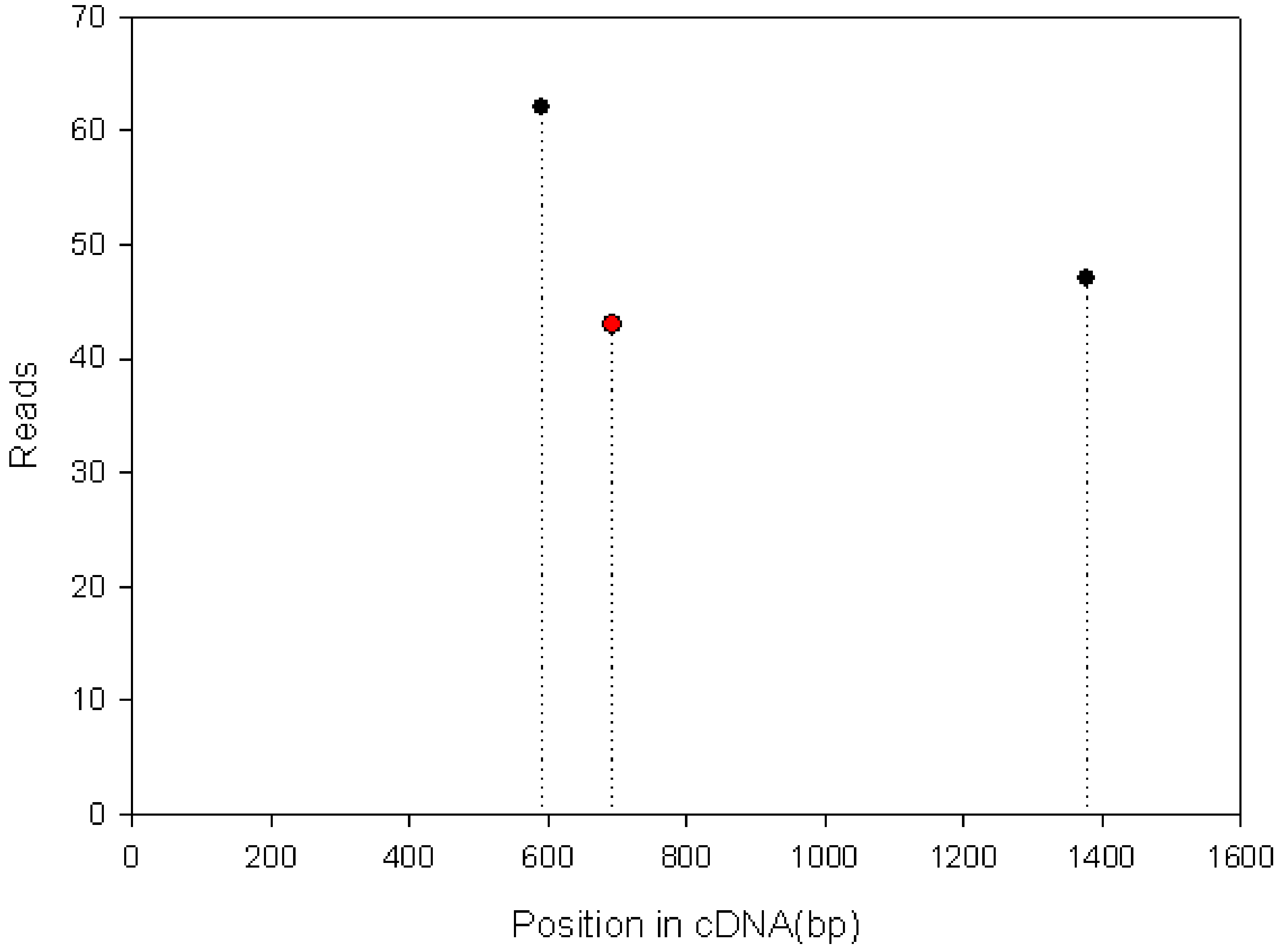


```
5' CUUGCUUCUGUCUCUUCUGUCAUCAC 3'        Orange1.1t01983.1
     ::: ::: ::::::::::::
3' -CACG-AGAGAGAGAAGACAGUU--- 5'        Csi-miR156h
```

Csi-miR159, target=Cs3g10900.1 gene=Cs3g10900
Category:3
Score=3.5
Cleavage Site=1442

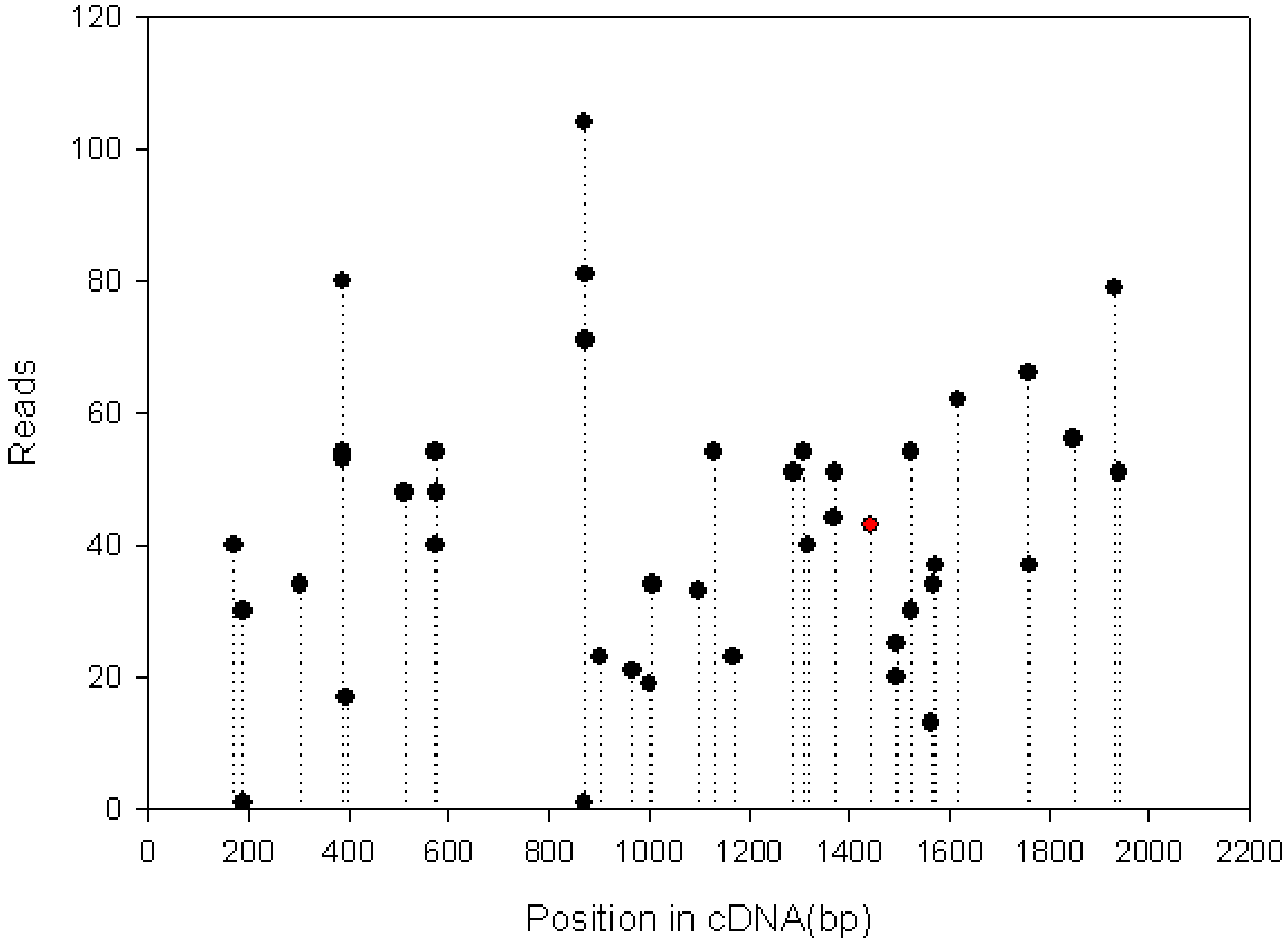


```
5' UGCAG-UUCCUUCAAUCCAAGGCACUU 3'       Cs3g10900.1
   :. :: :.:::::::::::::.
3' AUCUCGAGGGAAGUUAGGUUU------ 5'       Csi-miR159
```

Csi-miR159, target=Cs6g10950.1 gene=Cs6g10950
Category:3
Score=4
Cleavage Site=5461

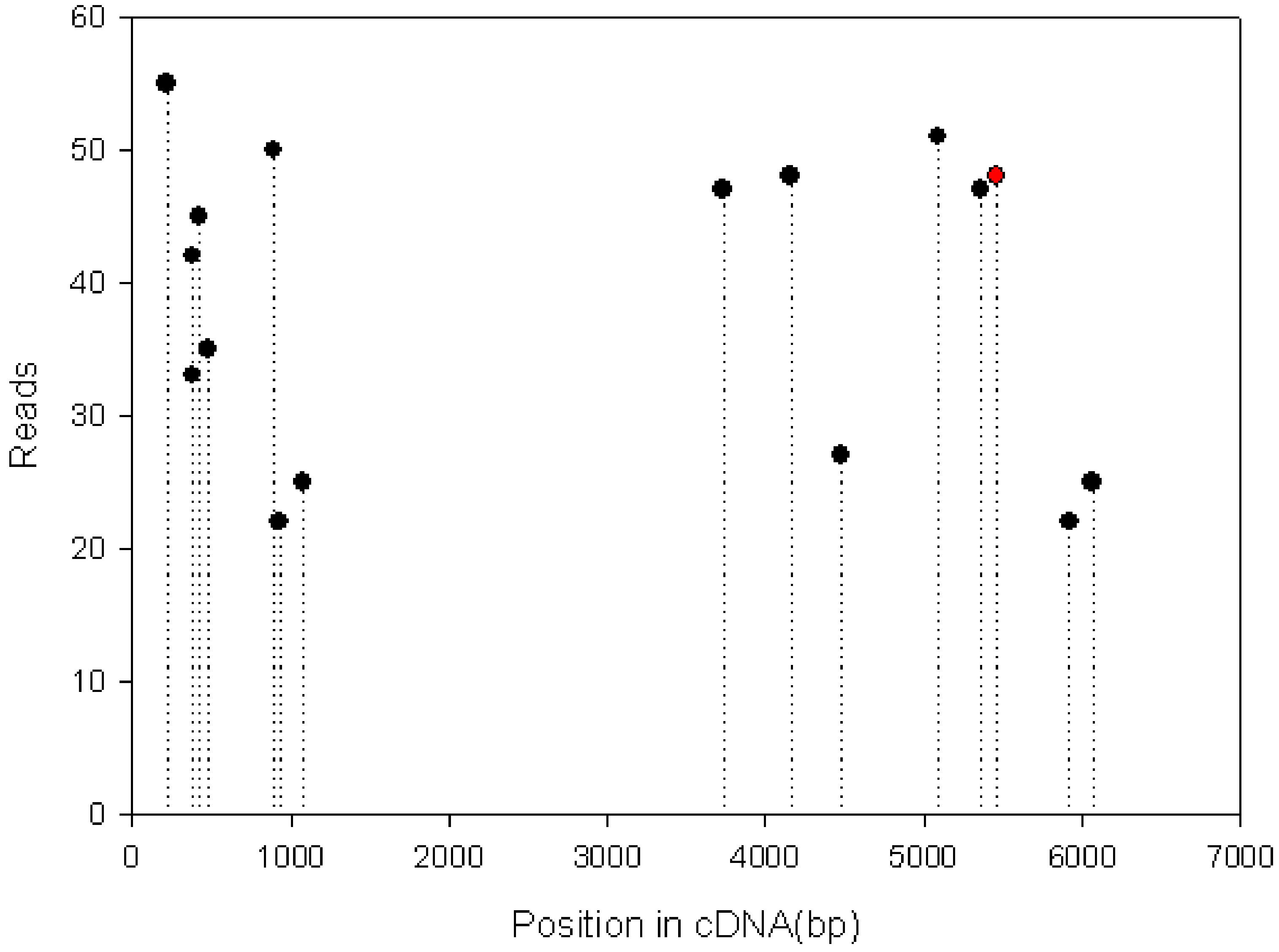


```
5' AAGAGCUCUACUUCAAUCCAAGCUUU 3'        Cs6g10950.1
    ::::::::. ::::::::::::.
3' AUCUCGAGG-GAAGUUAGGUUU---- 5'        Csi-miR159
```

Csi-miR159, target=Cs8g05120.1 gene=Cs8g05120
Category:1
Score=4
Cleavage Site=205

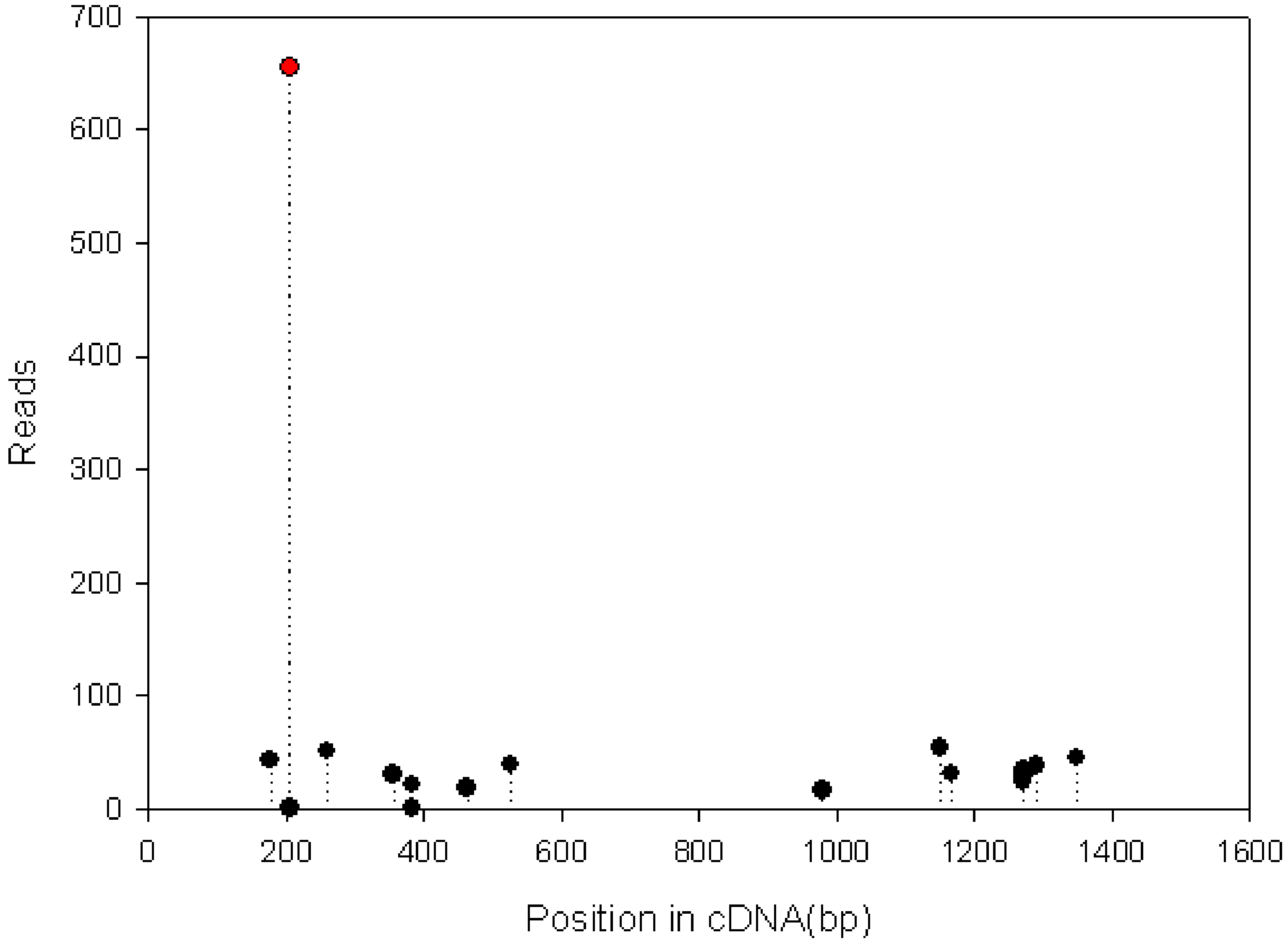


```
5' AUGAAGCUCUCUUCAAUCUCAAACGC 3'        Cs8g05120.1
    :.  ::::.:::::::::  ::::
3' -AUCUCGAGGGAAGUUAG-GUUU--- 5'        Csi-miR159
```

Csi-miR162-3p.2, target=Cs7g27400.1 gene=Cs7g27400
Category:3
Score=5
Cleavage Site=770

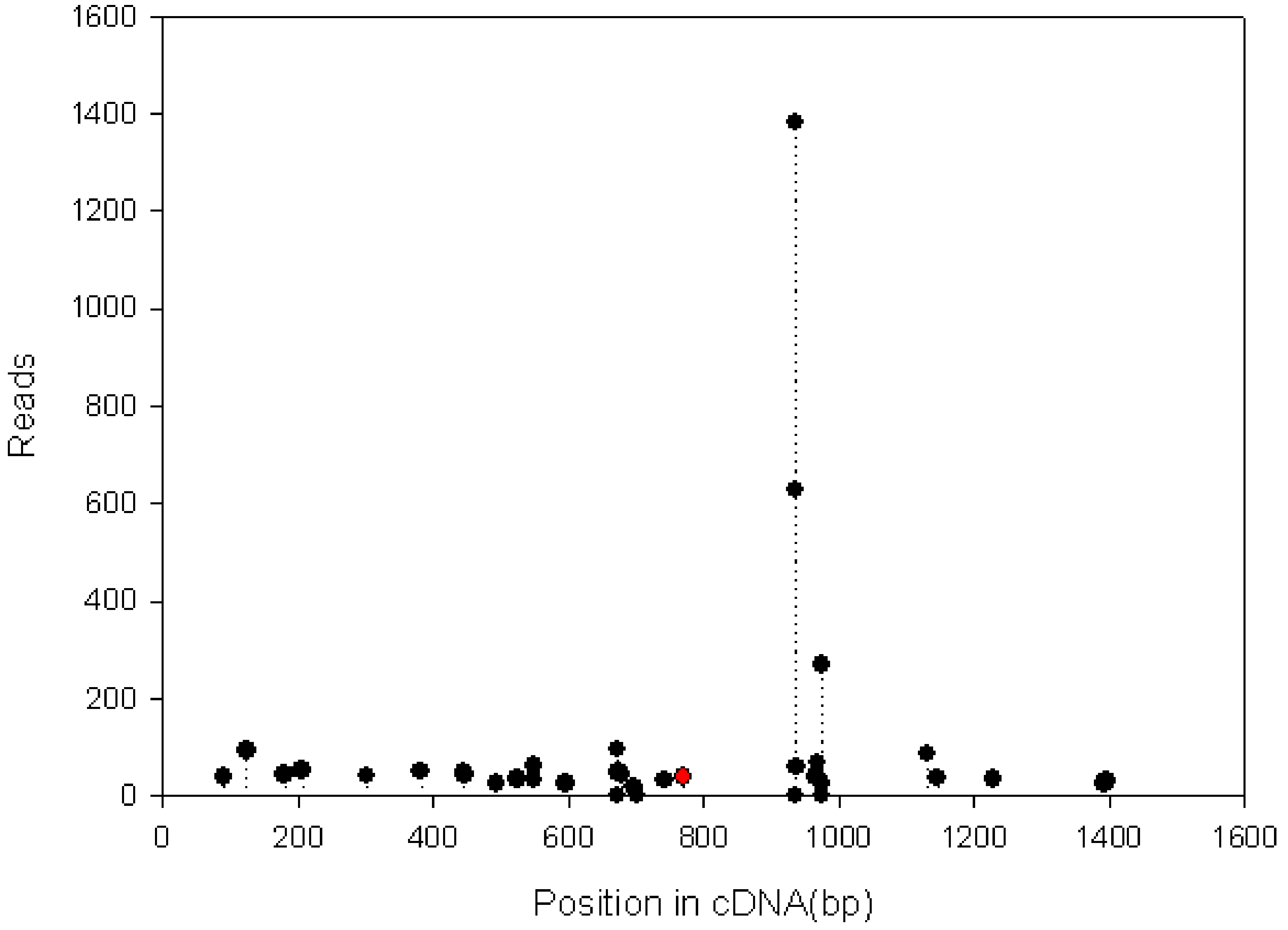


```
5' CCACUGGUUGCAAAGGUUUUUCAGCC 3'        Cs7g27400.1
       :::: :::: :::::: ::
3' ---GACCUACGUCUCCAAAUAG---- 5'        Csi-miR162-3p.2
```

Csi-miR164, target=Cs5g10870.1 gene=Cs5g10870
Category:1
Score=3.5
Cleavage Site=1062

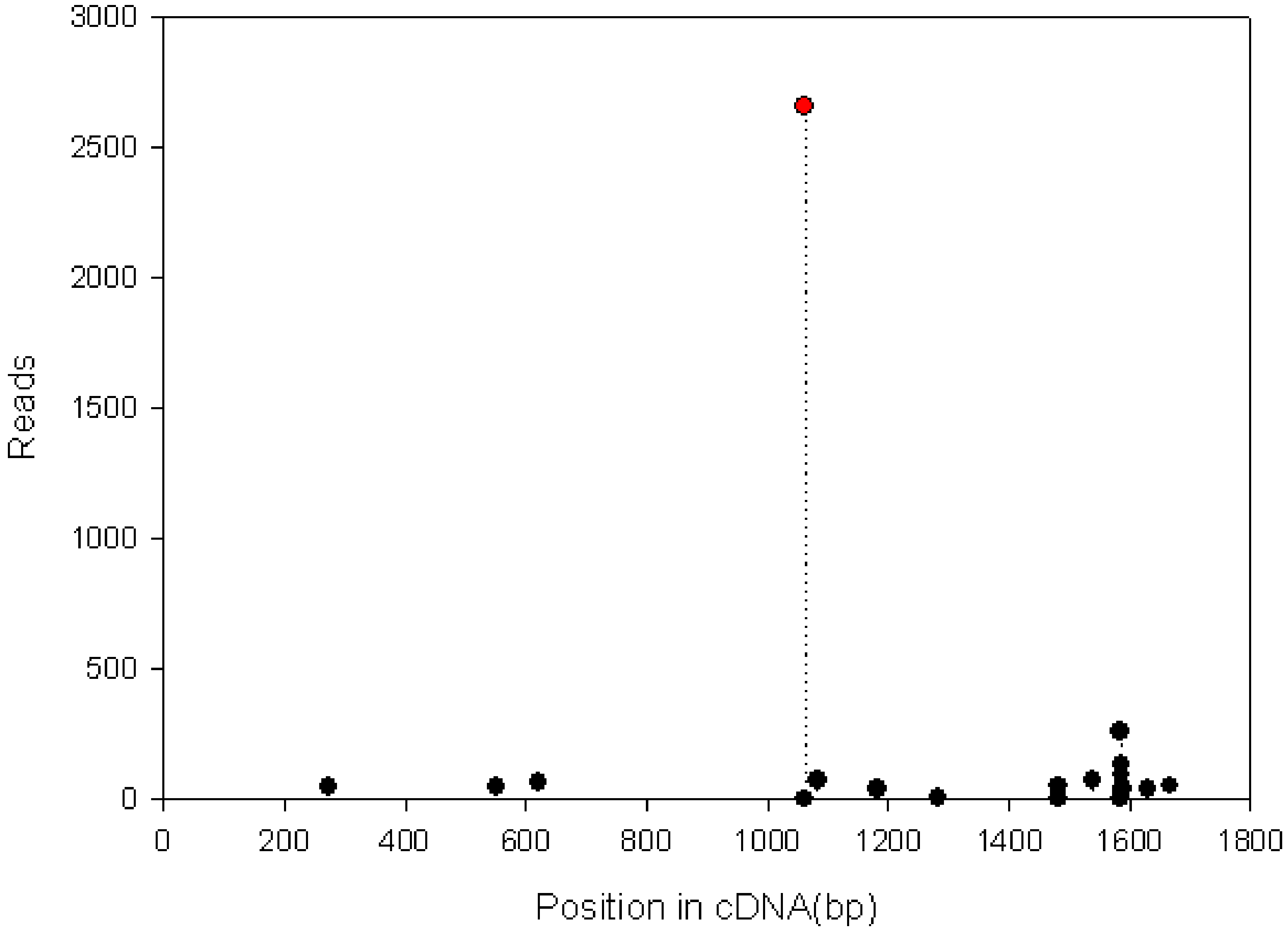


```
5' GCUUACGUGUCCUGCUUCUCCAAUUC 3'        Cs5g10870.1
      .:::::.:::::::::::::
3' -ACGUGCACGGGACGAAGAGGU---- 5'        Csi-miR164
```

Csi-miR164, target=Cs8g18140.1 gene=Cs8g18140
Category:3
Score=4.5
Cleavage Site=521

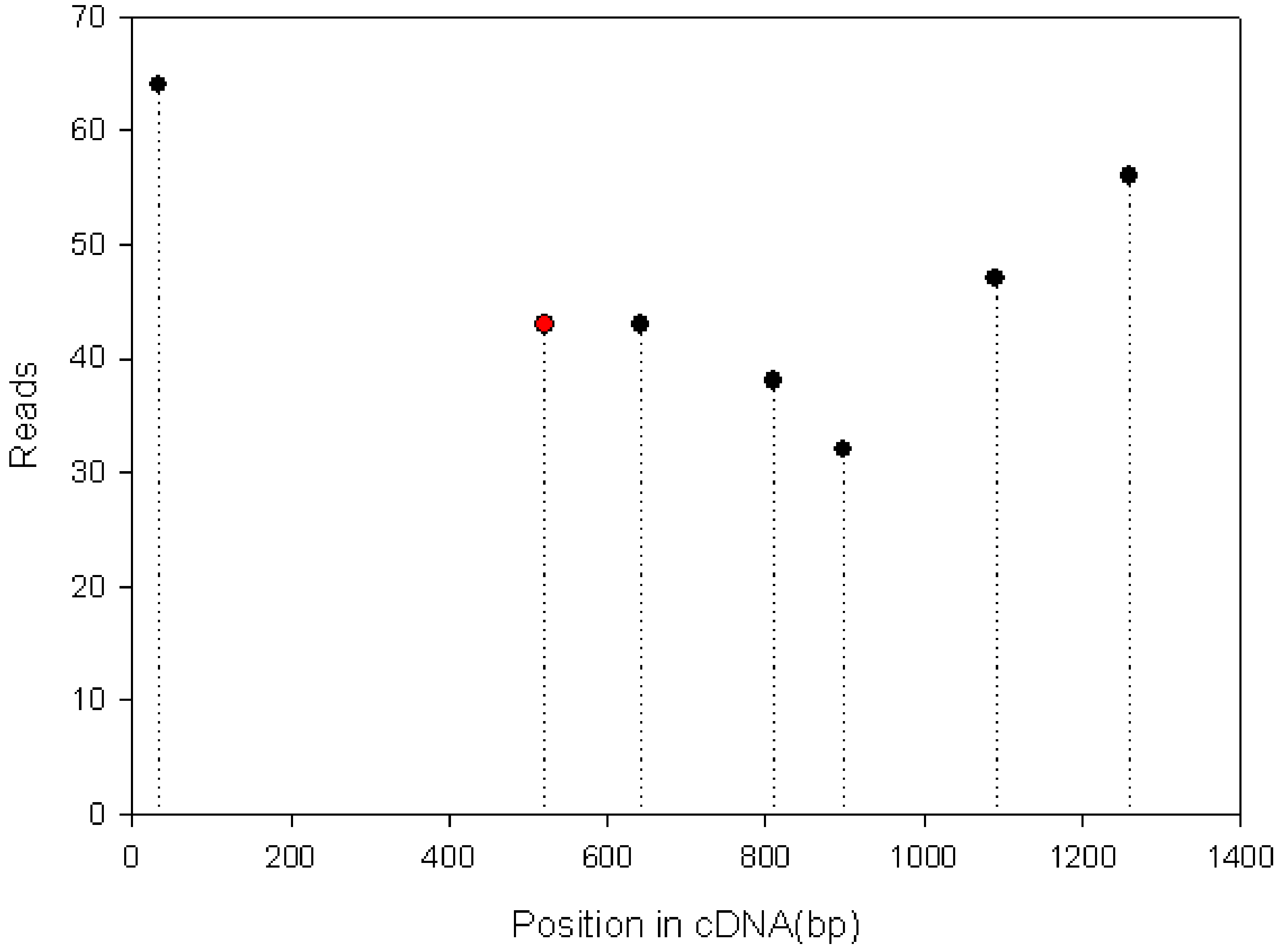


```
5' ACCUUGCAUGCCCUGCUUCUCCGAUU 3'        Cs8g18140.1
      ..: ::::::::::::::.
3' --ACGUGCACGGGACGAAGAGGU--- 5'        Csi-miR164
```

Csi-miR166a.1, target=Cs8g16510.1 gene=Cs8g16510
Category:1
Score=4
Cleavage Site=1434

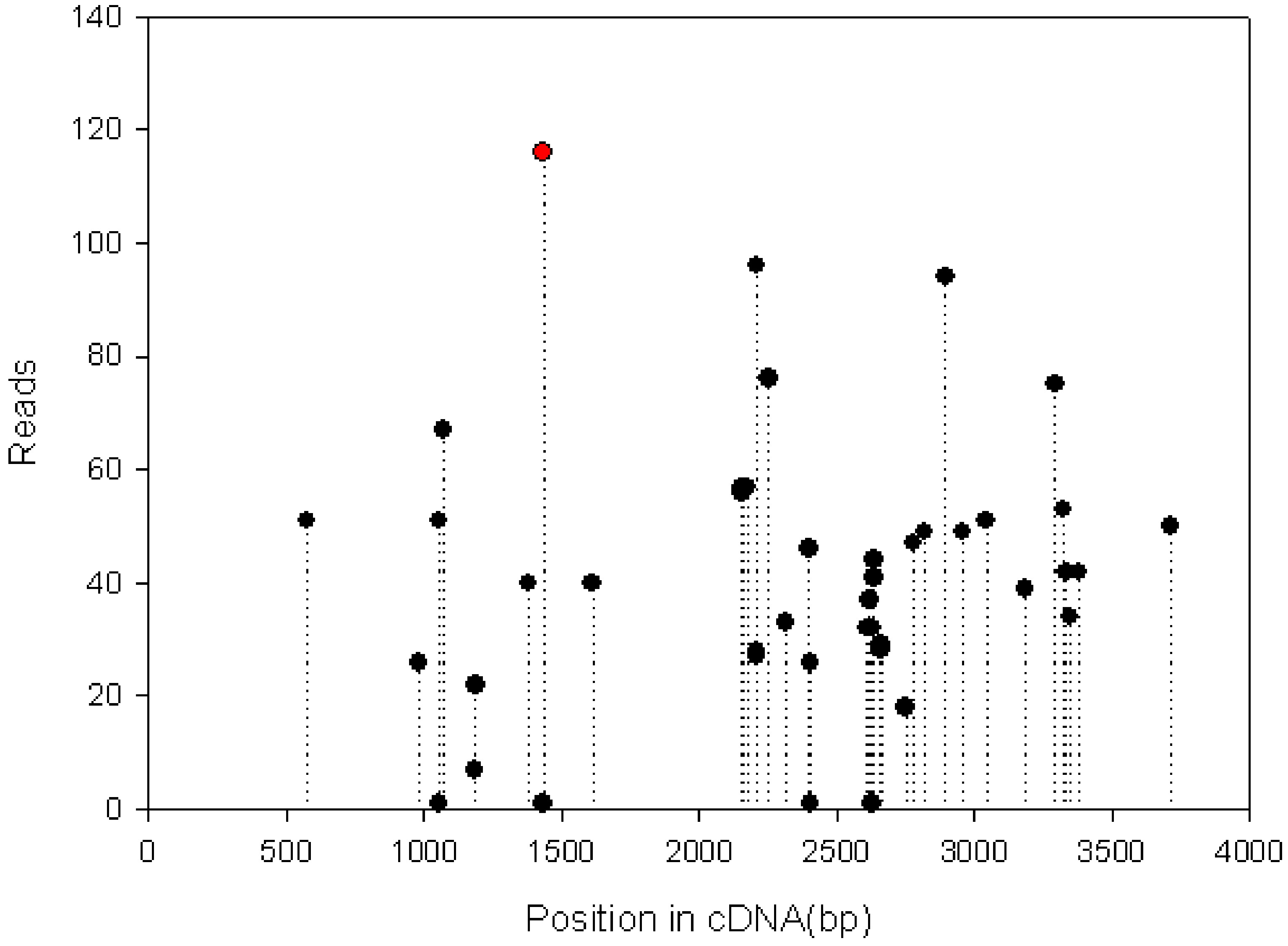


```
5' CCUGGGAUGAAGCCUGGUCCGGAUUC 3'        Cs8g16510.1
     ::.:::::::::::::::::.
3' CCCCCUUACUUCGGACCAGGCU---- 5'        Csi-miR166a.1
```

Csi-miR166a.2, target=Cs8g16510.1 gene=Cs8g16510
Category:1
Score=3
Cleavage Site=1434

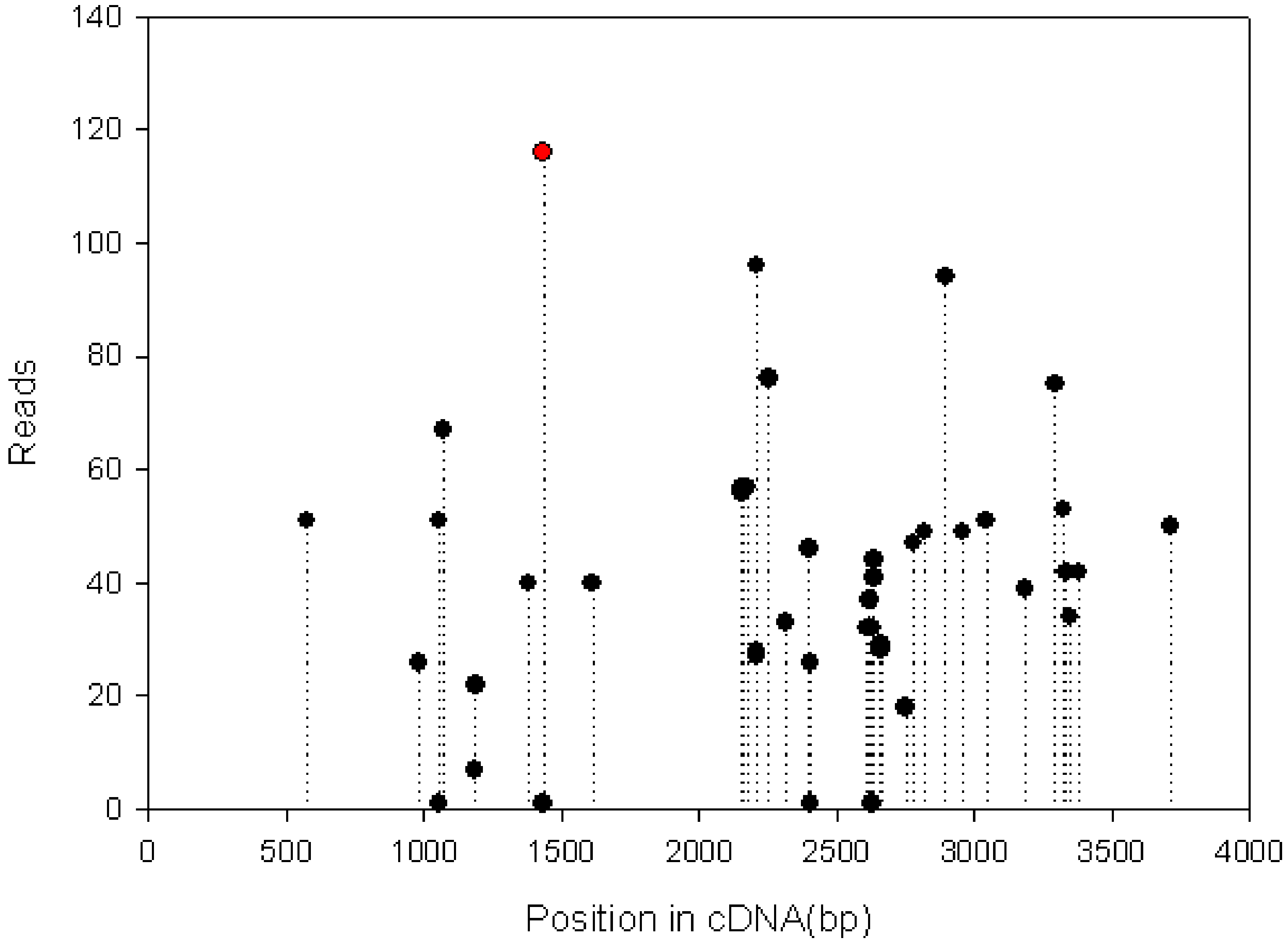


```
5' CCUGGGAUGAAGCCUGGUCCGGAUUC 3'        Cs8g16510.1
     ::.:::::::::::::::::.
3' -CCCCUUACUUCGGACCAGGCU---- 5'        Csi-miR166a.2
```

Csi-miR166b, target=Cs8g16510.1 gene=Cs8g16510
Category:1
Score=2
Cleavage Site=1434

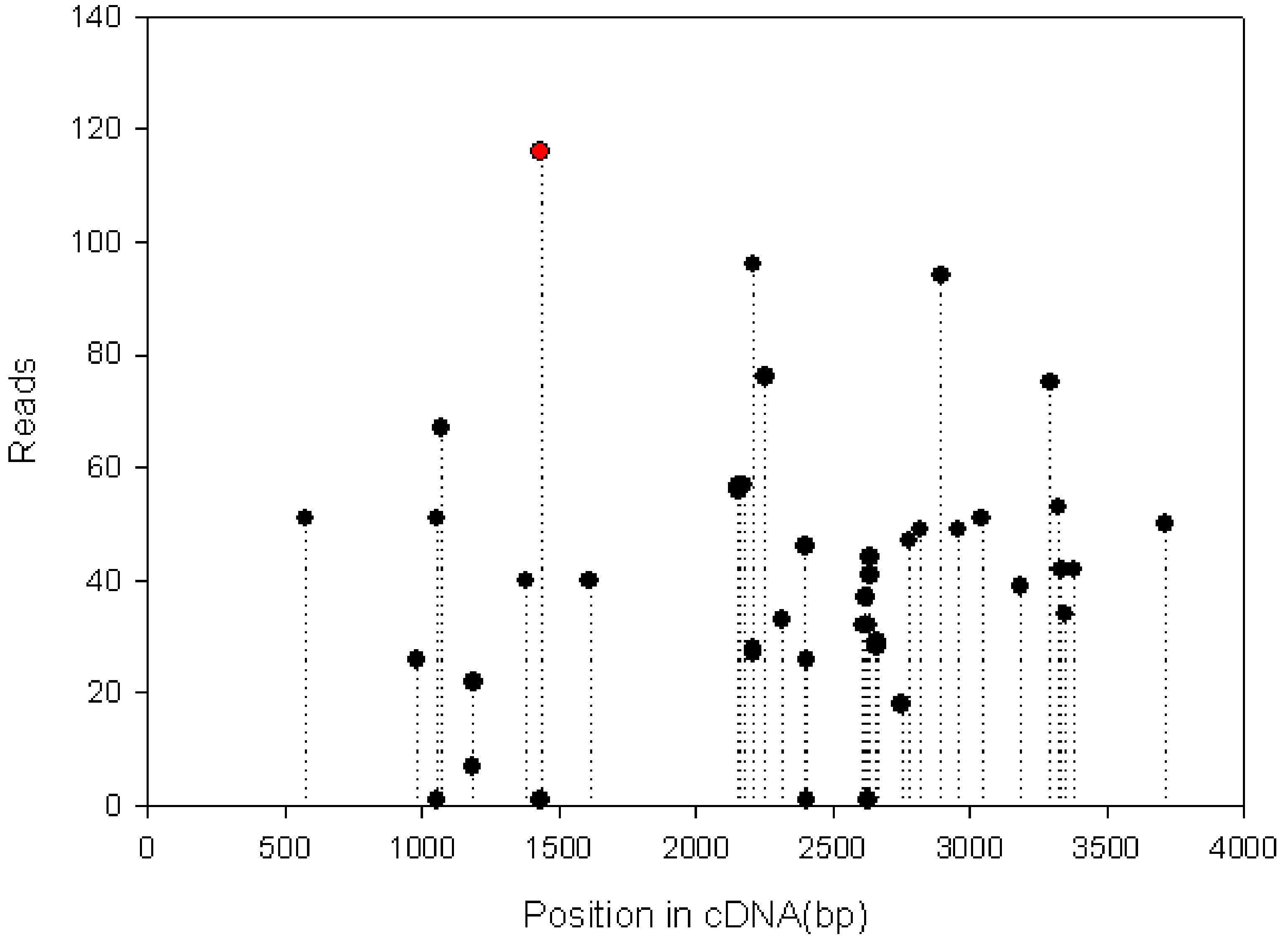


```
5' CCUGGGAUGAAGCCUGGUCCGGAUUC 3'        Cs8g16510.1
    :  ::.::::::::::::::::.
3' -GCCCUUACUUCGGACCAGGCU---- 5'        Csi-miR166b
```

Csi-miR166c.1, target=Cs8g16510.1 gene=Cs8g16510
Category:1
Score=1
Cleavage Site=1434

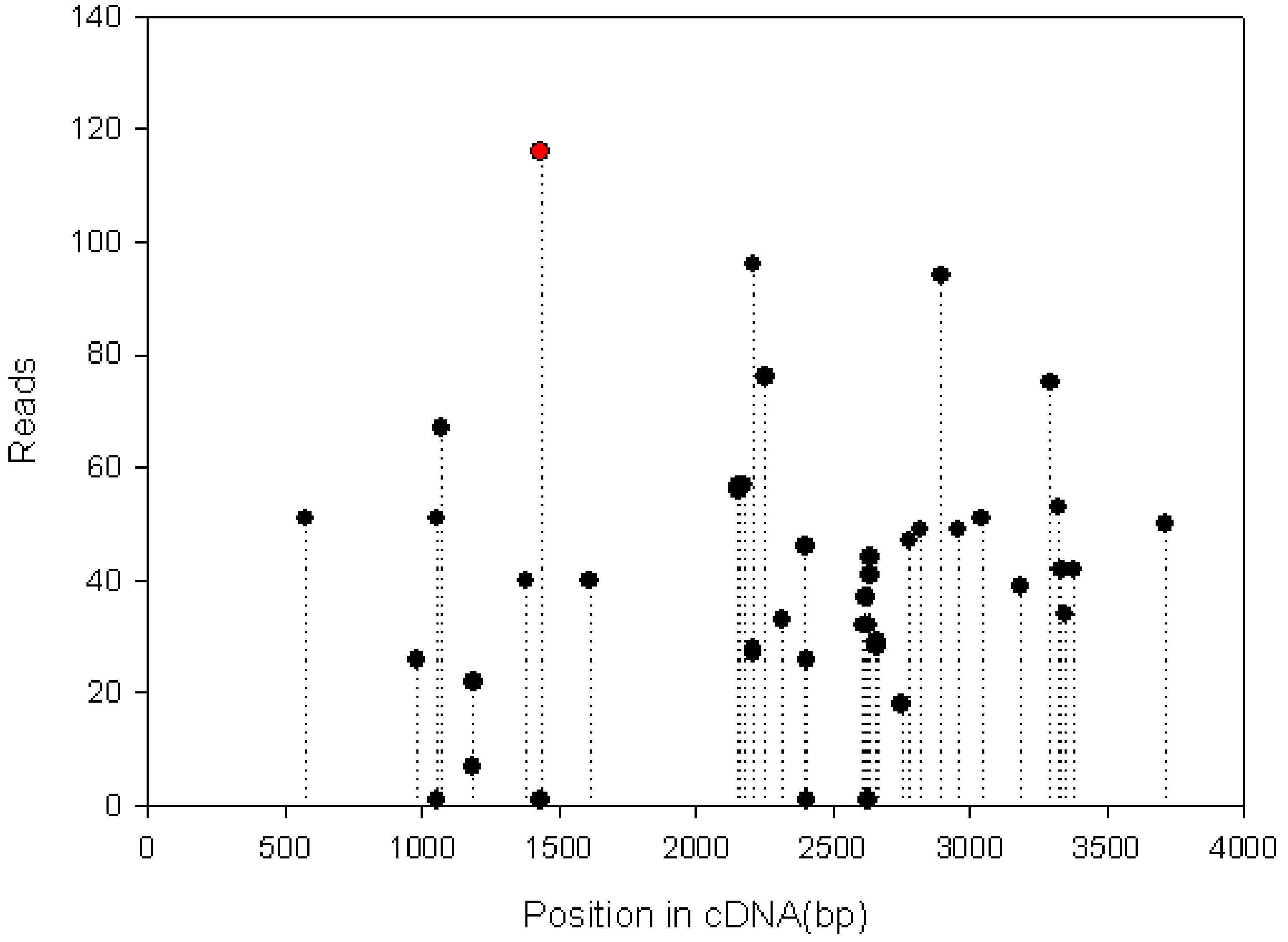


```
5' CCUGGGAUGAAGCCUGGUCCGGAUUC 3'        Cs8g16510.1
      ::.:::::::::::::::::.
3' ---CCUUACUUCGGACCAGGCU---- 5'        Csi-miR166c.1
```

Csi-miR166c.2, target=Cs4g06030.1 gene=Cs4g06030
Category:2
Score=4.5
Cleavage Site=2041

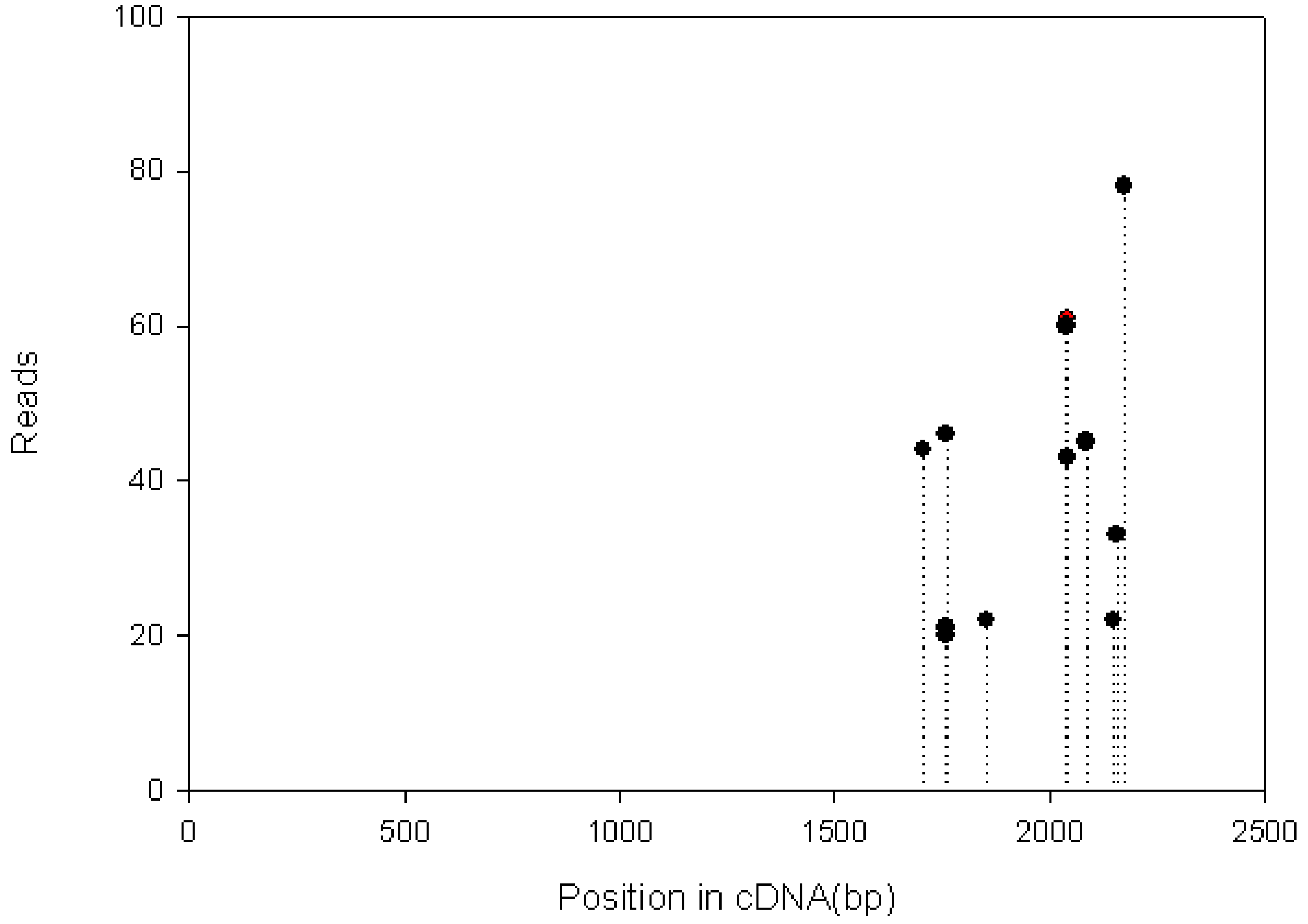


```
5' GCUAGGCUGAAU-AAGCCUGUUCUGGG 3'        Cs4g06030.1
          :::: ::::::: ::.:.
3' --------CUUACUUCGGACCAGGCU- 5'        Csi-miR166c.2
```

Csi-miR166c.2, target=Cs8g16510.1 gene=Cs8g16510
Category:1
Score=1
Cleavage Site=1434

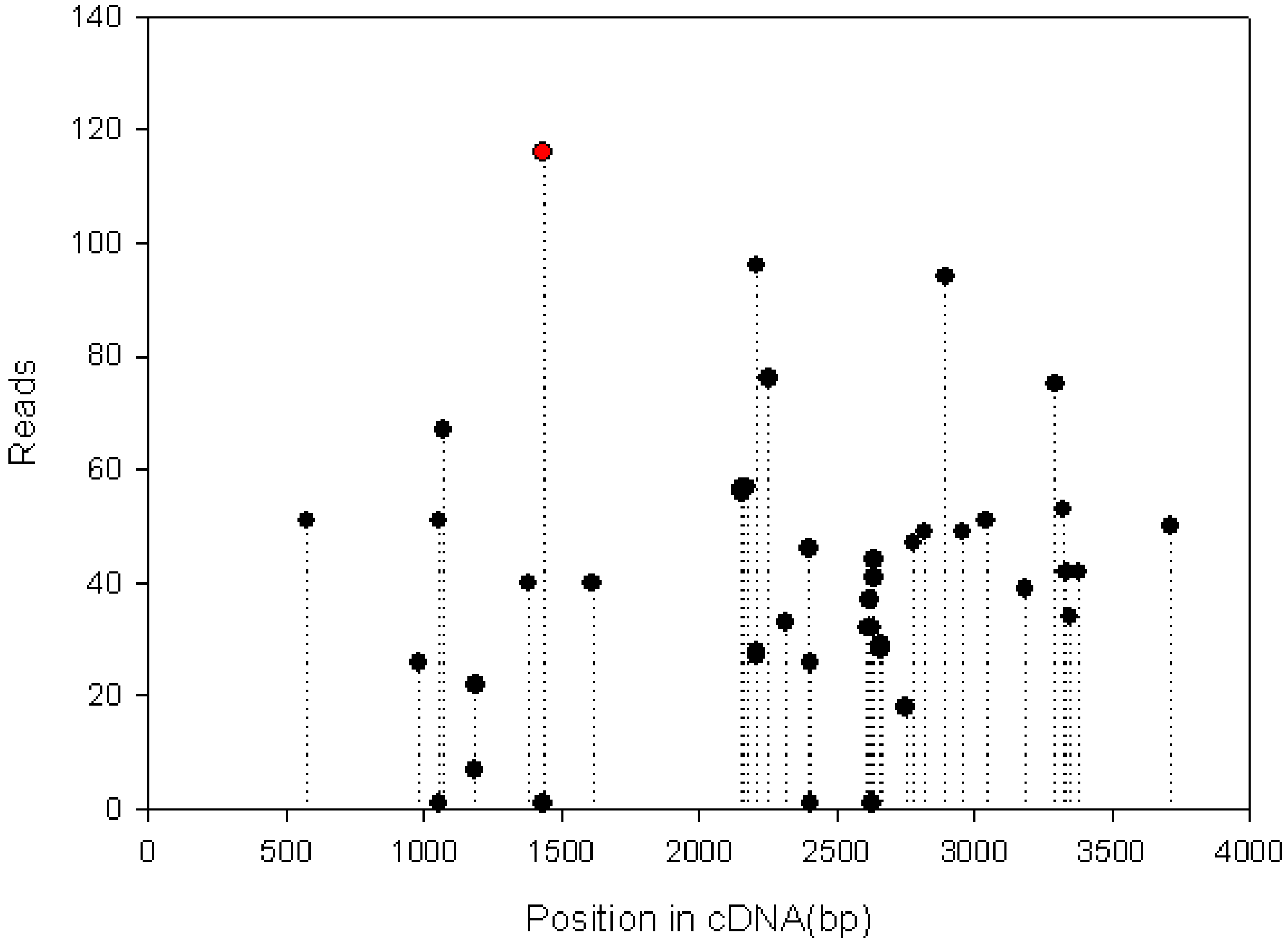


```
5' CCUGGGAUGAAGCCUGGUCCGGAUUC 3'        Cs8g16510.1
       :.:::::::::::::::::.
3' ----CUUACUUCGGACCAGGCU---- 5'        Csi-miR166c.2
```

Csi-miR166c.3, target=Cs8g16510.1 gene=Cs8g16510
Category:1
Score=2
Cleavage Site=1434

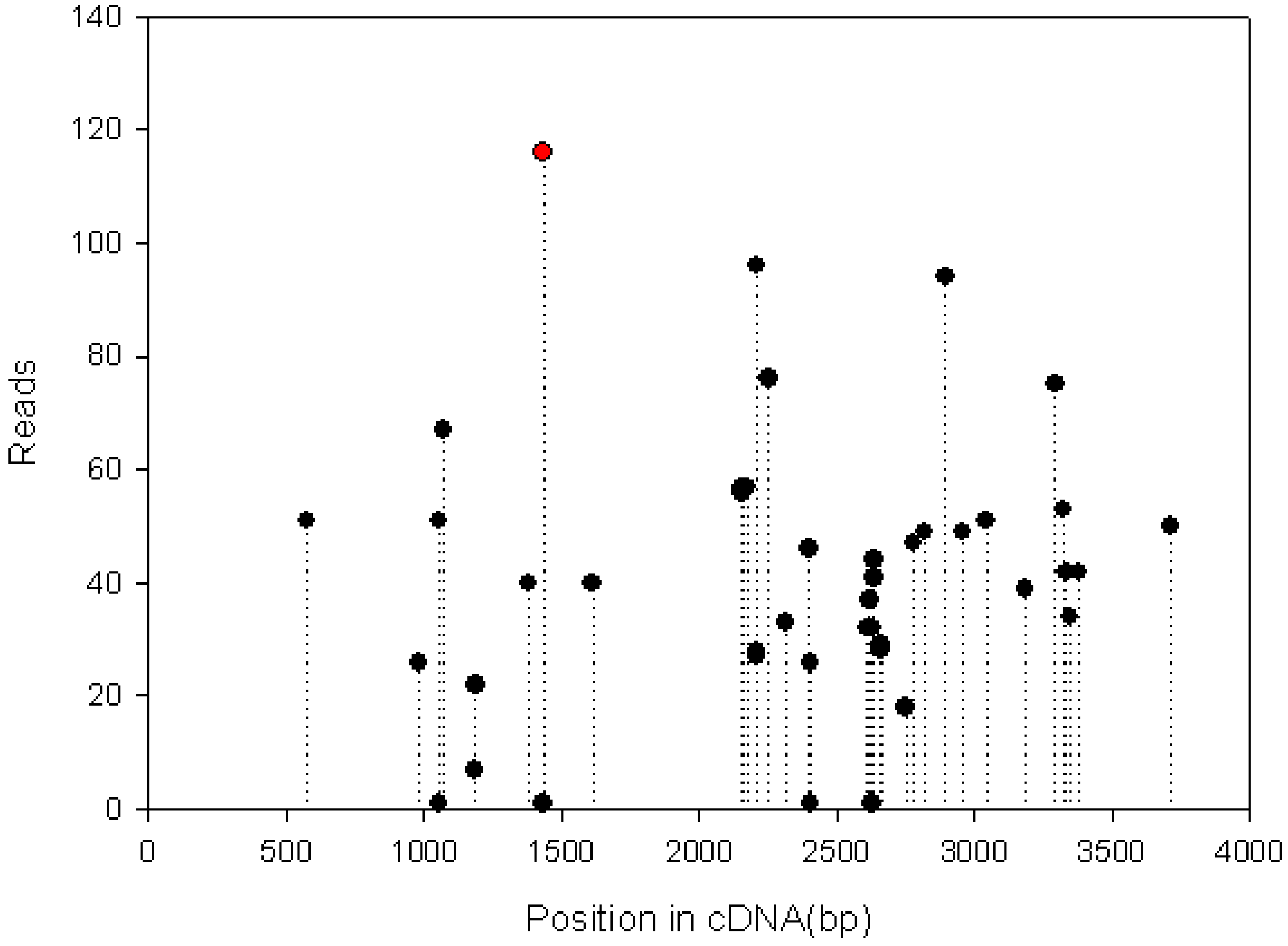


```
5' CCUGGGAUGAAGCCUGGUCCGGAUUC 3'        Cs8g16510.1
     ::.:::::::::::::::::.
3' --CCCUUACUUCGGACCAGGCU---- 5'        Csi-miR166c.3
```

Csi-miR166c.4, target=Cs8g16510.1 gene=Cs8g16510
Category:1
Score=3.5
Cleavage Site=1434

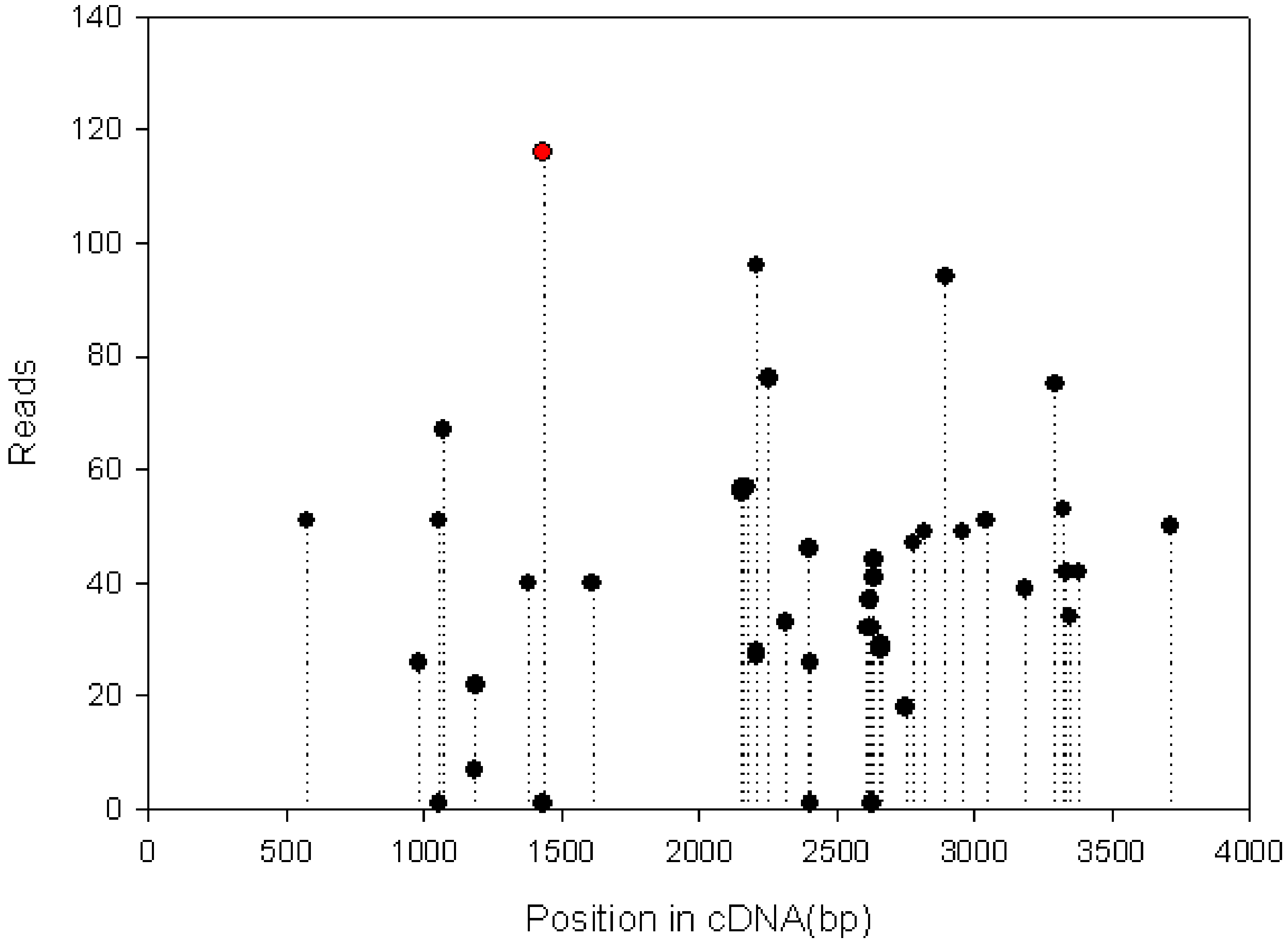


```
5' CCUGGGAUGAAGCCUGGUCCGGAUUC 3'        Cs8g16510.1
     ::.:::::::::::::::::.
3' --CCCUUACUUCGGACCAGGCUC--- 5'        Csi-miR166c.4
```

Csi-miR166c.5, target=Cs8g16510.1 gene=Cs8g16510
Category:1
Score=2.5
Cleavage Site=1434

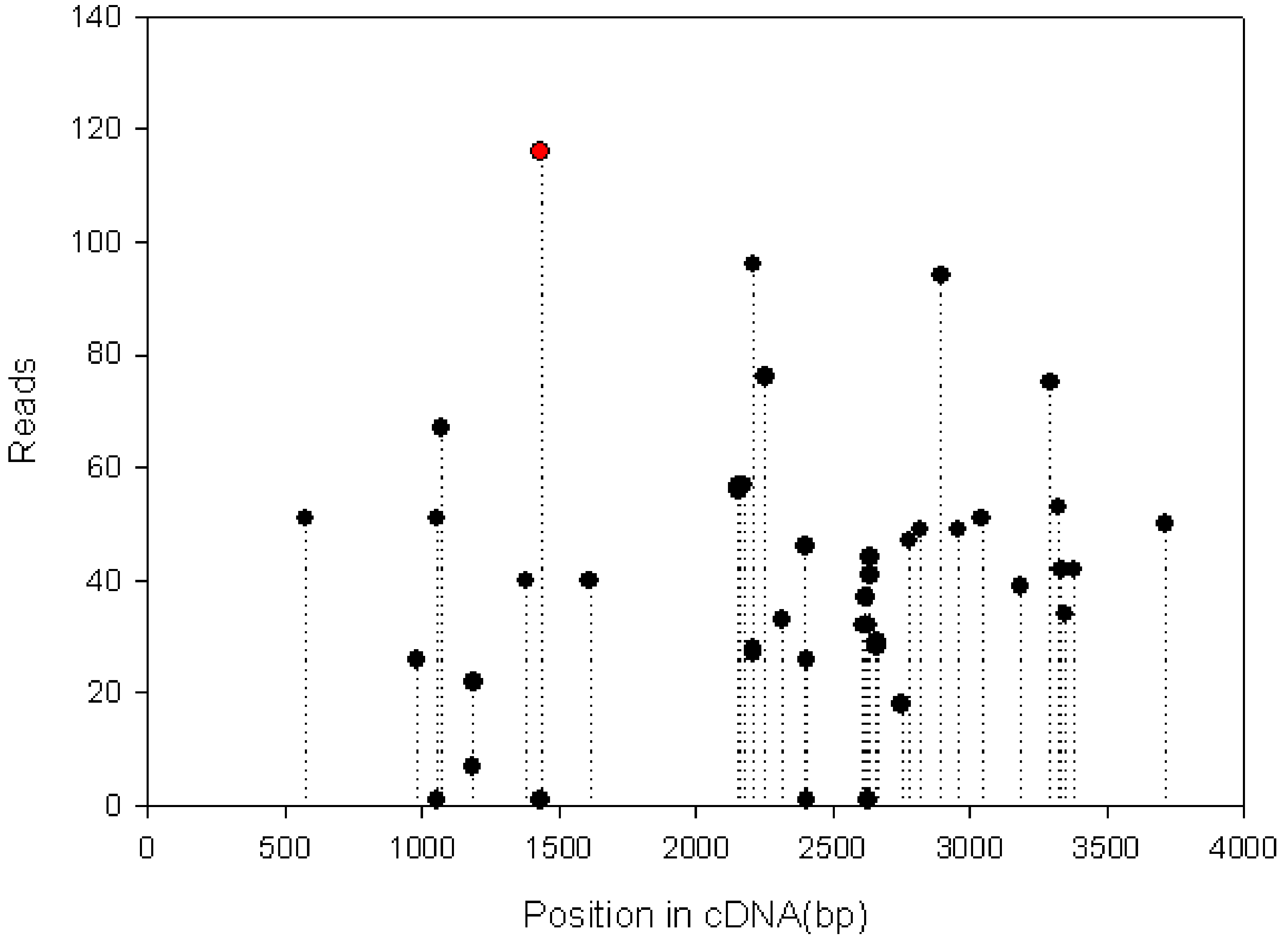


```
5' CCUGGGAUGAAGCCUGGUCCGGAUUC 3'        Cs8g16510.1
      ::.:::::::::::::::::.
3' ---CCUUACUUCGGACCAGGCUC--- 5'        Csi-miR166c.5
```

Csi-miR166d.1, target=Cs8g16510.1 gene=Cs8g16510
Category:1
Score=4
Cleavage Site=1434

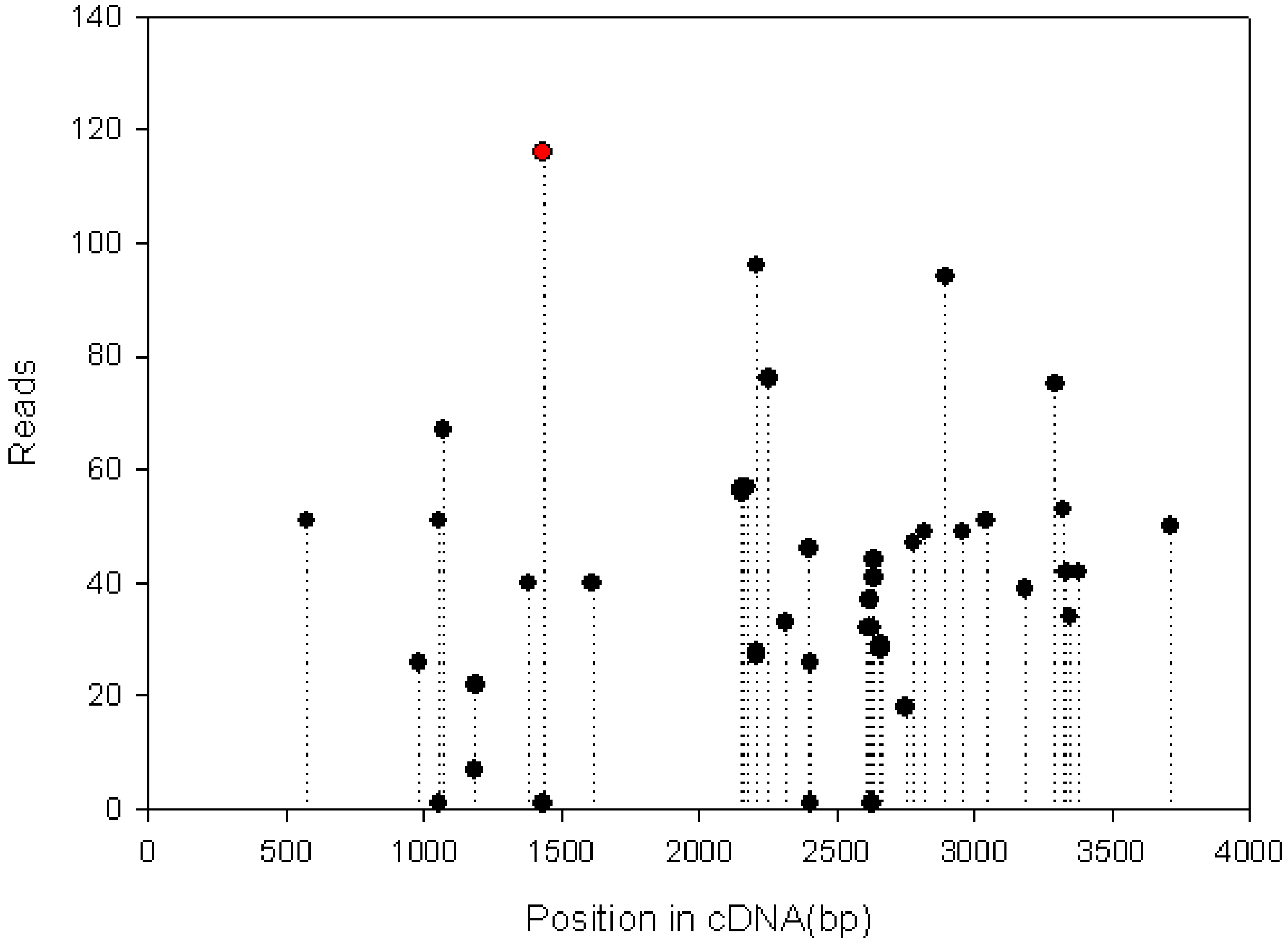


```
5' CCUGGGAUGAAGCCUGGUCCGGAUUC 3'        Cs8g16510.1
     ::.:::::::::::::::::.
3' UUCCCUUACUUCGGACCAGGCU---- 5'        Csi-miR166d.1
```

Csi-miR166d.2, target=Cs8g16510.1 gene=Cs8g16510
Category:1
Score=3
Cleavage Site=1434

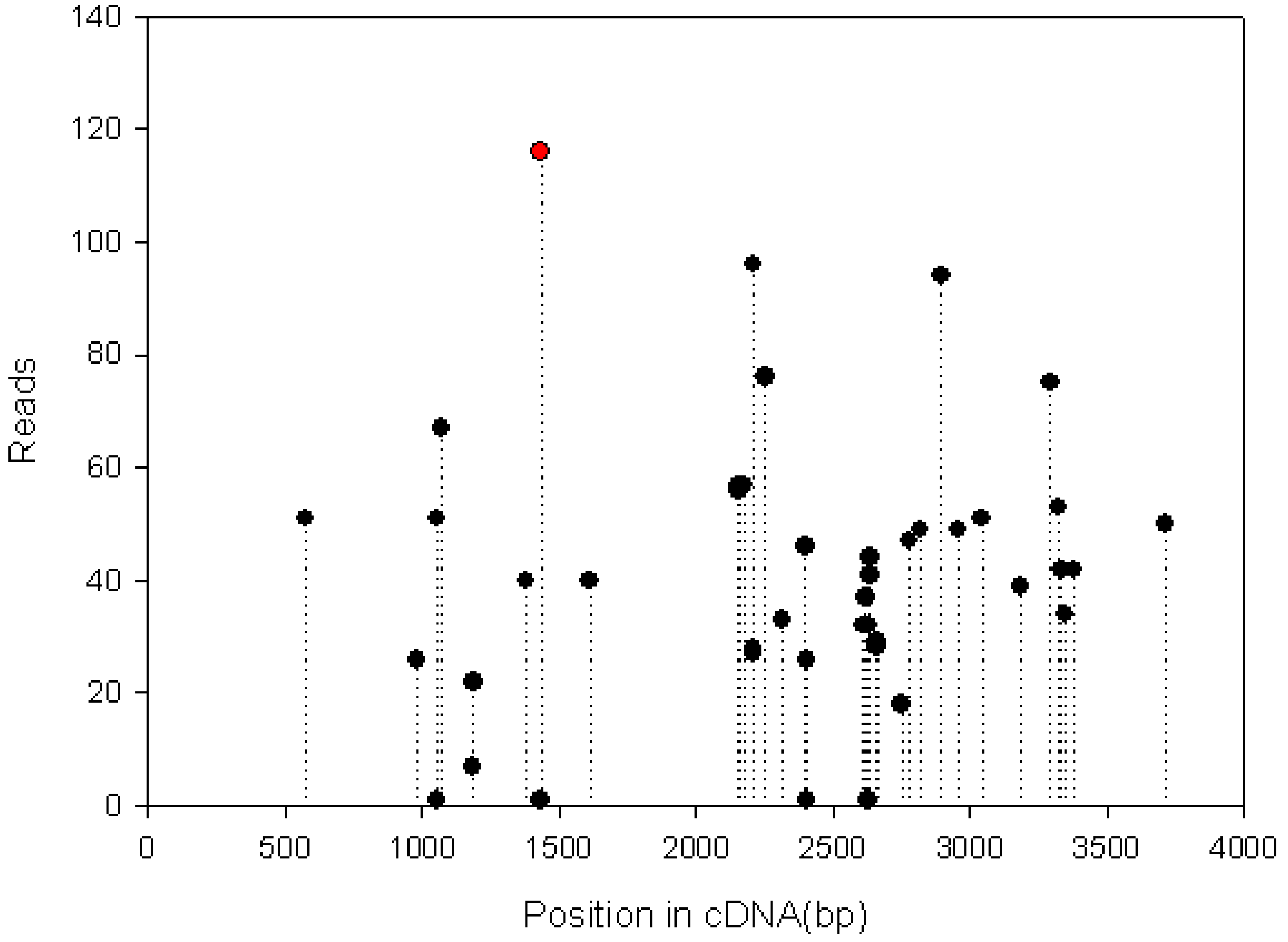


```
5' CCUGGGAUGAAGCCUGGUCCGGAUUC 3'        Cs8g16510.1
     ::.:::::::::::::::::.
3' -UCCCUUACUUCGGACCAGGCU---- 5'        Csi-miR166d.2
```

Csi-miR166g.1, target=Cs8g16510.1 gene=Cs8g16510
Category:1
Score=4.5
Cleavage Site=1434

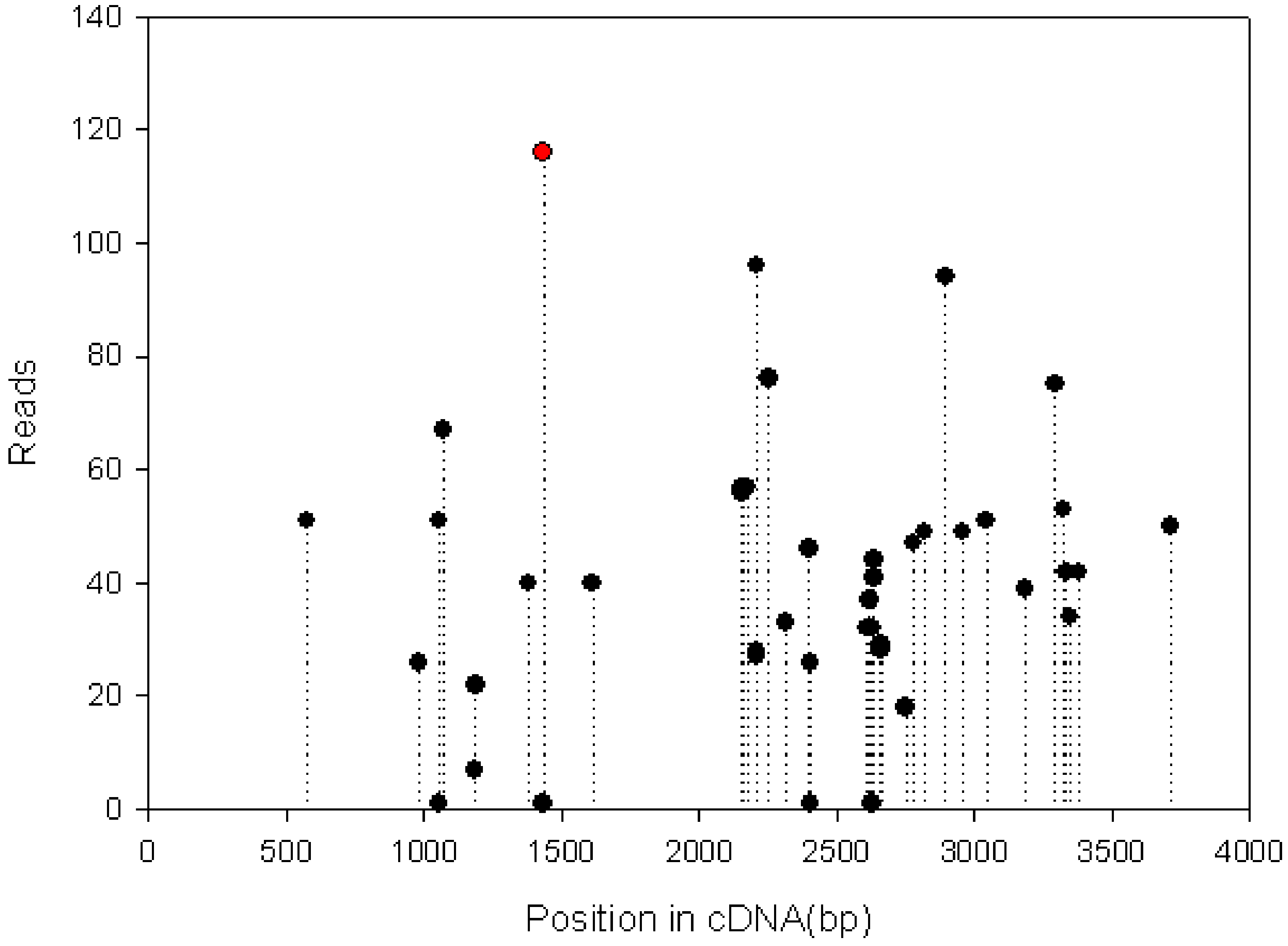


```
5' CCUGGGAUGAAGCCUGGUCCGGAUUC 3'        Cs8g16510.1
      ::.::::::::::::::::.
3' ---CCUUACUUCGGACCAGGCUCU-- 5'        Csi-miR166g.1
```

Csi-miR166i, target=Cs8g16510.1 gene=Cs8g16510
Category:1
Score=2.5
Cleavage Site=1434

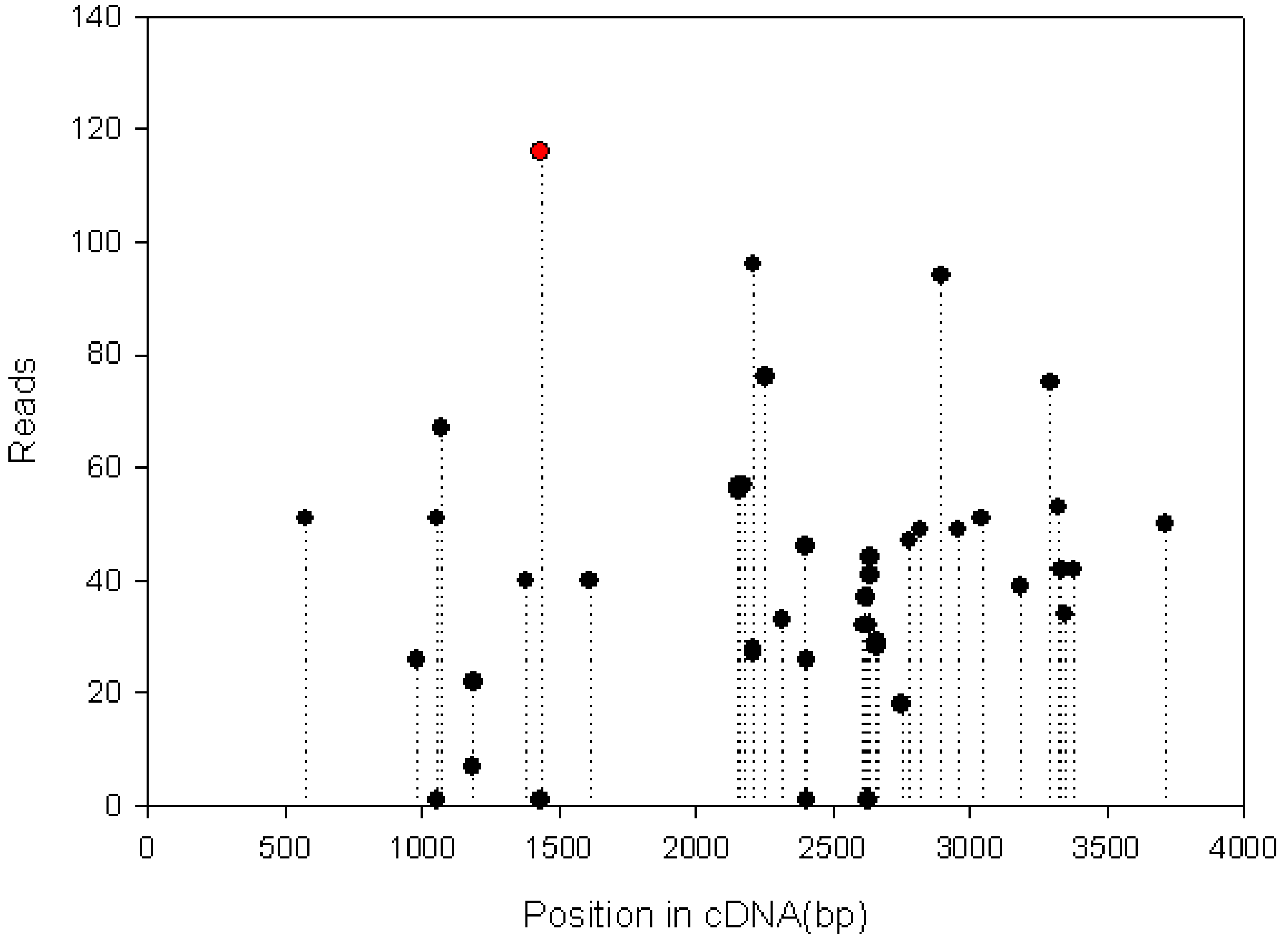


```
5' CCUGGGAUGAAGCCUGGUCCGGAUUC 3'        Cs8g16510.1
       ::.:::::::::::::::::.:
3' --CCCUUACUUCGGACCAGGCUU--- 5'        Csi-miR166i
```

Csi-miR166j.1, target=Cs8g16510.1 gene=Cs8g16510
Category:1
Score=4
Cleavage Site=1434

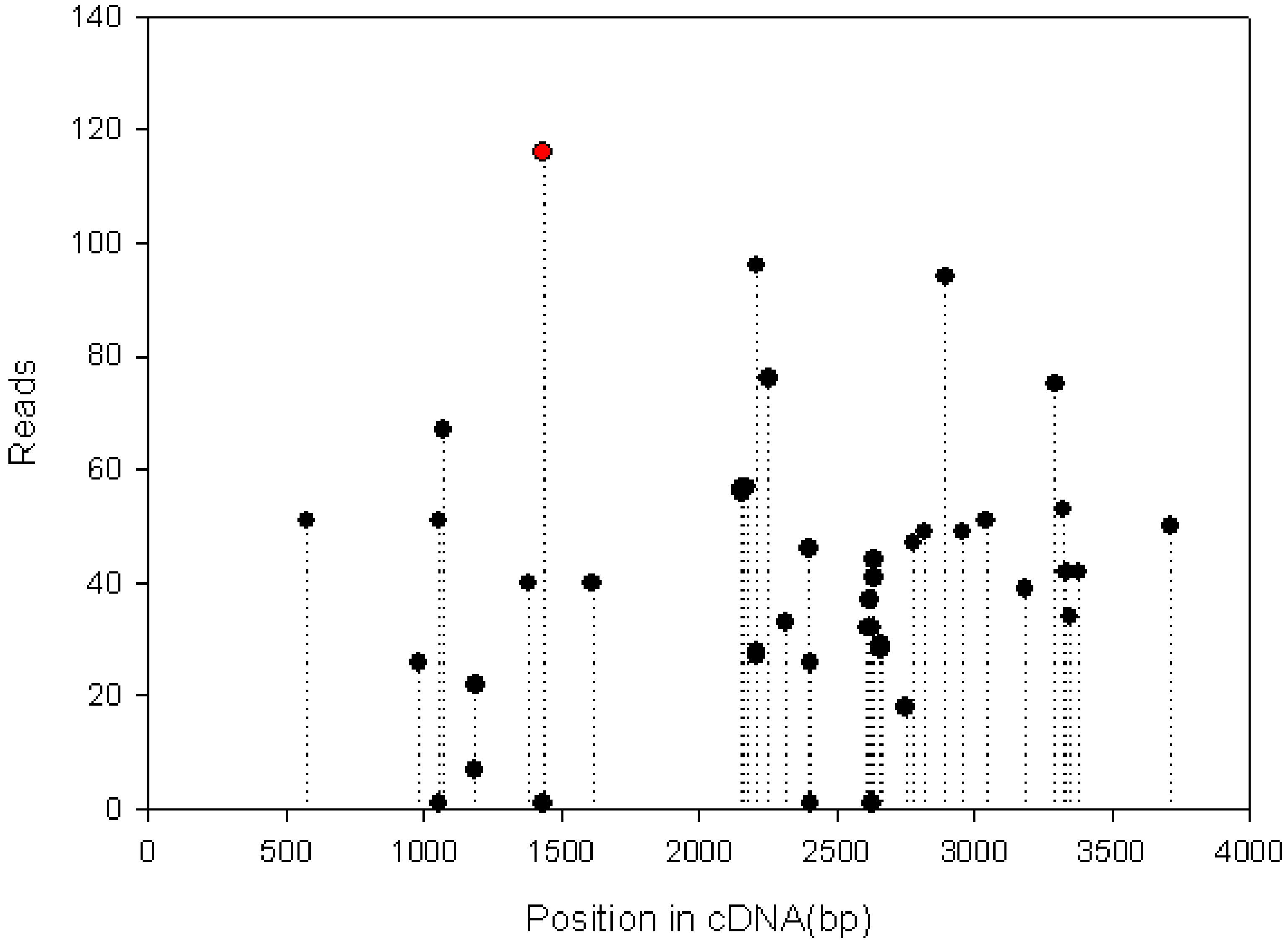


```
5' CCUGGGAUGAAGCCUGGUCCGGAUUC 3'        Cs8g16510.1
     ::.:::::::::::::::::..
3' -CUCCUUACUUCGGACCAGGUU---- 5'        Csi-miR166j.1
```

Csi-miR166j.3, target=Cs4g04520.1 gene=Cs4g04520
Category:3
Score=4.5
Cleavage Site=1936

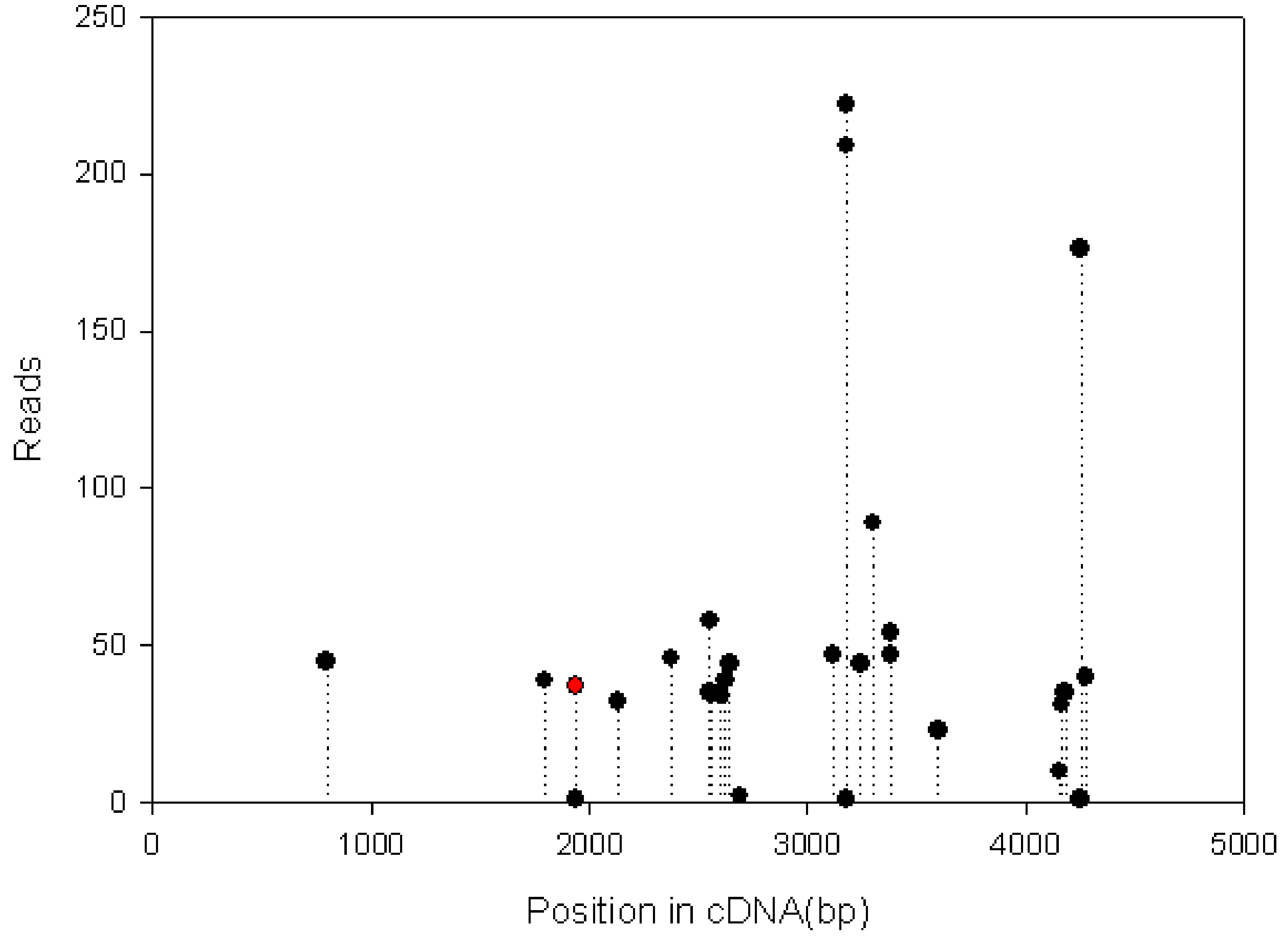


```
5' UCCUGGACU-AAGCUUGGUCCAGUGGA 3'        Cs4g04520.1
      :::: : ::::.::::::::.
3' --CACCUUACUUCGGACCAGGUU---- 5'        Csi-miR166j.3
```

Csi-miR166j.3, target=Cs8g16510.1 gene=Cs8g16510
Category:1
Score=3
Cleavage Site=1434

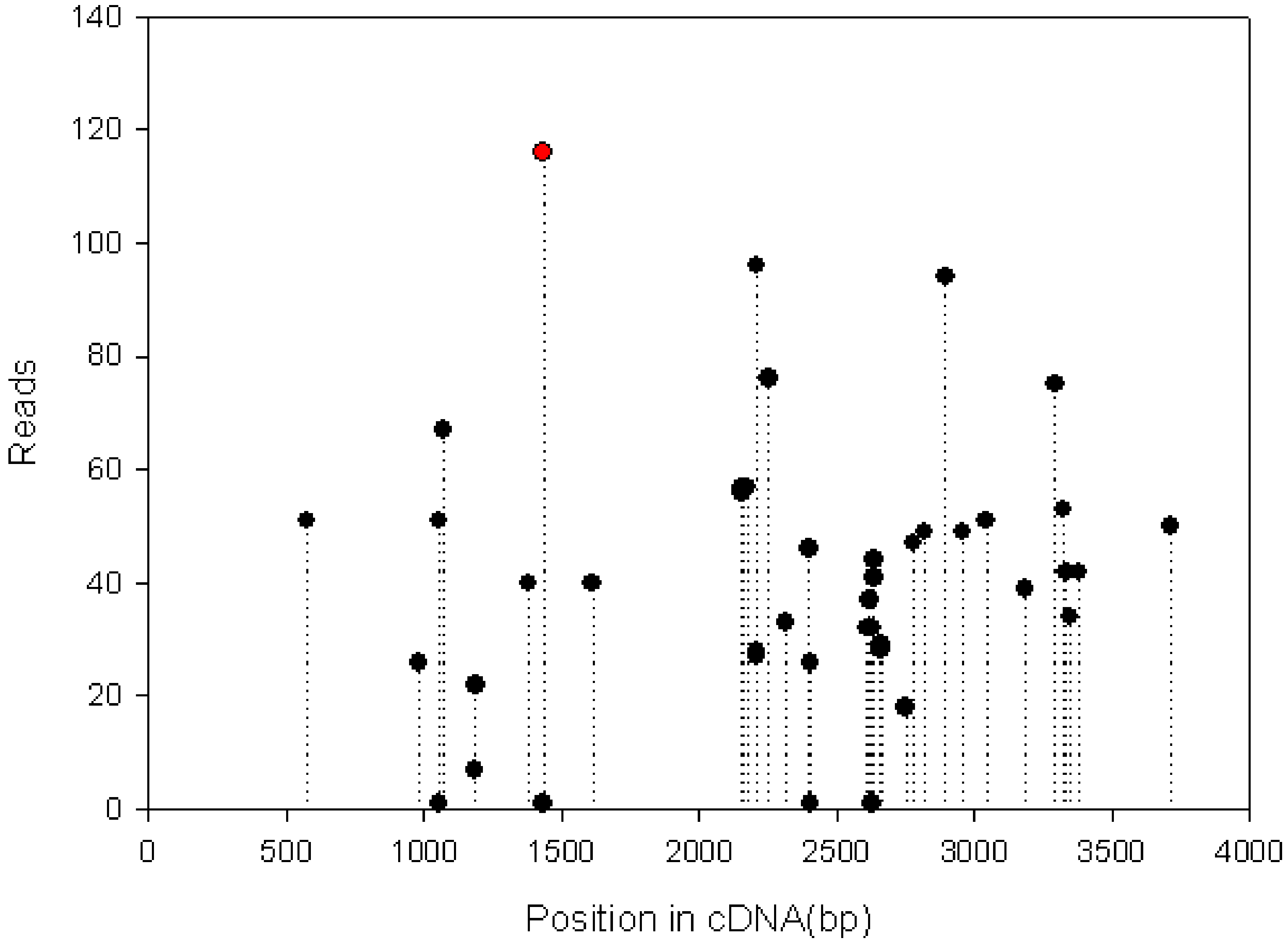


```
5' CCUGGGAUGAAGCCUGGUCCGGAUUC 3'        Cs8g16510.1
    :::.:::::::::::::::..
3' -CACCUUACUUCGGACCAGGUU---- 5'        Csi-miR166j.3
```

Csi-miR167a.1, target=Cs2g15130.1 gene=Cs2g15130
Category:2
Score=5
Cleavage Site=3444

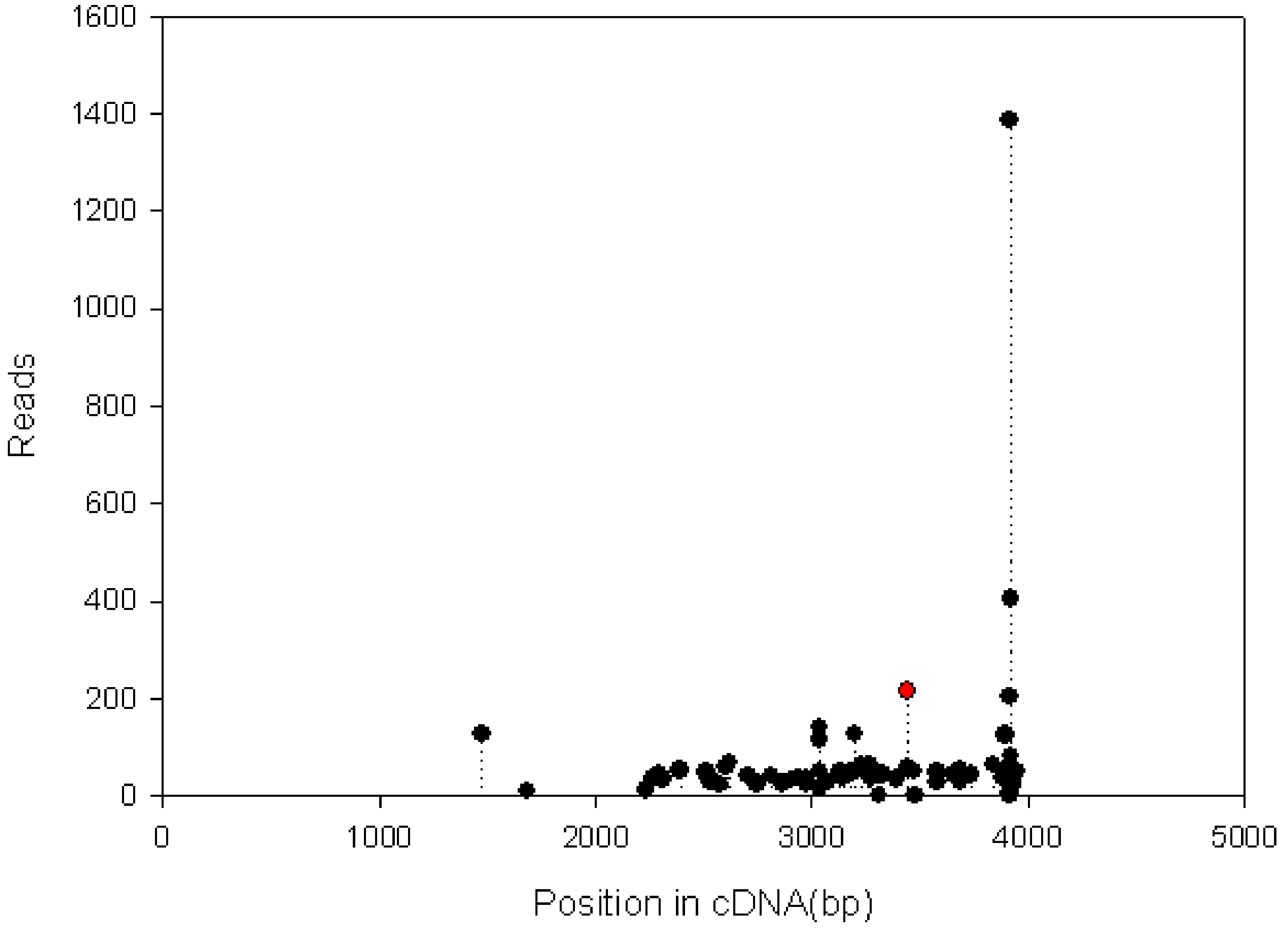


```
5' AGAGAUCAGGCUGGCAGCUUGUAUUU 3'        Cs2g15130.1
     :::::: ::::::::::: .:
3' -AUCUAGUACGACCGUCGAA-GU--- 5'        Csi-miR167a.1
```

Csi-miR167a.1, target=Cs8g08320.1 gene=Cs8g08320
Category:3
Score=4.5
Cleavage Site=585

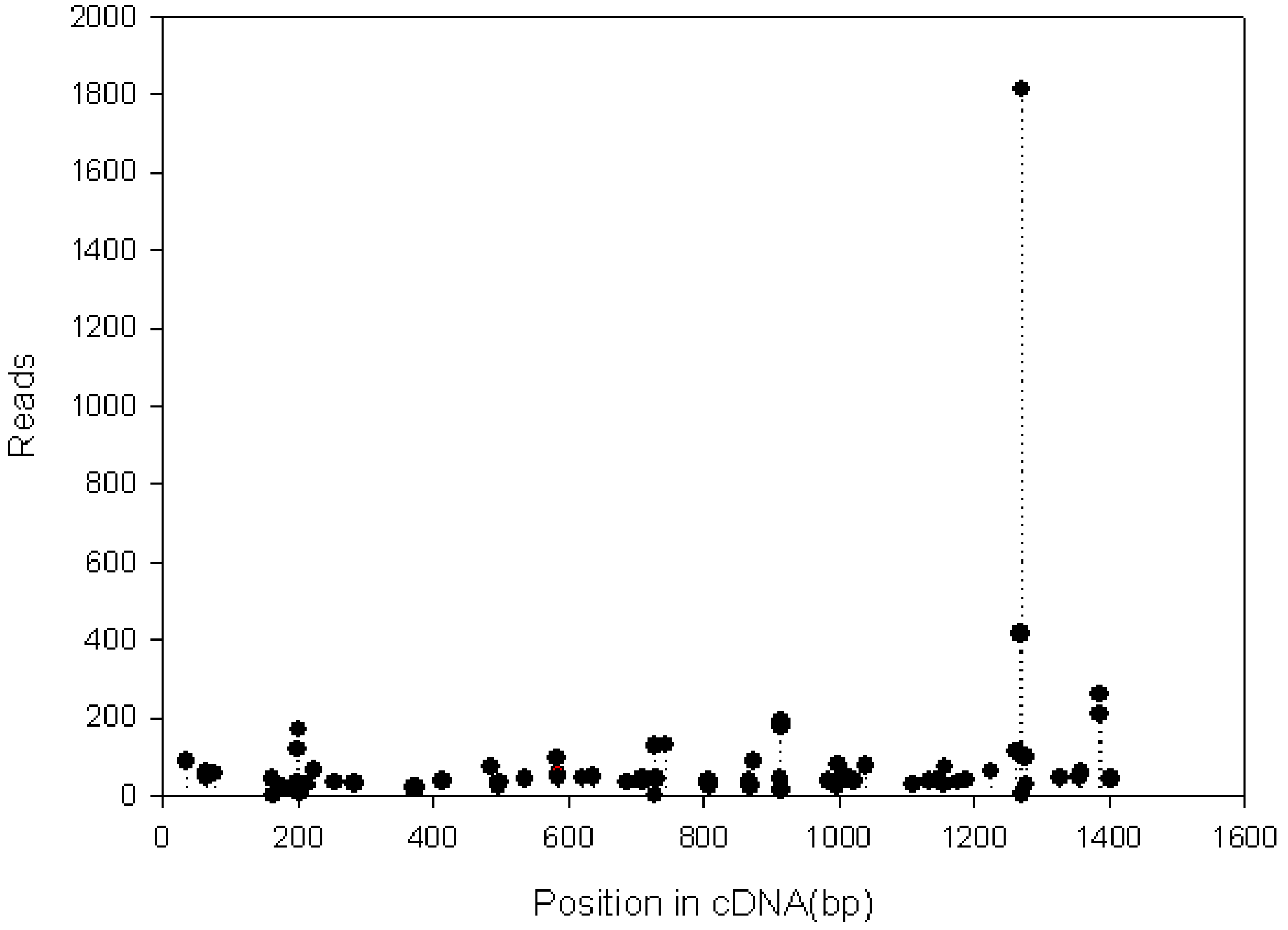


```
5' UCAUGCGGAUCAUGCUGG-AGUUUCAG 3'       Cs8g08320.1
        .:::::::::::: ::.::::
3' -----AUCUAGUACGACCGUCGAAGU- 5'       Csi-miR167a.1
```

Csi-miR167b.1, target=Cs2g09440.1 gene=Cs2g09440
Category:3
Score=4
Cleavage Site=3259

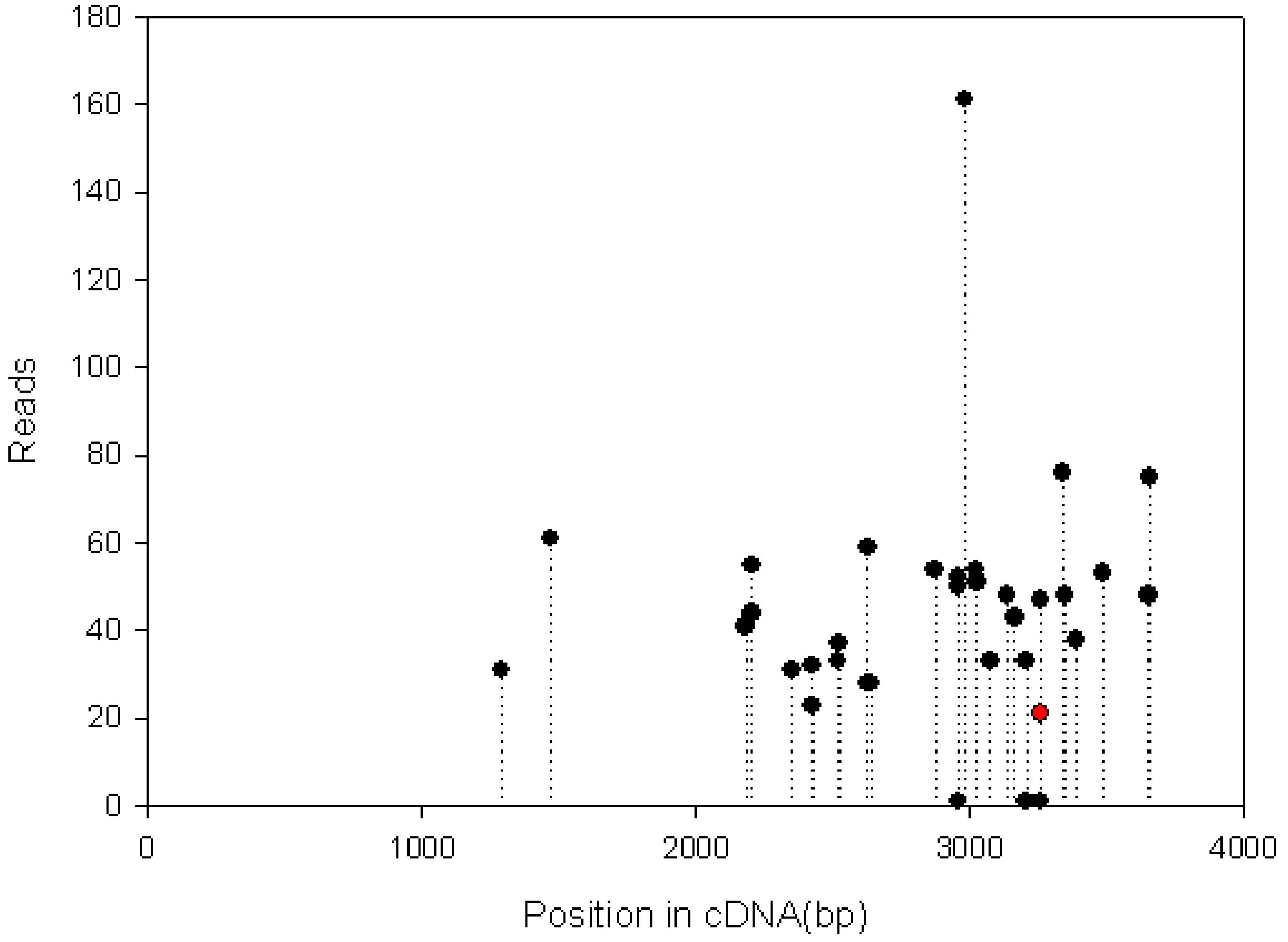


```
5' AGAUCAGGCUGGCAGCUUGUAUUCGU 3'        Cs2g09440.1
   :::::: ::::::::::: .:
3' UCUAGUACGACCGUCGAA-GU----- 5'        Csi-miR167b.1
```

Csi-miR167b.1, target=Cs2g15130.1 gene=Cs2g15130
Category:2
Score=4
Cleavage Site=3444

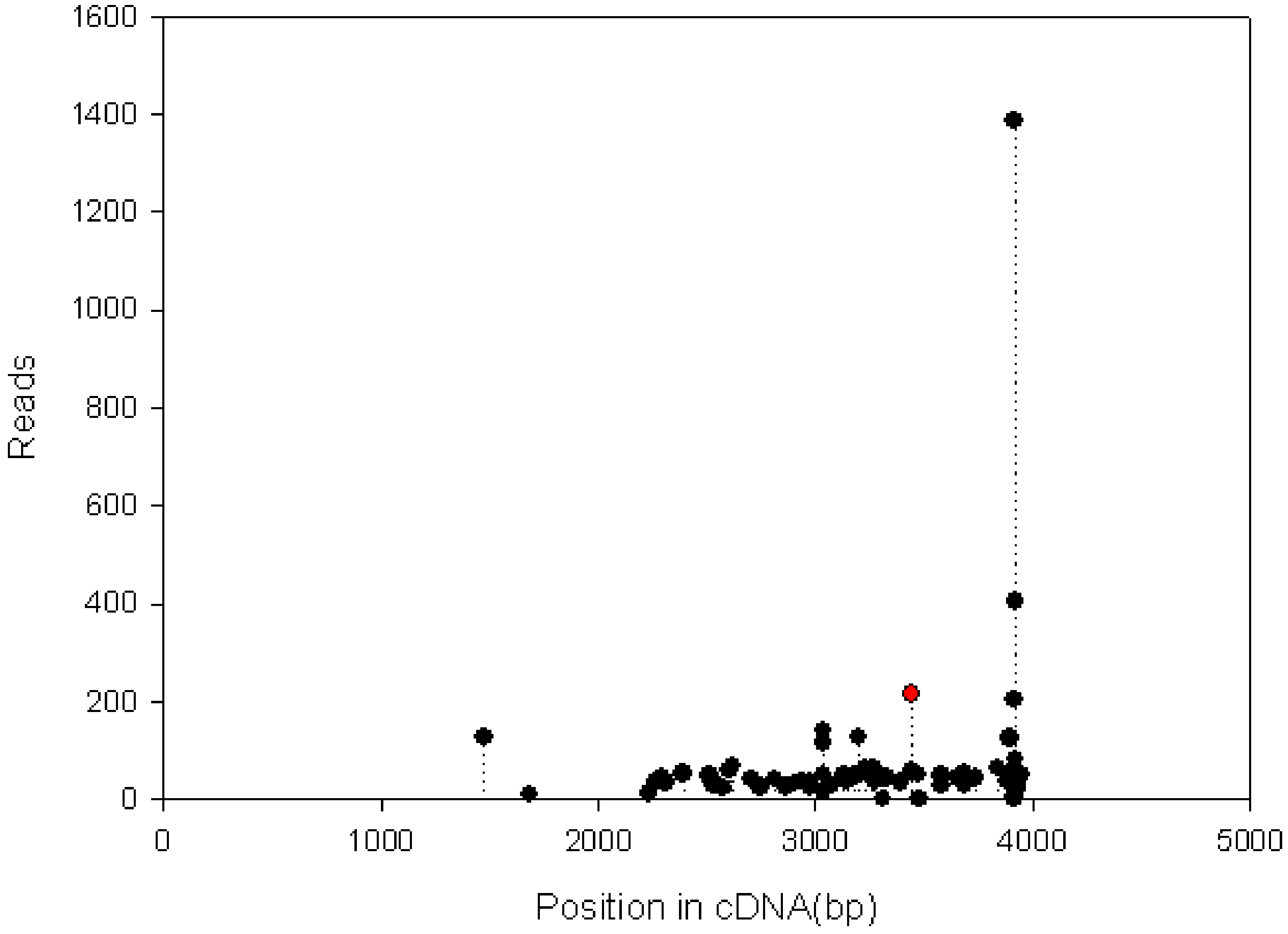


```
5' AGAGAUCAGGCUGGCAGCUUGUAUUU 3'        Cs2g15130.1
     :::::: ::::::::::: .:
3' --UCUAGUACGACCGUCGAA-GU--- 5'        Csi-miR167b.1
```

Csi-miR167b.1, target=Cs8g08320.1 gene=Cs8g08320
Category:3
Score=3.5
Cleavage Site=585

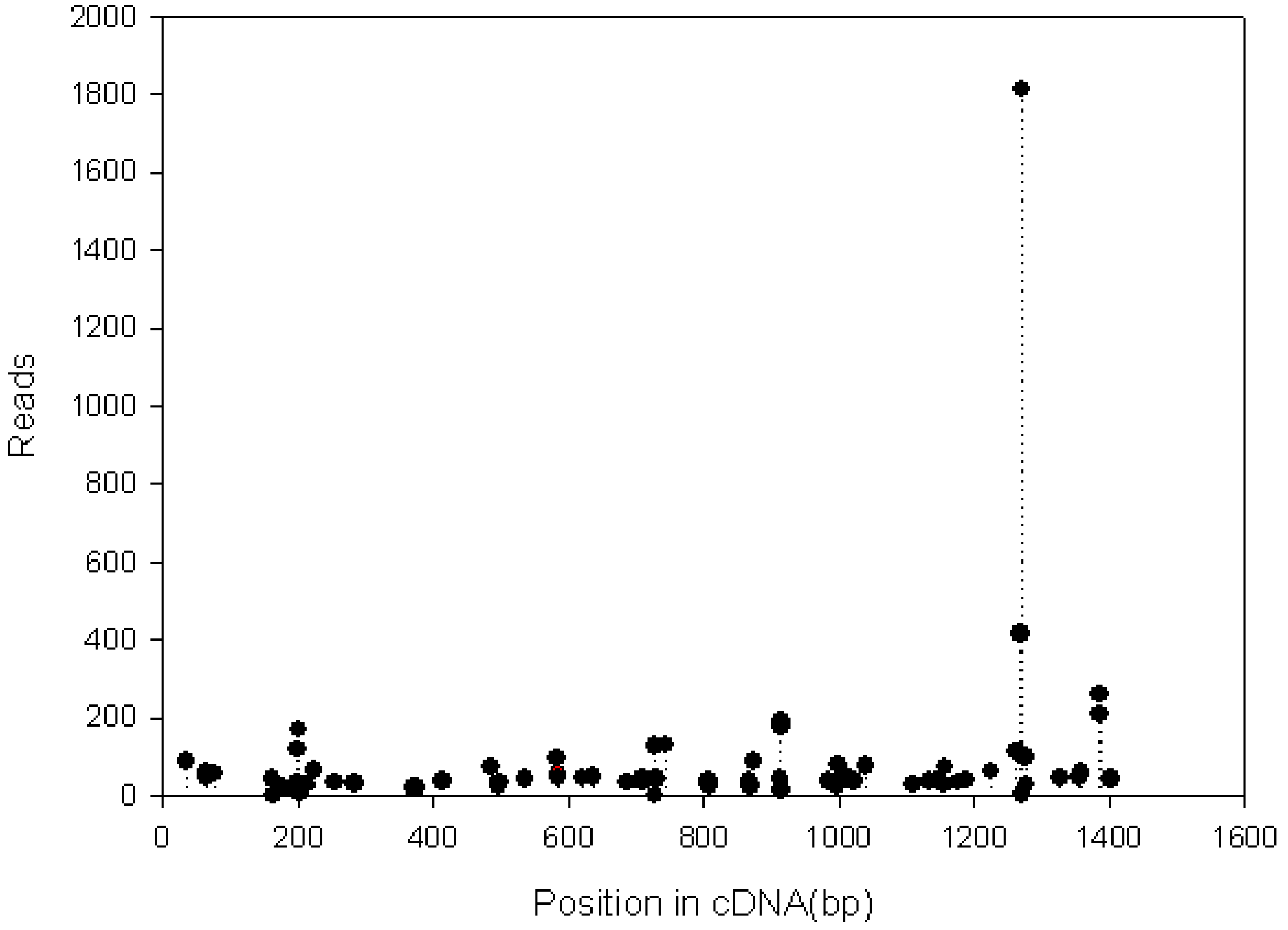


```
5' UCAUGCGGAUCAUGCUGG-AGUUUCAG 3'        Cs8g08320.1
         .:::::::::::: ::.::::
3' ------UCUAGUACGACCGUCGAAGU- 5'        Csi-miR167b.1
```

Csi-miR167b.3, target=Cs2g15130.1 gene=Cs2g15130
Category:2
Score=3.5
Cleavage Site=3444

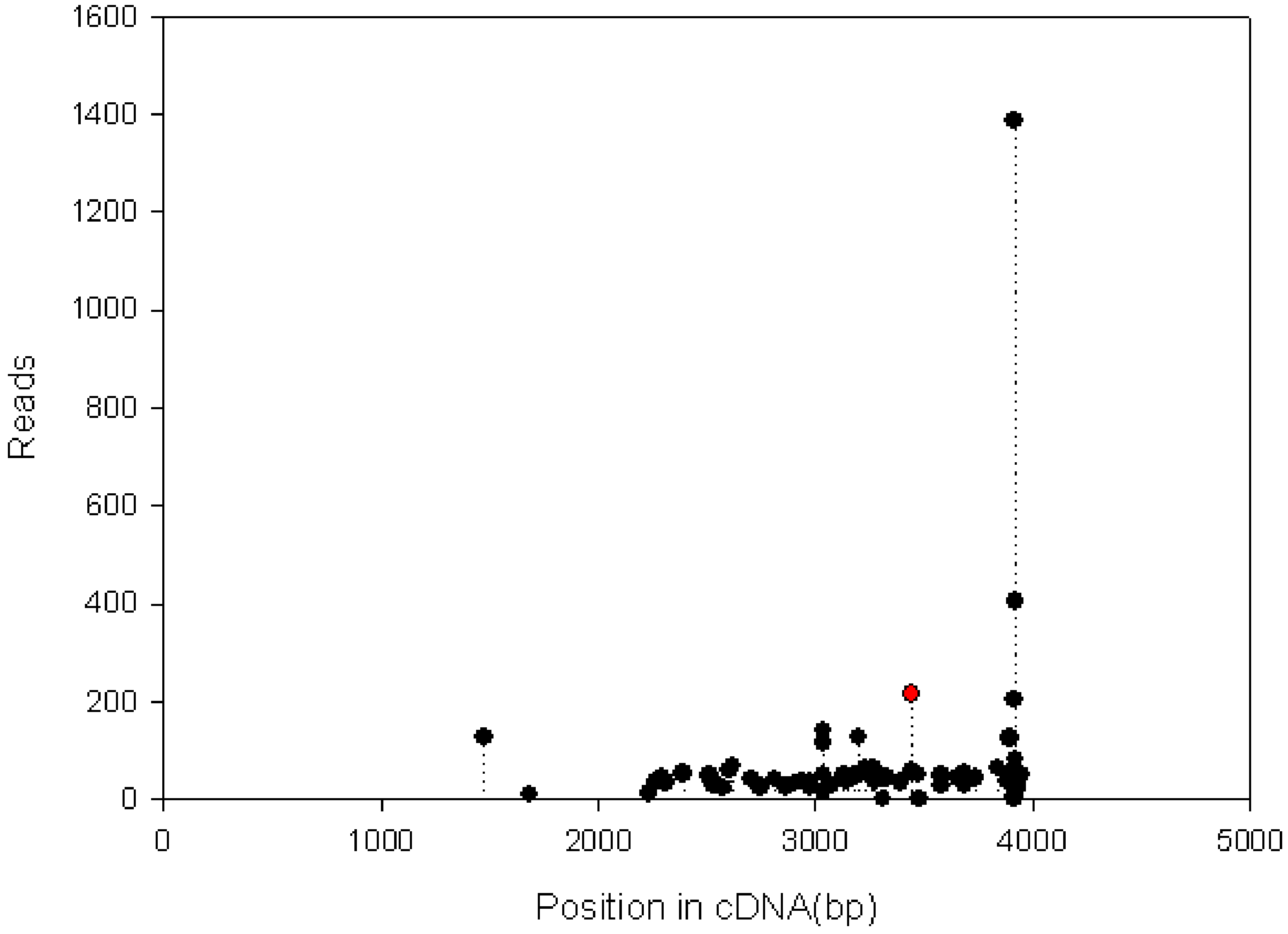


```
5' AGAGAUCAGGCUGGCAGCUUGUAUUU 3'        Cs2g15130.1
    .:::::: :::::::::::
3' AUUCUAGUACGACCGUCGAA------ 5'        Csi-miR167b.3
```

Csi-miR167b.3, target=Cs8g07440.1 gene=Cs8g07440
Category:3
Score=5
Cleavage Site=1606

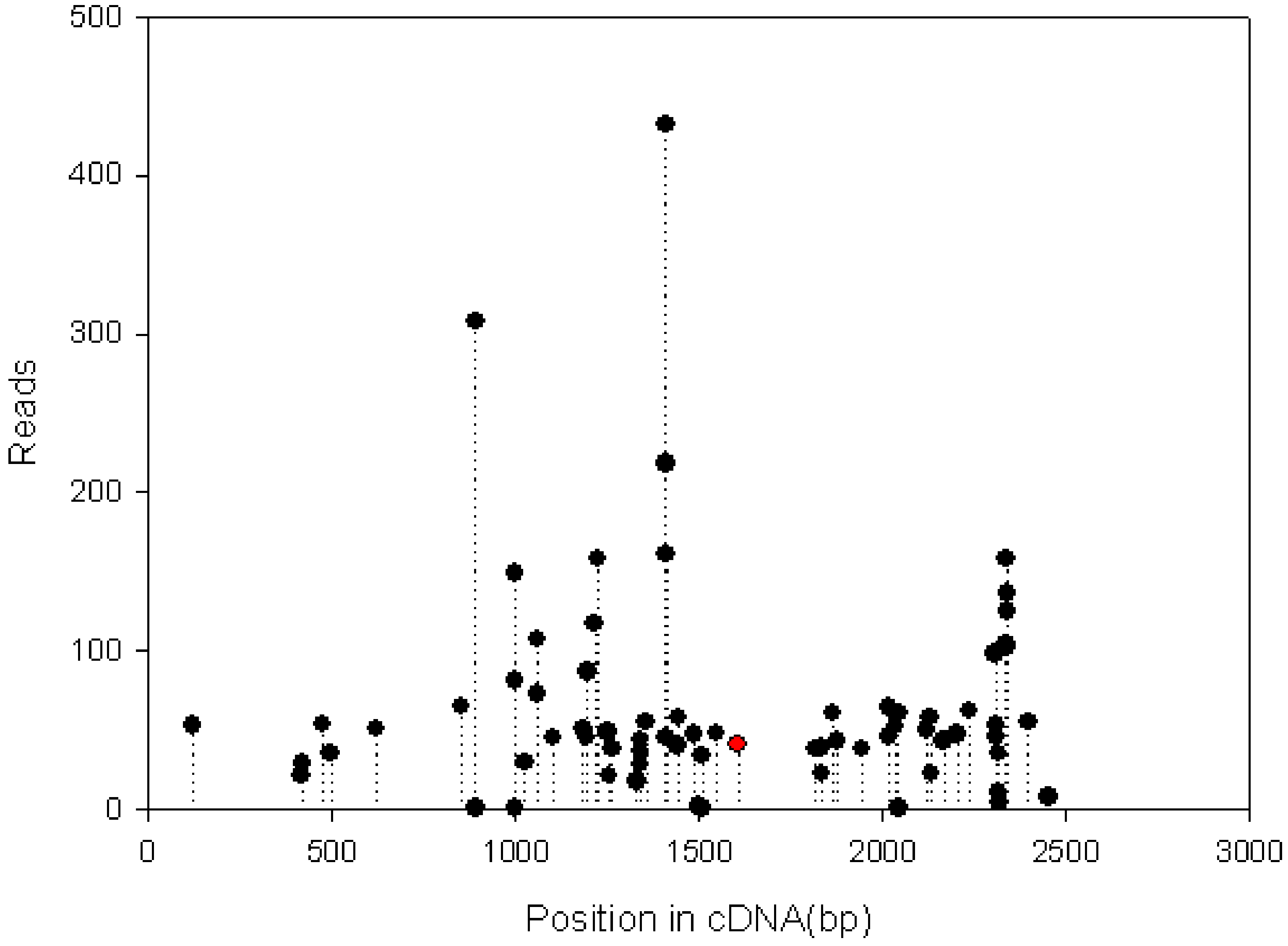


```
5' GGAAAACAAGCU-AUGCUGGAAGCUUC 3'        Cs8g07440.1
         ::: : ::::::: :::::
3' ------AUUCUAGUACGACCGUCGAA- 5'        Csi-miR167b.3
```

Csi-miR167b.4, target=Cs2g15130.1 gene=Cs2g15130
Category:2
Score=4.5
Cleavage Site=3444

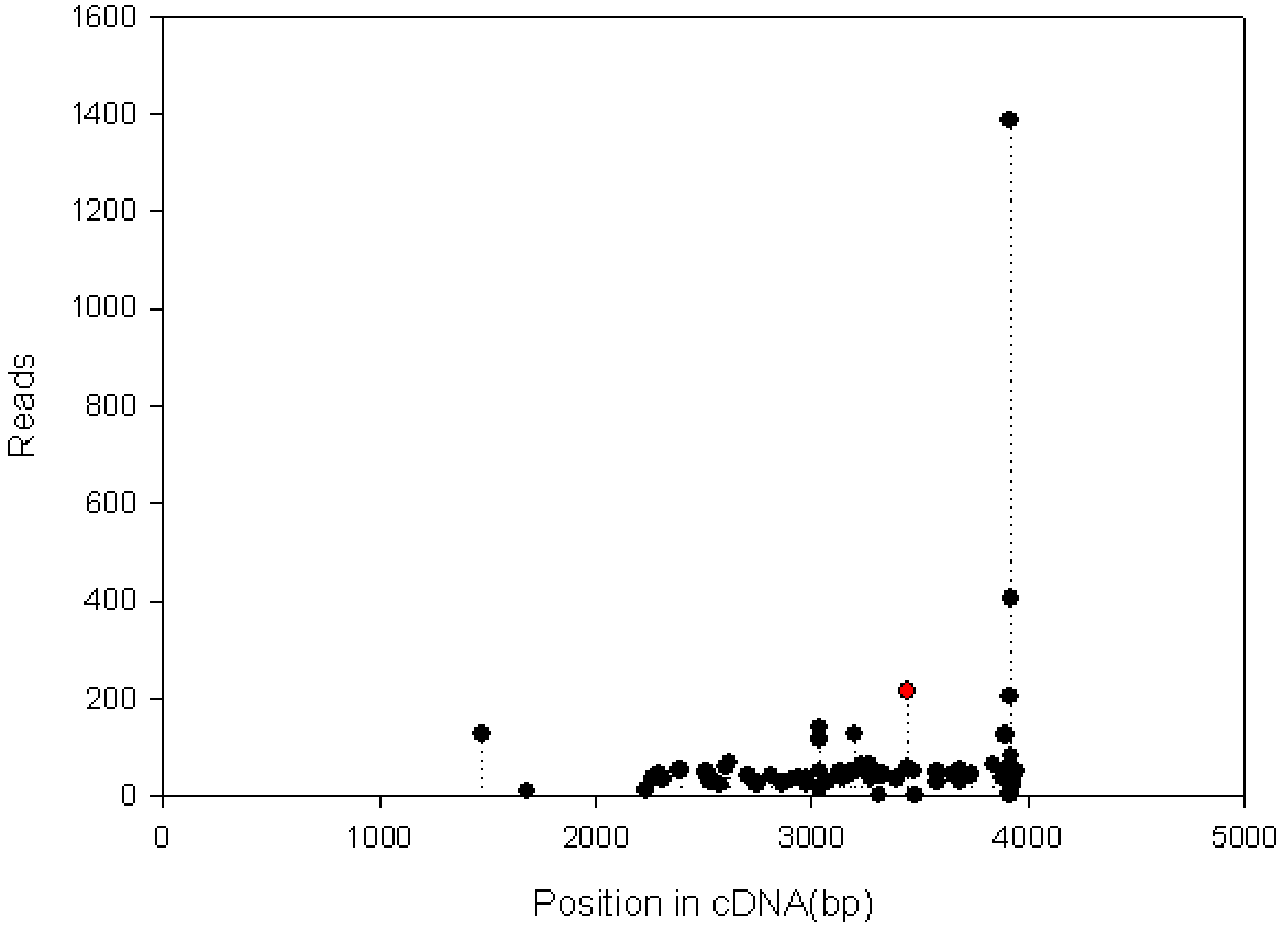


```
5' AGAGAUCAGGCUGGCAGCUUGUAUUU 3'        Cs2g15130.1
    .:::::: ::::::::::: .:
3' -UUCUAGUACGACCGUCGAA-GU--- 5'        Csi-miR167b.4
```

Csi-miR167b.4, target=Cs8g08320.1 gene=Cs8g08320
Category:3
Score=4.5
Cleavage Site=585

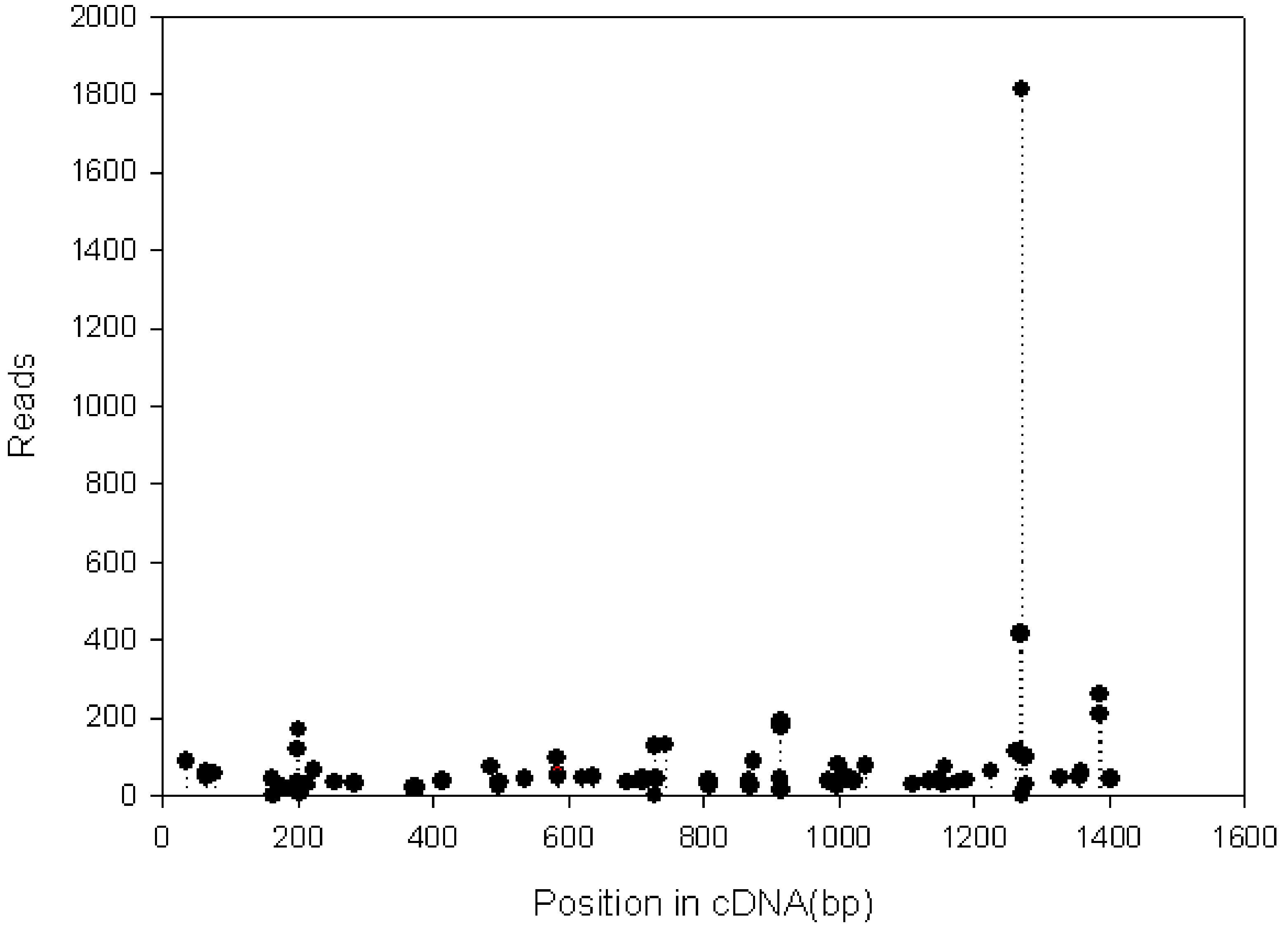


```
5' UCAUGCGGAUCAUGCUGG-AGUUUCAG 3'        Cs8g08320.1
        .:::::::::::: ::.::::
3' -----UUCUAGUACGACCGUCGAAGU- 5'        Csi-miR167b.4
```

Csi-miR167d.1, target=Cs8g08320.1 gene=Cs8g08320
Category:3
Score=4.5
Cleavage Site=585

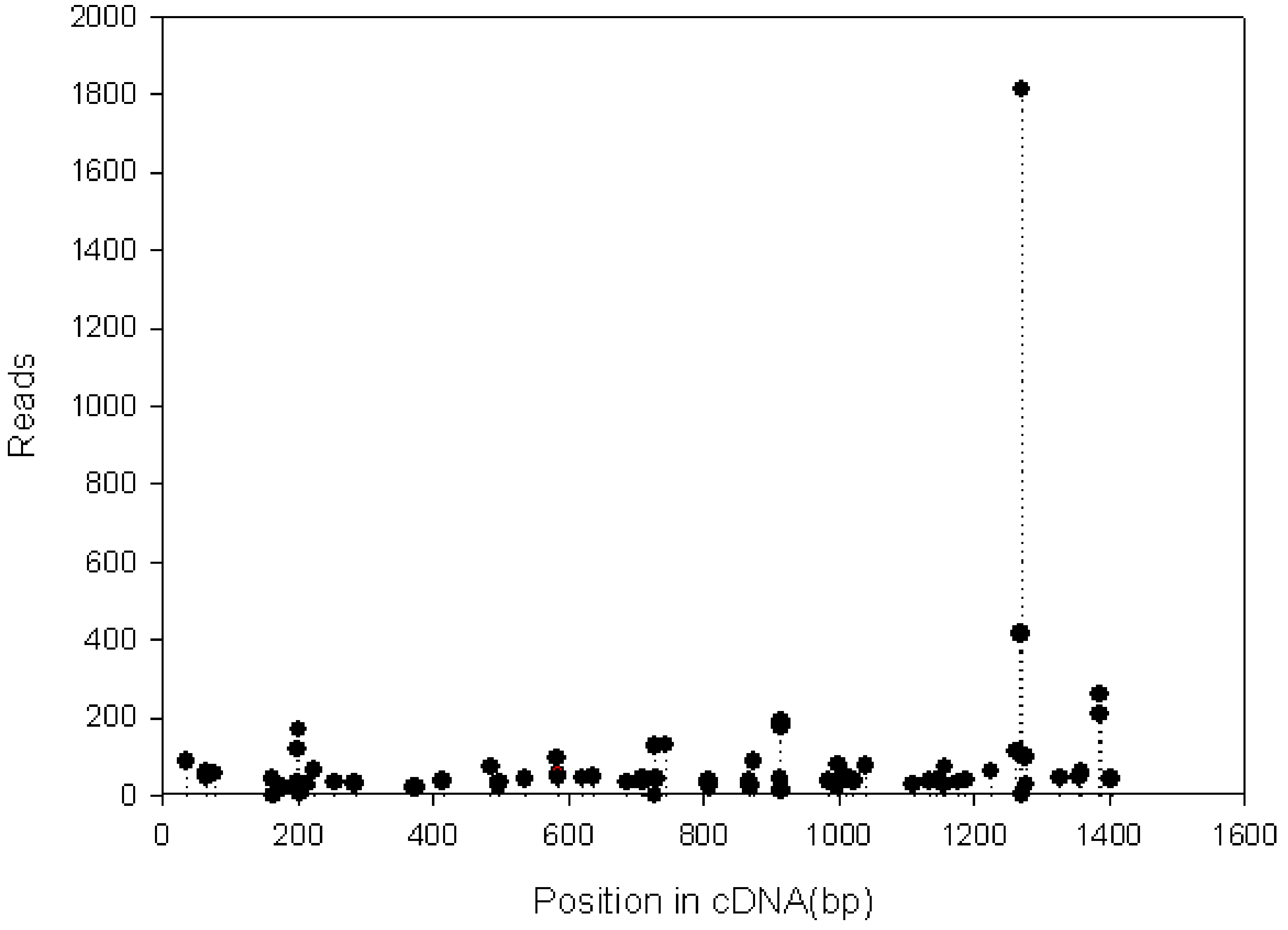


```
5' UCAUGCGGAUCAUGCUGG-AGUUUCAG 3'       Cs8g08320.1
       :.:::::::::::: ::.::::
3' ----AGUCUAGUACGACCGUCGAAGU- 5'       Csi-miR167d.1
```

Csi-miR167d.2, target=Cs2g15130.1 gene=Cs2g15130
Category:2
Score=5
Cleavage Site=3444

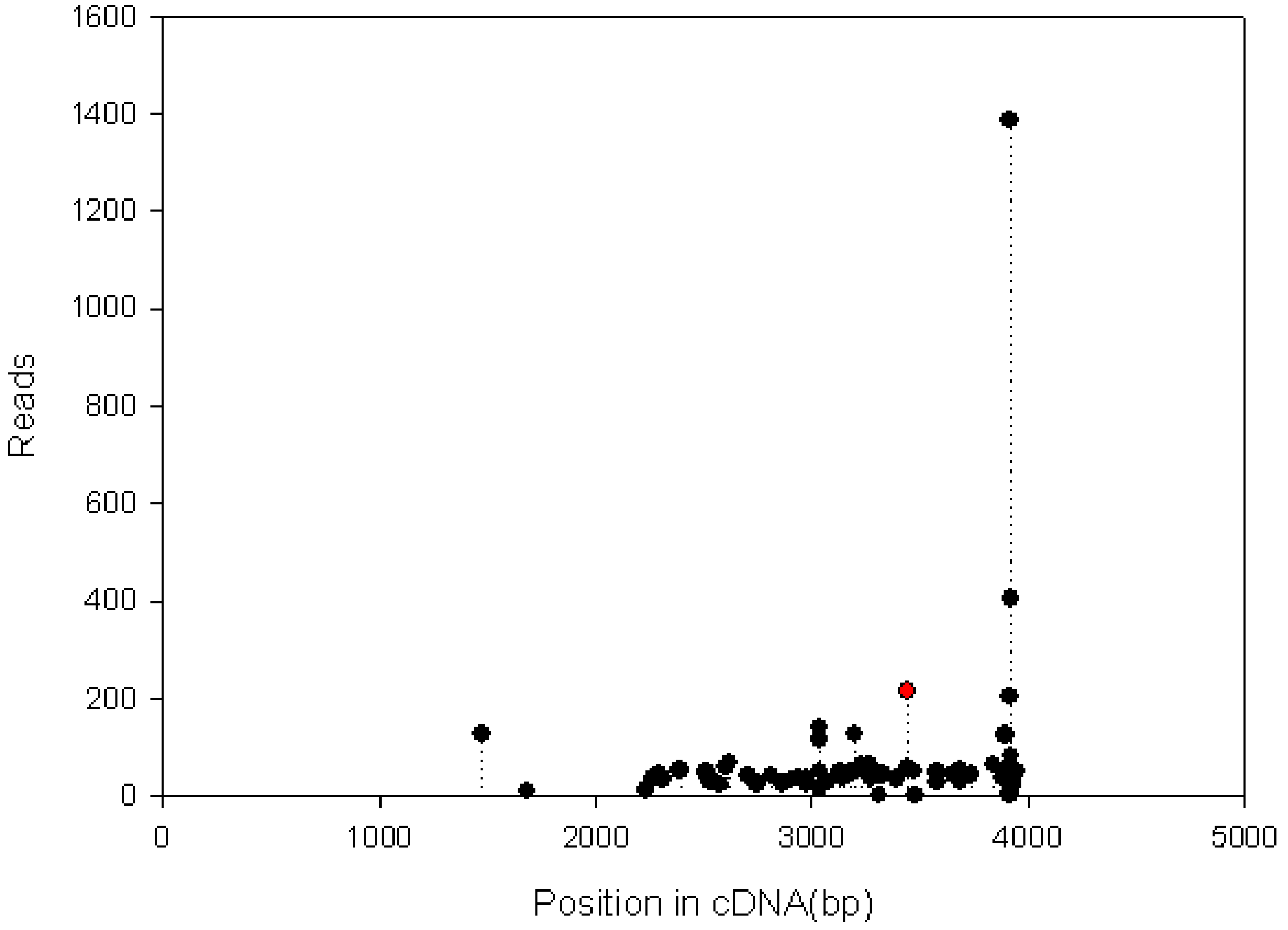


```
5' AGAGAUCAGGCUGGCAGCUUGUAUUU 3'        Cs2g15130.1
     :::::: :::::::::::: .:
3' -GUCUAGUACGACCGUCGAA-GU--- 5'        Csi-miR167d.2
```

Csi-miR167d.2, target=Cs8g08320.1 gene=Cs8g08320
Category:3
Score=3.5
Cleavage Site=585

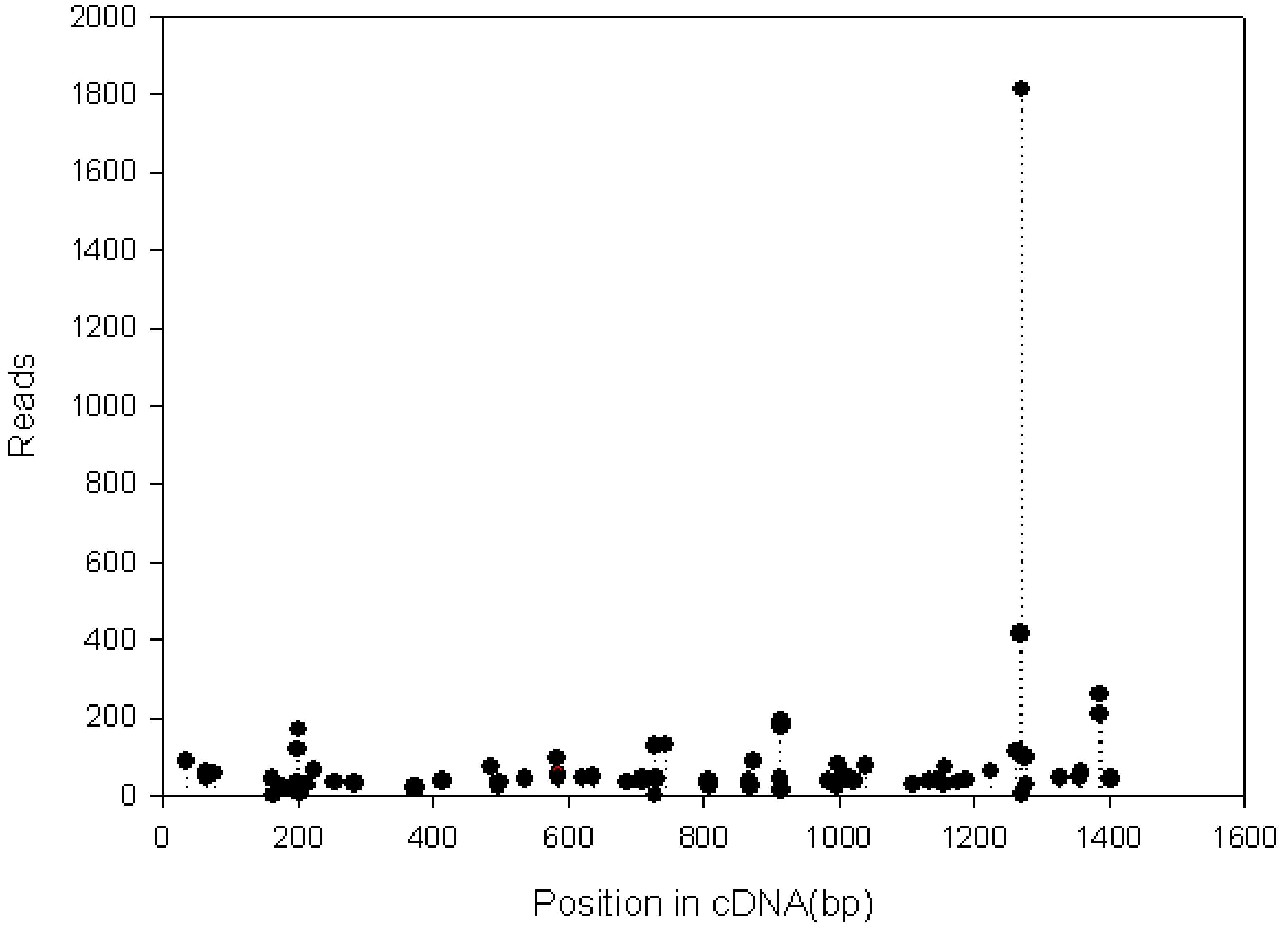


```
5' UCAUGCGGAUCAUGCUGG-AGUUUCAG 3'        Cs8g08320.1
         :.::::::::::: ::.::::
3' -----GUCUAGUACGACCGUCGAAGU- 5'        Csi-miR167d.2
```

Csi-miR168a, target=Cs5g05510.1 gene=Cs5g05510
Category:1
Score=5
Cleavage Site=1971

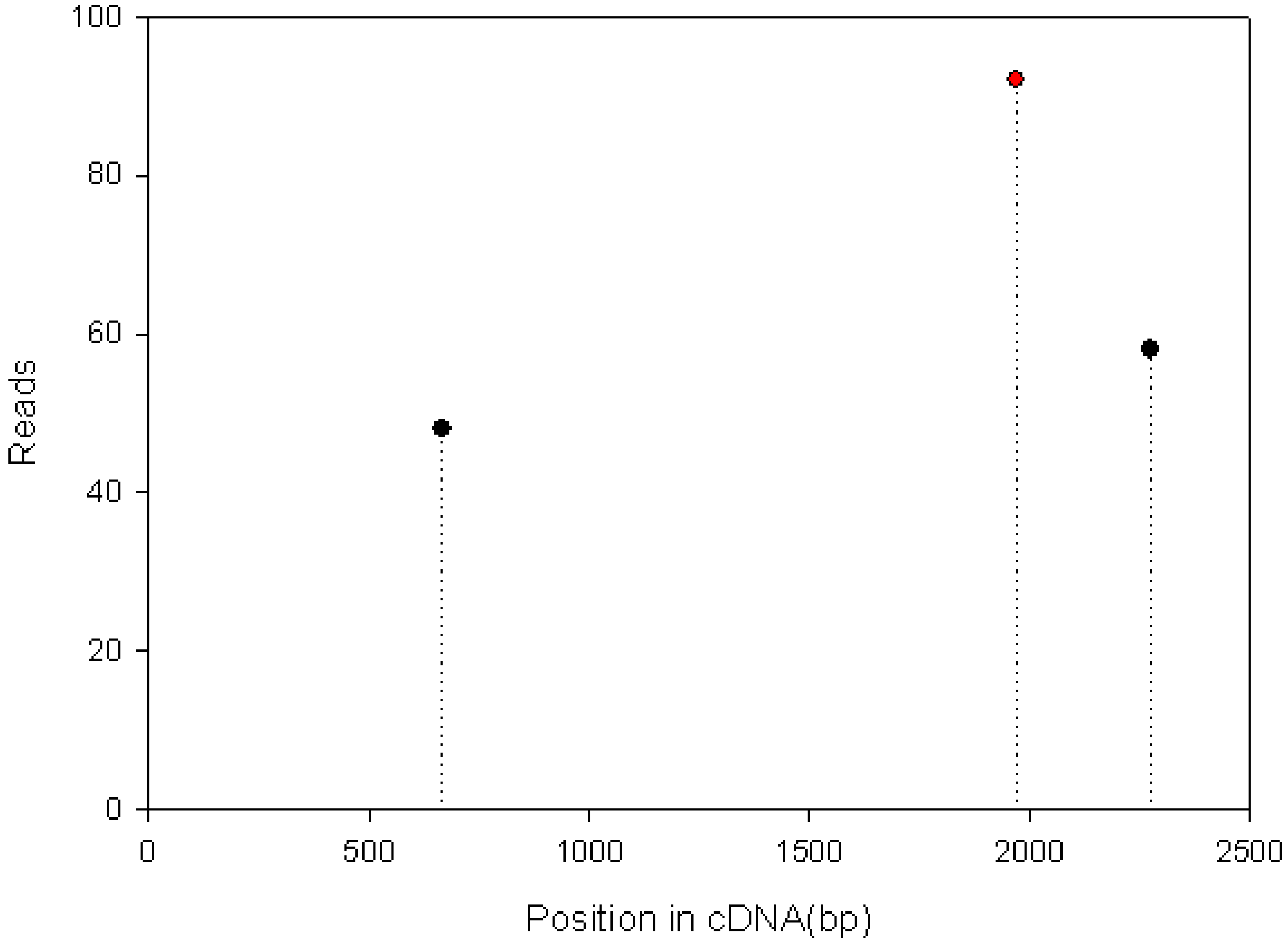


```
5' AUUCCCUCCCUGCACUAAACGAUAUA 3'        Cs5g05510.1
    :::::  :::::::.:: :::
3' -AAGGGCUGGACGUGGUUCGCU---- 5'        Csi-miR168a
```

Csi-miR168a, target=Cs5g16710.1 gene=Cs5g16710
Cagetory:2
Score=5
Cleavage Site=797

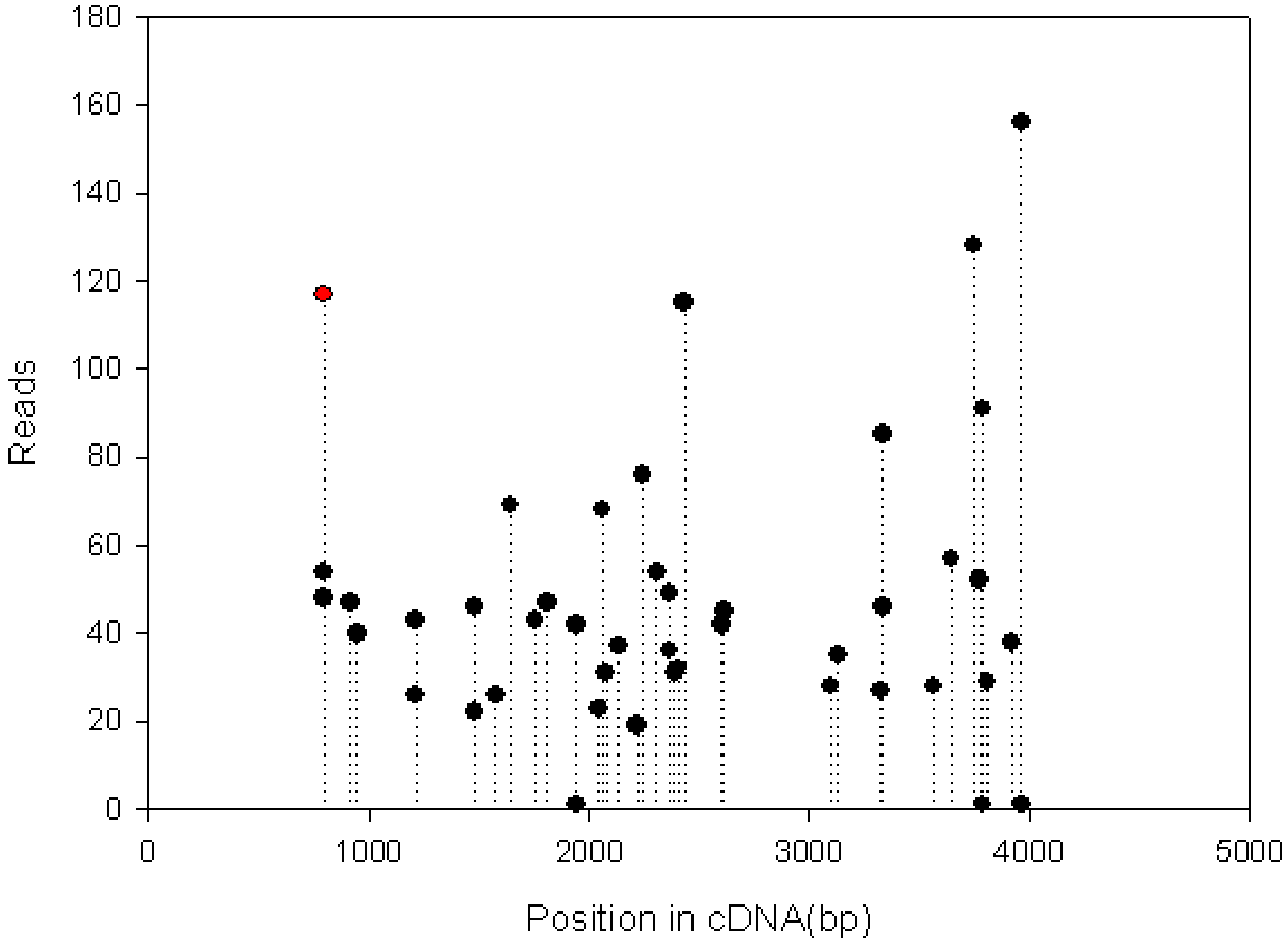


```
5' AUUCCCGAGCUACACCAAGCAACCCC 3'        Cs5g16710.1
    ::::::: :: :::::::: :
3' -AAGGGCUGGACGUGGUUCGCU---- 5'        Csi-miR168a
```

Csi-miR169b.1, target=Cs6g13560.1 gene=Cs6g13560
Cagetory:2
Score=3.5
Cleavage Site=1307

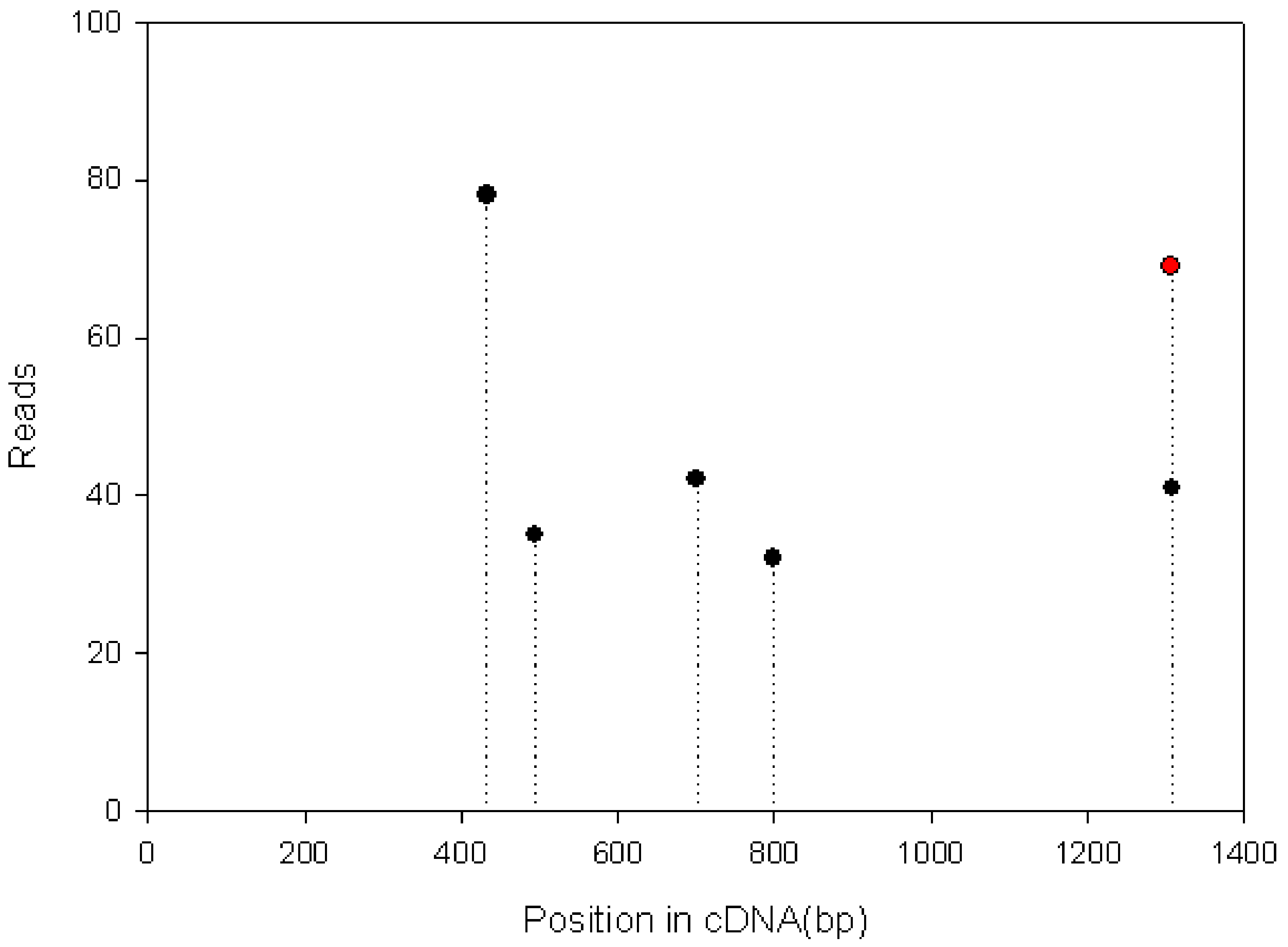


```
5' UUAGGCAAAUCAUUCUUGGCUCAUCU 3'        Cs6g13560.1
    .:::::: ::::.::::::::
3' -GUCCGUUCAGUAGGAACCGAU---- 5'        Csi-miR169b.1
```

Csi-miR169b.1-3p, target=Cs2g28180.1 gene=Cs2g28180
Category:2
Score=5
Cleavage Site=544

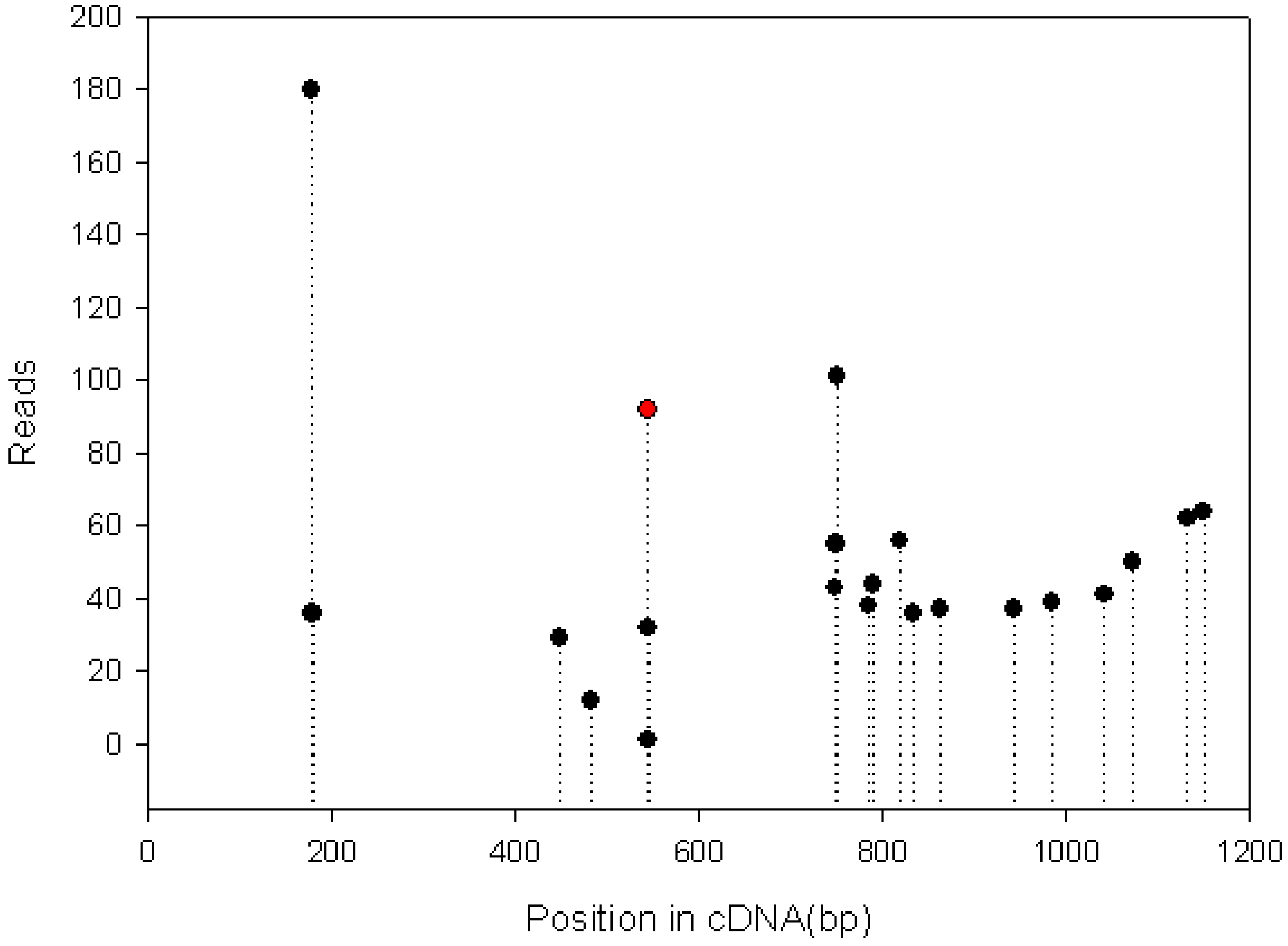


```
5' CCUUCCCCUU-GCCACUGAGACUGCCU 3'        Cs2g28180.1
          ::: ::::  ::::::::::
3' -------GAAUCGGUUCCUCUGACGGA 5'        Csi-miR169b.1-3p
```

Csi-miR169b.1-3p, target=Cs9g16380.1 gene=Cs9g16380
Category:3
Score=5
Cleavage Site=616

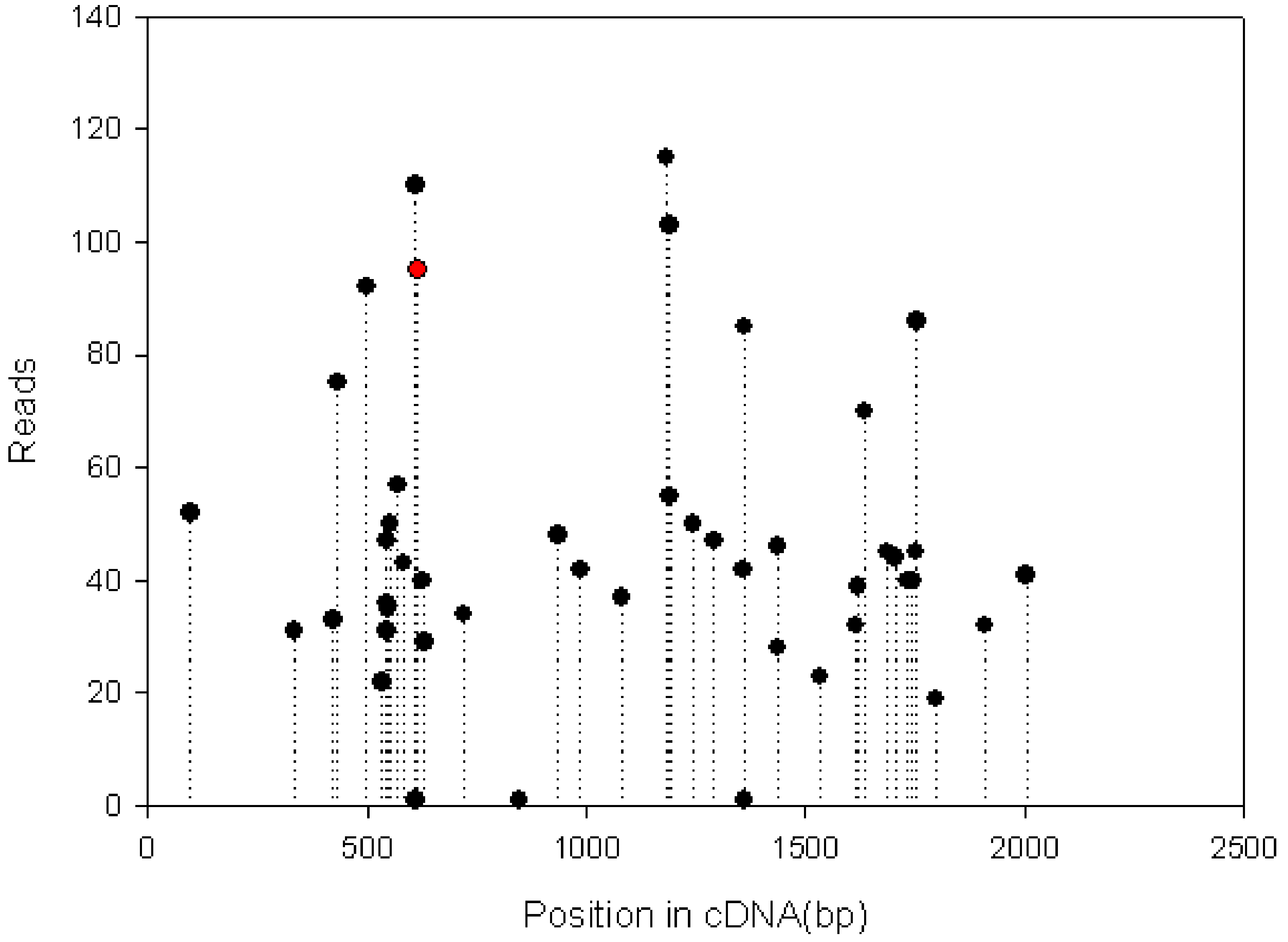


```
5' GUUGACAUCGCCAAGGGGAUUGCUUA 3'        Cs9g16380.1
        : : :::::::.::.:::.:
3' -----GAAUCGGUUCCUCUGACGGA- 5'        Csi-miR169b.1-3p
```

Csi-miR169h, target=Cs7g27875.1 gene=Cs7g27875
Category:1
Score=4.5
Cleavage Site=194

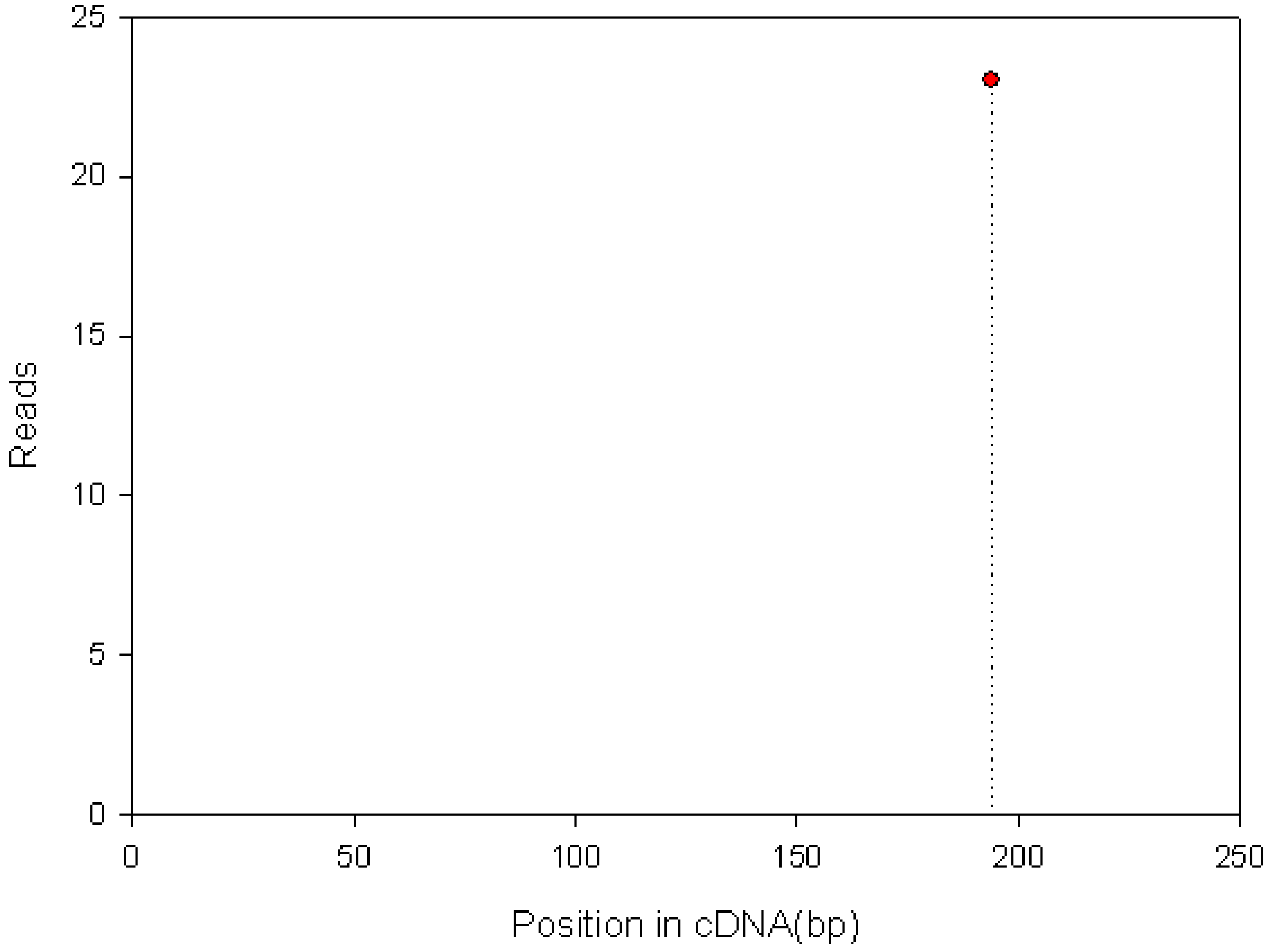


```
5' CAGGCAGGACAUCCUUGGUUUUCUAU 3'        Cs7g27875.1
   :::::::.: ::::::::::.:
3' GUCCGUUCAGUAGGAACCGAU----- 5'        Csi-miR169b.1
```

Csi-miR169i.1, target=Cs6g13560.1 gene=Cs6g13560
Category:2
Score=3.5
Cleavage Site=1307

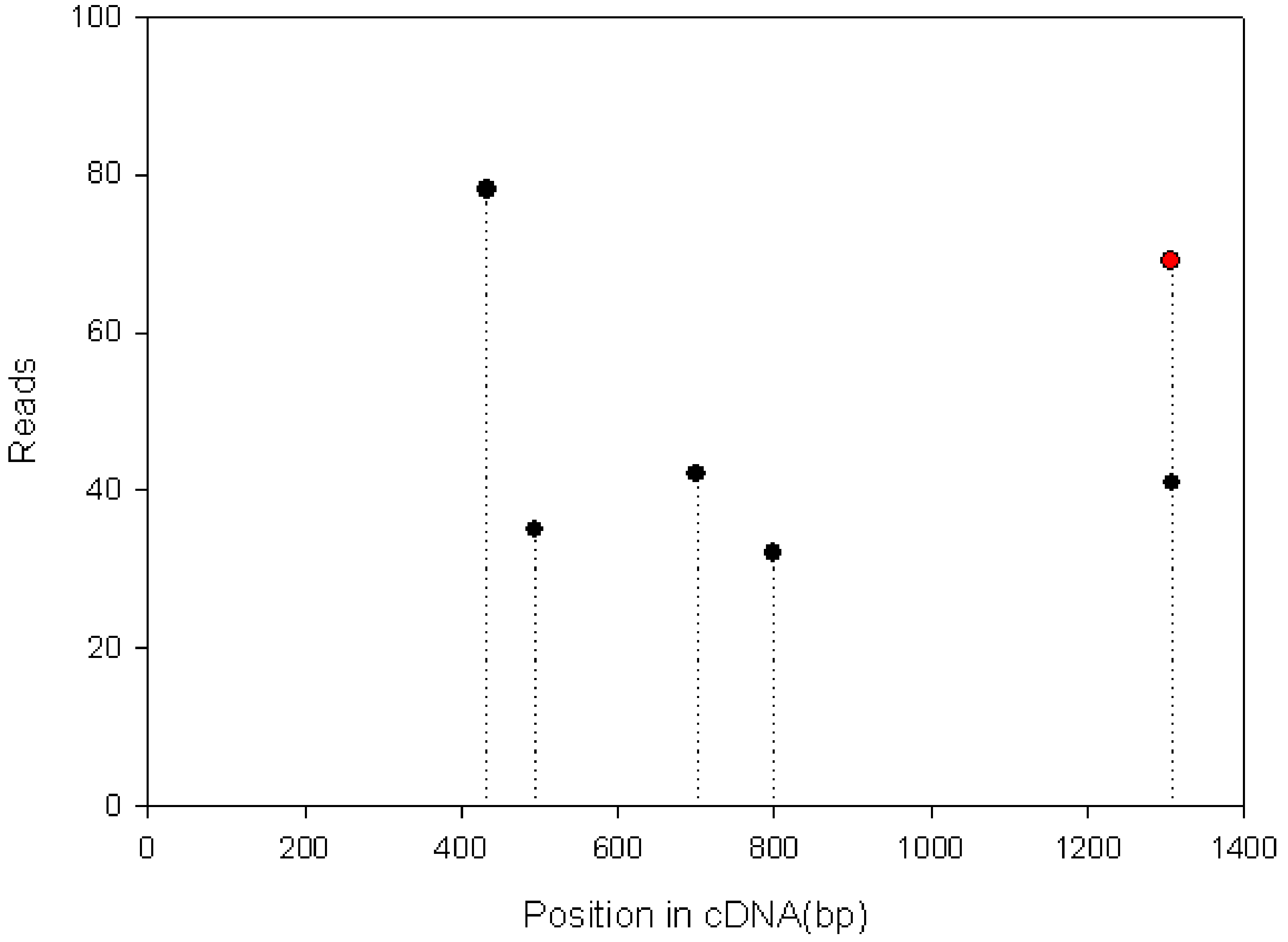


```
5' UUAGGCAAAUCAUUCUUGGCUCAUCU 3'        Cs6g13560.1
   :.:::::: ::::.::::::
3' AGUCCGUUCAGUAGGAACCGAU---- 5'        Csi-miR169i.1
```

Csi-miR169i.1-3p, target=Cs4g06620.1 gene=Cs4g06620
Category:3
Score=5
Cleavage Site=923

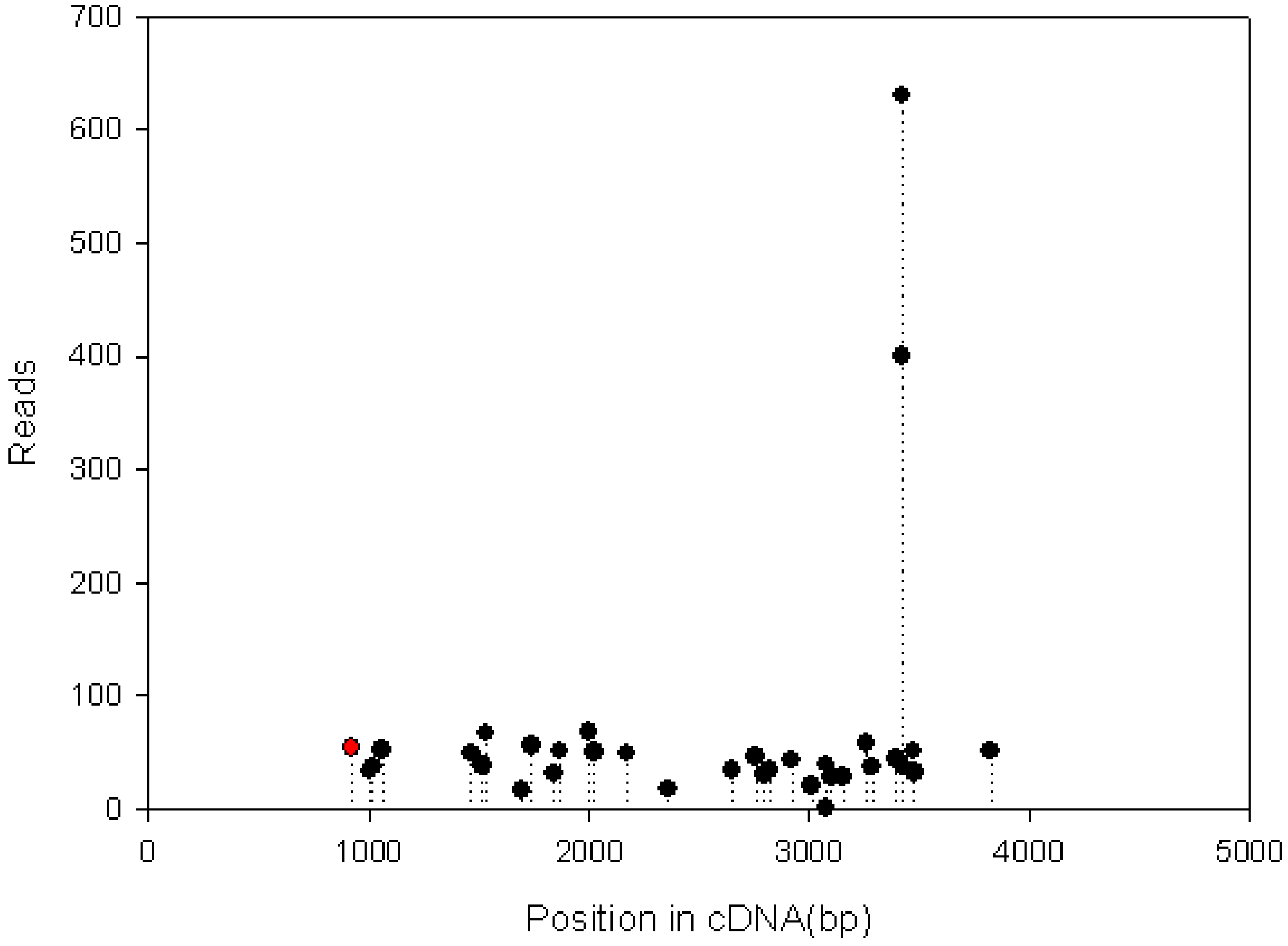


```
5' ACUUUUGUUGAGCCAAGUAGACUUCC 3'        Cs4g06620.1
          ::: ::::::: ::::: ::
3' ------CAA-UCGGUUCCUCUGACGG 5'        Csi-miR169i.1-3p
```

Csi-miR169i.1-3p, target=Cs7g12040.1 gene=Cs7g12040
Category:3
Score=5
Cleavage Site=1874

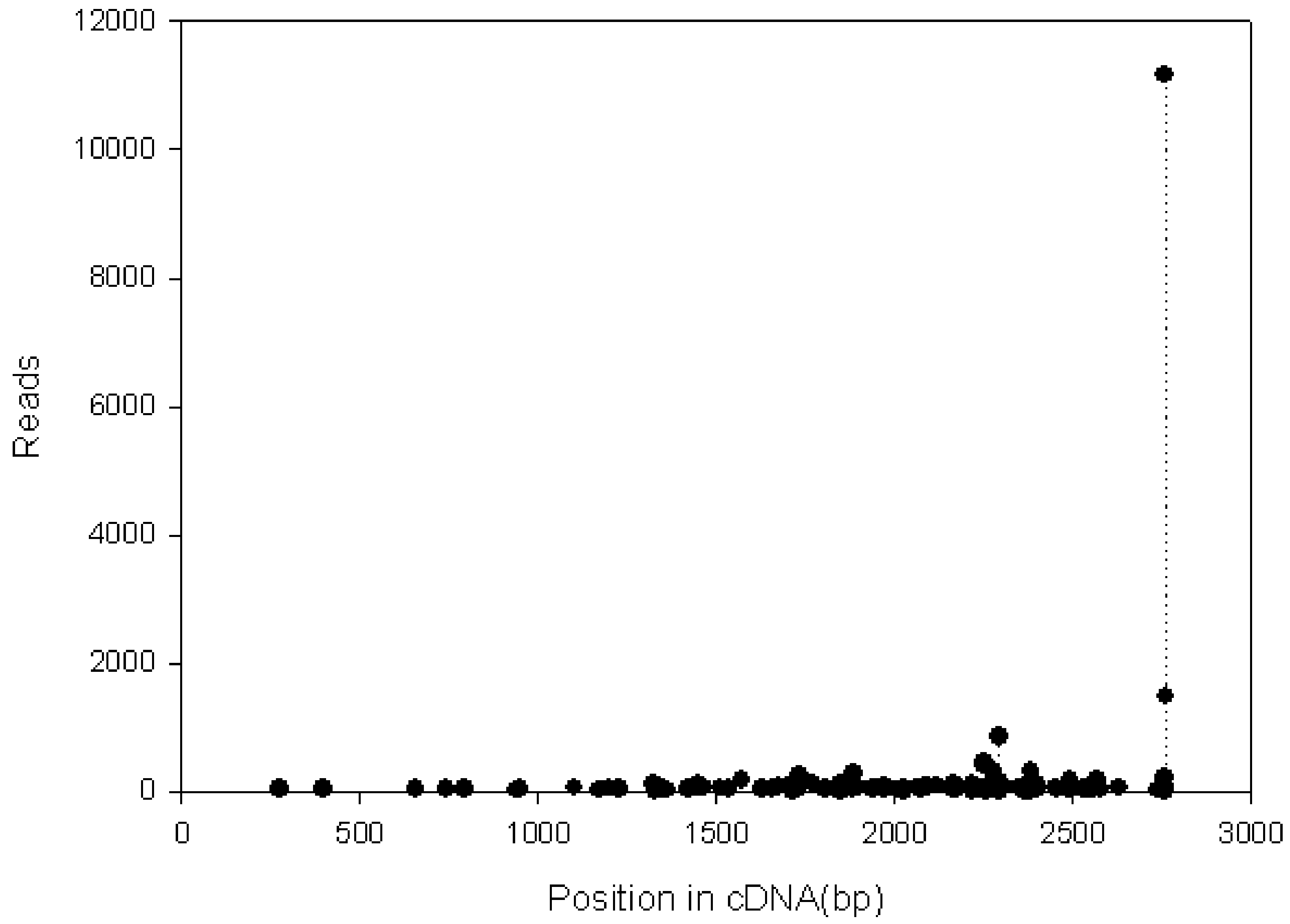


```
5' GCACAAUUUUUCUCAAGGAGGCUGCU 3'        Cs7g12040.1
           ::  .::::::::.::::.
3' -------CAAUCGGUUCCUCUGACGG 5'        Csi-miR169i.1-3p
```

Csi-miR169m.1, target=Cs6g13560.1 gene=Cs6g13560
Cagetory:2
Score=4.5
Cleavage Site=1307

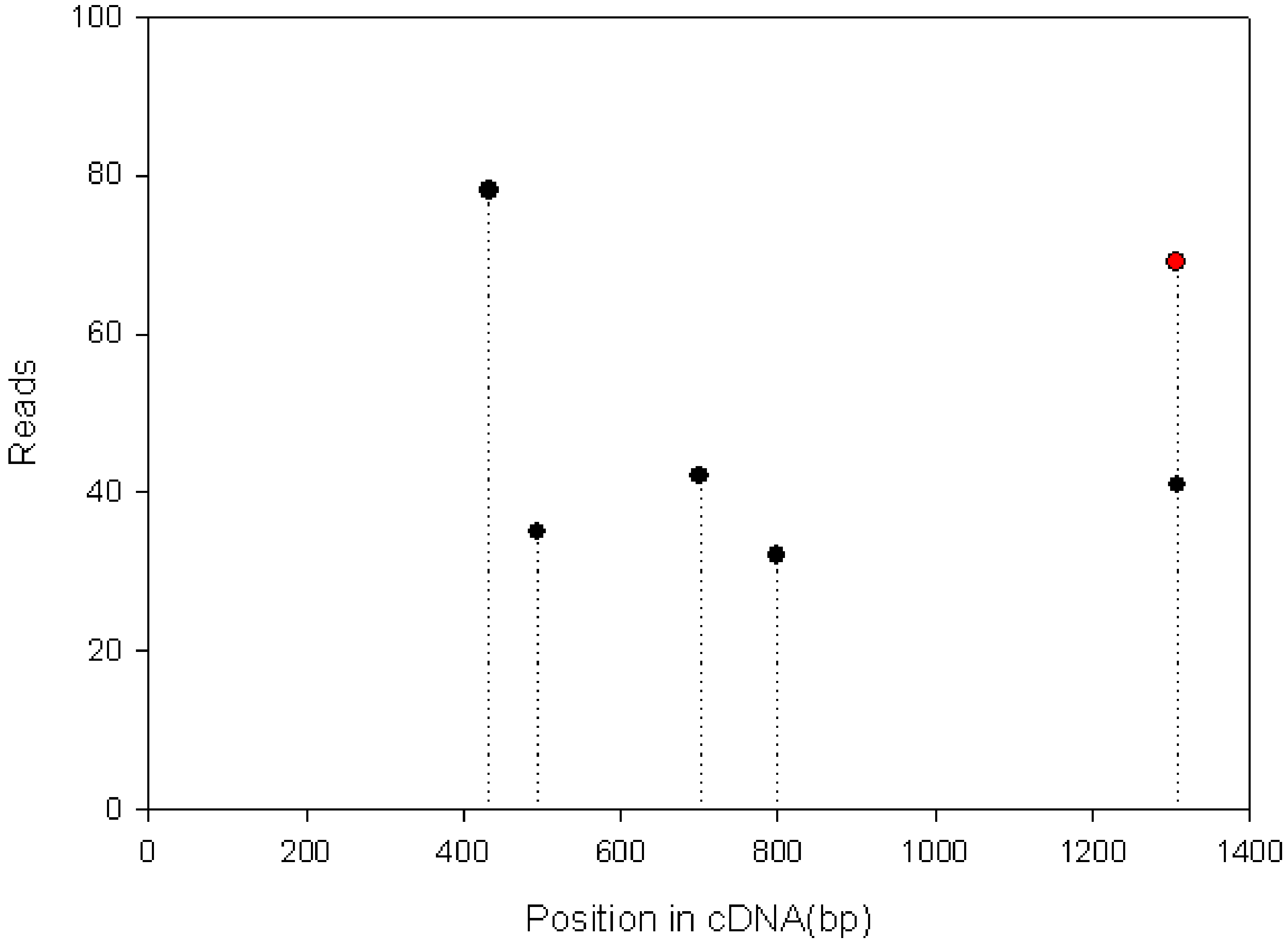


```
5' UUAGGCAAAUCAUUCUUGGCUCAUCU 3'          Cs6g13560.1
    .  ::::: :::::.:::::::
3' -GGCCGUUCAGUAGGAACCGAC---- 5'          Csi-miR169m.1
```

Csi-miR169m.1, target=Cs8g17280.1 gene=Cs8g17280
Category:3
Score=4.5
Cleavage Site=46

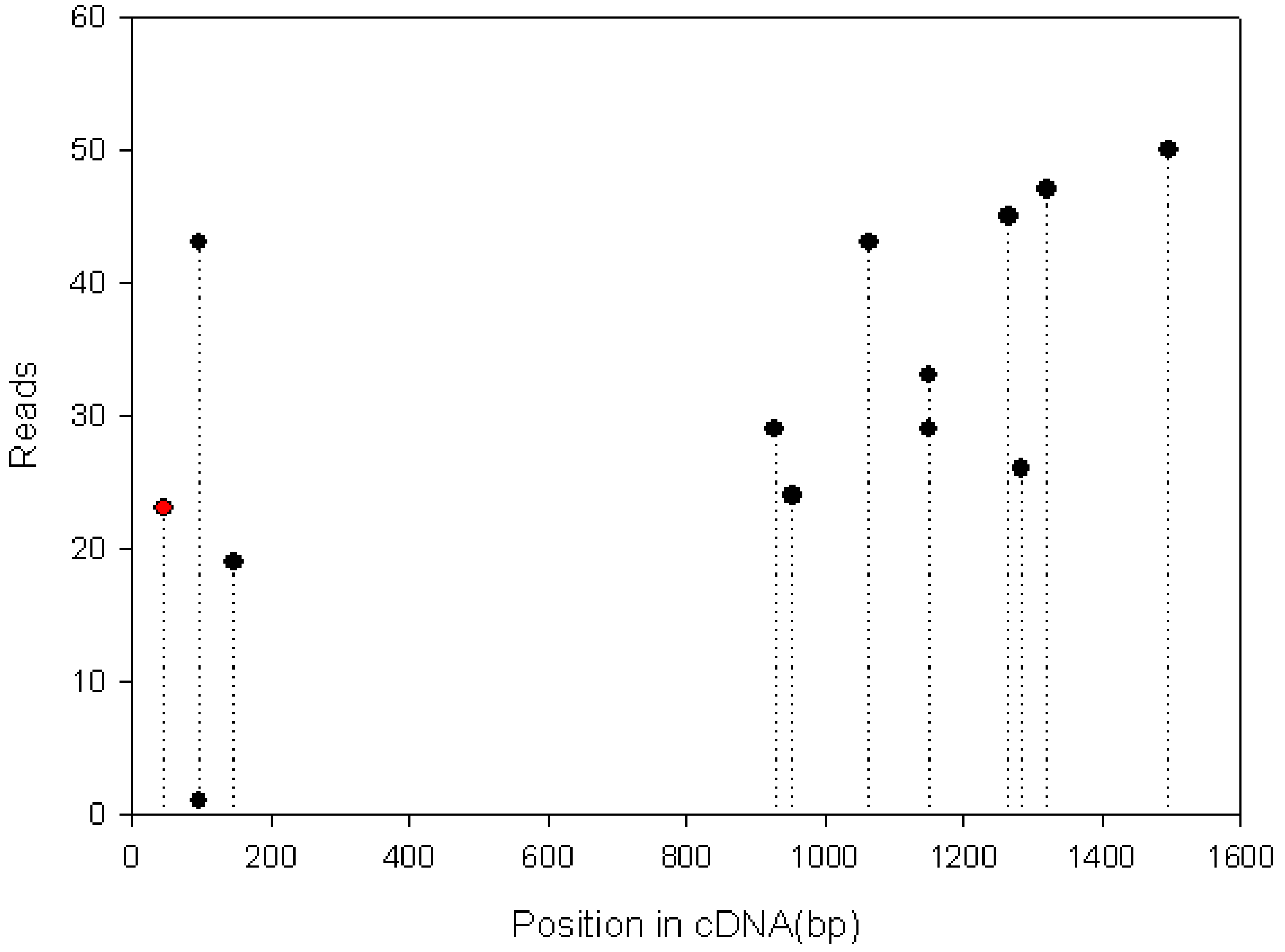


```
5' CGUCUGGCAAGUCACCCGUGGCUGAG 3'        Cs8g17280.1
       :.:::::::::: :: ::::::
3' ---GGCCGUUCAGUAGGAACCGAC-- 5'        Csi-miR169m.1
```

Csi-miR171b.1, target=Orange1.1t00200.1 gene=Orange1.1t00200
Category:1
Score=1
Cleavage Site=1514

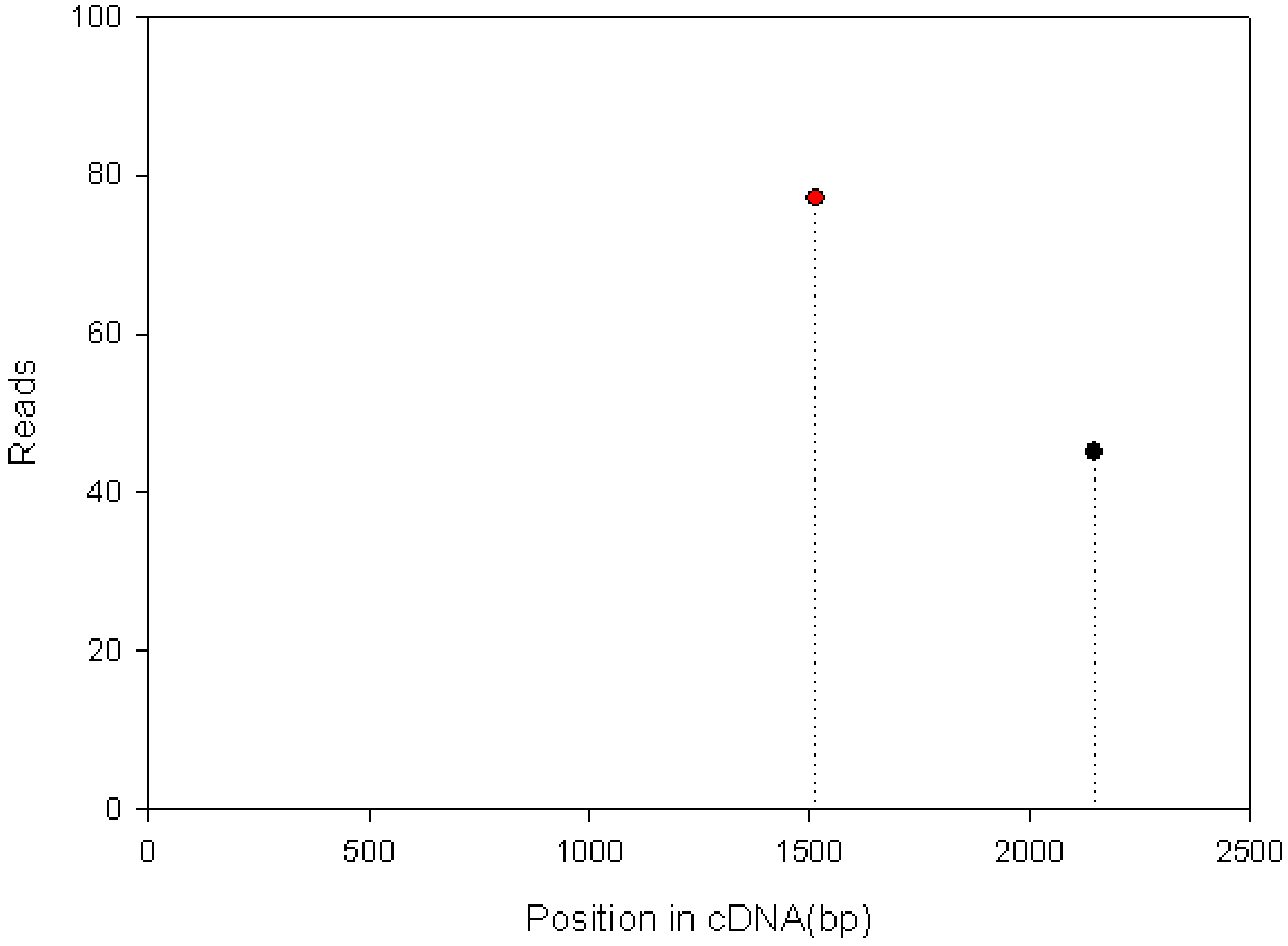


```
5' GGAUAUUGGCGCGGCUCAAUCACCAG 3'        Orange1.1t00200.1
     :::::::::.:::::::::::
3' -CUAUAACCGUGCCGAGUUAGU---- 5'        Csi-miR171g.1
```

Csi-miR171g.1, target=Orange1.1t00199.1 gene=Orange1.1t00199
Category:1
Score=1
Cleavage Site=1615

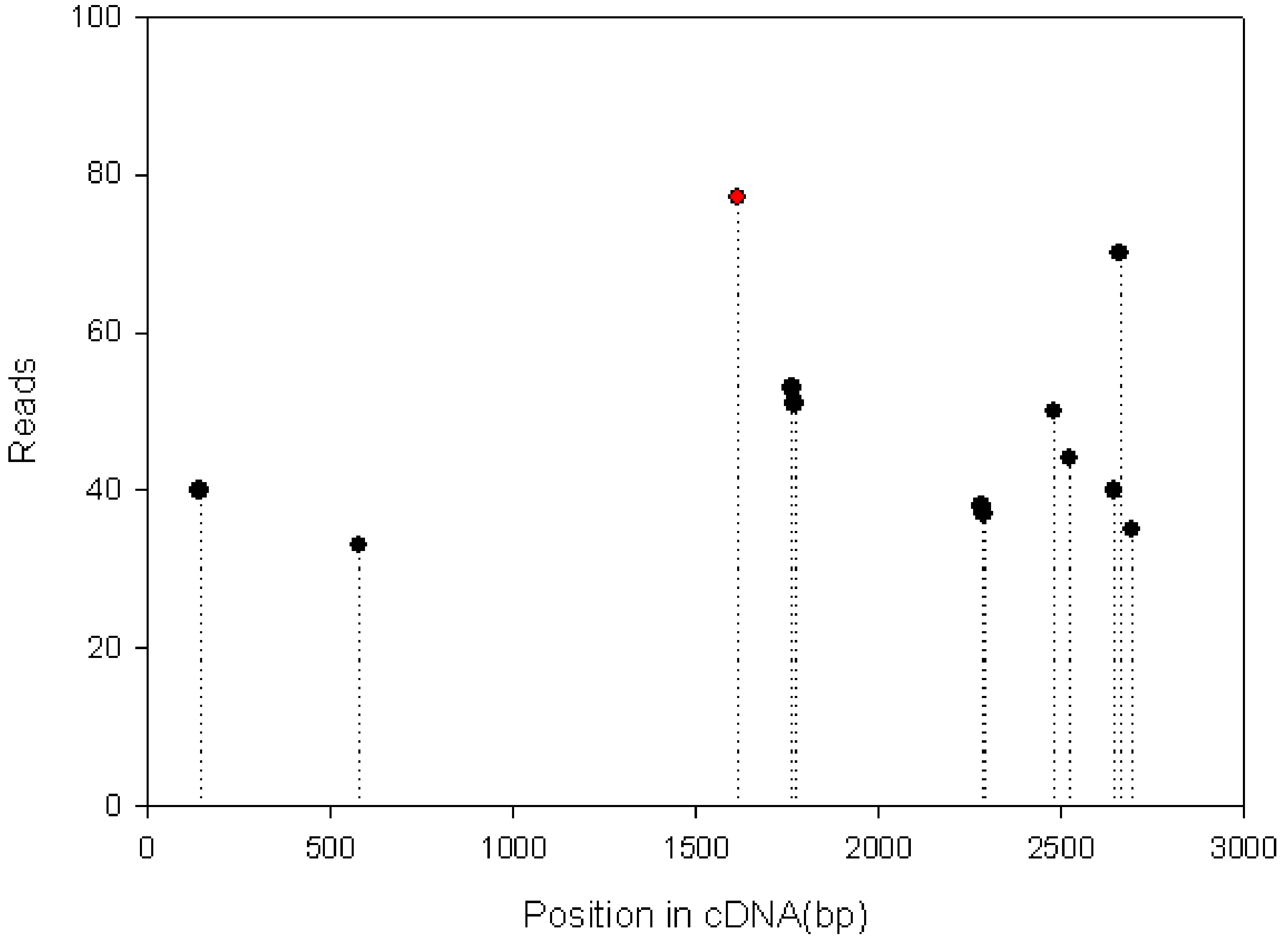


```
5' GGAUAUUGGCGCGGCUCAAUCACCAG 3'         Orange1.1t00199.1
    ::::::::.:::::::::::
3' -CUAUAACCGUGCCGAGUUAGU---- 5'         Csi-miR171g.1
```

Csi-miR172a-3p.1, target=Cs2g04660.1 gene=Cs2g04660
Category:2
Score=4.5
Cleavage Site=1098

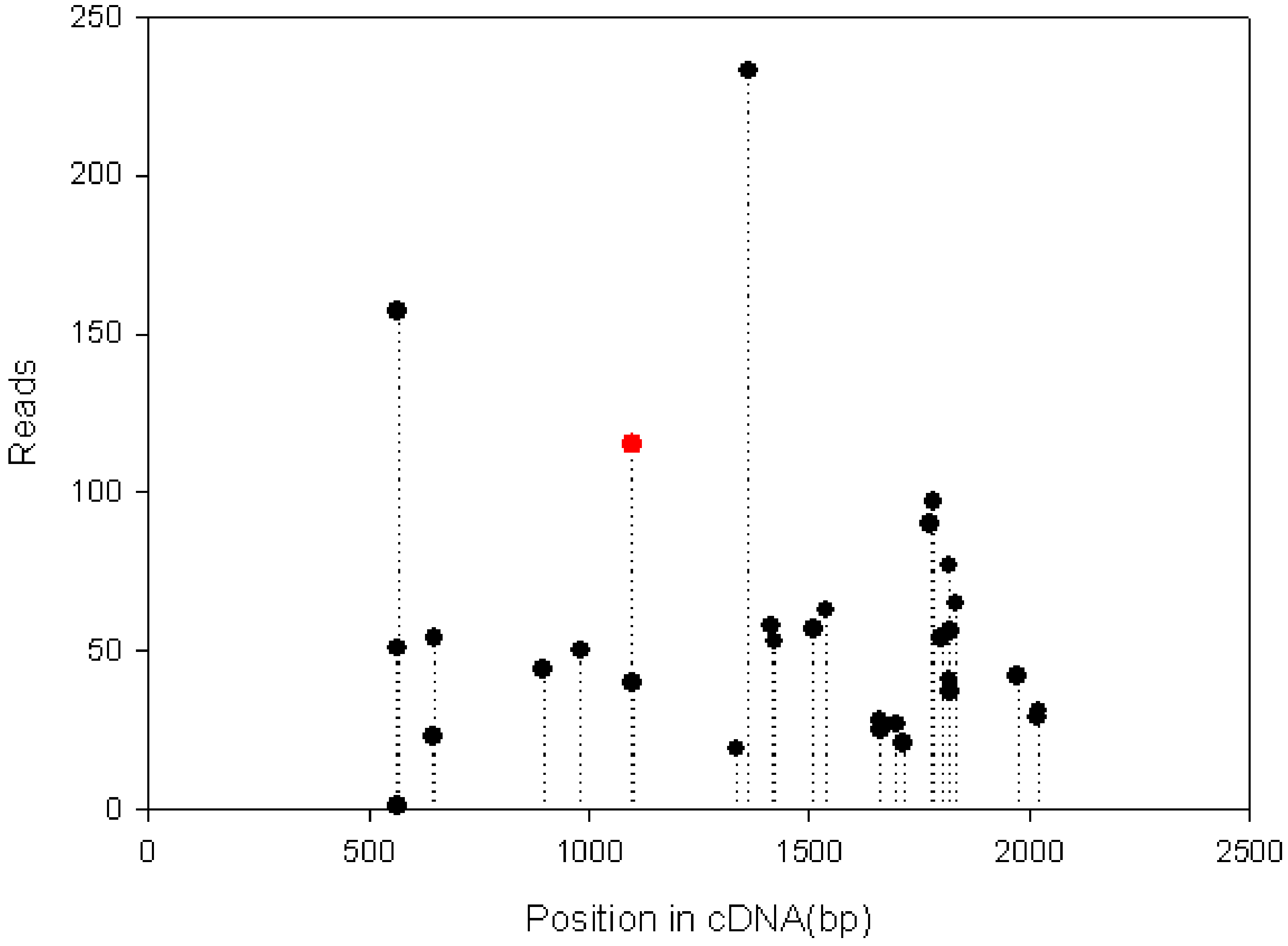


```
5' CUUGUAGAGAUUAUCAAGGUUCUCGG 3'        Cs2g04660.1
     ::.::  ::.:::::::.::::
3' --ACGUC-GUAGUAGUUCUAAGA--- 5'        Csi-miR172a-3p.1
```

Csi-miR172a-3p.1, target=Cs7g02590.1 gene=Cs7g02590
Category:3
Score=5
Cleavage Site=4764

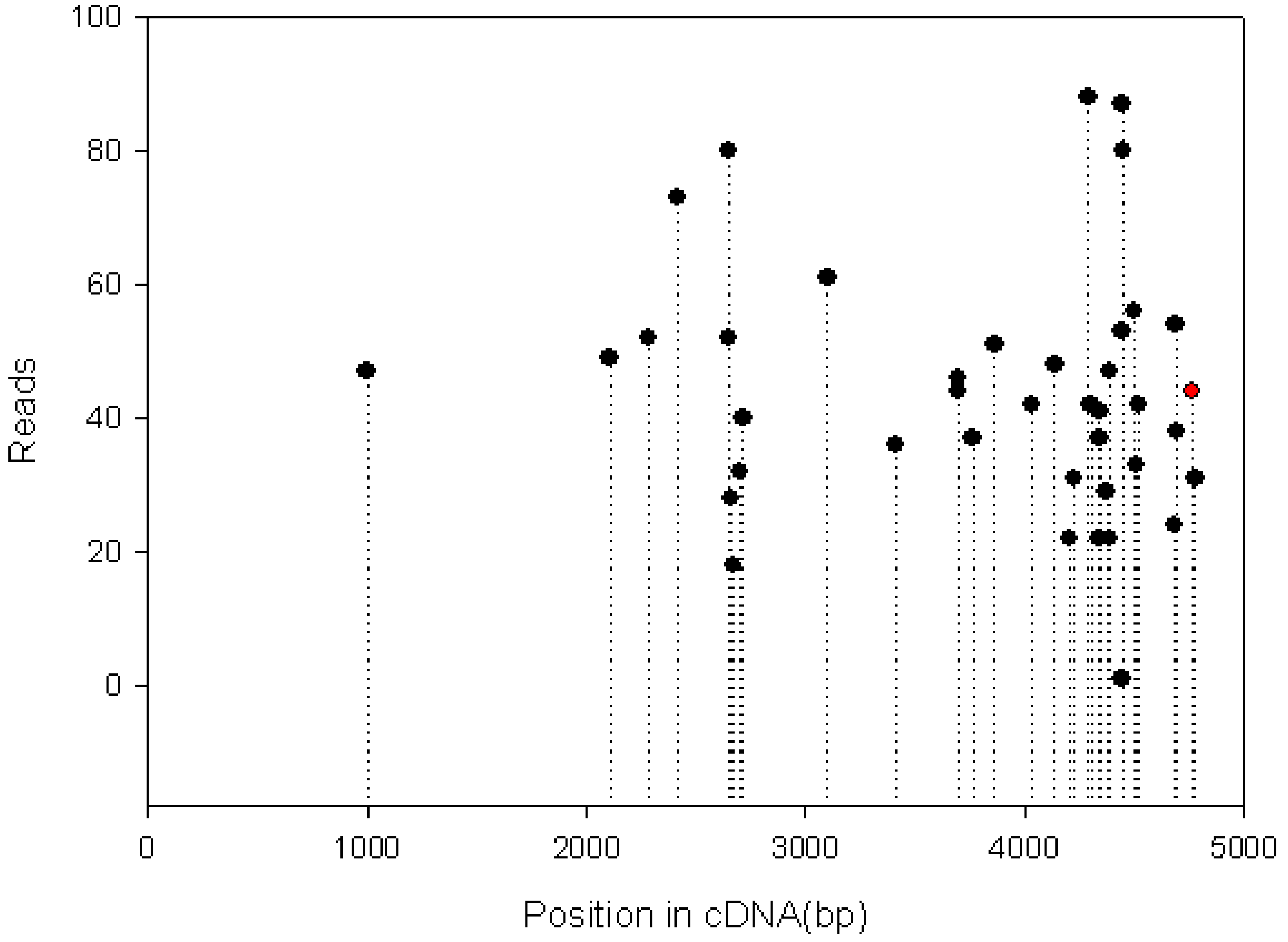


```
5' AGCGCAGCCU-AUCAAGAUUUUUCUUU 3'       Cs7g02590.1
       ::::: : ::::::::::.:
3' --ACGUCGUAGUAGUUCUAAGA----- 5'       Csi-miR172a-3p.1
```

Csi-miR172a-3p.1,target=Orange1.1t04055.1 gene=Orange1.1t04055
Category:3
Score=2
Cleavage Site=2049

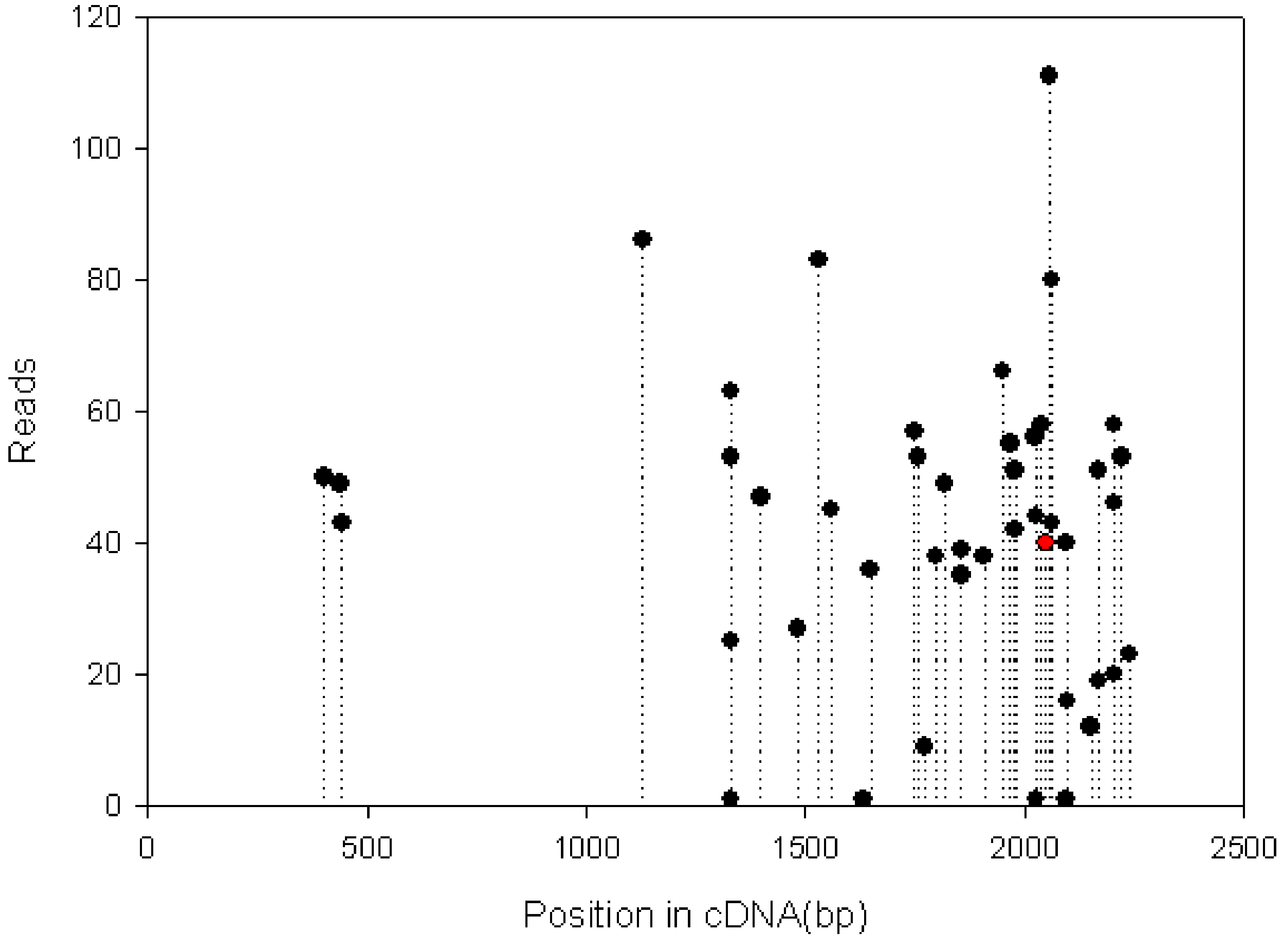


```
5' GCUGCAGCAUCAUCAGGAUUCCCUCA 3'        Orange1.1t04055.1
     ::::::::::::::.:::::
3' --ACGUCGUAGUAGUUCUAAGA---- 5'        Csi-miR172a-3p.1
```

Csi-miR172a-3p.2, target=Cs2g04660.1 gene=Cs2g04660
Category:2
Score=4.5
Cleavage Site=1098

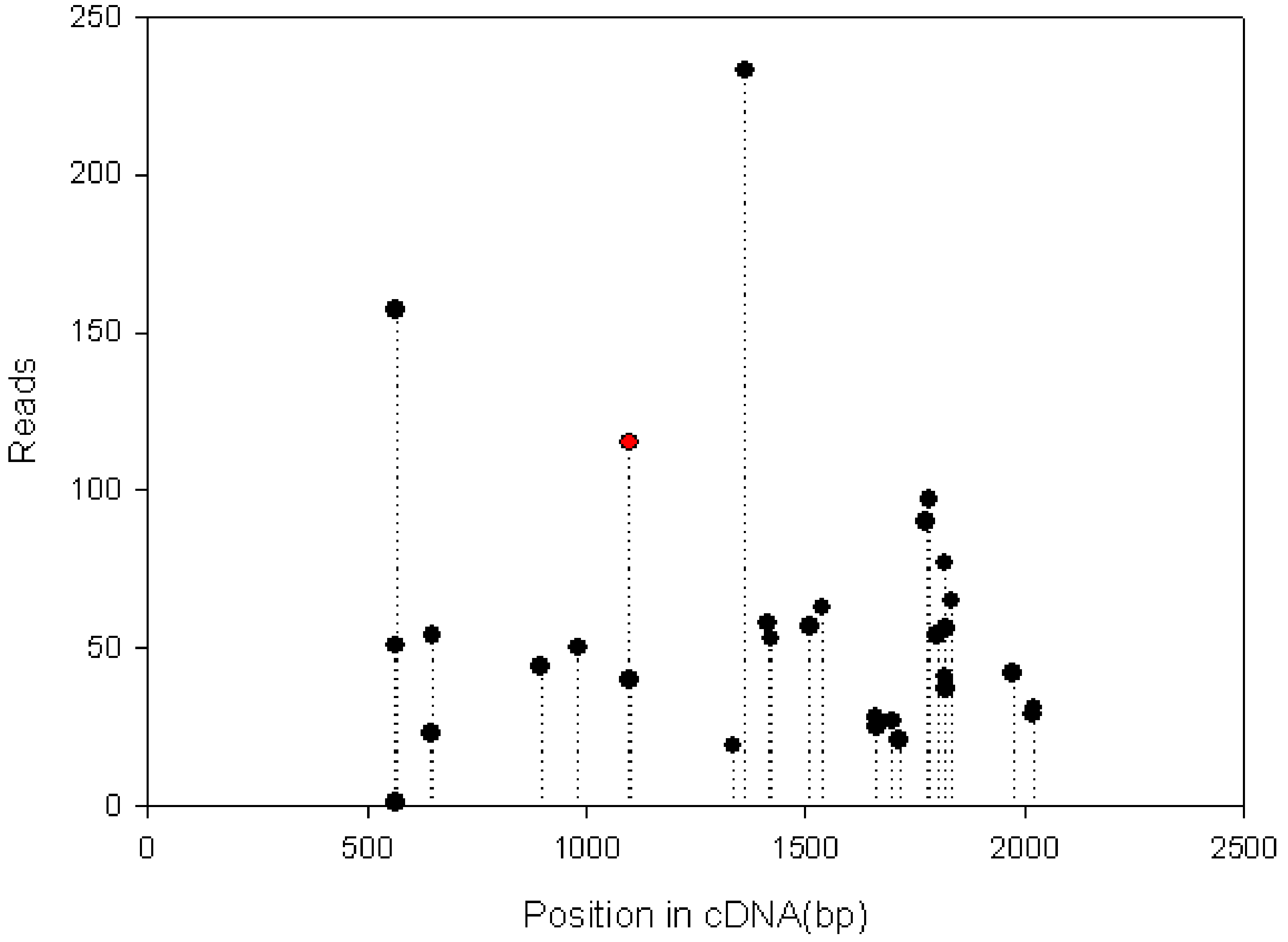


```
5' CUUGUAGAGAUUAUCAAGGUUCUCGG 3'        Cs2g04660.1
    :::.::  ::.::::::.::::
3' -AACGUC-GUAGUAGUUCUAAGA--- 5'        Csi-miR172a-3p.2
```

Csi-miR172a-3p.2,target=Orange1.1t04055.1 gene=Orange1.1t04055
Category:3
Score=3
Cleavage Site=2049

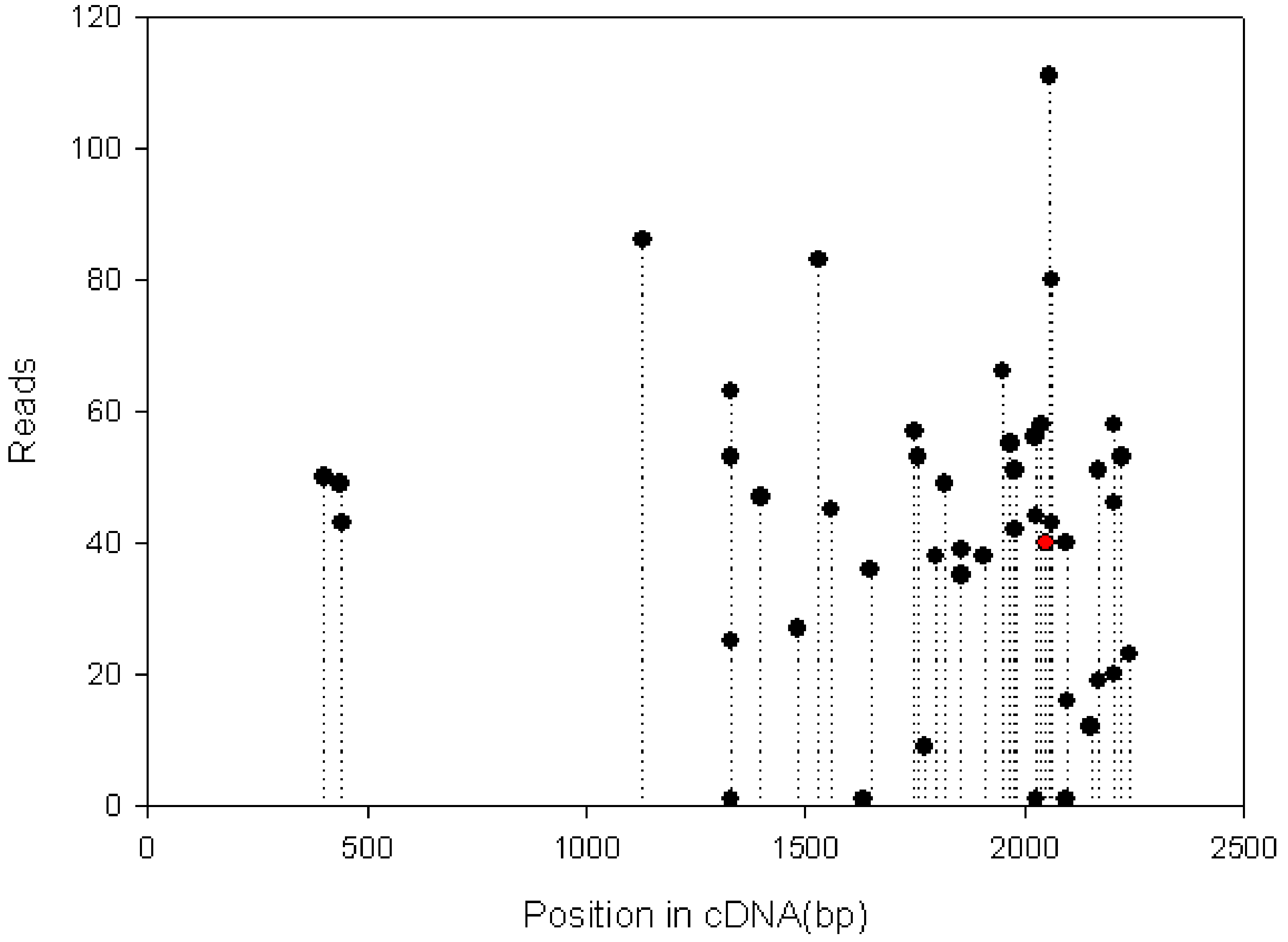


```
5' GCUGCAGCAUCAUCAGGAUUCCCUCA 3'        Orange1.1t04055.1
     ::::::::::::::.:::::
3' -AACGUCGUAGUAGUUCUAAGA---- 5'        Csi-miR172a-3p.2
```

Csi-miR172a-3p.3, target=Cs2g04660.1 gene=Cs2g04660
Category:2
Score=4.5
Cleavage Site=1098

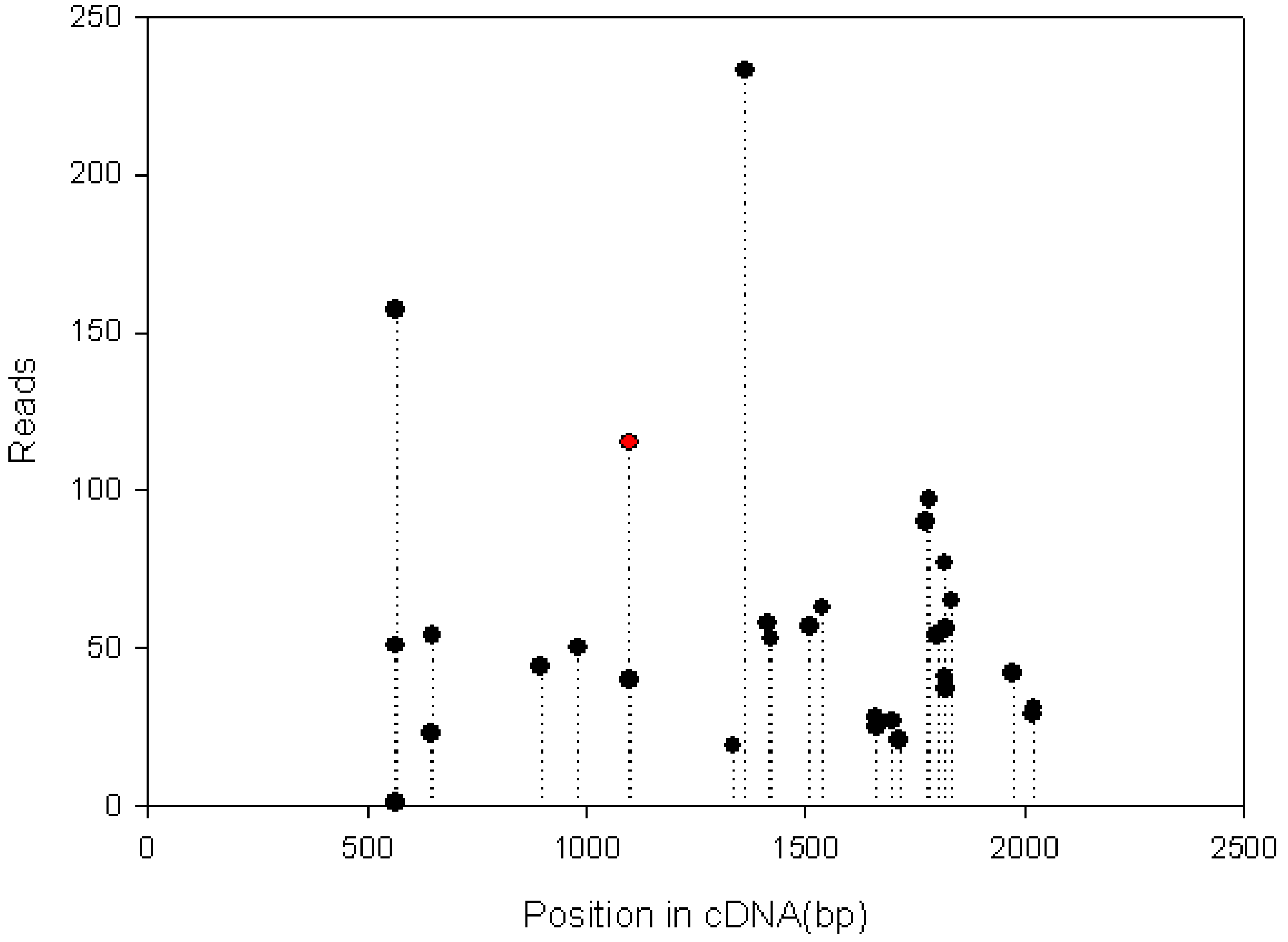


```
5' CUUGUAGAGAUUAUCAAGGUUCUCGG 3'        Cs2g04660.1
    :::.::  ::.:::::::.:::
3' -AACGUC-GUAGUAGUUCUAAG---- 5'        Csi-miR172a-3p.3
```

Csi-miR172a-3p.3, target=Cs2g21180.1 gene=Cs2g21180
Category:3
Score=5
Cleavage Site=438

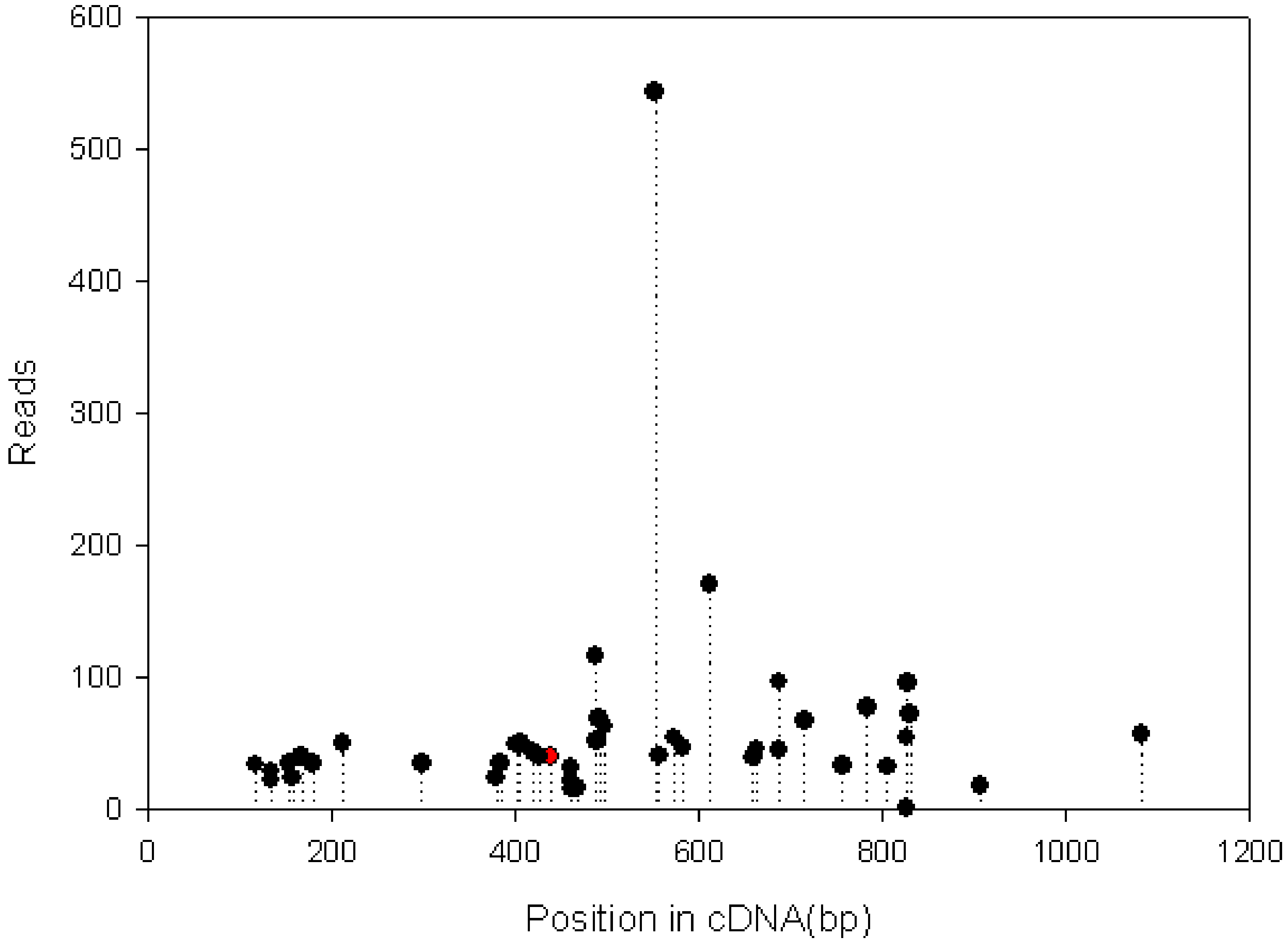


```
5' UUACAGCAUCCUCAAGAUCCAGCAAC 3'        Cs2g21180.1
   :: ::::::: ::::::: :
3' AACGUCGUAGUAGUUCUAAG------ 5'        Csi-miR172a-3p.3
```

Csi-miR172a-3p.3, target=Orange1.1t04055.1 gene=Orange1.1t04055
Category:3
Score=2
Cleavage Site=2049

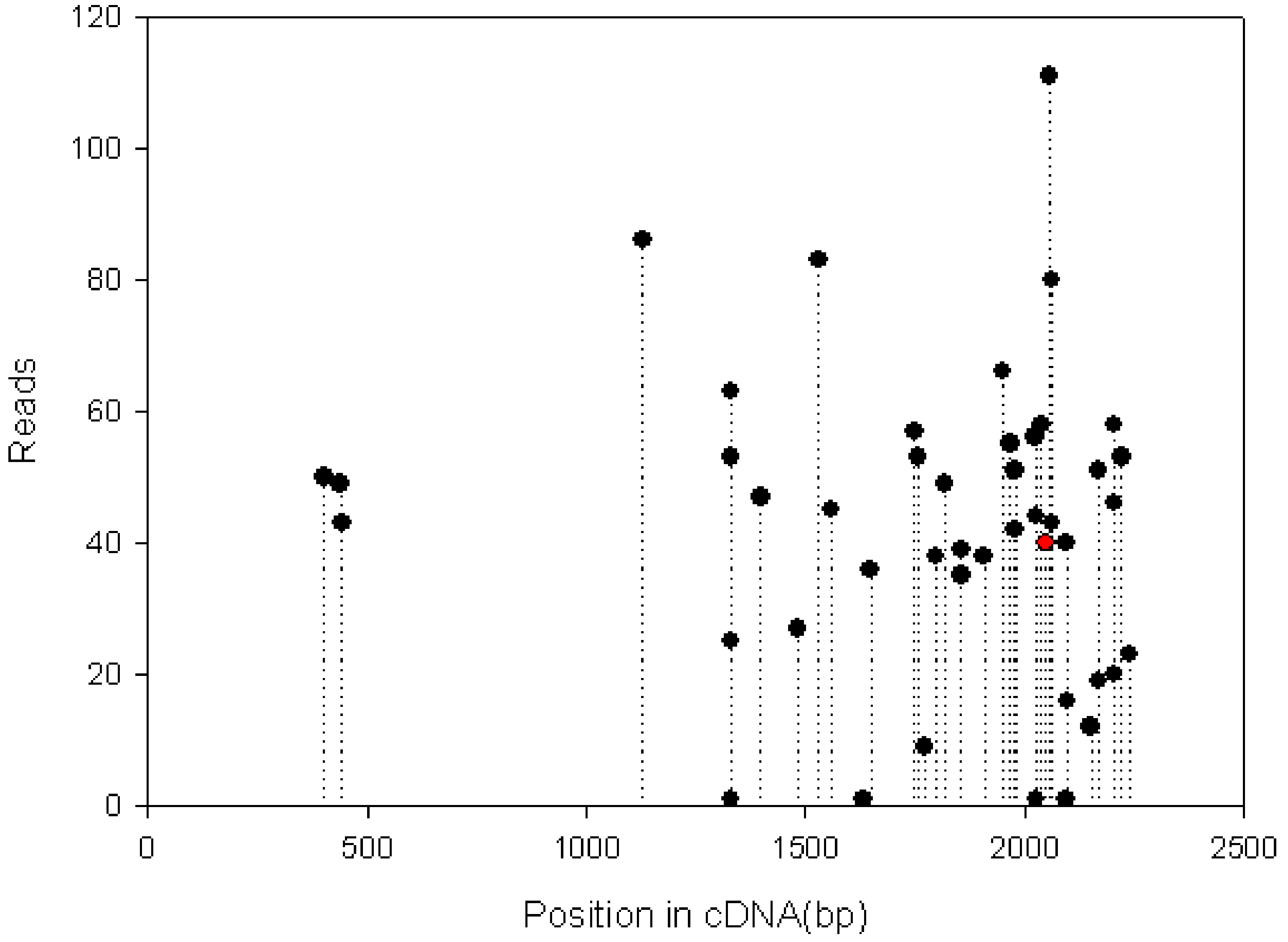


```
5' GCUGCAGCAUCAUCAGGAUUCCCUCA 3'        Orange1.1t04055.1
      ::::::::::::::.:::::
3' -AACGUCGUAGUAGUUCUAAG----- 5'        Csi-miR172a-3p.3
```

Csi-miR172c.1, target=Cs1g21180.1 gene=Cs1g21180
Category:3
Score=4
Cleavage Site=3251

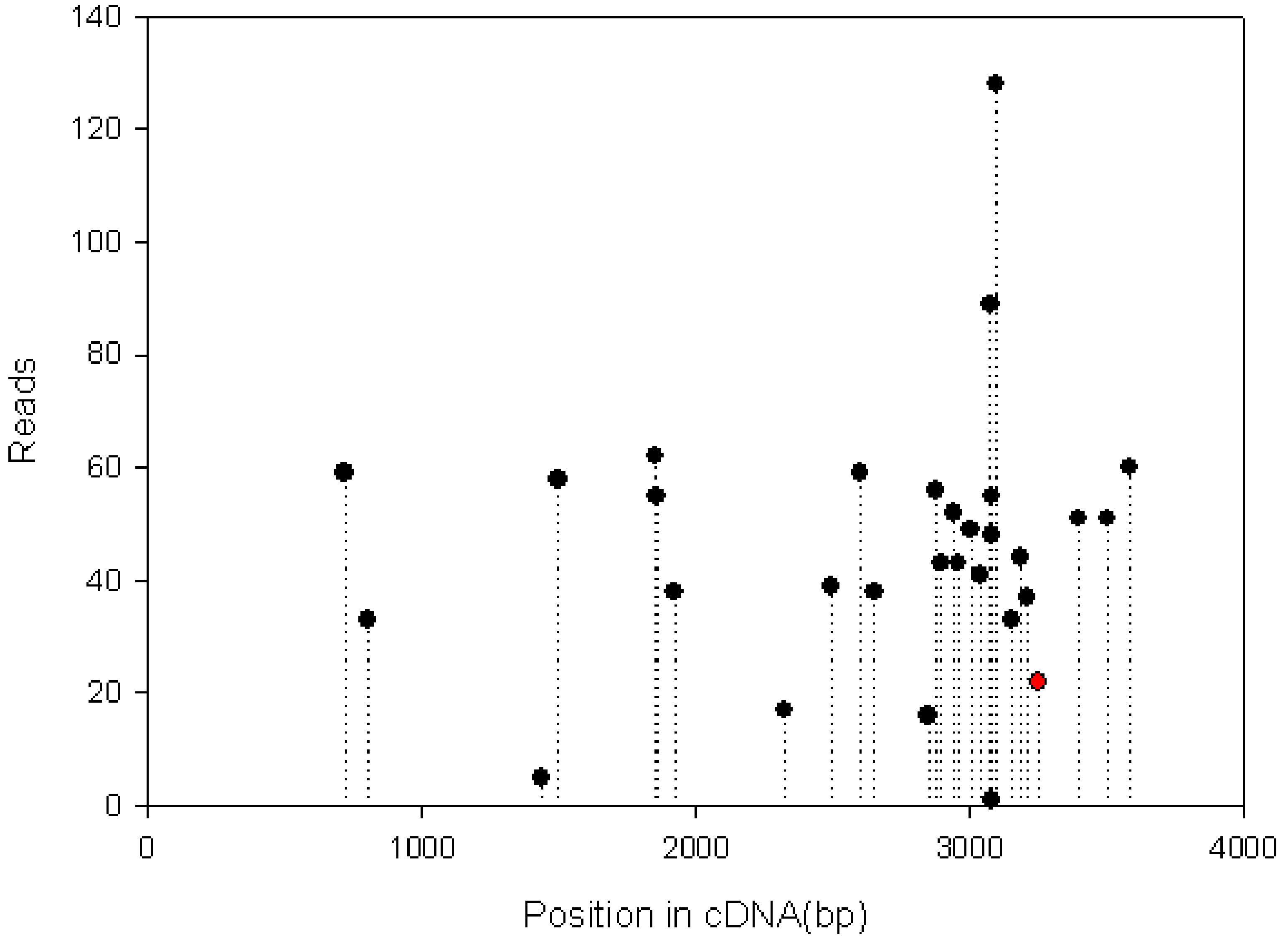


```
5' UUGUAGAUAAG-AUCAUUAAGAUUUCU 3'        Cs1g21180.1
             :: :::::.::::::.:
3' ----------UCGUAGUAGUUCUAAGGU 5'        Csi-miR172c.1
```

Csi-miR172c.1, target=Cs2g04660.1 gene=Cs2g04660
Category:2
Score=5
Cleavage Site=1098

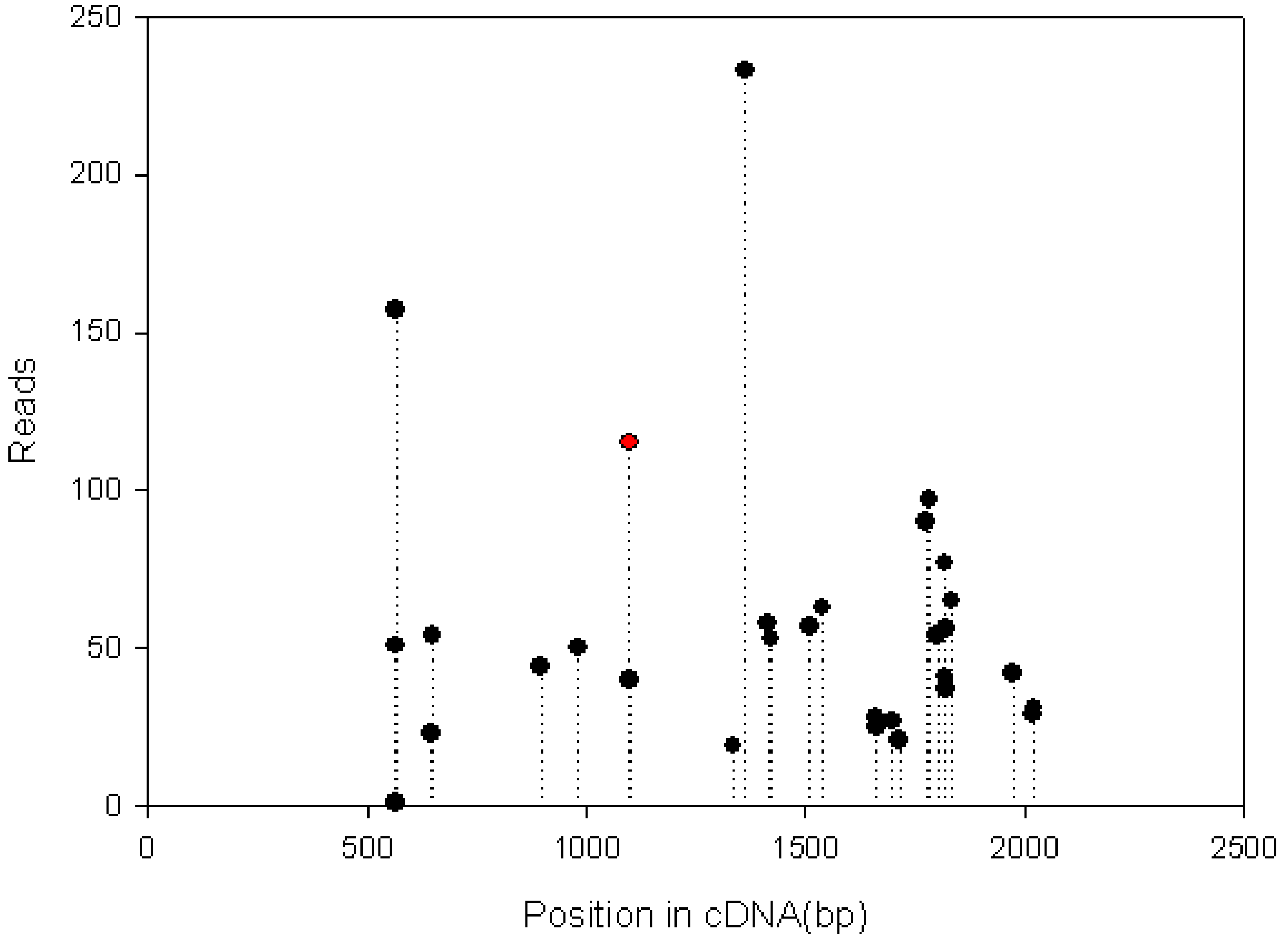


```
5' CUUGUAGAG-AUUAUCAAGGUUCUCGG 3'        Cs2g04660.1
          :: ::.::::::.:::.
3' -------UCGUAGUAGUUCUAAGGU-- 5'        Csi-miR172c.1
```

Csi-miR172c.1, target=Cs2g21180.1 gene=Cs2g21180
Category:3
Score=4
Cleavage Site=438

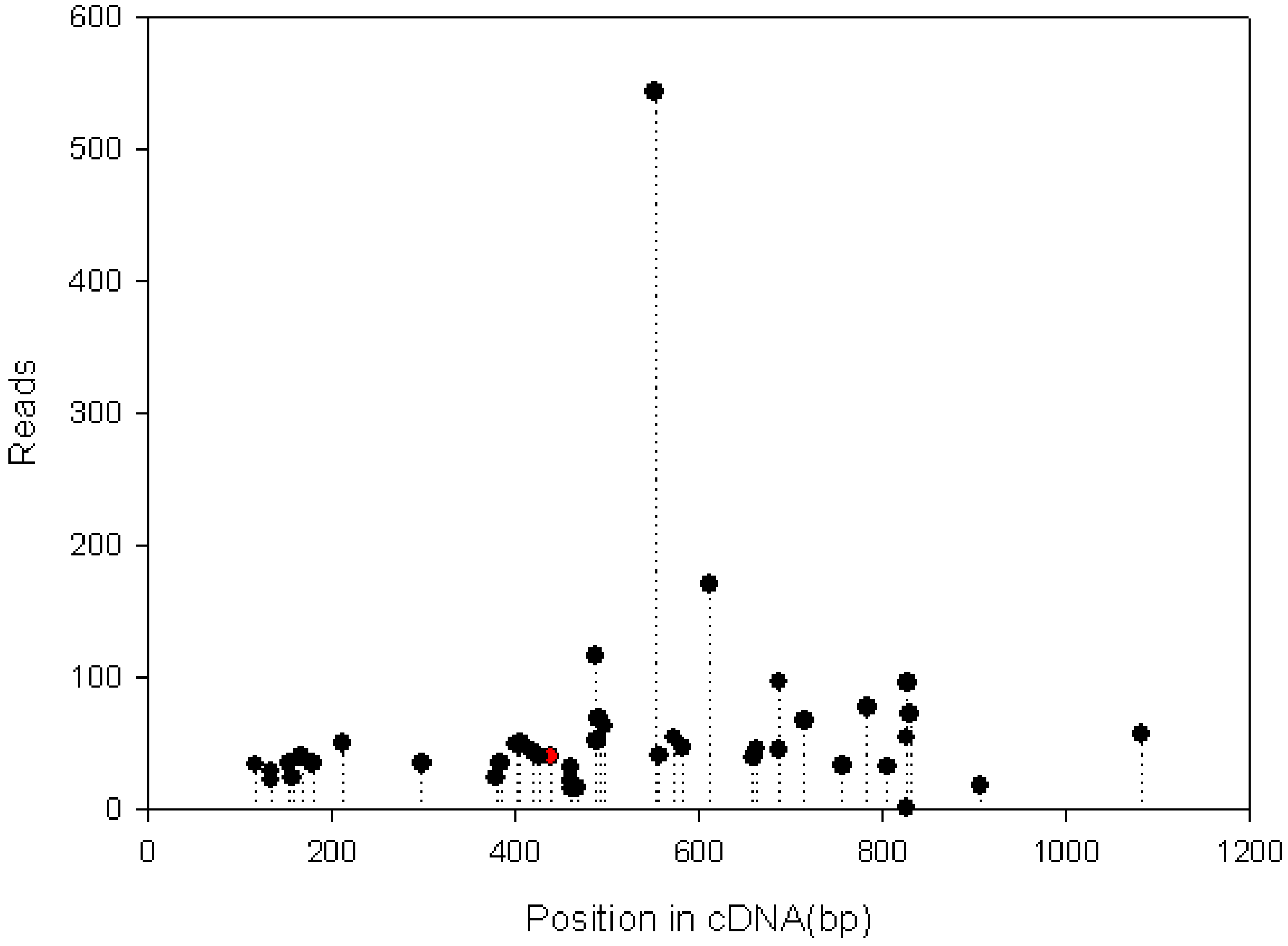


```
5' UUACAGCAUCCUCAAGA-UCCAGCAAC 3'        Cs2g21180.1
         :::::: :::::: ::::
3' ----UCGUAGUAGUUCUAAGGU----- 5'        Csi-miR172c.1
```

Csi-miR172c.1, target=Cs3g06140.1 gene=Cs3g06140
Category:1
Score=4.5
Cleavage Site=480

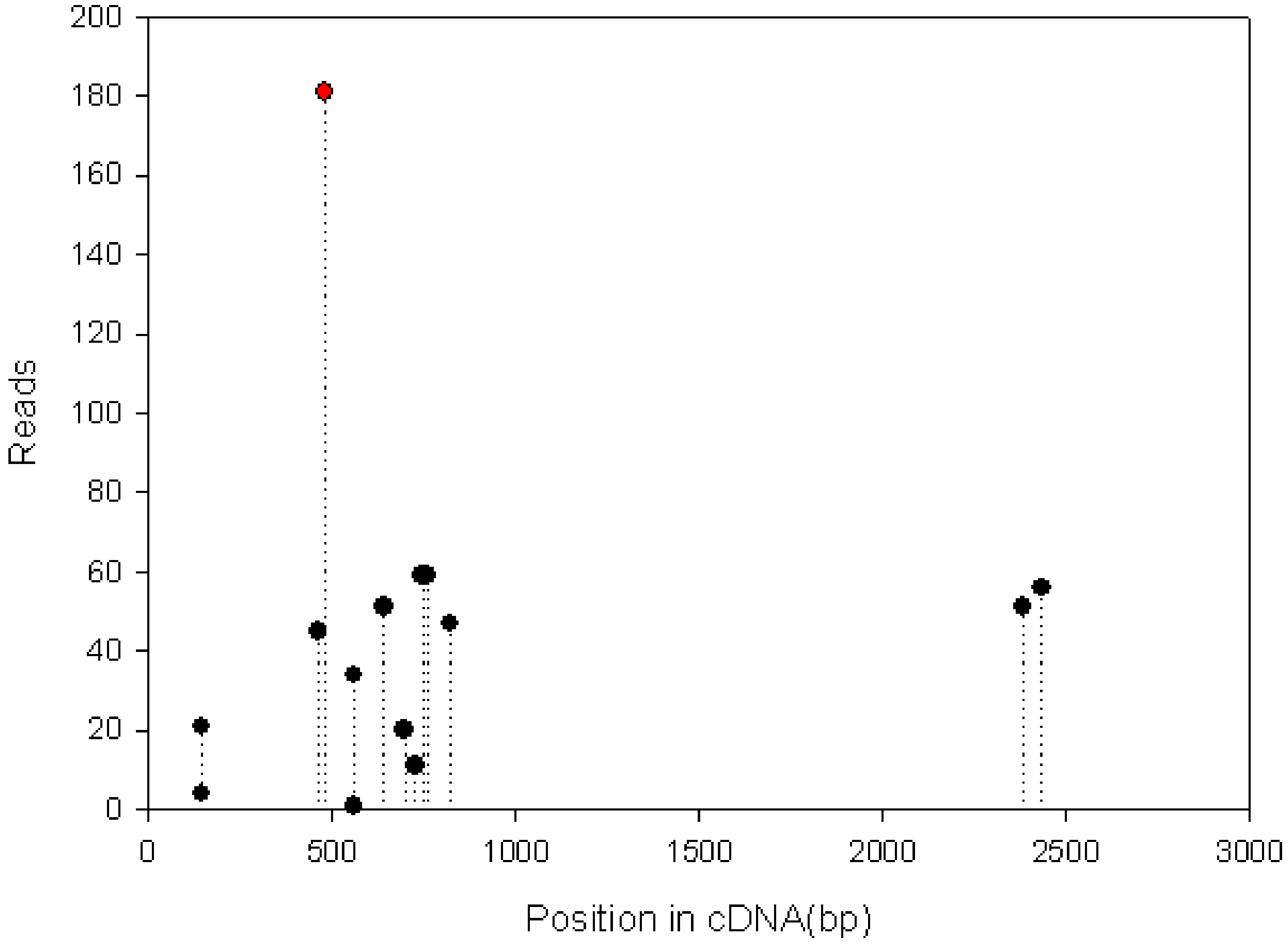


```
5' UGAGUCUGCCACCAUUAAGAUUCCUG 3'        Cs3g06140.1
          . :: :::.::::::::
3' -------UCGUAGUAGUUCUAAGGU- 5'        Csi-miR172c.1
```

Csi-miR172c.1, target=Cs7g19400.1 gene=Cs7g19400
Category:3
Score=5
Cleavage Site=1191

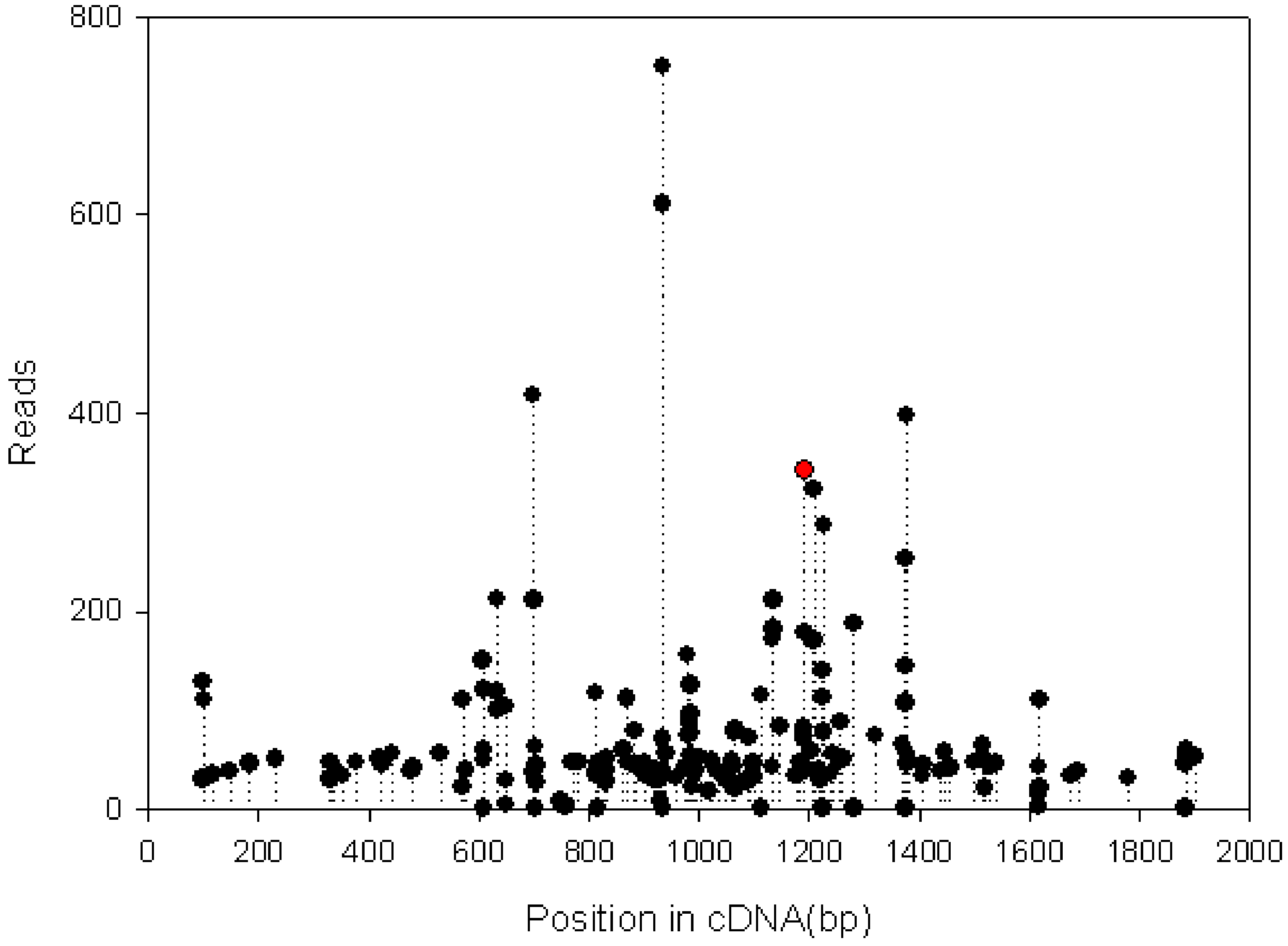


```
5' GUAUUUGGAAGCCUCAGC-AGAUUCCA 3'        Cs7g19400.1
             ::: ::: : ::::::::
3' ---------UCGUAGUAGUUCUAAGGU 5'        Csi-miR172c.1
```

Csi-miR172c.1, target=Cs7g32470.1 gene=Cs7g32470
Category:3
Score=5
Cleavage Site=623

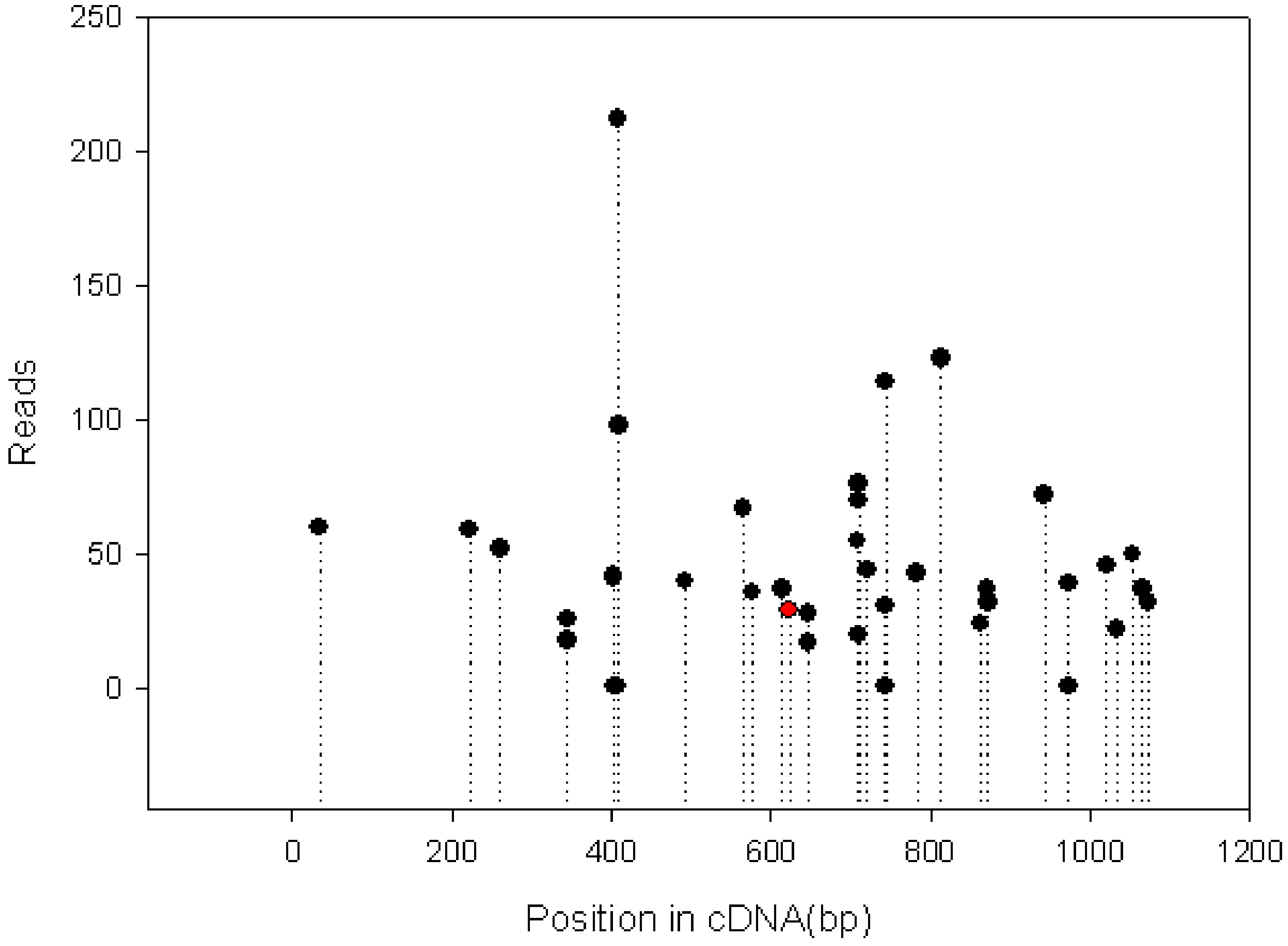


```
5' CAUCAUCCUCAAGGUUCUAGAAGCCG 3'        Cs7g32470.1
    : :::: :::::.::::.:
3' -UCGUAGUAGUUCUAAGGU------- 5'        Csi-miR172c.1
```

Csi-miR172c.1, target=Orange1.1t00903.1 gene=Orange1.1t00903
Category:3
Score=5
Cleavage Site=329

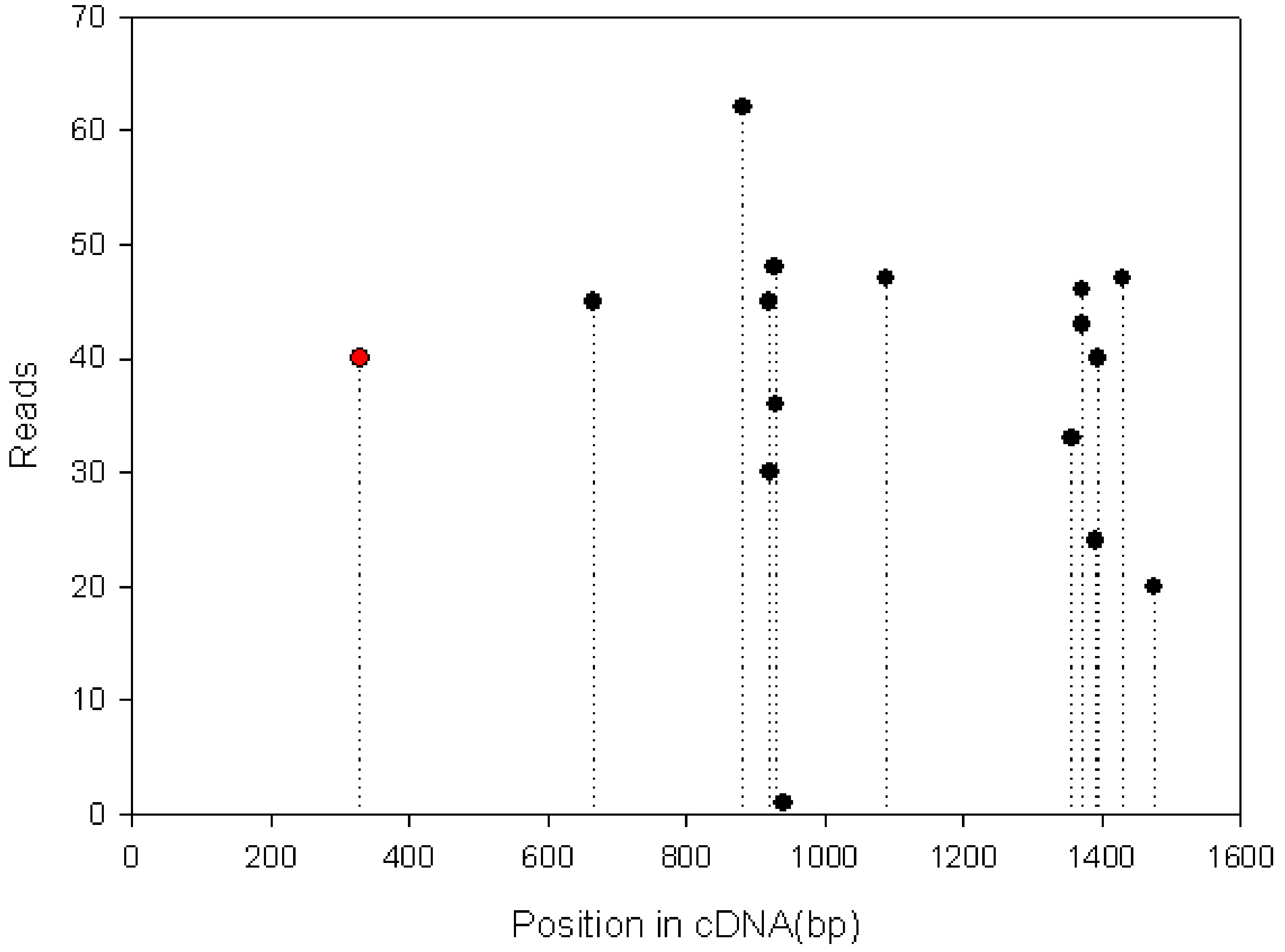


```
5' CAUCAUCAUCAAGG-UCUAGUAGAAAA 3'        Orange1.1t00903.1
    : ::::::::::. ::.:
3' -UCGUAGUAGUUCUAAGGU-------- 5'        Csi-miR172c.1
```

Csi-miR172c.1, target=Orange1.1t04055.1 gene=Orange1.1t04055
Category:3
Score=2
Cleavage Site=2049

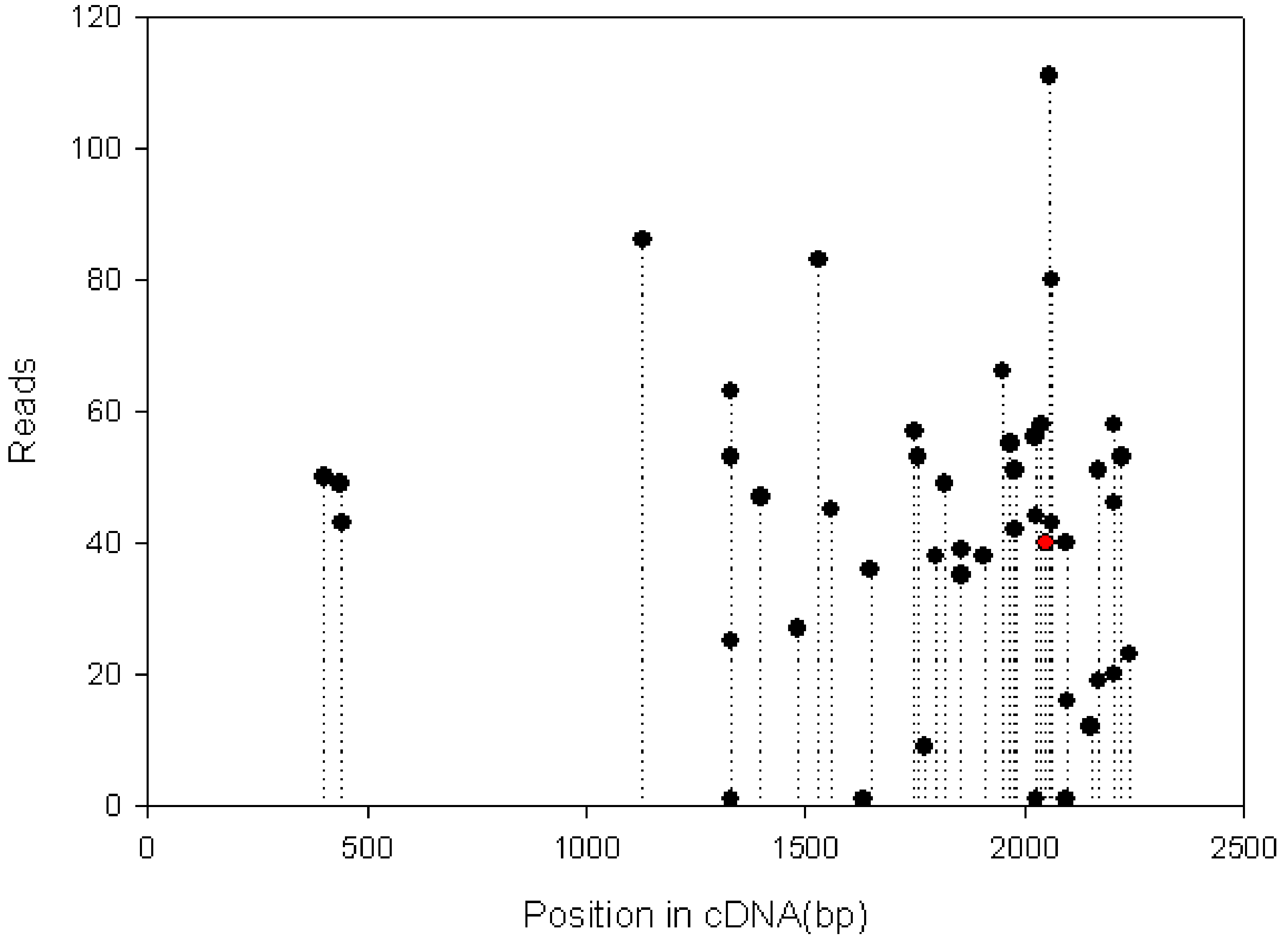


```
5' GCUGCAGCAUCAUCAGGAUUCCCUCA 3'        Orange1.1t04055.1
          :::::::::::.::::::
3' -----UCGUAGUAGUUCUAAGGU--- 5'        Csi-miR172c.1
```

Csi-miR172c.2, target=Orange1.1t04055.1 gene=Orange1.1t04055
Category:3
Score=2
Cleavage Site=2049

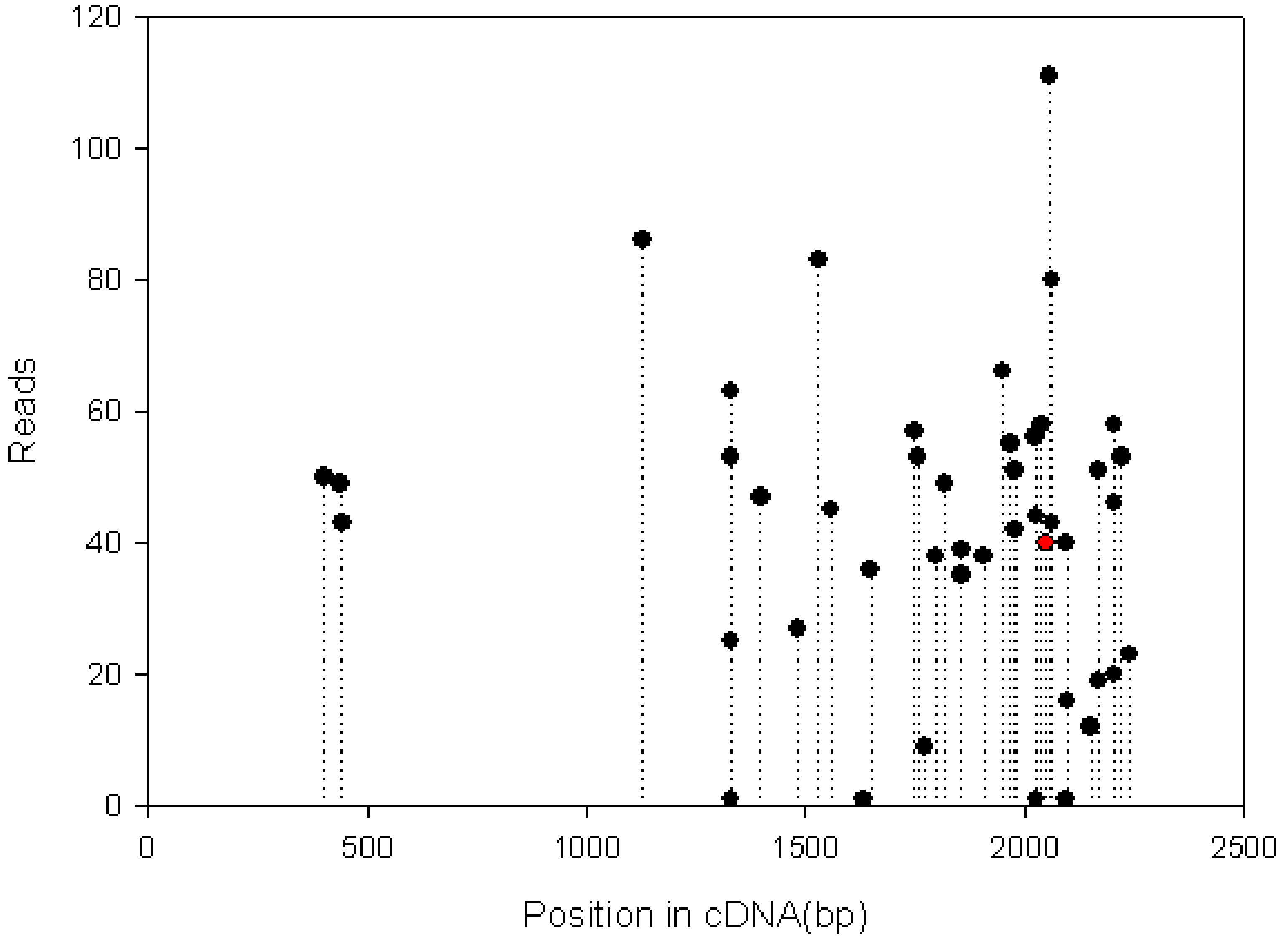


```
5' GCUGCAGCAUCAUCAGGAUUCCCUCA 3'         Orange1.1t04055.1
    ::::::::::::::::.:::::::
3' -GACGUCGUAGUAGUUCUAAGGU--- 5'         Csi-miR172c.2
```

Csi-miR172d, target=Orange1.1t04055.1 gene=Orange1.1t04055
Category:3
Score=3
Cleavage Site=2049

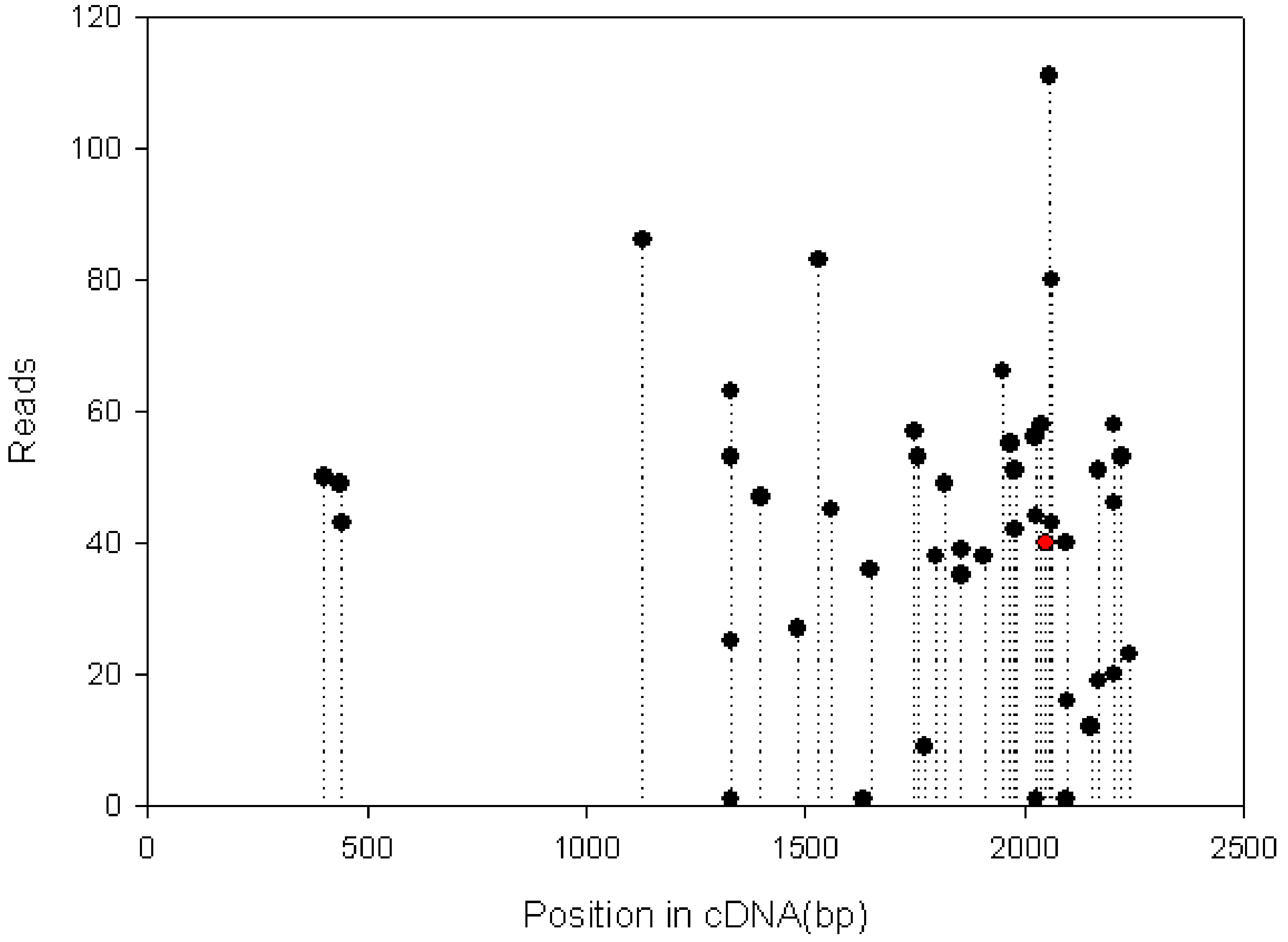


```
5' GCUGCAGCAUCAUCAGGAUUCCCUCA 3'        Orange1.1t04055.1
     ::::::::::::::.:::::
3' -UACGUCGUAGUAGUUCUAAGA---- 5'        Csi-miR172d
```

Csi-miR172d-3p, target=Cs5g08980.1 gene=Cs5g08980
Category:3
Score=4.5
Cleavage Site=1935

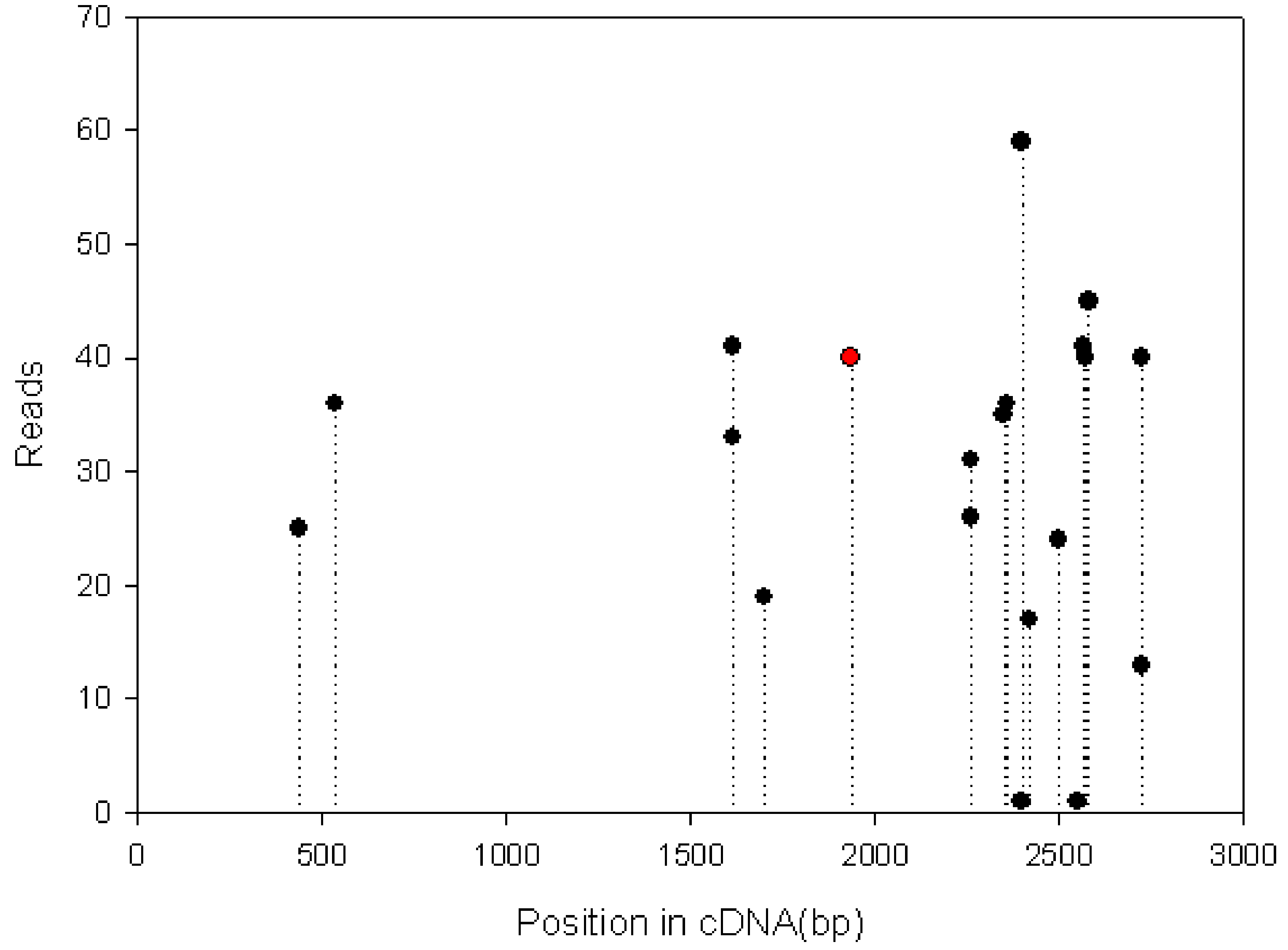


```
5' UUUAGAGGCUCUUGAUGAUGCUGACA 3'        Cs5g08980.1
      :  :. ::::::::::::::
3' ---ACACUUAGAACUACUACGACG-- 5'        Csi-miR172d-3p
```

Csi-miR172d-3p, target=Cs5g21000.1 gene=Cs5g21000
Category:3
Score=5
Cleavage Site=749

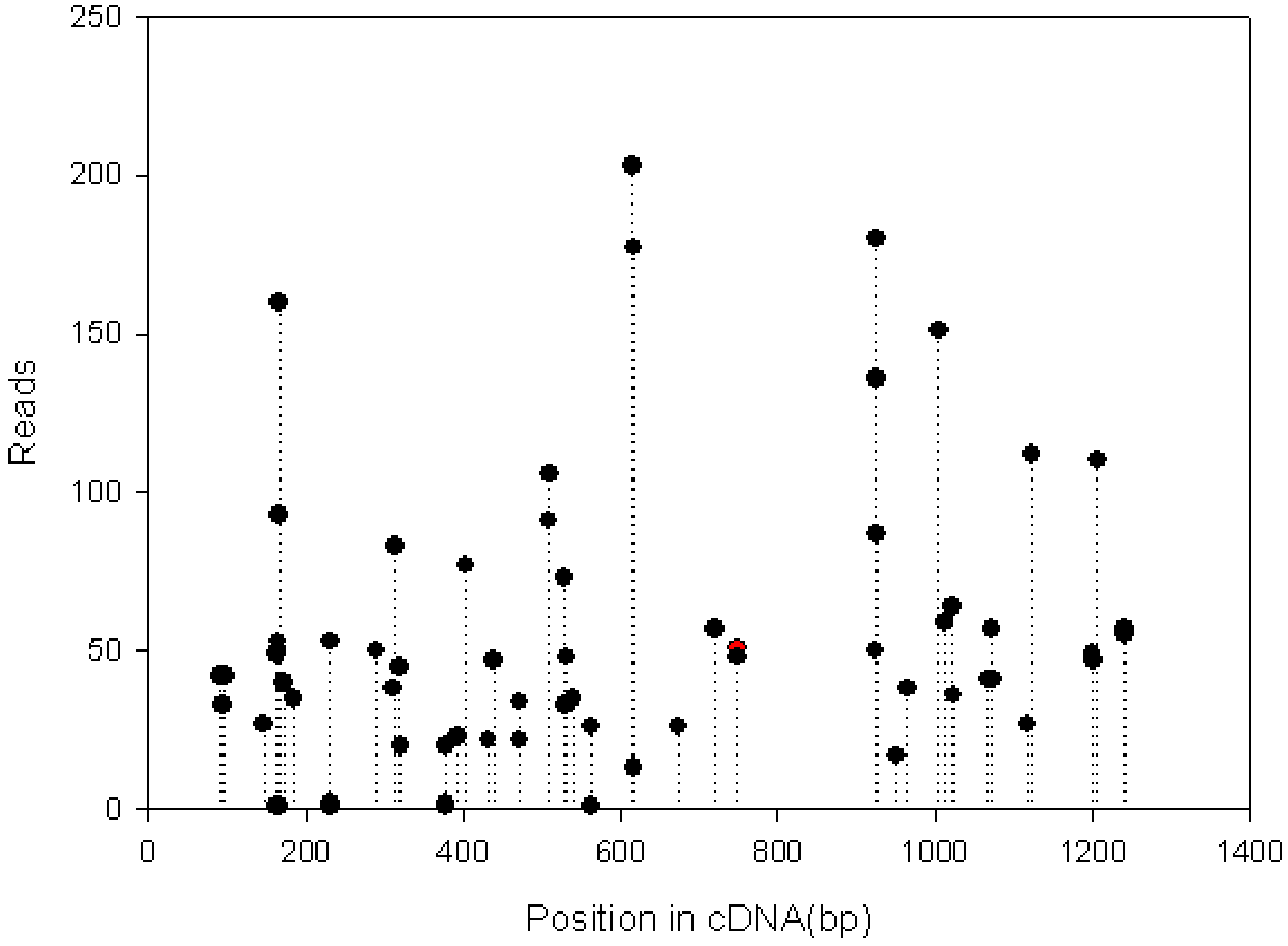


```
5' ACAAGUUUGGAU-UUGAUGAUGCUUUU 3'       Cs5g21000.1
         : ::.:: :::::::::: .
3' -----ACACUUAGAACUACUACGACG- 5'       Csi-miR172d-3p
```

Csi-miR172d-3p, target=Cs7g07030.1 gene=Cs7g07030
Category:3
Score=5
Cleavage Site=1972

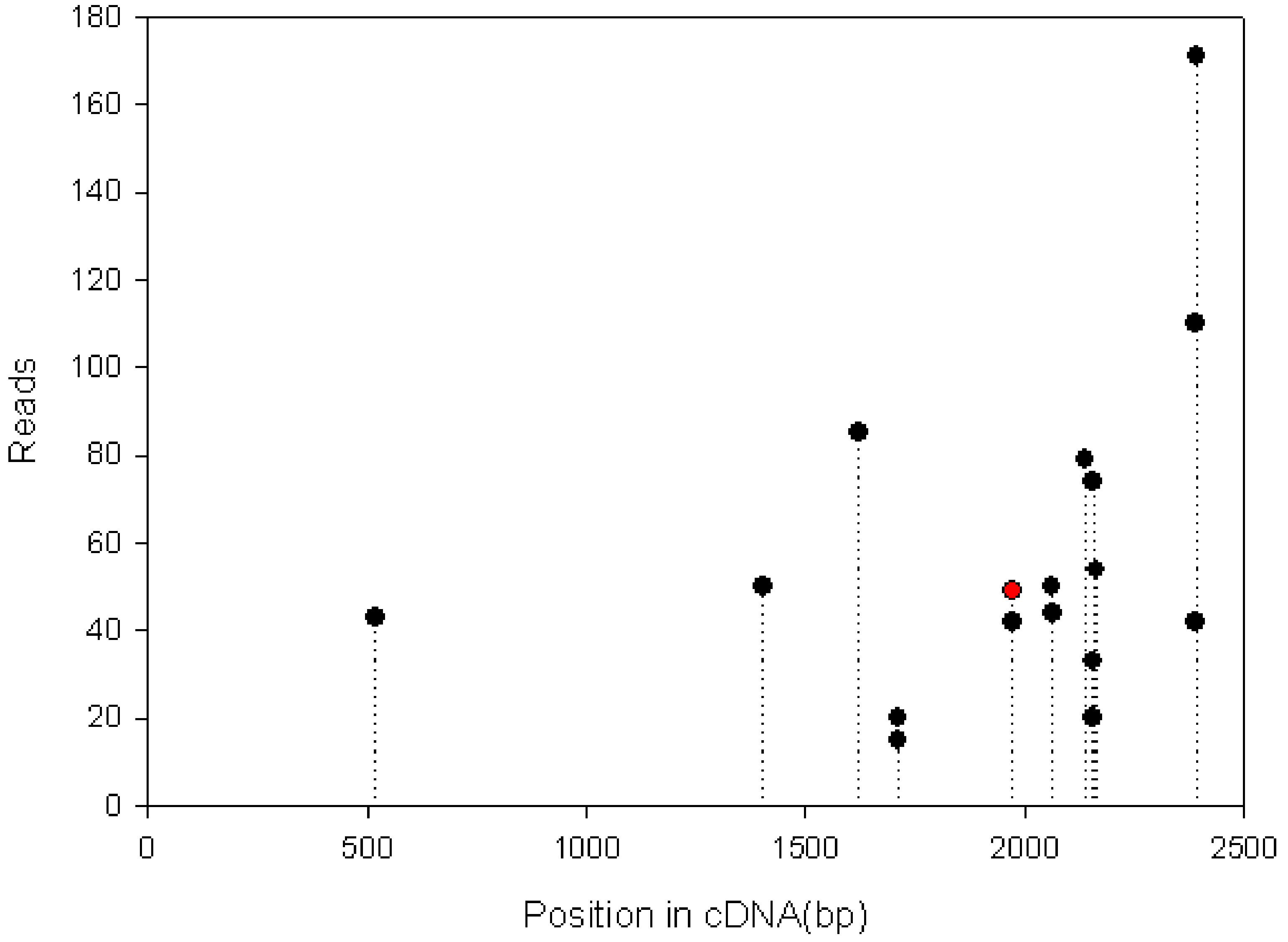


```
5' GAUGAUAUGAAUAUUGAUGAUCCUGA 3'        Cs7g07030.1
         : ::::: :::::::: :::
3' -----ACACUUAGAACUACUACGACG 5'        Csi-miR172d-3p
```

Csi-miR172d-3p, target=Cs9g03090.1 gene=Cs9g03090
Category:2
Score=4.5
Cleavage Site=2765

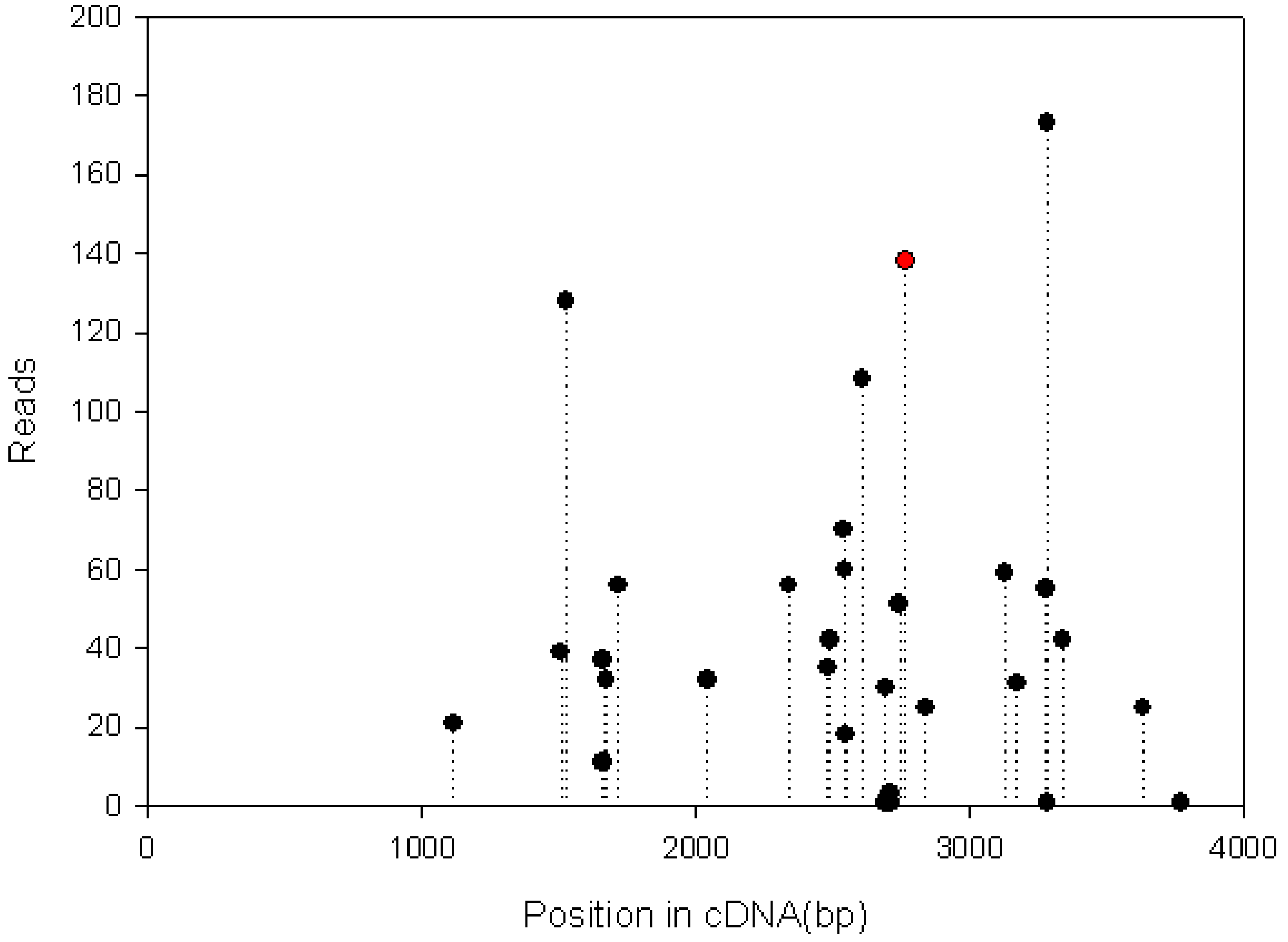


```
5' GCAAUCUGCAUUUUUGAUGAUGUUGC 3'        Cs9g03090.1
       :  ::  :  :.:::::::::.:::
3' ----ACAC-UUAGAACUACUACGACG 5'        Csi-miR172d-3p
```

Csi-miR172e.2, target=Cs1g23980.1 gene=Cs1g23980
Category:3
Score=5
Cleavage Site=1458

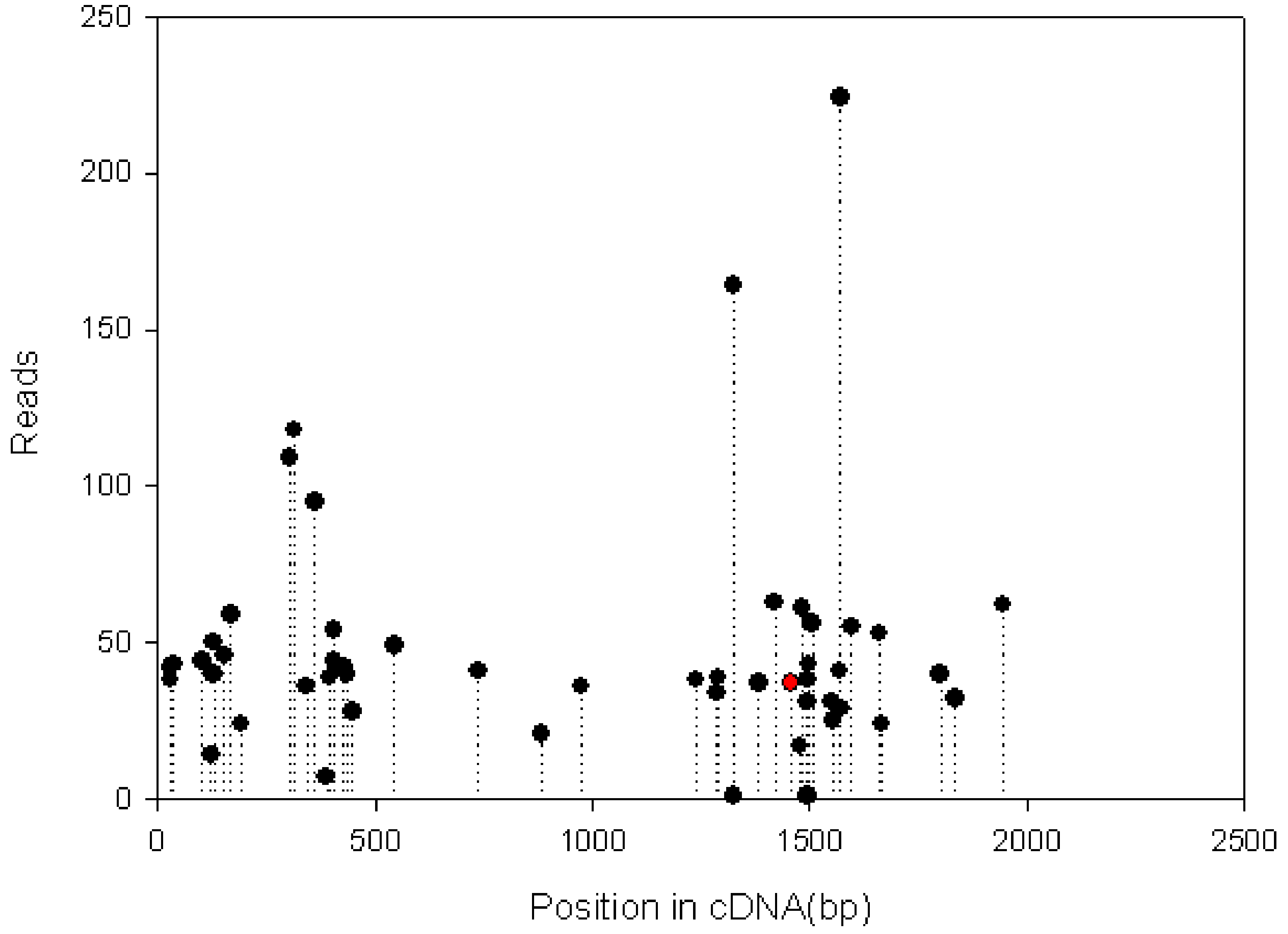


```
5' AACUAUG-AGCAUCAUCGGGGUUAUGG 3'        Cs1g23980.1
        ::: ::::::::::..:.::
3' ----UACGUCGUAGUAGUUCUAAG--- 5'        Csi-miR172e.2
```

Csi-miR172e.2, target=Cs6g08190.1 gene=Cs6g08190
Category:2
Score=5
Cleavage Site=1095

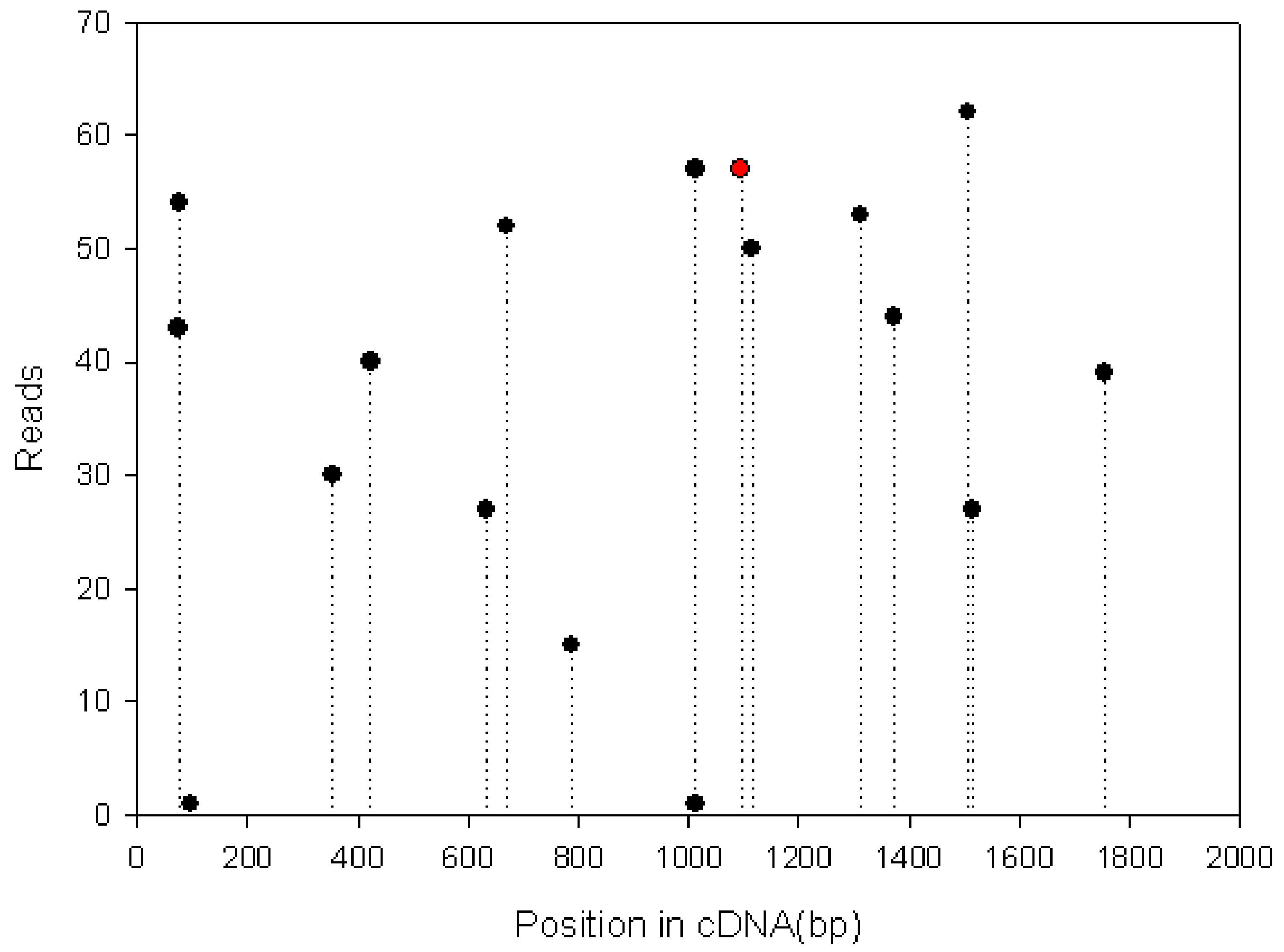


```
5' UCUGGGAAGCAGCAUGAUCAAGAAUC 3'        Cs6g08190.1
         : ::::::: ::::::: ::
3' ------UACGUCGUAGUAGUUCUAAG 5'        Csi-miR172e.2
```

Csi-miR172e.2, target=Orange1.1t04055.1 gene=Orange1.1t04055
Category:3
Score=2
Cleavage Site=2049

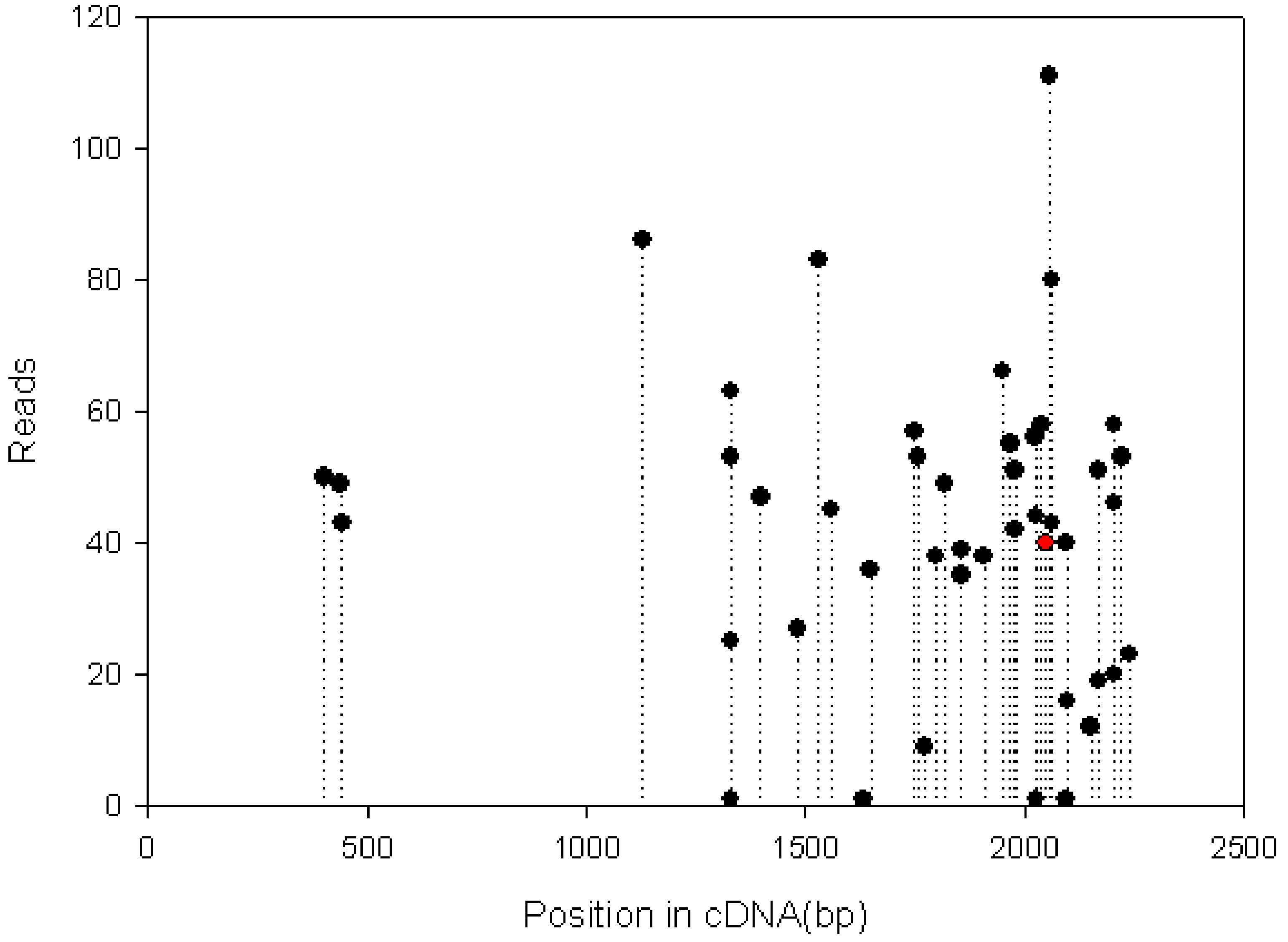


```
5' GCUGCAGCAUCAUCAGGAUUCCCUCA 3'        Orange1.1t04055.1
     :::::::::::::::.:::::
3' -UACGUCGUAGUAGUUCUAAG----- 5'        Csi-miR172e.2
```

Csi-miR172k, target=Cs4g17900.1 gene=Cs4g17900
Category:3
Score=5
Cleavage Site=454

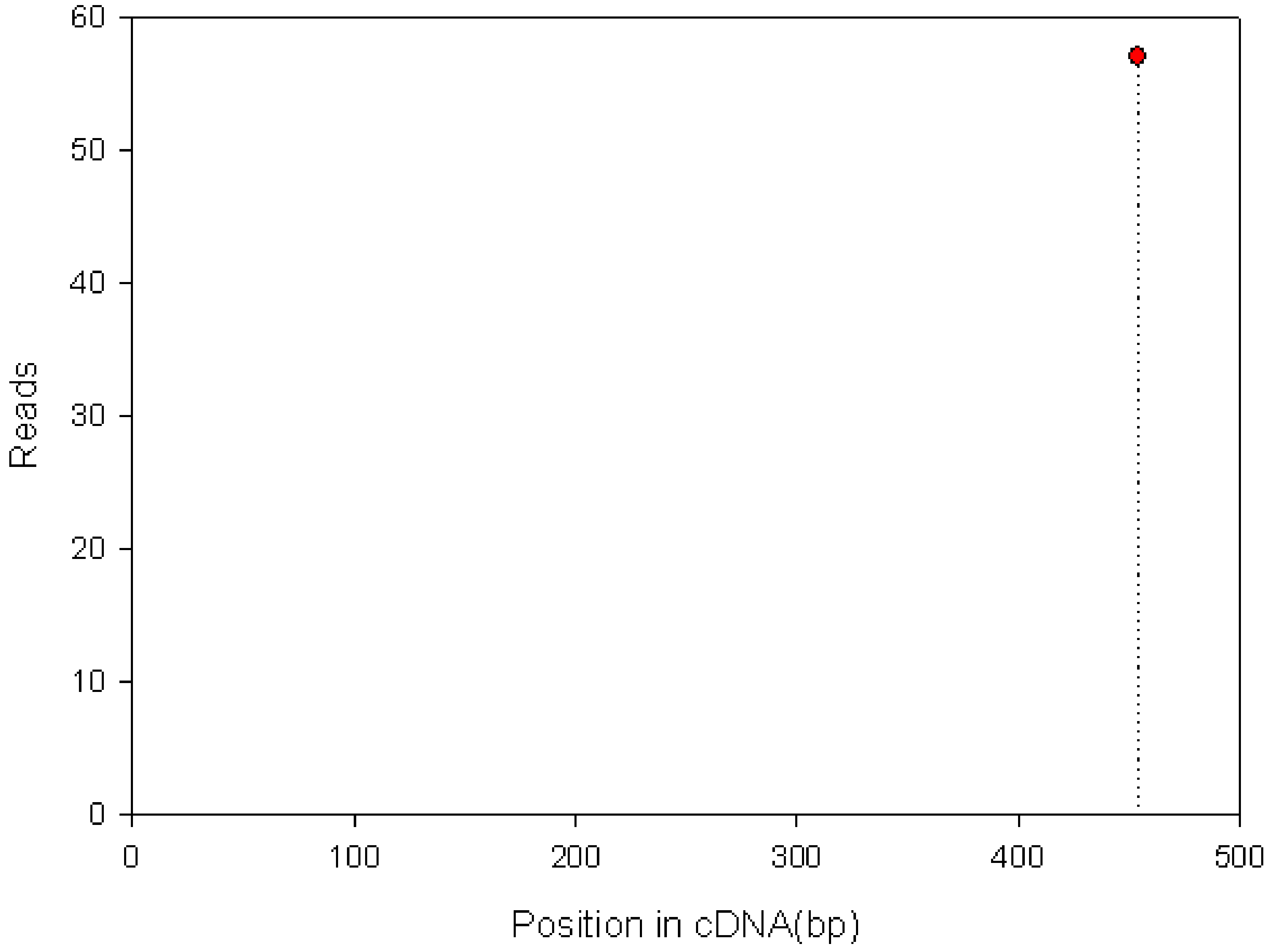


```
5' UUCAU-CAUCAUCUUCAAGGUUCAAGA 3'        Cs4g17900.1
      :: :: :::: ::::.::::
3' ---UACGUCGUAGUAGUUCUAAGU--- 5'        Csi-miR172k
```

Csi-miR172k, target=Orange1.1t00603.1 gene=Orange1.1t00603
Category:3
Score=5
Cleavage Site=1075

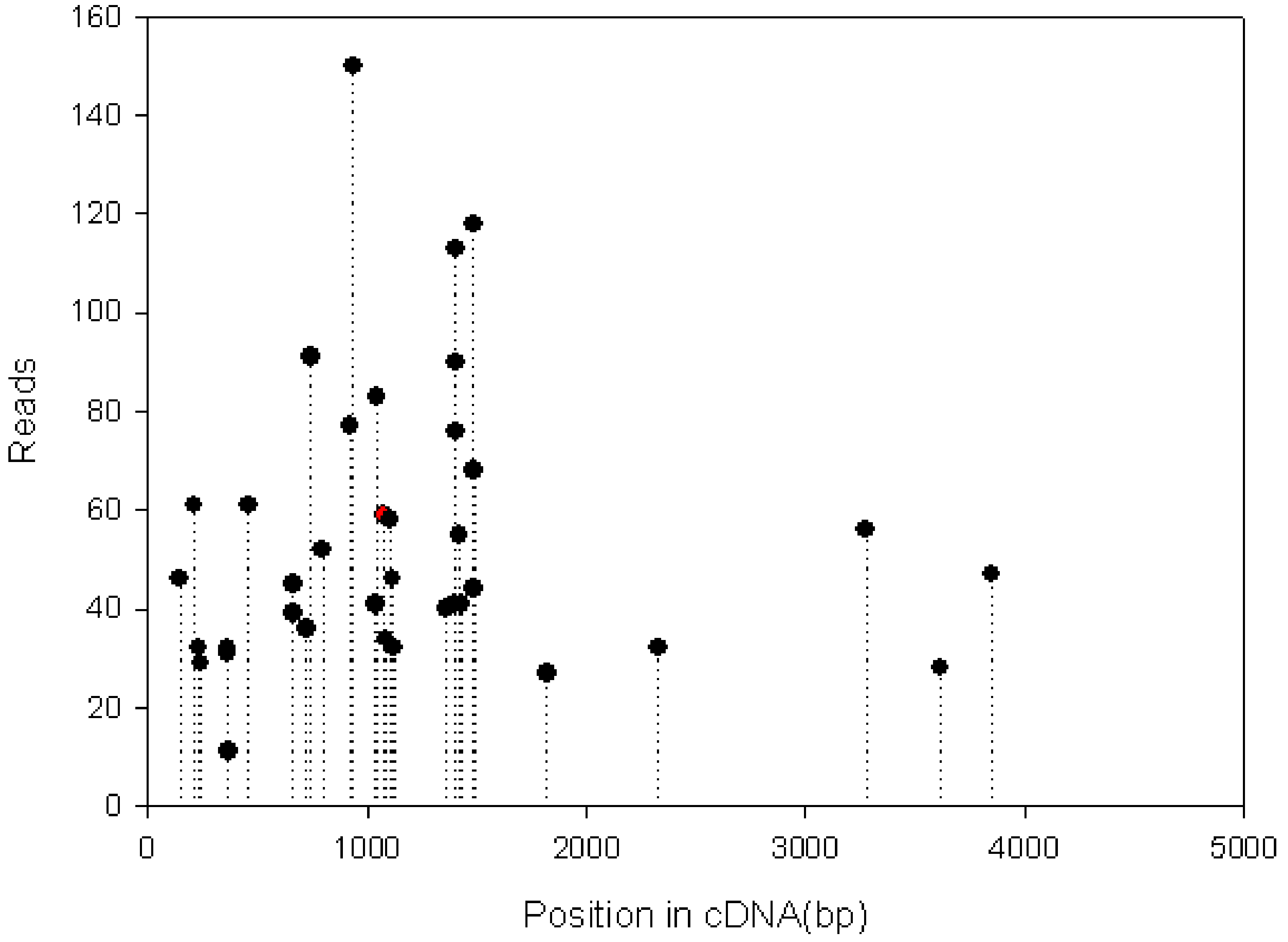


```
5' CGAUGCACCAACAUUAAGUAUUCAAA 3'        Orange1.1t00603.1
     ::::: :: :::.::: :::::
3' --UACGUCGUAGUAGUUC-UAAGU-- 5'        Csi-miR172k
```

Csi-miR172k, target=Orange1.1t04055.1 gene=Orange1.1t04055
Category:3
Score=3
Cleavage Site=2049

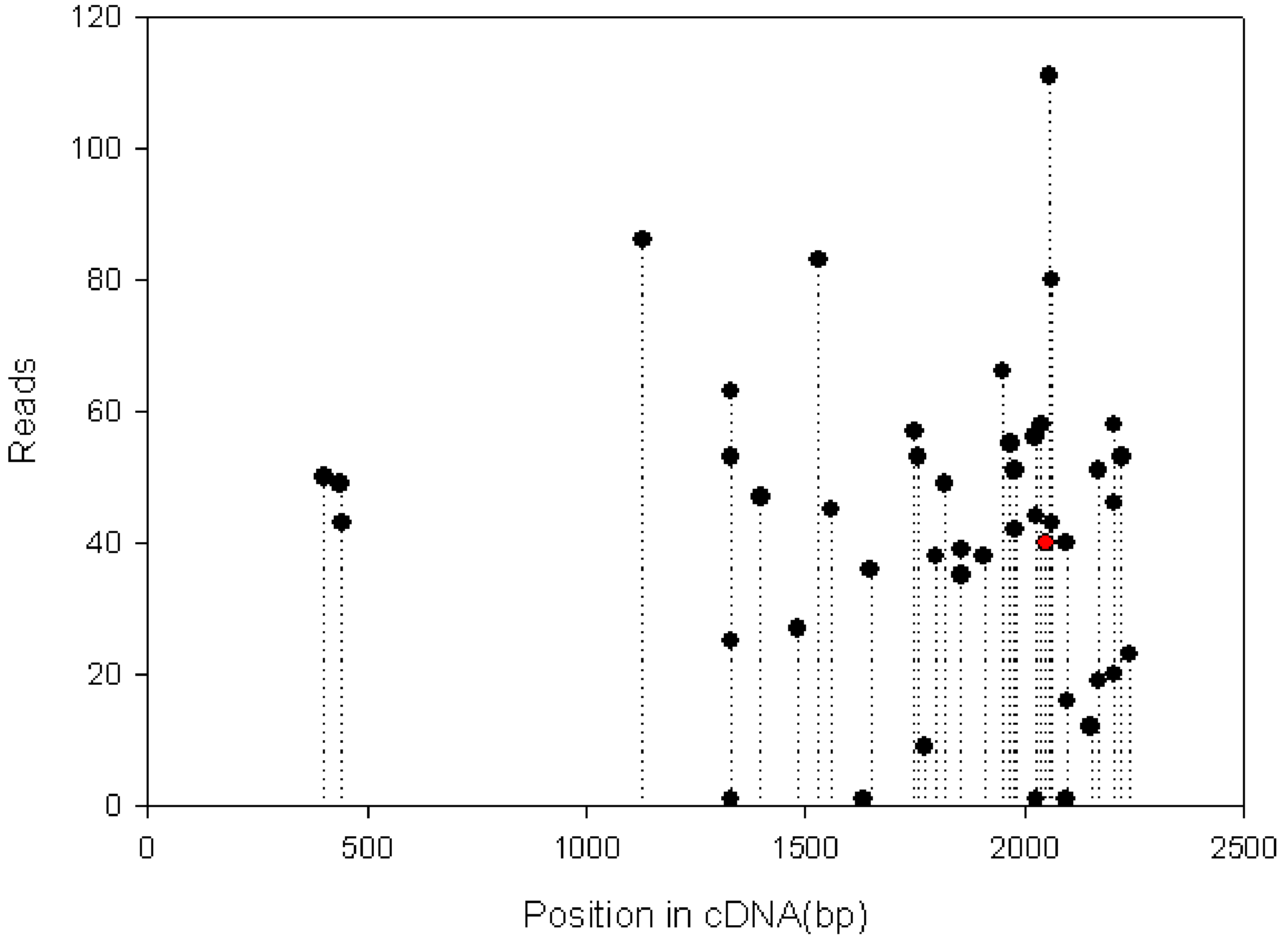


```
5' GCUGCAGCAUCAUCAGGAUUCCCUCA 3'        Orange1.1t04055.1
     :::::::::::::::::.:::::
3' -UACGUCGUAGUAGUUCUAAGU---- 5'        Csi-miR172k
```

Csi-miR2118.1, target=Orange1.1t00557.1 gene=Orange1.1t00557
Category:2
Score=4
Cleavage Site=131

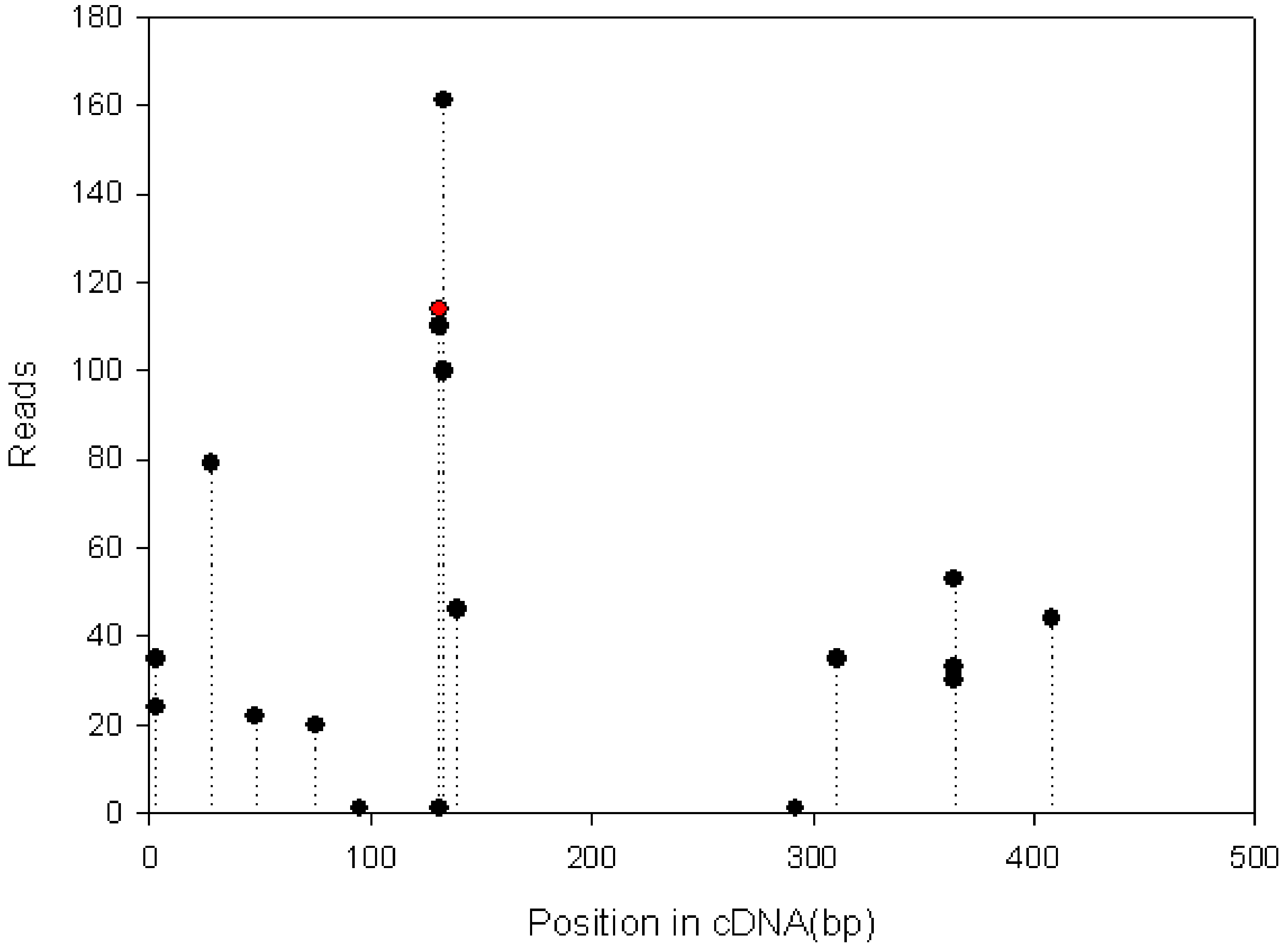


```
5' AAGACAUGGGUGGCAUAGGUCACAUC 3'        Orange1.1t00557.1
   :::  :::::::::::::: :
3' UUCCUUACCCACCGUAUCCAUU---- 5'        Csi-miR2118.1
```

Csi-miR2118.2, target=Cs3g12720.1 gene=Cs3g12720
Category:1
Score=5
Cleavage Site=547

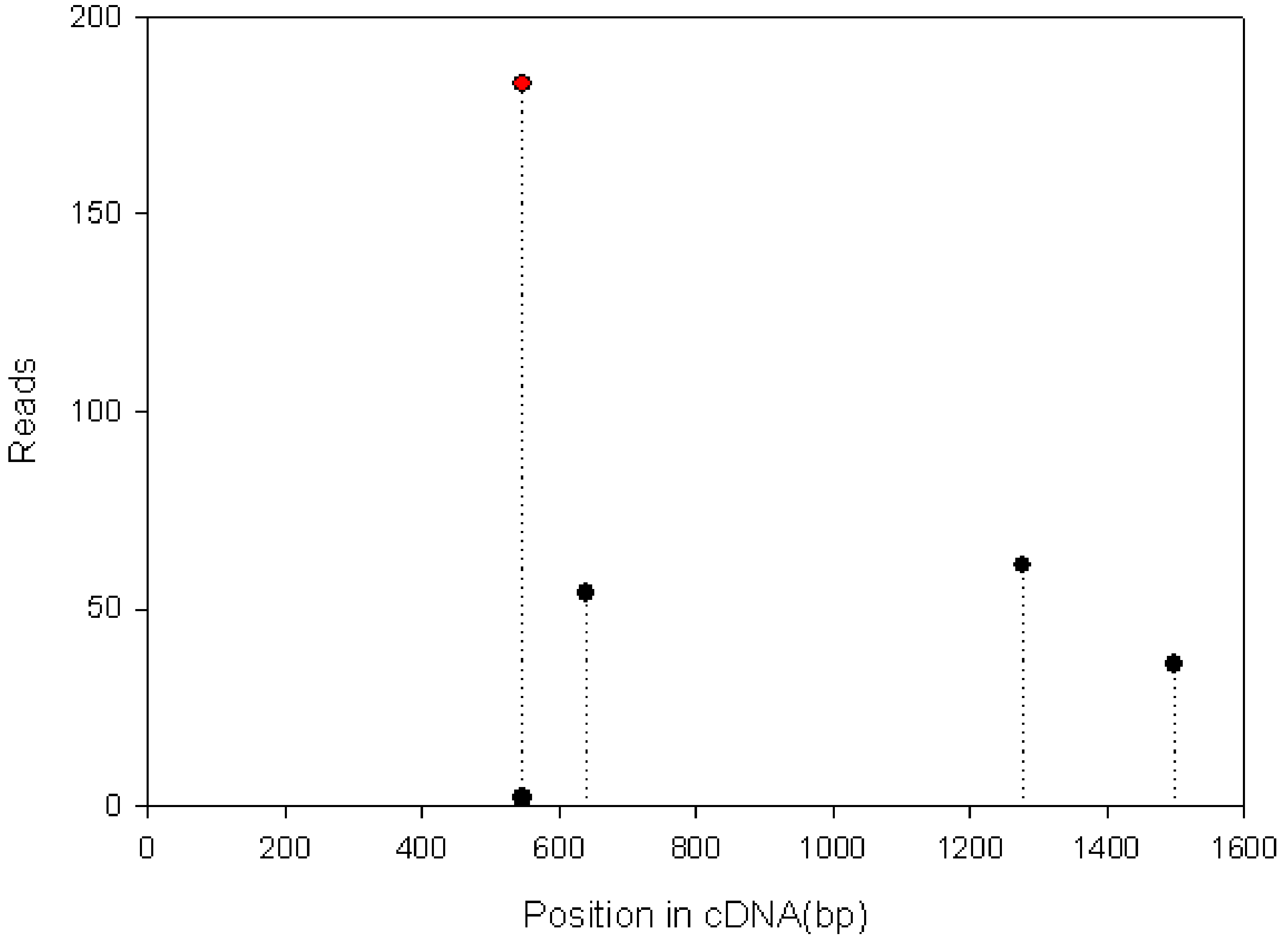


```
5' GGCAUGGGUGGCAUUGGAAAGACAAC 3'        Cs3g12720.1
   :: :::::::::::: :: ::
3' CCUUACCCACCGUAUCCAUU------ 5'        Csi-miR2118.2
```

Csi-miR2118.2, target=Cs3g12760.1 gene=Cs3g12760
Category:1
Score=4.5
Cleavage Site=1037

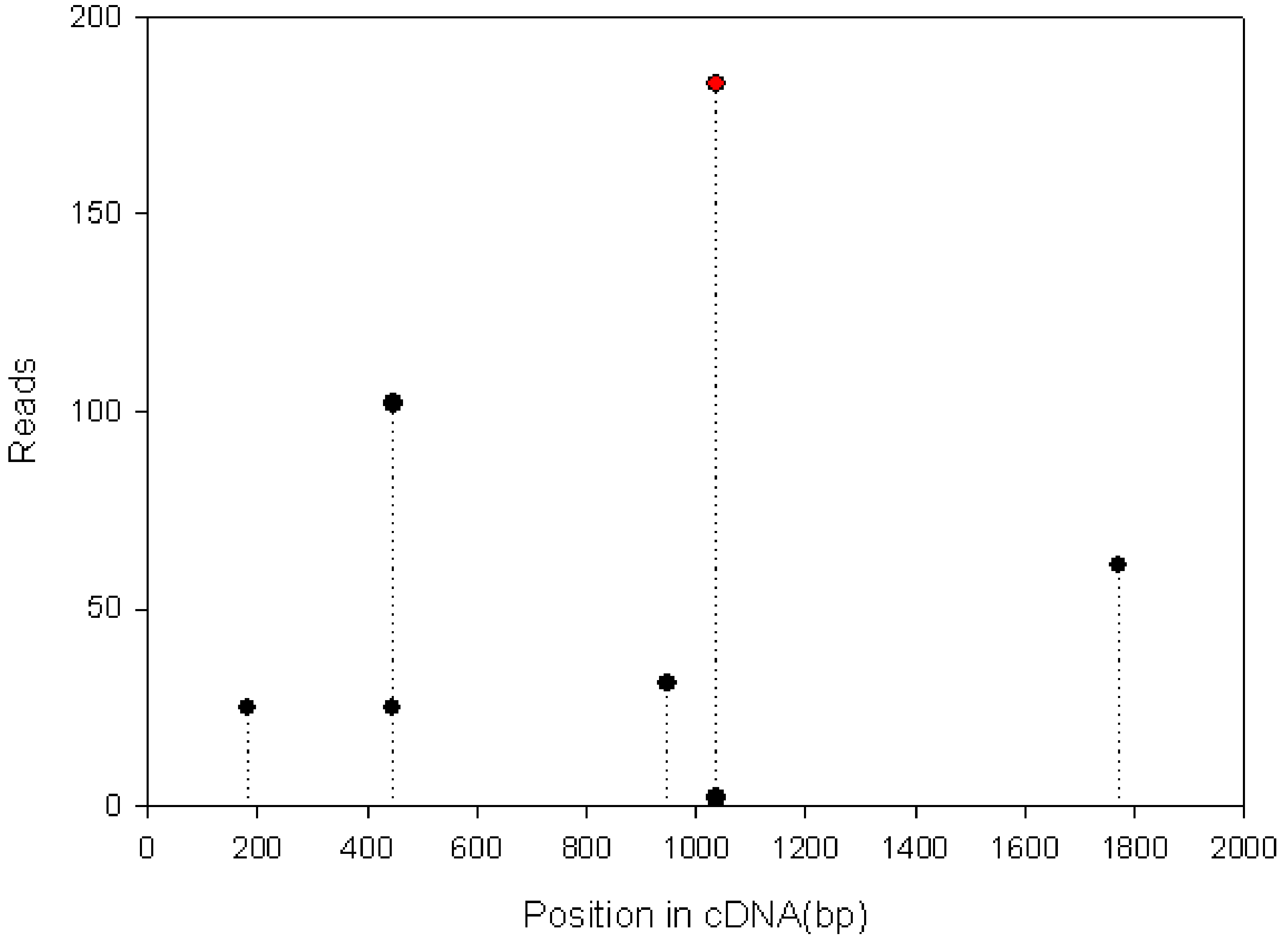


```
5' GGGAUGGGUGGCAUUGGAAAGACAAC 3'        Cs3g12760.1
   ::.:::::::::::: :: ::
3' CCUUACCCACCGUAUCCAUU------ 5'        Csi-miR2118.2
```

Csi-miR2118.2, target=Cs3g12850.1 gene=Cs3g12850
Category:1
Score=4.5
Cleavage Site=547

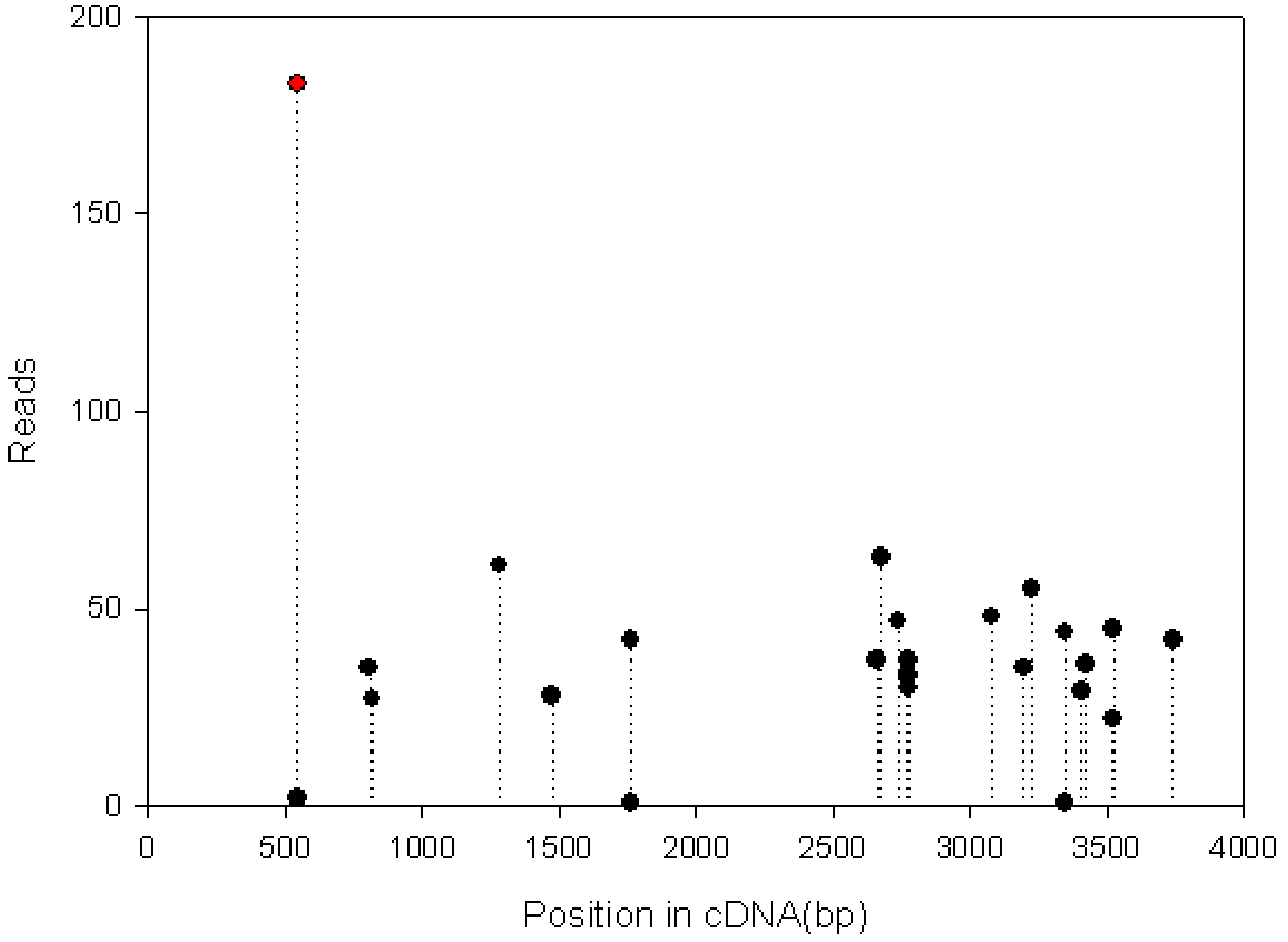


```
5'  GGGAUGGGUGGCAUUGGAAAGACAAC  3'         Cs3g12850.1
    ::.:::::::::::  :: ::
3'  CCUUACCCACCGUAUCCAUU------  5'         Csi-miR2118.2
```

Csi-miR2118.2, target=Cs3g13320.1 gene=Cs3g13320
Category:1
Score=4.5
Cleavage Site=643

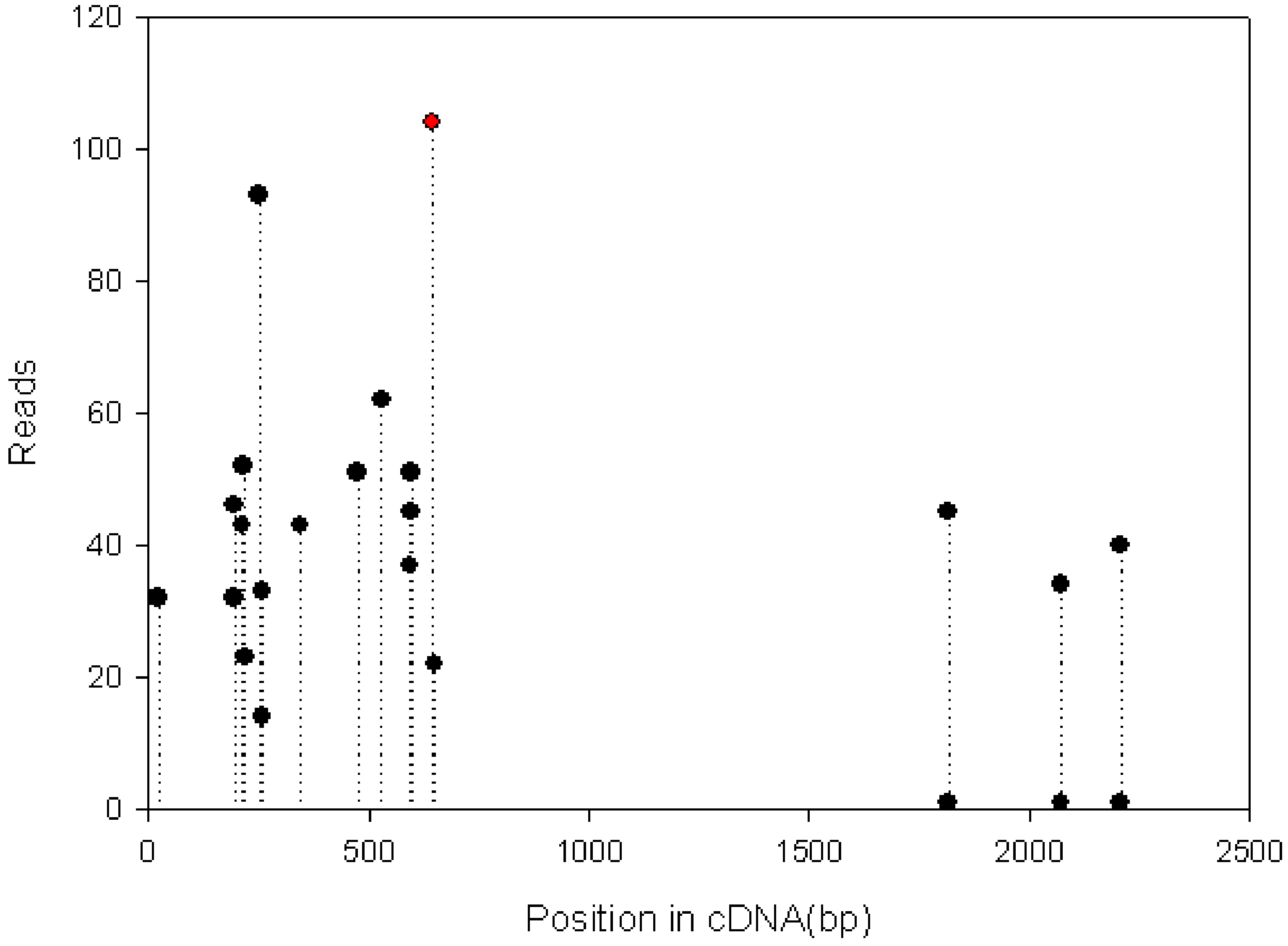


```
5' GGGAUGGGGGCAUAGGCAAAACUAC 3'        Cs3g13320.1
   ::.:::::: :::::::: ::
3' CCUUACCCACCGUAUCCAUU------ 5'       Csi-miR2118.2
```

Csi-miR2118.2, target=Cs3g13340.1 gene=Cs3g13340
Category:2
Score=4.5
Cleavage Site=702

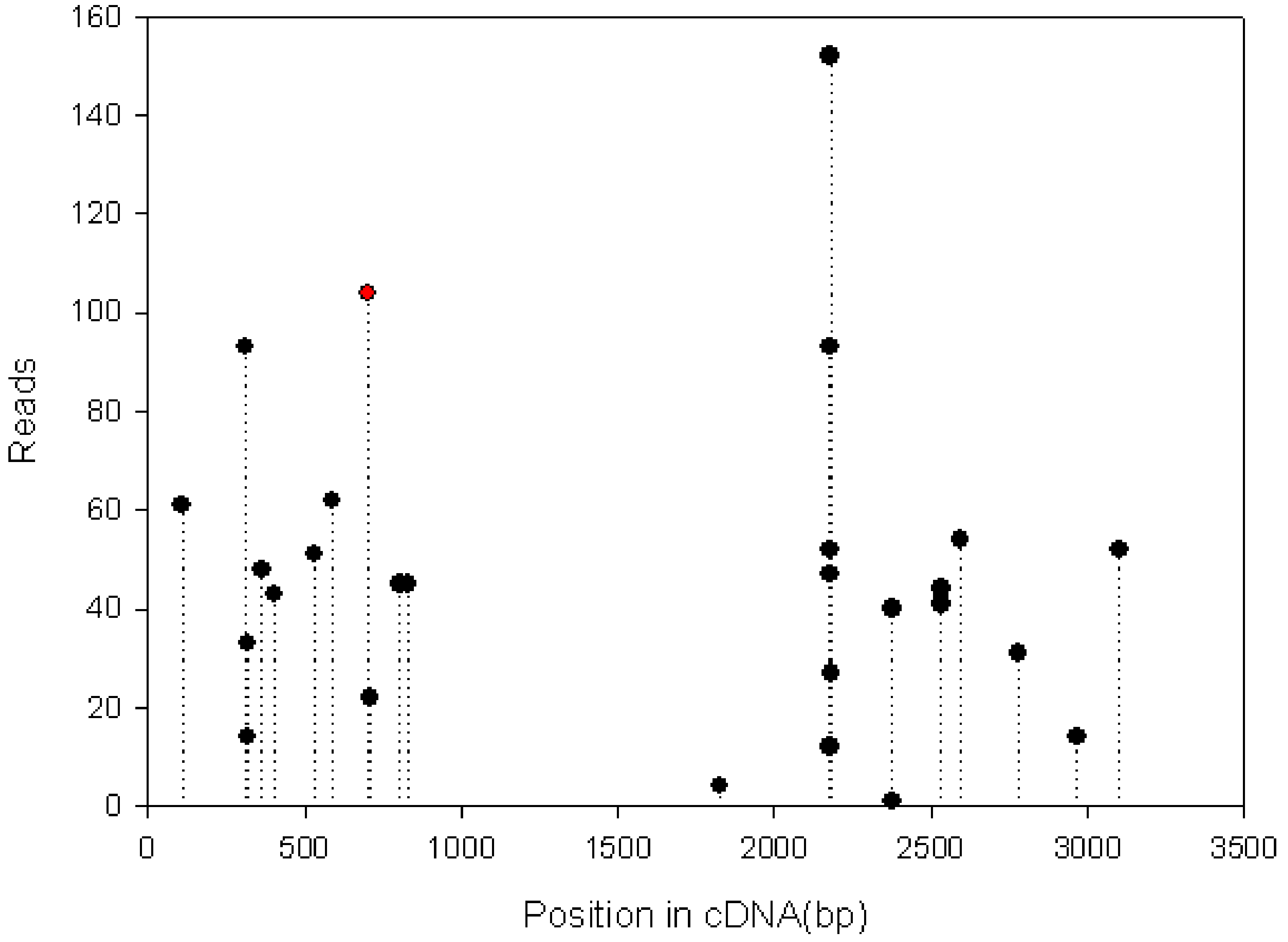


```
5' GGGAUGGGGGCAUAGGCAAAACUAC 3'        Cs3g13340.1
   ::.:::::: :::::::: ::
3' CCUUACCCACCGUAUCCAUU------ 5'       Csi-miR2118.2
```

Csi-miR2118.2, target=Cs3g13390.1 gene=Cs3g13390
Category:1
Score=4.5
Cleavage Site=800

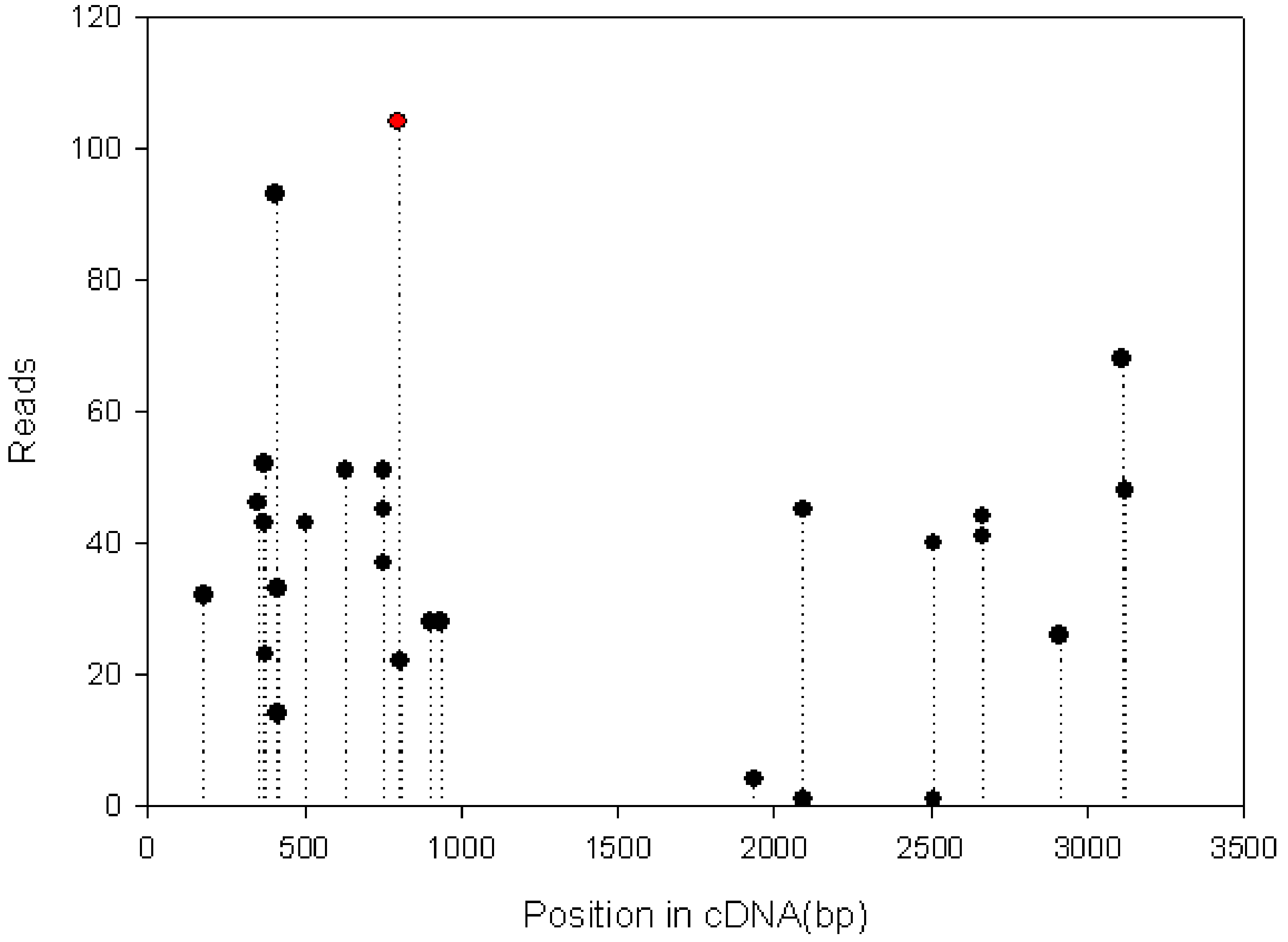


```
5' GGGAUGGGGGCAUAGGCAAAACUAC 3'        Cs3g13390.1
   ::.:::::: :::::::: ::
3' CCUUACCCACCGUAUCCAUU------ 5'        Csi-miR2118.2
```

Csi-miR2118.2, target=Cs3g13740.1 gene=Cs3g13740
Category:2
Score=4.5
Cleavage Site=686

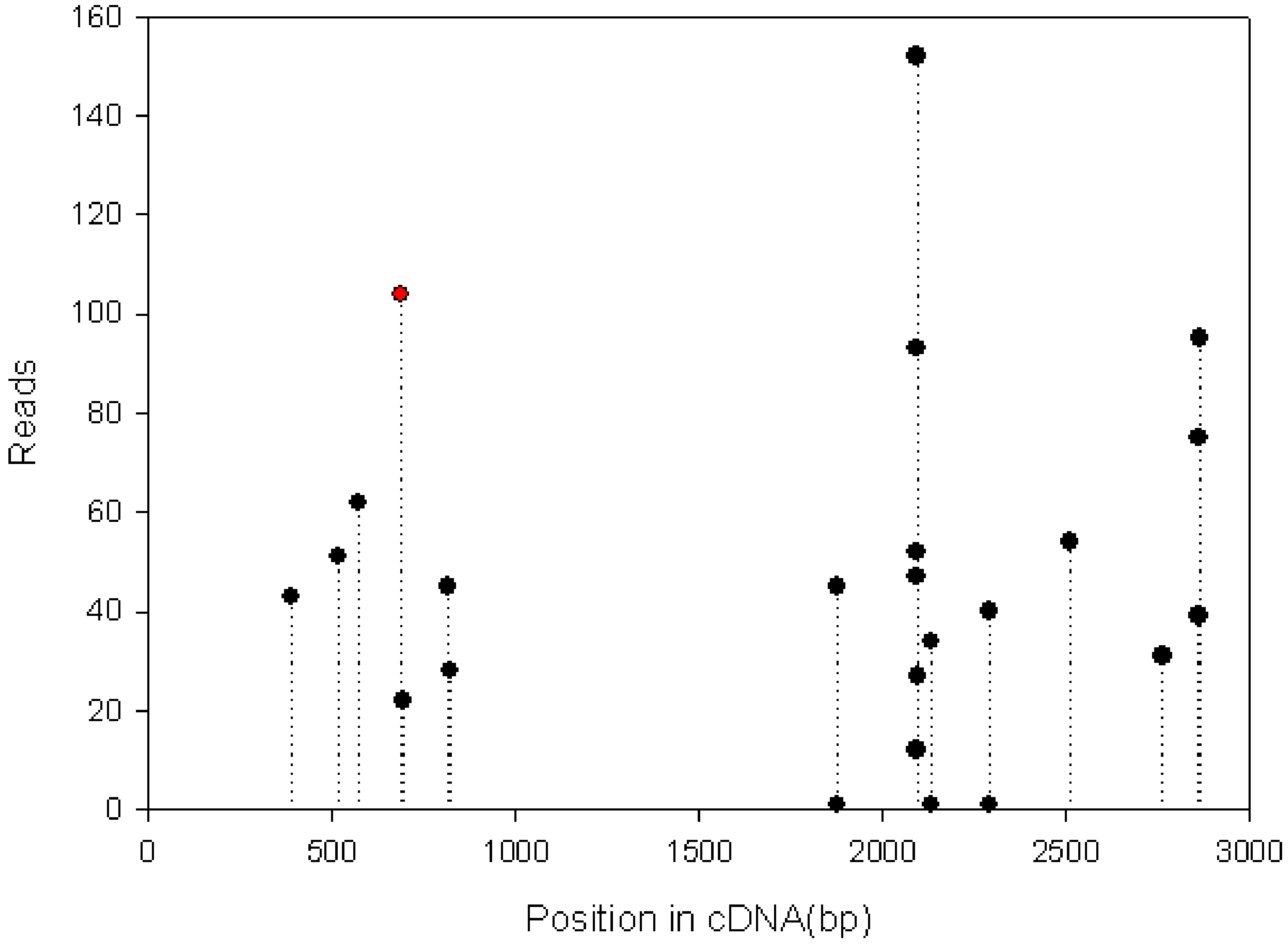


```
5' GGGAUGGGGGGCAUAGGCAAAACUAC 3'          Cs3g13740.1
   ::.::::: :::::::: ::
3' CCUUACCCACCGUAUCCAUU------ 5'          Csi-miR2118.2
```

Csi-miR2118.2, target=Cs5g19440.1 gene=Cs5g19440
Category:1
Score=4
Cleavage Site=364

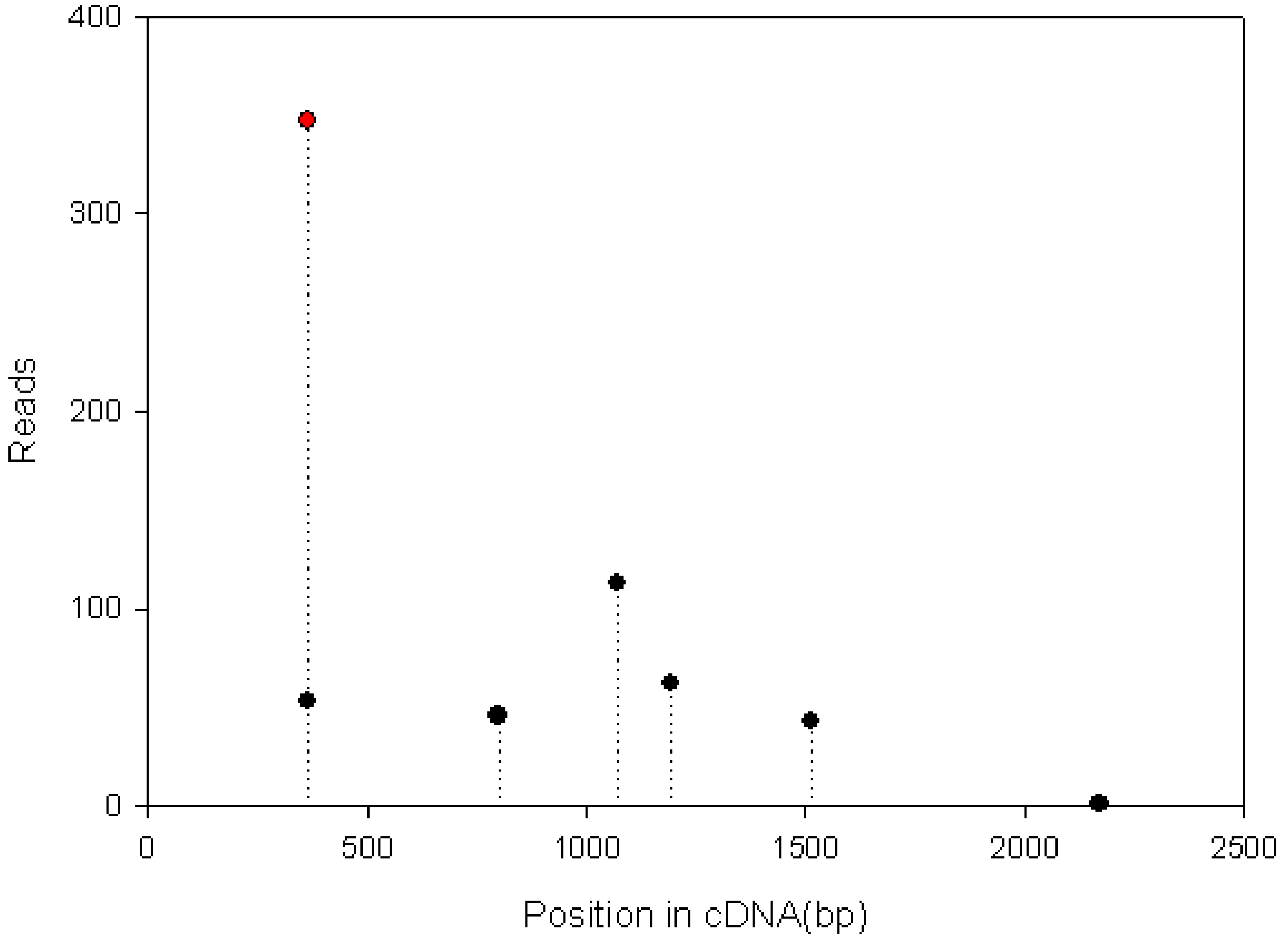


```
5' GGUAUGGGAGGUAUAGGUAAGACAAC 3'        Cs5g19440.1
   :: ::::: ::.::::::::
3' CCUUACCCACCGUAUCCAUU------ 5'        Csi-miR2118.2
```

Csi-miR2118.2, target=Orange1.1t00557.1 gene=Orange1.1t00557
Category:2
Score=4
Cleavage Site=131

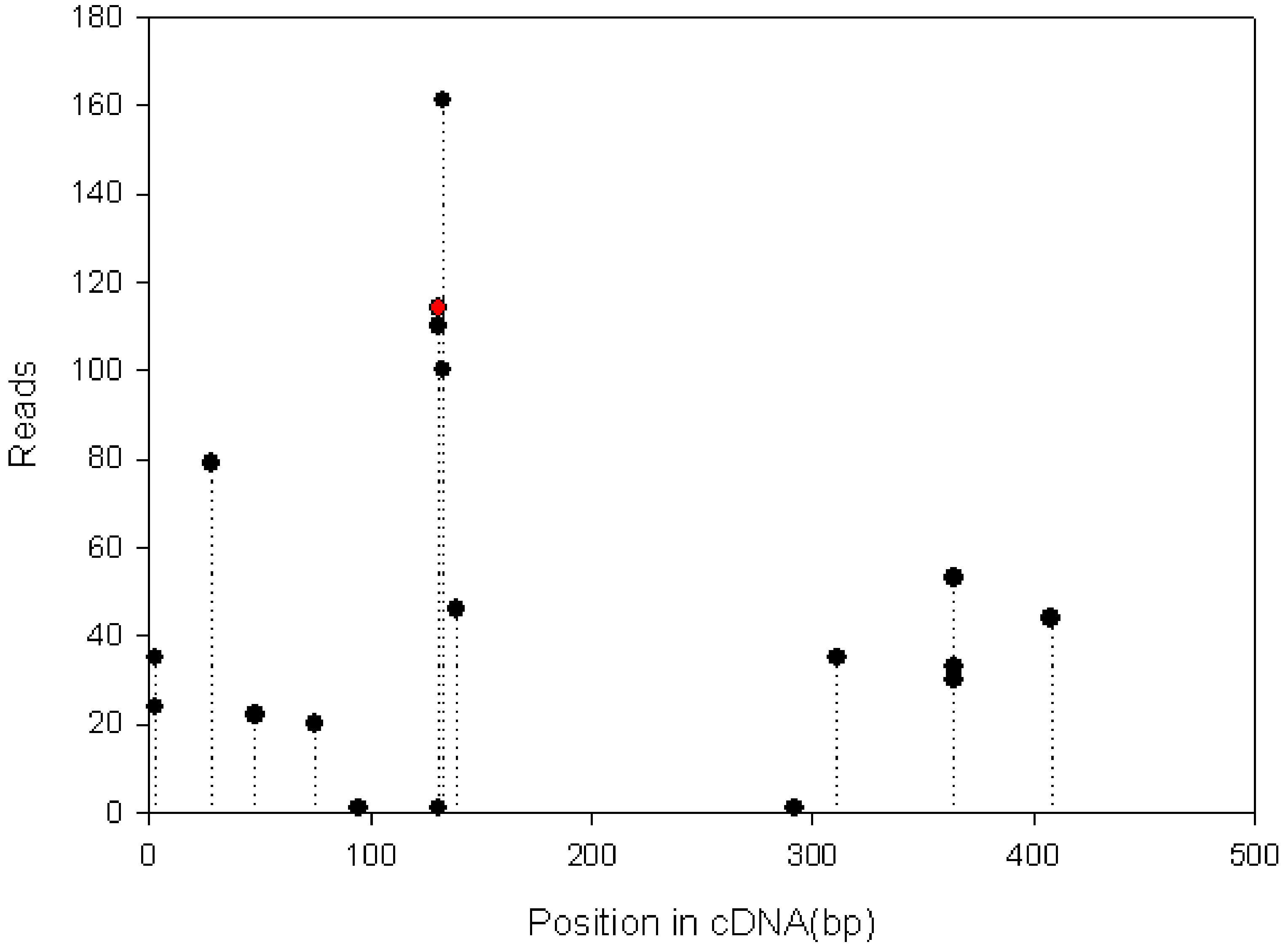


```
5' AAGACAUGGGUGGCAUAGGUCACAUC 3'        Orange1.1t00557.1
    :: ::::::::::::::: :
3' -CCU-UACCCACCGUAUCCAUU---- 5'        Csi-miR2118.2
```

Csi-miR2118.2, target=orange1.1t01829.1 gene=orange1.1t01829
Category:1
Score=4
Cleavage Site=810

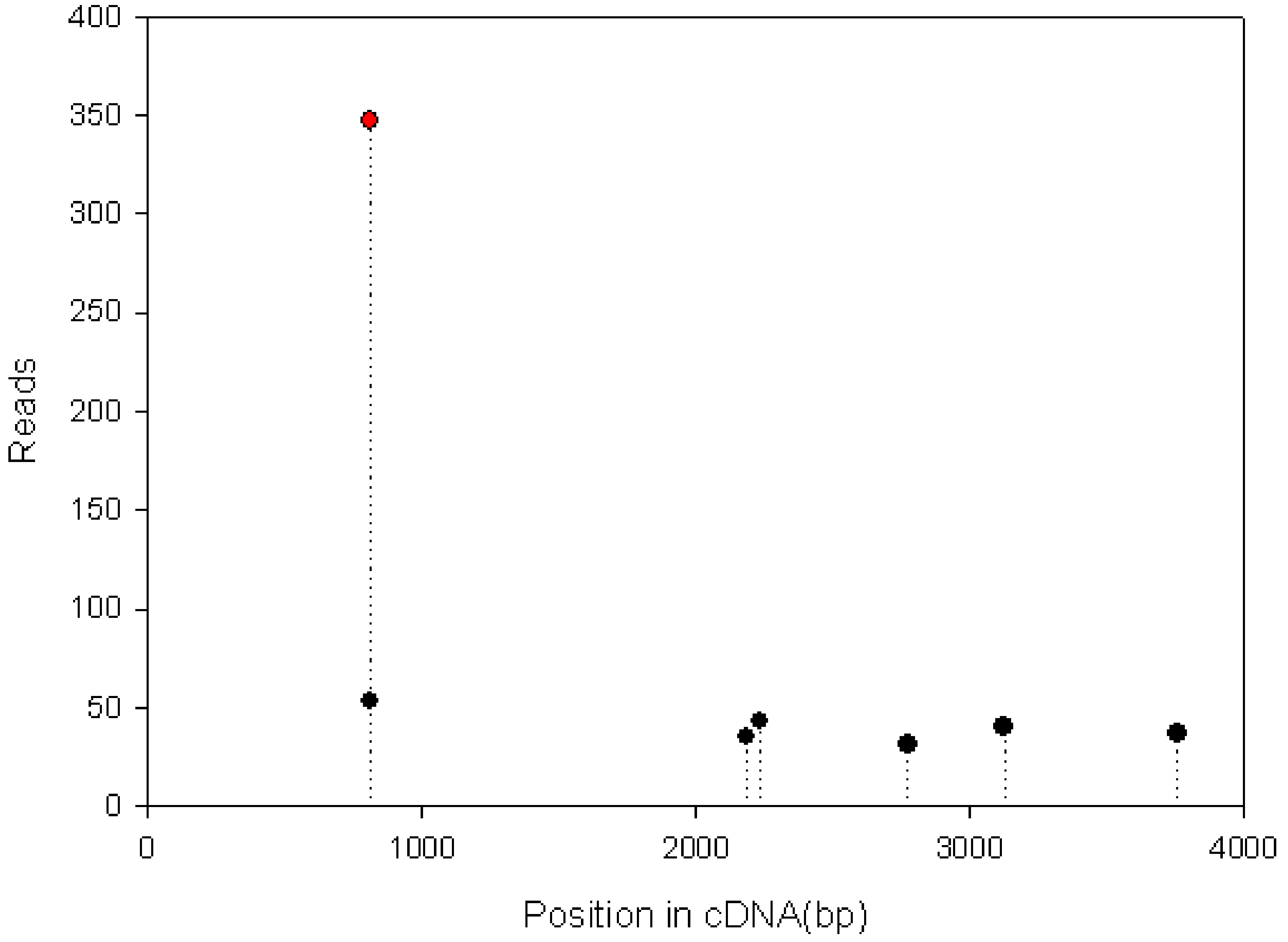


```
5' GGUAUGGGAGGUAUAGGUAAGACAAC 3'        Orange1.1t01829.1
   :: ::::: ::.:::::::::
3' CCUUACCCACCGUAUCCAUU------ 5'        Csi-miR2118.2
```

Csi-miR2118.2, target=Orange1.1t02518.1 gene=Orange1.1t02518
Category:1
Score=4.5
Cleavage Site=1369

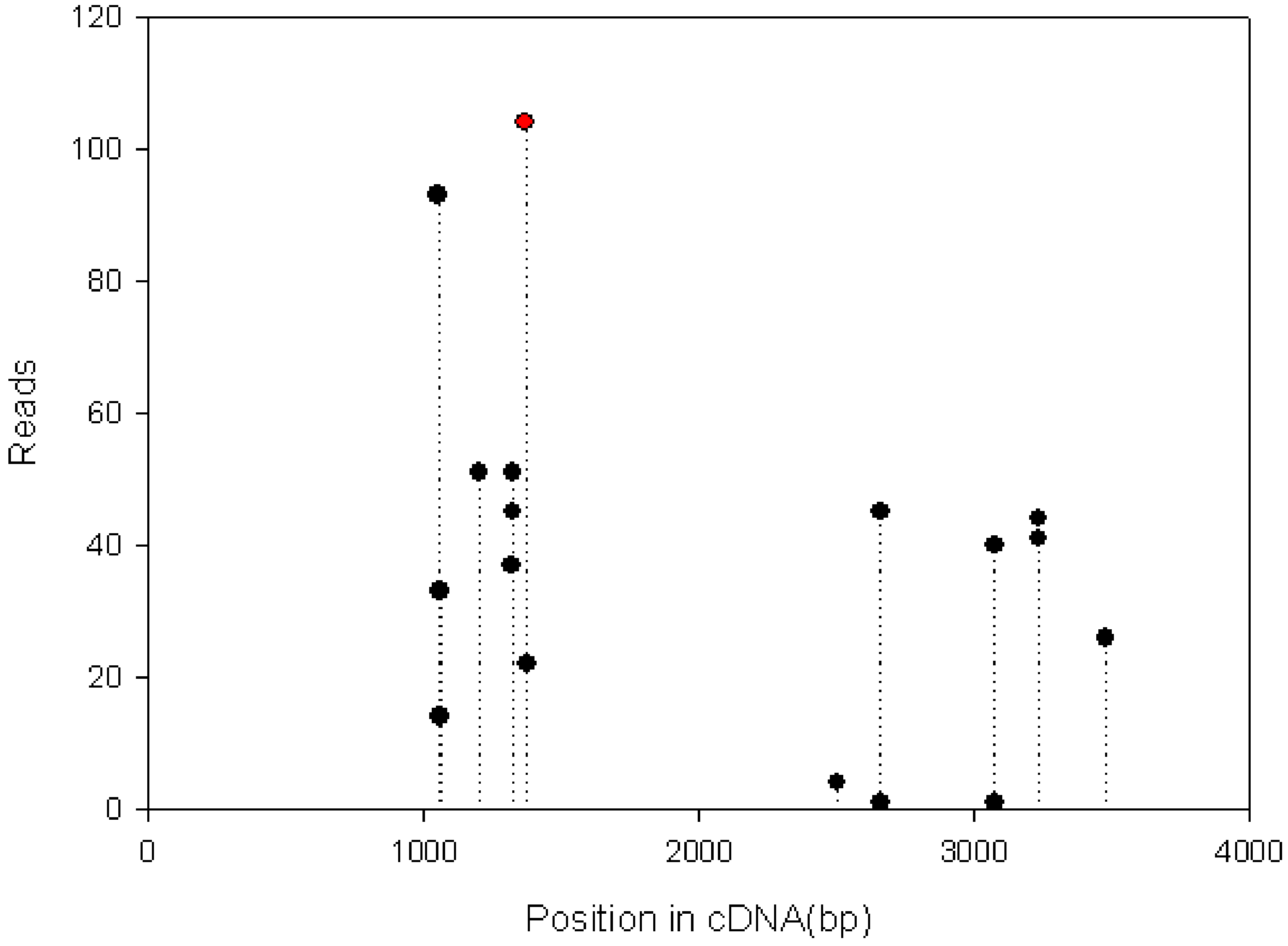


```
5' GGGAUGGGGGGCAUAGGCAAAACUAC 3'        Orange1.1t02518.1
   ::.::::: :::::::: ::
3' CCUUACCCACCGUAUCCAUU------ 5'        Csi-miR2118.2
```

Csi-miR390.1, target=Cs9g01780.1 gene=Cs9g01780
Category:2
Score=4.5
Cleavage Site=495

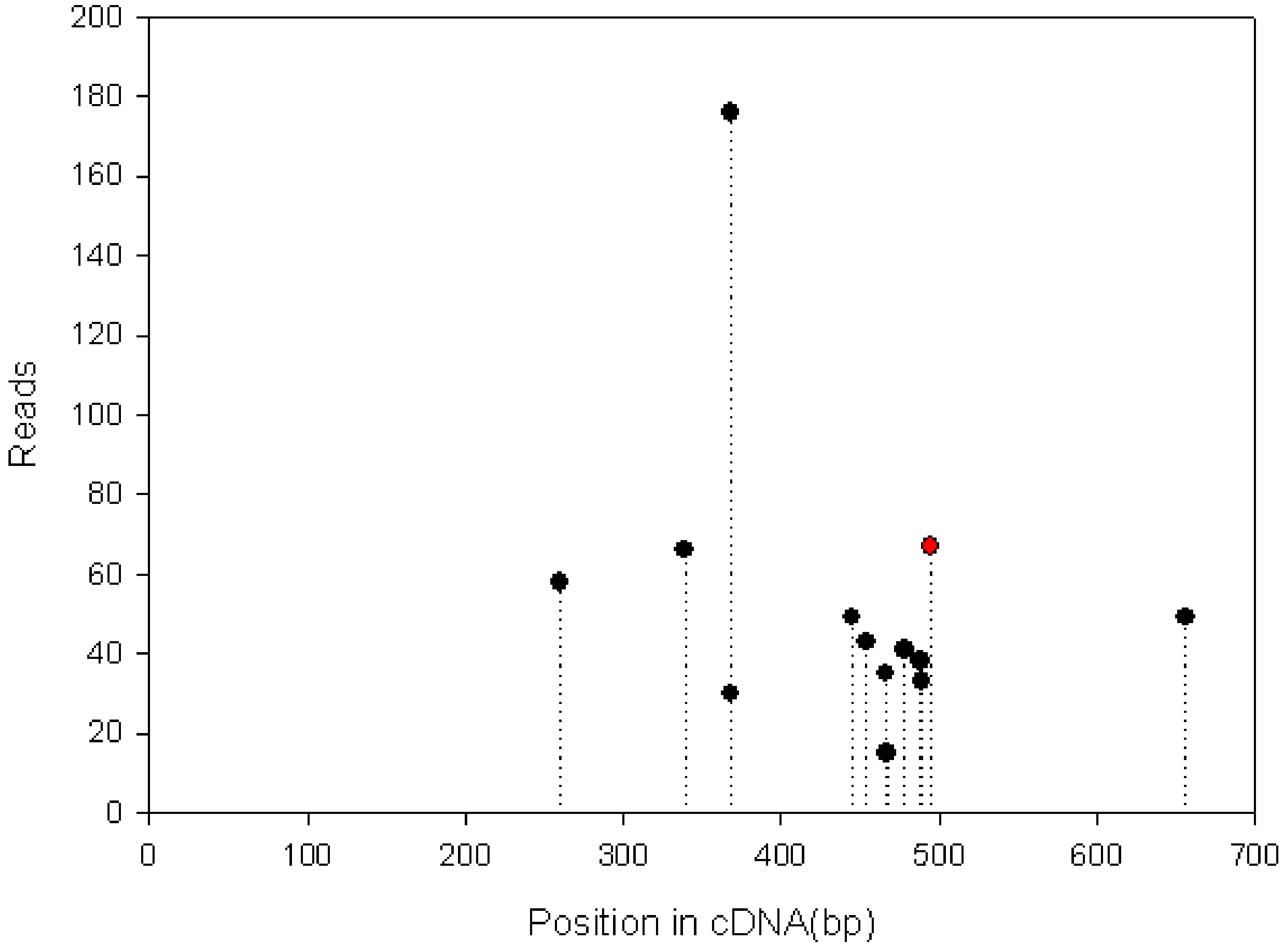


```
5' CUUCCUUGUCUAUCCCUCCUGAGCUG 3'        Cs9g01780.1
         .: ::::::::::::::::
3' ----CCGC-GAUAGGGAGGACUCGAA 5'        Csi-miR390.1
```

Csi-miR390.2, target=Cs9g01780.1 gene=Cs9g01780
Category:2
Score=3.5
Cleavage Site=495

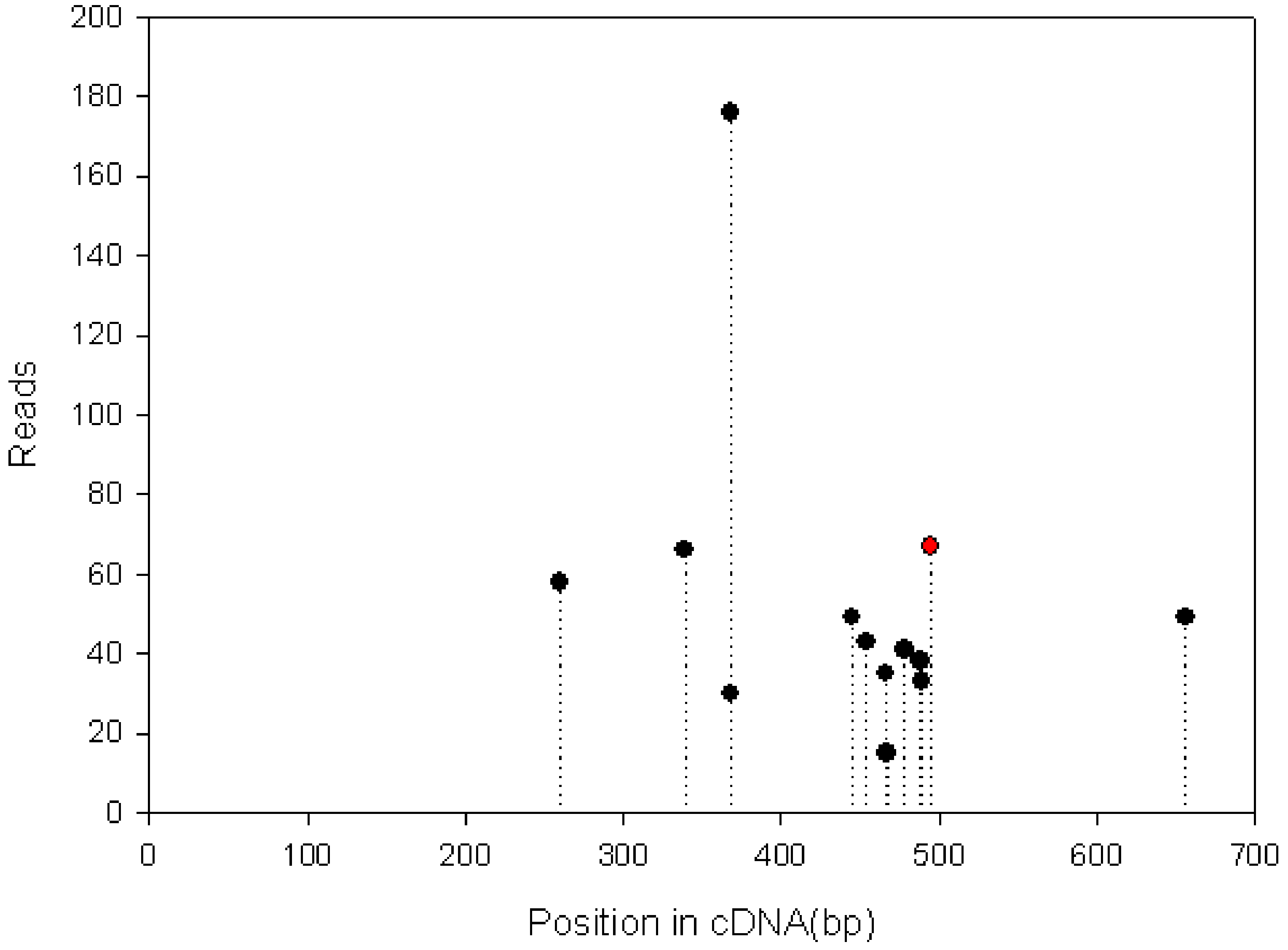


```
5' CUUCCUUGUCUAUCCCUCCUGAGCUG 3'        Cs9g01780.1
           .: ::::::::::::::::
3' ----CCGC-GAUAGGGAGGACUCGA- 5'        Csi-miR390.2
```

Csi-miR390b, target=Cs9g01780.1 gene=Cs9g01780
Category:2
Score=0
Cleavage Site=495

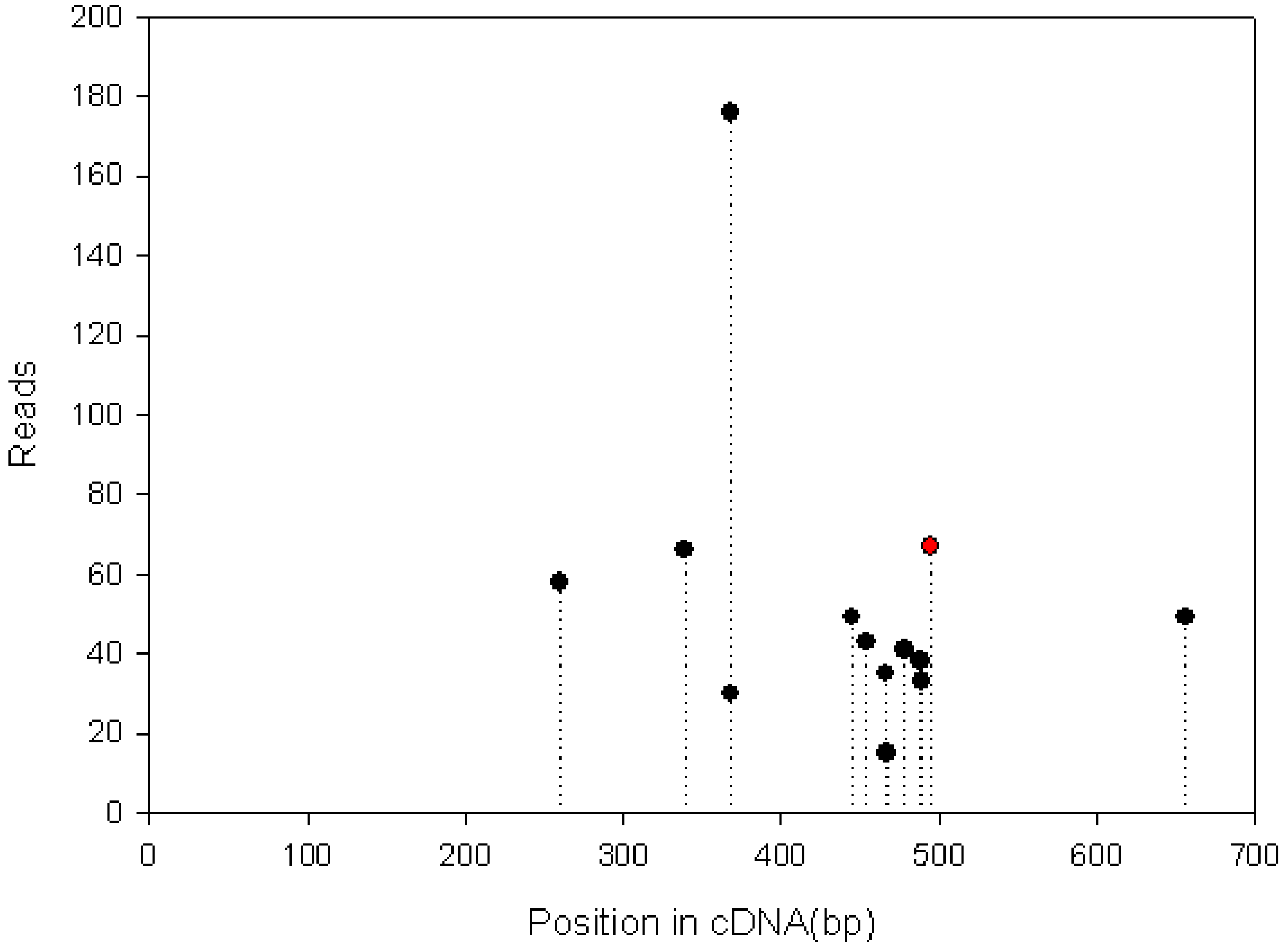


```
5' CUUCCUUGUCUAUCCCUCCUGAGCUG 3'        Cs9g01780.1
         :::::::::::::::::::::
3' -----AACAGAUAGGGAGGACUCGAC 5'        Csi-miR390b
```

Csi-miR391a.1, target=Cs5g17560.1 gene=Cs5g17560
Category:2
Score=4.5
Cleavage Site=2317

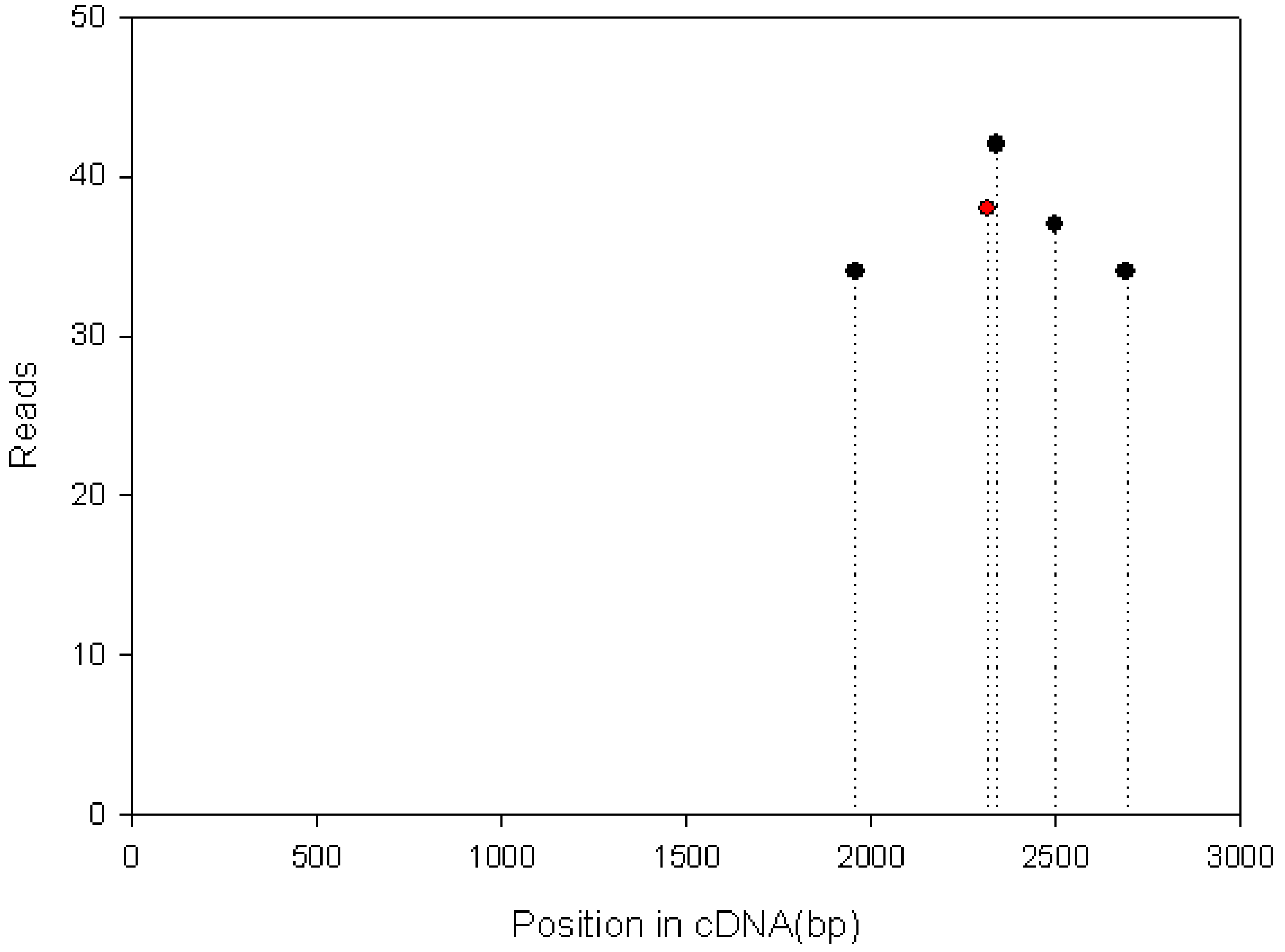


```
5' GCUUGC-AUUUUUCUGGUGACAAUCUC 3'        Cs5g17560.1
       ::: ::.:.::.::.:::::
3' ---ACGAUAGAGAGGCCGCUGUU---- 5'        Csi-miR391a.1
```

Csi-miR393a.2-3p, target=Orange1.1t01772.1 gene=Orange1.1t0177
Category:3
Score=3.5
Cleavage Site=1915

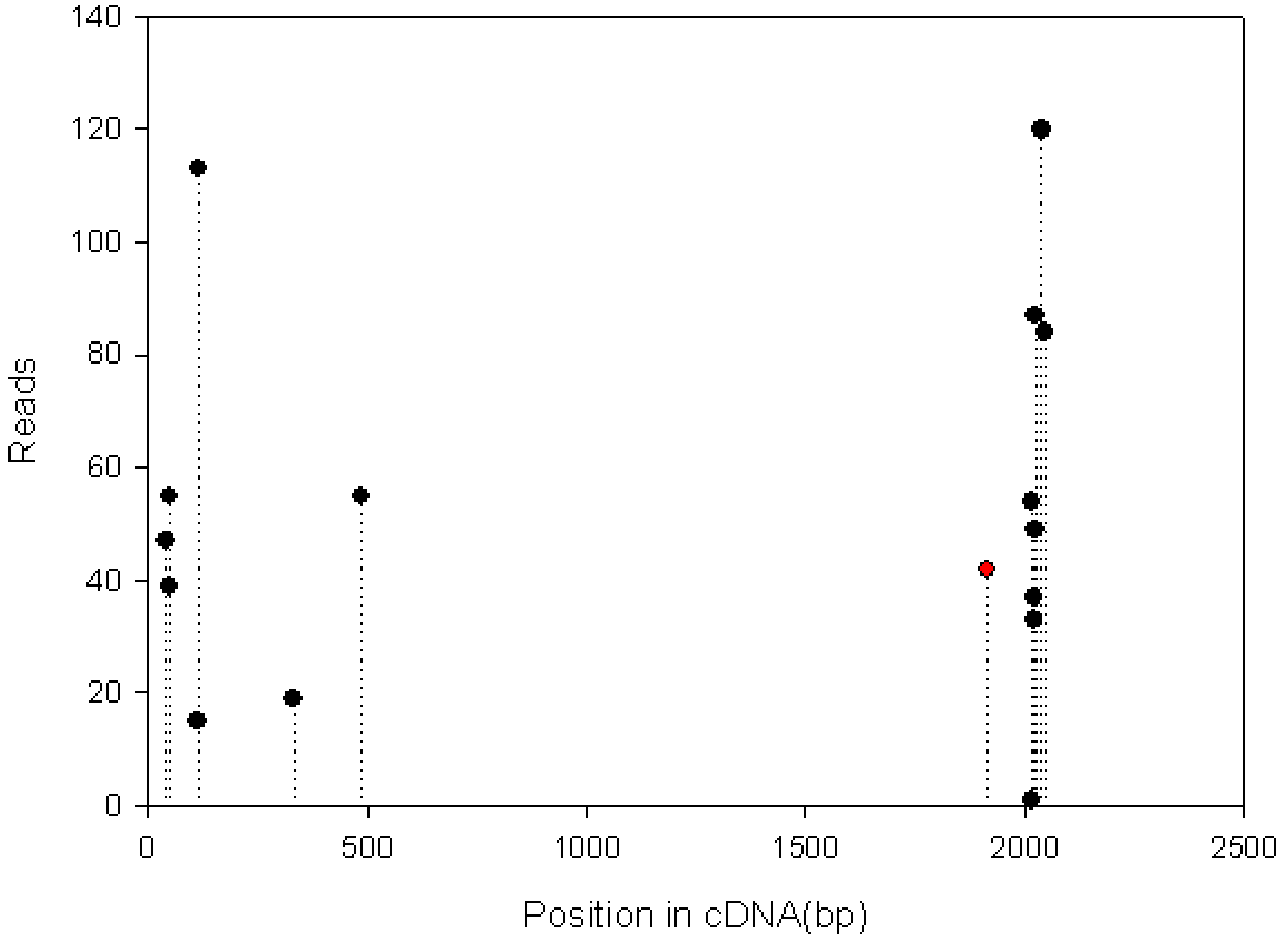


```
5' GGAAGAAUUCAAUGGAAUAGCAUGAU 3'        Orange1.1t01772.1
        :::.::: :: ::::::::::
3' -----UUAGGUUUCCCUAUCGUACUA 5'        Csi-miR393a.2-3p
```

Csi-miR394, target=Orange1.1t03542.1 gene=Orange1.1t03542
Category:3
Score=5
Cleavage Site=1135

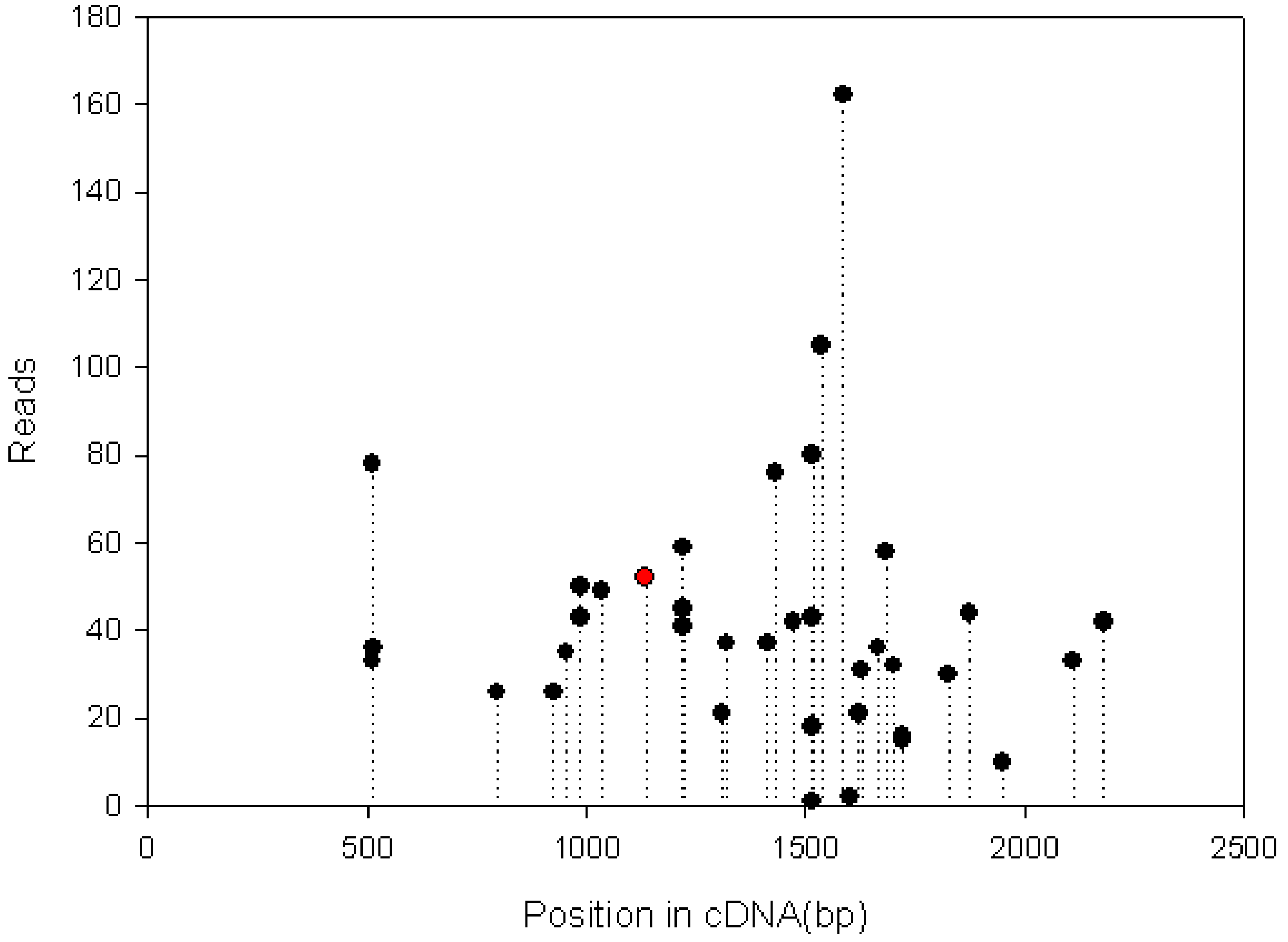


```
5' GUGAGG-GGACAGAAUGUAAAGCGCCA 3'        Orange1.1t03542.1
     :::: :::::::::::. ::
3' -CCUCCACCUGUCUUACGGUU------ 5'        Csi-miR394
```

Csi-miR394-3p.1, target=Cs3g09210.1 gene=Cs3g09210
Category:3
Score=5
Cleavage Site=1866

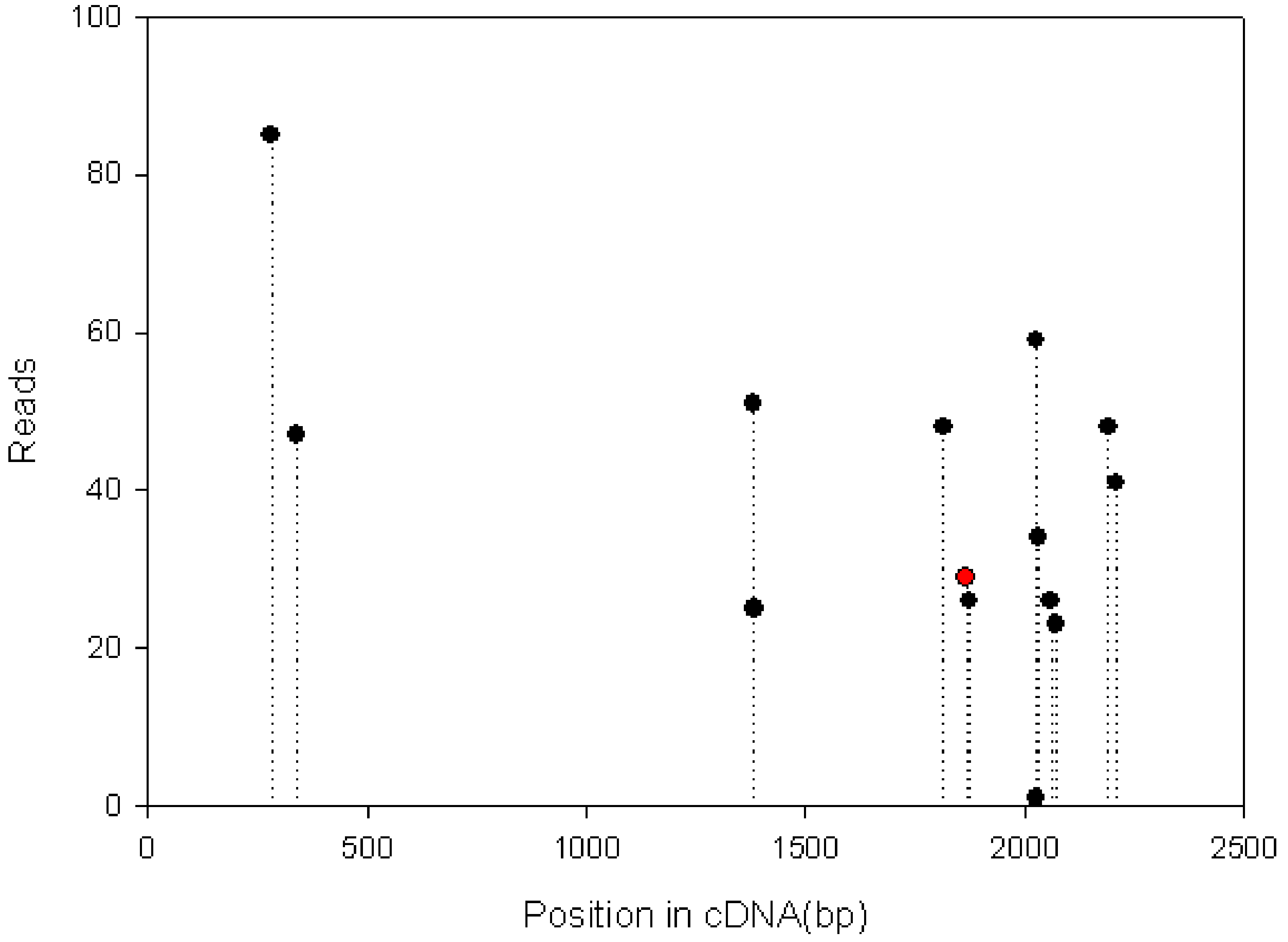


```
5' GUUUGUUUGGAAUUU-UCUAGCUGAUA 3'        Cs3g09210.1
     : ::::: ::::: ::.::::
3' --AUCAAACGUUAAACAGGUCGA---- 5'        Csi-miR394-3p.1
```

Csi-miR394-3p.2, target=Cs8g14600.1 gene=Cs8g14600
Category:3
Score=5
Cleavage Site=996

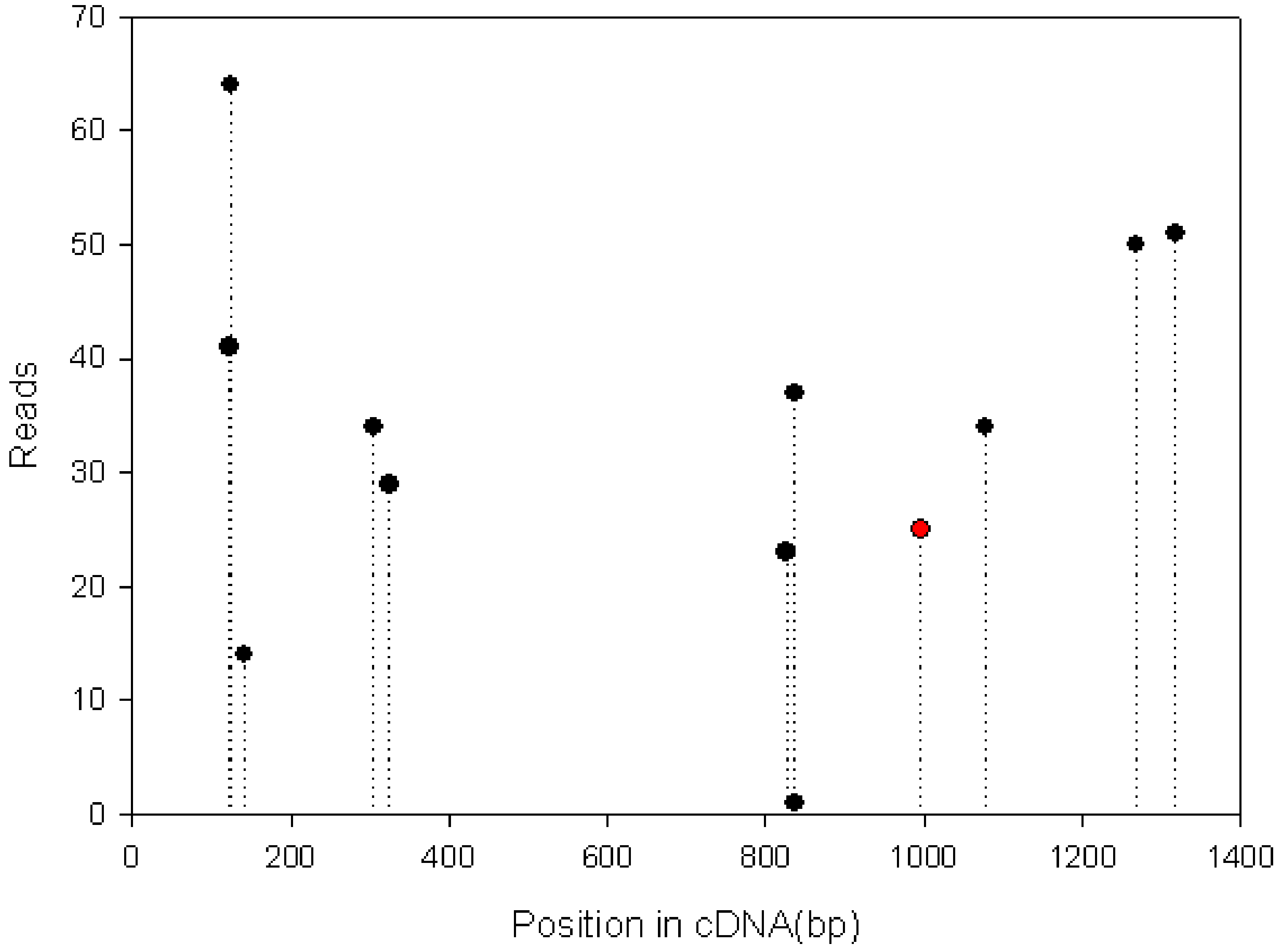


```
5' UGAAG-CUGCAGUUUGUCCAUCCAAAC 3'       Cs8g14600.1
   :: ::  ::::.:::::::::
3' ACAUCAAACGUUAAACAGGUC------ 5'       Csi-miR394-3p.2
```

Csi-miR3951, target=Cs1g06060.1 gene=Cs1g06060
Category:2
Score=2.5
Cleavage Site=174

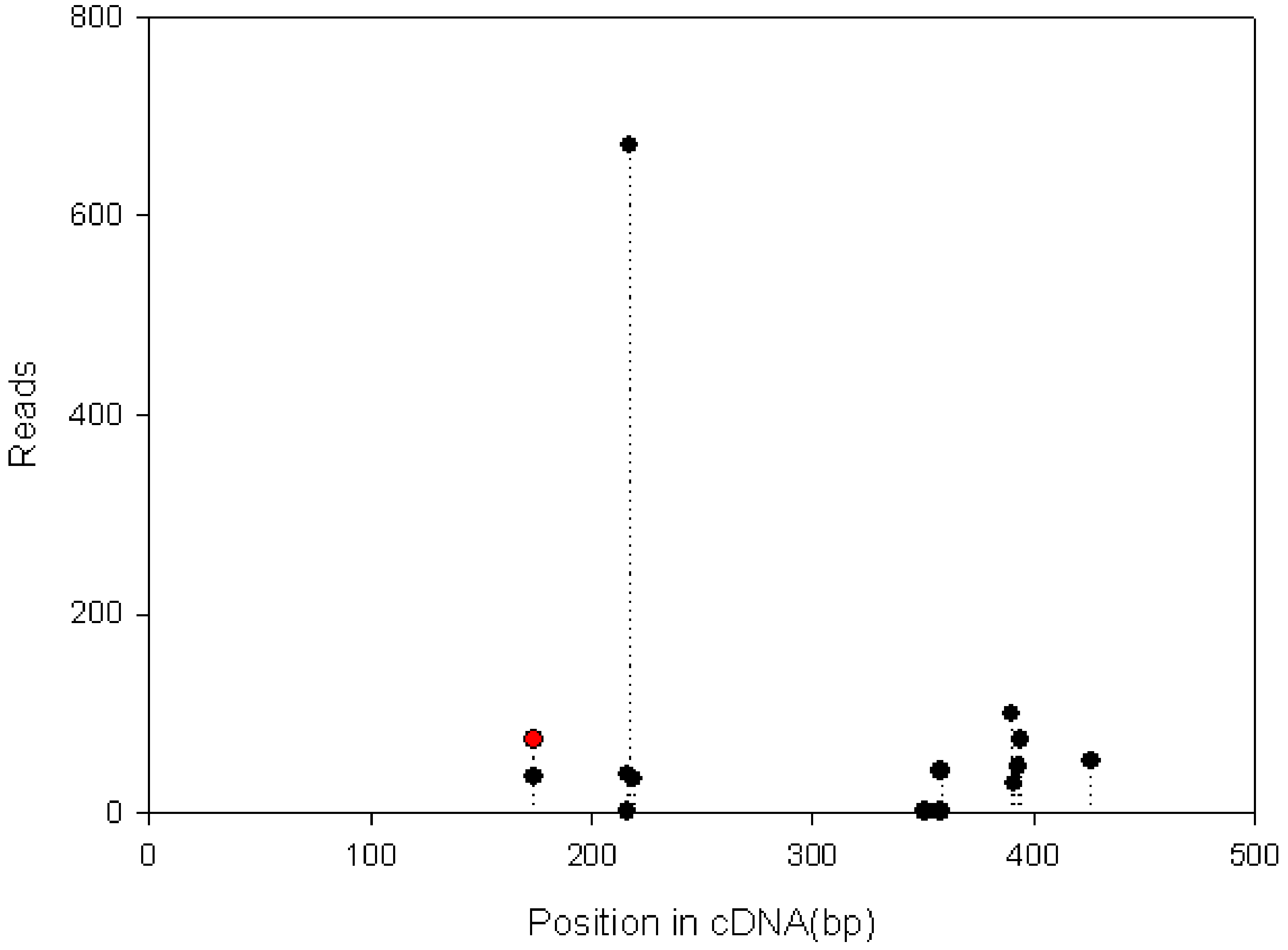


```
5' UUUUUUUCUCUC-UCUUUAUCUGUGGG 3'       Cs1g06060.1
     :::::::::: ::::::::.
3' --AAAAAGAGAGUAGAAAUAGAU---- 5'       Csi-miR3951
```

Csi-miR3951, target=Orange1.1t05622.1 gene=Orange1.1t05622
Category:2
Score=2.5
Cleavage Site=113

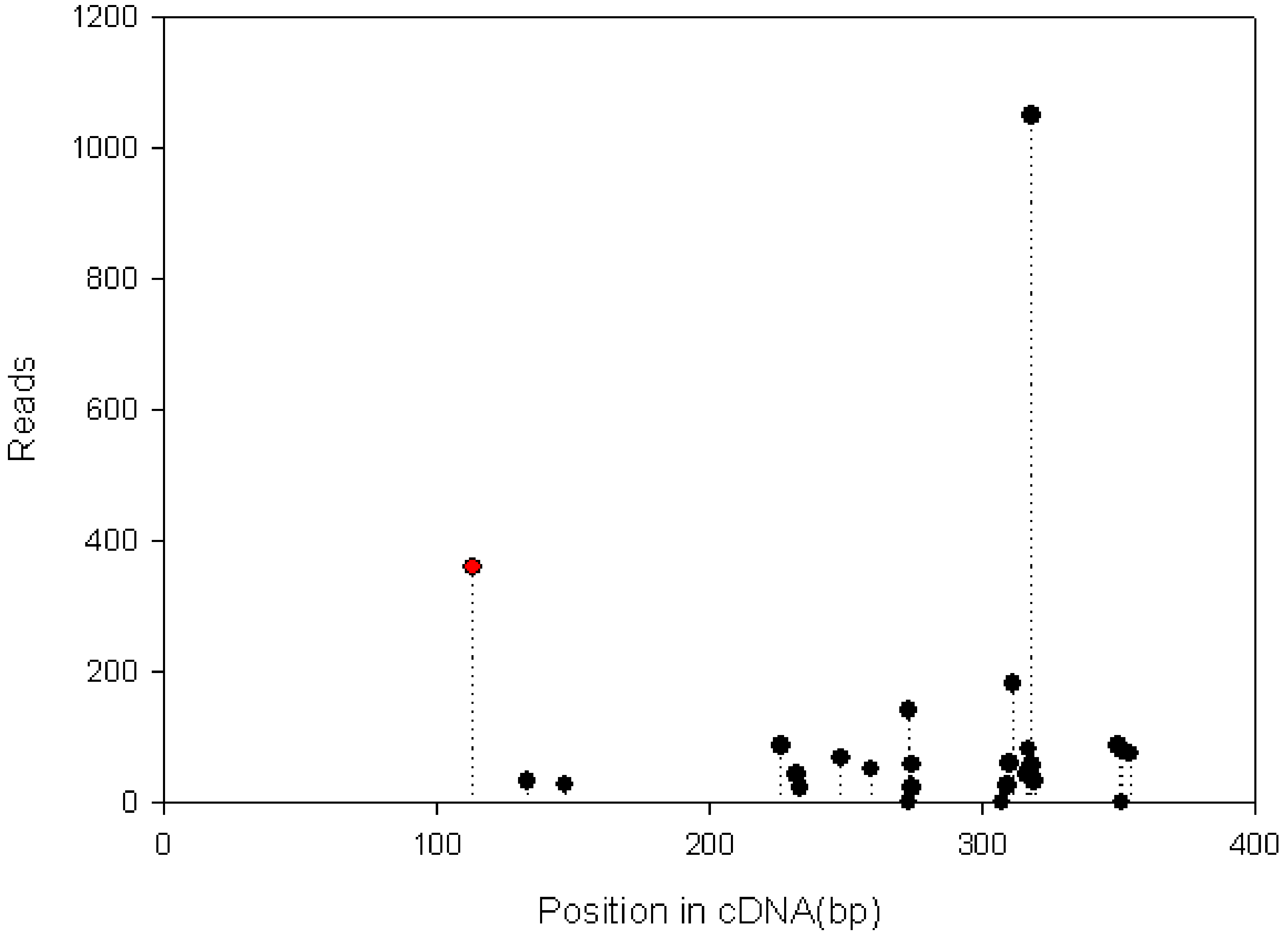


```
5' UUUUUUUCUCUC-UCUUUAUCUGUGGG 3'       Orange1.1t05622.1
     ::::::::::: ::::::::::.
3' --AAAAAGAGAGUAGAAAUAGAU---- 5'       Csi-miR3951
```

Csi-miR3951-3p, target=Cs6g09300.1 gene=Cs6g09300
Category:3
Score=5
Cleavage Site=352

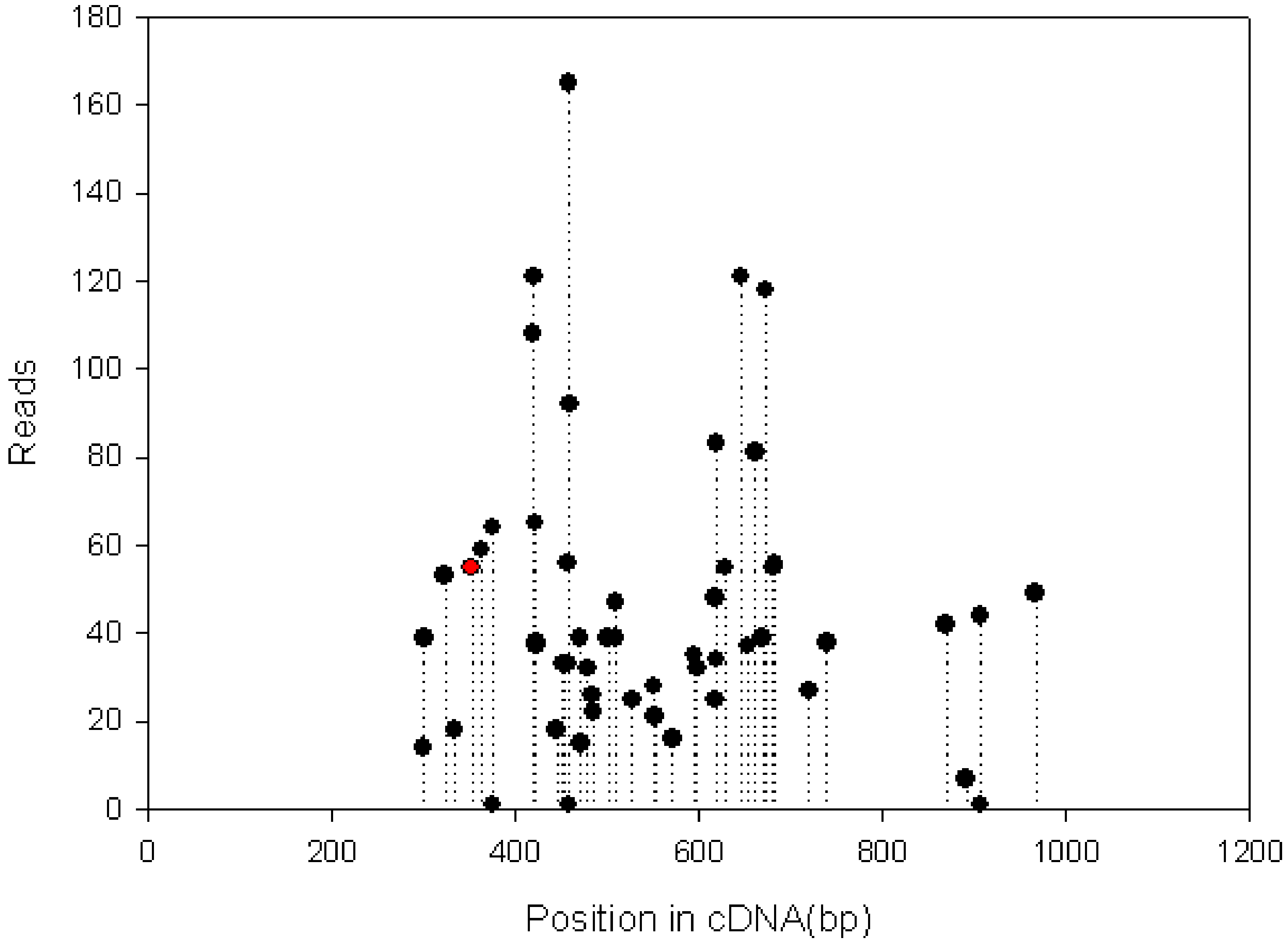


```
5' CCACCAAAGAACGAUAAGAAAAAGGU 3'        Cs6g09300.1
      :: : ::::::::: :::
3' ---UGUCUAUUGCUAUUCUCUUU--- 5'        Csi-miR3951-3p
```

Csi-miR3952.1, target=Cs8g13286.1 gene=Cs8g13286
Category:3
Score=3
Cleavage Site=304

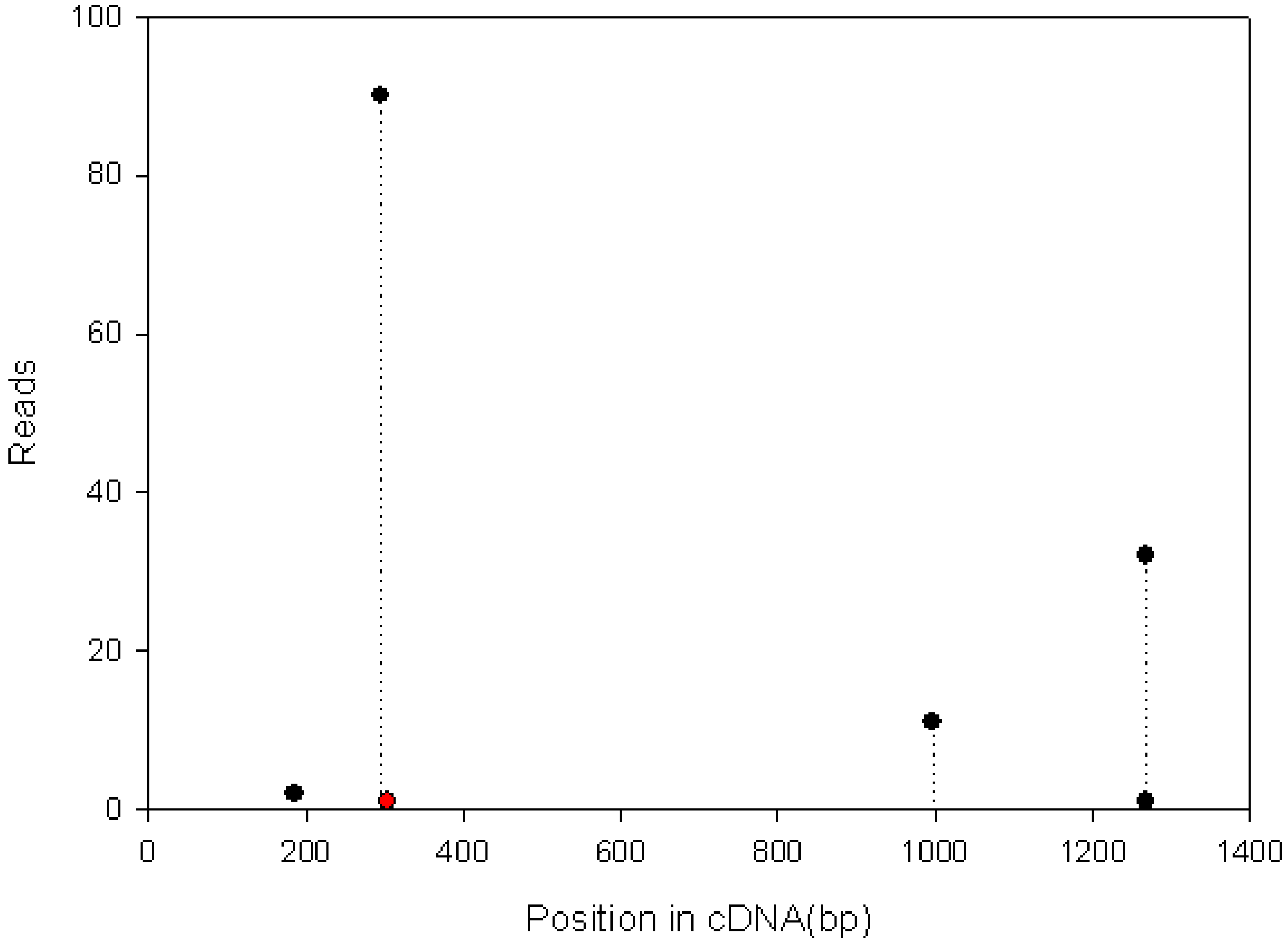


```
5' AGUUGAAGGGCCUUUCUAGAGCACUG 3'        Cs8g13286.1
     :::: ::::::::::: ::::
3' --AACUCCCCGGAAAGAUGUCGU--- 5'        Csi-miR3952.1
```

Csi-miR3952.2, target=Cs8g13286.1 gene=Cs8g13286
Category:3
Score=3
Cleavage Site=304

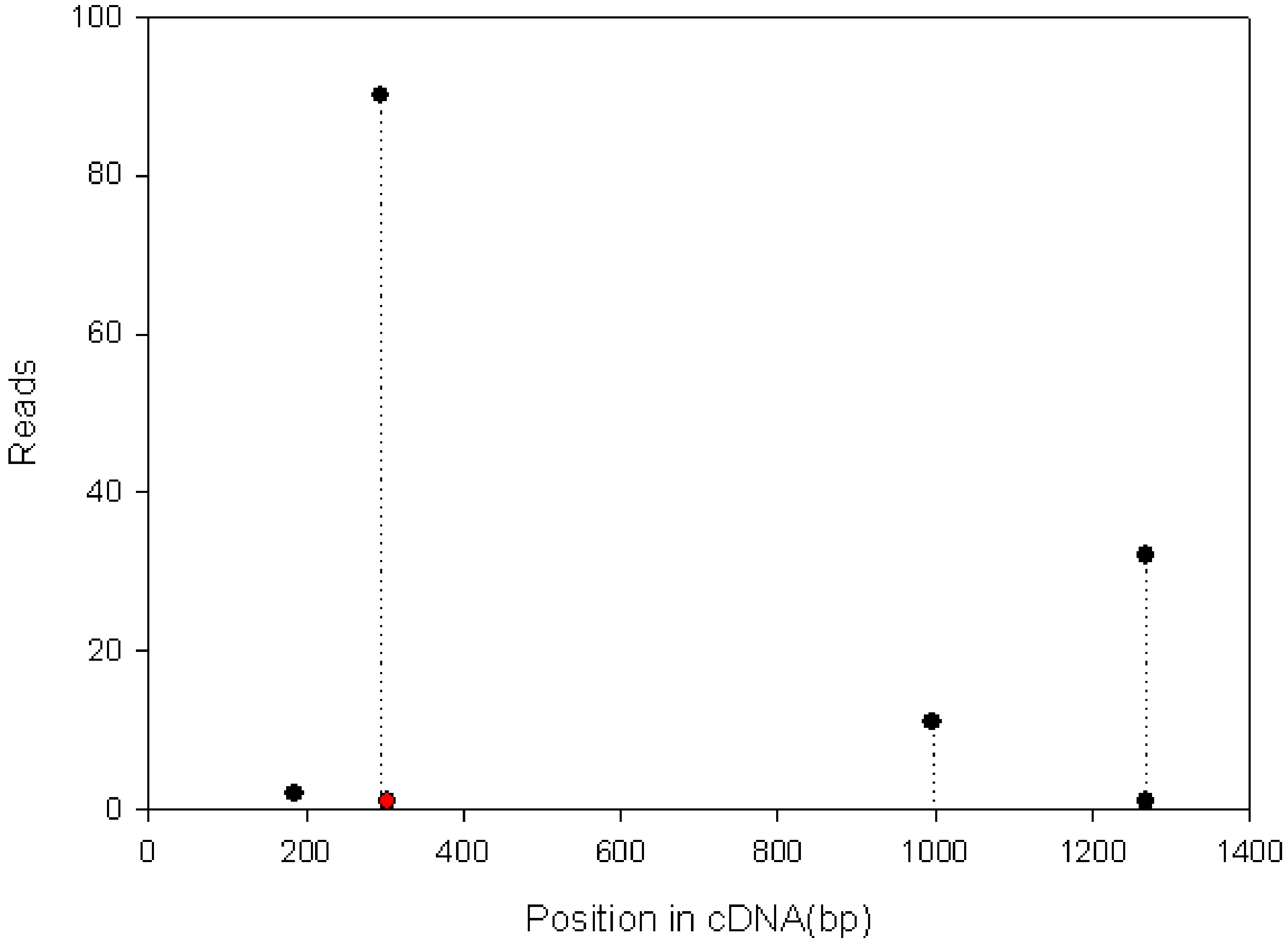


```
5' AGUUGAAGGGCCUUUCUAGAGCACUG 3'        Cs8g13286.1
   ::::: ::::::::::: :::
3' -CAACUCCCCGGAAAGAUGUCG---- 5'        Csi-miR3952.2
```

Csi-miR3952-3p, target=Cs8g13286.1 gene=Cs8g13286
Category:3
Score=3
Cleavage Site=185

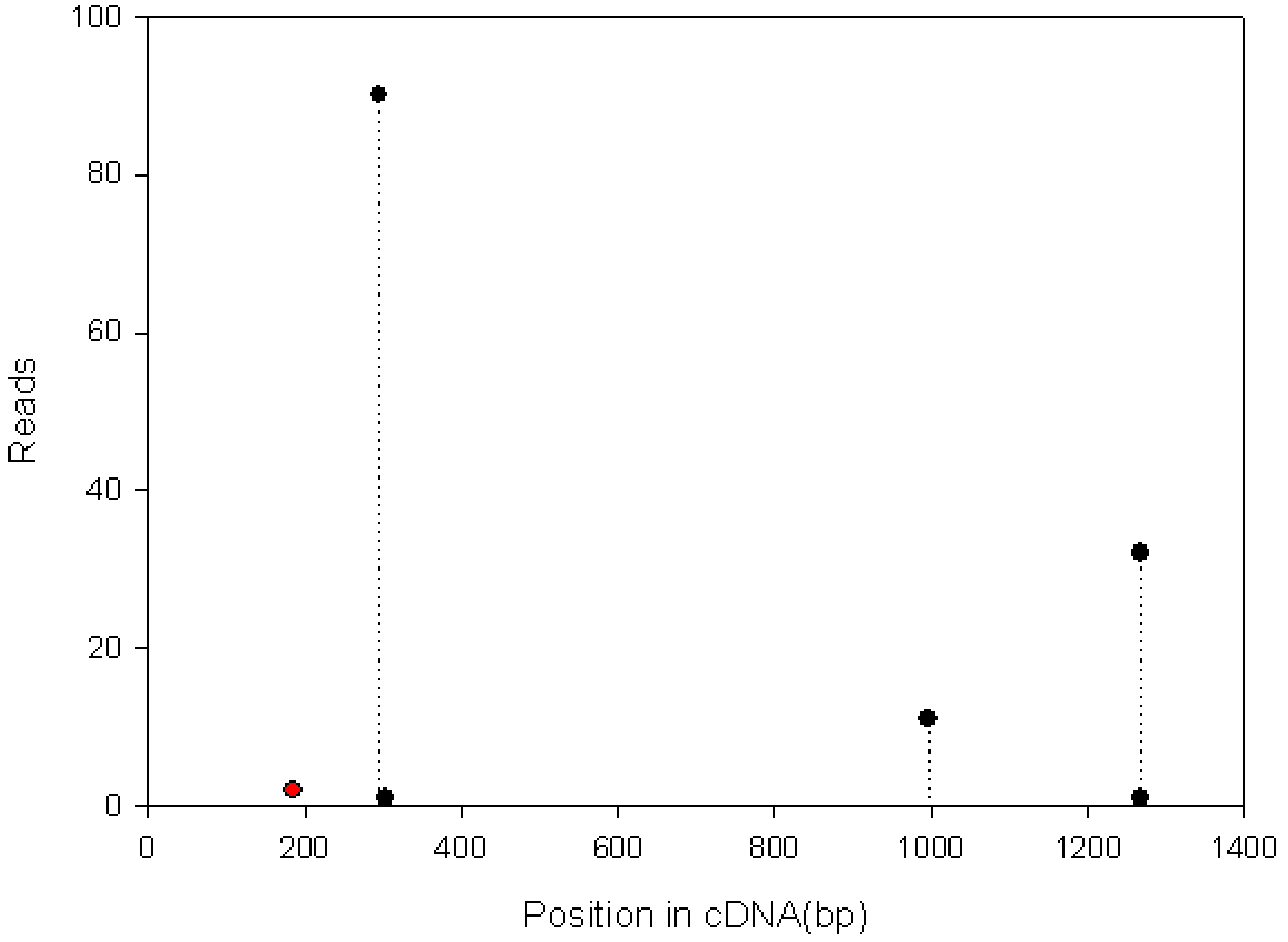


```
5' UGUGCUGUAGAAAGGCCCCUCAACCU 3'        Cs8g13286.1
    ::::: :::::::::::: :::
3' -CACGAGAUCUUUCCGGGAAGU---- 5'        Csi-miR3952-3p
```

Csi-miR3954a, target=Cs1g09600.1 gene=Cs1g09600
Category:3
Score=1
Cleavage Site=416

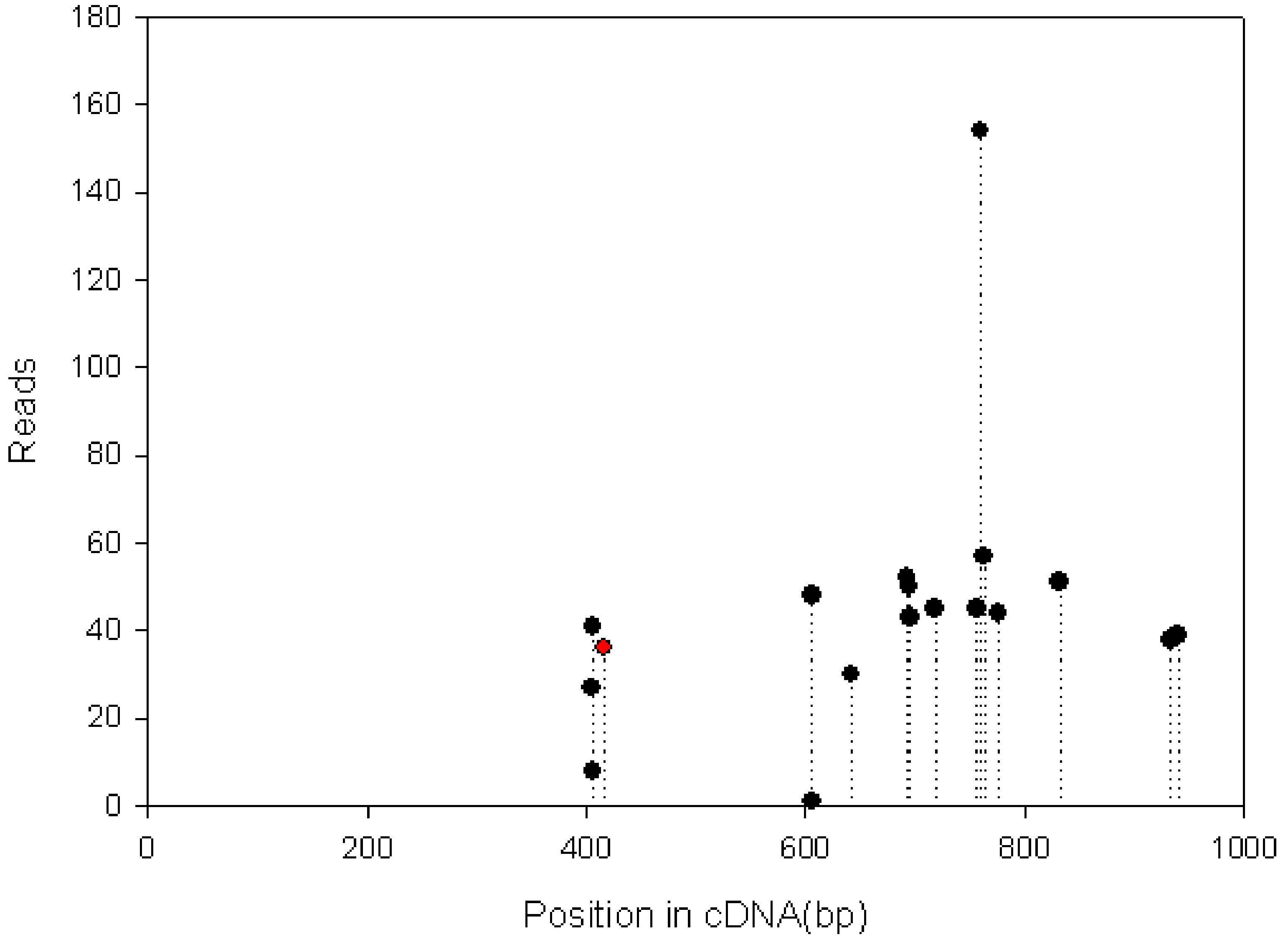


```
5' UGACCCUGAUUUCUCUGUCCAAGCAA 3'        Cs1g09600.1
   ::::: ::::::::::::::::
3' ACUGGCACUAAAGAGACAGGUU---- 5'        Csi-miR3954a
```

Csi-miR3954a, target=Cs5g04670.1 gene=Cs5g04670
Category:2
Score=1
Cleavage Site=142

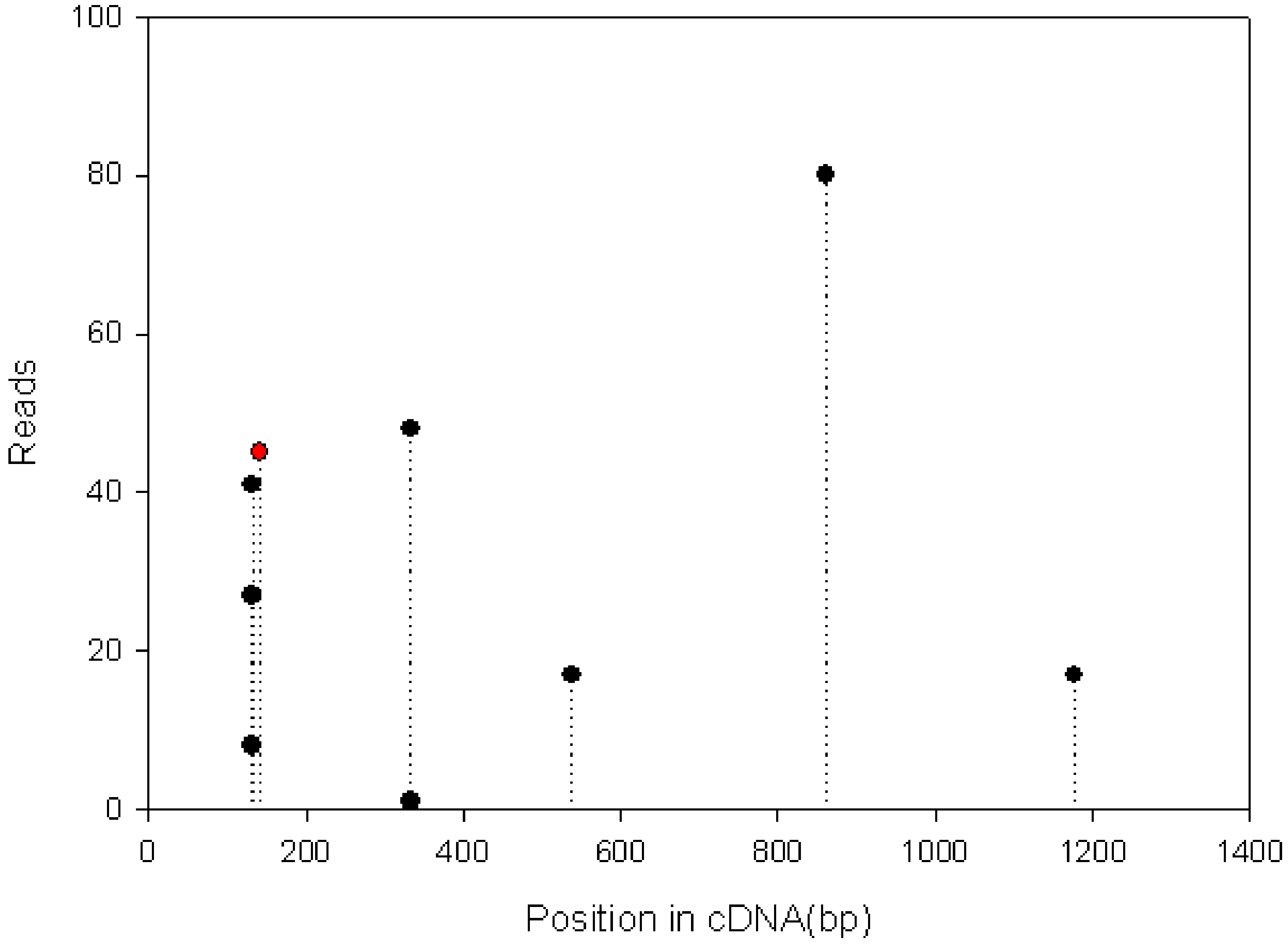


```
5' UGACCCUGAUUUCUCUGUCCAAACAA 3'        Cs5g04670.1
   ::::: ::::::::::::::::
3' ACUGGCACUAAAGAGACAGGUU---- 5'        Csi-miR3954a
```

Csi-miR3954b, target=Cs1g09600.1 gene=Cs1g09600
Category:3
Score=1
Cleavage Site=416

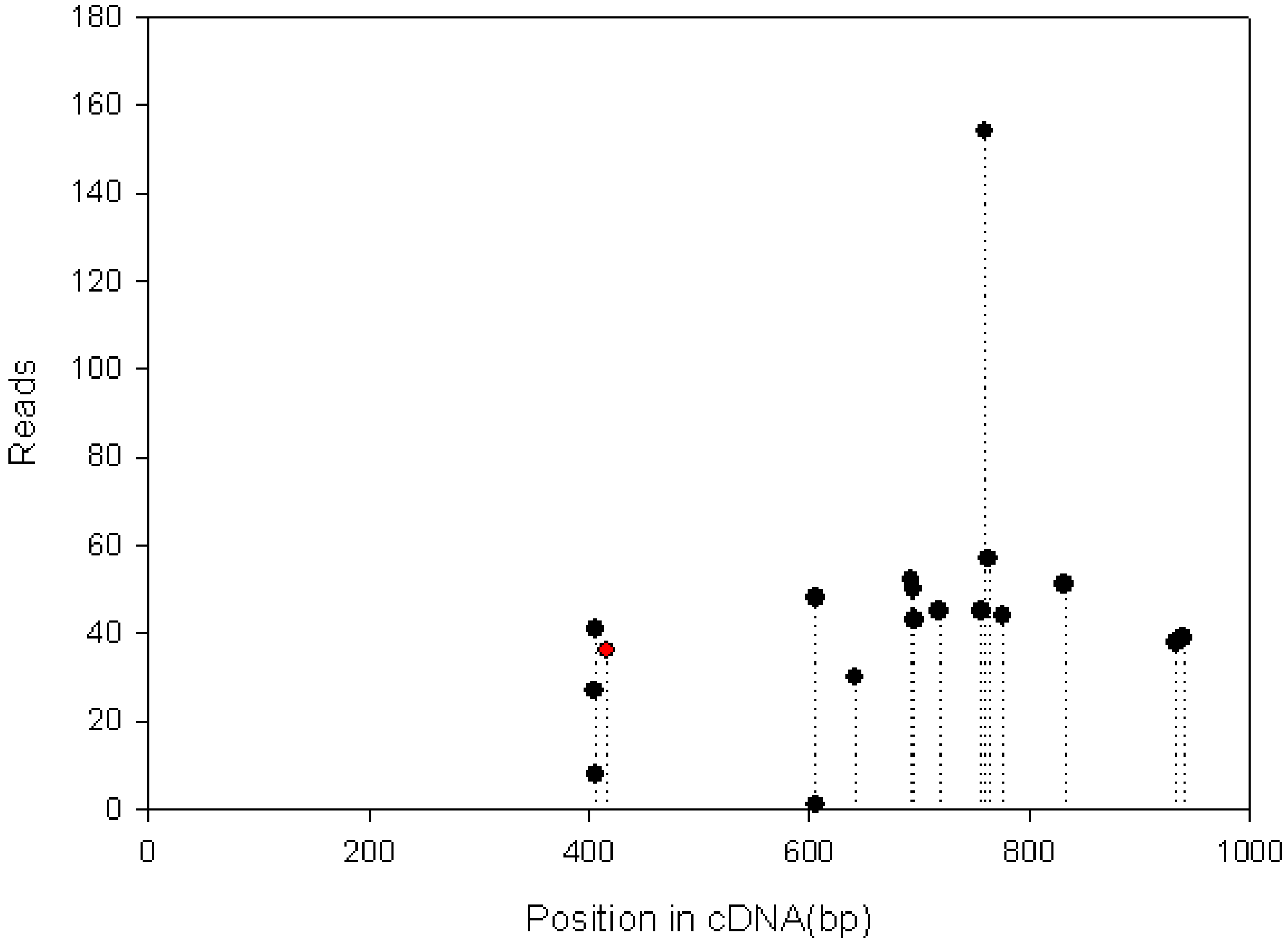


```
5' UGACCCUGAUUUCUCUGUCCAAGCAA 3'        Cs1g09600.1
   ::::: ::::::::::::::
3' ACUGGCACUAAAGAGACAGG------ 5'        Csi-miR3954b
```

Csi-miR3954b, target=Cs2g28990.1 gene=Cs2g28990
Category:3
Score=4.5
Cleavage Site=1505

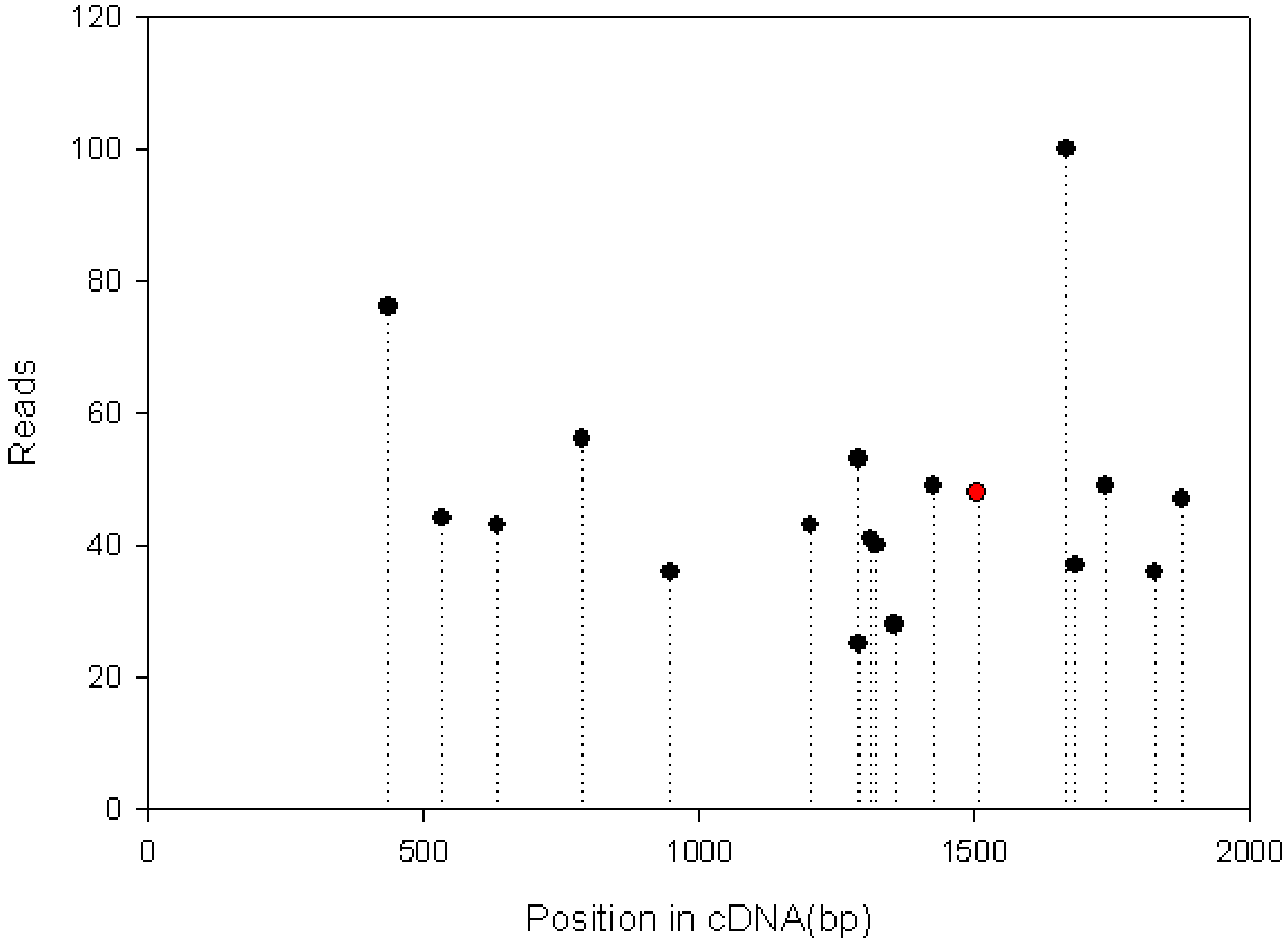


```
5' UGAGU-UGAUUUCUUUGUCAAAAGGAC 3'       Cs2g28990.1
   ::: . :::::::::.::::
3' ACUGGCACUAAAGAGACAGG------- 5'       Csi-miR3954b
```

Csi-miR3954b, target=Cs5g04670.1 gene=Cs5g04670
Category:2
Score=1
Cleavage Site=142

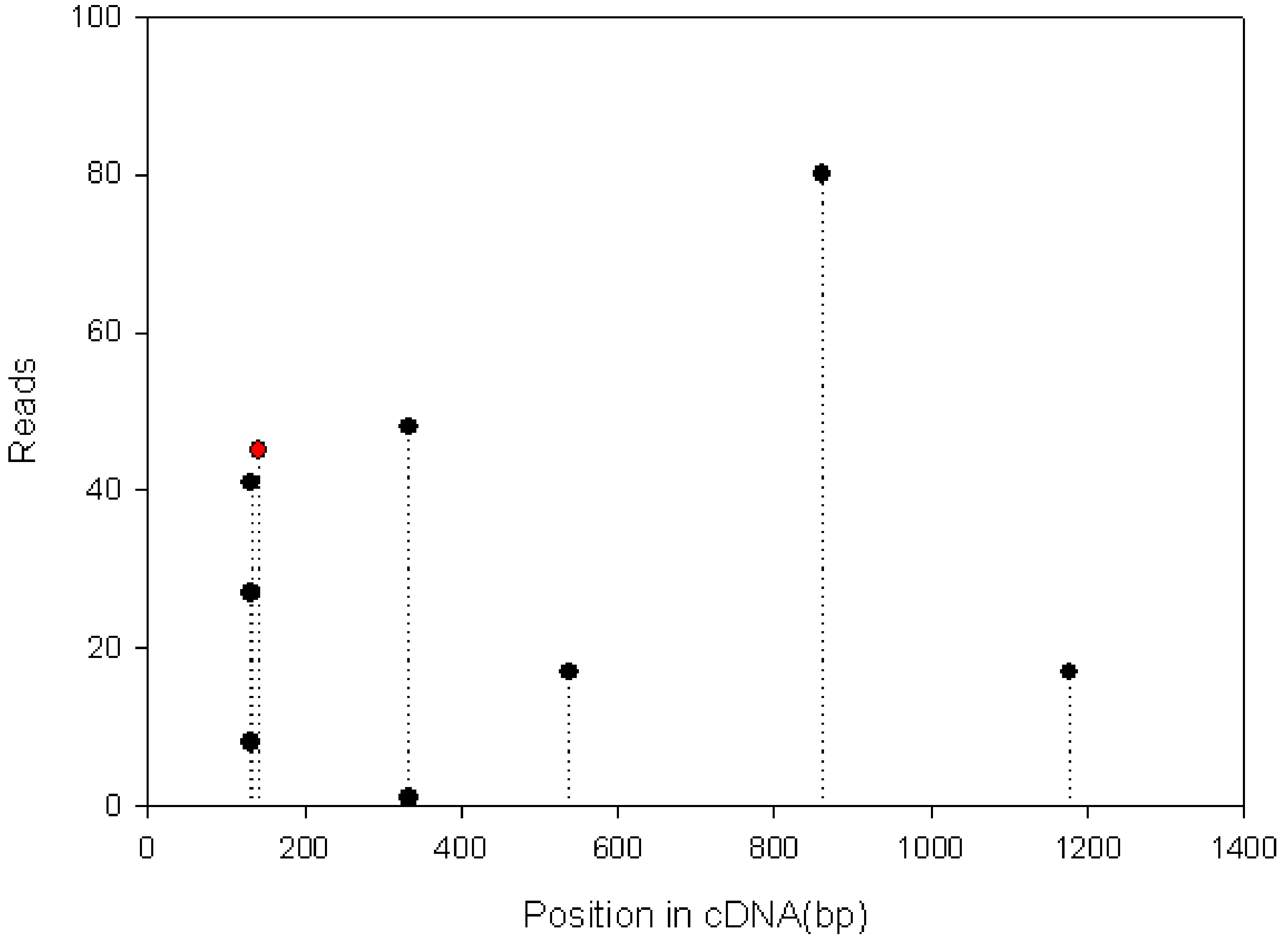


```
5' UGACCCUGAUUUCUCUGUCCAAACAA 3'        Cs5g04670.1
   ::::: ::::::::::::::
3' ACUGGCACUAAAGAGACAGG------ 5'        Csi-miR3954b
```

Csi-miR396a, target=Cs3g07260.1 gene=Cs3g07260
Category:3
Score=5
Cleavage Site=2418

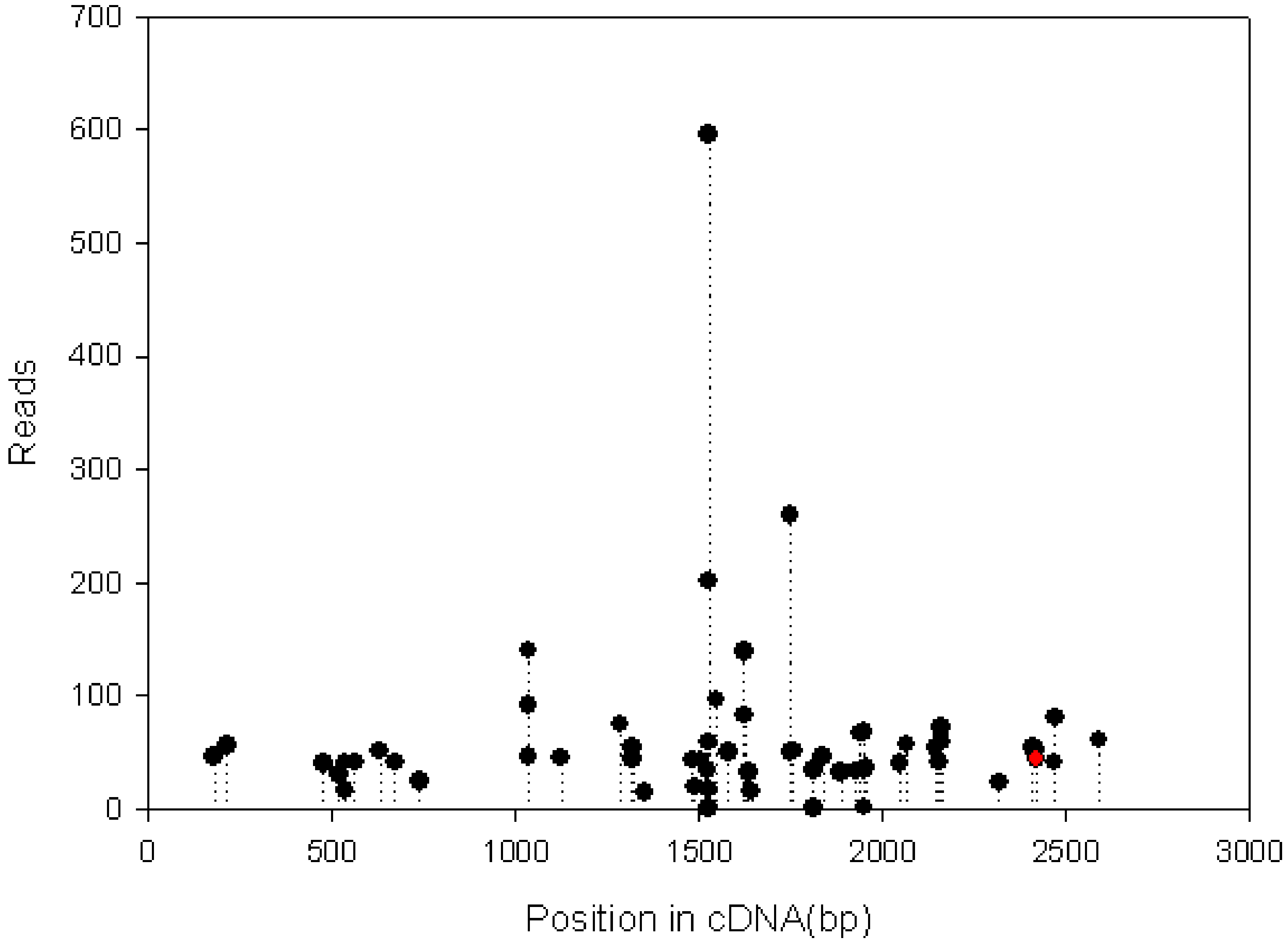


```
5' UUAUUCCAGGAAAGUUGUGGGAUAAA 3'         Cs3g07260.1
    .: :  ::.::::::.:::::.:
3' -GUCAAGUUCUUUCGACACCUU---- 5'         Csi-miR396a
```

Csi-miR396a, target=Cs3g23180.1 gene=Cs3g23180
Category: 3
Score=4
Cleavage Site=625

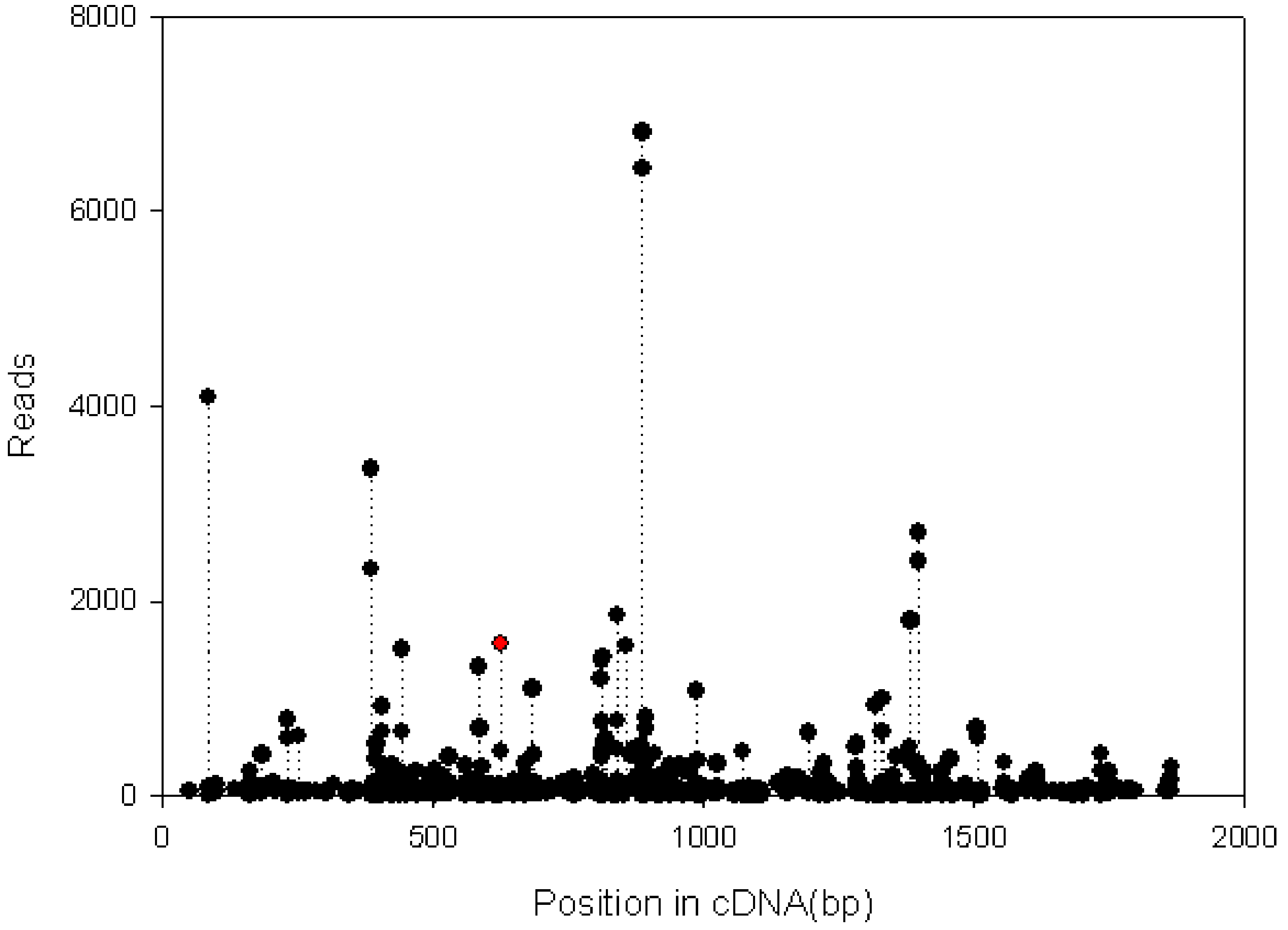


```
5' AAAGAUCAAGGAAGCUGUGGGAGUUG 3'         Cs3g23180.1
     :: :::::.:::::::::.:
3' -GUCAAGUUCUUUCGACACCUU---- 5'         Csi-miR396a
```

Csi-miR396a, target=Cs5g01380.1 gene=Cs5g01380
Category:1
Score=3
Cleavage Site=1181

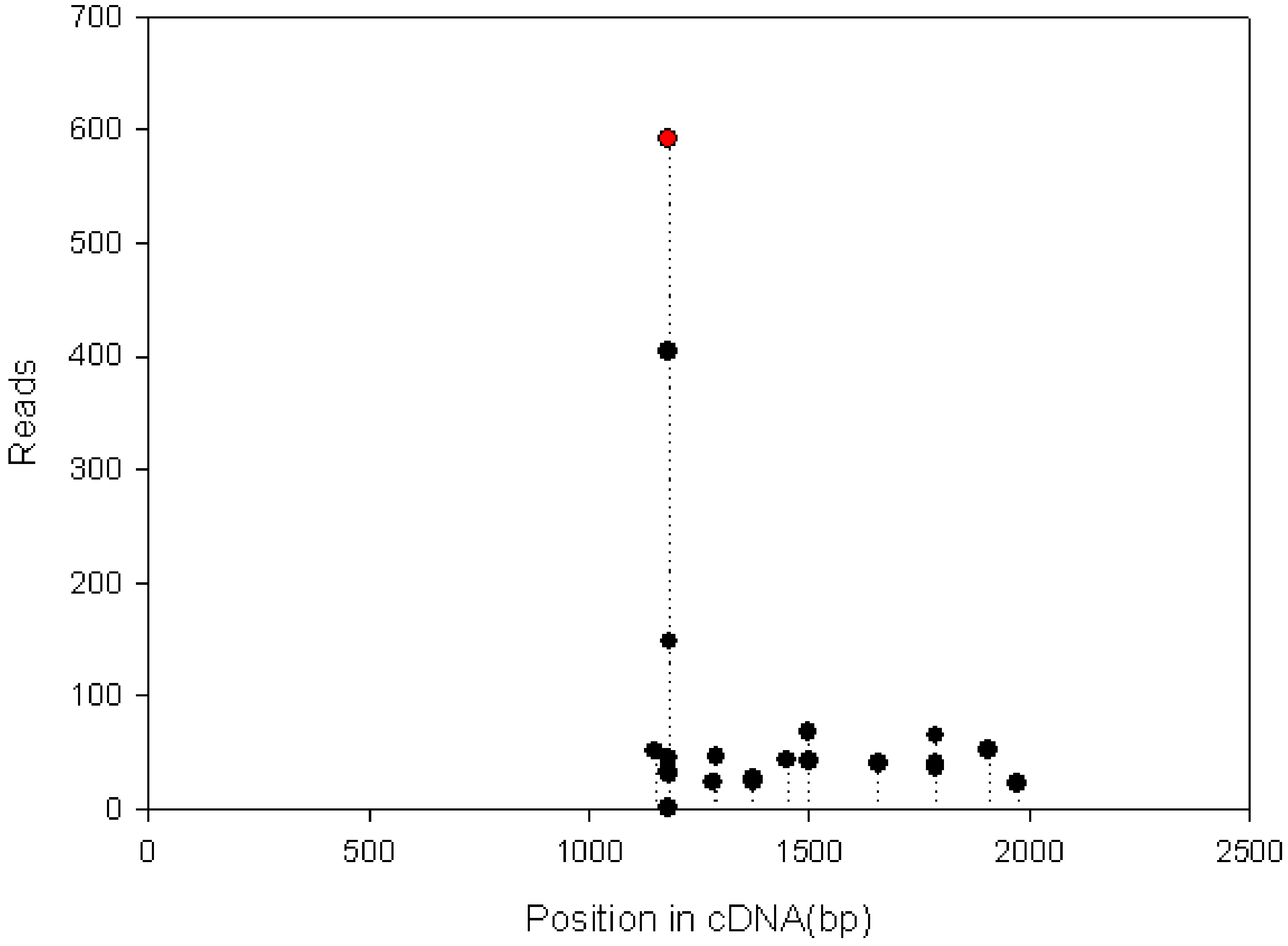


```
5' CCCGUUCAAGAAAGCCUGUGGAAUUA 3'        Cs5g01380.1
     : ::::::::::: ::::::::
3' -GUCAAGUUCUUUC-GACACCUU--- 5'        Csi-miR396a
```

Csi-miR396a, target=Cs5g19200.1 gene=Cs5g19200
Category:2
Score=4.5
Cleavage Site=201

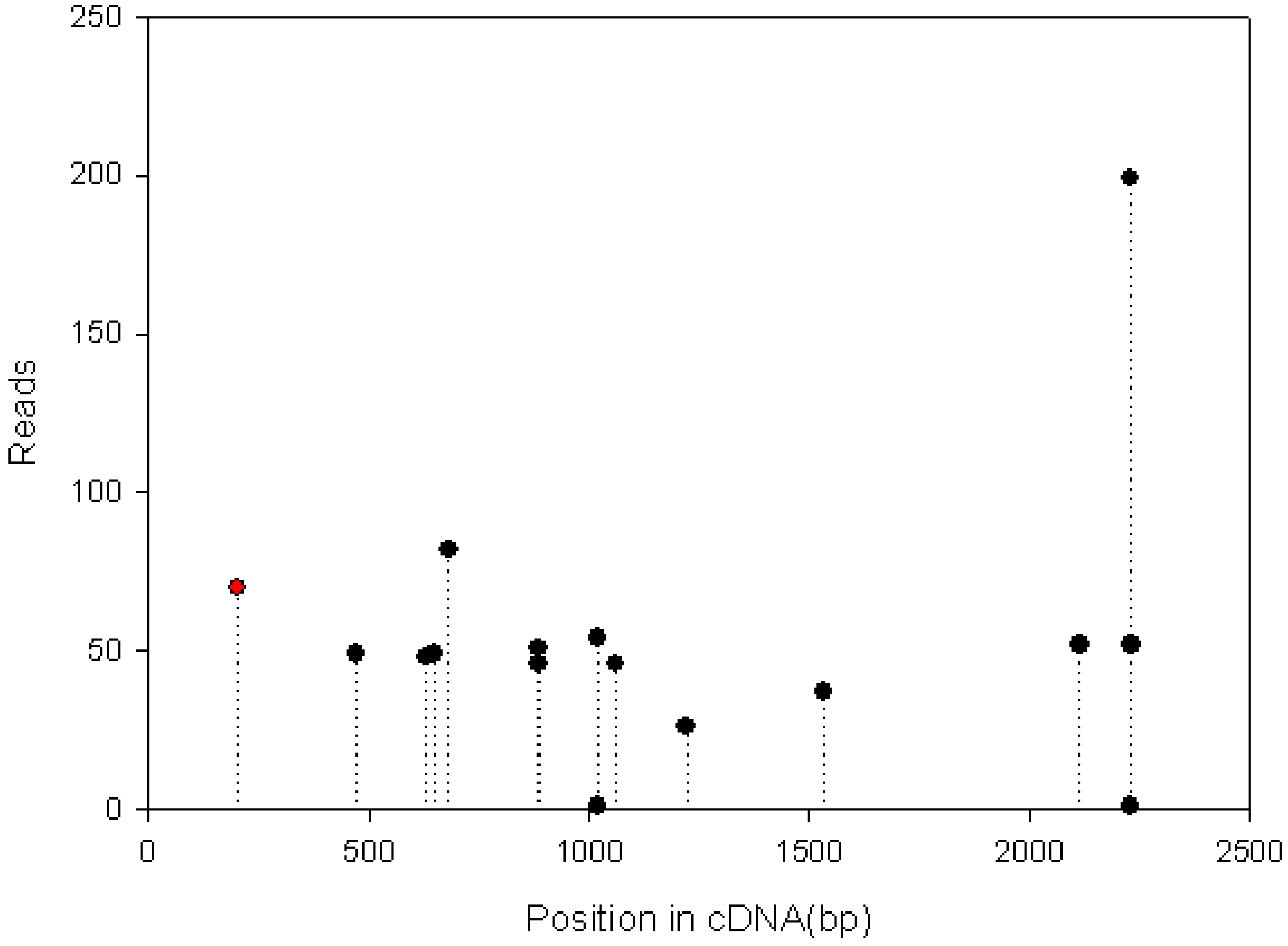


```
5' AAUGUUUAAGAAAGCAGUGGAAGCAA 3'        Cs5g19200.1
     :::.::::::::: ::::::
3' -GUCAAGUUCUUUCGACACCUU---- 5'        Csi-miR396a
```

Csi-miR396a, target=Cs7g12930.1 gene=Cs7g12930
Category:1
Score=4
Cleavage Site=1079

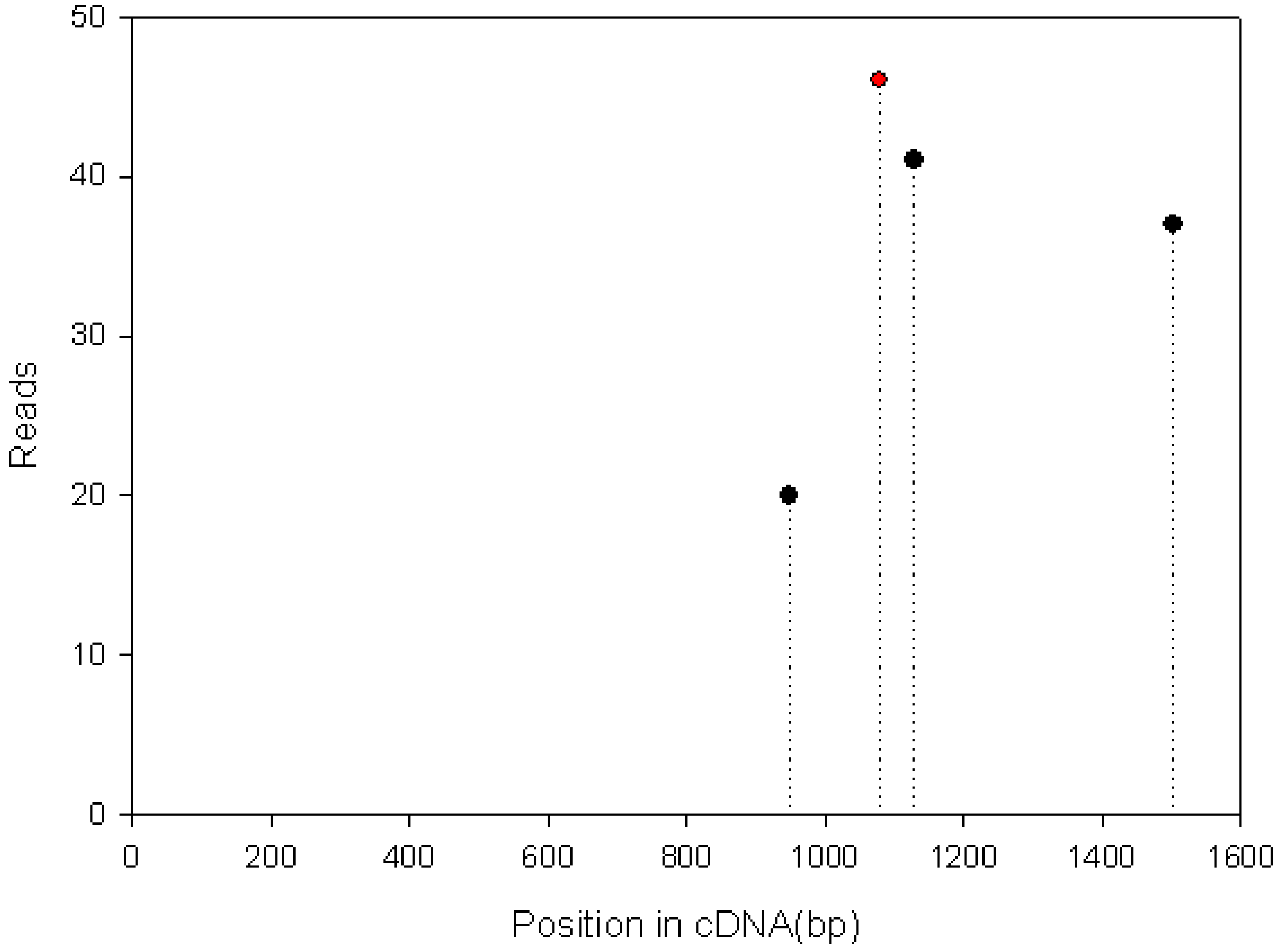


```
5' UAGUAUUAAGAAAGCUGAGGAAAAAG 3'        Cs7g12930.1
    .::: :.:::::::::: ::::
3' GUCA-AGUUCUUUCGACACCUU---- 5'        Csi-miR396a
```

Csi-miR396a, target=Cs7g15220.1 gene=Cs7g15220
Category:1
Score=3
Cleavage Site=377

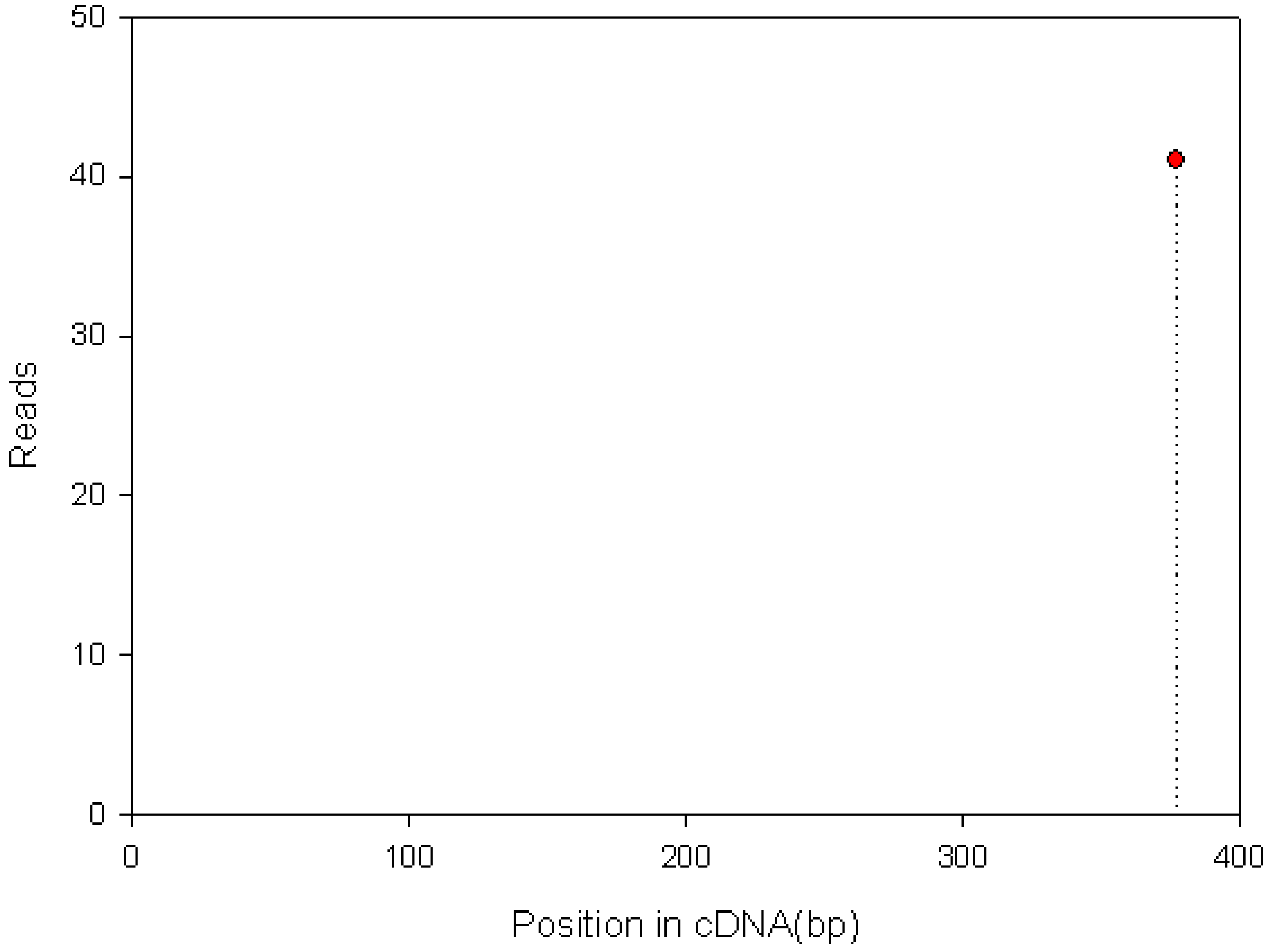


```
5' ACCGUUCAAGAAAGCCUGUGGAAUCA 3'        Cs7g15220.1
    : ::::::::::: ::::::::
3' -GUCAAGUUCUUUC-GACACCUU--- 5'        Csi-miR396a
```

Csi-miR396a, target=Orange1.1t02254.1 gene=Orange1.1t02254
Category:3
Score=4
Cleavage Site=1371

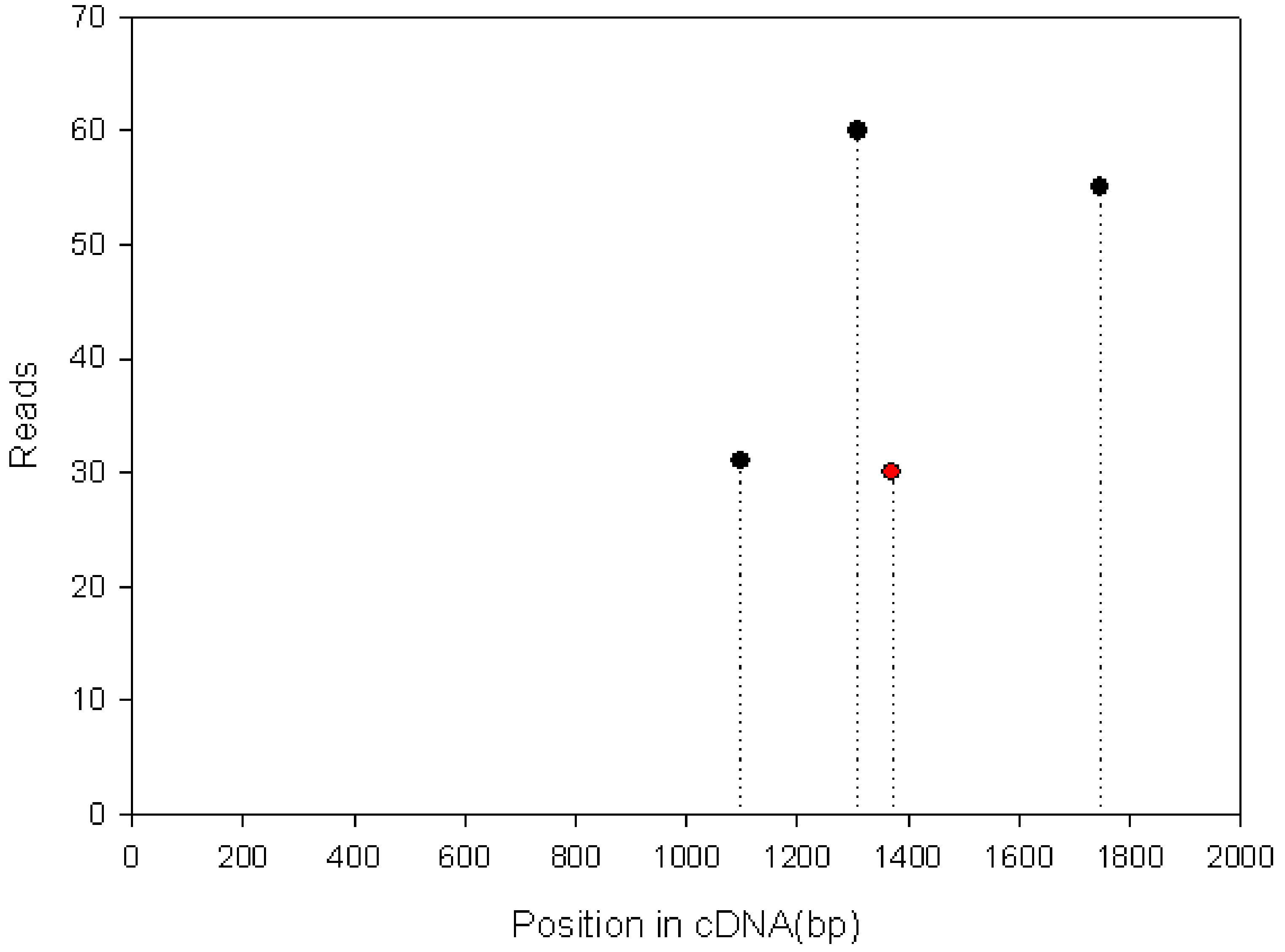


```
5' GUUGCUGAAGAAAGCUGUGGAGGAUC 3'        Orange1.1t02254.1
    . : : ::::::::::::::.
3' -GUCAAGUUCUUUCGACACCUU---- 5'        Csi-miR396a
```

Csi-miR396a, target=Orange1.1t03122.1 gene=Orange1.1t03122
Category:2
Score=3
Cleavage Site=583

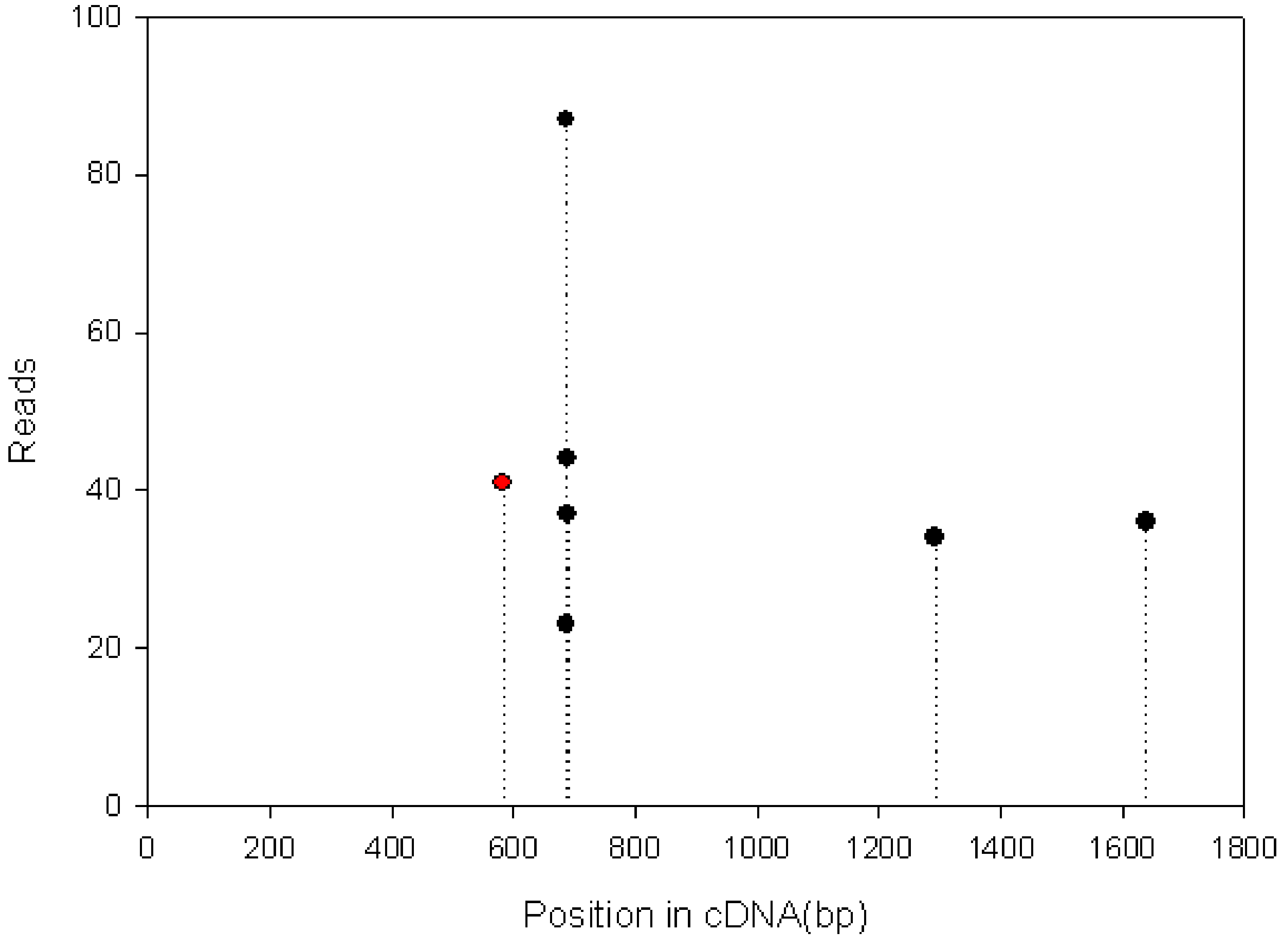


```
5' ACCGUUCAAGAAAGCCUGUGGAAUCA 3'        Orange1.1t03122.1
    : :::::::::::: ::::::::
3' -GUCAAGUUCUUUC-GACACCUU--- 5'        Csi-miR396a
```

Csi-miR396b.1, target=Cs3g07260.1 gene=Cs3g07260
Category:3
Score=4.5
Cleavage Site=2420

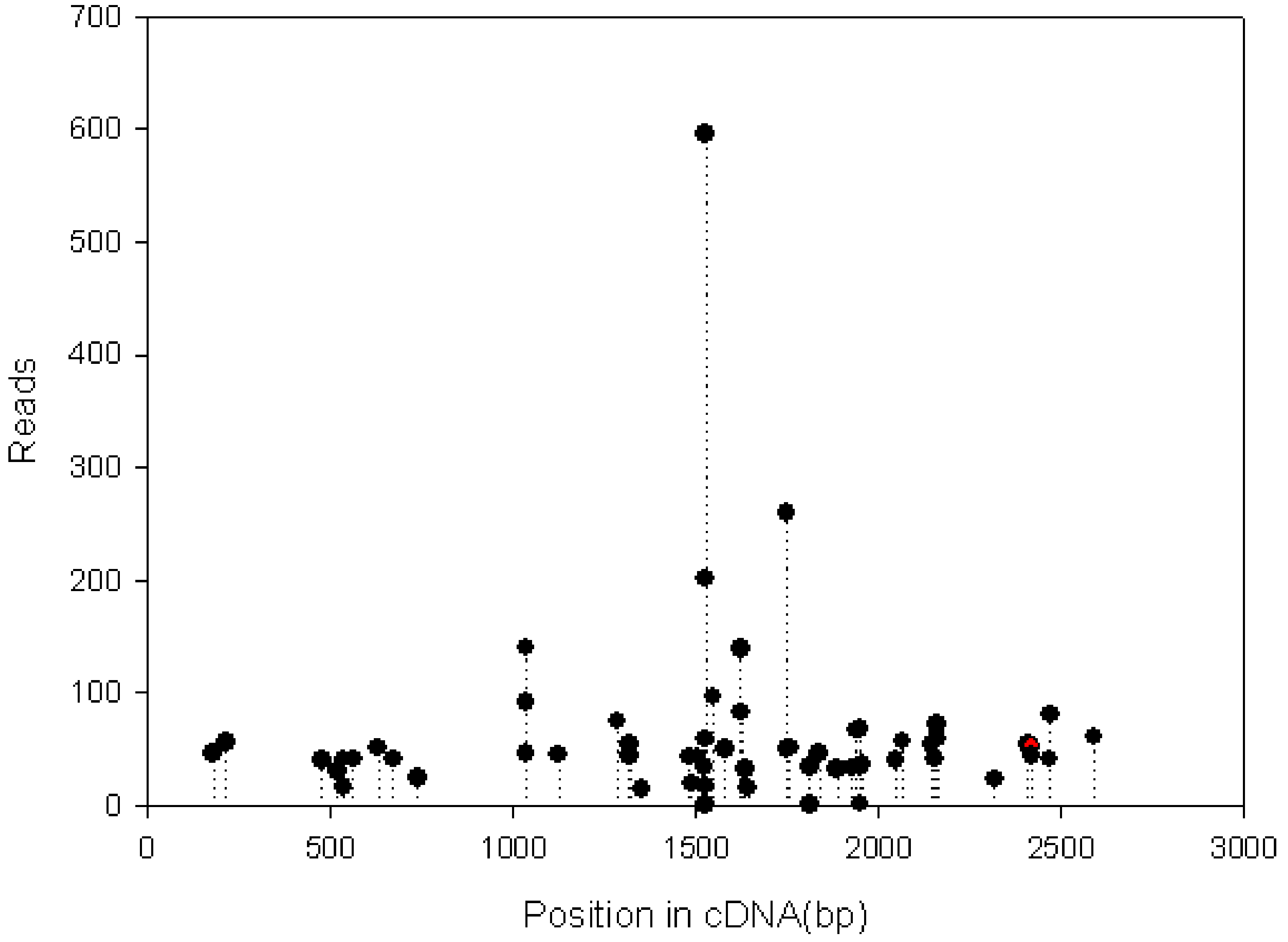


```
5' AUUCCAGGAAAGUUGUGGGAUAAAUA 3'        Cs3g07260.1
     : ::.::::.:::::.:
3' -CAAGUUCUUUCGACACCUU------ 5'        Csi-miR396b.1
```

Csi-miR396b.1, target=Cs3g23180.1 gene=Cs3g23180
Category: 3
Score=3
Cleavage Site=625

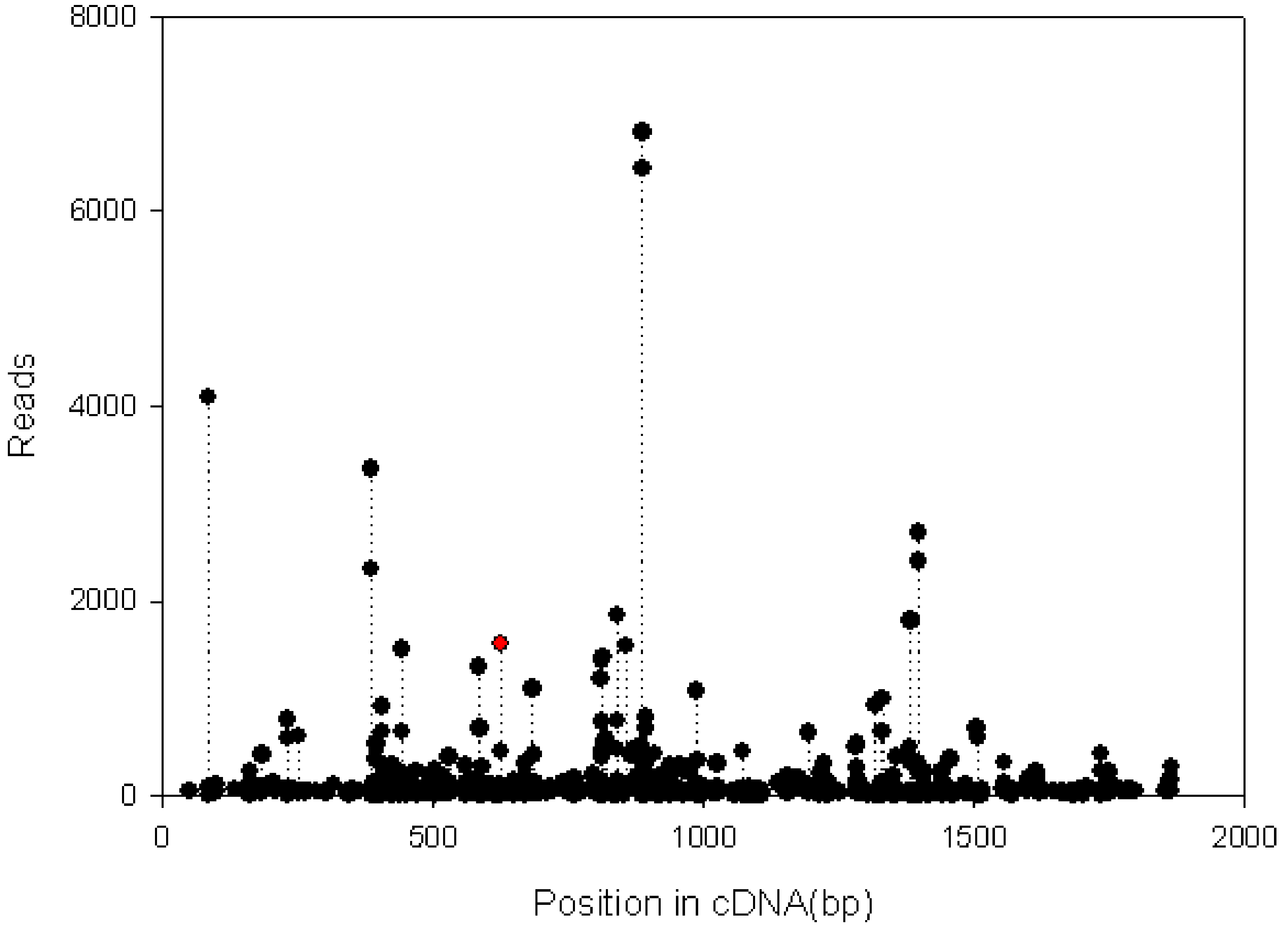


```
5' AAAGAUCAAGGAAGCUGUGGGAGUUG 3'        Cs3g23180.1
      : :::::.:::::::::.:
3' ---CAAGUUCUUUCGACACCUU---- 5'        Csi-miR396b.1
```

Csi-miR396b.1, target=Cs5g01380.1 gene=Cs5g01380
Category:1
Score=2
Cleavage Site=1181

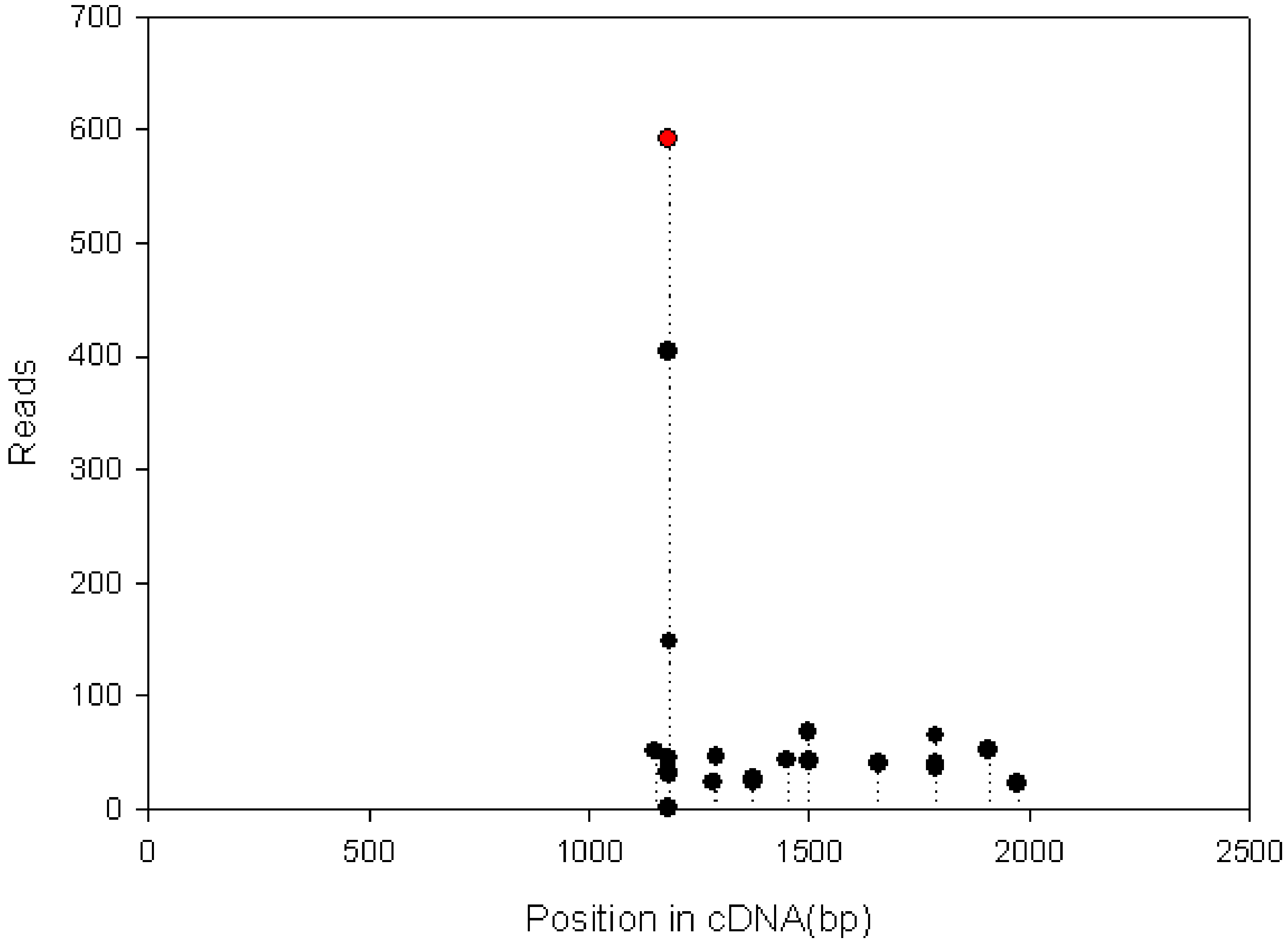


```
5' CCCGUUCAAGAAAGCCUGUGGAAUUA 3'        Cs5g01380.1
      ::::::::::: ::::::::
3' ---CAAGUUCUUUC-GACACCUU--- 5'        Csi-miR396b.1
```

Csi-miR396b.1, target=Cs5g19200.1 gene=Cs5g19200
Category:2
Score=2.5
Cleavage Site=201

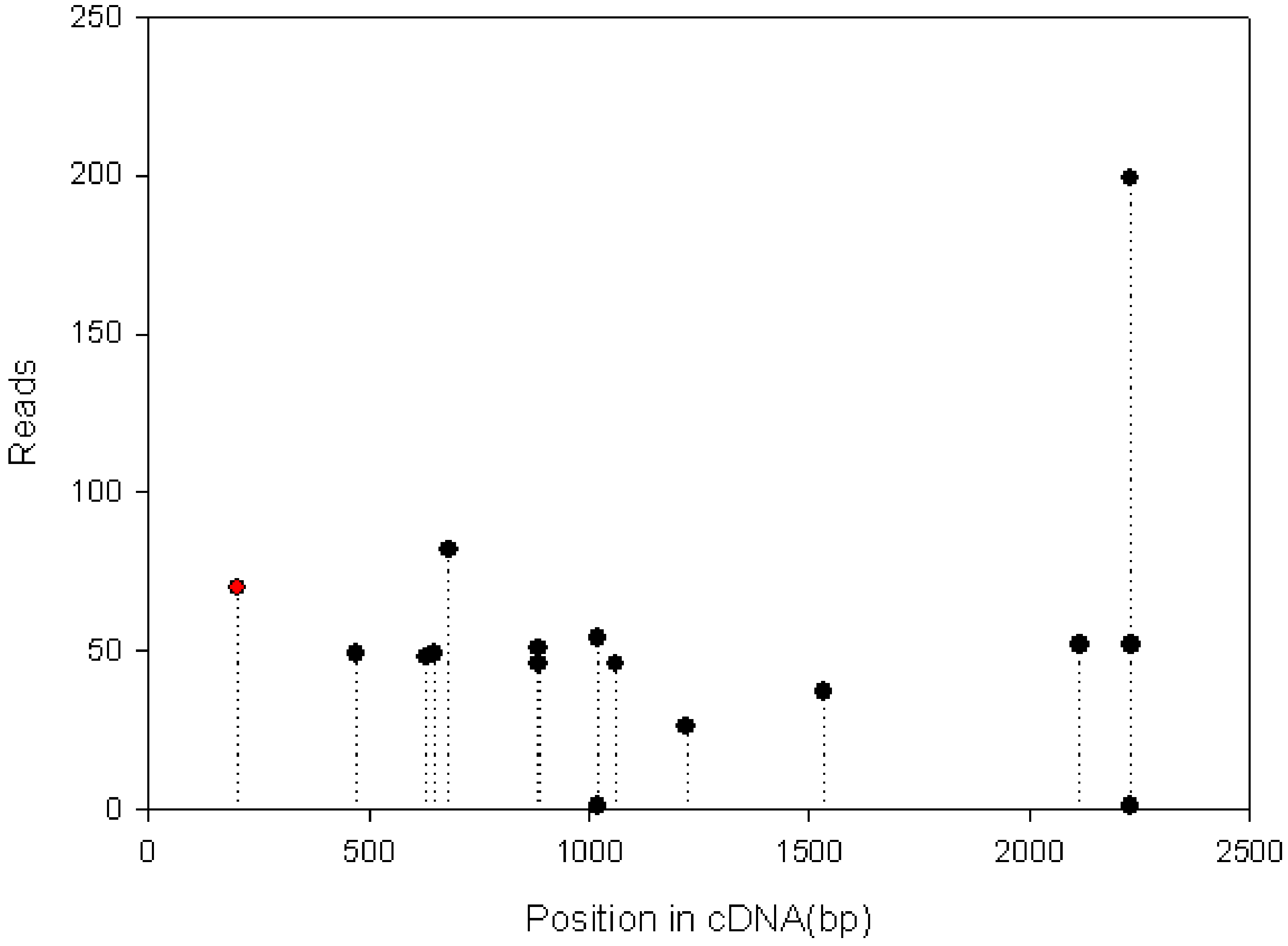


```
5' AAUGUUUAAGAAAGCAGUGGAAGCAA 3'        Cs5g19200.1
     :::.::::::::: ::::::
3' ---CAAGUUCUUUCGACACCUU---- 5'        Csi-miR396b.1
```

Csi-miR396b.1, target=Cs7g12930.1 gene=Cs7g12930
Category:1
Score=3.5
Cleavage Site=1079

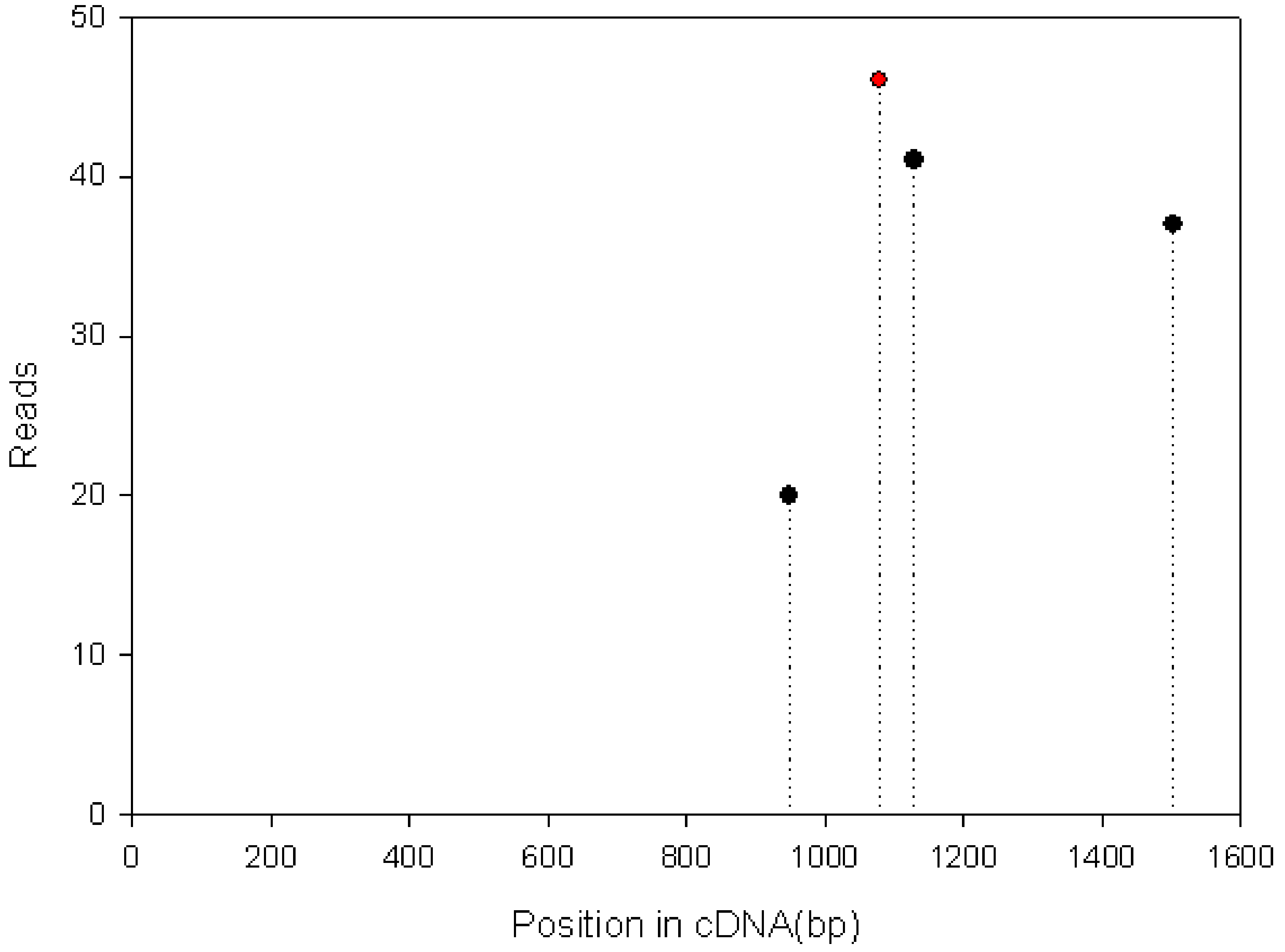


```
5' UAGUAUUAAGAAAGCUGAGGAAAAAG 3'        Cs7g12930.1
     :: :.:::::::::::: ::::
3' --CA-AGUUCUUUCGACACCUU---- 5'        Csi-miR396b.1
```

Csi-miR396b.1, target=Cs7g15220.1 gene=Cs7g15220
Category:1
Score=2
Cleavage Site=377

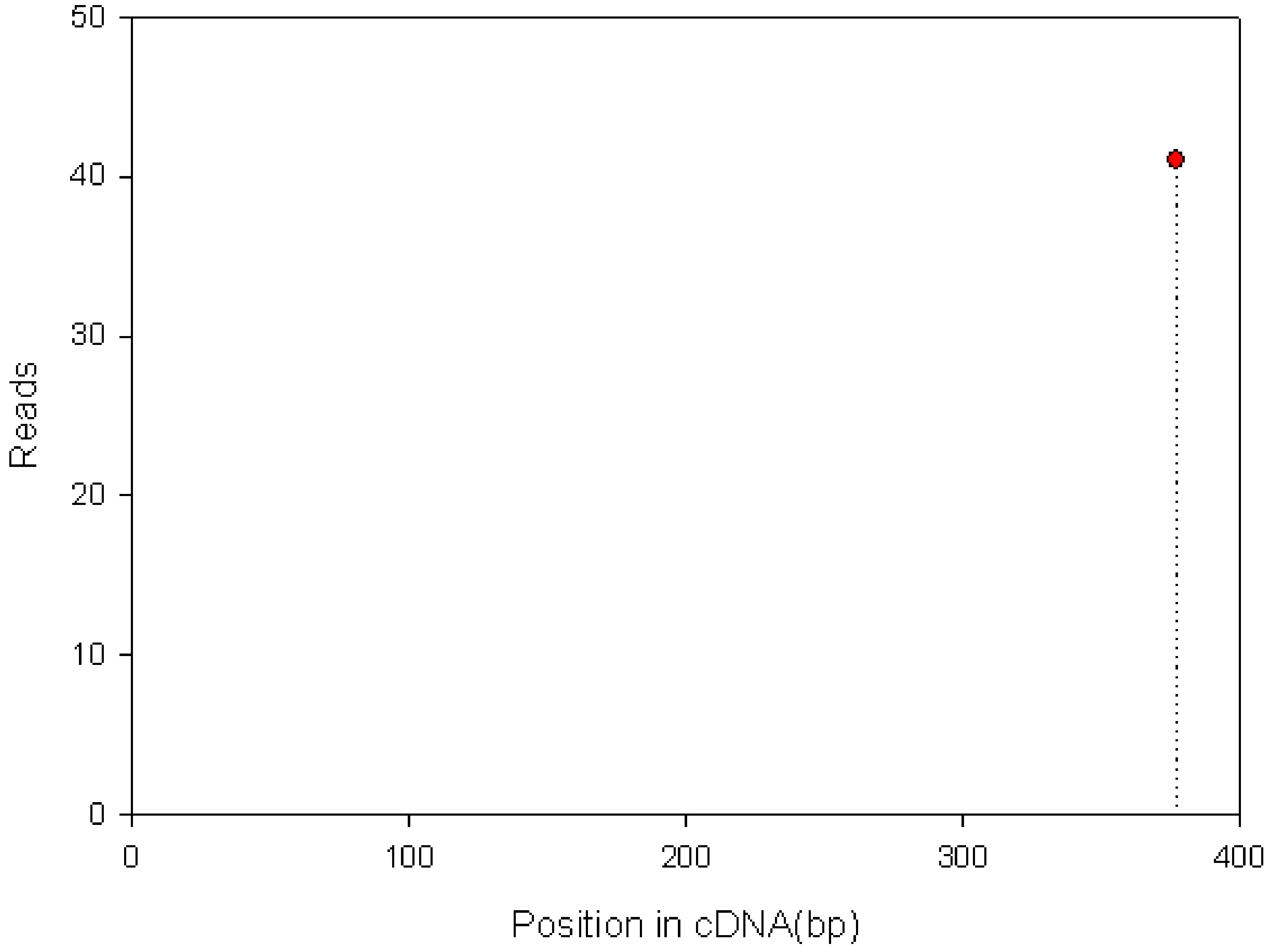


```
5' ACCGUUCAAGAAAGCCUGUGGAAUCA 3'        Cs7g15220.1
     ::::::::::: ::::::::
3' ---CAAGUUCUUUC-GACACCUU--- 5'        Csi-miR396b.1
```

Csi-miR396b.1, target=Orange1.1t02254.1 gene=Orange1.1t02254
Category:3
Score=2.5
Cleavage Site=1371

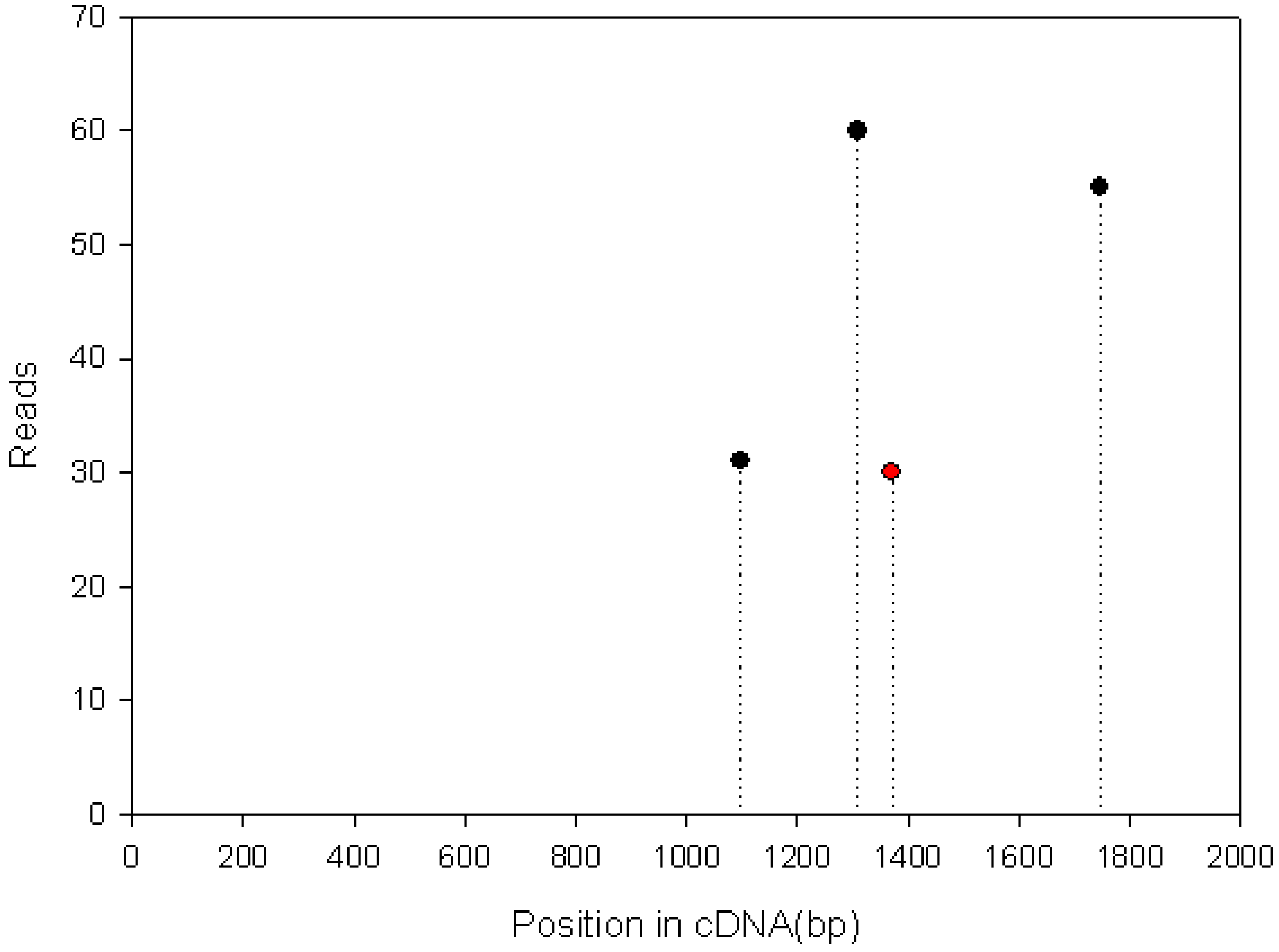


```
5' GUUGCUGAAGAAAGCUGUGGAGGAUC 3'        Orange1.1t02254.1
      : :  ::::::::::::::.
3' ---CAAGUUCUUUCGACACCUU---- 5'        Csi-miR396b.1
```

Csi-miR396b.1, target=Orange1.1t02555.1 gene=Orange1.1t02555
Category:1
Score=2
Cleavage Site=920

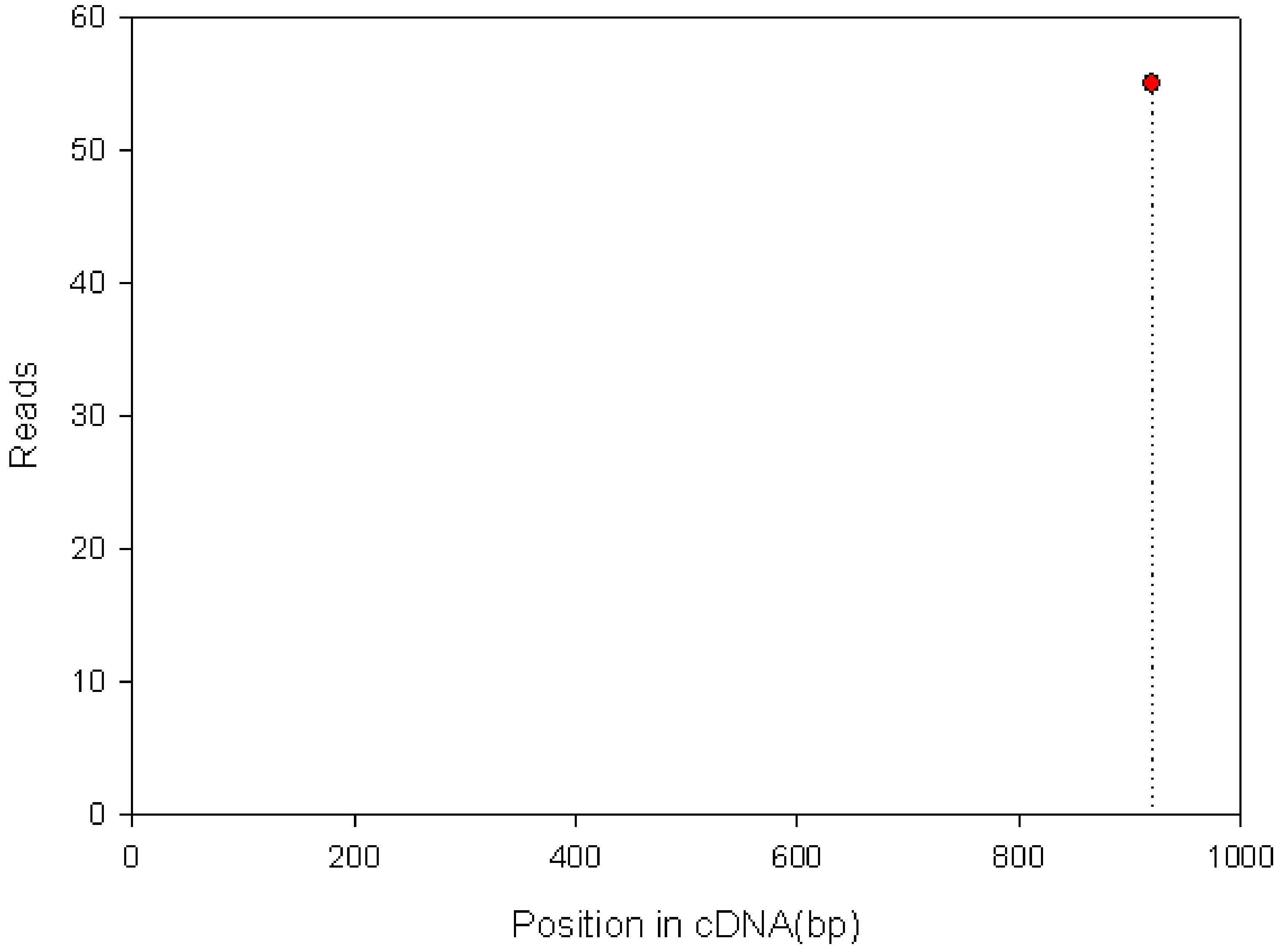


```
5' CACGUUCAAGAAAGCCUGUGGAACUU 3'        Orange1.1t02555.1
       ::::::::::: ::::::::
3' ---CAAGUUCUUUC-GACACCUU--- 5'        Csi-miR396b.1
```

Csi-miR396b.1, target=Orange1.1t03122.1 gene=Orange1.1t03122
Category:2
Score=2
Cleavage Site=583

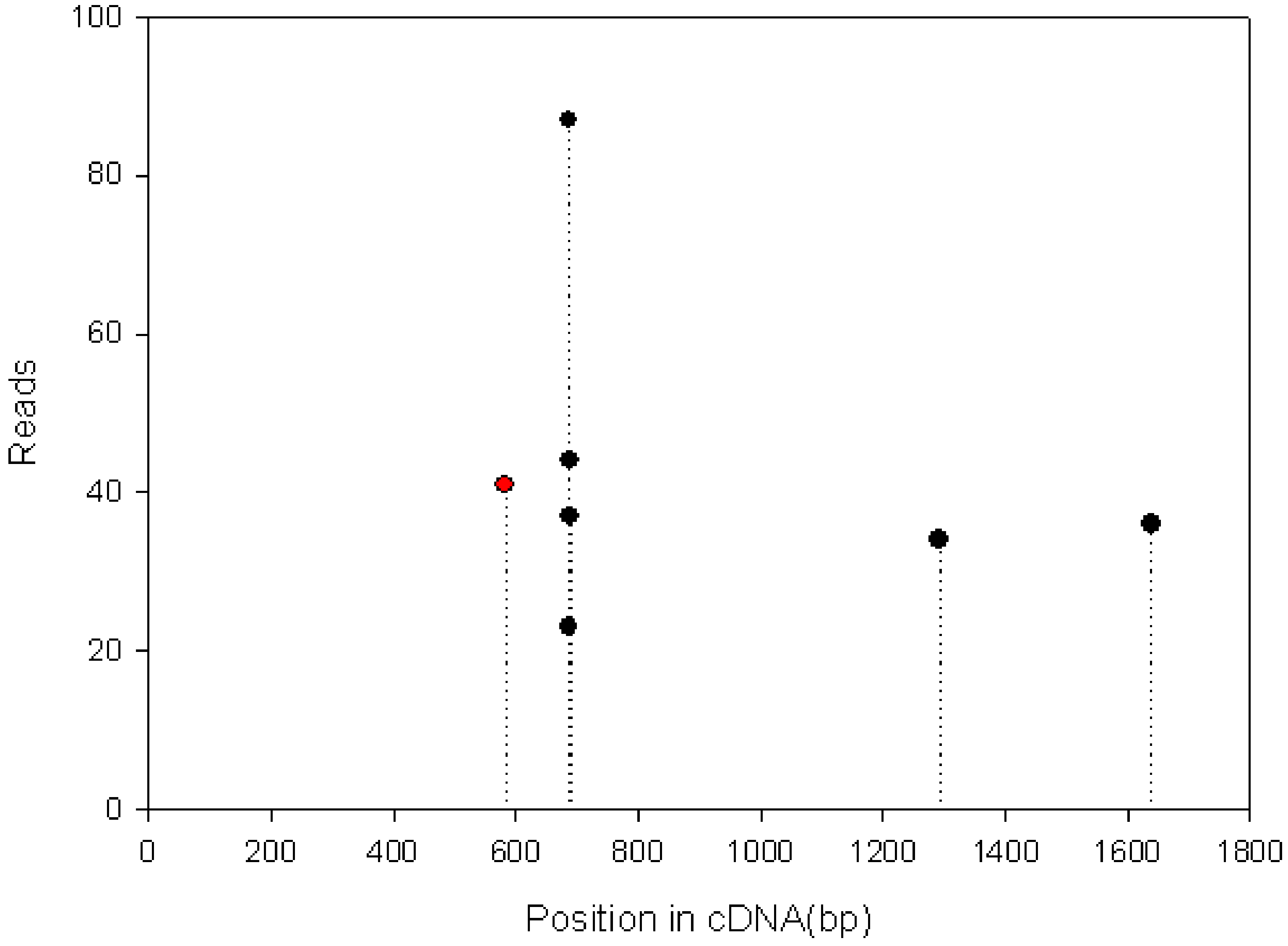


```
5' ACCGUUCAAGAAAGCCUGUGGAAUCA 3'        Orange1.1t03122.1
      ::::::::::: ::::::::
3' ---CAAGUUCUUUC-GACACCUU--- 5'        Csi-miR396b.1
```

Csi-miR396b.2, target=Cs1g21890.1 gene=Cs1g21890
Category:3
Score=4.5
Cleavage Site=1329

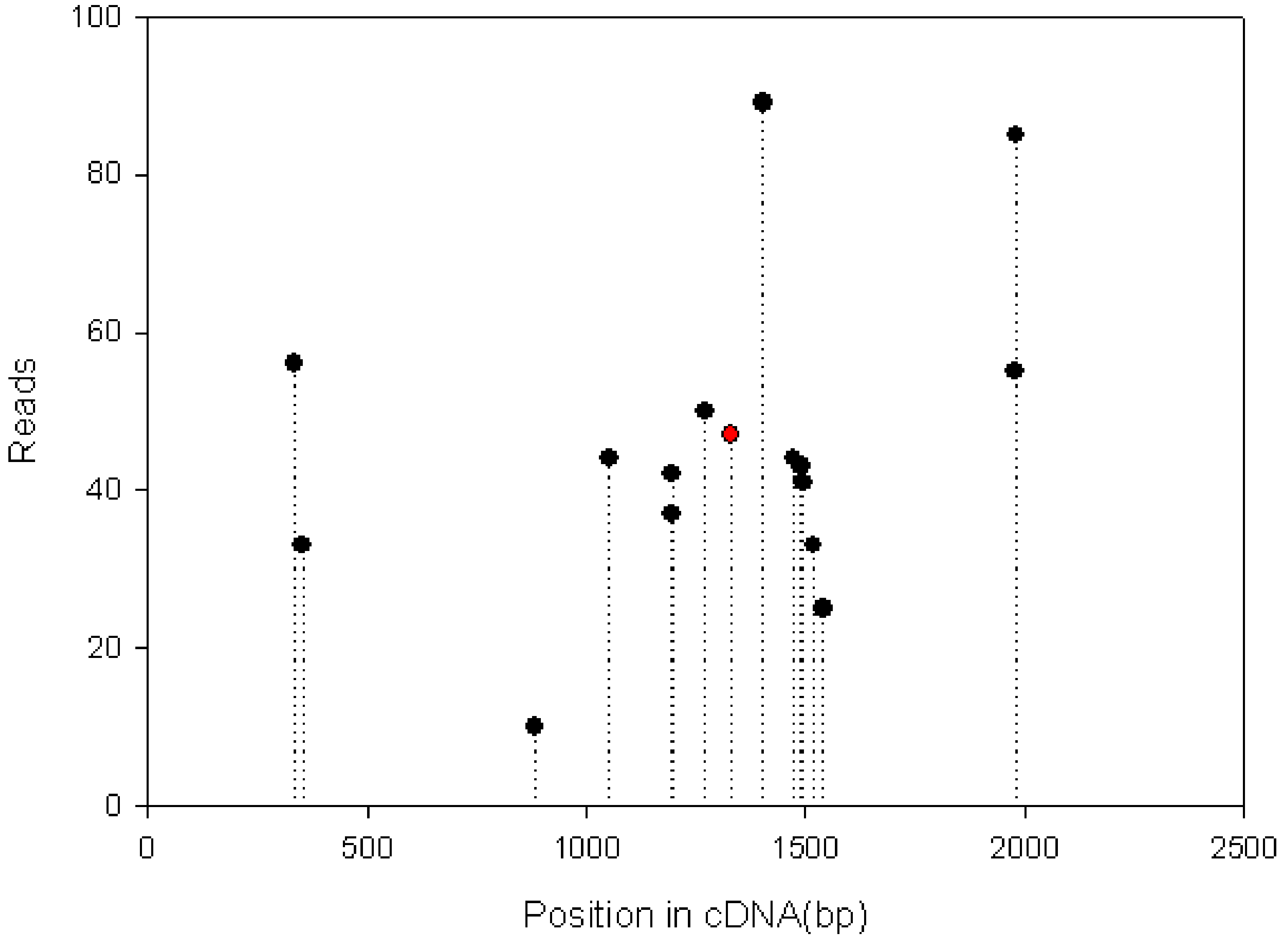


```
5' UAGGUUUCGAGGAAAUCUGUGGGAUU 3'        Cs1g21890.1
       ::: :.:::: :::::::.:
3' -----AAG-UUCUUUCGACACCUU-- 5'        Csi-miR396b.2
```

Csi-miR396b.2, target=Cs2g08090.1 gene=Cs2g08090
Category:3
Score=5
Cleavage Site=349

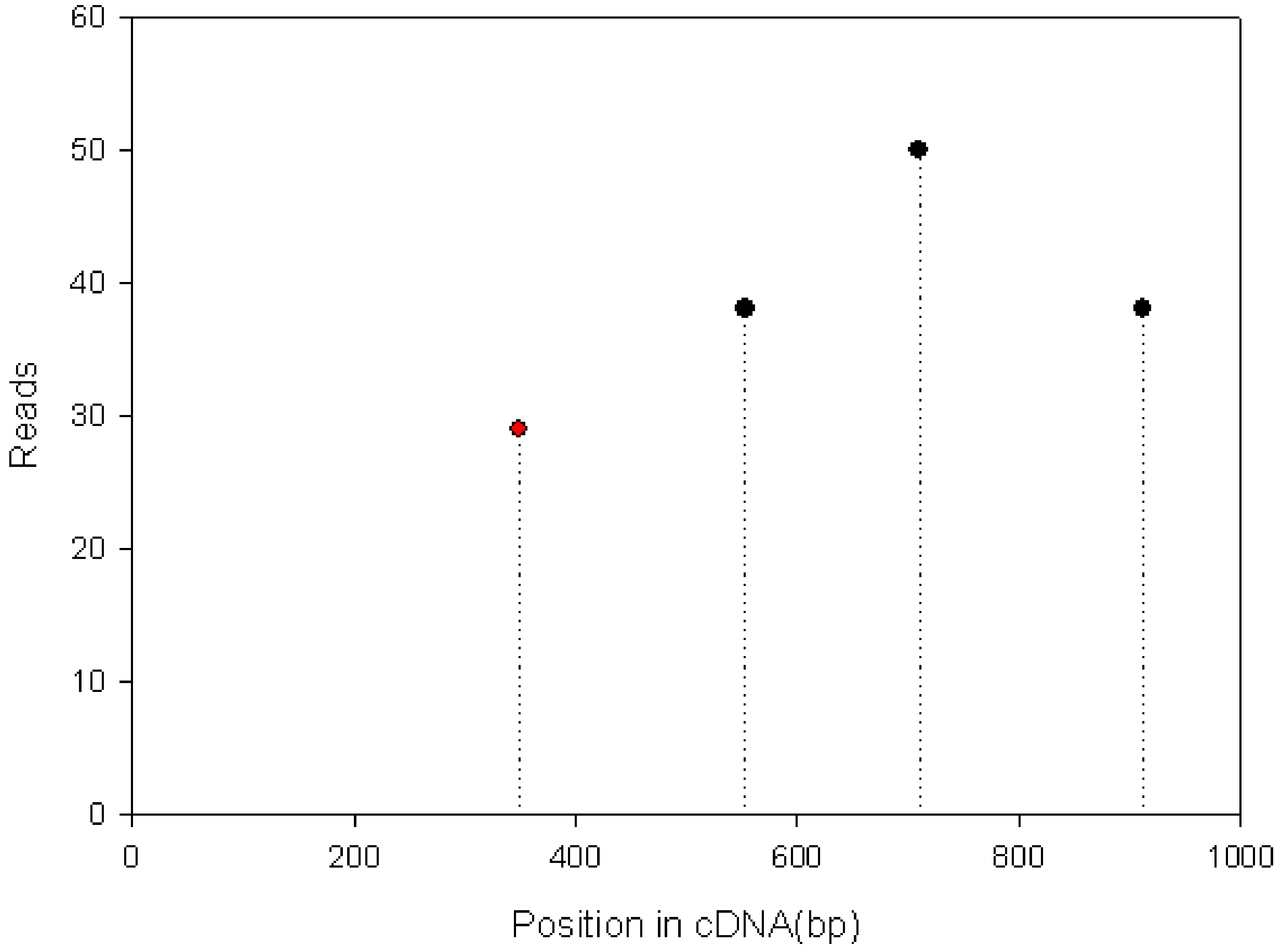


```
5' GGACUGUUCUA-AAAGUUGUGGACAAC 3'      Cs2g08090.1
         ::: : ::::.::::::
3' ------AAGUUCUUUCGACACCUU--- 5'      Csi-miR396b.2
```

Csi-miR396b.2, target=Cs3g07260.1 gene=Cs3g07260
Category:3
Score=3.5
Cleavage Site=2420

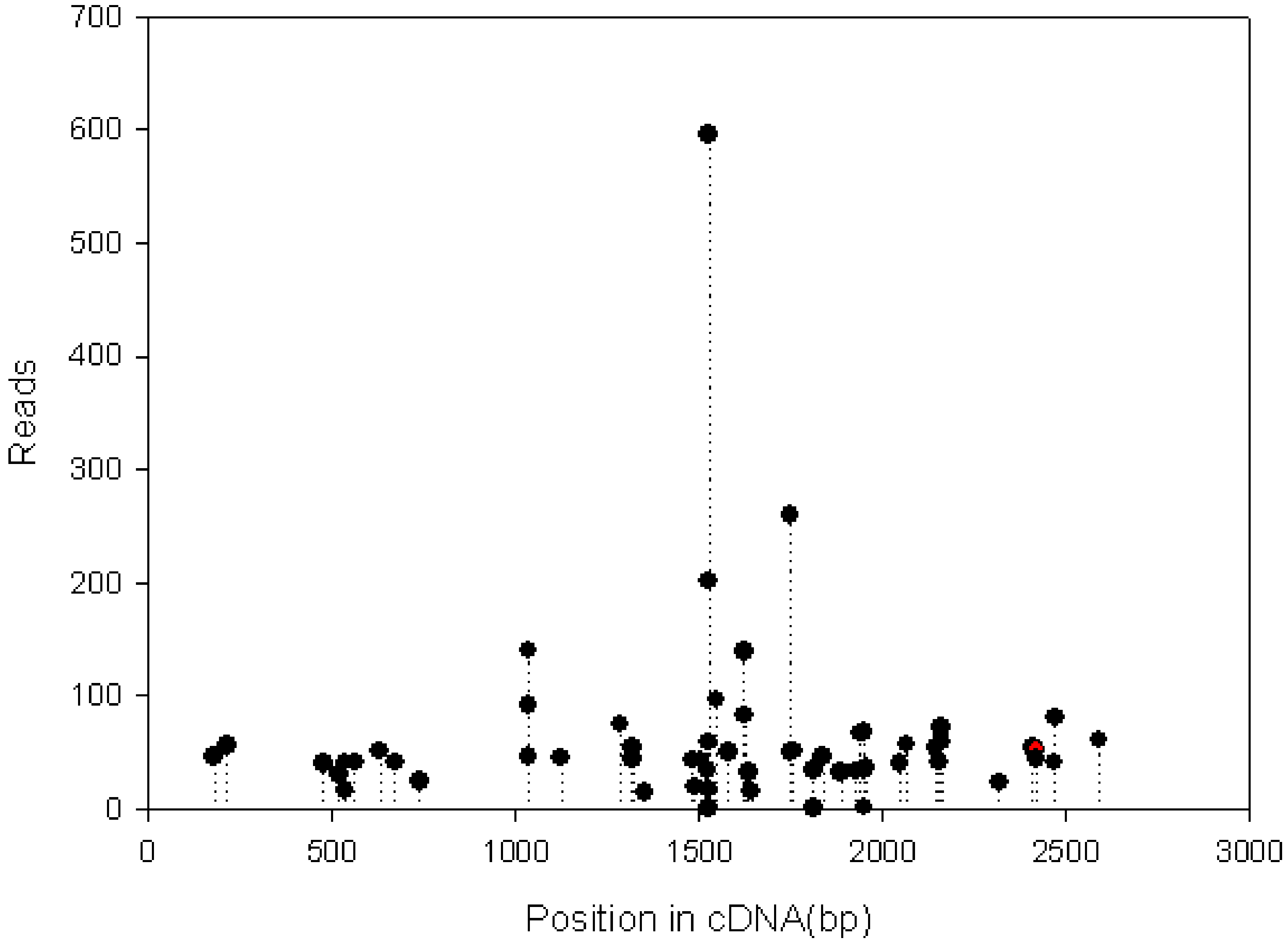


```
5' AUUCCAGGAAAGUUGUGGAUAAAUA 3'        Cs3g07260.1
     : ::.:::::.:::::.:
3' --AAGUUCUUUCGACACCUU------ 5'        Csi-miR396b.2
```

Csi-miR396b.2, target=Cs3g23180.1 gene=Cs3g23180
Category: 3
Score=3
Cleavage Site=625

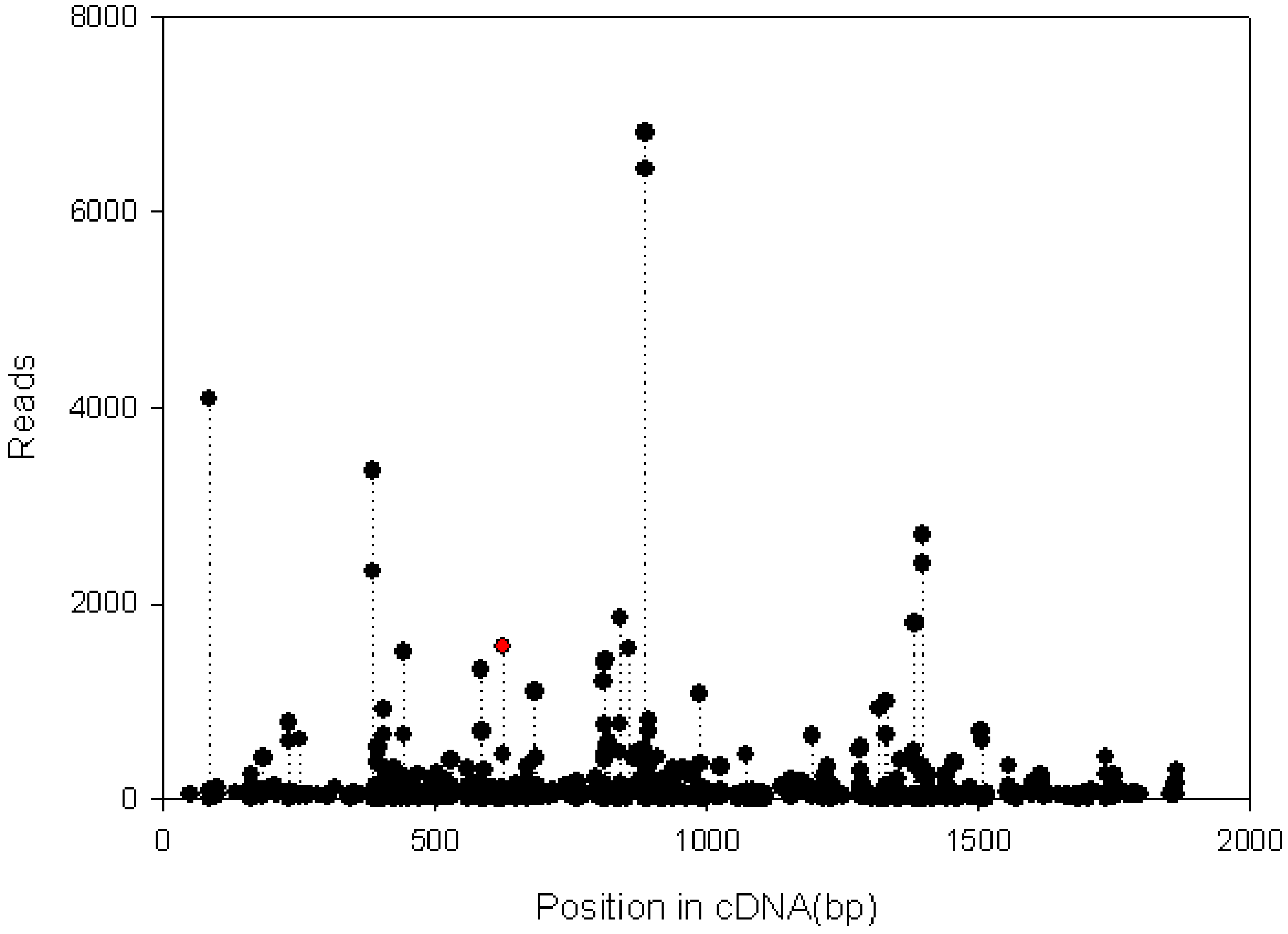


```
5' AAAGAUCAAGGAAGCUGUGGGAGUUG 3'        Cs3g23180.1
         :::::.:::::::::.:
3' ----AAGUUCUUUCGACACCUU---- 5'        Csi-miR396b.2
```

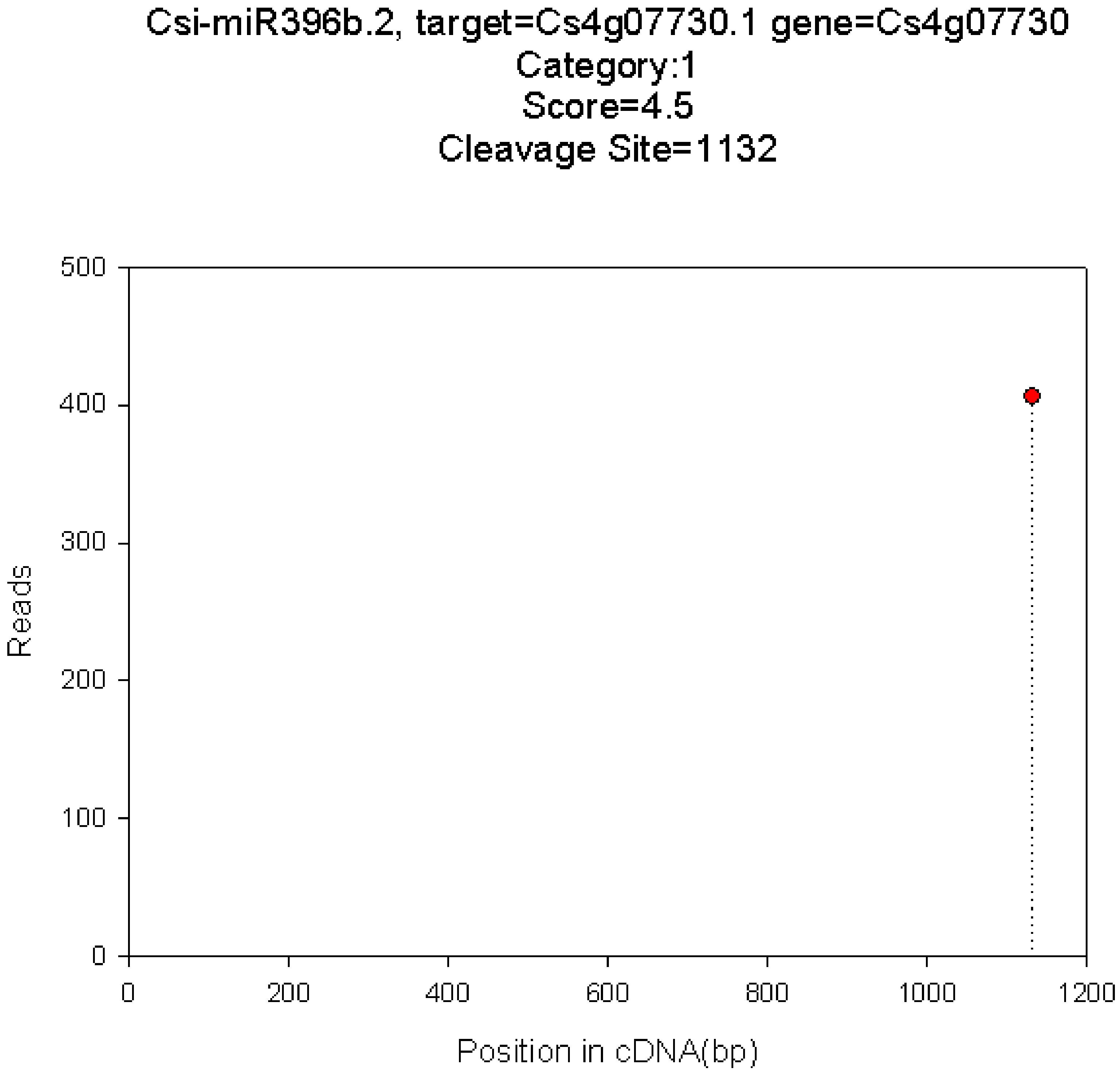


```
5' CGGAGGAAAGUUGUGGAAAAGUGUAA 3'        Cs4g07730.1
       :.:::::.:::::::
3' AAGUUCUUUCGACACCUU-------- 5'        Csi-miR396b.2
```

Csi-miR396b.2, target=Cs5g01380.1 gene=Cs5g01380
Category:1
Score=2
Cleavage Site=1181

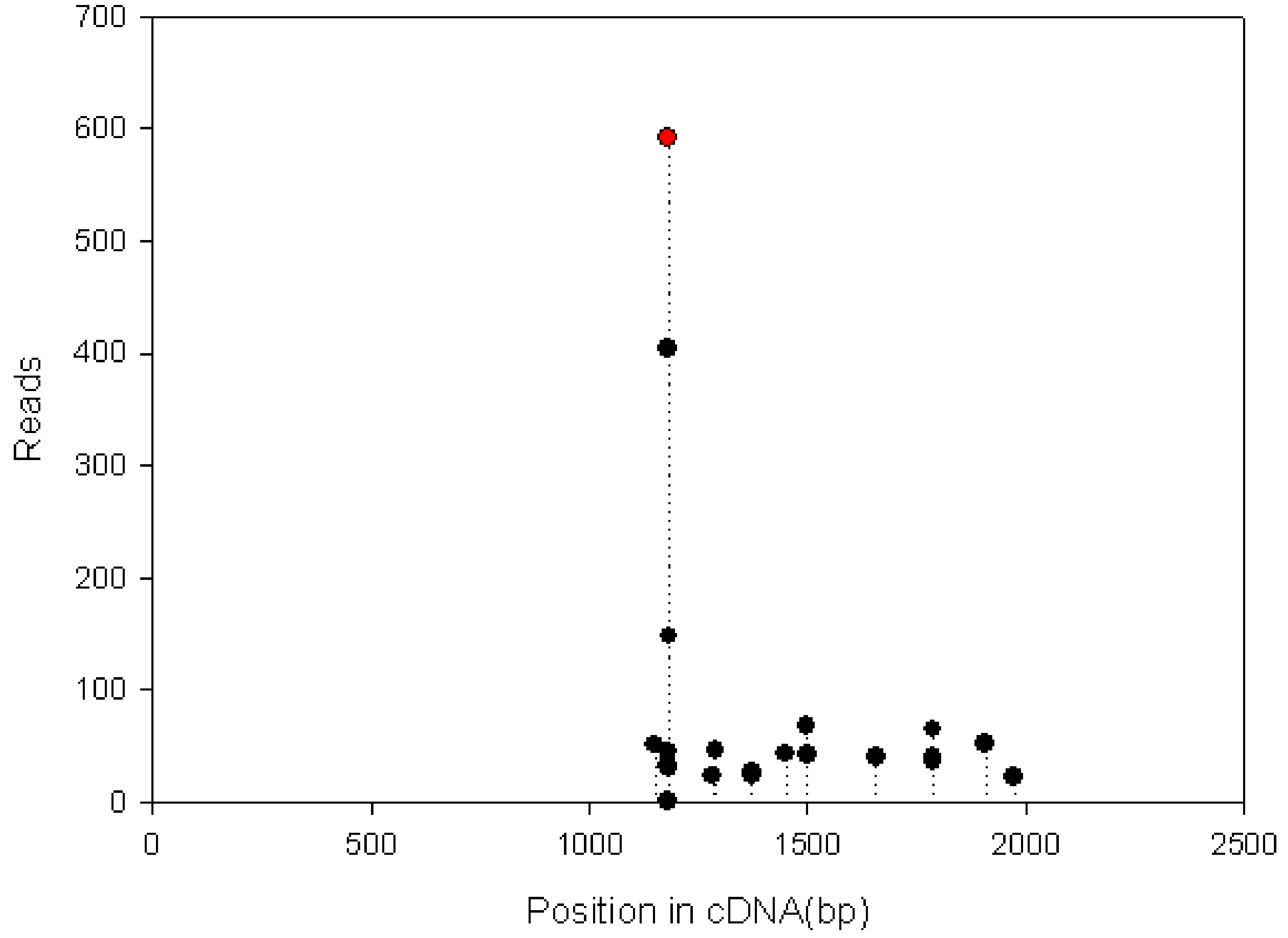


```
5' CCCGUUCAAGAAAGCCUGUGGAAUUA 3'        Cs5g01380.1
       :::::::::: ::::::::
3' ----AAGUUCUUUC-GACACCUU--- 5'        Csi-miR396b.2
```

Csi-miR396b.2, target=Cs5g05620.1 gene=Cs5g0562
Category:3
Score=5
Cleavage Site=404

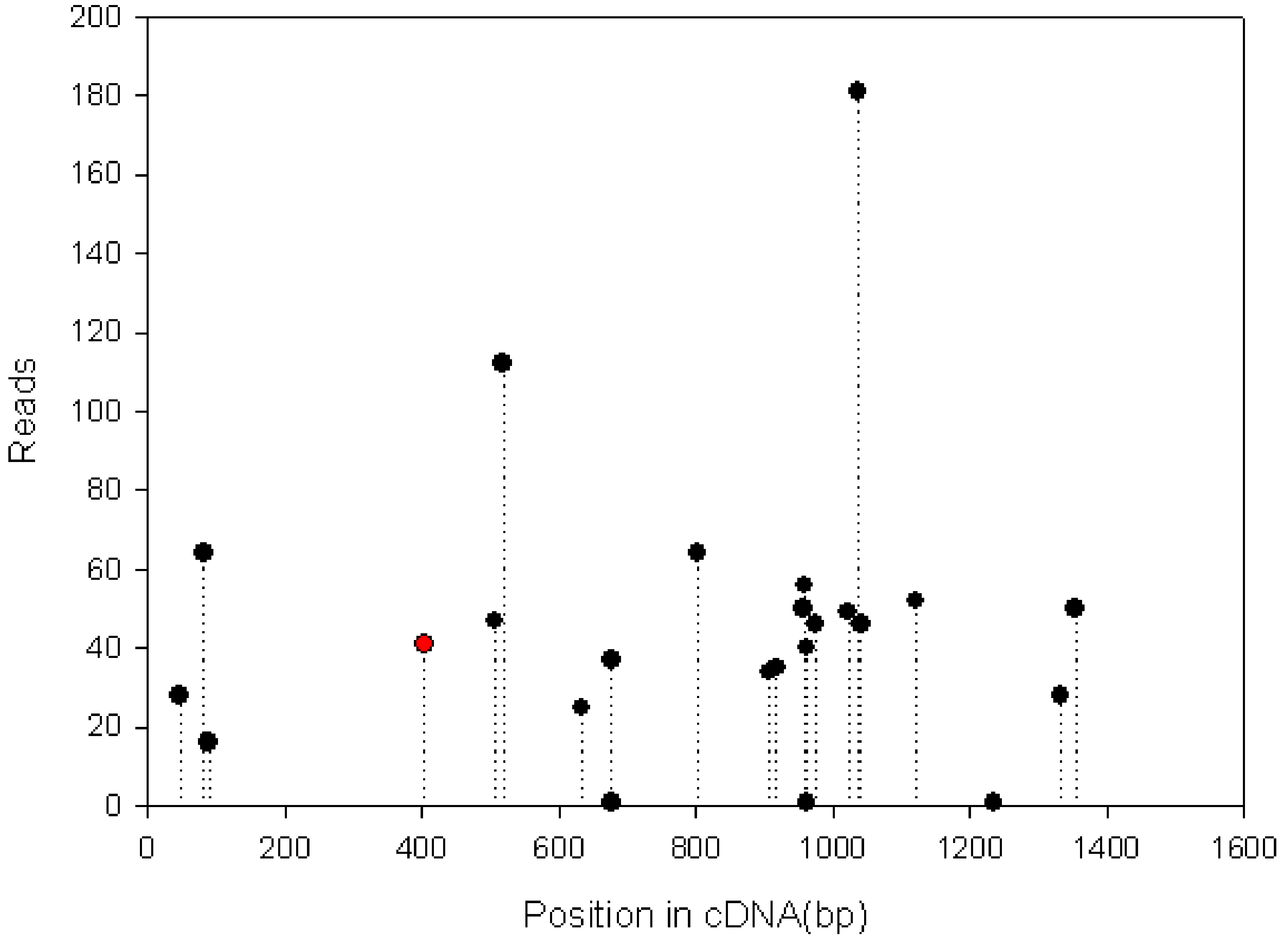


```
5' AAGGUGAGAAGAAGGUUGUGGAAAGU 3'        Cs5g05620.1
           :::::.:.:::::::
3' -----AAGUUCUUUCGACACCUU--- 5'        Csi-miR396b.2
```

Csi-miR396b.2, target=Cs5g19200.1 gene=Cs5g19200
Category:2
Score=2.5
Cleavage Site=201

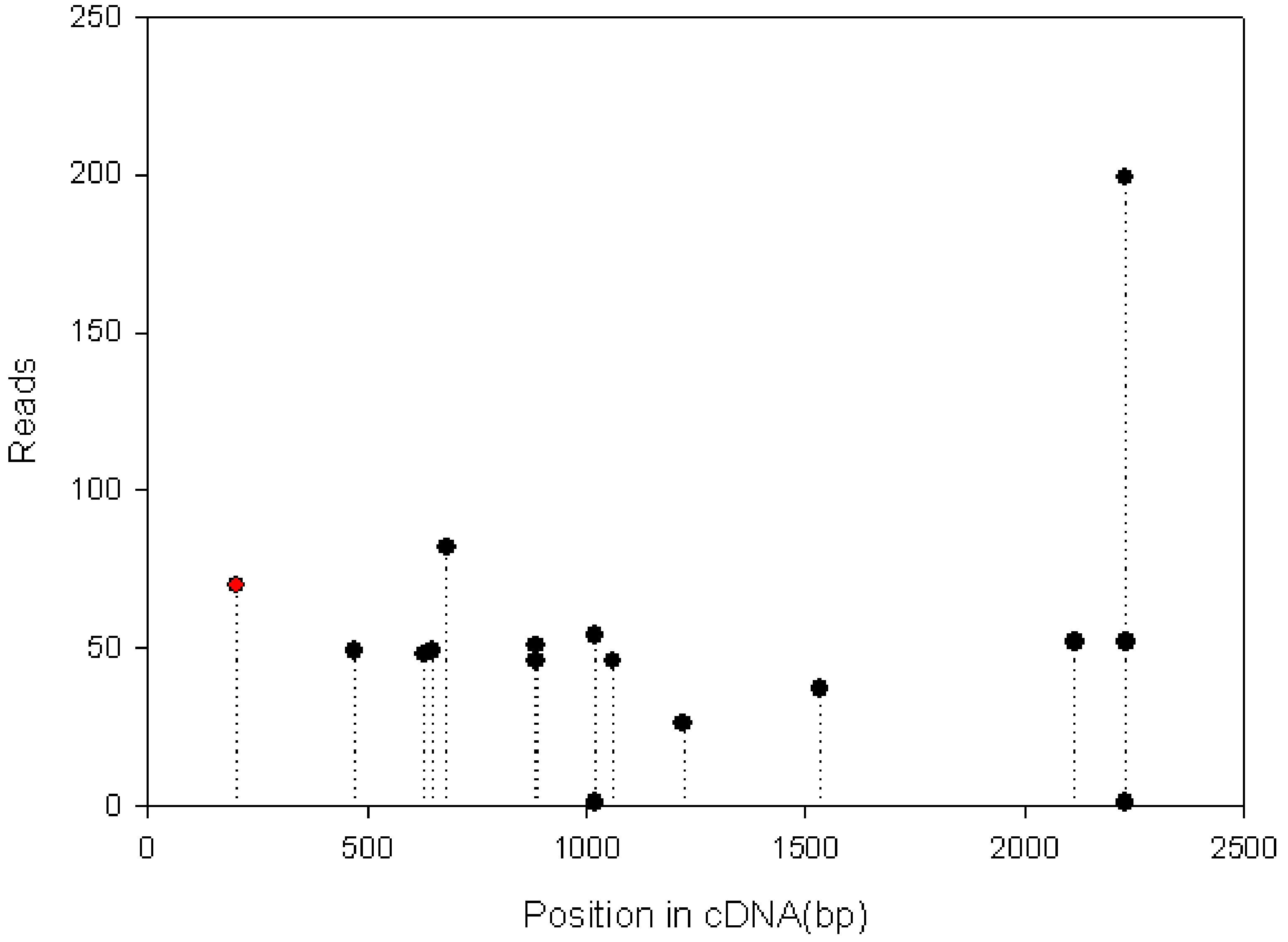


```
5' AAUGUUUAAGAAAGCAGUGGAAGCAA 3'        Cs5g19200.1
       ::.::::::::: ::::::
3' ----AAGUUCUUUCGACACCUU---- 5'        Csi-miR396b.2
```

Csi-miR396b.2, target=Cs5g23130.1 gene=Cs5g23130
Category:3
Score=4.5
Cleavage Site=3311

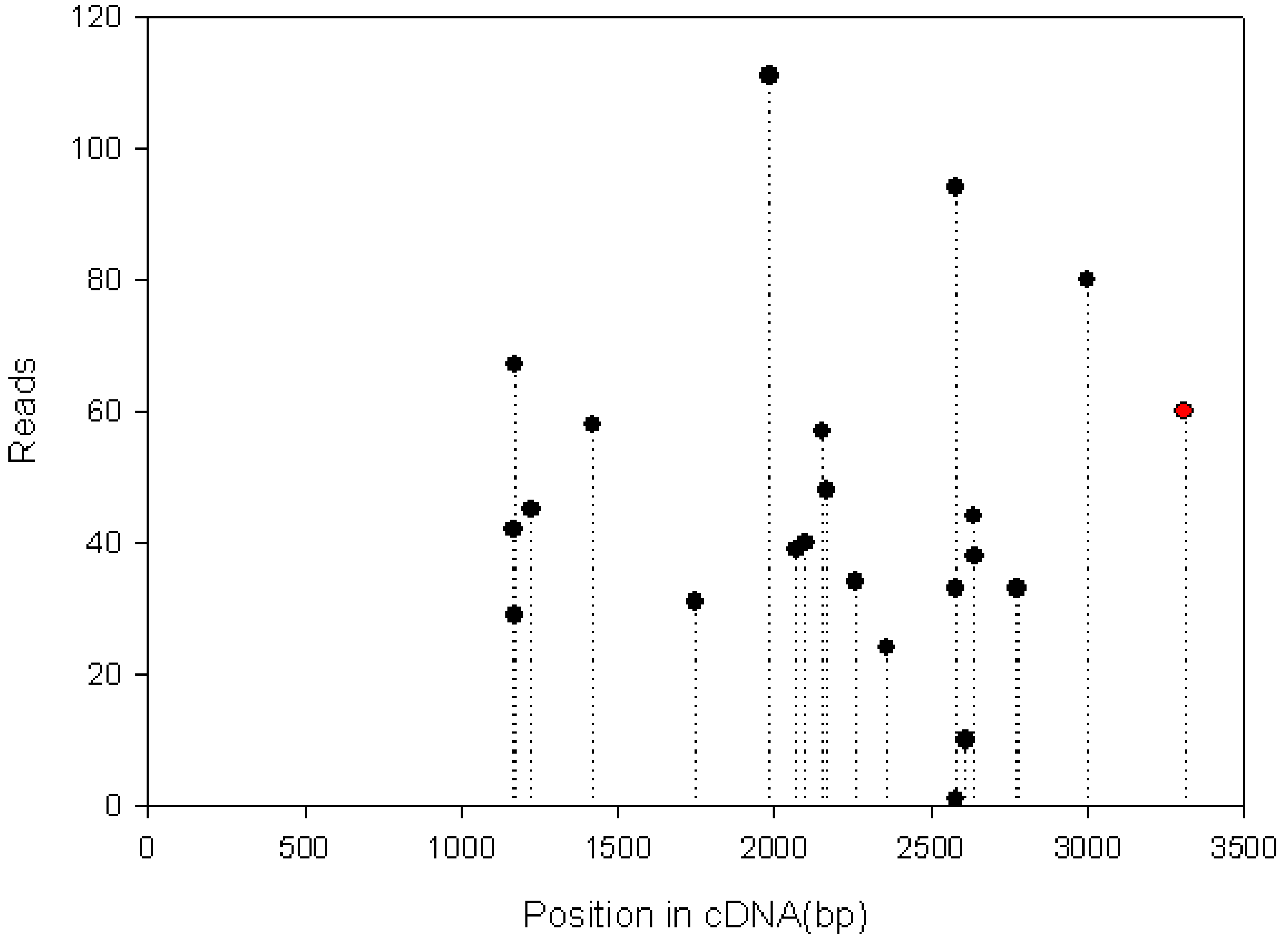


```
5' AACUUGUGGAGAAAGCUGUGGCAAAU 3'        Cs5g23130.1
          :  .:::::::::::: :
3' -----AAGUUCUUUCGACACCUU--- 5'        Csi-miR396b.2
```

Csi-miR396b.2, target=Cs5g24150.1 gene=Cs5g24150
Category:3
Score=5
Cleavage Site=3244

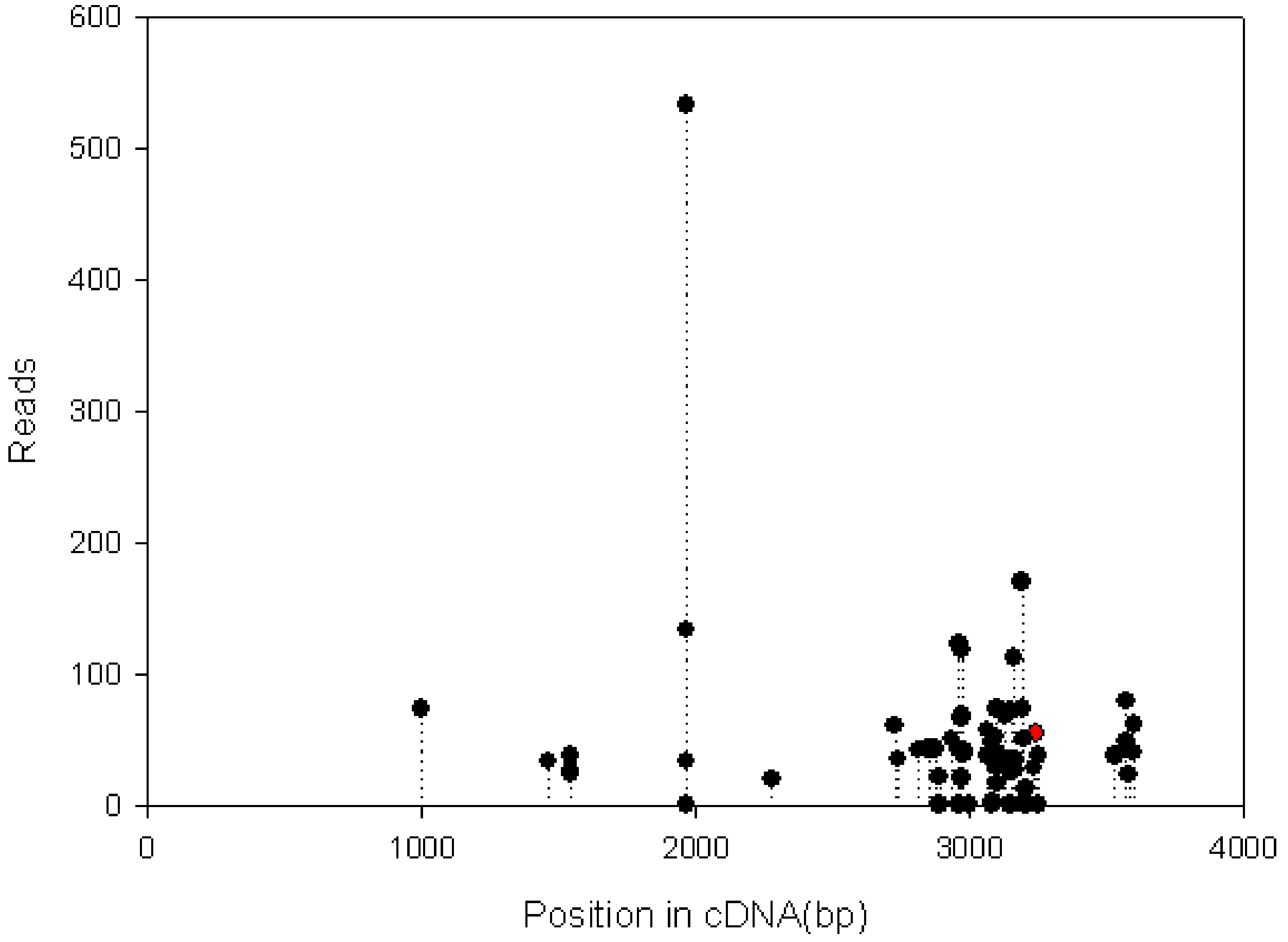


```
5' ACCAGAGCGAUCAA-AAAGCUGUAGAA 3'       Cs5g24150.1
               :::: :::::::: :::
3' ---------AAGUUCUUUCGACACCUU 5'       Csi-miR396b.2
```

Csi-miR396b.2, target=Cs6g04030.1 gene=Cs6g04030
Category:3
Score=4
Cleavage Site=857

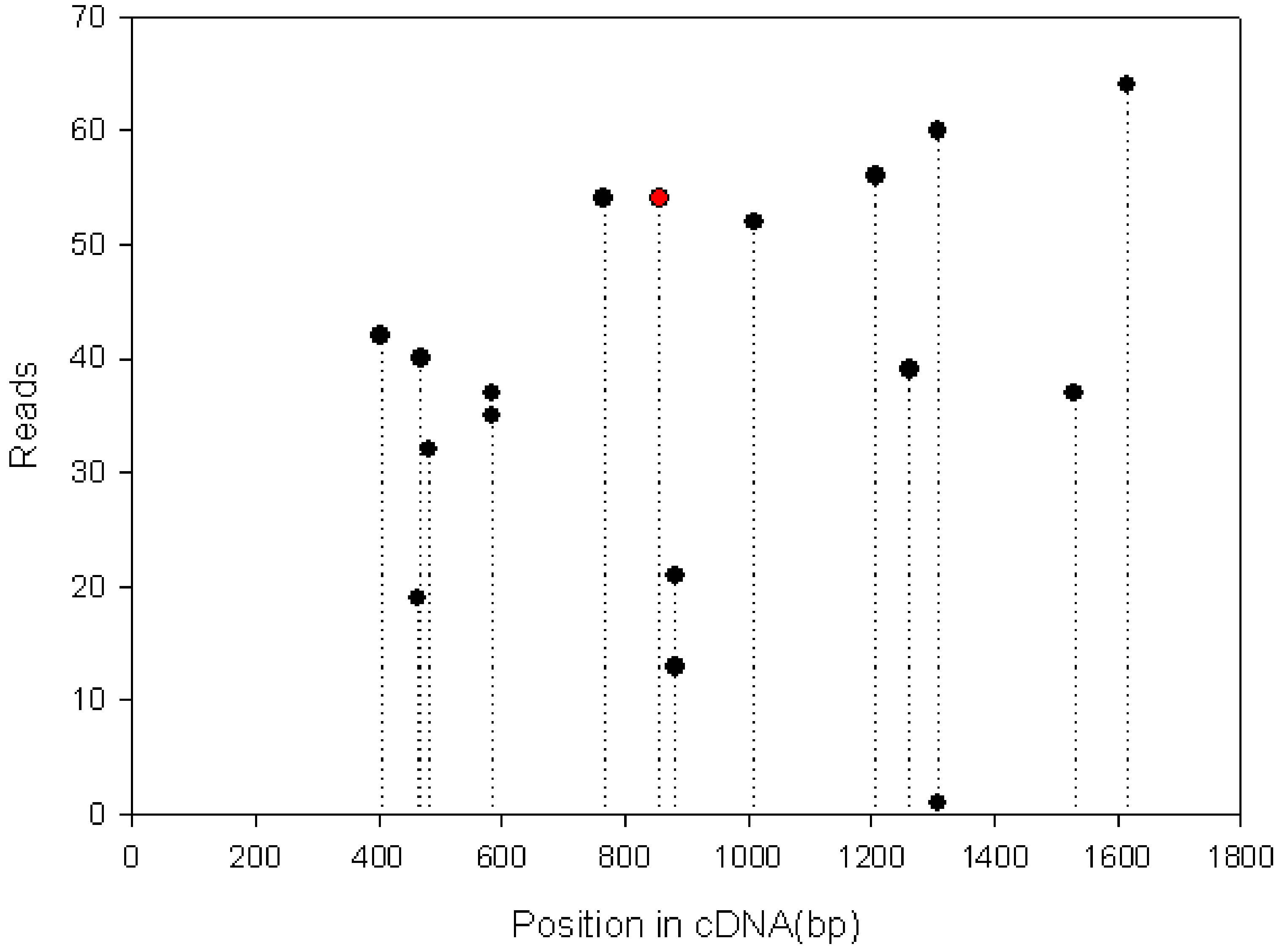


```
5' GUAAAUACUUUUUAG-AAGCUGUGGAG 3'        Cs6g04030.1
             ::. :: ::::::::::.
3' ---------AAGUUCUUUCGACACCUU 5'        Csi-miR396b.2
```

Csi-miR396b.2, target=Cs6g06540.1 gene=Cs6g06540
Category:3
Score=4
Cleavage Site=526

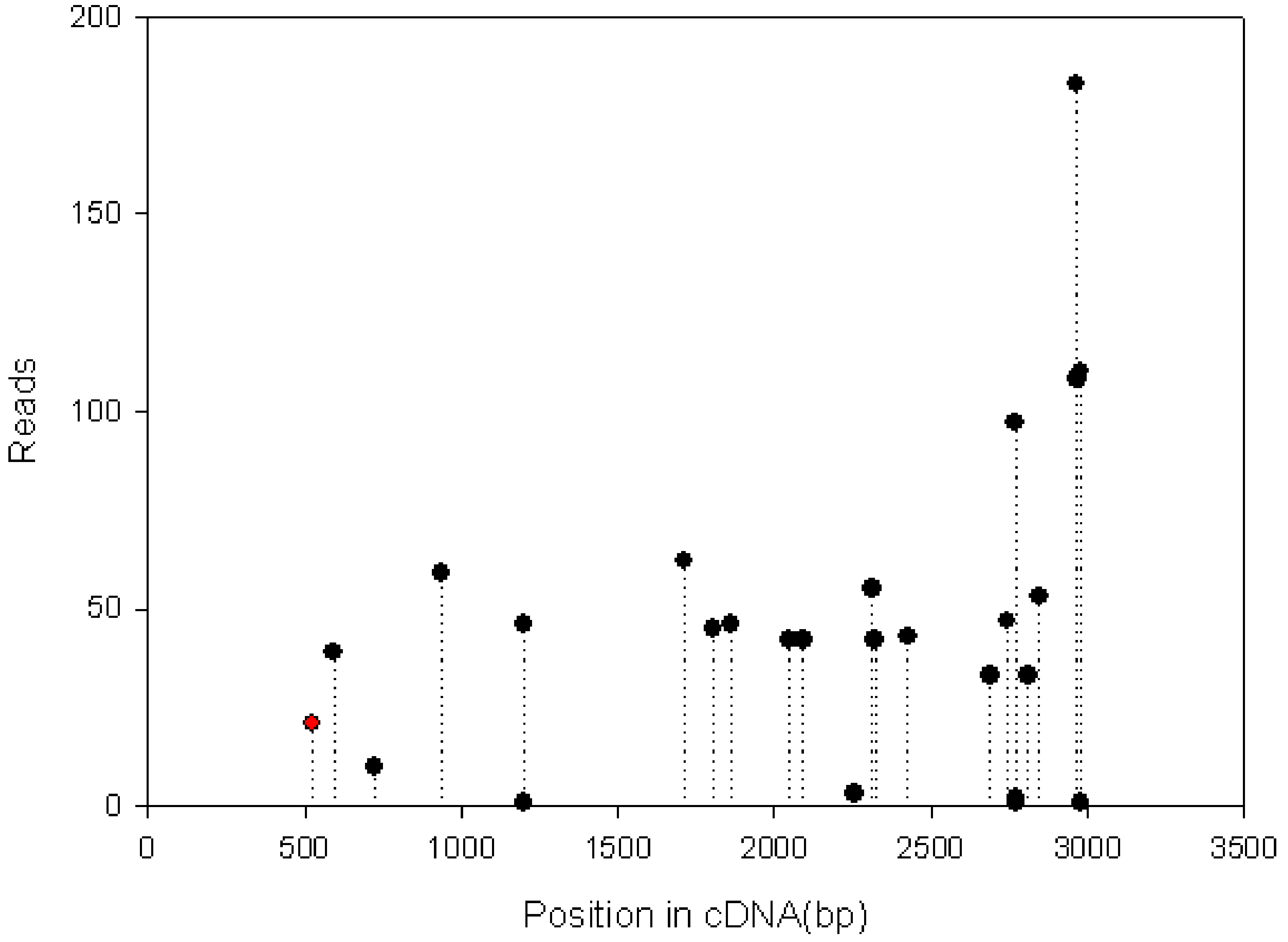


```
5' AACUUCCUUC-AGAAAGCUGAGGAUAA 3'        Cs6g06540.1
           ::: ::::::::: :::
3' -------AAGUUCUUUCGACACCUU-- 5'        Csi-miR396b.2
```

Csi-miR396b.2, target=Cs6g20570.1 gene=Cs6g20570
Category:3
Score=5
Cleavage Site=231

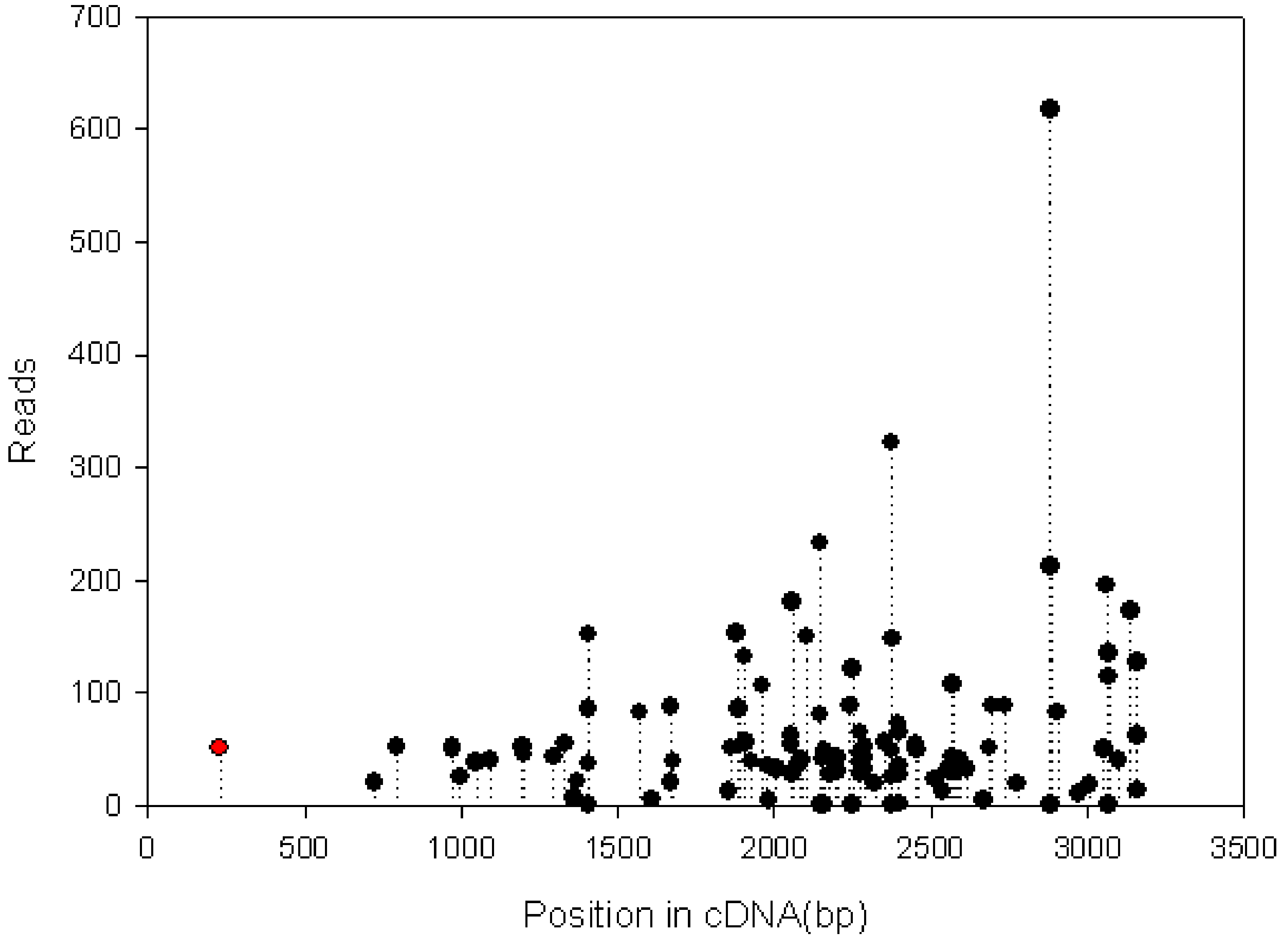


```
5' GCAGUGUUGAAGGAAGCUGUAGAUUU 3'        Cs6g20570.1
         :: :::.::::::: ::
3' ------AAGUUCUUUCGACACCUU-- 5'        Csi-miR396b.2
```

Csi-miR396b.2, target=Cs7g11670.1 gene=Cs7g11670
Category:3
Score=4.5
Cleavage Site=143

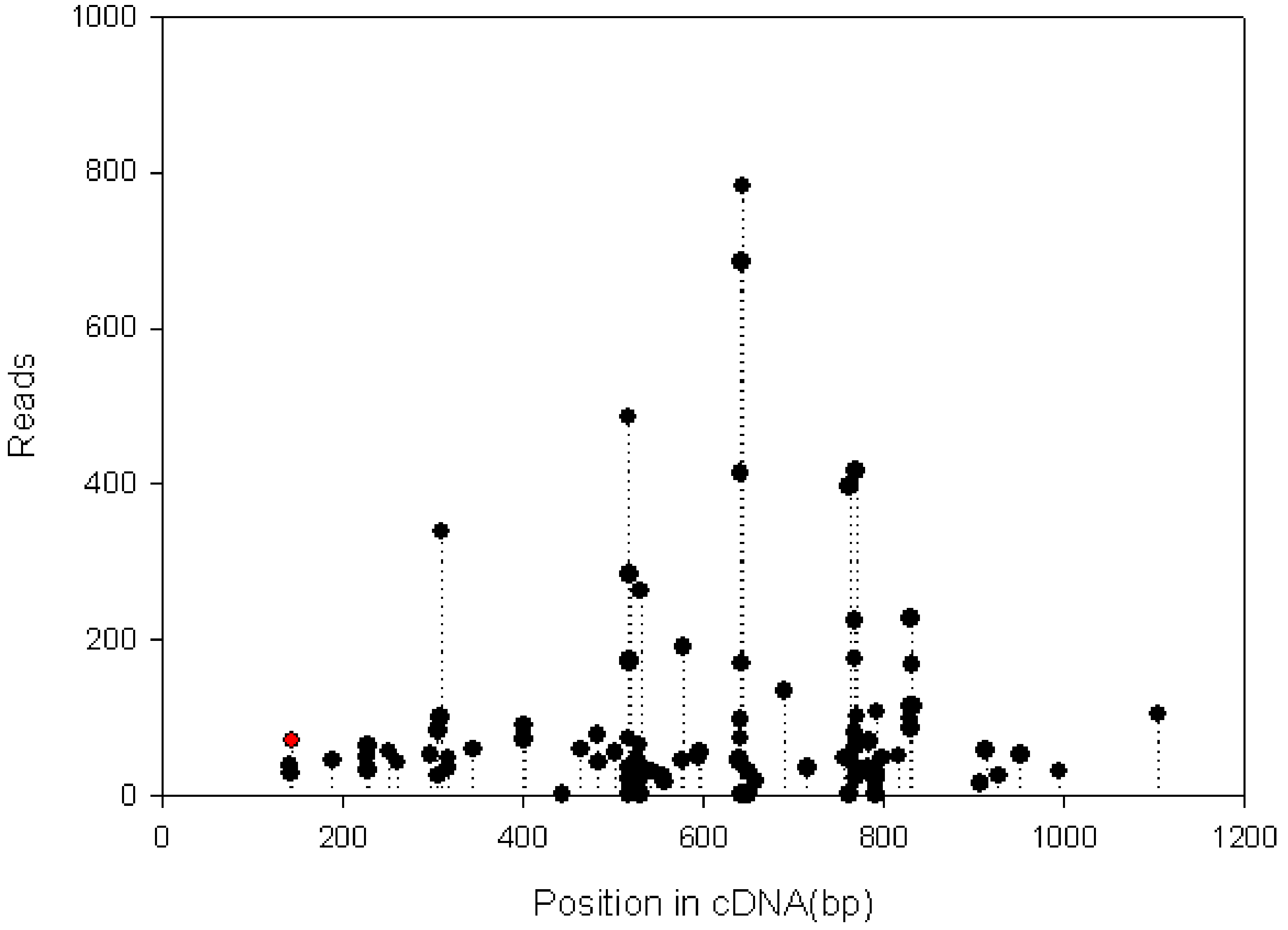


```
5' GGAUUCAUGAGAG-UGUGGAGGAGCUU 3'       Cs7g11670.1
       :::: ::.:: ::::::.
3' ---AAGUUCUUUCGACACCUU------ 5'       Csi-miR396b.2
```

Csi-miR396b.2, target=Cs7g12930.1 gene=Cs7g12930
Category:1
Score=3.5
Cleavage Site=1079

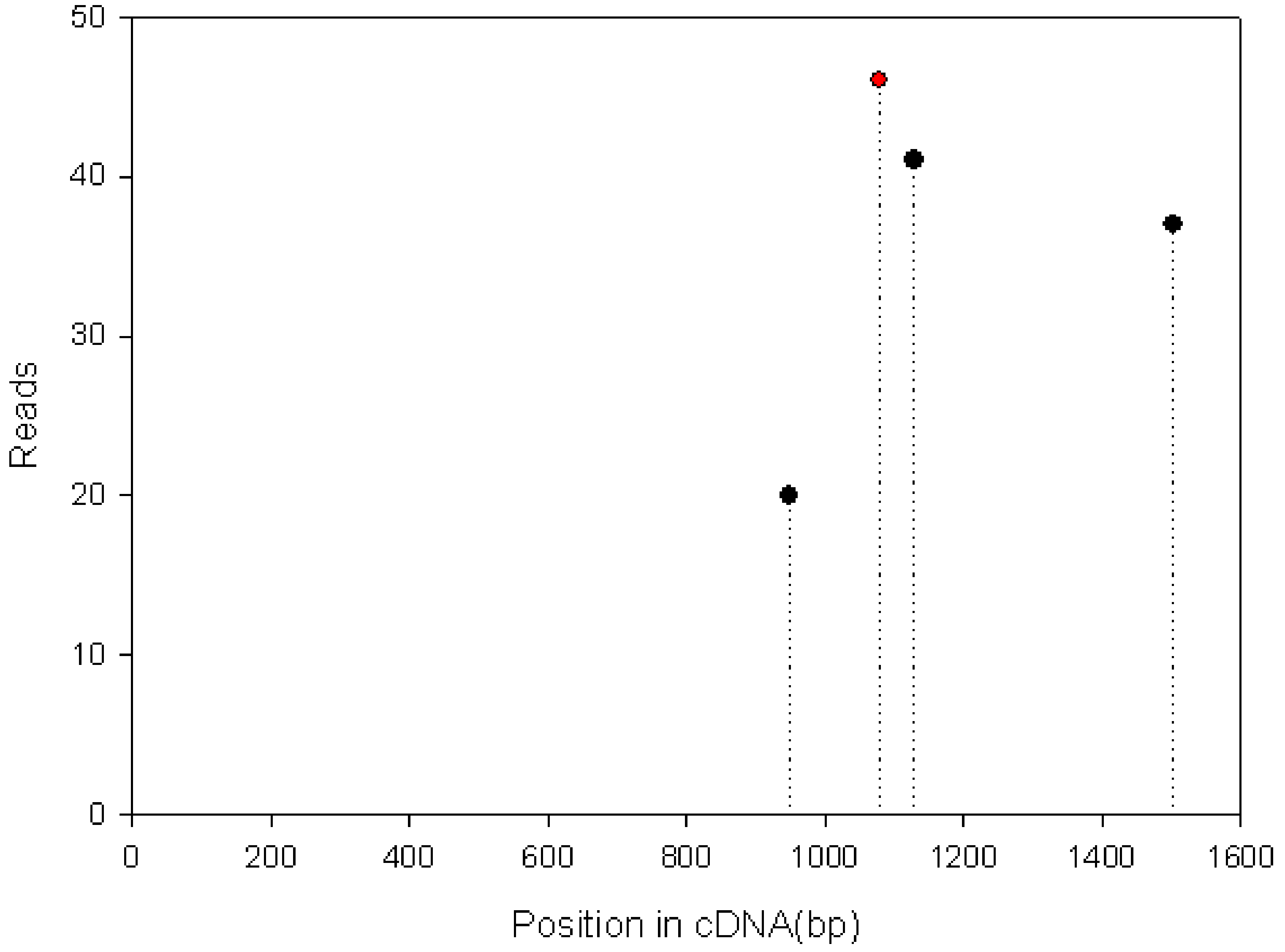


```
5' UAGUAUUAAGAAAGCUGAGGAAAAAG 3'        Cs7g12930.1
        :.:::::::::: ::::
3' ----AAGUUCUUUCGACACCUU---- 5'        Csi-miR396b.2
```

Csi-miR396b.2, target=Cs7g15220.1 gene=Cs7g15220
Category:1
Score=2
Cleavage Site=377

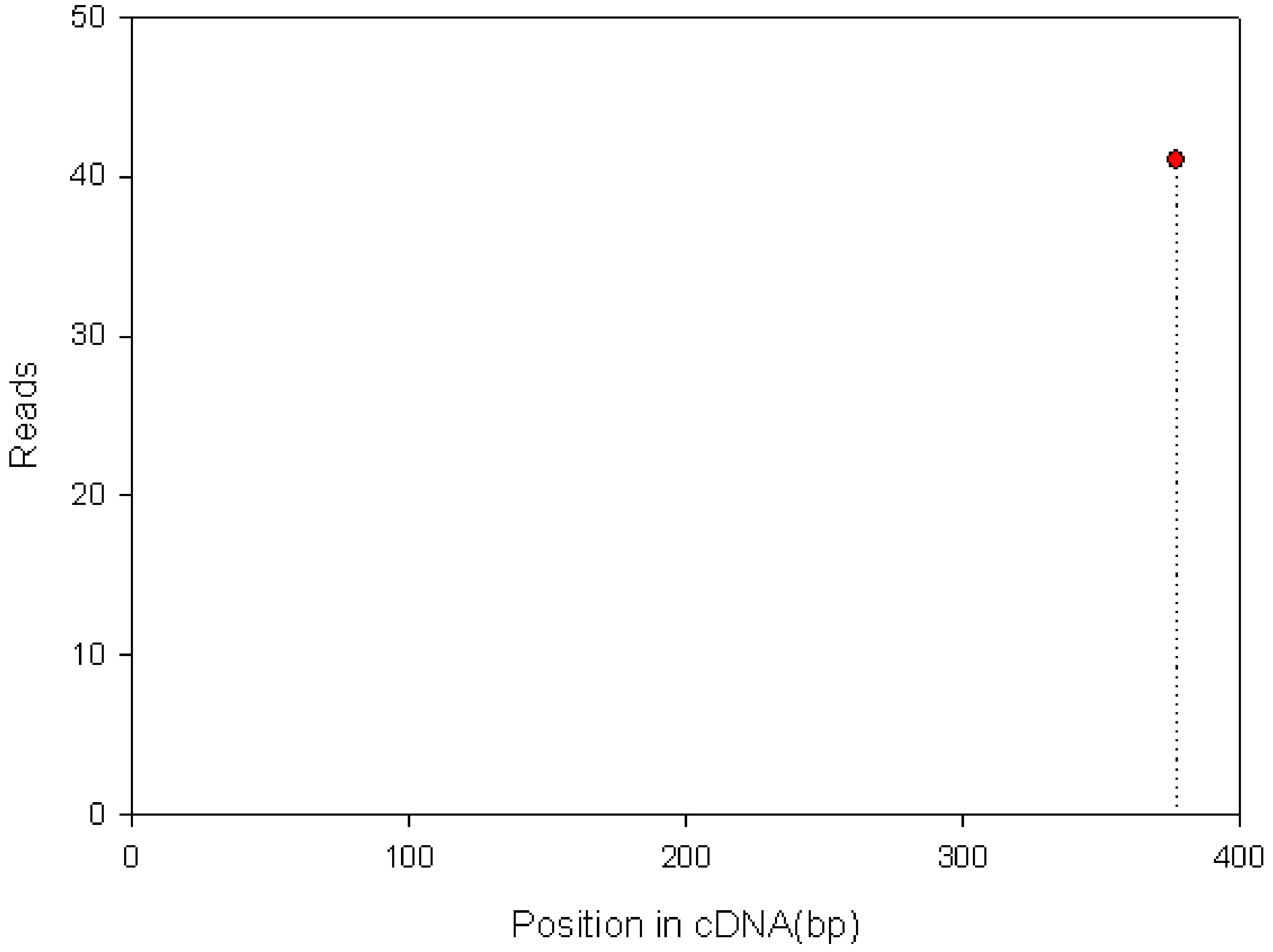


```
5' ACCGUUCAAGAAAGCCUGUGGAAUCA 3'        Cs7g15220.1
       :::::::::: ::::::::
3' ----AAGUUCUUUC-GACACCUU--- 5'        Csi-miR396b.2
```

Csi-miR396b.2, target=Cs7g18780.1 gene=Cs7g18780
Category:3
Score=5
Cleavage Site=1021

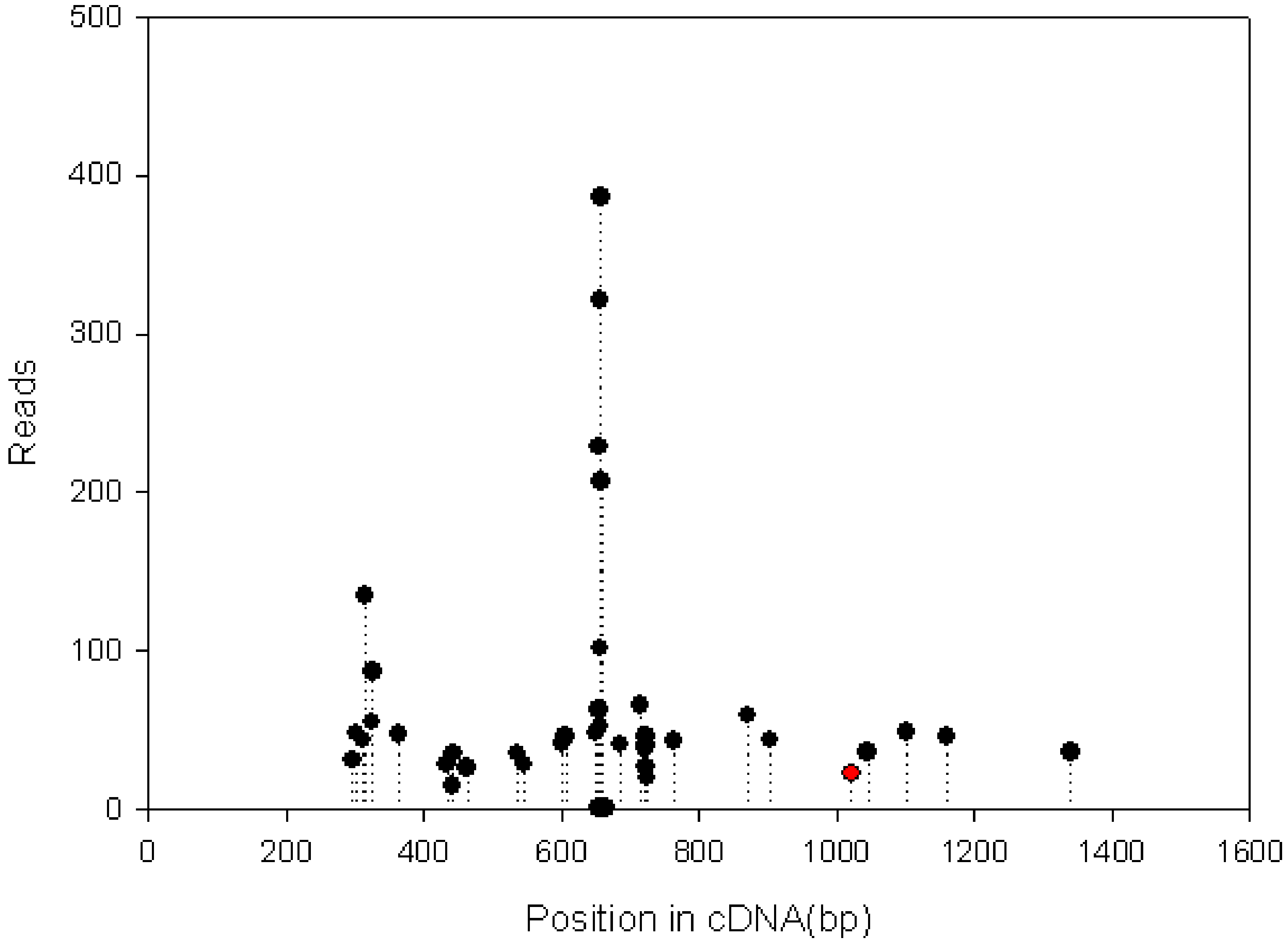


```
5' AGGGAACAAGAAAGUAUGUGGAAAGC 3'        Cs7g18780.1
         :::::::::. :::::::
3' ----AAGUUCUUUCG-ACACCUU--- 5'        Csi-miR396b.2
```

Csi-miR396b.2, target=Cs7g23200.1 gene=Cs7g23200
Category:2
Score=5
Cleavage Site=260

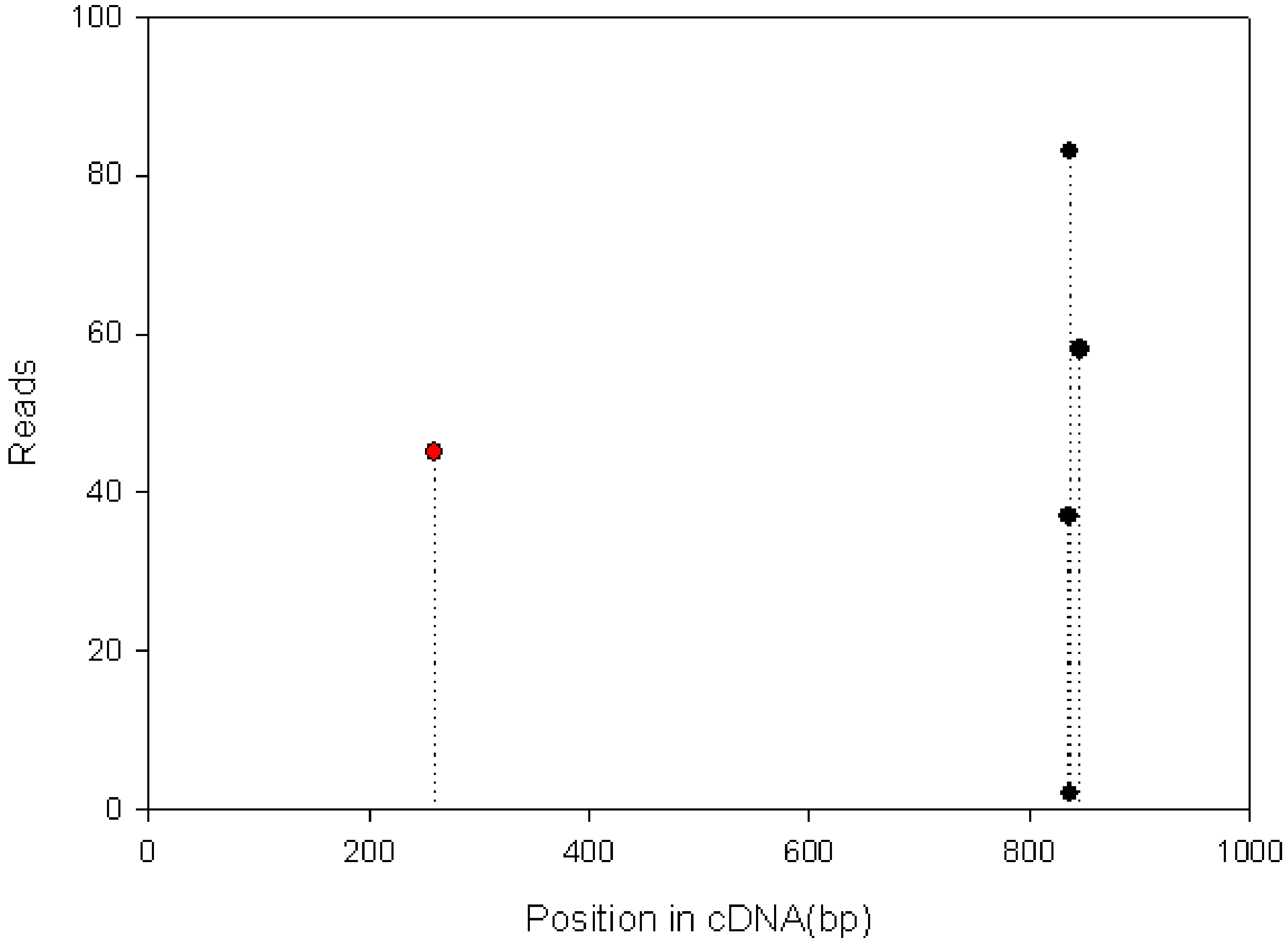


```
5' AAGAGUUCUGGAAGGCUGCGGAGGAC 3'        Cs7g23200.1
        ::: .:::.:::: :::.
3' -----AAGUUCUUUCGACACCUU--- 5'        Csi-miR396b.2
```

Csi-miR396b.2, target=Cs8g04510.1 gene=Cs8g04510
Category:3
Score=4.5
Cleavage Site=1239

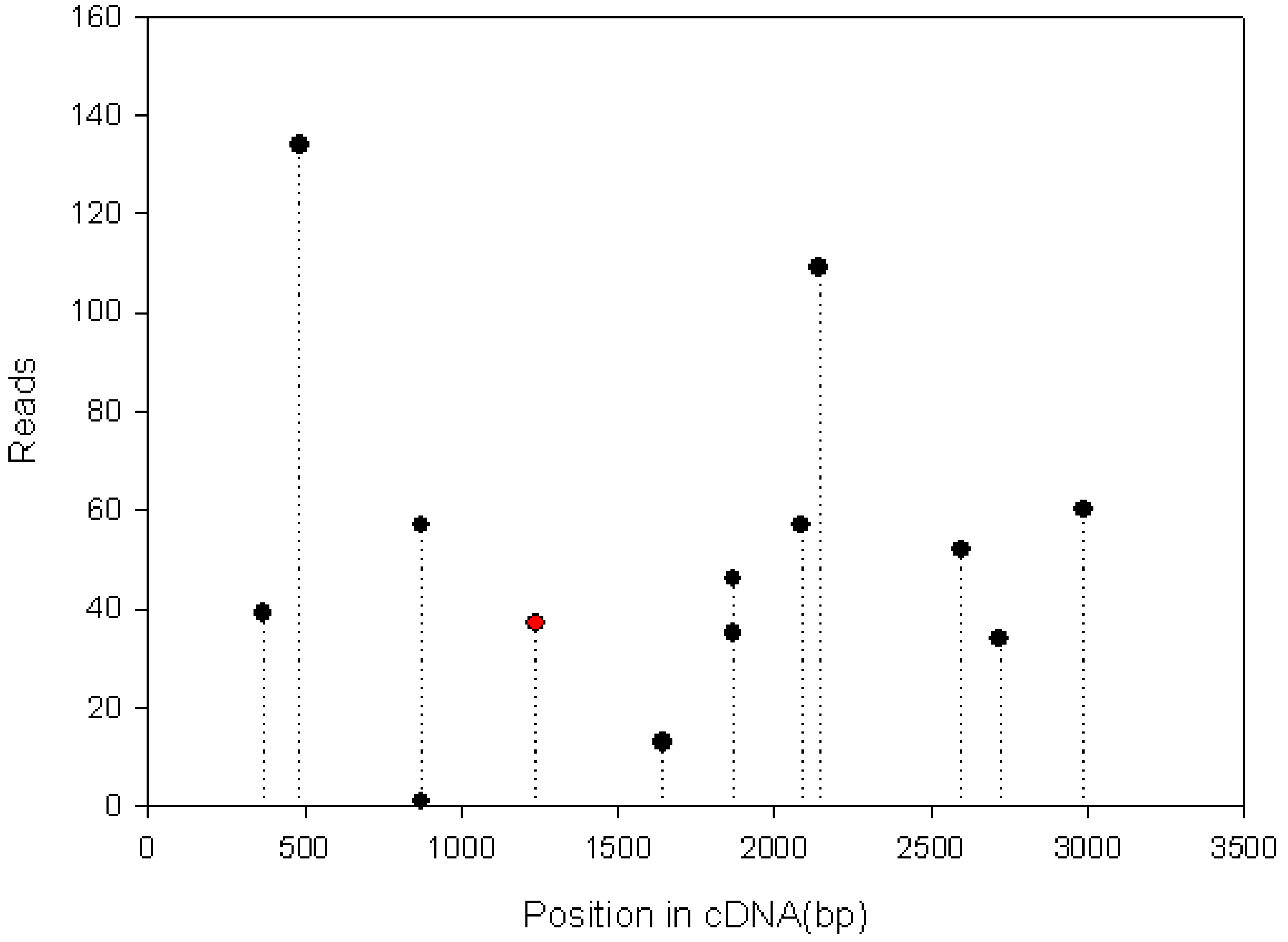


```
5' UCUGUUAAAGGAAGCUAUGGAGCUUA 3'        Cs8g04510.1
       :: :::.::::: ::::.
3' ----AAGUUCUUUCGACACCUU---- 5'        Csi-miR396b.2
```

Csi-miR396b.2, target=Cs8g17370.1 gene=Cs8g17370
Category:3
Score=4.5
Cleavage Site=137

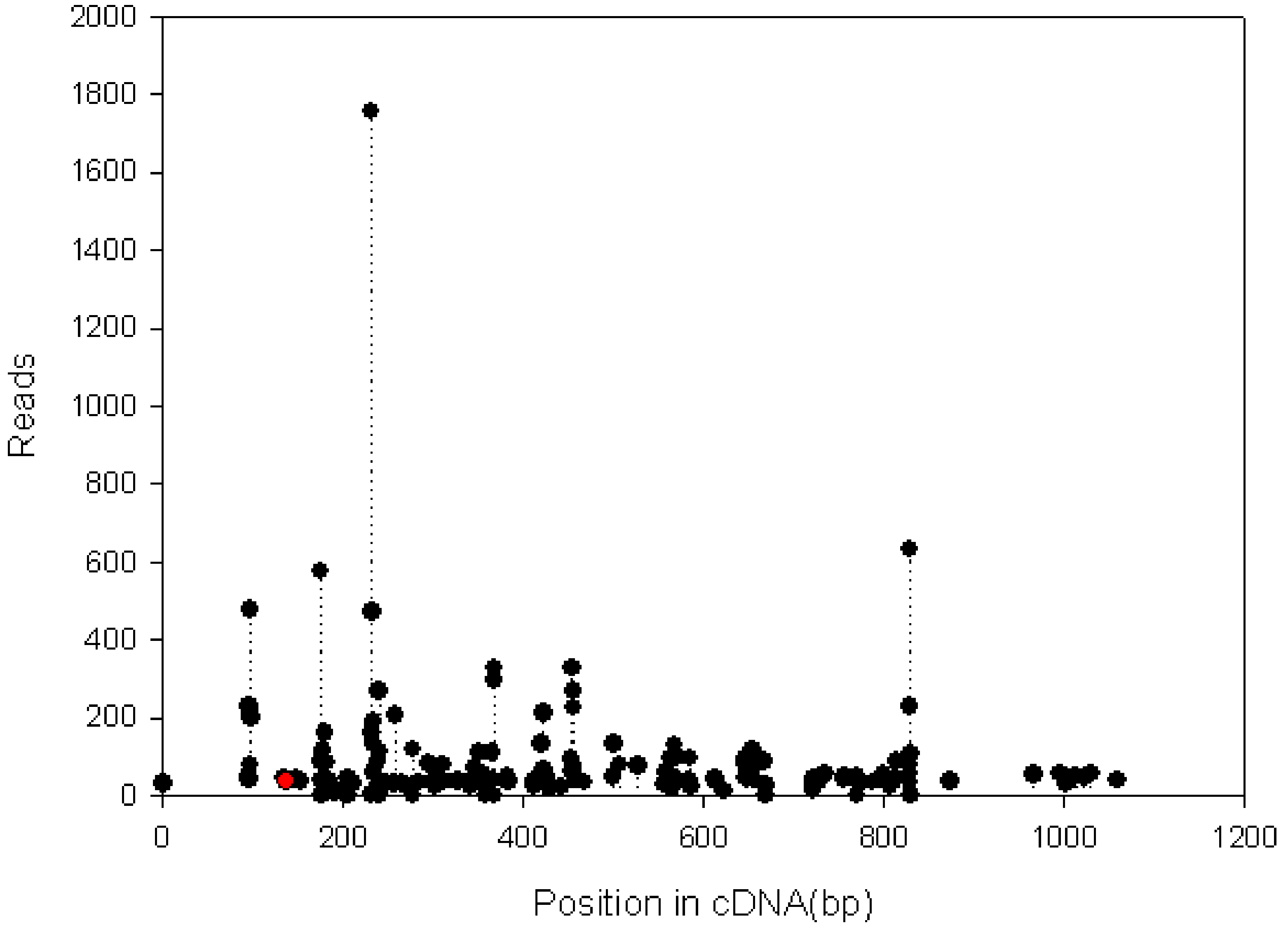


```
5' GCGAGGAUUACAAGAAGGCCGUGGAG 3'        Cs8g17370.1
           : ::::::.:: :::::.
3' --------AAGUUCUUUCGACACCUU 5'        Csi-miR396b.2
```

Csi-miR396b.2, target=Cs9g05650.1 gene=Cs9g0565
Category:2
Score=5
Cleavage Site=973

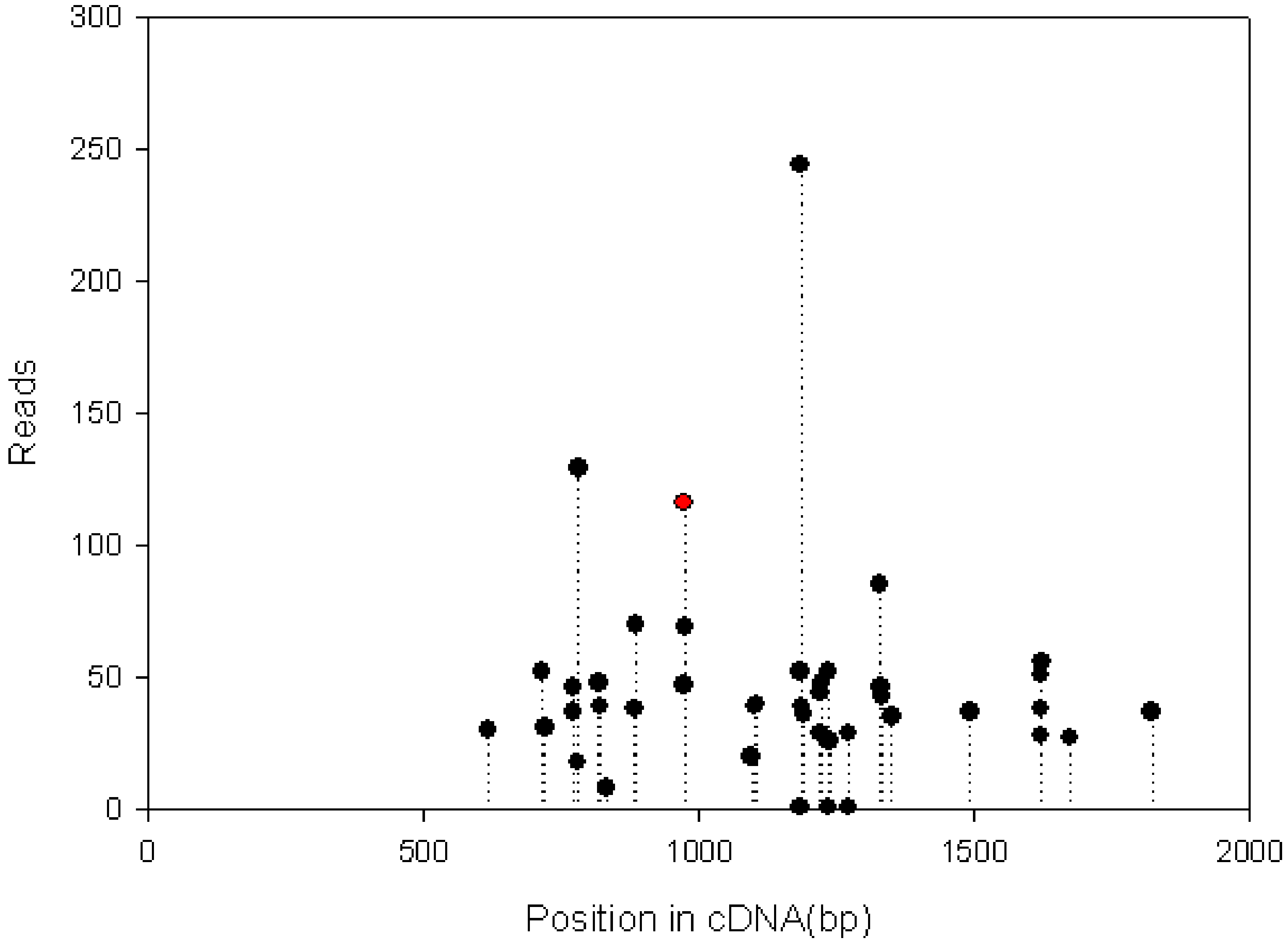


```
5' UUGUACUUGCAAUAAAGCU-UGGAAUG 3'       Cs9g05650.1
          : ::: :::::: :::::
3' -------AAGUUCUUUCGACACCUU-- 5'       Csi-miR396b.2
```

Csi-miR396b.2, target=Cs9g17470.1 gene=Cs9g17470
Category:3
Score=5
Cleavage Site=4344

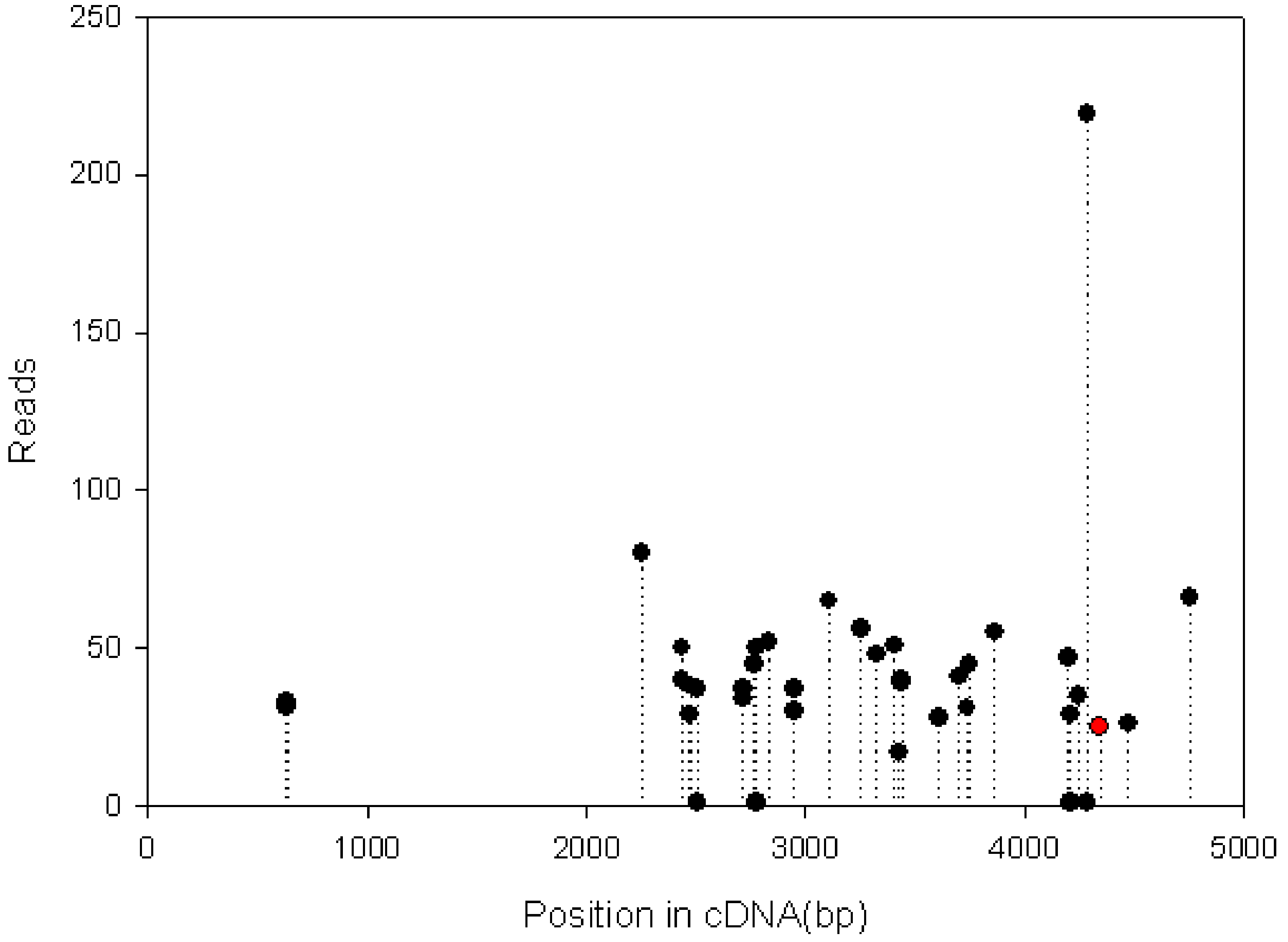


```
5' UAUGAGAAA-CUGUGGAUGCACGUGCG 3'        Cs9g17470.1
    :  ..::::: :::::::
3' AAGUUCUUUCGACACCUU---------- 5'        Csi-miR396b.2
```

Csi-miR396b.2, target=Cs9g19220.1 gene=Cs9g19220
Category:3
Score=3.5
Cleavage Site=1829

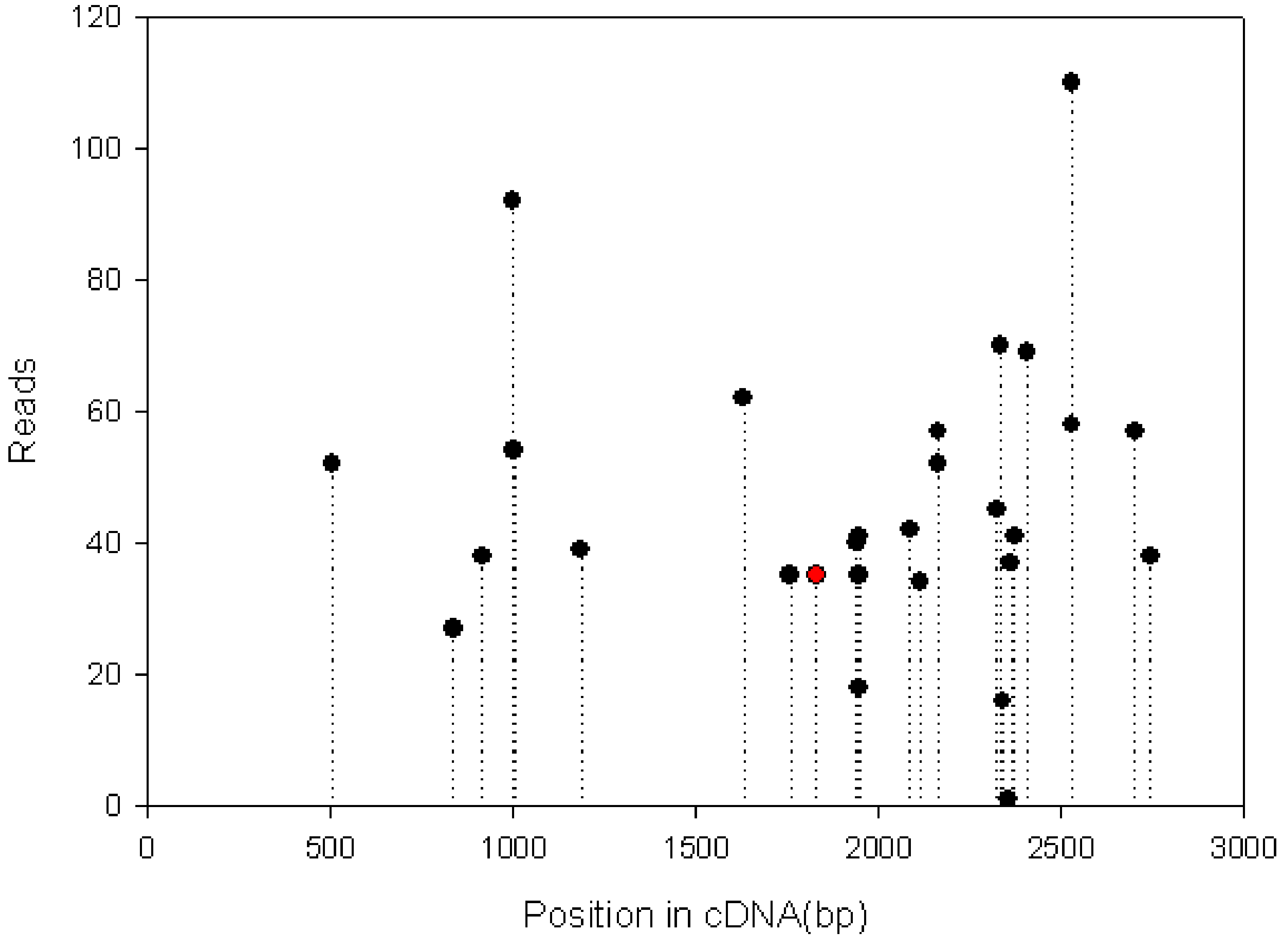


```
5' GGUGAGAGCAAGAAGGCUGUGGAGAA 3'        Cs9g19220.1
         ::::::.::::::::.
3' ------AAGUUCUUUCGACACCUU-- 5'        Csi-miR396b.2
```

Csi-miR396b.2, target=Orange1.1t01932.1 gene=Orange1.1t01932
Category:3
Score=5
Cleavage Site=968

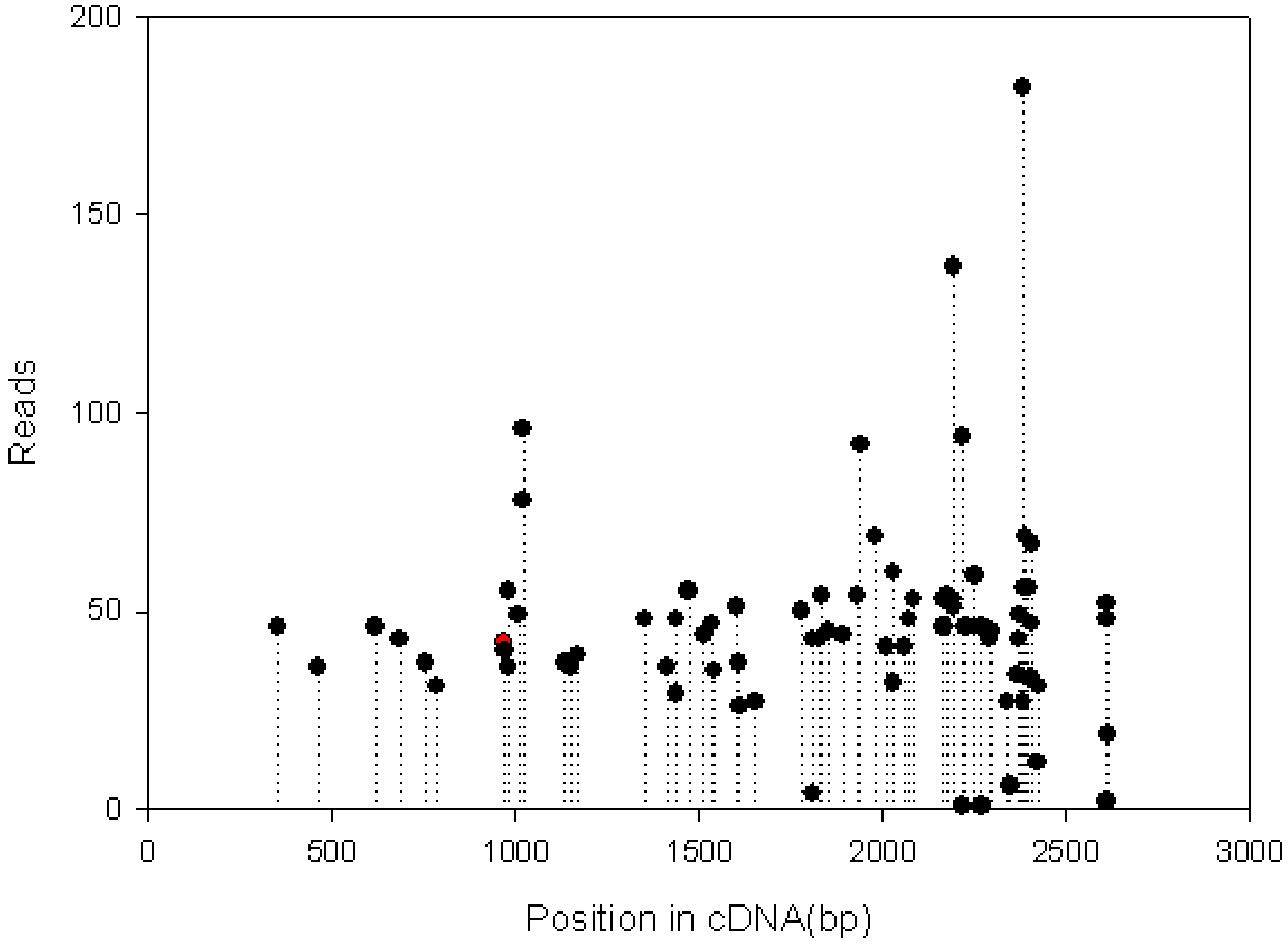


```
5' UCAGGUCUUGAGAAAGCUUGUGGAUG 3'        Orange1.1t01932.1
          :..:::::::: ::::::
3' ------AAGUUCUUUCG-ACACCUU- 5'        Csi-miR396b.2
```

Csi-miR396b.2, target=Orange1.1t02254.1 gene=Orange1.1t02254
Category:3
Score=2.5
Cleavage Site=1371

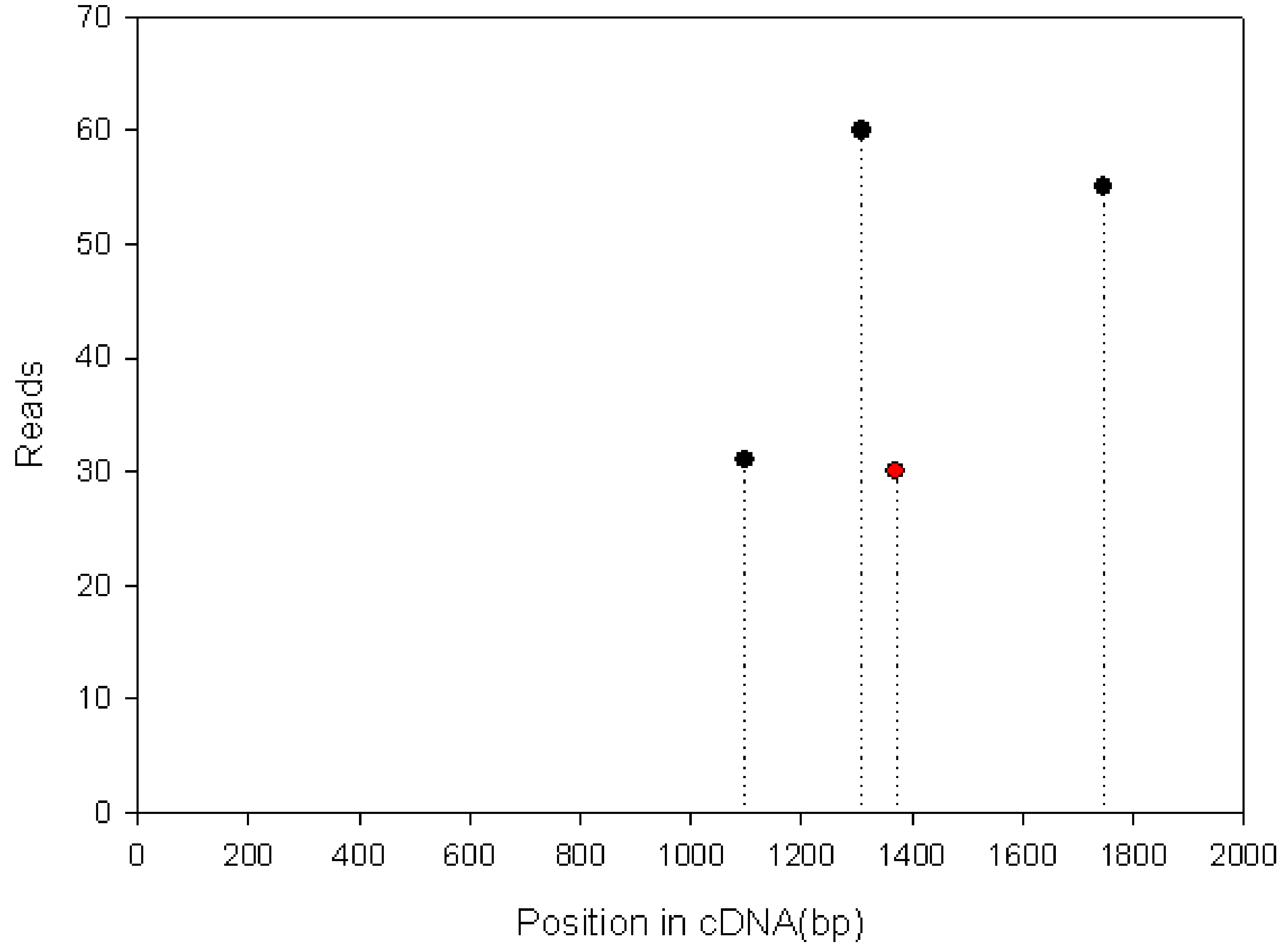


```
5' GUUGCUGAAGAAAGCUGUGGAGGAUC 3'        Orange1.1t02254.1
         : ::::::::::::::.
3' ----AAGUUCUUUCGACACCUU---- 5'        Csi-miR396b.2
```

Csi-miR396b.2, target=Orange1.1t02489.1 gene=Orange1.1t02489
Category:3
Score=5
Cleavage Site=182

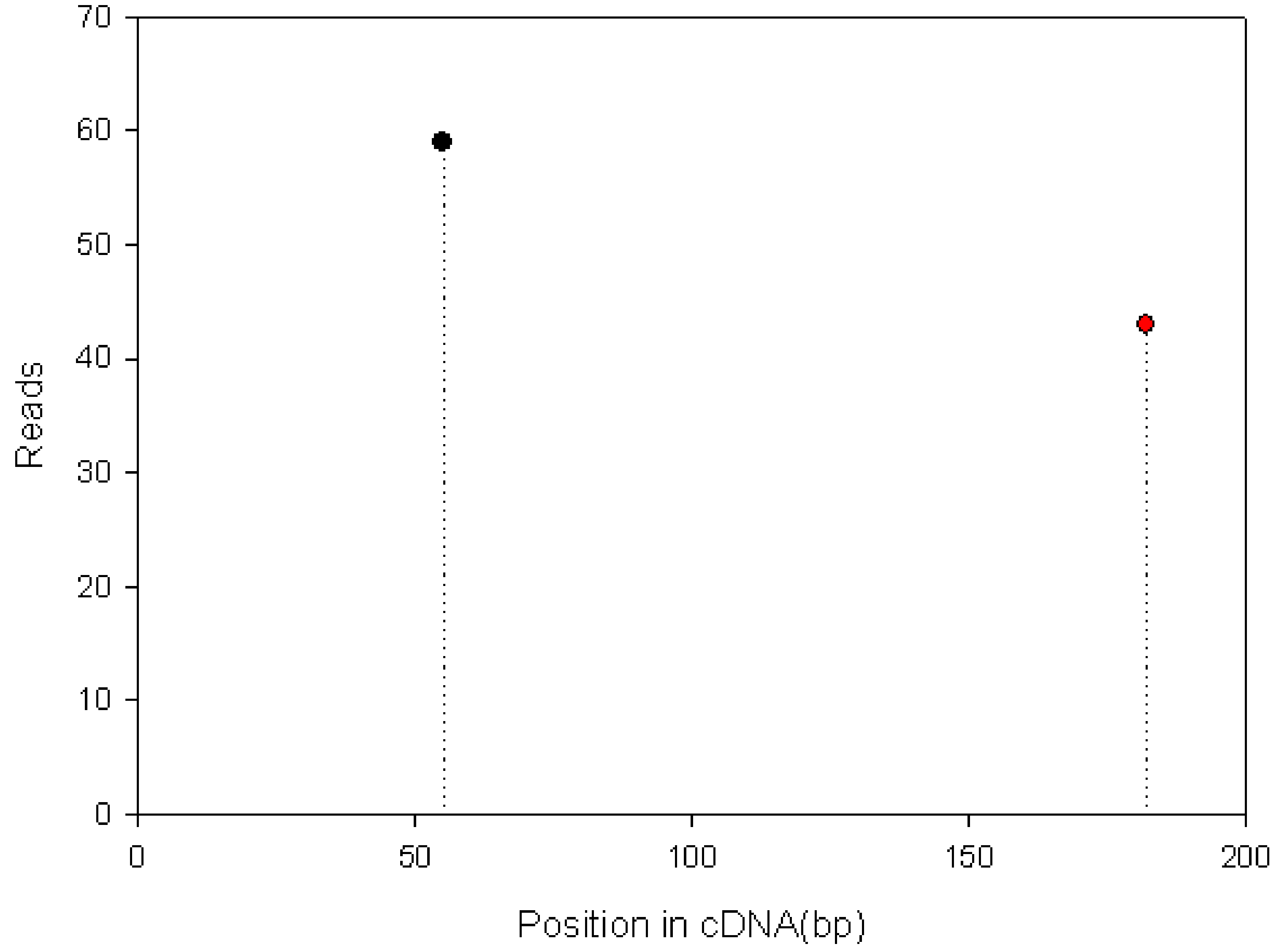


```
5' UUAUAUUCACUAAAGCUG-GGAAAAUC 3'        Orange1.1t02489.1
          ::::   ::::::: ::::
3' -----AAGUUCUUUCGACACCUU---- 5'        Csi-miR396b.2
```

Csi-miR396b.2, target=Orange1.1t02492.1 gene=Orange1.1t02492
Category:3
Score=5
Cleavage Site=1009

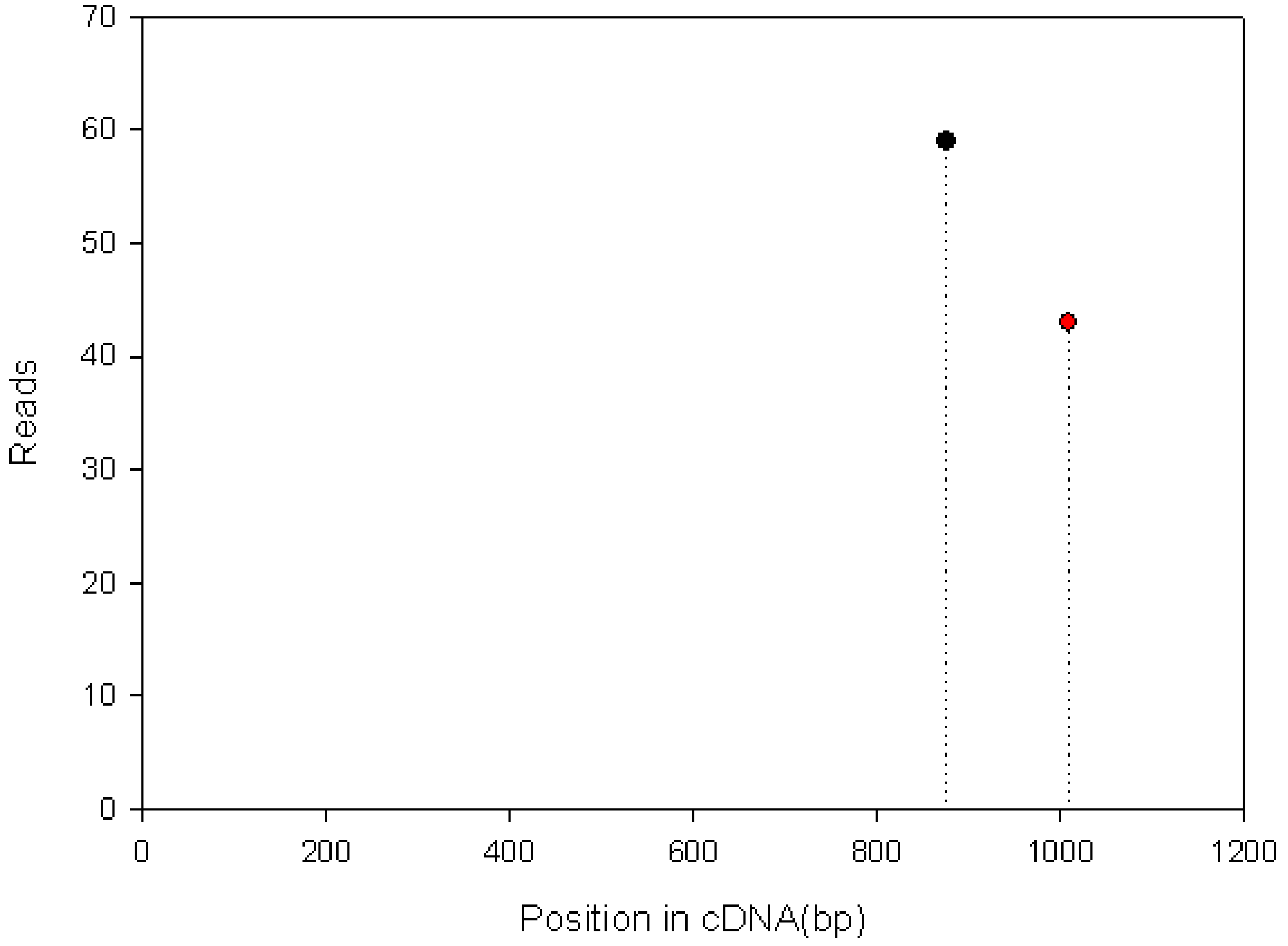


```
5' UUAUAUUCACUAAAGCUG-GGAAAAUC 3'        Orange1.1t02492.1
        ::::  ::::::: ::::
3' -----AAGUUCUUUCGACACCUU---- 5'        Csi-miR396b.2
```

Csi-miR396b.2, target=orange1.1t02555.1 gene=orange1.1t02555
Category:1
Score=2
Cleavage Site=920

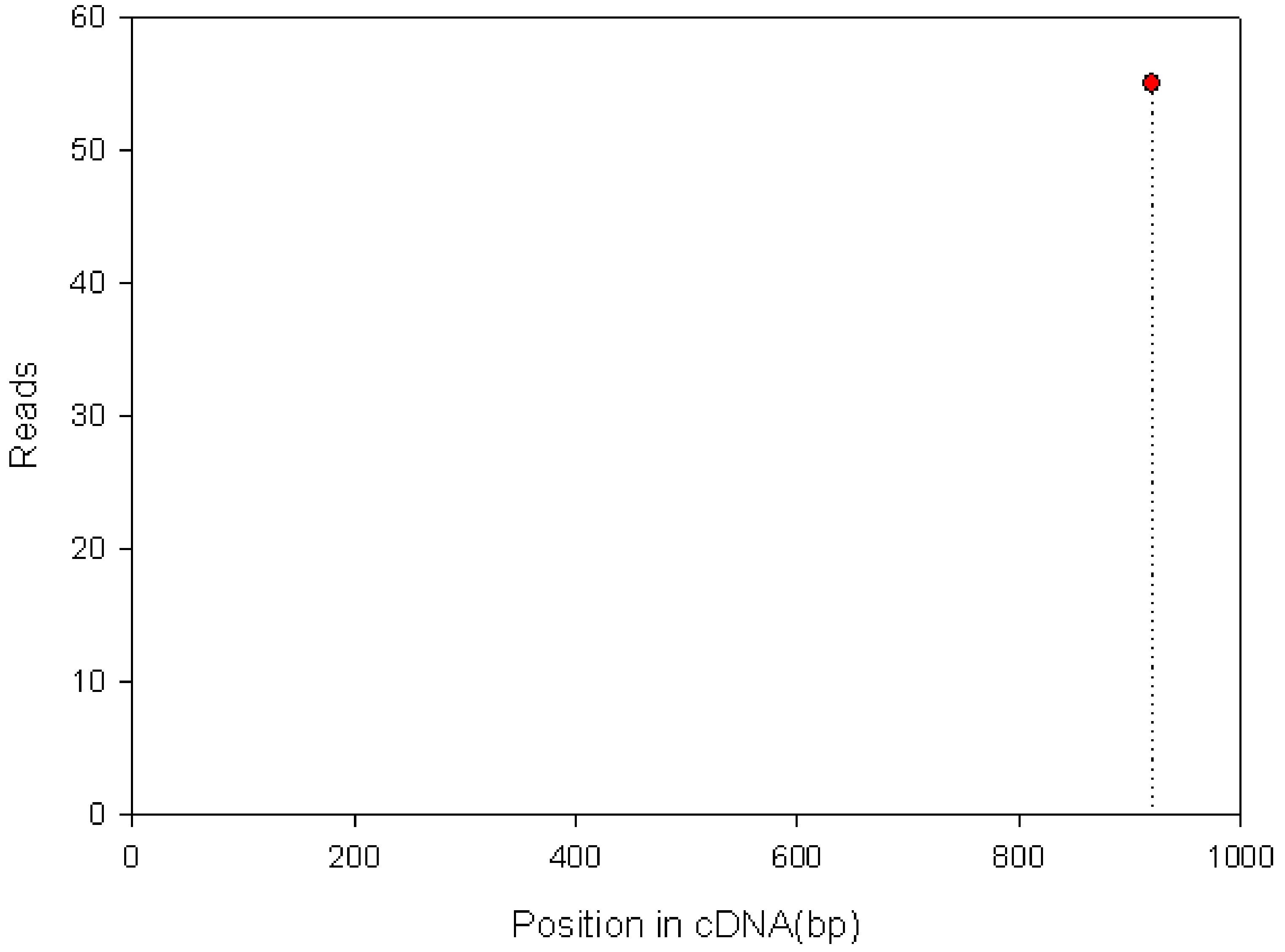


```
5' CACGUUCAAGAAAGCCUGUGGAACUU 3'        Orange1.1t02555.1
       :::::::::: ::::::::
3' ----AAGUUCUUUC-GACACCUU--- 5'        Csi-miR396b.2
```

Csi-miR396b.2, target=orange1.1t03122.1 gene=orange1.1t03122
Category:2
Score=2
Cleavage Site=583

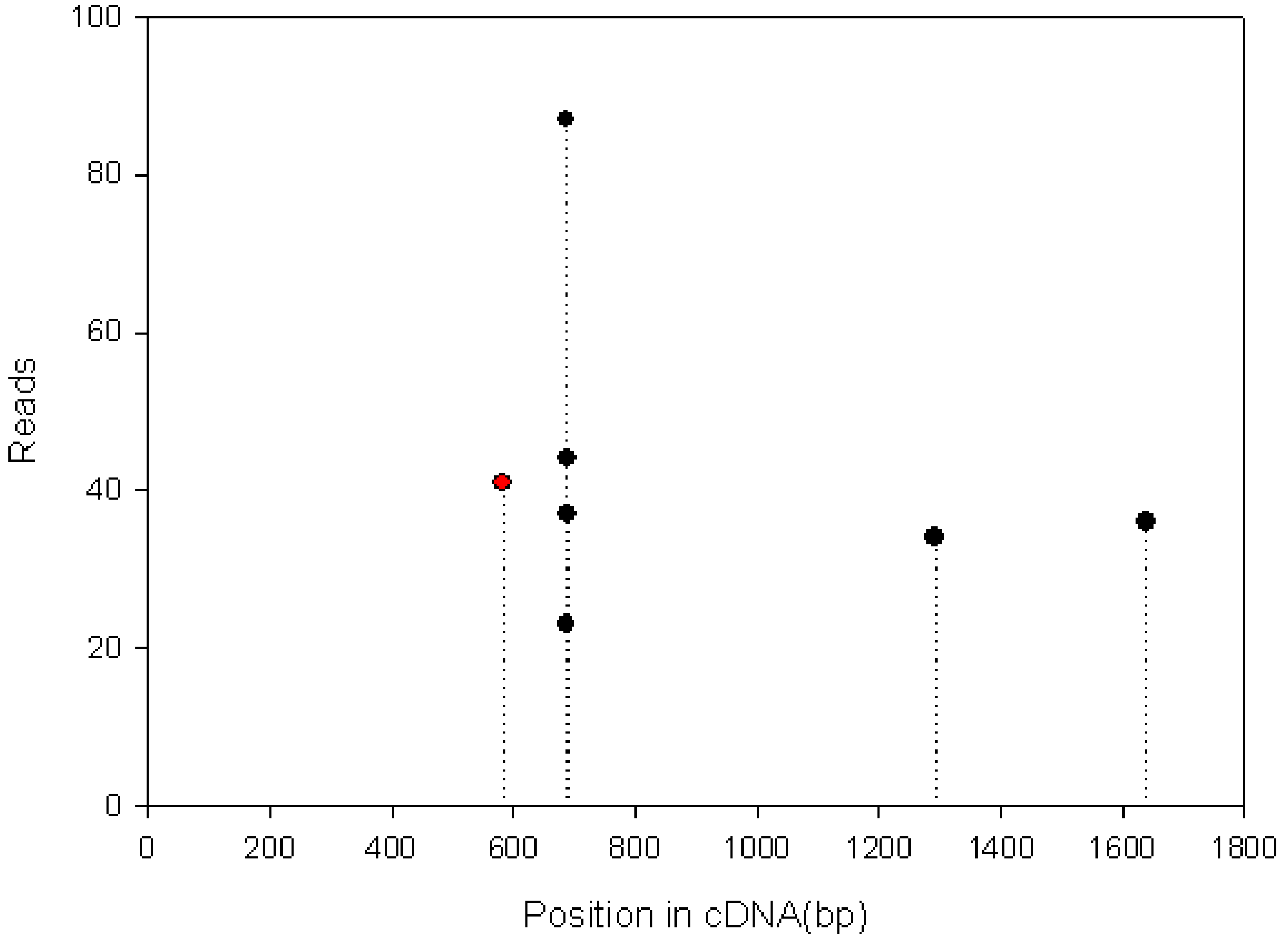


```
5' ACCGUUCAAGAAAGCCUGUGGAAUCA 3'        Orange1.1t03122.1
       :::::::::: ::::::::
3' ----AAGUUCUUUC-GACACCUU--- 5'        Csi-miR396b.2
```

Csi-miR396b.3, target=Cs3g23180.1 gene=Cs3g23180
Category: 3
Score=3
Cleavage Site=625

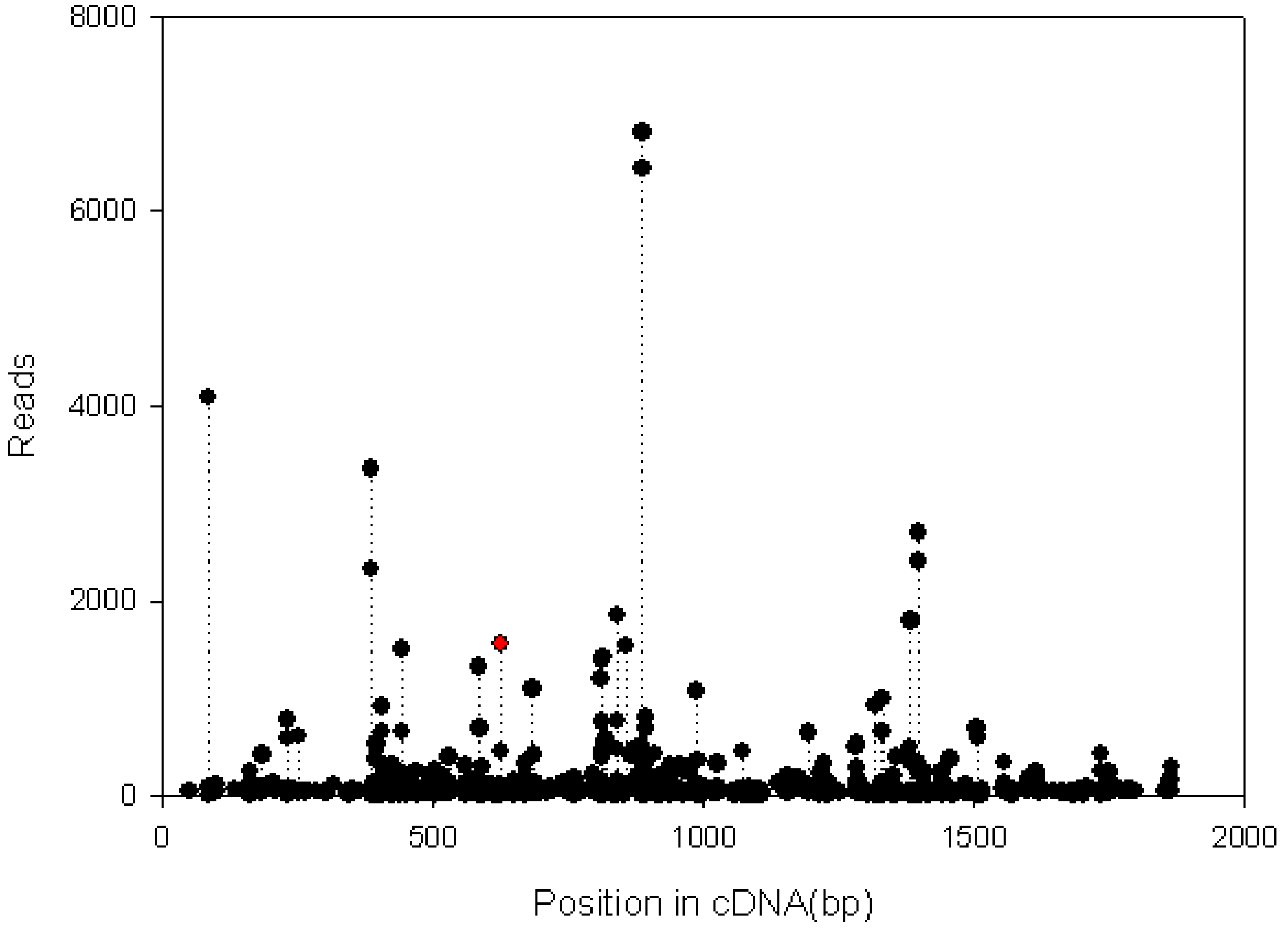


```
5' AAAGAUCAAGGAAGCUGUGGGAGUUG 3'        Cs3g23180.1
    ::: :::::.::::::::::.:
3' -UUCAAGUUCUUUCGACACCUU---- 5'        Csi-miR396b.3
```

Csi-miR396b.3, target=Cs5g01380.1 gene=Cs5g01380
Category:1
Score=4
Cleavage Site=1181

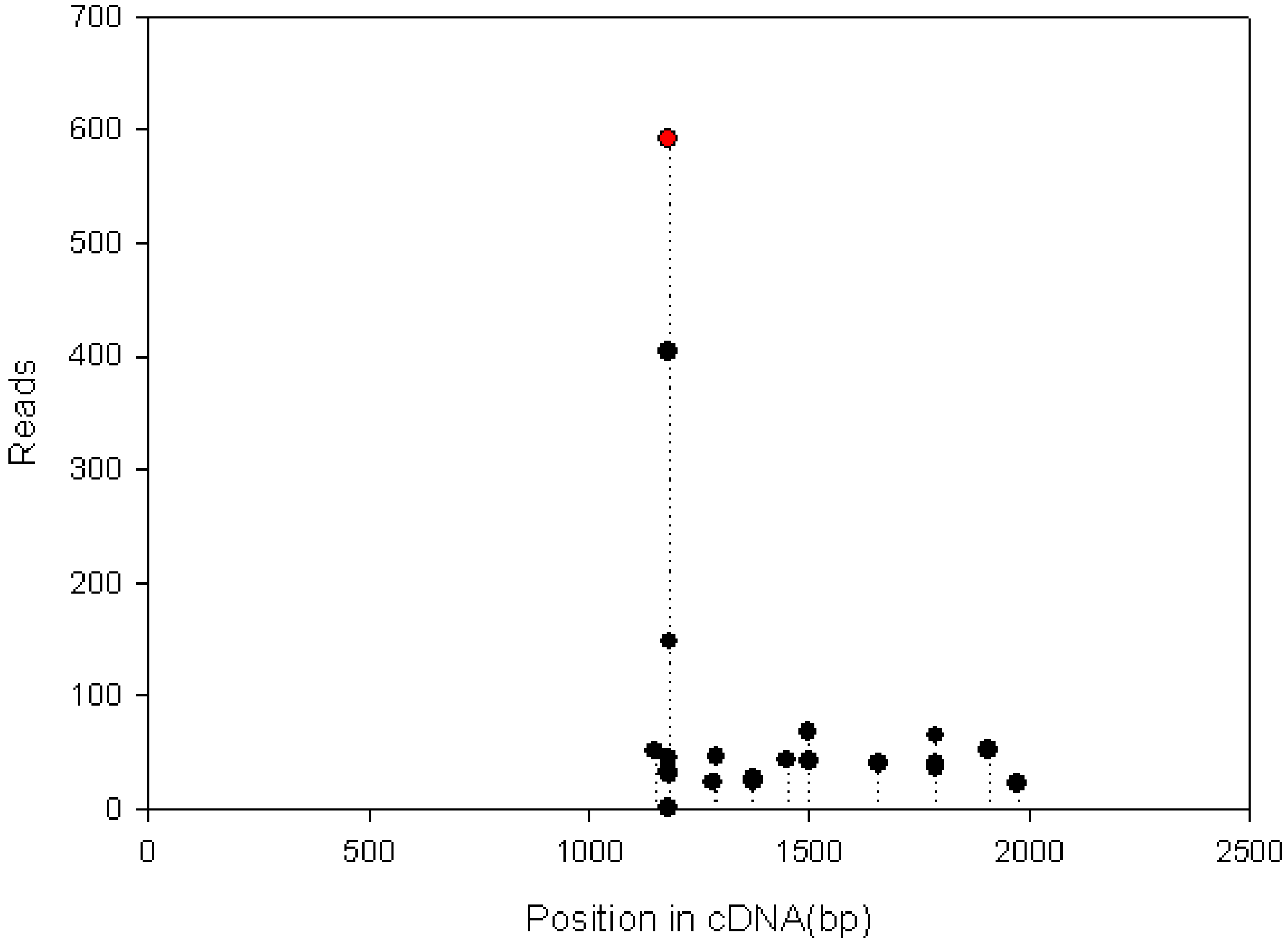


```
5' CCCGUUCAAGAAAGCCUGUGGAAUUA 3'        Cs5g01380.1
     :::::::::::: ::::::::
3' -UUCAAGUUCUUUC-GACACCUU--- 5'        Csi-miR396b.3
```

Csi-miR396b.3, target=Cs5g19200.1 gene=Cs5g19200
Category:2
Score=3.5
Cleavage Site=201

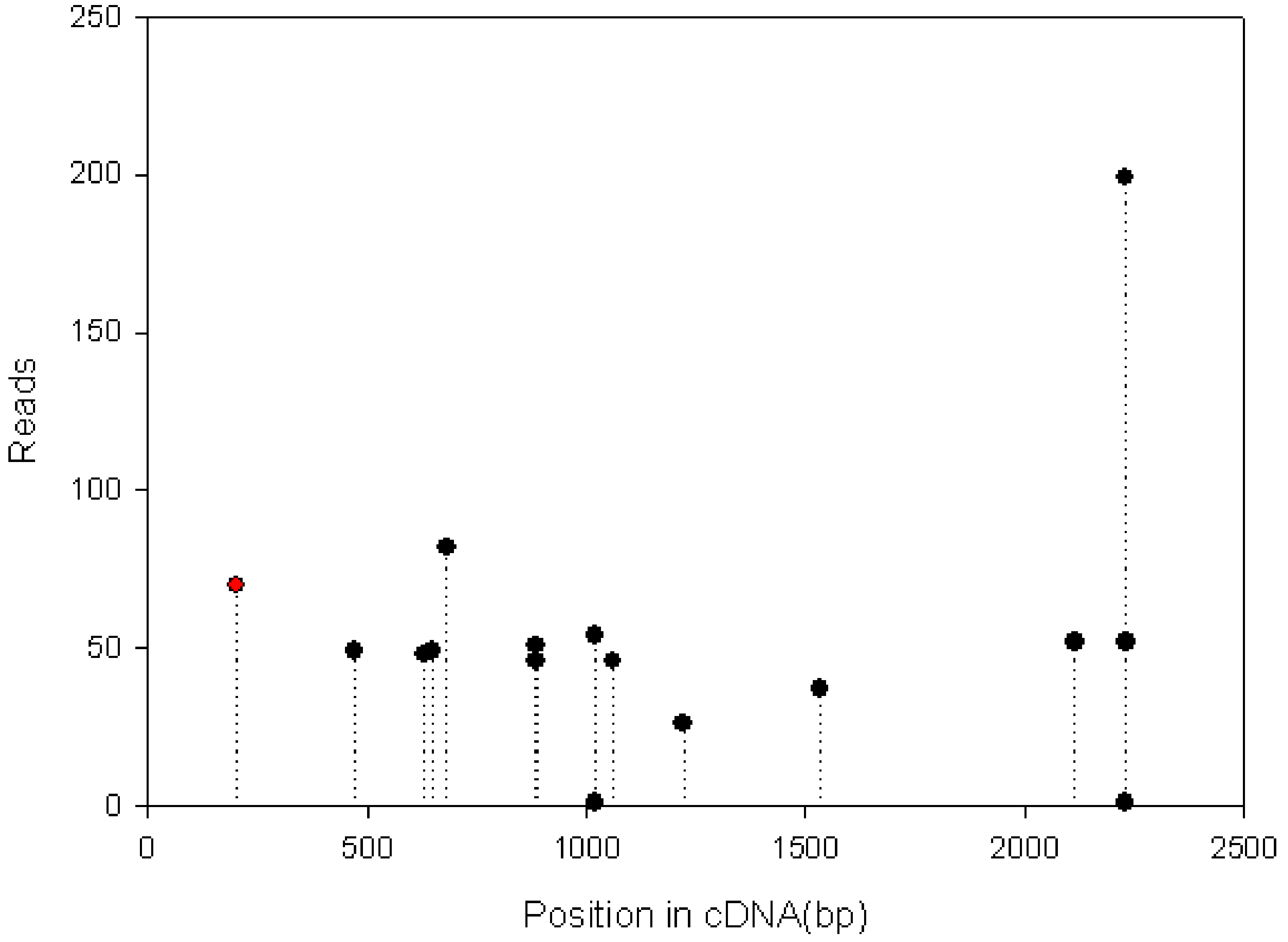


```
5' AAUGUUUAAGAAAGCAGUGGAAGCAA 3'        Cs5g19200.1
    :  :::.:::::::: ::::::
3' -UUCAAGUUCUUUCGACACCUU---- 5'        Csi-miR396b.3
```

Csi-miR396b.3, target=Cs7g12930.1 gene=Cs7g12930
Category:1
Score=4.5
Cleavage Site=1079

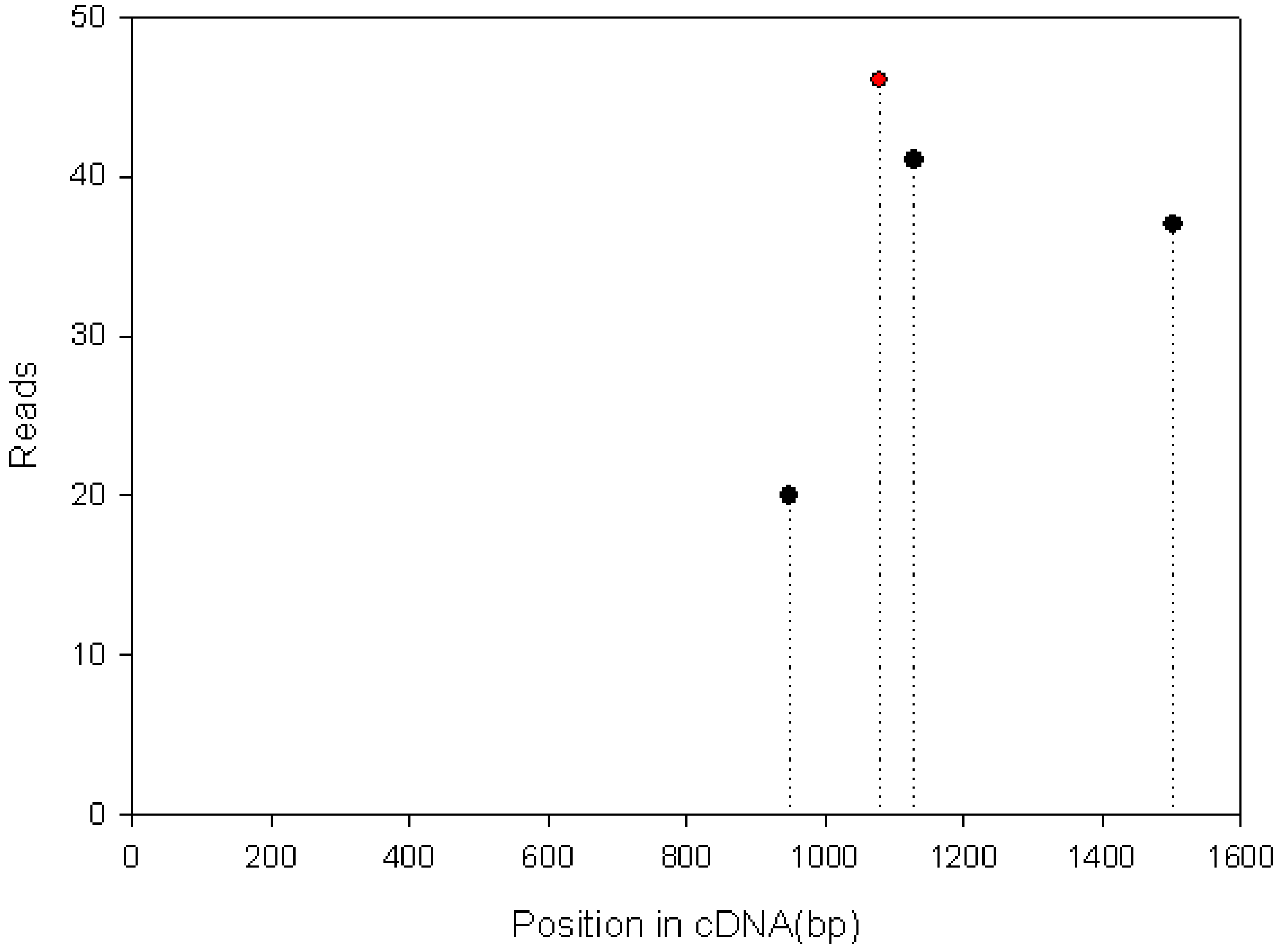


```
5' UAGUAUUAAGAAAGCUGAGGAAAAAG 3'        Cs7g12930.1
   ::: :.:::::::::: ::::
3' UUCA-AGUUCUUUCGACACCUU---- 5'        Csi-miR396b.3
```

Csi-miR396b.3, target=Cs7g15220.1 gene=Cs7g15220
Category:1
Score=4
Cleavage Site=377

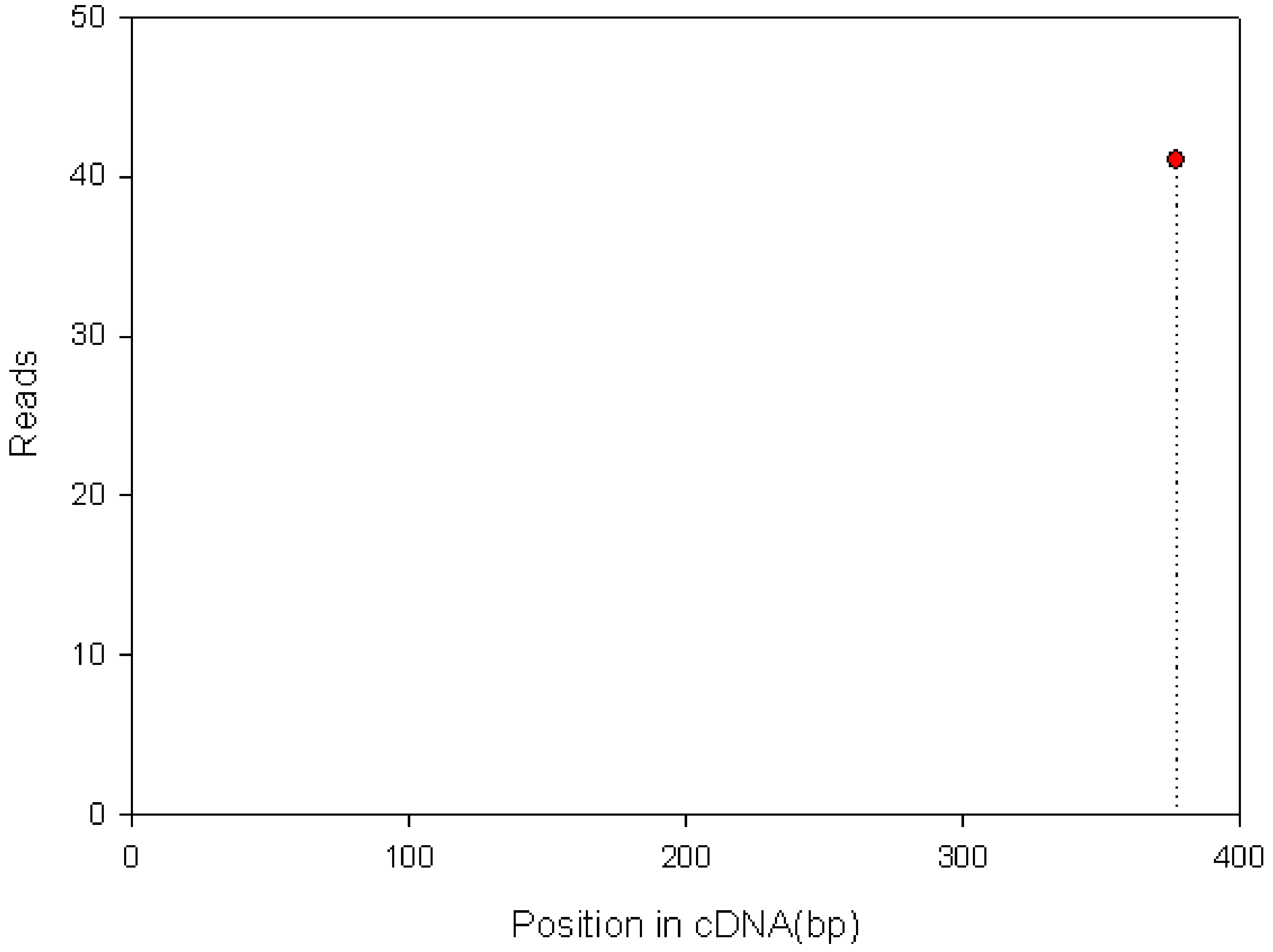


```
5' ACCGUUCAAGAAAGCCUGUGGAAUCA 3'        Cs7g15220.1
     ::::::::::: ::::::::
3' -UUCAAGUUCUUUC-GACACCUU--- 5'        Csi-miR396b.3
```

Csi-miR396b.3, target=Cs9g19220.1 gene=Cs9g19220
Category:3
Score=4
Cleavage Site=1829

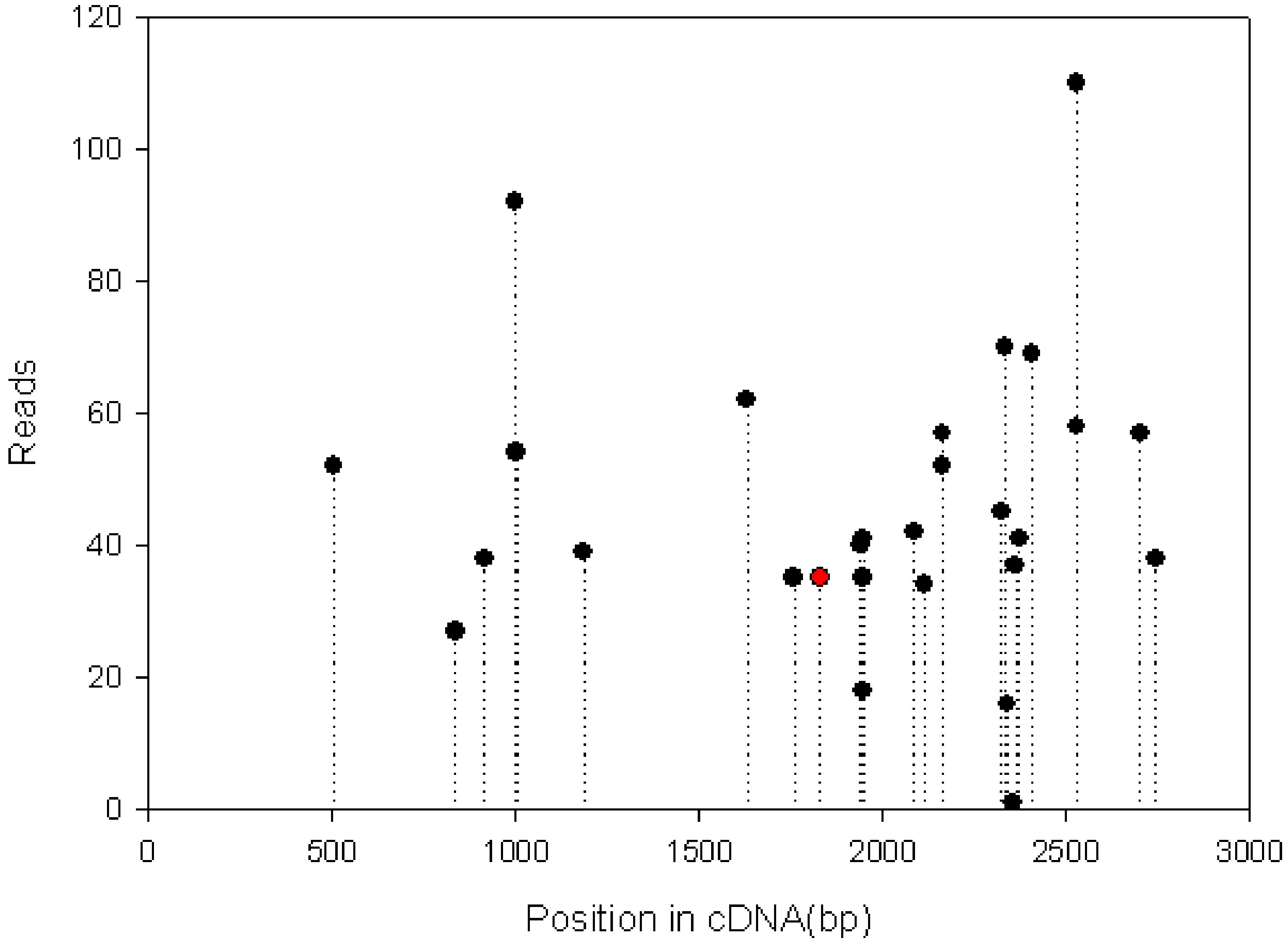


```
5' GGUGAGAGCAAGAAGGCUGUGGAGAA 3'        Cs9g19220.1
       .::   ::::::.::::::::.
3' ---UUCAAGUUCUUUCGACACCUU-- 5'        Csi-miR396b.3
```

Csi-miR396b.3, target=Orange1.1t02254.1 gene=Orange1.1t02254
Category:3
Score=4.5
Cleavage Site=1371

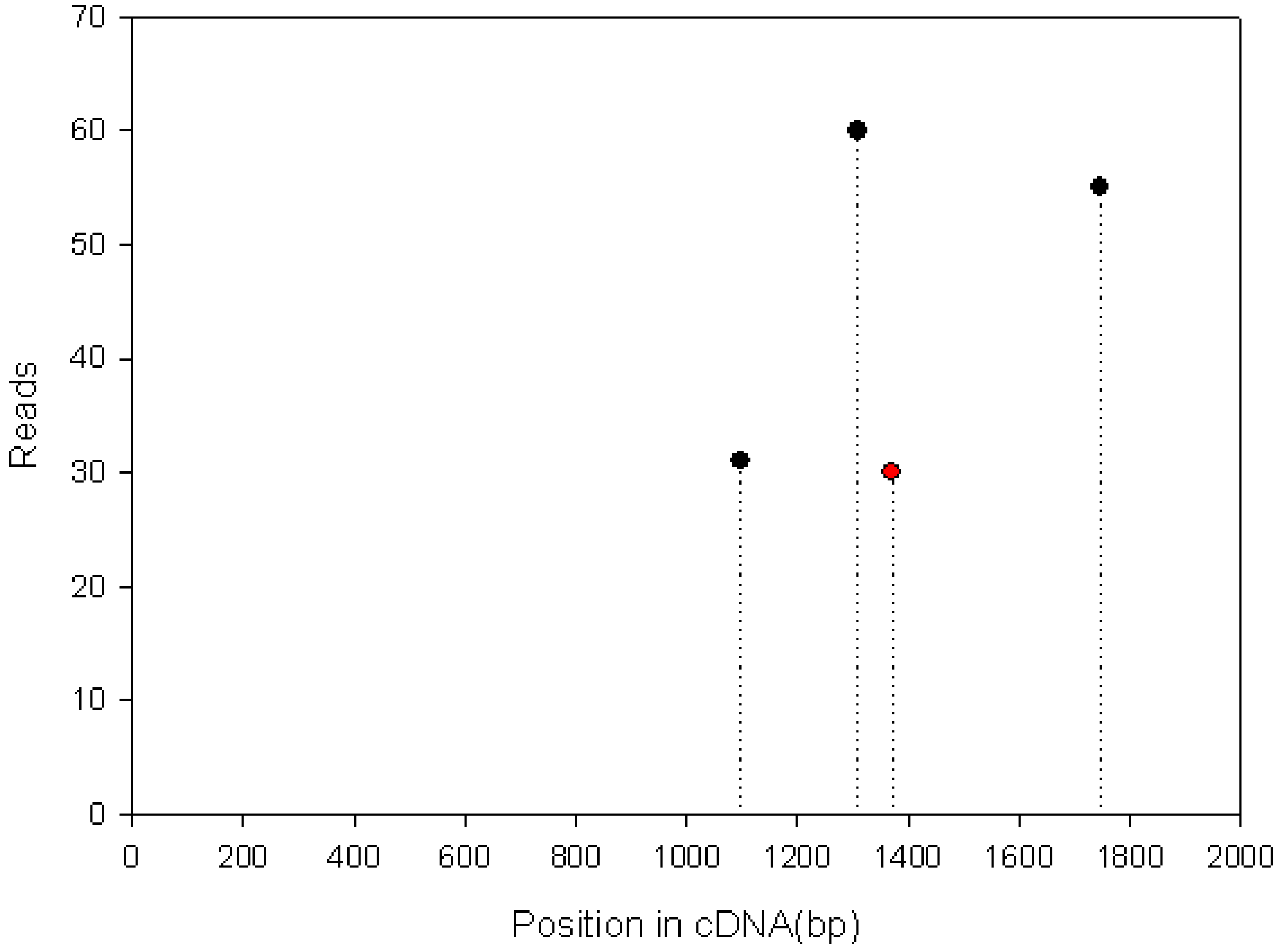


```
5' GUUGCUGAAGAAAGCUGUGGAGGAUC 3'        Orange1.1t02254.1
       : : ::::::::::::::.
3' -UUCAAGUUCUUUCGACACCUU---- 5'        Csi-miR396b.3
```

Csi-miR396b.3, target=Orange1.1t02555.1 gene=Orange1.1t02555
Category:1
Score=3
Cleavage Site=920

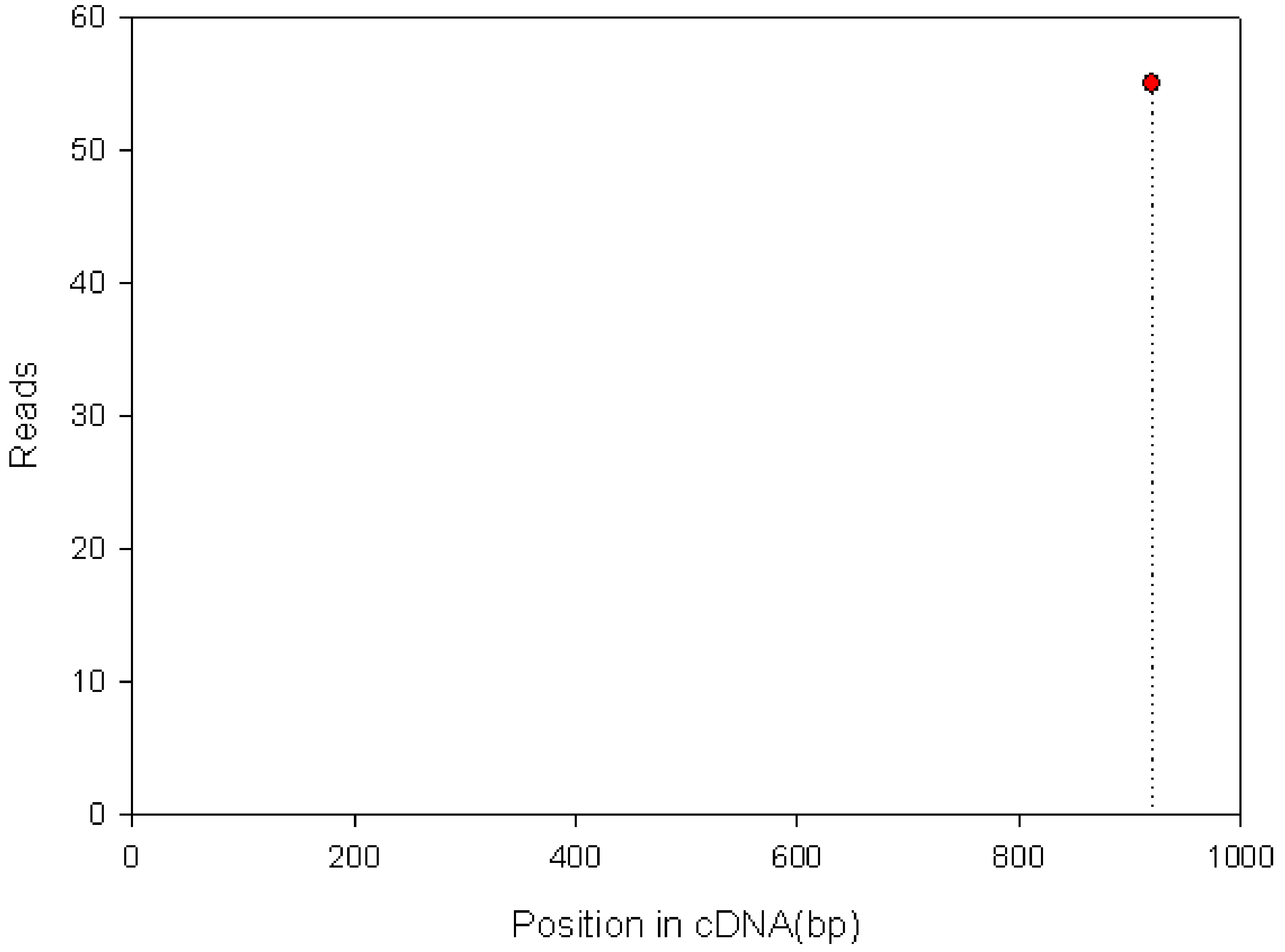


```
5' CACGUUCAAGAAAGCCUGUGGAACUU 3'        Orange1.1t02555.1
    : :::::::::: ::::::::
3' -UUCAAGUUCUUUC-GACACCUU--- 5'        Csi-miR396b.3
```

Csi-miR396b.3, target=Orange1.1t03122.1 gene=Orange1.1t03122
Category:2
Score=4
Cleavage Site=583

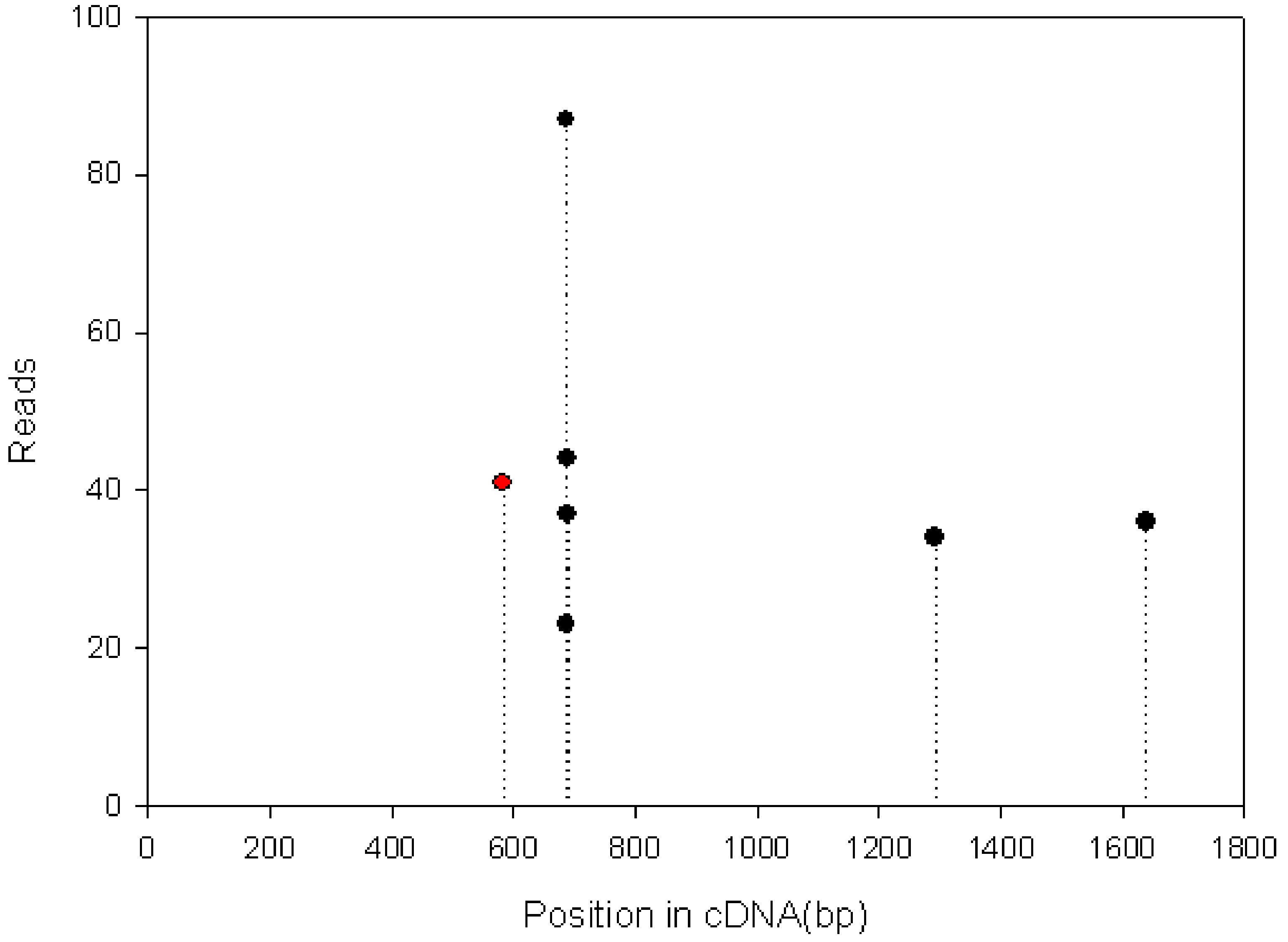


```
5' ACCGUUCAAGAAAGCCUGUGGAAUCA 3'        Orange1.1t03122.1
     ::::::::::: ::::::::
3' -UUCAAGUUCUUUC-GACACCUU--- 5'        Csi-miR396b.3
```

Csi-miR396b-3p.2, target=Cs8g05290.1 gene=Cs8g05290
Category:2
Score=3.5
Cleavage Site=160

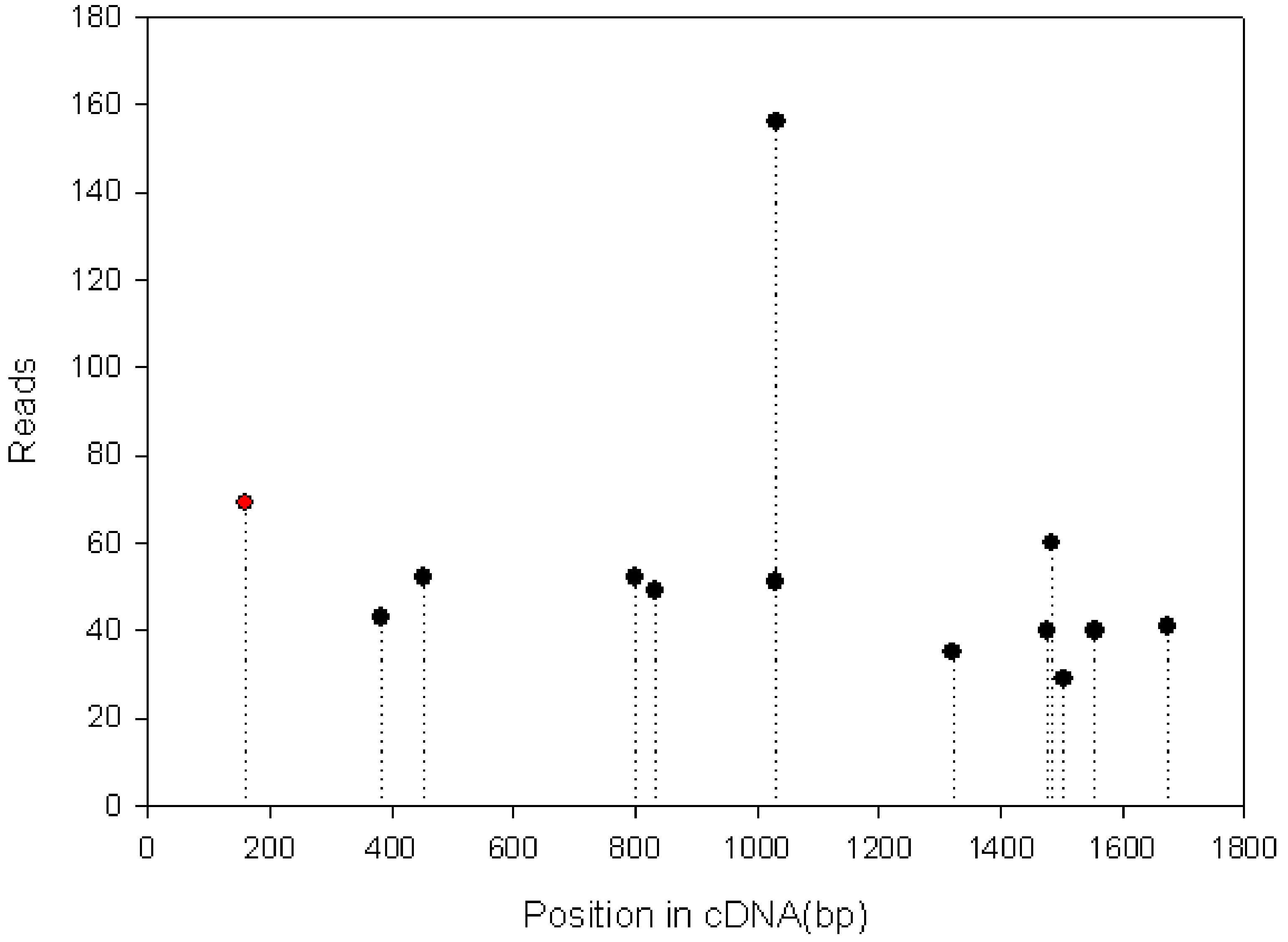


```
5' UAUAUUUUACGGCUUUCUUGAGUCCA 3'        Cs8g05290.1
     : :...::.:::::::::::
3' --AGAGGGUGUCGAAAGAACUC---- 5'        Csi-miR396b-3p.2
```

Csi-miR396c, target=Cs2g27200.1 gene=Cs2g27200
Category:3
Score=5
Cleavage Site=1455

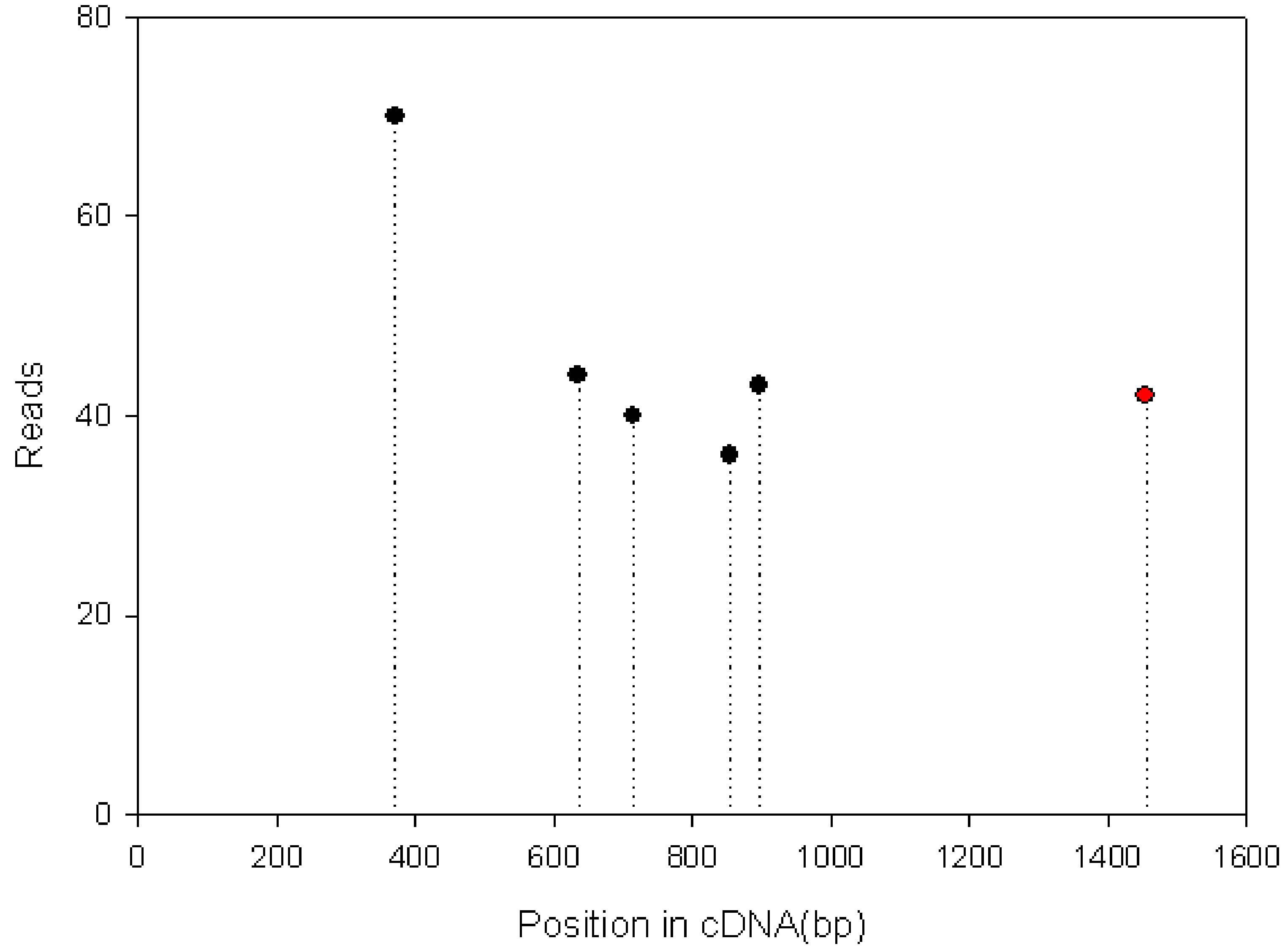


```
5' AUCUUCCUGCAUAUUUGUUGAACAAC 3'        Cs2g27200.1
     :::::..:: :::: :::::
3' --GAAGGGUGUCUAAAGAACUU---- 5'        Csi-miR396c
```

Csi-miR396c, target=Cs5g07360.1 gene=Cs5g07360
Category:2
Score=5
Cleavage Site=3325

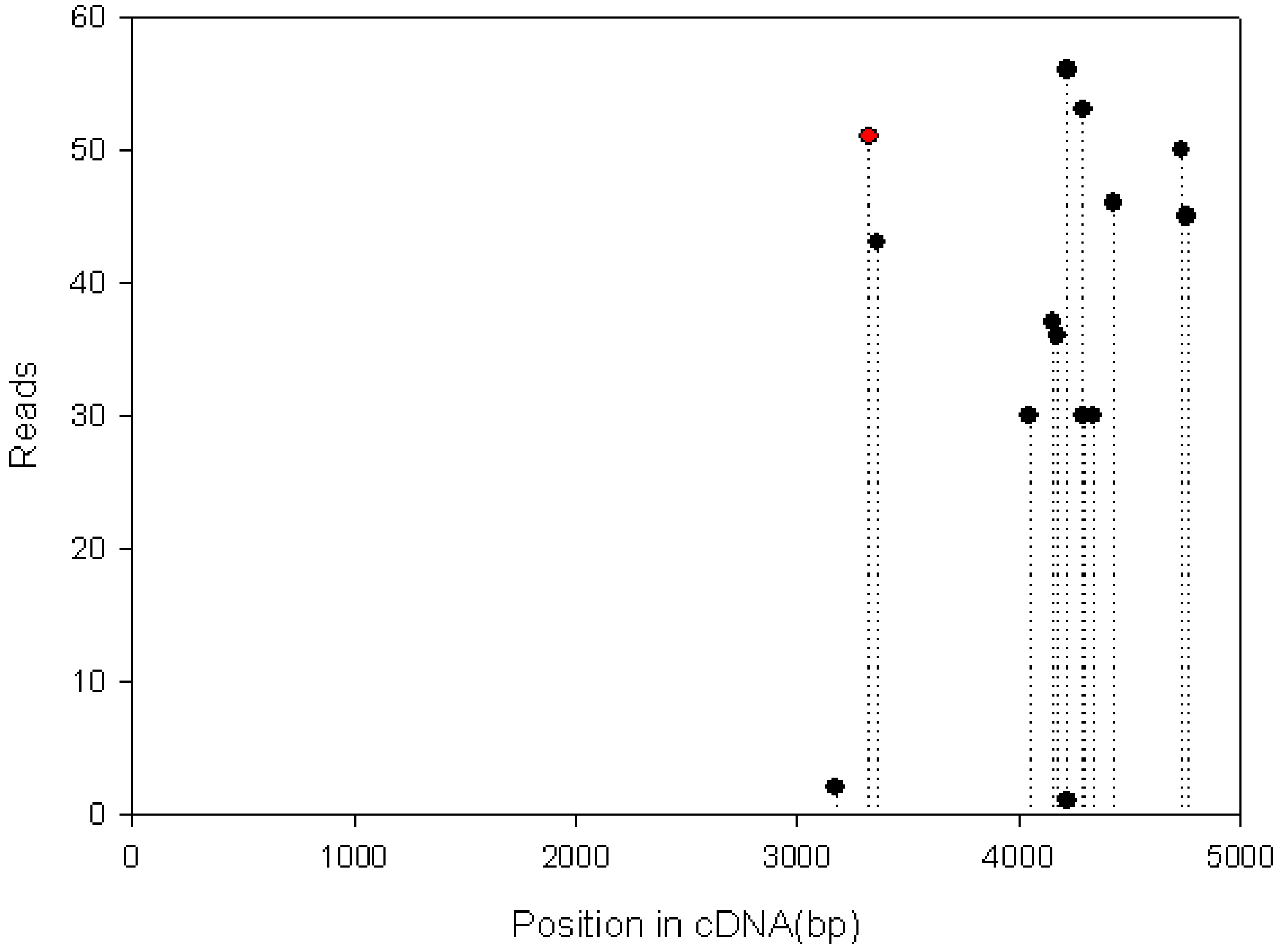


```
5' AUGCUUUUAACAGAUUUAUUGACGAG 3'        Cs5g07360.1
      ::::.. :::::::: ::::
3' ---GAAGGGUGUCUAAAGAACUU--- 5'        Csi-miR396c
```

Csi-miR396d.1, target=Cs3g23180.1 gene=Cs3g23180
Category: 3
Score=4.5
Cleavage Site=625

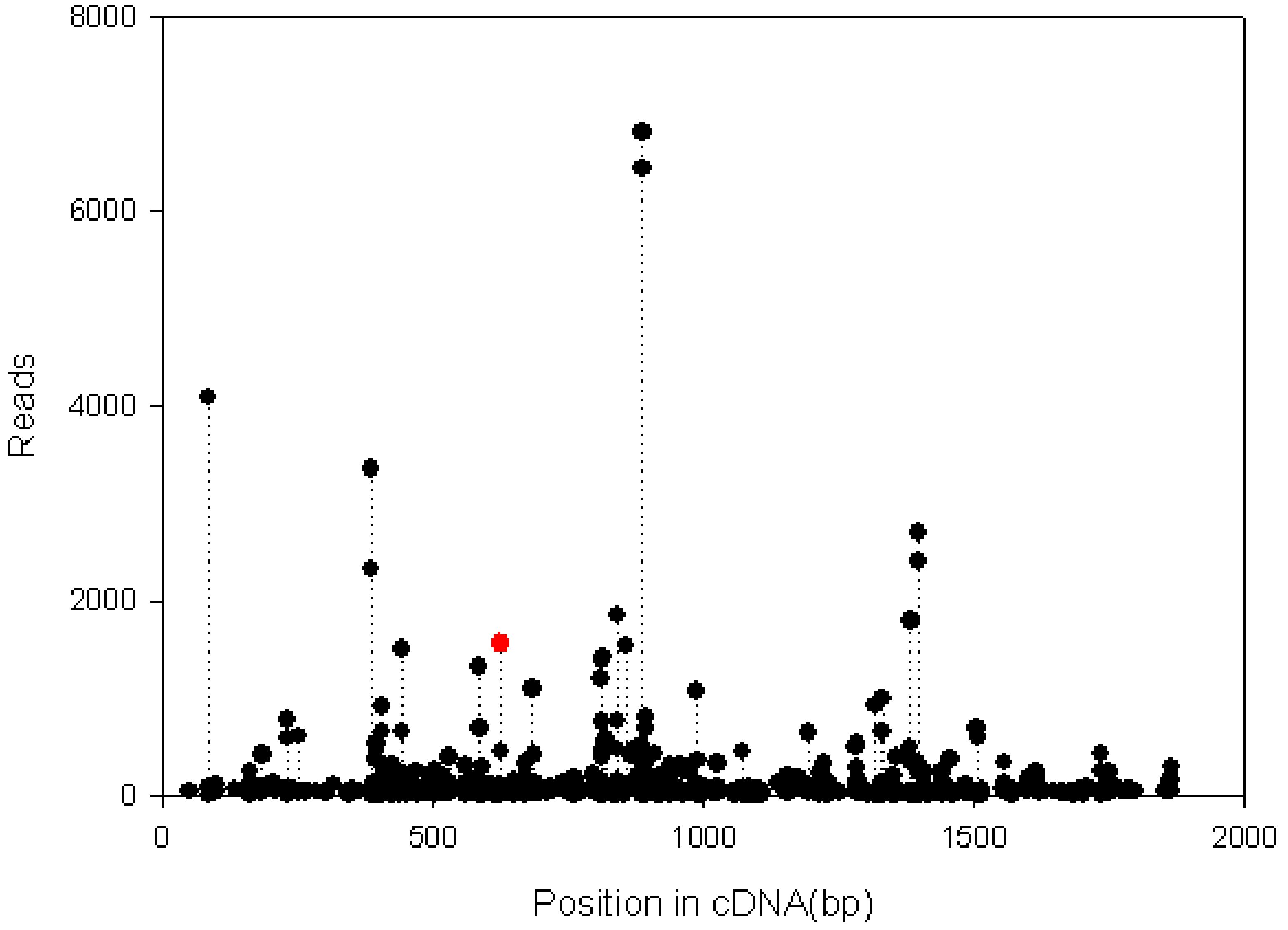


```
5' AAAGAUCAAGGAAGCUGUGGGAGUUG 3'        Cs3g23180.1
     :: :::::.::::.::::.:.
3' --UCAAGUUCUUUCGGCACCUUU--- 5'        Csi-miR396d.1
```

Csi-miR396d.1, target=Cs4g12200.1 gene=Cs4g12200
Category:2
Score=4.5
Cleavage Site=228

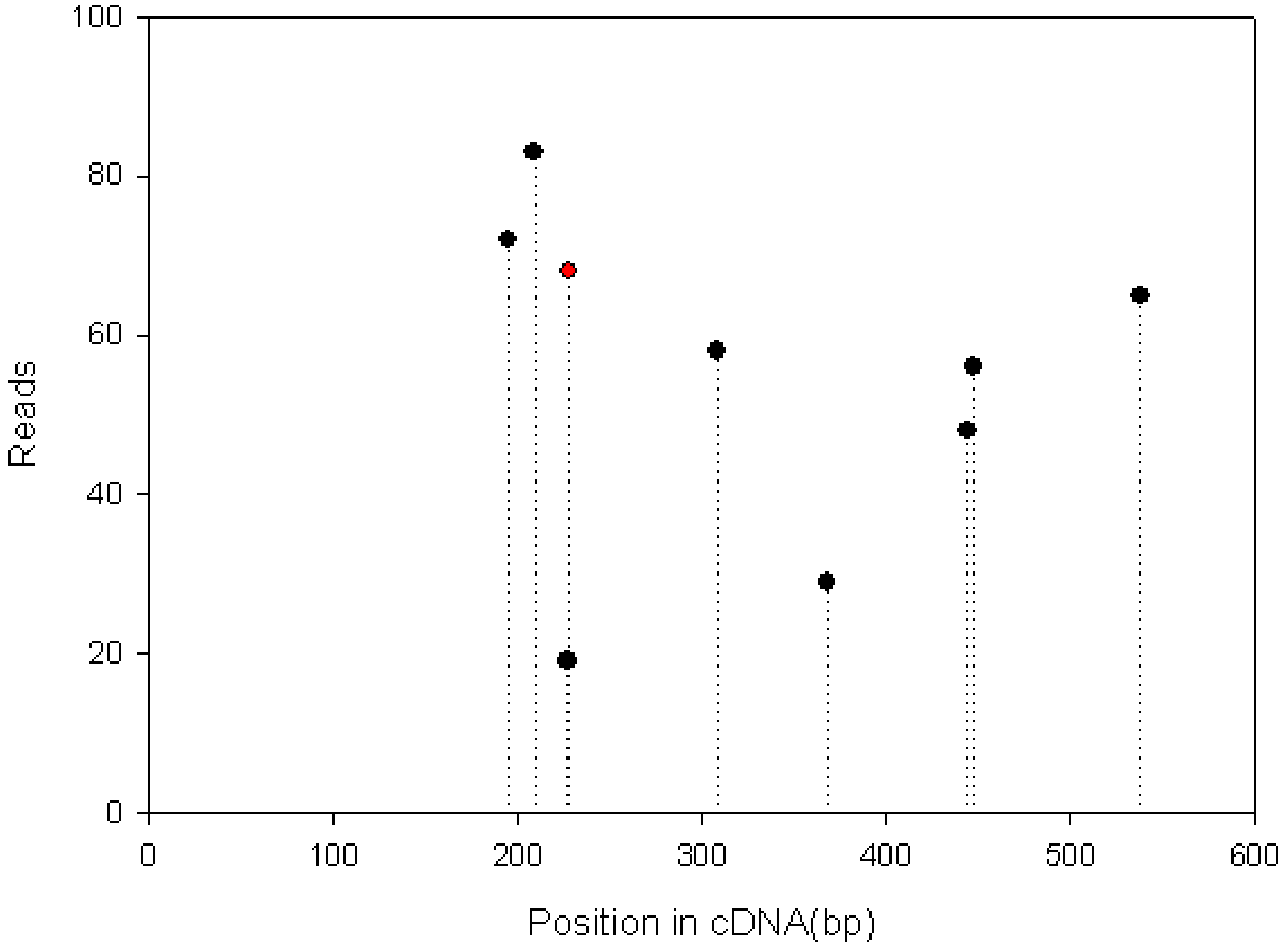


```
5' GGUUCCUGAAAGCCG-GGAAAUAUGCU 3'        Cs4g12200.1
   .::::   :::::::: :::::
3' UCAAGUUCUUUCGGCACCUUU------ 5'        Csi-miR396d.1
```

Csi-miR396d.1, target=Cs5g01380.1 gene=Cs5g01380
Category:1
Score=4
Cleavage Site=1181

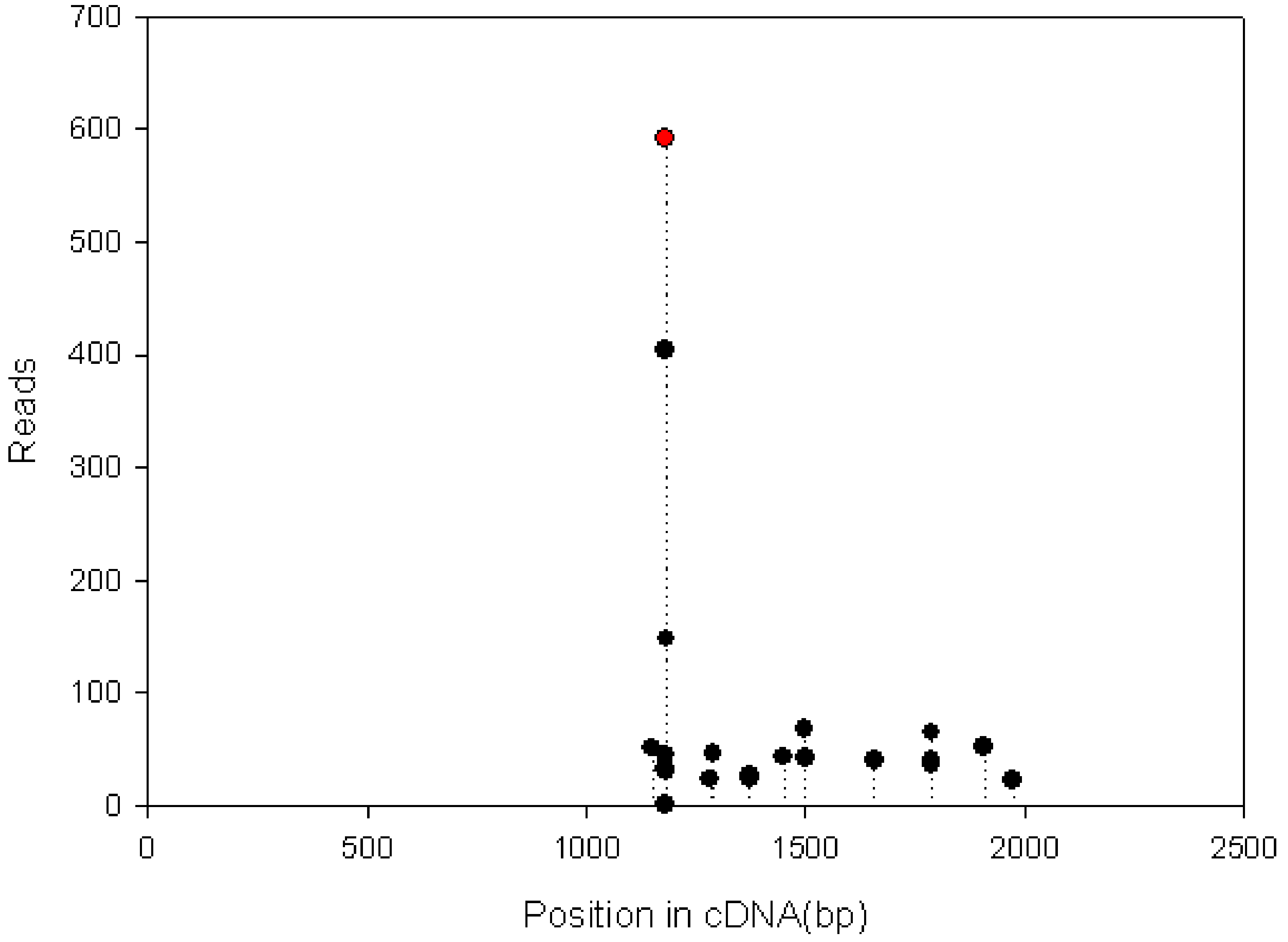


```
5' CCCGUUCAAGAAAGCCUGUGGAAUUA 3'        Cs5g01380.1
     ::::::::::::: ::::::
3' --UCAAGUUCUUUCGG-CACCUUU-- 5'        Csi-miR396d.1
```

Csi-miR396d.1, target=Cs5g19200.1 gene=Cs5g19200
Category:2
Score=4
Cleavage Site=201

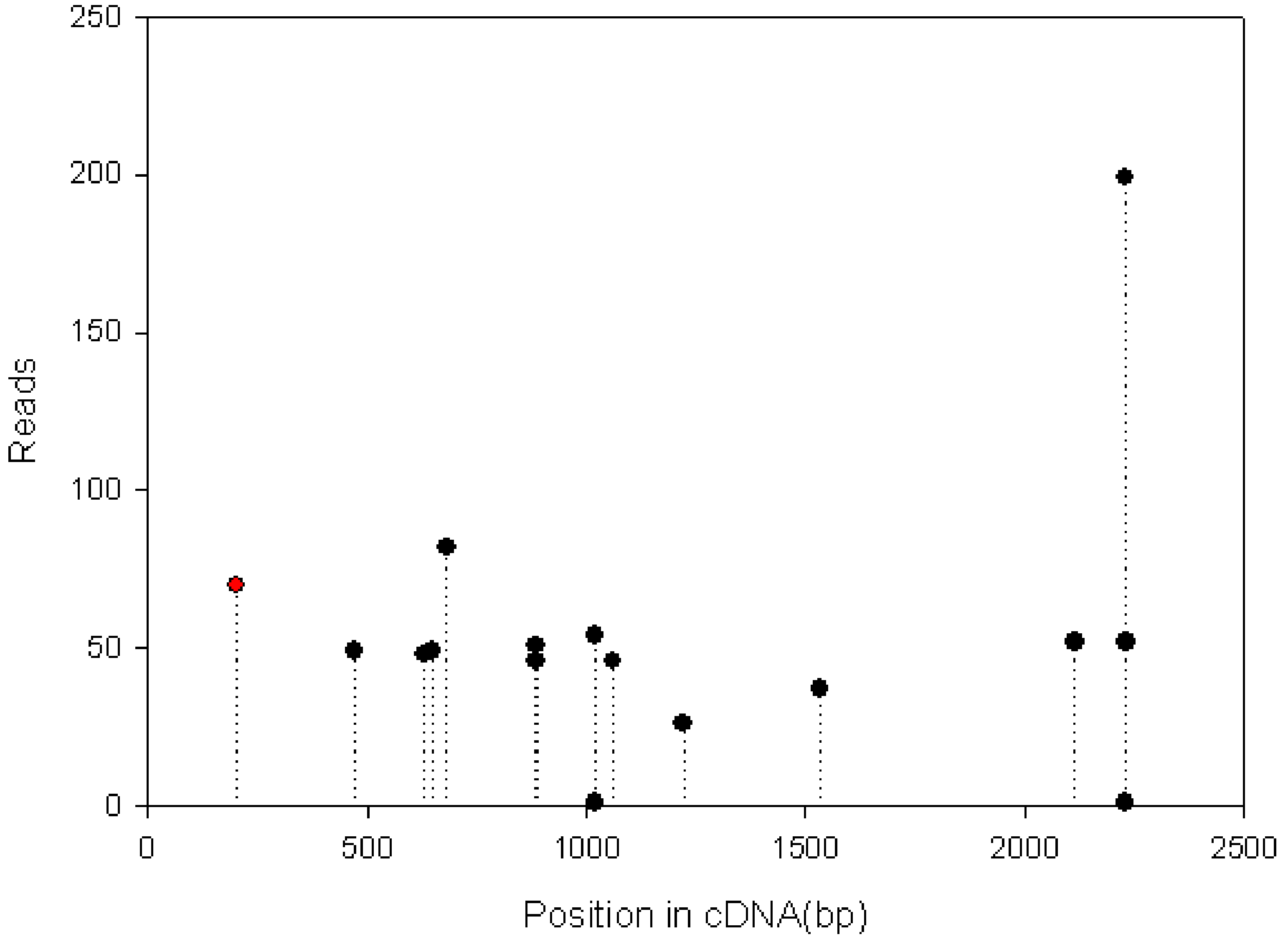


```
5' AAUGUUUAAGAAAGCAGUGGAAGCAA 3'        Cs5g19200.1
     :::.::::::::: ::::::.
3' --UCAAGUUCUUUCGGCACCUUU--- 5'        Csi-miR396d.1
```

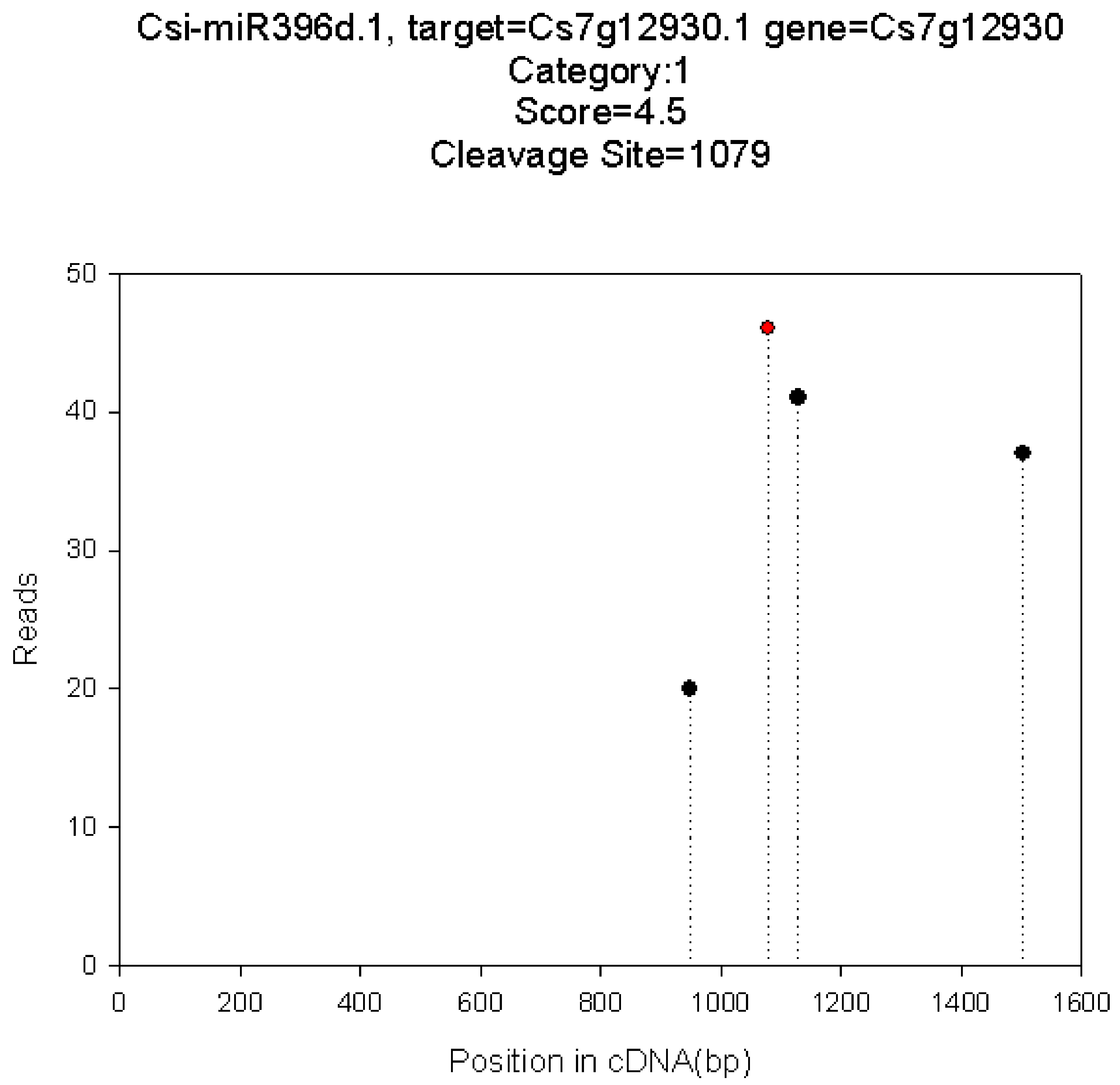


```
5' UAGUAUUAAGAAAGCUGAGGAAAAAG 3'        Cs7g12930.1
    ::: :.::::::::.: :::::
3' -UCA-AGUUCUUUCGGCACCUUU--- 5'        Csi-miR396d.1
```

Csi-miR396d.1, target=Cs7g15220.1 gene=Cs7g15220
Category:1
Score=4
Cleavage Site=377

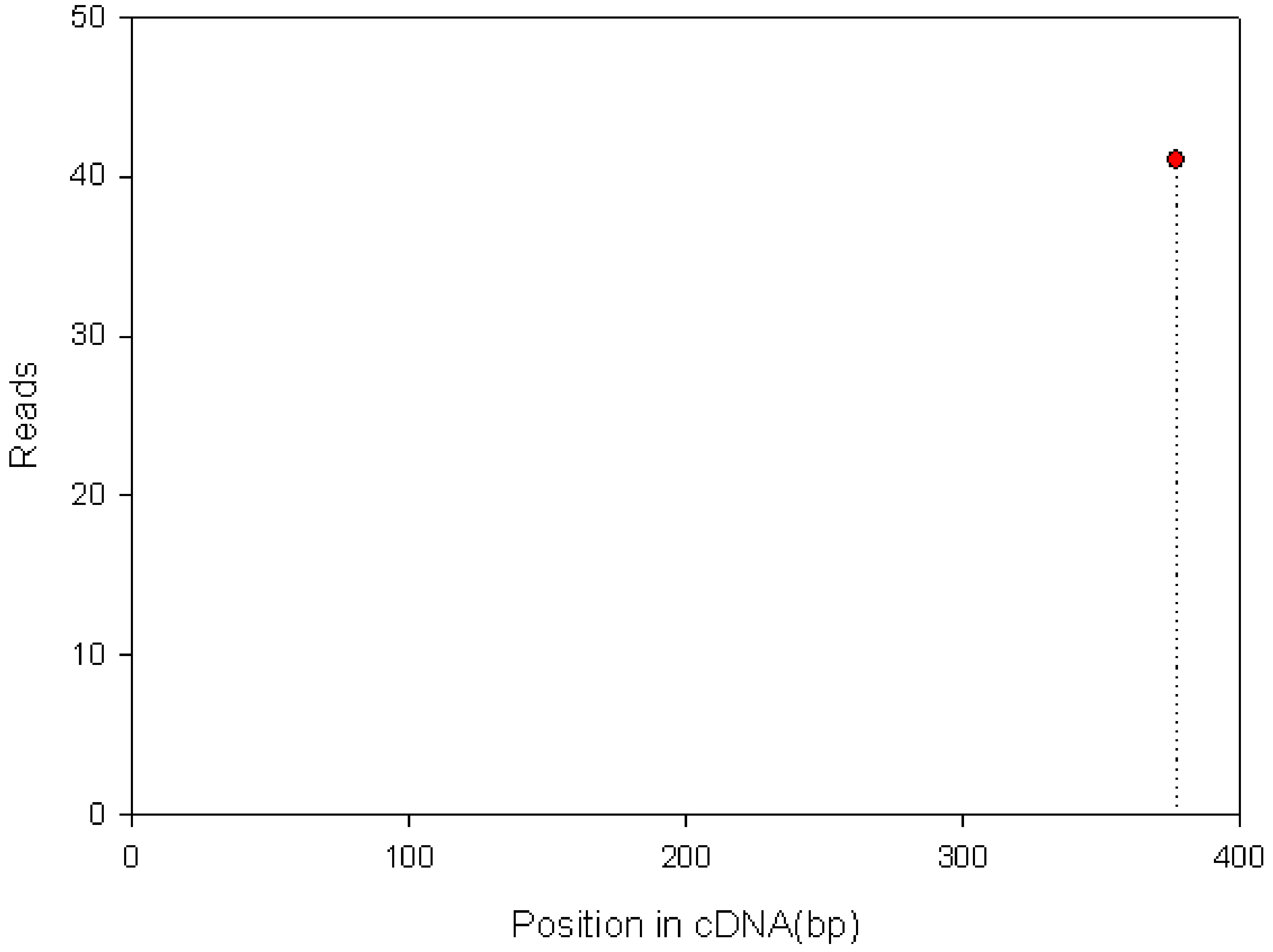


```
5' ACCGUUCAAGAAAGCCUGUGGAAUCA 3'        Cs7g15220.1
     ::::::::::::: ::::::
3' --UCAAGUUCUUUCGG-CACCUUU-- 5'        Csi-miR396d.1
```

Csi-miR396d.1, target=Cs9g19220.1 gene=Cs9g19220
Category:3
Score=5
Cleavage Site=1829

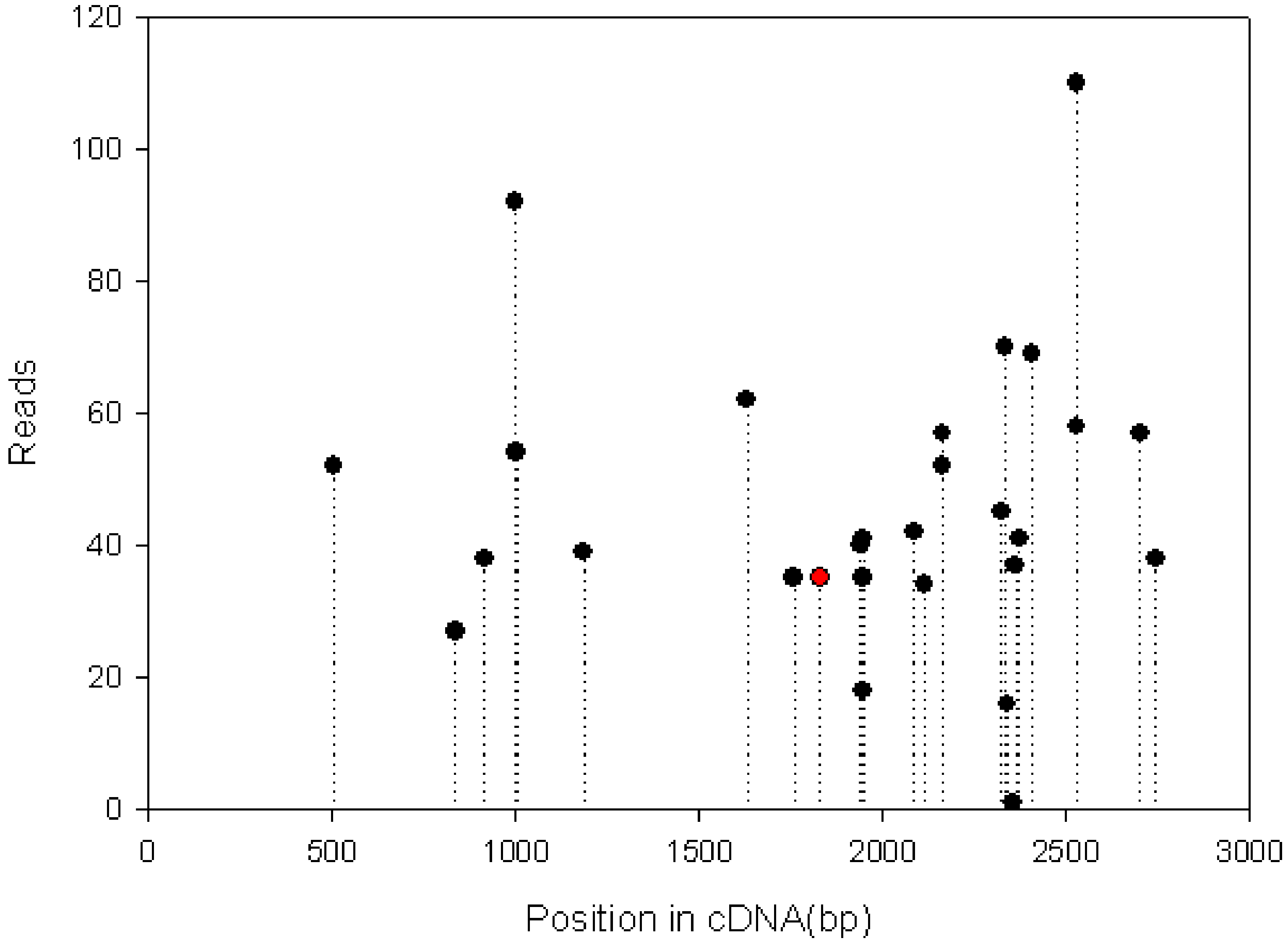


```
5' GGUGAGAGCAAGAAGGCUGUGGAGAA 3'        Cs9g19220.1
       ::   ::::::.::.:::::.:
3' ----UCAAGUUCUUUCGGCACCUUU- 5'        Csi-miR396d.1
```

Csi-miR396d.1, target=orange1.1t02555.1 gene=orange1.1t02555
Category:1
Score=4
Cleavage Site=920

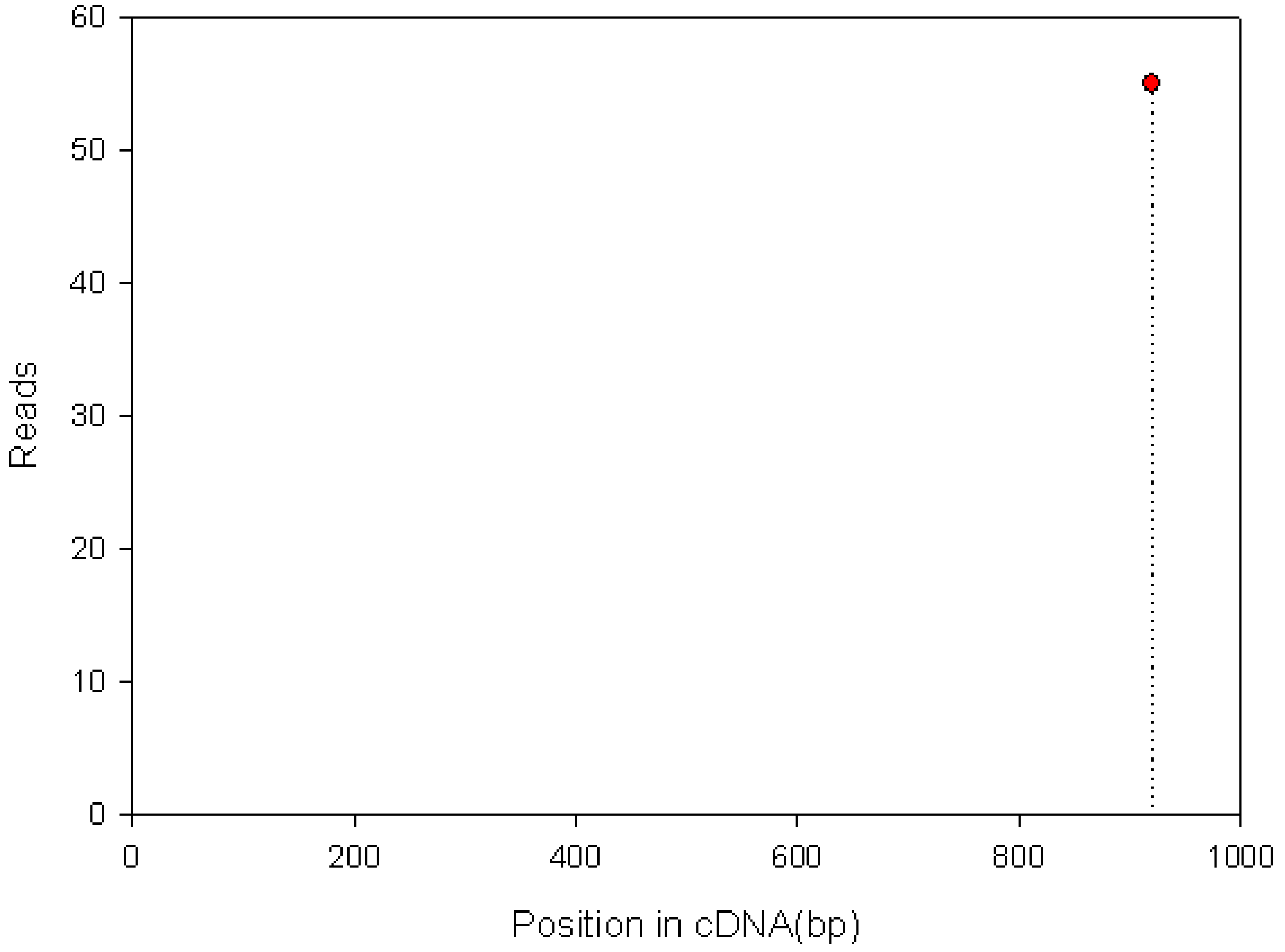


```
5' CACGUUCAAGAAAGCCUGUGGAACUU 3'        Orange1.1t02555.1
     ::::::::::::: ::::::
3' --UCAAGUUCUUUCGG-CACCUUU-- 5'        Csi-miR396d.1
```

Csi-miR396d.1, target=orange1.1t03122.1 gene=orange1.1t03122
Category:2
Score=4
Cleavage Site=583

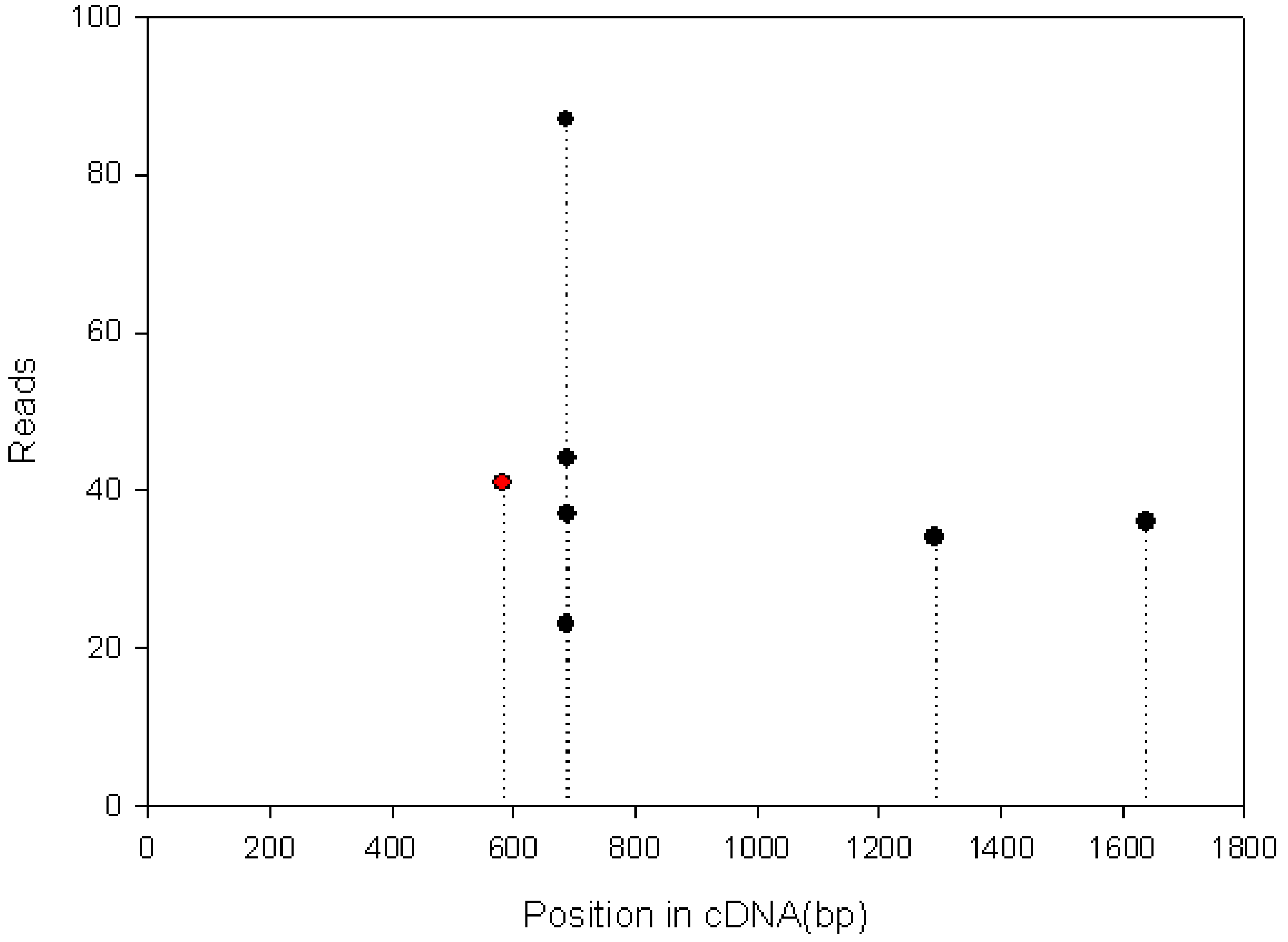


```
5' ACCGUUCAAGAAAGCCUGUGGAAUCA 3'        Orange1.1t03122.1
     ::::::::::::: ::::::
3' --UCAAGUUCUUUCGG-CACCUUU-- 5'        Csi-miR396d.1
```

Csi-miR396d.2, target=Cs2g09620.1 gene=Cs2g09620
Category:3
Score=5
Cleavage Site=3511

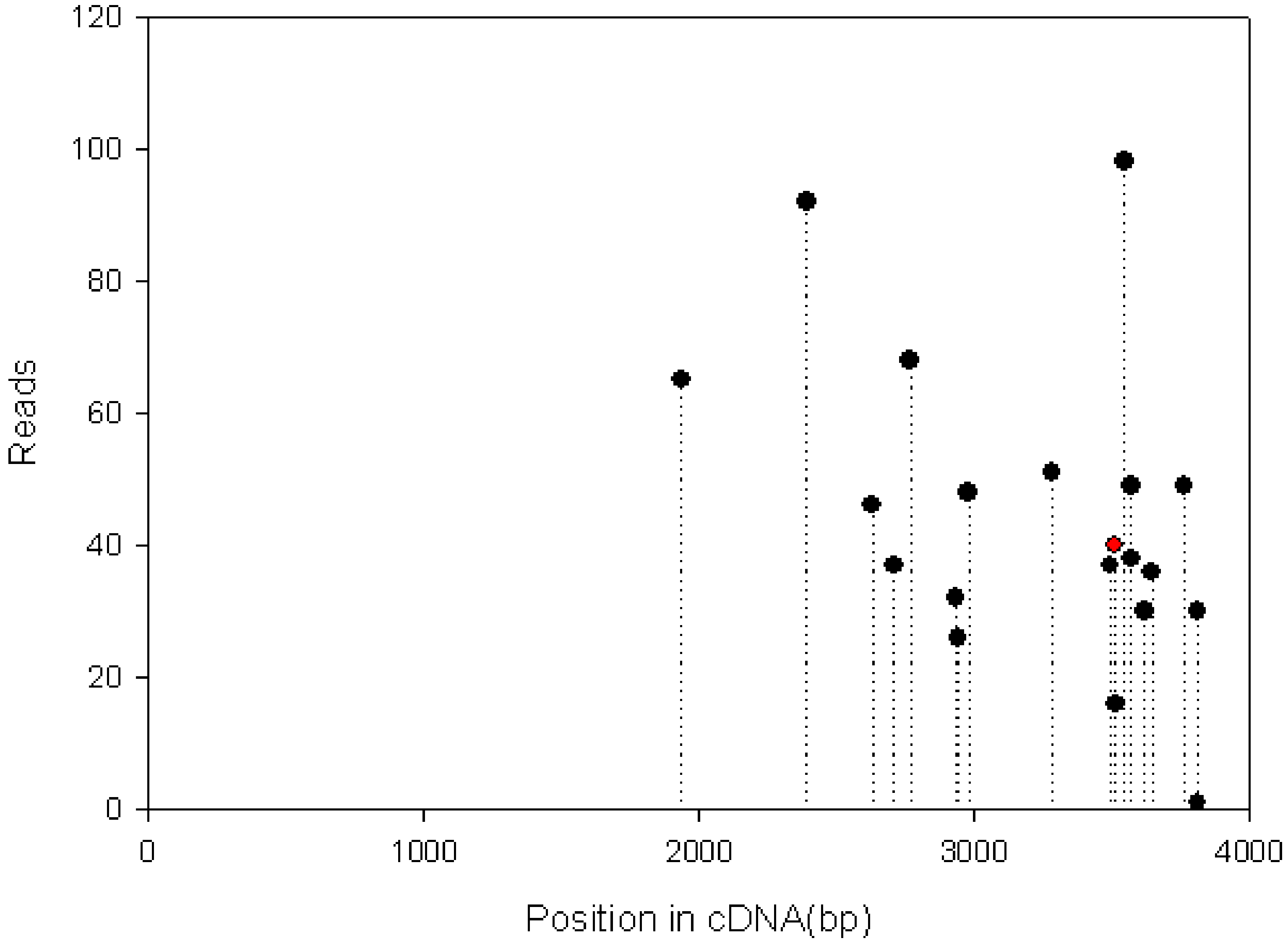


```
5' CCAAGUAC-UGAAAUCCGUGGAACUUU 3'        Cs2g09620.1
     :::: :  :::: ::::::::
3' --UUCAAGUUCUUUCGGCACCUU---- 5'        Csi-miR396d.2
```

Csi-miR396d.2, target=Cs3g23180.1 gene=Cs3g23180
Category: 3
Score=4
Cleavage Site=625

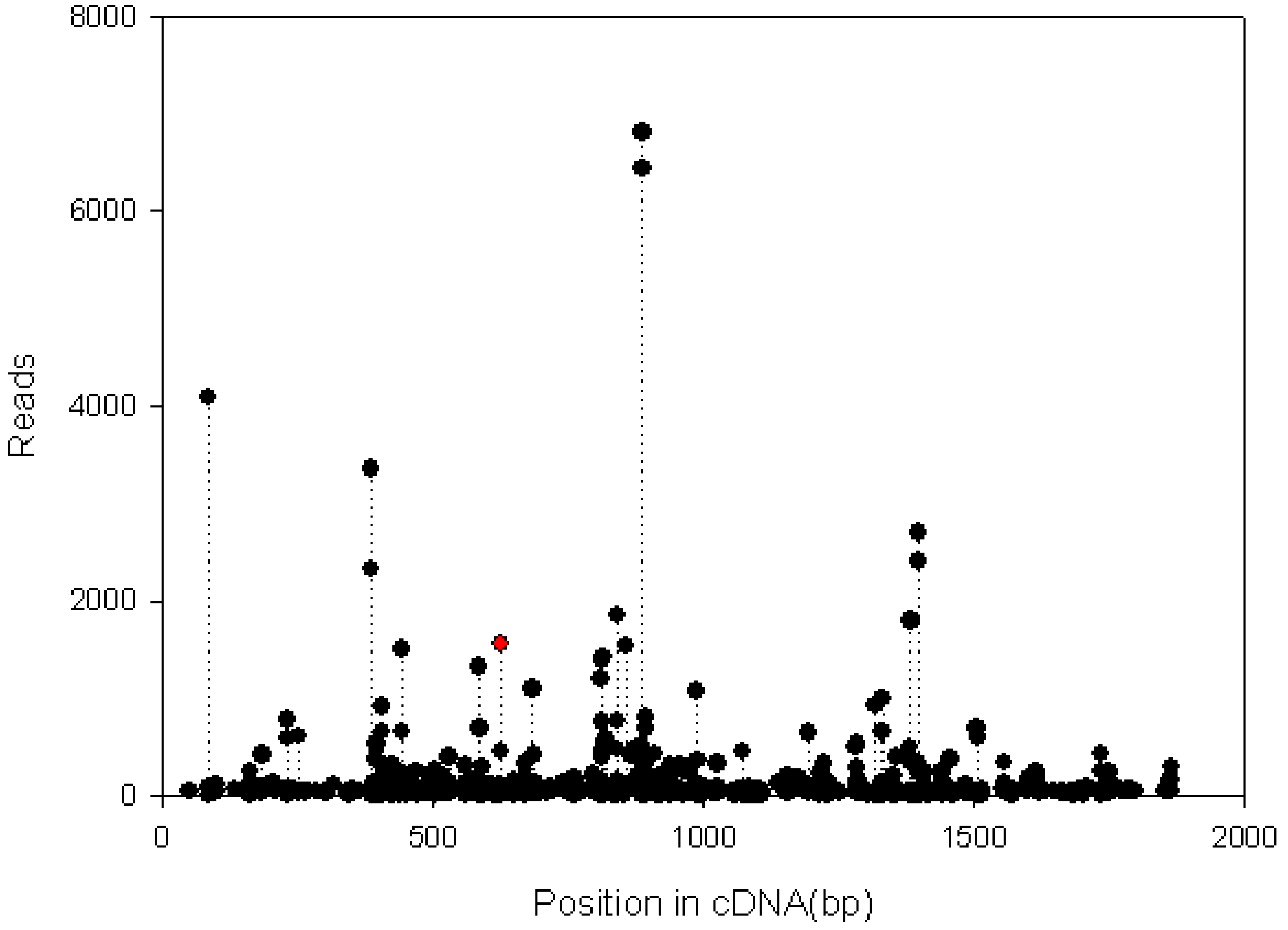


```
5' AAAGAUCAAGGAAGCUGUGGGAGUUG 3'        Cs3g23180.1
    ::: ::::::.::::.:::::.:
3' -UUCAAGUUCUUUCGGCACCUU---- 5'        Csi-miR396d.2
```

Csi-miR396d.2, target=Cs5g01380.1 gene=Cs5g01380
Category:1
Score=4
Cleavage Site=1181

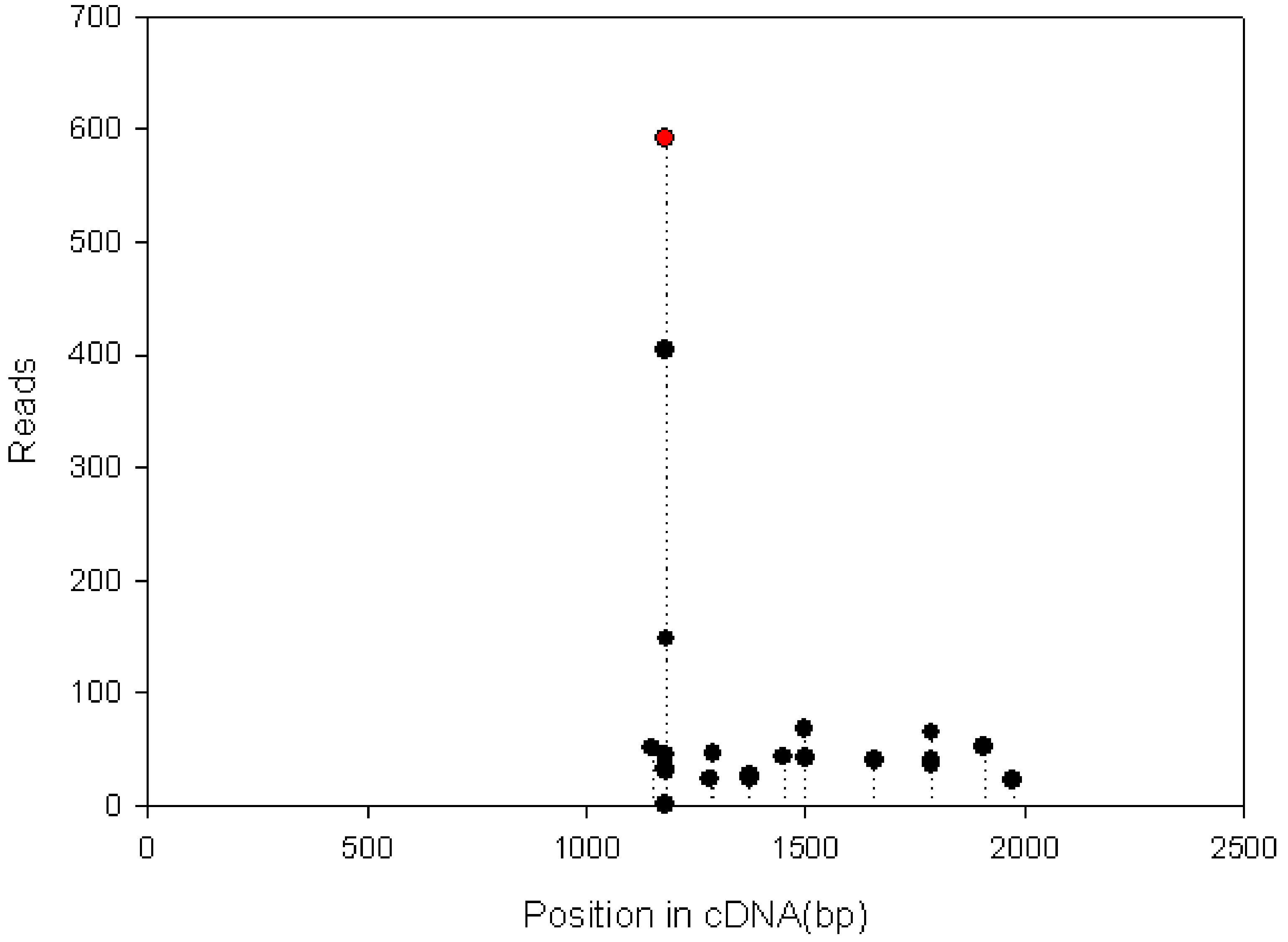


```
5' CCCGUUCAAGAAAGCCUGUGGAAUUA 3'        Cs5g01380.1
     ::::::::::::: ::::::
3' -UUCAAGUUCUUUCGG-CACCUU--- 5'        Csi-miR396d.2
```

Csi-miR396d.2, target=Cs5g19200.1 gene=Cs5g19200
Category:2
Score=3.5
Cleavage Site=201

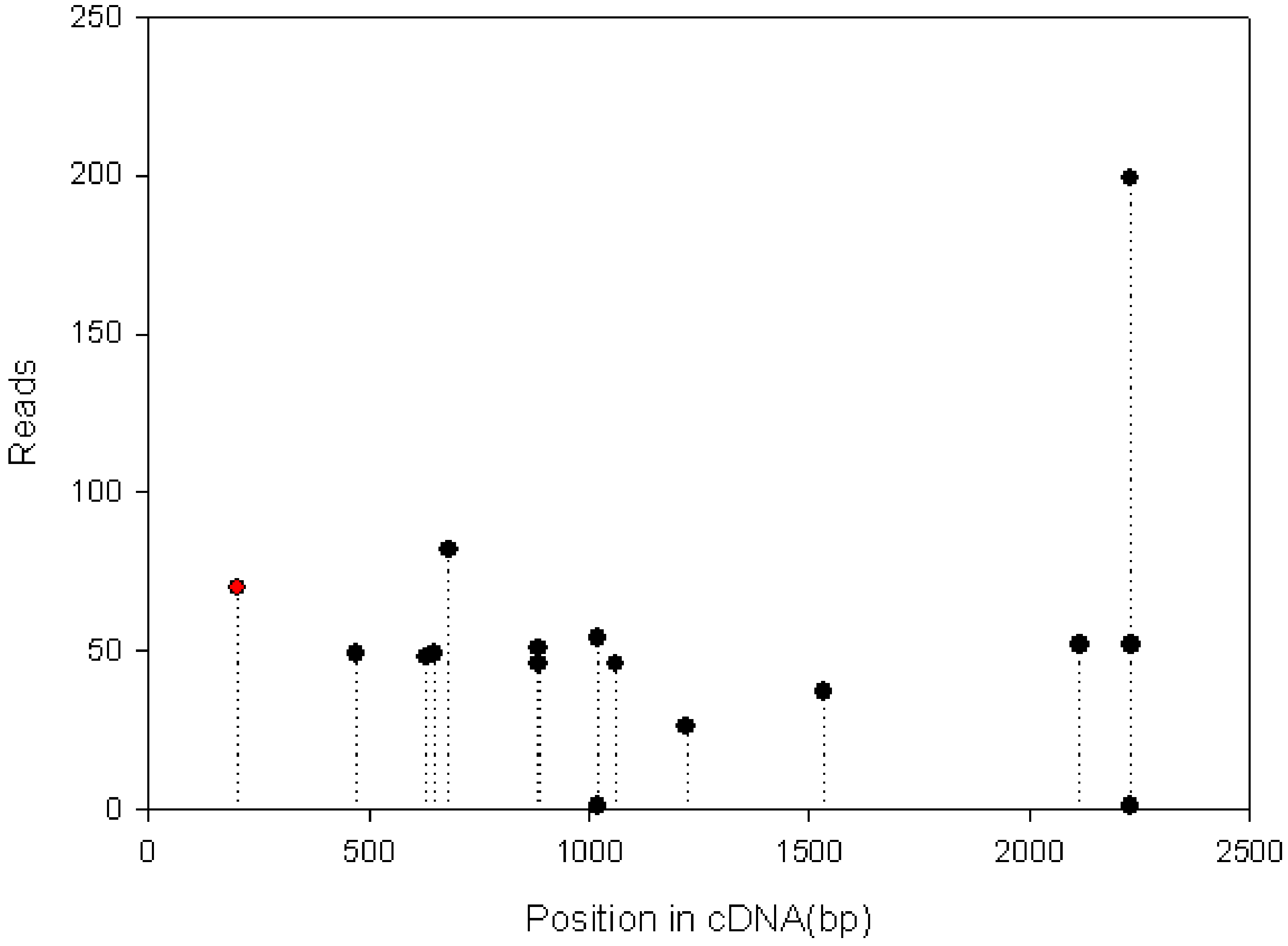


```
5' AAUGUUUAAGAAAGCAGUGGAAGCAA 3'        Cs5g19200.1
    : :::.:::::::: ::::::
3' -UUCAAGUUCUUUCGGCACCUU---- 5'        Csi-miR396d.2
```

Csi-miR396d.2, target=Cs7g15220.1 gene=Cs7g15220
Category:1
Score=4
Cleavage Site=377

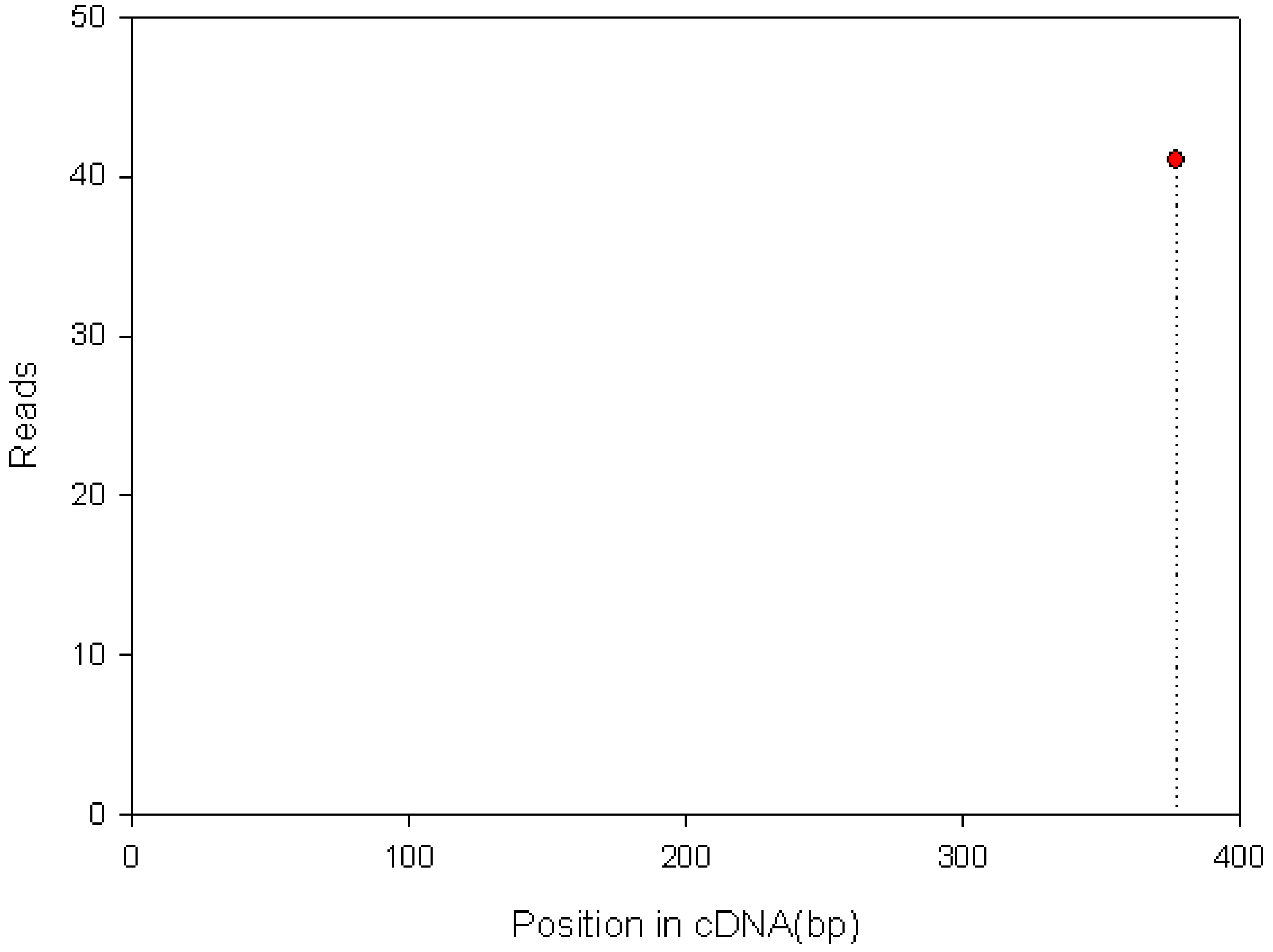


```
5' ACCGUUCAAGAAAGCCUGUGGAAUCA 3'        Cs7g15220.1
     ::::::::::::: ::::::
3' -UUCAAGUUCUUUCGG-CACCUU--- 5'        Csi-miR396d.2
```

Csi-miR396d.2, target=Cs8g17370.1 gene=Cs8g17370
Category:3
Score=4
Cleavage Site=137

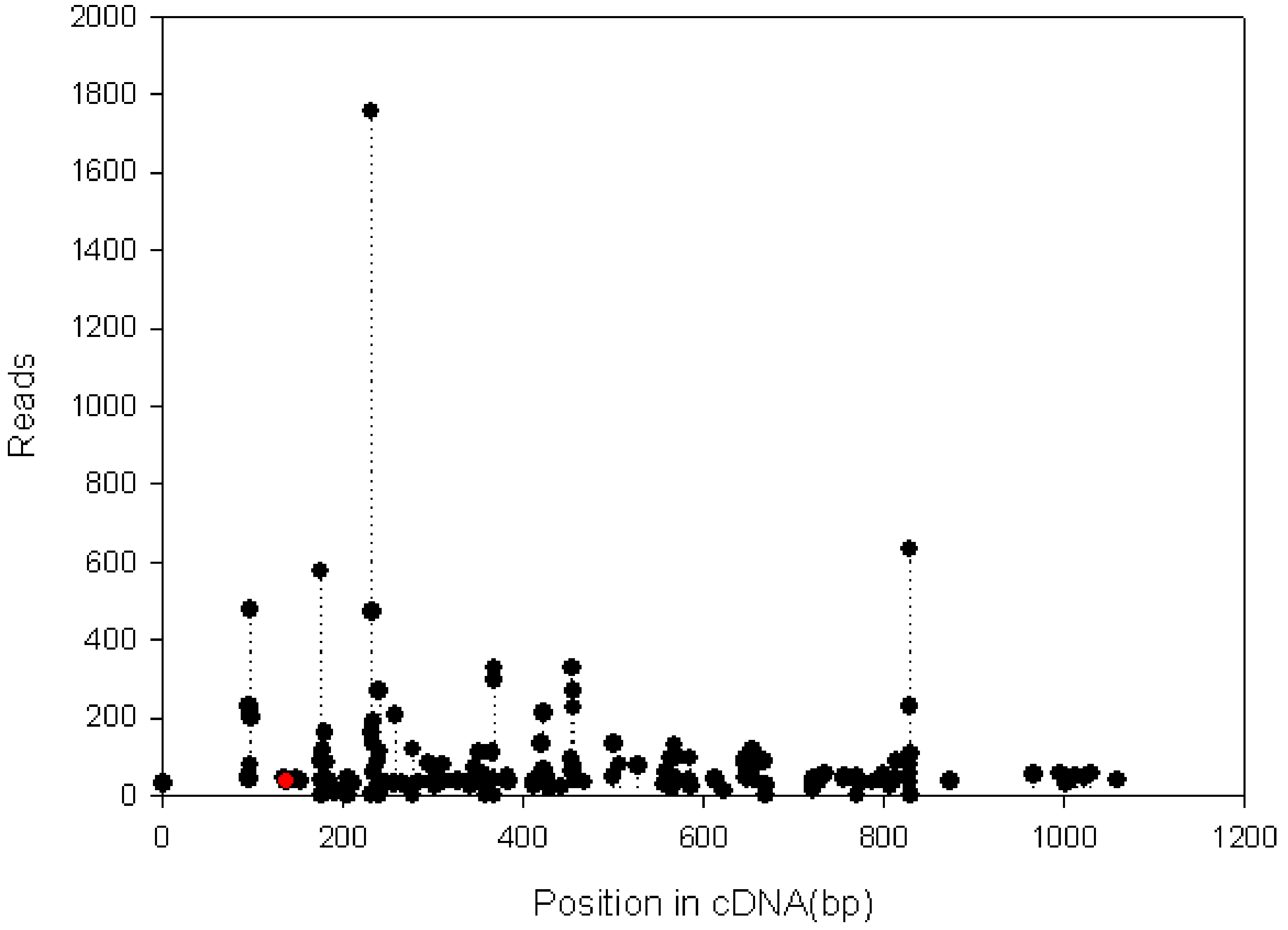


```
5' GCGAGGAUUACAAGAAGGCCGUGGAG 3'        Cs8g17370.1
          .: : ::::::.:::::::::.
3' -----UUCAAGUUCUUUCGGCACCUU 5'        Csi-miR396d.2
```

Csi-miR396d.2, target=Cs9g19220.1 gene=Cs9g19220
Category:3
Score=5
Cleavage Site=1829

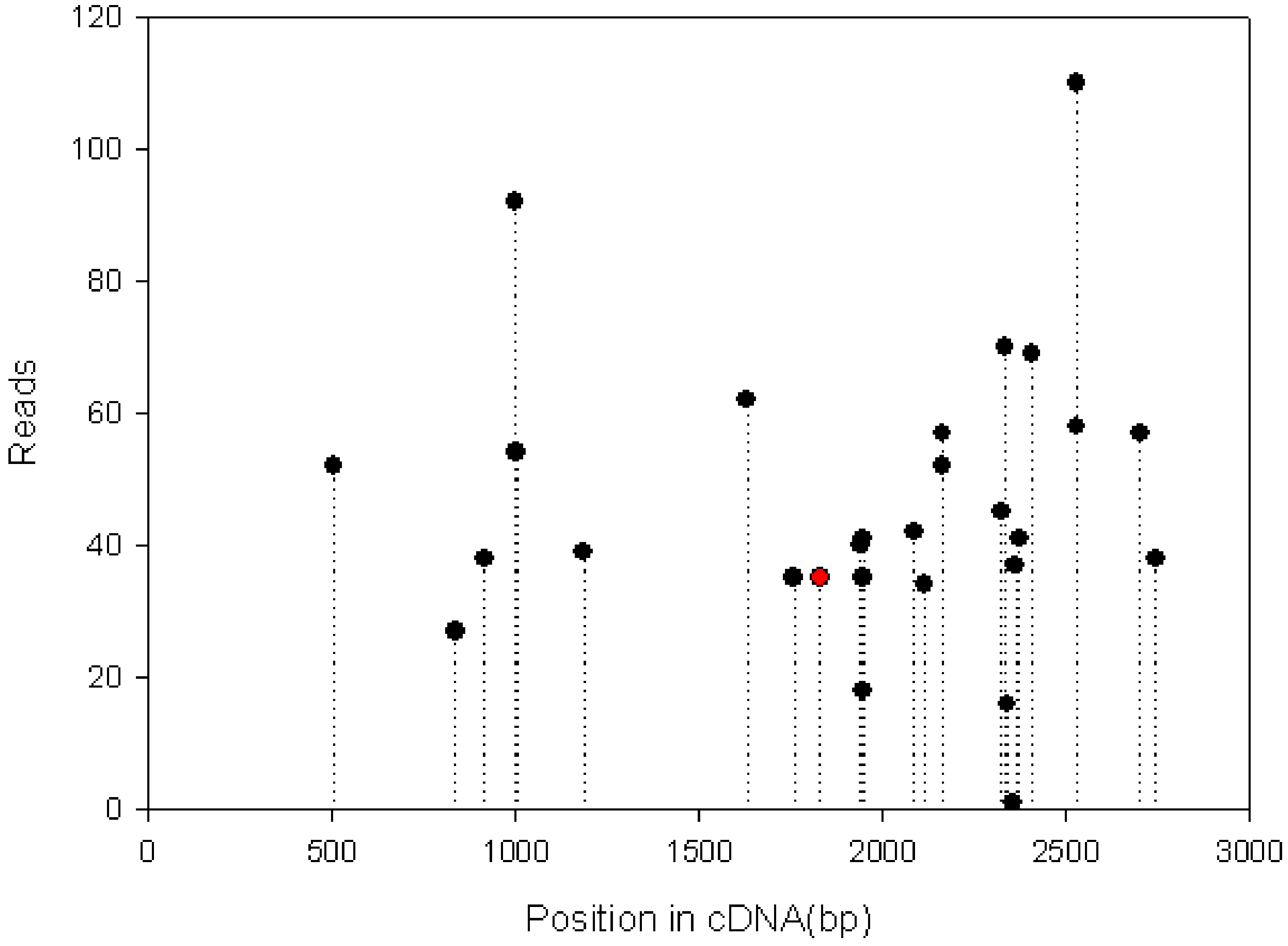


```
5' GGUGAGAGCAAGAAGGCUGUGGAGAA 3'        Cs9g19220.1
      .::  :::::::.::.:::::.
3' ---UUCAAGUUCUUUCGGCACCUU-- 5'        Csi-miR396d.2
```

Csi-miR396d.2, target=Orange1.1t02555.1 gene=Orange1.1t02555.1
Category:1
Score=3
Cleavage Site=920

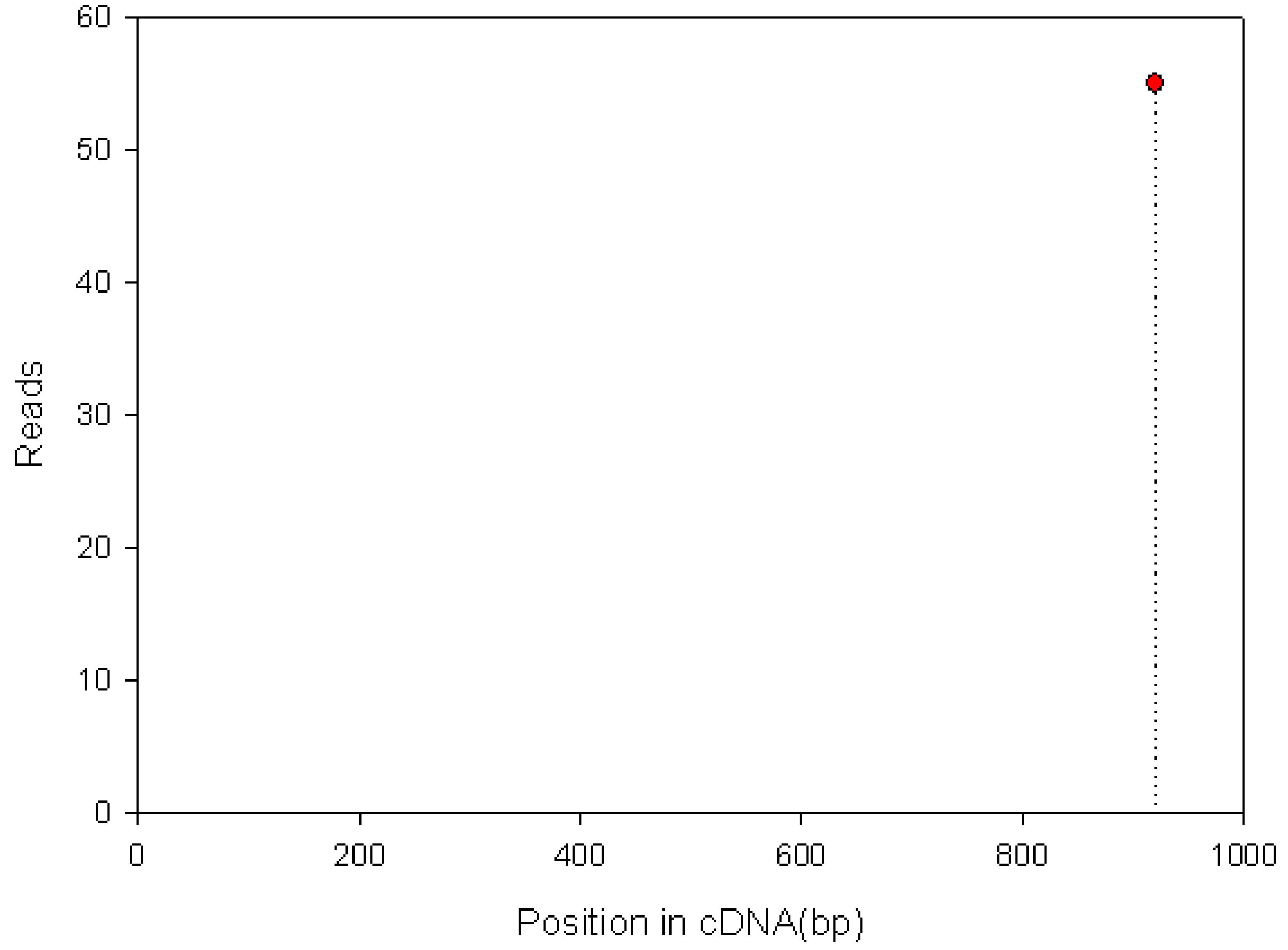


```
5' CACGUUCAAGAAAGCCUGUGGAACUU 3'        Orange1.1t02555.1
    : ::::::::::::: ::::::
3' -UUCAAGUUCUUUCGG-CACCUU--- 5'        Csi-miR396d.2
```

Csi-miR396d.2, target=Orange1.1t03122.1 gene=Orange1.1t03122
Category:2
Score=4
Cleavage Site=583

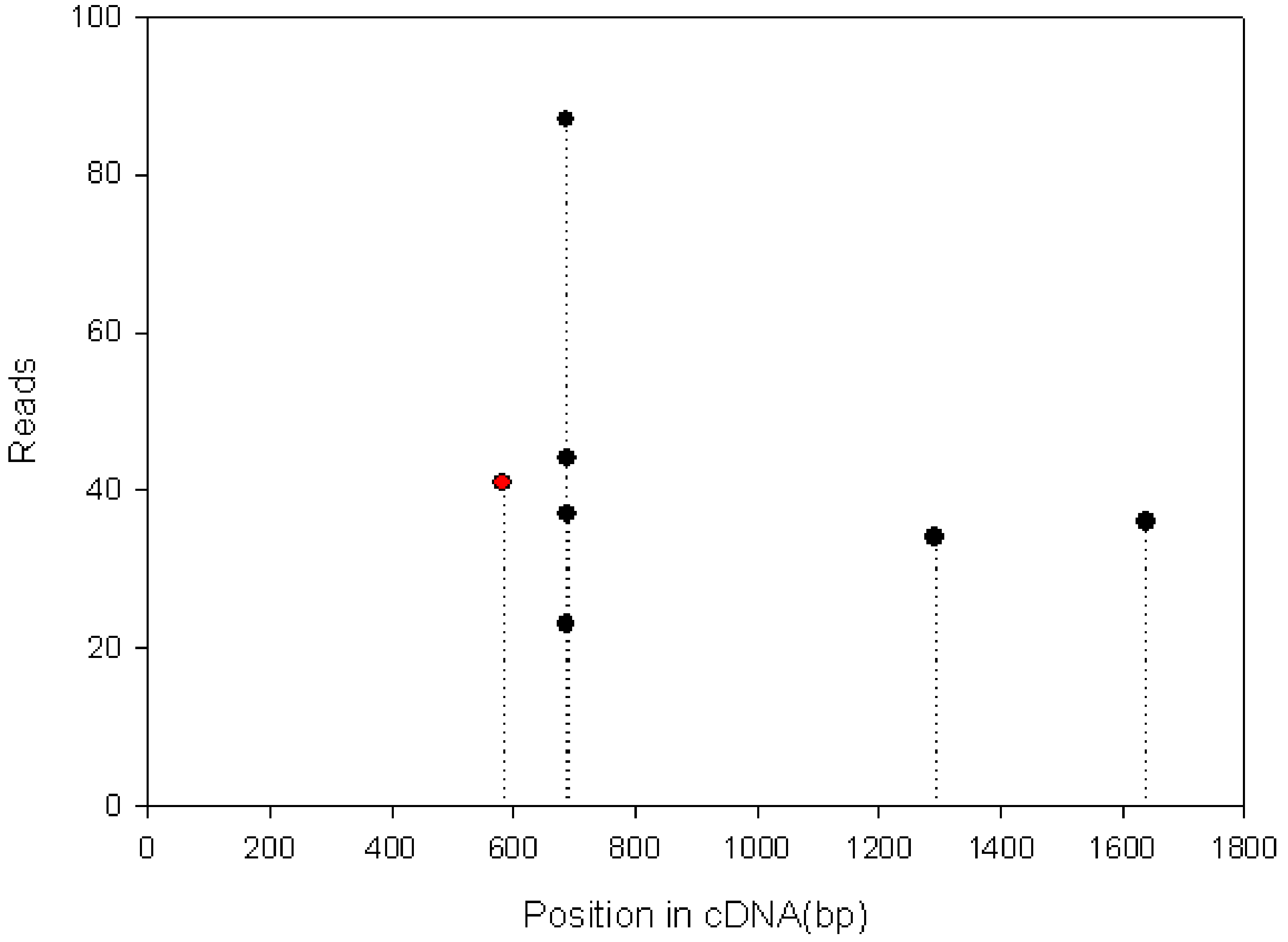


```
5' ACCGUUCAAGAAAGCCUGUGGAAUCA 3'        Orange1.1t03122.1
     ::::::::::::: ::::::
3' -UUCAAGUUCUUUCGG-CACCUU--- 5'        Csi-miR396d.2
```

Csi-miR396d.3, target=Cs1g15720.1 gene=Cs1g15720
Category:2
Score=5
Cleavage Site=1610

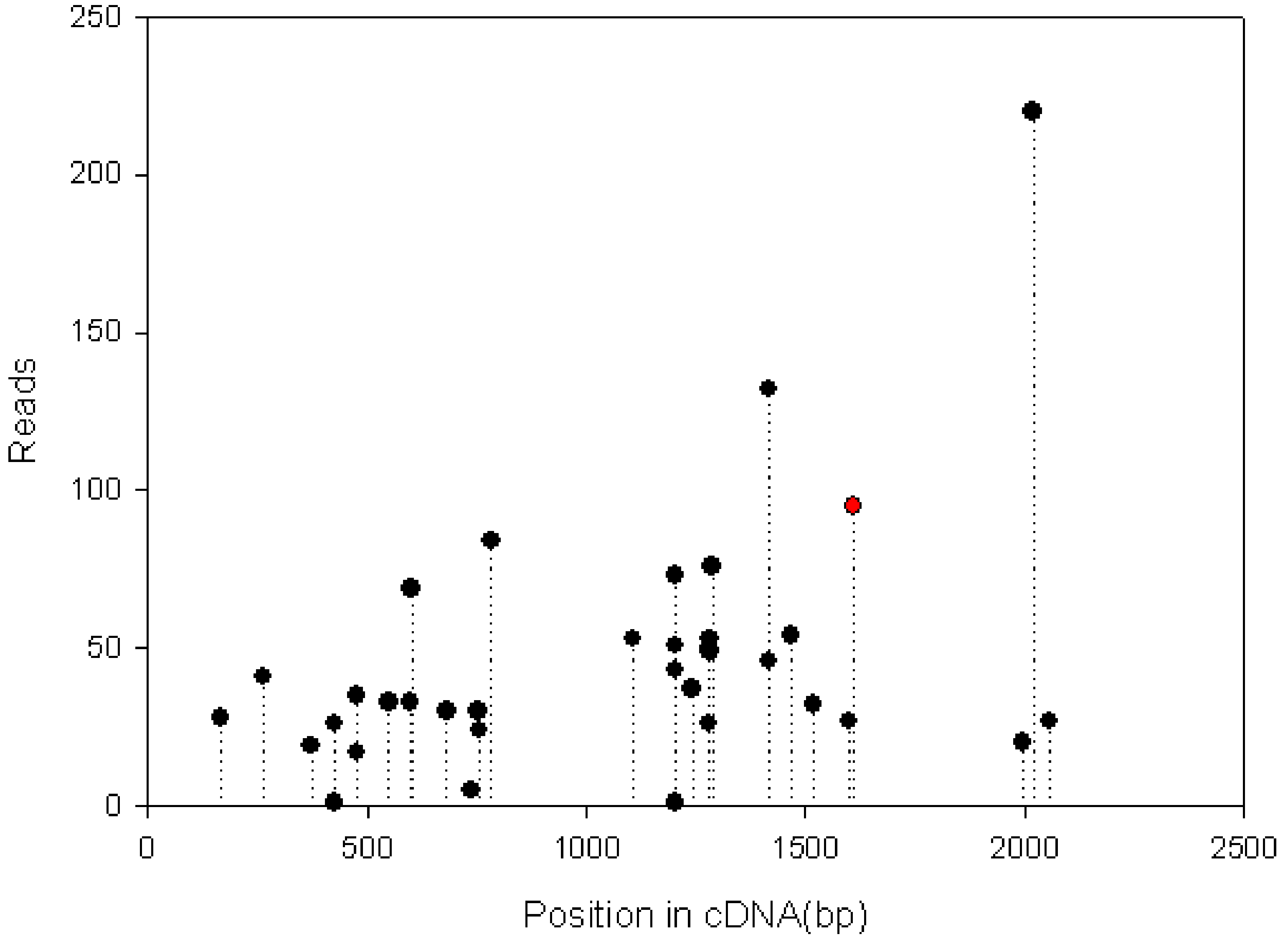


```
5' CAACAUUUCAA-AAAGCCGUGGGCUCA 3'       Cs1g15720.1
        ::::: ::::::::::.
3' -----CAAGUUCUUUCGGCACCUU--- 5'       Csi-miR396d.3
```

Csi-miR396d.3, target=Cs3g23180.1 gene=Cs3g23180
Category: 3
Score=4
Cleavage Site=625

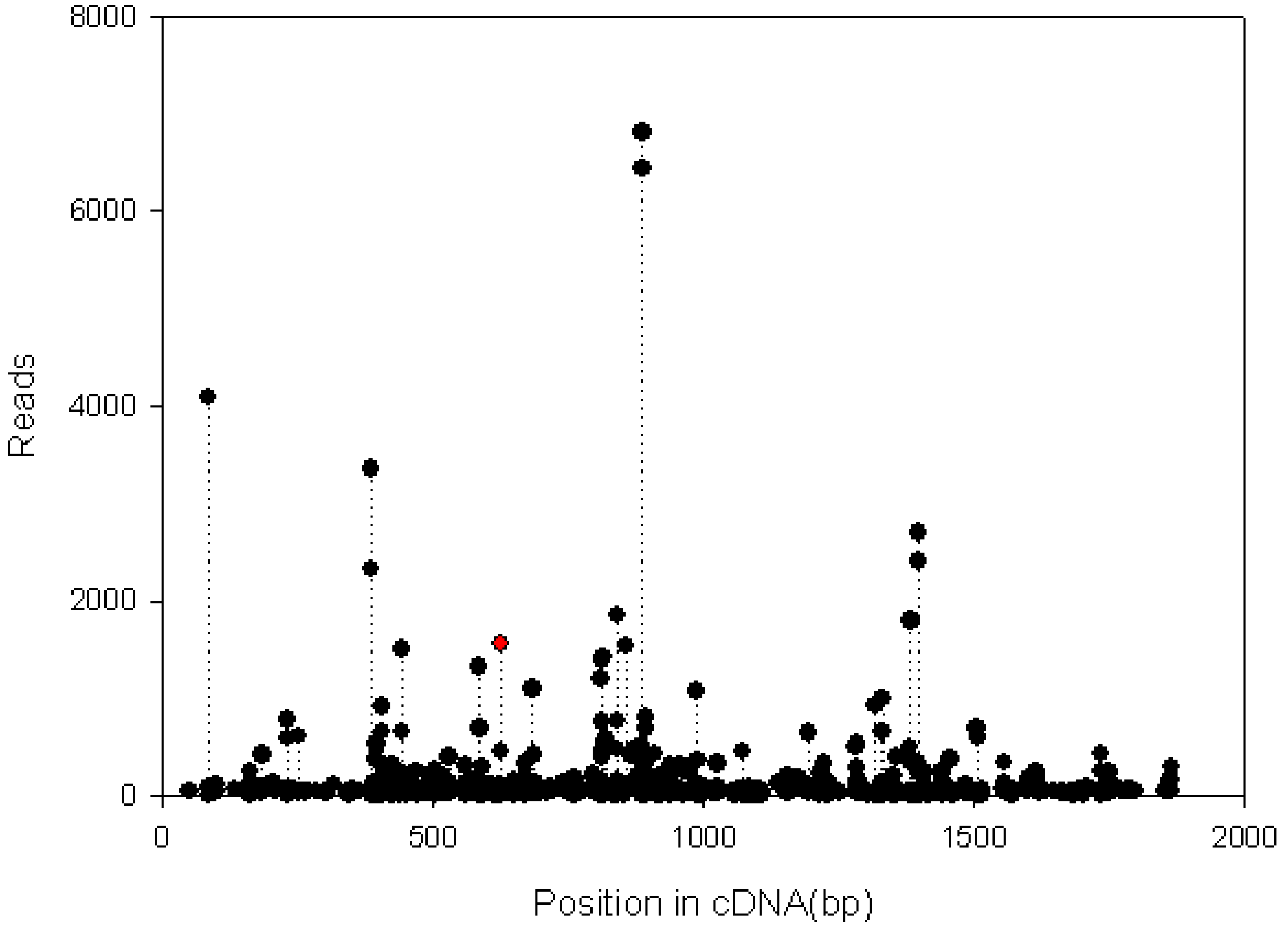


```
5' AAAGAUCAAGGAAGCUGUGGGAGUUG 3'        Cs3g23180.1
      :  :::::.::::.::::.:
3' ---CAAGUUCUUUCGGCACCUU---- 5'        Csi-miR396d.3
```

Csi-miR396d.3, target=Cs4g12200.1 gene=Cs4g12200
Category:2
Score=4
Cleavage Site=228

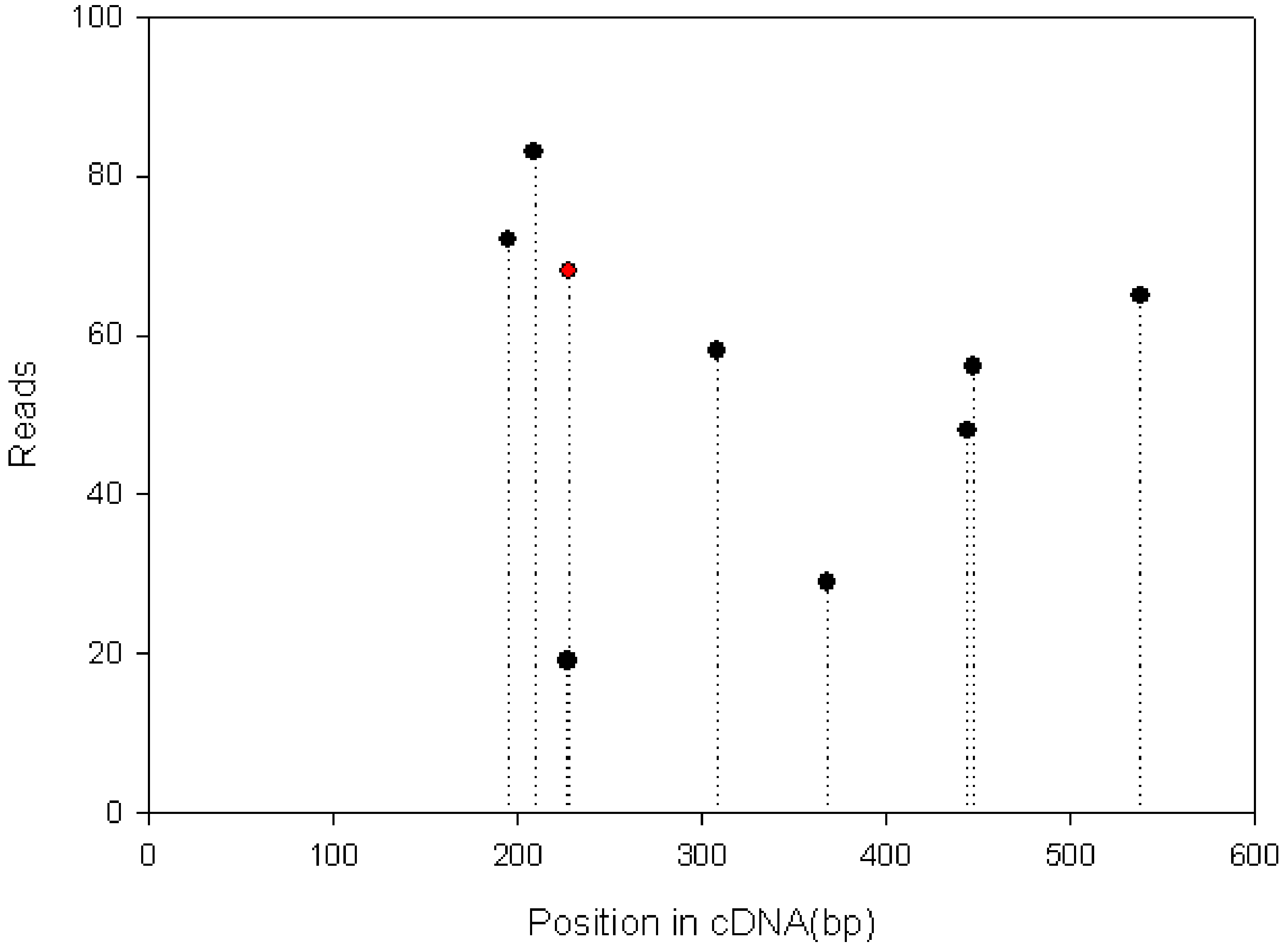


```
5' GGUUCCUGAAAGCCG-GGAAAUAUGCU 3'        Cs4g12200.1
    ::::  :::::::: ::::
3' -CAAGUUCUUUCGGCACCUU------- 5'        Csi-miR396d.3
```

Csi-miR396d.3, target=Cs5g01380.1 gene=Cs5g01380
Category:1
Score=2
Cleavage Site=1181

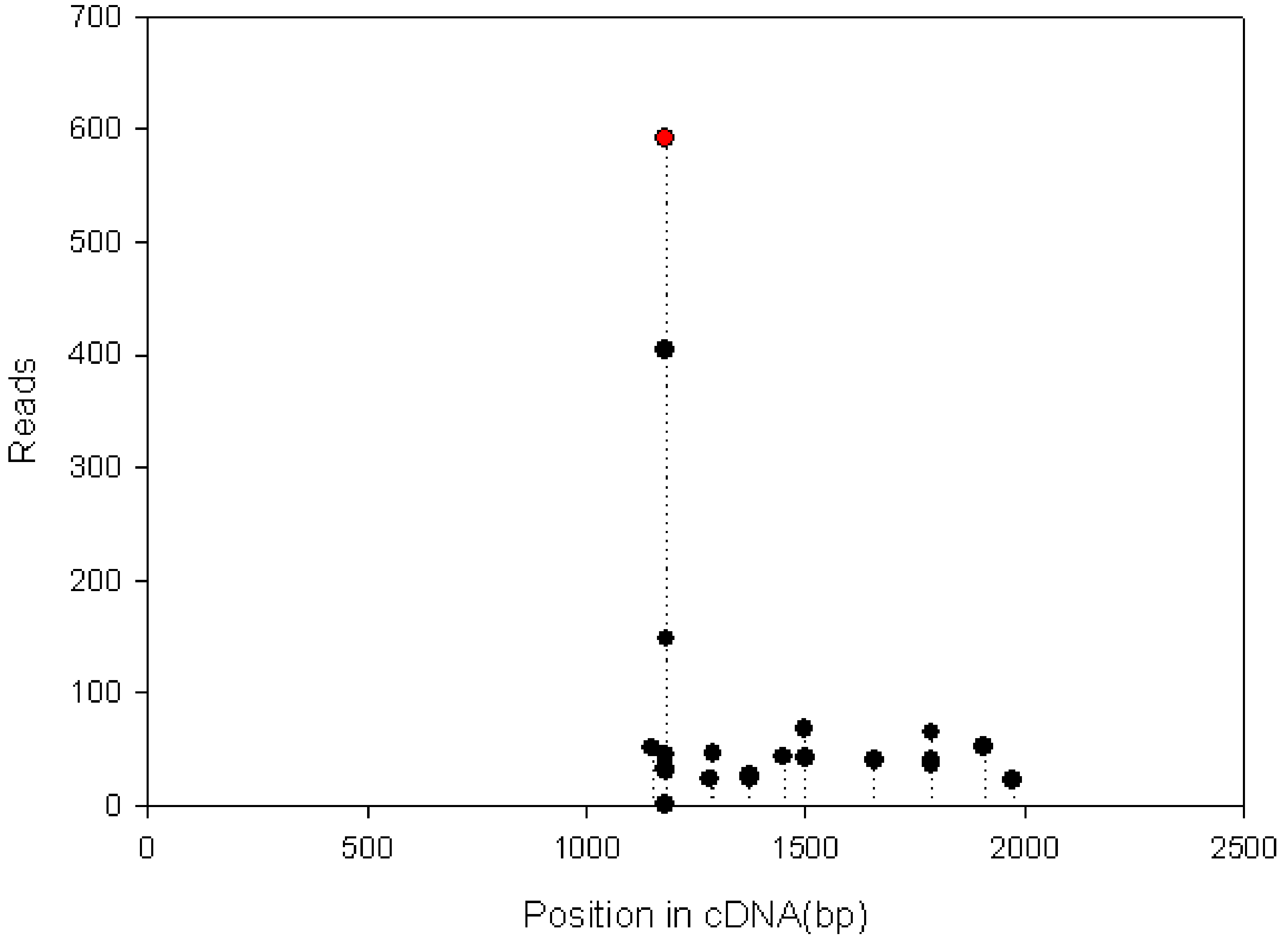


```
5' CCCGUUCAAGAAAGCCUGUGGAAUUA 3'        Cs5g01380.1
       ::::::::::::: ::::::
3' ---CAAGUUCUUUCGG-CACCUU--- 5'        Csi-miR396d.3
```

Csi-miR396d.3, target=Cs5g07120.1 gene=Cs5g07120
Category:2
Score=4.5
Cleavage Site=1257

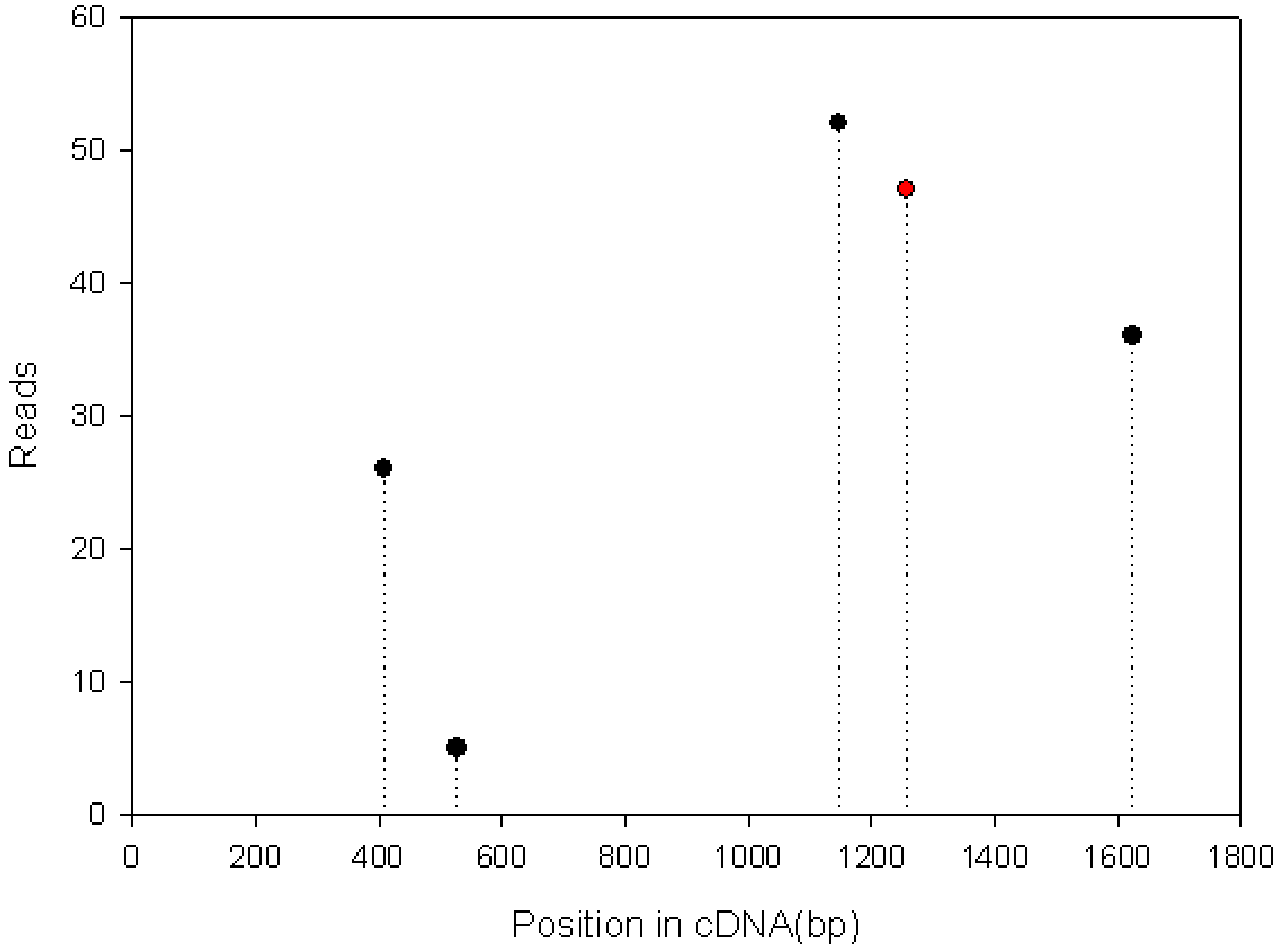


```
5' UGUUGGUAUGA-AAAGCCGUGGAGUCU 3'        Cs5g07120.1
         :: ..: ::::::::::::.
3' -----CAAGUUCUUUCGGCACCUU--- 5'        Csi-miR396d.3
```

Csi-miR396d.3, target=Cs5g19200.1 gene=Cs5g19200
Category:2
Score=2.5
Cleavage Site=201

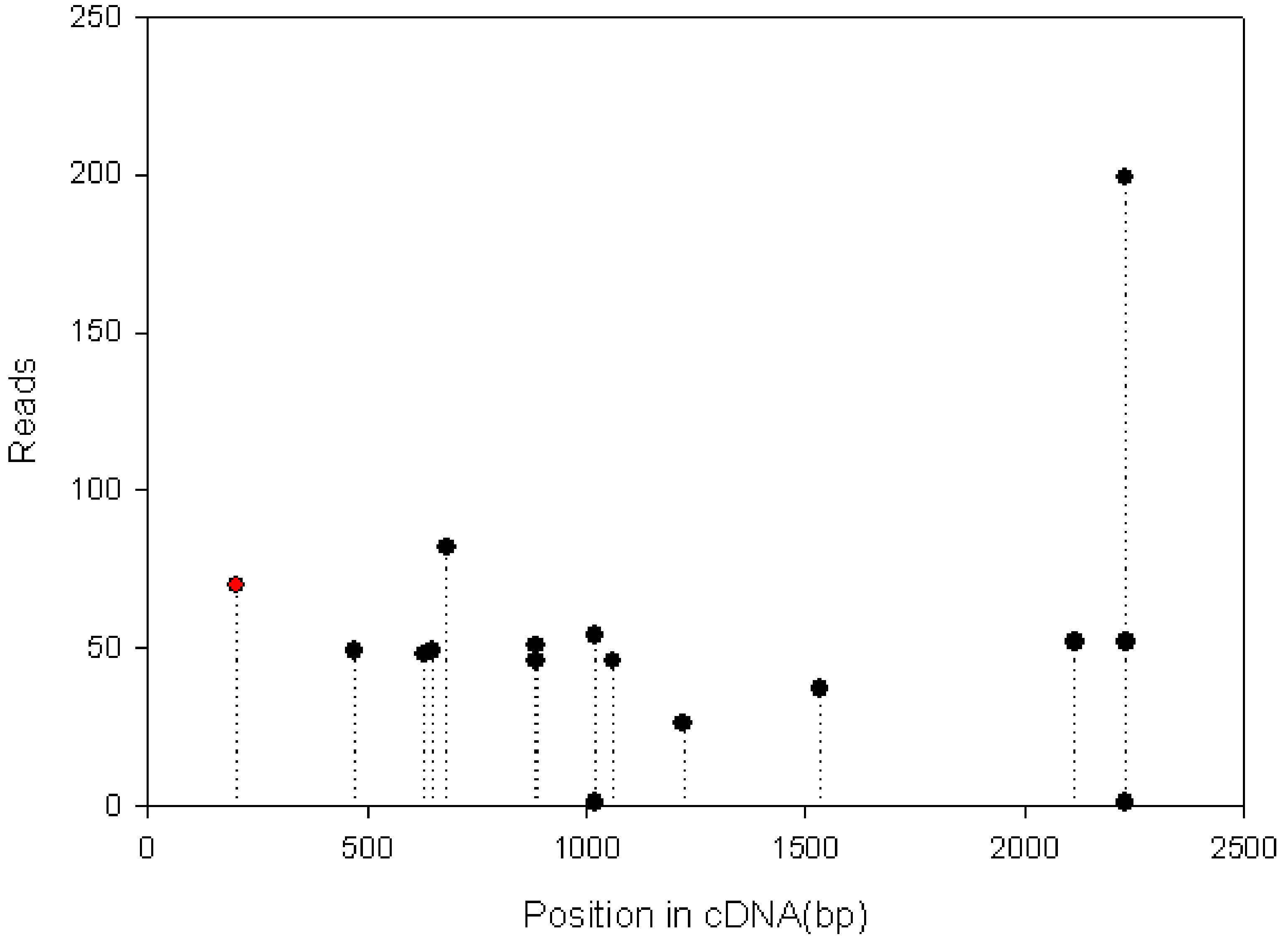


```
5' AAUGUUUAAGAAAGCAGUGGAAGCAA 3'        Cs5g19200.1
     :::.::::::::: ::::::
3' ---CAAGUUCUUUCGGCACCUU---- 5'        Csi-miR396d.3
```

Csi-miR396d.3, target=Cs7g12930.1 gene=Cs7g12930
Category:1
Score=4.5
Cleavage Site=1079

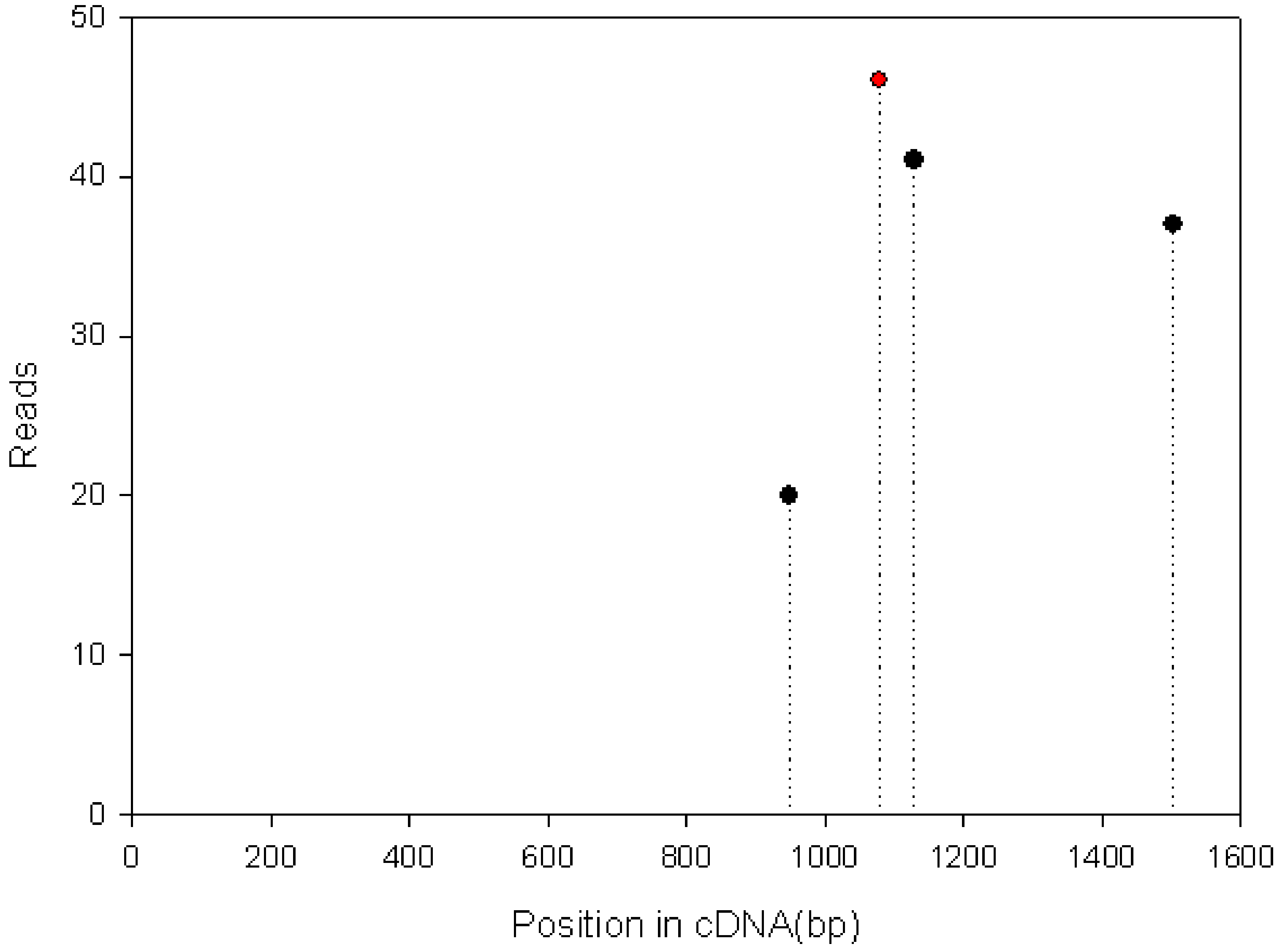


```
5' UAGUAUUAAGAAAGCUGAGGAAAAAG 3'        Cs7g12930.1
     :: :.:::::::.: ::::
3' --CA-AGUUCUUUCGGCACCUU---- 5'        Csi-miR396d.3
```

Csi-miR396d.3, target=Cs7g15220.1 gene=Cs7g15220
Category:1
Score=2
Cleavage Site=377

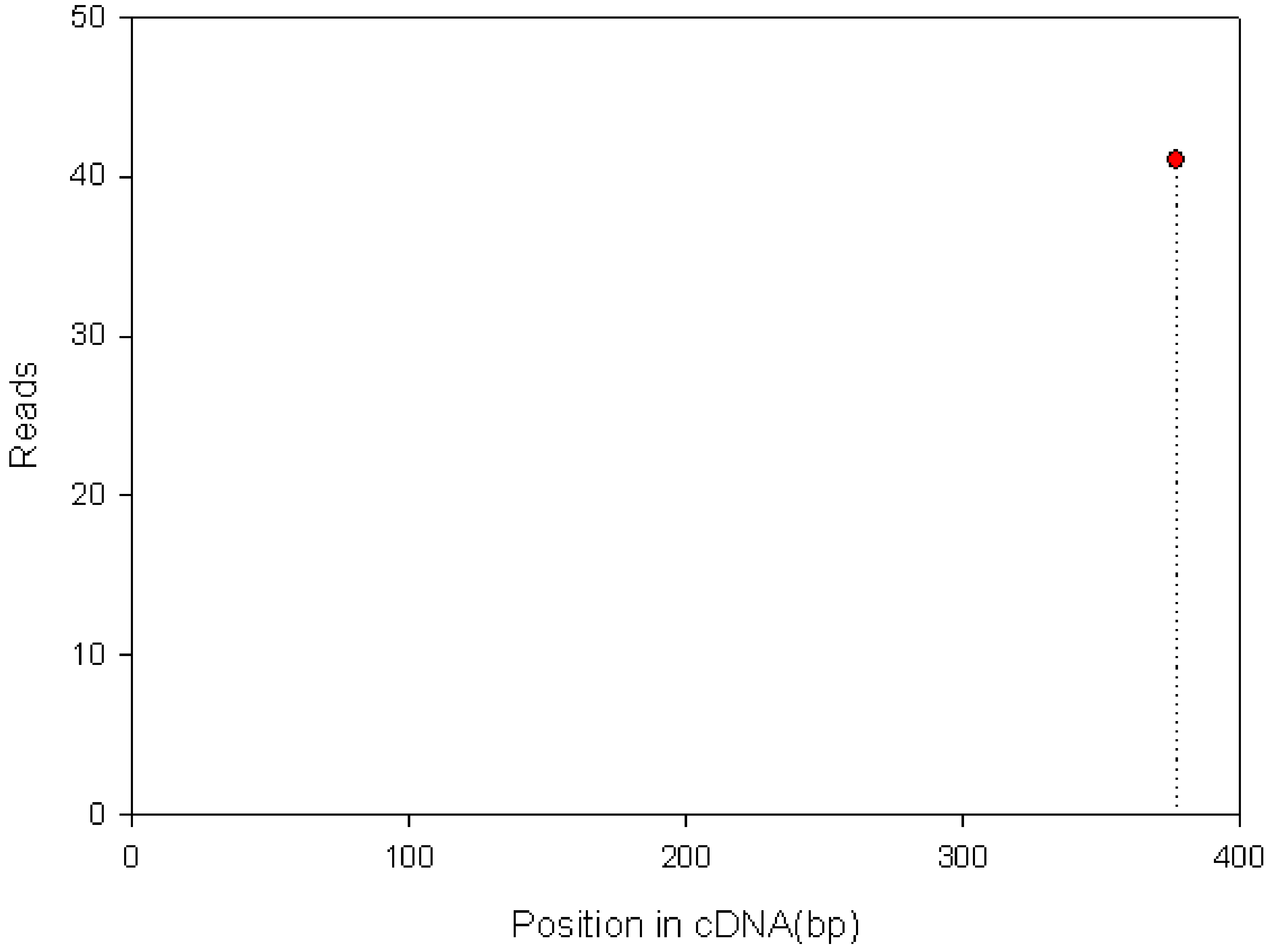


```
5' ACCGUUCAAGAAAGCCUGUGGAAUCA 3'        Cs7g15220.1
      ::::::::::::: ::::::
3' ---CAAGUUCUUUCGG-CACCUU--- 5'        Csi-miR396d.3
```

Csi-miR396d.3, target=Cs8g15450.1 gene=Cs8g15450
Category:3
Score=4.5
Cleavage Site=219

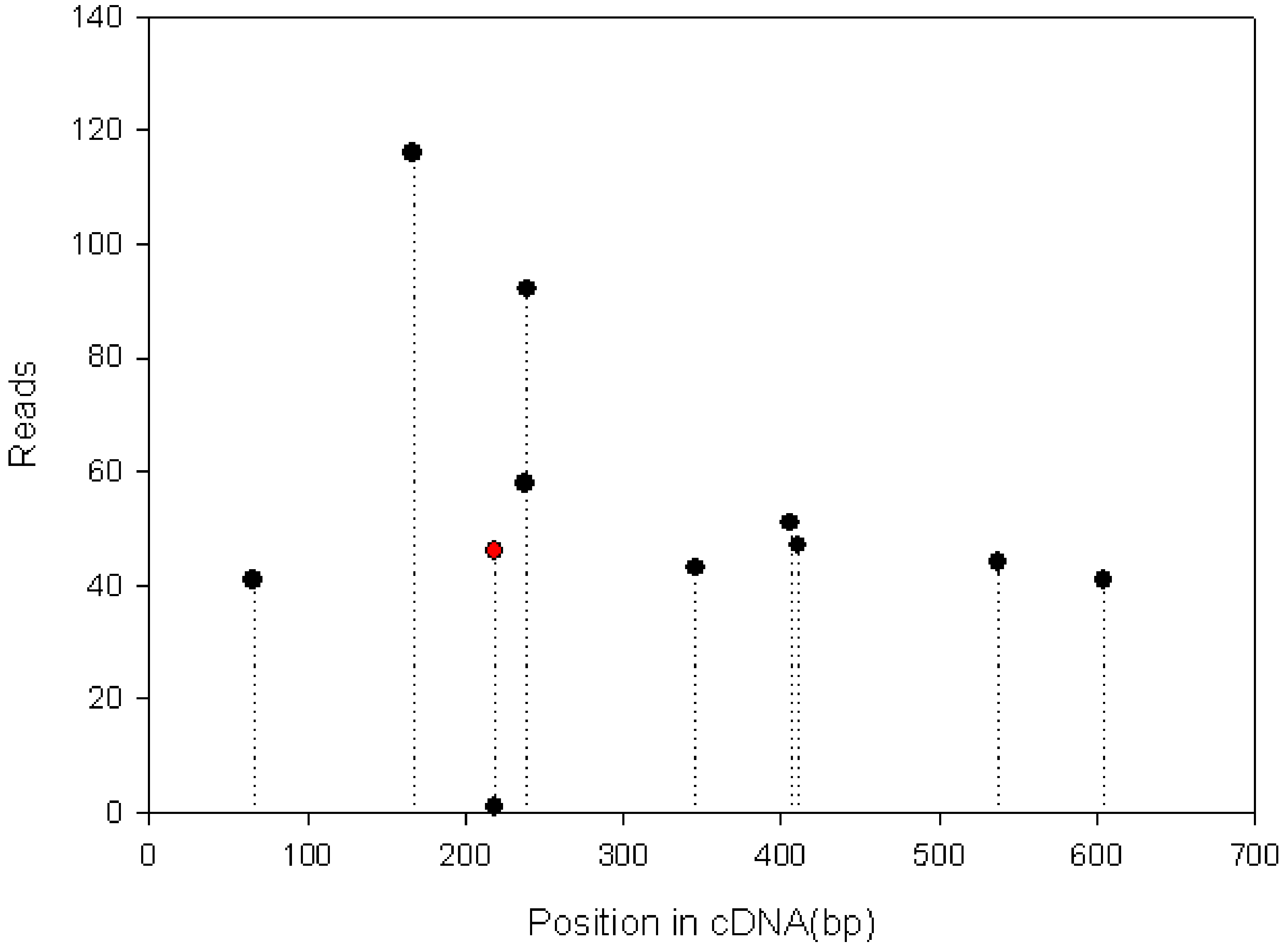


```
5' GUUCAGCAGAGCCGUGGAUGGGUUCA 3'         Cs8g15450.1
   :::::. :.:::::::::
3' CAAGUUCUUUCGGCACCUU------- 5'         Csi-miR396d.3
```

Csi-miR396d.3, target=Cs8g17370.1 gene=Cs8g17370
Category:3
Score=3.5
Cleavage Site=137

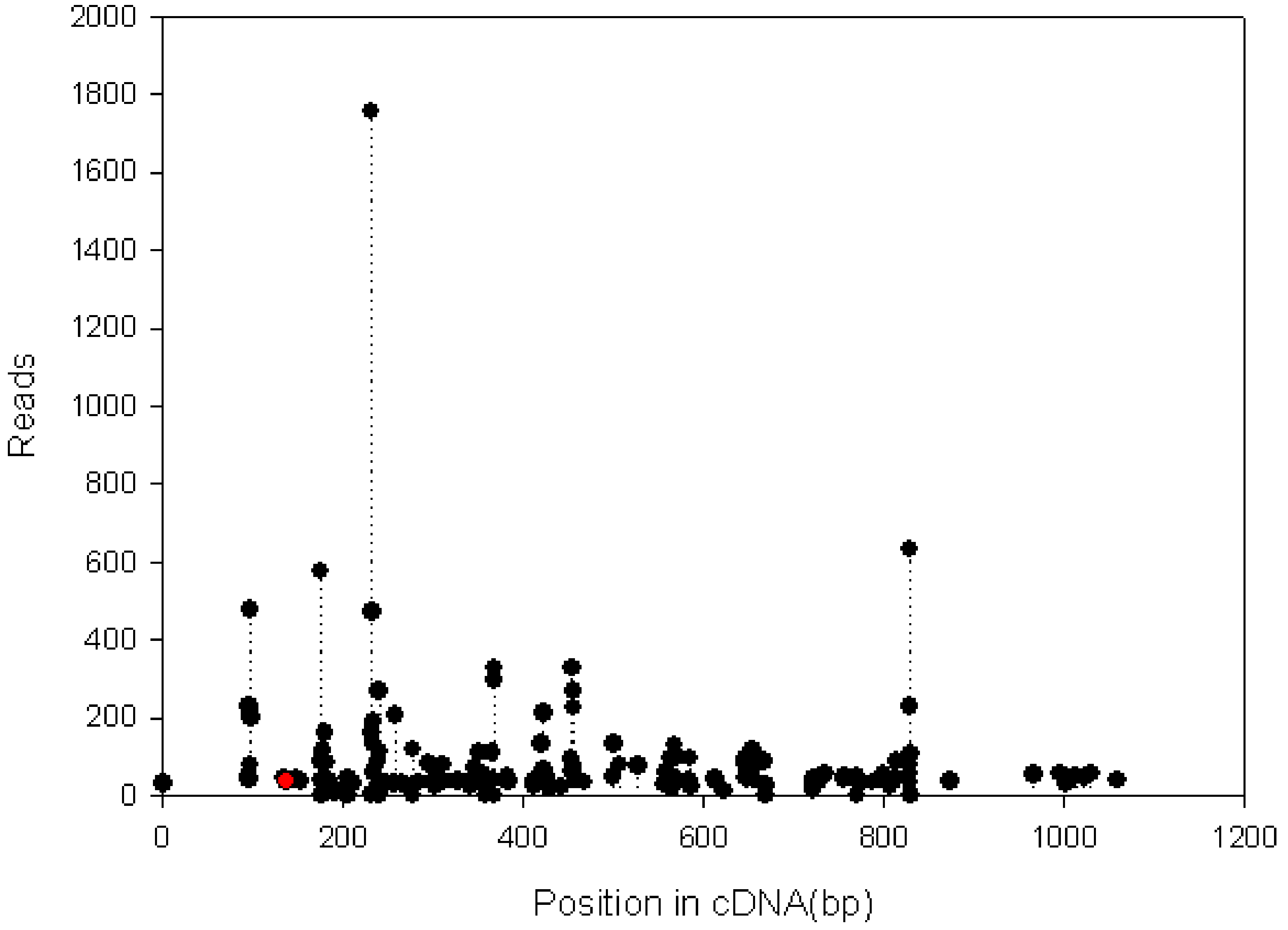


```
5' GCGAGGAUUACAAGAAGGCCGUGGAG 3'        Cs8g17370.1
          : ::::::.::::::::.
3' -------CAAGUUCUUUCGGCACCUU 5'        Csi-miR396d.3
```

Csi-miR396d.3, target=Cs9g14020.1 gene=Cs9g14020
Category:3
Score=5
Cleavage Site=639

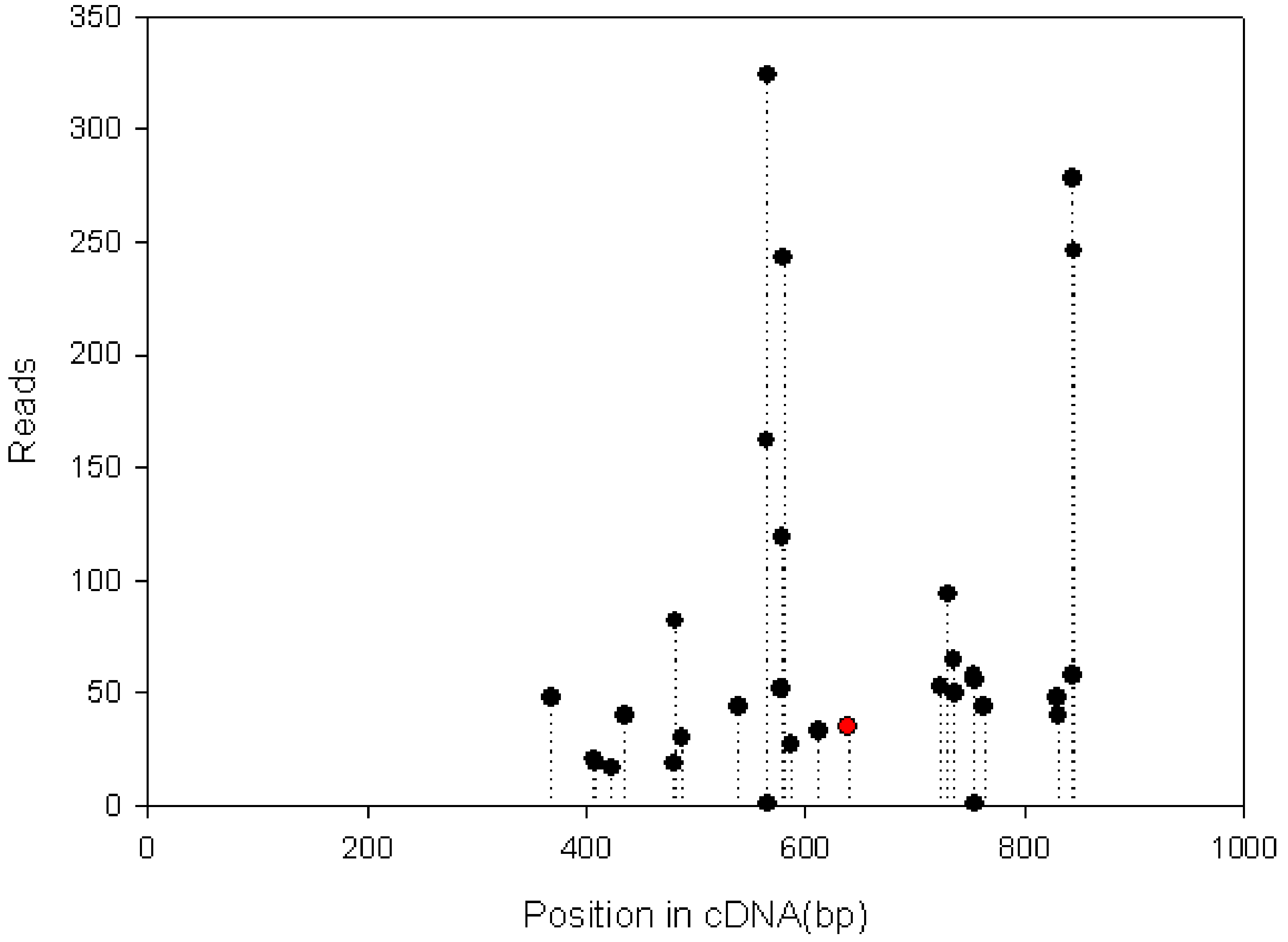


```
5' GGCCCAUC-AGAAAGCCCUGGAACGCC 3'        Cs9g14020.1
         :: :::::::: :::::
3' ----CAAGUUCUUUCGGCACCUU---- 5'        Csi-miR396d.3
```

Csi-miR396d.3, target=Cs9g19220.1 gene=Cs9g19220
Category:3
Score=4.5
Cleavage Site=1829

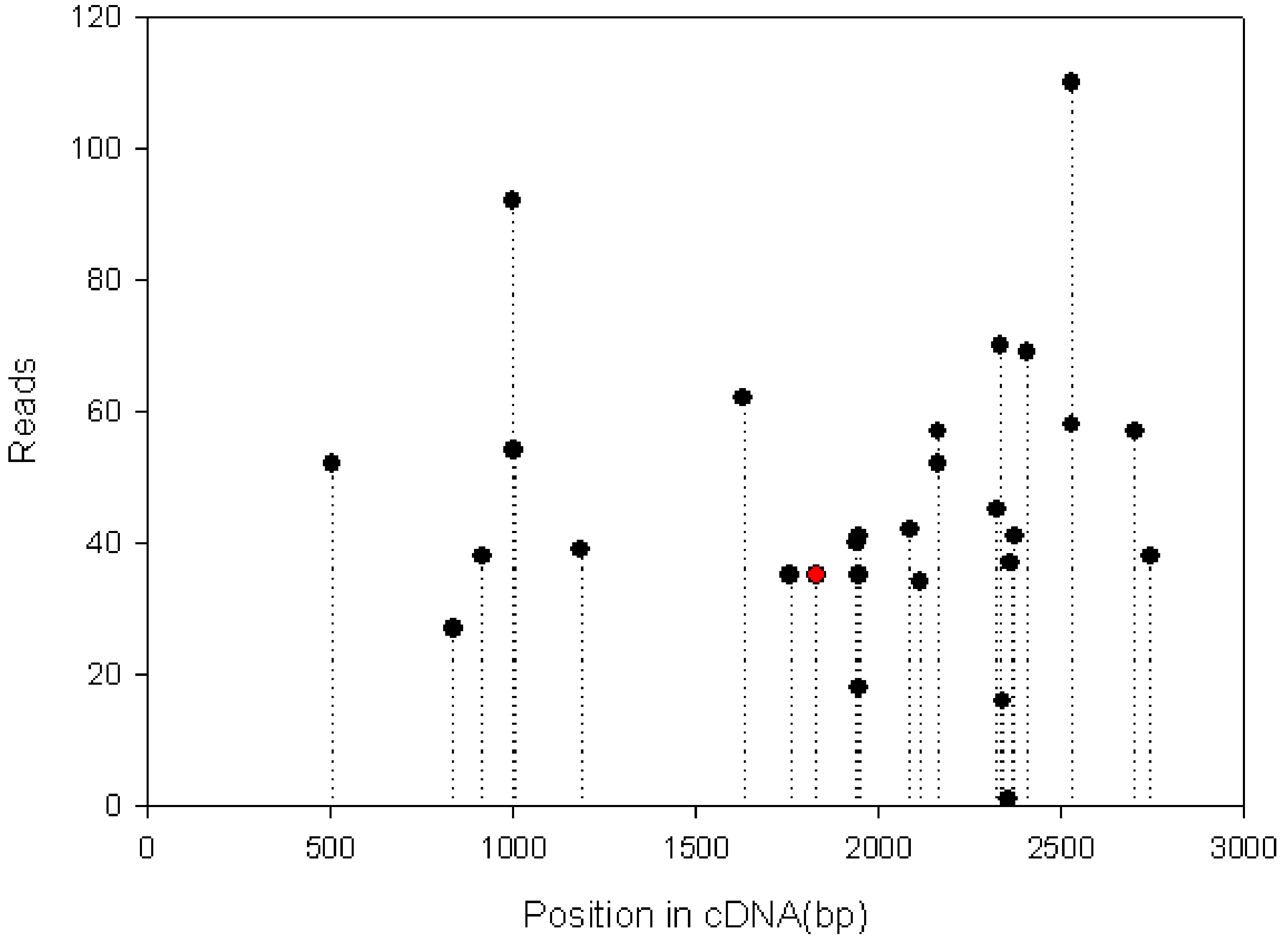


```
5' GGUGAGAGCAAGAAGGCUGUGGAGAA 3'        Cs9g19220.1
          :  ::::::.::.:::::.
3' -----CAAGUUCUUUCGGCACCUU-- 5'        Csi-miR396d.3
```

Csi-miR396d.3, target=Orange1.1t02254.1 gene=Orange1.1t02254
Category:3
Score=3.5
Cleavage Site=1371

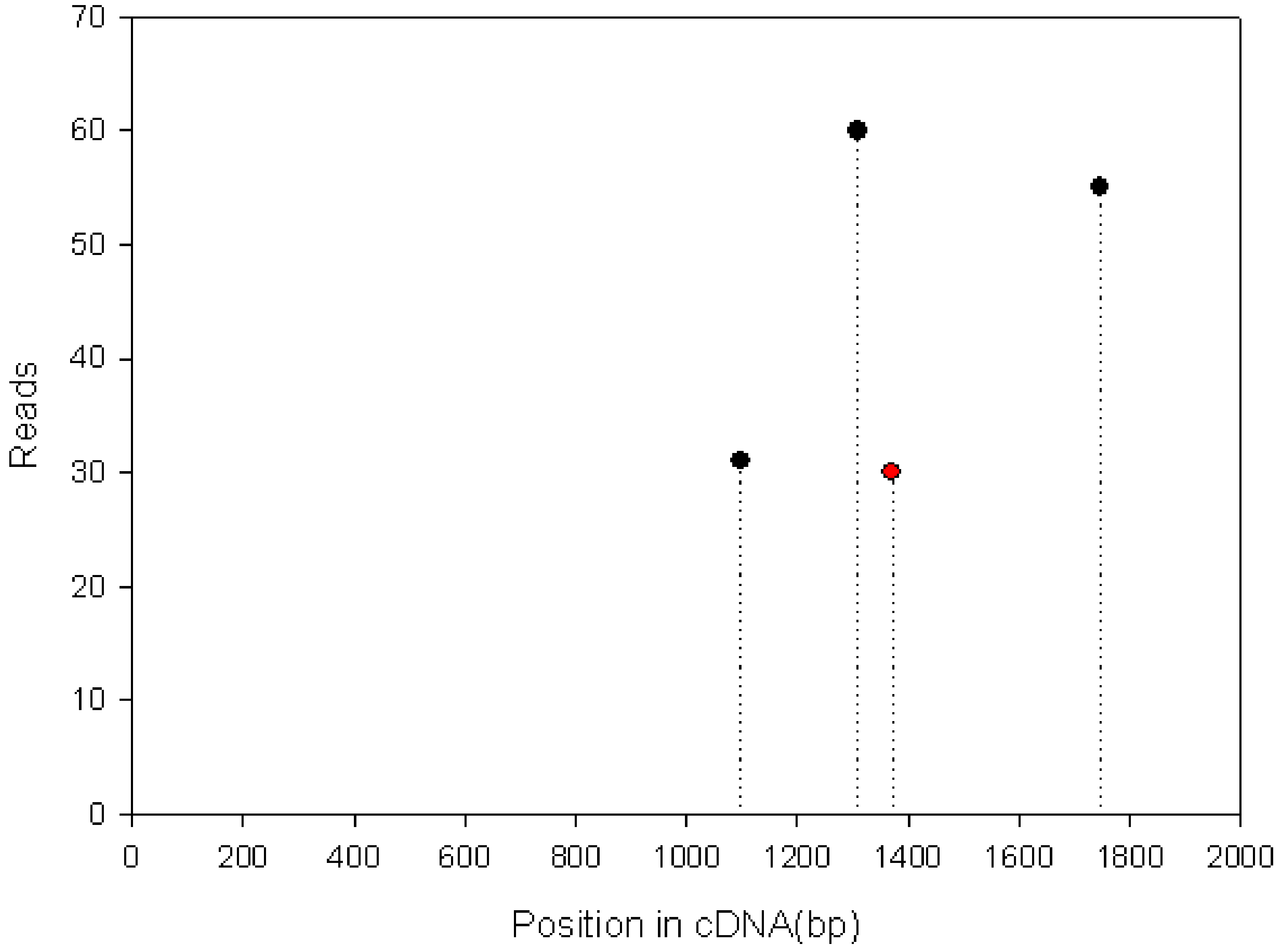


```
5' GUUGCUGAAGAAAGCUGUGGAGGAUC 3'        Orange1.1t02254.1
      :  :  :::::::::.:::::.
3' ---CAAGUUCUUUCGGCACCUU---- 5'        Csi-miR396d.3
```

Csi-miR396d.3, target=Orange1.1t02555.1 gene=Orange1.1t02555
Category:1
Score=2
Cleavage Site=920

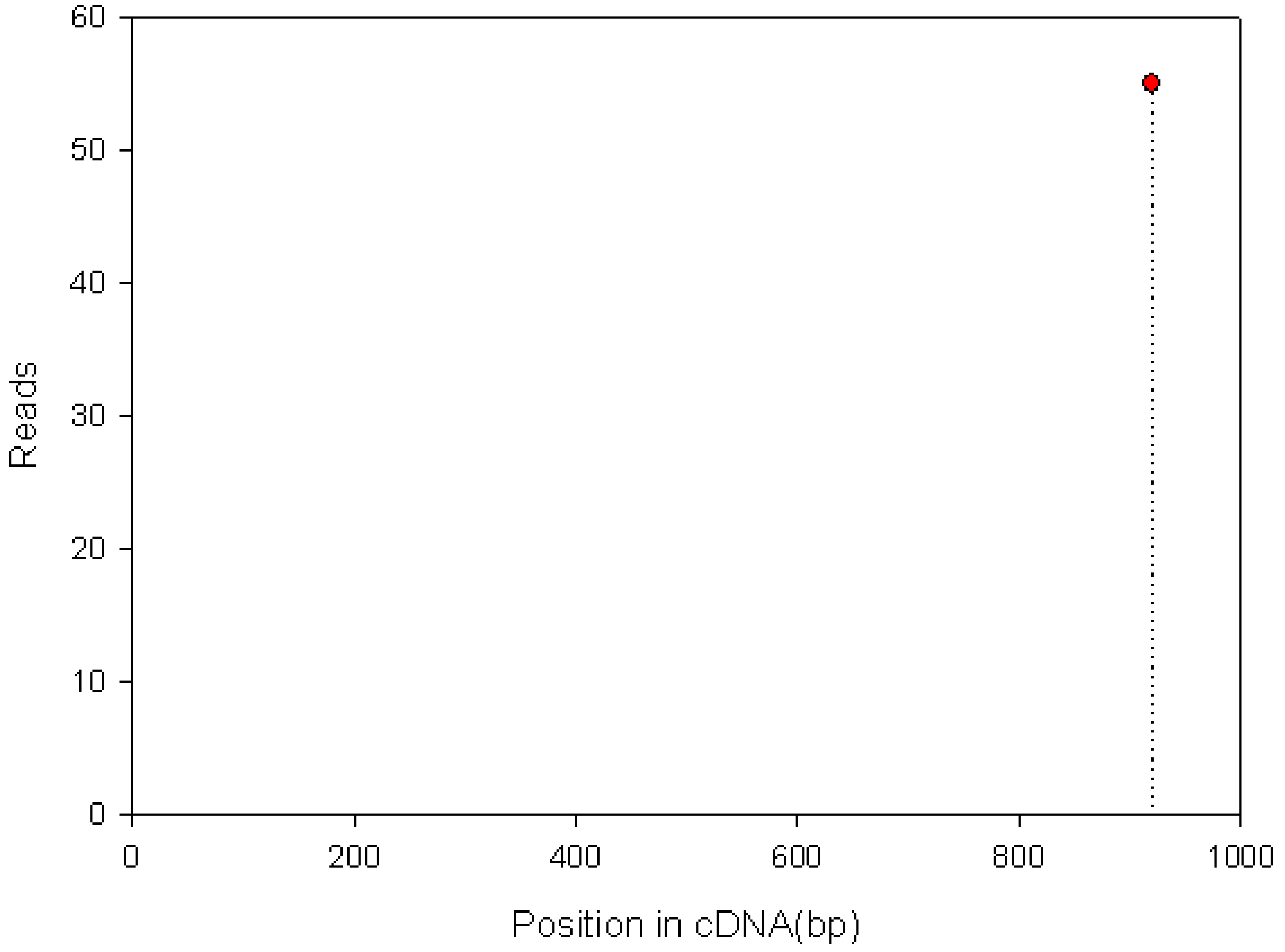


```
5' CACGUUCAAGAAAGCCUGUGGAACUU 3'        Orange1.1t02555.1
      ::::::::::::: ::::::
3' ---CAAGUUCUUUCGG-CACCUU--- 5'        Csi-miR396d.3
```

Csi-miR396d.3, target=Orange1.1t03122.1 gene=Orange1.1t03122
Category:2
Score=2
Cleavage Site=583

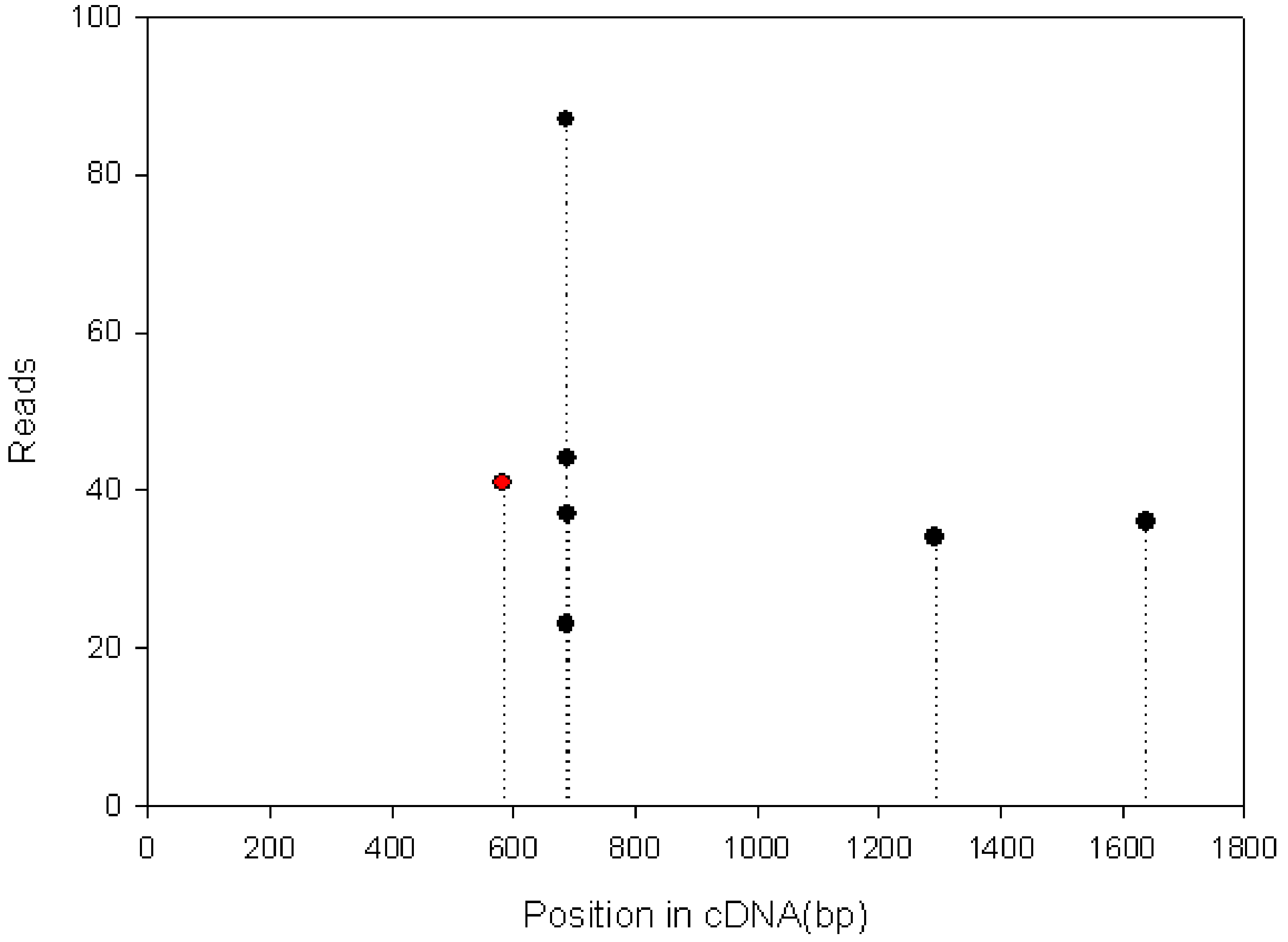


```
5' ACCGUUCAAGAAAGCCUGUGGAAUCA 3'        Orange1.1t03122.1
     ::::::::::::: ::::::
3' ---CAAGUUCUUUCGG-CACCUU--- 5'        Csi-miR396d.3
```

Csi-miR396d.4, target=Cs1g15720.1 gene=Cs1g15720
Category:2
Score=4
Cleavage Site=1610

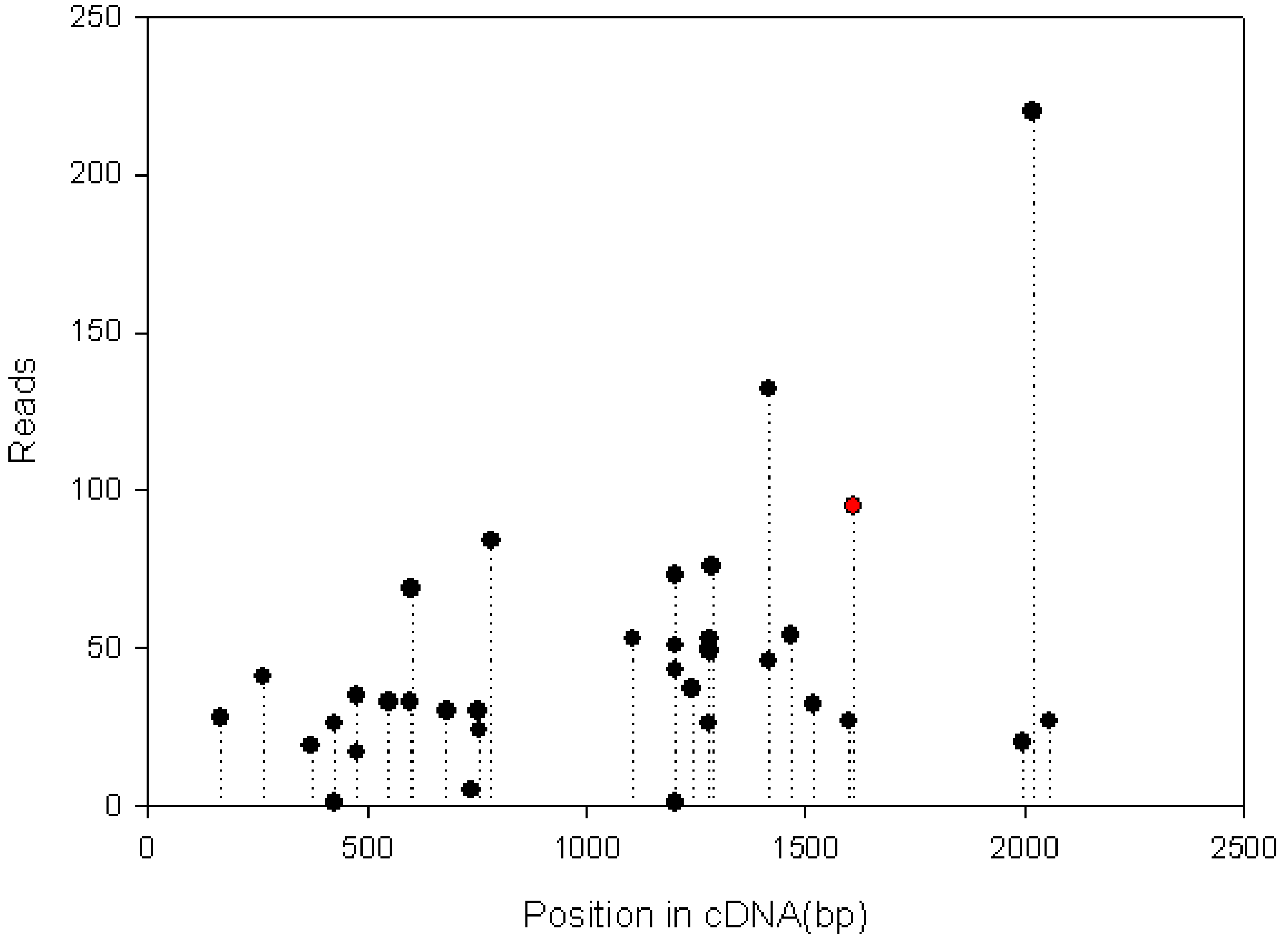


```
5' CAACAUUUCAA-AAAGCCGUGGGCUCA 3'       Cs1g15720.1
          ::::: :::::::::::.
3' ------AAGUUCUUUCGGCACCUU--- 5'       Csi-miR396d.4
```

Csi-miR396d.4, target=Cs1g16550.1 gene=Cs1g16550
Category:3
Score=5
Cleavage Site=418

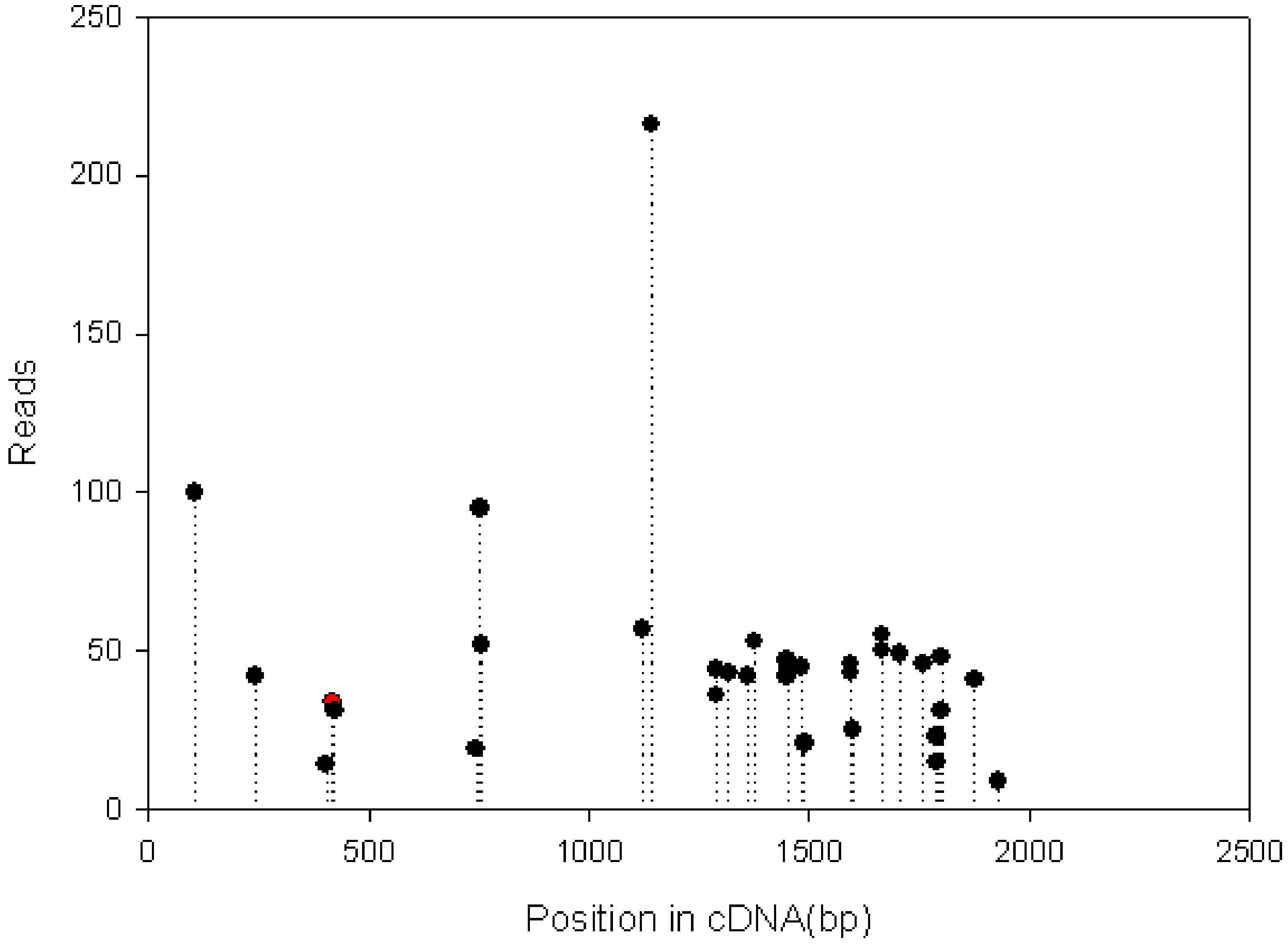


```
5' ACAUCACAACAACAGCCGUGGAACAC 3'         Cs1g16550.1
           ::: :: ::::::::::
3' ----AAGUUCUU-UCGGCACCUU--- 5'         Csi-miR396d.4
```

Csi-miR396d.4, target=Cs3g07260.1 gene=Cs3g07260
Category:3
Score=4.5
Cleavage Site=2420

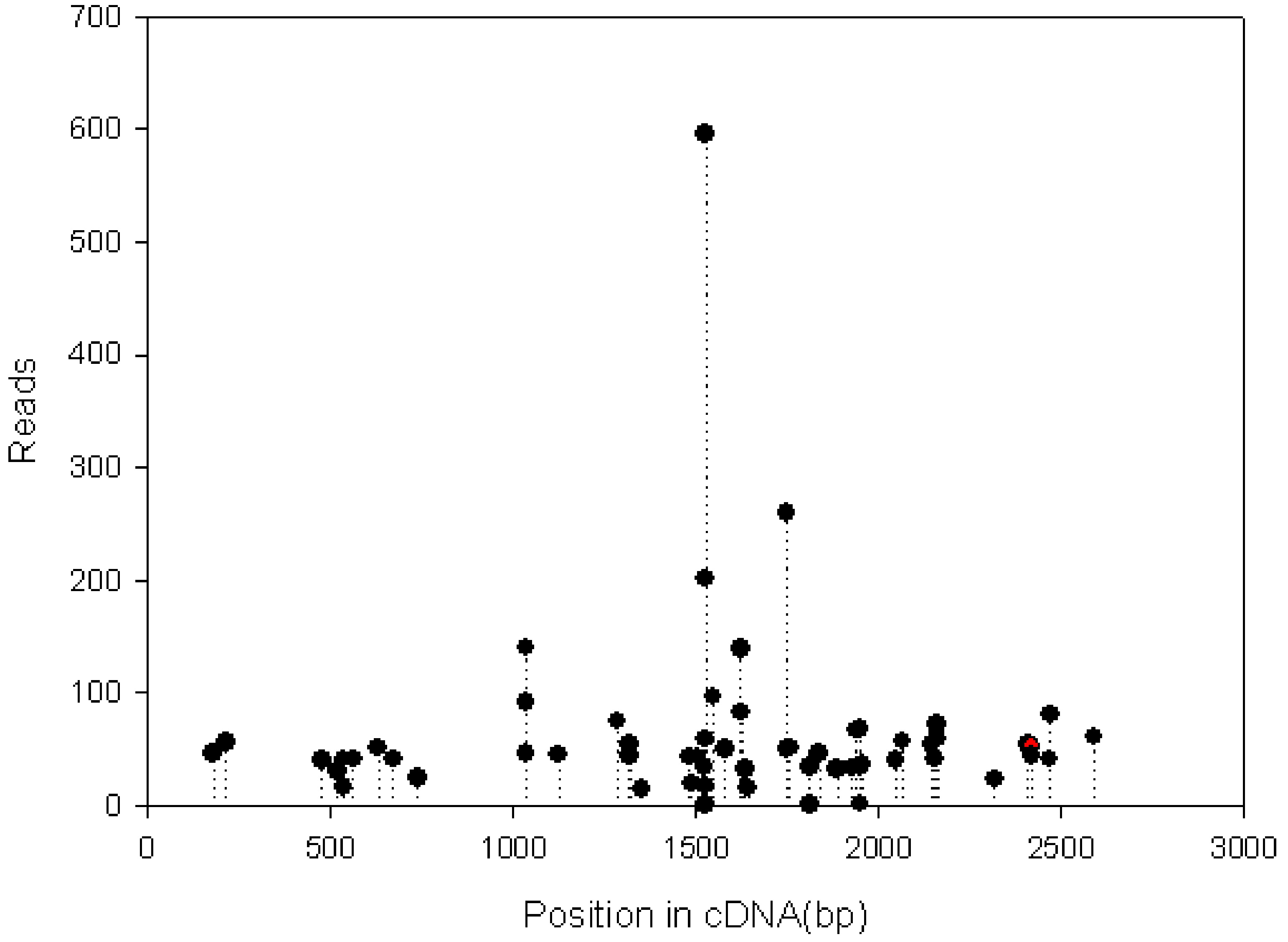


```
5' AUUCCAGGAAAGUUGUGGAUAAAUA 3'        Cs3g07260.1
     : ::.:::::..::::.:
3' --AAGUUCUUUCGGCACCUU------ 5'        Csi-miR396d.4
```

Csi-miR396d.4, target=Cs3g23180.1 gene=Cs3g23180
Category: 3
Score=4
Cleavage Site=625

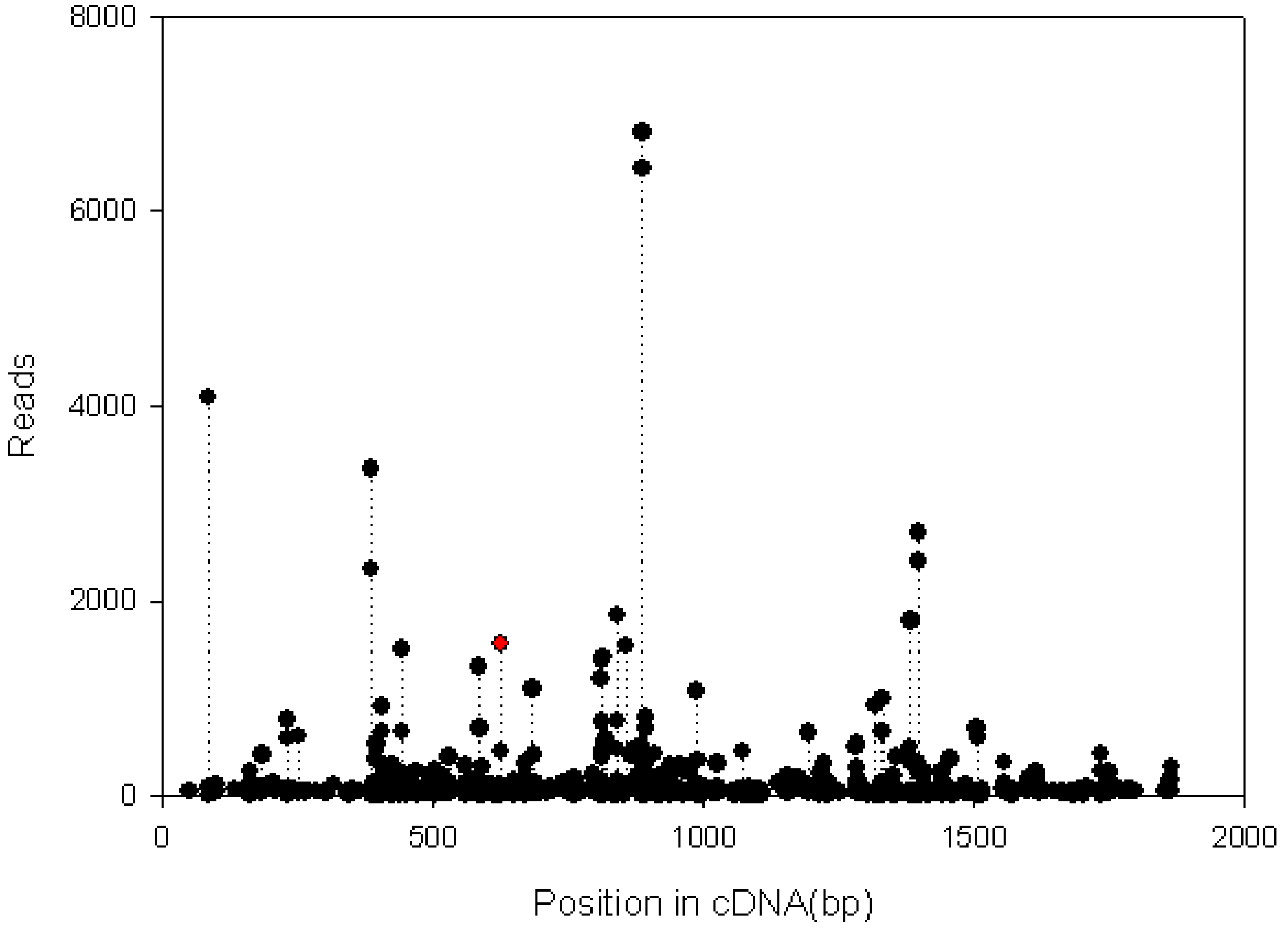


```
5' AAAGAUCAAGGAAGCUGUGGGAGUUG 3'        Cs3g23180.1
         ::::::.:::::.:::::.:
3' ----AAGUUCUUUCGGCACCUU---- 5'        Csi-miR396d.4
```

Csi-miR396d.4, target=Cs4g12200.1 gene=Cs4g12200
Category:2
Score=4
Cleavage Site=228

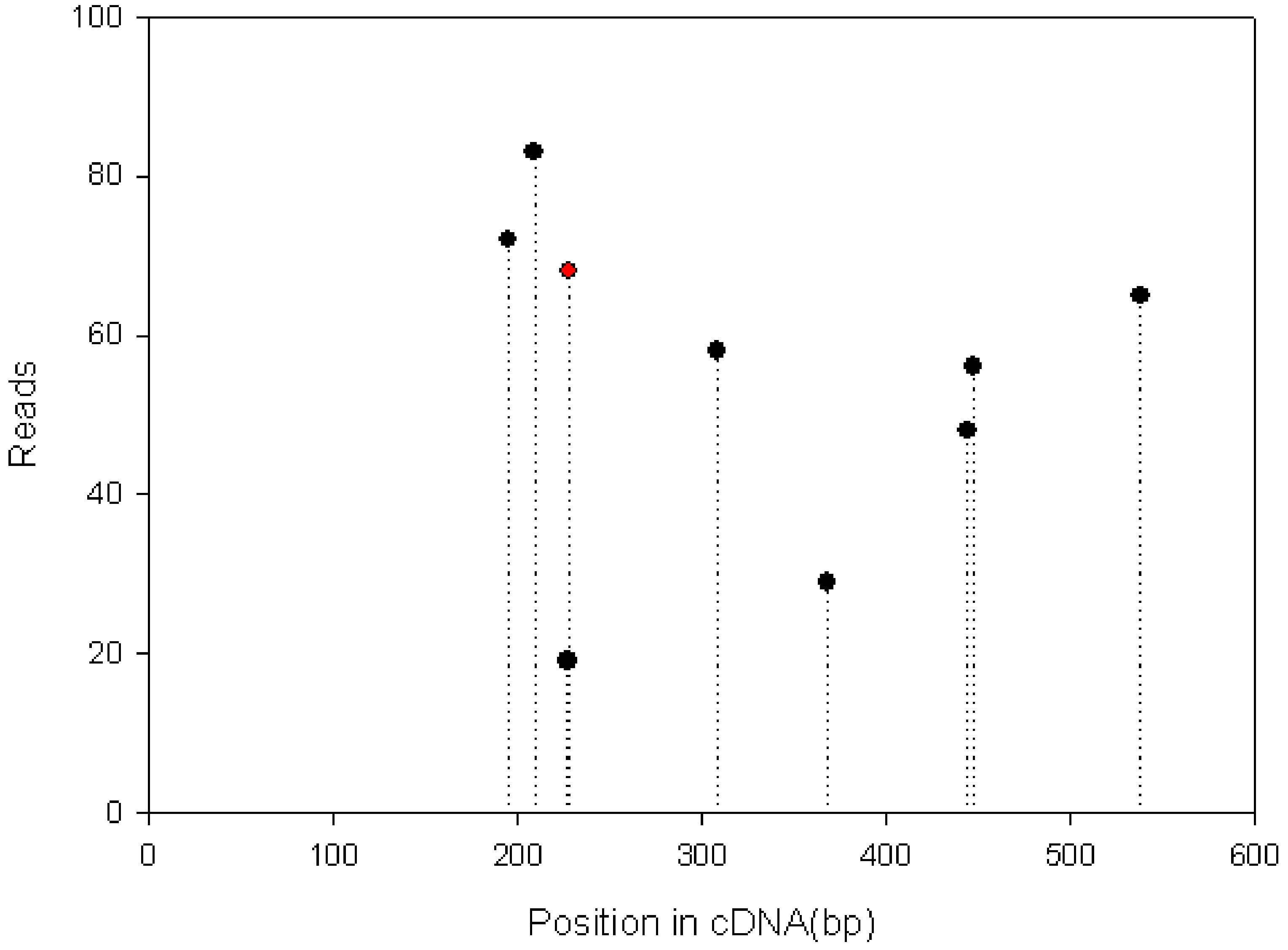


```
5' GGUUCCUGAAAGCCG-GGAAAUAUGCU 3'       Cs4g12200.1
     :::  :::::::: ::::
3' --AAGUUCUUUCGGCACCUU------- 5'       Csi-miR396d.4
```

Csi-miR396d.4, target=Cs4g18150.1 gene=Cs4g18150
Category:3
Score=5
Cleavage Site=506

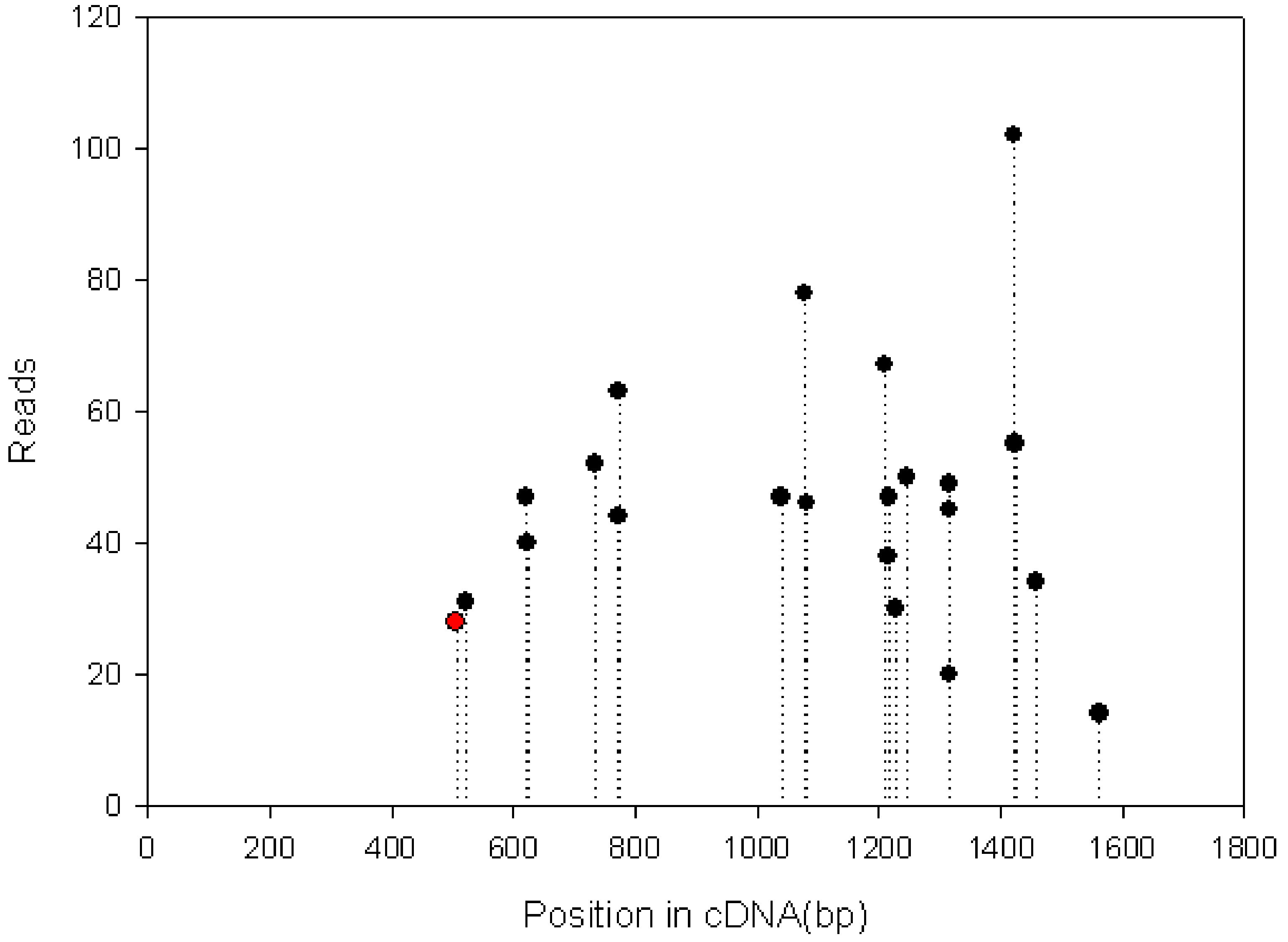


```
5' UUGUCUUGAAAGCCGAGGAAAUUCUC 3'        Cs4g18150.1
     ::   :::::::: ::::
3' --AAGUUCUUUCGGCACCUU------ 5'        Csi-miR396d.4
```

Csi-miR396d.4, target=Cs5g01380.1 gene=Cs5g01380
Category:1
Score=2
Cleavage Site=1181

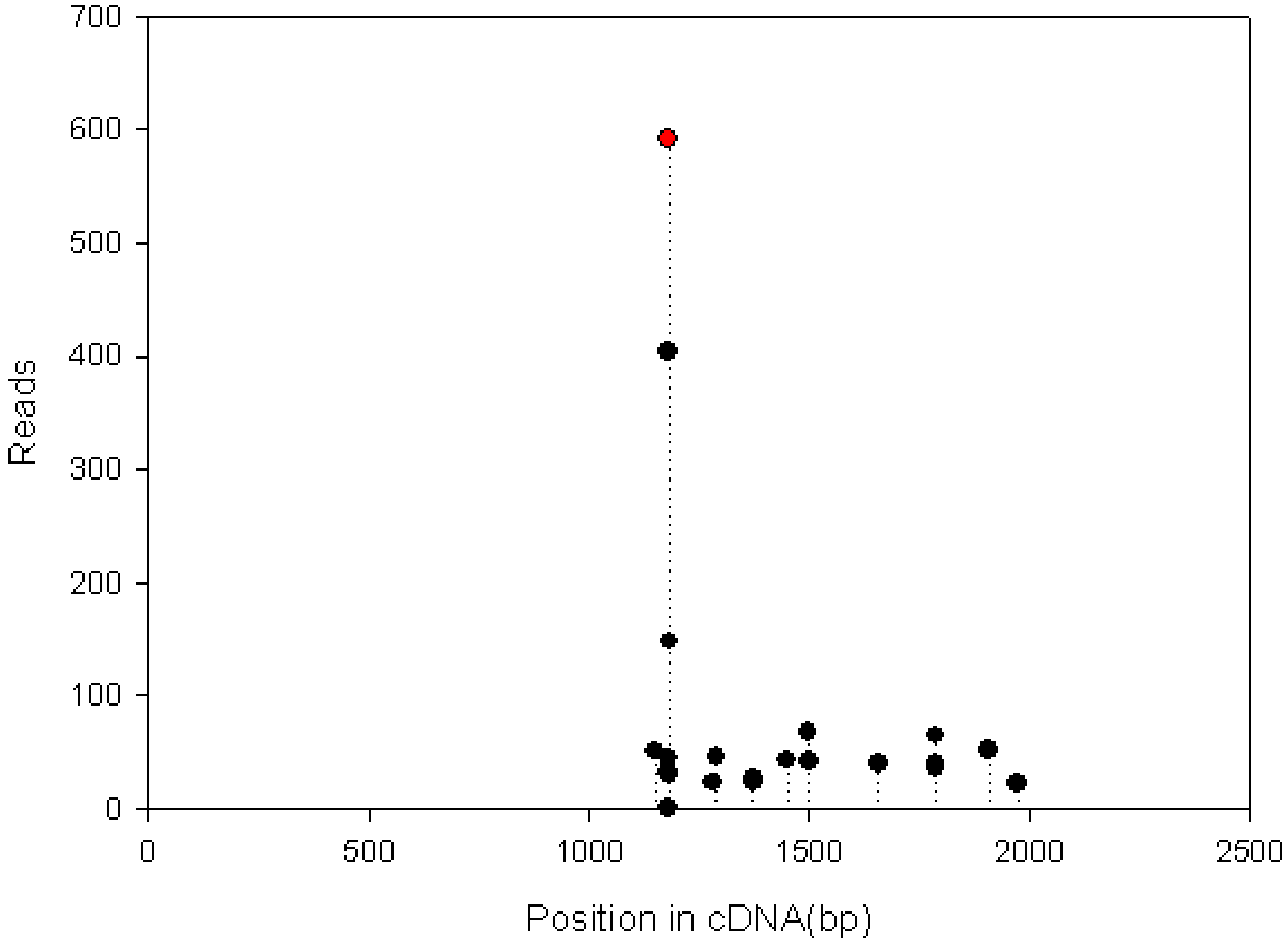


```
5' CCCGUUCAAGAAAGCCUGUGGAAUUA 3'        Cs5g01380.1
       :::::::::::: ::::::
3' ----AAGUUCUUUCGG-CACCUU--- 5'        Csi-miR396d.4
```

Csi-miR396d.4, target=Cs5g07120.1 gene=Cs5g07120
Category:2
Score=4.5
Cleavage Site=1257

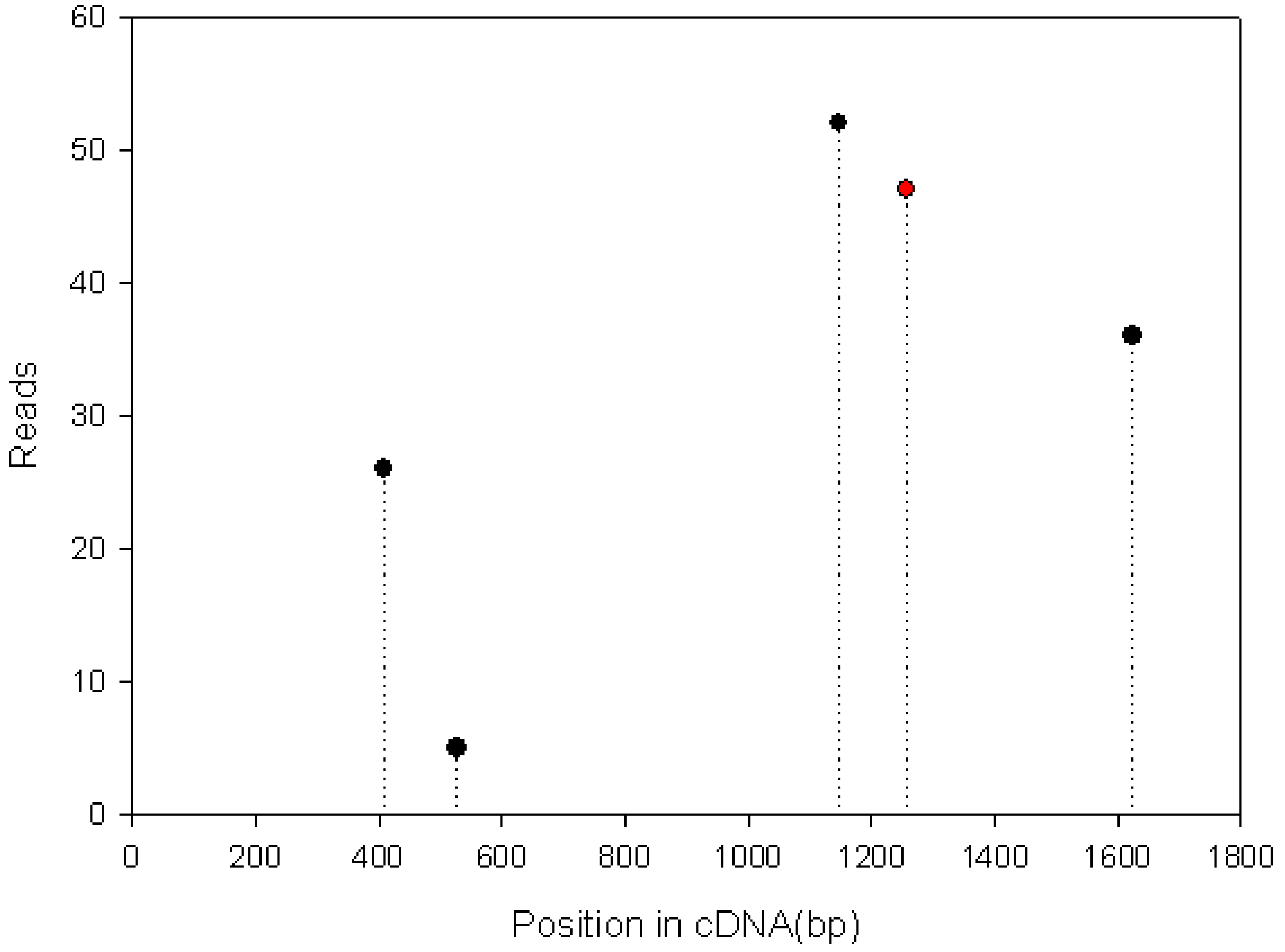


```
5' UGUUGGUAUGA-AAAGCCGUGGAGUCU 3'        Cs5g07120.1
          :  ..: :::::::::::.
3' ------AAGUUCUUUCGGCACCUU--- 5'        Csi-miR396d.4
```

Csi-miR396d.4, target=Cs5g19200.1 gene=Cs5g19200
Category:2
Score=2.5
Cleavage Site=201

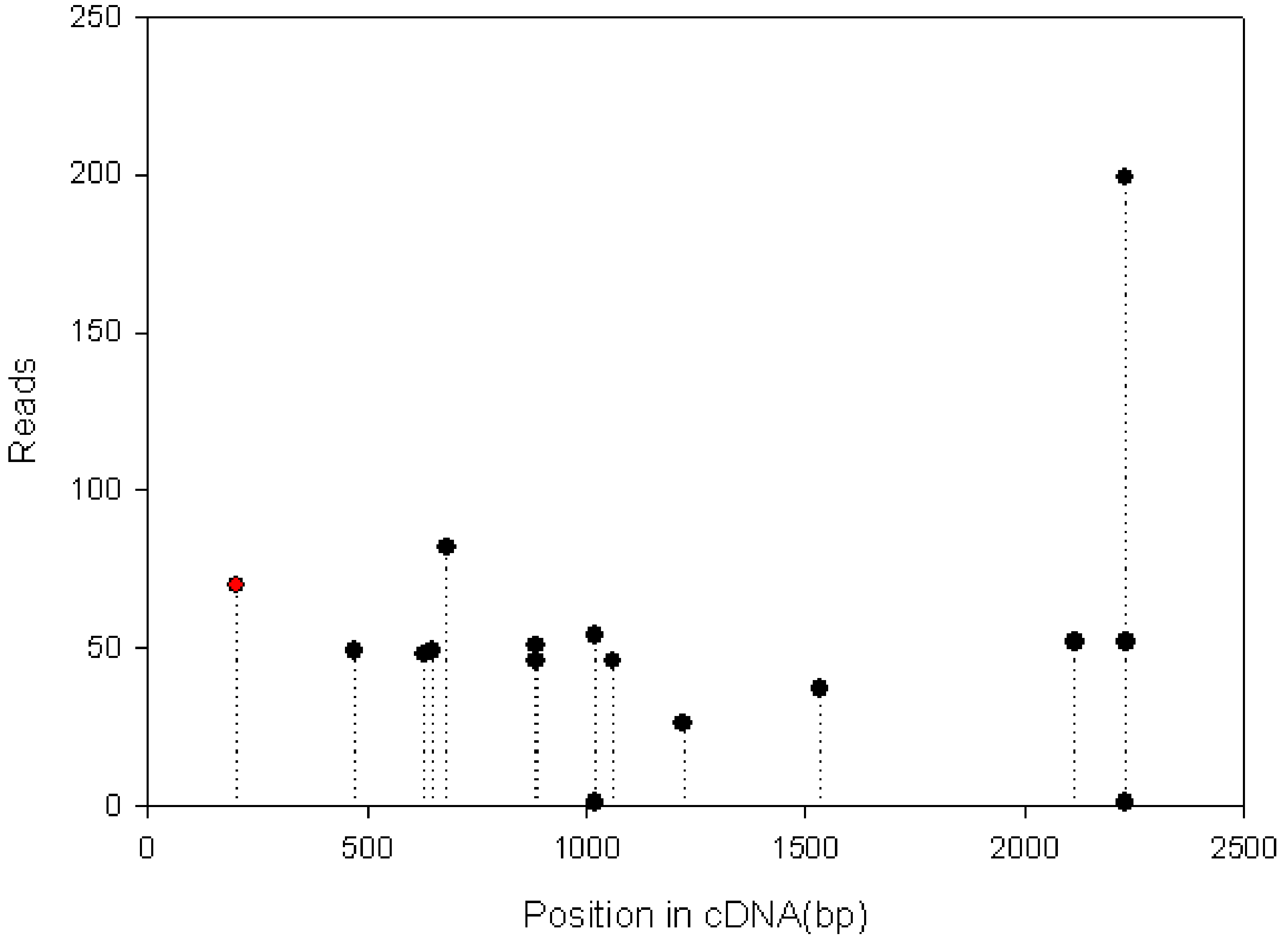


```
5' AAUGUUUAAGAAAGCAGUGGAAGCAA 3'        Cs5g19200.1
       ::.::::::::: ::::::
3' ----AAGUUCUUUCGGCACCUU---- 5'        Csi-miR396d.4
```

Csi-miR396d.4, target=Cs6g04030.1 gene=Cs6g04030
Category:3
Score=5
Cleavage Site=857

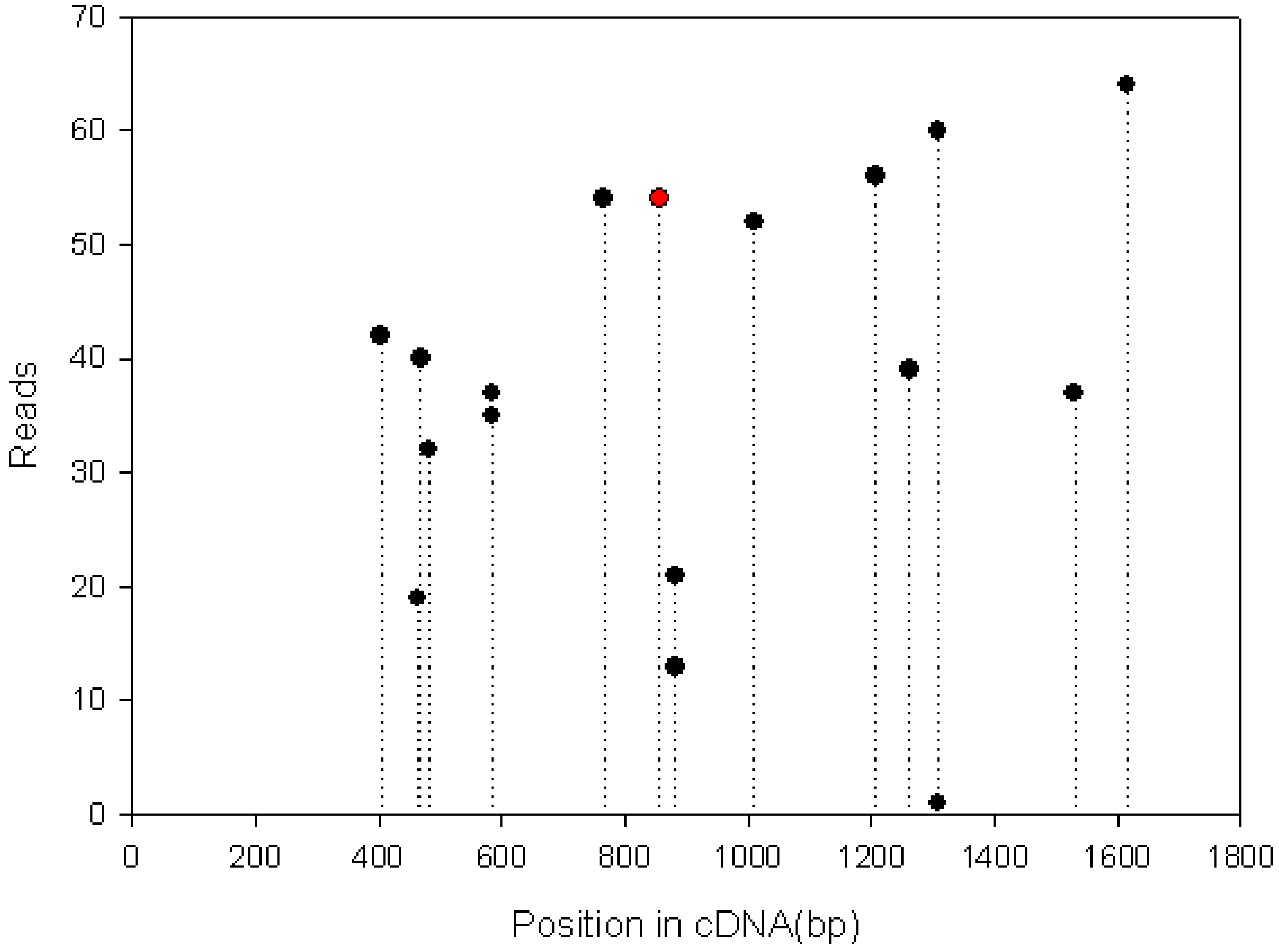


```
5' GUAAAUACUUUUUAG-AAGCUGUGGAG 3'      Cs6g04030.1
                ::. :: ::::.::::.
3' ---------AAGUUCUUUCGGCACCUU 5'      Csi-miR396d.4
```

Csi-miR396d.4, target=Cs6g06540.1 gene=Cs6g06540
Category:3
Score=5
Cleavage Site=526

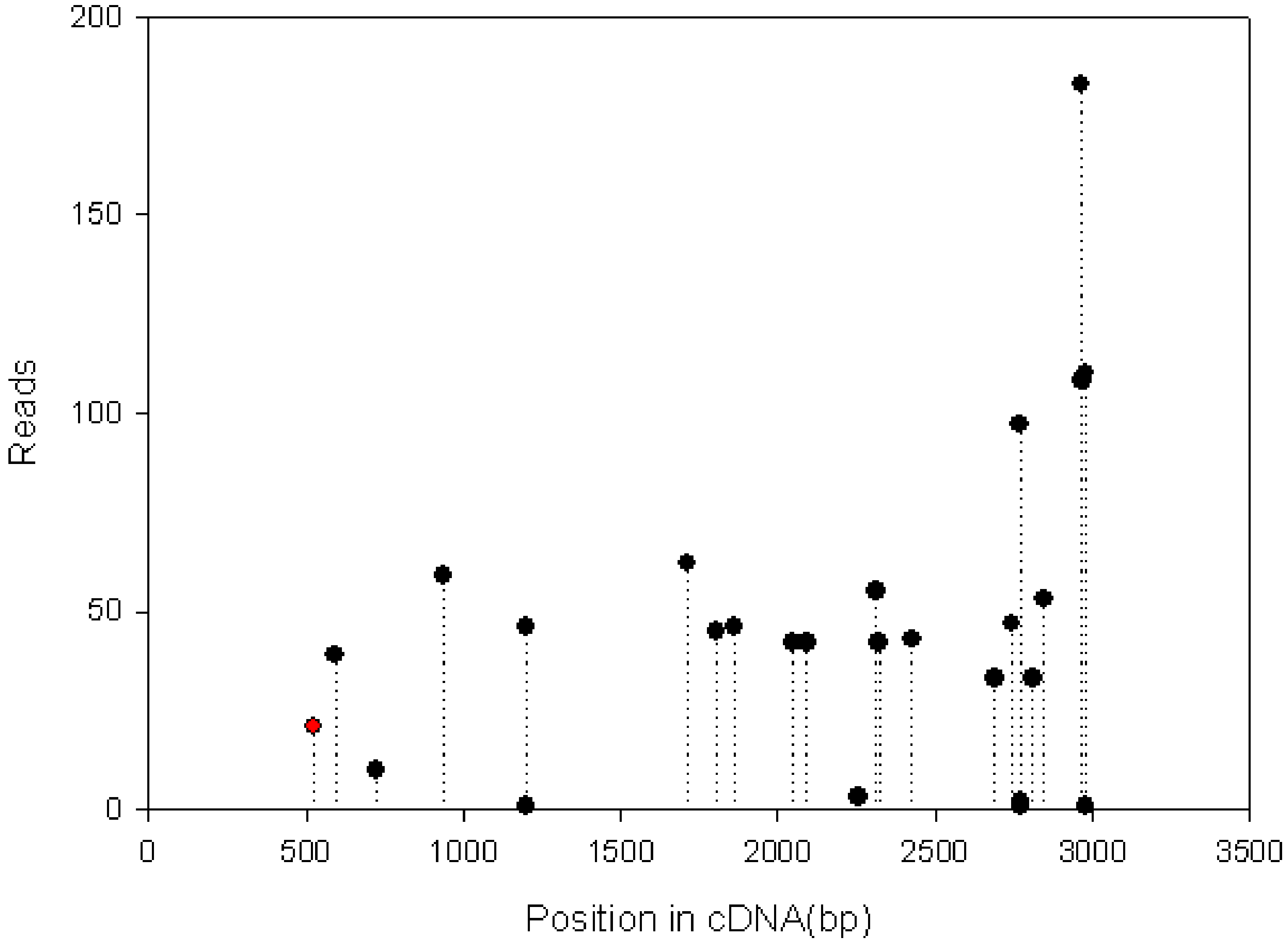


```
5' AACUUCCUUC-AGAAAGCUGAGGAUAA 3'        Cs6g06540.1
          ::: ::::::::.: :::
3' -------AAGUUCUUUCGGCACCUU-- 5'        Csi-miR396d.4
```

Csi-miR396d.4, target=Cs6g06560.1 gene=Cs6g06560
Category:3
Score=3.5
Cleavage Site=1538

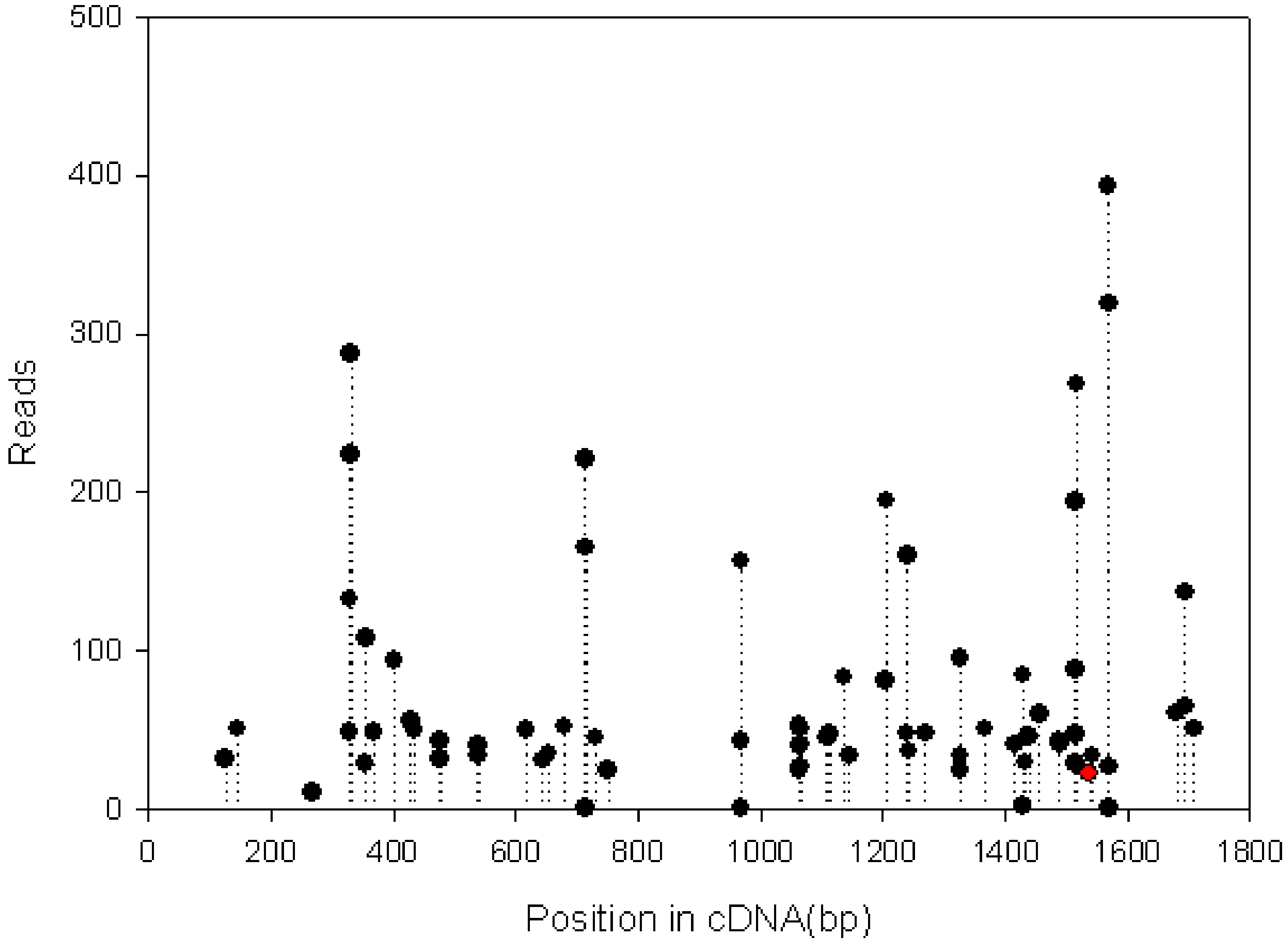


```
5' UUC-AGAAAGCCUUGGAGAGAUUAACC 3'        Cs6g06560.1
   ::: ::::::::: ::::.
3' AAGUUCUUUCGGCACCUU--------- 5'        Csi-miR396d.4
```

Csi-miR396d.4, target=Cs7g12930.1 gene=Cs7g12930
Category:1
Score=4.5
Cleavage Site=1079

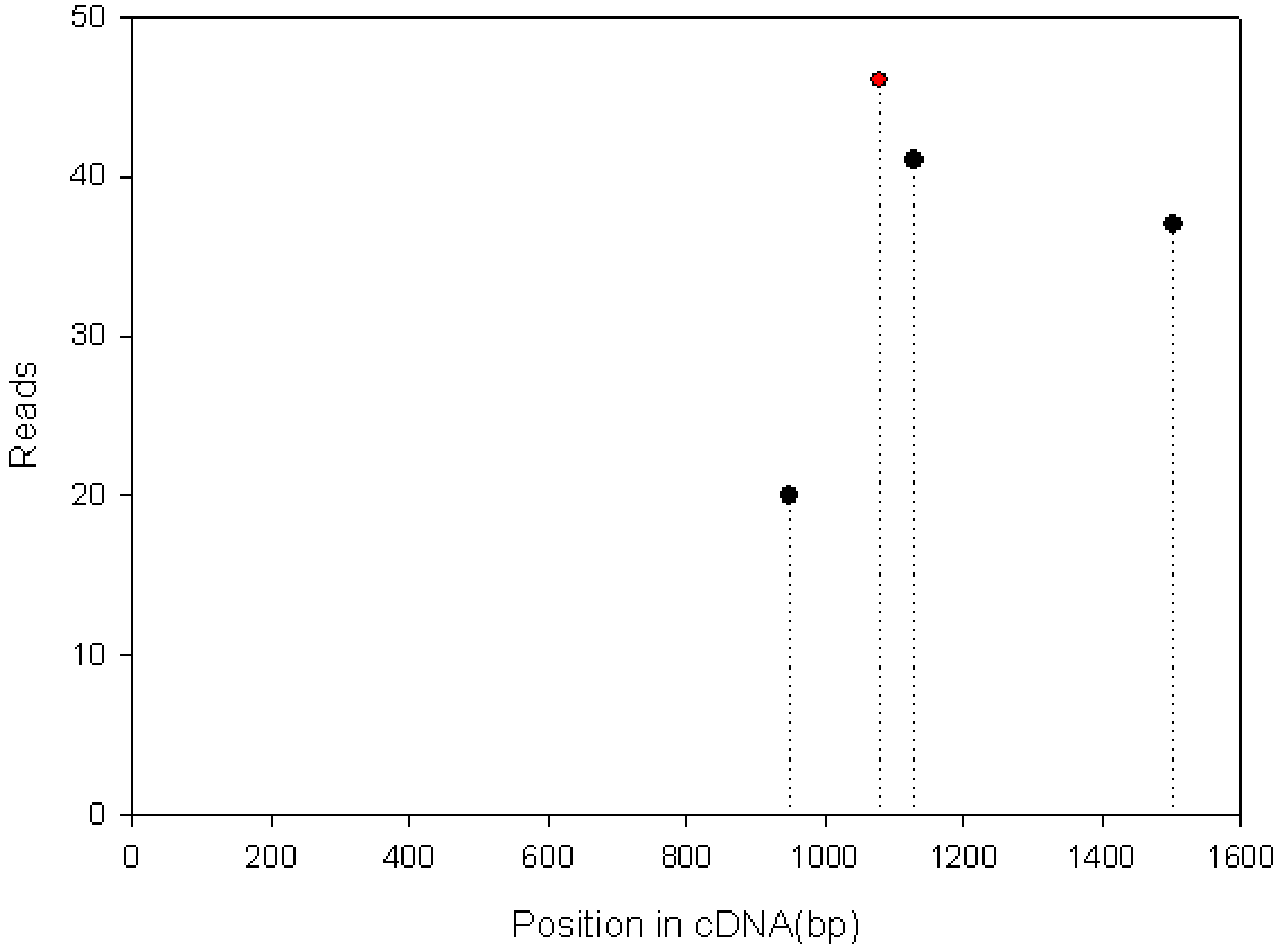


```
5' UAGUAUUAAGAAAGCUGAGGAAAAAG 3'        Cs7g12930.1
        :.::::::::.: ::::
3' ----AAGUUCUUUCGGCACCUU---- 5'        Csi-miR396d.4
```

Csi-miR396d.4, target=Cs7g15220.1 gene=Cs7g15220
Category:1
Score=2
Cleavage Site=377

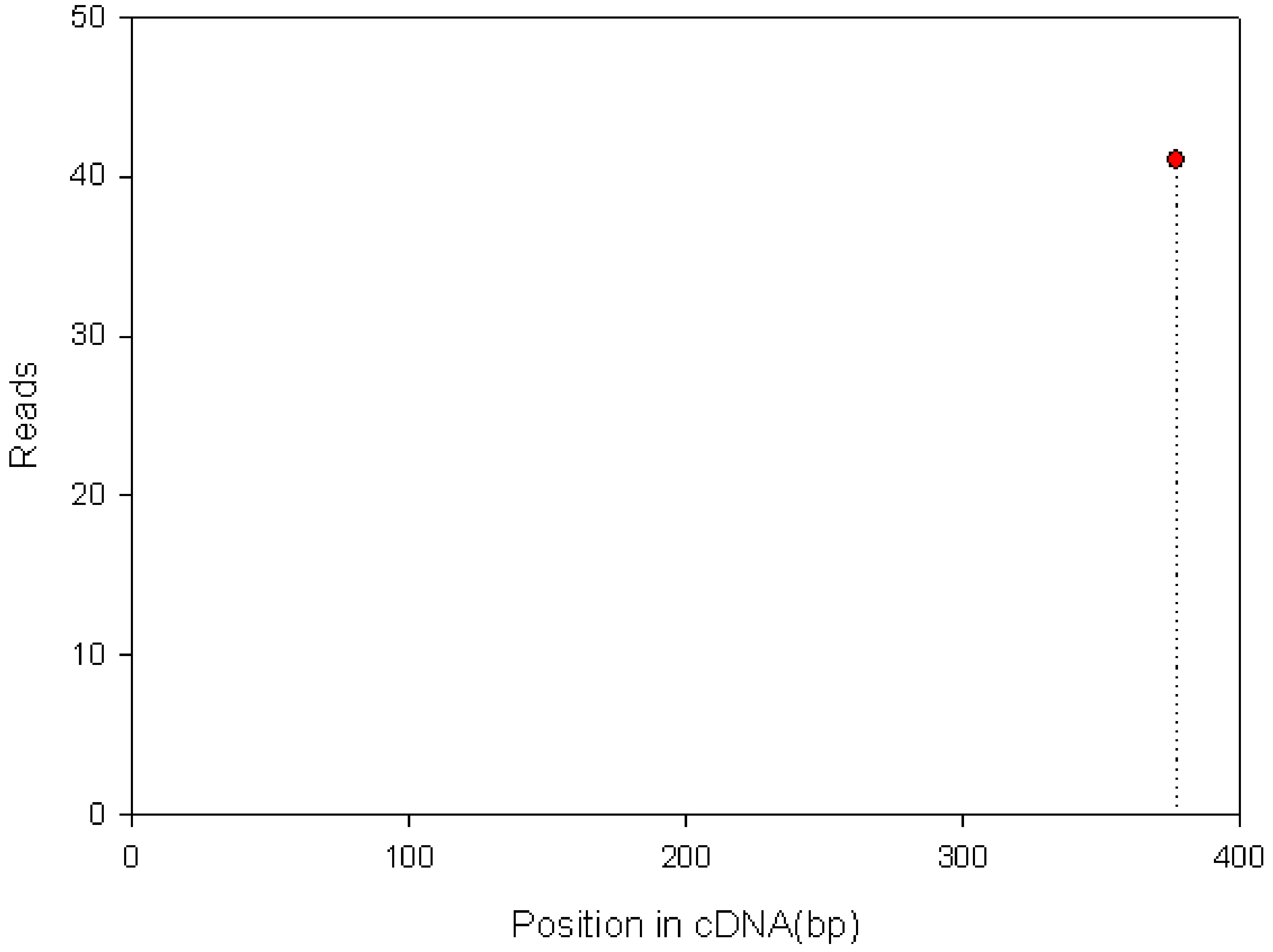


```
5' ACCGUUCAAGAAAGCCUGUGGAAUCA 3'        Cs7g15220.1
       :::::::::::: ::::::
3' ----AAGUUCUUUCGG-CACCUU--- 5'        Csi-miR396d.4
```

Csi-miR396d.4, target=Cs8g15450.1 gene=Cs8g15450
Category:3
Score=4.5
Cleavage Site=219

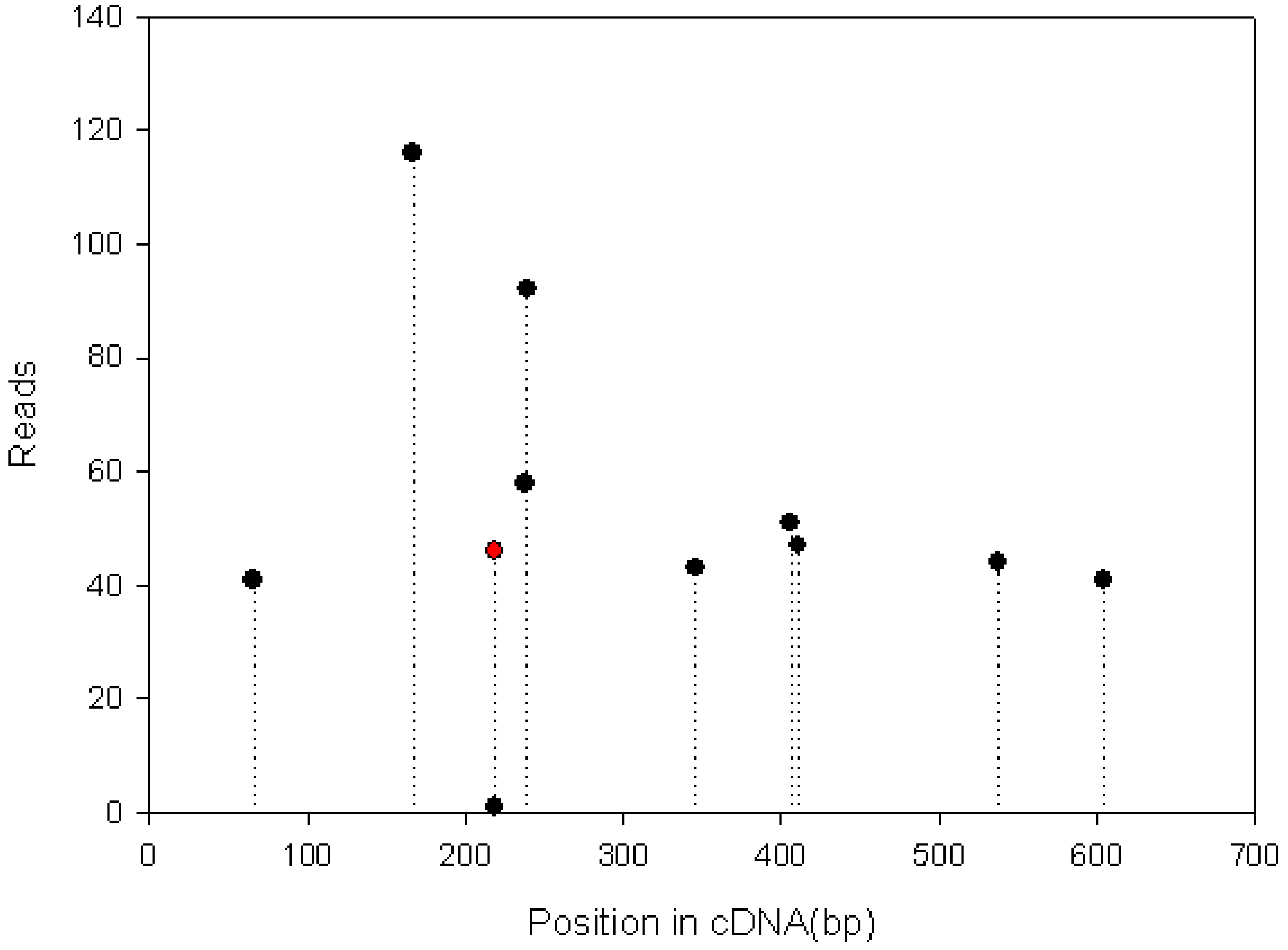


```
5' GUUCAGCAGAGCCGUGGAUGGGUUCA 3'        Cs8g15450.1
    ::::. :.::::::::::
3' -AAGUUCUUUCGGCACCUU------- 5'        Csi-miR396d.4
```

Csi-miR396d.4, target=Cs8g17370.1 gene=Cs8g17370
Category:3
Score=2.5
Cleavage Site=137

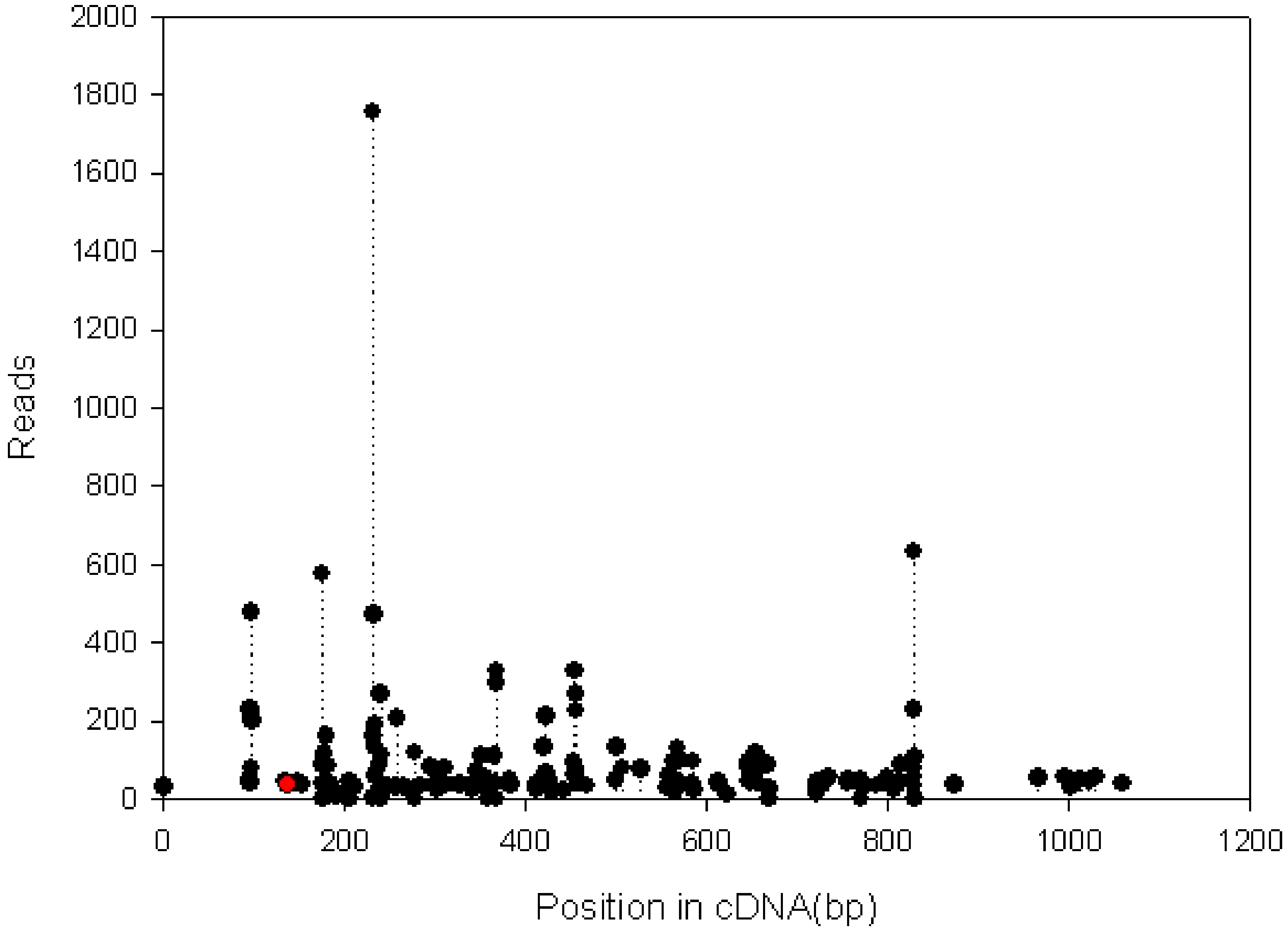


```
5' GCGAGGAUUACAAGAAGGCCGUGGAG 3'        Cs8g17370.1
            : :::::::.::::::::.
3' --------AAGUUCUUUCGGCACCUU 5'        Csi-miR396d.4
```

Csi-miR396d.4, target=Cs9g14020.1 gene=Cs9g14020
Category:3
Score=4
Cleavage Site=639

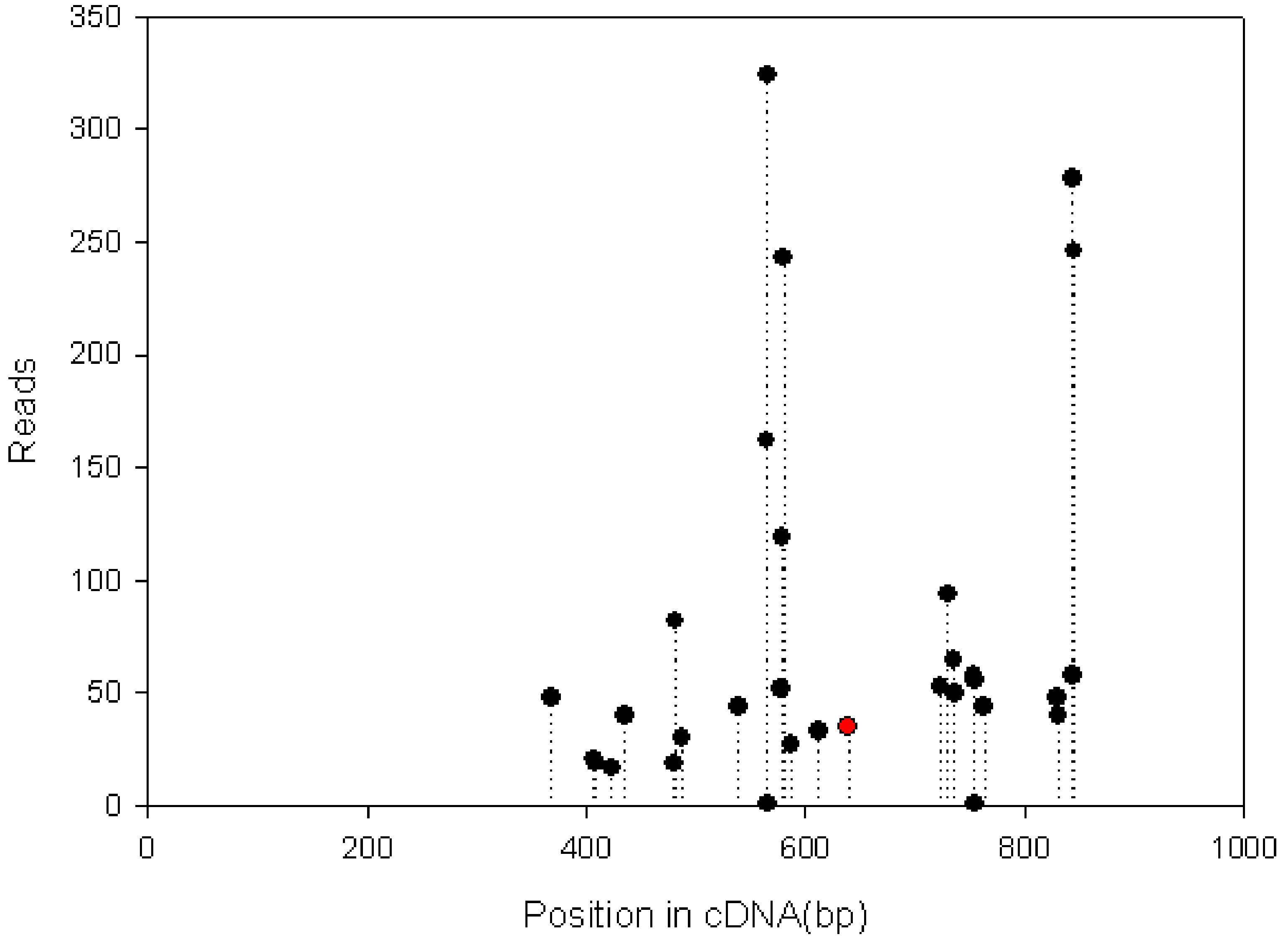


```
5' GGCCCAUC-AGAAAGCCCUGGAACGCC 3'       Cs9g14020.1
         :: :::::::: :::::
3' -----AAGUUCUUUCGGCACCUU---- 5'       Csi-miR396d.4
```

Csi-miR396d.4, target=Cs9g19220.1 gene=Cs9g19220
Category:3
Score=4.5
Cleavage Site=1829

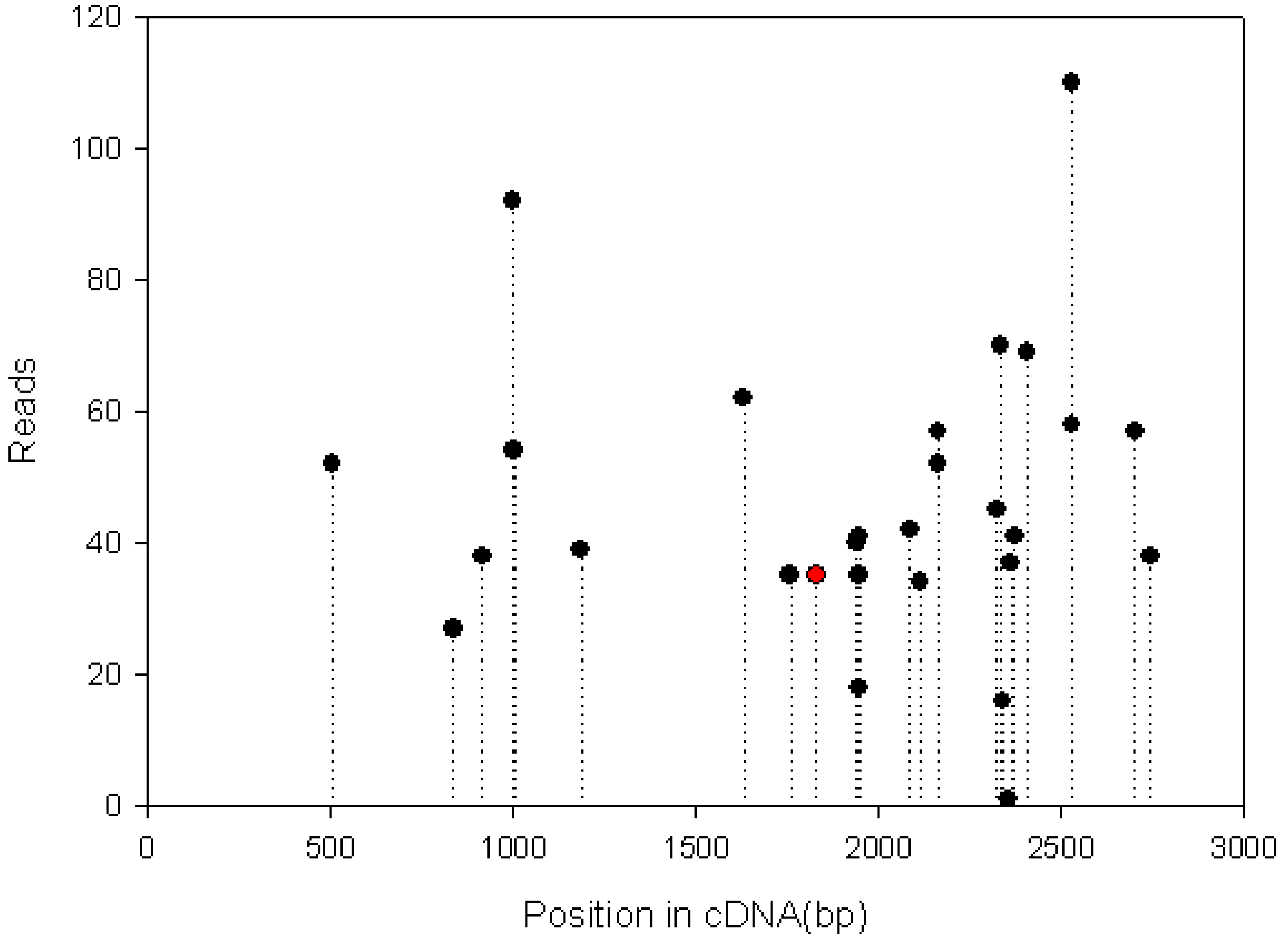


```
5' GGUGAGAGCAAGAAGGCUGUGGAGAA 3'        Cs9g19220.1
            ::::::.::.:::::.
3' ------AAGUUCUUUCGGCACCUU-- 5'        Csi-miR396d.4
```

Csi-miR396d.4, target=Orange1.1t02254.1 gene=Orange1.1t02254
Category:3
Score=3.5
Cleavage Site=1371

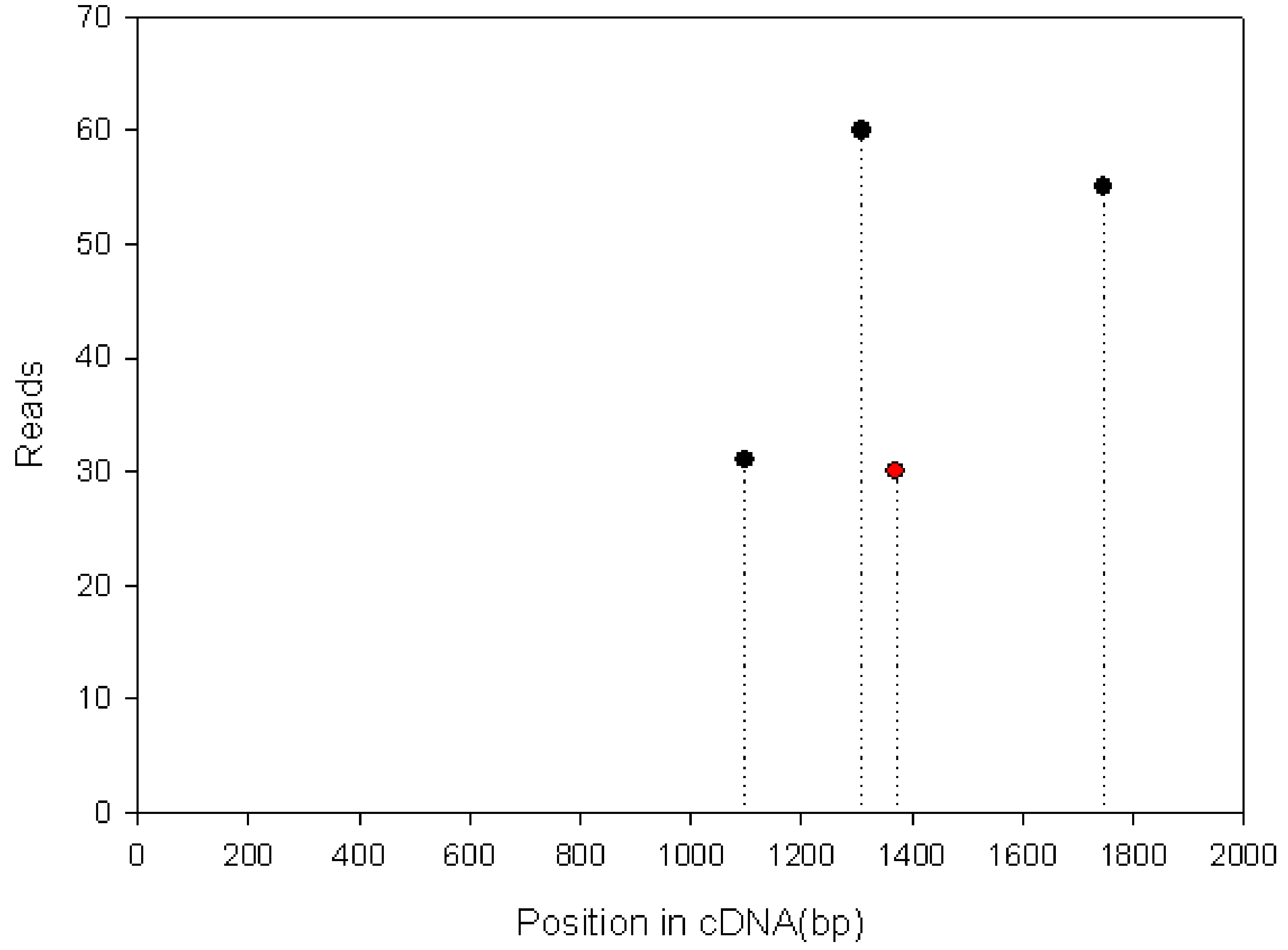


```
5' GUUGCUGAAGAAAGCUGUGGAGGAUC 3'        Orange1.1t02254.1
         :  ::::::::.:::::.
3' ----AAGUUCUUUCGGCACCUU---- 5'        Csi-miR396d.4
```

Csi-miR396d.4, target=Orange1.1t02555.1 gene=Orange1.1t02555
Category:1
Score=2
Cleavage Site=920

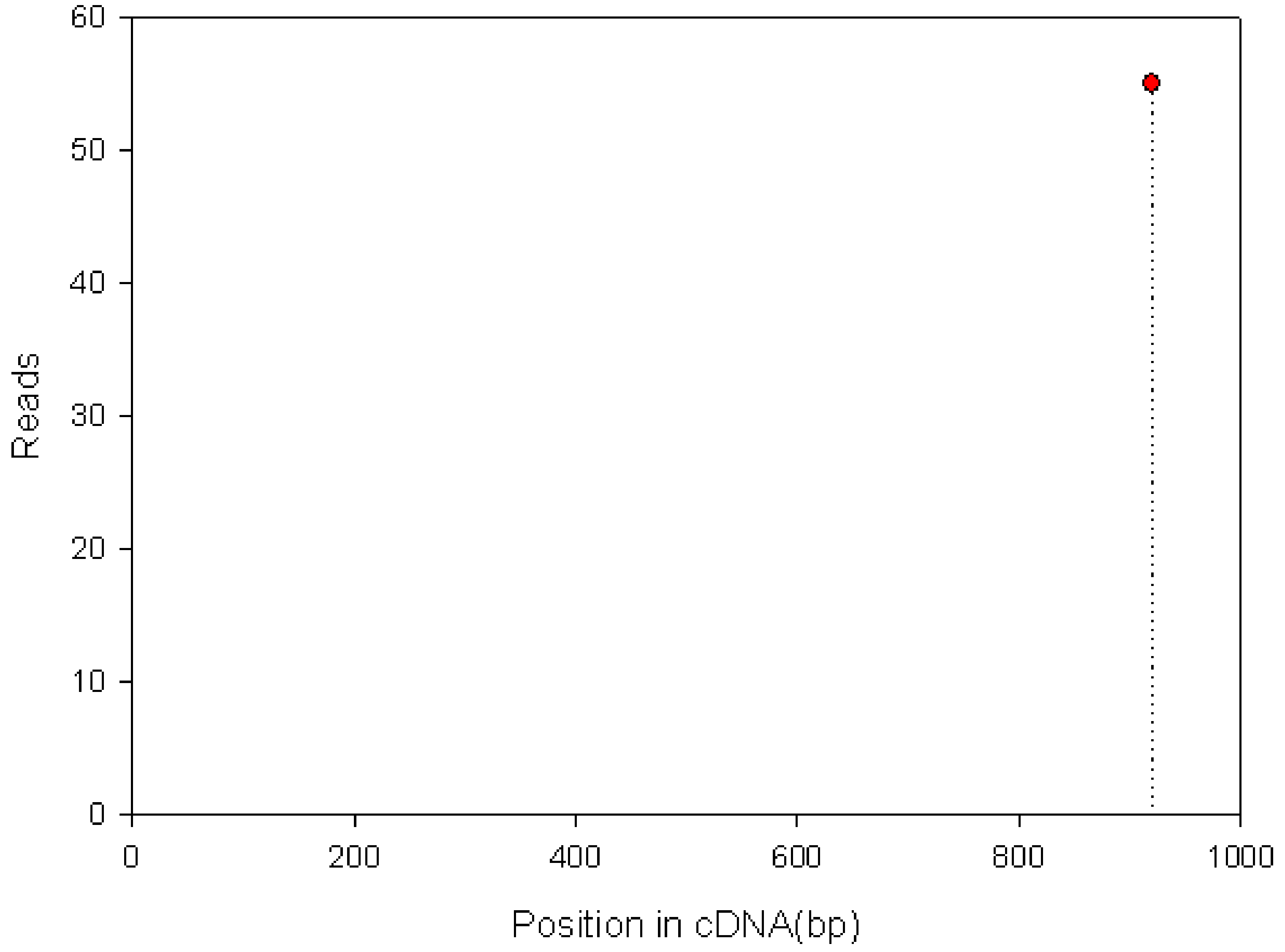


```
5' CACGUUCAAGAAAGCCUGUGGAACUU 3'        Orange1.1t02555.1
       :::::::::::: ::::::
3' ----AAGUUCUUUCGG-CACCUU--- 5'        Csi-miR396d.4
```

Csi-miR396d.4, target=Orange1.1t02739.1 gene=Orange1.1t02739
Category:2
Score=4.5
Cleavage Site=1251

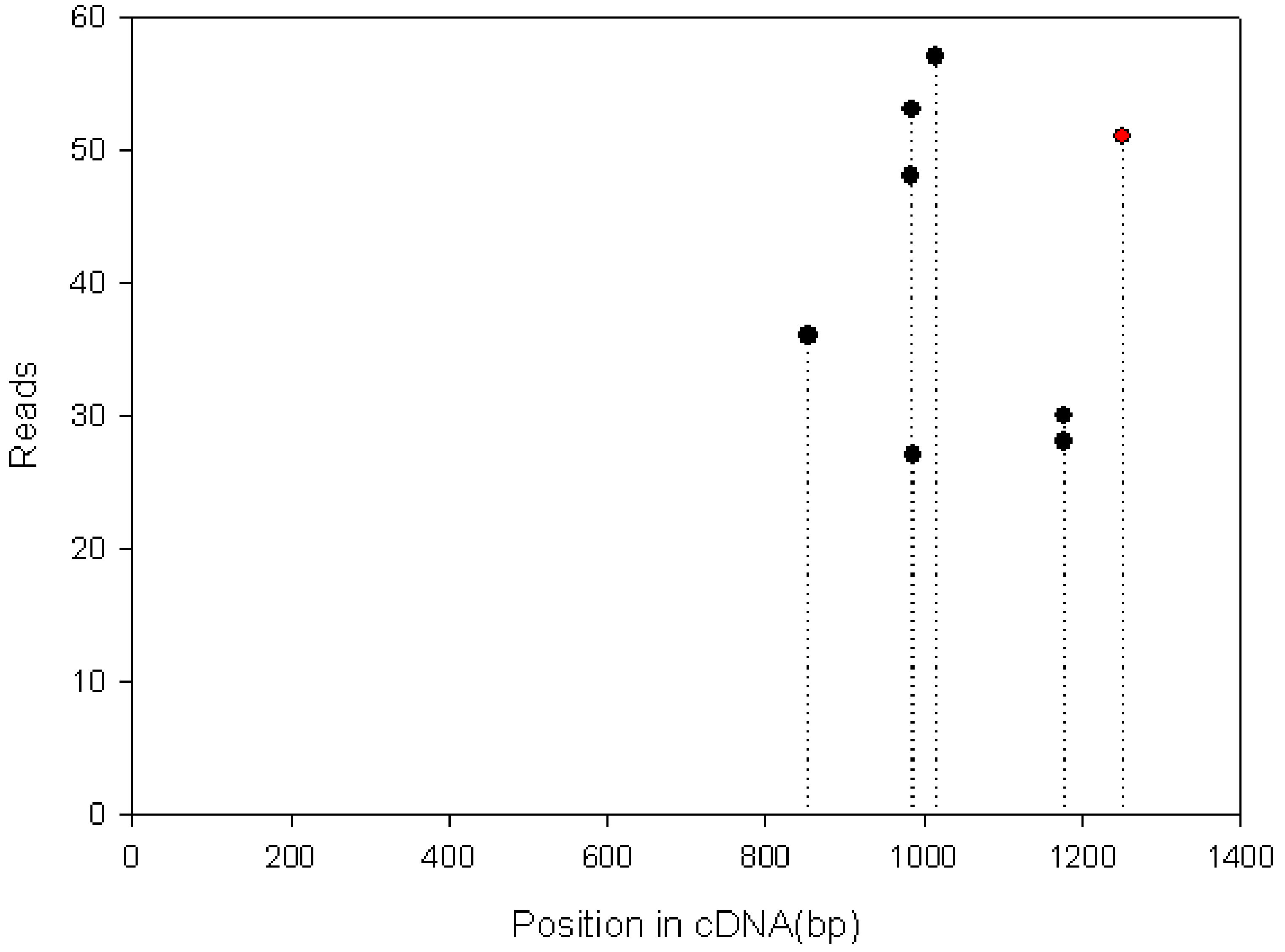


```
5' CUAUCAGAAAAGACCGUGGAAAUGGA 3'        Orange1.1t02739.1
     ::::. :::: ::::::::
3' --AAGUUCUUUC-GGCACCUU----- 5'        Csi-miR396d.4
```

Csi-miR396d.4, target=Orange1.1t03122.1 gene=Orange1.1t03122
Category:2
Score=2
Cleavage Site=583

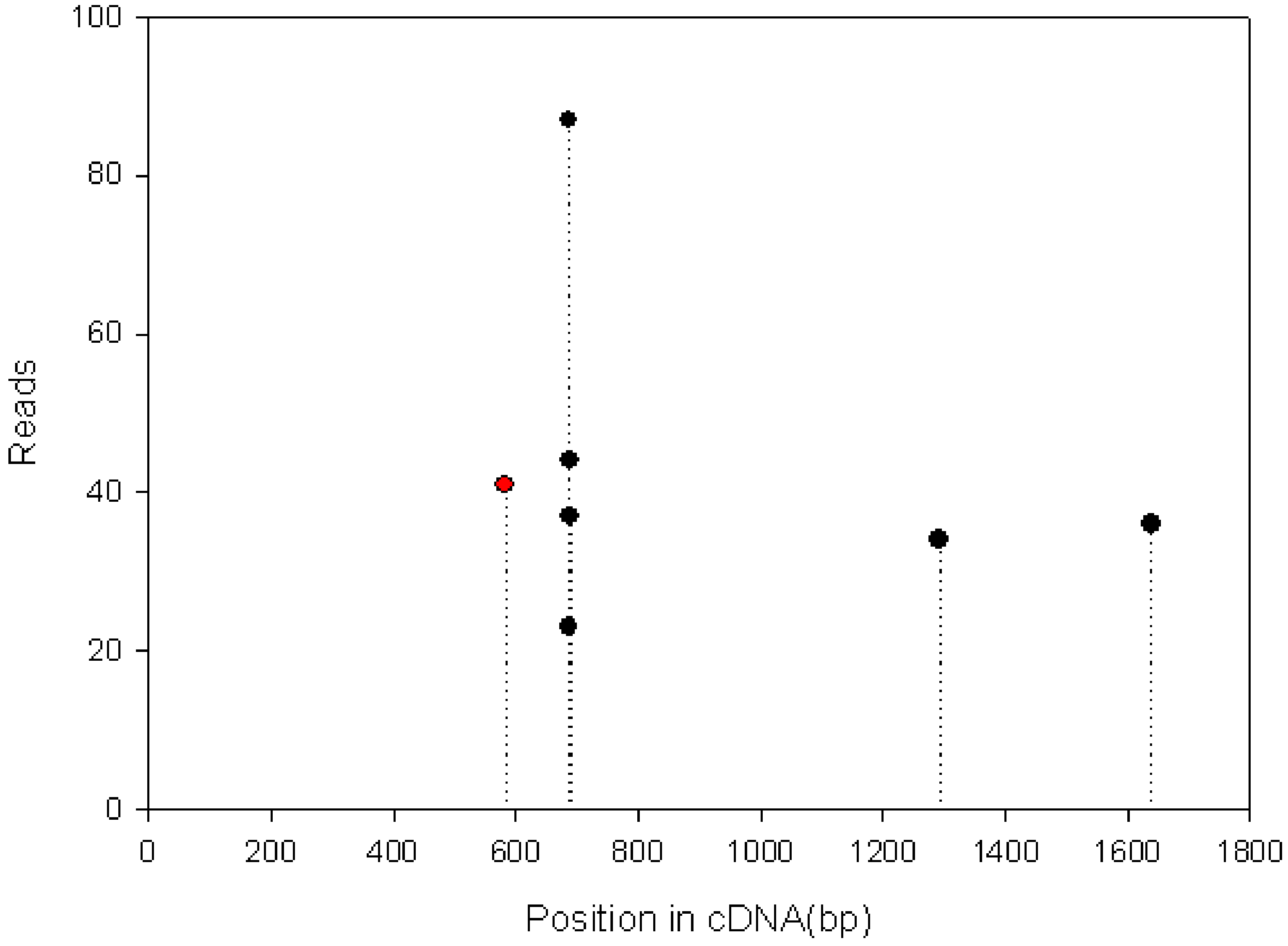


```
5' ACCGUUCAAGAAAGCCUGUGGAAUCA 3'        Orange1.1t03122.1
       :::::::::::: ::::::
3' ----AAGUUCUUUCGG-CACCUU--- 5'        Csi-miR396d.4
```

Csi-miR396d-3p, target=Cs8g05290.1 gene=Cs8g05290
Category:2
Score=5
Cleavage Site=160

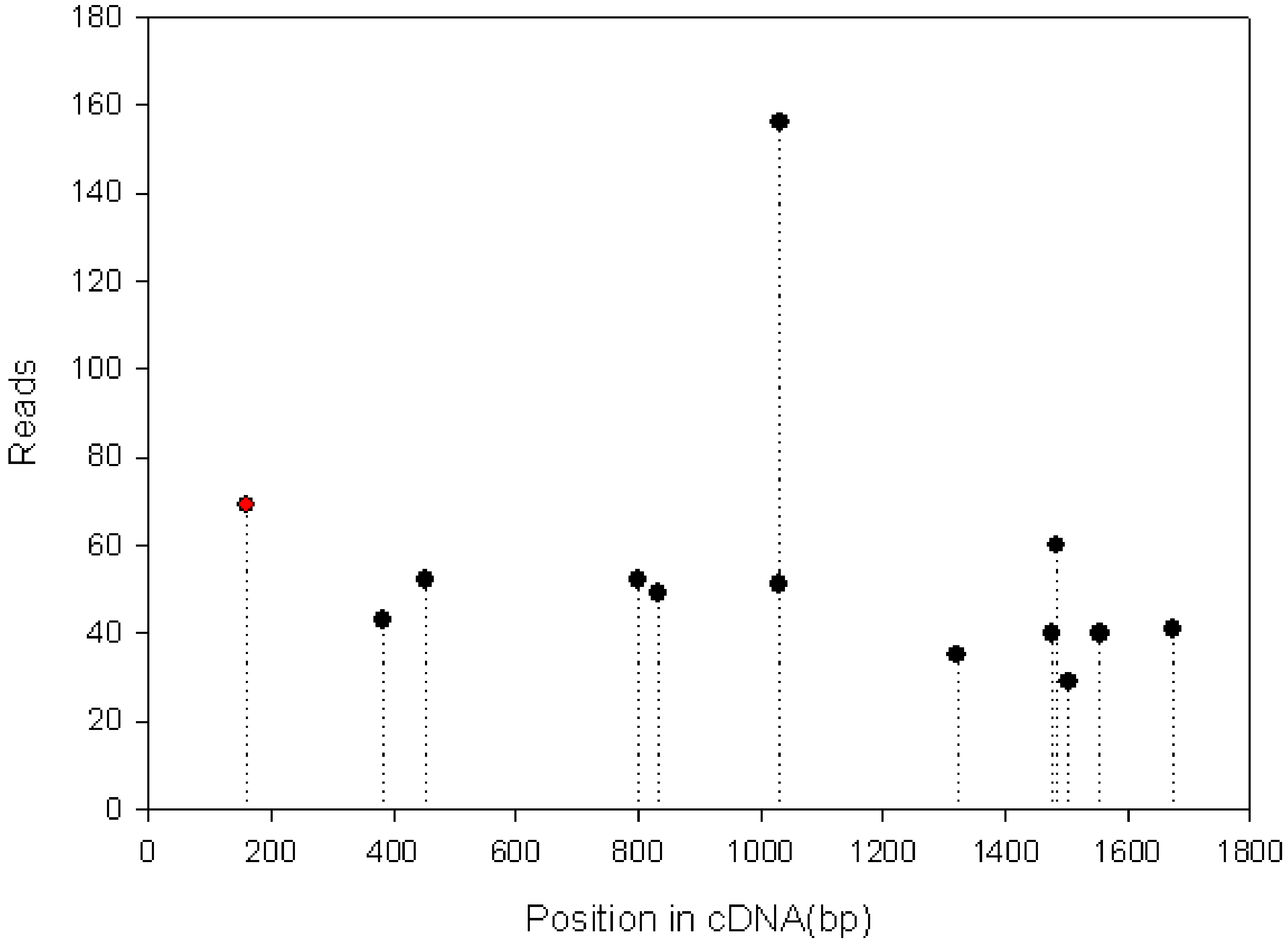


```
5' UAUAUUUUACGGCUUUCUUGAGUCCA 3'        Cs8g05290.1
     :  :...:::::  ::::::::.
3' --AAAGGGUGCCGUAAGAACUCG--- 5'        Csi-miR396d-3p
```

Csi-miR398b, target=Cs1g13240.1 gene=Cs1g13240
category:1
score=4
Cleavage Site=273

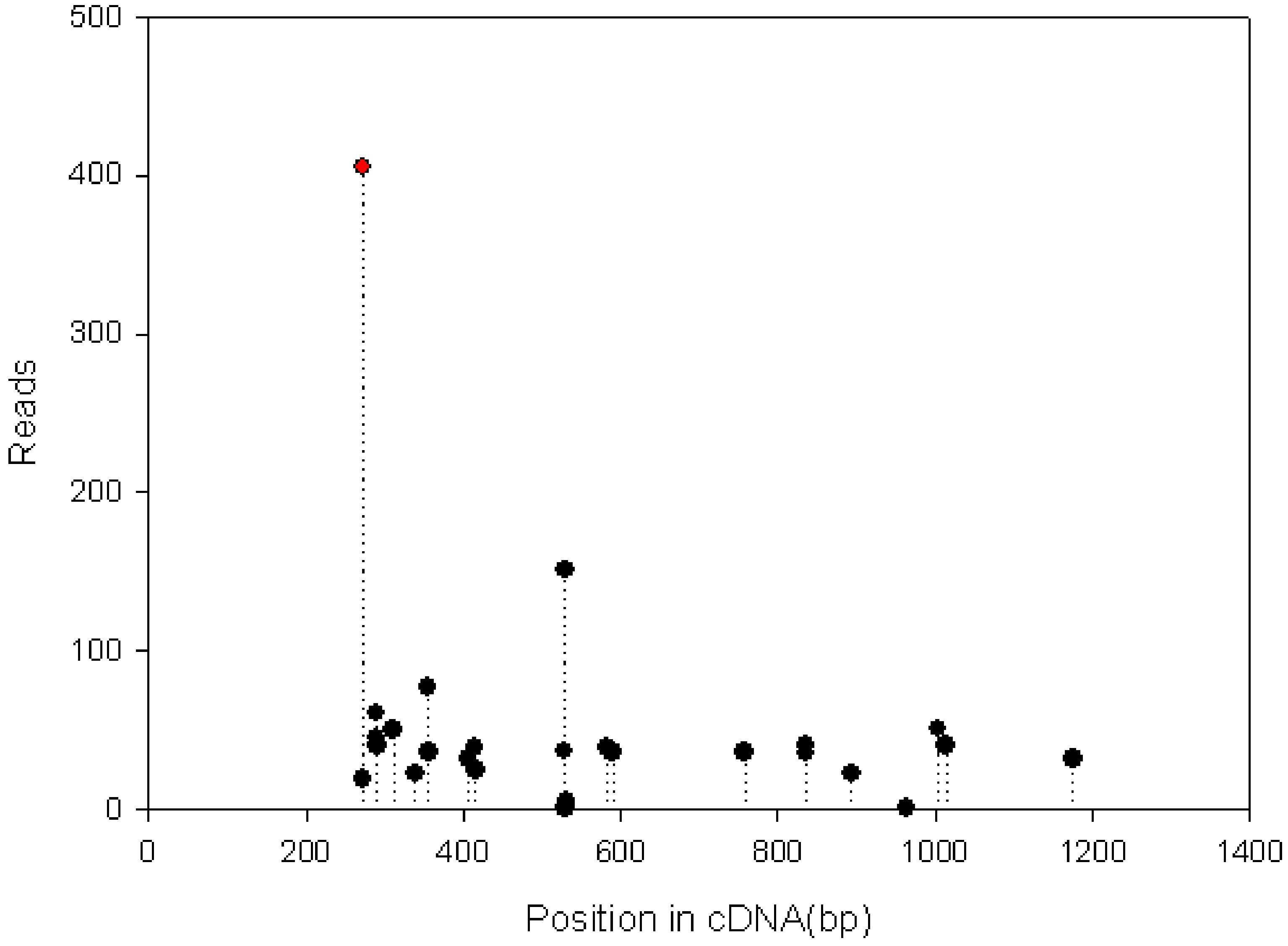


```
5' GAAAAAAAGGGGCGACCUGAGAAU-CA 3'        Cs1g13240.1
          ::::::::::::::::. ::
3' ------GUCCCCGCUGGACUCUUGUGU 5'        Csi-miR398b
```

Csi-miR399d, target=Cs4g08850.1 gene=Cs4g08850
Category:3
Score=4.5
Cleavage Site=560

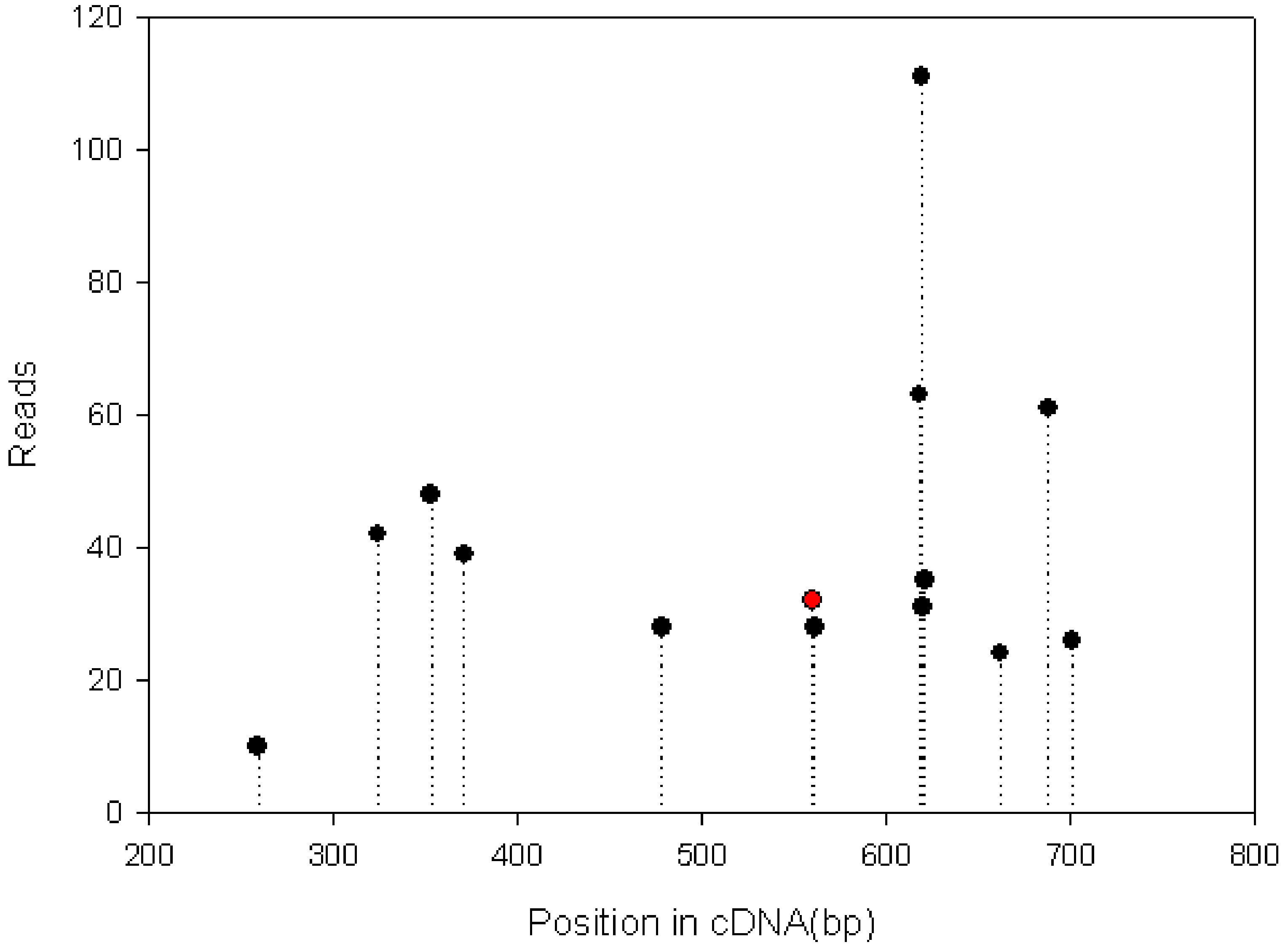


```
5' CACGGU-CCUCUUCUUUGGCAAAACCU 3'        Cs4g08850.1
   :: ::.  ::::.::::::::
3' GUCCCGUUGAGAGGAAACCGU------ 5'        Csi-miR399d
```

Csi-miR399e-5p, target=Orange1.1t03762.1 gene=Orange1.1t03762
Category:3
Score=5
Cleavage Site=500

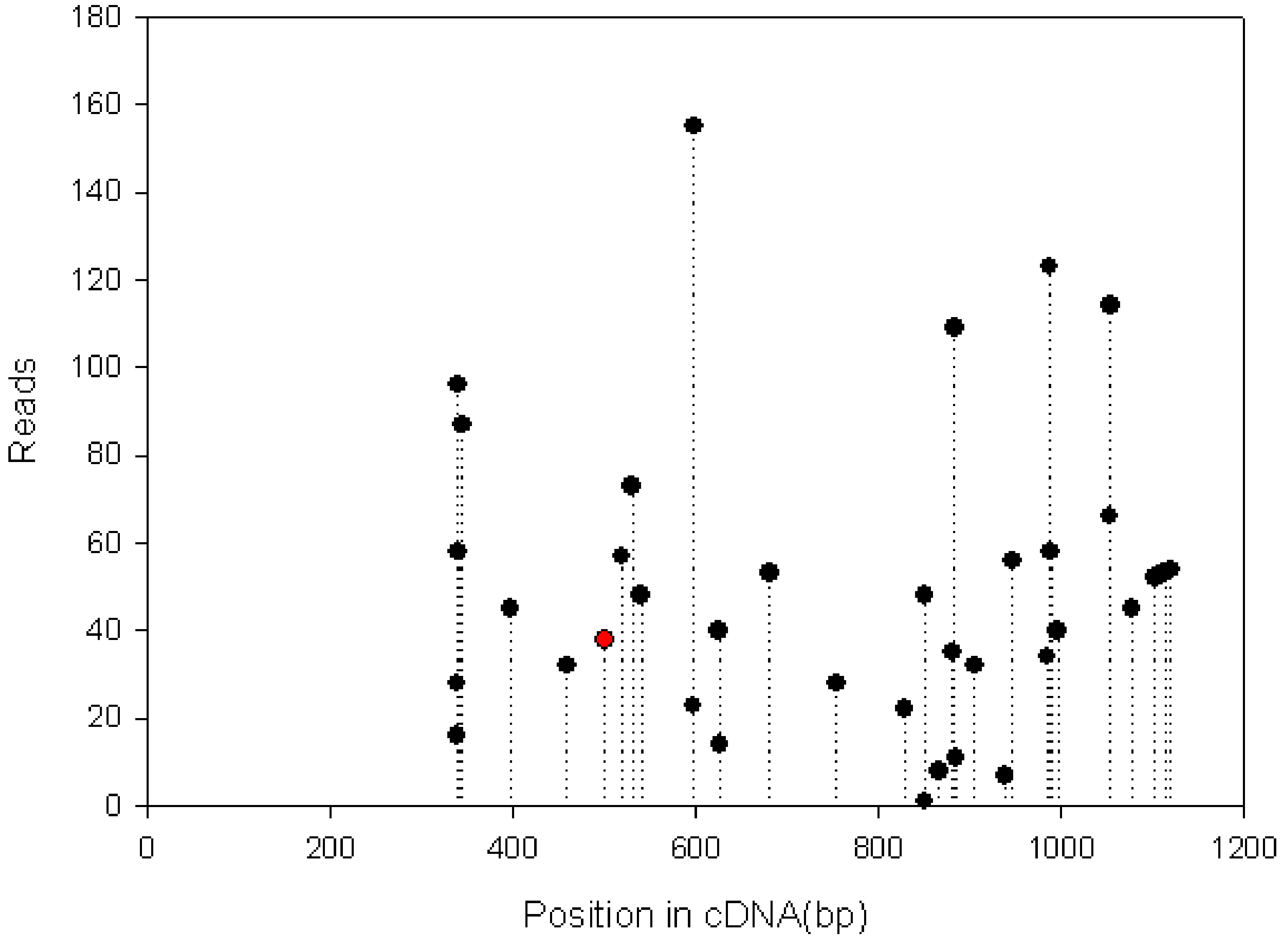


```
5' UGUUGGAAAGUCAAAGGAGGAC-GCAU 3'       Orange1.1t03762.1
         : :.::::::::::: :::.
3' ------GUGCGGUUUCCUCCUGACGUG 5'       Csi-miR399e-5p
```

Csi-miR403.3, target=Cs3g01650.1 gene=Cs3g01650
Category:3
Score=5
Cleavage Site=2429

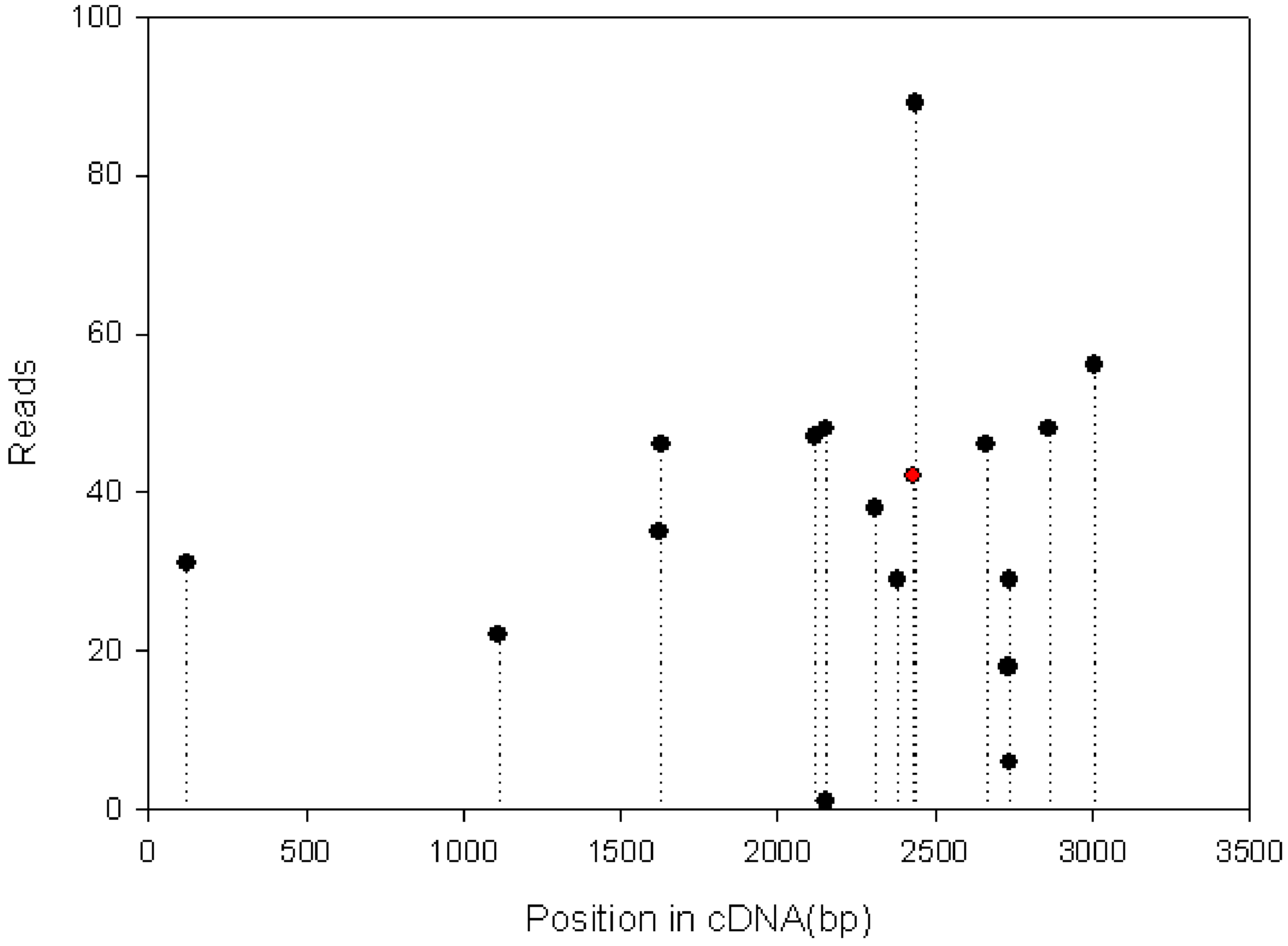


```
5' GUUU-UGUGAGAAUUUAAGUGGAGCUG 3'        Cs3g01650.1
   :::: ::.: ::::.:::
3' CAAACACGCACUUAGAUU---------- 5'        Csi-miR403.3
```

Csi-miR403.3, target=Cs8g07240.1 gene=Cs8g07240
Category:3
Score=5
Cleavage Site=530

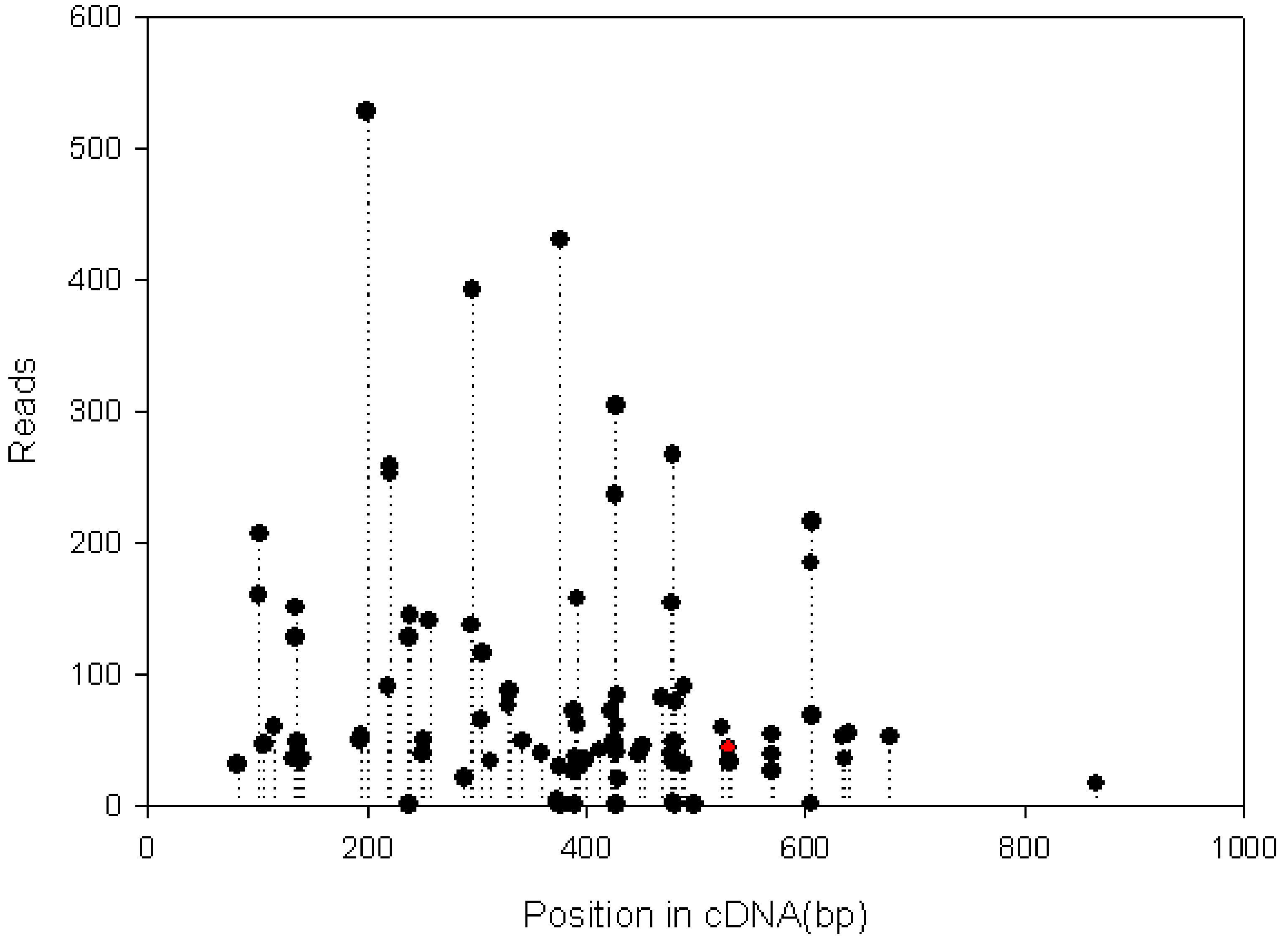


```
5' UUUUGUUUCU-UGUGAAUCUAUUUGAG 3'        Cs8g07240.1
       :::: : .:::::::::
3' ----CAAACACGCACUUAGAUU----- 5'        Csi-miR403.3
```

Csi-miR403.3, target=Cs9g07320.1 gene=Cs9g07320
Category:3
Score=5
Cleavage Site=582

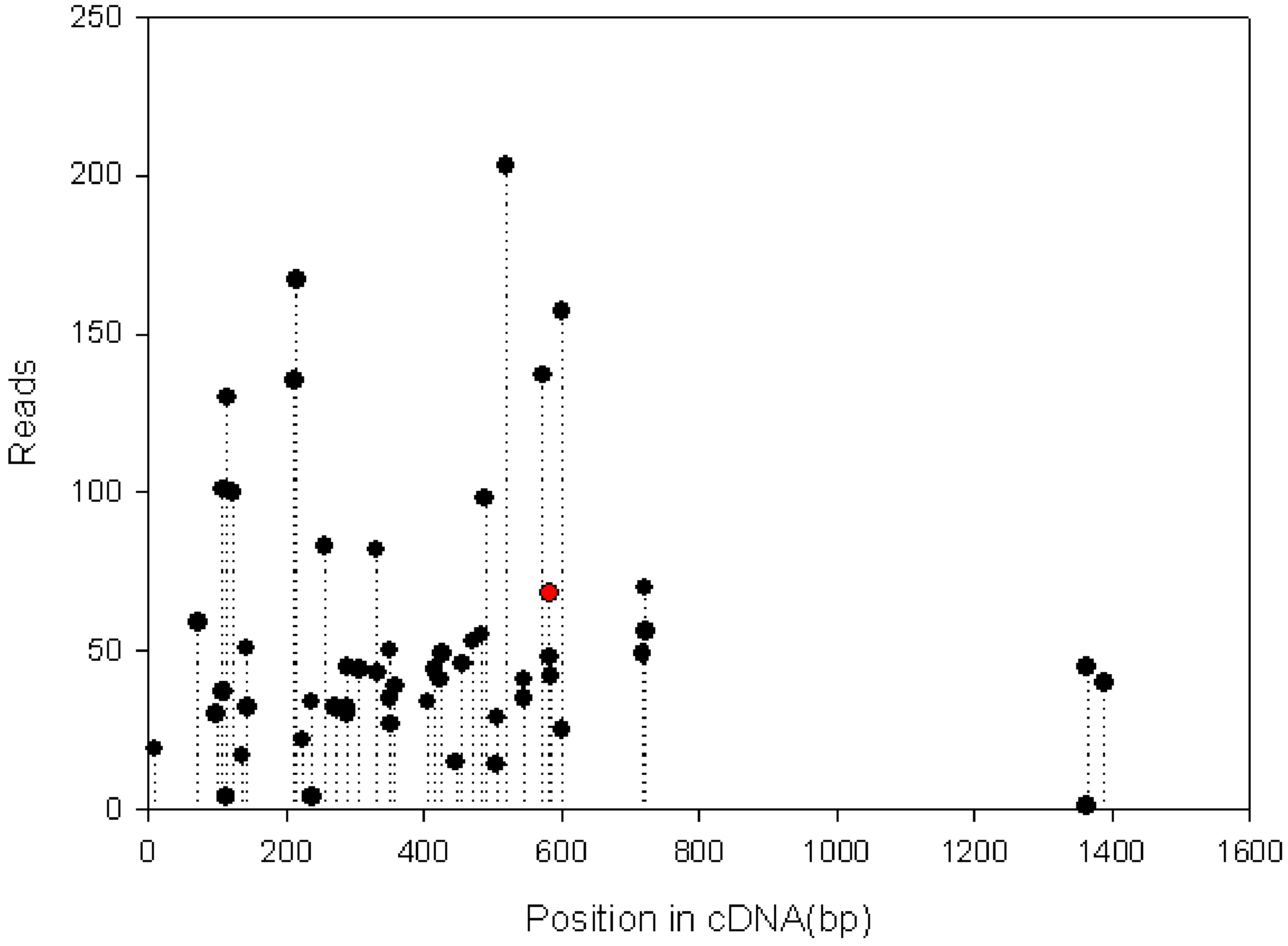


```
5' AGUAUGCUUG-GCGUGAAUCUGUCAAG 3'       Cs9g07320.1
           :  :::  ::::::::::.
3' -----CAAACACGCACUUAGAUU---- 5'       Csi-miR403.3
```

Csi-miR444a.1, target=Orange1.1t01329.1 gene=Orange1.1t01329
Category:2
Score=5
Cleavage Site=342

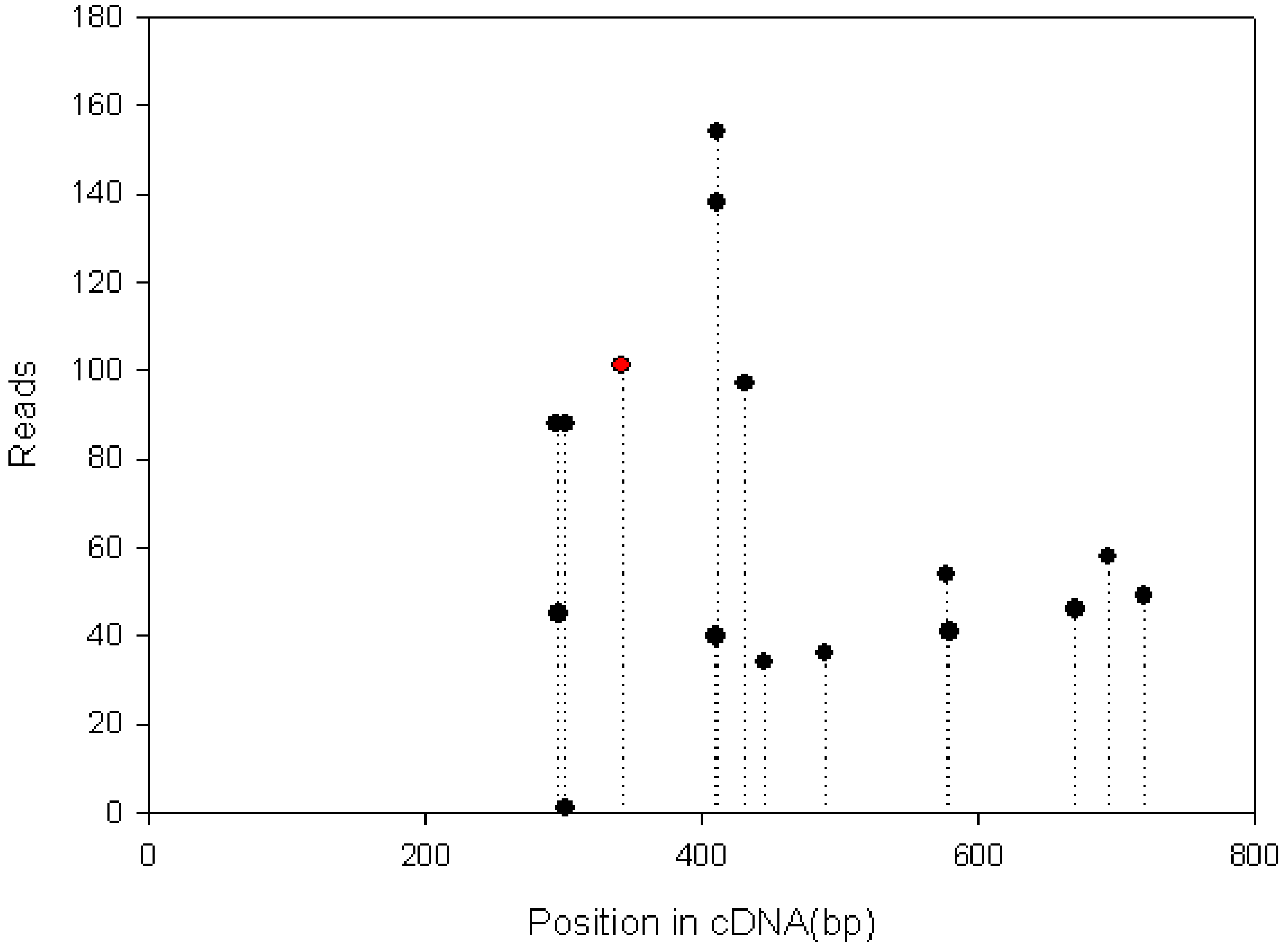


```
5' UG-AGGAAUUGUCUCAAGGAACCUUAC 3'        Orange1.1t01329.1
   :: :  :   ::::::::::.::
3' ACGUACAAAACAGAGUUCUUU------ 5'        Csi-miR444a.1
```

Csi-miR444a.2, target=Cs8g15380.1 gene=Cs8g15380
Category:3
Score=4.5
Cleavage Site=2635

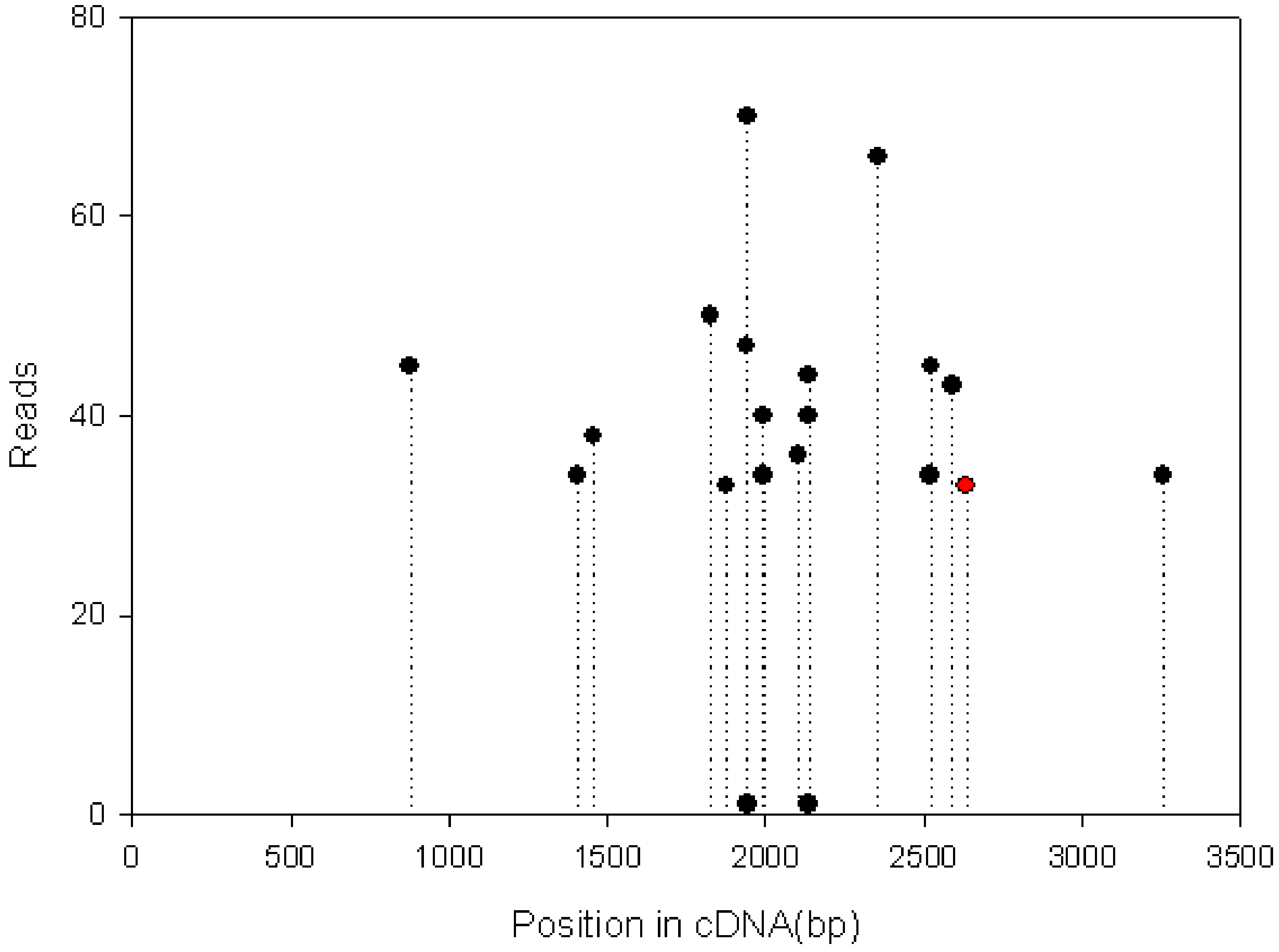


```
5' GGUGCAUCUUUUGGUCUCAAGGAAAG 3'        Cs8g15380.1
   :.::::: :::: :::::::::.::
3' CUACGUACAAAA-CAGAGUUCUUU-- 5'        Csi-miR444a.2
```

Csi-miR472, target=Cs1g13430.1 gene=Cs1g13430
Category:1
Score=5
Cleavage Site=700

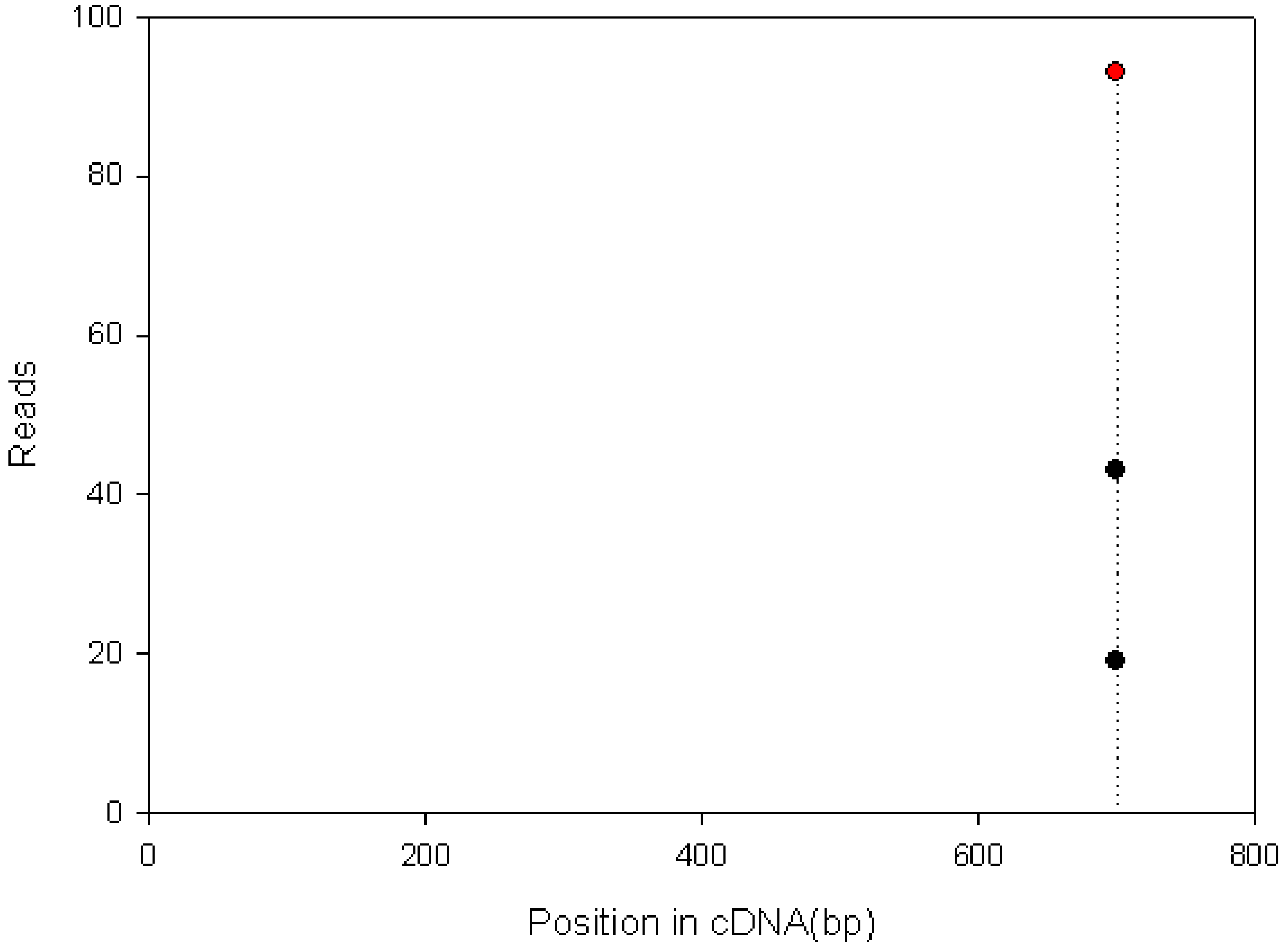


```
5' GGCAUGGGAGGGGUGGGUAAAACUAC 3'        Cs1g13430.1
   :: :::::::: ::::: :::
3' CCCUACCCUCCACACCCUUUU----- 5'        Csi-miR472
```

Csi-miR472, target=Cs2g30590.1 gene=Cs2g30590
Category:1
Score=5
Cleavage Site=889

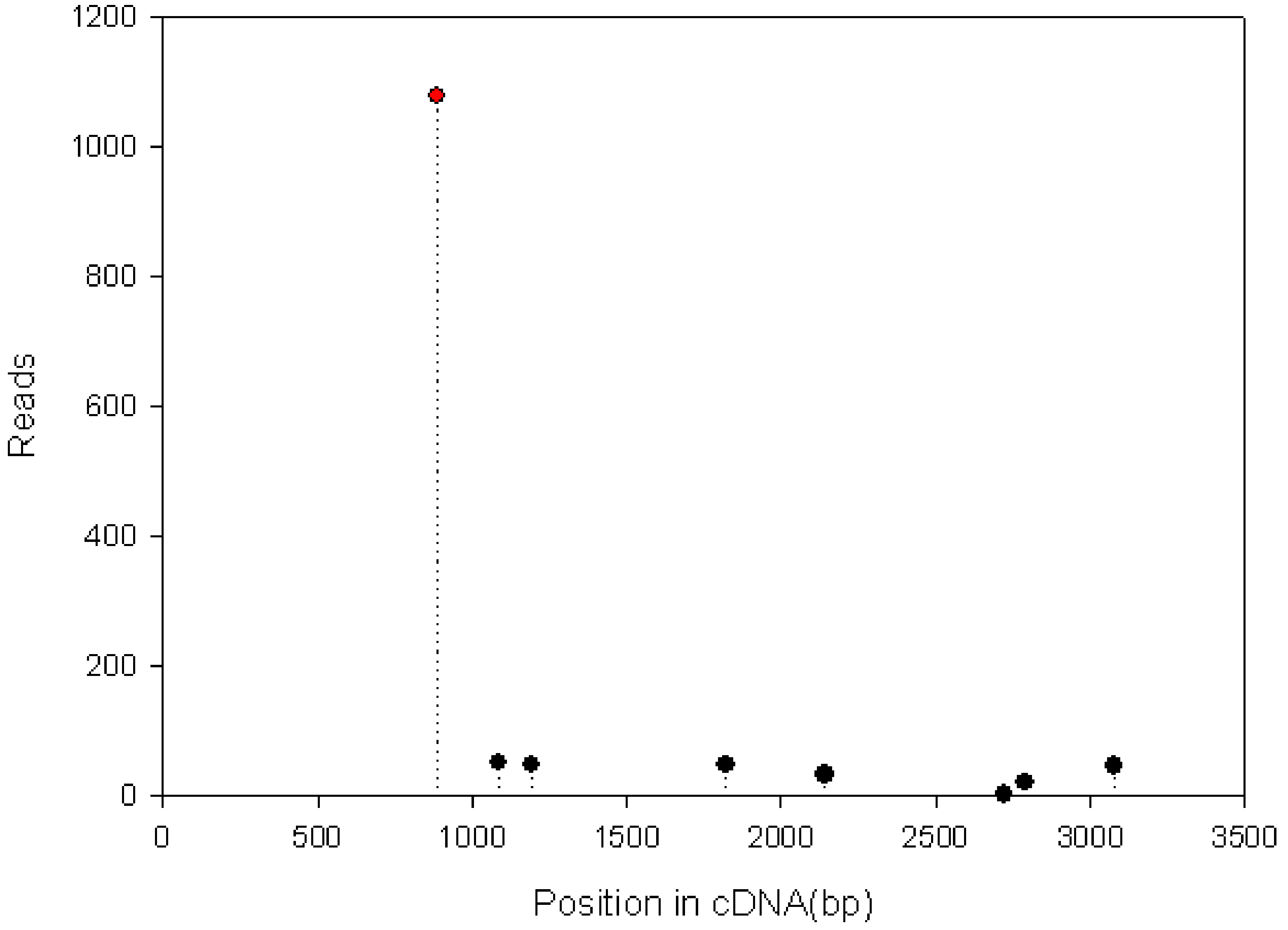


```
5' GGUAUGGGAGGUUUGGGCAAAACAAC 3'        Cs2g30590.1
   :: :::::::: :::: :::
3' CCCUACCCUCCACACCCUUUU----- 5'        Csi-miR472
```

Csi-miR472, target=Orange1.1t01918.1 gene=Orange1.1t01918
Category:1
Score=5
Cleavage Site=562

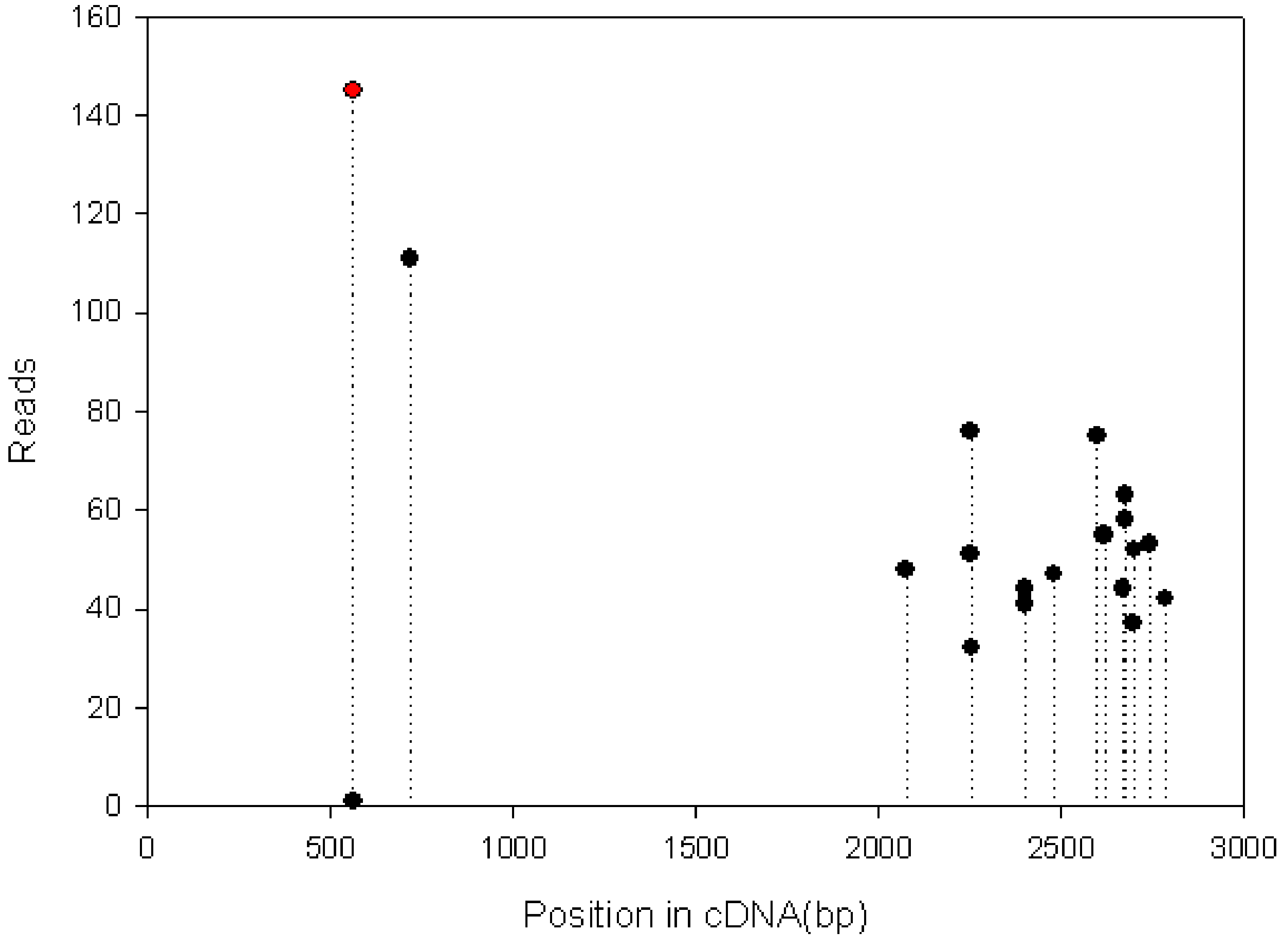


```
5' GGUAUGGGAGGUAUAGGGAAAACCAC 3'        Orange1.1t01918.1
   :: ::::::::: : :::::::
3' CCCUACCCUCCACA-CCCUUUU---- 5'        Csi-miR472
```

Csi-miR477a-3p, target=Cs6g19680.1 gene=Cs6g19680
Category:3
Score=4.5
Cleavage Site=260

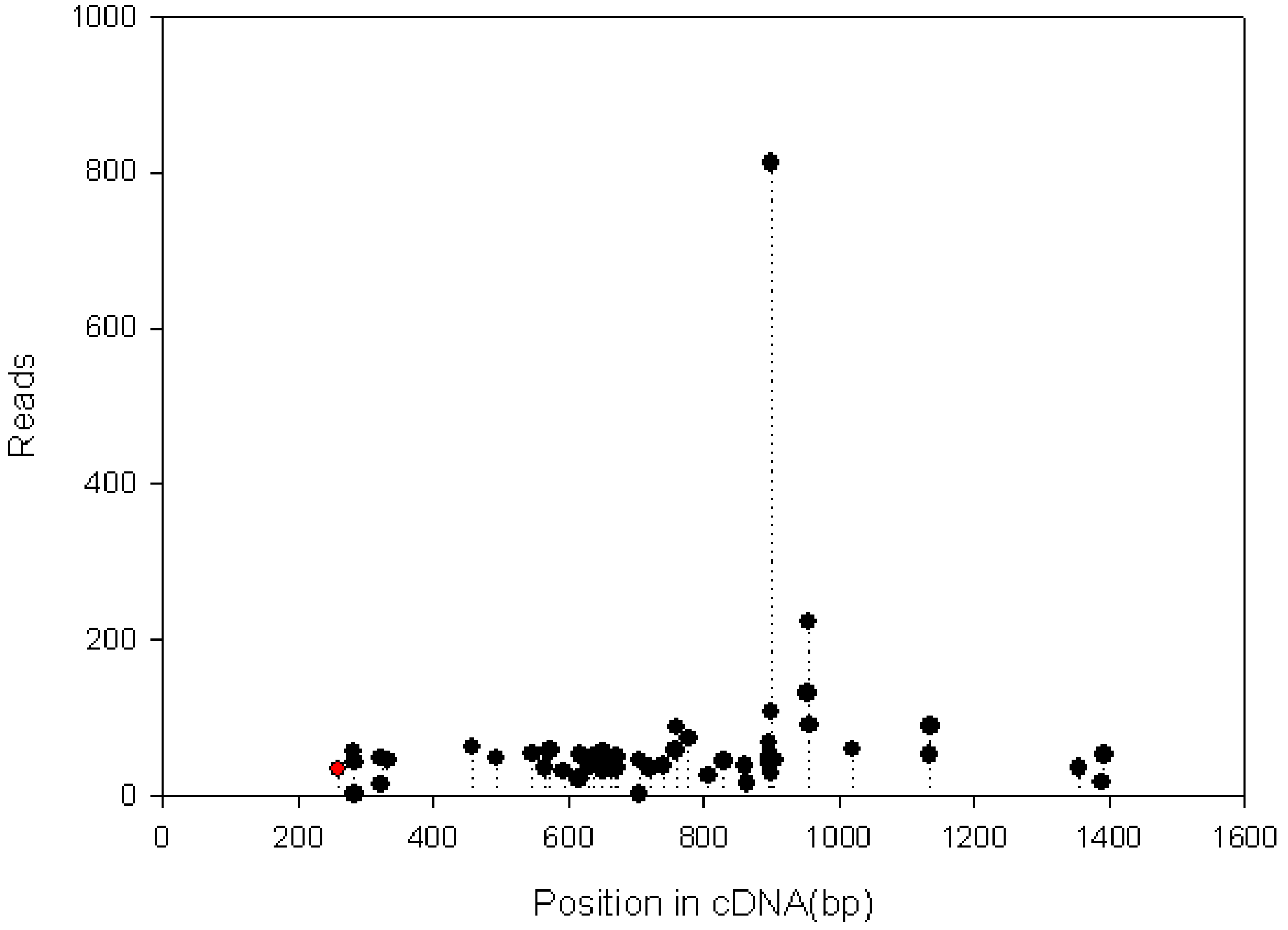


```
5' UUUUUUUUCUCUCUCUAGGGUUUCCA 3'        Cs6g19680.1
       .   .:::.:.:::::::::::
3' ----GCUGGAGGGGGAUCCCAAAGG- 5'        Csi-miR477a-3p
```

Csi-miR477b.2, target=Cs3g10900.1 gene=Cs3g10900
Category:3
Score=4.5
Cleavage Site=1317

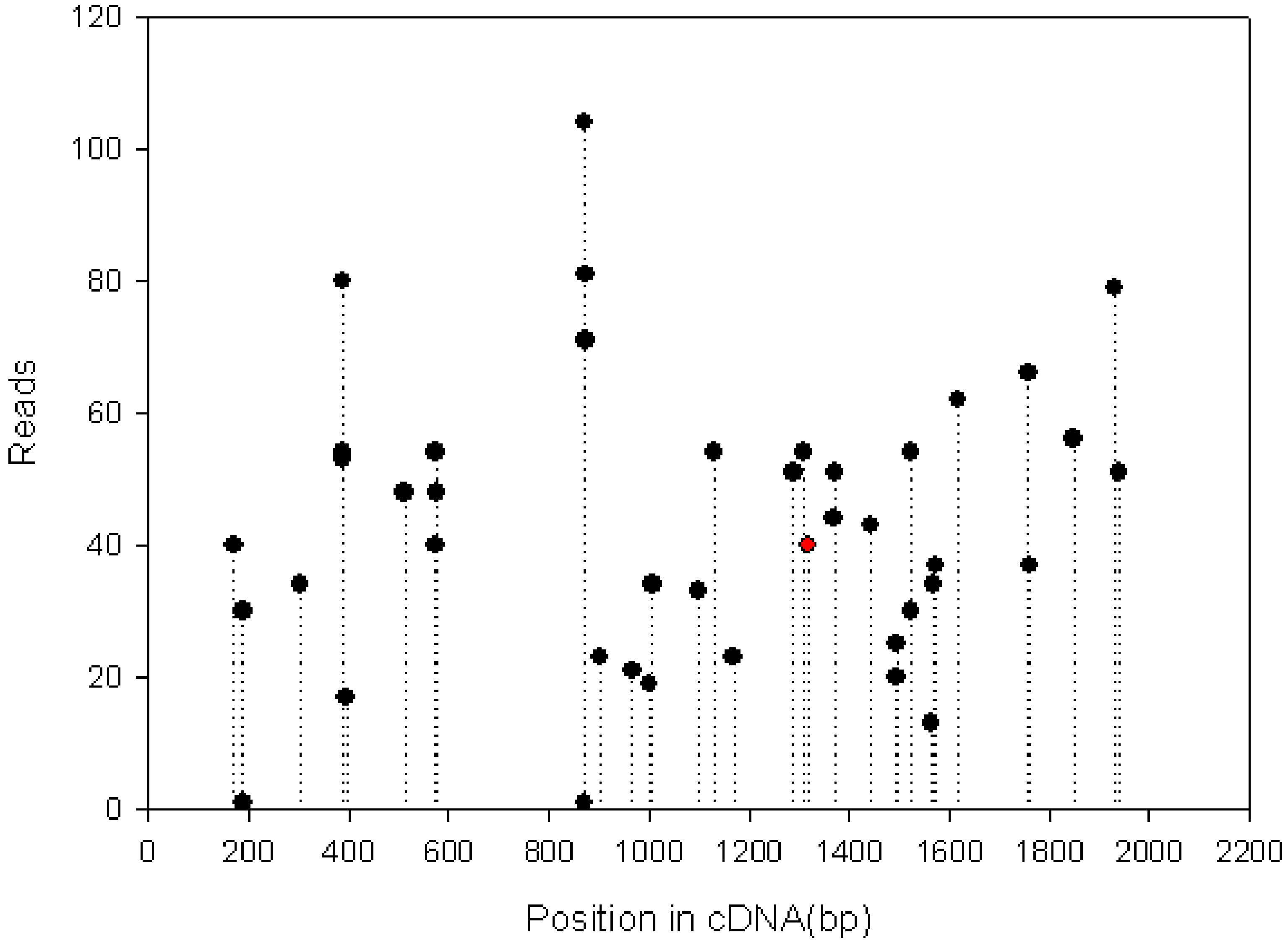


```
5' GUCAUCAGCUCUUGAGGGAGAGAGCA 3'        Cs3g10900.1
     :  :::.:::::::::::::
3' --CUCUUCGGGAACUCCCUCUCA--- 5'        Csi-miR477b.2
```

Csi-miR477d.2-5p, target=Cs3g10900.1 gene=Cs3g10900
Category:3
Score=3.5
Cleavage Site=1317

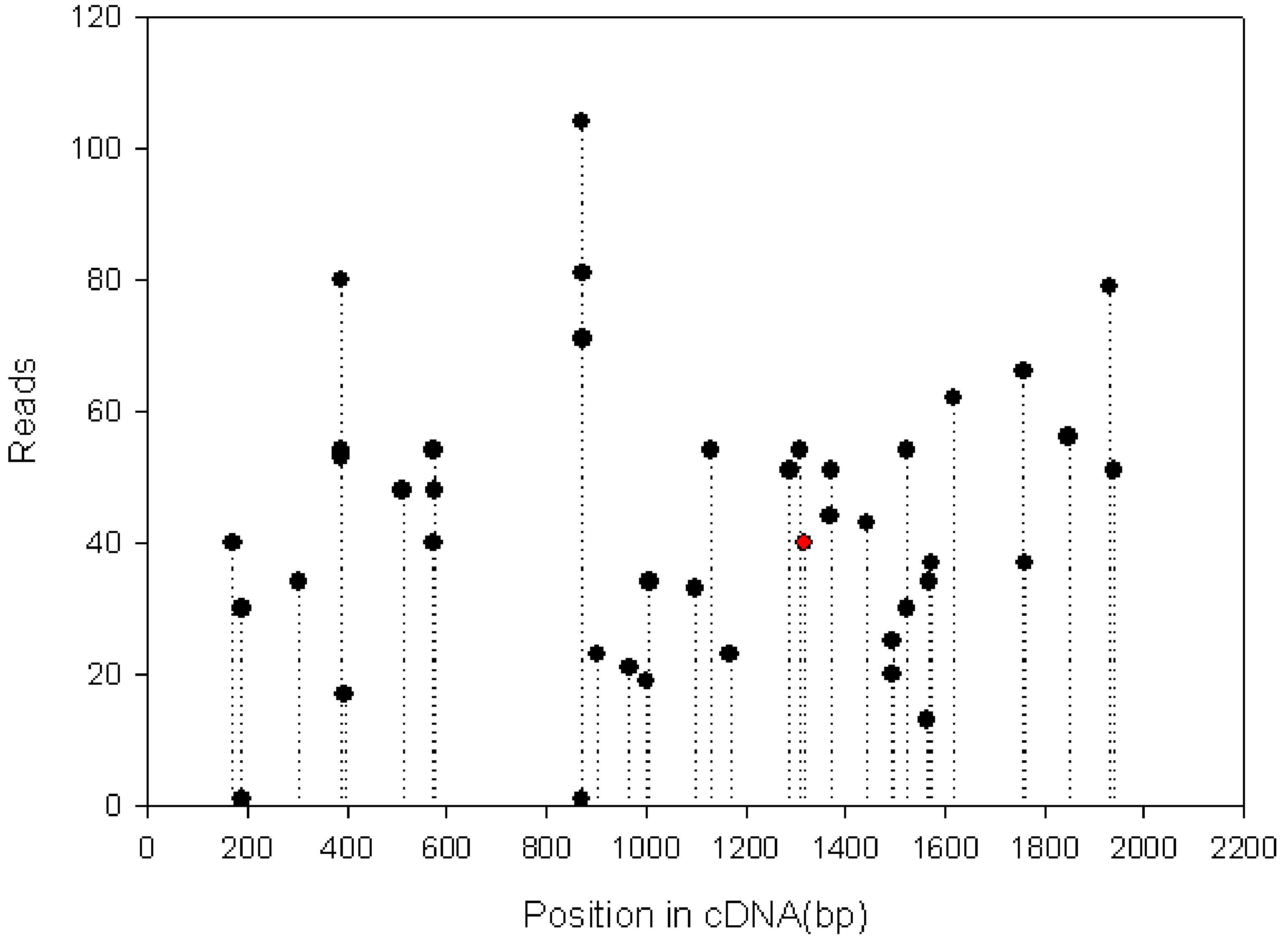


```
5' GUCAUCAGCUCUUGAGGGAGAGAGCA 3'        Cs3g10900.1
    :::  :::.:::::::::::::
3' -AGUCUUCGGGAACUCCCUCUCA--- 5'        Csi-miR477d.2-5p
```

Csi-miR482a-3p, target=Cs1g15550.1 gene=Cs1g15550
Category:1
Score=3
Cleavage Site=719

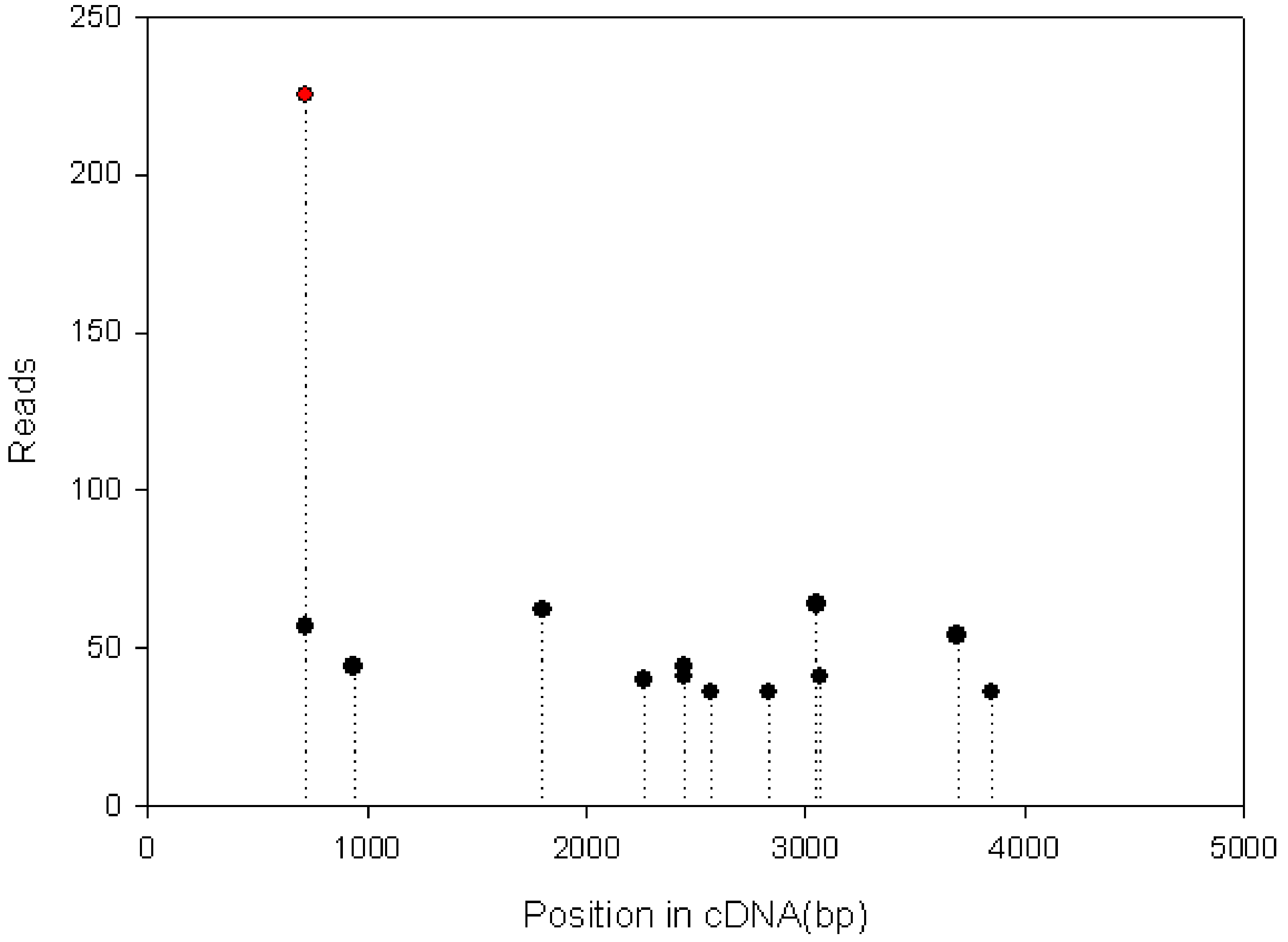


```
5' GGUAUGGGCGGUAUAGGGAAGACAAC 3'        Cs1g15550.1
   :: ::::: ::.::::::::::
3' CCUUACCCUCCGUAUCCCUUCU---- 5'        Csi-miR482a-3p
```

Csi-miR482a-3p, target=Cs3g13320.1 gene=Cs3g13320
Category:1
Score=5
Cleavage Site=643

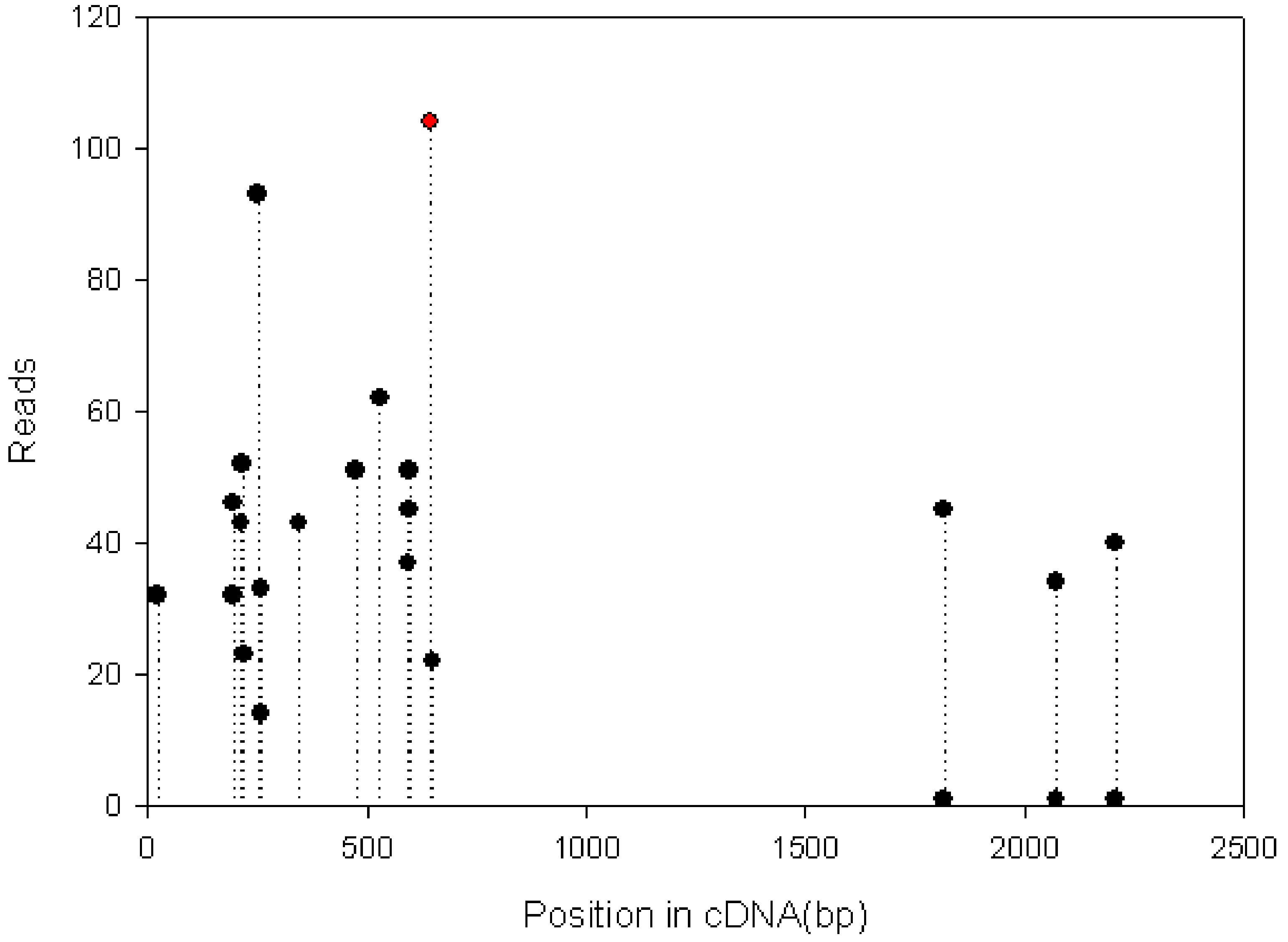


```
5' GGGAUGGGGGGCAUAGGCAAAACUAC 3'        Cs3g13320.1
   ::.:::::.::::::::  ::  :
3' CCUUACCCUCCGUAUCCCUUCU---- 5'        Csi-miR482a-3p
```

Csi-miR482a-3p, target=Cs3g13340.1 gene=Cs3g13340
Category:2
Score=5
Cleavage Site=702

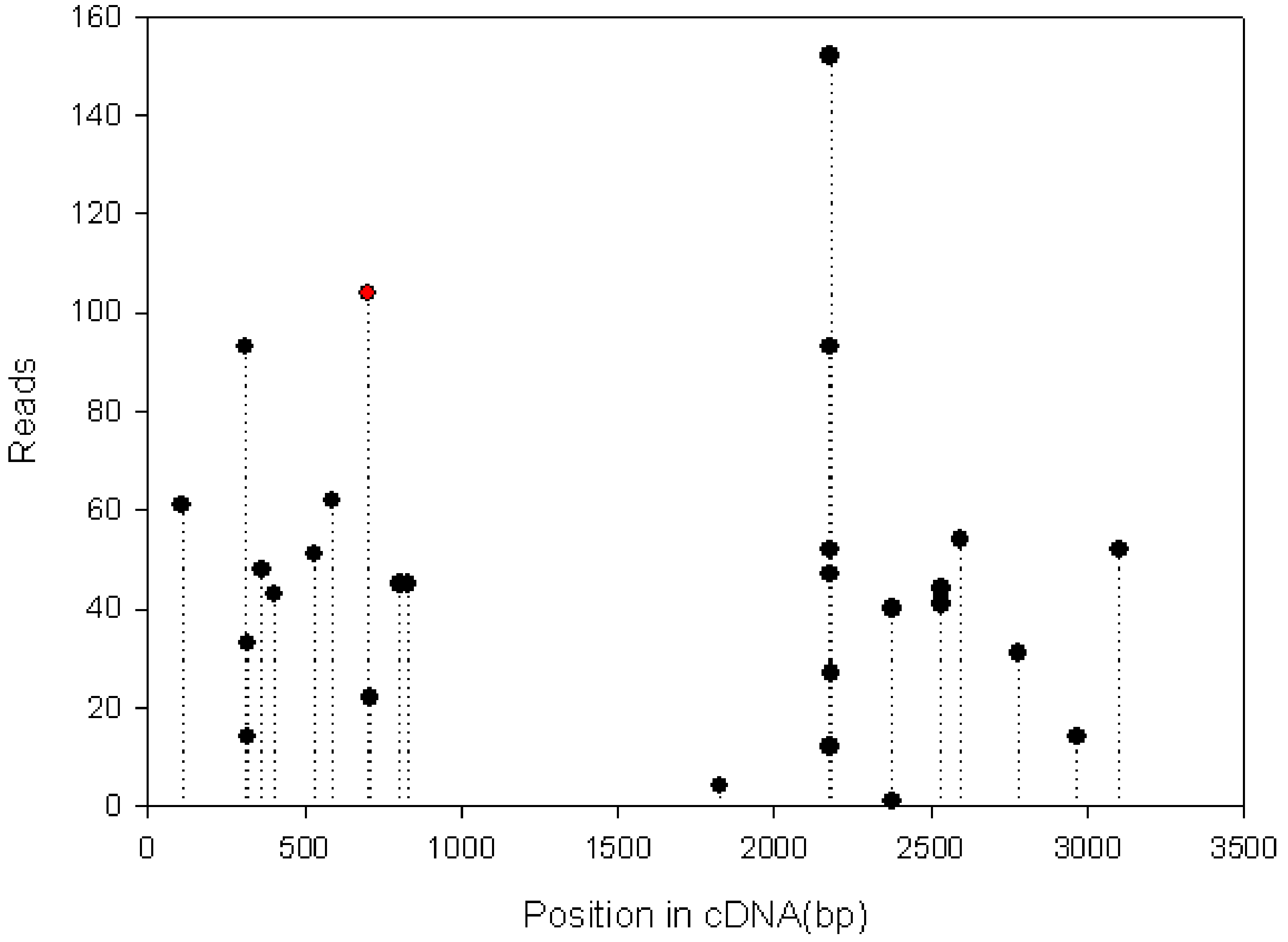


```
5' GGGAUGGGGGGCAUAGGCAAAACUAC 3'        Cs3g13340.1
   ::.:::::.:::::::: :: :
3' CCUUACCCUCCGUAUCCCUUCU---- 5'        Csi-miR482a-3p
```

Csi-miR482a-3p, target=Cs3g13390.1 gene=Cs3g13390
Category:1
Score=5
Cleavage Site=800

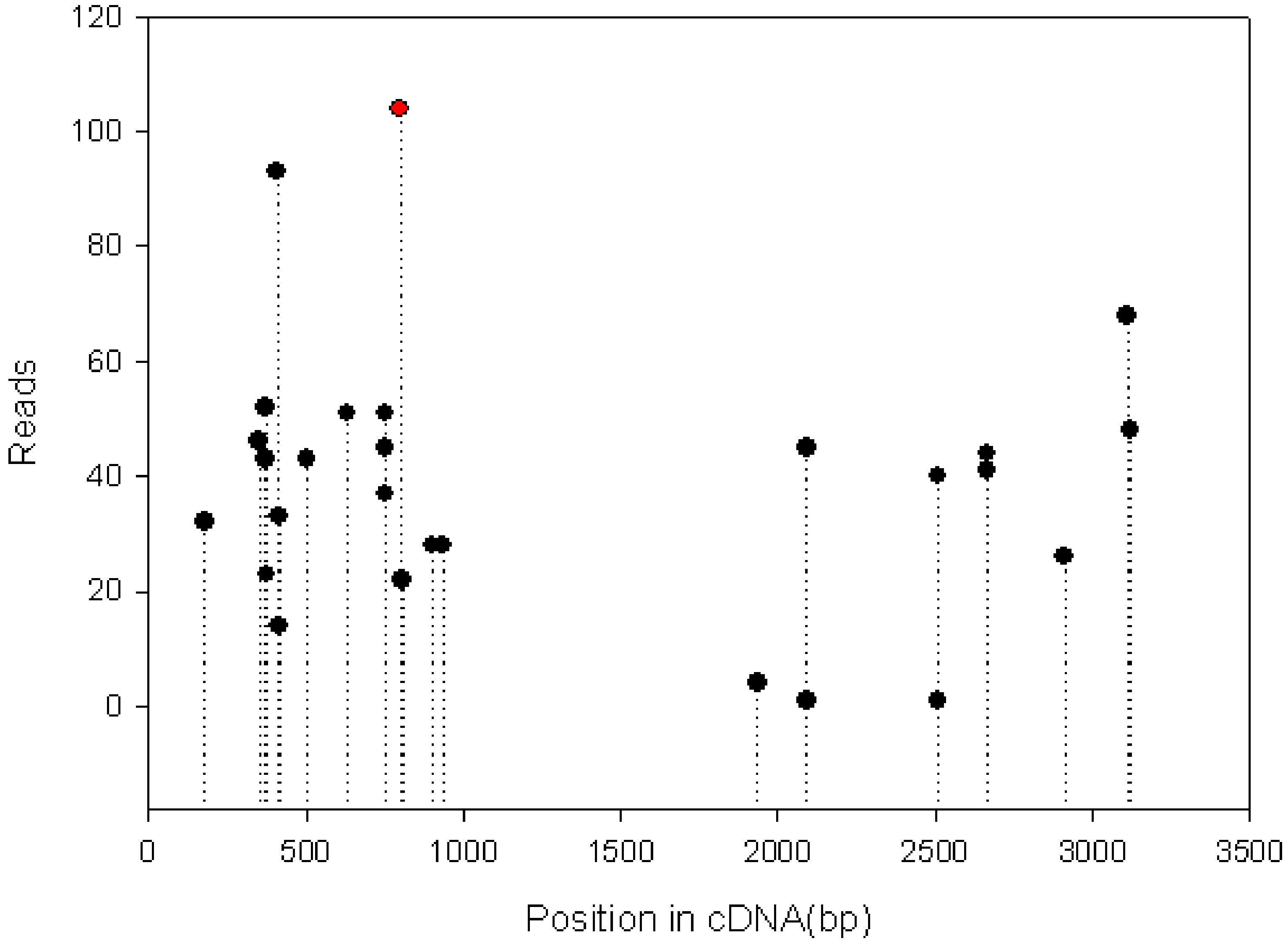


```
5'  GGGAUGGGGGGCAUAGGCAAAACUAC  3'         Cs3g13390.1
    ::.:::::.:::::::: :: :
3'  CCUUACCCUCCGUAUCCCUUCU----  5'         Csi-miR482a-3p
```

Csi-miR482a-3p, target=Cs3g13740.1 gene=Cs3g13740
Category:2
Score=5
Cleavage Site=686

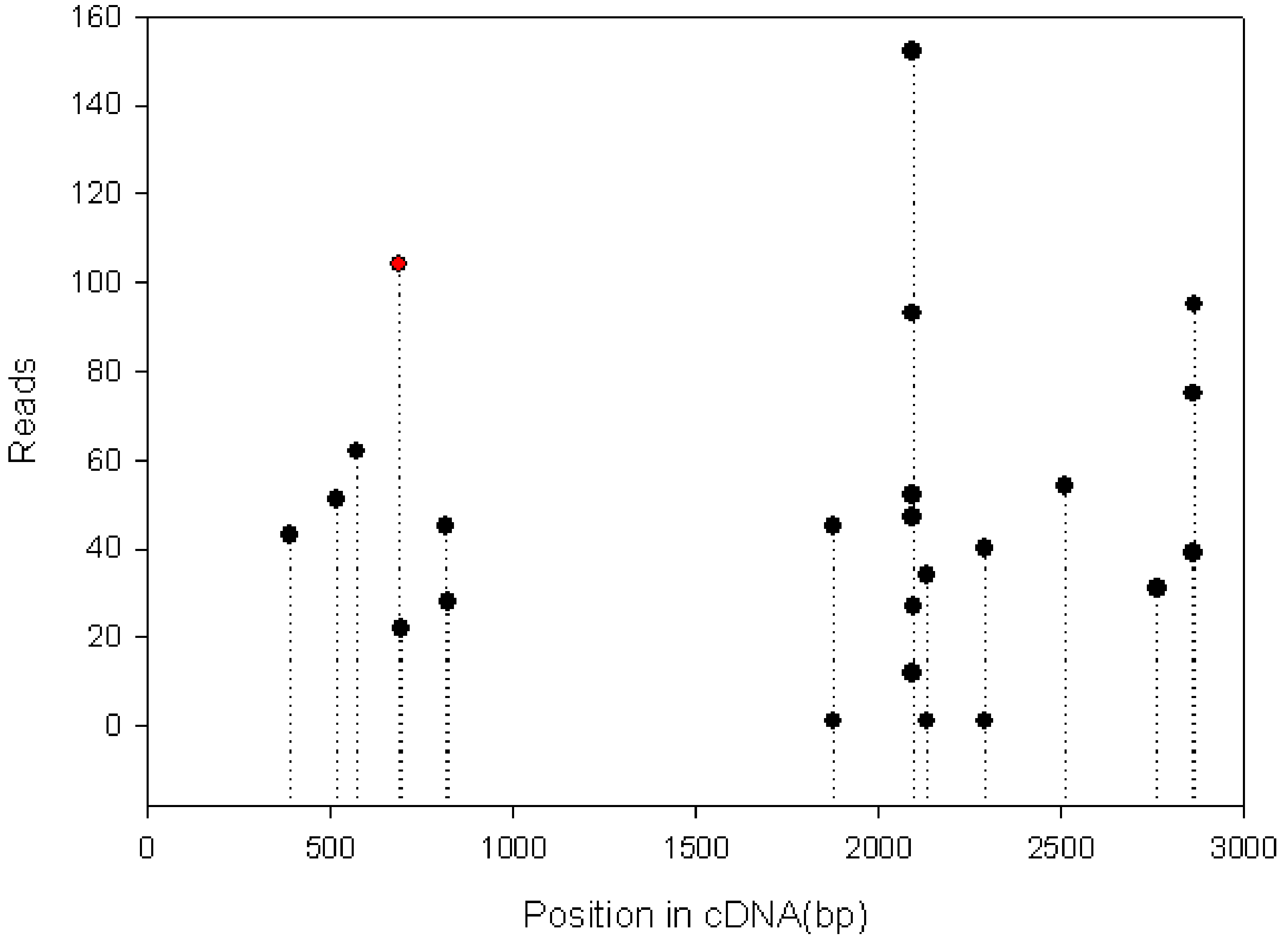


```
5'  GGGAUGGGGGGCAUAGGCAAAACUAC 3'        Cs3g13740.1
    ::.:::::.::::::::  :: :
3'  CCUUACCCUCCGUAUCCCUUCU---- 5'        Csi-miR482a-3p
```

Csi-miR482a-3p, target=Cs5g19440.1 gene=Cs5g19440
Category:1
Score=4
Cleavage Site=364

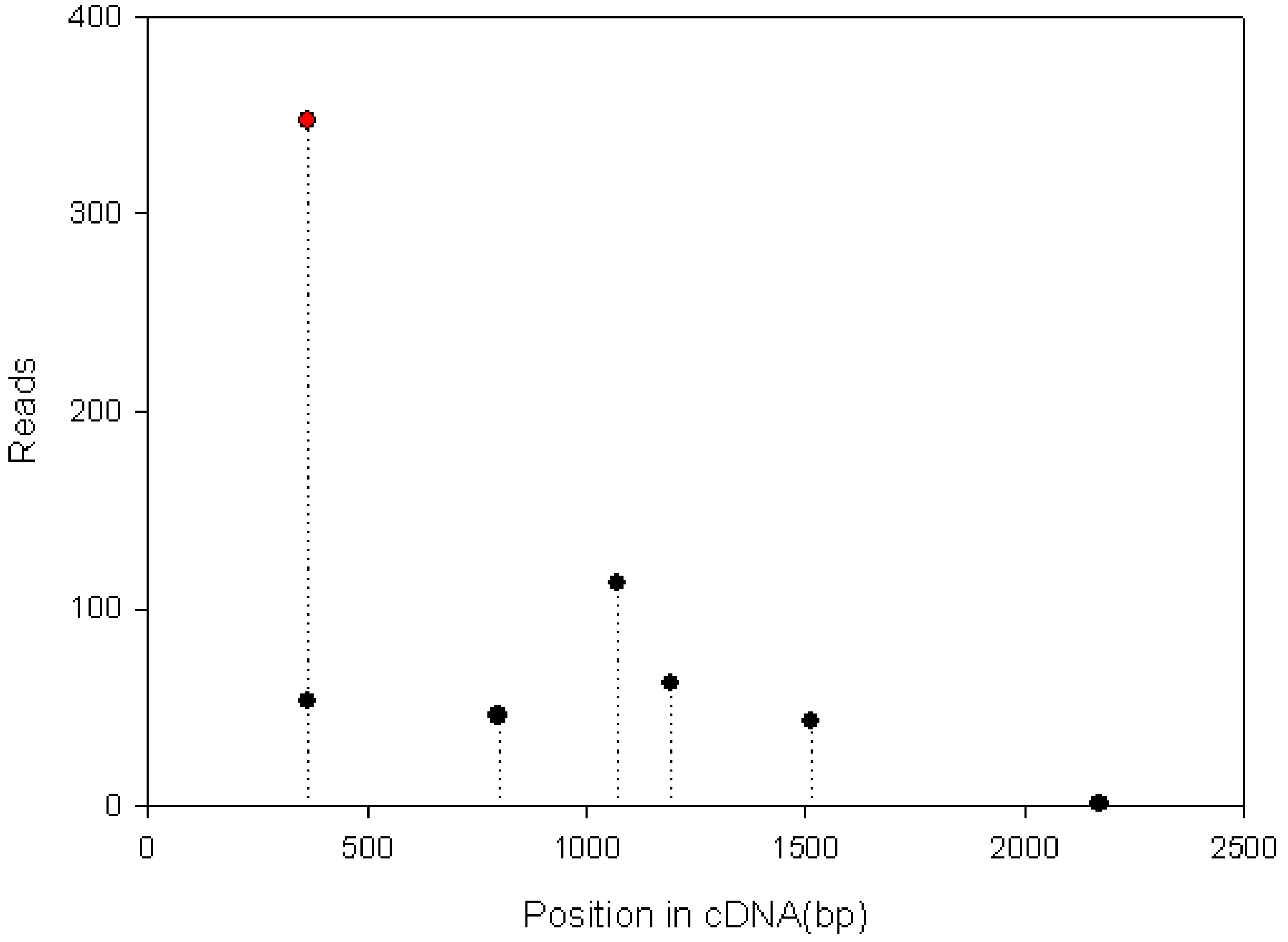


```
5'  GGUAUGGGAGGUAUAGGUAAGACAAC 3'        Cs5g19440.1
    :: ::::::::.::::: ::::
3'  CCUUACCCUCCGUAUCCCUUCU---- 5'        Csi-miR482a-3p
```

Csi-miR482a-3p, target=Orange1.1t01829.1 gene=Orange1.1t01829
Category:1
Score=4
Cleavage Site=810

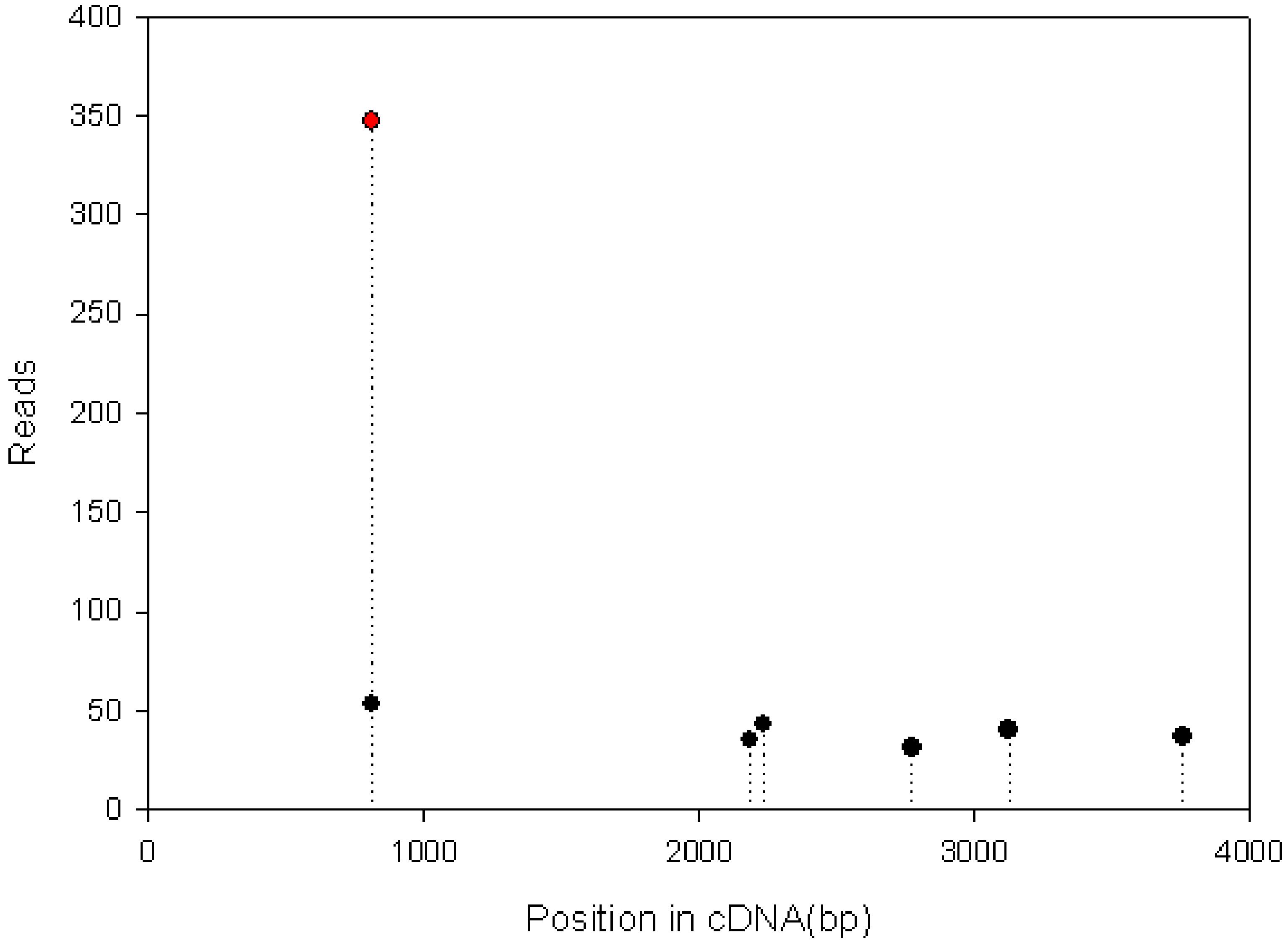


```
5' GGUAUGGGAGGUAUAGGUAAGACAAC 3'        Orange1.1t01829.1
   :: :::::::::.::::: ::::
3' CCUUACCCUCCGUAUCCCUUCU---- 5'        Csi-miR482a-3p
```

Csi-miR482a-3p, target=Orange1.1t01918.1 gene=Orange1.1t01918
Category:1
Score=4
Cleavage Site=562

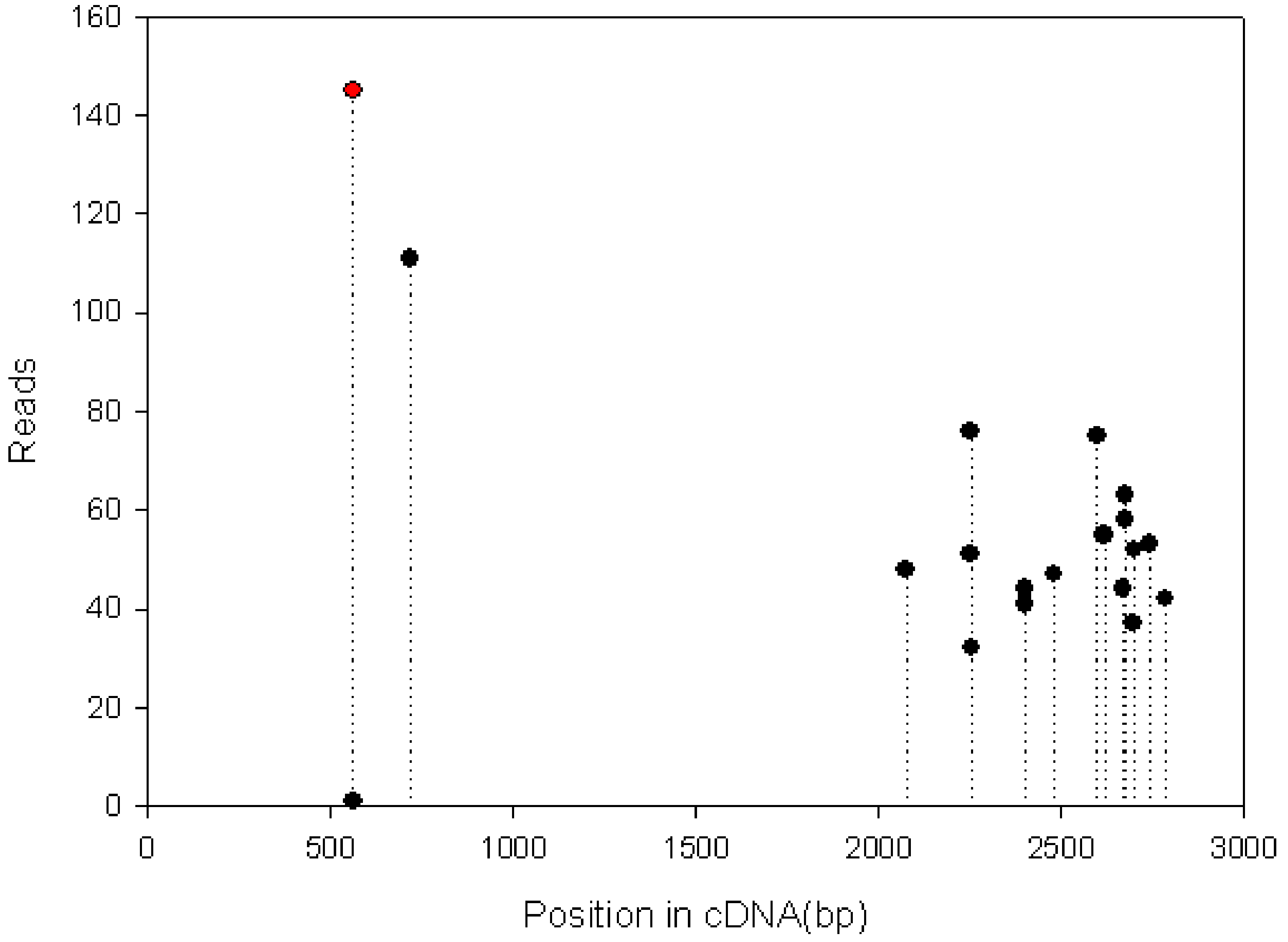


```
5' GGUAUGGGAGGUAUAGGGAAAACCAC 3'        Orange1.1t01918.1
   :: :::::::::.:::::::: :
3' CCUUACCCUCCGUAUCCCUUCU---- 5'        Csi-miR482a-3p
```

Csi-miR482a-3p, target=Orange1.1t02518.1 gene=Orange1.1t02518
Category:1
Score=5
Cleavage Site=1369

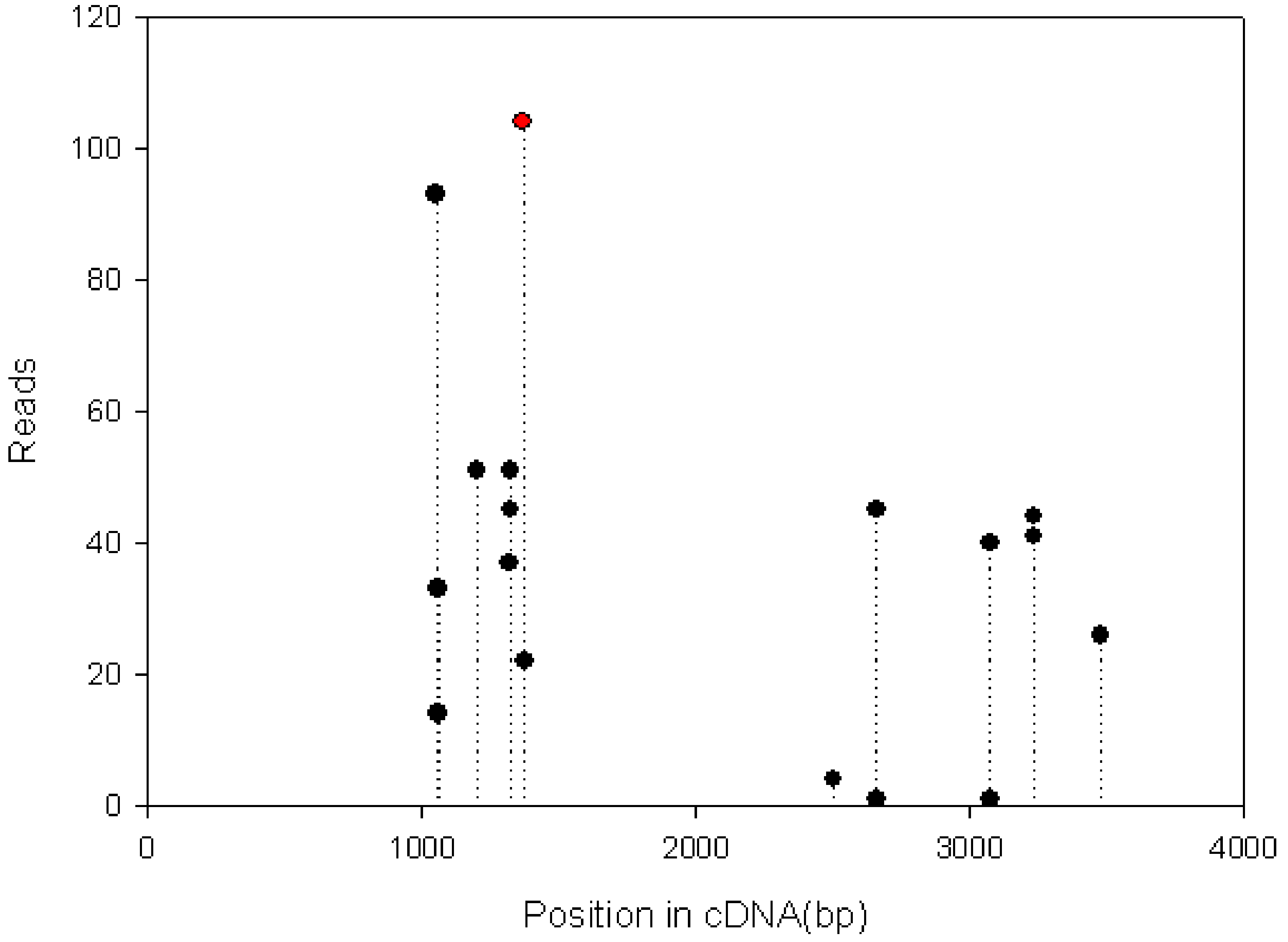


```
5' GGGAUGGGGGCAUAGGCAAAACUAC 3'        Orange1.1t02518.1
   ::.:::::.::::::::  :: :
3' CCUUACCCUCCGUAUCCCUUCU---- 5'       Csi-miR482a-3p
```

Csi-miR482b, target=Cs1g13430.1 gene=Cs1g13430
Category:1
Score=4
Cleavage Site=700

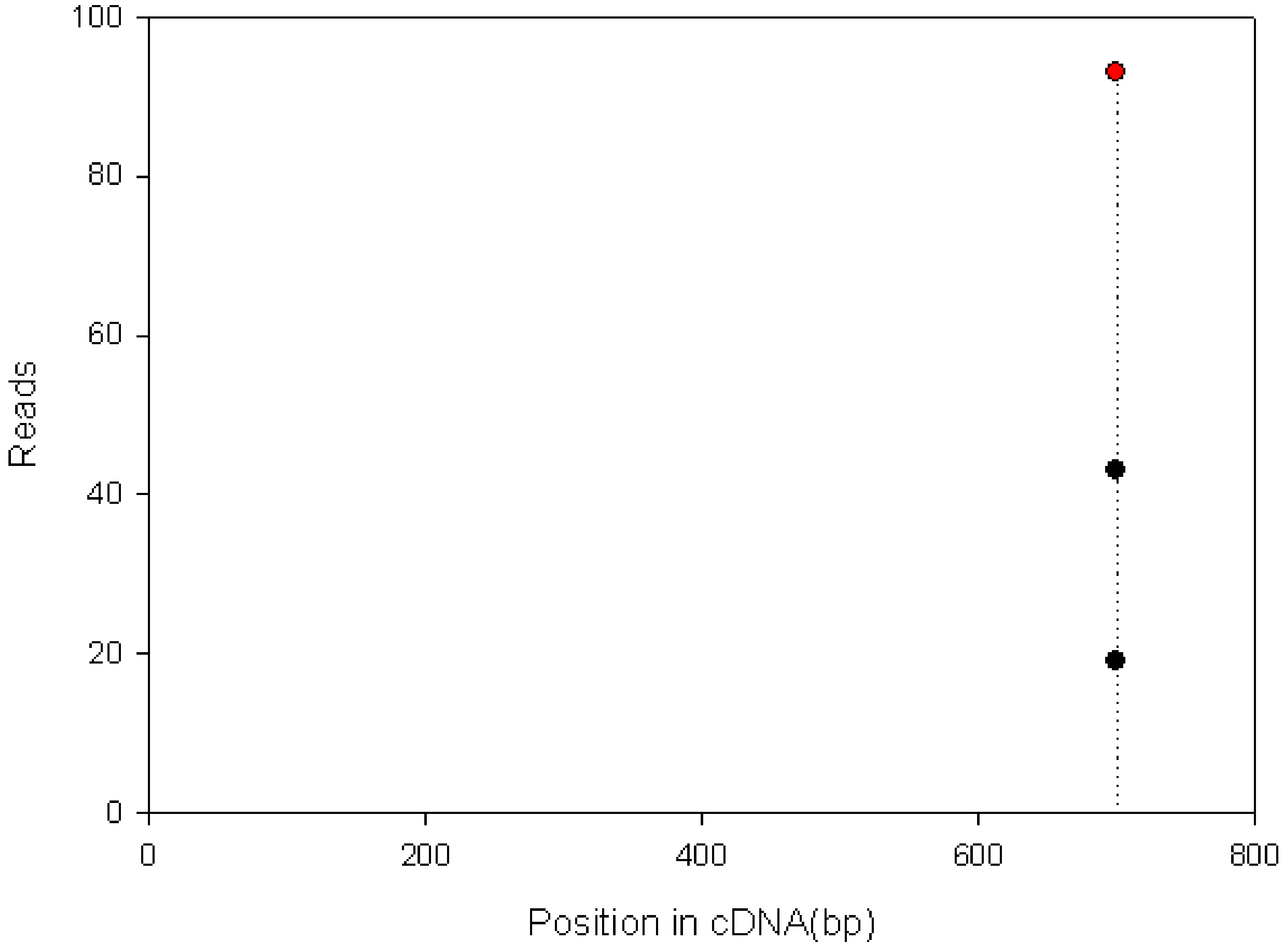


```
5' GGCAUGGGAGGGGUGGUAAAACUAC 3'        Cs1g13430.1
   :: ::::::::::::::.:: :
3' CCUUACCCUCCCCACCCGUUCU---- 5'       Csi-miR482b
```

Csi-miR482d, target=Cs5g21975.1 gene=Cs5g21975
Category:3
Score=5
Cleavage Site=616

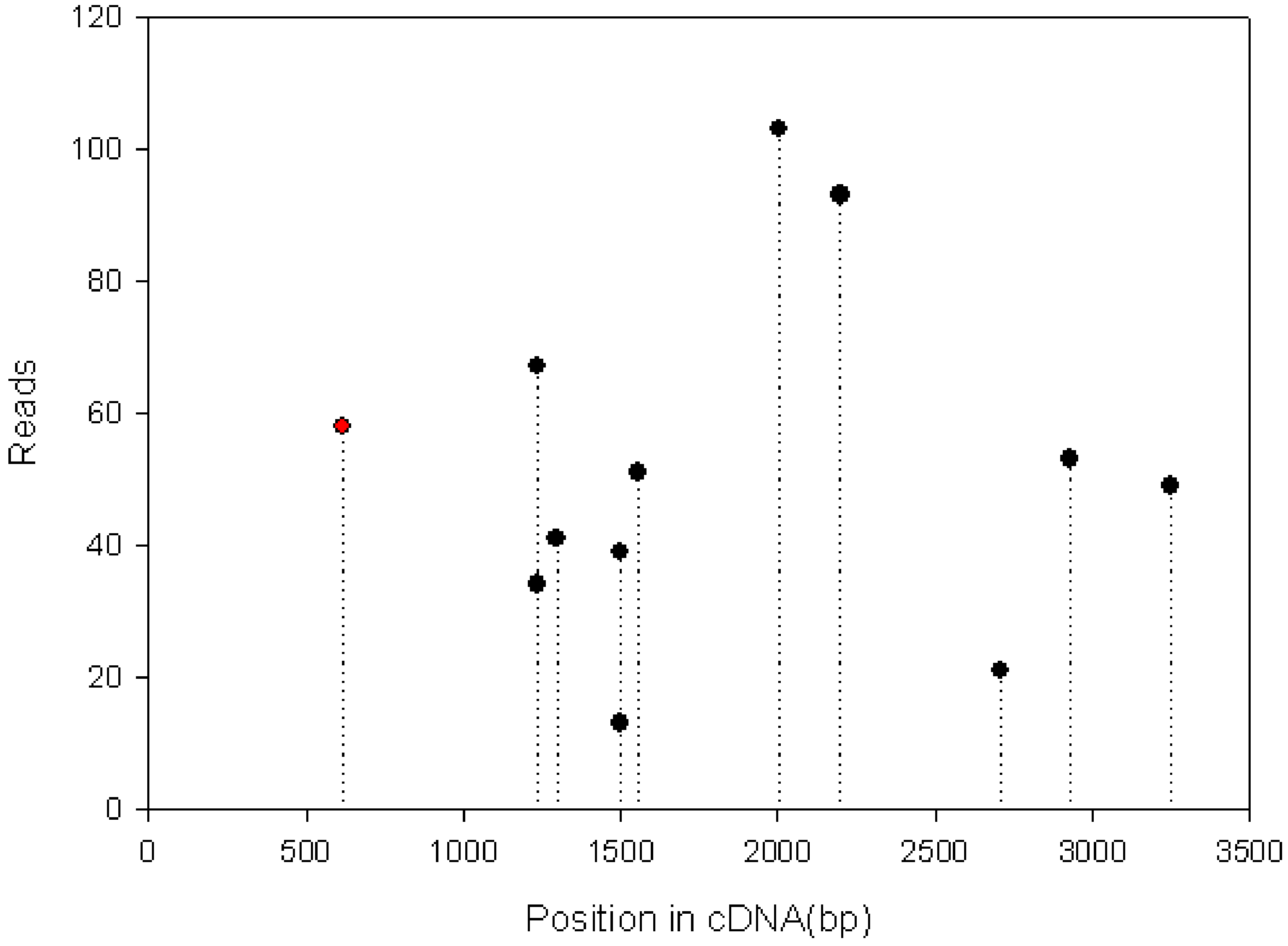


```
5' GGGAUGGGUGGAAUAGGAAAGACUAU 3'        Cs5g21975.1
   :: ::::::::: :::: ::::
3' CCGUACCCACCUCAUCCCUUCU---- 5'        Csi-miR482d
```

Csi-miR482d-5p.1, target=Cs1g02710.1 gene=Cs1g02710
Category:3
Score=5
Cleavage Site=1347

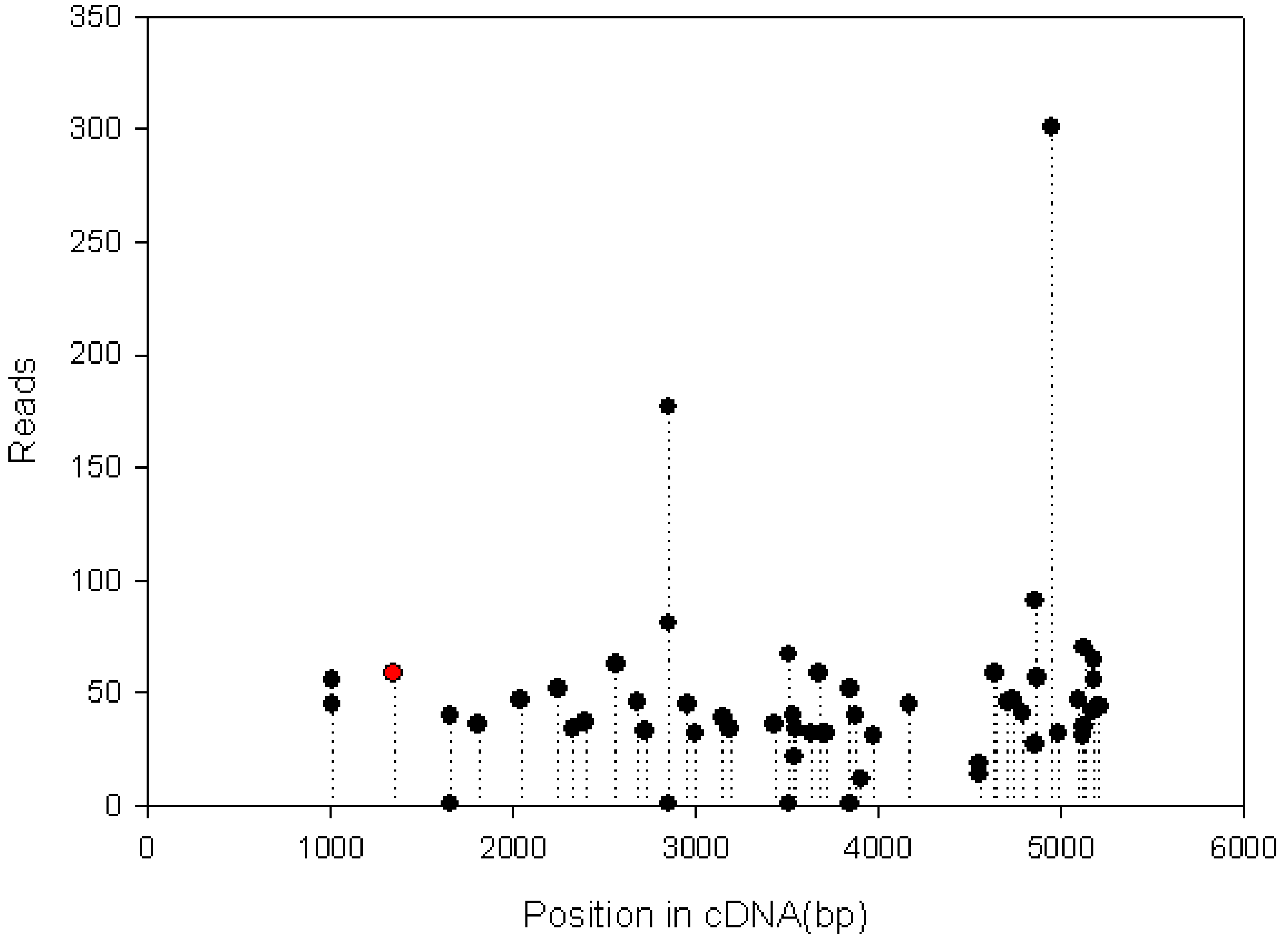


```
5' CUUUGAUAUCUCACCUAUACCGAAAG 3'        Cs1g02710.1
    :::.  :: ::::::.:::::.
3' GAAGGGAU-GAGUGGGUAUGGU---- 5'        Csi-miR482d-5p.1
```

Csi-miR5054.2, target=Cs2g09030.1 gene=Cs2g09030
Category:2
Score=5
Cleavage Site=2215

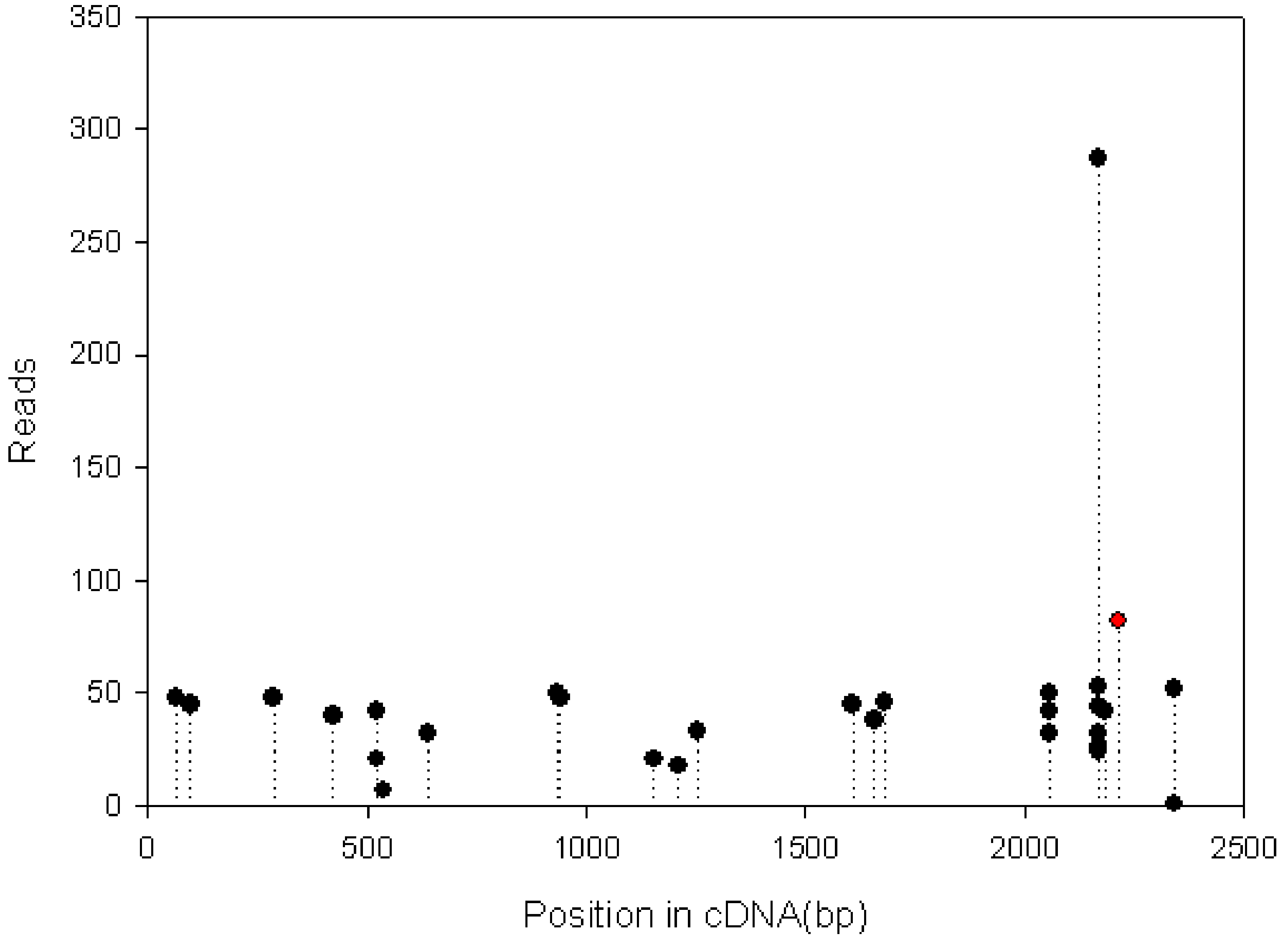


```
5' CUGUAUUGUUGUCGUUUGU-GGGAACC 3'        Cs2g09030.1
         :: .:.:::.::: ::::::
3' ------ACCGCGGCAGACACCCCUUG- 5'        Csi-miR5054.2
```

Csi-miR530b, target=Cs4g14880.1 gene=Cs4g14880
Category:3
Score=4.5
Cleavage Site=631

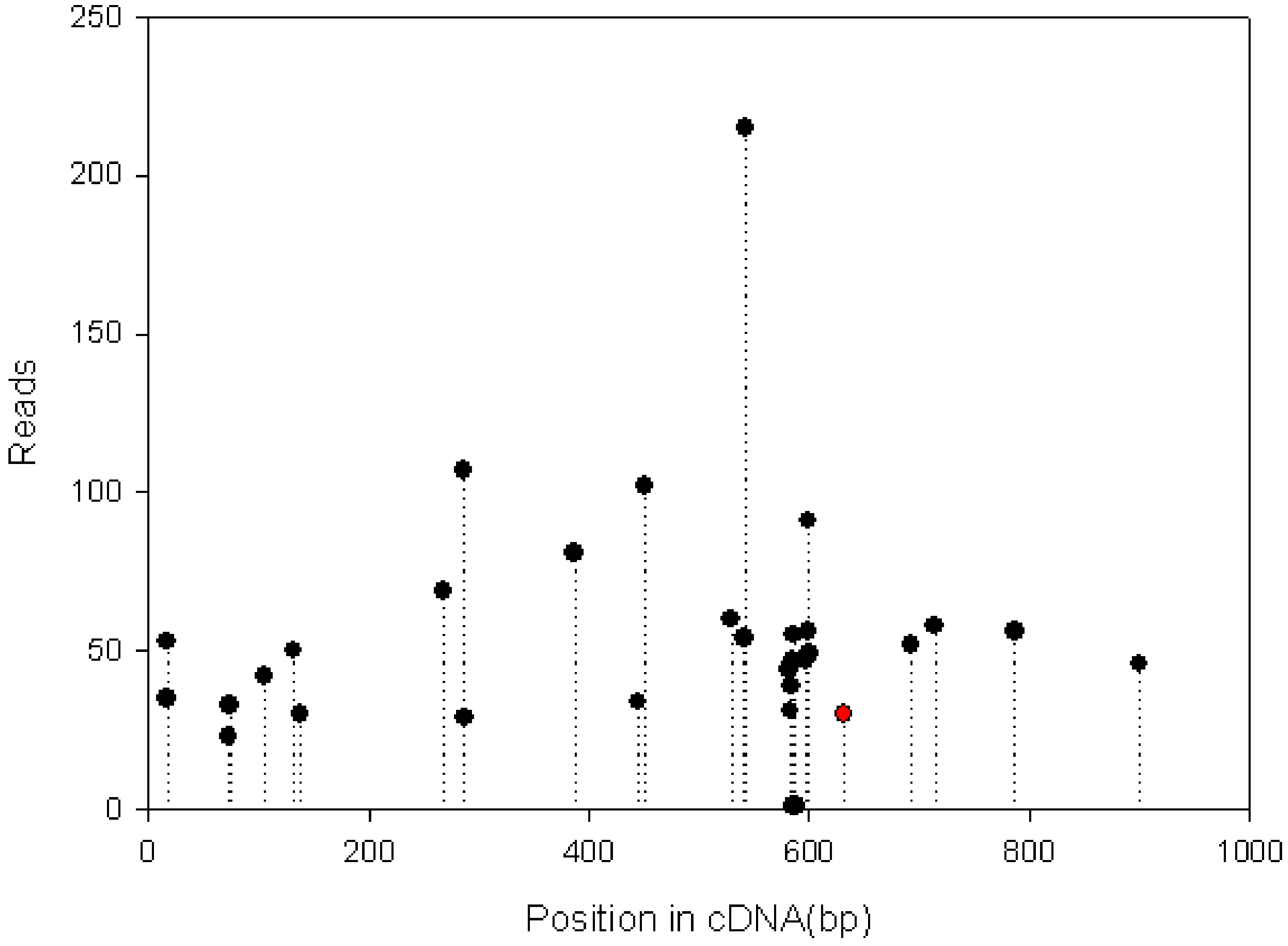


```
5' CGAGAUCCAGAUGCAAAUGCUUUAGA 3'        Cs4g14880.1
    :.:::: ::: :::::::::
3' GUUCUACGUCCACGUUUACGU----- 5'        Csi-miR530b
```

Csi-miR530b, target=Cs6g11650.1 gene=Cs6g11650
Category:3
Score=5
Cleavage Site=261

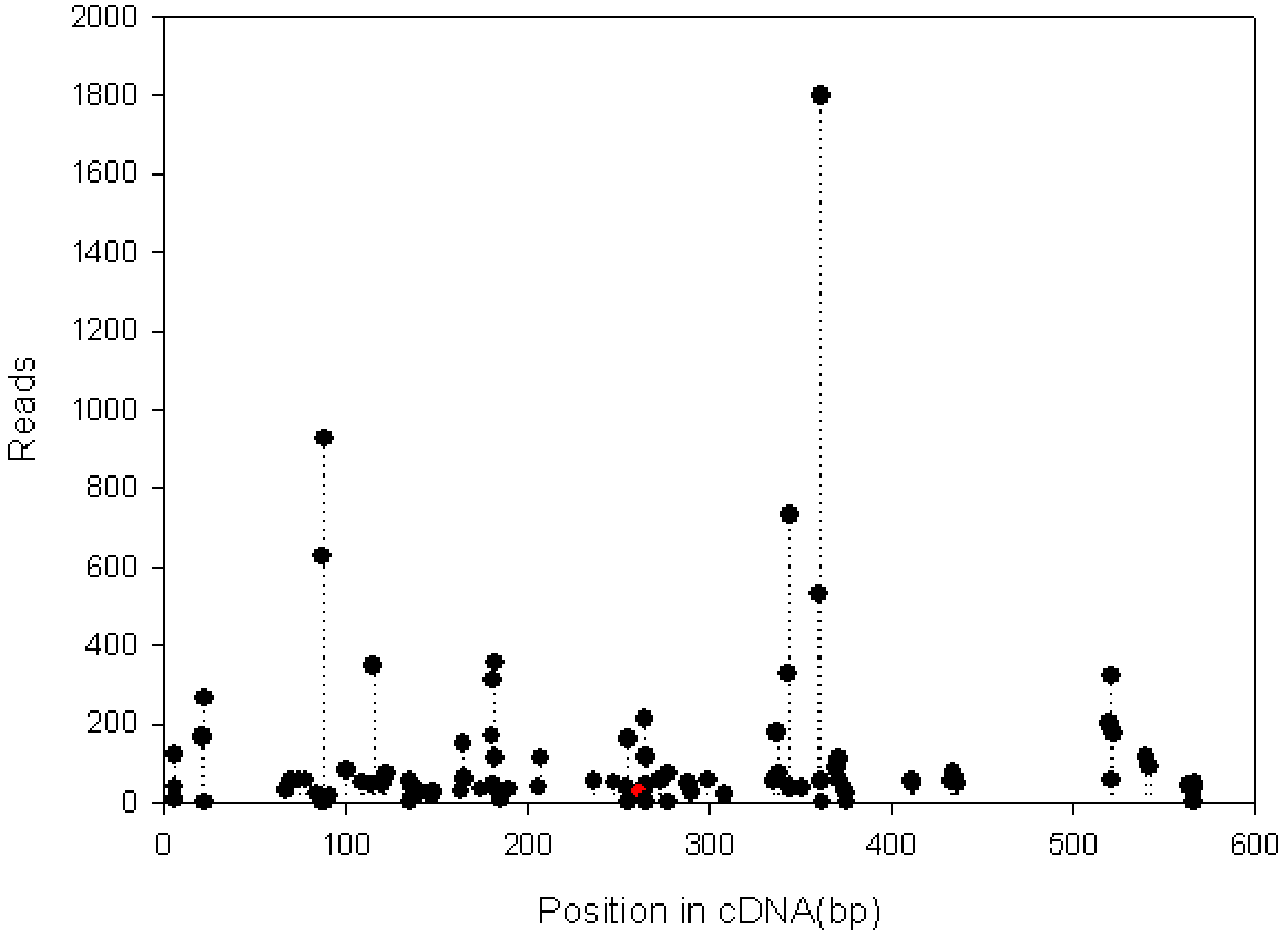


```
5' GGAGAUUCAGGUGCGAAUGUGAAGAG 3'        Cs6g11650.1
    .:::: ::::::::.:::::..
3' GUUCUACGUCCACGUUUACGU----- 5'        Csi-miR530b
```

Csi-miR535.2, target=Cs7g11770.1 gene=Cs7g11770
Category:1
Score=5
Cleavage Site=1487

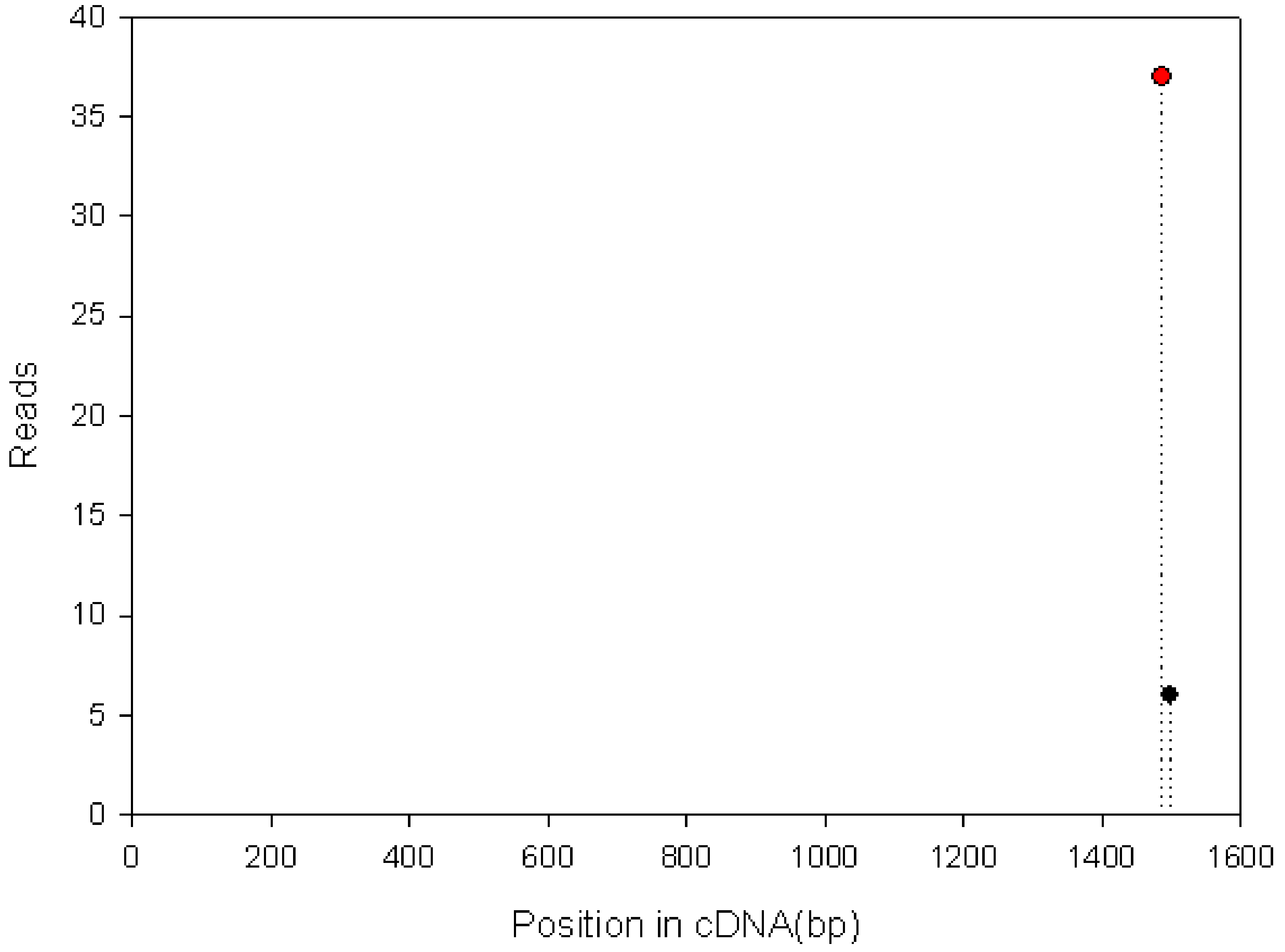


```
5' UGGUGCUCUCUCUCUUCUGUCAGCUC 3'        Cs7g11770.1
     ::::::::::::: : :::::
3' -ACACGAGAGAGAGUA-ACAGU---- 5'        Csi-miR535.2
```

Csi-miR535.2, target=Orange1.1t04075.1 gene=Orange1.1t04075
Category:3
Score=5
Cleavage Site=716

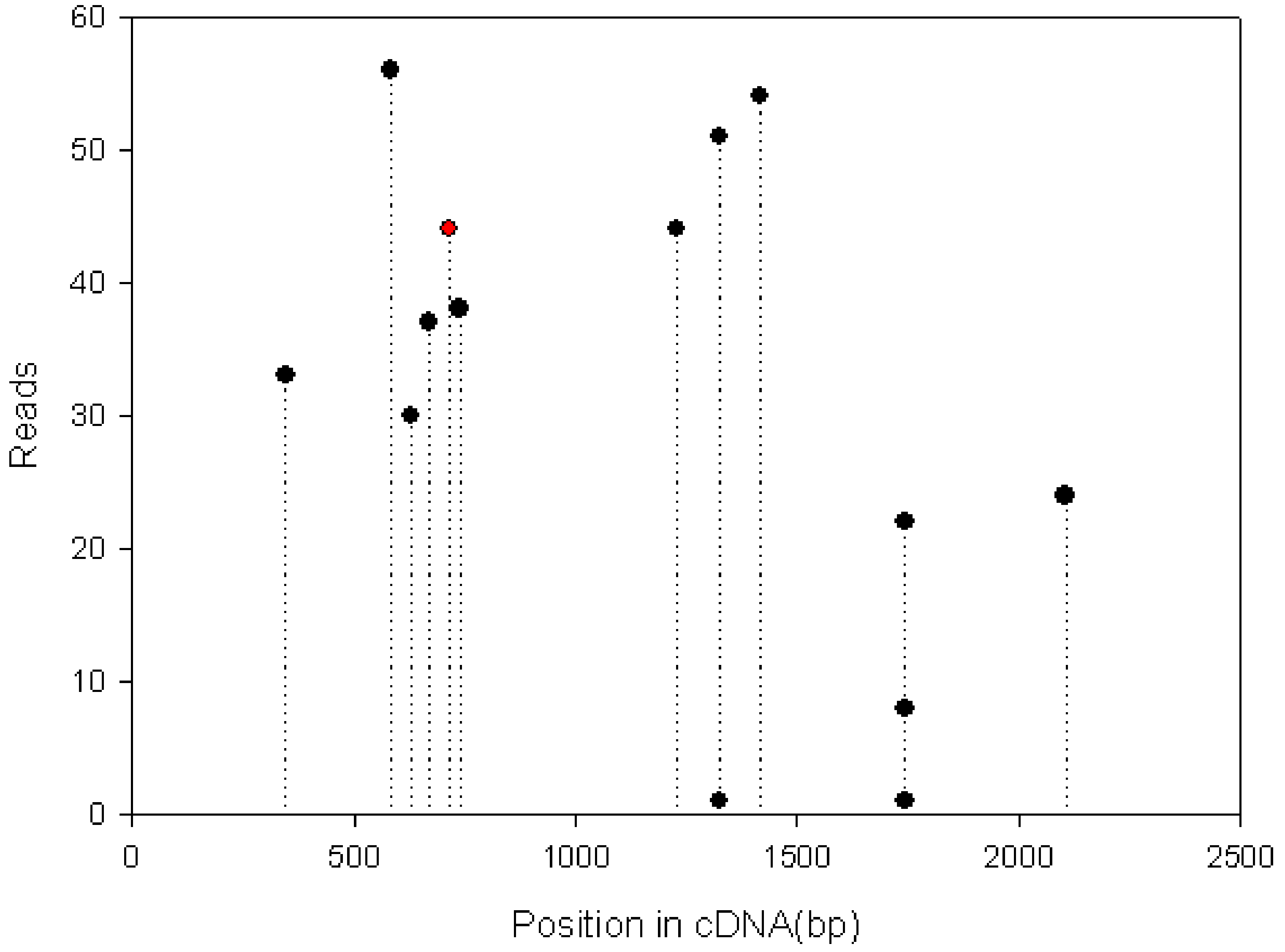


```
5' UUUUGAUCUCUCUUCGUUGUCAGAAA 3'        Orange1.1t04075.1
    : :: :::::: ::.::::::
3' -ACACGAGAGAG-AGUAACAGU---- 5'        Csi-miR535.2
```

Csi-miR827.1, target=Cs5g10180.1 gene=Cs5g10180
Category:3
Score=1
Cleavage Site=241

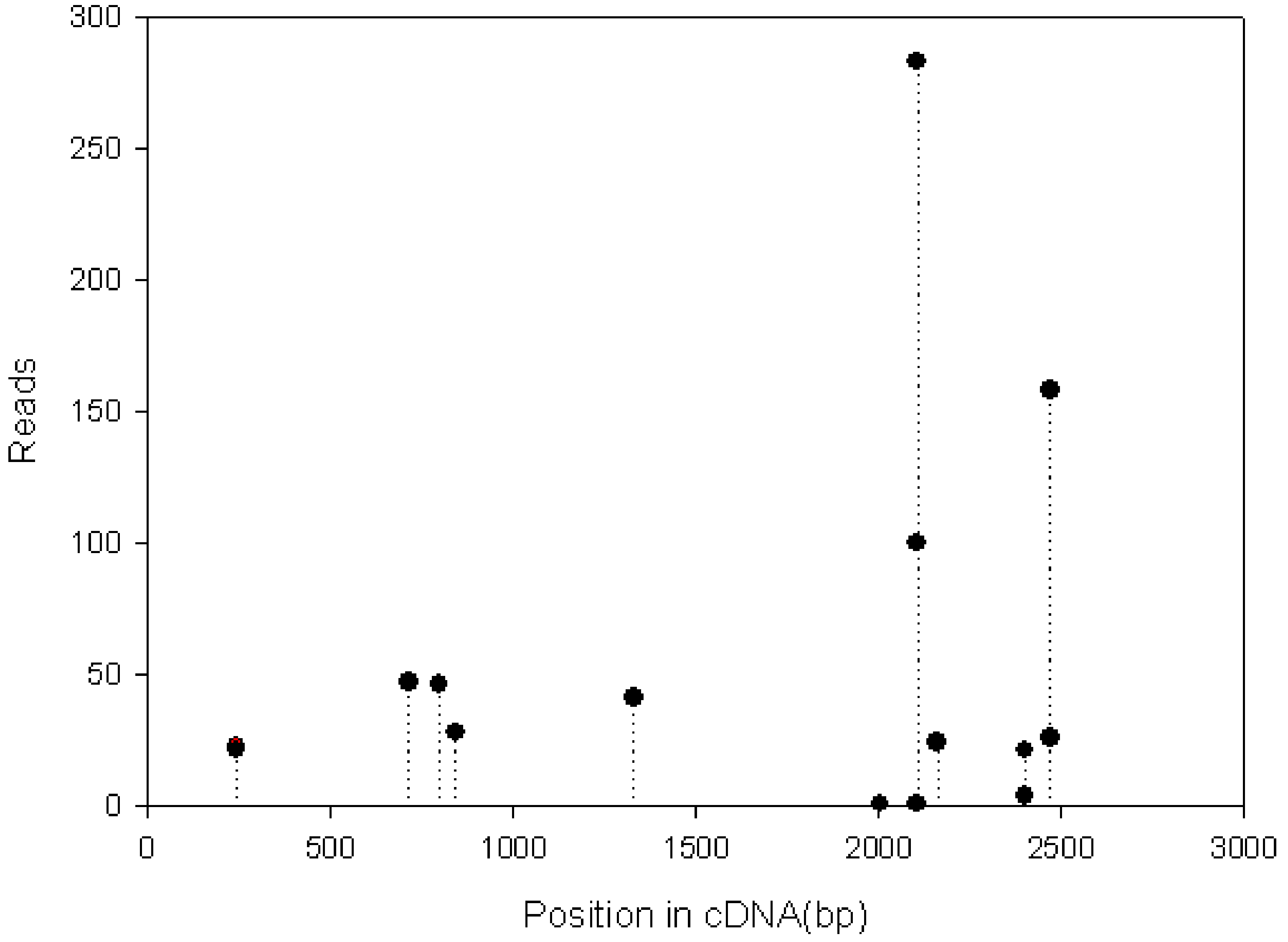


```
5' GUGUUCGUUGAUGGUCAUCUAAACUU 3'        Cs5g10180.1
    :::: ::::::::::::::::
3' -ACAAACAACUACCAGUAGAUU---- 5'        Csi-miR827.1
```

Csi-miR827.2, target=Cs5g10180.1 gene=Cs5g10180
Category:3
Score=1.5
Cleavage Site=241

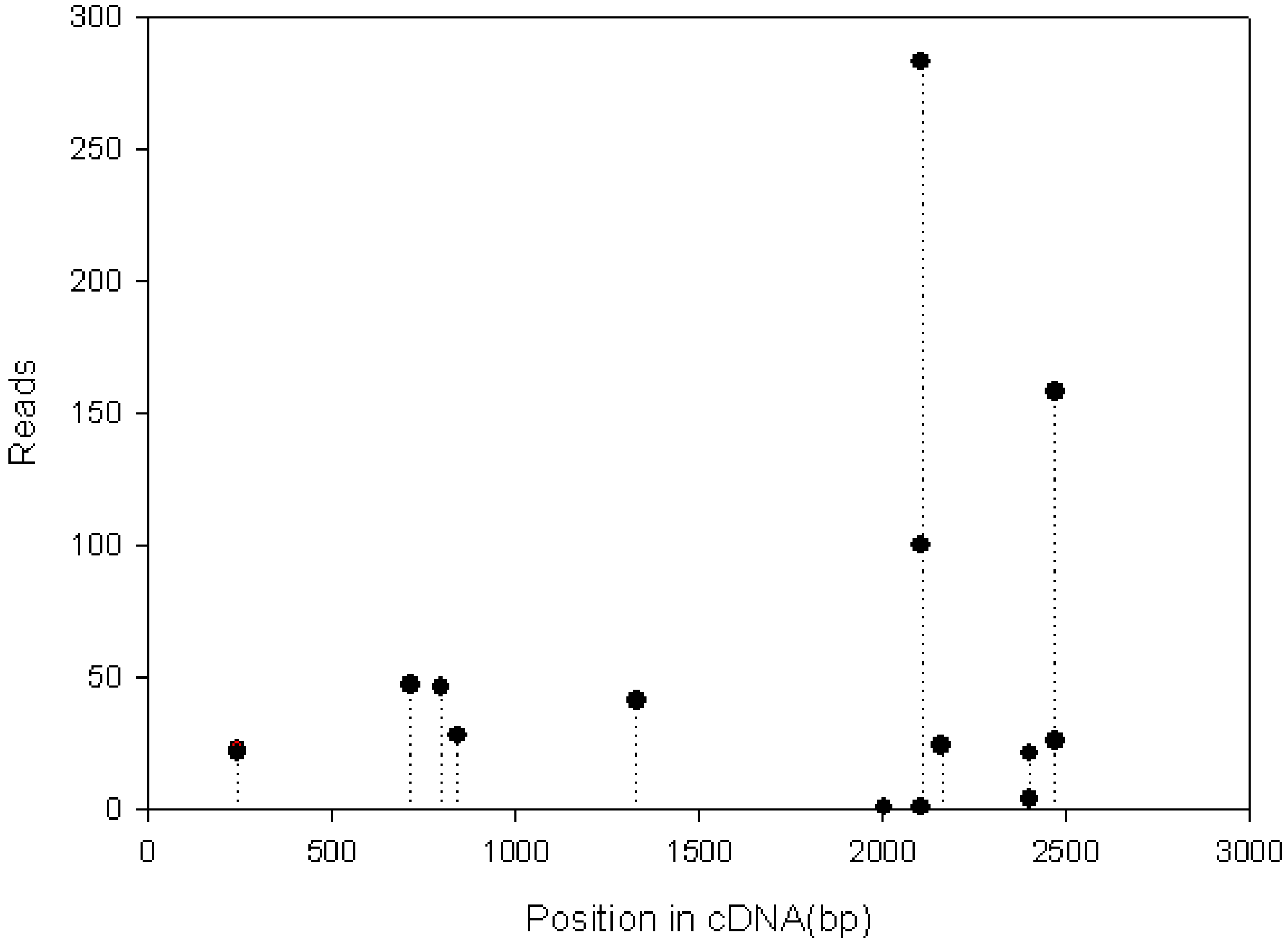


```
5' GUGUUCGUUGAUGGUCAUCUAAACUU 3'        Cs5g10180.1
   .:::: ::::::::::::::::
3' UACAAACAACUACCAGUAGAUU---- 5'        Csi-miR827.2
```

Csi-miR827-5p.1, target=Cs1g24800.1 gene=Cs1g24800
Category:3
Score=3.5
Cleavage Site=2093

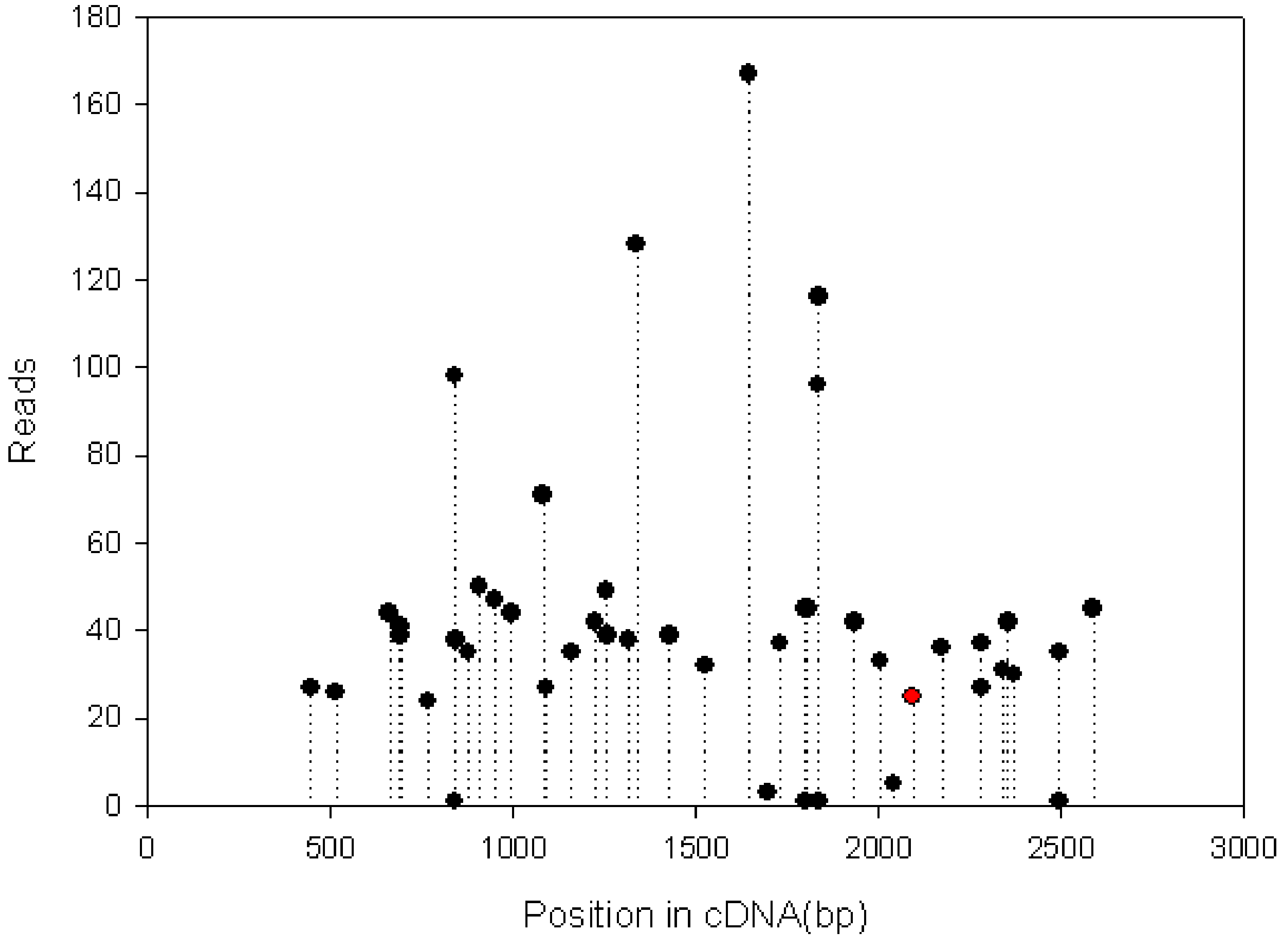


```
5' AUUGCGAGUGCAUGACAAUCAACAAA 3'        Cs1g24800.1
         :: :. ::::::::::::::
3' -----CUAAUCUACUGUUAGUUGUUC 5'        Csi-miR827-5p.1
```

Csi-miR827-5p.2, target=Cs8g15030.1 gene=Cs8g15030
Category:2
Score=5
Cleavage Site=963

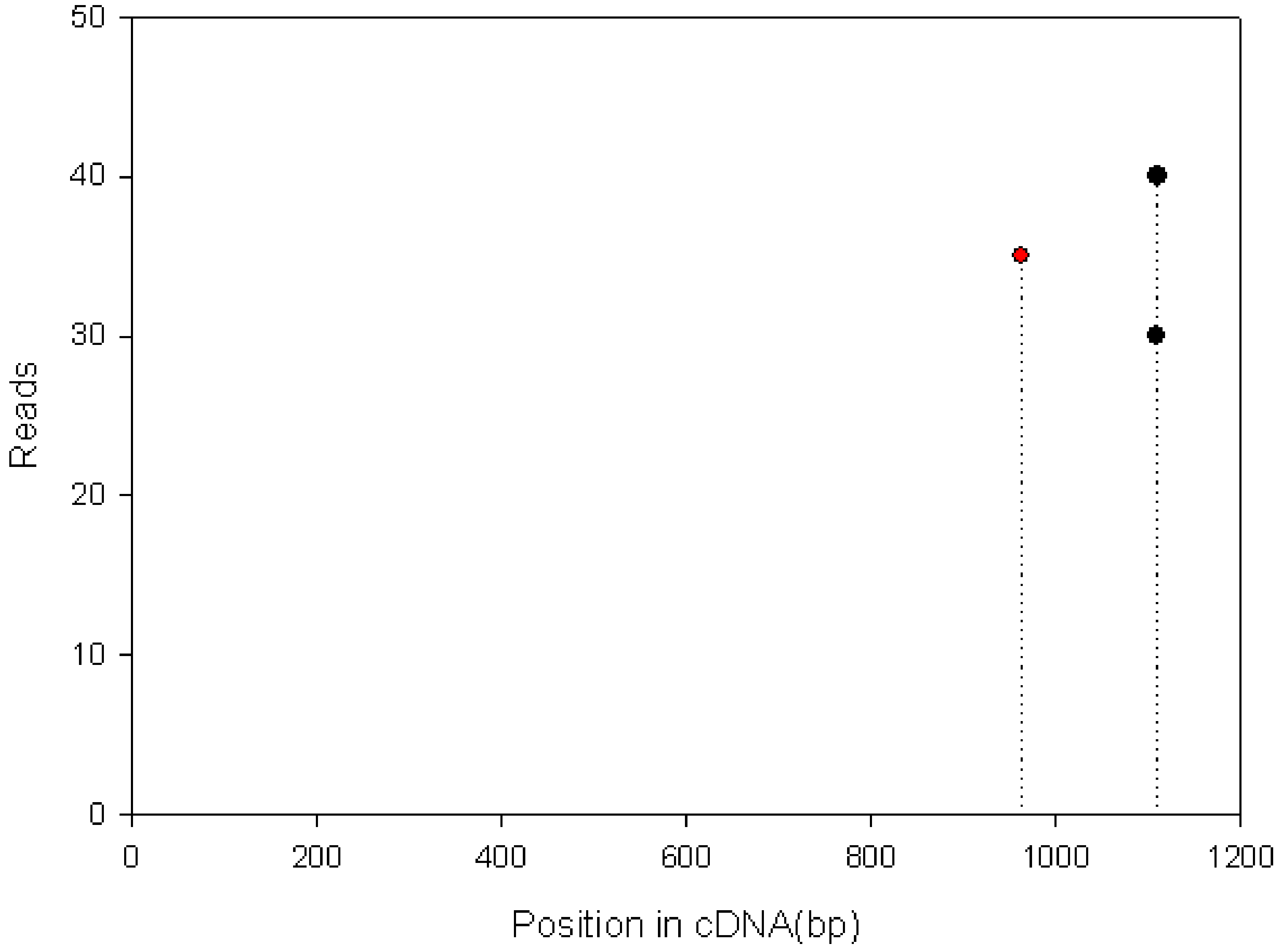


```
5' UUAGAGGACGAUCAAC-GGCACAUAUC 3'       Cs8g15030.1
   ::::: :::.:::::: .:::
3' AAUCUACUGUUAGUUGUUCGU------ 5'       Csi-miR827-5p.2
```

Csi-miR828, target=Orange1.1t00180.1 gene=Orange1.1t00180
Category:3
Score=5
Cleavage Site=652

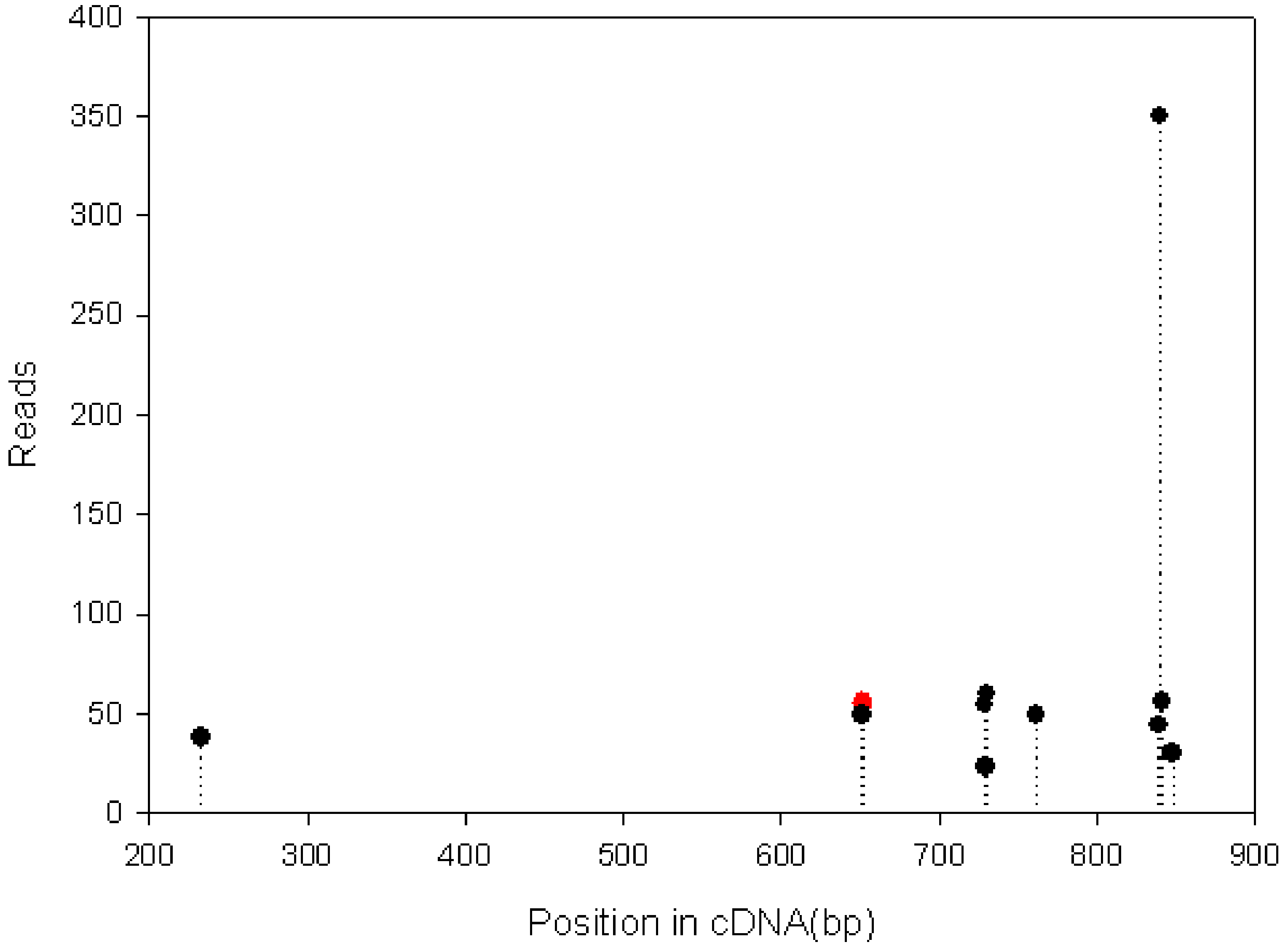


```
5' AGCUACUUG-CUAAAUGGGUAUGCUCU 3'       Orange1.1t00180.1
       :   :::: ::::::::.::::
3' --GCAGAACGGAUUUACUCAUAG---- 5'       Csi-miR828
```

Csi-miR833.1, target=Cs5g27500.1 gene=Cs5g27500
Category: 1
Score=5
Cleavage Site=620

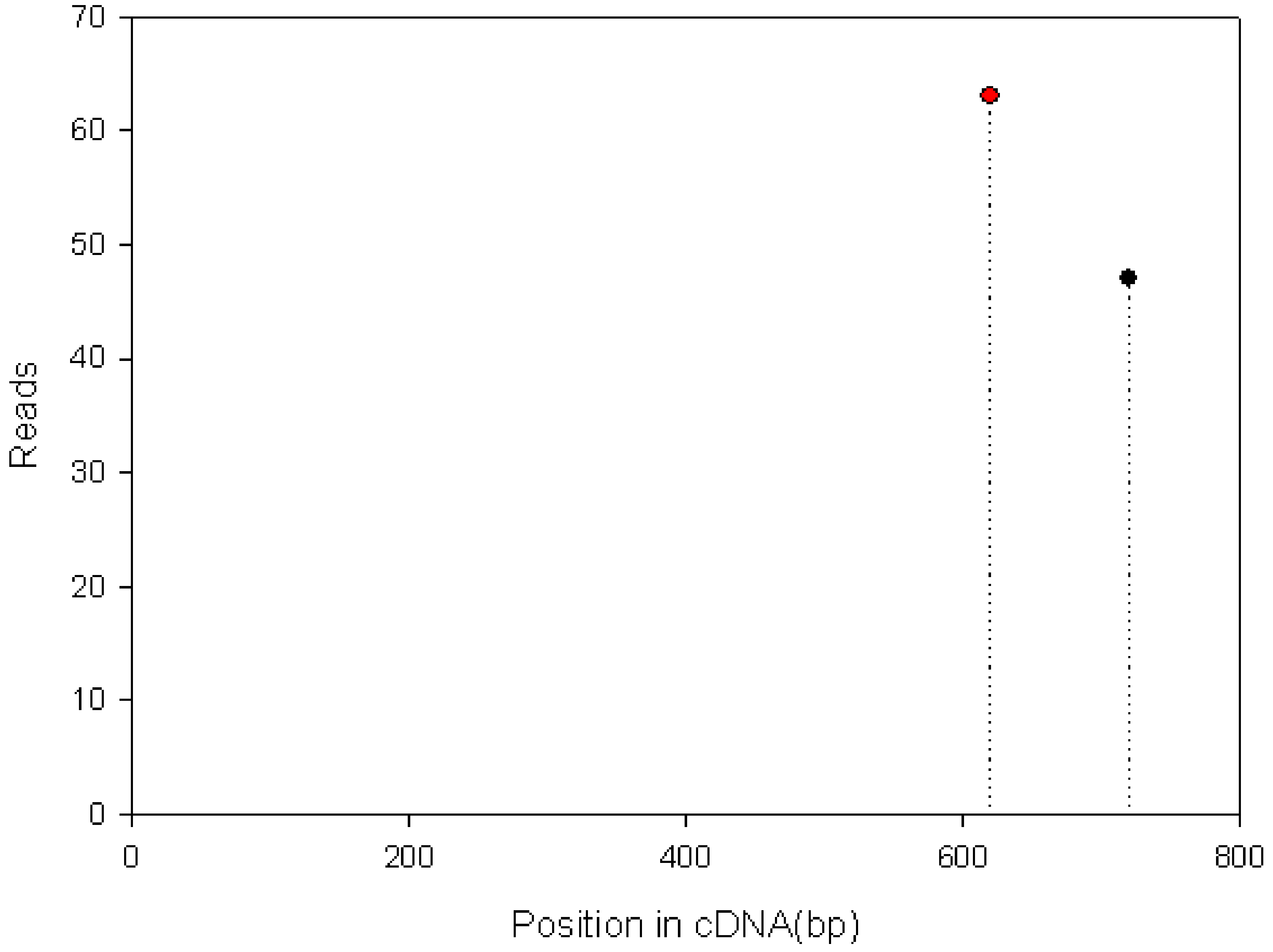


```
5' UUAUUGACUUA-AUCAACAAGAAGUCC 3'      Cs5g27500.1
       ::::..: :::::::: ::.:
3' ----ACUGGGUAUAGUUGUUAUUUA-- 5'      Csi-miR833.1
```

Csi-miR833.1, target=Cs9g02420.1 gene=Cs9g02420
Category:2
Score=4.5
Cleavage Site=935

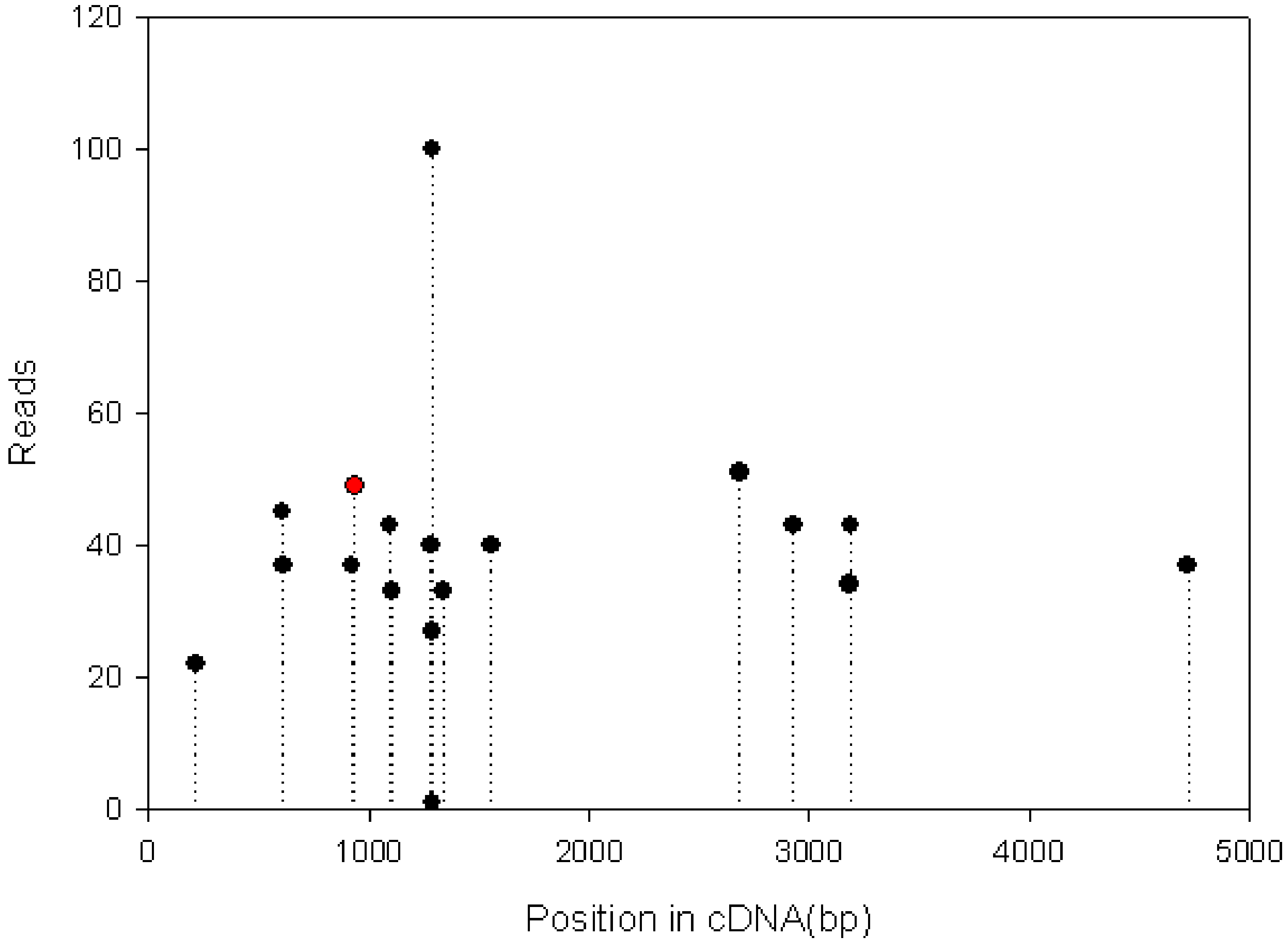


```
5' ACUGUUCUGUA-CAACAAUAAAUUUUC 3'        Cs9g02420.1
    ::  .:..::  :::::::::::
3' --ACUGGGUAUAGUUGUUAUUUA---- 5'        Csi-miR833.1
```

Csi-miRN01, target=Cs8g10430.1 gene=Cs8g10430
Category:2
Score=5
Cleavage Site=460

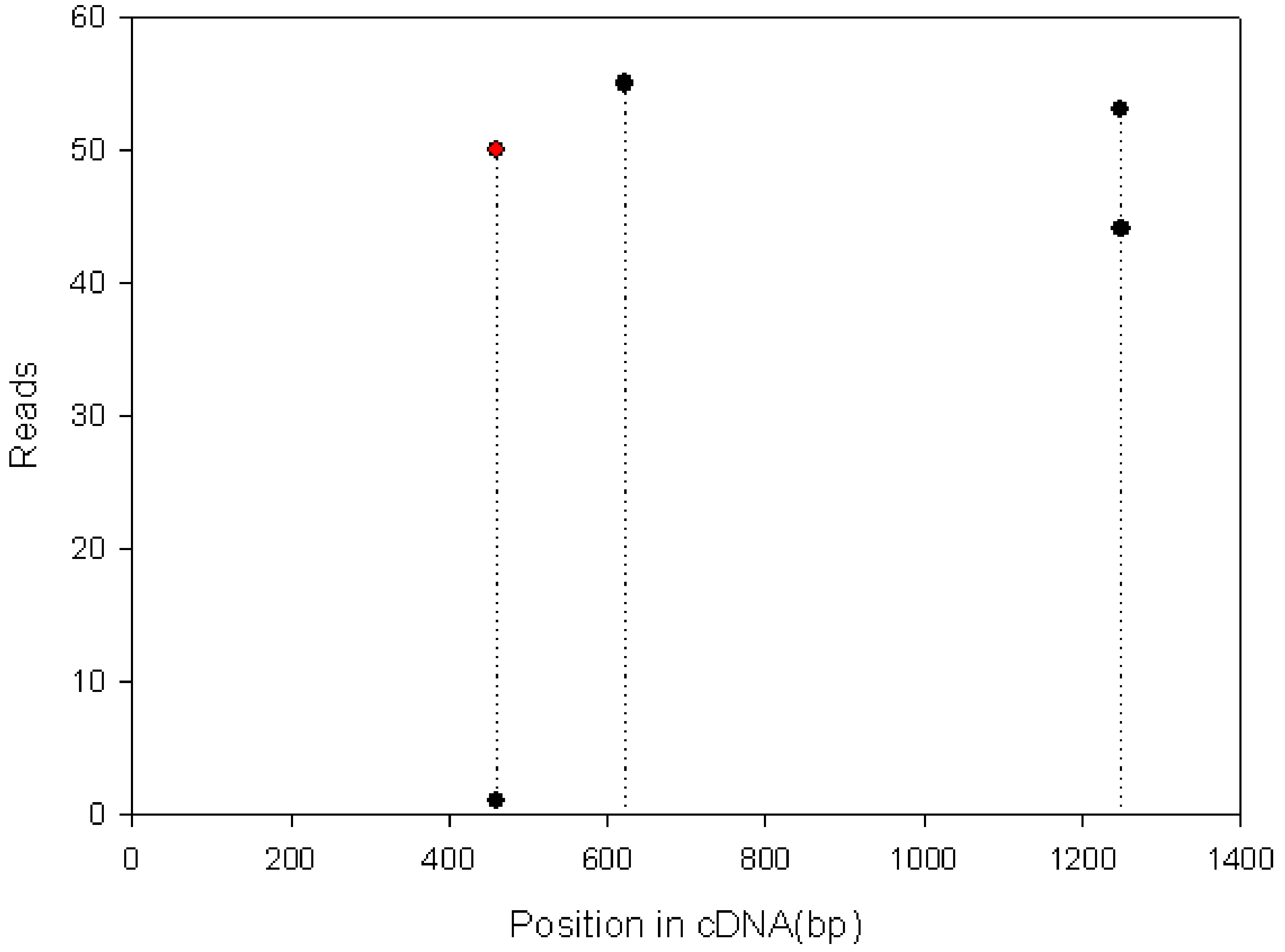


```
5' AAUUUUAAC-GAAGGGUGAUUUGUUUU 3'       Cs8g10430.1
        ::::: :::..::::.:.:::
3' ----AAUUGUCUUUUCACUGAGCAA-- 5'       Csi-miRN01
```

Csi-miRN02, target=Cs3g18790.1 gene=Cs3g18790
Category:1
Score=2
Cleavage Site=1818

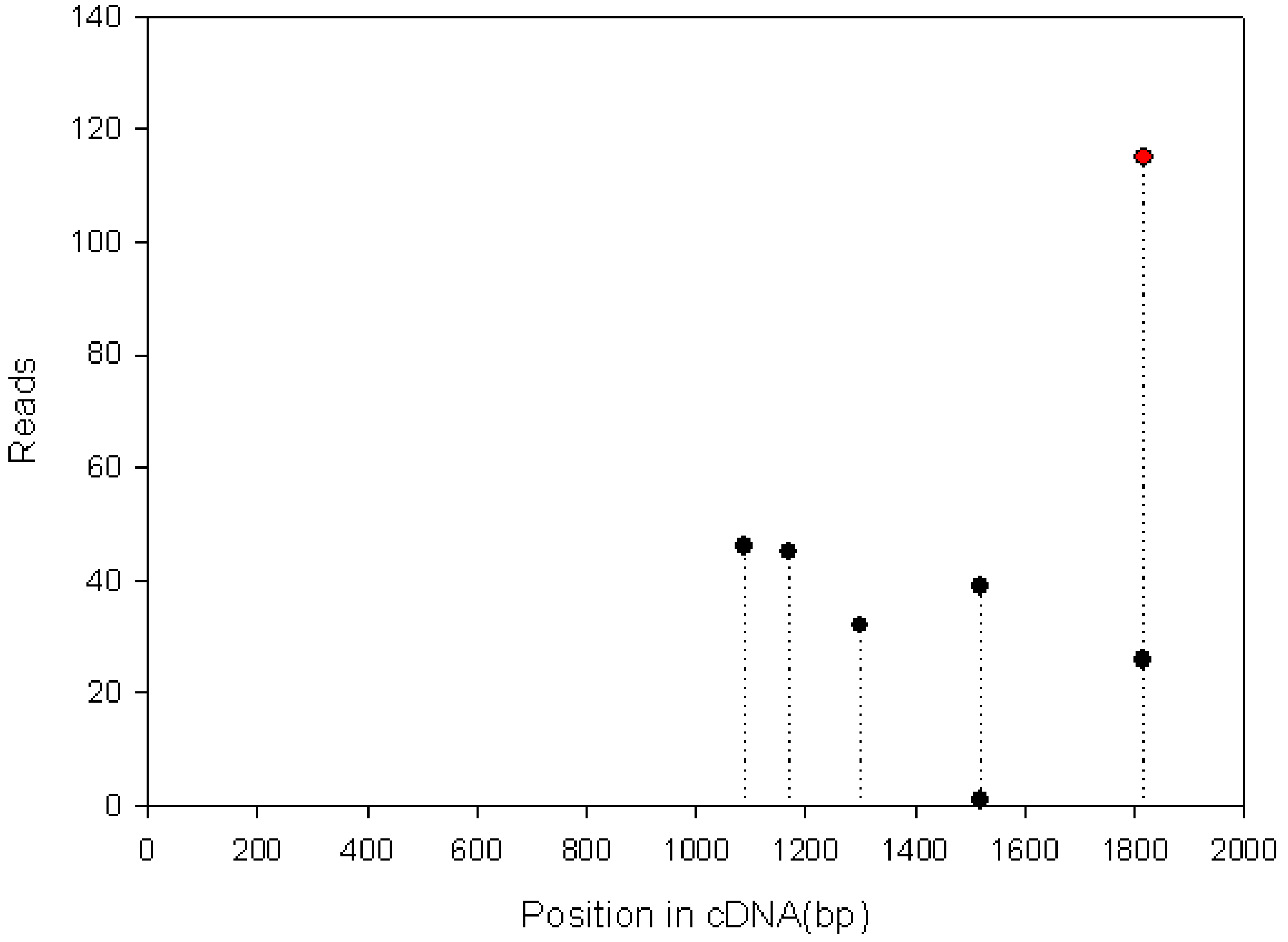


```
5' UGCAACUGUGGCACGGUACCACAGUU 3'        Cs3g18790.1
        :::::::::::::: ::::::
3' -----GACACCGUGCCAUGAUGUCAA 5'        Csi-miRN02
```

Csi-miRN02, target=Cs8g09160.1 gene=Cs8g09160
Category:3
Score=3.5
Cleavage Site=2245

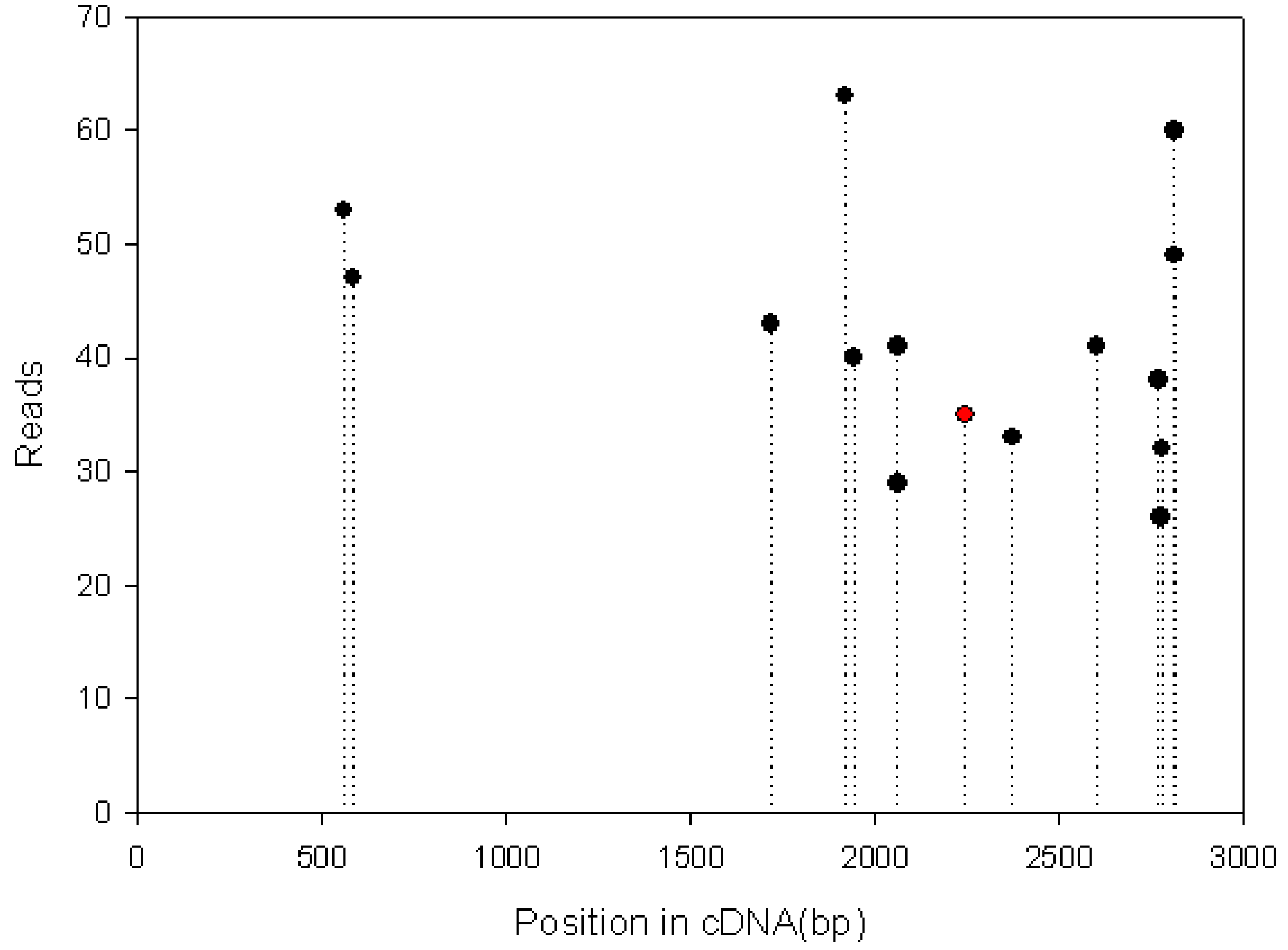


```
5' CGGUAAUACGGUACUACAGUUGUACC 3'        Cs8g09160.1
   : ::  .::::::::::::::
3' GACACCGUGCCAUGAUGUCAA----- 5'        Csi-miRN02
```

Csi-miRN03, target=Orange1.1t02280.1 gene=Orange1.1t02280
Category:3
Score=5
Cleavage Site=188

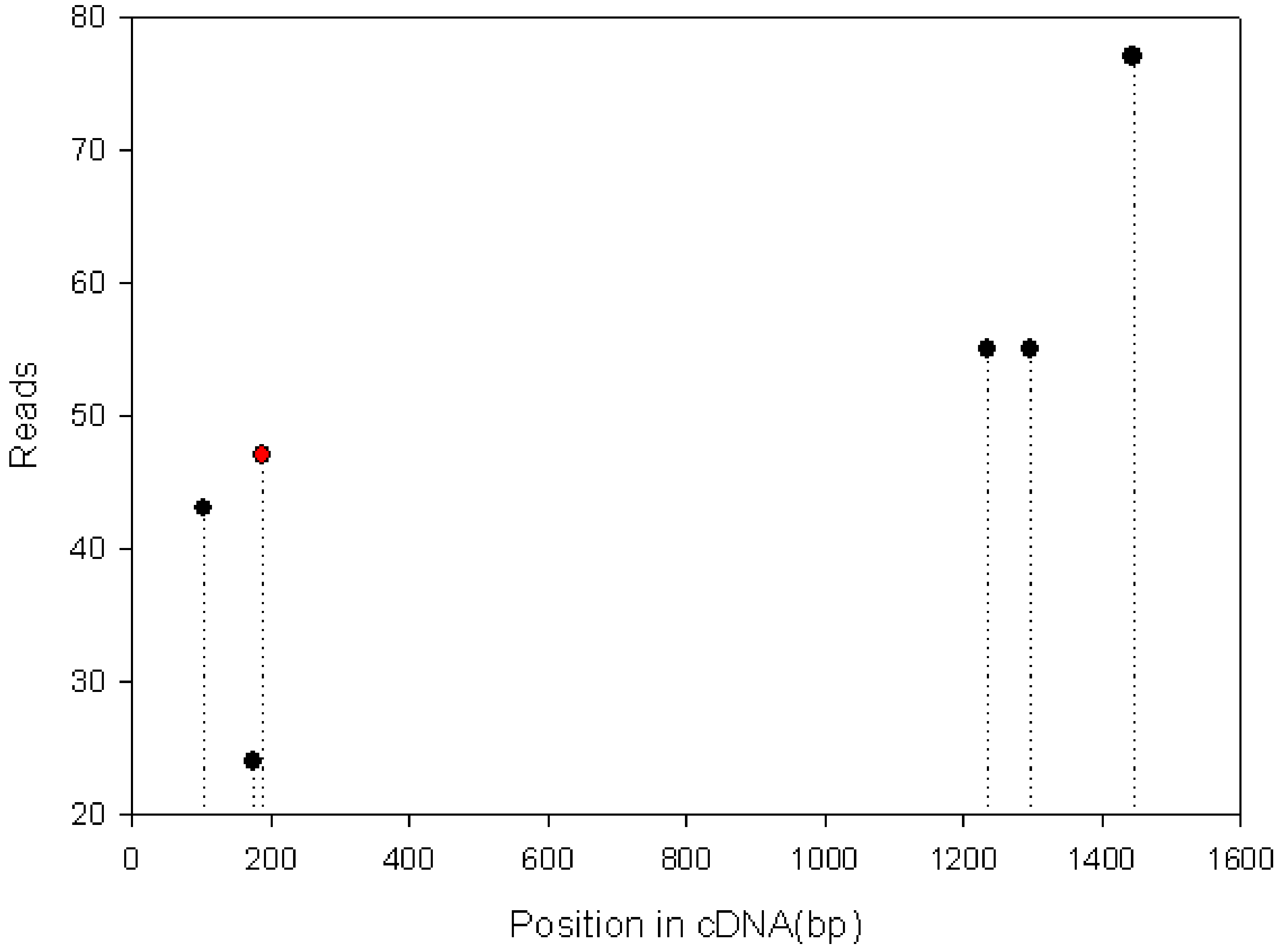


```
5' ACCAAAACCCGGUUCAACCAAAACUC 3'        Orange1.1t02280.1
      :: ::::: :::: :::::::
3' ---UUGUGGGCAAAGUGGGUUUUGU- 5'        Csi-miRN03
```

Csi-miRN04, target=Cs2g04030.2 gene=Cs2g04030
Category:3
Score=5
Cleavage Site=240

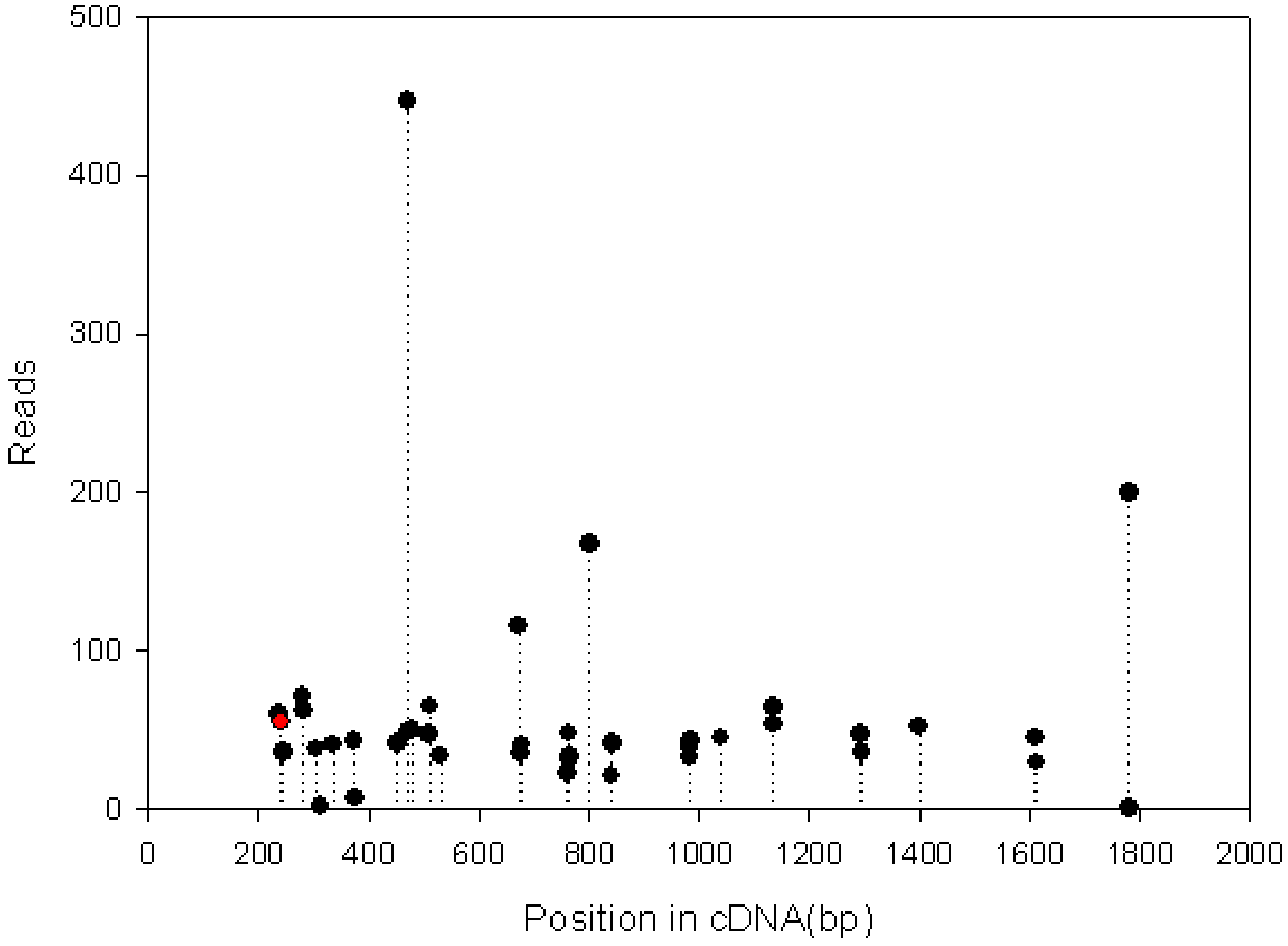


```
5' AAAAUCUAAAACAAACACACUCU-CAA 3'        Cs2g04030.2
         :   ::::  :::::::::  :::
3' ------ACAUUGUAUGUGUGAGAUGUU 5'        Csi-miRN04
```

Csi-miRN07, target=Cs1g18270.1 gene=Cs1g18270
Categroy:3
Score=4.5
Cleavage Site=1427

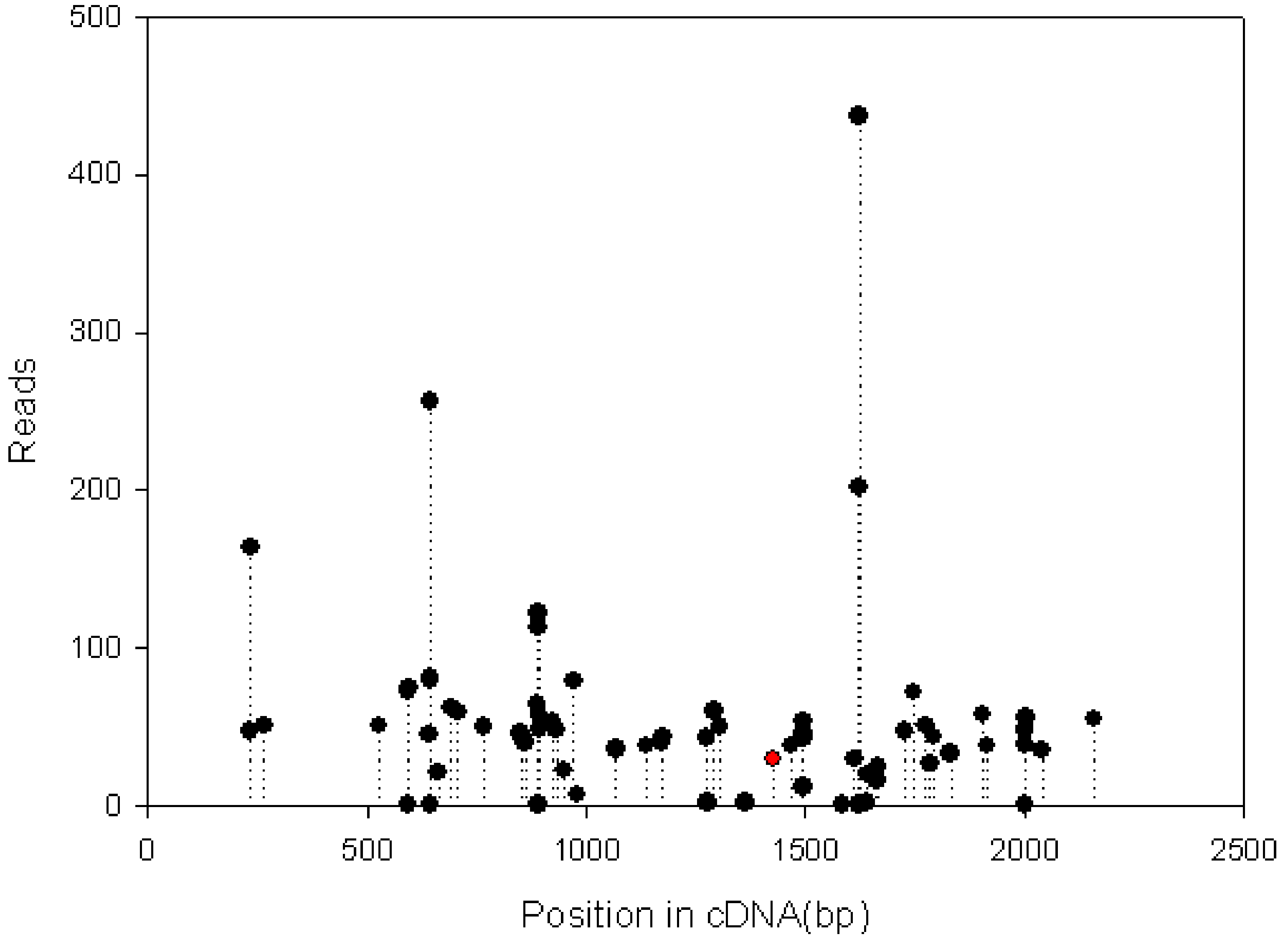


```
5' GACAAUGUUUGCUCUUGUUUUCCUAU 3'        Cs1g18270.1
      :: .::::: ::::::.::::
3' --GUGGCAAACUAGAACAGAAGG--- 5'        Csi-miRN07
```

Csi-miRN07, target=Cs6g11600.1 gene=Cs6g11600
Category:3
Score=5
Cleavage Site=2004

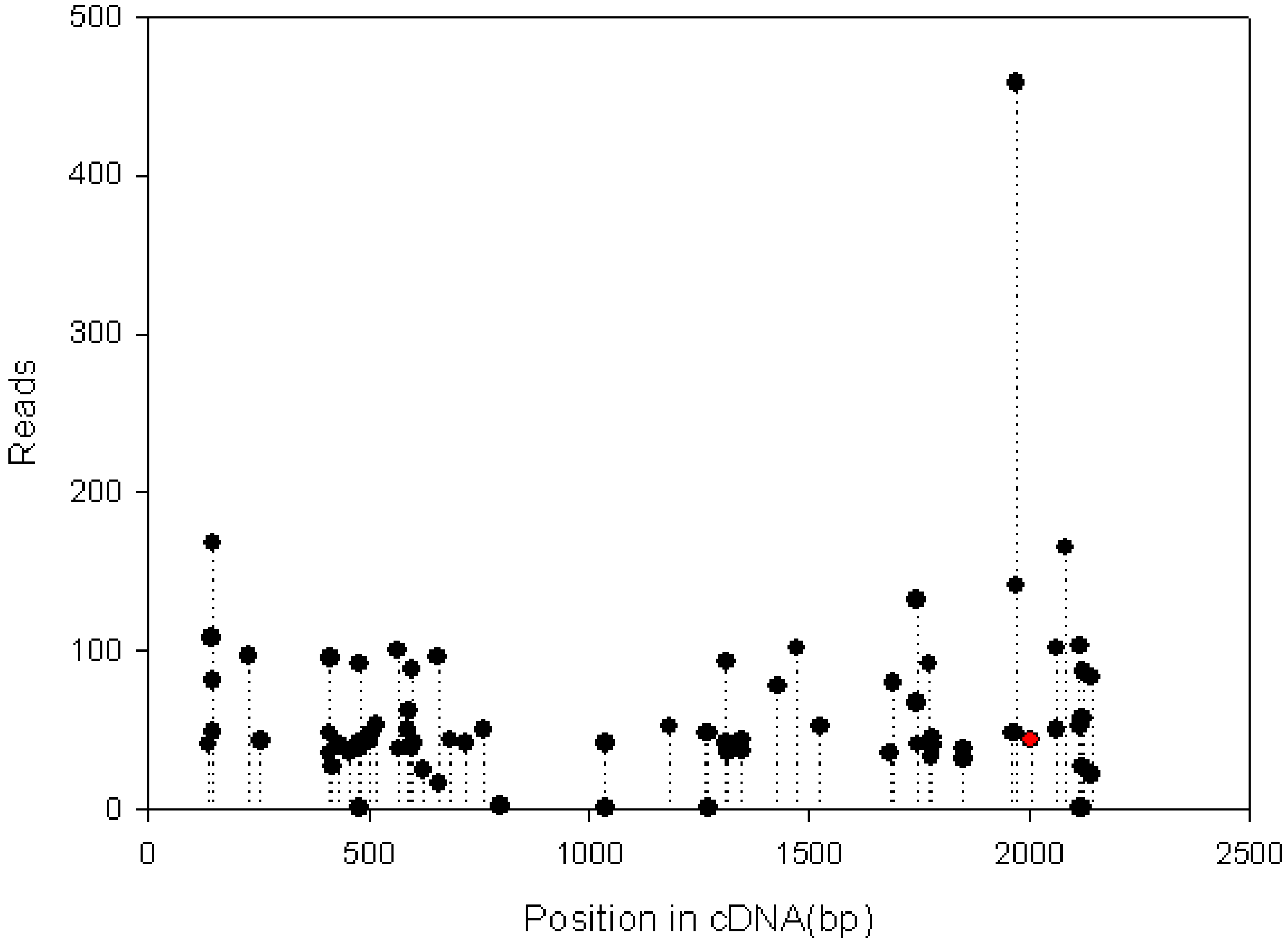


```
5' UGUUG-UUGAUGUUGUCUUCCAUAAAC 3'        Cs6g11600.1
   ....: ::::: :::::::::
3' GUGGCAAACUAGAACAGAAGG------ 5'        Csi-miRN07
```

Csi-miRN10, target=Cs4g05310.1 gene=Cs4g05310
Category:3
Score=4.5
Cleavage Site=1120

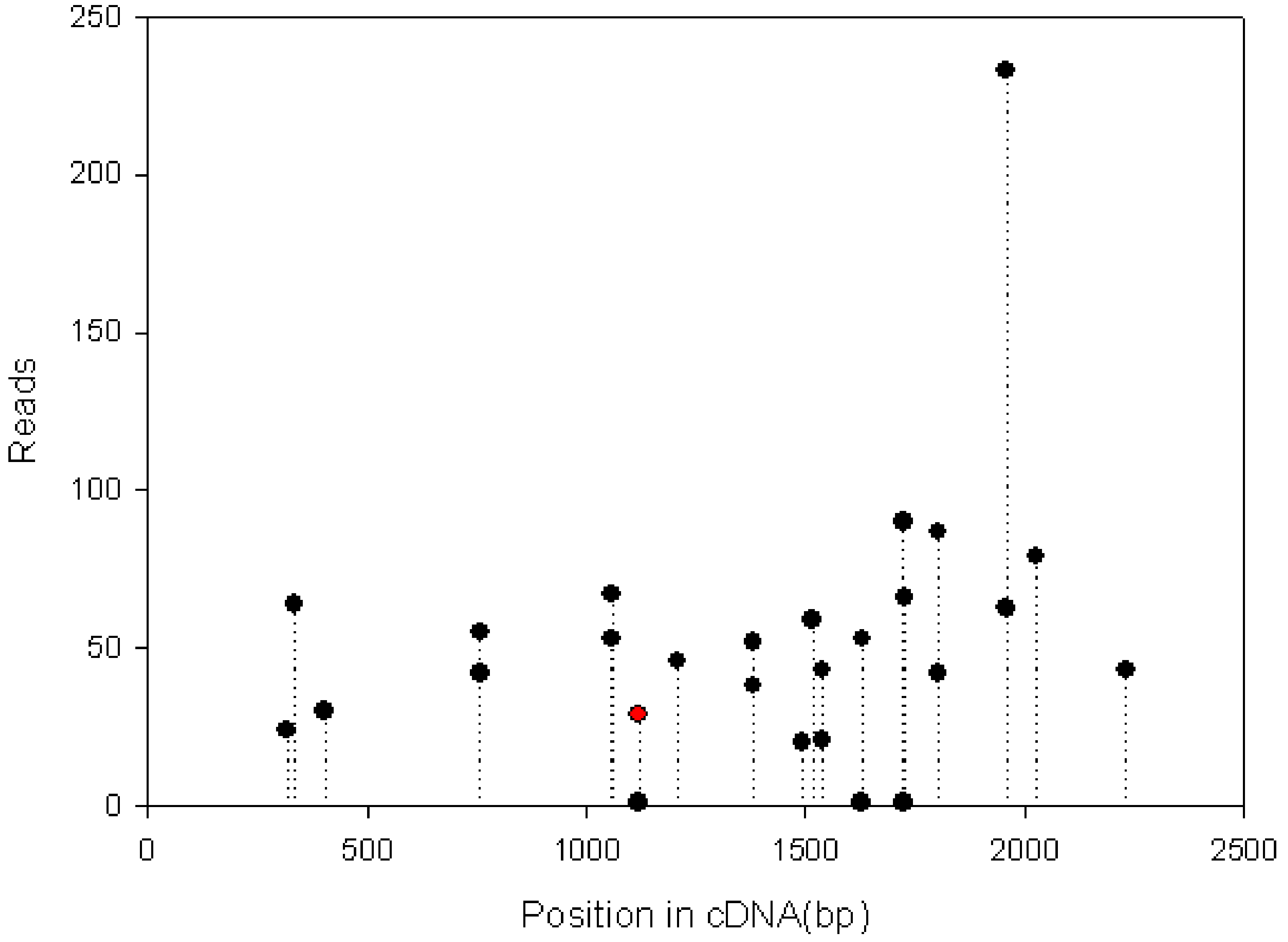


```
5' UGUAAAAUACACACCUCAAUGGGAGU 3'          Cs4g05310.1
   ::::: ::::::: :::::::..
3' ACAUUGUAUGUGU-GAGUUAUU---- 5'          Csi-miRN10
```

Csi-miRN11, target=Cs8g13560.1 gene=Cs8g13560
Category:1
Score=2
Cleavage Site=302

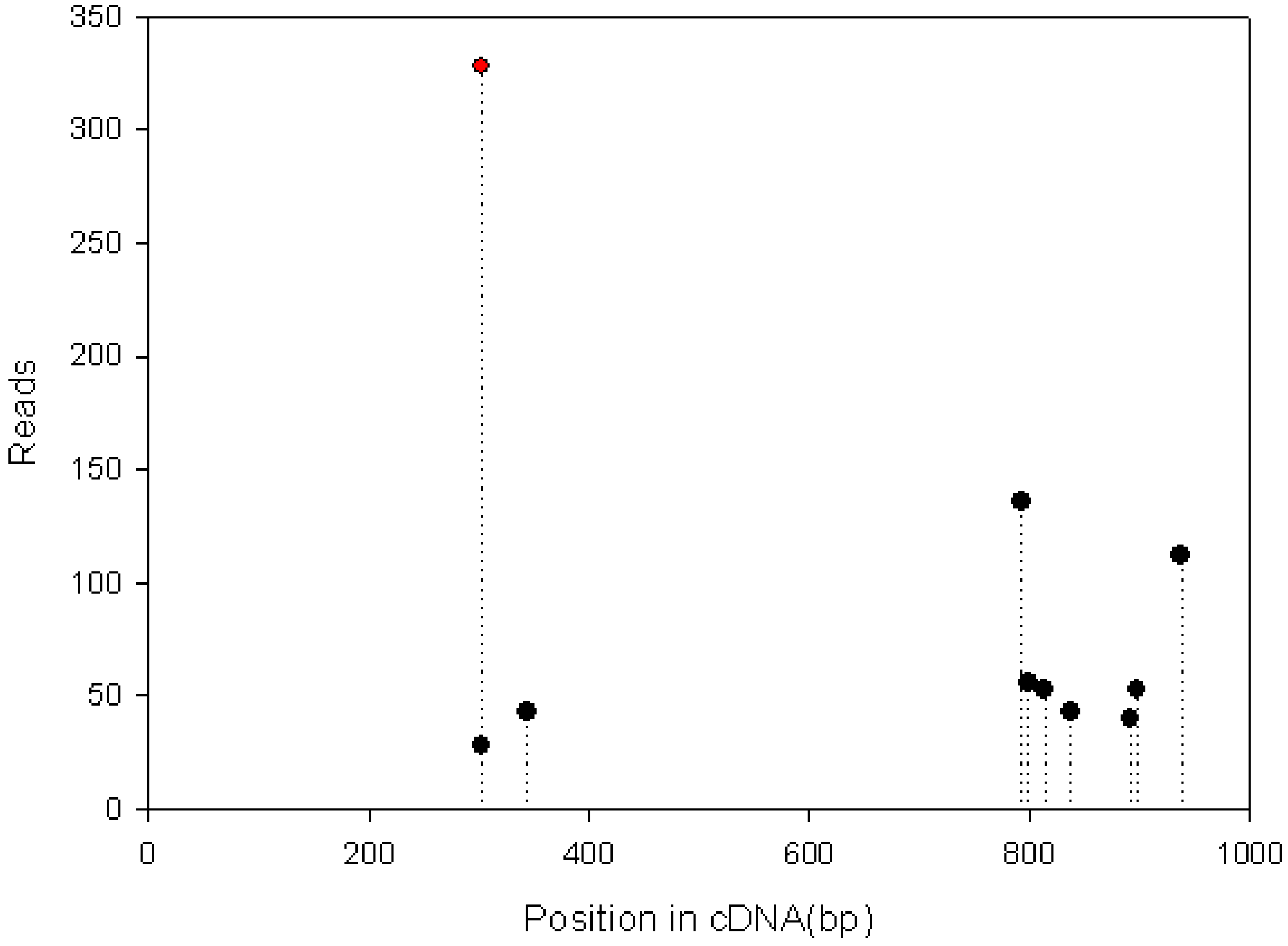


```
5'  GUACCGUAAAAAGCUCCUGCGACAUG 3'          Cs8g13560.1
       :::::: :::::::::::::
3'  -UUGGCAUCUUUCGAGGACGCU---- 5'          Csi-miRN11
```

Csi-miRN14, target=Cs7g14990.1 gene=Cs7g14990
Category:3
Score=4.5
Cleavage Site=2345

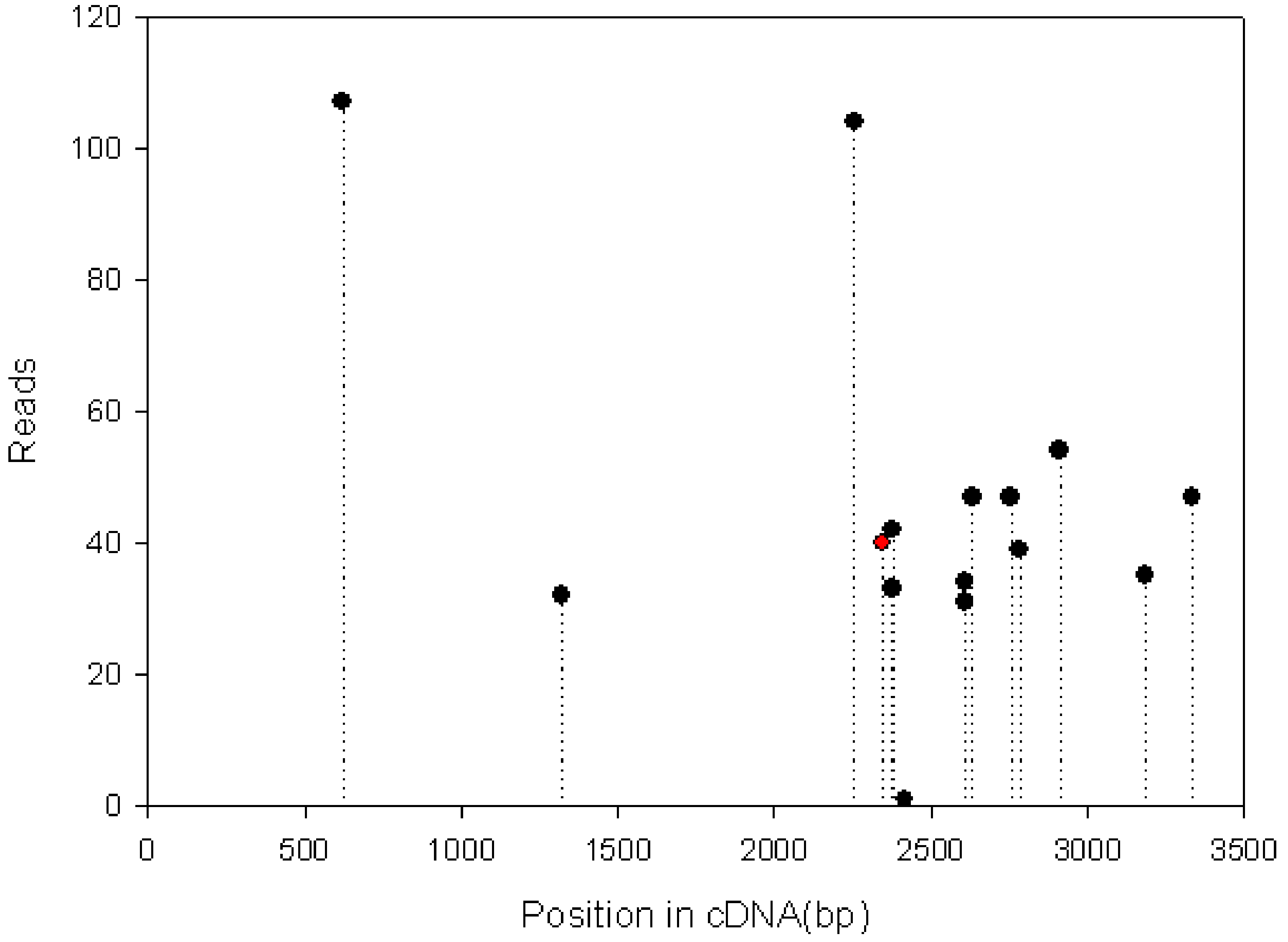


```
5' GAUGAAUAUUU-UUAUCAGGACUAUGA 3'       Cs7g14990.1
      :::: .:: :::::::.:::::::
3' ---CUUAAGAAUAAUAGUUCUGAUACG 5'       Csi-miRN14
```

Csi-miRN17, target=Cs5g04990.1 gene=Cs5g04990
Category:3
Score=5
Cleavage Site=2109

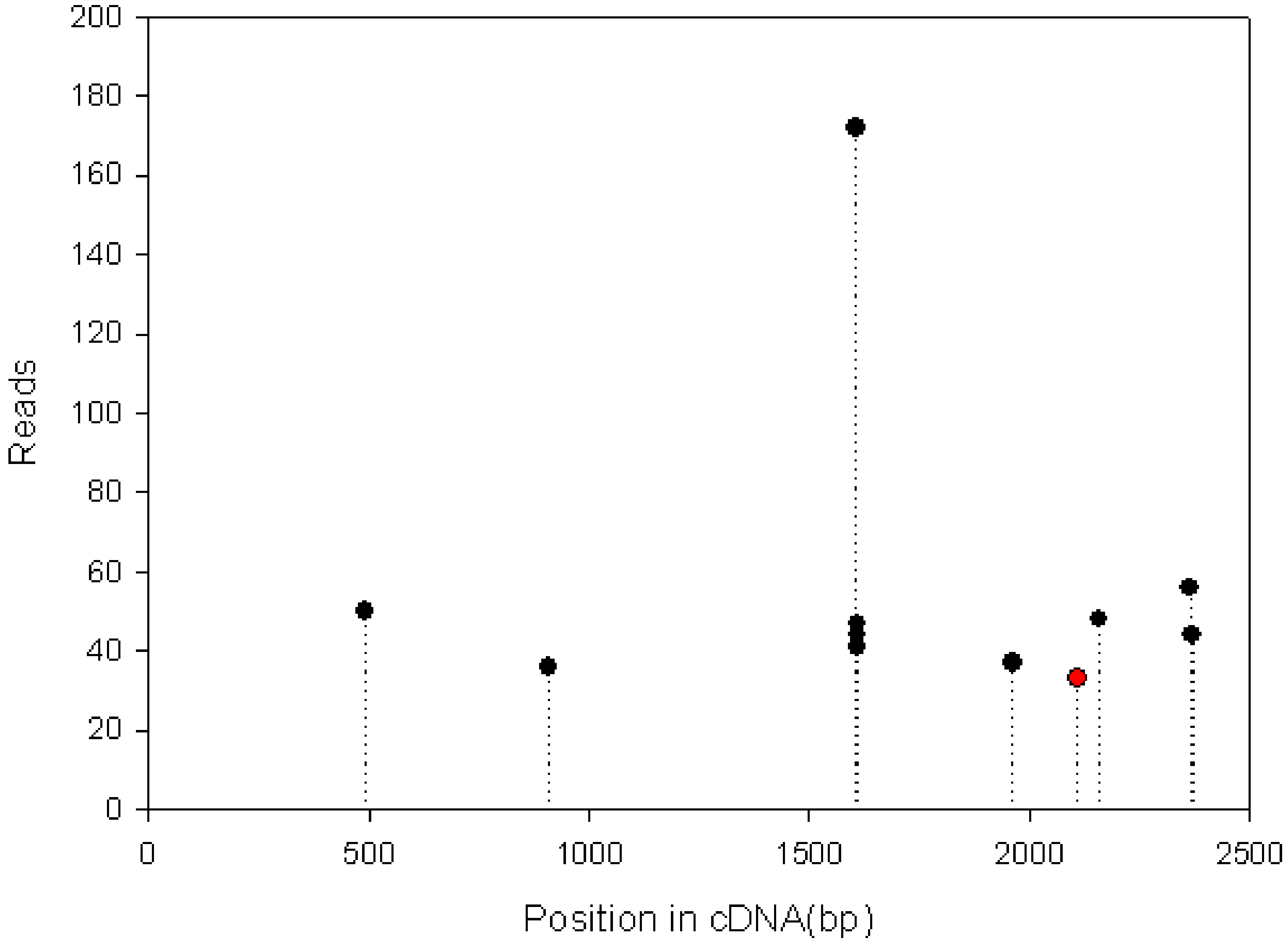


```
5' GGCUUCCUAAUG-ACACGCUCCACAAC 3'        Cs5g04990.1
          ::::.. ::::.::::::::
3' -----ACAUUGUAUGUGUGAGGUGUU- 5'        Csi-miRN17
```

Csi-miRN19, target=Cs5g32800.1 gene=Cs5g32800
Category:3
Score=5
Cleavage Site=1084

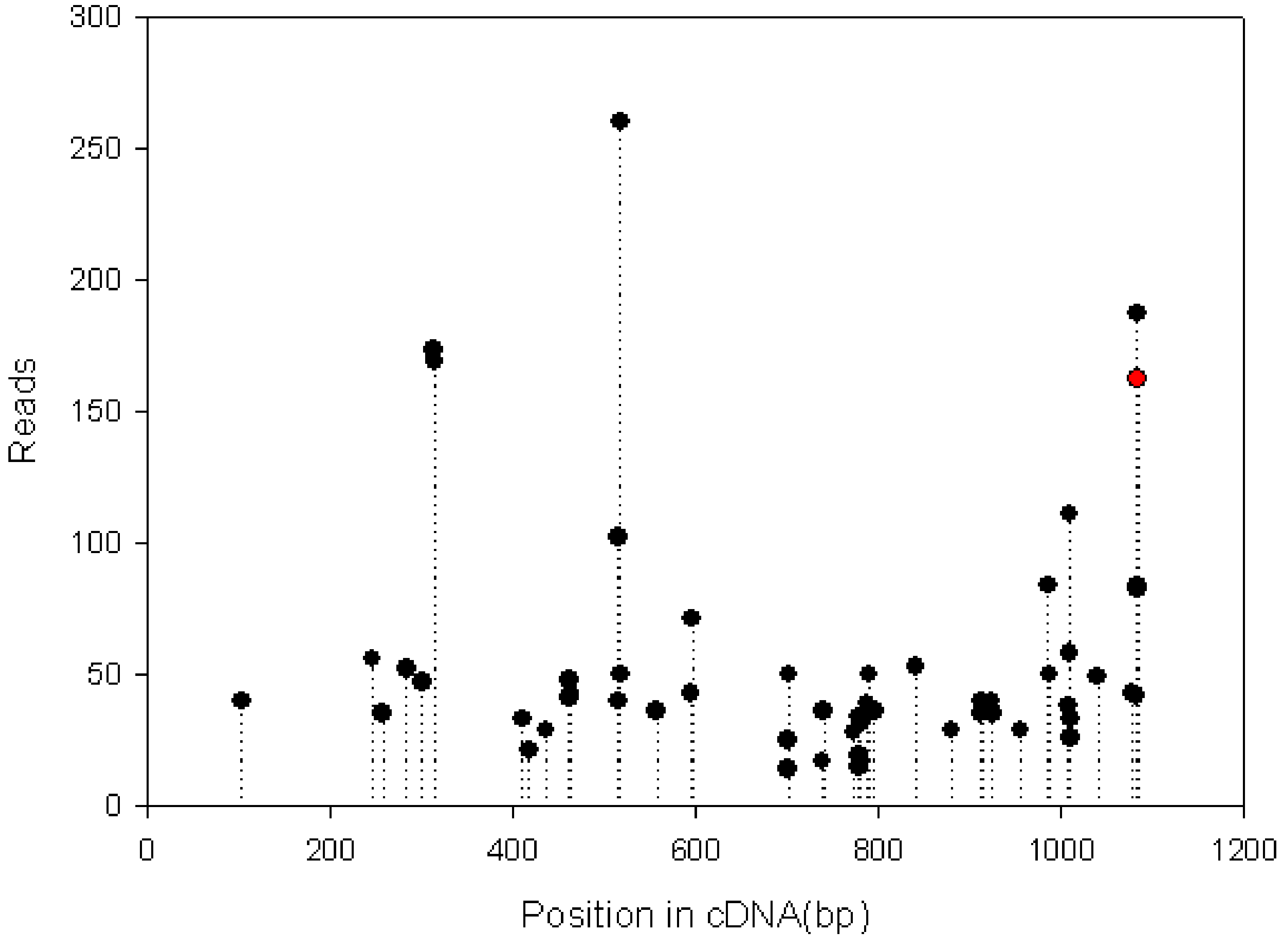


```
5' GUAAAAUUUAG-UAUUCAAUGAACGAC 3'        Cs5g32800.1
          :::::    :::::.::::::
3' ------AAAUCUCAAAGUUGCUUGCUU 5'        Csi-miRN19
```

Csi-miRN20, target=Cs1g12230.1 gene=Cs1g12230
category:1
score=4.5
Cleavage Site=1297

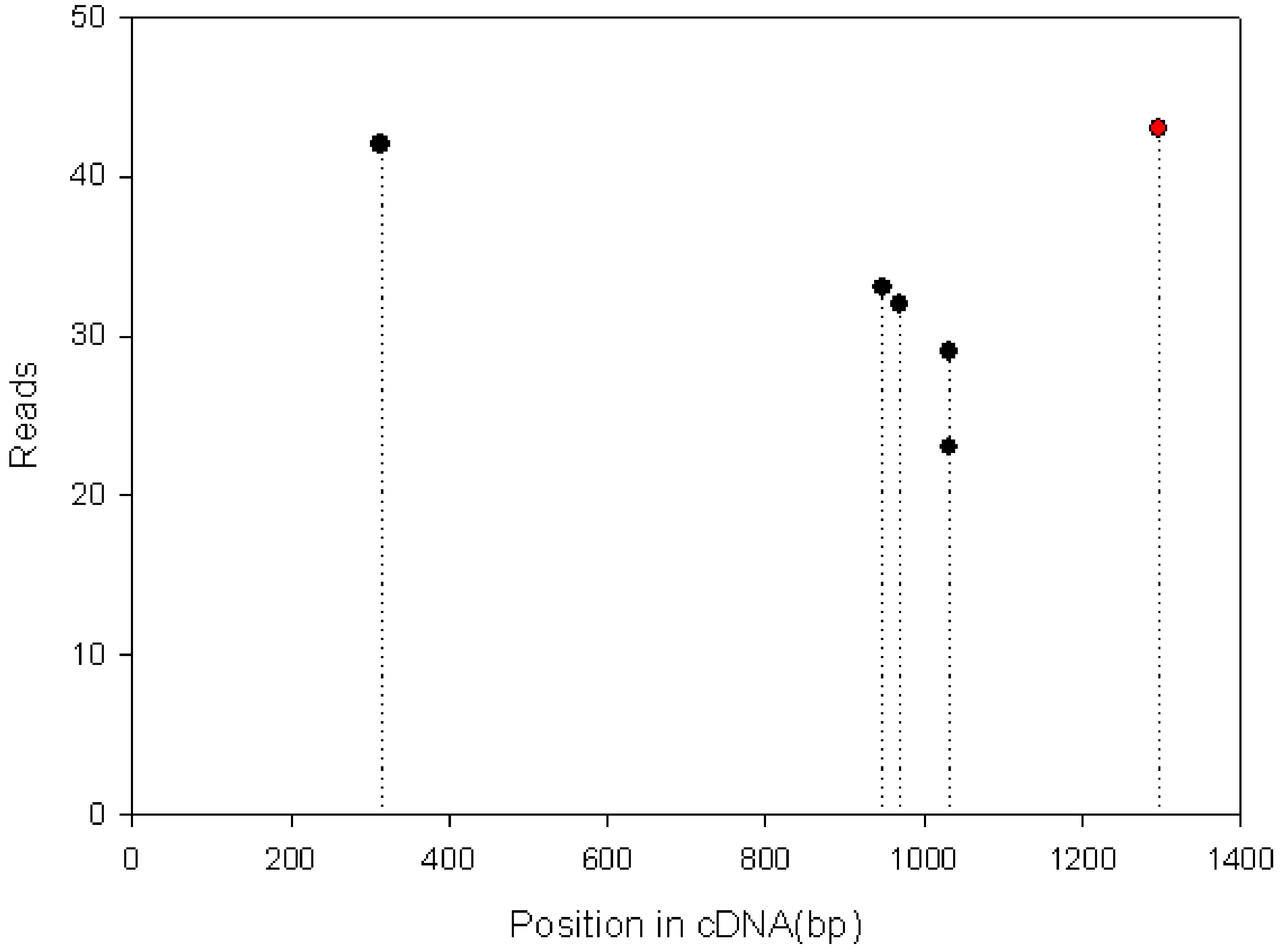


```
5' CAUUGUCAUGCAUCAUGCAAAAAAUU 3'        Cs1g12230.1
     ::.::: ::::::::::: :::
3' --AAUAGU-CGUAGUACGUUGUUUU- 5'        Csi-miRN20
```

Csi-miRN20, target=Cs3g05320.1 gene=Cs3g05320
Category:1
Score=0
Cleavage Site=451

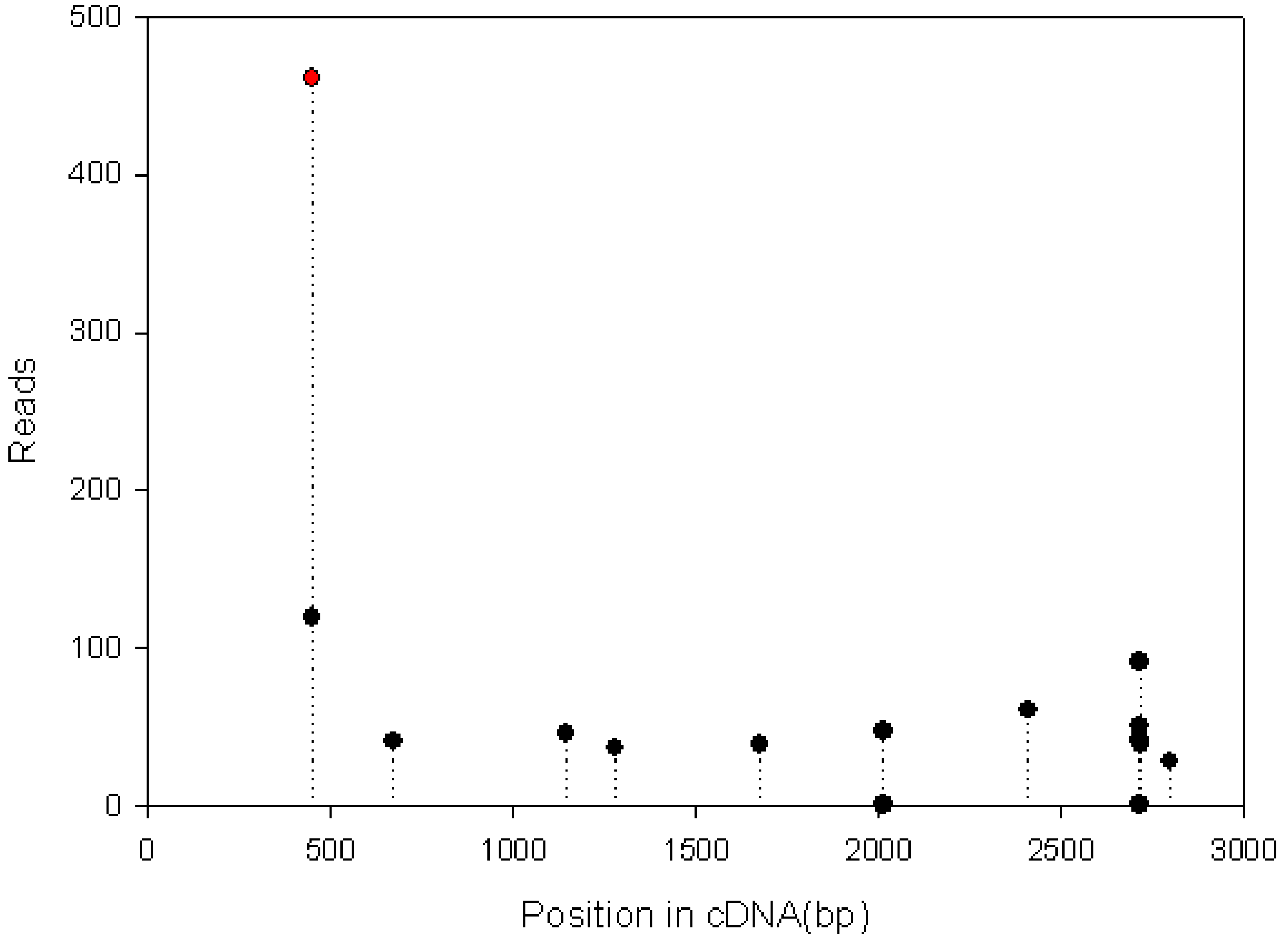


```
5' UUAUCAGCAUCAUGCAACAAAAUAGU 3'        Cs3g05320.1
   ::::::::::::::::::::::
3' AAUAGUCGUAGUACGUUGUUUU---- 5'        Csi-miRN20
```

Csi-miRN23, target=Cs1g07330.1 gene=Cs1g07330
Category:3
Score=5
Cleavage Site=130

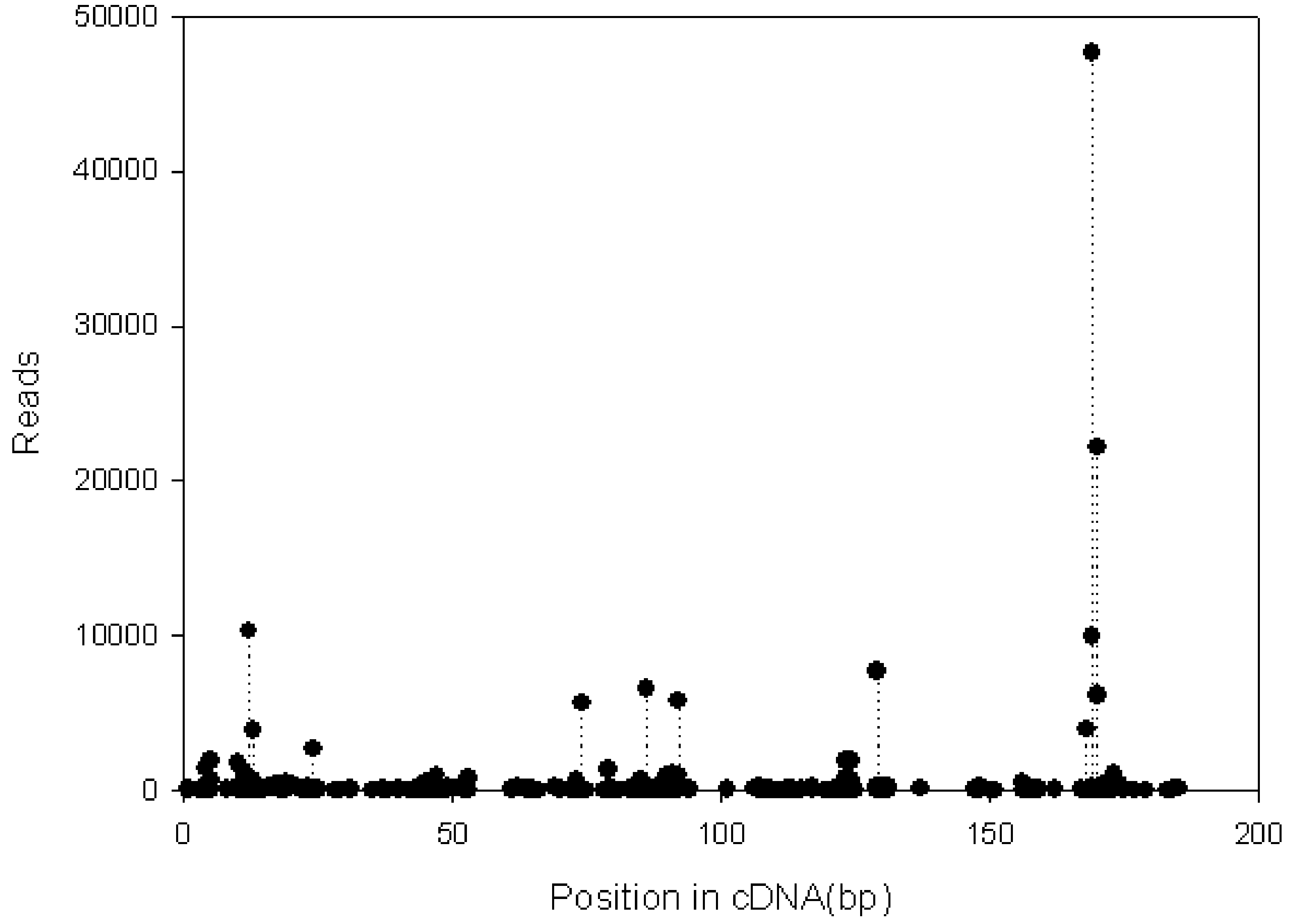


```
5' GUCAUGGACGUU-CAGGCUGCUGAGAC 3'        Cs1g07330.1
          ::::..  ::::::::::.:
3' ------CUGCGGCCUCCGACGACUUUC 5'        Csi-miRN23
```

Csi-miRN24, target=Cs1g01190.1 gene=Cs1g01190
Category:3
Score=4.5
Cleavage Site=103

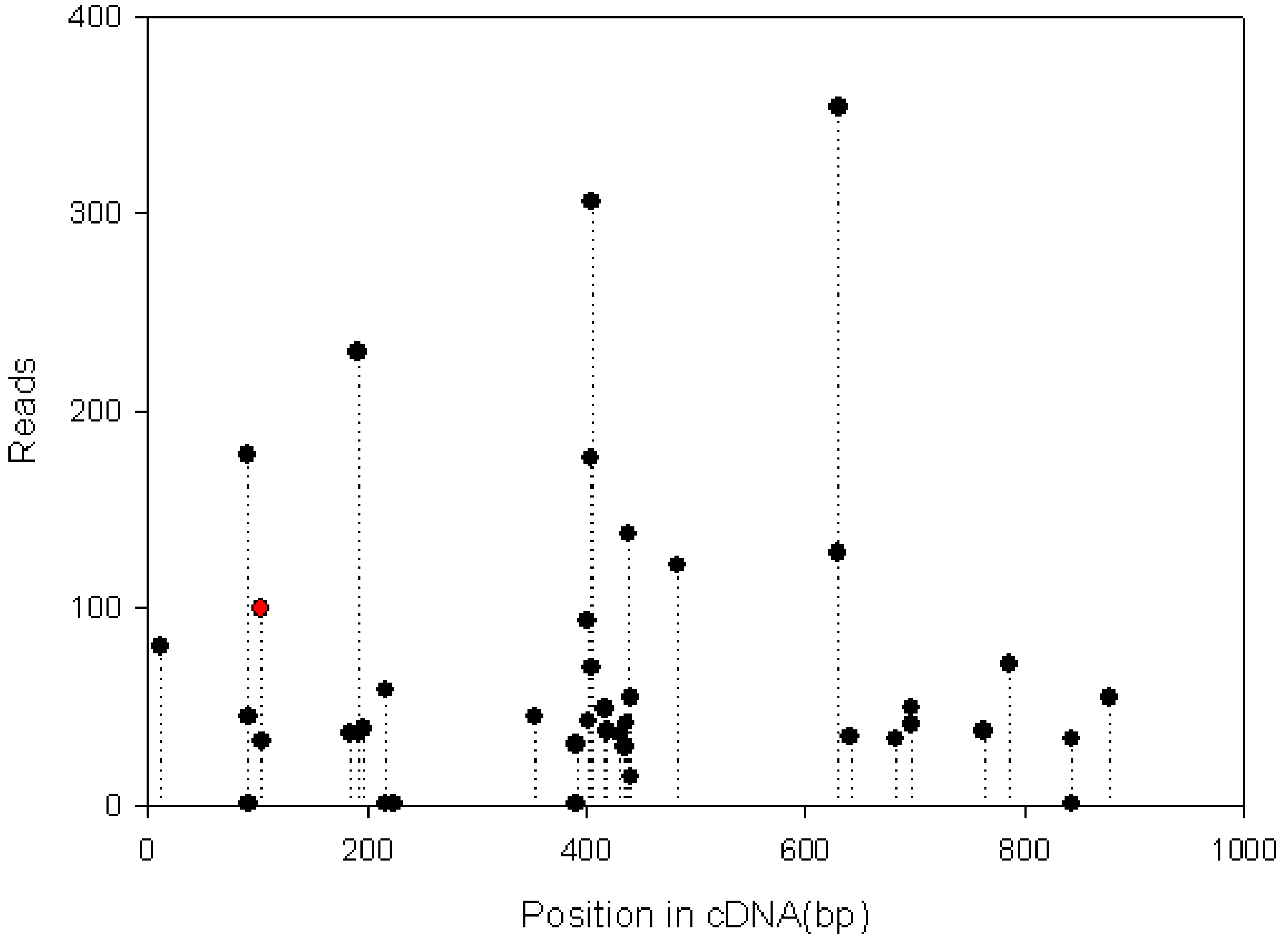


```
5' AUAGAGAAGAGCUCAGGAUGC-UGCUA 3'        Cs1g01190.1
          :   :::.:::::::: ::::
3' ------UAAUCGGGUCCUACGAACGA- 5'        Csi-miRN24
```

Csi-miRN27,target=Cs4g18880.1 gene=Cs4g18880
Category:3
Score=4.5
Cleavage Site=3257

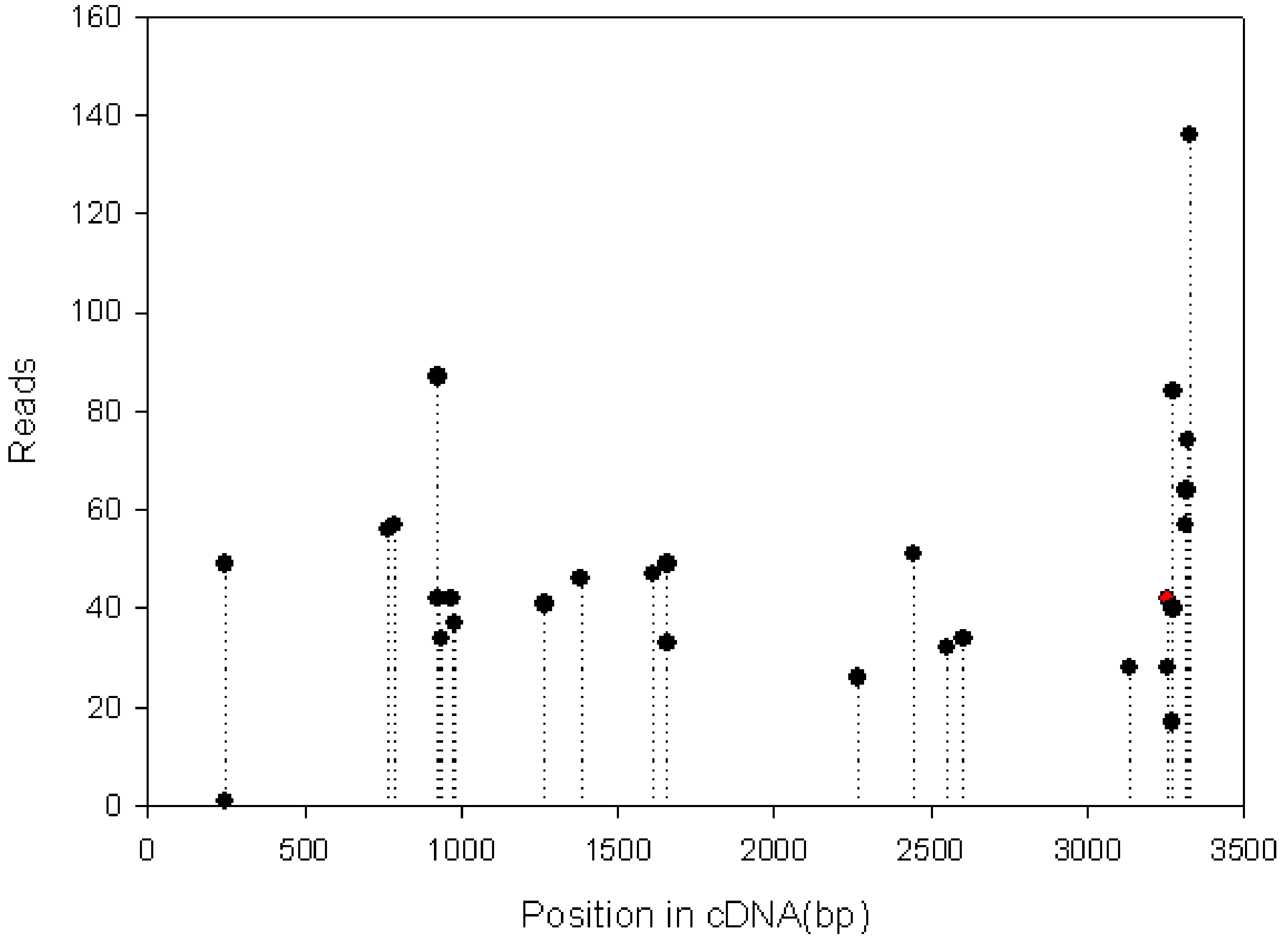


```
5' GCACGGUACUACAGUUGCAACAUAGC 3'        Cs4g18880.1
     ::  ::::.::  ::::::::::: 
3' -AUGGCAUGGUGACAACGUUGUAU-- 5'        Csi-miRN27
```

Csi-miRN28, target=Cs4g15010.1 gene=Cs4g15010
Category:2
Score=4.5
Cleavage Site=765

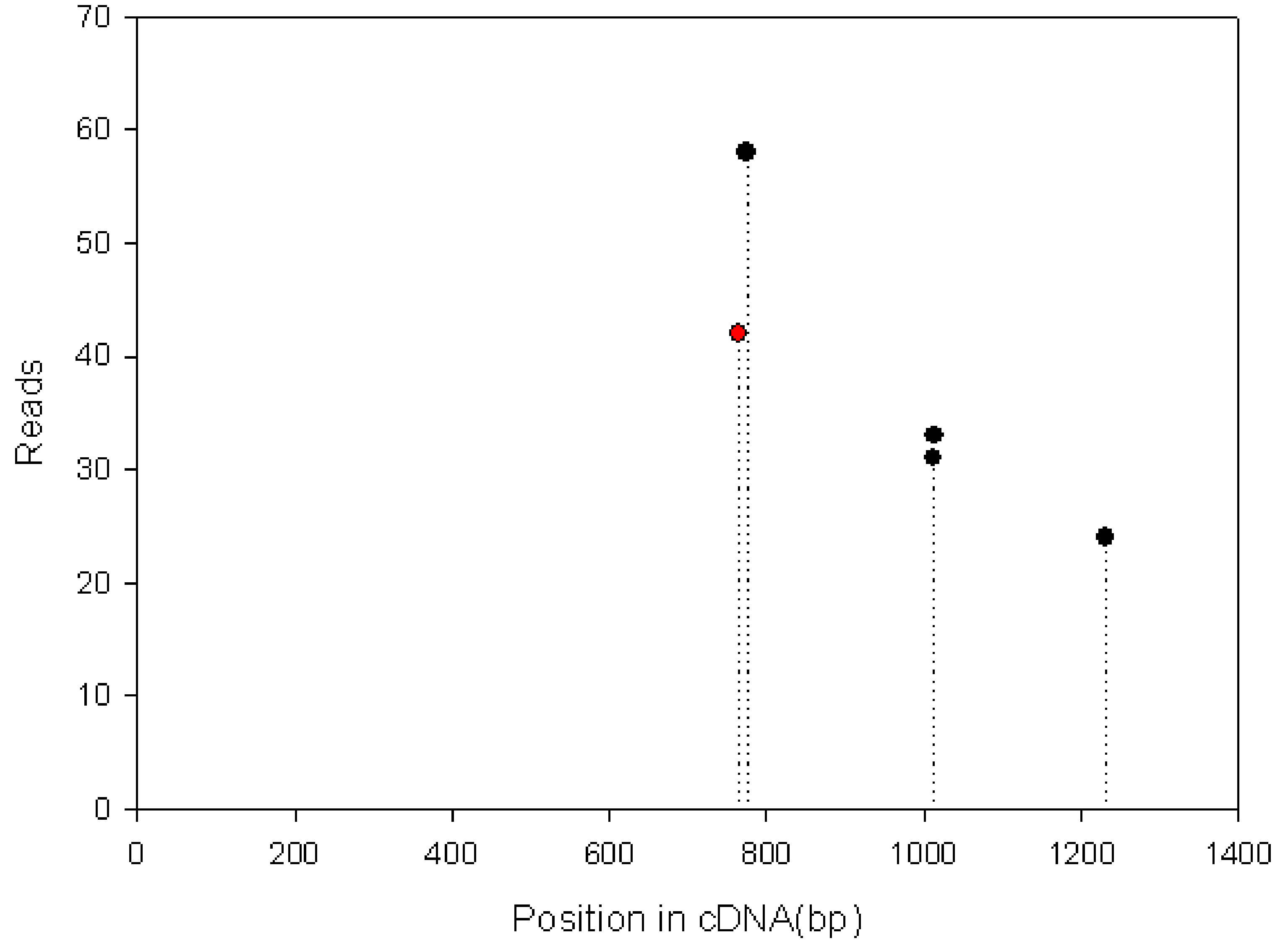


```
5' AGUACGAUCG-GCAGCUACUCCCAAUA 3'      Cs4g15010.1
         :.:: :::::::::::: ::
3' ------UGGCGCGUCGAUGAGGGGUAC 5'      Csi-miRN28
```

Csi-miRN37, target=Cs3g18880.2 gene=Cs3g18880
Category:2
Score=4.5
Cleavage Site=1309

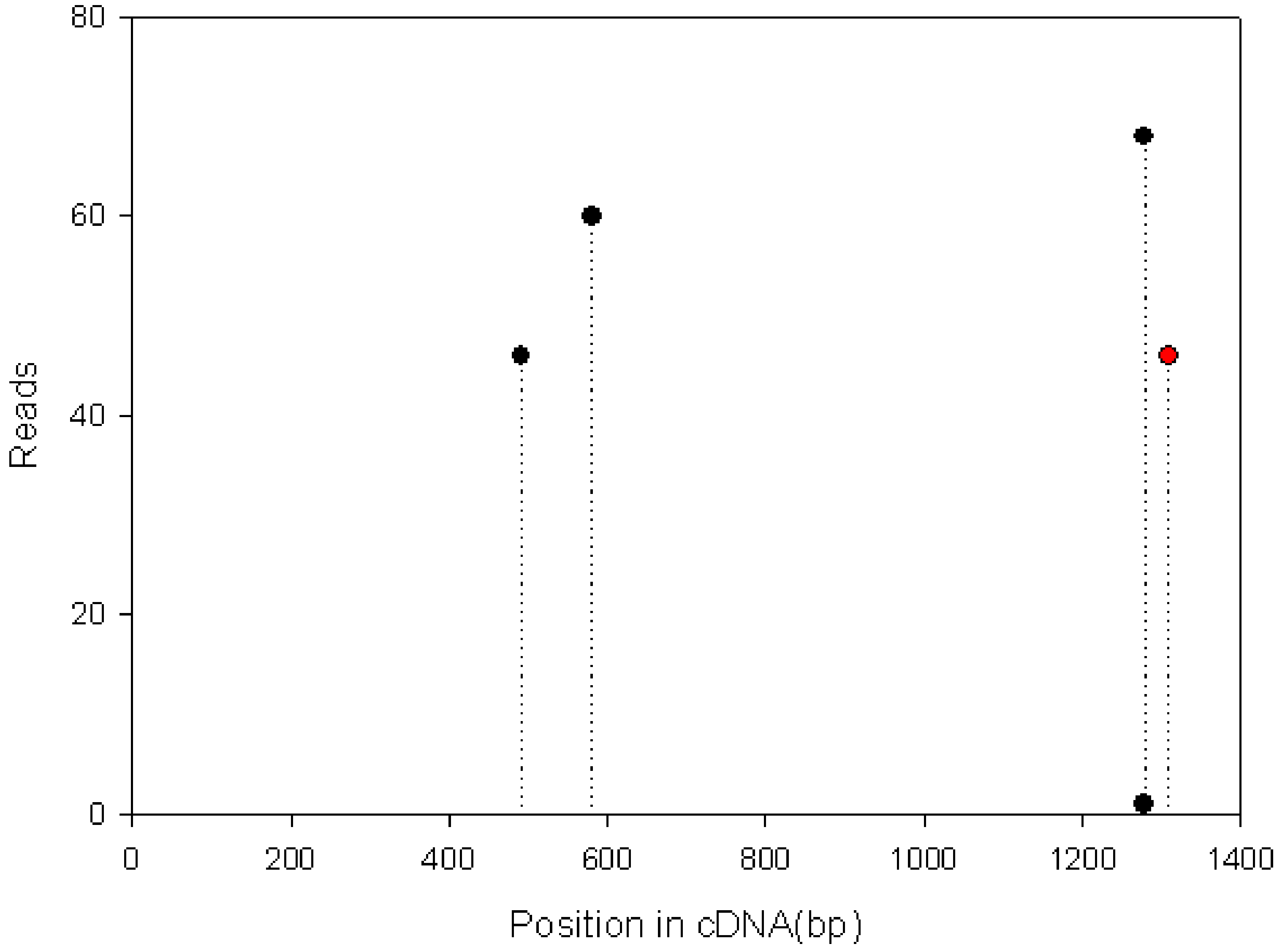


```
5' CACAACUCUCUCA-CUCUUCCCUCACU 3'        Cs3g18880.2
         :. ::: : :::::::::::
3' -----GGAAGAUUCGAGAAGGGAGU-- 5'        Csi-miRN37
```

Csi-miRN37, target=Cs7g08960.1 gene=Cs7g08960
Category:3
Score=5
Cleavage Site=442

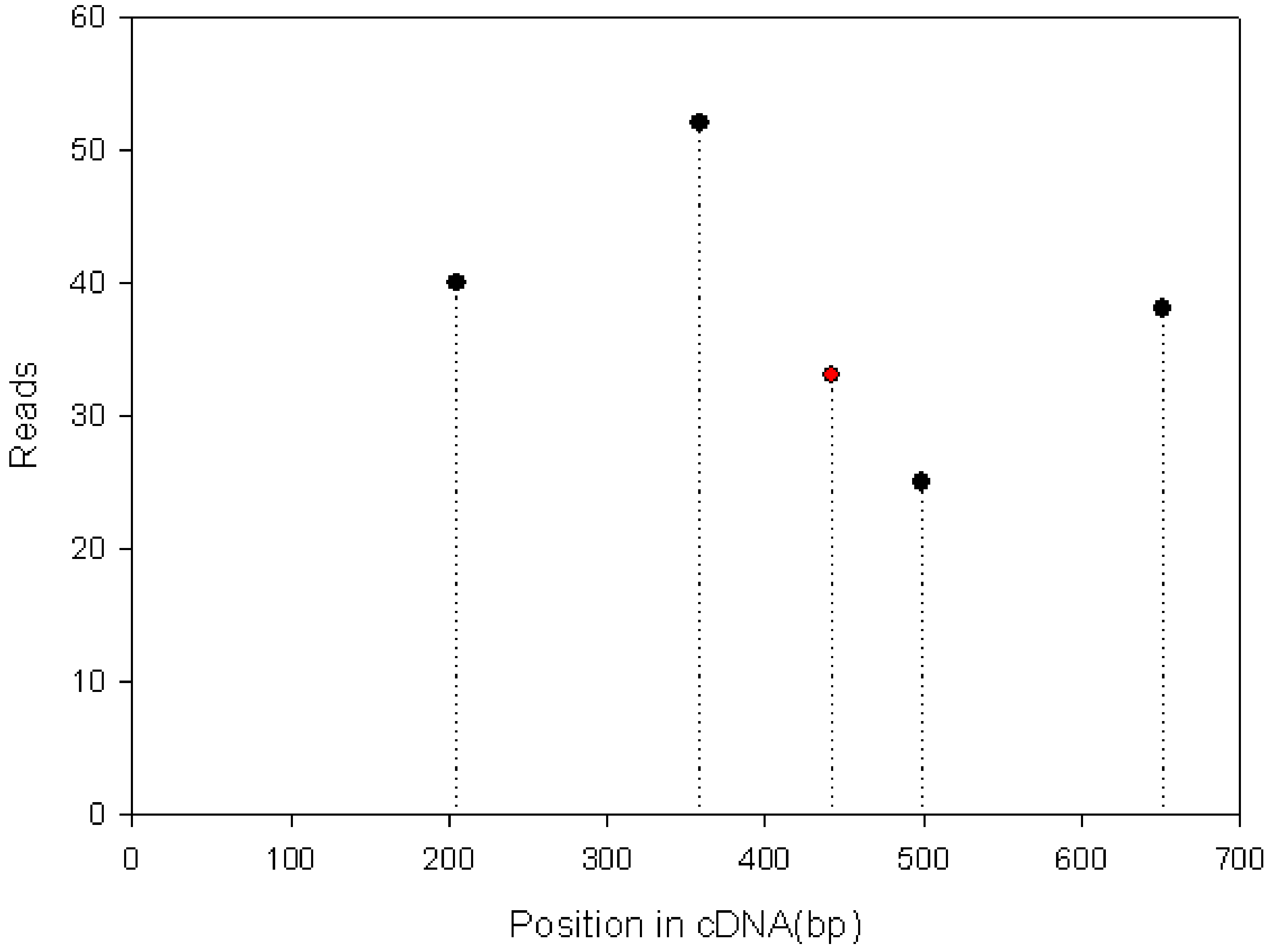


```
5' CUCUCGCCUUCCAAGCUC-UCCUUCUC 3'       Cs7g08960.1
          ::::: :::::: :::.::
3' ------GGAAGAUUCGAGAAGGGAGU- 5'       Csi-miRN37
```
