# Supplementary material for: Genome-wide comparison of microRNAs and their targeted transcripts among leaf, flower and fruit of sweet orange
Source: BMC Genomics. 2014 Aug 20;15(1):695. doi: 10.1186/1471-2164-15-695 (PMC4158063; doi:10.1186/1471-2164-15-695)

Additional file 12: Flow chart to identify the phased siRNAs and their target transcripts

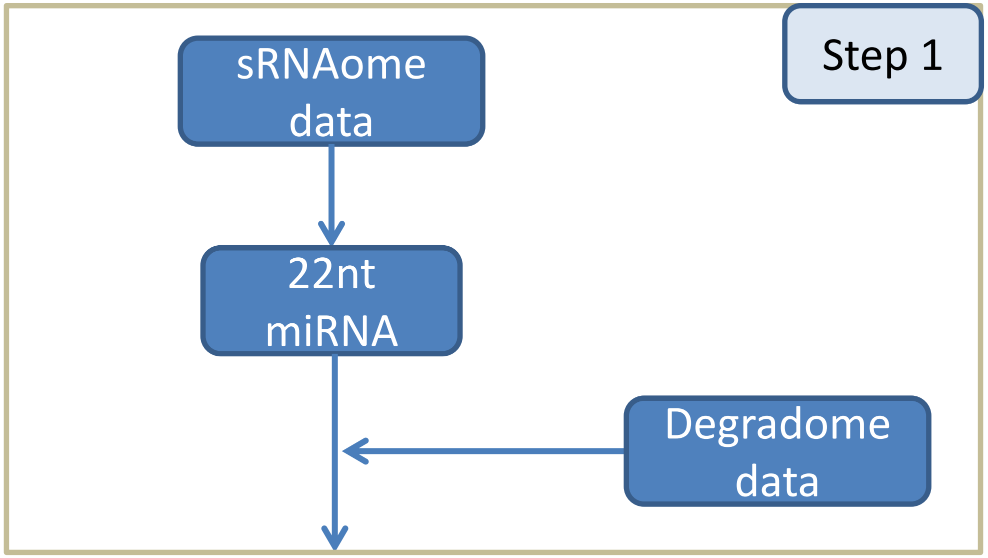

1. Obtain all miRNAs with length of 22 nt from sRNAome data and their target transcripts from degradome data.
2. Predict all 21 nt standard phased siRNAs generated from the cleavage site of target transcripts to 3’ end of targets one by one.
3. Check the expression level and target transcripts of phased siRNAs from sRNAome and degradome data.

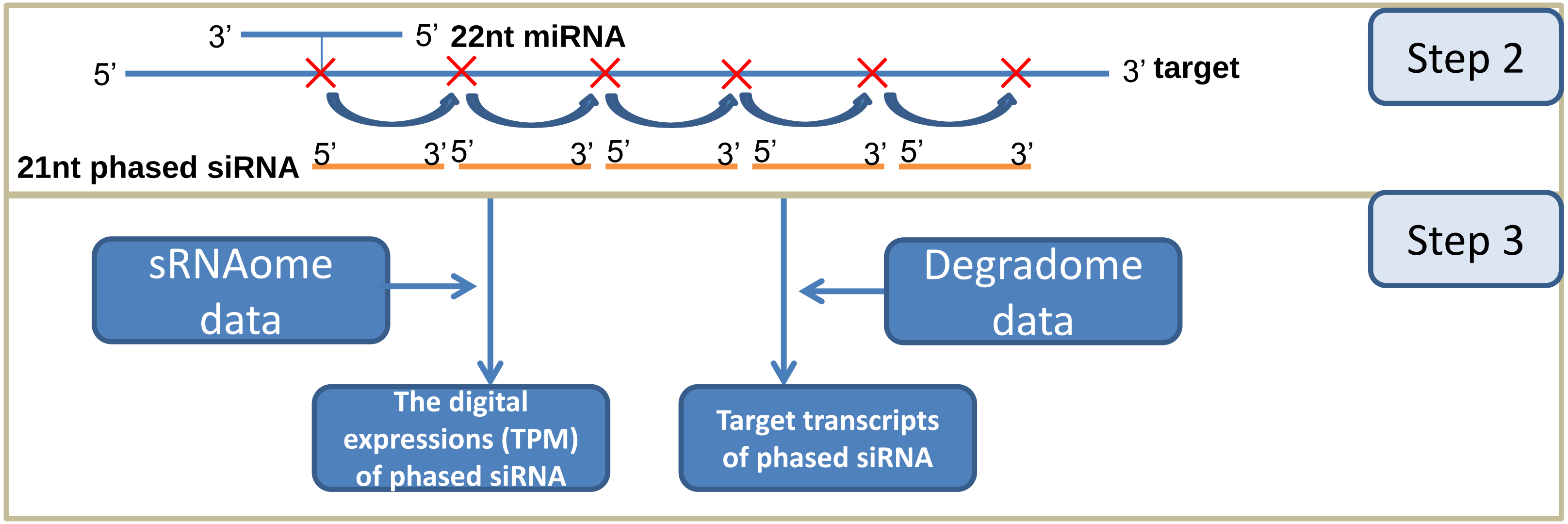

Supplement: Supplementary file 12 — Additional file 12: Flow chart to identify the phased siRNAs and their target transcripts. (PDF 227 KB) [file 12864_2014_6413_MOESM12_ESM.pdf]
